# Transcriptome-Wide Discovery of PASRs (Promoter-Associated Small RNAs) and TASRs (Terminus-Associated Small RNAs) in *Arabidopsis thaliana*

**DOI:** 10.1371/journal.pone.0169212

**Published:** 2017-01-03

**Authors:** Xiaoxia Ma, Ning Han, Chaogang Shao, Yijun Meng

**Affiliations:** 1 College of Life and Environmental Sciences, Hangzhou Normal University, Hangzhou, PR China; 2 Key Laboratory for Cell and Gene Engineering of Zhejiang Province, Institute of Genetics, College of Life Sciences, Zhejiang University, Hangzhou, Zhejiang, PR China; 3 College of Life Sciences, Huzhou University, Huzhou, PR China; Kunming University of Science and Technology, CHINA

## Abstract

Hints from animals point to the existence of two novel small RNA (sRNA) species surrounding the transcription start sites (TSSs) and the termini of the genes, respectively. In this study, we performed a comprehensive search for the two sRNA species named promoter-associated sRNAs (PASRs) and terminus-associated sRNAs (TASRs) in *Arabidopsis*. By using sRNA sequencing data from wild type plants and several mutants related to the sRNA biogenesis, Argonaute (AGO) 1- and AGO4-associated sRNA sequencing data, double-stranded RNA sequencing (dsRNA-seq) data, and DNA methylation profiling data, the biogenesis and action pathways of the PASRs and the TASRs were investigated. PASR and TASR peaks were identified on hundreds of the protein-coding genes. Deep analysis uncovered that some of the sRNA peaks were covered by dsRNA-seq reads, and these peaks were significantly repressed in specific mutants. Besides, certain PASRs and TASRs were preferentially recruited by AGO4, and site-specific DNA methylation signals encompassing the genomic loci of these sRNAs were also detected. Accordingly, we proposed a model that certain PASRs and TASRs were generated through a specific Pol IV-, RDR-, DCL-dependent pathway, and they were associated with AGO4 to perform site-specific DNA methylation on their host genes. The above results indicate the existence of PASRs and TASRs in plants. The proposed biogenesis pathway and action mode of the PASRs and TASRs could facilitate us to perform in-depth functional studies on these novel sRNA species.

## Introduction

At the beginning of the human genome sequencing project, researchers deduced that there should be thousands of protein-coding genes that made a great contribution to the complex human body structure performing highly ordered physiological functions [1]. However, subsequent gene annotations and transcriptome sequencing uncovered an unexpected phenomenon that more than 70% of the human genome produced transcription signals, and only less than 2% were originated from the protein-coding genes. In-depth analyses told us that a dominant portion of the transcribed regions on the human genome belonged to non-protein-coding genes (called non-coding genes for short). Increasing evidences are being obtained, indicating that non-coding RNAs, the transcribed products of the non-coding genes, possess essential regulatory functions, and could be one of the major contributors for the complex traits of the organisms [2].

In plants, a large number of non-coding genes have been discovered. Their transcripts were generally classified into two groups including long non-coding RNAs (longer than 200 nt) and small RNAs (sRNAs, shorter than 200 nt). To date, great progresses have been achieved on the plant sRNA research area [3]. In addition to the microRNAs (miRNAs) [4,5], several novel sRNA species have been defined in plants. For examples, natural antisense small interfering RNAs (nat-siRNAs) are processed from the highly complementary regions of the endogenous transcript pairs [6]. *Trans*-acting small interfering RNAs (ta-siRNAs) are initiated from the *TAS* transcripts cleaved by specific miRNAs [7,8]. Repeat-associated small interfering RNAs (ra-siRNAs) were generated from the repetitive element-containing loci such as transposons [9,10]. Current experimental results make clear that miRNAs, mostly 5’ U-started and 21 nt in length, are preferentially loaded into Argonaute 1 (AGO1)-associated silencing complexes [11]. The silencing complexes are guided by specific miRNAs to bind onto their target transcripts containing complementary miRNA recognition sites. Relying on the endonuclease activity of the AGO1 complexes, the targets are cleaved in the middle of the recognition sites, resulting in the post-transcriptional regulation of the target genes [4,5]. On the other hand, the 5’ A-started, 24-nt-long siRNAs are preferentially incorporated into AGO4-containing silencing complexes [11]. Also based on sequence complementarity, siRNA-guided AGO4 complexes are able to perform DNA methylation on specific genomic regions, defined as RNA-directed DNA methylation (RdDM). Different from the miRNAs, the AGO4-associated siRNAs regulate their target genes at transcriptional level, such as transposon silencing [3]. Facilitated by the genetic studies on the plant mutants, the biogenesis pathways of certain sRNAs were dissected. Most of the miRNA genes are transcribed by RNA polymerase (Pol) II, and Dicer-like 1 (DCL1), SERRATE and HYPONASTIC LEAVES 1 are implicated in the processing of miRNA precursors into mature miRNAs [4,5]. Differently, the biogenesis of ta-siRNAs involves DCL4 and RNA-dependent RNA polymerase 6 (RDR6), and ra-siRNAs are generated through an RNA Pol IV-, DCL3- and RDR2-dependent pathway [3,4]. Numerous pieces of experimental evidences demonstrate that miRNAs and some other sRNAs play important roles in plant organ development, and are involved in nearly all aspects of the physiological and metabolic processes [3,4]. Thus, discovery of novel sRNA species and depicting their biogenesis and action pathways become an essential issue for functional and mechanistic studies on plant development and gene regulation.

Recent years, sRNAs originated from the promoter regions of certain genes were successively discovered in animals, fungi and bacteria, and were named as promoter-associated small RNAs (PASRs) [12–20]. Pieces of experimental evidences indicate that these PASRs are involved in chromatin modifications within the promoter regions, thus modulating the transcription levels of their host genes [18,21–24]. However, we know little about PASRs in plants. In the review article by Taft *et al*. (2009), they performed a systemic search for the PASRs in *Arabidopsis* (*Arabidopsis thaliana*) and *Caenorhabditis elegans* by using sRNA high-throughput sequencing (HTS) data. As a result, PASR peaks were identified on the genes of *Caenorhabditis elegans*, but no significant signal was detected in *Arabidopsis*. Thus, they made a conclusion that PASRs did not conservatively exist in plants, or the plant PASRs were subjected to rapid degradation after their generation [25]. Two years later, Wang *et al*. (2011) revisited the scene of PASRs in *Arabidopsis* by using much more HTS data sets. As a result, the PASR peaks were observed surrounding the transcription start sites (TSSs) of the non-TE (transposable element) genes [26]. Unfortunately, no deeper view was provided to uncover the sequence characteristics and the biogenesis pathways of these PASRs. Another shortcoming in Wang *et al*.’s study is that a total of 17,000 non-TE genes were treated as a whole group for the detection of PASR peaks, and they did not tell us the exact genes encoding PASRs. Interestingly, another novel sRNA species termed TASRs (terminus-associated small RNAs) were recently identified in human and yeast [14,27–30]. According to the report by Kapranov *et al*. (2010), TASRs play a role in increasing the copy number of the transcripts of their host genes [29]. However, no related study on TASRs has been reported in plants.

In our study, by using sRNA HTS data, double-stranded RNA sequencing (dsRNA-seq) data and DNA methylation profiling data, the trace of both PASRs and TASRs was detected on hundreds of the *Arabidopsis* genes. Specifically, surrounding the TSSs, PASR peaks were identified on the sense strands of 233 protein-coding genes, and on the antisense strands of 231 protein-coding genes. Among the above peaks, a total of 65 paired PASR peaks distributed on both stands of the protein-coding genes were discovered. Surrounding the transcription termini, TASR peaks were identified on the sense strands of 287 protein-coding genes, and on the antisense strands of 265 protein-coding genes. Among the above peaks, a total of 132 paired TASR peaks distributed on both stands of the protein-coding genes were discovered. Interestingly, we observed that in many cases, the PASRs or the TASRs mapped to the transcription boundaries of the genes located on the chloroplast genome were highly abundant in the green organs such as leaves. We also showed that a portion of PASRs and TASRs potentially involved in site-specific DNA methylation might be generated through an RDR- and DCL-dependent pathway. Taken together, the above results indicate the existence of PASRs and TASRs in plants. The proposed biogenesis pathways and action modes of the PASRs and TASRs could facilitate us to perform in-depth functional studies on these novel sRNA species.

## Results

### Identification of PASR and TASR peaks on protein-coding genes

**Fig 1:**
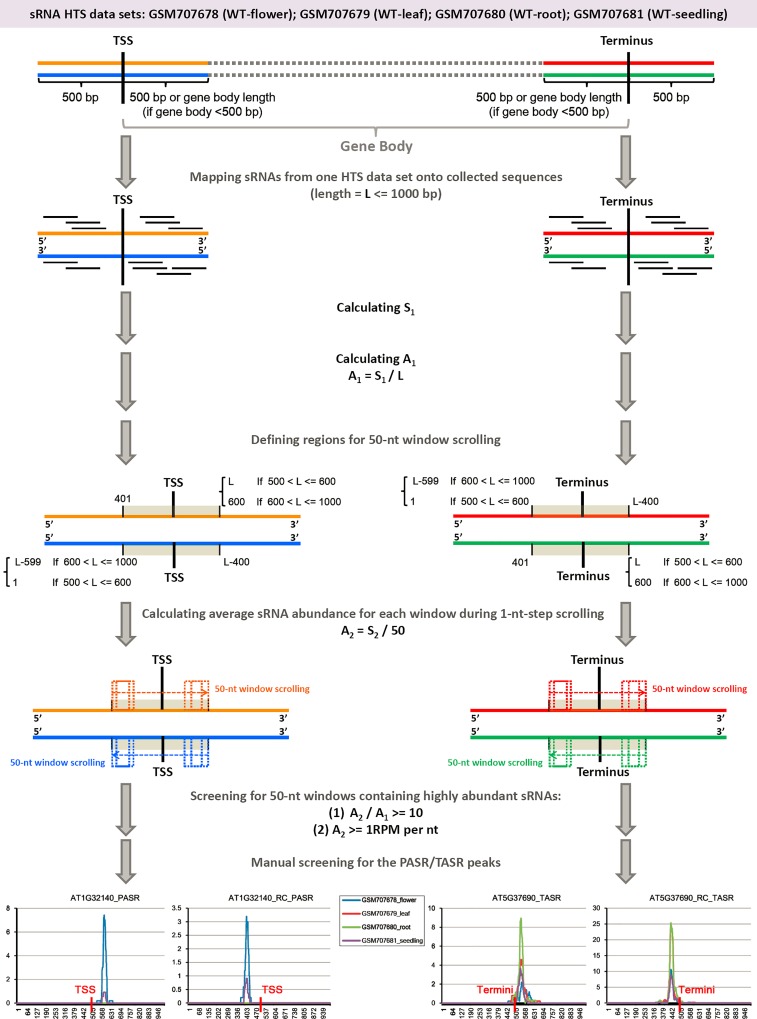
Analytical workflow for the transcriptome-wide identification of PASR (promoter-associated small RNA) and TASR (terminus-associated small RNA) peaks on protein-coding genes of *Arabidopsis*. Four small RNA (sRNA) high-throughput sequencing data sets including GSM707678 (the flowers of wild type plants), GSM707679 (the leaves of wild type plants), GSM707680 (the roots of wild type plants) and GSM707681 (the seedlings of wild type plants) were utilized for this analysis. Each step including the parameters for PASR and TASR identification was provided. TSS: transcription start site. Terminus: transcription terminus. RPM: reads per million. See detailed description in “Materials and Methods”.

**Fig 2:**
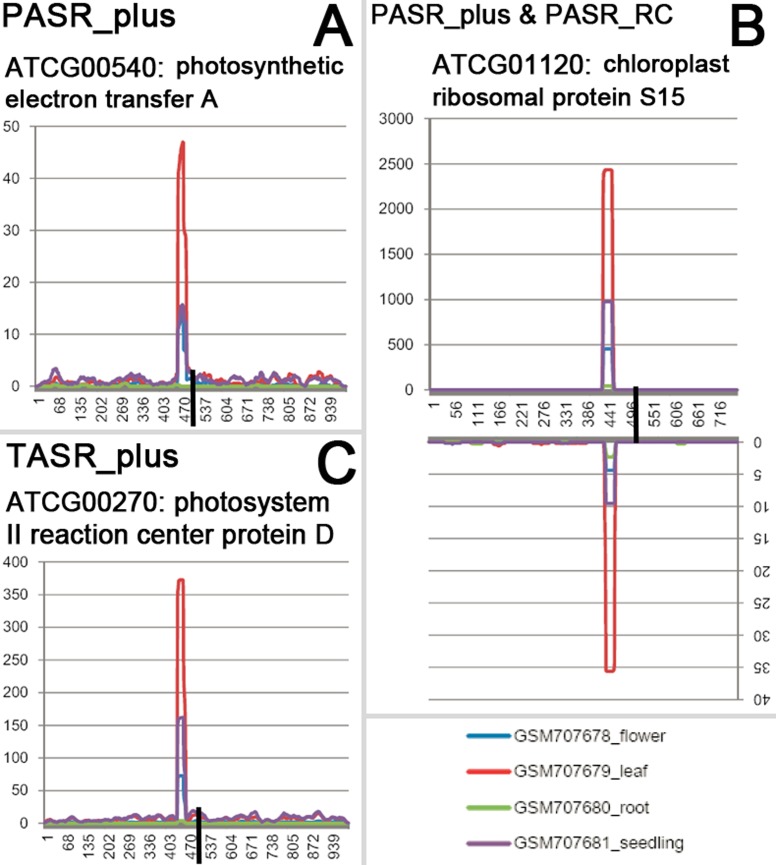
Examples of the chloroplast genes generating PASRs (promoter-associated small RNAs) or TASRs (terminus-associated small RNAs) dominantly in green organ (leaves) of *Arabidopsis*. (A) The PASR peak was identified on the sense strand of *ATCG00540* (encoding photosynthetic electron transfer A). The *x* axis measures the genomic positions surrounding the TSS (transcription start site, marked by a black vertical bar) of this gene. The *y* axis measures the abundance of the small RNAs perfectly mapped onto the genomic region surrounding the TSS, which is also applied to the other figure panels. (B) The PASR peak was identified on both strands of *ATCG01120* (chloroplast ribosomal protein S15). The *x* axis measures the genomic positions surrounding the TSS of this gene. (C) The TASR peak was identified on the sense strand of *ATCG00270* (photosystem II reaction center protein D). The *x* axis measures the genomic positions surrounding the transcription terminus (marked by a black vertical bar) of this gene.

As summarized in Fig 1 (also see details in Materials and Methods), we started a transcriptome-wide search for the PASR and TASR peaks on the protein-coding genes of *Arabidopsis*. After manual screening, PASR peaks were identified on the sense strands of 233 protein-coding genes [including 18 and four genes located on the chloroplast genome (named as chloroplast genes hereafter) and the mitochondrial genome (named as mitochondrial genes hereafter) respectively] (S1 Fig), and on the antisense strands of 231 protein-coding genes (including four chloroplast genes) (S2 Fig). TASR peaks were identified on the sense strands of 287 protein-coding genes (including six chloroplast genes and 15 mitochondrial genes) (S4 Fig), and on the antisense strands of 265 protein-coding genes (including one mitochondrial gene) (S5 Fig). Among the above identified genes, we observed that a total of 65 genes possessed paired PASR peaks symmetrically distributed on both sense and antisense strands (S3 Fig), and 132 genes possessed paired TASR peaks on both strands (S6 Fig). Besides, we also noticed that for a large portion of chloroplast genes possessing PASR or TASR peaks (such as *ATCG00140*, *ATCG00270* and *ATCG01120*), the PASRs and TASRs were prominently accumulated in leaves and young seedlings in which the chloroplasts were highly enriched (Fig 2 and S1, S2, S3 and S4 Figs).

**Fig 3:**
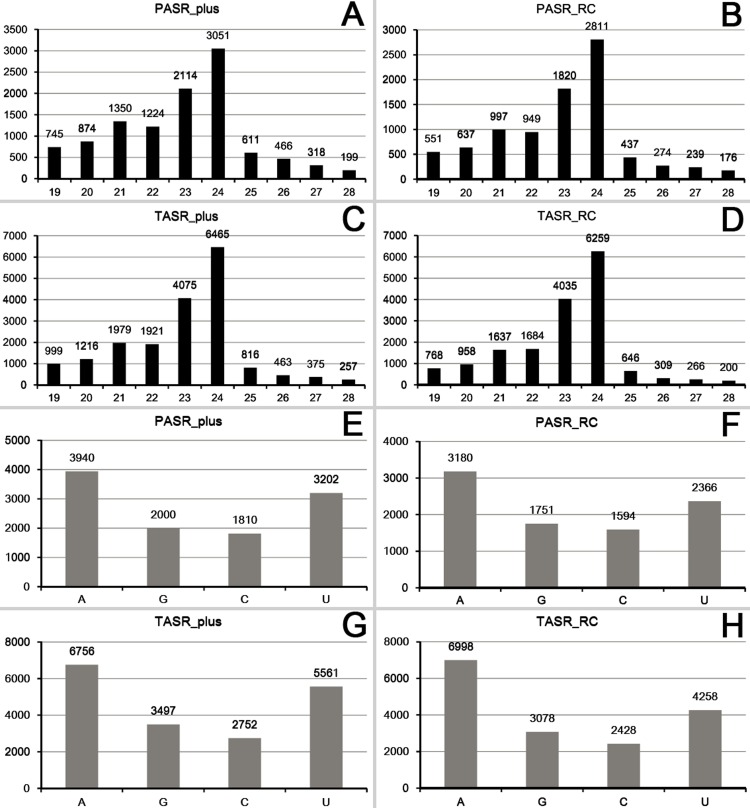
Sequence characteristics of the PASRs (promoter-associated small RNAs) and the TASRs (terminus-associated small RNAs). (A) Sequence length distribution of the PASRs identified on the sense strands of the protein-coding genes of *Arabidopsis*. (B) Sequence length distribution of the PASRs identified on the antisense strands of the protein-coding genes of *Arabidopsis*. (C) Sequence length distribution of the TASRs identified on the sense strands of the protein-coding genes of *Arabidopsis*. (D) Sequence length distribution of the TASRs identified on the antisense strands of the protein-coding genes of *Arabidopsis*. (E) 5’ terminal nucleotide compositions of the PASRs identified on the sense strands of the protein-coding genes of *Arabidopsis*. (F) 5’ terminal nucleotide compositions of the PASRs identified on the antisense strands of the protein-coding genes of *Arabidopsis*. (G) 5’ terminal nucleotide compositions of the TASRs identified on the sense strands of the protein-coding genes of *Arabidopsis*. (H) 5’ terminal nucleotide compositions of the antisense strands of the protein-coding genes of *Arabidopsis*.

Next, we set out to analyze the sequence characteristics of the PASRs and TASRs. The sRNAs located within or partially resided within the previously defined regions for 50-nt window scrolling (see Fig 1) were included in this analysis. We realized that these sRNAs were not only constituted by PASRs or TASRs, but also included the sRNAs surrounding the PASR or TASR peaks. However, this analysis is performed based on the following considerations: (1) The identification of the PASR and TASR peaks was assisted by computer programming combined with manual screening. Thus, it will be a hard task to extract the exact genomic region for each PASR or TASR peak since hundreds of the identified peaks have distinct locations. In contrast, it will be easier to extract the sRNAs located within or partially overlapped with the uniformly defined regions for 50-nt window scrolling. (2) Although the sRNAs regarded as “noise” were included in this analysis, a dominant portion of the analyzed sRNAs were constituted by those located within the PASR or TASR peaks. (3) The “noise” sRNAs distribute surrounding the TSSs or the transcription termini of the protein-coding genes. To some extent, these sRNAs outside the peaks could also be regarded as TSS- or transcription terminus-associated sRNAs. Thus, a total of 10,952 PASRs and 18,566 TASRs on the sense strands, and 8,891 PASRs and 16,762 TASRs on the antisense strands were included for the analysis, and were termed as “PASR_plus”, “PASR_RC”, “TASR_plus” and “TASR_RC”. Notably, for the four categories, 23- to 24-nt sRNAs occupied a dominant portion (47.16% of “PASR_plus”, 52.09% of “PASR_RC”, 56.77% of “TASR_plus” and 61.41% of “TASR_RC”) followed by 21- to 22-nt ones (23.50% of “PASR_plus”, 21.89% of “PASR_RC”, 21.00% of “TASR_plus” and 19.81% of “TASR_RC”). Moreover, the 5’ A-started sRNAs occupied a dominant portion of the four categories (35.98% of “PASR_plus”, 35.77% of “PASR_RC”, 36.39% of “TASR_plus” and 41.75% of “TASR_RC”), followed by the 5’ U-started ones (29.24% of “PASR_plus”, 26.61% of “PASR_RC”, 29.95% of “TASR_plus” and 25.40% of “TASR_RC”) (Fig 3).

### AGO loading preference of the PASRs and the TASRs

As introduced in the above section, sRNAs with different sequence features will be selectively recruited by distinct AGO-associated silencing complexes [11], leading to divergent pathways for sRNA actions [3–5]. Thus, it is prioritized to see the AGO loading preference of the PASRs and the TASRs before investigating their biological functions. According to the sequence features, a large portion of the PASRs and the TASRs are 5’ A-started and 23- to 24-nt in length, followed by 5’ U-started, 21- to 22-nt ones (Fig 3). The above characteristics are close to the sRNAs preferentially associated with AGO4 and AGO1 respectively [11]. In this consideration, four sRNA HTS data sets prepared from the sRNAs associated with AGO1 (GSM707682, GSM707683, GSM707684 and GSM707685; see detail in Materials and Methods), and four data sets from the sRNAs associated with AGO4 (GSM707686, GSM707687, GSM707688 and GSM707689; see detail in Materials and Methods) were included in this analysis. To investigate whether the AGO-associated sRNAs could also form peaks, the sRNA sequencing reads of the eight HTS data sets were mapped onto the genomic sequences containing TSSs or transcription terminus (the sequence range was described in Fig 1) of the protein-coding genes with PASR or TASR peaks (listed in S1, S2, S4 and S5 Figs), and the perfectly mapped reads were retained for “50-nt window” screening (refer to Materials and Methods). As a result, AGO-associated PASR peaks were identified on the sense strands of 145 protein-coding genes (62.23% of the 233 genes identified above) (S7 Fig), and on the antisense strands of 134 protein-coding genes (58% of the 231 genes identified above) (S8 Fig). AGO-associated TASR peaks were identified on the sense strands of 142 protein-coding genes (49.48% of the 287 genes identified above) (S10 Fig), and on the antisense strands of 128 protein-coding genes (48.30% of the 265 genes identified above) (S11 Fig). We also observed that a total of 56 genes (86.15% of the 65 genes with previously identified paired PASR peaks) possessed paired AGO-associated PASR peaks (S9 Fig), and 130 genes (98.48% of the 132 genes with previously identified paired TASR peaks) possessed paired AGO-associated TASR peaks (S12 Fig). To determine which AGO the sRNAs are preferentially loaded into, an accumulation level-based comparison between AGO1-associated and AGO4-associated sRNAs resided within the same peaked regions on specific genes was performed. The result showed that many sRNA peaks on certain genes exhibited specific AGO preference. For examples, the PASRs within the peaked regions on the genes *AT1G01073*, *AT1G08030*, *AT1G08035*, *AT1G09060*, *AT1G10820*, *AT2G23950*, *AT4G16460* and *AT5G35526* are preferentially incorporated into AGO4 (S7 and S8 Figs). The TASRs within the peaked regions on the genes *AT1G01180*, *AT1G20830*, *AT1G26400*, *AT1G28140*, *AT1G36950*, *AT2G14247*, *AT2G18465* and *AT5G26770* are also preferentially recruited by AGO4 (S10 and S11 Figs). On the other hand, the PASRs identified on the genes *AT3G06110*, *AT4G24026*, *AT5G07140*, *AT5G48830*, *ATCG00050* and *ATCG00065* are preferentially incorporated into AGO1 (S7 and S8 Figs). The TASRs identified on *AT1G16460*, *AT2G07771*, *AT2G28725*, *AT4G24030*, *ATMG00270* and *ATMG00570* are preferentially loaded into AGO1 (S10 and S11 Figs). Among the sRNAs preferentially loaded into one of the AGO proteins, we noticed that some of these sRNAs also showed organ-specific accumulation patterns. For examples, some of the PASRs preferentially associated with AGO1 were observed to be specifically accumulated in roots (for the PASRs identified on the genes *AT1G53542*, *AT1G64130*, *AT2G38025*, *AT3G18820*, *AT3G52570*, *AT5G02820* and *AT5G05950*), seedlings (*ATCG00280*), or flowers (*AT1G61030*, *AT3G16230* and *AT4G14810*). Some of the PASRs preferentially associated with AGO4 were specifically detected in roots (*AT5G48280*) or flowers (*AT2G24670* and *AT3G42550*) (S7 and S8 Figs). Moreover, some of the TASRs preferentially associated with AGO1 were specifically accumulated in roots (*AT1G18420*, *AT1G50110*, *AT4G25580*, *AT4G25590*, *AT4G30993*, *AT4G34030*, *AT5G06130*, *AT5G52360*, *AT5G60280* and *ATMG00070*) or seedlings (*ATCG00270*, *ATCG00420*, *ATCG00840*, *ATCG01300* and *ATMG00900*). Some of the TASRs preferentially associated with AGO4 were specifically detected in roots (*AT1G22720*) or seedlings (*AT2G02520*) (S10 and S11 Figs).

### PASR and TASR peaks covered by dsRNA-seq reads

To date, several sRNA species such as nat-siRNAs, ta-siRNAs, ra-siRNAs (or heterochromatic siRNAs) have been reported to be produced through RDR-dependent pathways [3,5–10]. In other words, the biogenesis of most of the currently known siRNAs requires RDR for dsRNA precursor synthesis. Thus, it is intriguing to see whether the PASRs and the TASRs are originated from double-stranded precursors. Recently, Zheng and his colleagues developed an approach called dsRNA-seq by marrying the classical nuclease-based structure mapping technique with HTS [31]. Before sequencing library construction, the ribosomal RNA (rRNA)-depleted RNA samples were treated with the single-strand-specific ribonuclease, thus enabling transcriptome-wide identification of the dsRNAs *in vivo*. In this consideration, dsRNA-seq data sets GSM575243 and GSM575244, a gift of Zheng *et al*.’s study, were recruited for this study. The dsRNA-seq reads were mapped onto the genomic sequences containing TSSs or transcription termini (described in Fig 1) of the protein-coding genes with the above identified PASR or TASR peaks, and the perfectly mapped reads were retained. As a result, PASR peaks on the sense strands of 30 protein-coding genes (S13 Fig) and on the antisense strands of 13 genes (S14 Fig), and TASR peaks on the sense strands of 29 protein-coding genes (S16 Fig) and on the antisense strands of nine genes (S17 Fig) were identified to be resided within the continuous regions of 100 nt or longer which were covered by dsRNA-seq reads. Notably, pairs of PASR peaks (on both sense and antisense strands), covered by dsRNA-seq reads, were identified on seven genes (*AT1G16820*, *AT1G53265*, *AT3G13857*, *AT3G43270*, *AT3G57770*, *AT4G16640* and *AT5G48000*) (S15 Fig), and pairs of TASR peaks were identified on 13 genes (*AT1G28304*, *AT1G66620*, *AT3G25130*, *AT3G41762*, *AT3G52830*, *AT4G04030*, *AT4G08160*, *AT4G14365*, *AT5G43525*, *AT5G50480*, *AT5G54410*, *AT5G54700* and *AT5G65005*) (S18 Fig). Summarily, the result indicates that a portion of PASRs and TASRs are likely to be processed from dsRNA precursors.

### DCL-, RDR- and Pol IV-dependent biogenesis pathway of certain PASRs and TASRs

The result that certain PASRs and TASRs were potentially produced from double-stranded precursors prompted us to interrogate the particular biogenesis pathway(s) that they were involved in. The dependence of the sRNAs on DCLs and RDRs was analyzed by using sRNA HTS data prepared from wild type plants, and *dcl* and *rdr* mutants (see details in Materials and Methods). According to the biogenesis model of the heterochromatic siRNAs which contribute to a great portion of the plant endogenous siRNAs, Pol IV is required for the transcription of the siRNA precursors. Relying on RDR2, the transcripts are converted to long dsRNAs which are then subjected to DCL3-mediated processing for siRNA production. A portion of the heterochromatic siRNAs was loaded into AGO4 silencing complexes to guide site-specific chromatin modifications [3]. In this study, the sRNA HTS data sets prepared from the *pol iv* mutants (including *nrpd1a* and *nrpd1b*) were also included. The sequencing reads of the above mentioned sRNA HTS data were mapped onto the genomic sequences containing TSSs or transcription termini of the protein-coding genes with PASR or TASR peaks, and the perfectly mapped reads were retained for “50-nt window” screening. After screening, we made a comparison of the signal intensity between the “wild type” peaks and the “mutant” peaks, in order to detect the dependence of PASRs and TASRs on DCL(s), RDR(s) and Pol IV. Based on the comparison, we could also see which DCL(s) and RDR(s) are specifically implicated in the biogenesis of the sRNAs forming a PASR or TASR peak. As a result, many PASR and TASR peaks show a clear dependence on specific DCLs, RDRs and Pol IV (S19 Fig). For examples, the accumulation level of the PASR peak on the sense strand of *AT1G53265* is greatly repressed in the mutants *dcl3*, *dcl234*, *rdr2* and *rdr6*. The abundance of the PASR peak on the antisense strand of *AT4G16640* is greatly reduced in *dcl234*, *rdr2*, *nrpd1a* and *nrpd1b*. The level of the TASR peak on the sense strand of *AT3G41762* is significantly repressed in *dcl2*, *dcl234*, *rdr2* and *nrpd1a*. The accumulation level of the TASR peak on the antisense strand of *AT3G41762* is significantly repressed in *dcl234*, *rdr2* and *nrpd1a*. Notably, the DCL/RDR/Pol IV dependence was detected for the paired PASR peaks on *AT1G53265*, *AT4G16640* and *AT5G48000*, and the paired TASR peaks on *AT1G28304*, *AT3G25130*, *AT3G41762*, *AT3G52830*, *AT4G04030*, *AT4G08160*, *AT4G14365*, *AT5G43525*, *AT5G50480*, *AT5G54700* and *AT5G65005* (S20 Fig).

### Site-specific DNA methylation signals detected on the genes with PASR or TASR peaks

**Fig 4:**
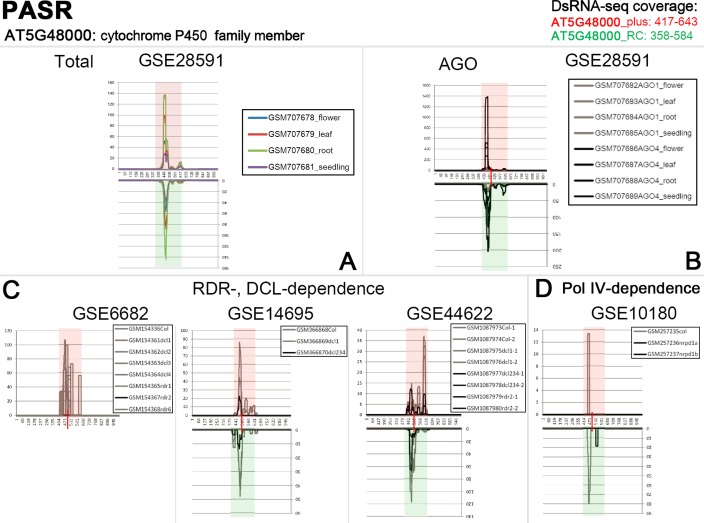
PASRs (promoter-associated small RNAs) identified on both strands of *AT5G48000*, and small RNA (sRNA) and double-stranded RNA (dsRNA) high-throughput sequencing (HTS)-based evidences showing potential Argonaute (AGO) loading preference and biogenesis pathways of PASRs. (A) Total: Initially, four sRNA HTS data sets belonging to GEO (Gene Expression Omnibus; www.ncbi.nlm.nih.gov/geo) accession ID GSE28591 were utilized to identify PASR peak near the TSS (transcription start site, marked by a red vertical bar) of the gene. Refer to Fig 1 for the analytical workflow. (B) AGO: Eight sRNA HTS data sets belonging to GSE28591 were divided into AGO1 data group (GSM707682, GSM707683, GSM707684 and GSM707685) and AGO4 data group (GSM707686, GSM707687, GSM707688 and GSM707689). To analyze the AGO loading preference of the PASRs, an accumulation level-based comparison was performed between the two AGO-associated sRNA HTS data groups. The higher accumulation levels of the PASRs were detected in the AGO4 data group, and were denoted by black lines. (C) RDR-, DCL-dependence: sRNA HTS data sets from different mutants (including *dcl* and *rdr* mutants) involved in sRNA biogenesis pathways were recruited for this analysis. Prominently repressed abundances of PASRs observed in specific mutants were denoted by black lines. (D) Pol IV-dependence: sRNA HTS data sets from two mutants (*nrpd1a* and *nrpd1b*, and both were denoted by black lines) of RNA polymerase (Pol) IV were used to analyze the dependence of PASR biogenesis on Pol IV. For all the figure panels, the dsRNA sequencing read-covered regions (the positions were provided on the top right) were highlighted in semitransparent red (for sense strand) and green (for antisense strand) background color. For the detailed explanation of the HTS data sets, please refer to “Data sources” within the “Materials and Methods” section.

**Fig 5:**
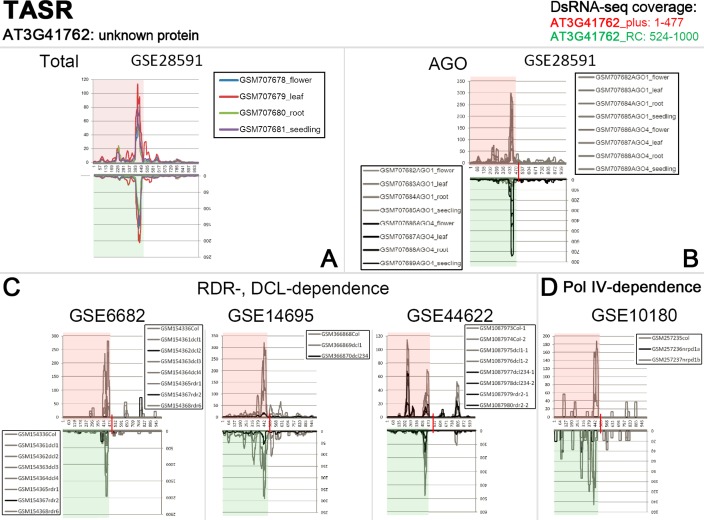
TASRs (terminus-associated small RNAs) identified on both strands of *AT3G41762*, and small RNA (sRNA) and double-stranded RNA (dsRNA) high-throughput sequencing (HTS)-based evidences showing potential Argonaute (AGO) loading preference and biogenesis pathways of TASRs. (A) Total: Initially, four sRNA HTS data sets belonging to GEO (Gene Expression Omnibus; www.ncbi.nlm.nih.gov/geo) accession ID GSE28591 were utilized to identify TASR peak near the transcription terminus (marked by a red vertical bar) of the gene. Refer to Fig 1 for the analytical workflow. (B) AGO: Eight sRNA HTS data sets belonging to GSE28591 were divided into AGO1 data group (GSM707682, GSM707683, GSM707684 and GSM707685) and AGO4 data group (GSM707686, GSM707687, GSM707688 and GSM707689). To analyze the AGO loading preference of the TASRs, an accumulation level-based comparison was performed between the two AGO-associated sRNA HTS data groups. The higher accumulation levels of the TASRs were detected in the AGO4 data group, and were denoted by black lines. (C) RDR-, DCL-dependence: sRNA HTS data sets from different mutants (including *dcl* and *rdr* mutants) involved in sRNA biogenesis pathways were recruited for this analysis. Prominently repressed abundances of TASRs observed in specific mutants were denoted by black lines. (D) Pol IV-dependence: sRNA HTS data sets from two mutants (*nrpd1a* and *nrpd1b*, and *nrpd1a* was denoted by black lines) of RNA polymerase (Pol) IV were used to analyze the dependence of TASR biogenesis on Pol IV. For all the figure panels, the dsRNA sequencing read-covered regions (the positions were provided on the top right) were highlighted in semitransparent red (for sense strand) and green (for antisense strand) background color. For the detailed explanation of the HTS data sets, please refer to “Data sources” within the “Materials and Methods” section.

**Fig 6:**
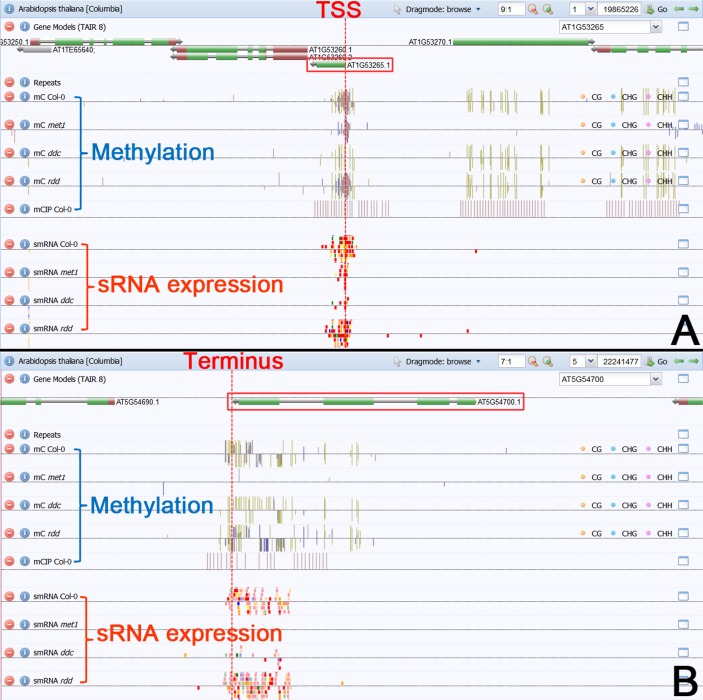
Examples of site-specific DNA methylation potentially mediated by PASRs (promoter-associated small RNAs) and TASRs (terminus-associated small RNAs) in *Arabidopsis*. (A) Site-specific DNA methylation signals were observed within the genomic region surrounding the TSS (transcription start site; marked by a vertical dashed line) of *AT1G53265*. Accordingly, abundant small RNAs (i.e. PASRs) were mapped onto this region. (B) Site-specific DNA methylation signals were detected within the genomic region surrounding the transcription terminus of *AT5G54700*. Accordingly, abundant small RNAs (i.e. TASRs) were mapped onto this region. *Arabidopsis* epigenome maps (neomorph.salk.edu/epigenome/epigenome.html) [32] were employed for this analysis.

According to the above results, interesting features were observed for several paired sRNA peaks. For example, PASR peaks surrounding the TSS were detected on both strands of *AT5G48000*. The paired peaks are covered by the dsRNA-seq reads (Fig 4A), and the PASRs are preferentially incorporated into AGO4 (Fig 4B). Moreover, the signal intensity of the PASR peaks is greatly repressed in *rdr2*, *dcl234*, *nrpd1a* and *nrpd1b* (Fig 4C and 4D). Another example was observed for *AT3G41762*, TASRs peaked surrounding its transcription terminus on both strands. The paired peaks are also covered by the dsRNA-seq reads (Fig 5A), and the TASRs on the antisense strand are preferentially incorporated into AGO4 (Fig 5B). Moreover, the signal intensity of the TASR peaks is greatly reduced in *rdr2*, *dcl2*, *dcl234* and *nrpd1a* (Fig 5C and 5D). The features of these PASRs and TASRs, including the association with AGO4 and the dependence on RDR2, DCL2/3/4 and Pol IV, led us to investigate whether these sRNAs could participate in chromatin modifications just as the heterochromatic siRNAs. In this regard, all of the protein-coding genes with PASR or TASR peaks (see gene lists in S1, S2, S3, S4, S5 and S6 Figs) were sent to the web server *Arabidopsis* epigenome maps (neomorph.salk.edu/epigenome/epigenome.html) [32] to discover site-specific DNA methylation signals. Fortunately, several methylated regions were observed, whose genomic positions correlated well with those of the PASR or TASR peaks on the host genes. Briefly, seven genes (*AT1G48660*, *AT1G67450*, *AT2G36180*, *AT3G17490*, *AT3G22730*, *AT3G56450* and *AT5G40320*) with PASR peaks on the sense strands and one gene (*AT2G29460*) with a PASR peak on the antisense strand were site-specifically methylated (S21 and S22 Figs). Three genes (*AT1G52180*, *AT5G36220* and *AT5G42203*) with TASR peaks on the sense strands and five genes (*AT1G59835*, *AT1G66540*, *AT2G04090*, *AT2G04830* and *AT5G48605*) with TASR peaks on the antisense strands were also site-specifically methylated (S24 and S25 Figs). Additionally, 13 genes with paired PASR peaks and 32 genes with paired TASR peaks were site-specifically methylated (S23 and S26 Figs). For example, PASR peaks were identified on both strands of *AT1G53265*. Accordingly, strong DNA methylation signals surrounding the TSS of *AT1G53265* were present on the *Arabidopsis* epigenome maps (Fig 6A). TASR peaks were identified on both strands of *AT5G54700*. Accordingly, DNA methylation signals surrounding the transcription terminus of *AT1G53265* were observed on the *Arabidopsis* epigenome maps (Fig 6B). Examples of paired PASR and TASR peaks potentially mediating site-specific DNA methylation are listed in Table 1 and S1 Table which also shows the potential pathways for their biogenesis and action.

**Table 1:** Examples of paired PASR (promoter-associated small RNA) and TASR (terminus-associated small RNA) peaks potentially mediating site-specific DNA methylation, and analysis of their biogenesis and action pathways.

	Gene ID	Site-specific methylation^1^	DsRNA-seq coverage^2^	AGO preference^3^	Dependence^4^		
					DCL2/3/4	RDR2/6	Pol IV
**Paired PASR peaks**	AT1G53265	D^5^	D	AGO4_flower; AGO4_leaf; AGO4_root	Repressed in *dcl3* and *dcl234*	Repressed in *rdr2* and *rdr6*	ND^6^
	AT5G48000	D	D	AGO4_flower; AGO4_leaf; AGO4_root; AGO4_seedling	Repressed in *dcl234*	Repressed in *rdr2*	Repressed in *nrpd1a* and *nrpd1b*
**Paired TASR peaks**	AT3G25130	D	D	AGO4_flower	Repressed in *dcl234*	Repressed in *rdr2*	ND
	AT4G14365	D	D	AGO4_flower	Repressed in *dcl234*	Repressed in *rdr2*	Repressed in *nrpd1a* and *nrpd1b*
	AT5G50480	D	D	AGO4_flower; AGO4_leaf; AGO4_root	Repressed in *dcl4* and *dcl234*	Repressed in *rdr2*	Repressed in *nrpd1a* and *nrpd1b*
	AT5G54700	D	D	AGO4_flower; AGO4_leaf; AGO4_root; AGO1_root	Repressed in *dcl234*	Repressed in *rdr2*	Repressed in *nrpd1a* and *nrpd1b*

^**1**^Based on the information provided by *Arabidopsis* epigenome maps (http://neomorph.salk.edu/epigenome/epigenome.html), DNA methylation signals were detected on the genomic positions well corresponding to those of the PASR or TASR peaks.

^**2**^The PASR or TASR peaks were observed to be covered by dsRNA-seq (double-stranded sequencing) reads.

^**3**^High-throughput sequencing (HTS) data from AGO (Argonaute)-associated small RNA (sRNA) population was utilized, including AGO1 data group and AGO4 data group. A comparison of accumulation levels was made between AGO1 (GSM707682, GSM707683, GSM707684 and GSM707685) and AGO4 (GSM707686, GSM707687, GSM707688 and GSM707689) groups, which facilitated us to deduce the preference of PASRs and TASRs when loading into specific AGO complexes.

^**4**^Dependence of PASR and TASR biogenesis on the activities of DCL2 (Dicer-like 2), DCL3, DCL4, RDR2 (RNA-dependent RNA polymerase 2), RDR6, Pol IV (RNA polymerase IV) in *Arabidopsis*. The accumulation of PASRs and TASRs was observed to be repressed in several mutants, such as *dcl3*, *dcl4*, *dcl234* (triple mutant of *DCL2*, *DCL3* and *DCL4*), *rdr2*, *rdr6*, *nrpd1a* (NRPD1 is the subunit of Pol IV), *nrpd1b*.

^**5**^D: Detected.

^**6**^ND: Not detected.

## Discussion

### A biogenesis and action model proposed for certain PASRs and TASRs

In this study, by using sRNA HTS data, dsRNA-seq data and DNA methylation profiling data, we reported the following interesting discoveries: (1) For hundreds of the protein-coding genes in *Arabidopsis*, sRNAs peaked within the genomic regions neighboring to or covering the TSSs or the transcription termini, which were termed as PASR and TASR peaks respectively. (2) Some of the PASRs and TASRs might be processed from dsRNA precursors, the biogenesis of which relies on RNA Pol IV and specific RDR(s). (3) The processing of these PASRs and TASRs from the double-stranded precursors depends on the activity of specific DCL(s). (4) A portion of the PASRs and TASRs are preferentially loaded into AGO1 or AGO4. (5) For some of the sRNAs associated with AGO4, site-specific DNA methylation signals were detected within the genomic regions covering the PASR or TASR peaks.

**Fig 7:**
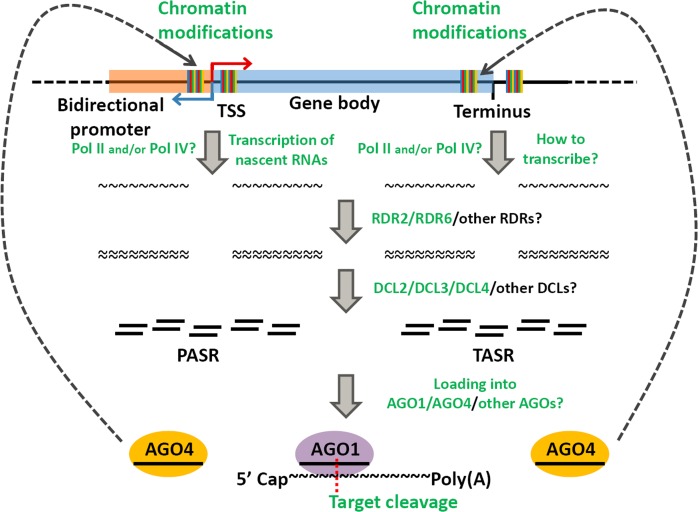
Proposed biogenesis pathways, action modes of the PASRs (promoter-associated small RNAs) and the TASRs (terminus-associated small RNAs) identified in *Arabidopsis*. For a protein-coding gene possessing a bidirectional promoter, the precursors of the PASRs could be transcribed either upstream or downstream of the TSS (transcription start site), which needs experimental investigations. However, the underlying mechanism of the transcription of the TASR precursors could not be deduced. Although several pieces of high-throughput sequencing (HTS) data-based evidences pointed to the dependence of the biogenesis of the PASRs and the TASRs on RNA polymerase (Pol) IV, RDR2 (RNA-dependent RNA polymerase 2), RDR6, DCL2 (Dicer-like 2), DCL3 and DCL4, other key factors implicated in the accumulation of the two small RNA (sRNA) species might have not been uncovered owing to the limited sRNA HTS data utilized in this study. Based on the HTS data from the Argonaute (AGO)-associated sRNAs, many Pol IV-, RDR- and DCL-dependent PASRs and TASRs were preferentially loaded into AGO4 silencing complexes. And, site-specific DNA methylation was observed on the genomic regions well corresponding to the origins of the PASRs and the TASRs. Thus, the AGO4-associated PASRs and TASRs were proposed to mediate *cis*-methylation. Besides, the AGO1-associated PASRs and TASRs were proposed to mediate target cleavages similar to the plant microRNAs.

Based on the above results, a biogenesis and action model was proposed for certain PASRs and TASRs (Fig 7). However, several key points should be discussed here for this model. First, in this study, the sRNA HTS data from the *pol iv* mutant was used to investigate the dependence of the transcription of the PASR and TASR precursors. Whether the other RNA polymerases, such as Pol II and Pol V are also involved in the transcription needs further investigations. Second, for different genes, PASRs and TASRs peak at distinct positions related to their TSSs and transcription termini respectively. Some peak upstream and some peak downstream of the TSSs or the transcription termini, while the other peaks cover the TSSs or the transcription termini (S1, S2, S4 and S5 Figs). It will be interesting to see whether these sRNA peaks with different locations are produced through distinct pathways. Third, only AGO1 and AGO4 were analyzed in the AGO association study. In fact, a portion of the sRNA peaks are not associated with AGO1 or AGO4. Whether the PASRs and the TASRs are associated with other AGOs for their actions remains to be investigated. Fourth, the levels of certain PASRs and TASRs were intensively repressed in the triple mutant *dcl234*, but not in the single mutant *dcl2*, *dcl3* and *dcl4* (see Fig 4C for example). Thus, the functional redundancy of the three DCLs in the processing of the PASRs and the TASRs needs to be further analyzed. On the other hand, in addition to RDR2 and RDR6, whether other RDRs are implicated in the production of the PASRs and the TASRs requires further investigations. A recent study in murine embryonic stem cells uncovered a class of sRNAs peaked surrounding TSSs. These sRNAs called TSSa-RNAs (transcription start site-associated RNAs) range from 20 to 90 nt in length [15]. In human, chicken and *Drosophila*, Taft and his colleagues identified 18-nt tiny RNAs, which reside within the genomic regions ranging from 60 nt upstream to 120 nt downstream of the TSSs [17]. In bacteria, sRNAs associated with TSSs were also discovered. But, these sRNAs have an average size of 45 nt [19]. In our study, both the PASRs and the TASRs range from 19 to 28 nt (Fig 3A, 3B, 3C and 3D). Thus, in the following studies, it is necessary to clarify whether the observed length range of the PASRs and the TASRs is resulted from the sRNA HTS data used in our analysis (since all the sRNA reads of the HTS data sets are shorter than 40 nt), or is the fact resulting from the nature of the PASRs and the TASRs specifically existed in plants.

In order to clearly depict the biogenesis and action pathway(s) of certain transcription boundary-associated sRNAs, some fine-scale experimental approaches are proposed here. First, the dependence of the PASR or TASR production on specific RNA polymerase, RDR(s) and DCL(s) should be detected in the corresponding *pol*, *rdr* and *dcl* mutants by Northern blotting, taking wild type plants as a control. Second, to investigate the action mode of AGO4-associated sRNAs, DNA methylation sequencing should be carried out for both wild type plants and the *ago4* mutant, to compare the methylation patterns on the specific genomic regions (e.g. the sRNA-coding regions if *cis*-DNA methylation is mediated by the sRNAs) between the two plant materials. Third, target prediction and degradome sequencing could be performed for the AGO1-associated sRNAs, in order to identify the downstream target genes of specific sRNAs. Besides, transgenic experiments could be carried out for both the AGO1-associated sRNAs (over-expression) and their downstream target genes (knock down), in order to see the biological outputs of the sRNA-mediated regulatory pathways.

### What are the biological outputs of the PASRs and the TASRs?

Our study shows that certain AGO4-associated PASRs and TASRs are likely to mediate site-specific DNA methylation. However, further in-depth analyses are needed to elucidate the influence of the PASR- or TASR-mediated DNA methylation on the expression of their host genes. As described above, some of the AGO1- or AGO4-associated PASRs and TASRs were observed to be accumulated with organ-specific patterns (S7, S8, S10 and S11 Figs). Thus, whether the host genes could be regulated by specific PASRs or TASRs in an organ-specific way remains to be an open question. In further studies, degradome sequencing data could be employed for identification of the target genes regulated by AGO1-associated PASRs and TASRs.

Another interesting phenomenon is that the PASRs and the TASRs from certain chloroplast genes were abundantly accumulated in the green organ such as leaves and the young seedlings (see Fig 2 for example). We questioned whether these sRNAs were implicated in the regulation of the chloroplast genes. However, no methylation signal was detected surrounding the TSSs or the transcription termini of the chloroplast genes. Perhaps, other regulatory mechanisms underlie the regulation of the chloroplast genes encoding PASRs or TASRs. Or, the accumulation of PASRs and TASRs might be the result of the transcription of their host genes in chloroplasts. Or, a feedback regulatory circuit might exist between PASRs/TASRs and their host genes.

A recent report by Kapranov and his colleagues revealed an intriguing regulatory function for the termini-associated sRNAs in human cells. These novel sRNAs antisense to the 3’ ends of the annotated transcripts could increase the RNA copy numbers through a yet uncharacterized endogenous biochemical pathway [29]. In our study, a large portion of TASRs were discovered on the antisense strands of certain protein-coding genes. Thus, whether these antisense TASRs play a role in increasing the transcript copy numbers of their host genes is worth investigating in depth.

## Materials and Methods

### Data sources

Based on the genomic information provided by TAIR (The *Arabidopsis* Information Resource; release 10; www.arabidopsis.org/index.jsp) [33], 500 bp upstream and 499 bp (if the length of the gene body is shorter than 500 nt, then the whole gene body from the TSS to the transcription terminus was collected) downstream of the TSSs (a total of 1000 bp, or 500 bp plus the length of the gene body) of the protein-coding genes, and 499 bp upstream (if the length of the gene body is shorter than 500 nt, then the whole gene body from the TSS to the transcription terminus was collected) and 500 bp downstream of the transcription terminus (a total of 1000 bp, or 500 bp plus the length of the gene body) of the protein-coding genes were collected for this study. For the protein-coding genes with multiple transcription models, only the TSSs and the transcription termini of the longest models were used for sequence collection.

Small RNA HTS data sets and dsRNA-seq data sets utilized in this study were retrieved from GEO (Gene Expression Omnibus; www.ncbi.nlm.nih.gov/geo) [34], and their accession IDs with brief introductions are provided in S2 Table.

To investigate site-specific DNA methylation potentially mediated by PASRs or TASRs, the web server *Arabidopsis* epigenome maps (neomorph.salk.edu/epigenome/epigenome.html) [32] were utilized.

### Transcriptome-wide identification of PASR and TASR peaks

Four sRNA HTS data sets (GSM707678, GSM707679, GSM707680 and GSM707681) were utilized to identify the PASR and TASR peaks. To allow accumulation level-based comparison among different data sets, normalized read counts (in RPM, reads per million) of the sRNAs were calculated for each data set as follows: the raw read count of each sRNA was divided by the total raw counts of this data set, and then multiplied by 10^6^.

Firstly, the sequences surrounding TSSs and transcription termini (include sense and antisense strands) were collected from TAIR (see “Data sources”). And, the sRNAs from the above four HTS data sets were mapped onto the collected sequences by using in-house Perl script. Only the perfectly mapped sRNAs were retained. For each sequence encompassing TSSs or transcription termini, “**A**_**1**_” (average sRNA count of this sequence) was calculated by dividing the “**S**_**1**_” (summed sRNA count of this sequence) by “**L**” (the length of the sequence) (see Fig 1). Secondly, we defined a specific region for 50-nt window scrolling on this sequence (please refer to Fig 1 for the parameters for defining scrolling regions). Subsequently, a 50-nt window was scrolled within the defined region. And, for each 1-nt scrolling step, “**A**_**2**_” (average sRNA count of the scrolling window at this step) was calculated by dividing the “**S**_**2**_” (summed sRNA count of the scrolling window at this step) by 50 nt. Thirdly, computer-assisted screening was performed to search for the sequences containing 50-nt windows accommodating highly abundant sRNAs by employing the following criterion: “**A**_**2**_” should be ten times or more than “**A**_**1**_”, and “**A**_**2**_” should be 1 RPM/nt or higher. Finally, the retained 50-nt windows on specific sequences were subjected to manual screening to identify PASR or TASR peaks by adopting the following criterion: sRNAs should peaked within the regions defined for 50-nt window scrolling, and the peaks should not be submerged in the surrounding sRNA “noise”.

## Supporting Information

S1 Fig PASR peaks identified on the sense strands of the protein-coding genes of Arabidopsis. For the chloroplast genes, sRNAs dominantly detected in leaves and seedlings were marked by green arrows.
(PDF)

S2 Fig PASR peaks identified on the antisense strands of the protein-coding genes of Arabidopsis. For the chloroplast genes, sRNAs dominantly detected in leaves and seedlings were marked by green arrows.
(PDF)

S3 Fig Paired PASR peaks identified on both strands of the protein-coding genes of Arabidopsis. For the chloroplast genes, sRNAs dominantly detected in leaves and seedlings were marked by green arrows.
(PDF)

S4 Fig TASR peaks identified on the sense strands of the protein-coding genes of Arabidopsis. For the chloroplast genes, sRNAs dominantly detected in leaves and seedlings were marked by green arrows.
(PDF)

S5 Fig TASR peaks identified on the antisense strands of the protein-coding genes of Arabidopsis. (PDF)

S6 Fig Paired TASR peaks identified on both strands of the protein-coding genes of Arabidopsis. (PDF)

S7 Fig AGO-associated PASR peaks identified on the sense strands of the protein-coding genes of Arabidopsis. (PDF)

S8 Fig AGO-associated PASR peaks identified on the antisense strands of the protein-coding genes of Arabidopsis. (PDF)

S9 Fig AGO-associated paired PASR peaks identified on both strands of the protein-coding genes of Arabidopsis. (PDF)

S10 Fig AGO-associated TASR peaks identified on the sense strands of the protein-coding genes of Arabidopsis. (PDF)

S11 Fig AGO-associated TASR peaks identified on the antisense strands of the protein-coding genes of Arabidopsis. (PDF)

S12 Fig AGO-associated paired TASR peaks identified on both strands of the protein-coding genes of Arabidopsis. (PDF)

S13 Fig DsRNA-seq read-covered PASR peaks identified on the sense strands of the protein-coding genes of Arabidopsis. (PDF)

S14 Fig DsRNA-seq read-covered PASR peaks identified on the antisense strands of the protein-coding genes of Arabidopsis. (PDF)

S15 Fig DsRNA-seq read-covered paired PASR peaks identified on both strands of the protein-coding genes of Arabidopsis. (PDF)

S16 Fig DsRNA-seq read-covered TASR peaks identified on the sense strands of the protein-coding genes of Arabidopsis. (PDF)

S17 Fig DsRNA-seq read-covered TASR peaks identified on the antisense strands of the protein-coding genes of Arabidopsis. (PDF)

S18 Fig DsRNA-seq read-covered paired TASR peaks identified on both strands of the protein-coding genes of Arabidopsis. (PDF)

S19 Fig Results showing the dependence of certain PASR and TASR peaks on the activities of specific DCL(s), RDR(s) and Pol IV, and the loading preference of the PASRs and TASRs into specific AGO(s). PASR_plus: PASR peaks on the sense strands of the protein-coding genes of Arabidopsis. PASR_RC: PASR peaks on the antisense strands of the protein-coding genes of Arabidopsis. TASR_plus: TASR peaks on the sense strands of the protein-coding genes of Arabidopsis. TASR__RC: TASR peaks on the antisense strands of the protein-coding genes of Arabidopsis.
(PDF)

S20 Fig Results showing the dependence of certain paired PASR and TASR peaks on the activities of specific DCL(s), RDR(s) and Pol IV, and the loading preference of the PASRs and TASRs into specific AGO(s). (PDF)

S21 Fig Site-specific DNA methylation signals were detected at the genomic positions well corresponding to those of the PASR peaks identified on the sense strands of the protein-coding genes in Arabidopsis. (PDF)

S22 Fig Site-specific DNA methylation signals were detected at the genomic positions well corresponding to those of the PASR peaks identified on the antisense strands of the protein-coding genes in Arabidopsis. (PDF)

S23 Fig Site-specific DNA methylation signals were detected at the genomic positions well corresponding to those of the paired PASR peaks identified on both strands of the protein-coding genes in Arabidopsis. (PDF)

S24 Fig Site-specific DNA methylation signals were detected at the genomic positions well corresponding to those of the TASR peaks identified on the sense strands of the protein-coding genes in Arabidopsis. (PDF)

S25 Fig Site-specific DNA methylation signals were detected at the genomic positions well corresponding to those of the TASR peaks identified on the antisense strands of the protein-coding genes in Arabidopsis. (PDF)

S26 Fig Site-specific DNA methylation signals were detected at the genomic positions well corresponding to those of the paired TASR peaks identified on both strands of the protein-coding genes in Arabidopsis. (PDF)

S1 Table Full list of AGO4-associated PASR and TASR peaks potentially mediating site-specific DNA methylation, and analysis of their biogenesis and action pathways. (PDF)

S2 Table High-throughput sequencing data sets used in this study. (PDF)

## Abbreviations

AGO: Argonaute
DCL: Dicer-like
dsRNA-seq: double-stranded RNA sequencing
GEO: Gene Expression Omnibus
HTS: high-throughput sequencing
miRNA: microRNA
nat-siRNA: natural antisense small interfering RNA
PASR: promoter-associated small RNA
Pol: RNA polymerase
ra-siRNA: repeat-associated small interfering RNA
RdDM: RNA-directed DNA methylation
RDR: RNA-dependent RNA polymerase
RPM: reads per million
rRNA: ribosomal RNA
sRNA: small RNA
TAIR: The Arabidopsis Information Resource
TASR: terminus-associated small RNA
ta-siRNA: trans-acting small interfering RNA
TE: transposable element
TSS: transcription start site
TSSa-RNA: transcription start site-associated RNA
