# Supplementary material for: Transcriptome-Wide Discovery of PASRs (Promoter-Associated Small RNAs) and TASRs (Terminus-Associated Small RNAs) in Arabidopsis thaliana
Source: PLoS One. 2017 Jan 3;12(1):e0169212. doi: 10.1371/journal.pone.0169212 (PMC5207706; doi:10.1371/journal.pone.0169212)

Figure S1 PASR peaks identified on the sense strands of the protein-coding genes of *Arabidopsis*. For each plot, x axis measures the position of the sense strand, and y axis measures the abundance (in RPM, reads per million) of sRNAs. For the chloroplast genes, sRNAs dominantly detected in leaves and seedlings were marked by green arrows.

AT1G01020

Arv1-like protein

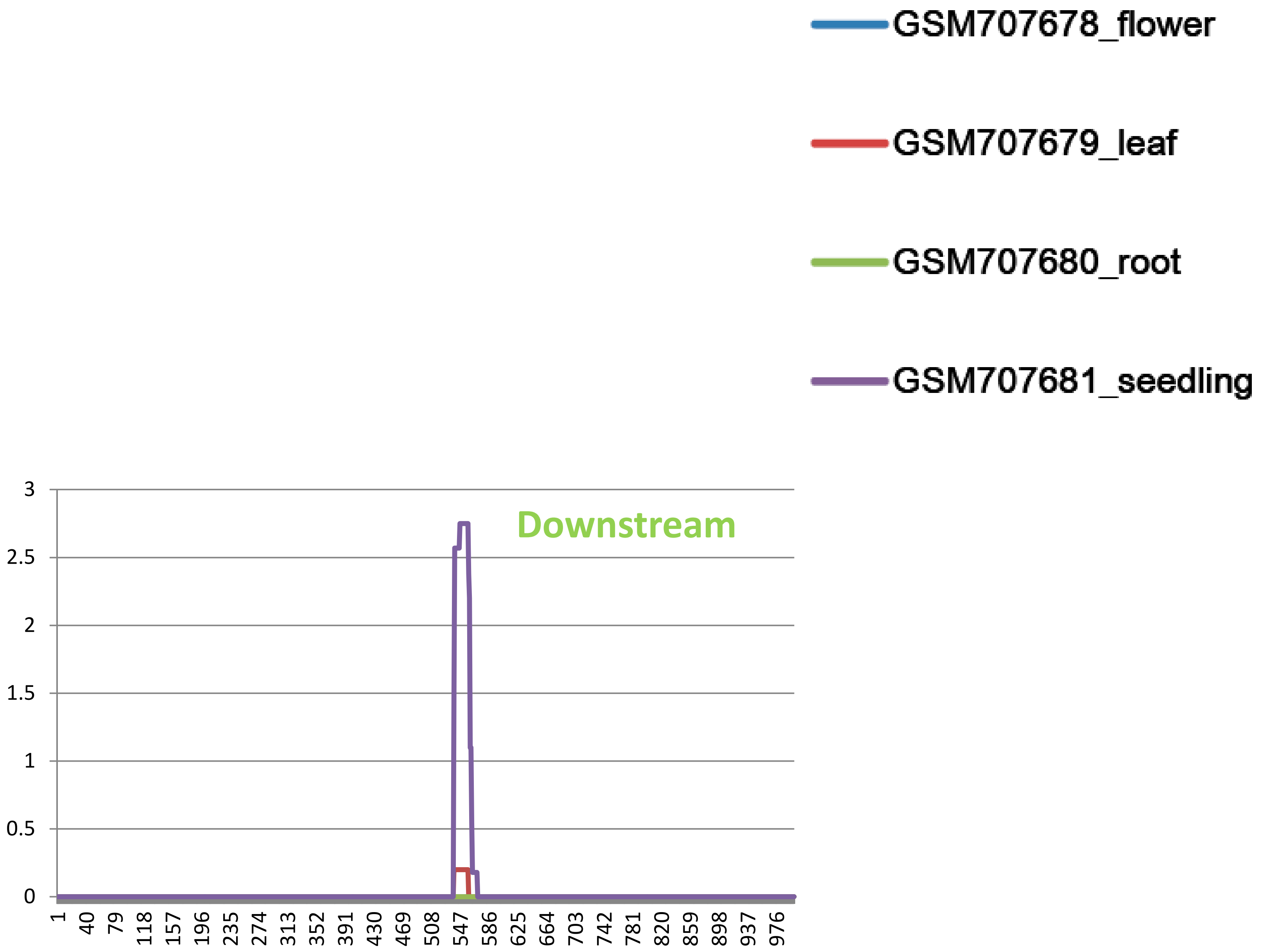

AT1G01073

unknown protein

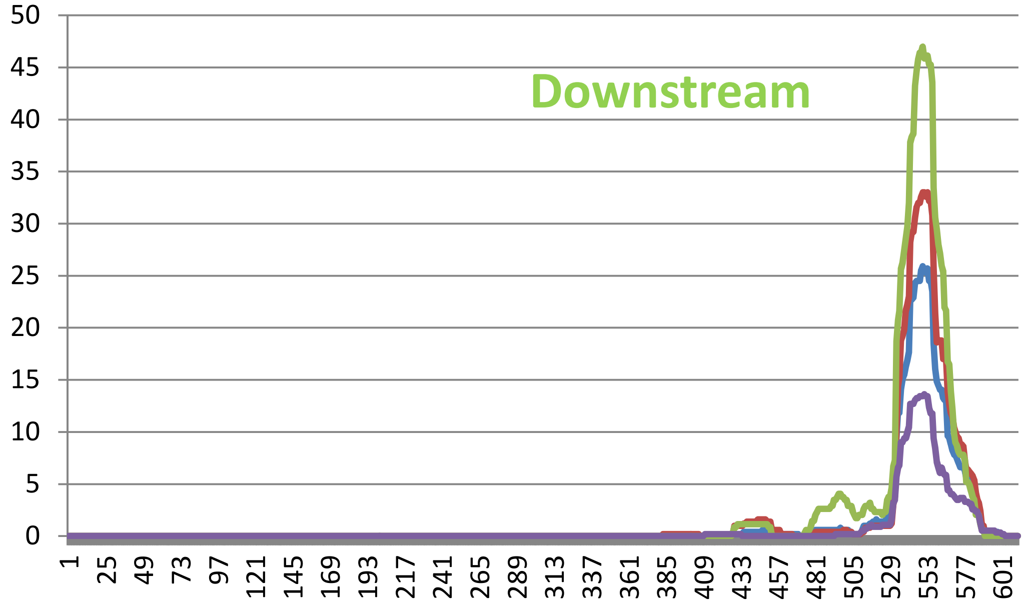

AT1G03810

Nucleic acid-binding, OB-fold-like protein

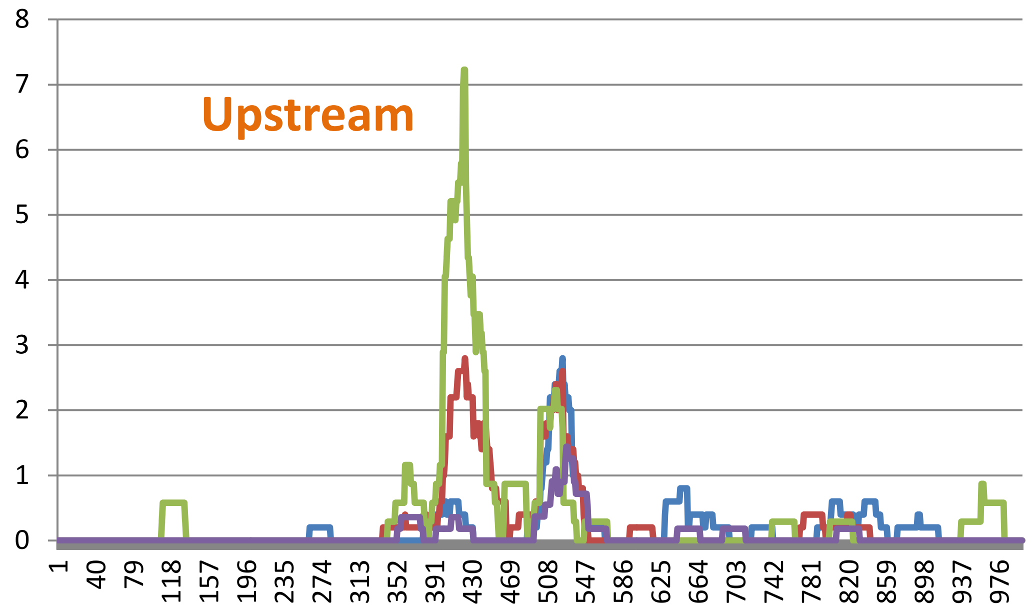

AT1G06850

Basic-leucine zipper (bZIP) transcription factor family protein

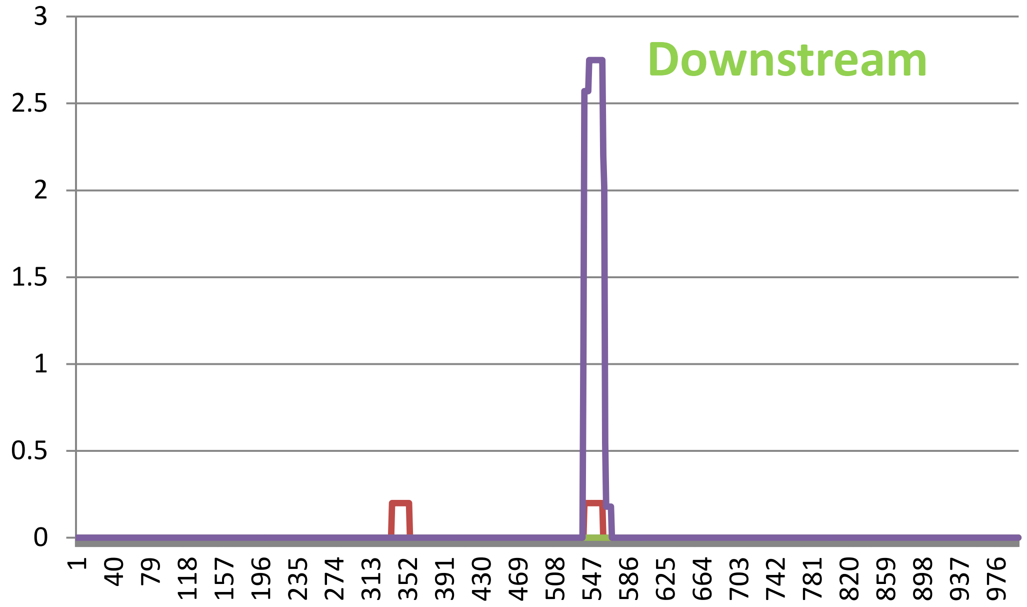

AT1G07660

Histone superfamily protein

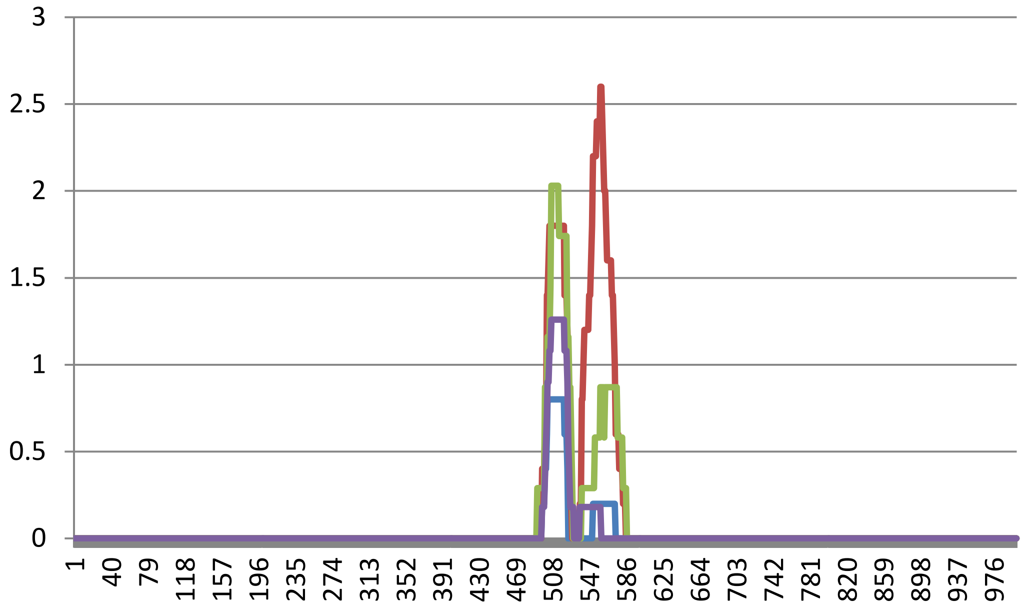

AT1G08030

Encodes a tyrosylprotein sulfotransferase (TPST). TPST is expressed throughout the plant body, and the highest levels of expression are in the root apical meristem. TPST acts in the auxin pathway to maintain postembryonic root stem cell niche by defining the expression of the PLETHORA stem cell transcription factor genes. A loss-of-function mutant TPST displayed a marked dwarf phenotype accompanied by stunted roots, pale green leaves, reduction in higher order veins, early senescence, and a reduced number of flowers and siliques.

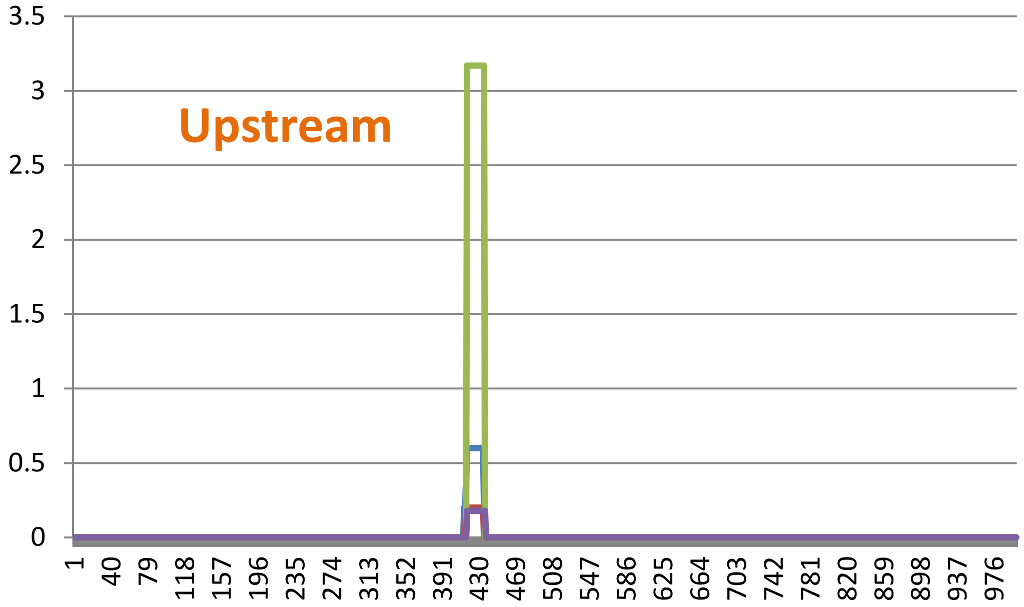

AT1G08035

unknown protein

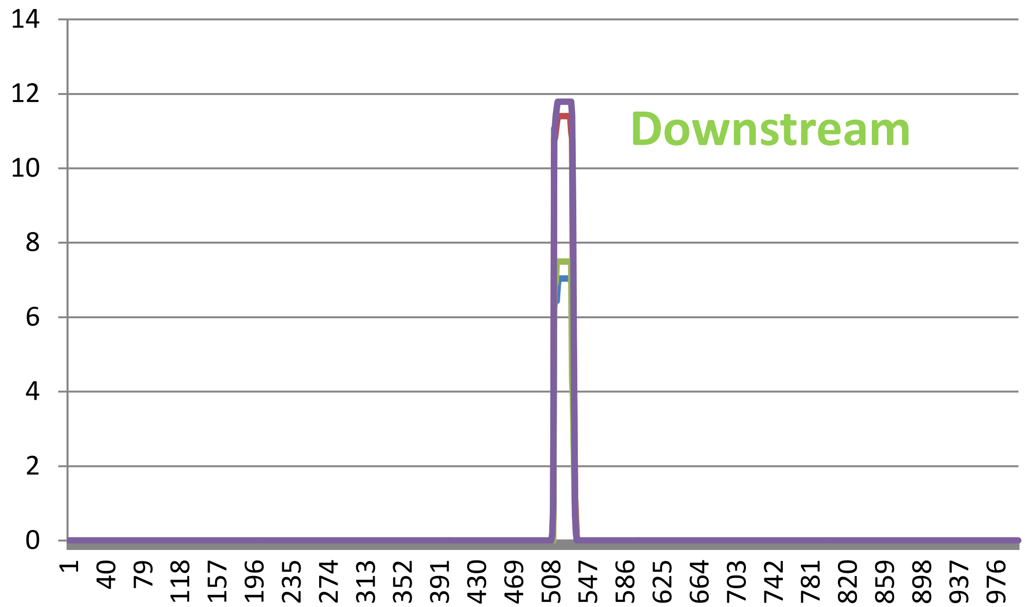

AT1G09060

Zinc finger, RING-type;Transcription factor jumonji/aspartyl beta-hydroxylase.

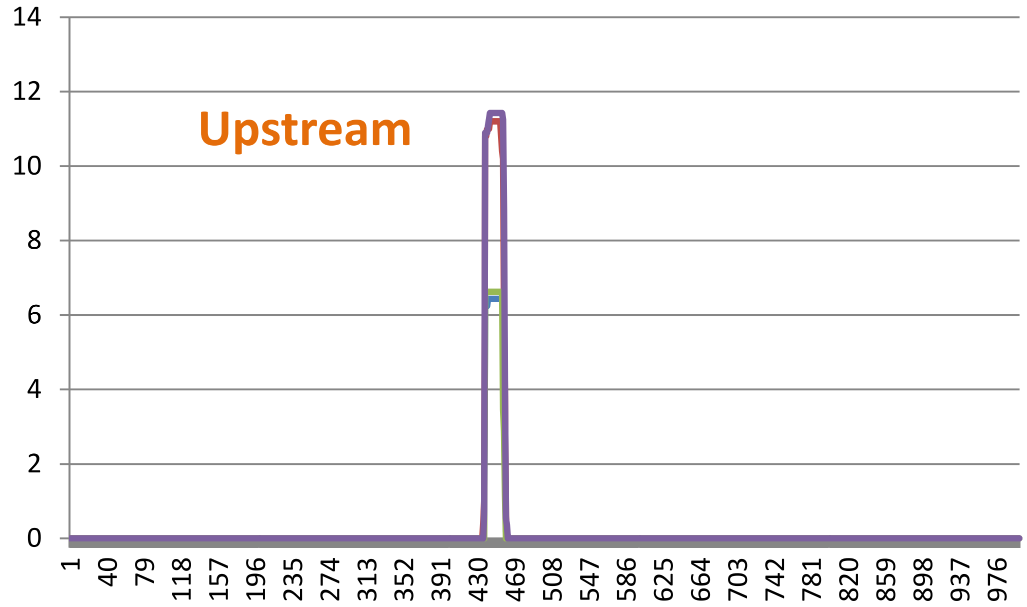

AT1G10820

Protein of unknown function (DUF3755)

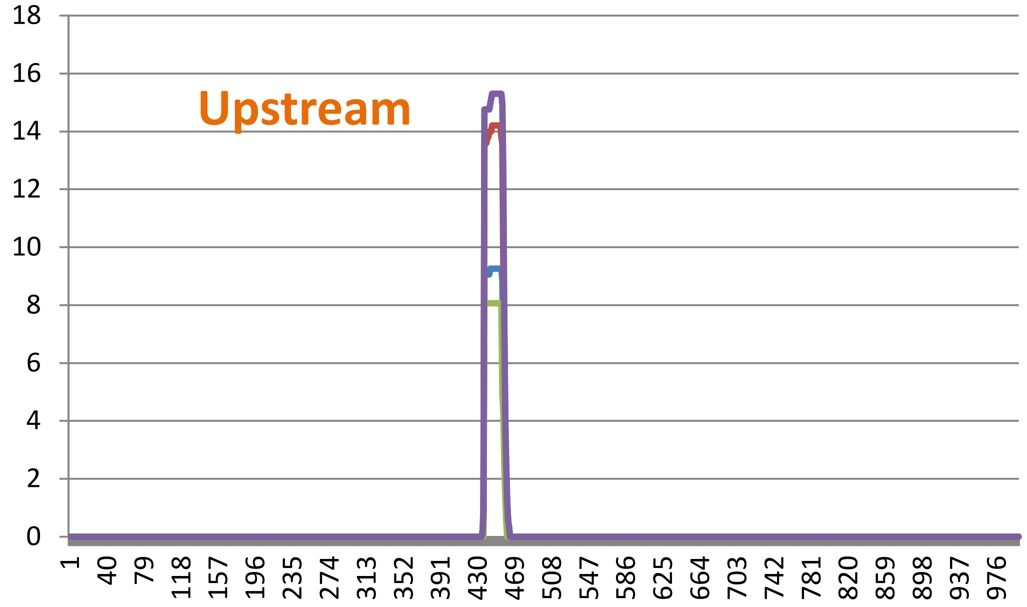

AT1G11020

RING/FYVE/PHD zinc finger superfamily protein

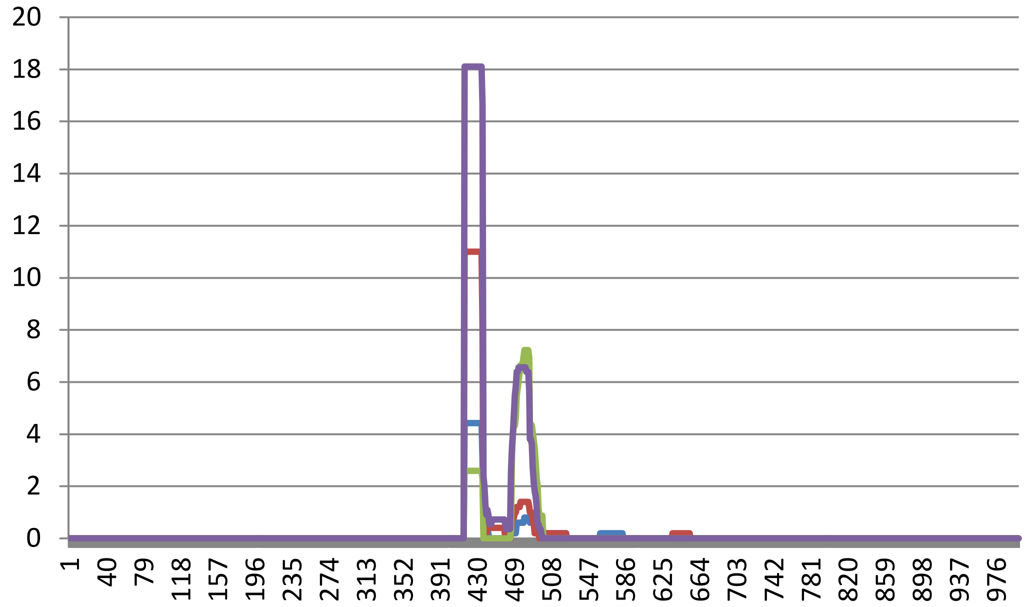

AT1G11785

unknown protein

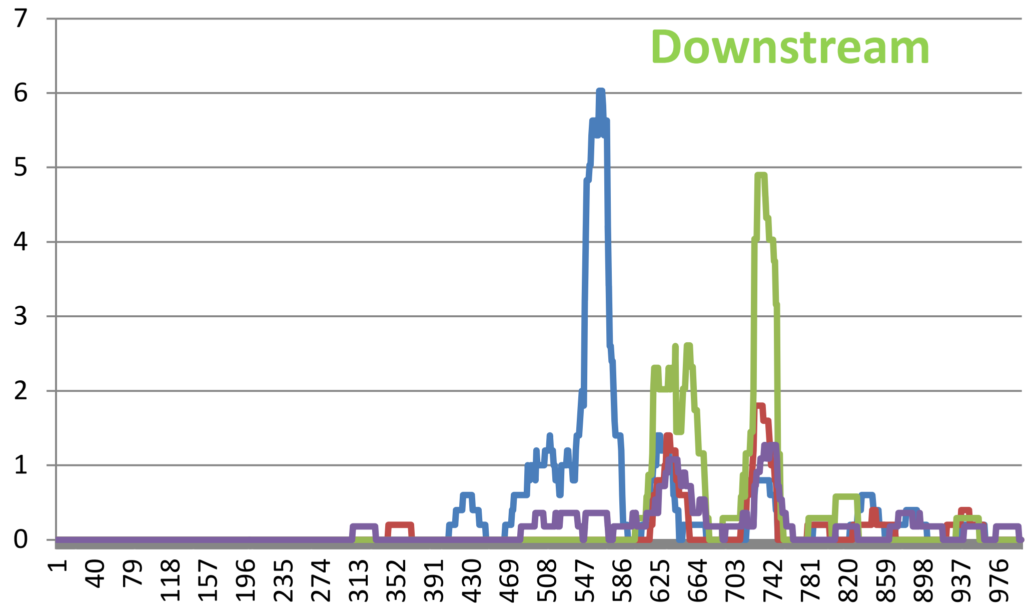

AT1G16760

Protein kinase protein with adenine nucleotide alpha hydrolases-like domain

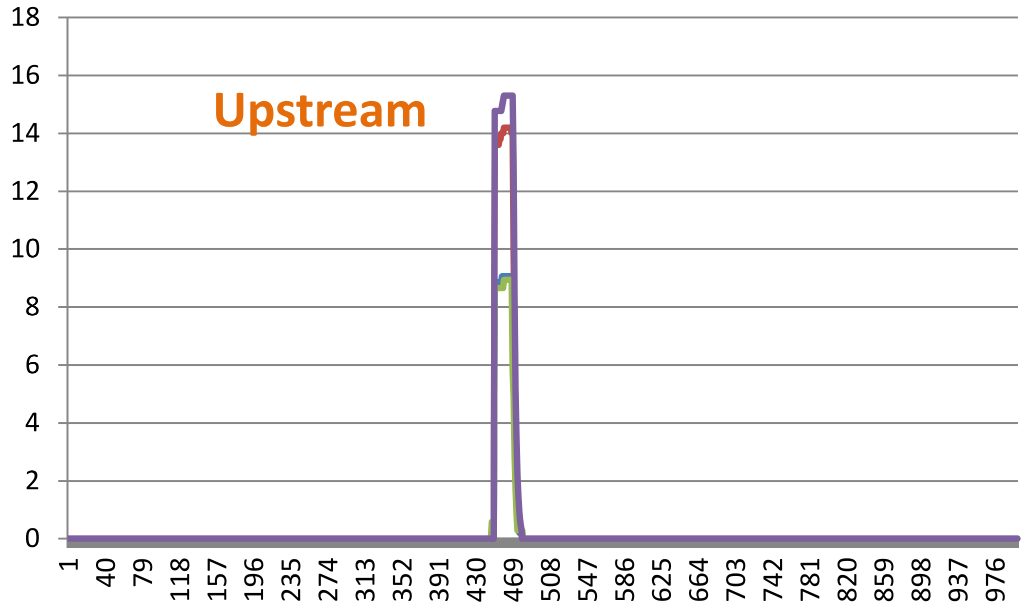

AT1G16820

vacuolar ATP synthase catalytic subunit-related / V-ATPase-related / vacuolar proton pump-related

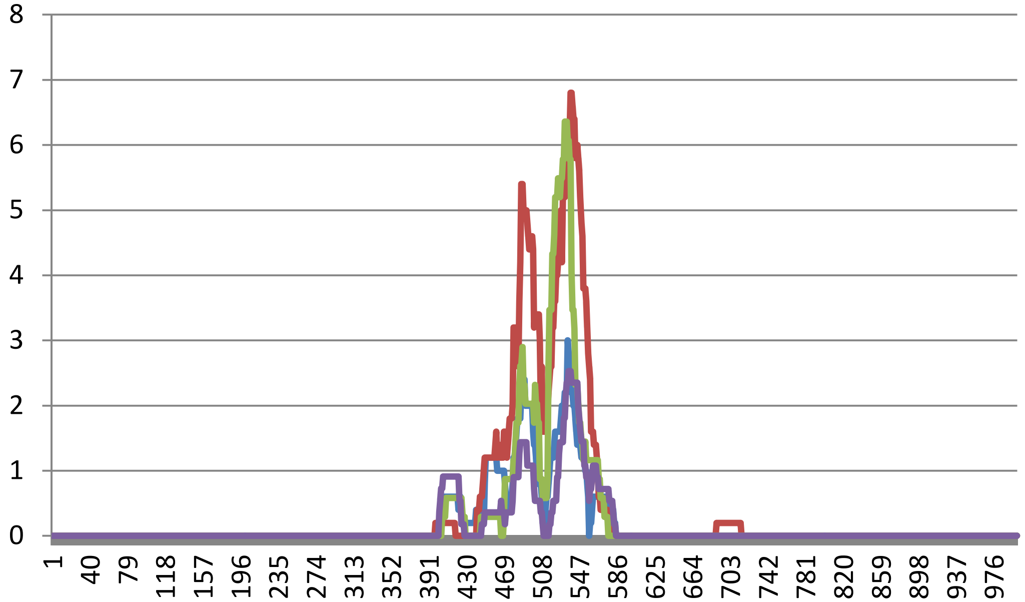

AT1G17270

O-fucosyltransferase family protein

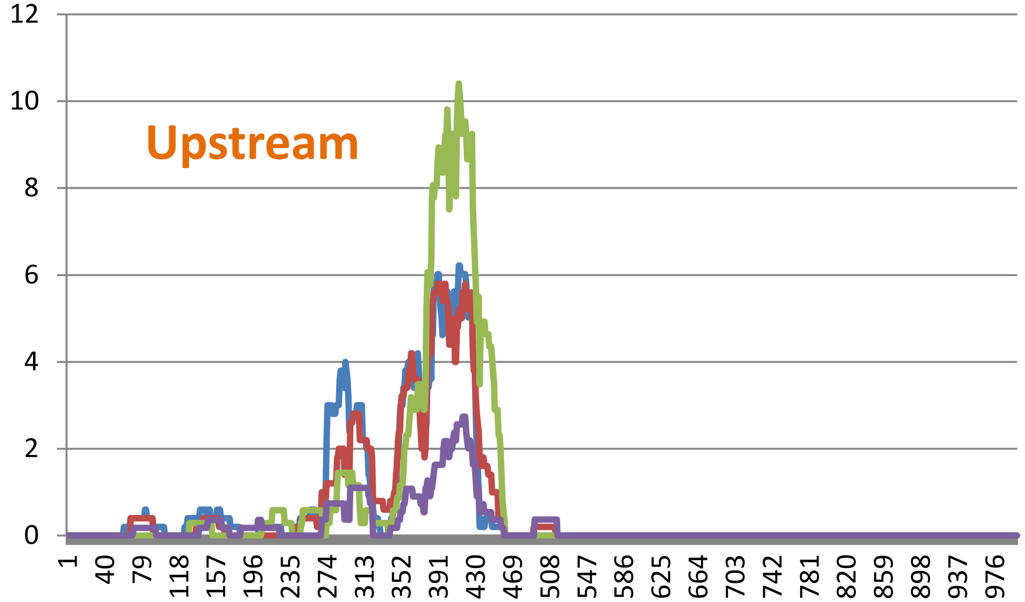

AT1G17720

type 2A protein serine/threonine phosphatase

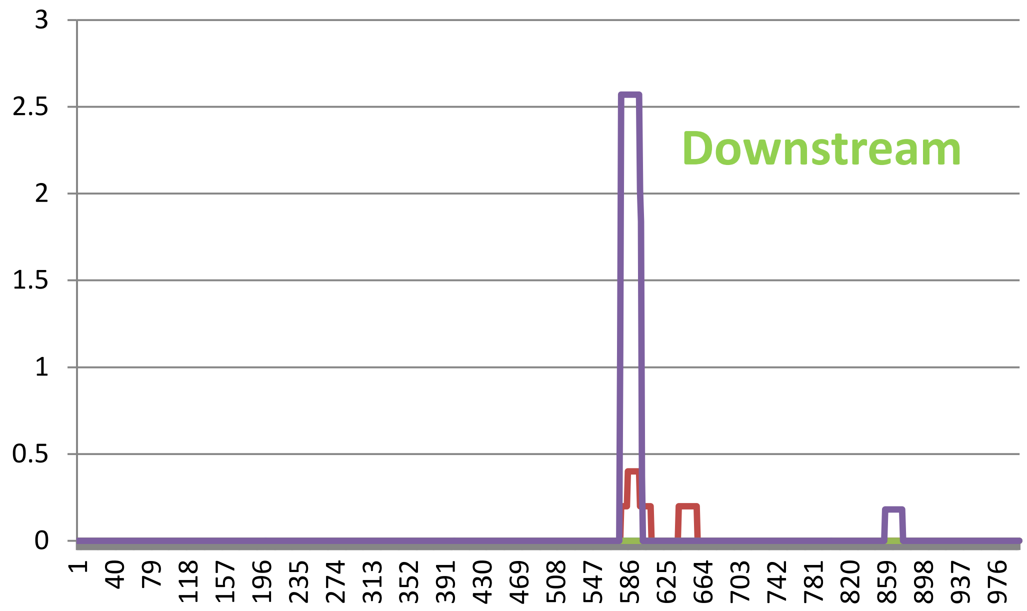

AT1G20967

unknown protein

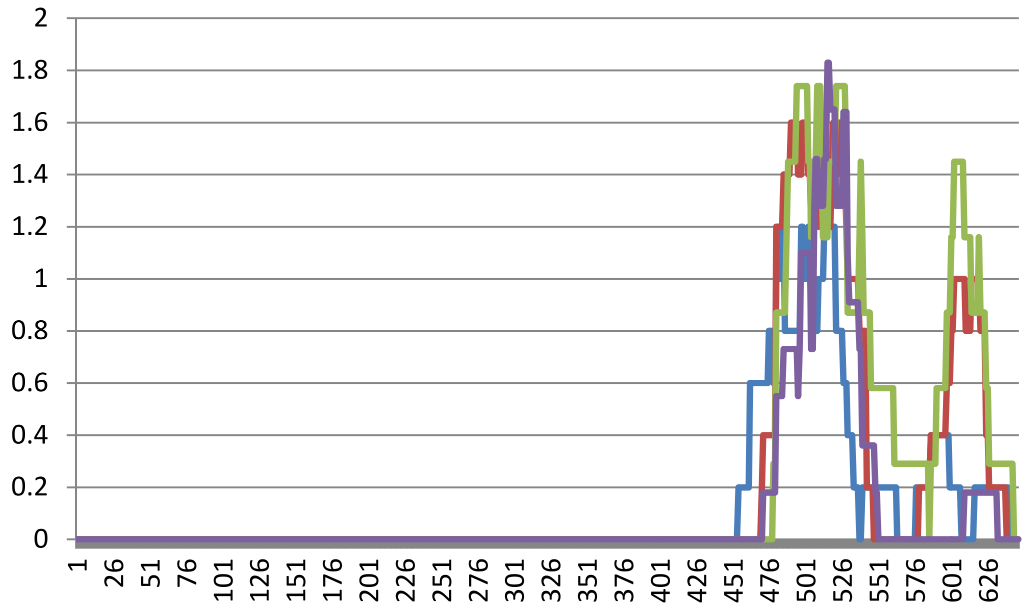

AT1G24388

unknown protein

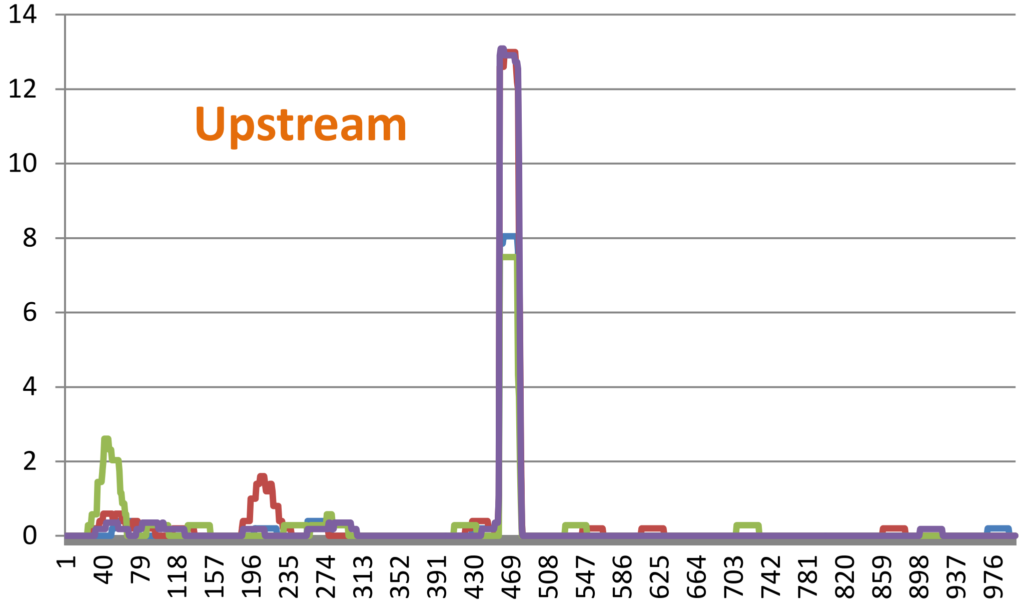

AT1G26640

Amino acid kinase family protein

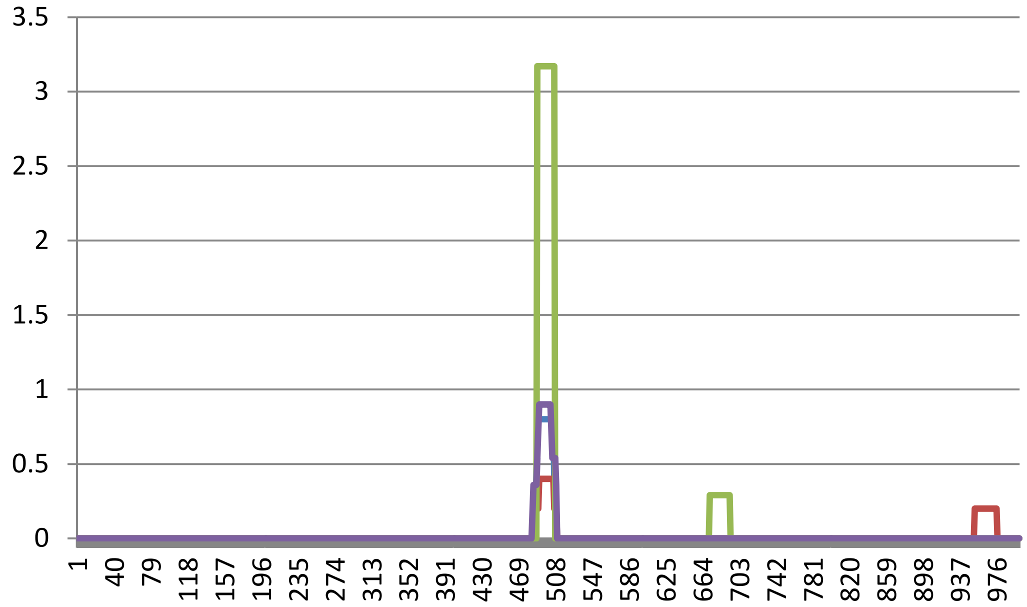

AT1G27160

valyl-tRNA synthetase / valine--tRNA ligase-related

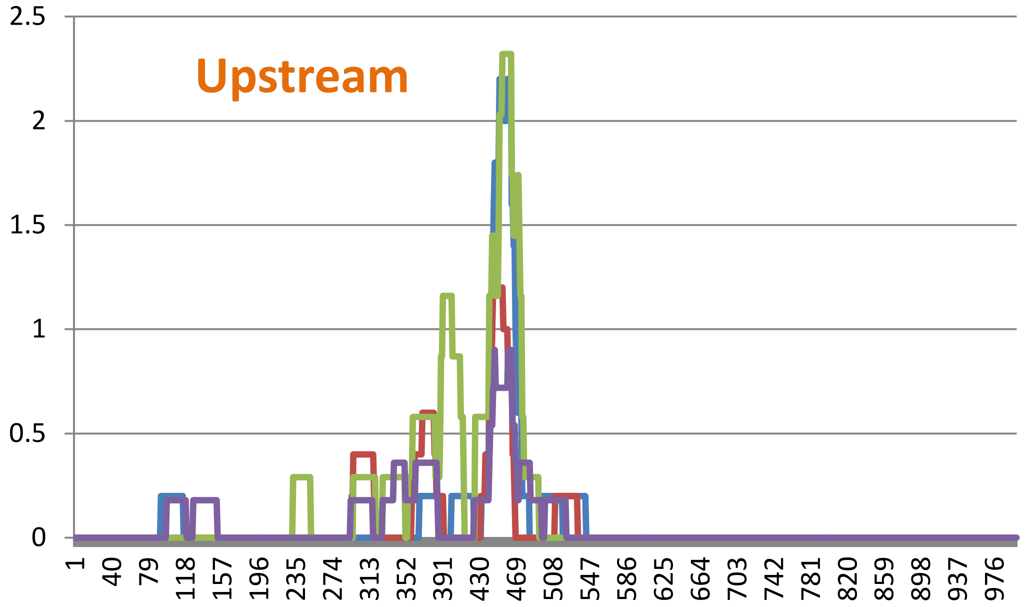

AT1G28281

unknown protein

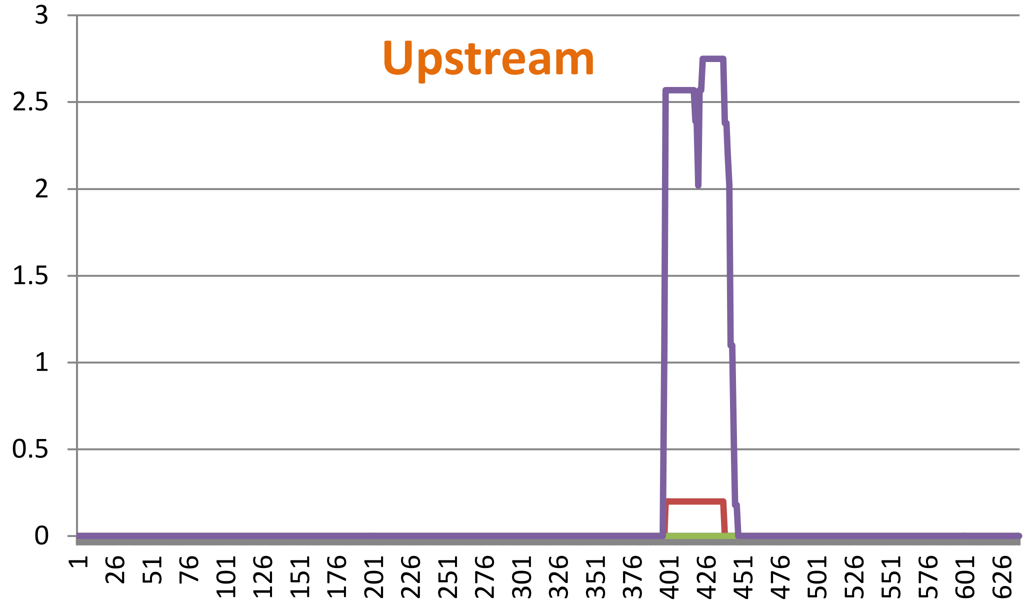

AT1G31960

unknown protein

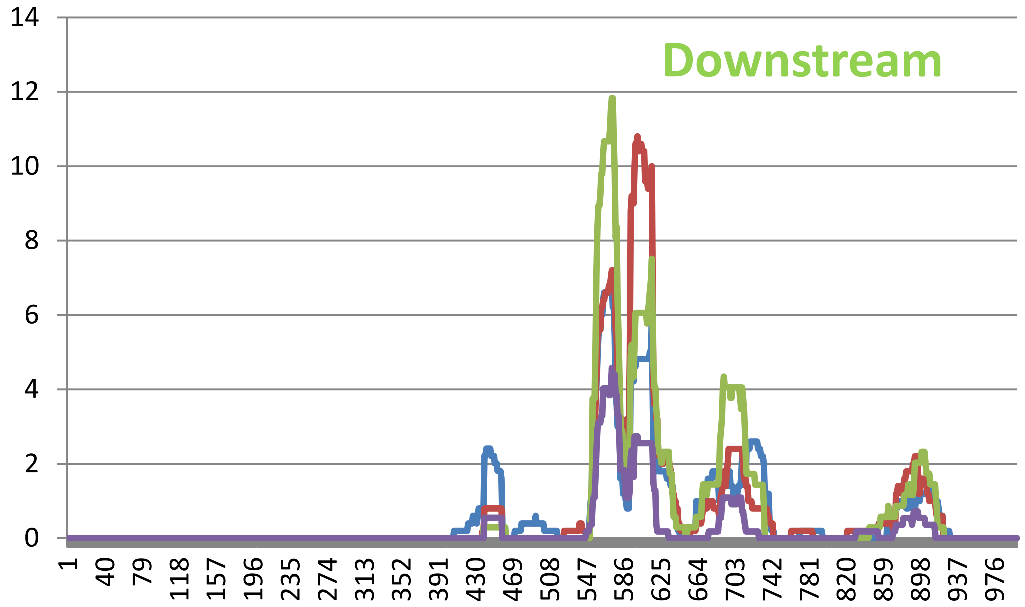

AT1G32140

F-box family protein

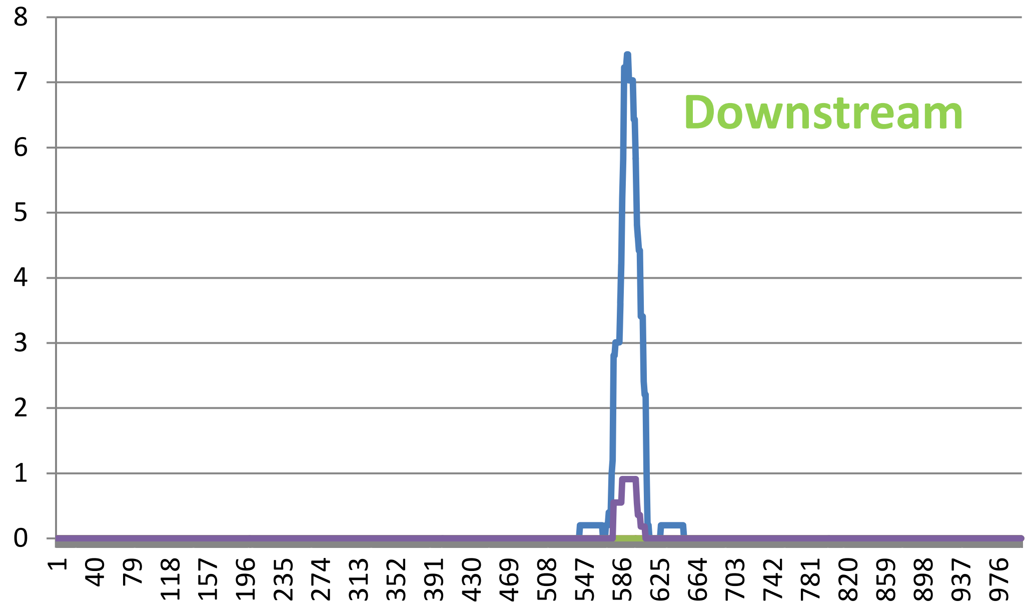

AT1G32610

hydroxyproline-rich glycoprotein family protein

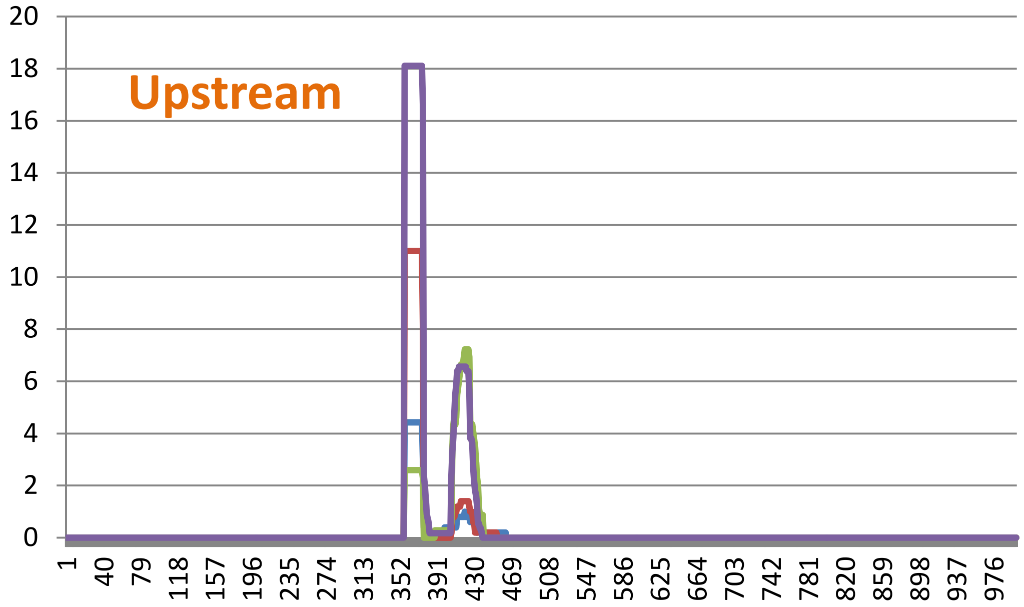

AT1G47280

unknown protein

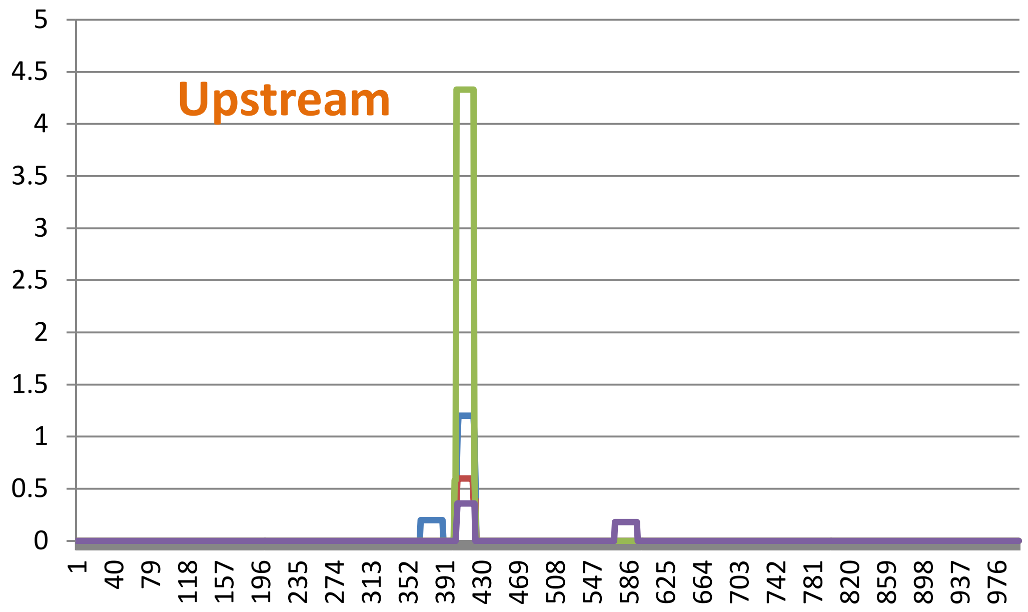

AT1G47765

F-box and associated interaction domains-containing protein

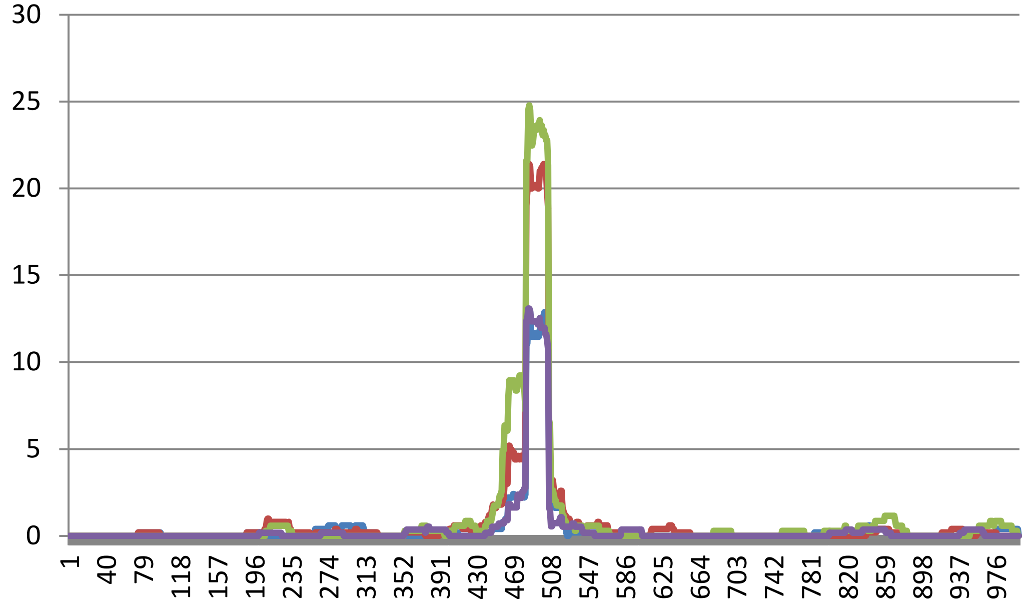

AT1G48660

Auxin-responsive GH3 family protein

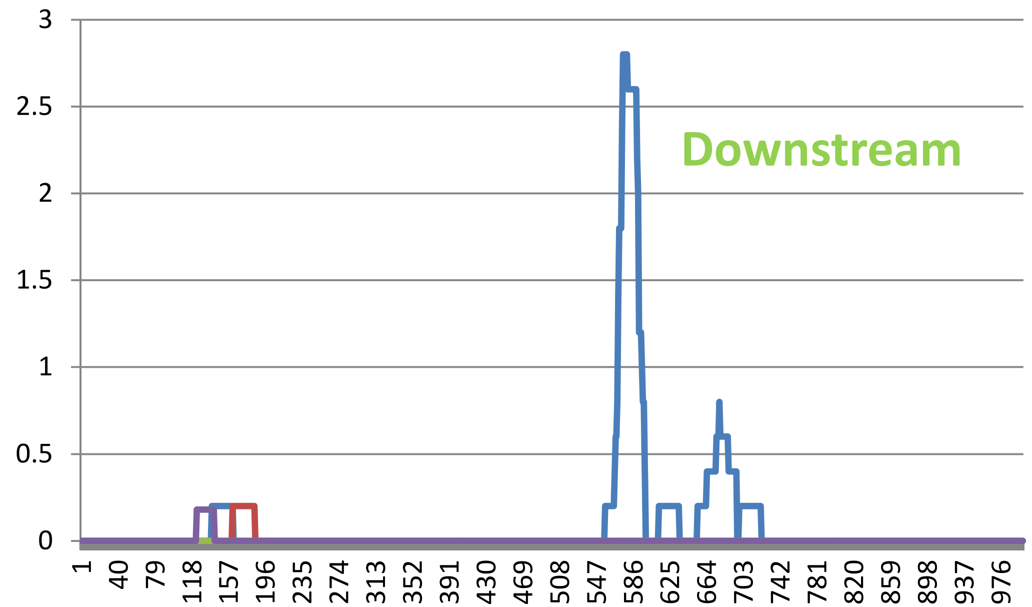

AT1G50160

Polynucleotidyl transferase, ribonuclease H-like superfamily protein

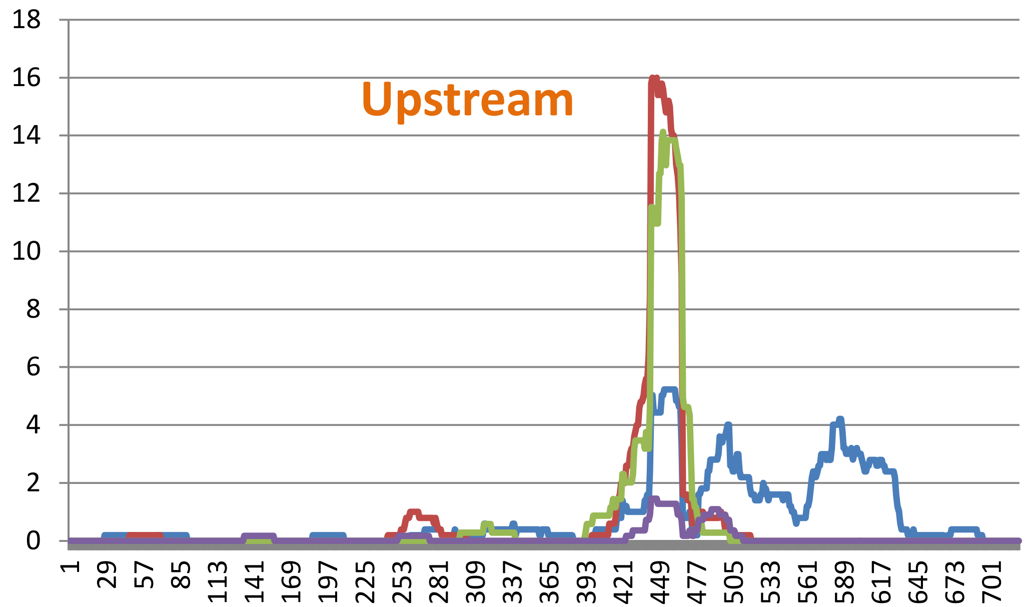

AT1G52110

Mannose-binding lectin superfamily protein

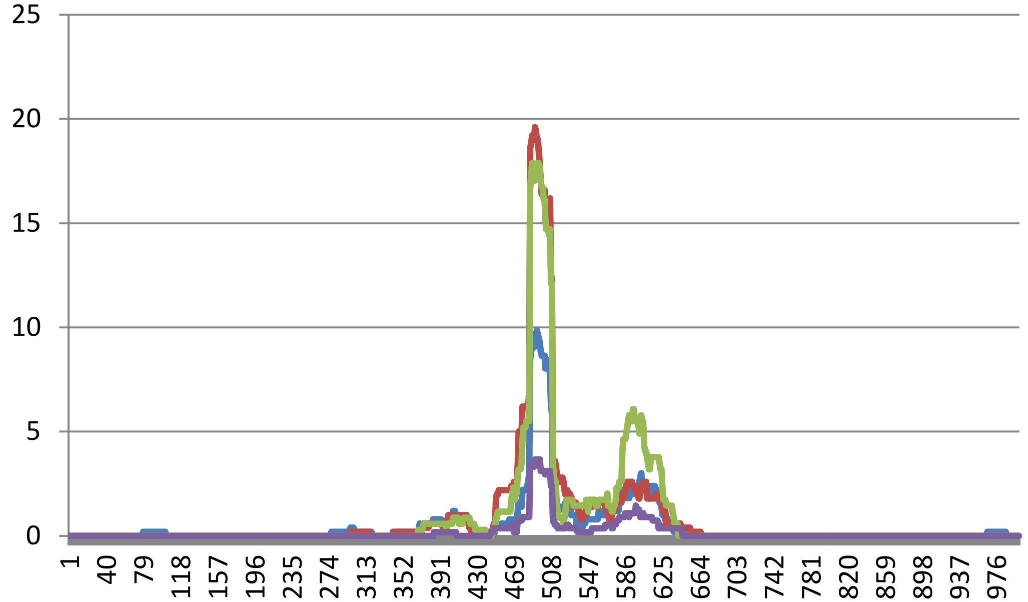

AT1G52940

purple acid phosphatase 5 (PAP5)

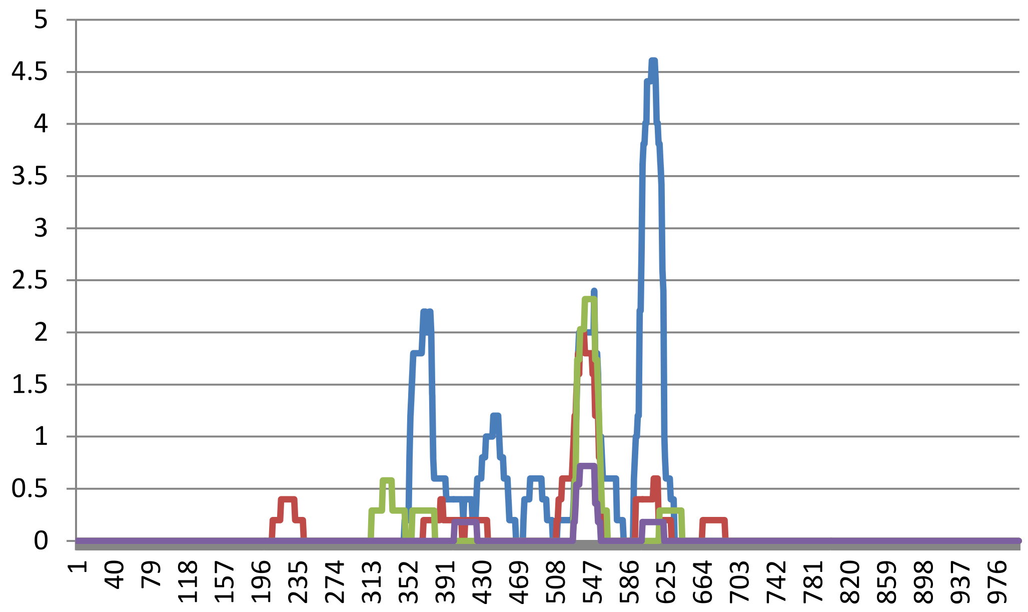

AT1G53265

unknown protein

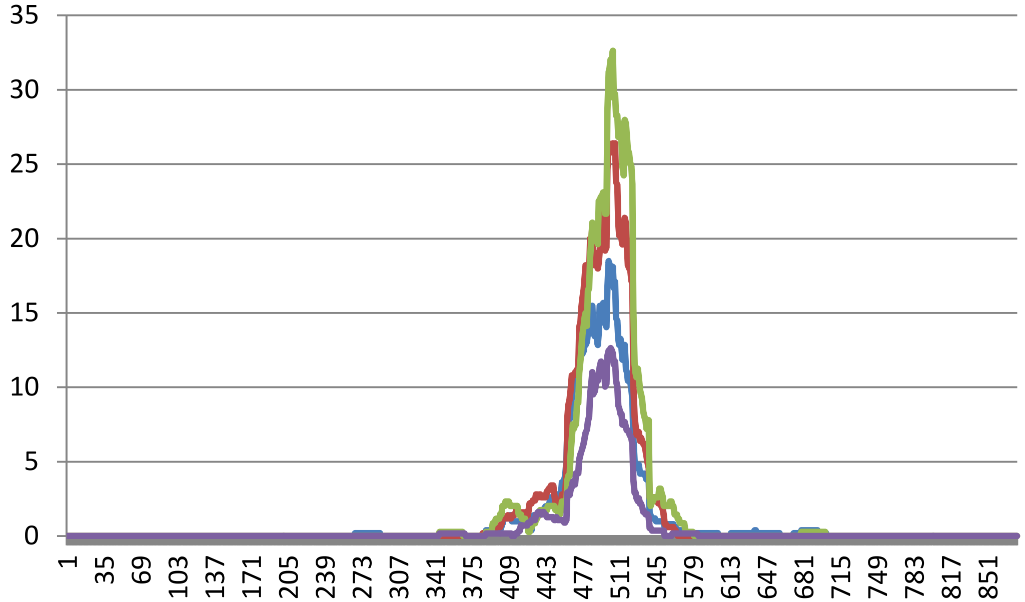

AT1G53400

Ubiquitin domain-containing protein

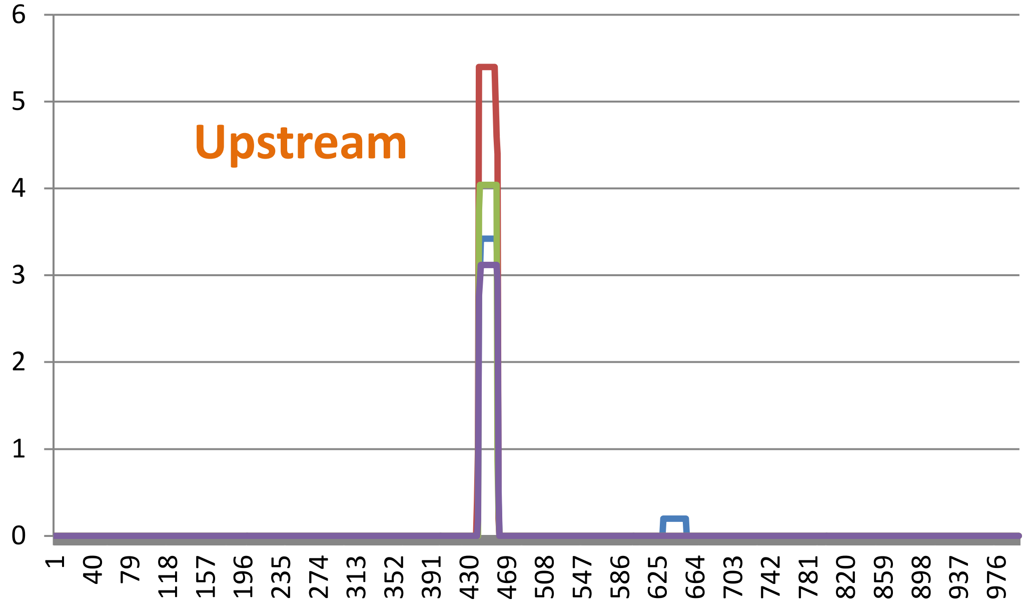

AT1G53542

unknown protein

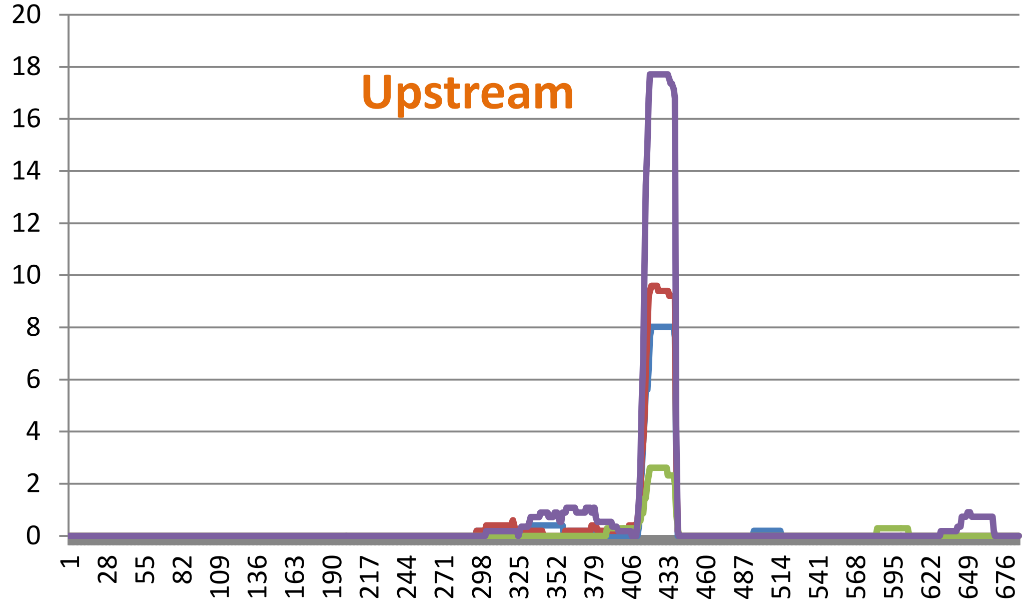

AT1G59680

embryo sac development arrest 1 (EDA1)

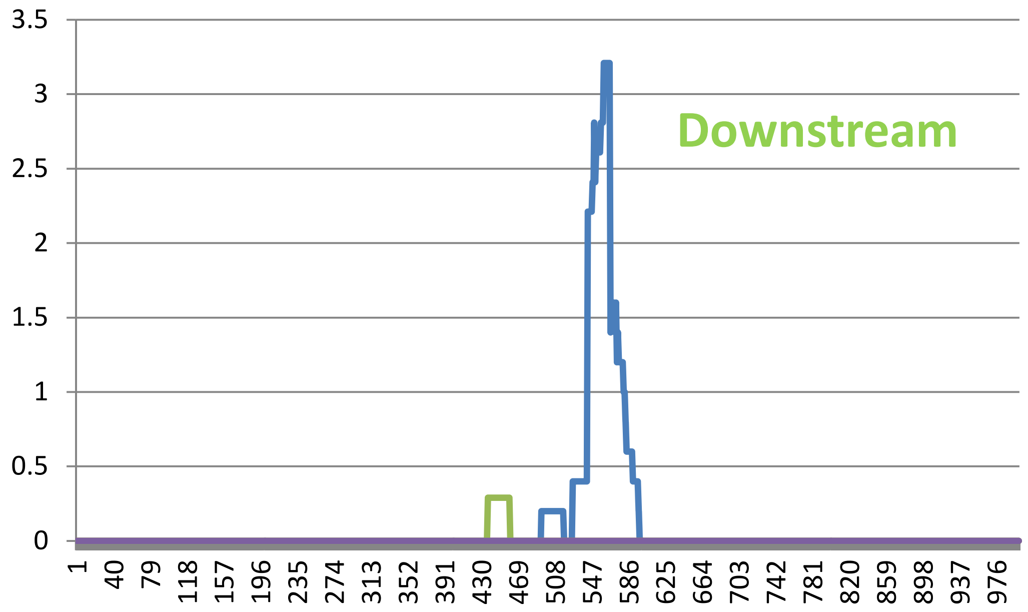

AT1G60720

RNA-directed DNA polymerase (reverse transcriptase)-related family protein

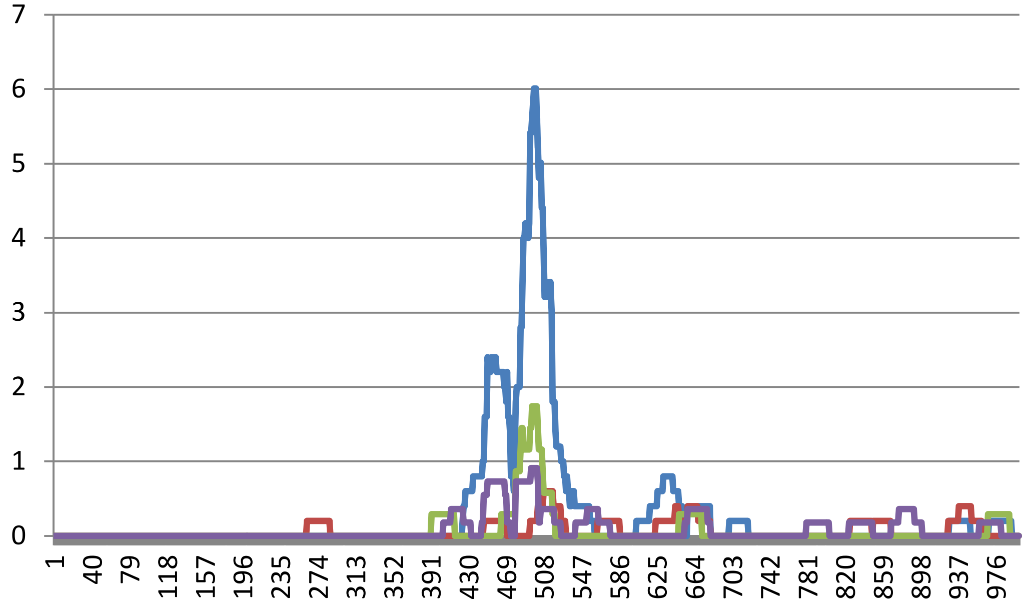

AT1G60940

Encodes a member of SNF1-related protein kinases (SnRK2) whose activity is activated by ionic (salt) and non-ionic (mannitol) osmotic stress.

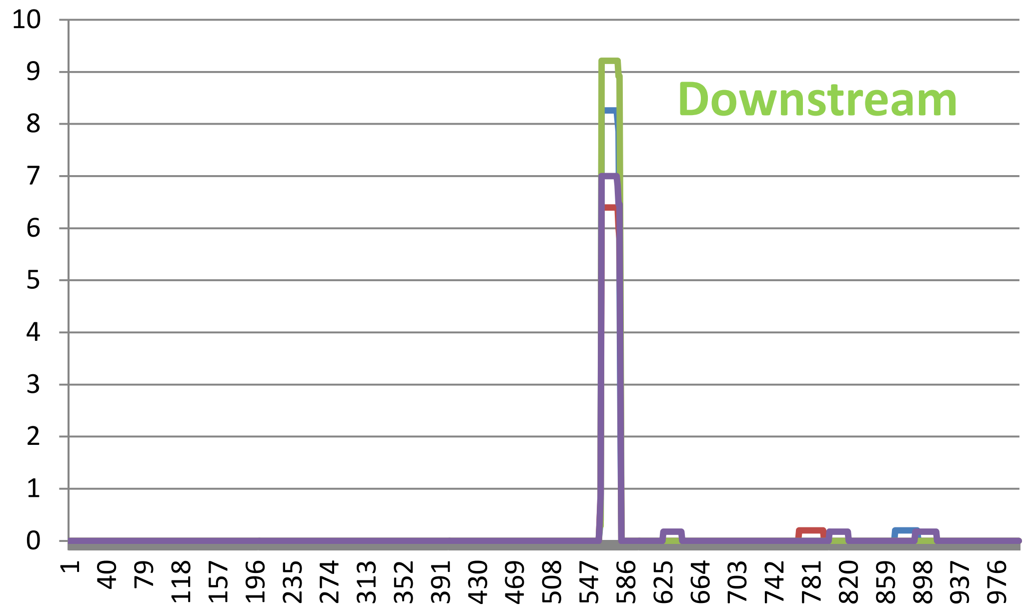

AT1G61820

beta glucosidase 46 (BGLU46)

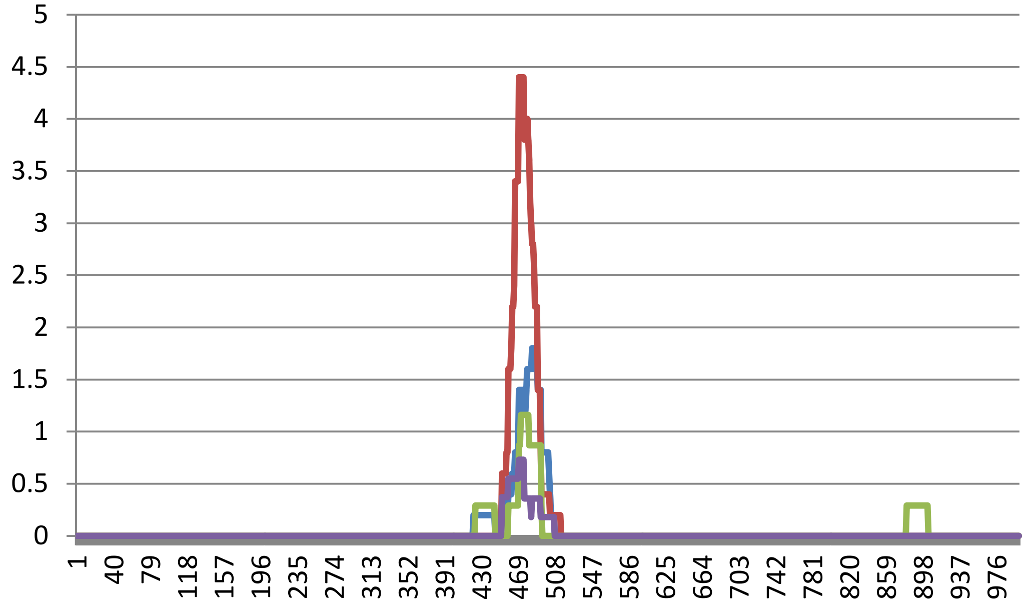

AT1G63210

SPT6L encodes a putative WG/GW-repeat protein involved in the regulation of apical/basal polarity of embryo

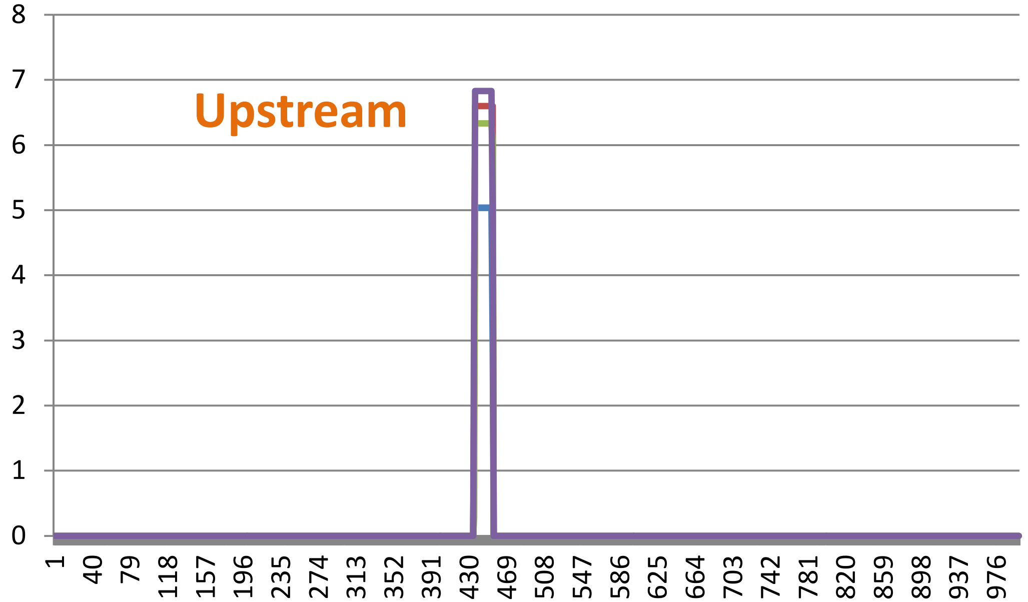

AT1G63800

ubiquitin-conjugating enzyme 5 (UBC5)

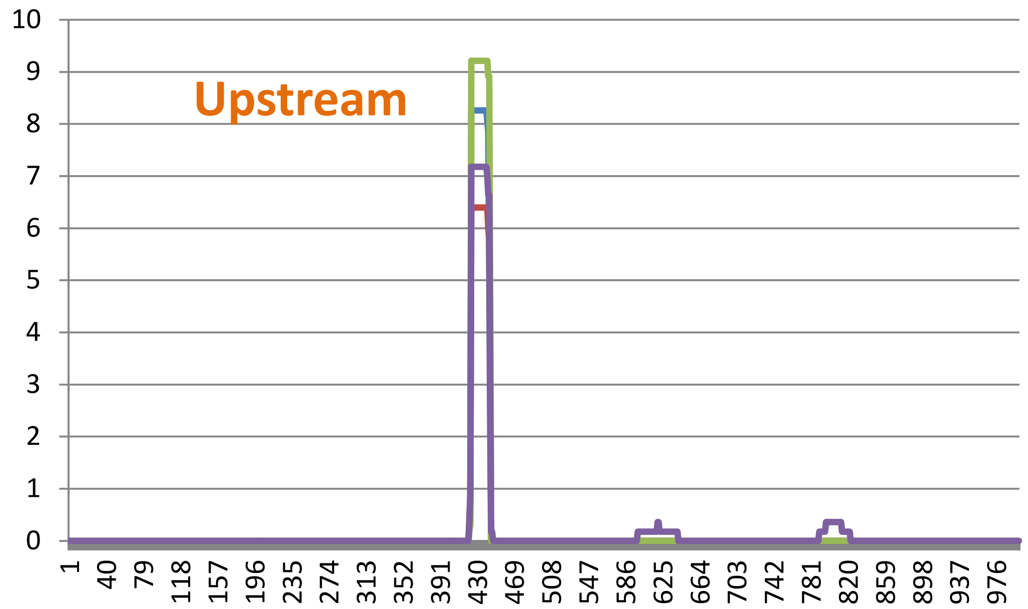

AT1G64130

Polyketide cyclase/dehydrase and lipid transport superfamily protein

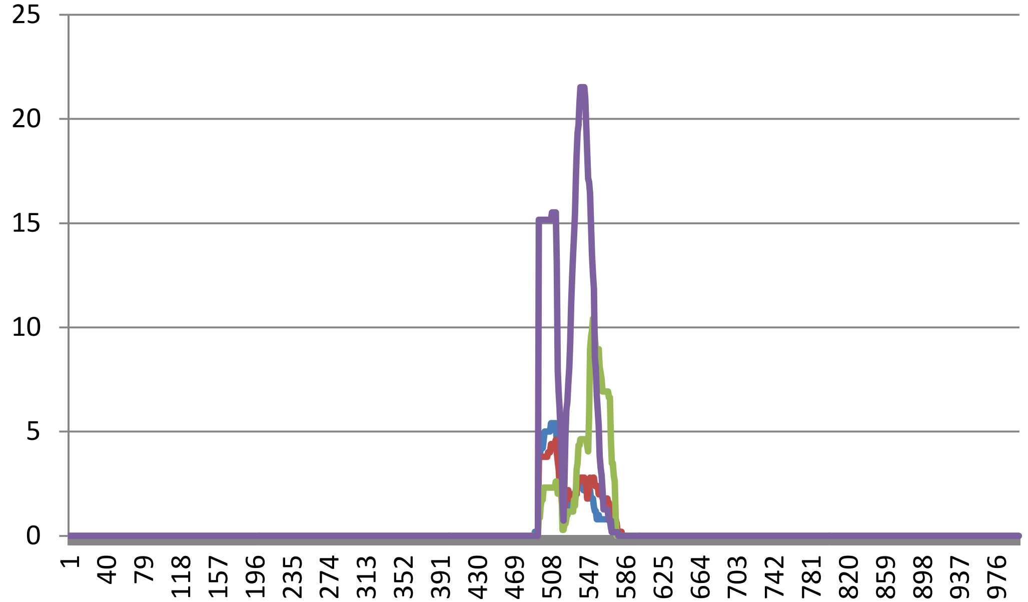

AT1G64770

Encodes a novel subunit of the chloroplast NAD(P)H dehydrogenase complex, involved in cyclic electron flow around photosystem I to produce ATP.

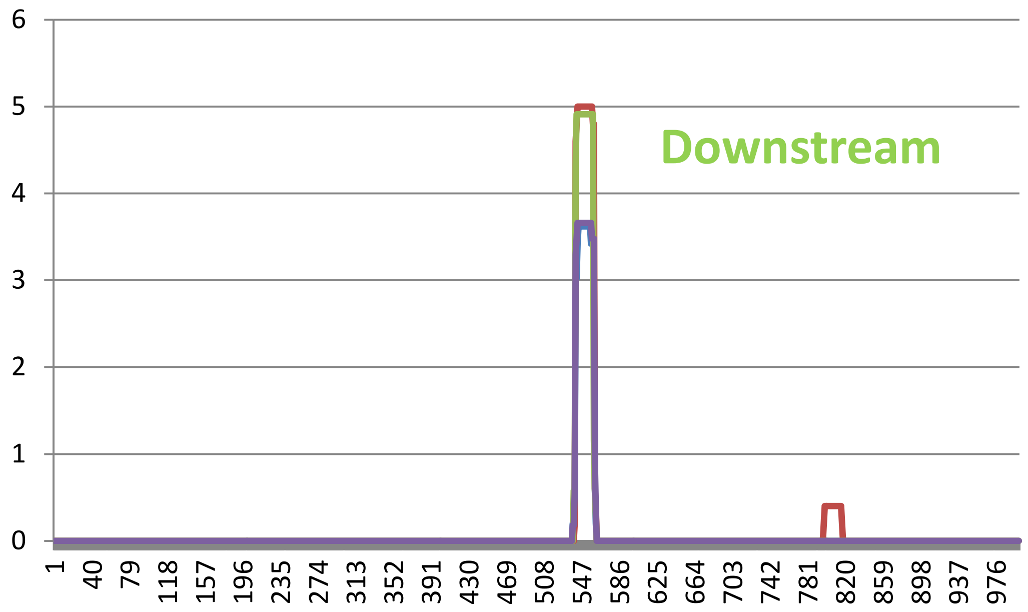

AT1G66290

F-box/RNI-like superfamily protein

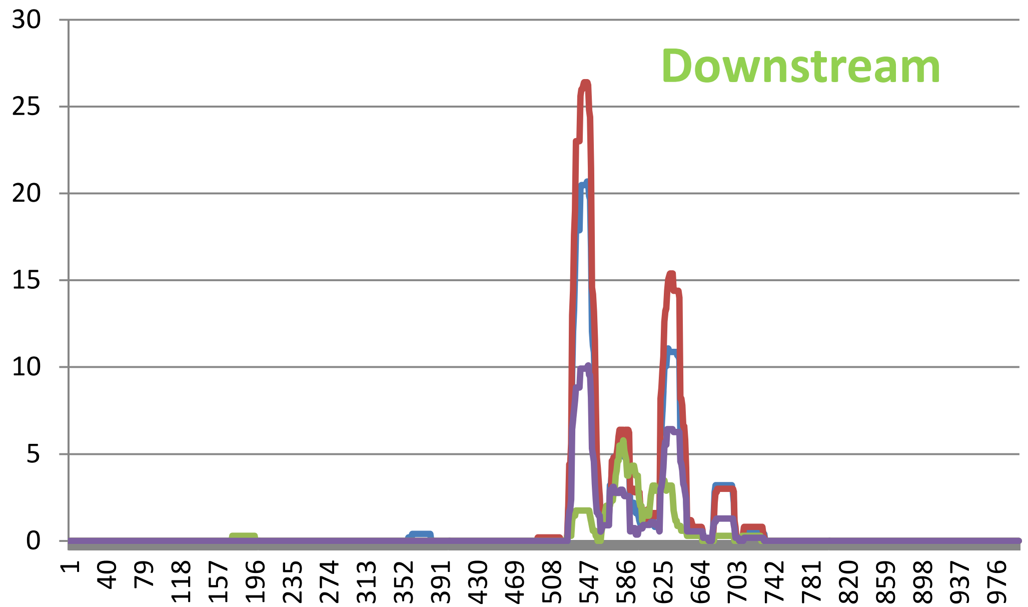

AT1G66490

F-box and associated interaction domains-containing protein

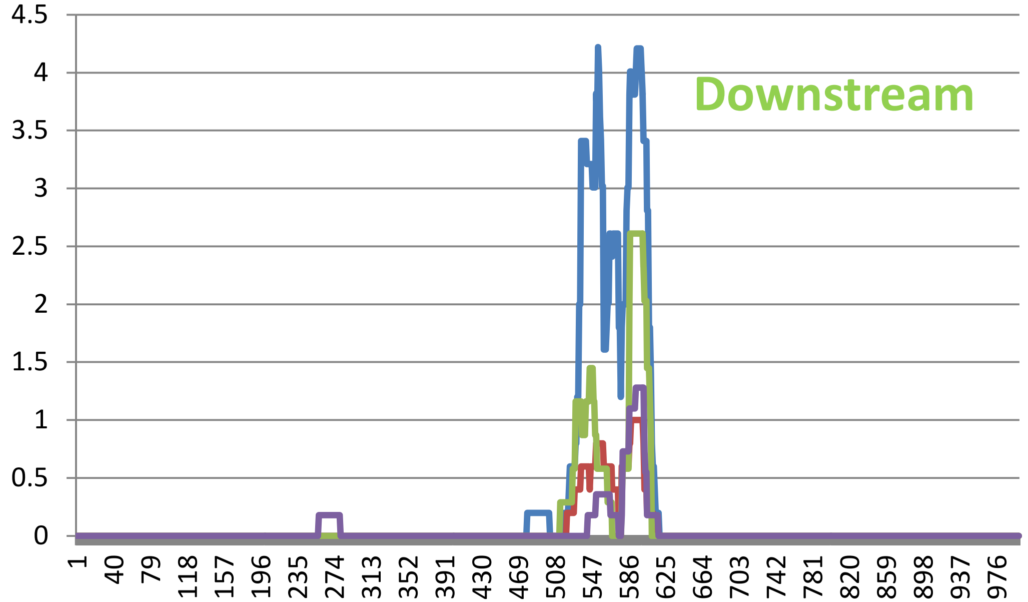

AT1G66553

unknown protein

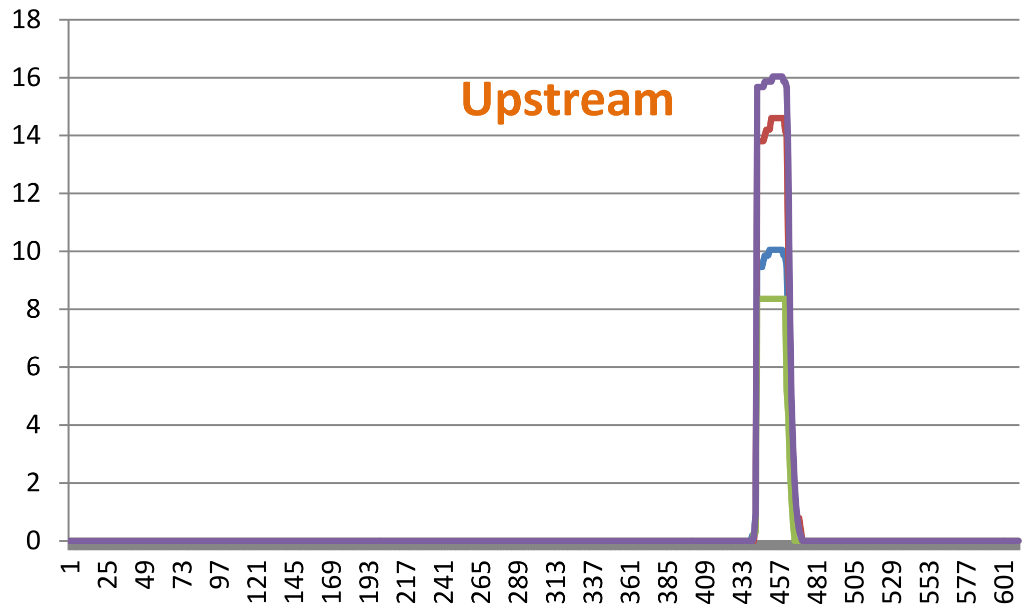

AT1G66640

RNI-like superfamily protein

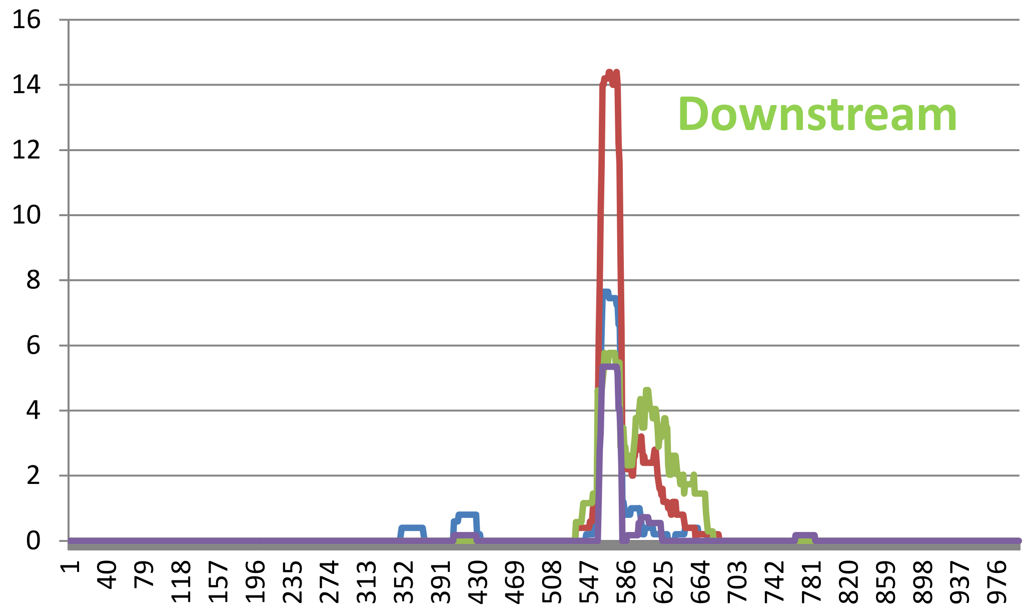

AT1G66740

Located on the SSL2 region of Arabidopsis thaliana, which is homeologous to the Brassica S locus for self incompatibility. Expressed in both vegetative and reproductive organs suggesting AtSP7 might not be involved in self incompatibility.

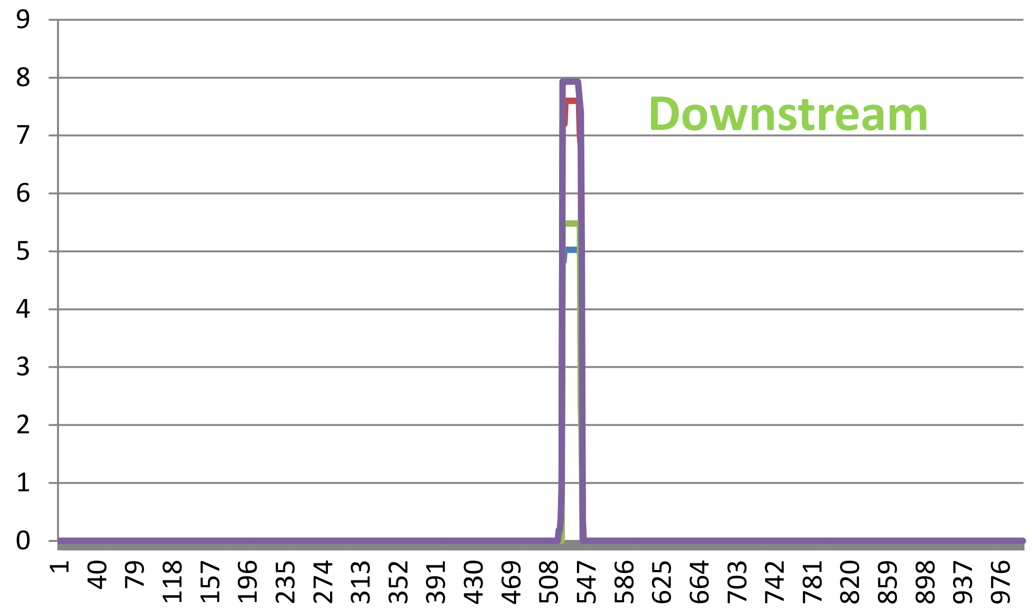

AT1G67450

F-box and associated interaction domains-containing protein

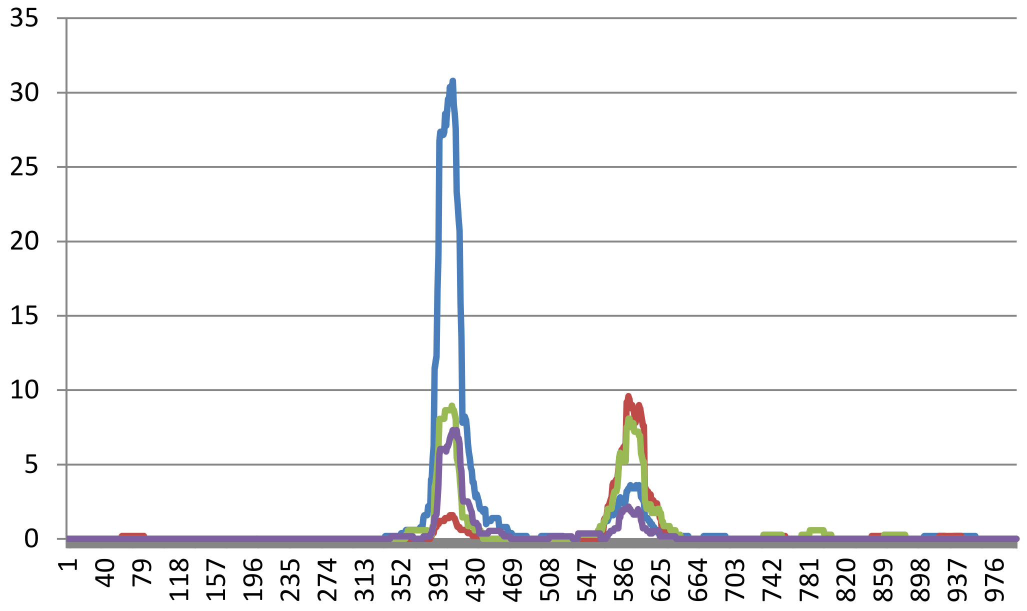

AT1G68830

STN7 protein kinase; required for state transitions, phosphorylation of the major antenna complex (LHCII) between PSII and PSI, and light adaptation

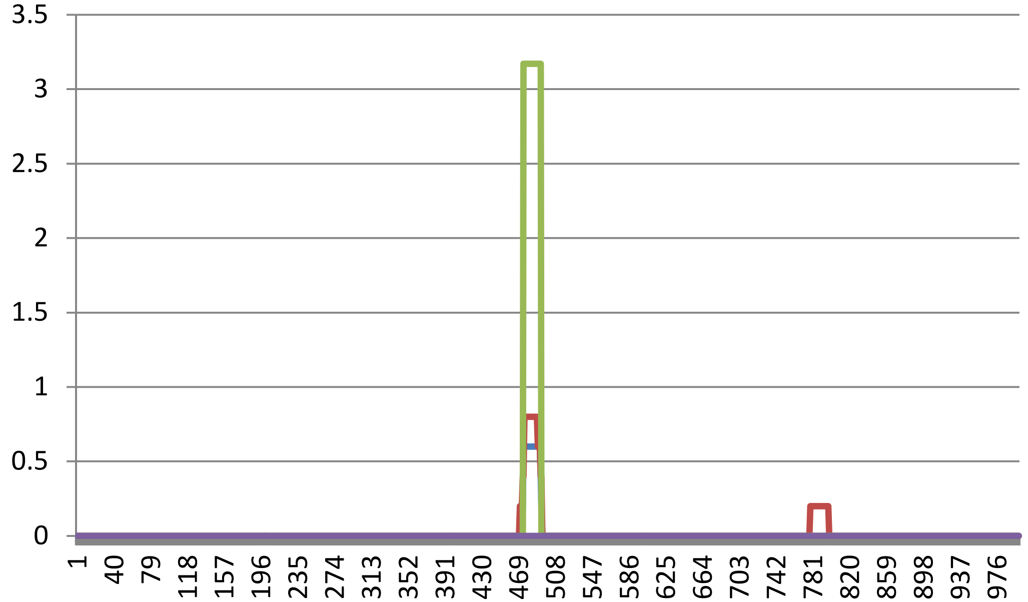

AT1G70650

Ran BP2/NZF zinc finger-like superfamily protein

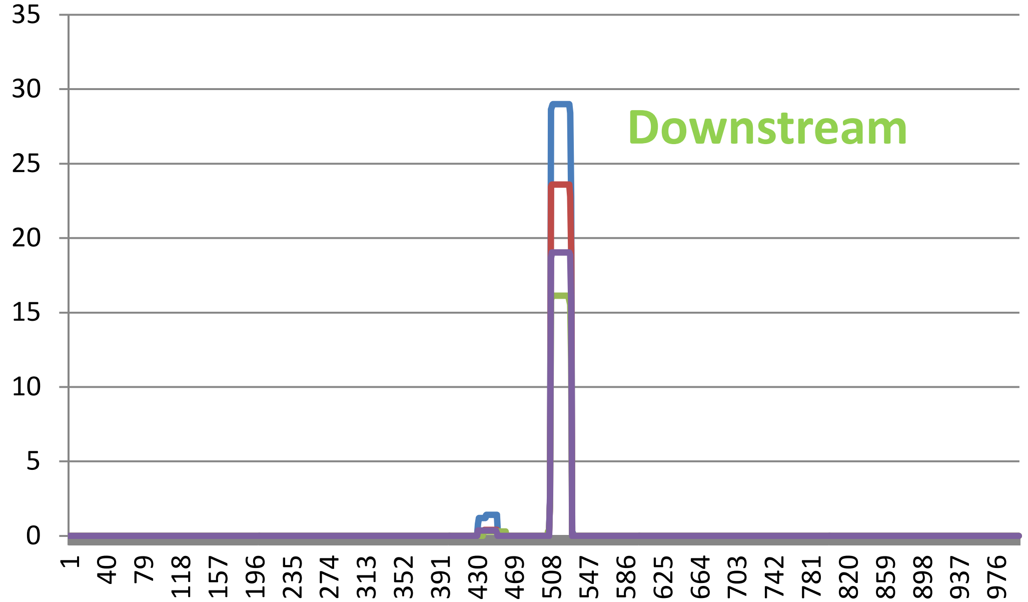

AT1G71820

Encodes a member of the exocyst complex gene family. The exocyst is a protein complex involved in tethering vesicles to the plasma membrane during regulated or polarized secretion.

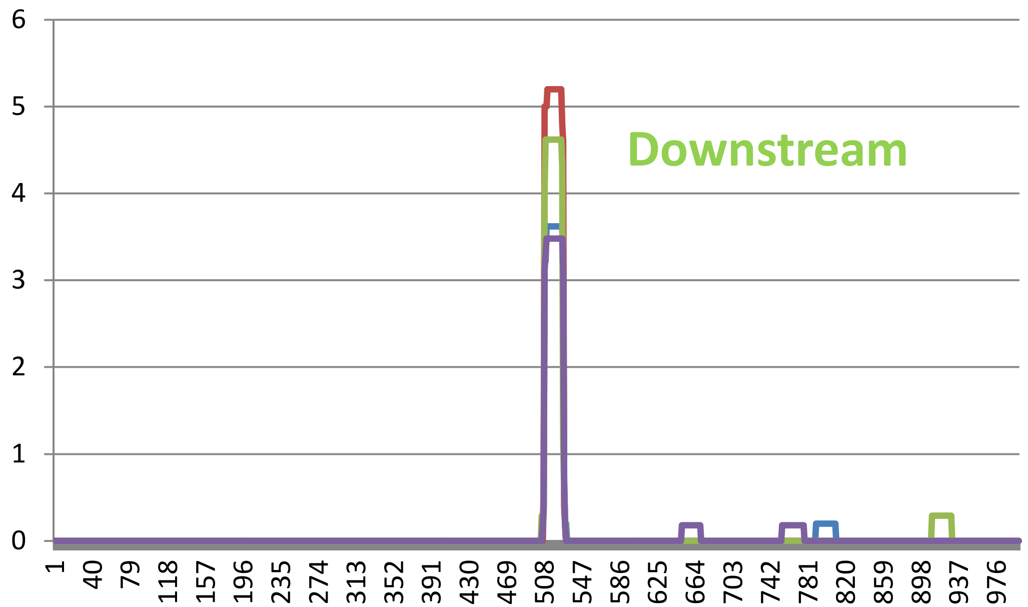

AT1G73710

Pentatricopeptide repeat (PPR) superfamily protein

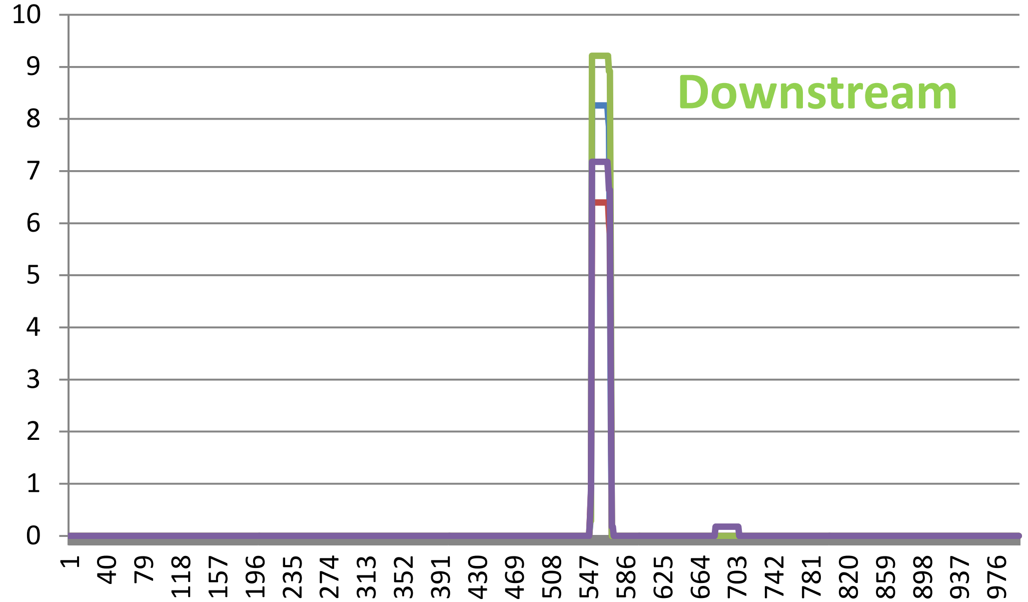

AT1G75050

Pathogenesis-related thaumatin superfamily protein

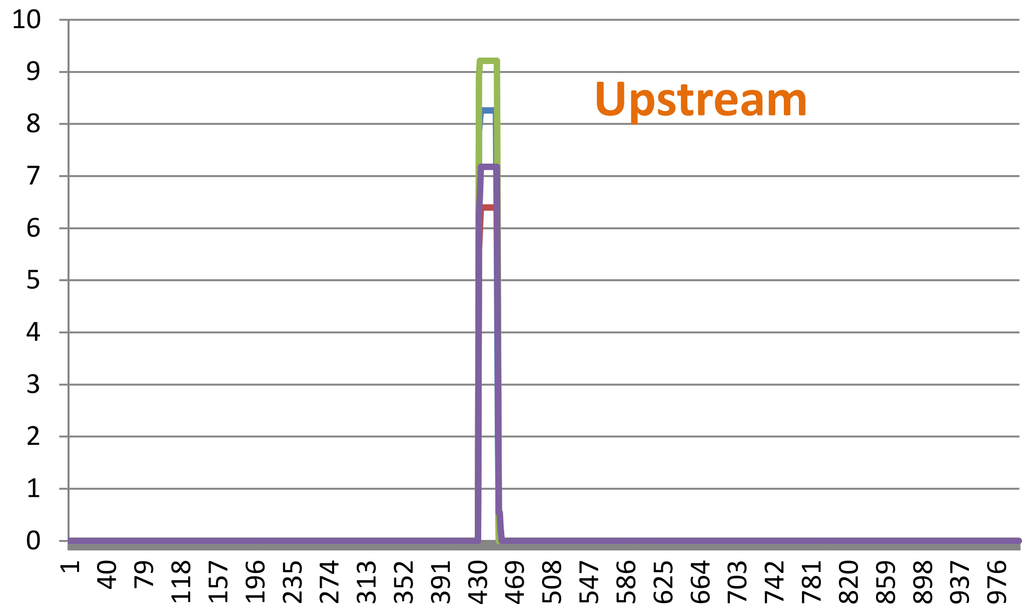

AT1G78820

D-mannose binding lectin protein with Apple-like carbohydrate-binding domain

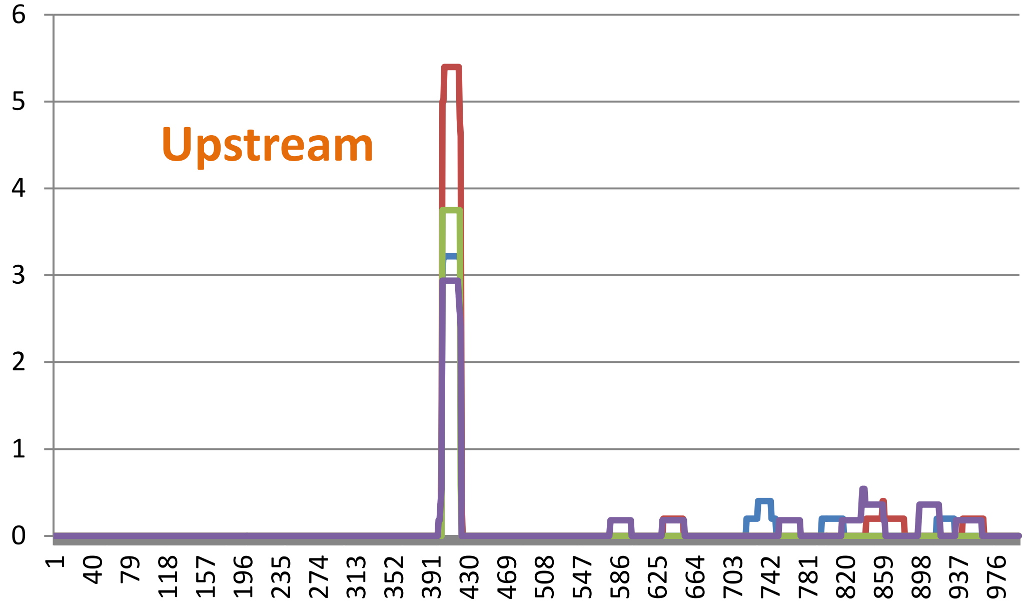

AT1G79490

Embryo defective 2217 (EMB2217)

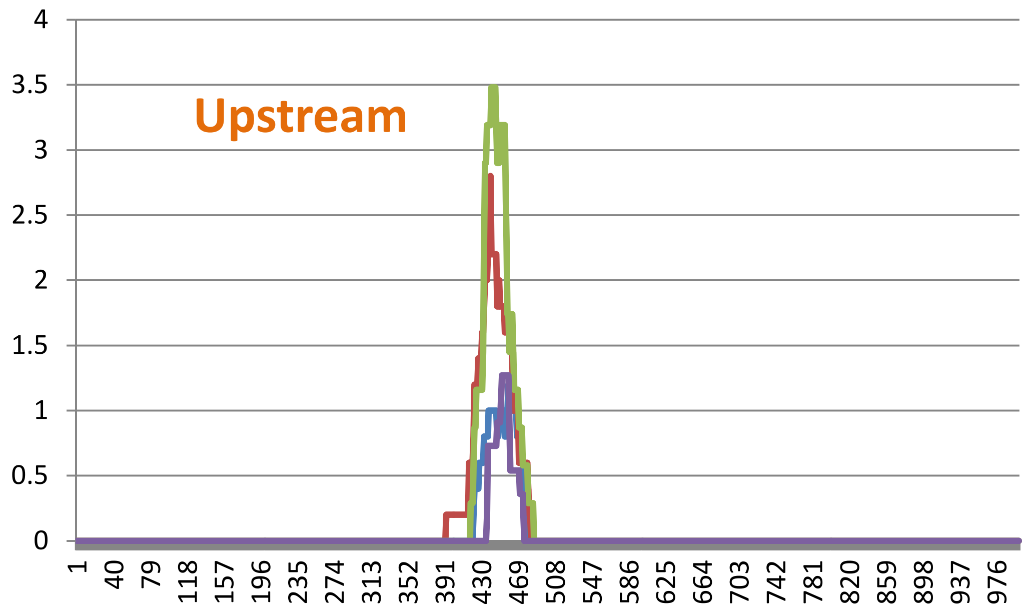

AT1G79800

Early nodulin-like protein 7 (ENODL7)

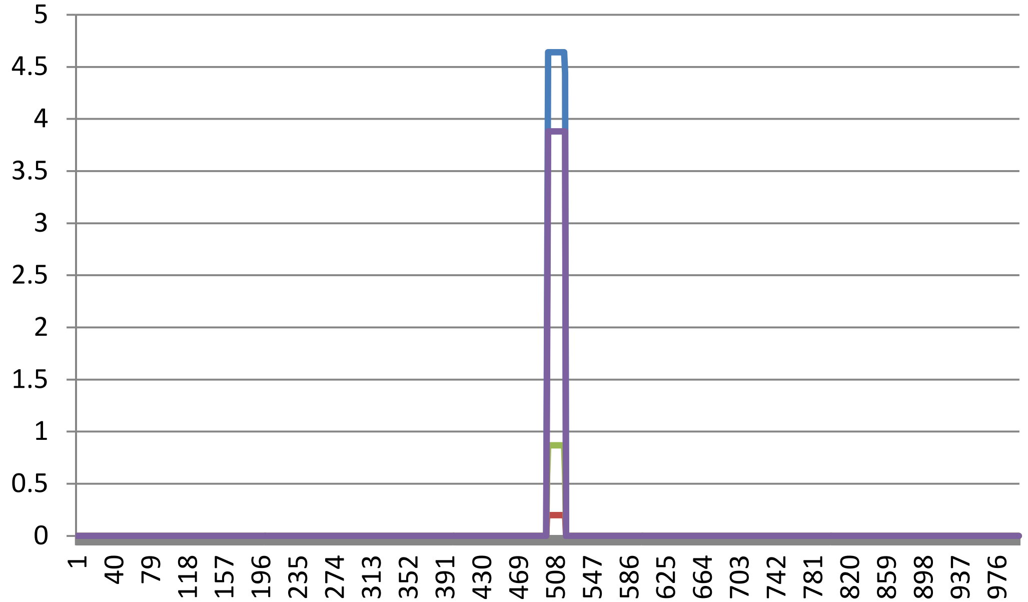

AT1G80490

TOPLESS-related 1 (TPR1)

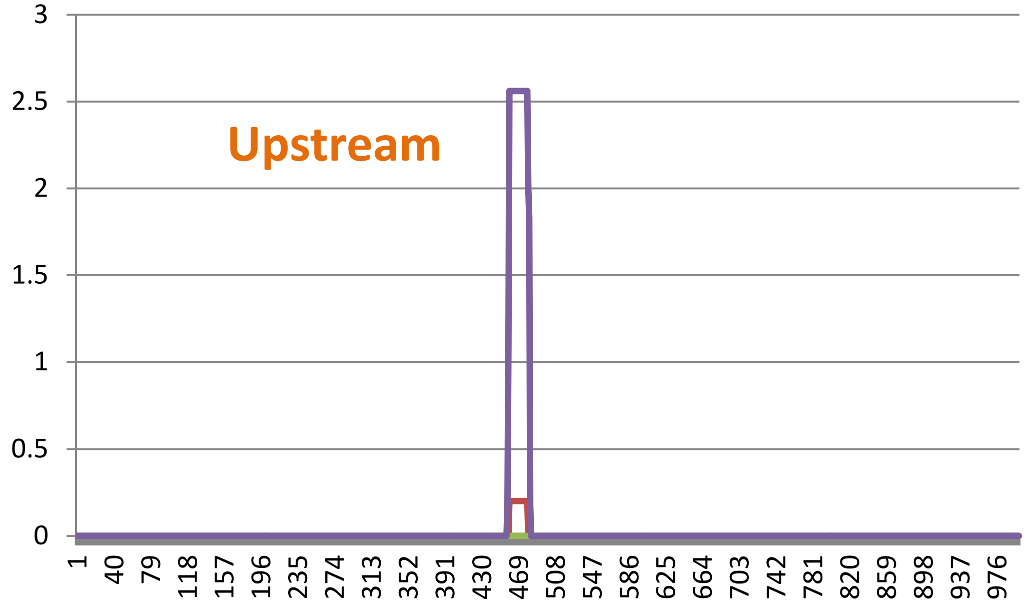

AT1G80890

unknown protein

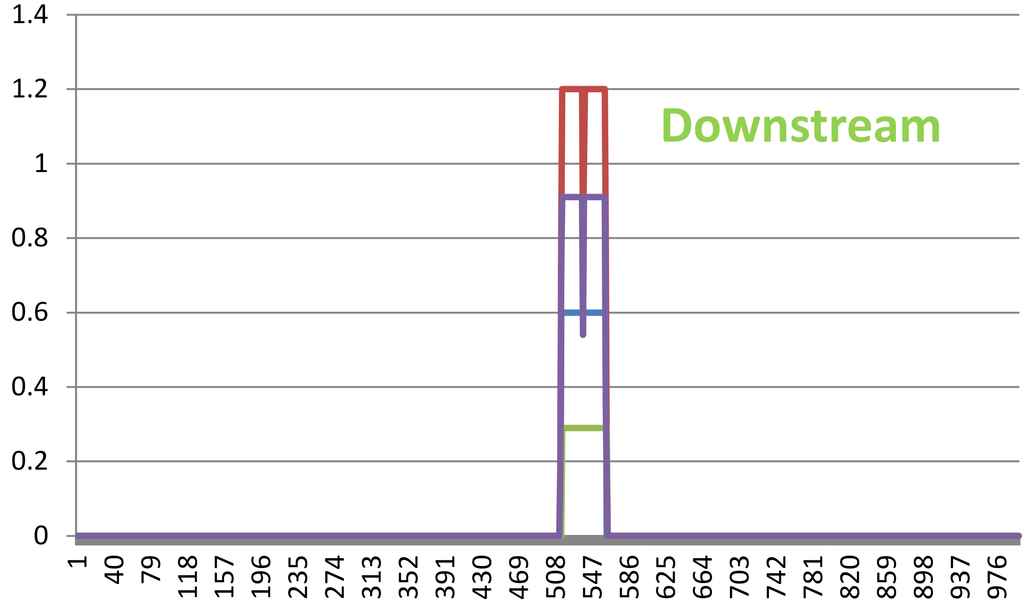

AT2G04620

Cation efflux family protein

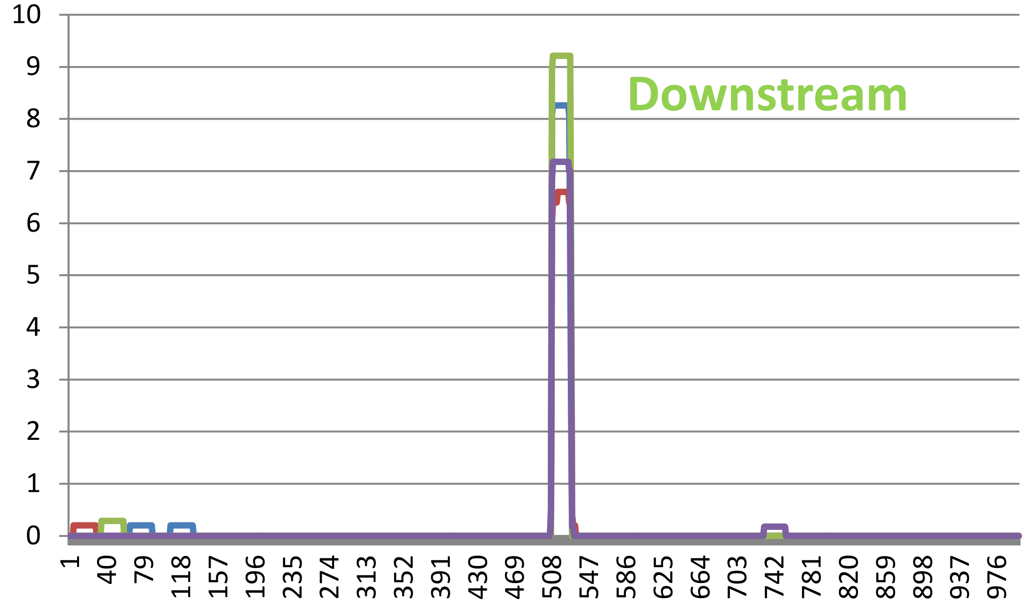

AT2G05380

Glycine-rich protein 3 short isoform (GRP3S) mRNA, complete

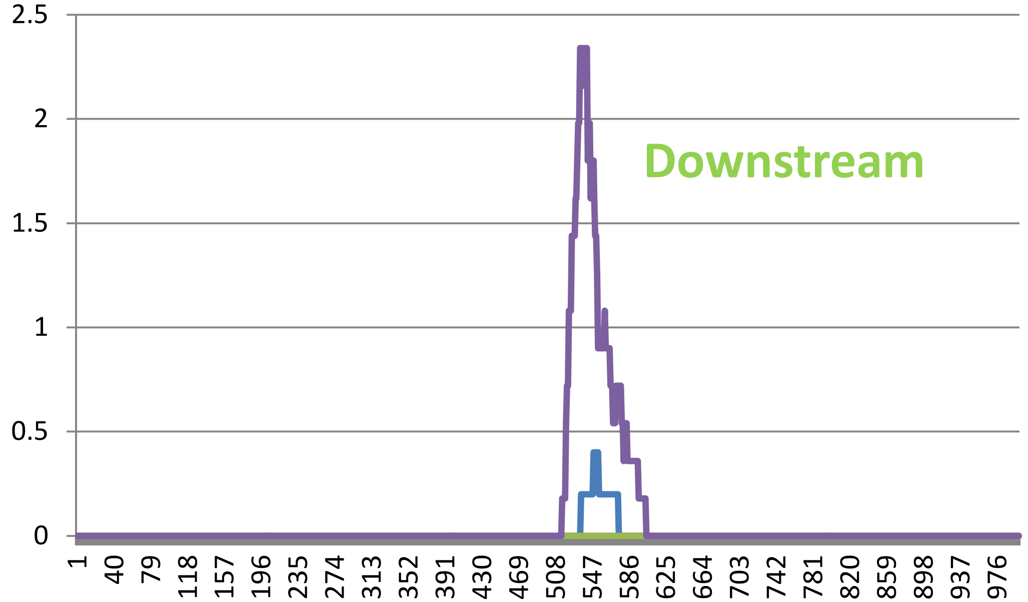

AT2G06020

Homeodomain-like superfamily protein

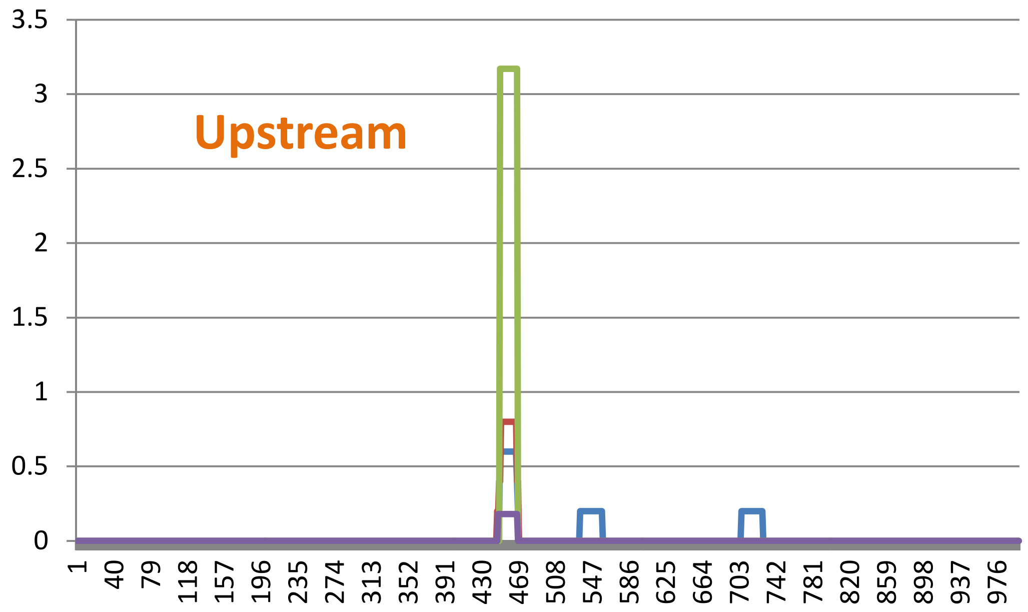

AT2G06850

Endoxyloglucan transferase (EXGT-A1) gene

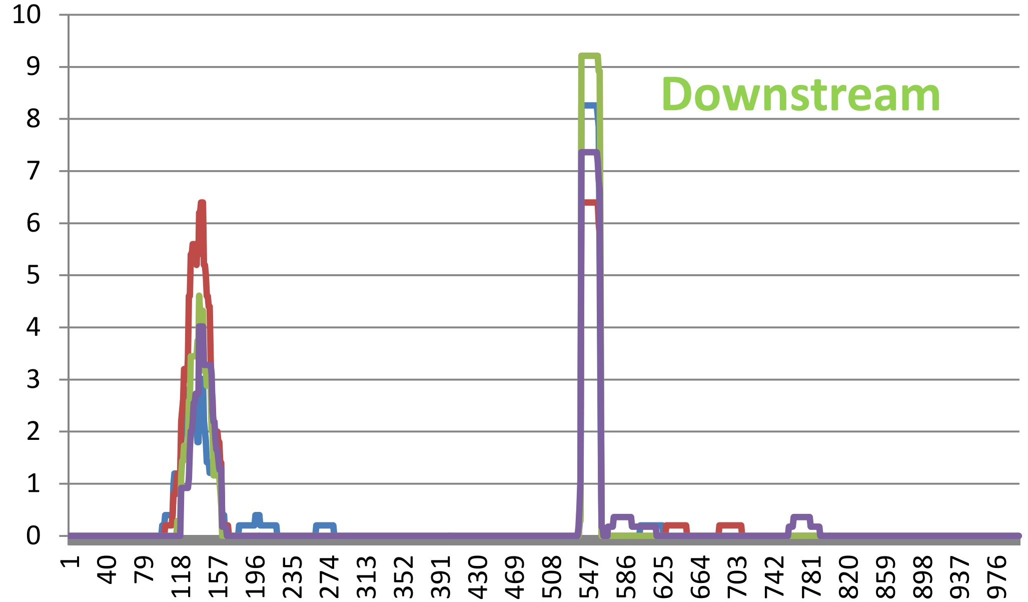

AT2G07000

unknown protein

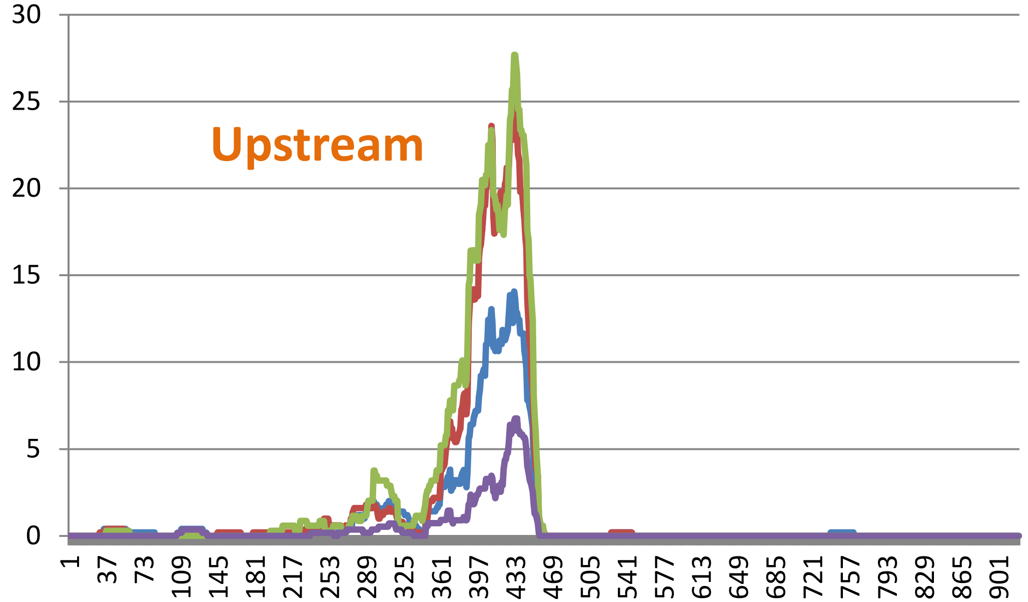

AT2G07708

unknown protein

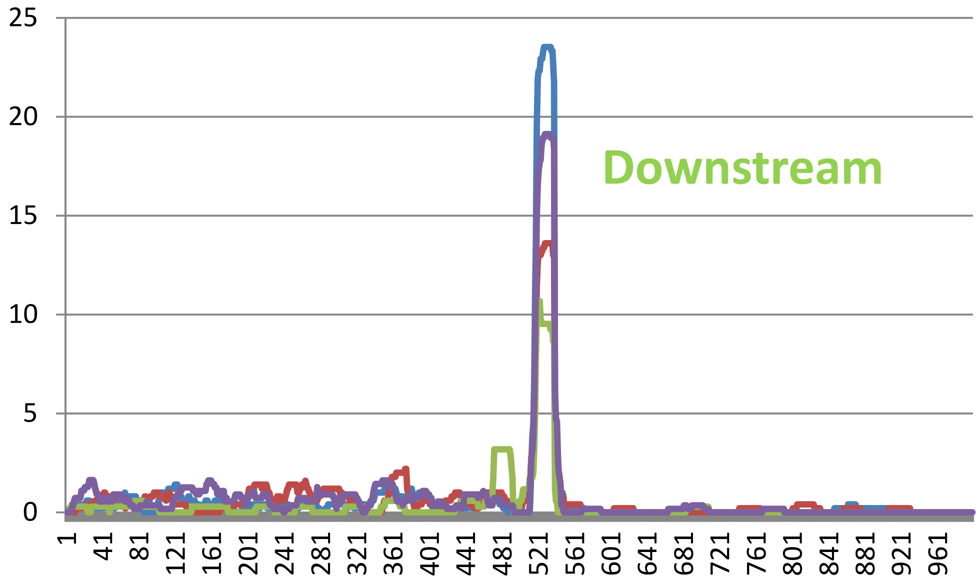

AT2G07738

unknown protein

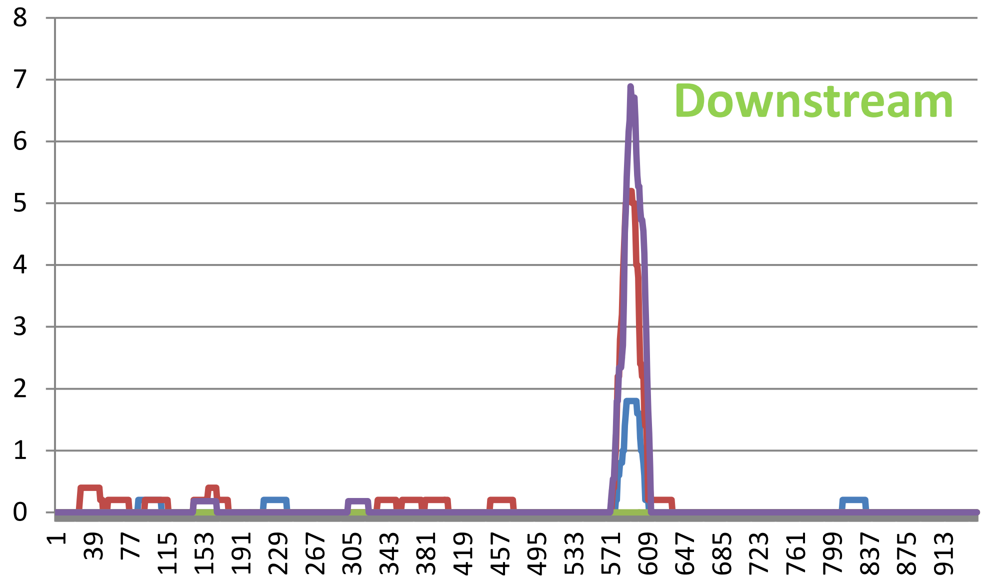

AT2G13440

Glucose-inhibited division family A protein

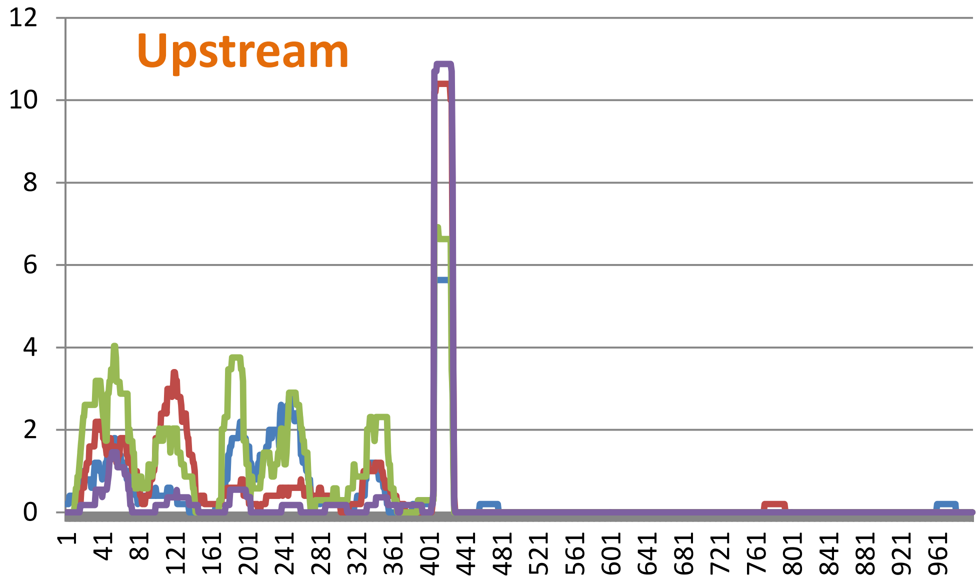

AT2G13540

Encodes a nuclear cap-binding protein that forms a heterodimeric complex with CBP20 and is involved in ABA signaling and flowering. Mutants are early flowering and exhibit hypersensitive response to ABA in germination inhibition. Loss of ABH1 function results in abnormal processing of mRNAs for several important floral regulators (FLC, CO, FLM). Analysis of loss of function mutations suggests a role in pri-miRNA processing and mRNA splicing.

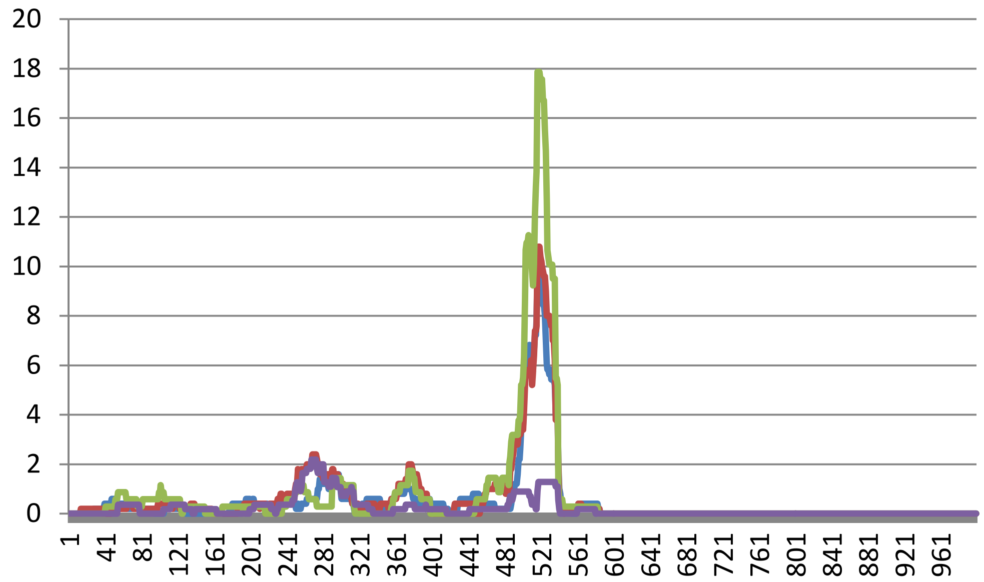

AT2G16018

unknown protein

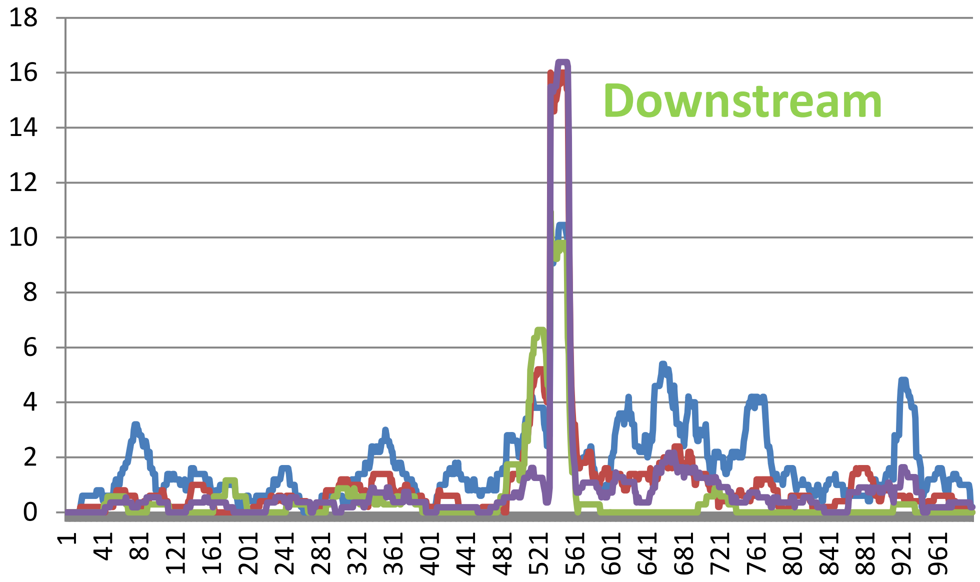

AT2G16090

ARIADNE 2 (ARI2)

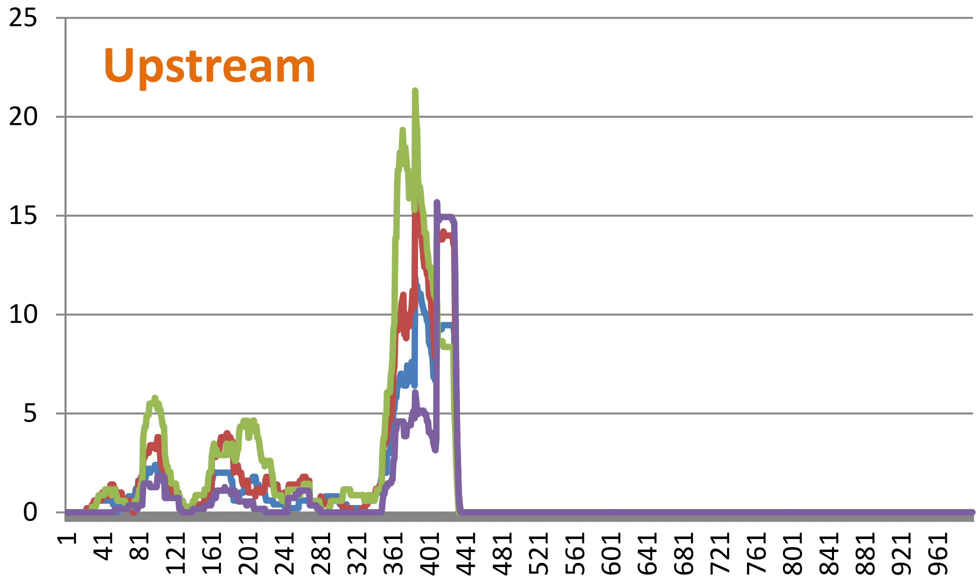

AT2G18600

Ubiquitin-conjugating enzyme family protein

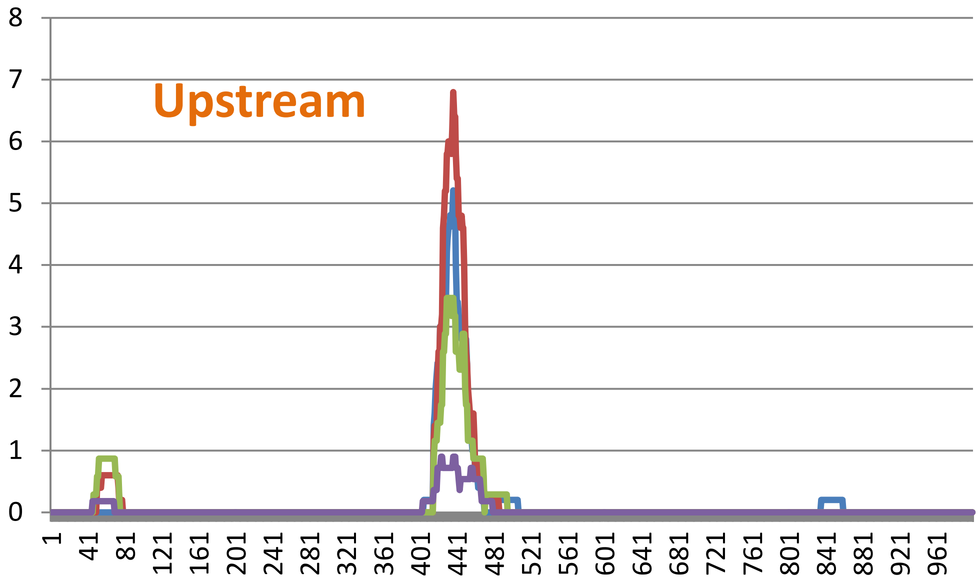

AT2G22440

Ribonuclease H-like superfamily protein

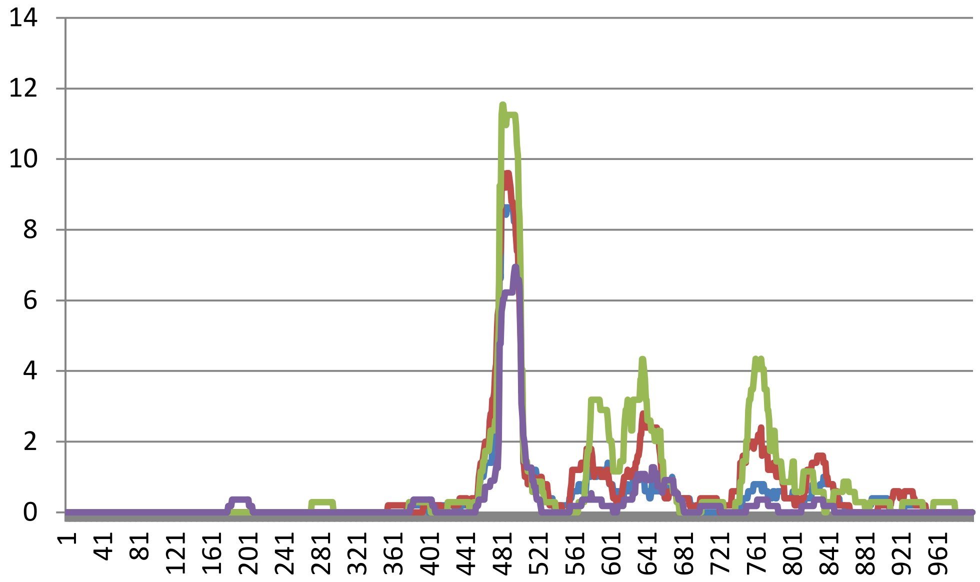

AT2G23950

Leucine-rich repeat protein kinase family protein

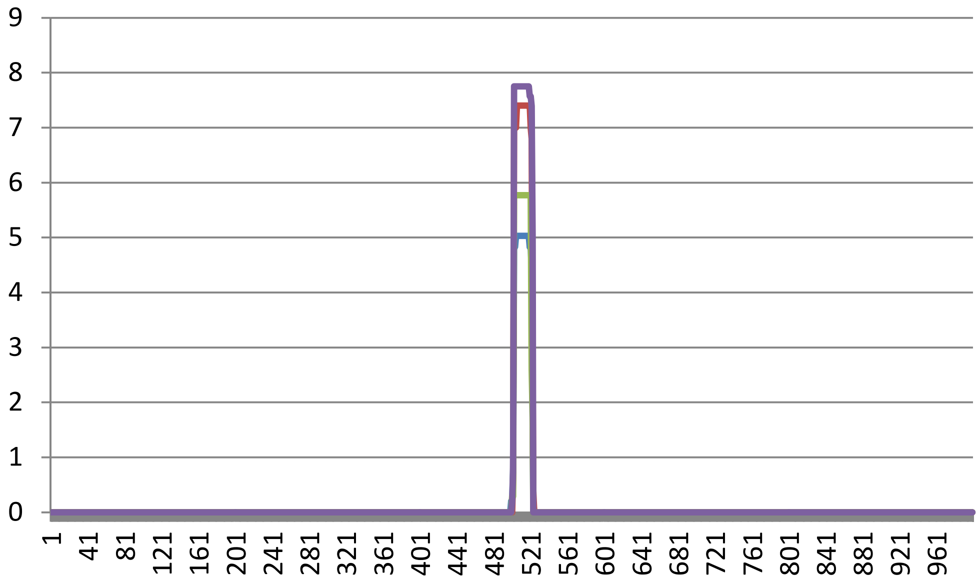

AT2G24010

serine carboxypeptidase-like 23 (scpl23)

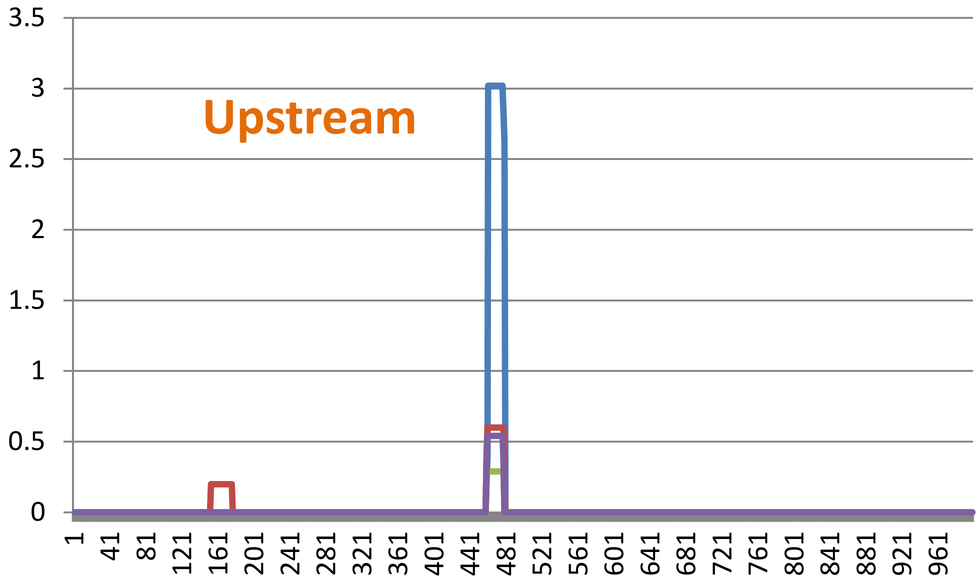

AT2G24670

Domain of unknown function (DUF313)

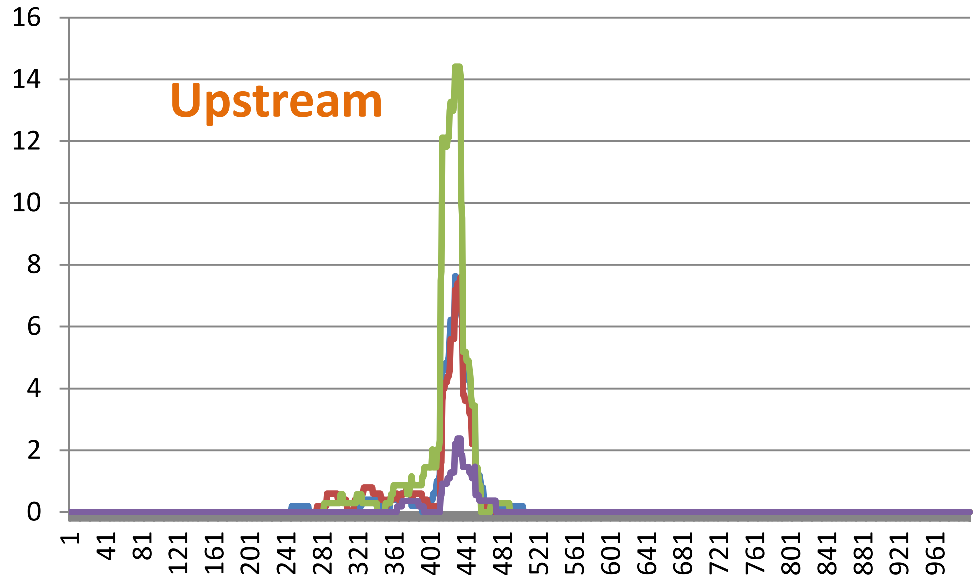

AT2G26740

Encodes a soluble epoxide hydrolase whose expression is induced by auxin and water stress.

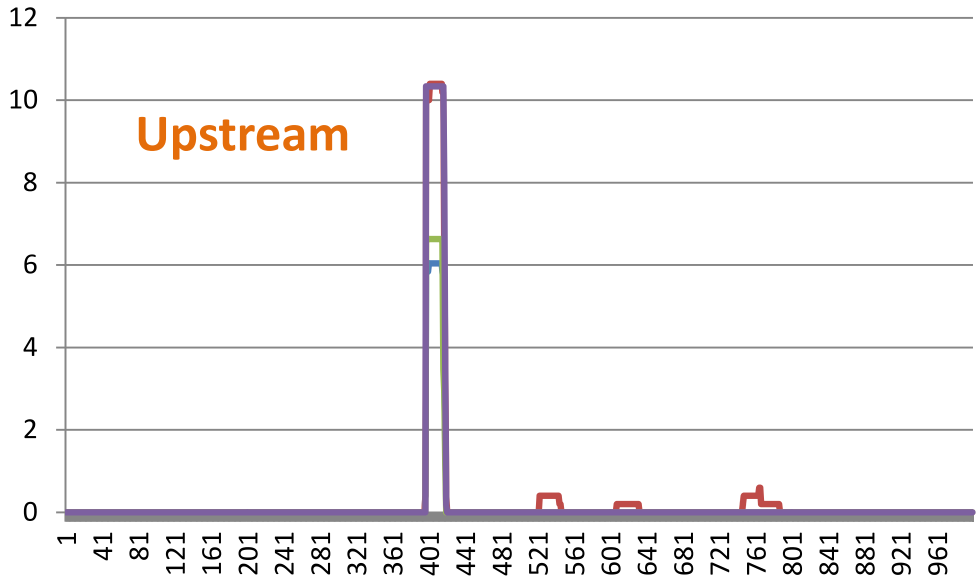

AT2G27130

Bifunctional inhibitor/lipid-transfer protein/seed storage 2S albumin superfamily protein

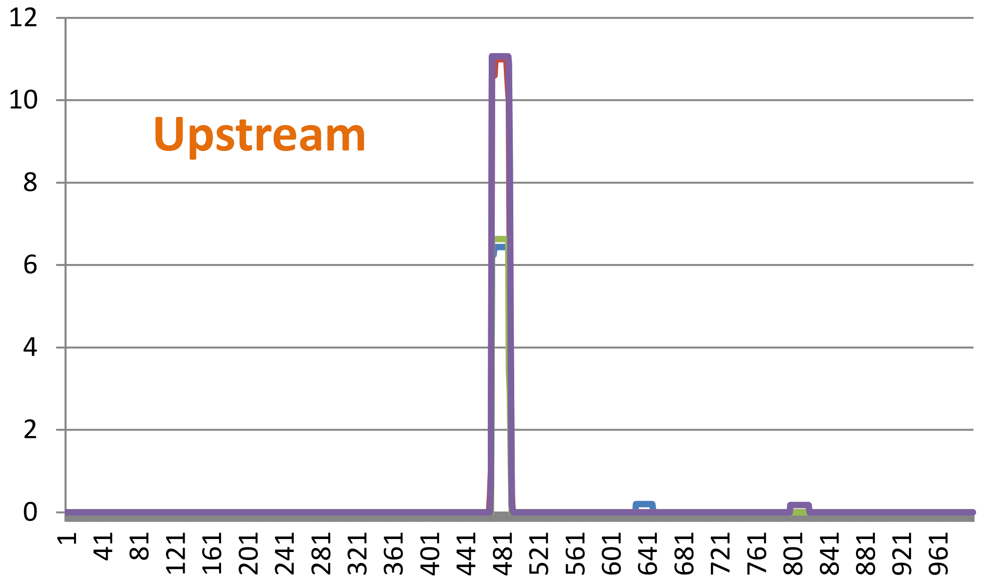

AT2G28310

Protein of unknown function (DUF707)

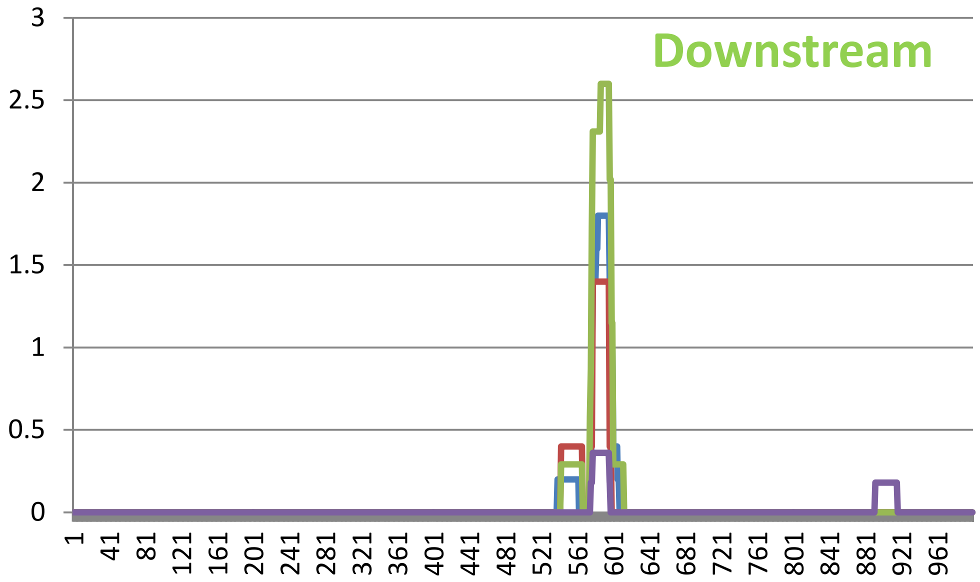

AT2G30910

Actin-related protein C1A (ARPC1A)

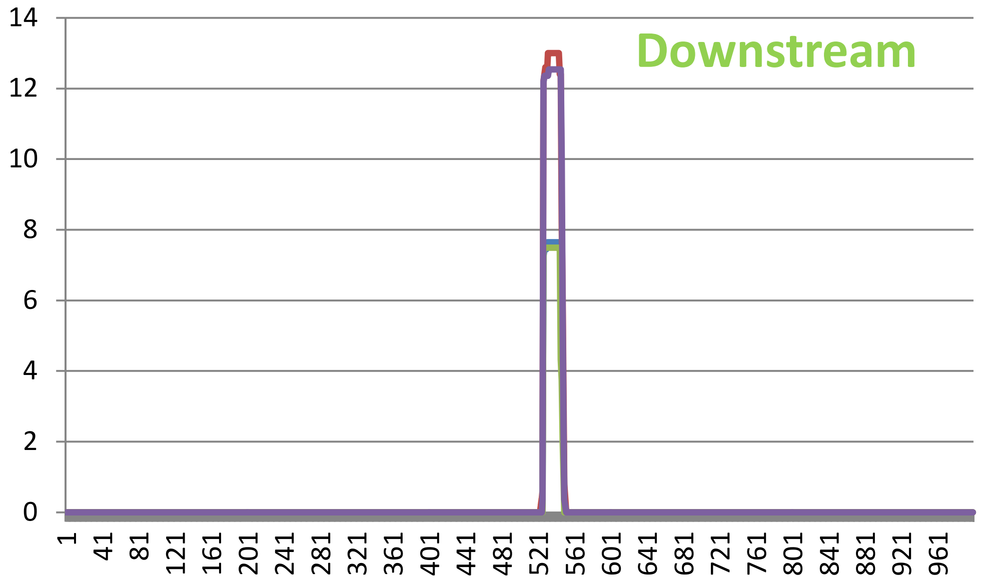

AT2G30940

Protein kinase superfamily protein

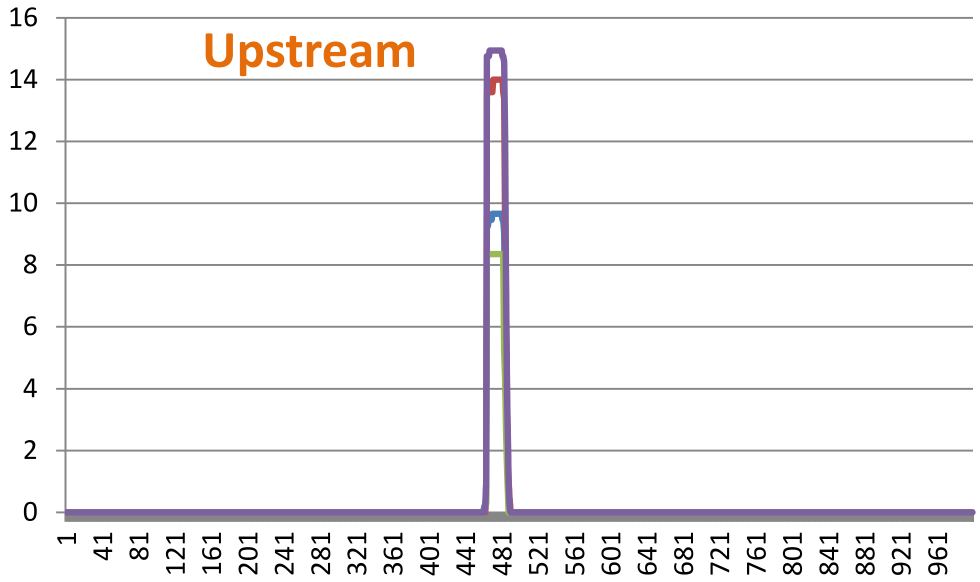

AT2G31230

Encodes a member of the ERF (ethylene response factor) subfamily B-3 of ERF/AP2 transcription factor family. The protein contains one AP2 domain.

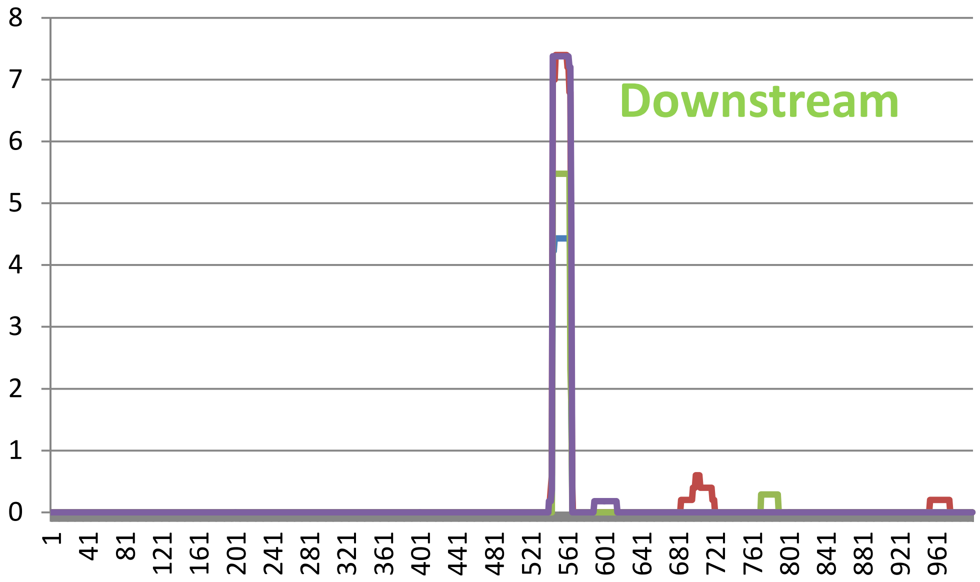

AT2G32130

Plant protein of unknown function (DUF641)

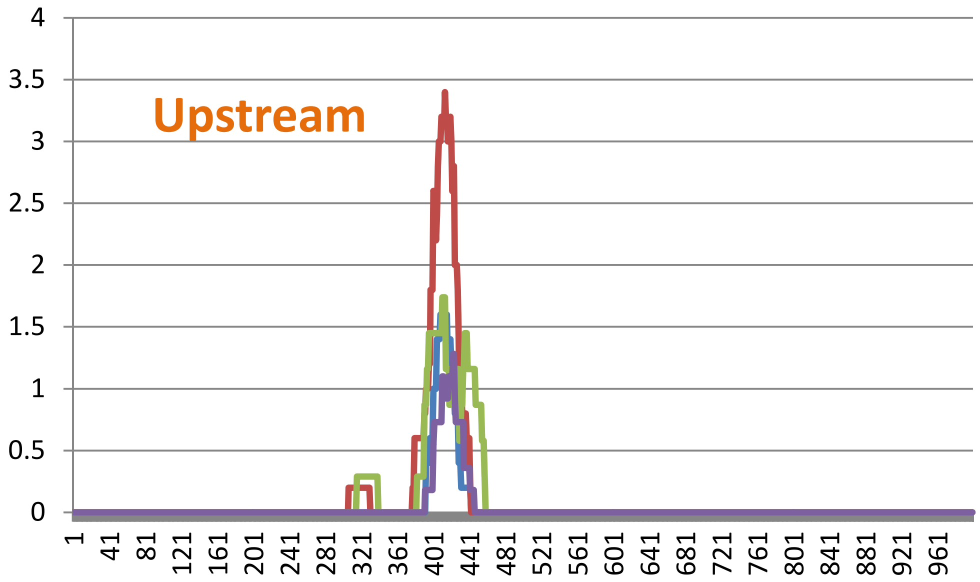

AT2G32710

Kip-related protein (KRP) gene, encodes CDK (cyclin-dependent kinase) inhibitor (CKI). A member of seven KRP genes found in Arabidopsis thaliana. Negative regulator of cell division. Expressed in actively dividing cells.

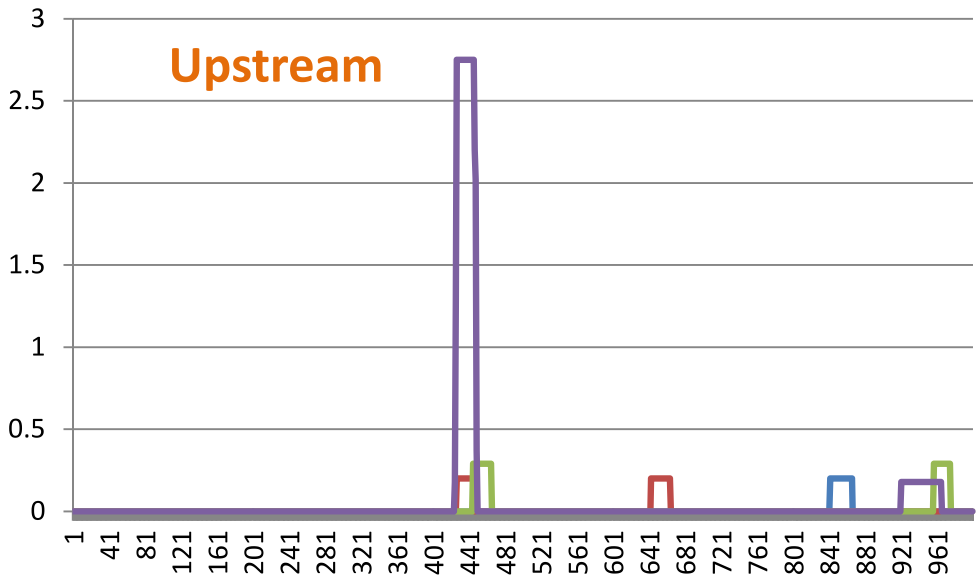

AT2G34655

Unknown protein

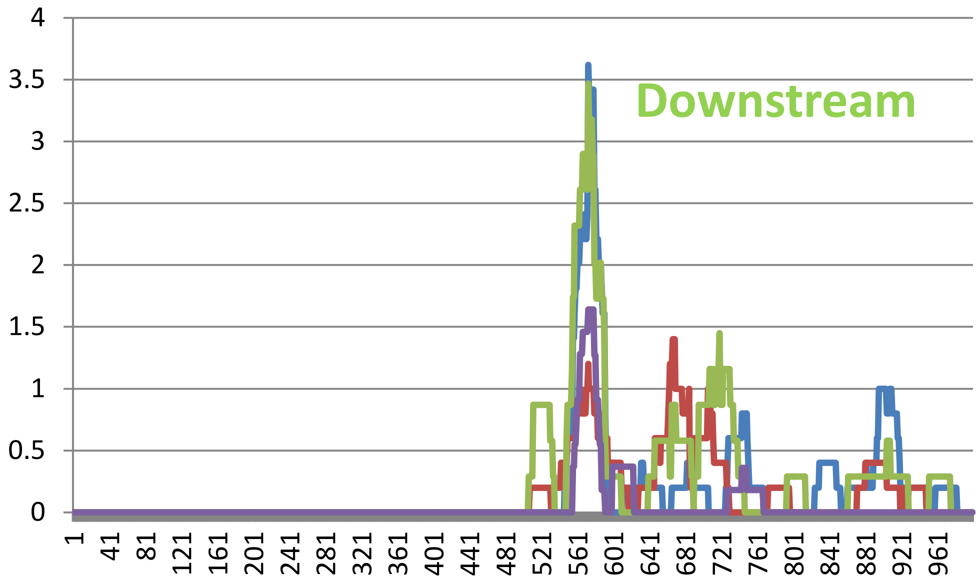

AT2G34840

Coatomer epsilon subunit

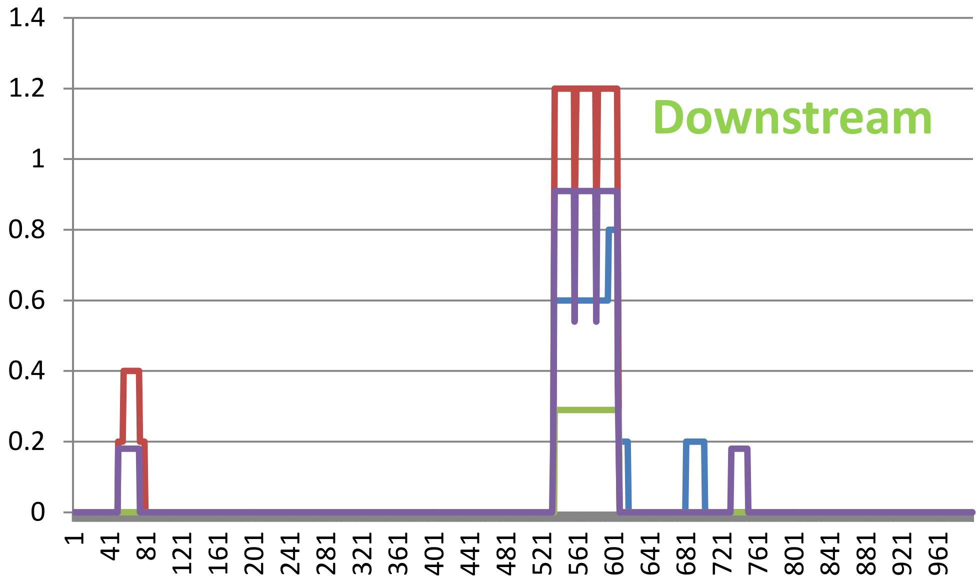

AT2G35650

A member of Glycosyltransferase- Family 2 and encodes a beta-mannan synthase based on in vitro enzyme assays from heterologously expressed protein. Mutants exhibit defects in pollen tube growth and embryo development. The defective embryonic development was associated with reduced proliferation and failed cellularization of the endosperm.

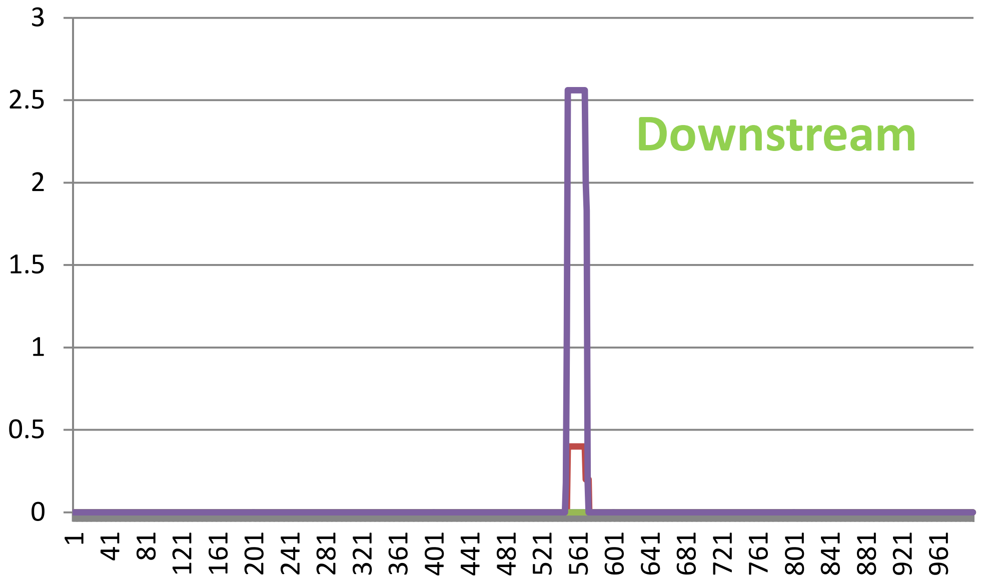

AT2G35800

Encodes a predicted calcium-dependent S-adenosyl methionine carrier.

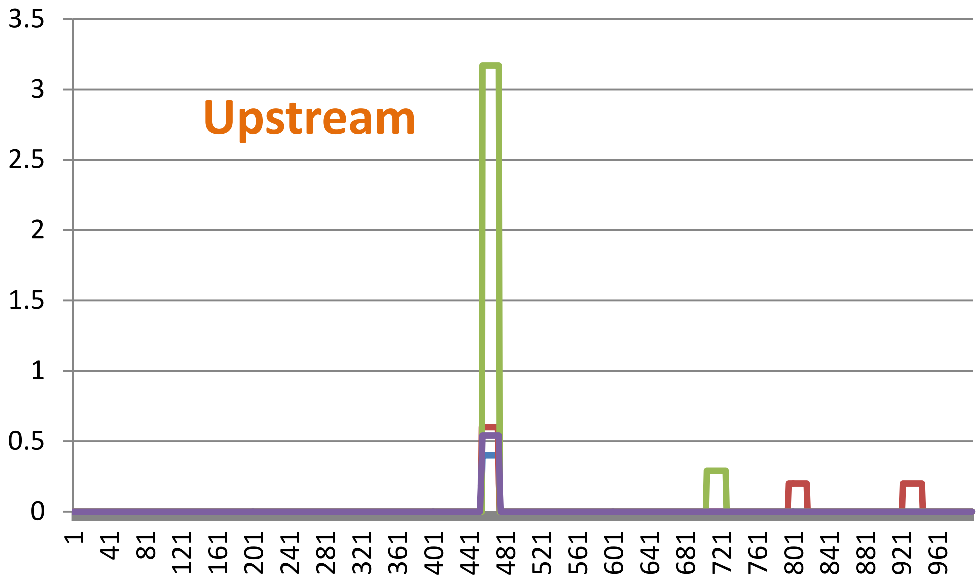

AT2G36180

EF hand calcium-binding protein family

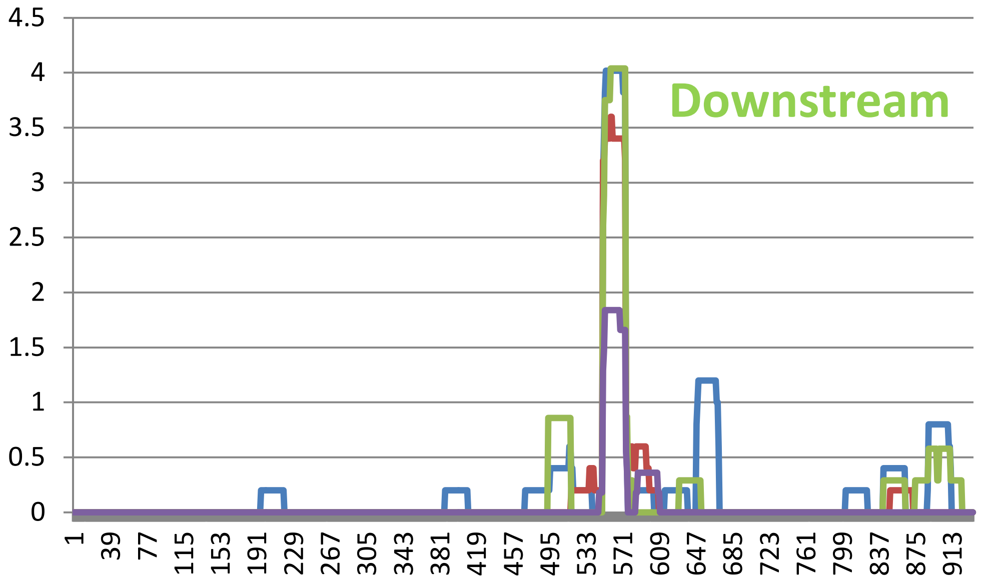

AT2G36460

Aldolase superfamily protein

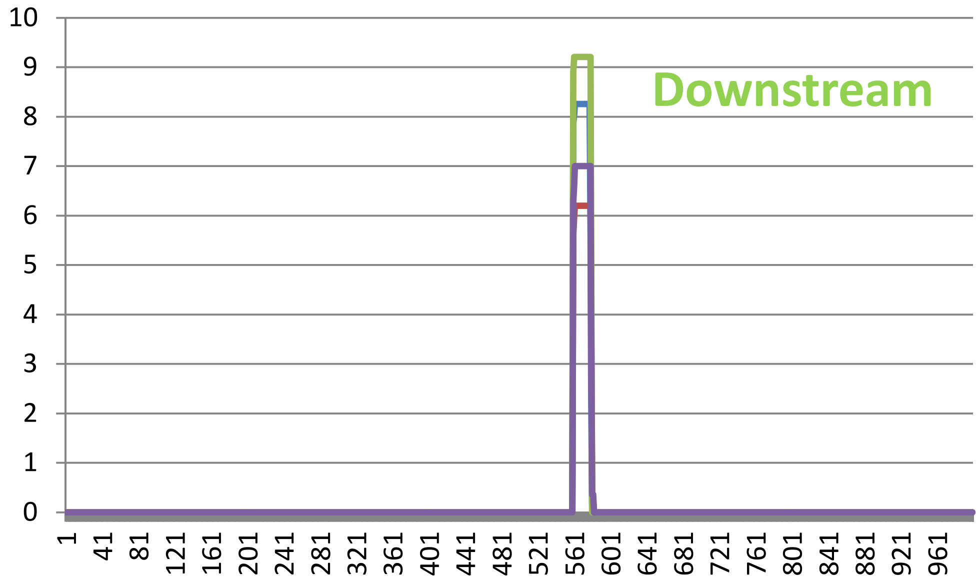

AT2G38380

Peroxidase superfamily protein

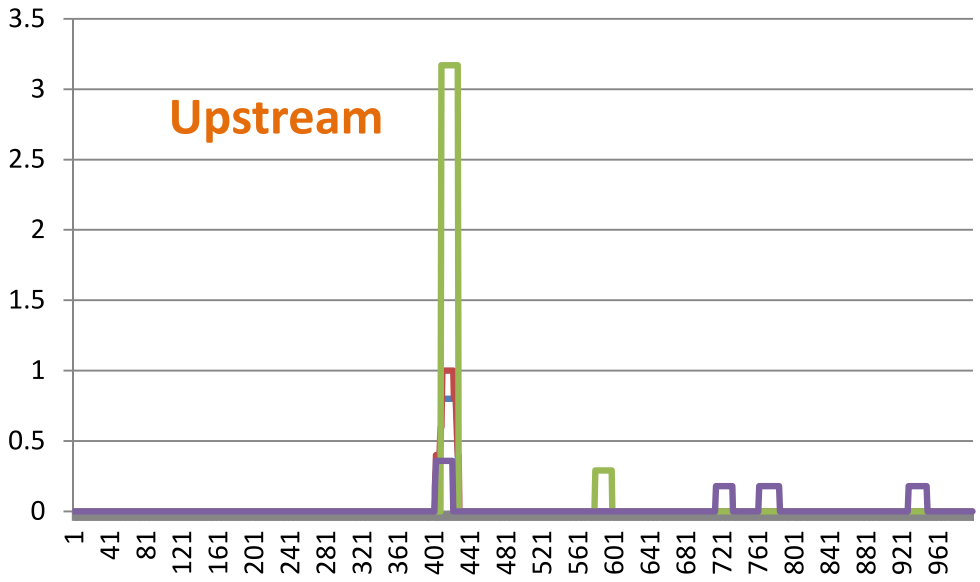

AT2G38630

Transducin/WD40 repeat-like superfamily protein

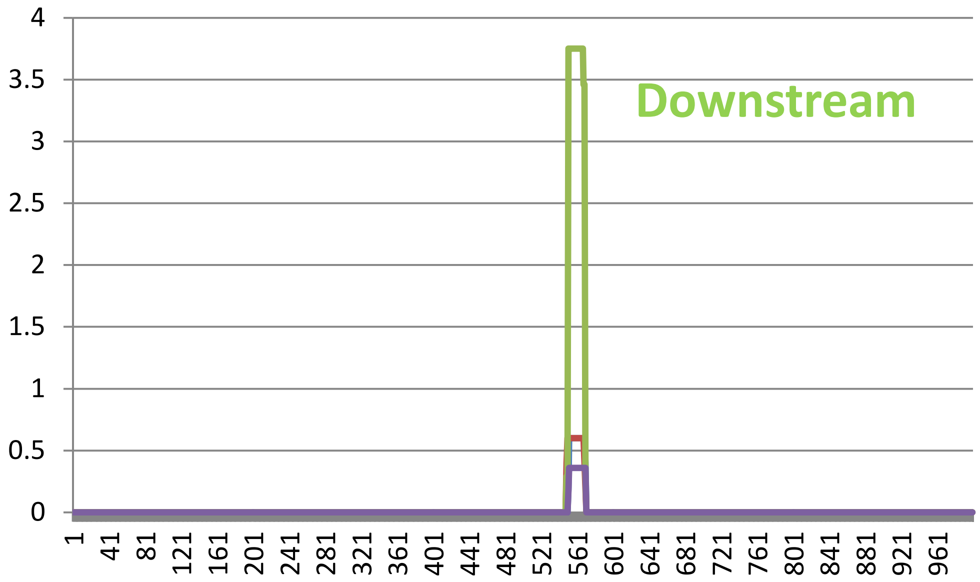

AT2G39450

Encodes a Golgi-localized manganese transporter that is involved in Mn tolerance.

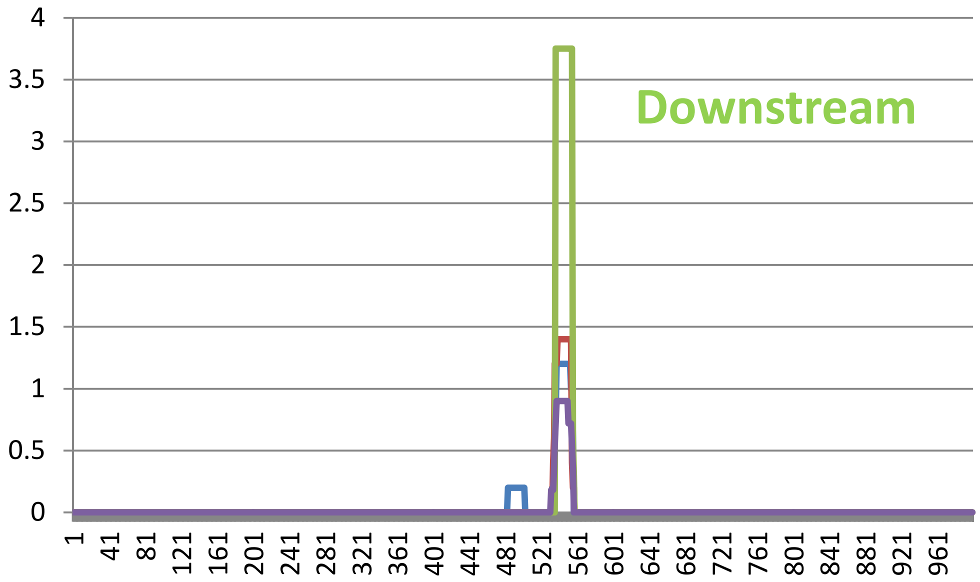

AT2G40004

Unknown protein

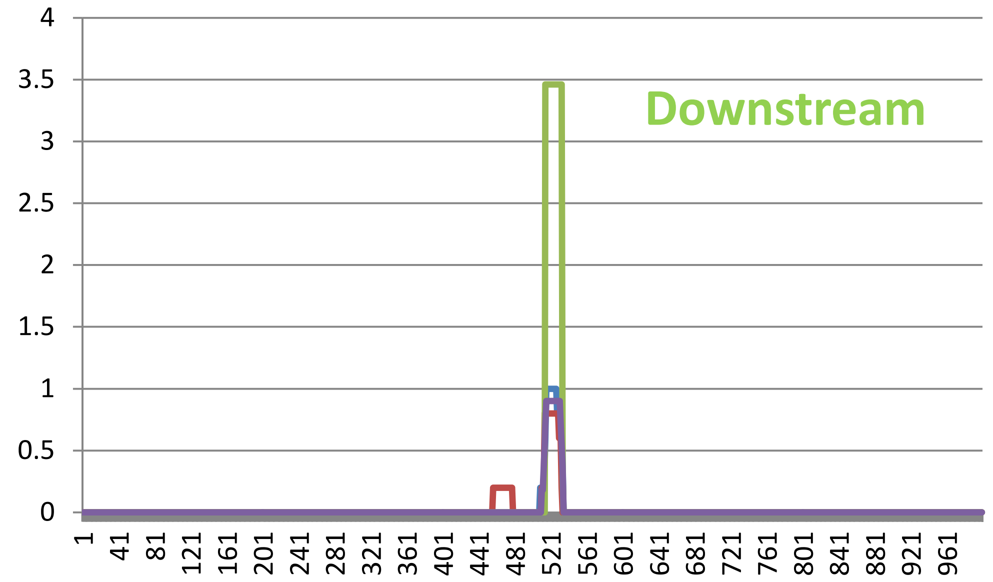

AT2G40510

Ribosomal protein S26e family protein

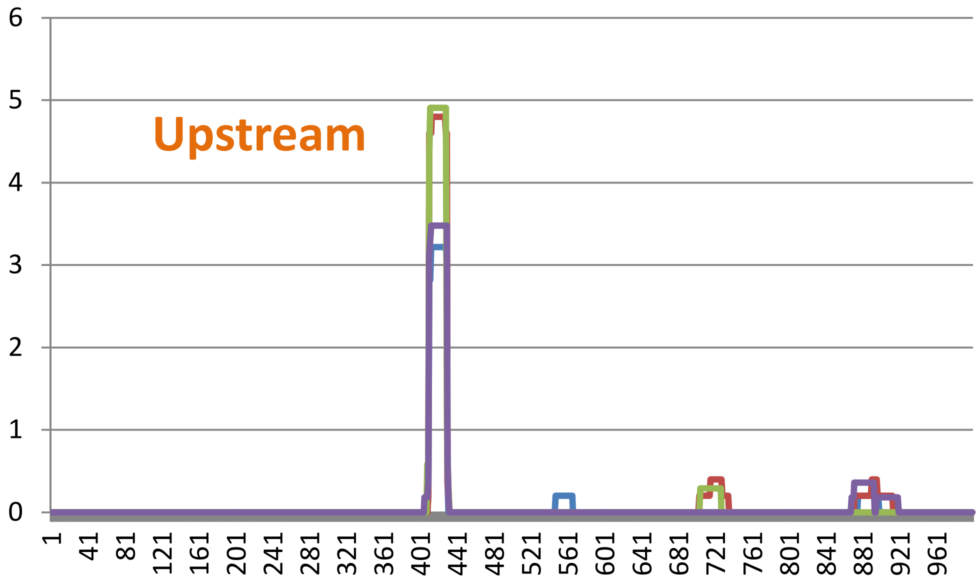

AT2G42500

Encodes one of the isoforms of the catalytic subunit of protein phosphatase 2A

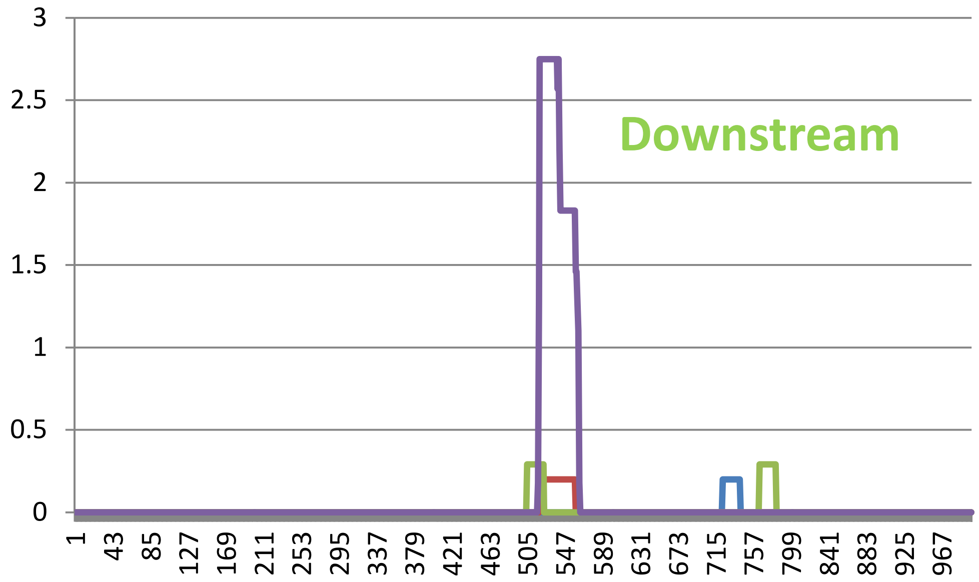

AT2G43790

Encodes a MAP kinase induced by pathogens, ethylene biosynthesis, oxidative stress and osmotic stress. Also involved in ovule development.

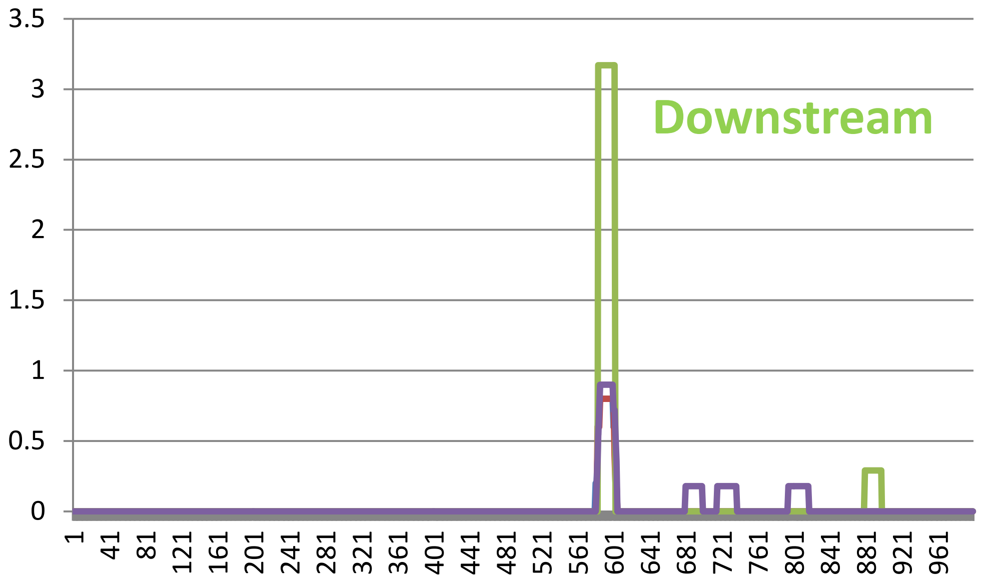

AT2G43865

Unknown protein

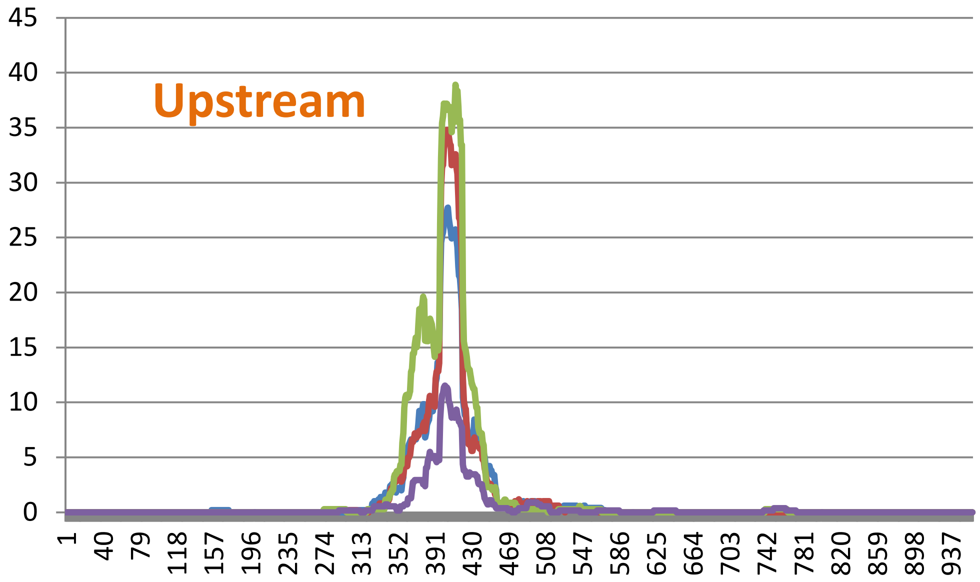

AT2G47050

Plant invertase/pectin methylesterase inhibitor superfamily protein

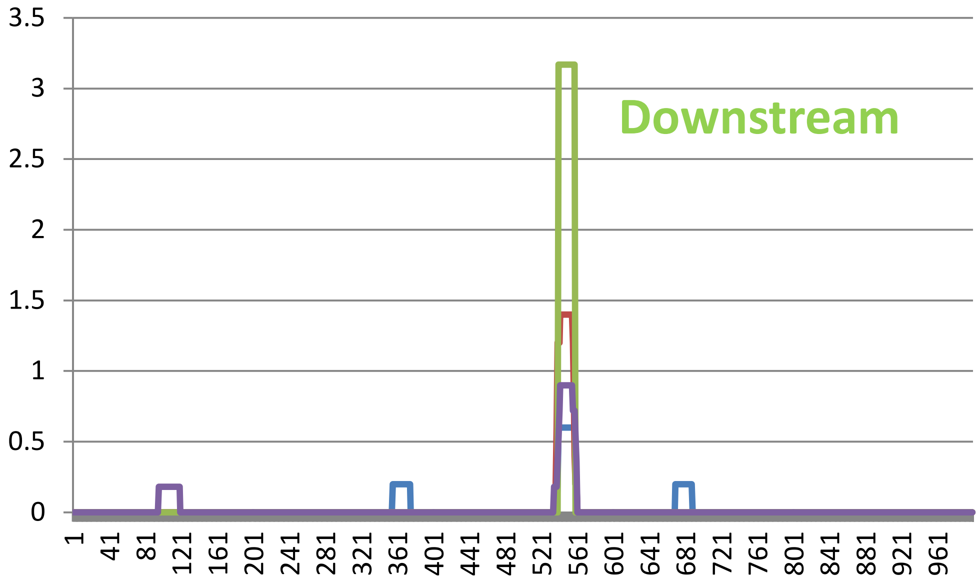

AT2G47570

Ribosomal protein L18e/L15 superfamily protein

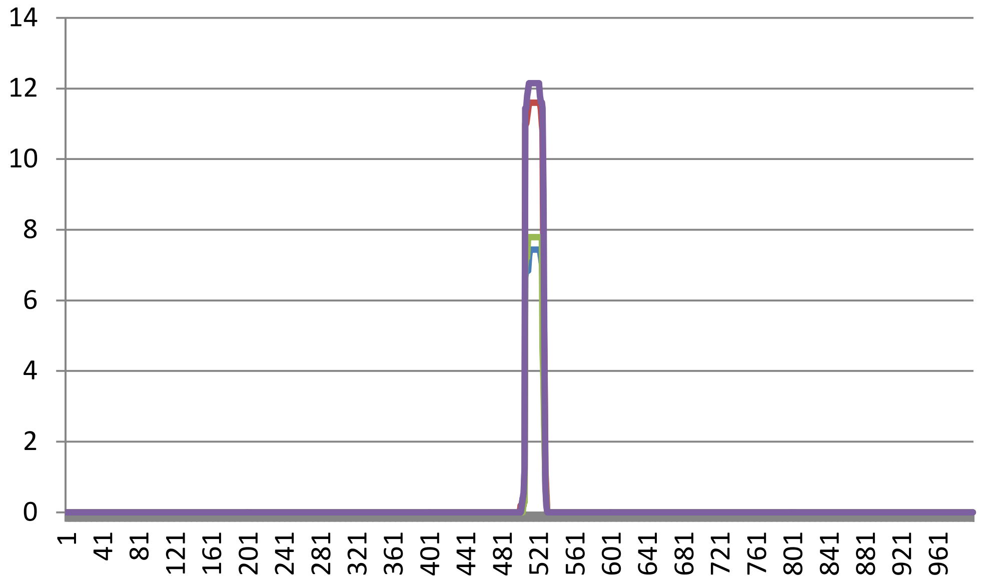

AT3G02890

RING/FYVE/PHD zinc finger superfamily protein

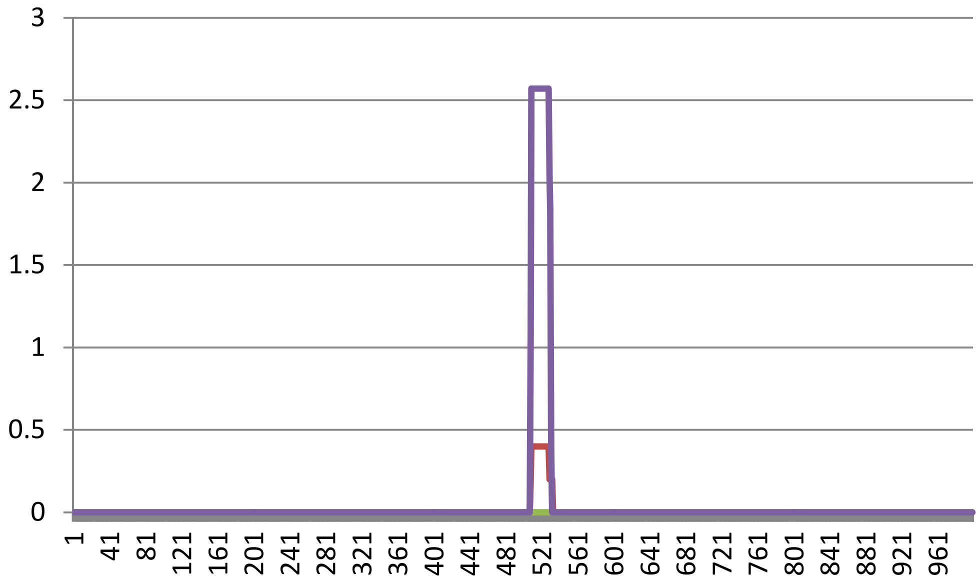

AT3G03340

Unfertilized embryo sac 6 (UNE6)

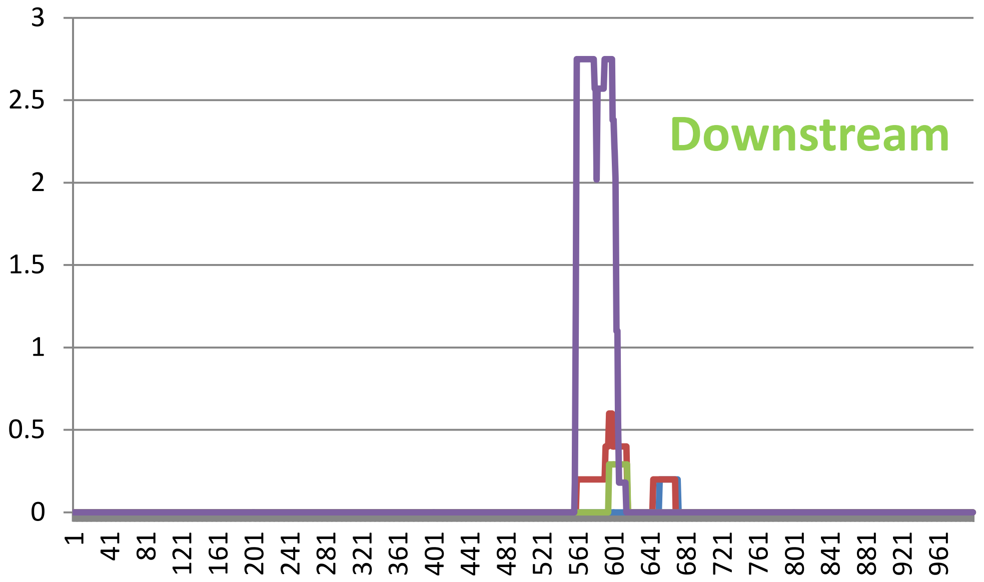

AT3G04290

Li-tolerant lipase 1 (LTL1)

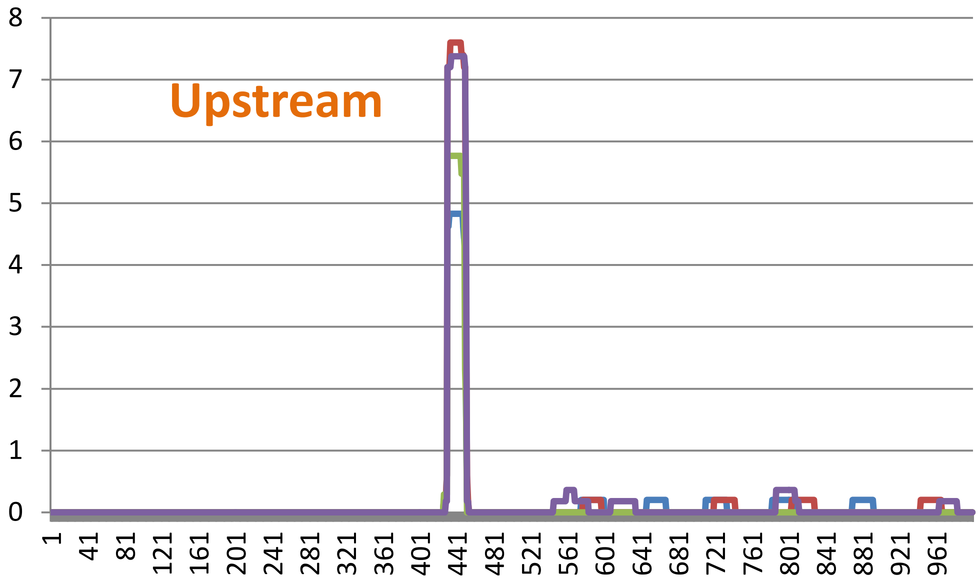

AT3G04630

Member of a small gene family which have a KLEEK domain which may be involved in protein- protein interactions. Over expression of WDL1 results in abnormal root development.

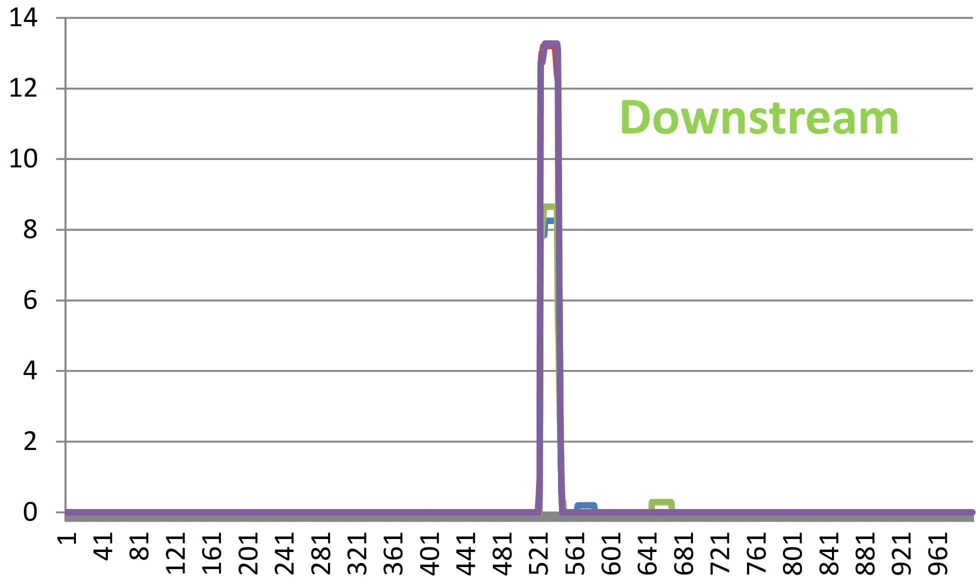

AT3G05770

Unknown protein

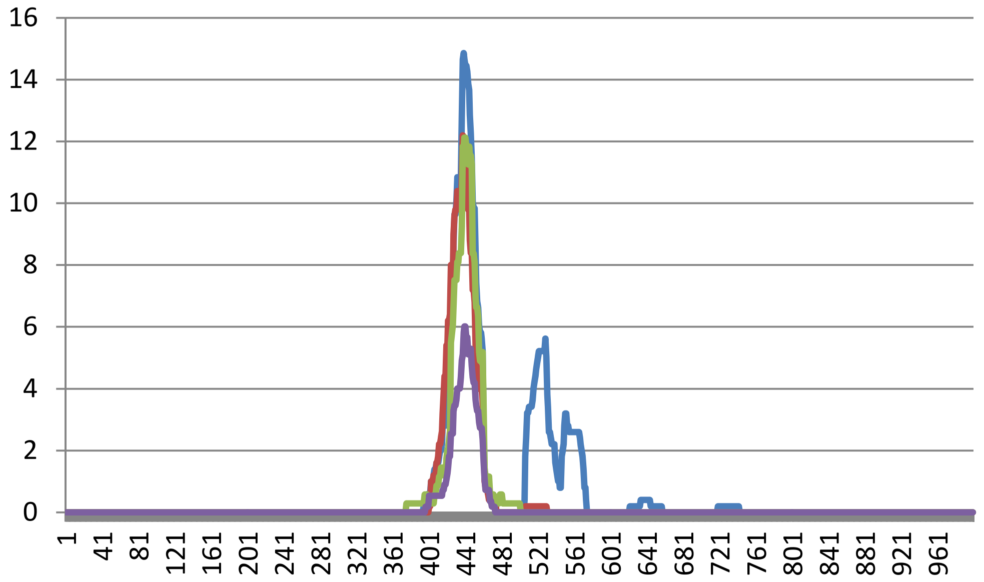

AT3G06490

Putative transcription factor MYB108 (MYB108) mRNA

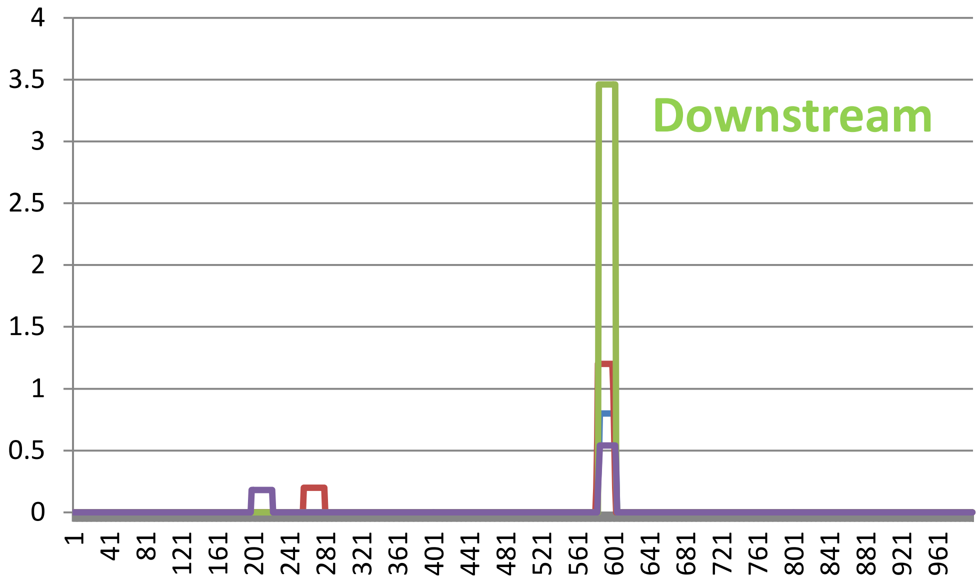

AT3G09980

Family of unknown function (DUF662)

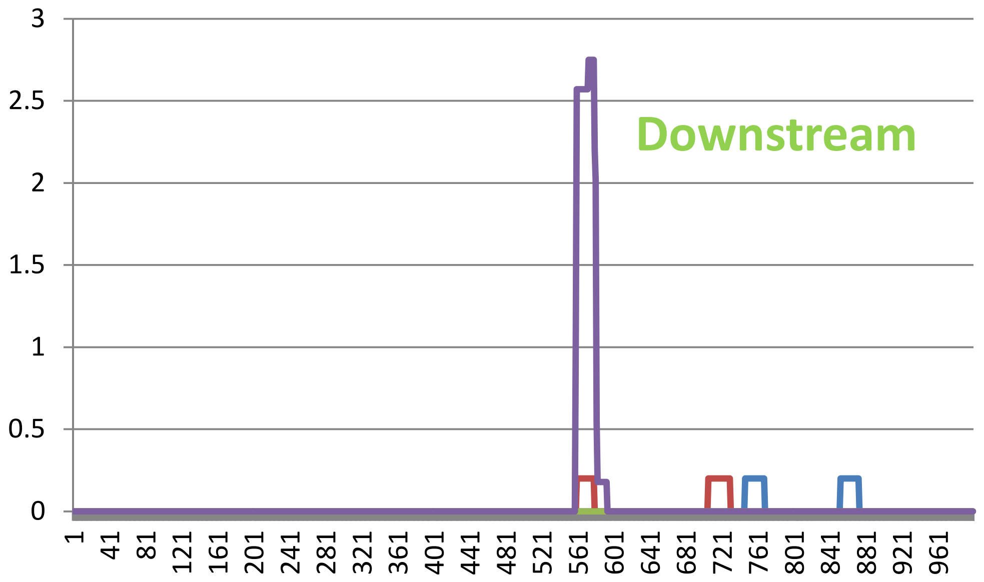

AT3G10760

Homeodomain-like superfamily protein

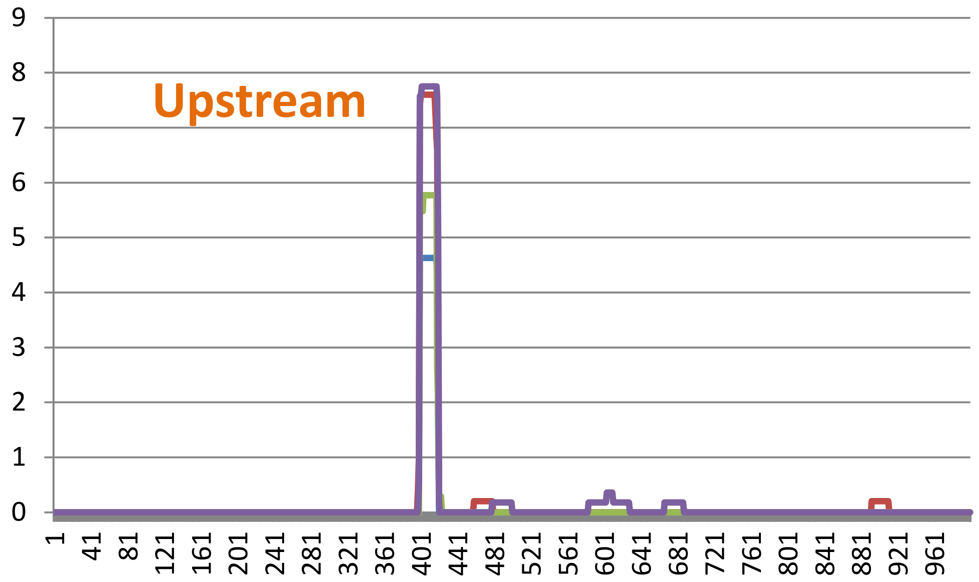

AT3G12955

SAUR-like auxin-responsive protein family

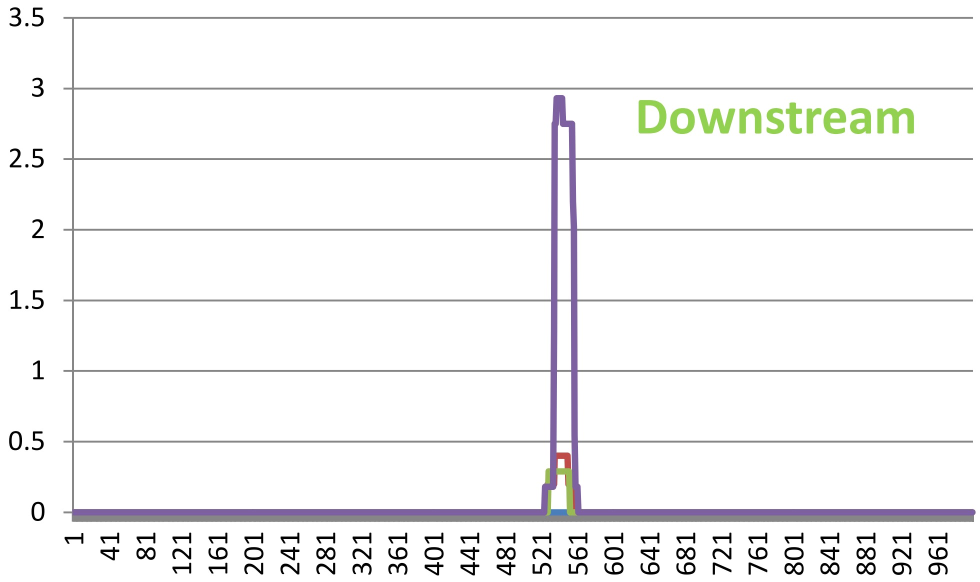

AT3G13620

Encodes POLYAMINE UPTAKE TRANSPORTER 4, an amino acid permease family protein.

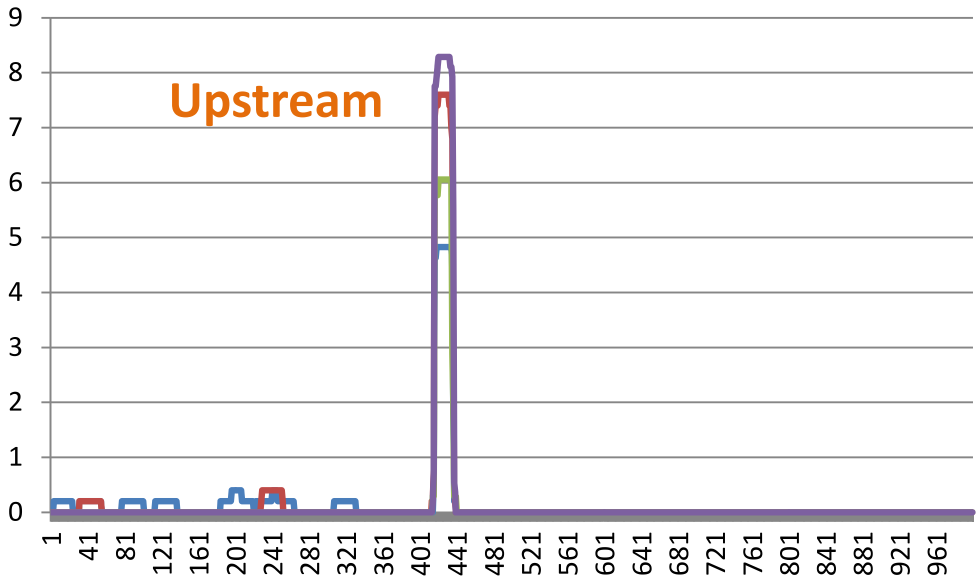

AT3G13857

Unknown protein

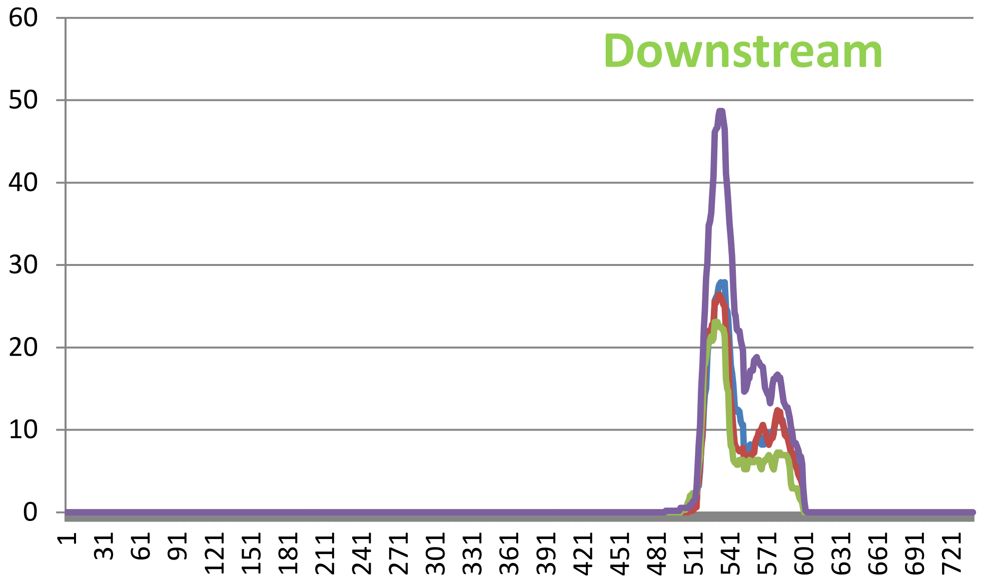

AT3G13960

Growth regulating factor encoding transcription activator. One of the nine members of a GRF gene family, containing nuclear targeting domain. Involved in leaf development and expressed in root, shoot and flower.

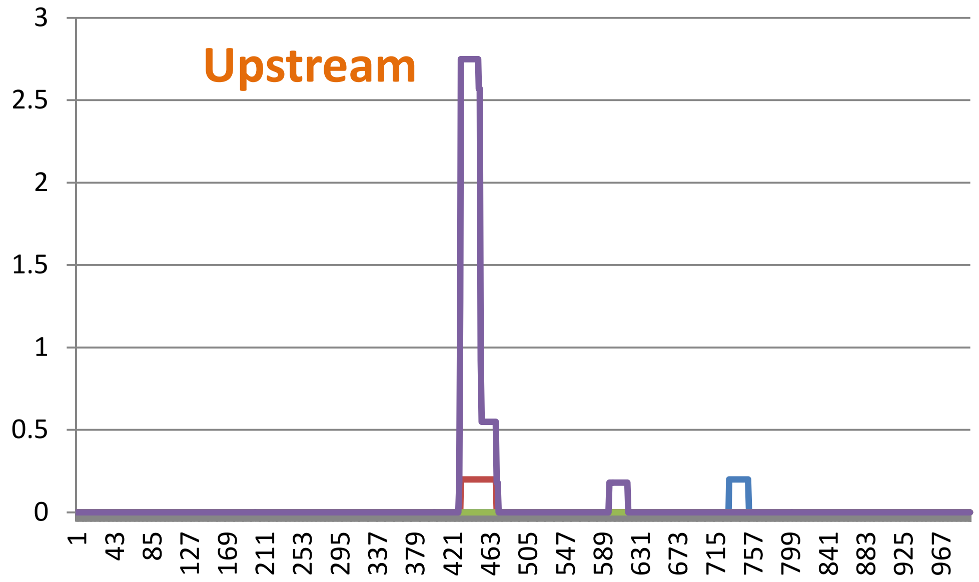

AT3G16370

GDSL-like Lipase/Acylhydrolase superfamily protein

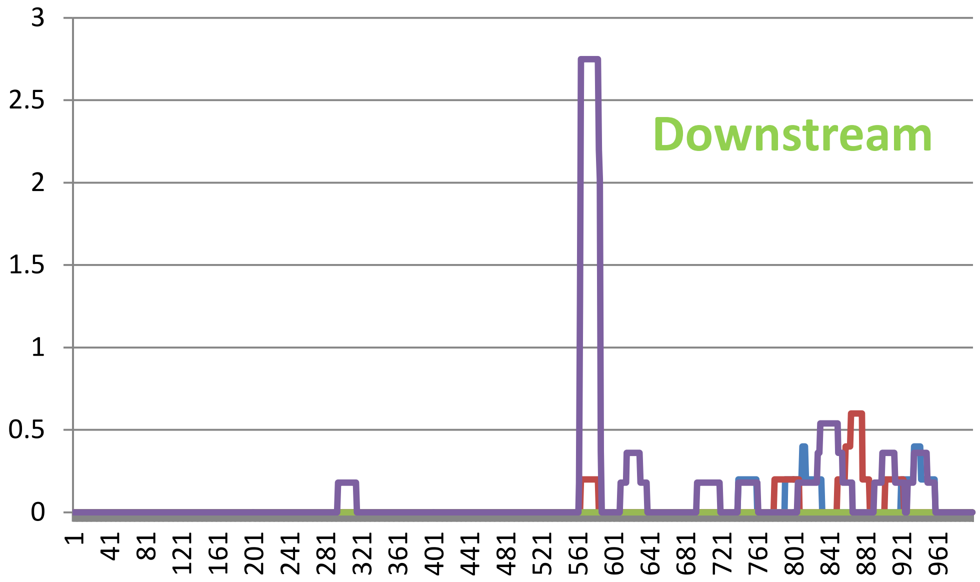

AT3G17190

Unknown protein

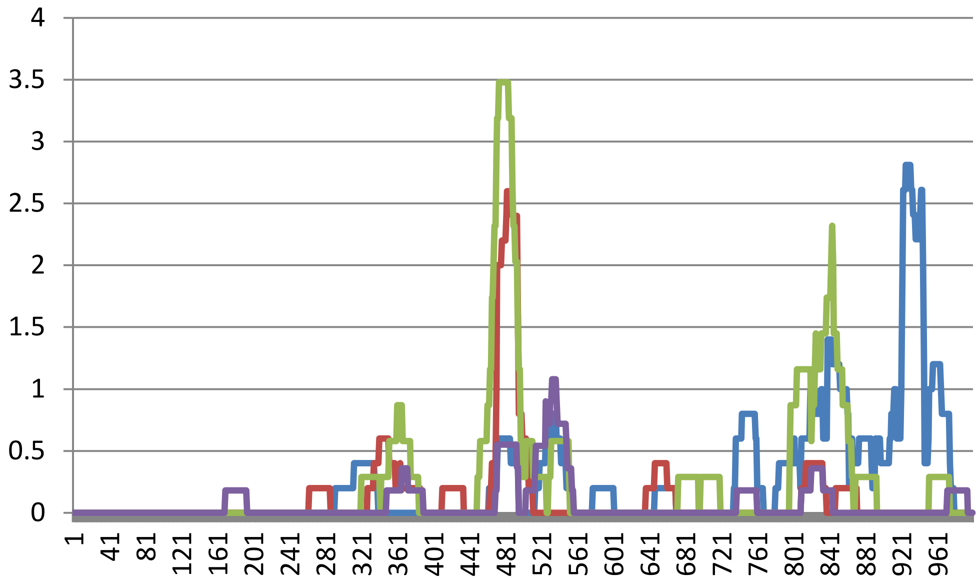

AT3G17490

F-box and associated interaction domains-containing protein

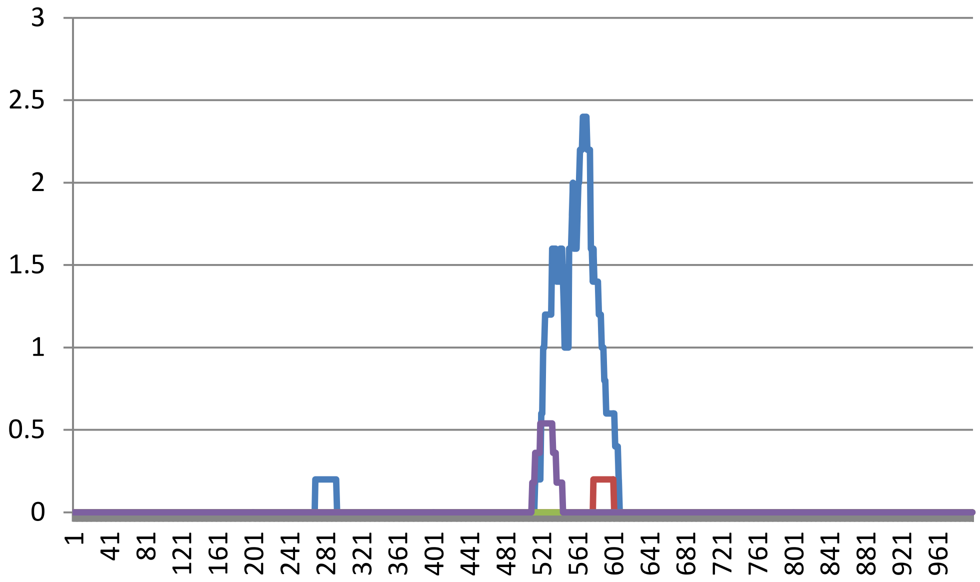

AT3G17500

F-box family protein

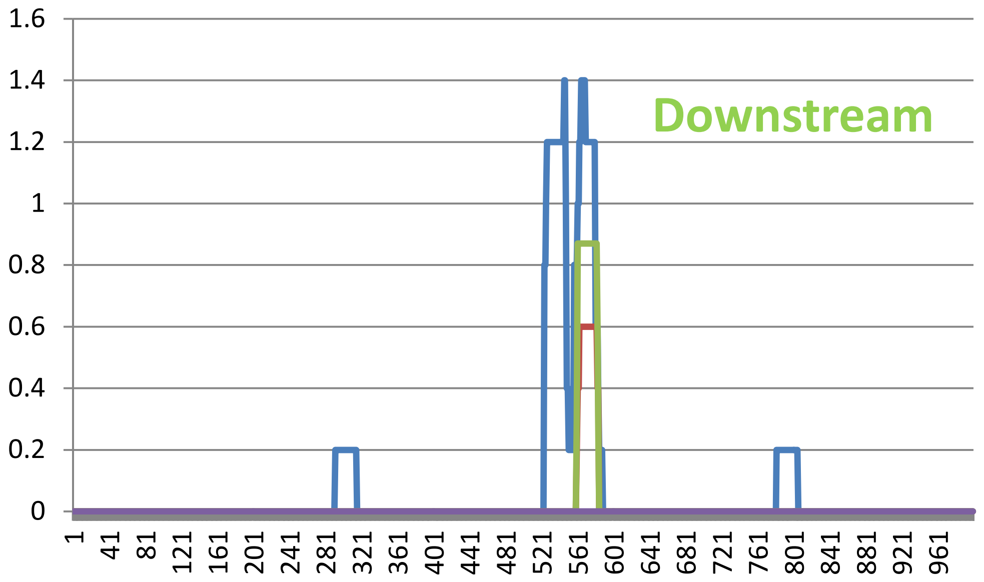

AT3G19710

Belongs to the branched-chain amino acid aminotransferase gene family. Encodes a methionine-oxo-acid transaminase. Involved in the methionine chain elongation pathway that leads to the ultimate biosynthesis of methionine-derived glucosinolates.

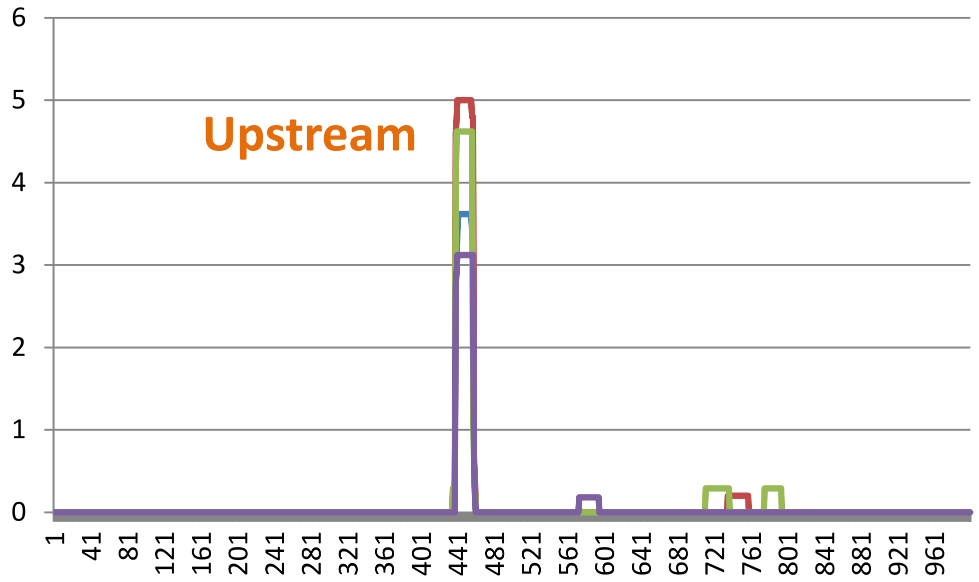

AT3G19880

F-box and associated interaction domains-containing protein

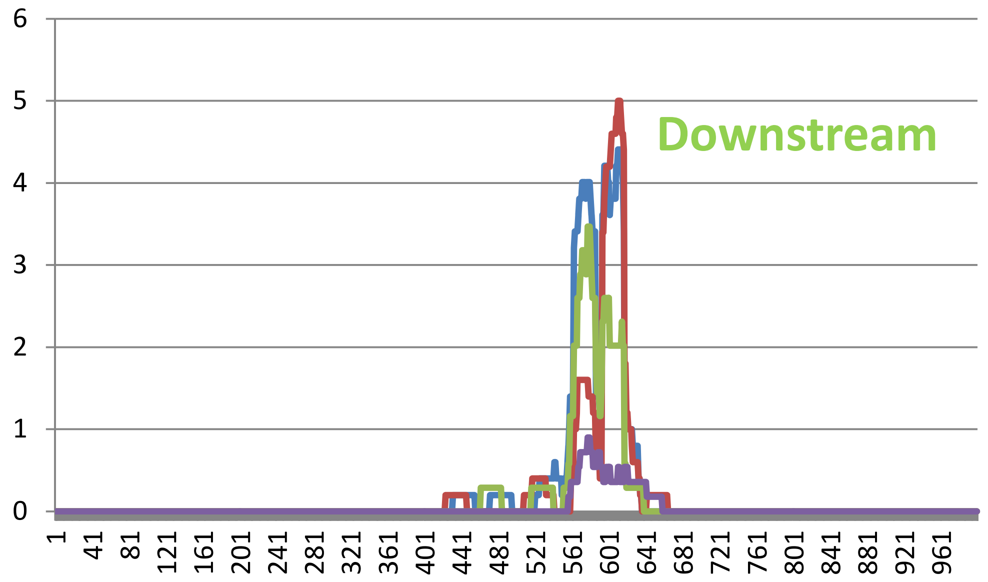

AT3G20760

Nse4, component of Smc5/6 DNA repair complex

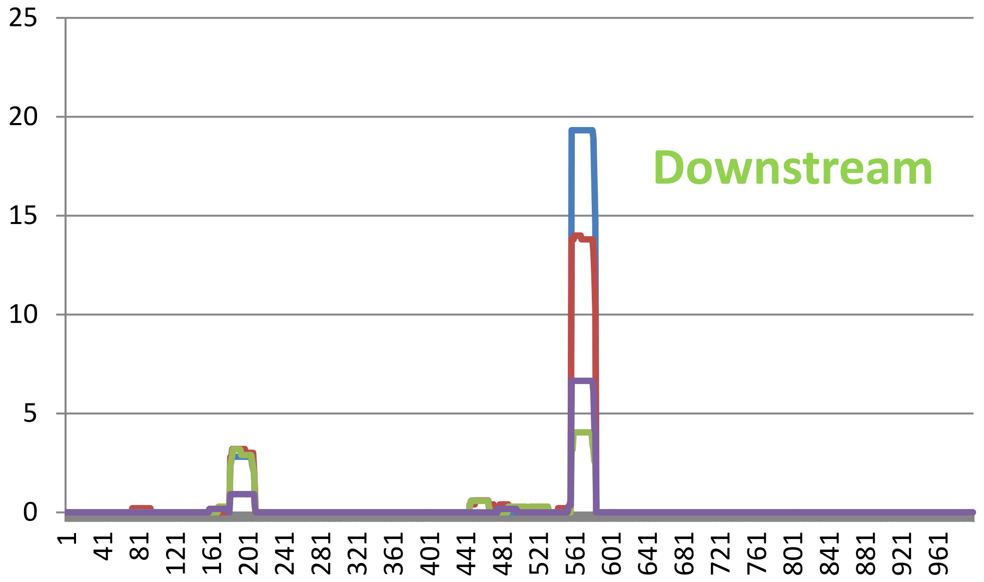

AT3G22350

F-box and associated interaction domains-containing protein

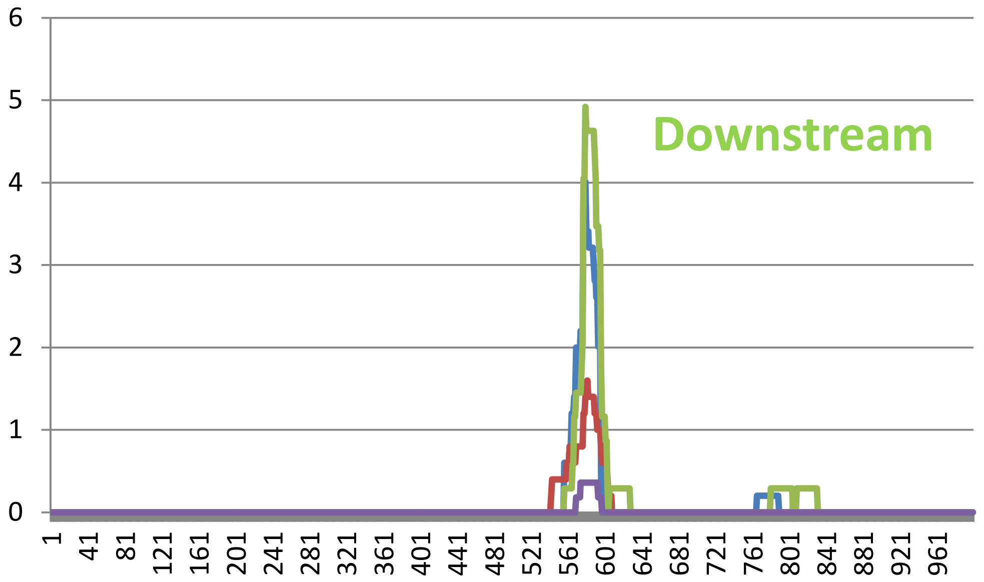

AT3G22730

F-box and associated interaction domains-containing protein

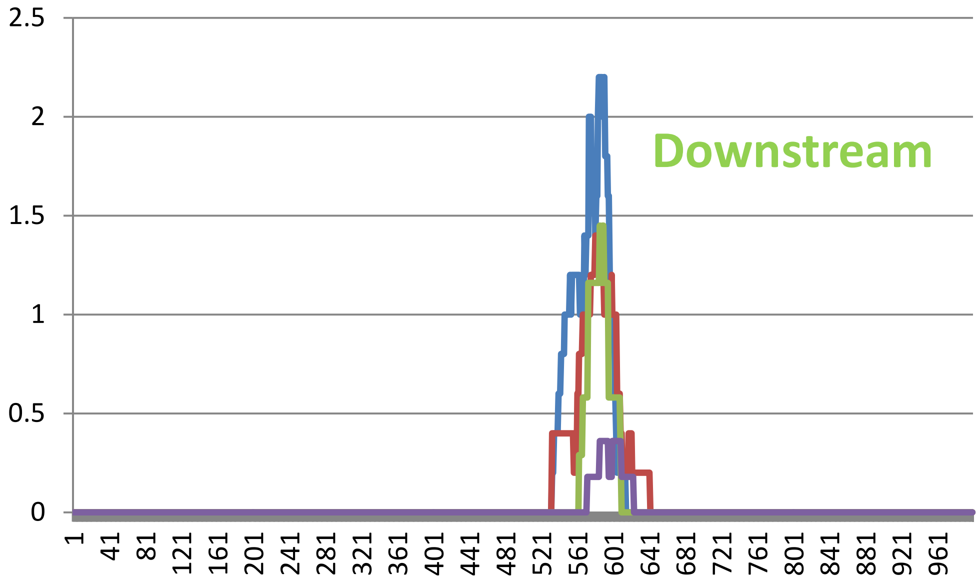

AT3G22770

F-box associated ubiquitination effector family protein

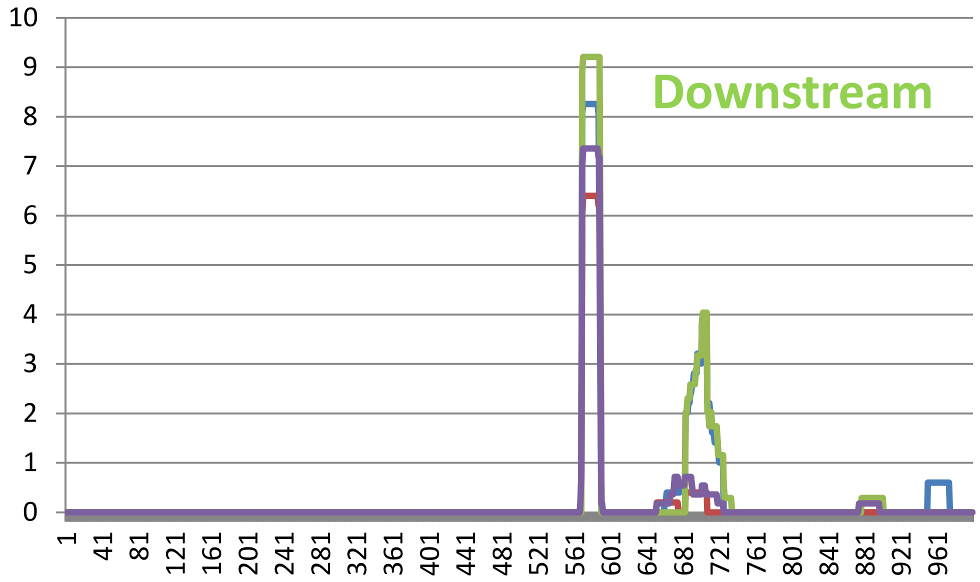

AT3G23040

Unknown protein

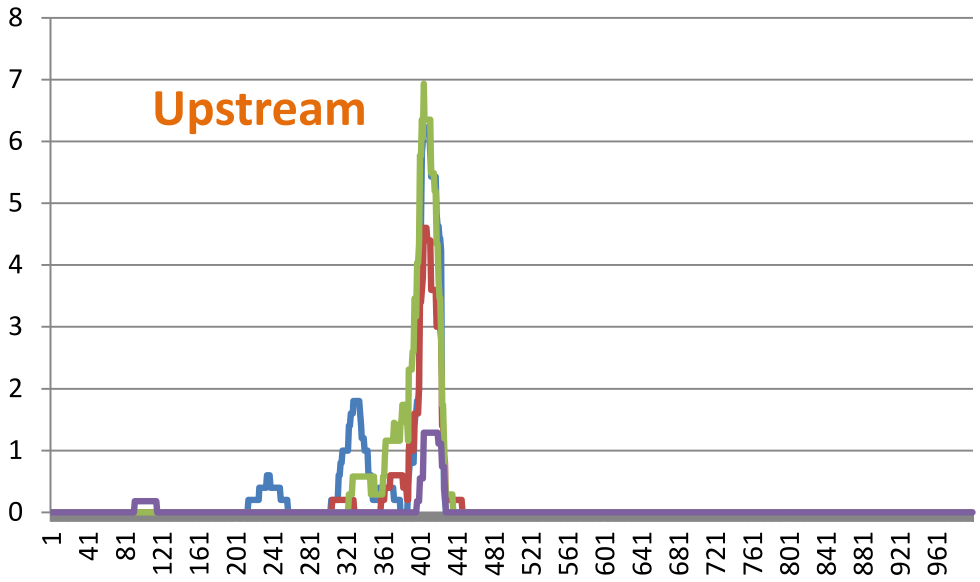

AT3G24542

Beta-galactosidase related protein

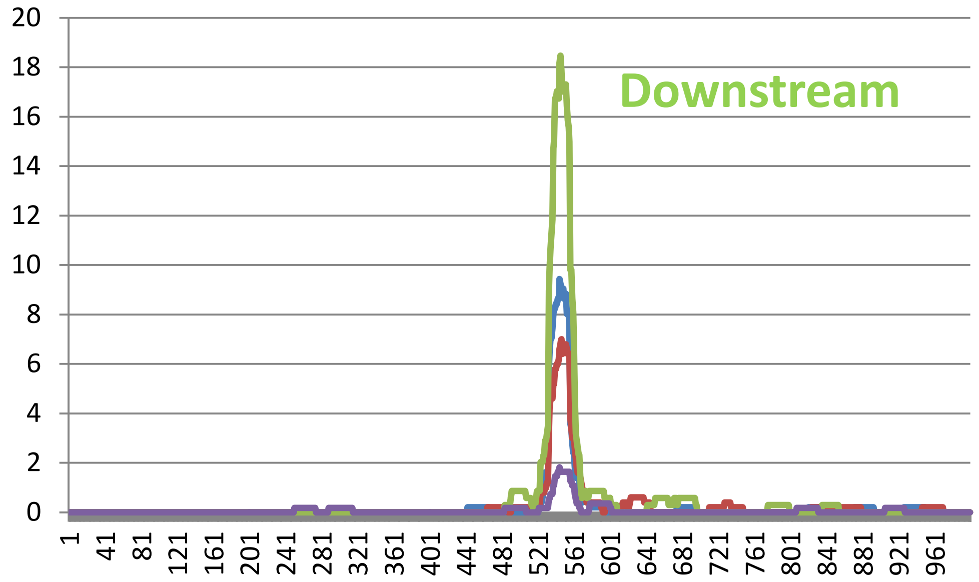

AT3G26616

Unknown protein

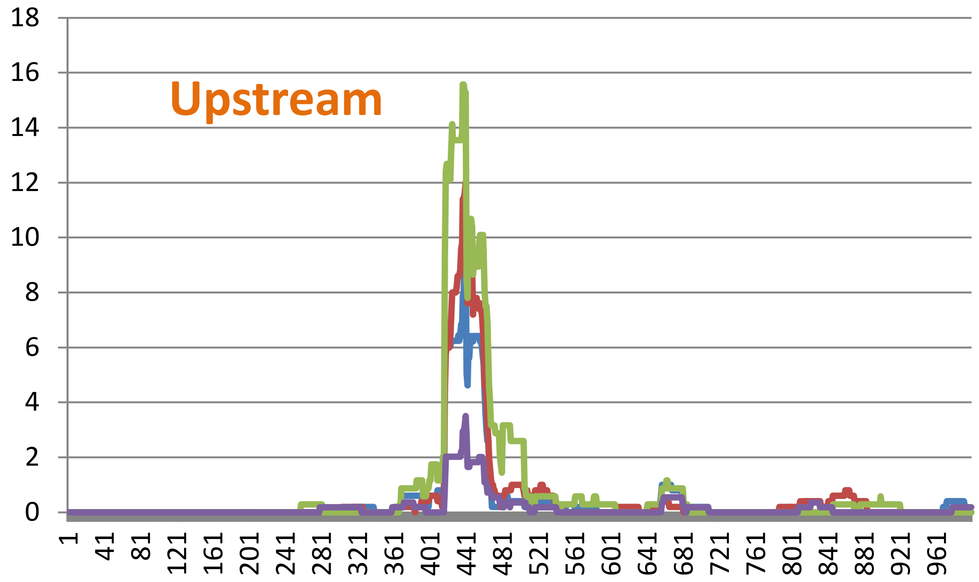

AT3G28840

Protein of unknown function (DUF1216)

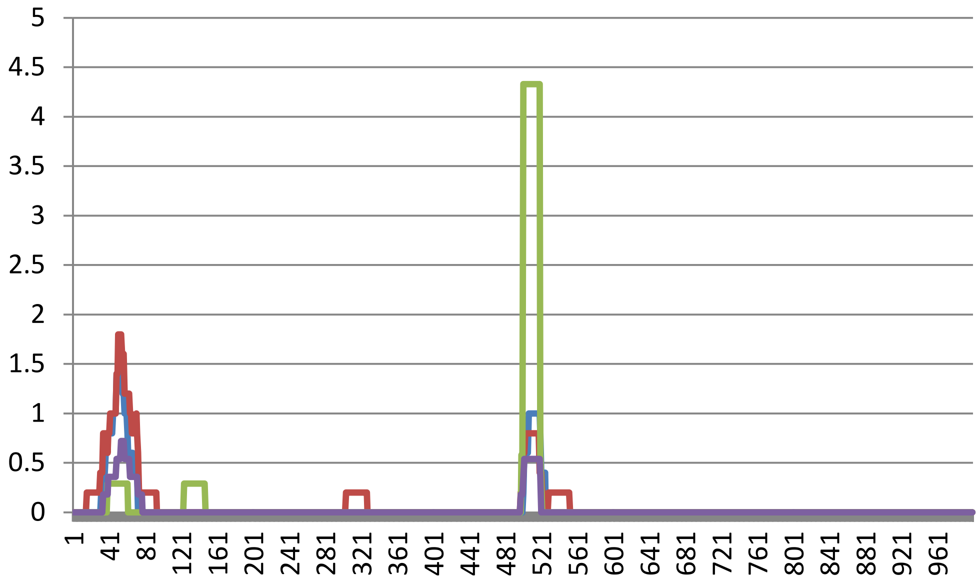

AT3G28980

Protein of unknown function (DUF1216)

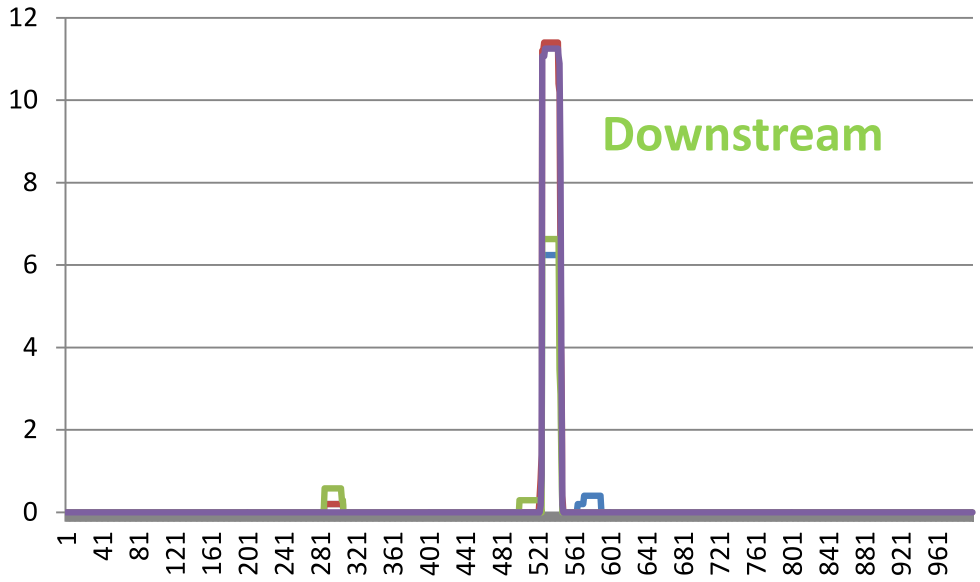

AT3G43270

Plant invertase/pectin methylesterase inhibitor superfamily

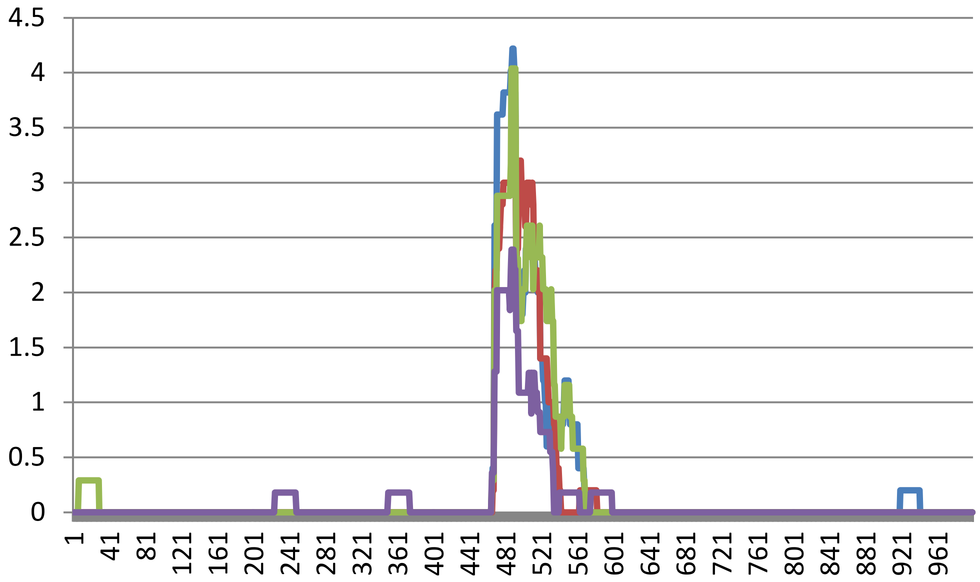

AT3G46640

Encodes a myb family transcription factor with a single Myb DNA-binding domain (type SHAQKYF) that is unique to plants and is essential for circadian rhythms, specifically for transcriptional regulation within the circadian clock.

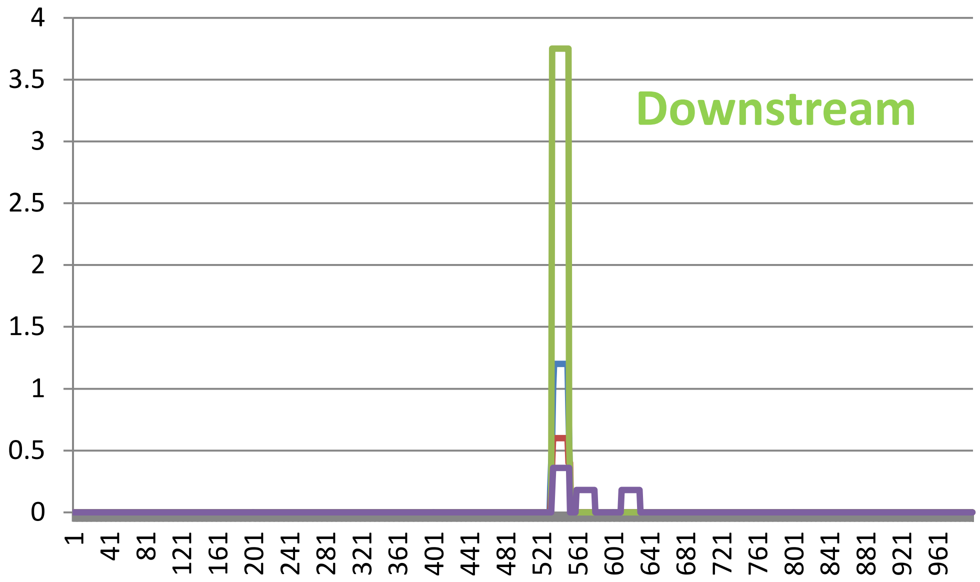

AT3G47030

F-box and associated interaction domains-containing protein

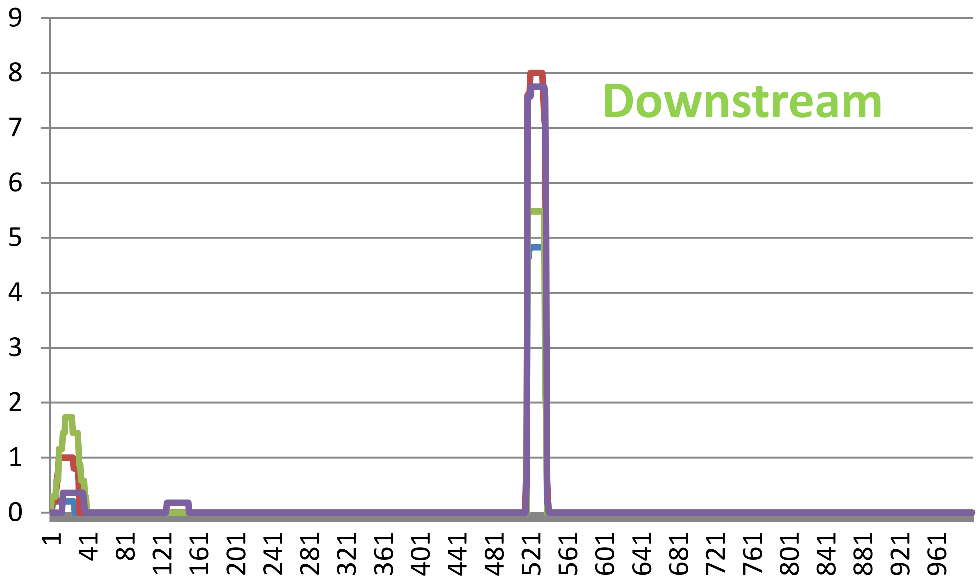

AT3G47300

SELT-like protein precursor (SELT)

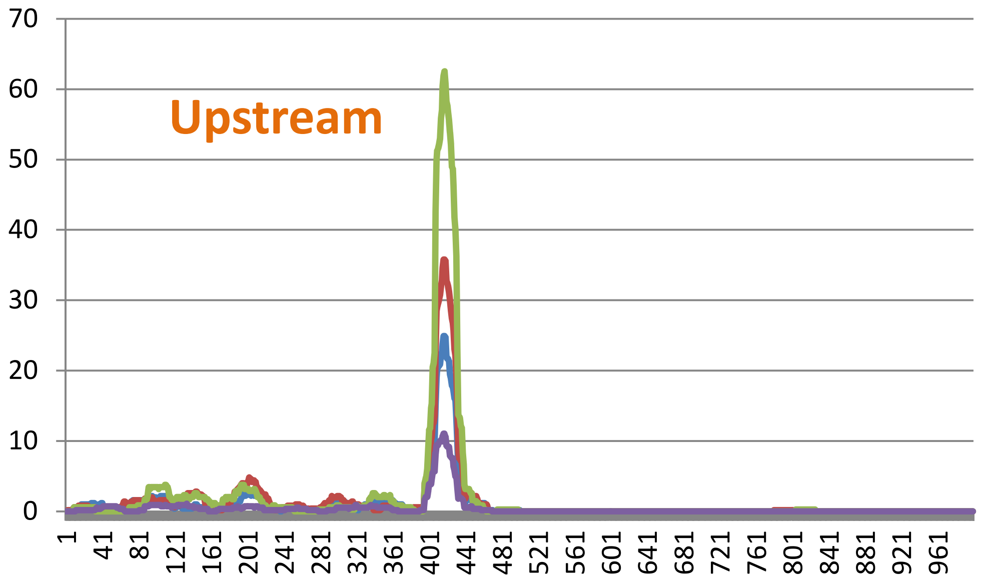

AT3G49645

Unknown protein

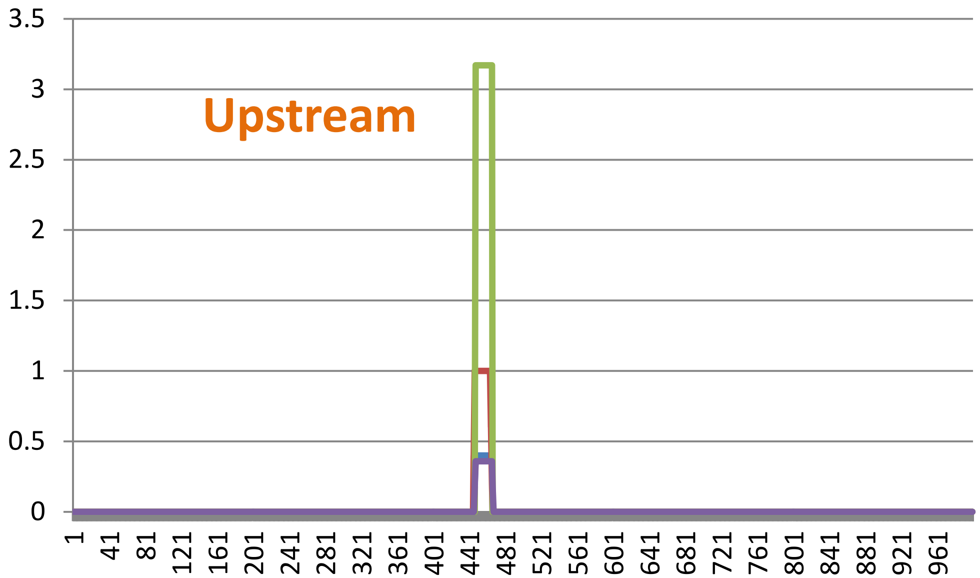

AT3G52570

alpha/beta-Hydrolases superfamily protein

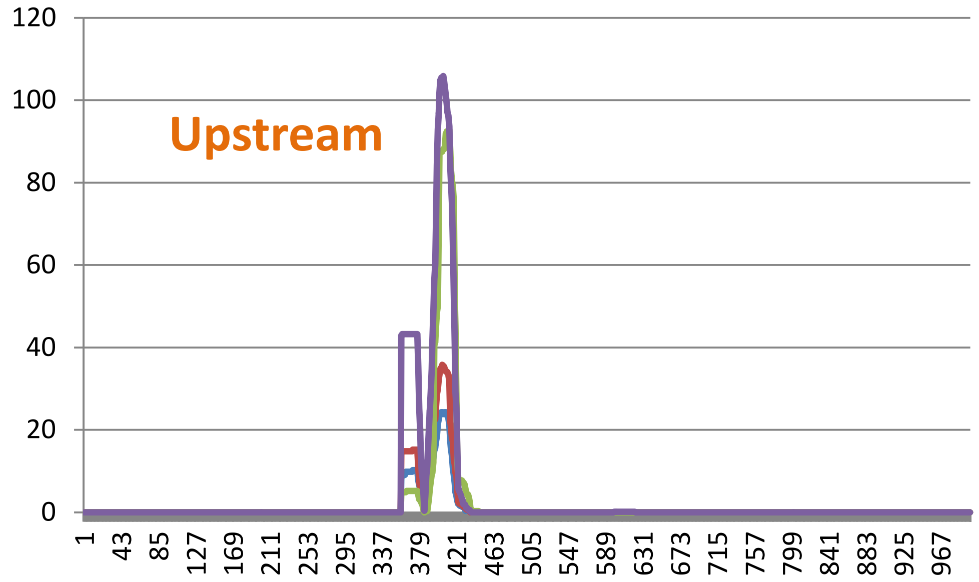

AT3G52700

Unknown protein

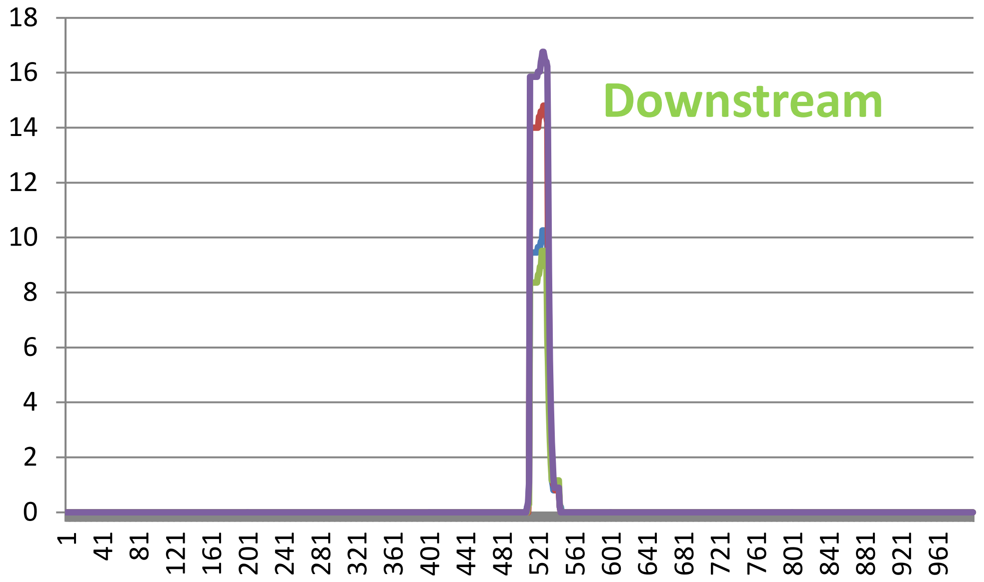

AT3G53090

Encodes a ubiquitin-protein ligase containing a HECT domain.

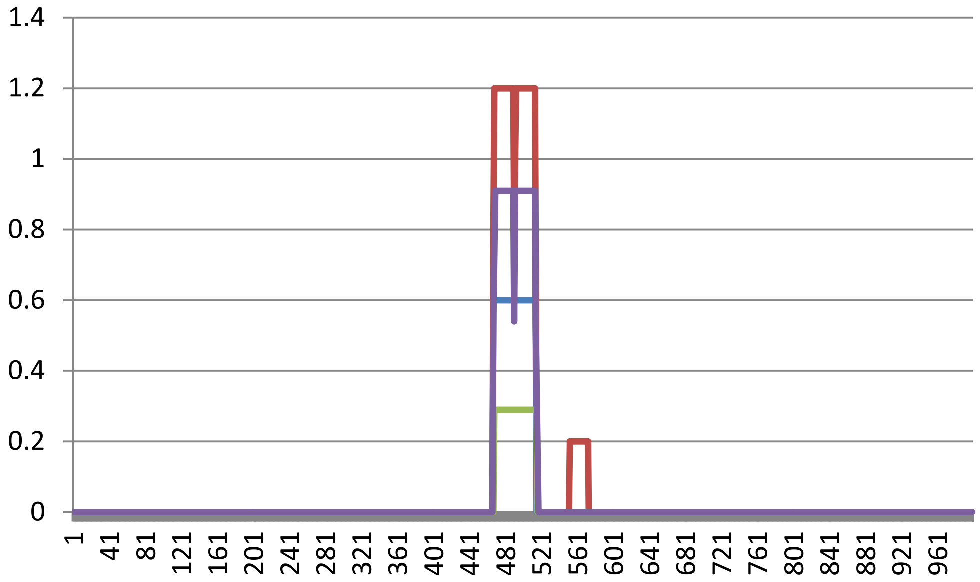

AT3G53920

Encodes a sigma-like transcription factor, Sigma 3 (SIG3 or SIGC). As a subunit of chloroplast RNA polymerase, SIG3 confers the ability to recognize promoter sequences on the core enzyme. SIG3 transcribes specifically the psbN gene in plastids.

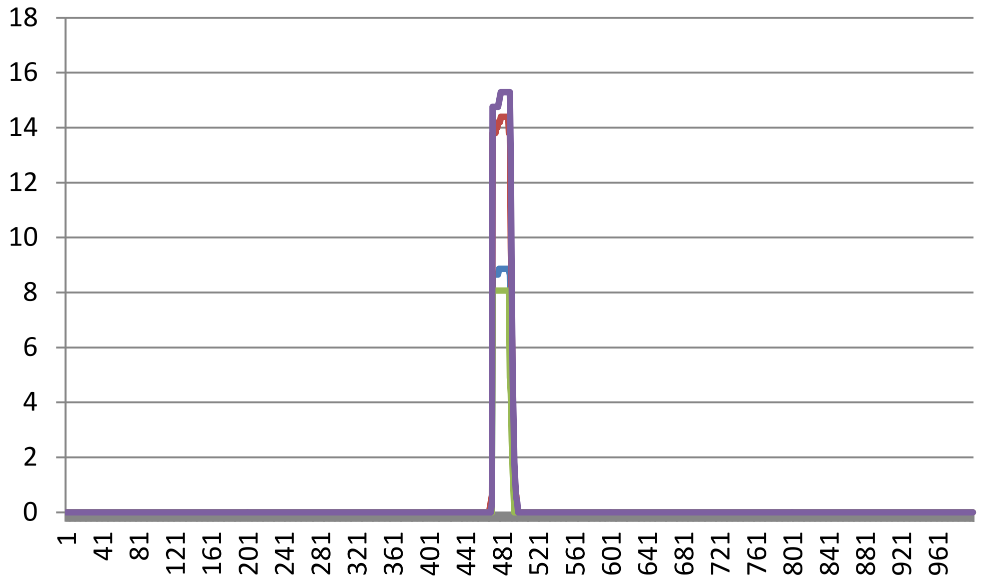

AT3G54360

Zinc ion binding

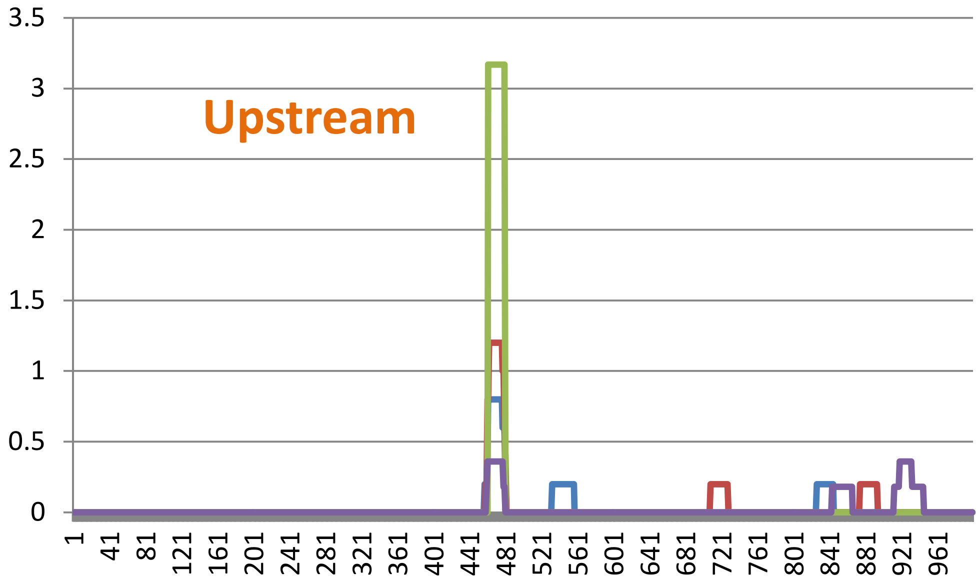

AT3G55020

Ypt/Rab-GAP domain of gyp1p superfamily protein

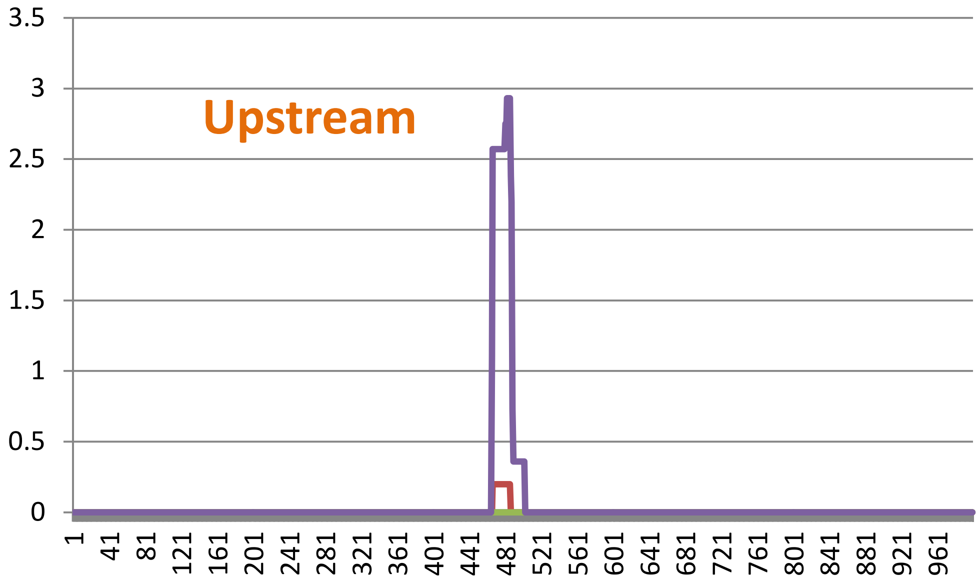

AT3G55850

Encodes a product that might regulate nucleo-cytoplasmic trafficking of an intermediate(s) involved in phyA signal transduction.

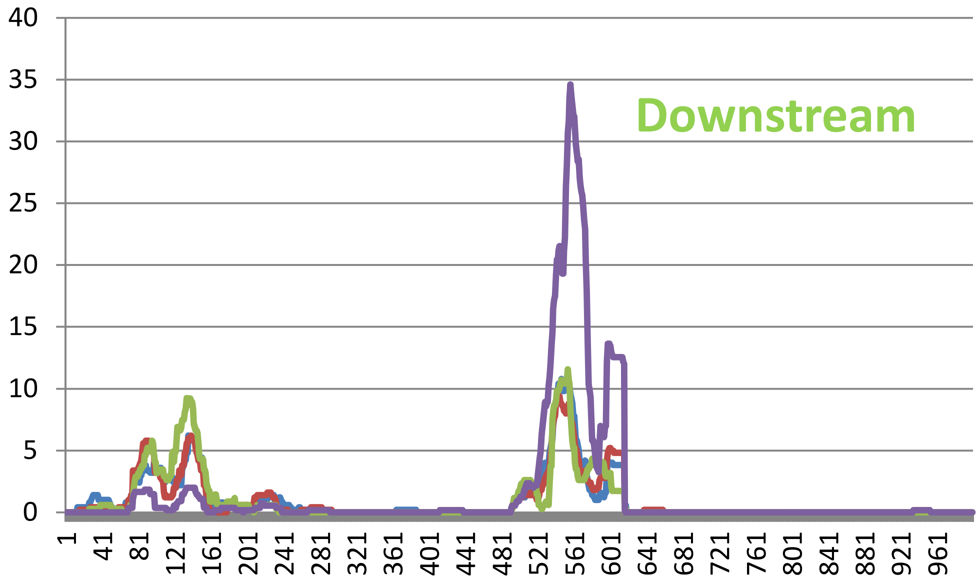

AT3G55860

Unknown protein

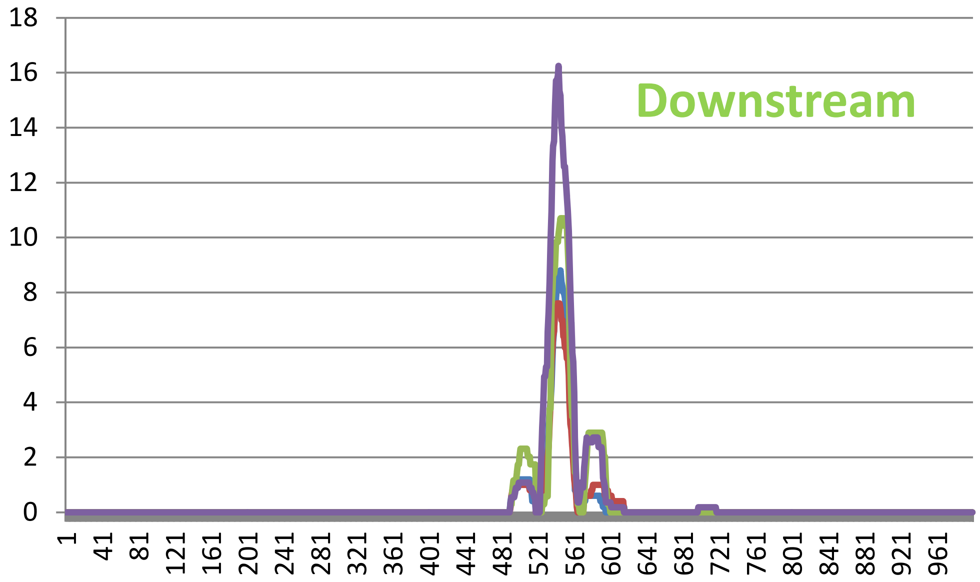

AT3G56450

Member of alpha-SNAP Gene Family

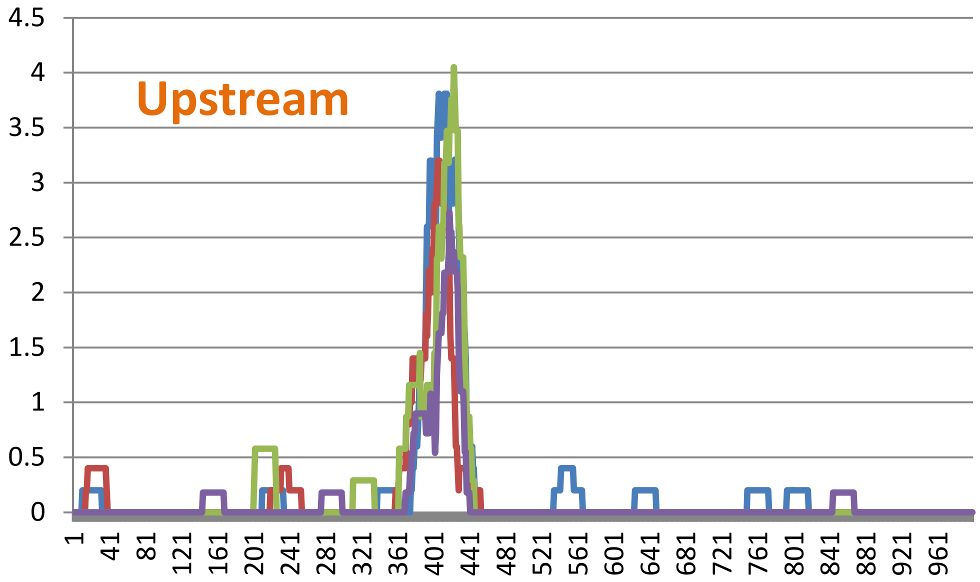

AT3G56460

GroES-like zinc-binding alcohol dehydrogenase family protein

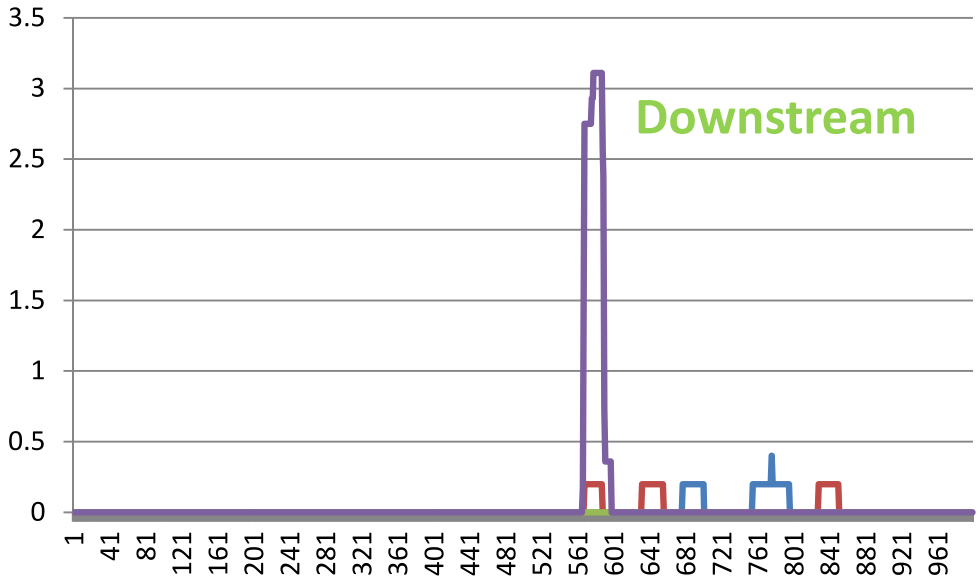

AT3G57770

Protein kinase superfamily protein

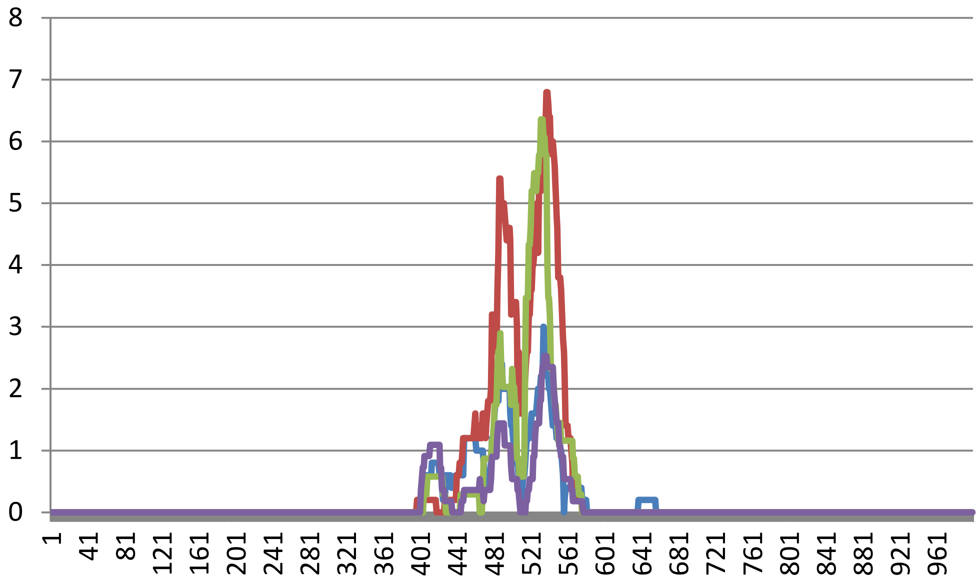

AT3G59220

Encodes a cupin-domain containing protein that is similar to pirins which interact with a CCAAT box binding transcription factor. The protein interacts with GPA1 (G protein alpha-subunit) in vitro. Mutants in the gene are affected in germination and early seedling development.

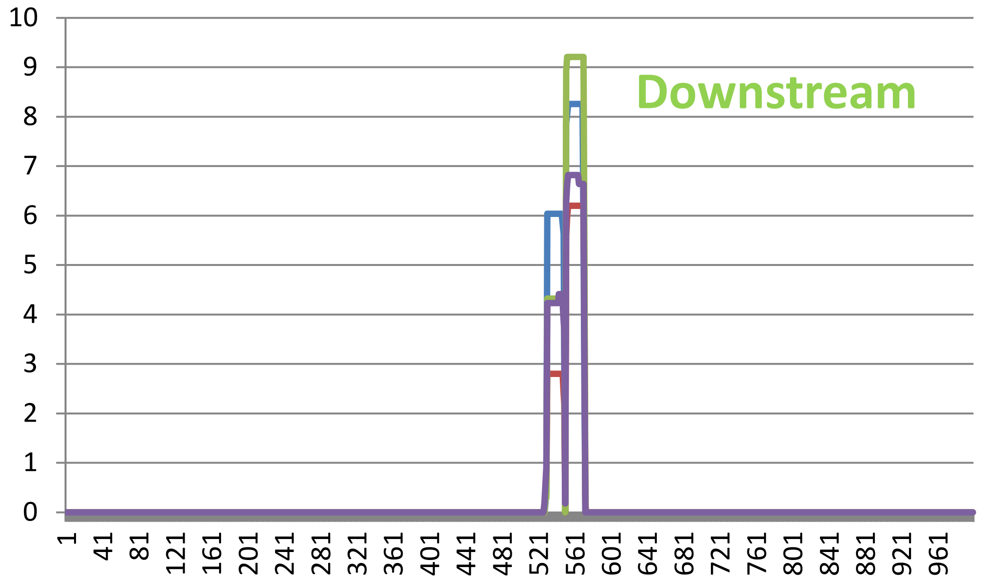

AT3G60790

F-box family protein

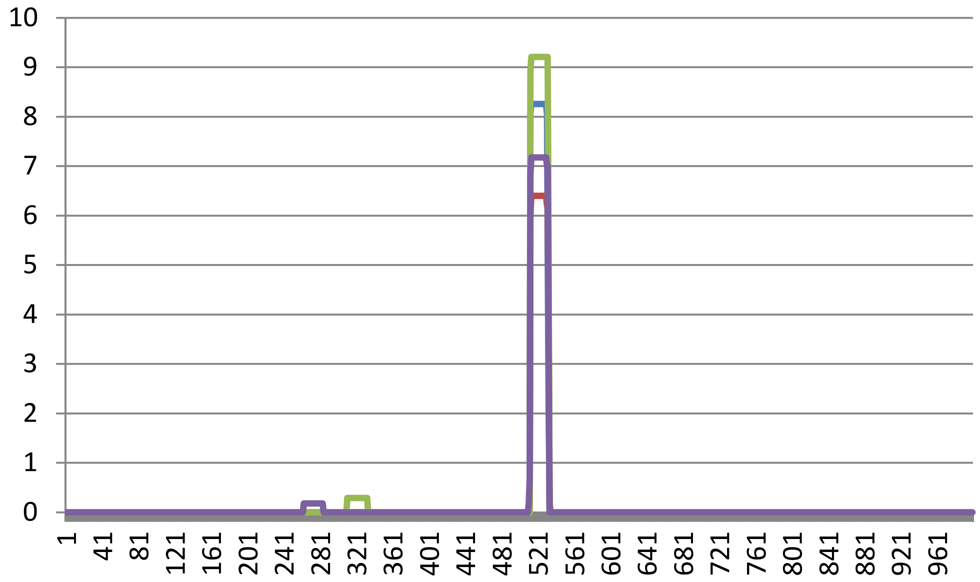

AT3G63470

Serine carboxypeptidase-like 40 (scpl40)

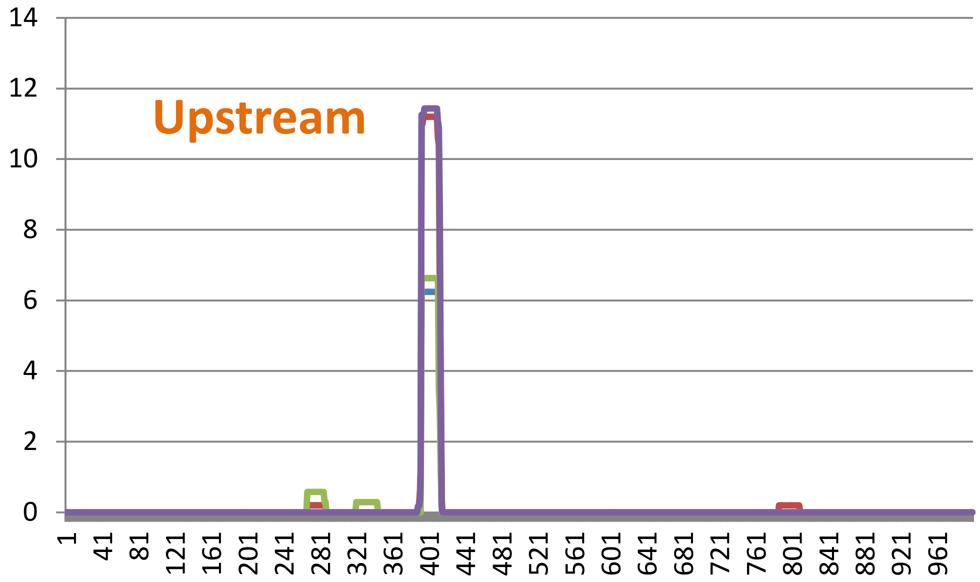

AT4G00580

COP1-interacting protein-related

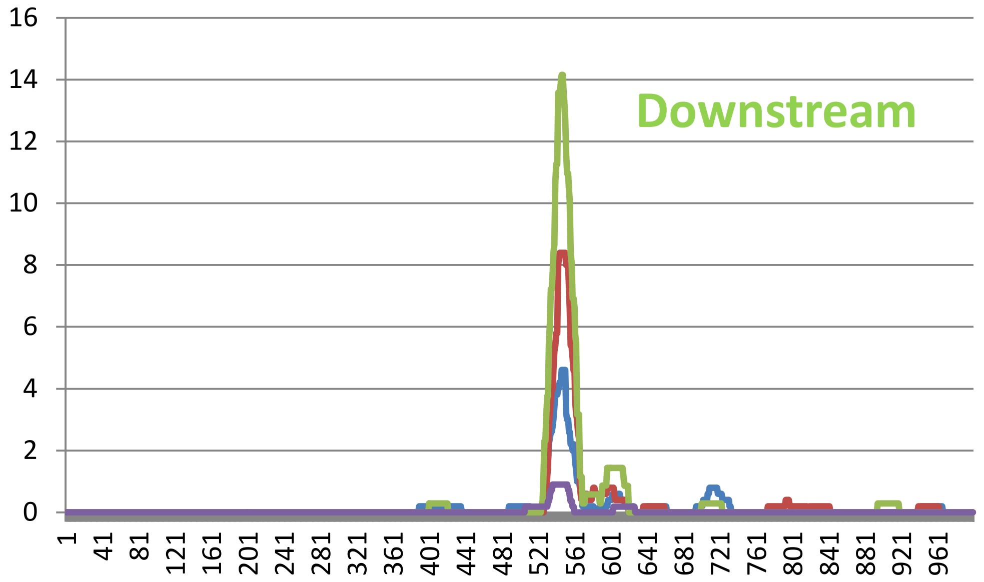

AT4G00950

Maternal effect embryo arrest 47 (MEE47)

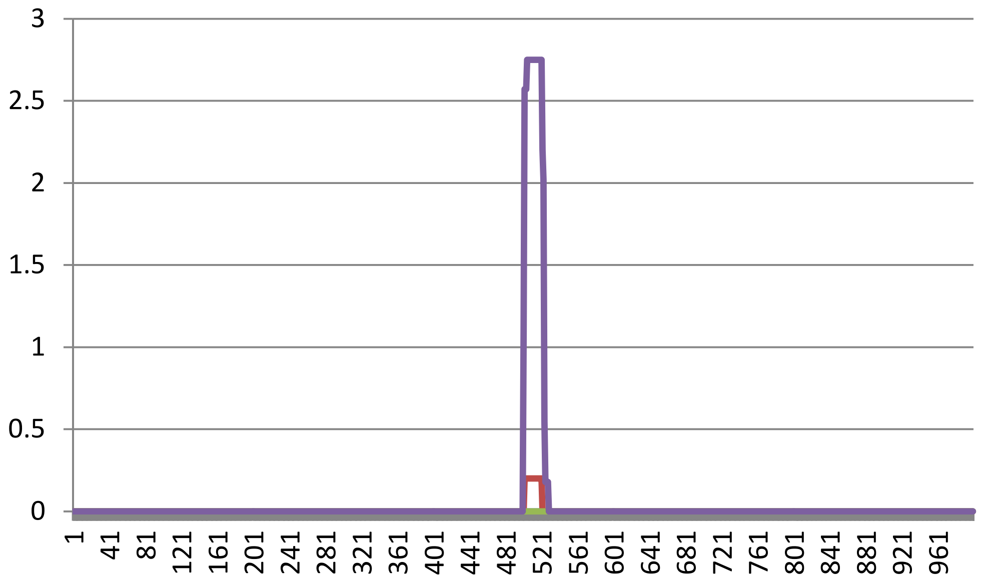

AT4G03940

Unknown protein

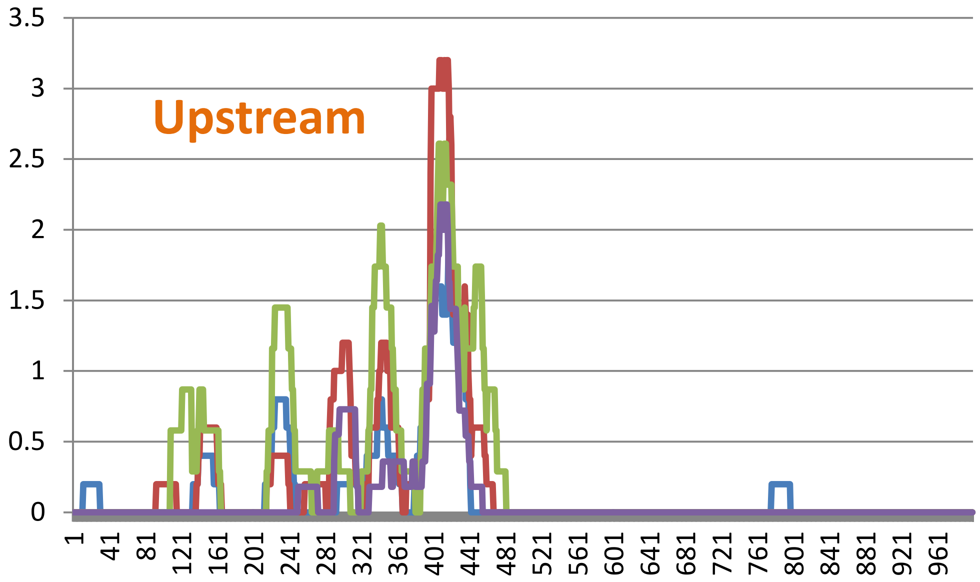

AT4G05030

Copper transport protein family

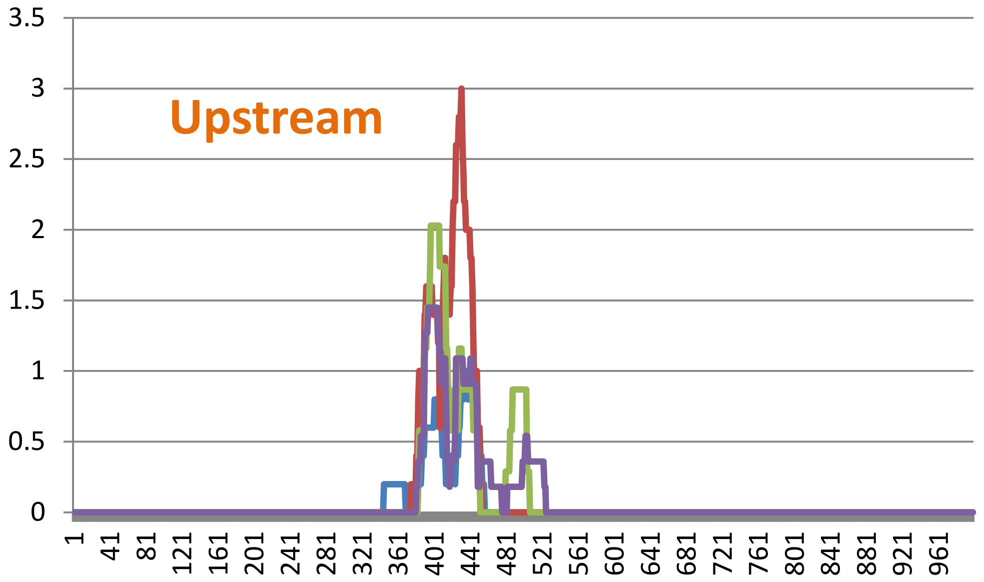

AT4G06740

Unknown protein

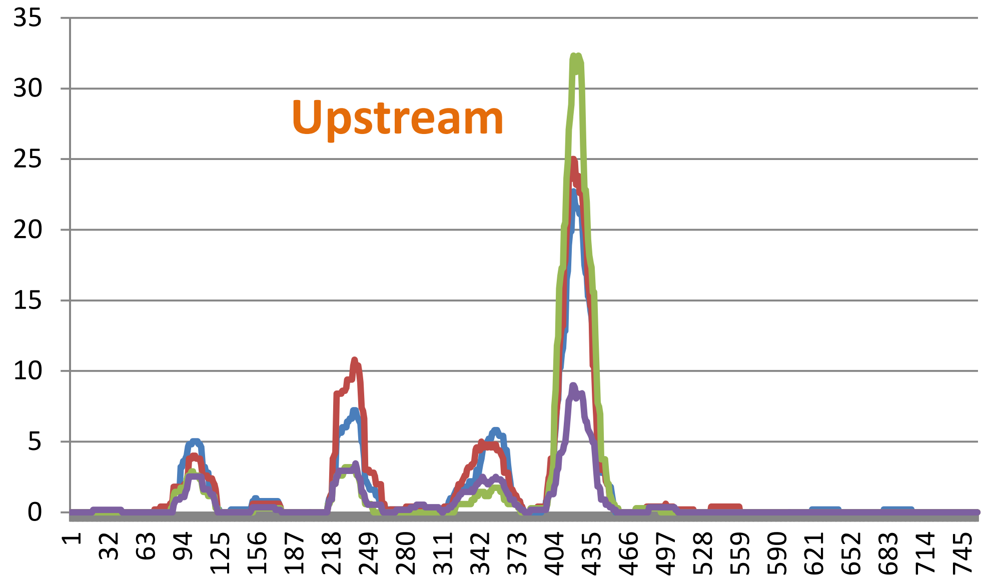

AT4G07526

Unknown protein

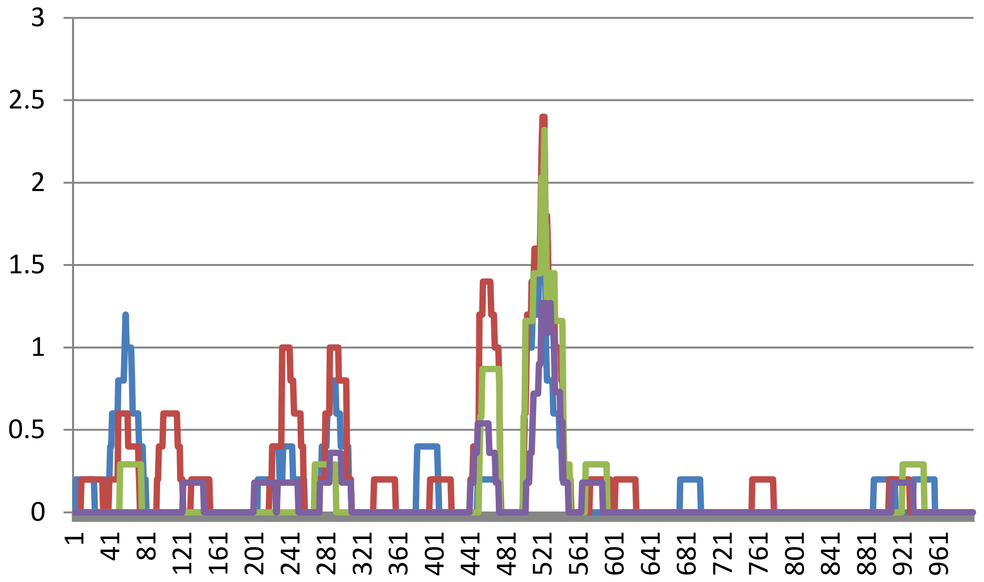

AT4G07960

Encodes a gene similar to cellulose synthase

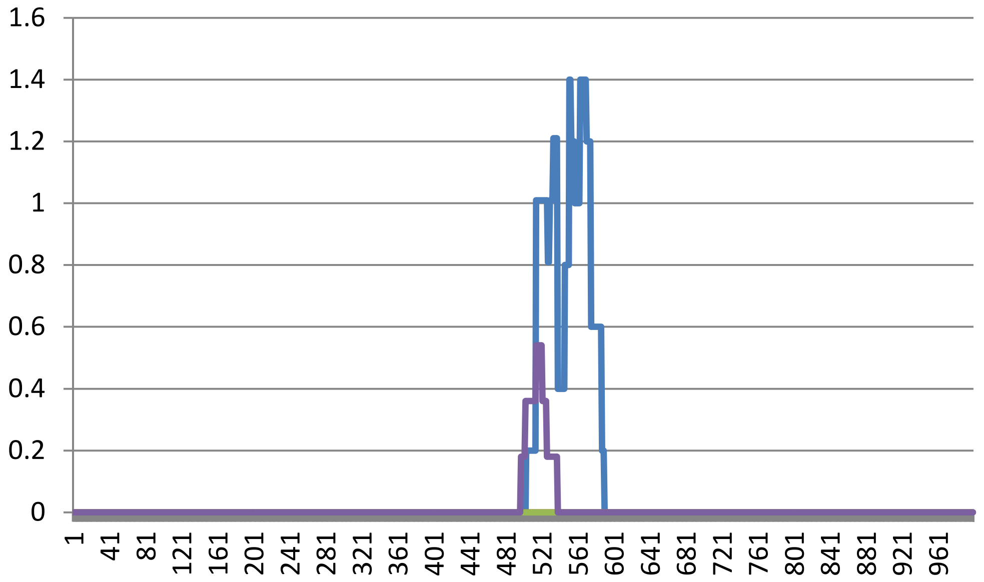

AT4G10596

This gene encodes a small protein and has either evidence of transcription or purifying selection.

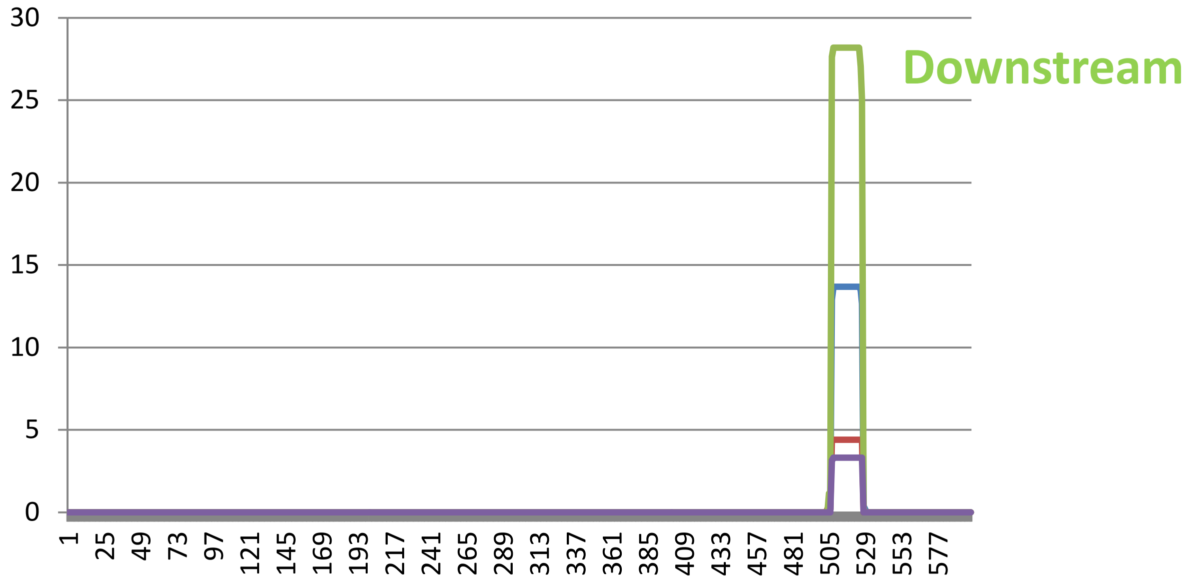

AT4G11485

Encodes a member of a family of small, secreted, cysteine rich protein with sequence similarity to the PCP (pollen coat protein) gene family.

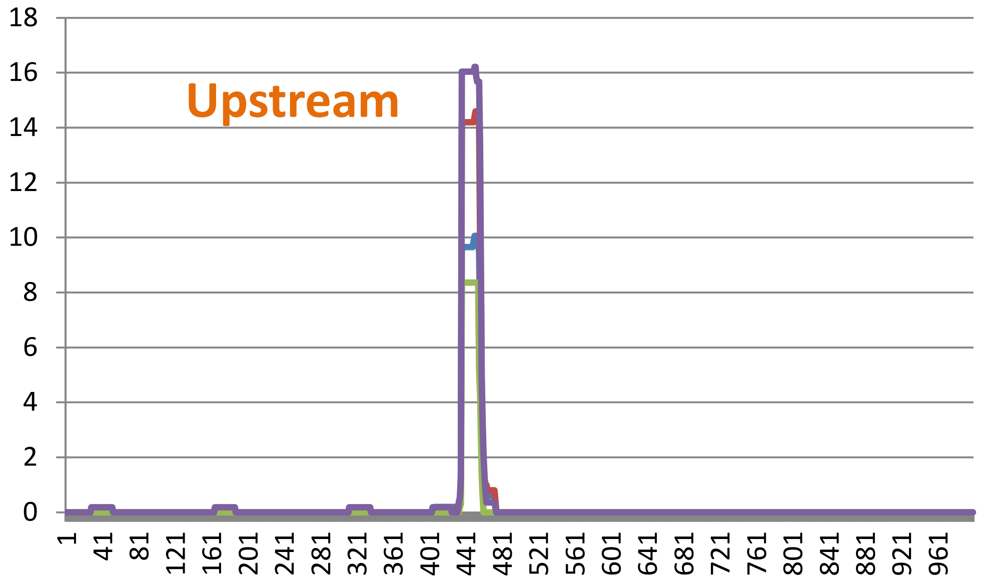

AT4G13420

Encodes a protein of the KUP/HAK/KT potassium channel class that is upregulated in the roots by K levels.

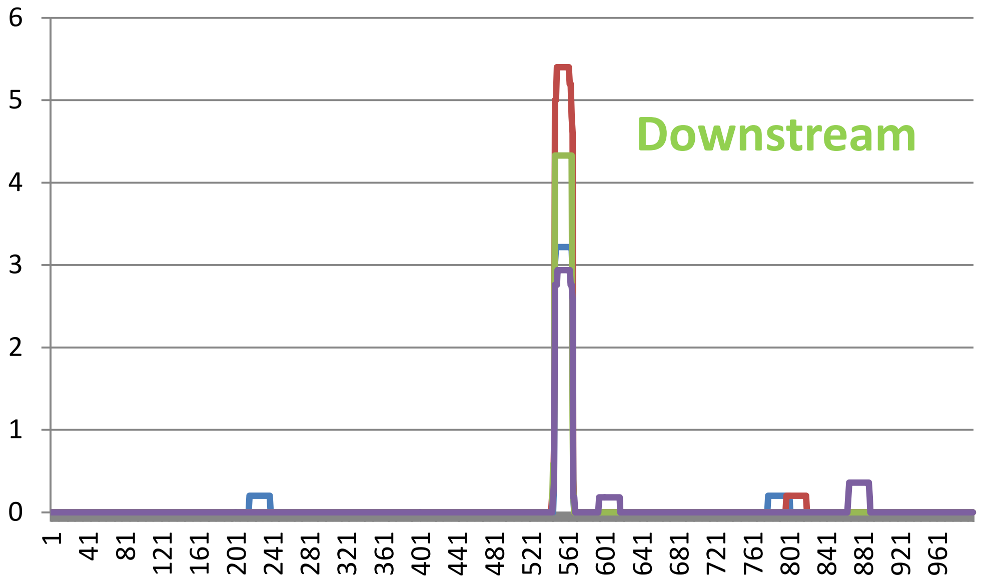

AT4G13992

Cysteine/Histidine-rich C1 domain family protein

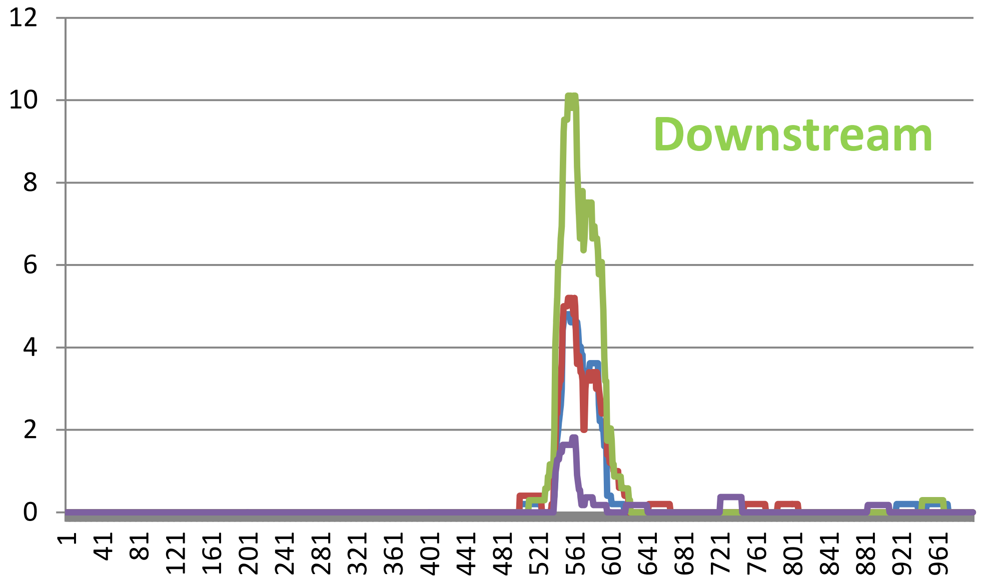

AT4G14130

Xyloglucan endotransglycosylase-related protein (XTR7)

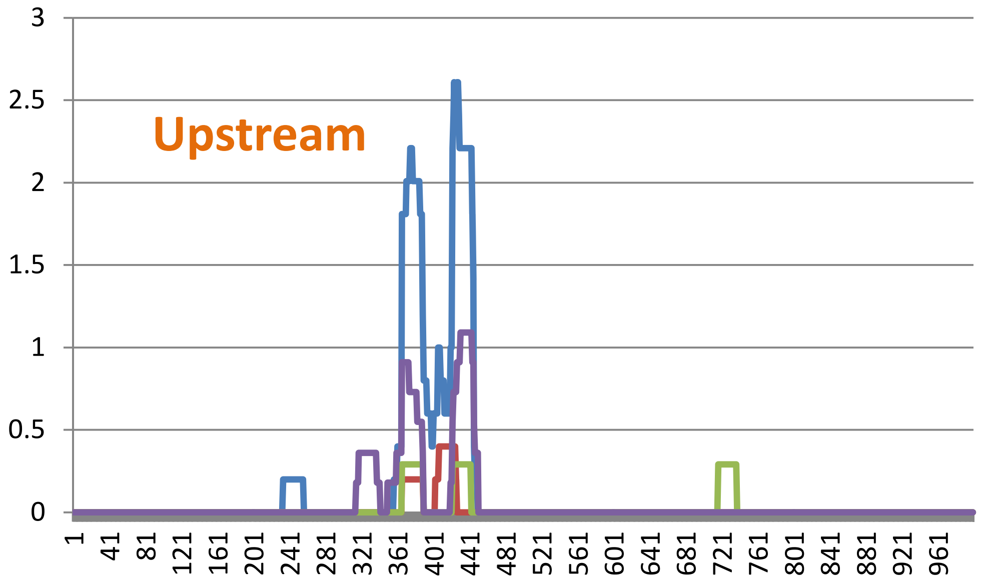

AT4G16240

Unknown protein

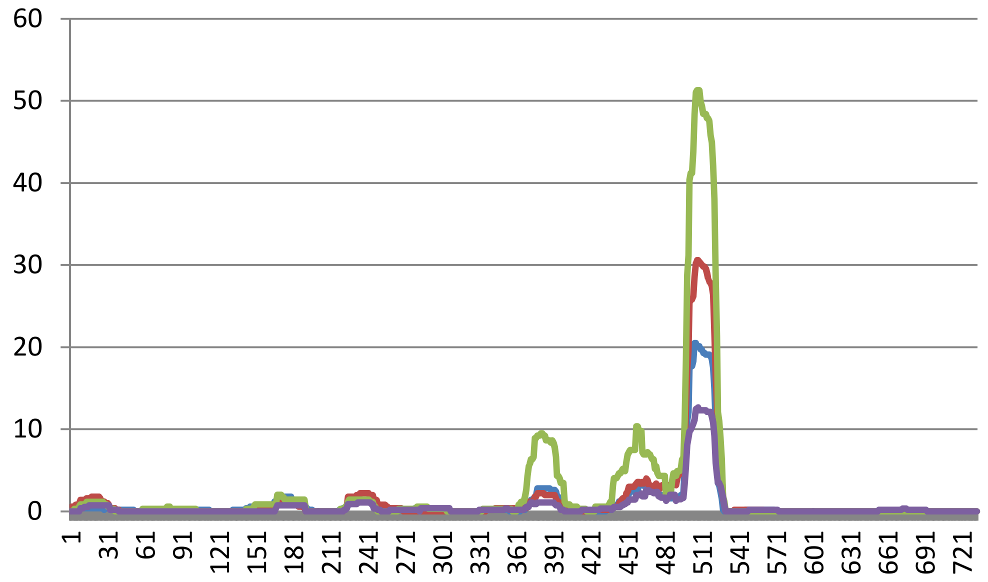

AT4G16640

Matrixin family protein

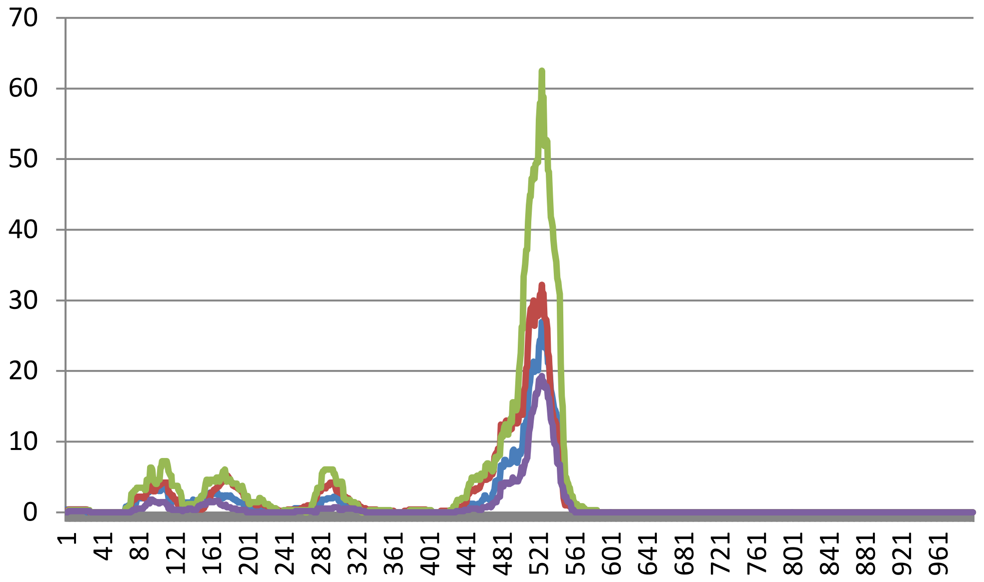

AT4G16790

Hydroxyproline-rich glycoprotein family protein

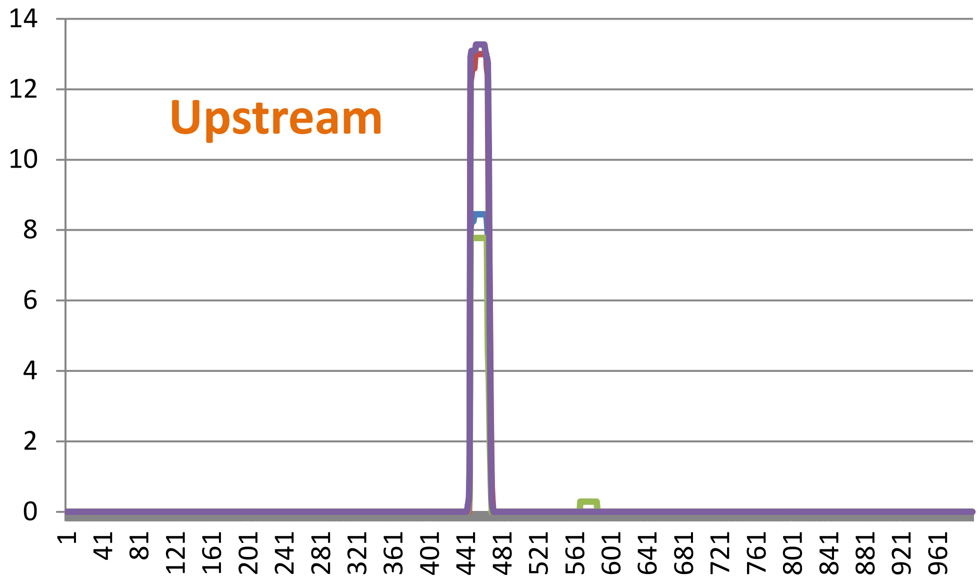

AT4G17950

AT hook motif DNA-binding family protein

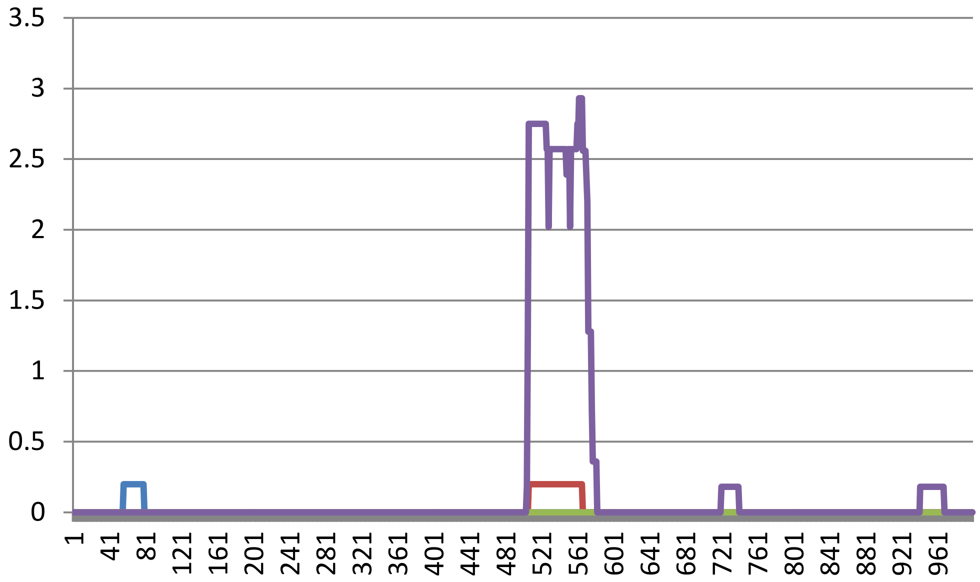

AT4G21210

Encodes a PPDK regulatory protein that has both protein kinase and protein phosphatase activities towards PPDK (pyruvate orthophosphate dikinase).

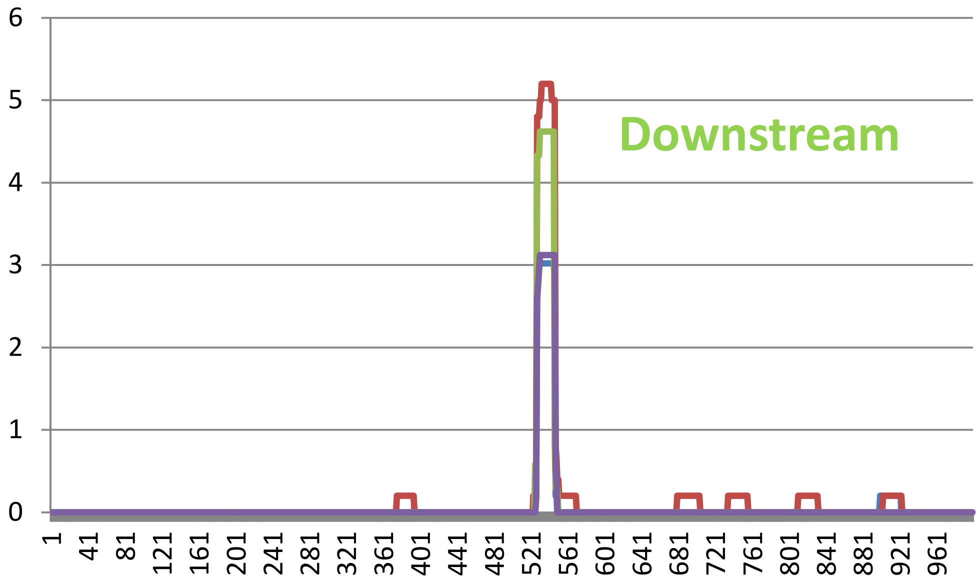

AT4G22150

Arabidopsis thaliana CDC48-interacting UBX-domain protein (PUX3)

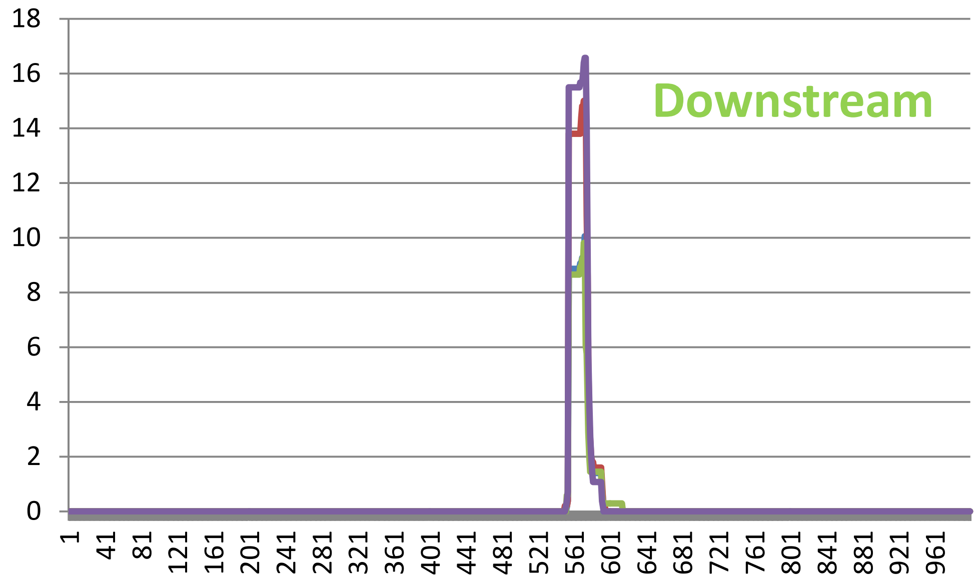

AT4G22890

Encodes PGRL1A, a transmembrane protein present in thylakoids.

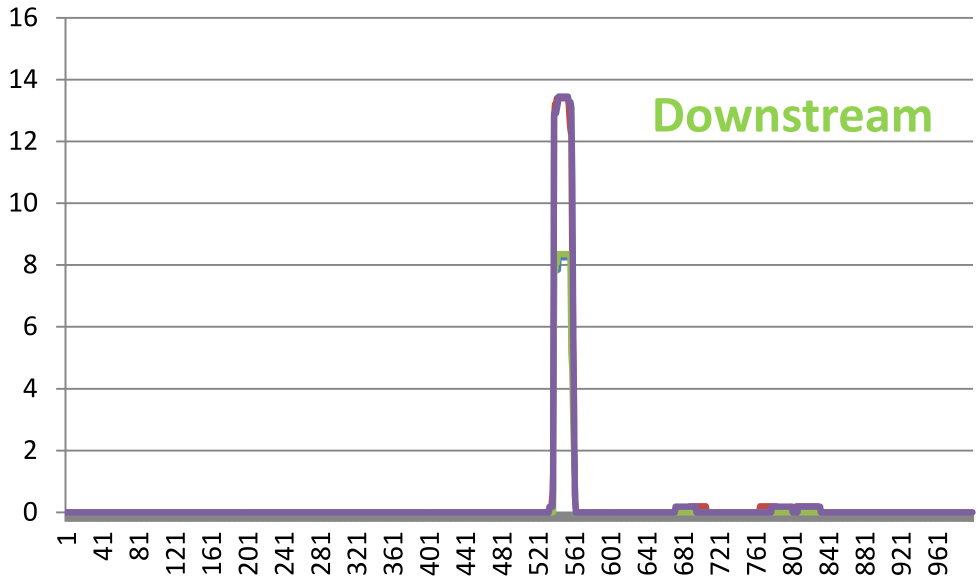

AT4G25240

Encodes GPI-anchored SKU5-like protein.

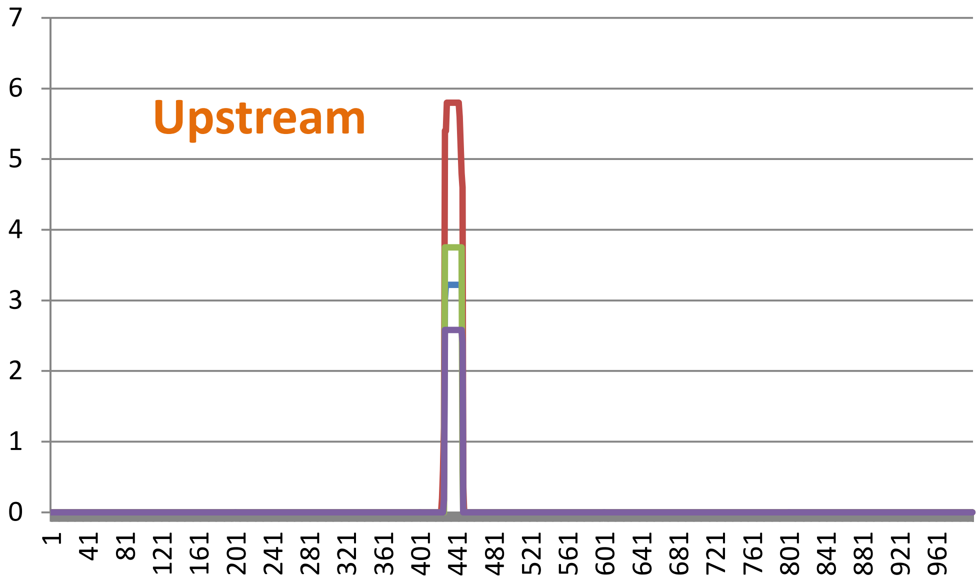

AT4G29033

Encodes a defensin-like (DEFL) family protein.

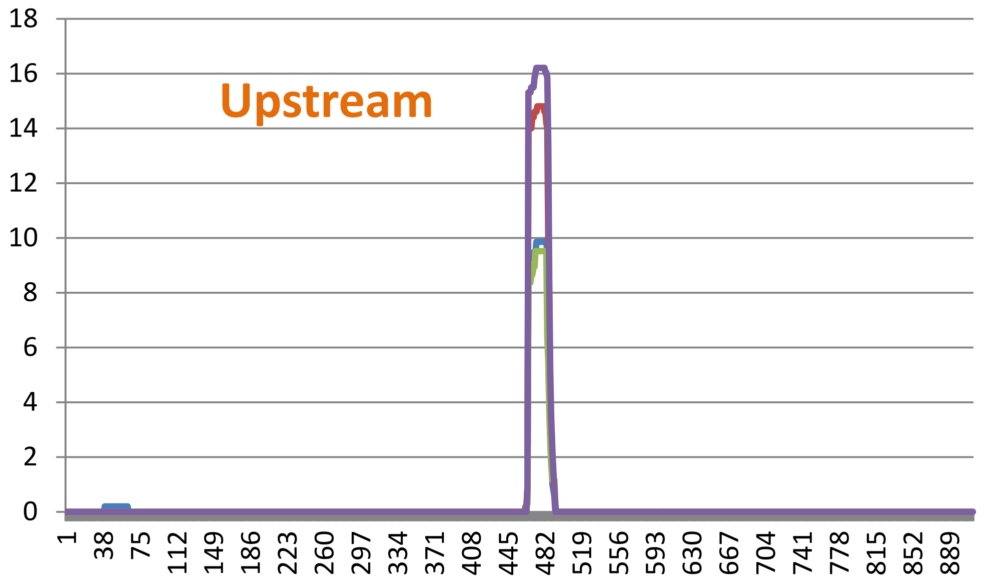

AT4G29090

Ribonuclease H-like superfamily protein

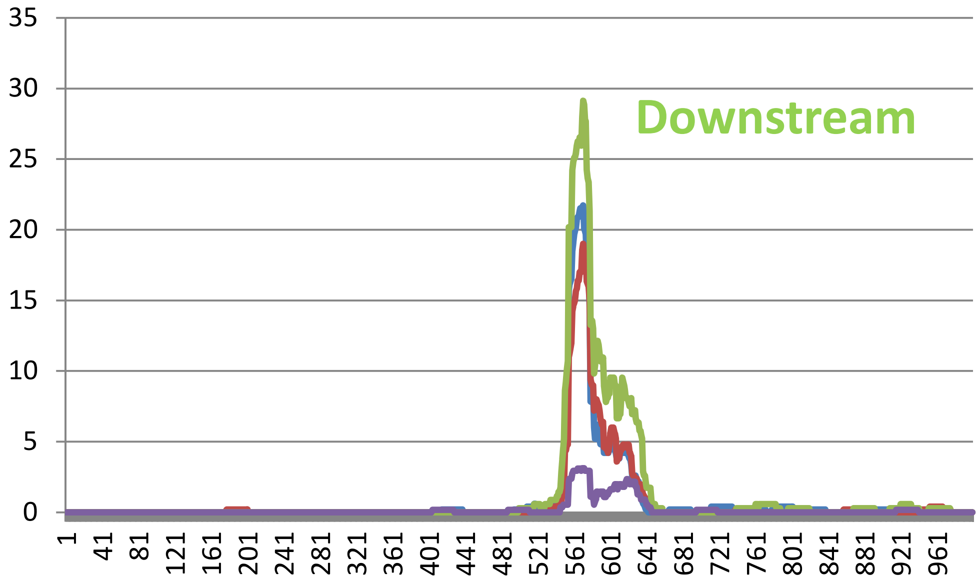

AT4G31350

Core-2/I-branching beta-1,6-N-acetylglucosaminyltransferase family protein

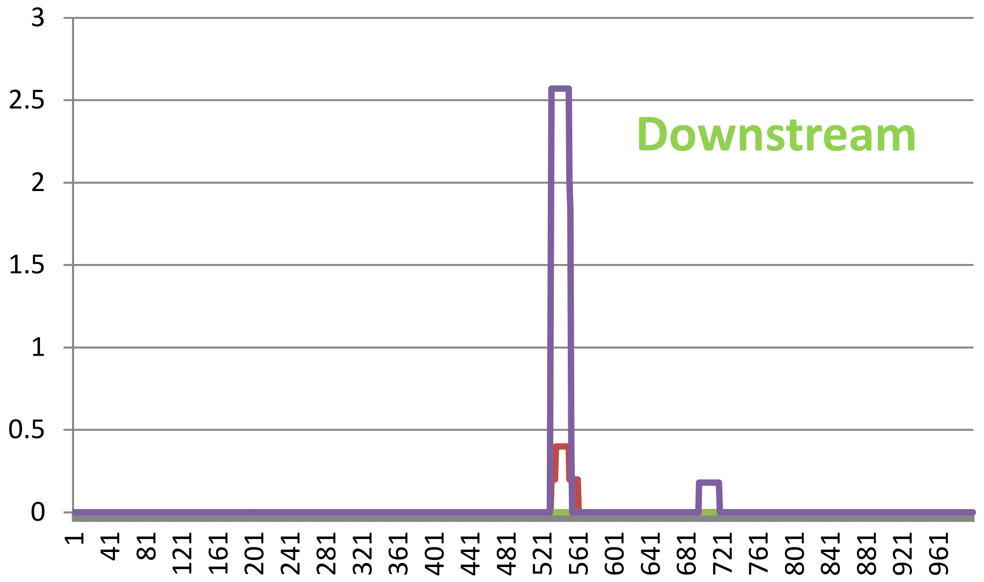

AT4G31980

Unknown protein

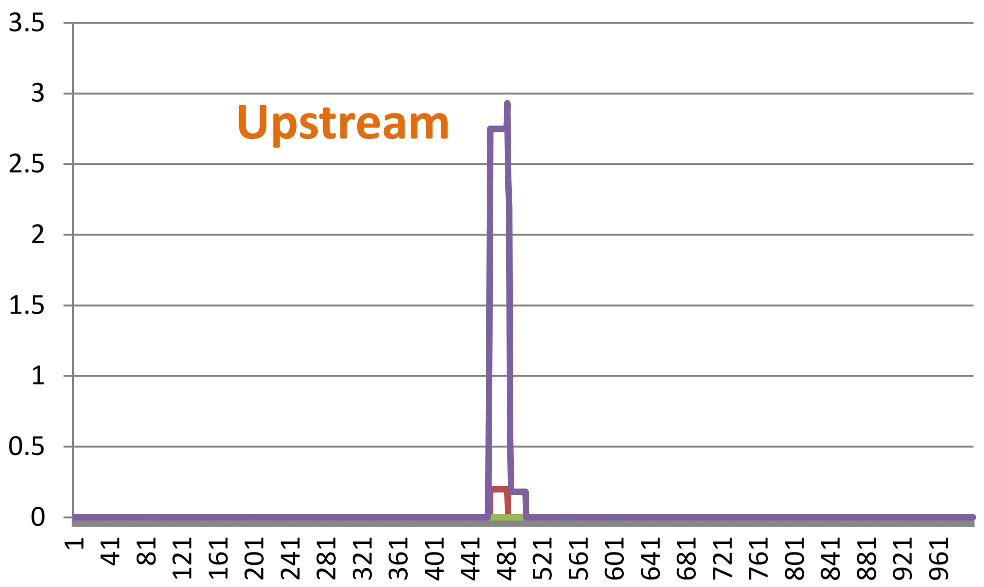

AT4G32800

Encodes a member of the DREB subfamily A-4 of ERF/AP2 transcription factor family.

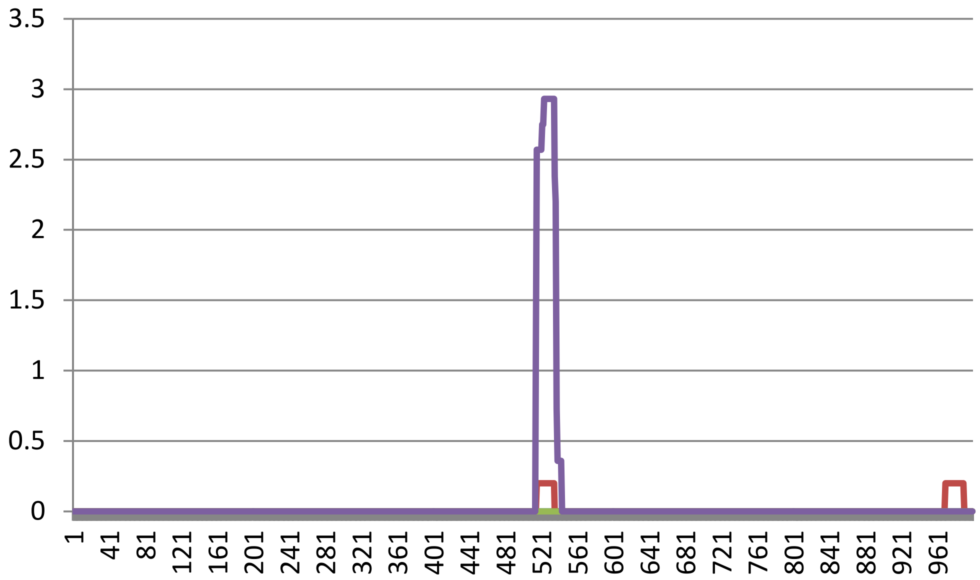

AT4G32950

Protein phosphatase 2C family protein

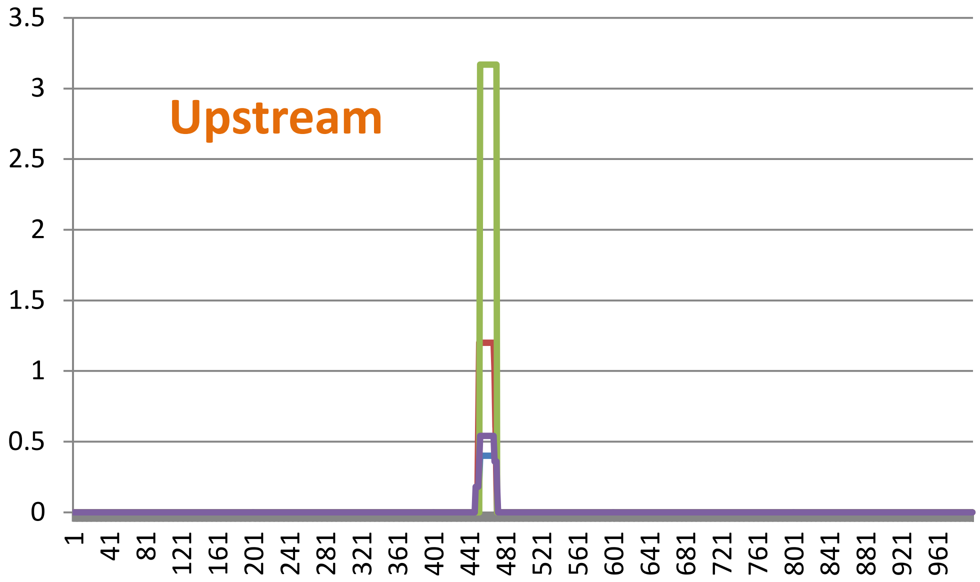

AT4G33840

Glycosyl hydrolase family 10 protein

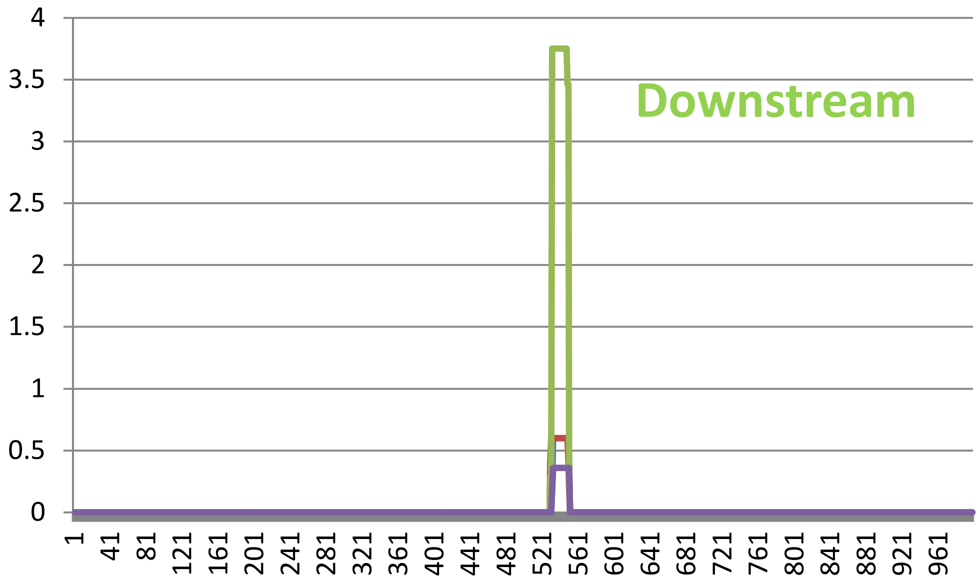

AT5G02700

F-box/RNI-like superfamily protein

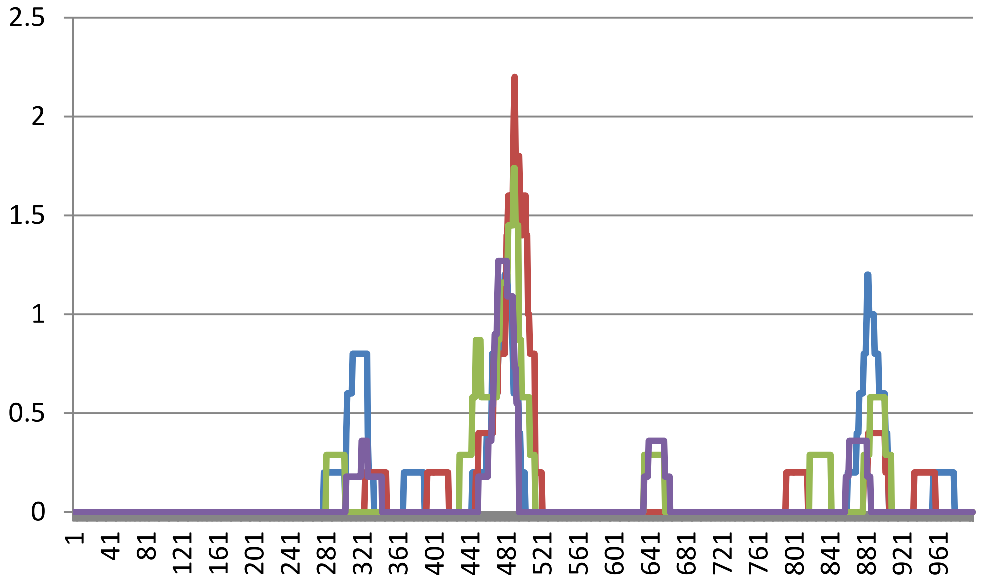

AT5G03340

ATPase, AAA-type, CDC48 protein

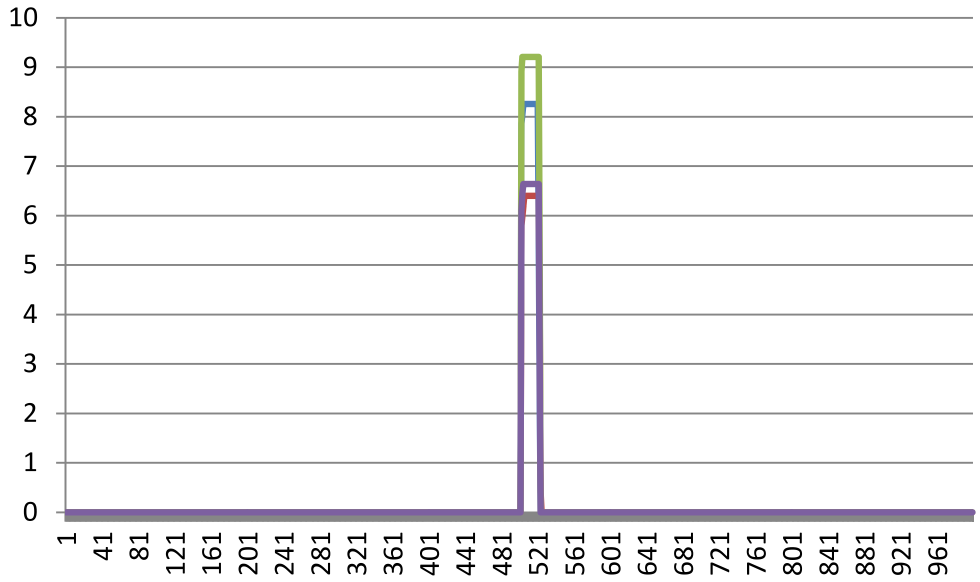

AT5G03730

Homologous to the RAF family of serine/threonine protein kinases. Negative regulator in the ethylene signal transduction pathway. Interacts with the putative ethylene receptors ETR1 and ERS. Constitutively expressed.

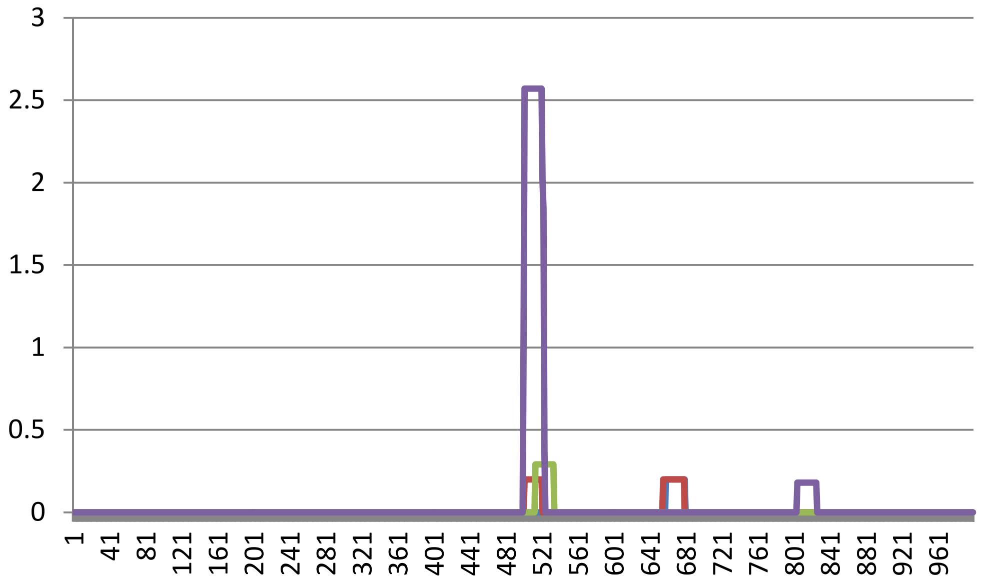

AT5G05100

Single-stranded nucleic acid binding R3H protein

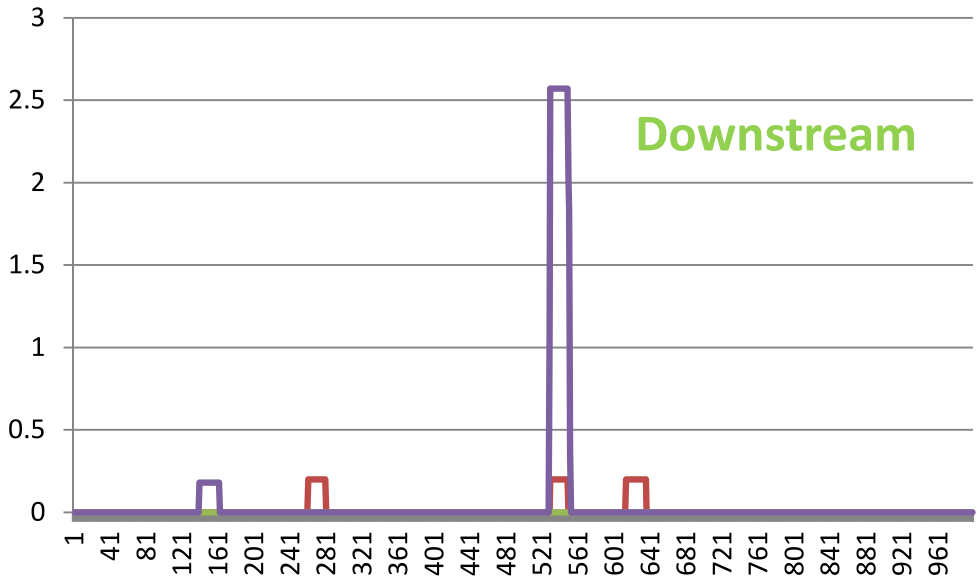

AT5G05950

Maternal effect embryo arrest 60 (MEE60)

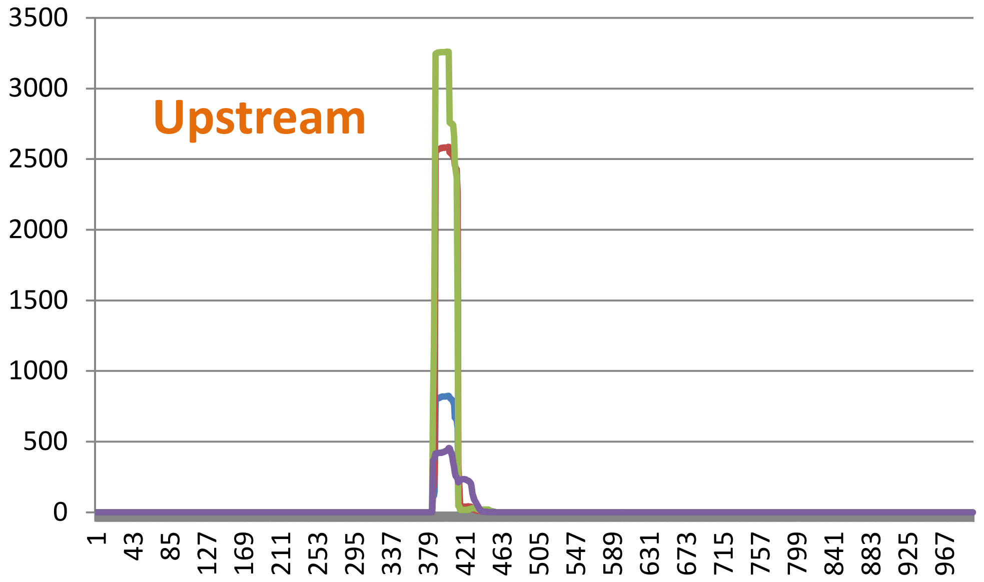

AT5G08550

Encodes a transcriptional repressor that is homologous to the C-terminal region of mammalian GC binding factor. It regulates endoreduplication through control of CYC2A expression.

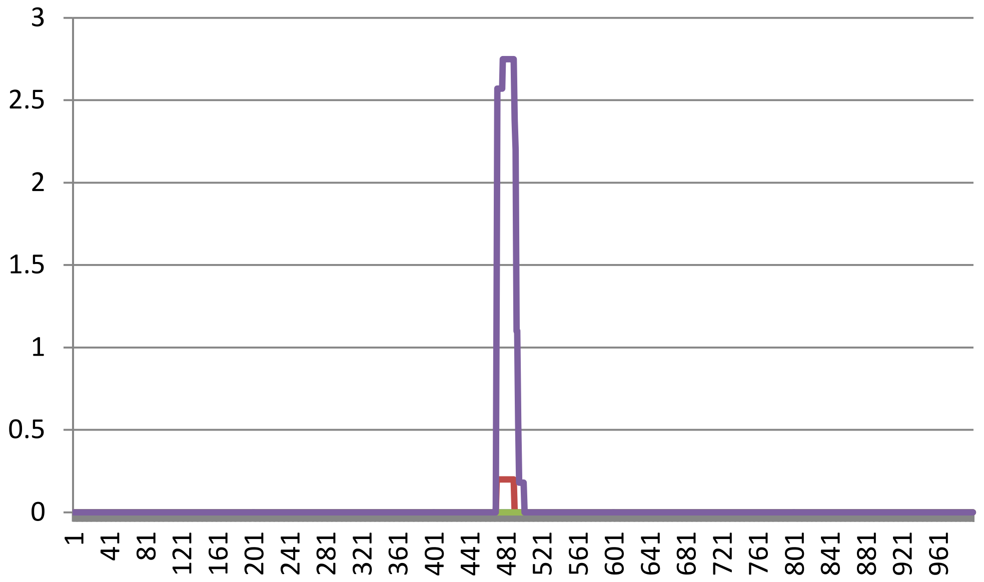

AT5G10340

F-box family protein

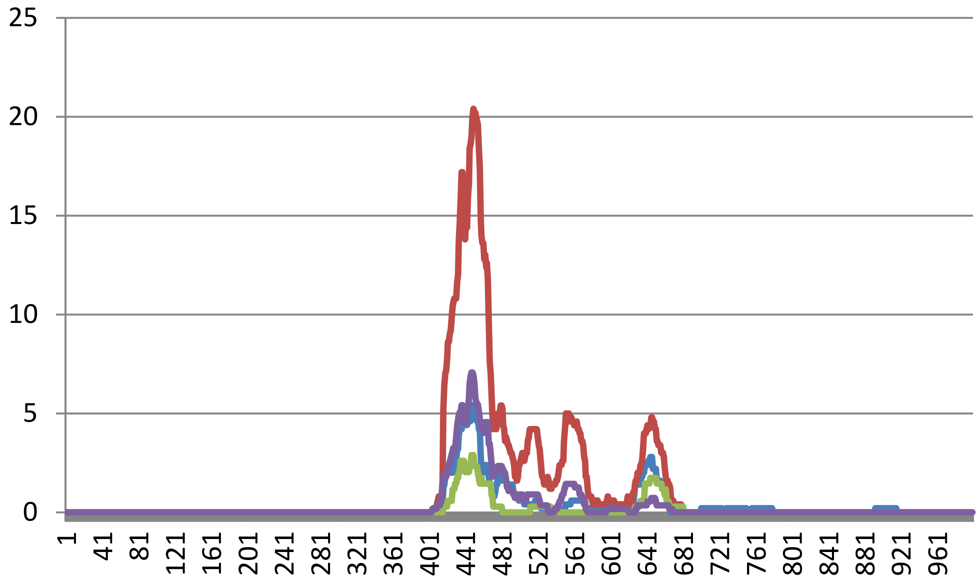

AT5G11510

Arabidopsis thaliana putative c-myb-like transcription factor MYB3R-4. Functions in powdery mildew induced host endoreduplication at the site of infection.

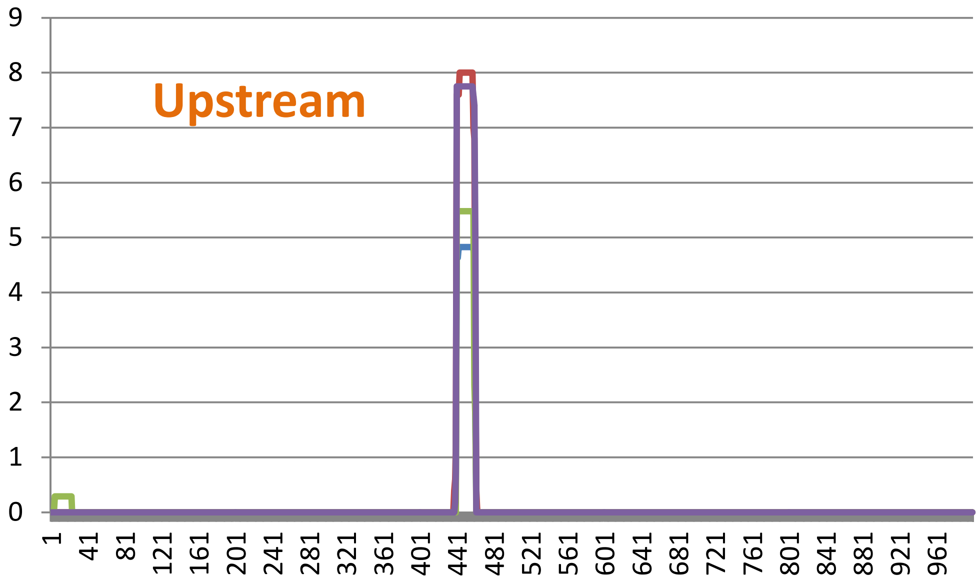

AT5G15420

Unknown protein

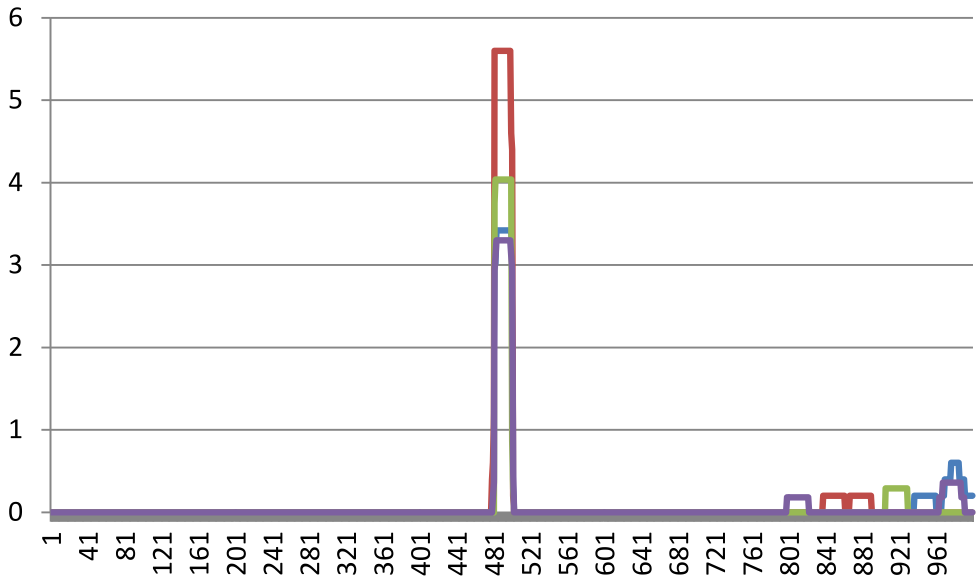

AT5G18260

RING/U-box superfamily protein

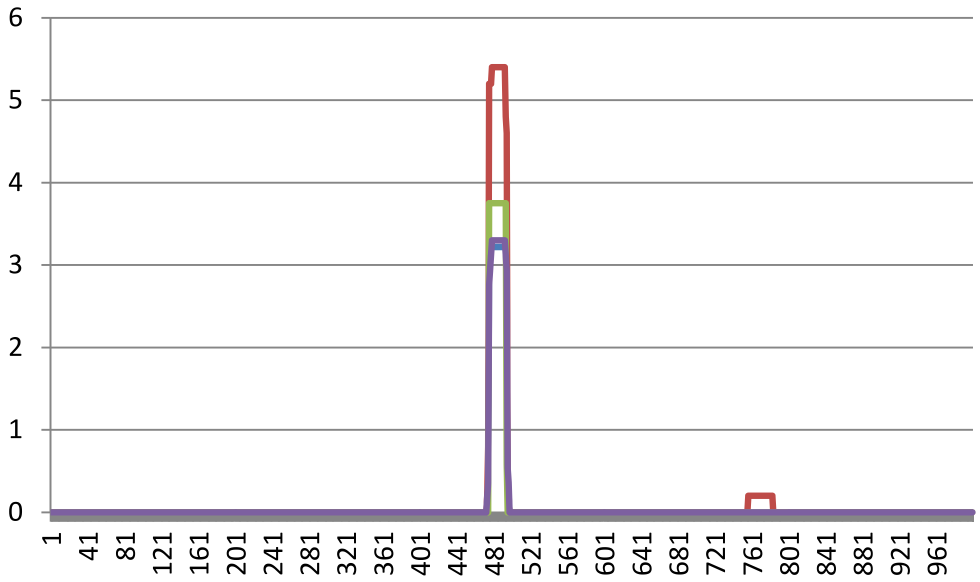

AT5G18610

Protein kinase superfamily protein

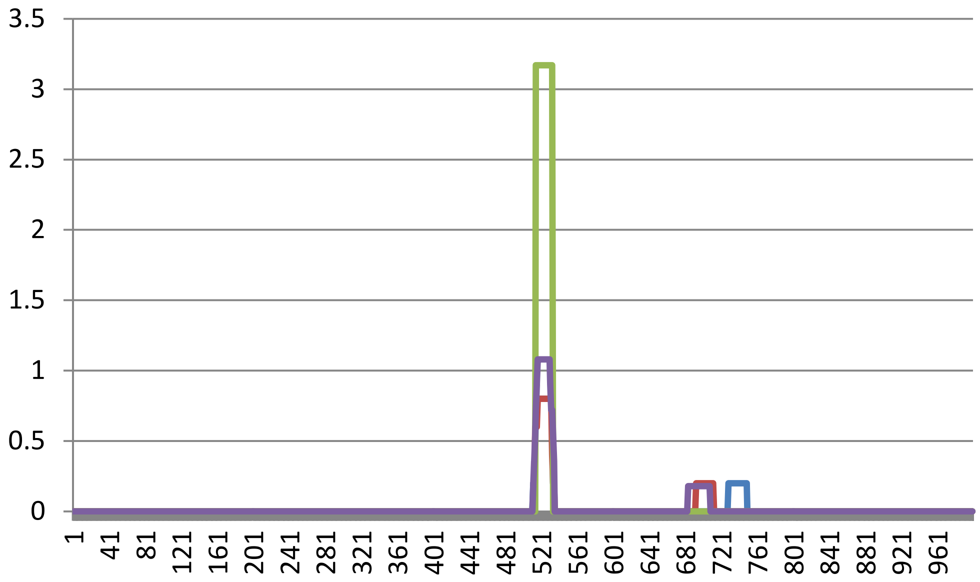

AT5G22590

Leucine Rich Repeat protein family

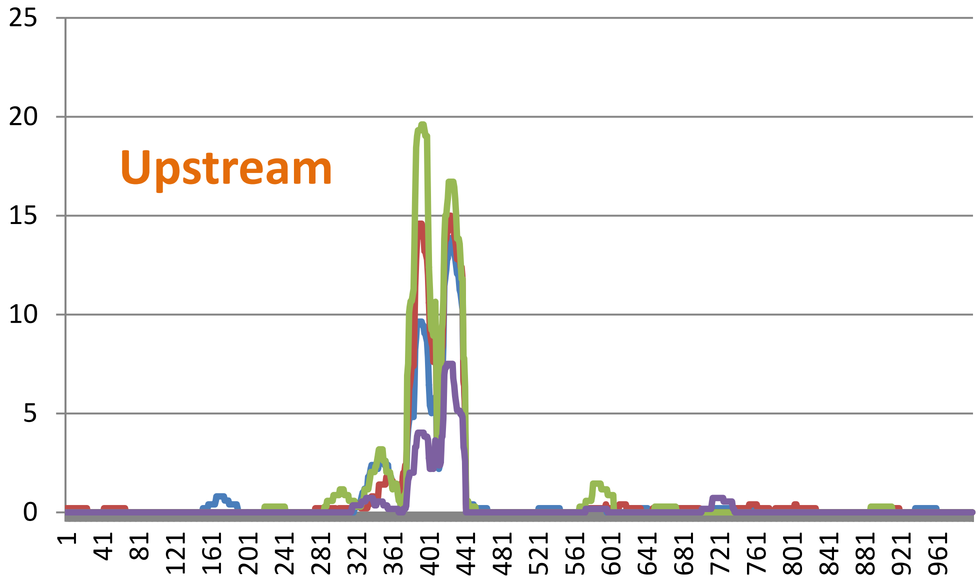

AT5G24240

Phosphatidylinositol 3- and 4-kinase; Ubiquitin family protein.

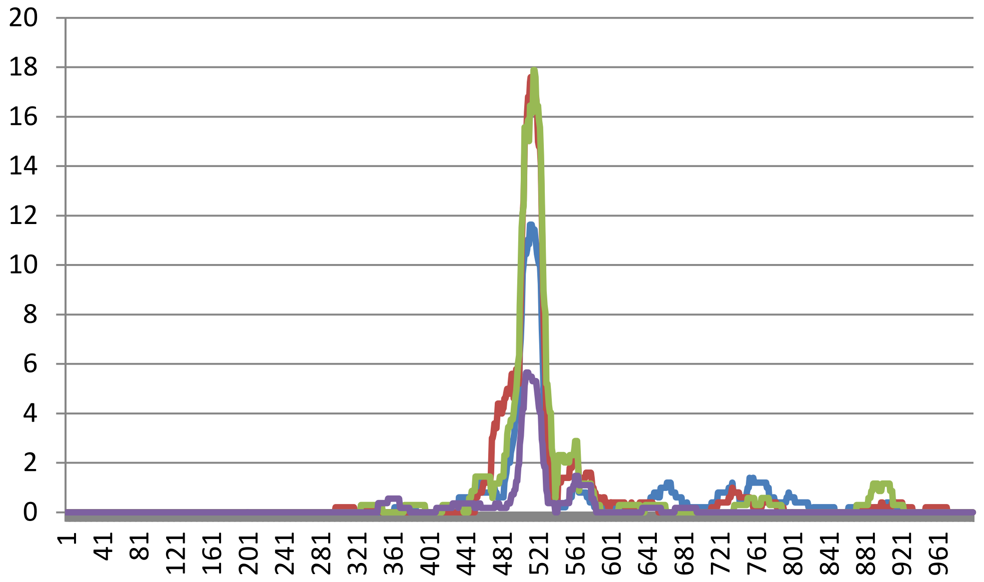

AT5G26700

RmlC-like cupins superfamily protein

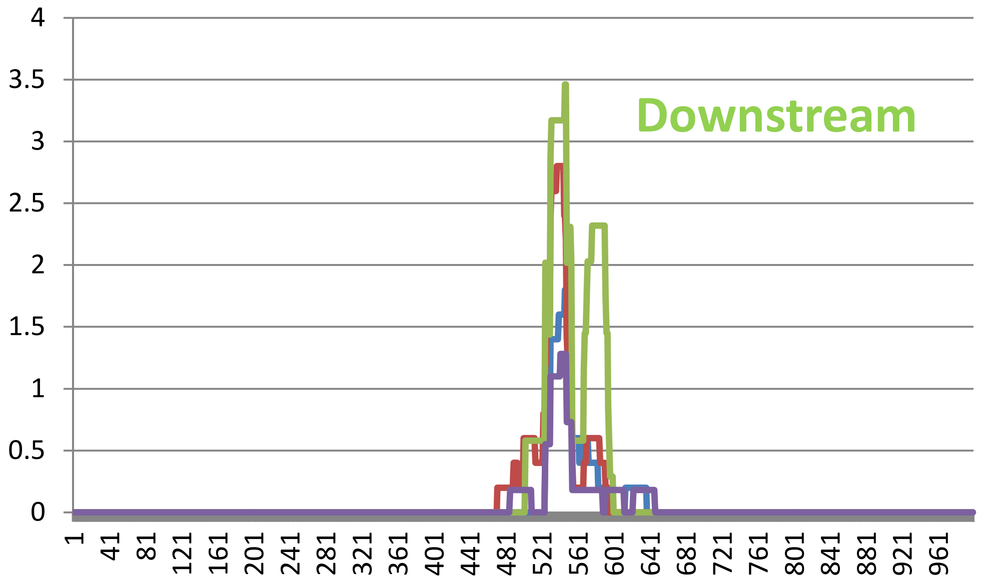

AT5G26840

Unknown protein

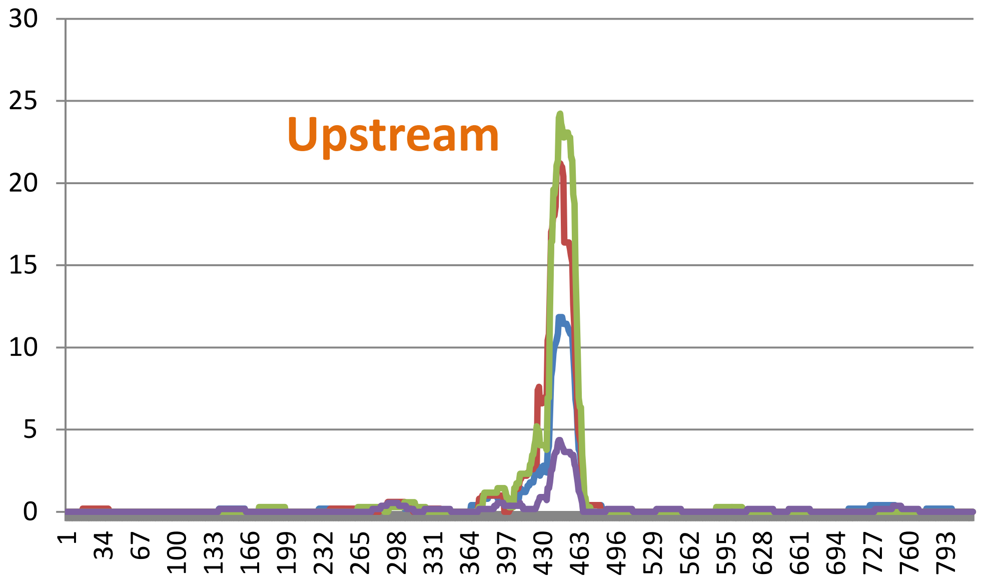

AT5G27870

Plant invertase/pectin methylesterase inhibitor superfamily

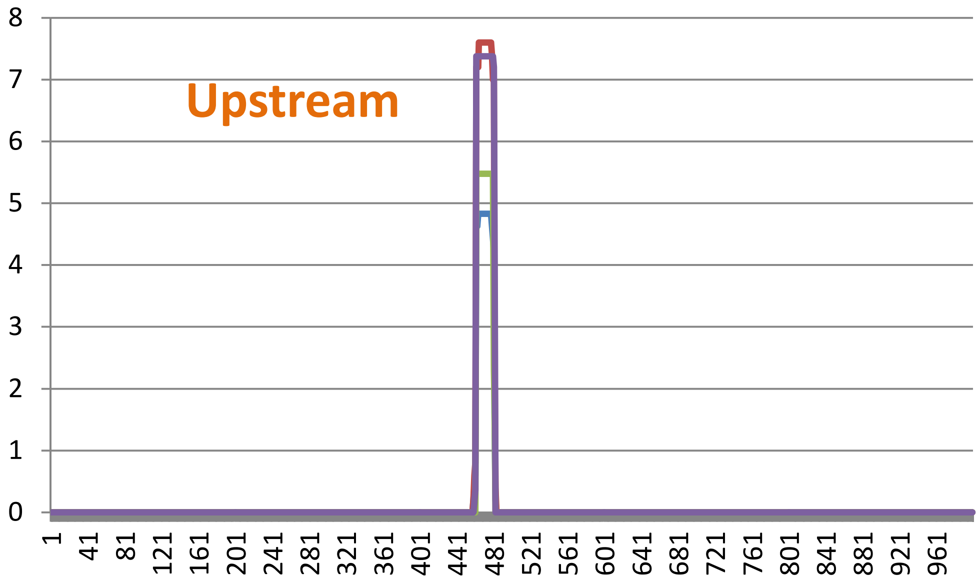

AT5G28465

This gene encodes a small protein and has either evidence of transcription or purifying selection.

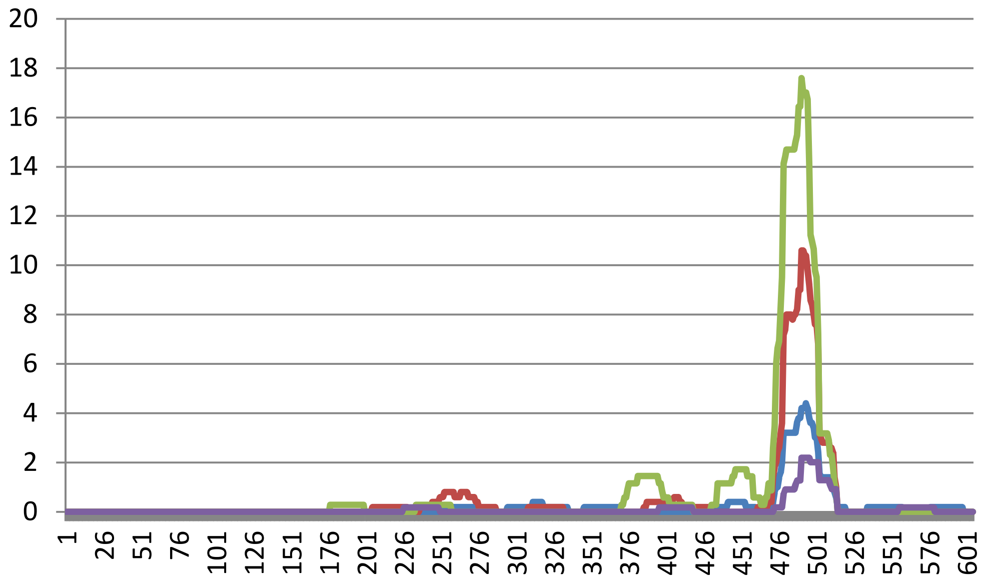

AT5G35930

AMP-dependent synthetase and ligase family protein

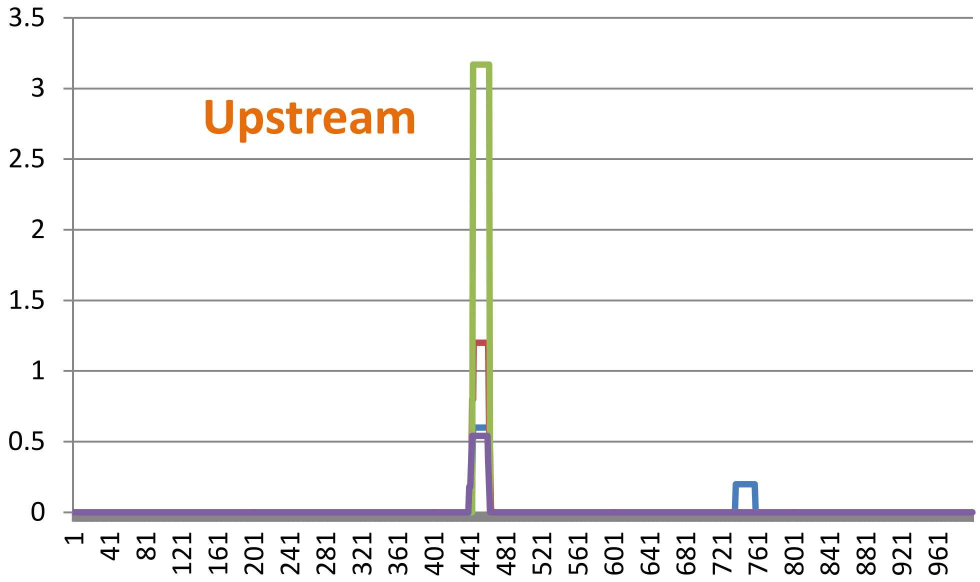

AT5G39645

Encodes a Defensin-like (DEFL) family protein

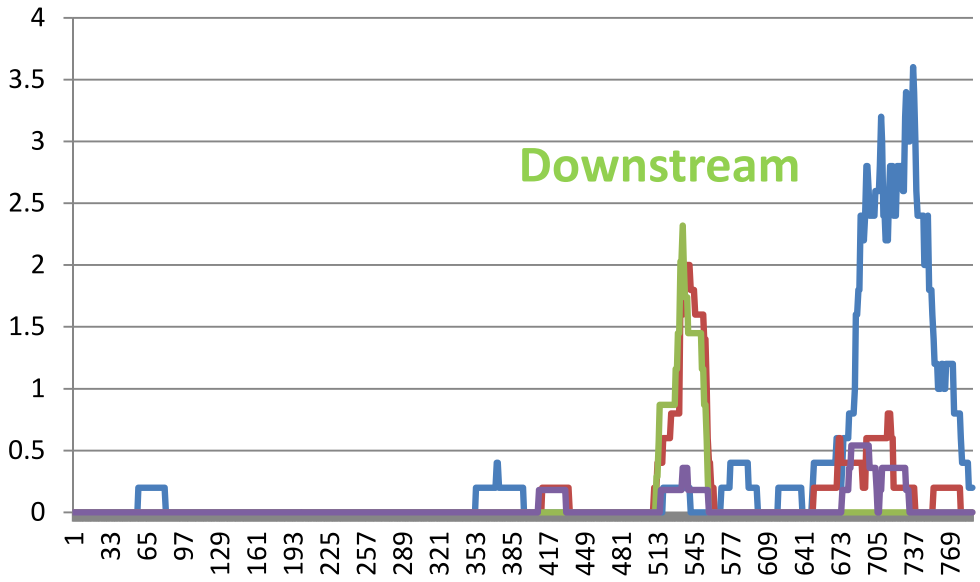

AT5G40320

Cysteine/Histidine-rich C1 domain family protein

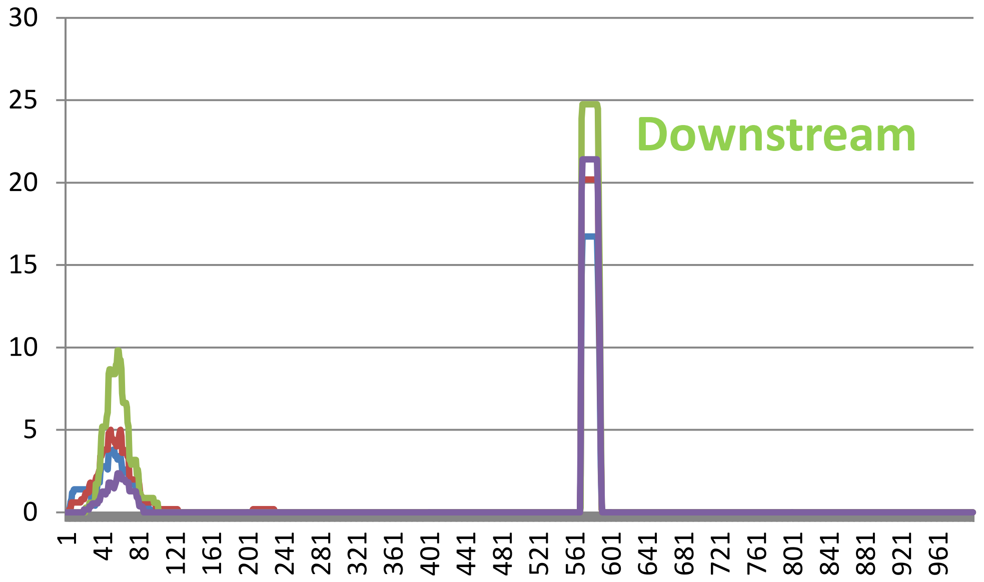

AT5G41330

BTB/POZ domain with WD40/YVTN repeat-like protein

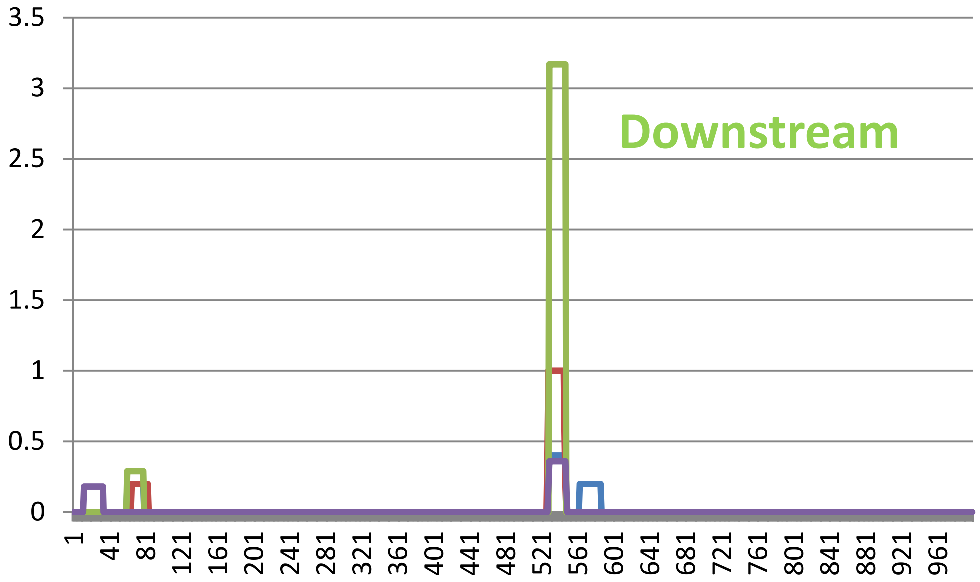

AT5G42146

Unknown protein

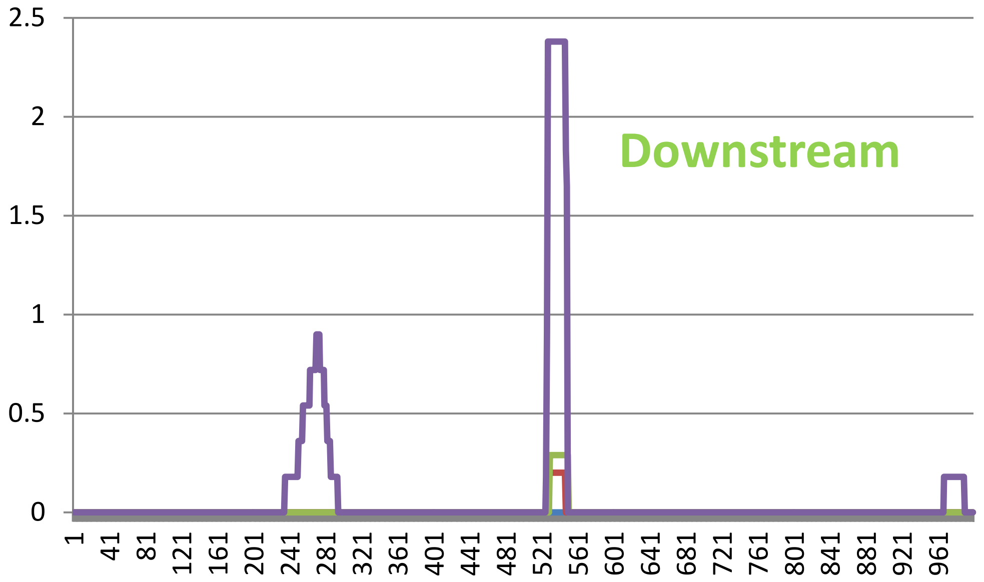

AT5G42620

Metalloendopeptidases; Zinc ion binding

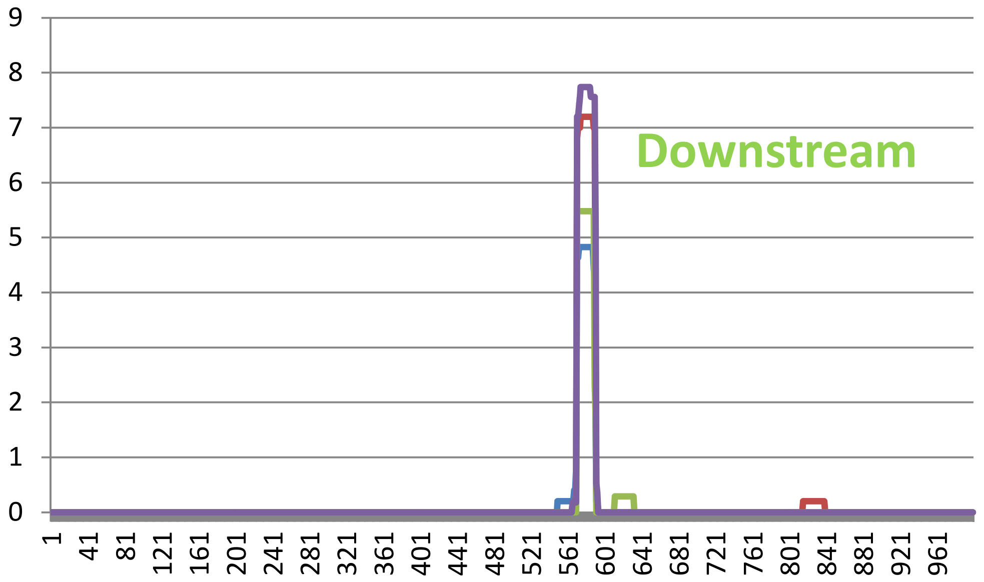

AT5G42970

Encodes subunit 4 of COP9 signalosome complex.

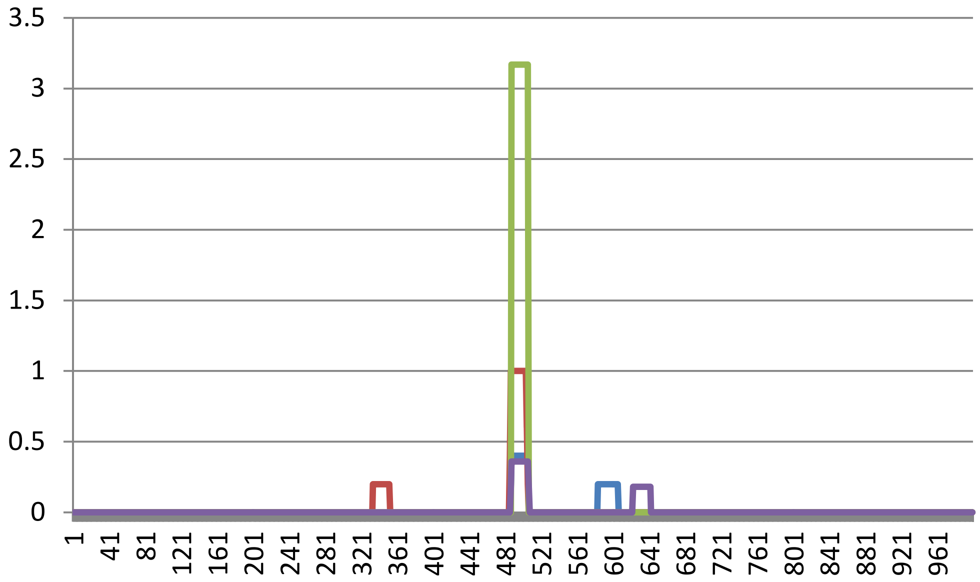

AT5G43180

Protein of unknown function, DUF599

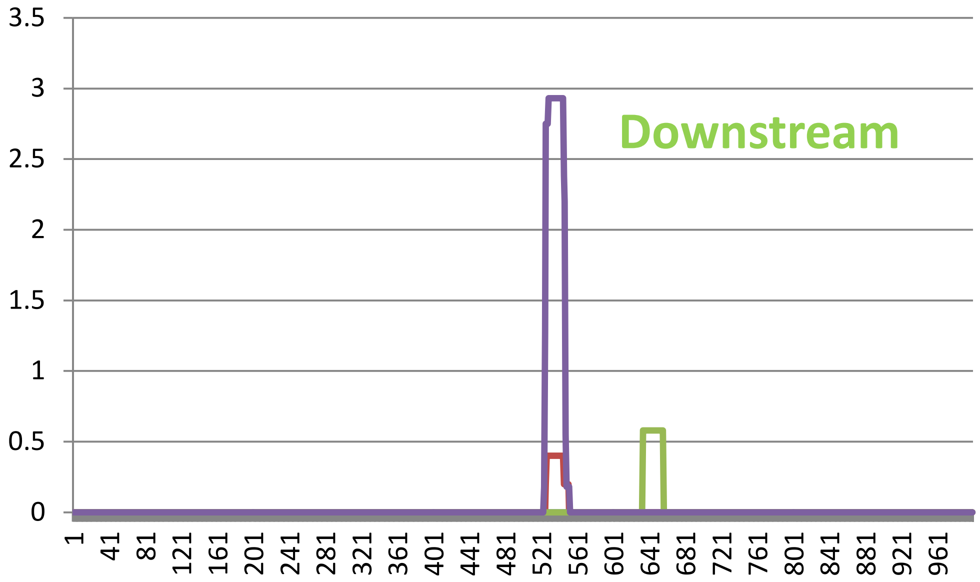

AT5G43500

Encodes a protein whose sequence is similar to actin-related proteins (ARPs) in other organisms.

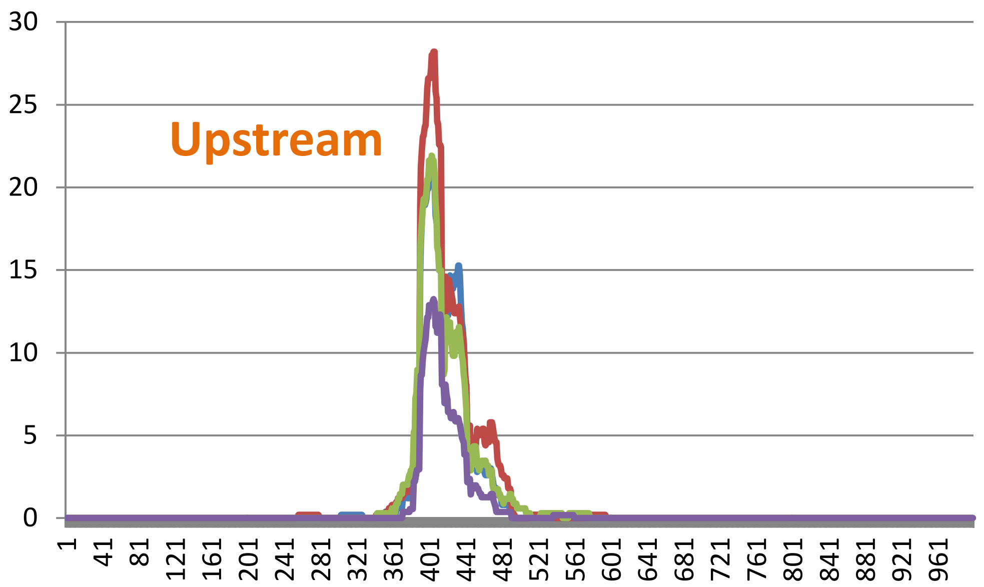

AT5G43810

Encodes Argonaute10, a member of the EIF2C (elongation initiation factor 2c)/Argonaute class of proteins. Required to establish the central-peripheral organization of the embryo apex. Along with WUS and CLV genes, controls the relative organization of central zone and peripheral zone cells in meristems. Acts in embryonic provascular tissue potentiating WUSCHEL function during meristem development in the embryo. AGO10 specifically sequesters miR166/165 to regulate shoot apical meristem development.

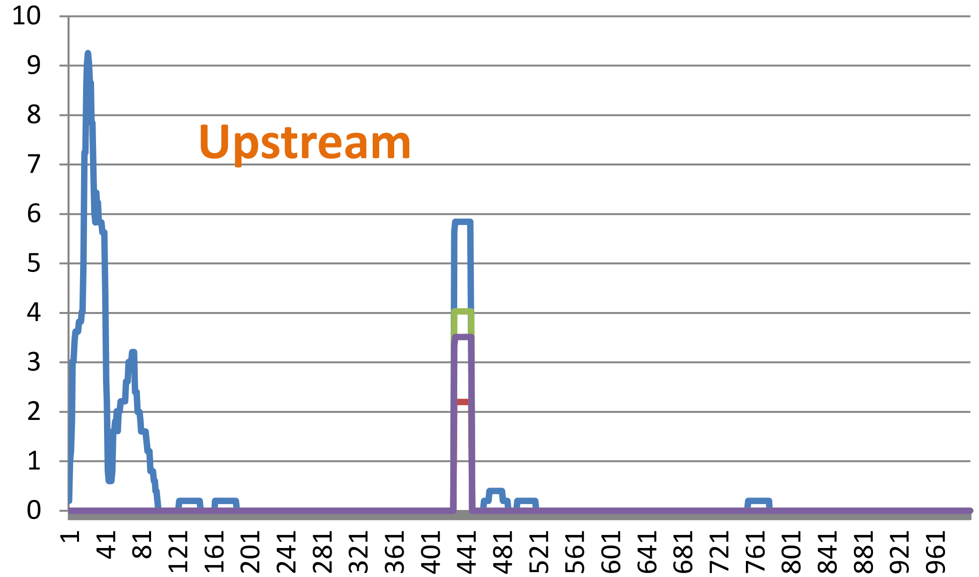

AT5G44930

Encodes a putative arabinosyltransferase.

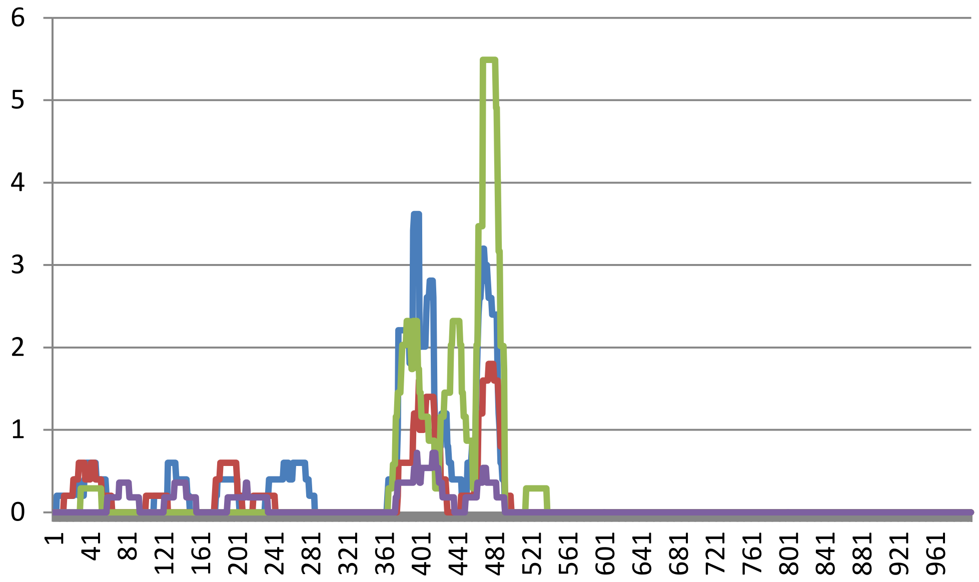

AT5G45428

Upstream open reading frames (uORFs) are small open reading frames found in the 5' UTR of a mature mRNA, and can potentially mediate translational regulation of the largest, or major, ORF (mORF).

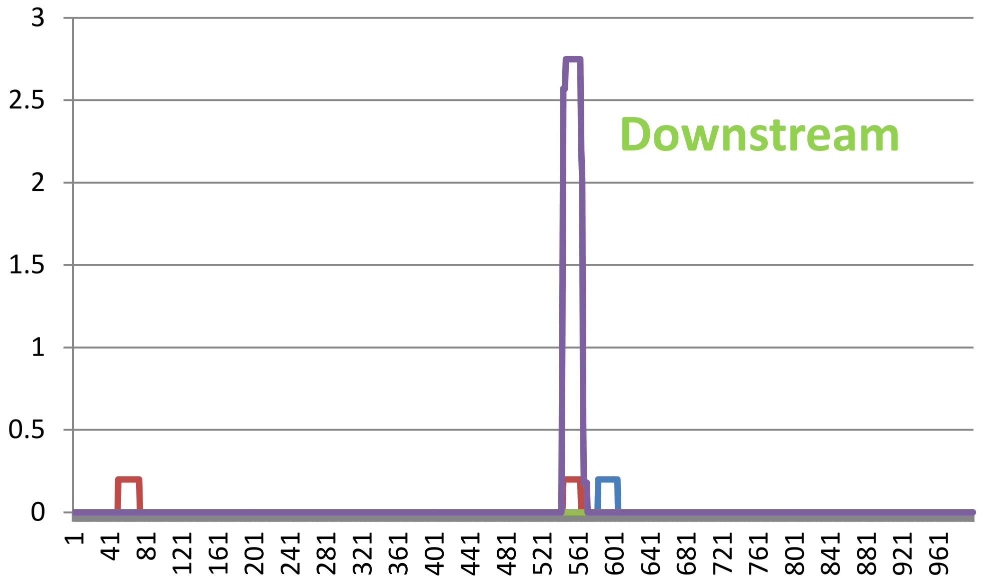

AT5G45430

Protein kinase superfamily protein

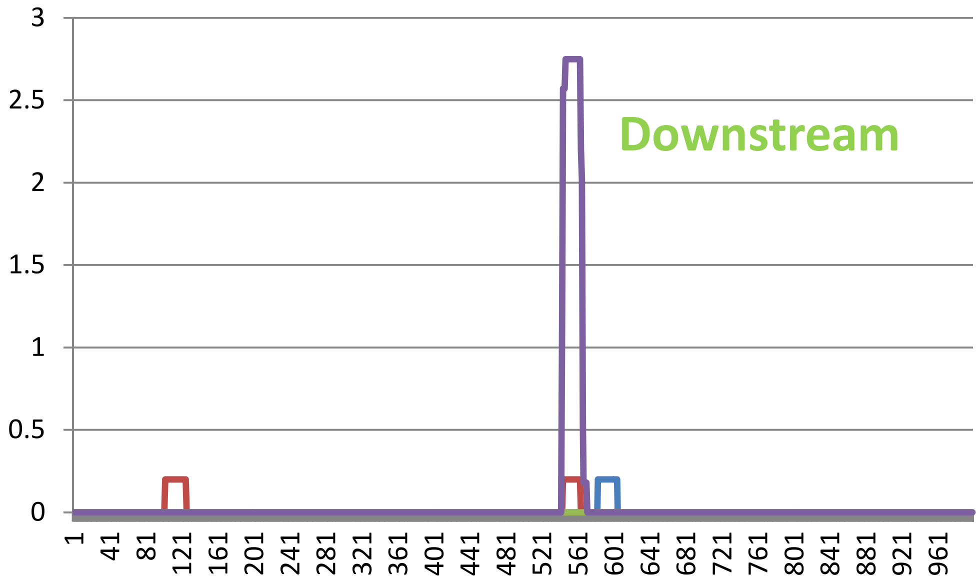

AT5G46930

Plant invertase/pectin methylesterase inhibitor superfamily protein

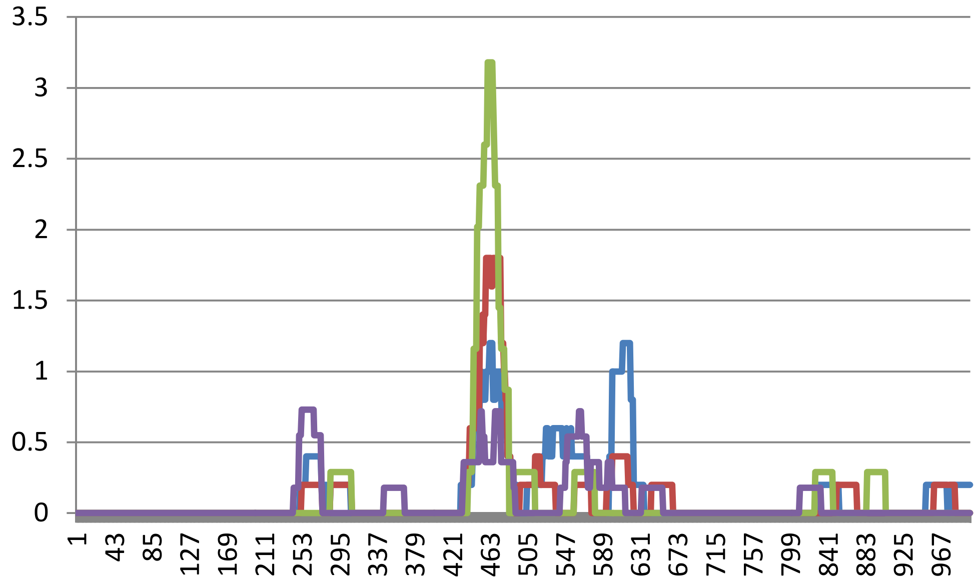

AT5G47110

Chlorophyll A-B binding family protein

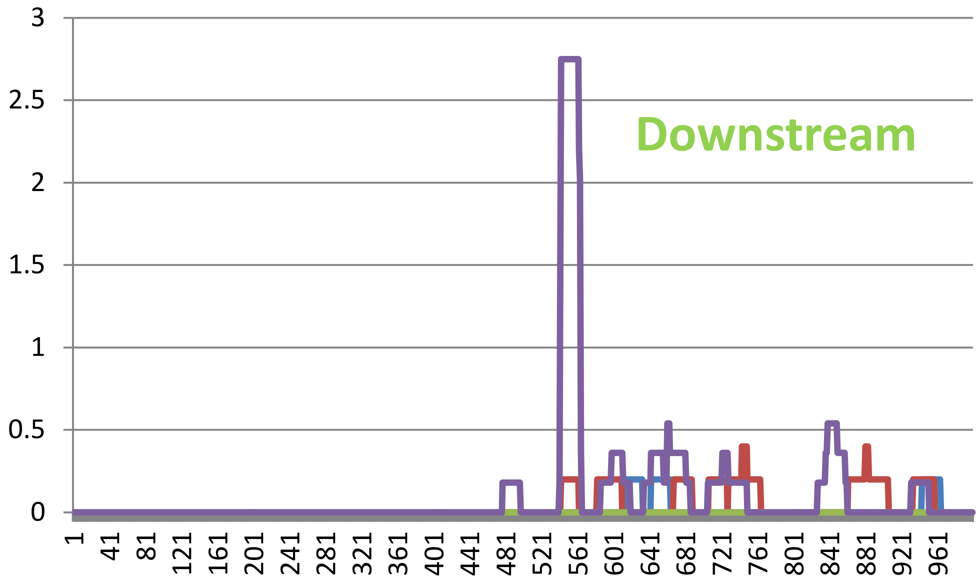

AT5G47260

ATP binding; GTP binding; nucleotide binding; nucleoside-triphosphatases

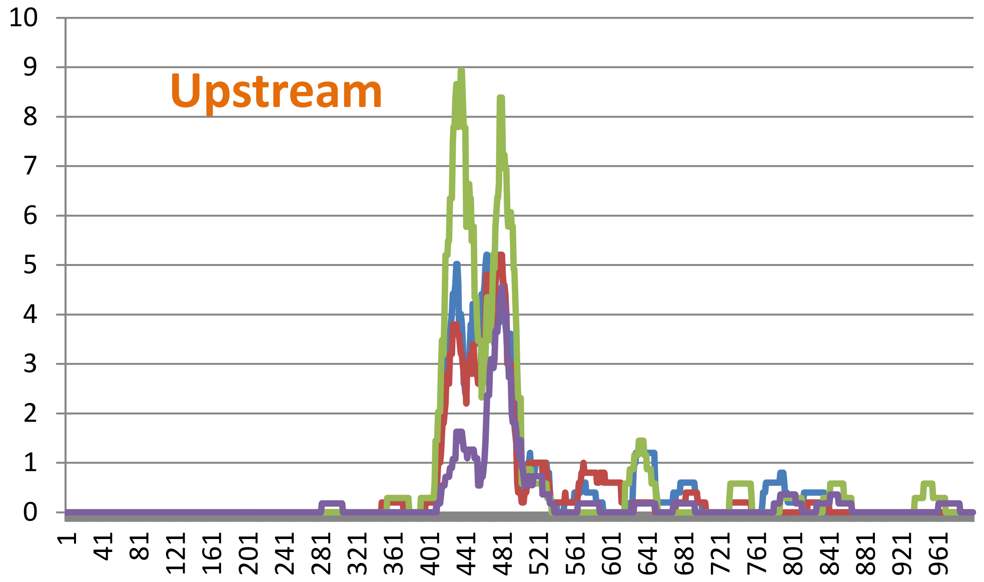

AT5G47880

Encodes a eukaryotic release factor 1 homolog.

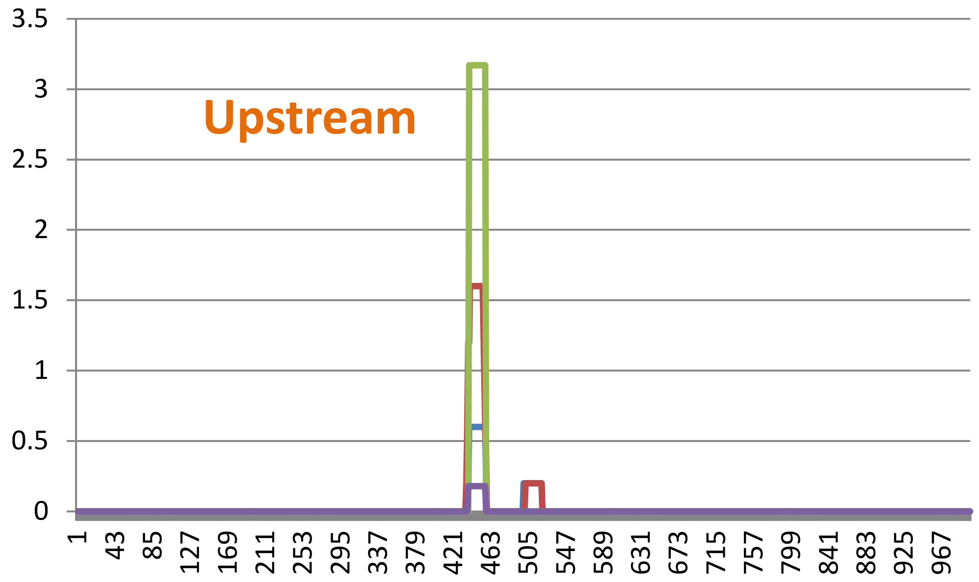

AT5G48000

Encodes a member of the CYP708A family of cytochrome P450 enzymes.

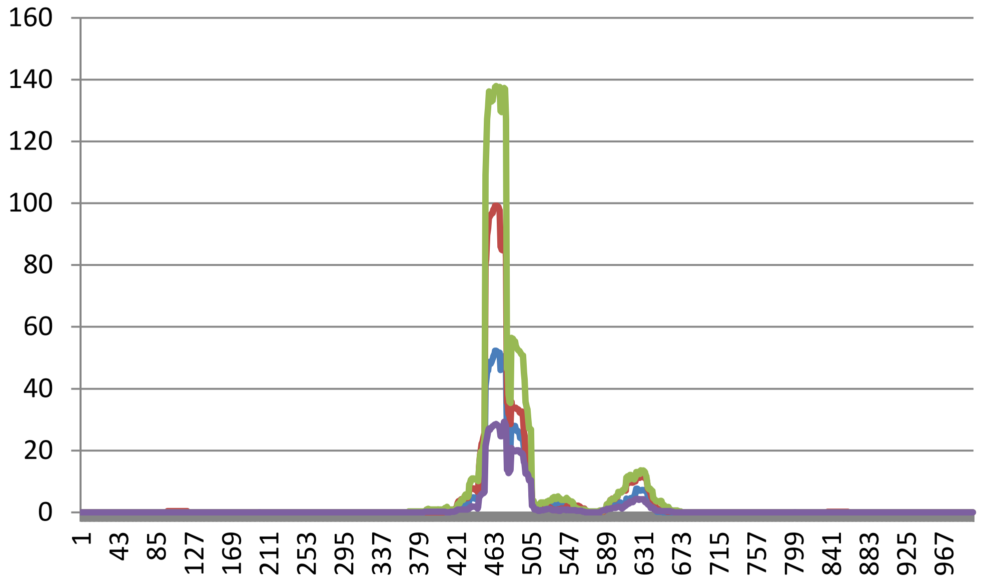

AT5G48280

Unknown protein

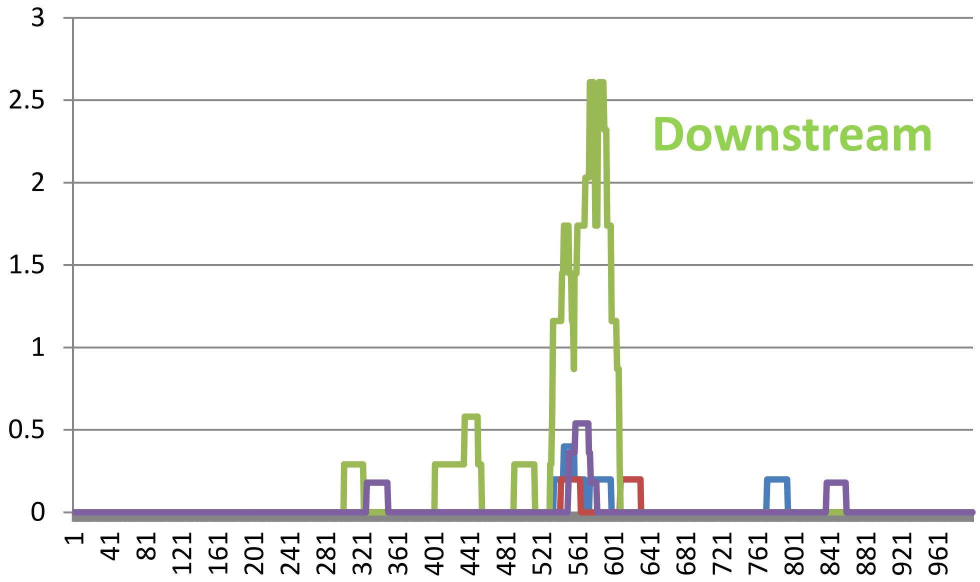

AT5G49240

Member of Response Regulator: Pseudo

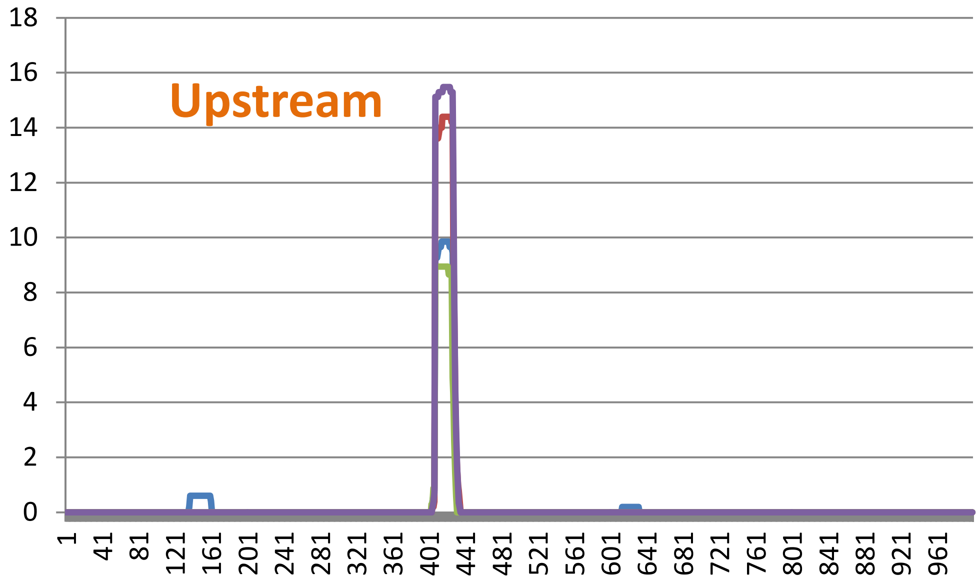

AT5G59780

Encodes a putative transcription factor (MYB59).

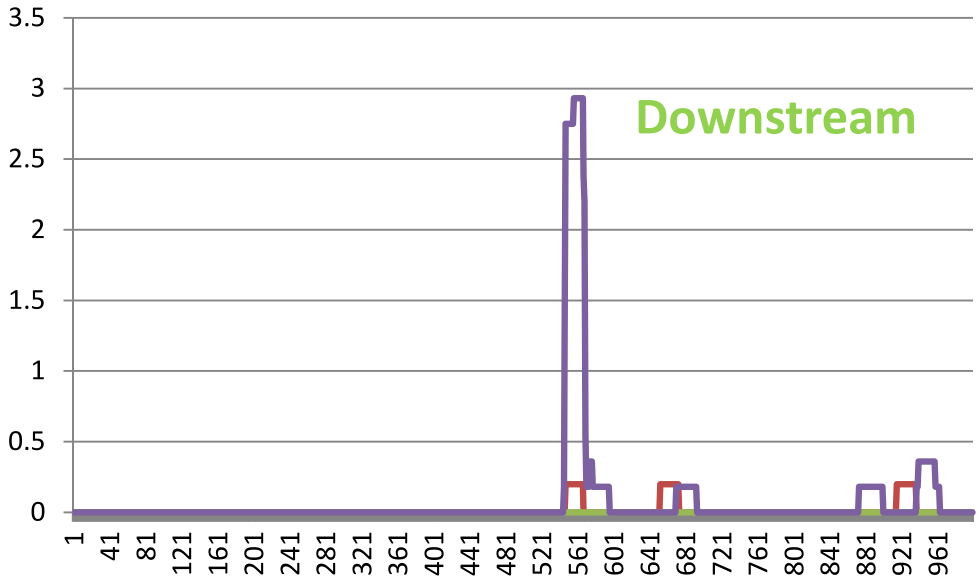

AT5G61420

Encodes a nuclear localized member of the MYB transcription factor family. Involved in positive regulation of aliphatic glucosinolate biosynthesis. Expression is induced by touch, wounding and glucose.

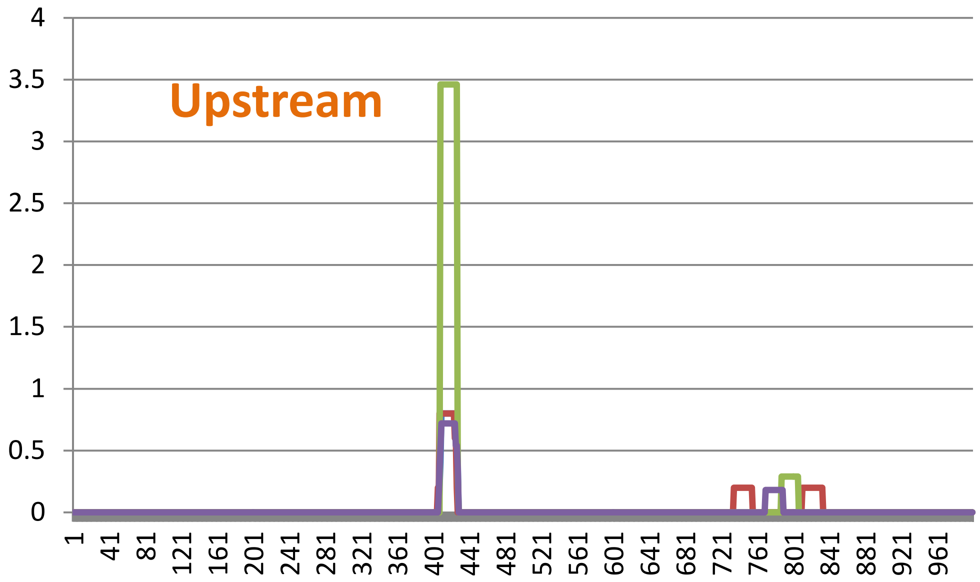

AT5G61510

GroES-like zinc-binding alcohol dehydrogenase family protein

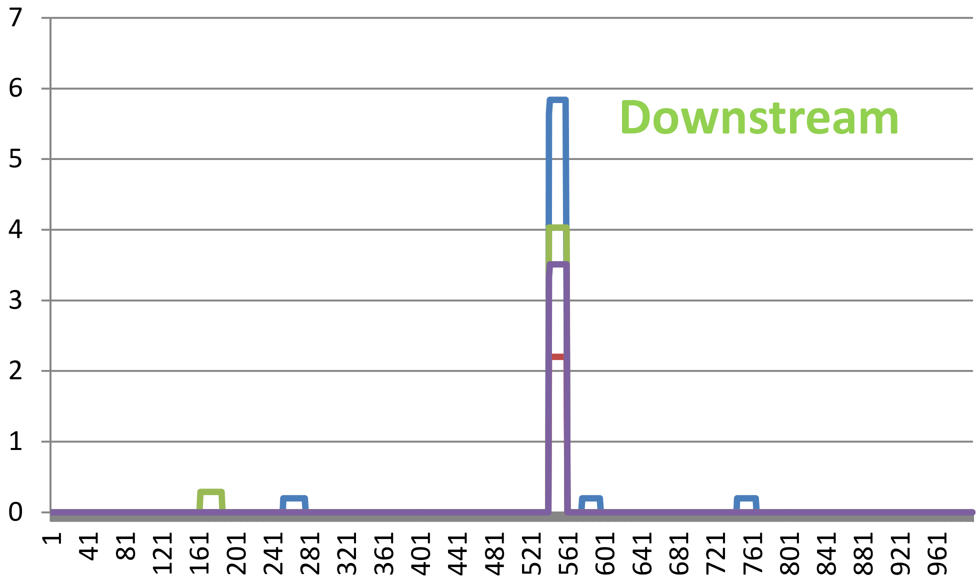

AT5G65050

Originally published as Agamous like MADS-box protein AGL31. One of a group of MADS box genes involved in control of flowering time. Four variant sequences have been identified for this locus but have not been characterized for differences in expression pattern and/or function.

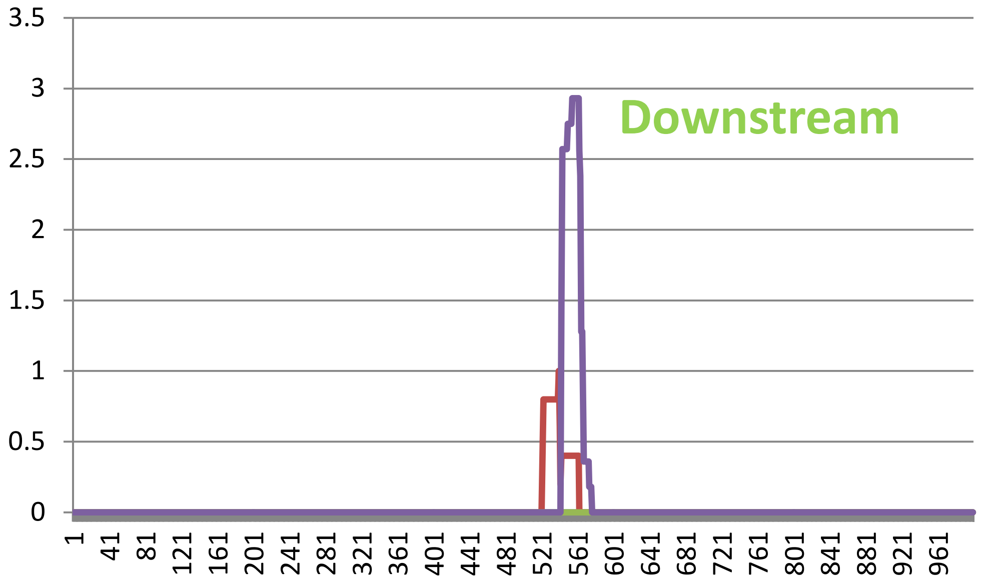

AT5G67110

Encodes a myc/bHLH transcription factor-like protein. Gene product is involved in fruit dehiscence. Mutant siliques fail to dehisce.

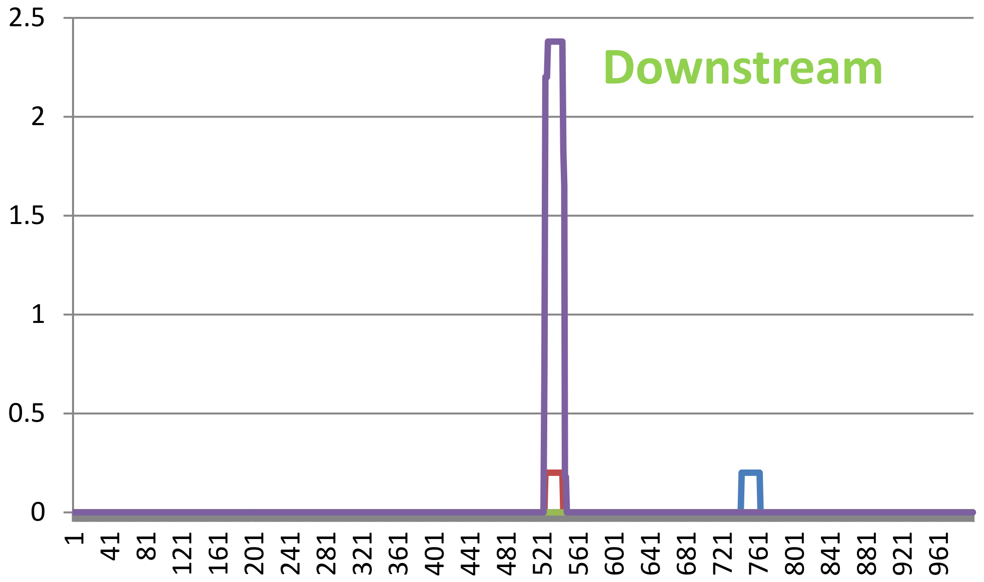

ATCG00050

Homologous to the bacterial ribosomal protein S16

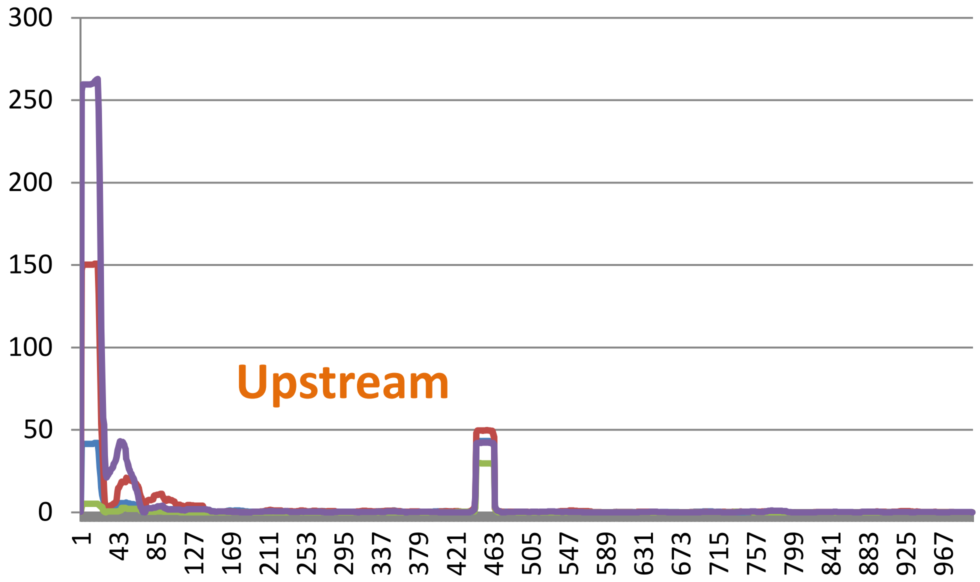

ATCG00065

Chloroplast gene encoding ribosomal protein s12. The gene is located in three distinct loci on the chloroplast genome and is transpliced to make one transcript.

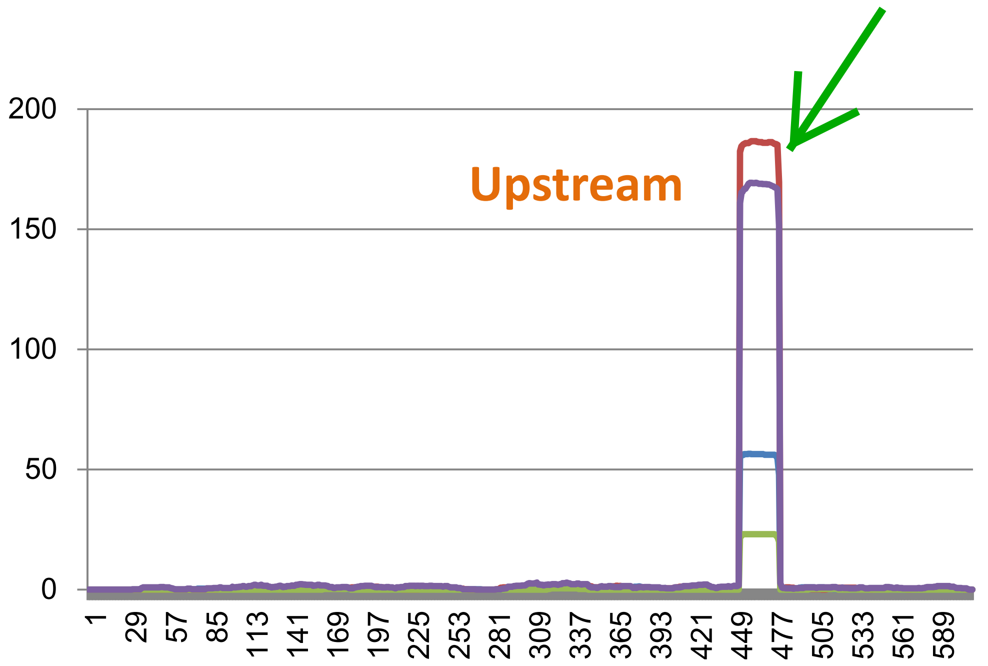

ATCG00120

Encodes the ATPase alpha subunit, which is a subunit of ATP synthase and part of the CF1 portion which catalyzes the conversion of ADP to ATP using the proton motive force. This complex is located in the thylakoid membrane of the chloroplast.

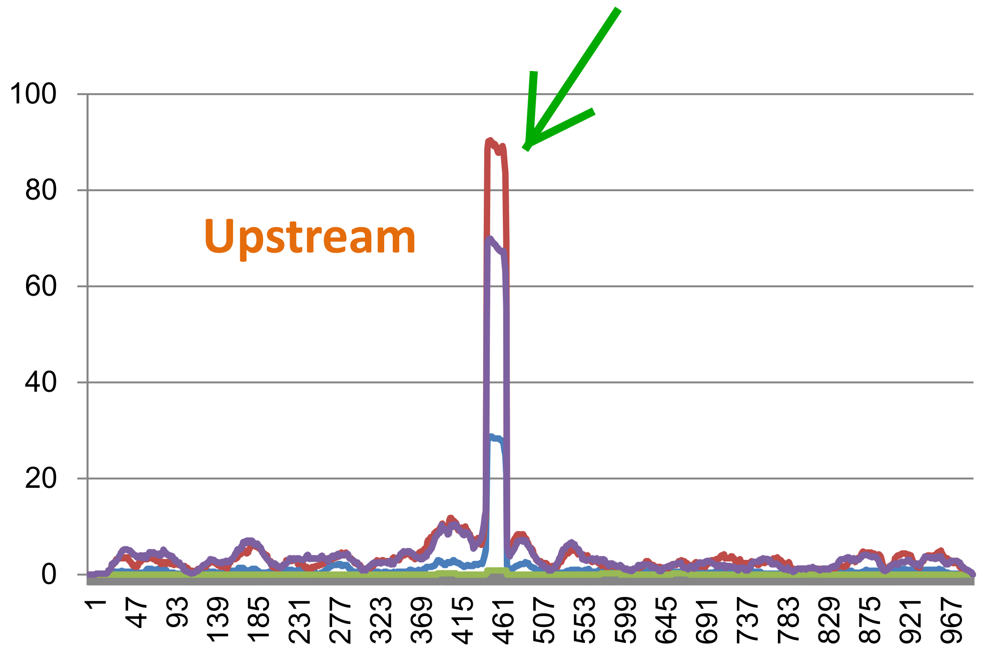

ATCG00140

ATPase III subunit

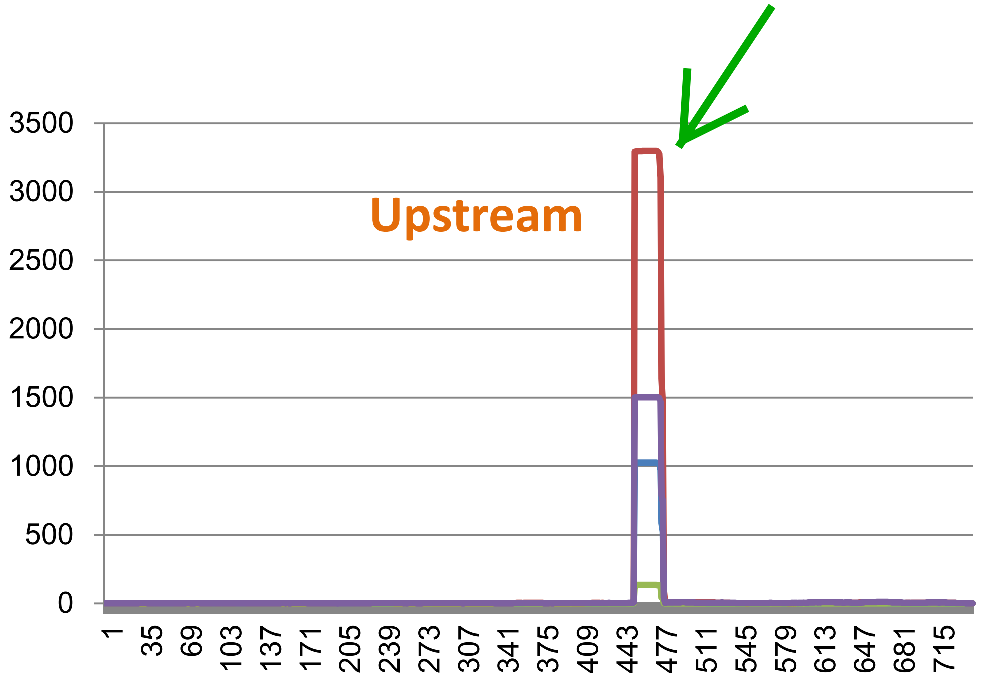

ATCG00150

Encodes a subunit of ATPase complex CF0, which is a proton channel that supplies the proton motive force to drive ATP synthesis by CF1 portion of the complex.

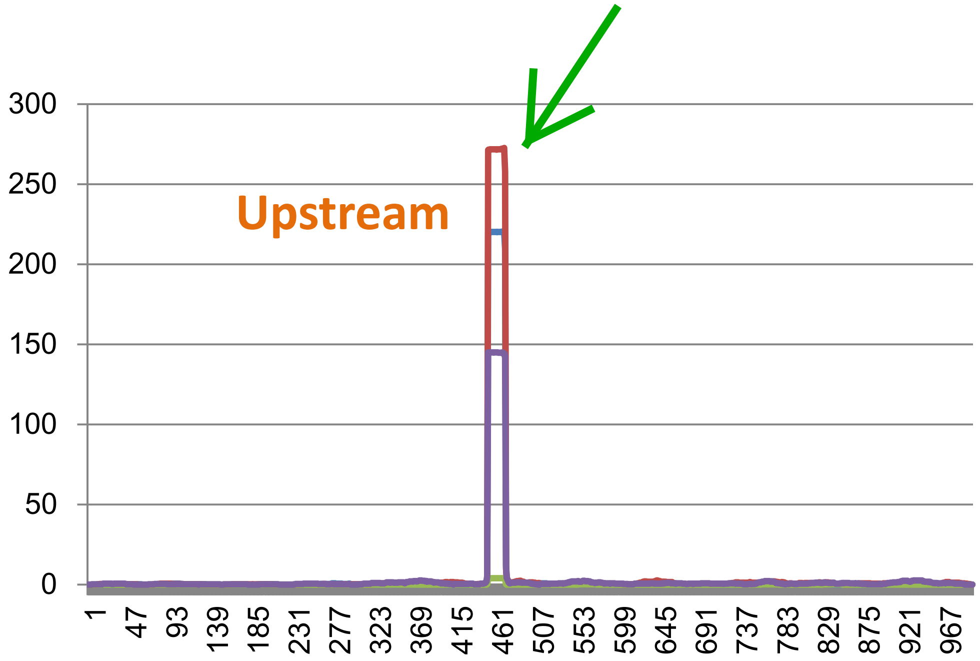

ATCG00170

RNA polymerase beta' subunit-2

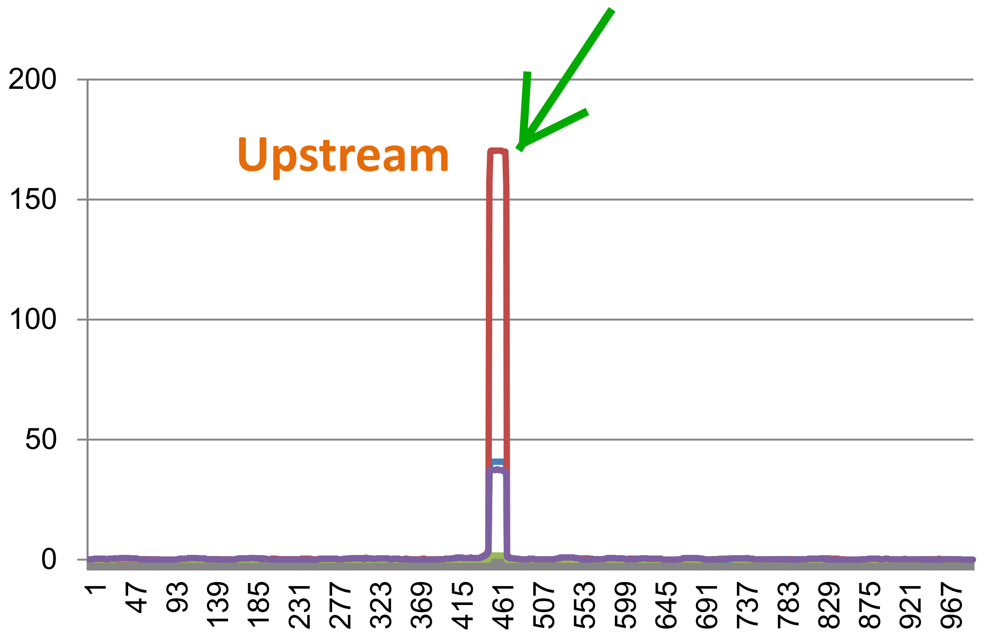

ATCG00280

Chloroplast gene encoding a CP43 subunit of the photosystem II reaction center.
Promoter contains a blue-light responsive element.

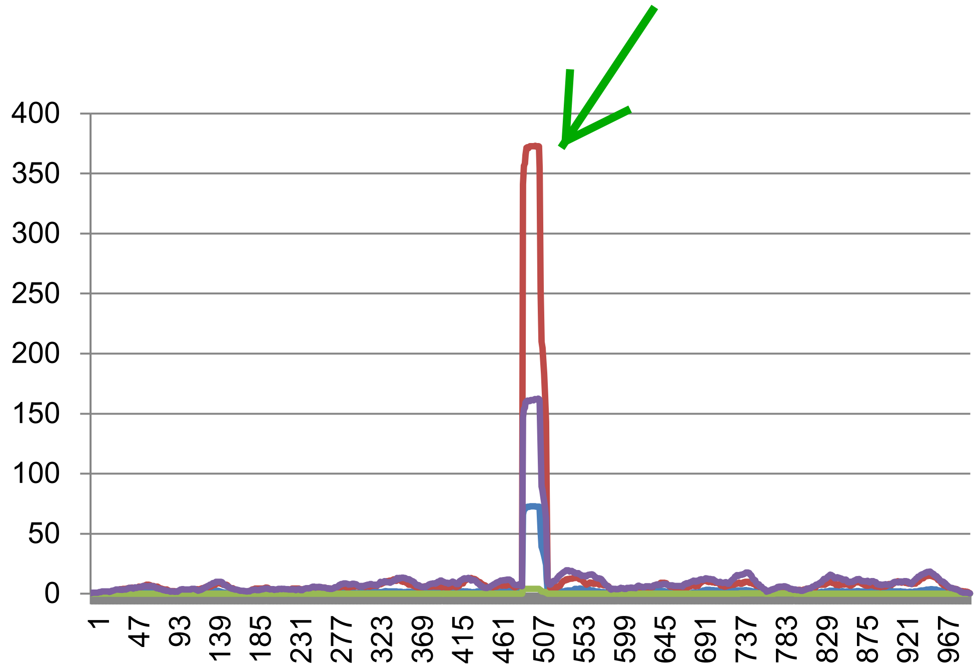

ATCG00500

Encodes the carboxytransferase beta subunit of the Acetyl-CoA carboxylase (ACCase) complex in plastids. This complex catalyzes the carboxylation of acetyl-CoA to produce malonyl-CoA, the first committed step in fatty acid synthesis.

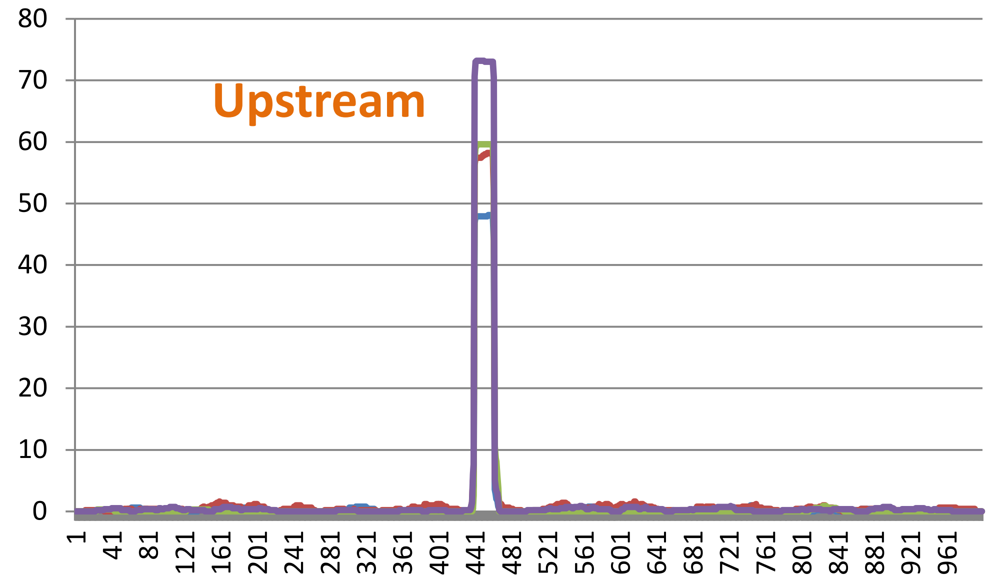

ATCG00540

Encodes cytochrome f apoprotein; involved in photosynthetic electron transport chain; encoded by the chloroplast genome and is transcriptionally repressed by a nuclear gene HCF2.

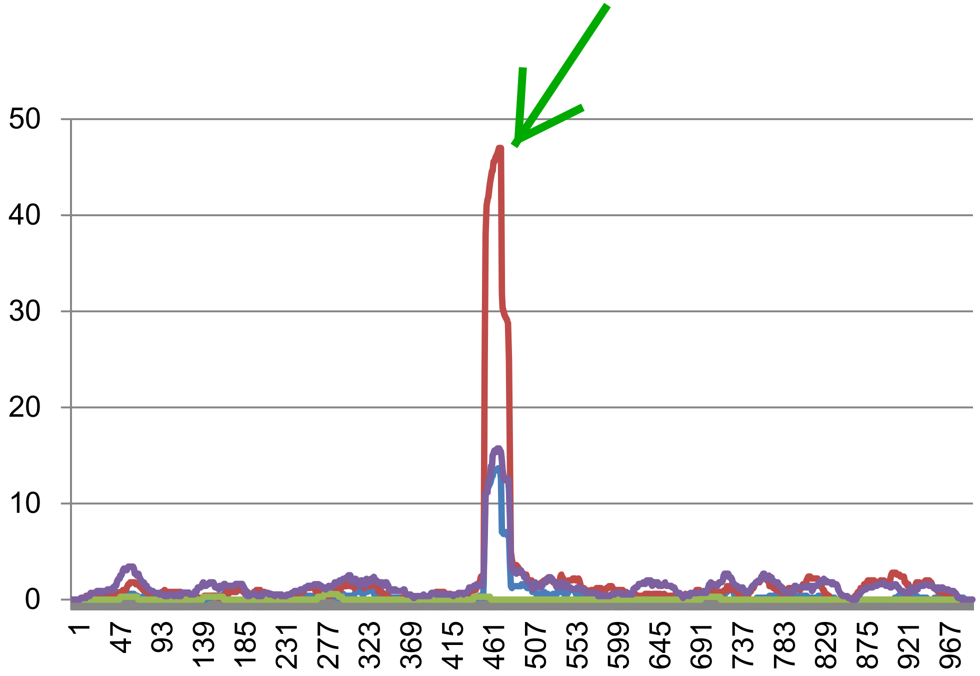

ATCG00590

Hypothetical protein

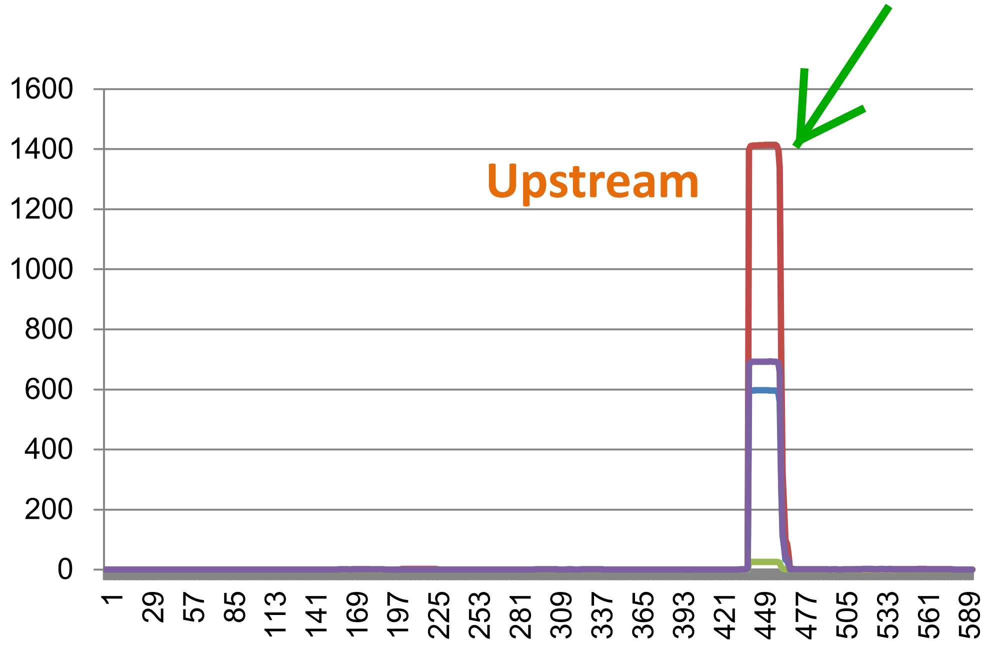

ATCG00670

Encodes the only ClpP (caseinolytic protease) encoded within the plastid genome.

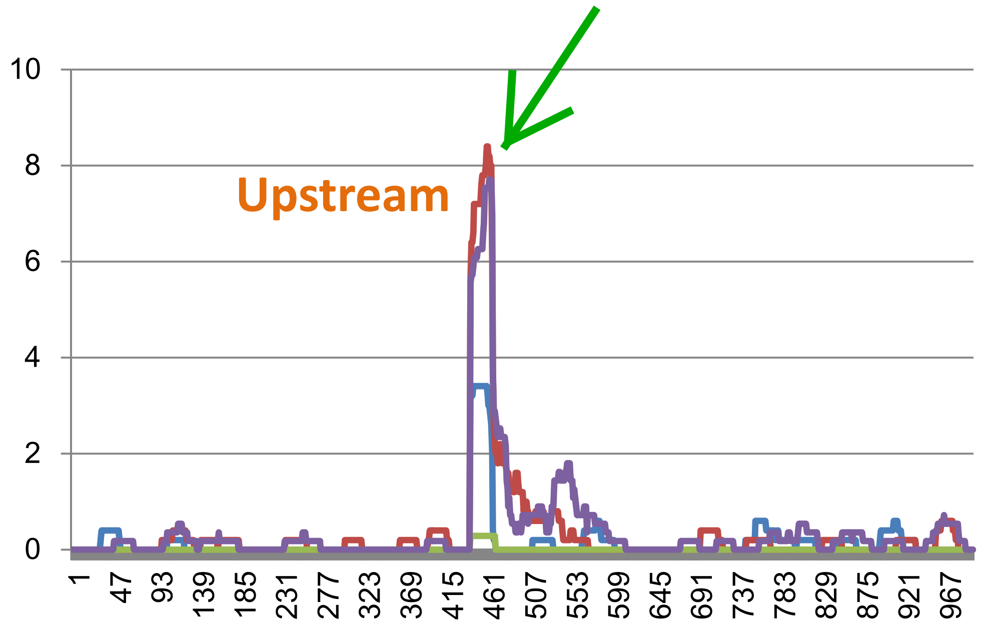

ATCG00720

Encodes the cytochrome b(6) subunit of the cytochrome b6f complex.

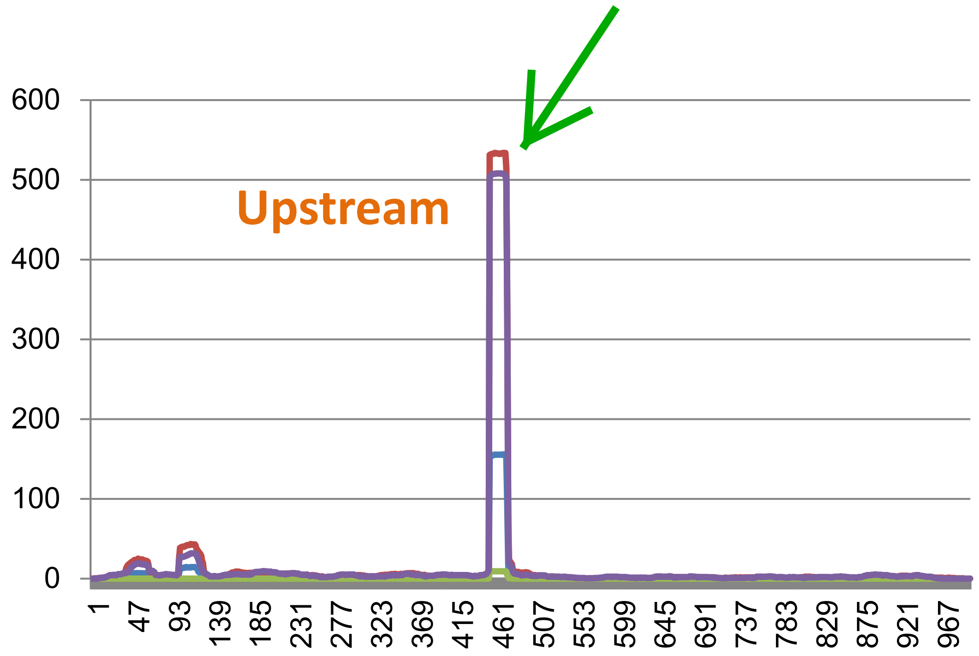

ATCG00830

Encodes a chloroplast ribosomal protein L2, a constituent of the large subunit of the ribosomal complex

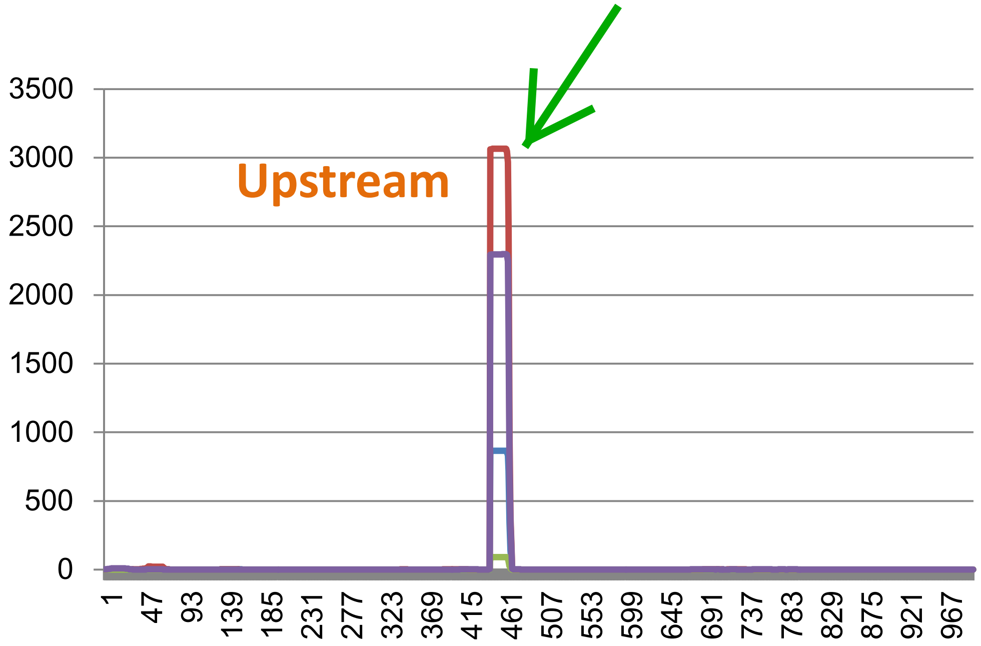

ATCG00870

Hypothetical protein

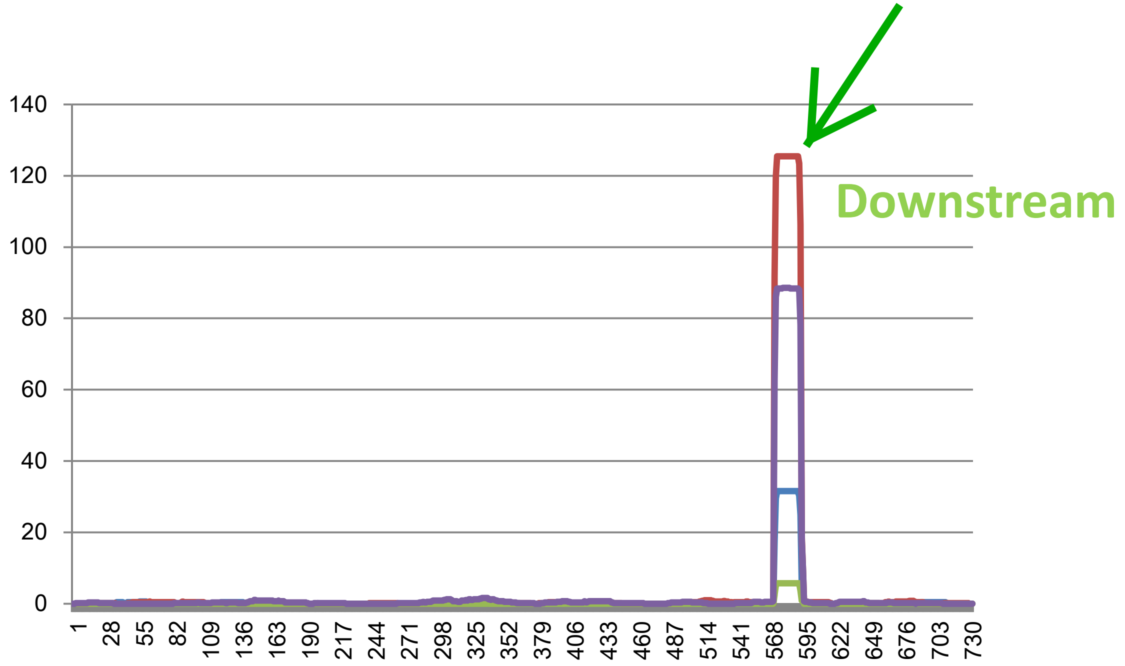

ATCG01100

NADH dehydrogenase ND1

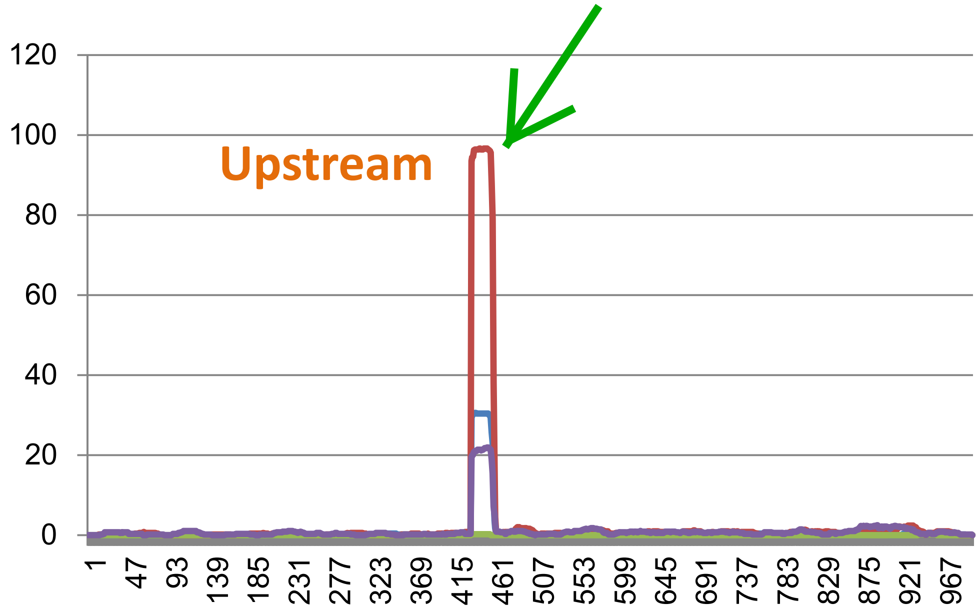

ATCG01120

Encodes a chloroplast ribosomal protein S15, a constituent of the small subunit of the ribosomal complex

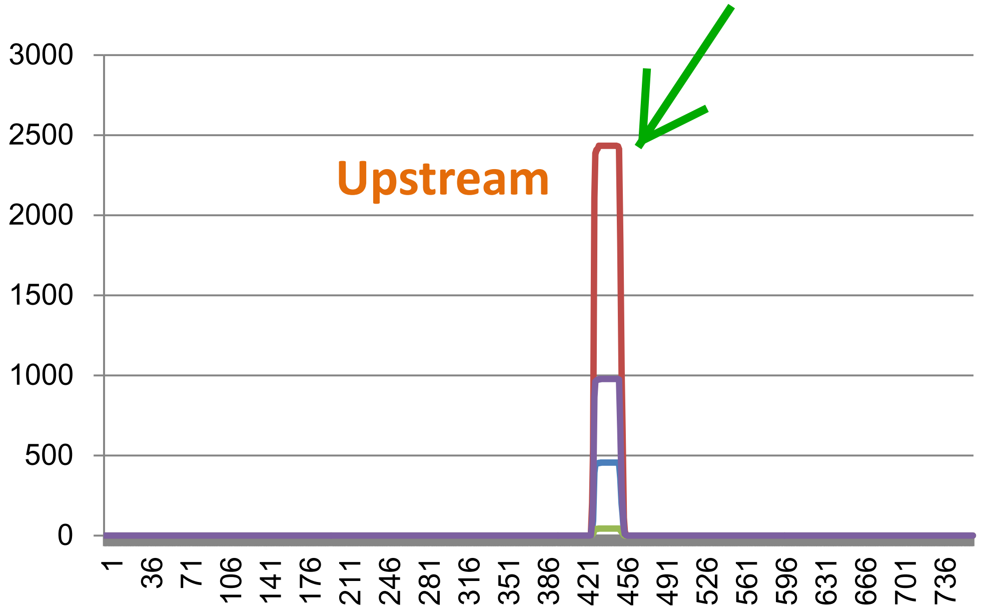

ATCG01270

Hypothetical protein

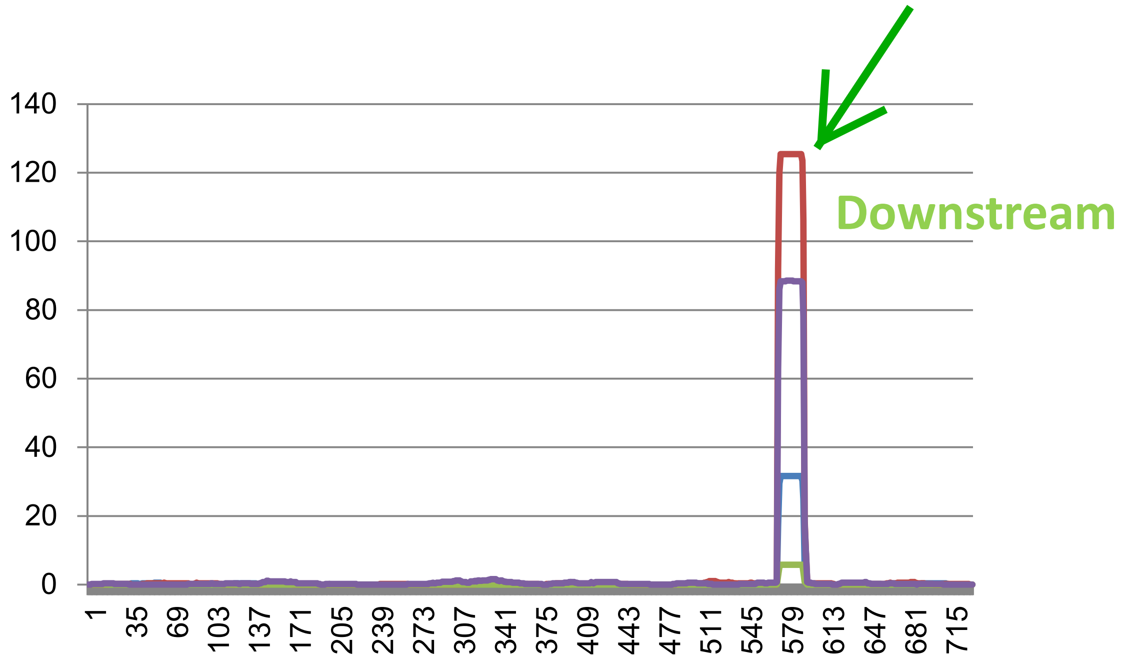

ATCG01310

Encodes a chloroplast ribosomal protein L2, a constituent of the large subunit of the ribosomal complex

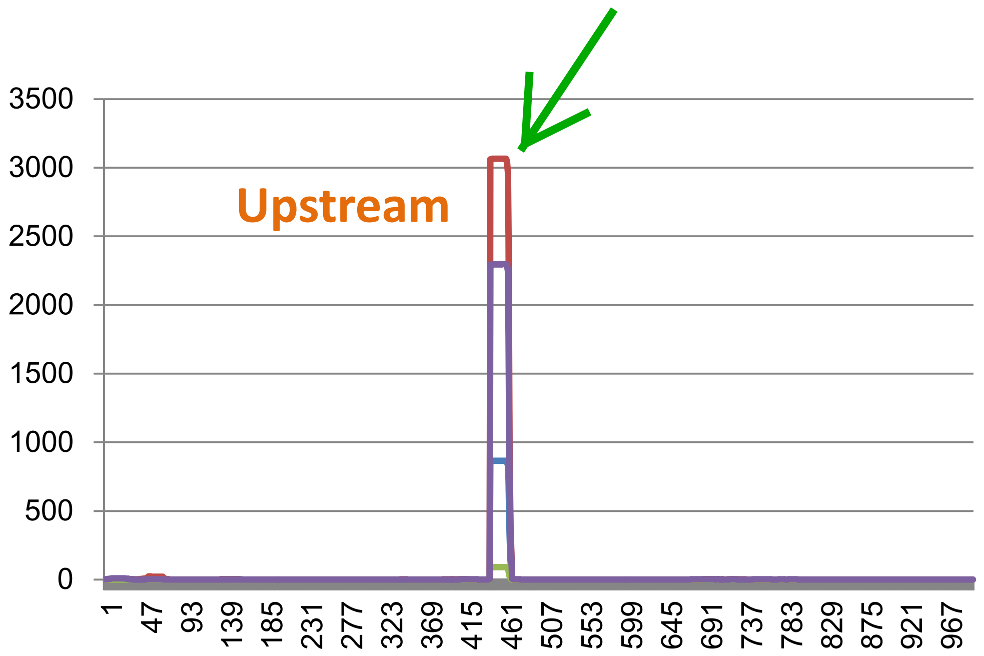

ATMG00570

Encodes a protein of unknown function. The transcript has extensive RNA editing at the 3' end.

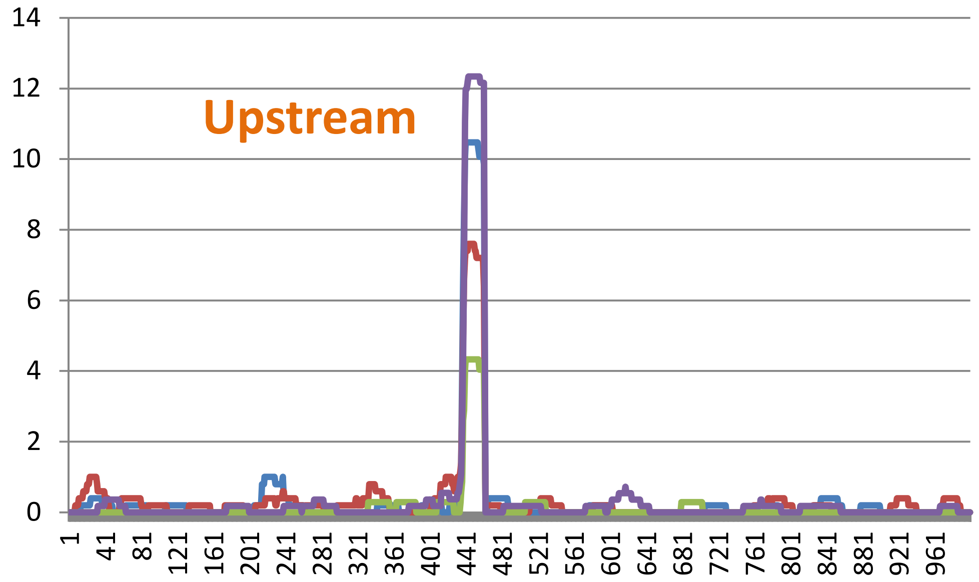

ATMG00690

Hypothetical protein

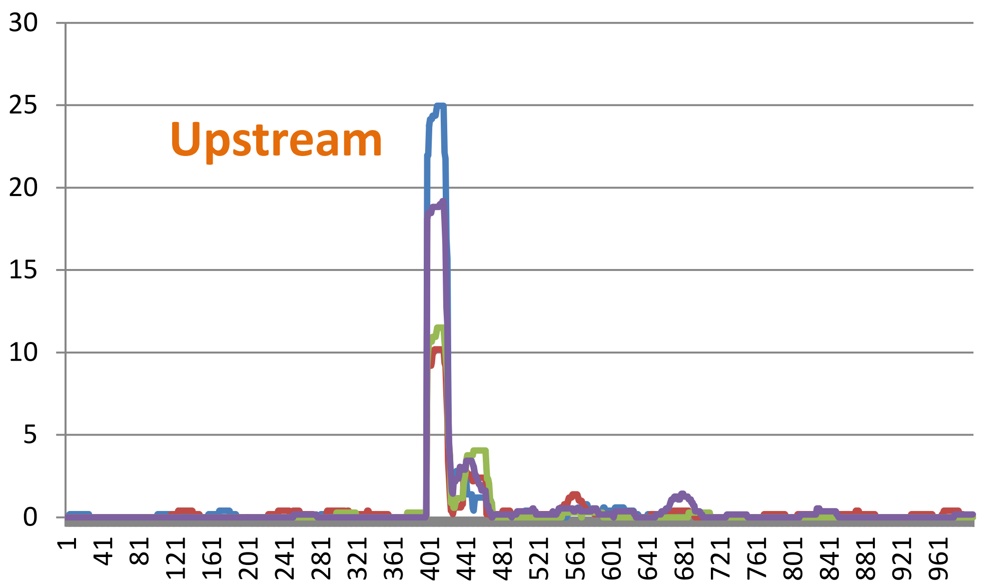

ATMG00890

Hypothetical protein

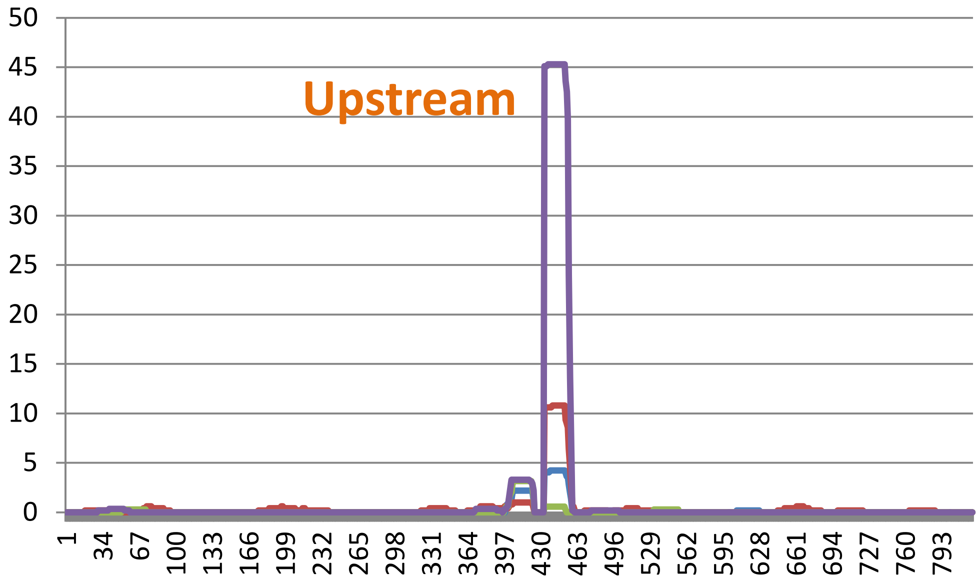

ATMG01350

Hypothetical protein

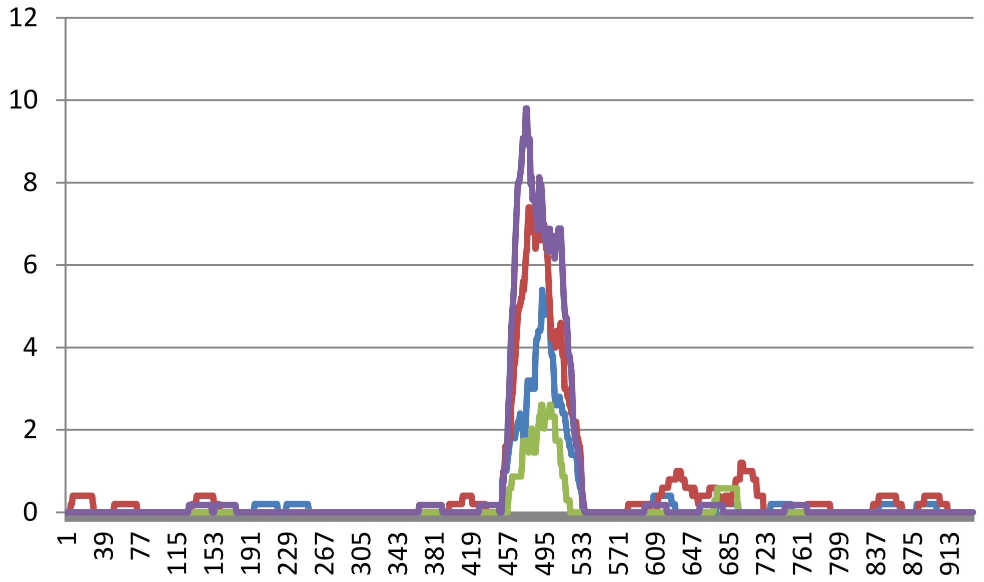

Supplement: S1 Fig — For the chloroplast genes, sRNAs dominantly detected in leaves and seedlings were marked by green arrows. (PDF) [file pone.0169212.s001.pdf]
