# Supplementary material for: Transcriptome-Wide Discovery of PASRs (Promoter-Associated Small RNAs) and TASRs (Terminus-Associated Small RNAs) in Arabidopsis thaliana
Source: PLoS One. 2017 Jan 3;12(1):e0169212. doi: 10.1371/journal.pone.0169212 (PMC5207706; doi:10.1371/journal.pone.0169212)

Figure S2 PASR peaks identified on the antisense strands of the protein-coding genes of *Arabidopsis*. For each plot, x axis measures the position of the antisense strand, and y axis measures the abundance (in RPM, reads per million) of sRNAs. For the chloroplast genes, sRNAs dominantly detected in leaves and seedlings were marked by green arrows.

AT1G01073

Unknown protein

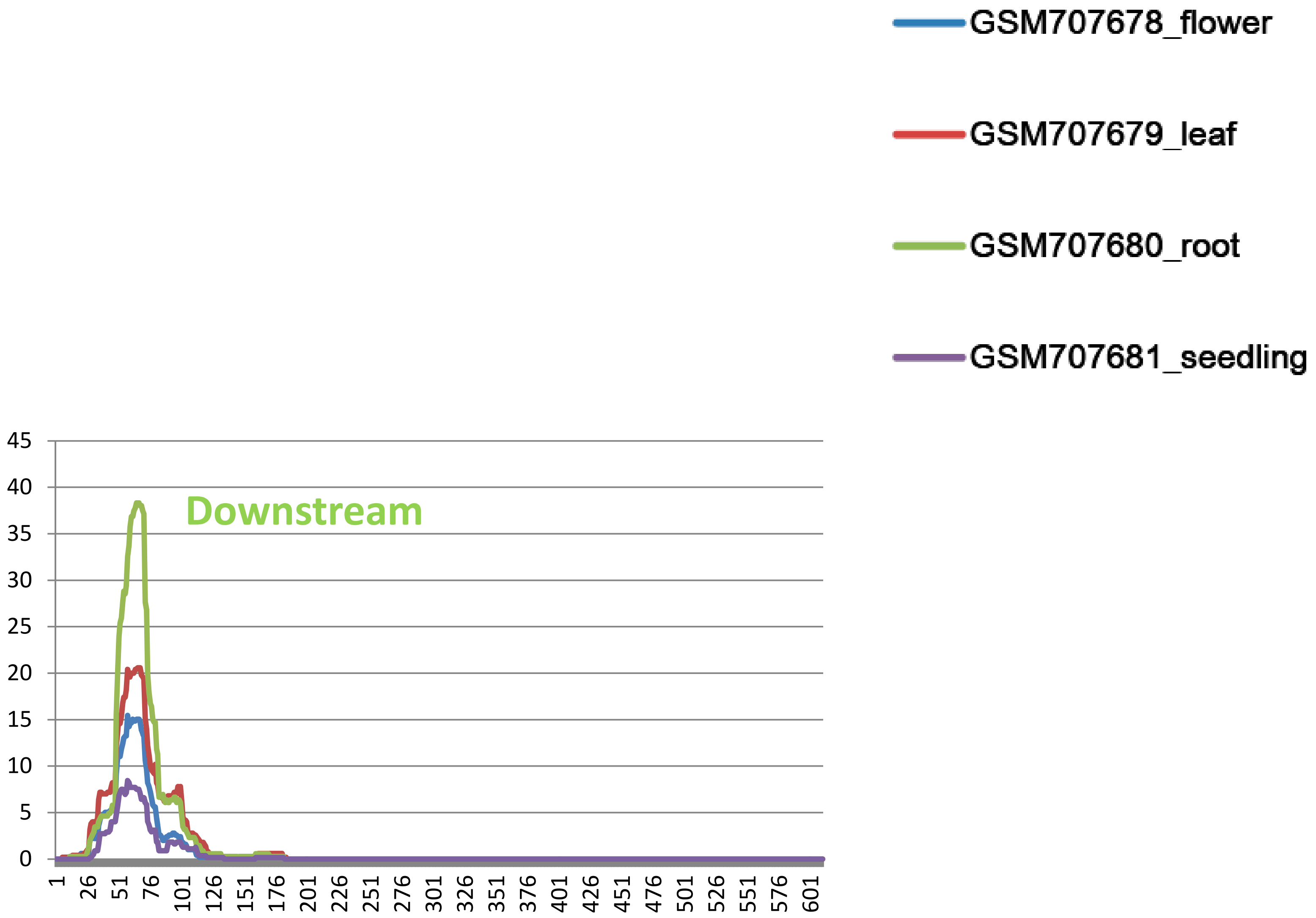

AT1G02475

Polyketide cyclase/dehydrase and lipid transport superfamily protein

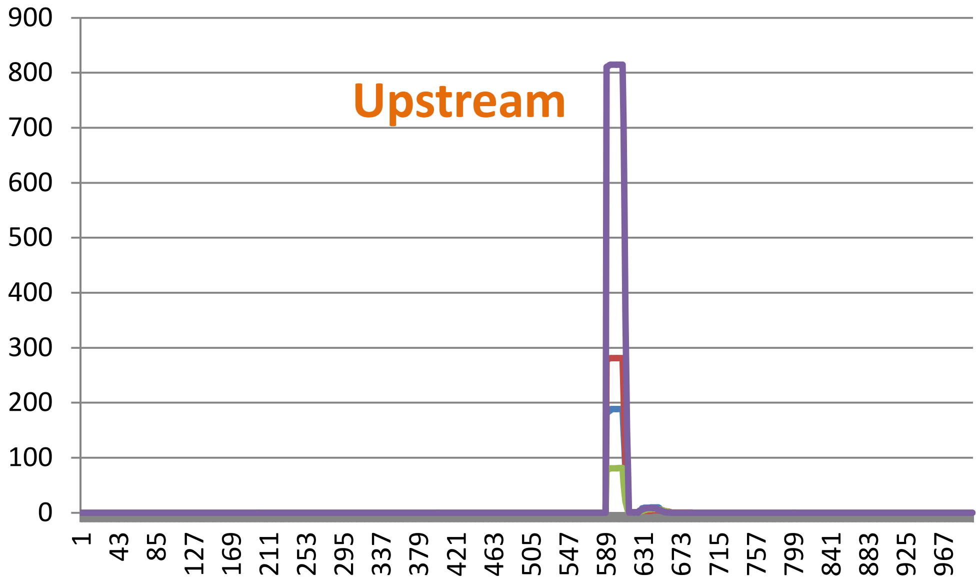

AT1G03810

Nucleic acid-binding, OB-fold-like protein

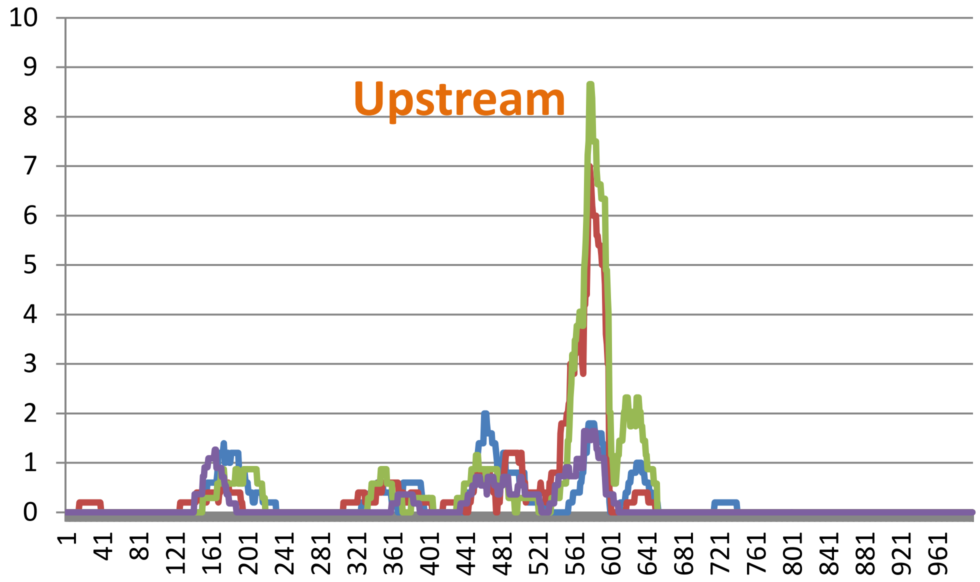

AT1G04310

Encodes an ethylene receptor related to bacterial two-component histidine kinases

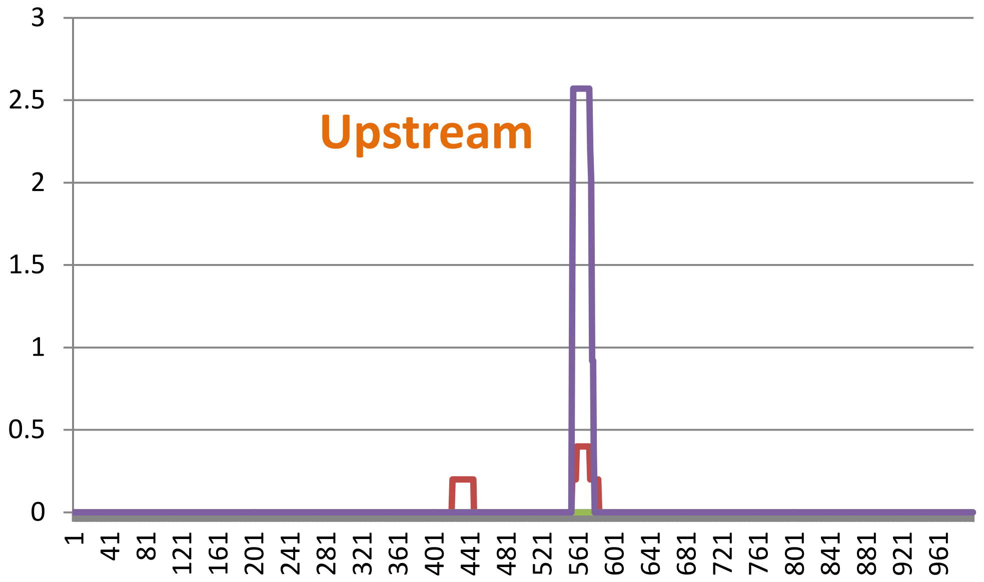

AT1G07660

Histone superfamily protein

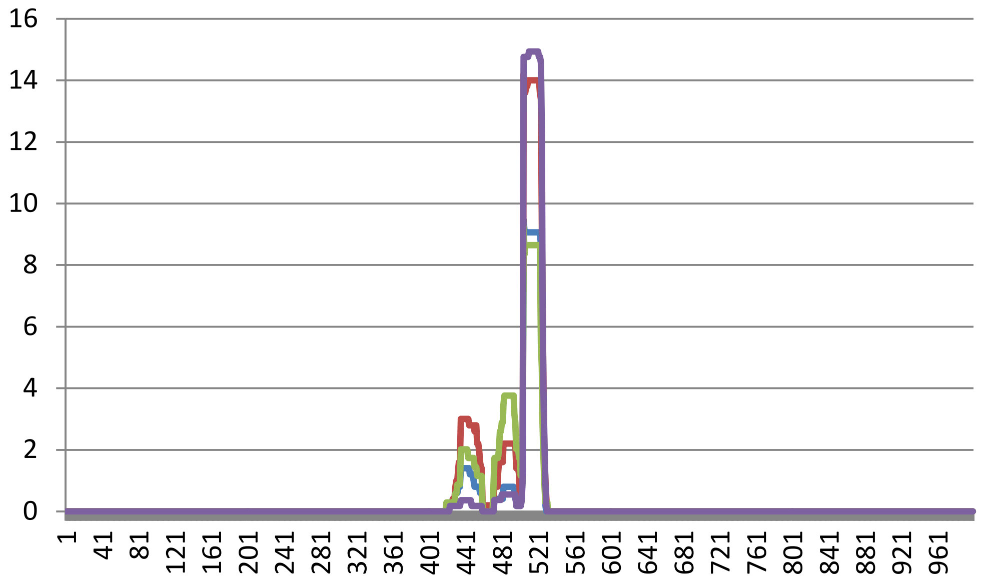

AT1G08940

Phosphoglycerate mutase family protein

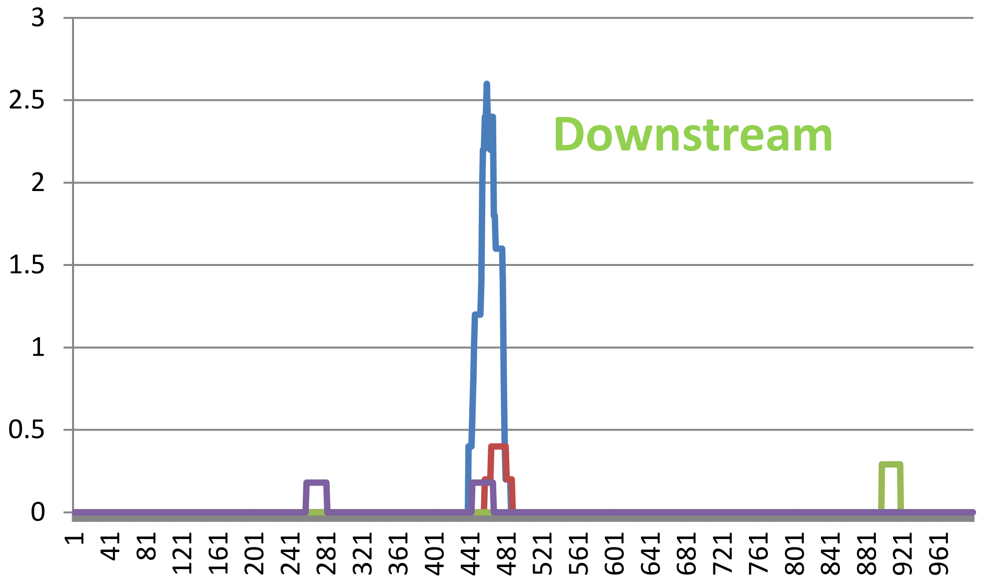

AT1G09290

Unknown protein

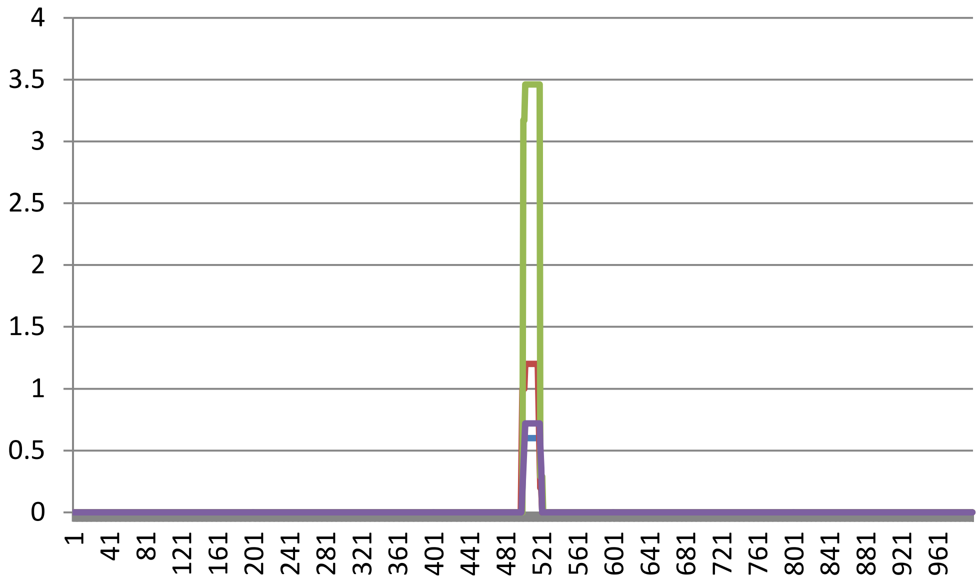

AT1G09910

Rhamnogalacturonate lyase family protein

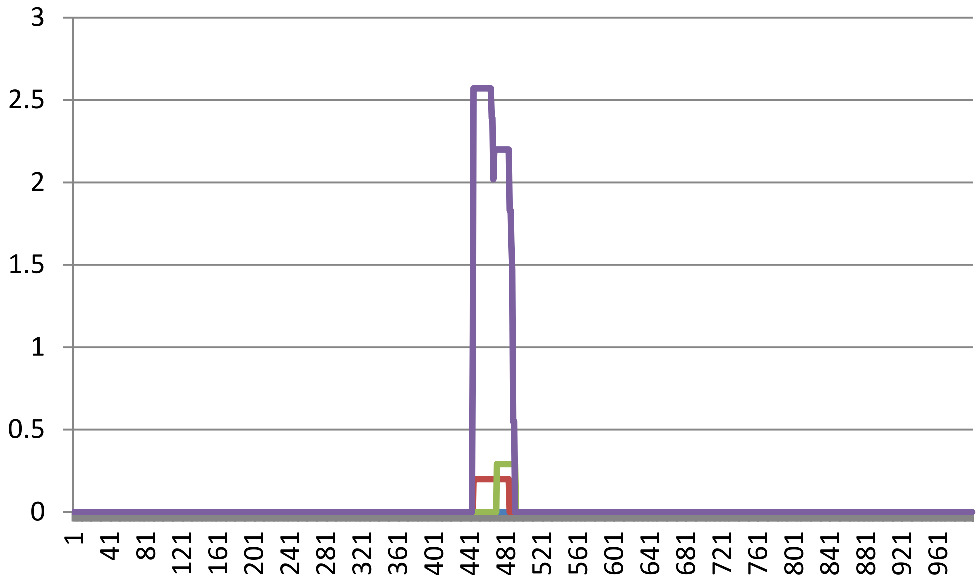

AT1G12870

F-box and associated interaction domains-containing protein

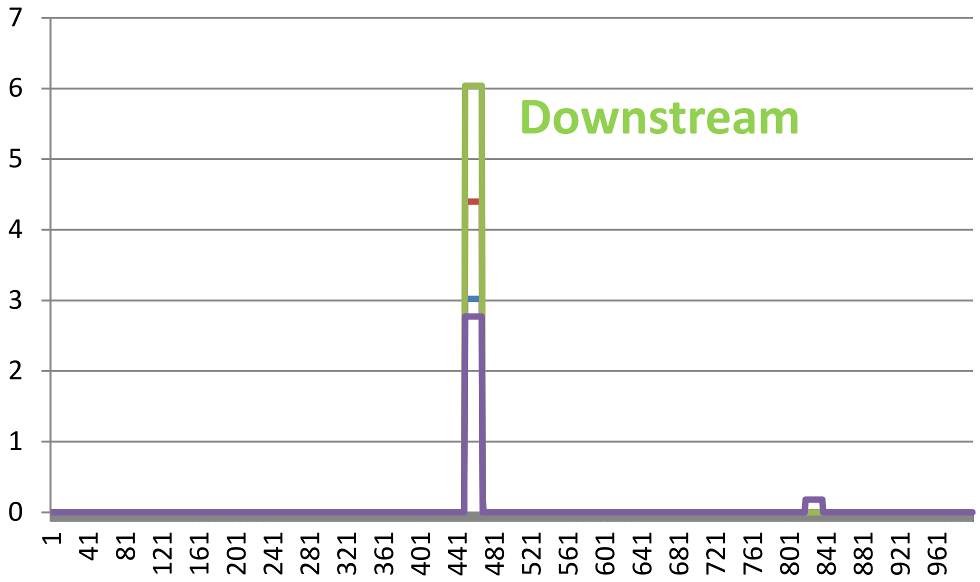

AT1G16820

Vacuolar ATP synthase catalytic subunit-related/V-ATPase-related/vacuolar proton pump-related

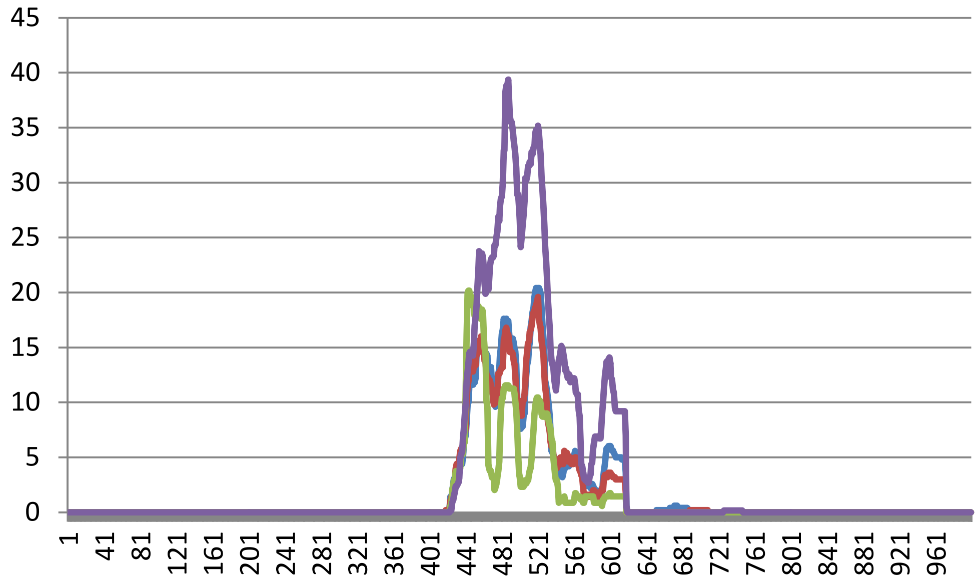

AT1G17270

O-fucosyltransferase family protein

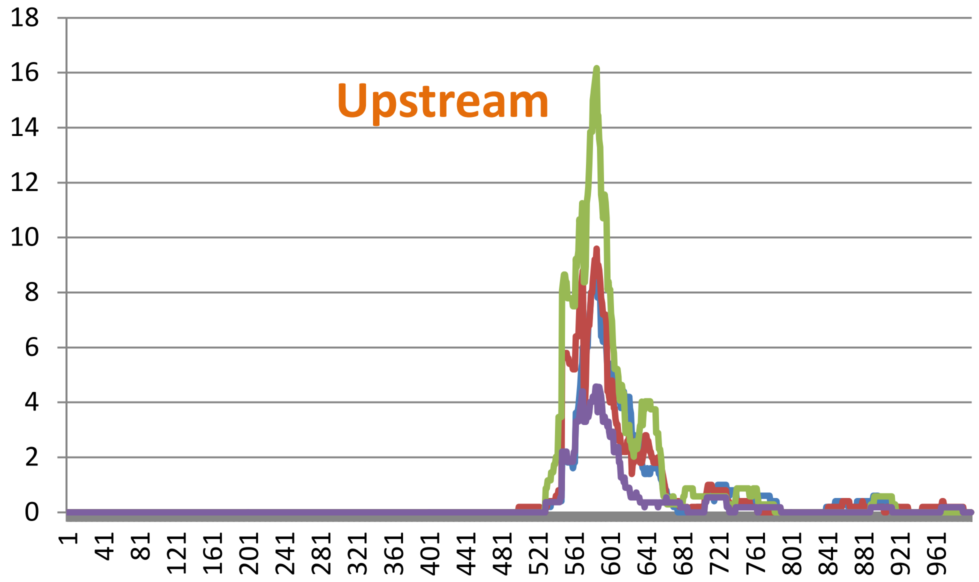

AT1G17680

Tetratricopeptide repeat (TPR)-containing protein

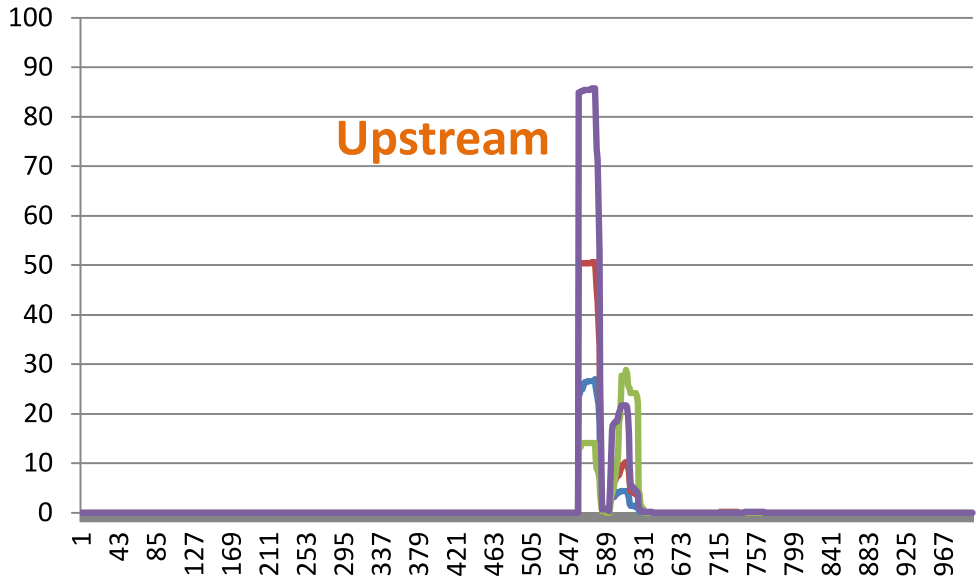

AT1G17830

Protein of unknown function (DUF789)

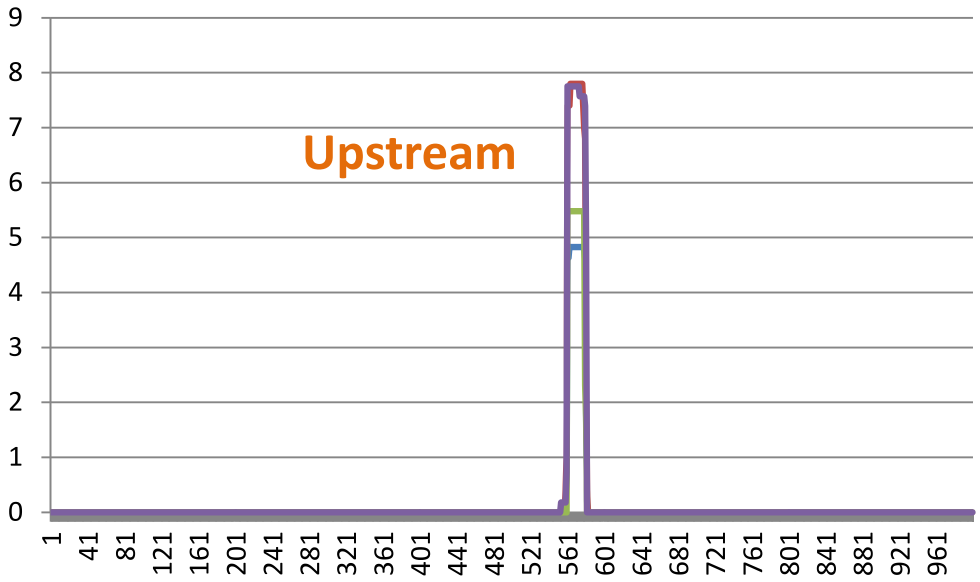

AT1G22067

Unknown protein

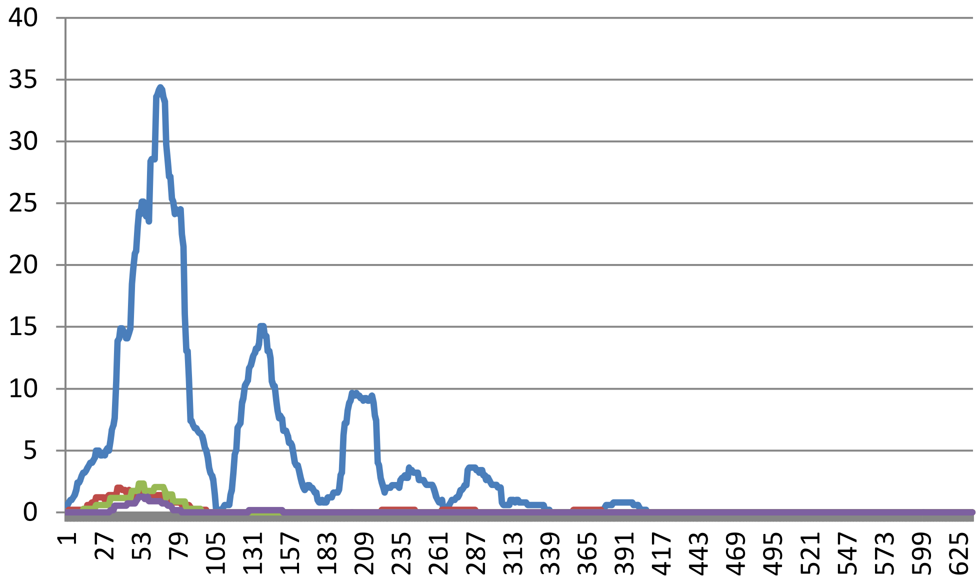

AT1G24625

Encodes a zinc finger protein containing only a single zinc finger

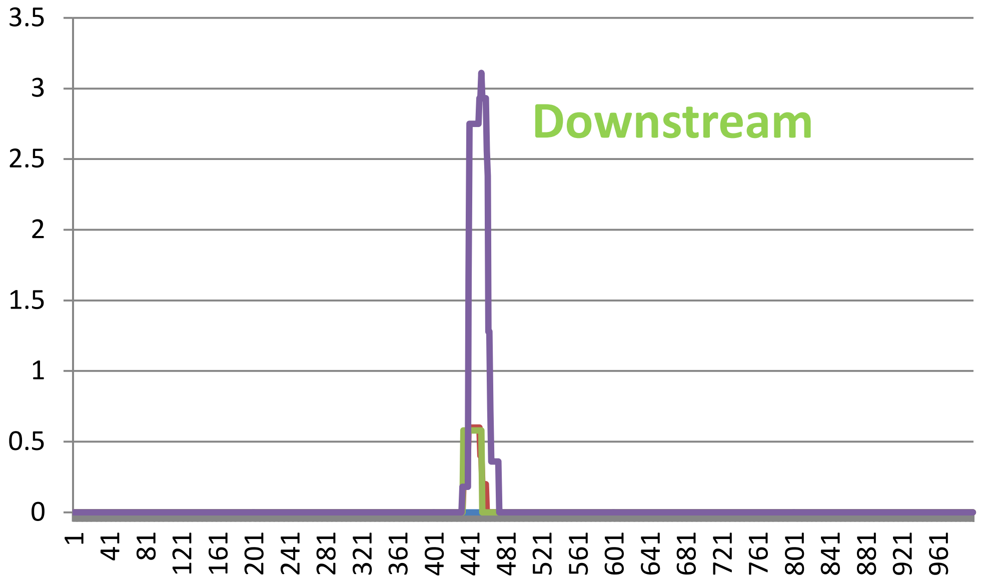

AT1G26110

Encodes decapping 5, required for mRNA decapping, P-body formation and translational repression during postembryonic development.

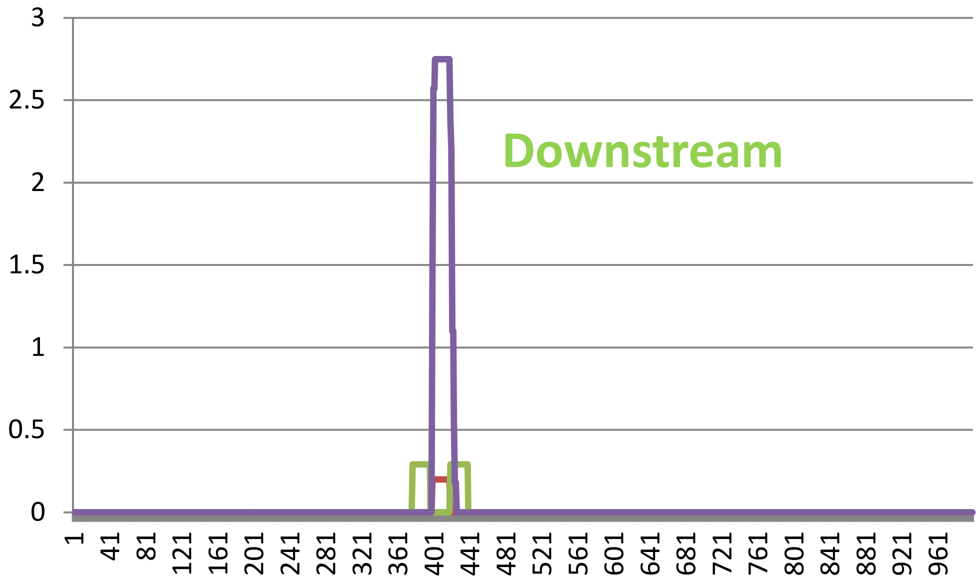

AT1G28280

VQ motif-containing protein

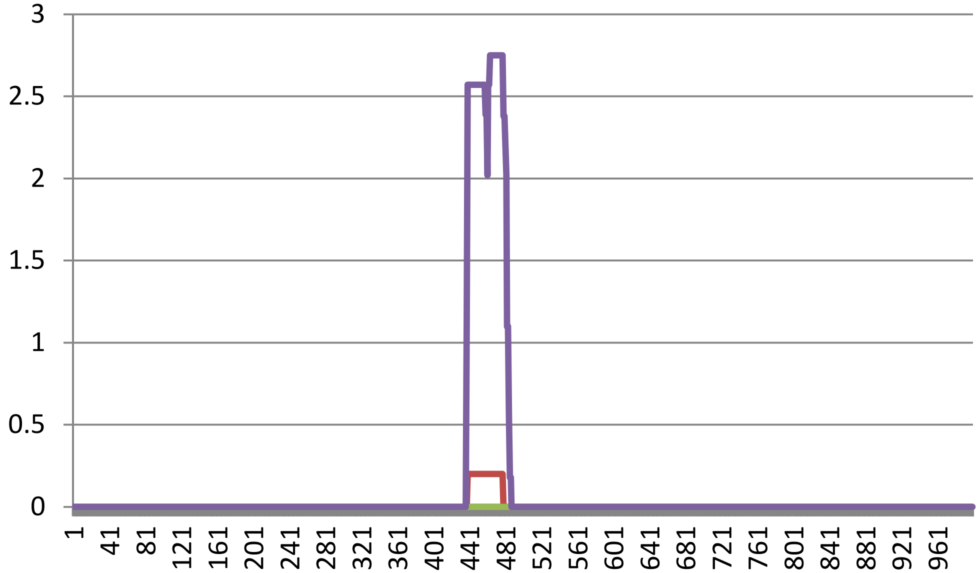

AT1G28290

Encodes an atypical arabinogalactan protein that is localized to the plasma membrange. AGP31 is highly expressed in flowers and vascular tissue and is repressed by jasmonic acid. AGP31 may play a role in vascular tissue function during defense and development.

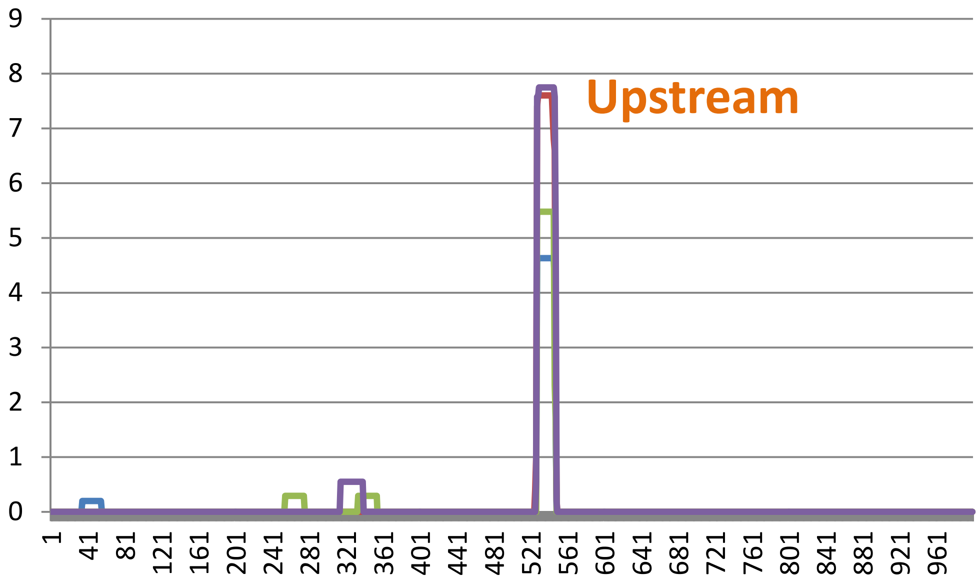

AT1G30370

Encodes a mitochondria-localized class III phospholipase A1 that plays a role in seed viability.

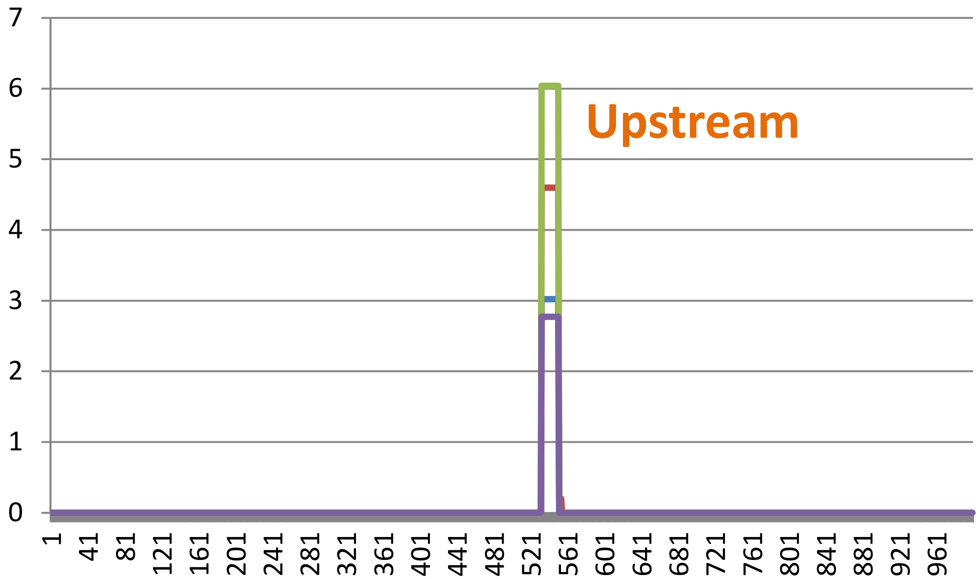

AT1G32140

F-box family protein

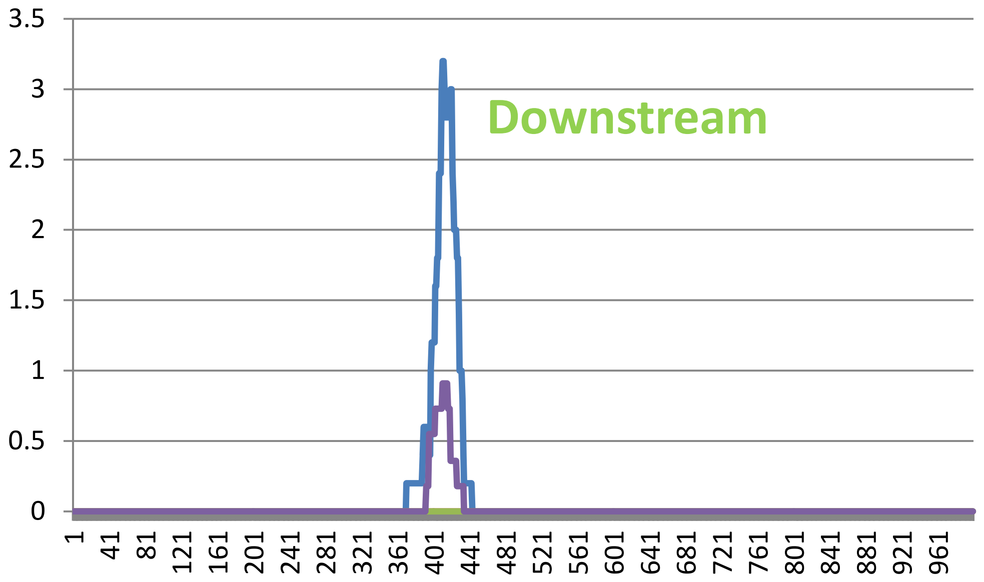

AT1G32630

Unknown protein

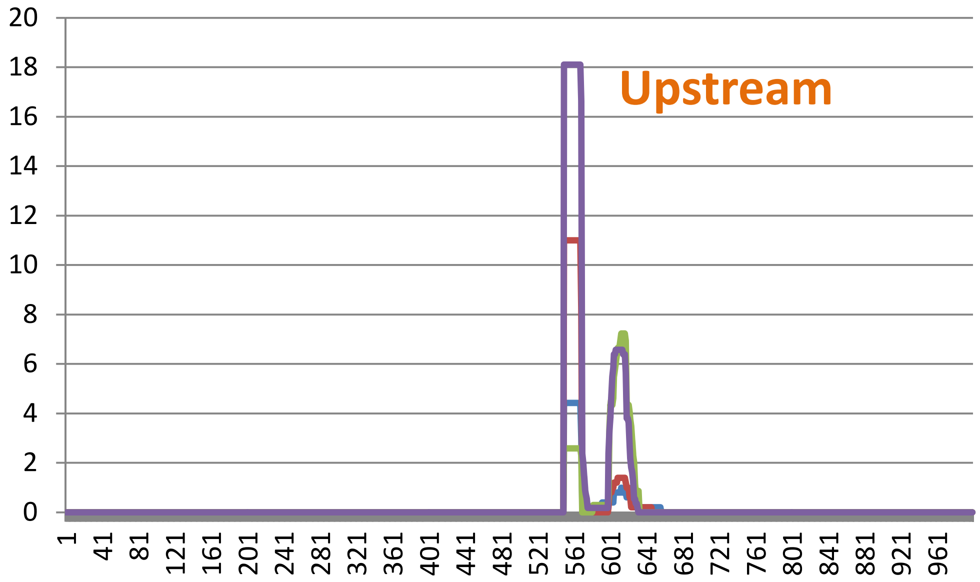

AT1G33350

Pentatricopeptide repeat (PPR) superfamily protein

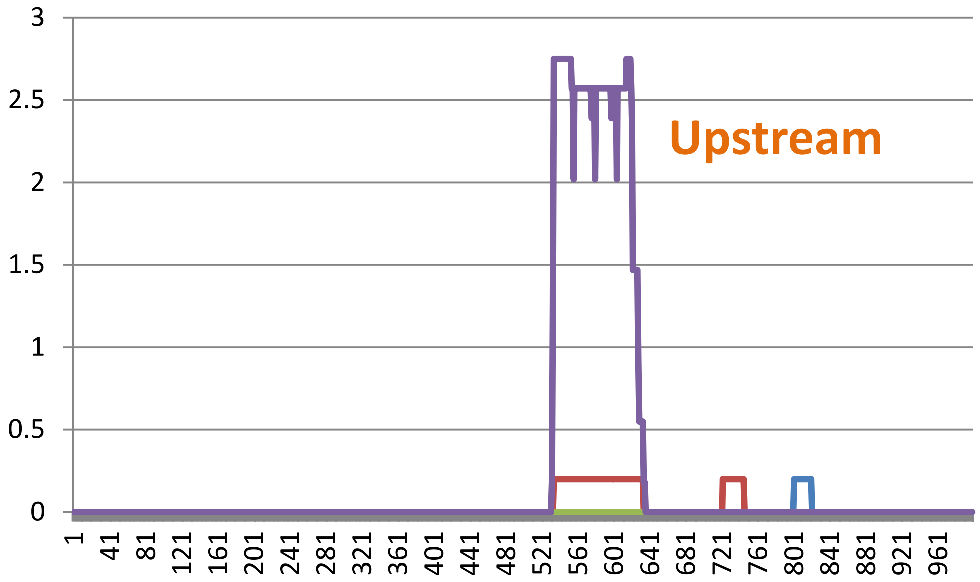

AT1G42540

Member of Putative ligand-gated ion channel subunit family

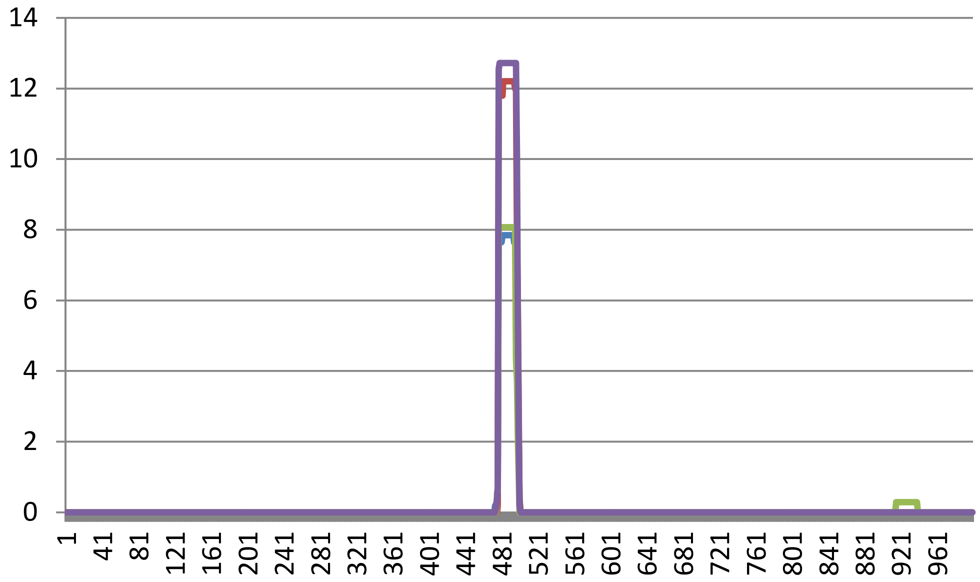

AT1G43624

Unknown protein

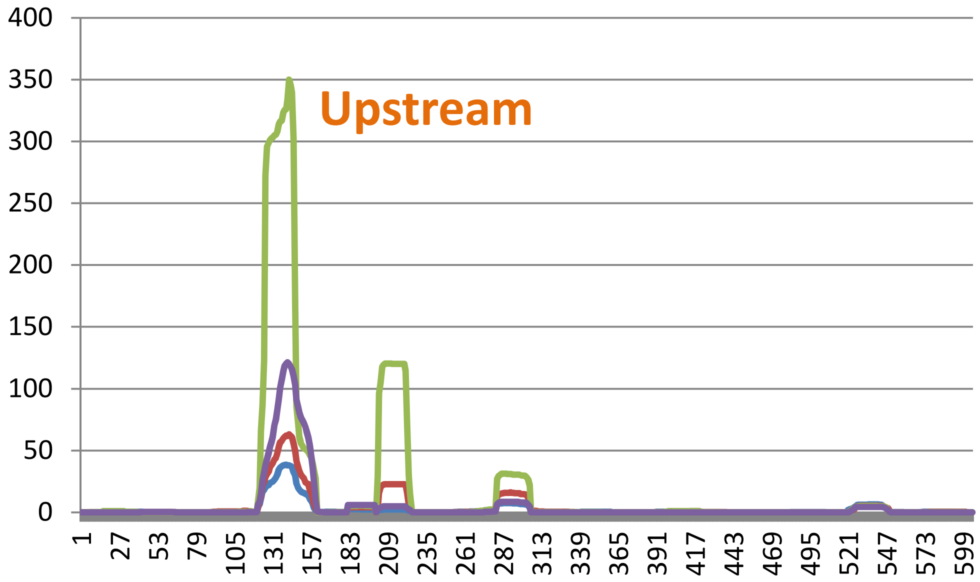

AT1G45233

Encodes a component of the putative Arabidopsis THO/TREX complex: THO1 or HPR1 (At5g09860), THO2 (At1g24706), THO3 or TEX1 (At5g56130), THO5 (At5g42920, At1g45233), THO6 (At2g19430), and THO7 (At5g16790, At3g02950). THO/TREX complexes in animals have been implicated in the transport of mRNA precursors. Mutants of THO3/TEX1, THO1, THO6 accumulate reduced amount of small interfering (si)RNA, suggesting a role of the putative Arabidopsis THO/TREX in siRNA biosynthesis.

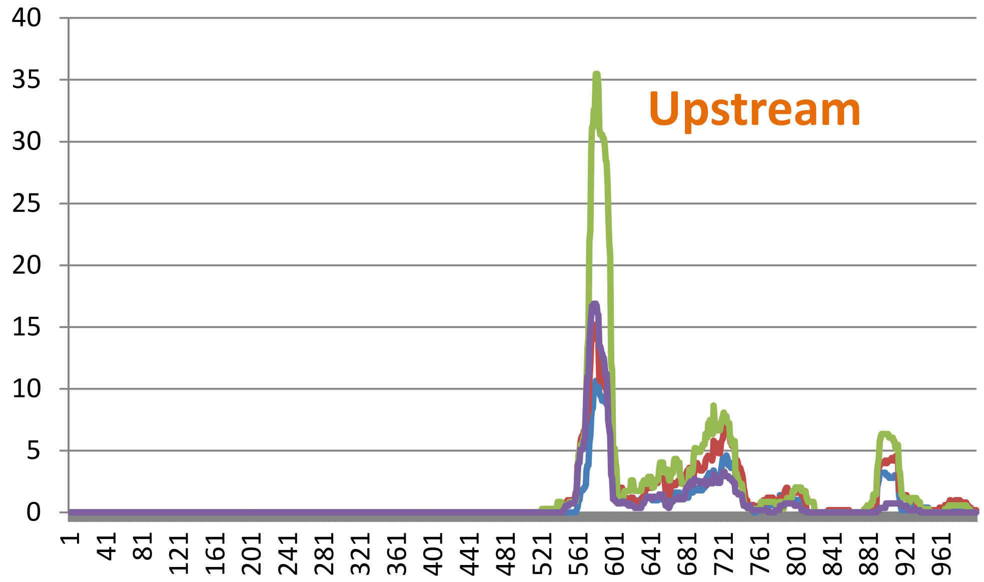

AT1G47765

F-box and associated interaction domains-containing protein

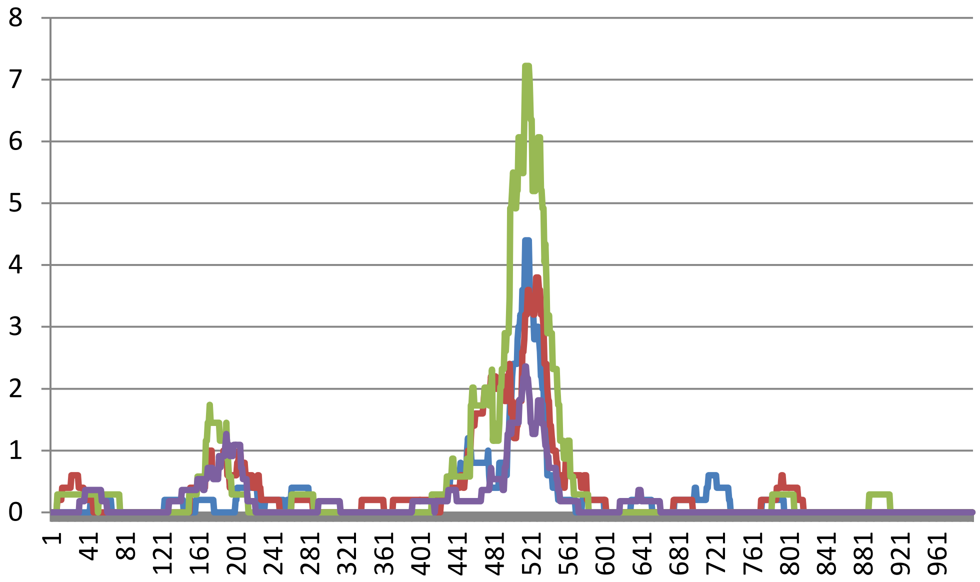

AT1G48598

Upstream open reading frames (uORFs) are small open reading frames found in the 5' UTR of a mature mRNA, and can potentially mediate translational regulation of the largest, or major, ORF (mORF).

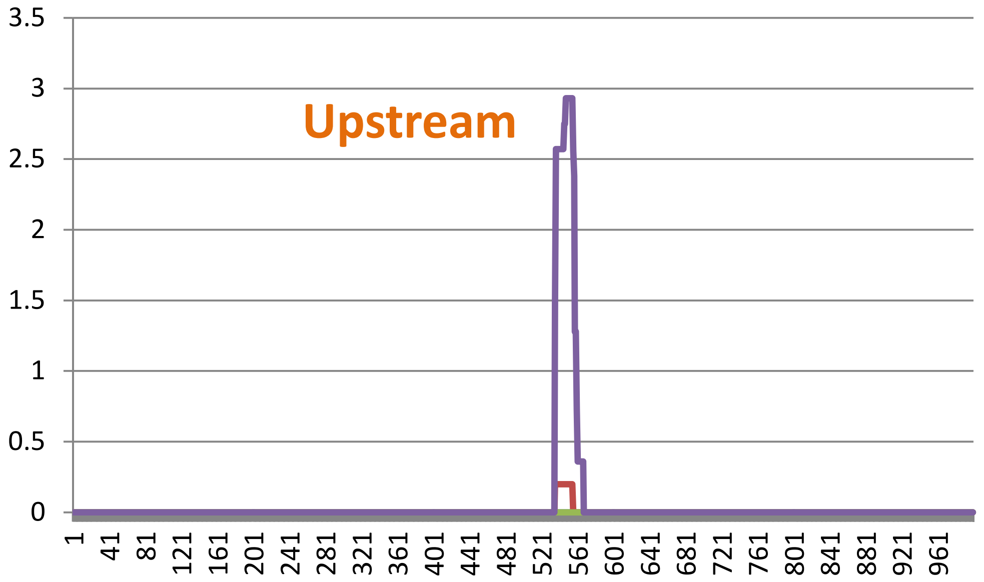

AT1G48600

Encodes a phosphoethanolamine N-methyltransferase that catalyses the last two methylation steps of the three sequential methylations of phosphoethanolamine (PEA) that are required for the synthesis of phosphocholine (PCho) in plants.

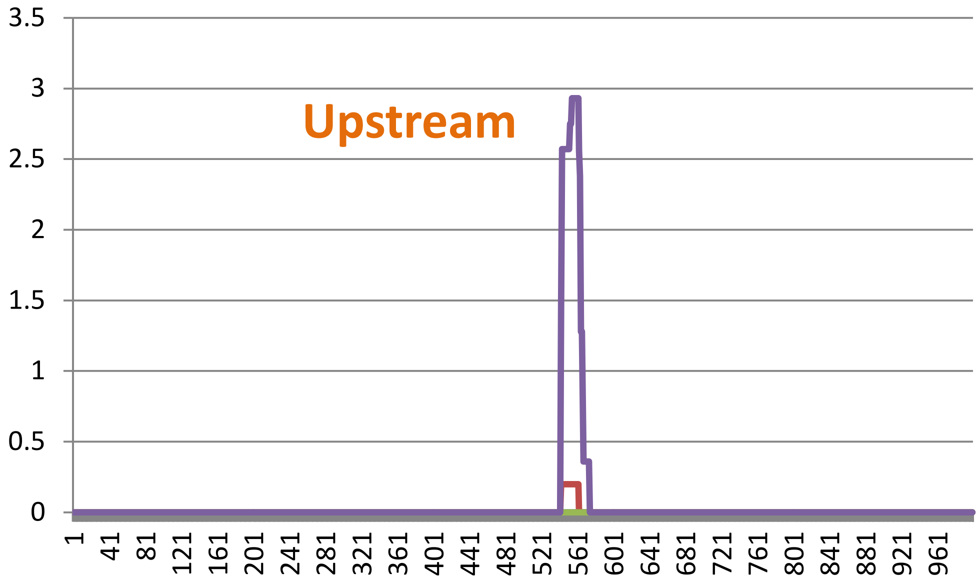

AT1G50160

Polynucleotidyl transferase, ribonuclease H-like superfamily protein

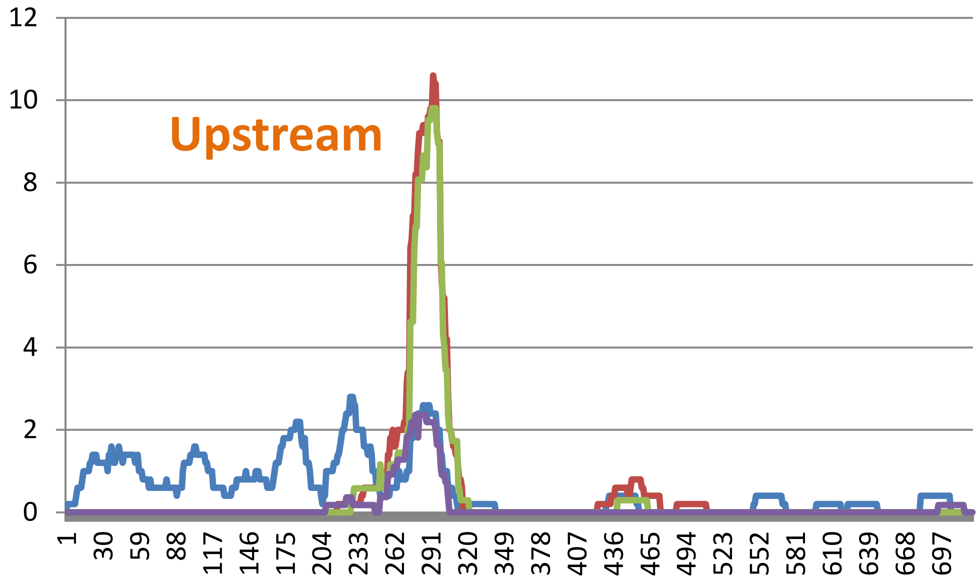

AT1G52110

Mannose-binding lectin superfamily protein

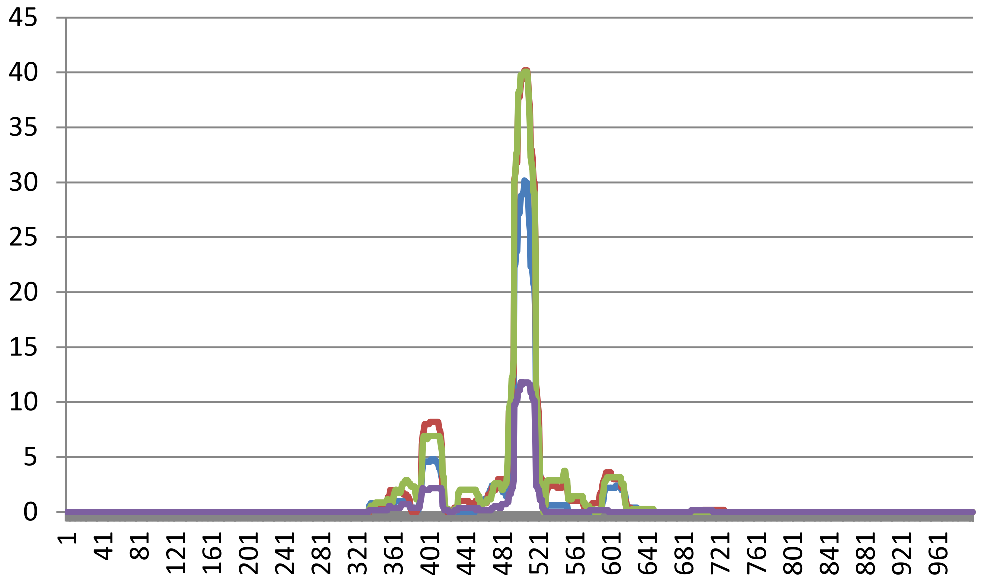

AT1G52618

Unknown protein

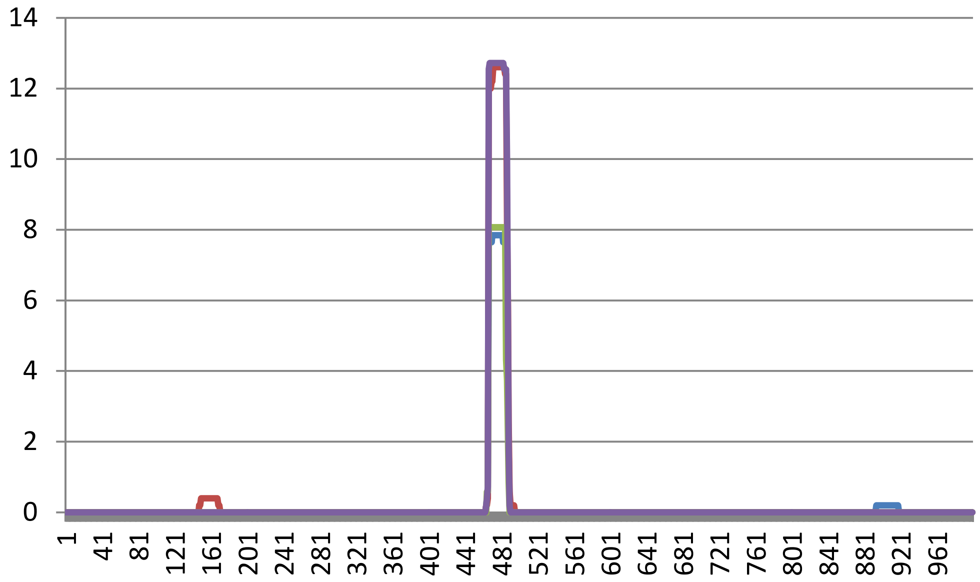

AT1G52940

Purple acid phosphatase 5 (PAP5)

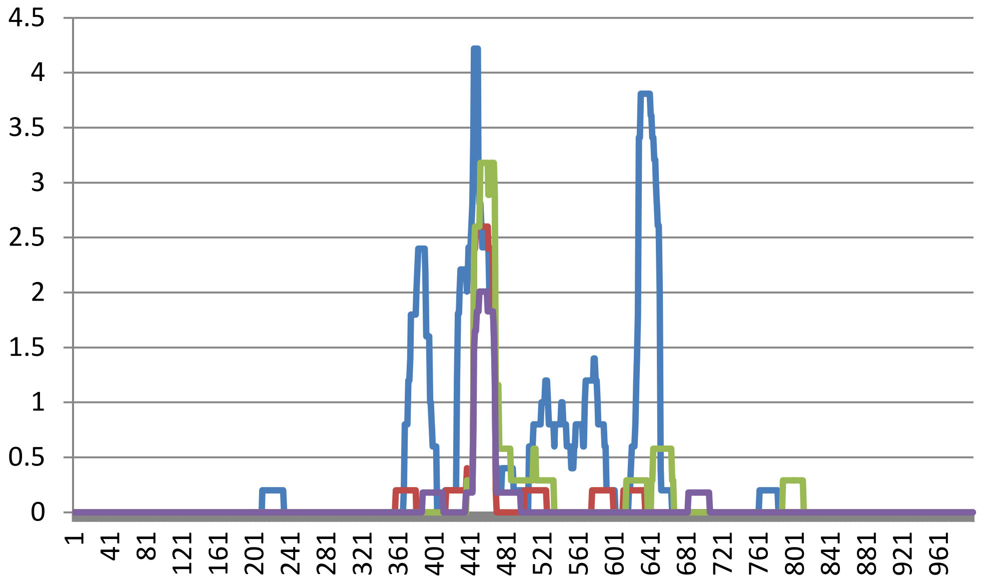

AT1G53250

Unknown protein

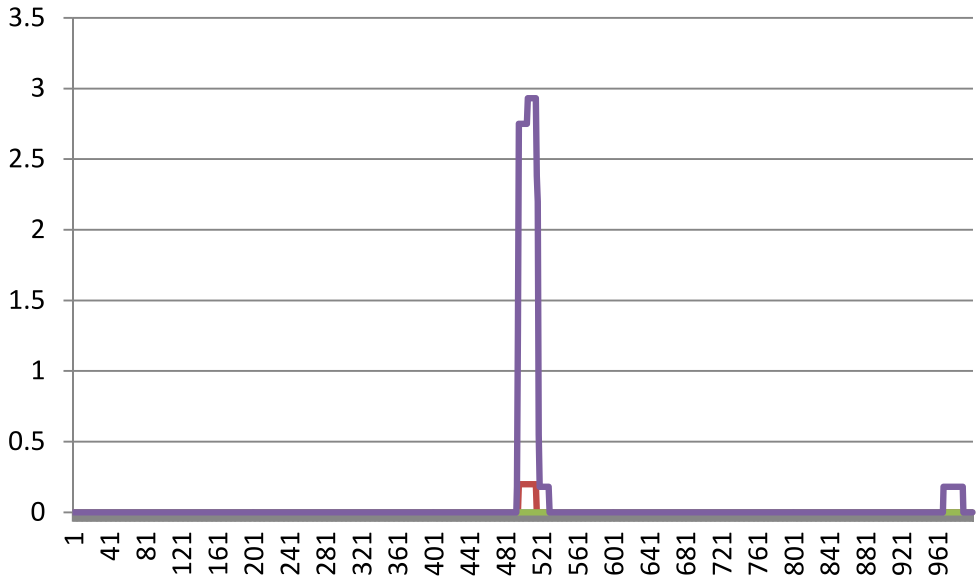

AT1G53265

Unknown protein

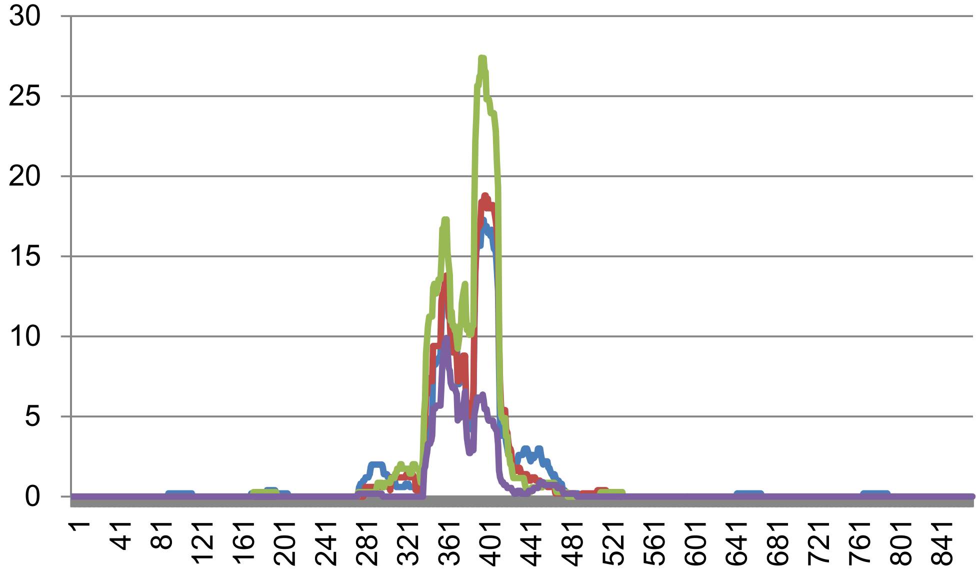

AT1G53541

Unknown protein

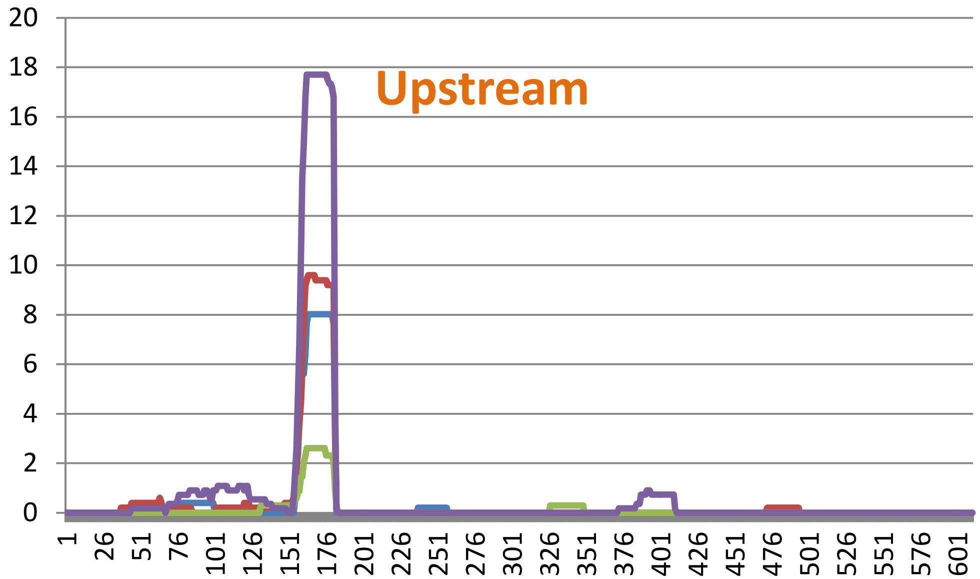

AT1G54030

Encodes a vacuolar protein. Mutation causes organizational defects in the endoplasmic reticulum and aberrant protein trafficking in the plant secretory pathway.

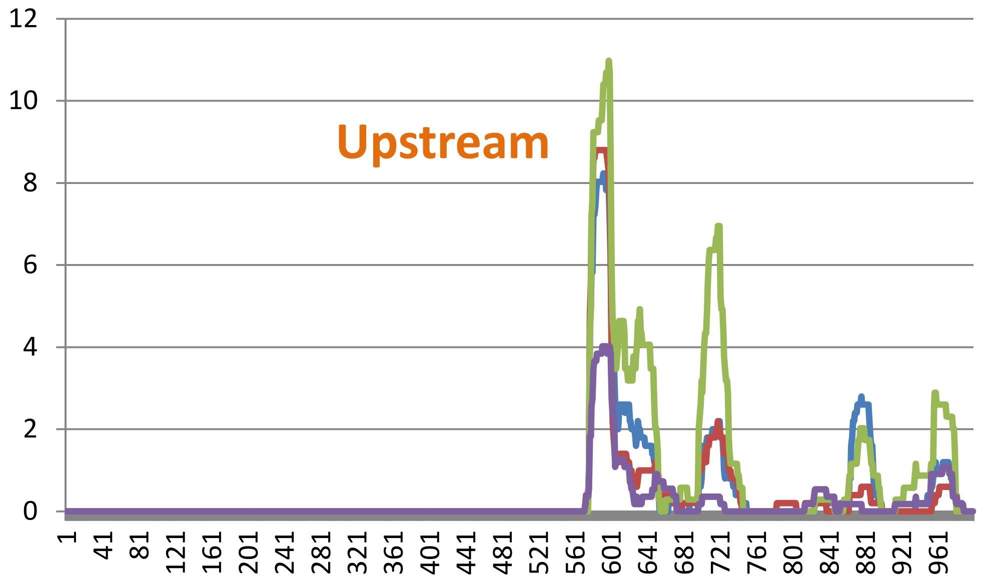

AT1G54775

Encodes a Plant thionin family protein

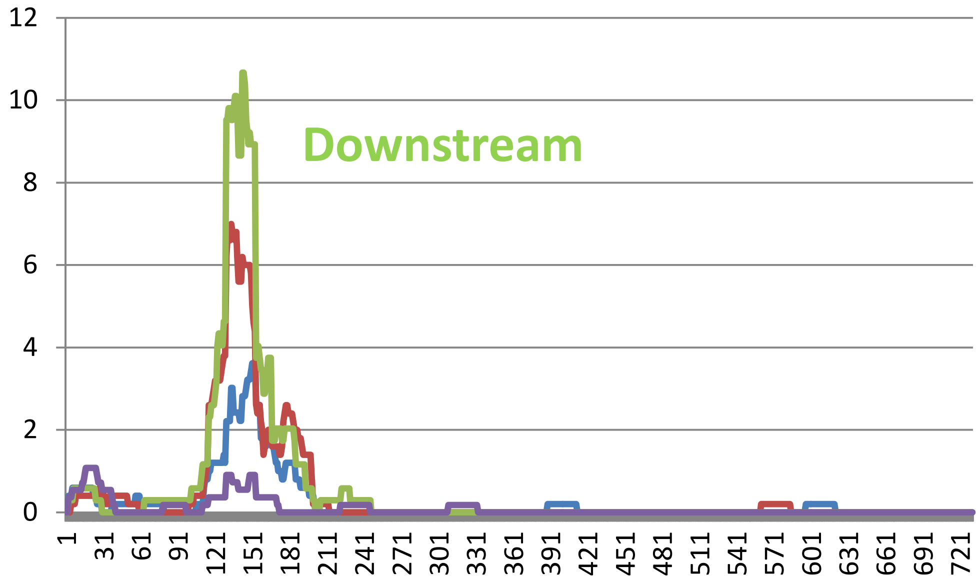

AT1G55300

TBP-associated factor 7 (TAF7)

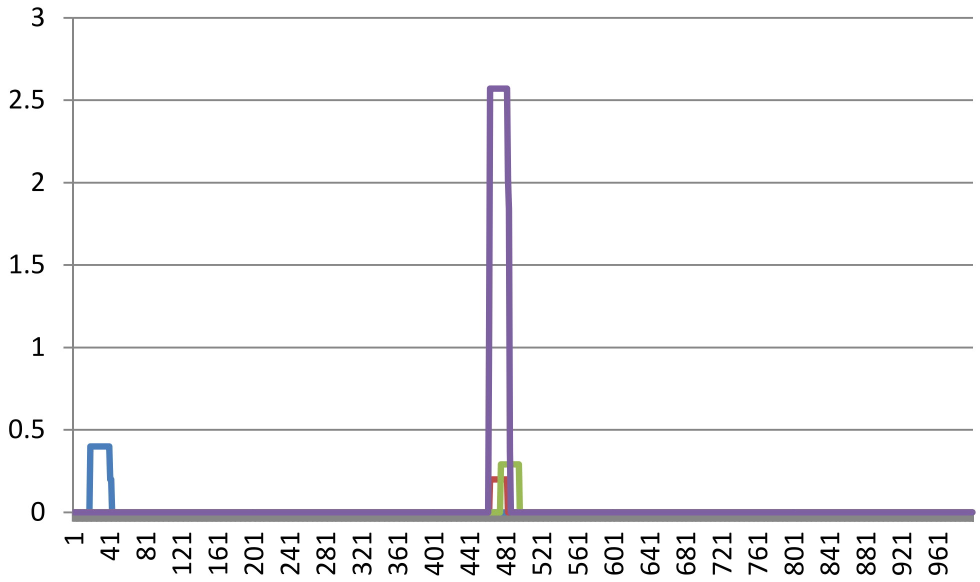

AT1G55680

Transducin/WD40 repeat-like superfamily protein

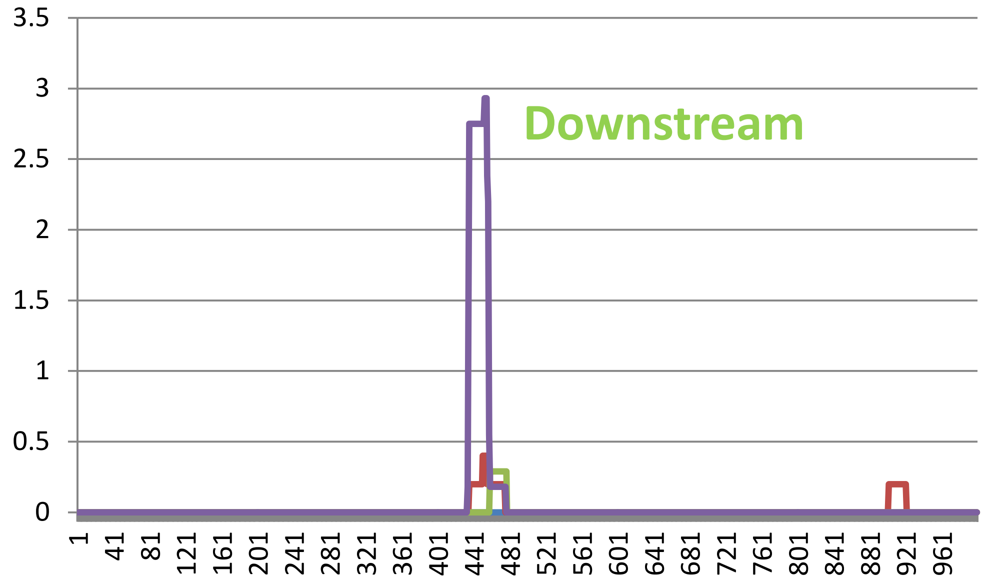

AT1G58245

Encodes a Plant thionin family protein

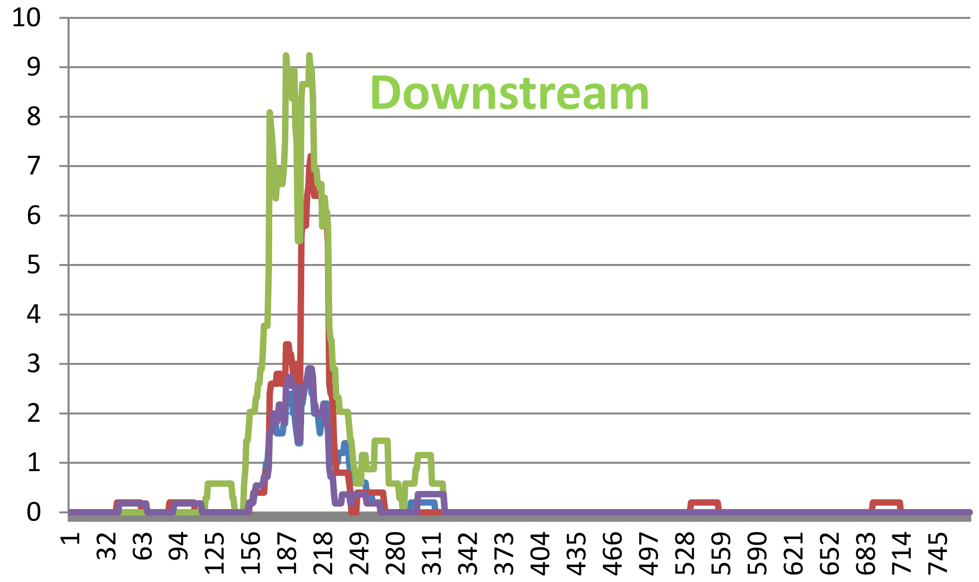

AT1G58248

Encodes a Plant thionin family protein

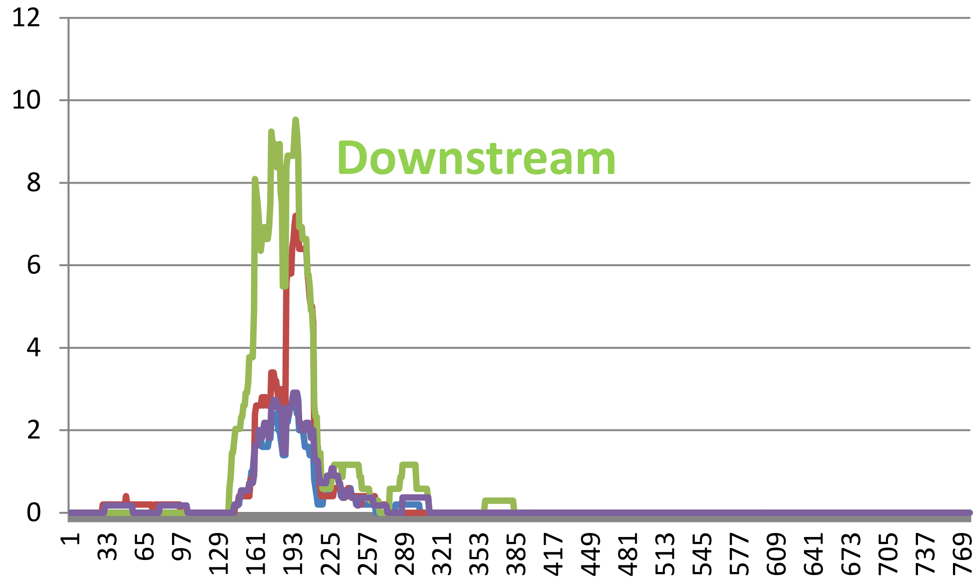

AT1G58390

Disease resistance protein (CC-NBS-LRR class) family

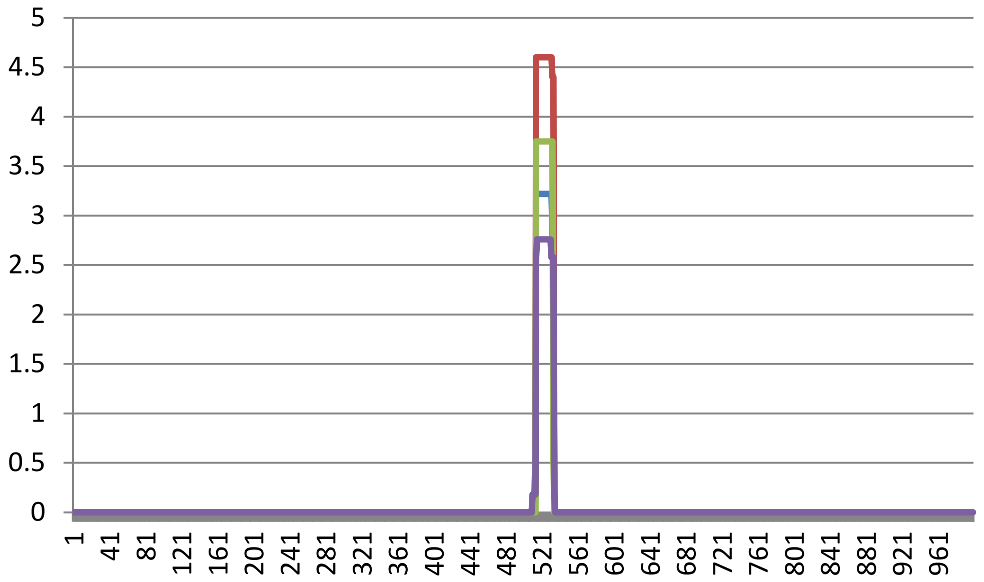

AT1G59680

Embryo sac development arrest 1 (EDA1)

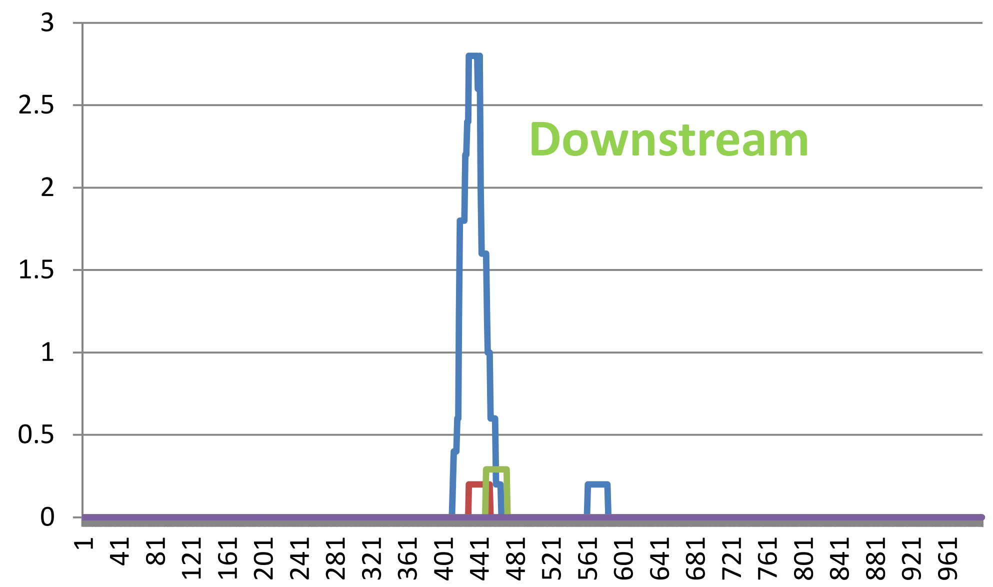

AT1G60720

RNA-directed DNA polymerase (reverse transcriptase)-related family protein

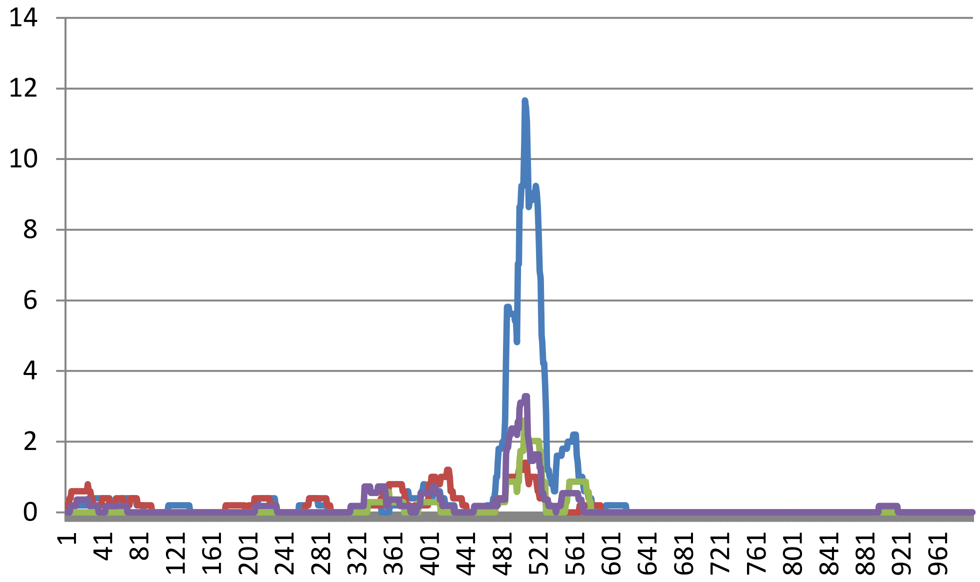

AT1G60940

Encodes a member of SNF1-related protein kinases (SnRK2) whose activity is activated by ionic (salt) and non-ionic (mannitol) osmotic stress.

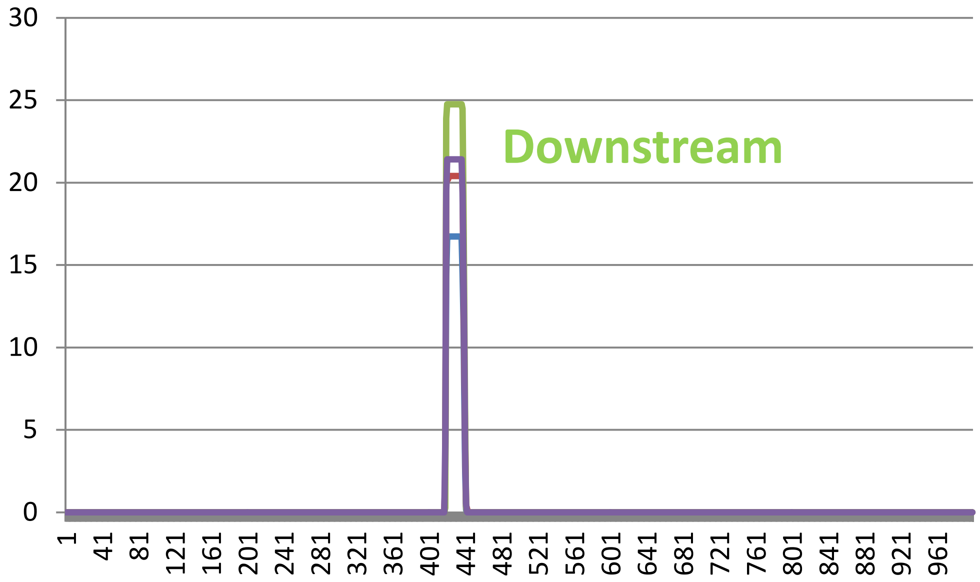

AT1G61030

WAPL (Wings apart-like protein regulation of heterochromatin) protein

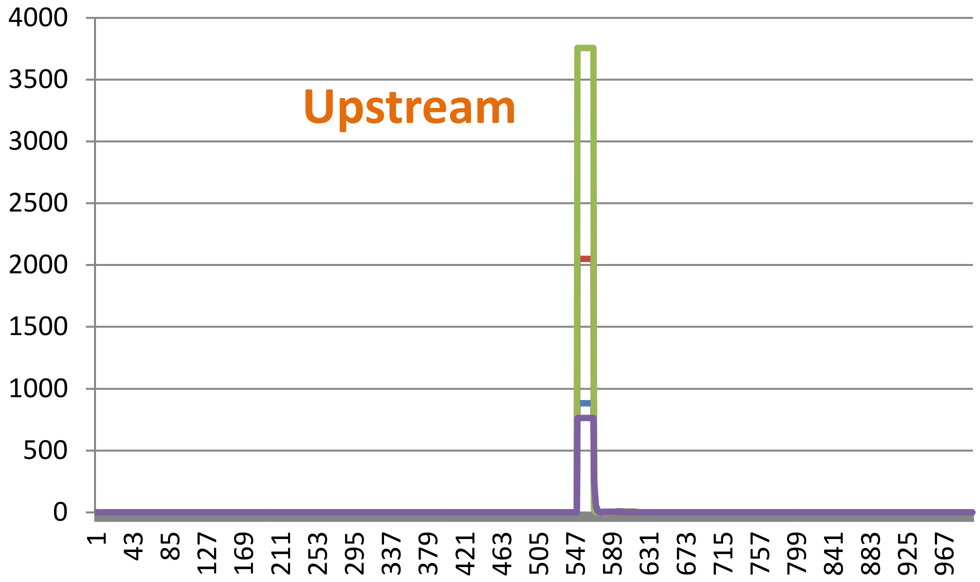

AT1G61820

Beta glucosidase 46 (BGLU46)

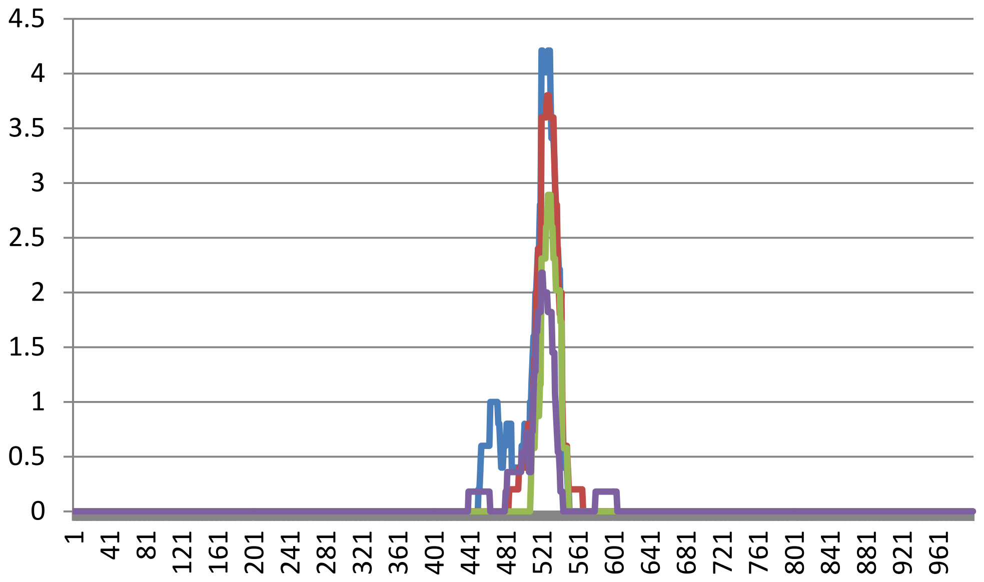

AT1G63210

SPT6L encodes a putative WG/GW-repeat protein involved in the regulation of apical/basal polarity of embryo

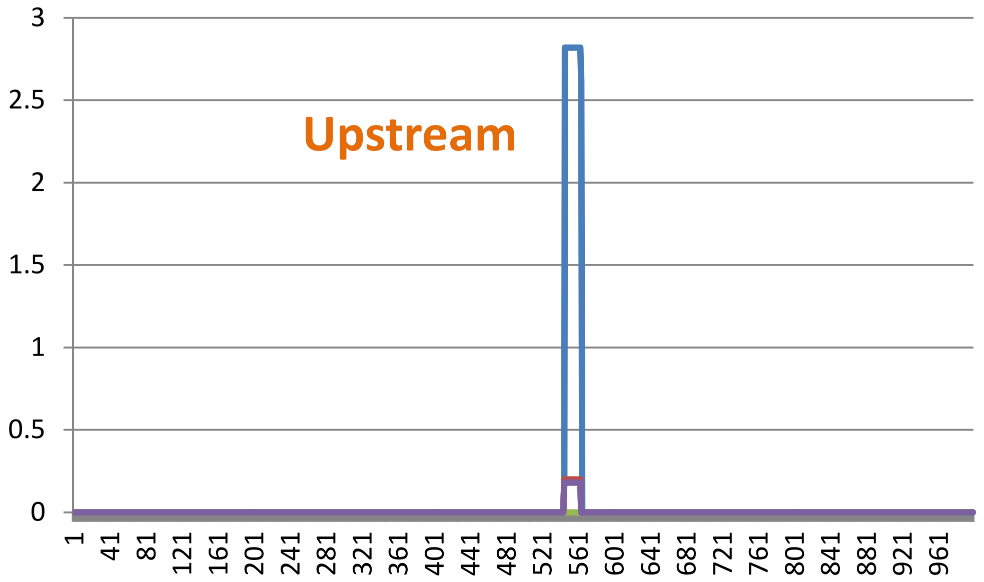

AT1G63480

AT hook motif DNA-binding family protein

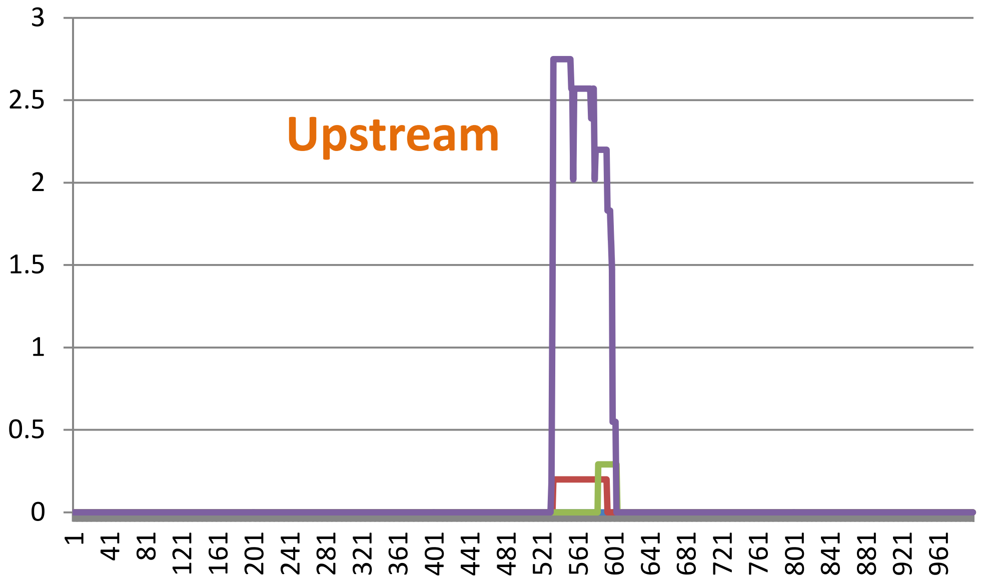

AT1G63800

Ubiquitin-conjugating enzyme 5 (UBC5)

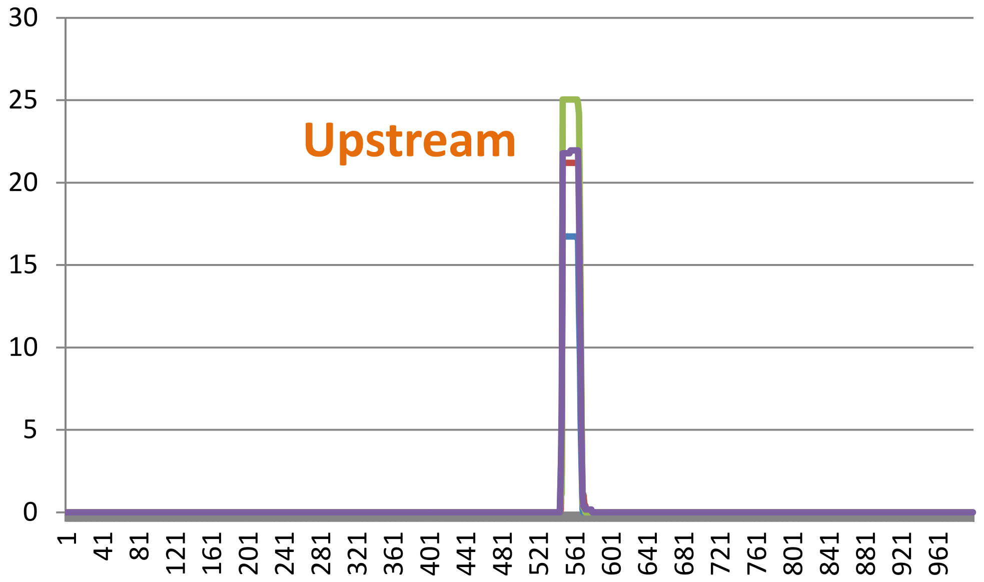

AT1G66290

F-box/RNI-like superfamily protein

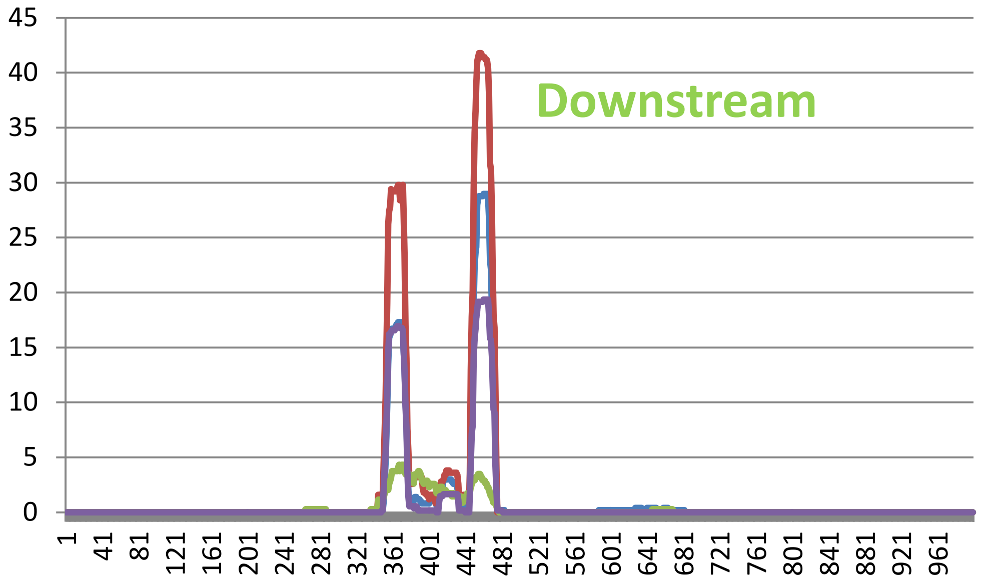

AT1G66490

F-box and associated interaction domains-containing protein

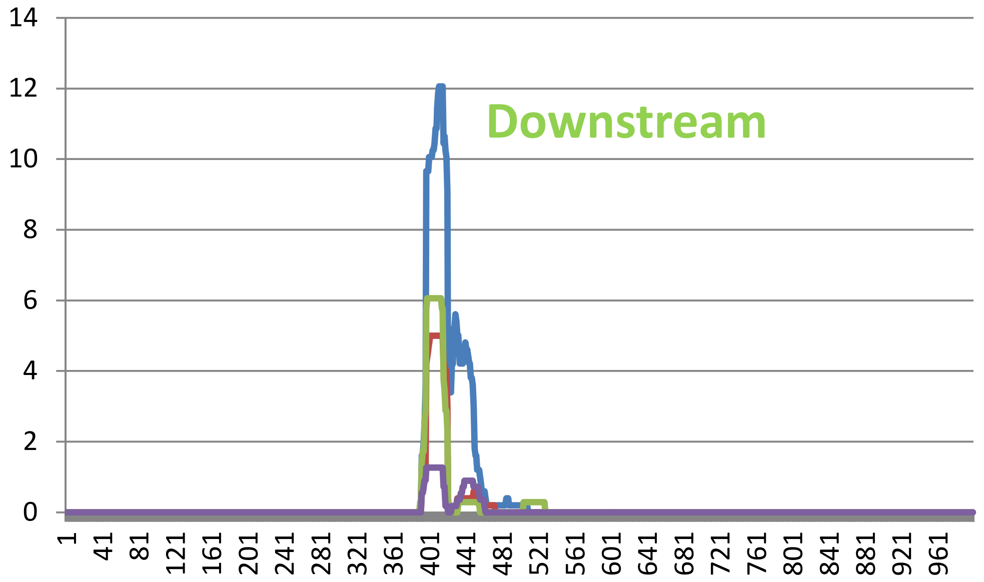

AT1G66640

RNI-like superfamily protein

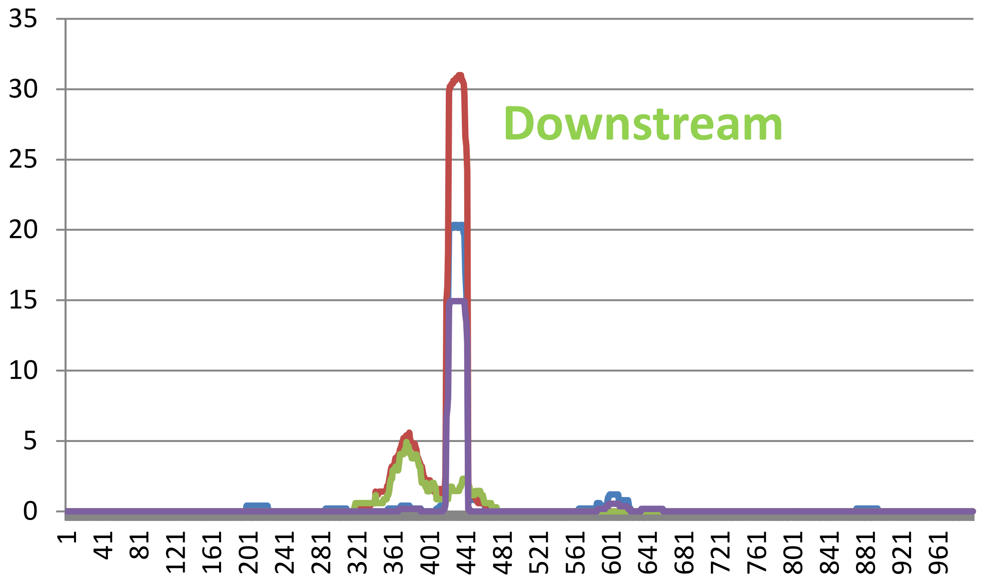

AT1G68945

Unknown protein

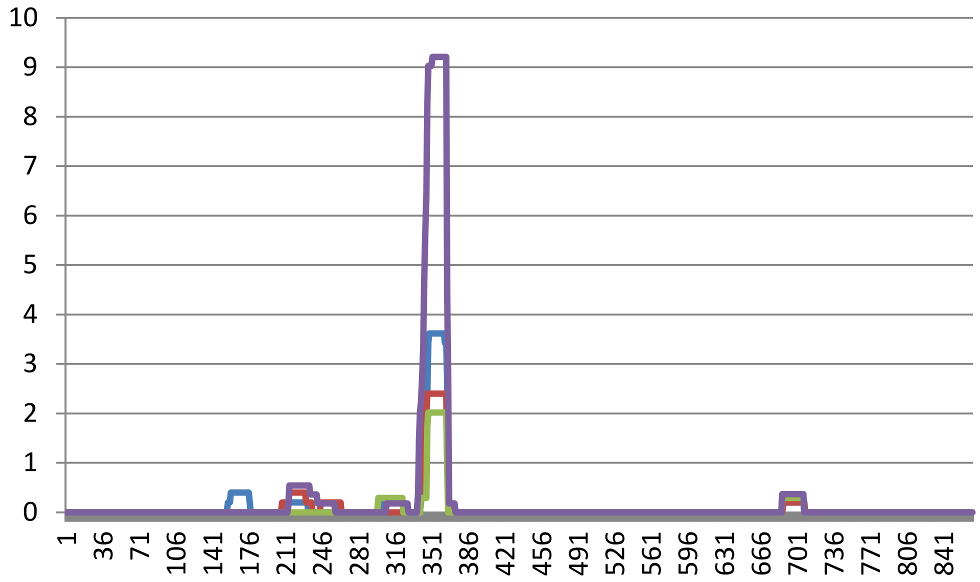

AT1G73710

Pentatricopeptide repeat (PPR) superfamily protein

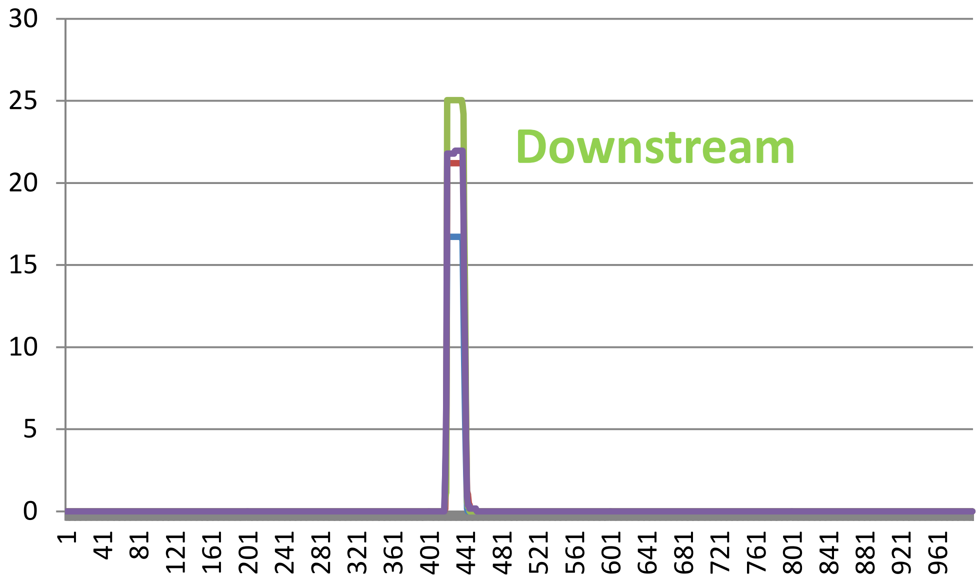

AT1G75050

Pathogenesis-related thaumatin superfamily protein

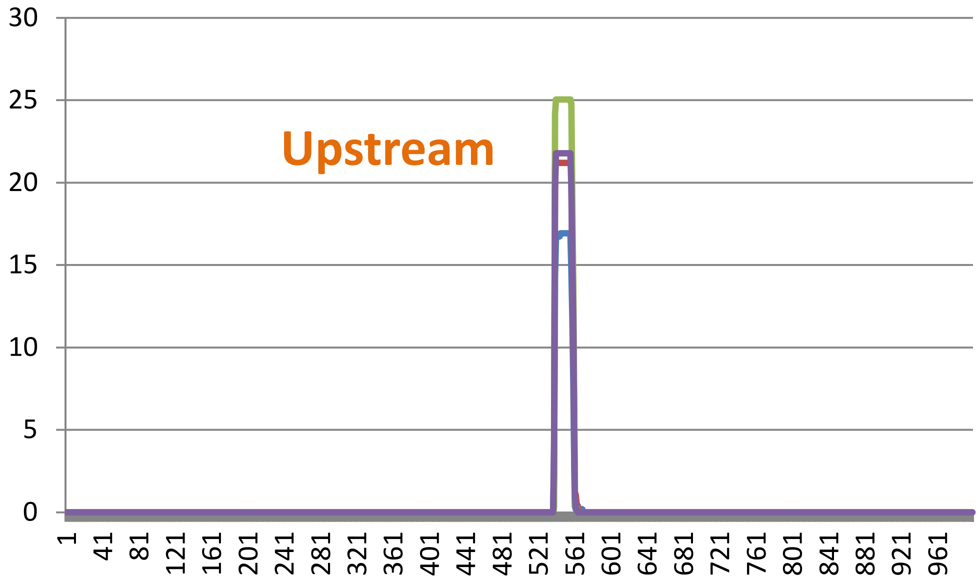

AT1G76810

Eukaryotic translation initiation factor 2 (eIF-2) family protein

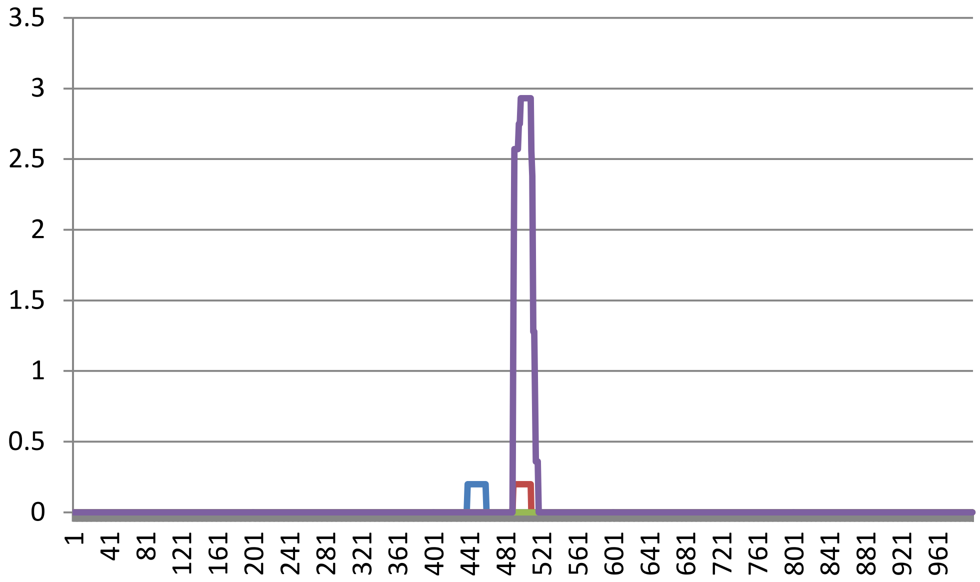

AT1G77210

AtSTP14 belongs to the family of sugar transport proteins (AtSTPs) involved in monosaccharide transport.

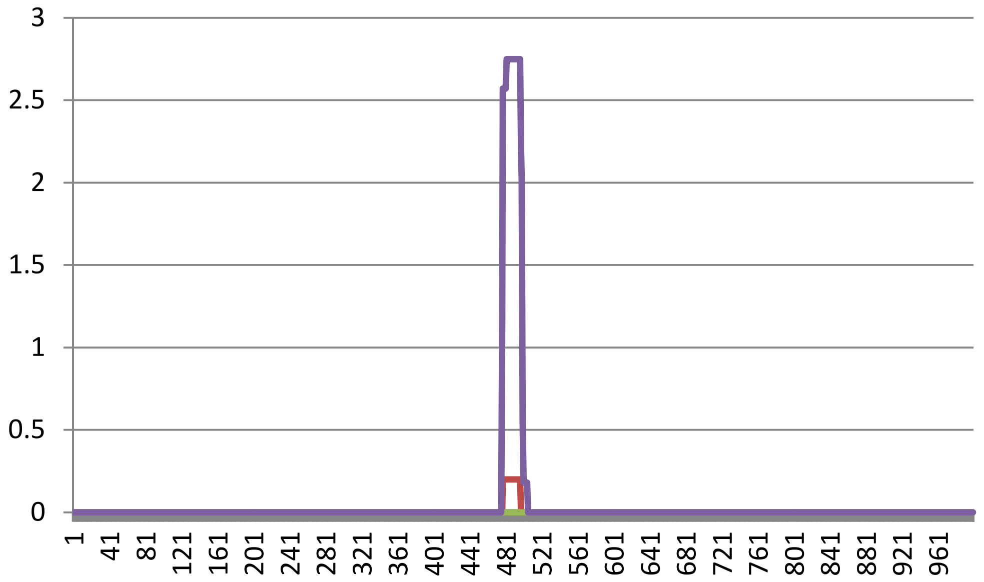

AT1G77950

AGAMOUS-like 67 (AGL67)

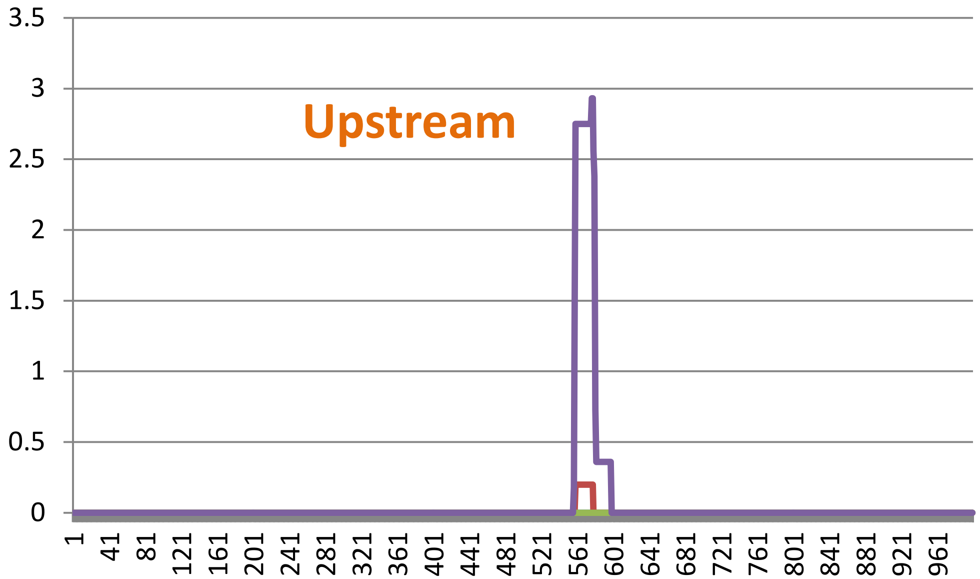

AT1G78420

RING/U-box superfamily protein

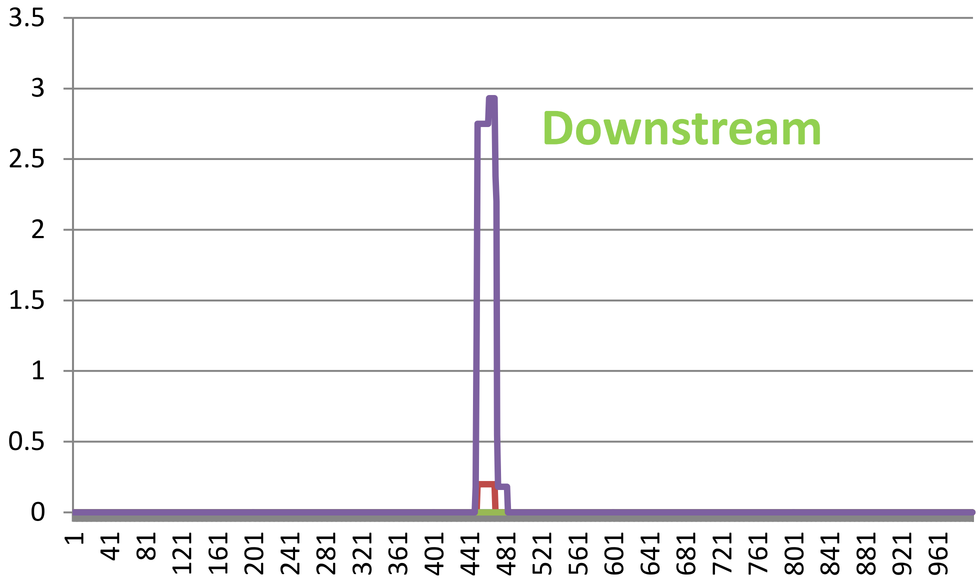

AT1G79490

Embryo defective 2217 (EMB2217)

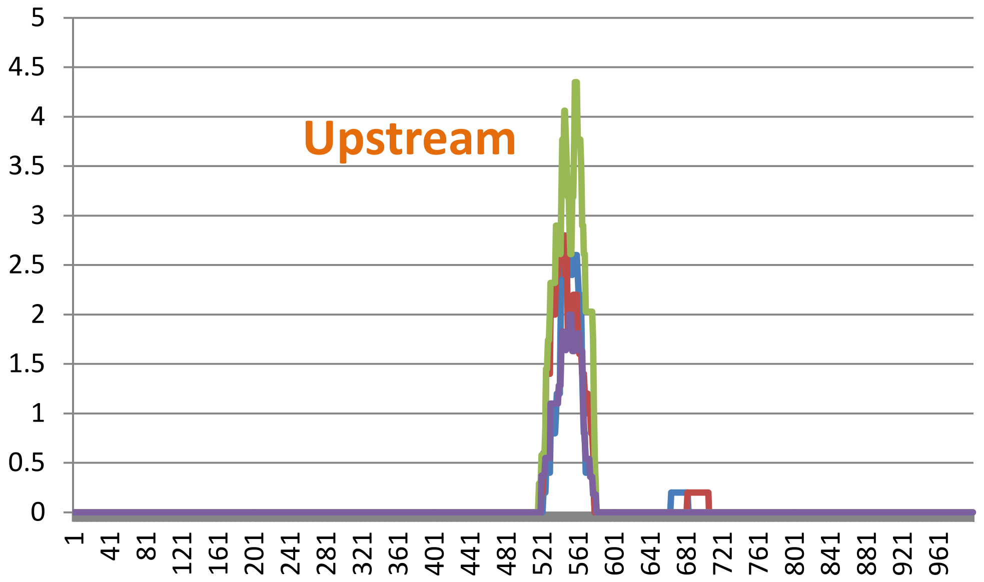

AT1G79800

Early nodulin-like protein 7 (ENODL7)

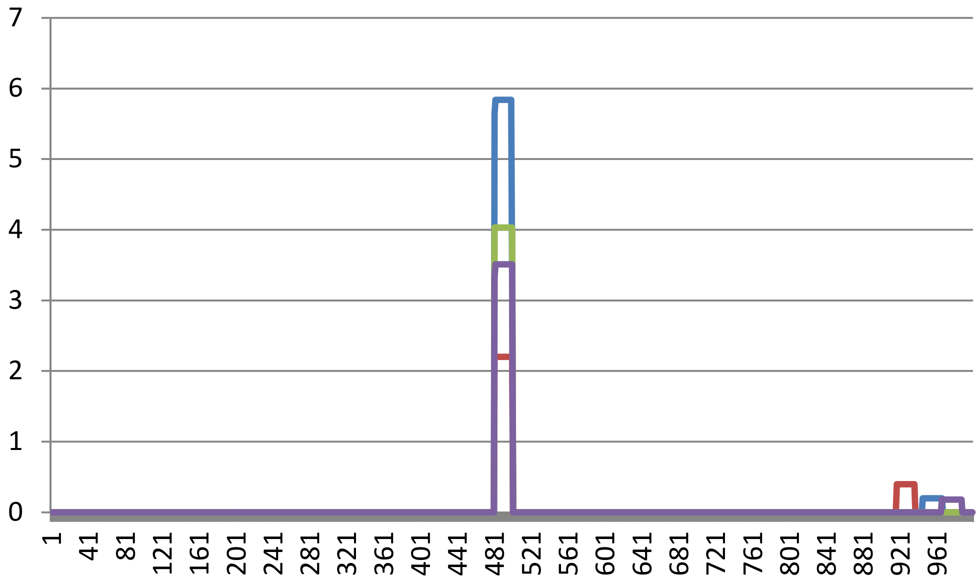

AT1G79990

Structural molecules

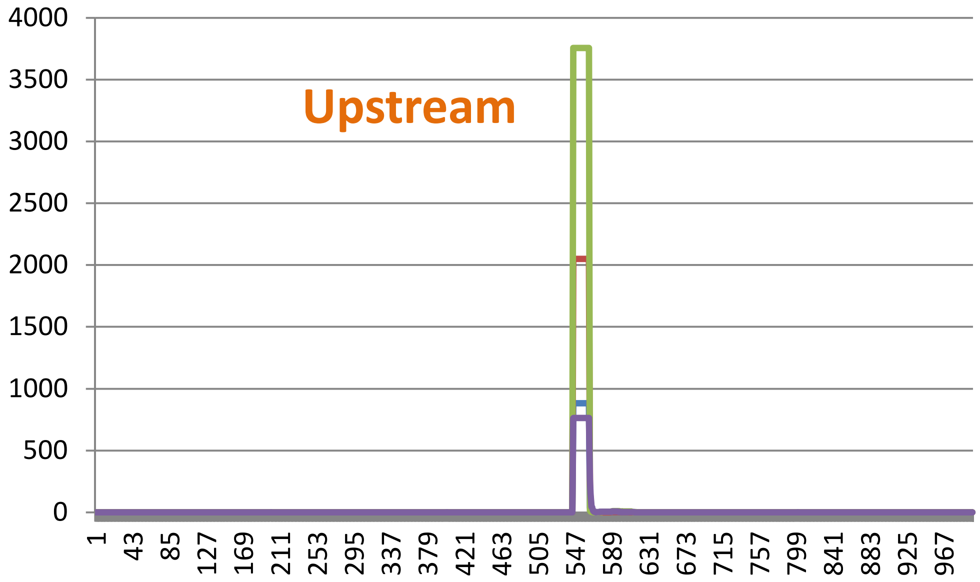

AT2G02540

Zinc finger homeobox protein. Expressed in vascular tissue.

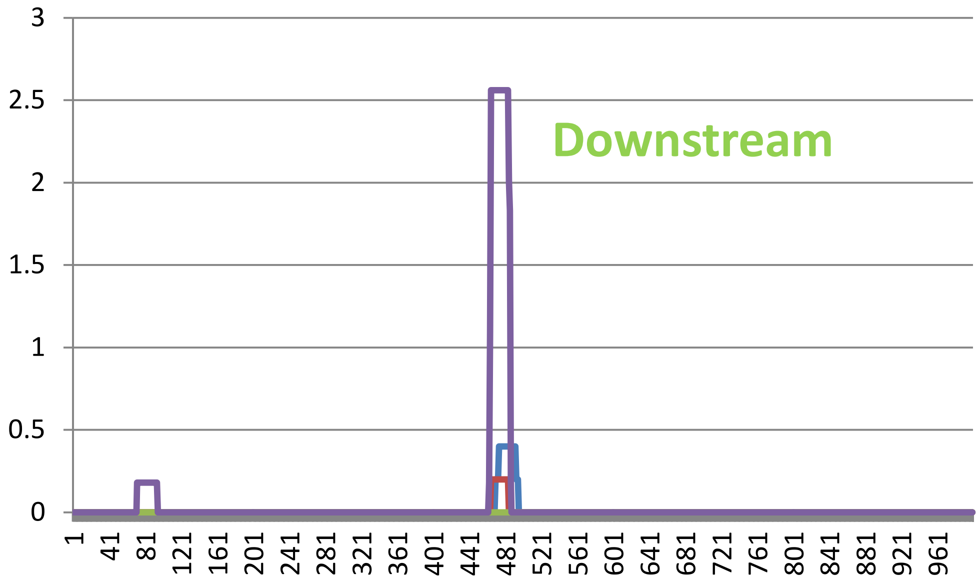

AT2G03667

Asparagine synthase family protein

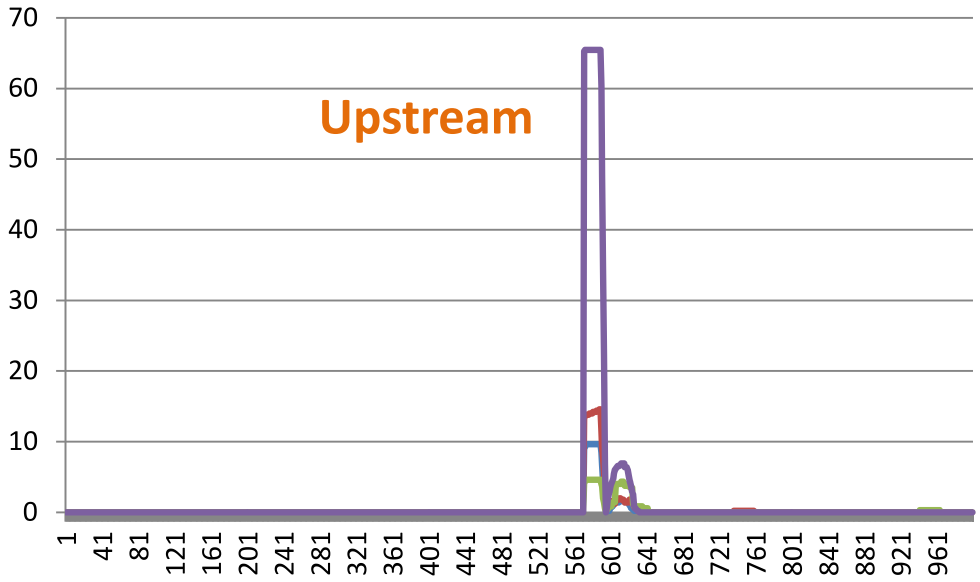

AT2G04620

Cation efflux family protein

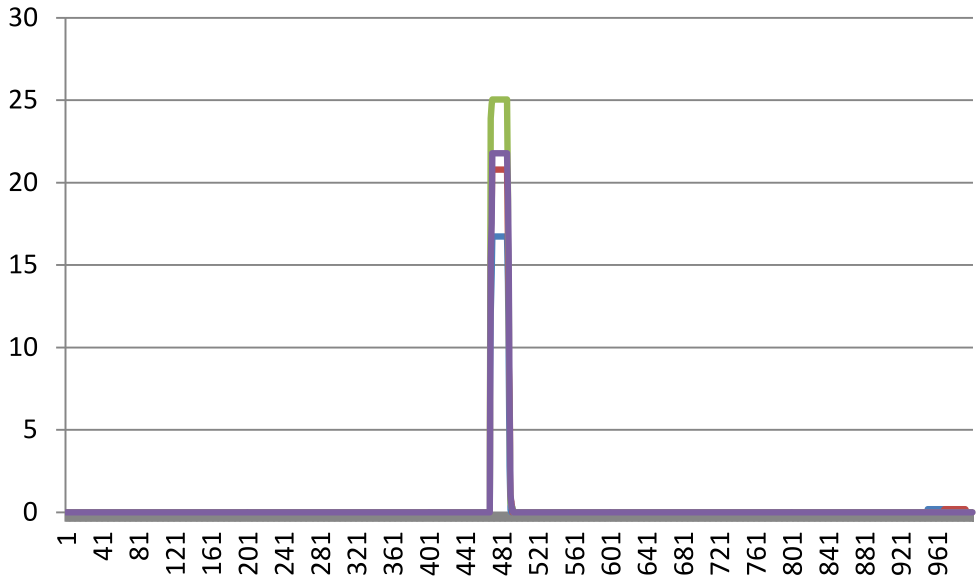

AT2G06850

Endoxyloglucan transferase (EXGT-A1) gene

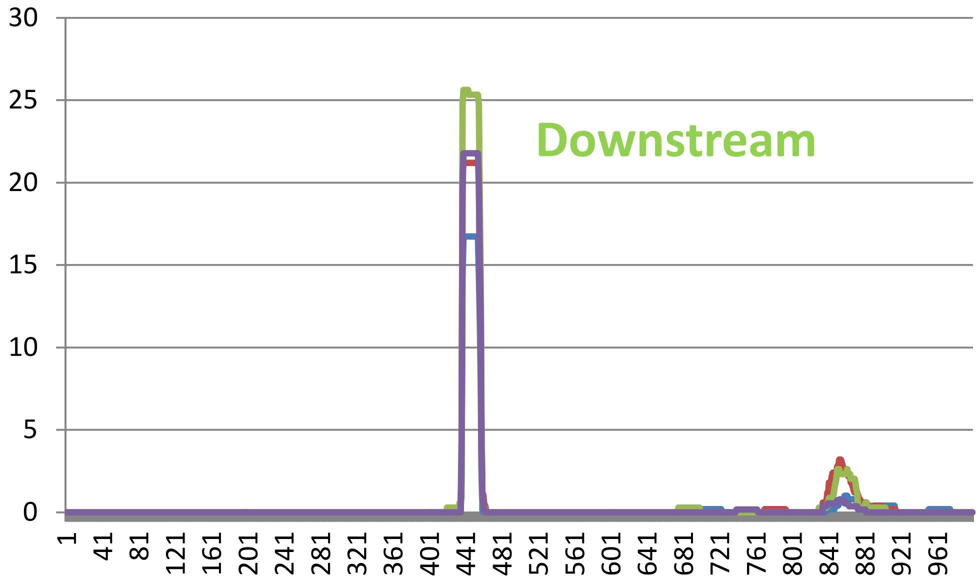

AT2G07000

Unknown protein

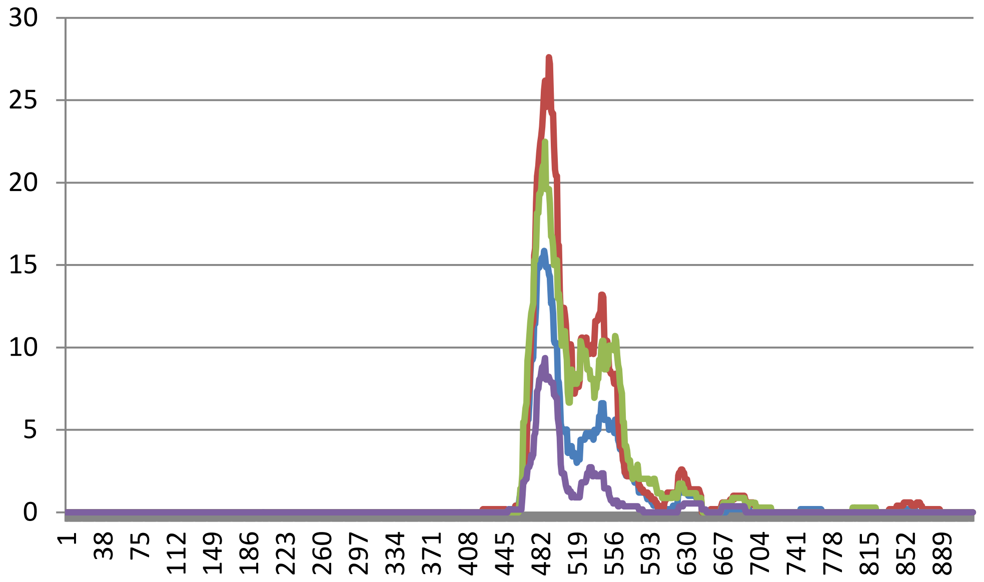

AT2G13540

Encodes a nuclear cap-binding protein that forms a heterodimeric complex with CBP20 and is involved in ABA signaling and flowering. Mutants are early flowering and exhibit hypersensitive response to ABA in germination inhibition. Loss of ABH1 function results in abnormal processing of mRNAs for several important floral regulators (FLC, CO, FLM). Analysis of loss of function mutations suggests a role in pri-miRNA processing and mRNA splicing.

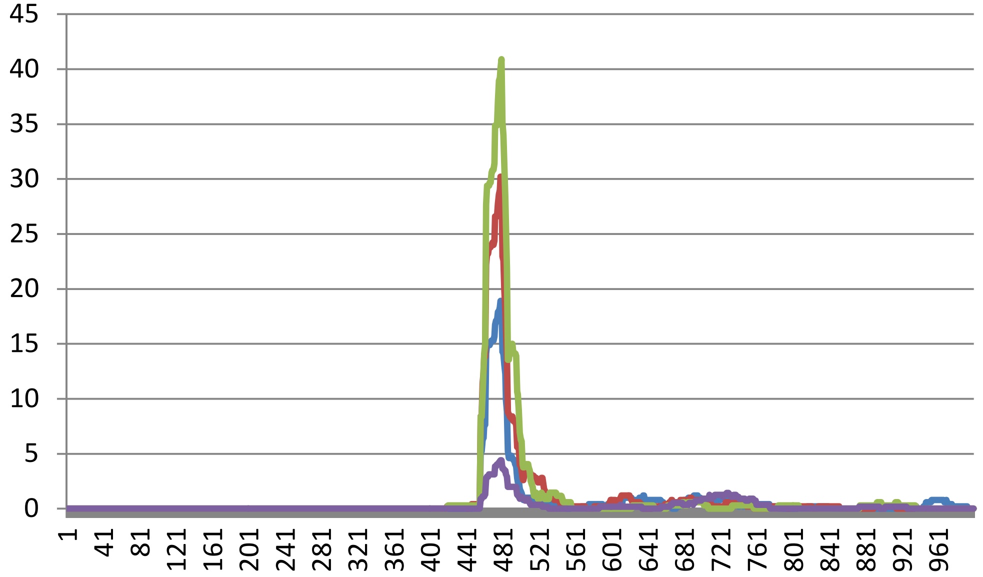

AT2G15790

SQN encodes the Arabidopsis homolog of cyclophilin 40 (CyP40).

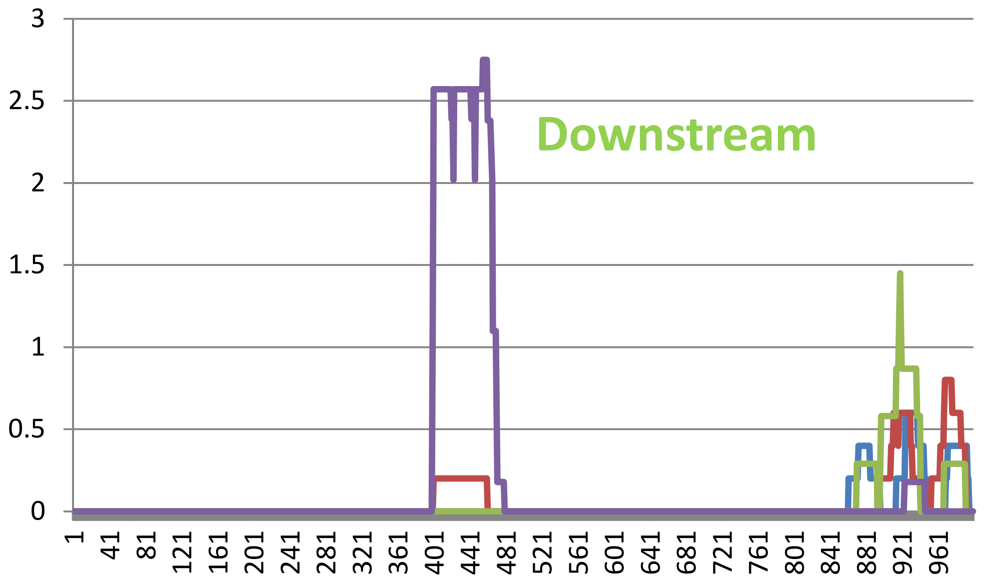

AT2G16365

F-box family protein

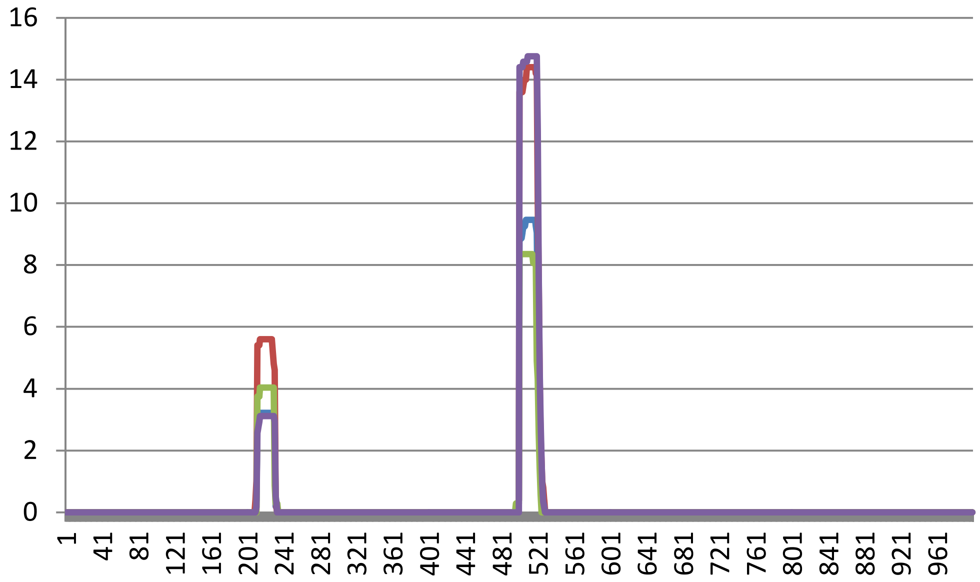

AT2G16640

Multimeric translocon complex in the outer envelope membrane 132 (TOC132)

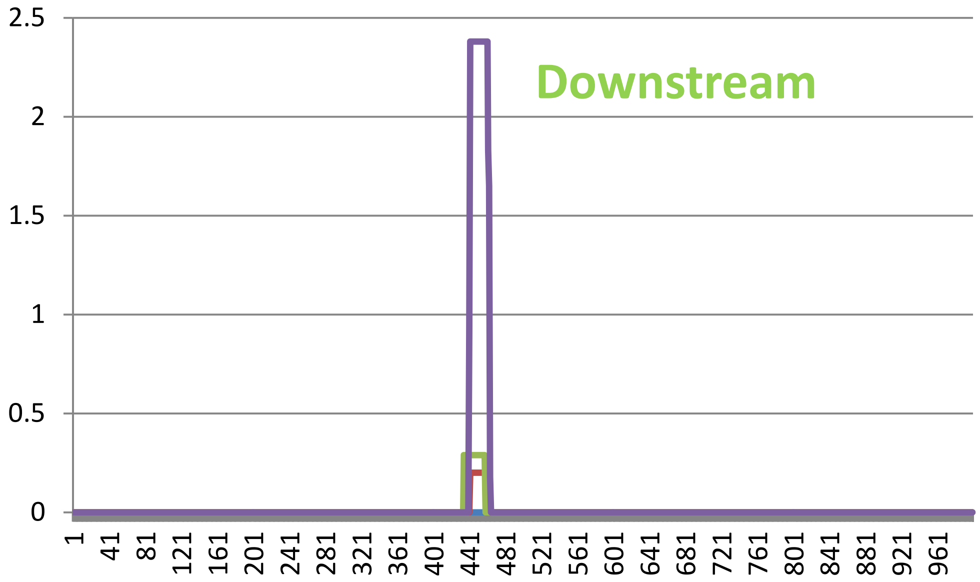

AT2G17510

EMBRYO DEFECTIVE 2763 (EMB2763)

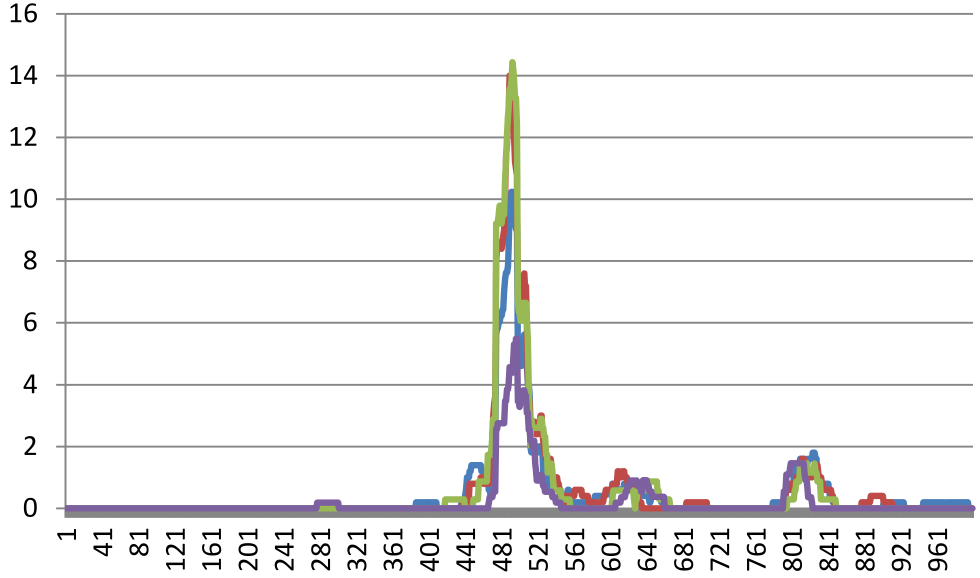

AT2G18600

Ubiquitin-conjugating enzyme family protein

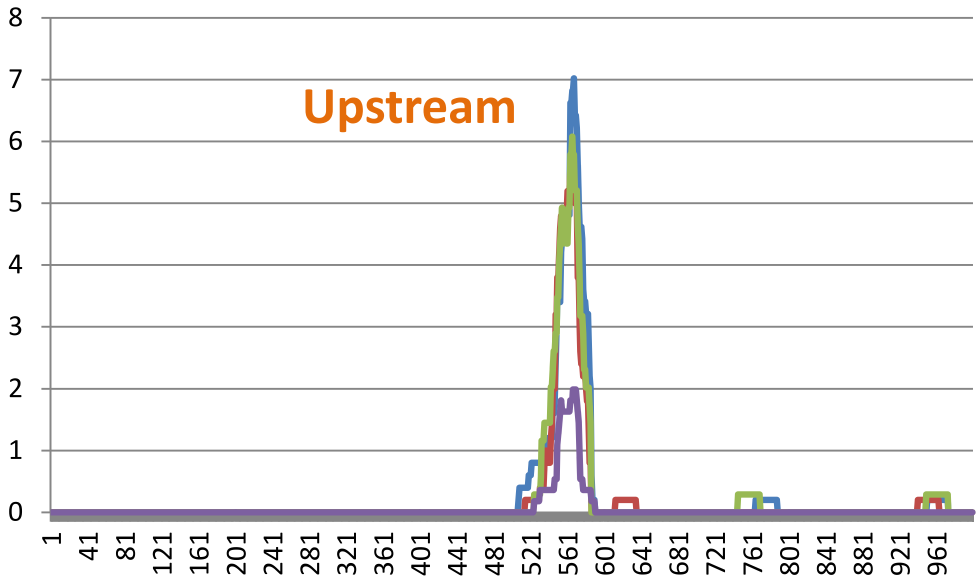

AT2G18870

Vernalization5/VIN3-like (VEL3)

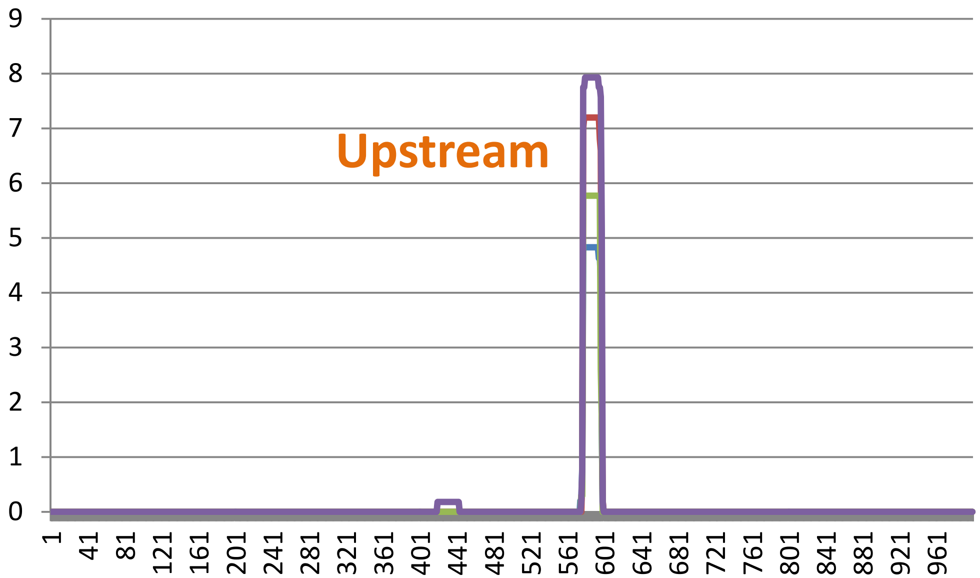

AT2G19270

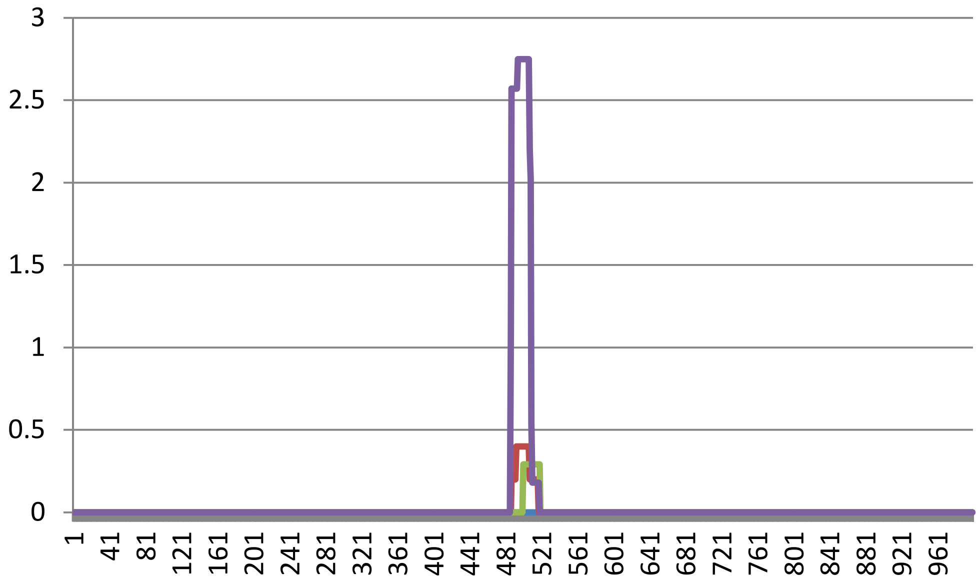

3
2.5
2
1.5
1
0.5
0
1
41
81
121
161
201
241
281
321
361
401
441
481
521
561
601
641
681
721
761
801
841
881
921
961

AT2G21420

IBR domain containing protein

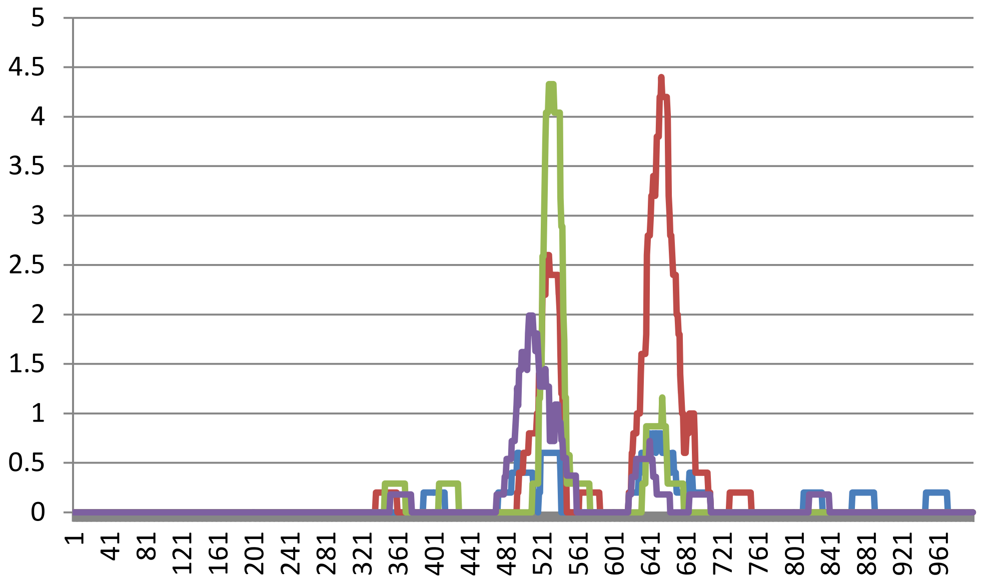

AT2G24010

Serine carboxypeptidase-like 23 (scpl23)

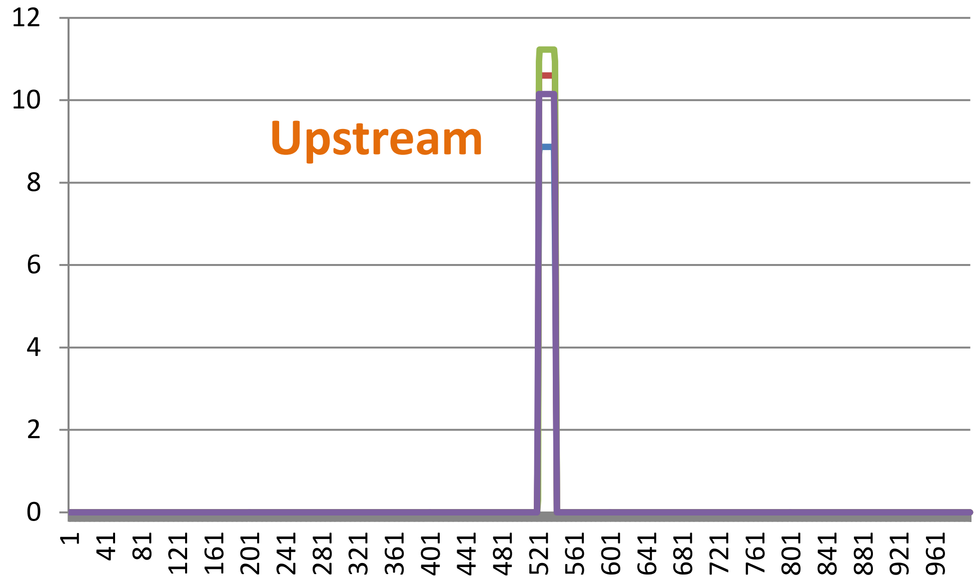

AT2G24670

Domain of unknown function (DUF313)

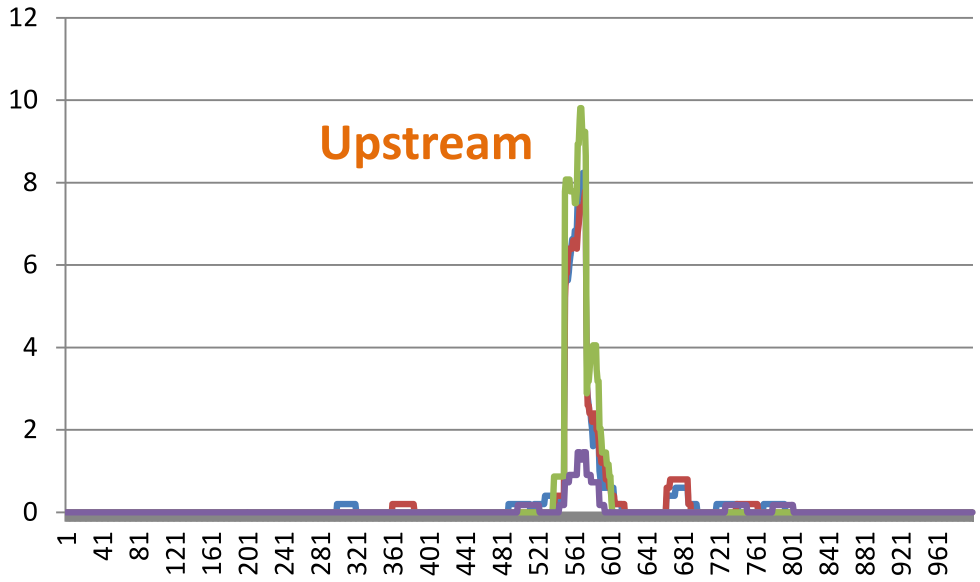

AT2G26210

Ankyrin repeat family protein

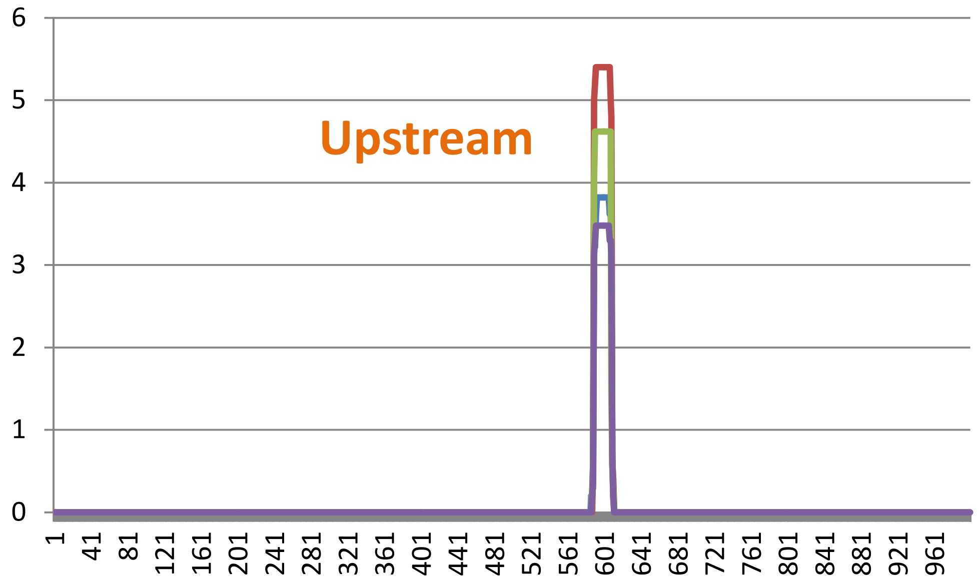

AT2G27050

Ethylene-insensitive3-like1 (EIL1)

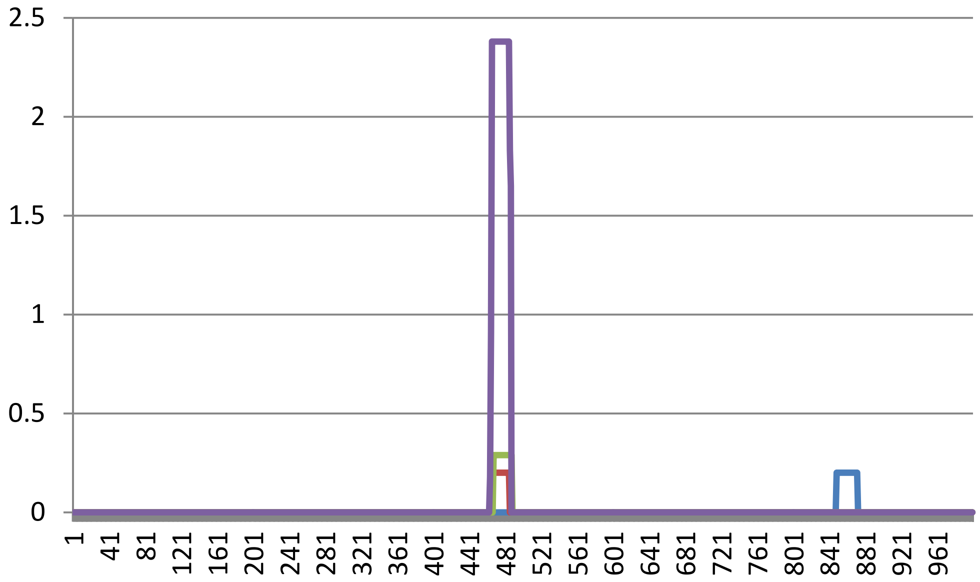

AT2G28710

C2H2-type zinc finger family protein

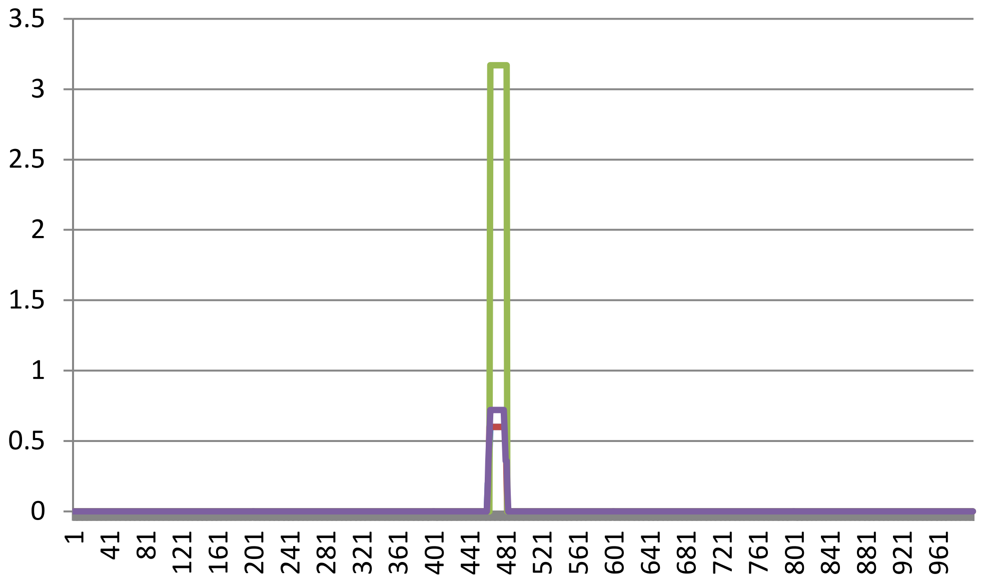

AT2G29460

Encodes glutathione transferase belonging to the tau class of GSTs.

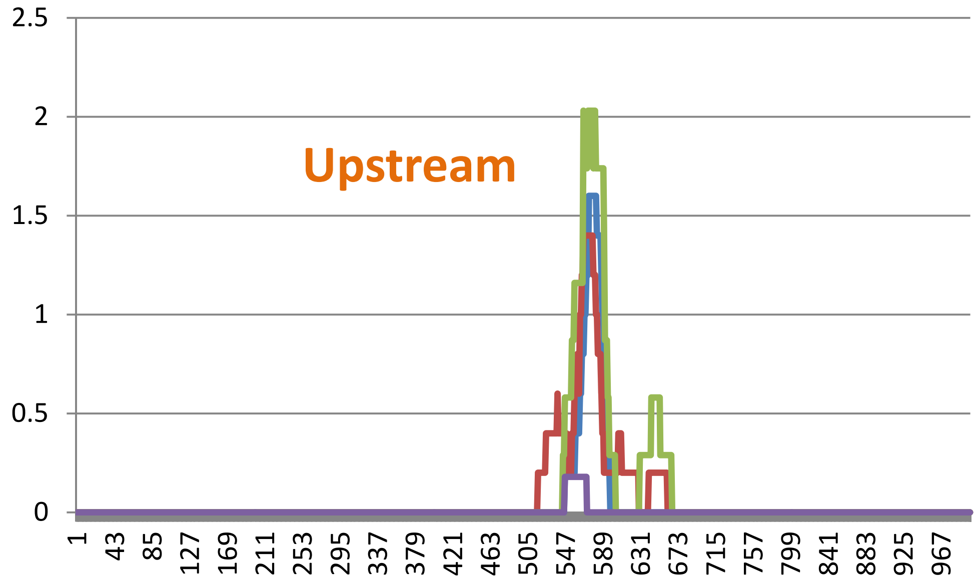

AT2G29605

Plant protein 1589 of unknown function

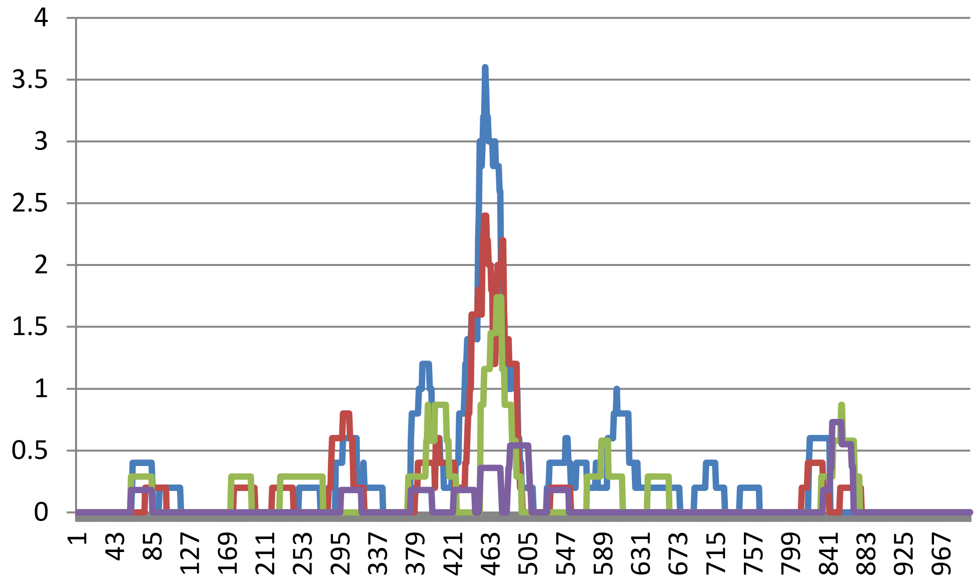

AT2G31305

Encodes inhibitor-3 (Inh3), a regulatory subunit of protein phosphatase 1 (PP1). Inh3 inhibits the phosphatase activity of the PP1 catalytic subunit (PP1c). Biochemical analyses demonstrate that Inh3 binds to PP1c via the RVxF motif of Inh3, a consensus PP1c-binding sequence both in vitro and in vivo.

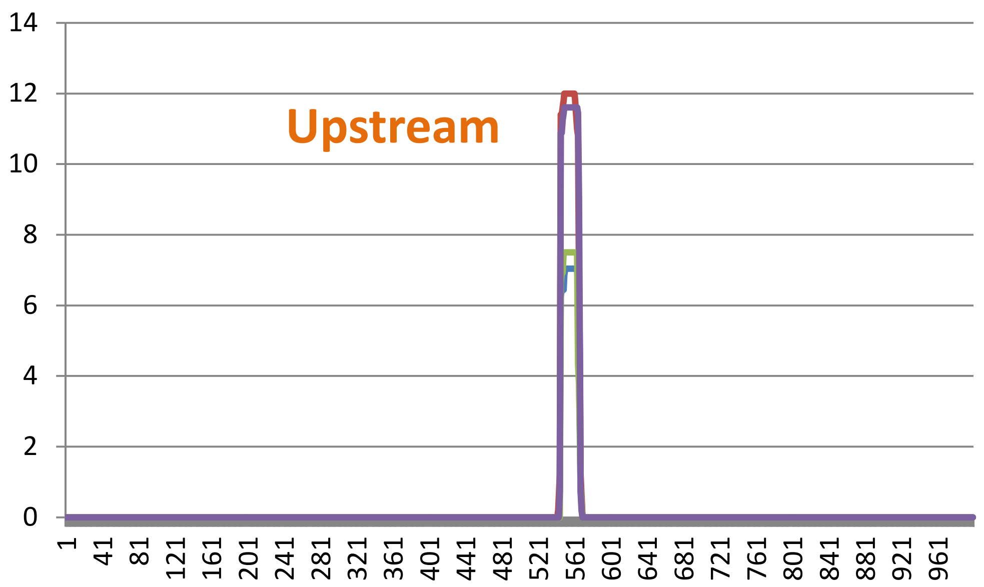

AT2G32130

Plant protein of unknown function (DUF641)

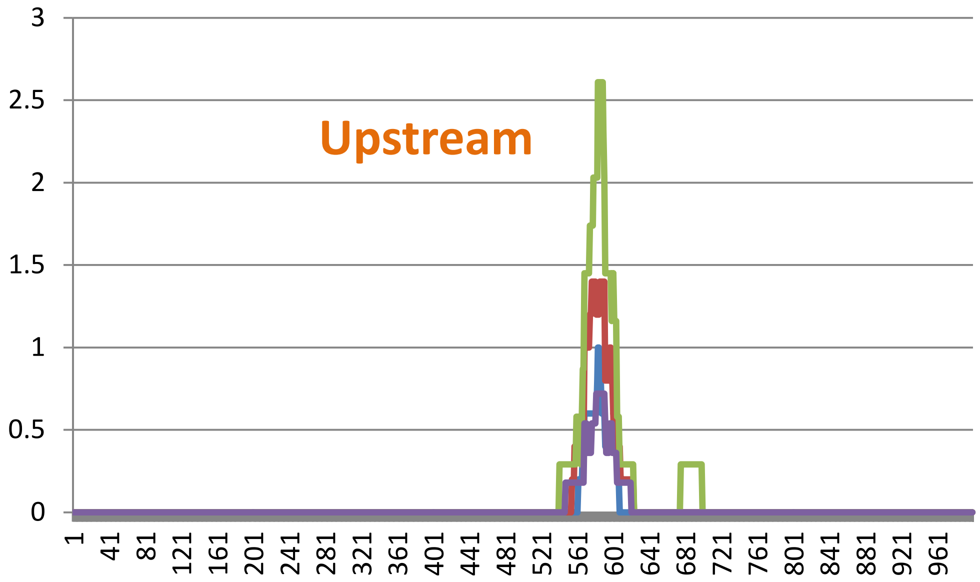

AT2G32410

AXR1-like (AXL)

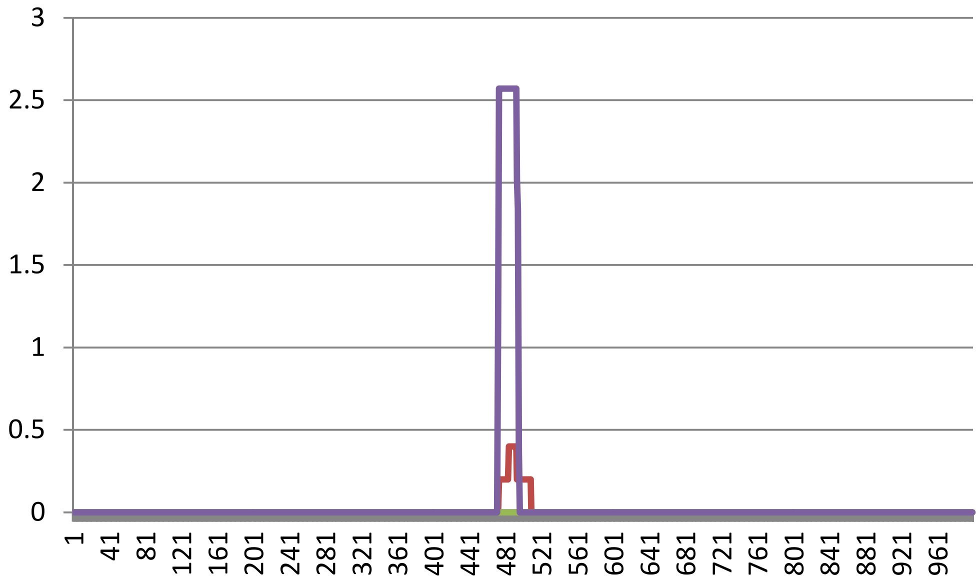

AT2G35830

Unknown protein

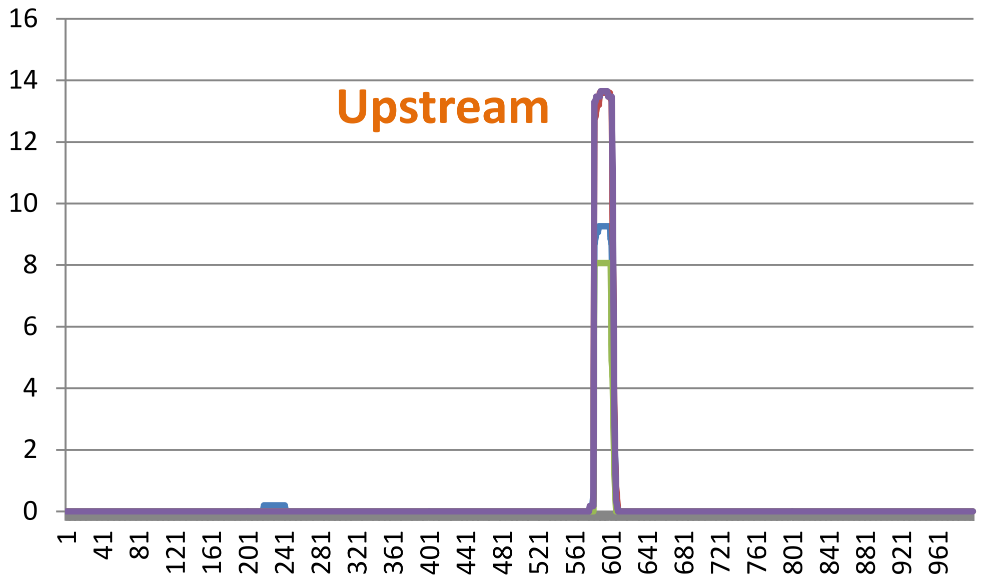

AT2G36460

Aldolase superfamily protein

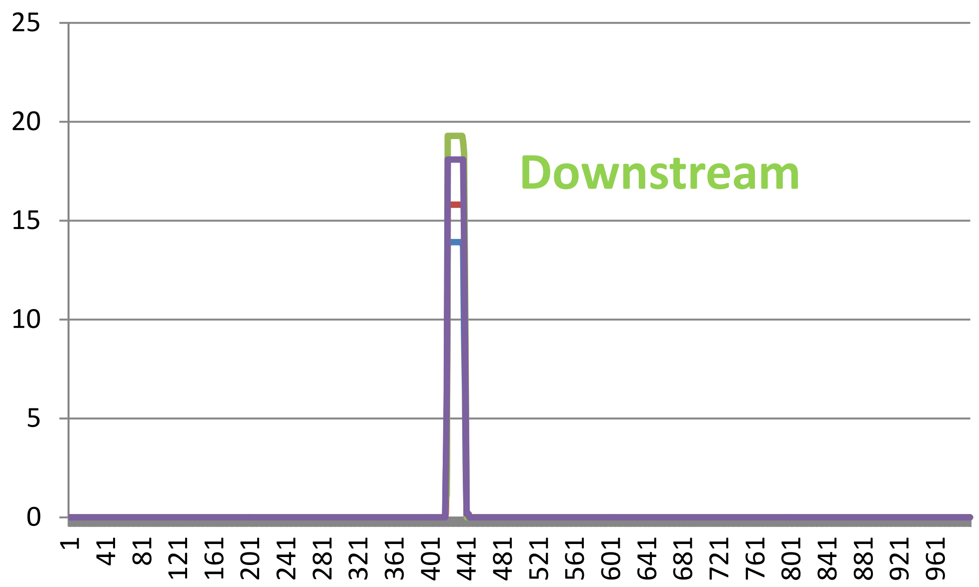

AT2G38025

Cysteine proteinases superfamily protein

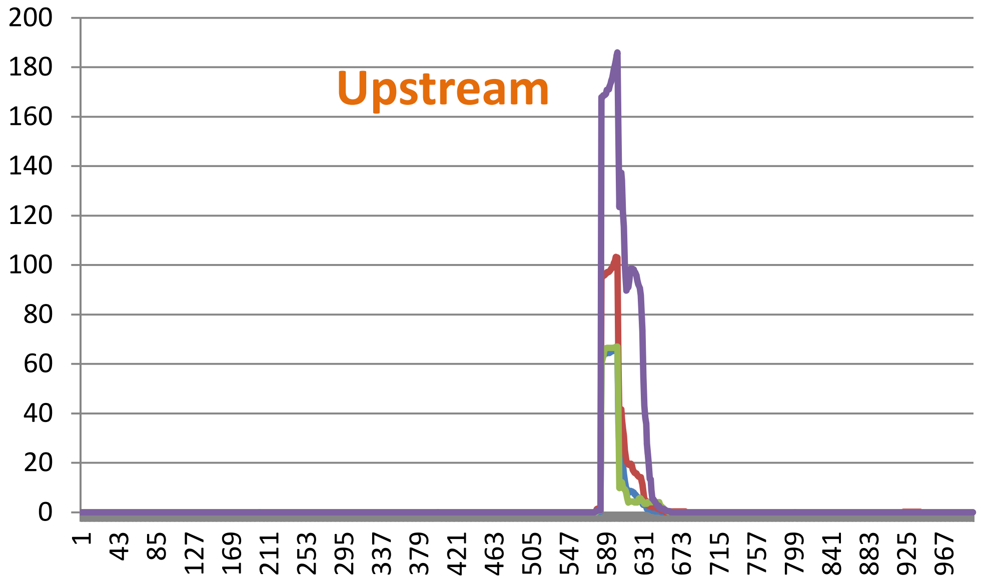

AT2G38110

Encodes a protein with glycerol-3-phosphate acyltransferase activity.

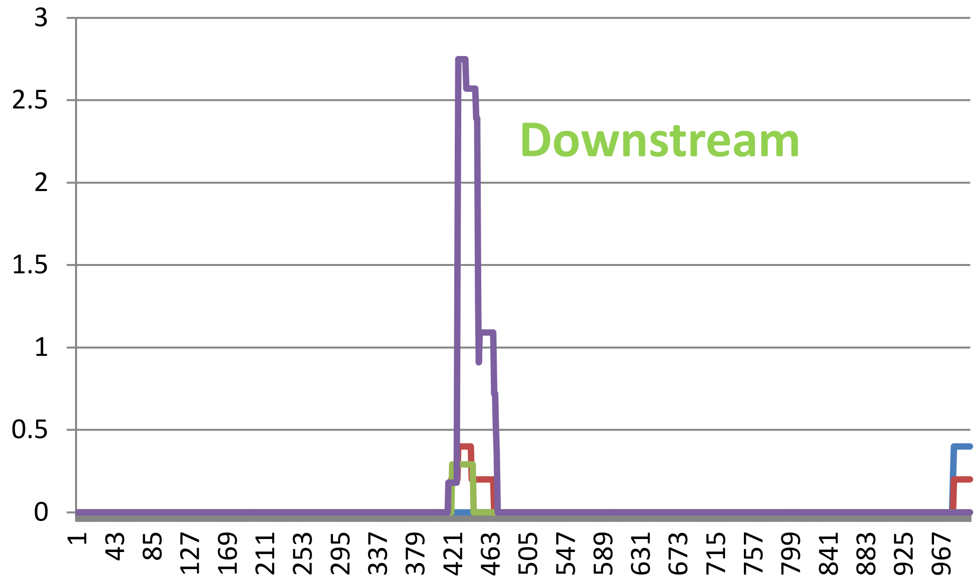

AT2G39310

Jacalin-related lectin 22 (JAL22)

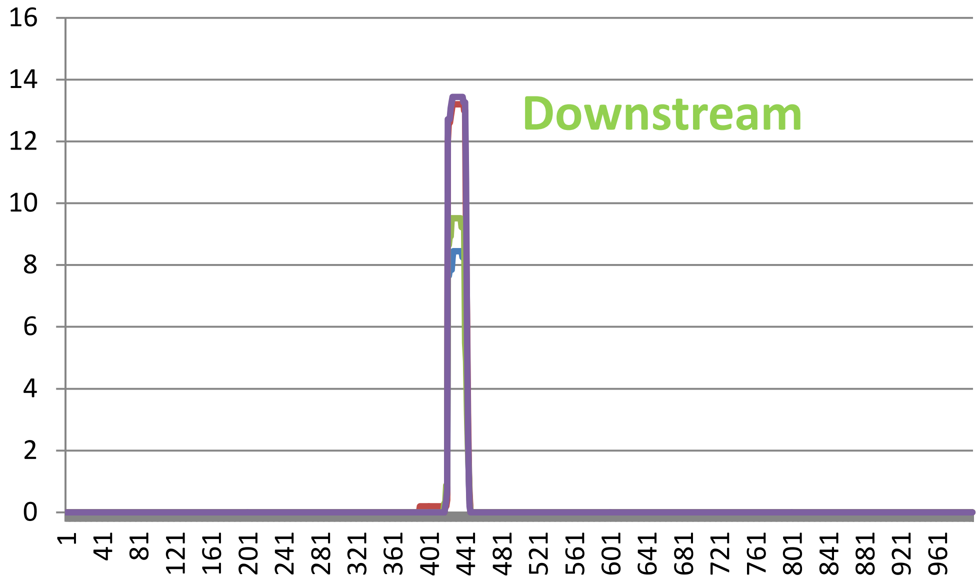

AT2G42880

Member of MAP Kinase

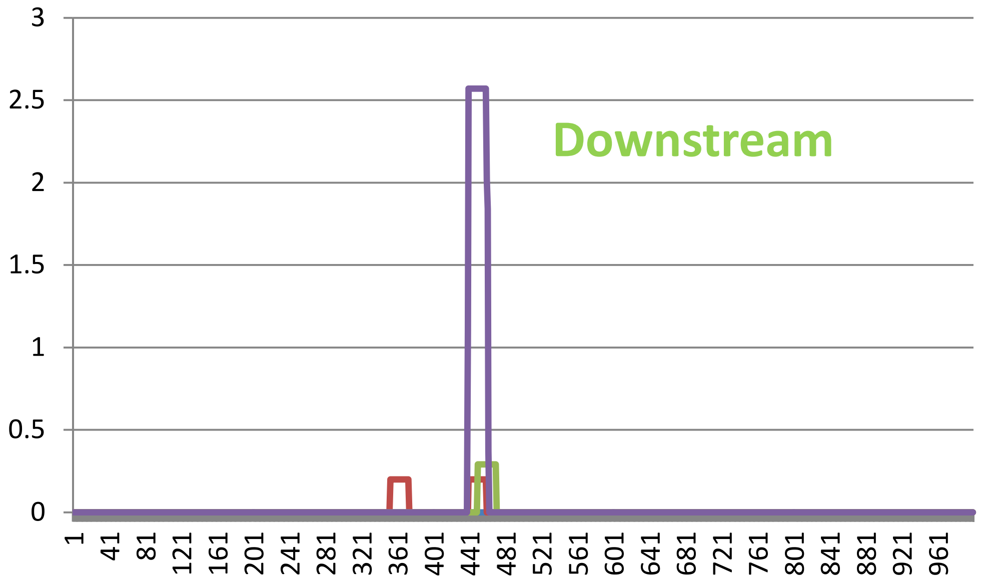

AT2G46920

Pol mutations are recessive, partial suppressors of meristem defects in strong clv1 and clv3 mutants, and nearly complete suppressors of weak clv1 mutants. Single mutants appear normal. Acts downstream of the CLV signaling pathway in meristem development and is required together with PLL1 for stem-cell maintenance through the regulation of WUS.

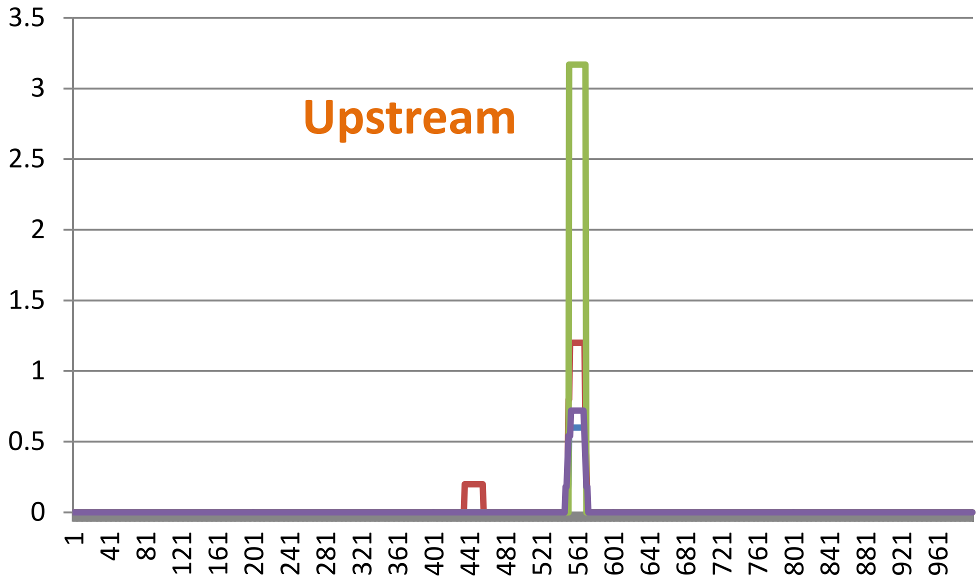

AT2G47090

Zinc ion binding; nucleic acid binding

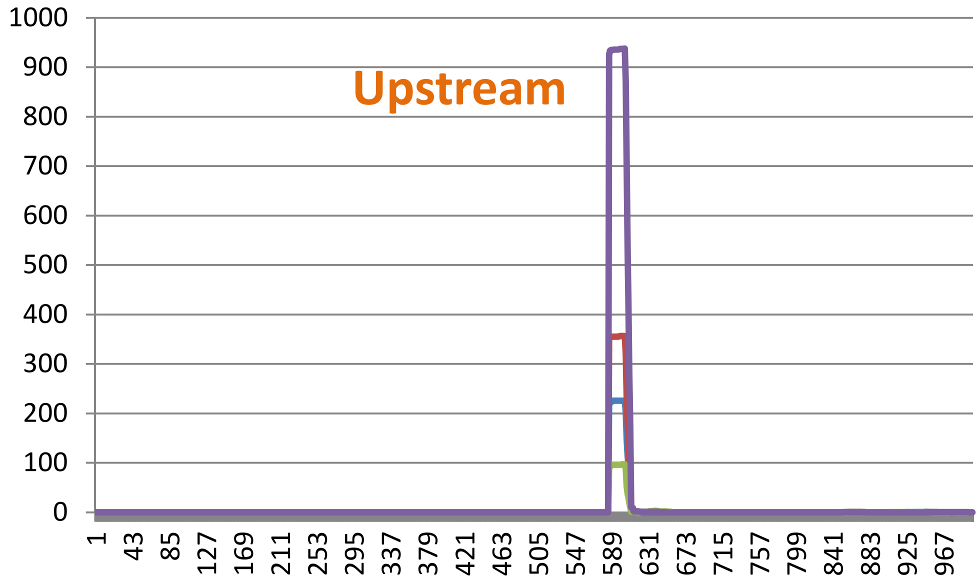

AT3G01890

SWIB/MDM2 domain superfamily protein

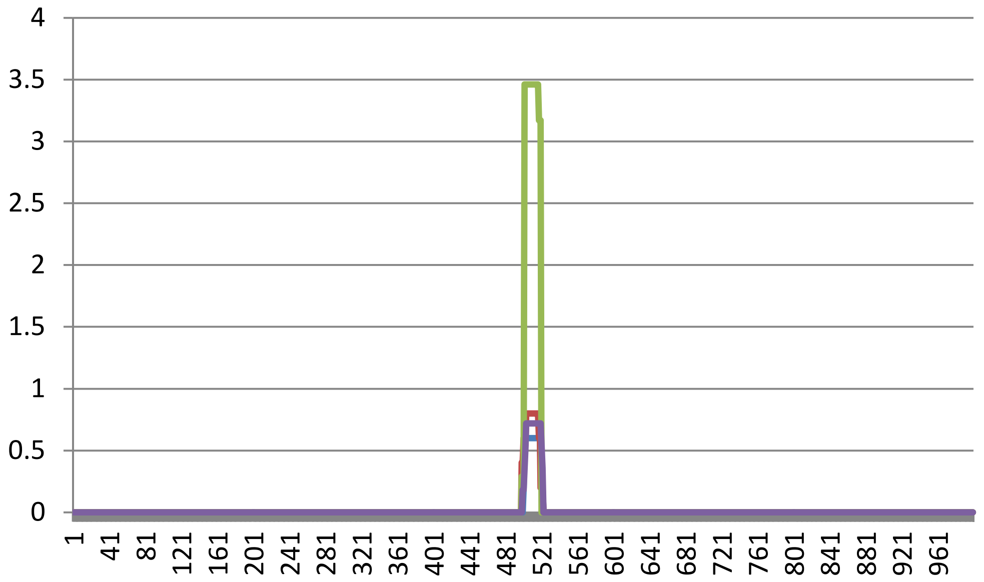

AT3G02110

Serine carboxypeptidase-like 25 (scpl25)

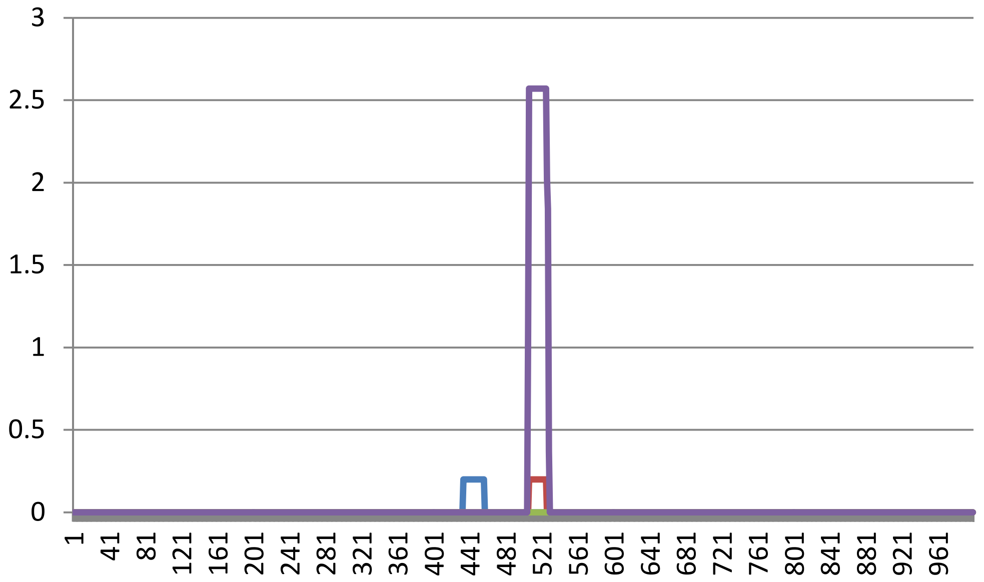

AT3G04740

Encodes a protein with similarities to subunits of the Mediator complex, required for RNA polymerase II recruitment at target promoters in response to specific activators. Lines carrying loss of function mutations in the gene have reduced cell numbers in aerial organs. On the other hand, lines overexpressing the gene have increased number of small cells in clusters, suggesting cell division is more unsynchronized in the overexpressors.

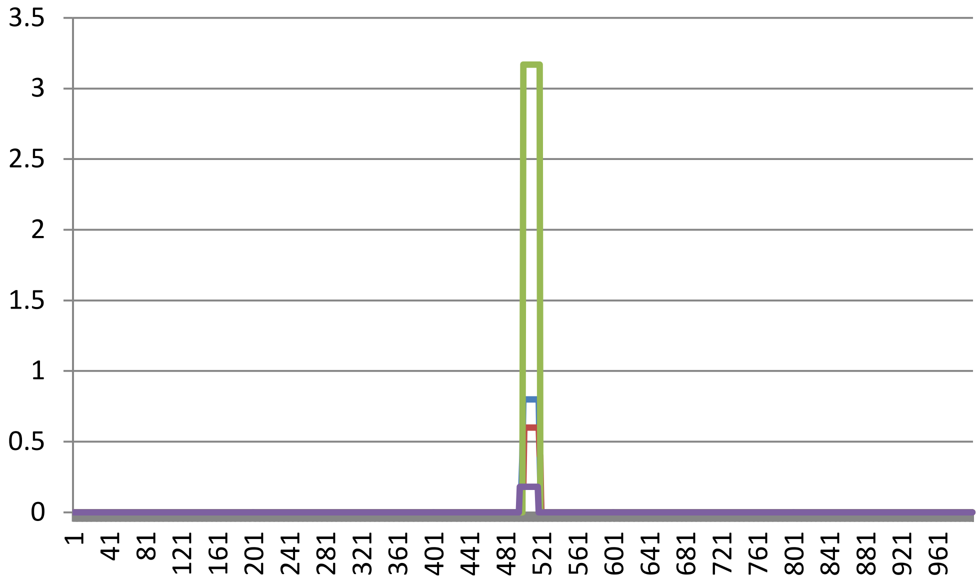

AT3G05320

O-fucosyltransferase family protein

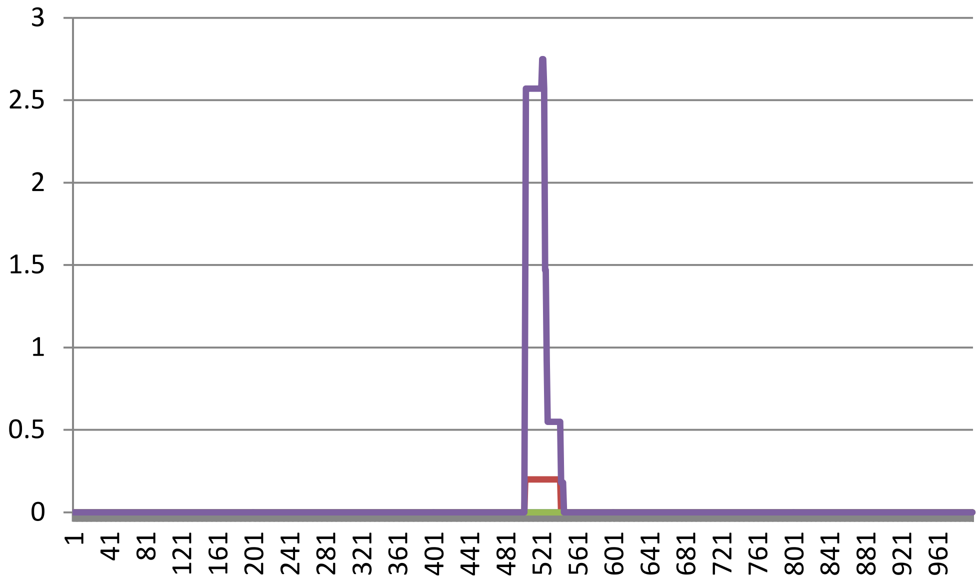

AT3G05710

Encodes a member of SYP4 Gene Family that is a plant ortholog of the Tlg2/syntaxin16 Qa-SNARE. Together with SYP42, it regulates the secretory and vacuolar transport pathways in the post-Golgi network and maintains the morphology of the Golgi apparatus and TGN and is required for extracellular resistance responses to a fungal pathogen.

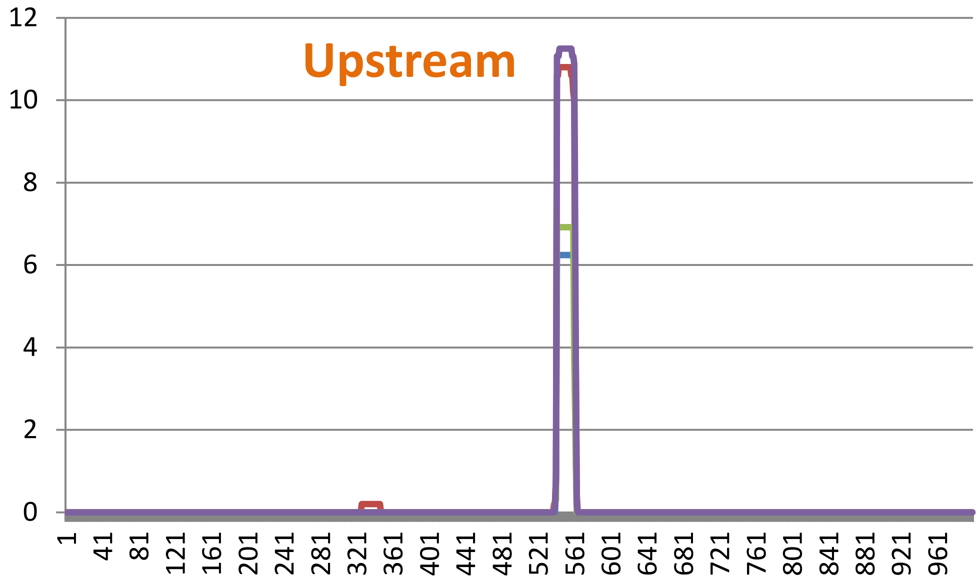

AT3G05760

C2H2 and C2HC zinc fingers superfamily protein

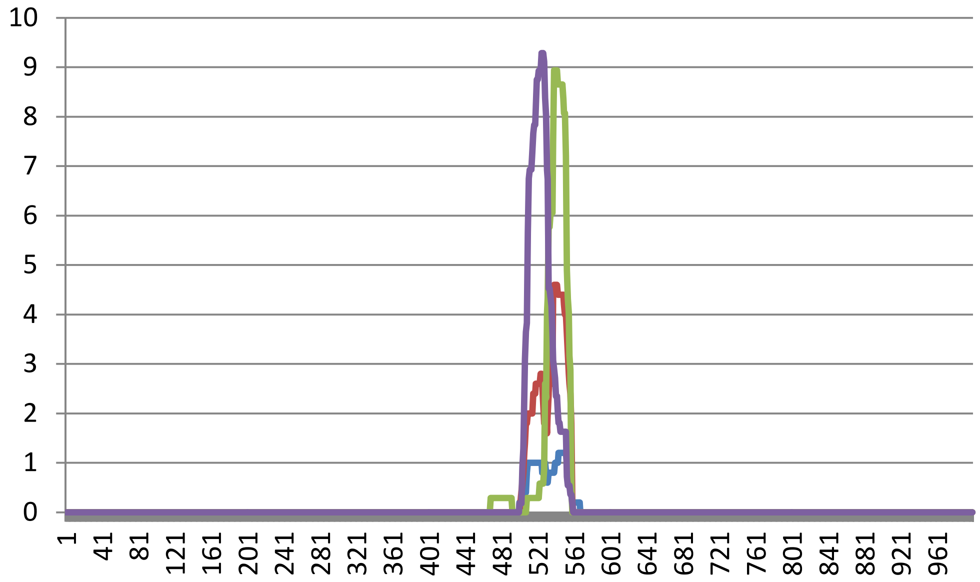

AT3G05770

Unknown protein

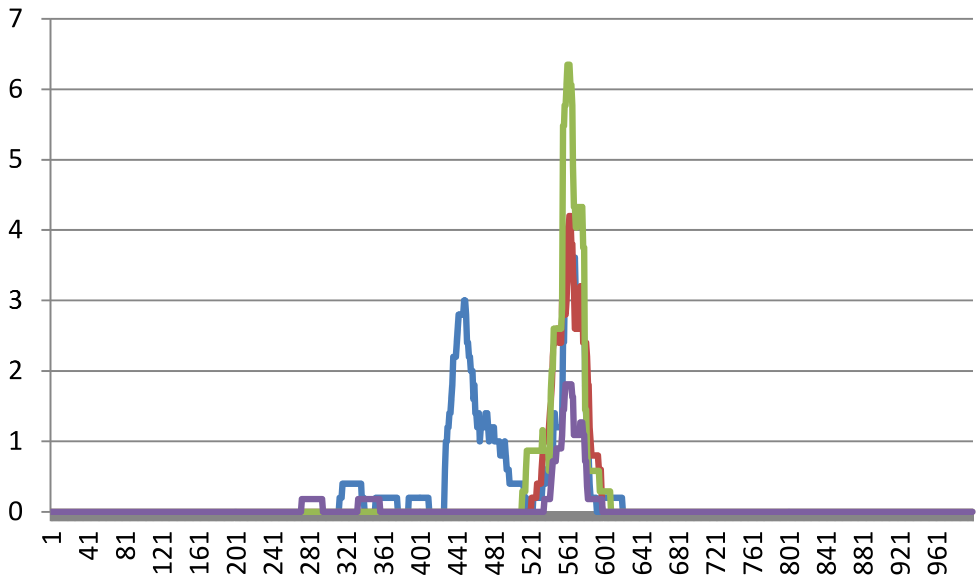

AT3G06110

Encodes a nuclear-localized MAP kinase phosphatase. Plants with reduced levels of MKP2 transcripts are hypersensitive to ozone and ozone-mediated activation of MPK3 and MPK6 is prolonged in these plants.

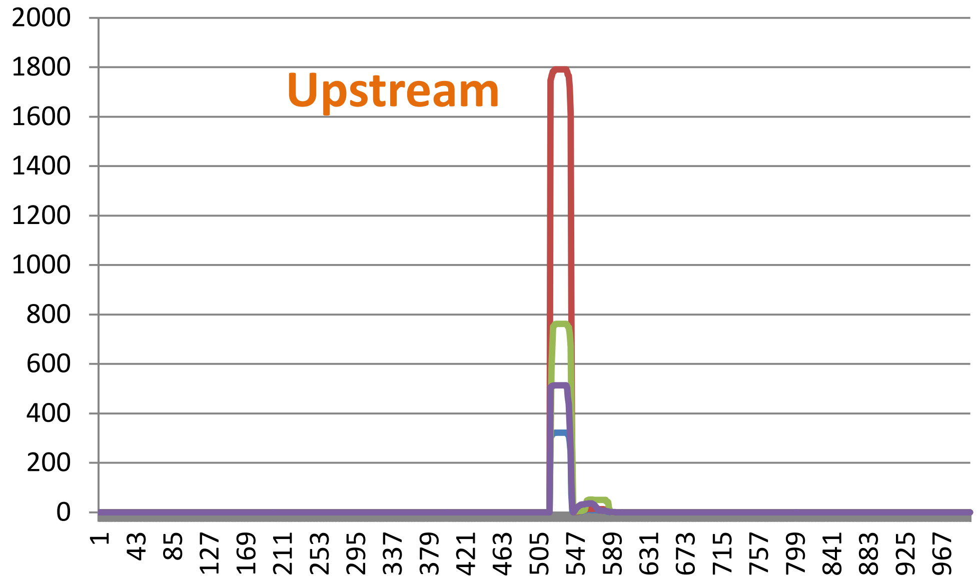

AT3G06490

Putative transcription factor MYB108 (MYB108) mRNA

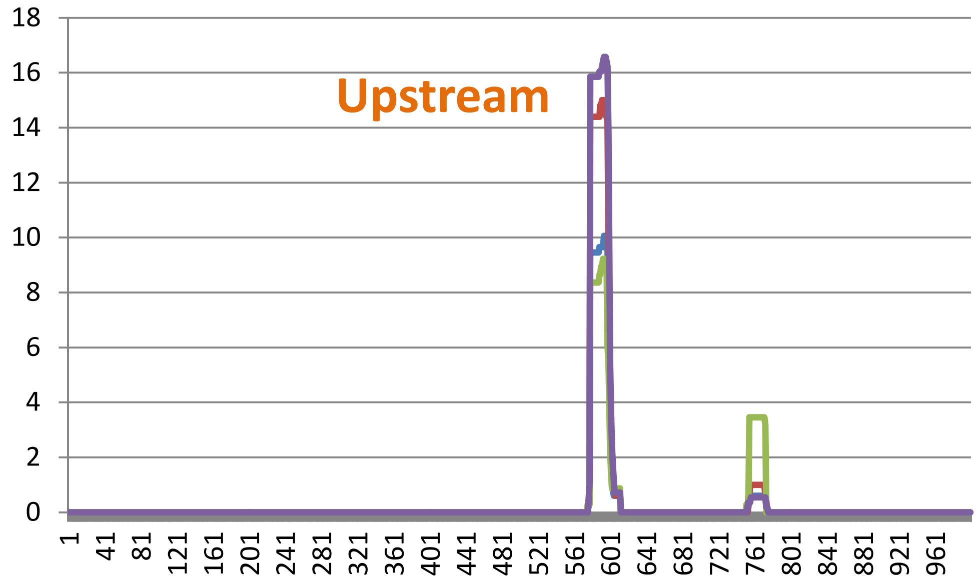

AT3G07060

Embryo defective 1974 (emb1974)

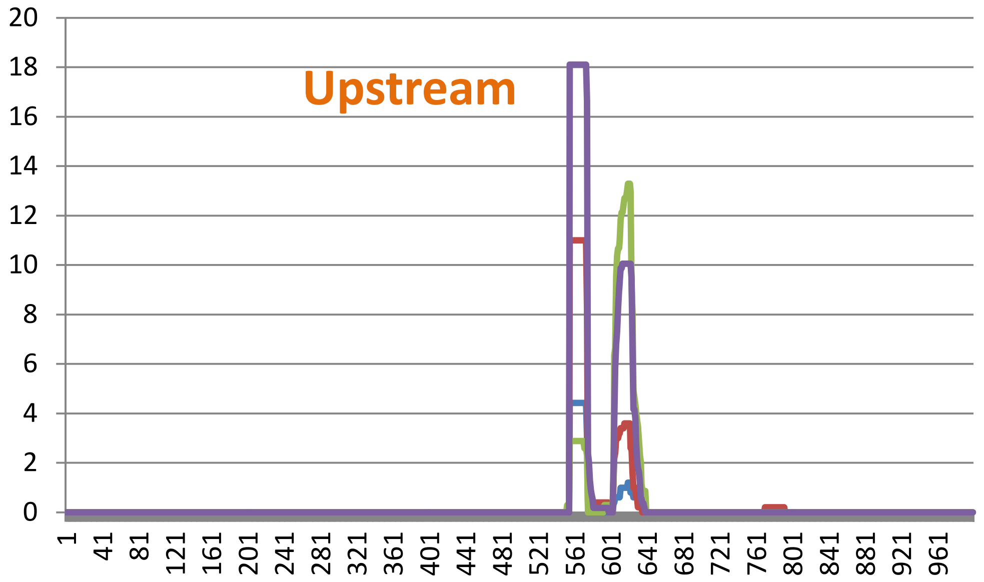

AT3G07180

GPI transamidase component PIG-S-related

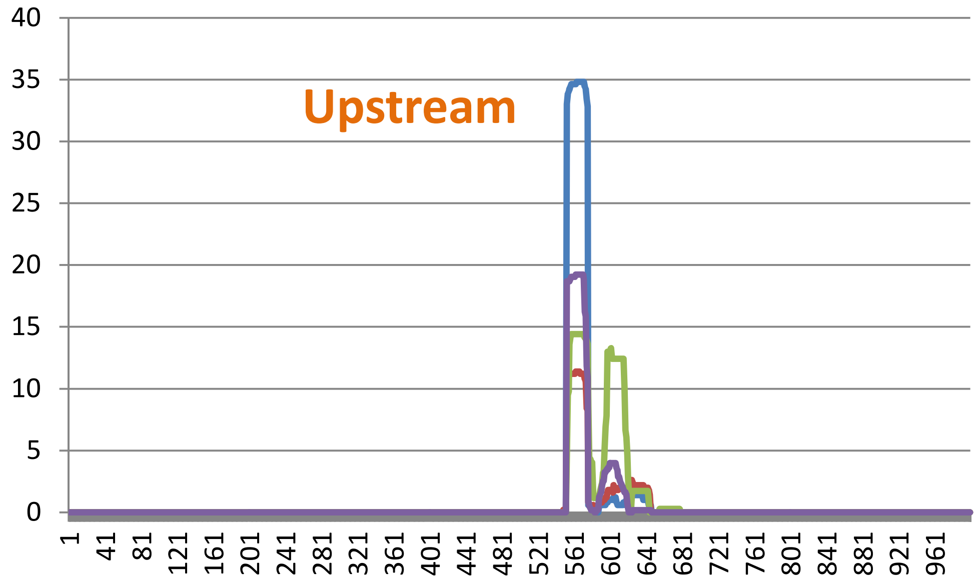

AT3G07810

RNA-binding (RRM/RBD/RNP motifs) family protein

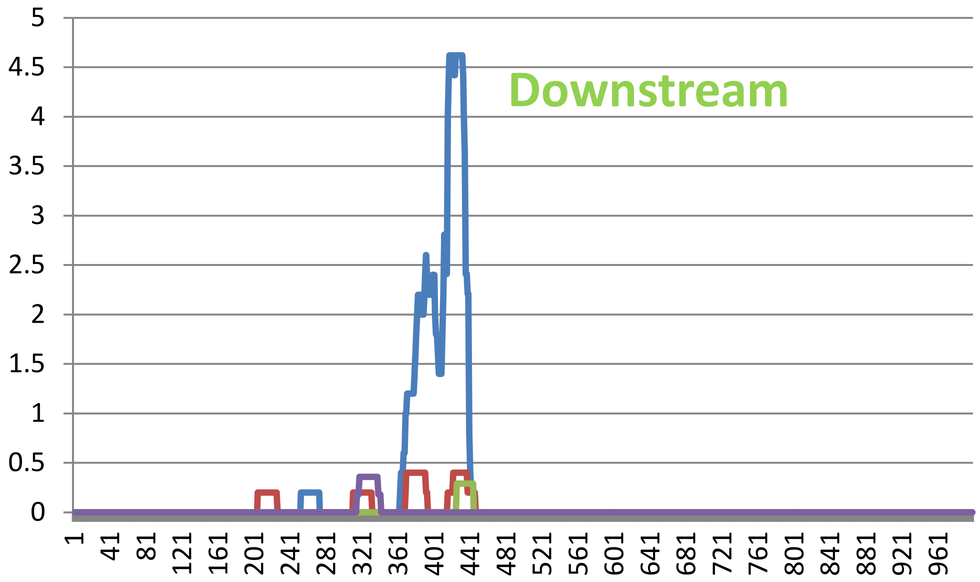

AT3G11760

Unknown protein

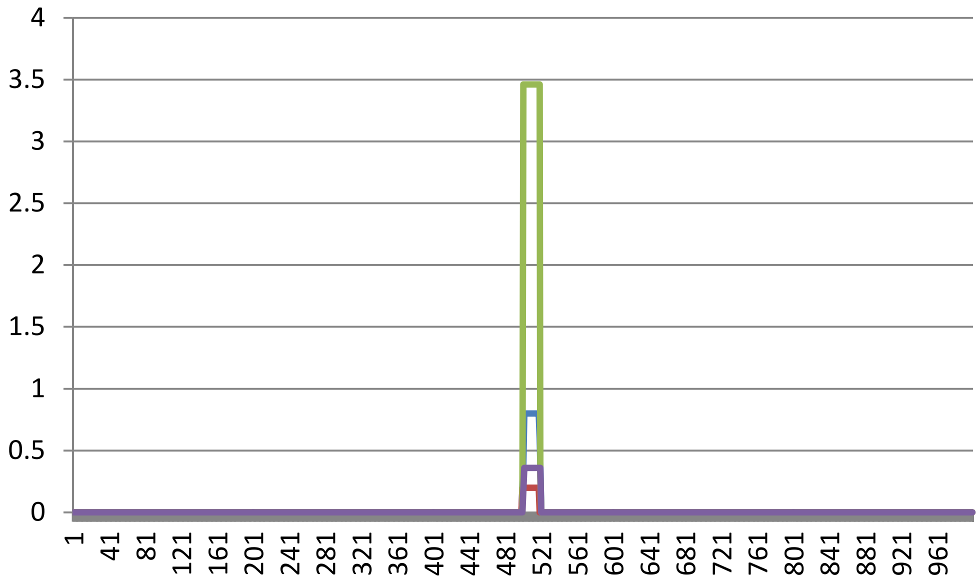

AT3G13440

S-adenosyl-L-methionine-dependent methyltransferases superfamily protein

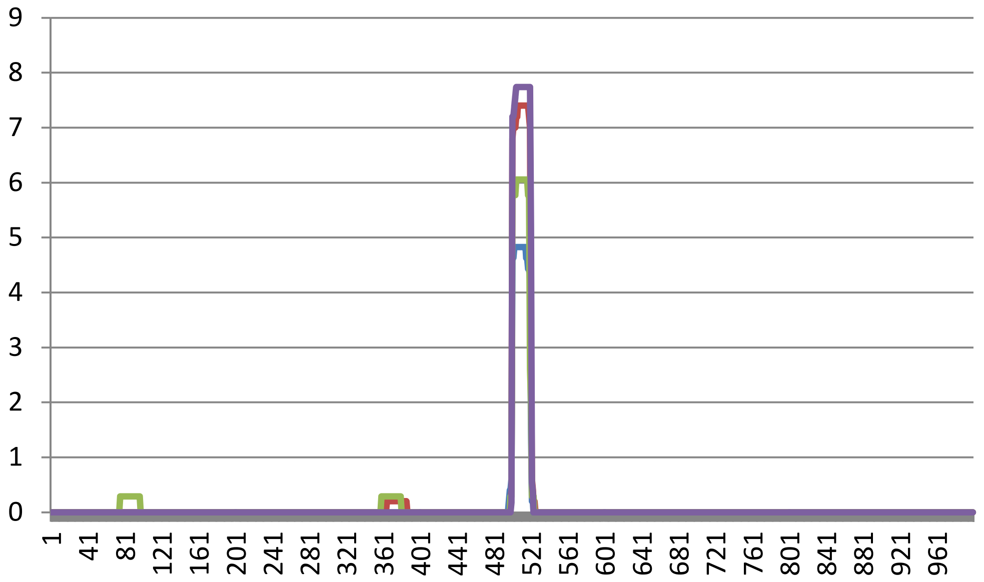

AT3G13480

Unknown protein

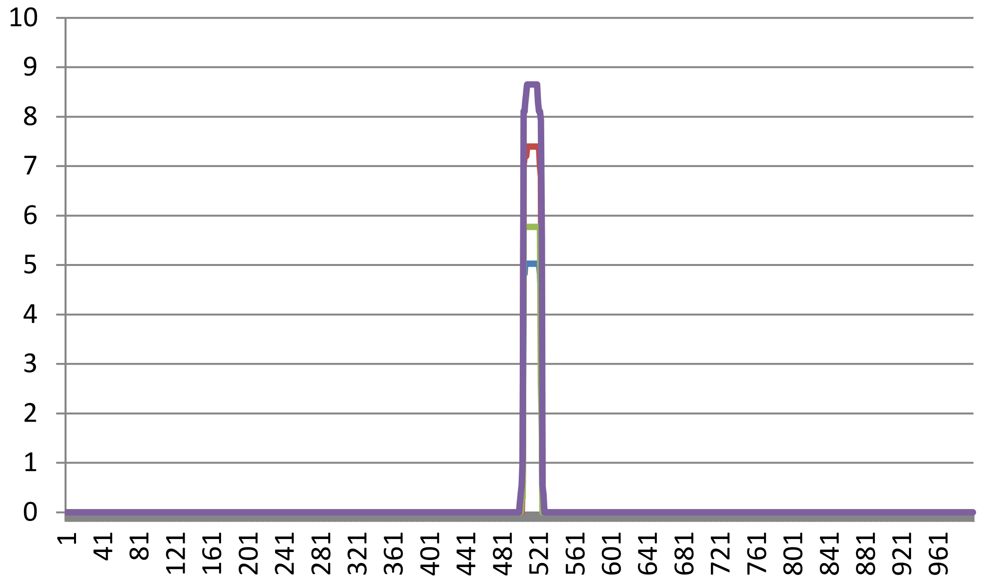

AT3G13857

Unknown protein

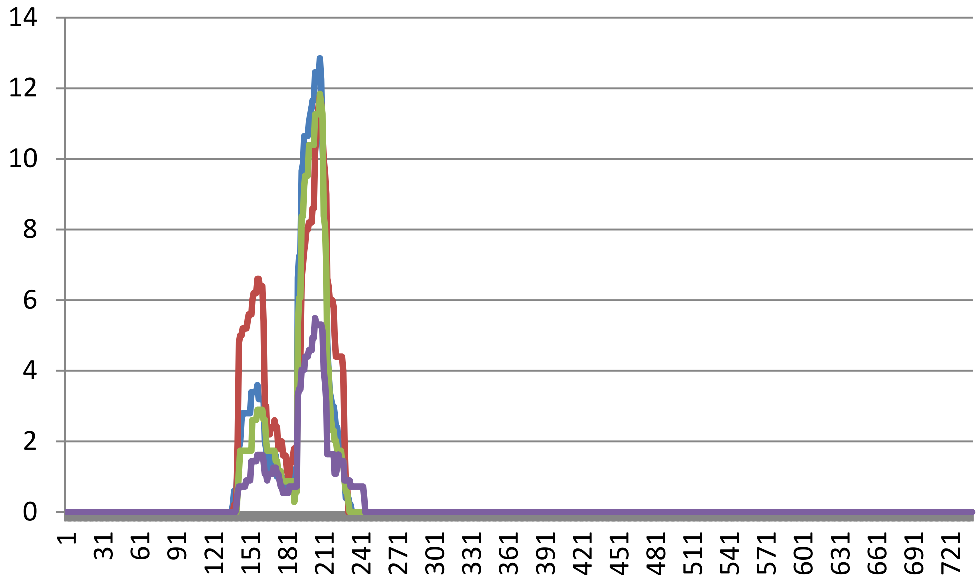

AT3G15580

Encodes APG8, a component of autophagy conjugation pathway. Delivered to the lumens of vacuole under nitrogen-starvation condition.

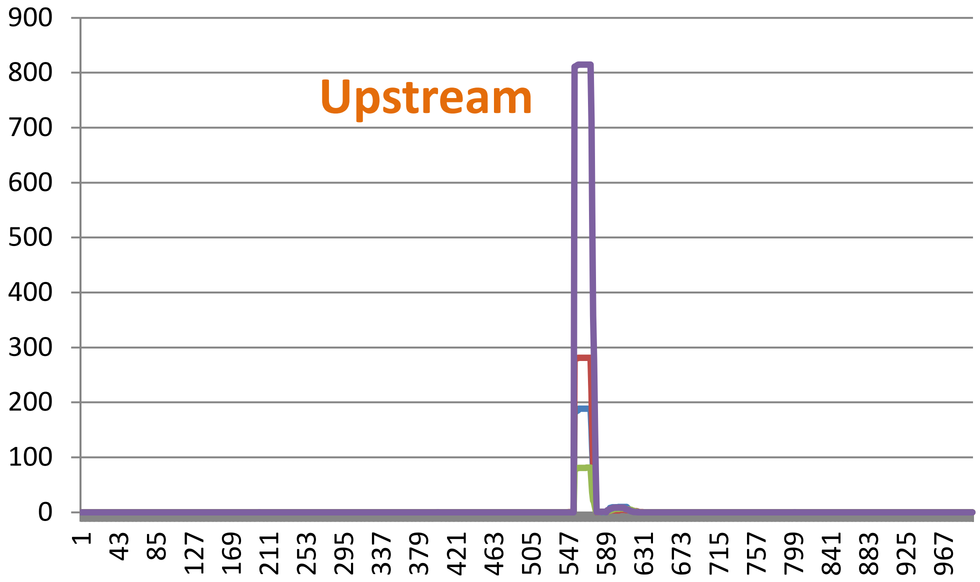

AT3G16230

Predicted eukaryotic LigT

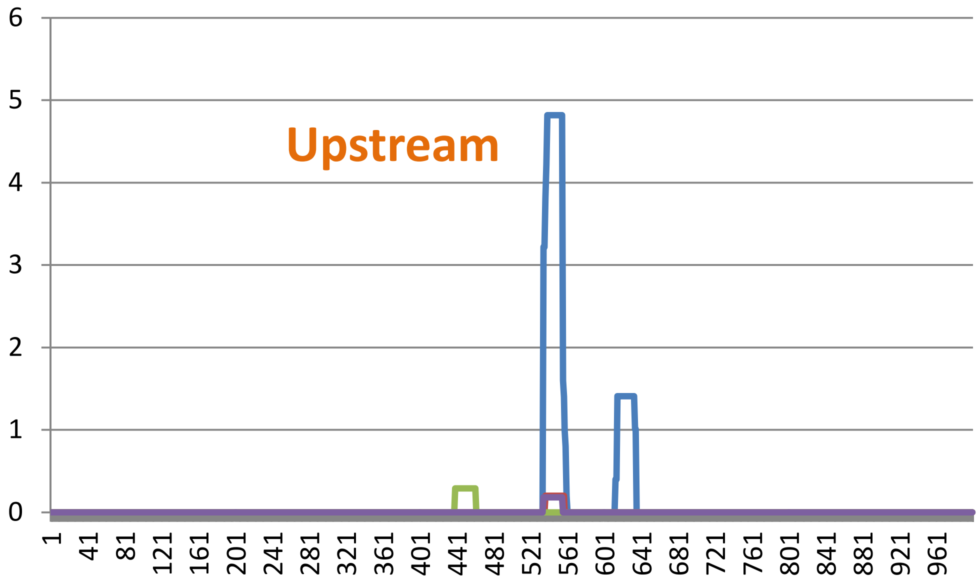

AT3G17500

F-box family protein

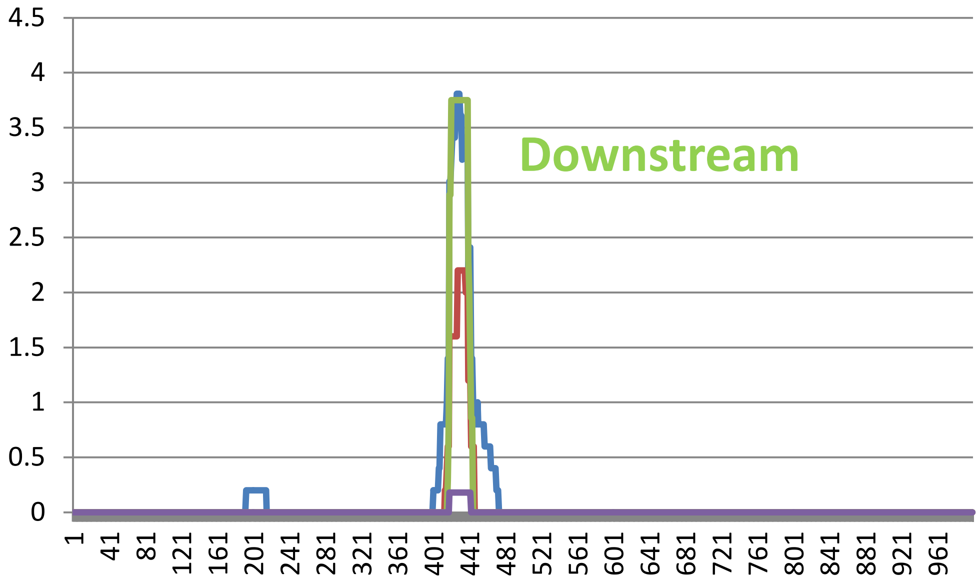

AT3G18820

RAB GTPase homolog G3F (RAB7B)

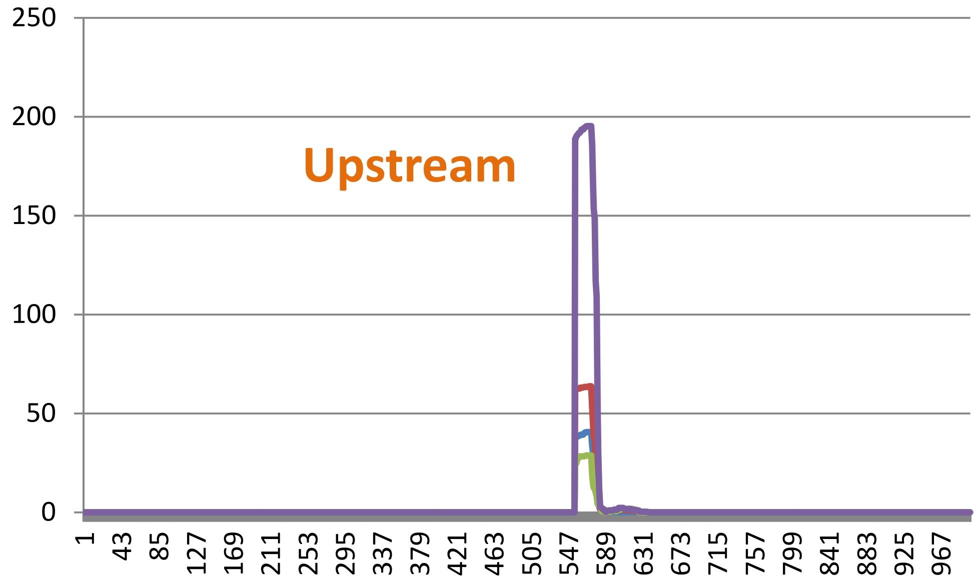

AT3G19880

F-box and associated interaction domains-containing protein

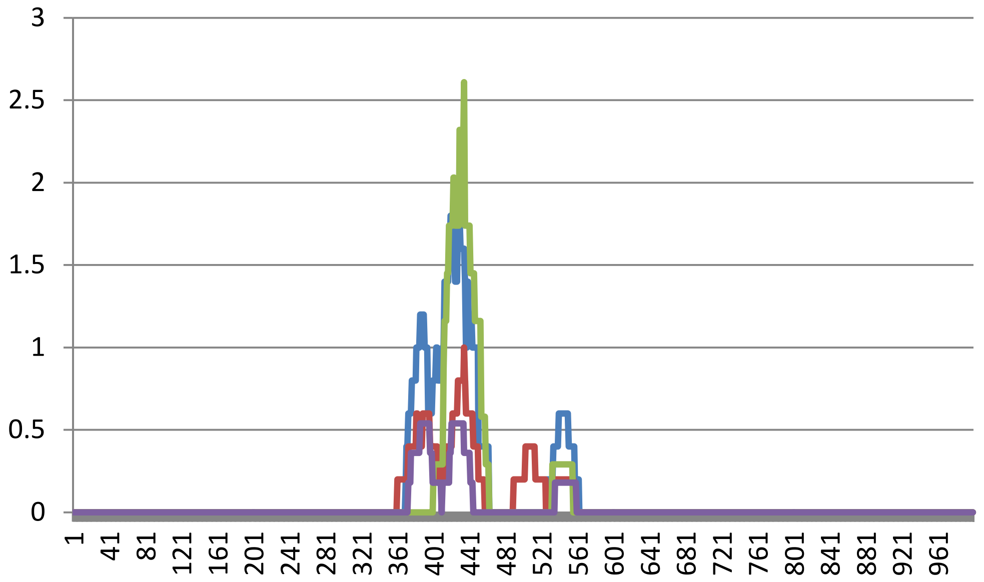

AT3G20090

Member of CYP705A

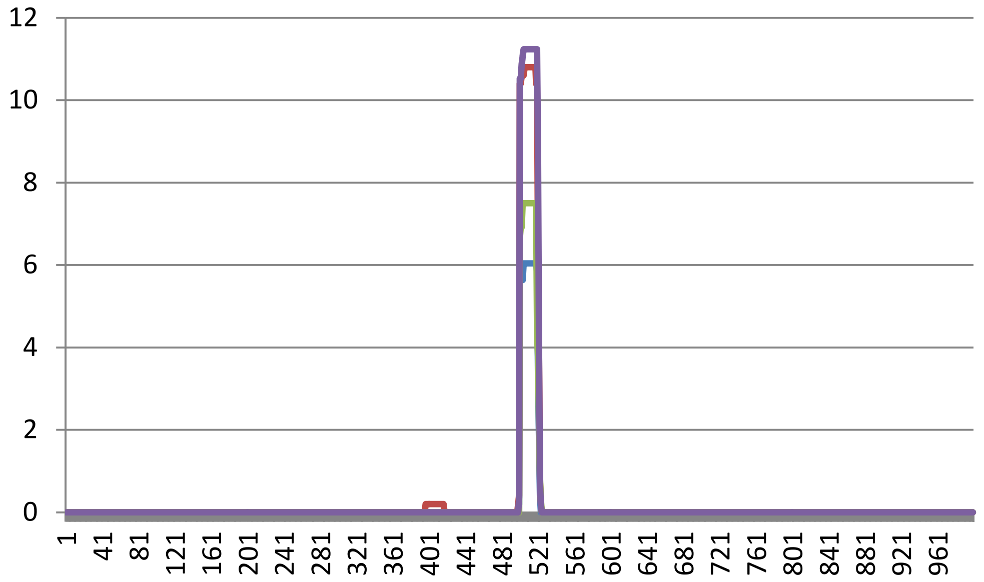

AT3G20290

Encodes AtEHD1, one of the Arabidopsis Eps15 homology domain proteins involved in endocytosis (AtEHD2, At4g05520).

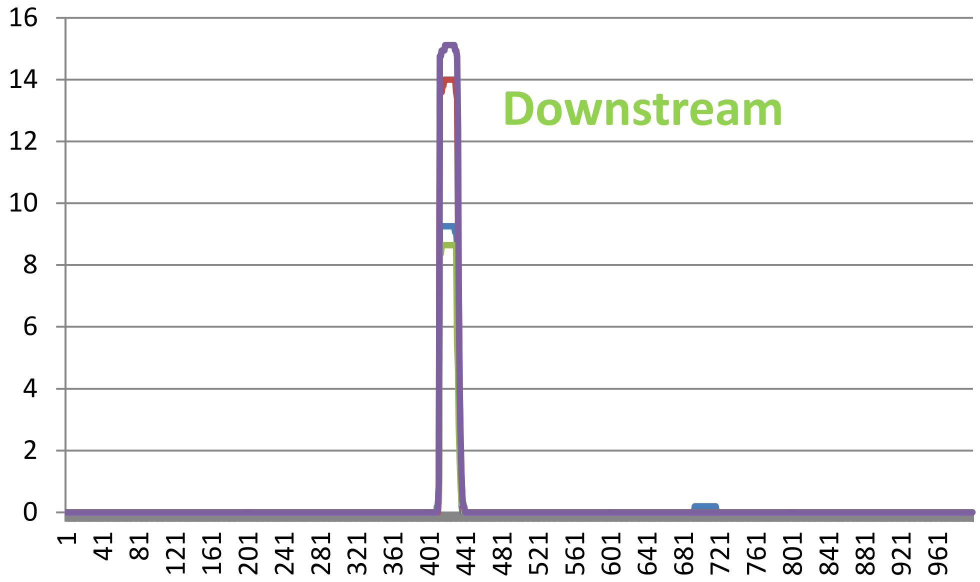

AT3G20720

Unknown protein

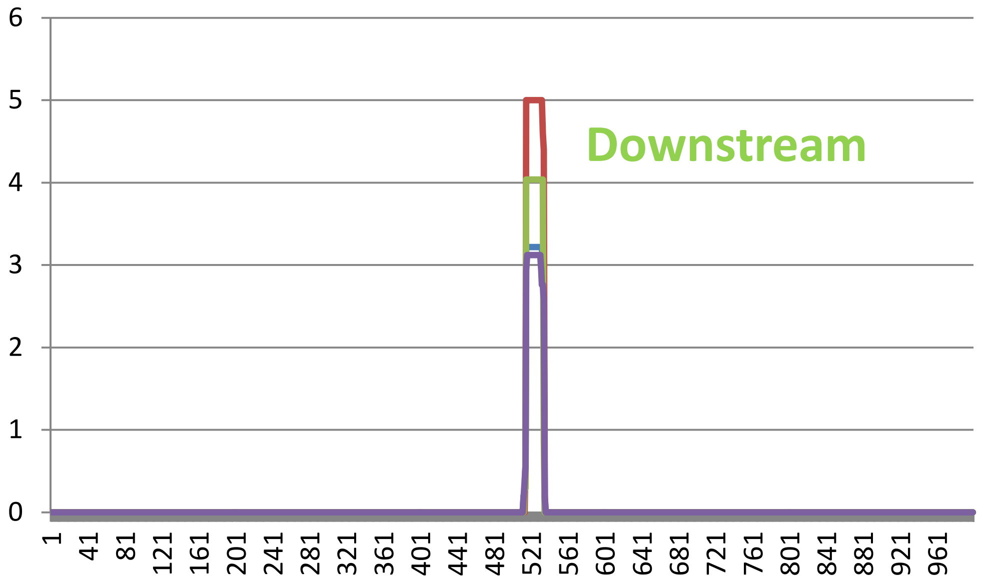

AT3G22350

F-box and associated interaction domains-containing protein

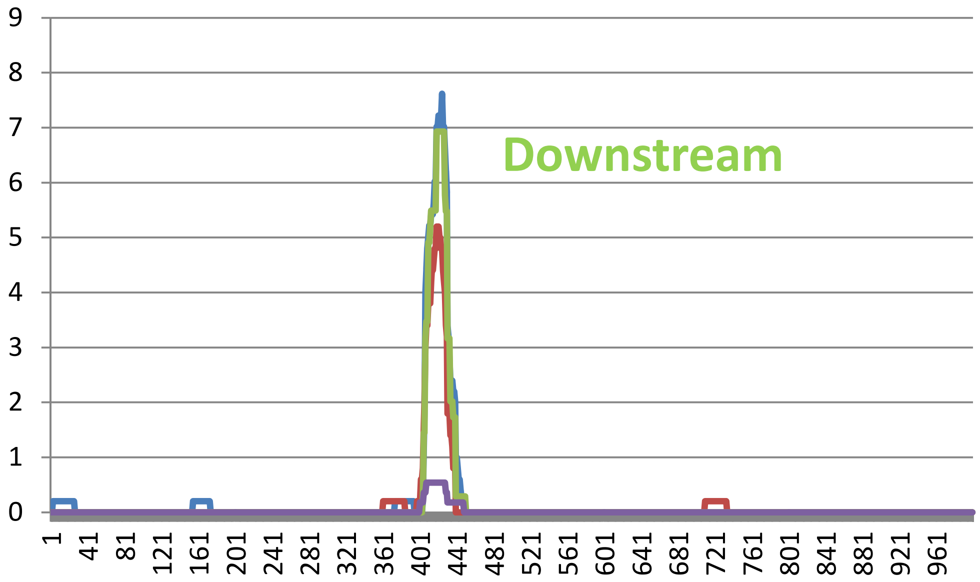

AT3G22730

F-box and associated interaction domains-containing protein

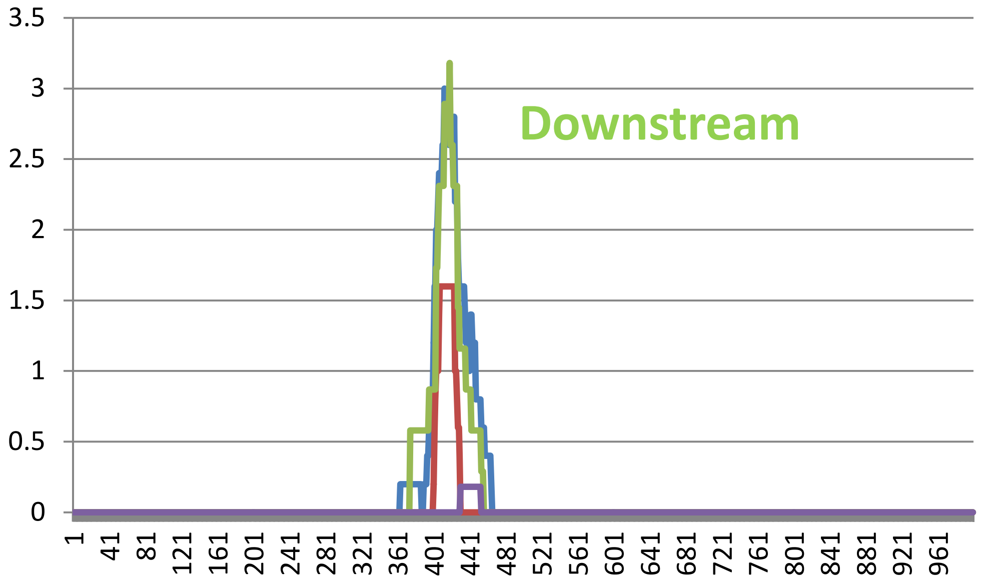

AT3G22770

F-box associated ubiquitination effector family protein

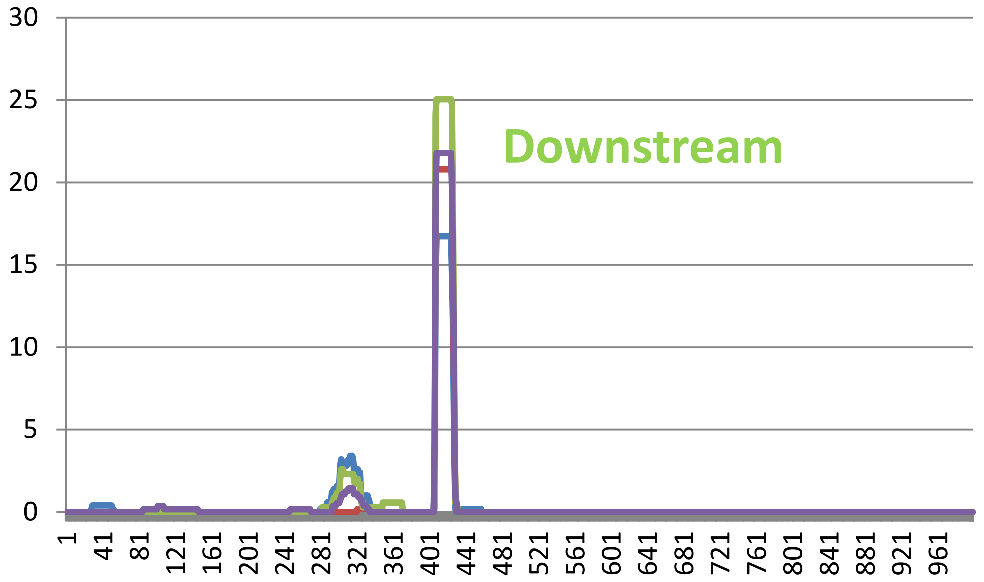

AT3G23740

Unknown protein

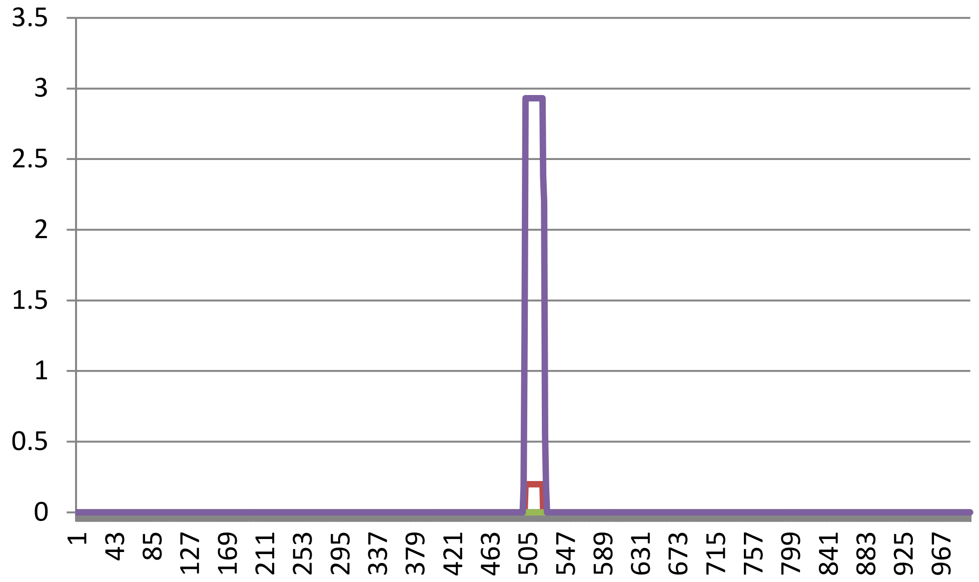

AT3G23890

Encodes a topoisomerase II that is highly expressed in young seedlings. The protein is localized in the nucleus and gene expression levels are increased in proliferative tissues.

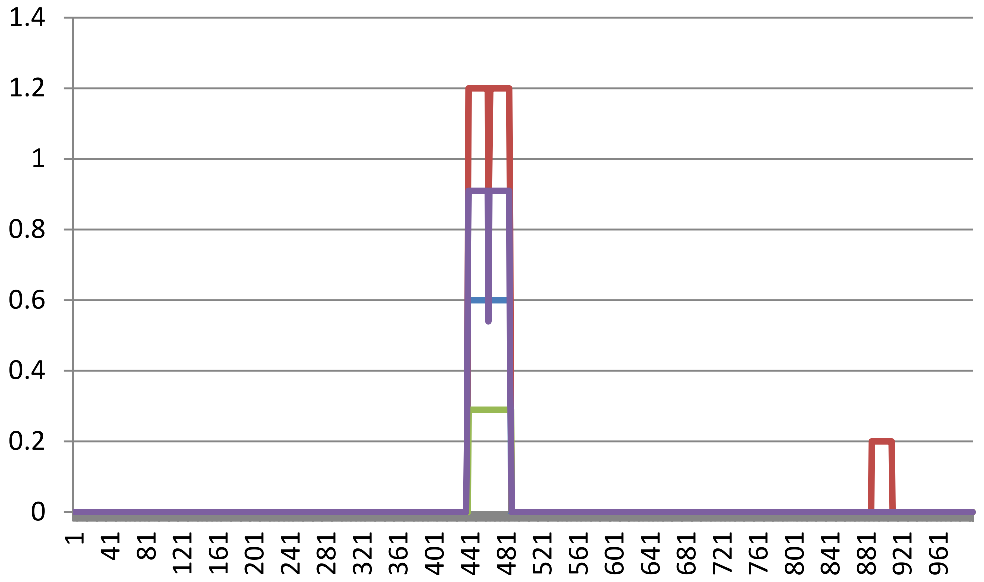

AT3G25460

F-box and associated interaction domains-containing protein

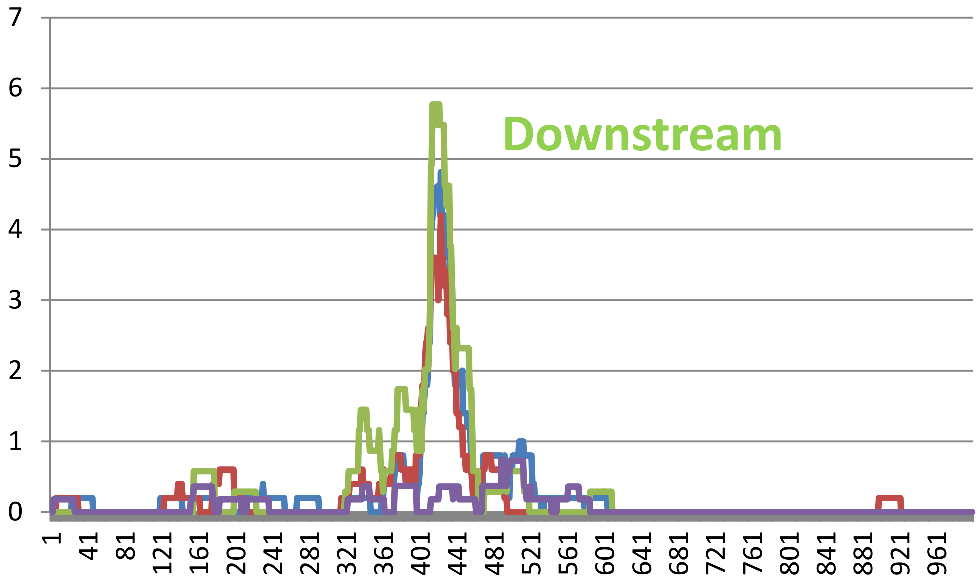

AT3G25585

Aminoalcoholphosphotransferase (AAPT2) mRNA, complete CDS

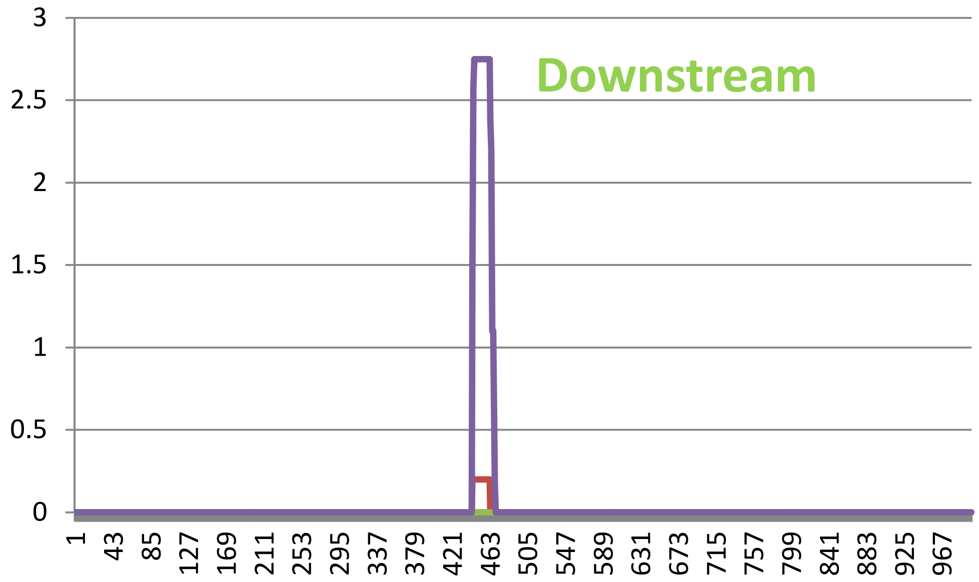

AT3G26616

Unknown protein

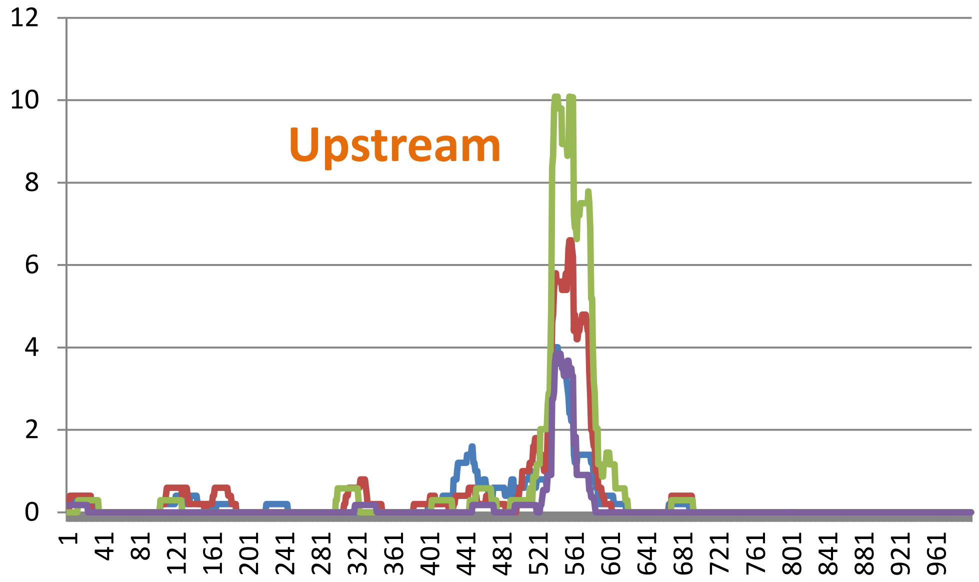

AT3G29560

Unknown protein

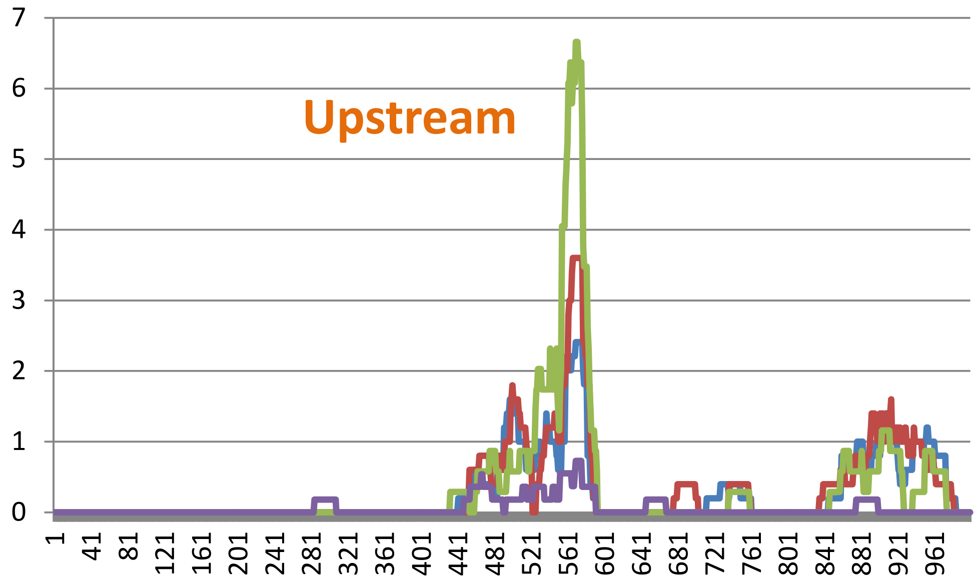

AT3G30290

A member of cytochrome P450 gene family

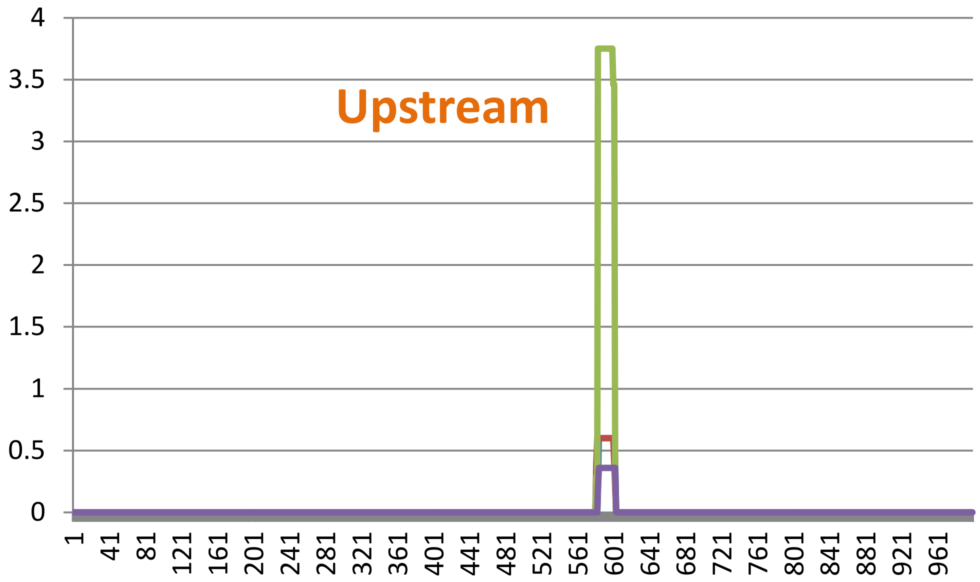

AT3G30580

Unknown protein

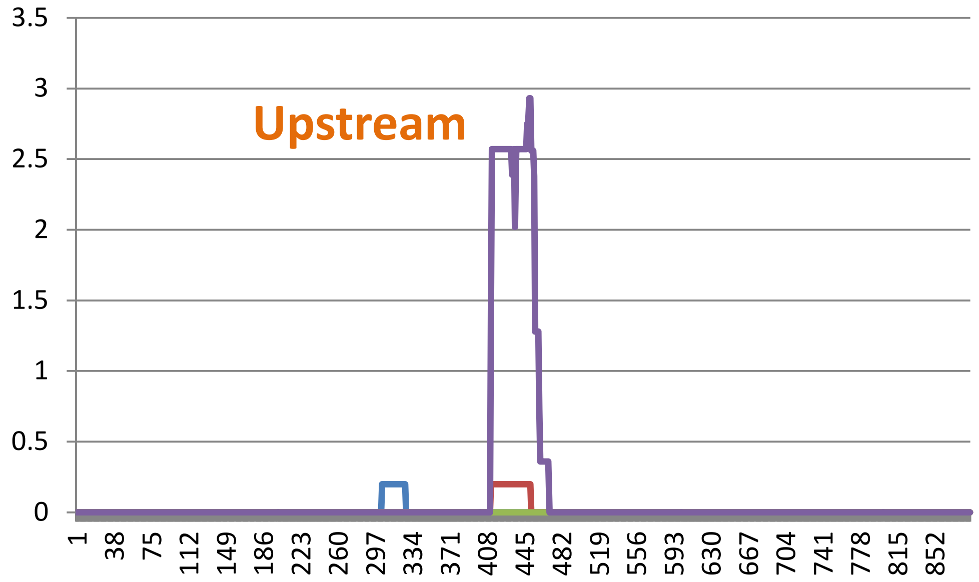

AT3G42550

Eukaryotic aspartyl protease family protein

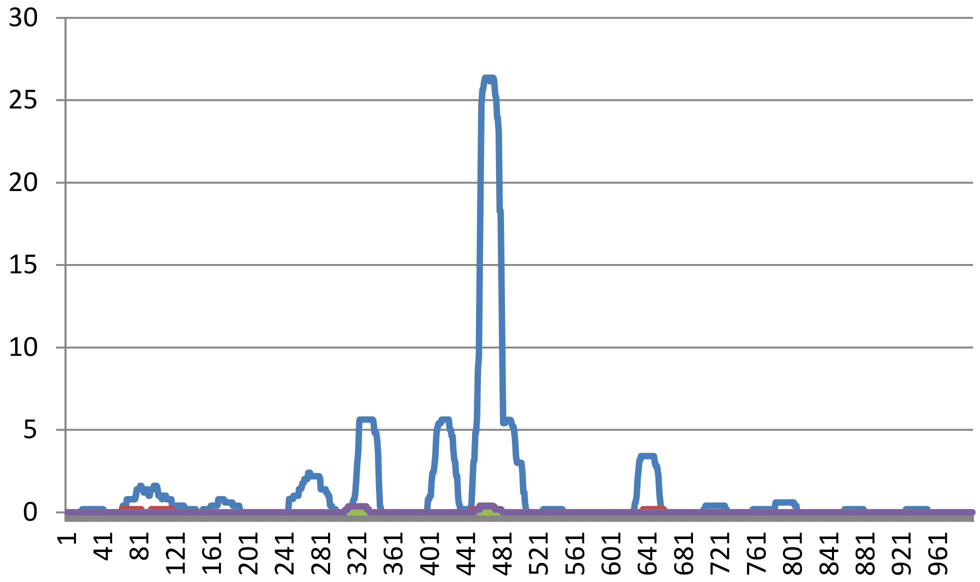

AT3G42723

Aminoacyl-tRNA ligases;ATP binding;nucleotide binding

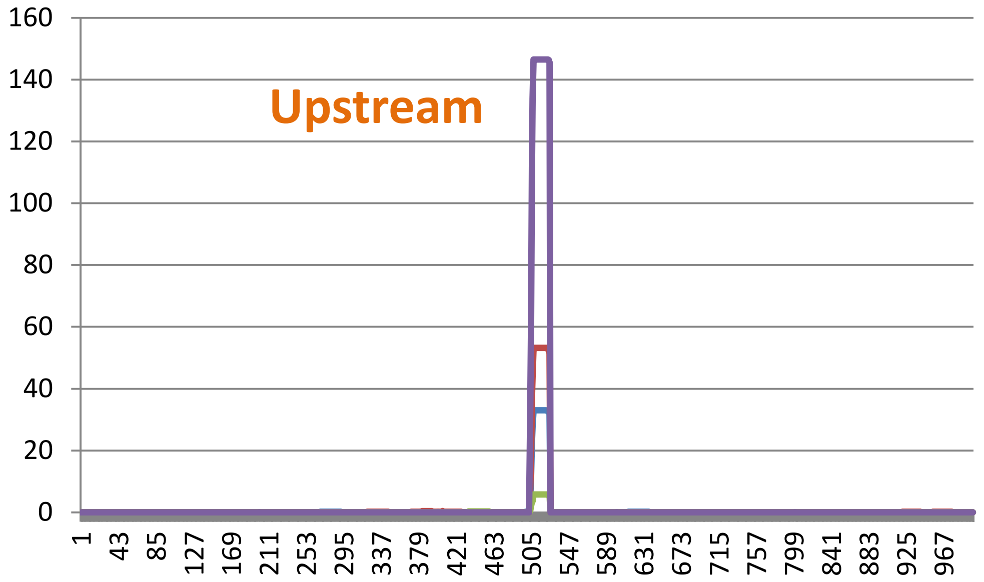

AT3G43270

Plant invertase/pectin methylesterase inhibitor superfamily

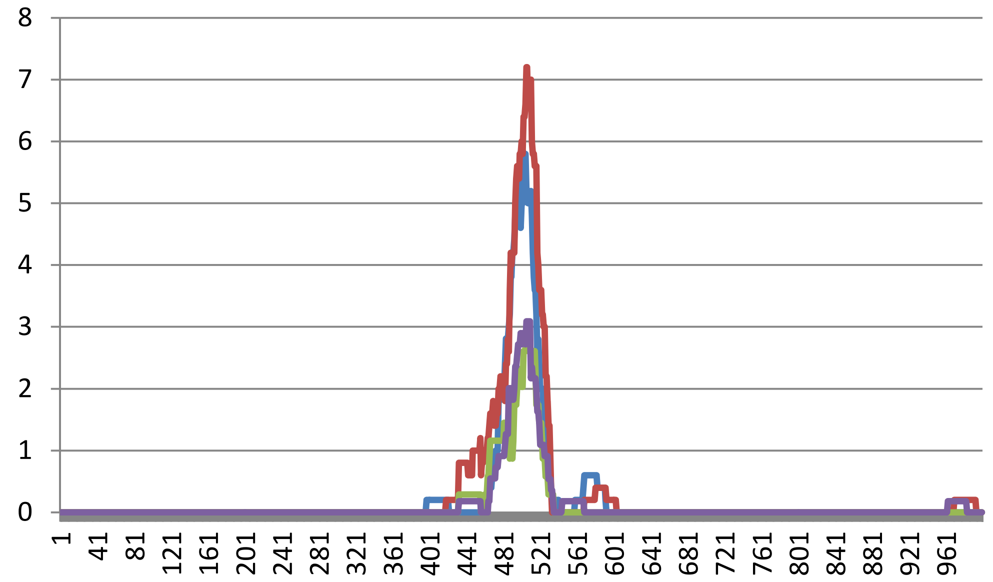

AT3G44020

Thylakoid lumenal P17.1 protein

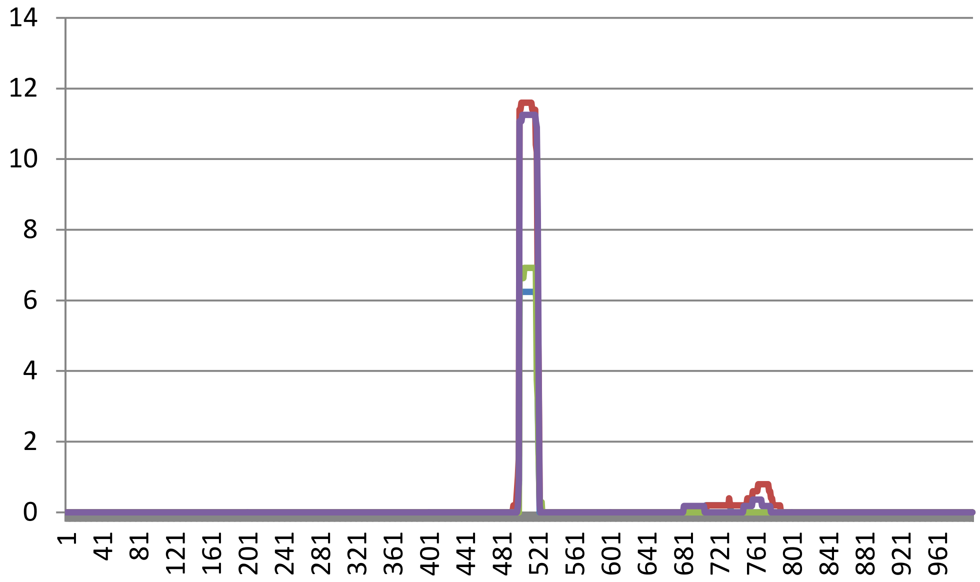

AT3G44230

Unknown protein

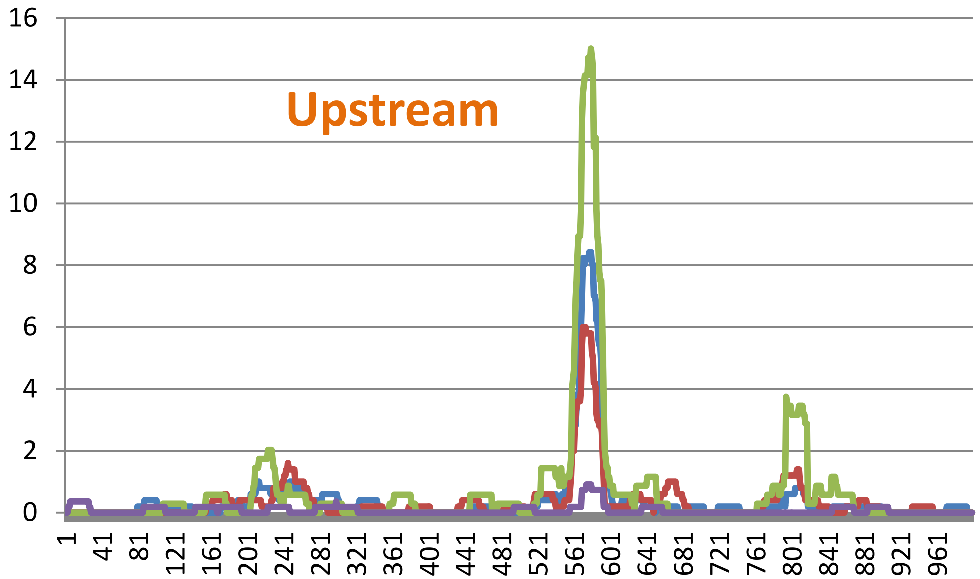

AT3G45190

SIT4 phosphatase-associated family protein

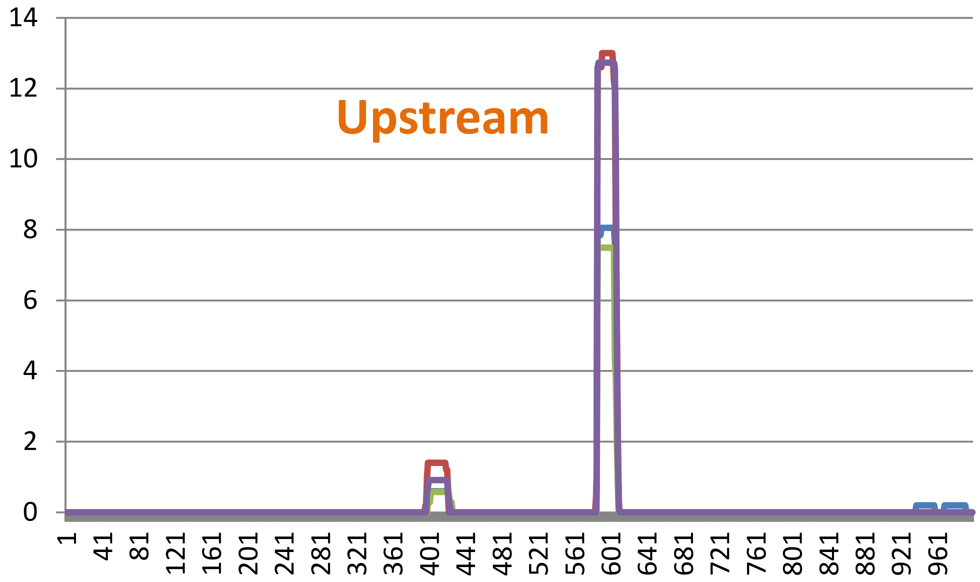

AT3G45577

tRNA-intron endonucleases

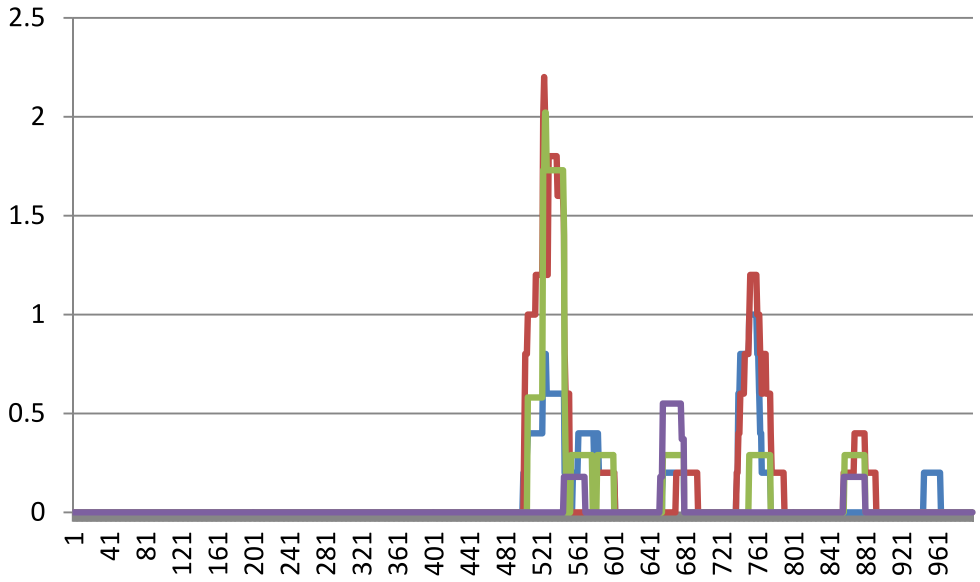

AT3G45800

Plant protein 1589 of unknown function

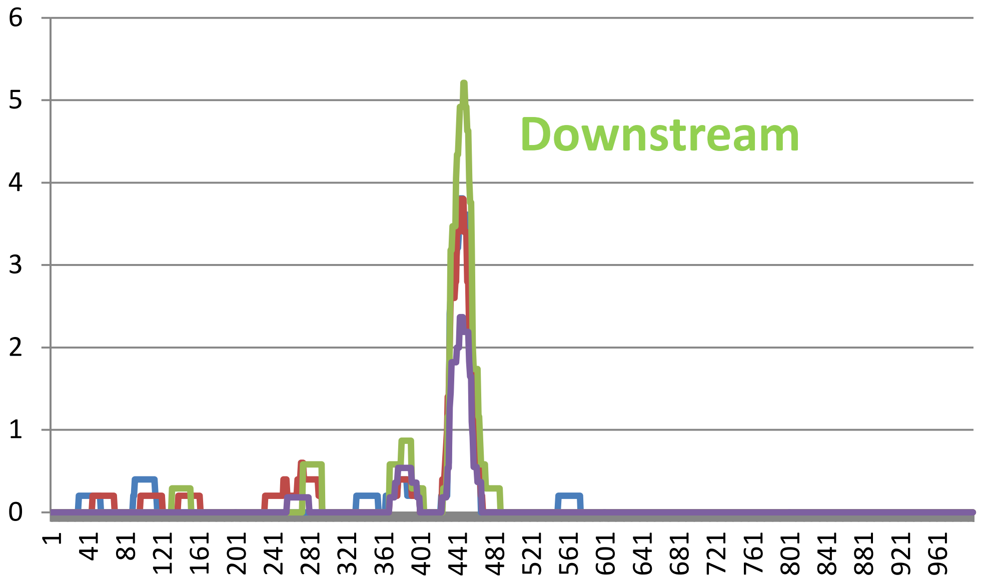

AT3G47300

SELT-like protein precursor (SELT)

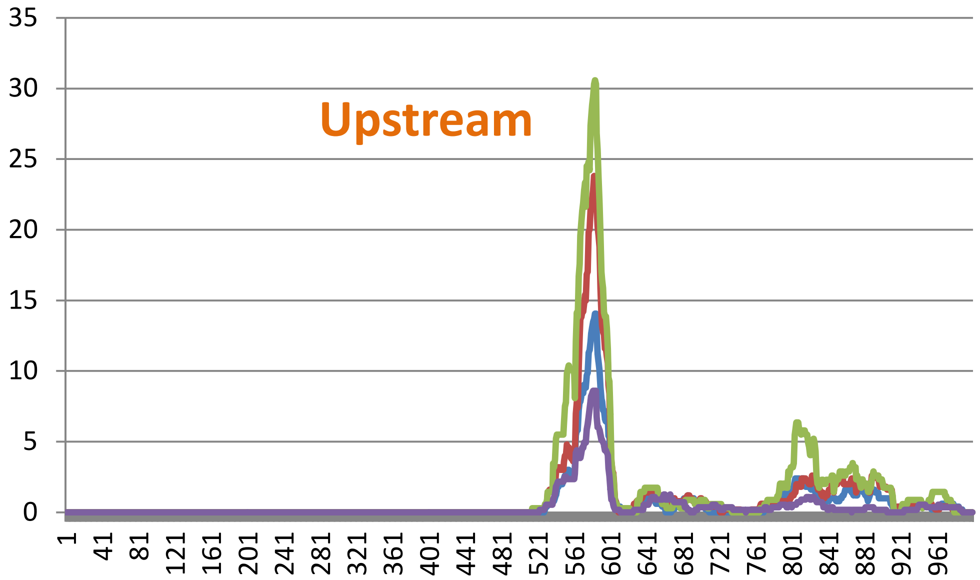

AT3G47600

Encodes a putative transcription factor (MYB94).

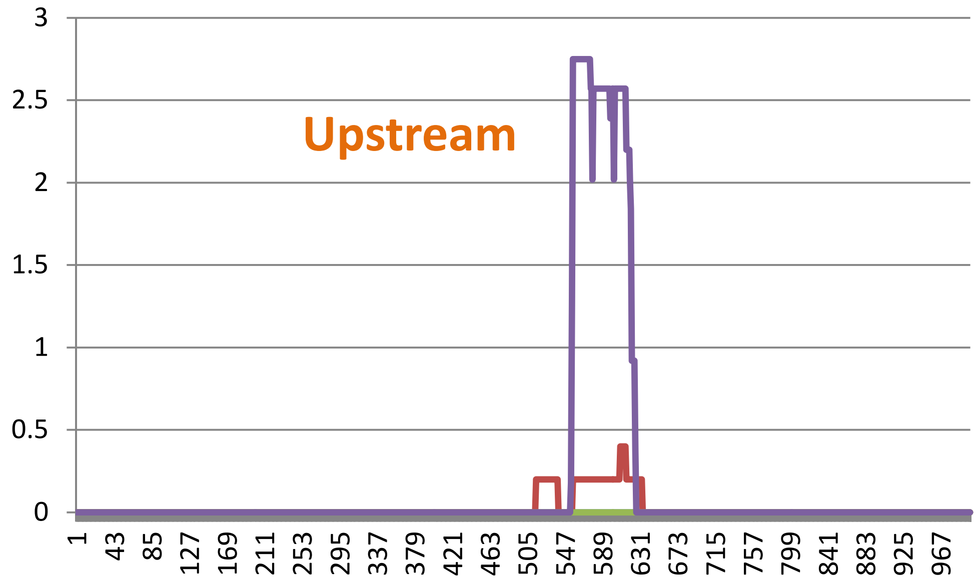

AT3G49520

F-box and associated interaction domains-containing protein

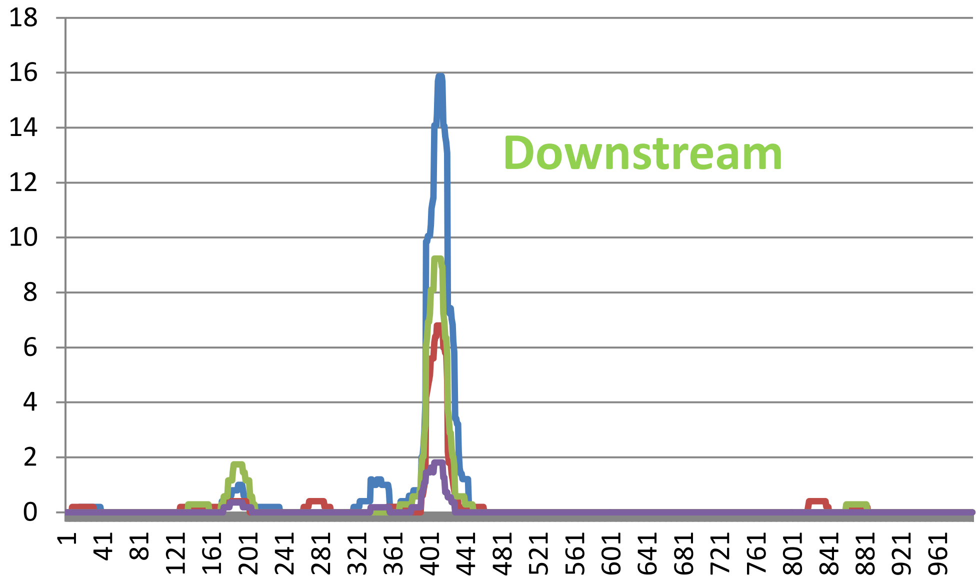

AT3G49920

Encodes a voltage-dependent anion channel.

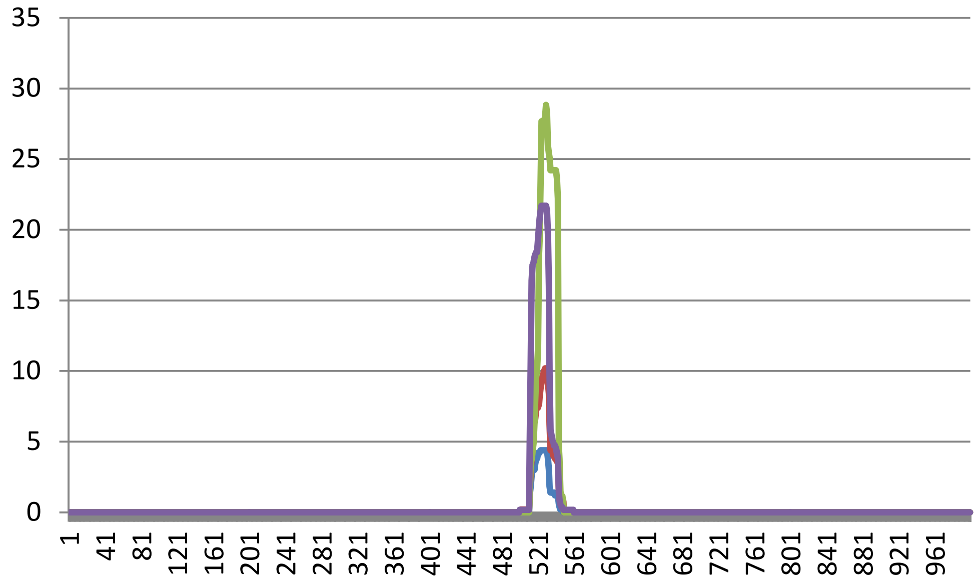

AT3G50370

Unknown protein

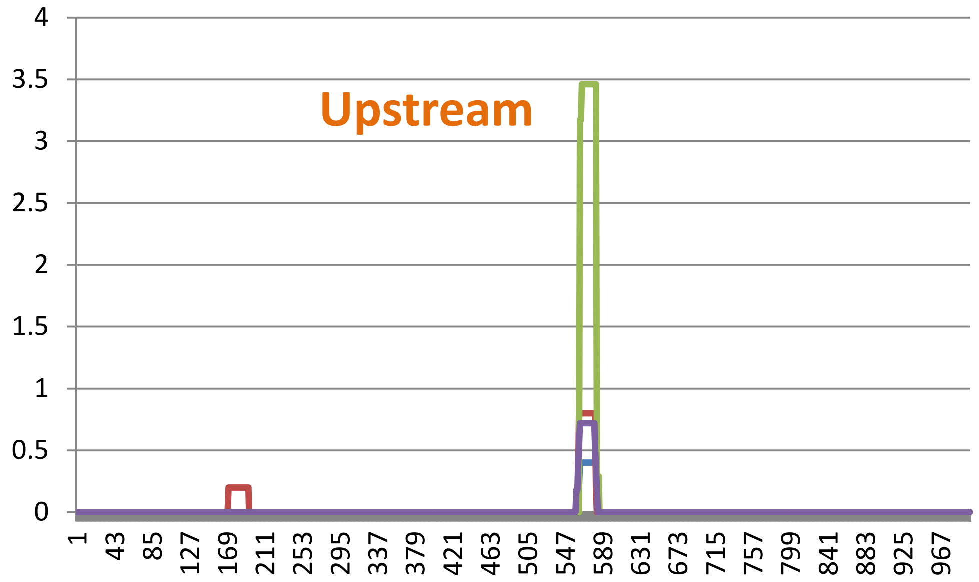

AT3G51140

Protein of unknown function (DUF3353)

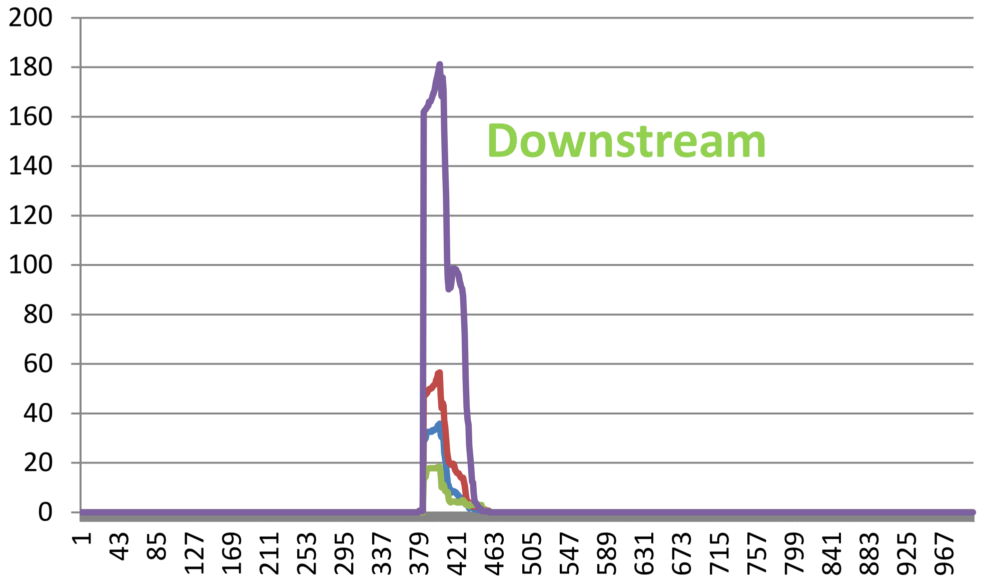

AT3G51270

Protein serine/threonine kinases; ATP binding; catalytics

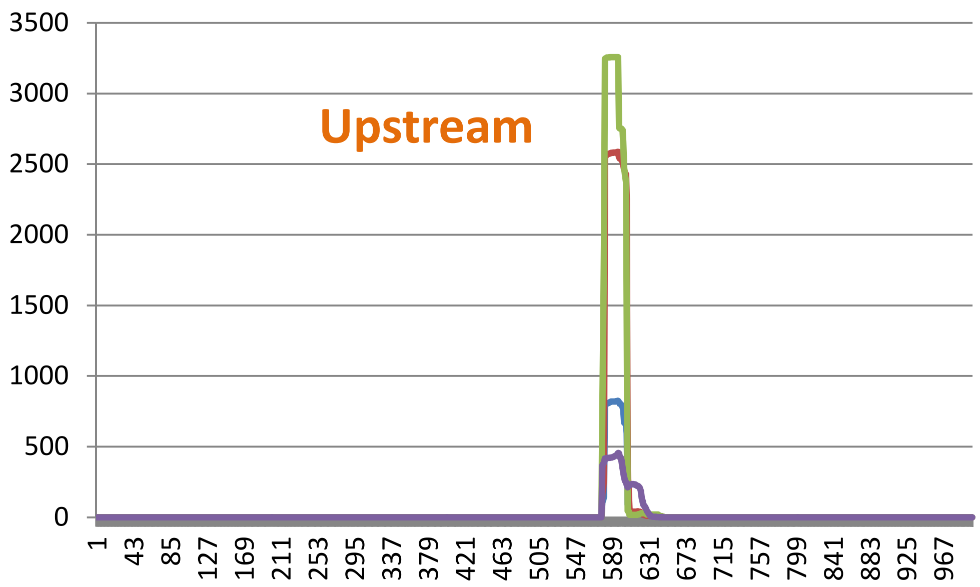

AT3G51390

DHHC-type zinc finger family protein

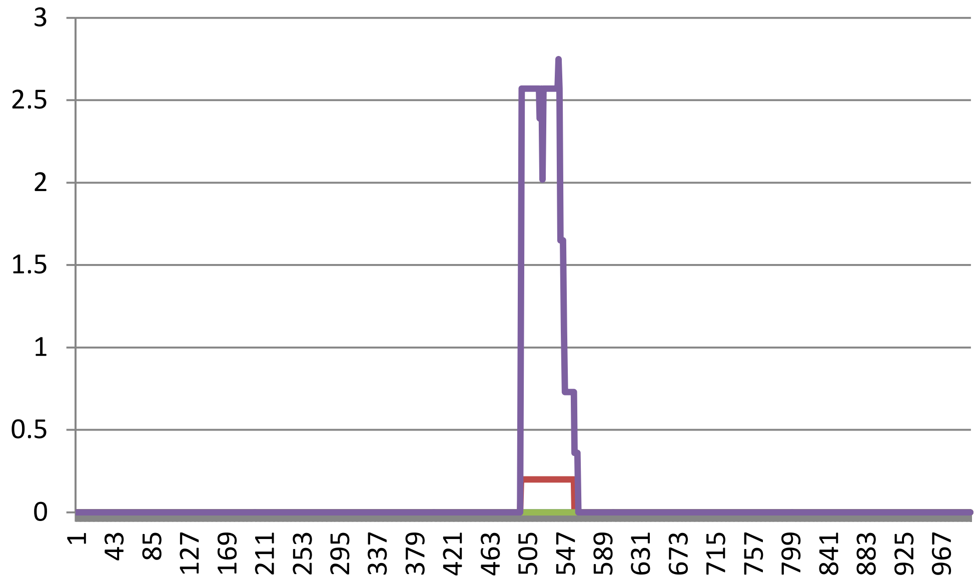

AT3G54960

Encodes a protein disulfide isomerase-like (PDIL) protein, a member of a multigene family within the thioredoxin (TRX) superfamily.

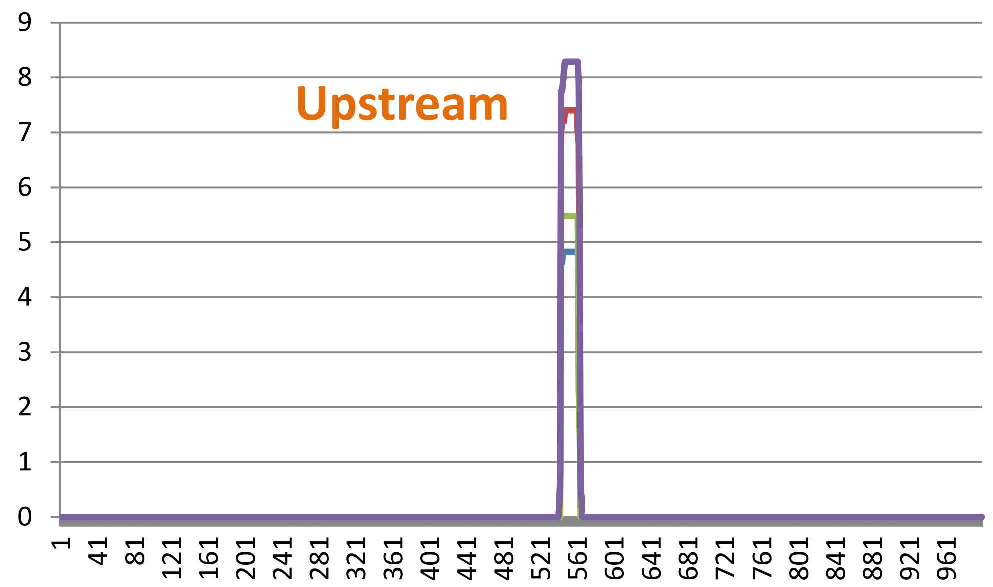

AT3G55370

Encodes a nuclear localized Dof domain containing transcription factor expressed primarily in roots. Responsive to salicylic acid. Transgenic overexpressors have yellow leaves and short, defective roots.

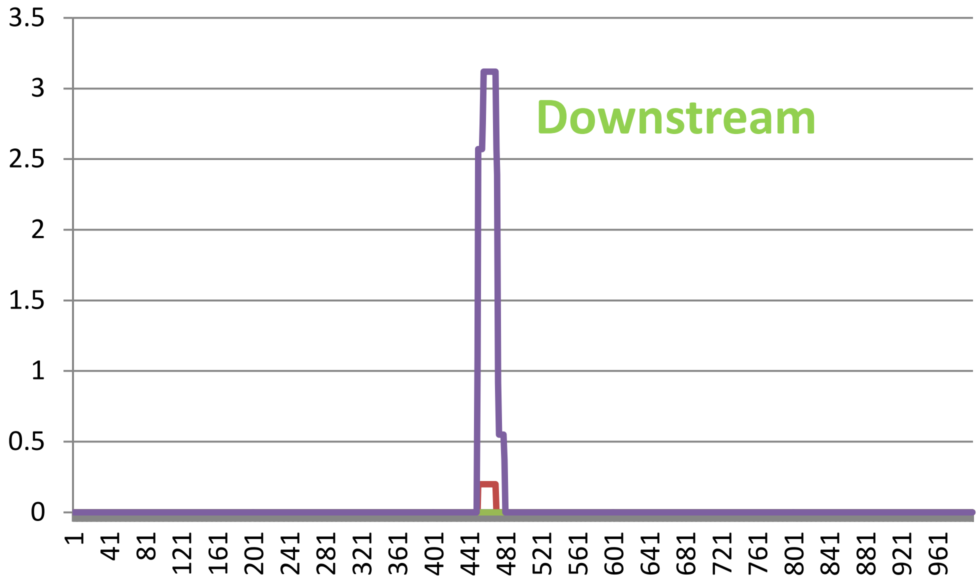

AT3G55740

Encodes a proline transporter with affinity for gly betaine, proline, and GABA. Protein is expressed most highly in the roots.

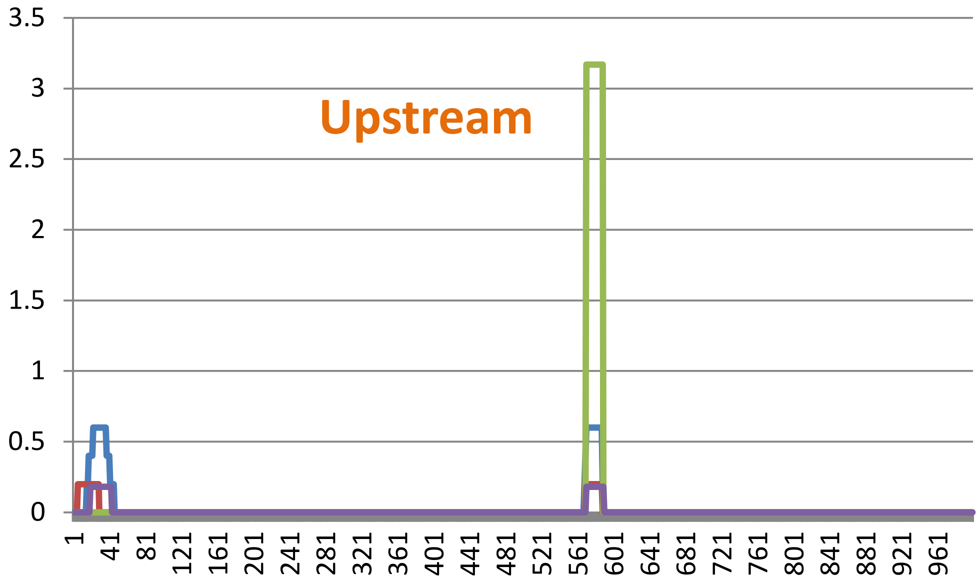

AT3G55960

Haloacid dehalogenase-like hydrolase (HAD) superfamily protein

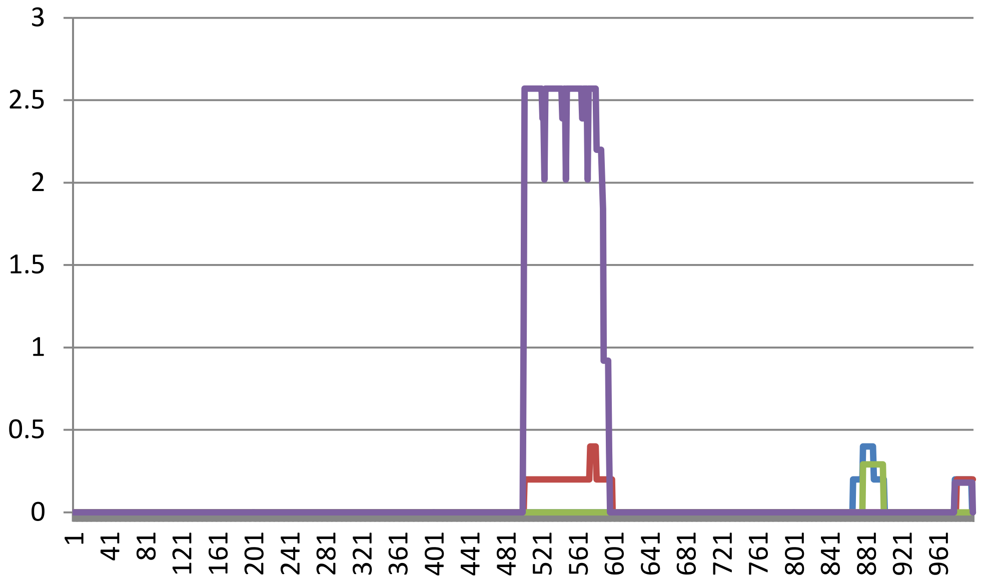

AT3G56450

Member of alpha-SNAP Gene Family

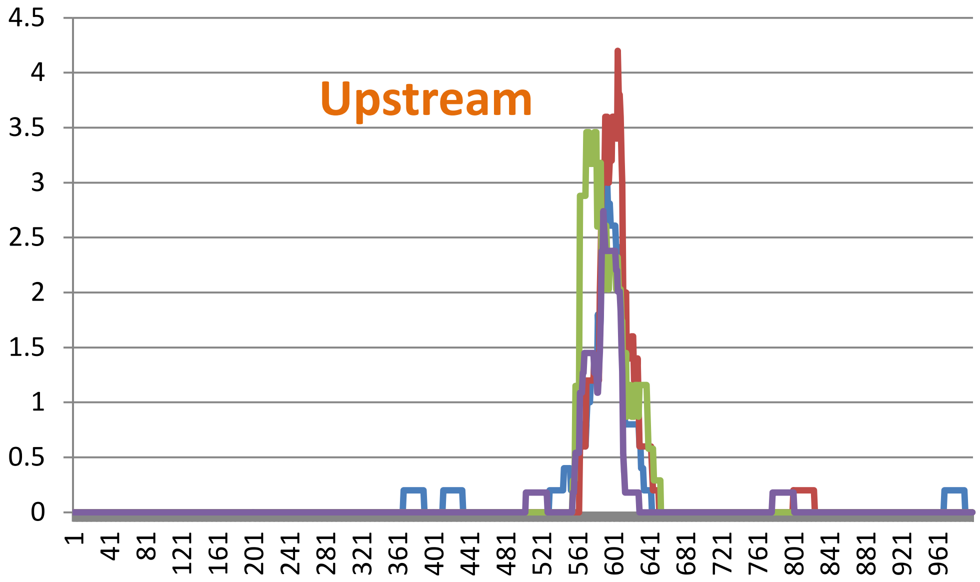

AT3G57770

Protein kinase superfamily protein

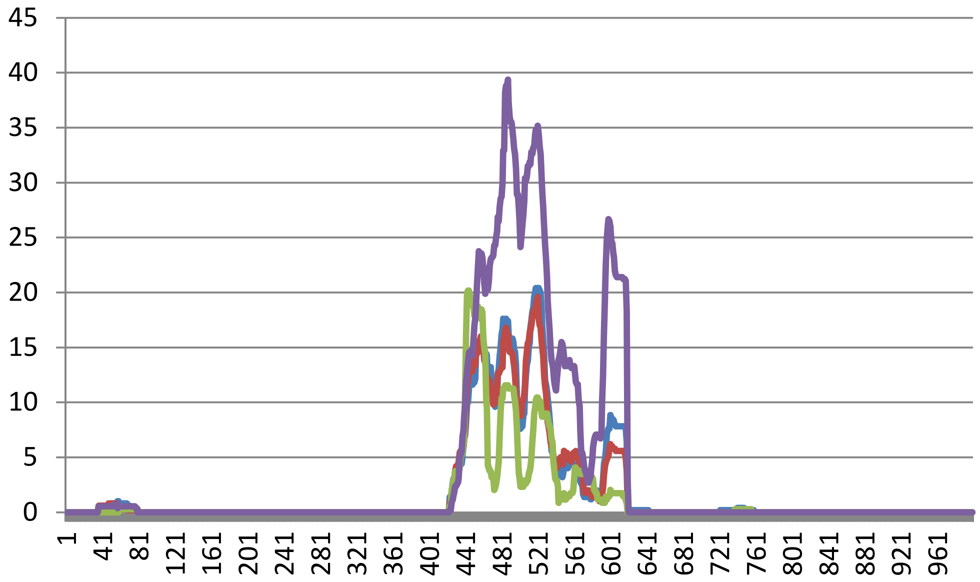

AT3G58820

F-box/RNI-like superfamily protein

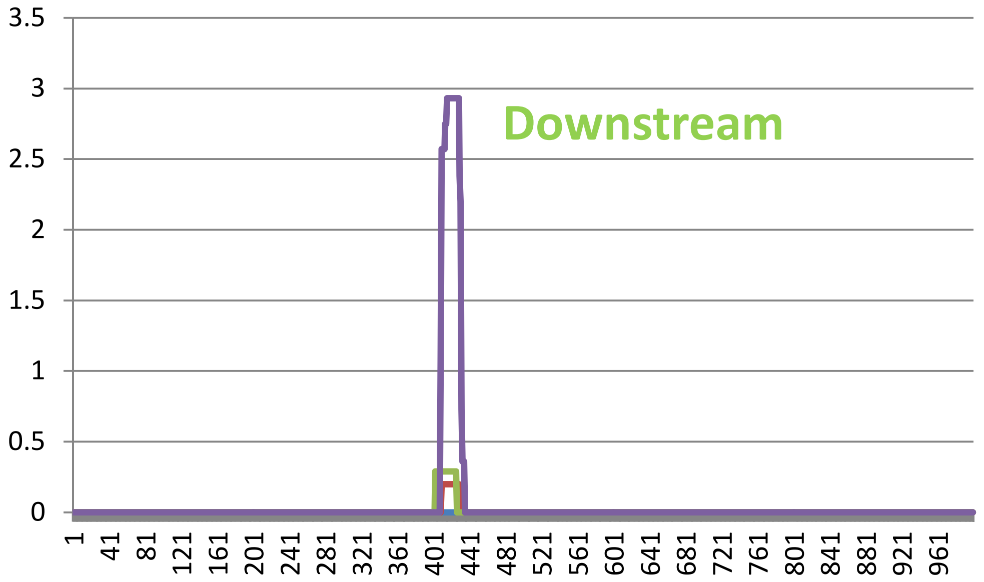

AT3G59220

Encodes a cupin-domain containing protein that is similar to pirins which interact with a CCAAT box binding transcription factor. The protein interacts with GPA1 (G protein alpha-subunit) in vitro. Mutants in the gene are affected in germination and early seedling development.

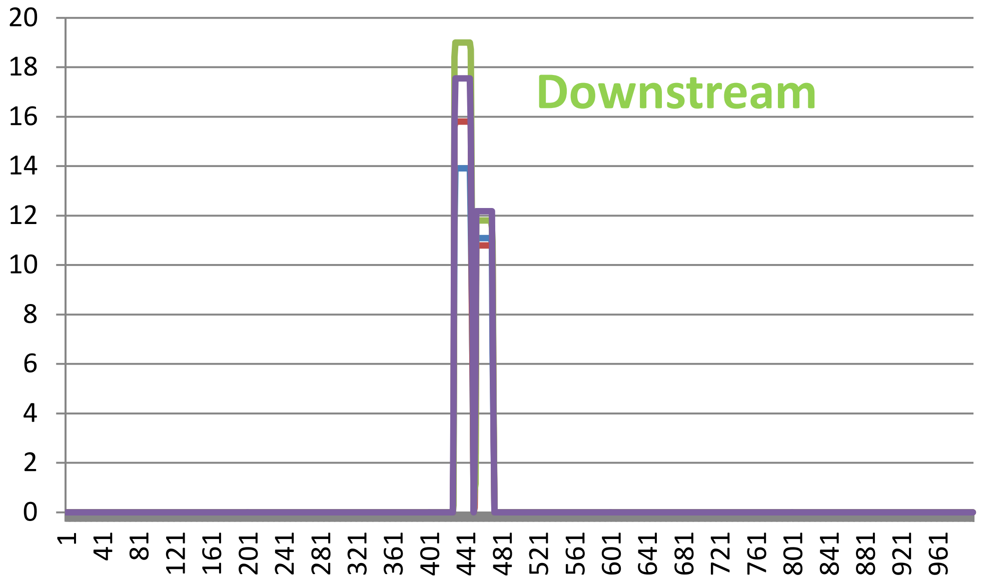

AT3G60790

F-box family protein

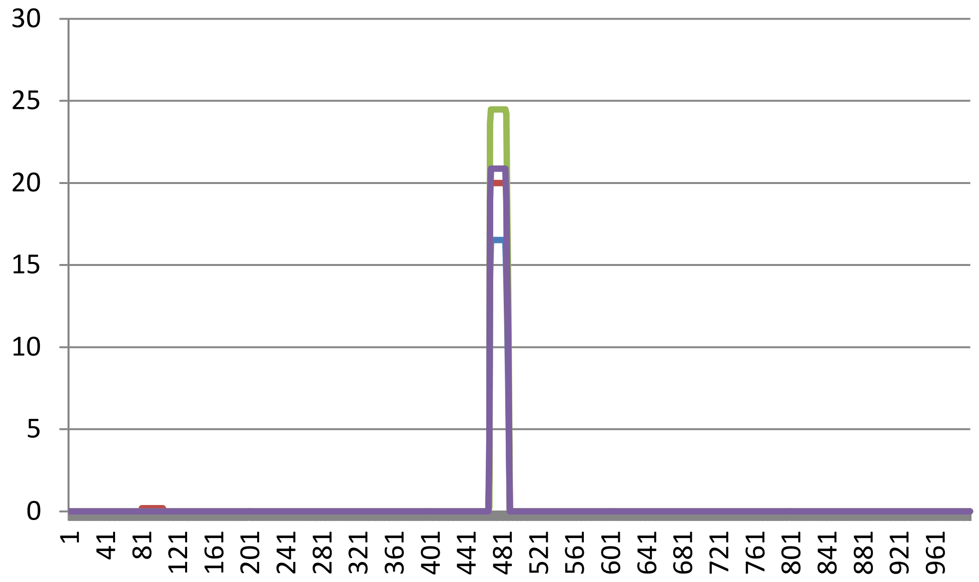

AT3G62220

Protein kinase superfamily protein

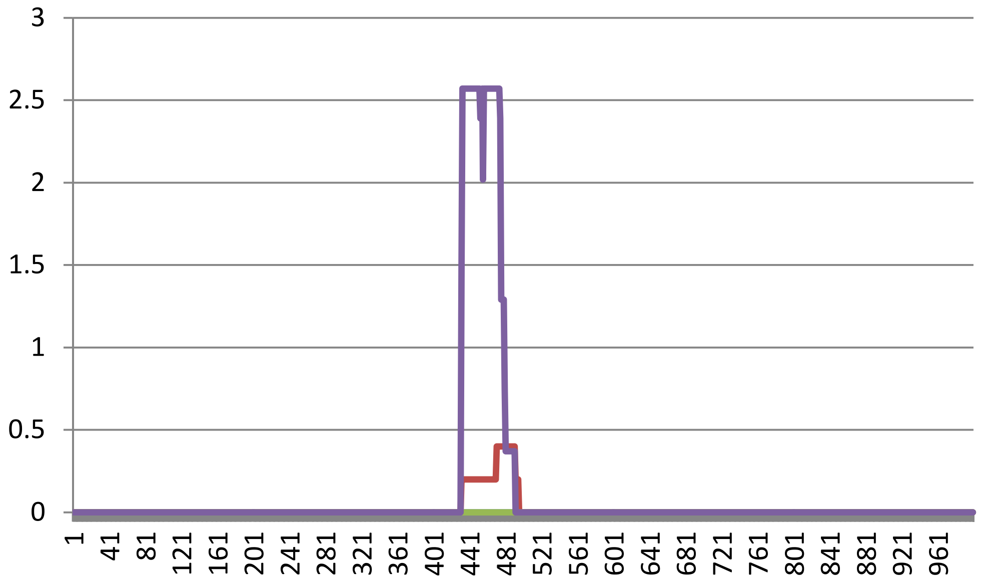

AT3G62290

A member of ARF GTPase family.

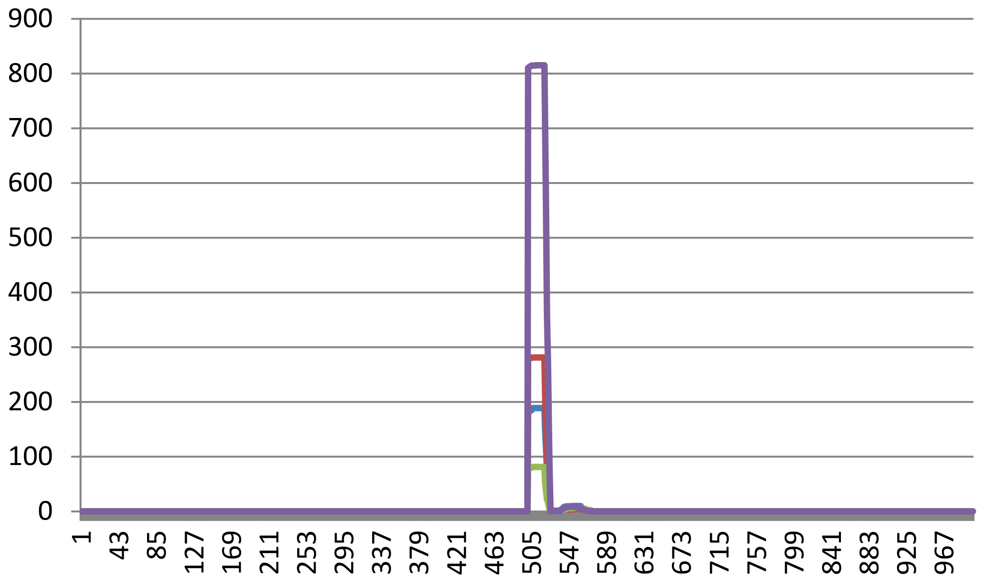

AT3G62570

Tetratricopeptide repeat (TPR)-like superfamily protein

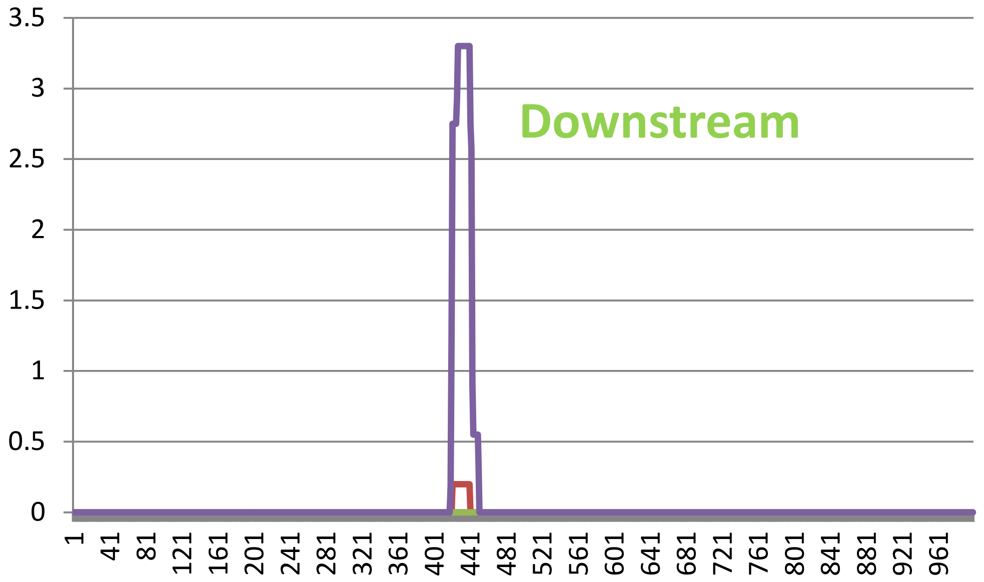

AT4G00580

COP1-interacting protein-related

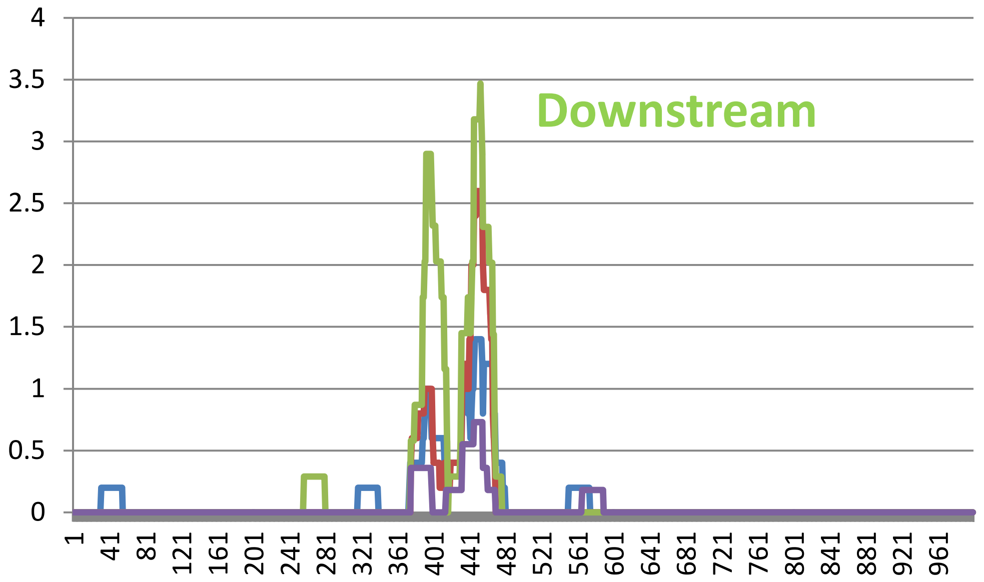

AT4G05520

Encodes AtEHD2, one of the Arabidopsis Eps15 homology domain proteins involved in endocytosis (AtEHD1, At3g20290).

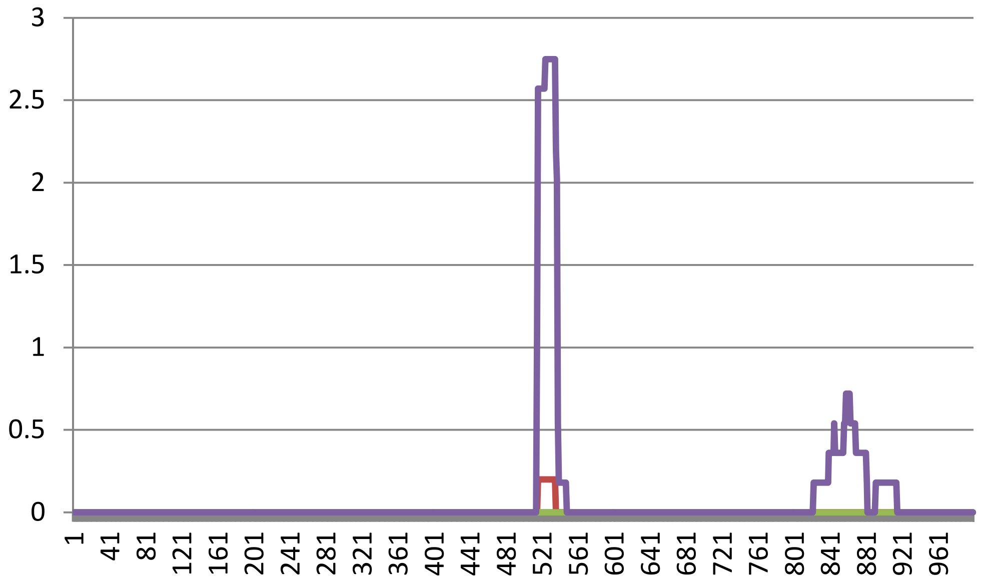

AT4G13992

Cysteine/Histidine-rich C1 domain family protein

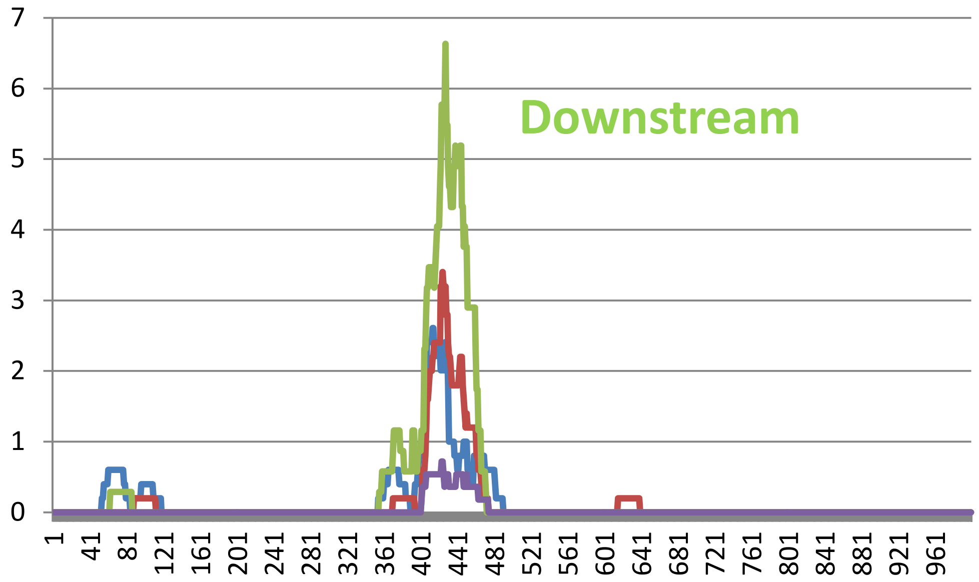

AT4G14130

Xyloglucan endotransglycosylase-related protein (XTR7)

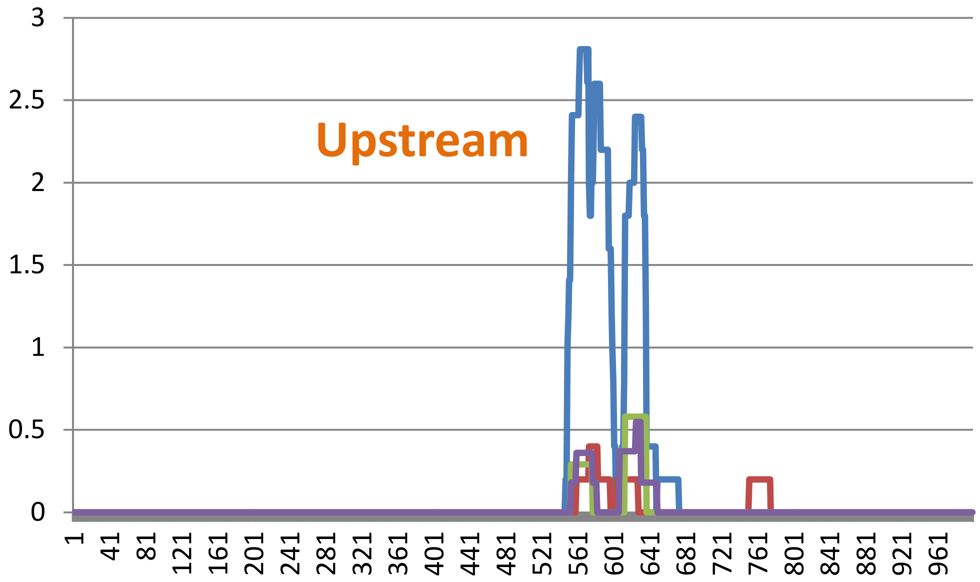

AT4G14810

Unknown protein

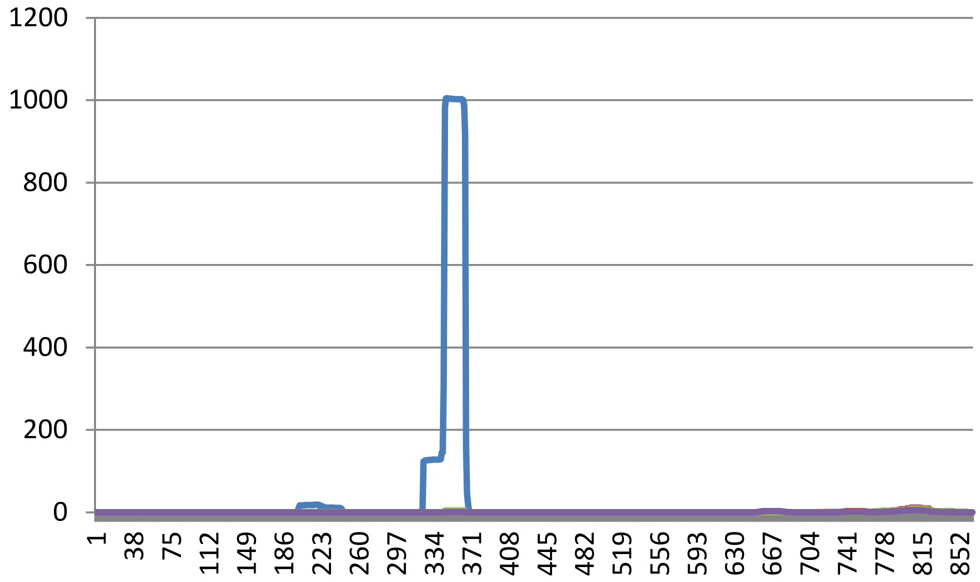

AT4G14910

Encodes a protein that is predicted to act as a imidazoleglycerol-phosphate dehydratase involved in histidine biosynthesis.

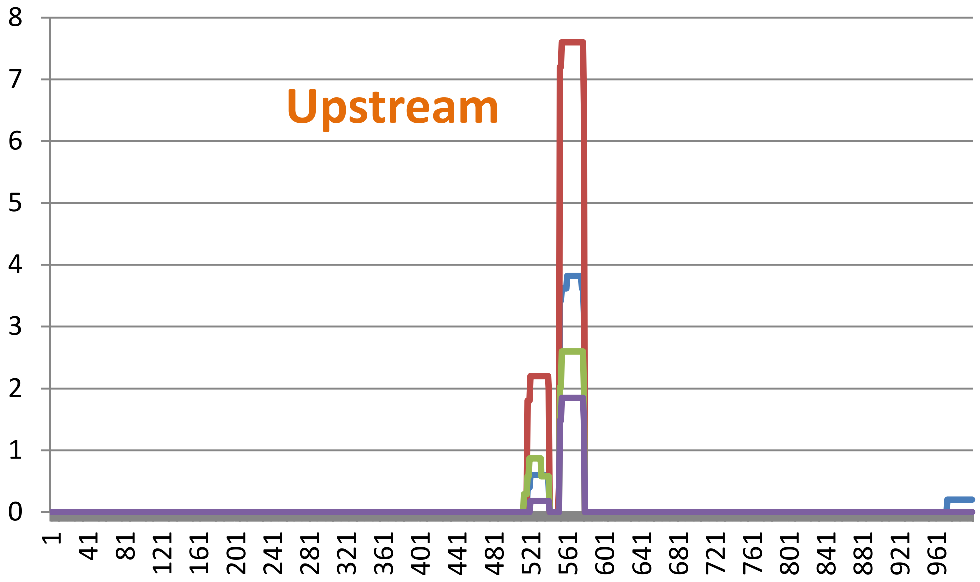

AT4G15820

BEST Arabidopsis thaliana protein match is: embryo defective 1703 (TAIR:AT3G61780.1).

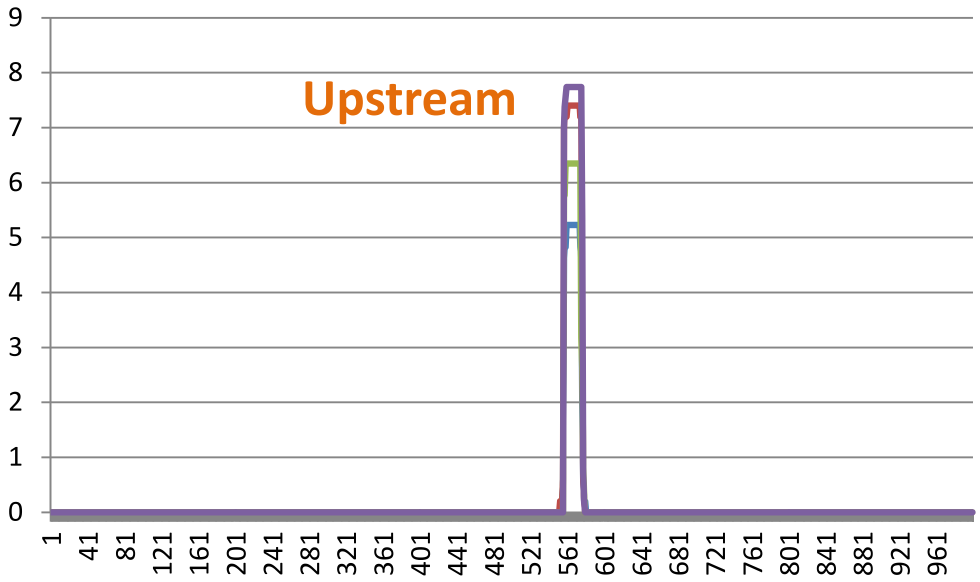

AT4G16460

Unknown protein

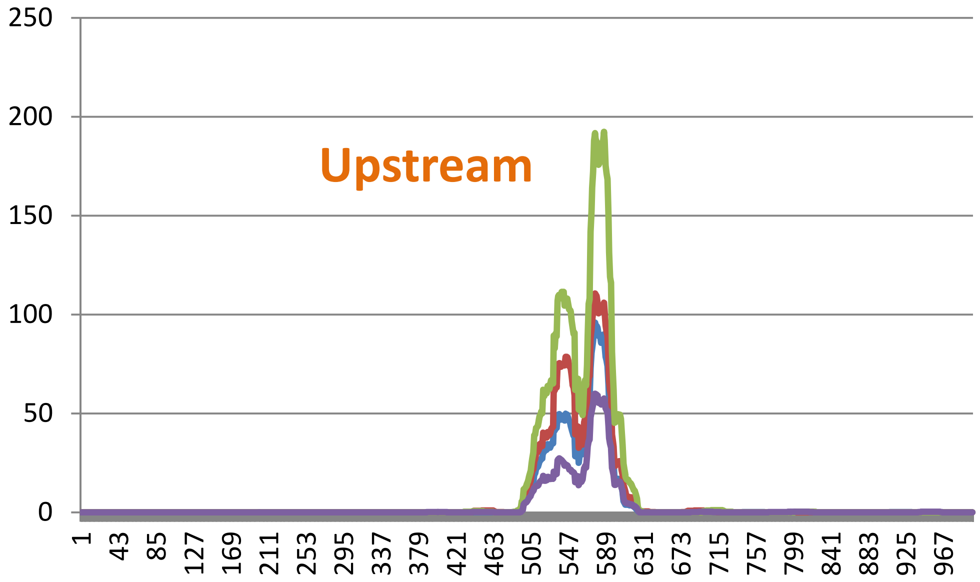

AT4G16640

Matrixin family protein

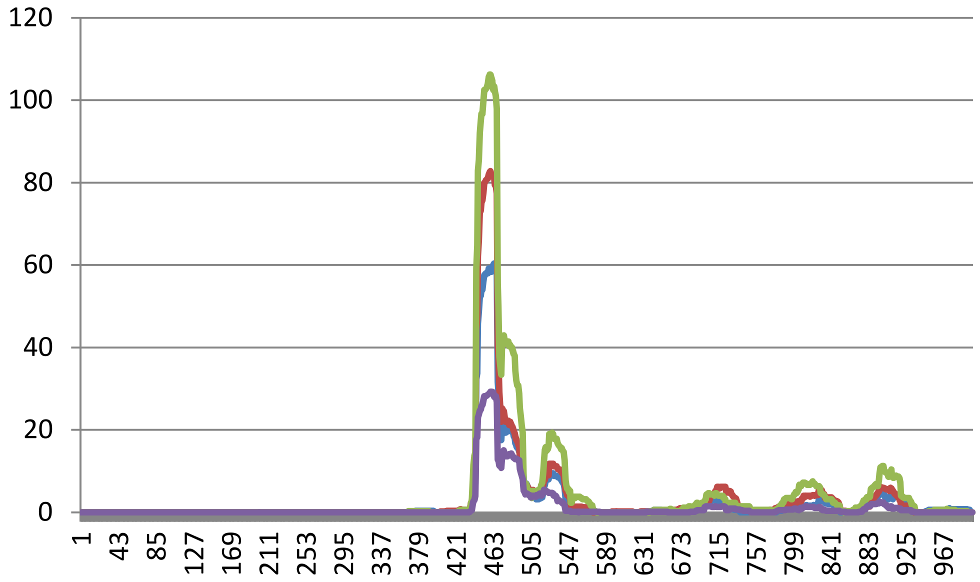

AT4G21160

ADP-ribosylation factor GTPase-activating protein containing zinc finger and C2 domains and a novel PI-3-P-binding protein region. Binds PI-3-P. Highest expression levels in flowering tissue, rosettes and roots.

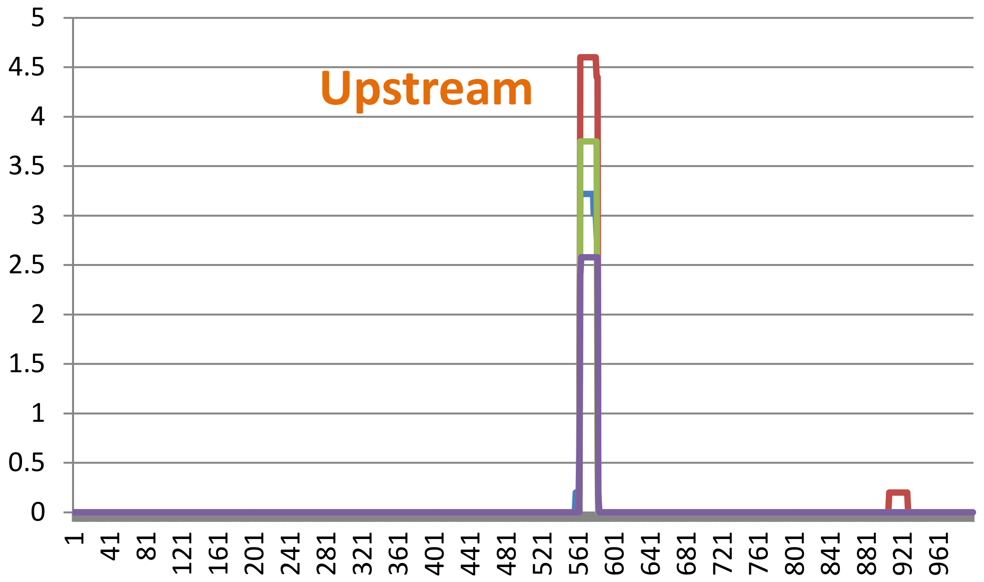

AT4G24026

Unknown protein

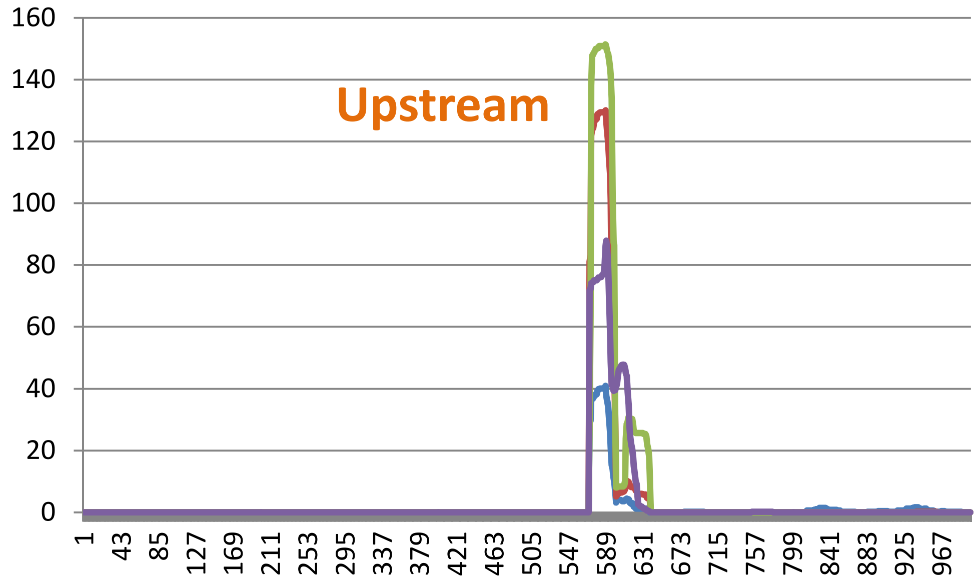

AT4G24060

Dof-type zinc finger DNA-binding family protein

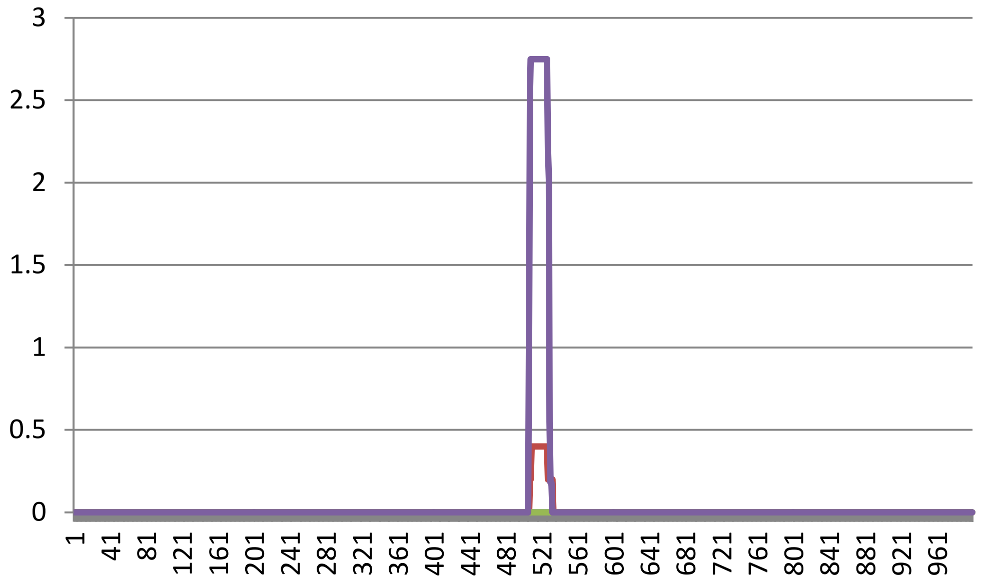

AT4G25870

Core-2/I-branching beta-1,6-N-acetylglucosaminyltransferase family protein

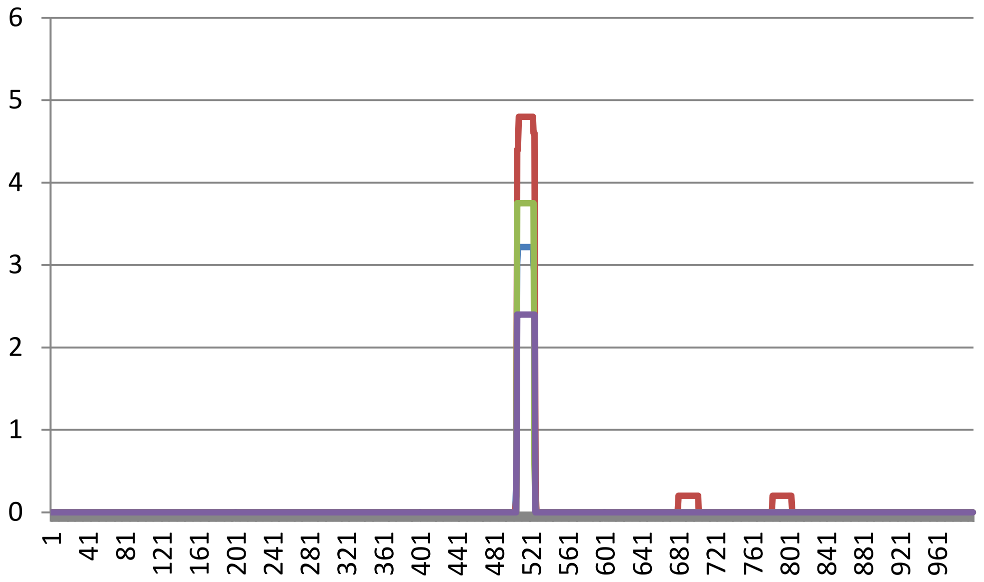

AT4G26100

Encodes a member of the casein kinase 1 protein family that is expressed in punctate particles at the cell periphery suggesting possible plasmodesmatal localization.

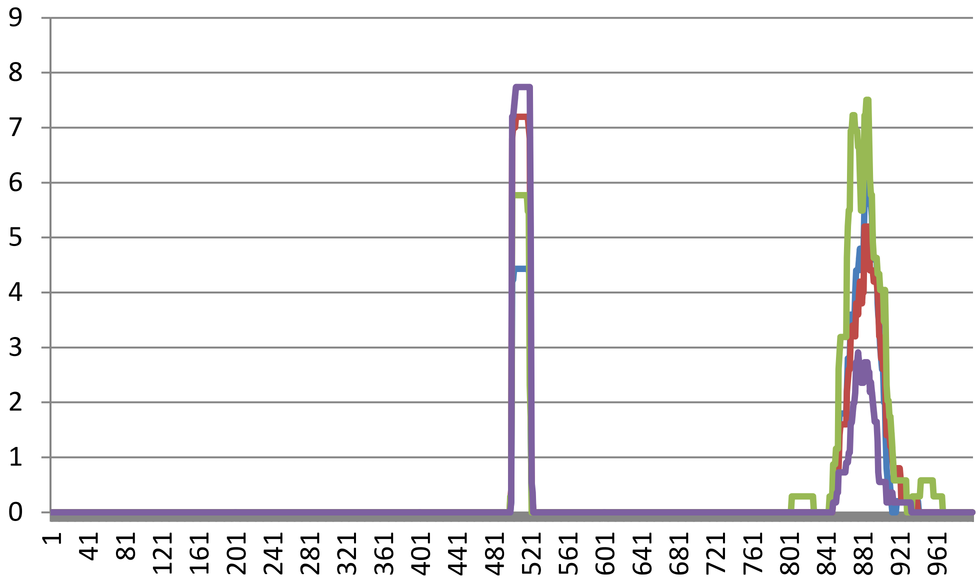

AT4G26370

Antitermination NusB domain-containing protein

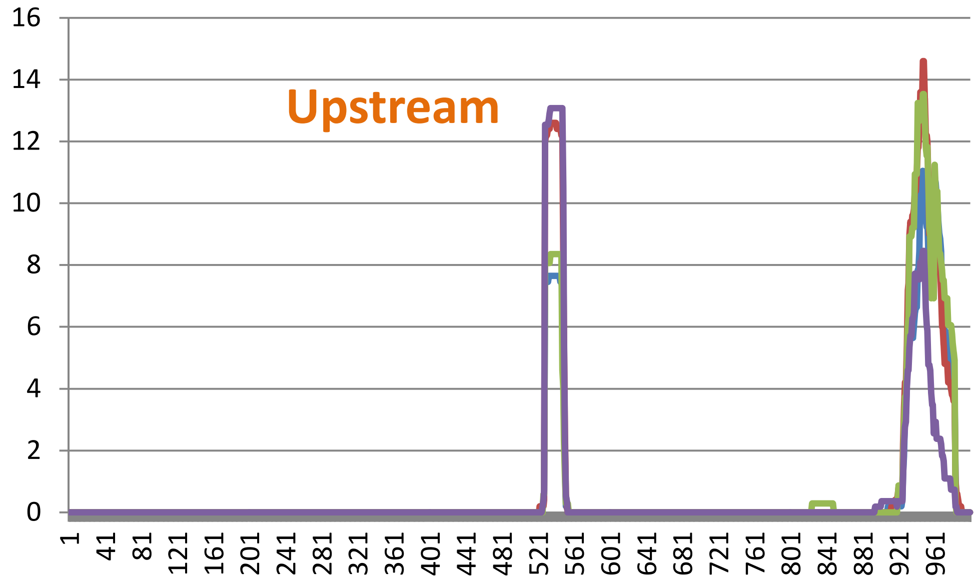

AT4G27390

Unknown protein

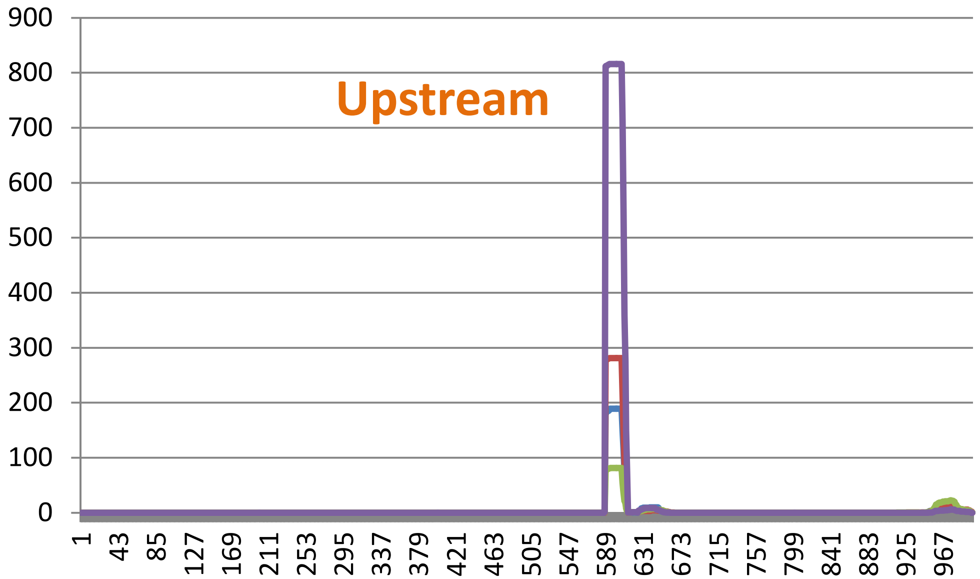

AT4G29090

Ribonuclease H-like superfamily protein

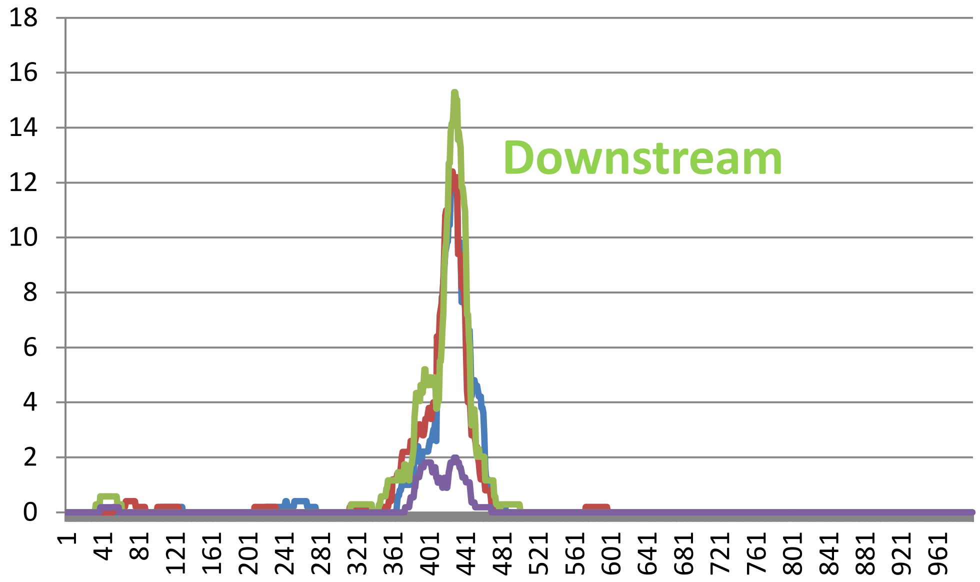

AT4G29360

O-Glycosyl hydrolases family 17 protein

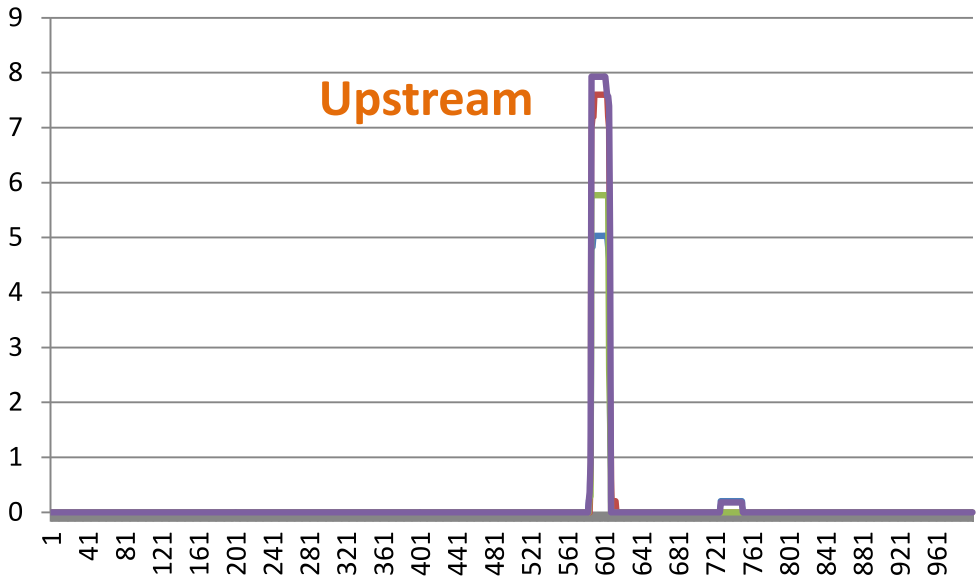

AT4G30110

Encodes a protein similar to Zn-ATPase, a P1B-type ATPases transport zinc

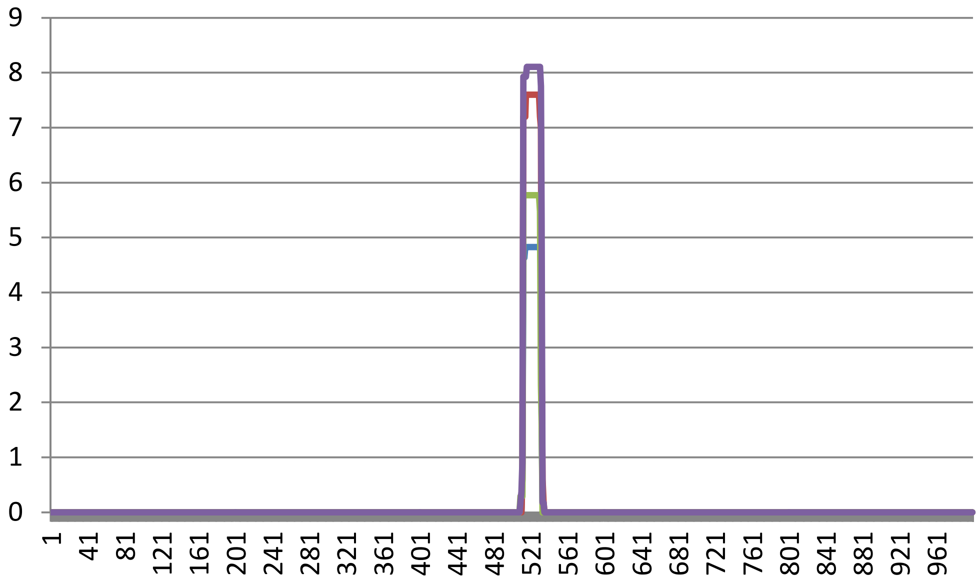

AT4G30740

Unknown protein

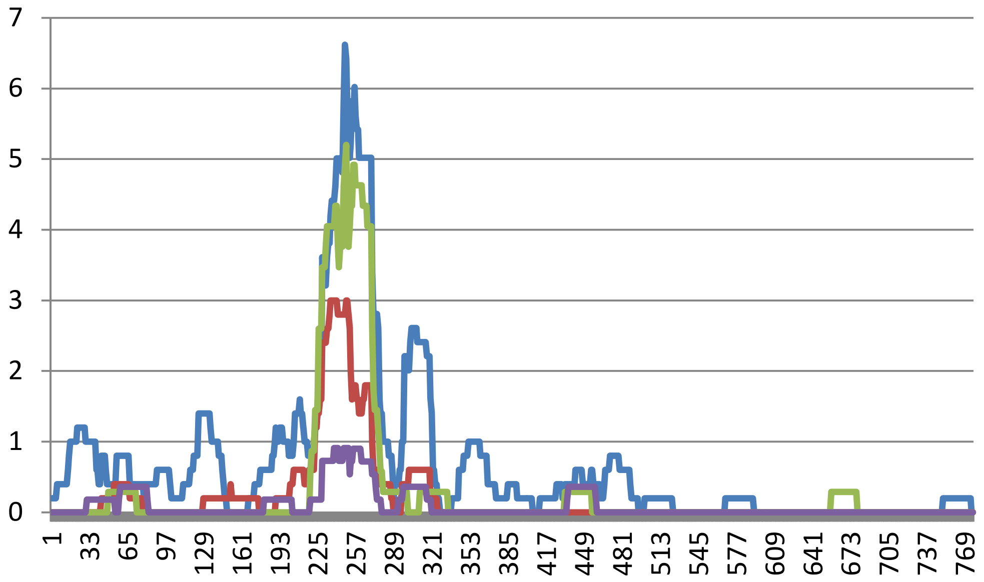

AT4G38760

Protein of unknown function (DUF3414).

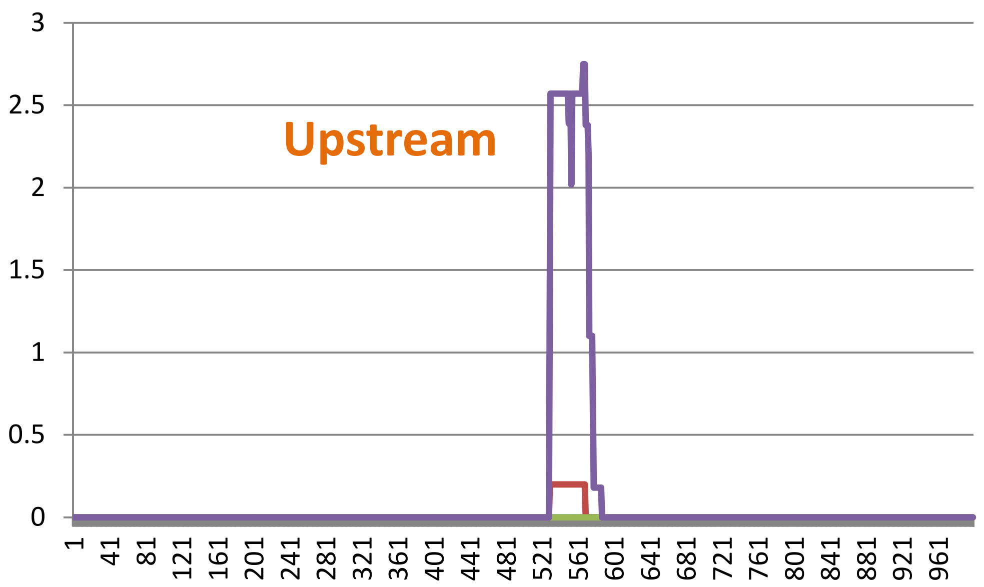

AT4G39900

Unknown protein

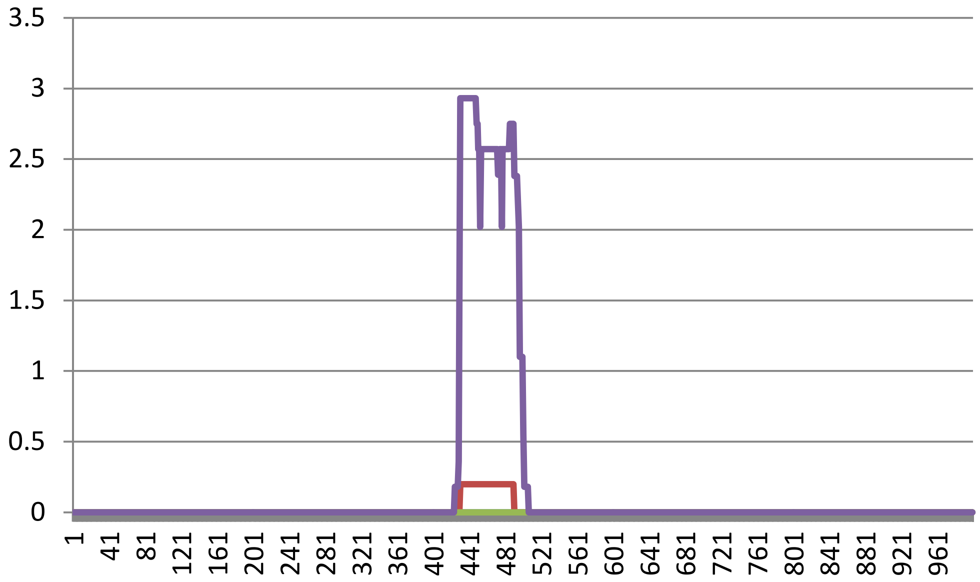

AT4G40060

Encodes a homeodomain leucine zipper class I (HD-Zip I) protein.

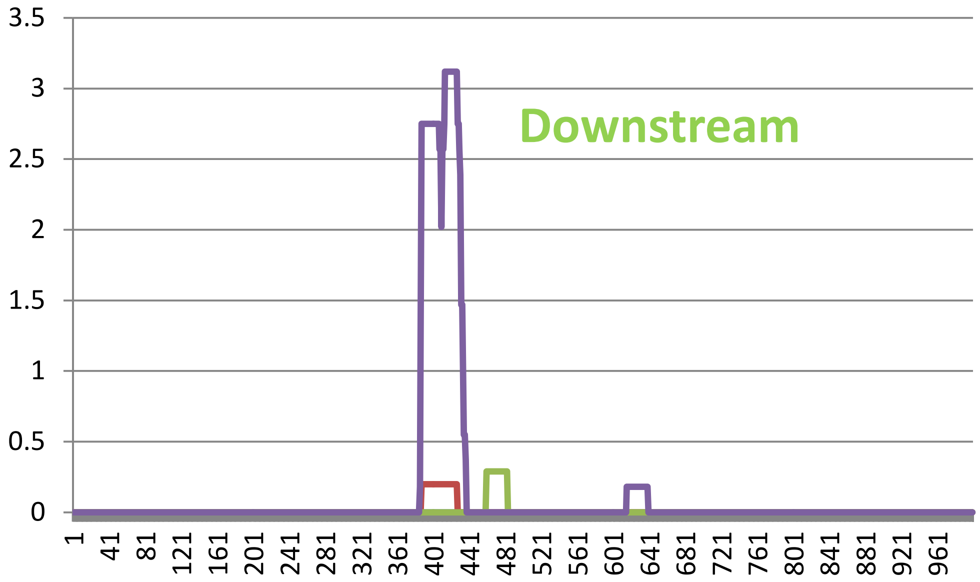

AT5G02820

Involved in the patterning and shape of leaf trichomes. Encodes the DNA topoisomerase VI SPO11-3, involved in endoreduplication.

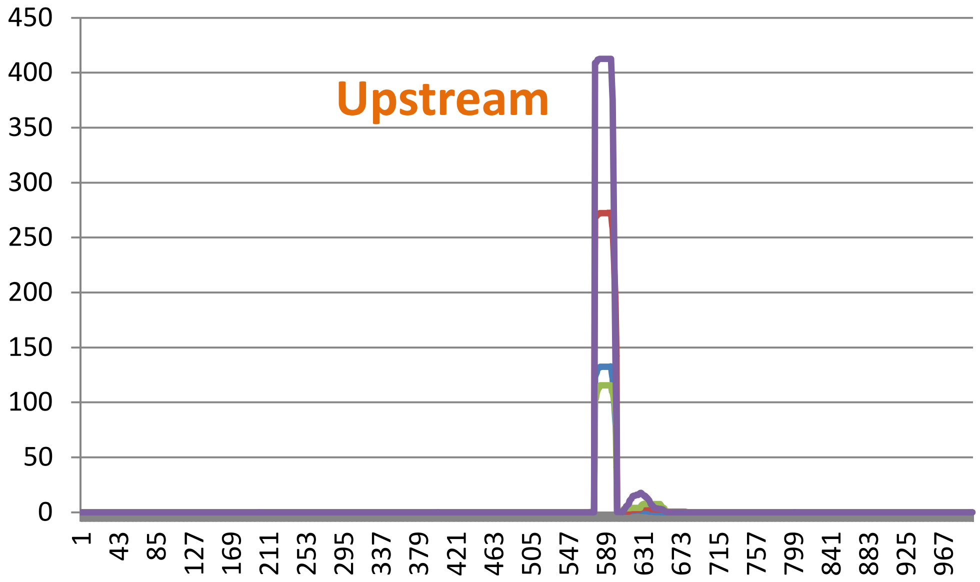

AT5G03340

ATPase, AAA-type, CDC48 protein.

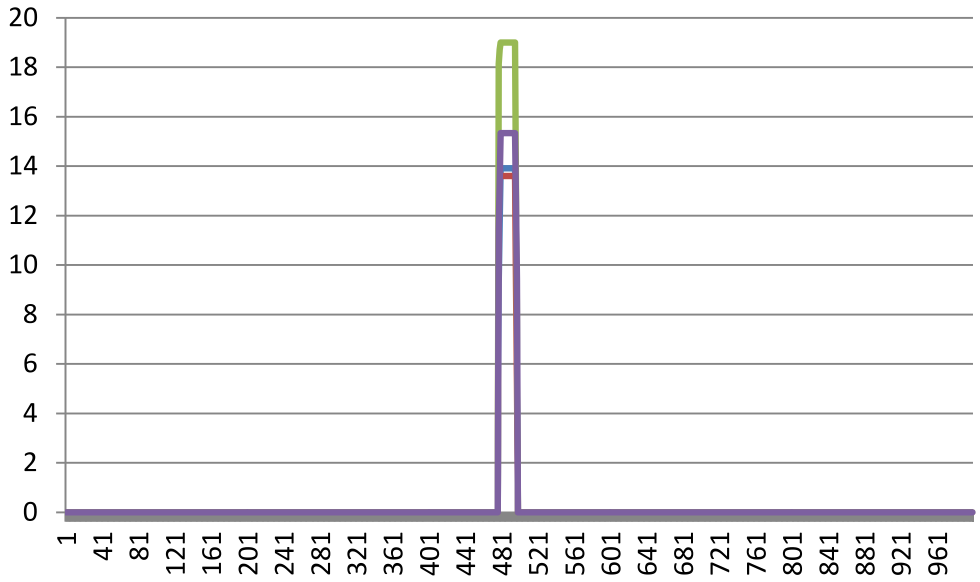

AT5G03455

Encodes a homolog of yeast cell cycle regulator CDC25.

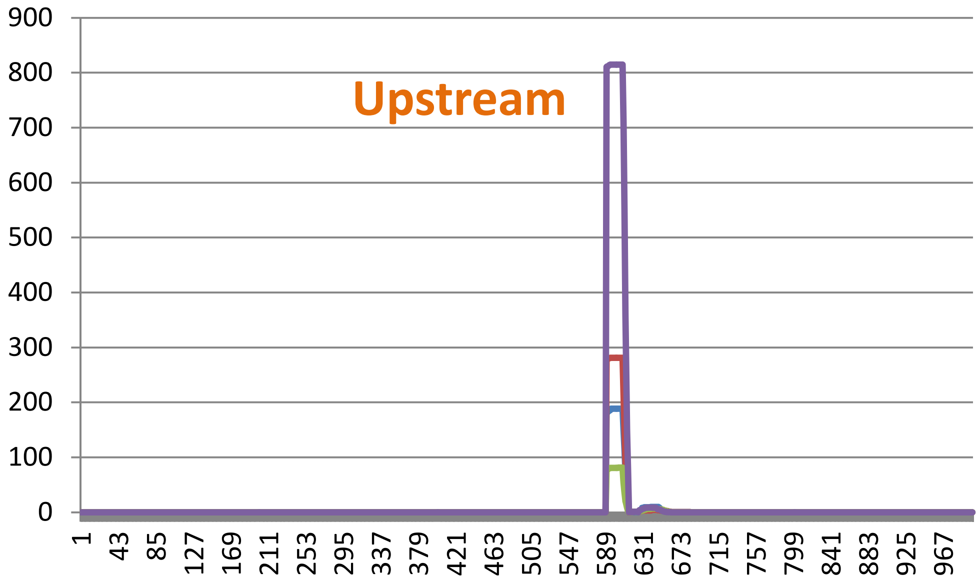

AT5G03570

Encodes FPN2, a tonoplast localized nickel transport protein.

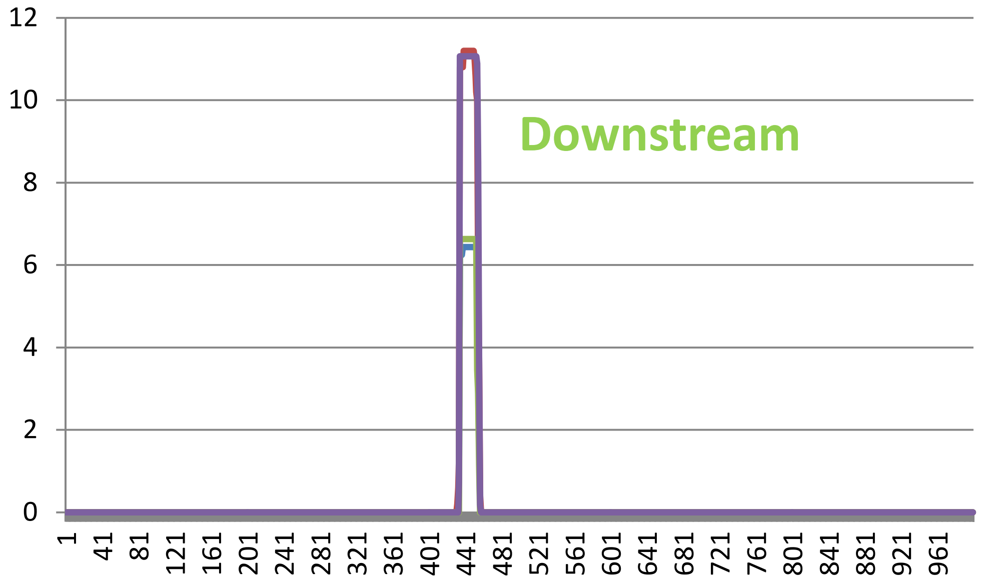

AT5G07140

Protein kinase superfamily protein

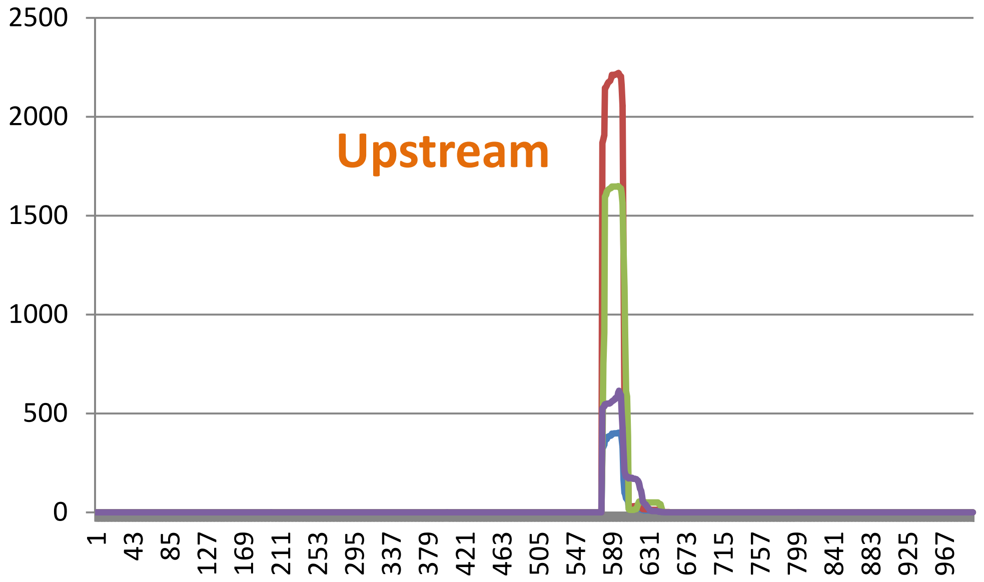

AT5G08430

SWIB/MDM2 domain; Plus-3; GYF.

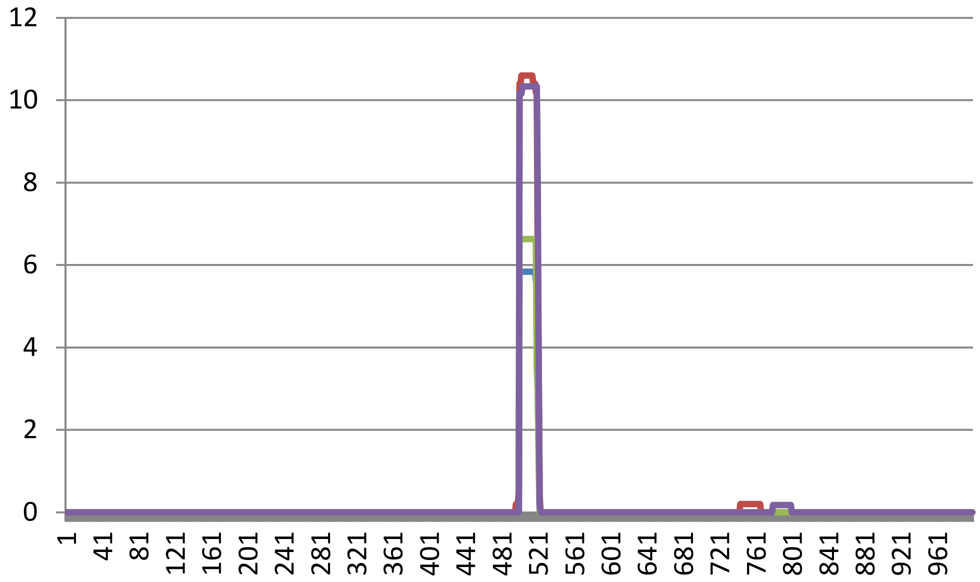

AT5G15170

Tyrosyl-DNA phosphodiesterase-related

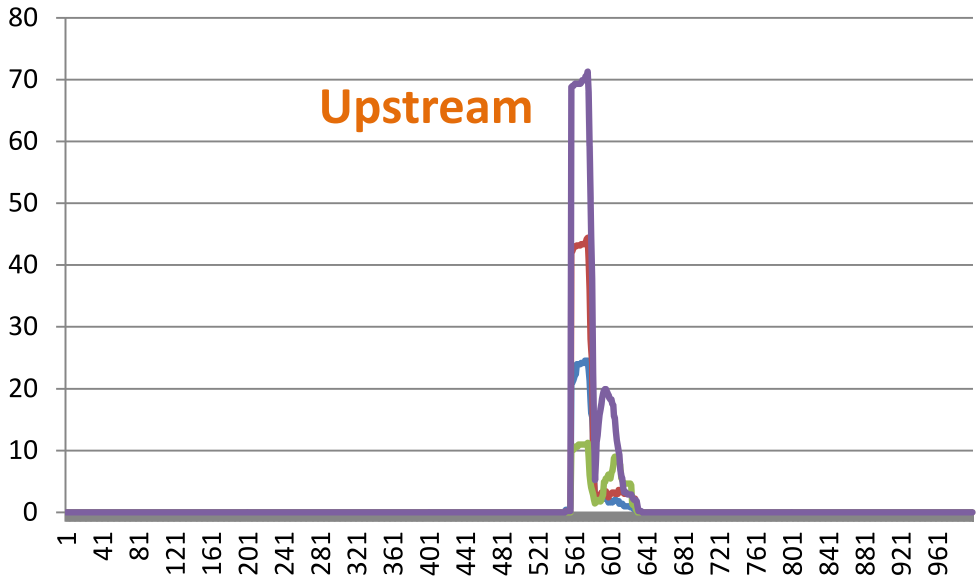

AT5G16280

Tetratricopeptide repeat (TPR)-like superfamily protein.

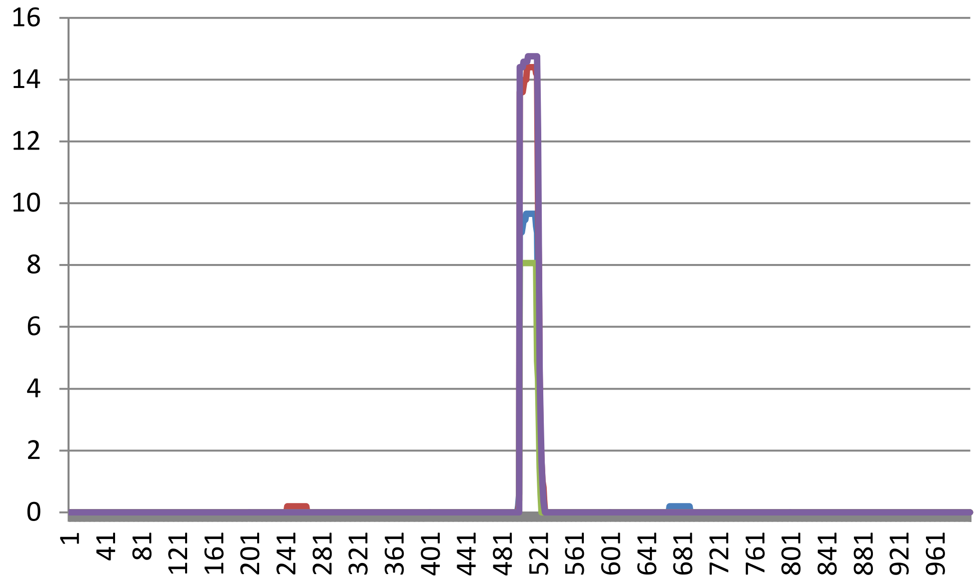

AT5G16520

Unknown protein

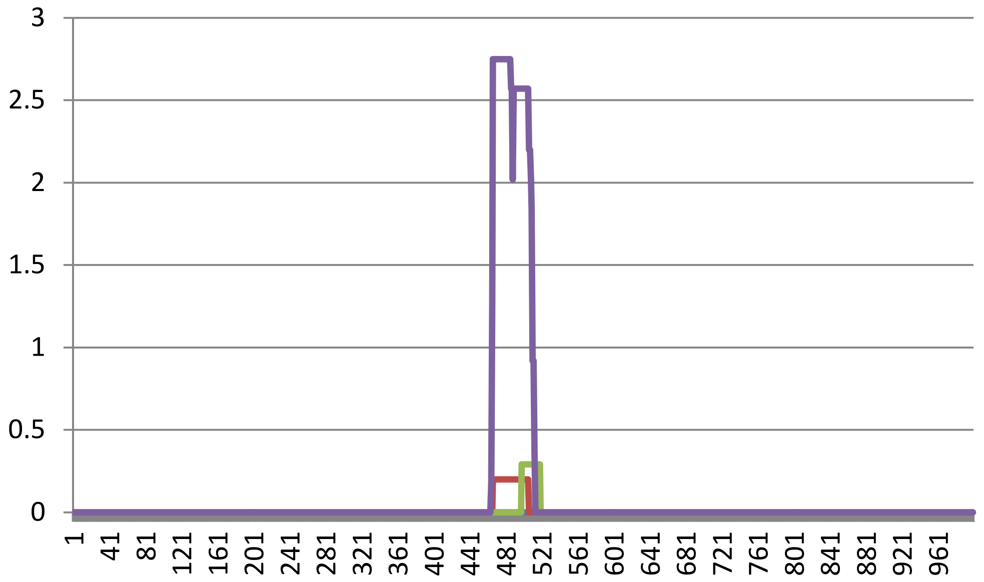

AT5G16720

Protein of unknown function, DUF593.

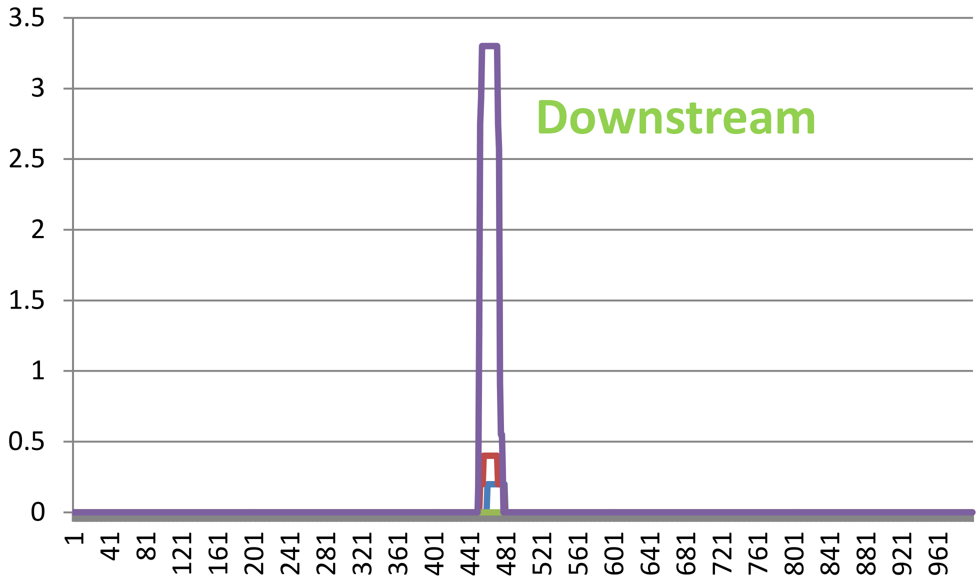

AT5G22590

Leucine Rich Repeat protein family.

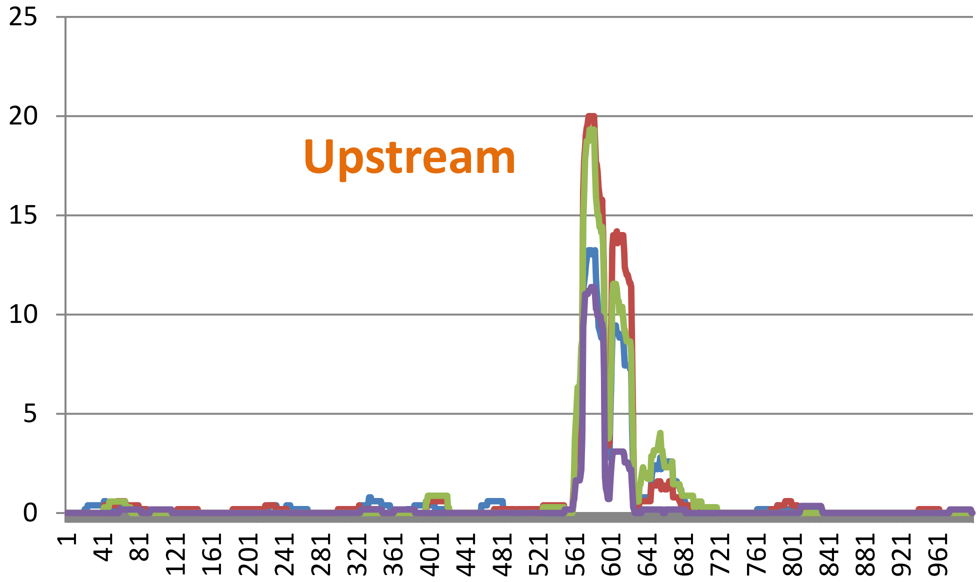

AT5G23580

Unique family of enzymes containing a single polypeptide chain with a kinase domain at the amino terminus and a putative calcium-binding EF hands structure at the carboxyl terminus; recombinant protein is fully active and induced by Ca2+.

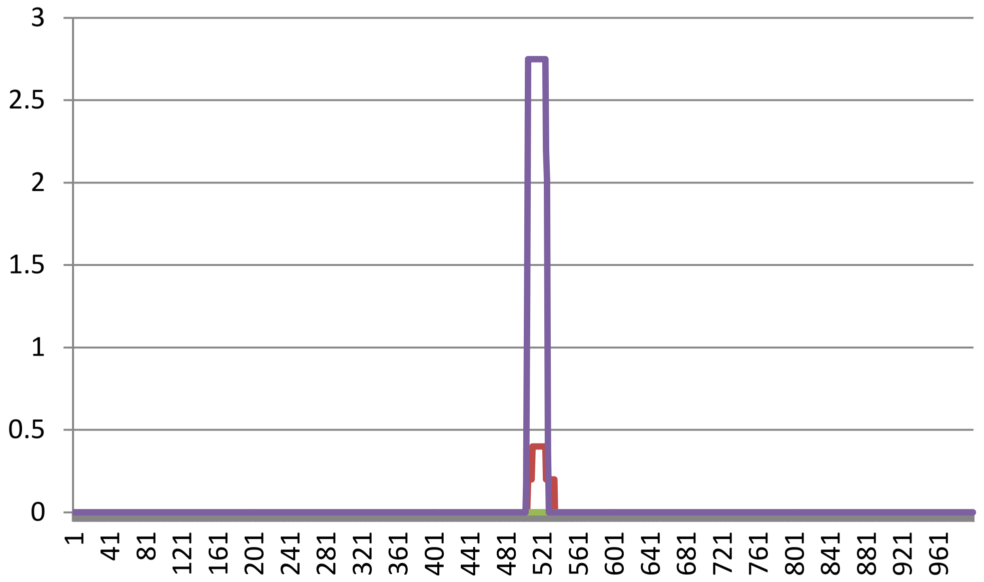

AT5G24240

Phosphatidylinositol 3- and 4-kinase; Ubiquitin family protein.

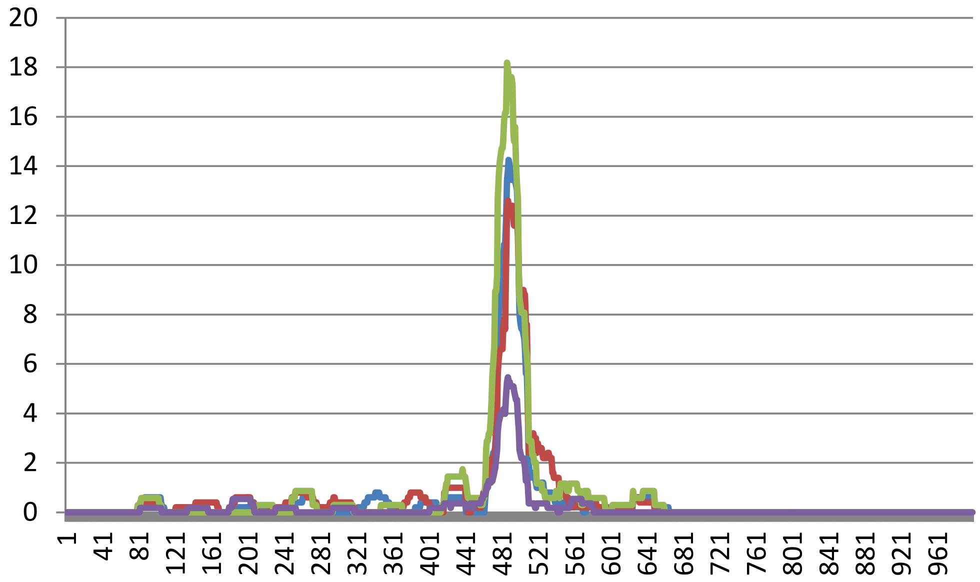

AT5G25760

PEROXIN4 (PEX4). Mutant displays sucrose-dependent seedling development and reduced lateral root production. PEX4 interacts with PEX22 in a yeast two-hybrid. Necessary for peroxisome biogenesis. The PEX4 and PEX22 pair may be important during the remodeling of peroxisome matrix contents as glyoxysomes transition to leaf peroxisomes.

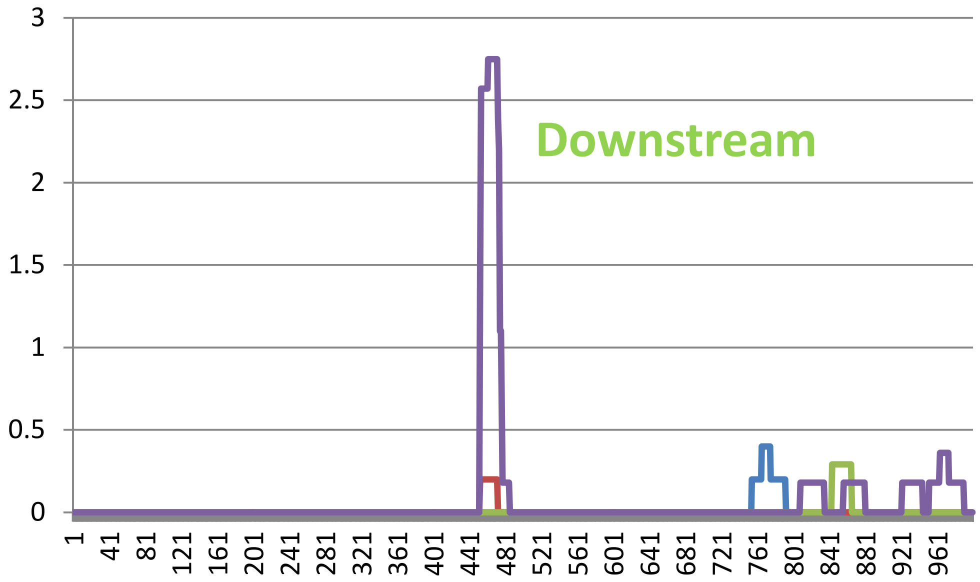

AT5G26700

RmlC-like cupins superfamily protein.

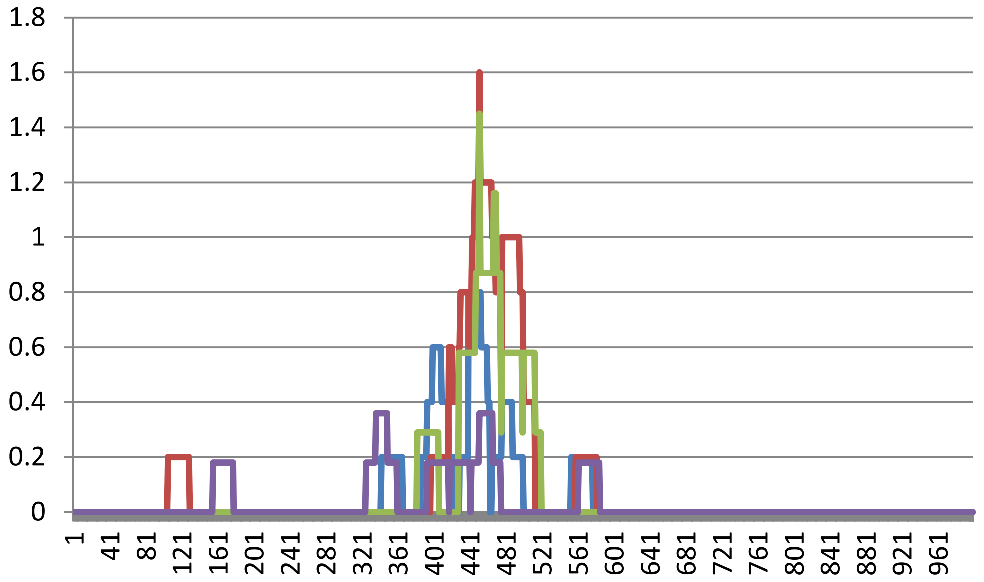

AT5G26840

Unknown protein

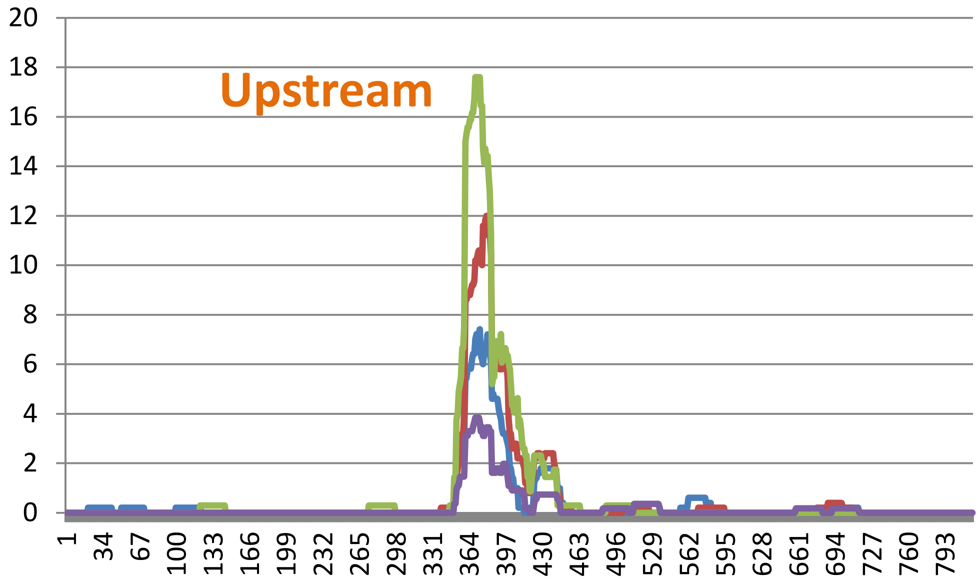

AT5G27720

Embryo defective 1644 (emb1644).

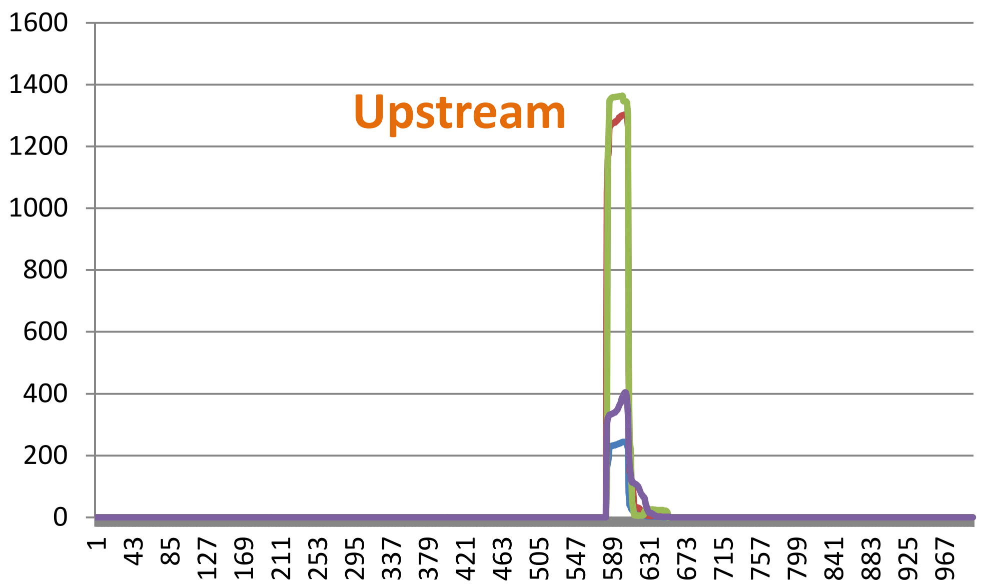

AT5G34581

Hydroxyproline-rich glycoprotein family protein

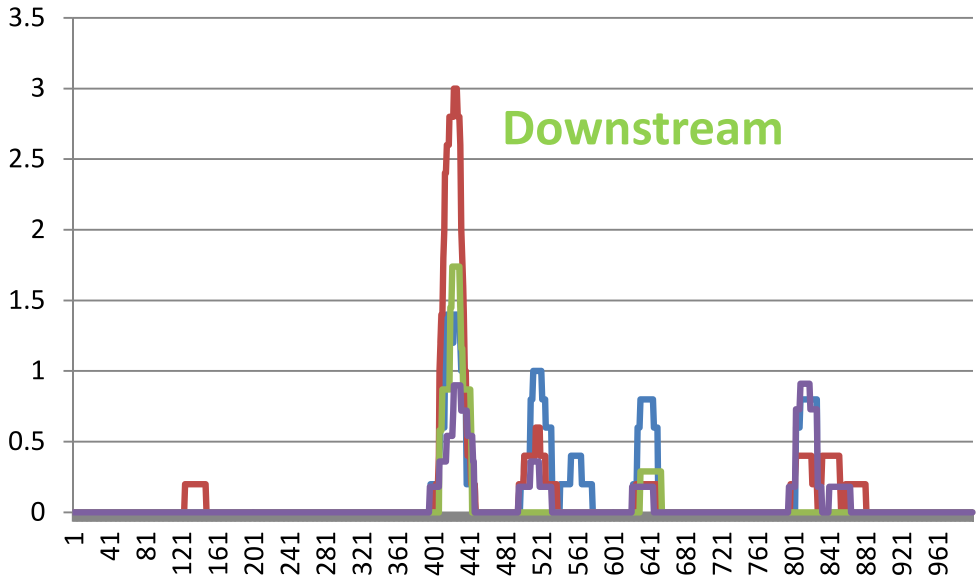

AT5G35526

This gene encodes a small protein and has either evidence of transcription or purifying selection.

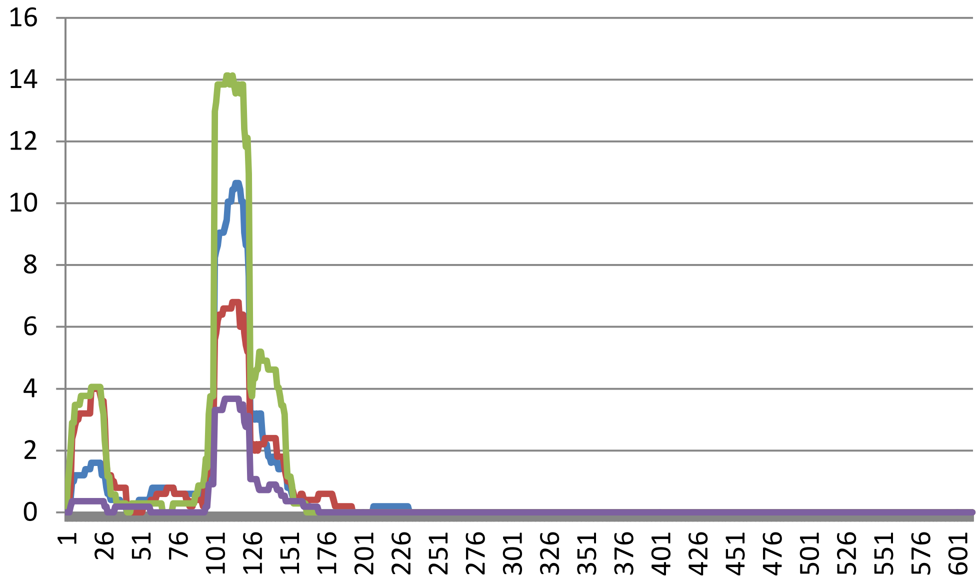

AT5G38270

F-box family protein.

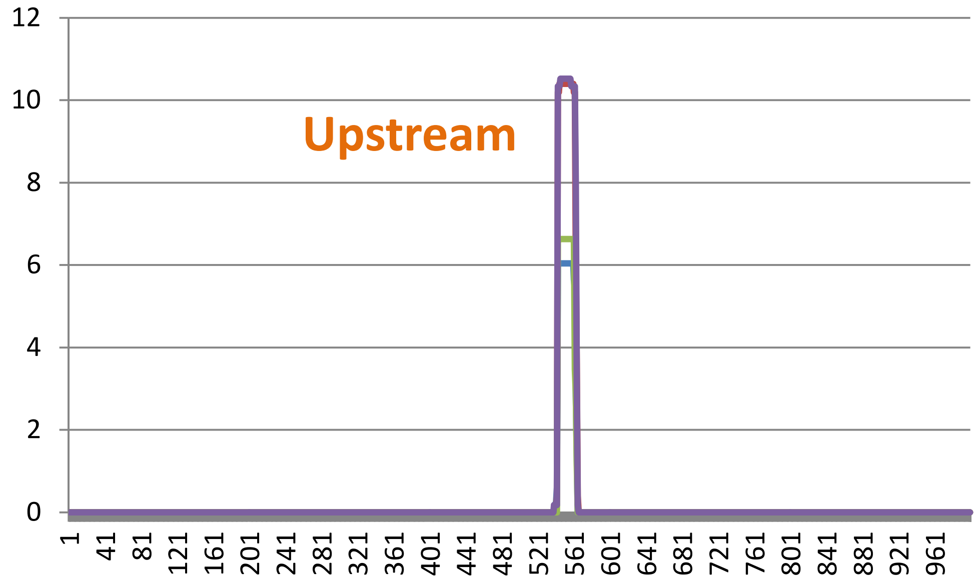

AT5G39560

Galactose oxidase/kelch repeat superfamily protein

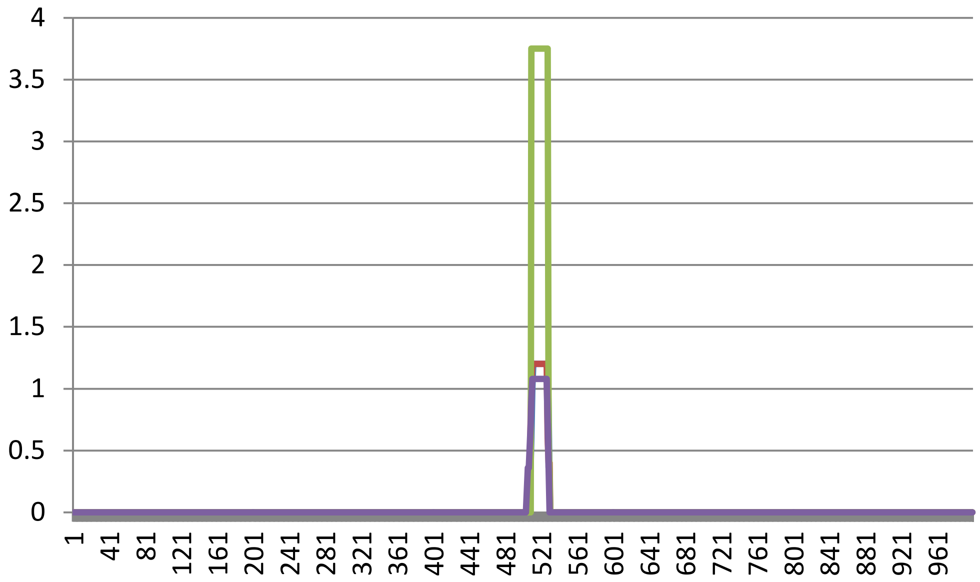

AT5G39645

Encodes a Defensin-like (DEFL) family protein.

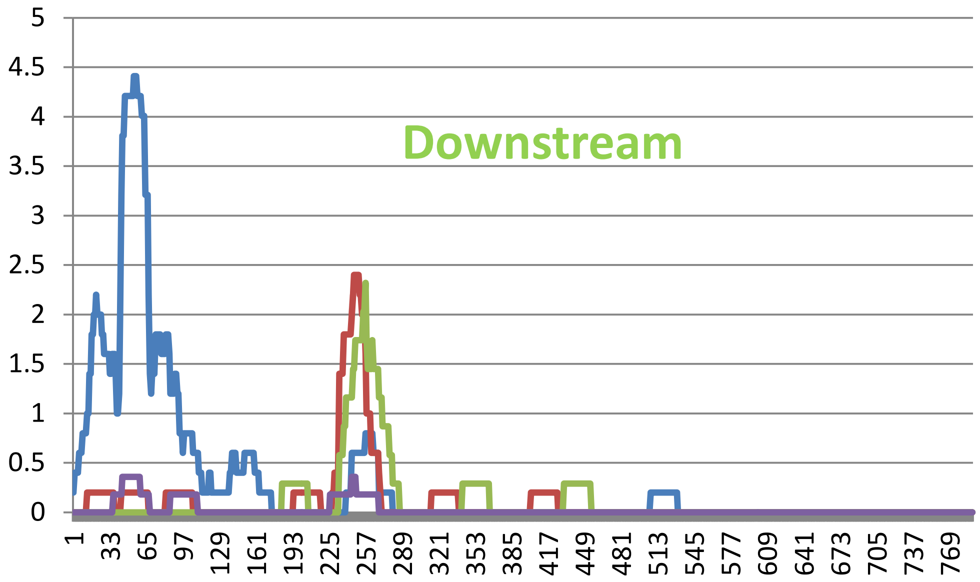

AT5G40320

Cysteine/Histidine-rich C1 domain family protein

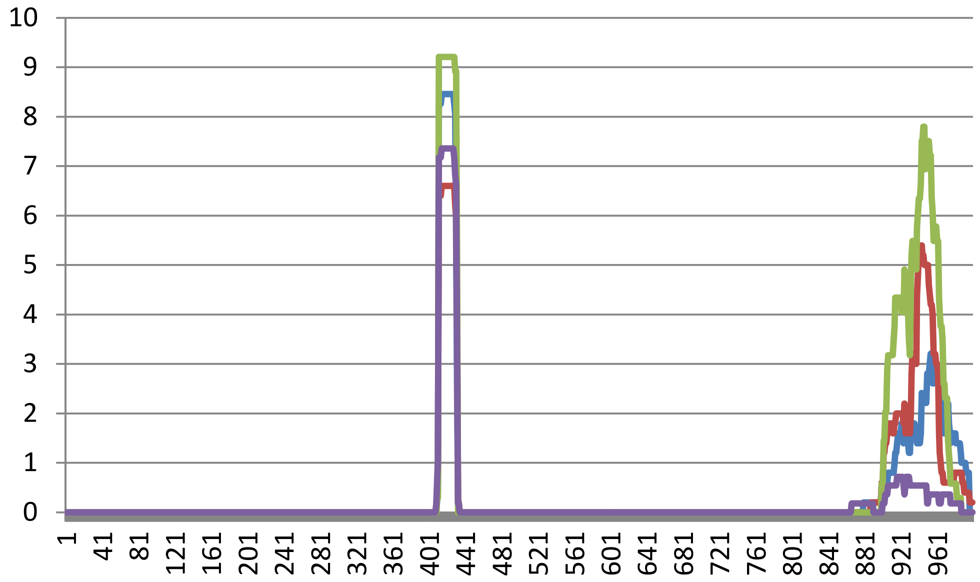

AT5G40330

Encodes a MYB gene that, when overexpressed ectopically, can induce ectopic trichome formation.

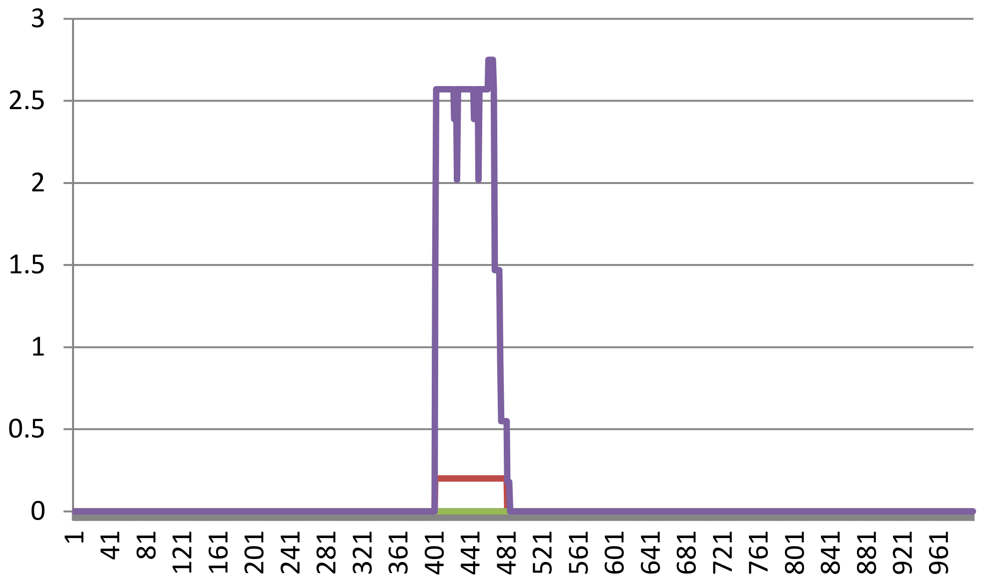

AT5G40820

Encodes an Arabidopsis ortholog of the ATR protein kinase that is involved in a wide range of responses to DNA damage and plays a central role in cell-cycle regulation.

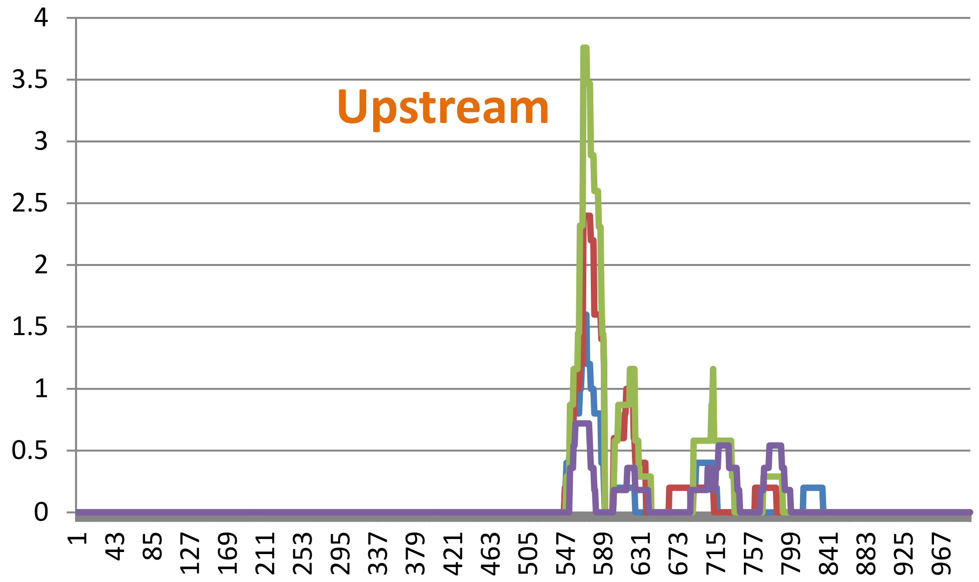

AT5G42635

Glycine-rich protein

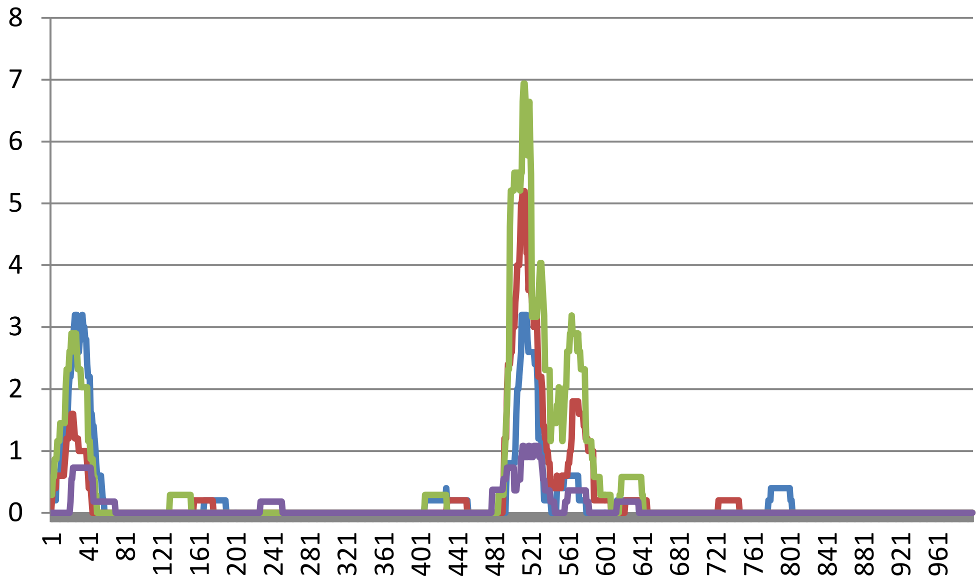

AT5G42960

Unknown protein

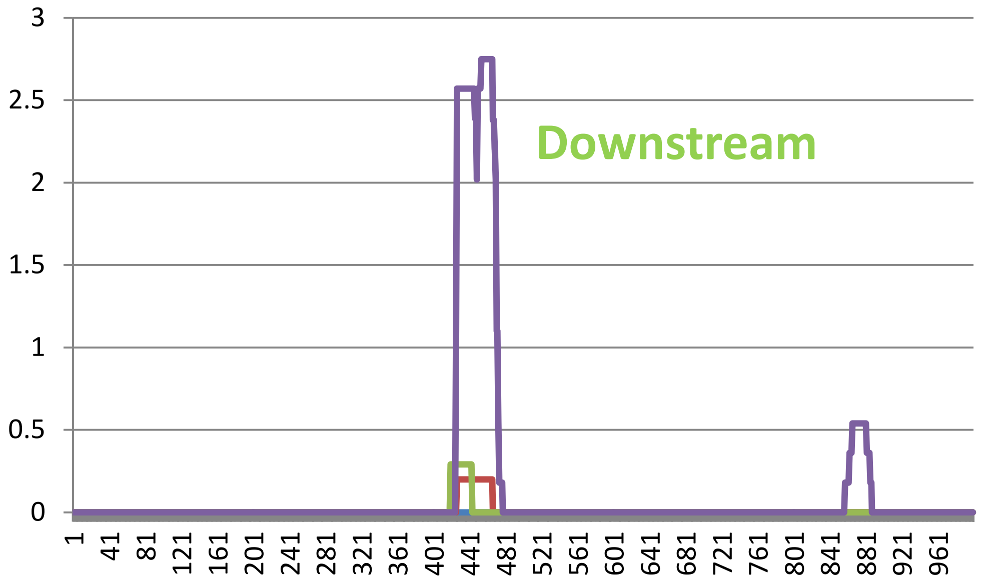

AT5G43270

SQUAMOSA PROMOTER BINDING PROTEIN-LIKE 2 (SPL2). Member of the SPL (squamosa-promoter binding protein-like) gene family, a novel gene family encoding DNA binding proteins and putative transcription factors. In conjunction with SPL10 and SPL11, SPL2 redundantly controls proper development of lateral organs in association with shoot maturation in the reproductive phase.

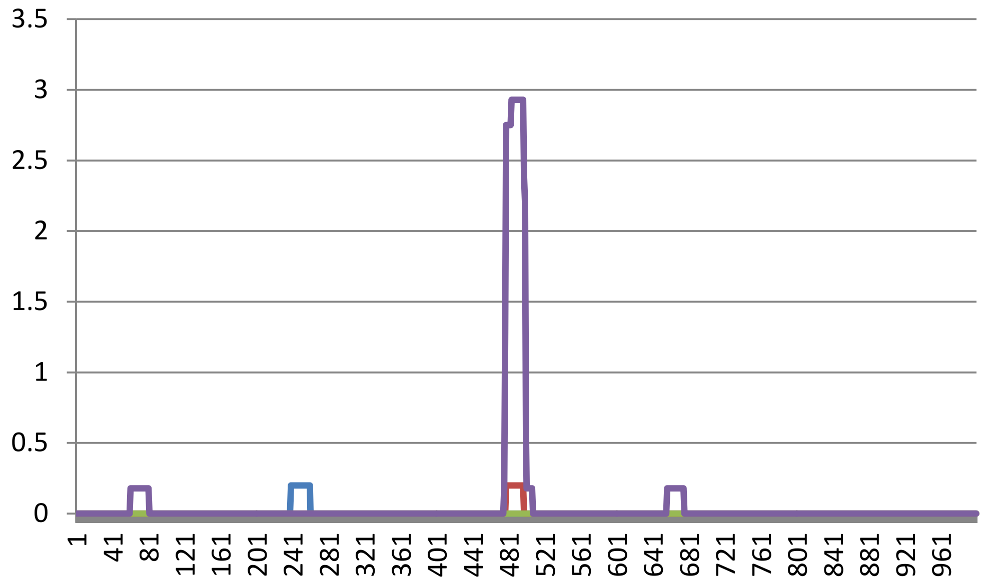

AT5G43500

Encodes a protein whose sequence is similar to actin-related proteins (ARPs) in other organisms. Member of nuclear ARP family of genes.

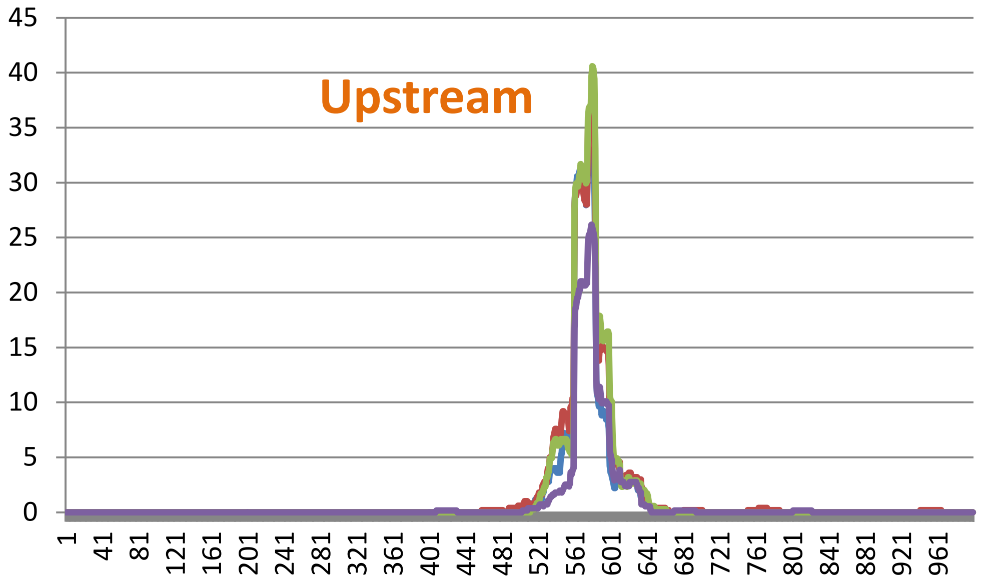

AT5G43810

Encodes Argonaute10, a member of the EIF2C (elongation initiation factor 2c)/Argonaute class of proteins. Required to establish the central-peripheral organization of the embryo apex. Along with WUS and CLV genes, controls the relative organization of central zone and peripheral zone cells in meristems. Acts in embryonic provascular tissue potentiating WUSCHEL function during meristem development in the embryo. AGO10 specifically sequesters miR166/165 to regulate shoot apical meristem development.

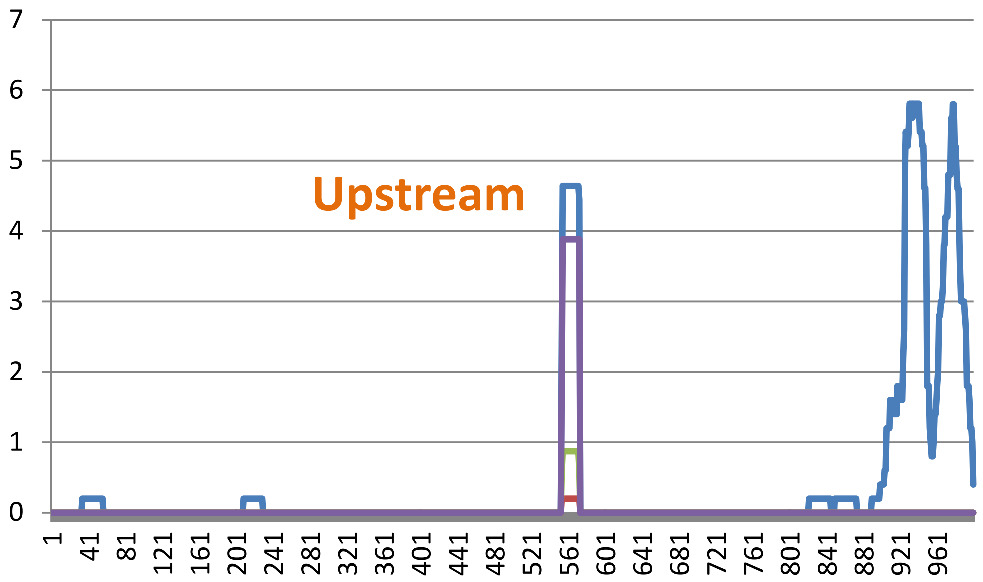

AT5G44930

Encodes a putative arabinosyltransferase.

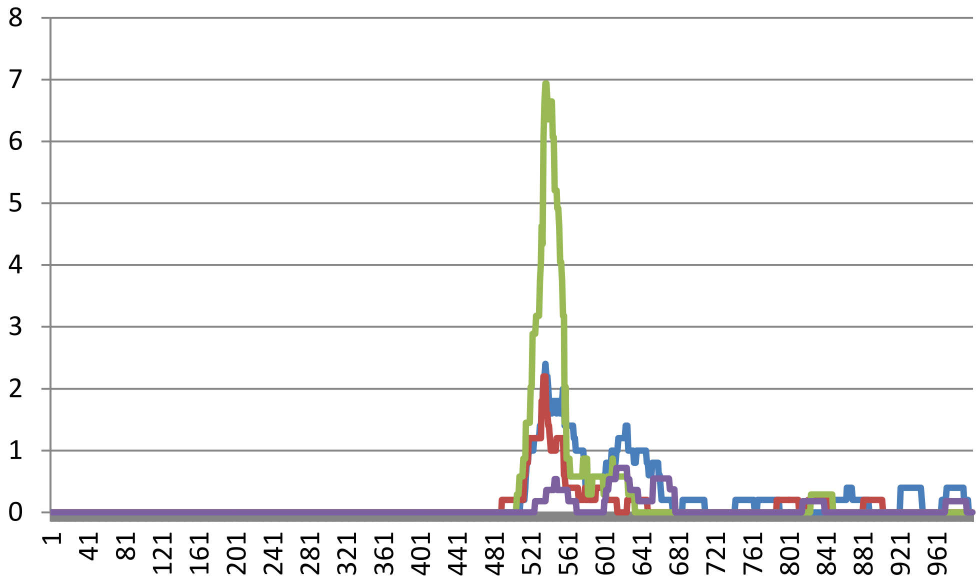

AT5G47260

ATP binding; GTP binding; nucleotide binding; nucleoside-triphosphatases

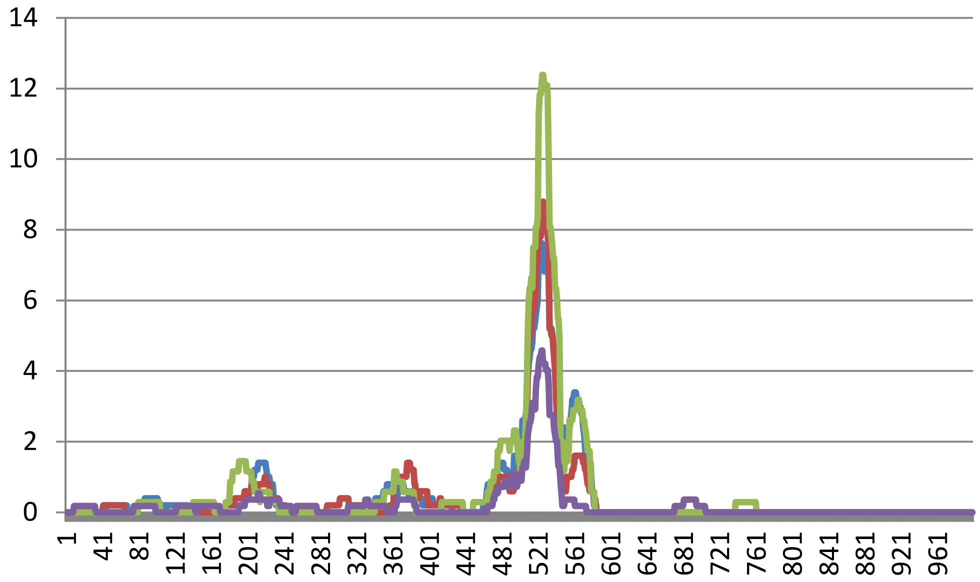

AT5G48000

Encodes a member of the CYP708A family of cytochrome P450 enzymes. THAH appears to add a hydroxyl group to the triterpene thalianol. thah1 mutants have an elevated accumulation of thalianol. thah1-1 mutants have longer roots than wild type plants.

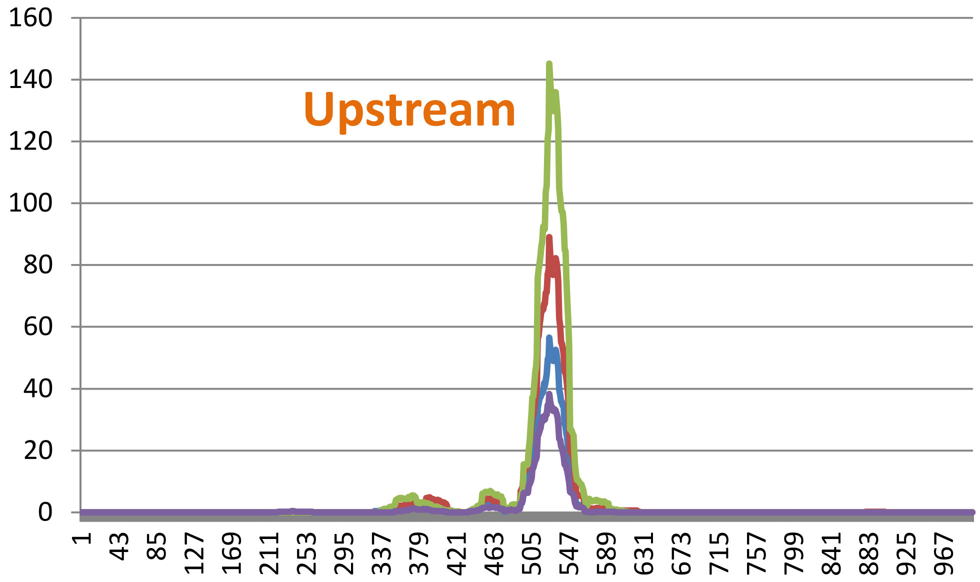

AT5G48830

Unknown protein

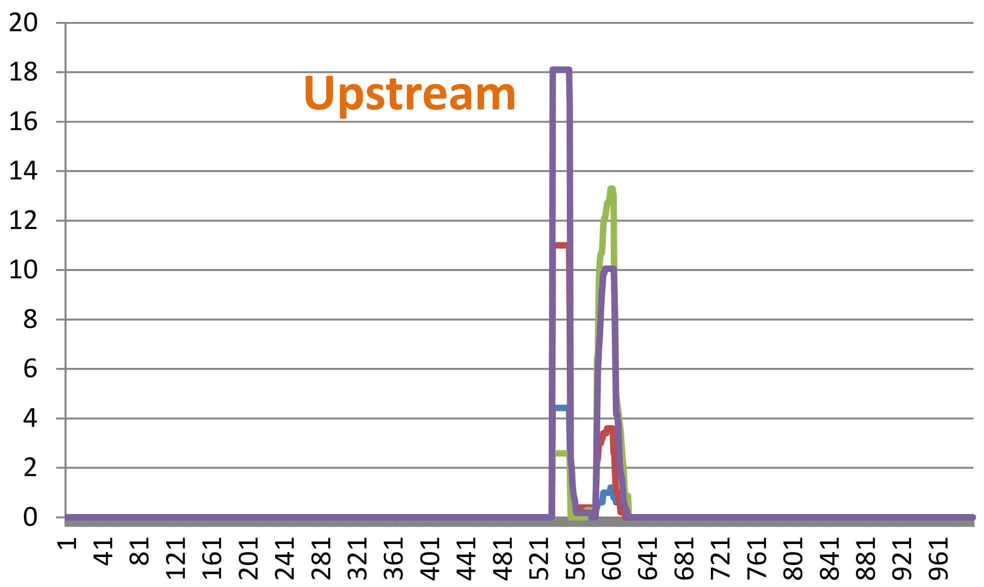

AT5G50530

CBS/octicosapeptide/Phox/Bemp1 (PB1) domains-containing protein

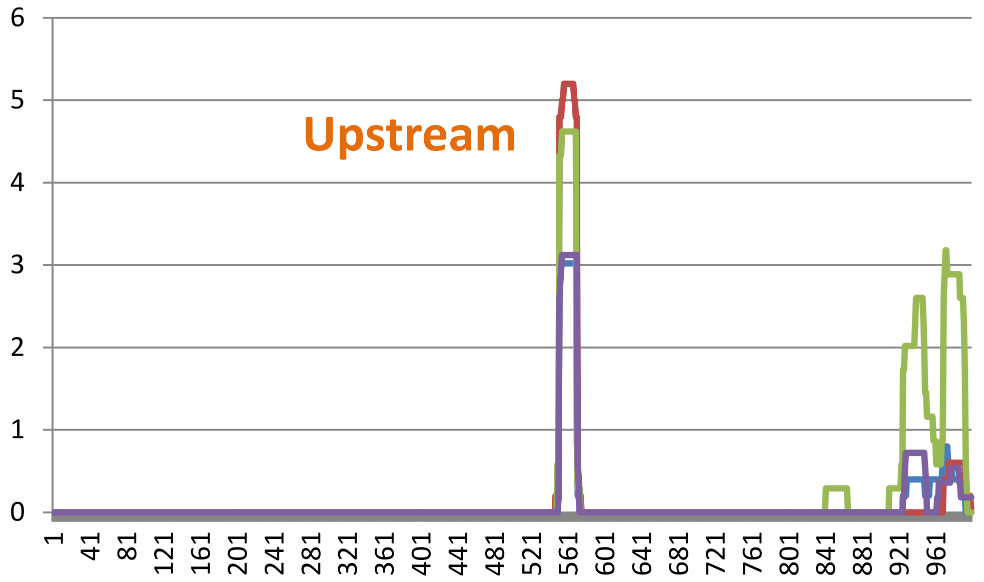

AT5G50640

CBS/octicosapeptide/Phox/Bemp1 (PB1) domains-containing protein

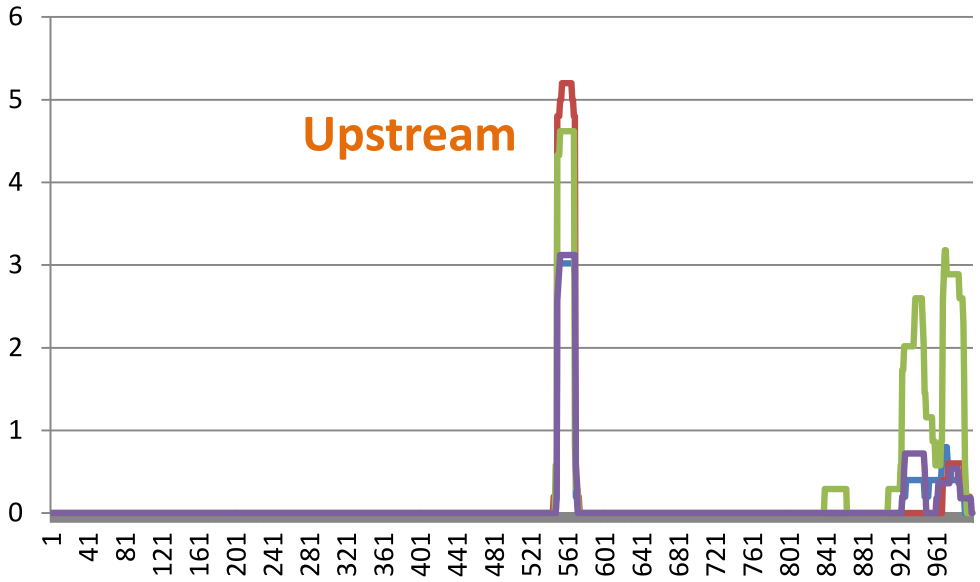

AT5G52690

Copper transport protein family

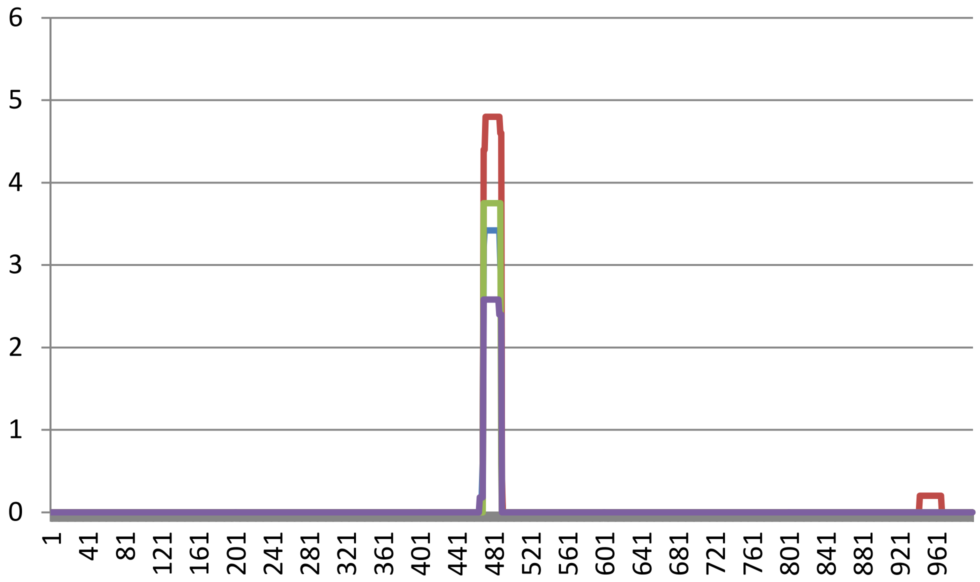

AT5G53120

SPERMIDINE SYNTHASE 3 (SPDS3). Encodes a novel spermine synthase and is a paralog of previously characterized spermidine synthases, SPDS1 and SPDS2. SPDS3 forms heterodimers with SDPS2, which in turn forms heterodimers with SDPS1 in vivo. The gene does not complement speDelta3 deficiency of spermidine synthase in yeast but DOES complement speDelta4 deficiency.

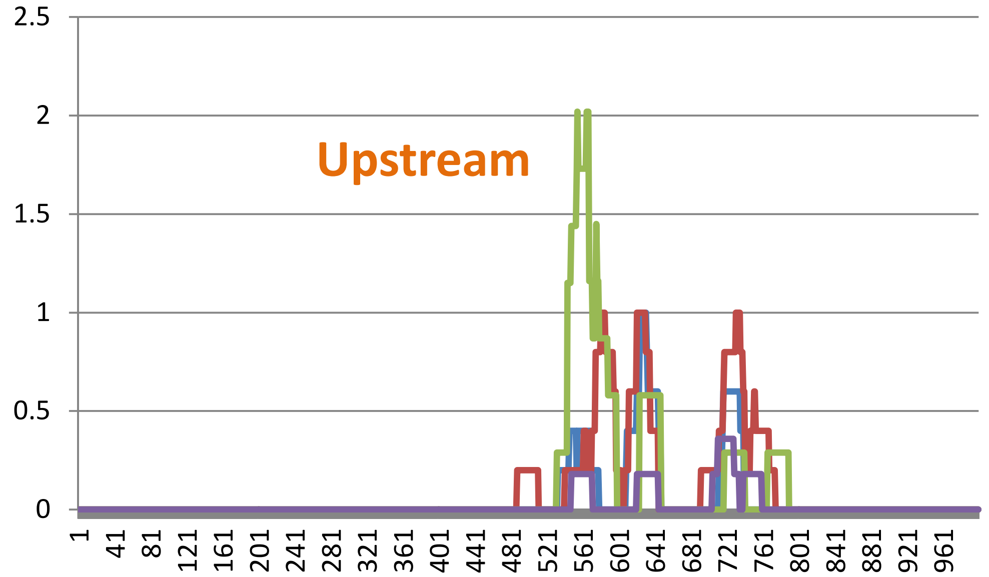

AT5G55480

Glycerophosphoryl diester phosphodiesterase-like protein involved in cell wall cellulose accumulation and pectin linking. Impacts root hair, trichome and epidermal cell development.

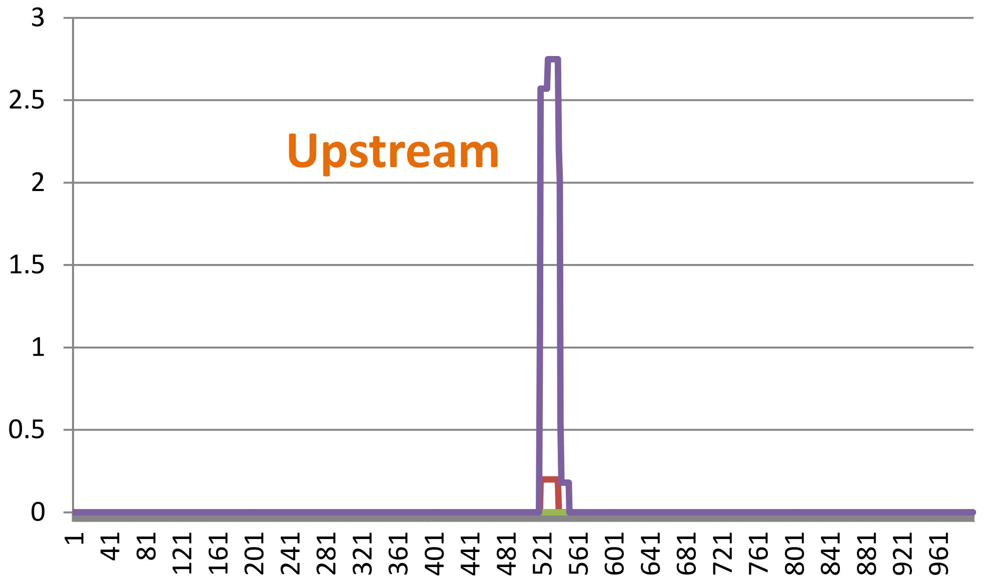

AT5G57655

Xylose isomerase family protein

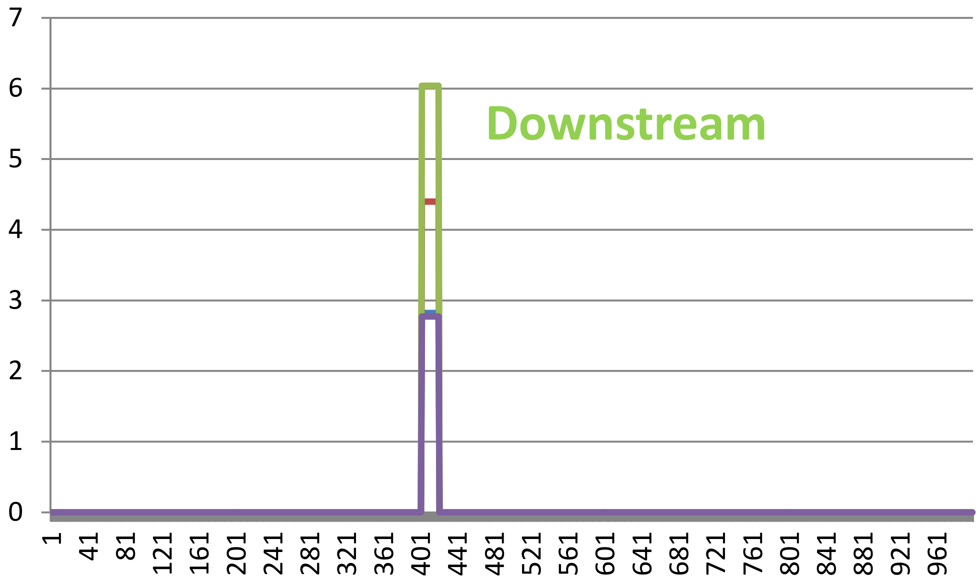

AT5G58375

Methyltransferase-related protein.

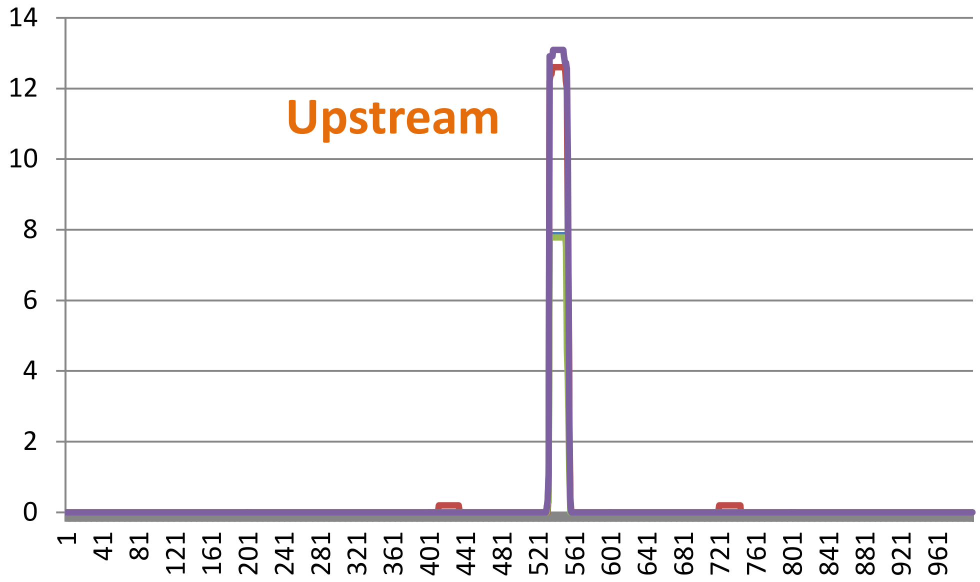

AT5G58550

ETO1-LIKE 2 (EOL2). Encodes a paralog of ETO1, which is a negative regulator of ACS5 (a key enzyme in ethylene biosynthesis pathway). EOL2 also interacts with and inhibits the activity of ACS5.

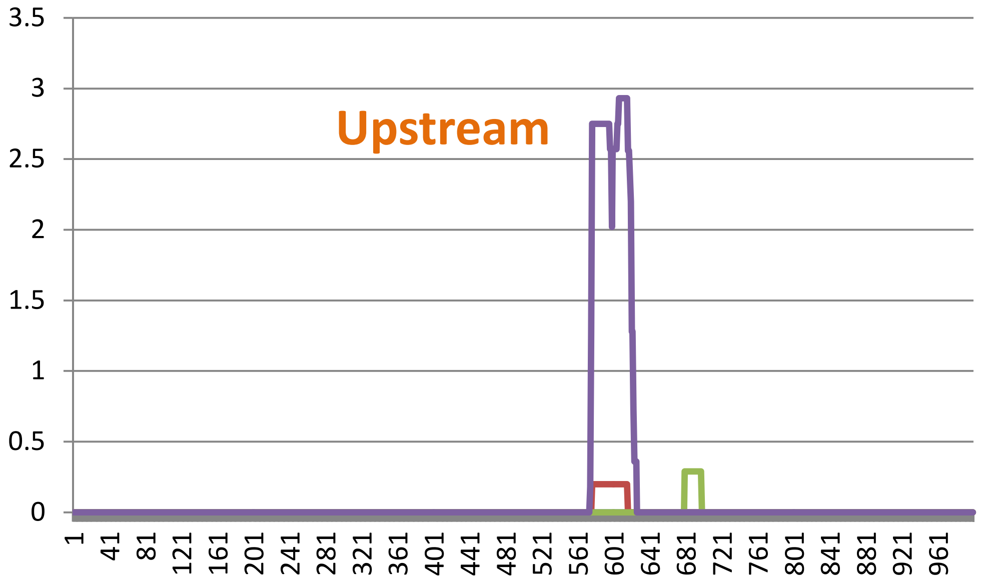

AT5G59030

Encodes a putative copper transport protein that contains copper-binding motif and functionally complements in copper-transport defective yeast strains

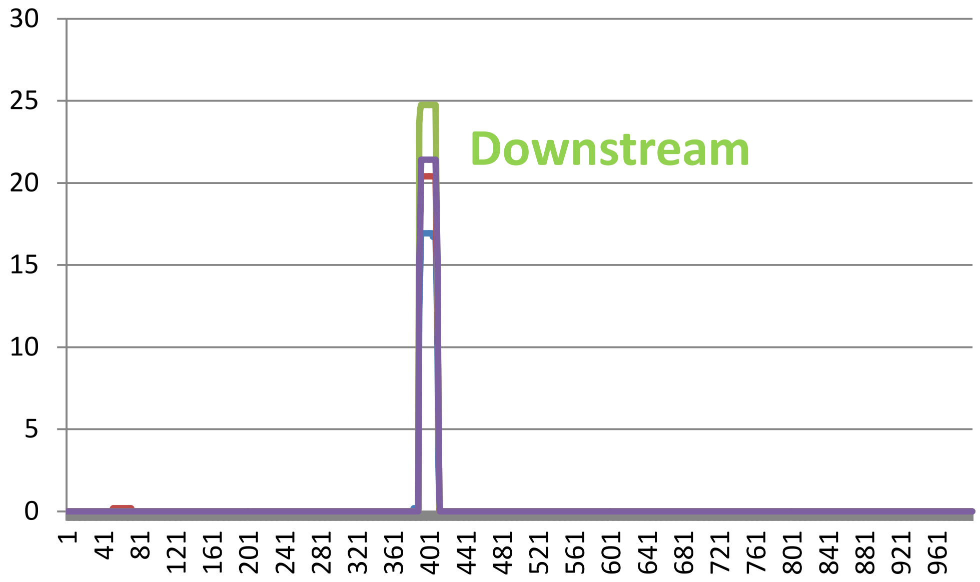

AT5G59950

RNA-binding (RRM/RBD/RNP motifs) family protein

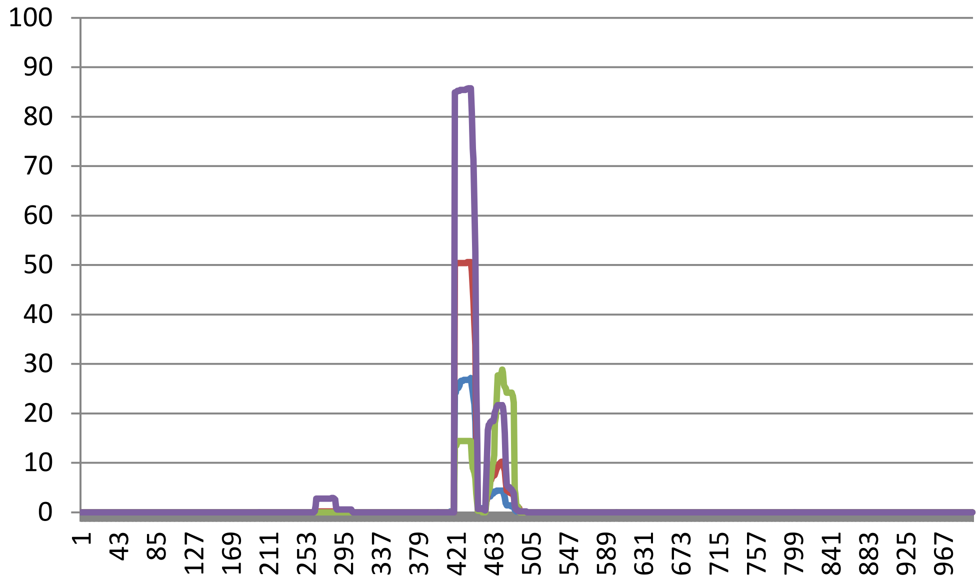

AT5G61510

GroES-like zinc-binding alcohol dehydrogenase family protein.

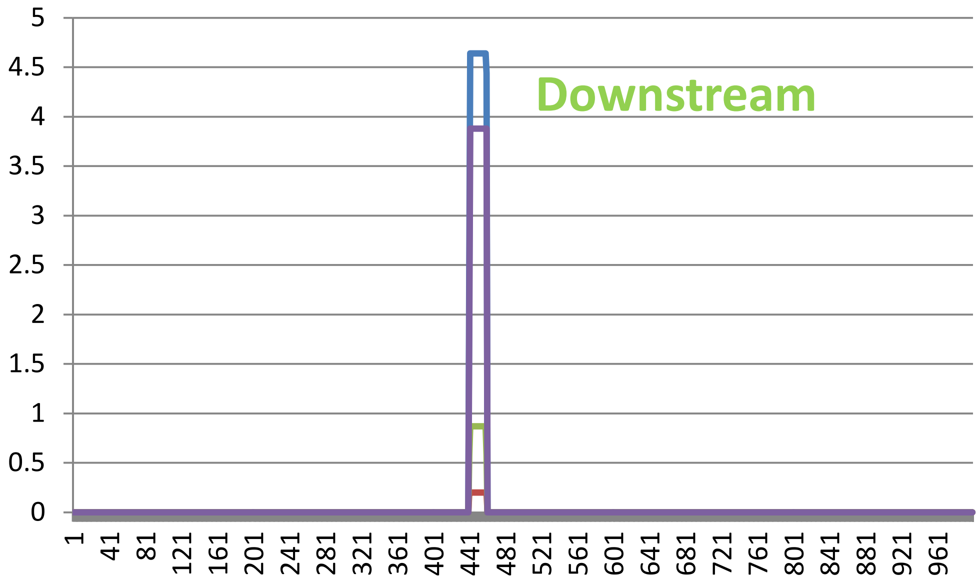

AT5G62130

Per1-like family protein.

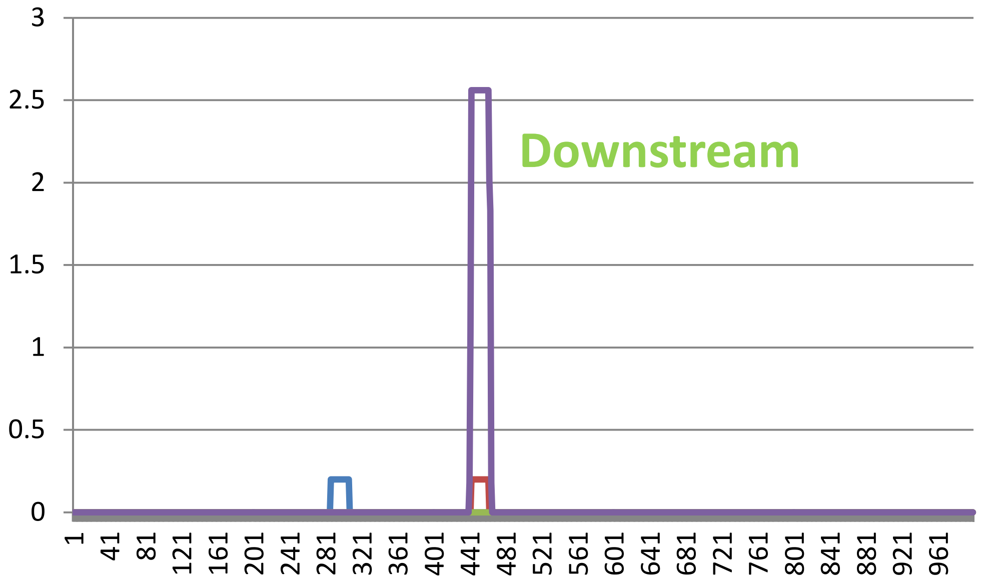

ATCG00790

Chloroplast gene encoding a ribosomal protein L16, which is a constituent of 50S large ribosomal subunit.

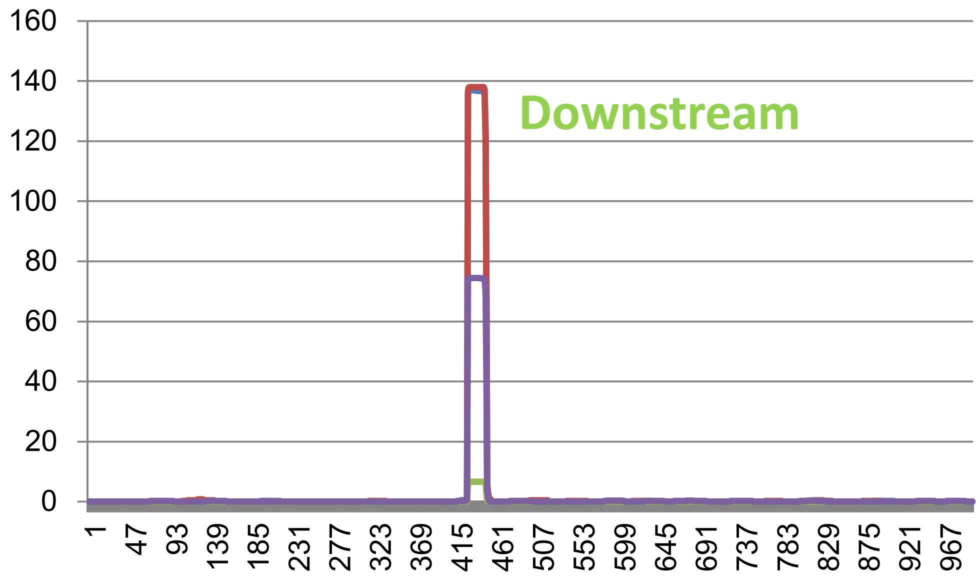

ATCG00840

One of two chloroplast genes that encode chloroplast ribosomal protein L23, a constituent of the large subunit of the ribosomal complex.

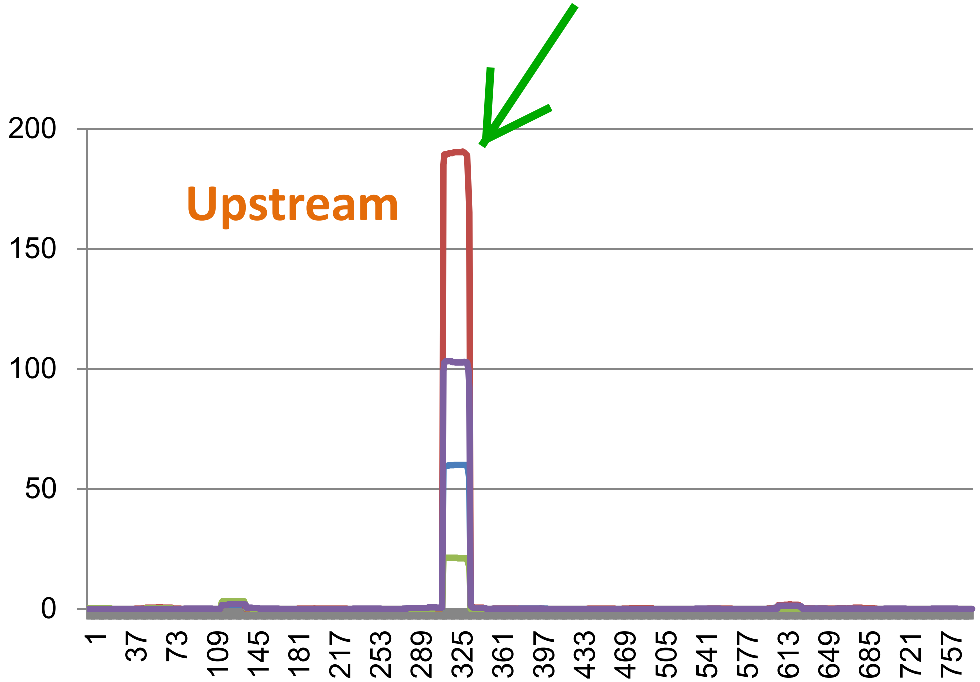

ATCG01120

Encodes a chloroplast ribosomal protein S15, a constituent of the small subunit of the ribosomal complex.

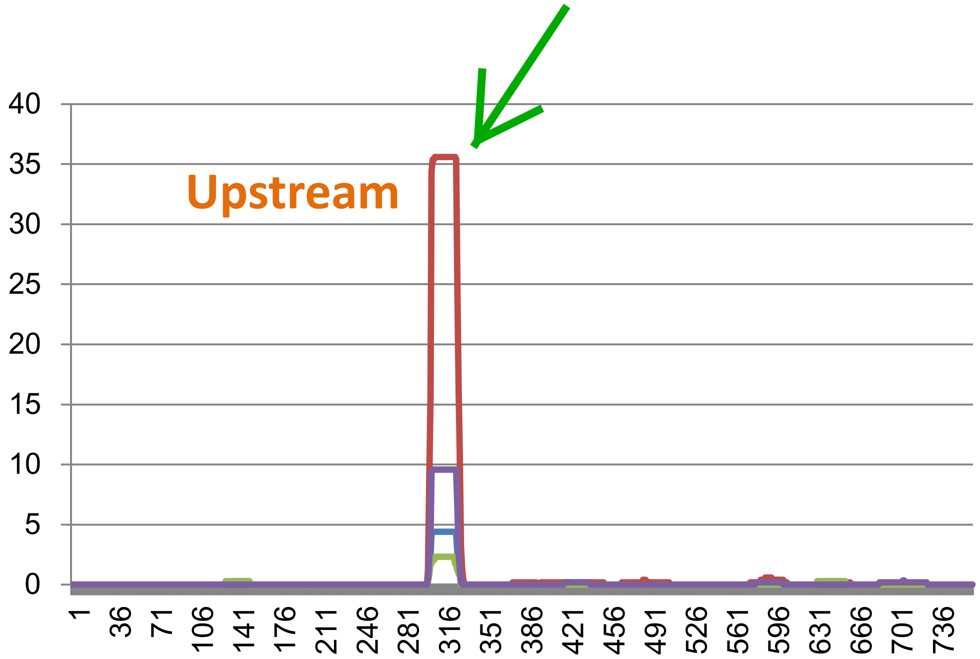

ATCG01300

One of two chloroplast genes that encode chloroplast ribosomal protein L23, a constituent of the large subunit of the ribosomal complex.

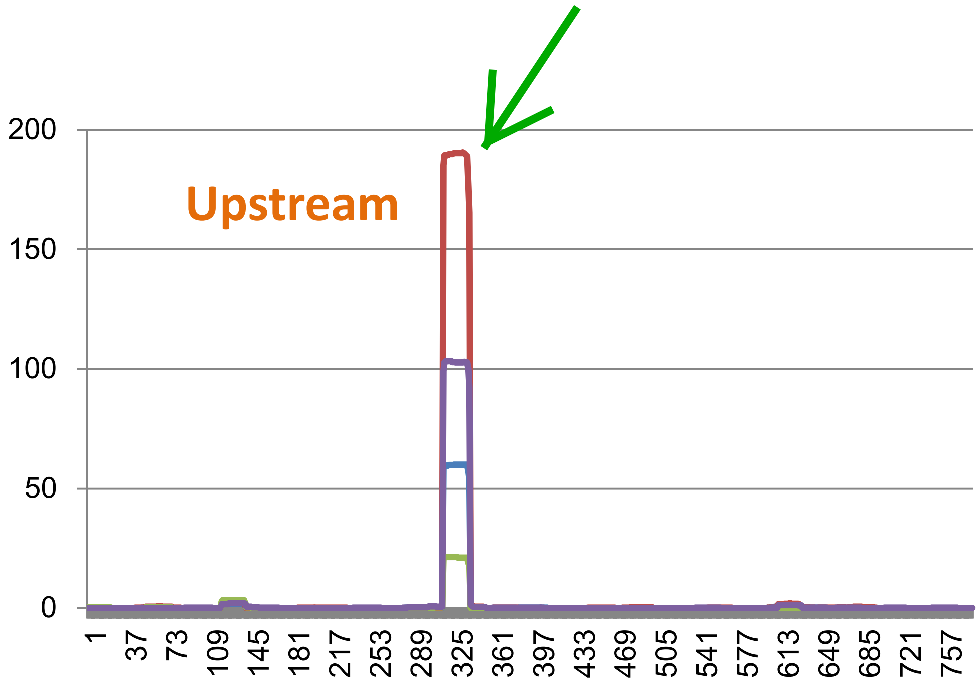

Supplement: S2 Fig — For the chloroplast genes, sRNAs dominantly detected in leaves and seedlings were marked by green arrows. (PDF) [file pone.0169212.s002.pdf]
