# Supplementary material for: Transcriptome-Wide Discovery of PASRs (Promoter-Associated Small RNAs) and TASRs (Terminus-Associated Small RNAs) in Arabidopsis thaliana
Source: PLoS One. 2017 Jan 3;12(1):e0169212. doi: 10.1371/journal.pone.0169212 (PMC5207706; doi:10.1371/journal.pone.0169212)

Figure S4 TASR peaks identified on the sense strands of the protein-coding genes of *Arabidopsis*. For each plot, x axis measures the position of the sense strand, and y axis measures the abundance (in RPM, reads per million) of sRNAs. For the chloroplast genes, sRNAs dominantly detected in leaves and seedlings were marked by green arrows.

AT1G01073

Unknown protein

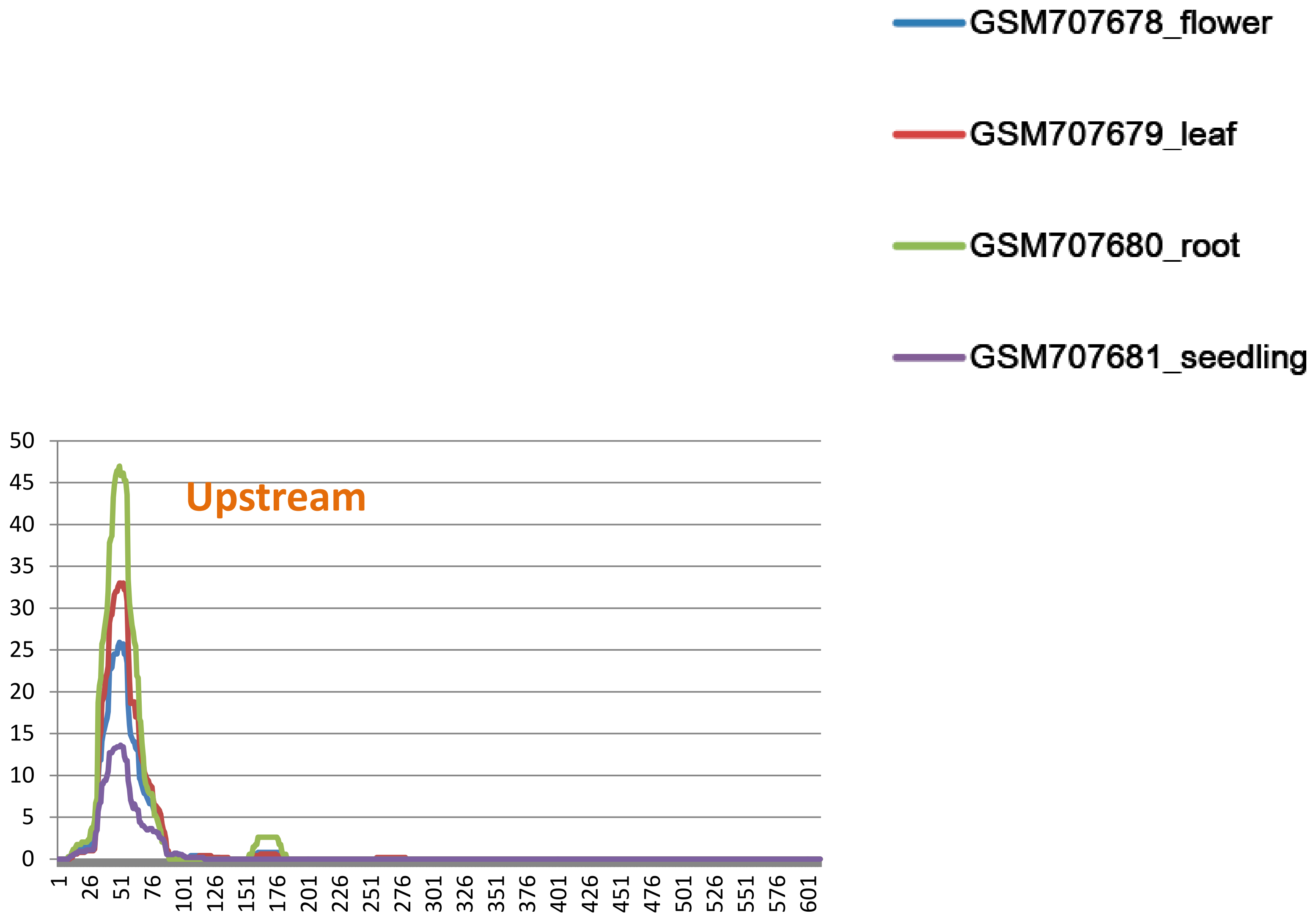

AT1G01180

S-adenosyl-L-methionine-dependent methyltransferases superfamily protein.

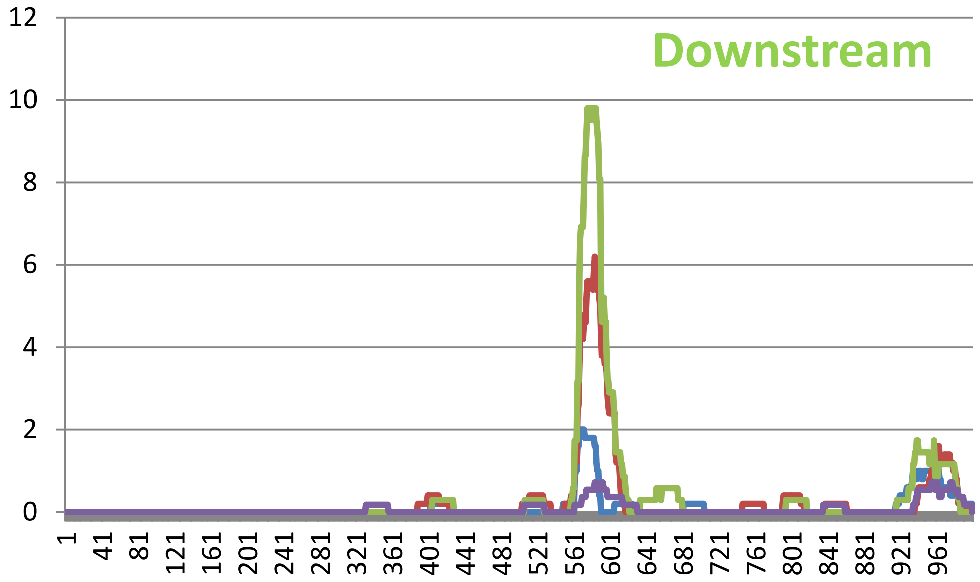

AT1G09026

Unknown protein

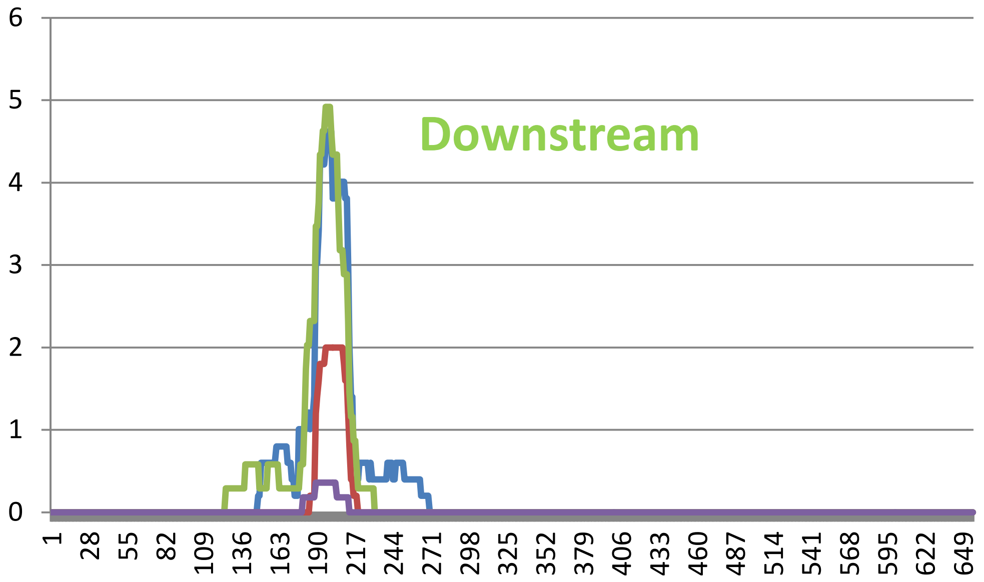

AT1G10000

Ribonuclease H-like superfamily protein

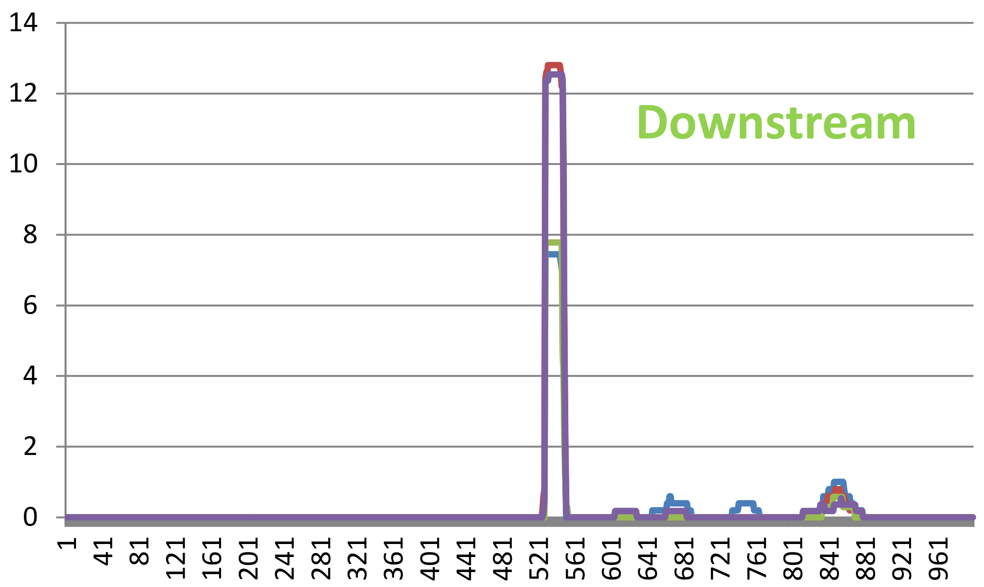

AT1G10745

Encodes a Maternally expressed gene (MEG) family protein.

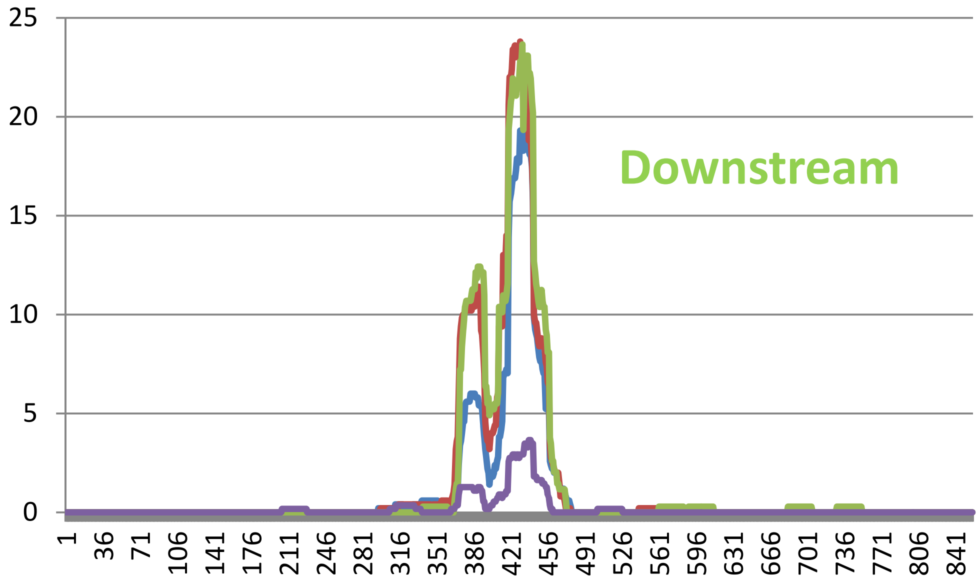

AT1G12520

COPPER CHAPERONE FOR SOD1 (CCS). Copper-zinc superoxide dismutase copper chaperone (delivers copper to the Cu-Zn superoxide dismutase). Localized to the chloroplast. Expressed in roots and shoots. Up-regulated in response to copper and senescence. The AtACC activates all three CuZnSOD activities located in three different subcellular compartments. Contains three domains, central, ATX-1 like and C-terminal. ATX-1 like domain essential for the copper chaperone function of AtCCS in planta.

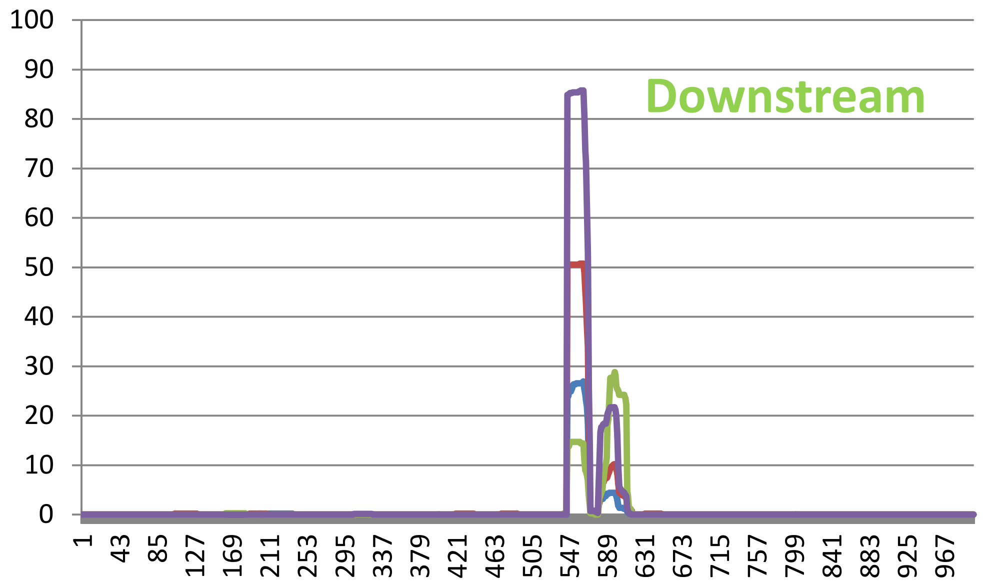

AT1G14580

C2H2-like zinc finger protein

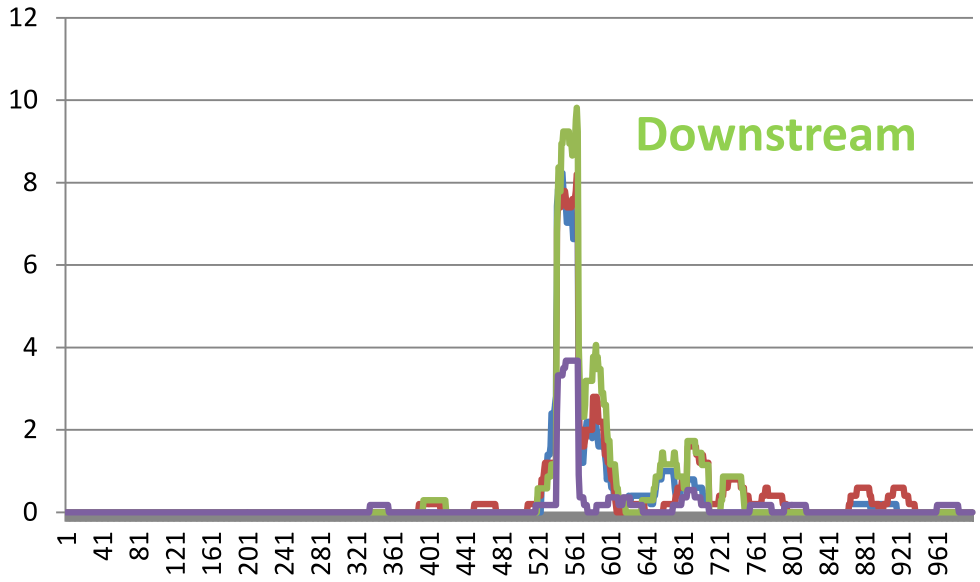

AT1G15130

Endosomal targeting BRO1-like domain-containing protein

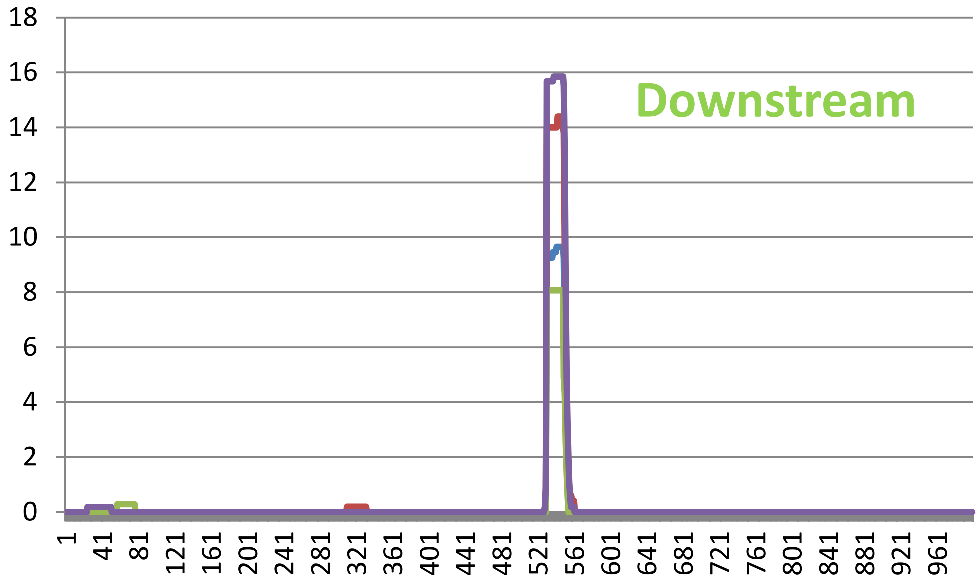

AT1G15670

Galactose oxidase/kelch repeat superfamily protein

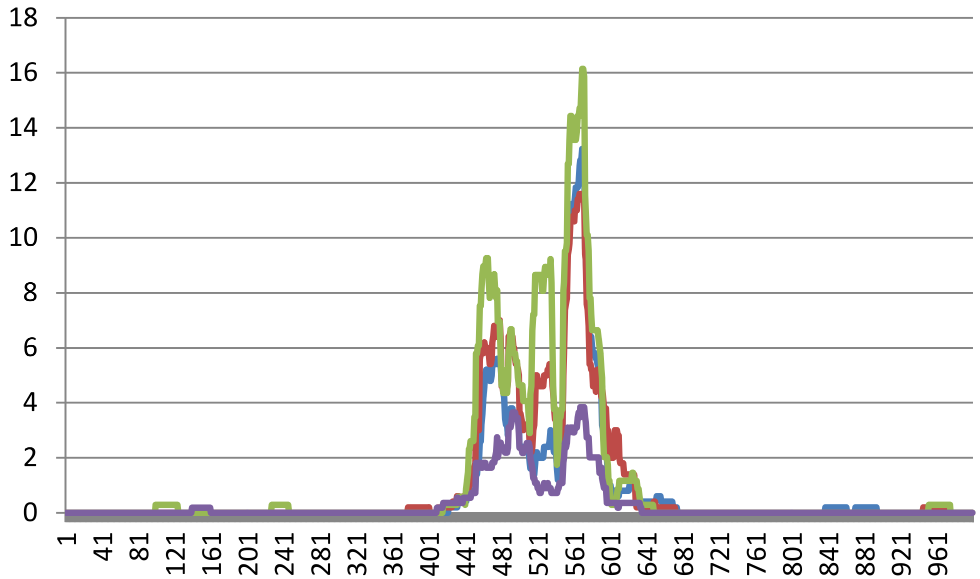

AT1G16270

Protein kinase superfamily protein with octicosapeptide/Phox/Bem1p domain

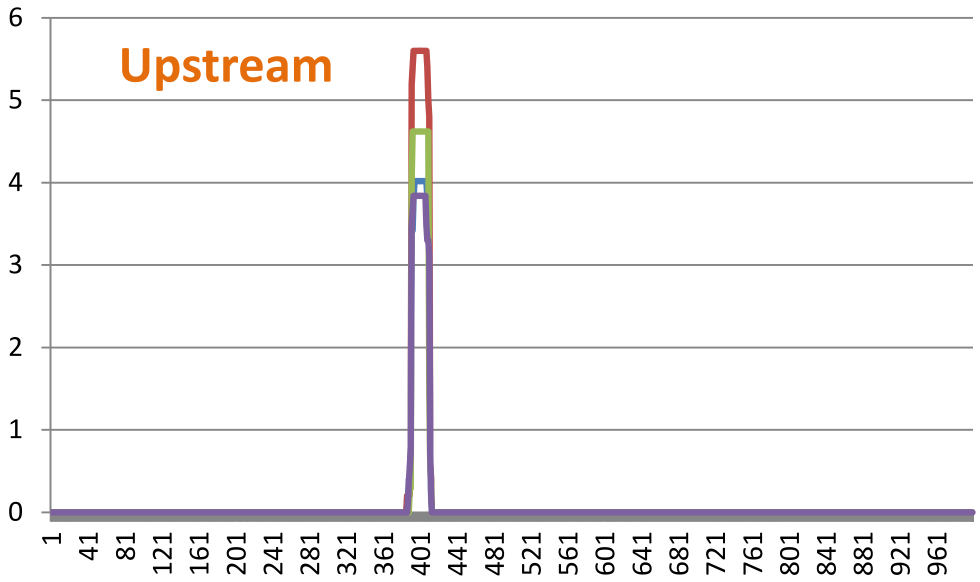

AT1G16460

RHODANESE HOMOLOGUE 2 (RDH2). Encodes a cytoplasmic thiosulfate:cyanide sulfurtransferase, activity of which increased the rhodanese activity of transgenic yeast. Can also act as a mercaptopyruvate sulfurtransferase.

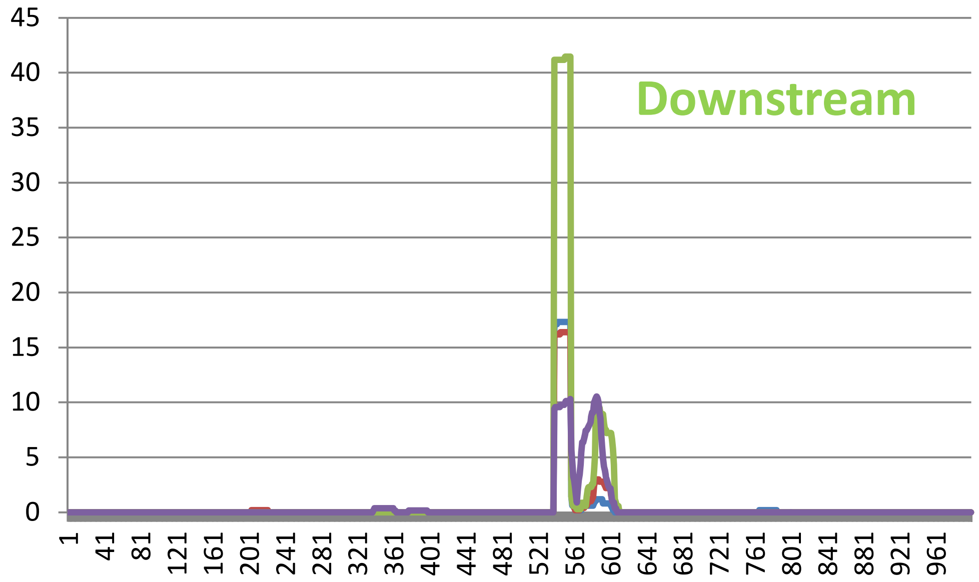

AT1G16610

Encodes SR45, a member of the highly conserved family of serine/arginine-rich (SR) proteins, which play key roles in pre-mRNA splicing and other aspects of RNA metabolism. SR45 is a spliceosome protein, interacts with SR33 and the U1-70K protein of the U1 snRNP. Also involved in plant sugar response. sr45-1 mutation confers hypersensitivity to glucose during early seedling growth.

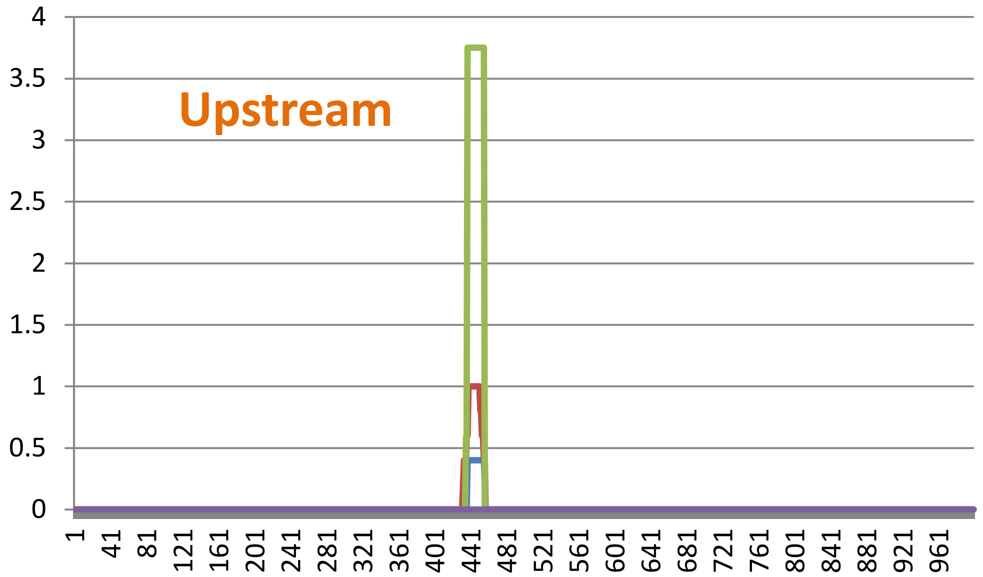

AT1G18060

Unknown protein

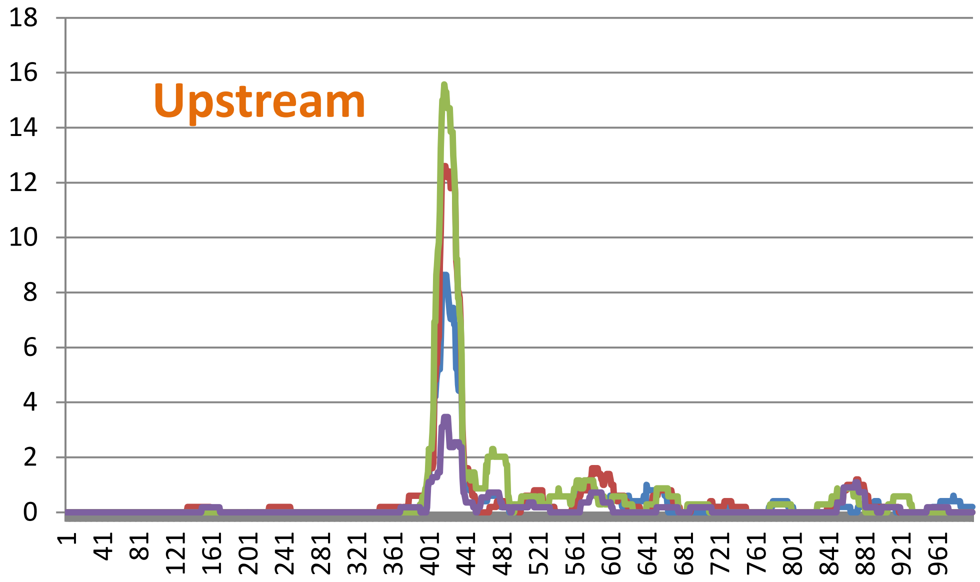

AT1G18420

Aluminium activated malate transporter family protein

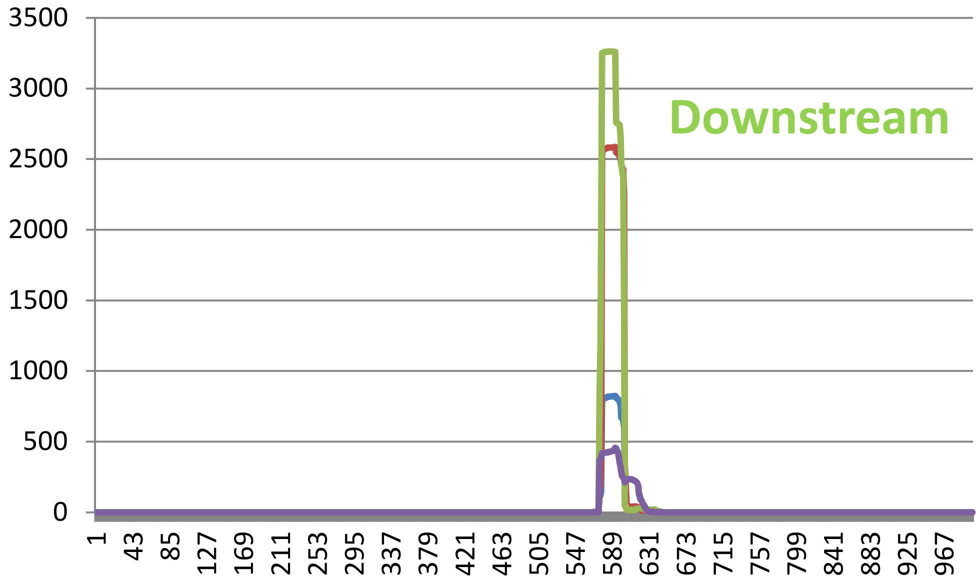

AT1G18760

Zinc finger, C3HC4 type (RING finger) family protein

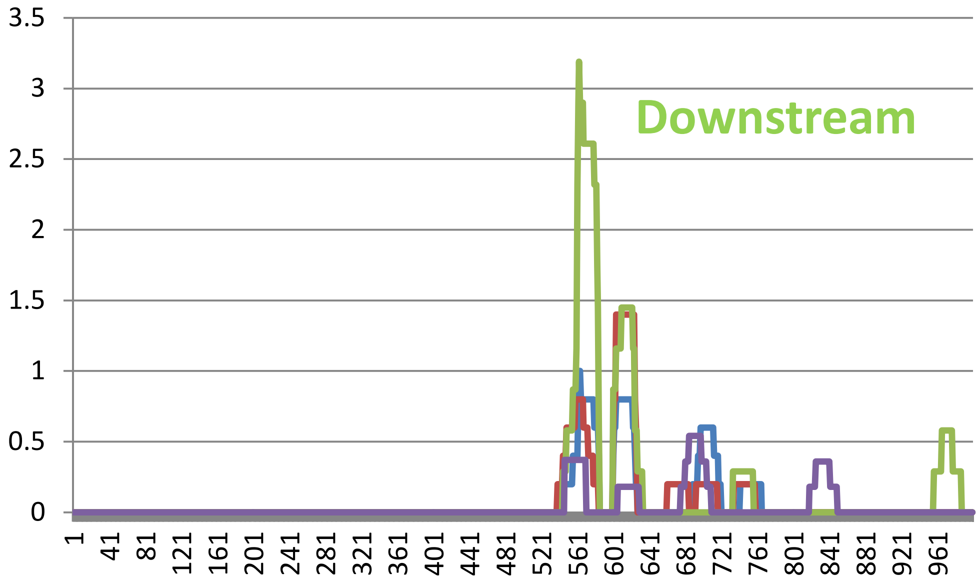

AT1G18770

RING/U-box superfamily protein

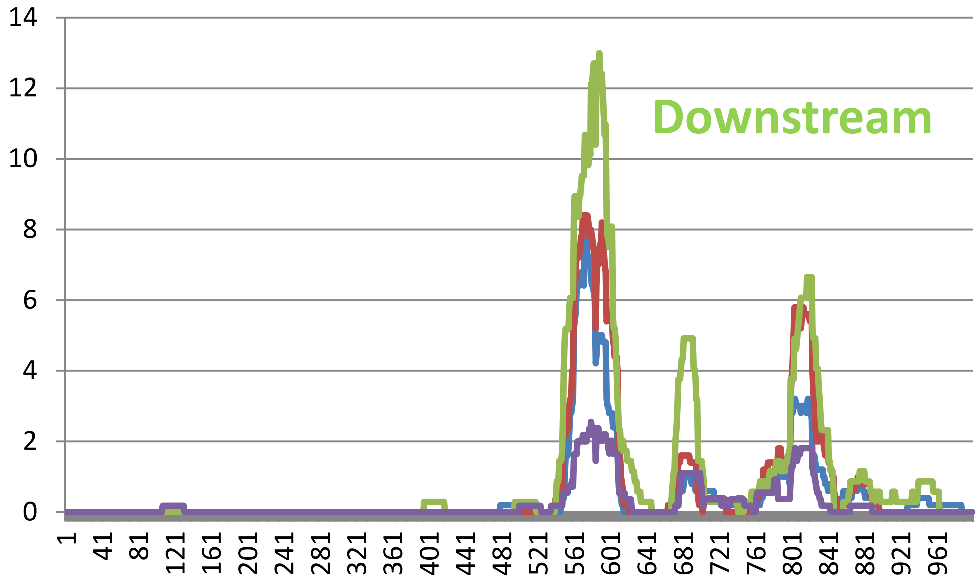

AT1G19830

SAUR-like auxin-responsive protein family

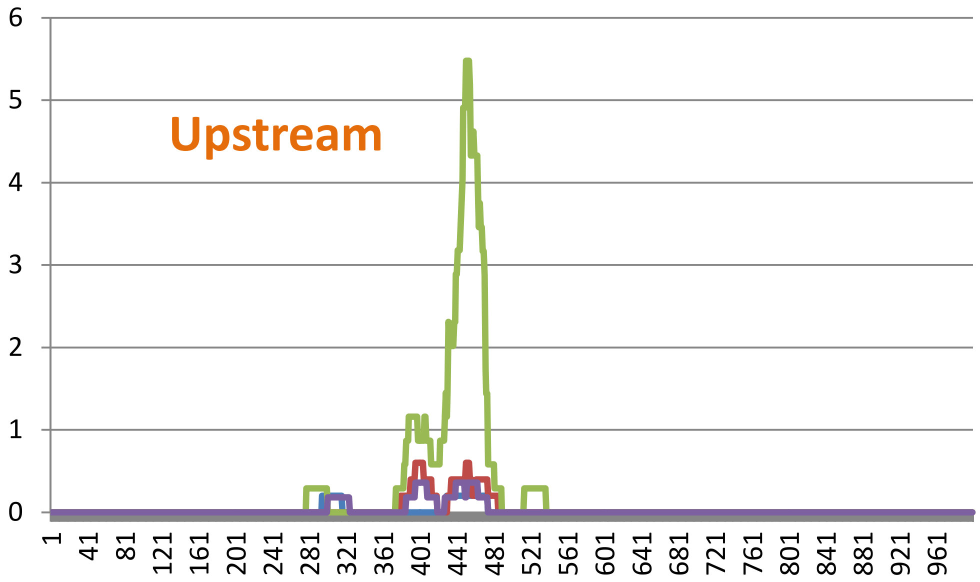

AT1G20830

Encodes MCD1 (MULTIPLE CHLOROPLAST DIVISION SITE 1). Determines the site of chloroplast division in concert with MinD (AT5G24020).

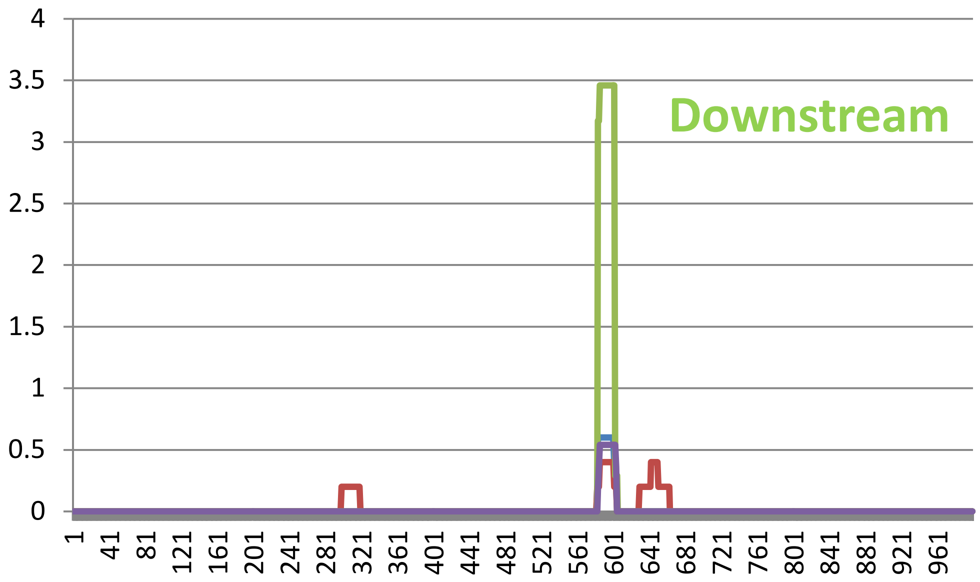

AT1G22720

Protein kinase superfamily protein

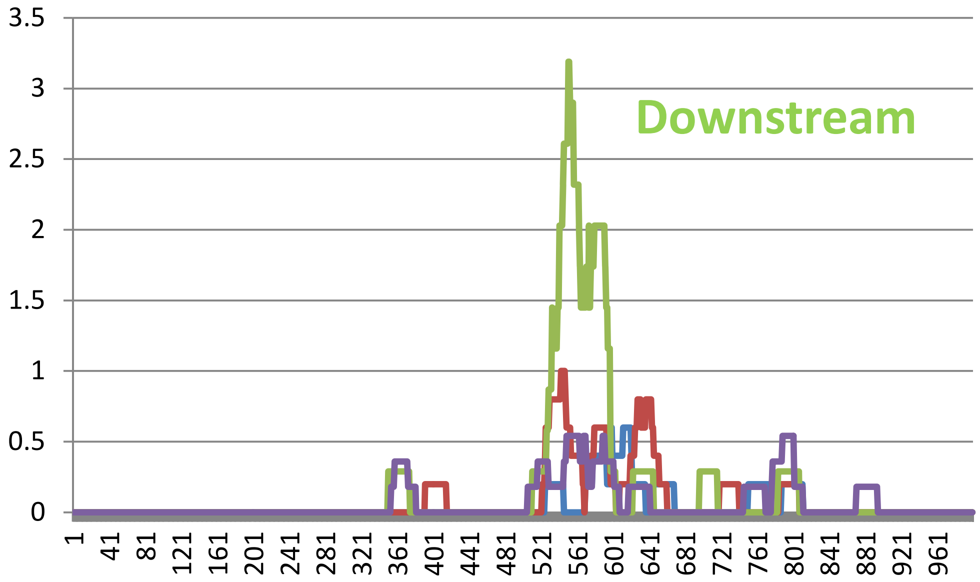

AT1G23650

Unknown protein

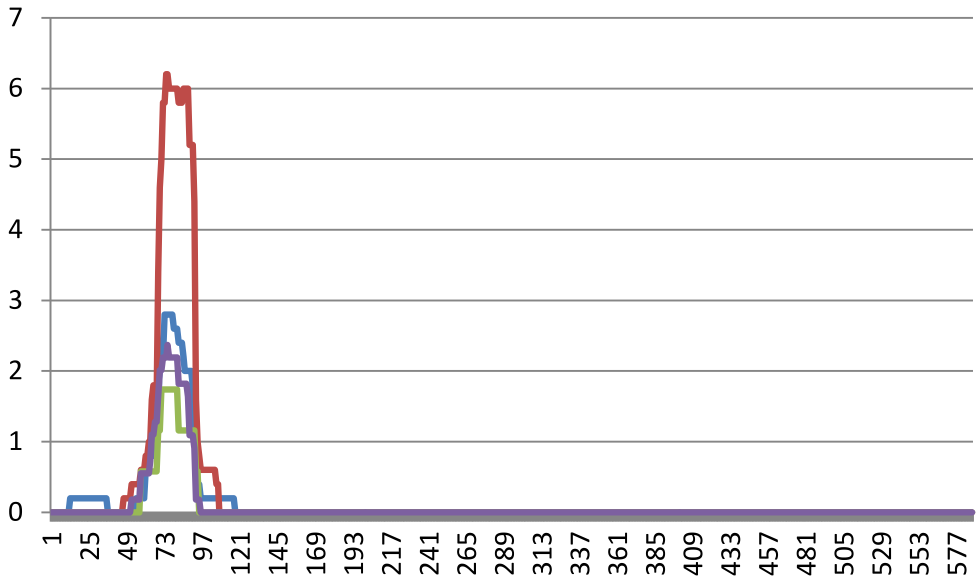

AT1G24388

Unknown protein

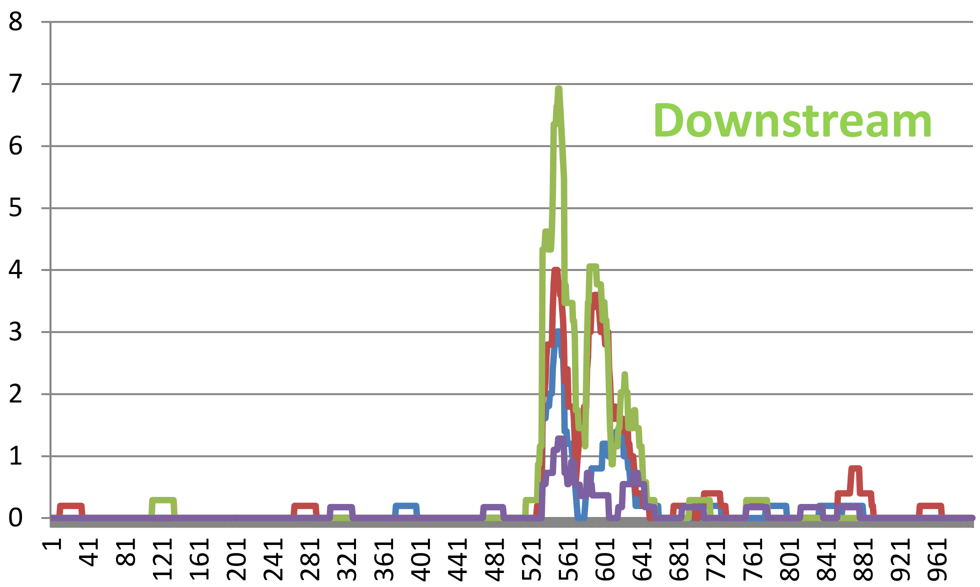

AT1G26400

FAD-binding Berberine family protein

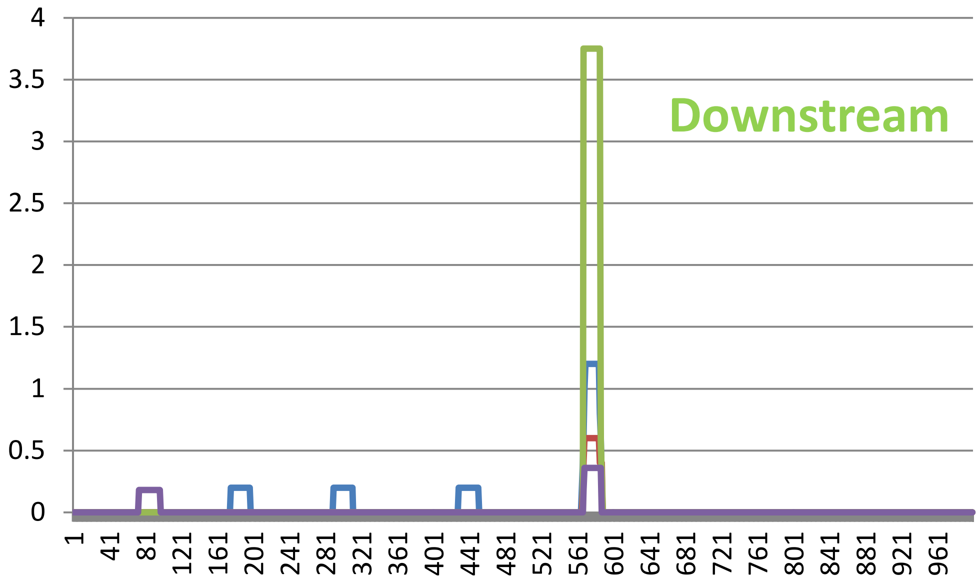

AT1G26762

Unknown protein

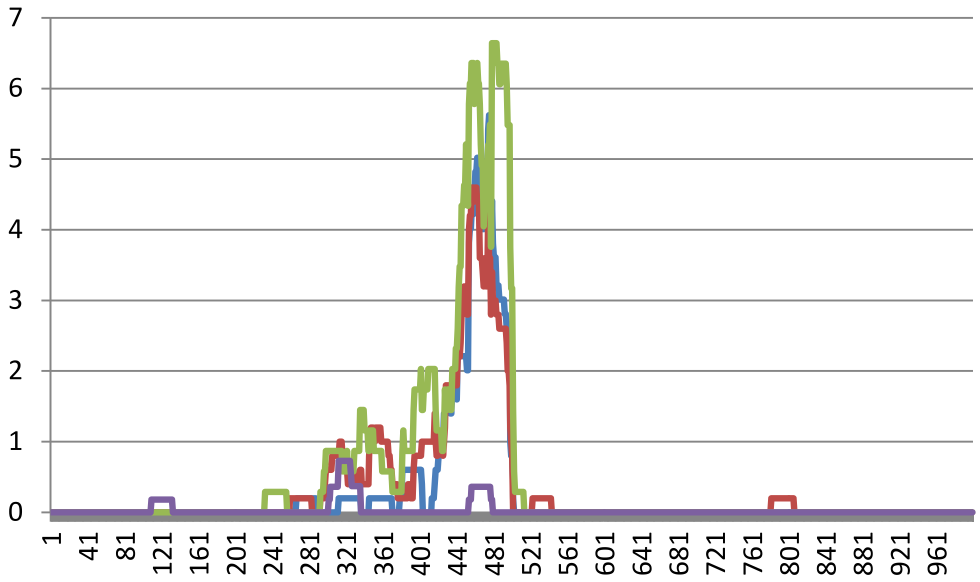

AT1G28140

Unknown protein

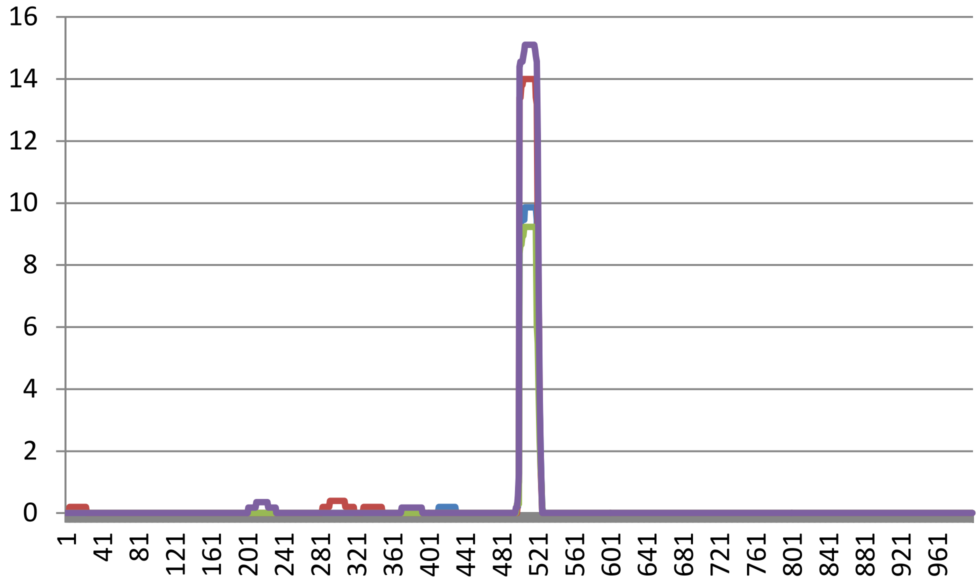

AT1G28304

Unknown protein

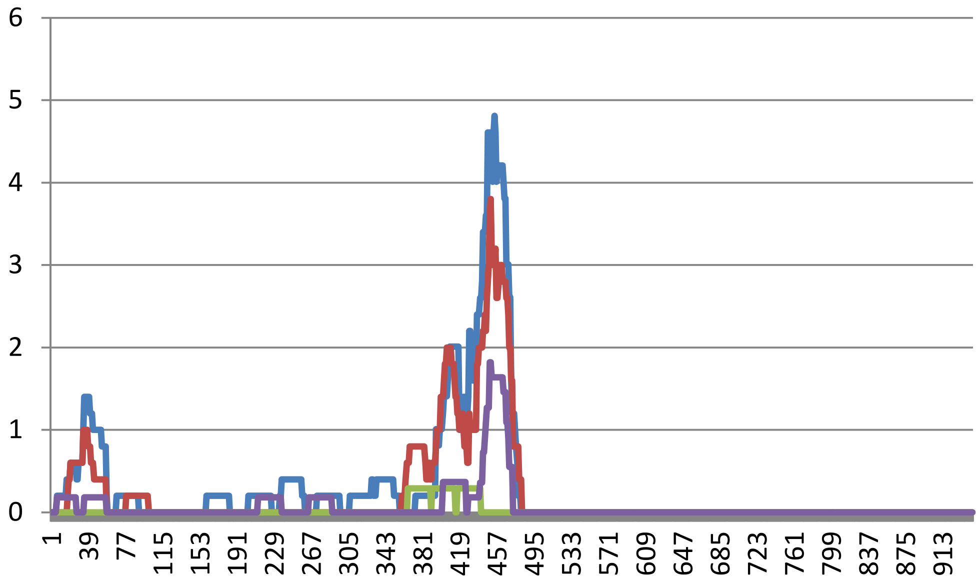

AT1G28670

Arabidopsis thaliana lipase

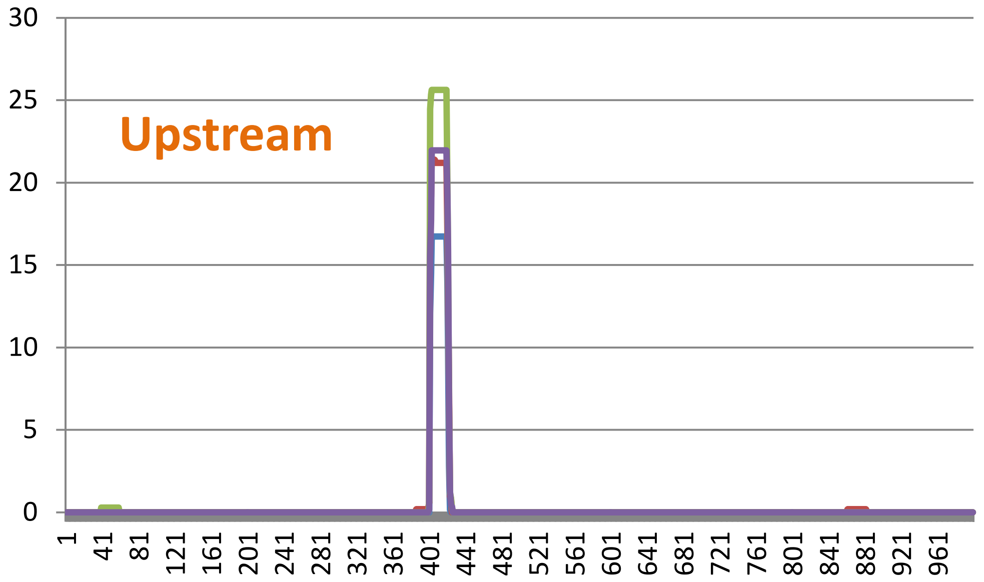

AT1G30974

Encodes a plant thionin family protein

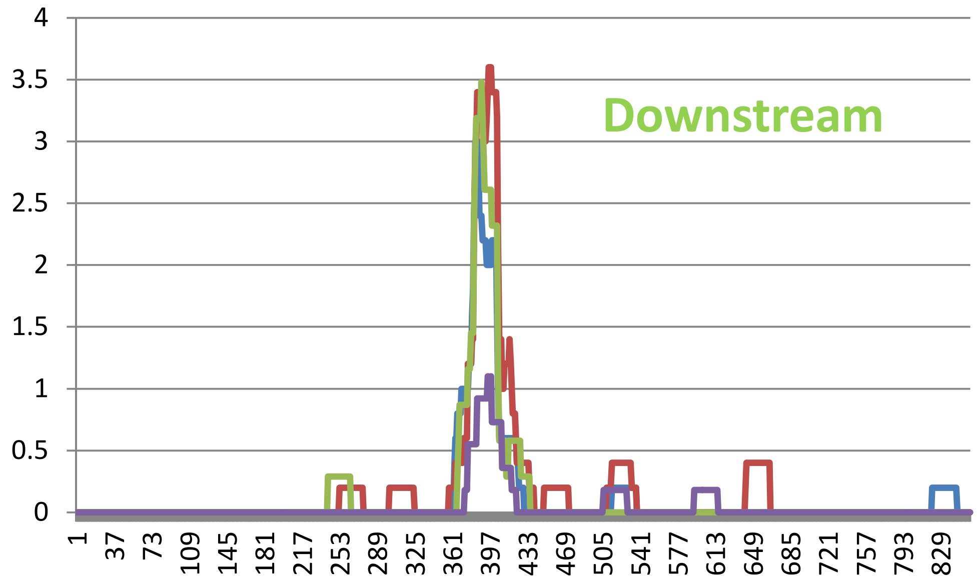

AT1G32583

BEST Arabidopsis thaliana protein match is: tapetum determinant 1 (TAIR:AT4G24972.1).

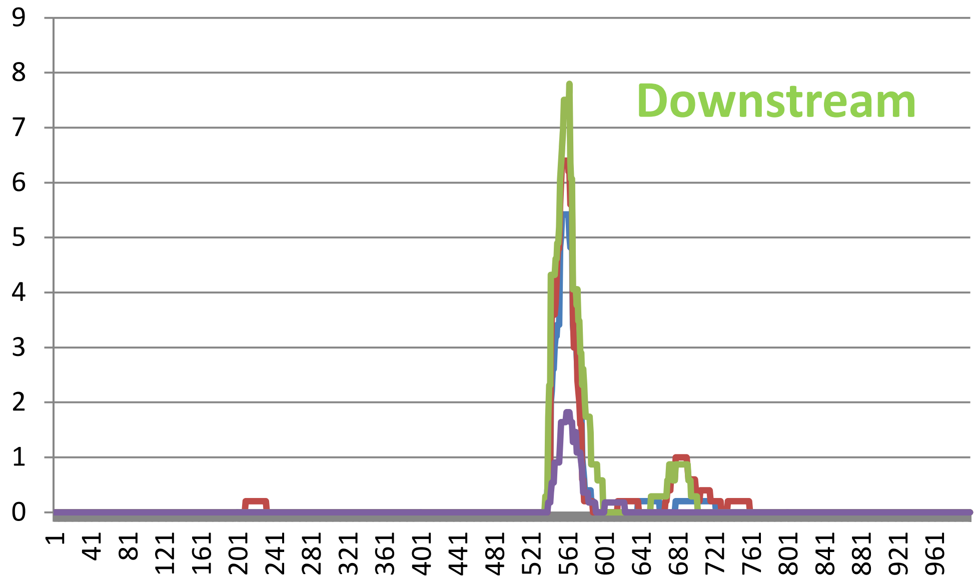

AT1G35400

BEST Arabidopsis thaliana protein match is: Protein of unknown function (DUF1184) (TAIR:AT1G35410.1)

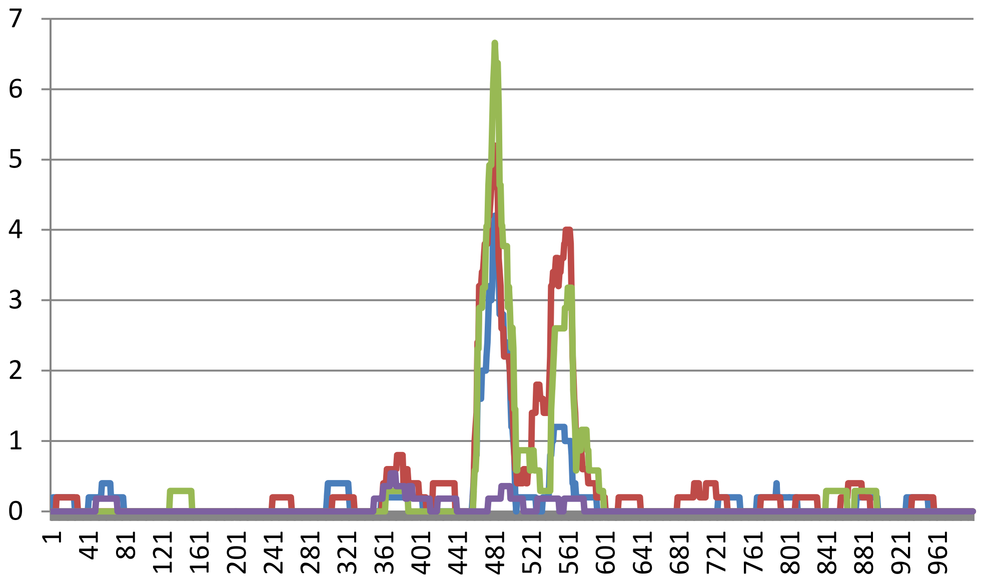

AT1G35516

MYB-like transcription factor family protein

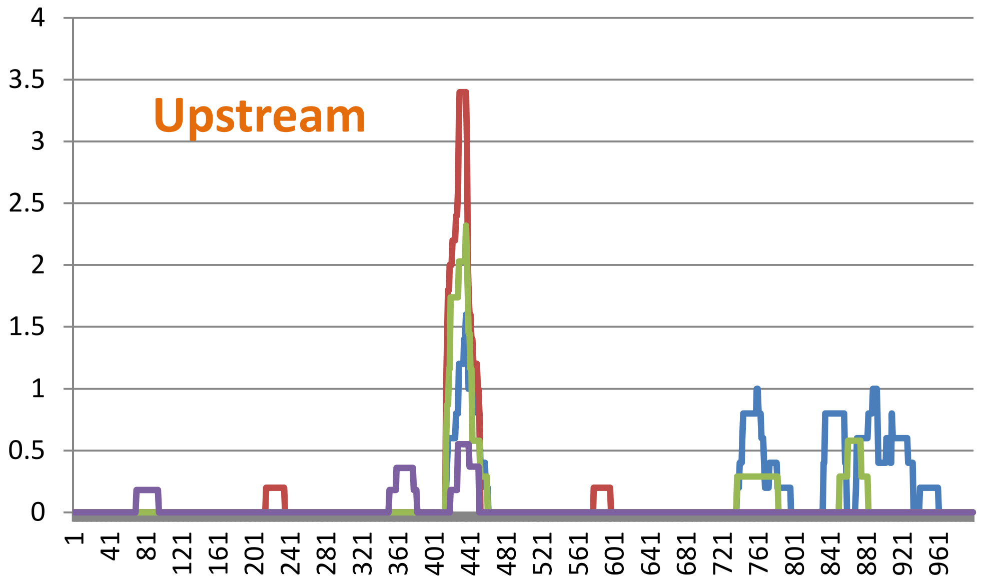

AT1G35710

Protein kinase family protein with leucine-rich repeat domain.

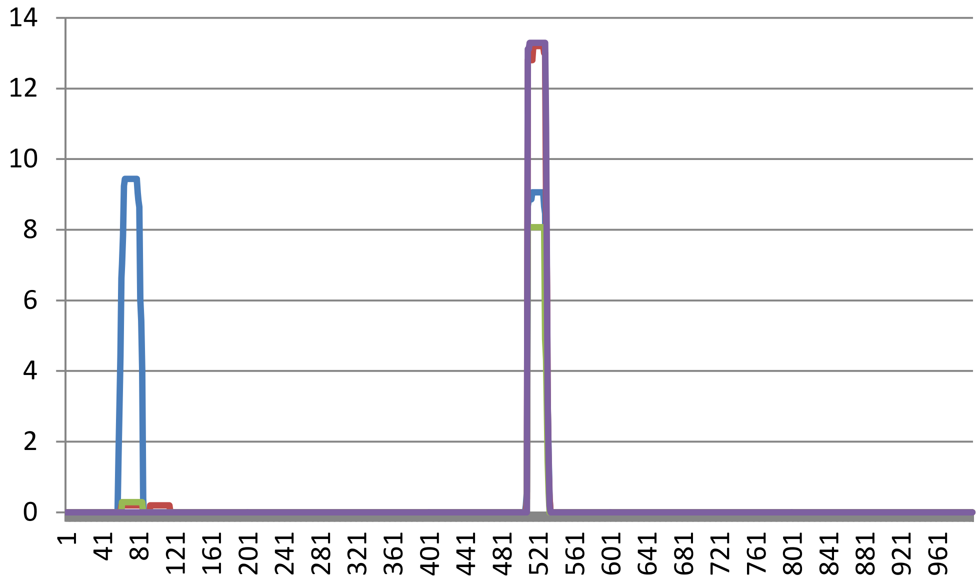

AT1G36640

Unknown protein

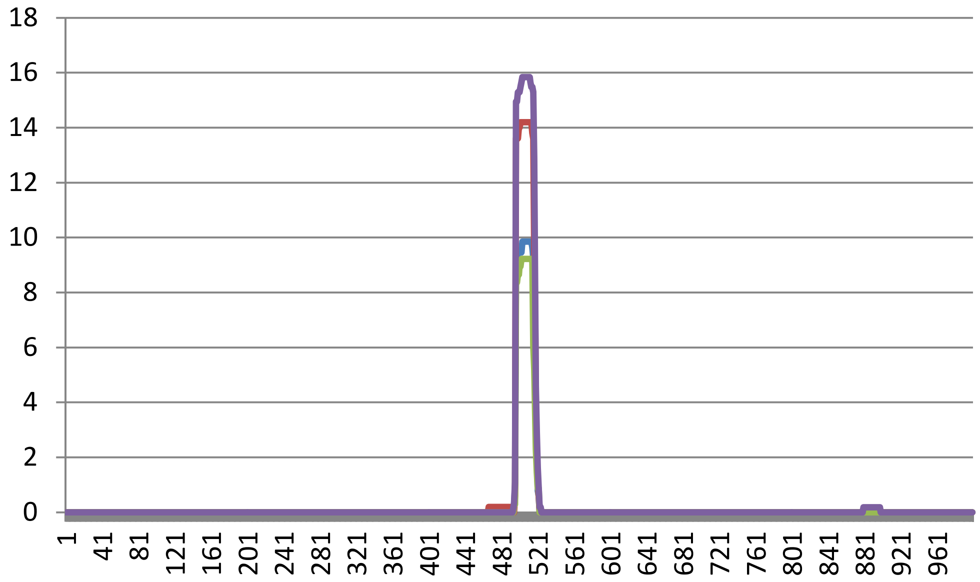

AT1G36950

RING/U-box superfamily protein

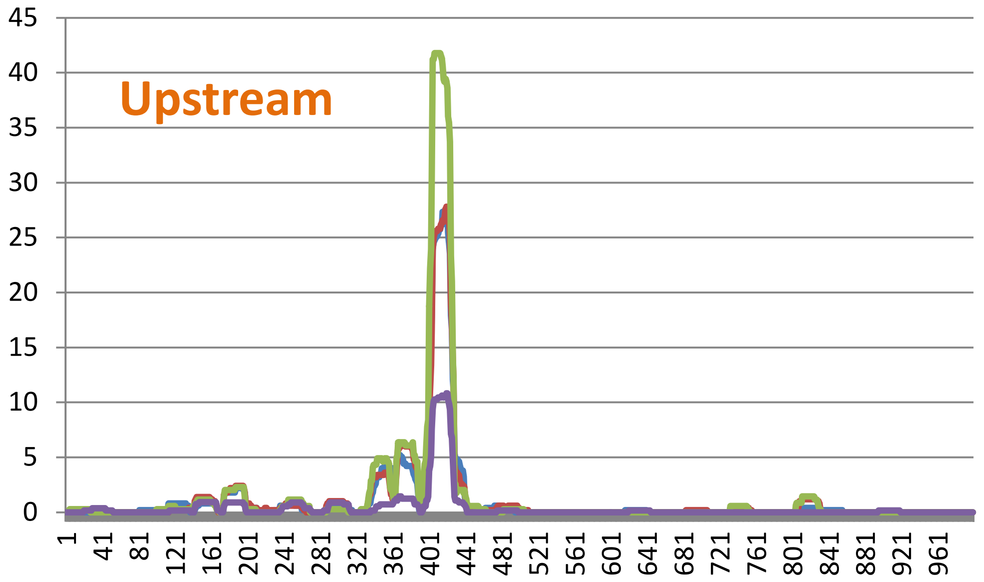

AT1G38790

Unknown protein

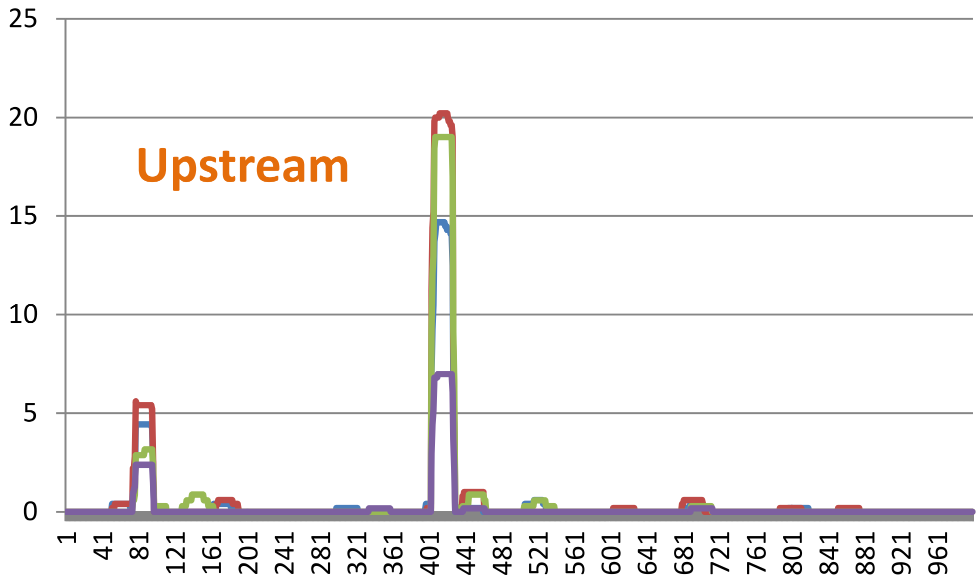

AT1G40129

Unknown protein

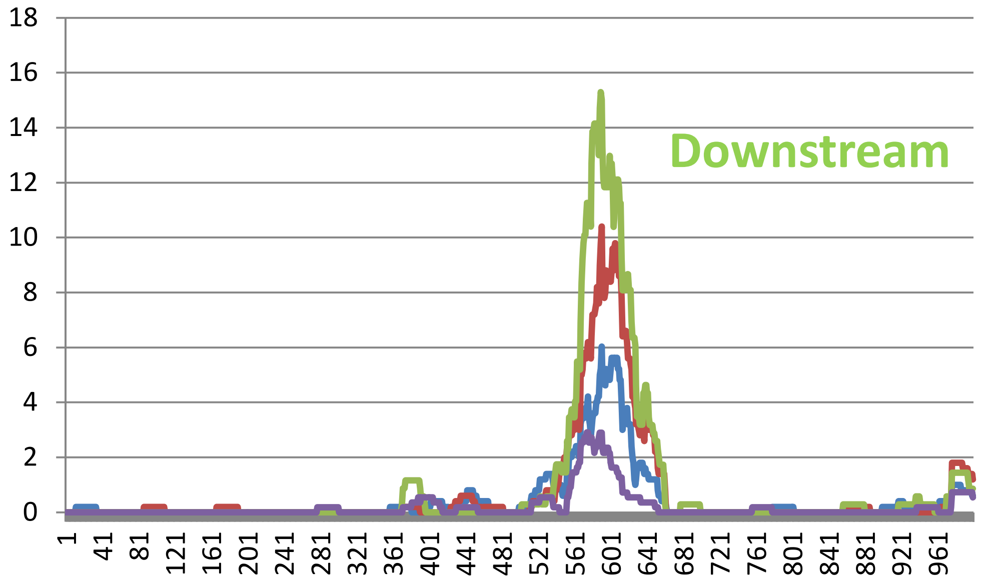

AT1G41820

Unknown protein

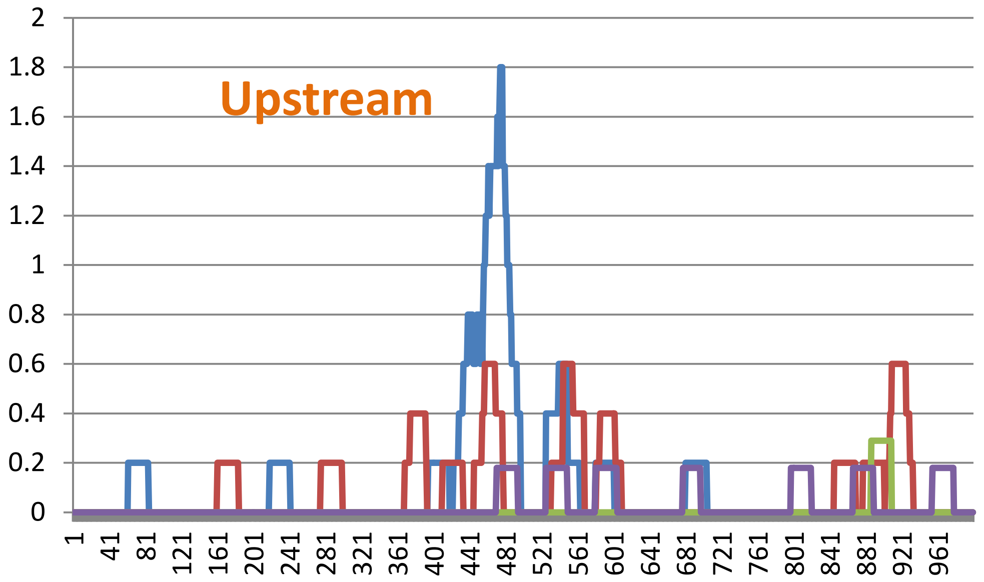

AT1G43160

Encodes a member of the ERF (ethylene response factor) subfamily B-4 of ERF/AP2 transcription factor family (RAP2.6). The protein contains one AP2 domain. There are 7 members in this subfamily.

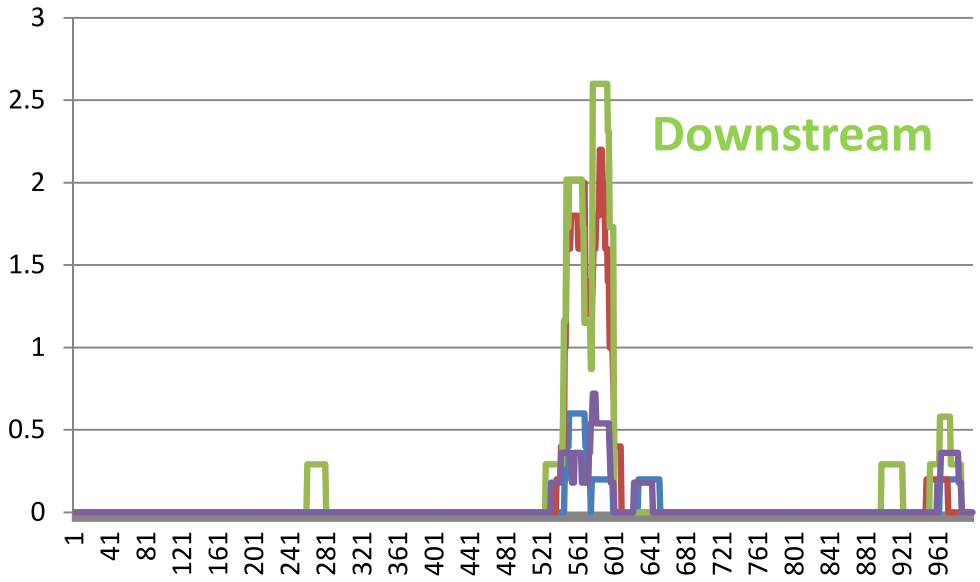

AT1G44542

Cyclase family protein

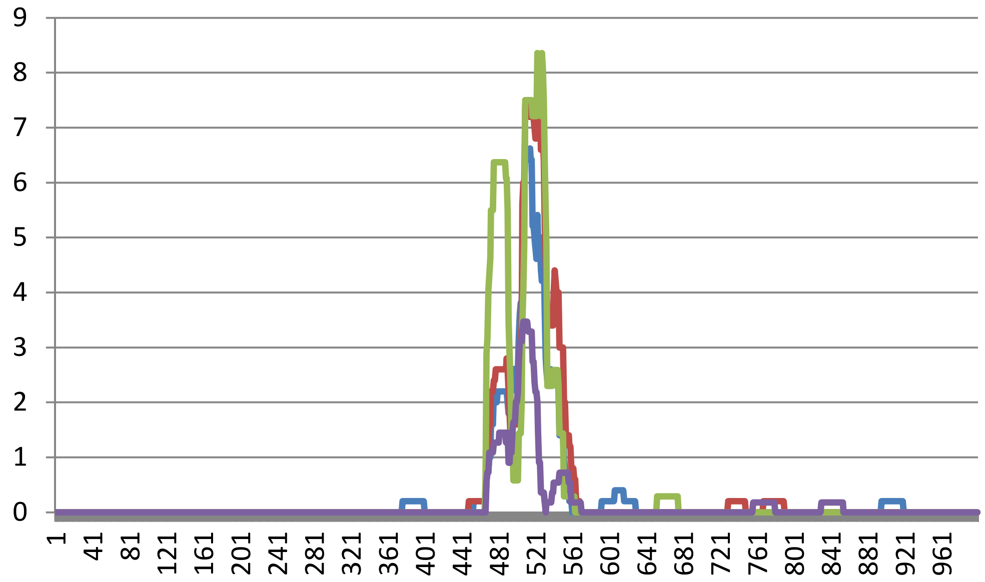

AT1G47265

Unknown protein

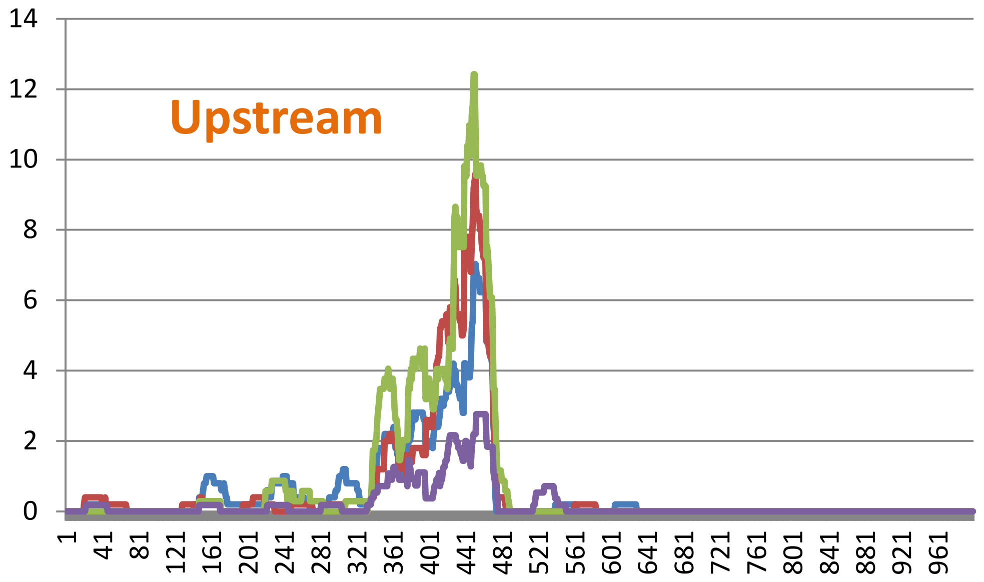

AT1G47450

Protein of unknown function (DUF784)

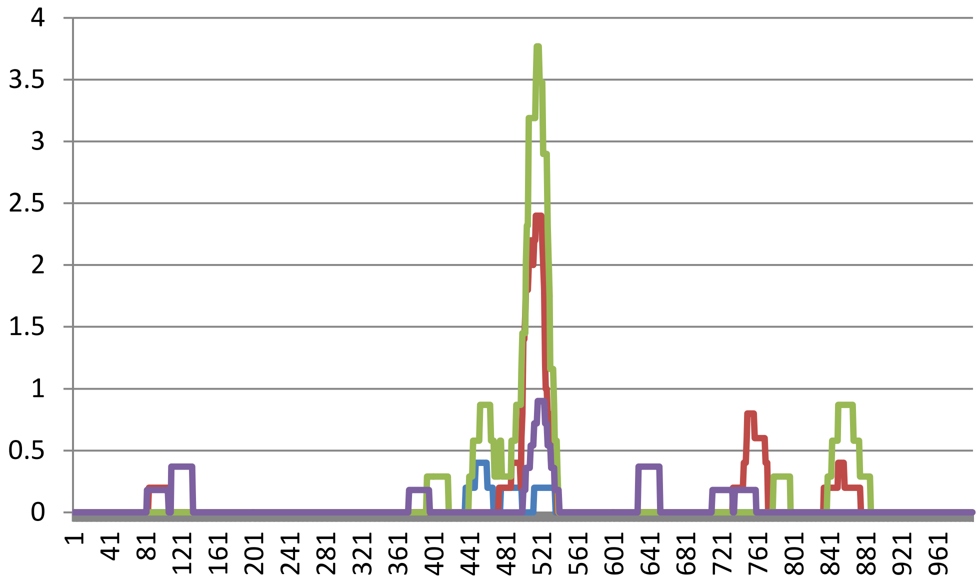

AT1G49700

Plant protein 1589 of unknown function

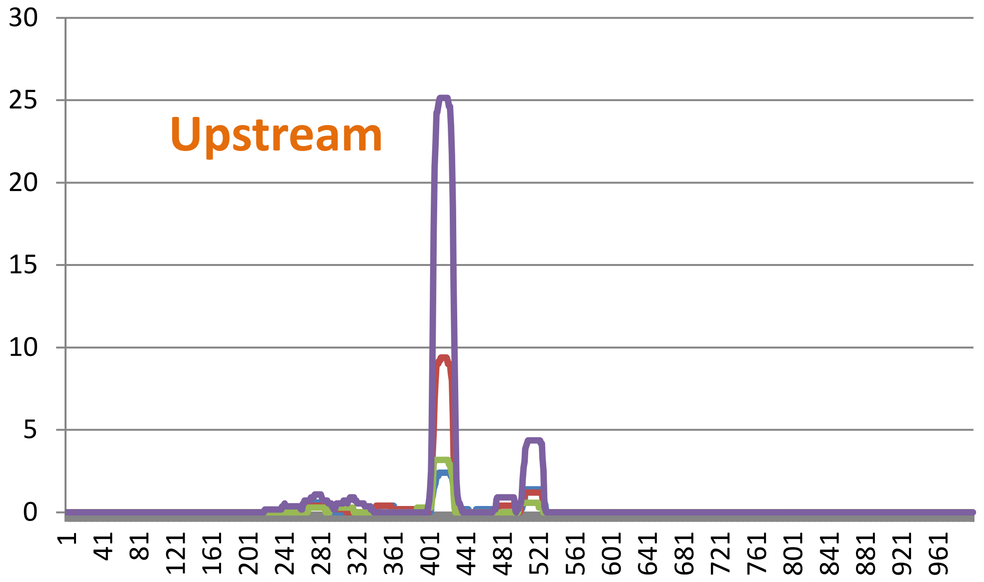

AT1G51150

Encodes a putative DegP protease.

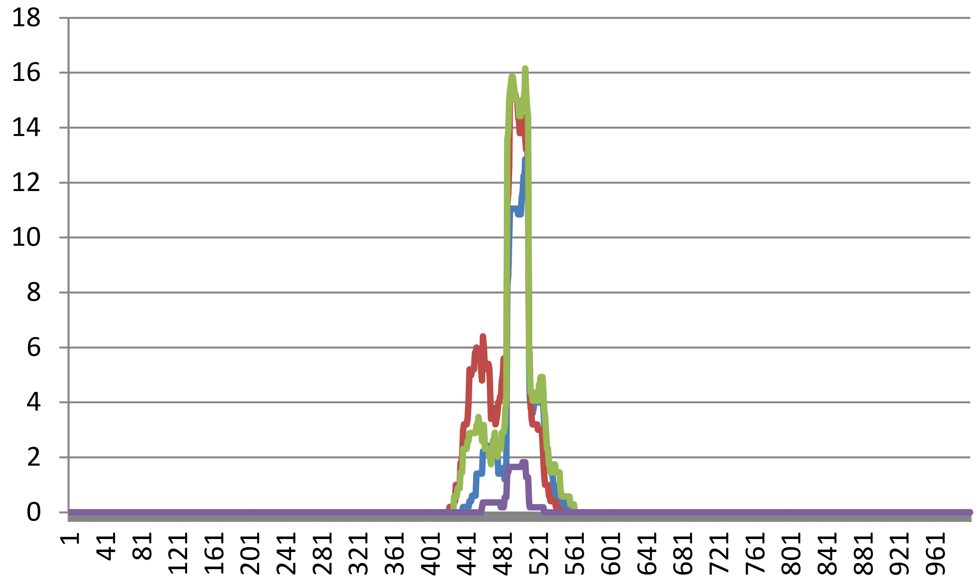

AT1G51820

Leucine-rich repeat protein kinase family protein

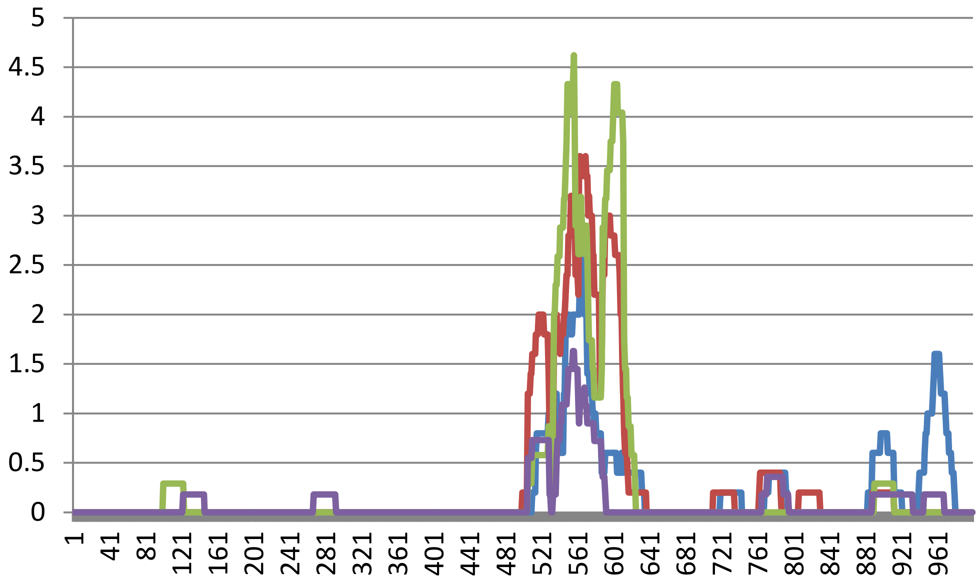

AT1G52160

Encodes a tRNase Z.

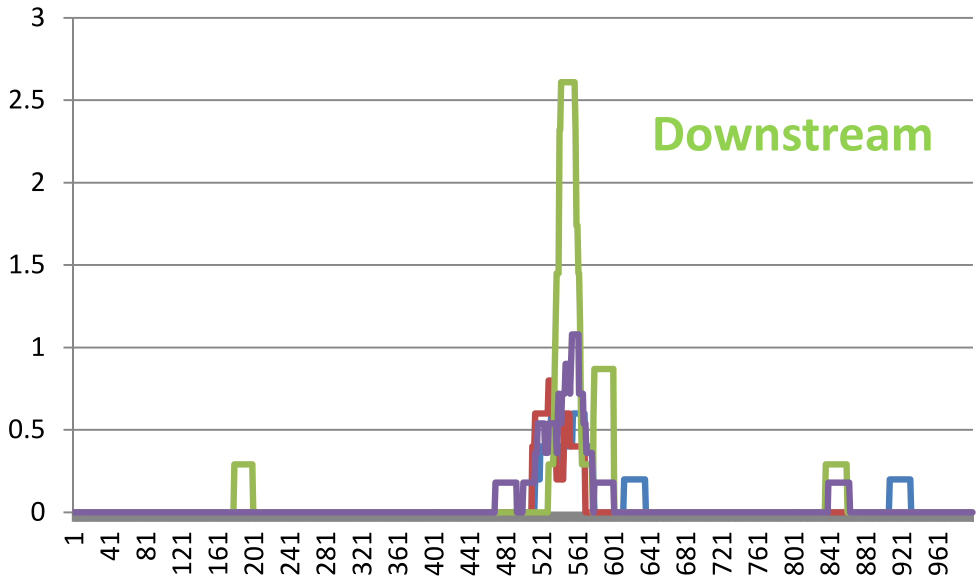

AT1G52180

Aquaporin-like superfamily protein

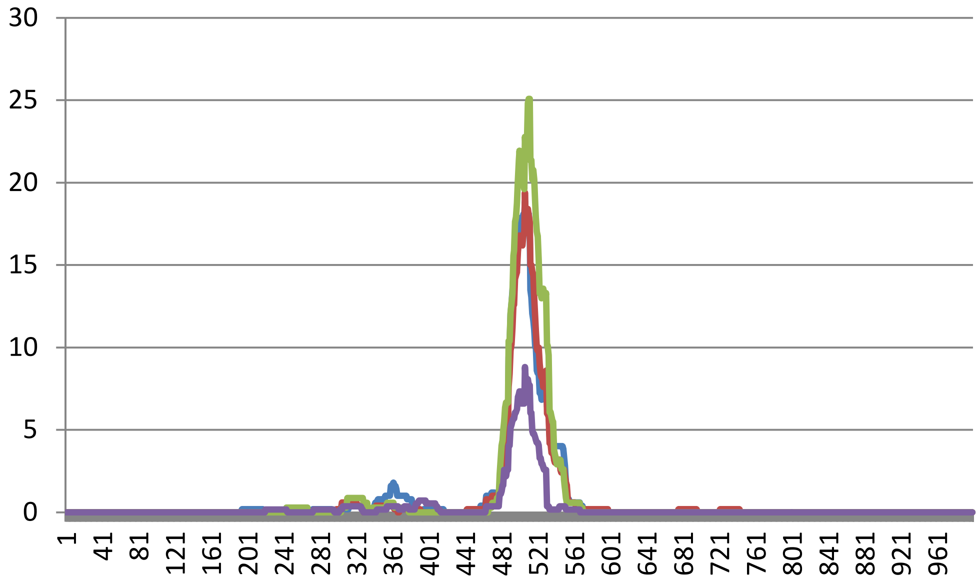

AT1G54000

GDSL-like Lipase/Acylhydrolase superfamily protein

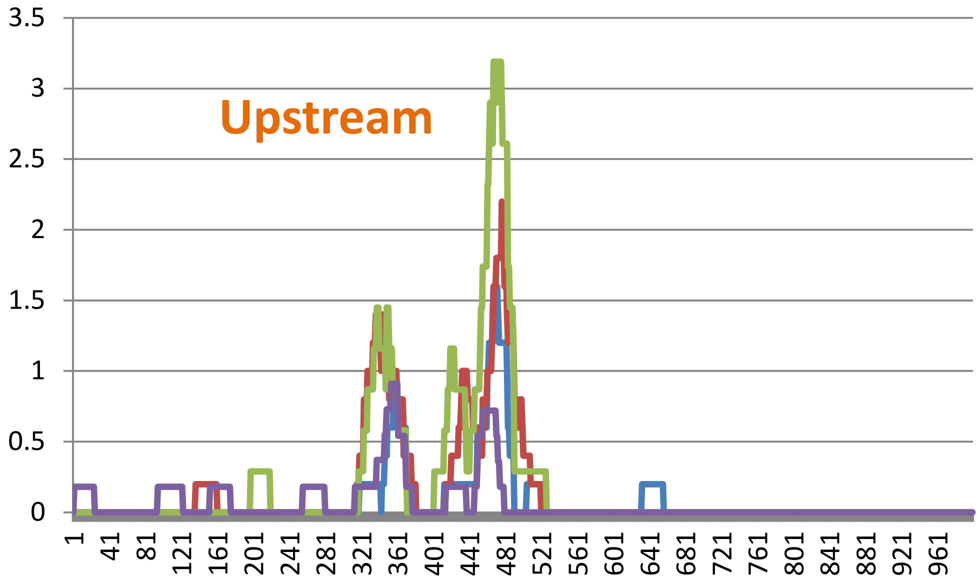

AT1G54990

AUXIN RESISTANT 4 (AXR4)

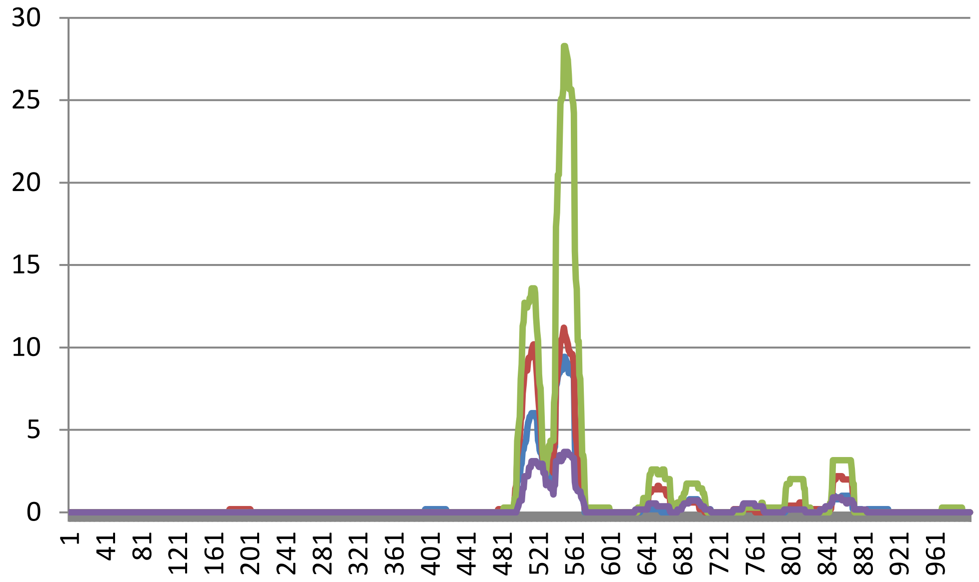

AT1G55700

Cysteine/Histidine-rich C1 domain family protein

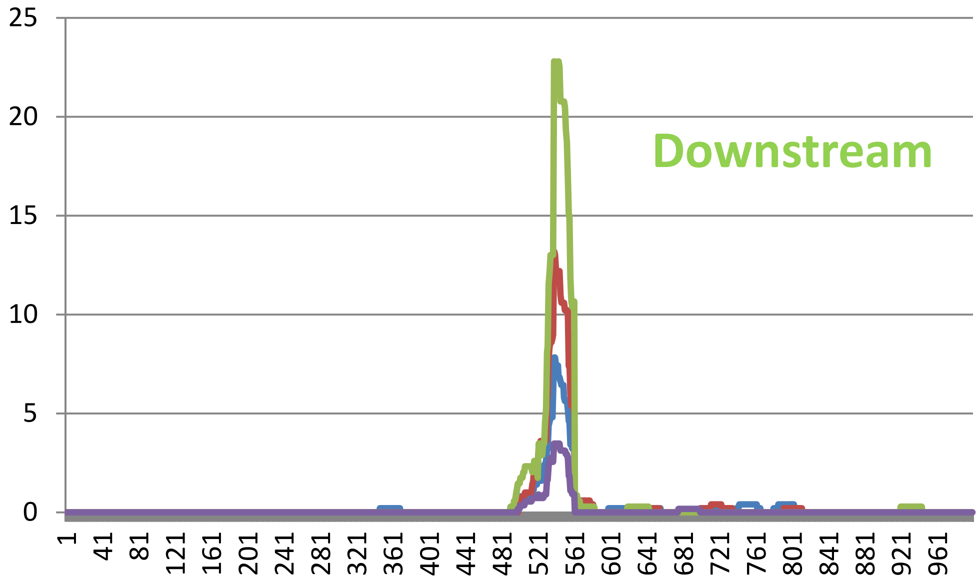

AT1G57770

FAD/NAD(P)-binding oxidoreductase family protein

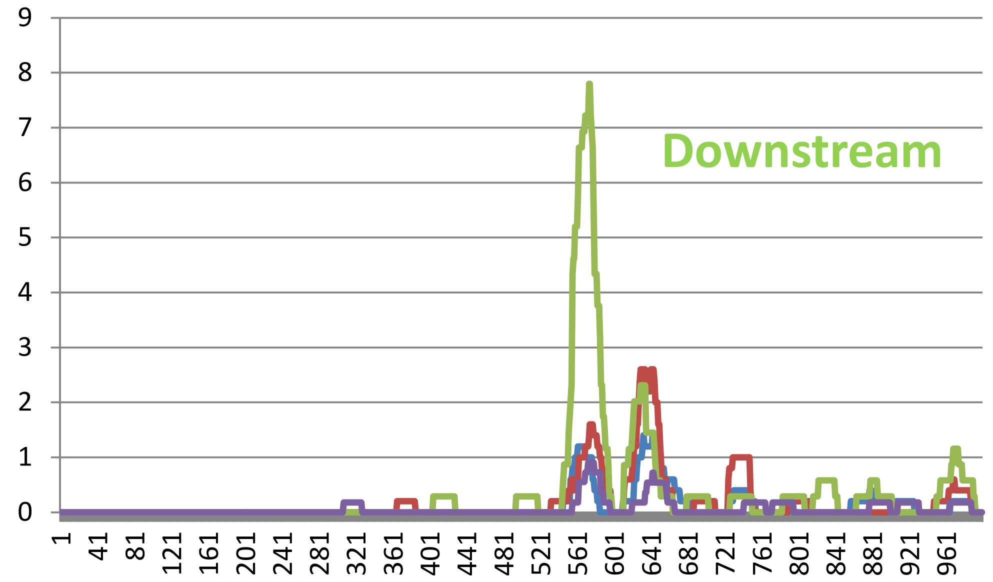

AT1G59885

Unknown protein

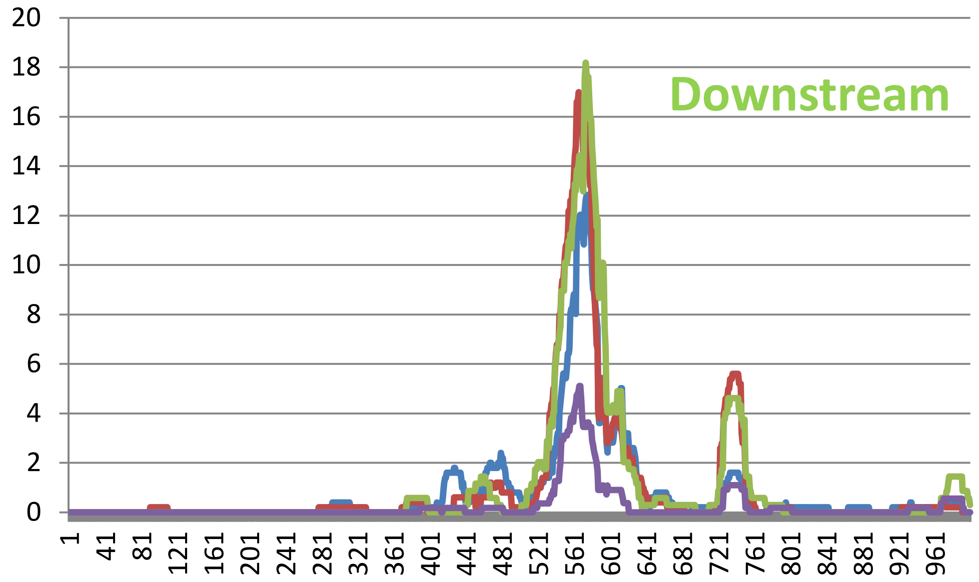

AT1G60986

Encodes a member of a family of small, secreted, cysteine rich proteins with sequence similarity to SCR (S locus cysteine-rich protein).

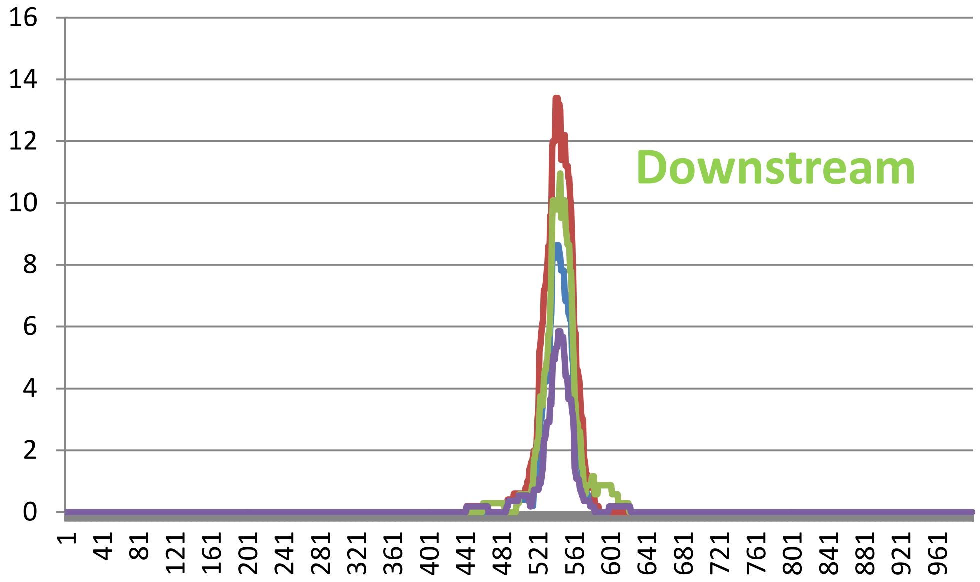

AT1G61255

BEST Arabidopsis thaliana protein match is: glycine-rich protein (TAIR:AT4G21620.2)

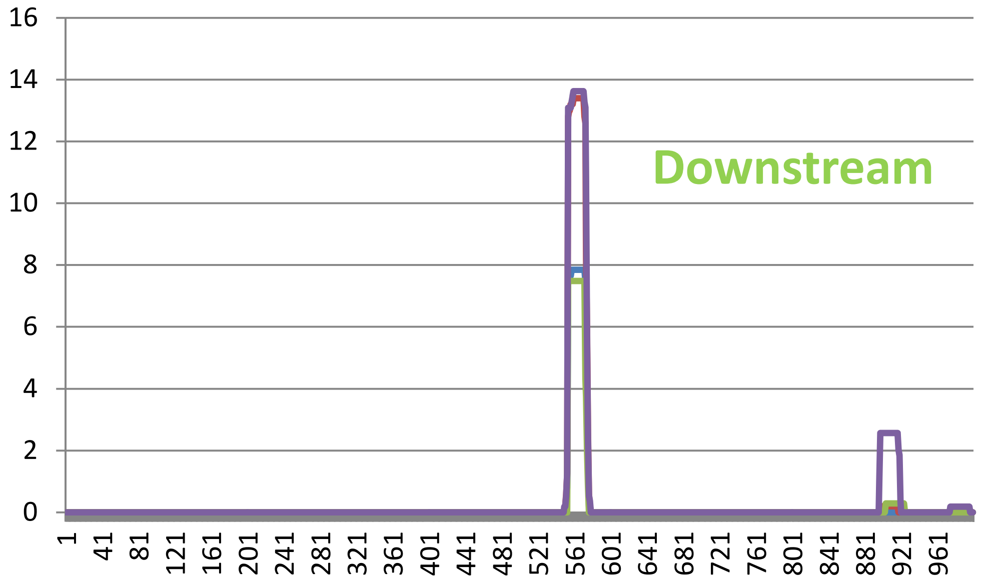

AT1G61320

FBD/Leucine Rich Repeat domains containing protein

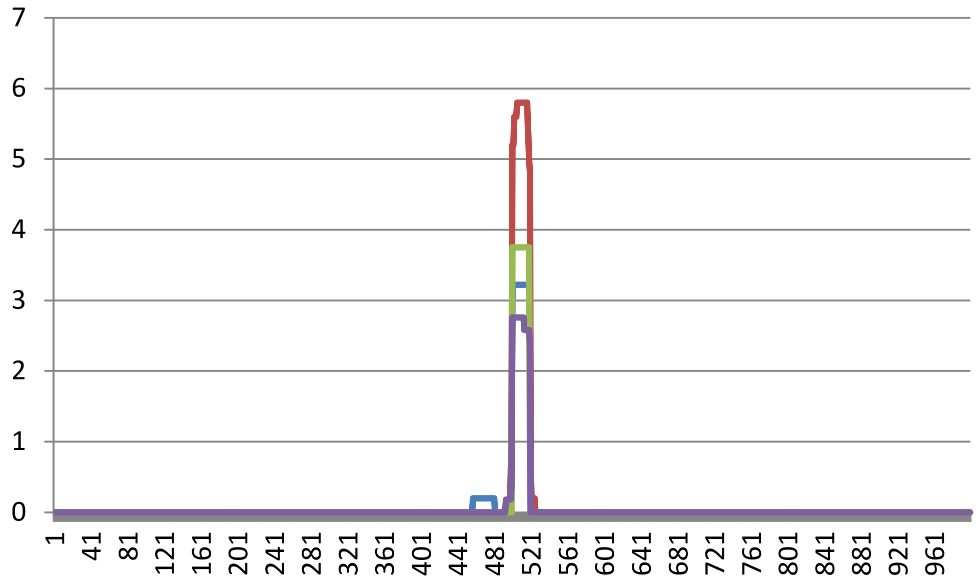

AT1G62180

Encodes a adenosine 5'-phosphosulfate reductase, involved in sulfate assimilation. Is a major effect locus for natural variation of shoot sulfate content in Arabidopsis.

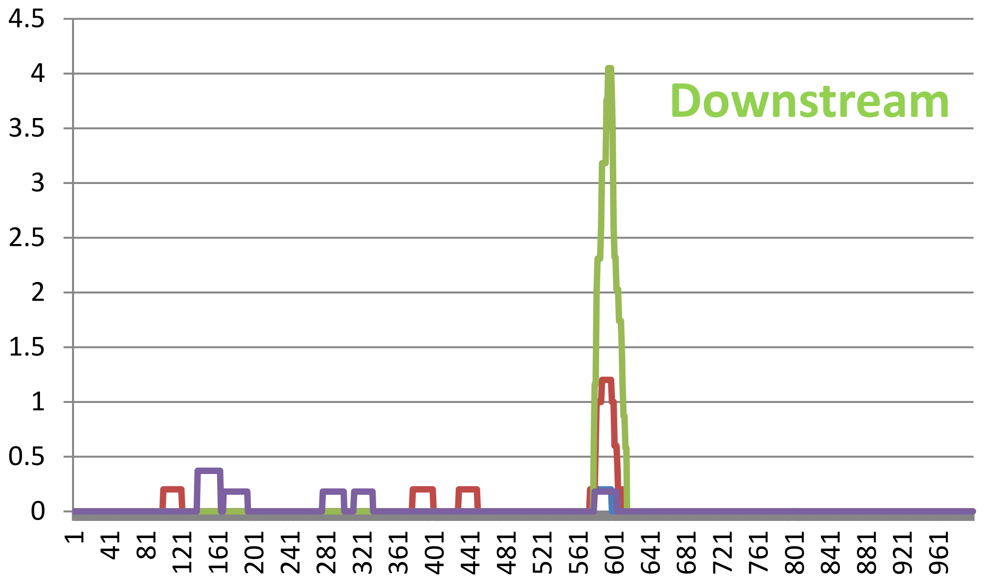

AT1G62410

MIF4G domain-containing protein

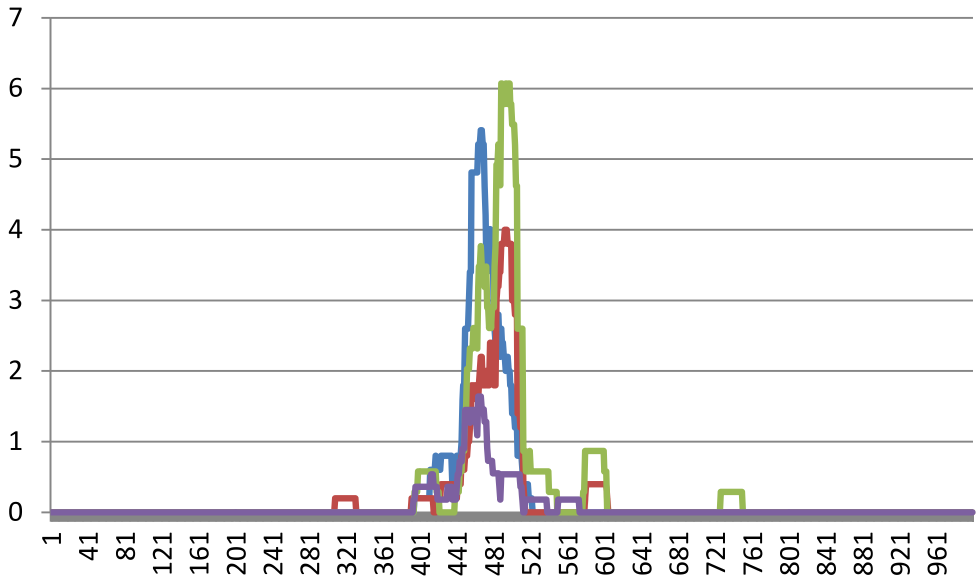

AT1G62560

Belongs to the flavin-monooxygenase (FMO) family, encodes a glucosinolate S-oxygenase that catalyzes the conversion of methylthioalkyl glucosinolates to methylsulfinylalkyl glucosinolates.

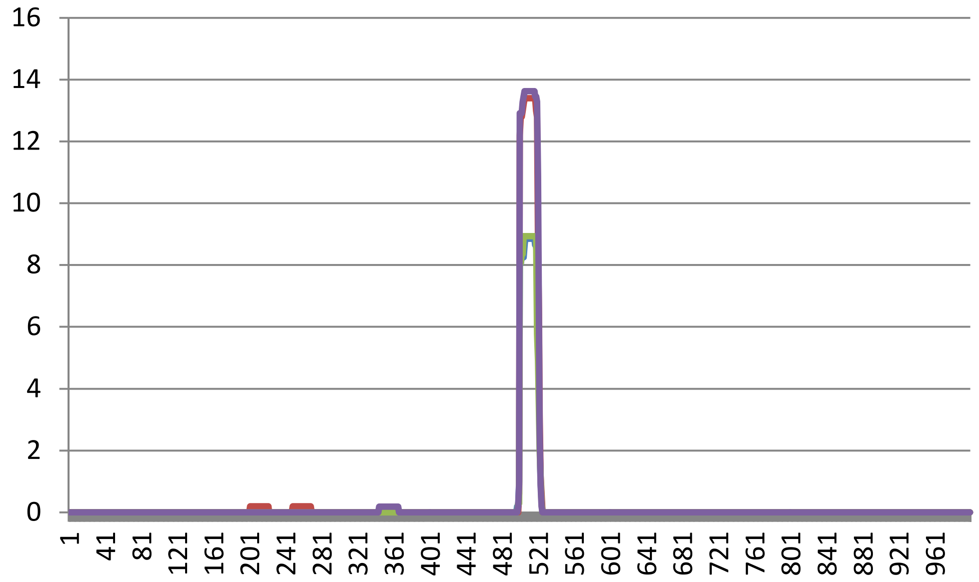

AT1G63520

Protein of unknown function (DUF3527)

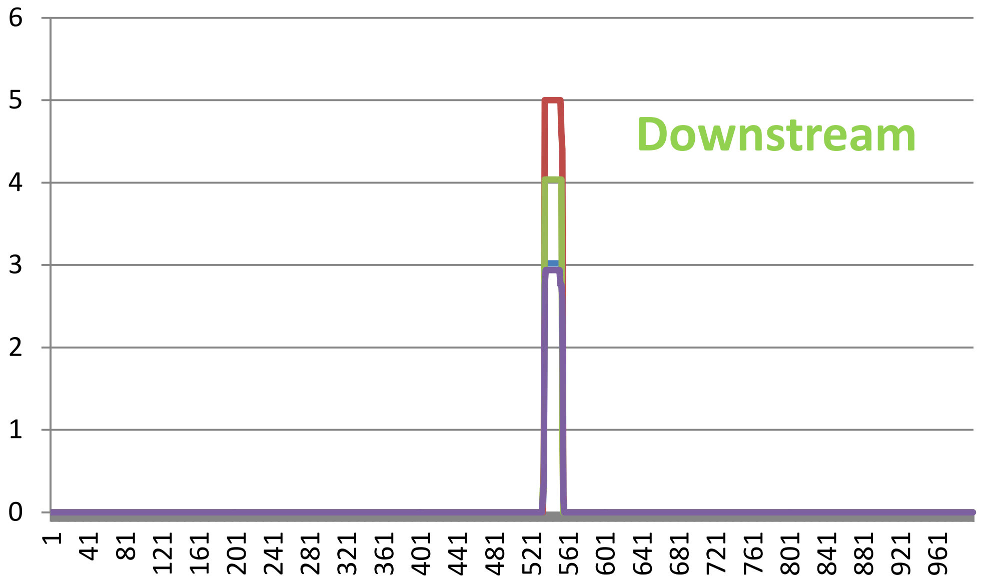

AT1G63800

Ubiquitin-conjugating enzyme 5 (UBC5)

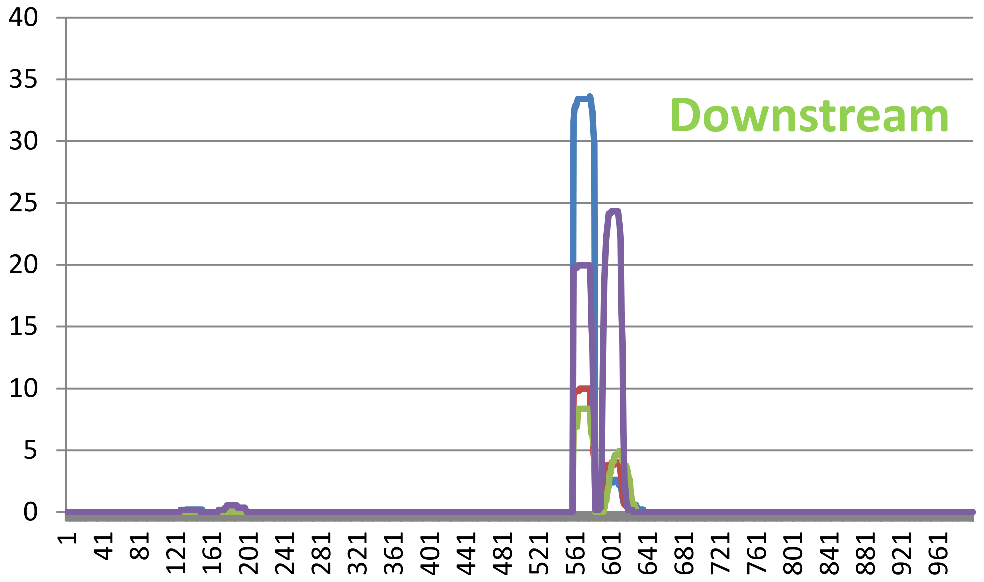

AT1G65860

Belongs to the flavin-monooxygenase (FMO) family, encodes a glucosinolate S-oxygenase that catalyzes the conversion of methylthioalkyl glucosinolates to methylsulfinylalkyl glucosinolates.

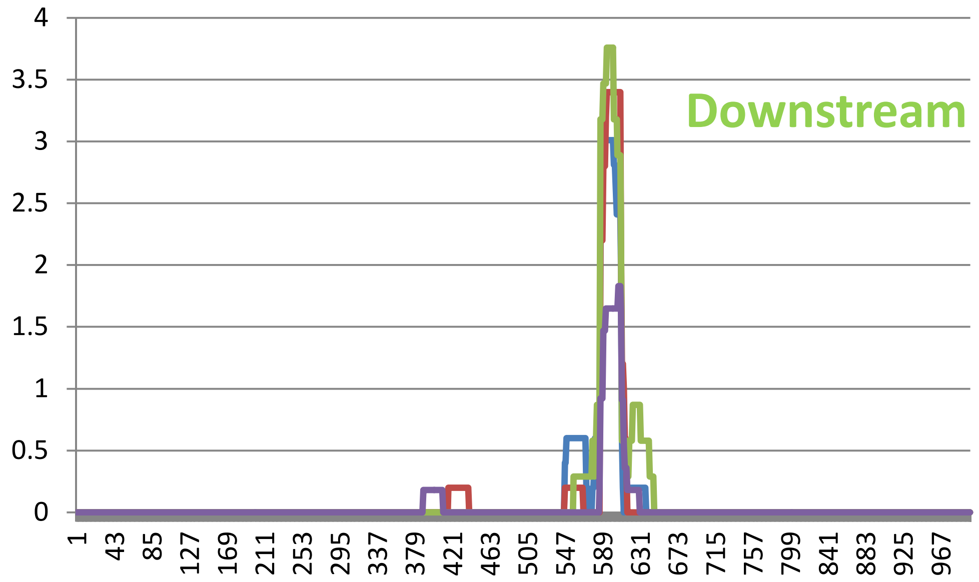

AT1G66620

Protein with RING/U-box and TRAF-like domains

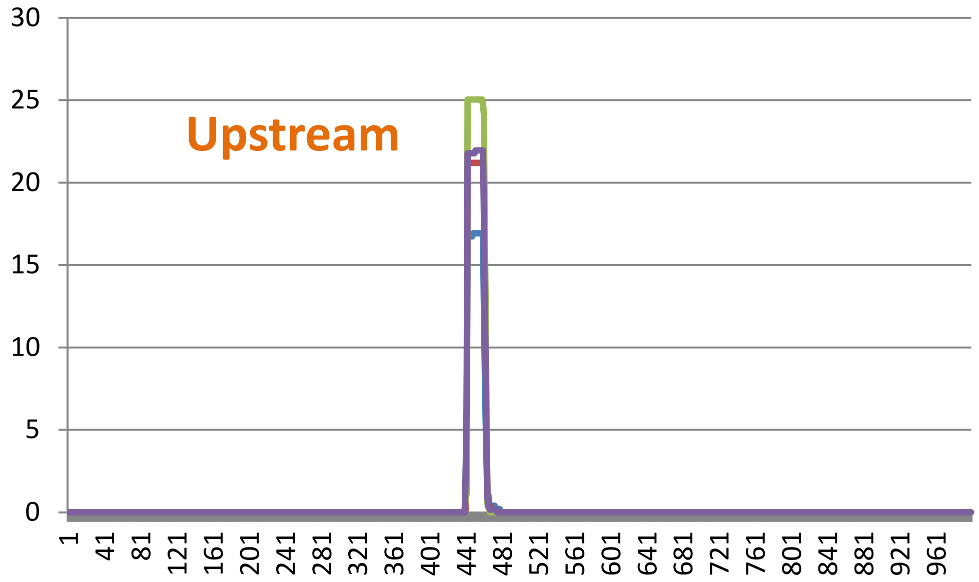

AT1G68040

S-adenosyl-L-methionine-dependent methyltransferases superfamily protein.

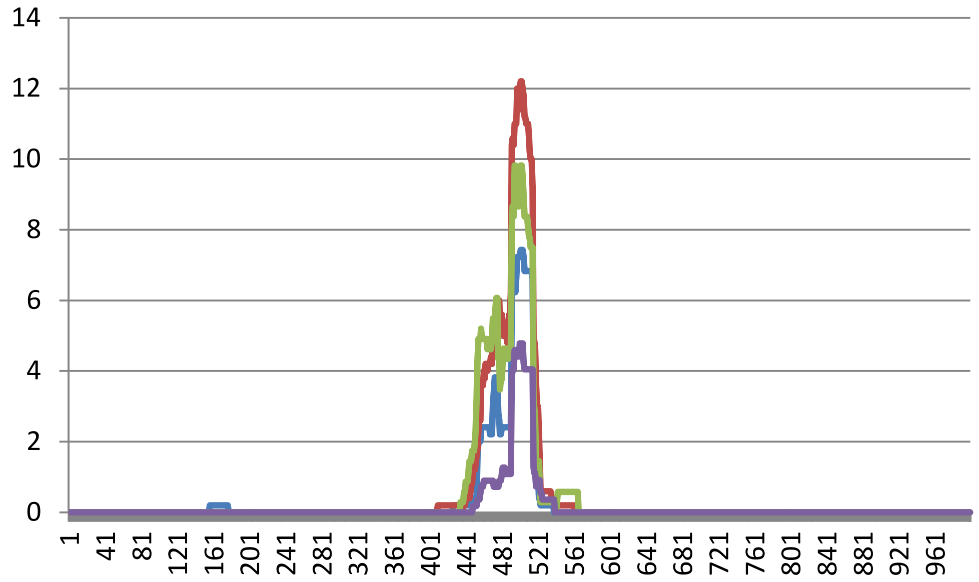

AT1G69280

Unknown protein

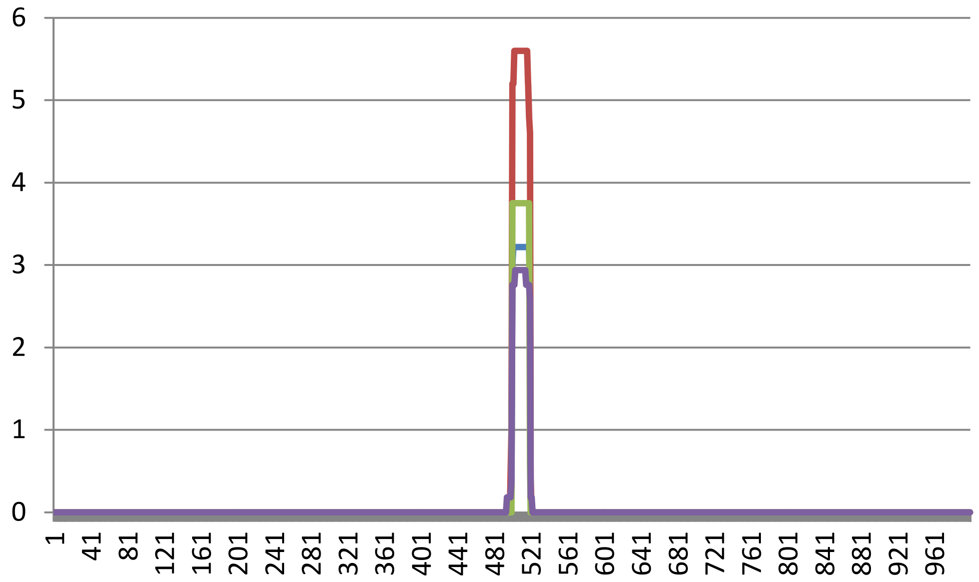

AT1G71697

Encodes choline kinase. mRNA levels are increased in response to wounding.

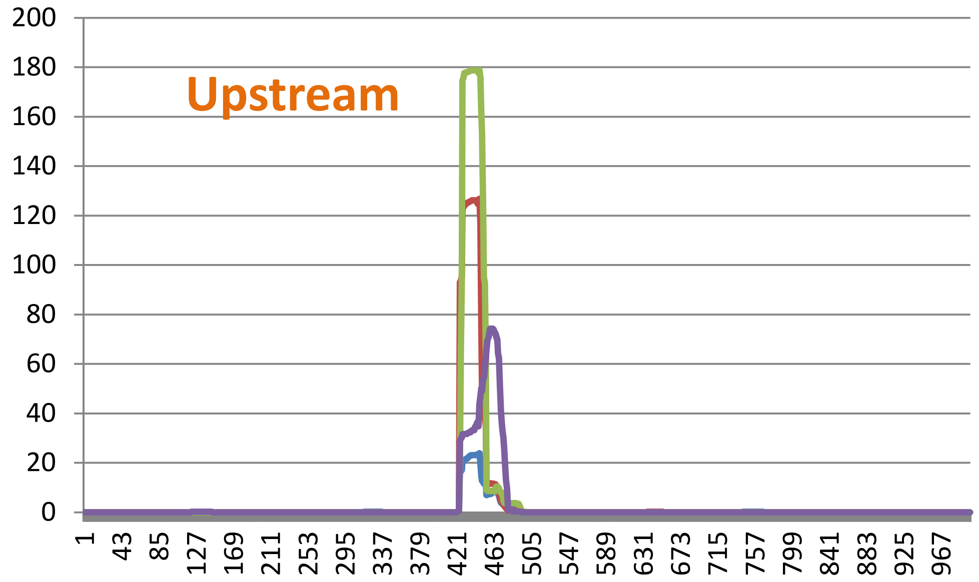

AT1G72140

Major facilitator superfamily protein

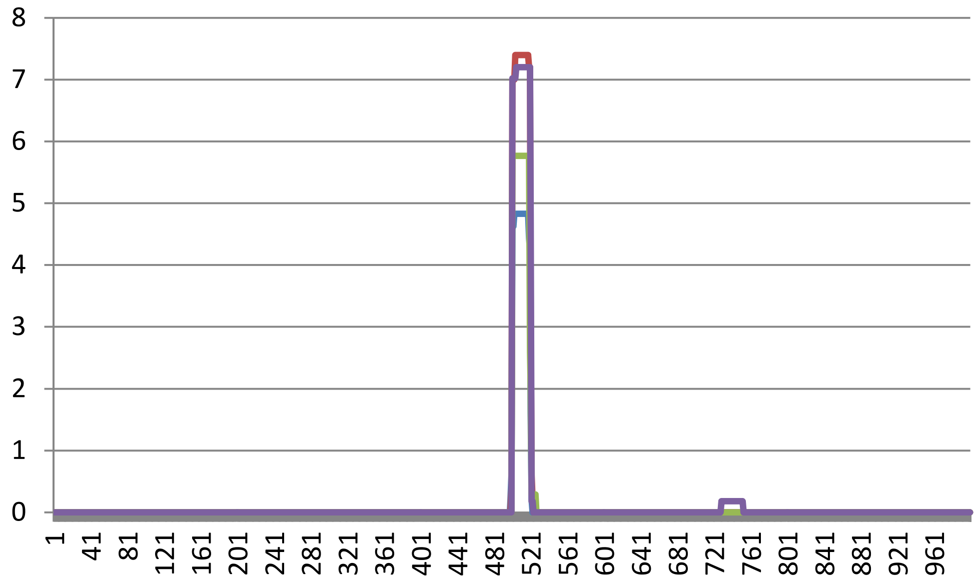

AT1G72580

Unknown protein

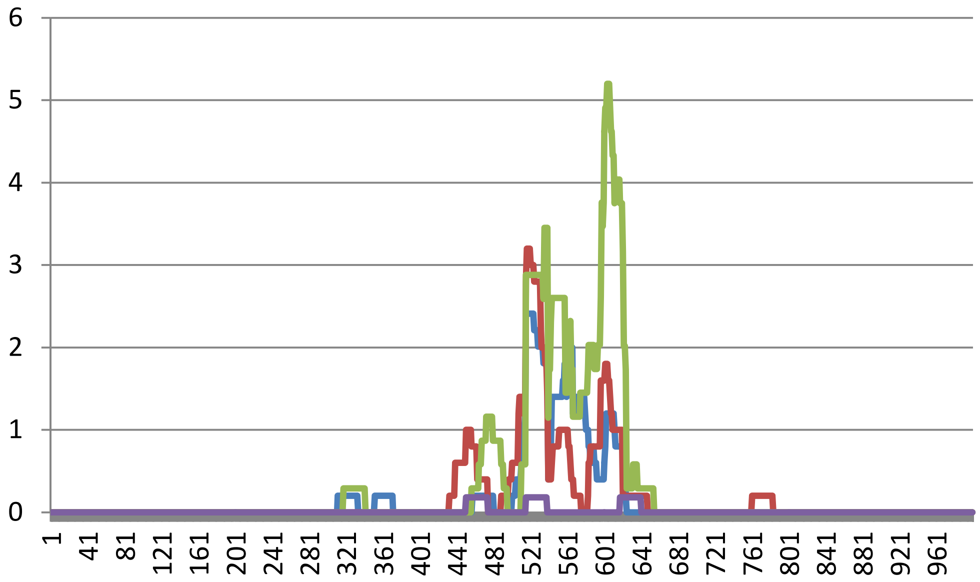

AT1G73640

RAB GTPase homolog A6A (RABA6a)

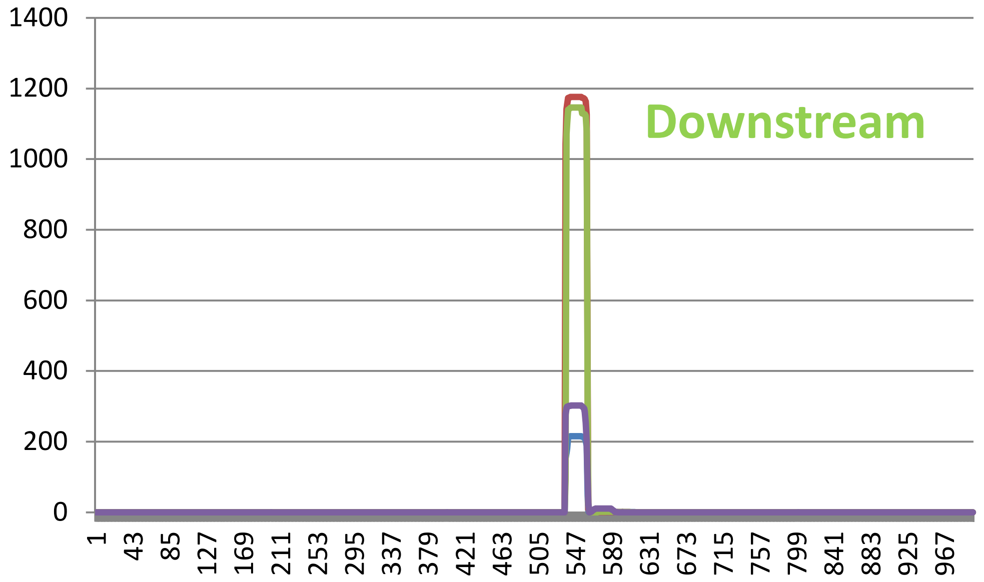

AT1G79170

Unknown protein

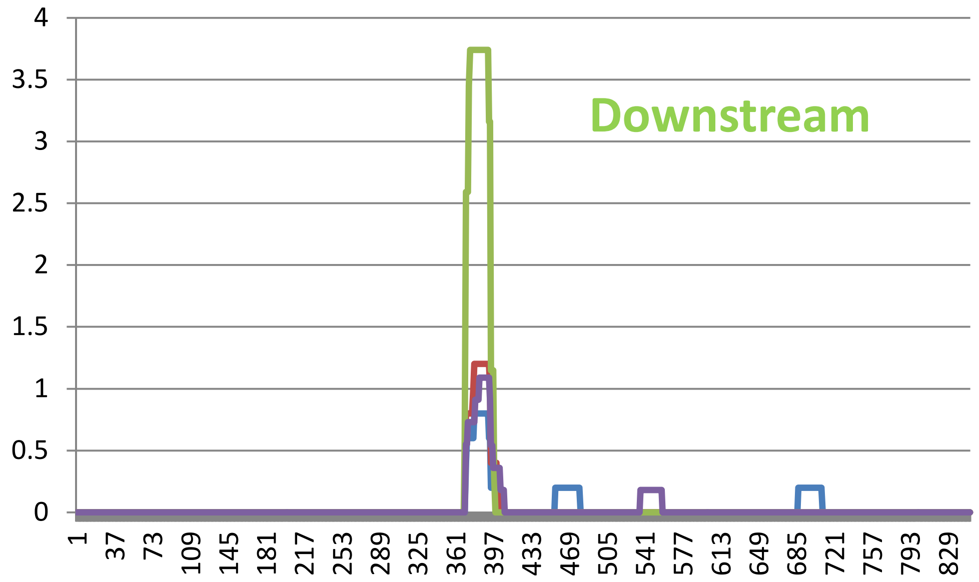

AT1G80690

PPPDE putative thiol peptidase family protein

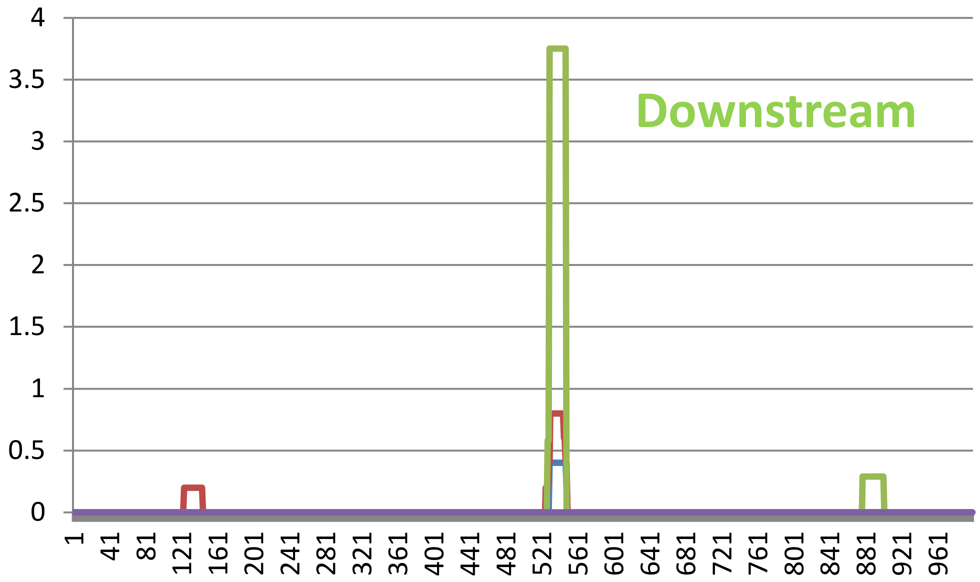

AT2G01950

Encodes a leucine rich repeat receptor kinase and associated with provascular/procambial cells. Similar to BRI, brassinosteroid receptor protein.

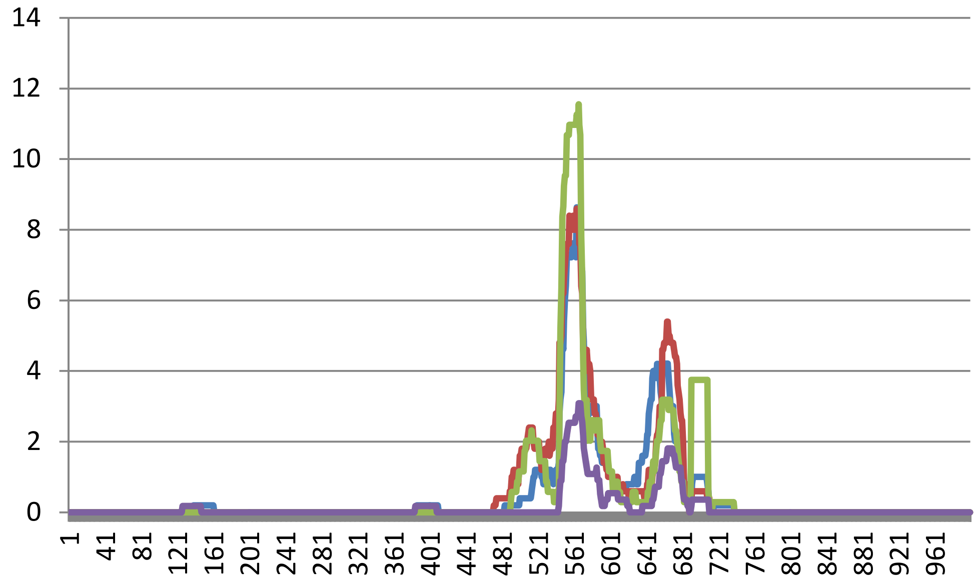

AT2G02390

Encodes glutathione transferase belonging to the zeta class of GSTs.

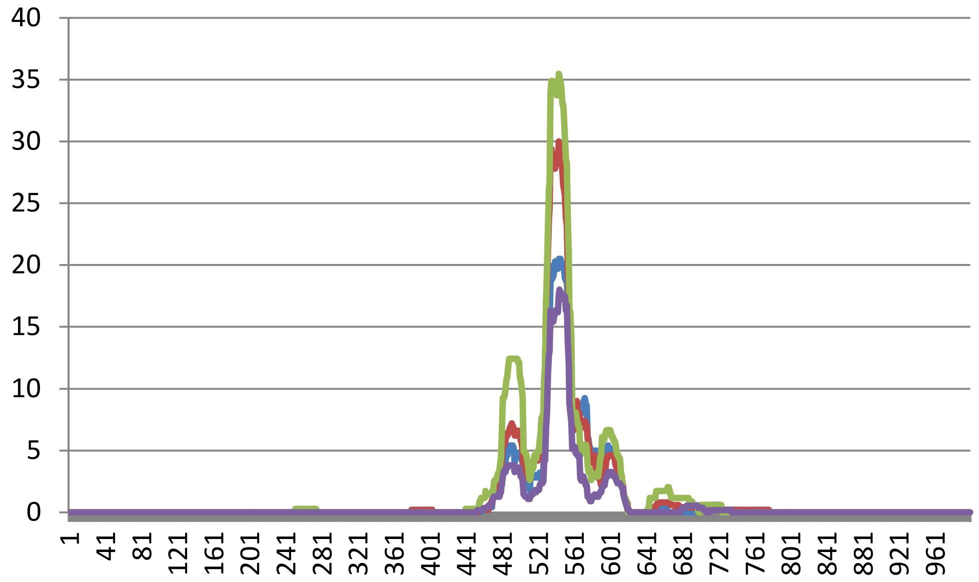

AT2G04220

Plant protein of unknown function (DUF868)

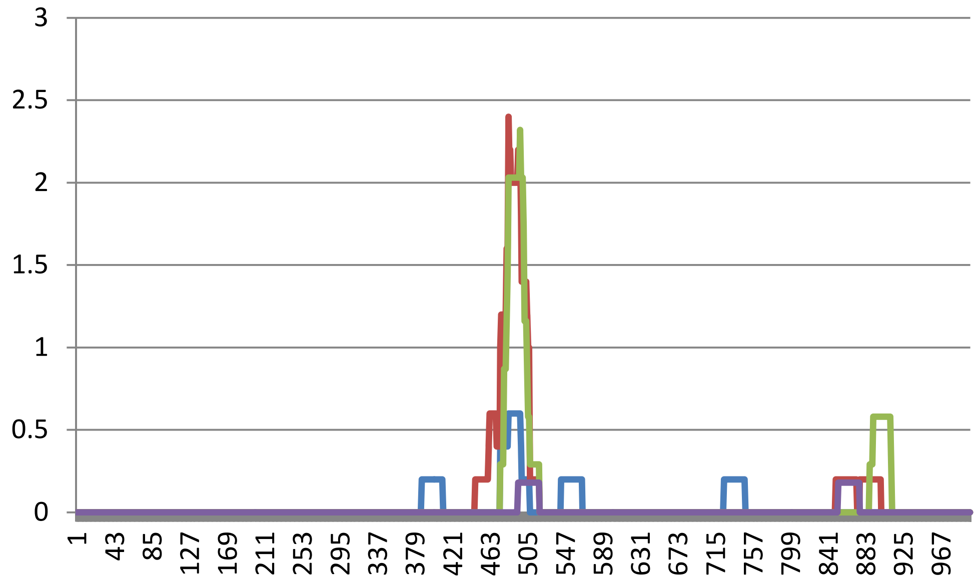

AT2G05335

Encodes a member of a family of small, secreted, cysteine rich proteins with sequence similarity to SCR (S locus cysteine-rich protein).

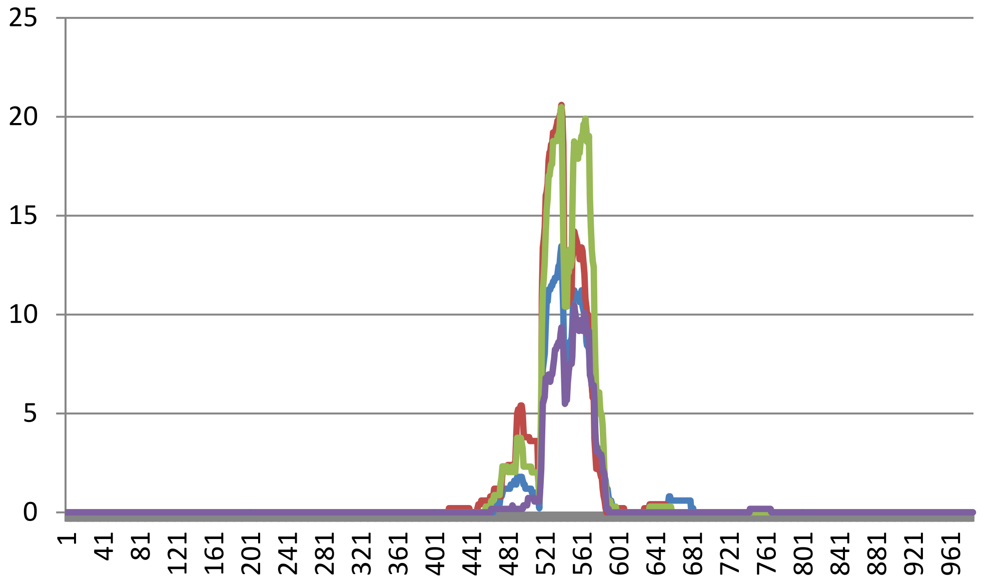

AT2G05540

Glycine-rich protein family

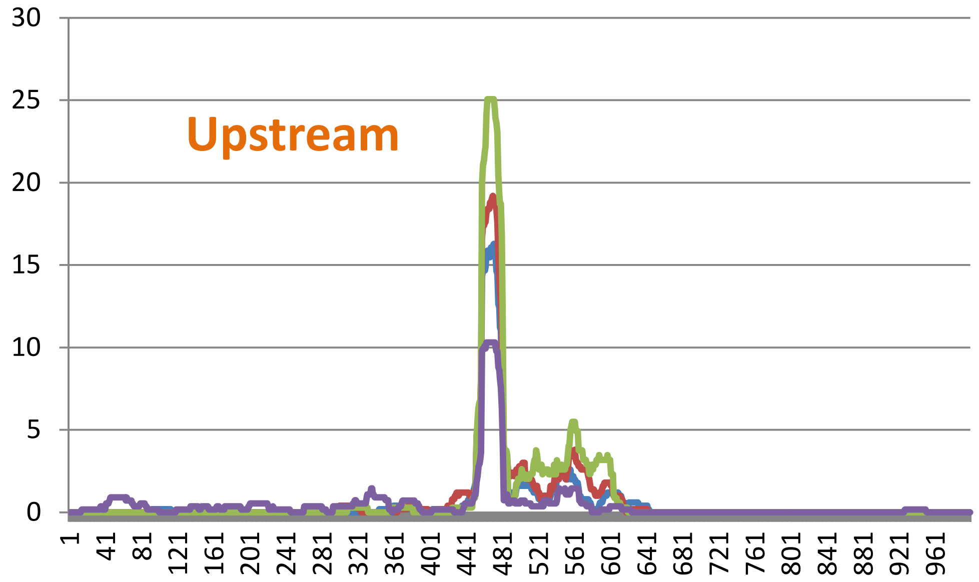

AT2G06255

ELF4-like 3 (ELF4-L3)

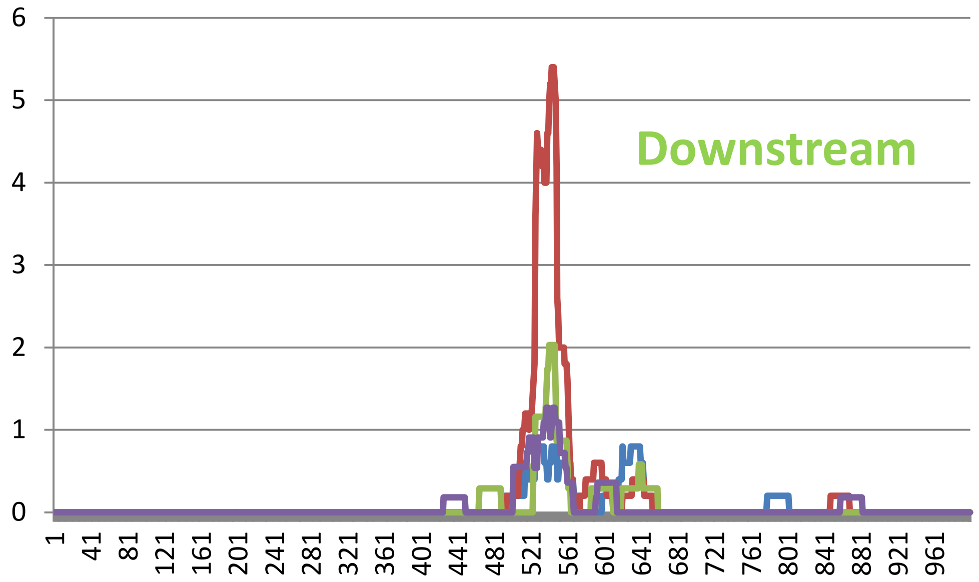

AT2G06541

TTF-type zinc finger protein with HAT dimerisation domain

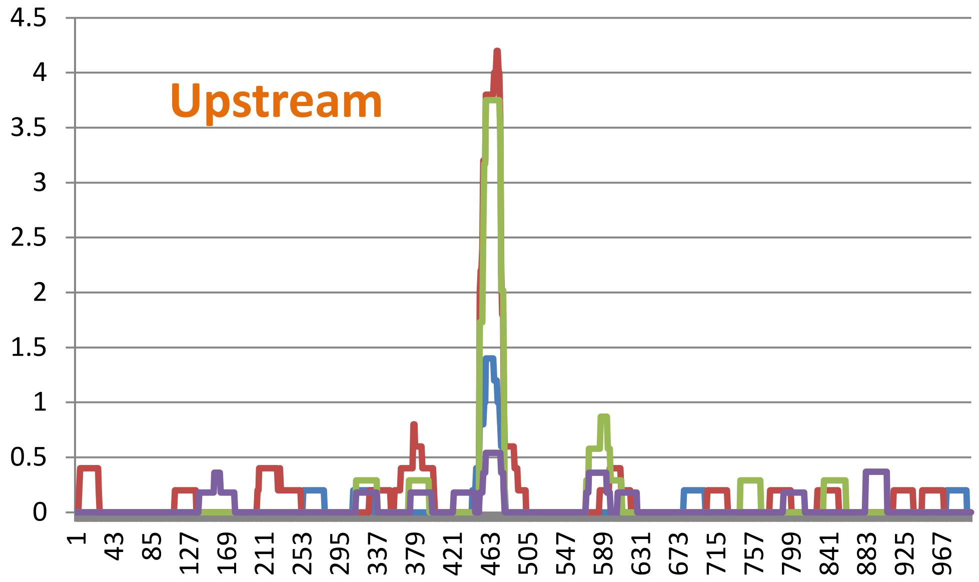

AT2G07771

Cytochrome C assembly protein

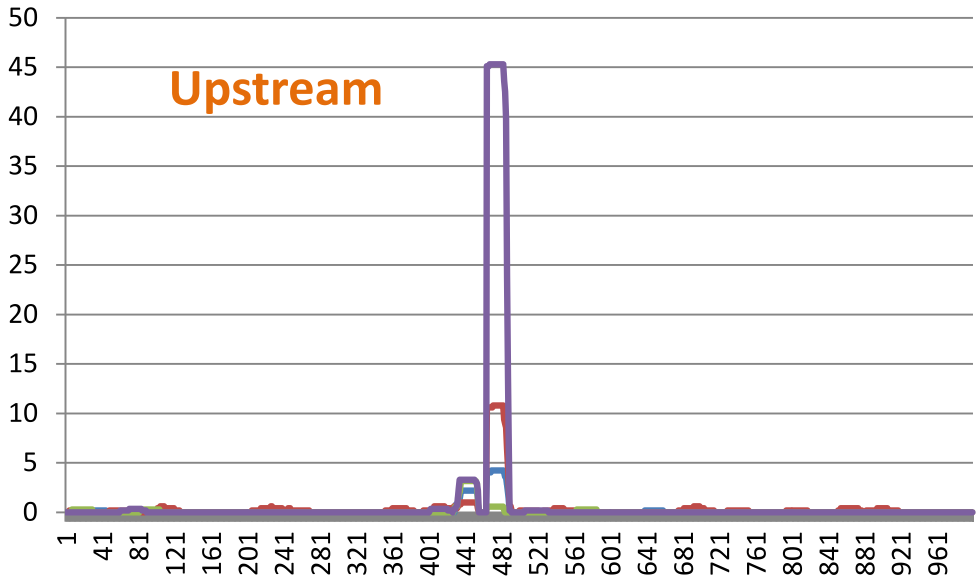

AT2G07777

ATP synthase 9 mitochondrial

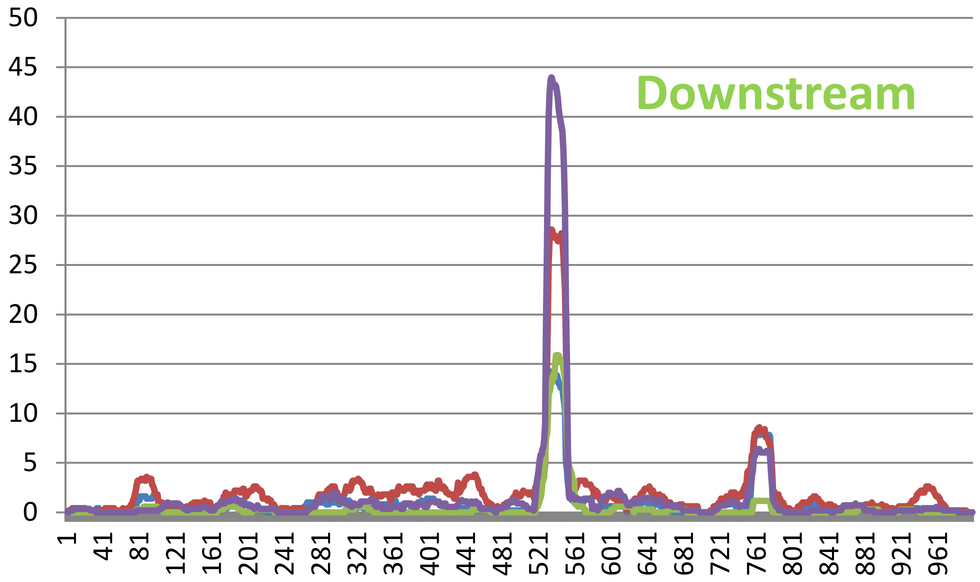

AT2G10608

Unknown protein

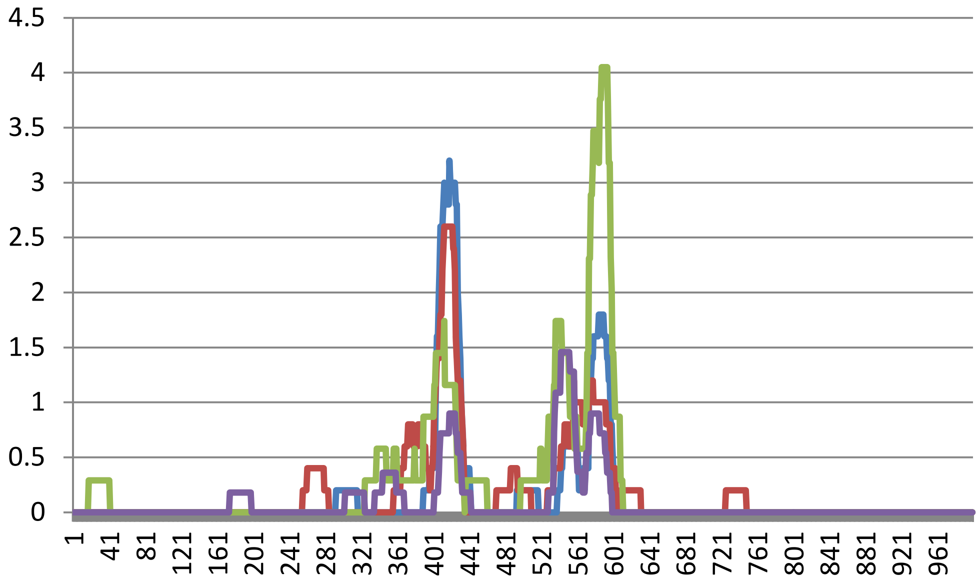

AT2G13960

Homeodomain-like superfamily protein

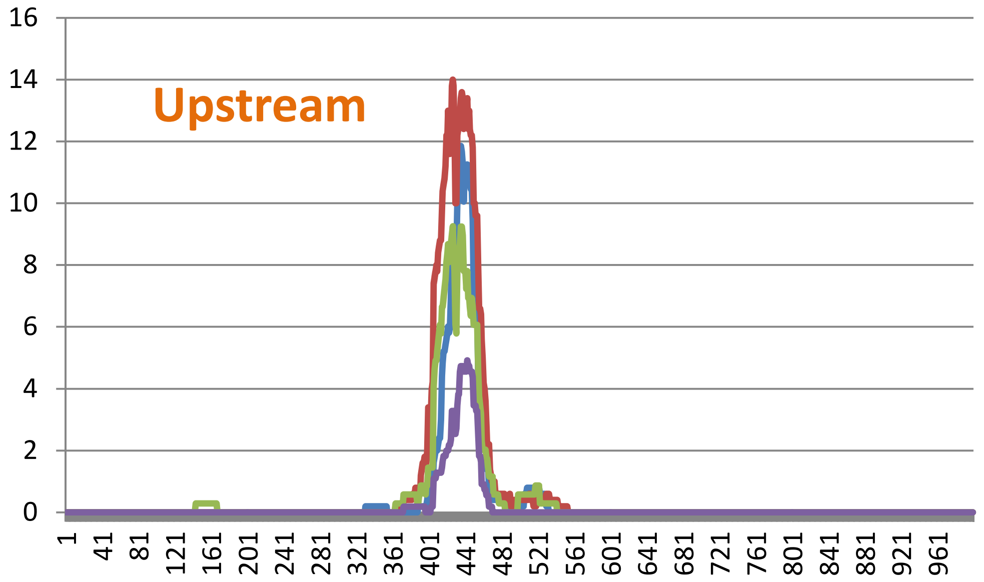

AT2G14247

Expressed protein

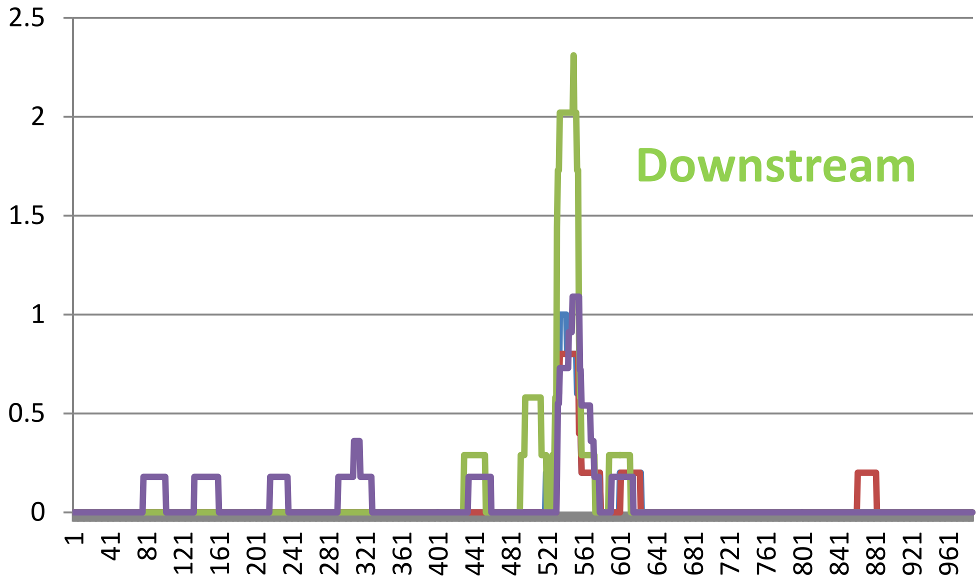

AT2G14560

Encodes LURP1, a member of the LURP cluster (late upregulated in response to Hyaloperonospora parasitica) which exhibits a pronounced upregulation after recognition of the pathogenic oomycte H. parasitica. LURP1 is required for full basal defense to H. parasitica and resistance to this pathogen mediated by the R-proteins RPP4 and RPP5.

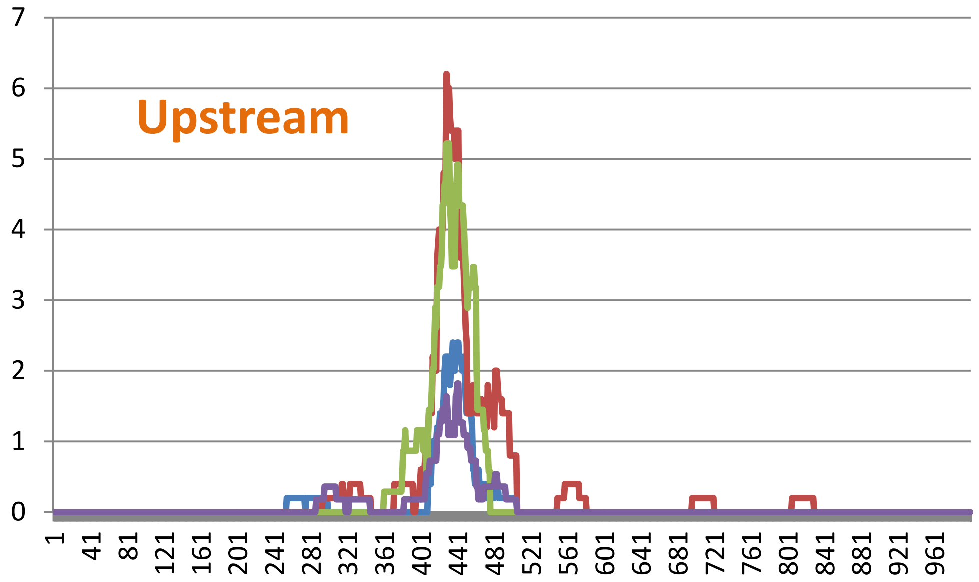

AT2G15050

Predicted to encode a PR (pathogenesis-related) protein.

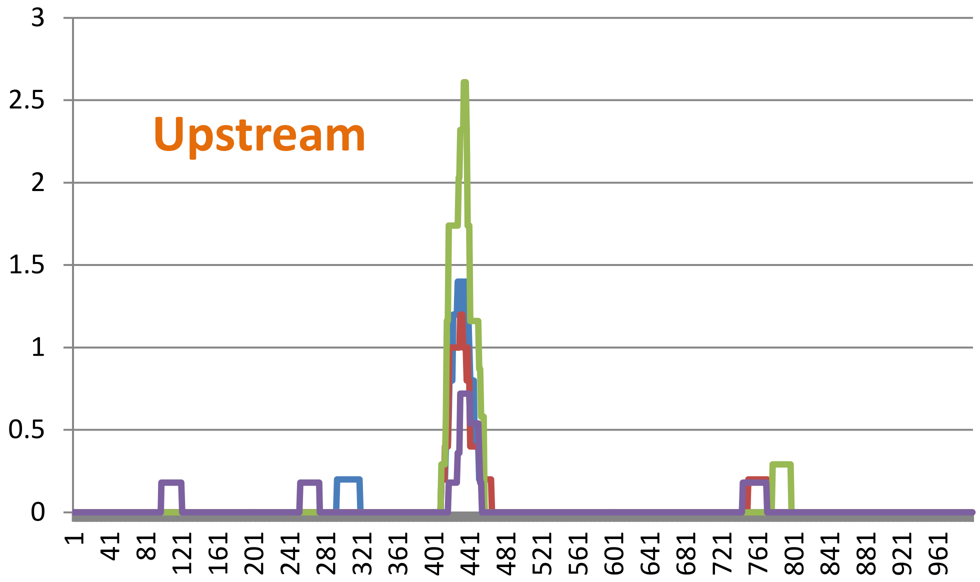

AT2G16030

S-adenosyl-L-methionine-dependent methyltransferases superfamily protein.

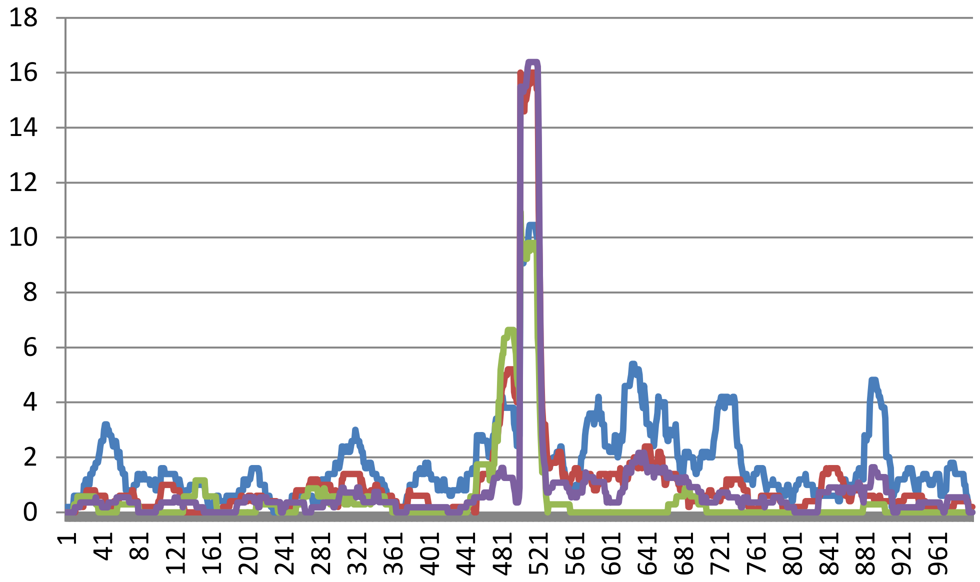

AT2G16340

Unknown protein

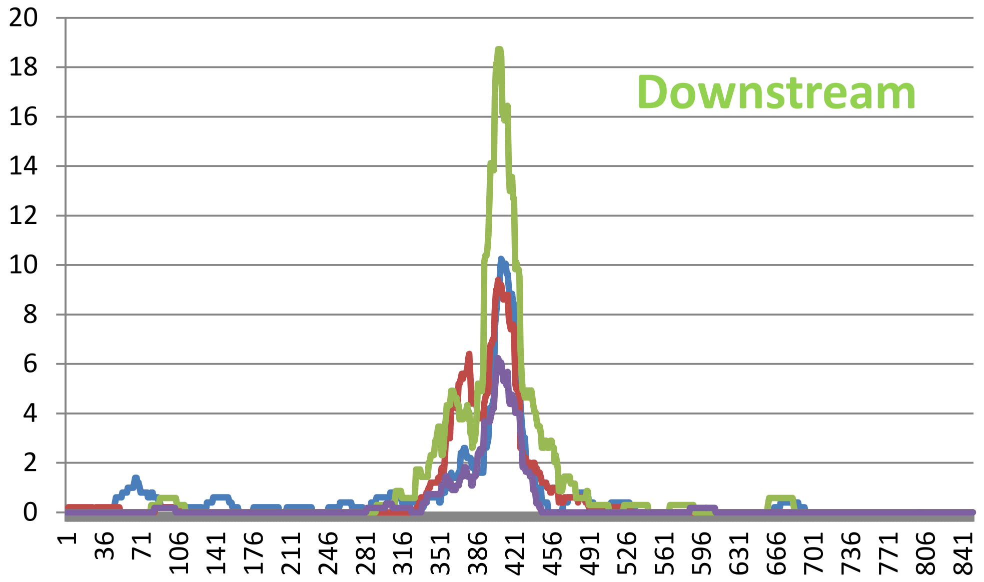

AT2G16380

Sec14p-like phosphatidylinositol transfer family protein.

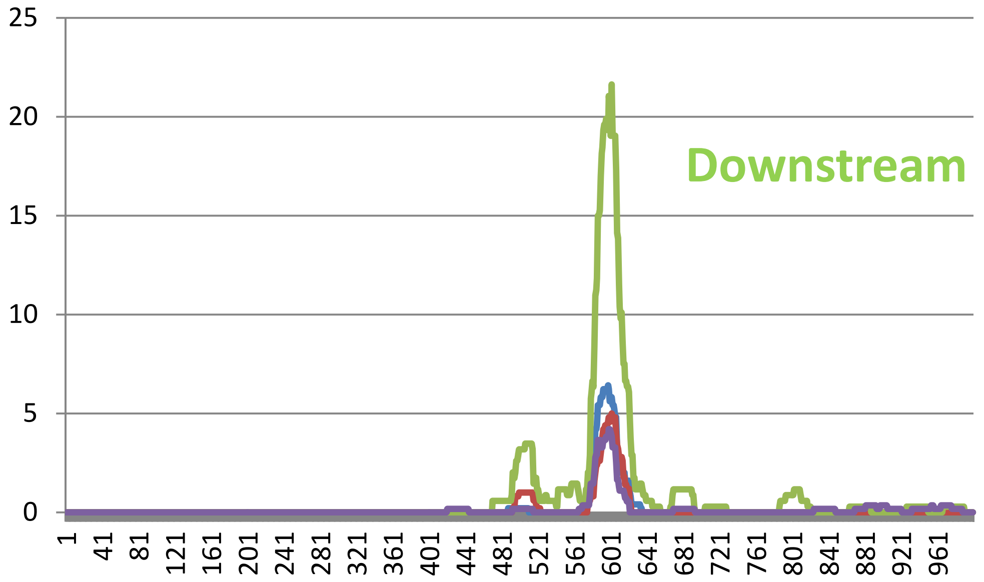

AT2G18465

Chaperone DnaJ-domain superfamily protein

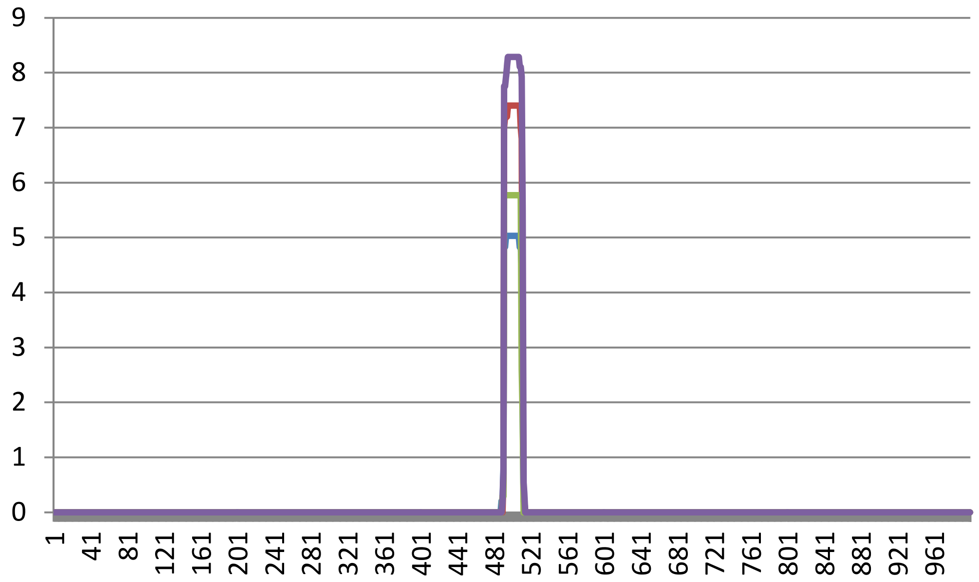

AT2G18980

Peroxidase superfamily protein

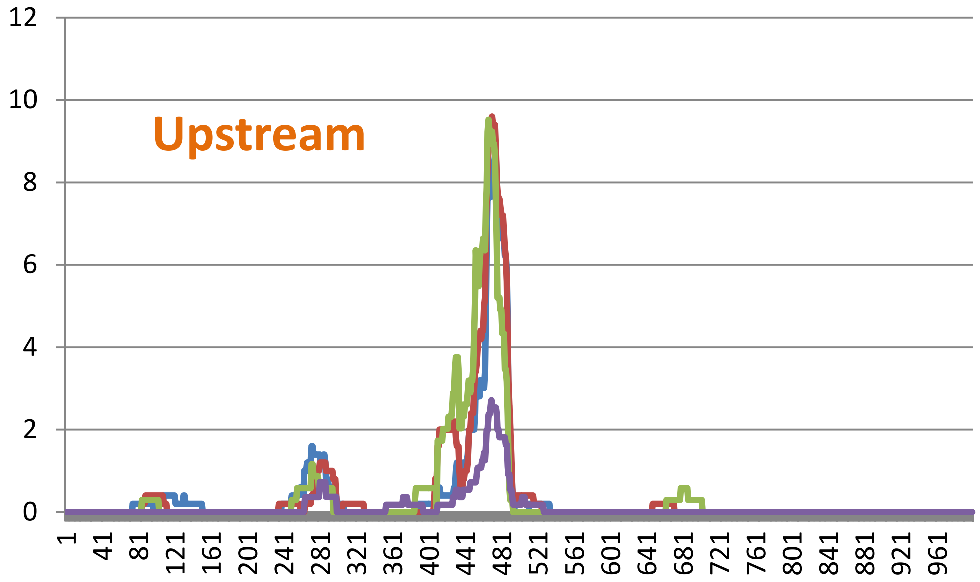

AT2G20190

Encodes a microtubule-associated protein that is involved in both cell division and cell expansion. It likely promotes microtubule stability.

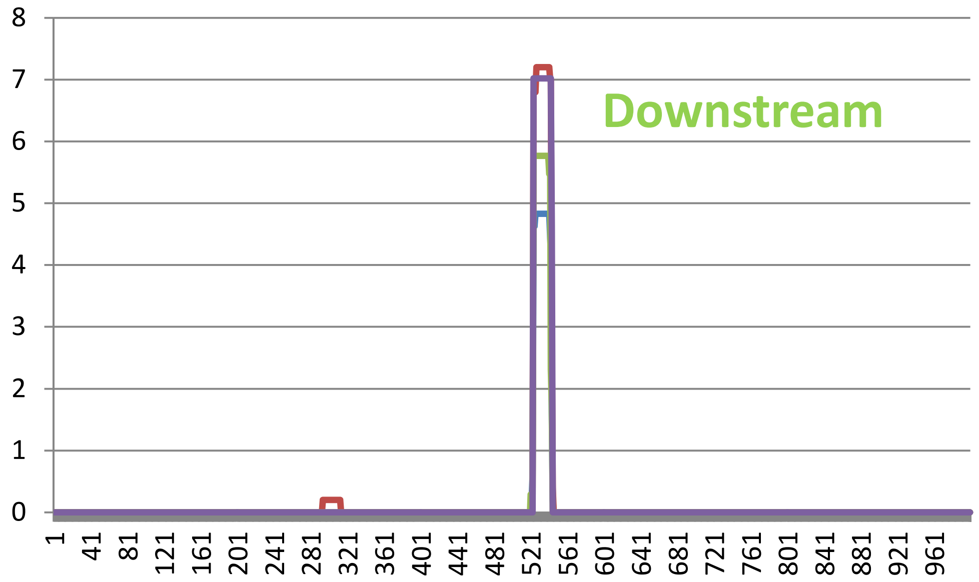

AT2G22890

Kua-ubiquitin conjugating enzyme hybrid localisation domain

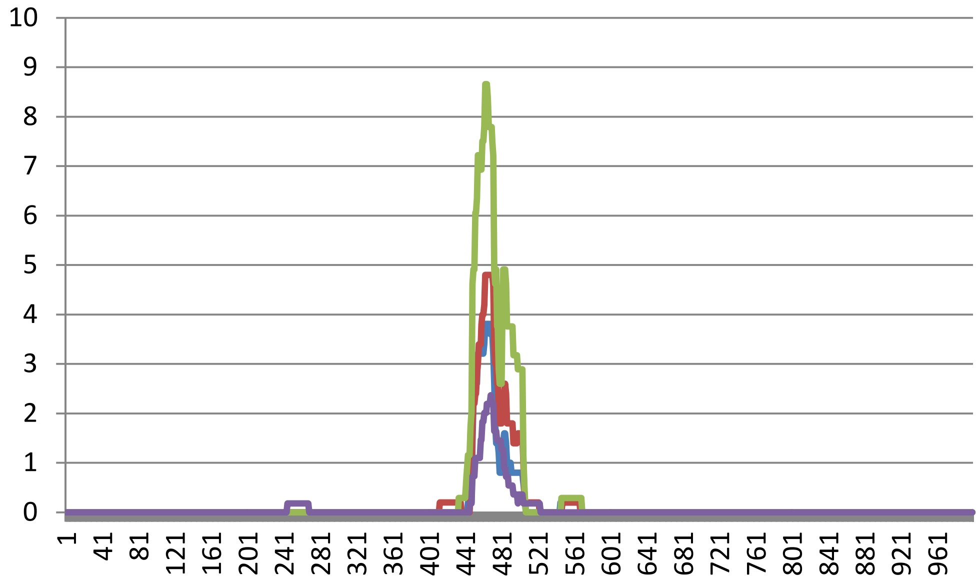

AT2G23640

Encodes RTNLB13, a reticulon protein integral to the endoplasmic reticulum (ER) membrane that have the ability to shape the ER into tubules.

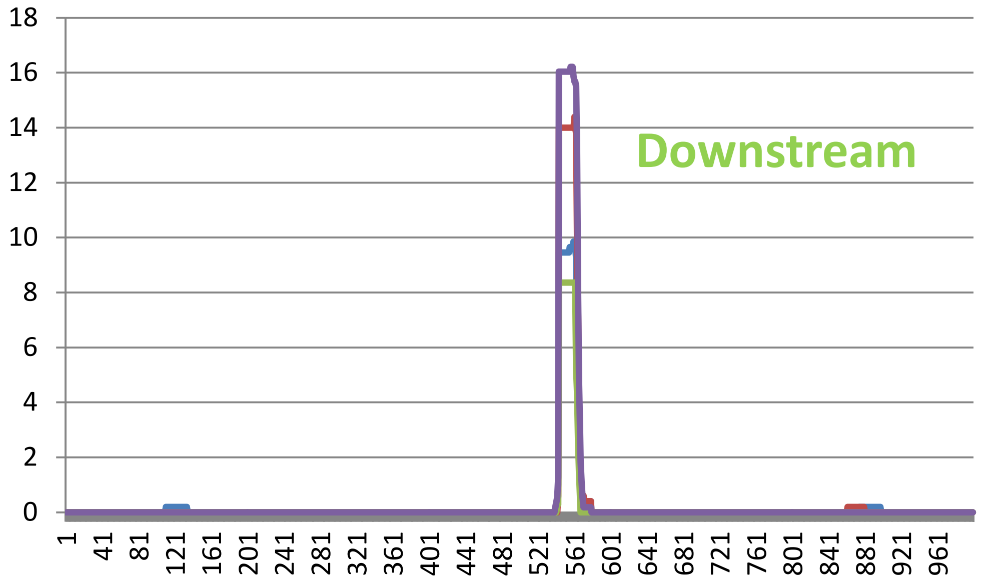

AT2G23890

HAD-superfamily hydrolase, subfamily IG, 5'-nucleotidase

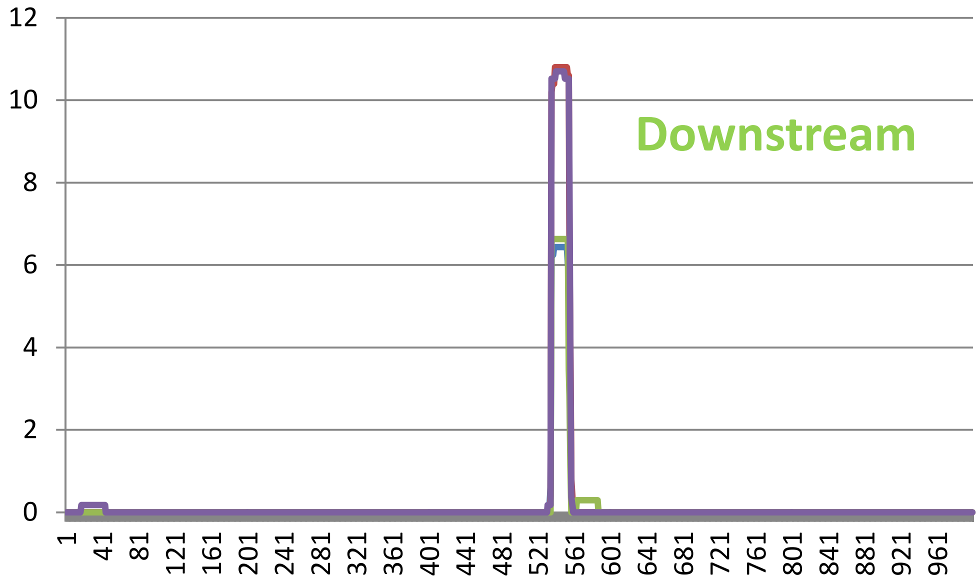

AT2G24740

Encodes a SU(VAR)3-9 homolog, a SET domain protein (Homology Subgroup V; Orthology Group 1). Known SET domain proteins are involved in epigenetic control of gene expression. There are 10 SUVH genes in Arabidopsis and members of this subfamily of the SET proteins have an additional conserved SRA domain. This protein is a putative histone methyltransferase (predicted to methylate H3K9/20) related to the the Drosophila Su(var)3-9 and mammalian G9a proteins.

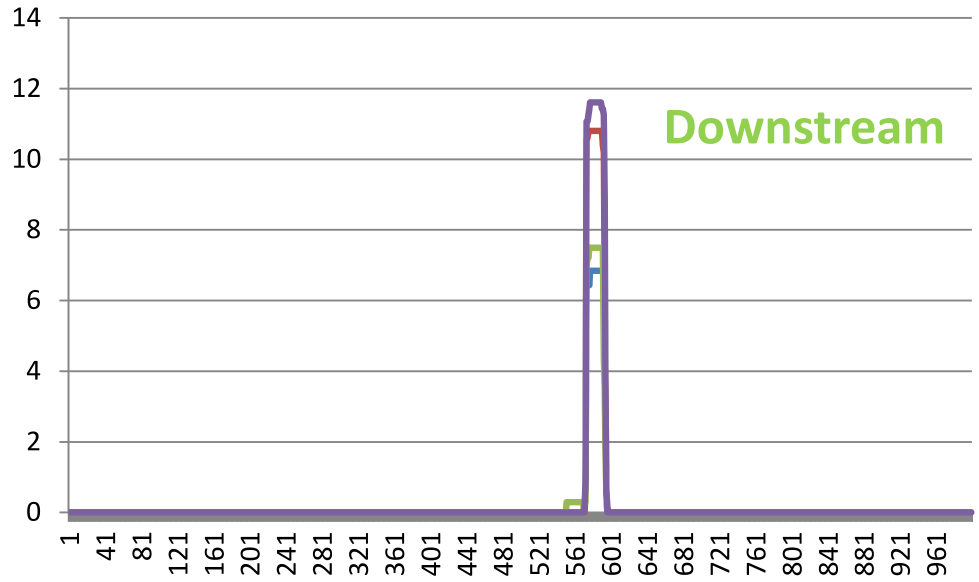

AT2G24880

Plant self-incompatibility protein S1 family

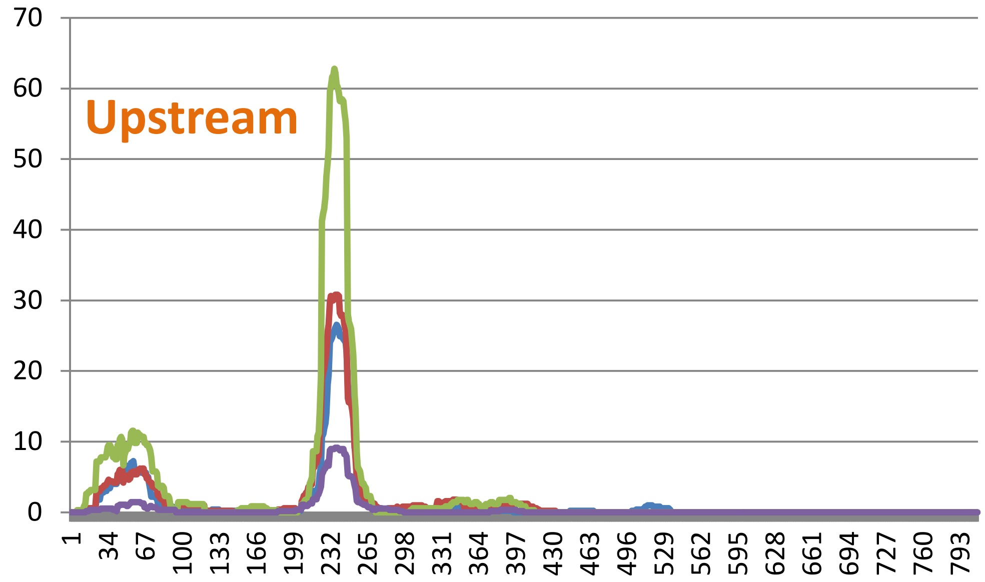

AT2G27420

Cysteine proteinases superfamily protein

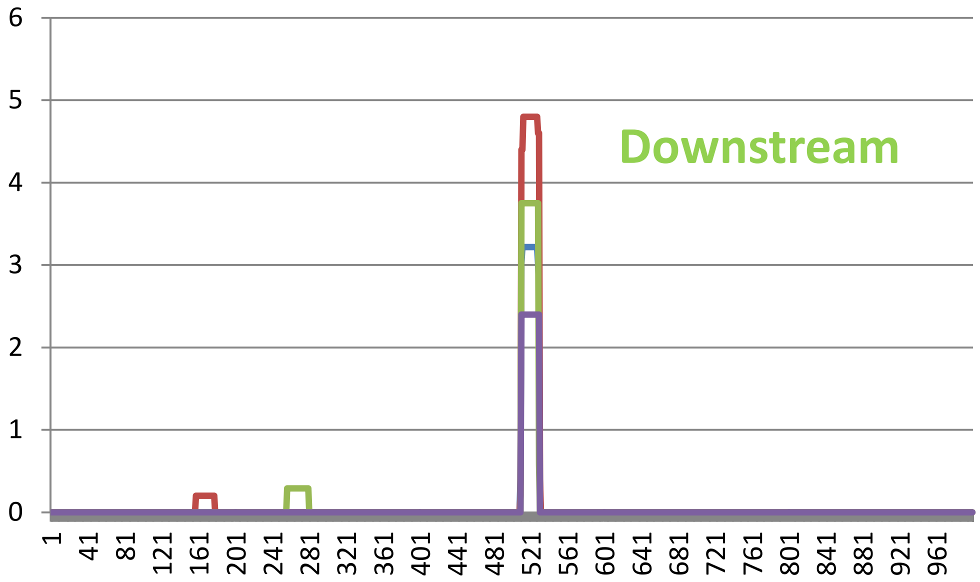

AT2G28725

Unknown protein

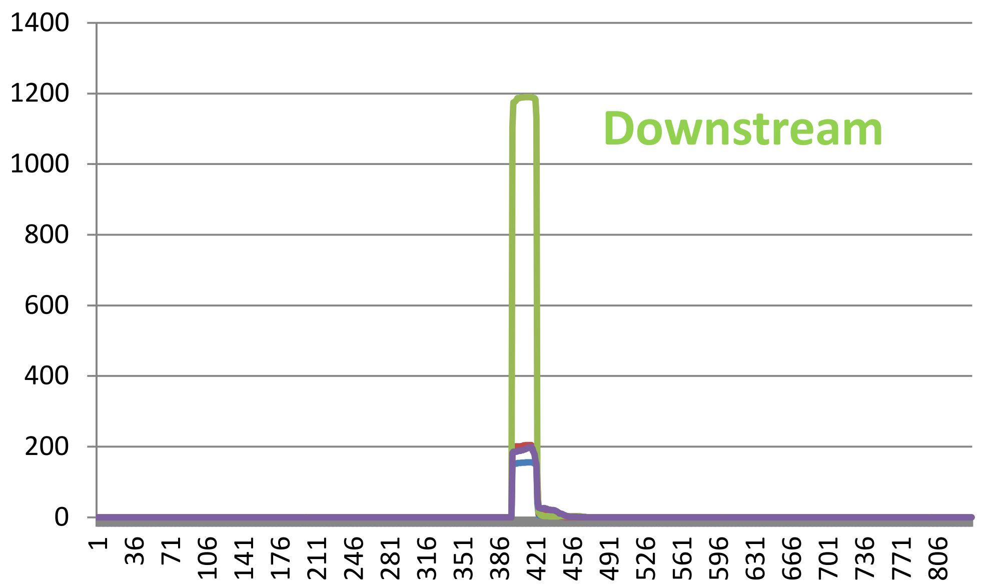

AT2G31270

Encodes a cyclin-dependent protein kinase. Involved in nuclear DNA replication and plastid division. Located in nucleus and chloroplast.

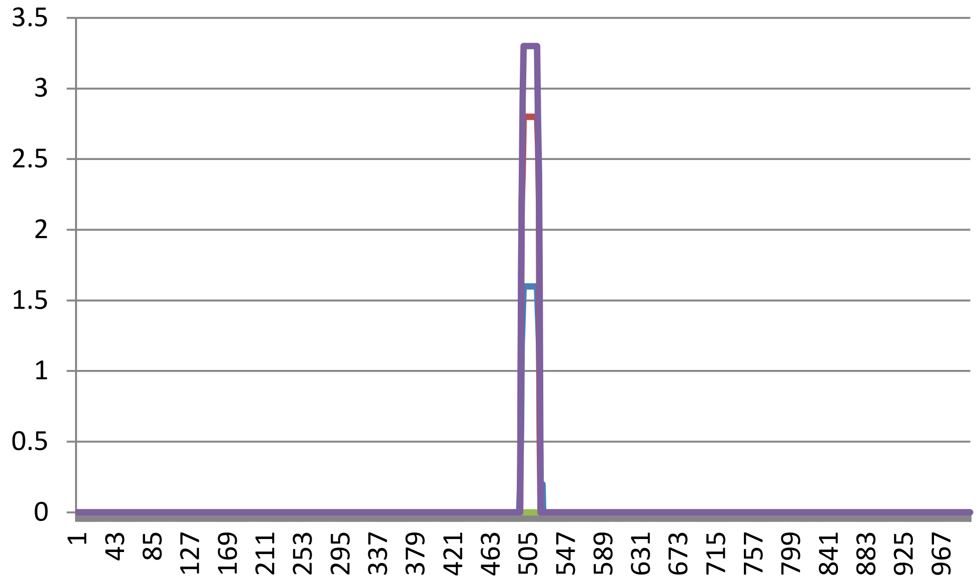

AT2G32140

Transmembrane receptors

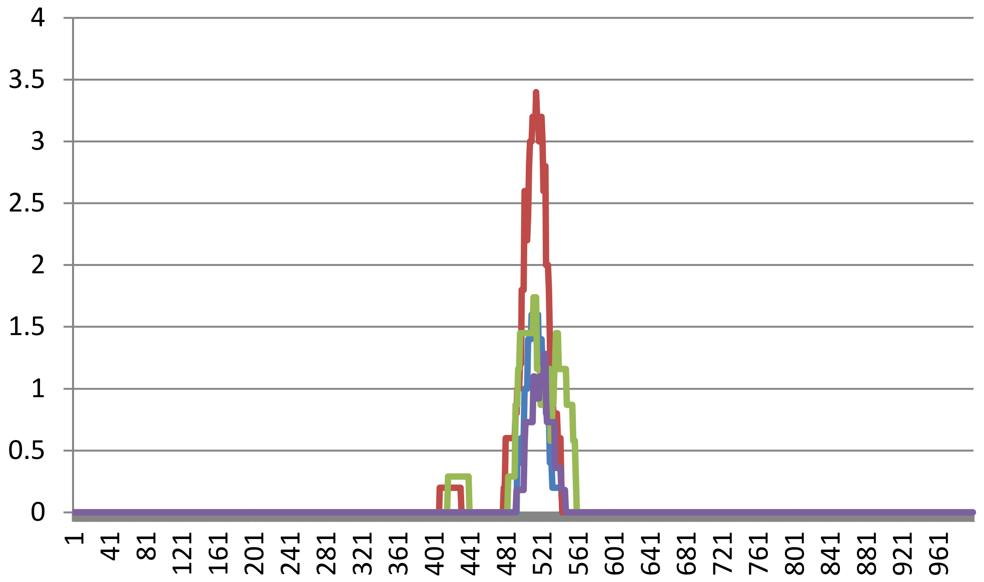

AT2G33150

Encodes an organellar (peroxisome, glyoxysome) 3-ketoacyl-CoA thiolase, involved in fatty acid b-oxidation during germination and subsequent seedling growth. Mutants have defects in glyoxysomal fatty acid beta-oxidation.

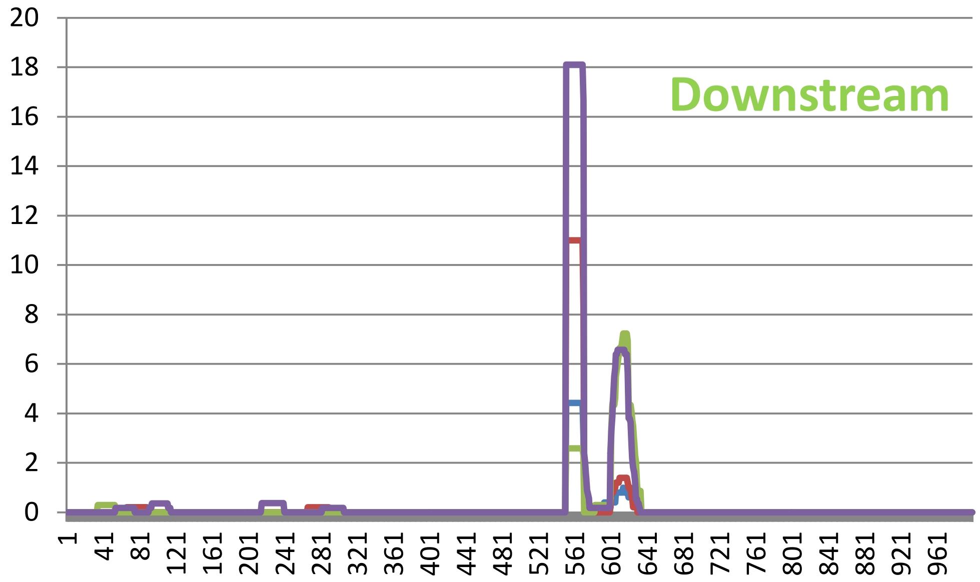

AT2G34655

Unknown protein

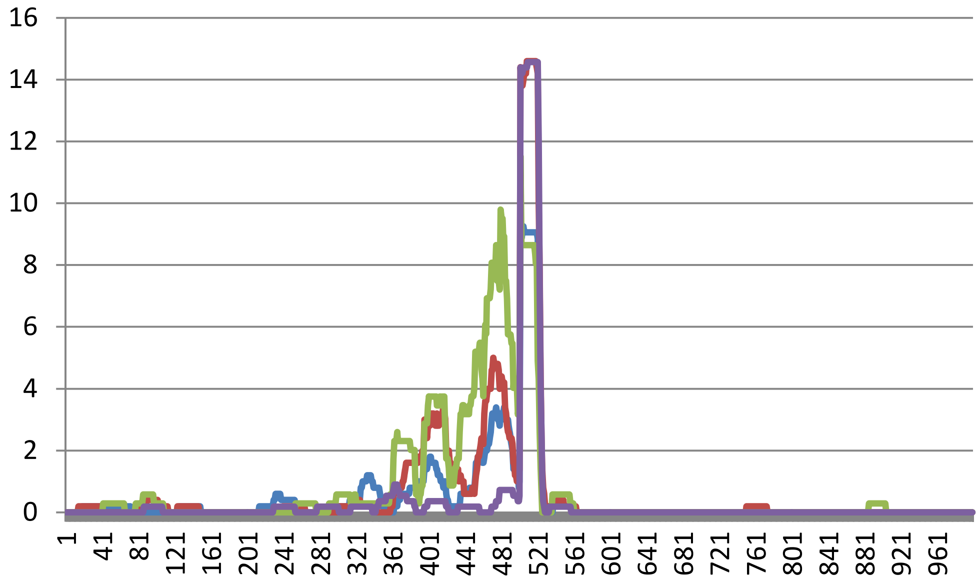

AT2G35250

Protein of Unknown Function (DUF239)

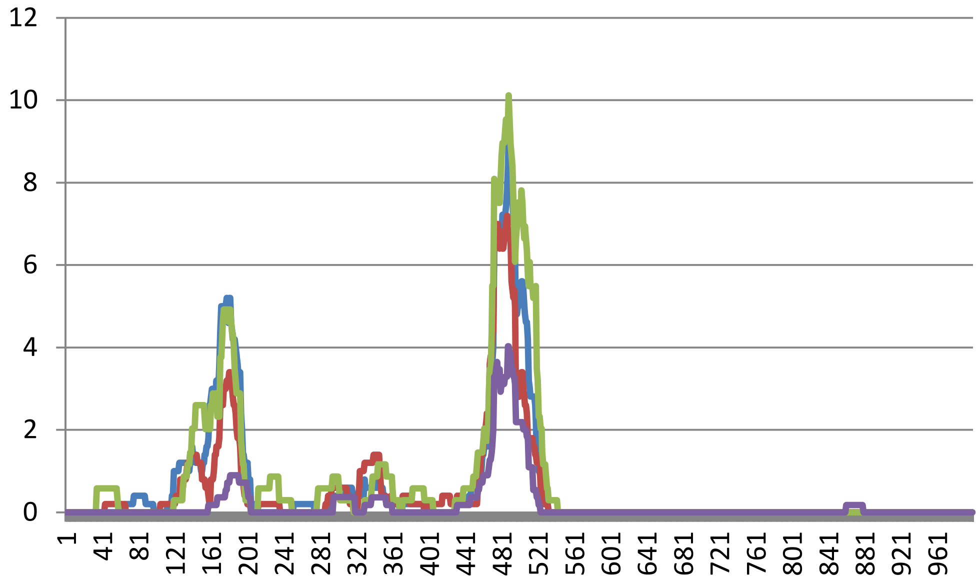

AT2G36940

Unknown protein

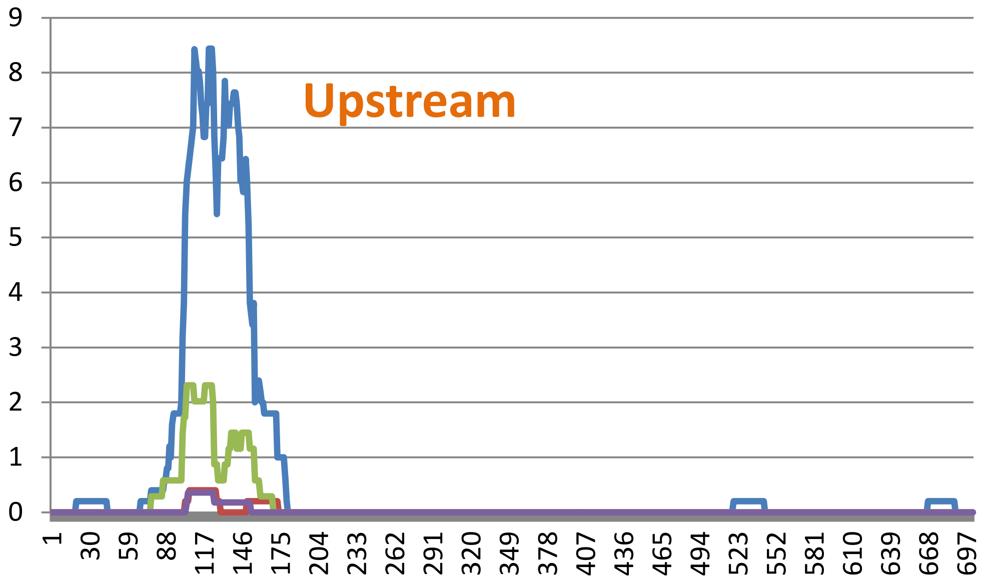

AT2G37810

Cysteine/Histidine-rich C1 domain family protein

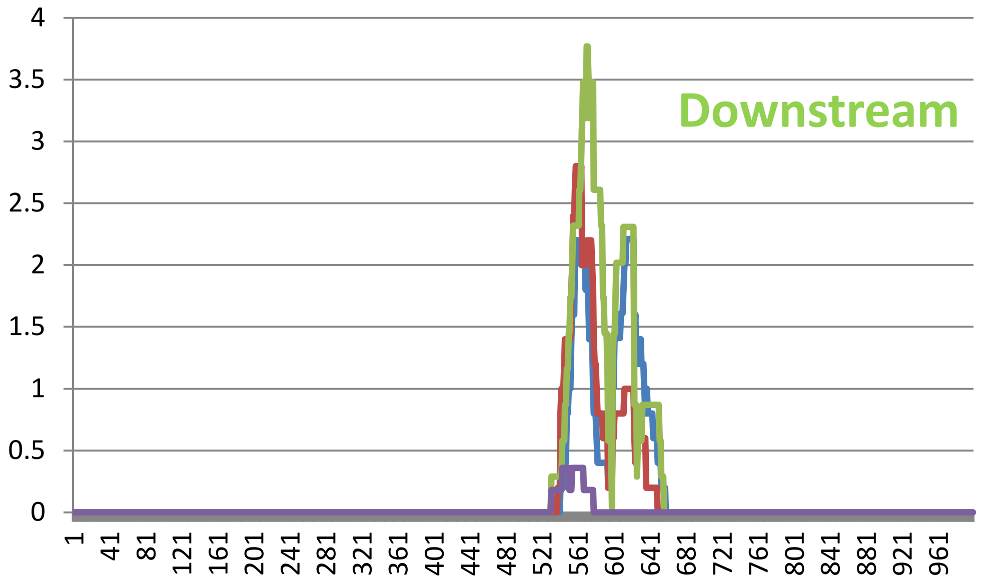

AT2G39630

Encodes a putative dolichyl-phosphate β-glucosyltransferase.

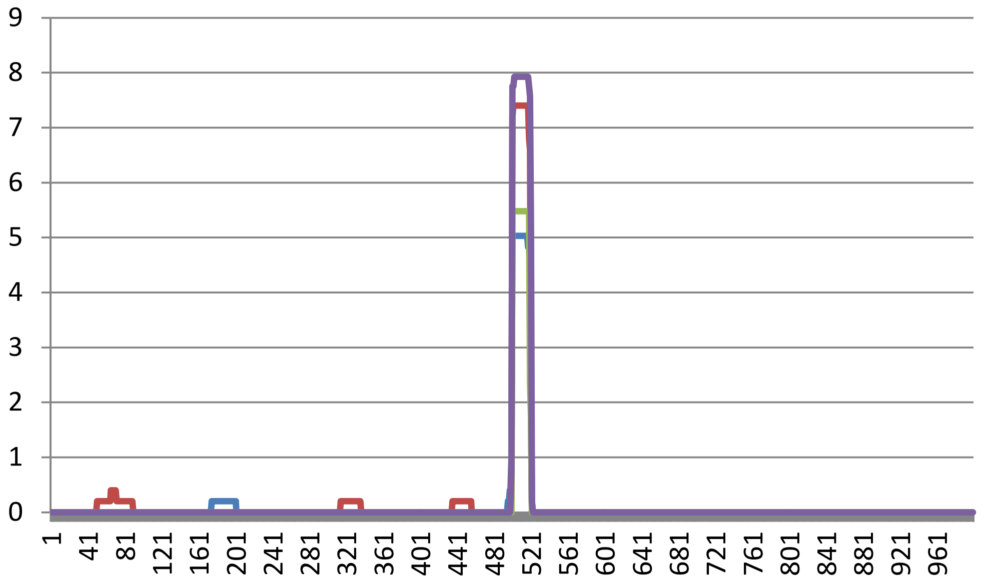

AT2G41920

Protein kinase superfamily protein

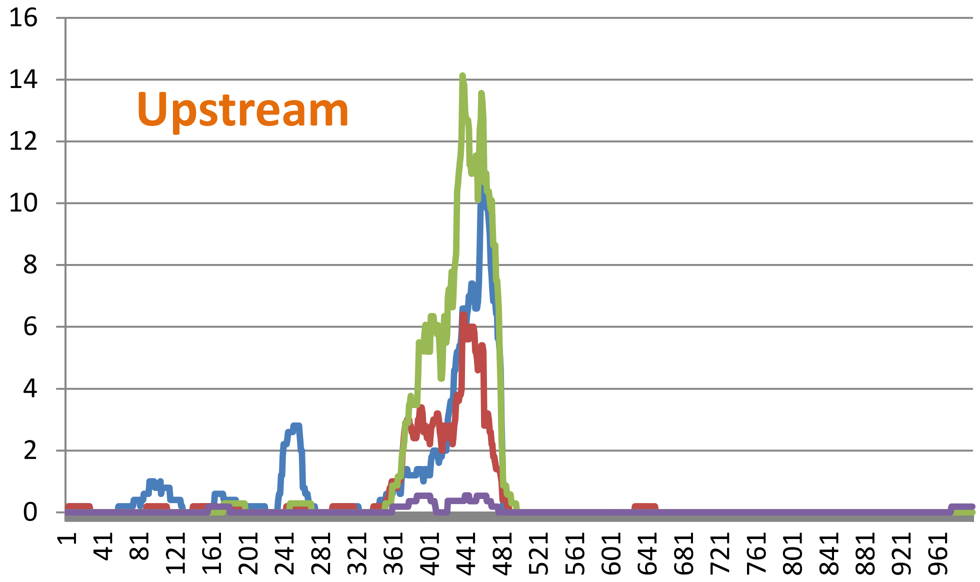

AT2G41980

Protein with RING/U-box and TRAF-like domains

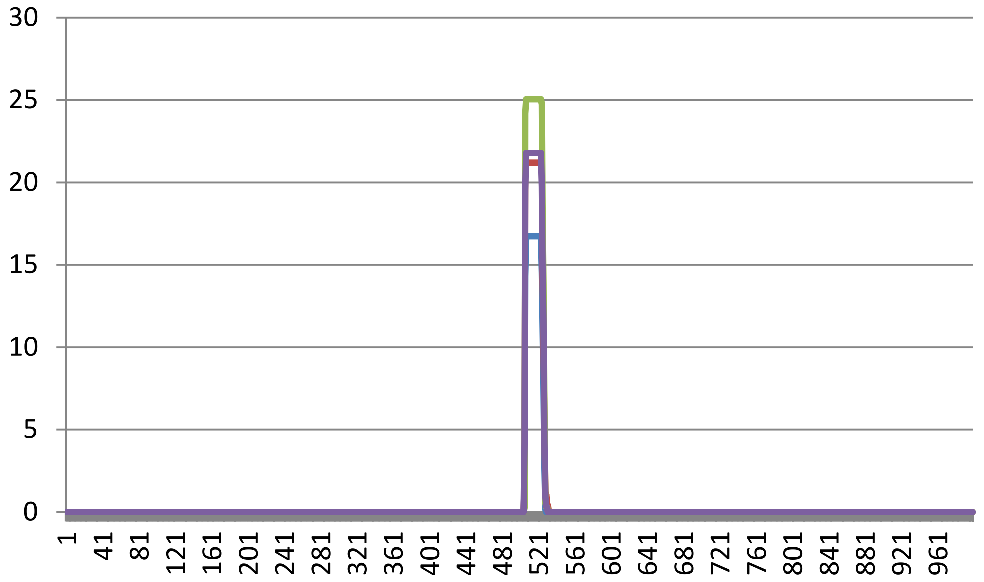

AT2G45010

PLAC8 family protein

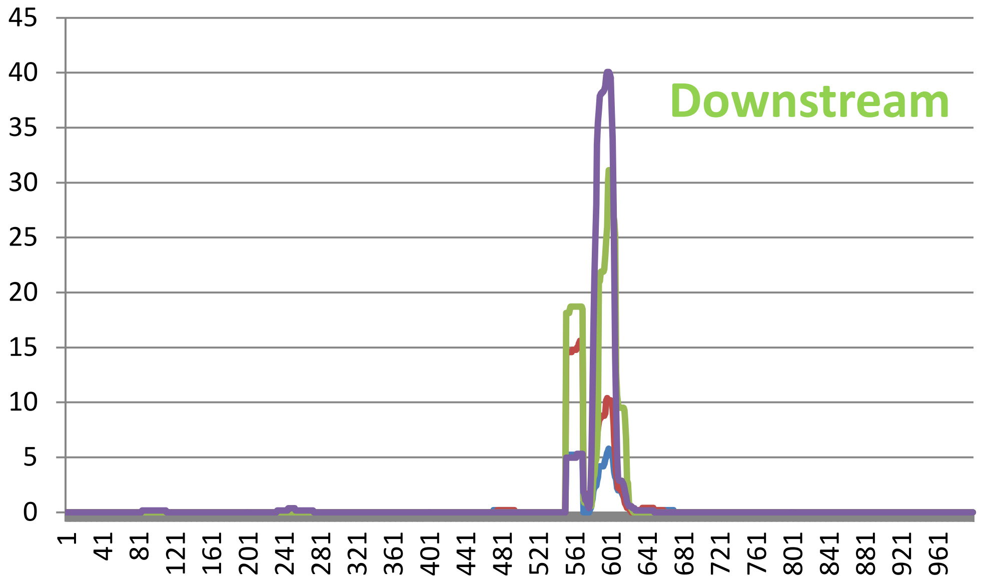

AT3G01240

Unknown protein

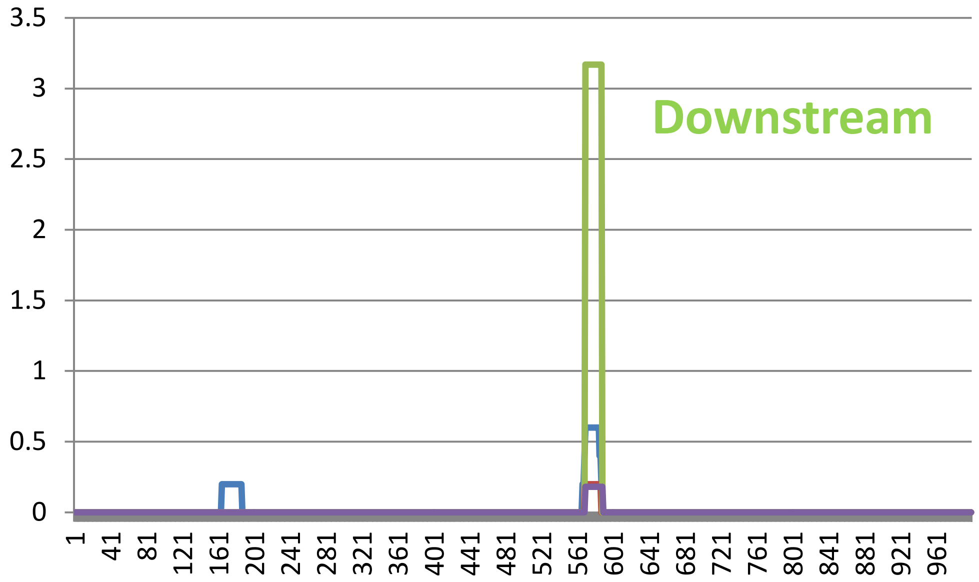

AT3G01700

ARABINOGALACTAN PROTEIN 11 (AGP11). Encodes an arabinogalactan protein that is expressed in pollen, pollen sac and pollen tube. Loss of AGP11 function results in decreased fertility due to defects in pollen tube growth.

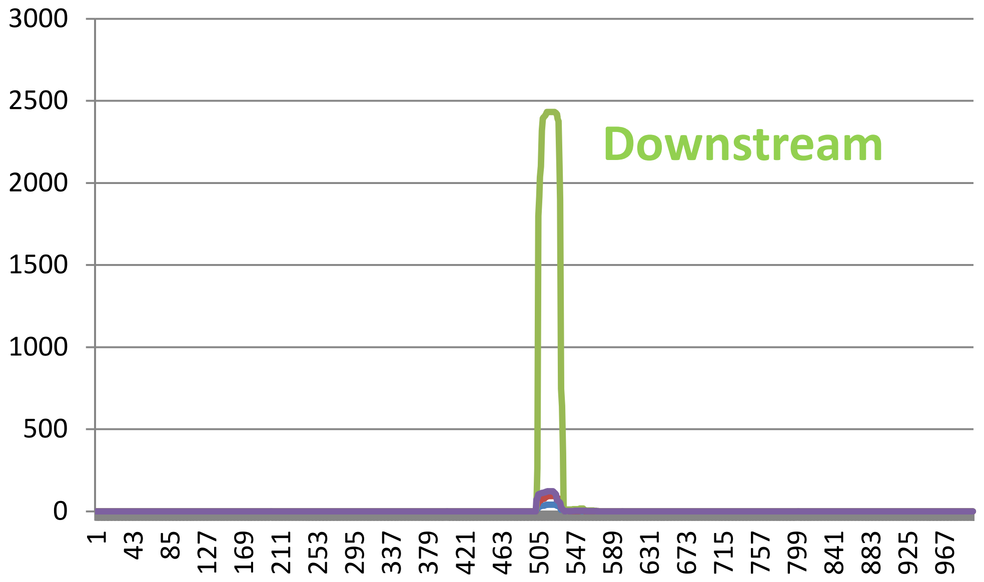

AT3G02420

Unknown protein

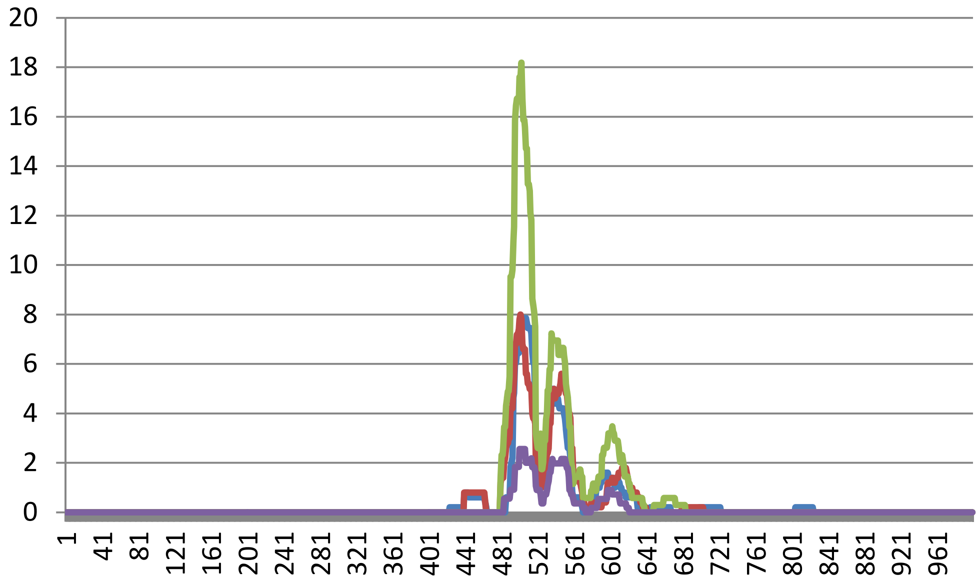

AT3G05660

Receptor like protein 33 (RLP33)

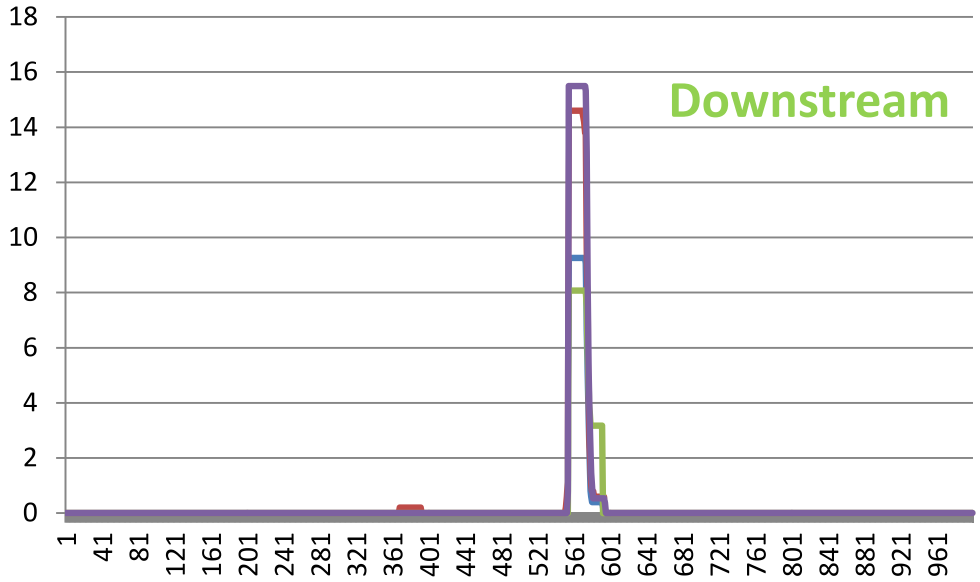

AT3G08720

Encodes a ribosomal-protein S6 kinase. Gene expression is induced by cold and salt (NaCl).

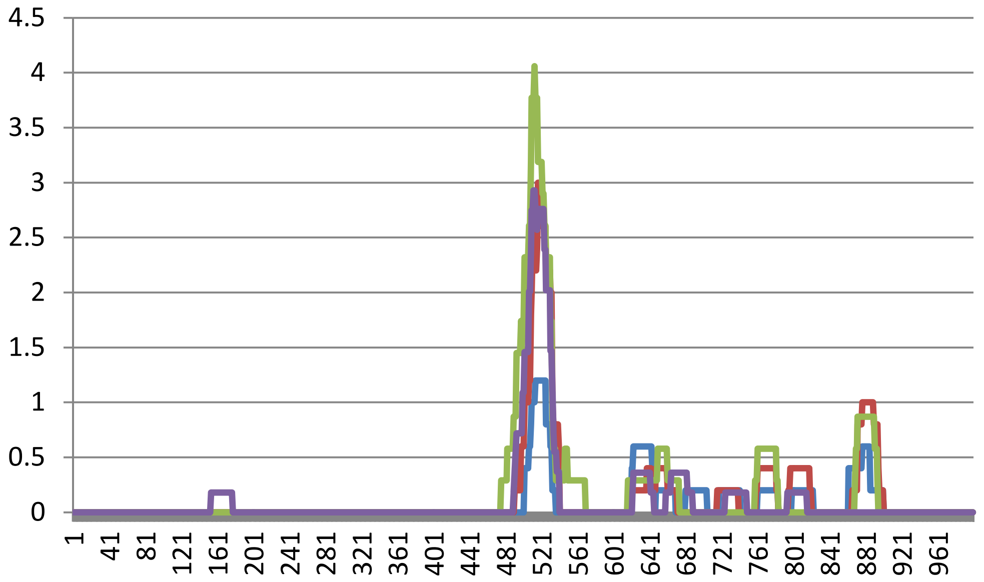

AT3G09960

Calcineurin-like metallo-phosphoesterase superfamily protein

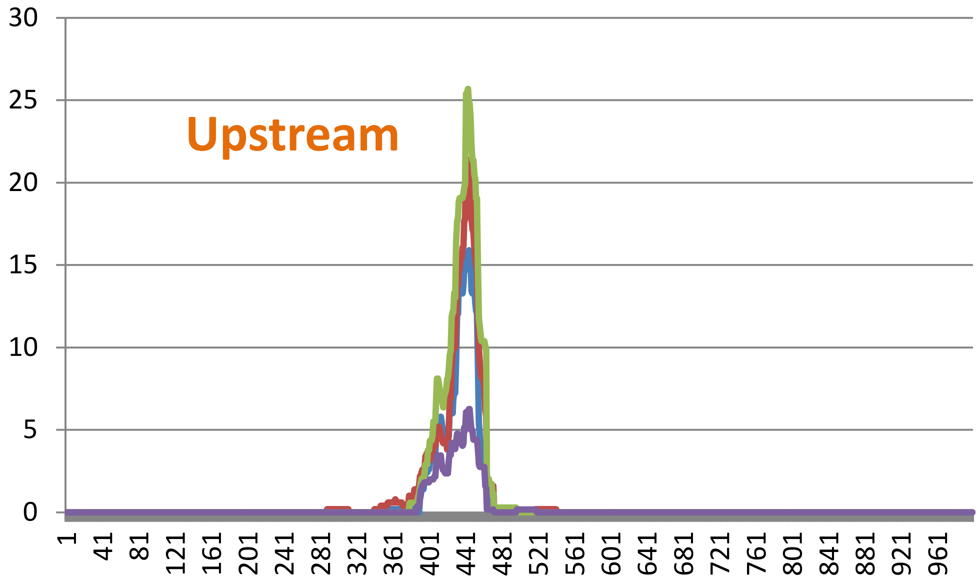

AT3G10900

Glycosyl hydrolase superfamily protein

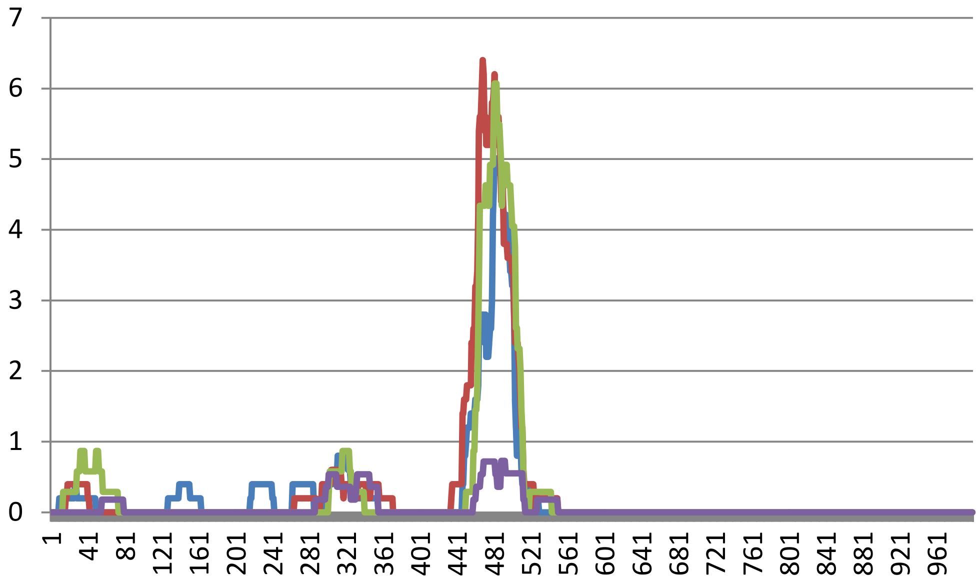

AT3G11310

Unknown protein

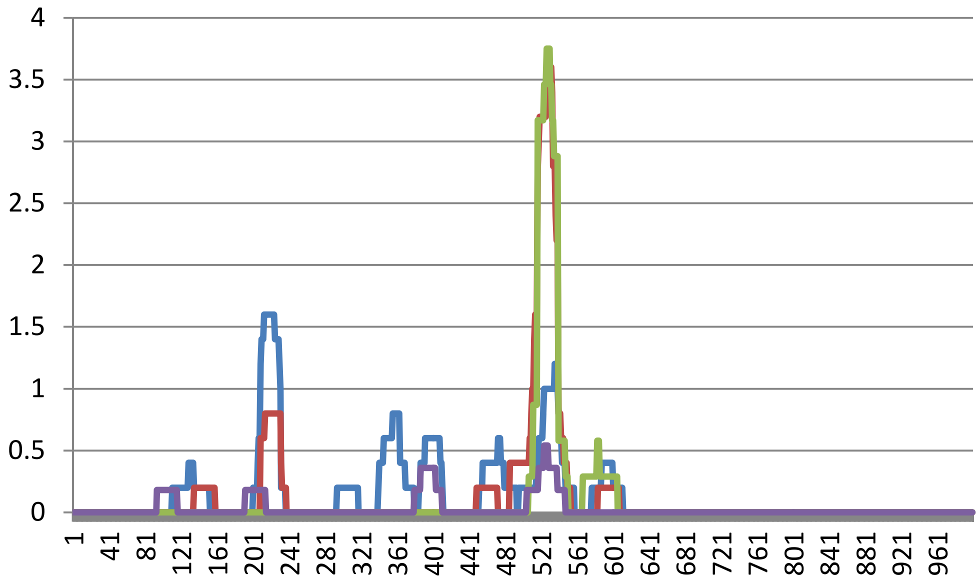

AT3G16400

Encodes a nitrile-specifier protein NSP1 responsible for constitutive and herbivore-induced simple nitrile formation in rosette leaves.

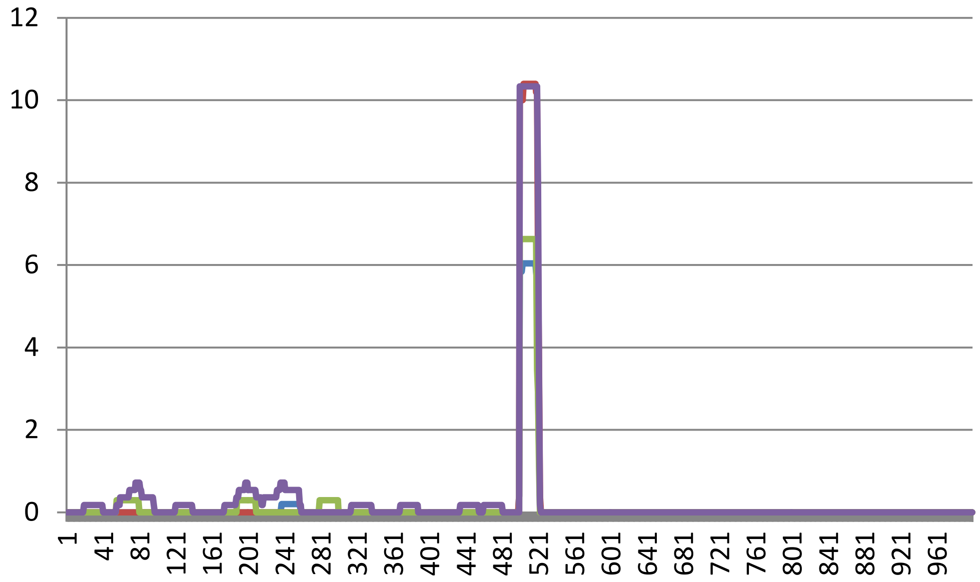

AT3G21870

Cyclin p2;1 (CYCP2;1)

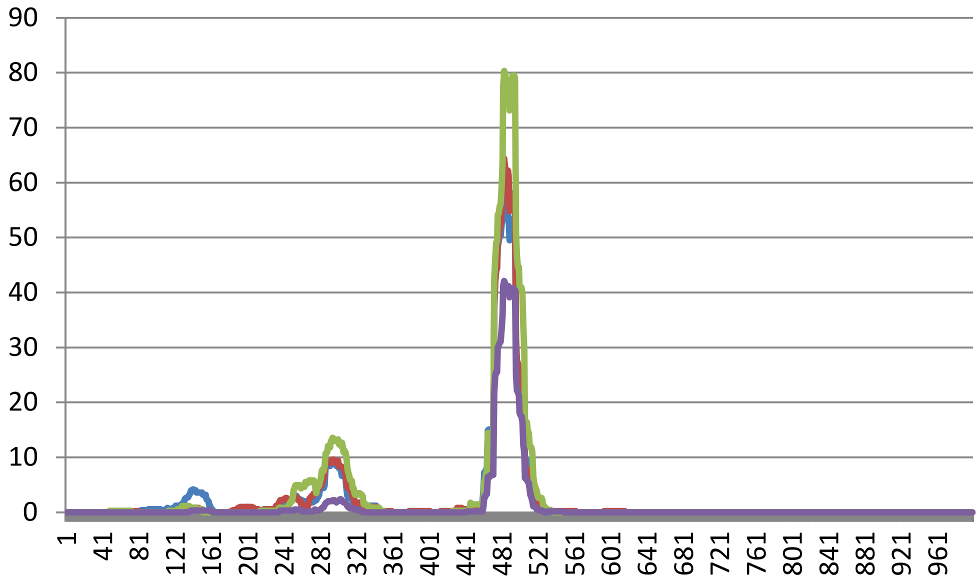

AT3G21960

Receptor-like protein kinase-related family protein

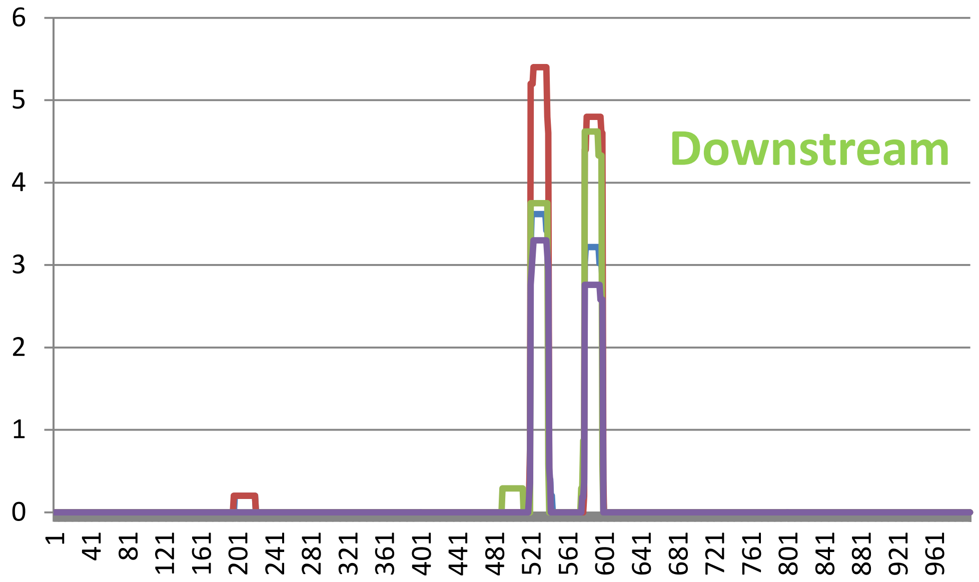

AT3G22710

F-box family protein

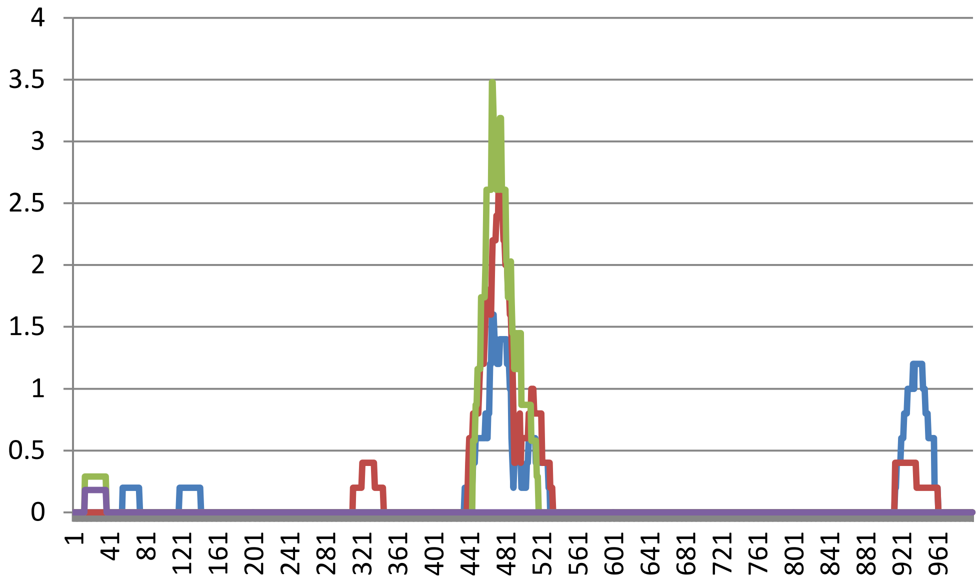

AT3G23310

AGC (cAMP-dependent, cGMP-dependent and protein kinase C) kinase family protein

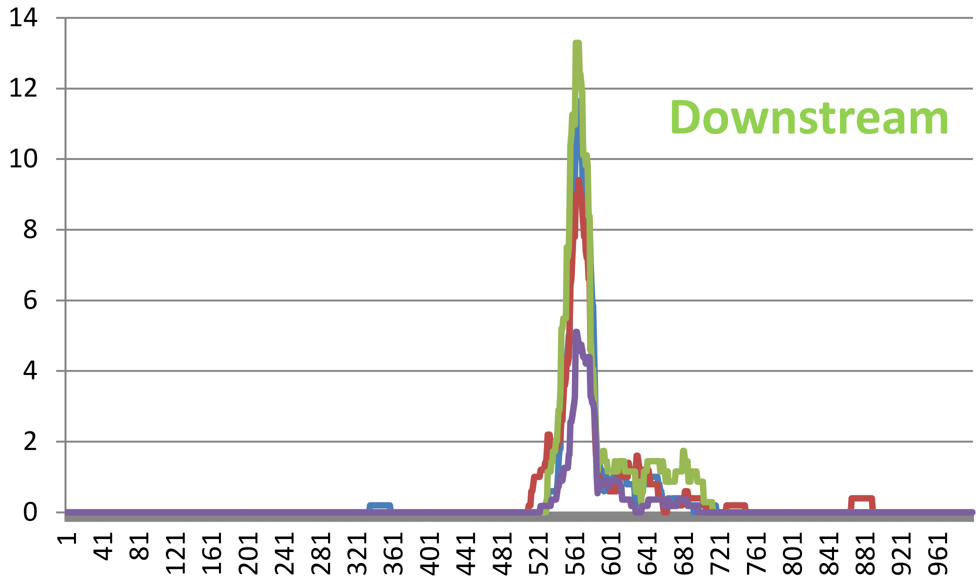

AT3G23440

EMBRYO SAC DEVELOPMENT ARREST 6 (EDA6)

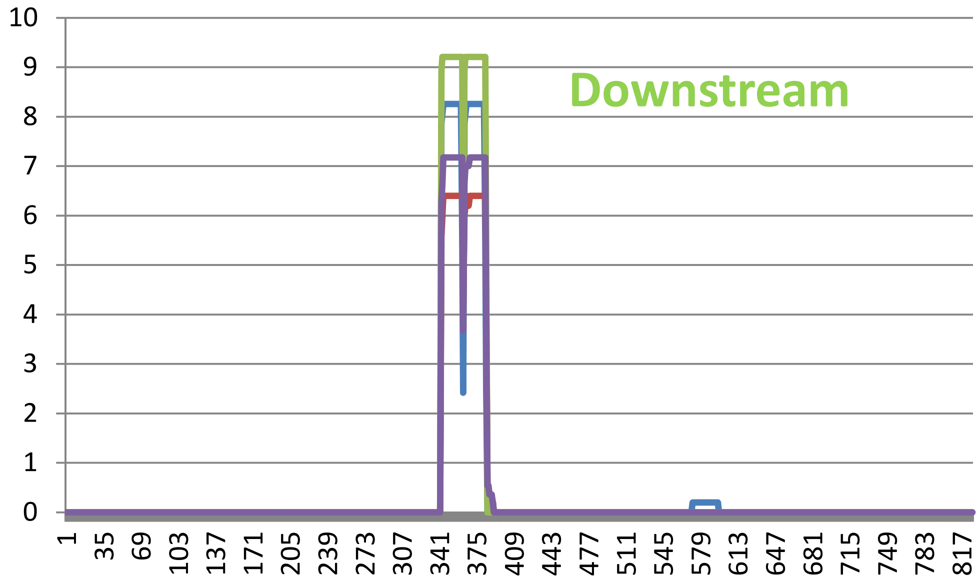

AT3G23590

Encodes a protein shown to physically associate with the conserved transcriptional coregulatory complex, Mediator, and is involved in the regulation of phenylpropanoid homeostasis.

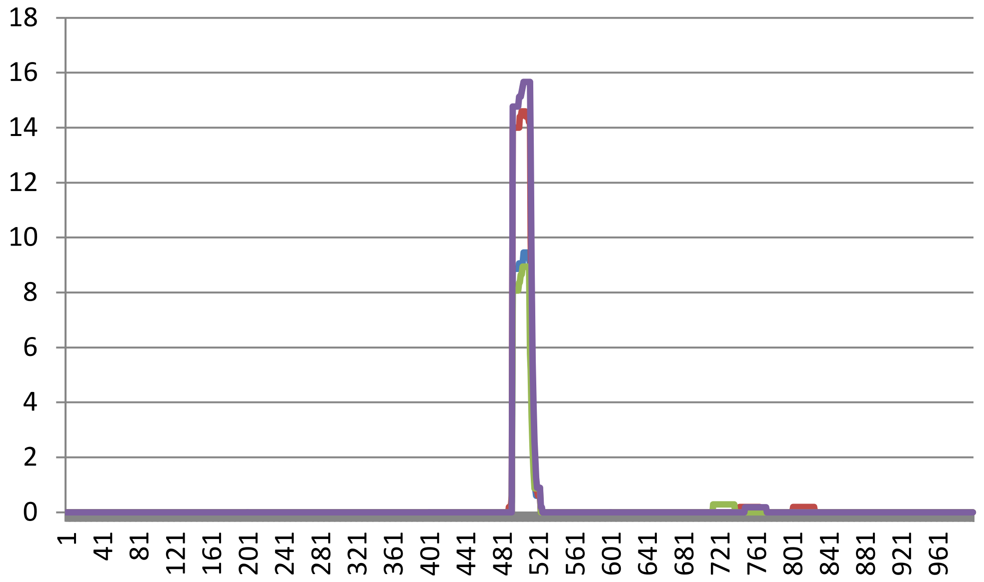

AT3G25130

Unknown protein

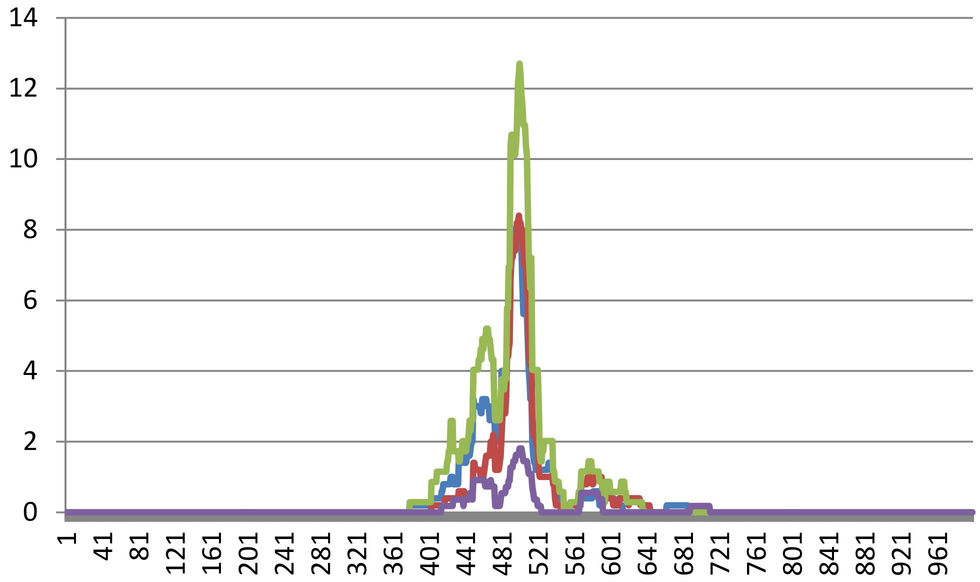

AT3G25585

Aminoalcoholphosphotransferase (AAPT2) mRNA, complete cds

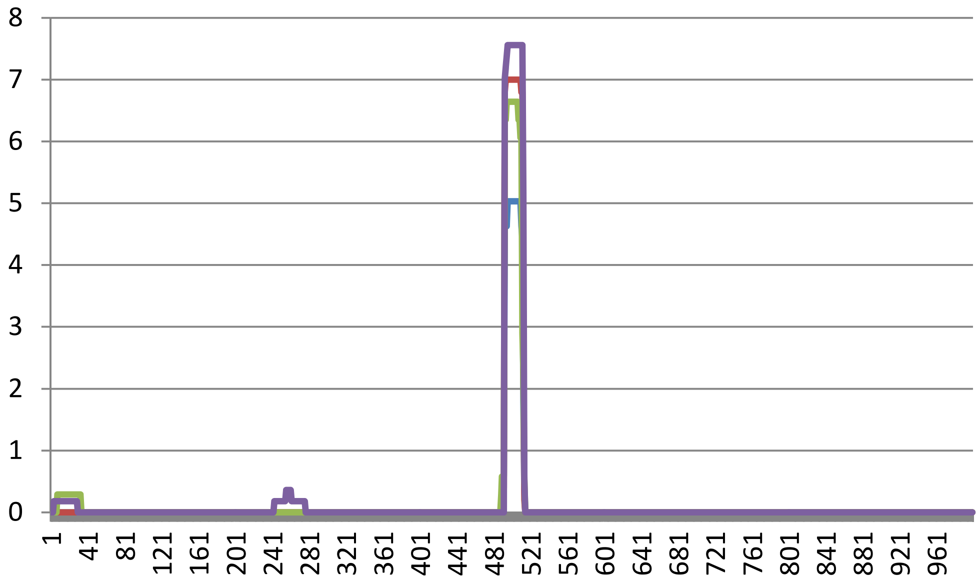

AT3G25720

RNA-directed DNA polymerase (reverse transcriptase)-related family protein

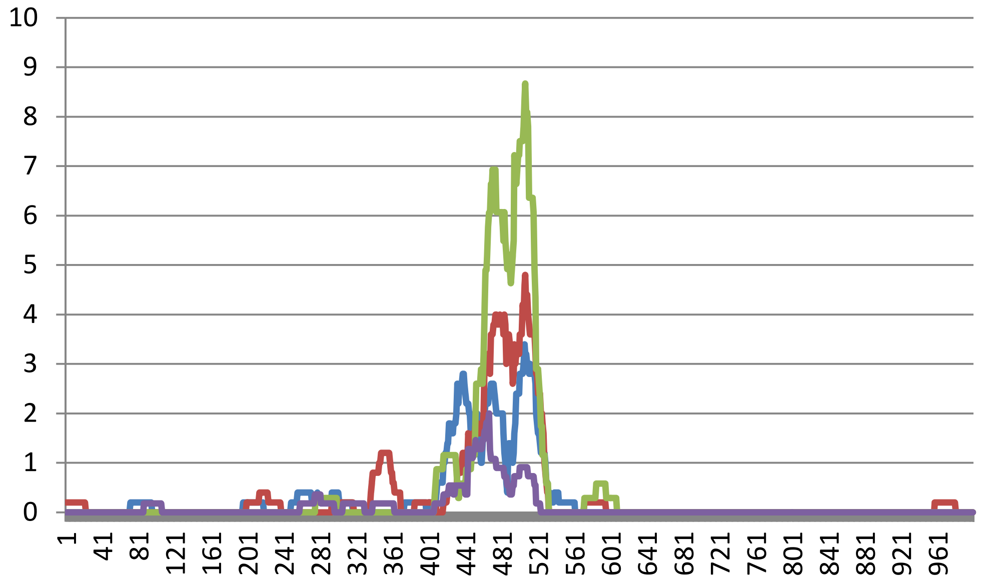

AT3G25855

Copper transport protein family

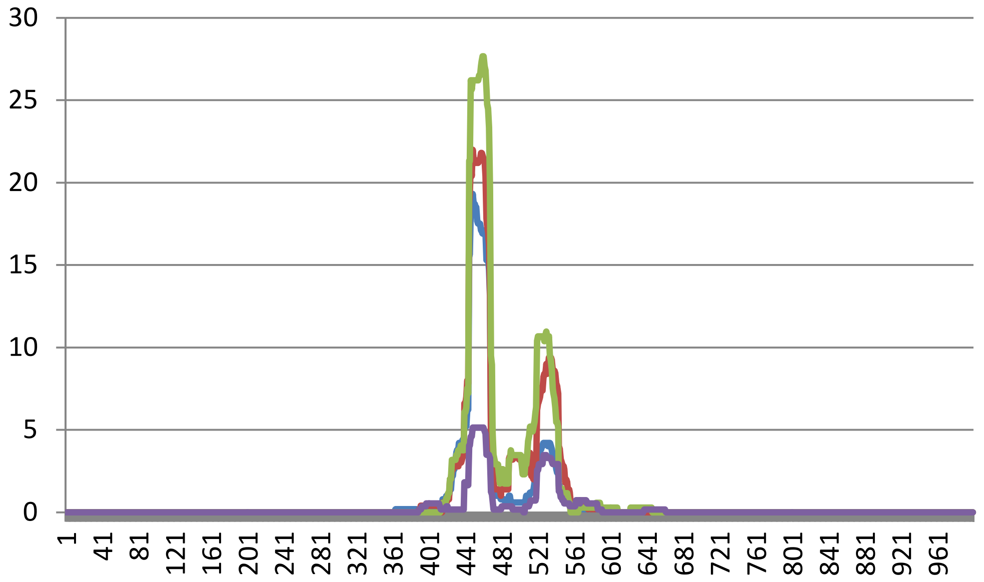

AT3G26100

Regulator of chromosome condensation (RCC1) family protein

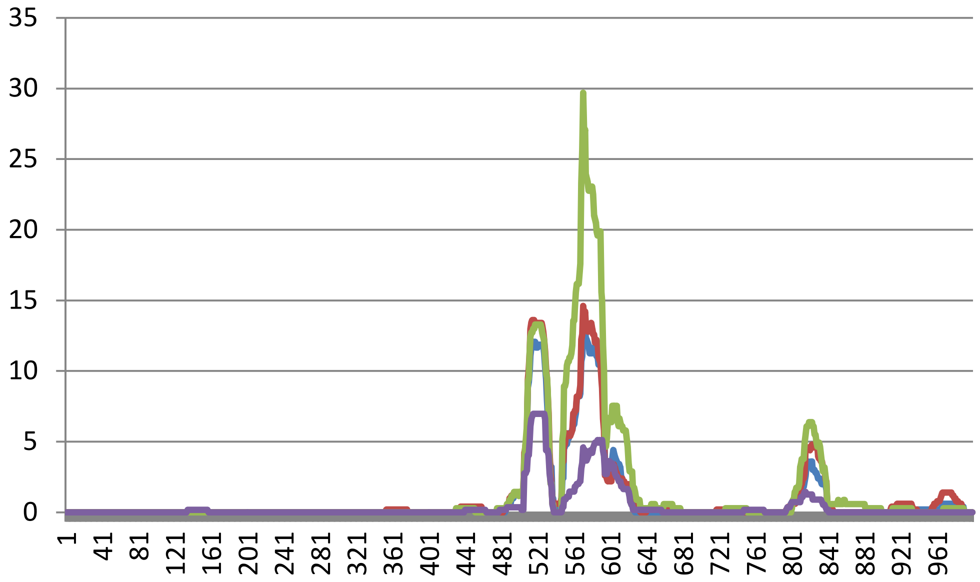

AT3G26140

Cellulase (glycosyl hydrolase family 5) protein

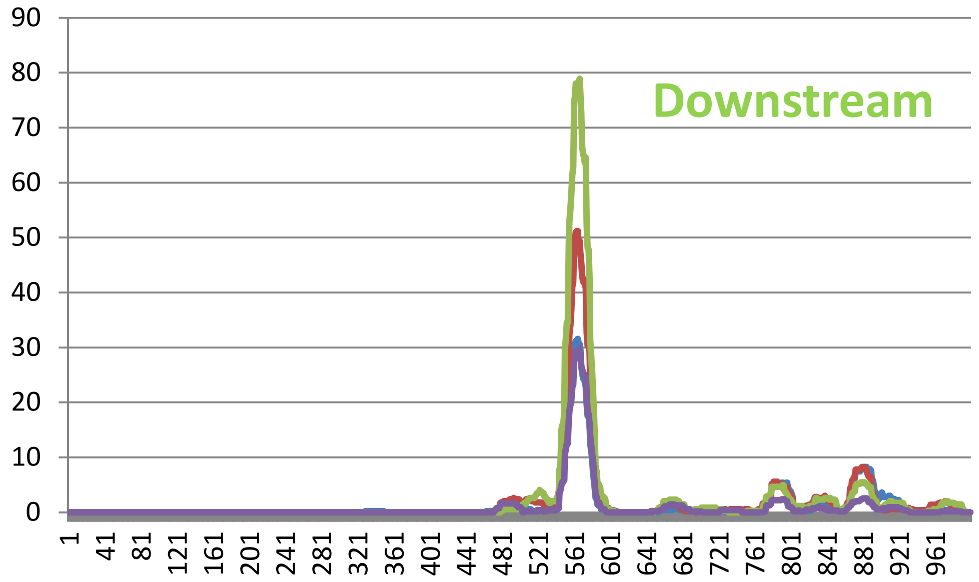

AT3G26280

Cytochrome P450 monooxygenase

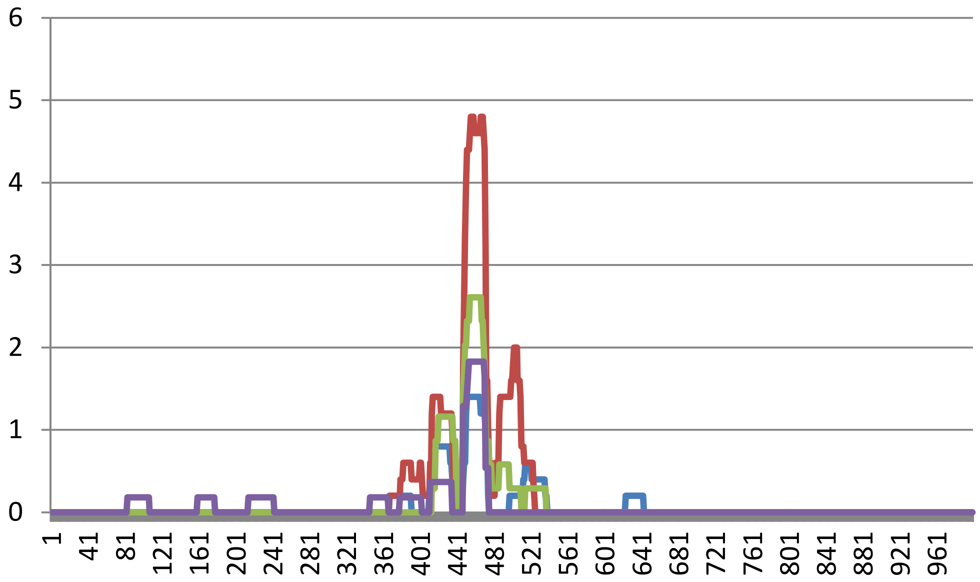

AT3G27250

Unknown protein

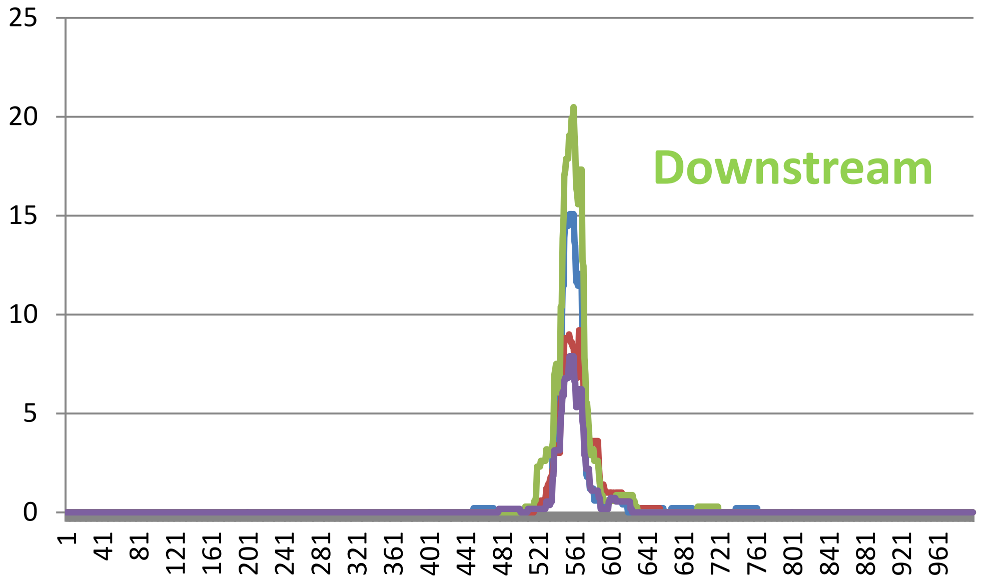

AT3G27590

Unknown protein

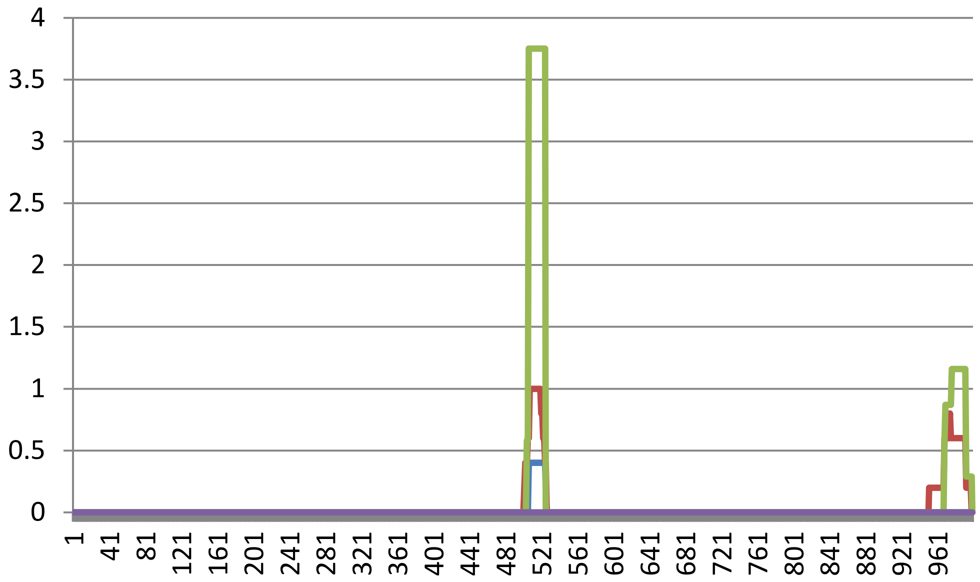

AT3G30340

Nodulin MtN21-like transporter family protein

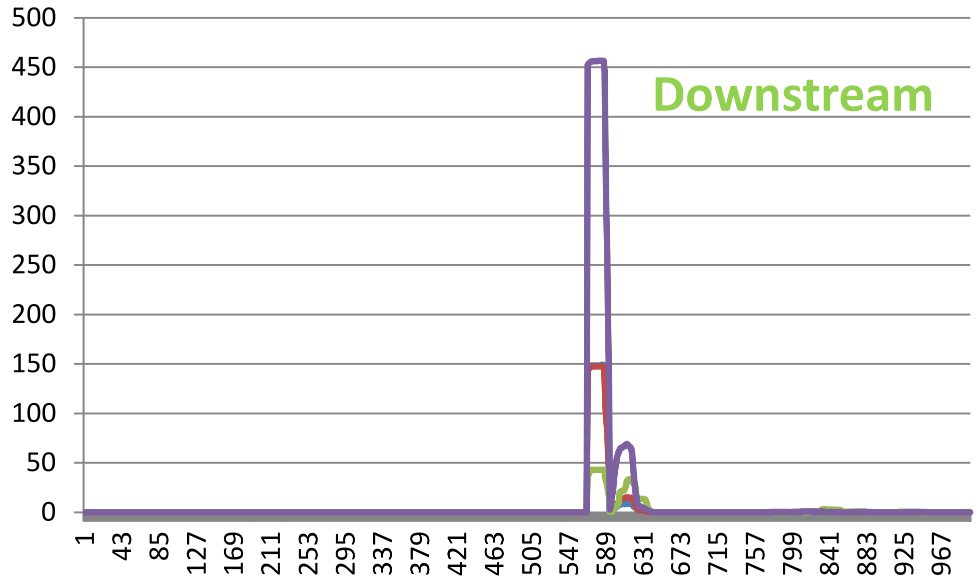

AT3G30385

Encodes a ECA1 gametogenesis related family protein

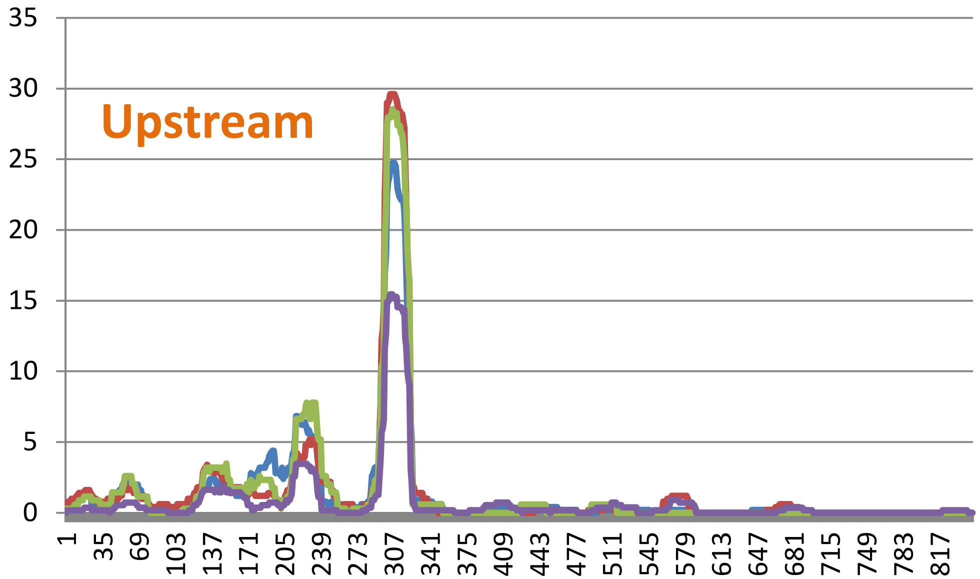

AT3G30387

Encodes a ECA1 gametogenesis related family protein

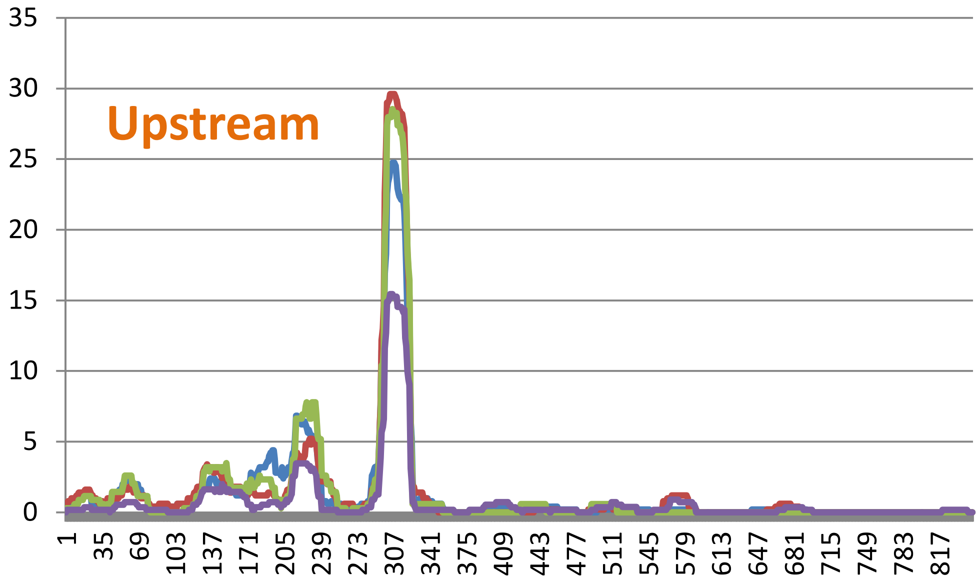

AT3G31402

General transcription factor 2-related zinc finger protein

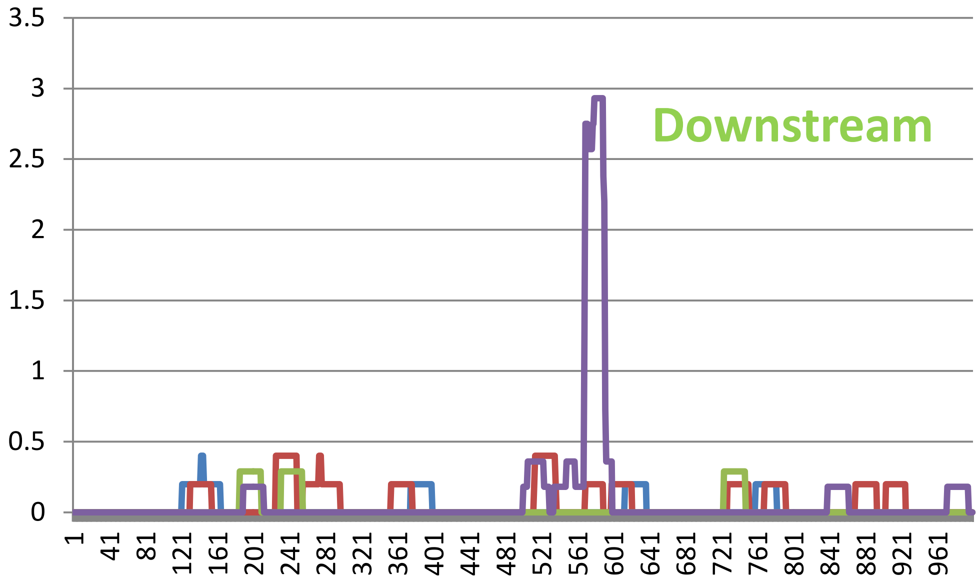

AT3G32050

Unknown protein

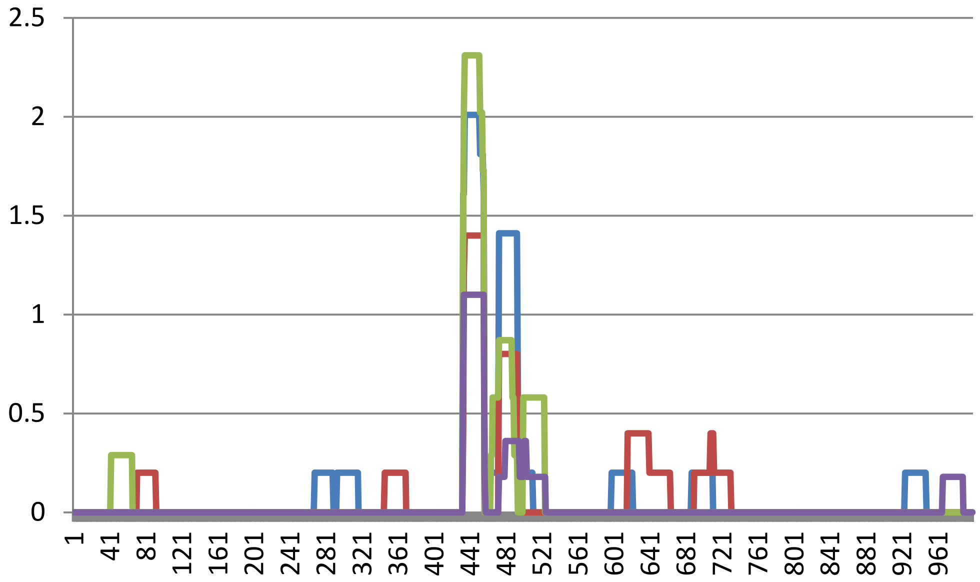

AT3G41762

Unknown protein

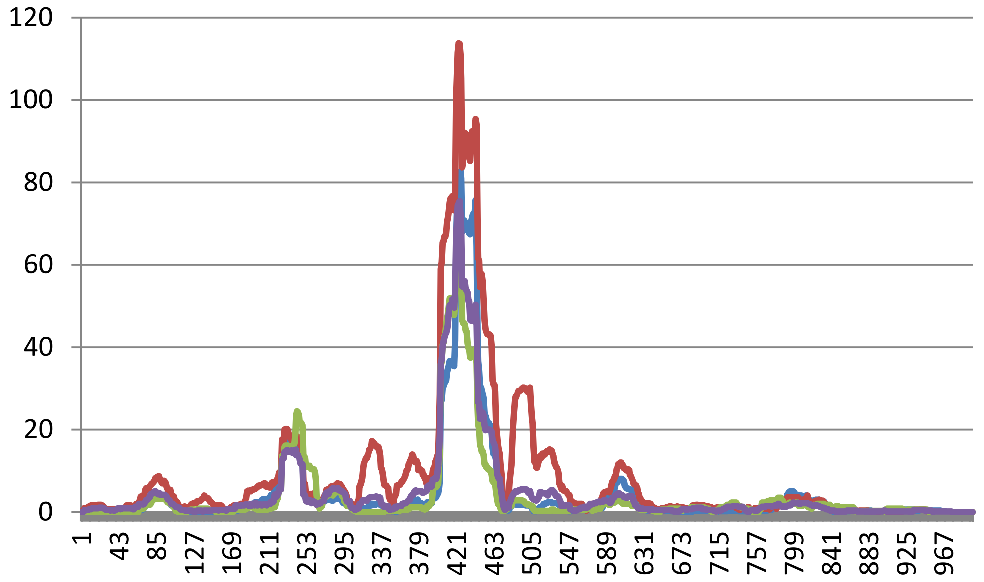

AT3G43420

Unknown protein

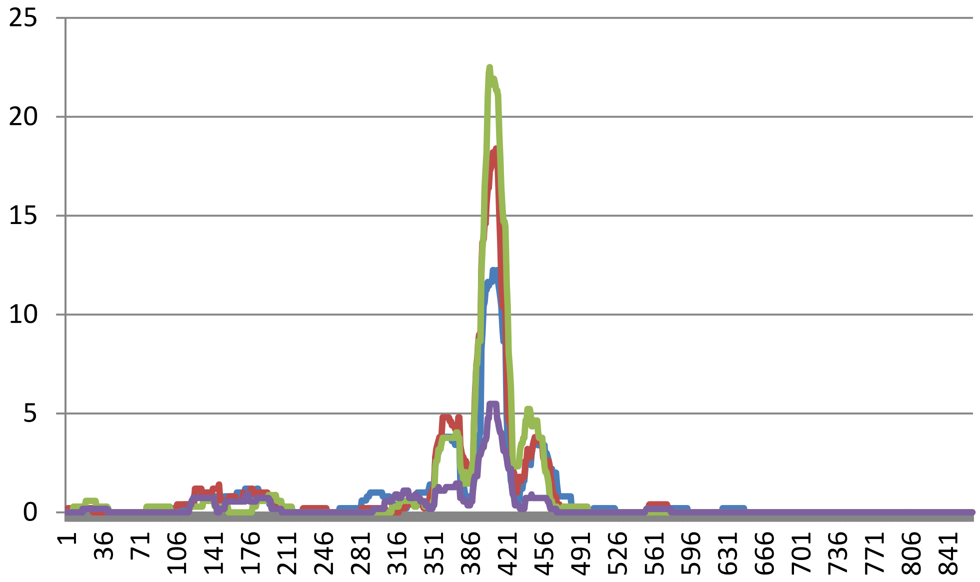

AT3G44810

F-box family protein

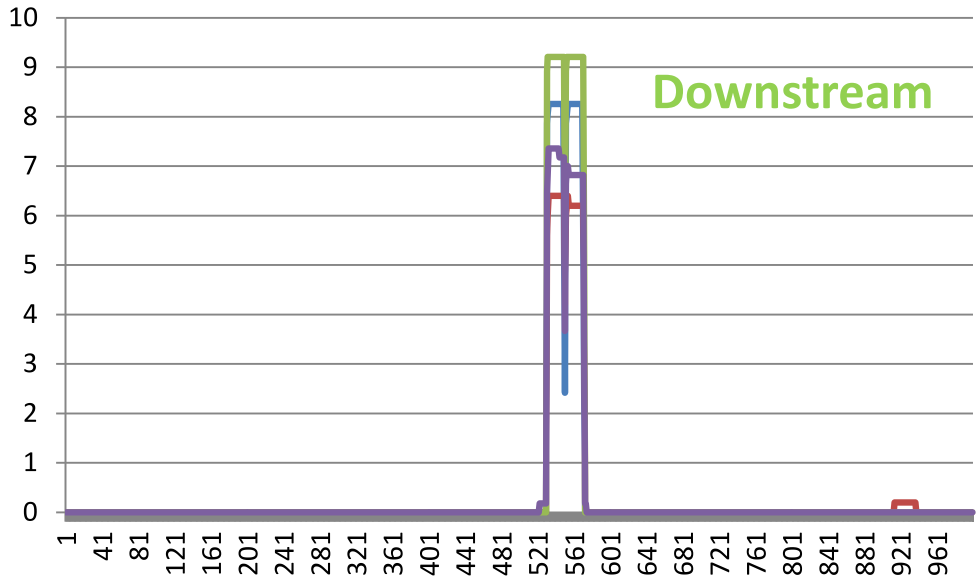

AT3G44980

Unknown protein

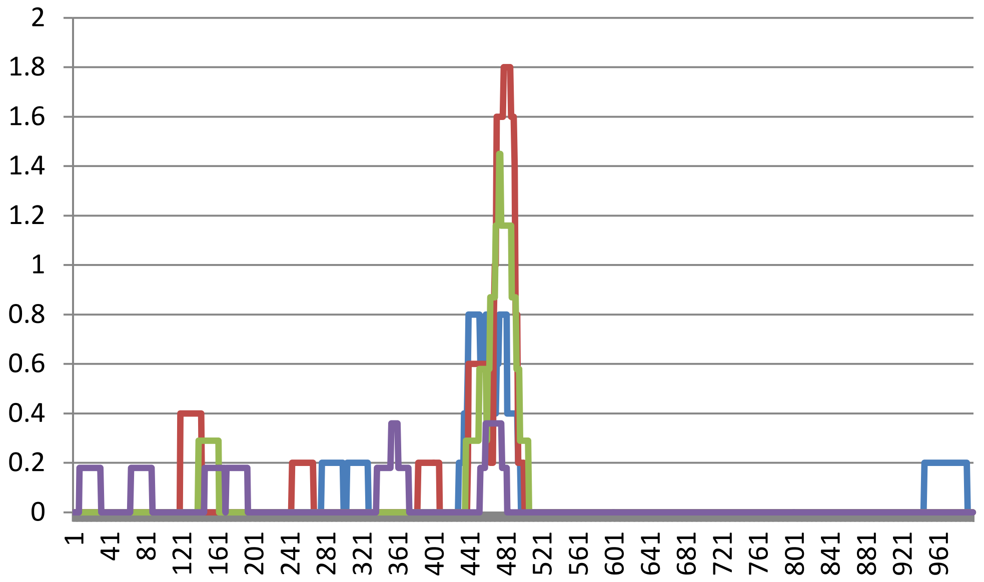

AT3G45260

C2H2-like zinc finger protein

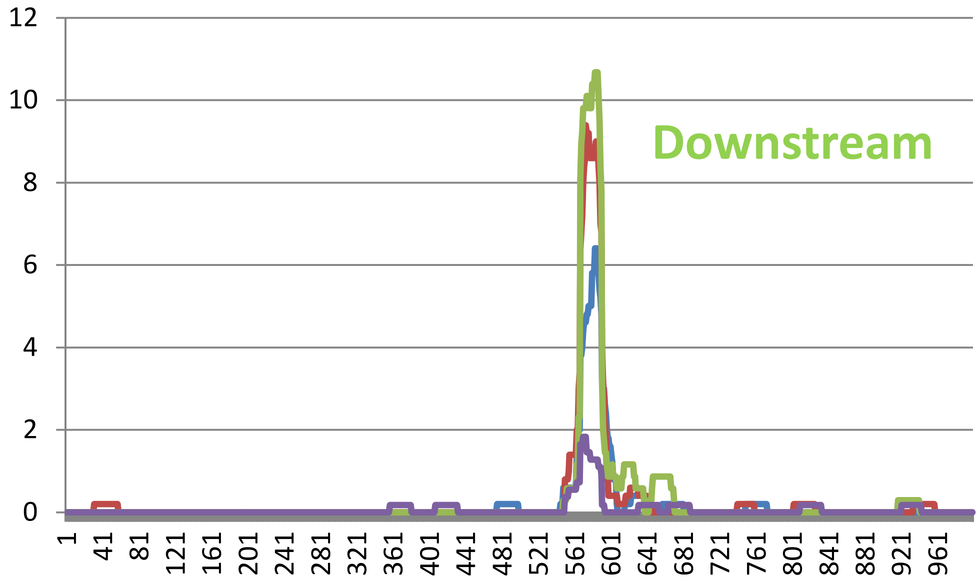

AT3G45460

IBR domain containing protein

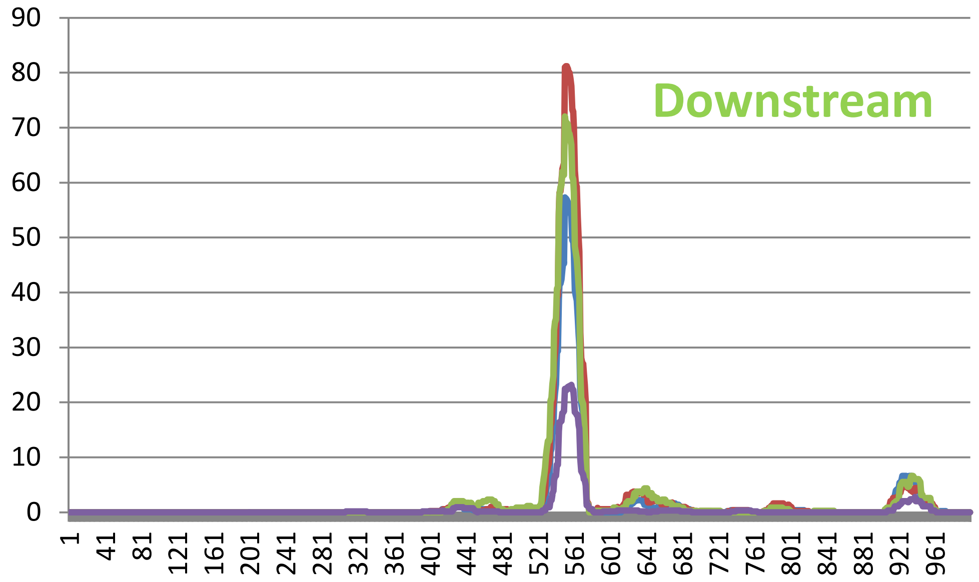

AT3G47920

Unknown protein

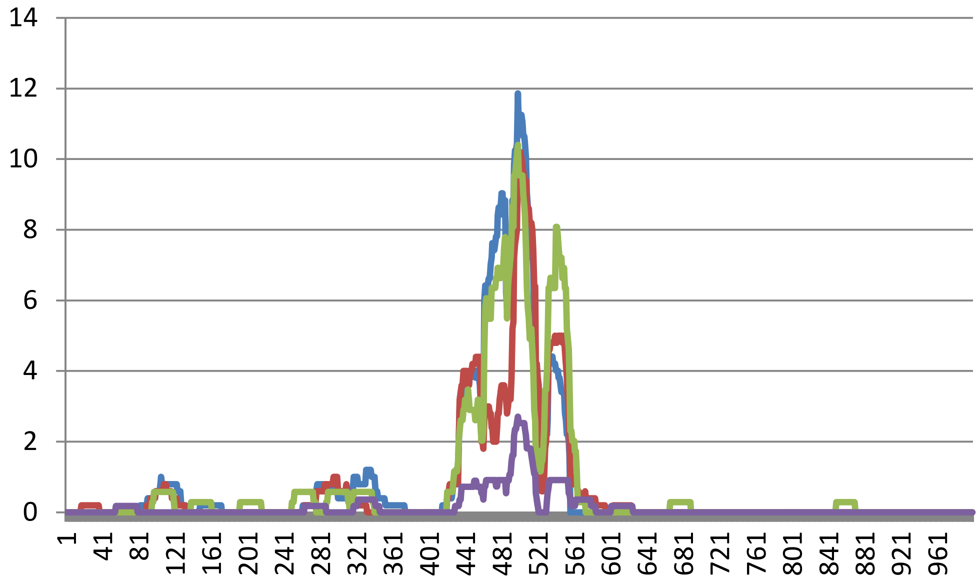

AT3G49810

ARM repeat superfamily protein

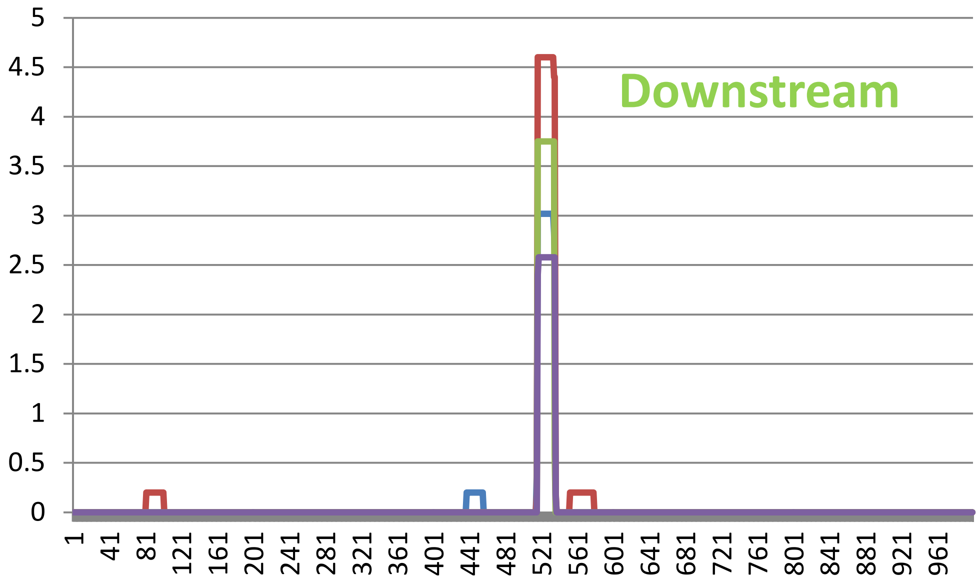

AT3G50300

HXXXD-type acyl-transferase family protein

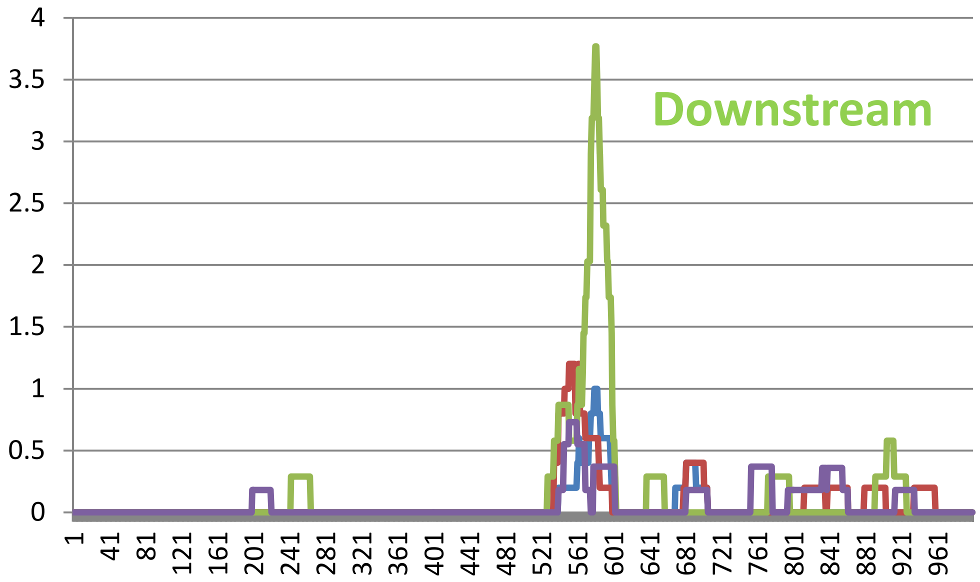

AT3G50510

LOB domain-containing protein 28 (LBD28)

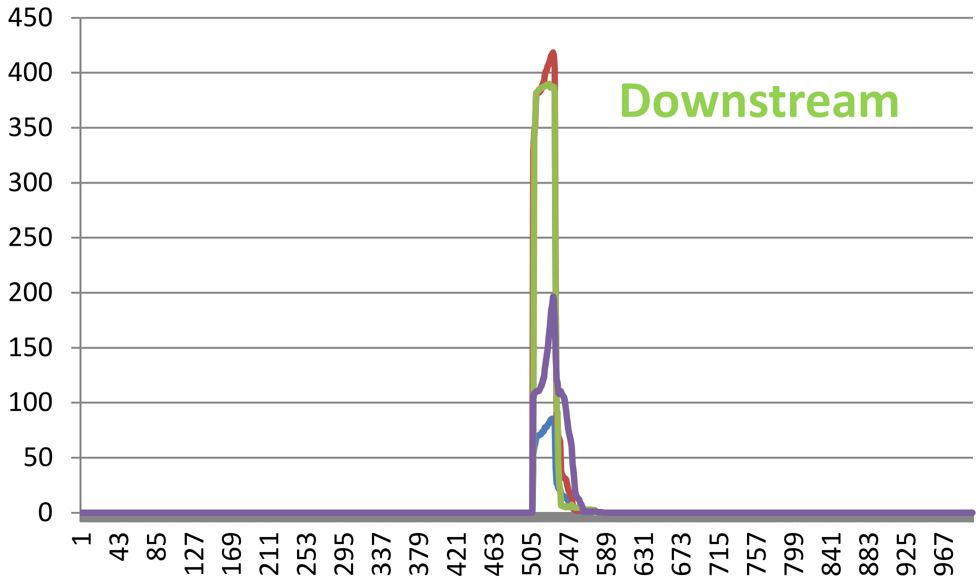

AT3G50540

Unknown protein

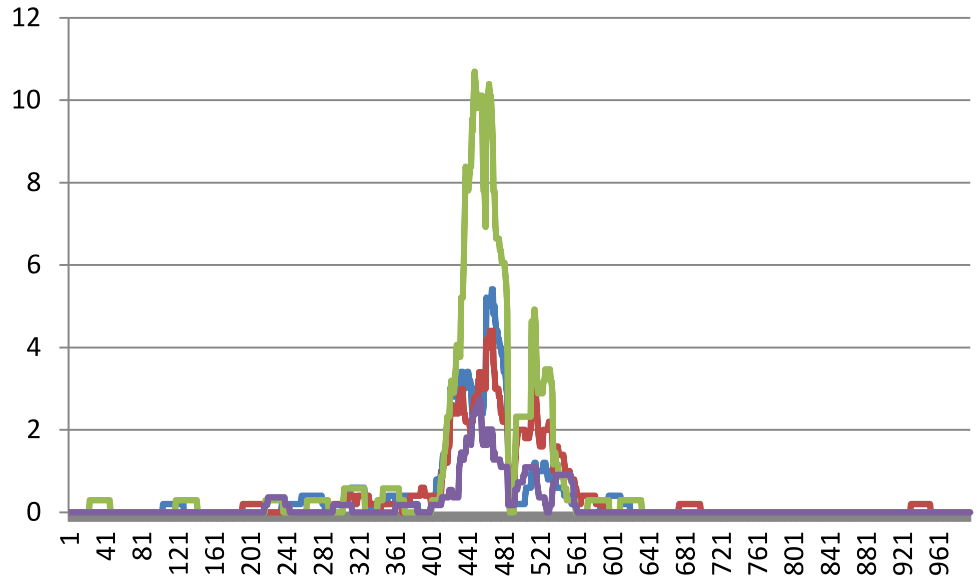

AT3G50840

Phototropic-responsive NPH3 family protein

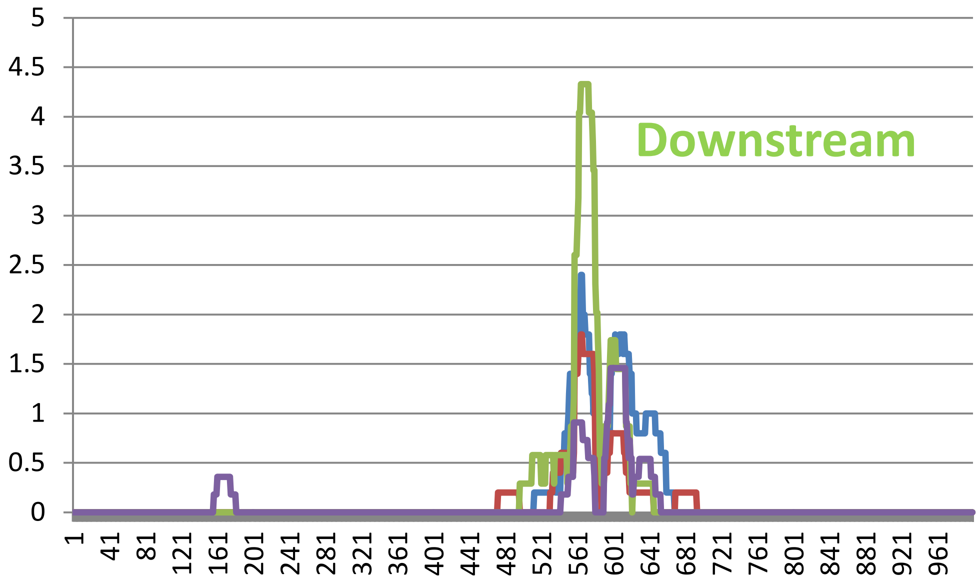

AT3G52830

BEST Arabidopsis thaliana protein match is: Ankyrin repeat family protein (TAIR:AT5G54700.1)

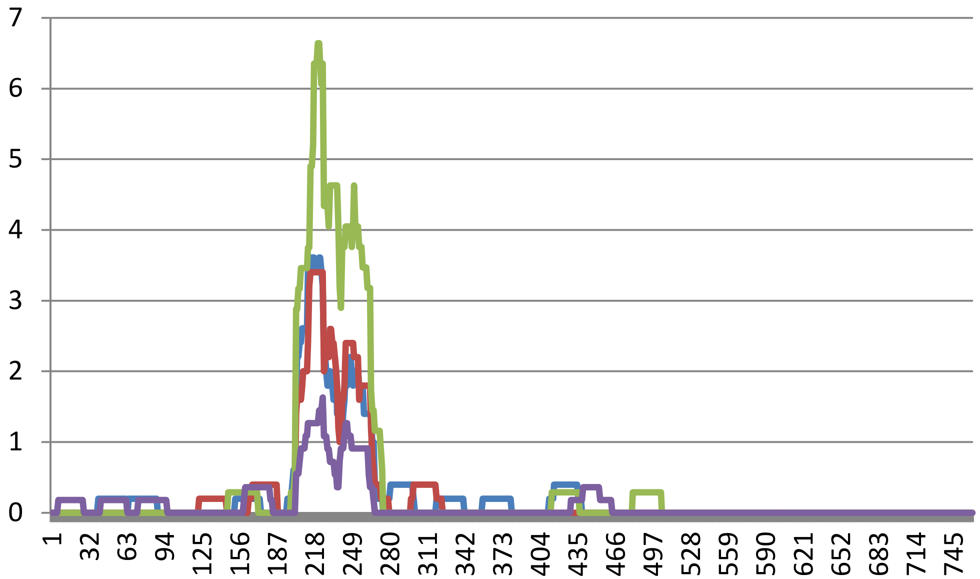

AT3G53280

Cytochrome P450 monooxygenase

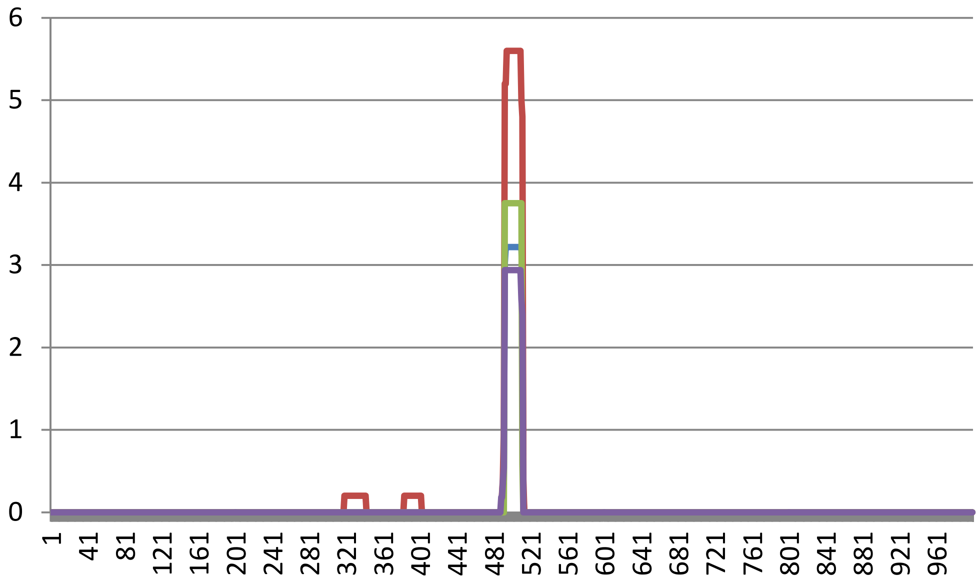

AT3G54150

S-adenosyl-L-methionine-dependent methyltransferases superfamily protein

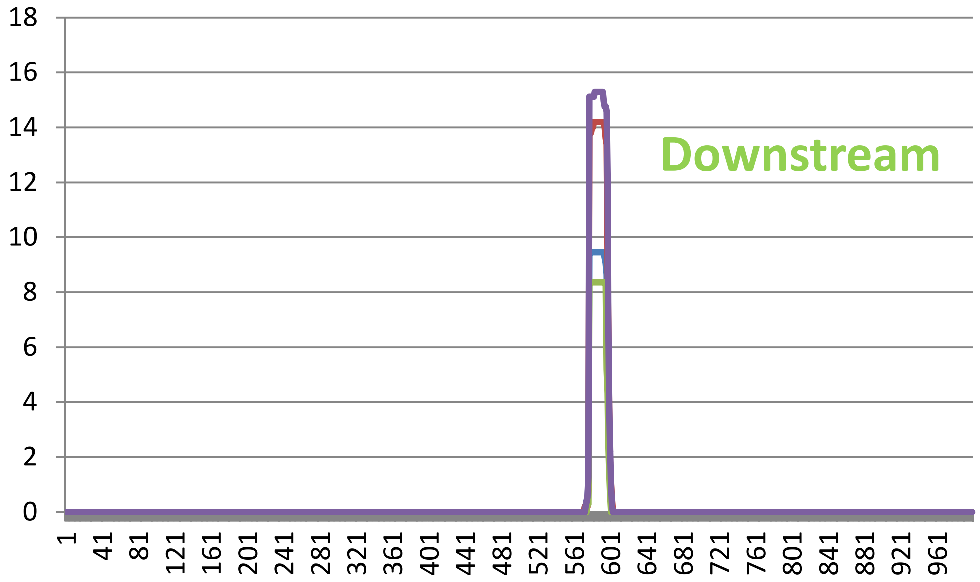

AT3G54730

BEST Arabidopsis thaliana protein match is: ovate family protein 9 (TAIR:AT4G04030.1)

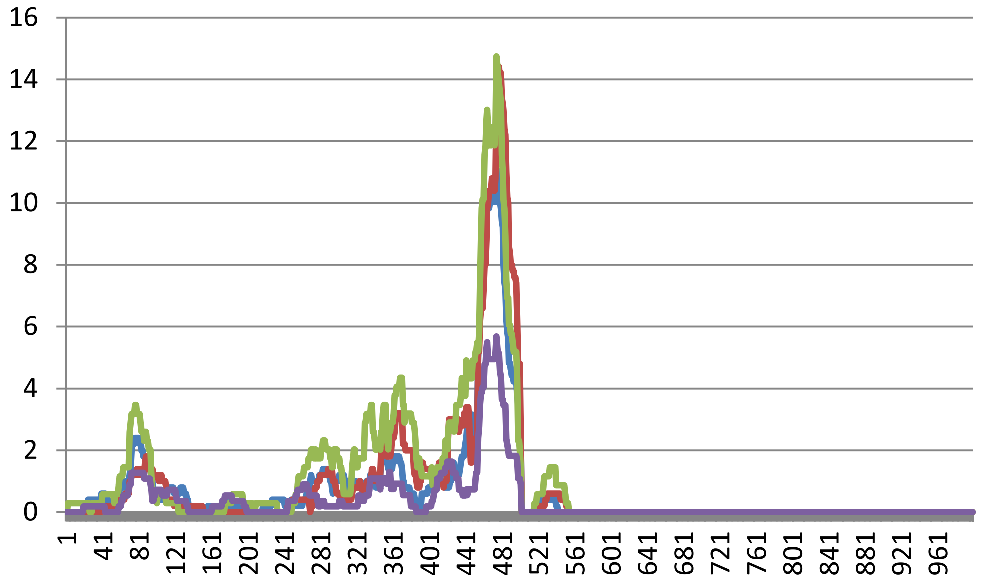

AT3G55672

Plant self-incompatibility protein S1 family

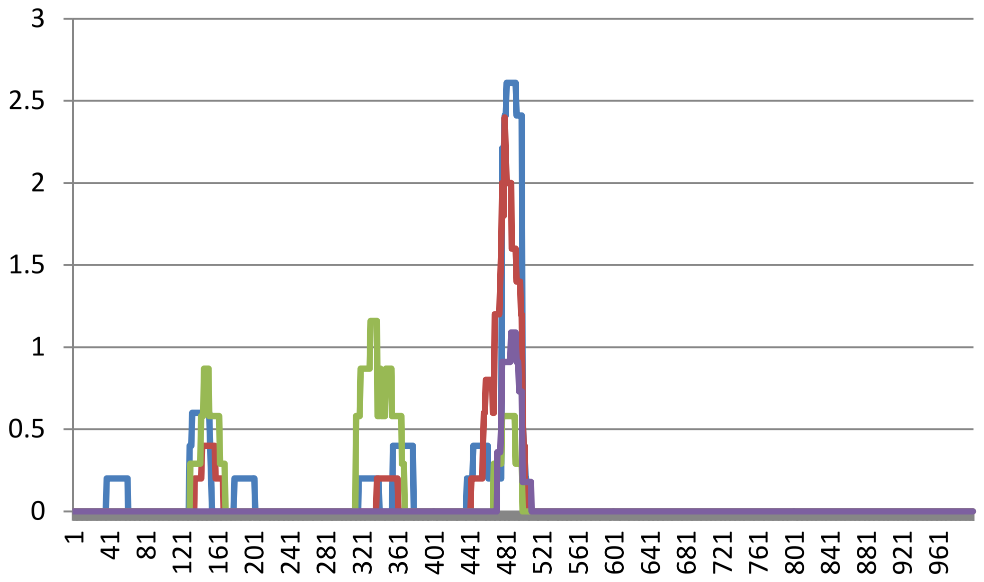

AT3G55730

Putative transcription factor MYB109 (MYB109) mRNA

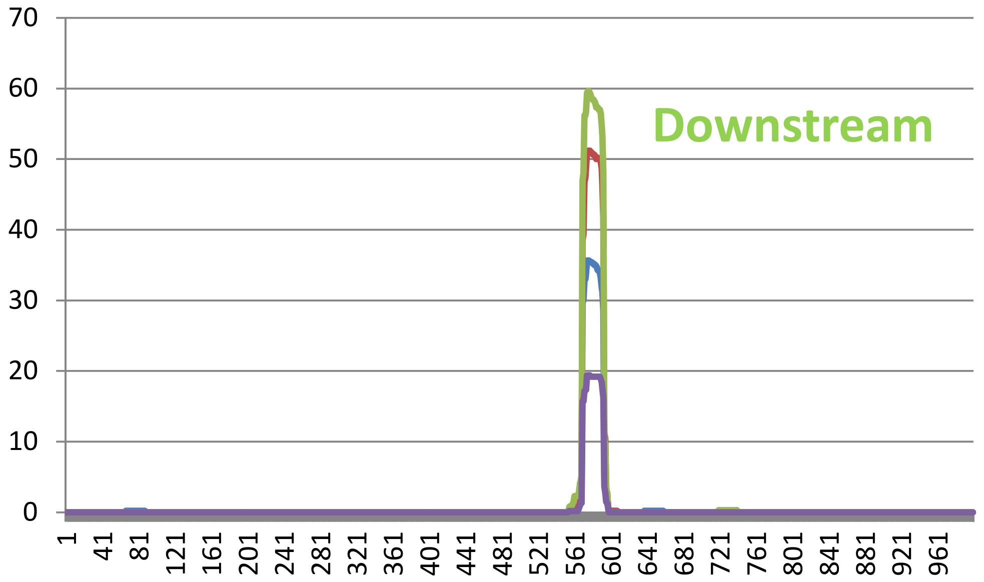

AT3G59580

Plant regulator RWP-RK family protein

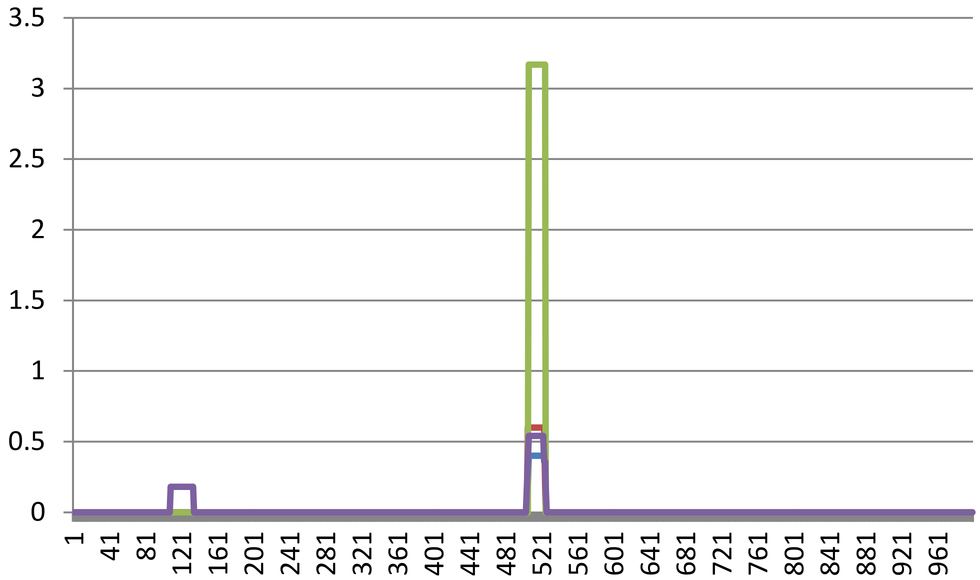

AT4G00080

Unfertilized embryo sac 11 (UNE11)

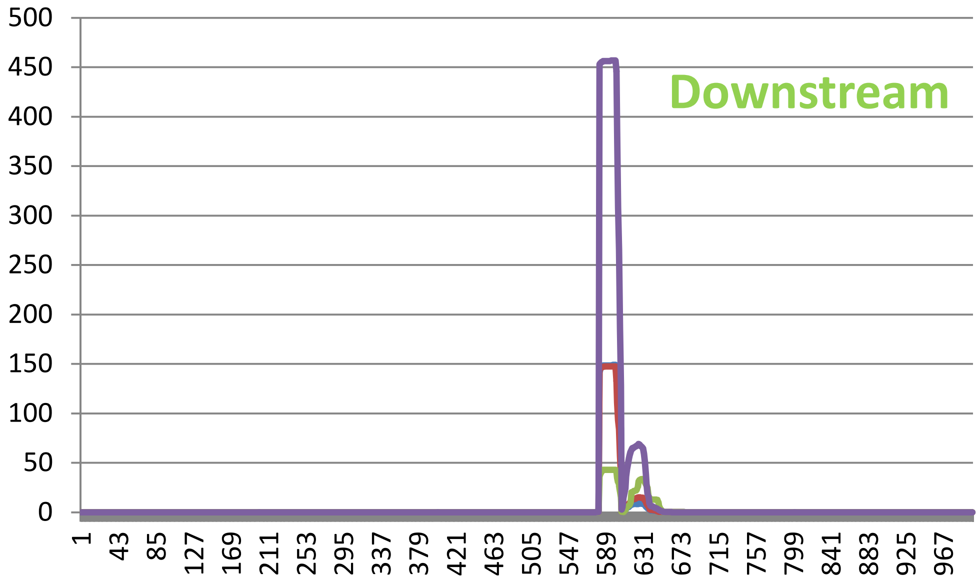

AT4G00120

INDEHISCENT (IND)

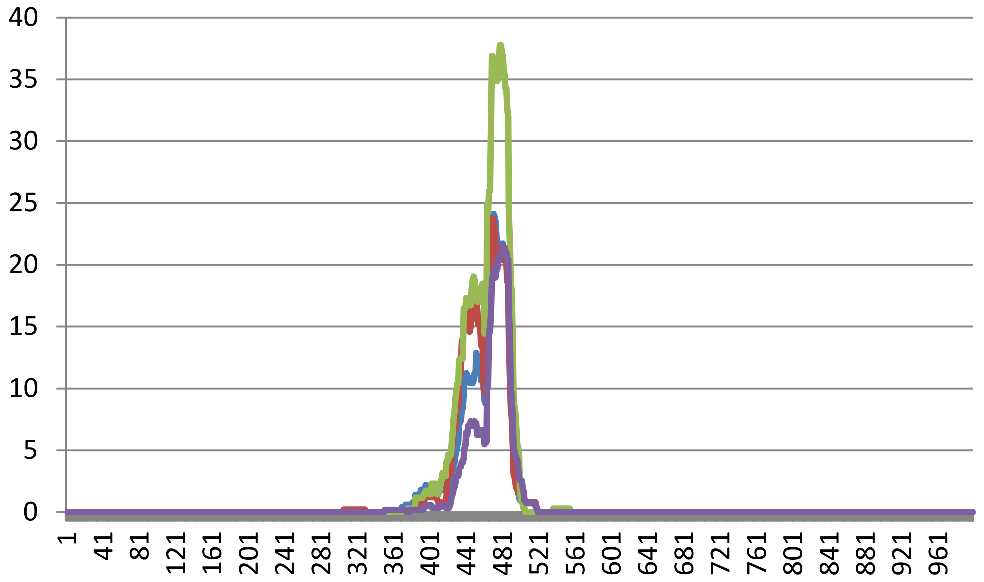

AT4G01870

tolB protein-related

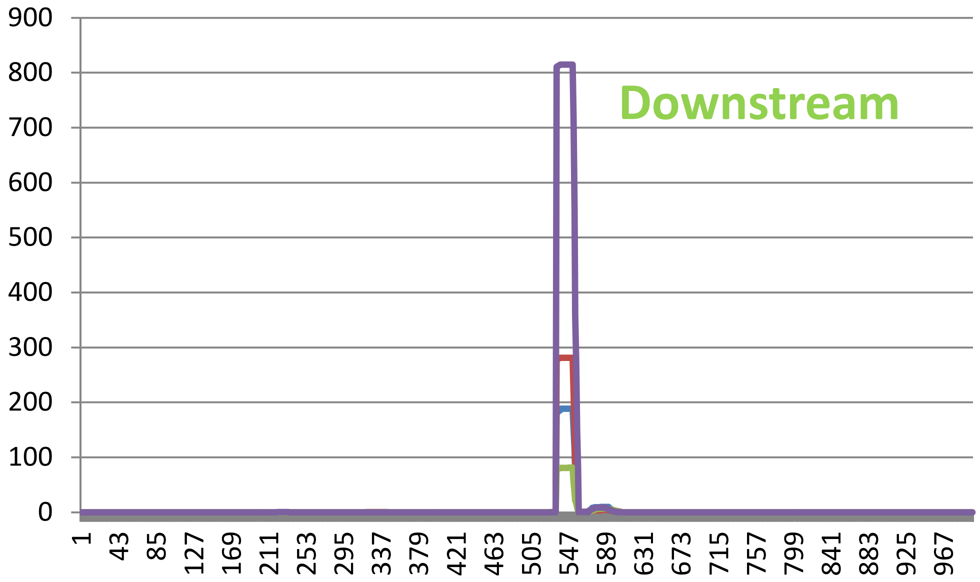

AT4G01900

Encodes a PII protein that may function as part of a signal transduction network involved in perceiving the status of carbon and organic nitrogen. Forms a protein complex with N-acetylglutamate kinase and regulates the kinase activity by relieving the feedback inhibition of the kinase by arginine. Regulates acetyl-CoA carboxylase activity.

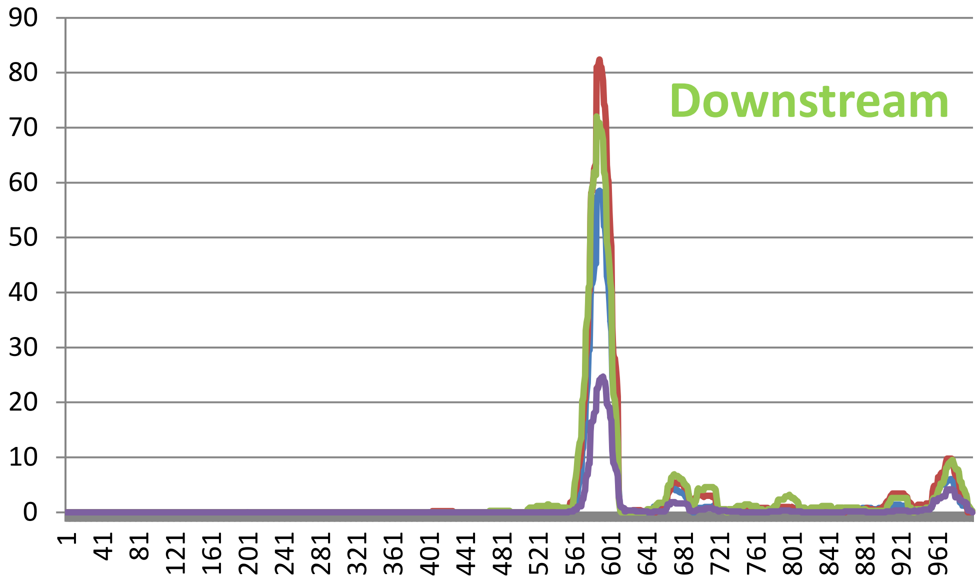

AT4G03160

BEST Arabidopsis thaliana protein match is: AP2/B3-like transcriptional factor family protein (TAIR:AT4G03170.1)

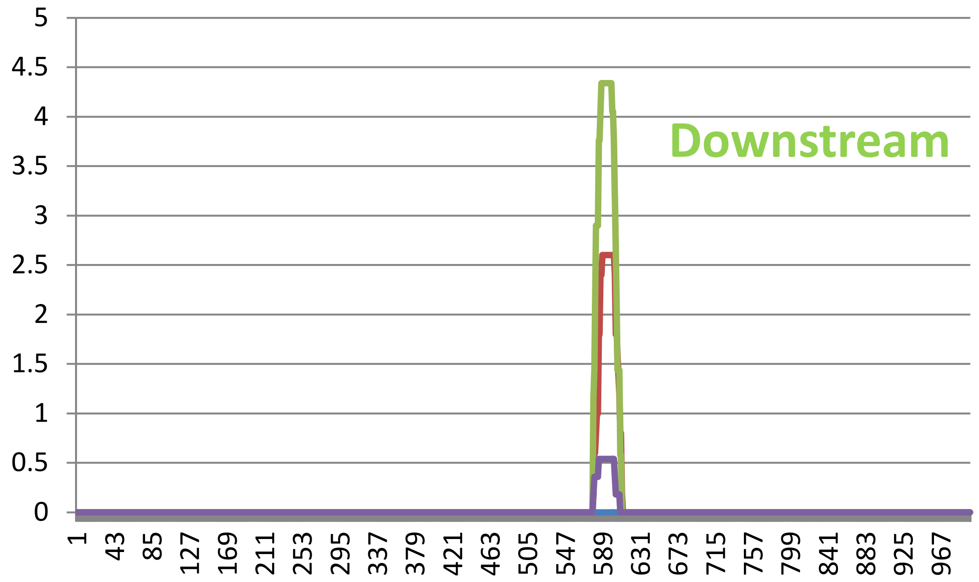

AT4G03165

Unknown protein

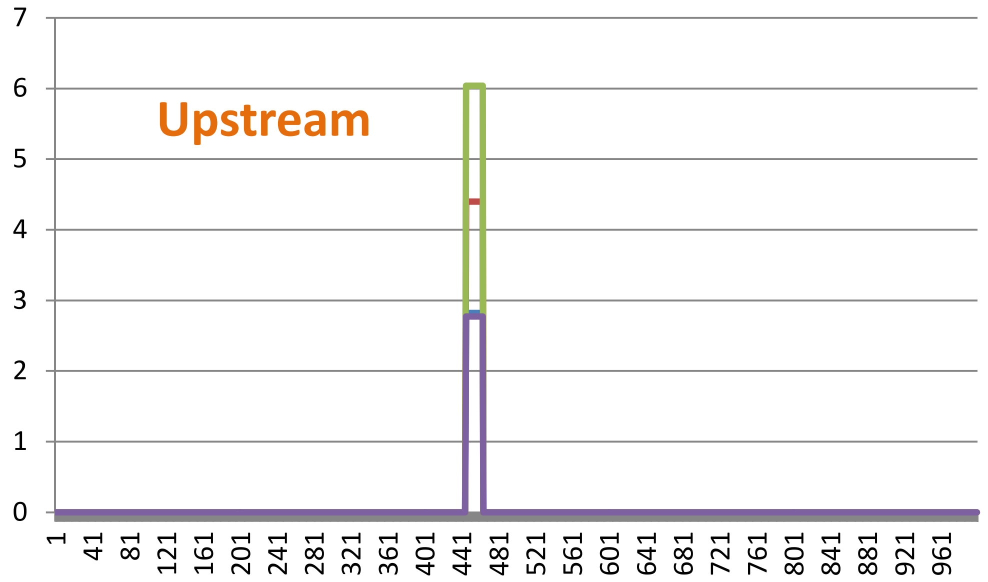

AT4G03930

Plant invertase/pectin methylesterase inhibitor superfamily

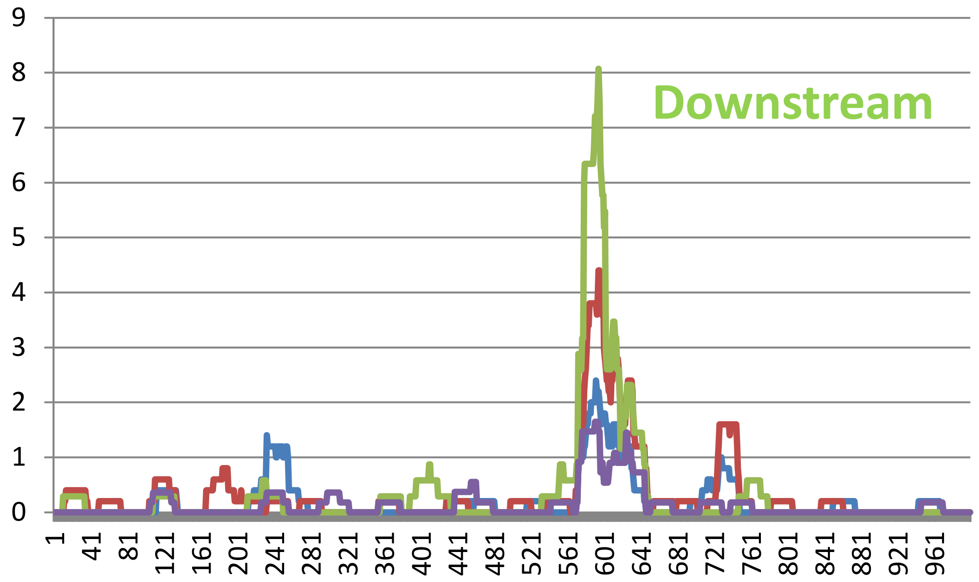

AT4G04030

Ovate family protein 9 (OFP9)

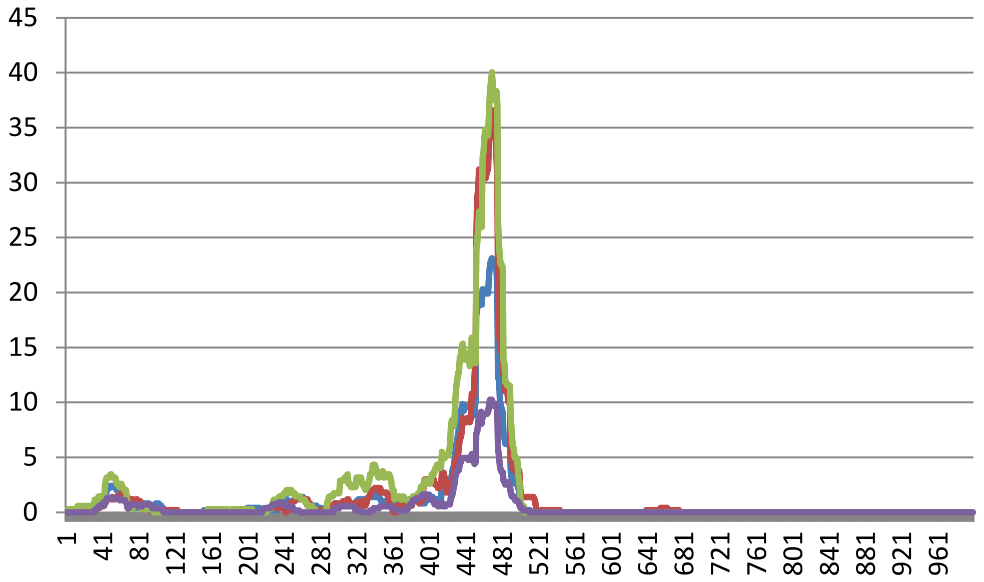

AT4G04925

Unknown protein

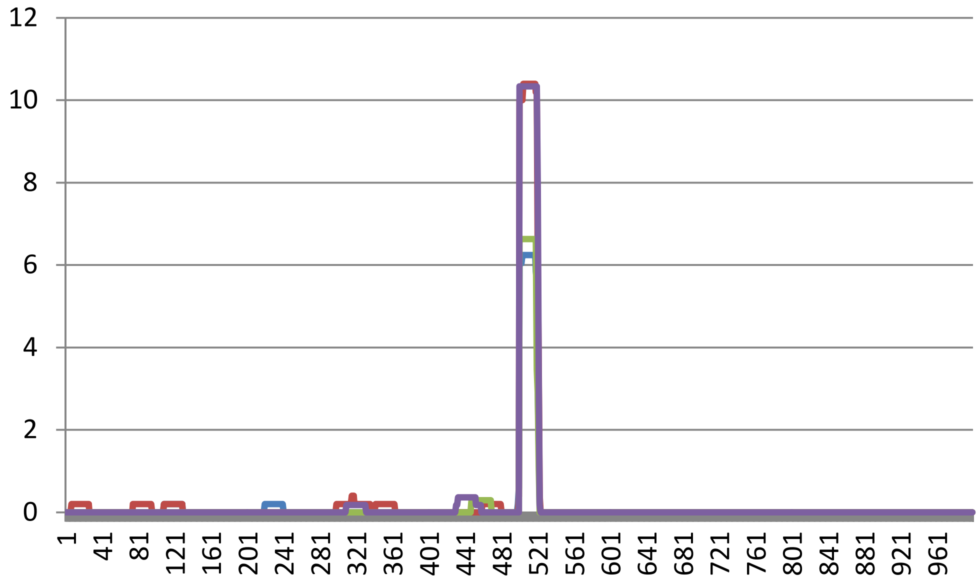

AT4G04970

Encodes a gene similar to callose synthase

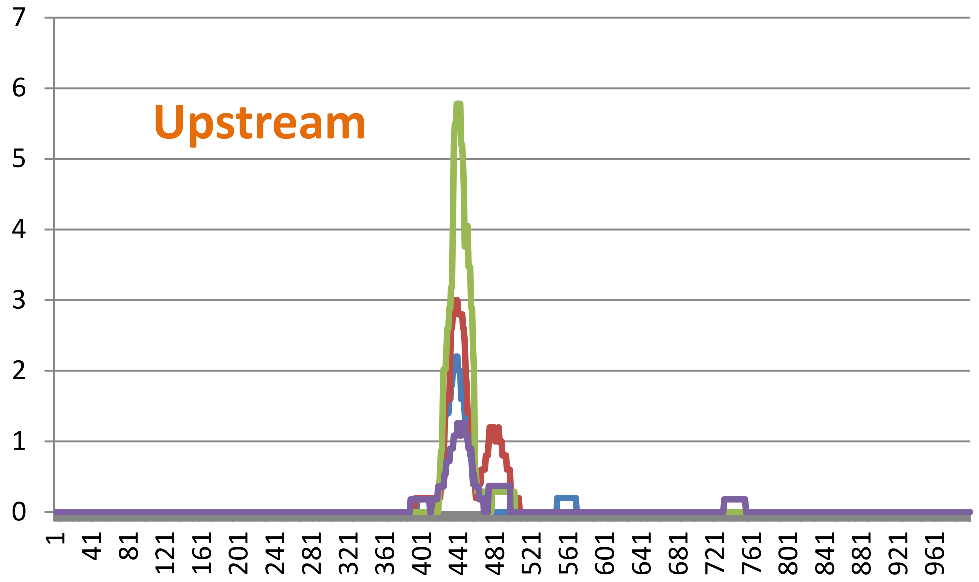

AT4G08039

Encodes a defensin-like (DEFL) family protein.

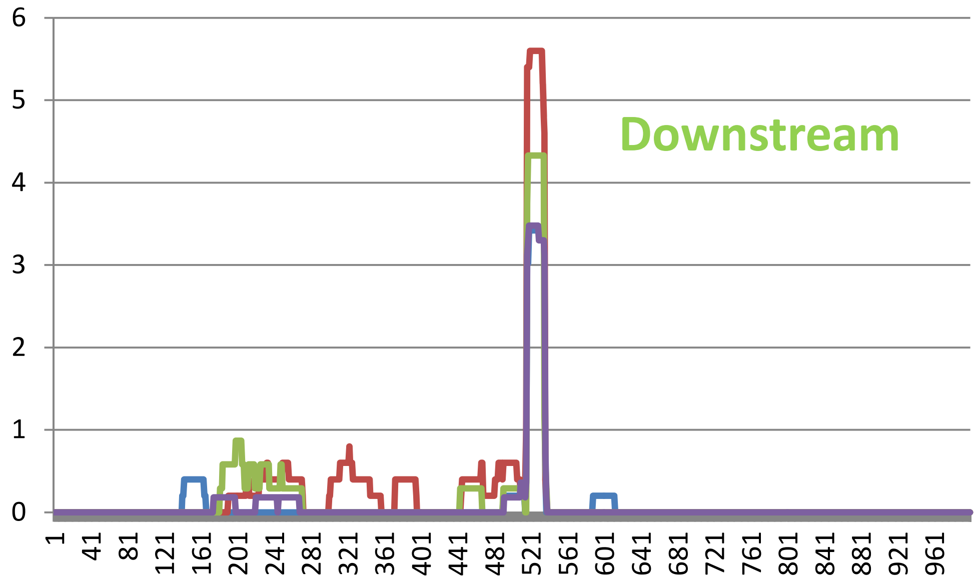

AT4G08160

Encodes a putative glycosyl hydrolase family 10 protein (xylanase).

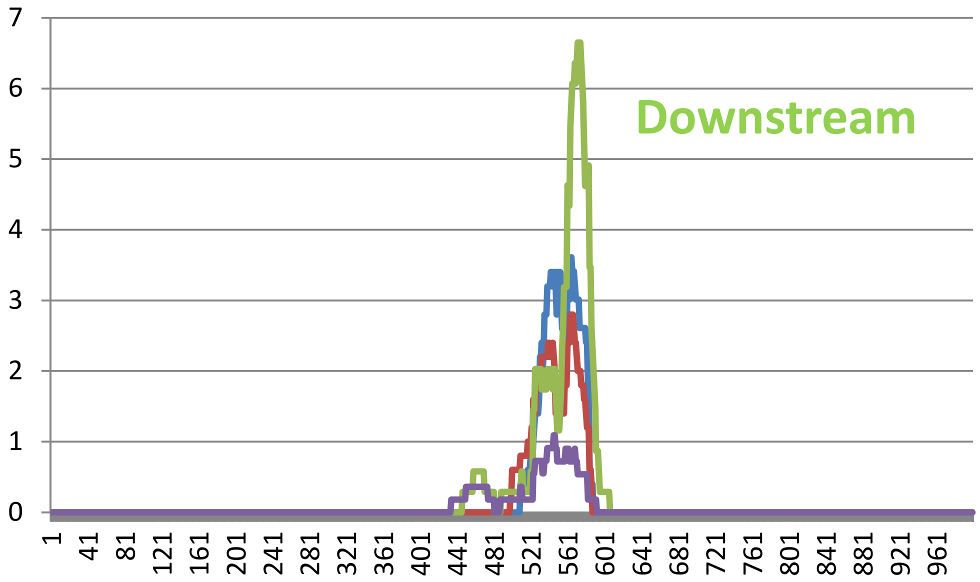

AT4G08850

Leucine-rich repeat receptor-like protein kinase family protein

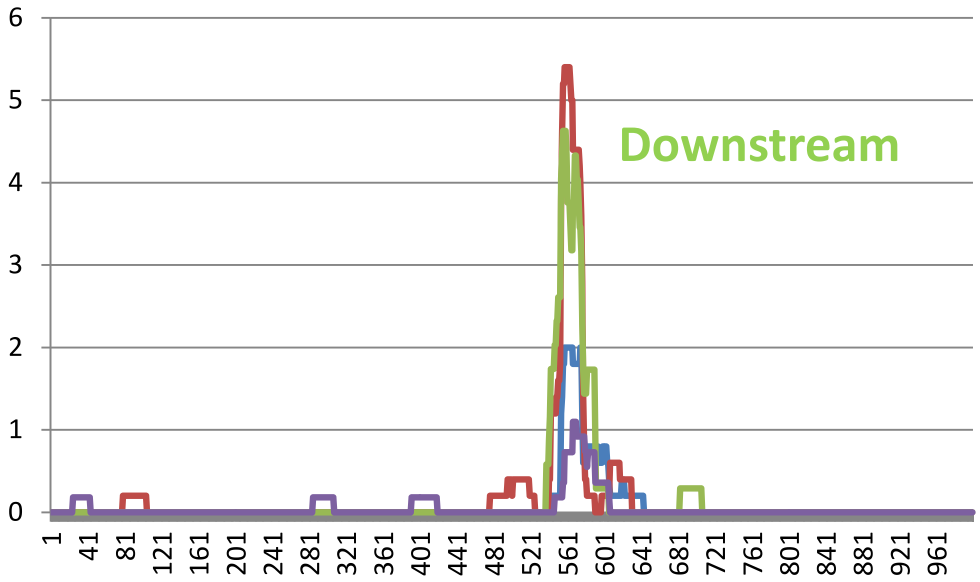

AT4G10596

This gene encodes a small protein and has either evidence of transcription or purifying selection.

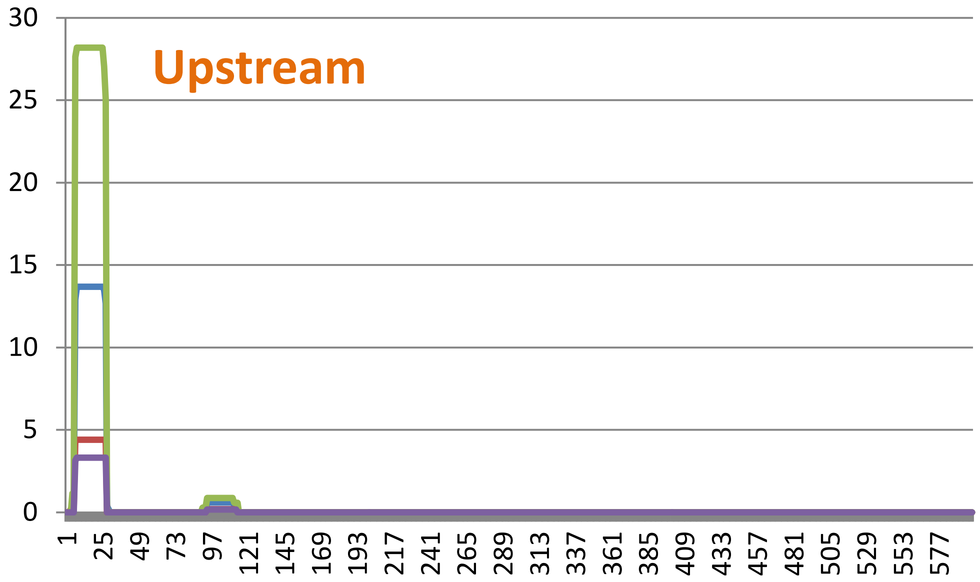

AT4G11370

Encodes a putative RING-H2 finger protein RHA1a.

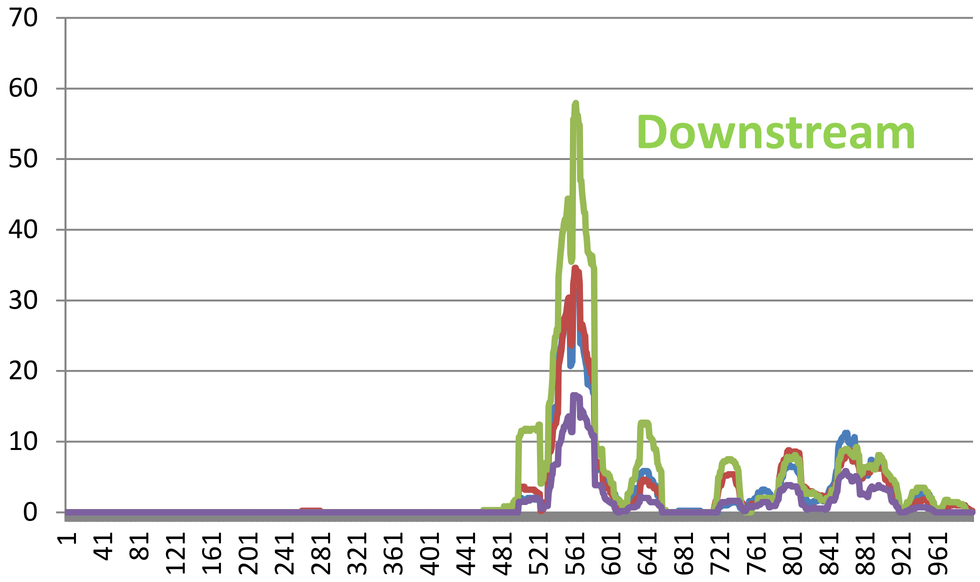

AT4G11900

S-locus lectin protein kinase family protein

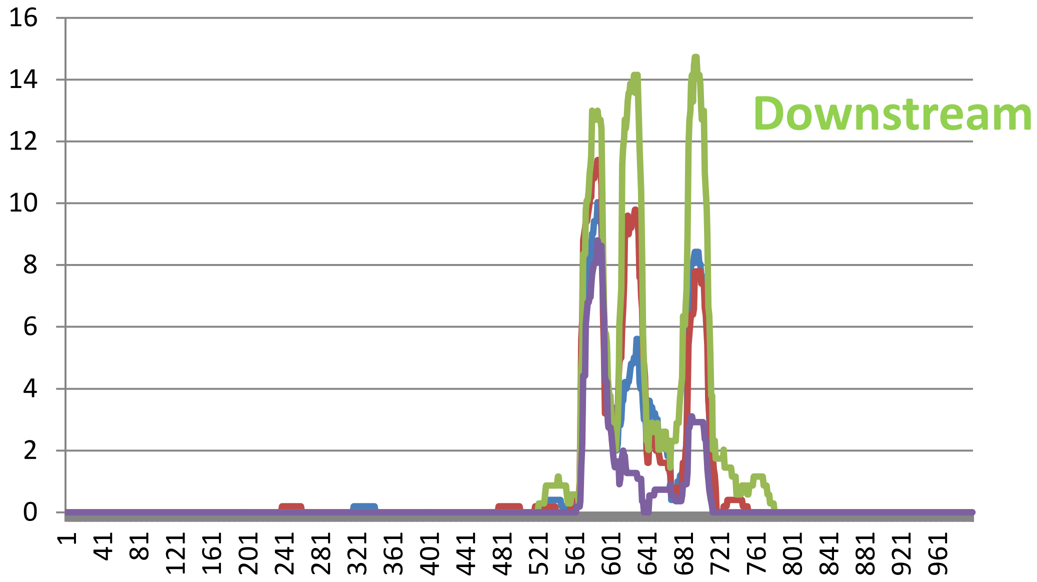

AT4G12110

Encodes a member of the SMO1 family of sterol 4alpha-methyl oxidases.

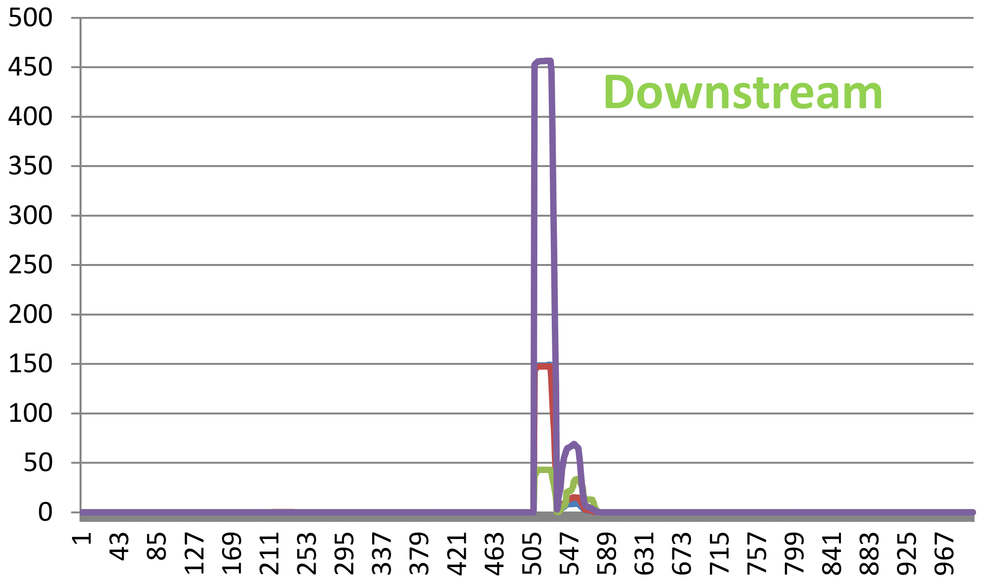

AT4G14365

XB3 ortholog 4 in Arabidopsis thaliana (XBAT34).

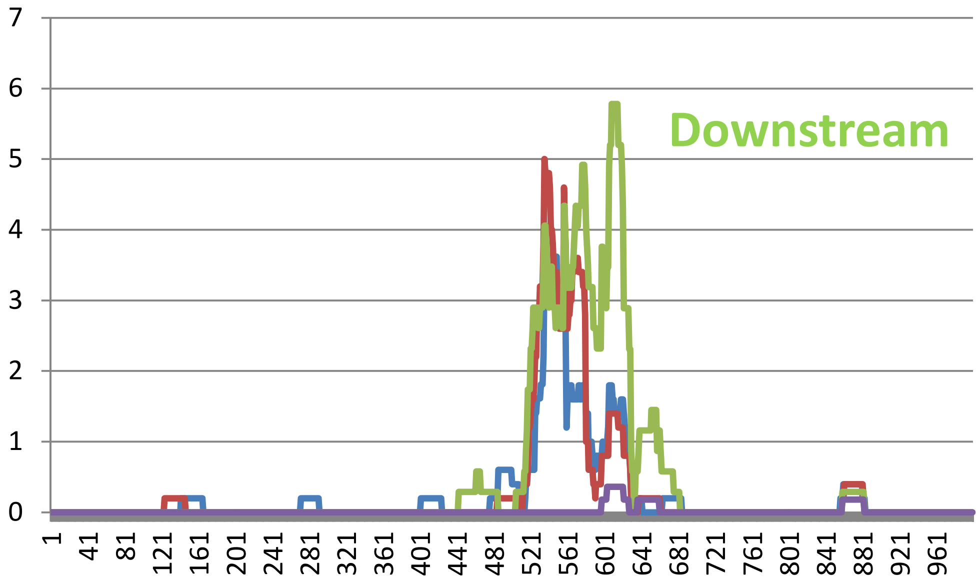

AT4G14940

AMINE OXIDASE 1 (AO1). Atao1 gene of Arabidopsis thaliana encodes an extracellular copper amine oxidase expressed during early stages of vascular tissue development.

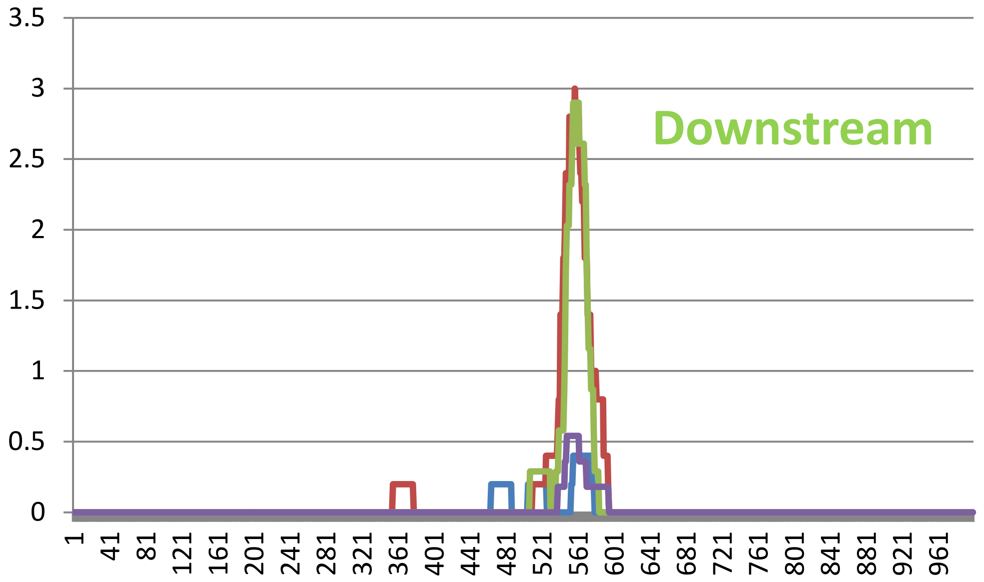

AT4G15570

MAGATAMA 3 (MAA3). Similar to yeast Sen1 (splicing endonuclease 1)helicase protein. Involved in female gametophyte development.

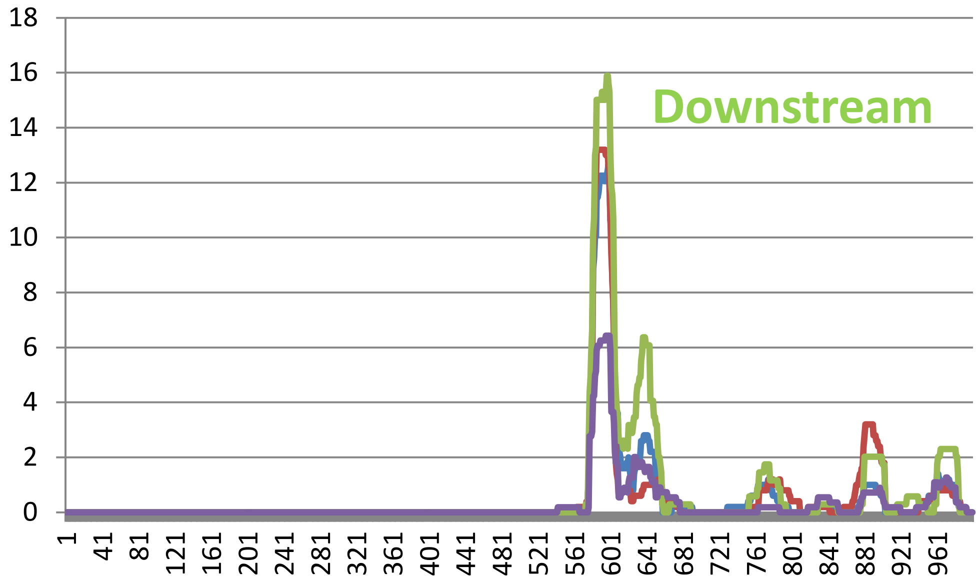

AT4G16295

Self-incompatibility (S) protein homolog, expressed at very low levels in floral buds

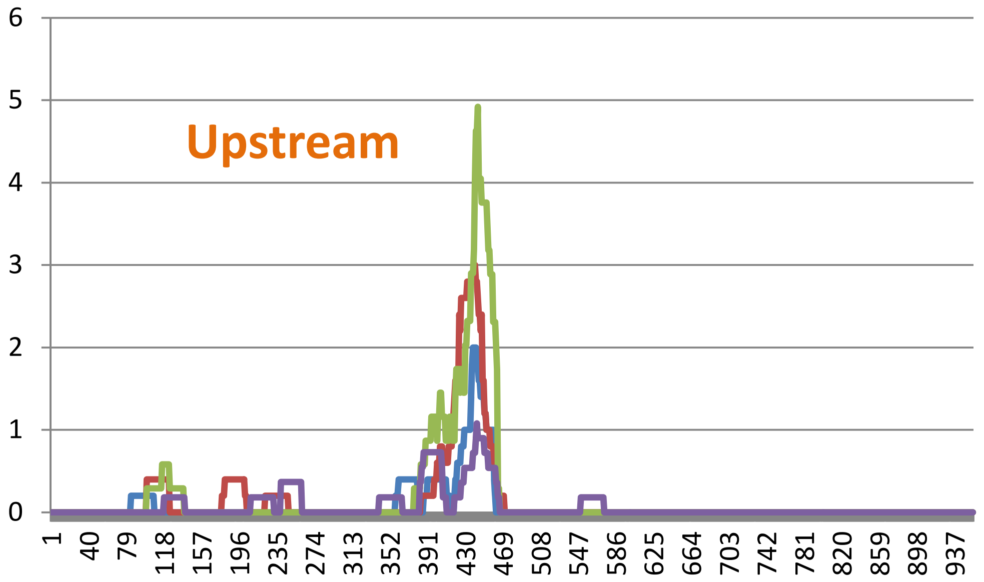

AT4G18150

Kinase-related protein of unknown function (DUF1296)

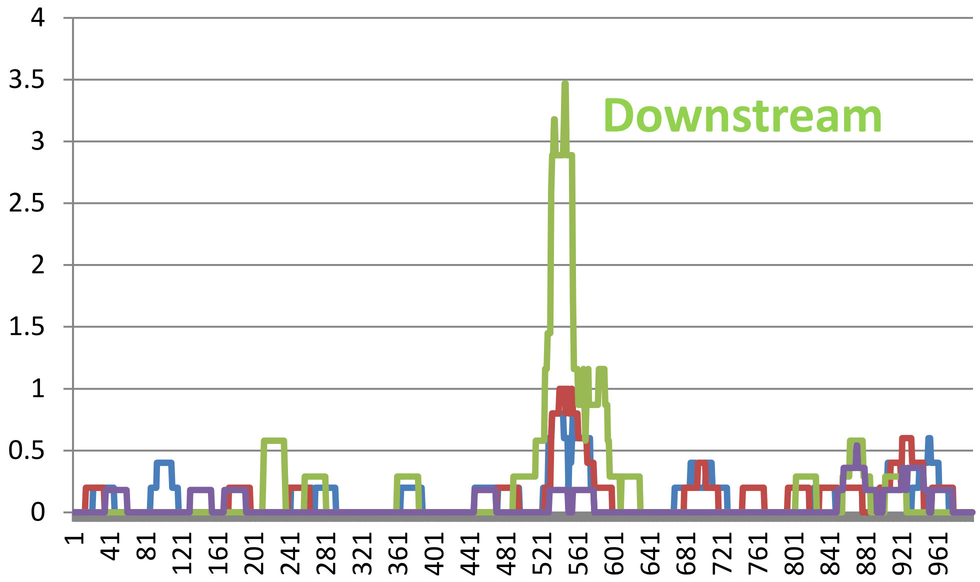

AT4G18690

Unknown protein

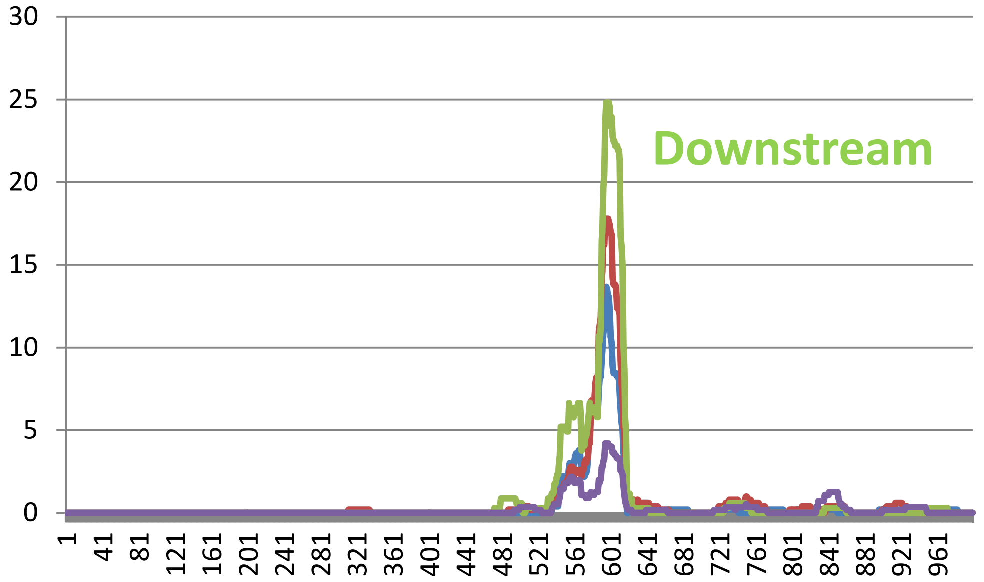

AT4G19090

Protein of unknown function (DUF594)

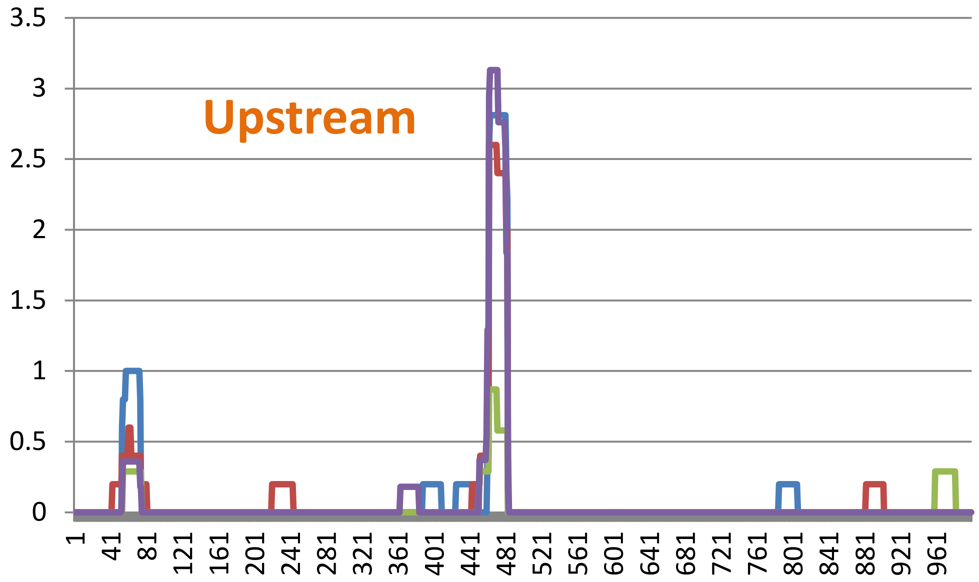

AT4G21820

Binding; calmodulin binding

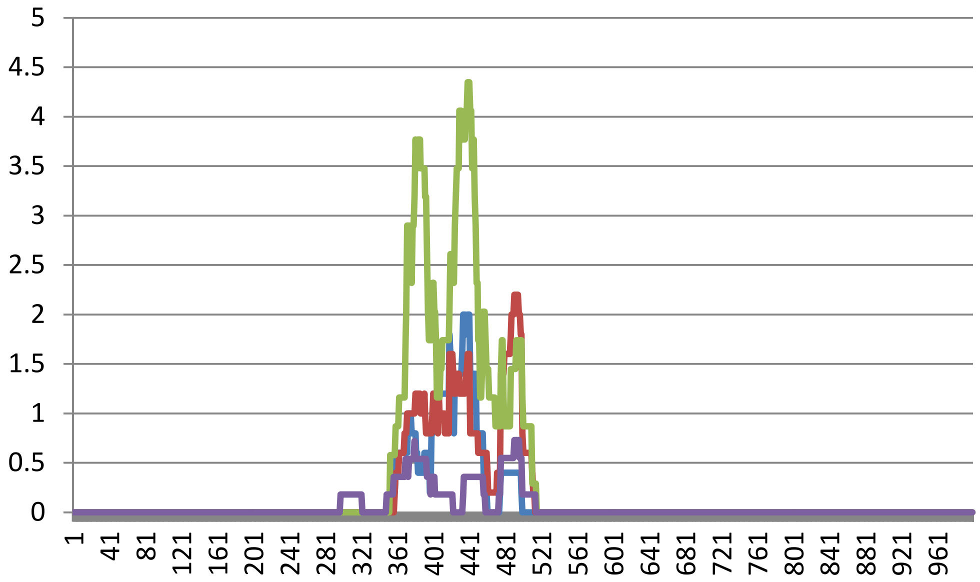

AT4G22650

BEST Arabidopsis thaliana protein match is: Bifunctional inhibitor/lipid-transfer protein/seed storage 2S albumin superfamily protein (TAIR:AT4G22640.1).

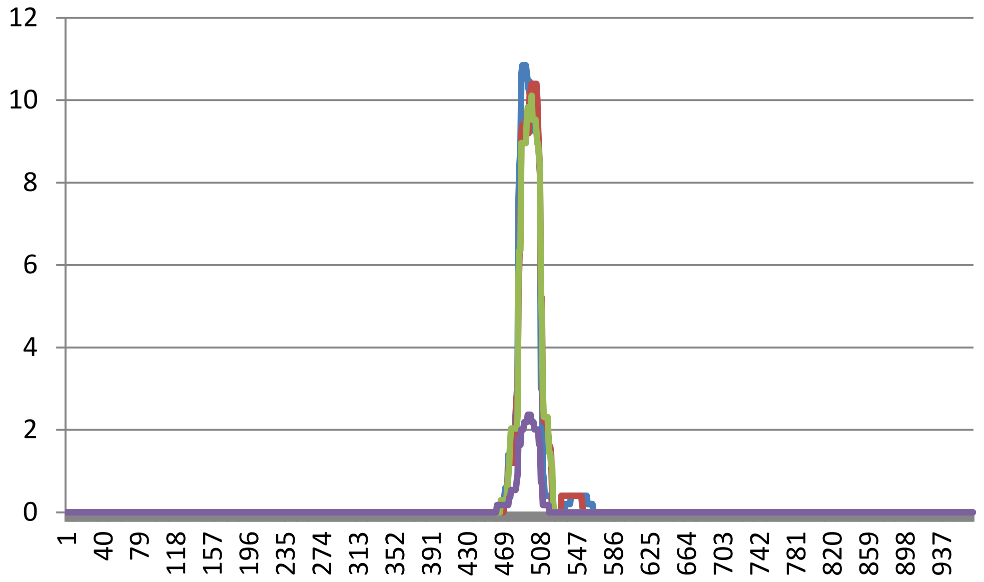

AT4G22756

Encodes a member of the SMO1 family of sterol 4alpha-methyl oxidases.

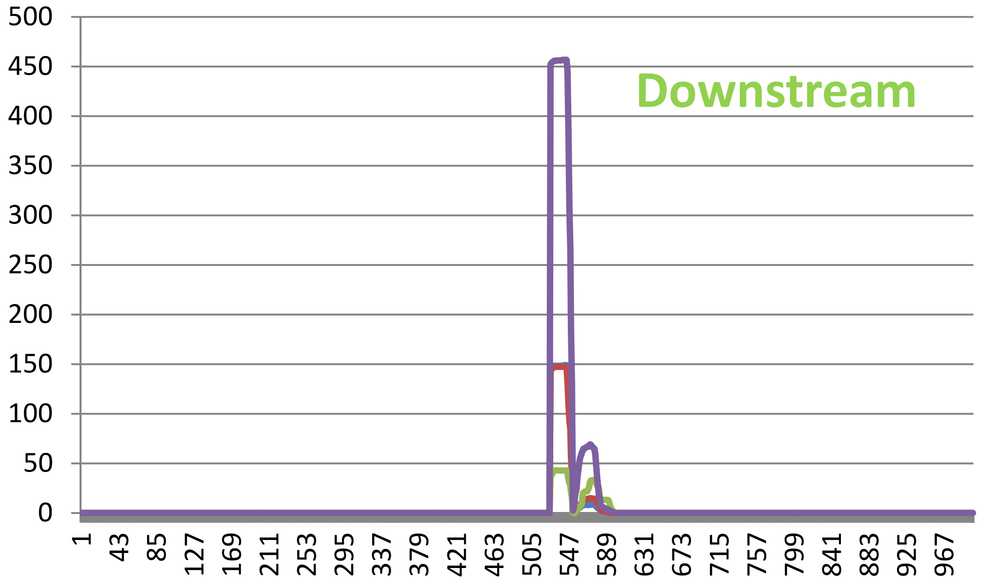

AT4G24030

Unknown protein

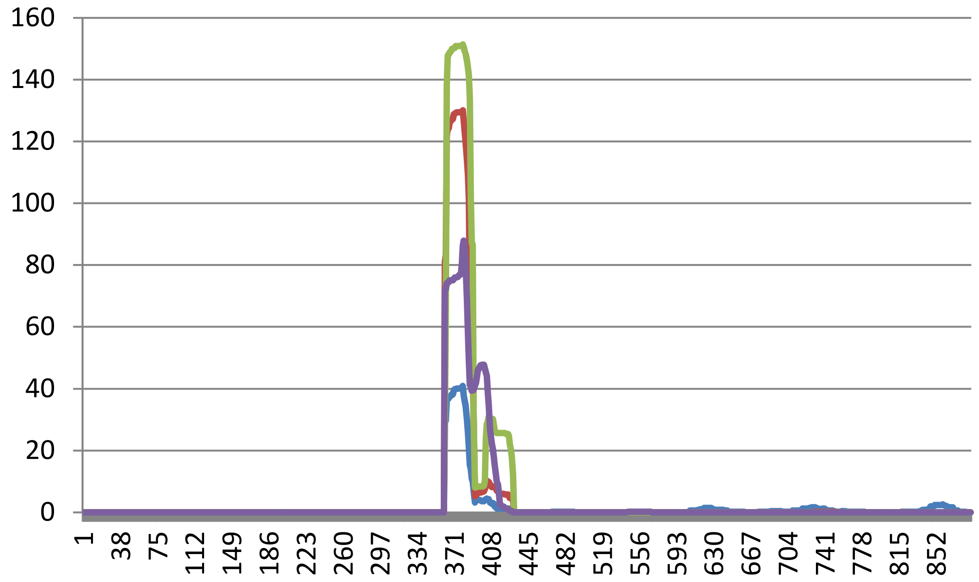

AT4G25590

Actin depolymerizing factor 7 (ADF7)

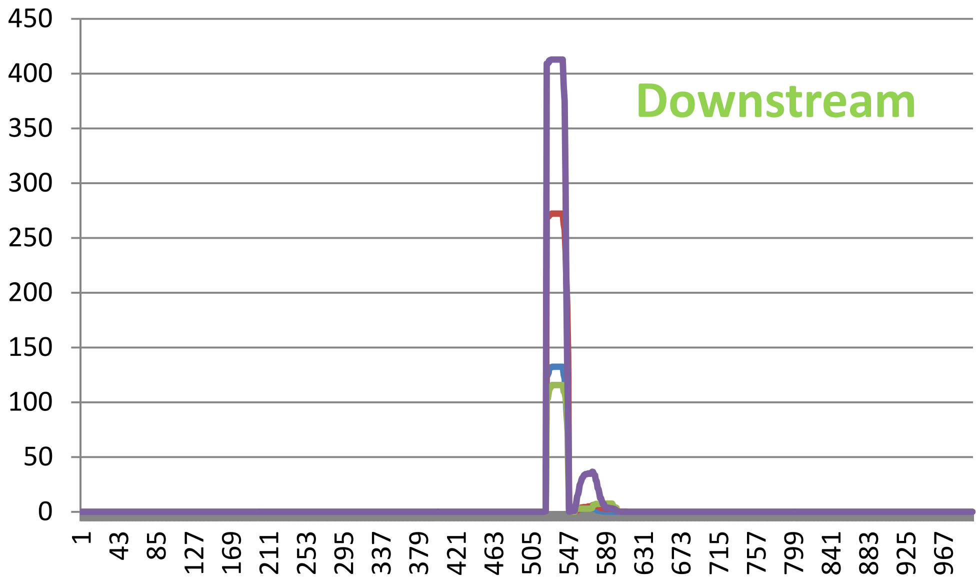

AT4G28850

Xyloglucan endotransglucosylase/hydrolase 26 (XTH26)

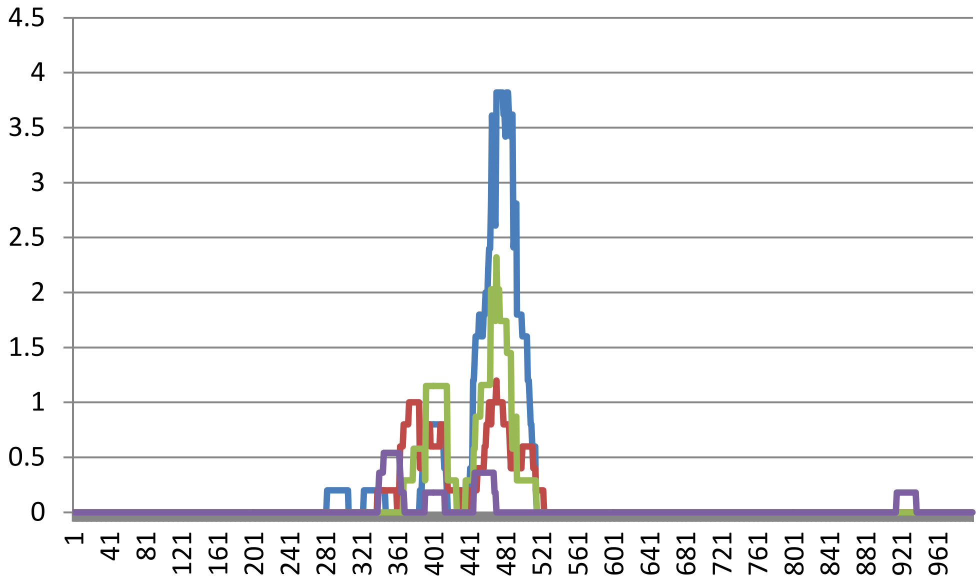

AT4G29090

Ribonuclease H-like superfamily protein

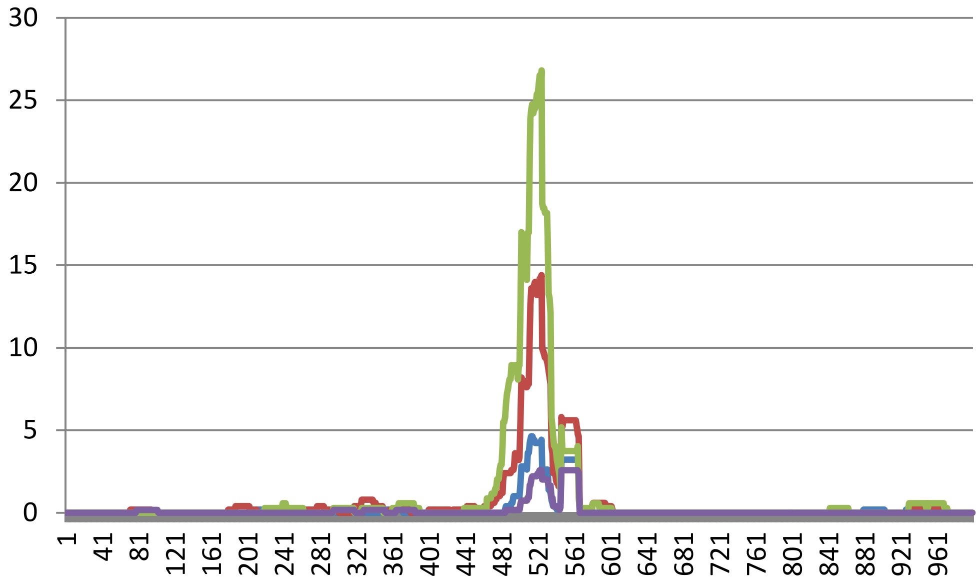

AT4G29200

Beta-galactosidase related protein

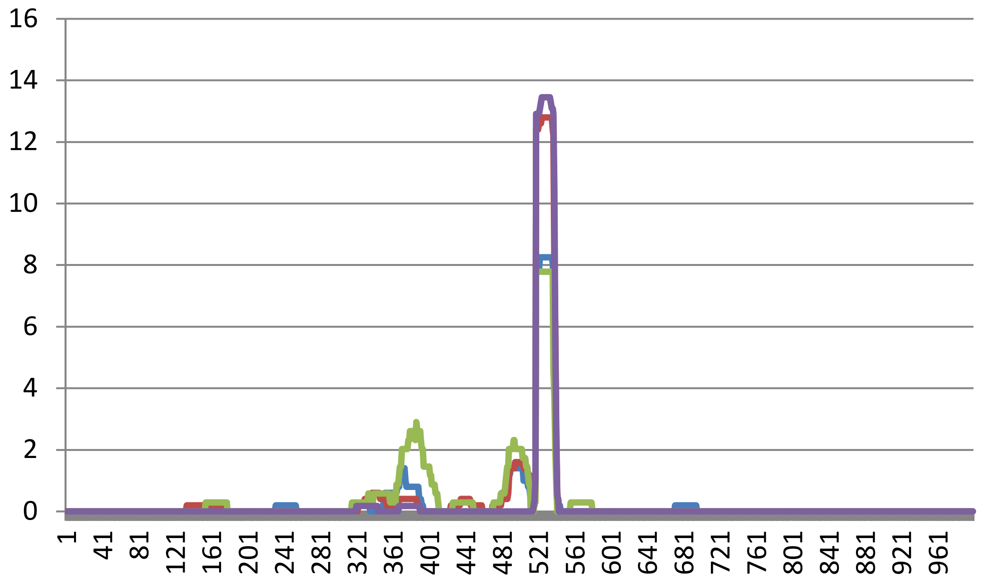

AT4G29290

Encodes a member of a family of small, secreted, cysteine rich protein with sequence similarity to the PCP (pollen coat protein) gene family.

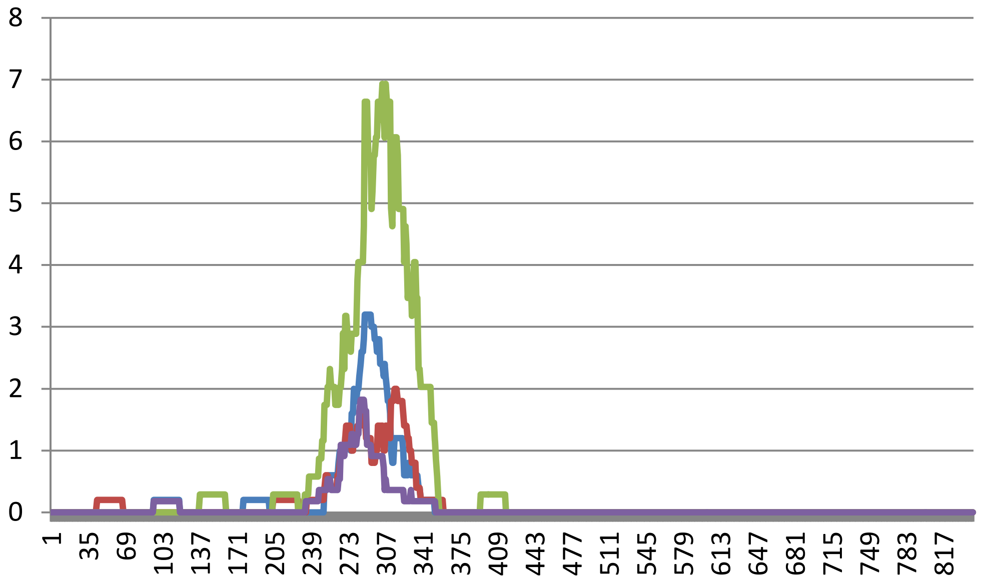

AT4G29305

Encodes a member of a family of small, secreted, cysteine rich protein with sequence similarity to the PCP (pollen coat protein) gene family.

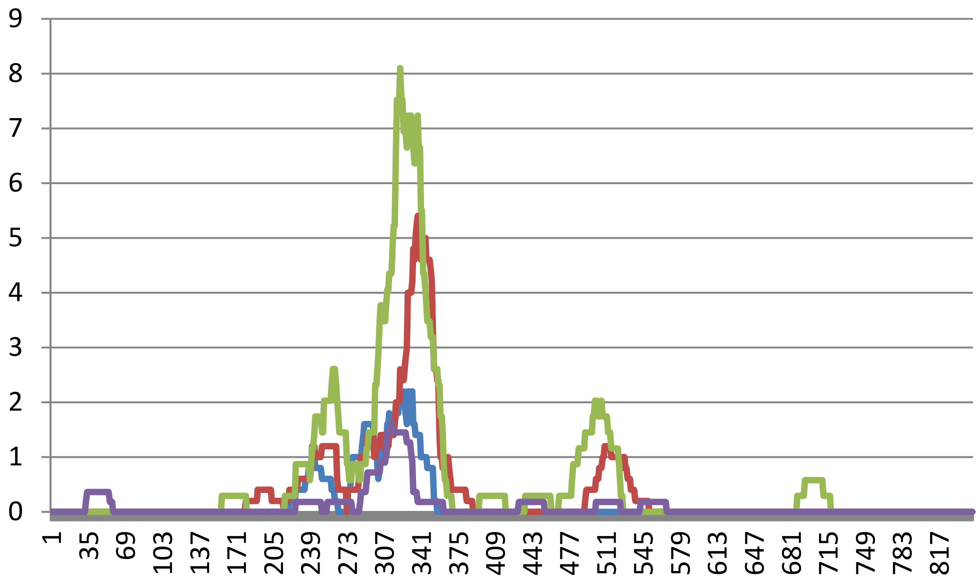

AT4G29740

It encodes a protein whose sequence is similar to cytokinin oxidase/dehydrogenase, which catalyzes the degradation of cytokinins.

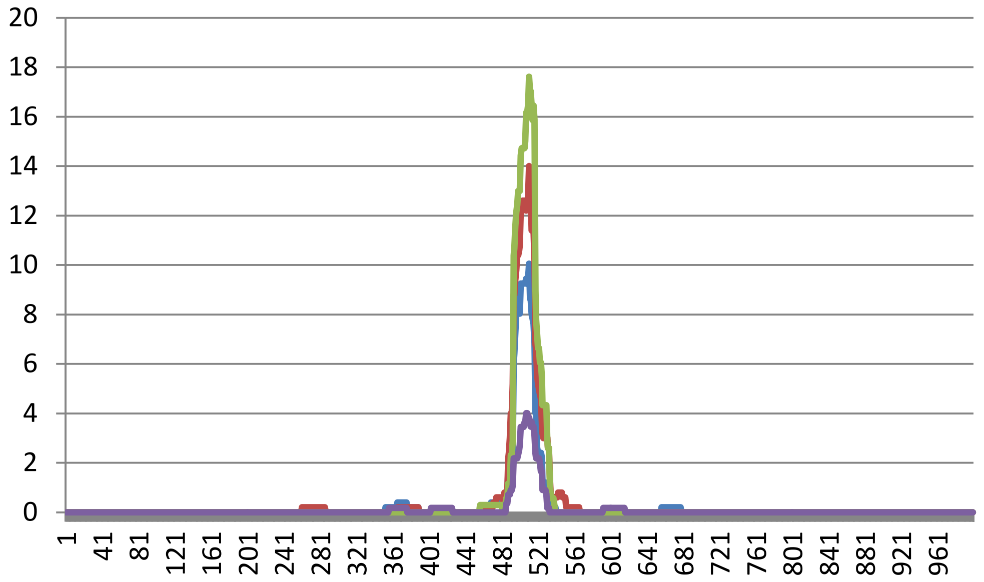

AT4G30993

Calcineurin-like metallo-phosphoesterase superfamily protein

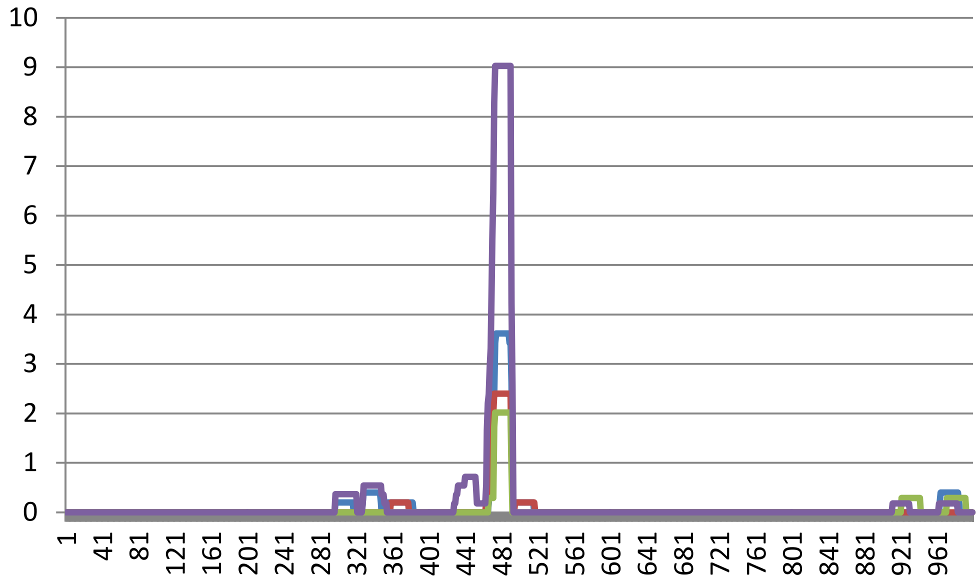

AT4G33820

Glycosyl hydrolase superfamily protein

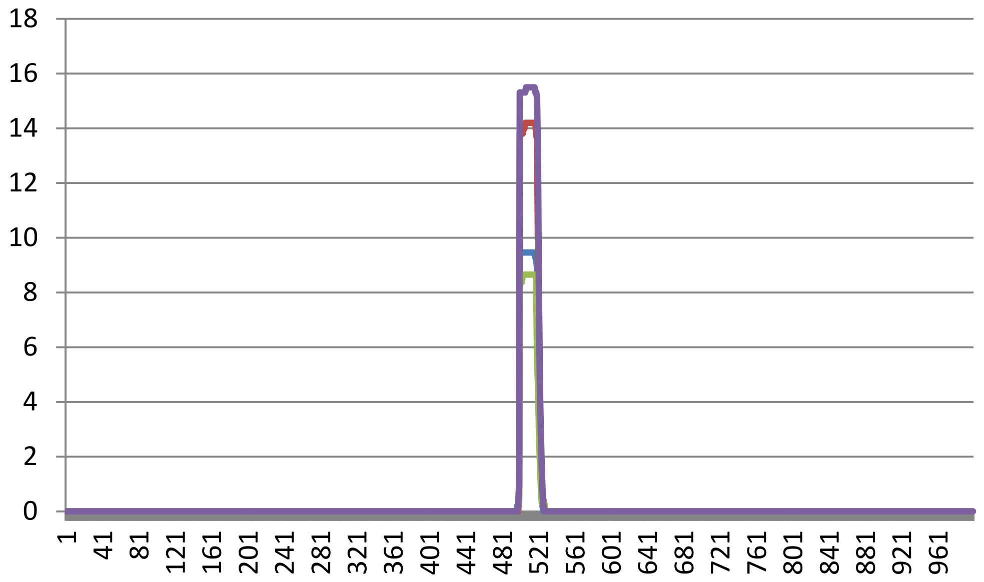

AT4G34030

3-METHYLCROTONYL-COA CARBOXYLASE (MCCB). MCC-B is involved in leucine degradation in mitochondria.

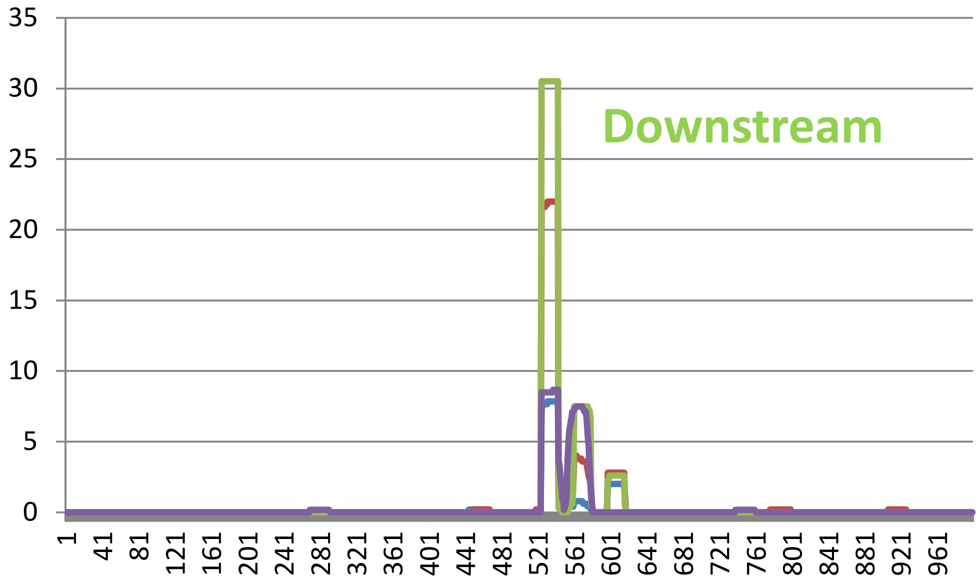

AT4G34400

AP2/B3-like transcriptional factor family protein

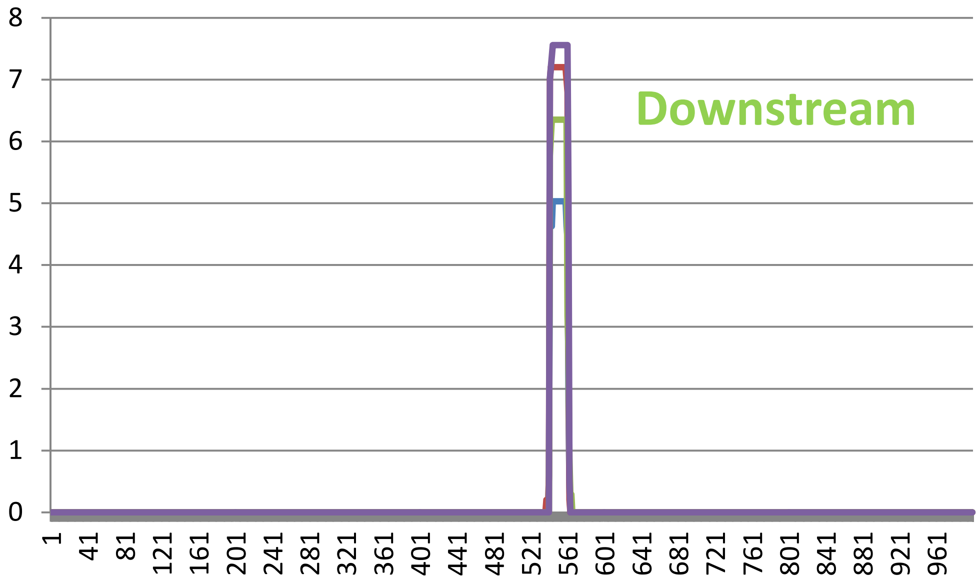

AT4G36190

Serine carboxypeptidase S28 family protein

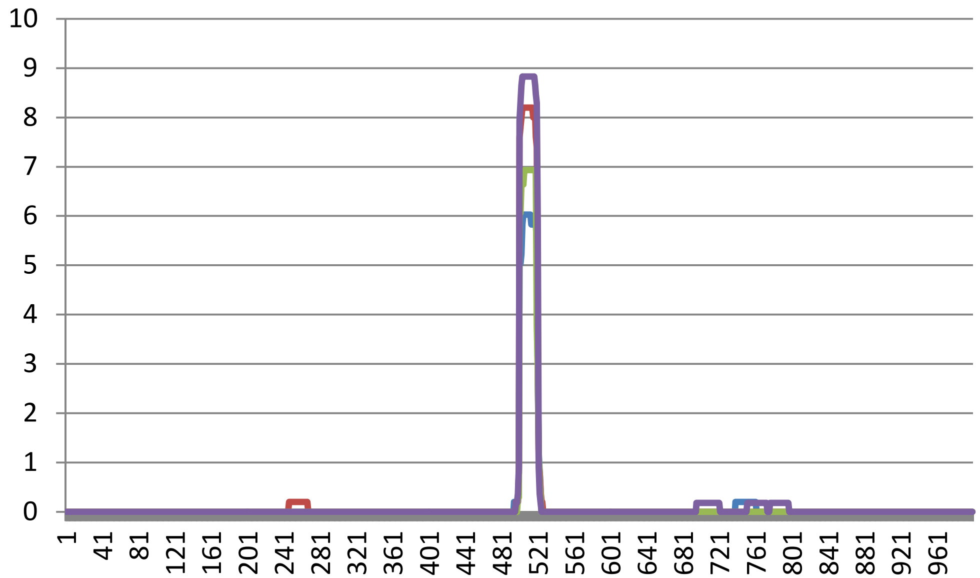

AT4G37130

Hydroxyproline-rich glycoprotein family protein

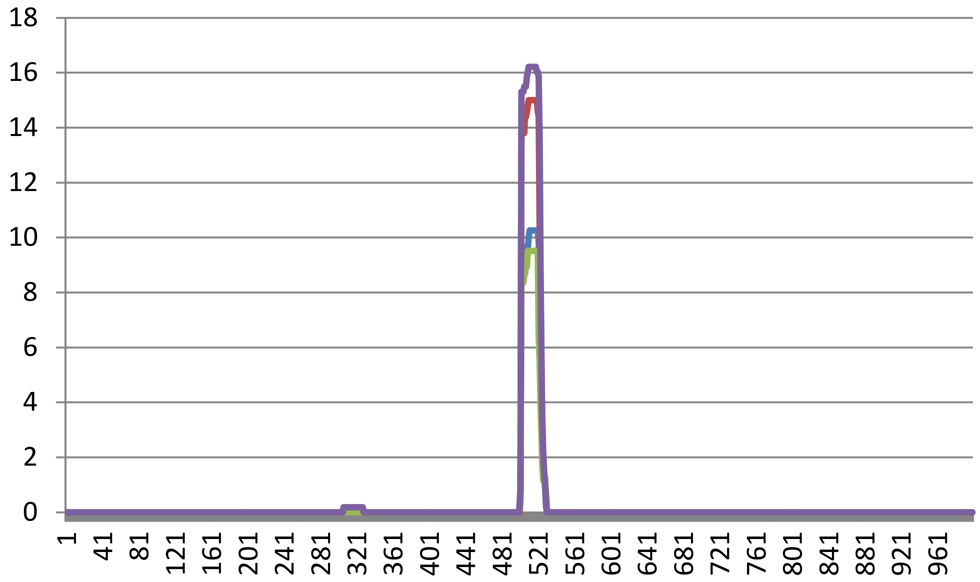

AT4G39130

Dehydrin family protein

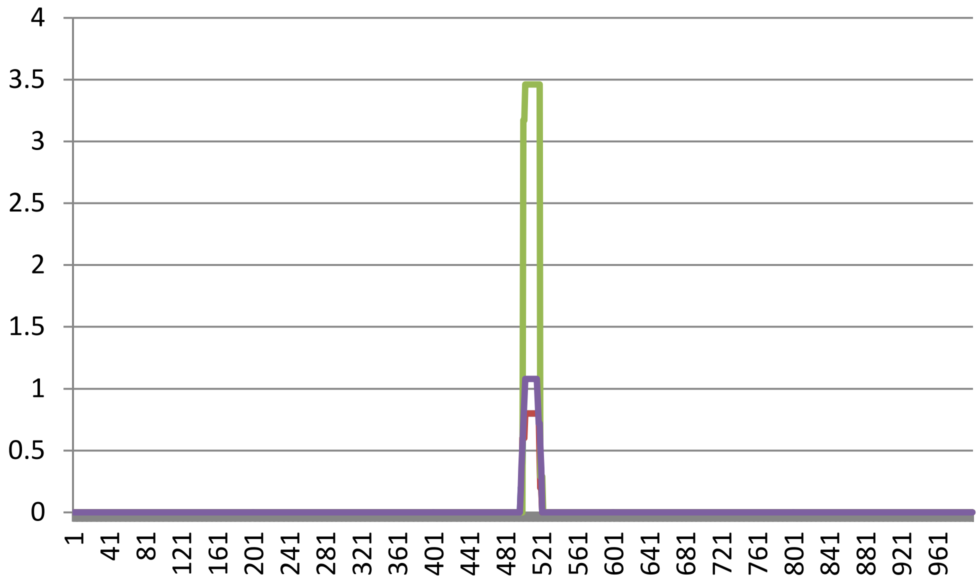

AT4G39980

Encodes a 2-deoxy-D-arabino-heptulosonate 7-phosphate (DAHP) synthase, which catalyzes the first committed step in aromatic amino acid biosynthesis. Gene expression is induced by wounding and pathogenic bacteria Pseudomonas syringae.

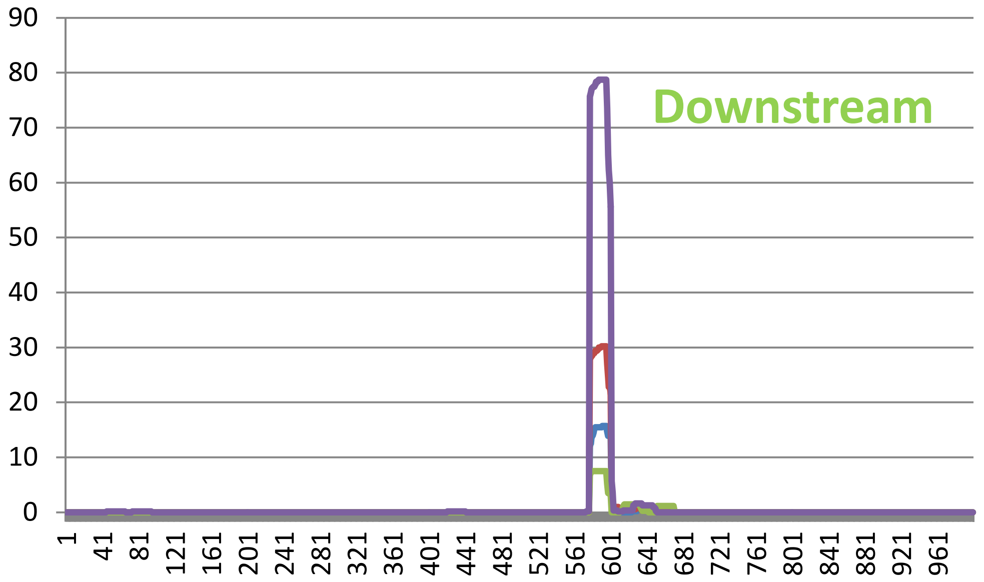

AT5G01015

Unknown protein

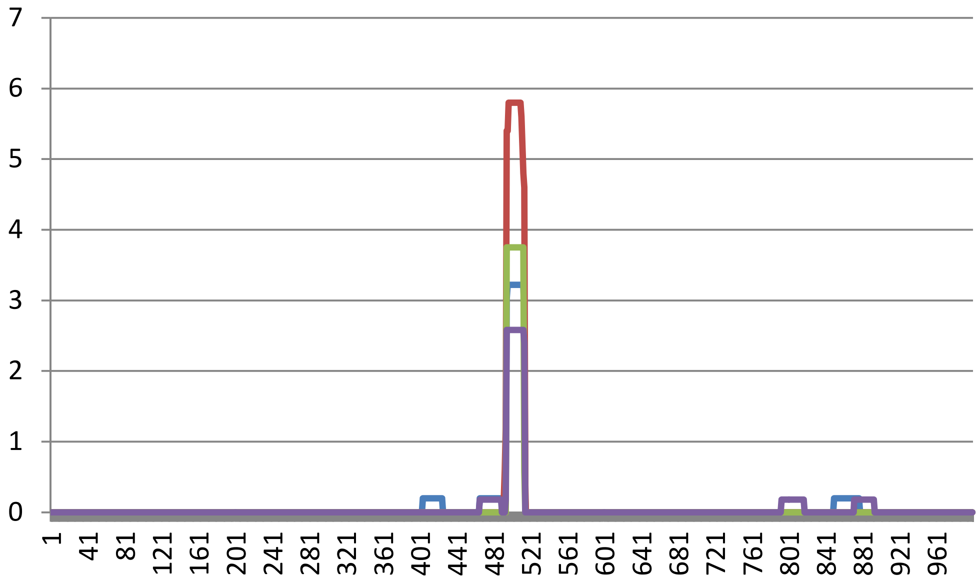

AT5G01080

Beta-galactosidase related protein

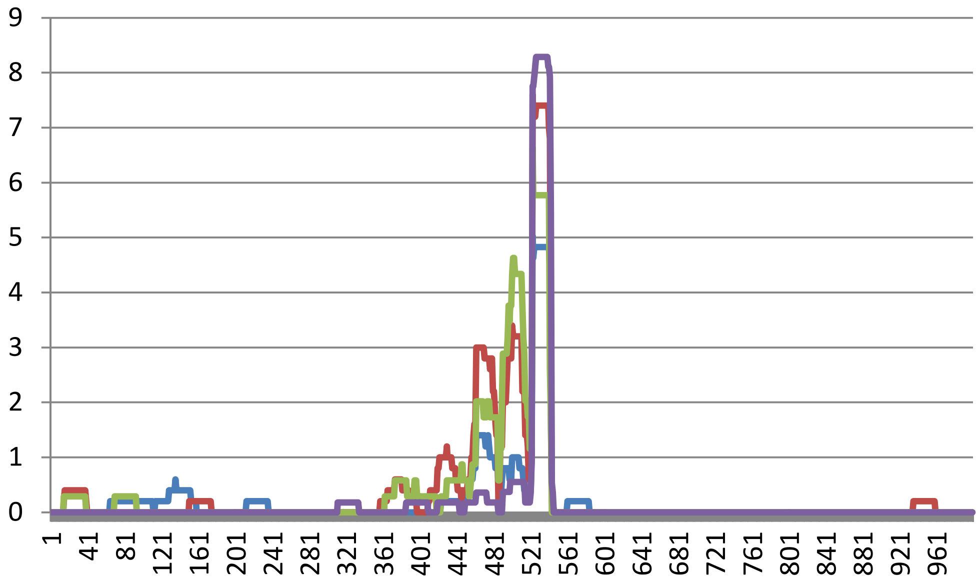

AT5G01260

Carbohydrate-binding-like fold

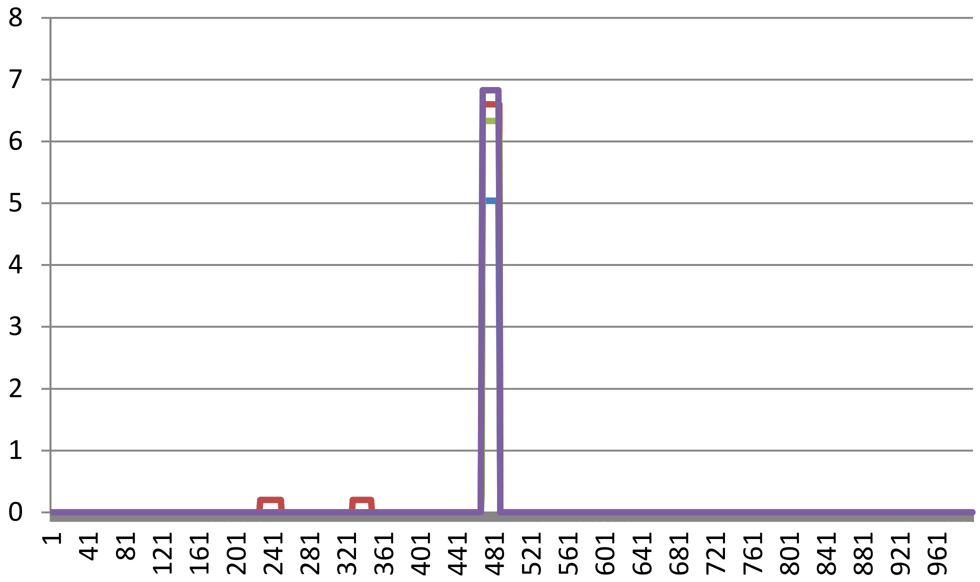

AT5G02990

Galactose oxidase/kelch repeat superfamily protein

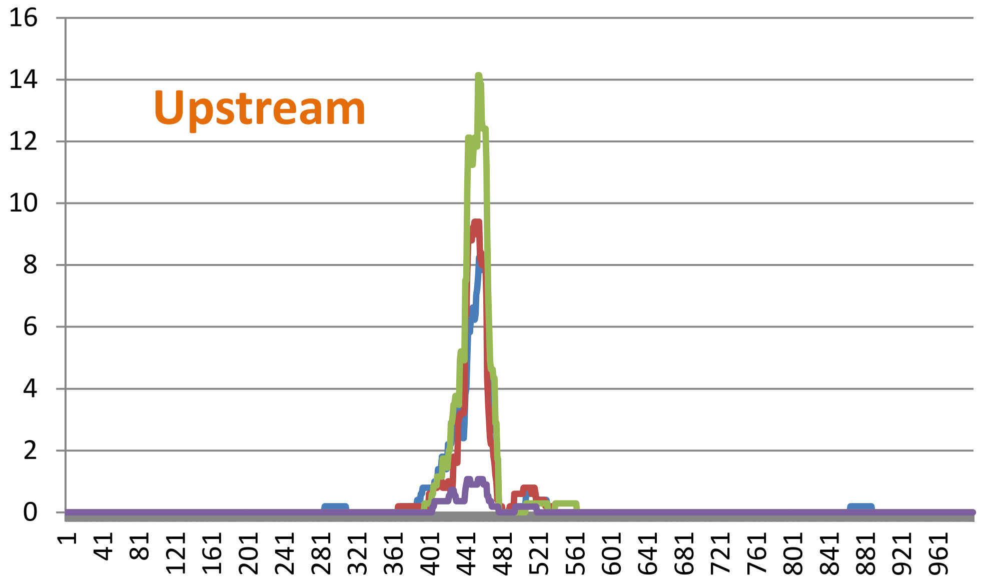

AT5G03060

Unknown protein

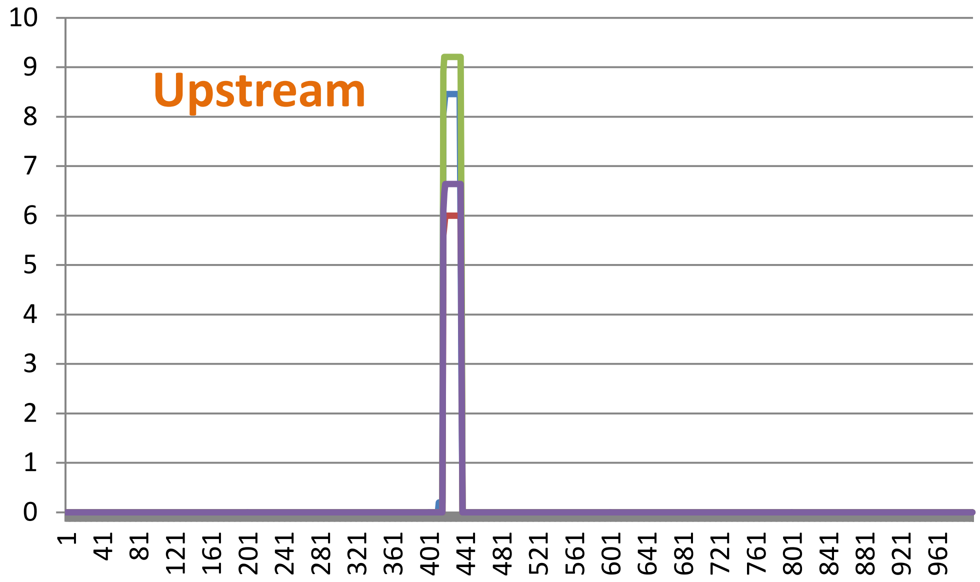

AT5G04950

Encodes a nicotianamide synthase.

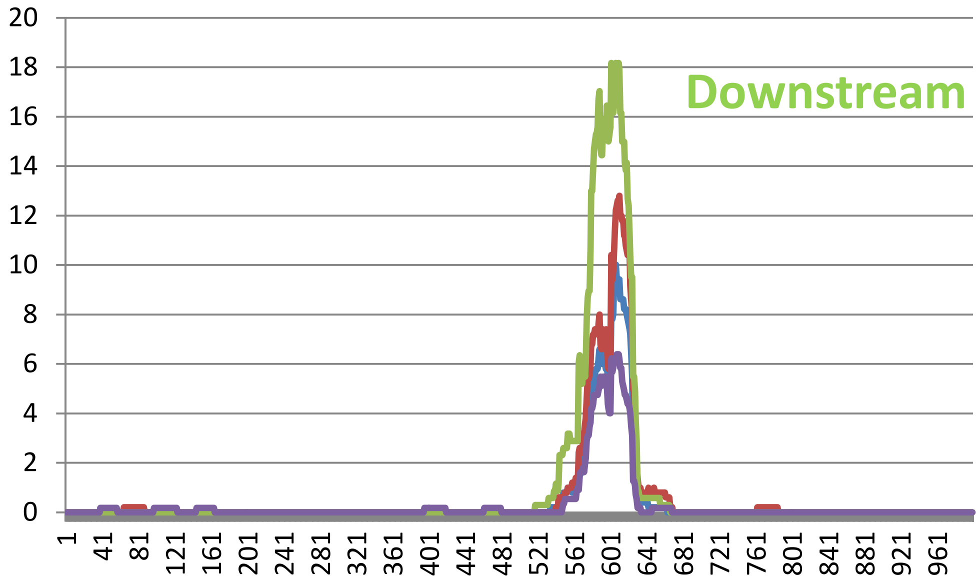

AT5G04970

Plant invertase/pectin methylesterase inhibitor superfamily

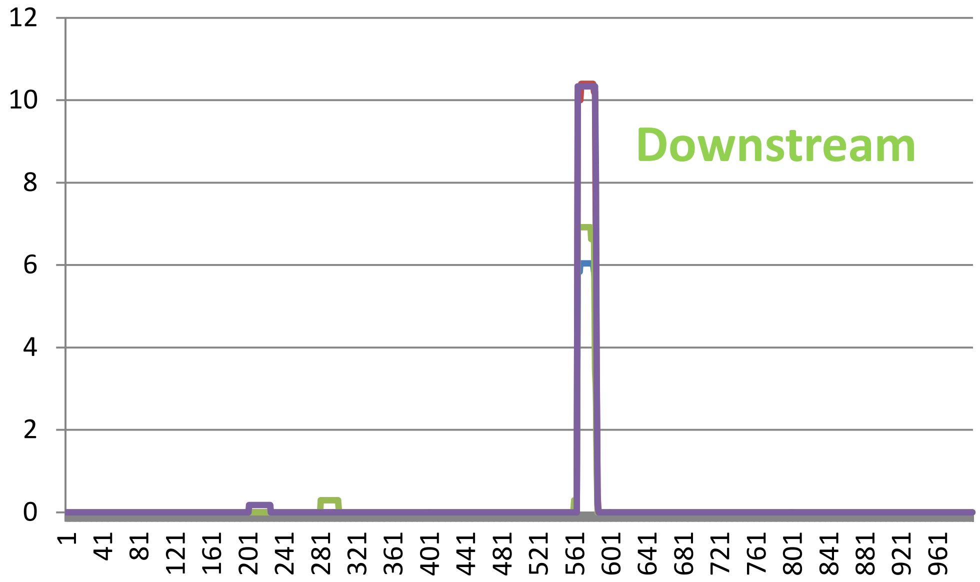

AT5G06130

Chaperone protein dnaJ-related

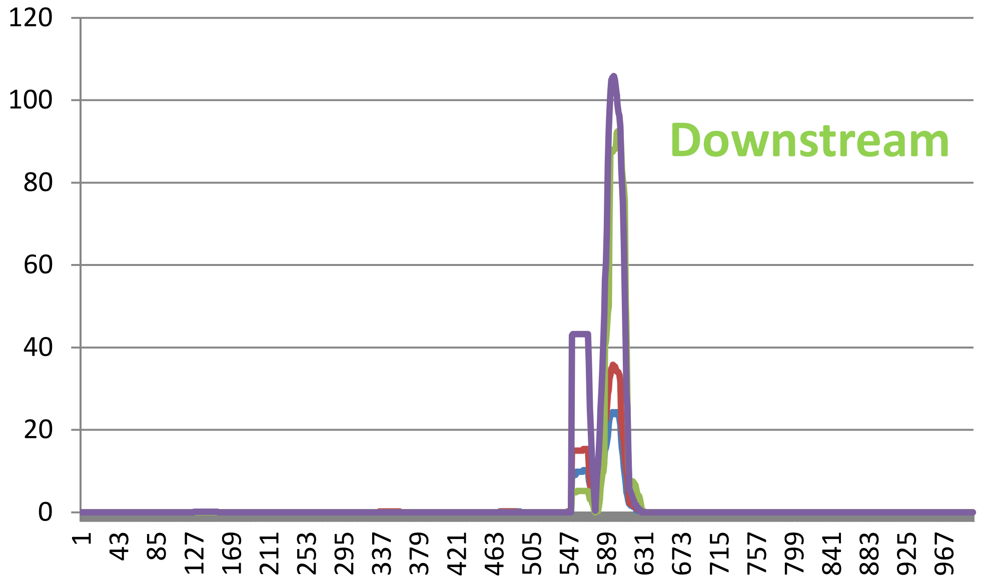

AT5G07140

Protein kinase superfamily protein

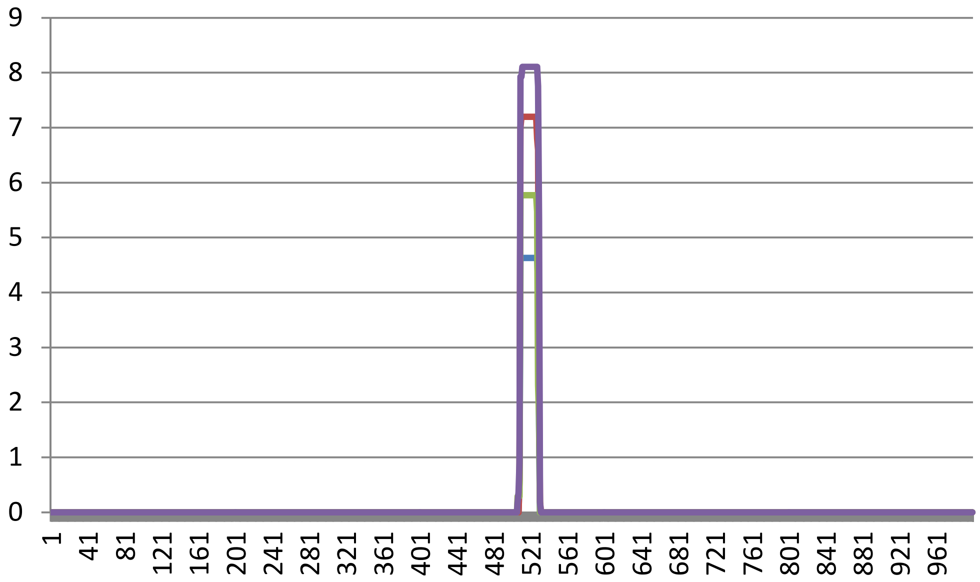

AT5G10100

Haloacid dehalogenase-like hydrolase (HAD) superfamily protein

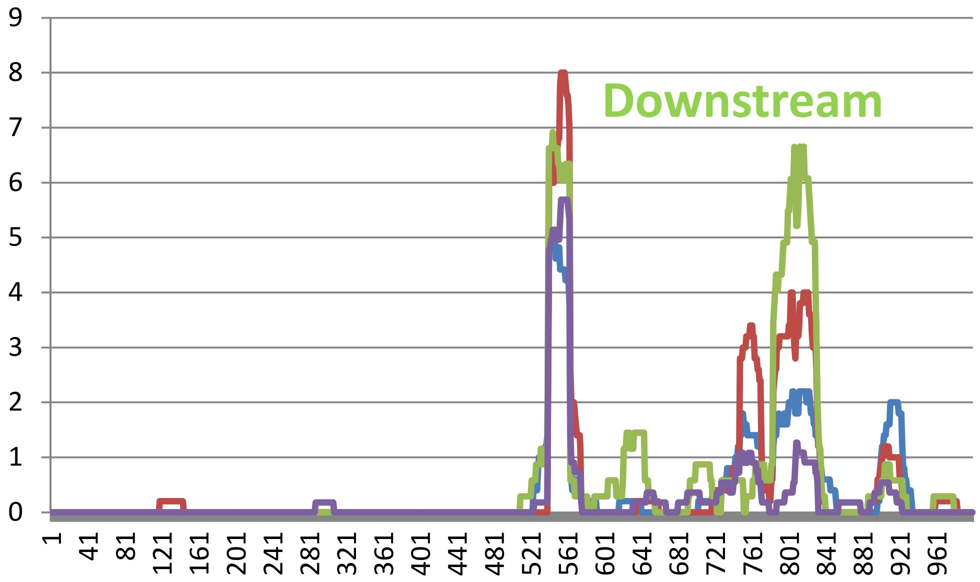

AT5G10660

Calmodulin-binding protein-related

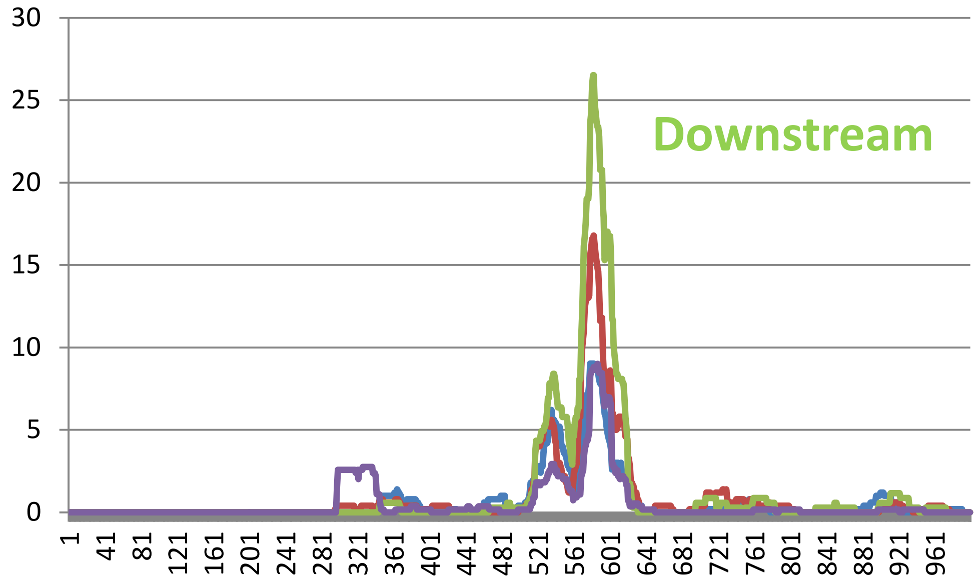

AT5G13825

Unknown protein

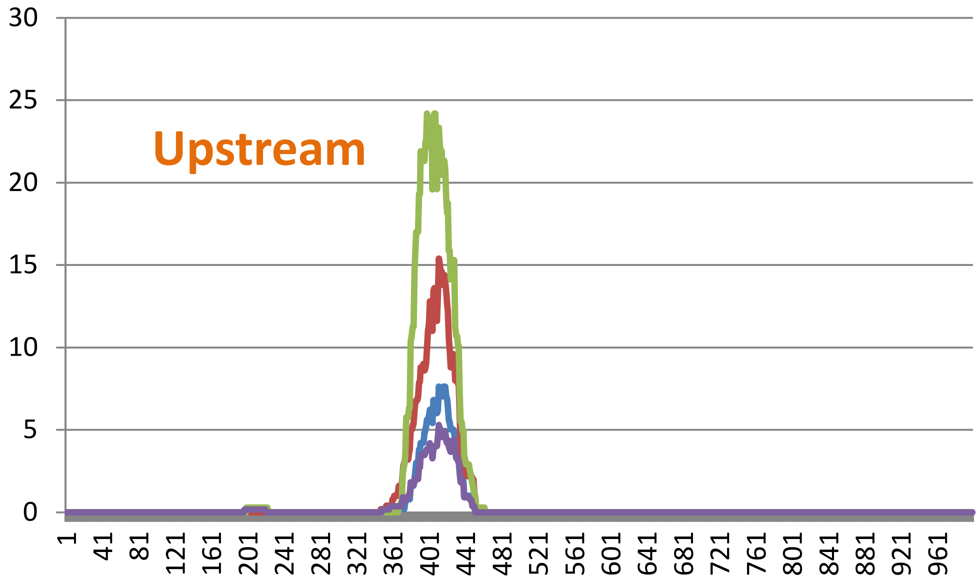

AT5G18570

Encodes AtObgC, a plant ortholog of bacterial Obg. AtObgC is a chloroplast-targeting GTPase essential for early embryogenesis. Mutations in this locus result in embryo lethality. The protein is dually localized in the stroma and the inner envelope membrane and is involved in thylakoid membrane biogenesis and functions primarily in plastid ribosome biogenesis during chloroplast development.

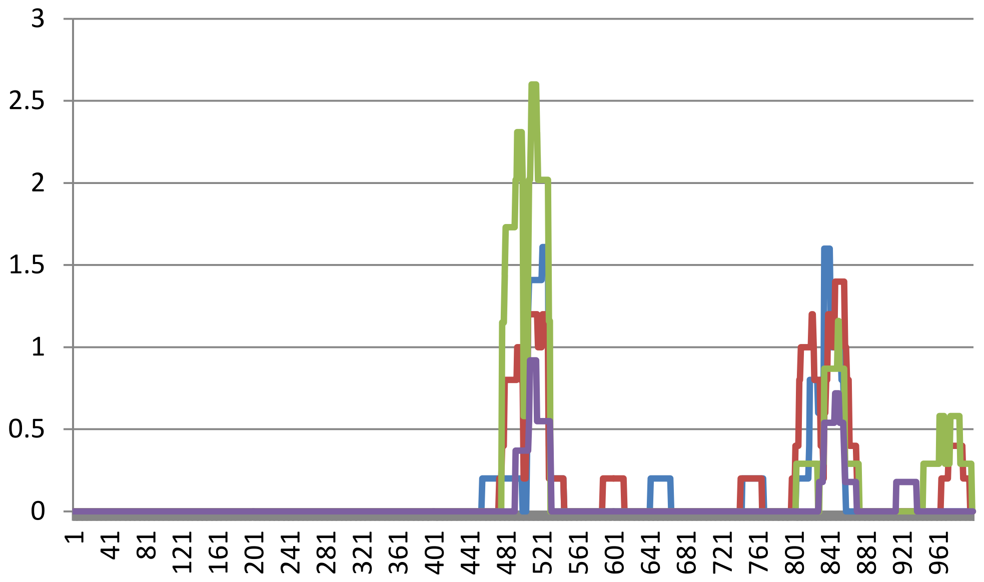

AT5G19160

Encodes a member of the TBL (TRICHOME BIREFRINGENCE-LIKE) gene family containing a plant-specific DUF231 (domain of unknown function) domain.

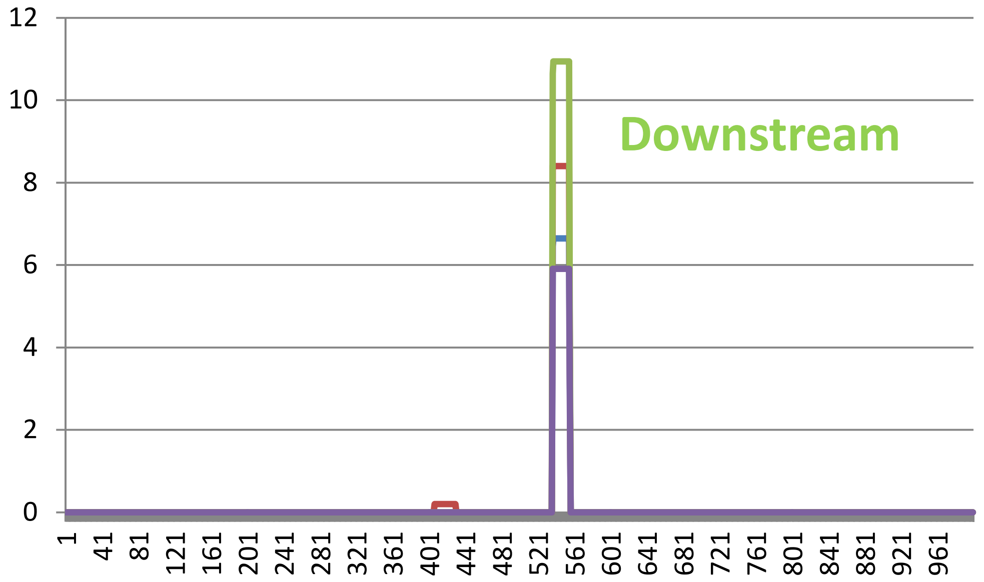

AT5G19170

Protein of Unknown Function (DUF239)

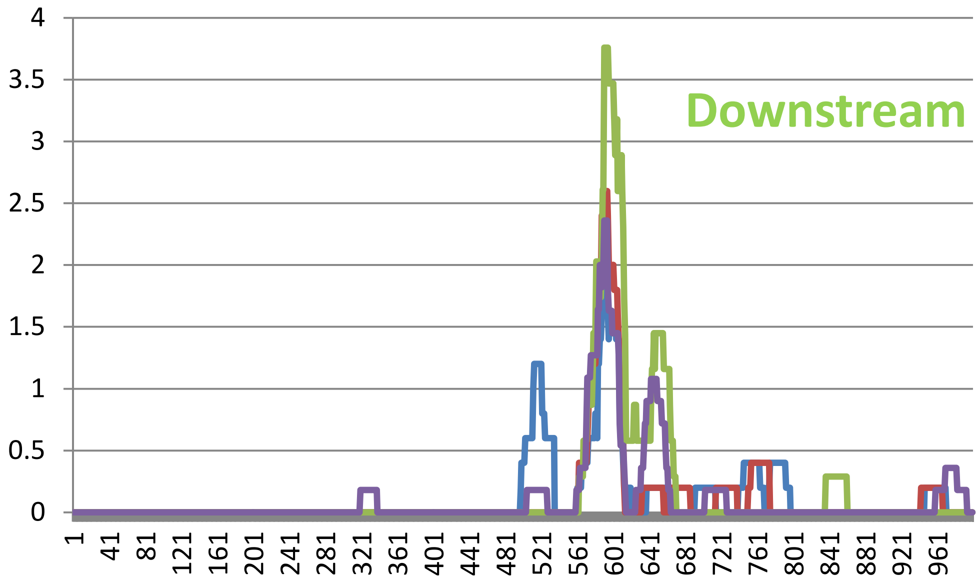

AT5G21125

Unknown protein

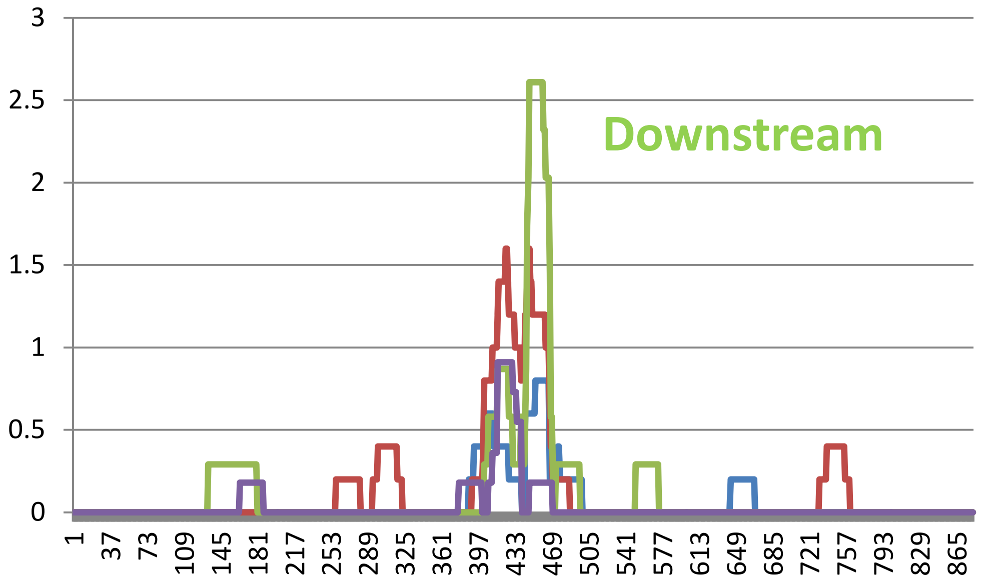

AT5G23270

Sugar transporter 11 (STP11)

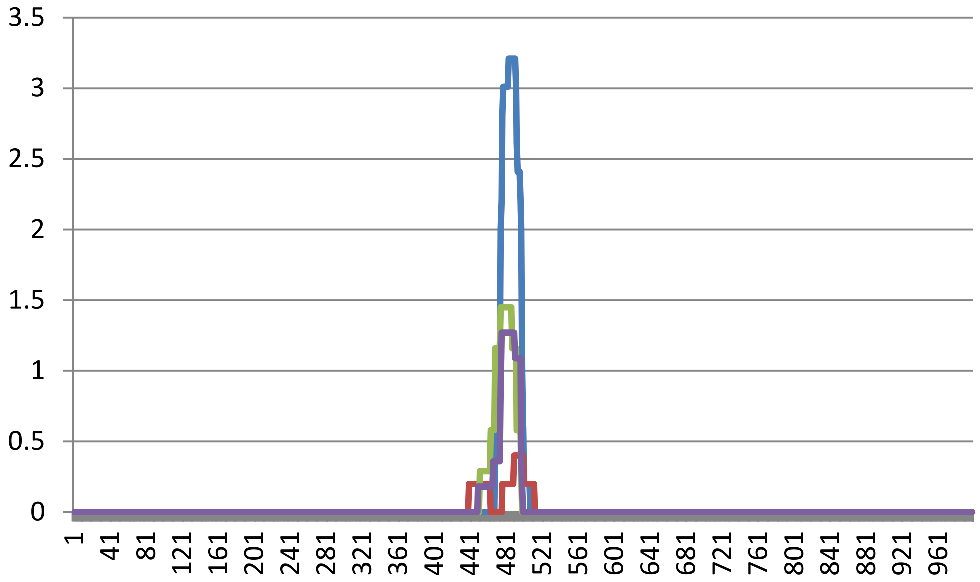

AT5G24290

Vacuolar iron transporter (VIT) family protein

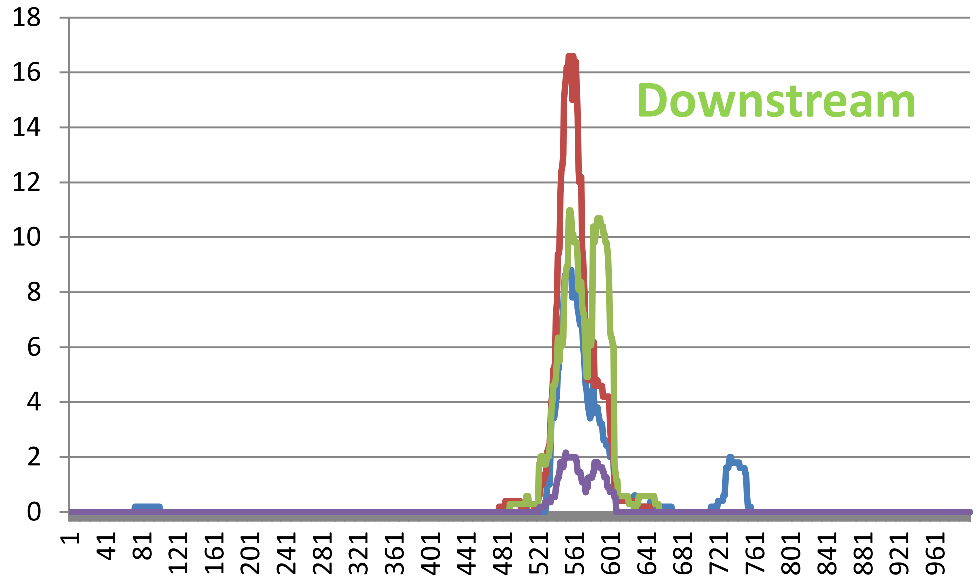

AT5G26270

Unknown protein

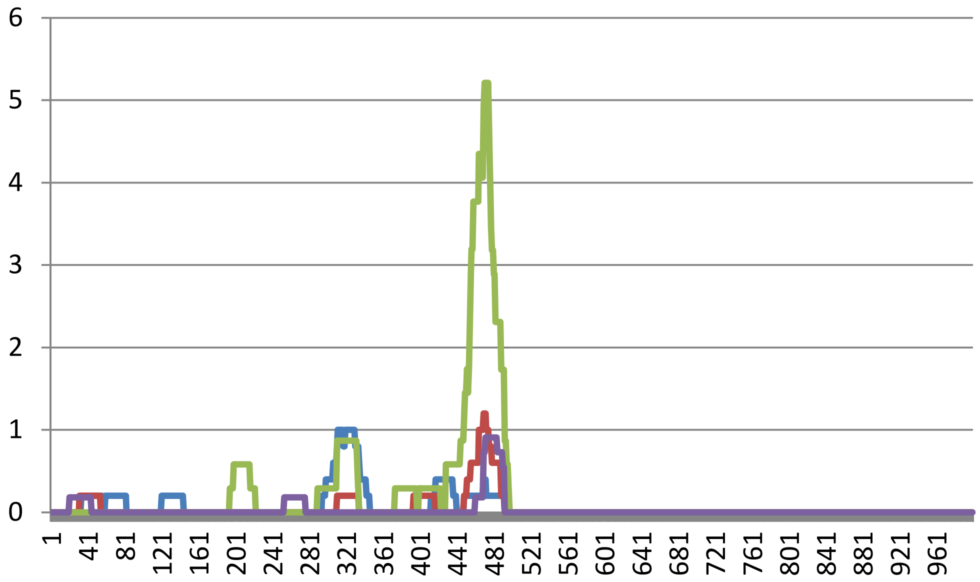

AT5G26673

Encodes a Plant thionin family protein

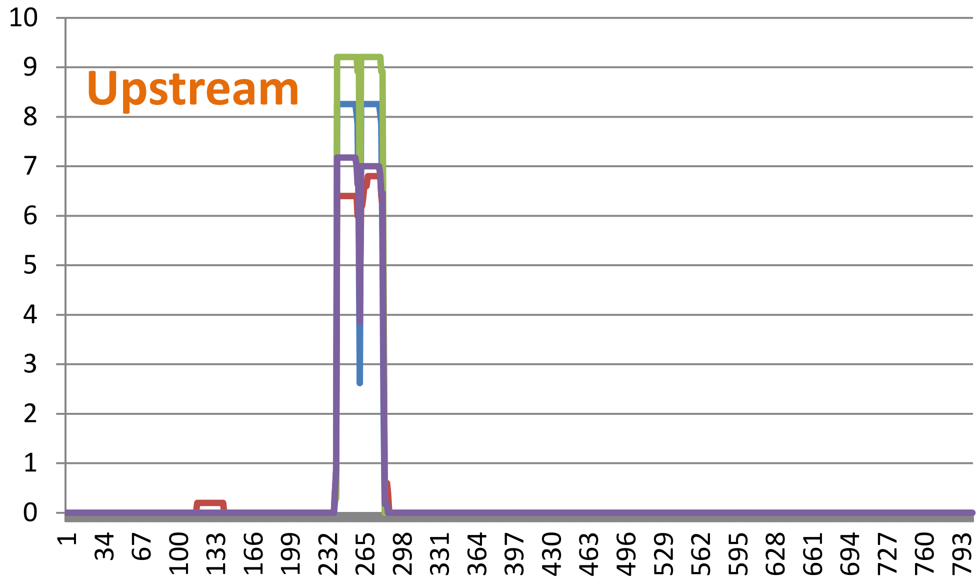

AT5G26770

Unknown protein

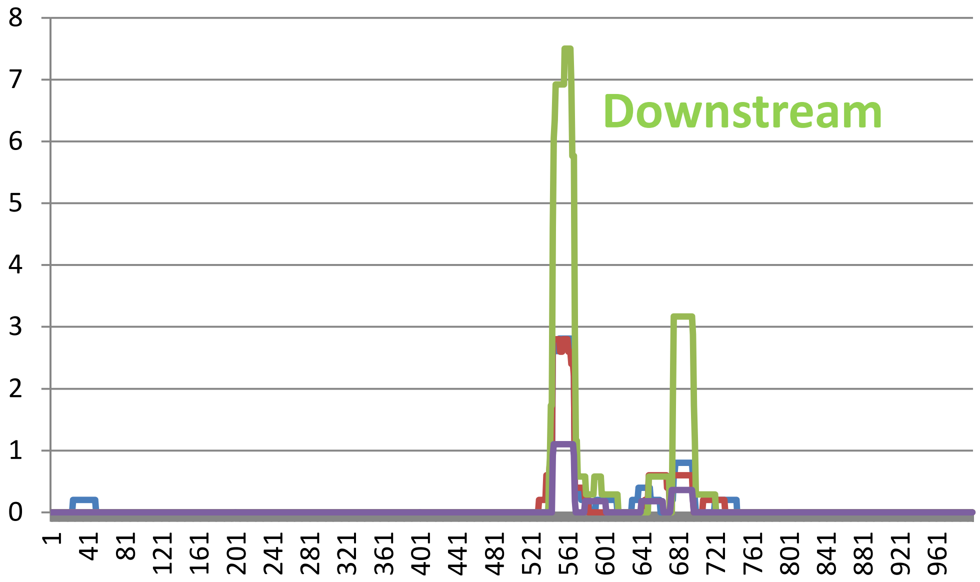

AT5G27660

Trypsin family protein with PDZ domain

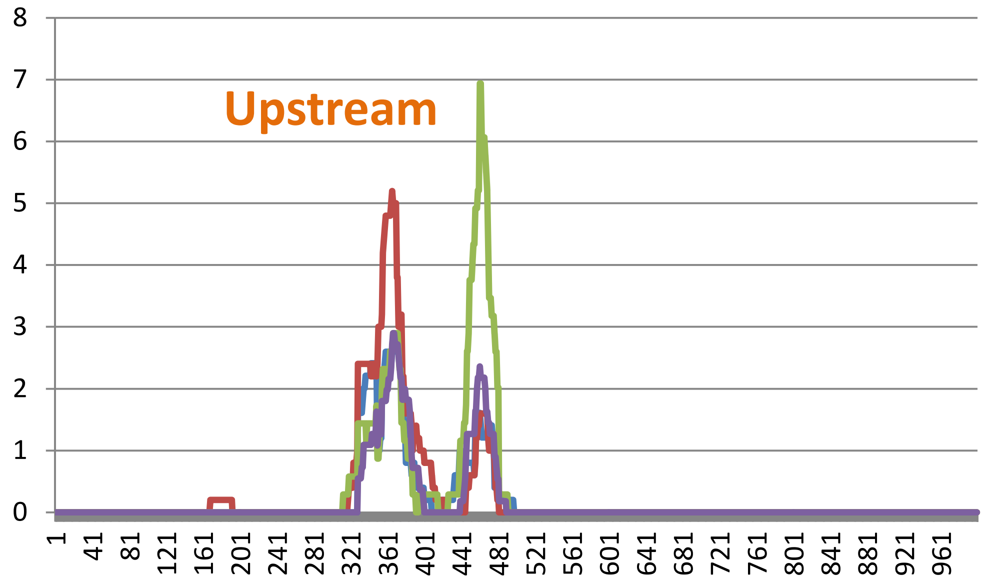

AT5G27870

Plant invertase/pectin methylesterase inhibitor superfamily

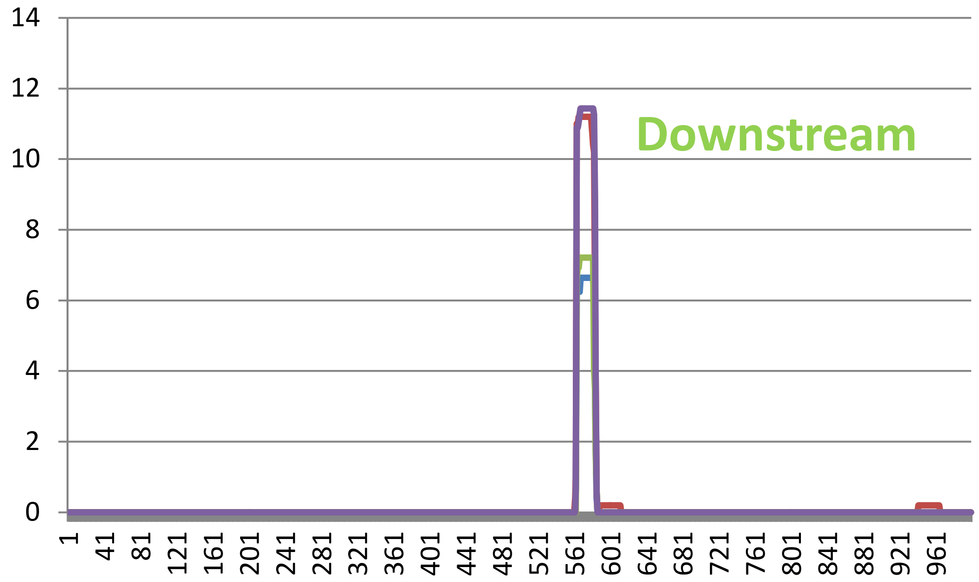

AT5G27880

C2H2 and C2HC zinc fingers superfamily protein

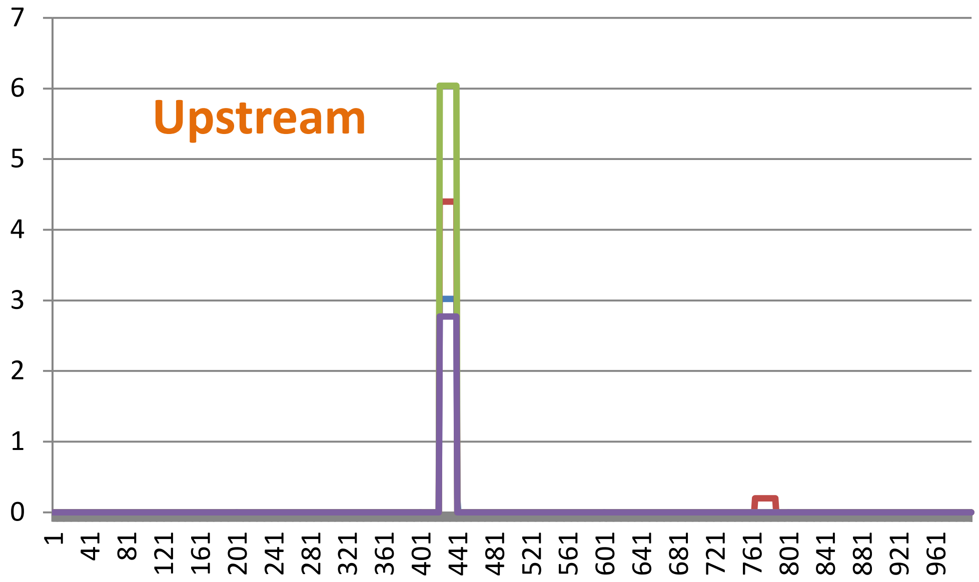

AT5G28442

BEST Arabidopsis thaliana protein match is: Quinoprotein amine dehydrogenase, beta chain-like; RIC1-like guanyl-nucleotide exchange factor (TAIR:AT5G28350.1).

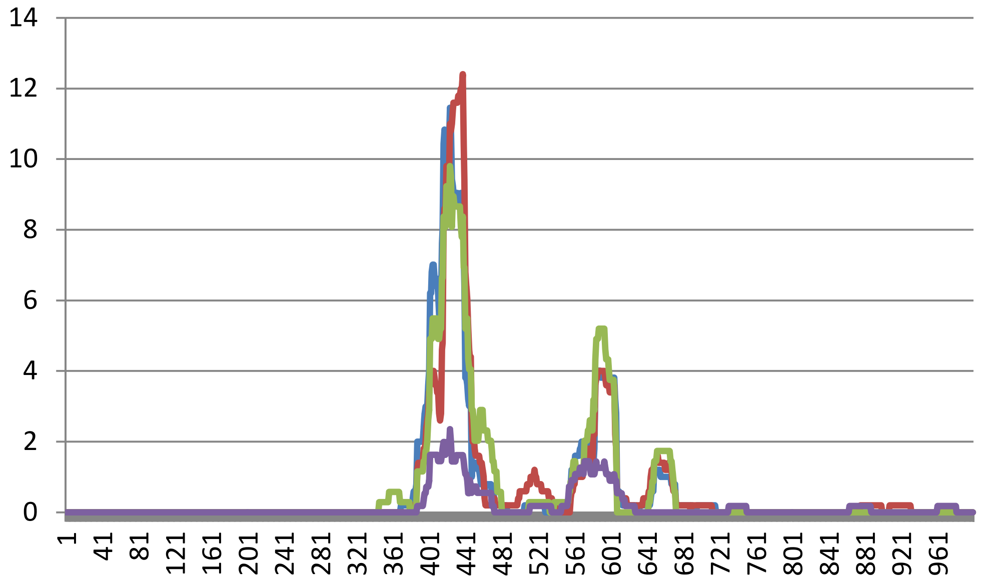

AT5G29000

Homeodomain-like superfamily protein

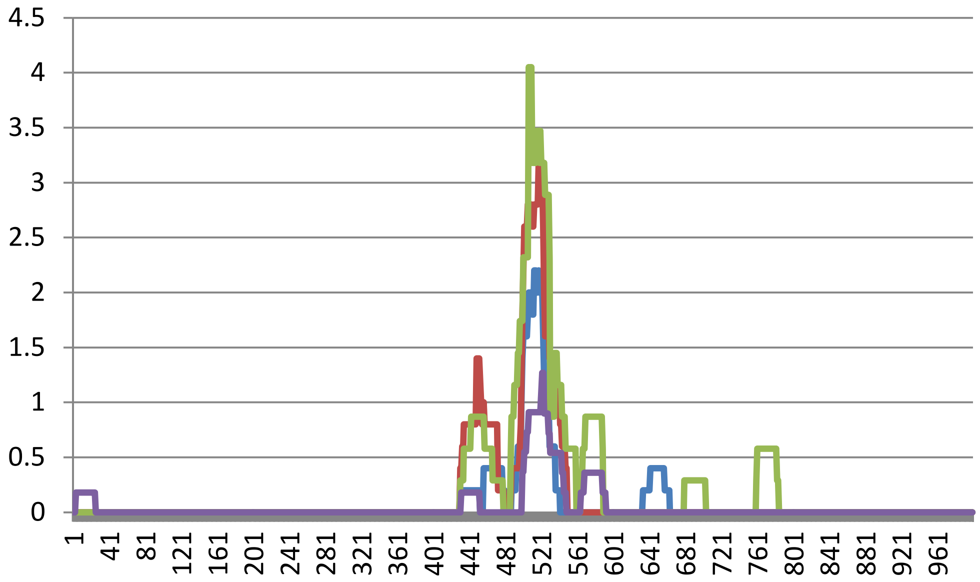

AT5G34882

Encodes a ECA1 gametogenesis related family protein

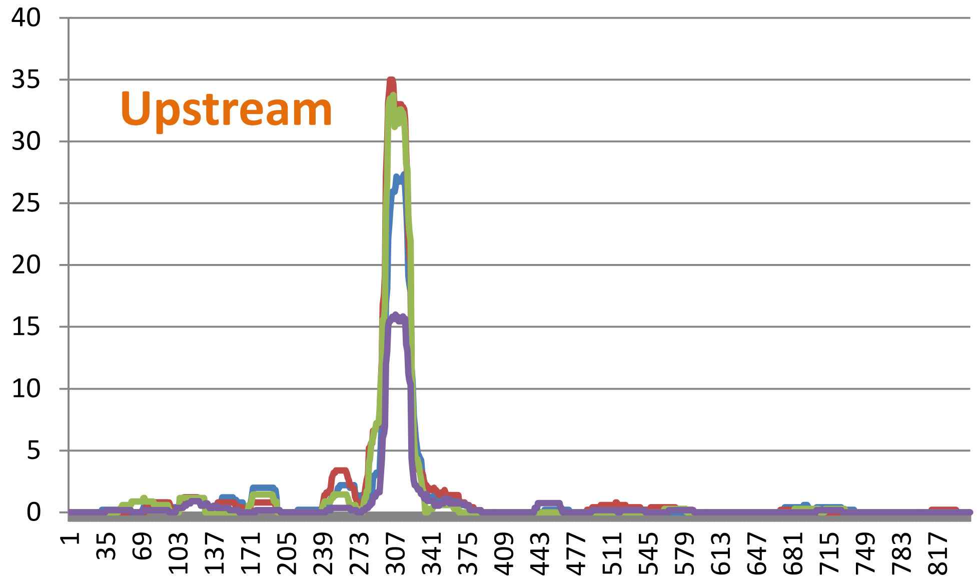

AT5G34883

Protein of unknown function (DUF784)

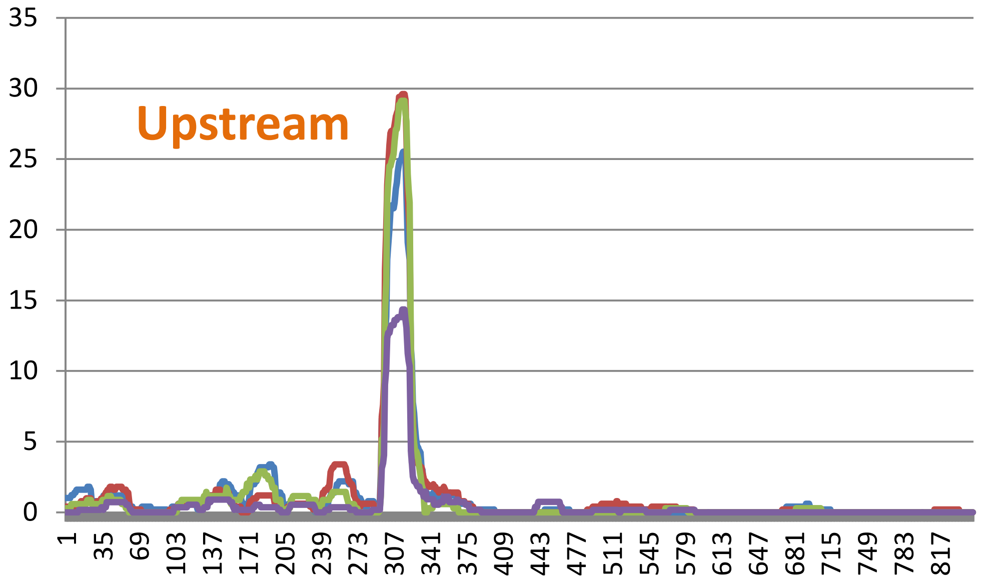

AT5G36140

CYTOCHROME P450, FAMILY 716, SUBFAMILY A, POLYPEPTIDE 2 (CYP716A2).
Member of CYP716A.

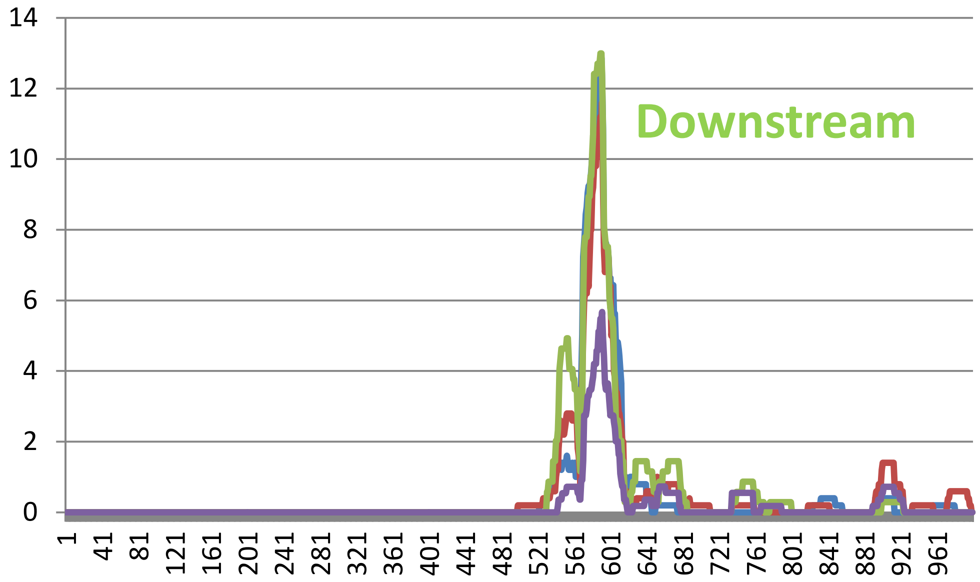

AT5G36220

CYTOCHROME P450, FAMILY 81, SUBFAMILY D, POLYPEPTIDE 1 (CYP81D1). Member of CYP81D family of cytochrome p450s.

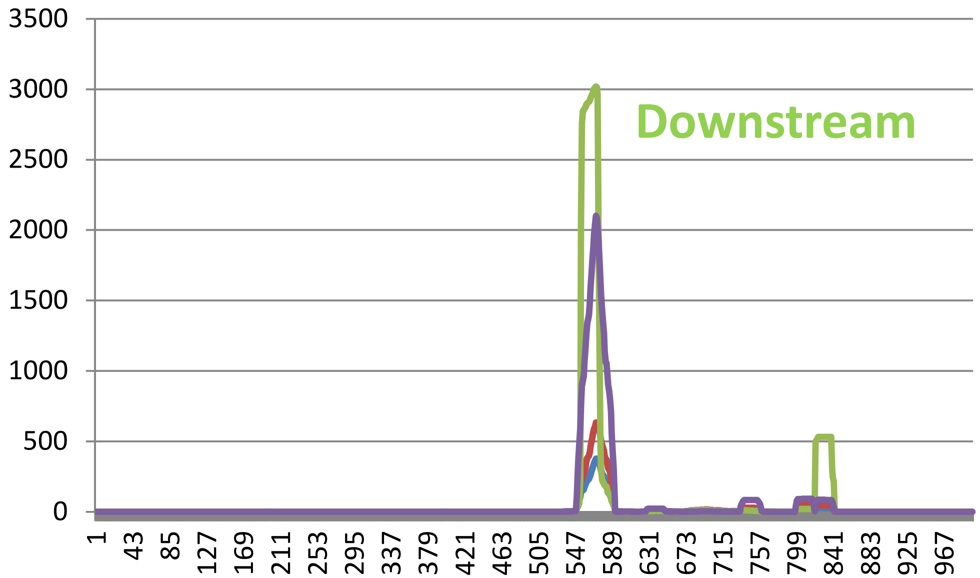

AT5G37420

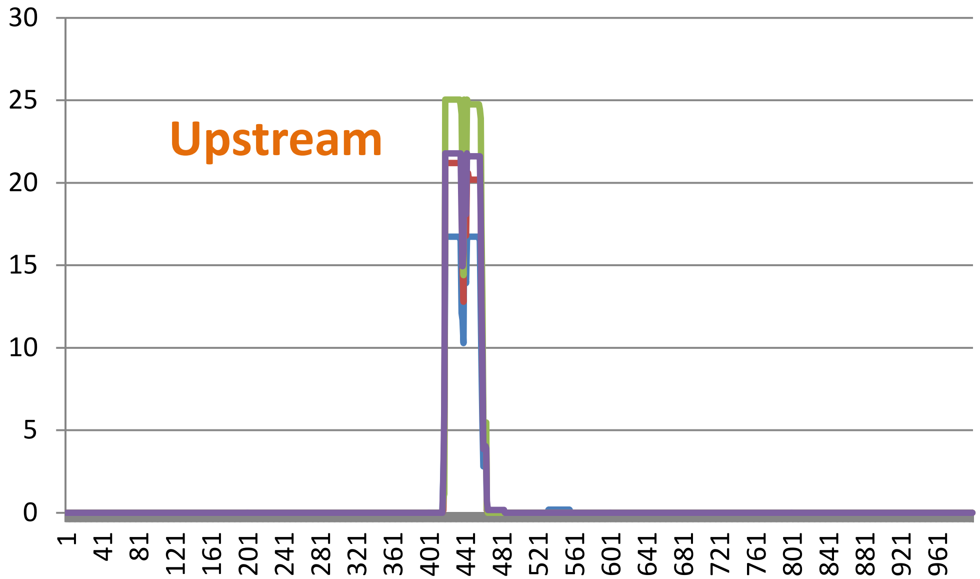

30
25
20
15
10
5
0
Upstream
1
41
81
121
161
201
241
281
321
361
401
441
481
521
561
601
641
681
721
761
801
841
881
921
961

AT5G37430

Family of unknown function (DUF577)

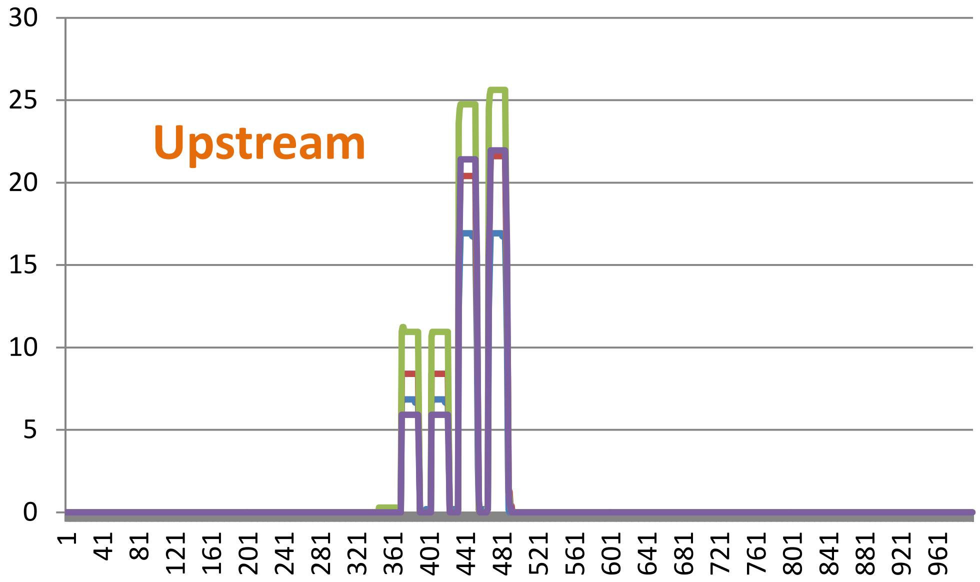

AT5G37440

Chaperone DnaJ-domain superfamily protein

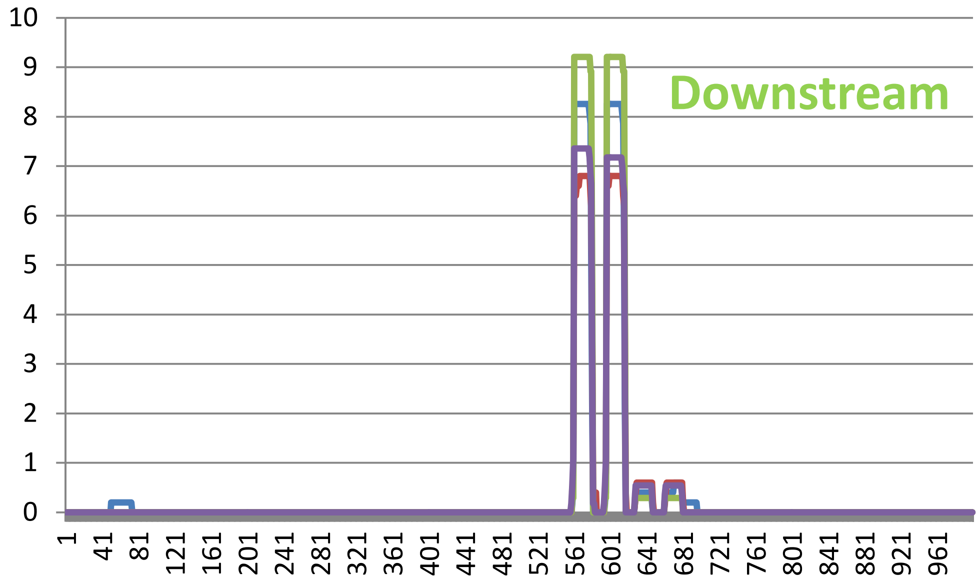

AT5G37690

SGNH hydrolase-type esterase superfamily protein

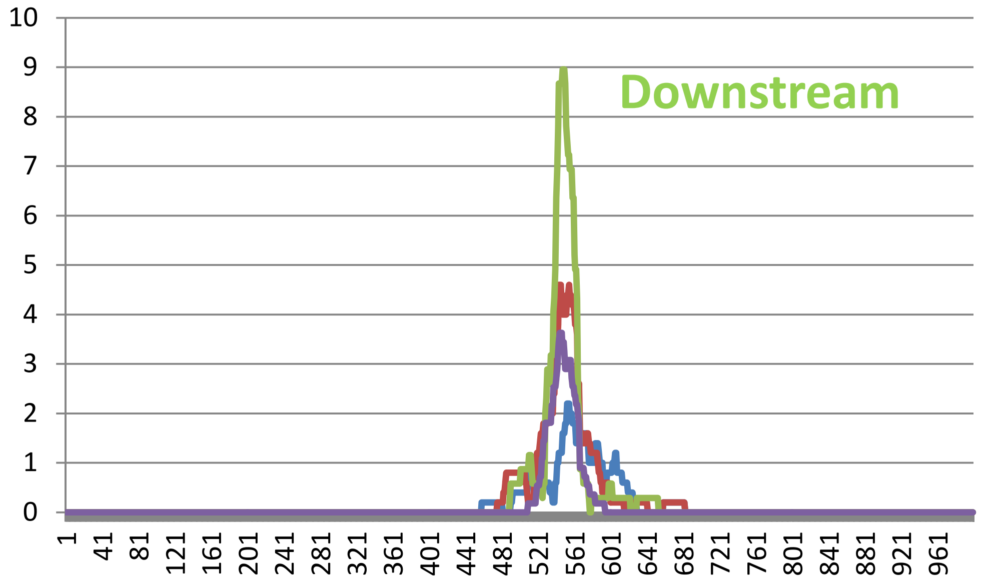

AT5G38440

Plant self-incompatibility protein S1 family

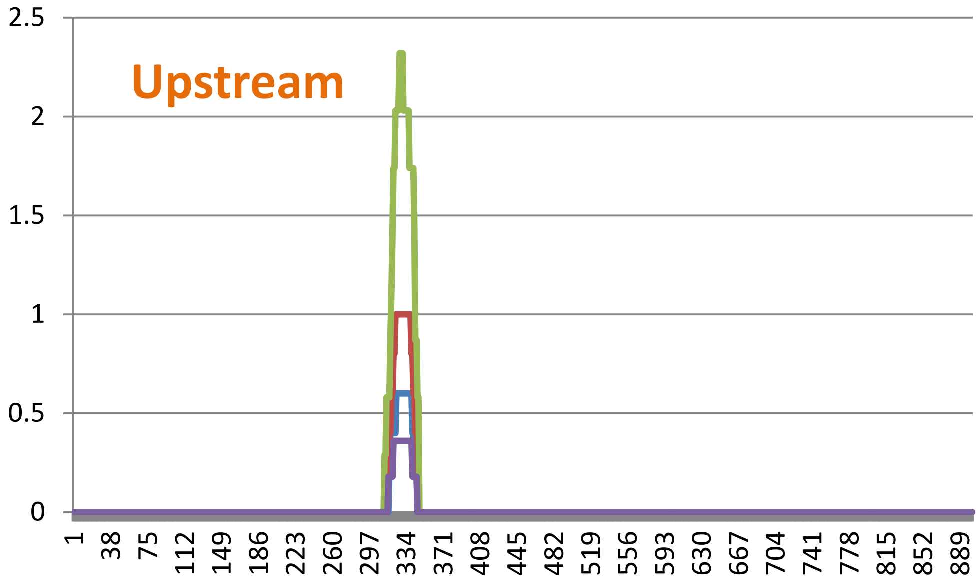

AT5G39080

HXXXD-type acyl-transferase family protein

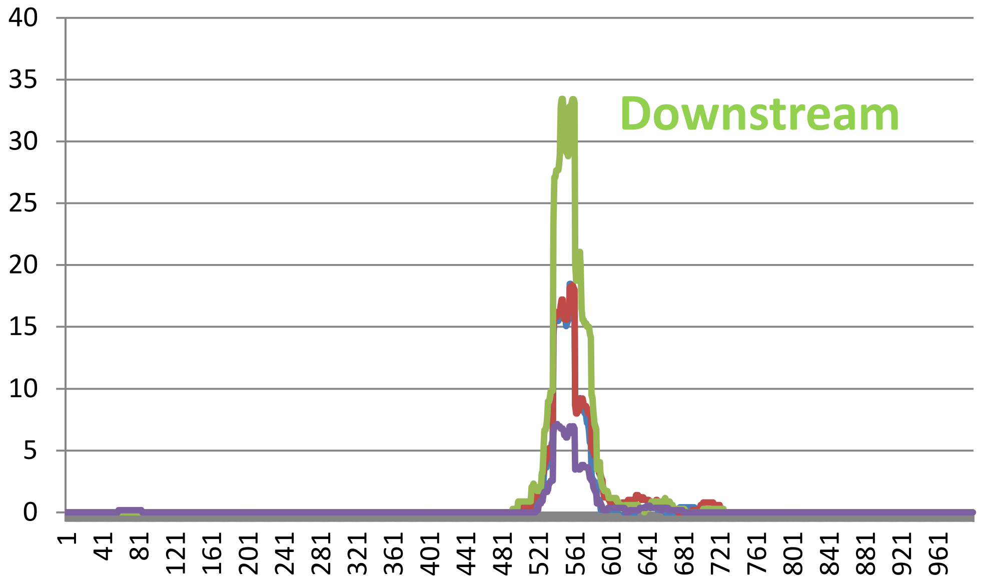

AT5G39720

Avirulence induced gene 2 like protein (AIG2L)

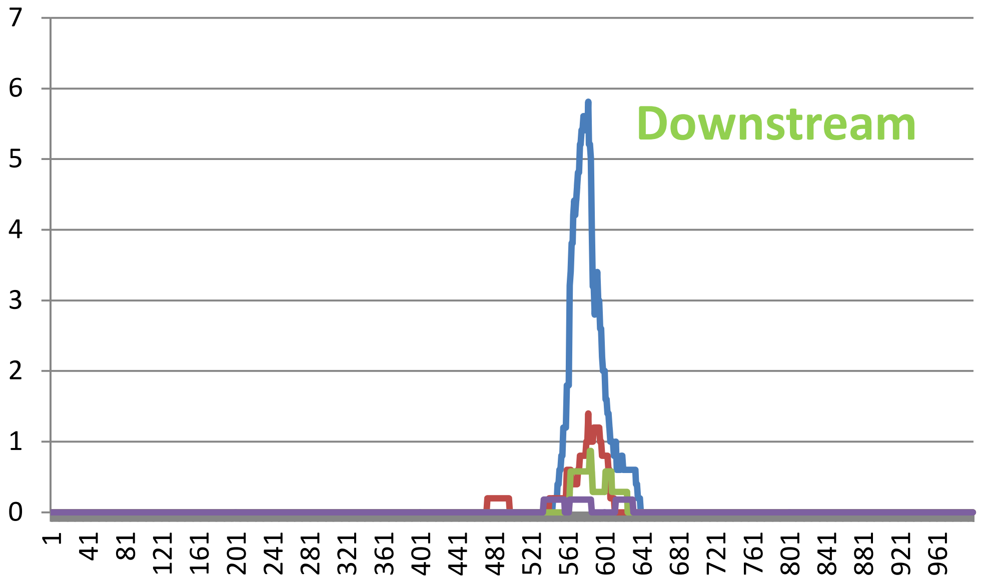

AT5G39890

Protein of unknown function (DUF1637)

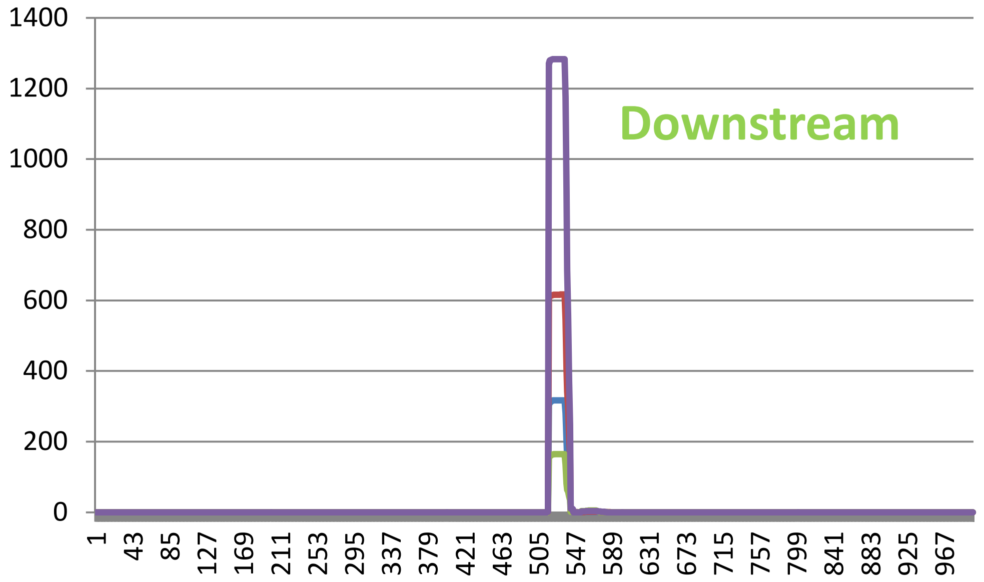

AT5G41150

Confers resistance to UV radiation. Homolog of the human xeroderma pigmentosum group F DNA repair and yeast Rad1 proteins

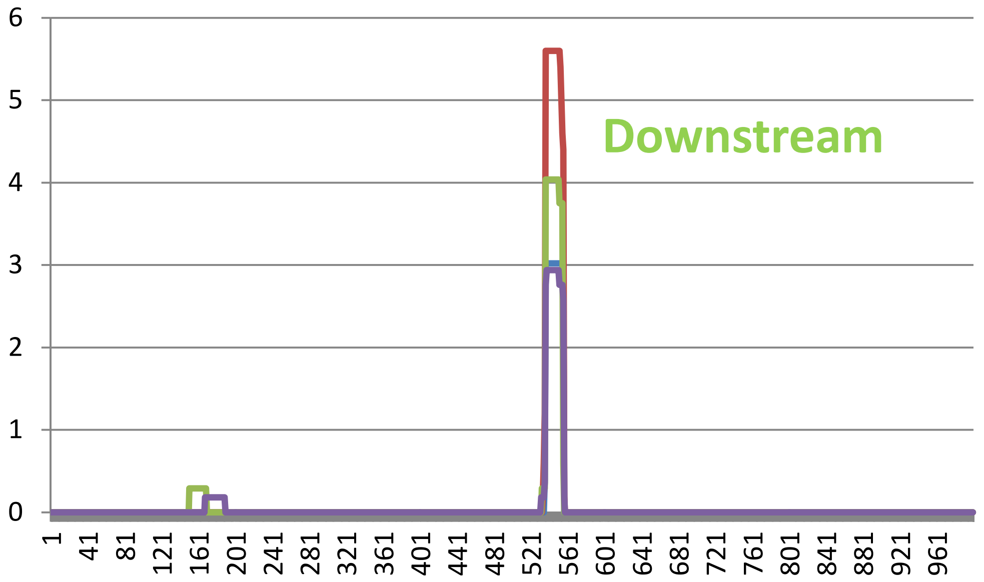

AT5G42203

This gene encodes a small protein and has either evidence of transcription or purifying selection.

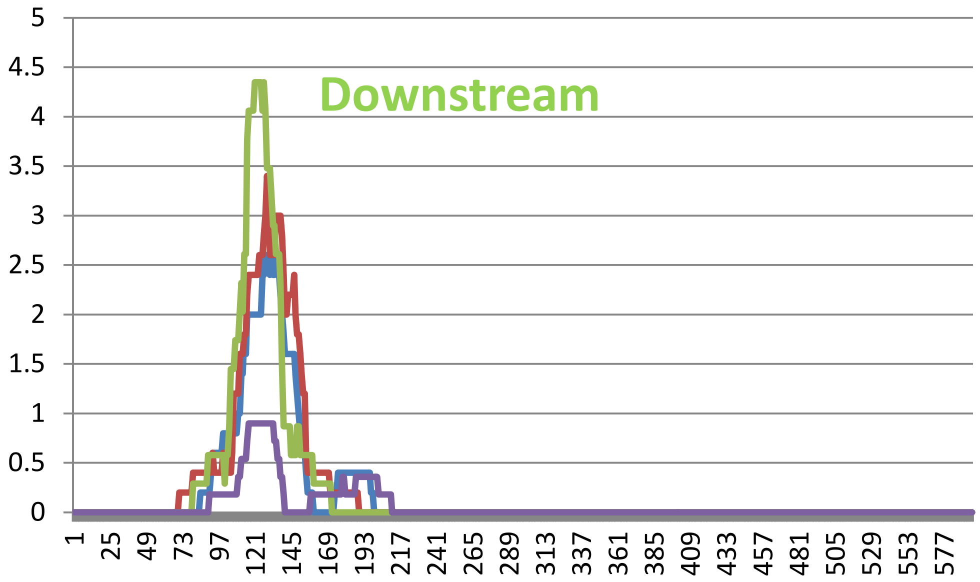

AT5G42567

Encodes a ECA1 gametogenesis related family protein

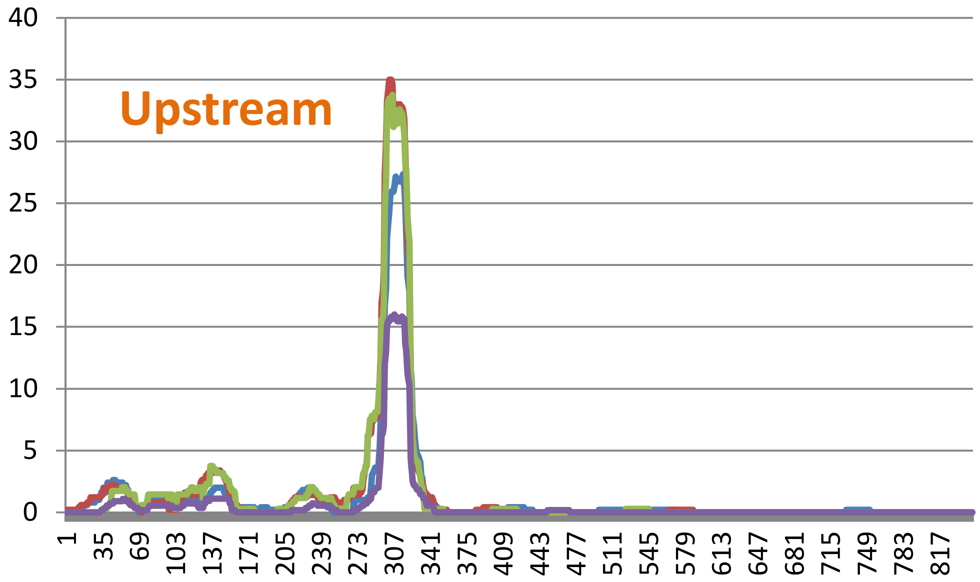

AT5G42635

Glycine-rich protein

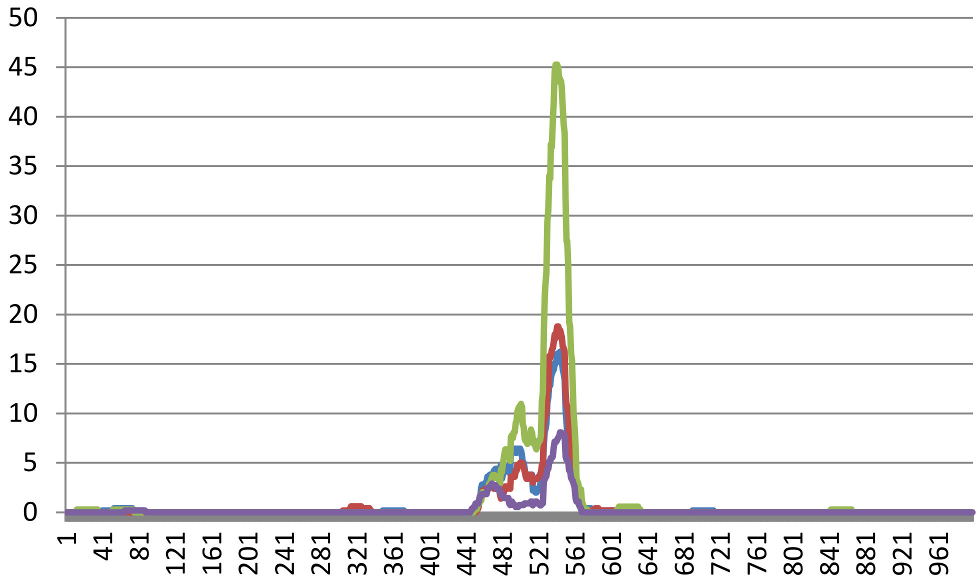

AT5G42930

Alpha/beta-Hydrolases superfamily protein

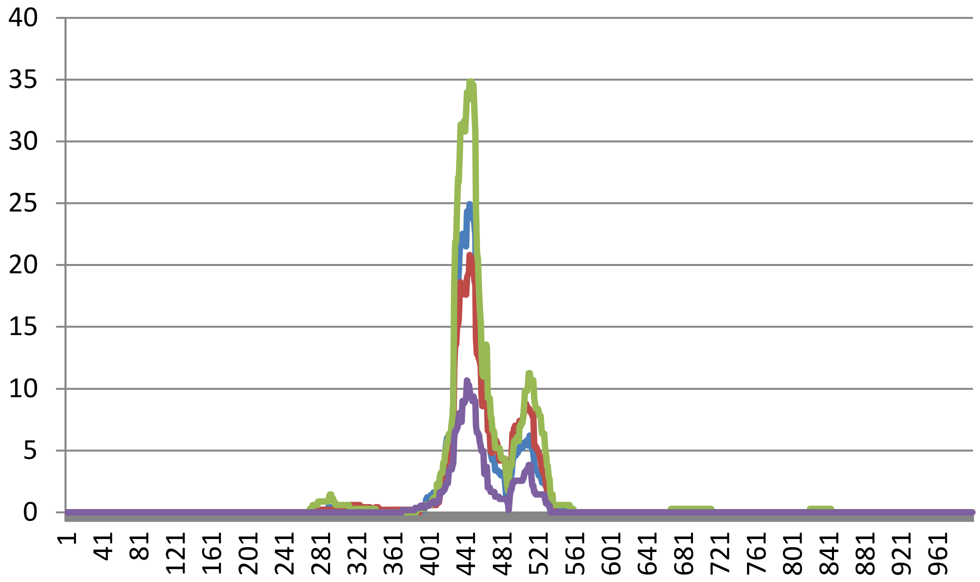

AT5G43285

Encodes a defensin-like (DEFL) family protein.

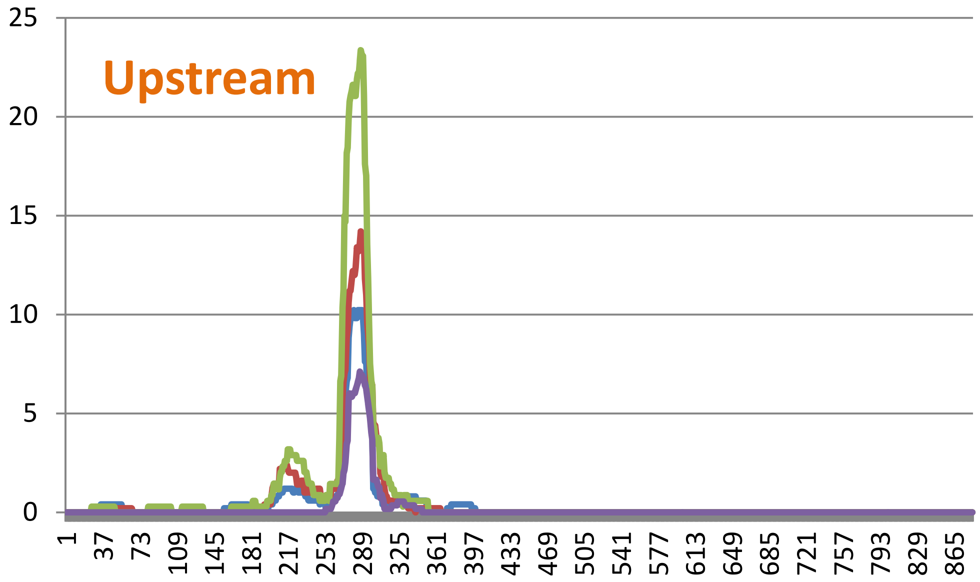

AT5G43525

Encodes a defensin-like (DEFL) family protein.

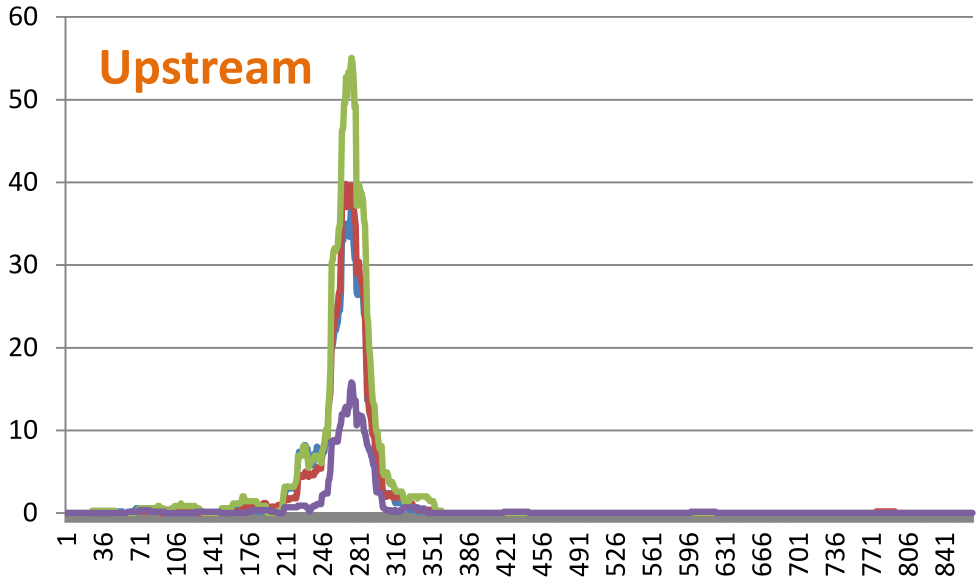

AT5G43755

BEST Arabidopsis thaliana protein match is: Polynucleotidyl transferase, ribonuclease H-like superfamily protein (TAIR:AT2G04420.1).

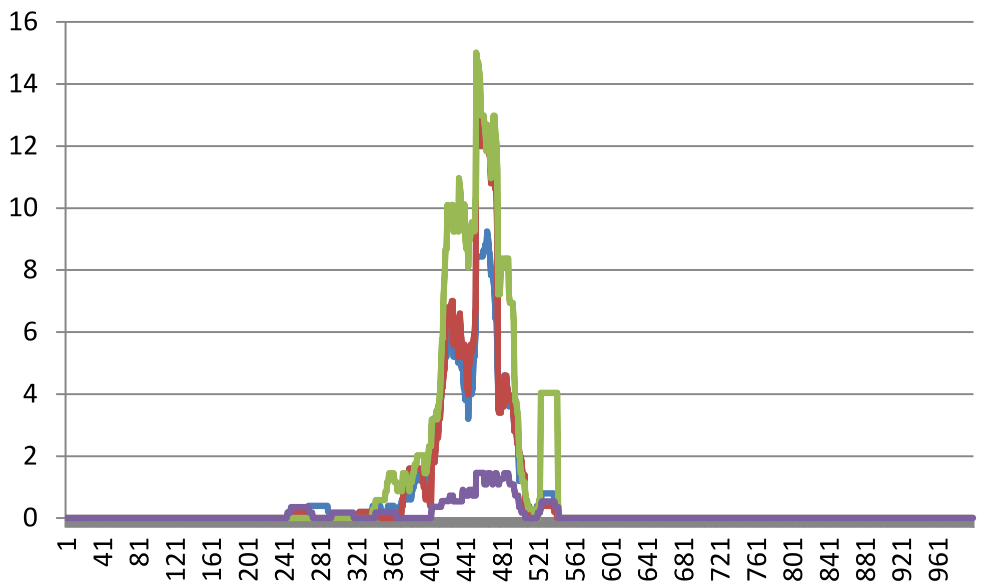

AT5G44870

Encodes LAZ5, a TIR-class NB-LRR R protein of unknown pathogen specificity with sequence similarity to RPS4, an R protein conferring resistance to Pseudomonas syringae expressing the effector AvrRPS4. Overexpression of LAZ5 results in hypersensitive cell death (plants did not survive to set seeds).

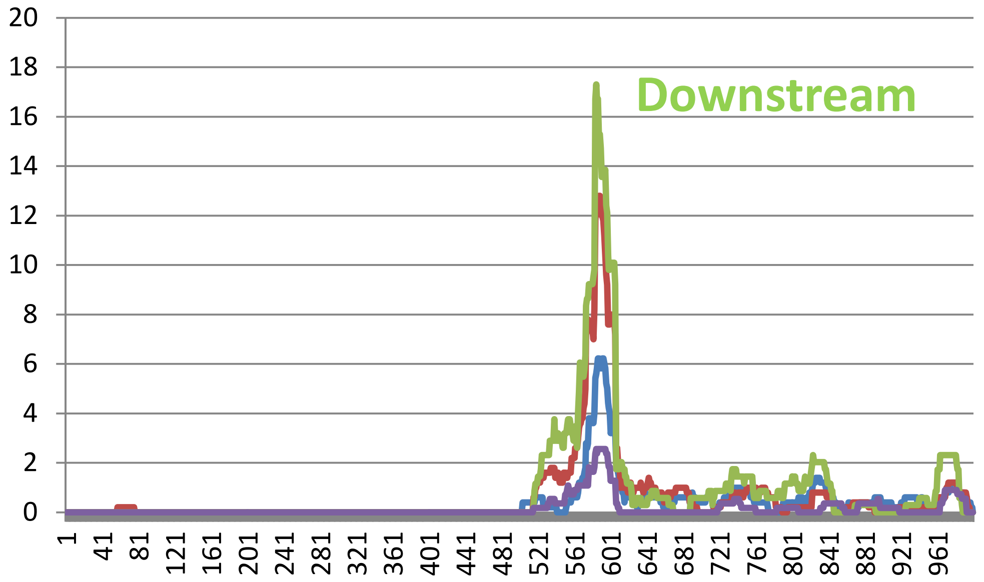

AT5G46750

A member of ARF GAP domain (AGD), A thaliana has 15 members, grouped into four classes.

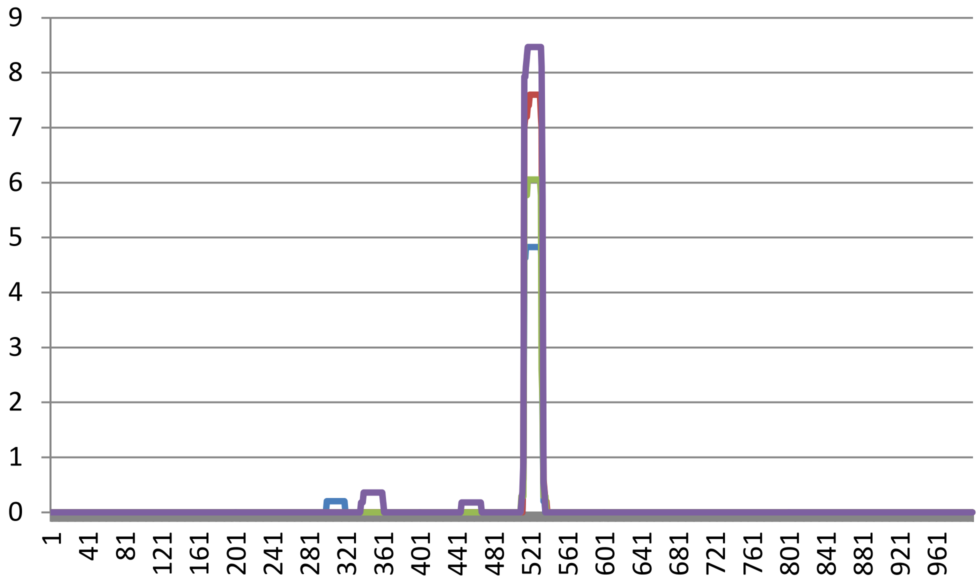

AT5G48515

Encodes a defensin-like (DEFL) family protein.

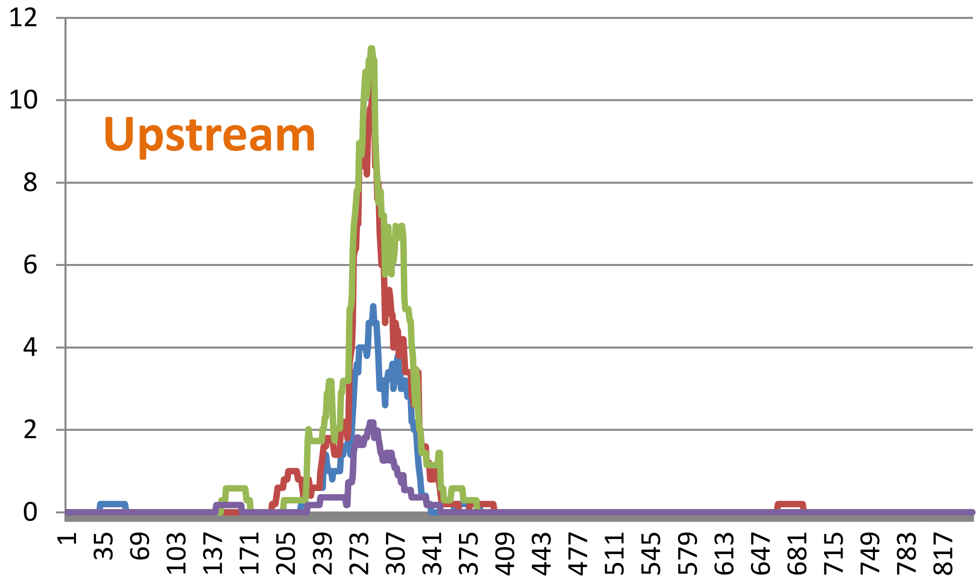

AT5G48595

Encodes a defensin-like (DEFL) family protein.

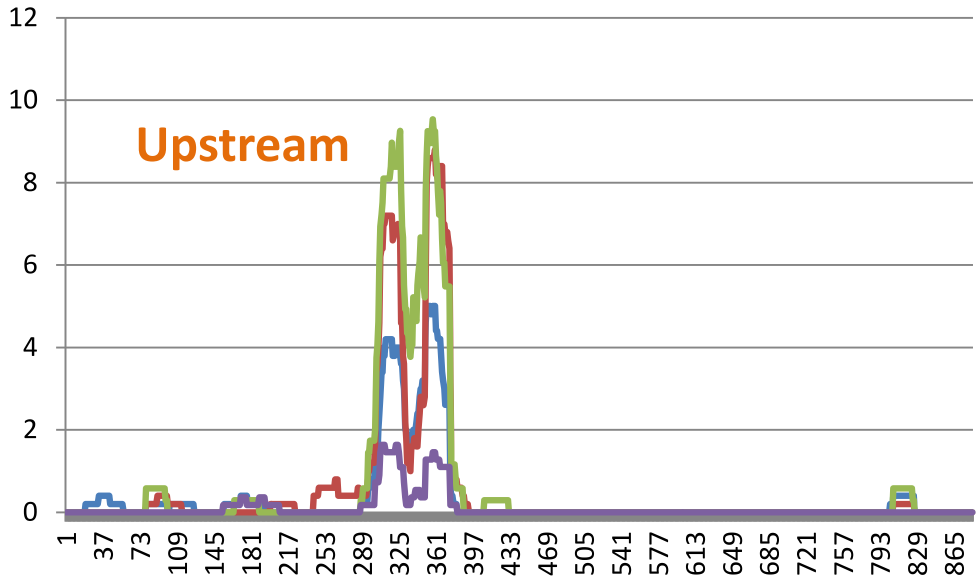

AT5G49420

MADS-box transcription factor family protein.

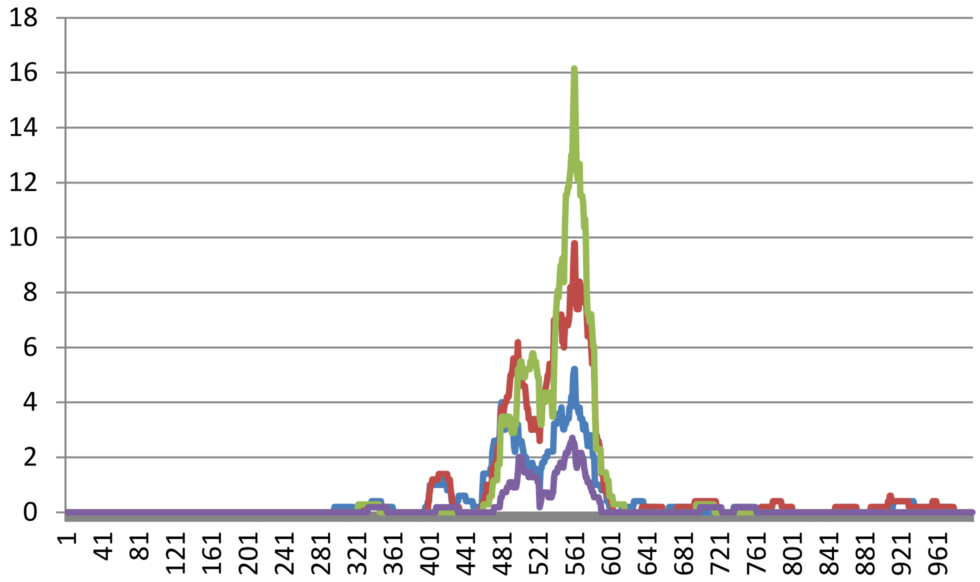

AT5G49440

Unknown protein

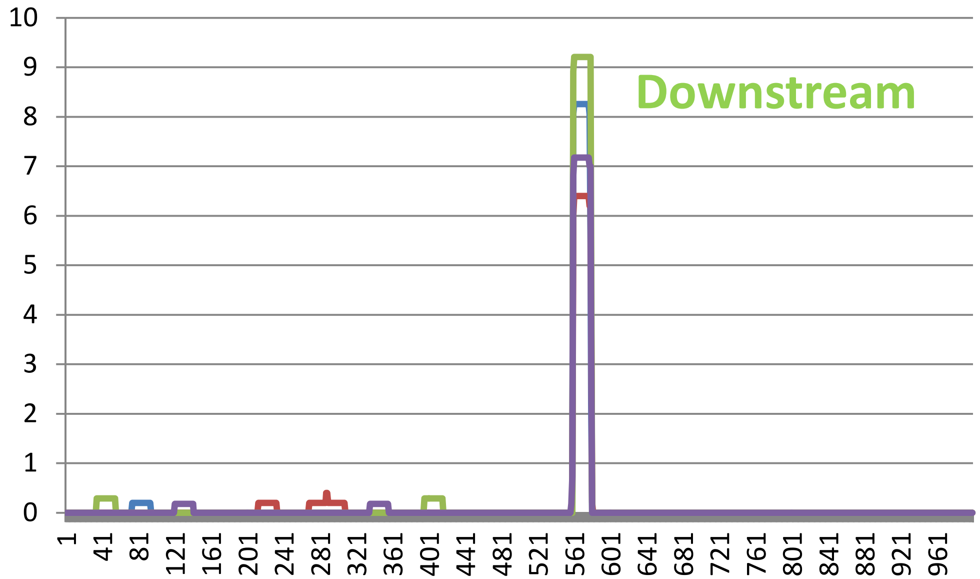

AT5G50480

Nuclear factor Y, subunit C6 (NF-YC6)

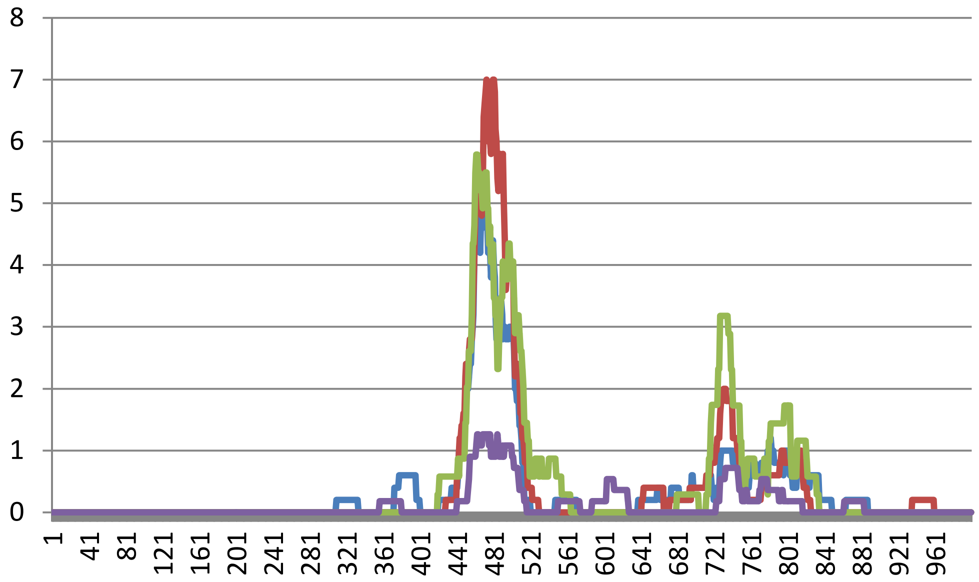

AT5G52360

Actin depolymerizing factor 10 (ADF10)

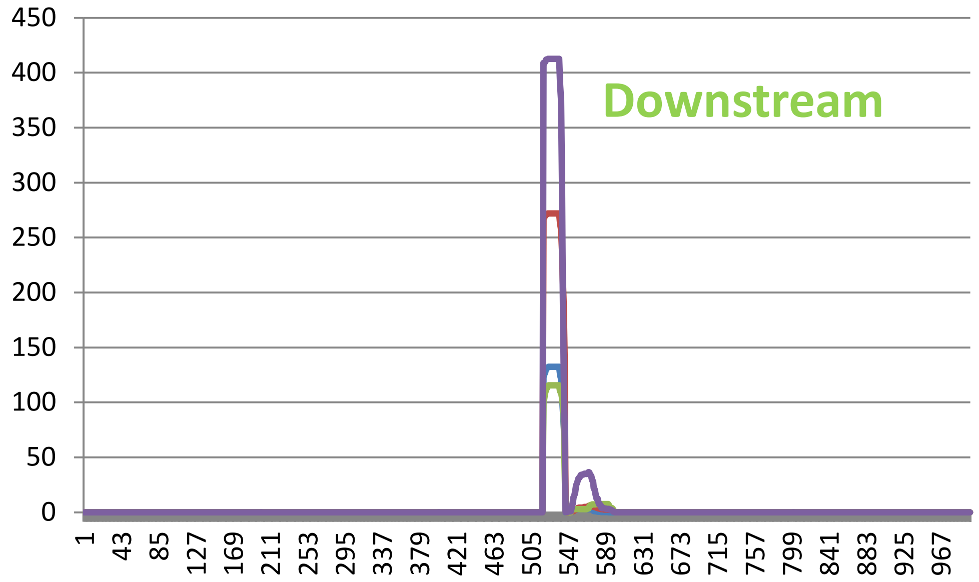

AT5G54070

A member of Heat Stress Transcription Factor (Hsf) family. Not responding to heat stress. Is regulated by the seed-specific transcription factor ABI3. In turn, it regulates other heat stress proteins including Hsp17.4-CI, Hsp17.7-CII and Hsp101 during seed maturation.

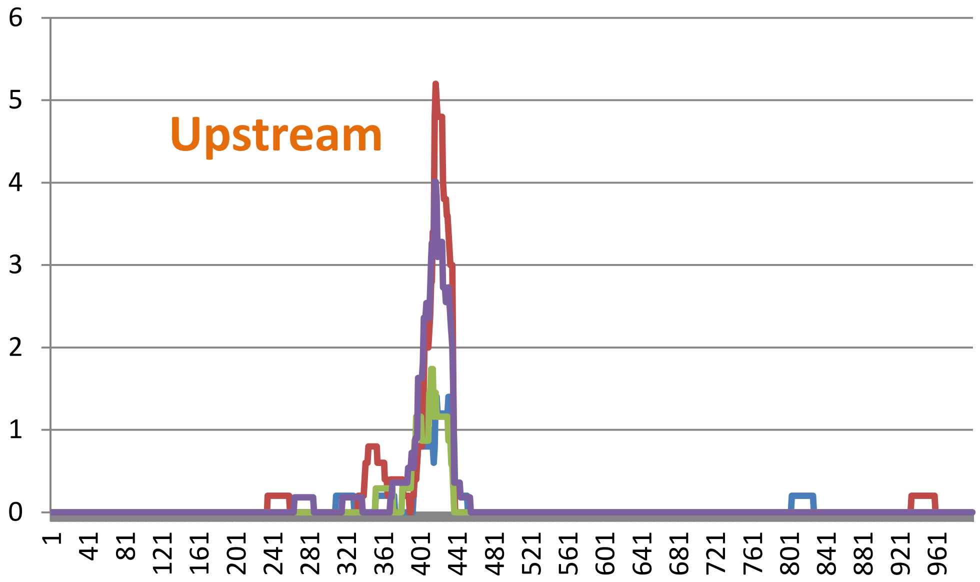

AT5G54370

Late embryogenesis abundant (LEA) protein-related.

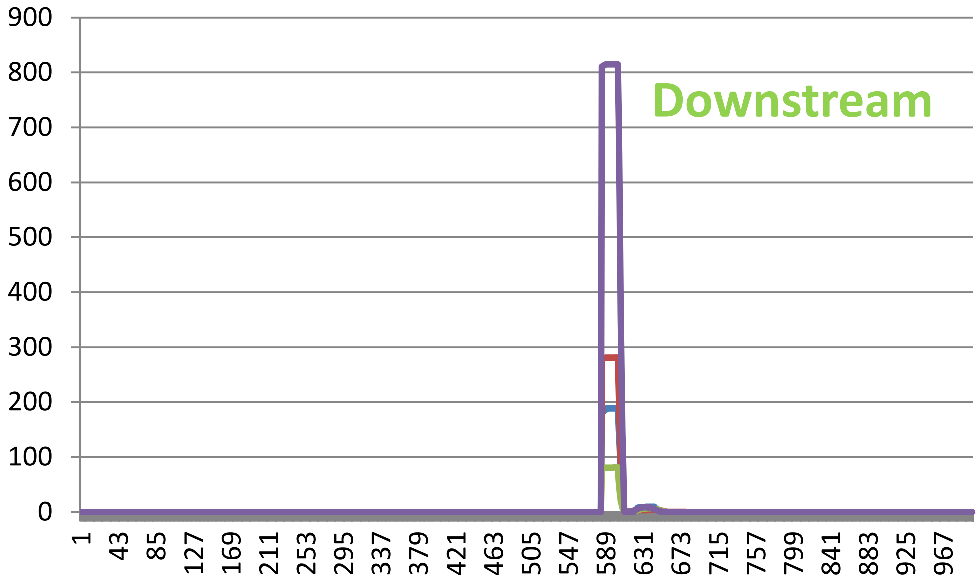

AT5G54410

Unknown protein

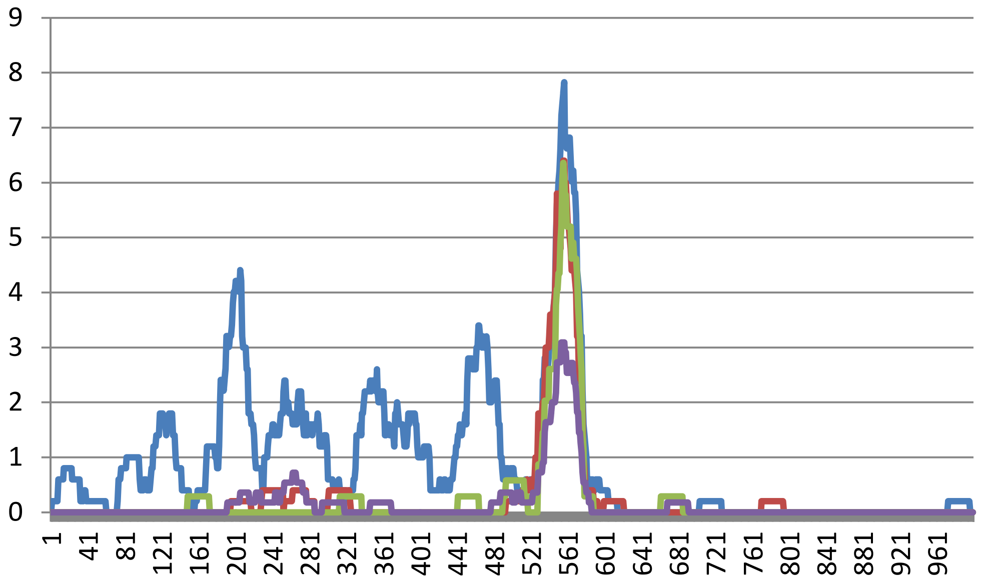

AT5G54700

Ankyrin repeat family protein

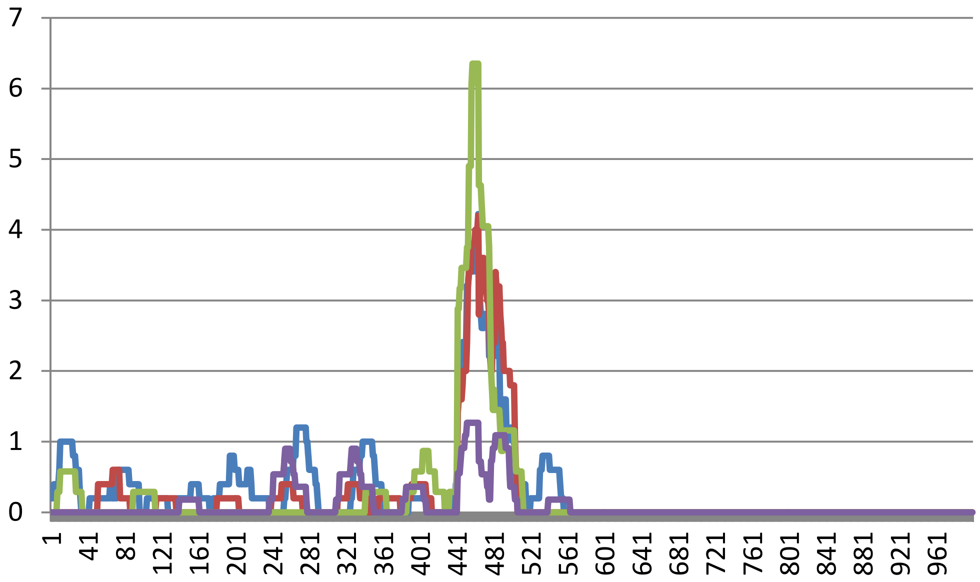

AT5G55110

Stigma-specific Stig1 family protein

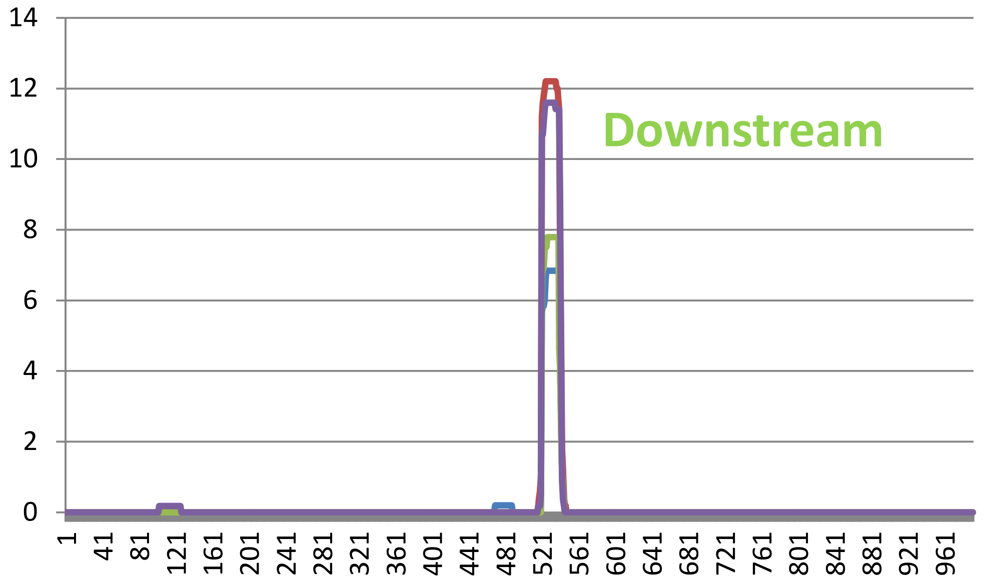

AT5G60280

Concanavalin A-like lectin protein kinase family protein

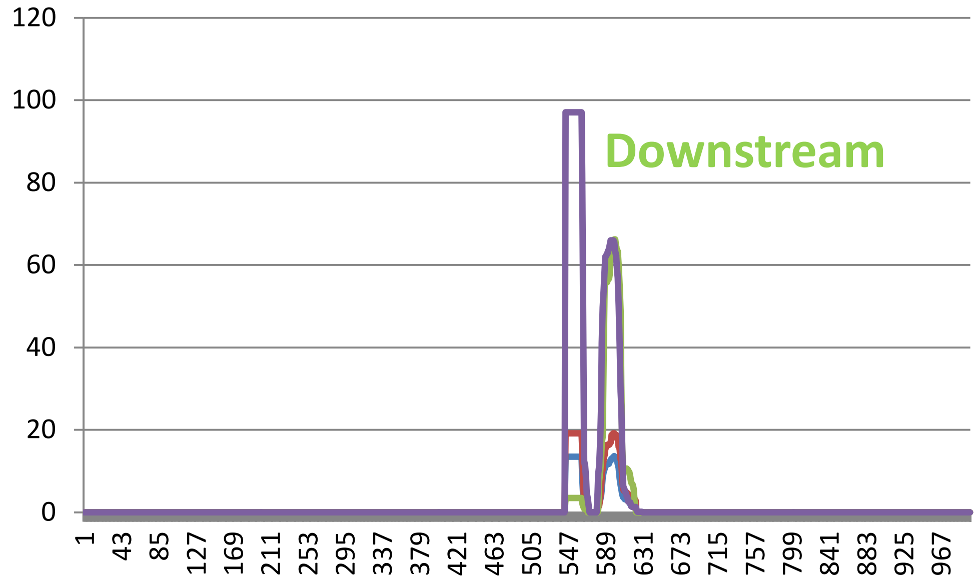

AT5G60548

Upstream open reading frames (uORFs) are small open reading frames found in the 5' UTR of a mature mRNA, and can potentially mediate translational regulation of the largest, or major, ORF (mORF).

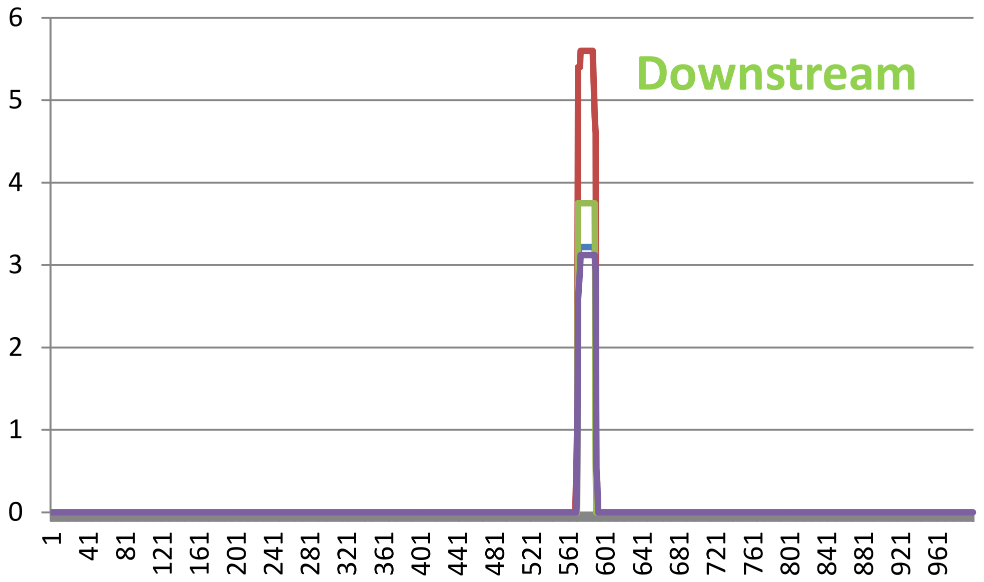

AT5G60550

GEMINIVIRUS REP INTERACTING KINASE 2 (GRIK2). Encodes a geminivirus Rep interacting kinase (GRIK; GRIK1/AT3G45240, GRIK2/AT5G60550). GRIKs are SnRK1 (SNF1-related kinases) activating kinases. Both GRIKs specifically bind to the SnRK1 catalytic subunit and phosphorylate the equivalent threonine residue in its activation loop in vitro.

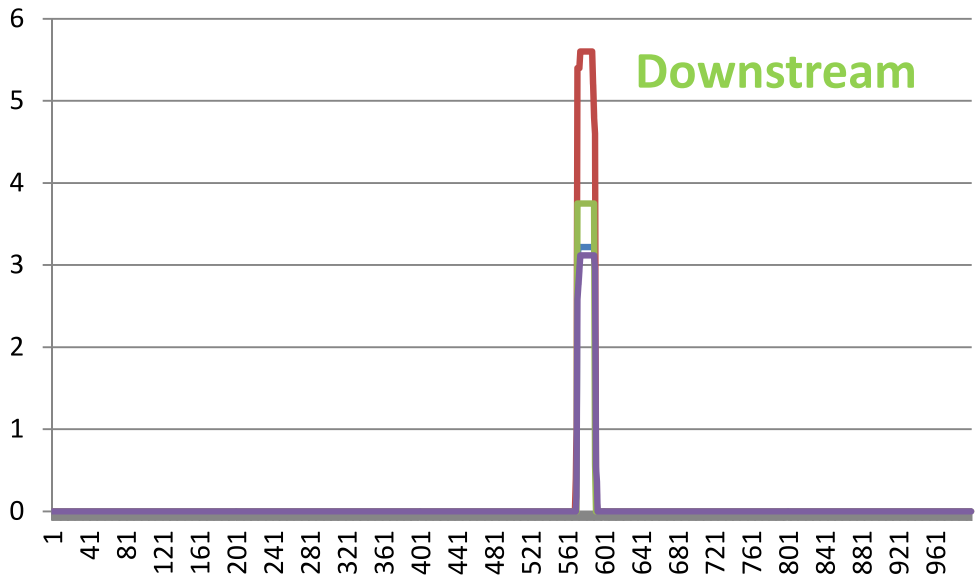

AT5G60553

Encodes a defensin-like (DEFL) family protein.

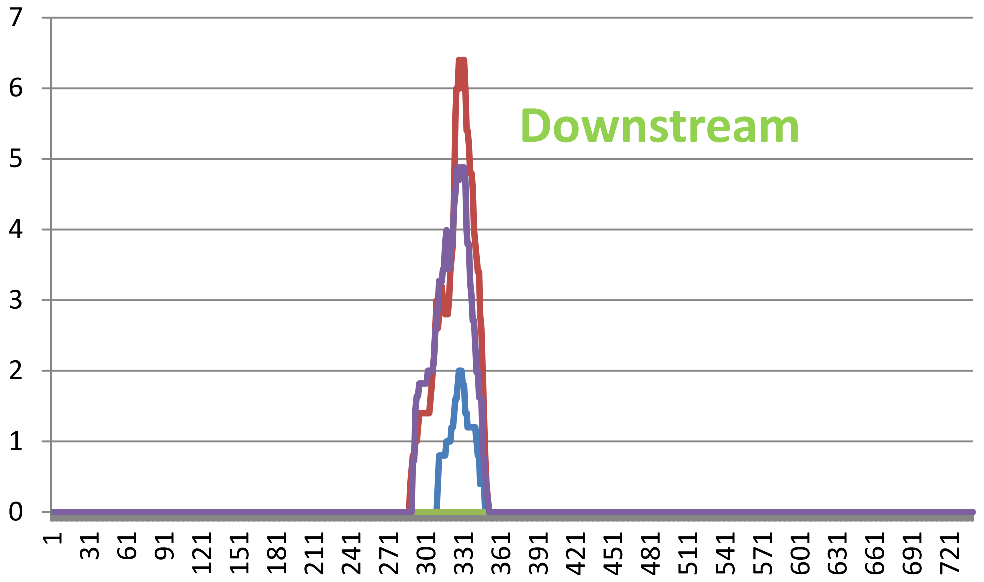

AT5G61520

Major facilitator superfamily protein

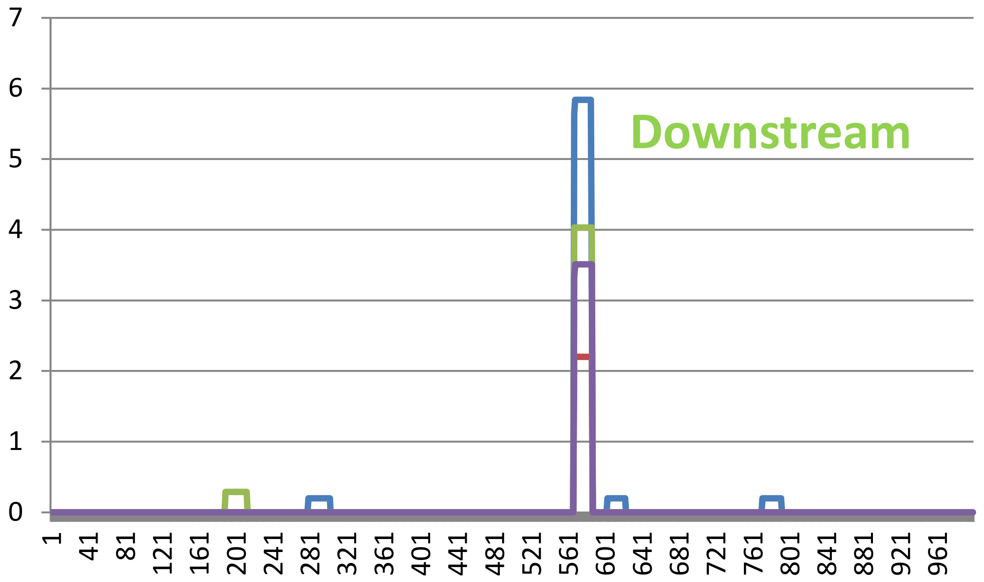

AT5G61830

NAD(P)-binding Rossmann-fold superfamily protein

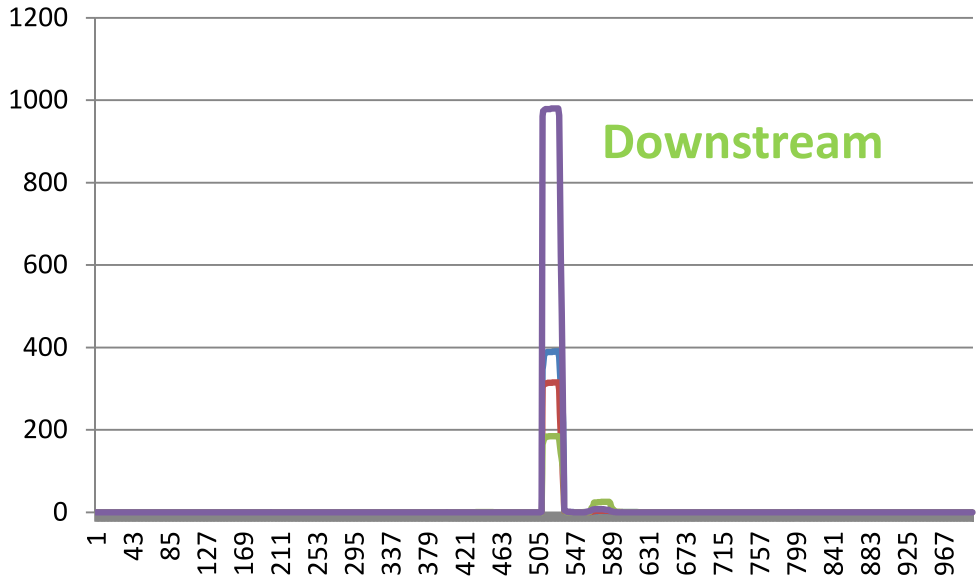

AT5G63950

Chromatin remodeling 24 (CHR24)

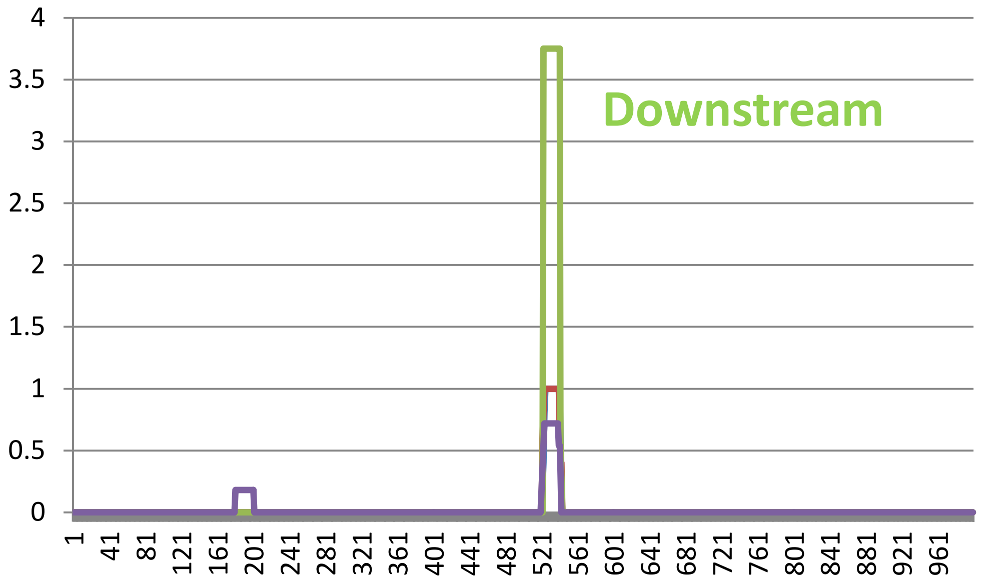

AT5G65005

Polynucleotidyl transferase, ribonuclease H-like superfamily protein.

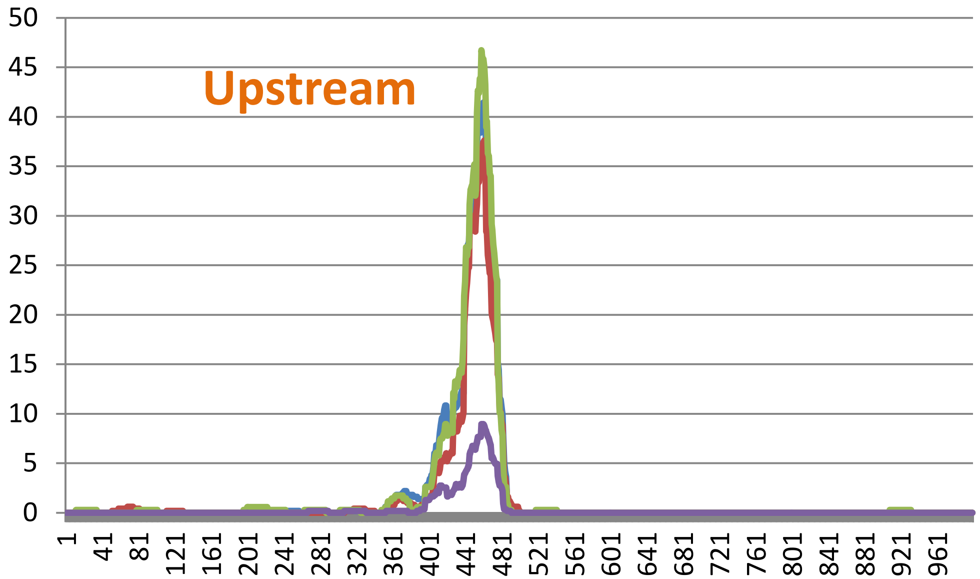

AT5G65430

GENERAL REGULATORY FACTOR 8 (GRF8). Member of 14-3-3 proteins. This protein is reported to interact with the BZR1 transcription factor involved in brassinosteroid signaling and may affect the nucleocytoplasmic shuttling of BZR1.

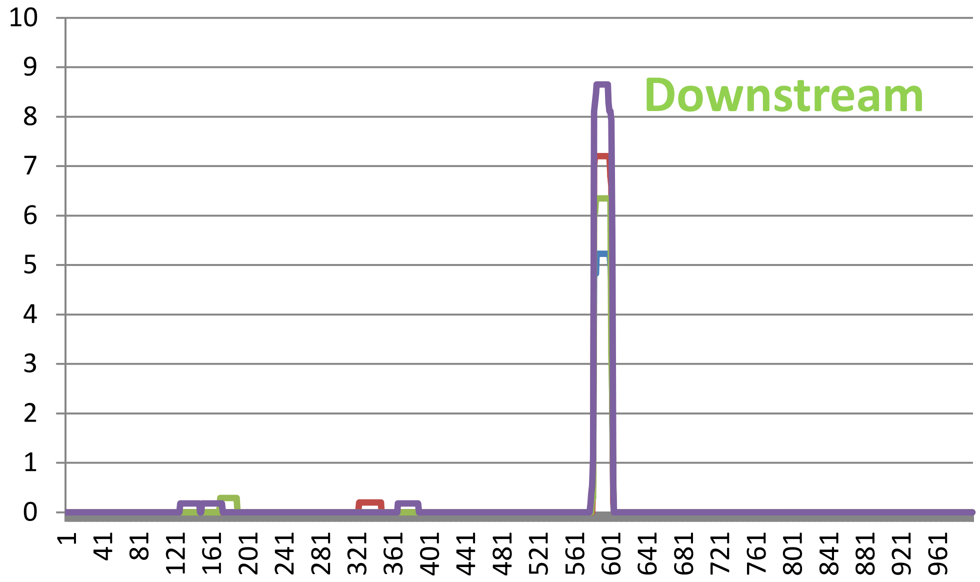

AT5G66090

Unknown protein

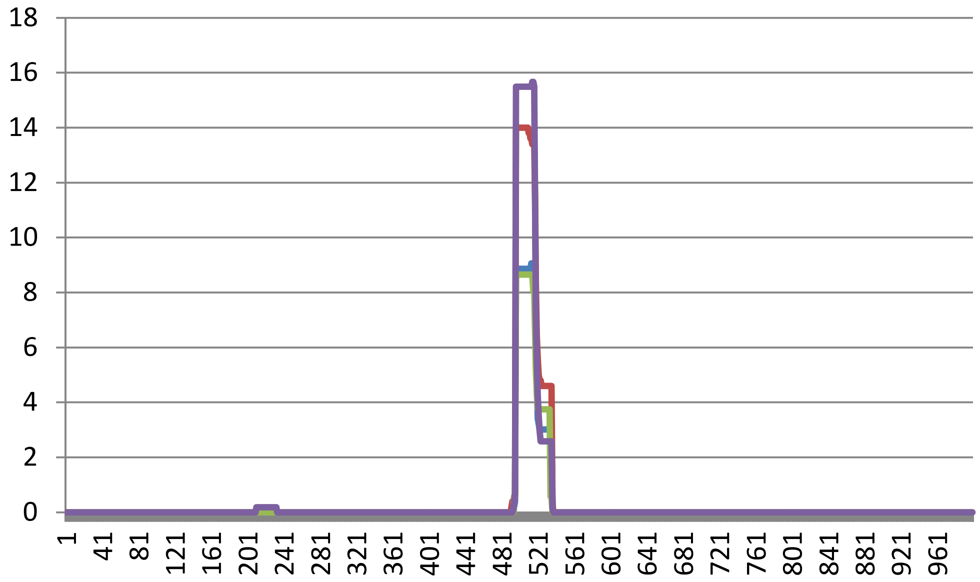

ATCG00130

ATPase F subunit.

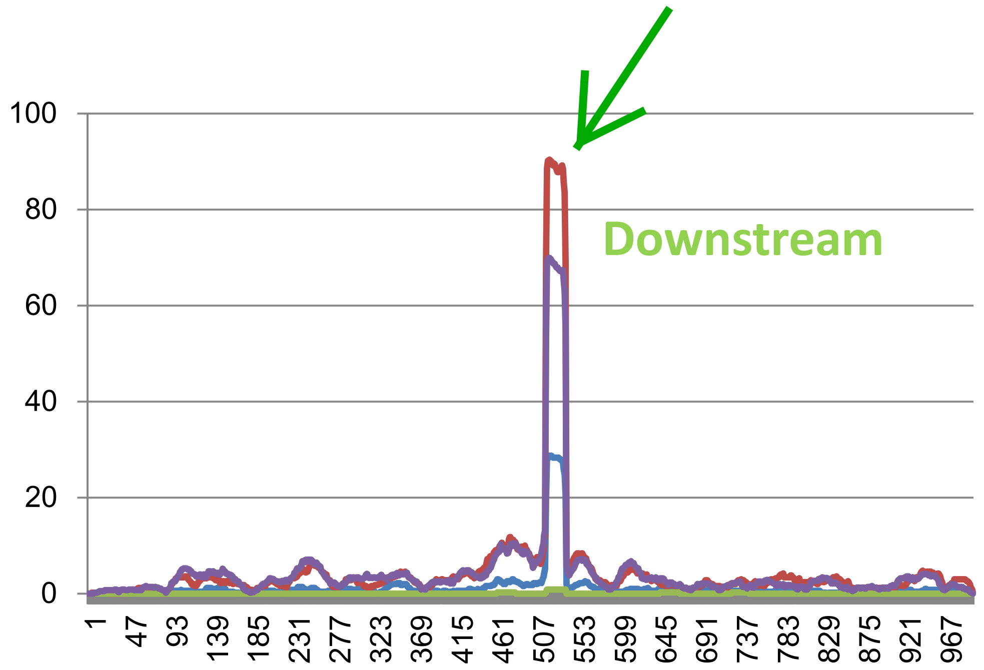

ATCG00270

PSII D2 protein

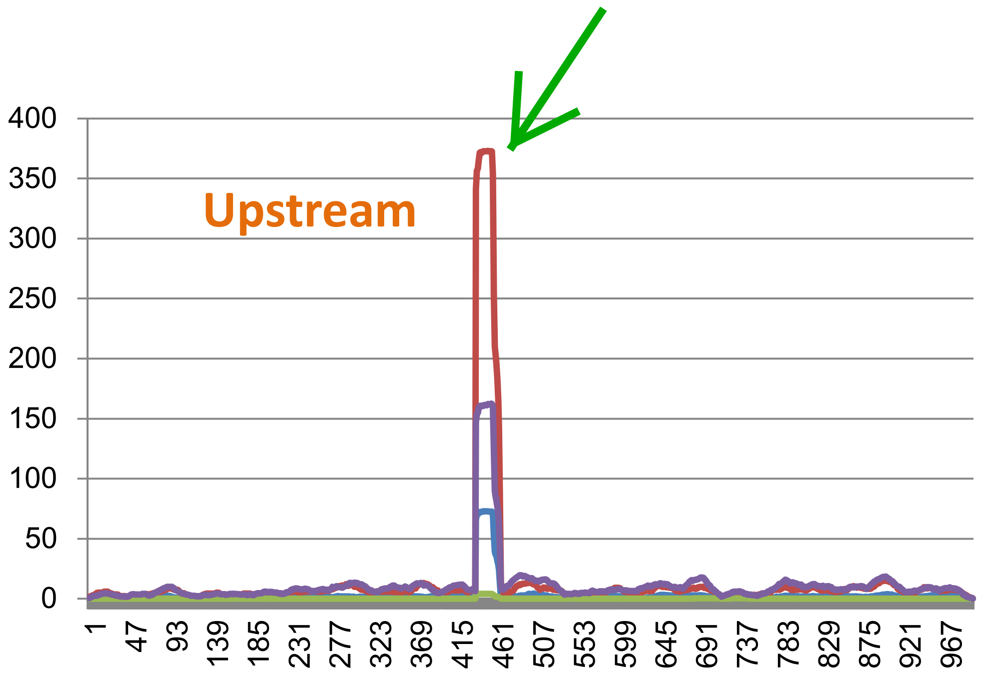

ATCG00420

Encodes NADH dehydrogenase subunit J. Its transcription is increased upon sulfur depletion.

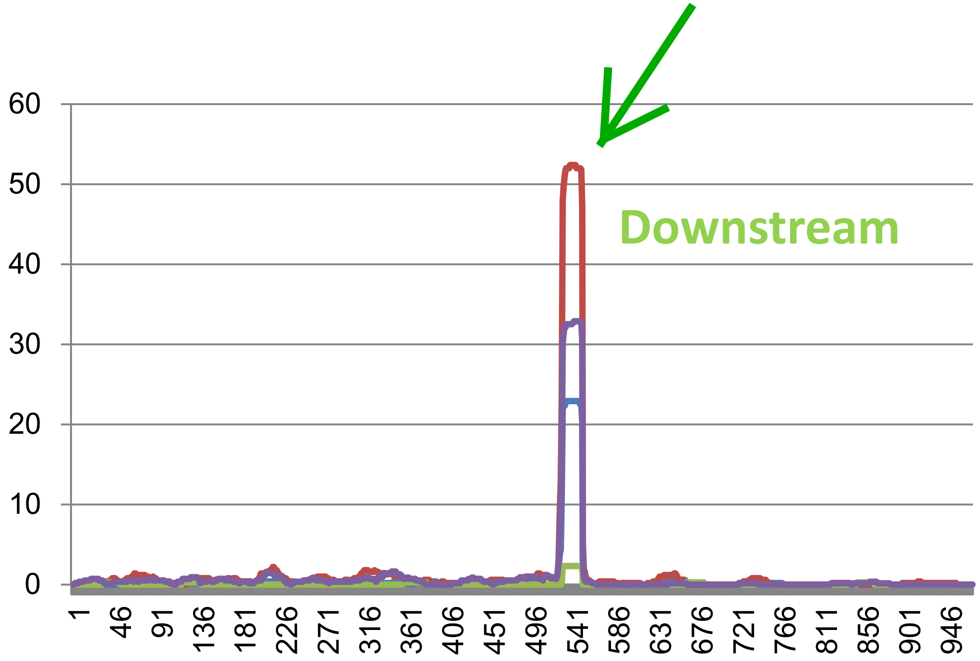

ATCG00840

One of two chloroplast genes that encode chloroplast ribosomal protein L23, a constituent of the large subunit of the ribosomal complex.

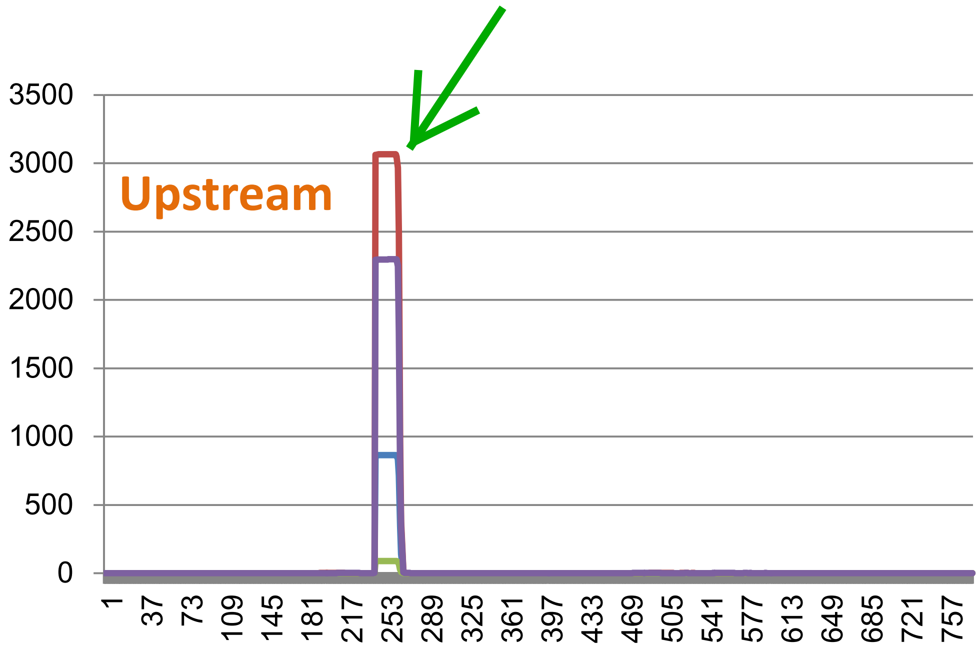

ATCG01110

Encodes the 49KDa plastid NAD(P)H dehydrogenase subunit H protein. Its transcription is regulated by an ndhF-specific plastid sigma factor, SIG4.

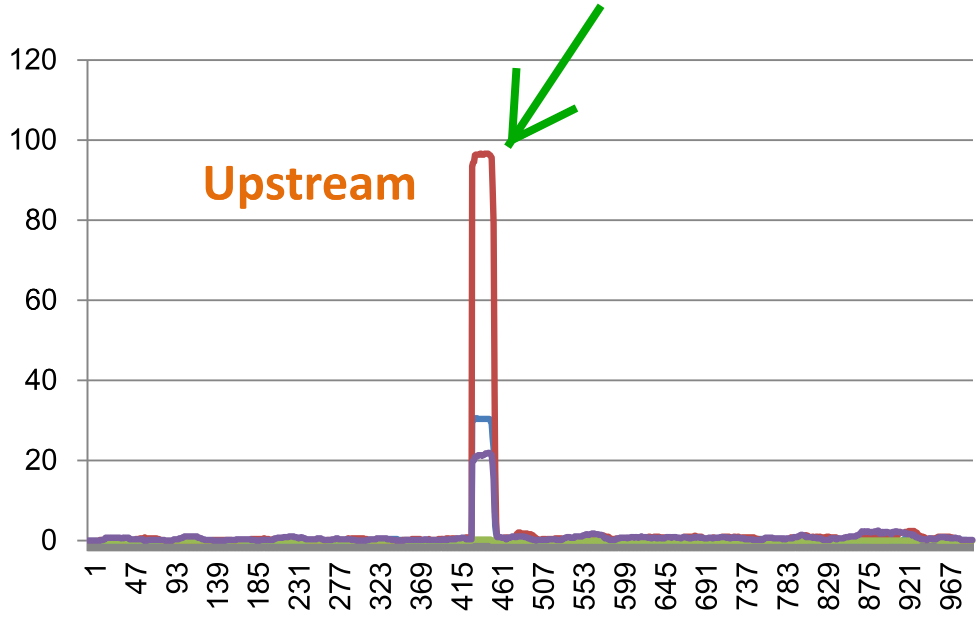

ATCG01300

One of two chloroplast genes that encode chloroplast ribosomal protein L23, a constituent of the large subunit of the ribosomal complex.

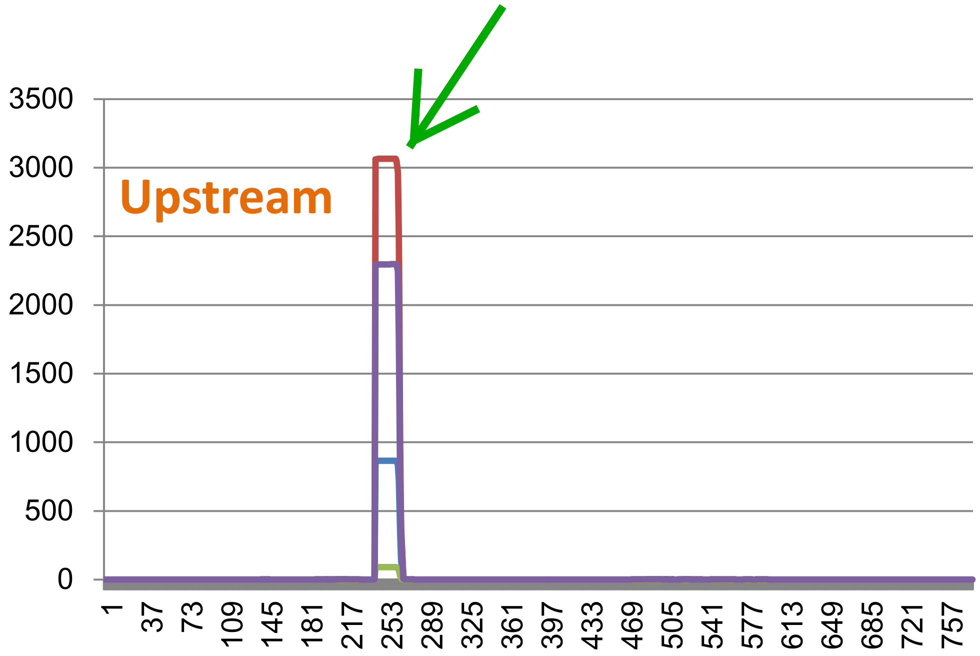

ATMG00060

Mitochondrial NADH dehydrogenase subunit 5. The gene is trans-spliced from the three different pre-cursors, NAD5a, NAD5b and NAD5c.

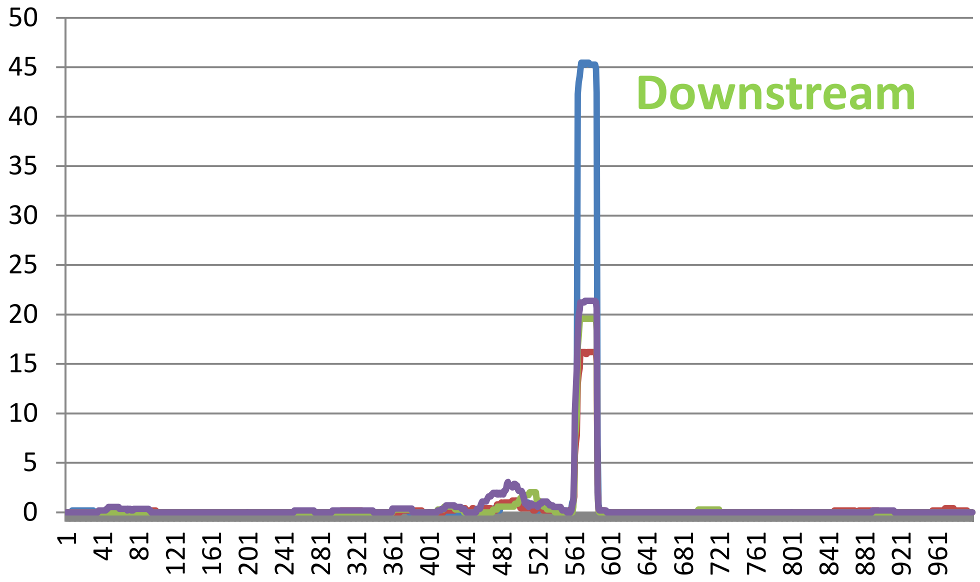

ATMG00070

NADH dehydrogenase subunit 9

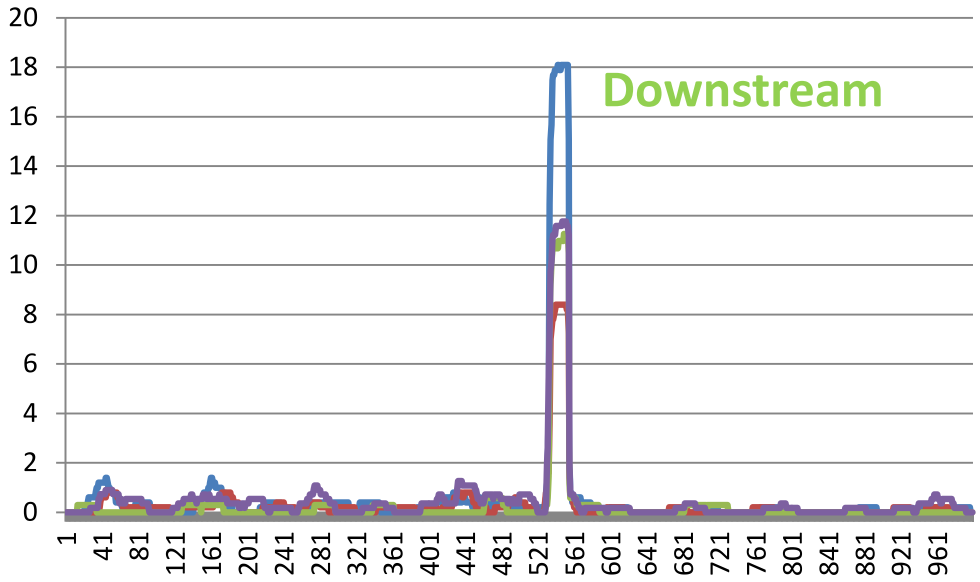

ATMG00080

Encodes a mitochondrial ribosomal protein L16, which is a constituent of the large ribosomal subunit.

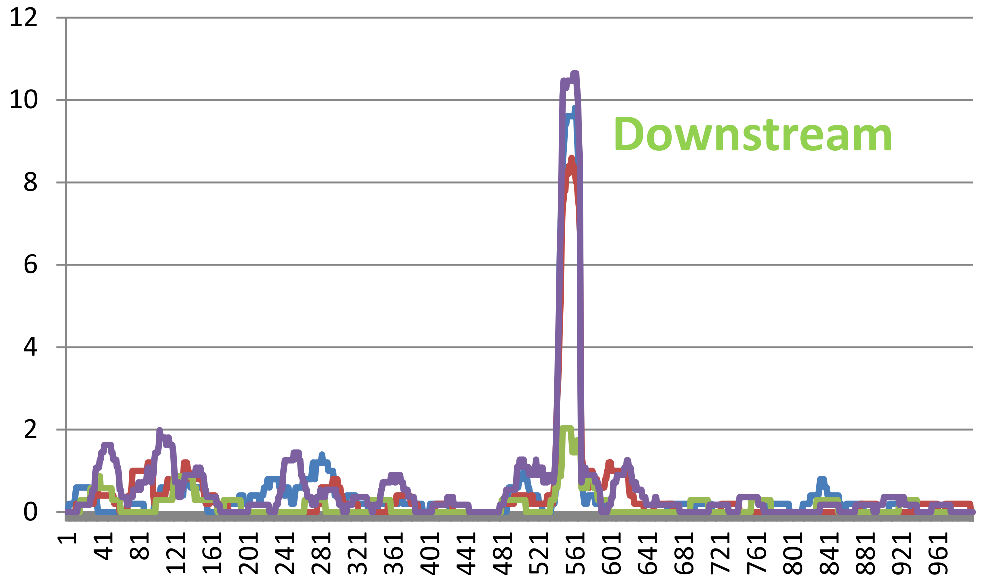

ATMG00110

Encodes a mitochondria-encoded cytochrome c biogenesis protein.

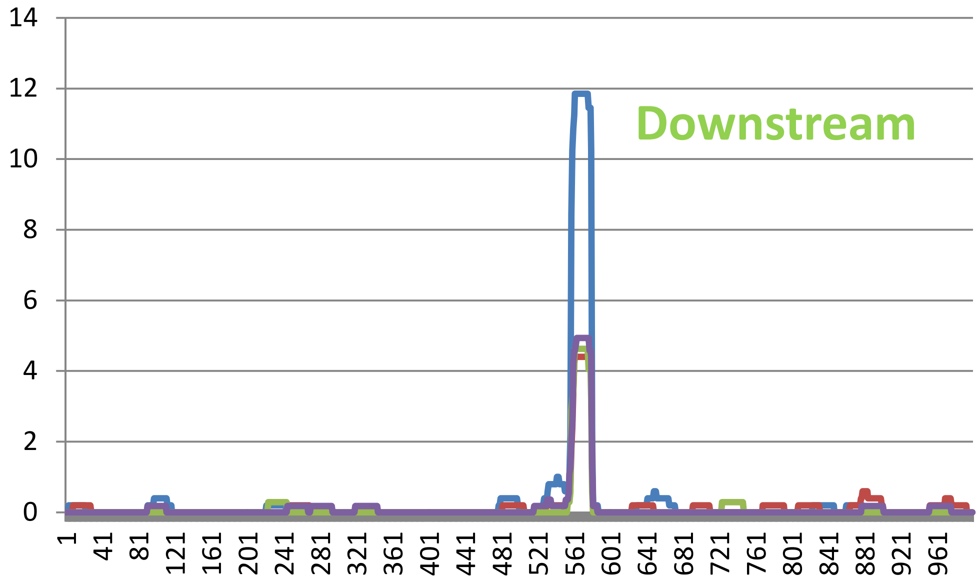

ATMG00270

NADH dehydrogenase subunit 6

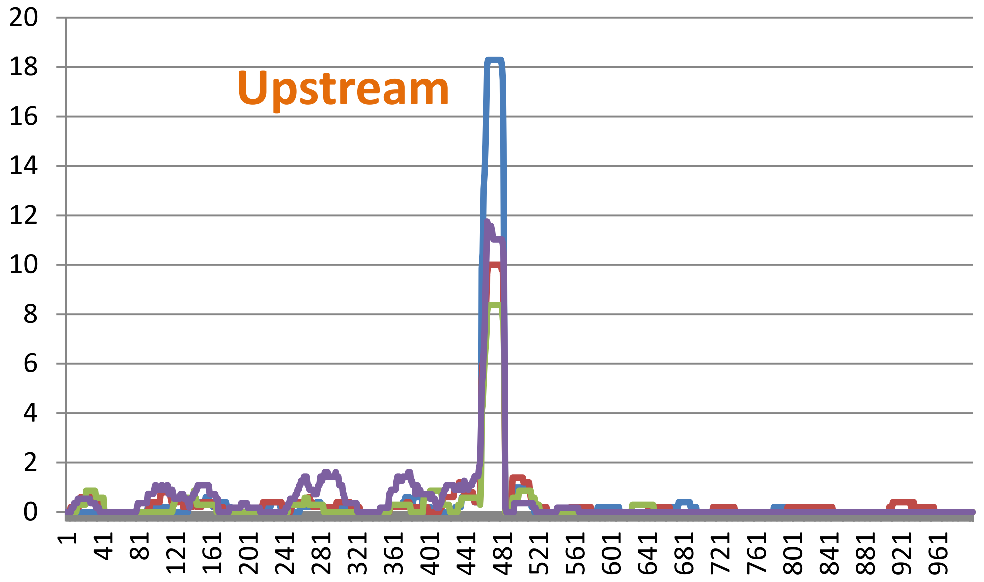

ATMG00510

NADH dehydrogenase subunit 7

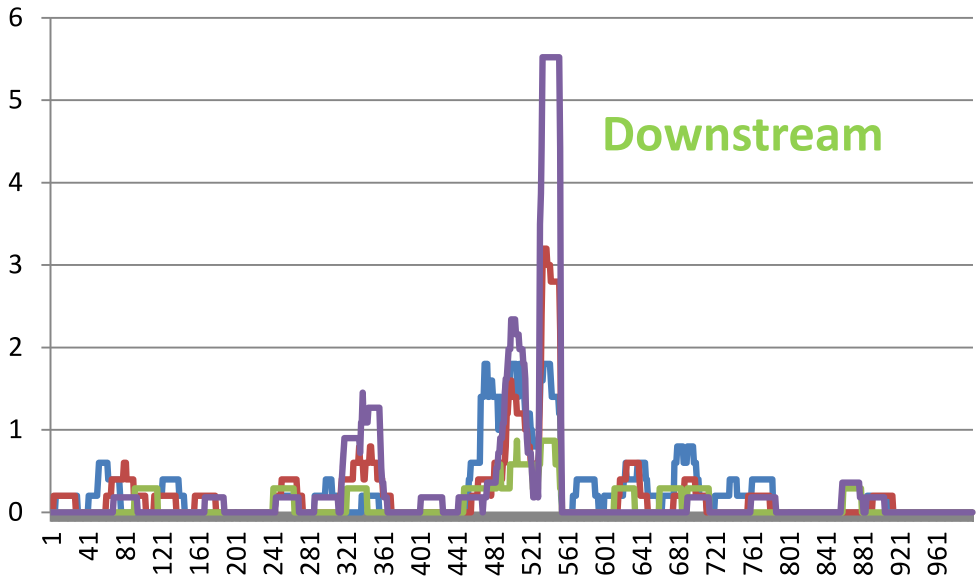

ATMG00516

Encodes subunit of mitochondrial NAD(P)H dehydrogenase that is trans-spliced from three precursors, NAD1A, NAD1B, and NAD1C.

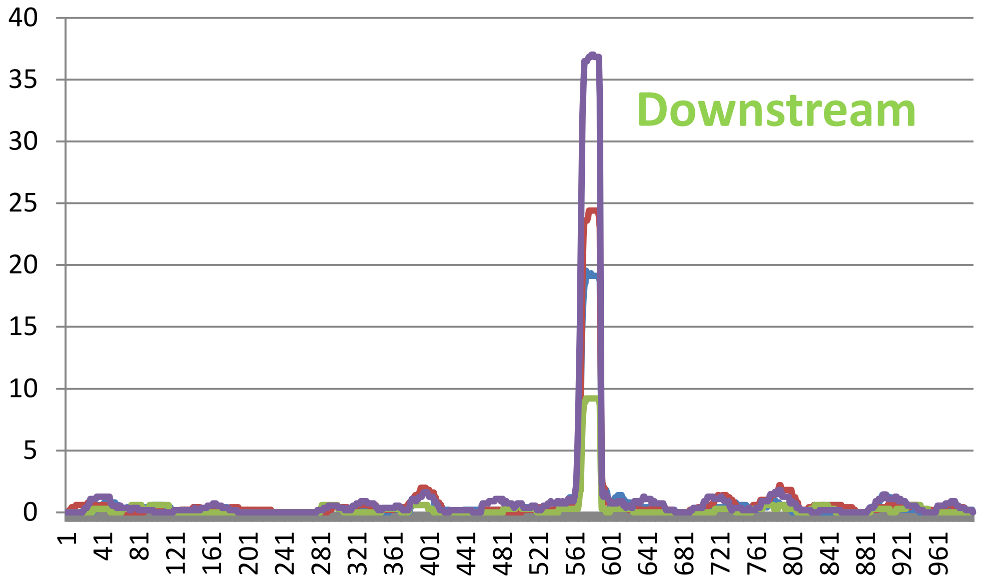

ATMG00560

Encodes a mitochondrial ribosomal protein L2, a constituent of the large subunit of the ribosomal complex.

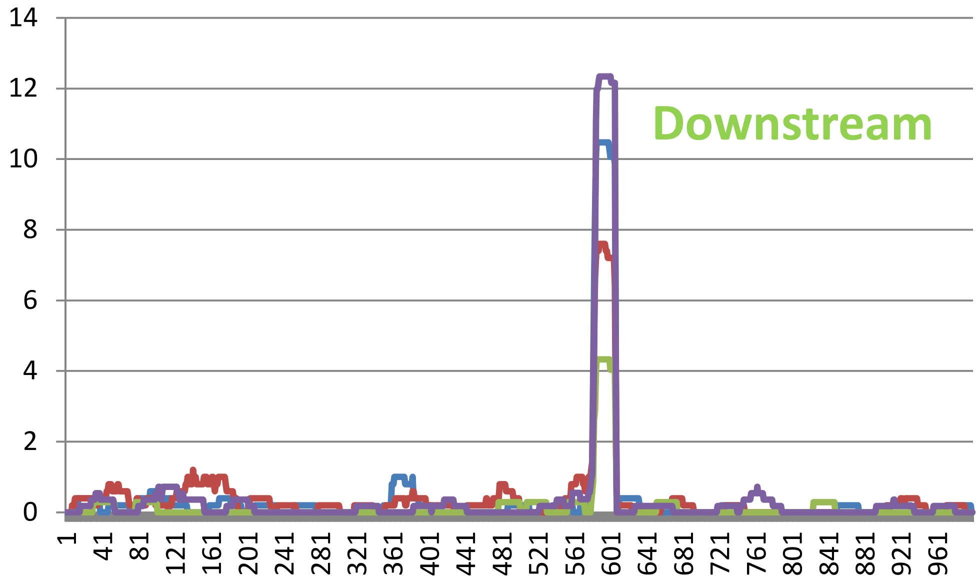

ATMG00570

Encodes a protein of unknown function. The transcript has extensive RNA editing at the 3' end. Protein has orthologous in other plants and sequence is similar to E. coli ORFs orf154 and orf131, both of unknown function. So far, similar proteins are found only in plants and prokaryotes.

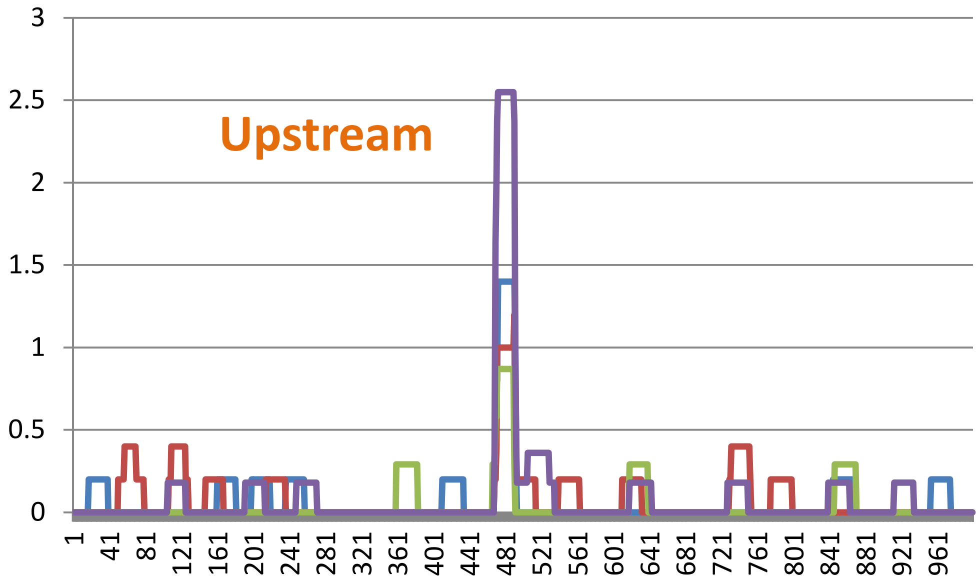

ATMG00580

NADH dehydrogenase subunit 4

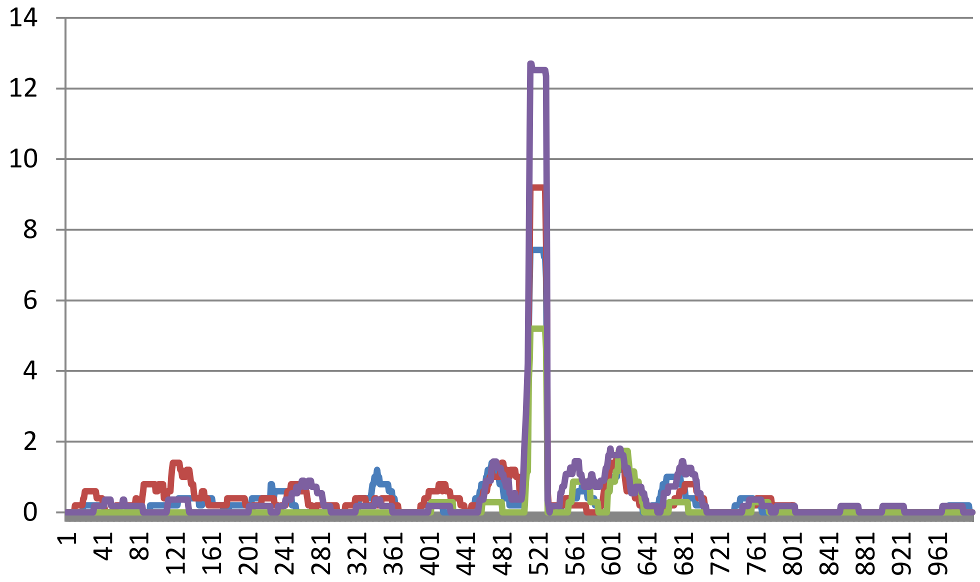

ATMG00640

Encodes a plant b subunit of mitochondrial ATP synthase based on structural similarity and the presence in the F(0) complex.

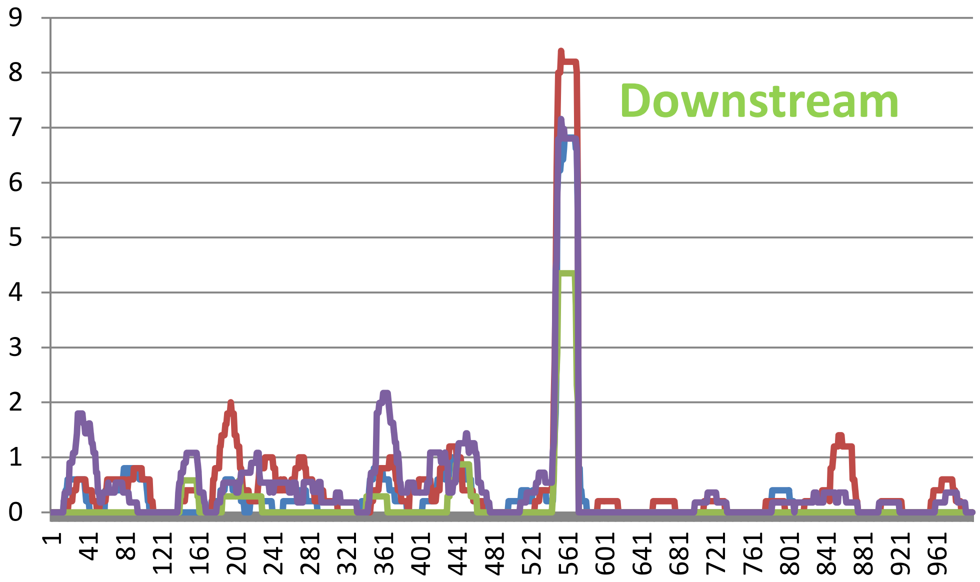

ATMG00900

Cytochrome c biogenesis orf256

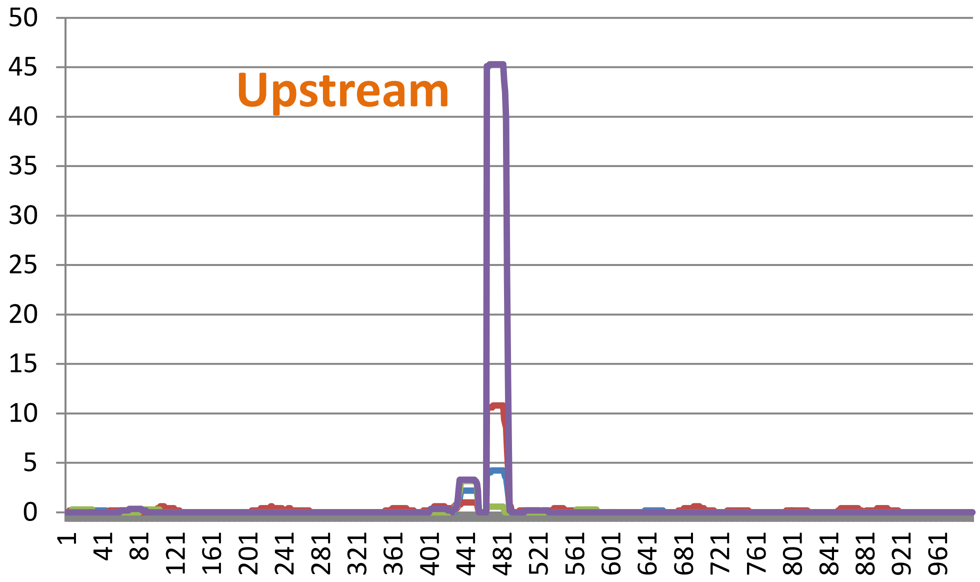

ATMG01090

Hypothetical protein

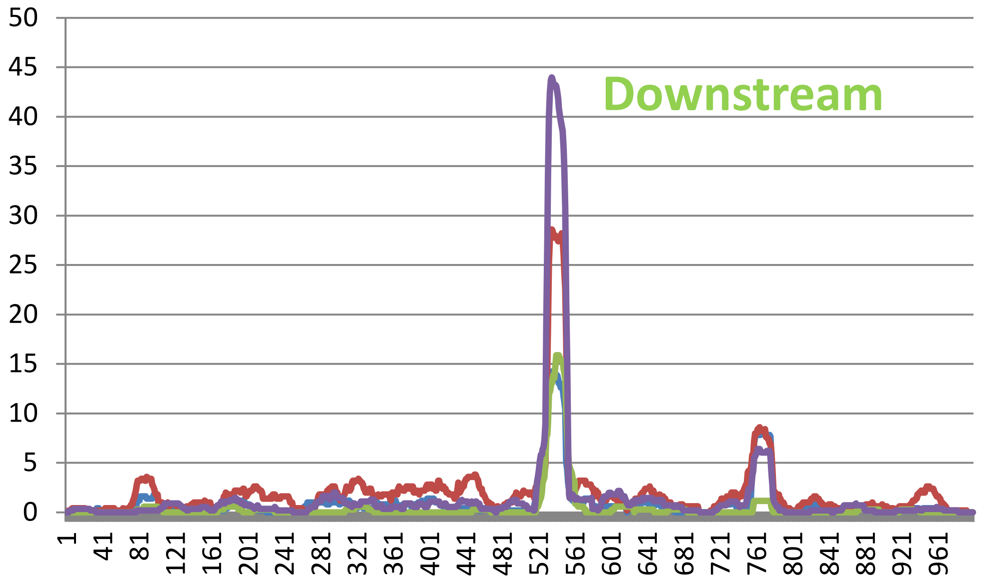

ATMG01320

Encodes subunit of mitochondrial NAD(P)H dehydrogenase that is trans-spliced from two precursors, NAD2A and NAD2B.

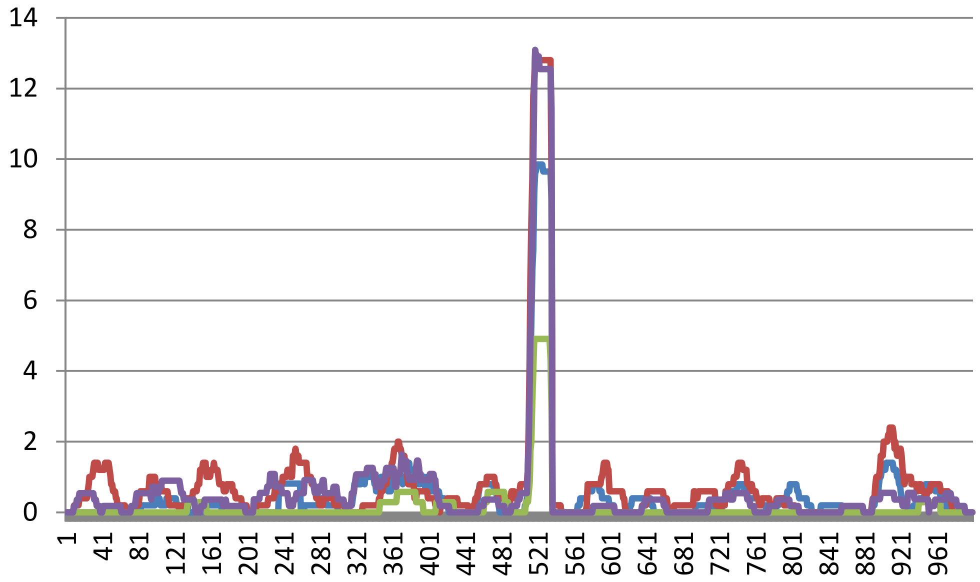

ATMG01360

Cytochrome c oxidase subunit 1

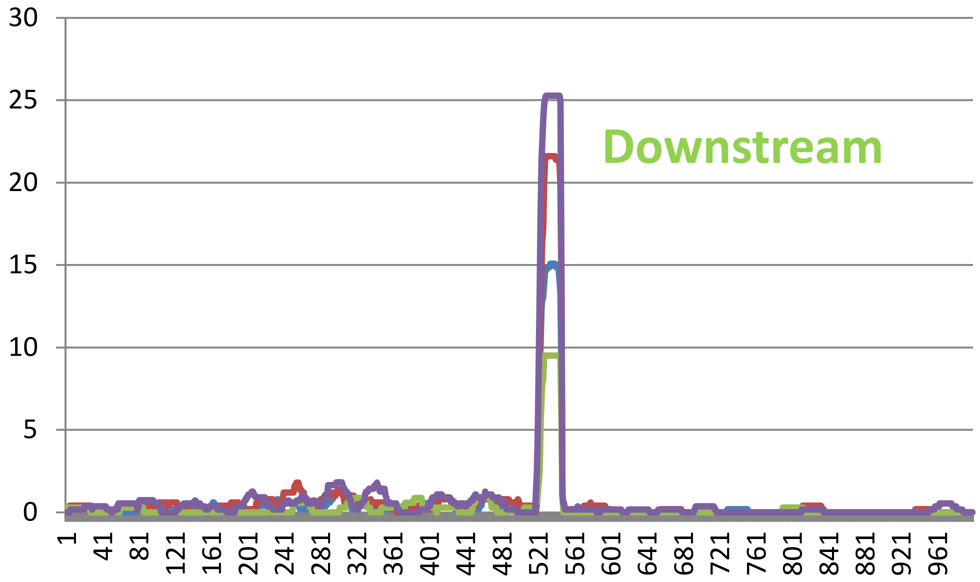

Supplement: S4 Fig — For the chloroplast genes, sRNAs dominantly detected in leaves and seedlings were marked by green arrows. (PDF) [file pone.0169212.s004.pdf]
