# Supplementary material for: Transcriptome-Wide Discovery of PASRs (Promoter-Associated Small RNAs) and TASRs (Terminus-Associated Small RNAs) in Arabidopsis thaliana
Source: PLoS One. 2017 Jan 3;12(1):e0169212. doi: 10.1371/journal.pone.0169212 (PMC5207706; doi:10.1371/journal.pone.0169212)

**Figure S5** TASR peaks identified on the antisense strands of the protein-coding genes of *Arabidopsis*. For each plot, x axis measures the position of the antisense strand, and y axis measures the abundance (in RPM, reads per million) of sRNAs.

AT1G01880

5'-3' exonuclease family protein

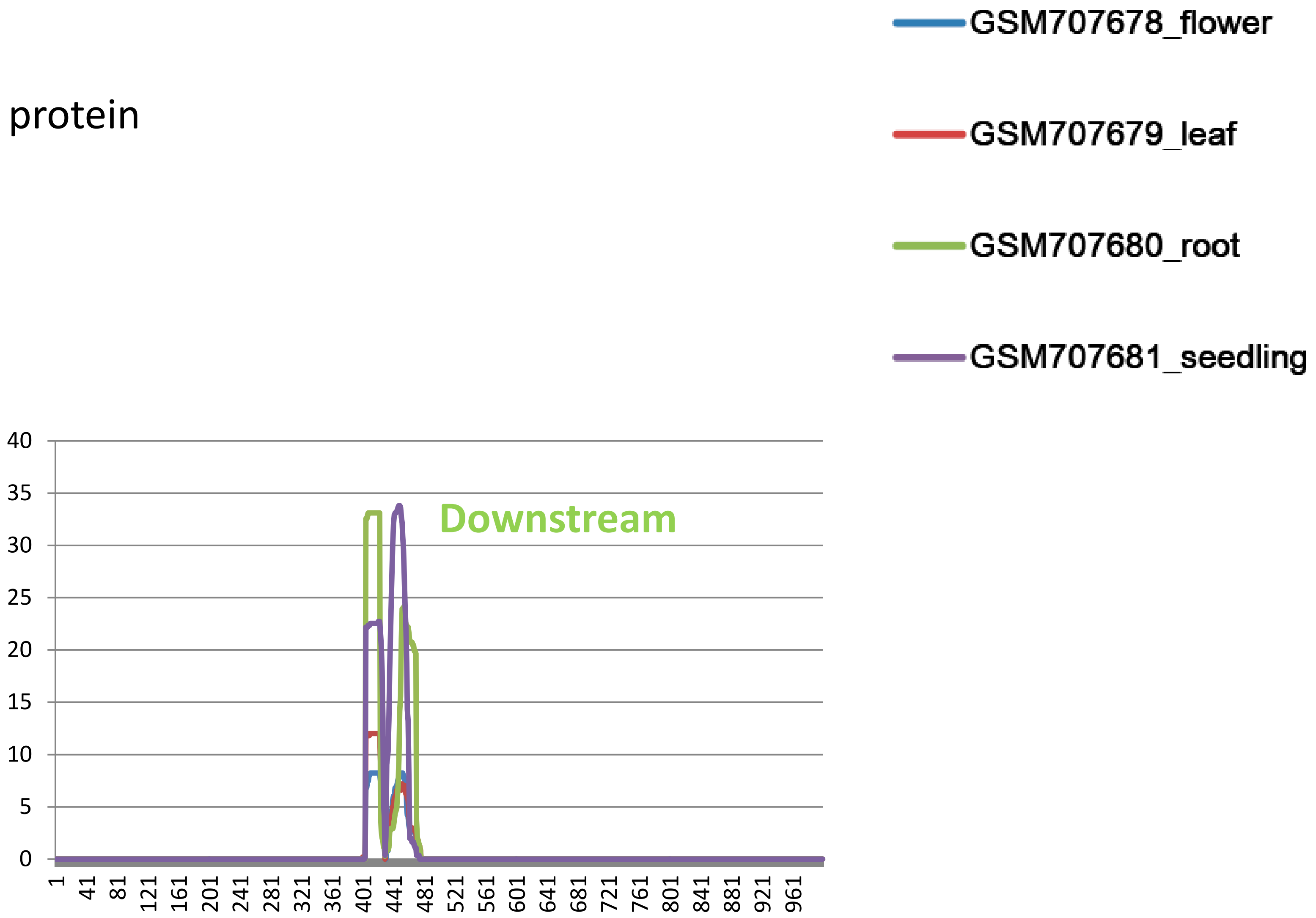

# AT1G09026

## Unknown protein

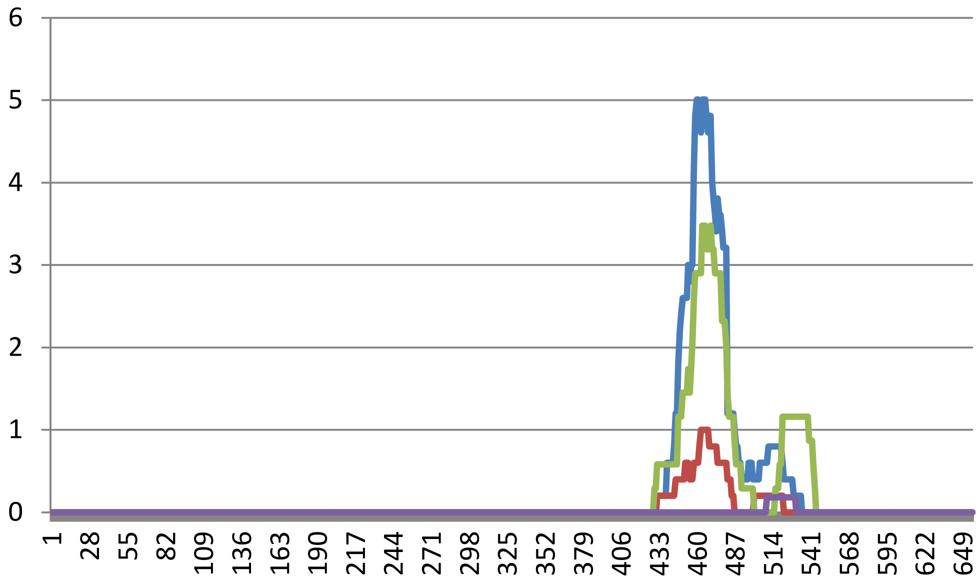

AT1G10095

Protein prenylyltransferase superfamily protein

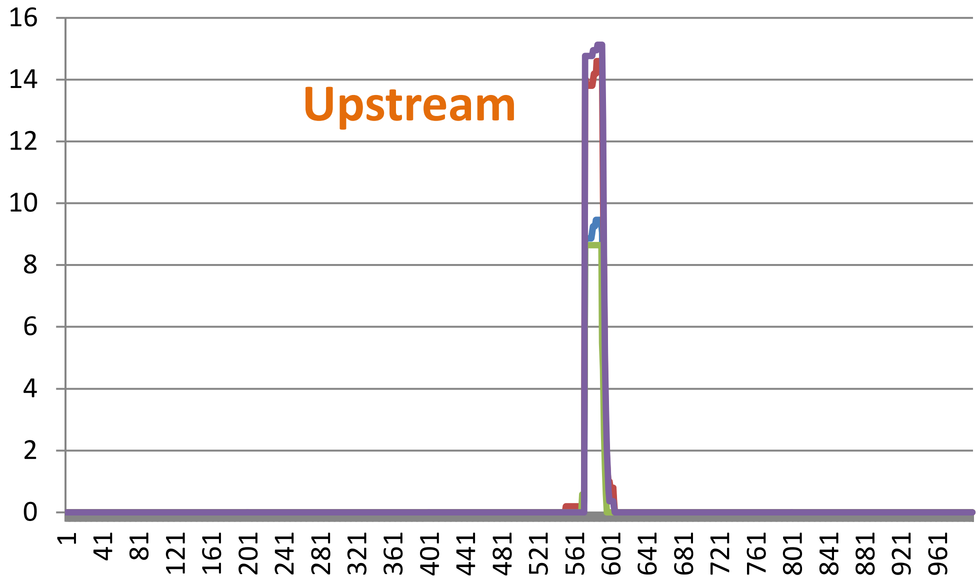

AT1G10745

Encodes a Maternally expressed gene (MEG) family protein.

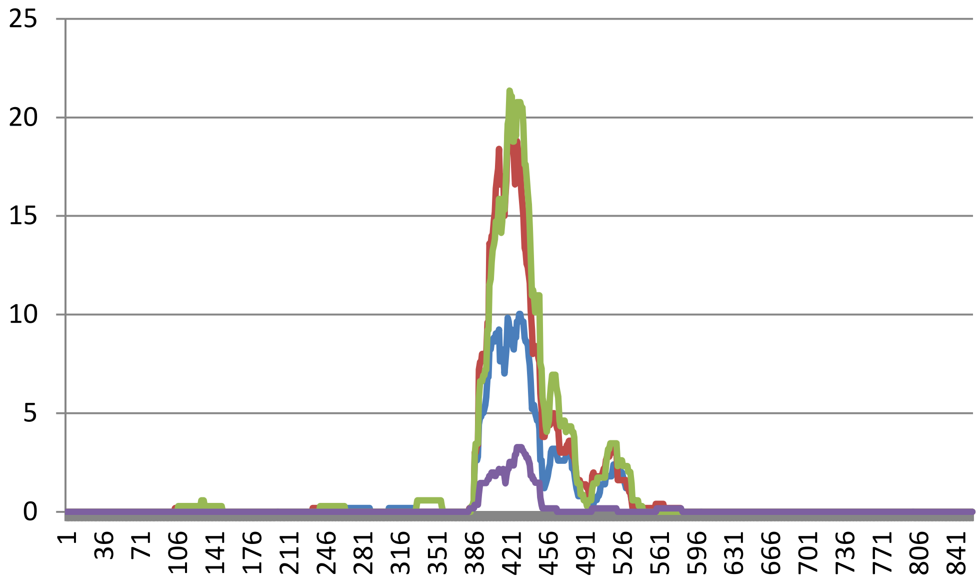

AT1G13860

Encodes QUASIMODO2 LIKE1 (QUL1), a paralog of QUASIMODO2 (QUA2). AT1G78240 (QUA2), AT1G13860 (QUL1) and AT2G03480 (QUL2) form a clade with a possible role in plant vasculature development.

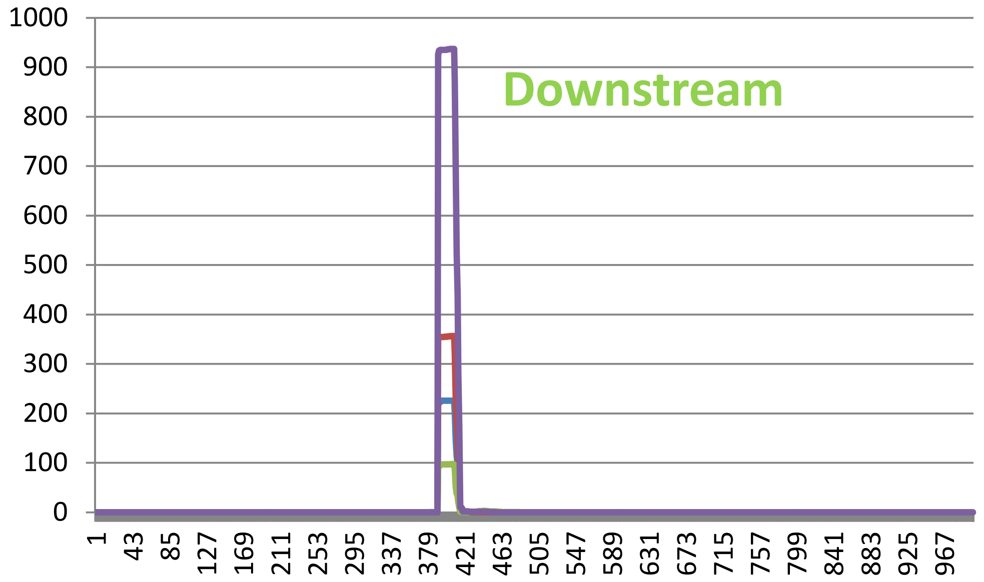

AT1G14580

C2H2-like zinc finger protein

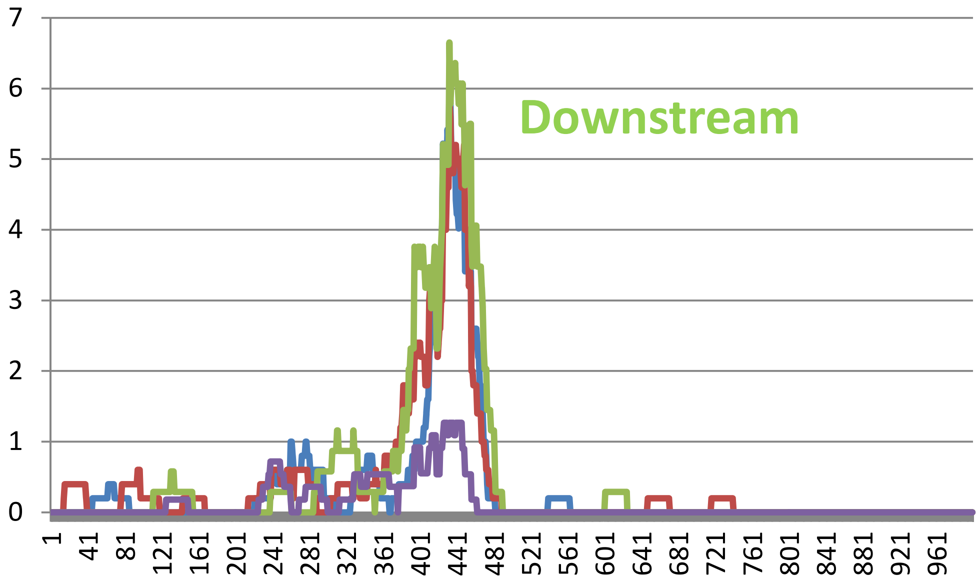

# AT1G14890

Plant invertase/pectin methylesterase inhibitor superfamily protein.

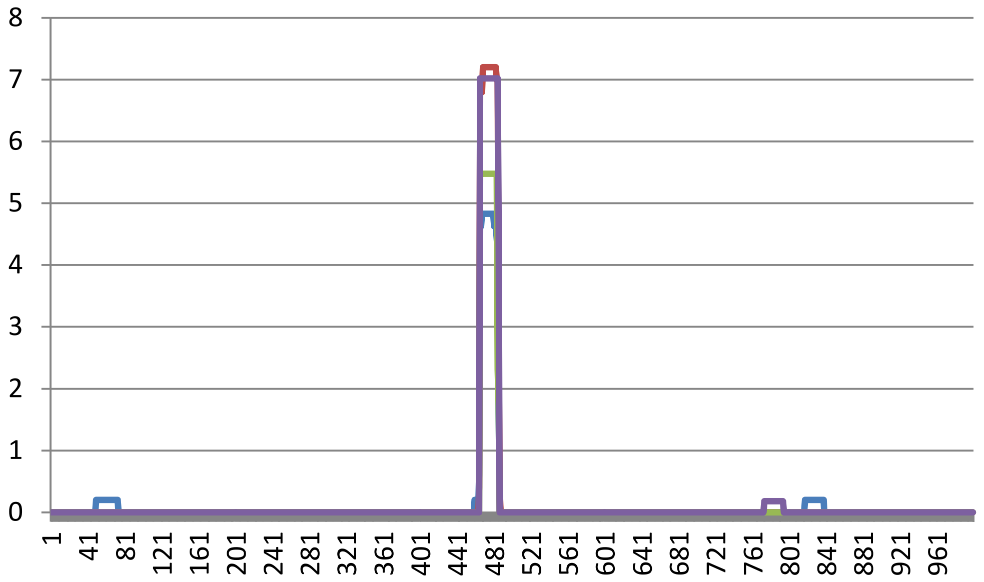

AT1G15080

Encodes phosphatidic acid phosphatase. Involved in ABA signaling. Functions as a negative regulator upstream of ABI4. Expressed during germination and seed development. Expressed overall in young seedlings, in roots, hypocotyls, and vascular cells of cotyledons and leaves of 10 day-old seedlings, in flower filaments and stem elongation zones. Not expressed in anthers, pollen nor petals.

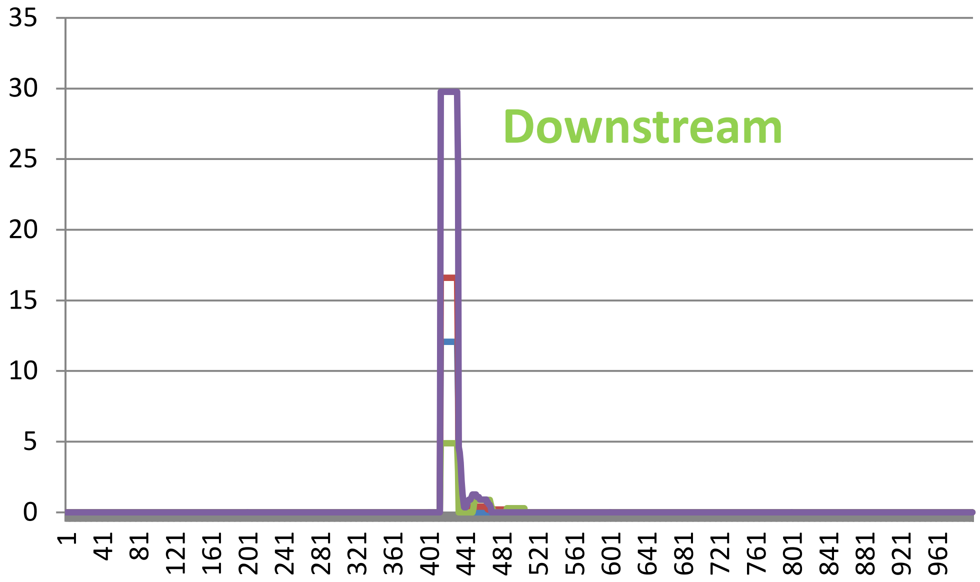

# AT1G15125

S-adenosyl-L-methionine-dependent methyltransferases superfamily protein.

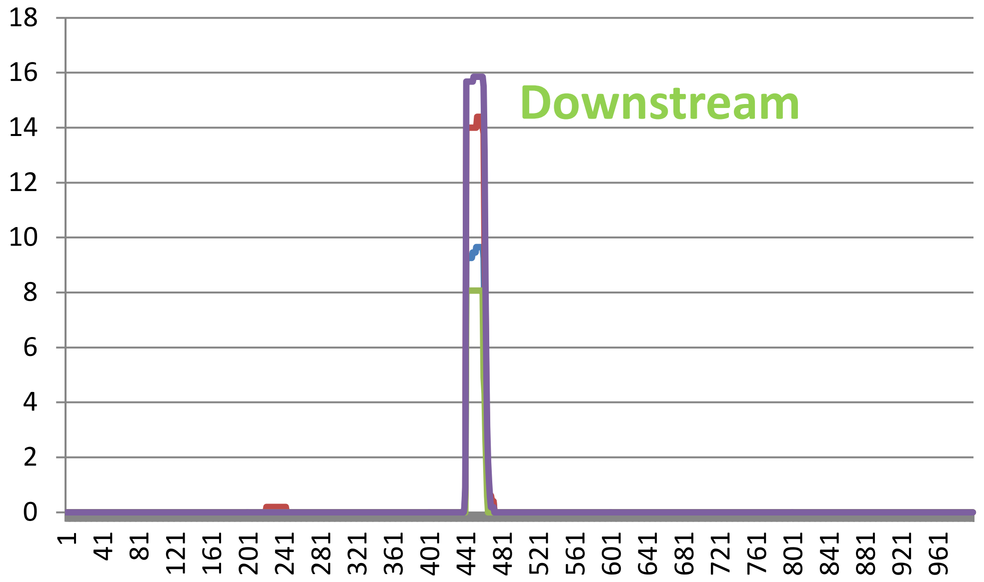

# AT1G15530

## Concanavalin A-like lectin protein kinase family protein

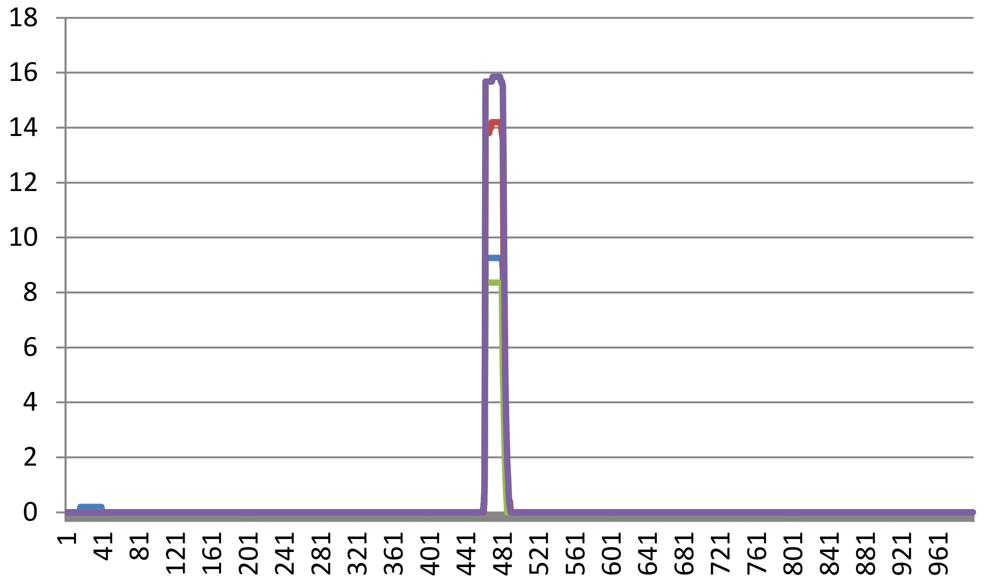

# AT1G15670

## Galactose oxidase/kelch repeat superfamily protein

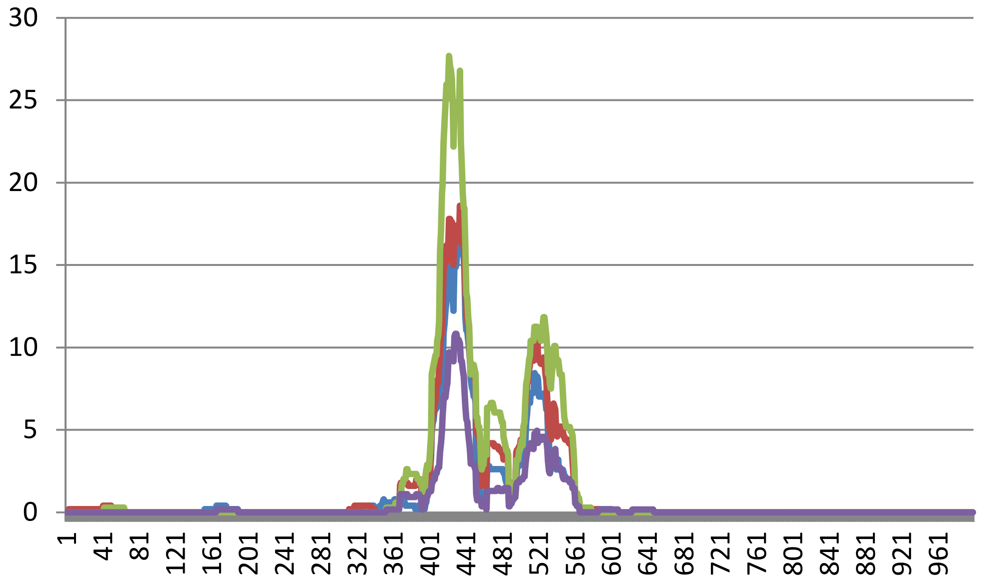

# AT1G18060

## Unknown protein

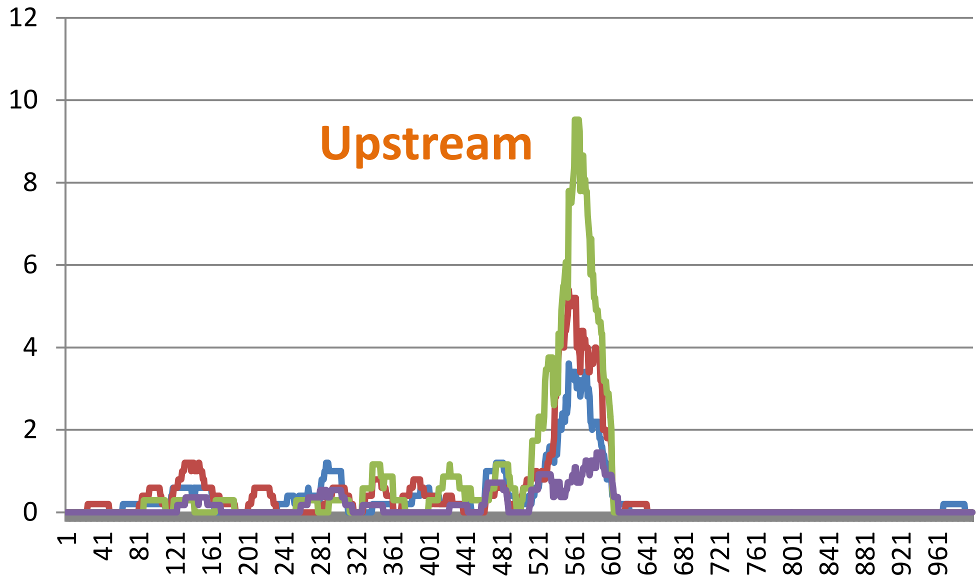

# AT1G18770

## RING/U-box superfamily protein

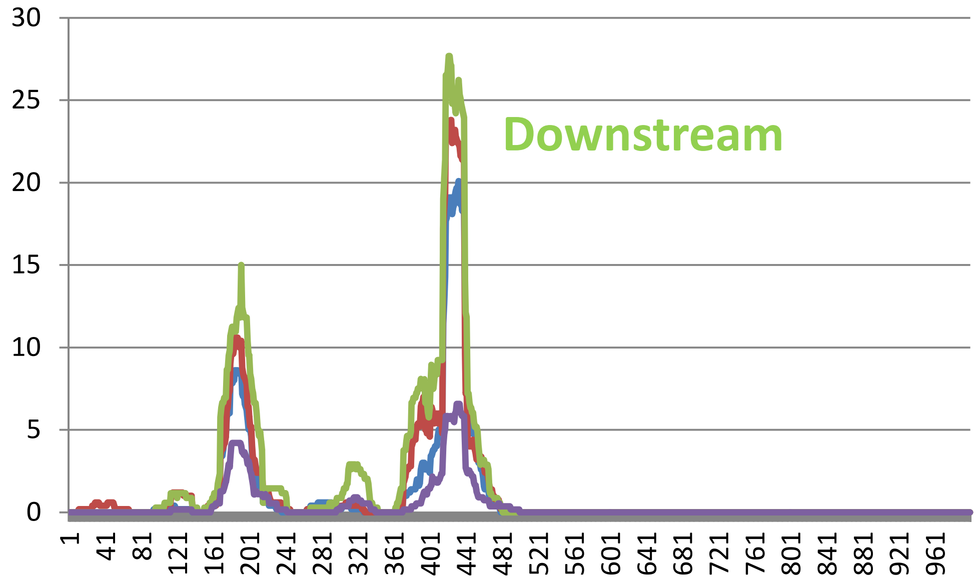

# AT1G19830

## SAUR-like auxin-responsive protein family

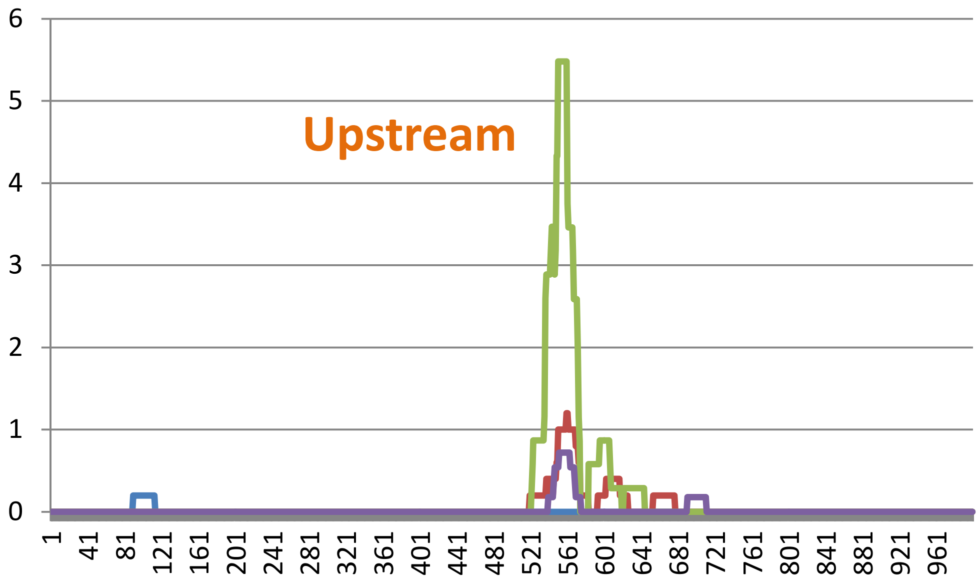

AT1G20290

SWI-SNF-related chromatin binding protein

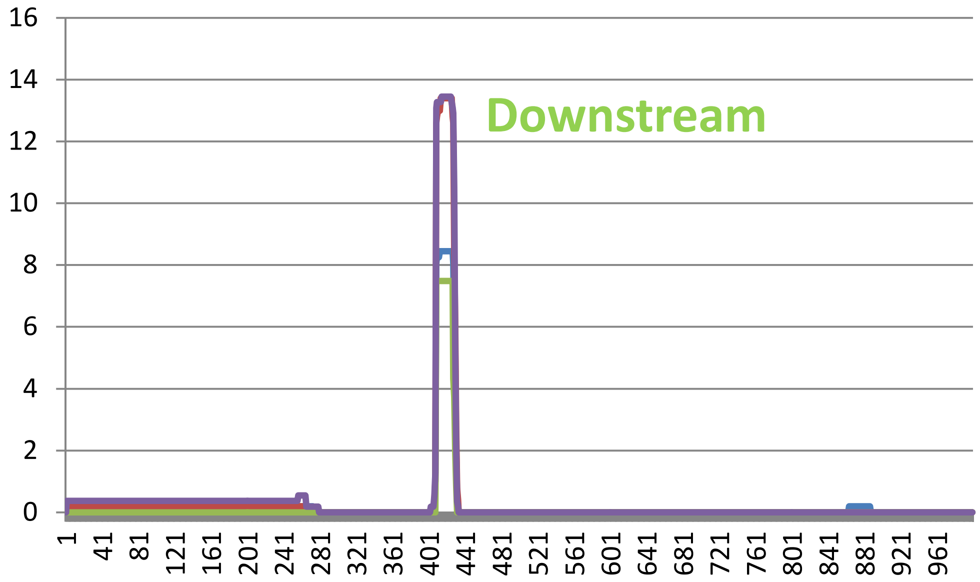

AT1G20840

The protein encoded by this gene is found in the tonoplast (vacuole membrane) of Arabidopsis cells. The gene is expressed at highest levels in juvenile (sink) and adult (source) leaves, followed by flower tissues.

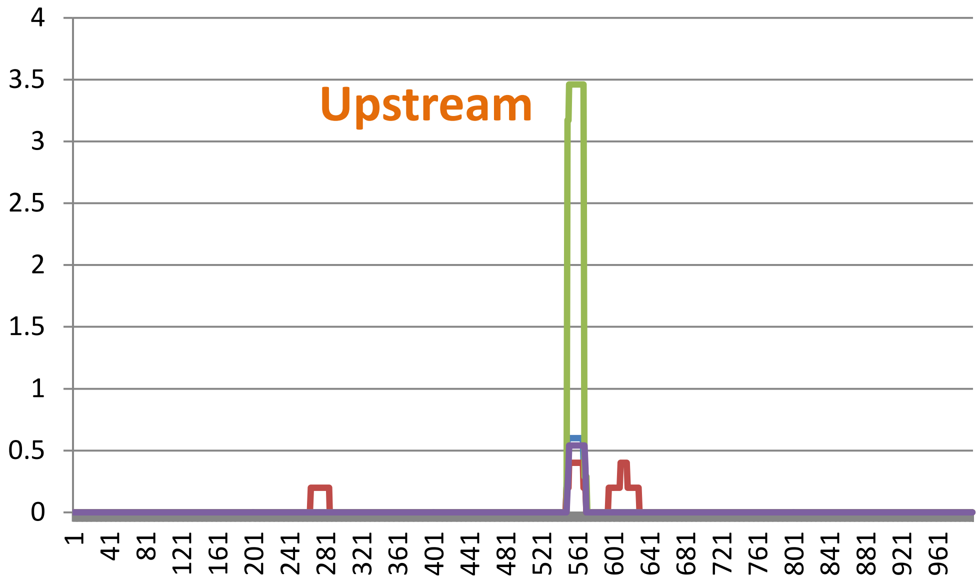

# AT1G22360

## UDP-glucosyl transferase 85A2 (UGT85A2)

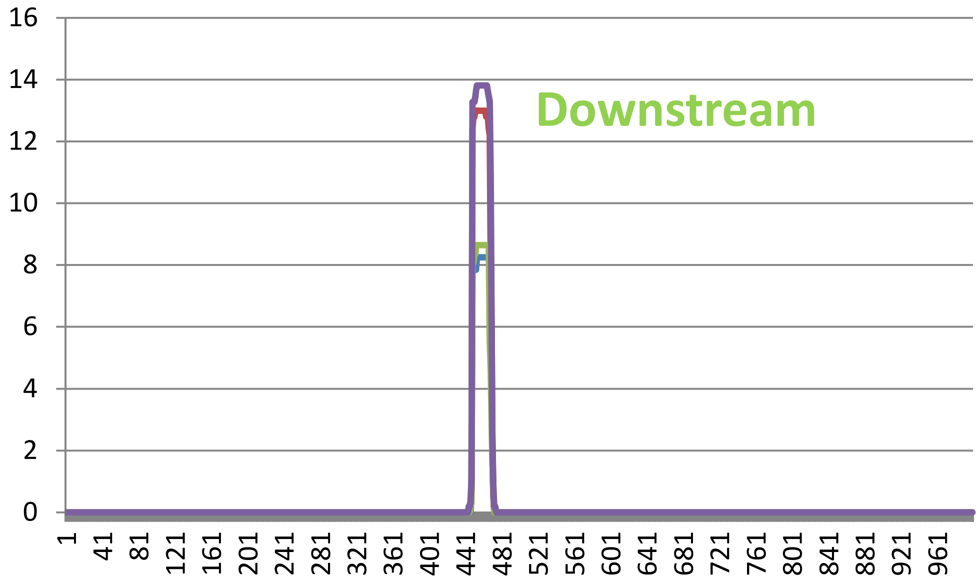

# AT1G23650

## Unknown protein

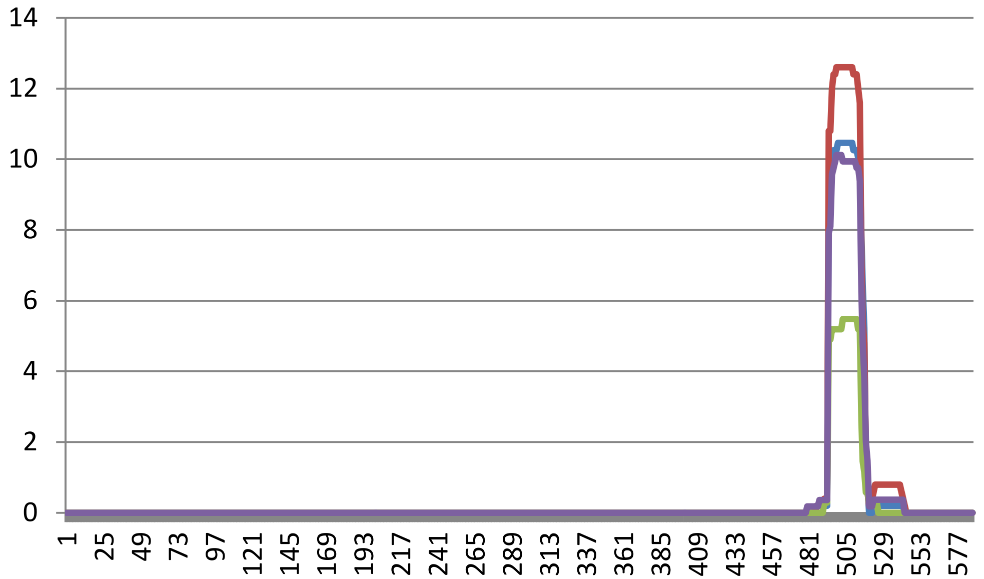

# AT1G24388

## Unknown protein

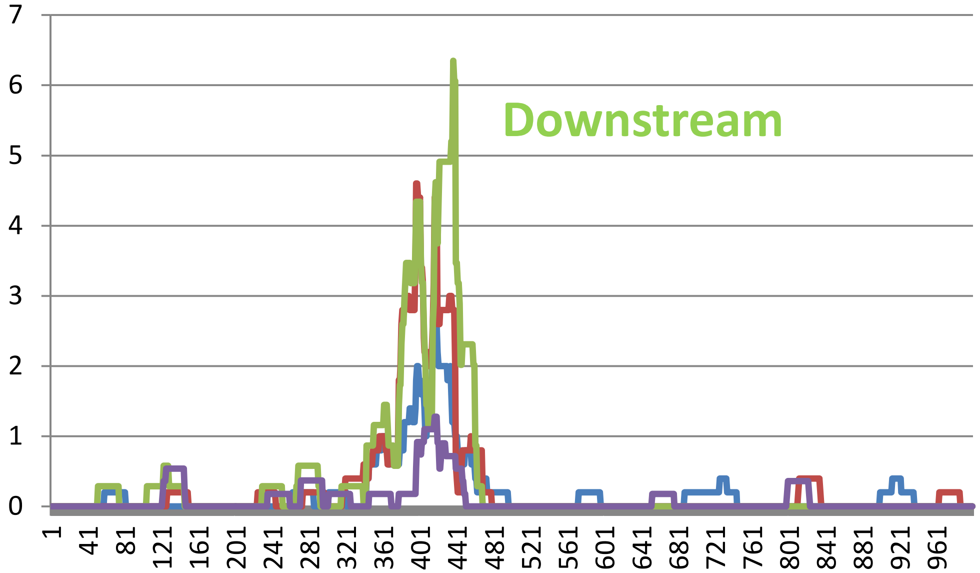

AT1G25375

Metallo-hydrolase/oxidoreductase superfamily protein

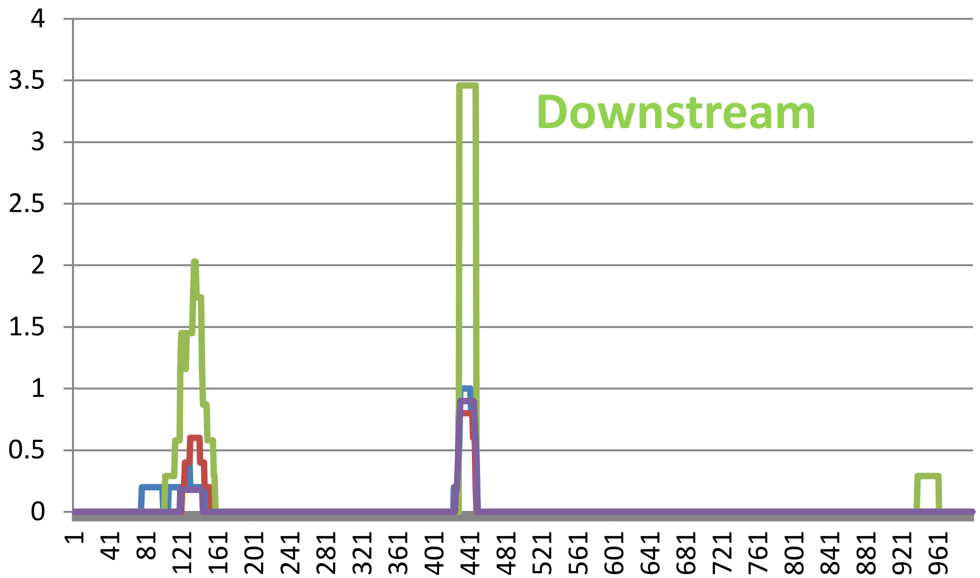

AT1G26762

Unknown protein

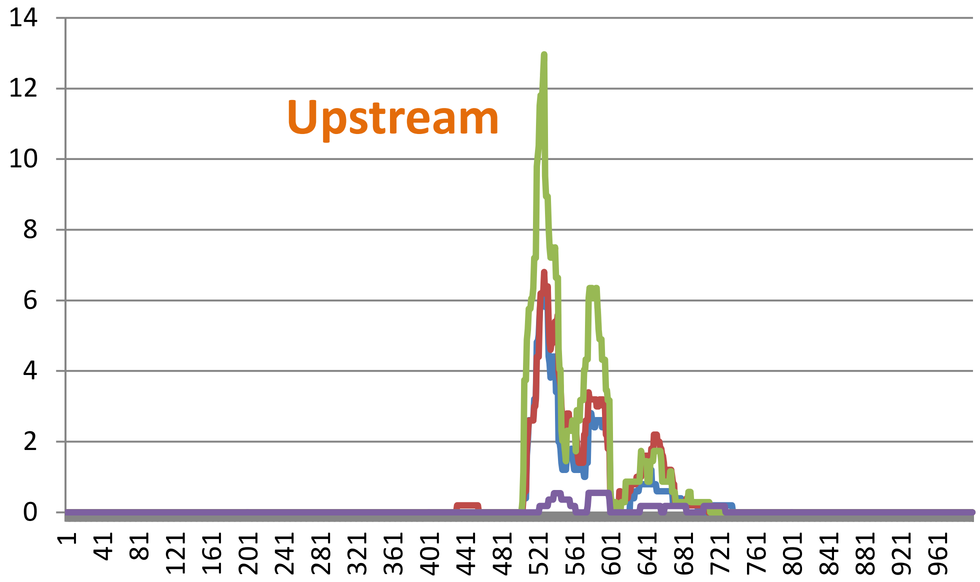

AT1G28304

Unknown protein

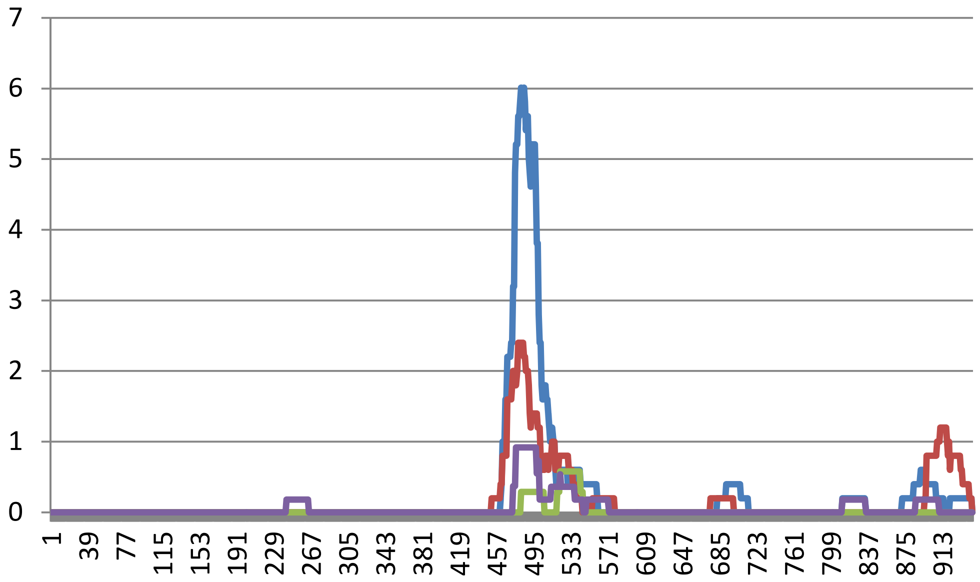

# AT1G28670

## Arabidopsis thaliana lipase

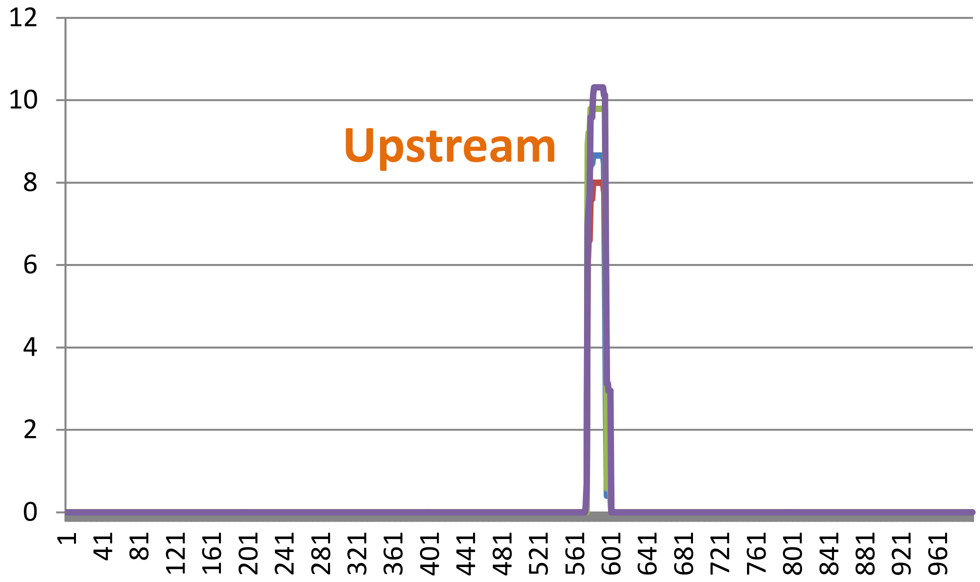

# AT1G29660

## GDSL-like Lipase/Acylhydrolase superfamily protein

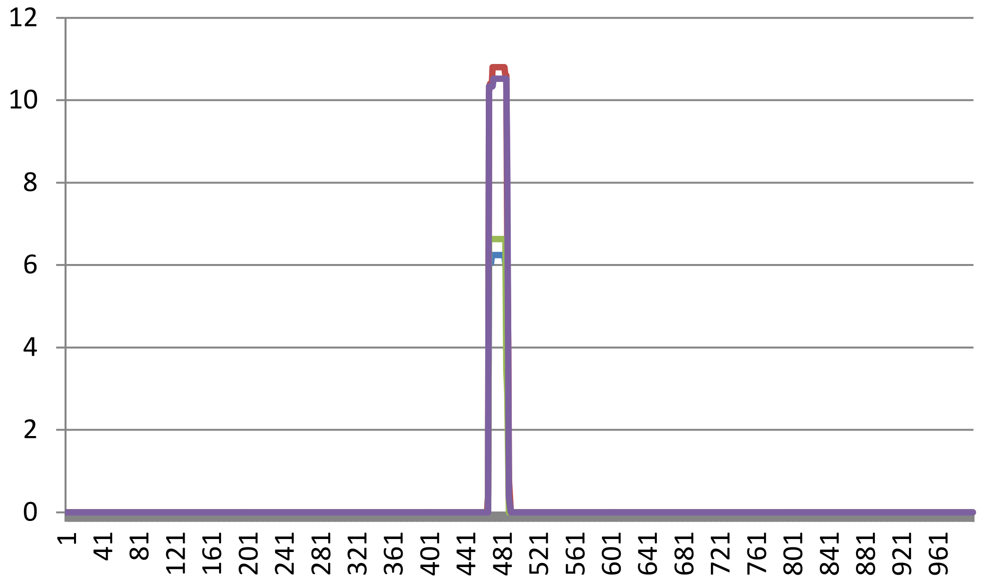

# AT1G30974

Encodes a Plant thionin family protein

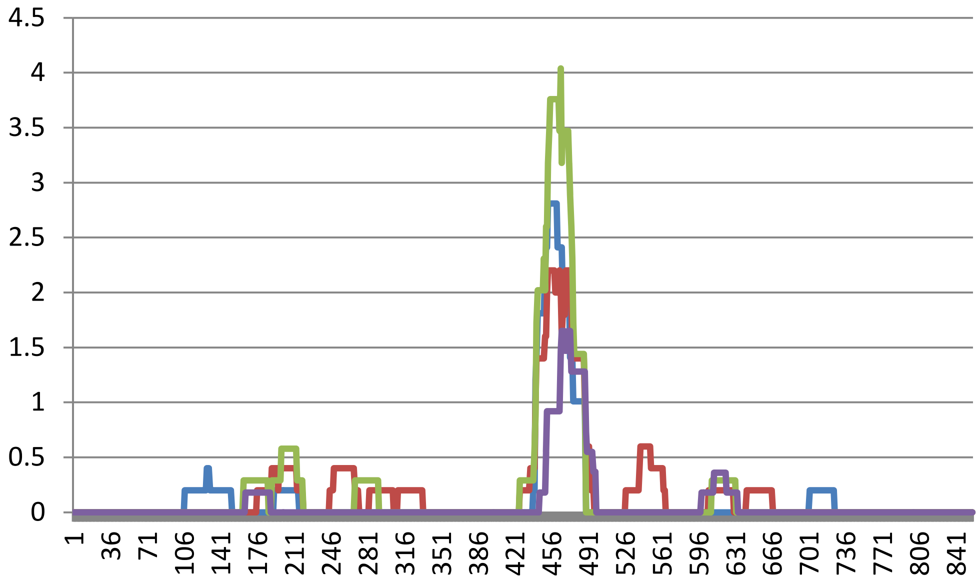

AT1G32583

BEST Arabidopsis thaliana protein match is: tapetum determinant 1 (TAIR:AT4G24972.1)

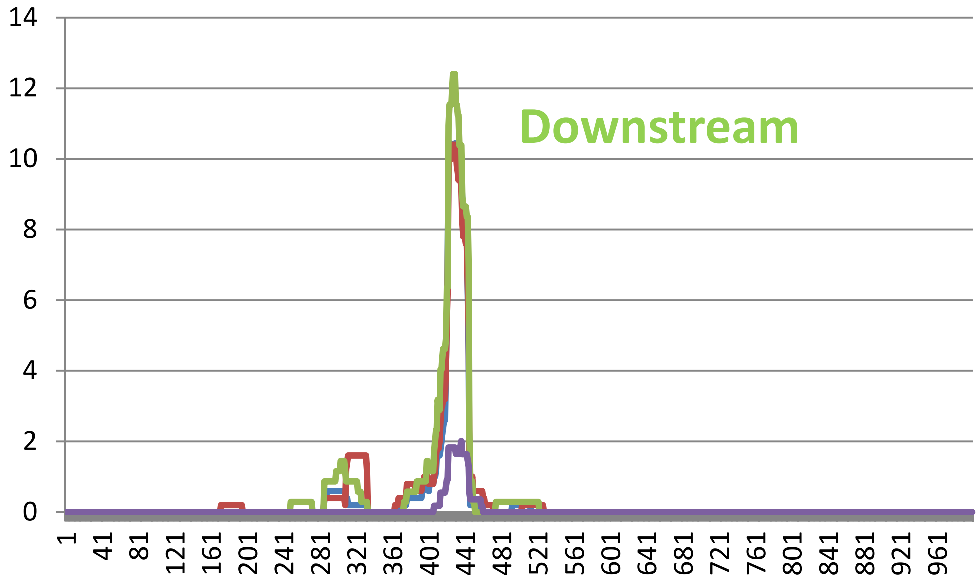

AT1G33680

KH domain-containing protein

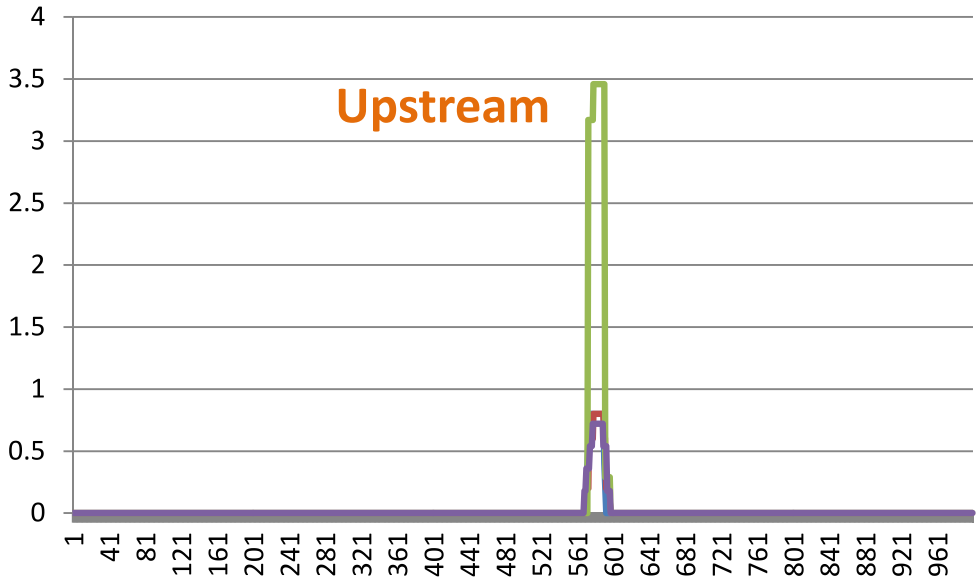

AT1G35400

BEST Arabidopsis thaliana protein match is: Protein of unknown function (DUF1184) (TAIR:AT1G35410.1)

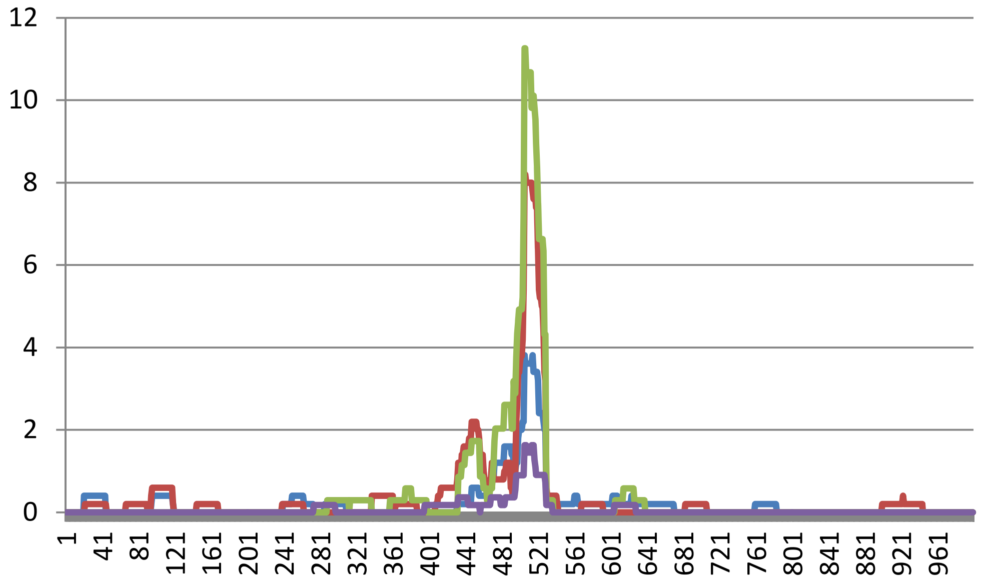

AT1G35516

MYB-like transcription factor family protein

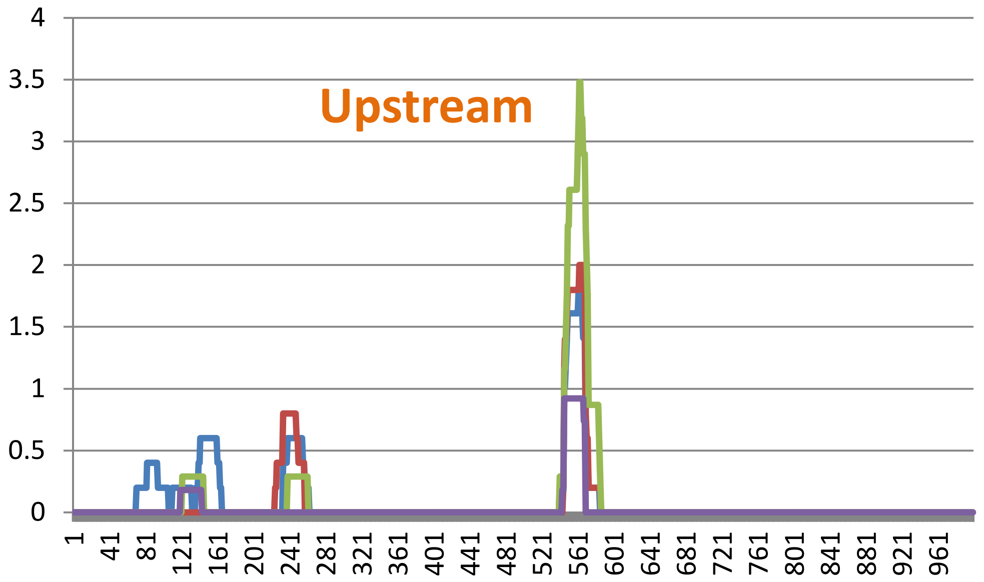

AT1G36310

S-adenosyl-L-methionine-dependent methyltransferases superfamily protein

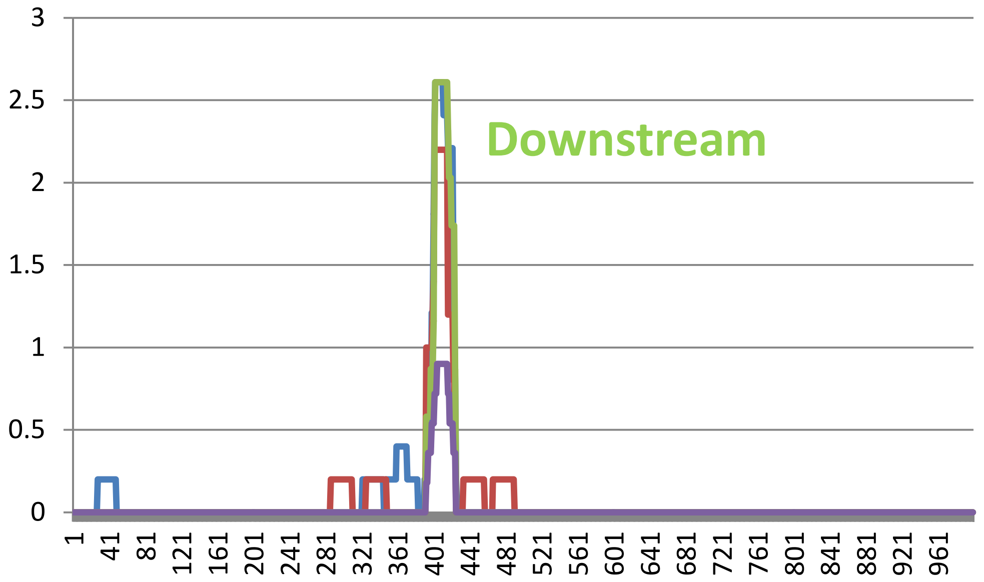

# AT1G40129

## Unknown protein

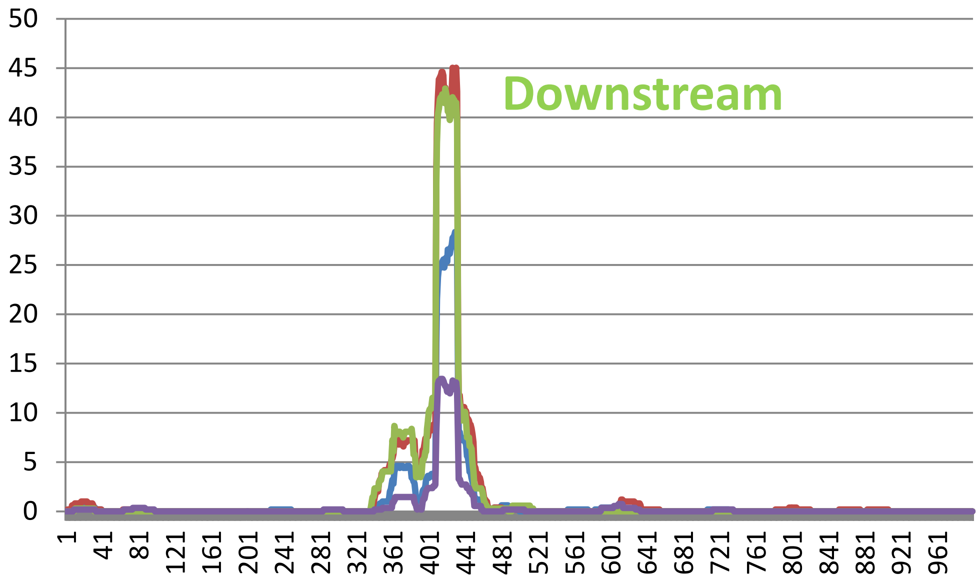

# AT1G43160

Encodes a member of the ERF (ethylene response factor) subfamily B-4 of ERF/AP2 transcription factor family (RAP2.6). The protein contains one AP2 domain. There are 7 members in this subfamily.

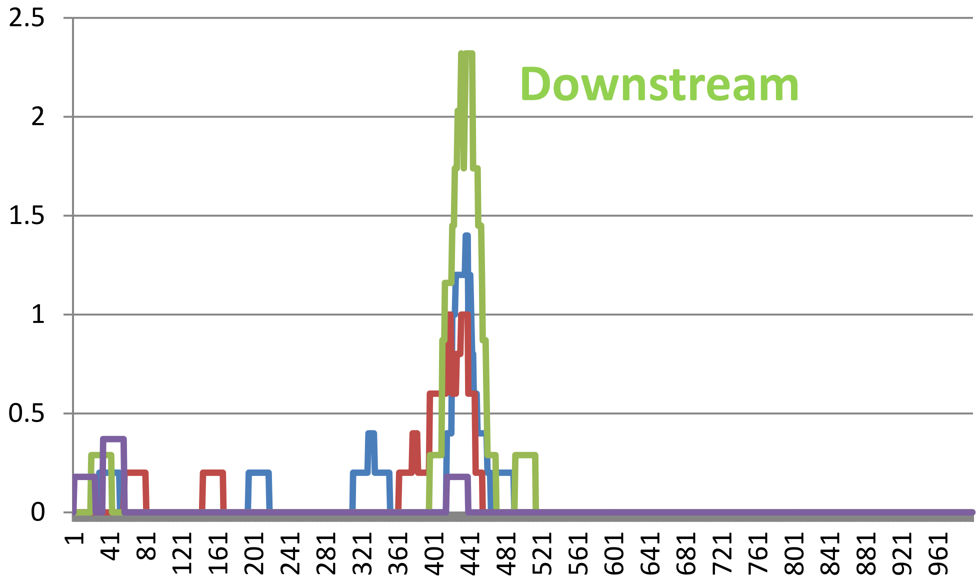

# AT1G44542

## Cyclase family protein

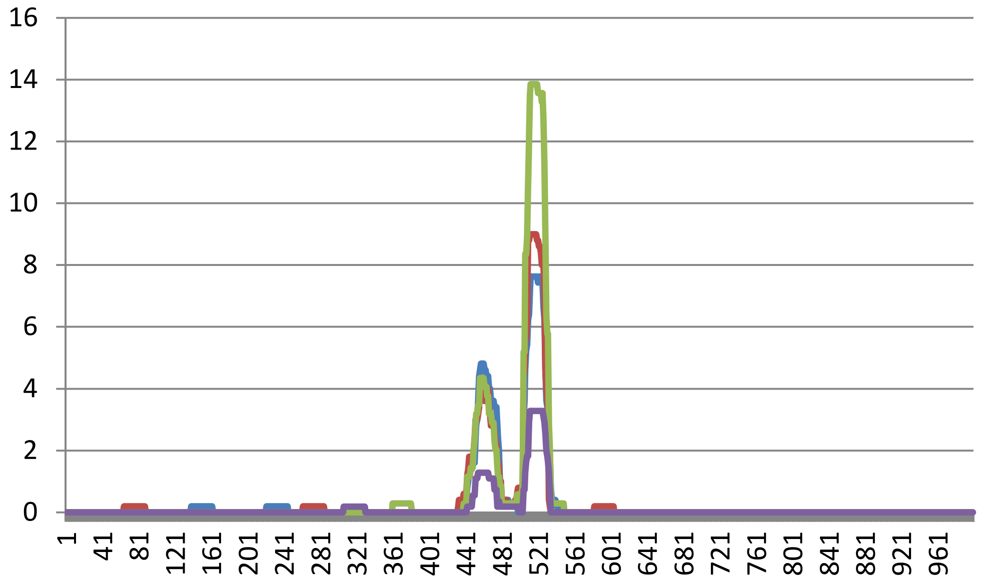

# AT1G47265

## Unknown protein

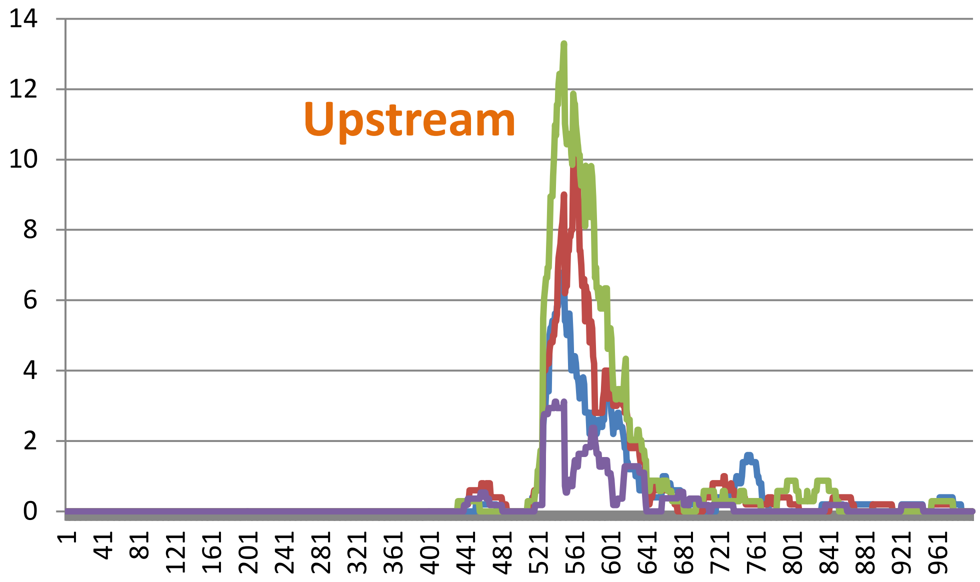

# AT1G47300

## F-box family protein

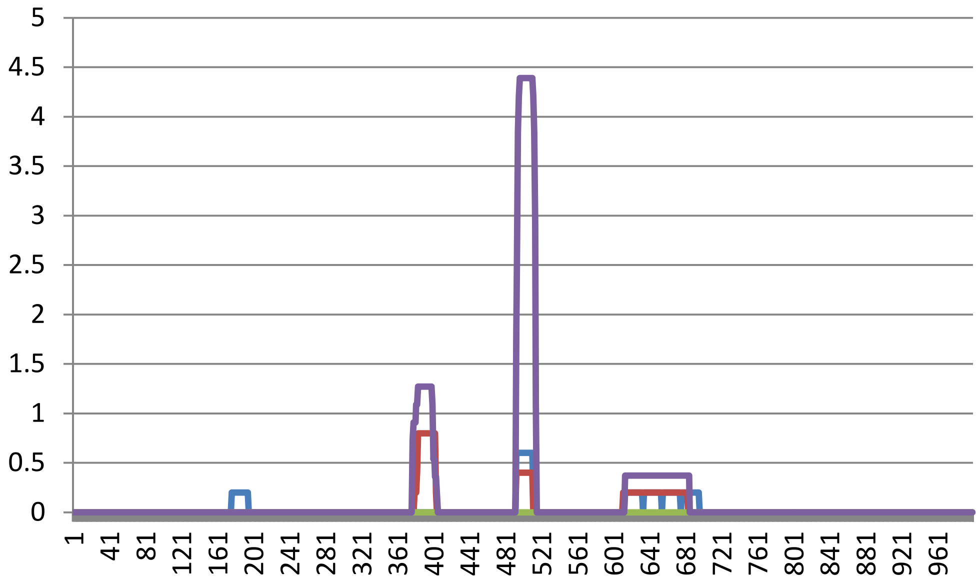

AT1G47450

Protein of unknown function (DUF784)

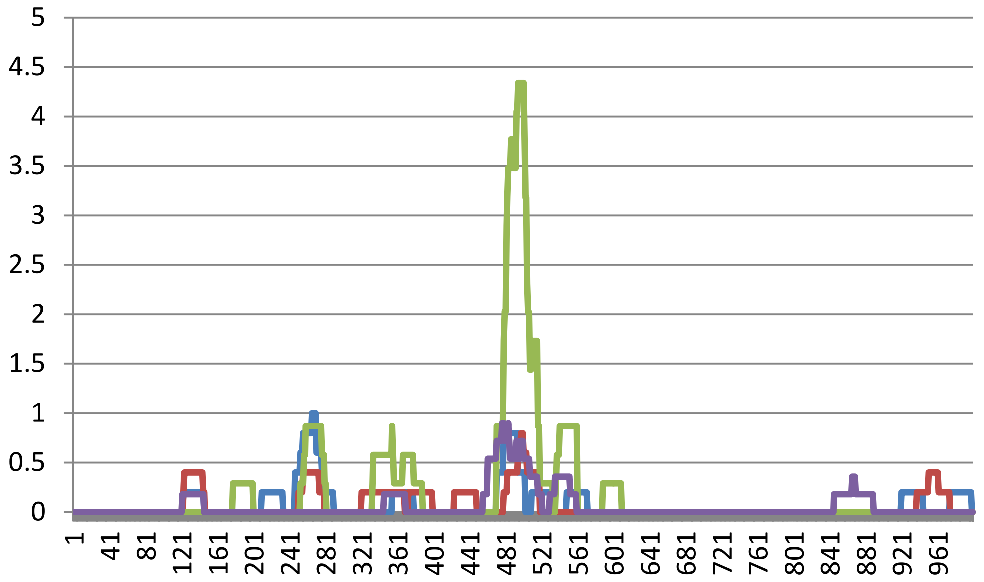

# AT1G48990

## Oleosin family protein

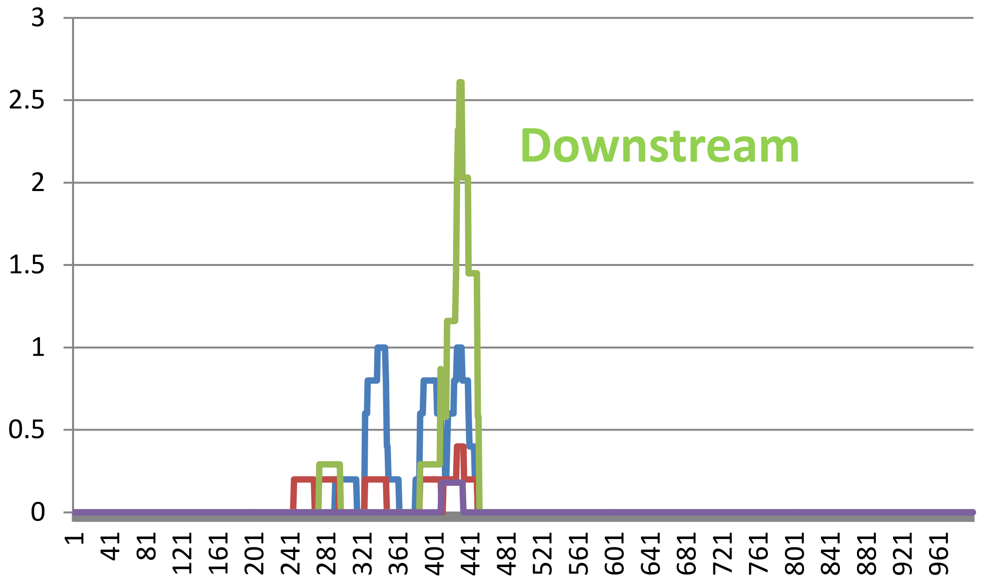

AT1G50110

D-aminoacid aminotransferase-like PLP-dependent enzymes superfamily protein

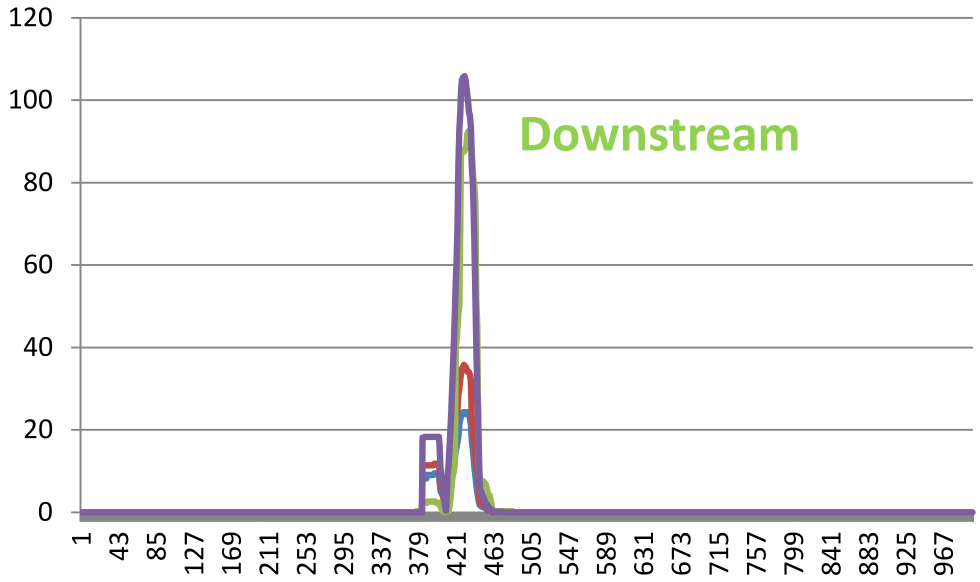

AT1G50970

Membrane trafficking VPS53 family protein

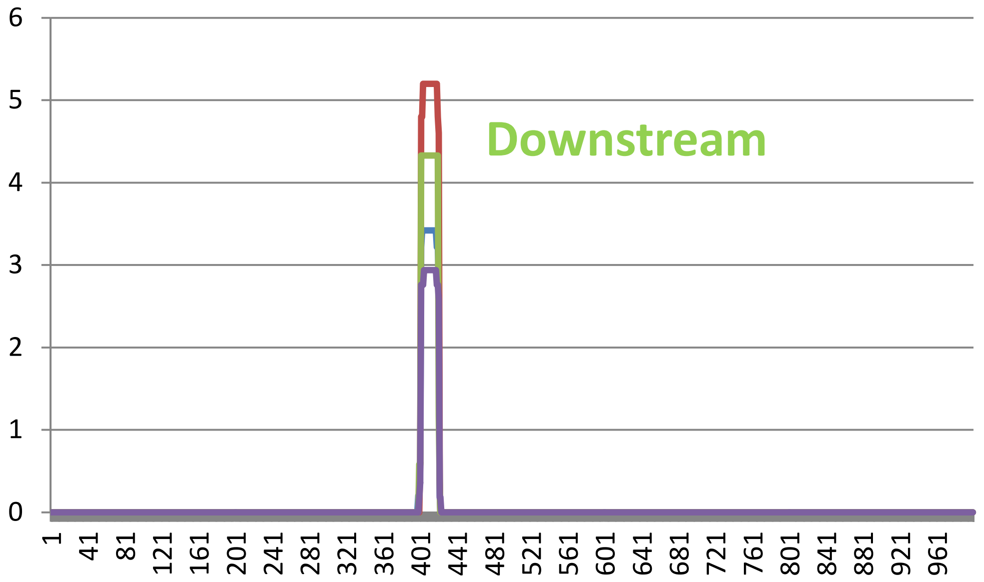

# AT1G51150

Encodes a putative DegP protease.

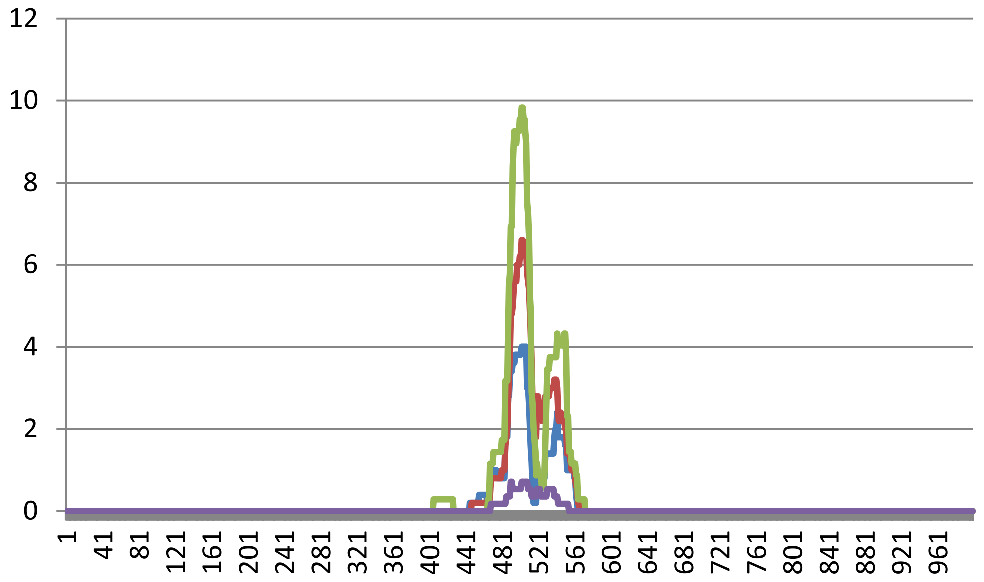

AT1G51820

Leucine-rich repeat protein kinase family protein

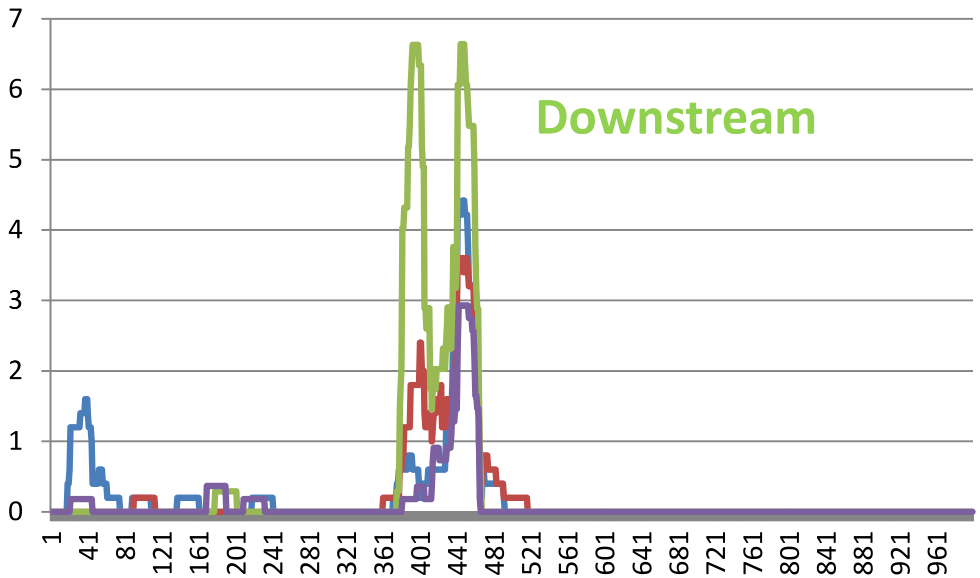

# AT1G52160

Encodes a tRNase Z.

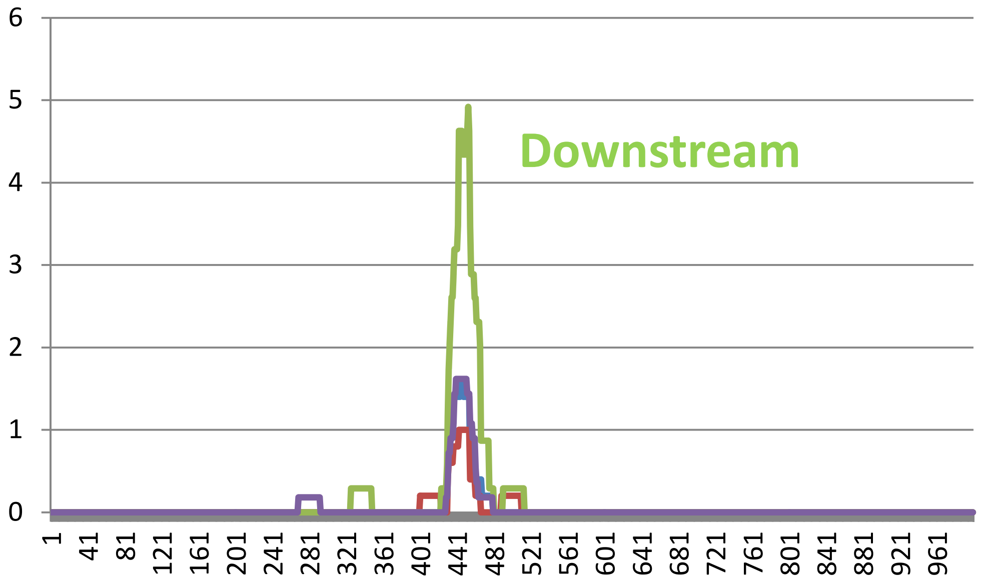

# AT1G55700

## Cysteine/Histidine-rich C1 domain family protein

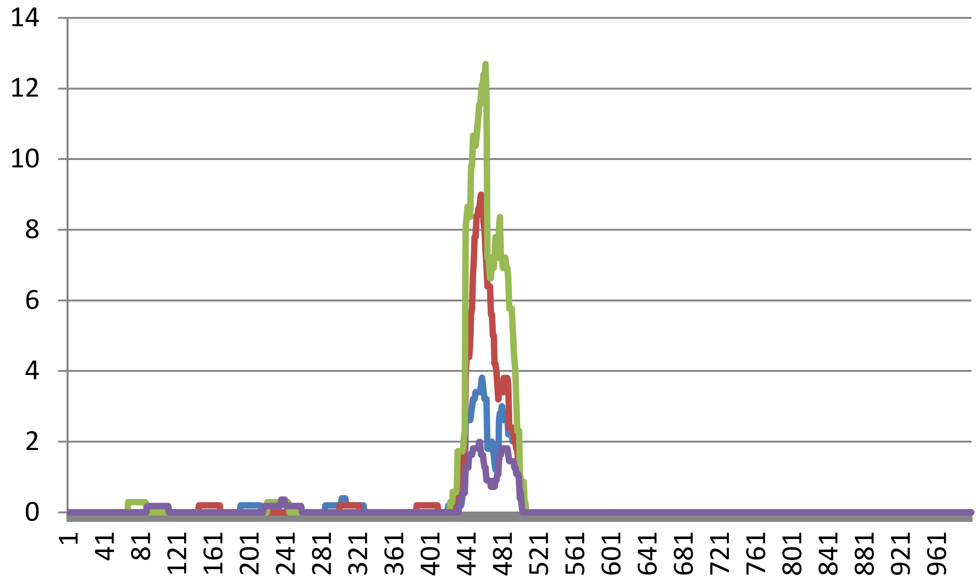

# AT1G57770

## FAD/NAD(P)-binding oxidoreductase family protein

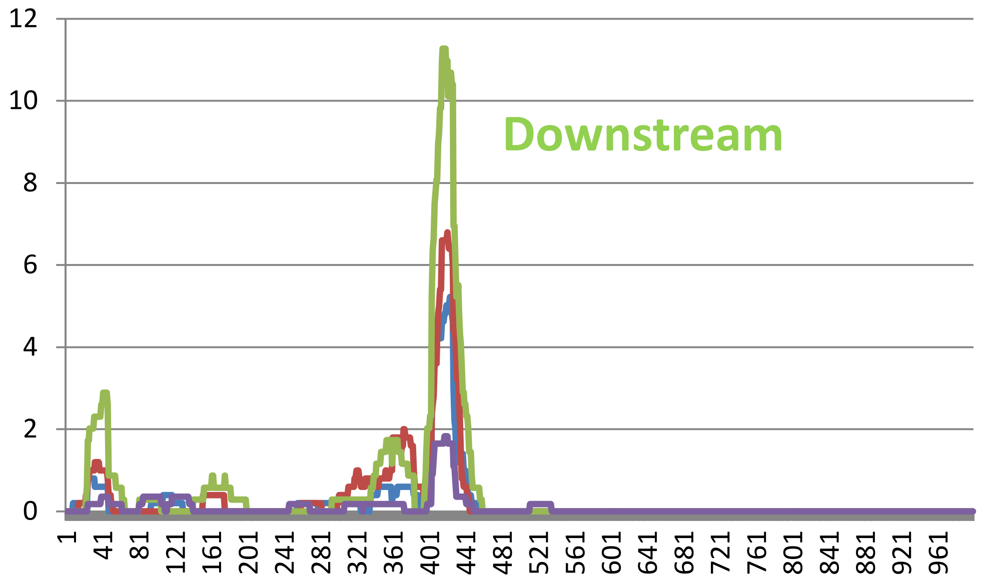

# AT1G58025

## DNA-binding bromodomain-containing protein

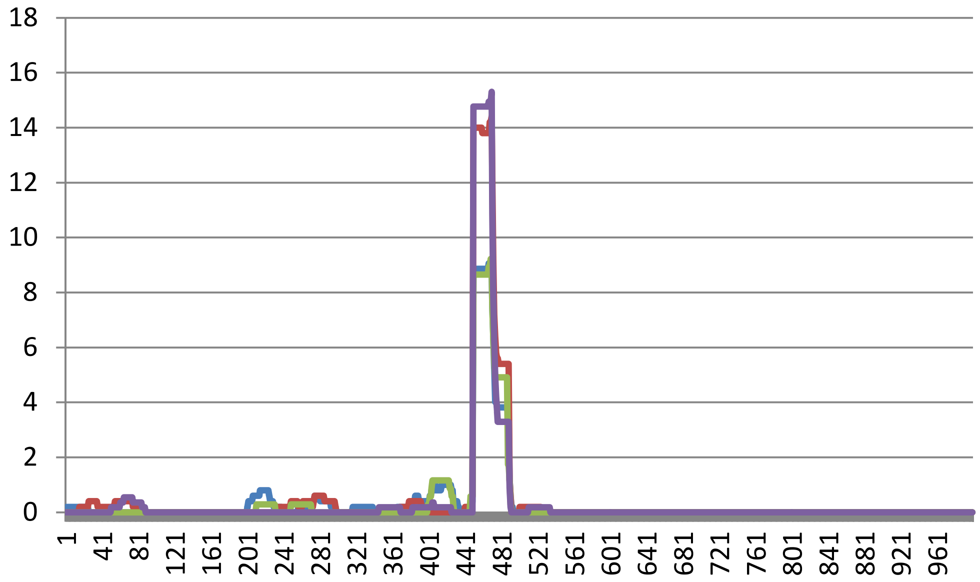

AT1G59835

Unknown protein

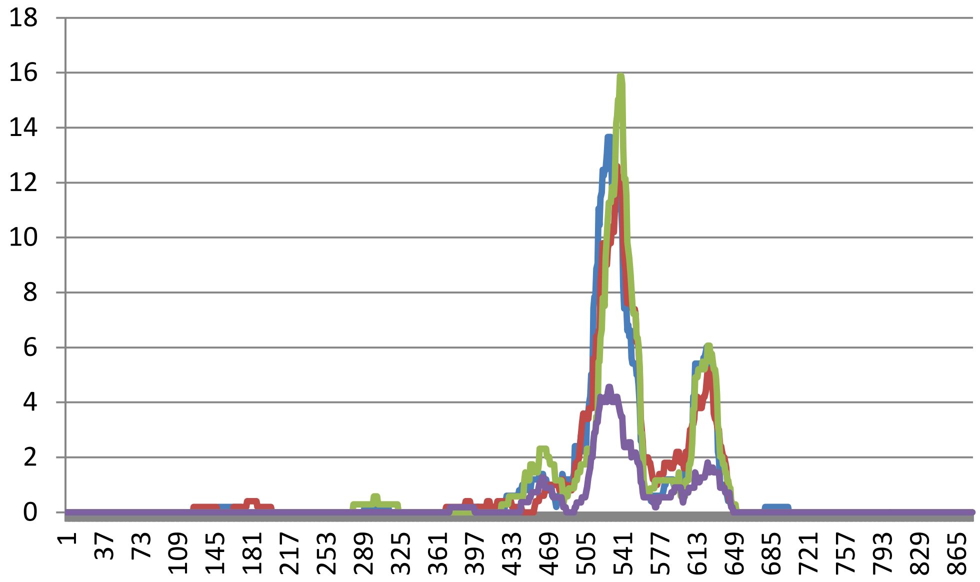

# AT1G59885

## Unknown protein

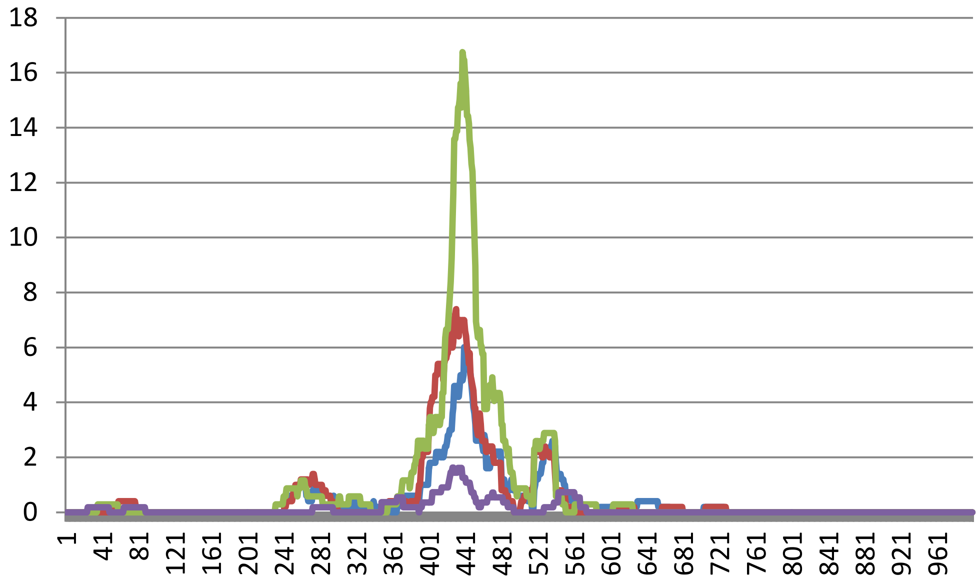

AT1G60986

Encodes a member of a family of small, secreted, cysteine rich proteins with sequence similarity to SCR (S locus cysteine-rich protein).

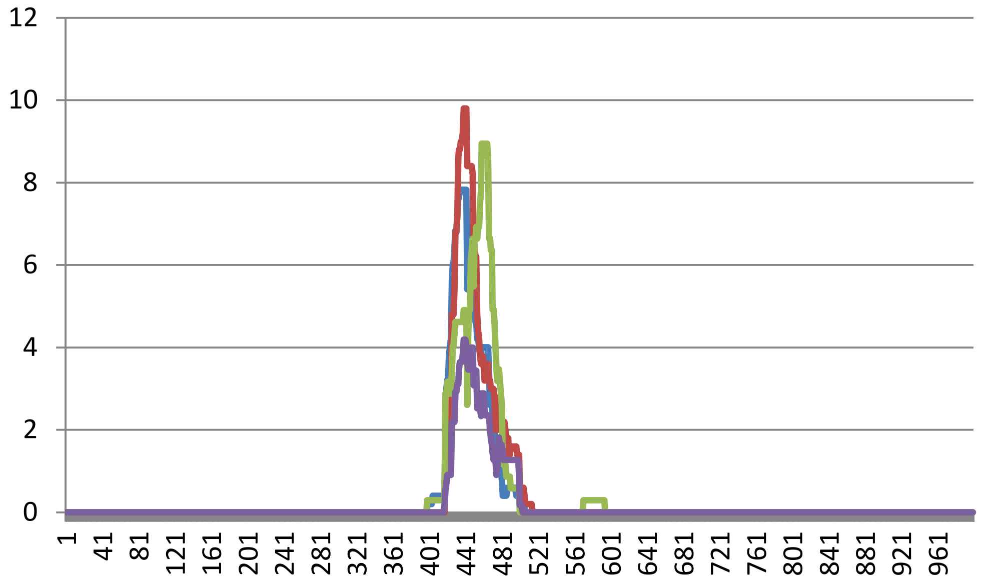

# AT1G62410

## MIF4G domain-containing protein

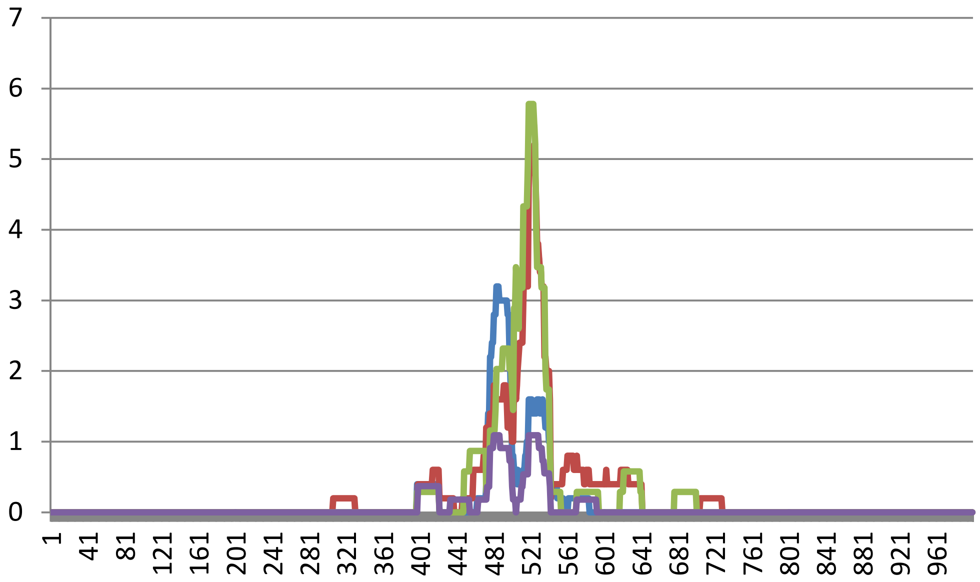

# AT1G63522

Encodes a defensin-like (DEFL) family protein.

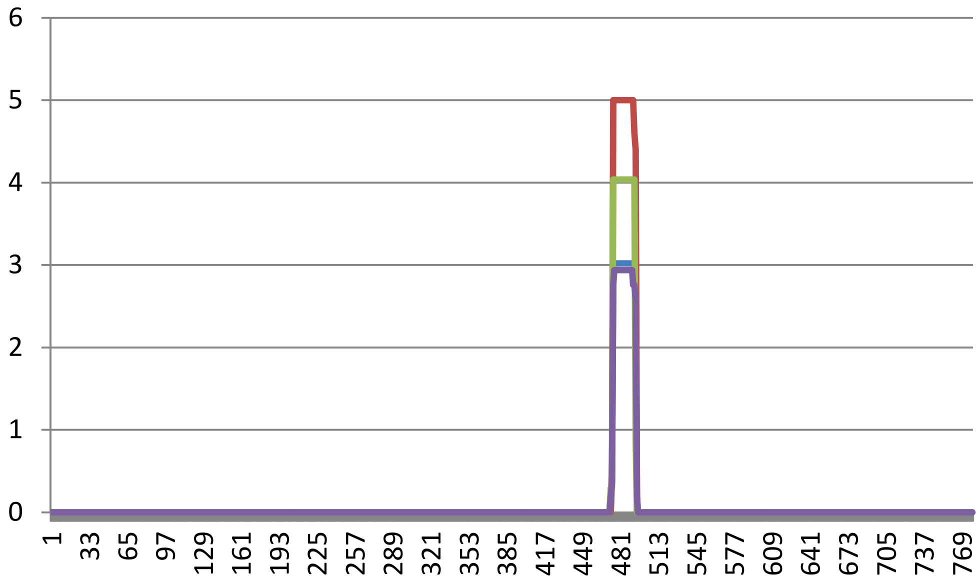

# AT1G65210

## Galactose-binding protein

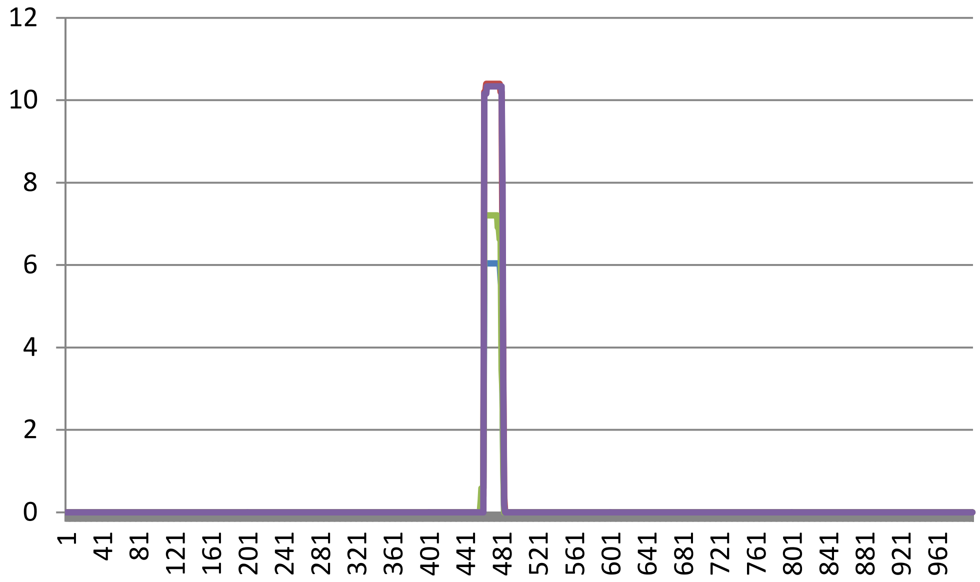

# AT1G66540

## Cytochrome P450 superfamily protein

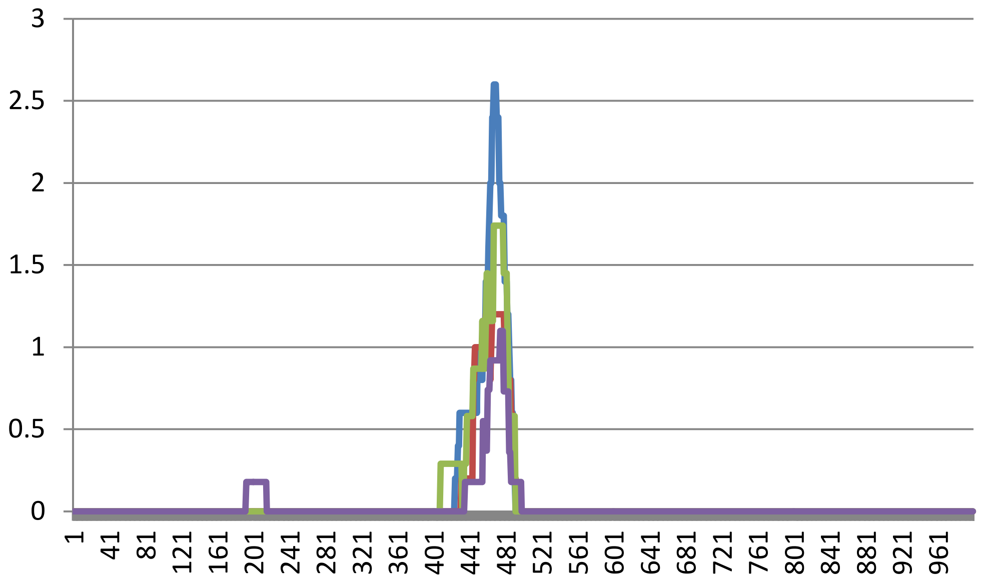

AT1G66570

Sucrose-proton symporter 7 (SUC7)

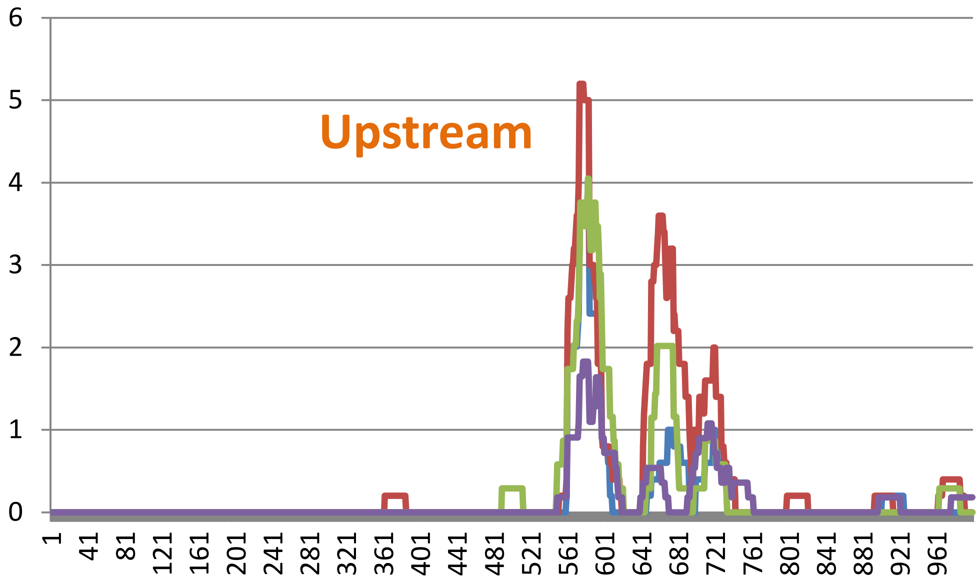

# AT1G66620

## Protein with RING/U-box and TRAF-like domains

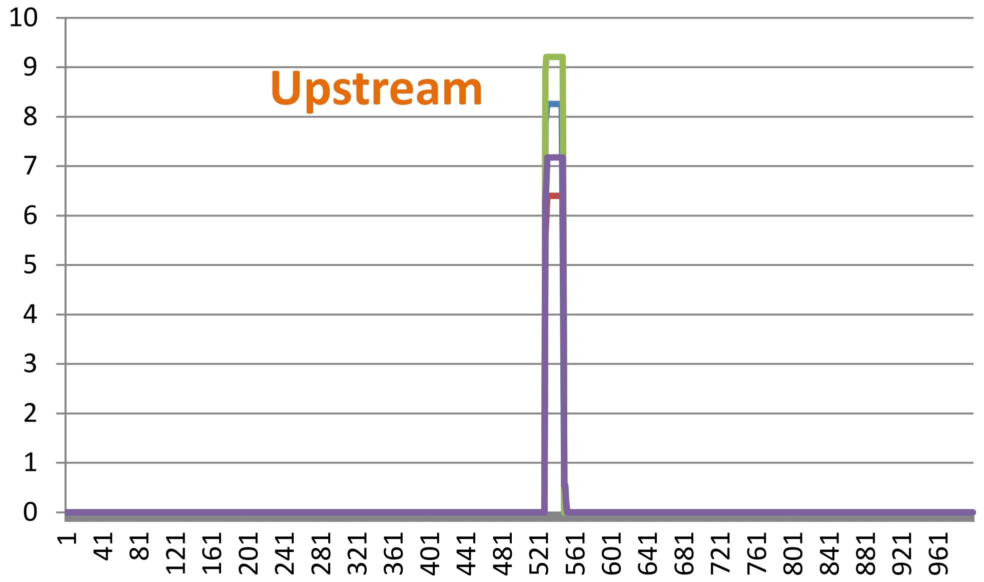

AT1G68040

S-adenosyl-L-methionine-dependent methyltransferases superfamily protein

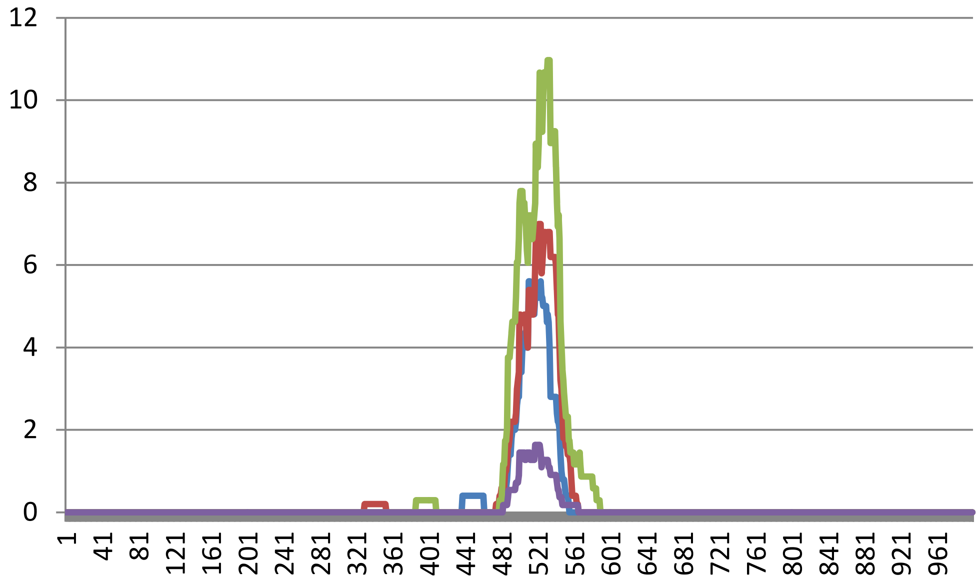

# AT1G70040

## Protein of unknown function (DUF1163)

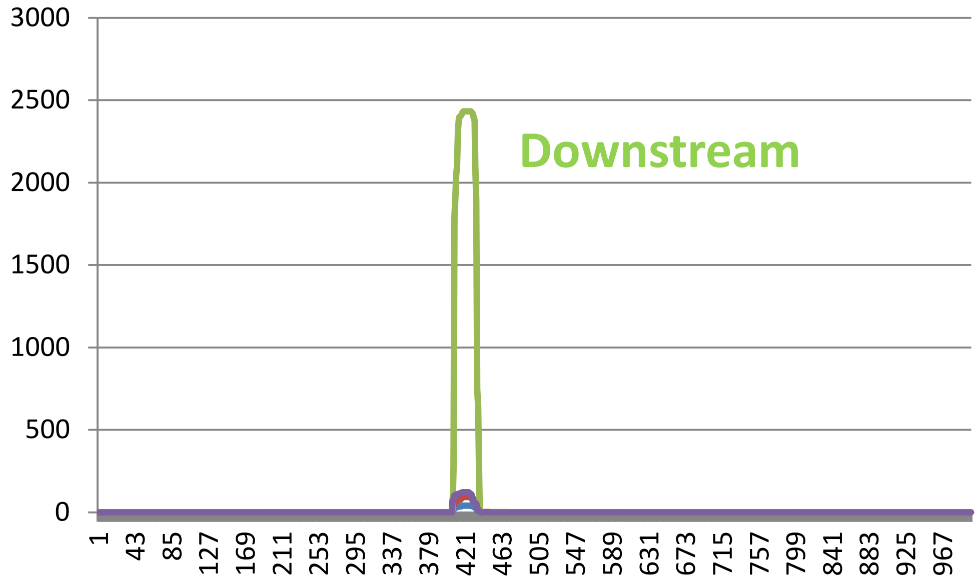

AT1G72580

Unknown protein

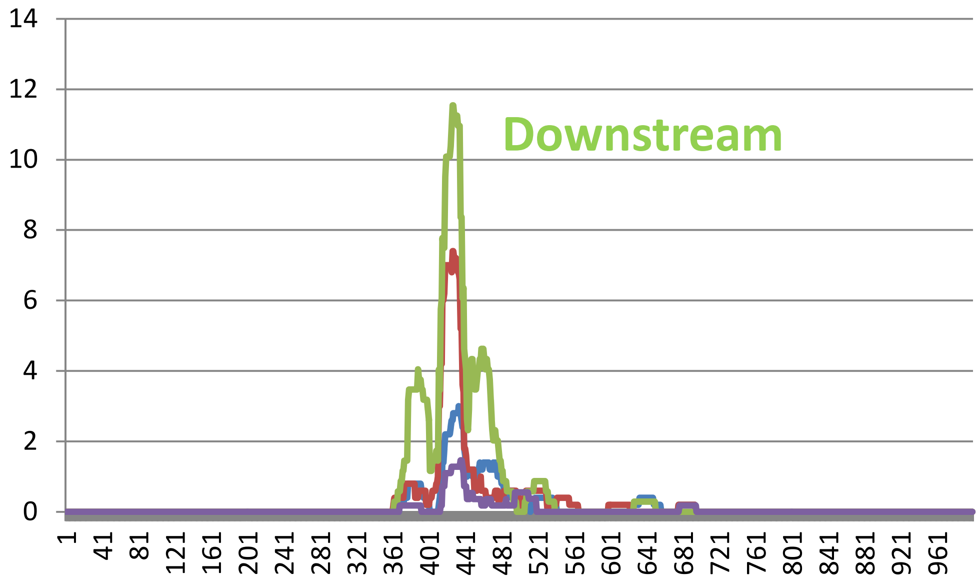

AT1G73360

Encodes a homeobox-leucine zipper family protein belonging to the HD-ZIP IV family. It is involved in trichome branching.

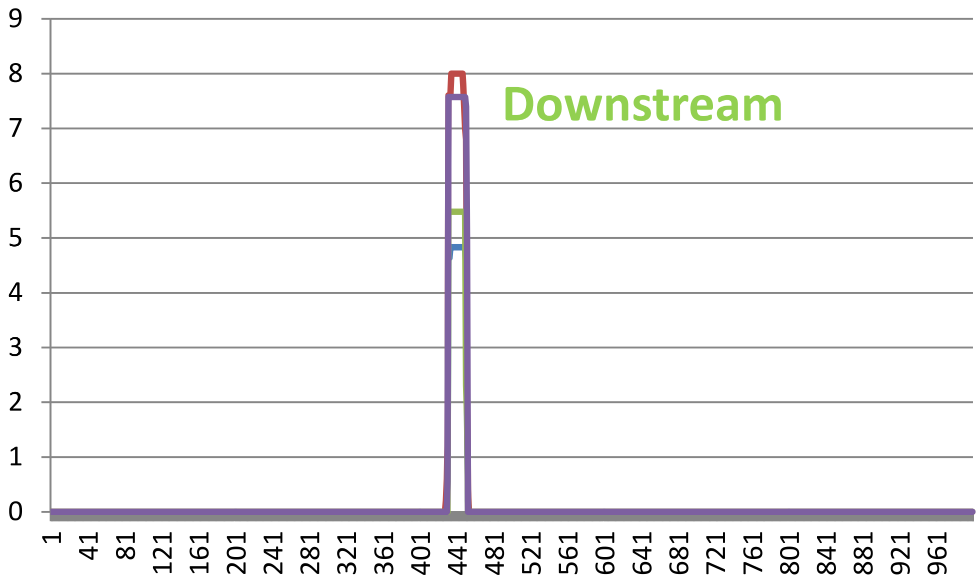

AT1G73650

BEST Arabidopsis thaliana protein match is: Protein of unknown function (DUF1295) (TAIR:AT1G18180.1)

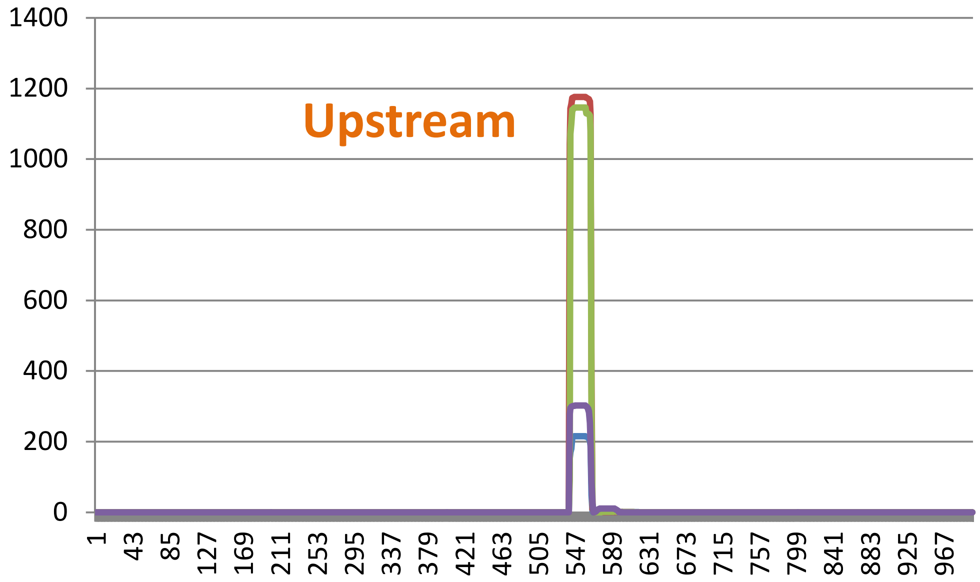

# AT1G77450

## NAC domain containing protein 32 (NAC032)

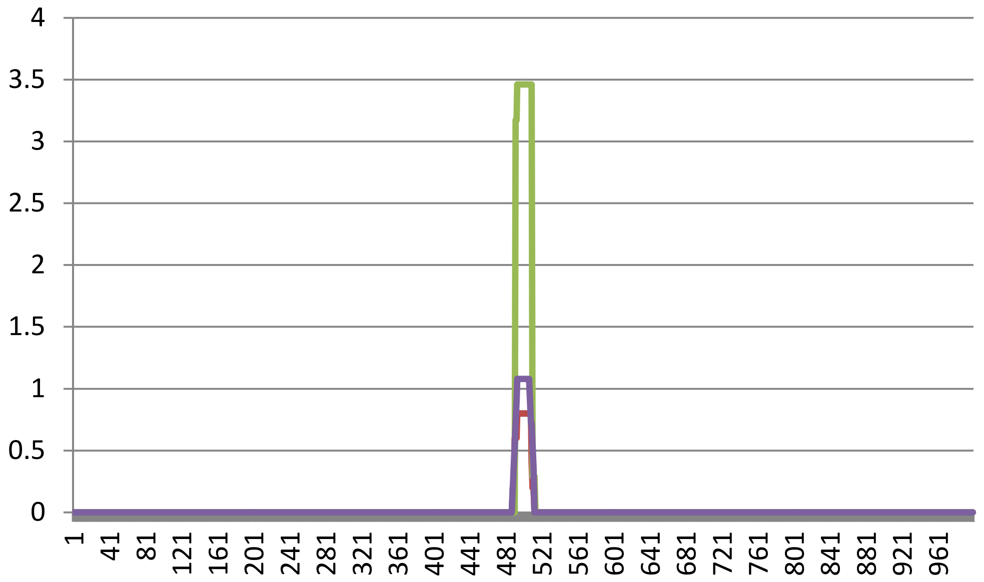

# AT2G01950

Encodes a leucine rich repeat receptor kinase and associated with provascular/procambial cells. Similar to BRI, brassinosteroid receptor protein.

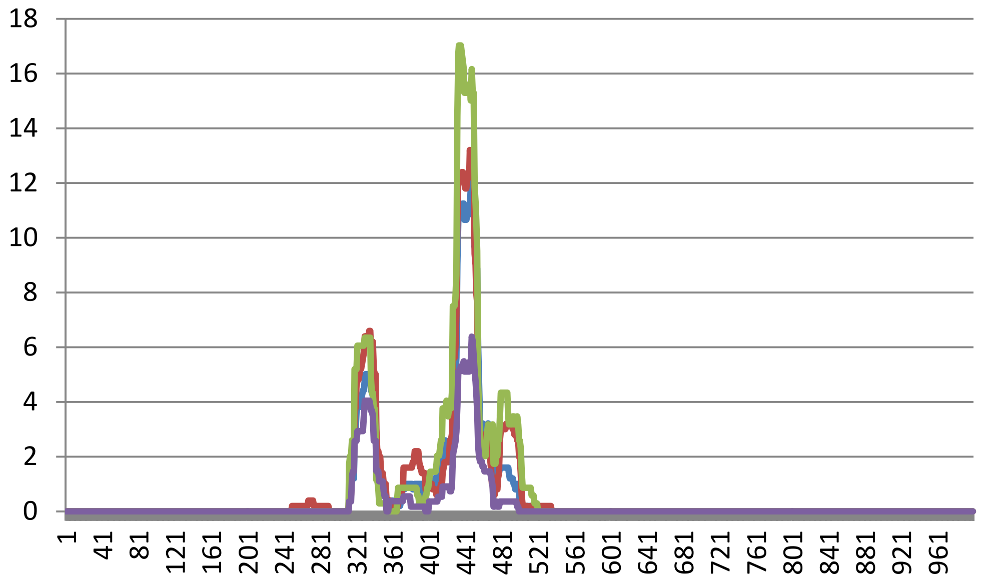

# AT2G02400

## NAD(P)-binding Rossmann-fold superfamily protein

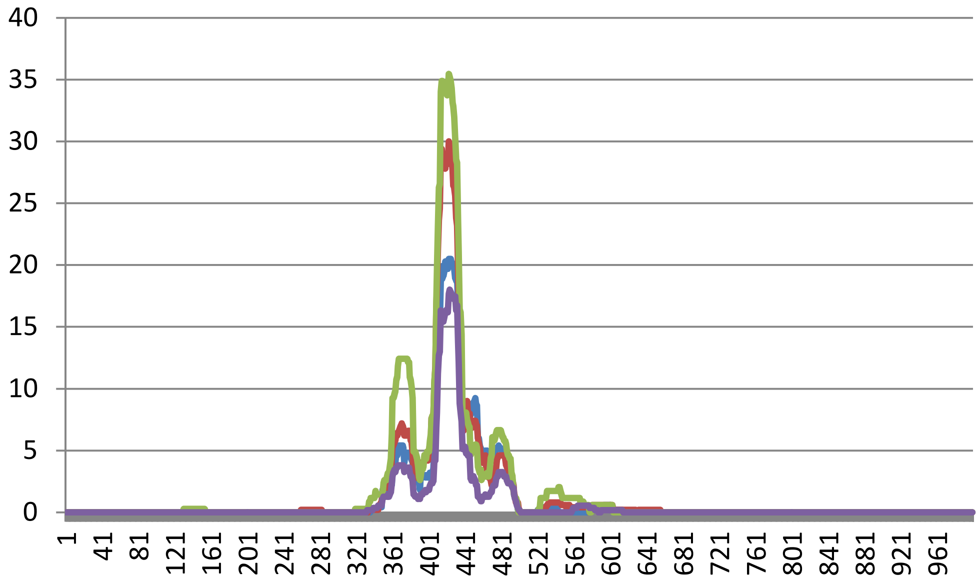

AT2G02520

RNA-directed DNA polymerase (reverse transcriptase)-related family protein

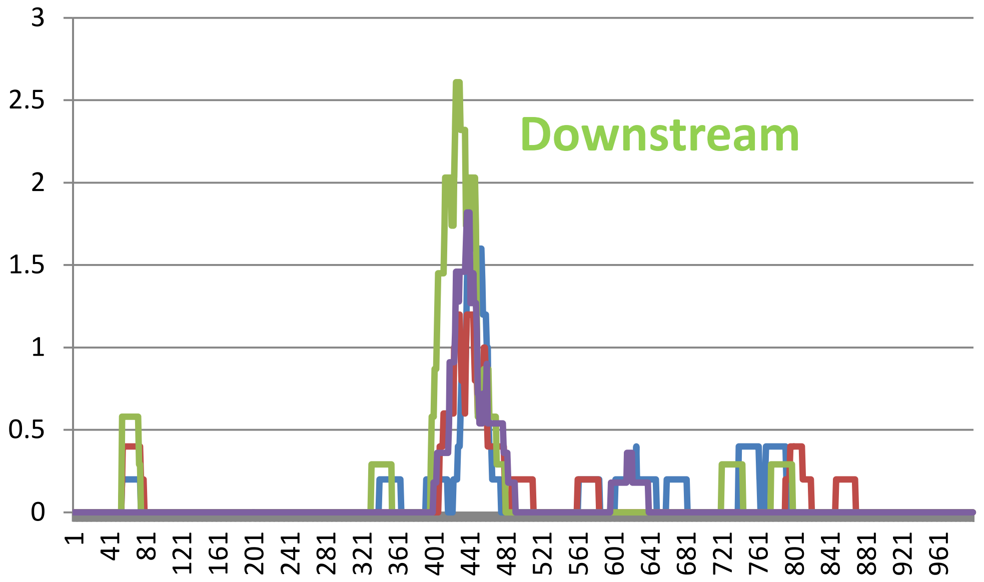

# AT2G03370

## Glycosyltransferase family 61 protein

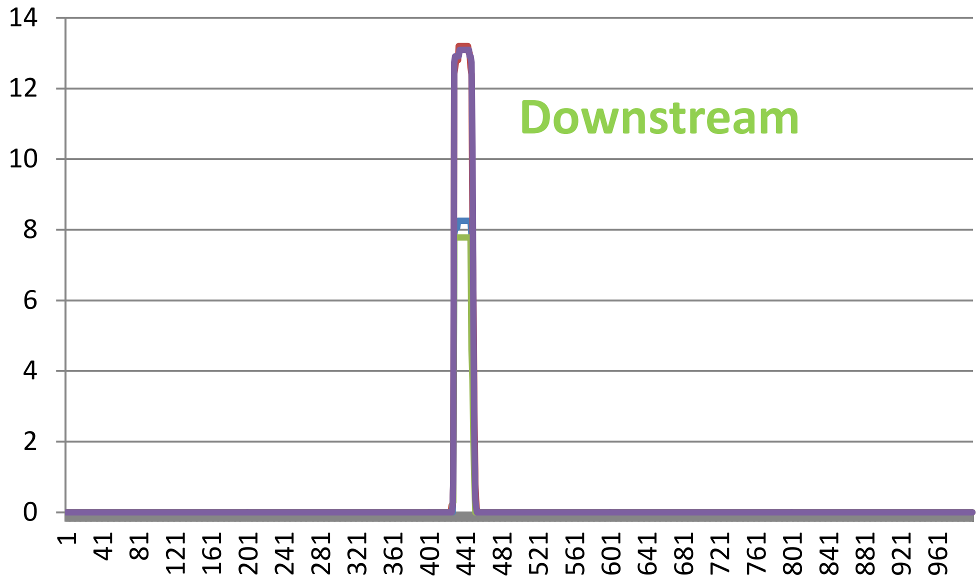

AT2G04090

MATE efflux family protein

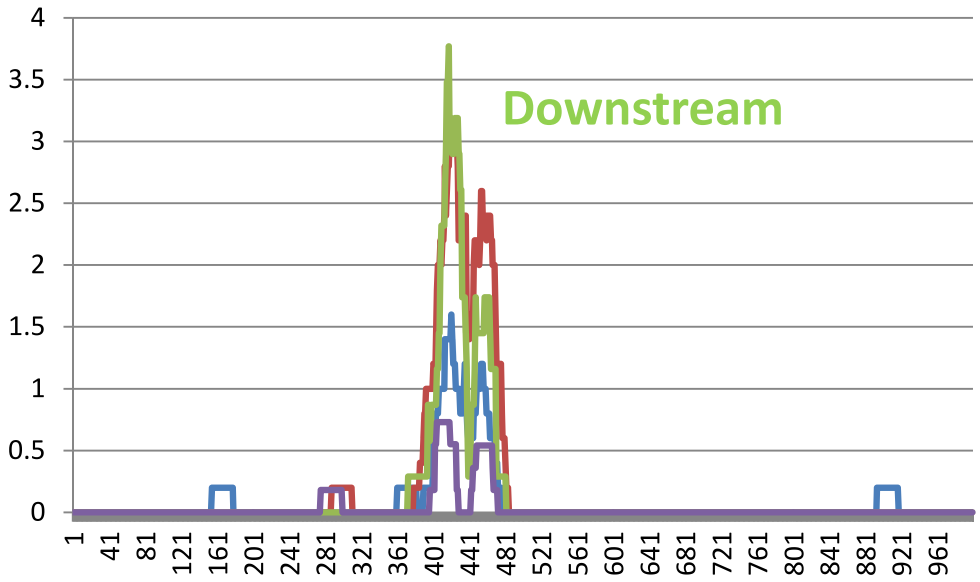

# AT2G04115

## Plant self-incompatibility protein S1 family

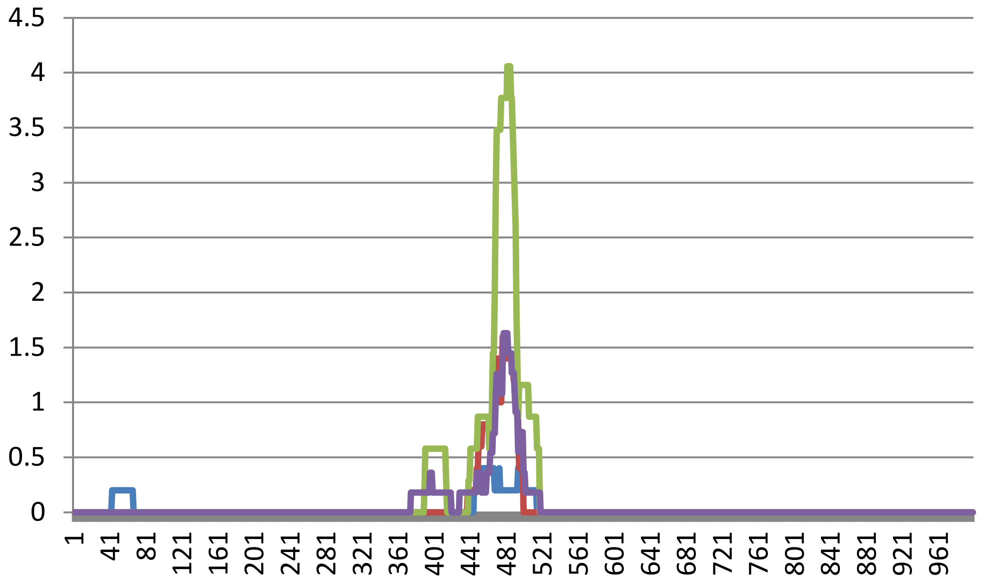

AT2G04220

Plant protein of unknown function (DUF868)

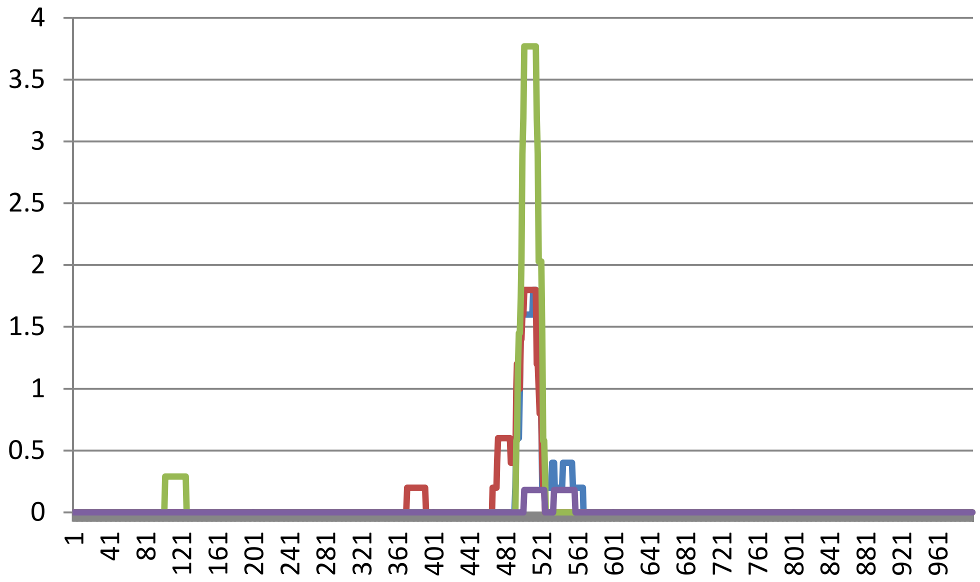

# AT2G04830

## Protein of unknown function (DUF295)

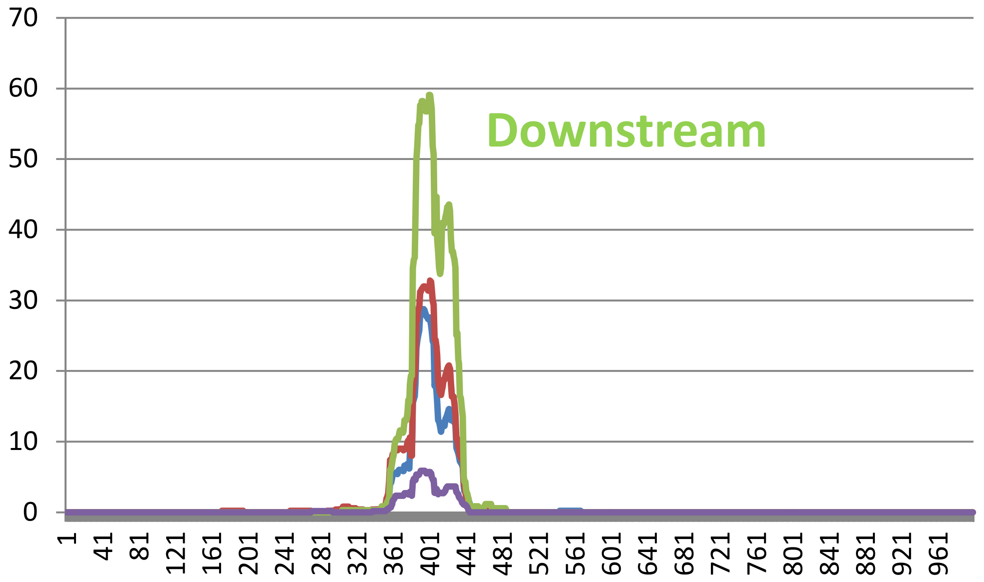

AT2G05335

Encodes a member of a family of small, secreted, cysteine rich proteins with sequence similarity to SCR (S locus cysteine-rich protein).

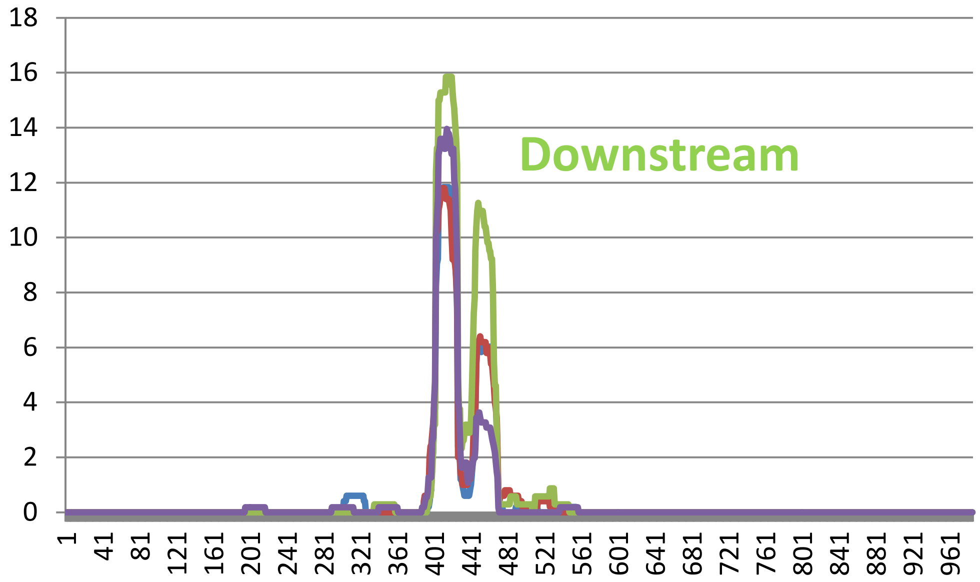

AT2G06255

ELF4-like 3 (ELF4-L3)

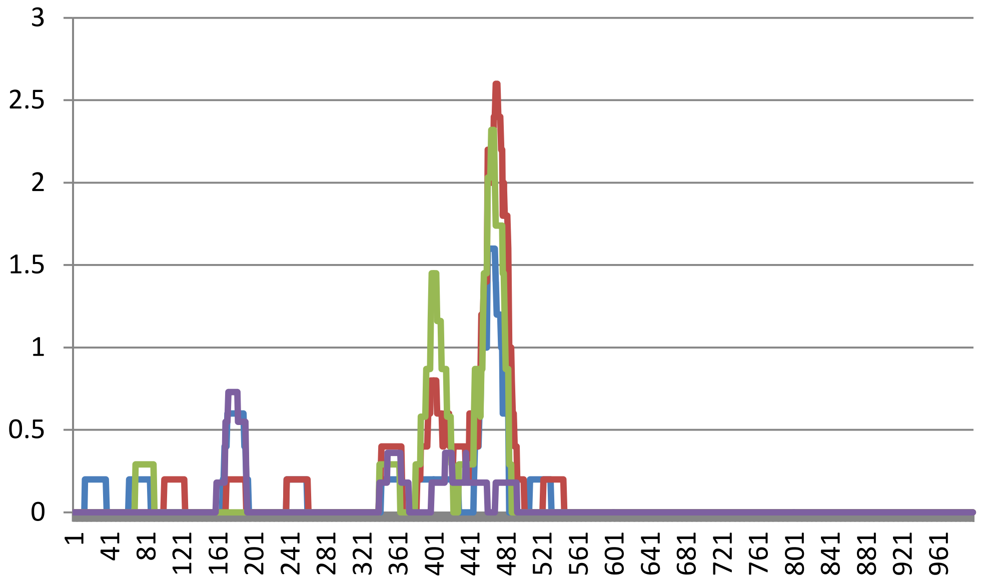

# AT2G06541

## TTF-type zinc finger protein with HAT dimerisation domain

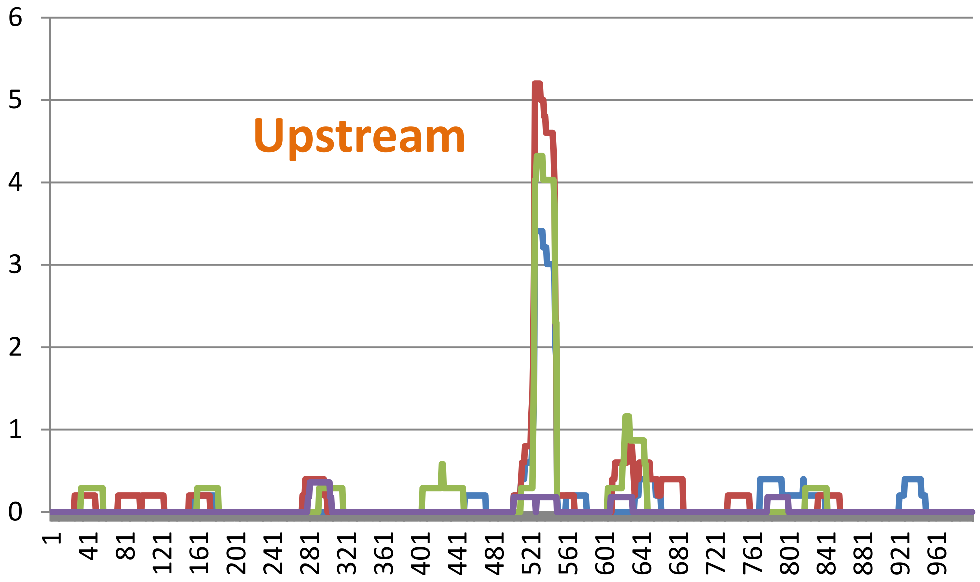

AT2G07360

SH3 domain-containing protein

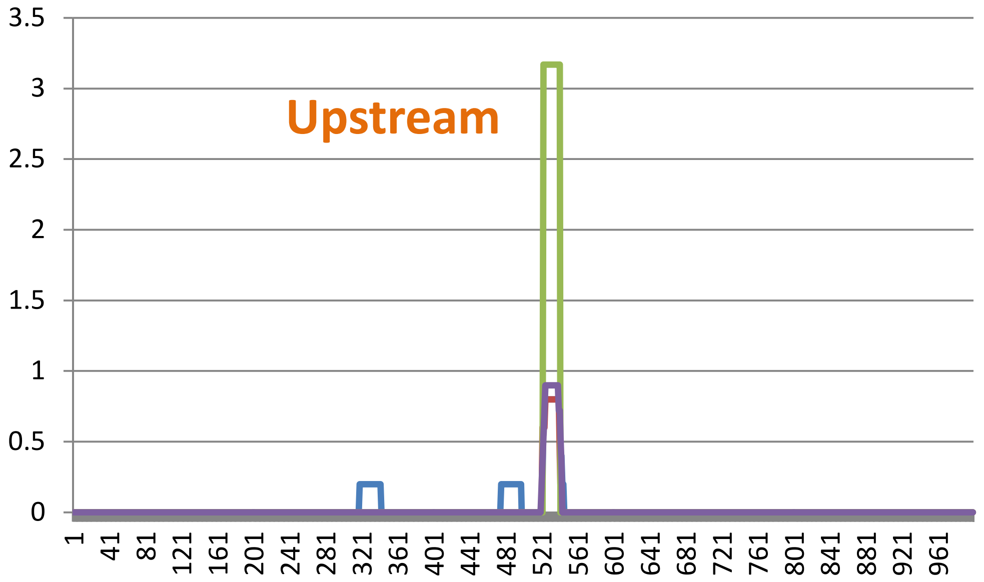

AT2G10608

Unknown protein

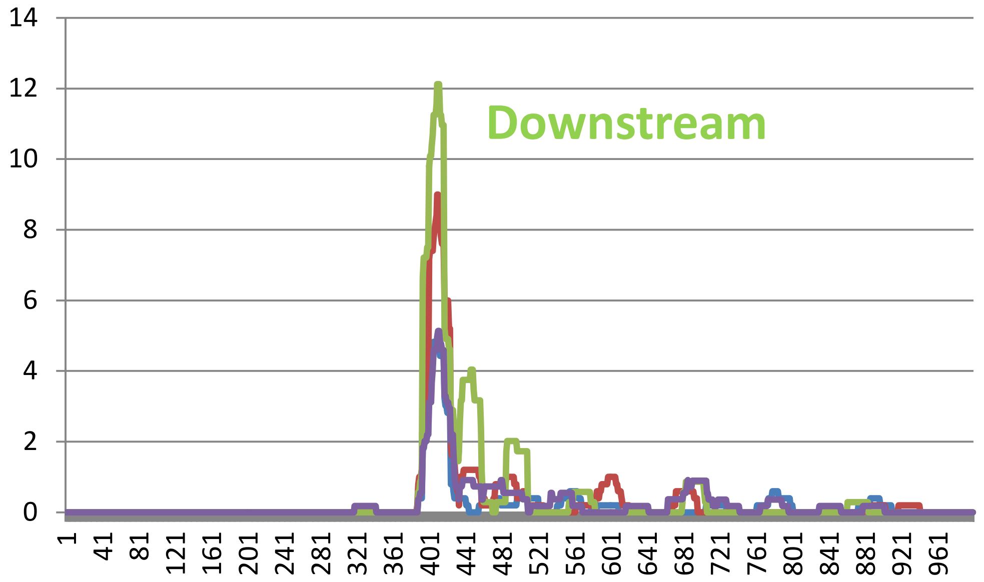

AT2G13960

Homeodomain-like superfamily protein

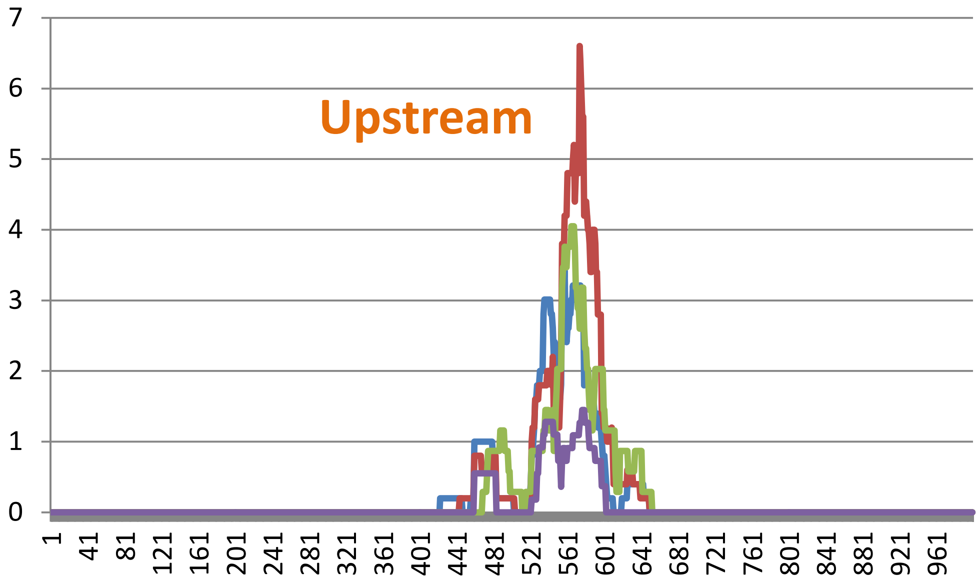

AT2G14560

Encodes LURP1, a member of the LURP cluster (late upregulated in response to Hyaloperonospora parasitica) which exhibits a pronounced upregulation after recognition of the pathogenic oomycte H. parasitica.  LURP1 is required for full basal defense to H. parasitica and resistance to this pathogen mediated by the R-proteins RPP4 and RPP5.

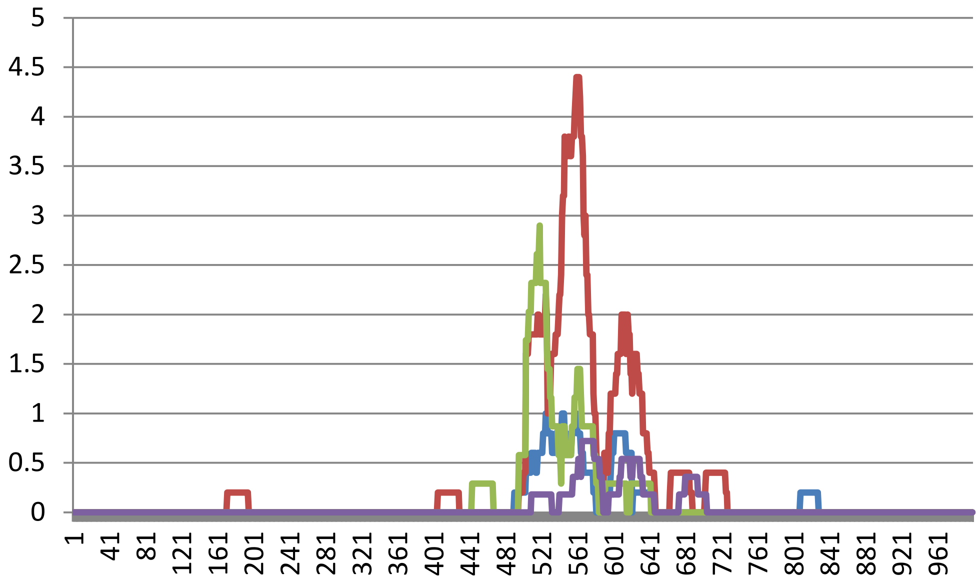

# AT2G15050

Predicted to encode a PR (pathogenesis-related) protein.

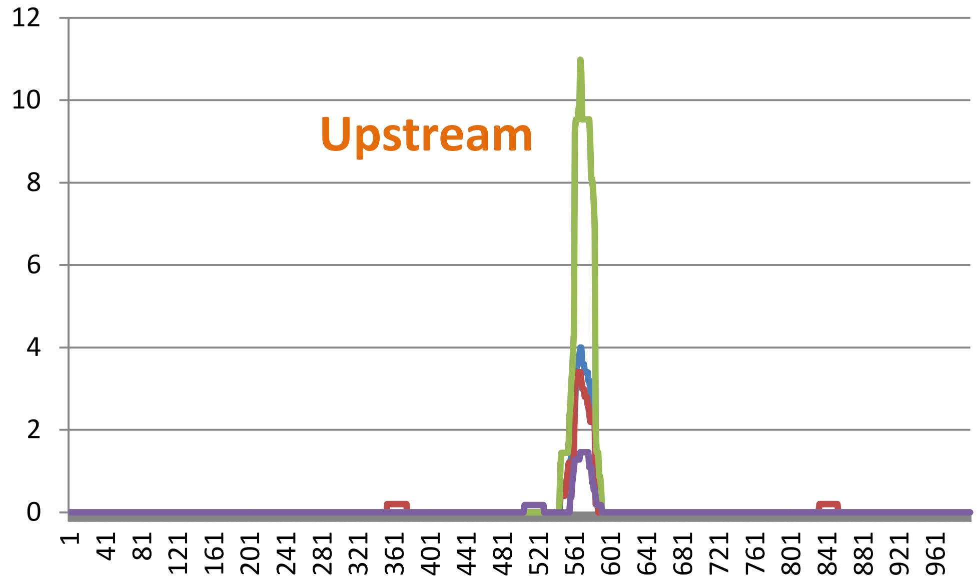

AT2G15535

Low-molecular-weight cysteine-rich 10 (LCR10)

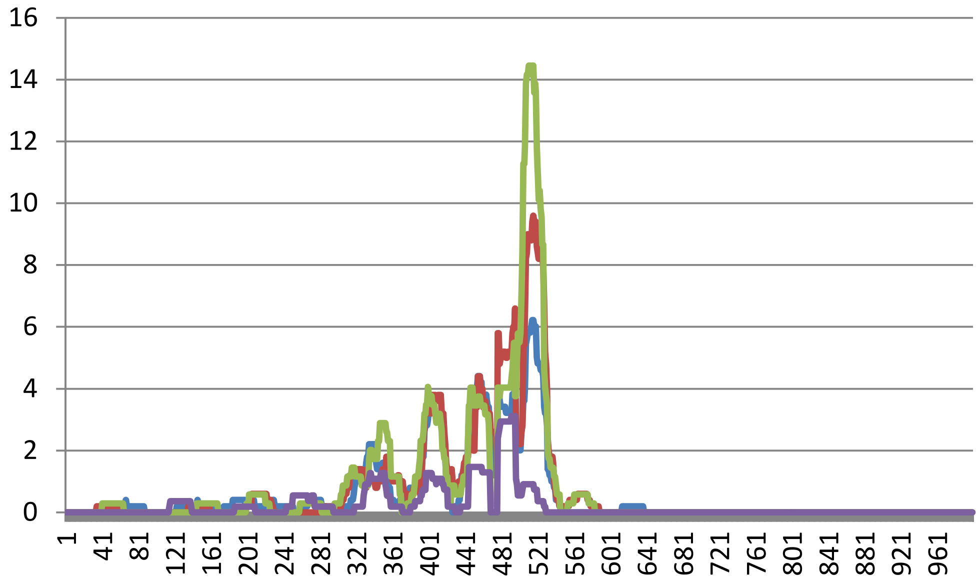

AT2G16340

Unknown protein

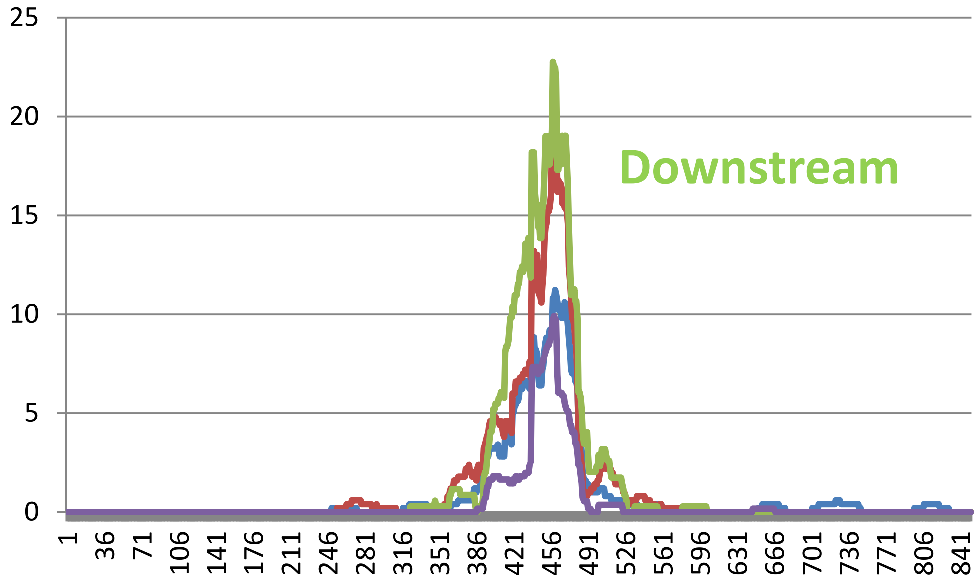

AT2G16870

Disease resistance protein (TIR-NBS-LRR class) family

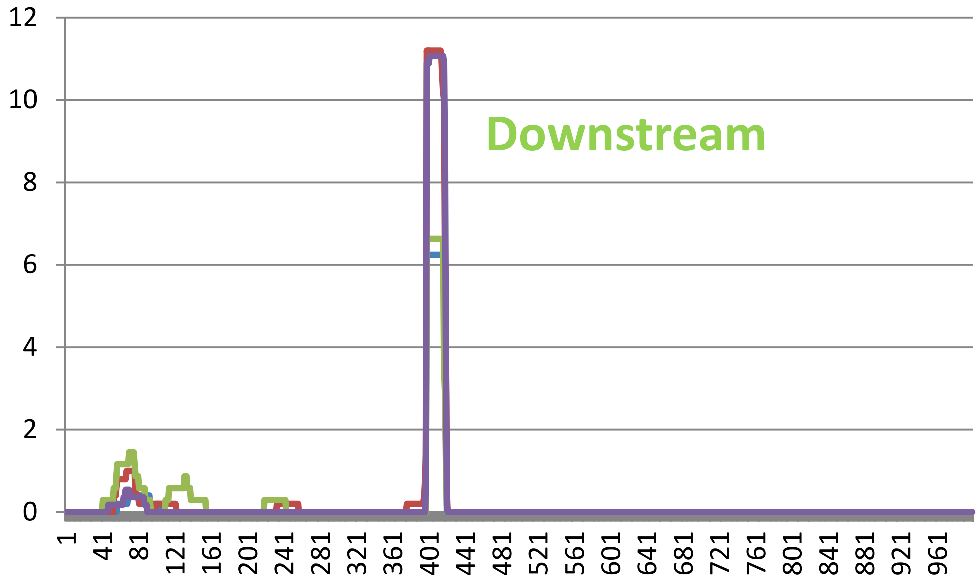

AT2G18980

Peroxidase superfamily protein

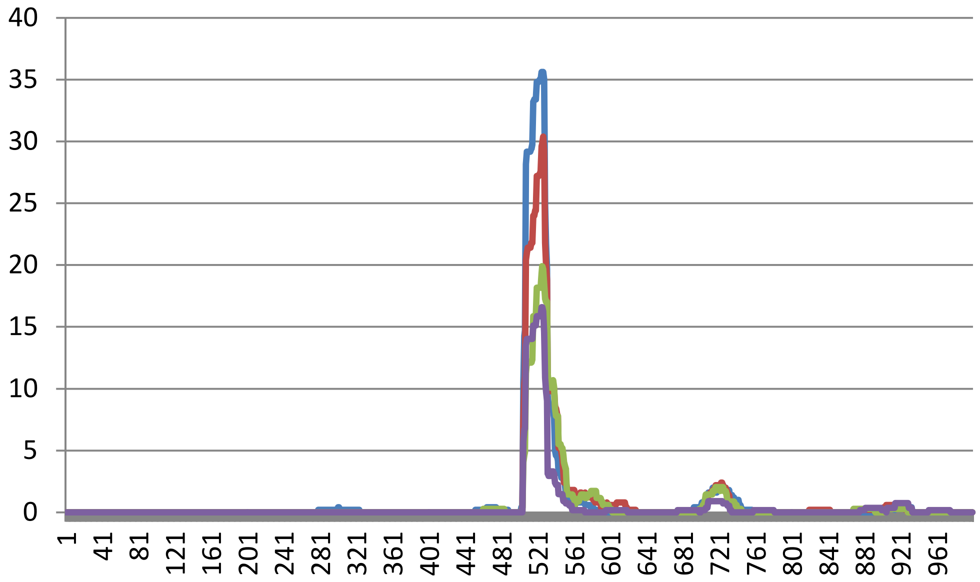

AT2G19360

Protein of Unknown Function (DUF239)

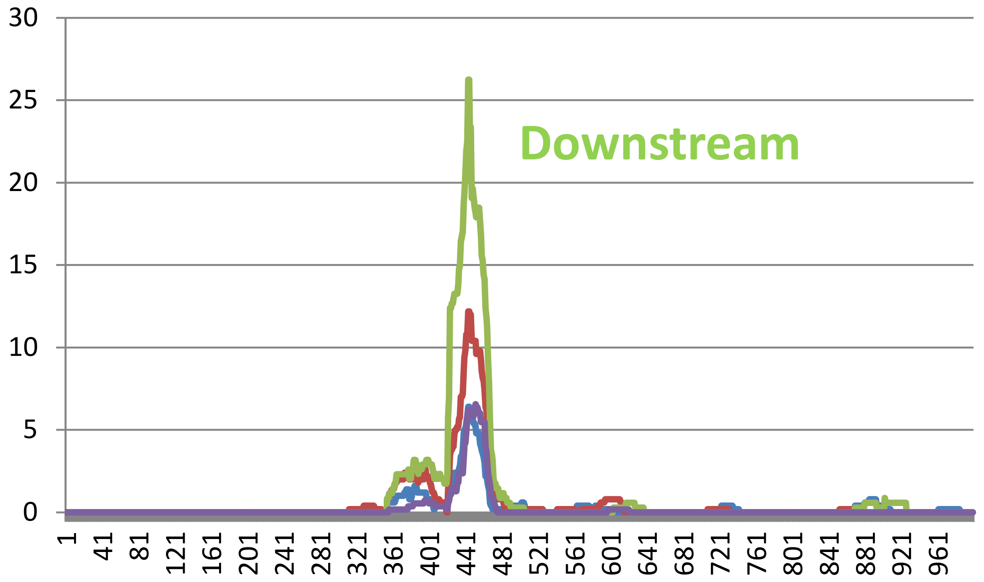

AT2G20580

Encoding the RPN subunits of the 26S proteasome

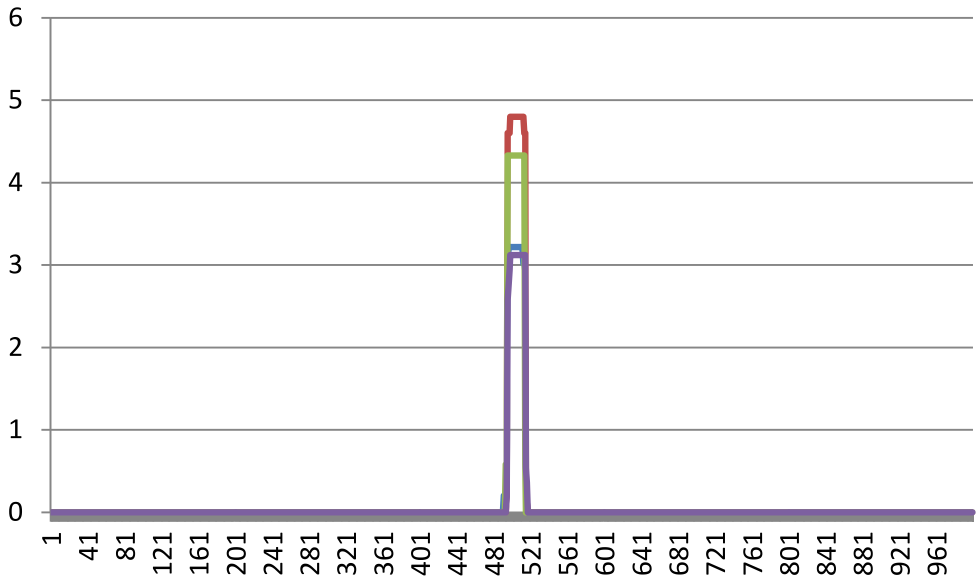

AT2G21370

Although this gene has a sequence similar to xylulose kinases, several lines of experimental evidence suggest that it does not act on xylulose or deoxy-xylulose.

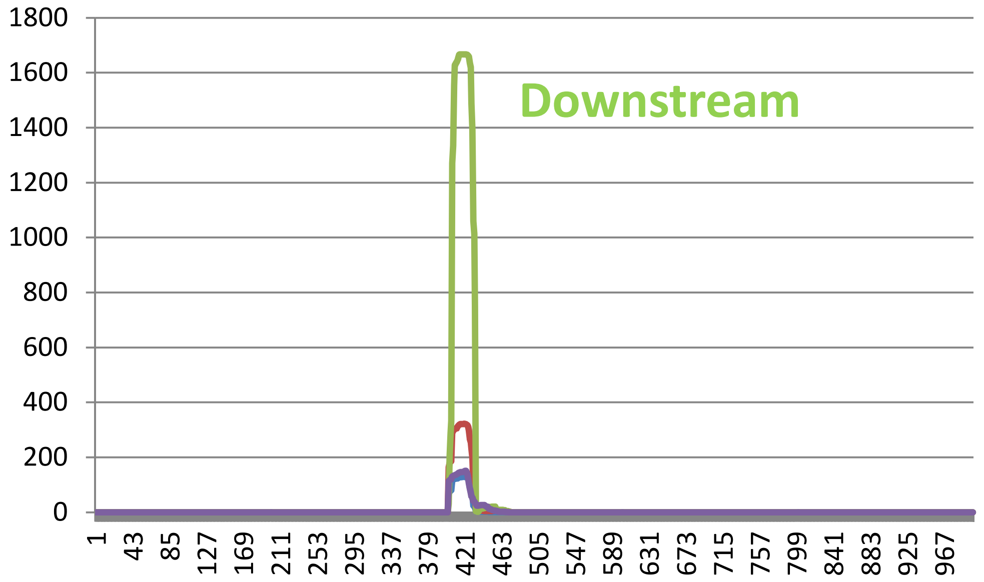

AT2G22890

Kua-ubiquitin conjugating enzyme hybrid localisation domain

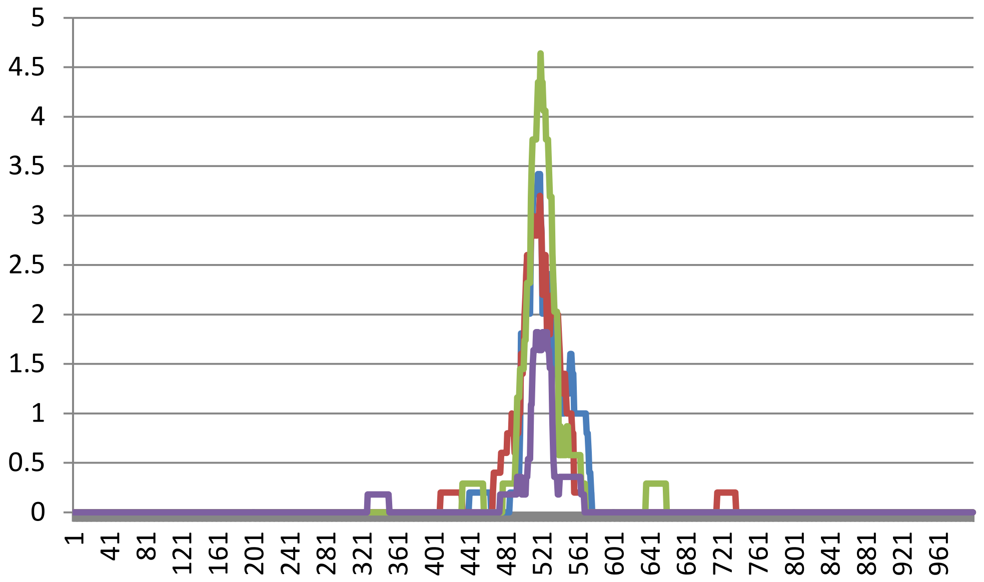

AT2G23770

Protein kinase family protein/peptidoglycan-binding LysM domain-containing protein

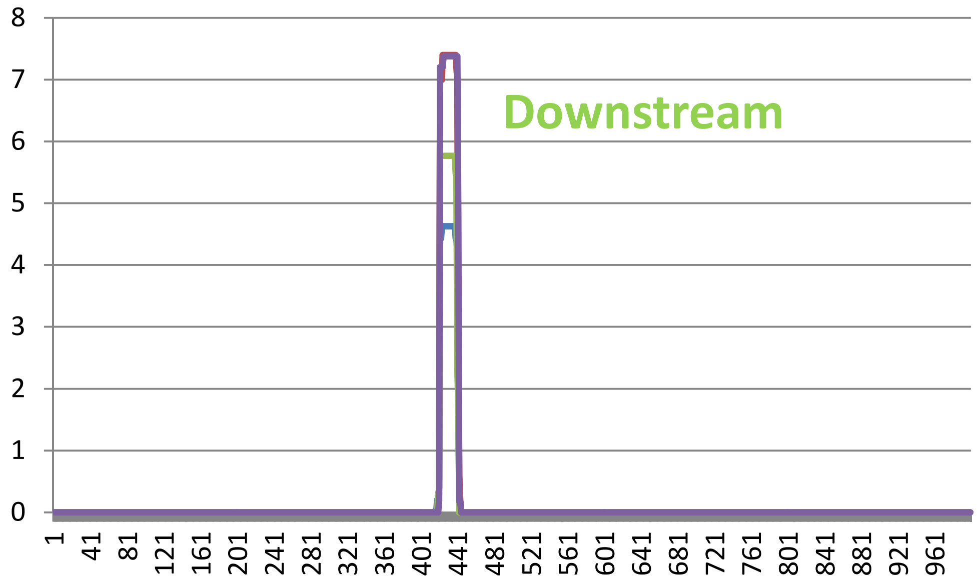

AT2G24880

Plant self-incompatibility protein S1 family

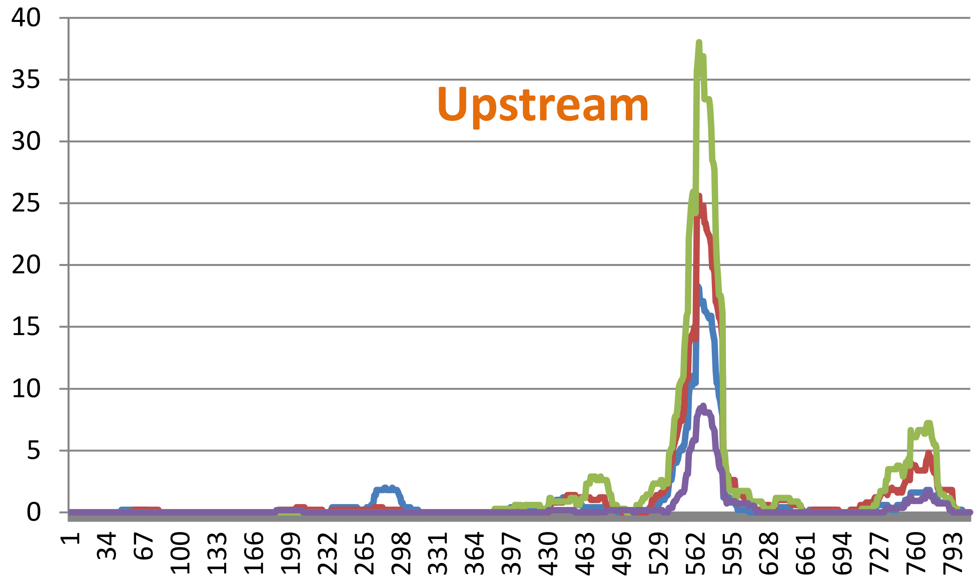

# AT2G32140

## Transmembrane receptors

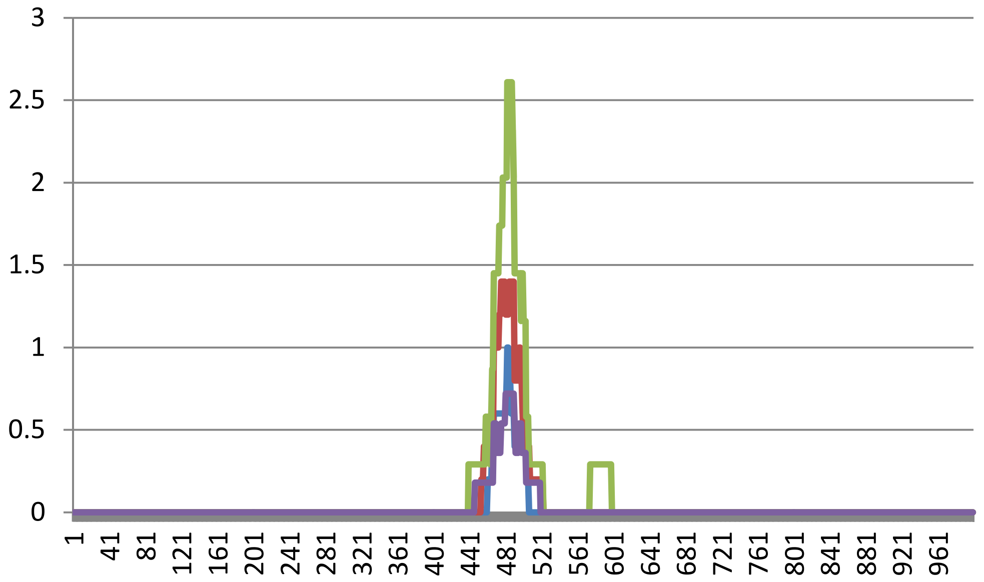

AT2G32310

CCT motif family protein

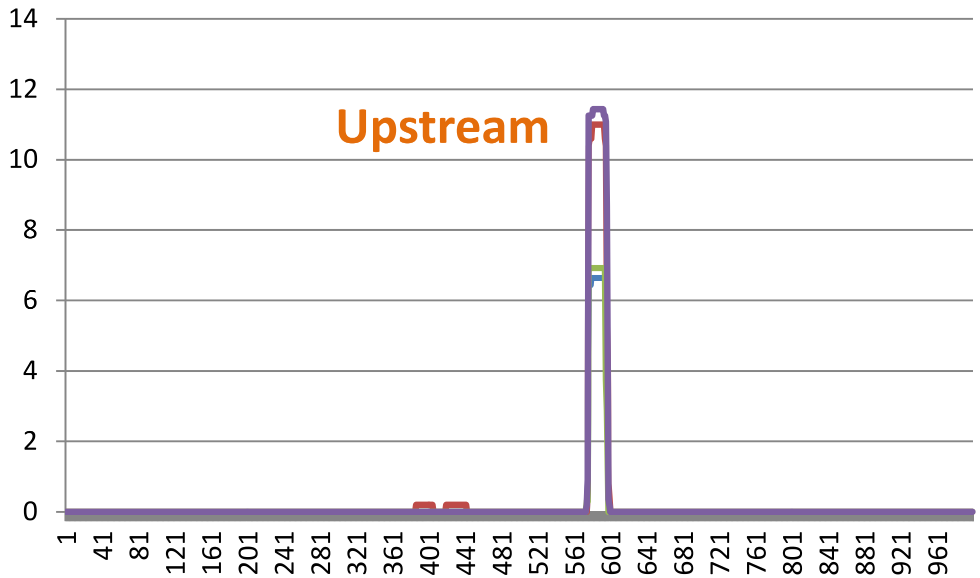

# AT2G32785

## Encodes a Rapid ALkalinization Factor (RALF) family protein

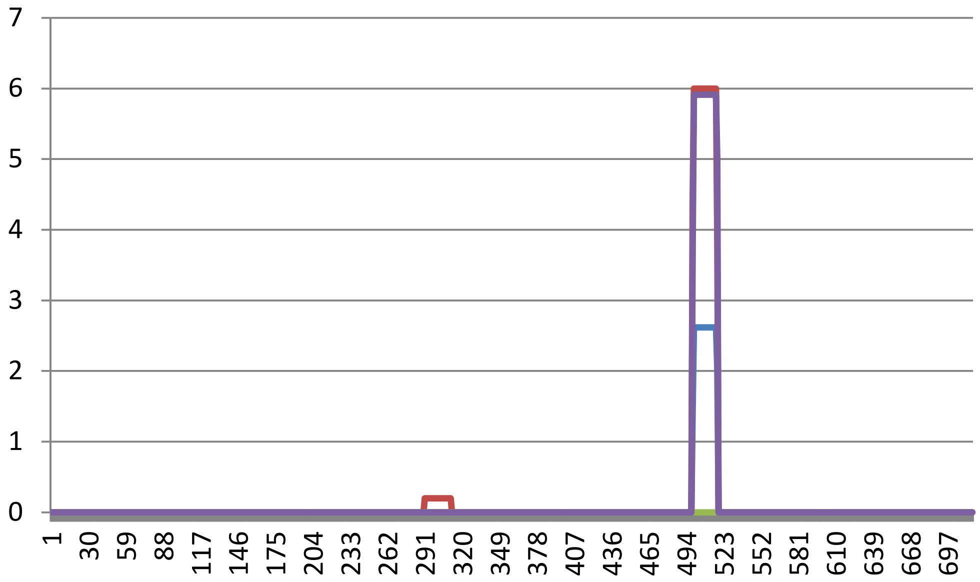

AT2G32790

Ubiquitin-conjugating enzyme family protein

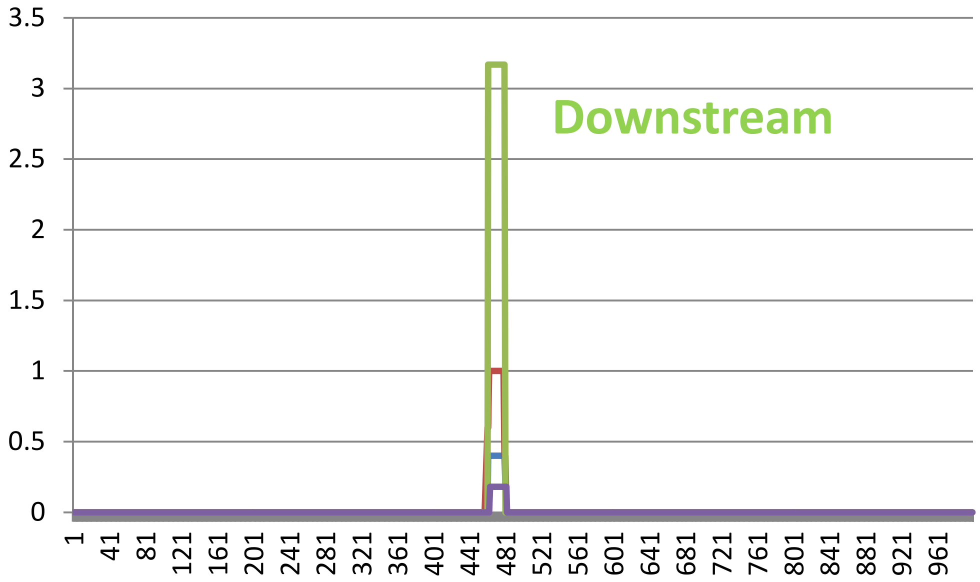

# AT2G34655

## Unknown protein

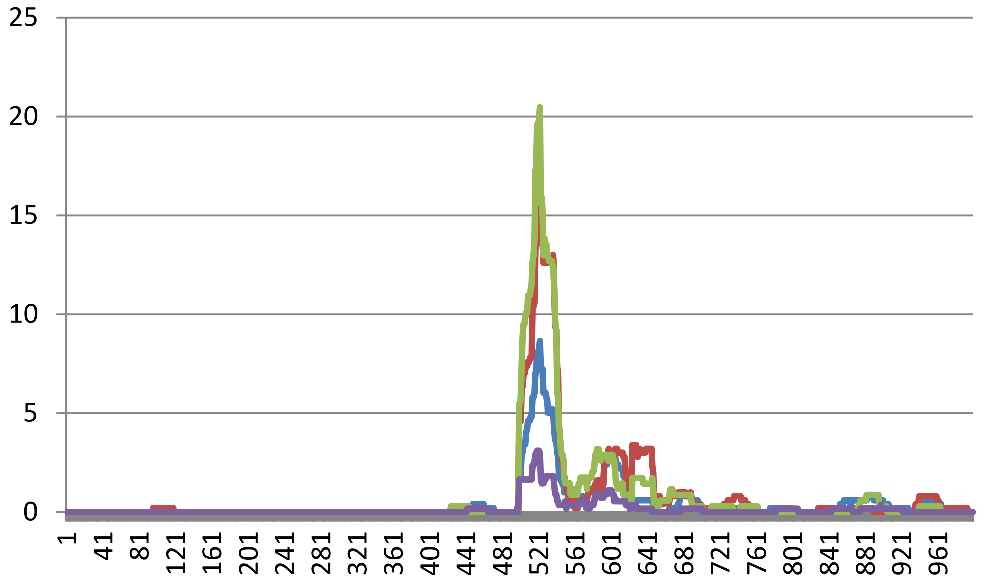

# AT2G35250

## Protein of Unknown Function (DUF239)

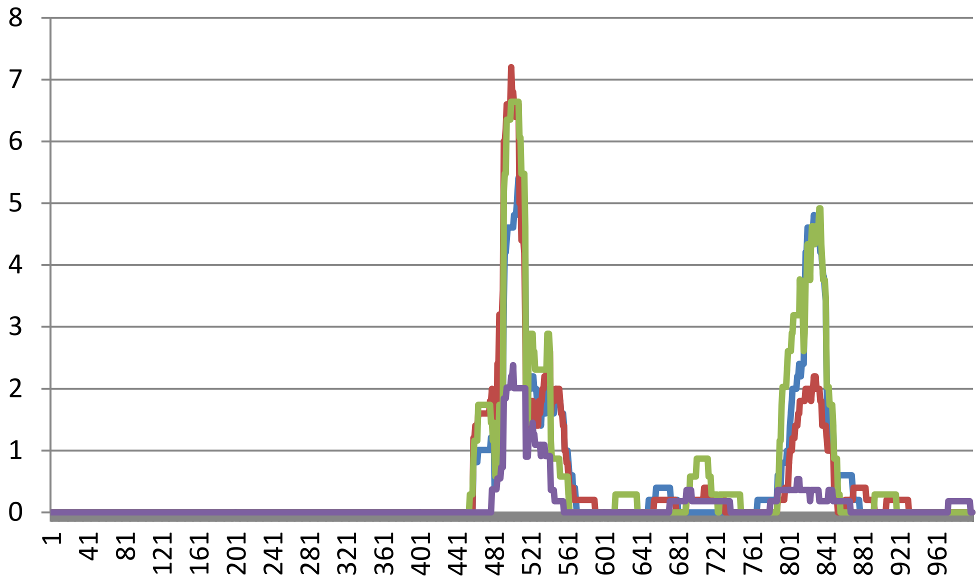

AT2G36940

Unknown protein

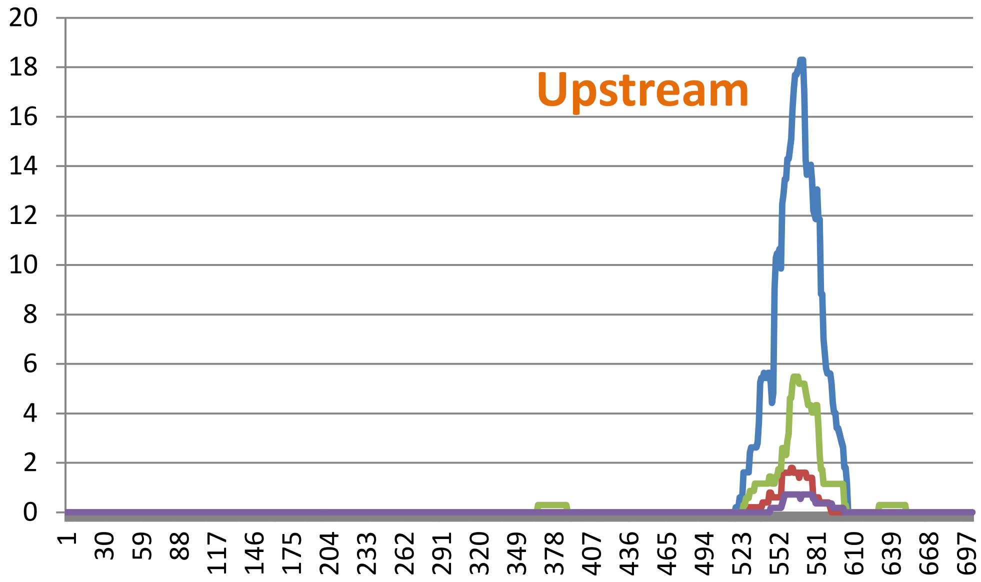

AT2G37780

Cysteine/Histidine-rich C1 domain family protein

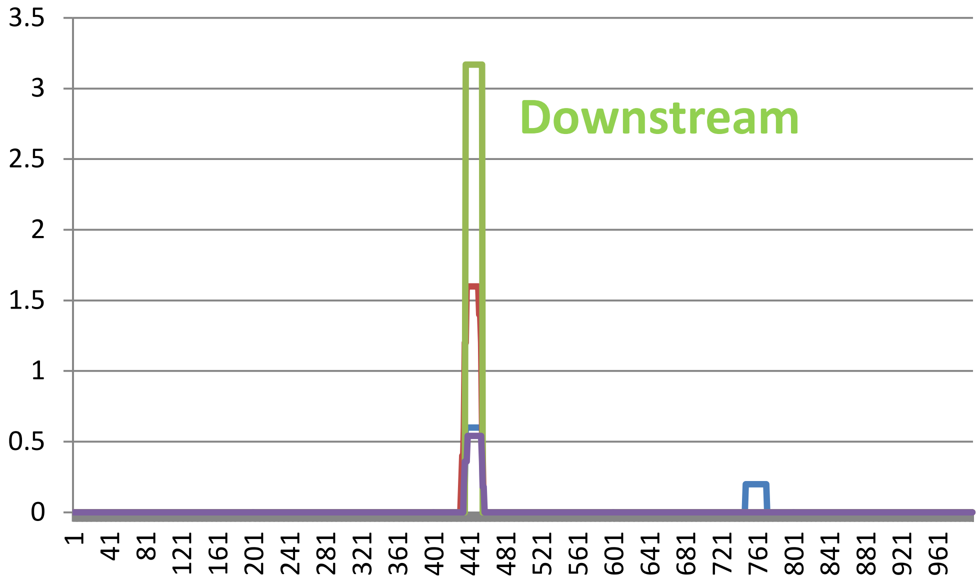

AT2G37810

Cysteine/Histidine-rich C1 domain family protein

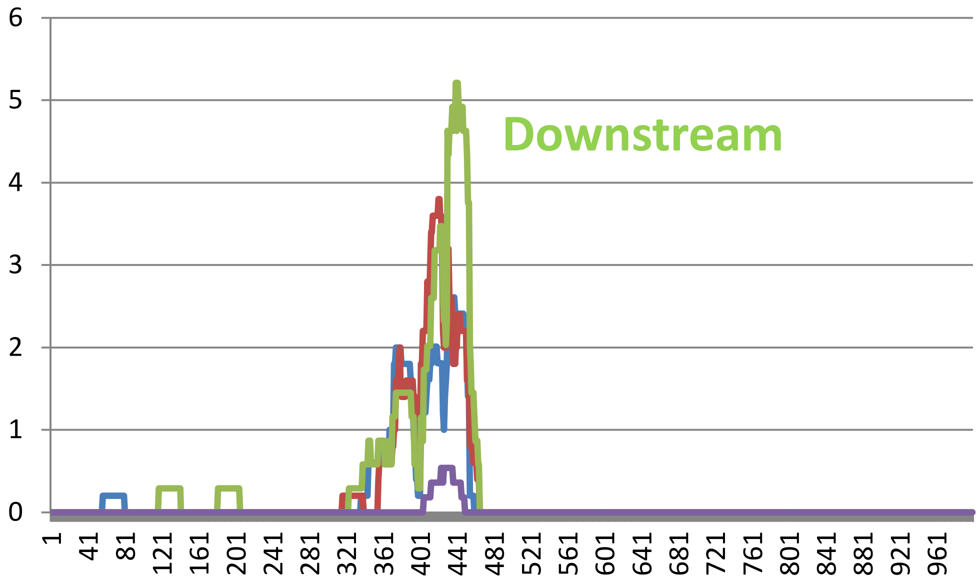

# AT2G40920

F-box and associated interaction domains-containing protein

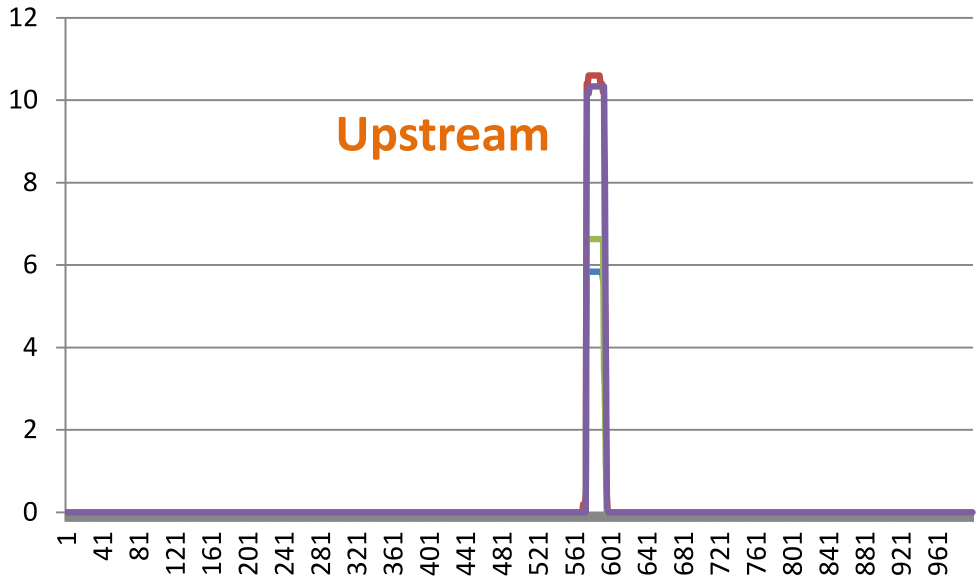

# AT2G41980

## Protein with RING/U-box and TRAF-like domains

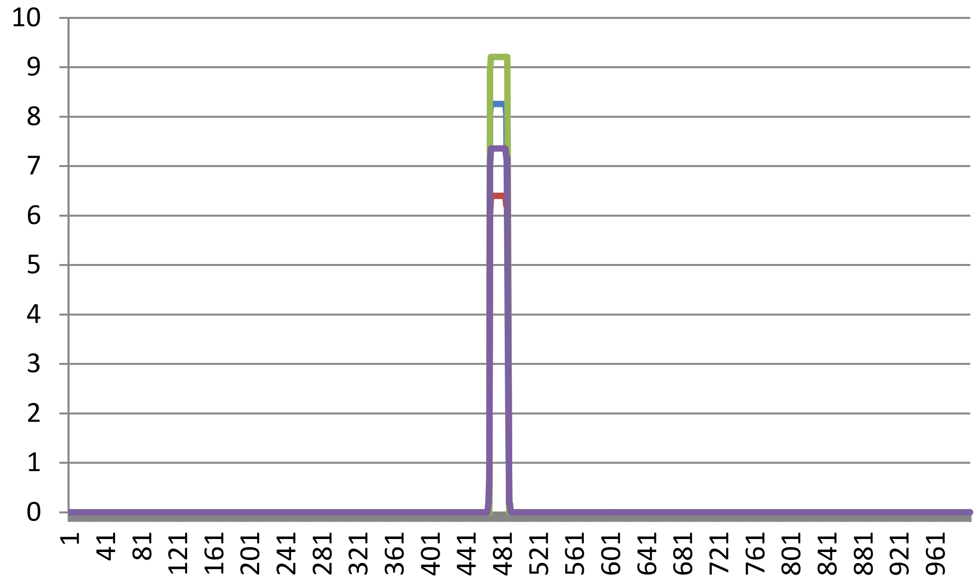

AT2G43580

Chitinase family protein

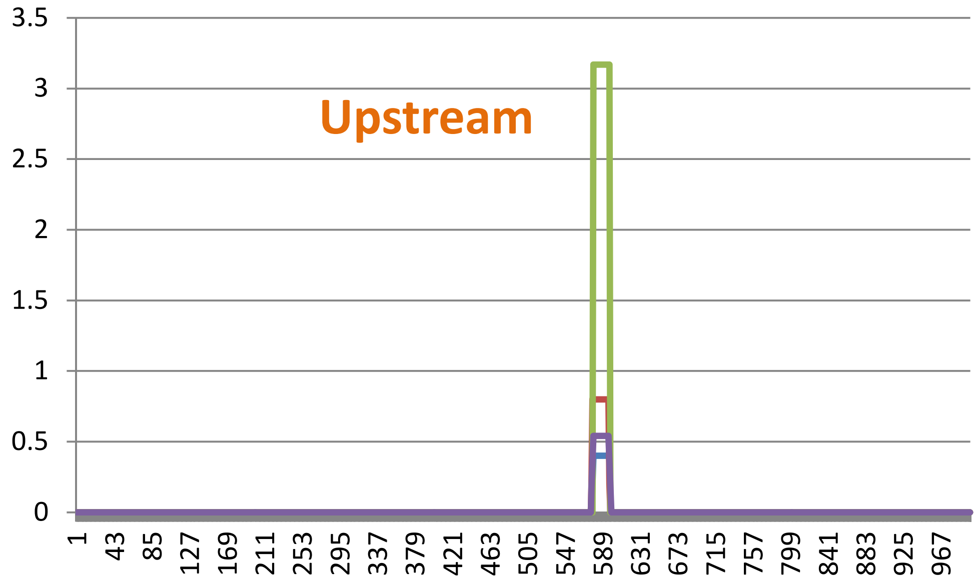

# AT2G44925

## Unknown protein

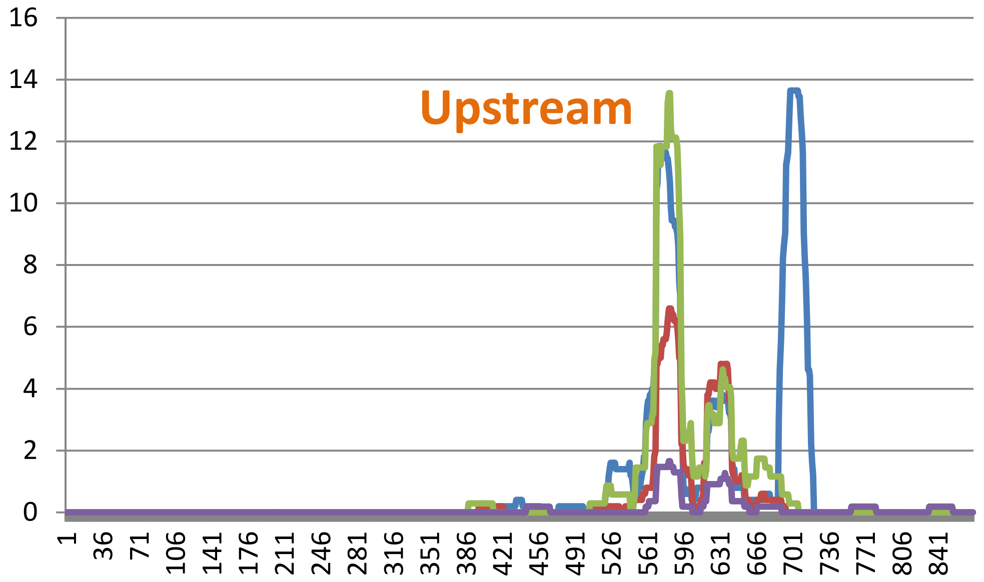

AT2G46460

Polynucleotidyl transferase, ribonuclease H-like superfamily protein

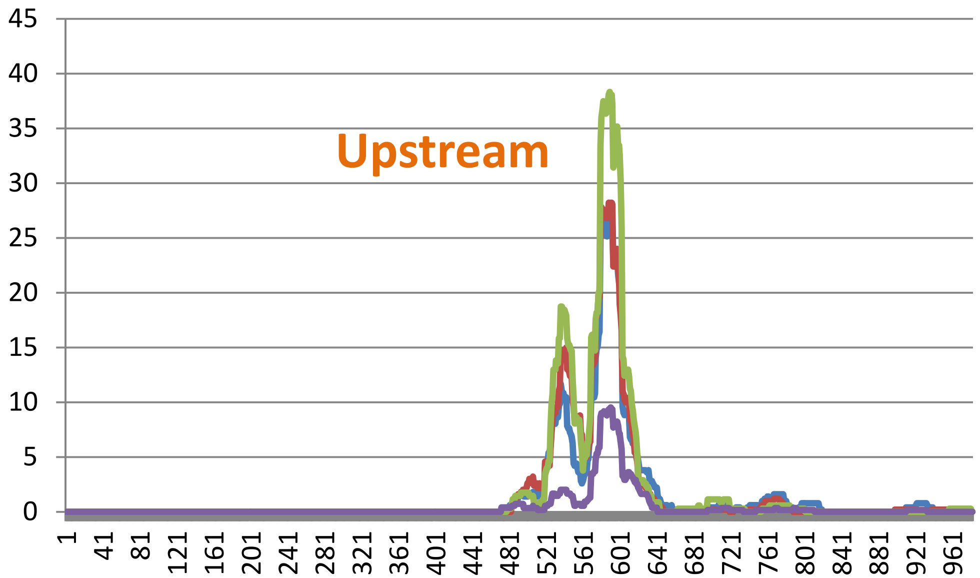

# AT2G47660

## Unknown protein

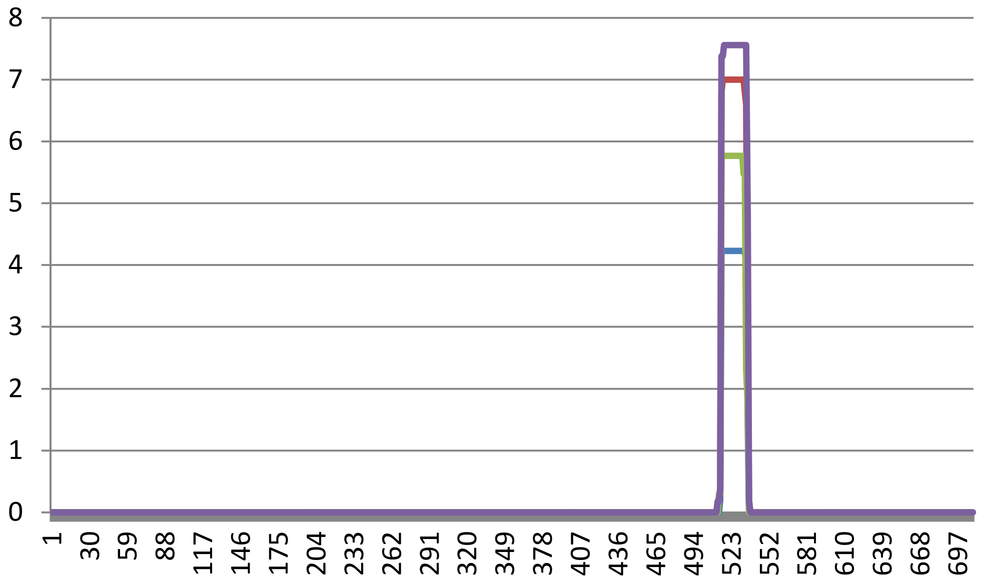

AT2G47750

Encodes GH3.9, a member of the GH3 family auxin-responsive genes. gh3.9-1 mutants had greater primary root length, increased sensitivity to indole-3-acetic acid (IAA)-mediated root growth inhibition, but no obvious effects on apical dominance or leaf morphology.

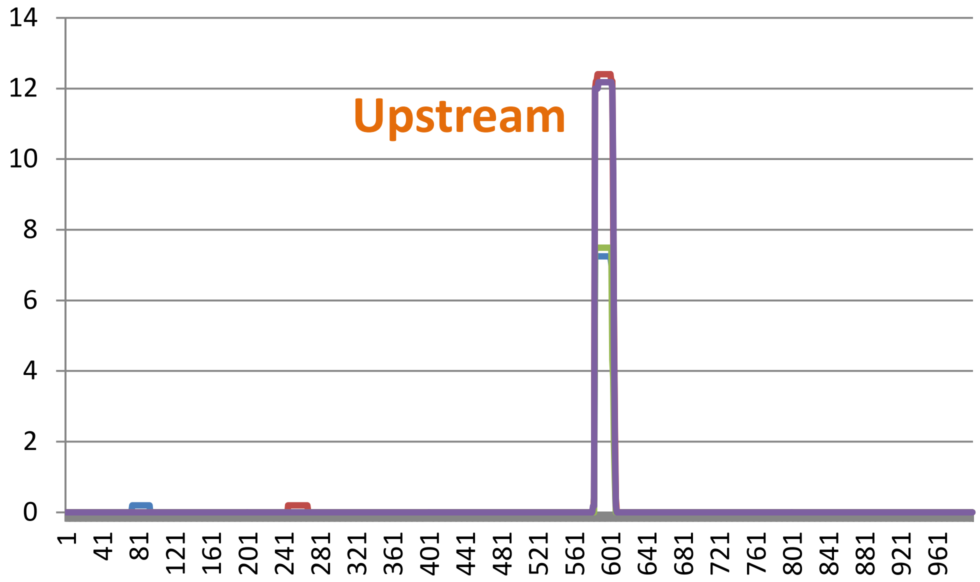

AT3G01710

TPX2 (targeting protein for Xklp2) protein family

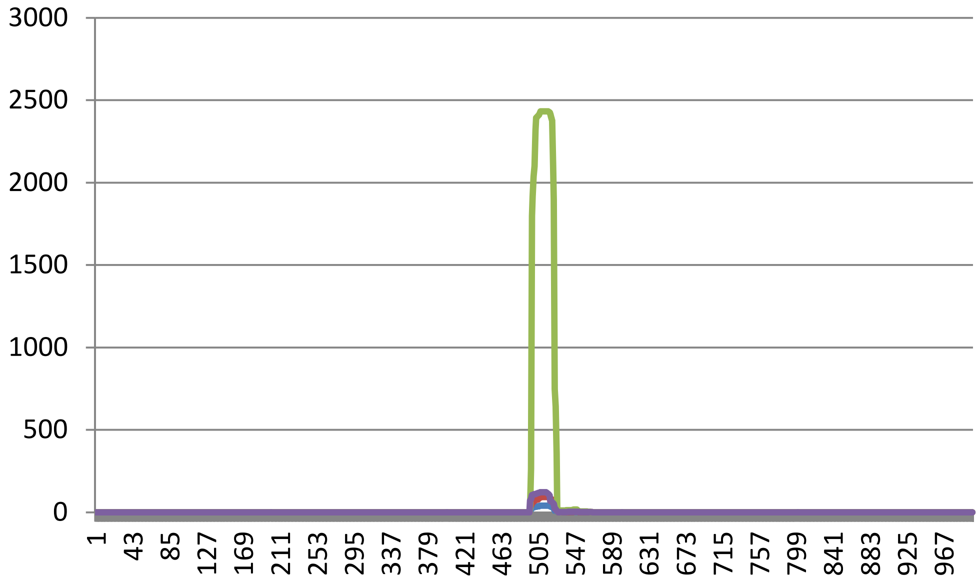

AT3G02350

Encodes a protein with  putative galacturonosyltransferase activity.

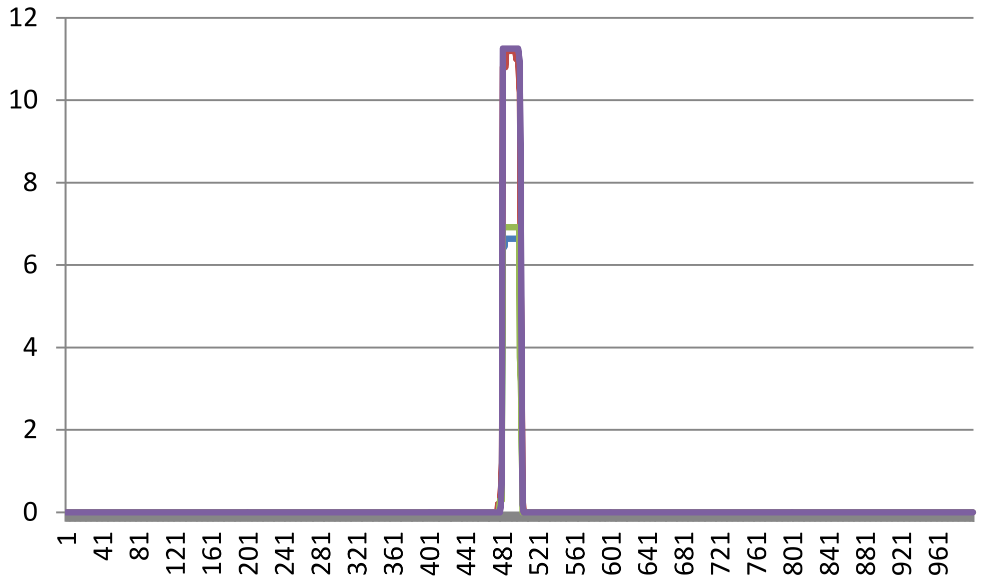

# AT3G02420

## Unknown protein

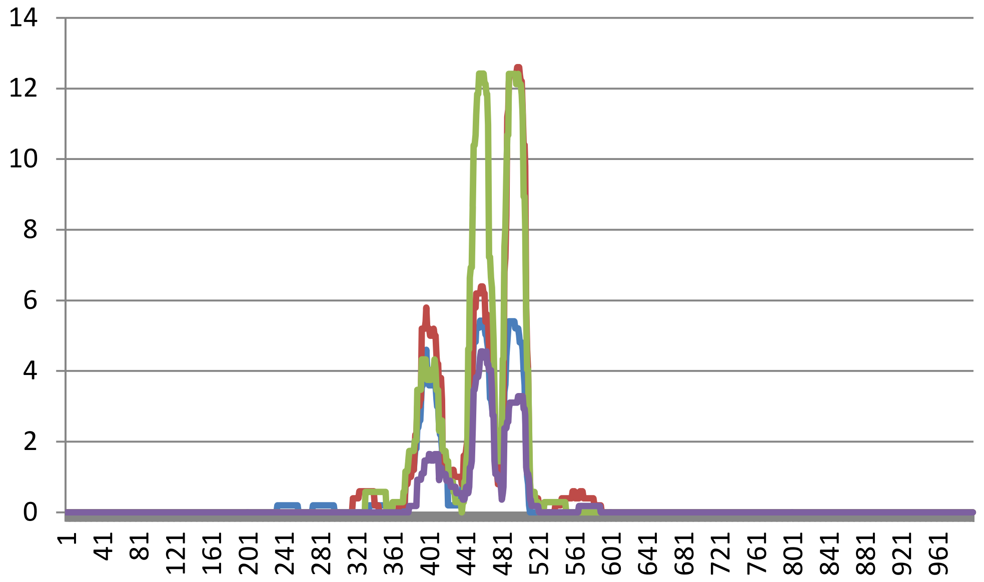

AT3G05520

Subunits of heterodimeric actin filament capping protein Capz superfamily

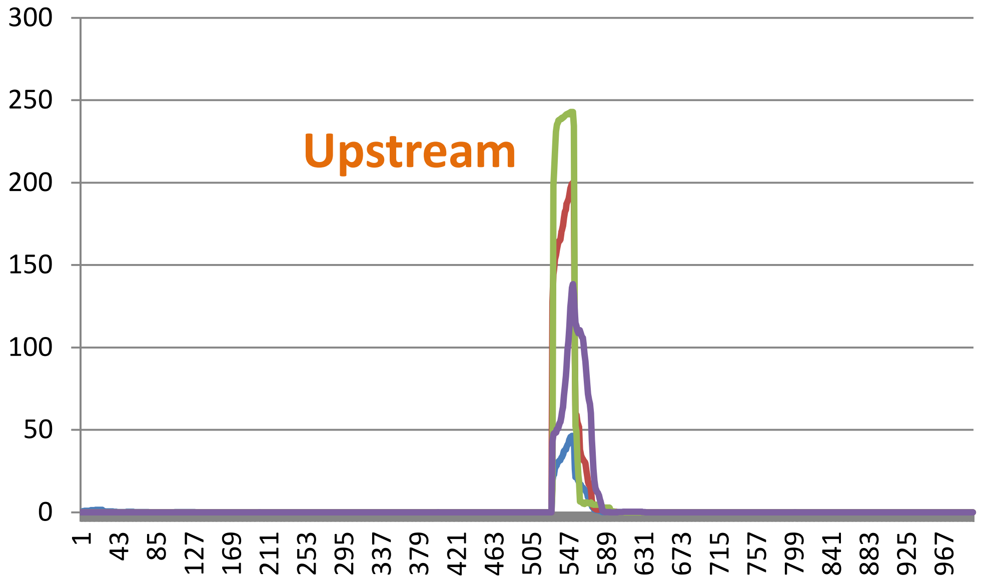

# AT3G06660

## PAPA-1-like family protein / zinc finger (HIT type) family protein

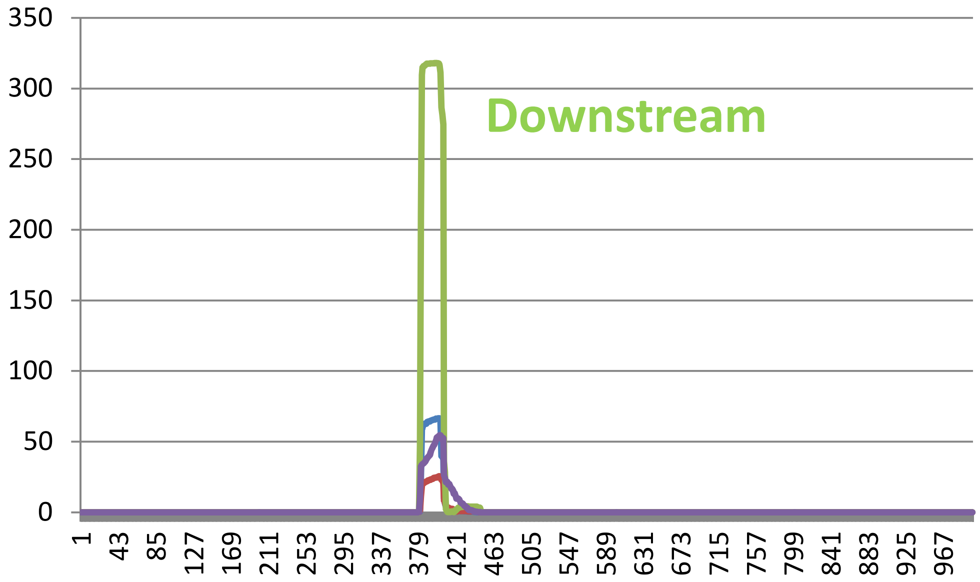

AT3G07050

Arabidopsis NSN1 encodes a nucleolar GTP-binding protein and is required for maintenance of inflorescence meristem identity and floral organ development.

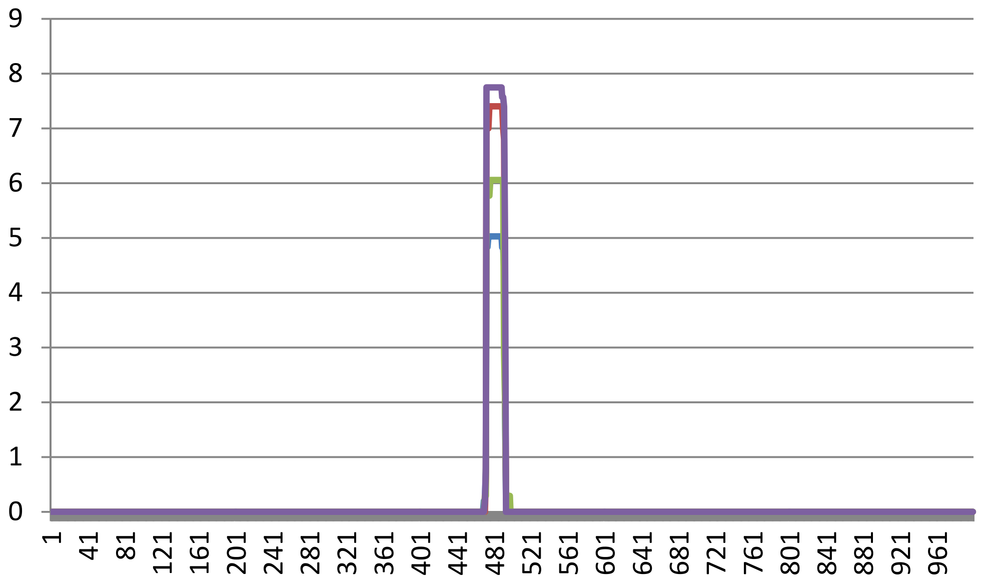

# AT3G09510

## Ribonuclease H-like superfamily protein

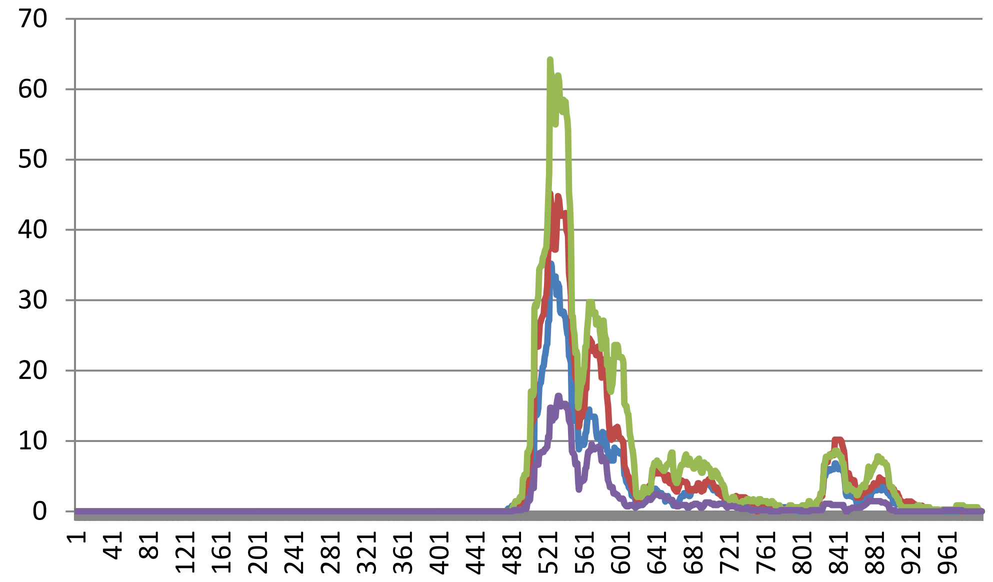

AT3G09960

Calcineurin-like metallo-phosphoesterase superfamily protein

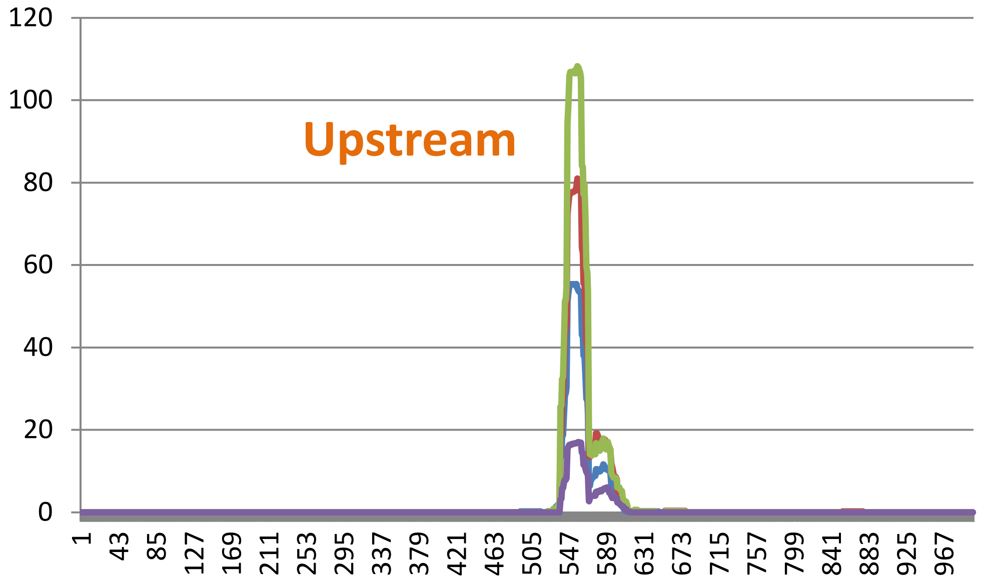

AT3G10090

Nucleic acid-binding, OB-fold-like protein

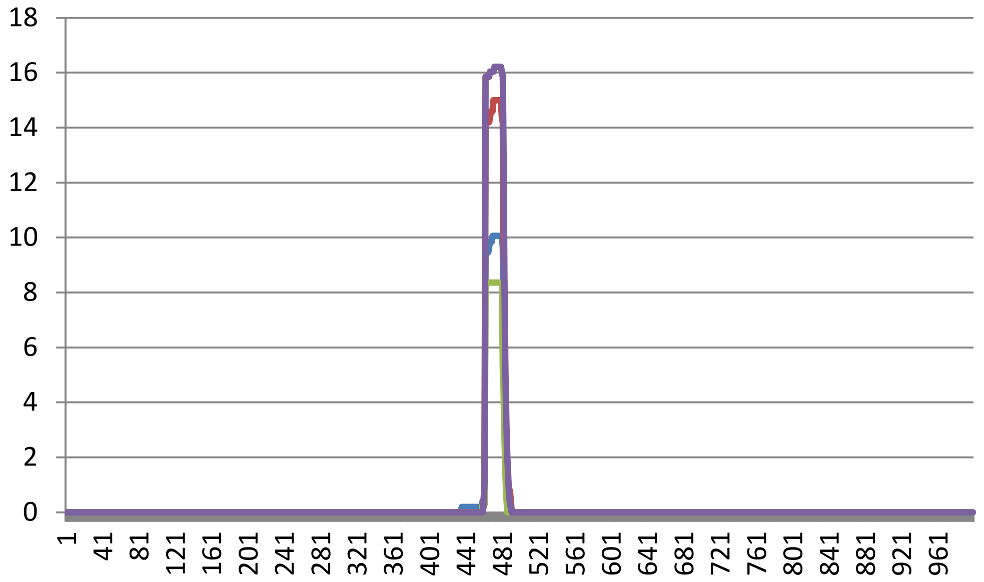

# AT3G10900

## Glycosyl hydrolase superfamily protein

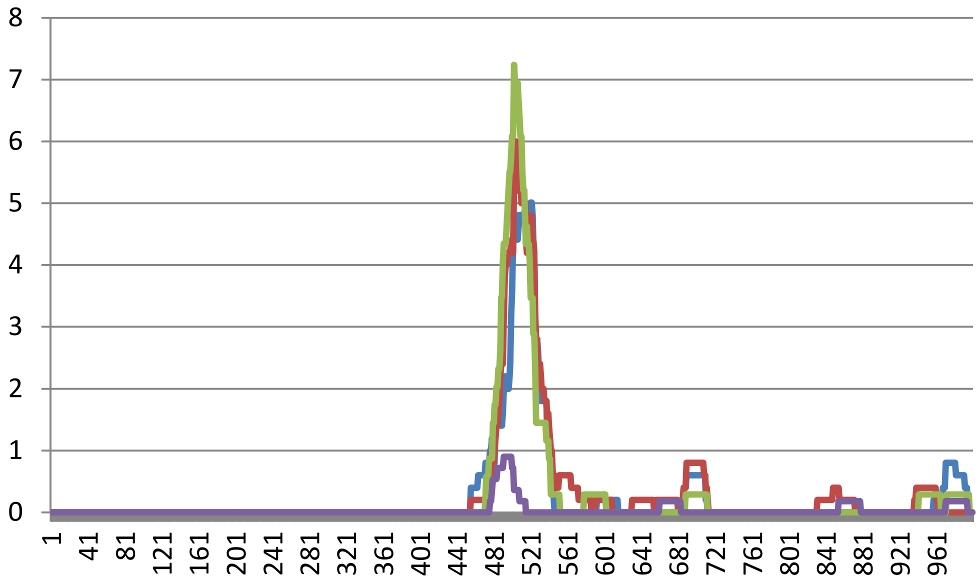

# AT3G11310

## Unknown protein

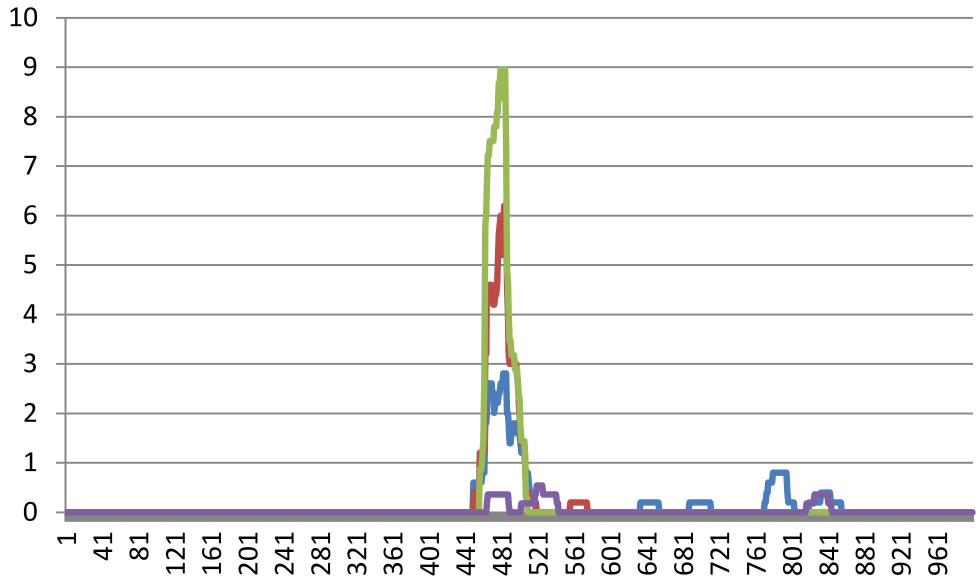

# AT3G17890

## Unknown protein

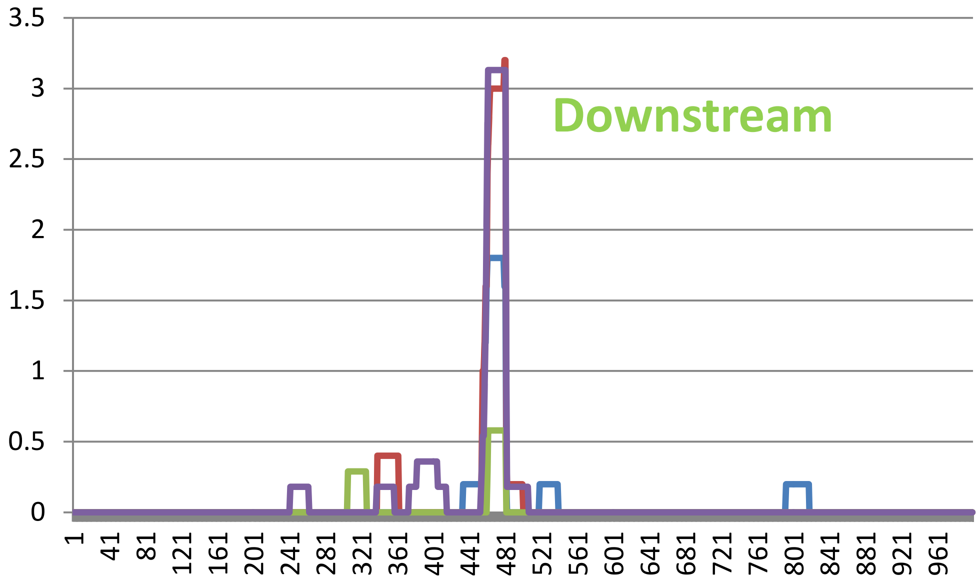

AT3G18770

Autophagy-related protein 13

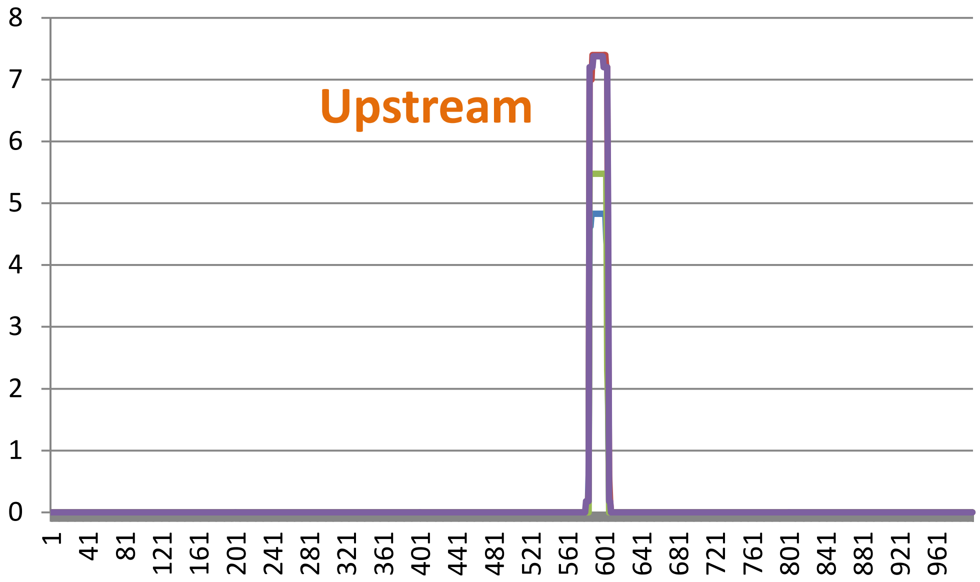

AT3G20640

Basic helix-loop-helix (bHLH) DNA-binding superfamily protein

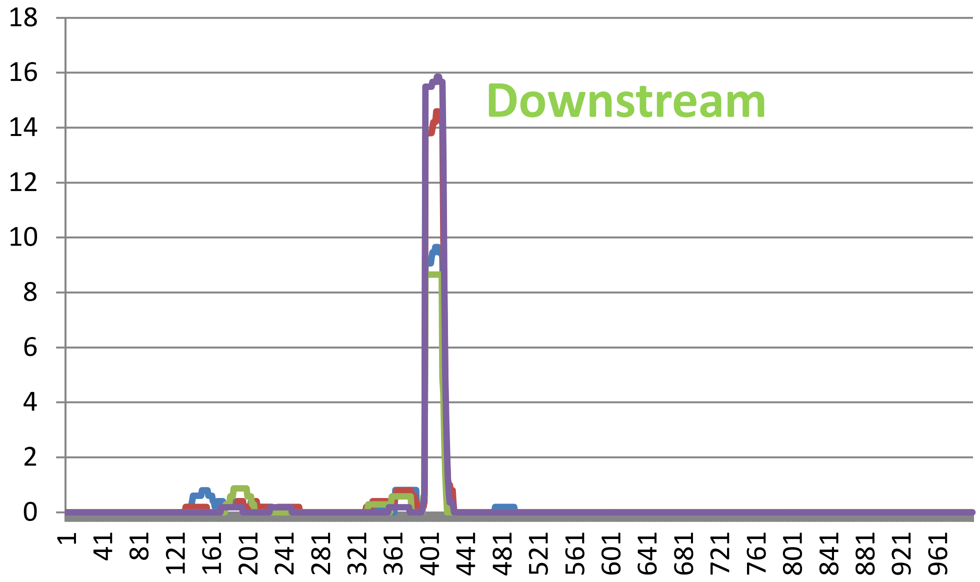

AT3G21730

FOLB3

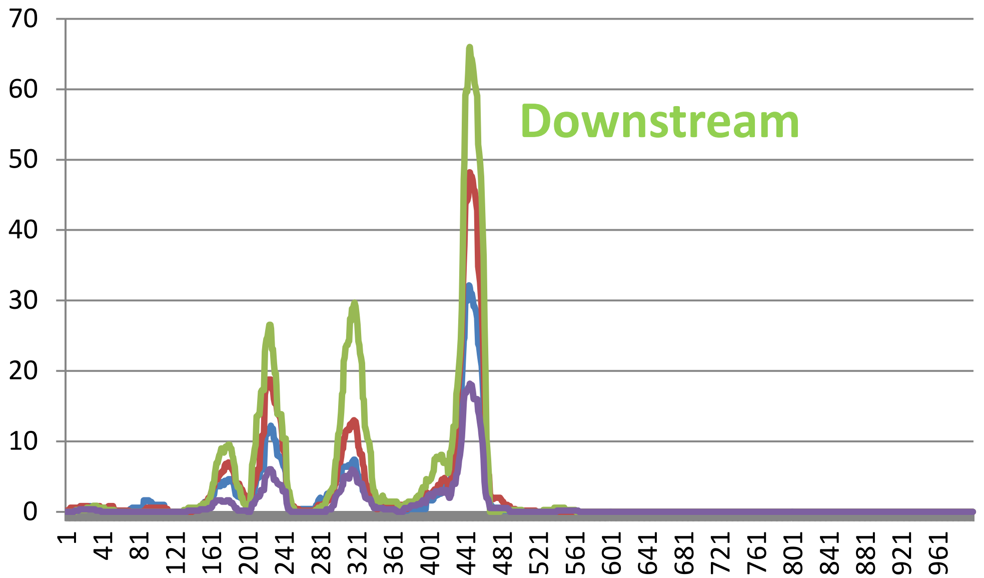

# AT3G21870

## Cyclin p2;1 (CYCP2;1)

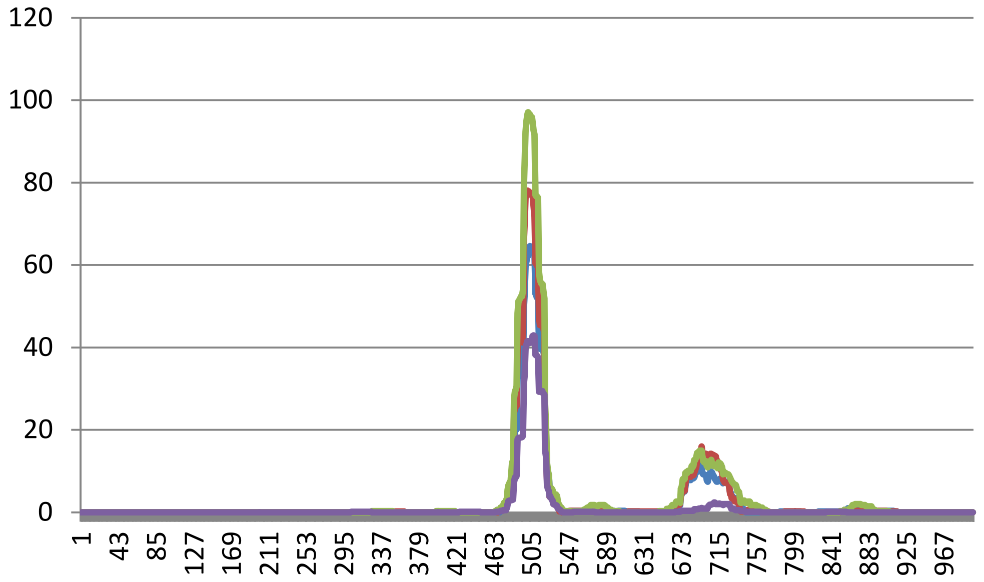

AT3G22050

Domain of unknown function (DUF26)

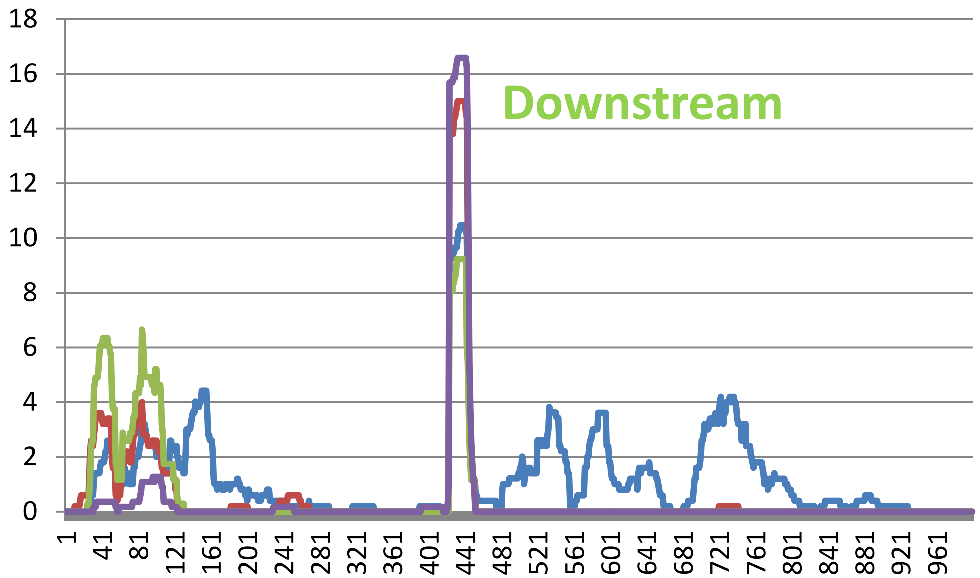

# AT3G22710

## F-box family protein

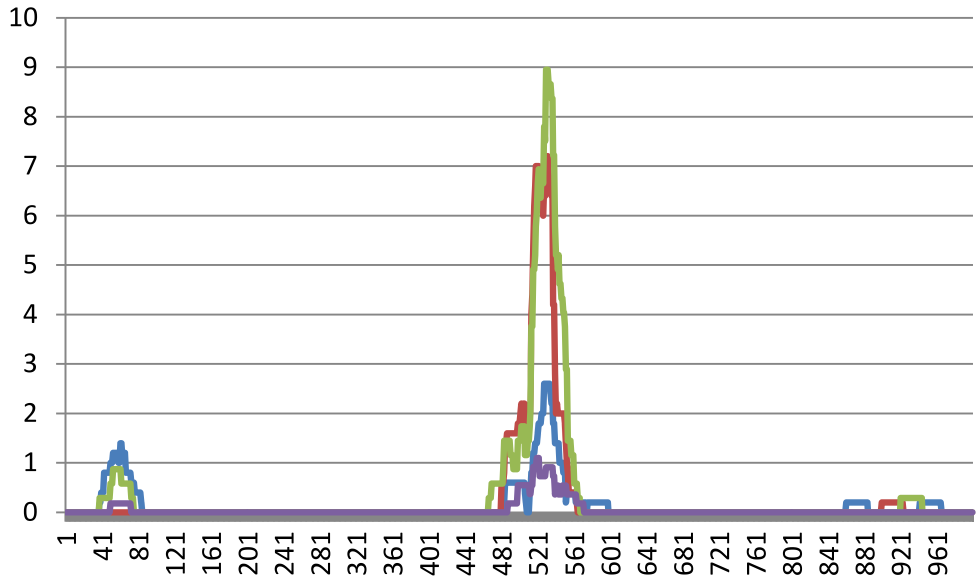

AT3G23440

EMBRYO SAC DEVELOPMENT ARREST 6 (EDA6)

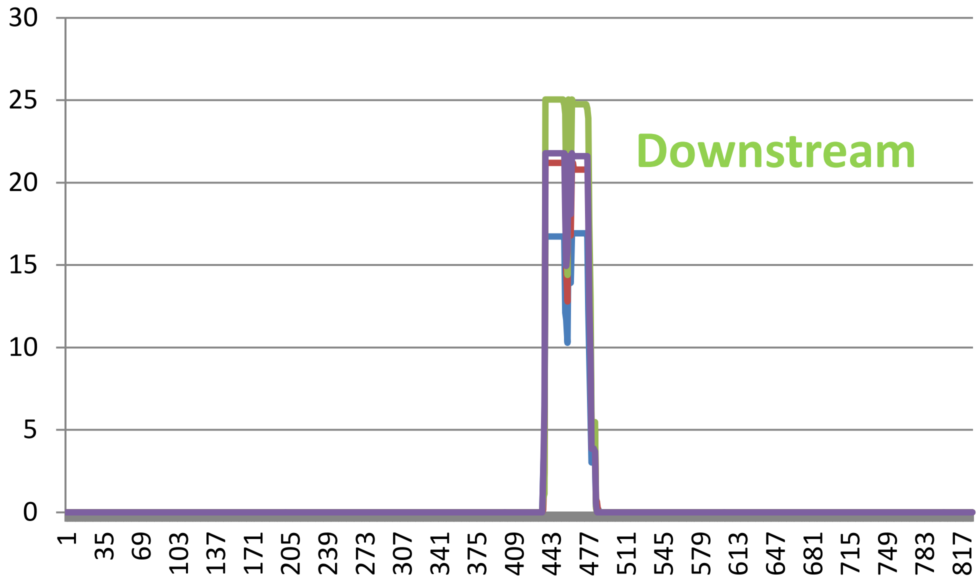

AT3G25130

Unknown protein

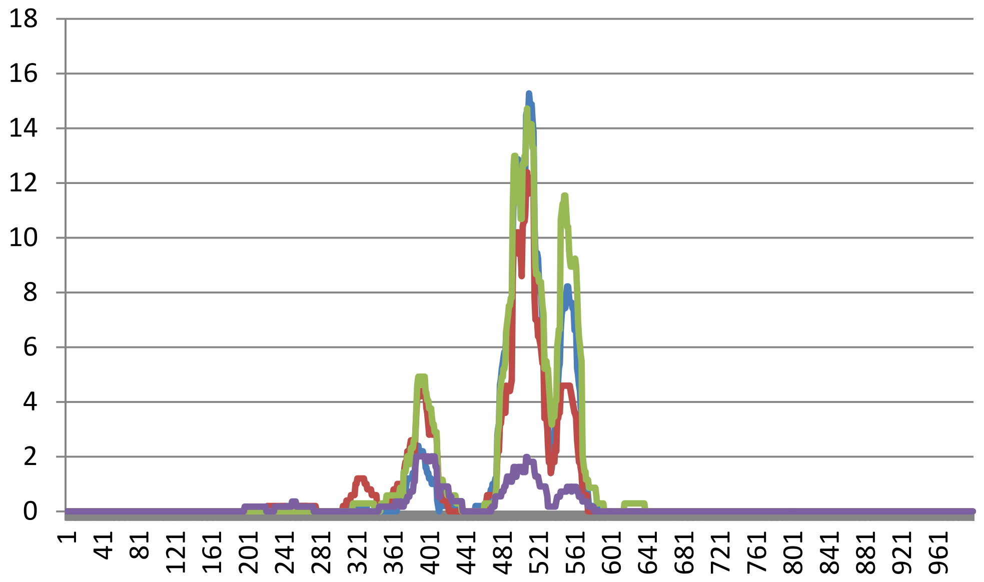

AT3G25720

RNA-directed DNA polymerase (reverse transcriptase)-related family protein

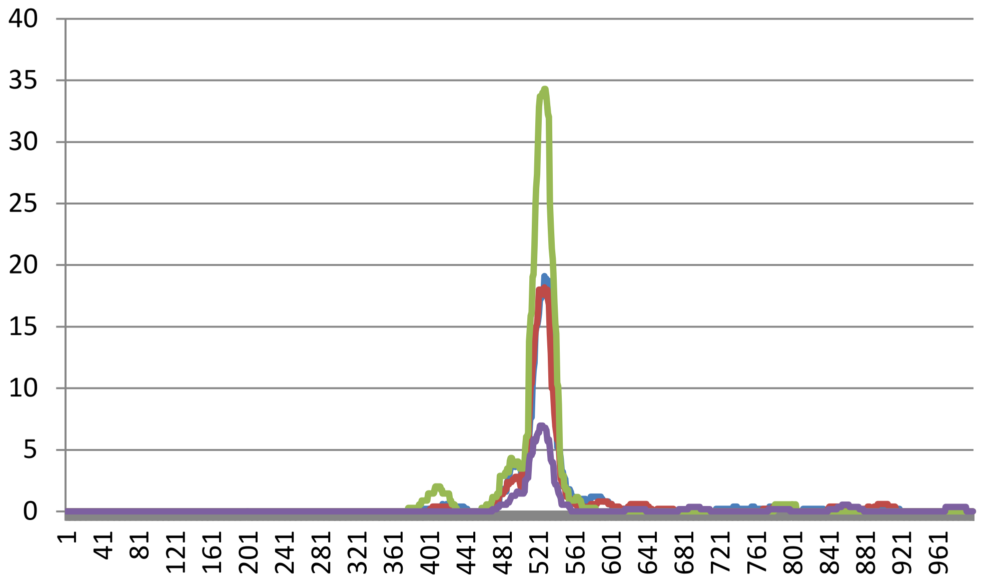

AT3G25855

Copper transport protein family

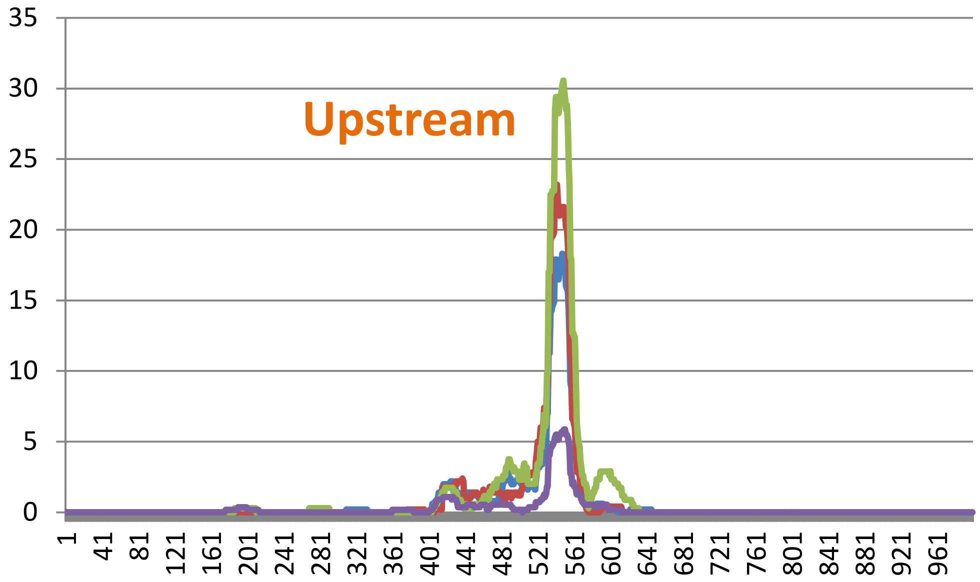

# AT3G26100

## Regulator of chromosome condensation (RCC1) family protein

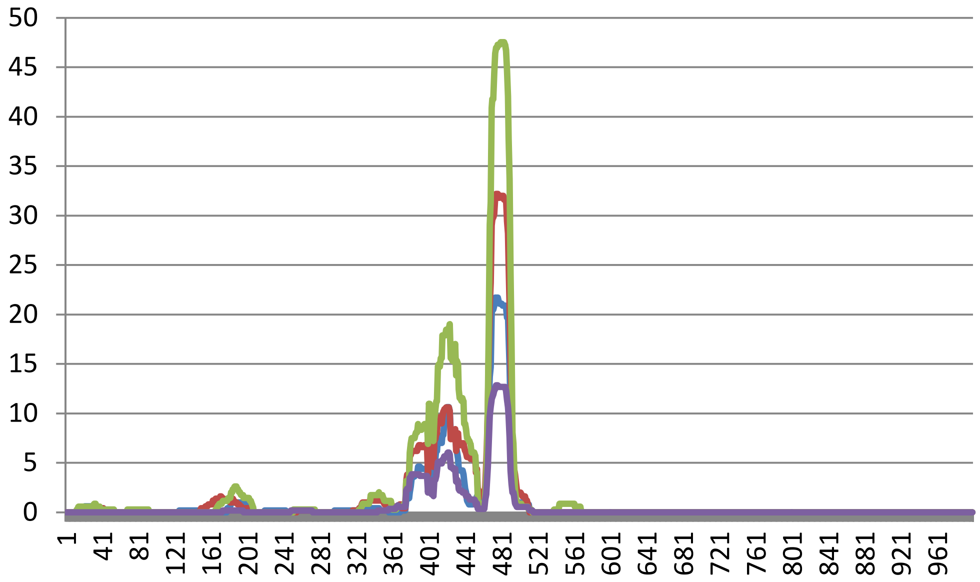

AT3G26140

Cellulase (glycosyl hydrolase family 5) protein

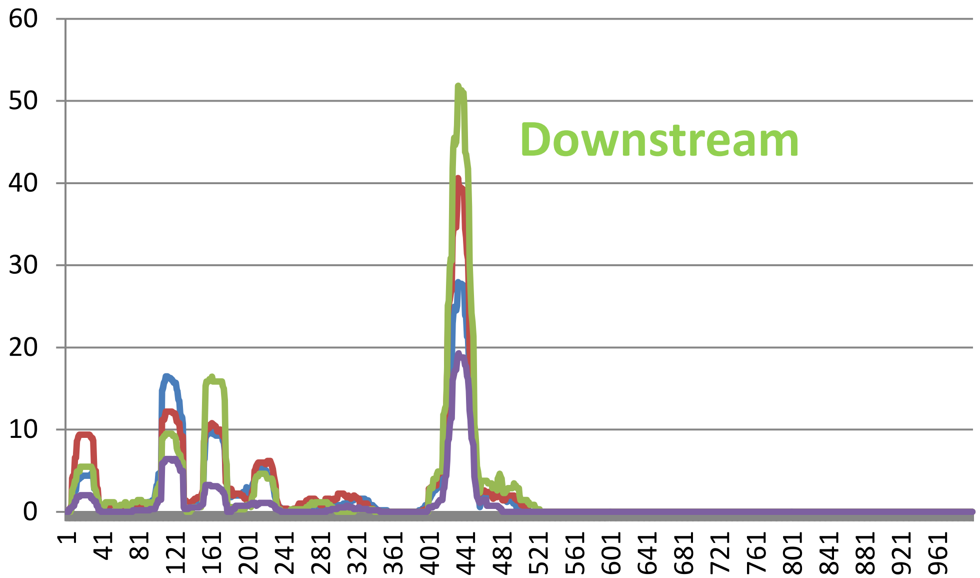

# AT3G26280

## Cytochrome P450 monooxygenase

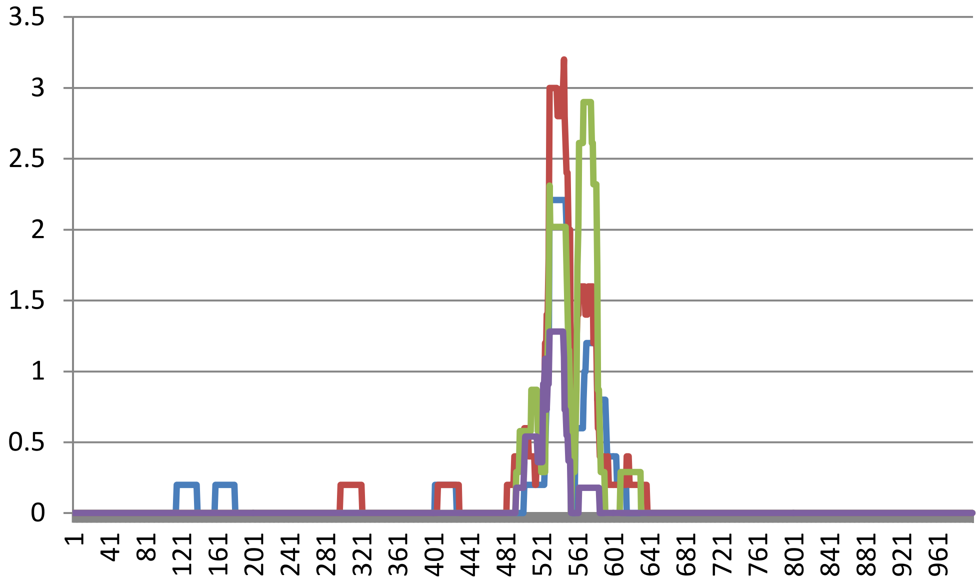

AT3G27040

Meprin and TRAF (MATH) homology domain-containing protein

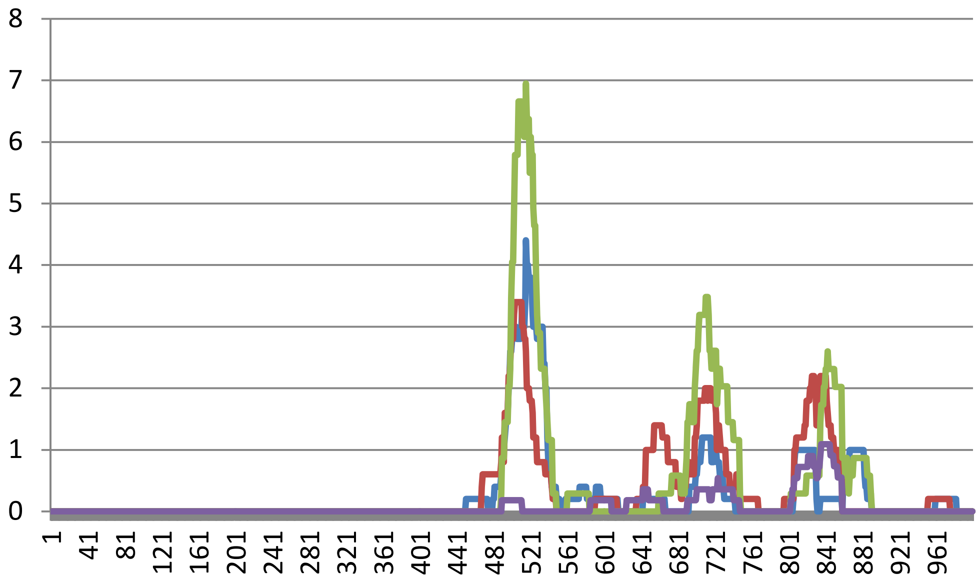

# AT3G27250

## Unknown protein

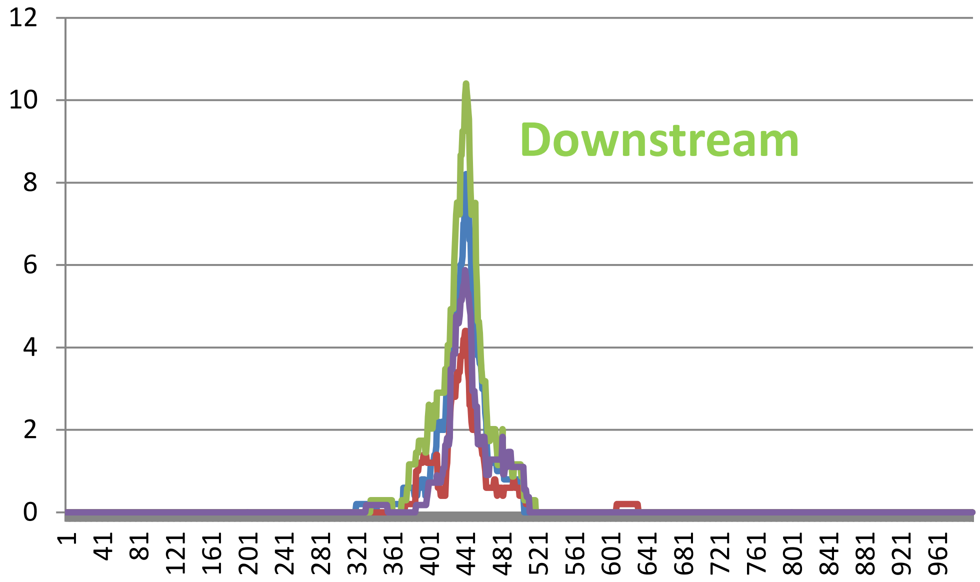

# AT3G27510

## Cysteine/Histidine-rich C1 domain family protein

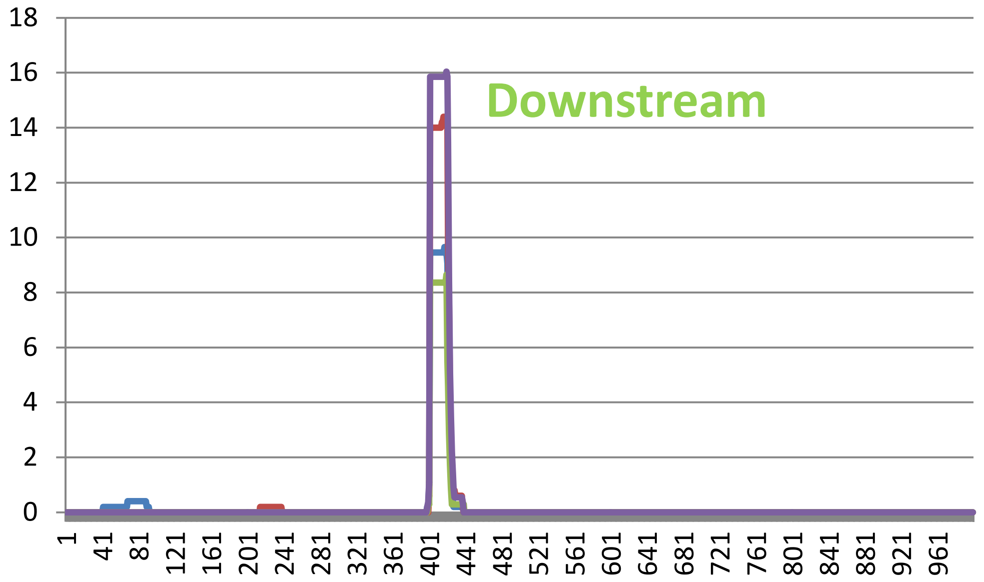

AT3G27560

Encodes a protein with kinase domains, including catalytic domains for serine/threonine as well as tyrosine kinases. A member of multi-gene family and is expressed in all tissues examined.

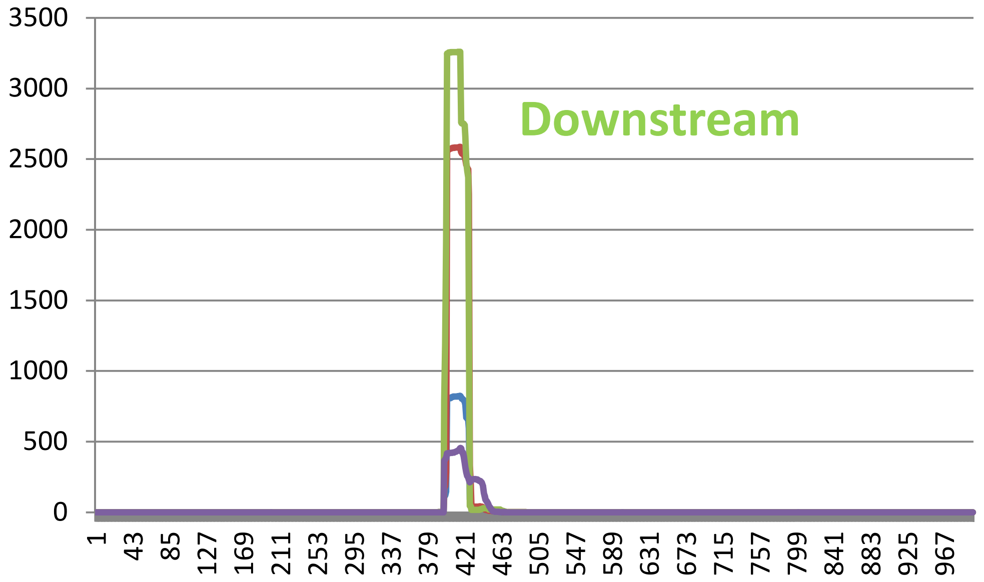

AT3G32050

Unknown protein

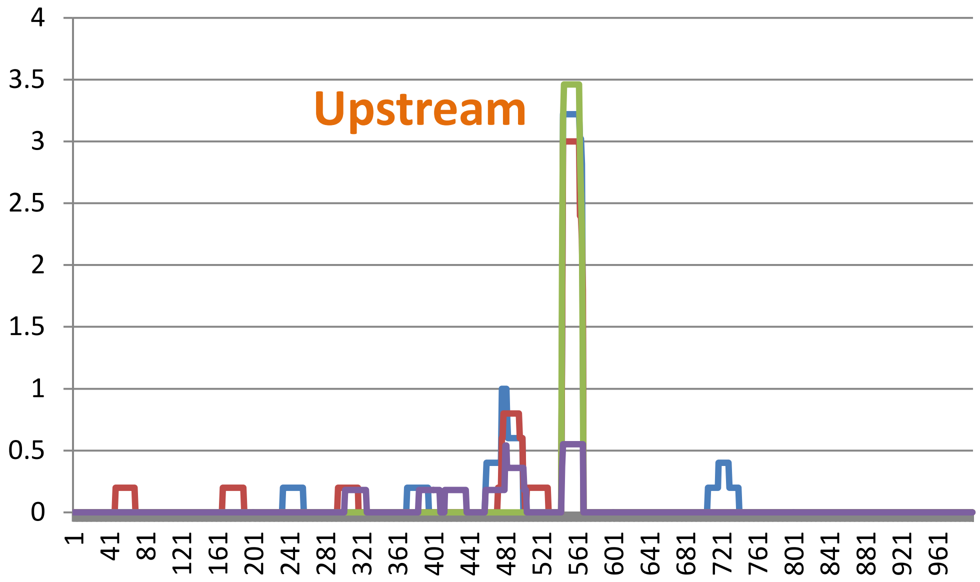

AT3G41762

Unknown protein

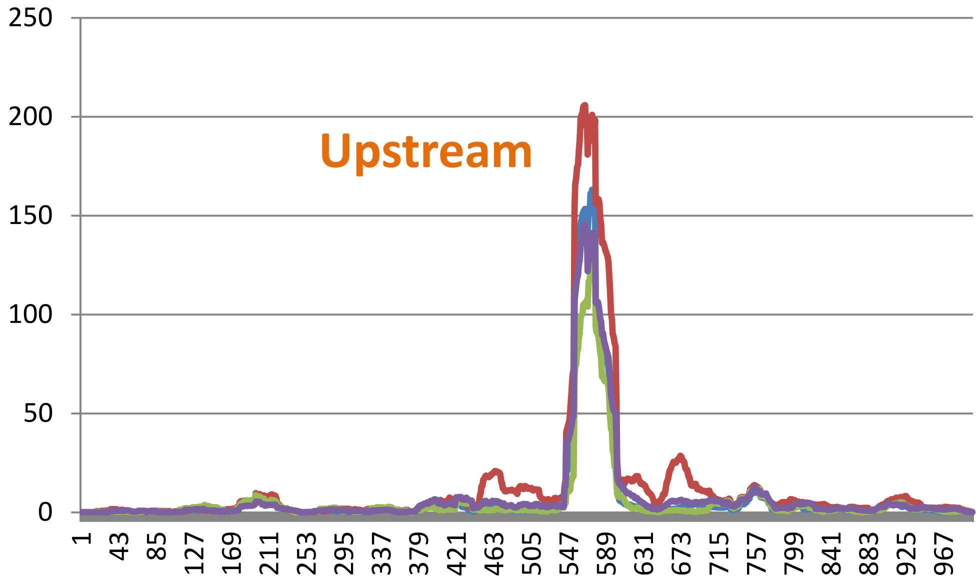

# AT3G43153

## cAMP-dependent protein kinase inhibitor-related

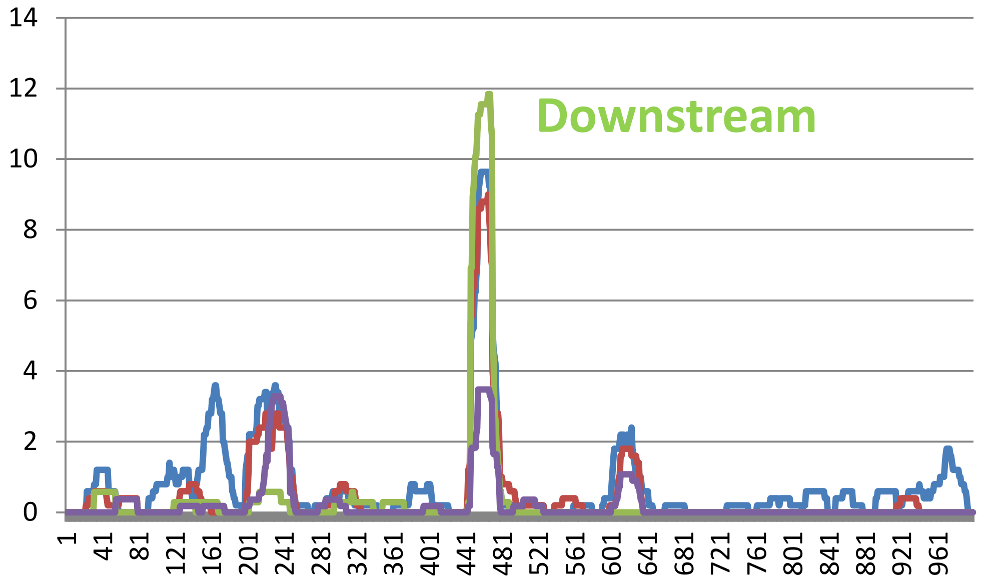

# AT3G43420

## Unknown protein

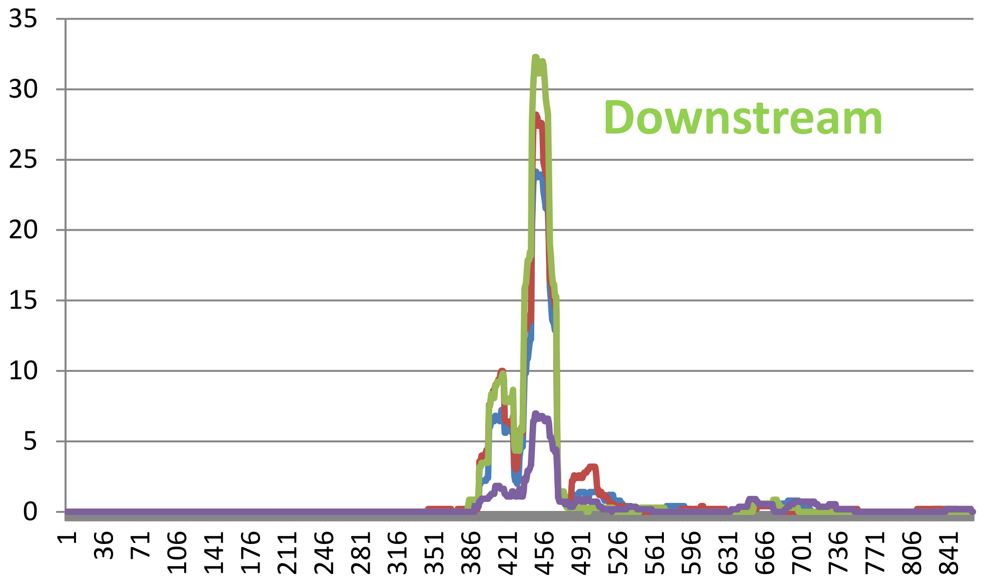

# AT3G44210

## Unknown protein

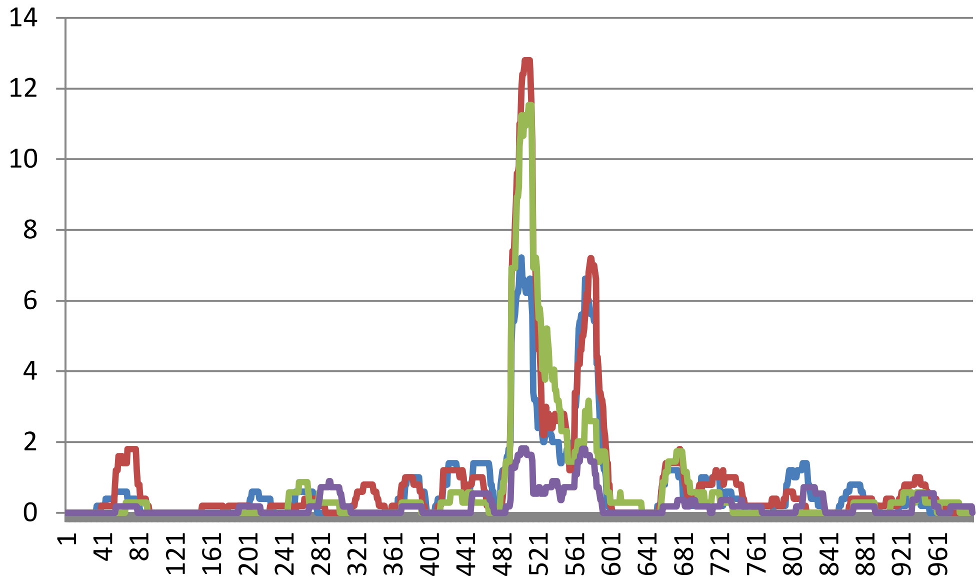

AT3G44810

F-box family protein

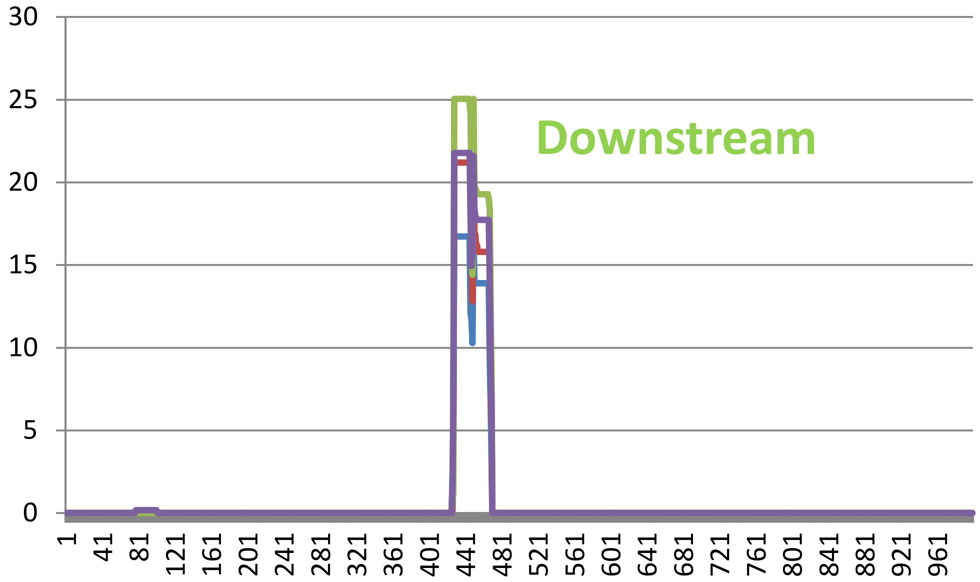

# AT3G45260

## C2H2-like zinc finger protein

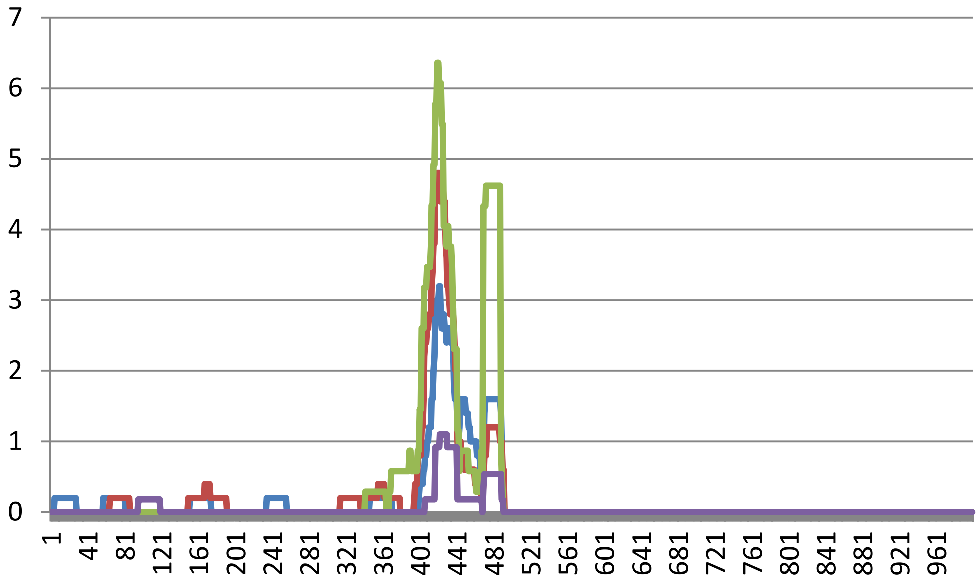

# AT3G45460

## IBR domain containing protein

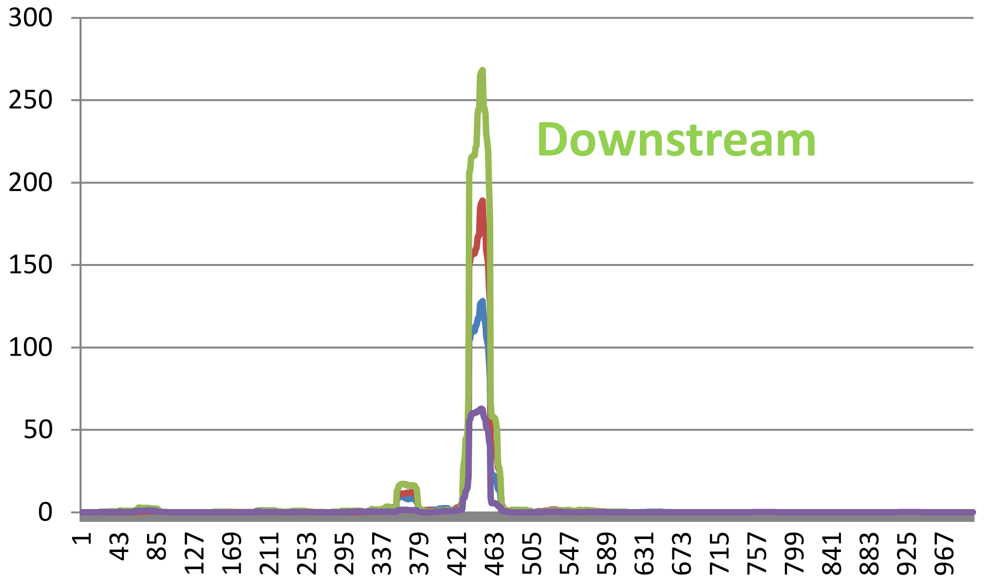

AT3G46270

Receptor protein kinase-related

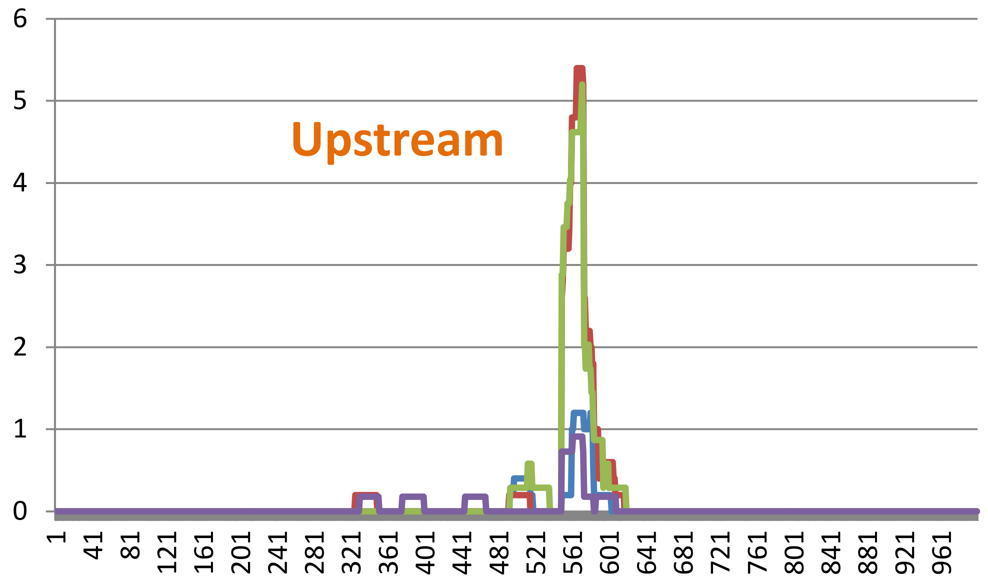

AT3G47920

Unknown protein

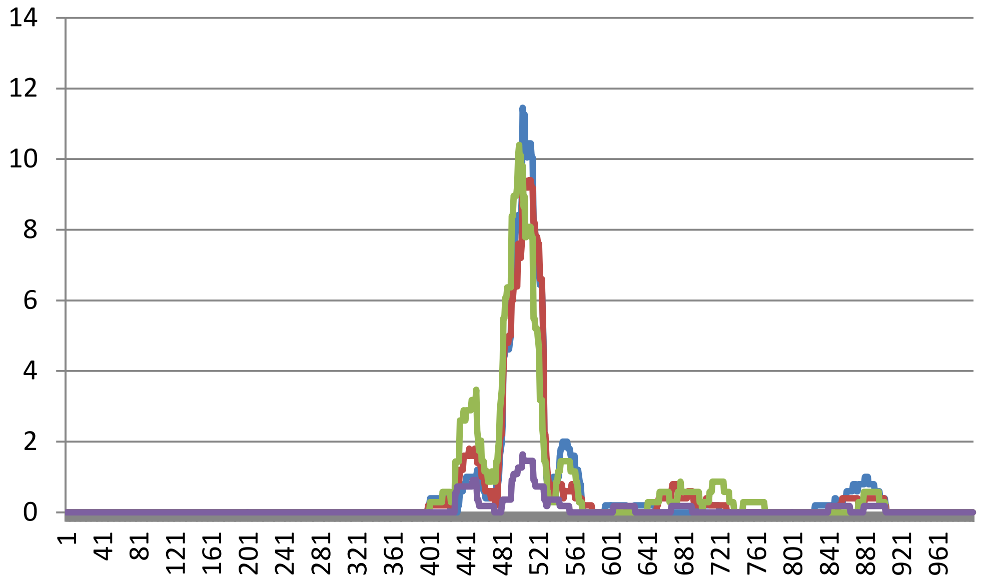

# AT3G50090

## Exonuclease family protein

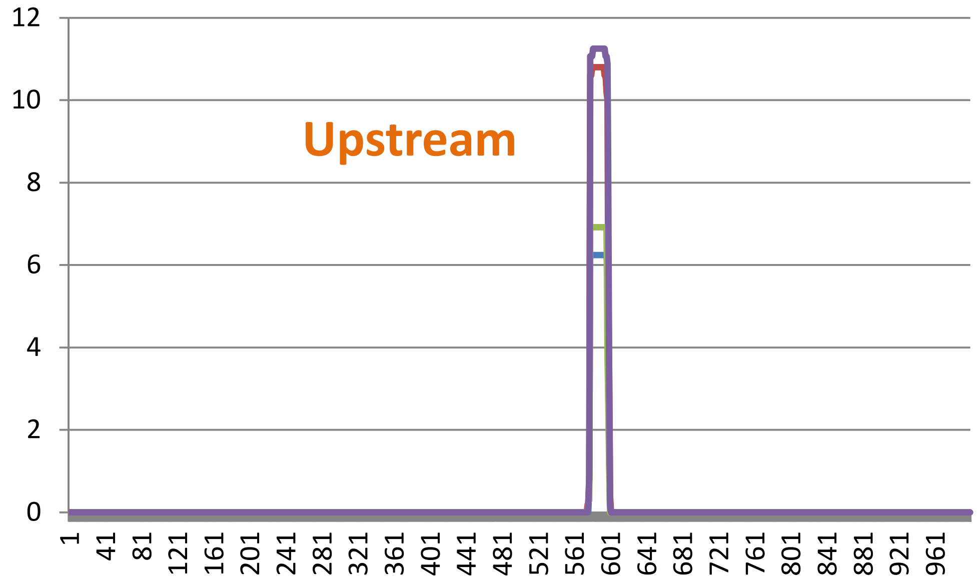

# AT3G50300

## HXXXD-type acyl-transferase family protein

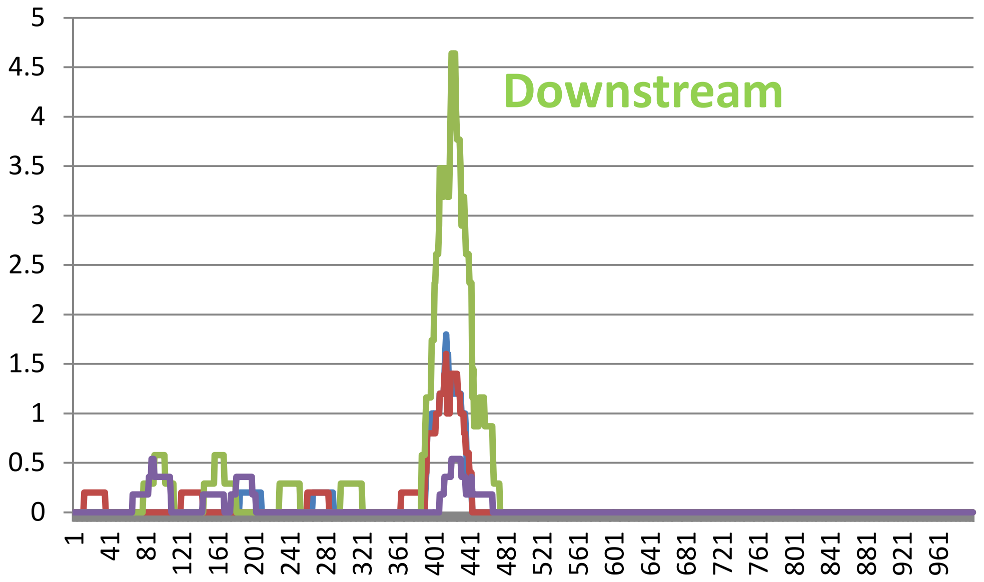

# AT3G50540

## Unknown protein

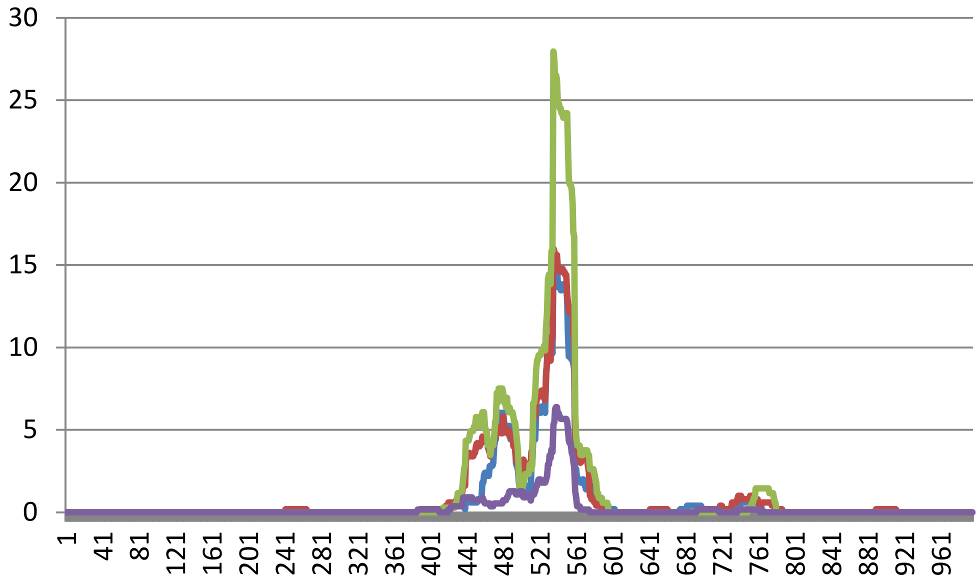

AT3G50840

Phototropic-responsive NPH3 family protein

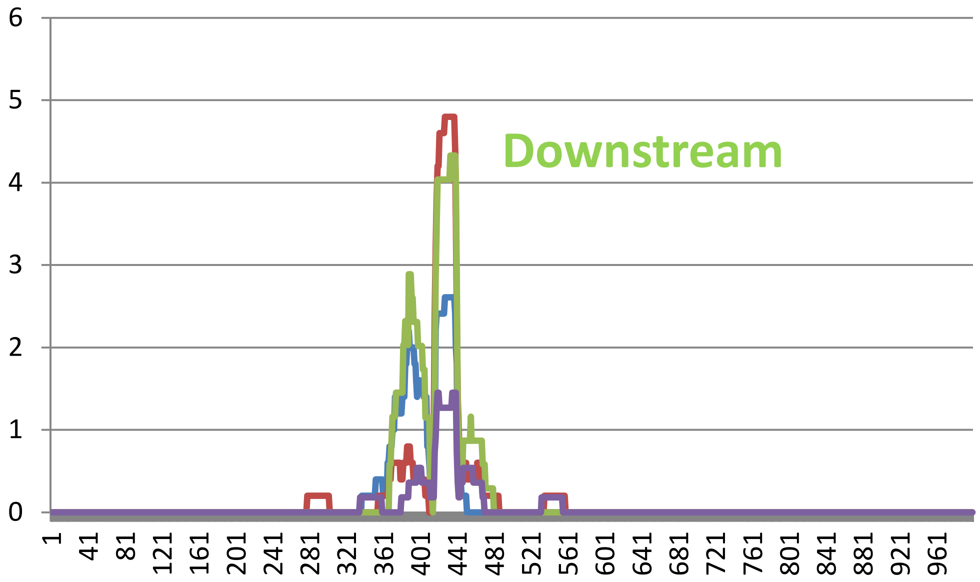

# AT3G51760

## Protein of unknown function (DUF688)

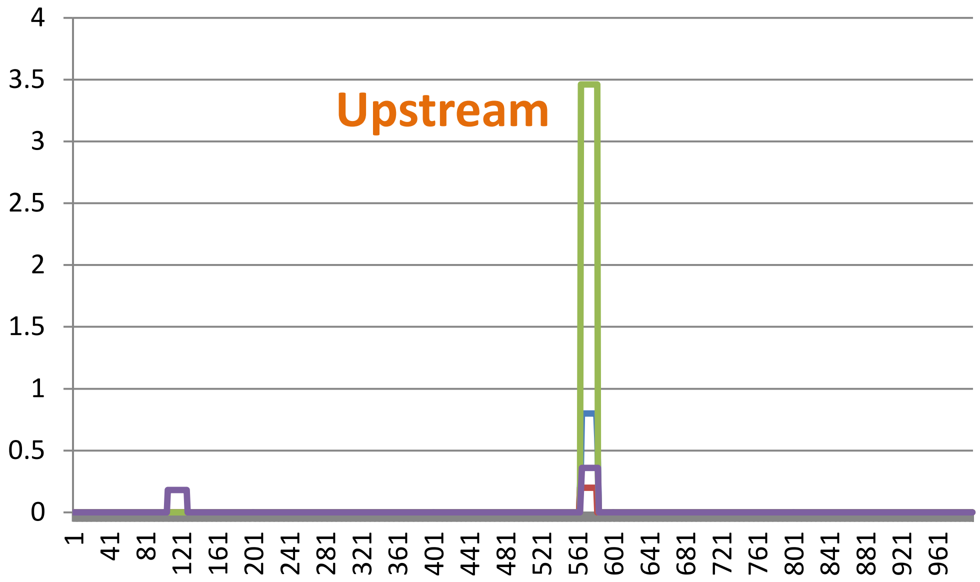

AT3G52830

BEST Arabidopsis thaliana protein match is: Ankyrin repeat family protein (TAIR:AT5G54700.1)

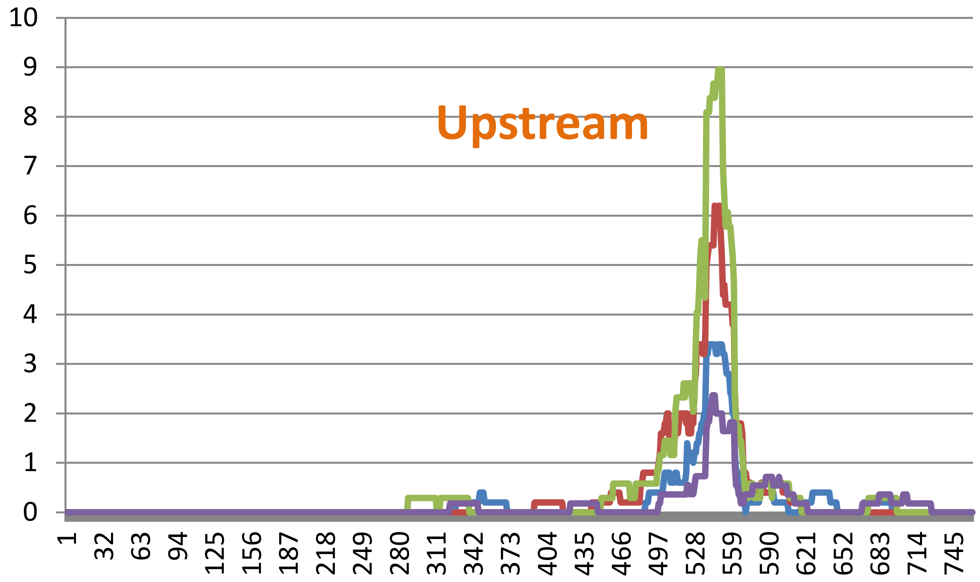

# AT3G55672

## Plant self-incompatibility protein S1 family

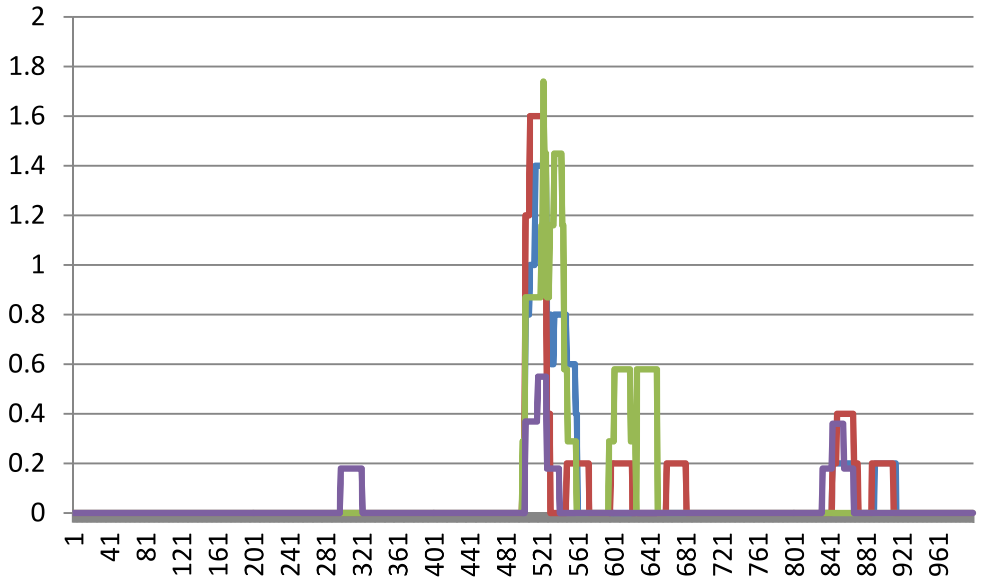

AT3G55730

Putative transcription factor MYB109 (MYB109) mRNA

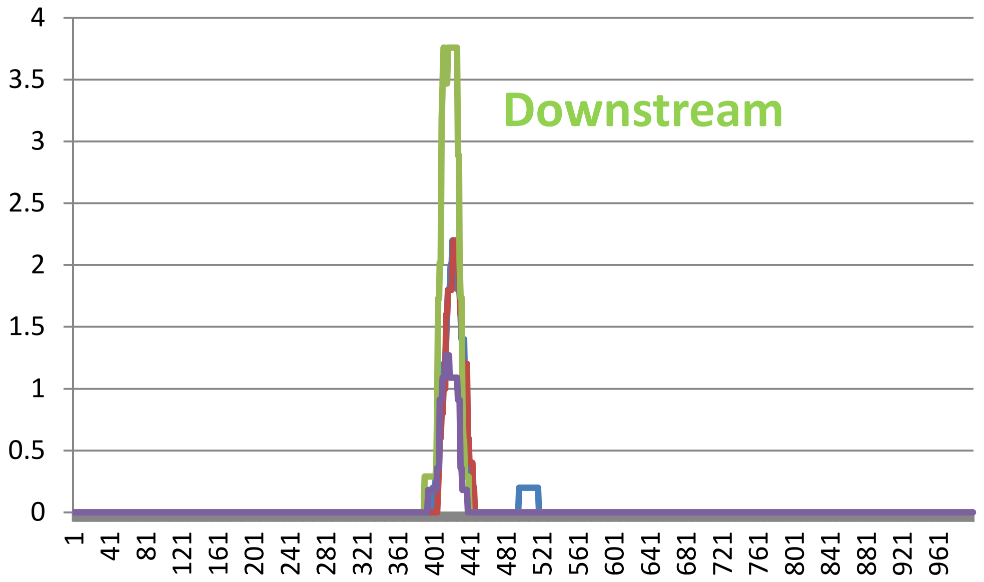

AT3G56380

RESPONSE REGULATOR 17 (RR17)

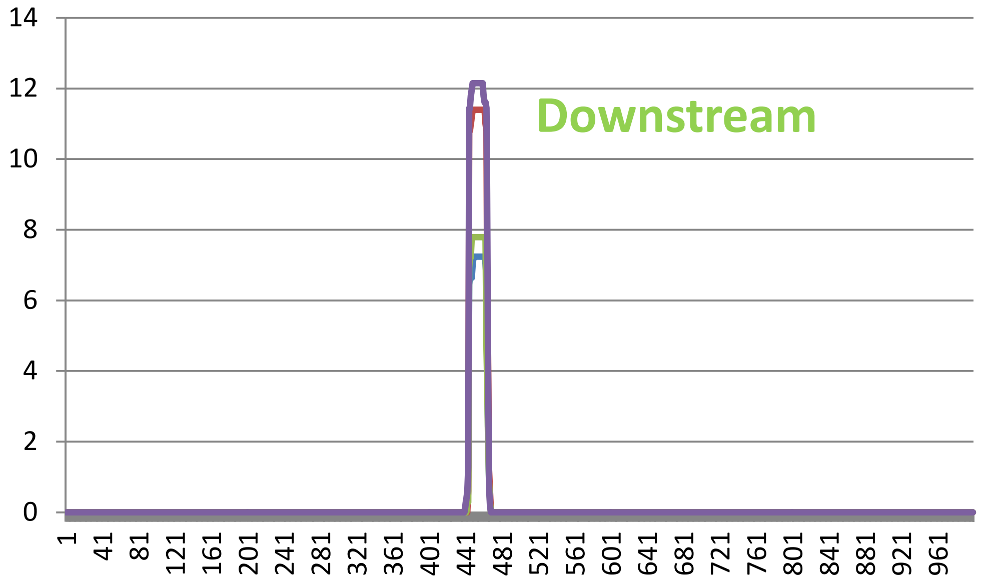

AT3G58170

BET1P/SFT1P-LIKE PROTEIN 14A (BS14A)

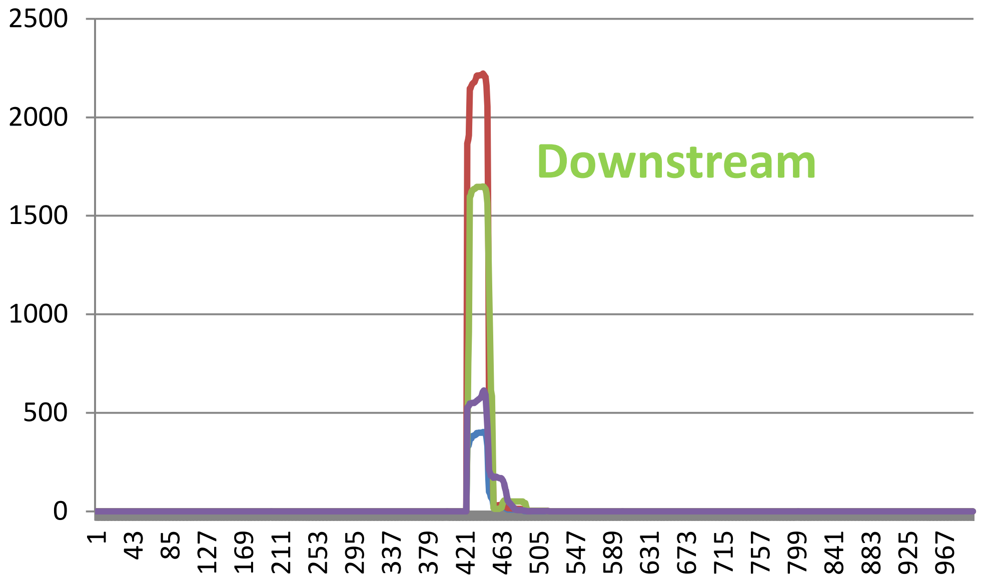

AT3G60550

CYCLIN P3;2 (CYCP3;2)

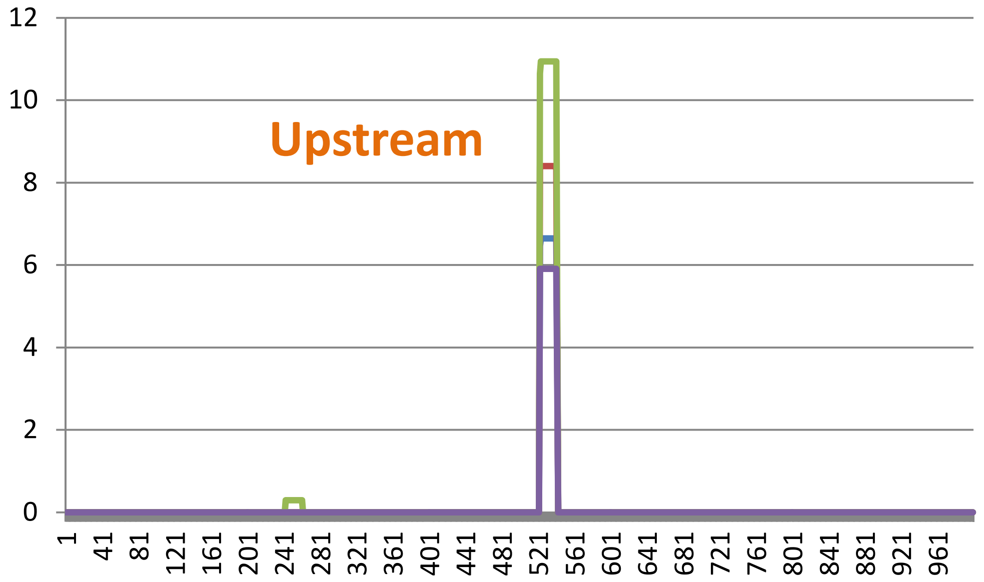

AT4G00120

INDEHISCENT (IND)

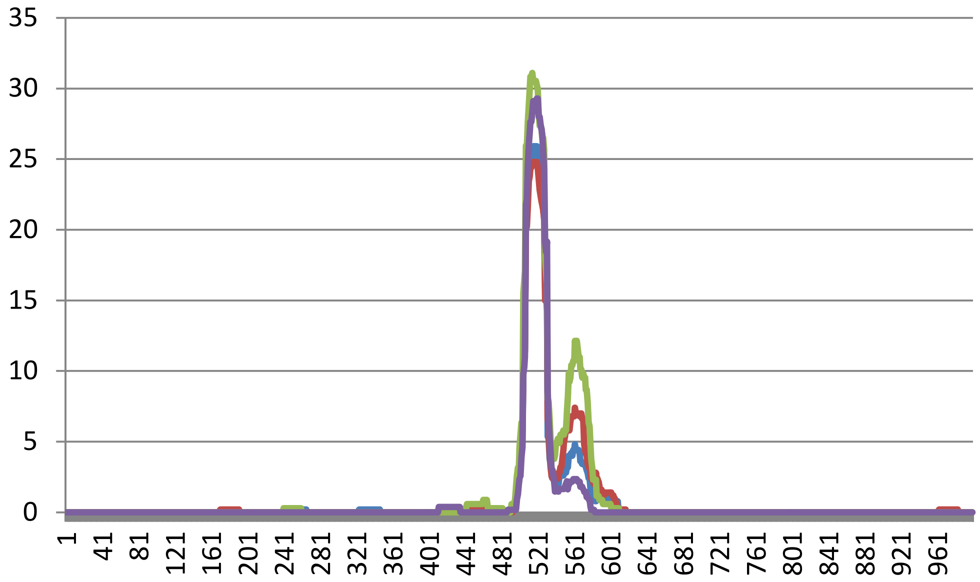

AT4G01900

Encodes a PII protein that may function as part of a signal transduction network involved in perceiving the status of carbon and organic nitrogen. Forms a protein complex with N-acetylglutamate kinase and regulates the kinase activity by relieving the feedback inhibition of the kinase by arginine. Regulates acetyl-CoA carboxylase activity.

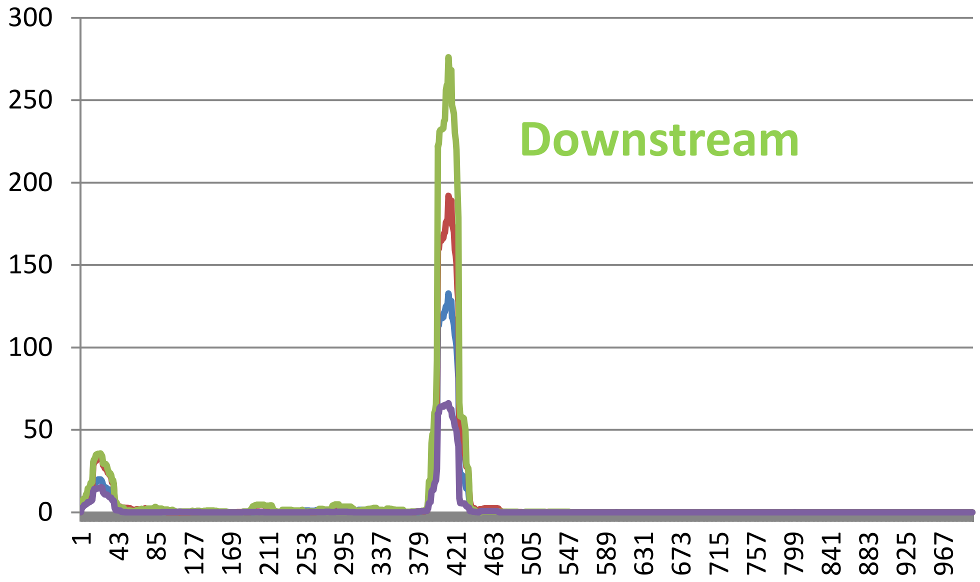

AT4G03160

BEST Arabidopsis thaliana protein match is: AP2/B3-like transcriptional factor family protein (TAIR:AT4G03170.1).

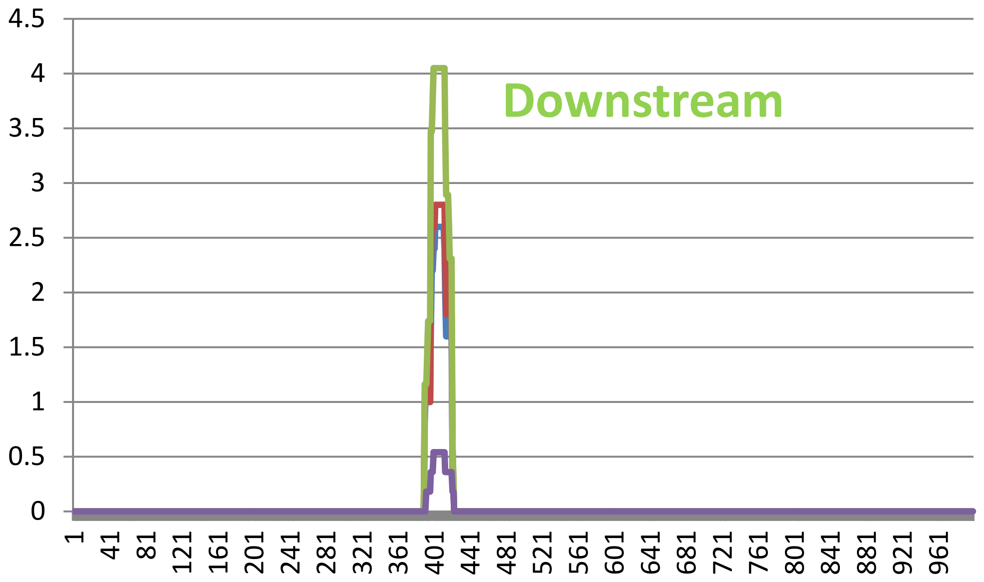

AT4G04030

Ovate family protein 9 (OFP9)

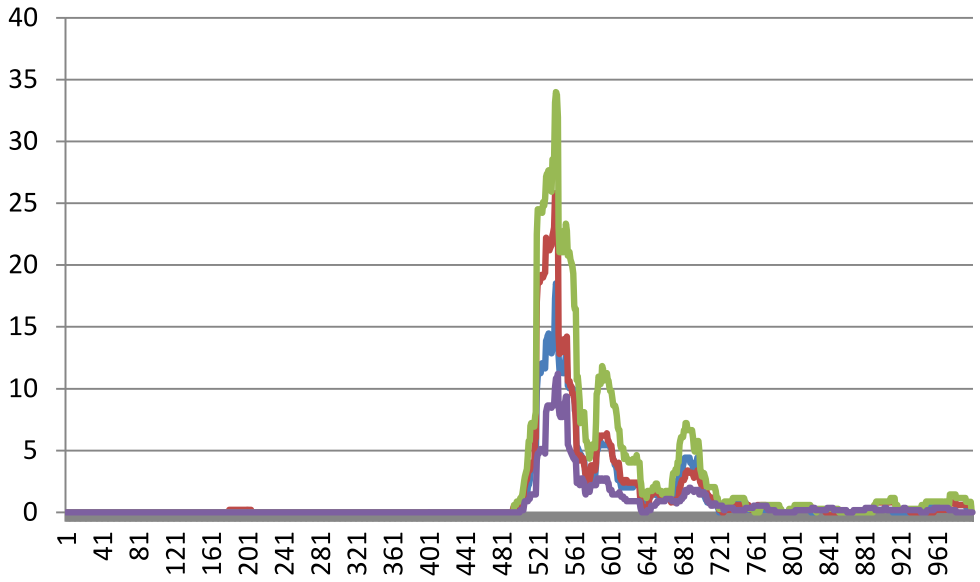

# AT4G04510

Encodes a cysteine-rich receptor-like protein kinase.

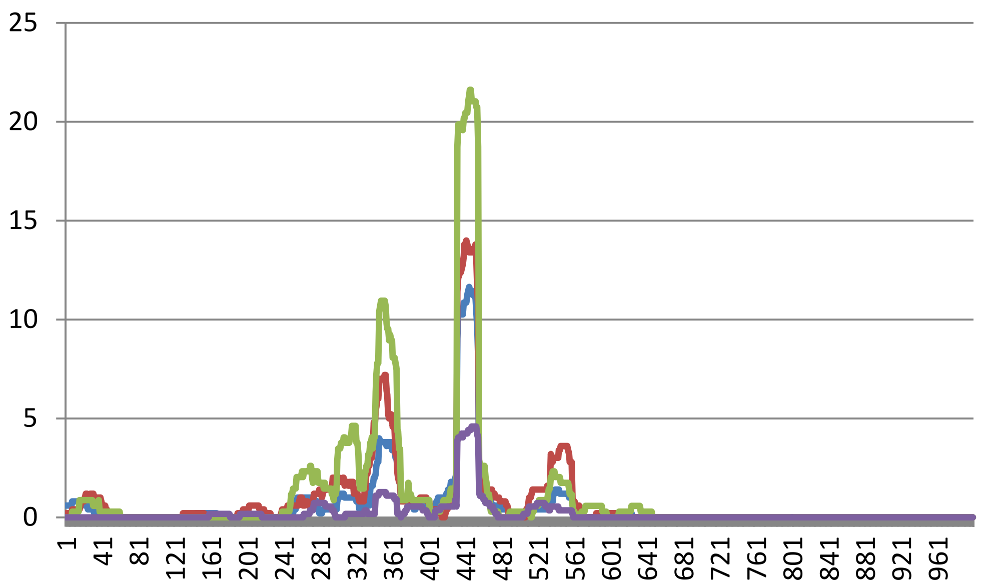

AT4G04970

Encodes a gene similar to callose synthase

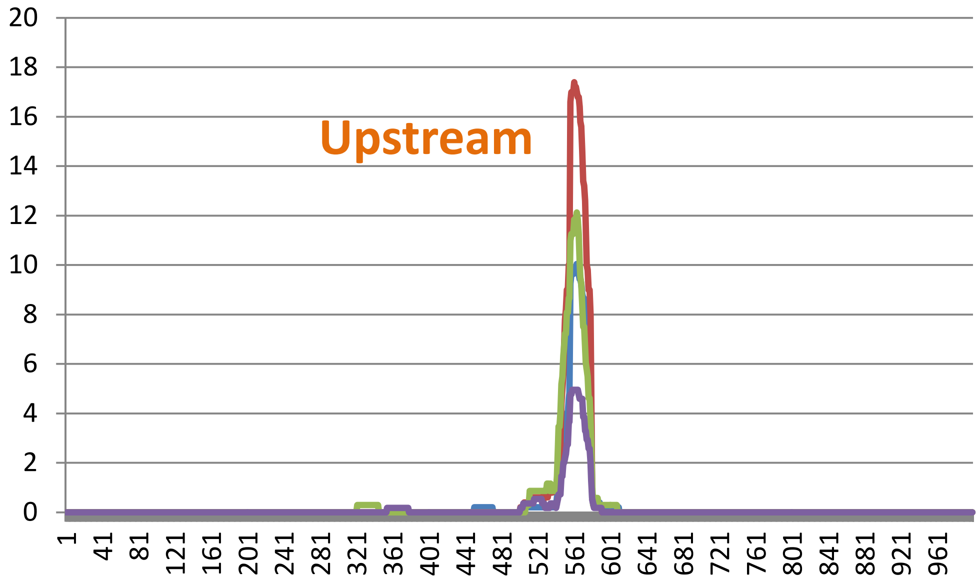

AT4G08160

Encodes a putative glycosyl hydrolase family 10 protein (xylanase).

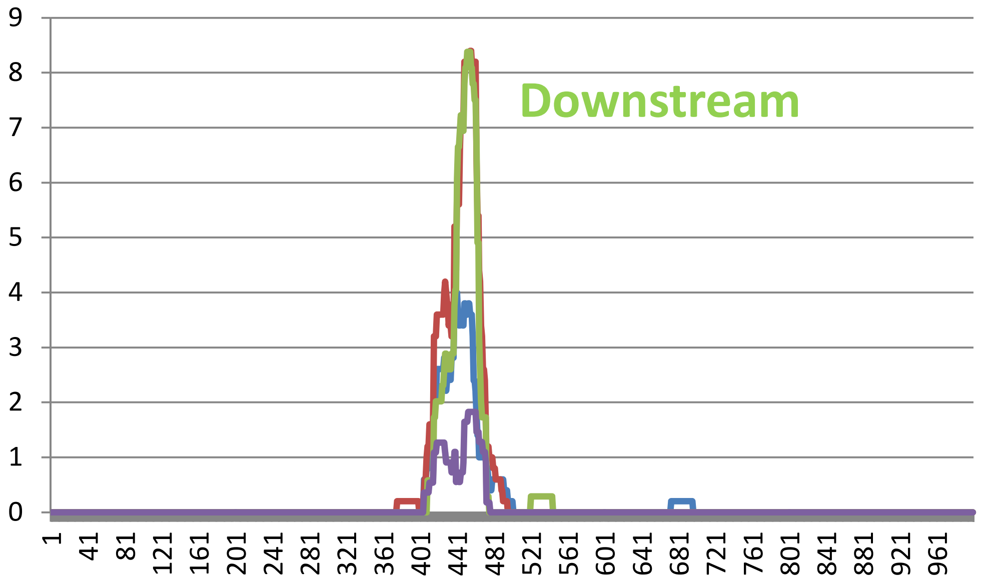

AT4G08300

Nodulin MtN21-like transporter family protein

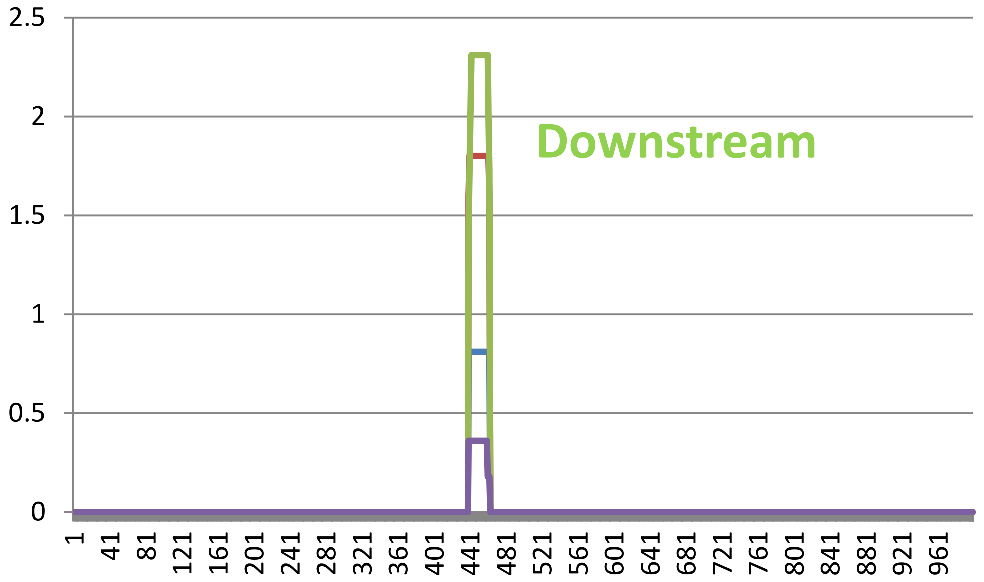

AT4G08850

Leucine-rich repeat receptor-like protein kinase family protein

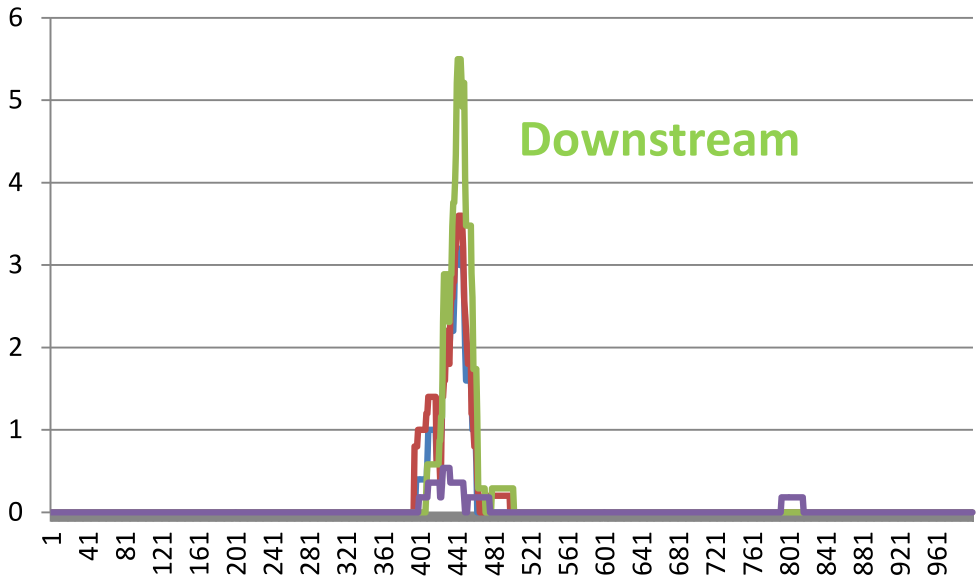

AT4G09770

TRAF-like family protein

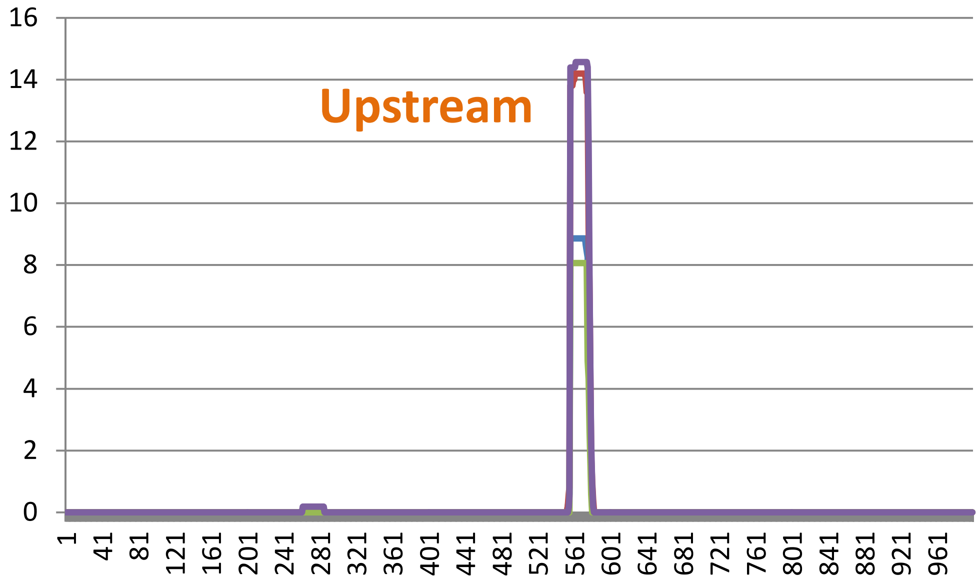

# AT4G11040

## Protein phosphatase 2C family protein

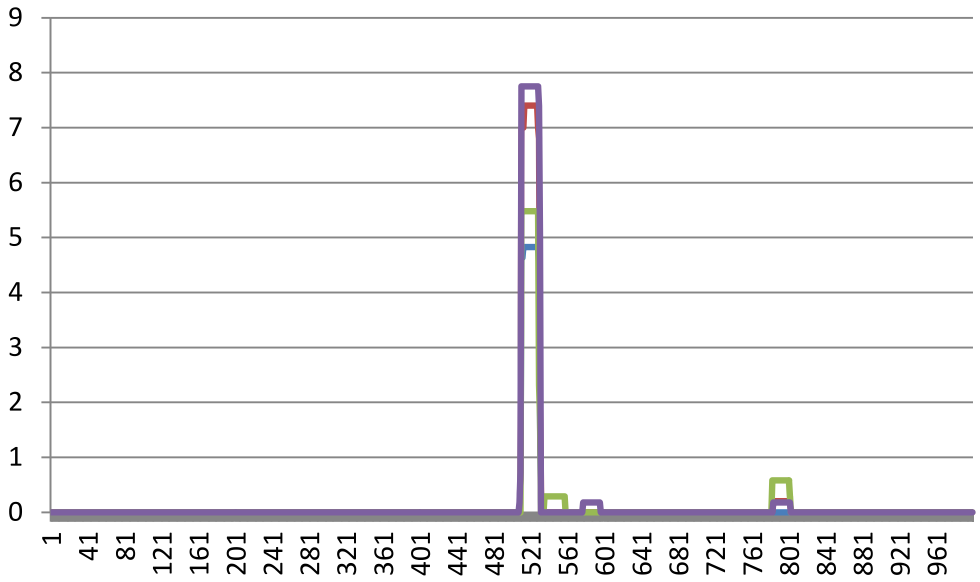

# AT4G11950

## Protein of unknown function (DUF1191)

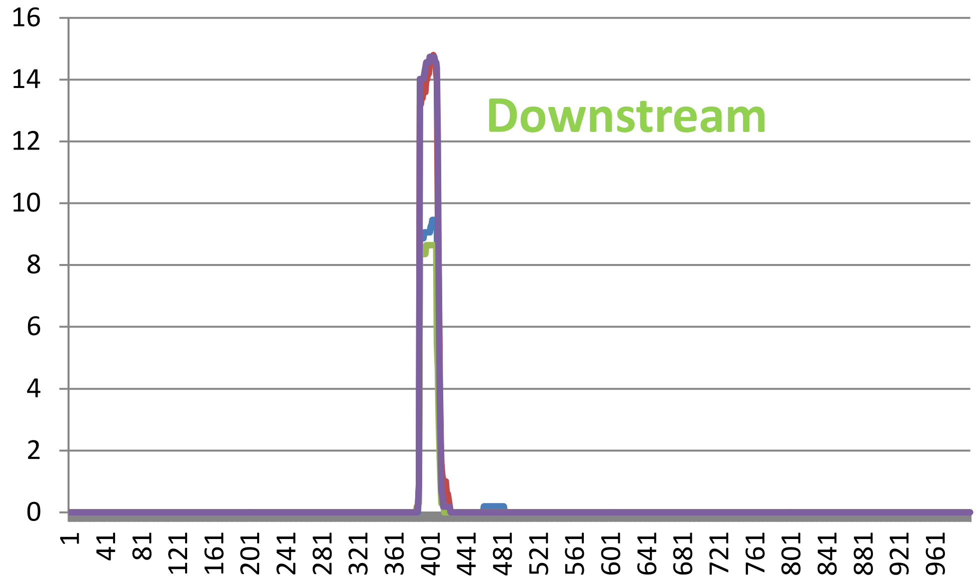

# AT4G13261

## Protein of unknown function (DUF784)

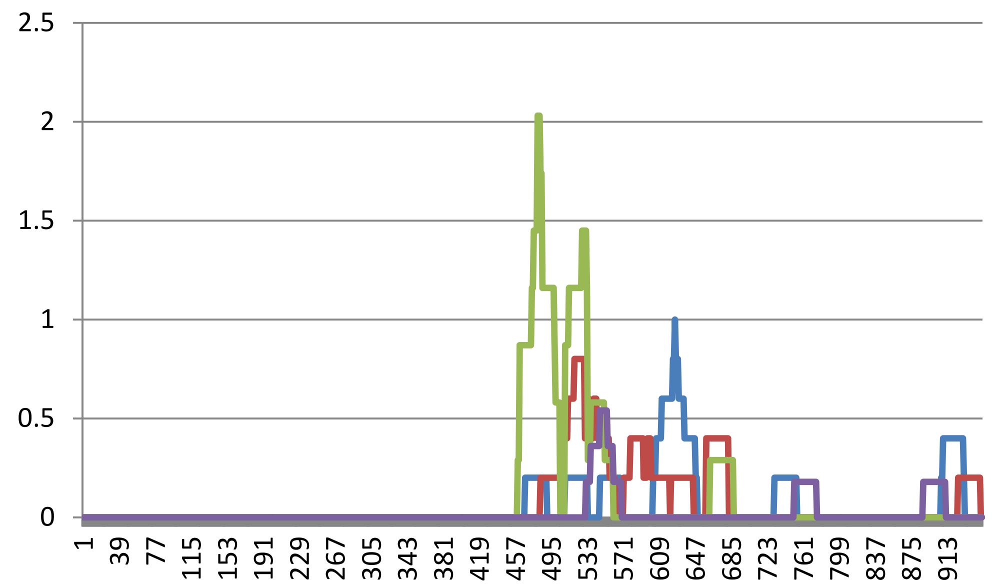

# AT4G14365

## XB3 ortholog 4 in Arabidopsis thaliana (XBAT34)

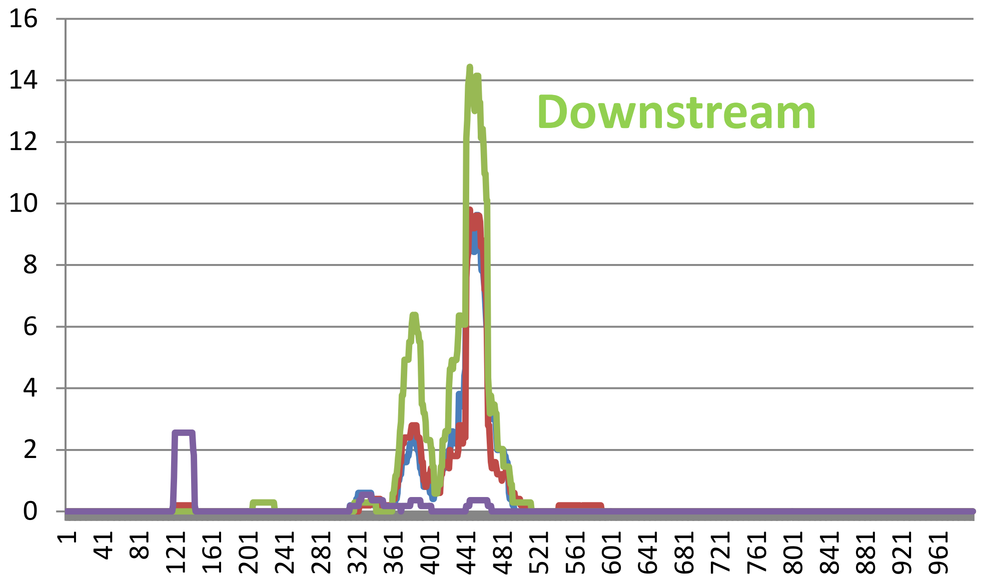

AT4G14940

Atao1 gene of Arabidopsis thaliana encodes an extracellular copper amine oxidase expressed during early stages of vascular tissue development.

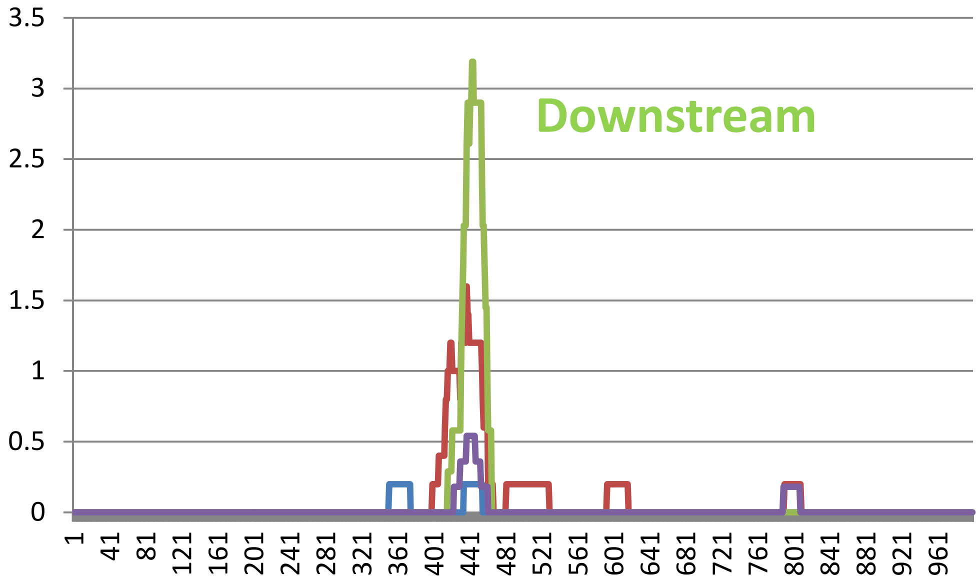

# AT4G15570

MAGATAMA 3 (MAA3). Similar to yeast Sen1 (splicing endonuclease 1) helicase protein. Involved in female gametophyte development.

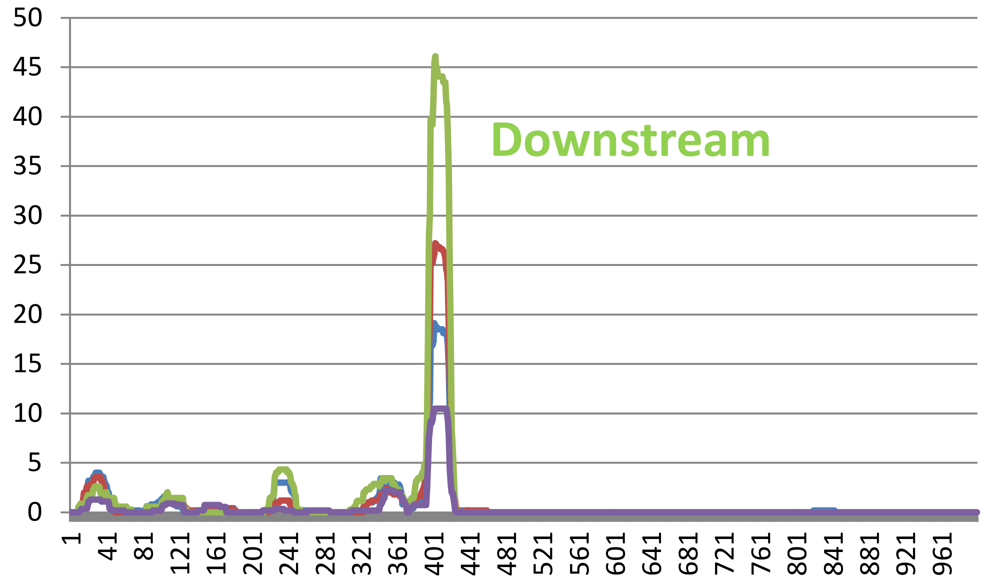

AT4G18690

Unknown protein

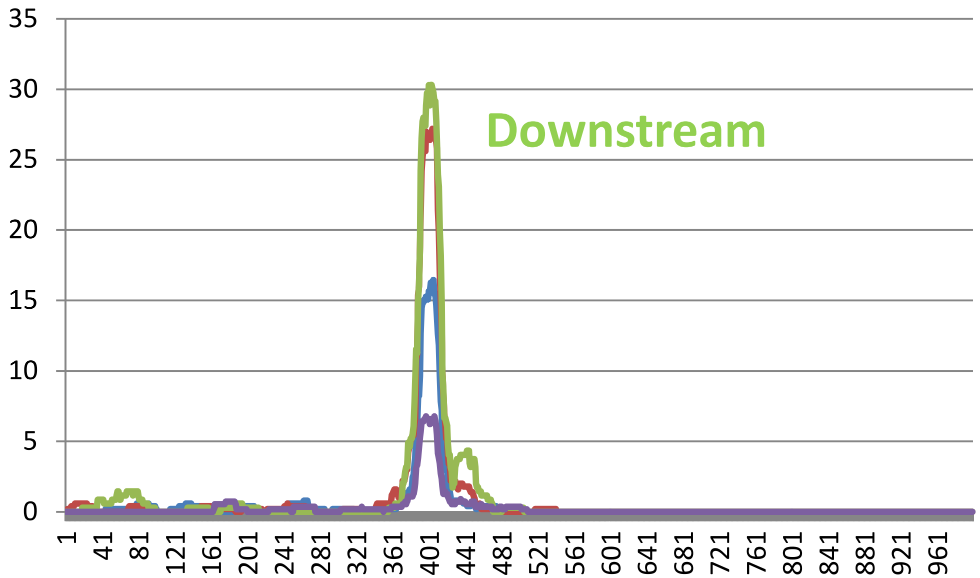

AT4G19330

Galactose oxidase/kelch repeat superfamily protein

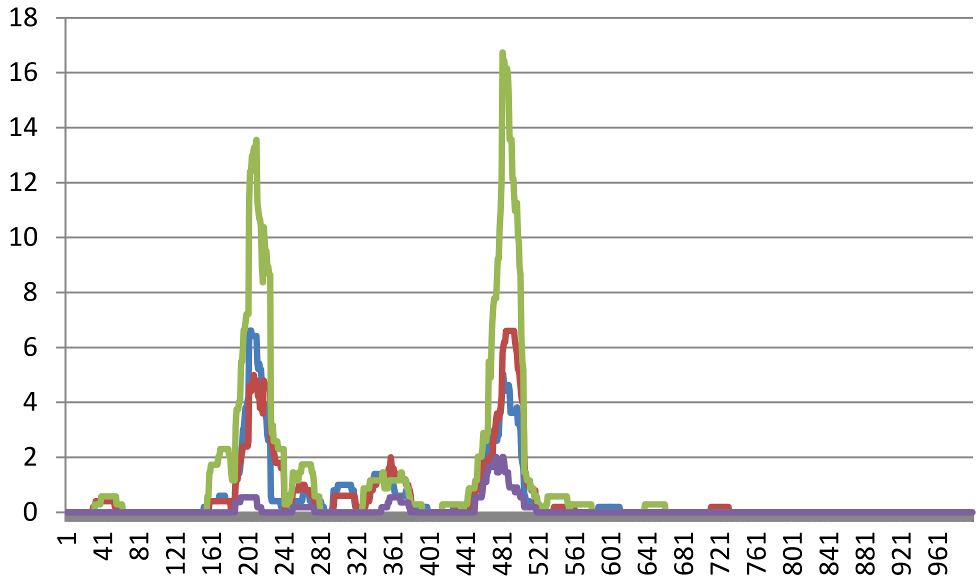

# AT4G20110

## VACUOLAR SORTING RECEPTOR 7 (VSR7)

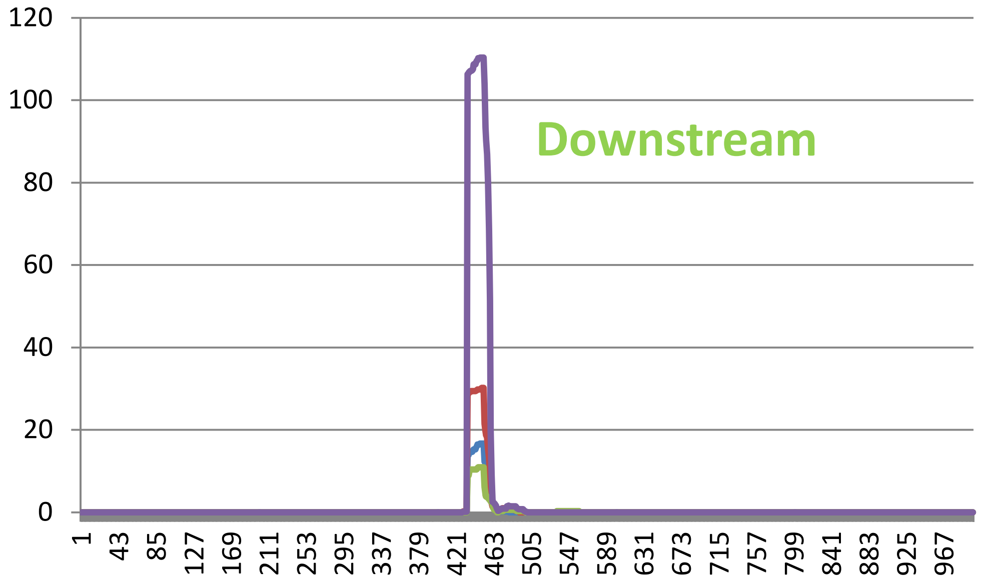

AT4G21585

Encodes a putative endonuclease but no demonstrable endonuclease activity, either towards single stranded DNA or mismatches, has been seen in vitro.

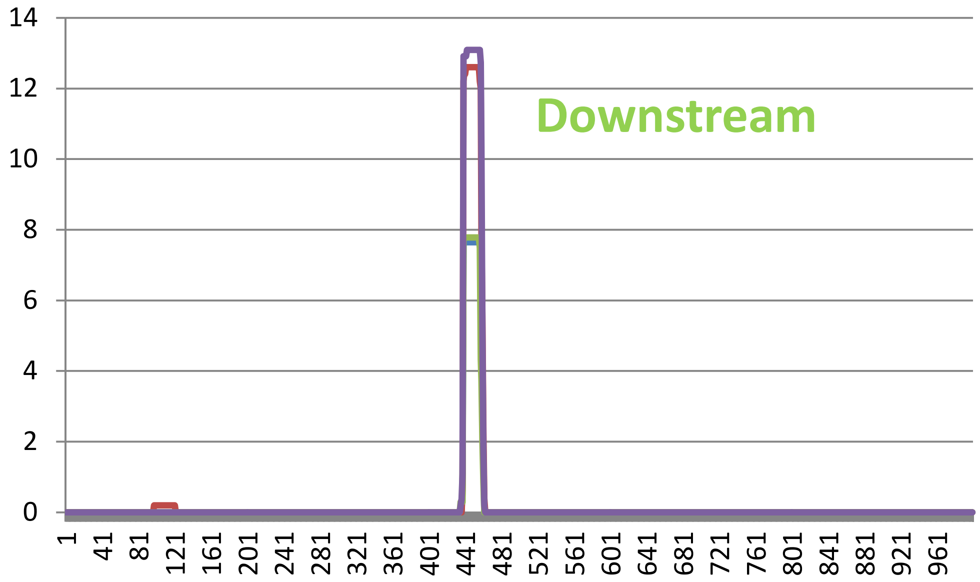

AT4G21820

Binding; calmodulin binding.

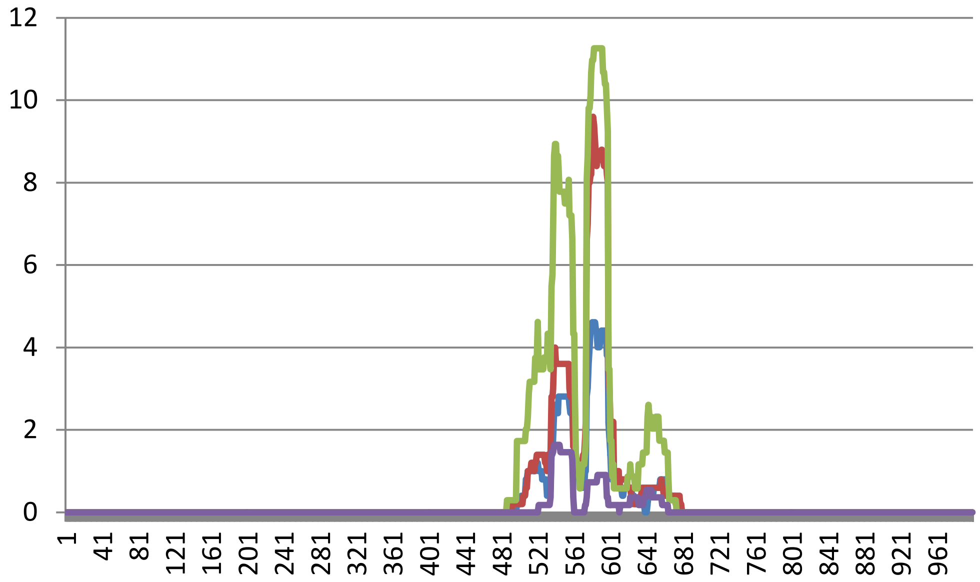

AT4G22650

BEST Arabidopsis thaliana protein match is: Bifunctional inhibitor/lipid-transfer protein/seed storage 2S albumin superfamily protein (TAIR:AT4G22640.1).

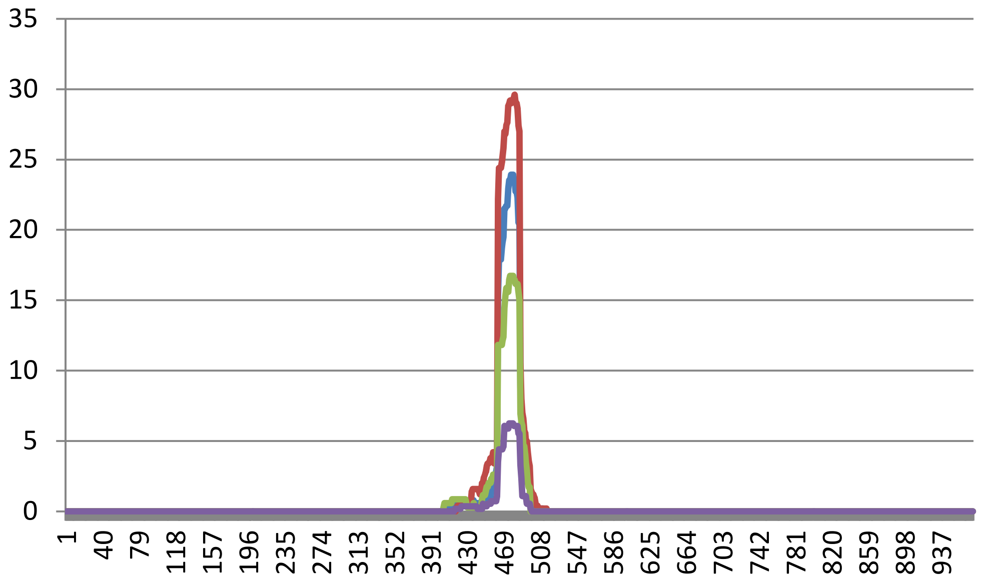

AT4G22790

MATE efflux family protein

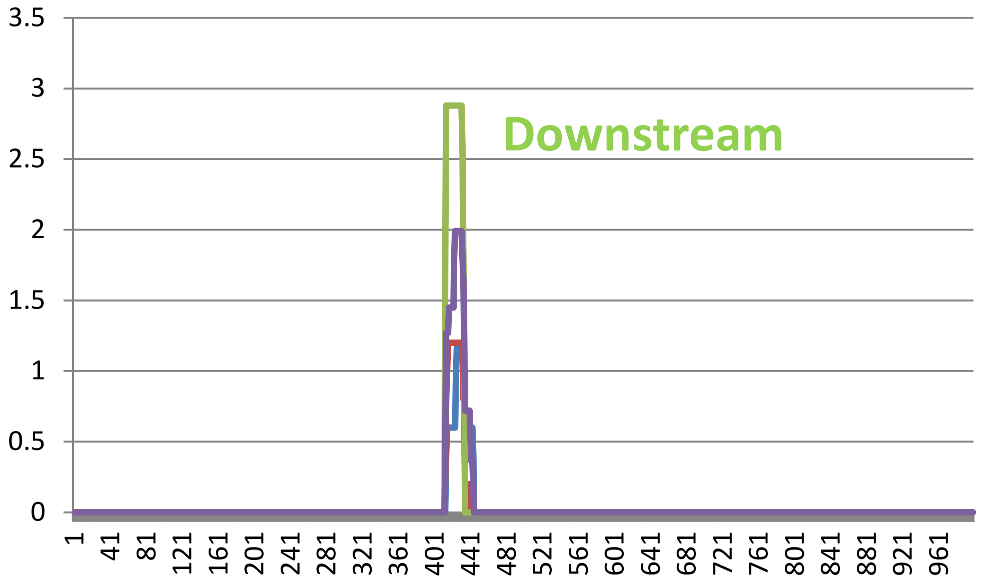

AT4G23120

Bromo-adjacent homology (BAH) domain-containing protein.

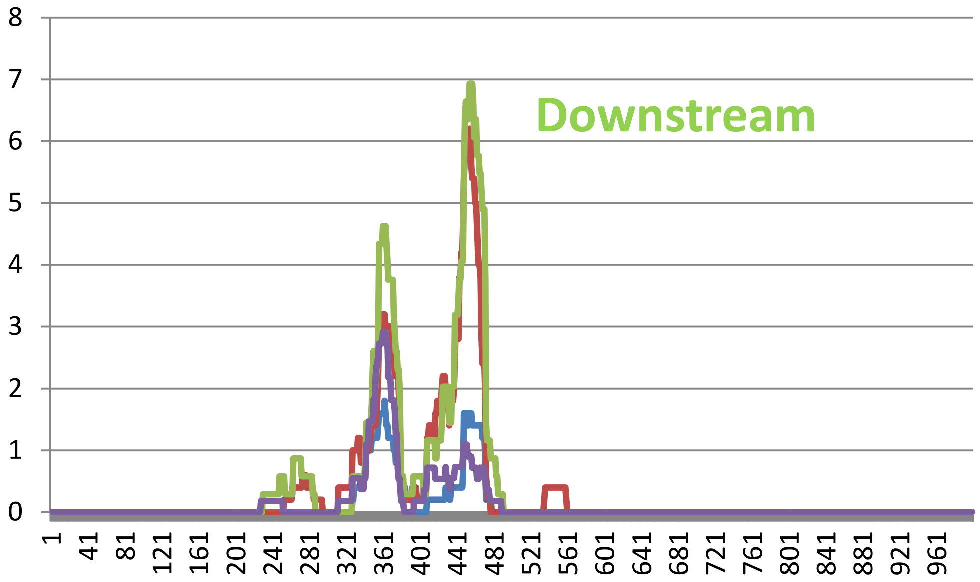

AT4G23640

TINY ROOT HAIR 1 (TRH1). Functions as a potassium transporter and is required for the establishment of root tip growth.

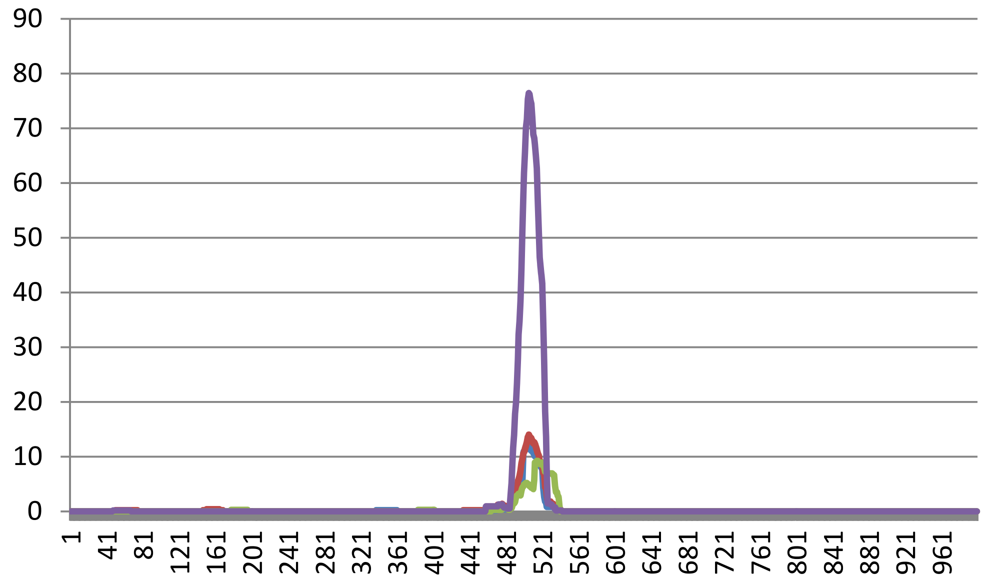

AT4G23730

Galactose mutarotase-like superfamily protein

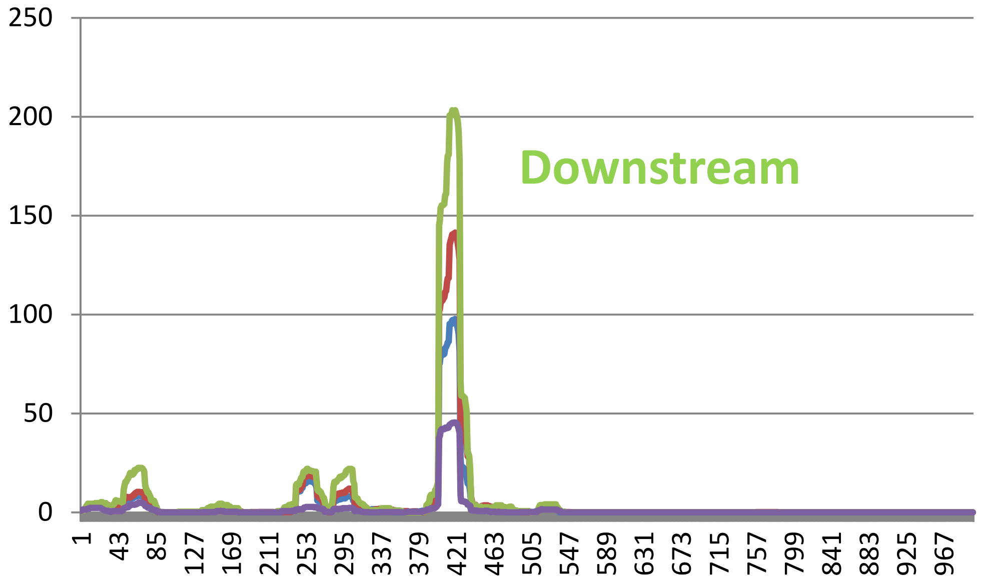

AT4G24440

Transcription initiation factor IIA gamma chain/TFIIA-gamma (TFIIA-S)

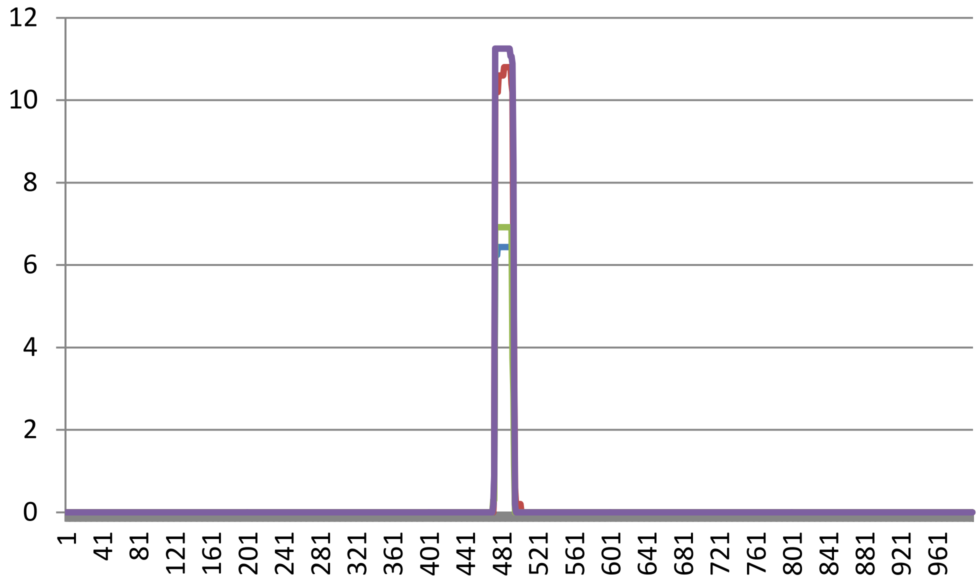

AT4G24644

This gene encodes a small protein and has either evidence of transcription or purifying selection.

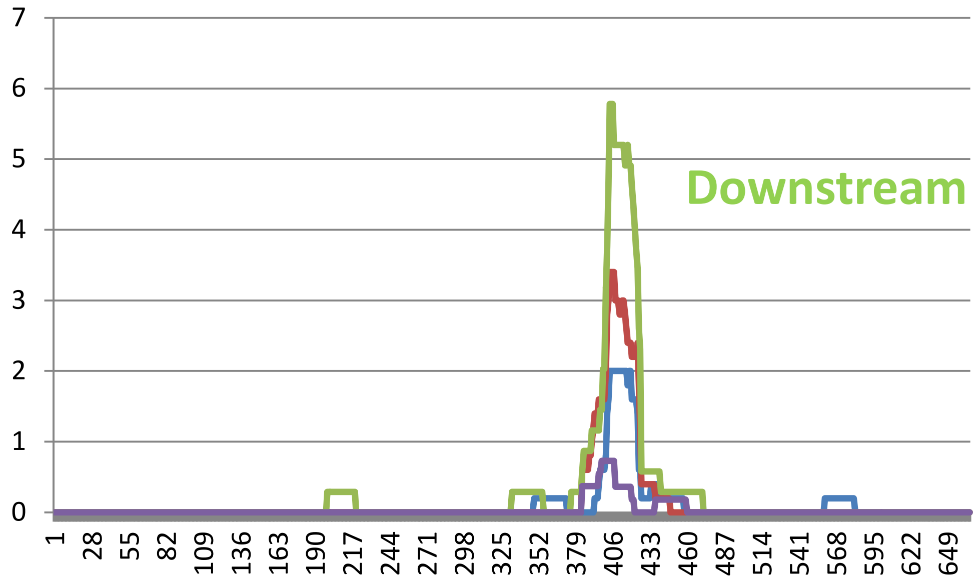

AT4G25580

CAP160 protein

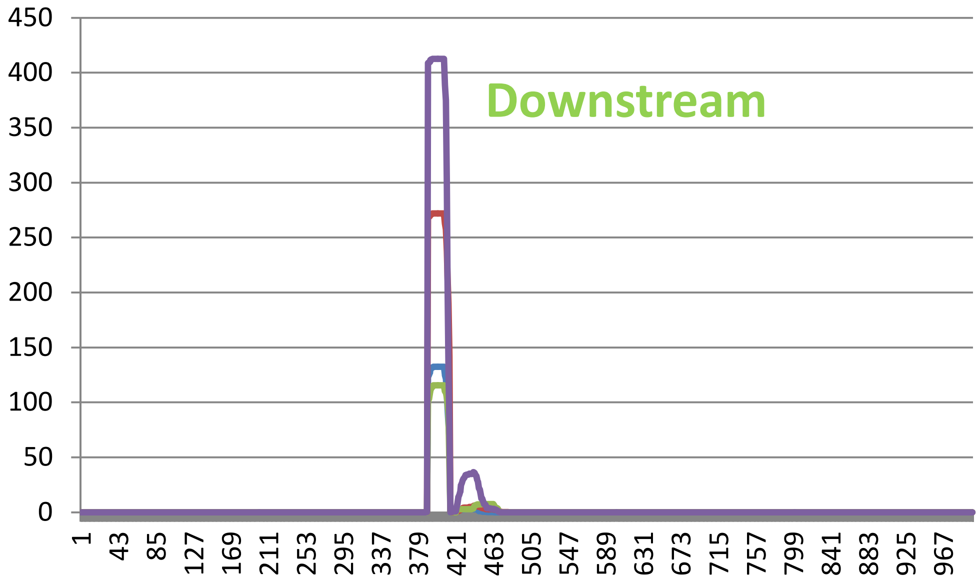

# AT4G26380

## Cysteine/Histidine-rich C1 domain family protein

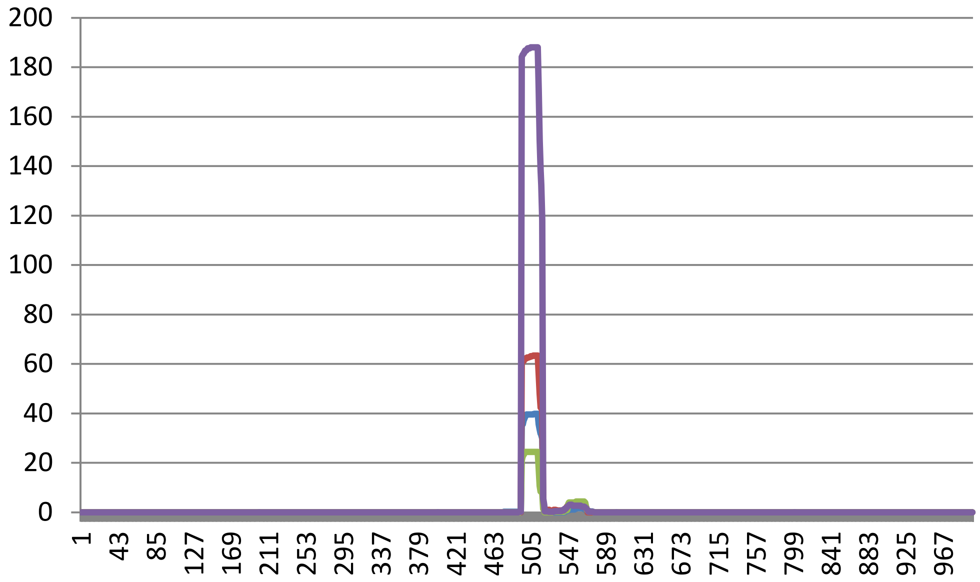

AT4G26900

Encodes a glutamine amidotransferase and cyclase, catalyzes the fifth and sixth steps of the histidine biosynthetic pathway.

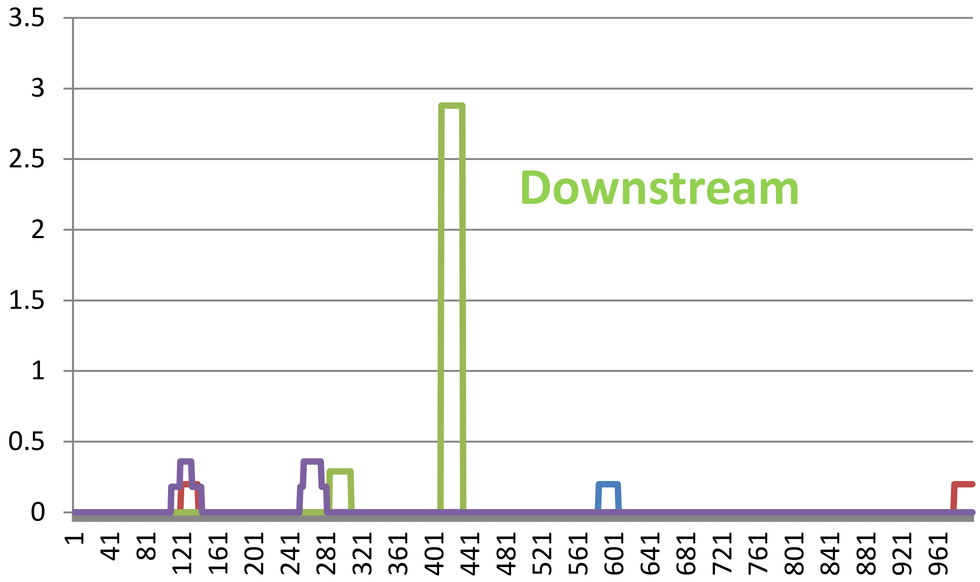

AT4G27870

Vacuolar iron transporter (VIT) family protein

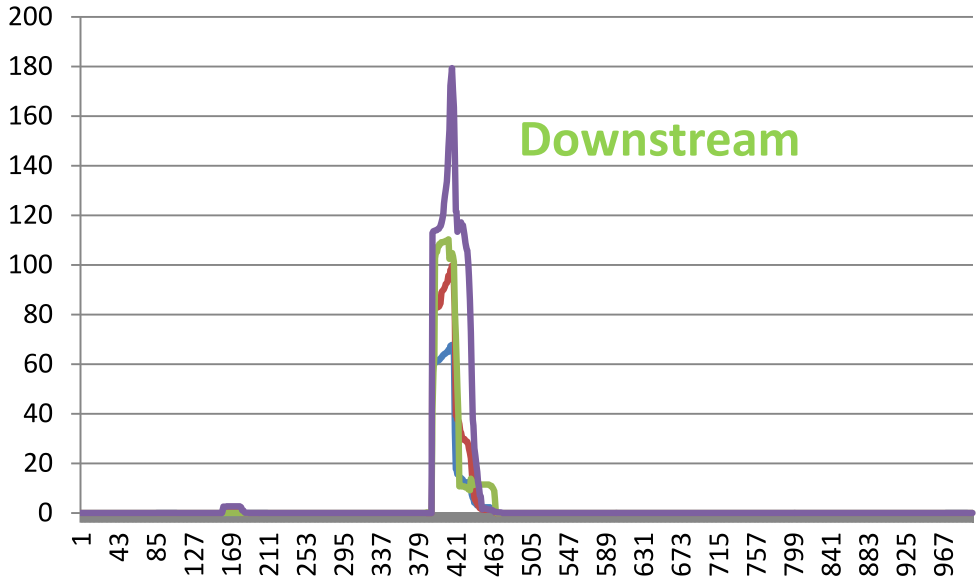

AT4G28850

Xyloglucan endotransglucosylase/hydrolase 26 (XTH26)

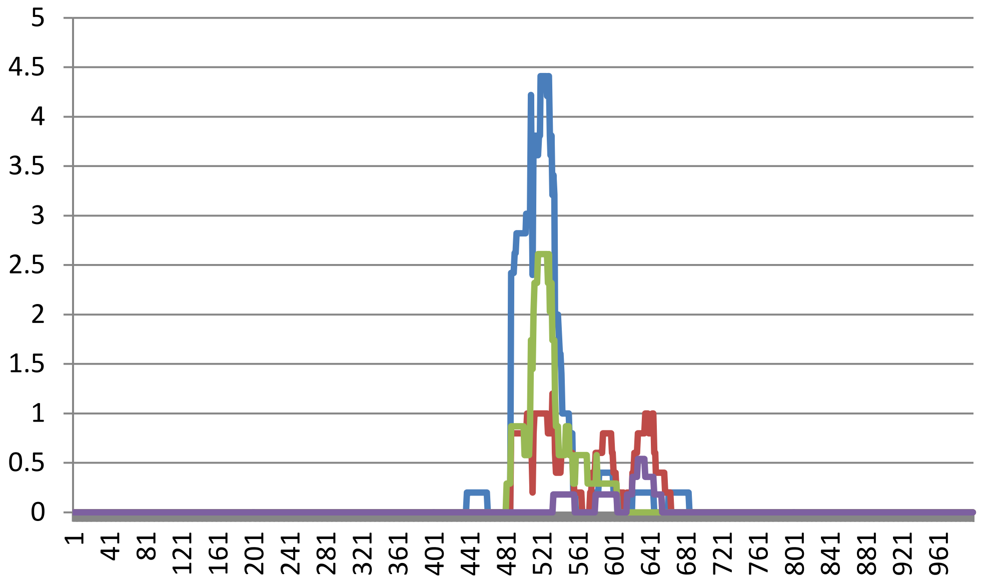

# AT4G29090

## Ribonuclease H-like superfamily protein

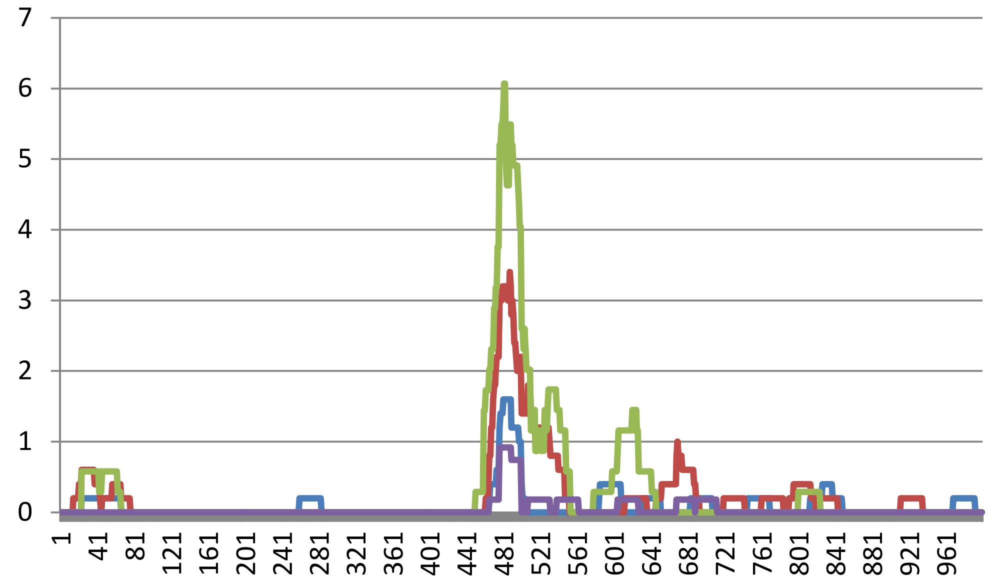

# AT4G29290

Encodes a member of a family of small, secreted, cysteine rich protein with sequence similarity to the PCP (pollen coat protein) gene family.

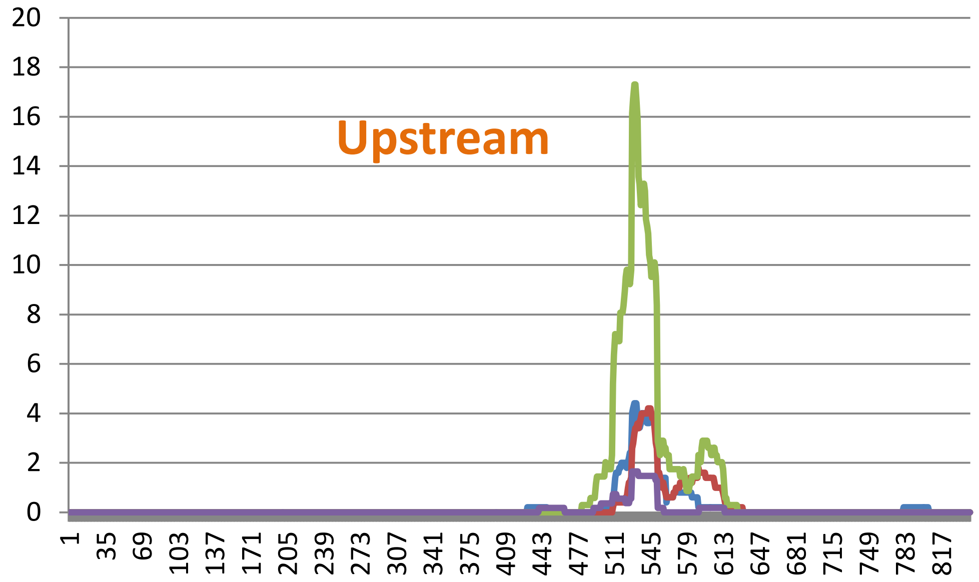

AT4G29305

Encodes a member of a family of small, secreted, cysteine rich protein with sequence similarity to the PCP (pollen coat protein) gene family.

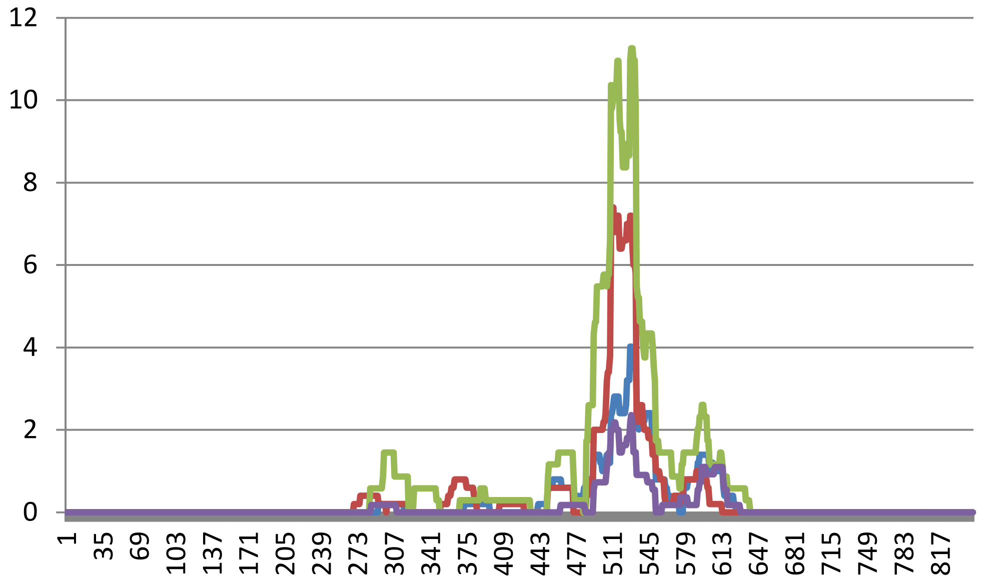

AT4G29740

It encodes a protein whose sequence is similar to cytokinin oxidase/dehydrogenase, which catalyzes the degradation of cytokinins.

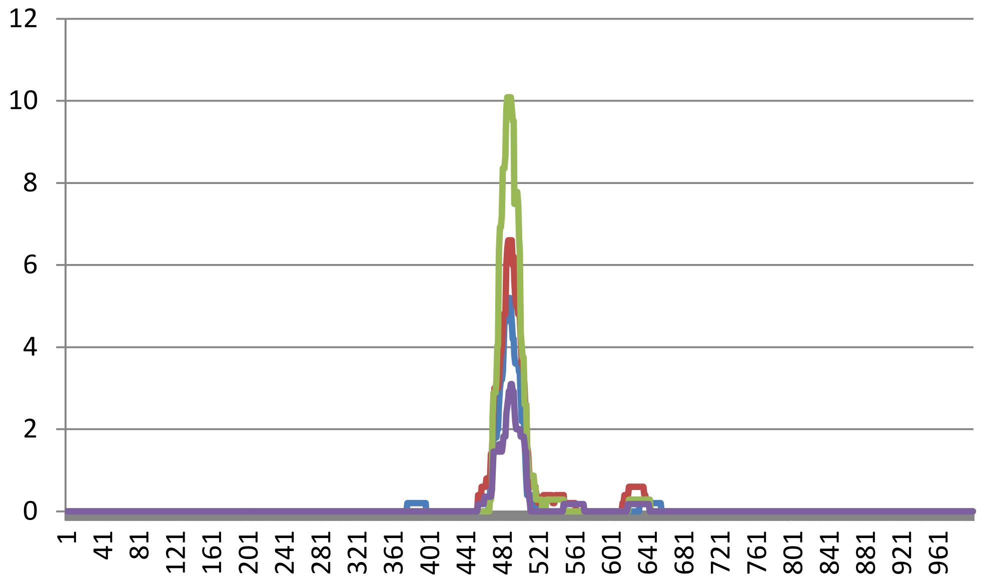

# AT4G31270

## Sequence-specific DNA binding transcription factors

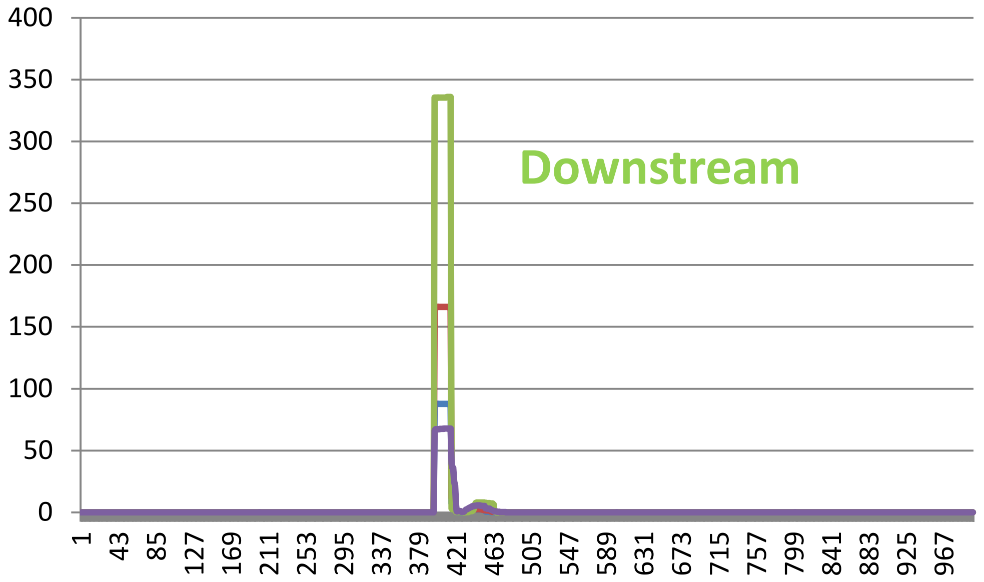

AT4G32200

Meiotic asynaptic mutant 2, homologue of ASY1

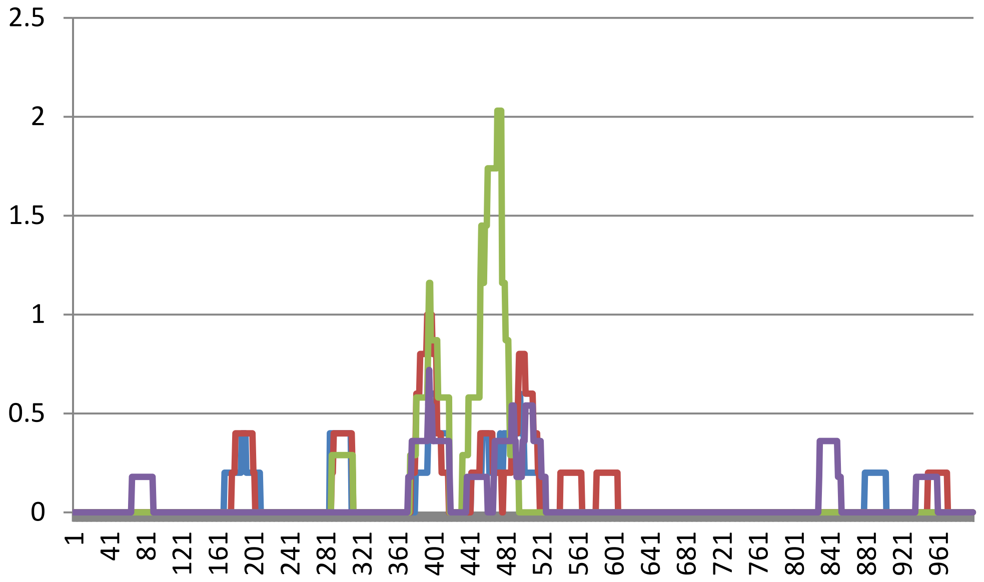

# AT4G32860

## Unknown protein

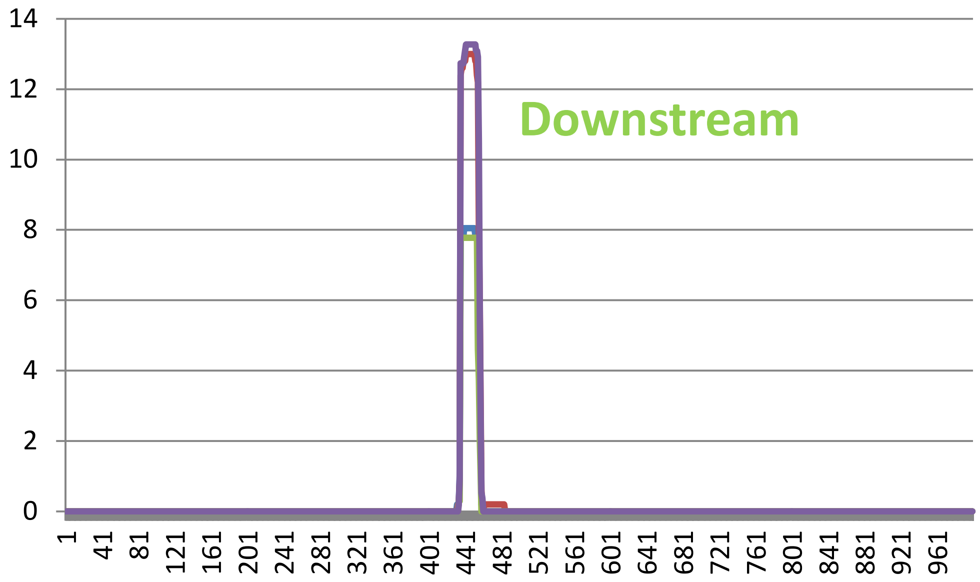

AT4G36925

Unknown protein

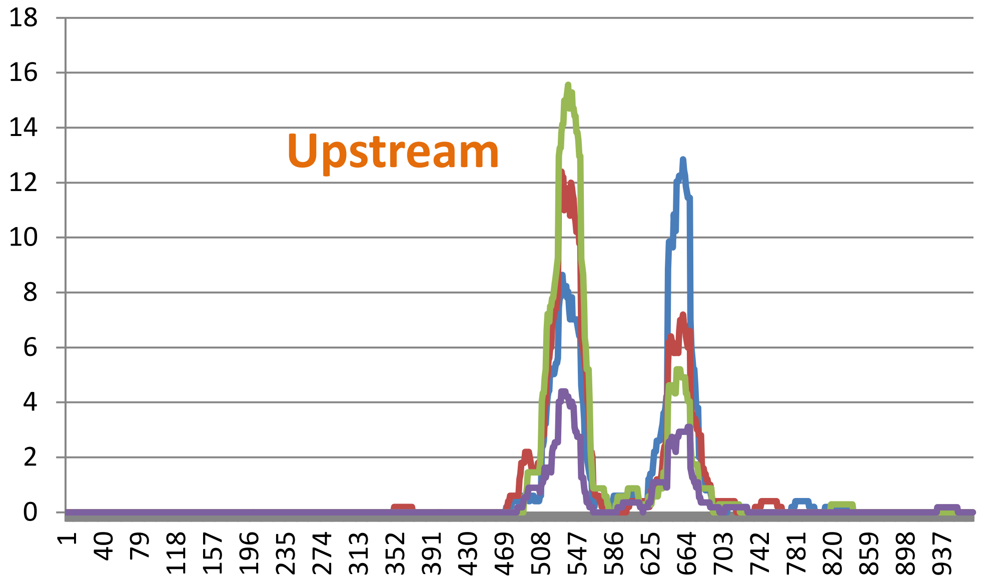

AT4G37140

Encodes a protein with similarity to SABP2, a methyl salicylate esterase from tobacco. However, this protein is truncated and lacks two of the residues of the predicted catalytic triad, suggesting that it does not have this enzymatic activity.

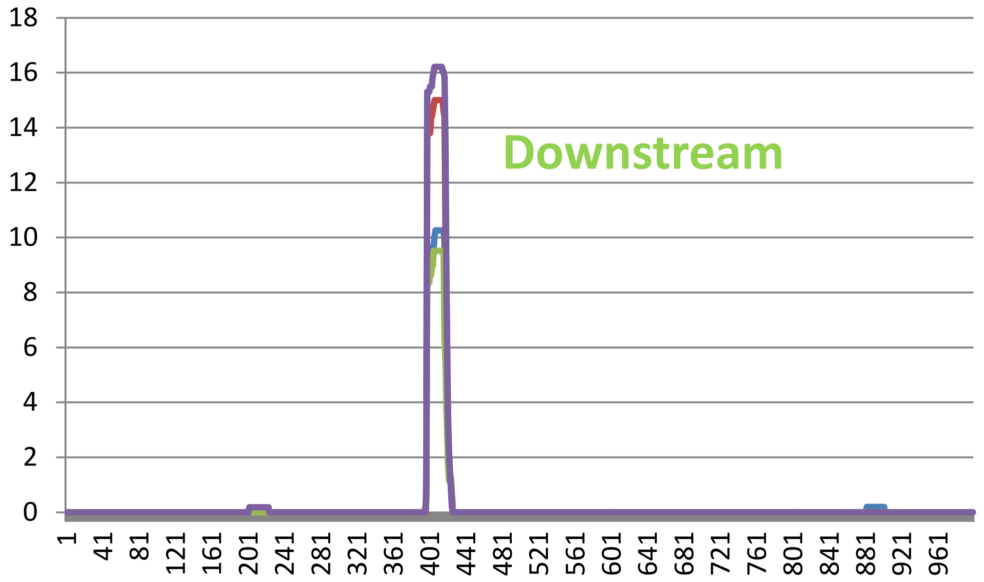

AT4G37180

MYB family transcription factor

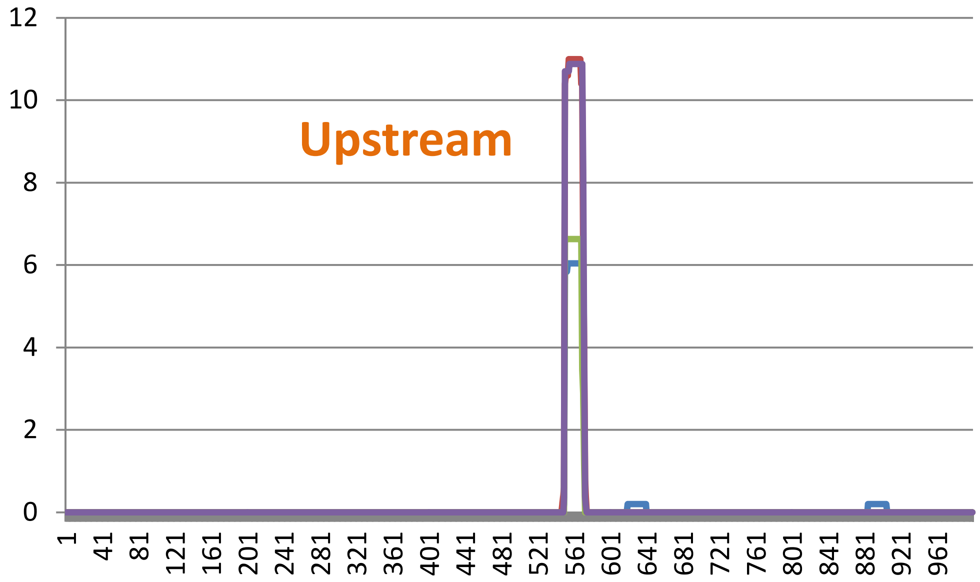

# AT4G38930

## Ubiquitin fusion degradation UFD1 family protein

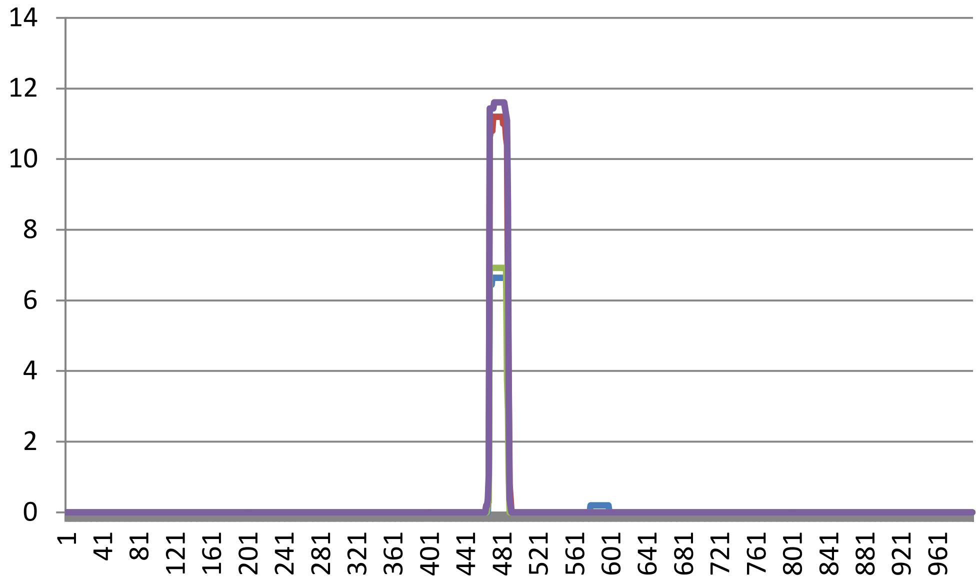

# AT4G39756

## Galactose oxidase/kelch repeat superfamily protein

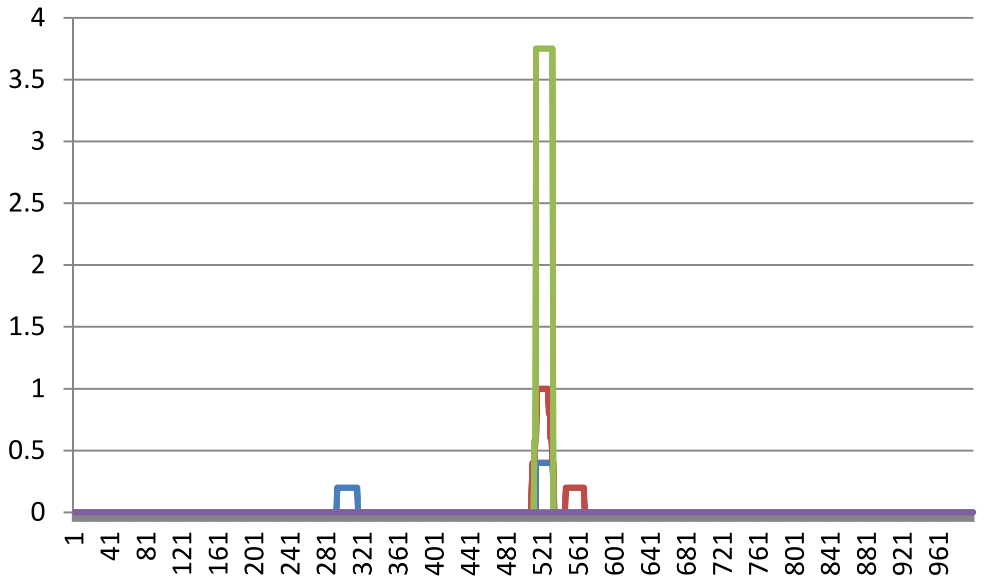

# AT5G01080

## Beta-galactosidase related protein

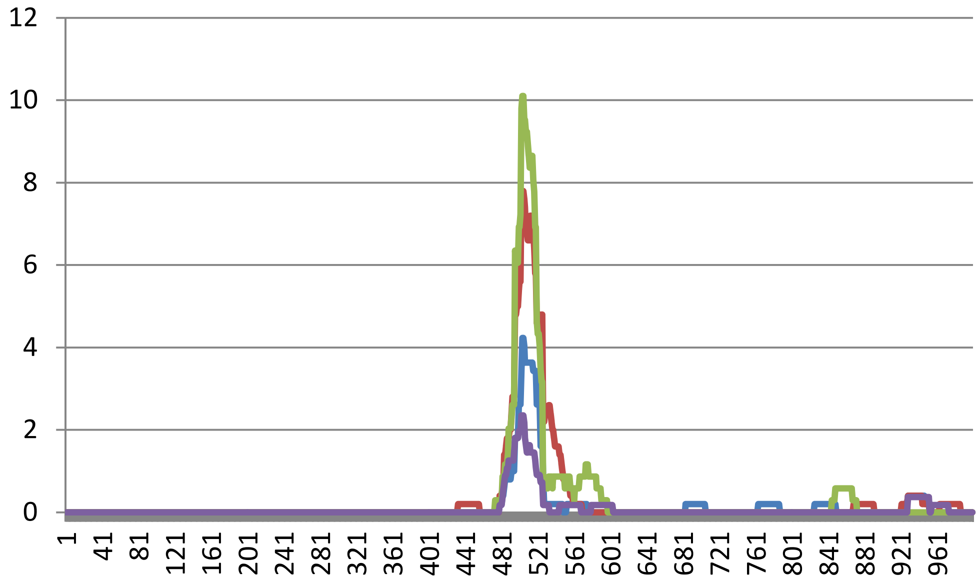

# AT5G01090

## Concanavalin A-like lectin family protein

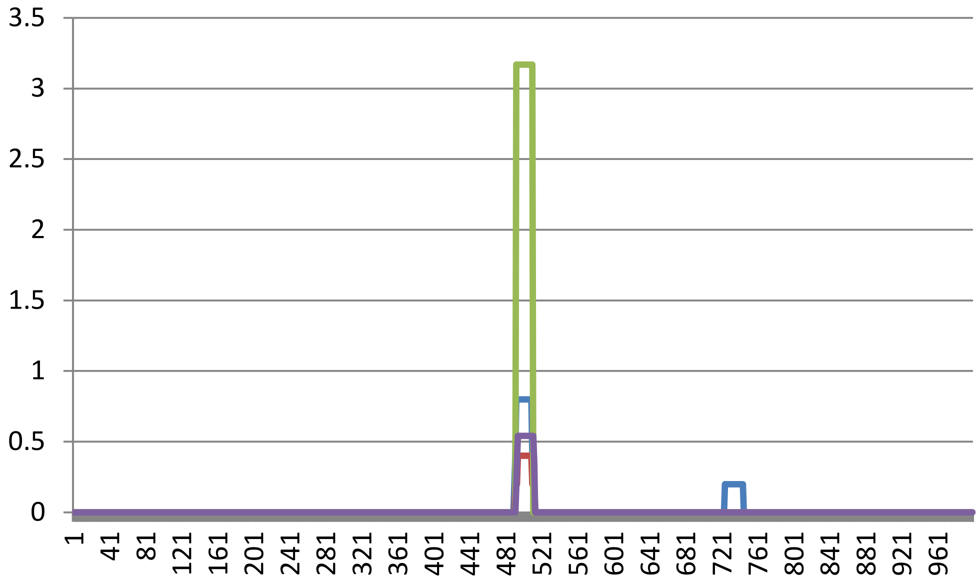

# AT5G01260

## Carbohydrate-binding-like fold

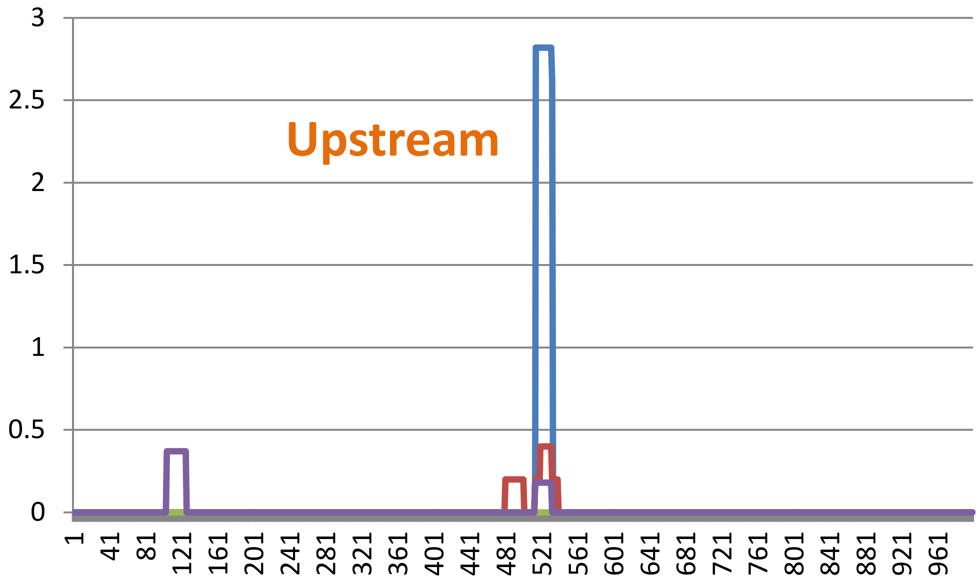

AT5G02990

Galactose oxidase/kelch repeat superfamily protein

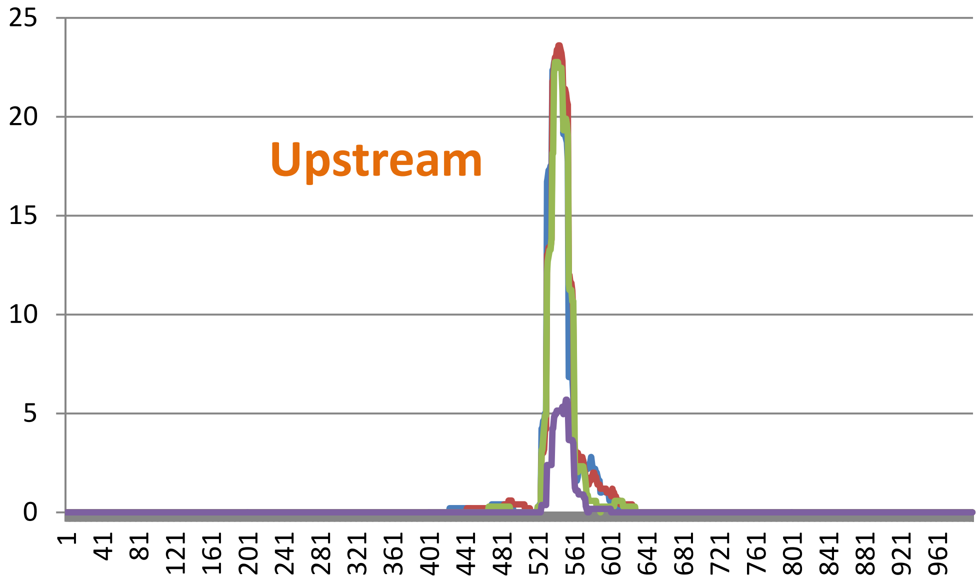

# AT5G03060

## Unknown protein

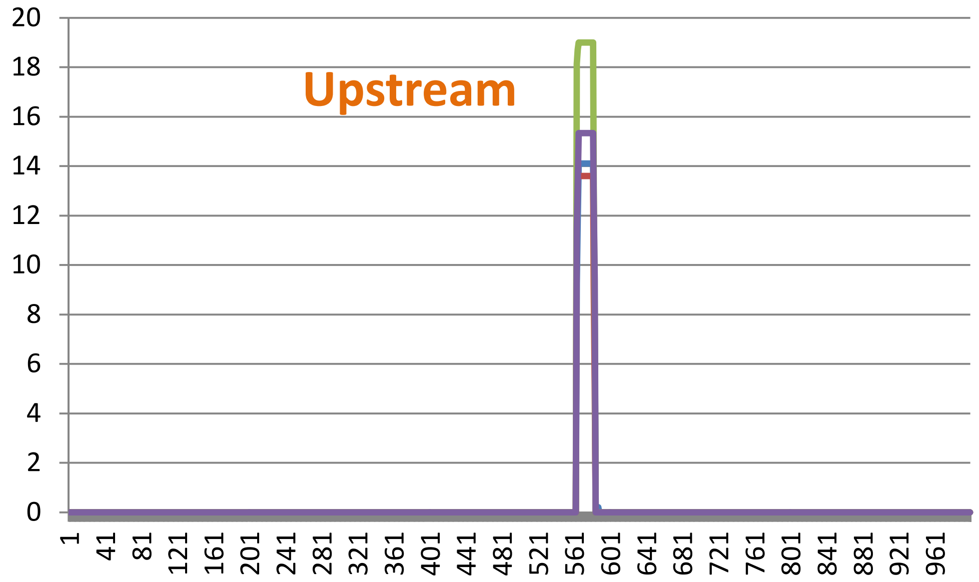

AT5G04950

Encodes a nicotianamide synthase.

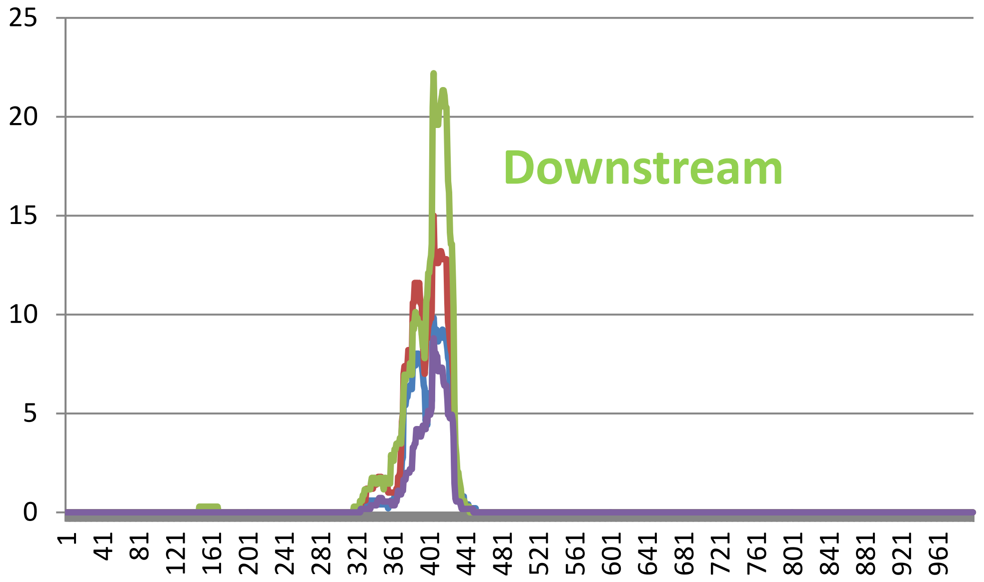

# AT5G04960

## Plant invertase/pectin methylesterase inhibitor superfamily

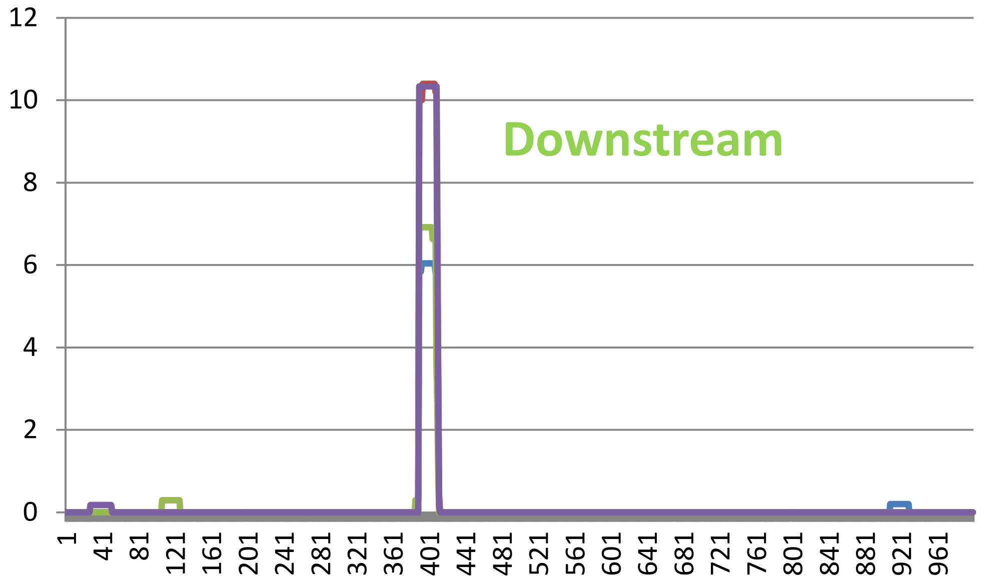

AT5G06043

Unknown protein

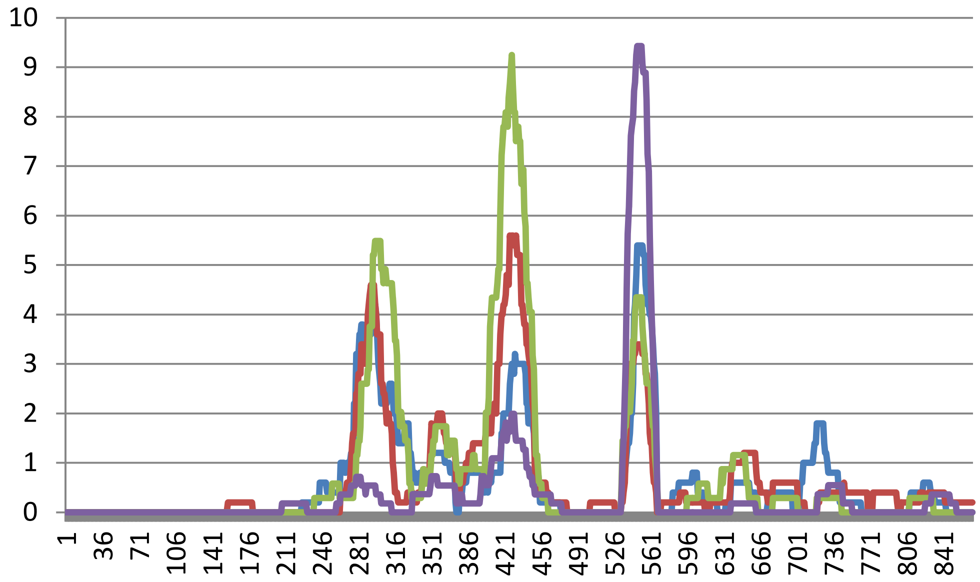

AT5G09660

Encodes a microbody NAD-dependent malate dehydrogenase encodes an peroxisomal NAD-malate dehydrogenase that is involved in fatty acid beta-oxidation through providing NAD to the process of converting fatty acyl CoA to acetyl CoA.

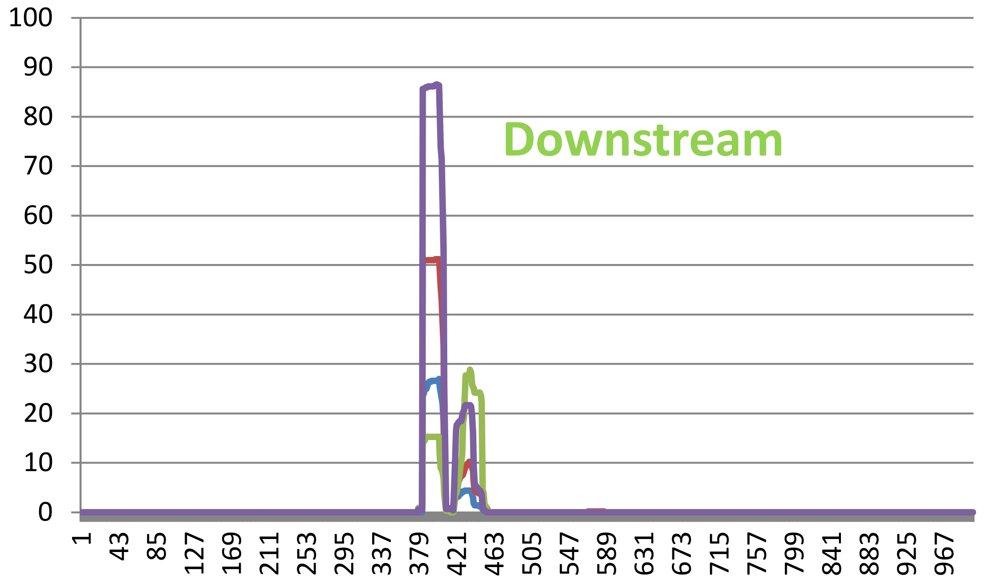

AT5G09910

Ras-related small GTP-binding family protein

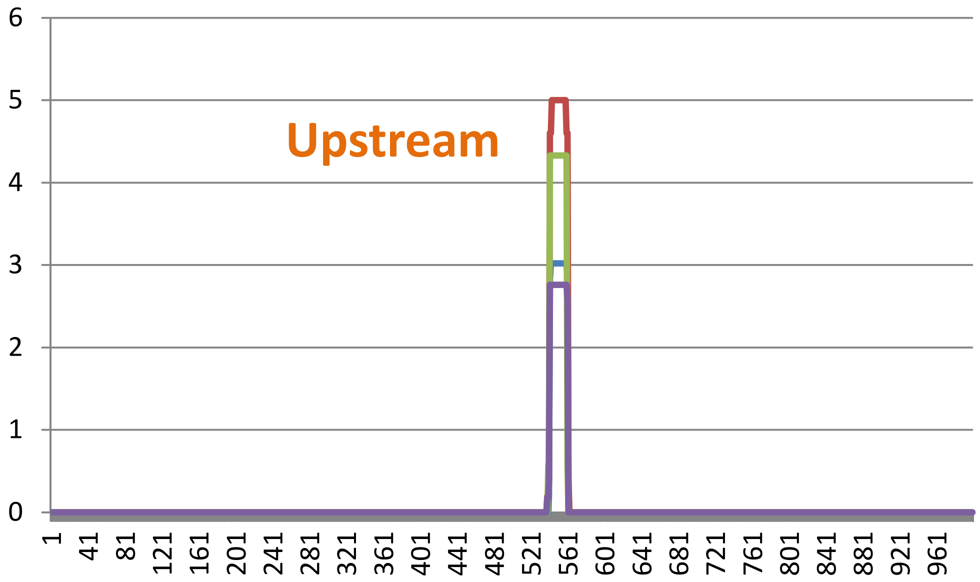

AT5G11590

Encodes a member of the DREB subfamily A-4 of ERF/AP2 transcription factor family. The protein contains one AP2 domain. There are 17 members in this subfamily including TINY.

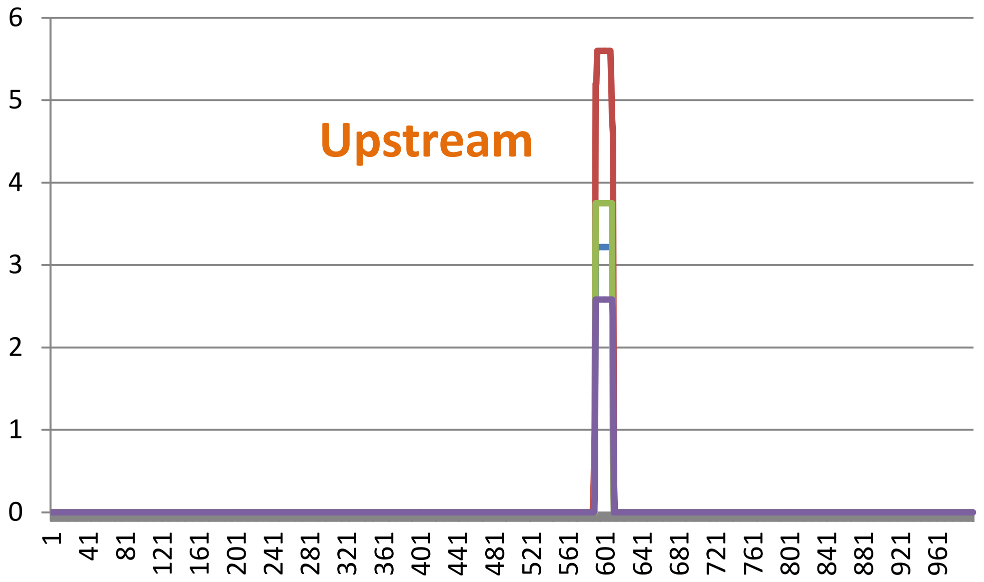

# AT5G13825

## Unknown protein

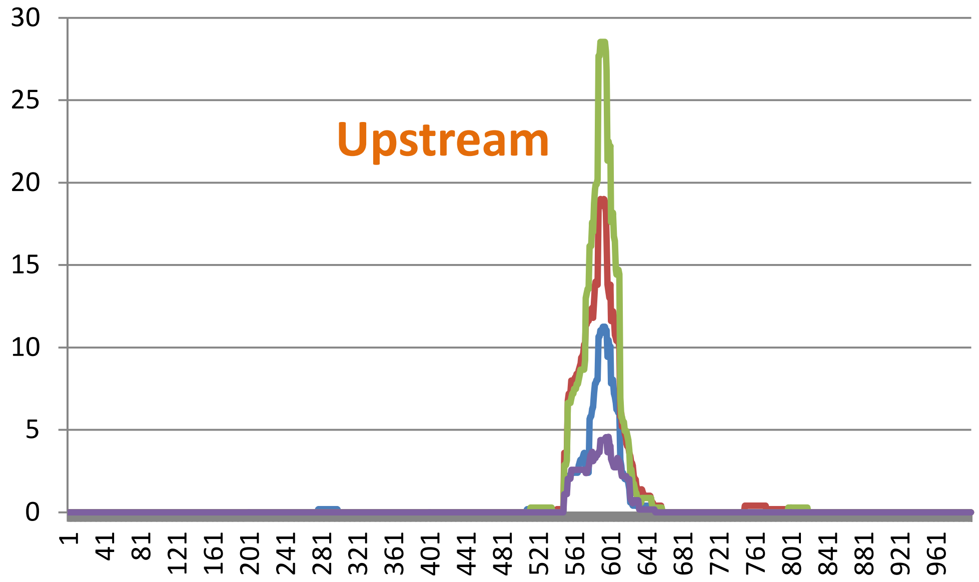

AT5G14550

Core-2/I-branching beta-1,6-N-acetylglucosaminyltransferase family protein

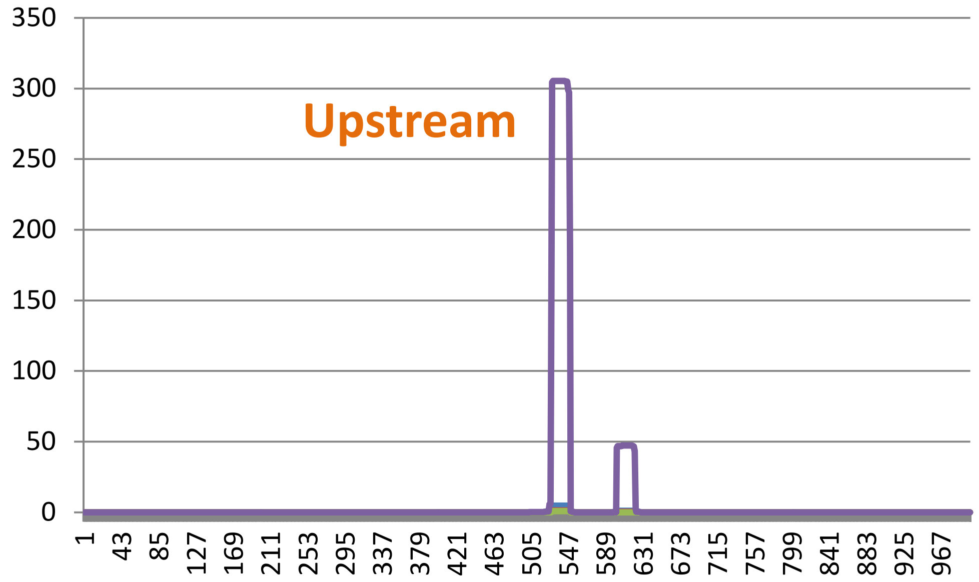

# AT5G15270

## RNA-binding KH domain-containing protein

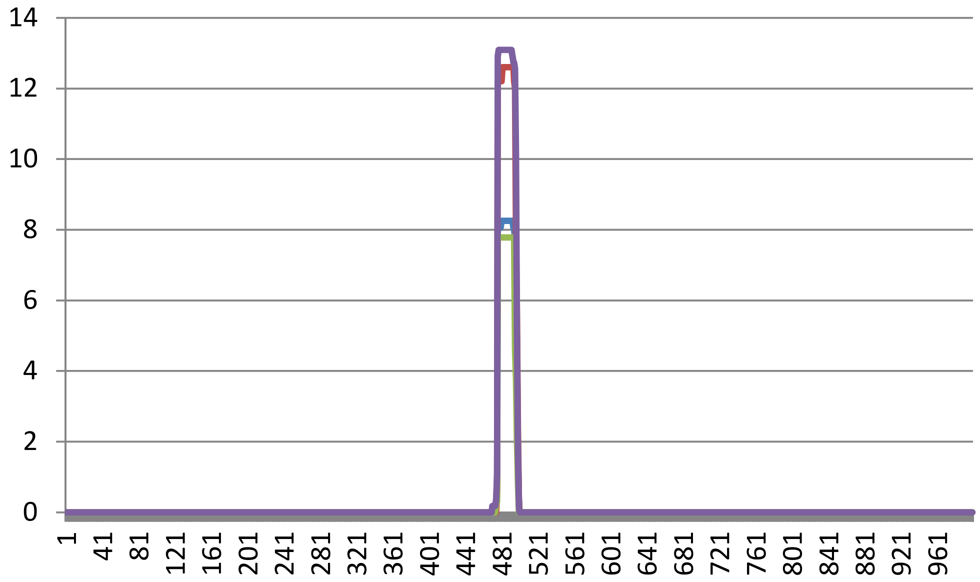

AT5G18570

Encodes AtObgC, a plant ortholog of bacterial Obg. AtObgC is a chloroplast-targeting GTPase essential for early embryogenesis. Mutations in this locus result in embryo lethality. The protein is dually localized in the stroma and the inner envelope membrane and is involved in thylakoid membrane biogenesis and functions primarily in plastid ribosome biogenesis during chloroplast development.

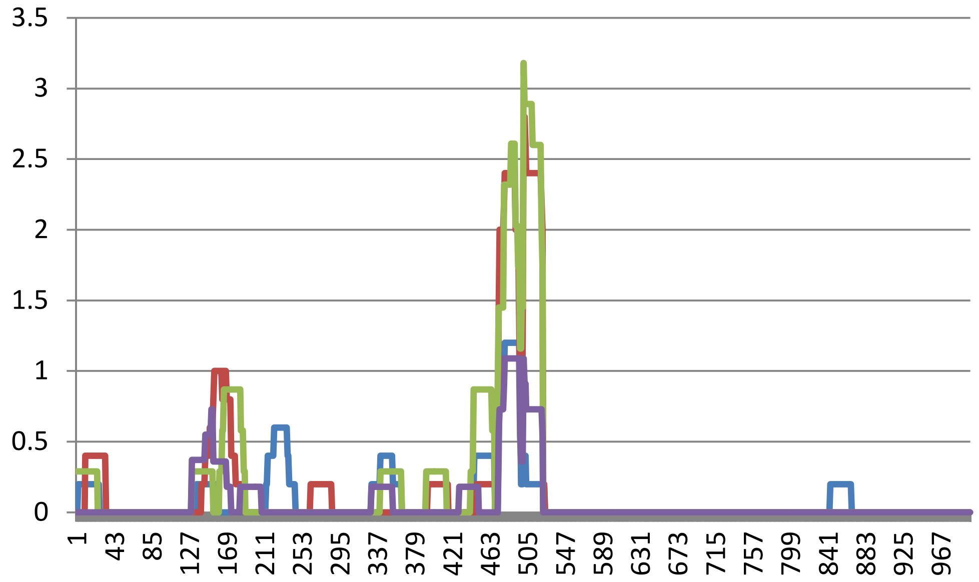

# AT5G18610

## Protein kinase superfamily protein

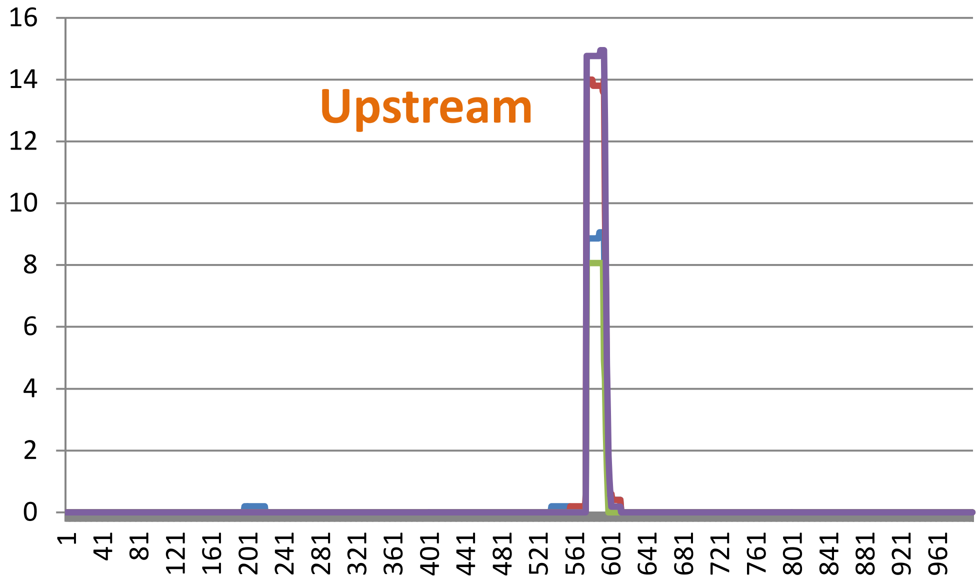

# AT5G22170

## Unknown protein

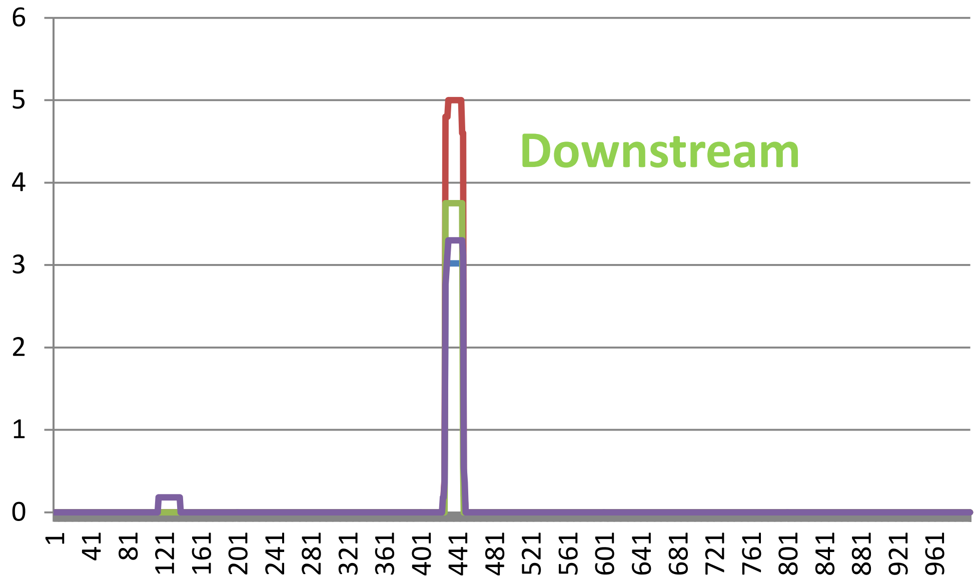

AT5G22320

Leucine-rich repeat (LRR) family protein

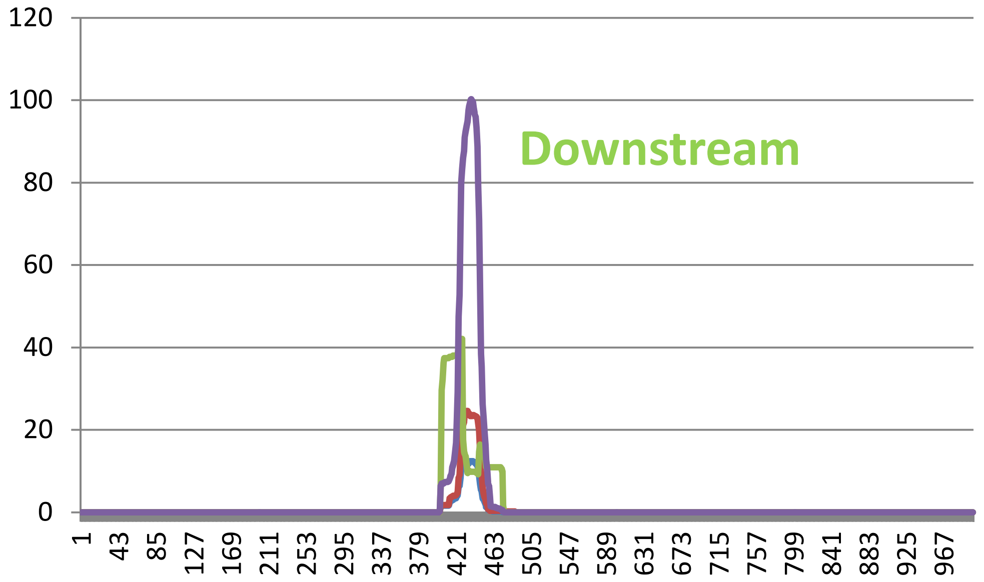

AT5G23380

Protein of unknown function (DUF789)

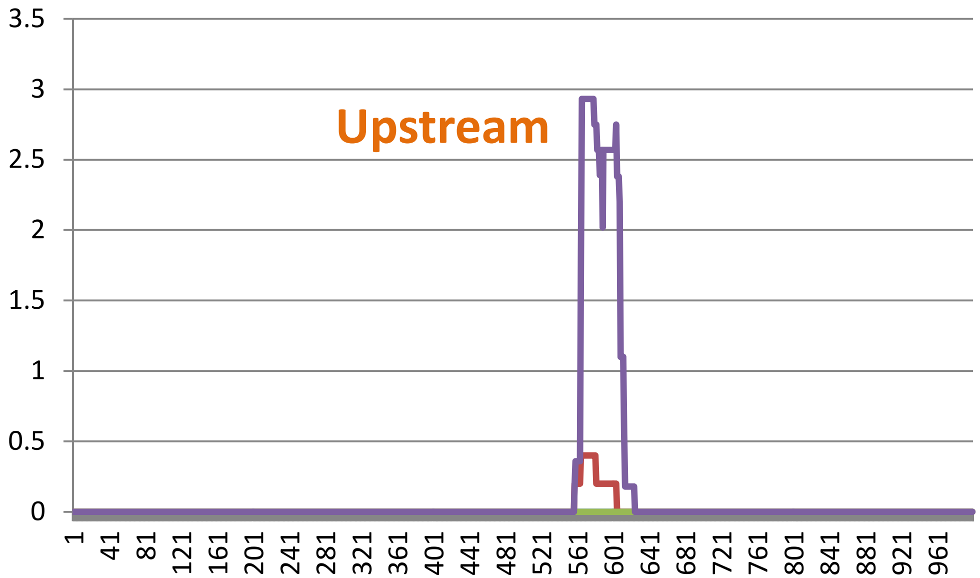

AT5G24290

Vacuolar iron transporter (VIT) family protein

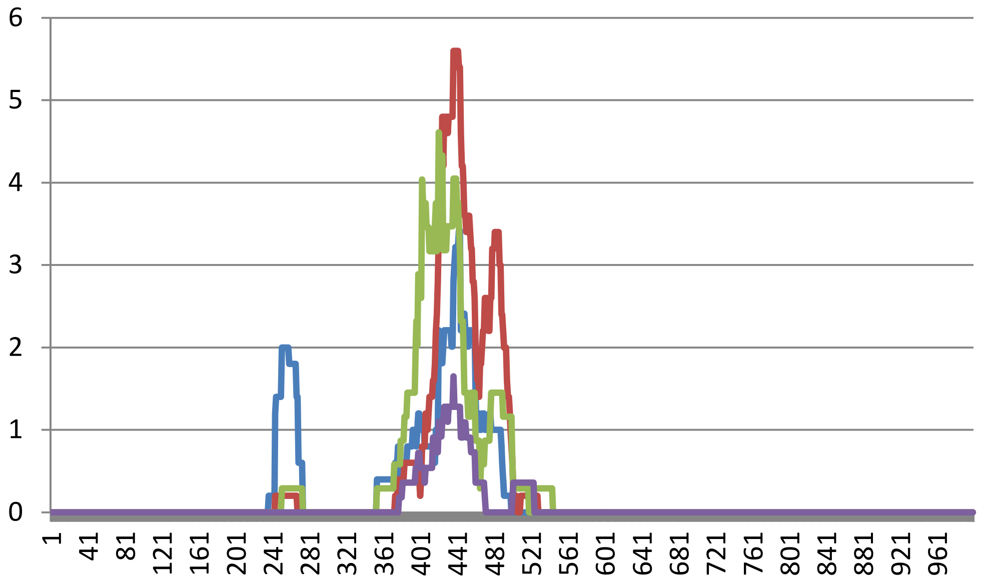

# AT5G24593

## Unknown protein

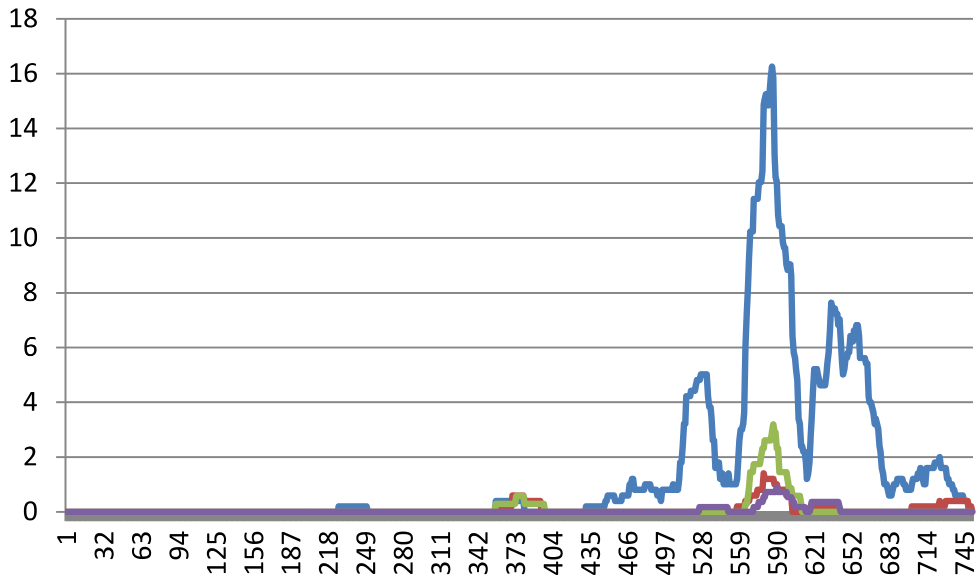

# AT5G26270

## Unknown protein

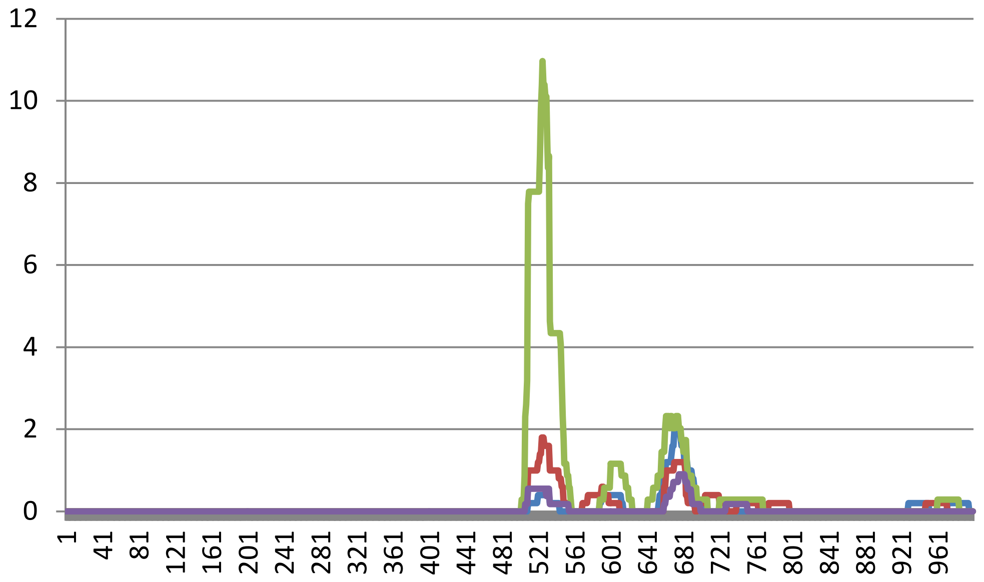

AT5G26673

Encodes a Plant thionin family protein

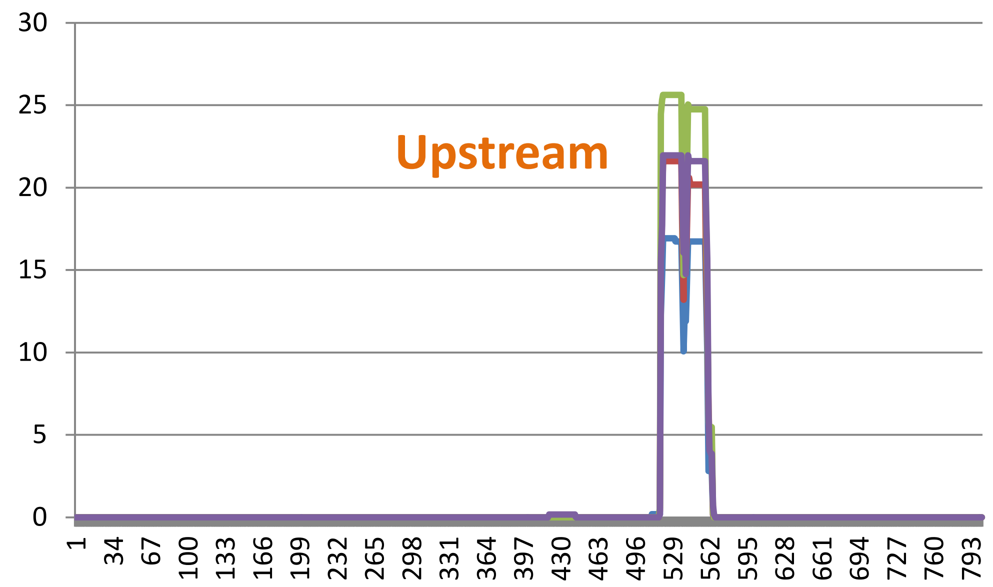

AT5G27660

Trypsin family protein with PDZ domain

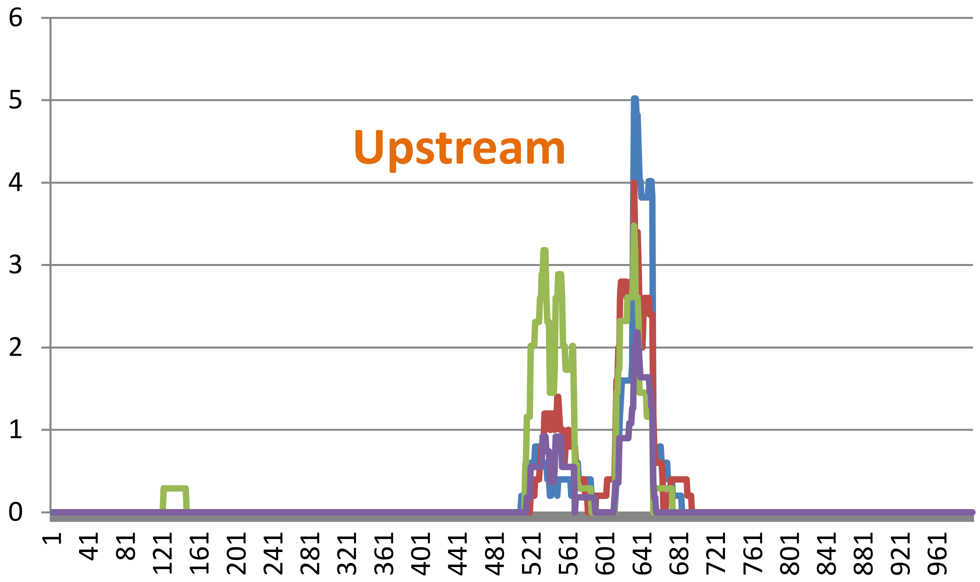

# AT5G27710

## Unknown protein

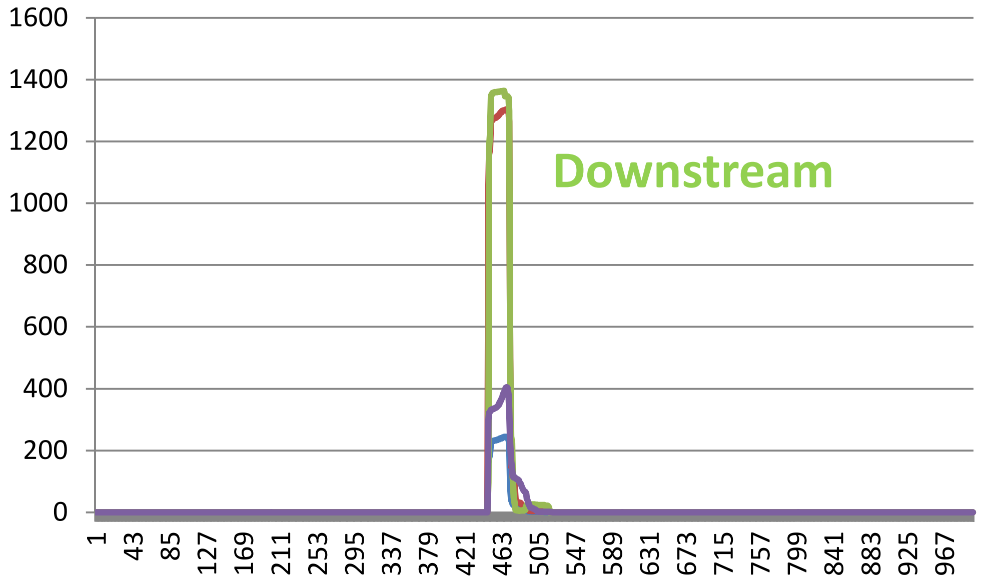

AT5G28442

BEST Arabidopsis thaliana protein match is: Quinoprotein amine dehydrogenase, beta chain-like; RIC1-like guanyl-nucleotide exchange factor (TAIR:AT5G28350.1).

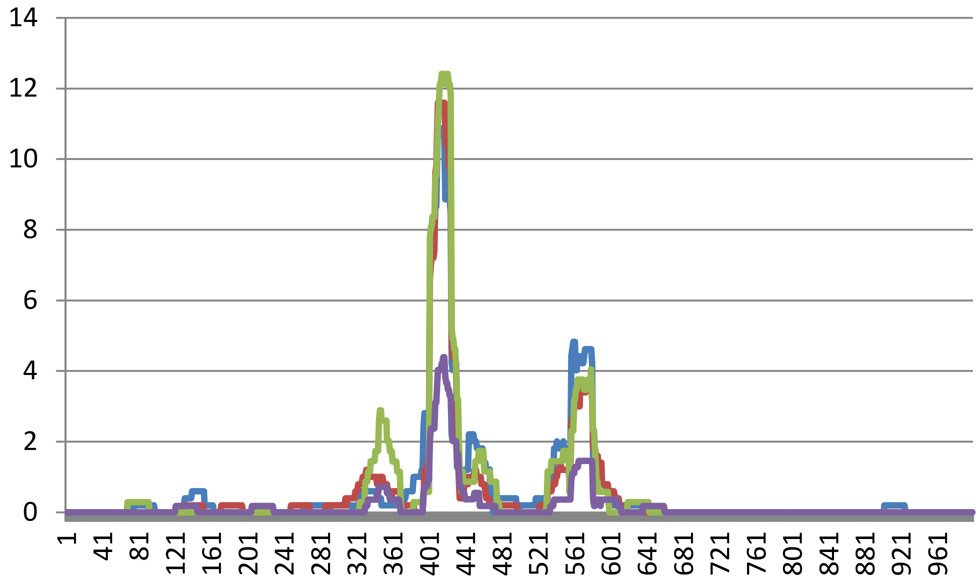

# AT5G29000

## Homeodomain-like superfamily protein

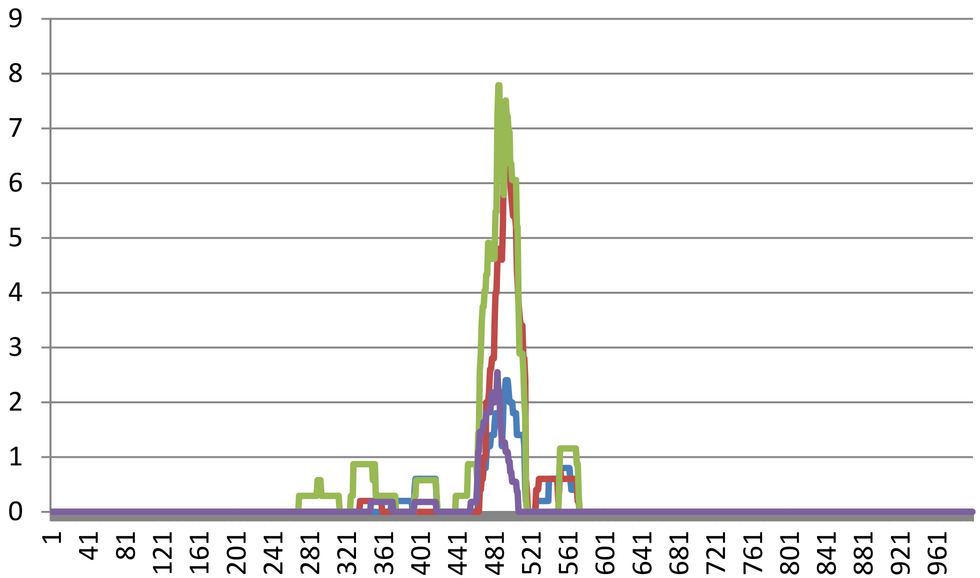

AT5G34882

Encodes a ECA1 gametogenesis related family protein

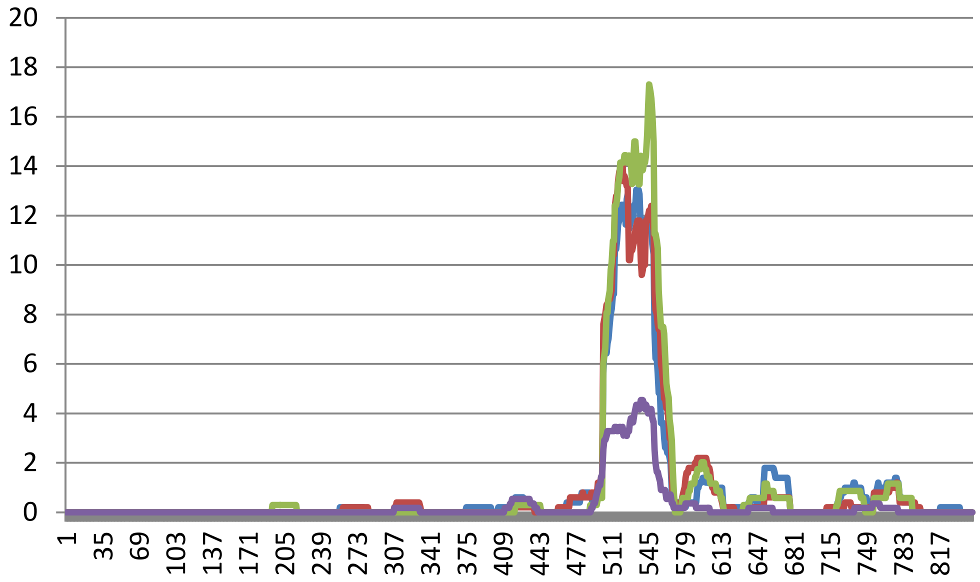

AT5G34883

Protein of unknown function (DUF784)

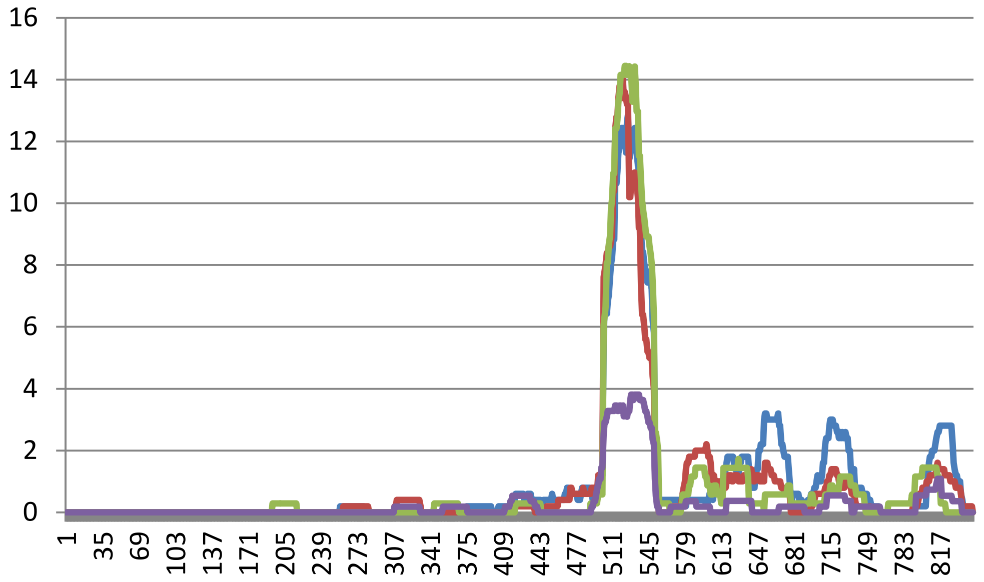

AT5G35526

This gene encodes a small protein and has either evidence of transcription or purifying selection.

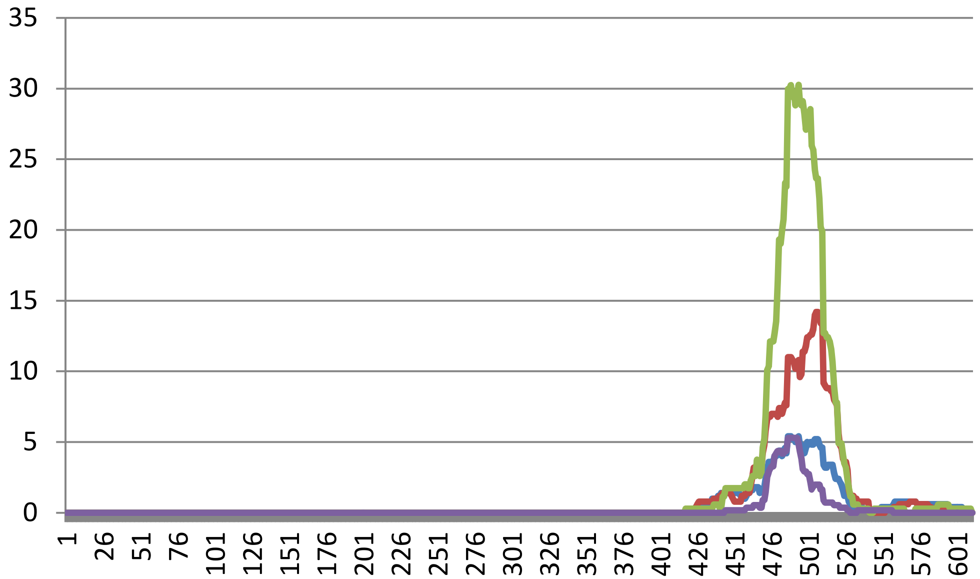

AT5G36140

Member of CYP716A

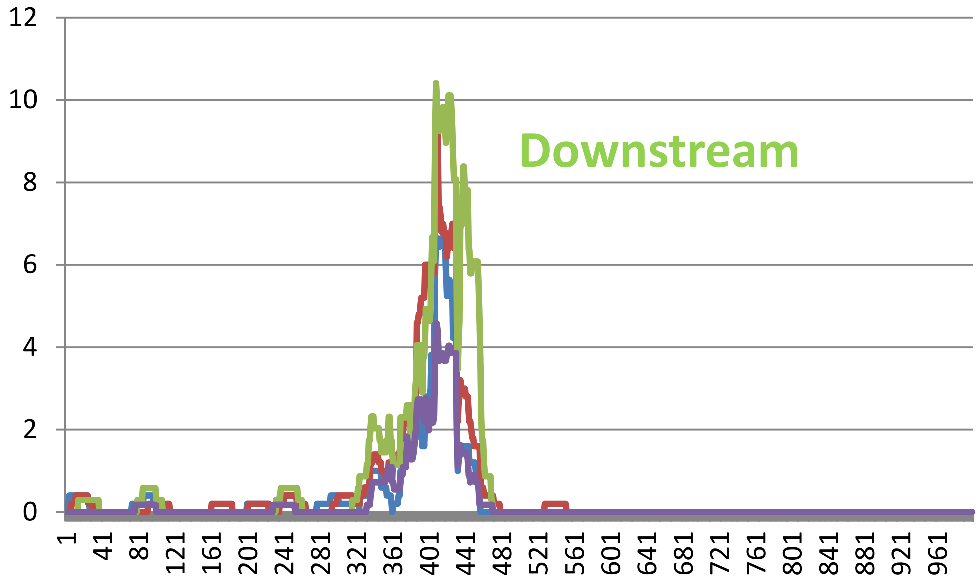

AT5G37420

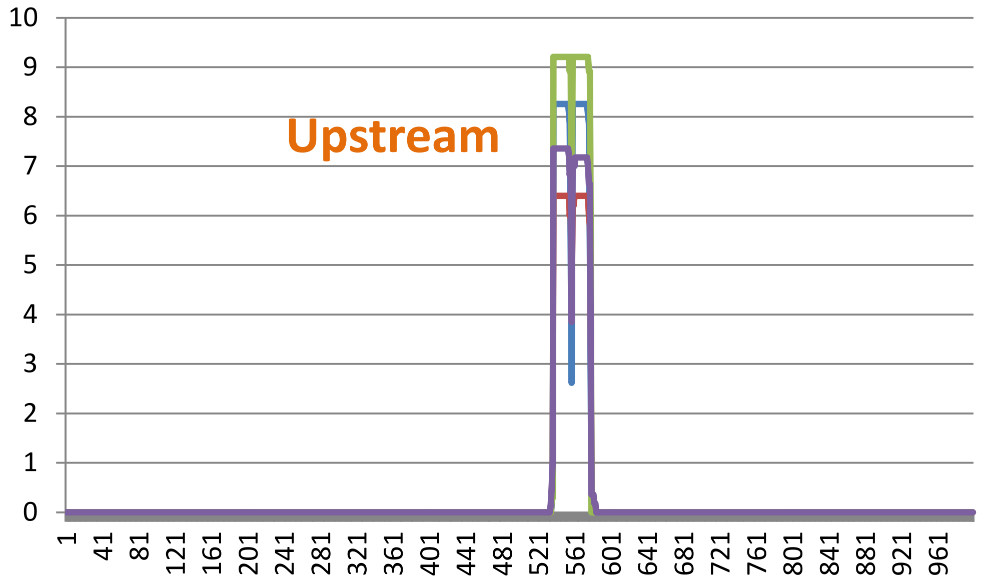

10
9
8
7
6
5
4
3
2
1
0
Upstream
1
41
81
121
161
201
241
281
321
361
401
441
481
521
561
601
641
681
721
761
801
841
881
921
961

# AT5G37430

## Family of unknown function (DUF577)

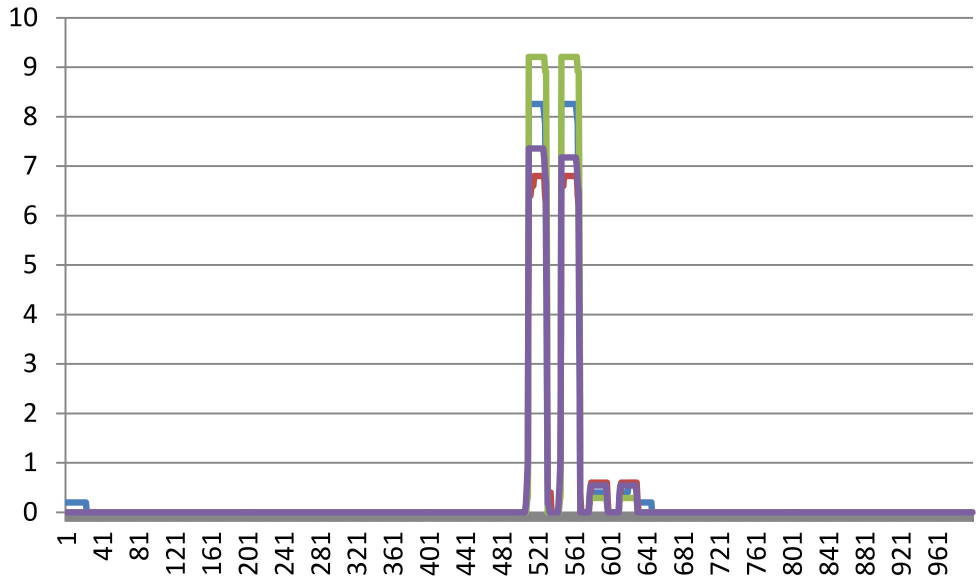

AT5G37690

SGNH hydrolase-type esterase superfamily protein

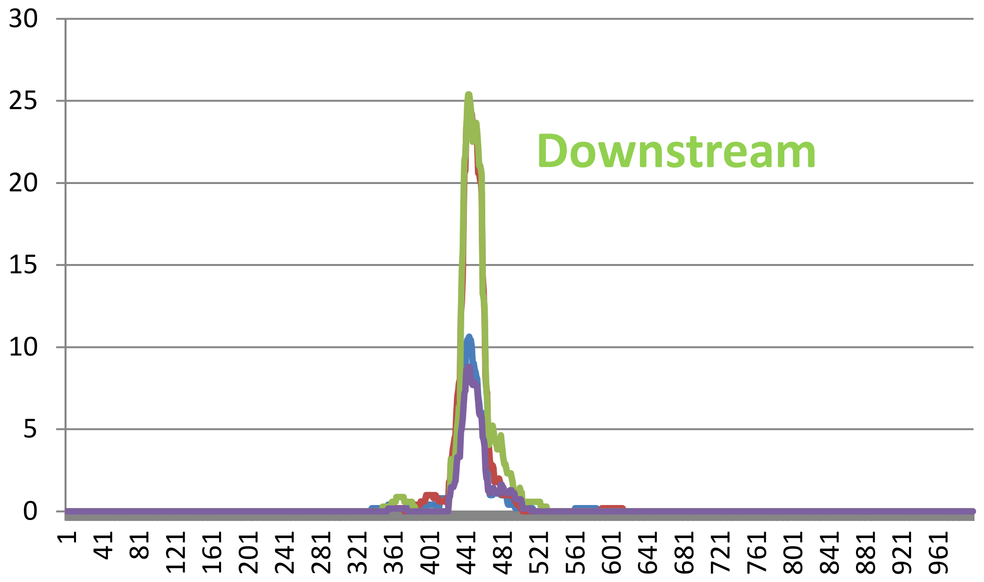

AT5G37980

Zinc-binding dehydrogenase family protein

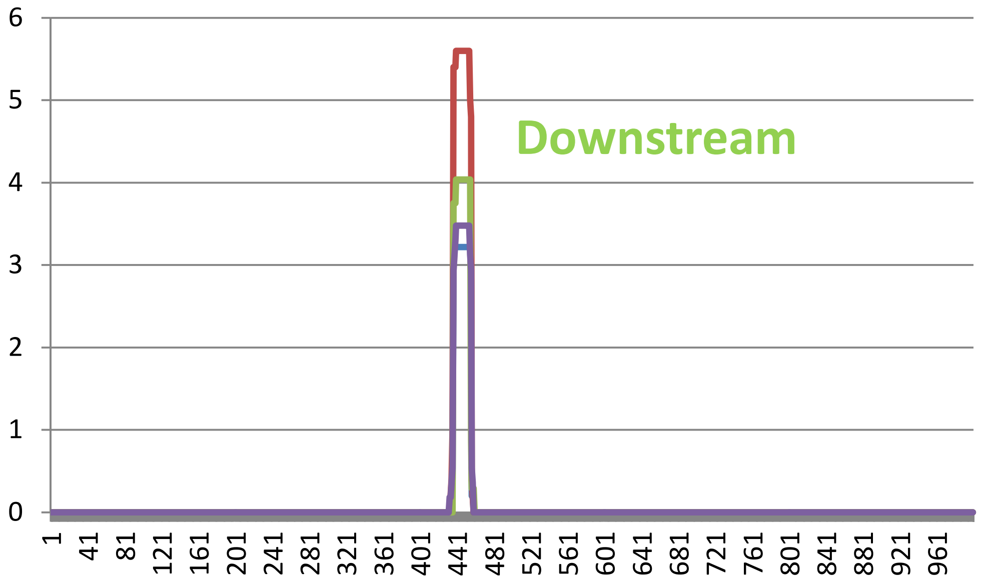

AT5G39080

HXXXD-type acyl-transferase family protein

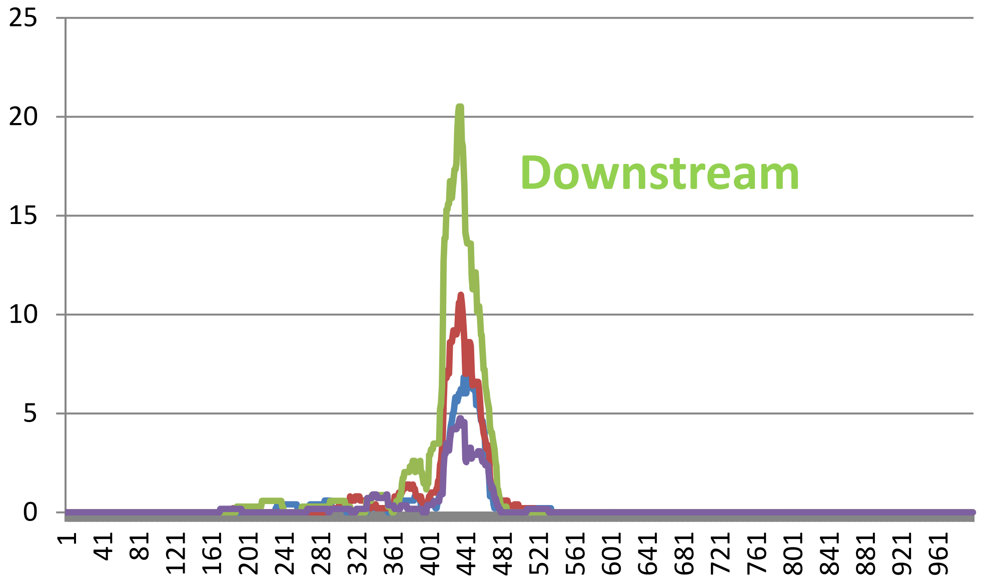

AT5G39720

Avirulence induced gene 2 like protein (AIG2L)

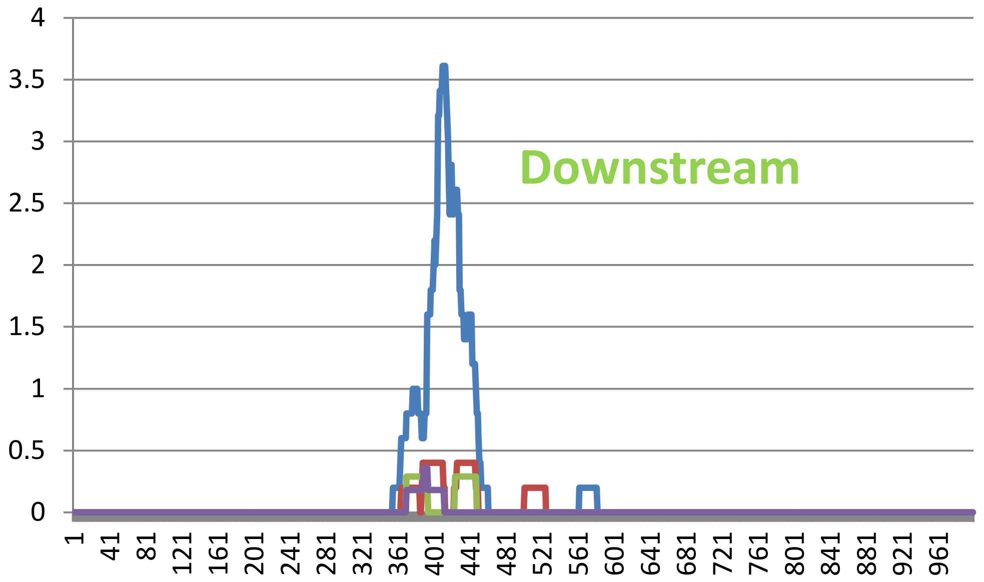

# AT5G40540

## Protein kinase superfamily protein

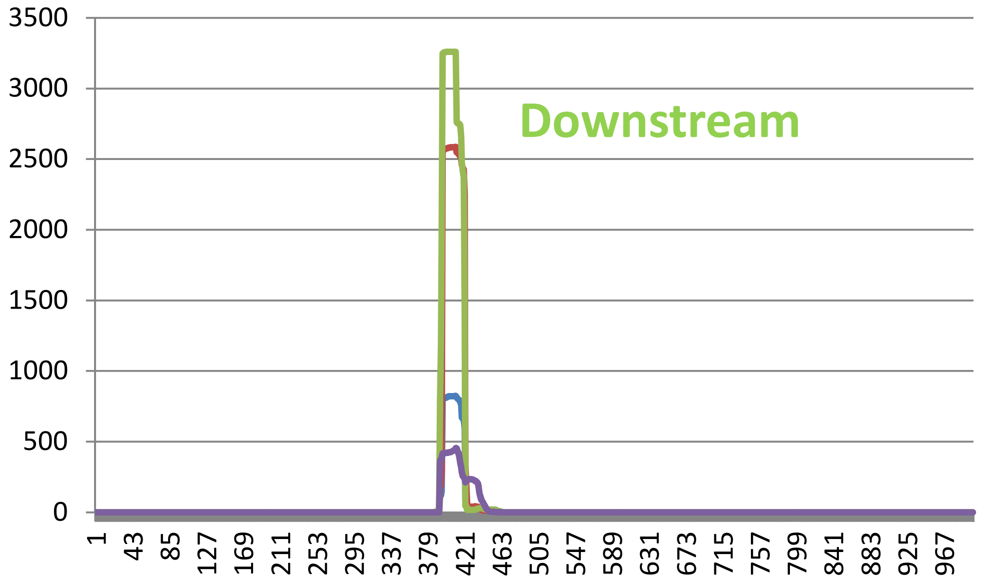

# AT5G41140

## Myosin heavy chain-related protein

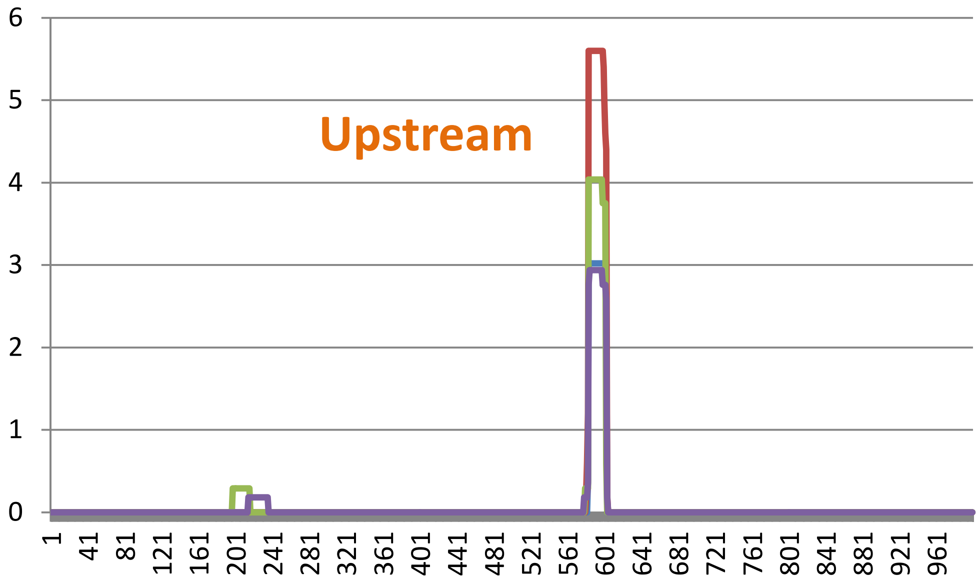

AT5G41900

Alpha/beta-Hydrolases superfamily protein

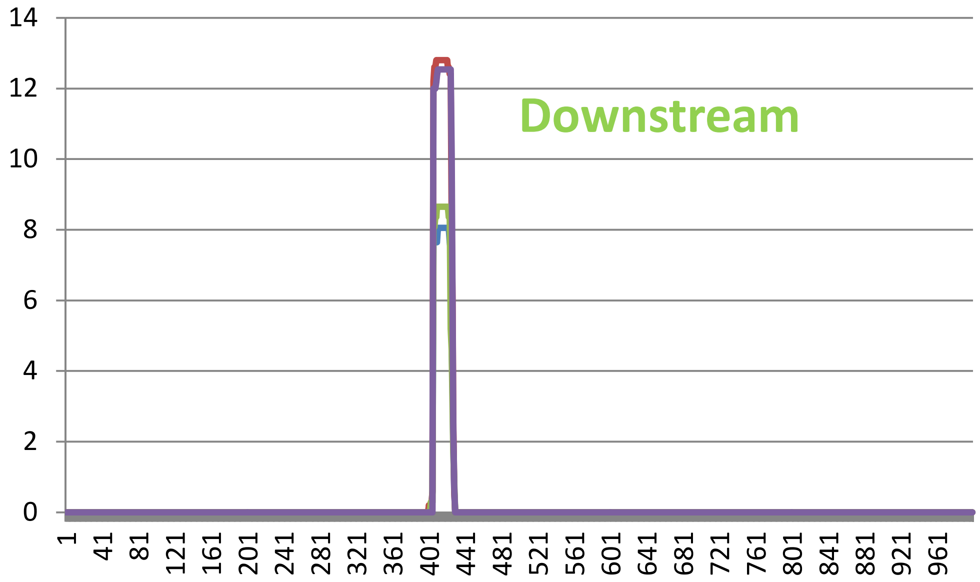

AT5G42100

Encodes a plasmodesmal (Pd)-associated membrane protein involved in plasmodesmal callose degradation

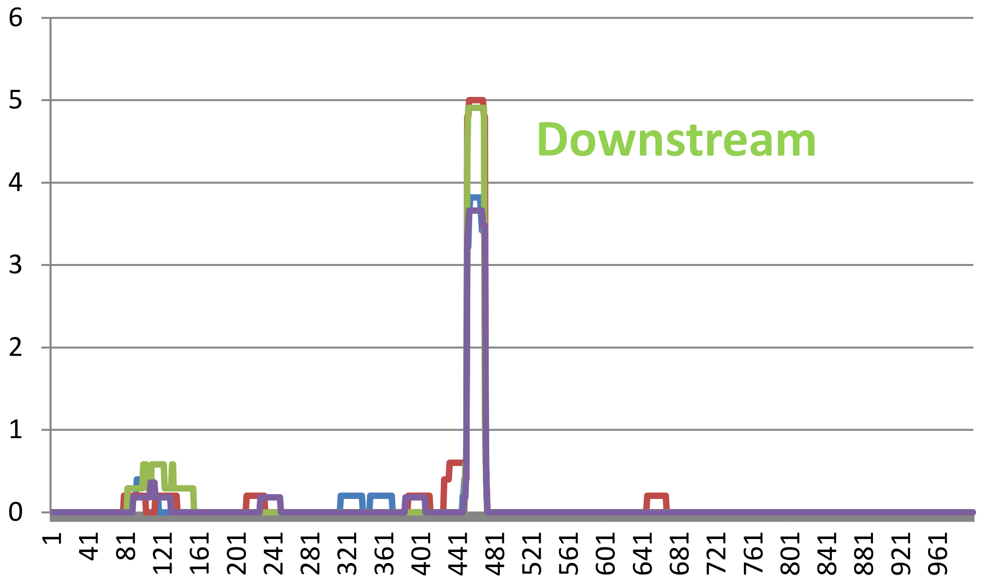

AT5G42203

This gene encodes a small protein and has either evidence of transcription or purifying selection.

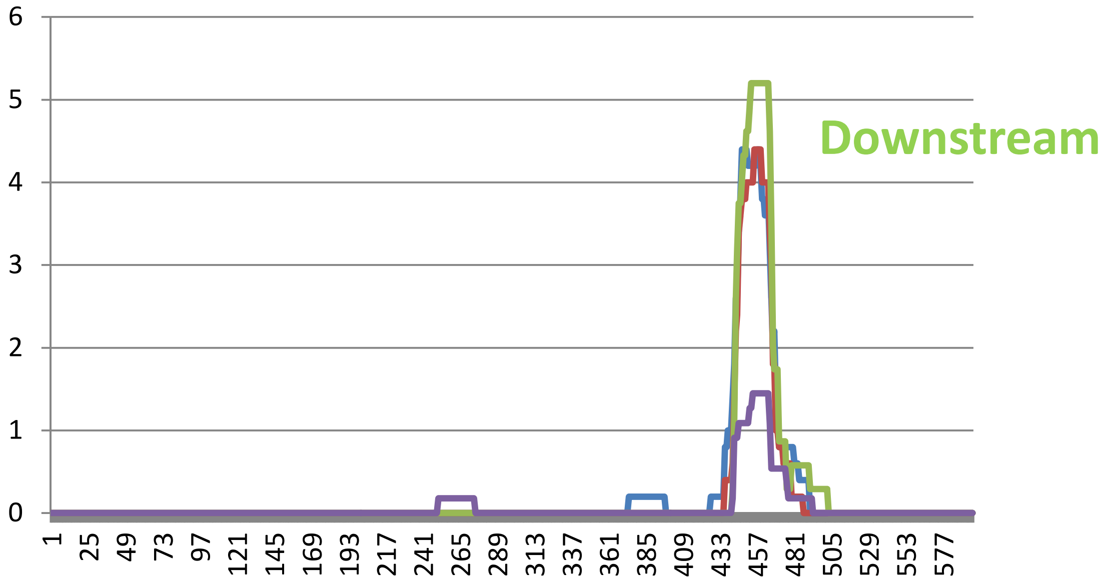

AT5G42567

Encodes a ECA1 gametogenesis related family protein

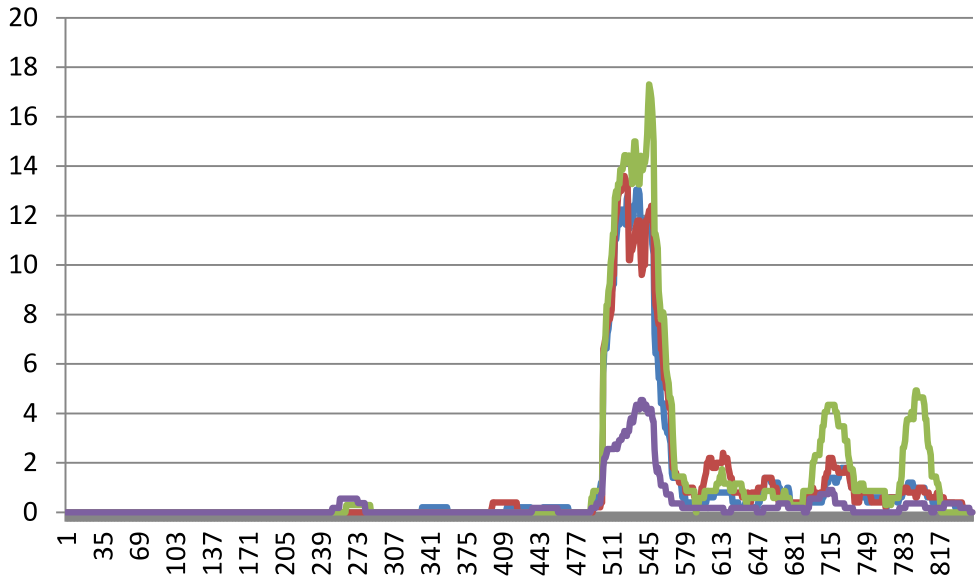

# AT5G42635

## Glycine-rich protein

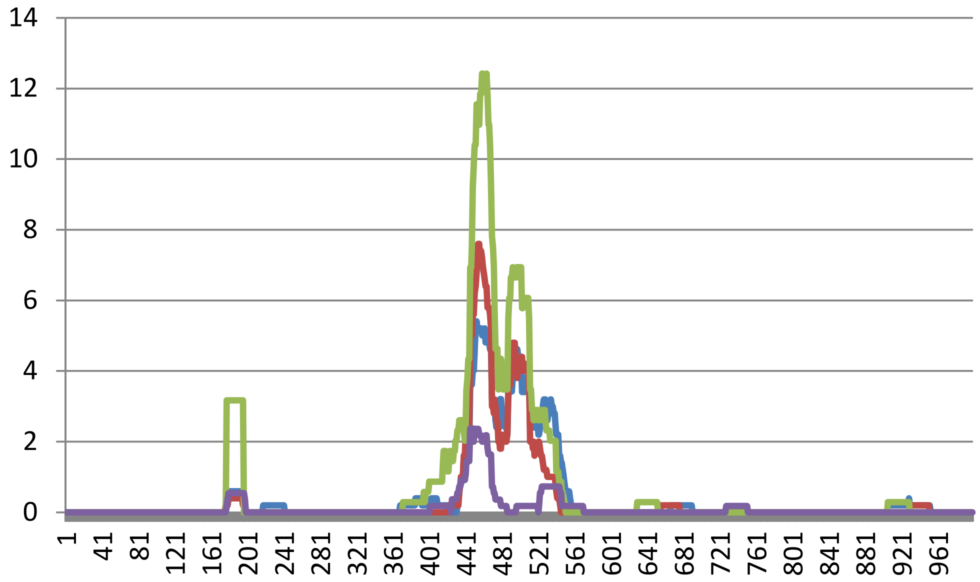

AT5G42930

Alpha/beta-Hydrolases superfamily protein

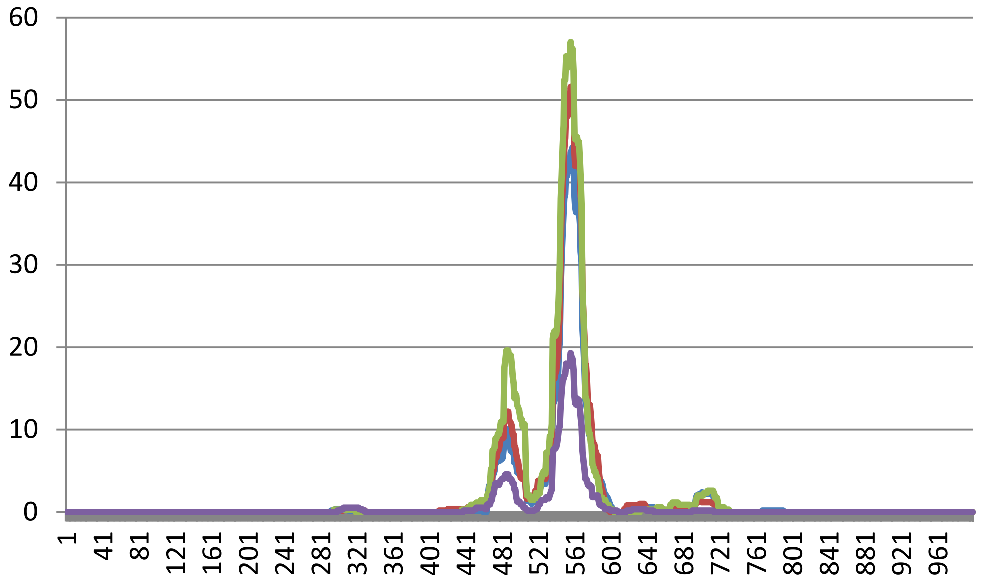

AT5G43513

Encodes a defensin-like (DEFL) family protein.

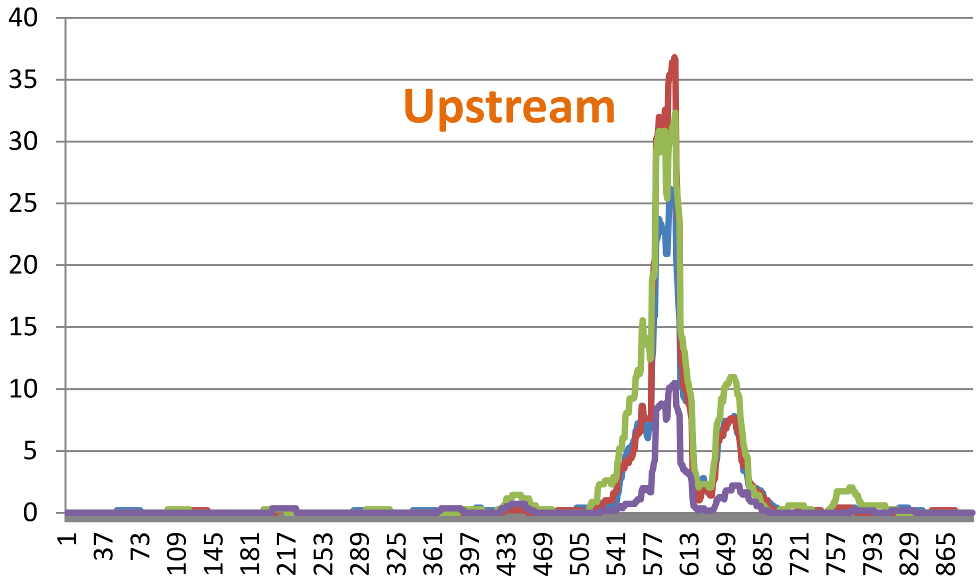

# AT5G43525

Encodes a defensin-like (DEFL) family protein.

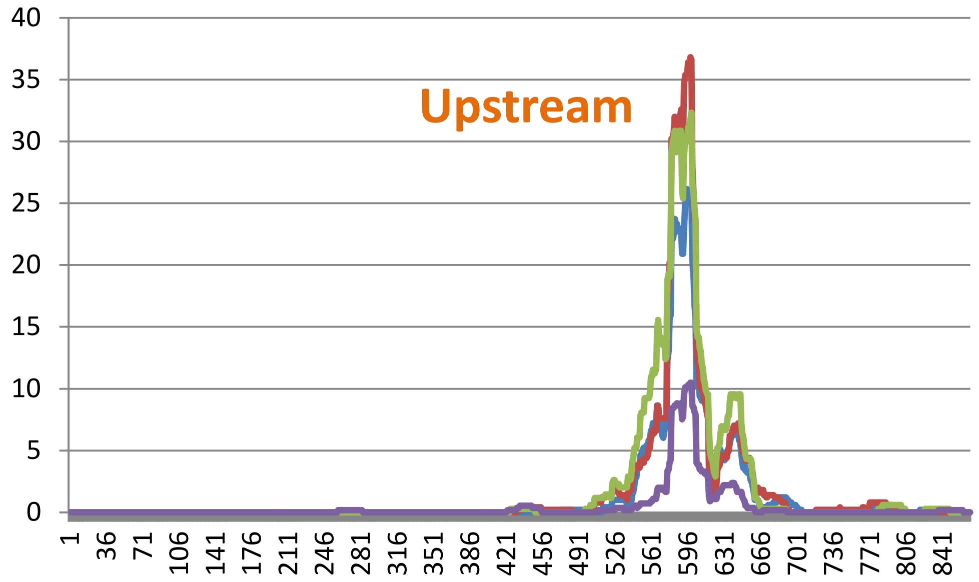

AT5G43610

Sucrose-proton symporter 6 (SUC6)

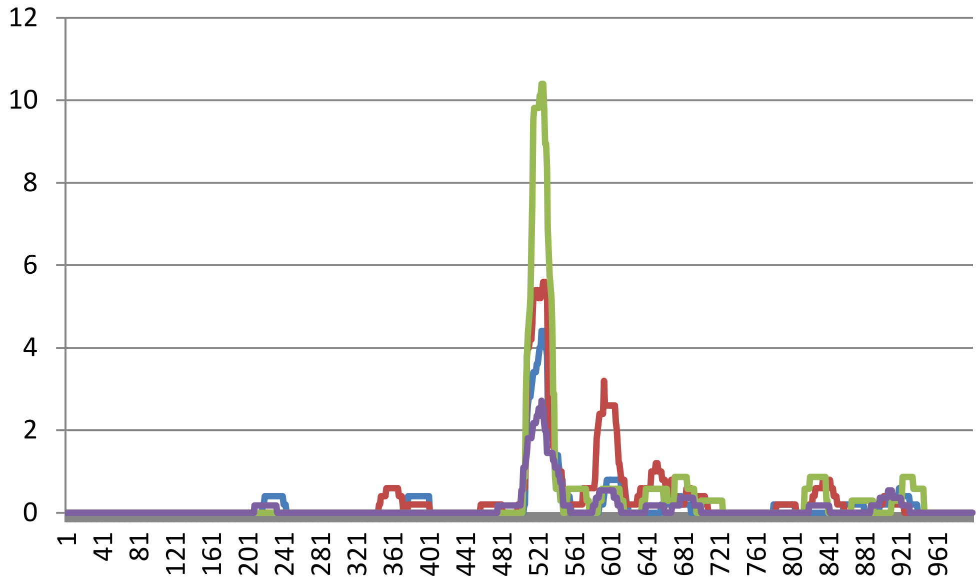

AT5G43755

BEST Arabidopsis thaliana protein match is: Polynucleotidyl transferase, ribonuclease H-like superfamily protein (TAIR:AT2G04420.1).

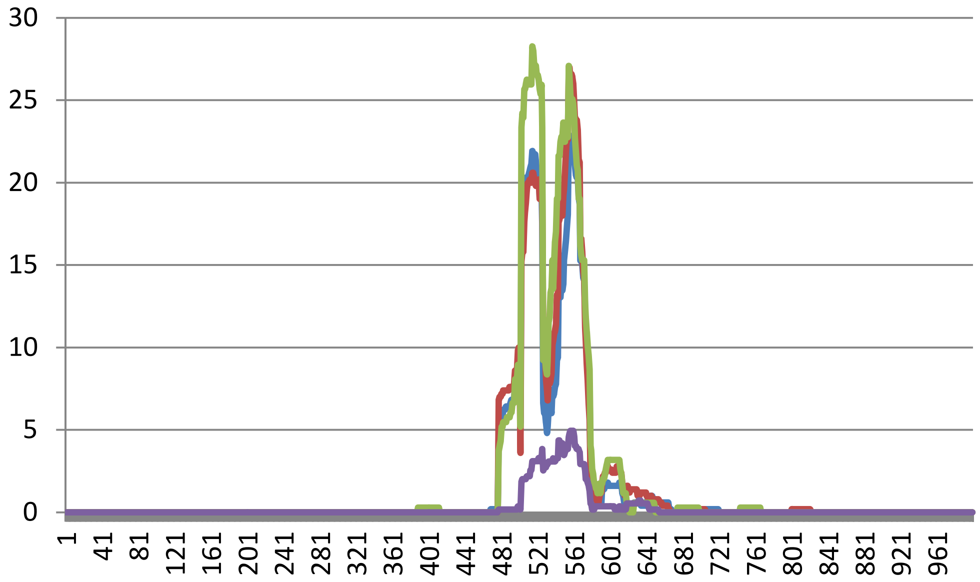

# AT5G48515

Encodes a defensin-like (DEFL) family protein.

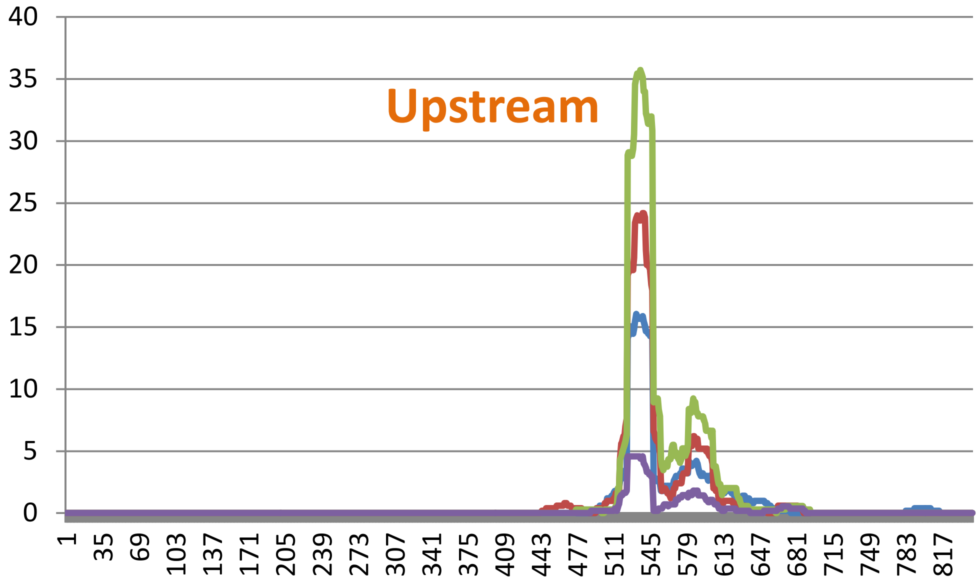

AT5G48595

Encodes a defensin-like (DEFL) family protein.

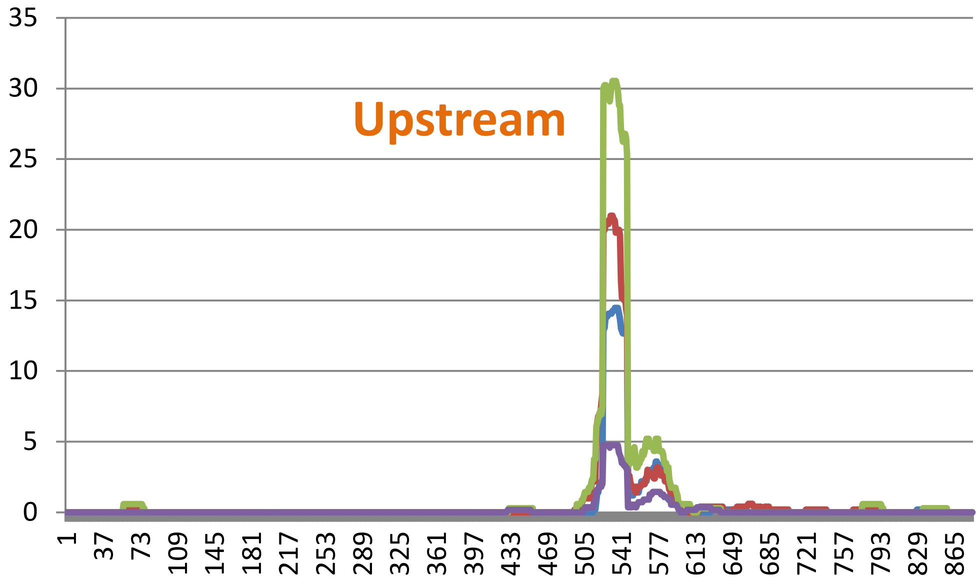

# AT5G48605

Encodes a defensin-like (DEFL) family protein.

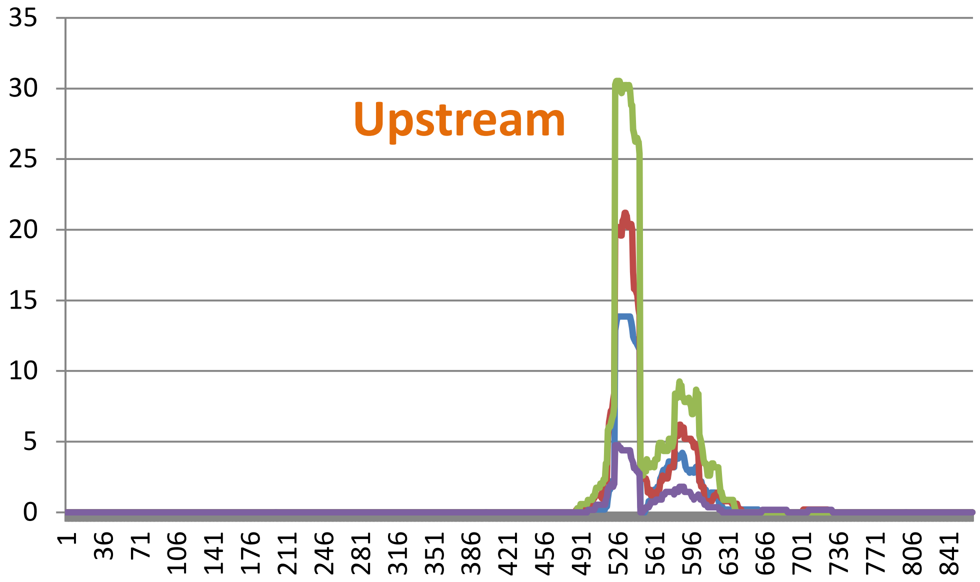

AT5G49420

MADS-box transcription factor family protein

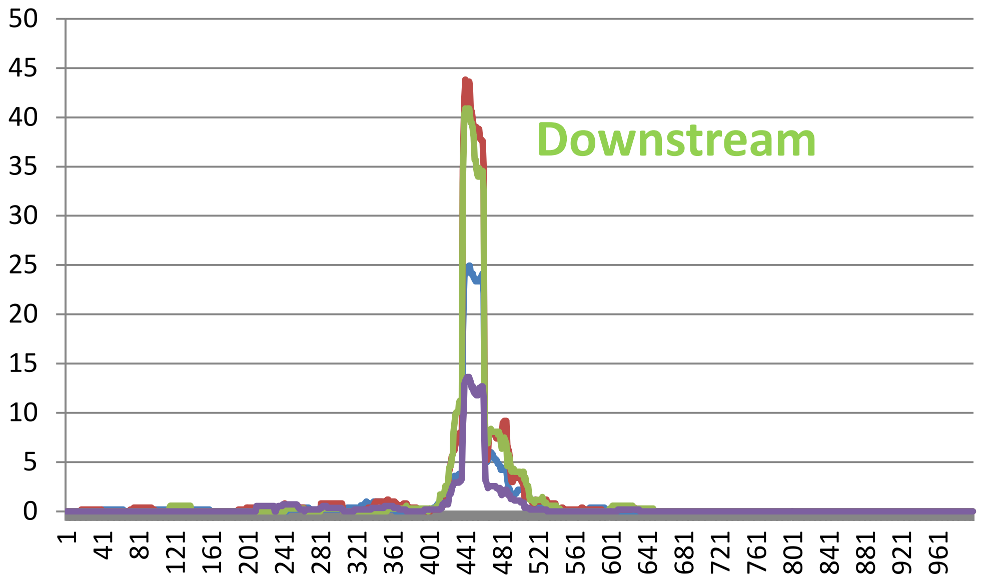

AT5G49440

Unknown protein

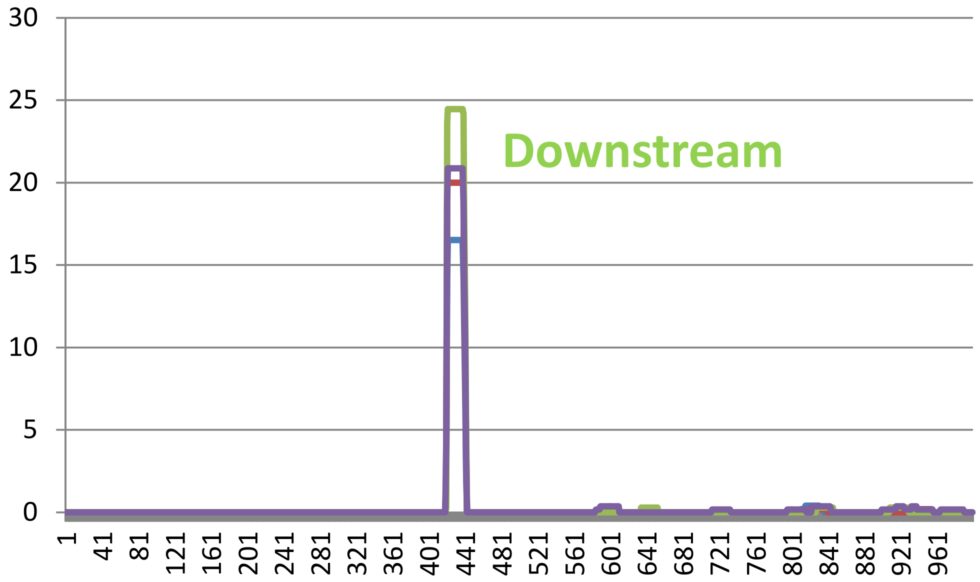

# AT5G49900

## Beta-glucosidase, GBA2 type family protein

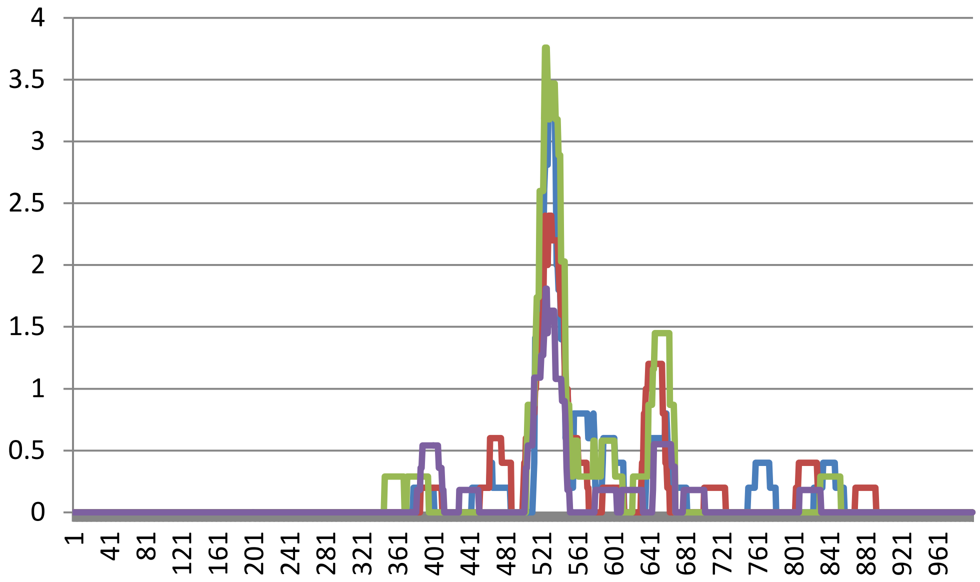

# AT5G50480

## Nuclear factor Y, subunit C6 (NF-YC6)

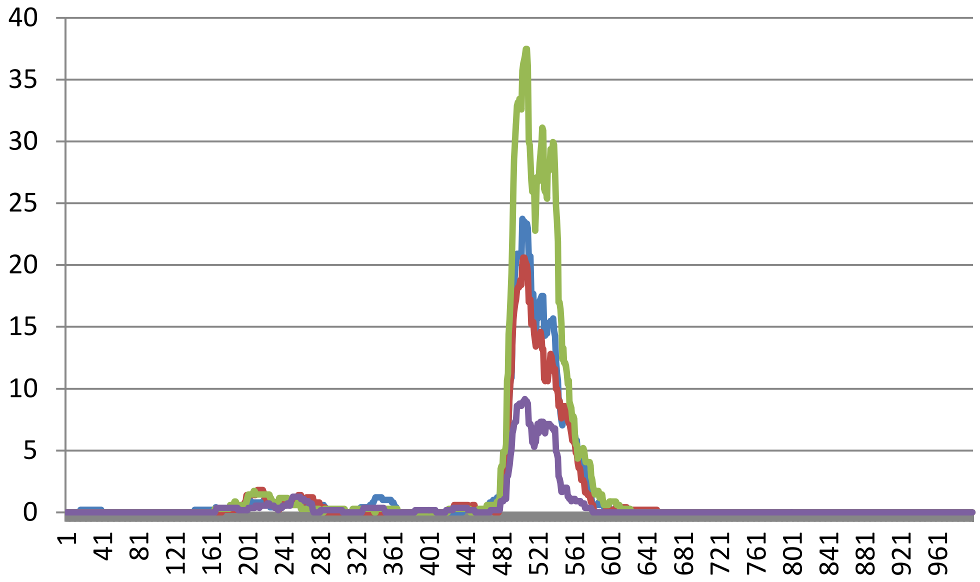

# AT5G50880

## Unknown protein

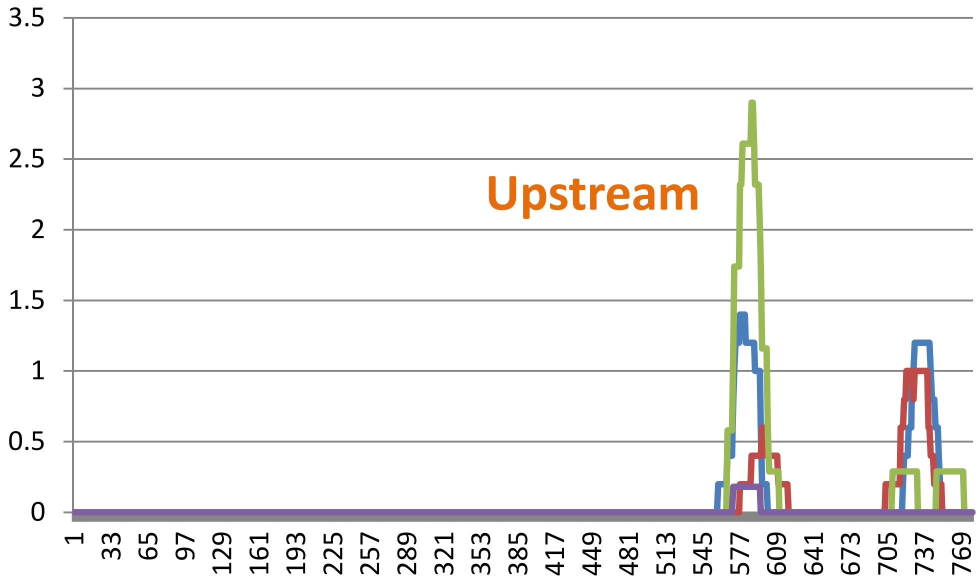

# AT5G53742

Encodes a ECA1 gametogenesis related family protein.

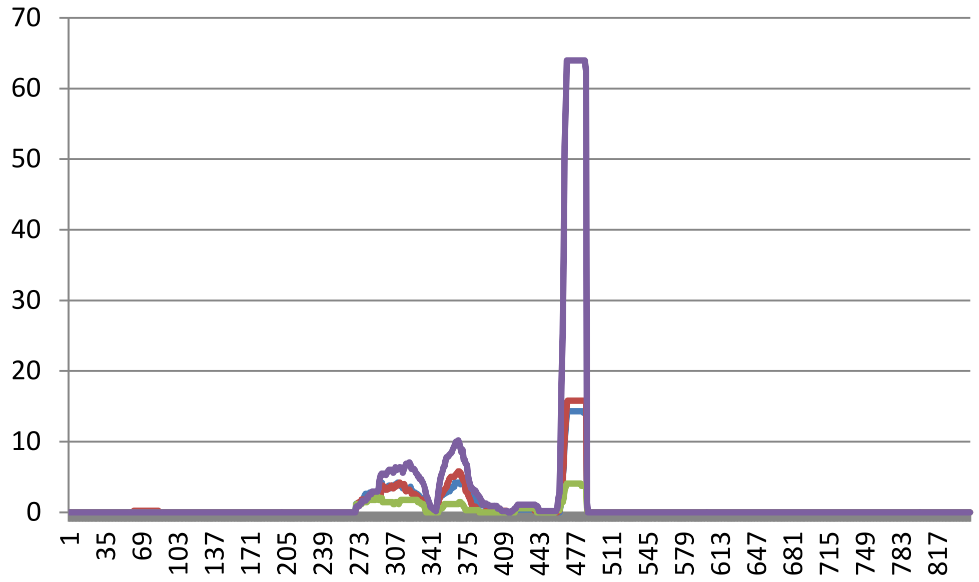

# AT5G54410

## Unknown protein

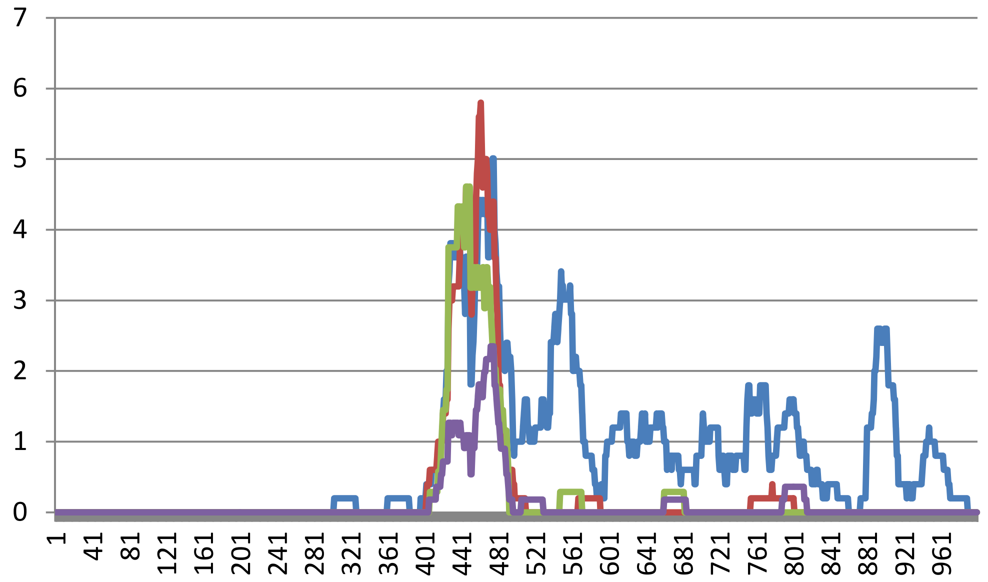

AT5G54700

Ankyrin repeat family protein

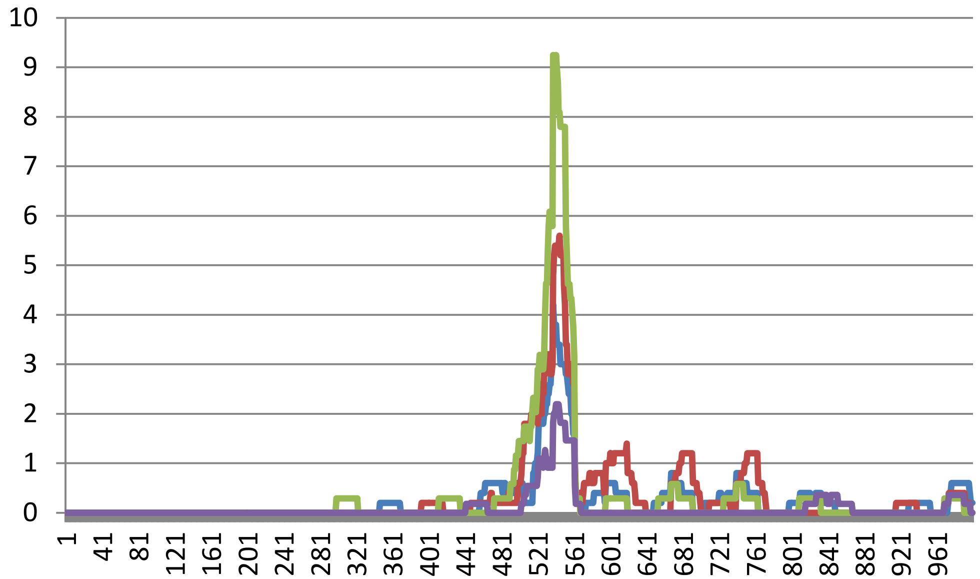

# AT5G58390

## Peroxidase superfamily protein

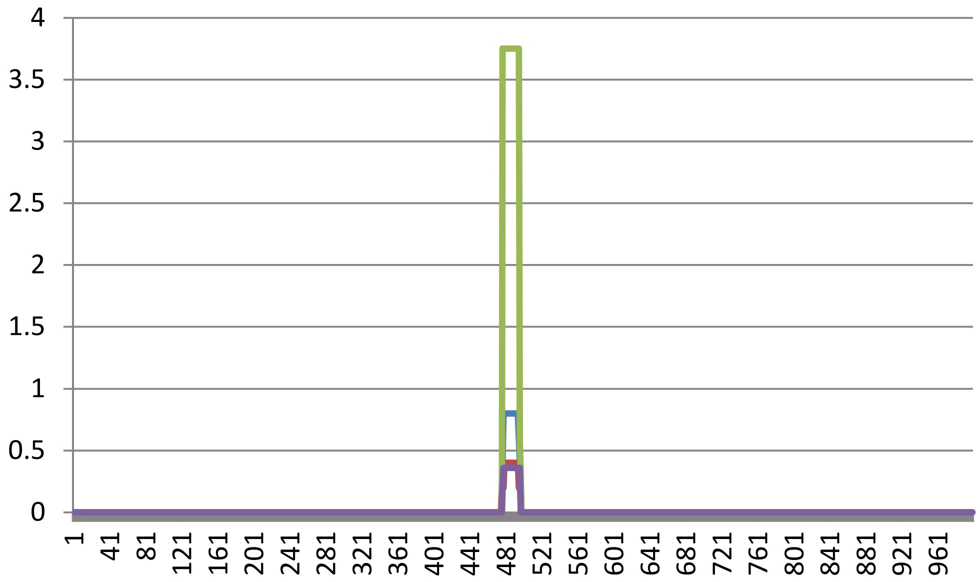

AT5G59060

BEST Arabidopsis thaliana protein match is: RNA-directed DNA polymerase (reverse transcriptase)-related family protein (TAIR:AT5G18880.1).

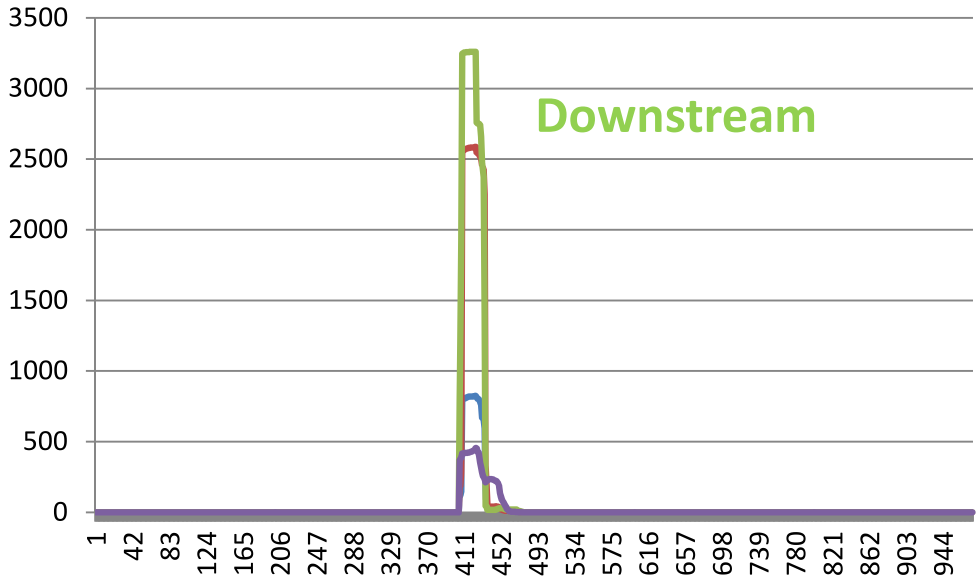

AT5G61520

Major facilitator superfamily protein

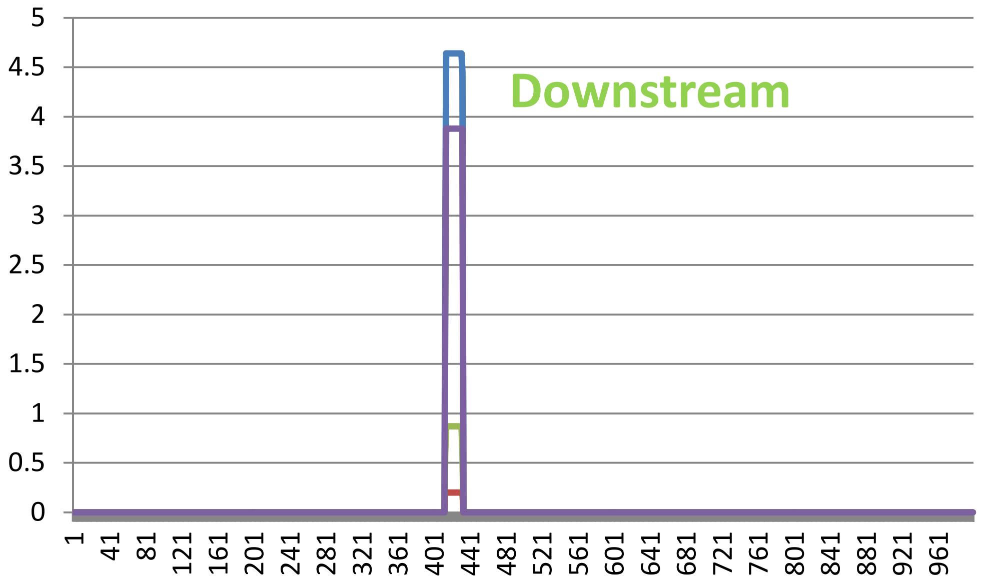

AT5G62280

Protein of unknown function (DUF1442)

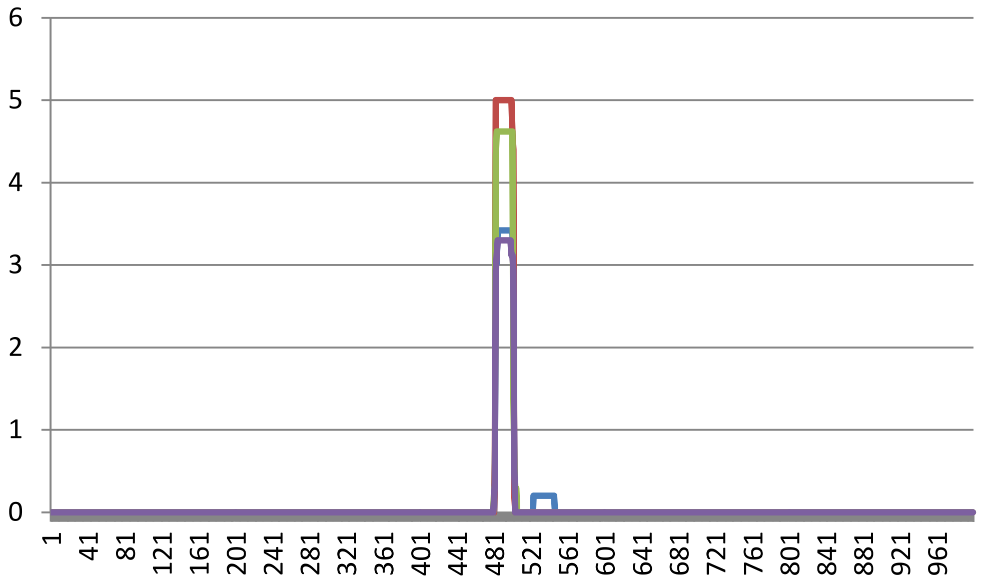

# AT5G64860

Encodes a maltotriose-metabolizing enzyme with chloroplastic glucanotransferase activity. Mutant has altered starch degradation.

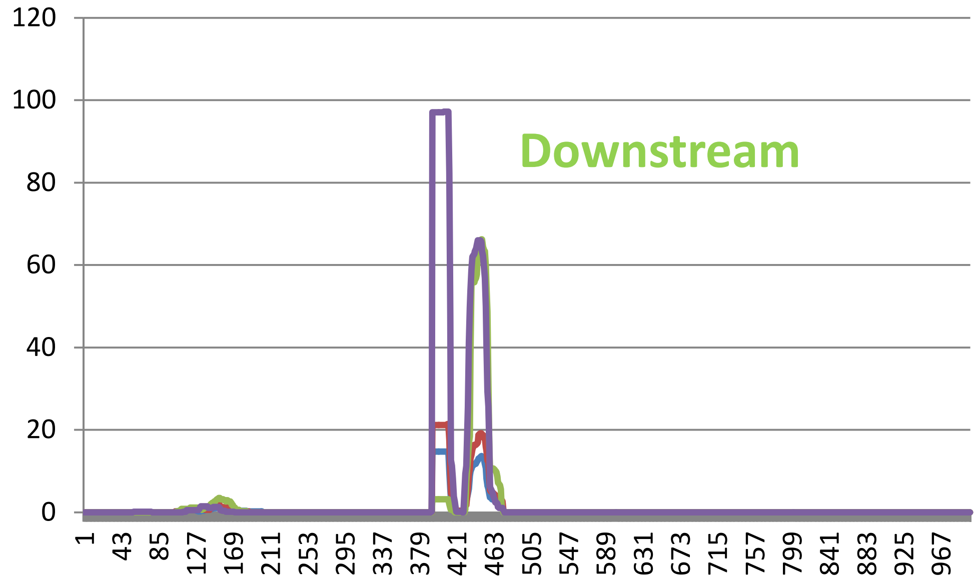

# AT5G65005

Polynucleotidyl transferase, ribonuclease H-like superfamily protein.

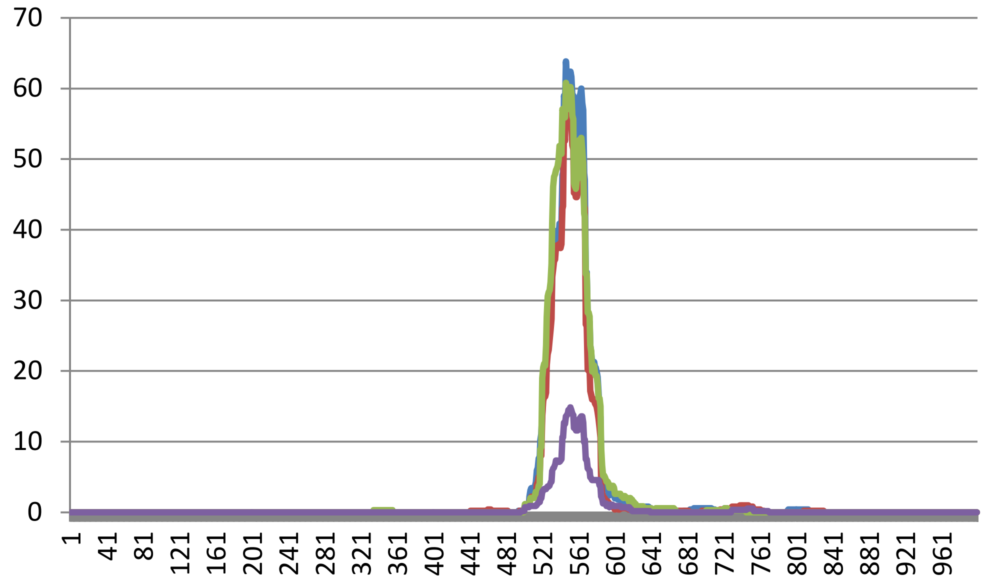

# AT5G67190

Encodes a member of the DREB subfamily A-5 of ERF/AP2 transcription factor family. The protein contains one AP2 domain.

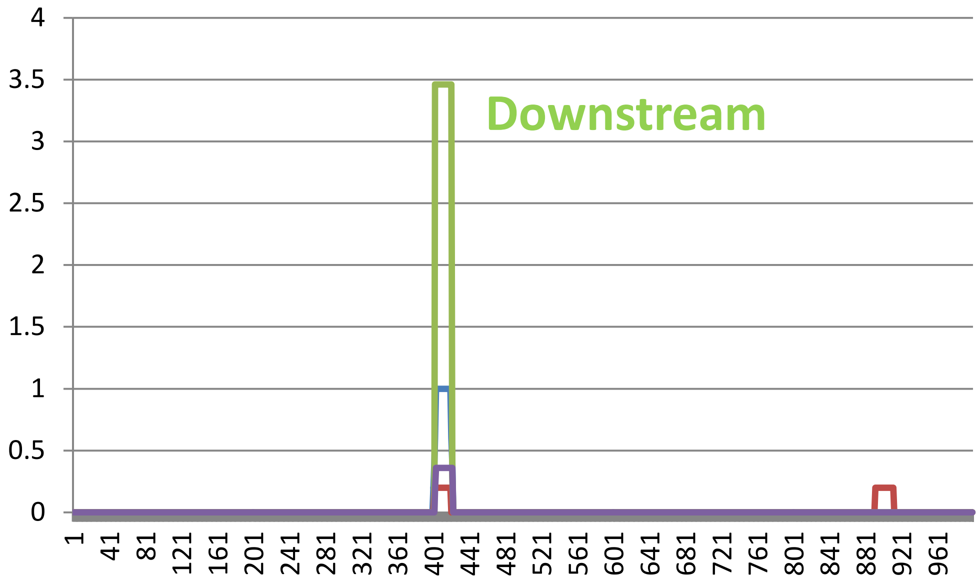

ATMG00810

Hypothetical protein

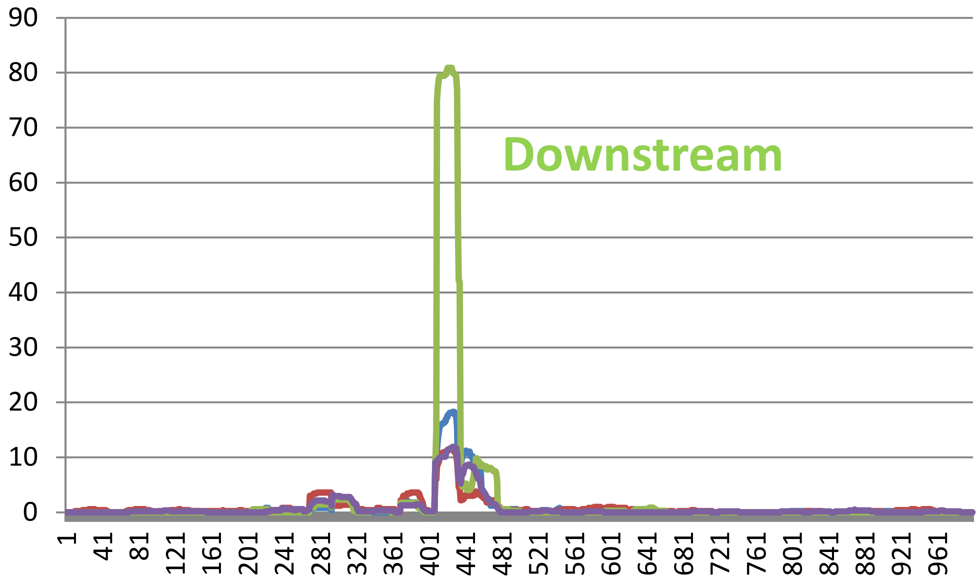

Supplement: S5 Fig — (PDF) [file pone.0169212.s005.pdf]
