# Supplementary material for: Transcriptome-Wide Discovery of PASRs (Promoter-Associated Small RNAs) and TASRs (Terminus-Associated Small RNAs) in Arabidopsis thaliana
Source: PLoS One. 2017 Jan 3;12(1):e0169212. doi: 10.1371/journal.pone.0169212 (PMC5207706; doi:10.1371/journal.pone.0169212)

Figure S6 Paired TASR peaks identified on both strands of the protein-coding genes of *Arabidopsis*. For each plot, x axis measures the position on the paired strands, and y axis measures the abundance (in RPM, reads per million) of sRNAs.

AT1G09026

unknown protein

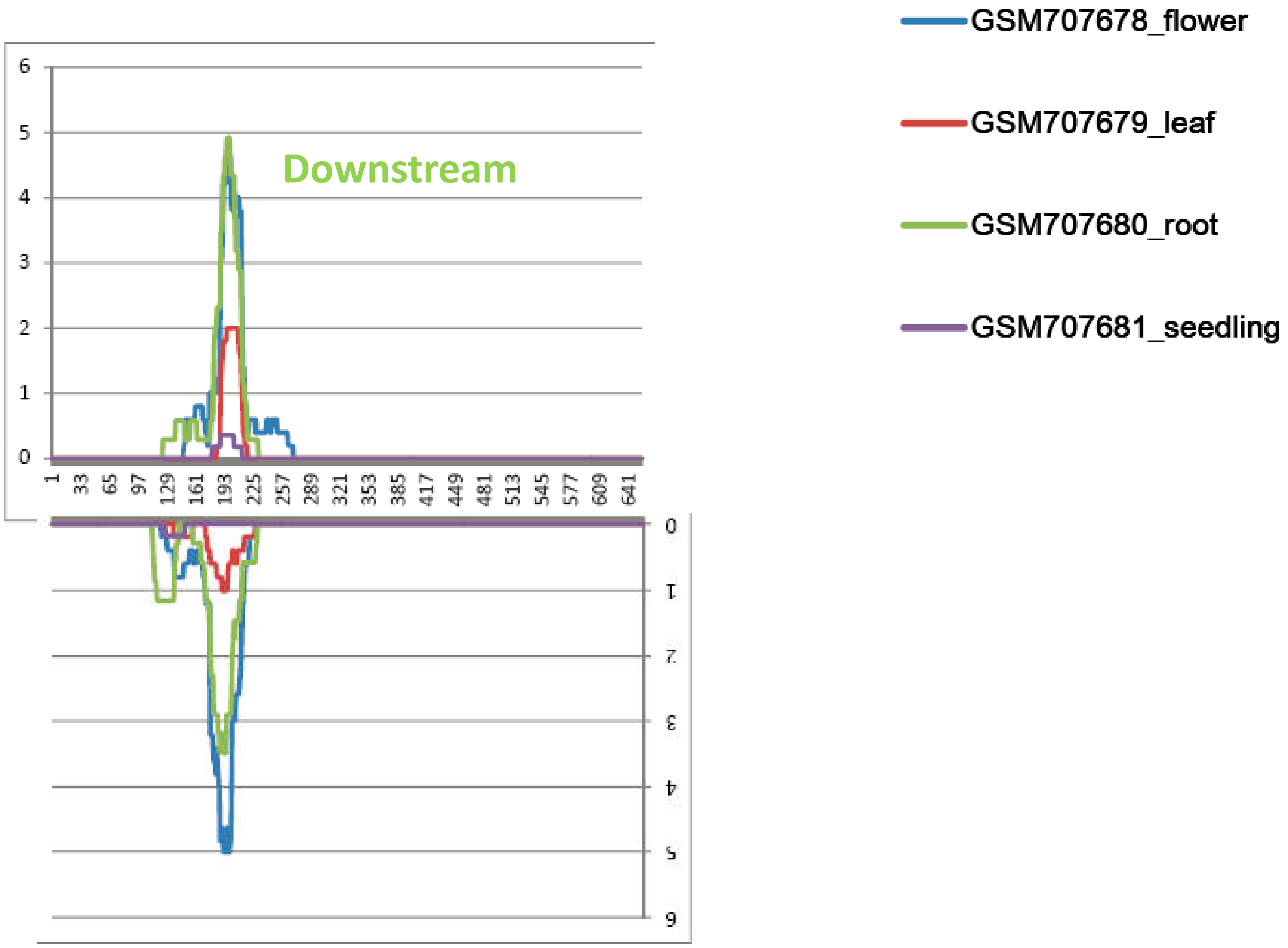

AT1G10745

Encodes a maternally expressed gene (MEG) family protein

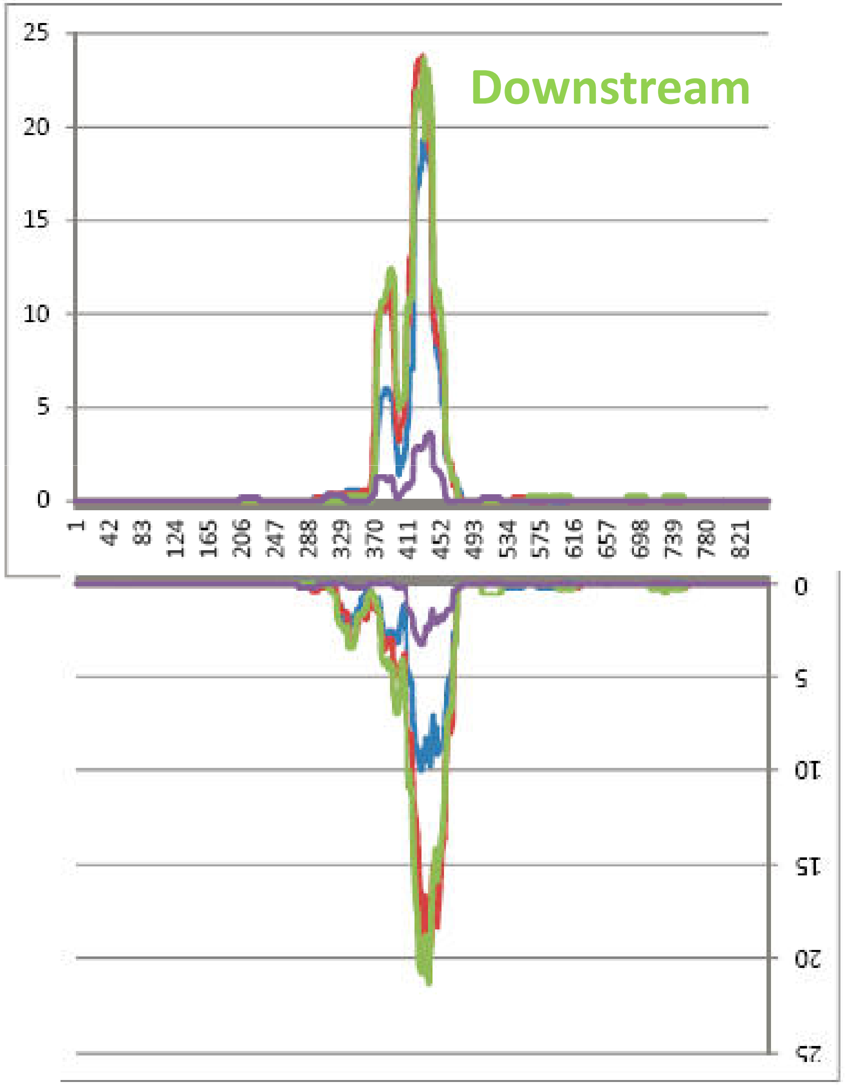

AT1G14580

C2H2-like zinc finger protein

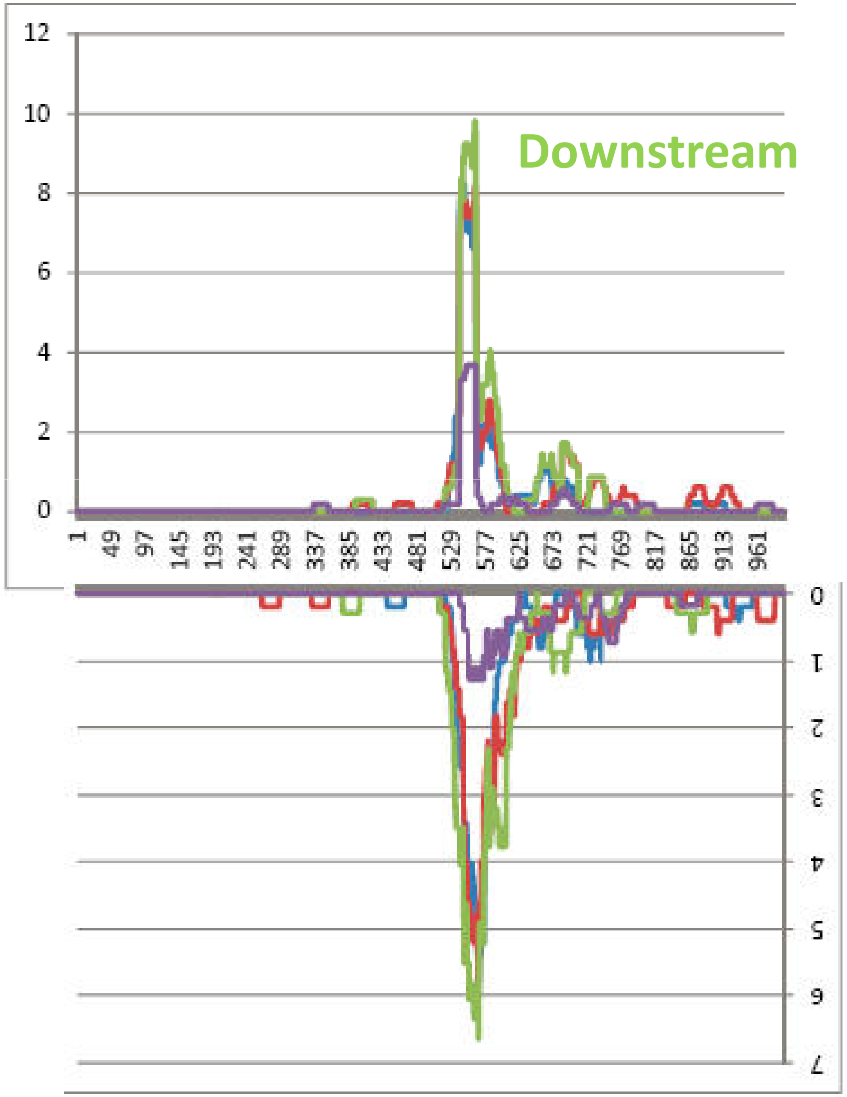

AT1G15670

Encodes a member of a family of F-box proteins, called the KISS ME DEADLY (KMD) family, that targets type-B ARR proteins for degradation and is involved in the negative regulation of the cytokinin response. Also named as KFB1, a member of a group of Kelch repeat F-box proteins that negatively regulate phenylpropanoid biosynthesis by targeting the phenypropanoid biosynthesis enzyme phenylalanine ammonia-lyase.

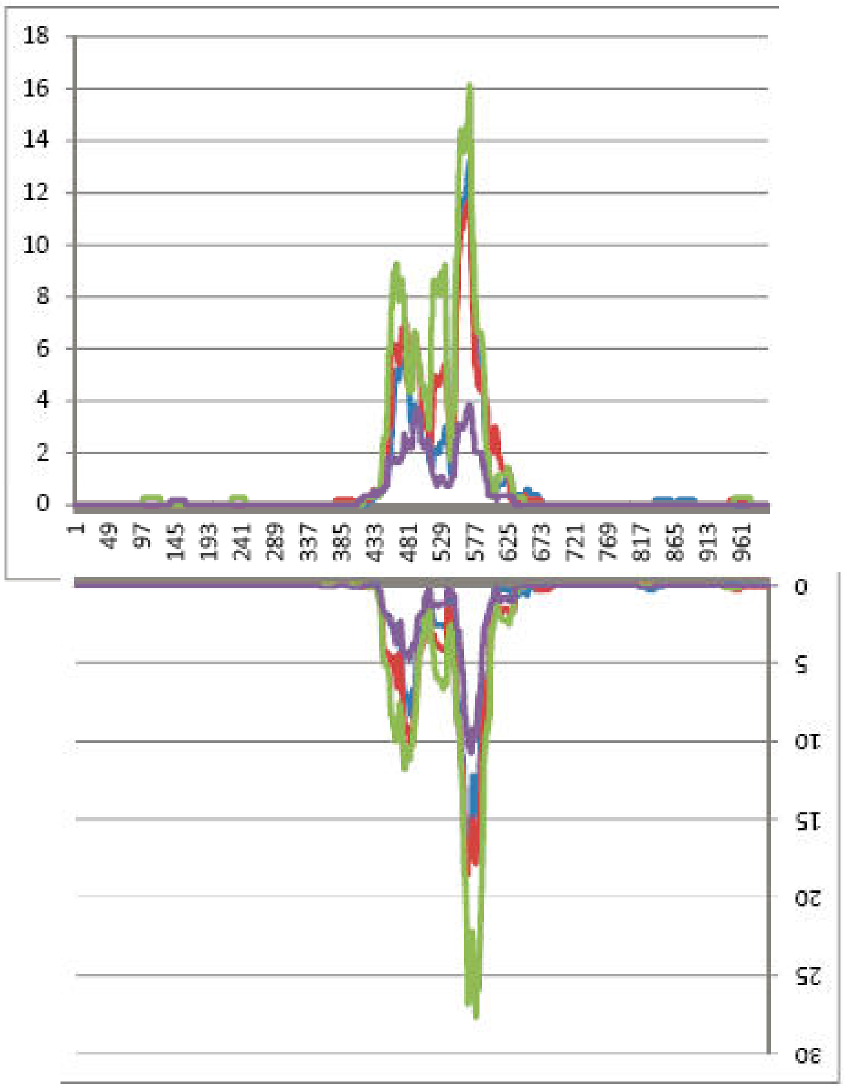

AT1G18060

unknown protein

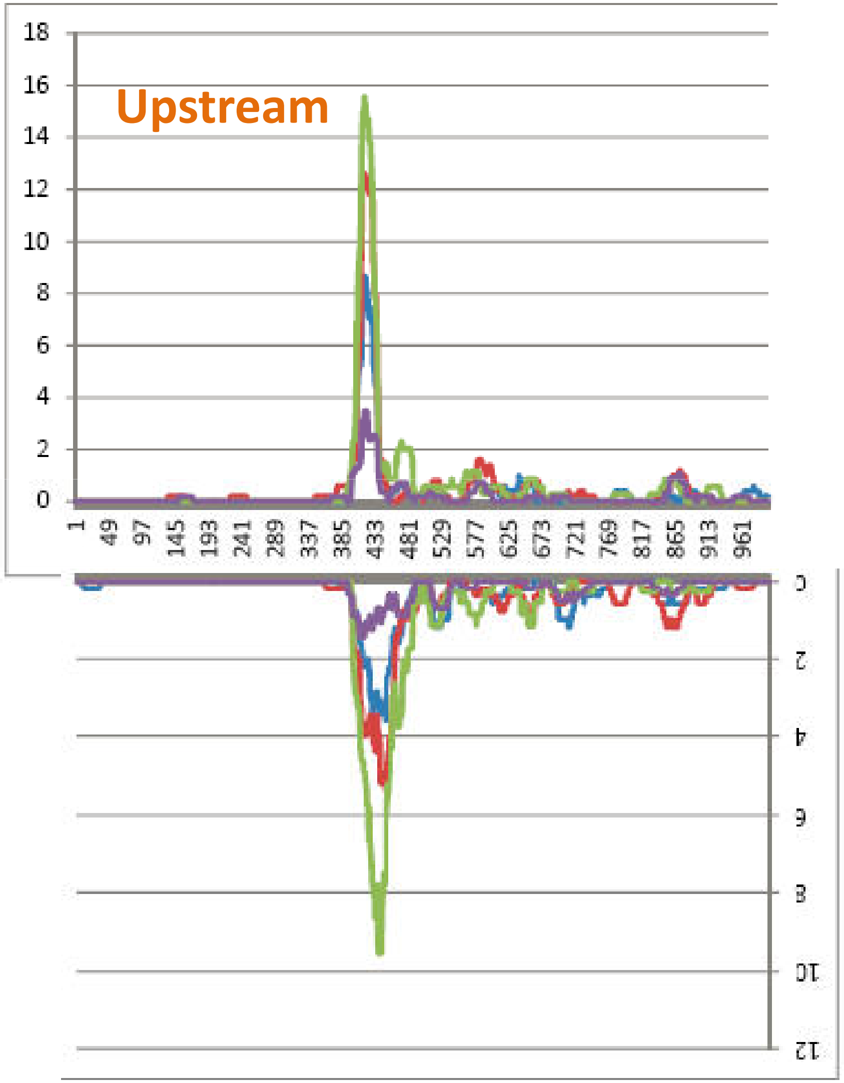

AT1G18770

RING/U-box superfamily protein

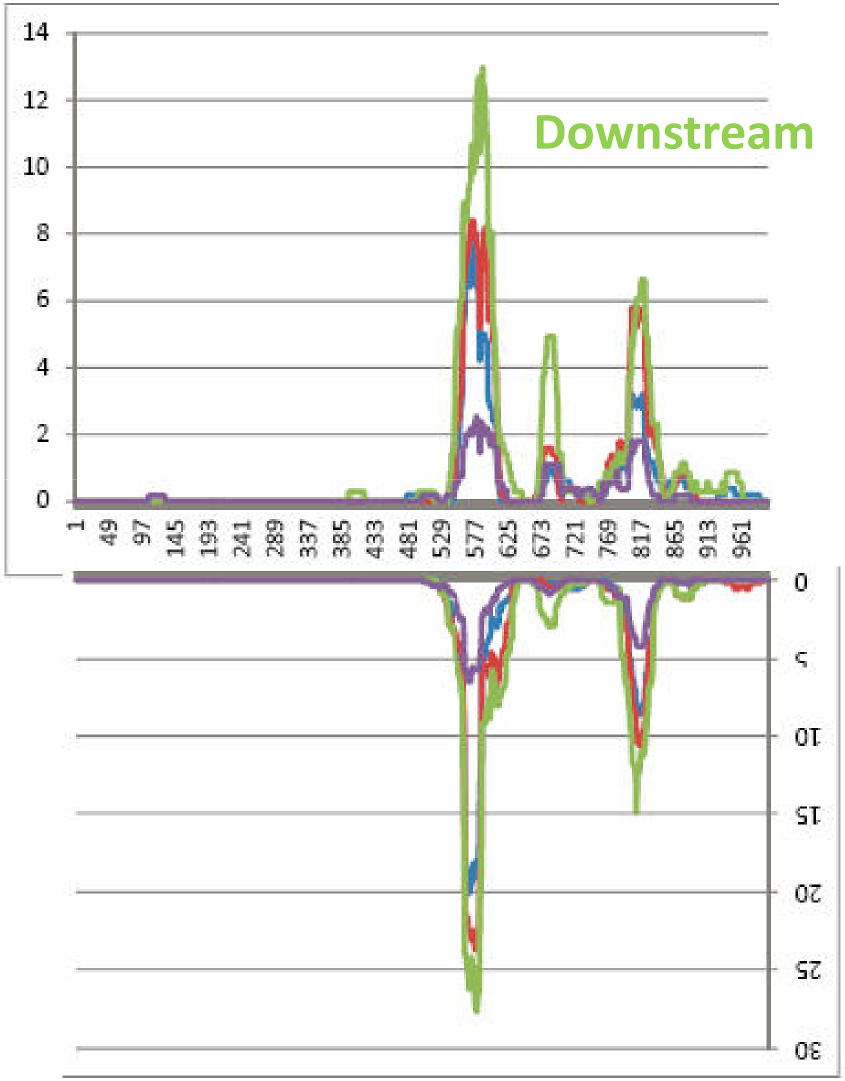

AT1G19830

SAUR-like auxin-responsive protein family

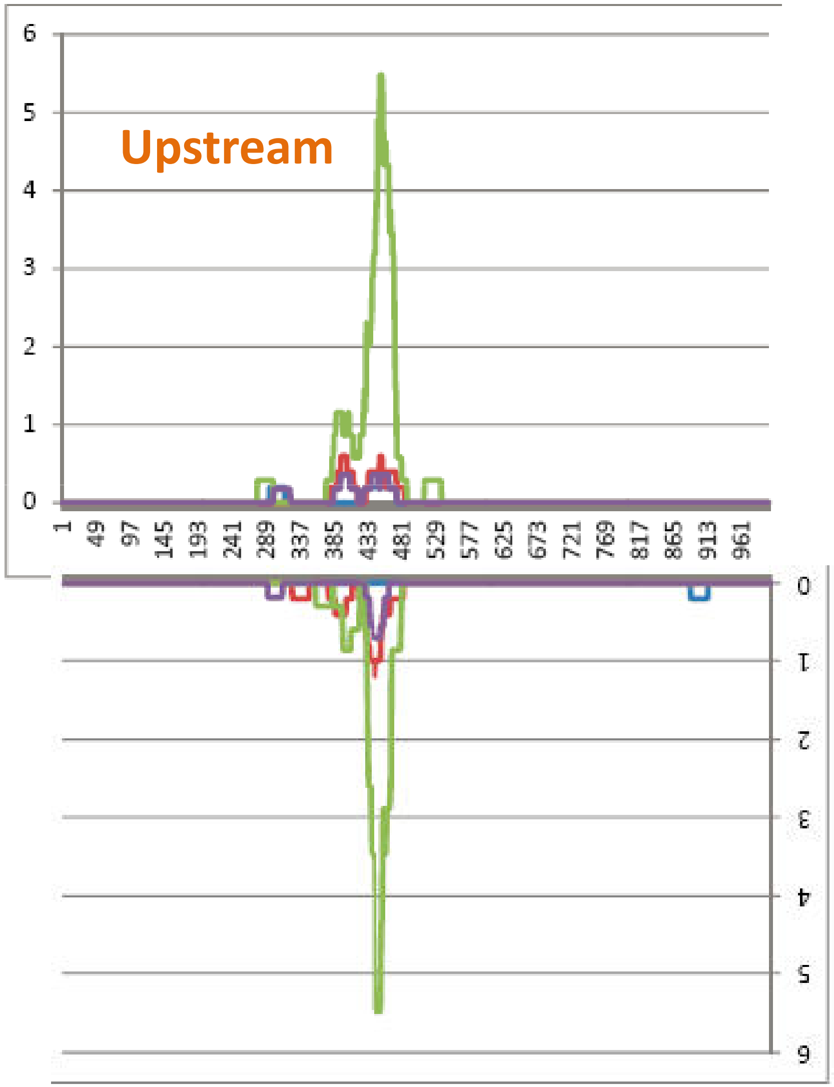

AT1G23650

unknown protein

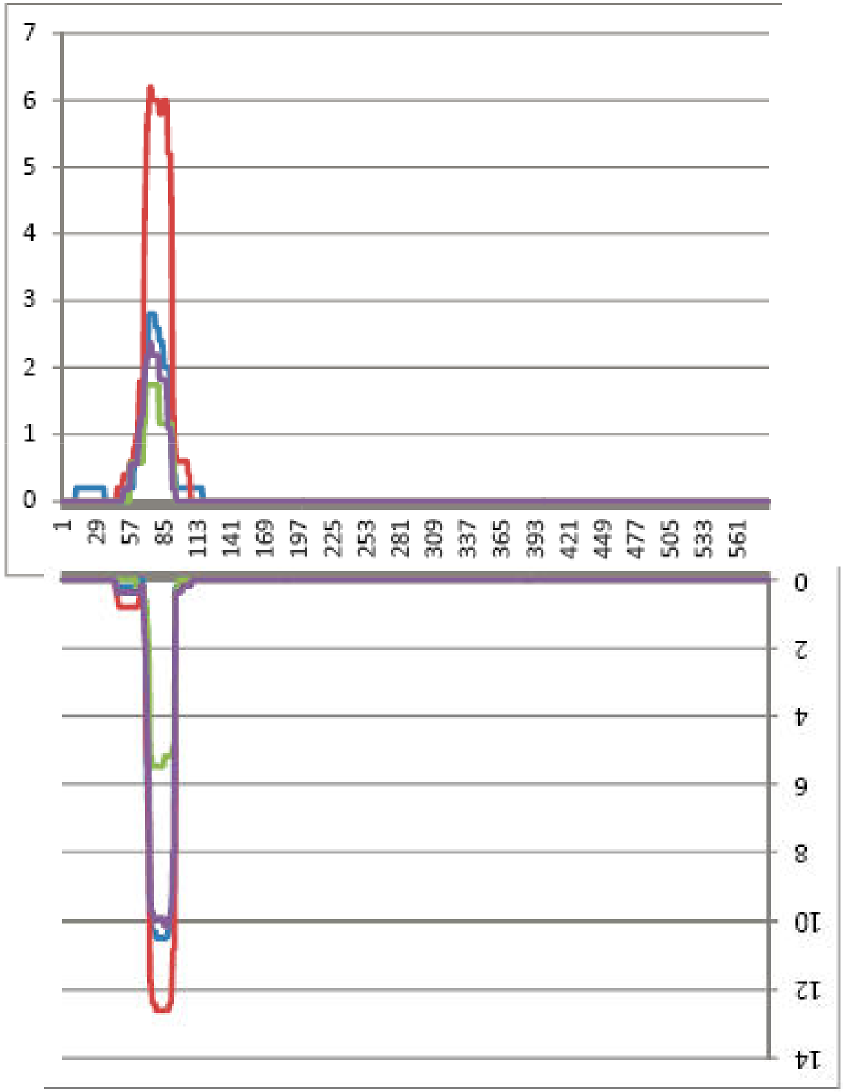

AT1G24388

unknown protein

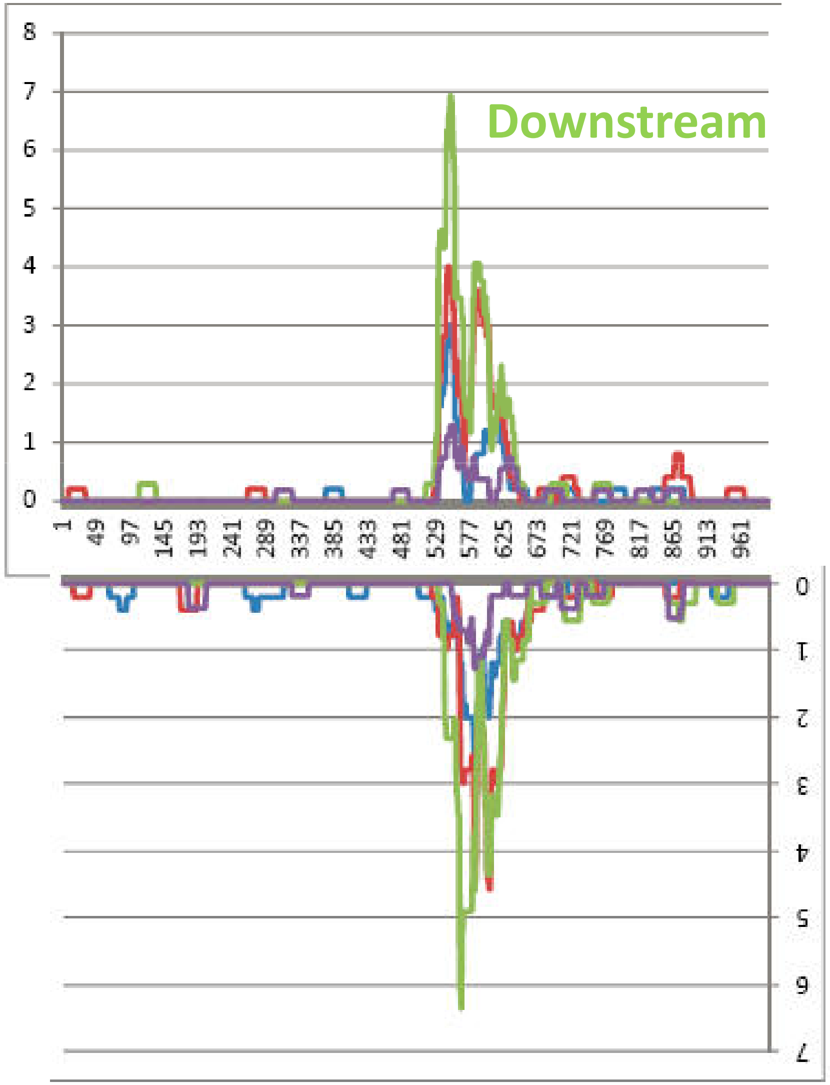

AT1G26762

unknown protein

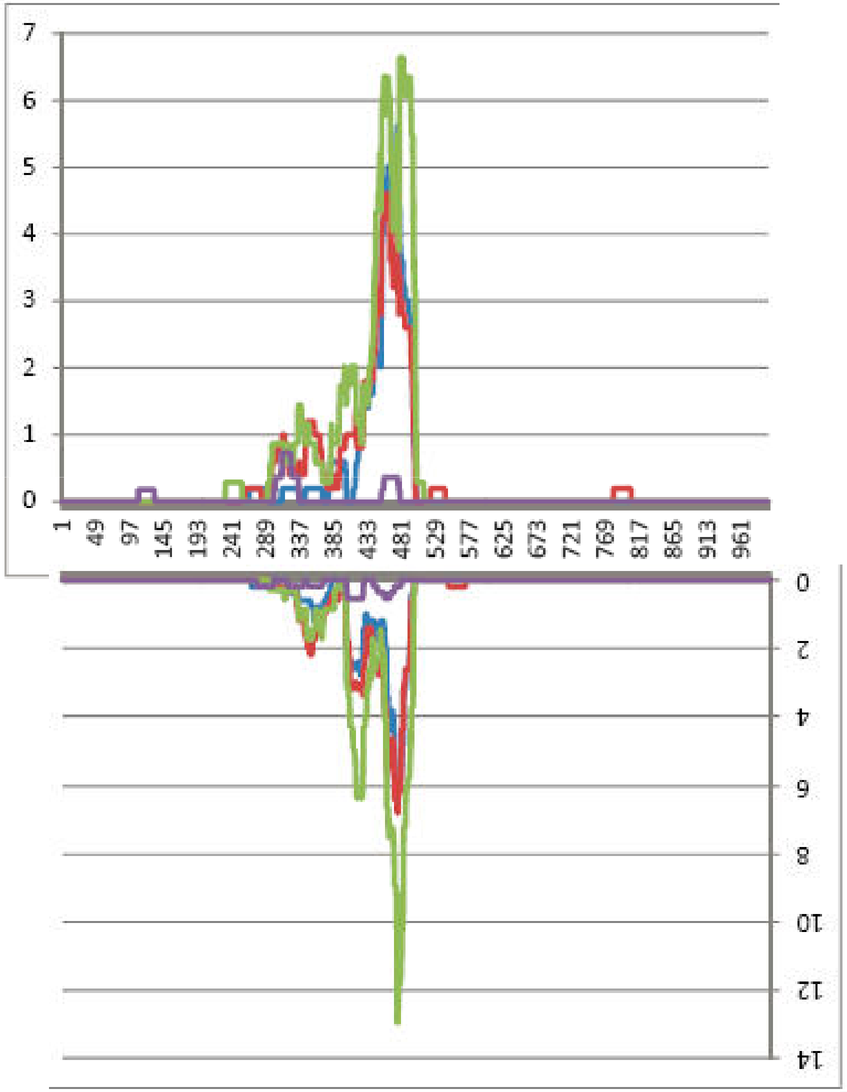

AT1G28304

unknown protein

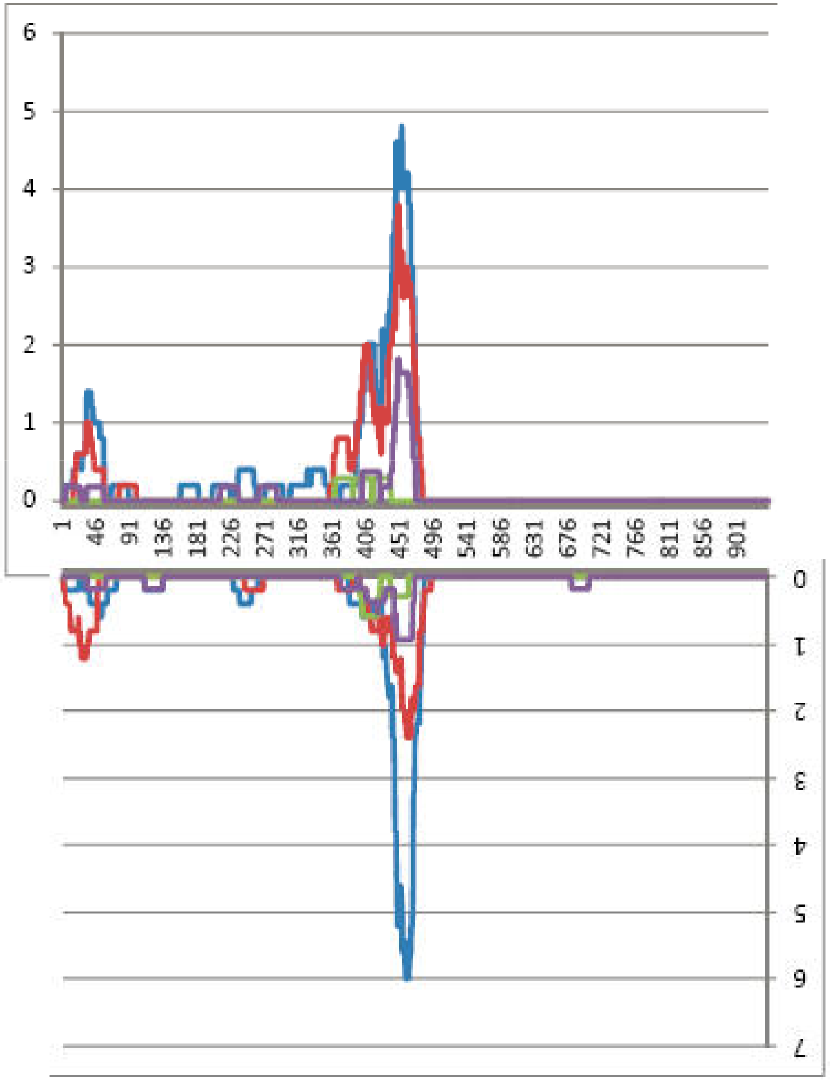

AT1G28670

Arabidopsis thaliana lipase

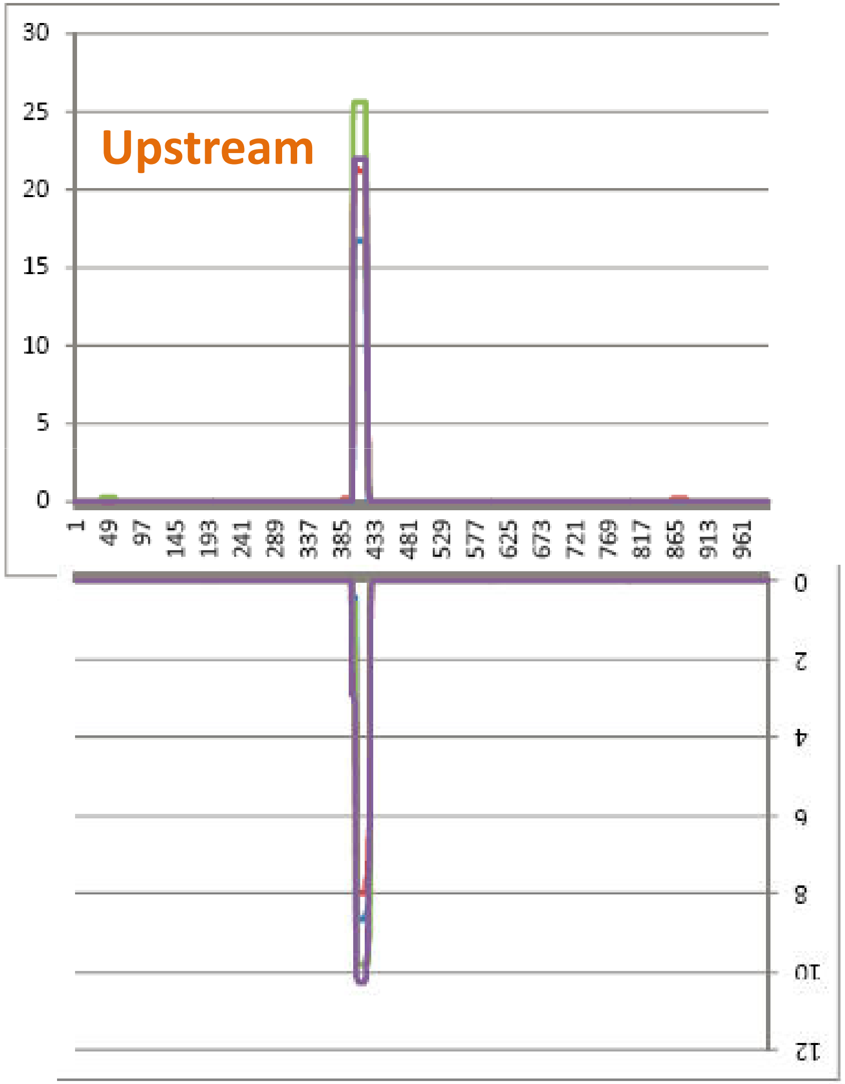

AT1G30974

Encodes a Plant thionin family protein

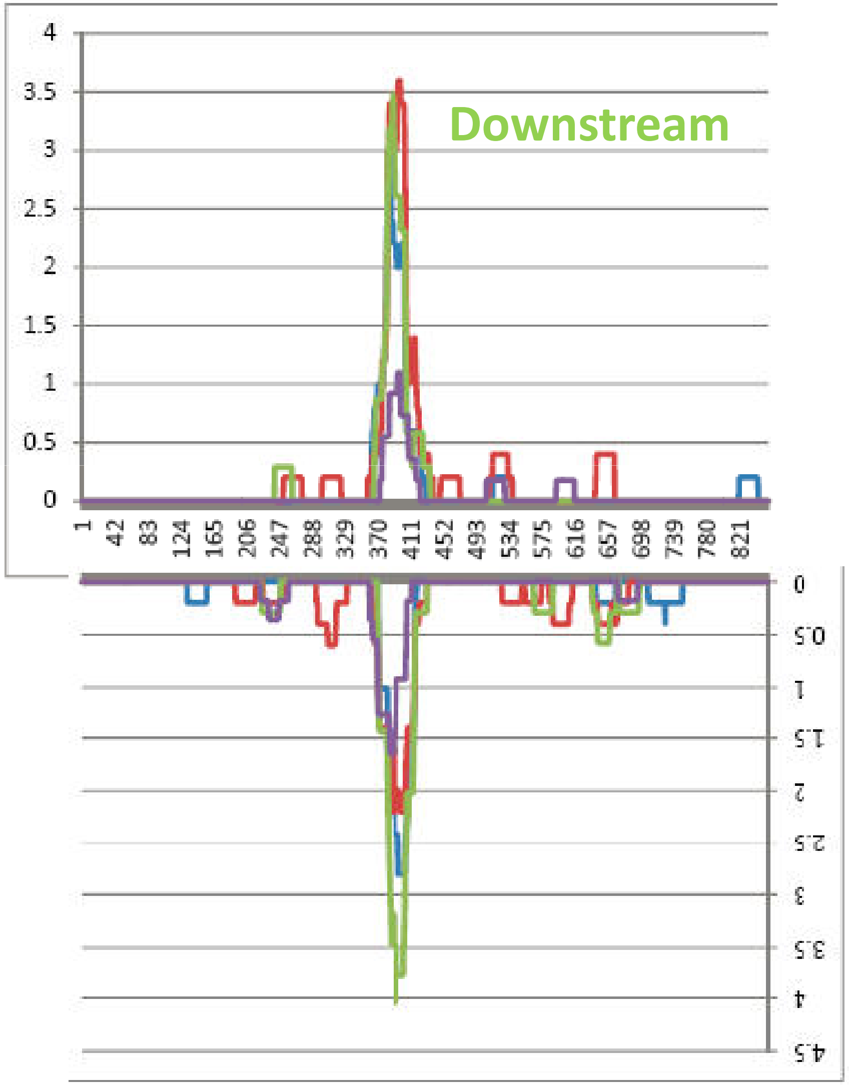

AT1G32583

A microRNA MIR400 is derived from the first intron of At1g32583 in the 5'UTR. A stress-induced alternative splicing event in At1g32583 resulted in greater accumulation of miR400 primary transcripts and a low level of mature miR400.

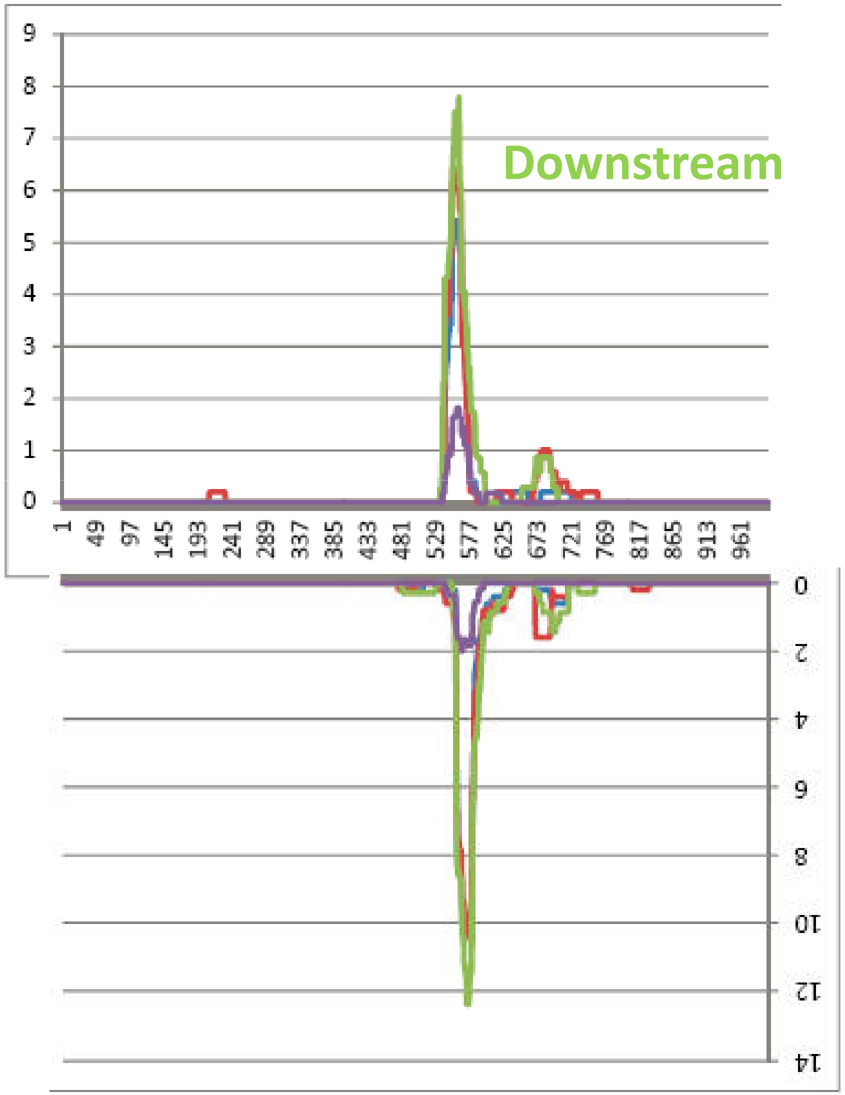

AT1G35400

CONTAINS InterPro DOMAIN/s: Protein of unknown function DUF1184 (InterPro:IPR009568), Uncharacterised conserved protein UCP031143 (InterPro:IPR016970); BEST Arabidopsis thaliana protein match is: Protein of unknown function (DUF1184) (TAIR:AT1G35410.1).

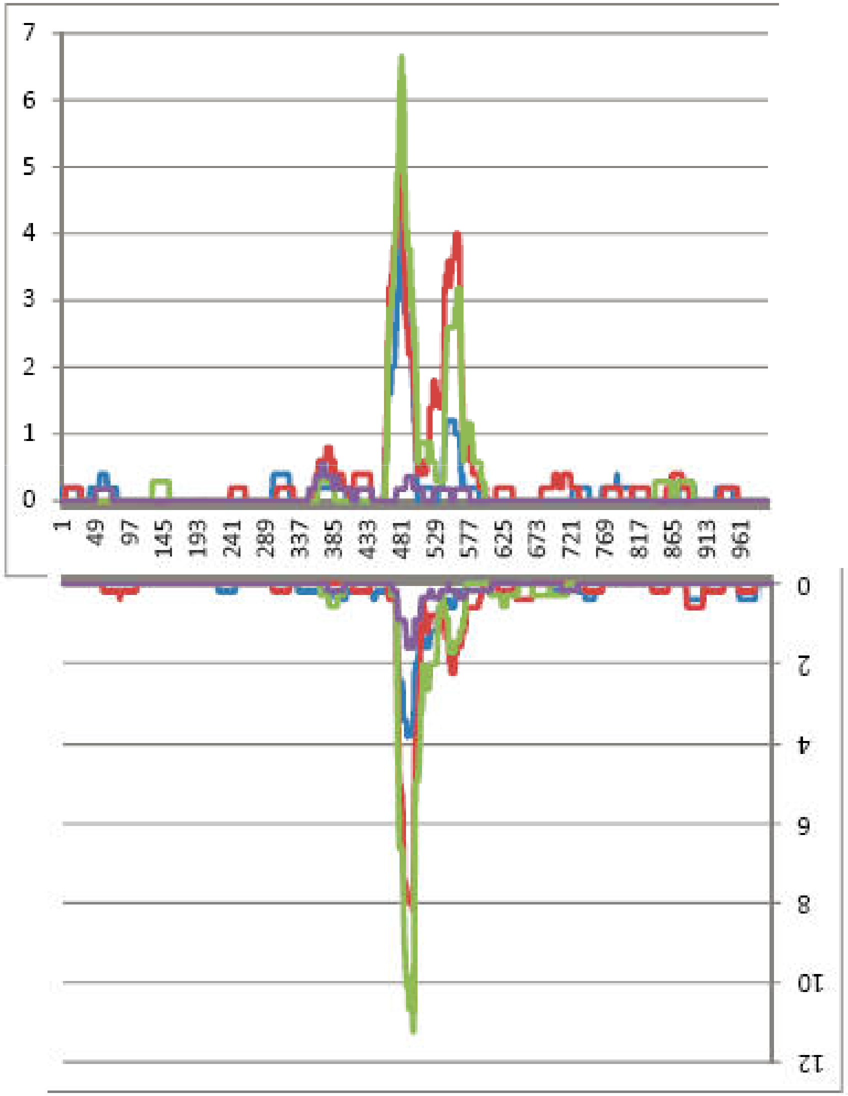

AT1G35516

myb-like transcription factor family protein

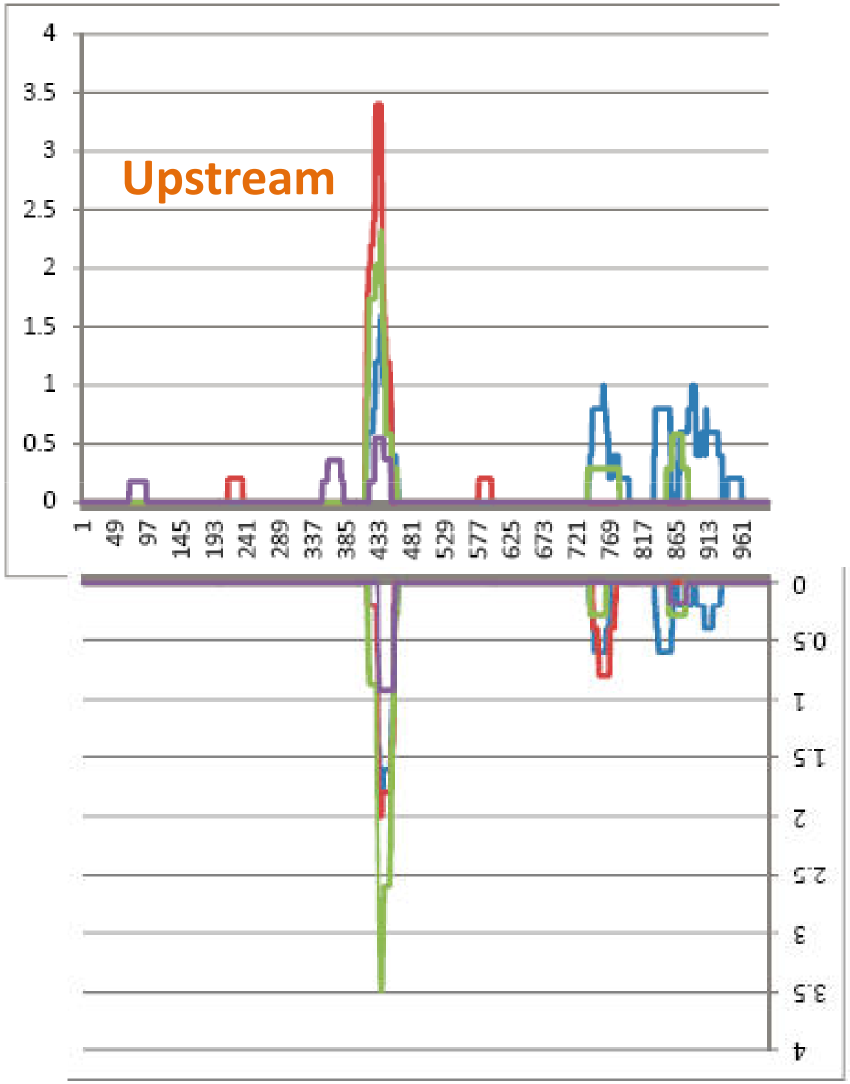

AT1G40129

unknown protein

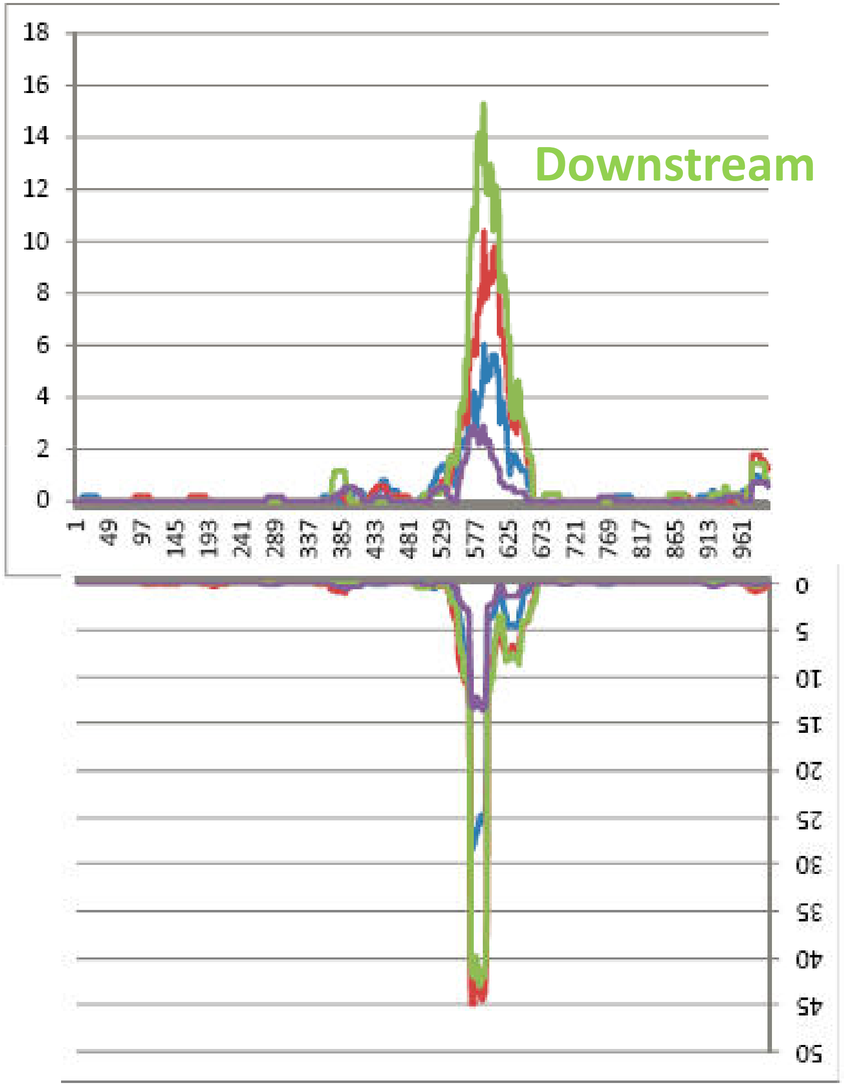

AT1G43160

encodes a member of the ERF (ethylene response factor) subfamily B-4 of ERF/AP2 transcription factor family (RAP2.6). The protein contains one AP2 domain. There are 7 members in this subfamily.

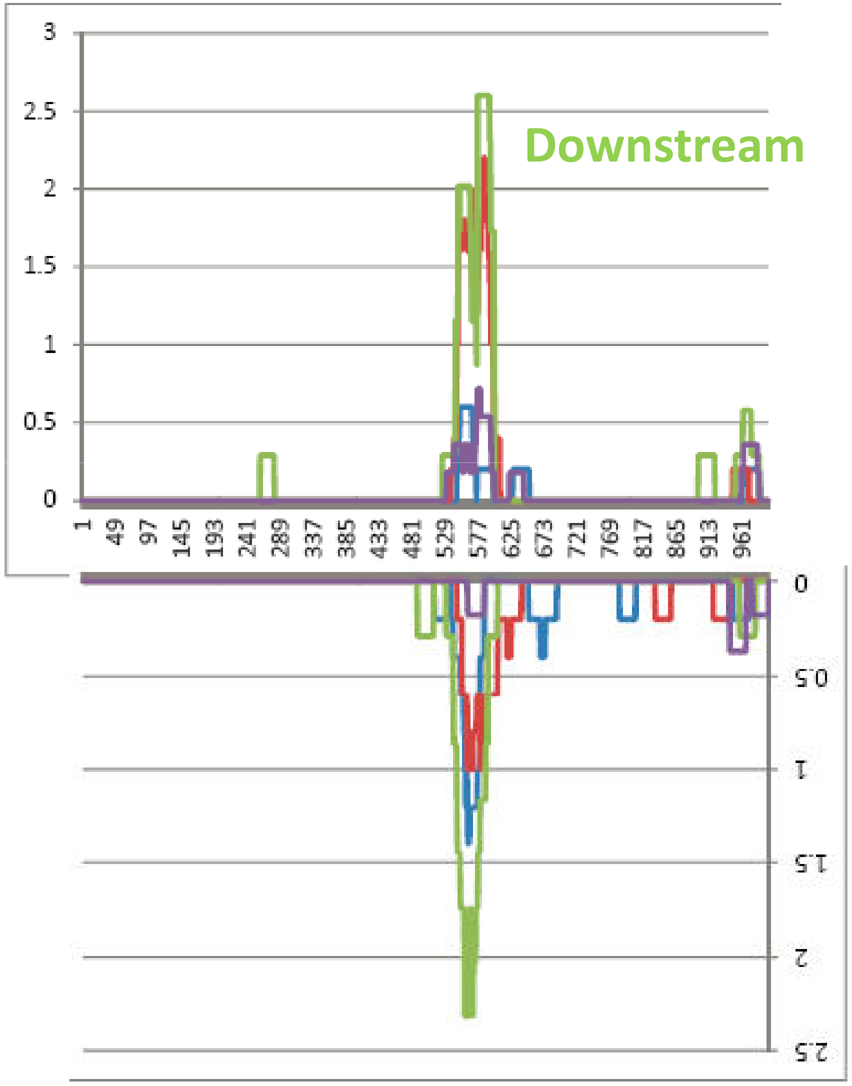

AT1G44542

Cyclase family protein

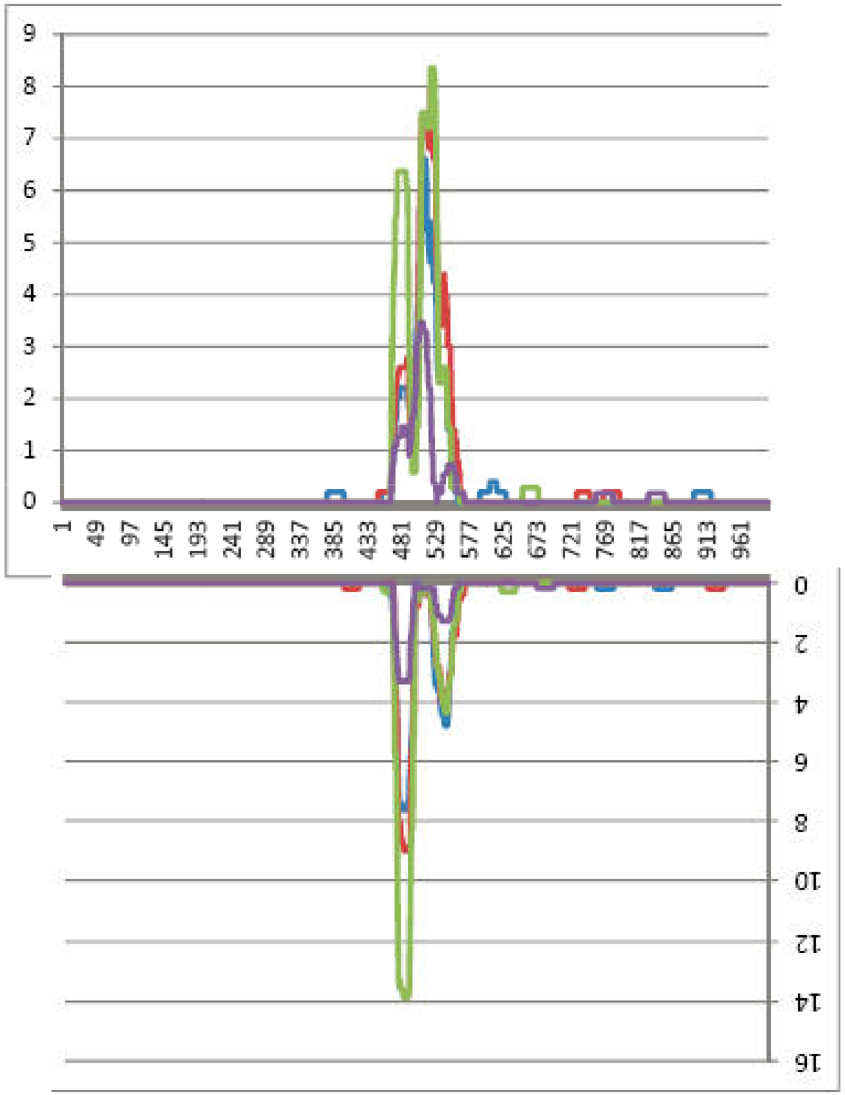

AT1G47265

unknown protein

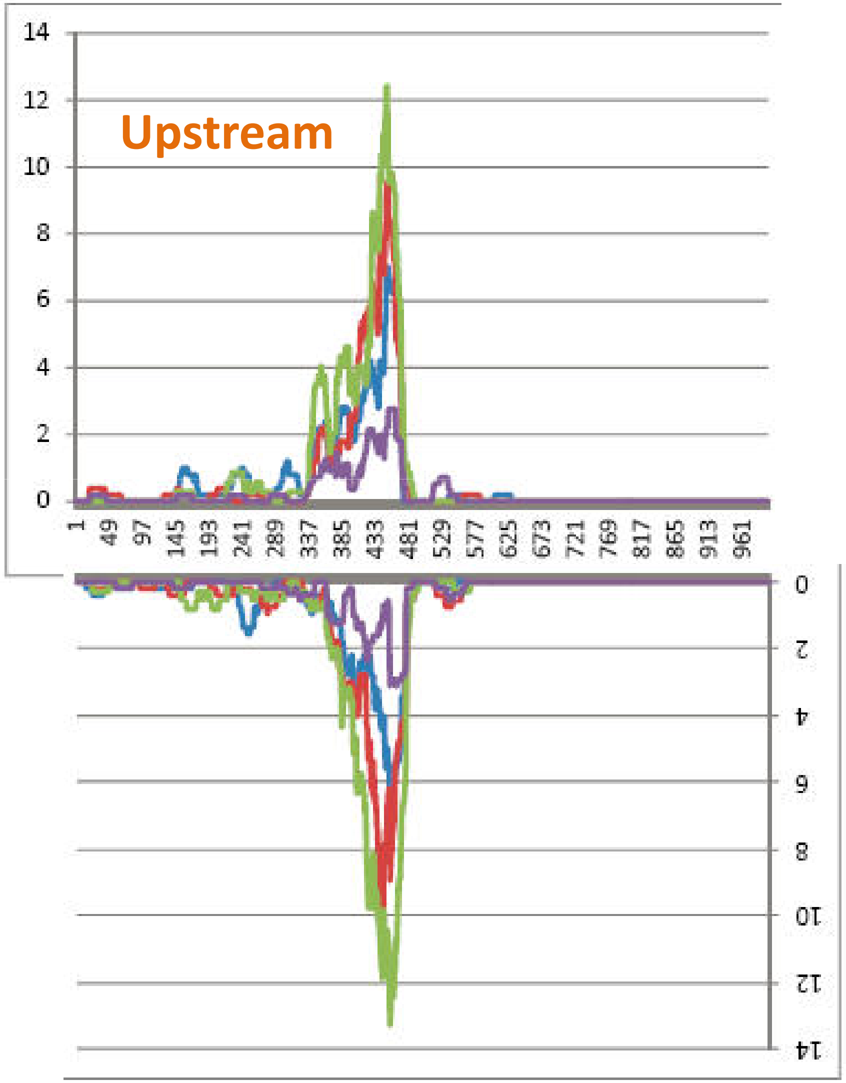

AT1G47450

Protein of unknown function (DUF784)

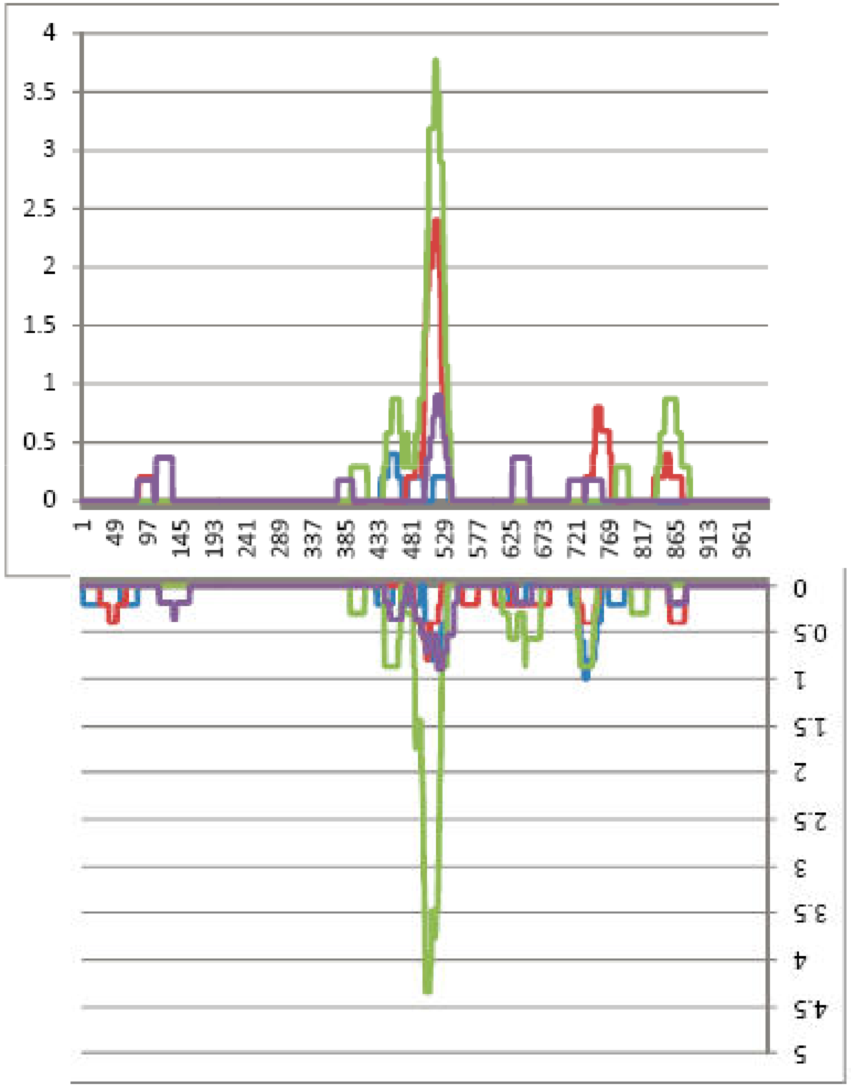

AT1G51150

Encodes a putative DegP protease

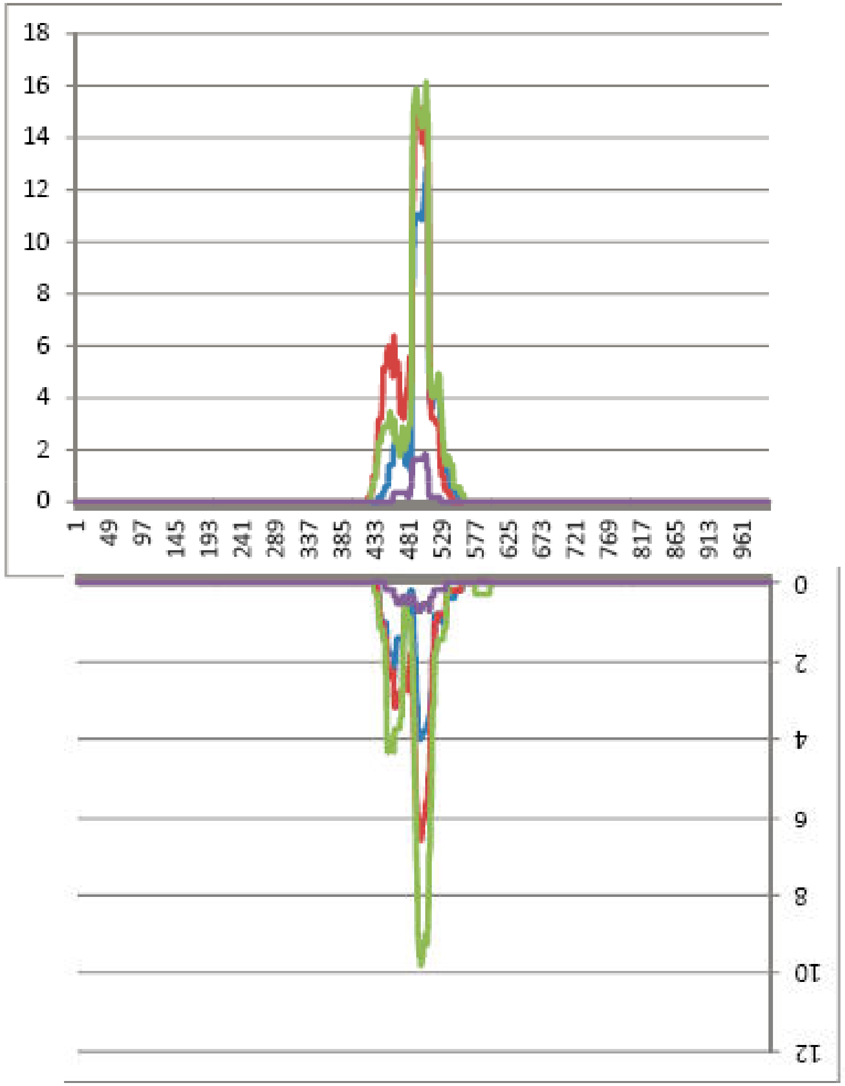

AT1G51820

Leucine-rich repeat protein kinase family protein

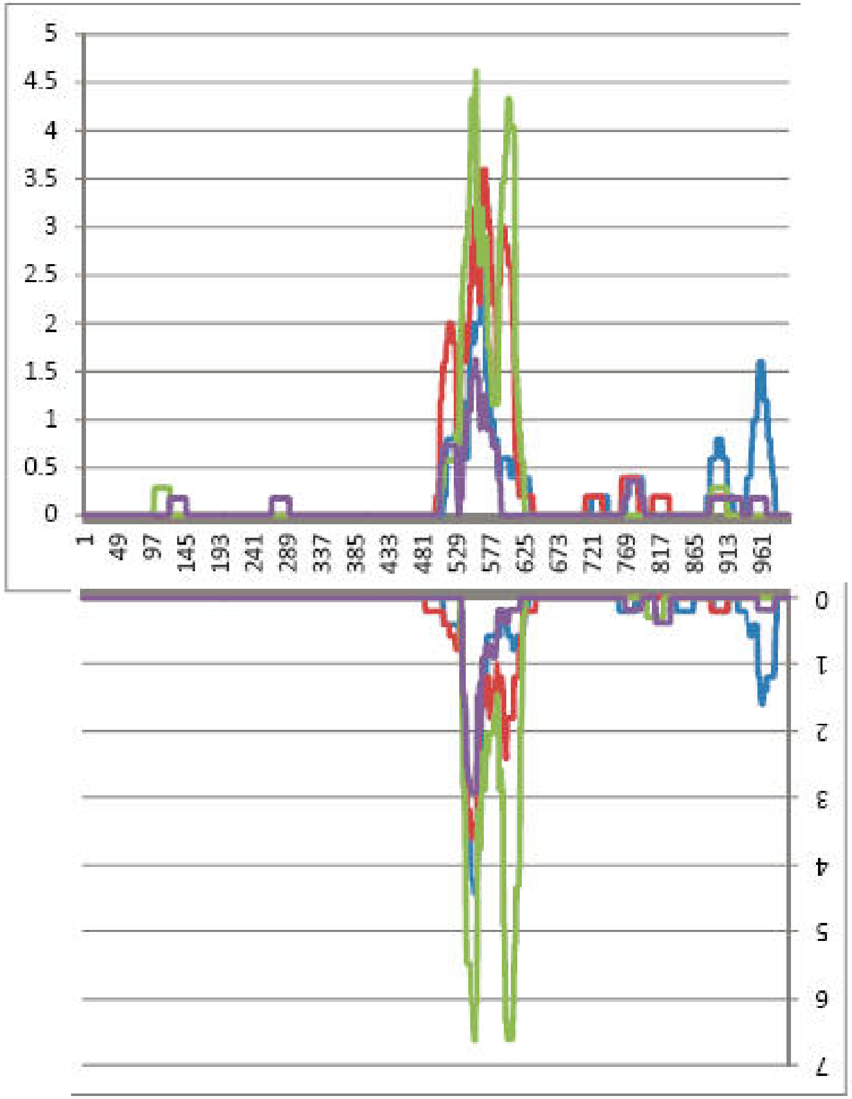

AT1G52160

Encodes a tRNase Z

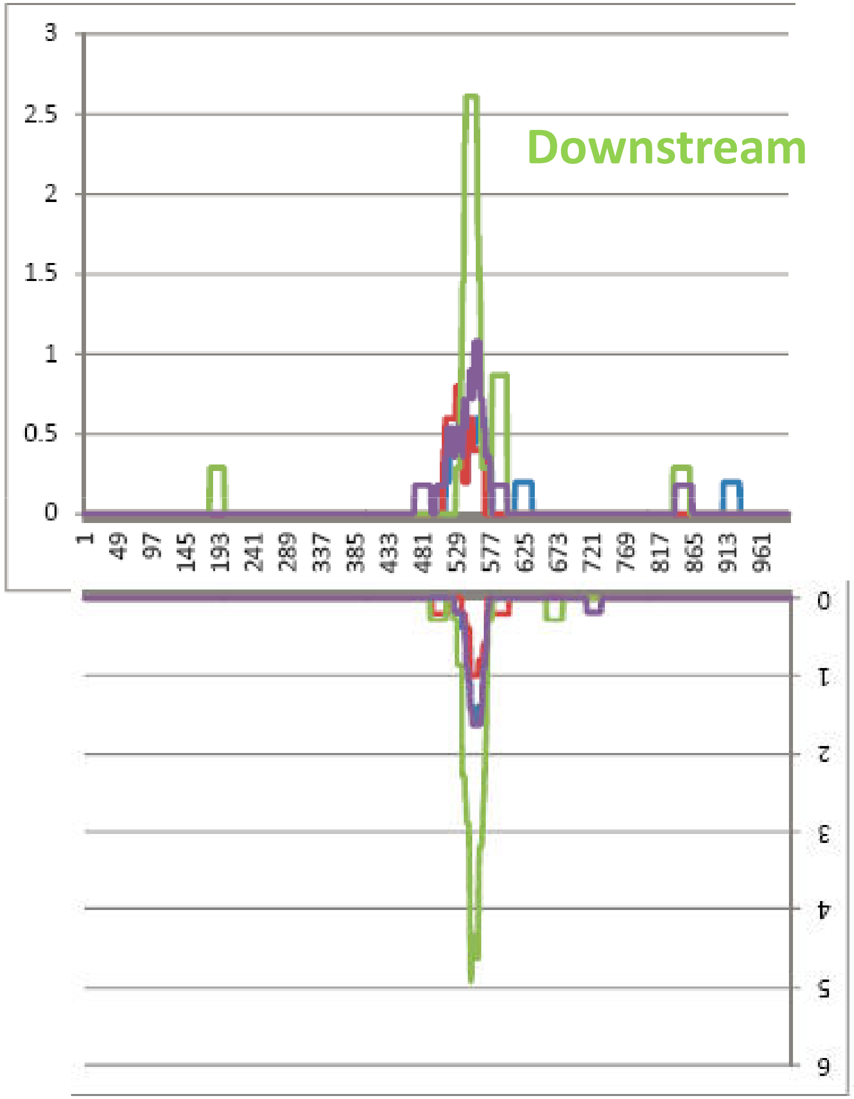

AT1G55700

Cysteine/Histidine-rich C1 domain family protein

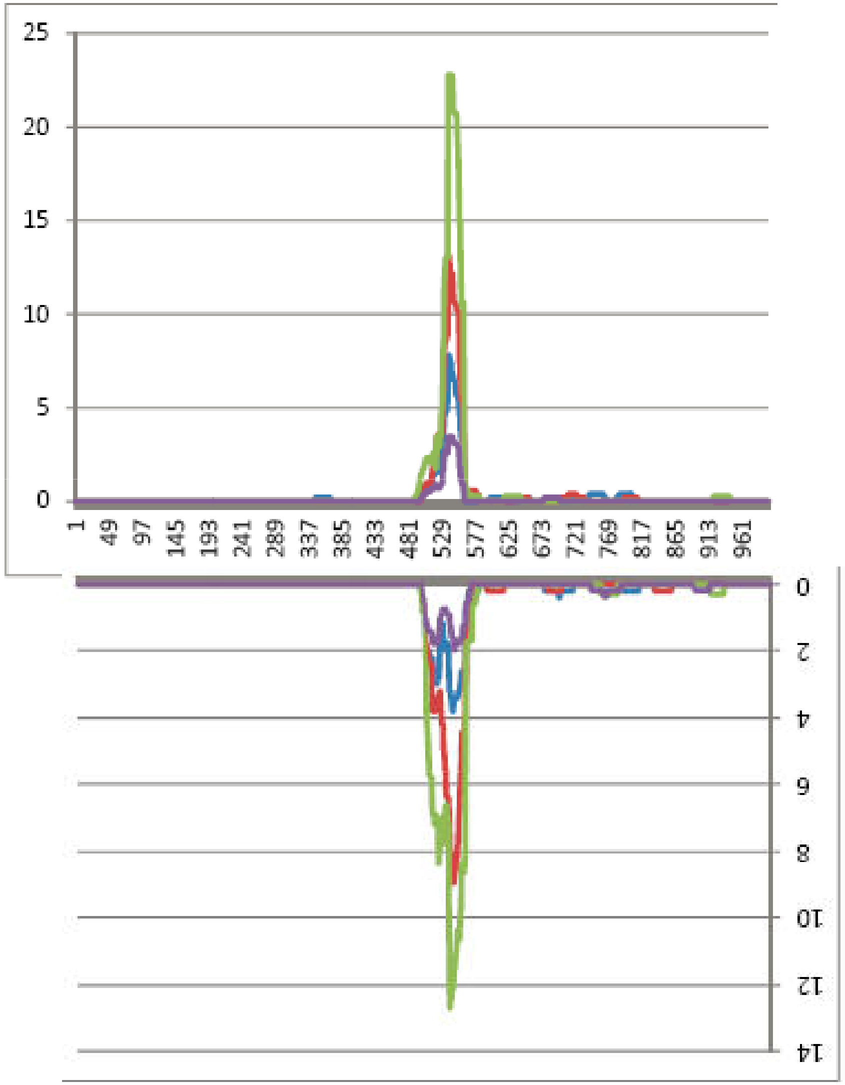

AT1G57770

FAD/NAD(P)-binding oxidoreductase family protein

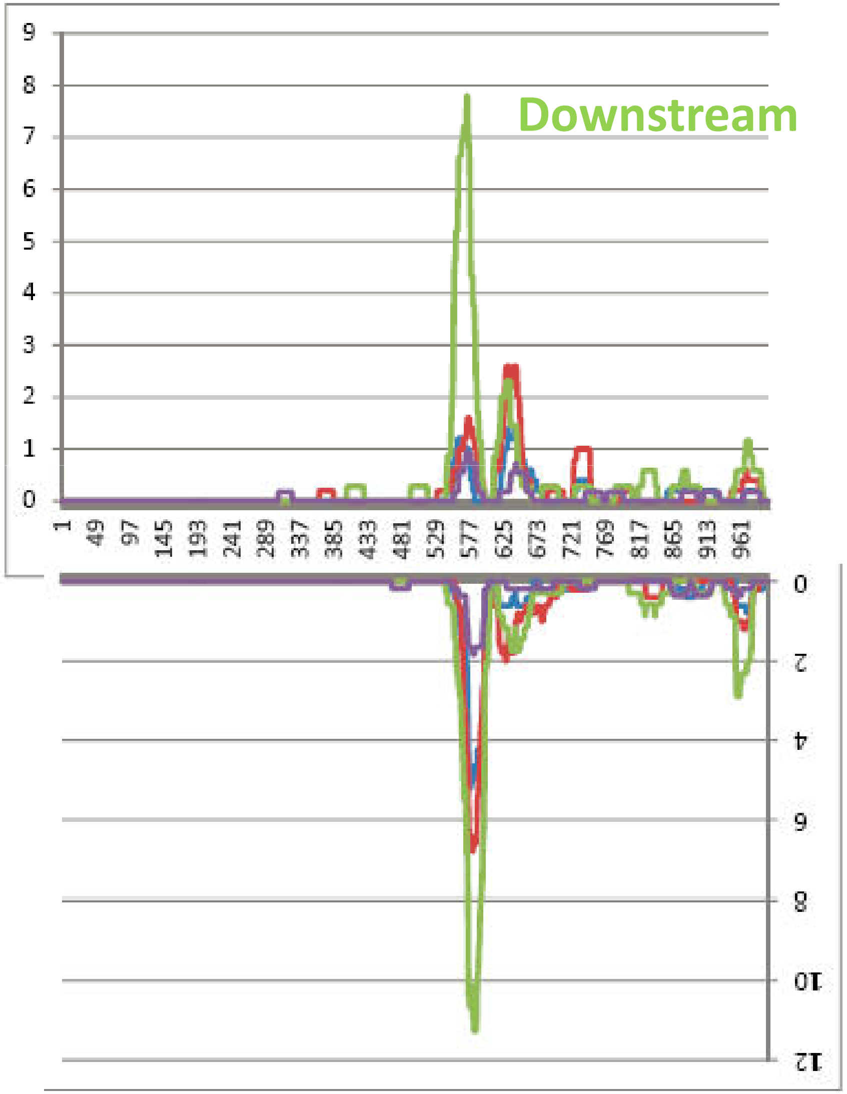

AT1G59885

unknown protein

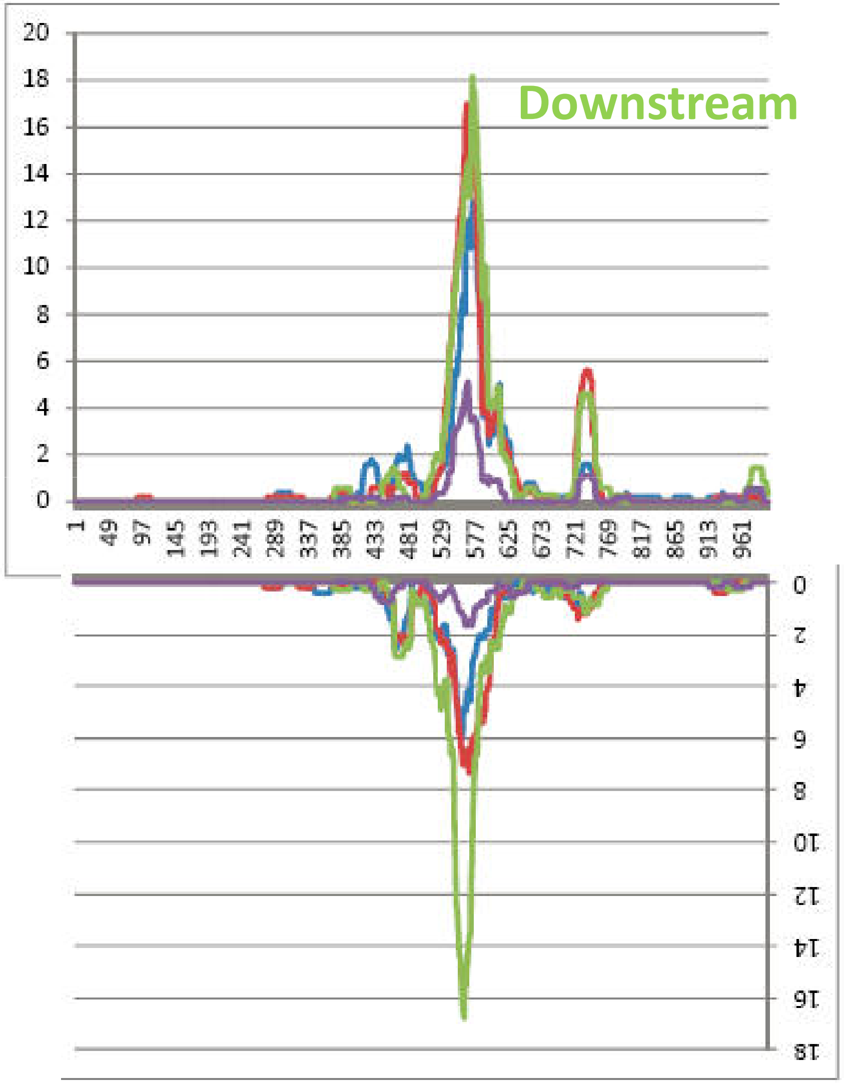

AT1G60986

Encodes a member of a family of small, secreted, cysteine rich proteins with sequence similarity to SCR (S locus cysteine-rich protein).

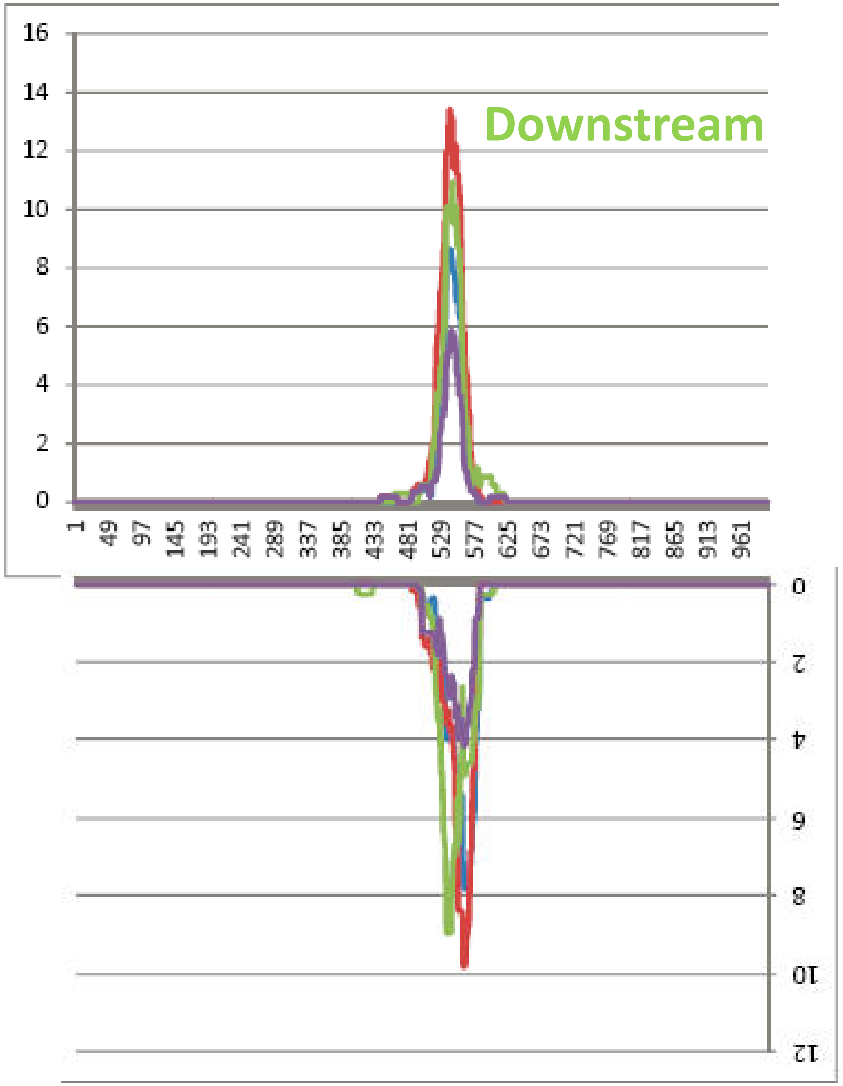

AT1G62410

MIF4G domain-containing protein

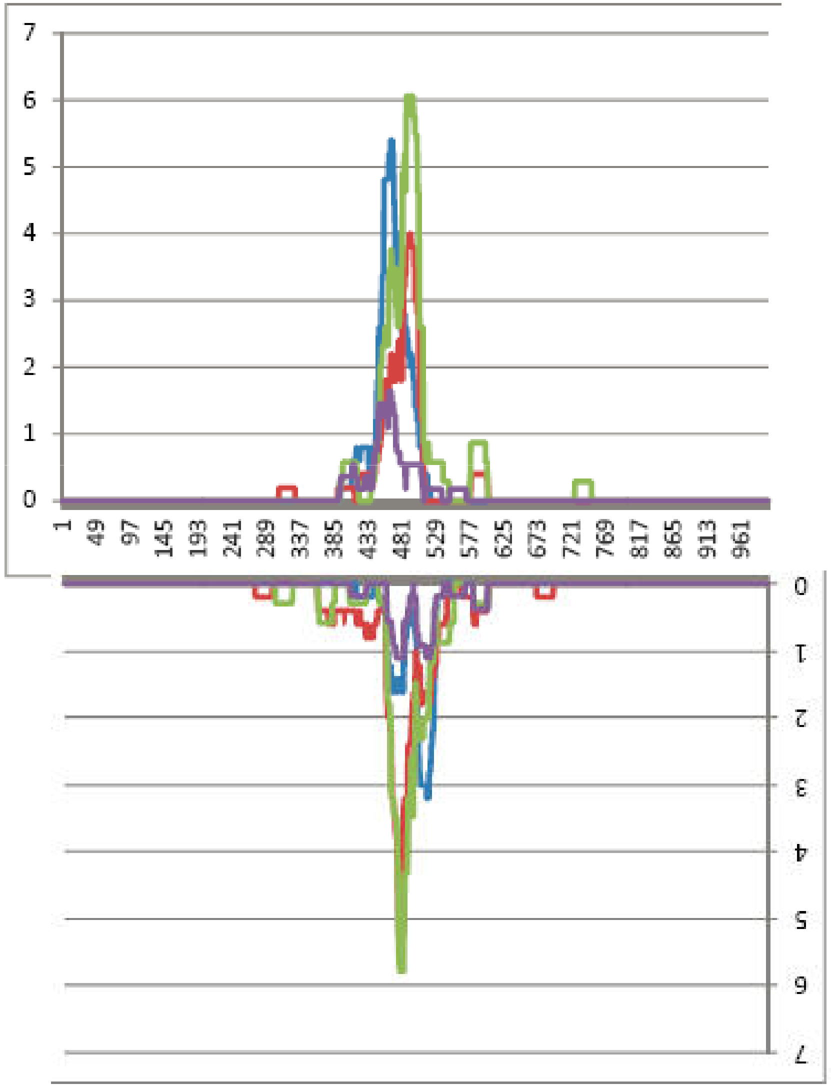

AT1G66620

Protein with RING/U-box and TRAF-like domains

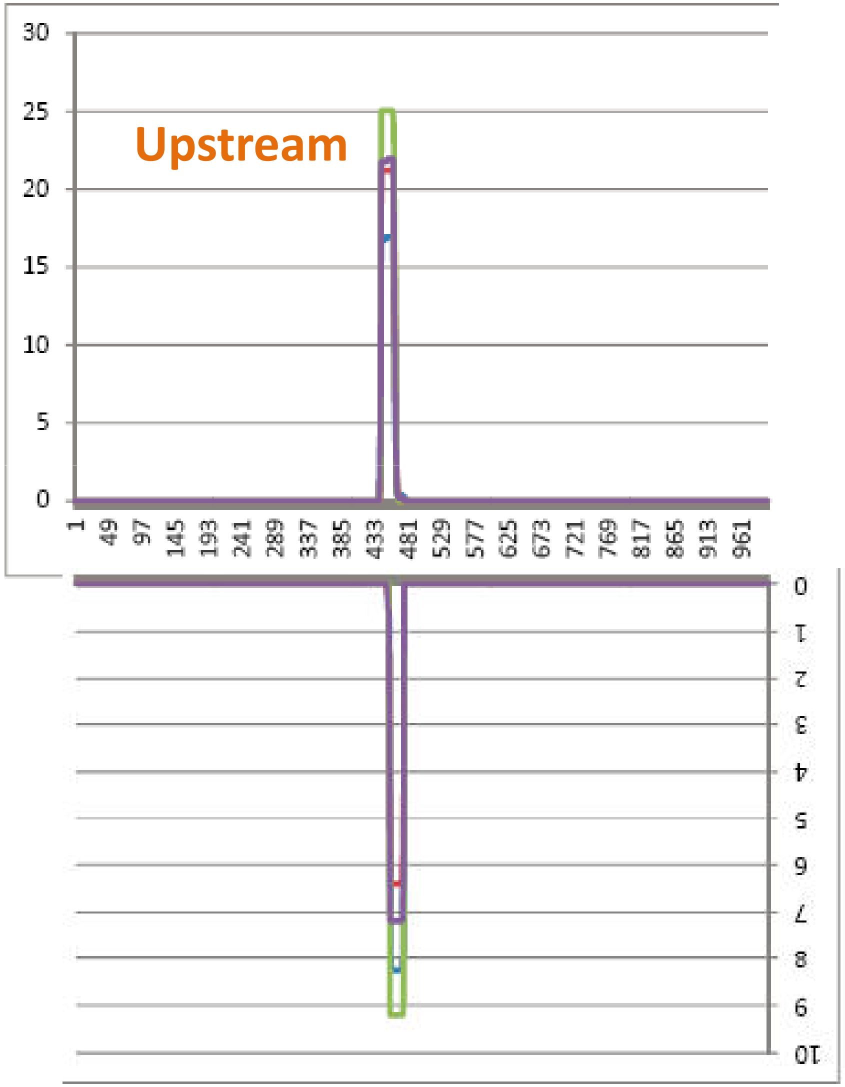

AT1G68040

S-adenosyl-L-methionine-dependent methyltransferases superfamily protein

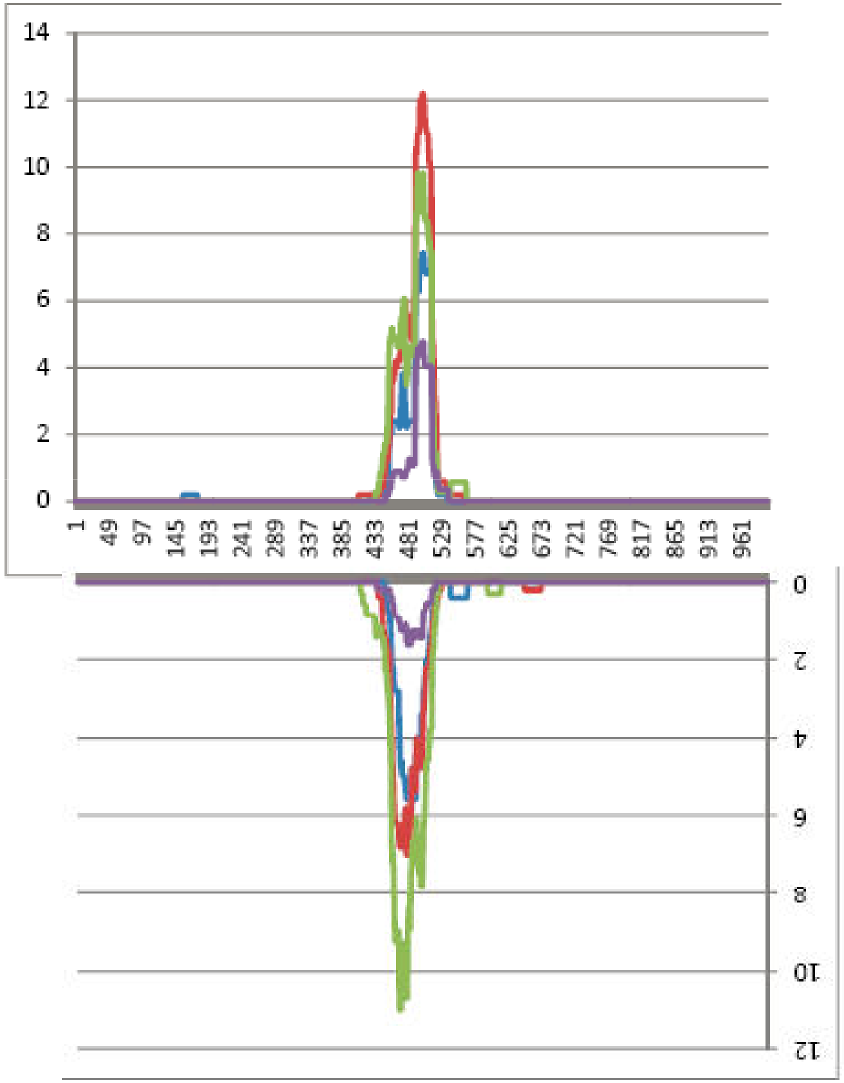

AT1G72580

unknown protein

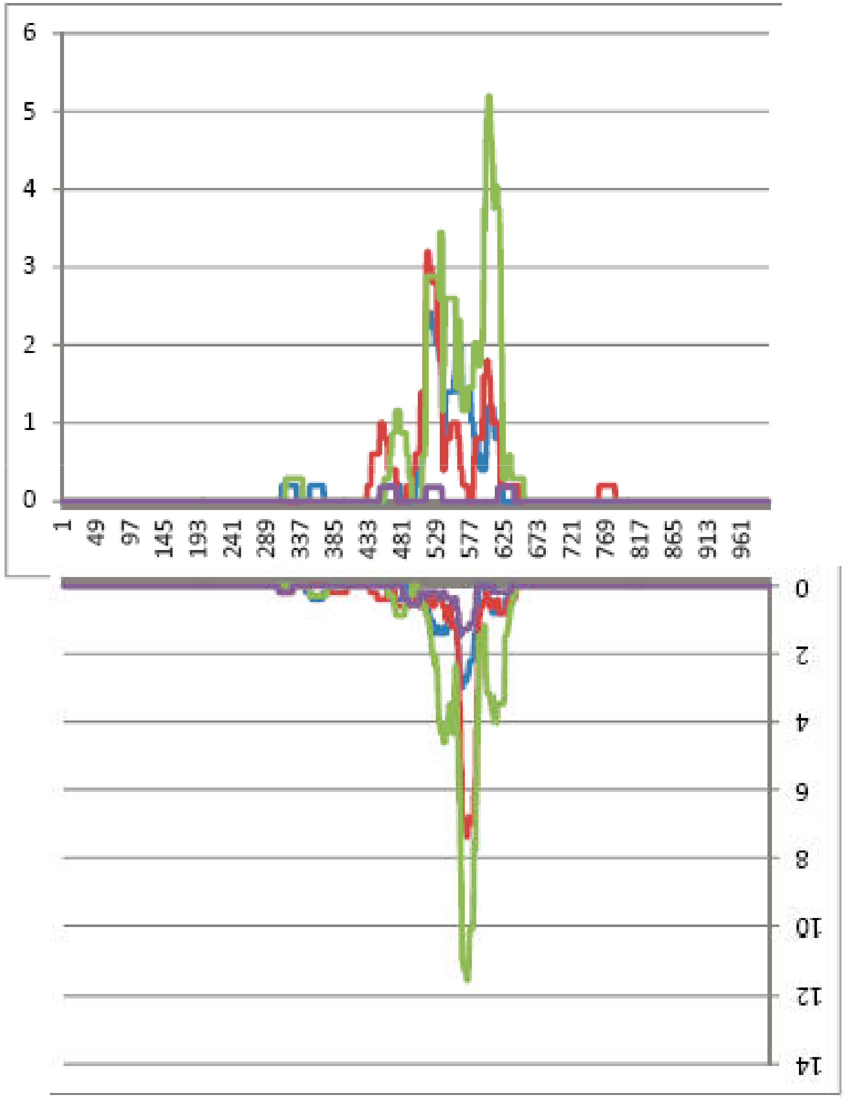

AT2G01950

Encodes a leucine rich repeat receptor kinase and associated with provascular/procambial cells. Similar to BRI, brassinosteroid receptor protein.

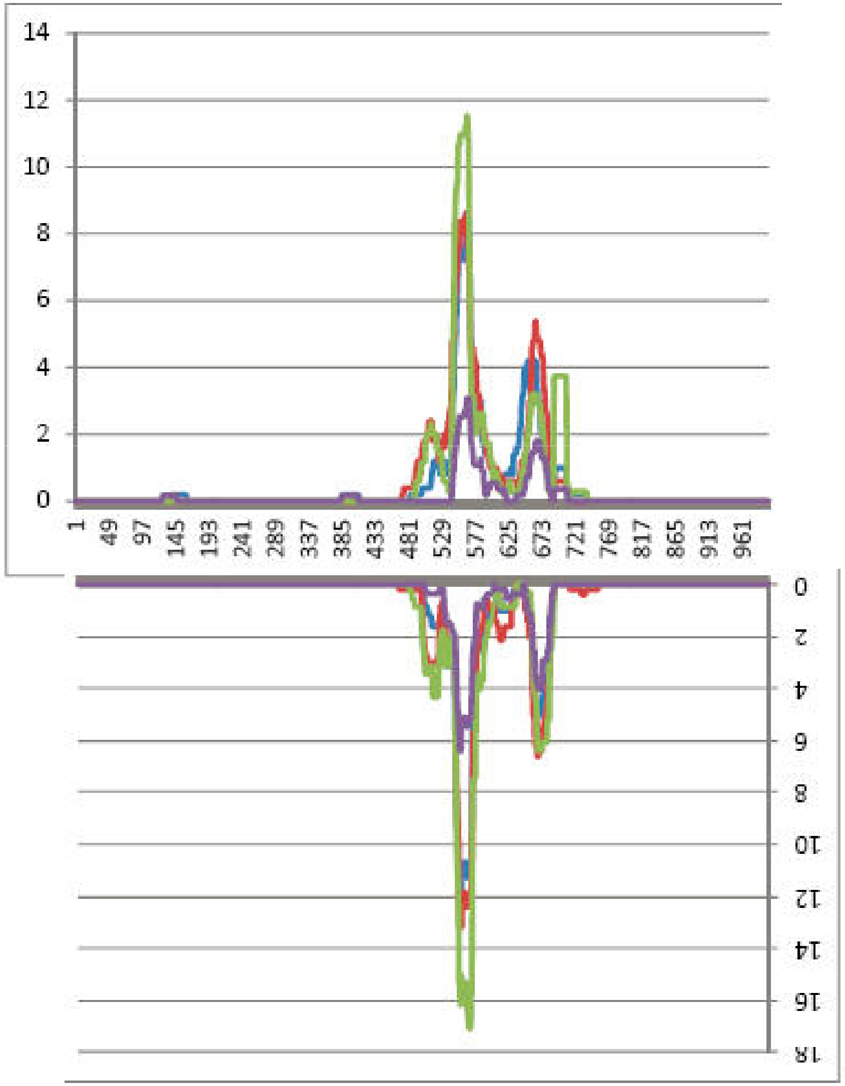

AT2G04220

Plant protein of unknown function (DUF868)

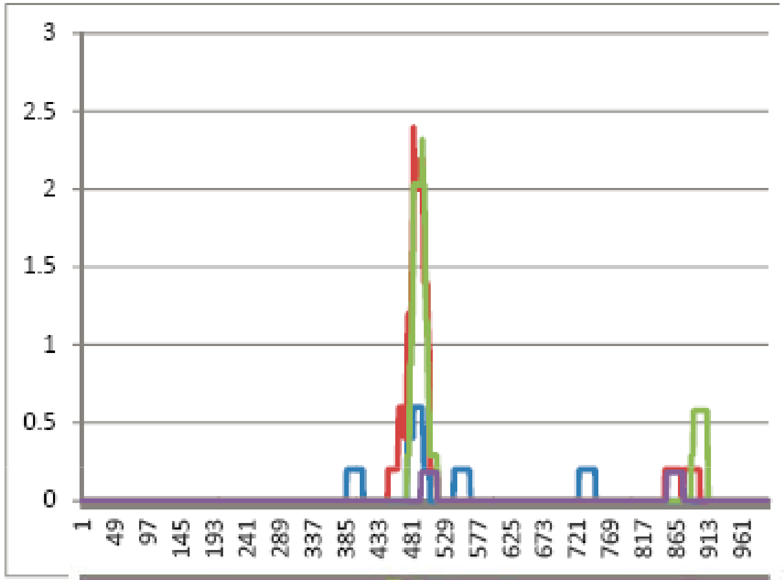

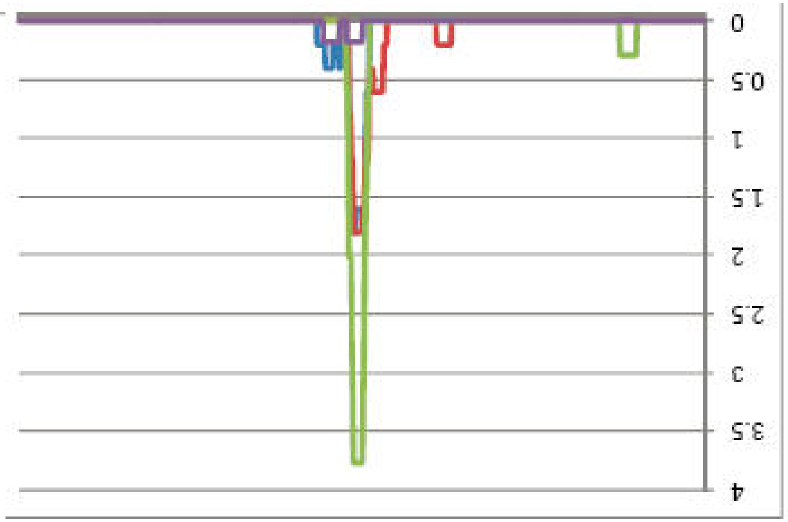

AT2G05335

Encodes a member of a family of small, secreted, cysteine rich proteins with sequence similarity to SCR (S locus cysteine-rich protein).

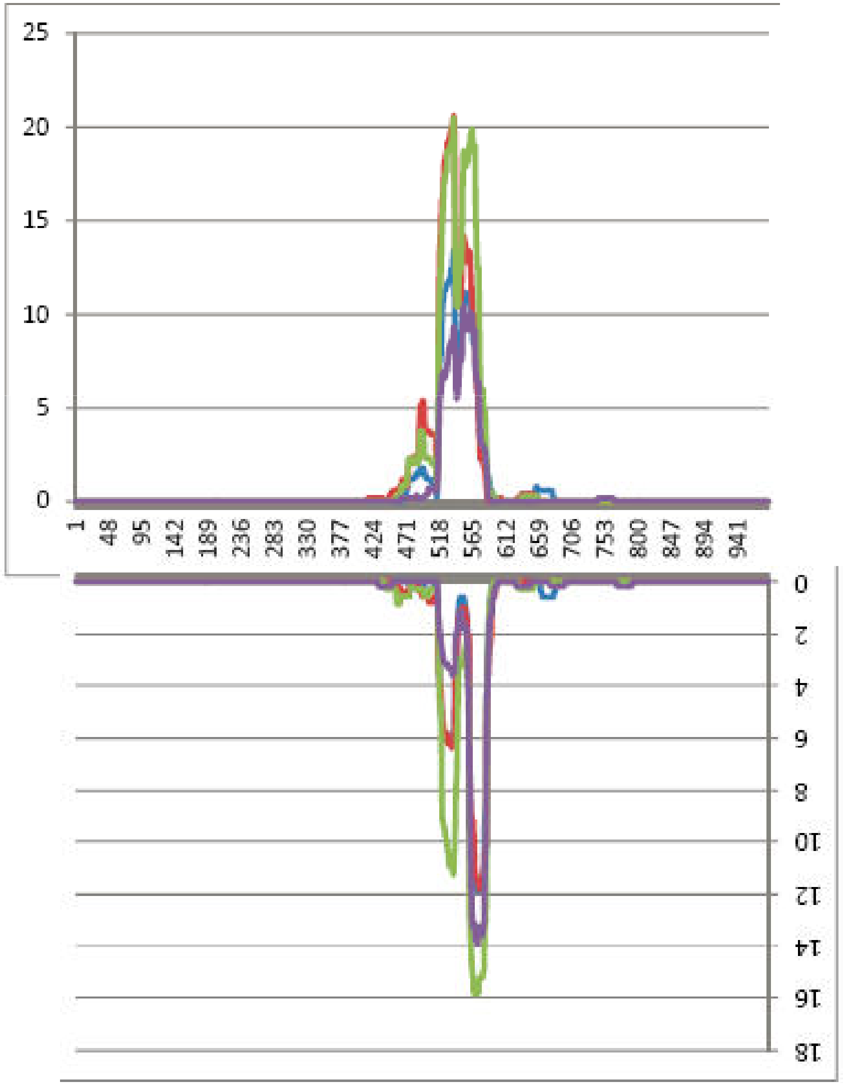

AT2G06255

ELF4-like 3 (ELF4-L3)

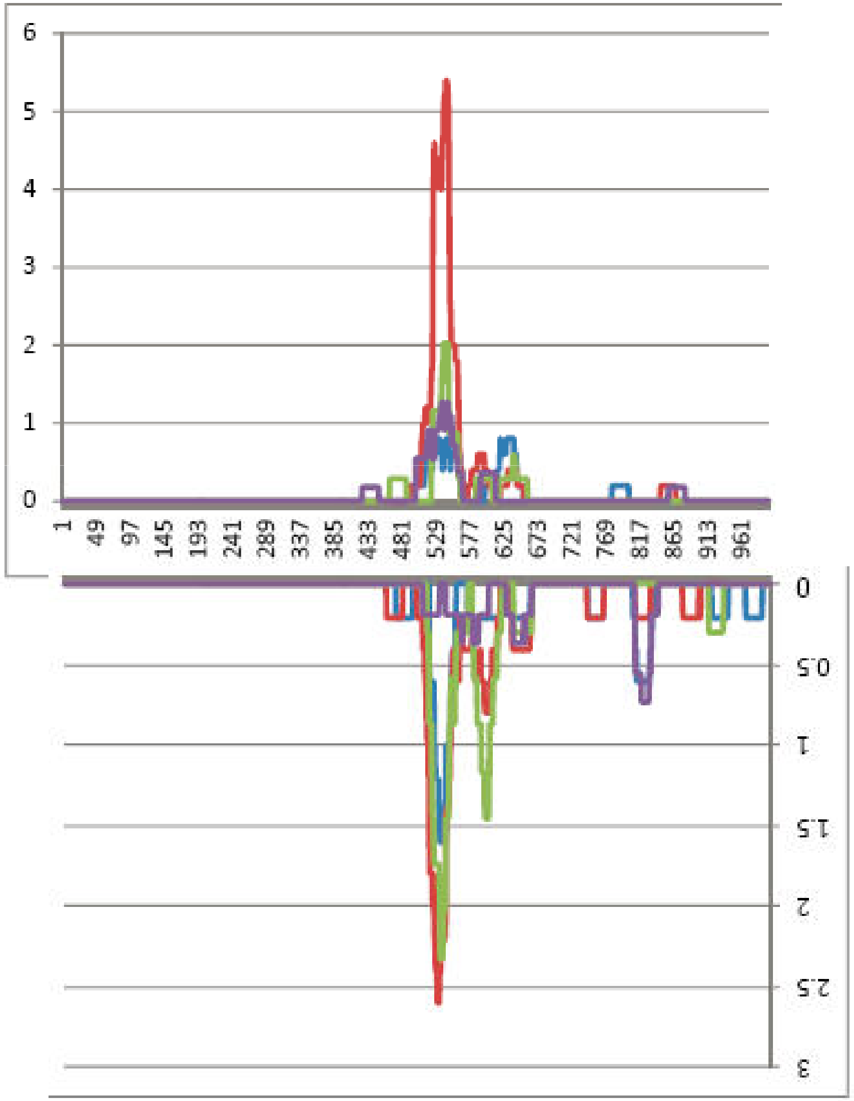

AT2G06541

TTF-type zinc finger protein with HAT dimerisation domain

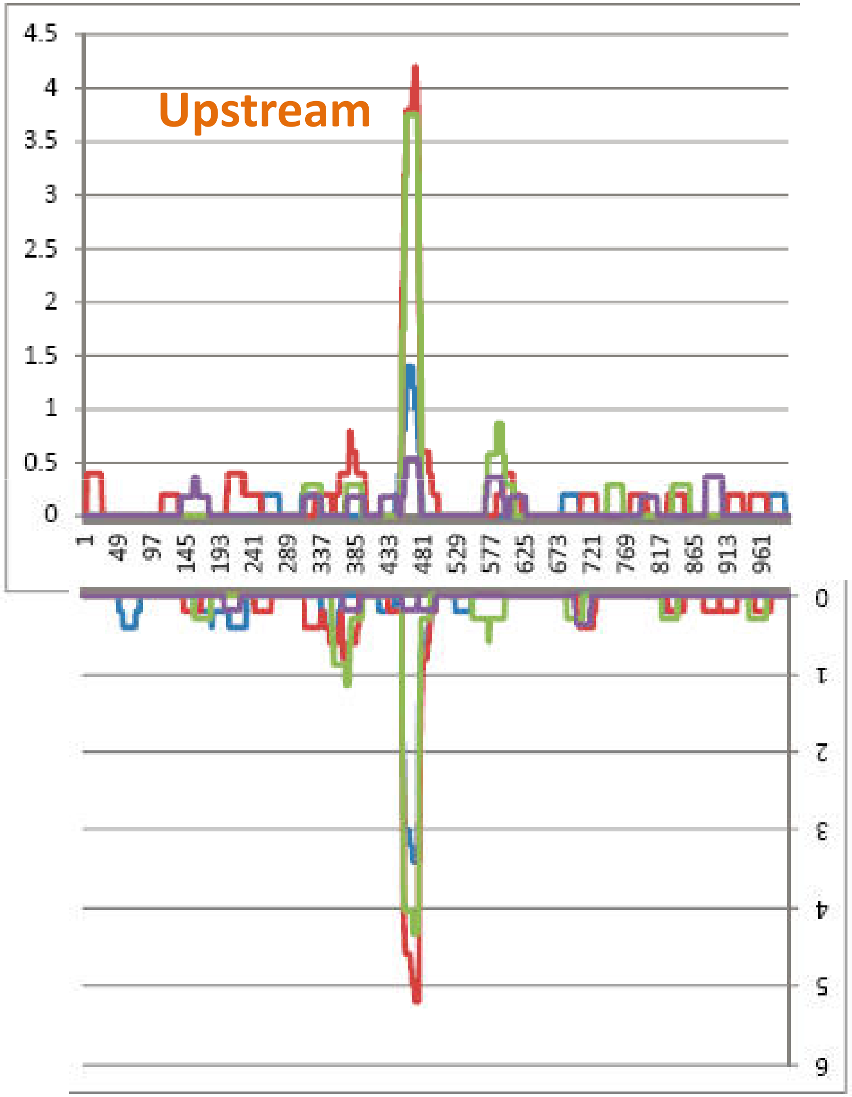

AT2G10608

unknown protein

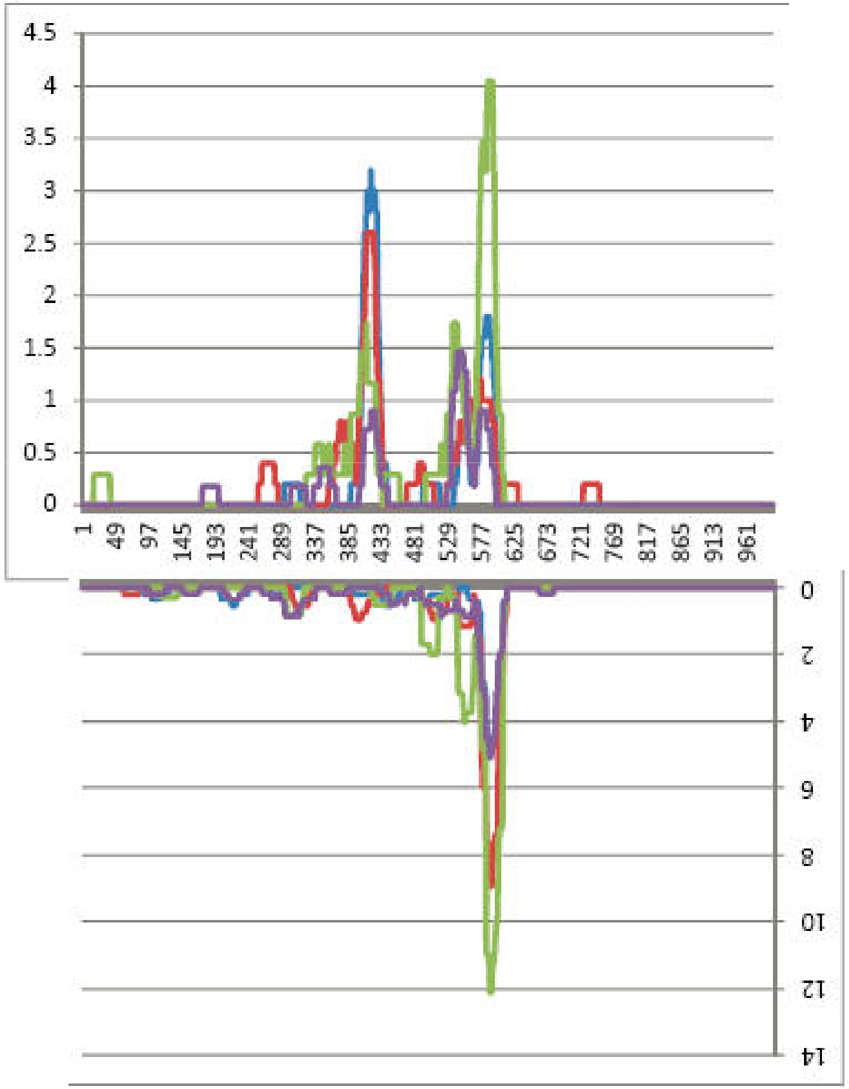

AT2G13960

Homeodomain-like superfamily protein

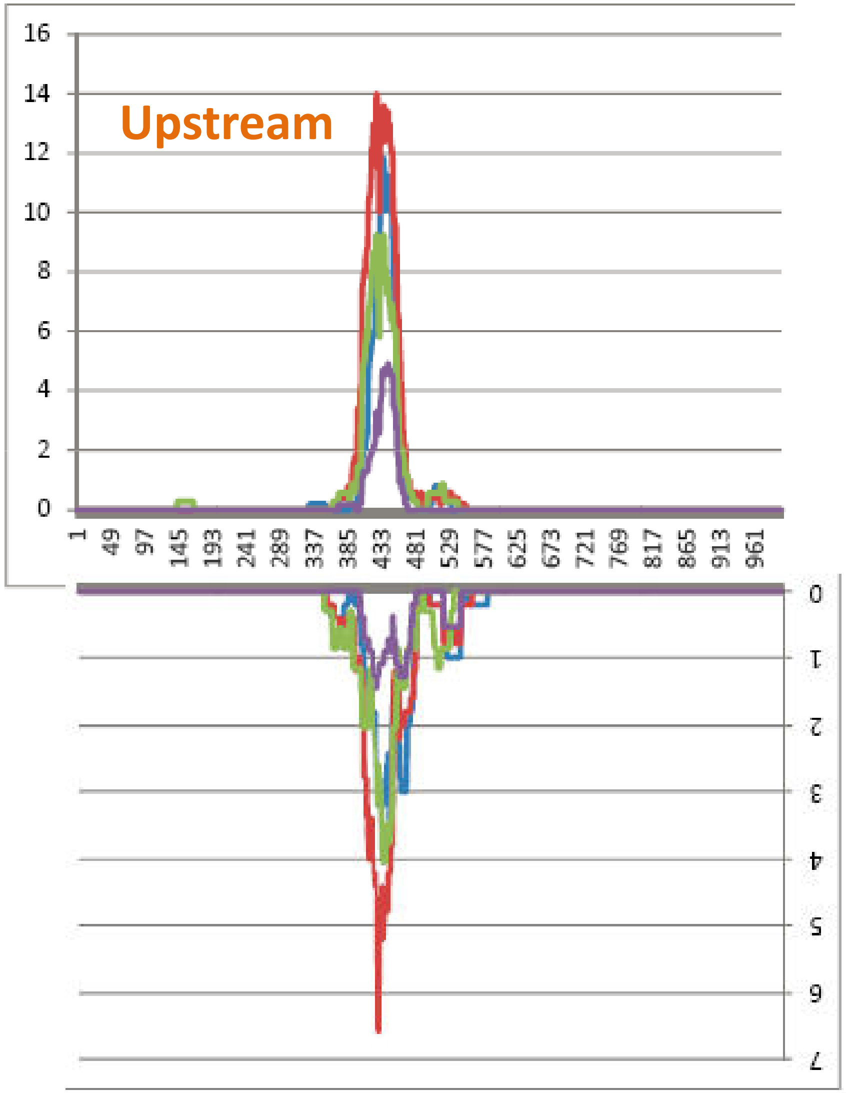

AT2G14560

Encodes LURP1, a member of the LURP cluster (late upregulated in response to Hyaloperonospora parasitica) which exhibits a pronounced upregulation after recognition of the pathogenic oomycte H. parasitica. LURP1 is required for full basal defense to H. parasitica and resistance to this pathogen mediated by the R-proteins RPP4 and RPP5.

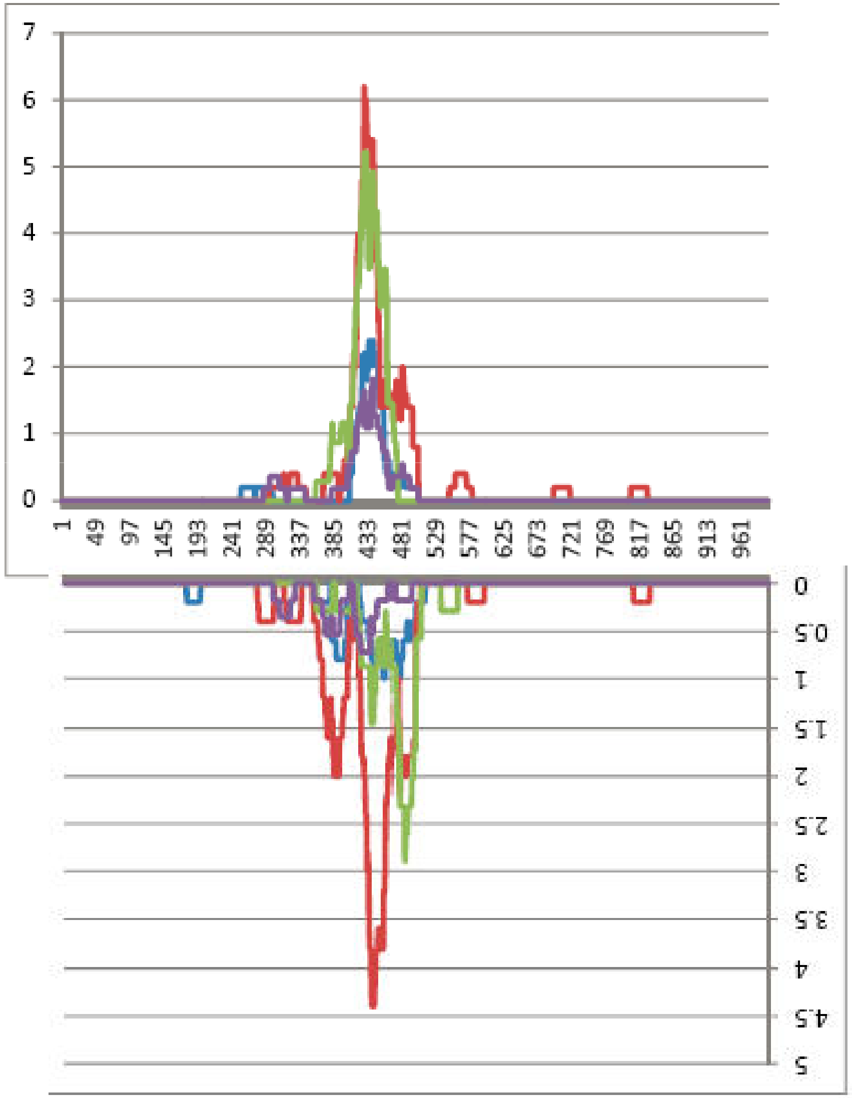

AT2G15050

Predicted to encode a PR (pathogenesis-related) protein.

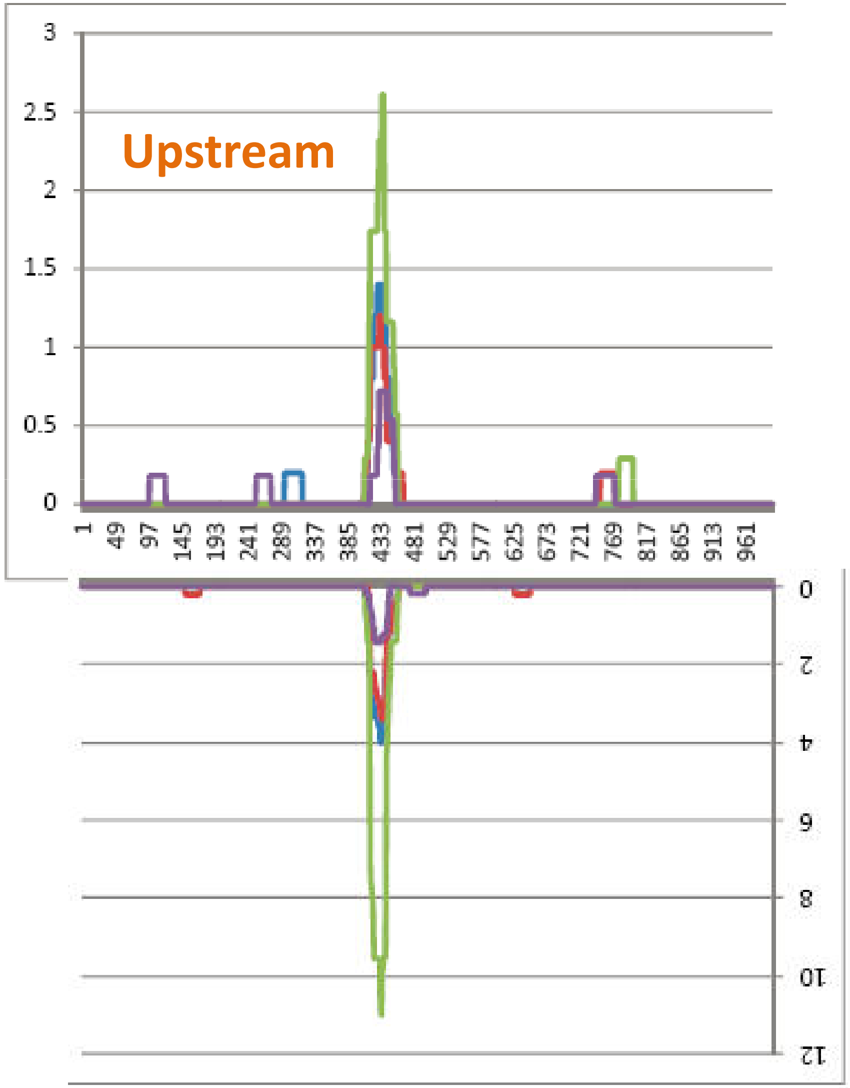

AT2G16340

unknown protein

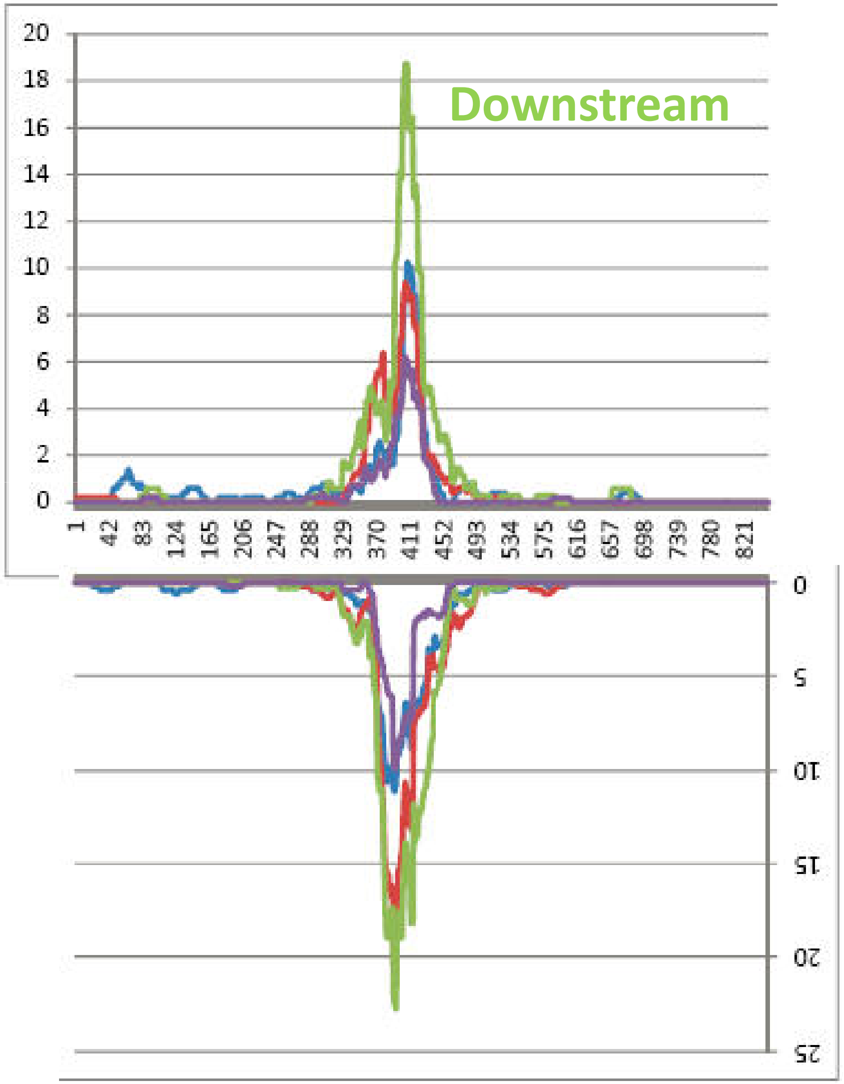

AT2G18980

Peroxidase superfamily protein

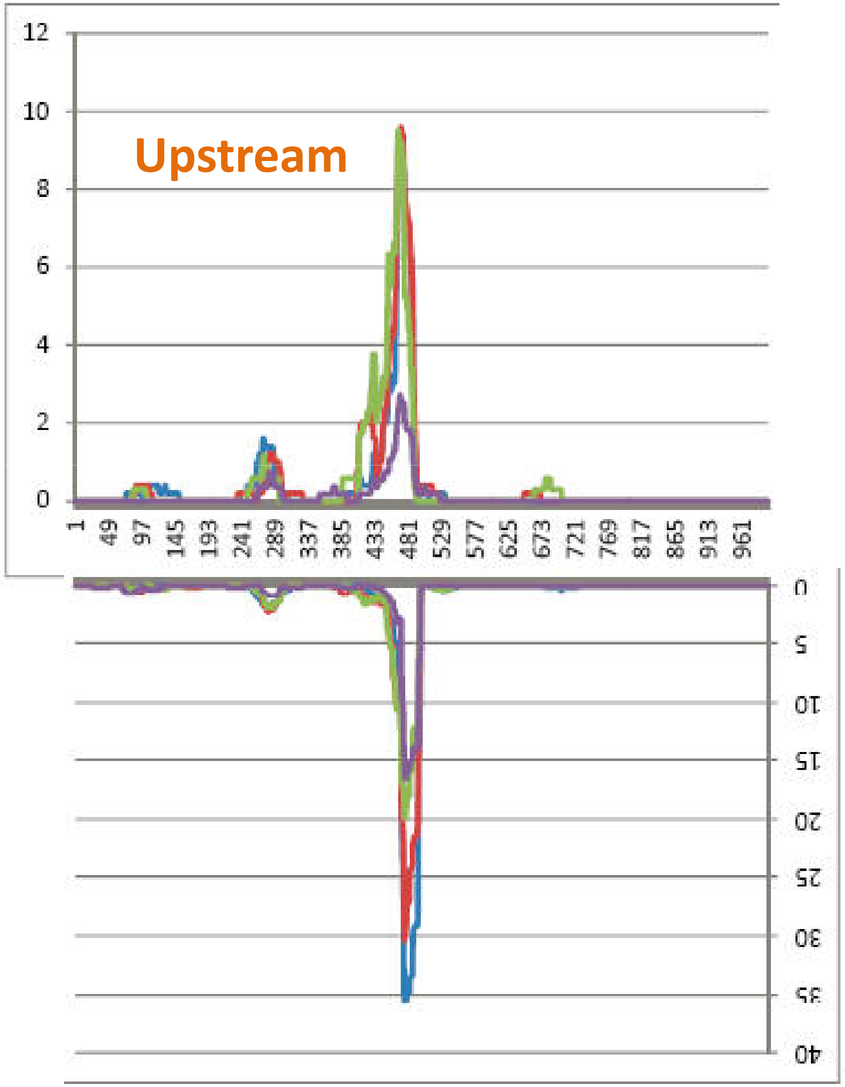

AT2G22890

Kua-ubiquitin conjugating enzyme hybrid localisation domain; LOCATED IN: chloroplast; EXPRESSED IN: 21 plant structures.

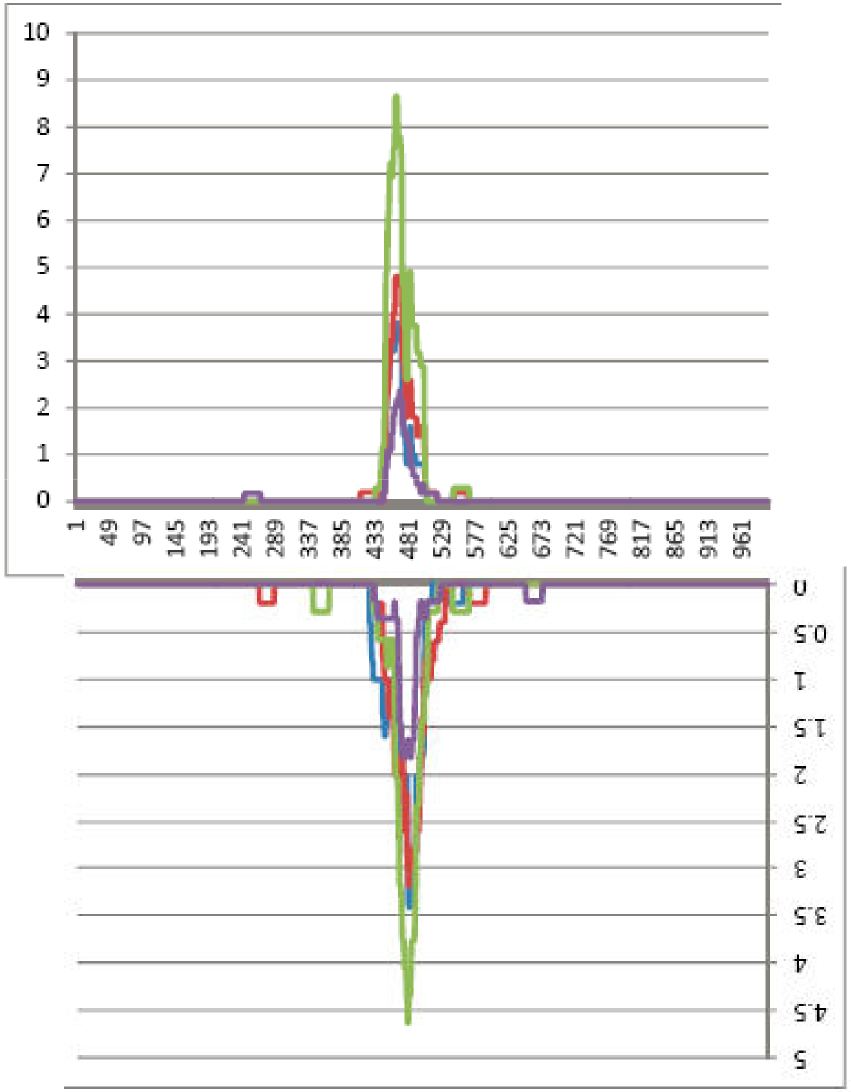

AT2G24880

Plant self-incompatibility protein S1 family

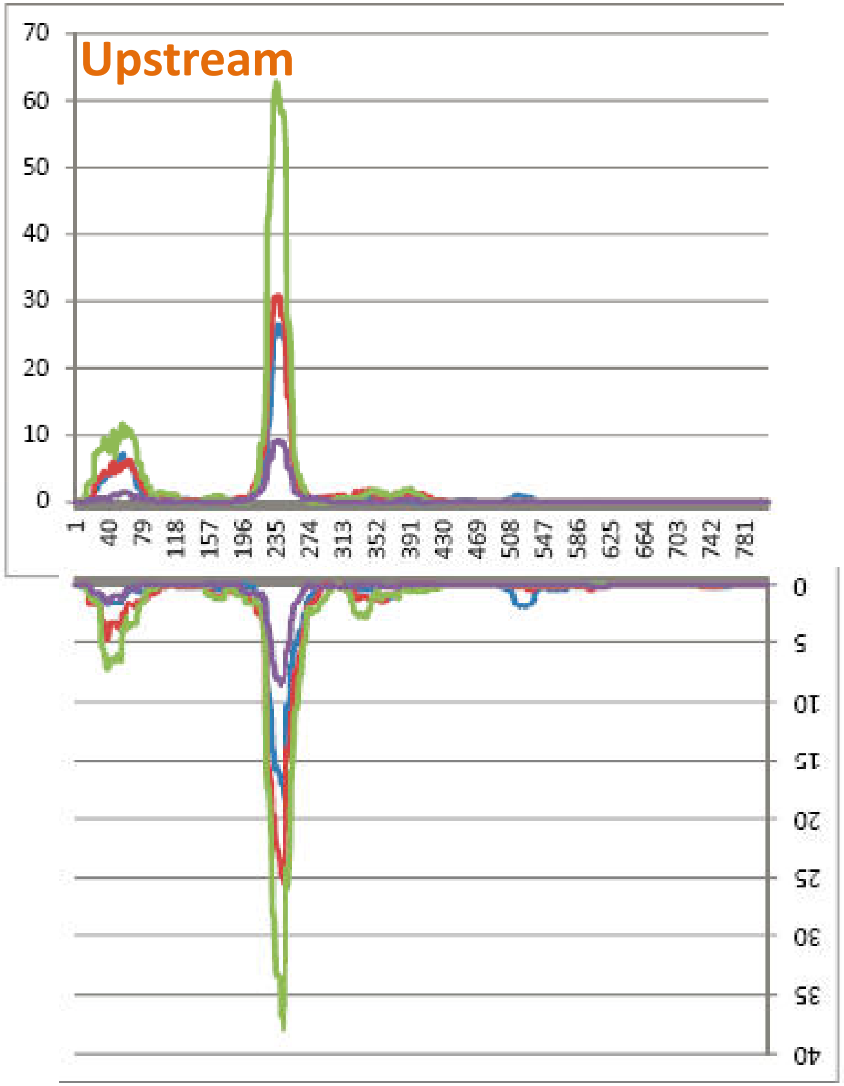

AT2G32140

transmembrane receptors

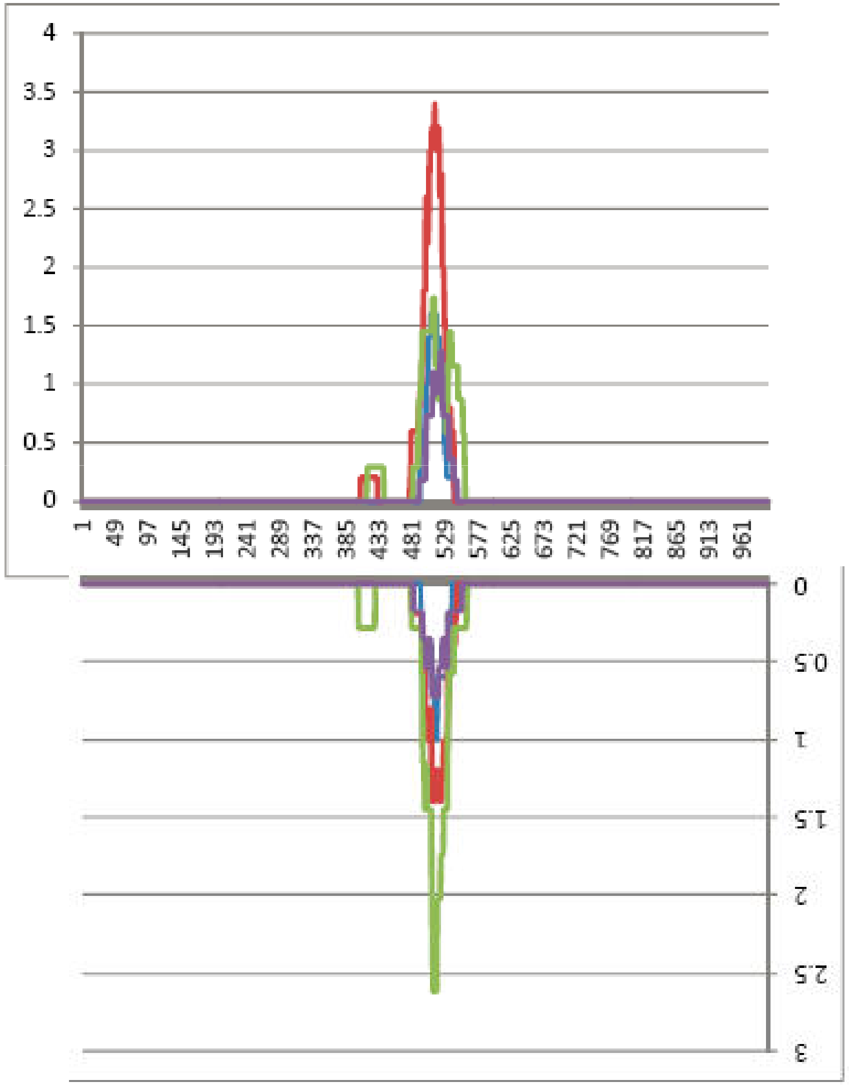

AT2G34655

unknown protein

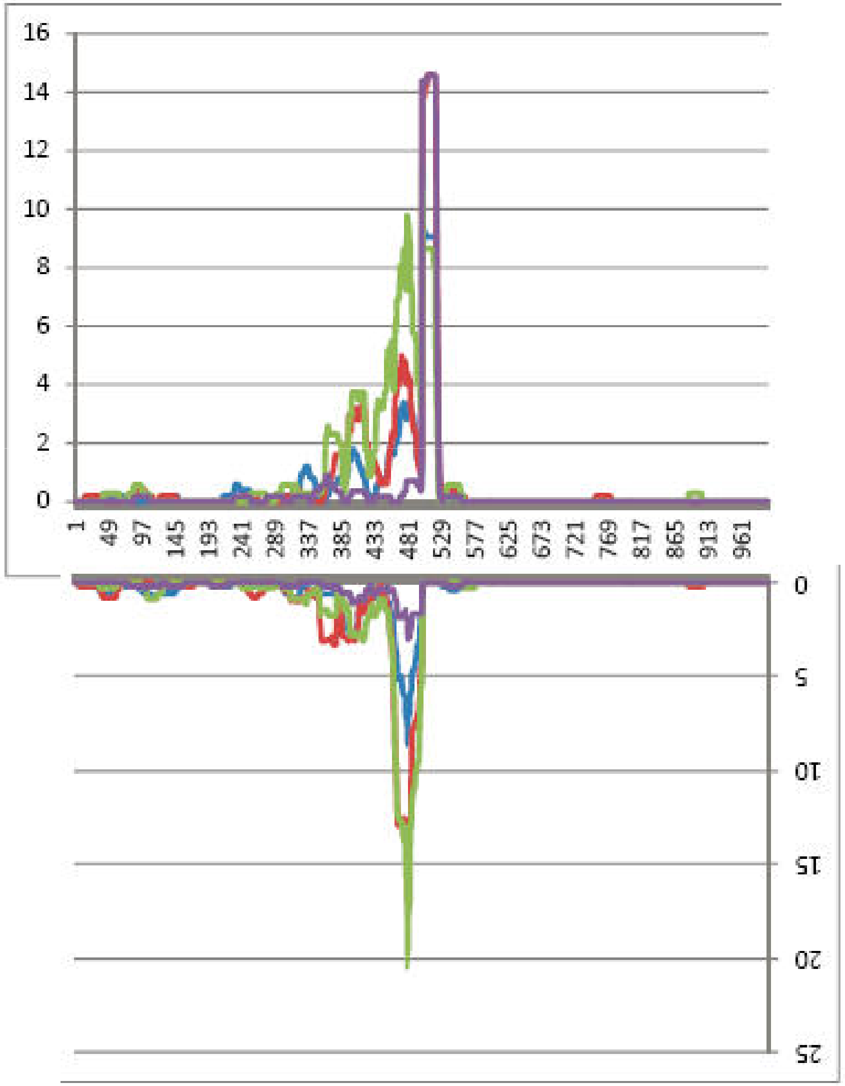

AT2G35250

Protein of Unknown Function (DUF239)

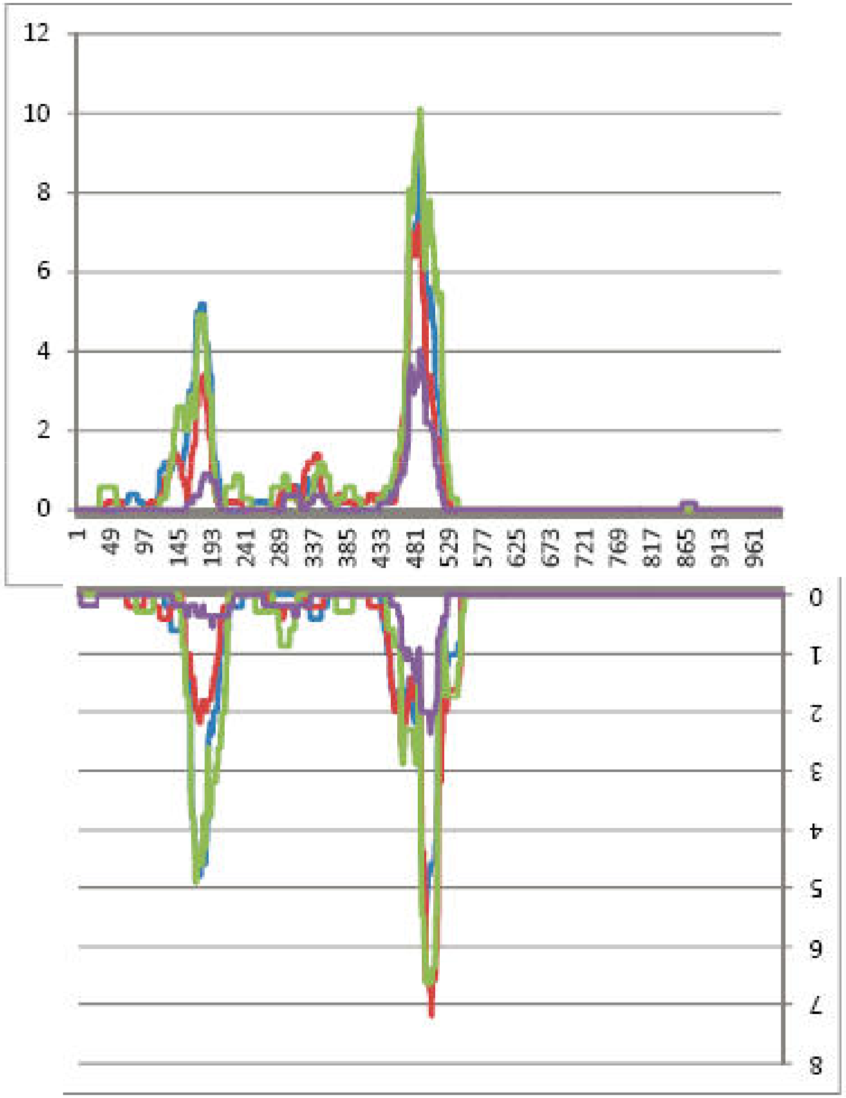

AT2G36940

unknown protein

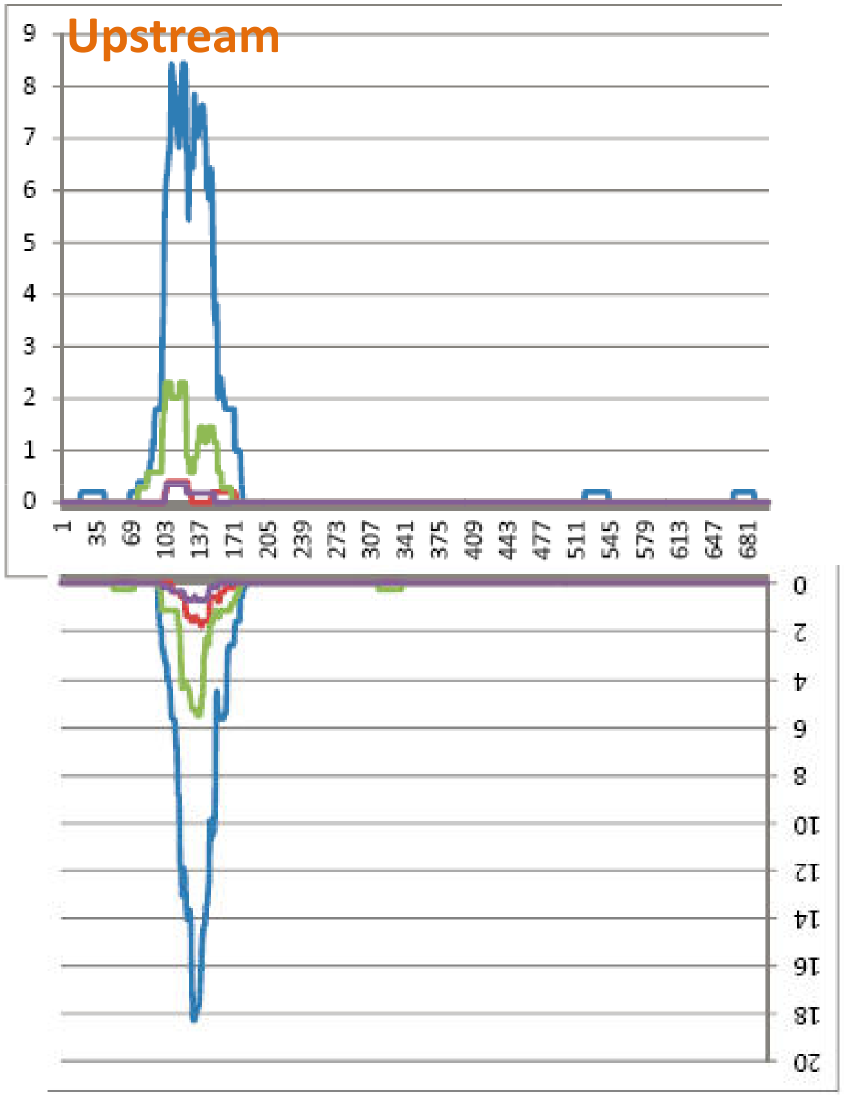

AT2G37810

Cysteine/Histidine-rich C1 domain family protein

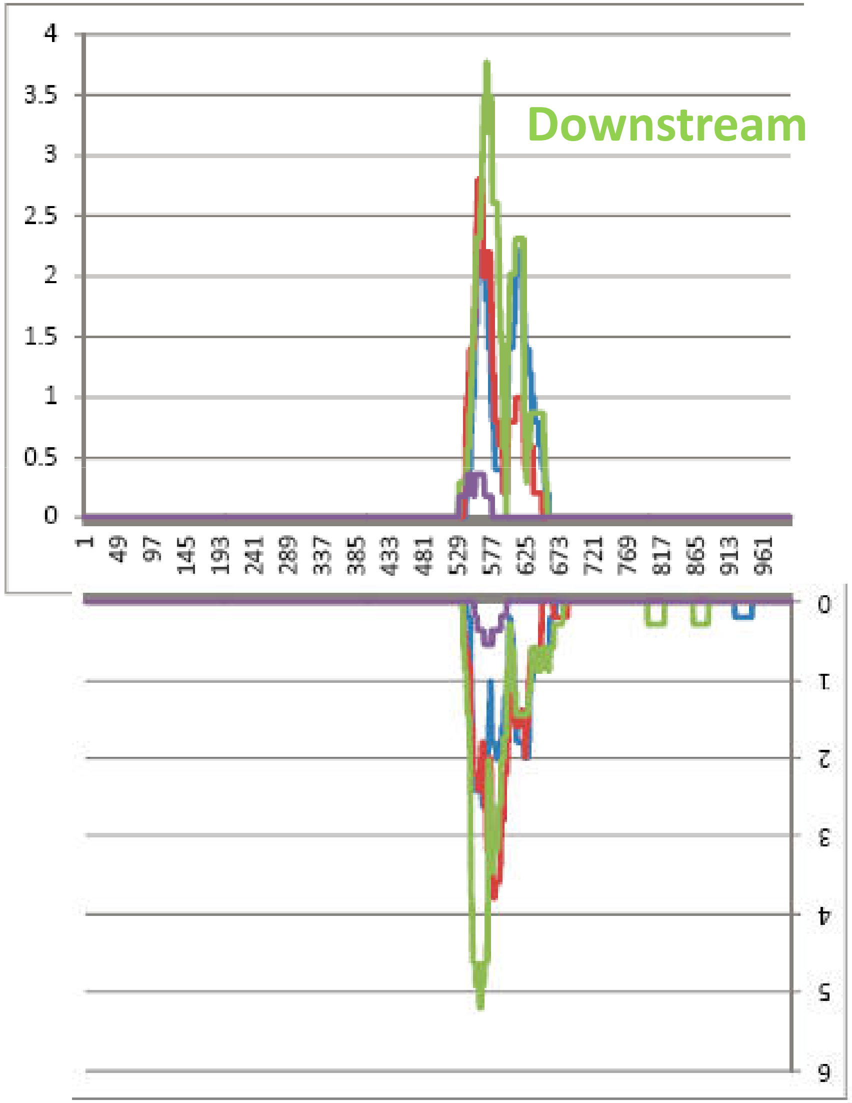

AT2G41980

Protein with RING/U-box and TRAF-like domains

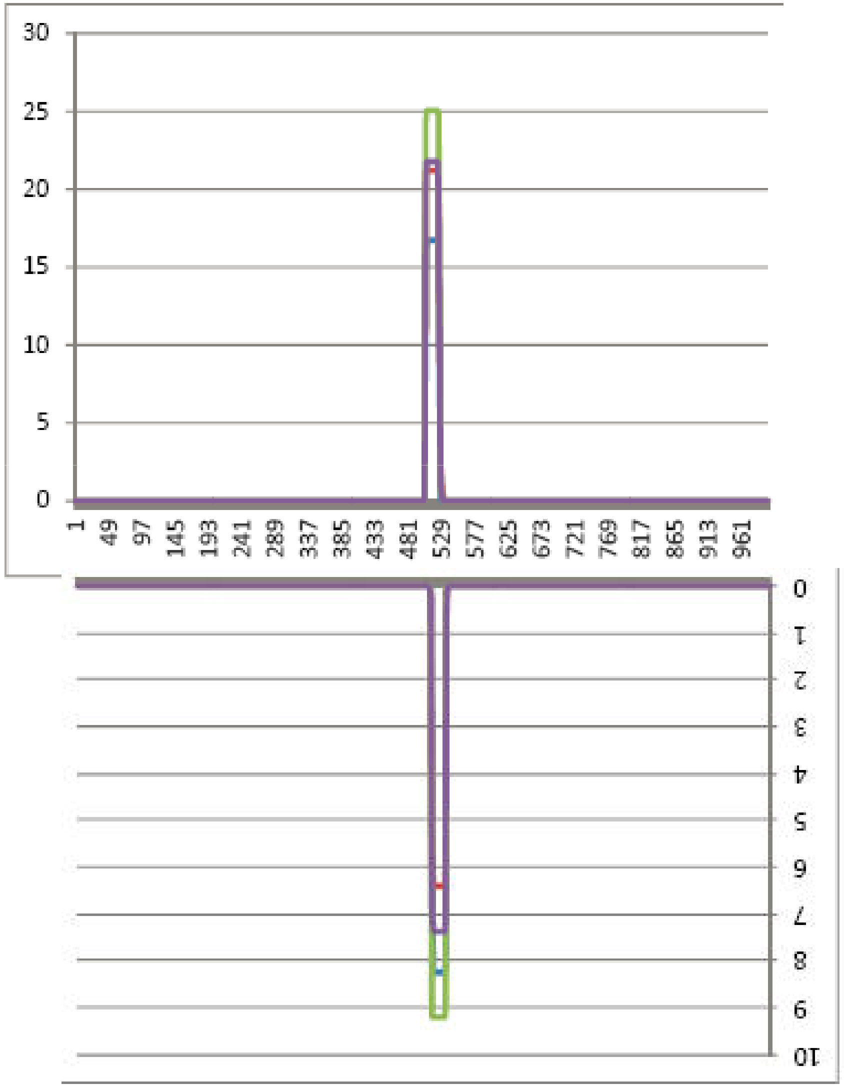

AT3G02420

unknown protein

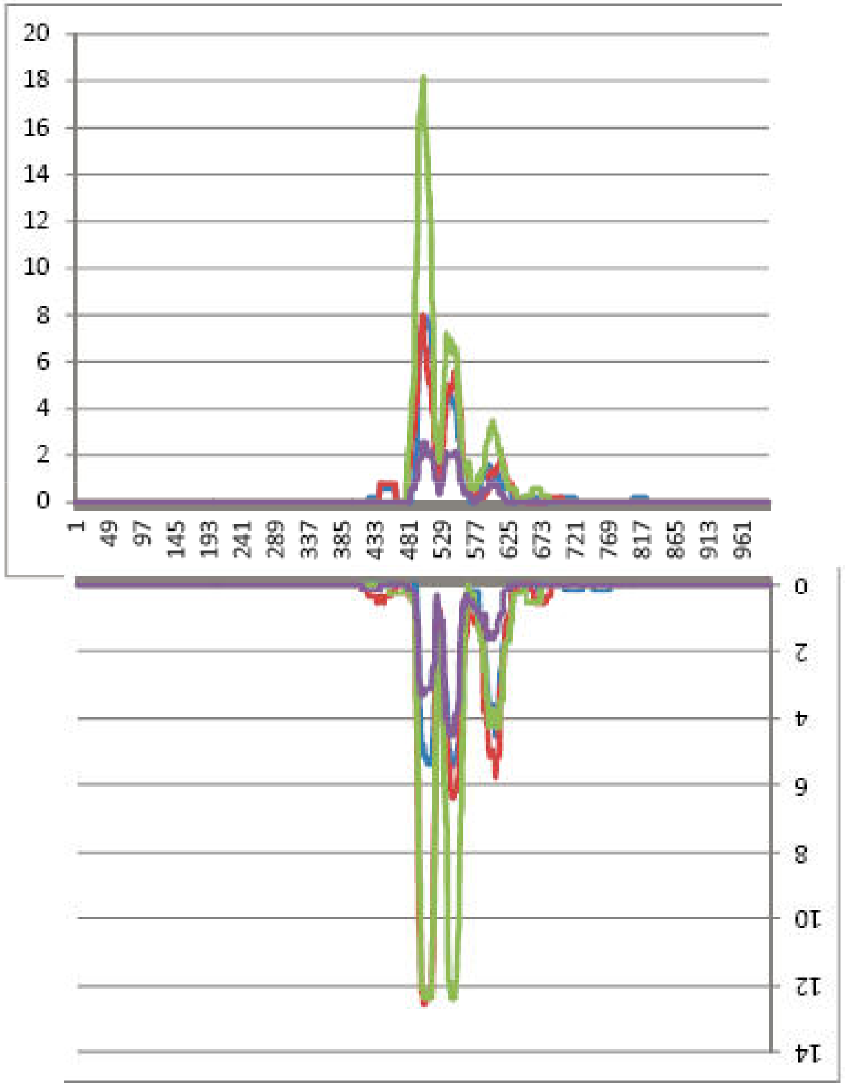

AT3G09960

Calcineurin-like metallo-phosphoesterase superfamily protein

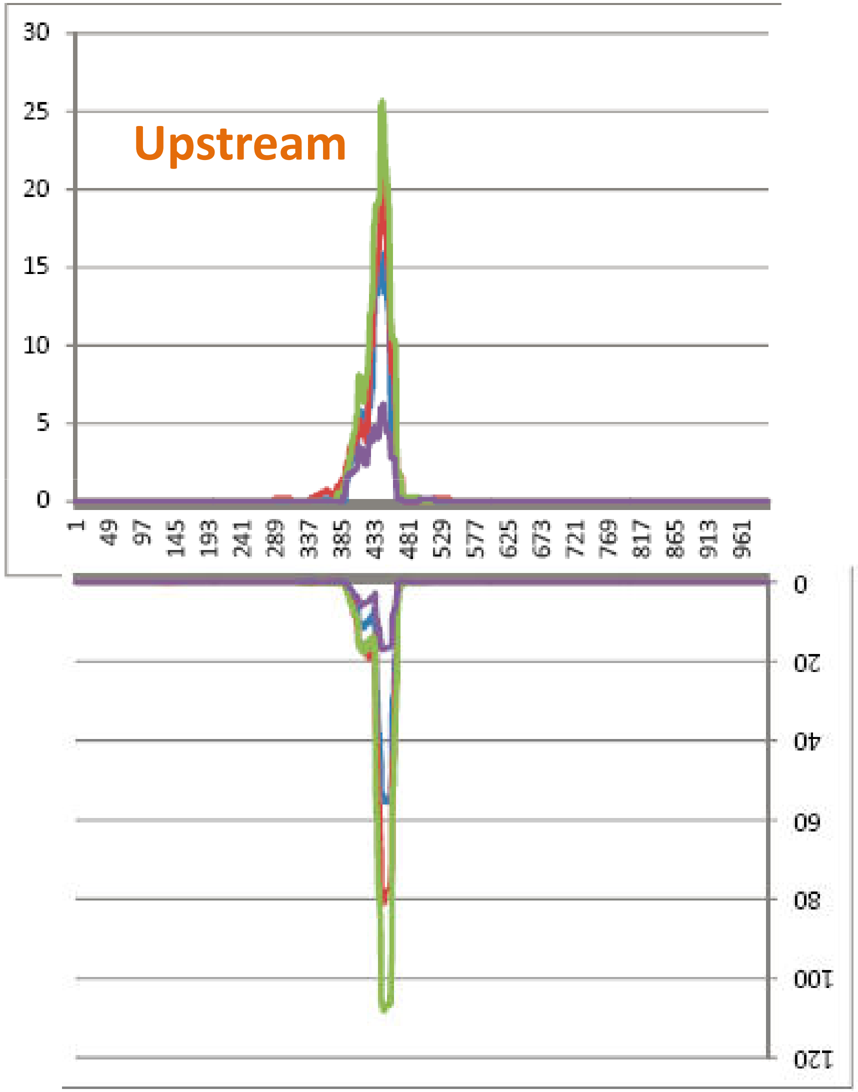

AT3G10900

Glycosyl hydrolase superfamily protein

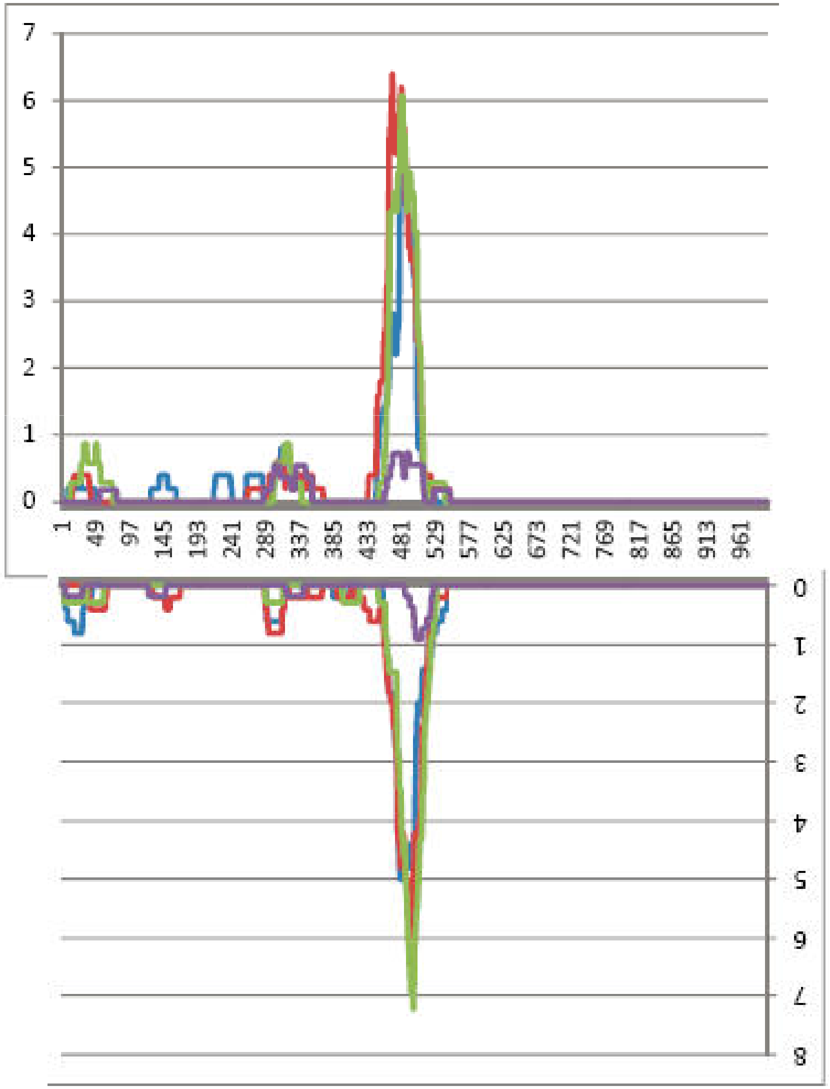

AT3G11310

unknown protein

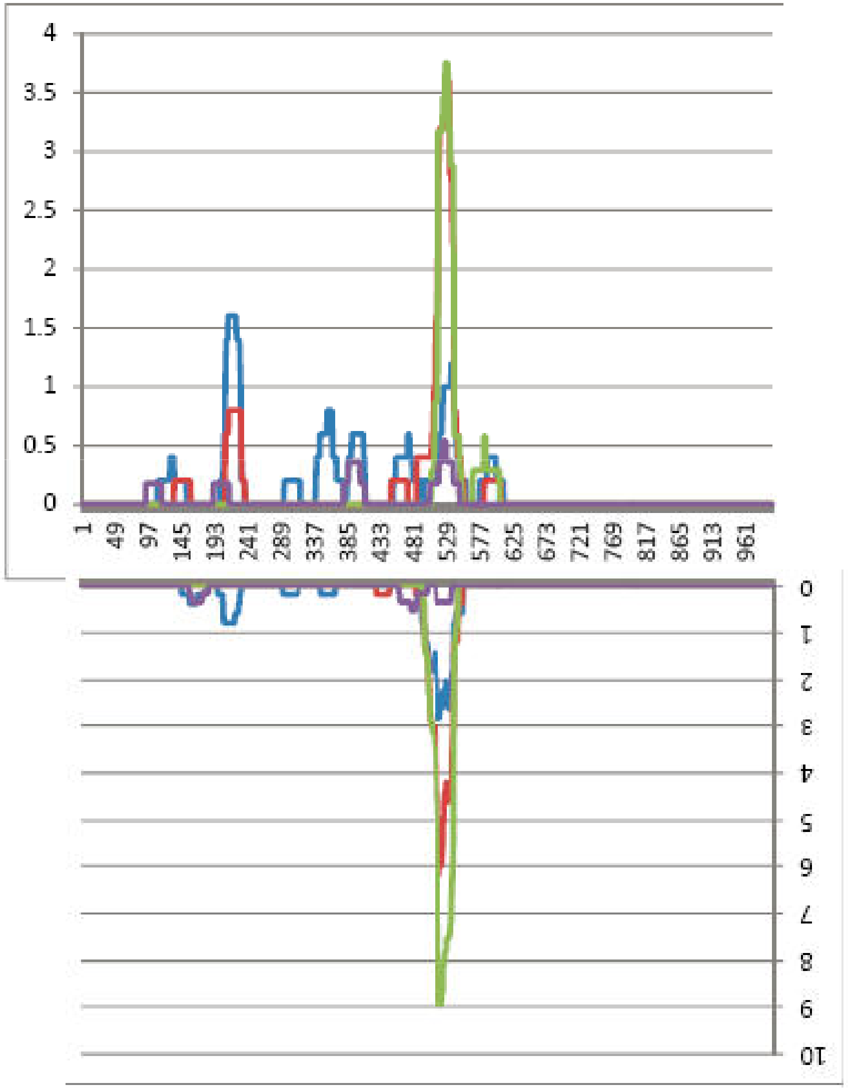

AT3G21870

cyclin p2;1
(CYCP2;1)

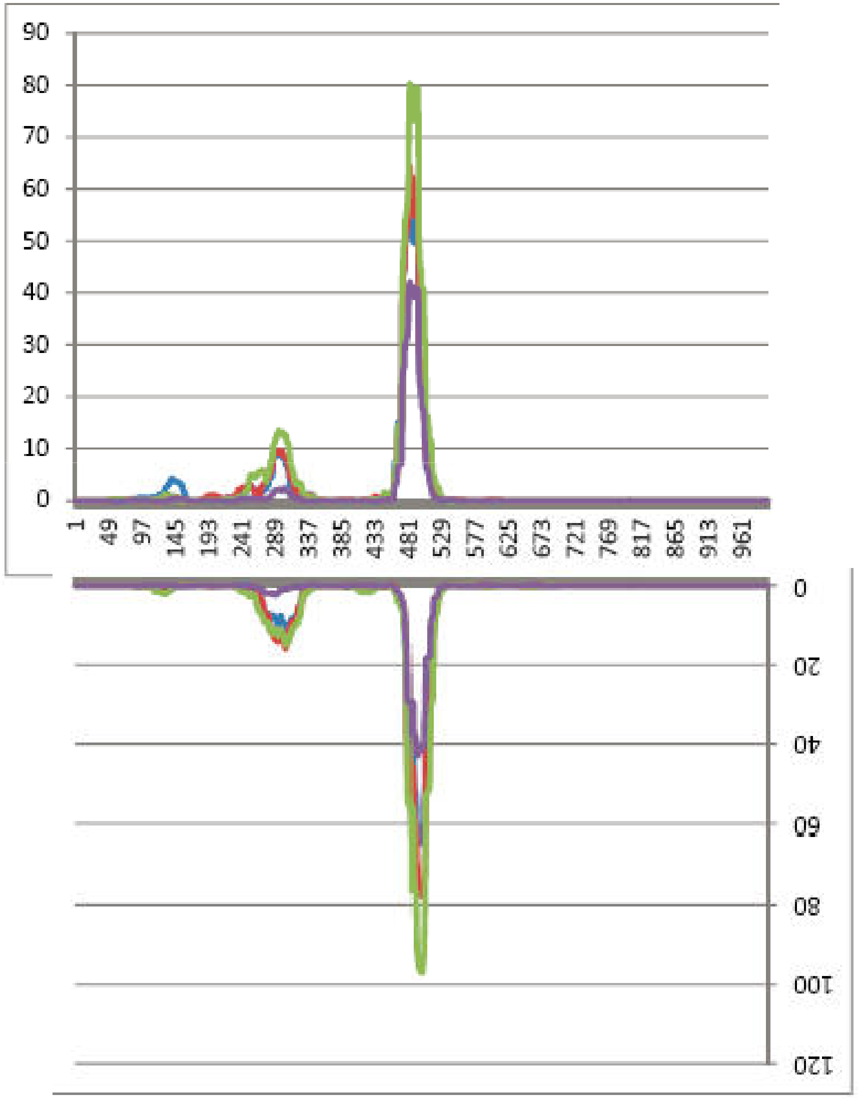

AT3G22710

F-box family protein

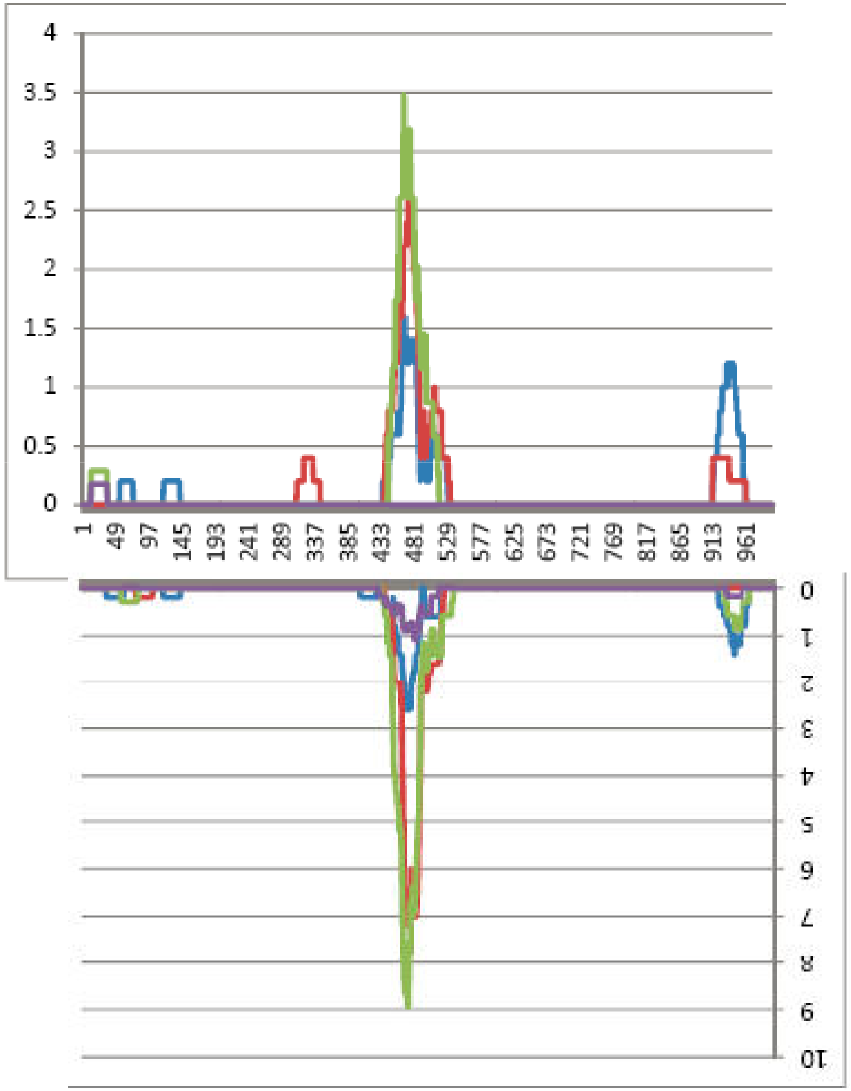

AT3G23440

EMBRYO SAC DEVELOPMENT ARREST 6 (EDA6)

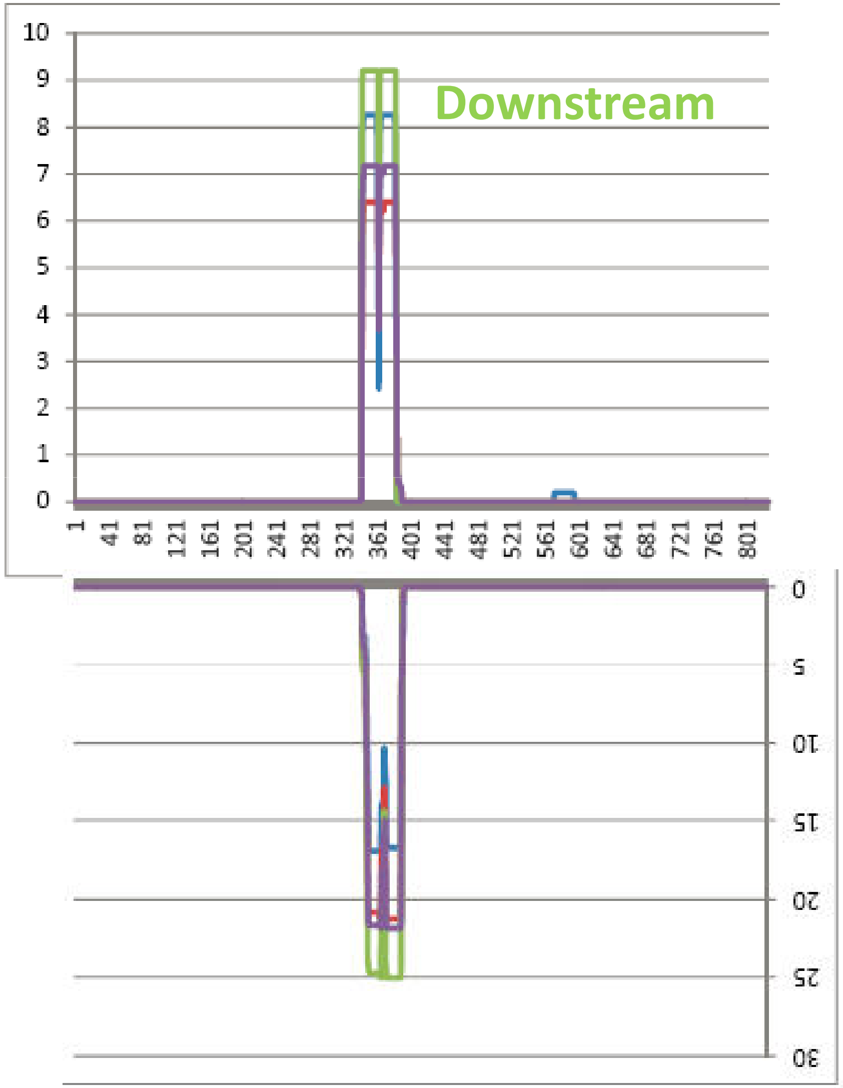

AT3G25130

unknown protein

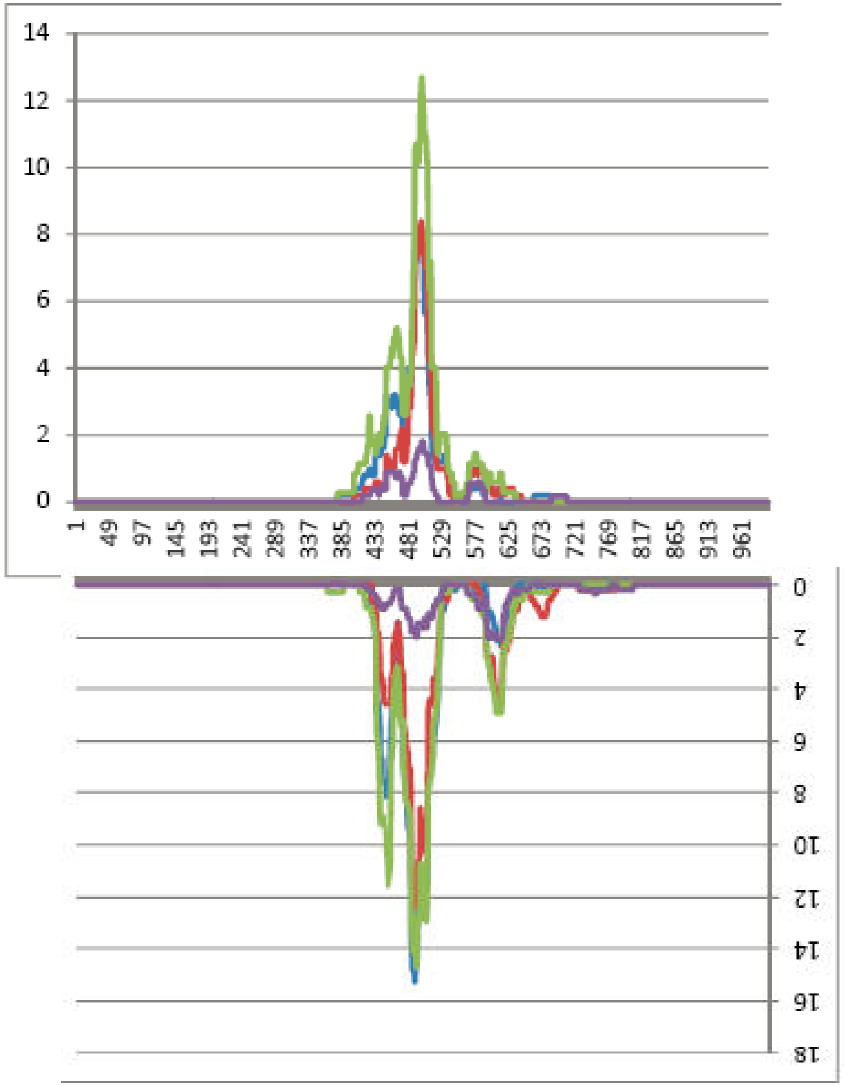

AT3G25720

RNA-directed DNA polymerase (reverse transcriptase)-related family protein

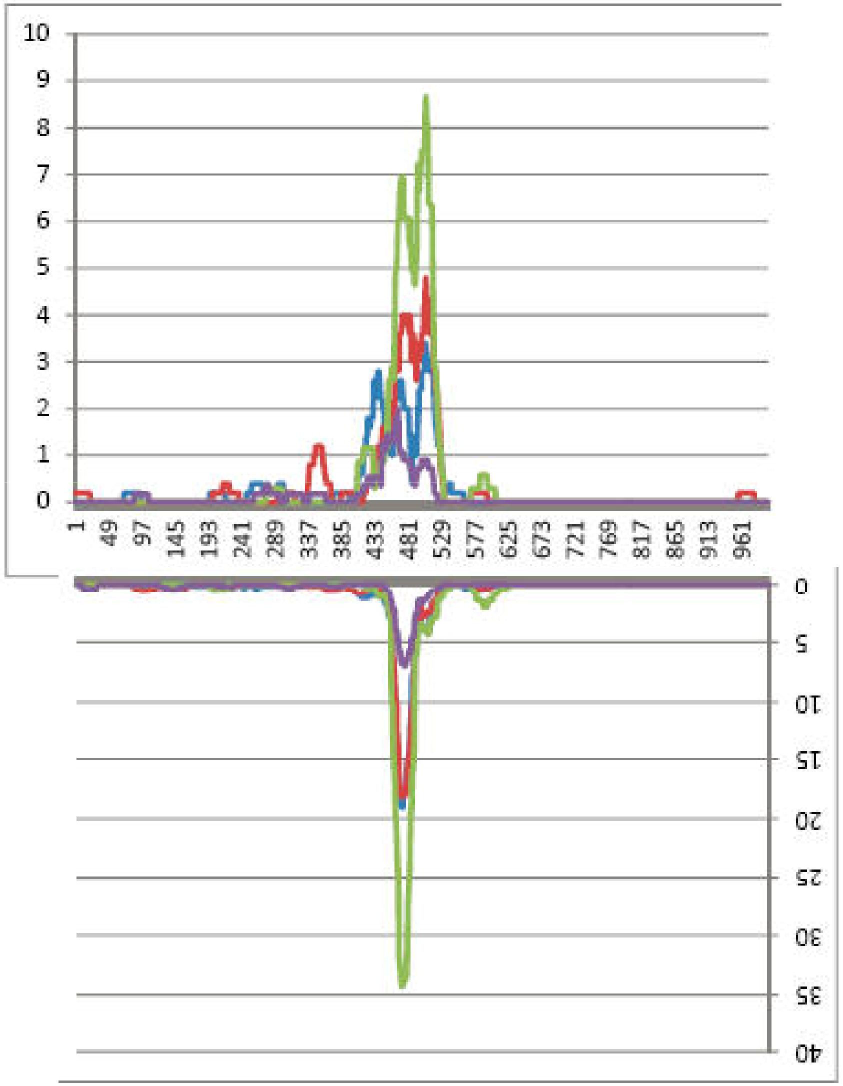

AT3G25855

Copper transport protein family

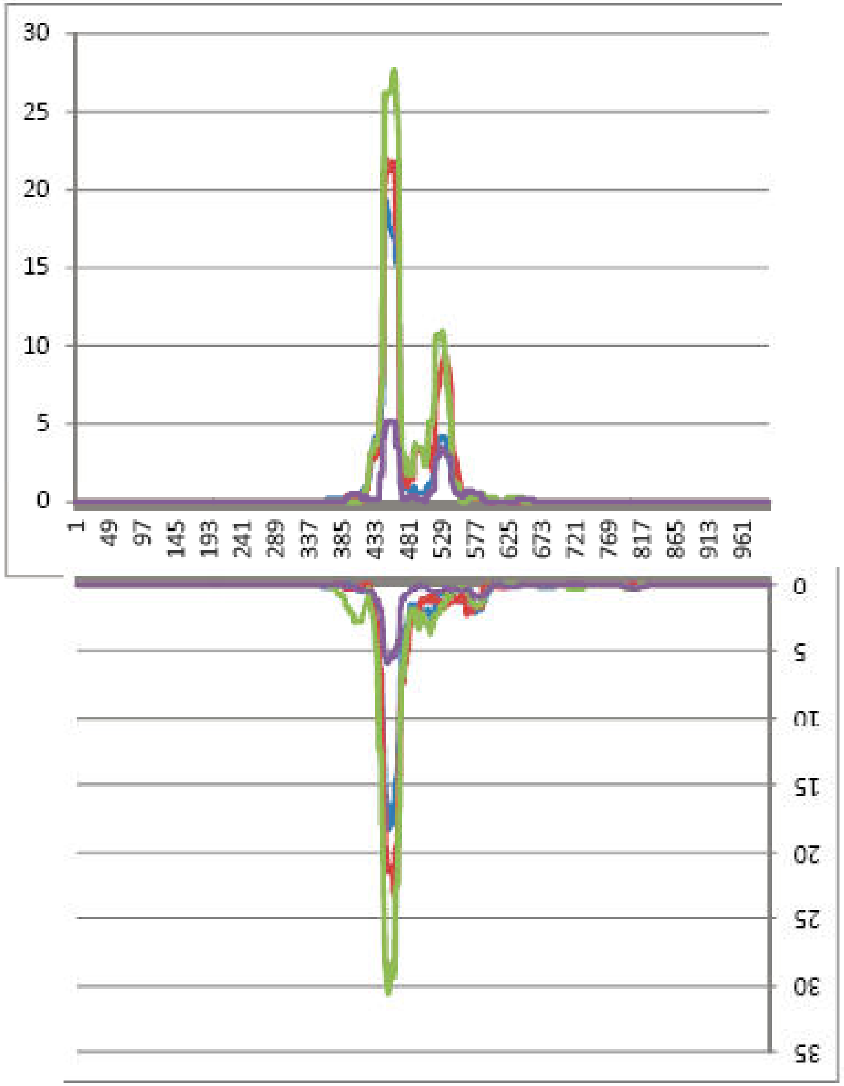

AT3G26100

Regulator of chromosome condensation (RCC1) family protein

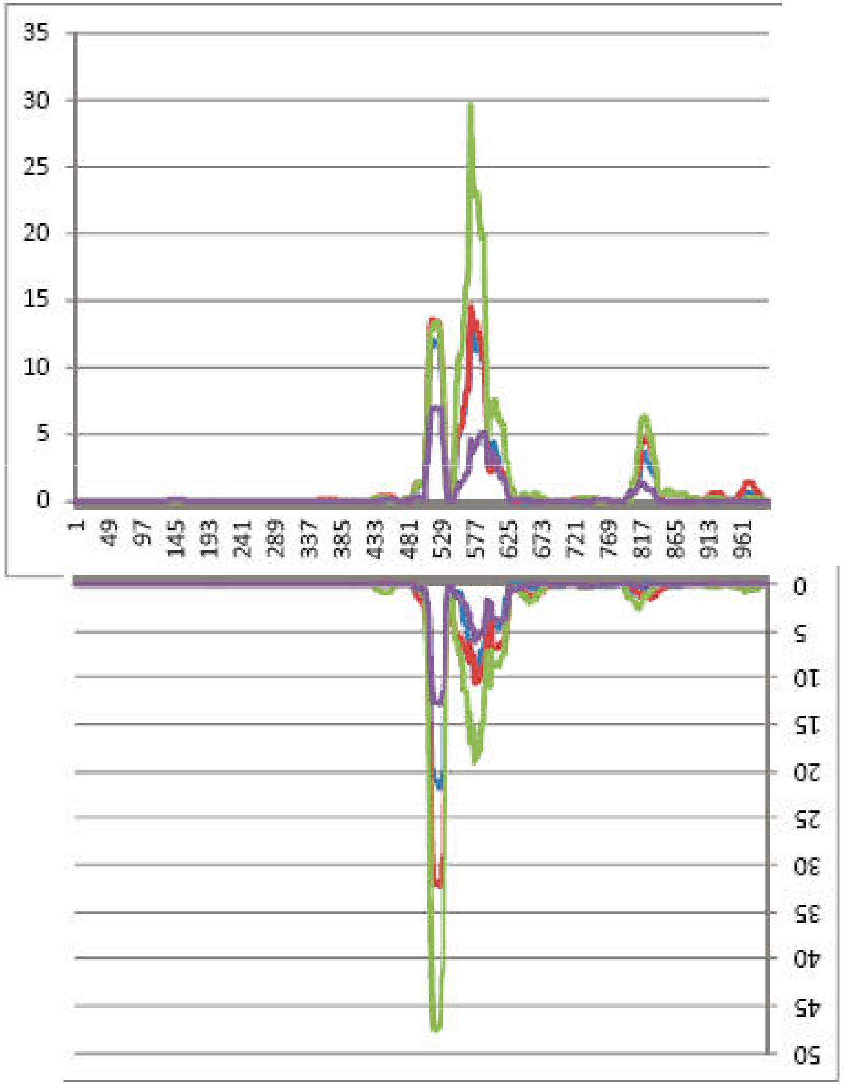

AT3G26140

Cellulase (glycosyl hydrolase family 5) protein

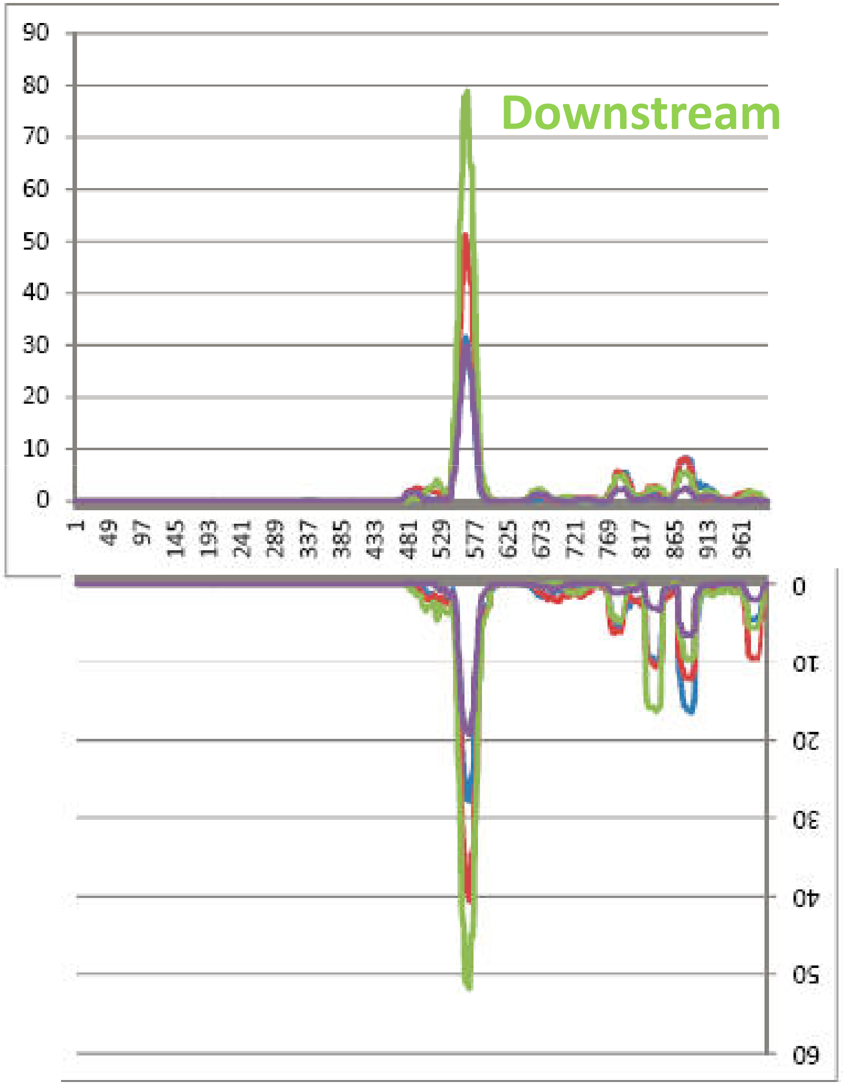

AT3G26280

Cytochrome P450 monooxygenase

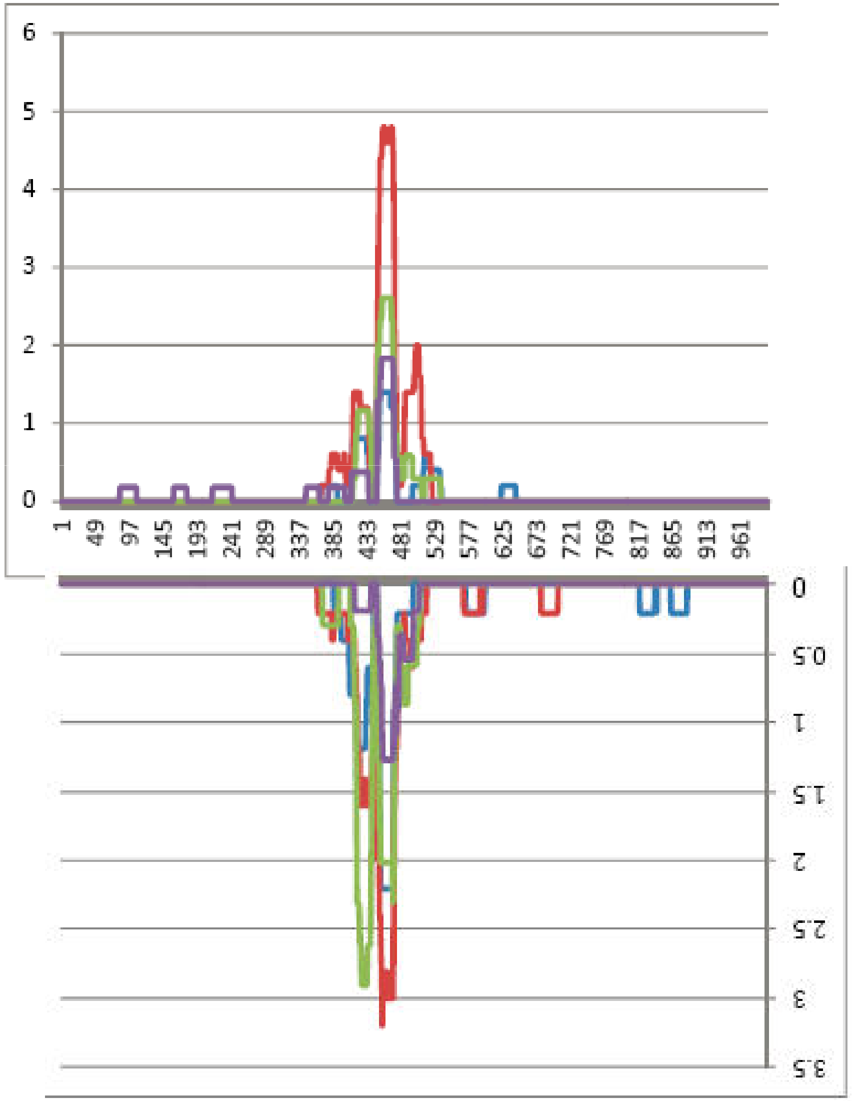

AT3G27250

unknown protein

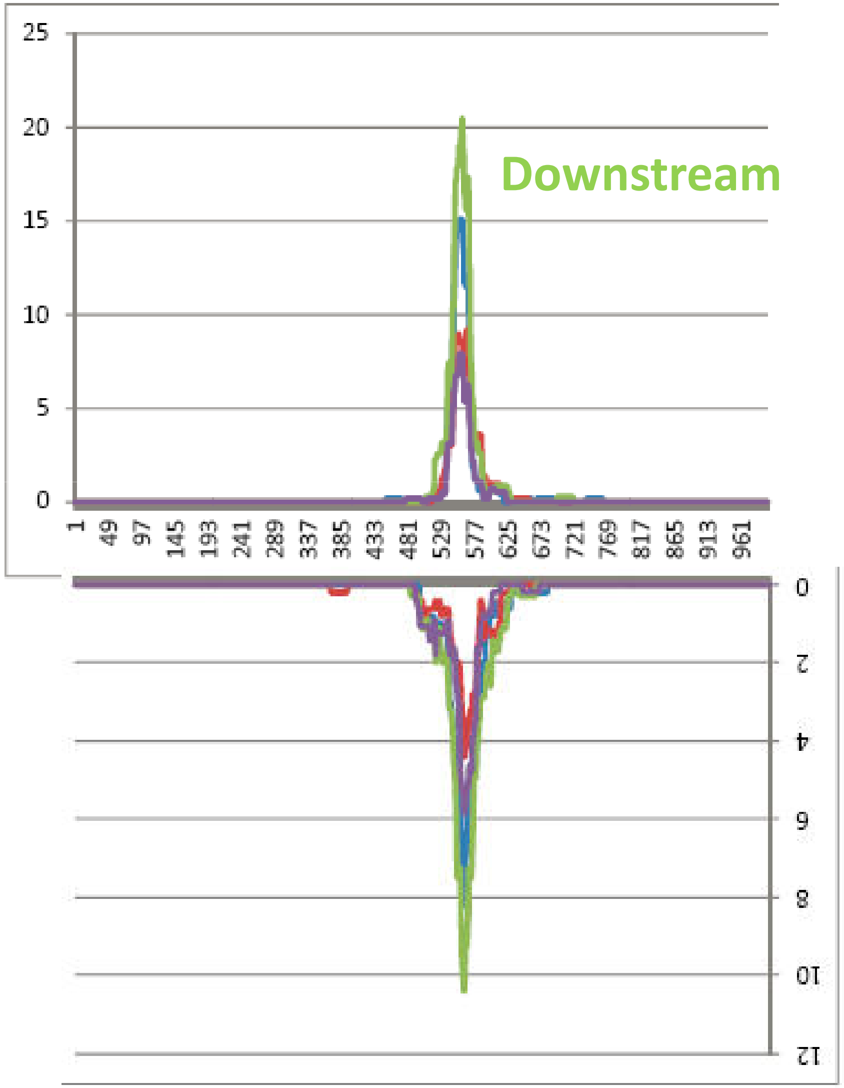

AT3G32050

unknown protein

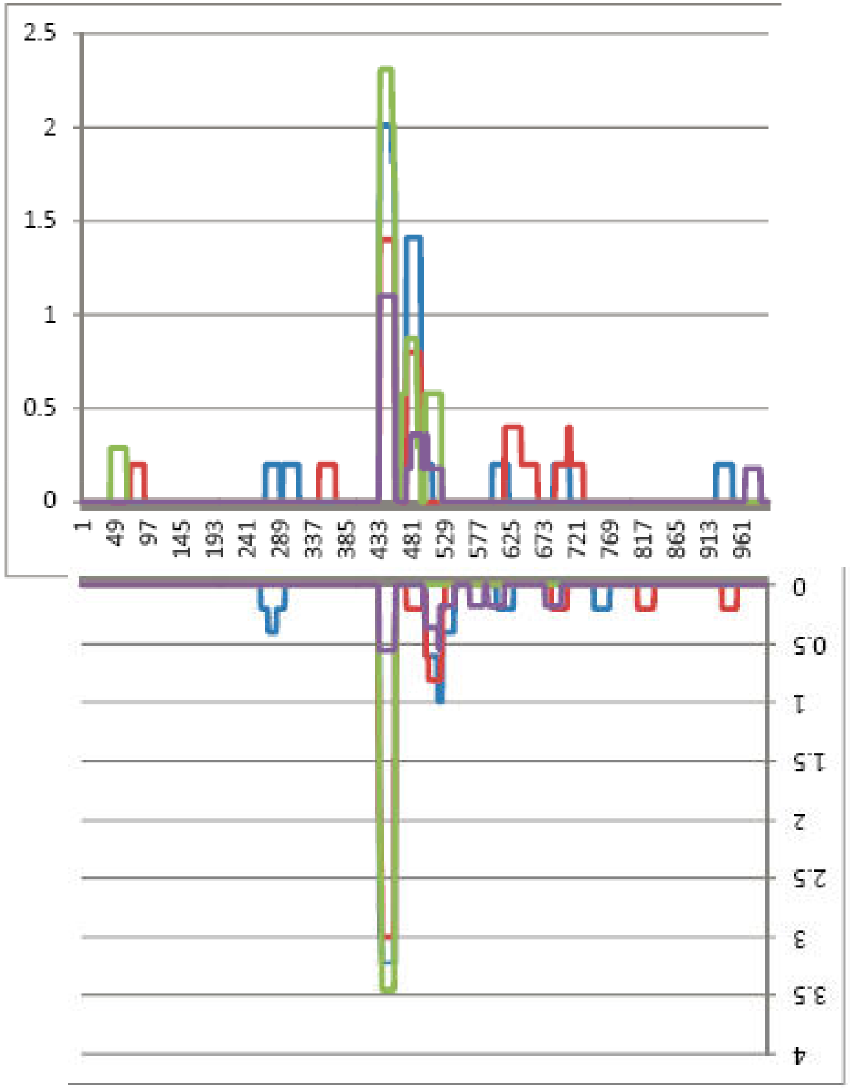

AT3G41762

unknown protein

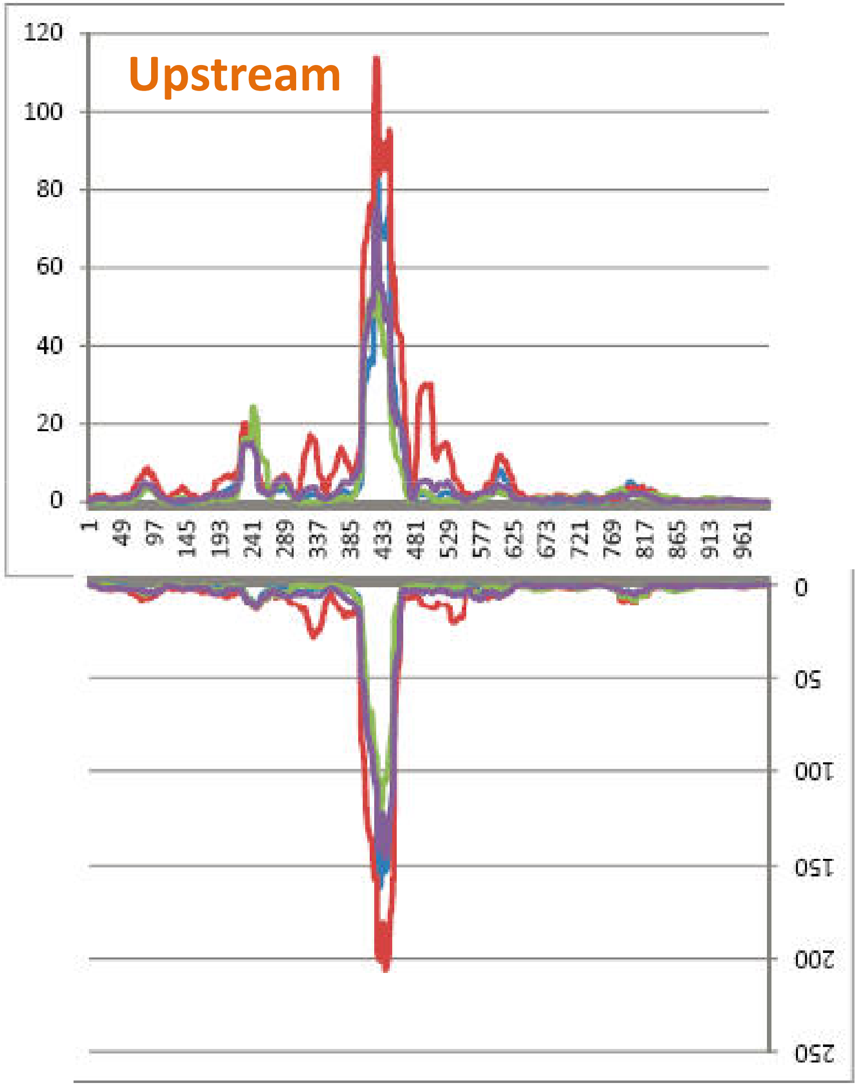

AT3G43420

unknown protein

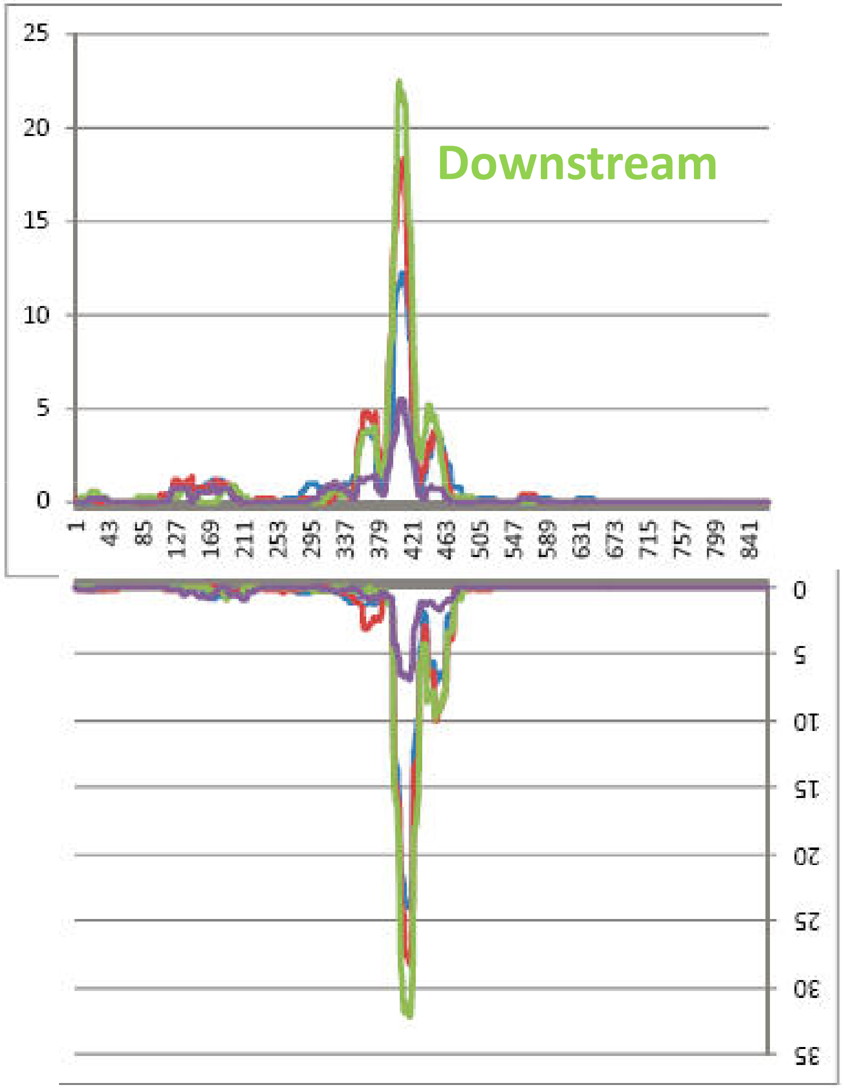

AT3G44810

F-box family protein

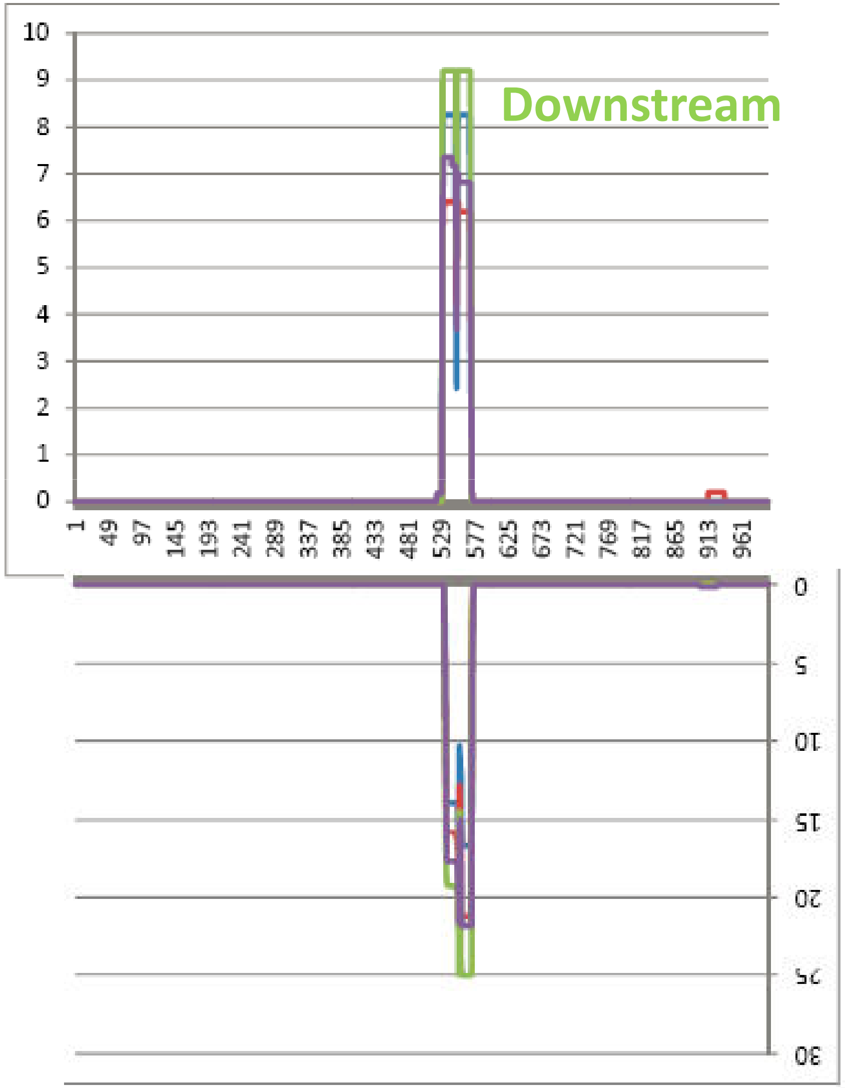

AT3G45260

C2H2-like zinc finger protein

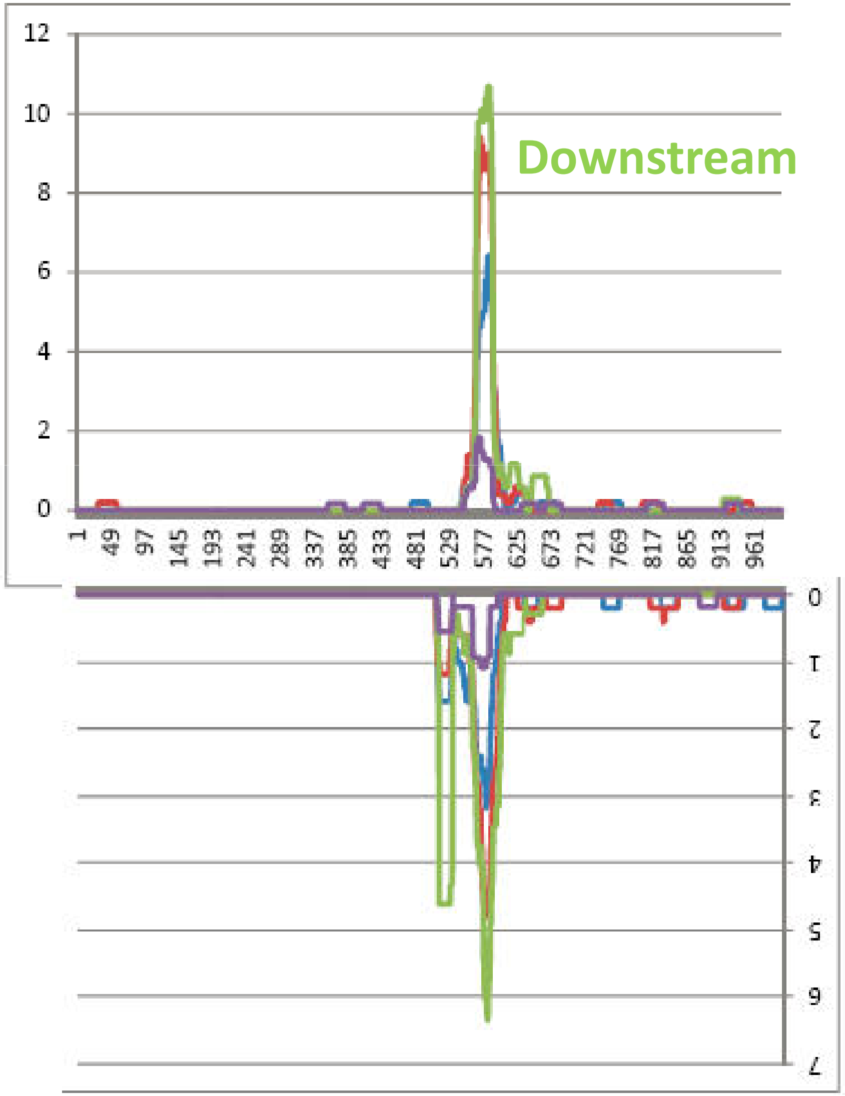

AT3G45460

IBR domain containing protein

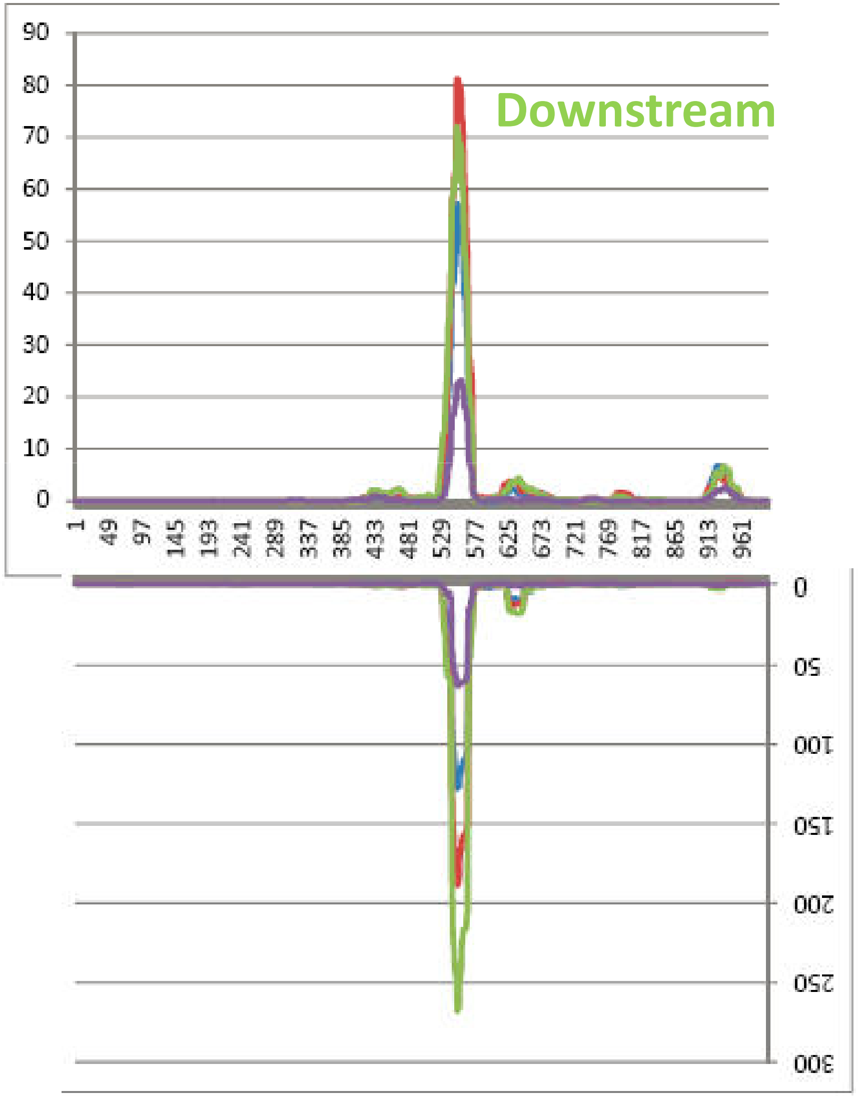

AT3G47920

unknown protein

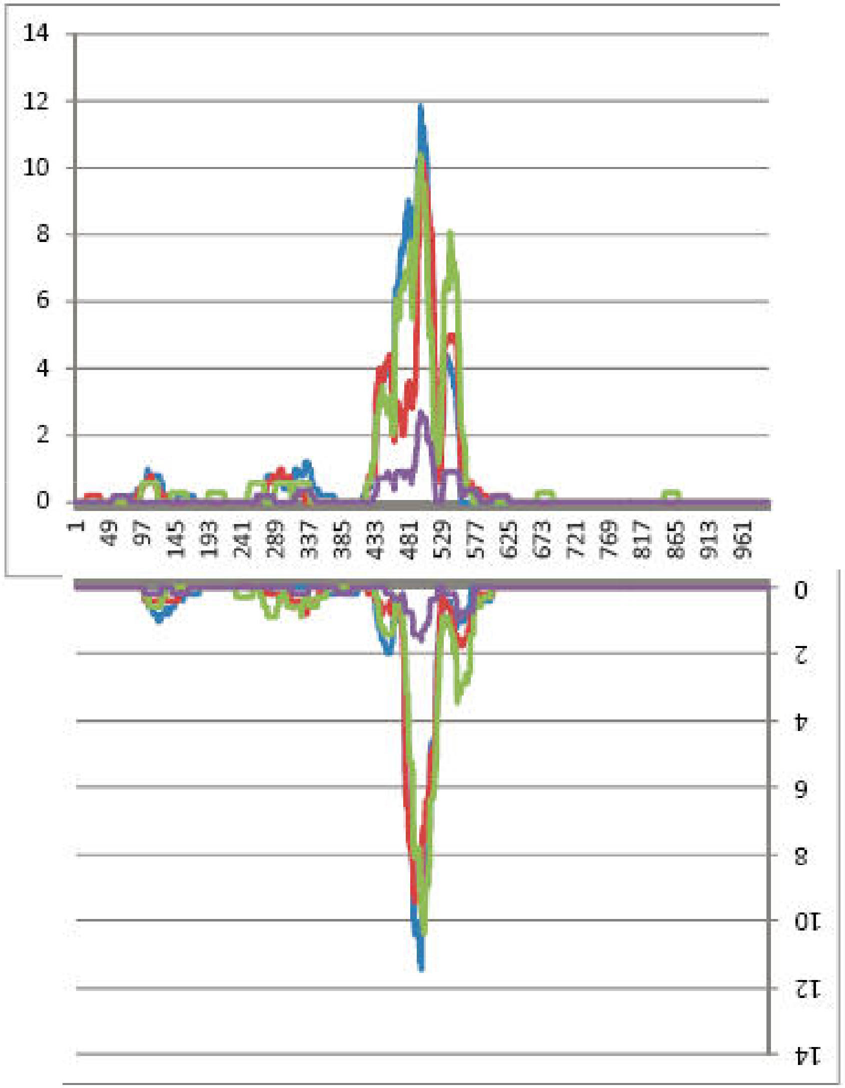

AT3G50300

HXXXD-type acyl-transferase family protein; EXPRESSED IN: hypocotyl, root.

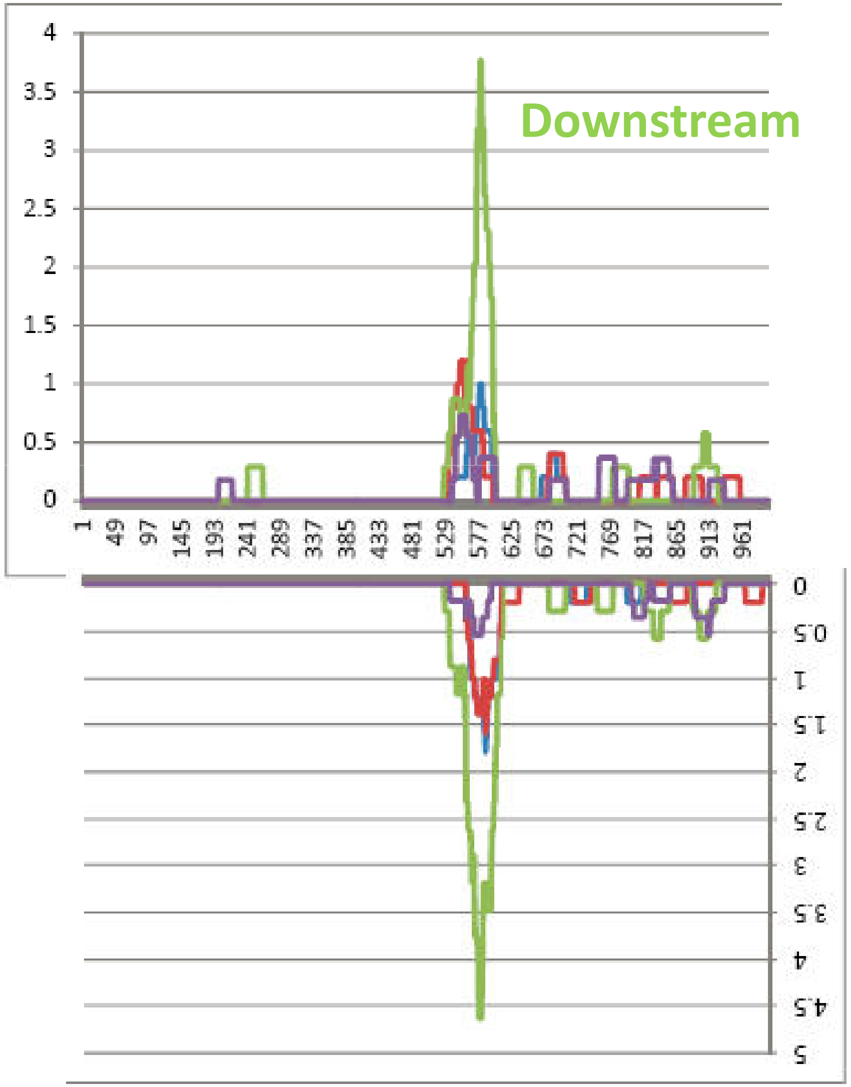

AT3G50540

unknown protein

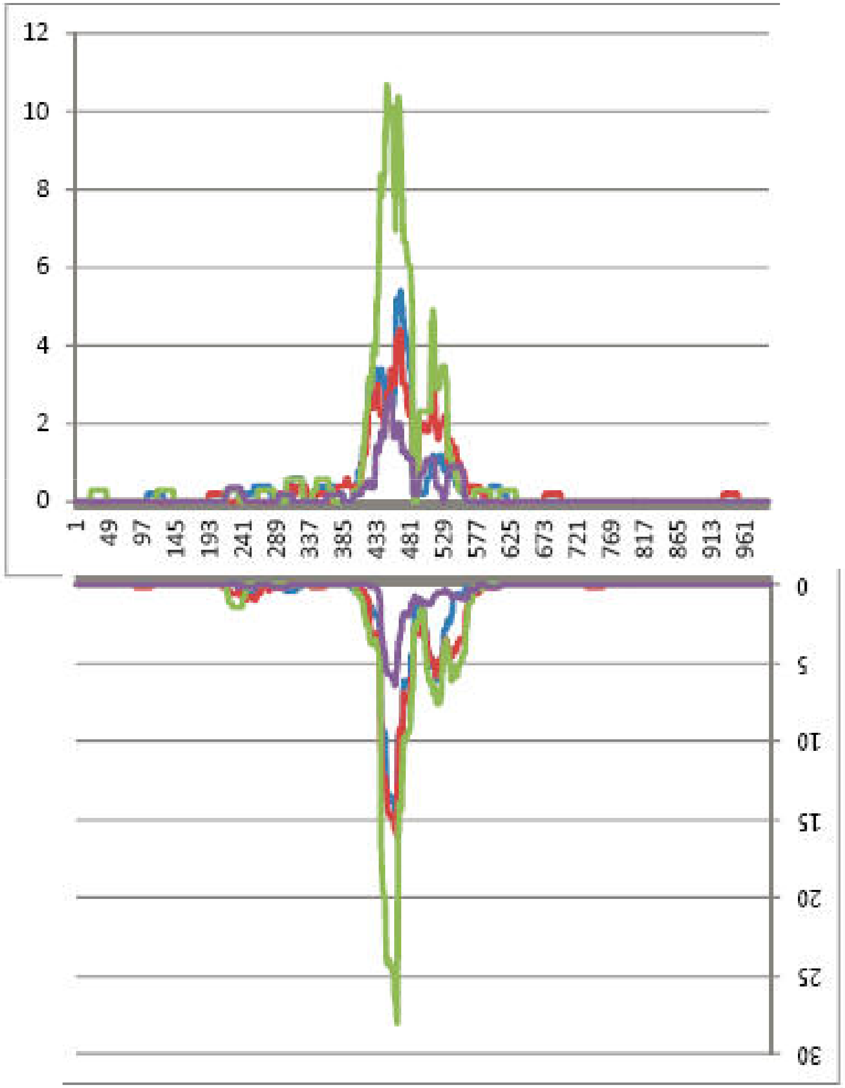

AT3G50840

Phototropic-responsive NPH3 family protein

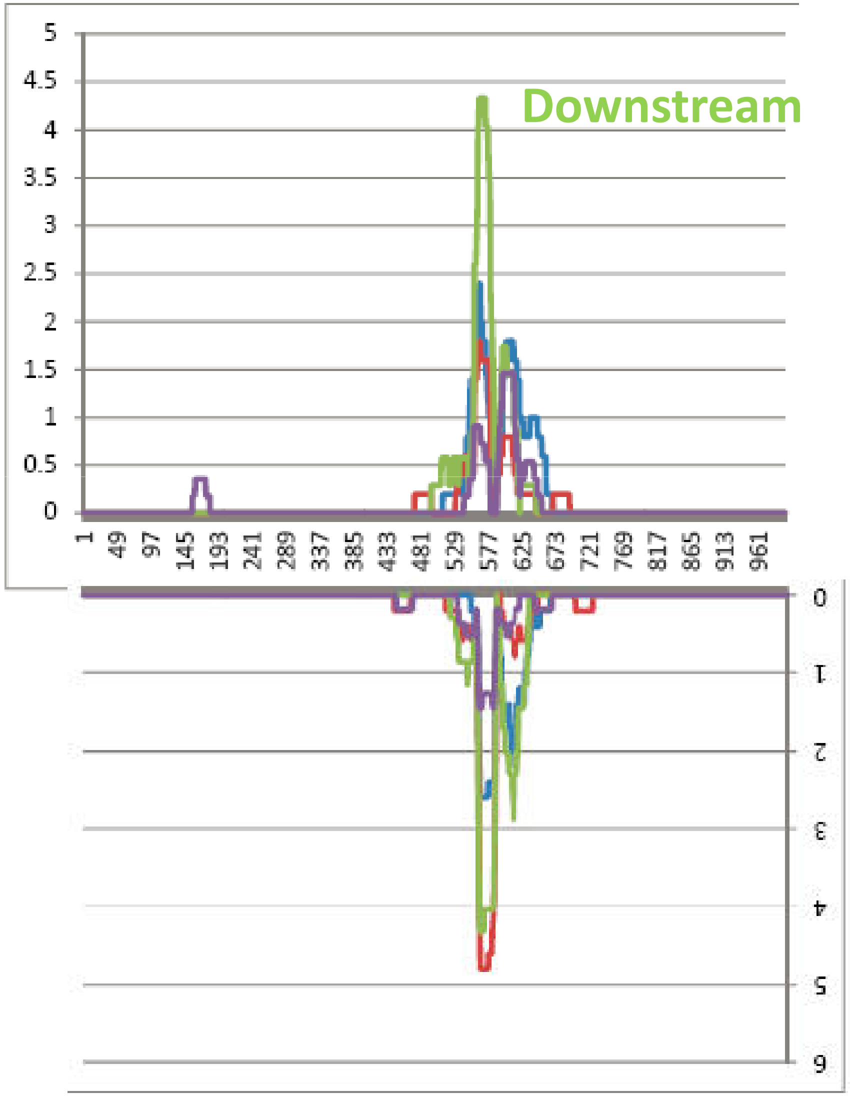

AT3G52830

BEST Arabidopsis thaliana protein match is: Ankyrin repeat family protein (TAIR:AT5G54700.1)

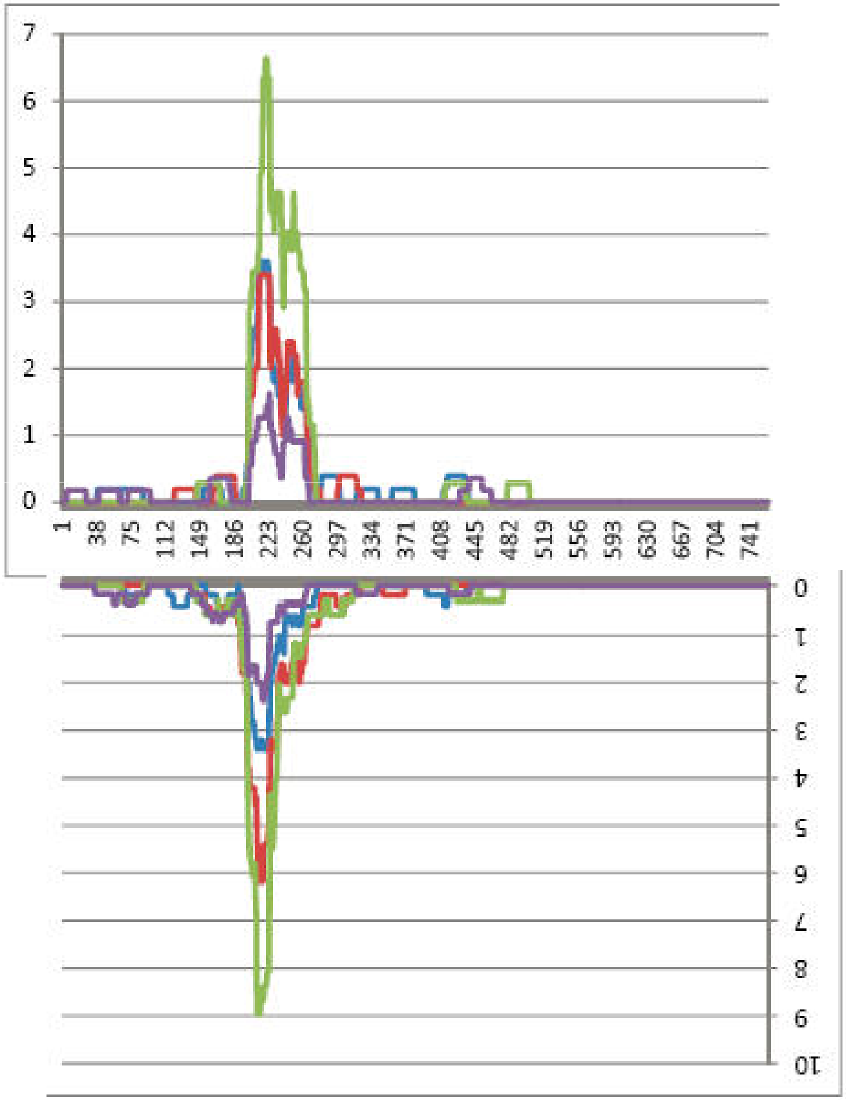

AT3G55672

Plant self-incompatibility protein S1 family

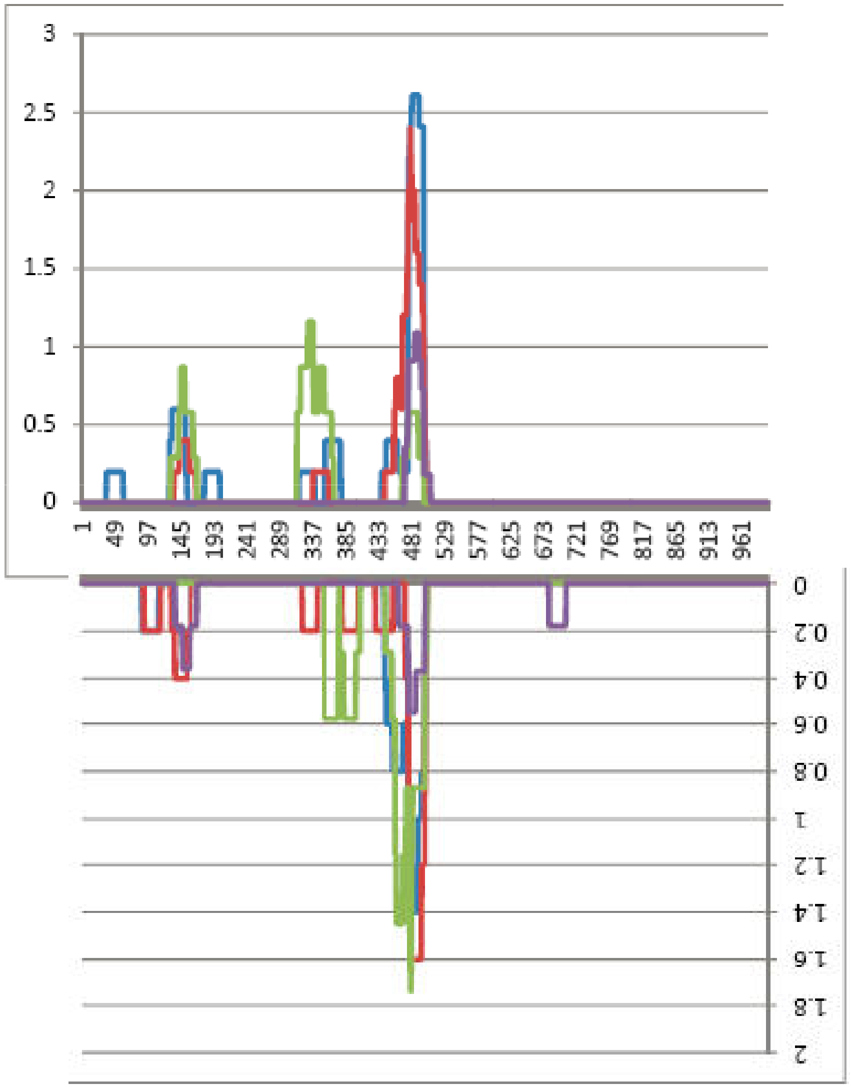

AT3G55730

Putative transcription factor MYB109 (MYB109)

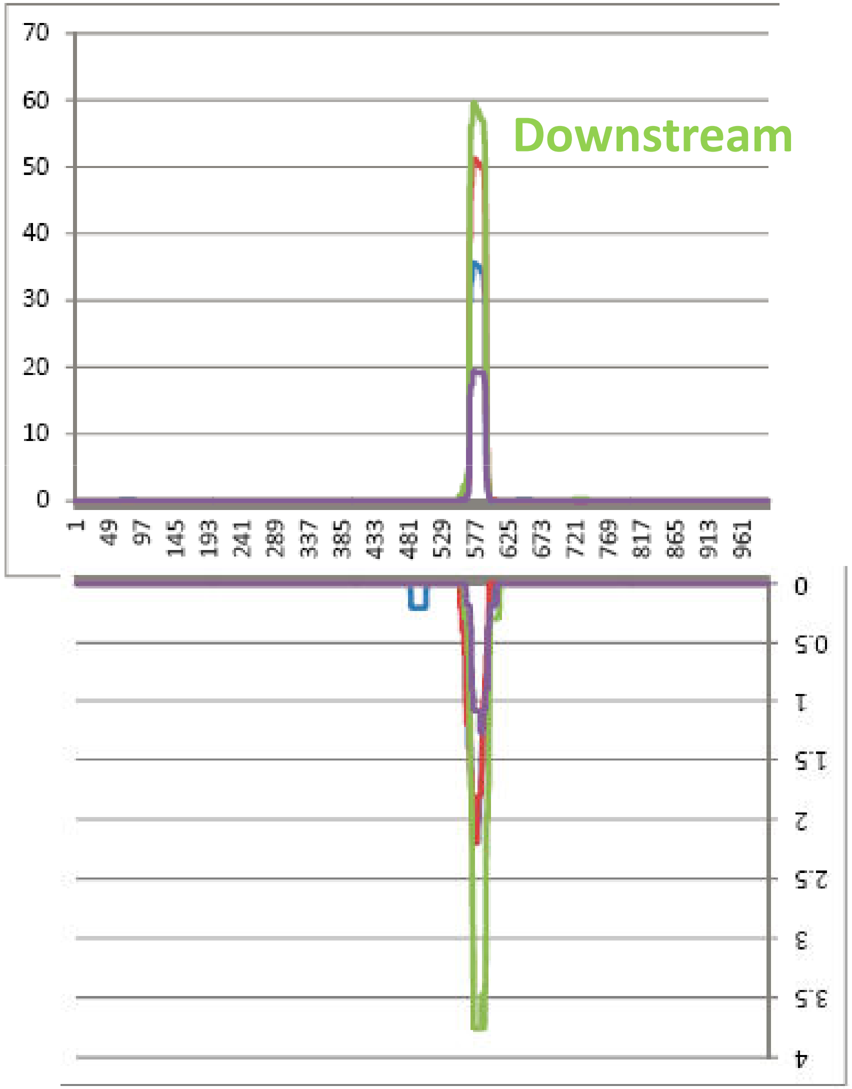

AT4G00120

INDEHISCENT (IND)

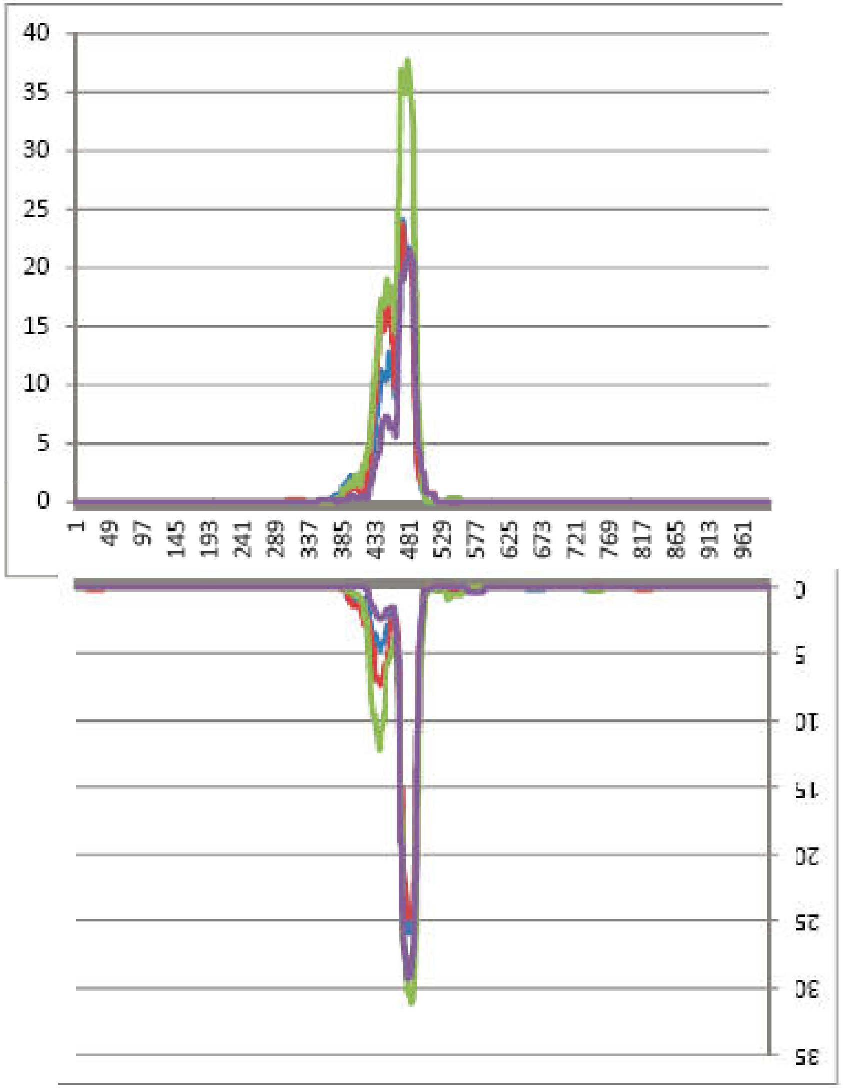

AT4G01900

Encodes a PII protein that may function as part of a signal transduction network involved in perceiving the status of carbon and organic nitrogen. Forms a protein complex with N-acetylglutamate kinase and regulates the kinase activity by relieving the feedback inhibition of the kinase by arginine. Regulates acetyl-CoA carboxylase activity.

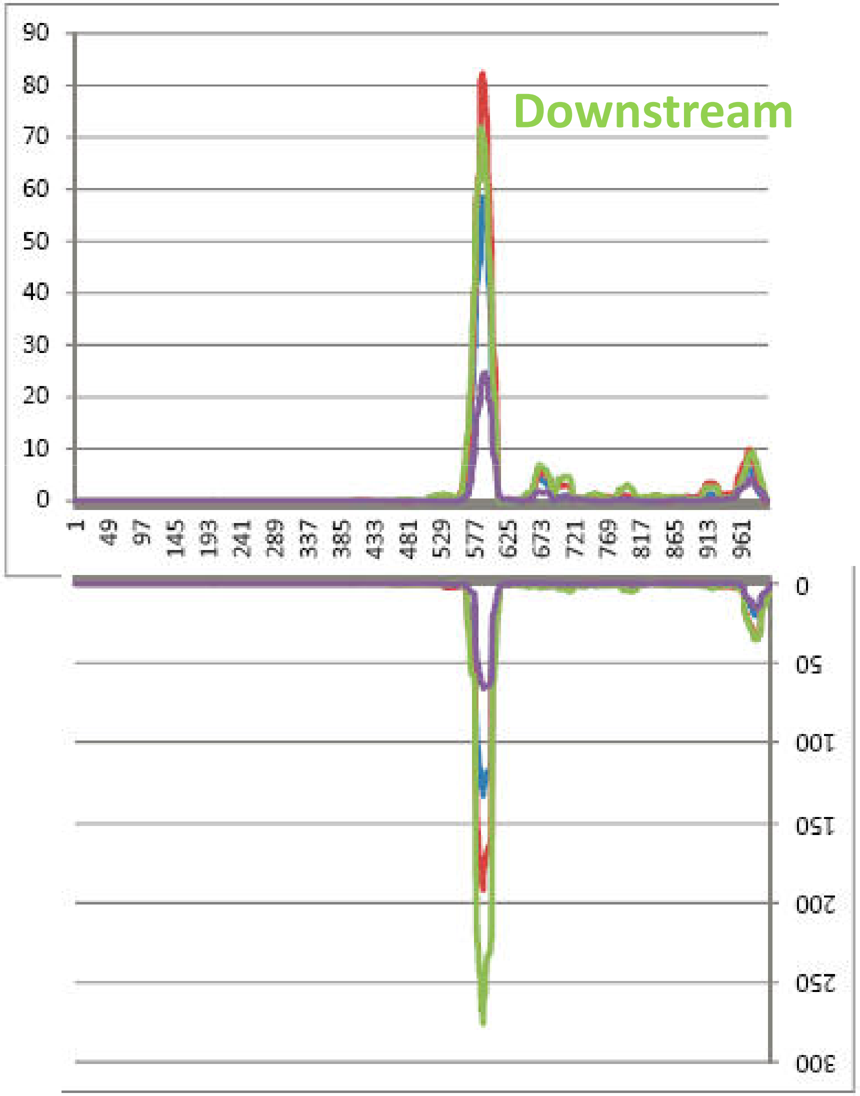

AT4G03160

BEST Arabidopsis thaliana protein match is: AP2/B3-like transcriptional factor family protein (TAIR:AT4G03170.1).

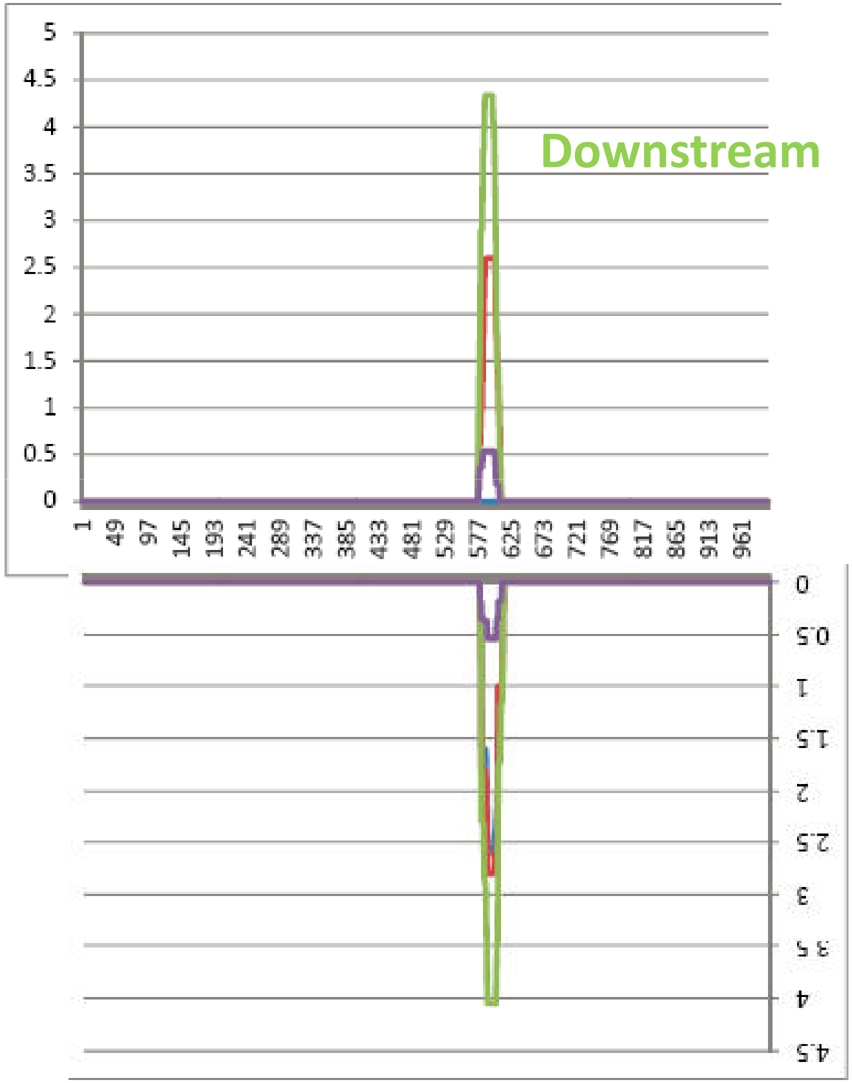

AT4G04030

Ovate family protein 9 (OFP9)

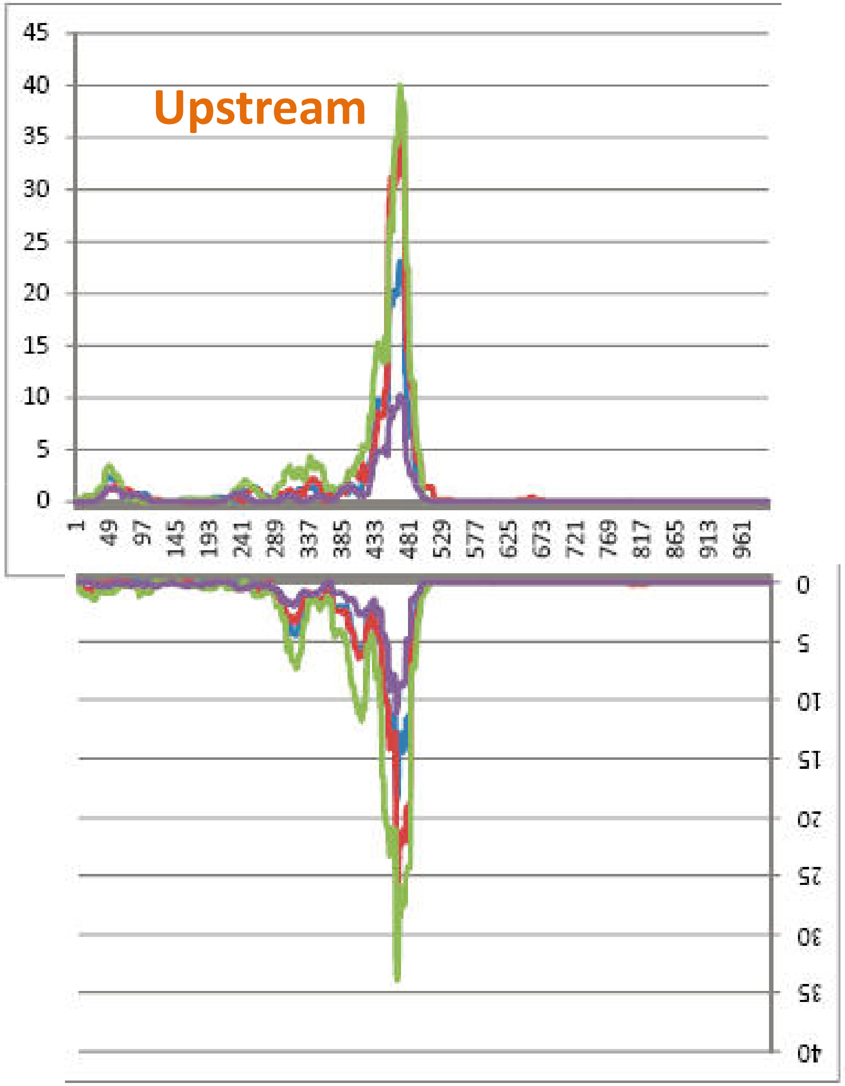

AT4G04970

Encodes a gene similar to callose synthase

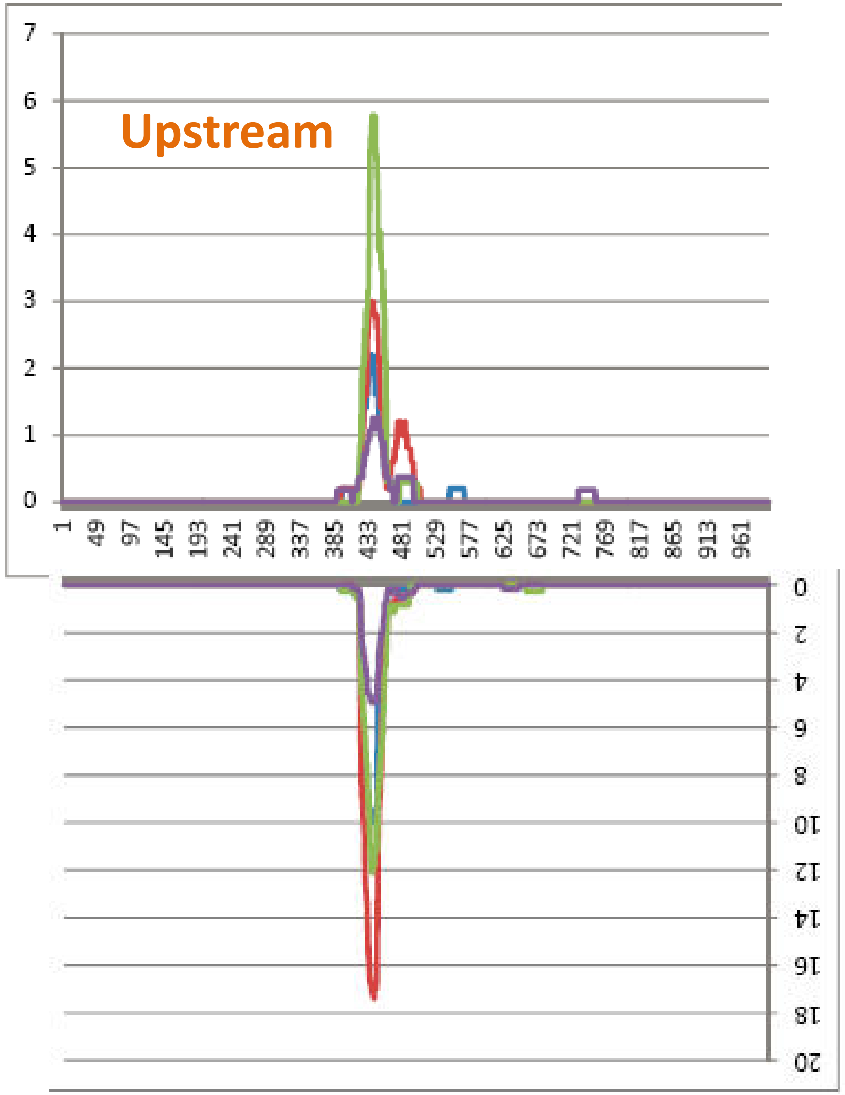

AT4G08160

Encodes a putative glycosyl hydrolase family 10 protein (xylanase).

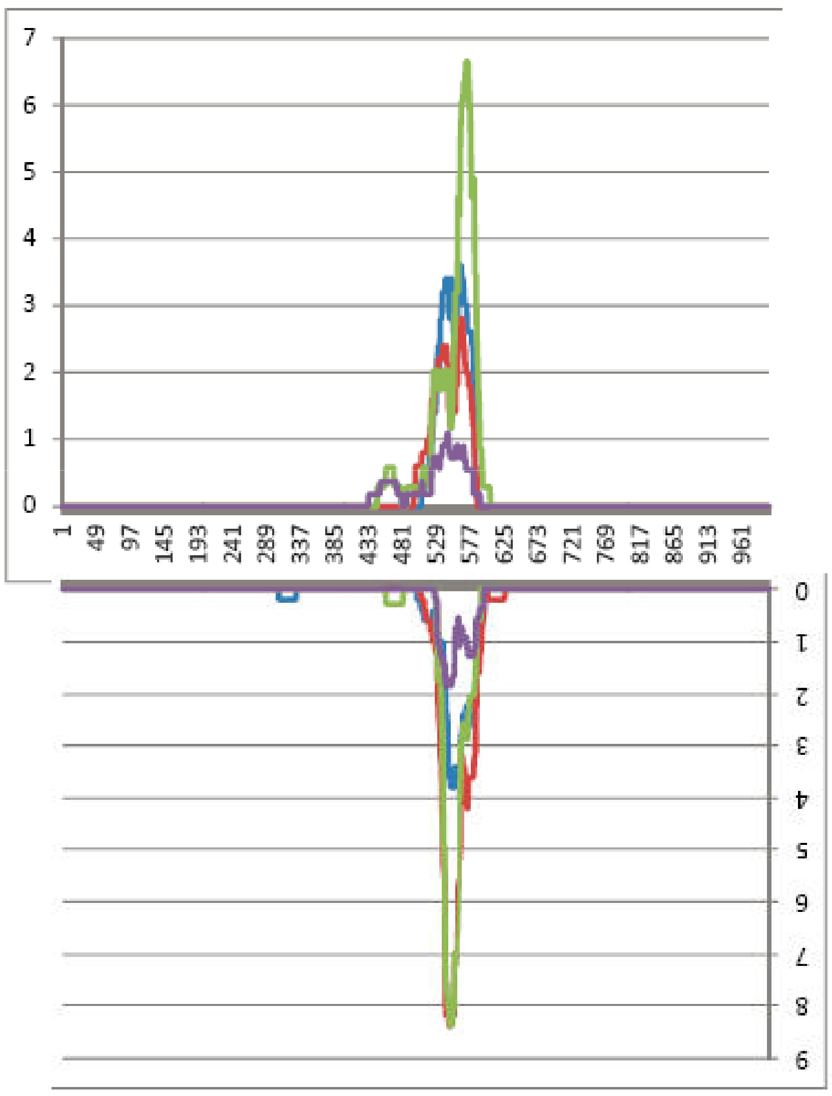

AT4G08850

Leucine-rich repeat receptor-like protein kinase family protein

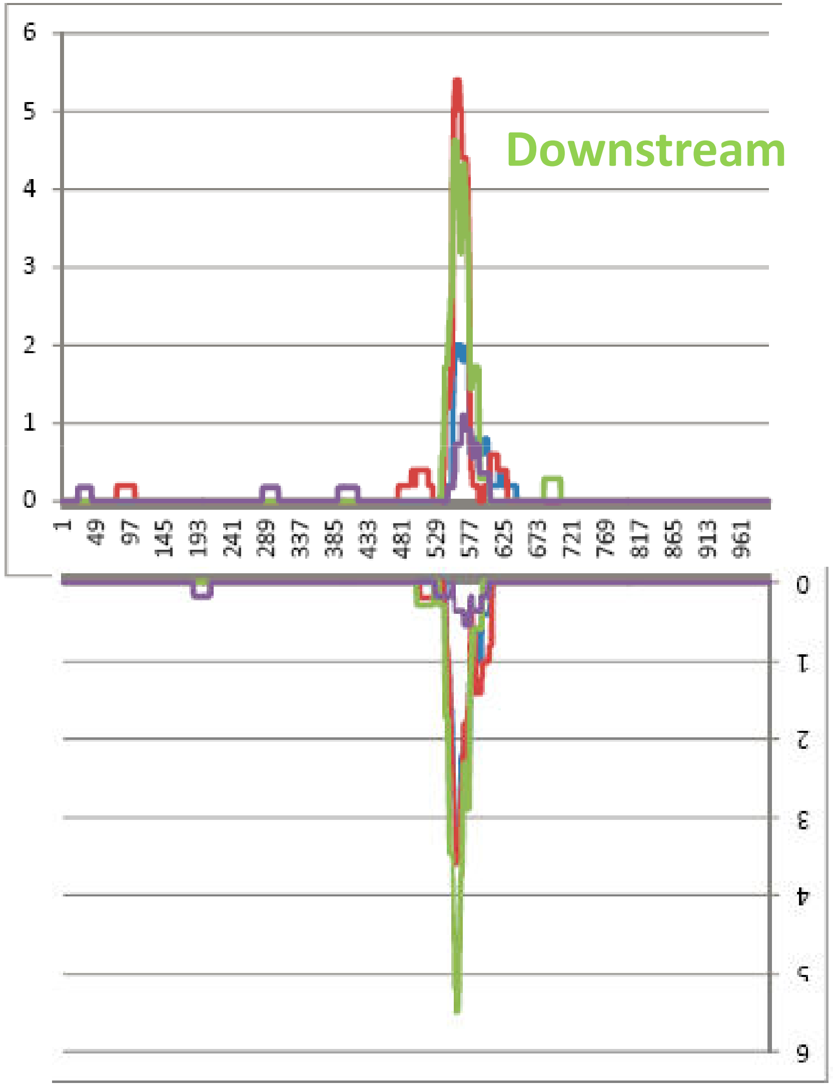

AT4G14365

XB3 ortholog 4 in Arabidopsis thaliana (XBAT34)

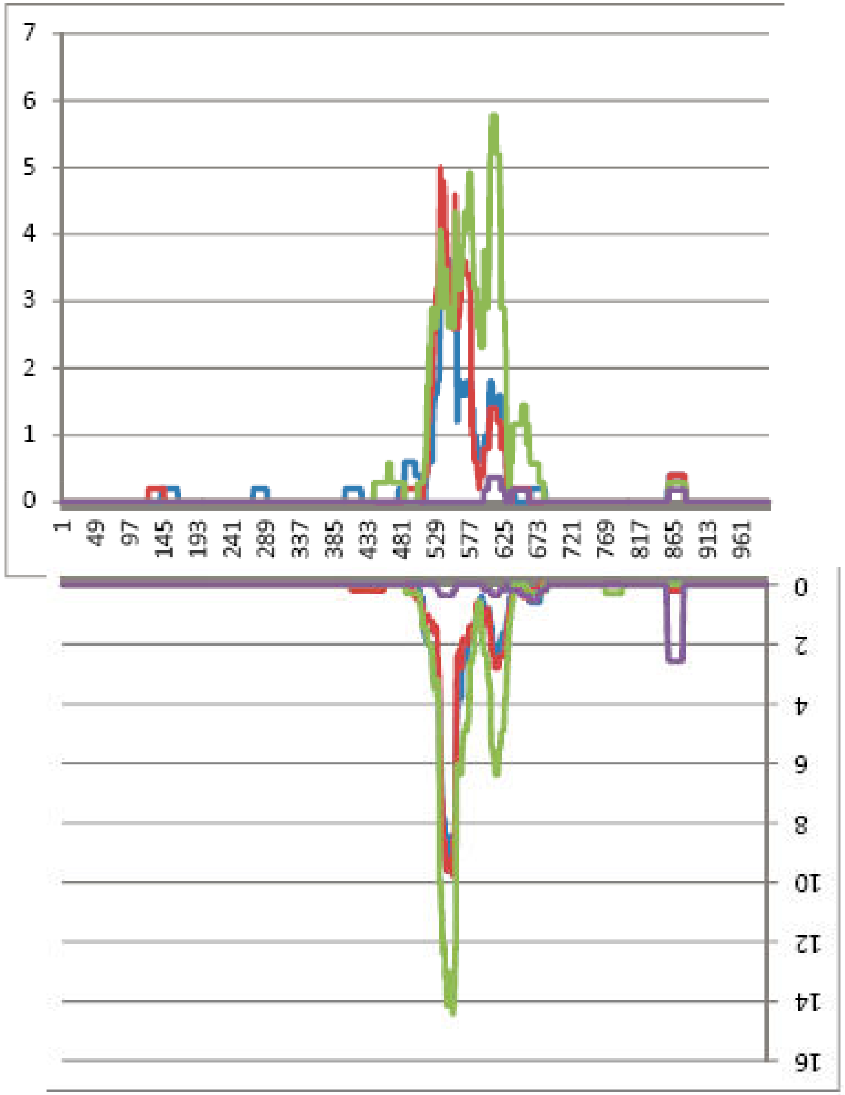

AT4G14940

atao1 gene of Arabidopsis thaliana encodes an extracellular copper amine oxidase expressed during early stages of vascular tissue development.

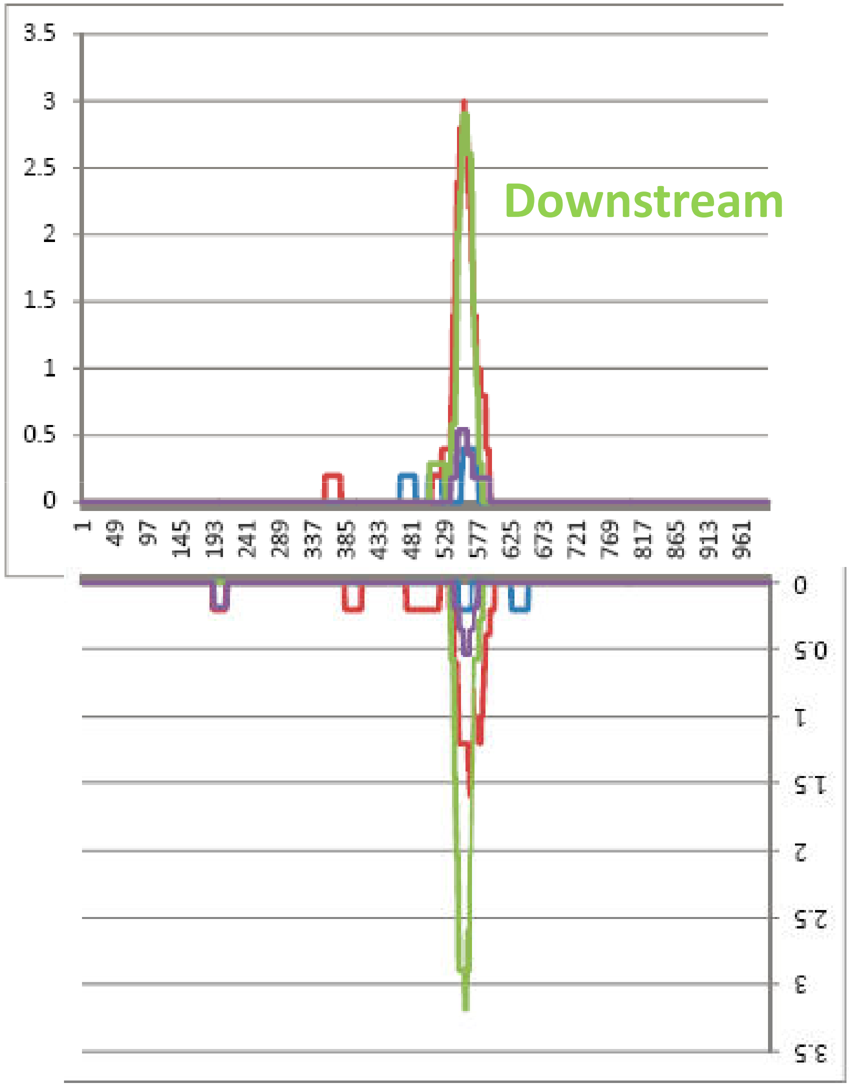

AT4G15570

Similar to yeast Sen1 (splicing endonuclease 1) helicase protein. Involved in female gametophyte development.

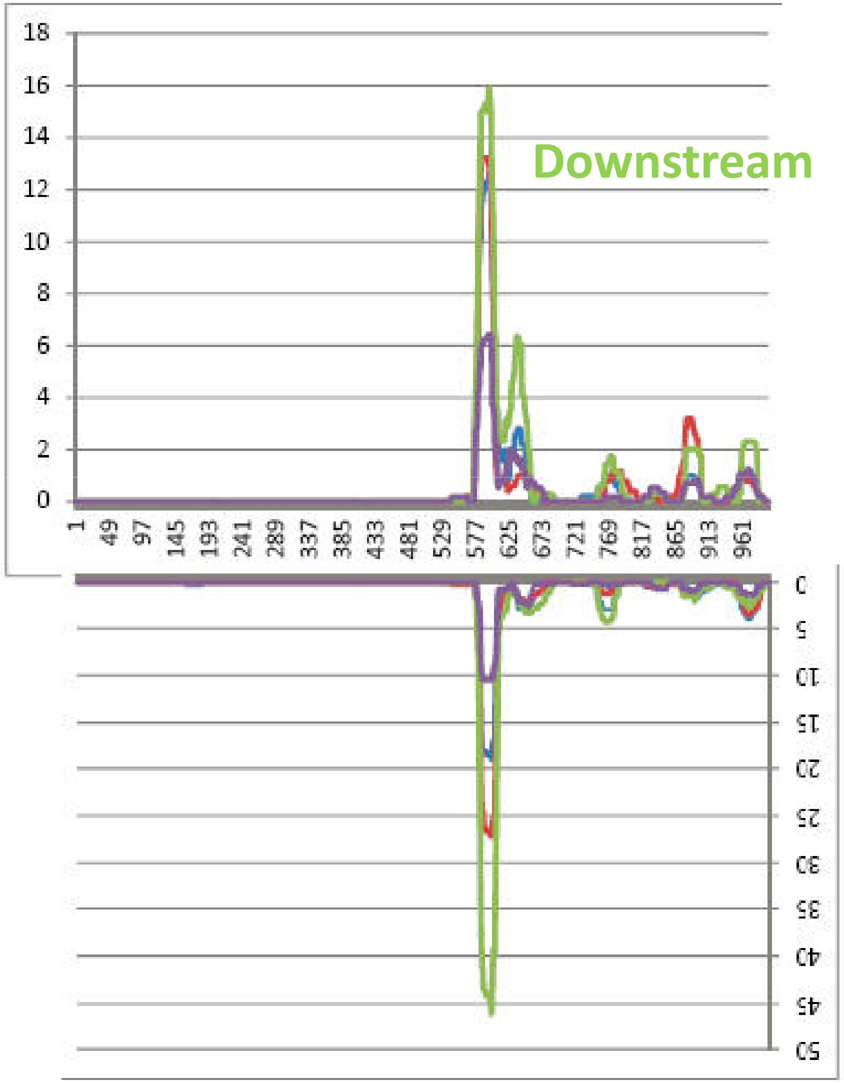

AT4G18690

unknown protein

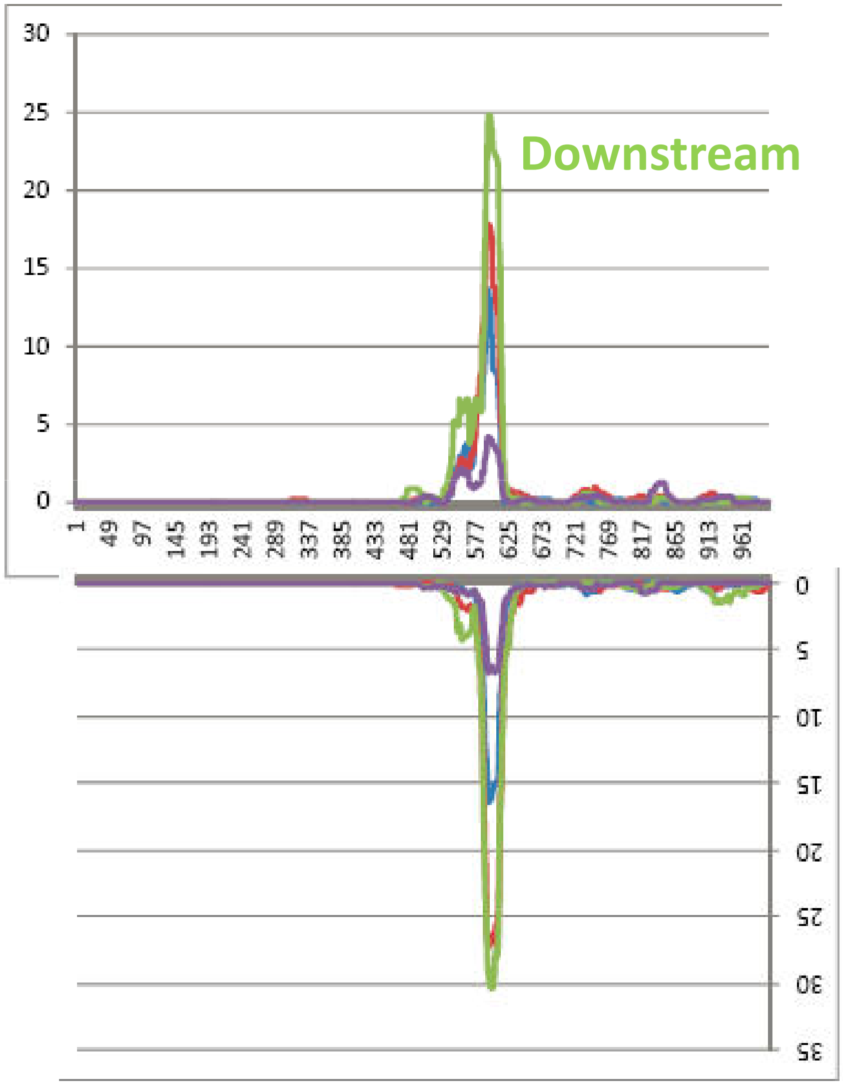

AT4G21820

Calmodulin binding

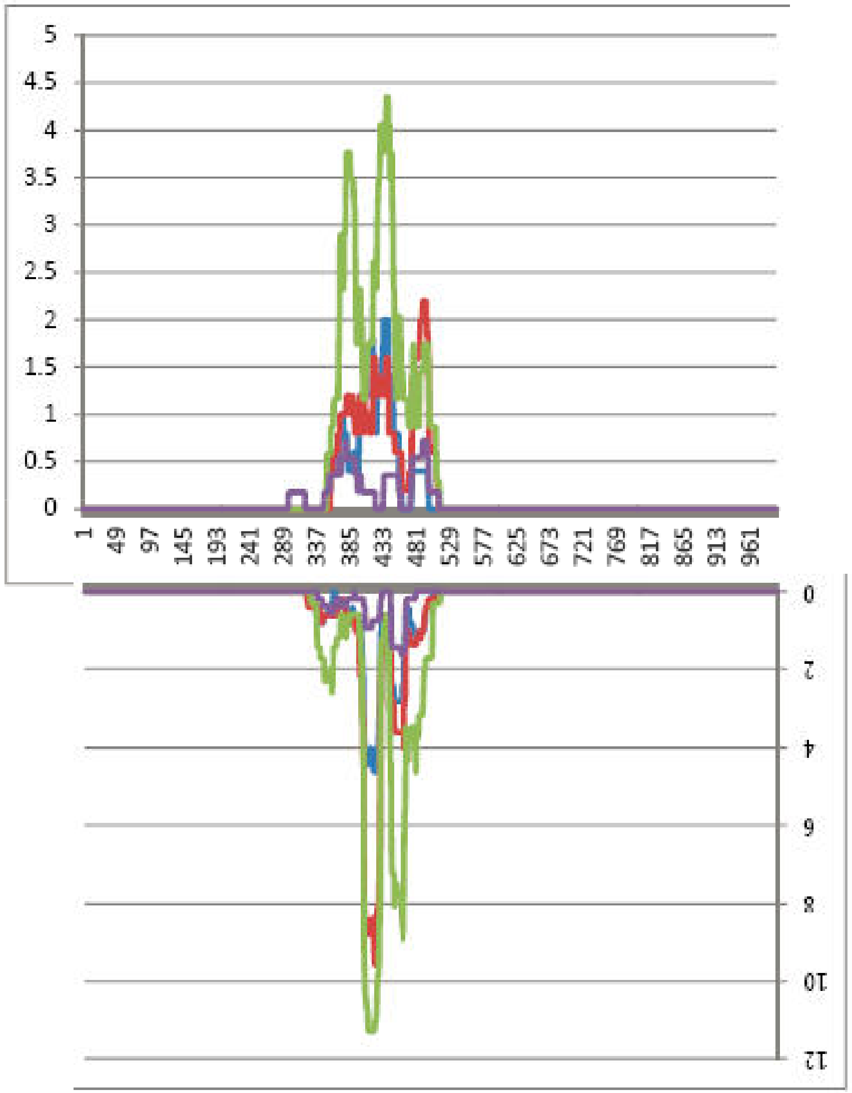

AT4G22650

BEST Arabidopsis thaliana protein match is: Bifunctional inhibitor/lipid-transfer protein/seed storage 2S albumin superfamily protein (TAIR:AT4G22640.1).

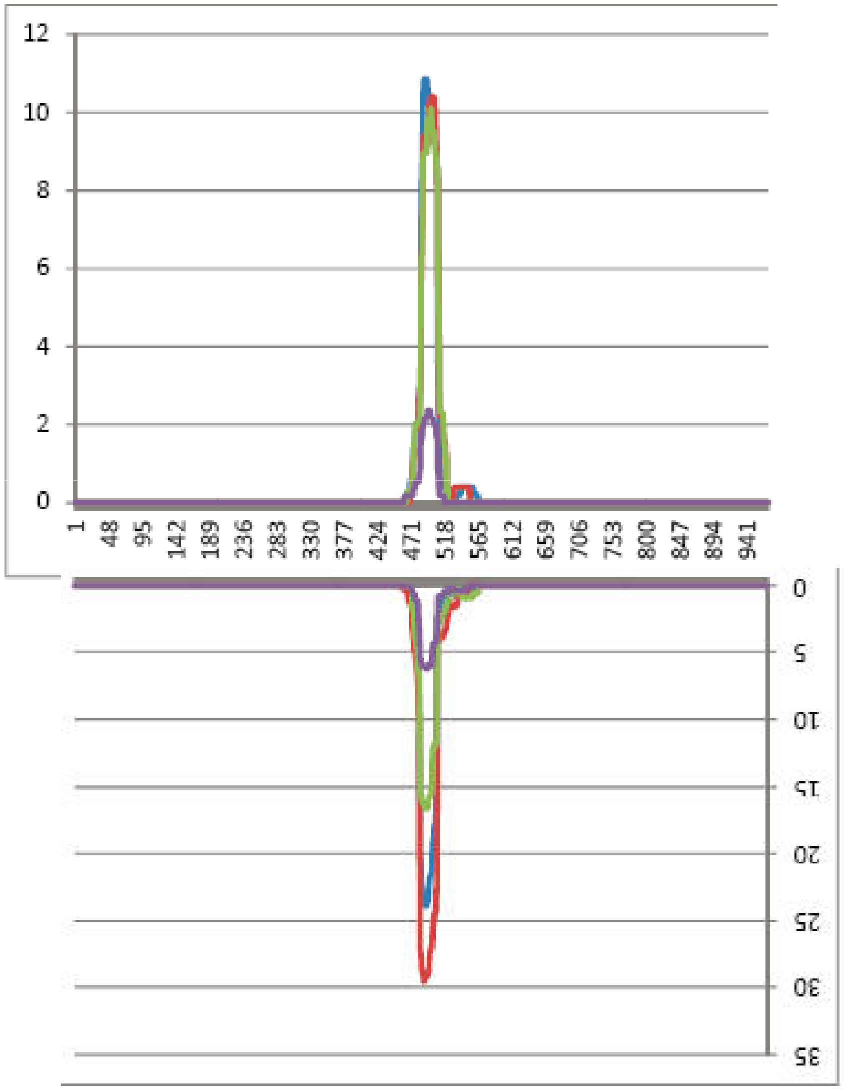

AT4G28850

Xyloglucan endotransglucosylase /hydrolase 26 (XTH26)

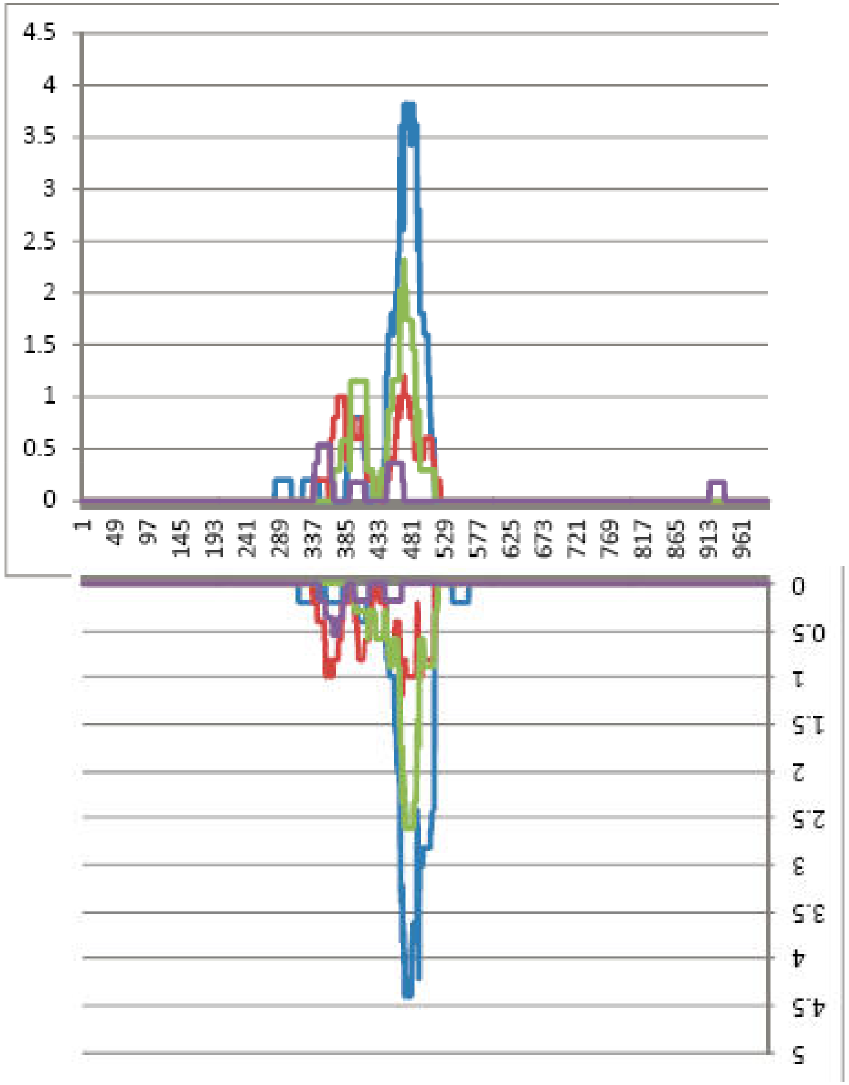

AT4G29090

Ribonuclease H-like superfamily protein

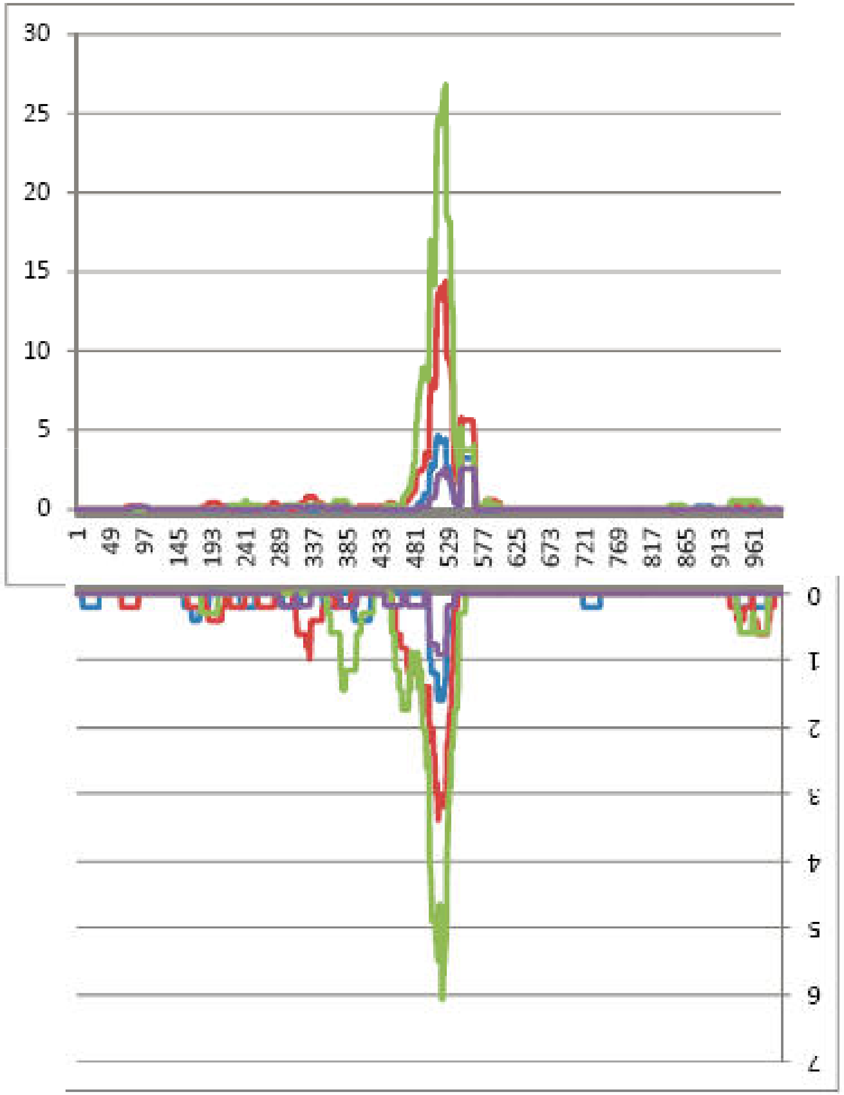

AT4G29290

Encodes a member of a family of small, secreted, cysteine rich protein with sequence similarity to the PCP (pollen coat protein) gene family.

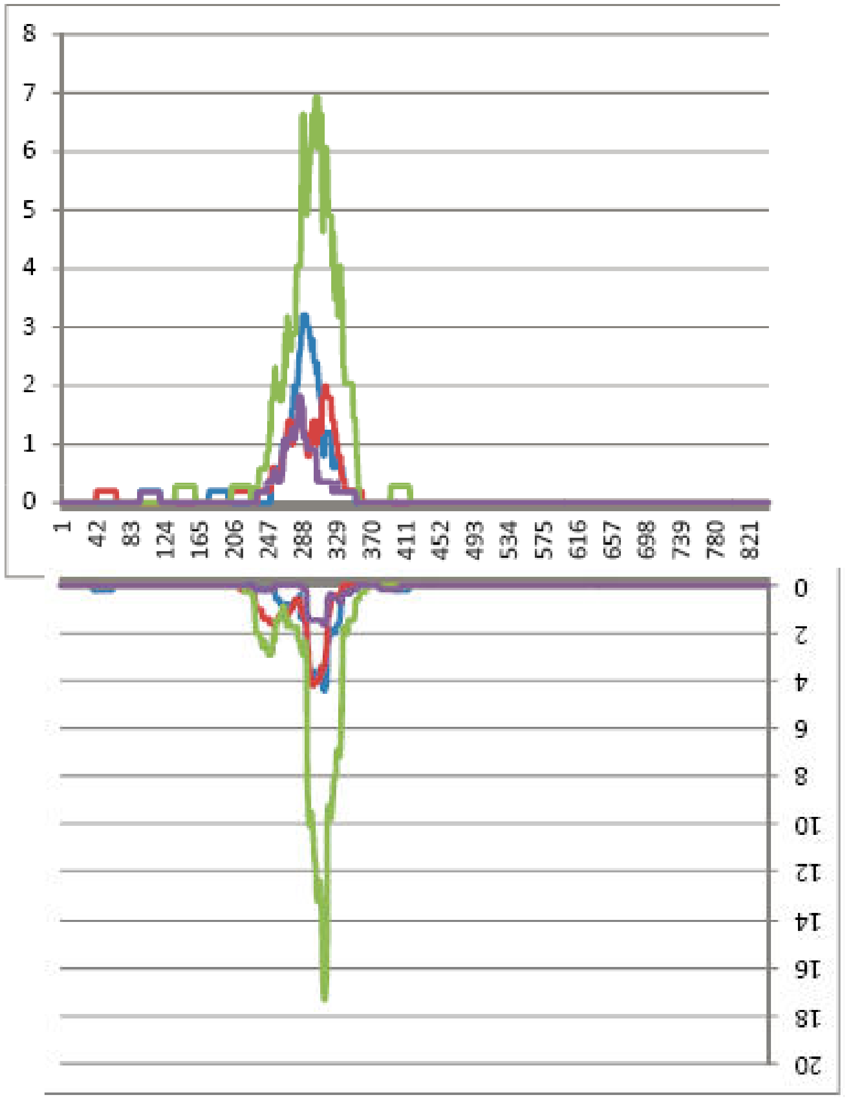

AT4G29305

Encodes a member of a family of small, secreted, cysteine rich protein with sequence similarity to the PCP (pollen coat protein) gene family.

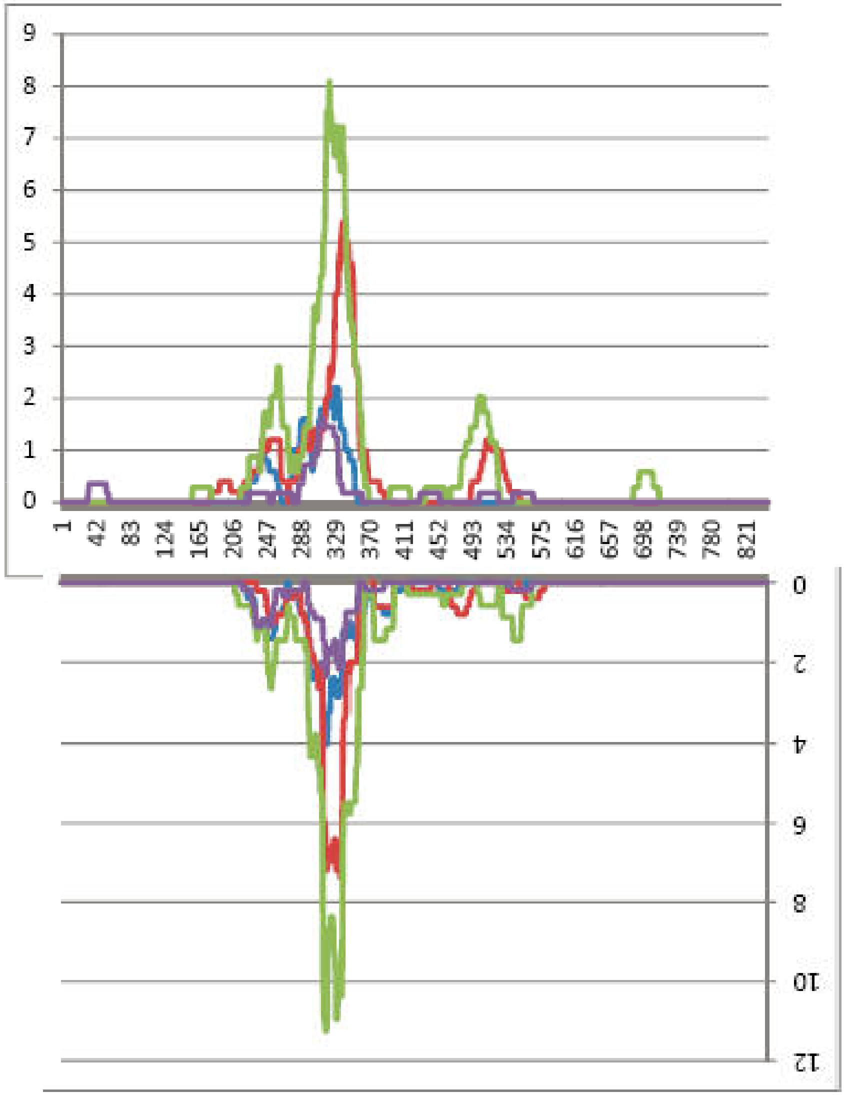

AT4G29740

It encodes a protein whose sequence is similar to cytokinin oxidase/dehydrogenase, which catalyzes the degradation of cytokinins.

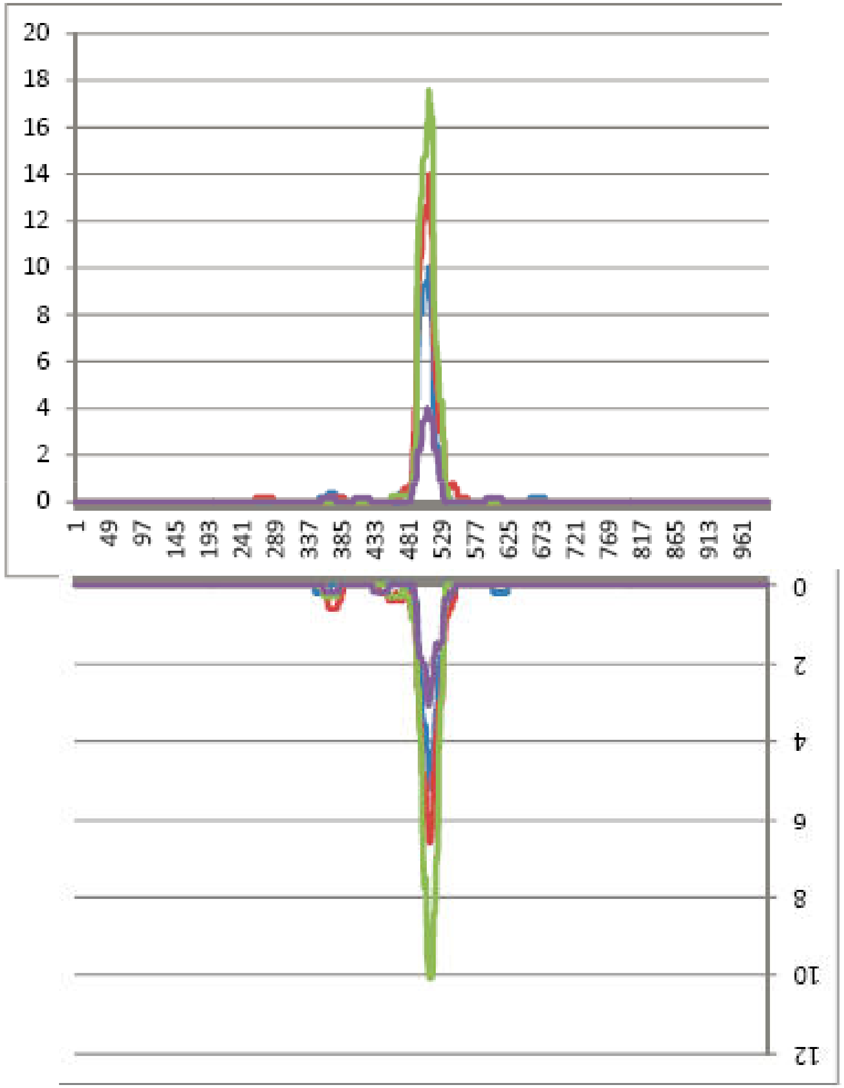

AT5G01080

Beta-galactosidase related protein

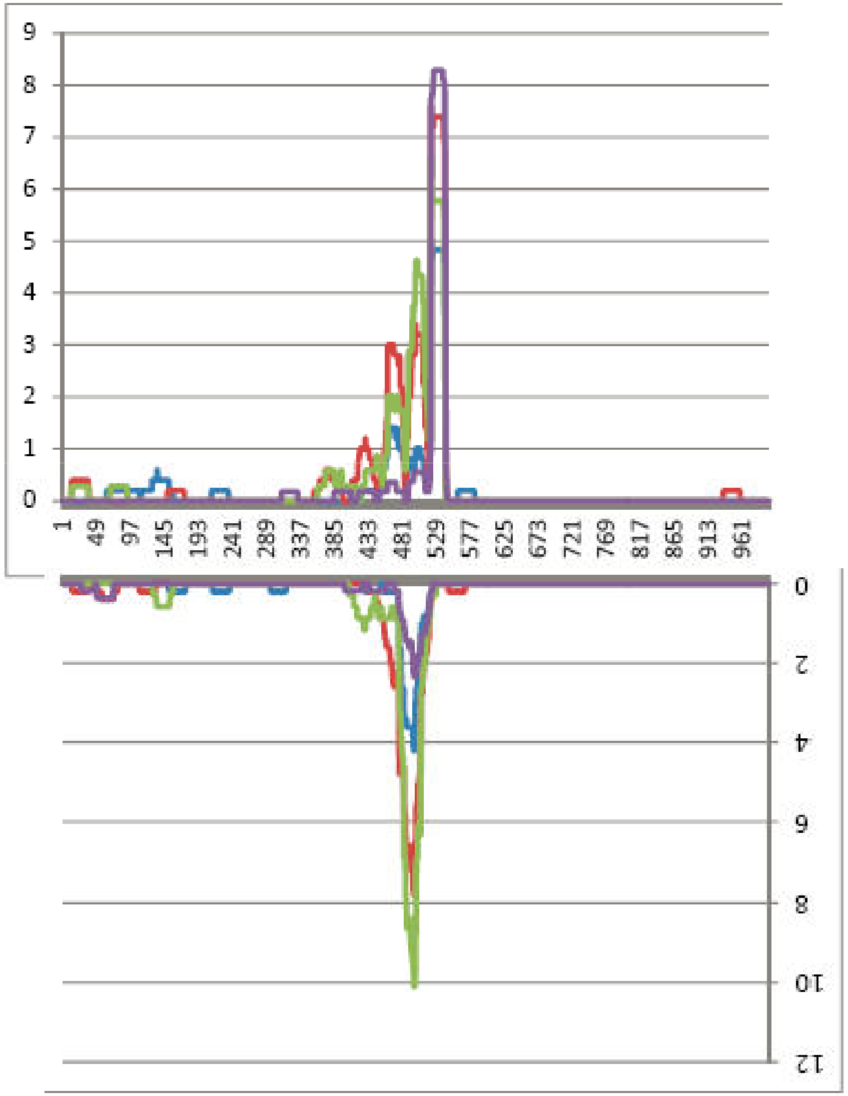

AT5G01260

Carbohydrate-binding-like fold

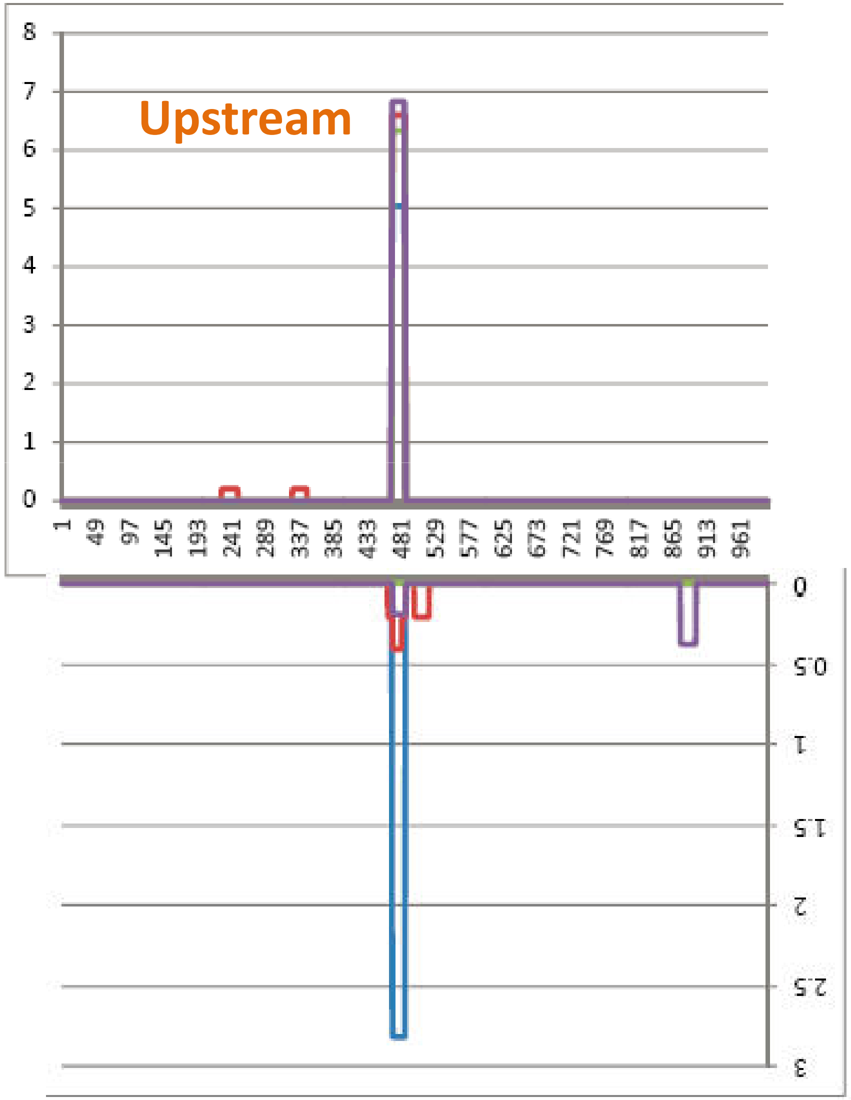

AT5G02990

Galactose oxidase/kelch repeat superfamily protein

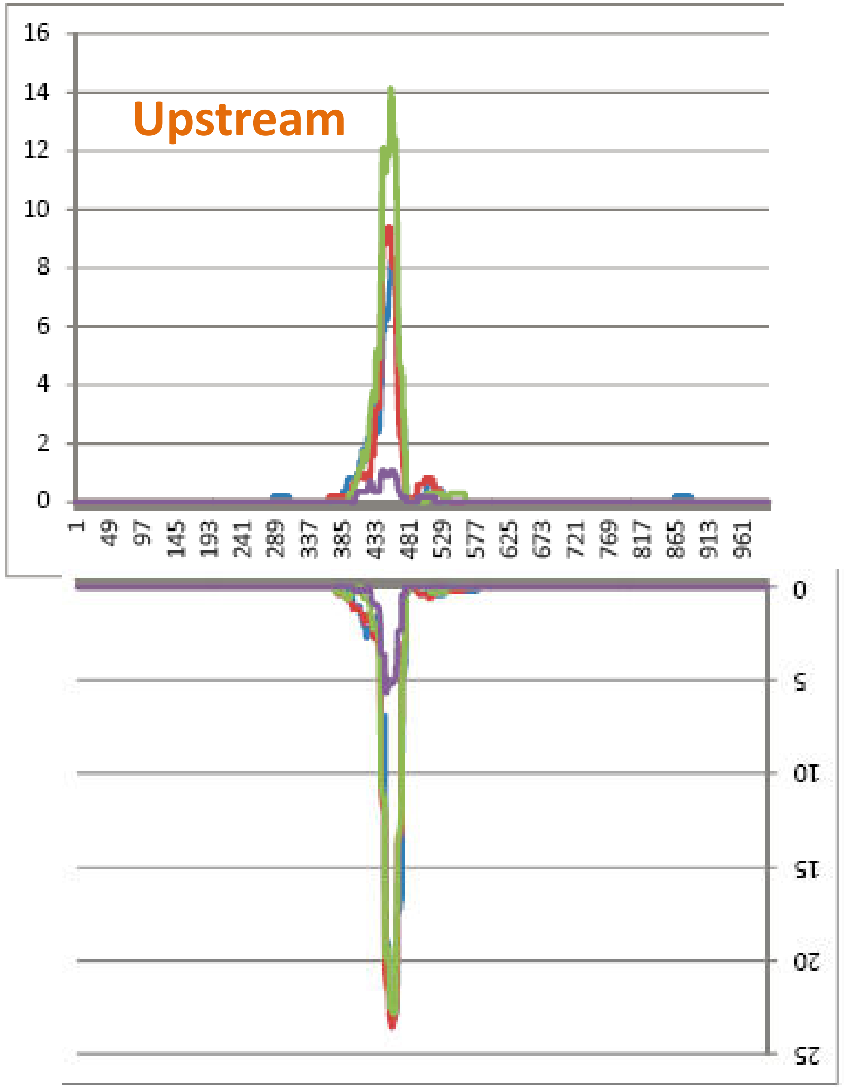

AT5G03060

unknown protein

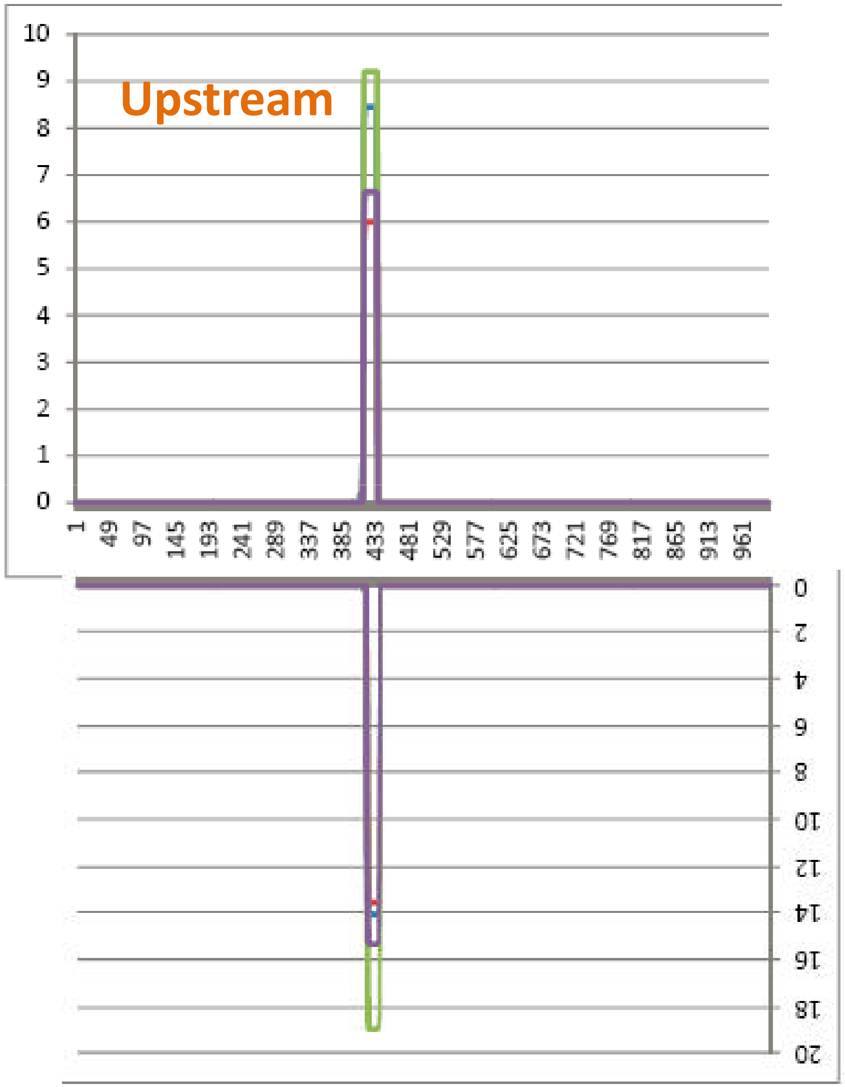

AT5G04950

Encodes a nicotianamide synthase.

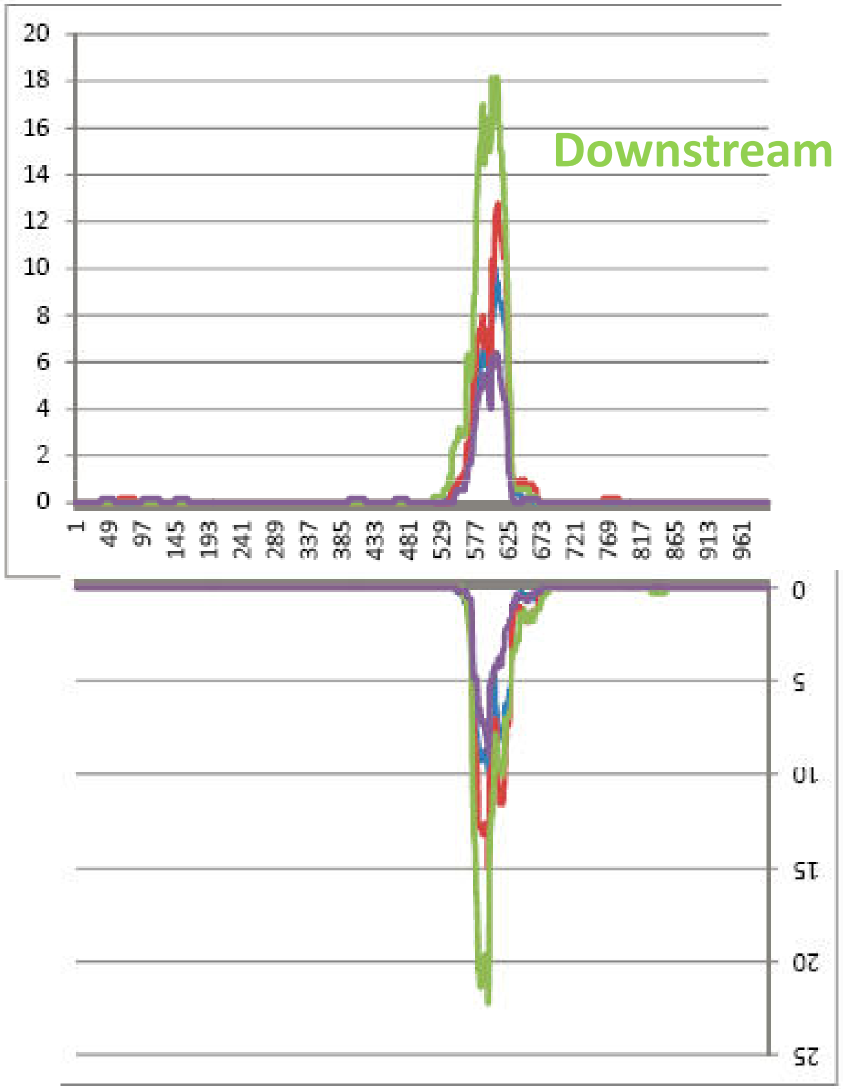

AT5G13825

unknown protein

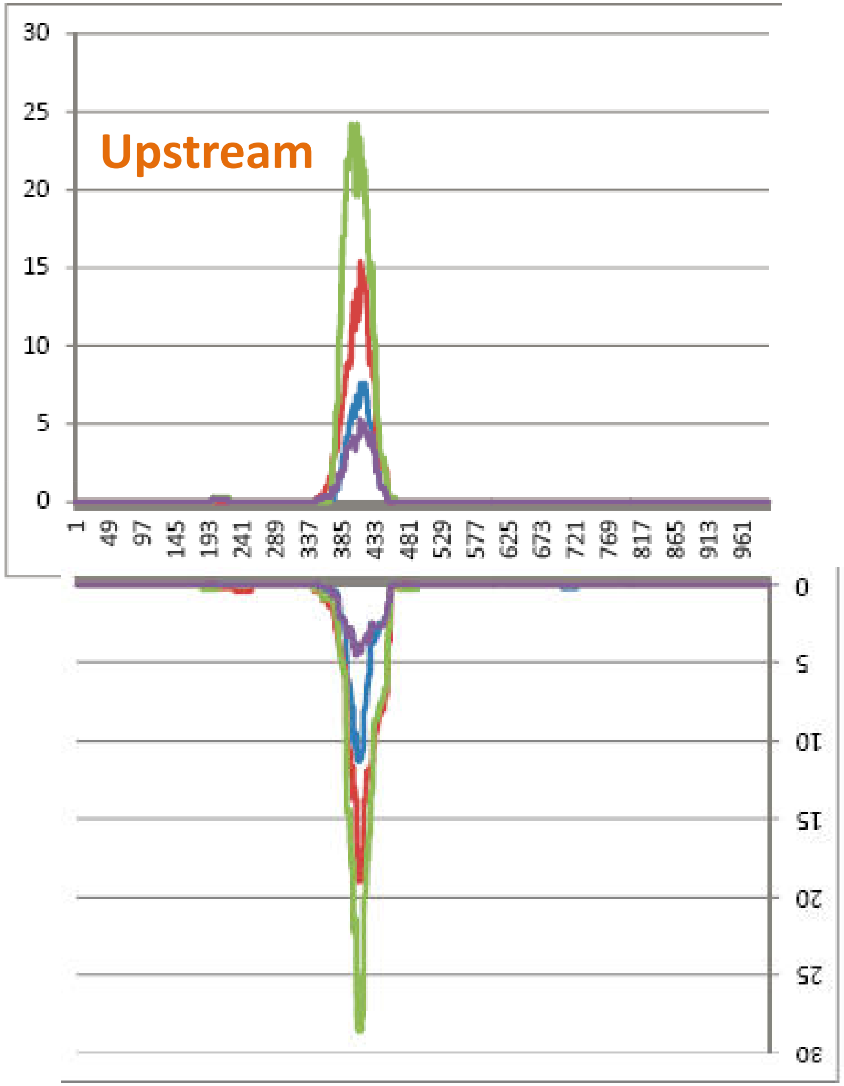

AT5G18570

Encodes AtObgC, a plant ortholog of bacterial Obg. AtObgC is a chloroplast-targeting GTPase essential for early embryogenesis. Mutations in this locus result in embryo lethality. The protein is dually localized in the stroma and the inner envelope membrane and is involved in thylakoid membrane biogenesis and functions primarily in plastid ribosome biogenesis during chloroplast development.

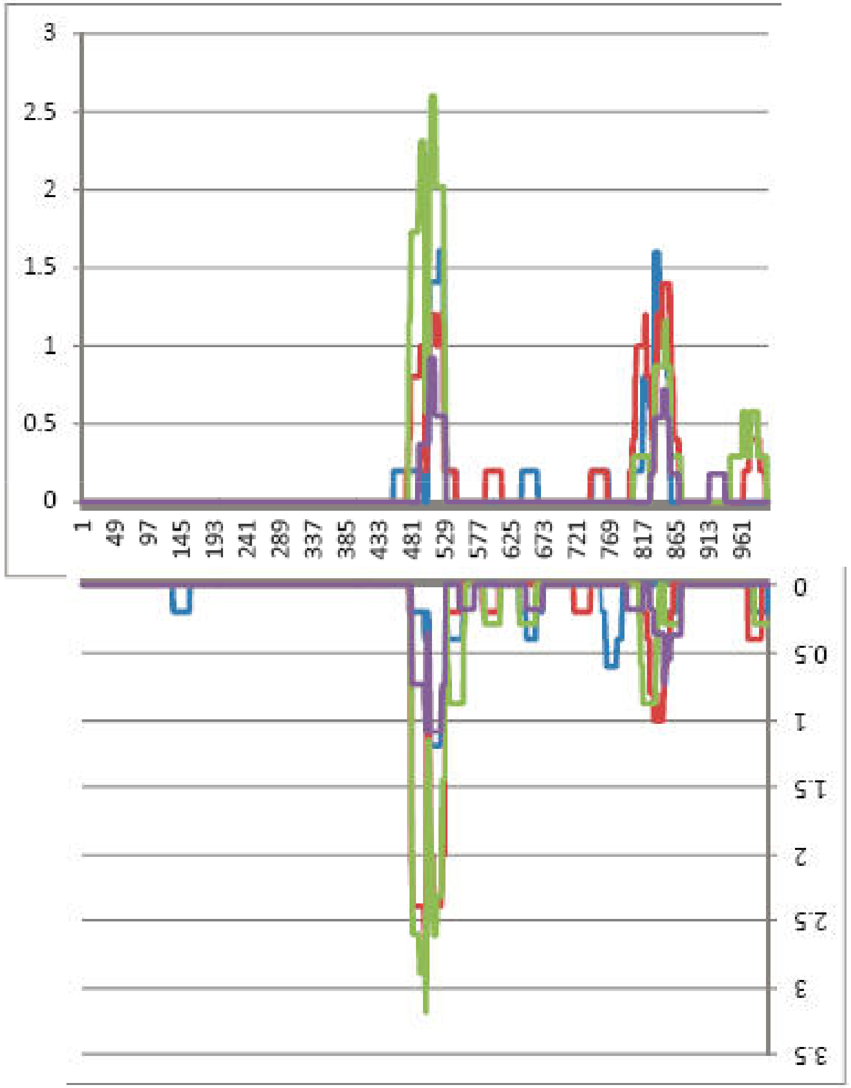

AT5G24290

Vacuolar iron transporter (VIT) family protein

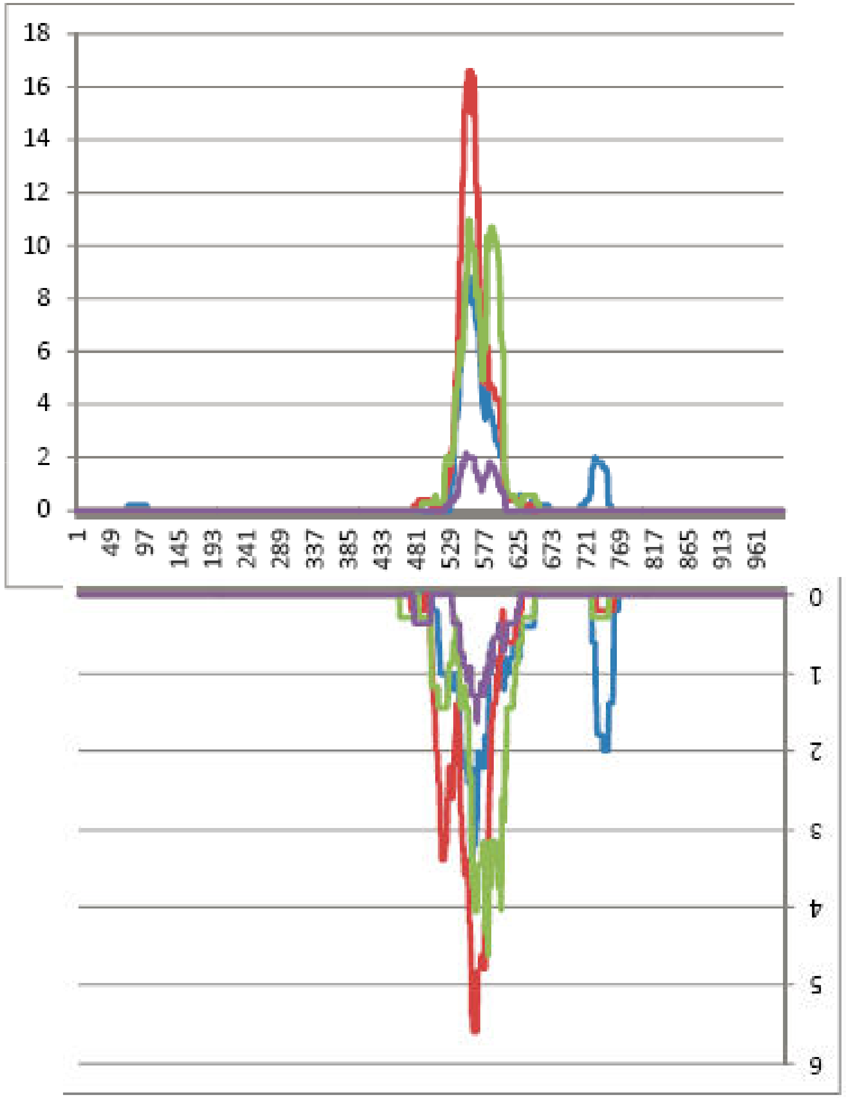

AT5G26270

unknown protein

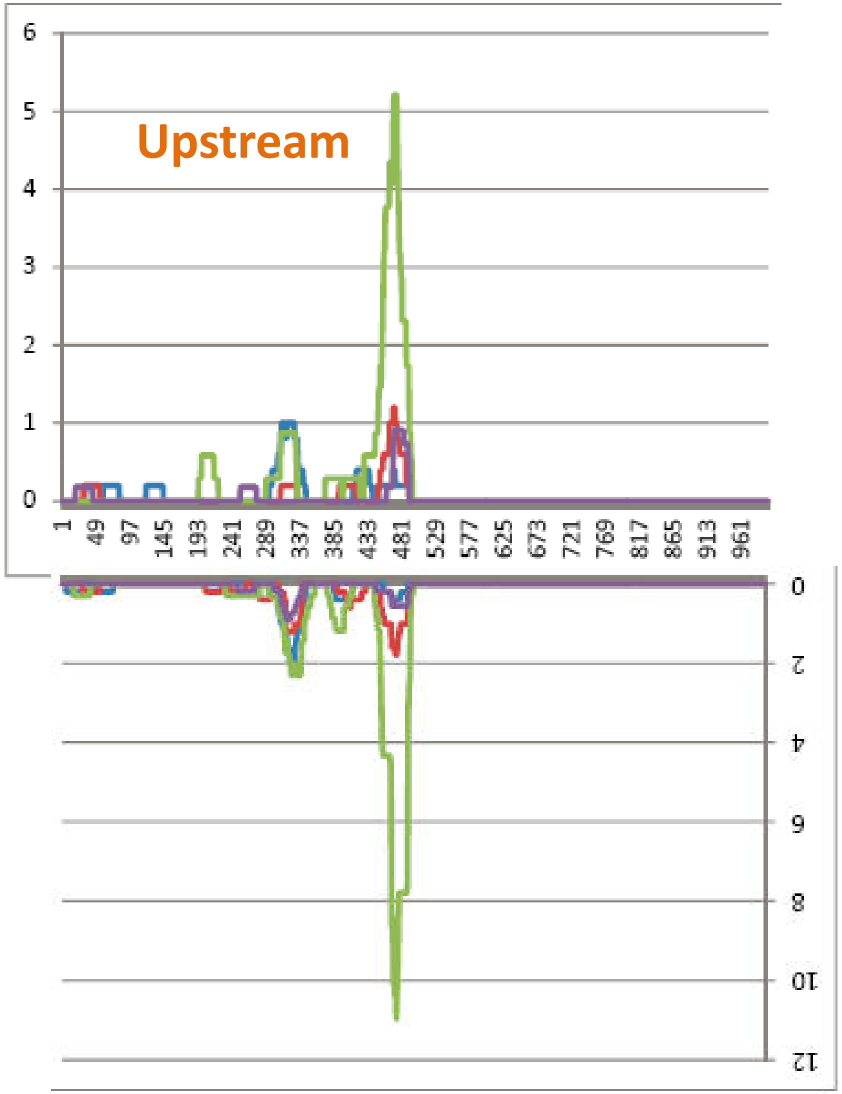

AT5G26673

Encodes a Plant thionin family protein

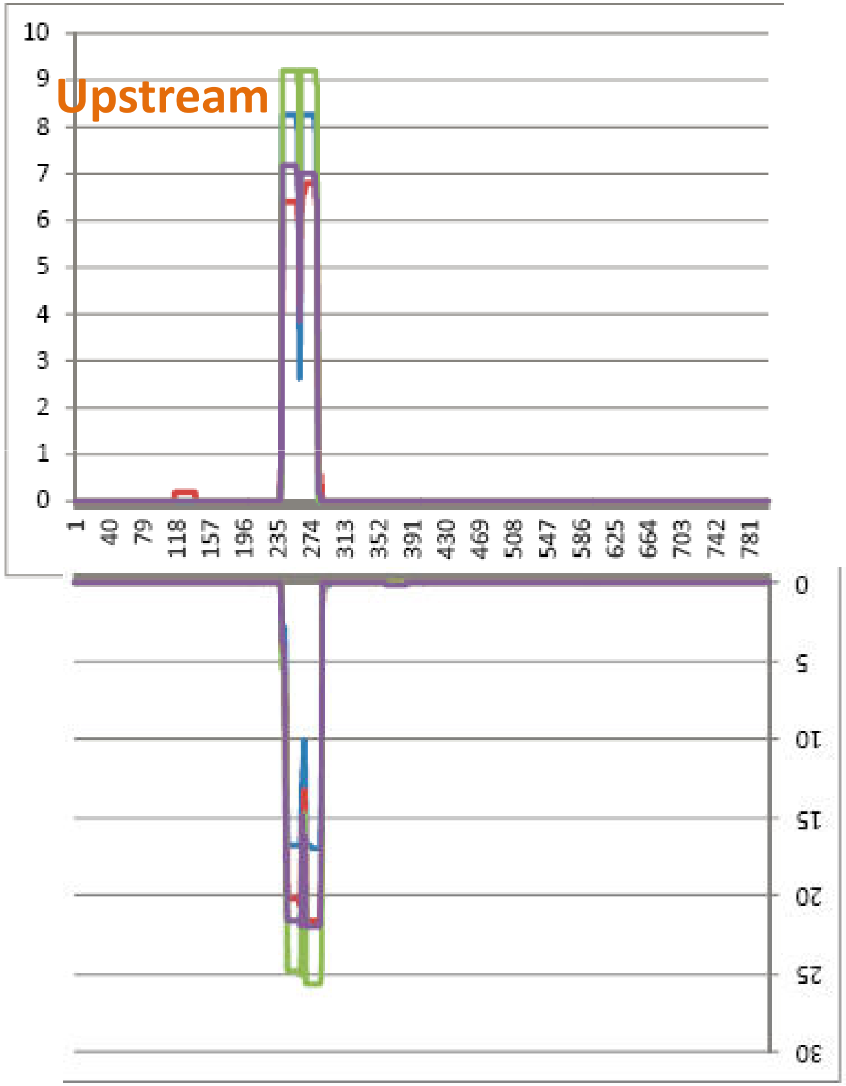

AT5G27660

Encodes a protein with similarity to human PARK13, a mitochondrial protease implicated in Parkinson disease. DEG14 is induced by heat stress and involved in degradation of misfolded proteins.

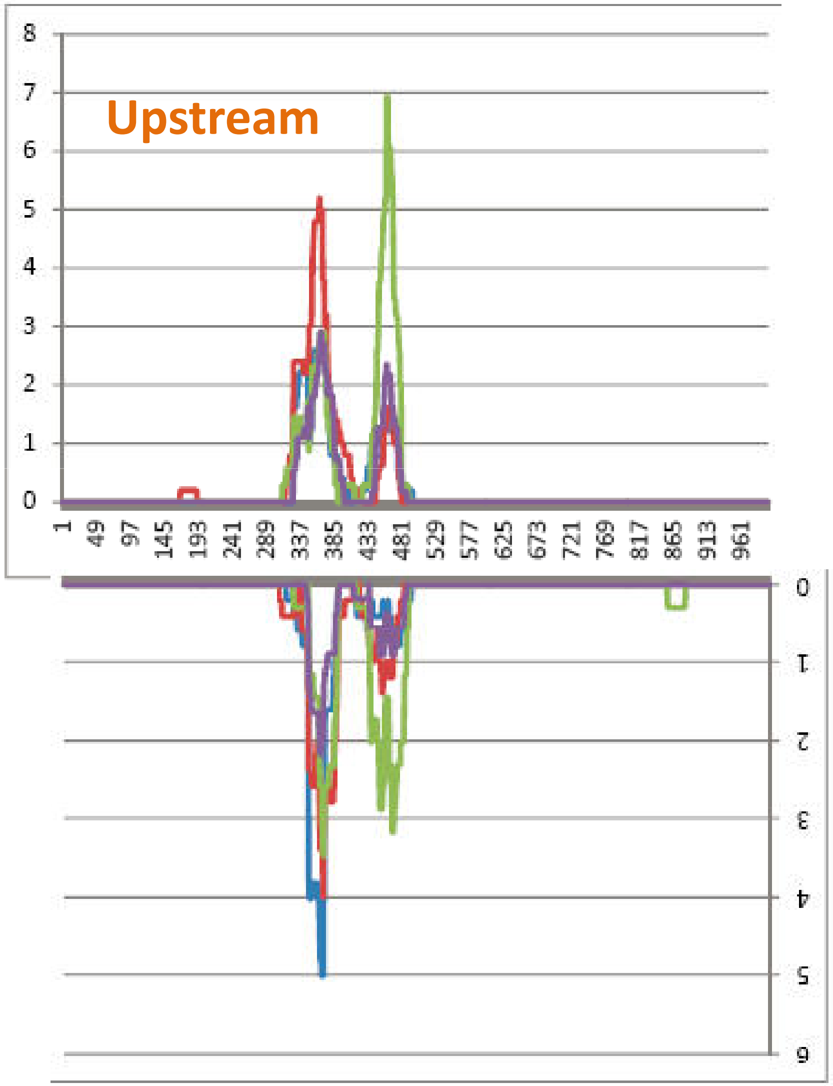

AT5G28442

BEST Arabidopsis thaliana protein match is: Quinoprotein amine dehydrogenase, beta chain-like; RIC1-like guanyl-nucleotide exchange factor (TAIR:AT5G28350.1).

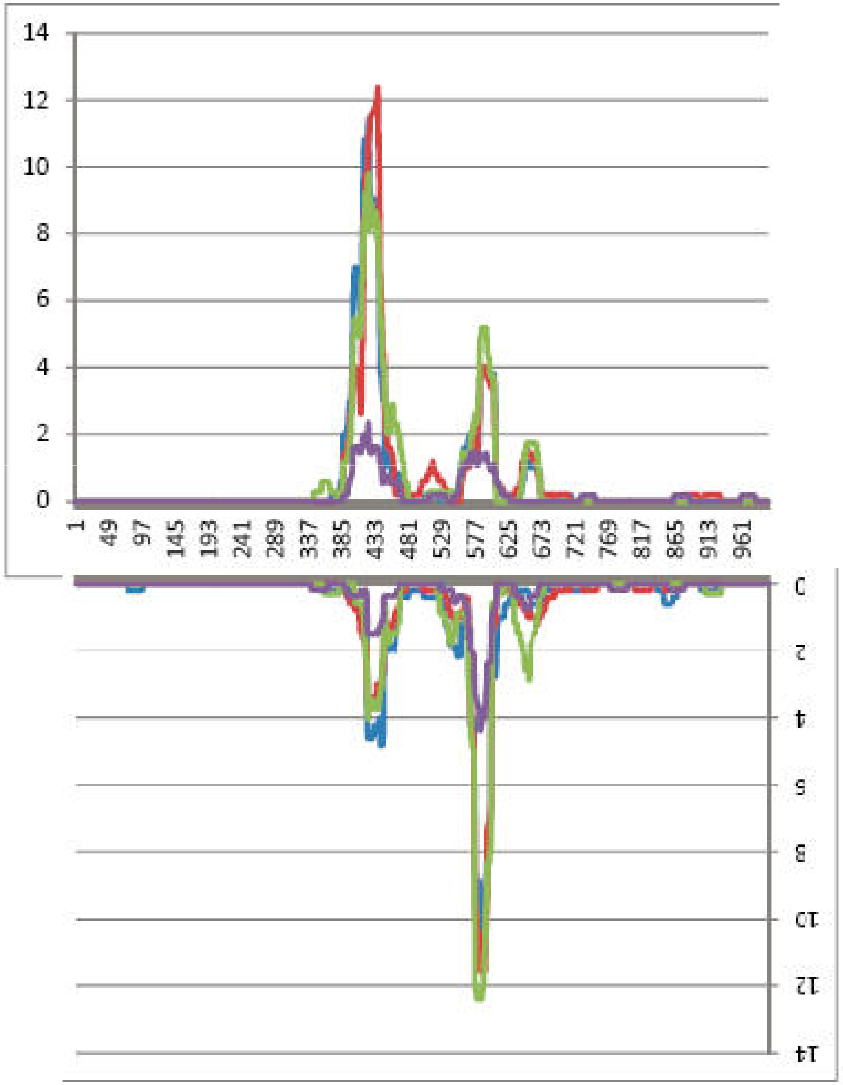

AT5G29000

Homeodomain-like superfamily protein

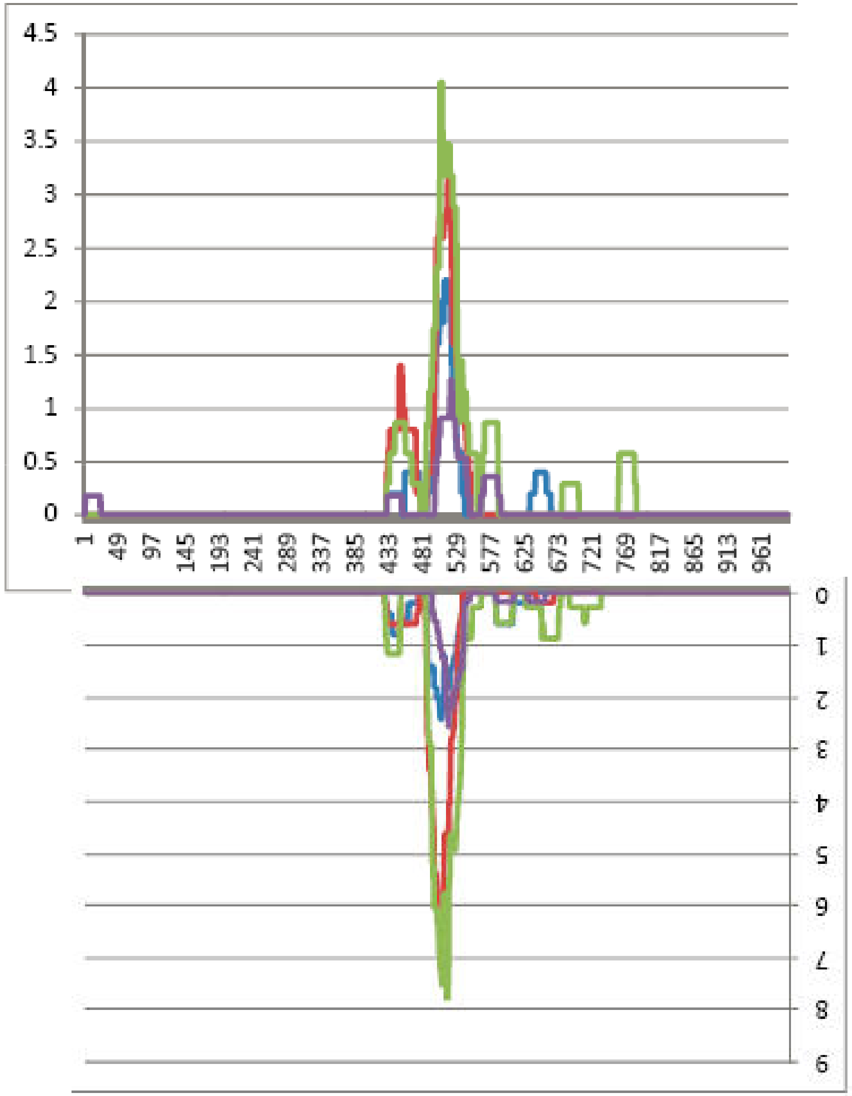

AT5G34882

Encodes a ECA1 gametogenesis related family protein.

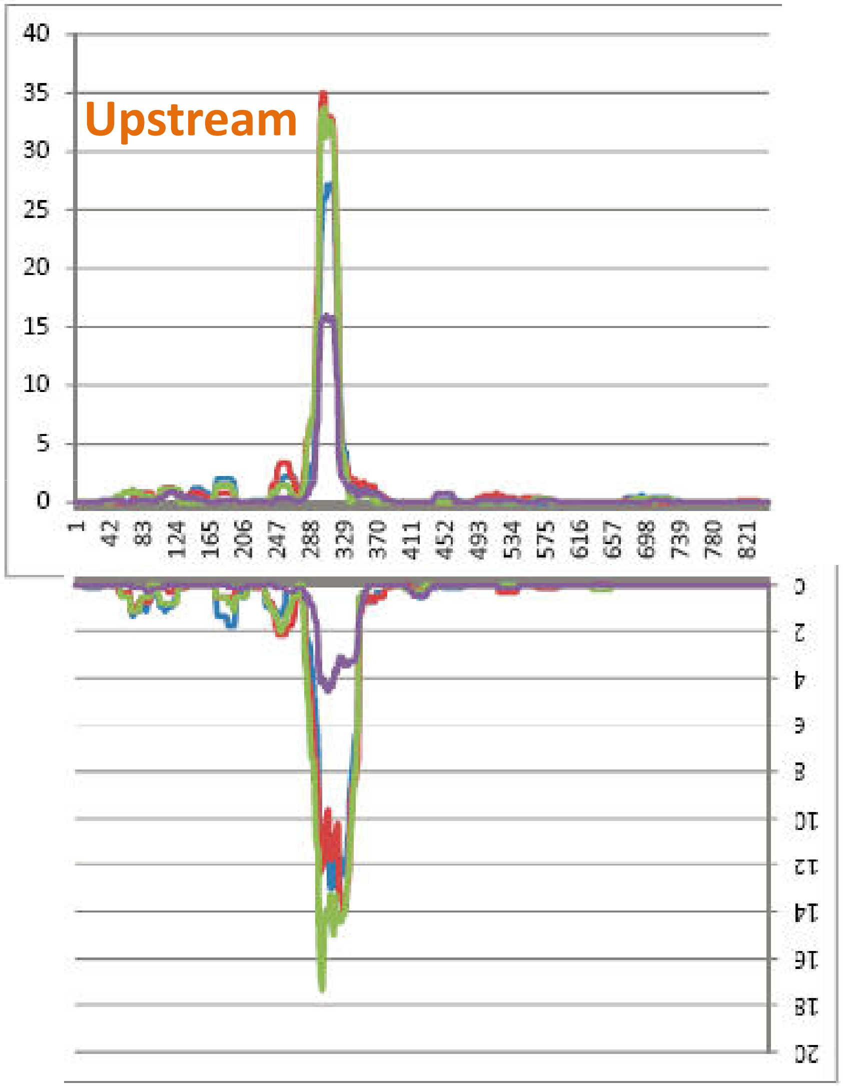

AT5G34883

Protein of unknown function (DUF784)

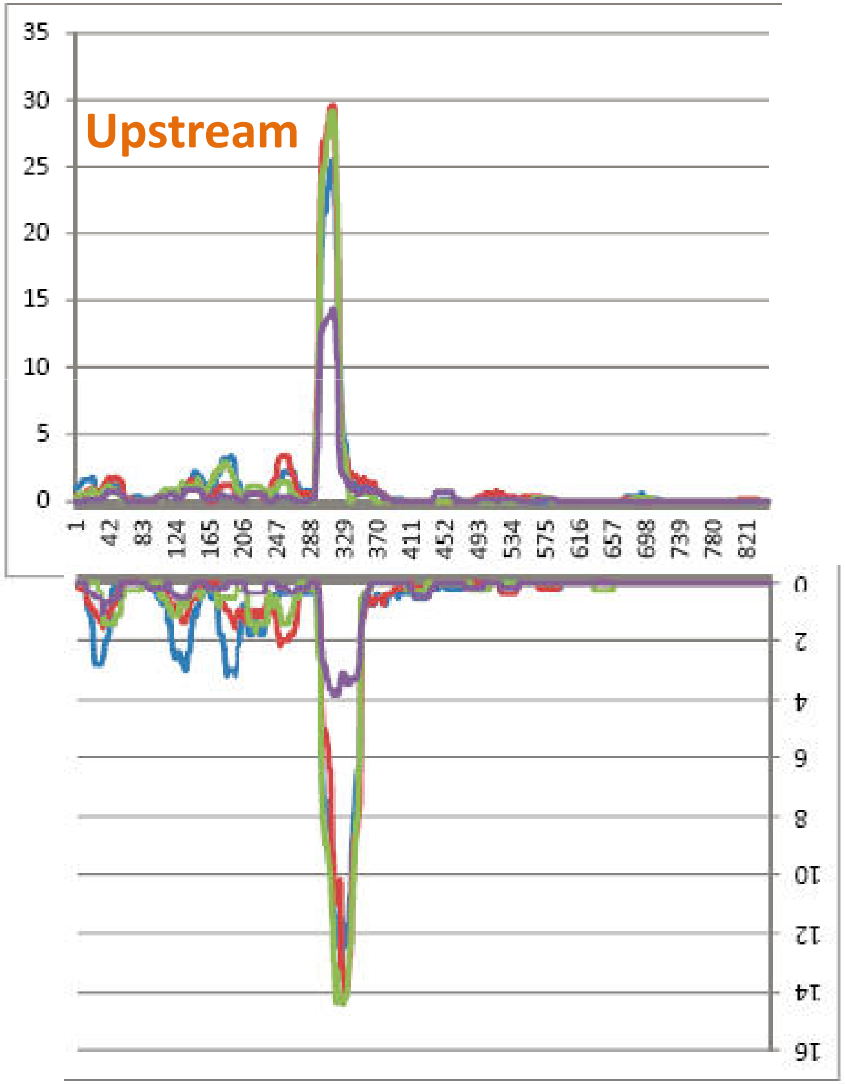

AT5G36140

Member of
CYP716A

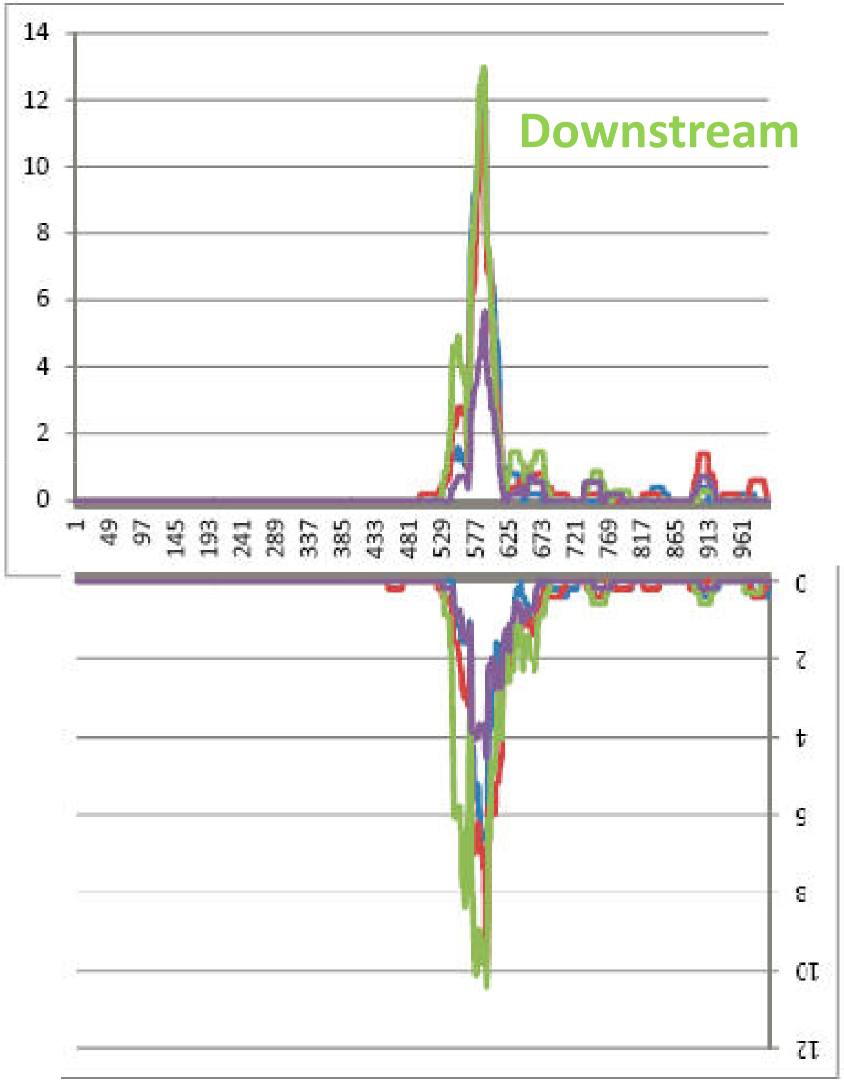

AT5G37420

Note that previous reports (Plant Cell 2003,15:1538; PNAS 2003, 100:13407) have incorrectly named AT5G37420 as AGL105. AT5G37415 has now been named as AGL105 based on Plant Cell 2003, 15:1538 where the GenBank accession number given for AGL105 is AY141227 (Supplemental Table 3), which corresponds to AT5G37415.

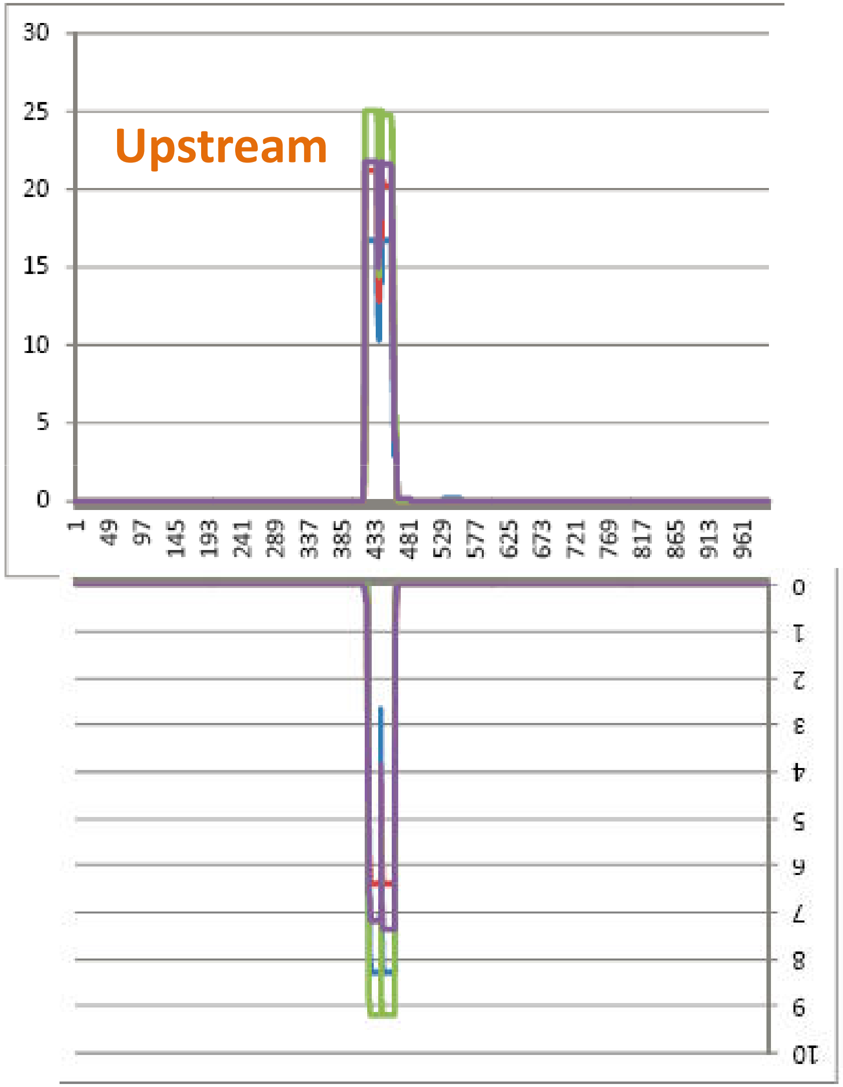

AT5G37430

Family of unknown function (DUF577)

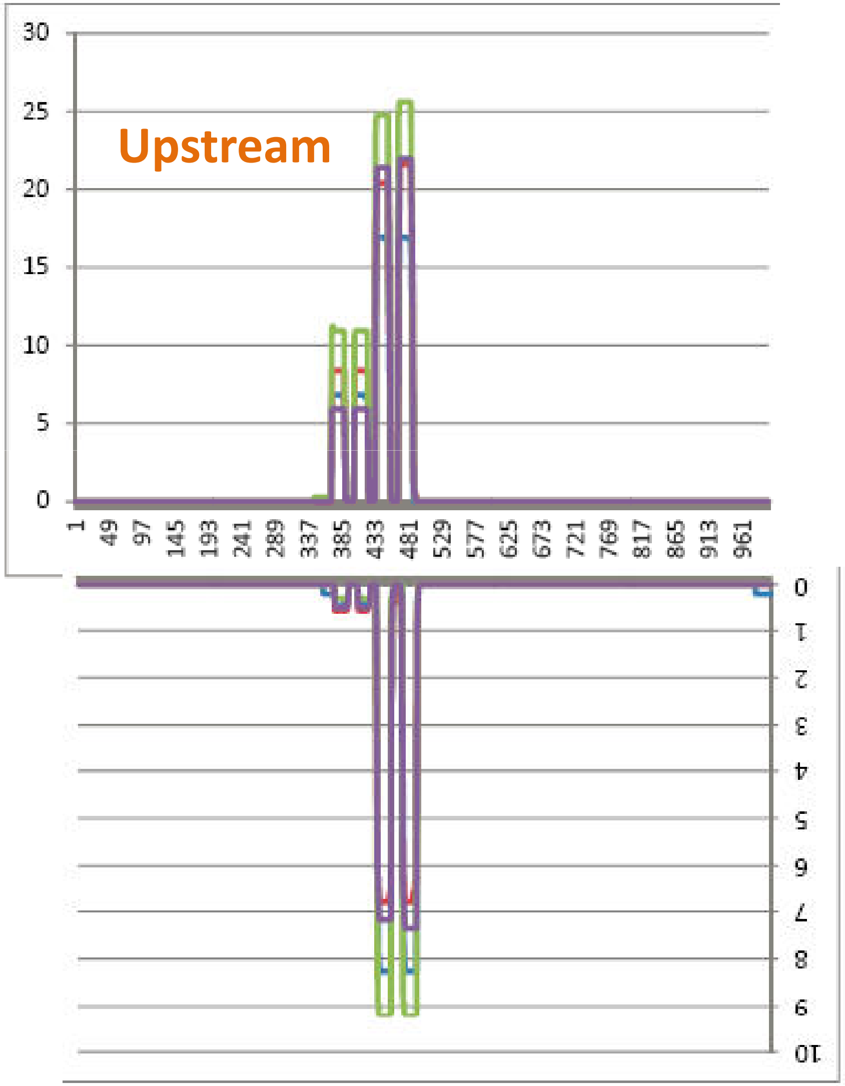

AT5G37690

SGNH hydrolase-type esterase superfamily protein

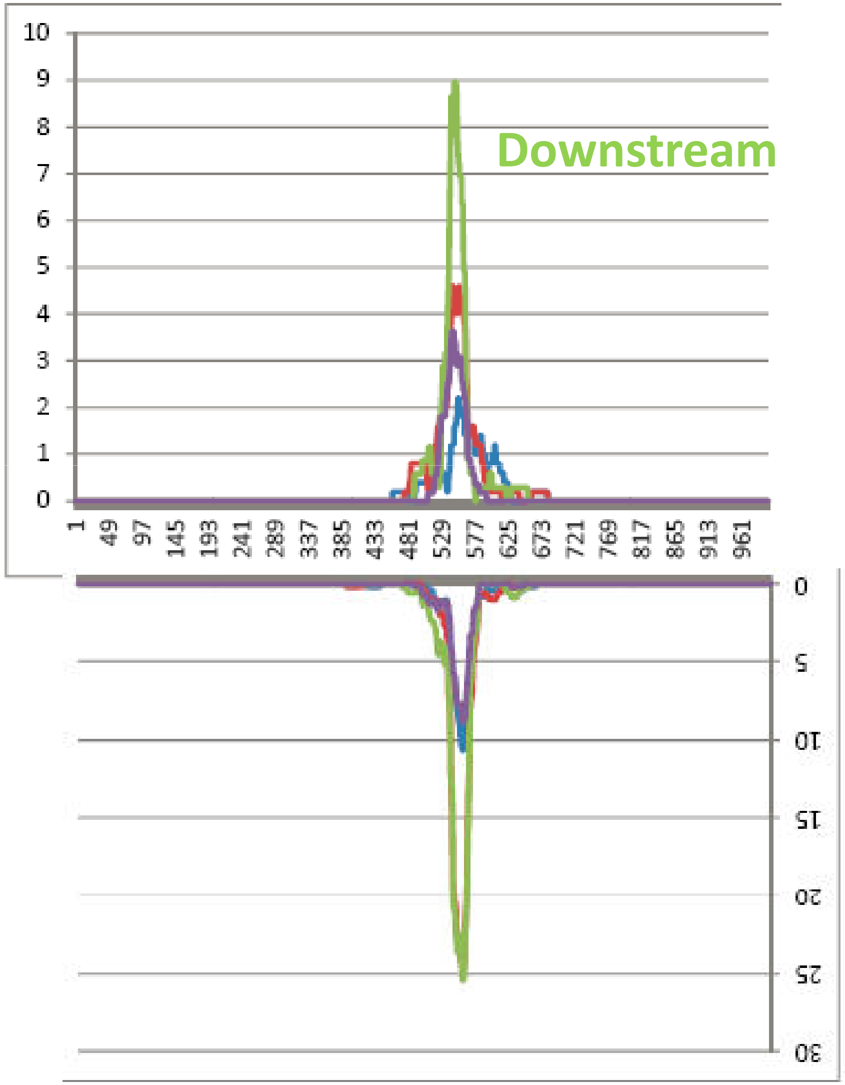

AT5G39080

HXXXD-type acyl-transferase family protein

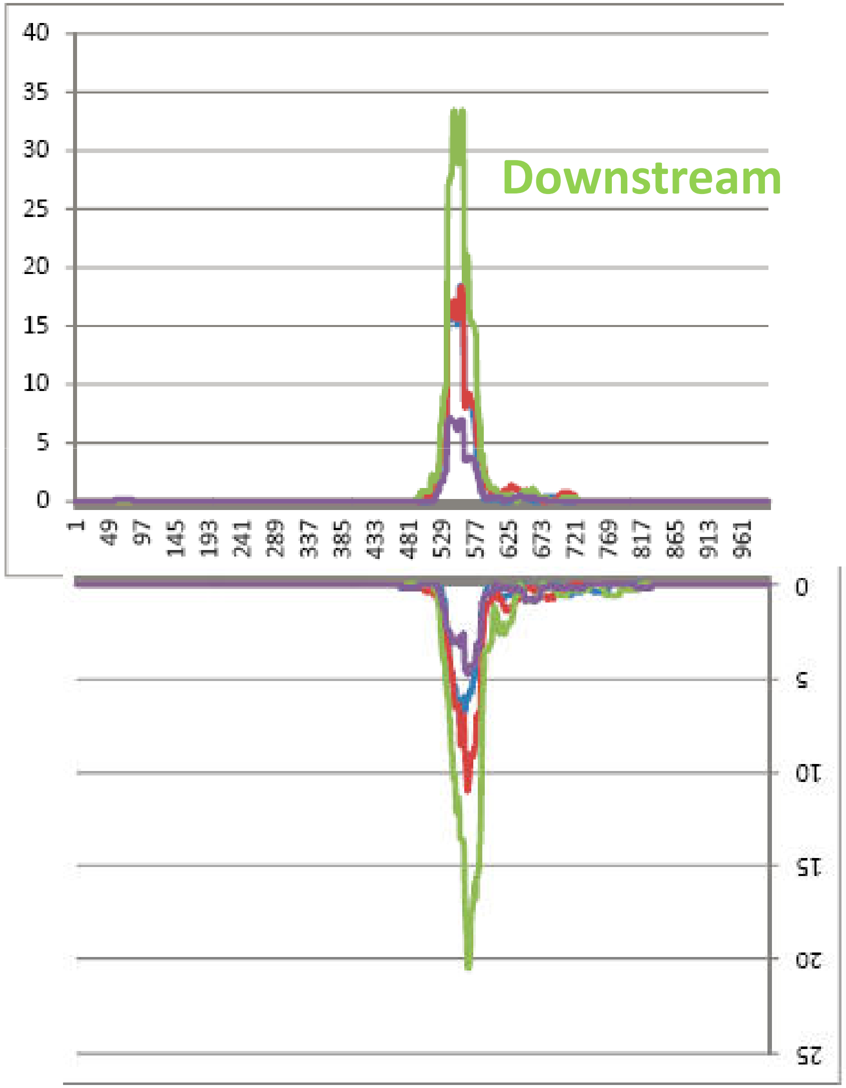

AT5G39720

Avirulence induced gene 2 like protein (AIG2L).

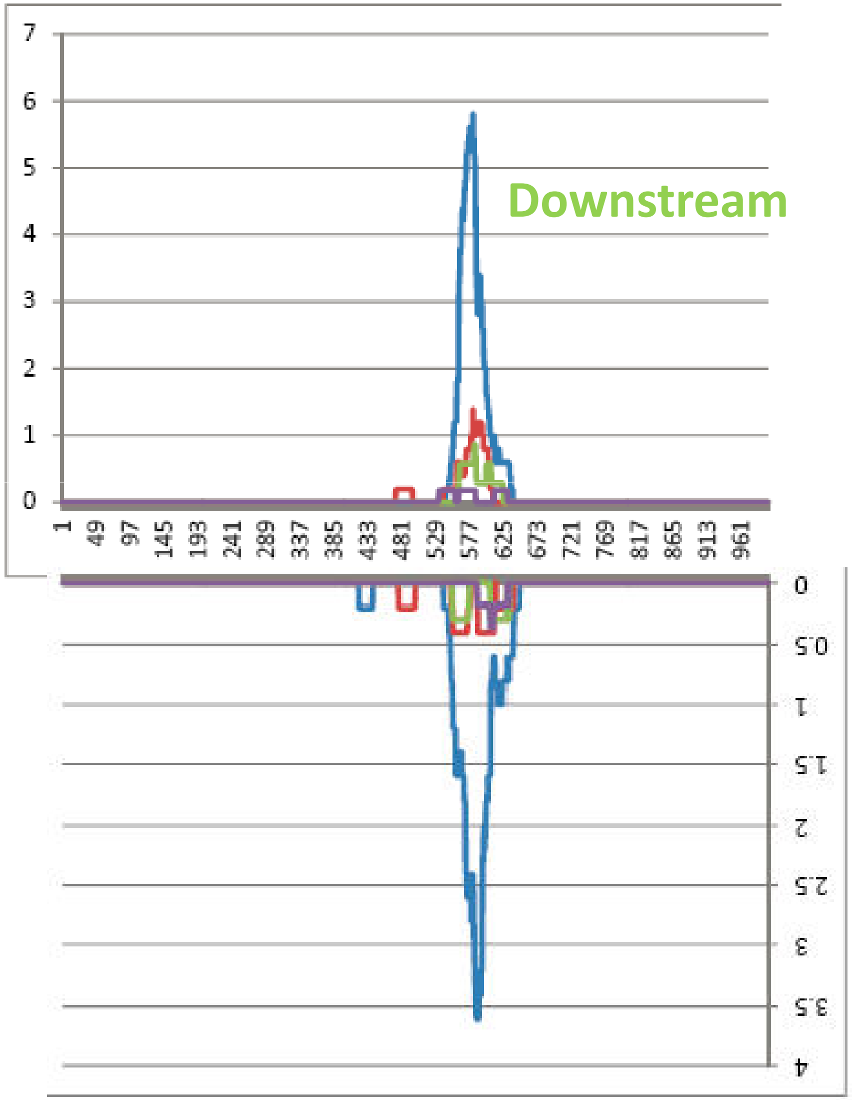

AT5G42203

This gene encodes a small protein and has either evidence of transcription or purifying selection.

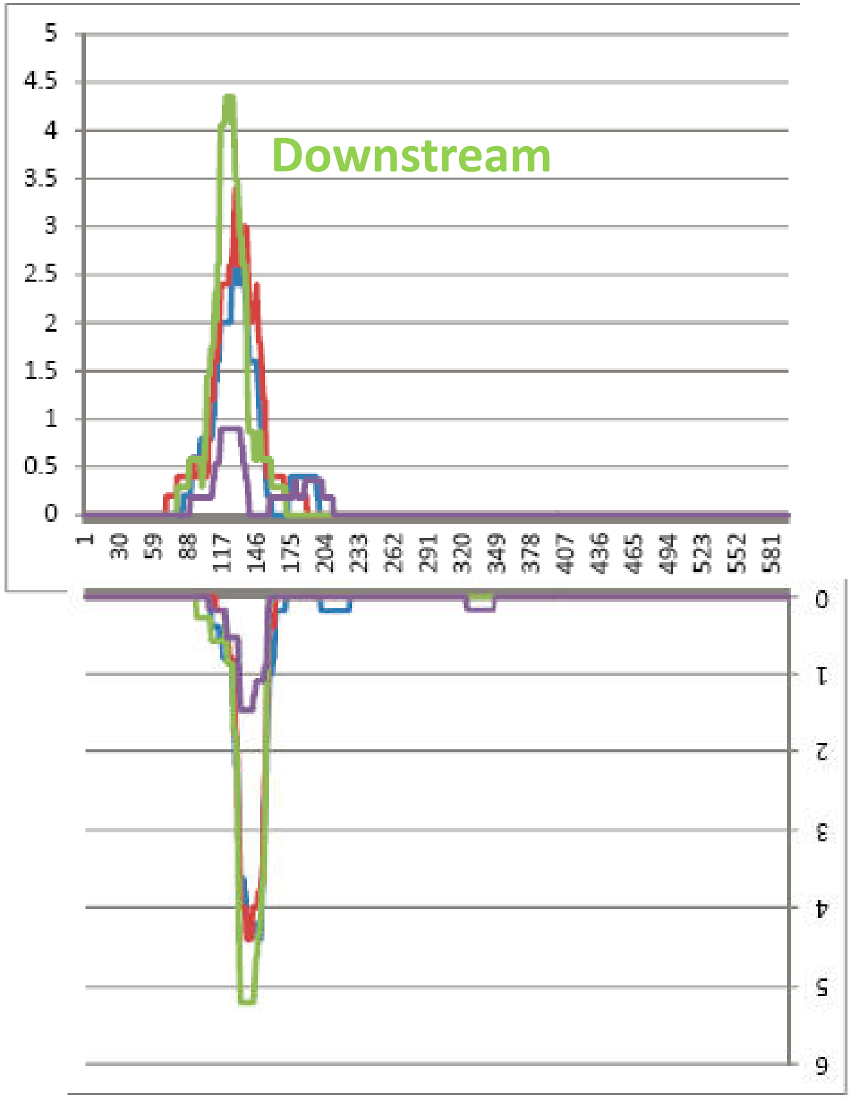

AT5G42567

Encodes a ECA1 gametogenesis related family protein

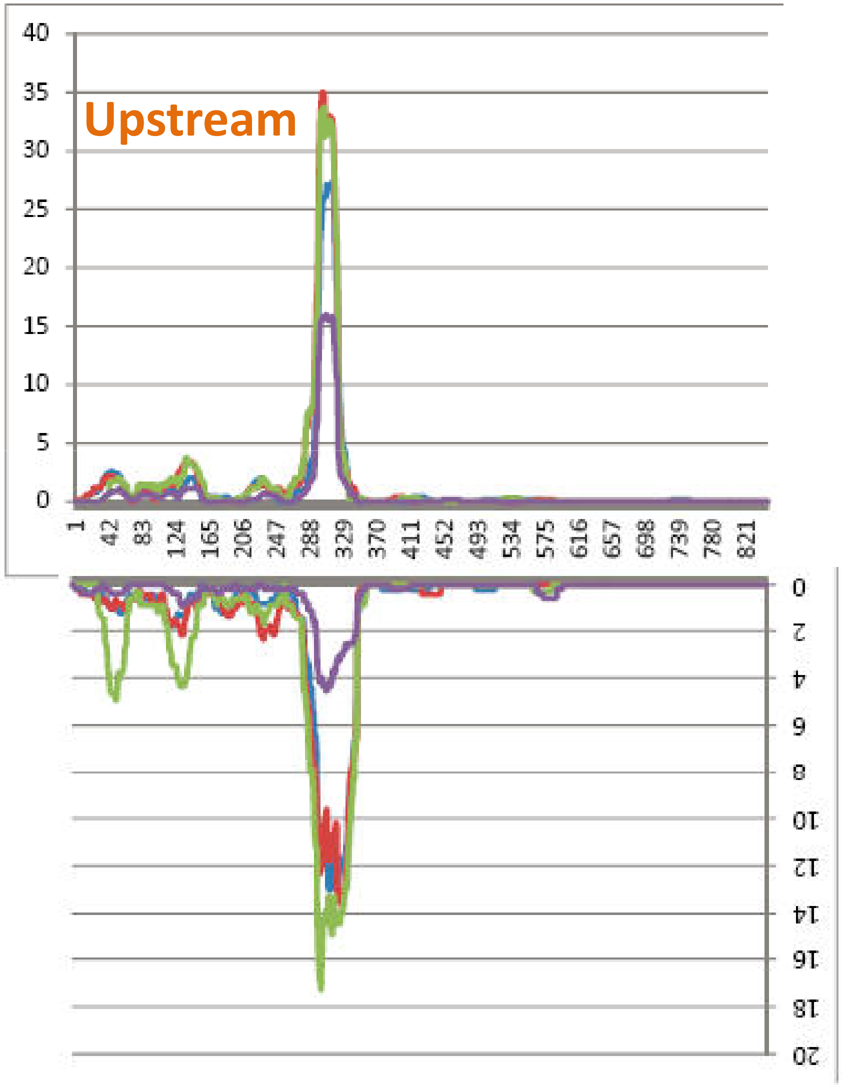

AT5G42635

Glycine-rich protein

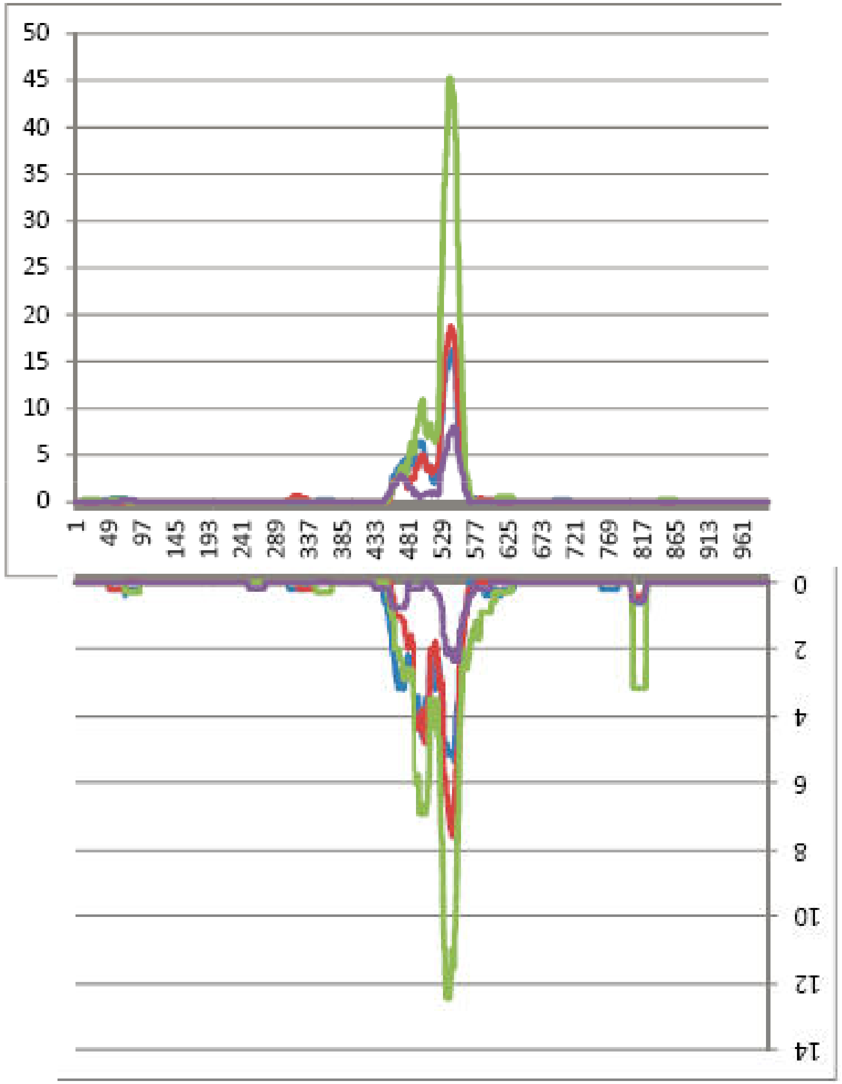

AT5G42930

Alpha/beta-Hydrolases superfamily protein

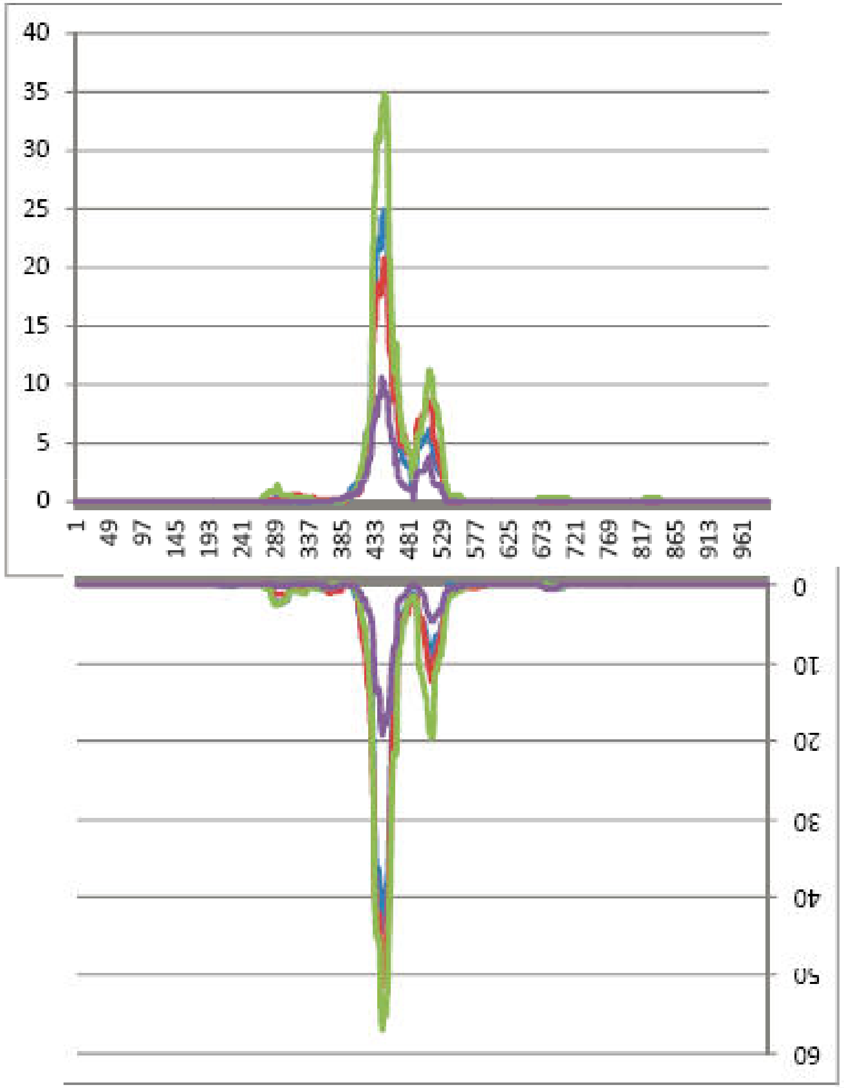

AT5G43525

Encodes a cysteine-rich peptide that is expressed in the synergid cell and appears to be secreted toward the funicular surface through the micropyle. Its closely related family members are involved in pollen tube guidance but this particular peptide lacks a conserved cysteine residue that appears critical for this function and does not affect pollen tube guidance.

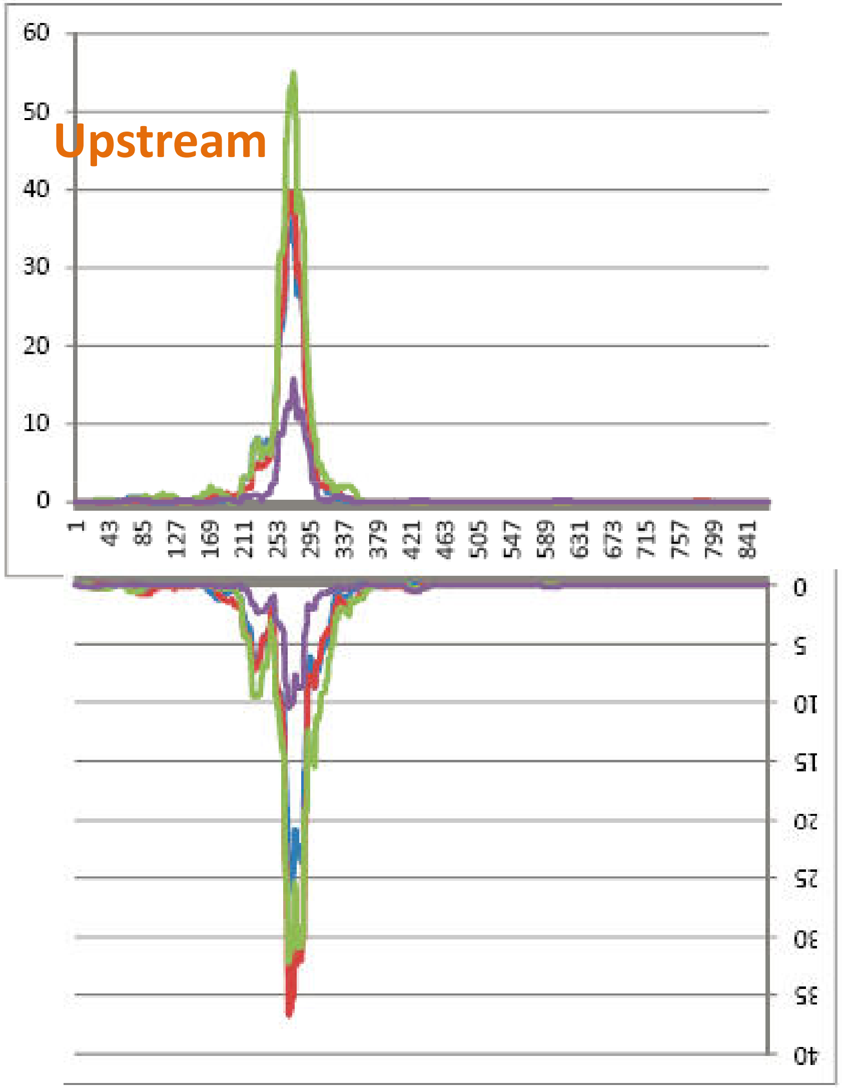

AT5G43755

BEST Arabidopsis thaliana protein match is: Polynucleotidyl transferase, ribonuclease H-like superfamily protein (TAIR:AT2G04420.1).

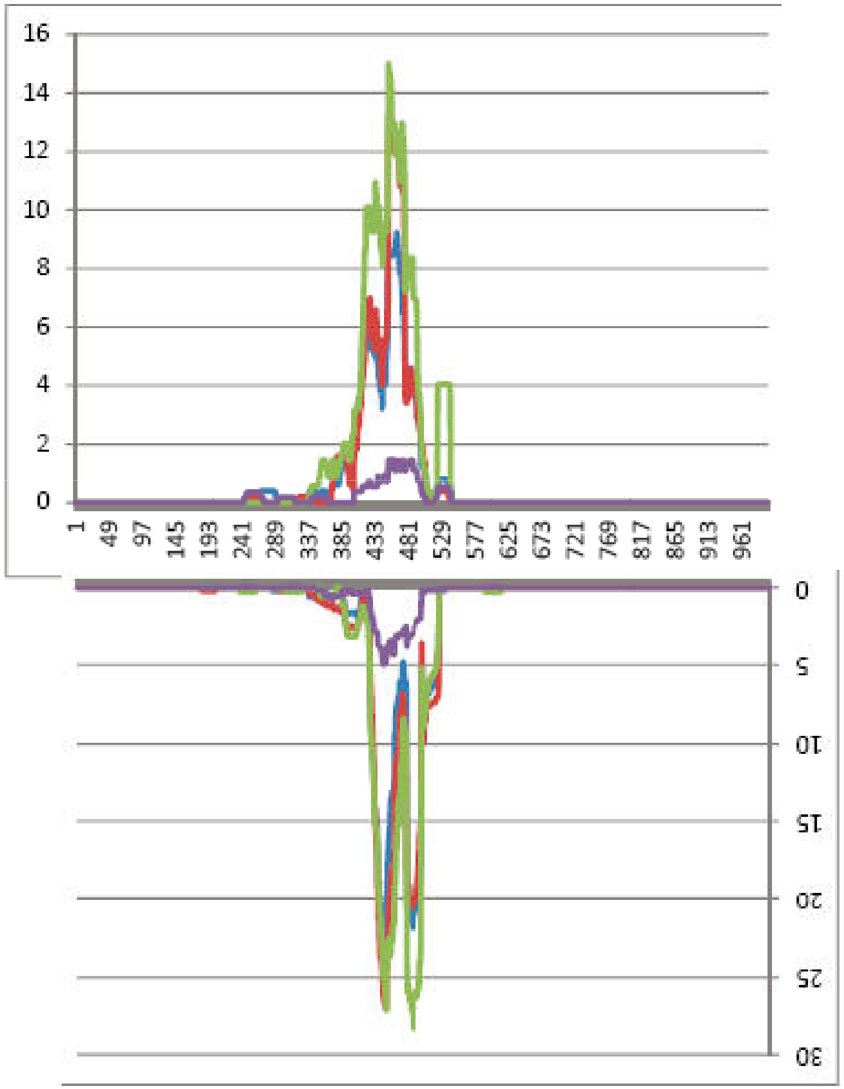

AT5G48515

Encodes a defensin-like (DEFL) family protein.

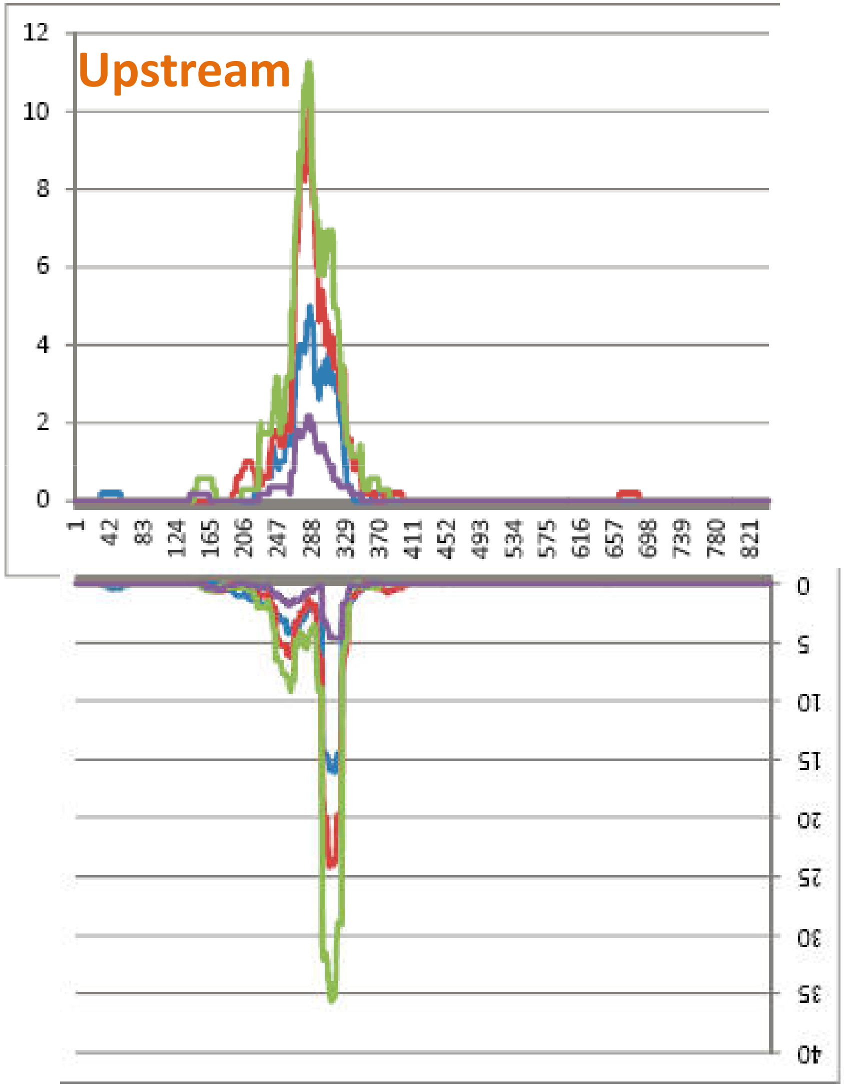

AT5G48595

Encodes a defensin-like (DEFL) family protein.

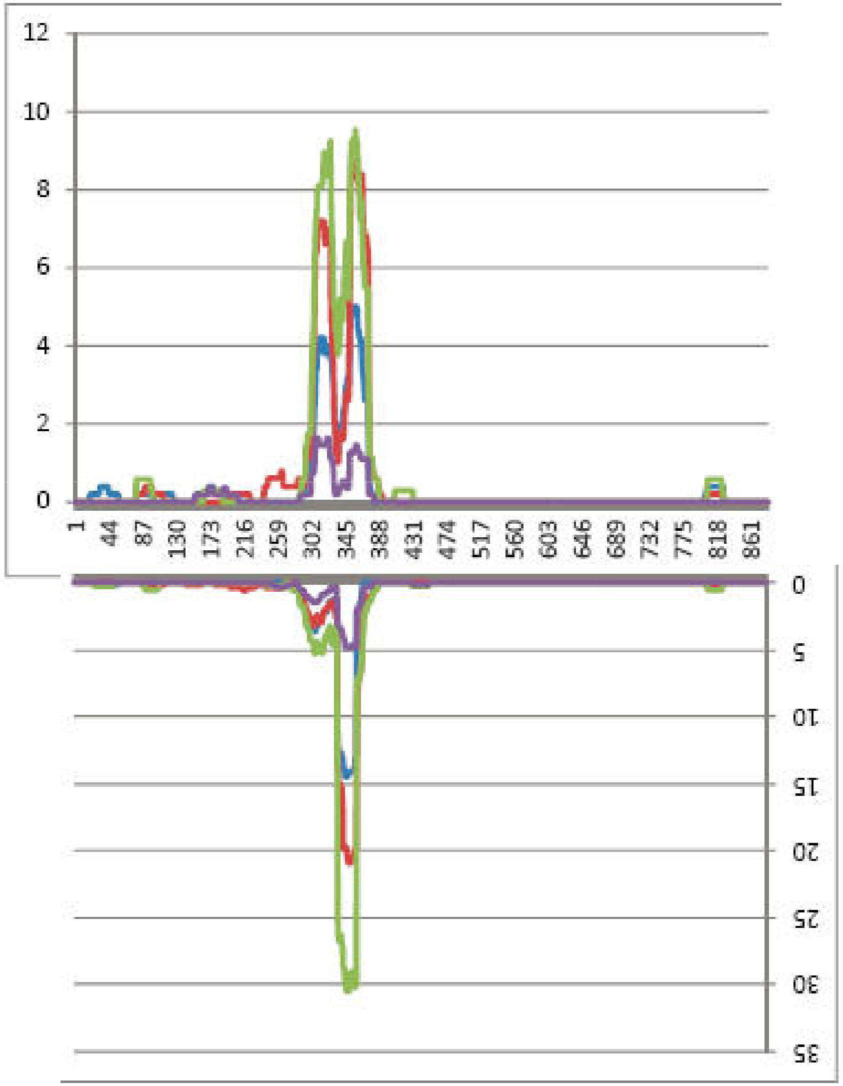

AT5G49420

MADS-box transcription factor family protein

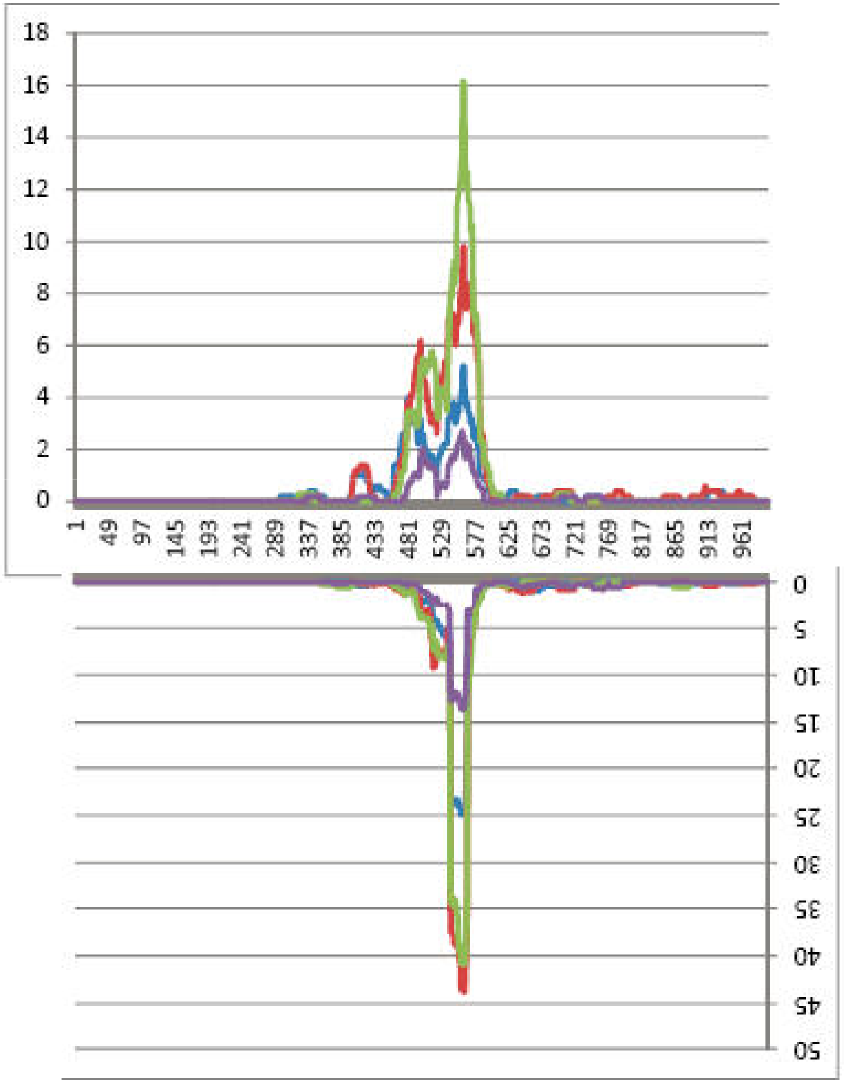

AT5G49440

unknown protein

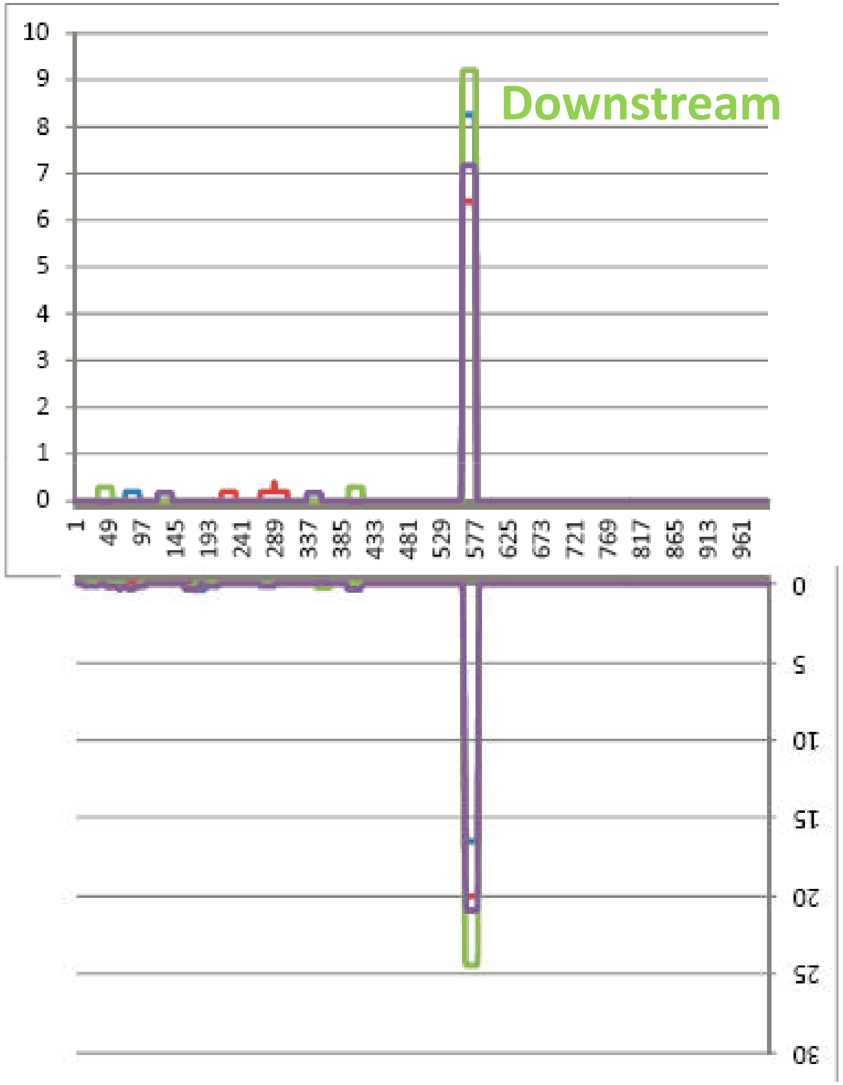

AT5G50480

nuclear factor Y,
subunit C6 (NF-YC6)

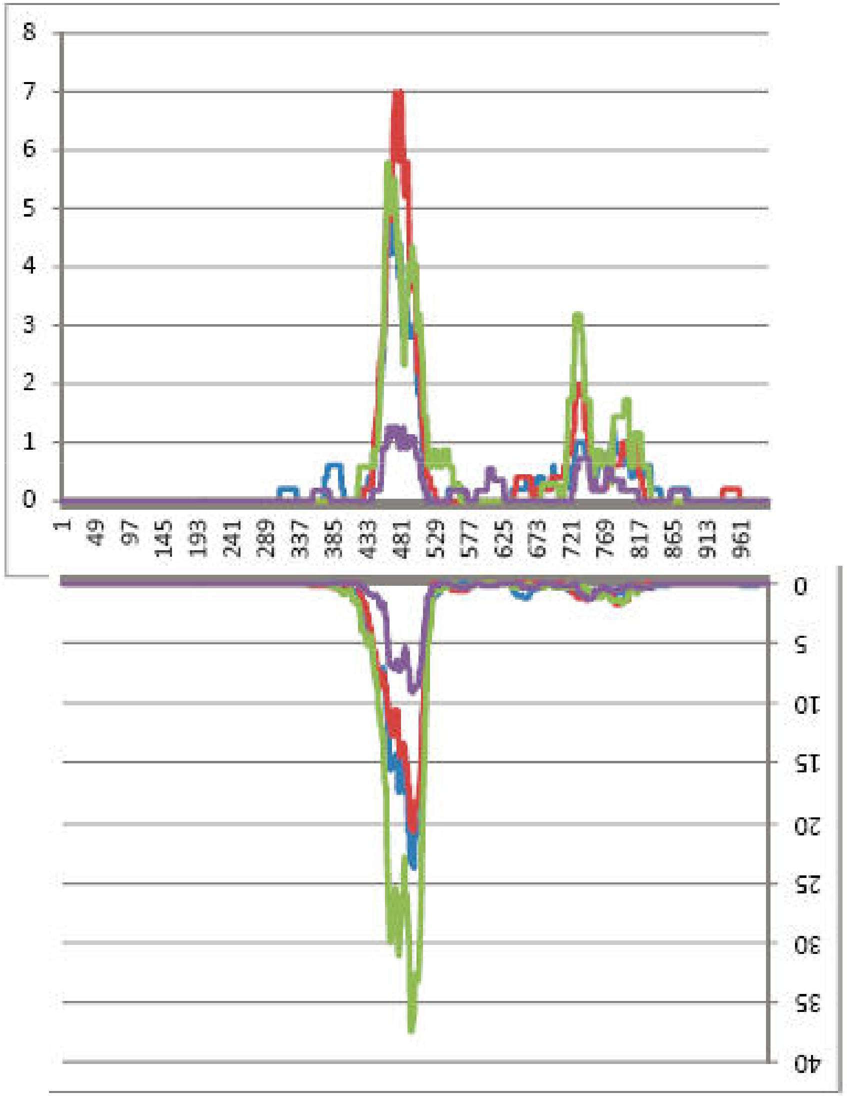

AT5G54410

unknown protein

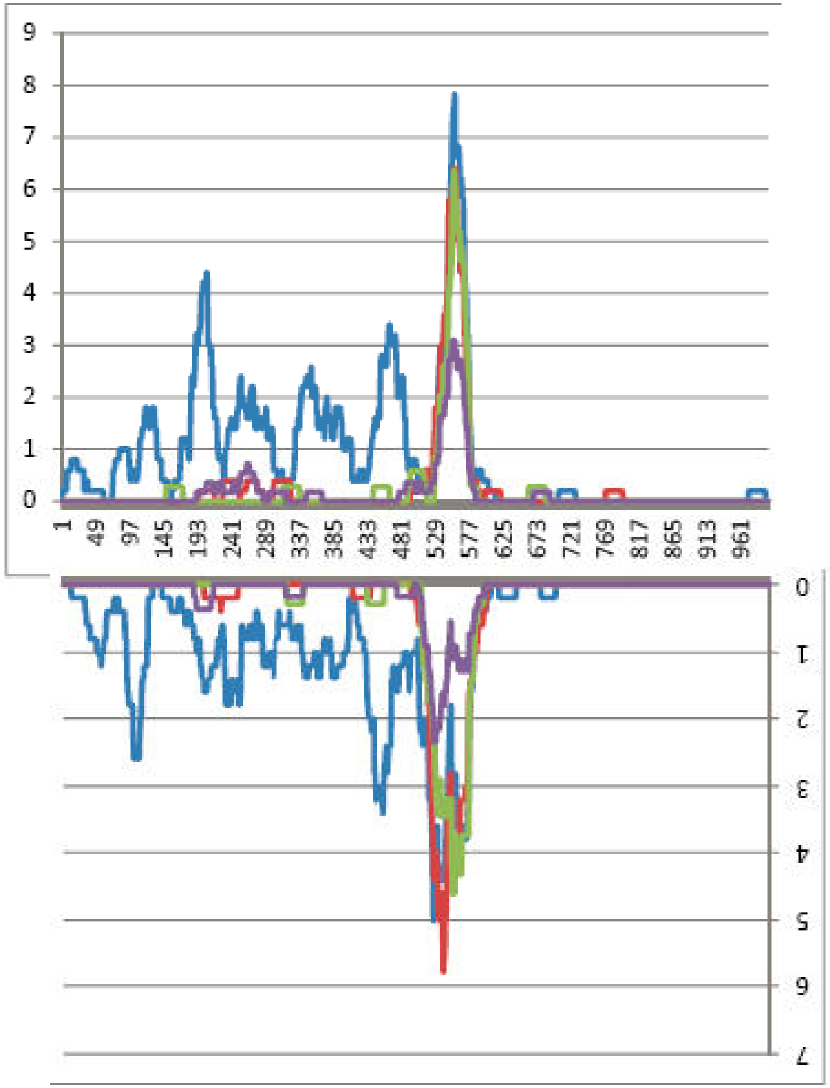

AT5G54700

Ankyrin repeat family protein

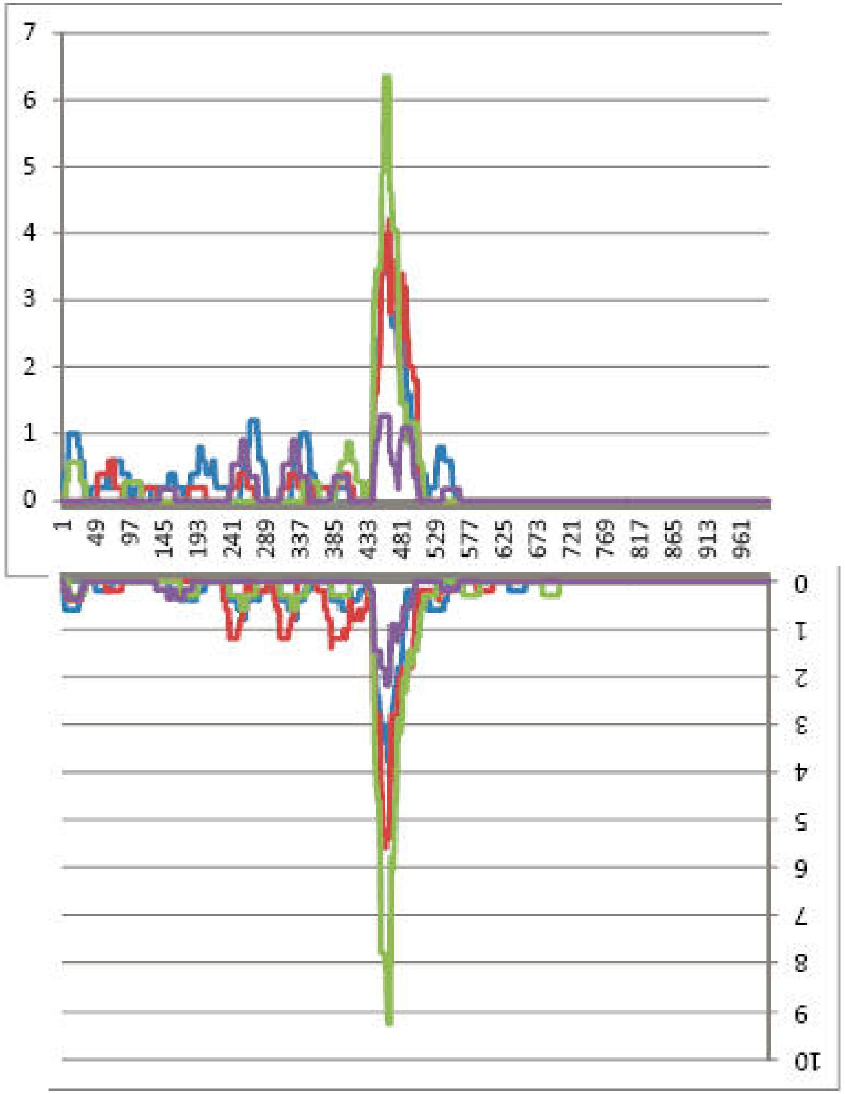

AT5G61520

Major facilitator
superfamily protein

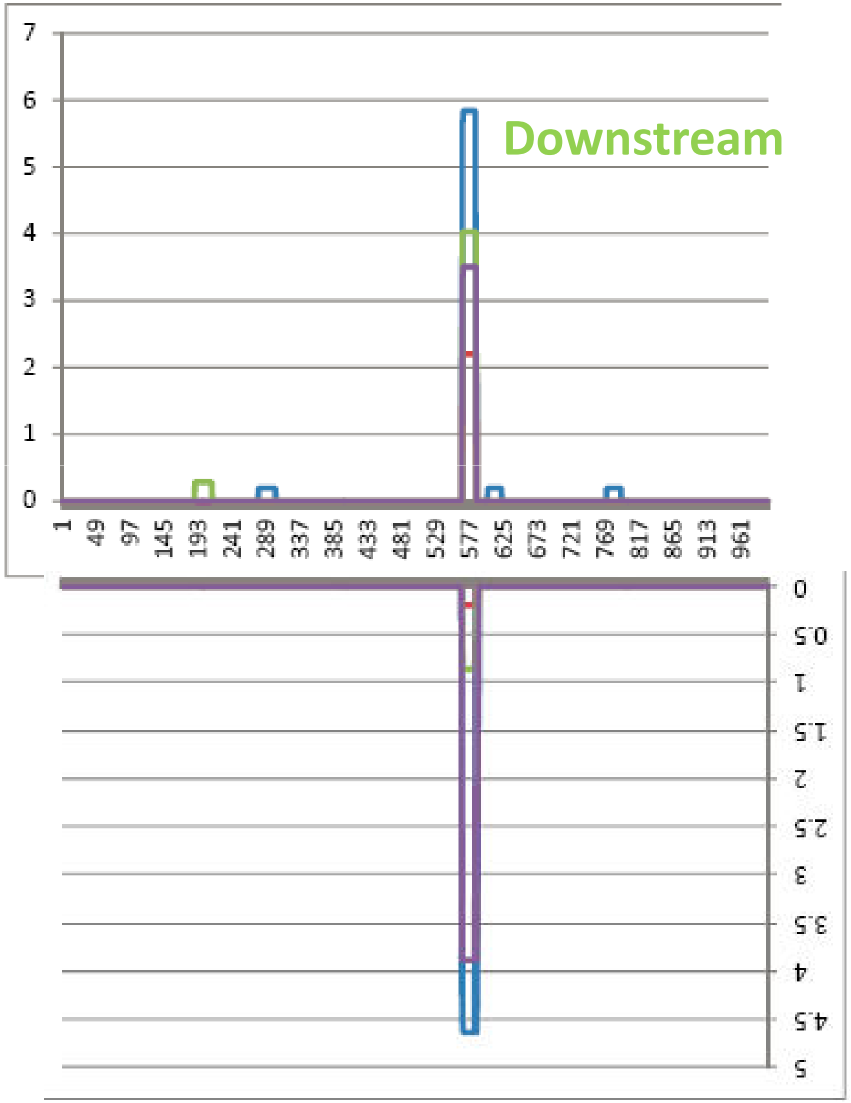

AT5G65005

Polynucleotidyl transferase, ribonuclease H-like superfamily protein

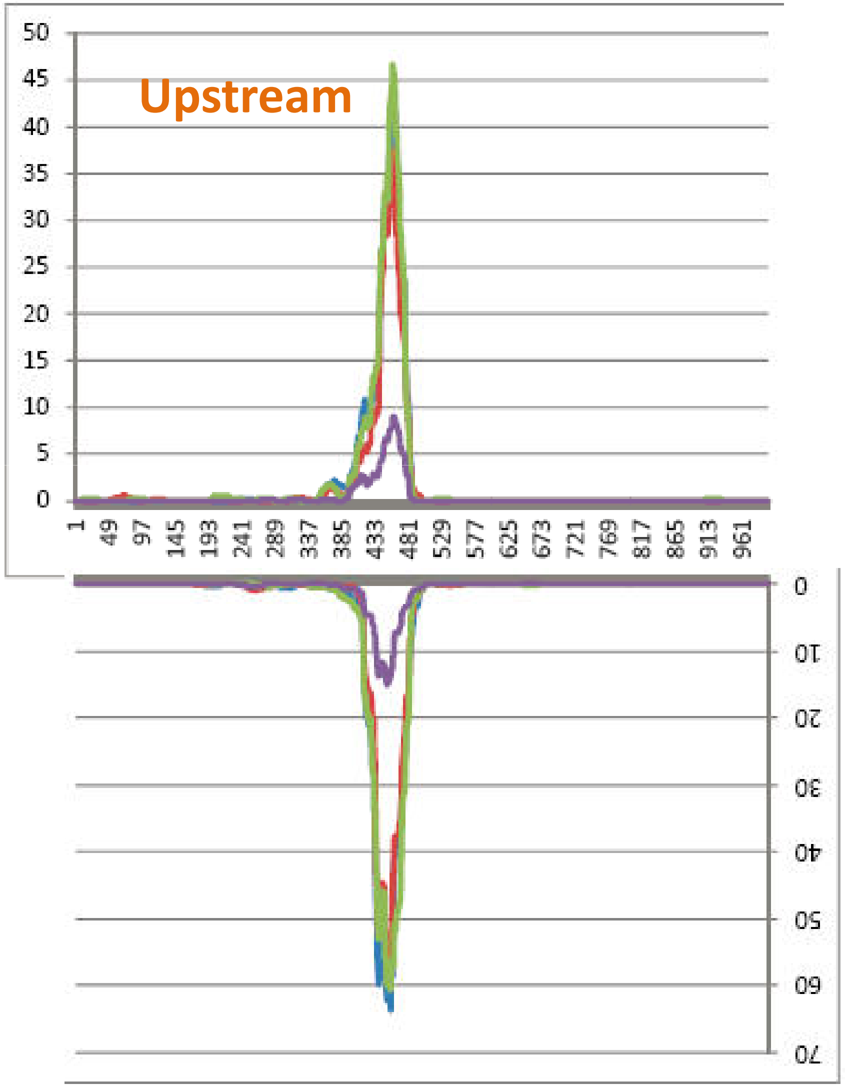

Supplement: S6 Fig — (PDF) [file pone.0169212.s006.pdf]
