# Supplementary material for: Transcriptome-Wide Discovery of PASRs (Promoter-Associated Small RNAs) and TASRs (Terminus-Associated Small RNAs) in Arabidopsis thaliana
Source: PLoS One. 2017 Jan 3;12(1):e0169212. doi: 10.1371/journal.pone.0169212 (PMC5207706; doi:10.1371/journal.pone.0169212)
Supplement: S7 Fig — (PDF) [file pone.0169212.s007.pdf]

Figure S7 AGO-associated PASR peaks identified on the sense strands of the protein-coding genes of *Arabidopsis*. For each plot, x axis measures the position of the sense strand, and y axis measures the abundance (in RPM, reads per million) of sRNAs.

AT1G01020_root

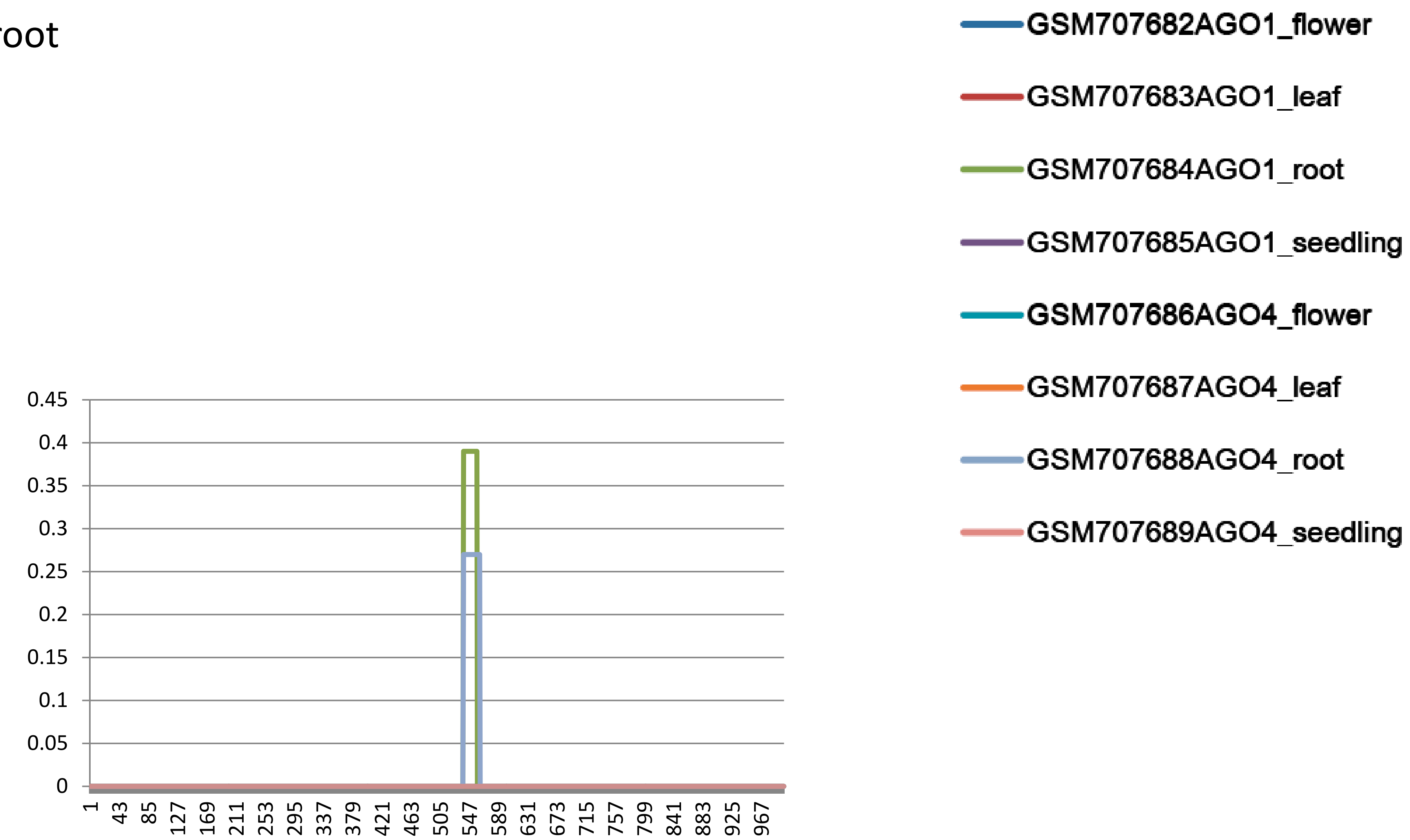

AT1G01073_AGO4

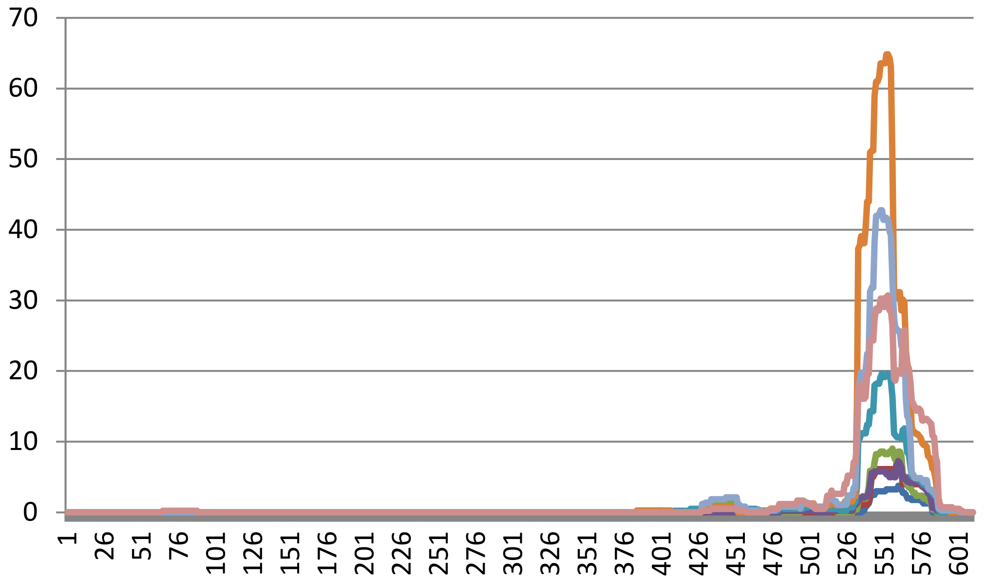

AT1G06850

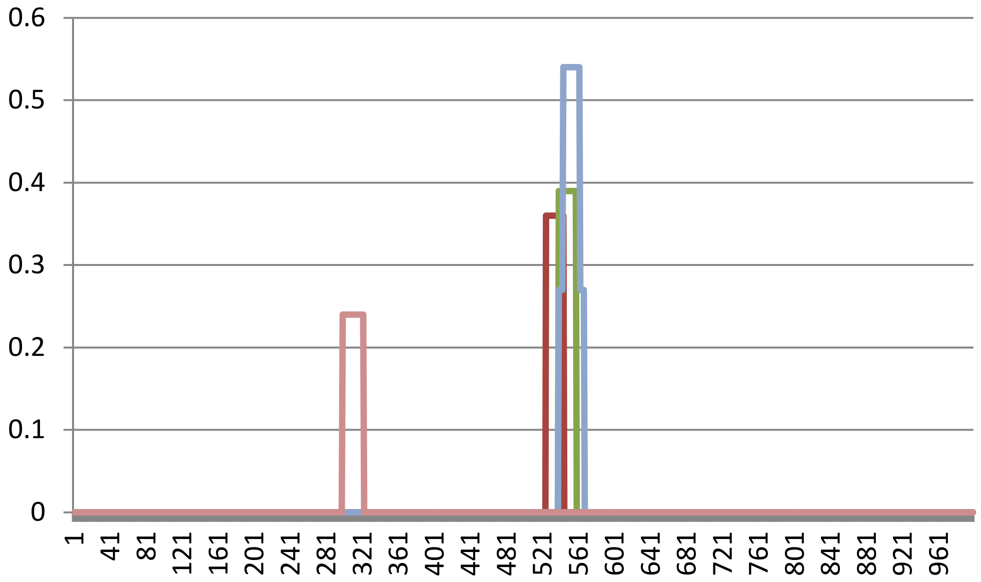

0.6
0.5
0.4
0.3
0.2
0.1
0
1
41
81
121
161
201
241
281
321
361
401
441
481
521
561
601
641
681
721
761
801
841
881
921
961

AT1G08030_AGO4

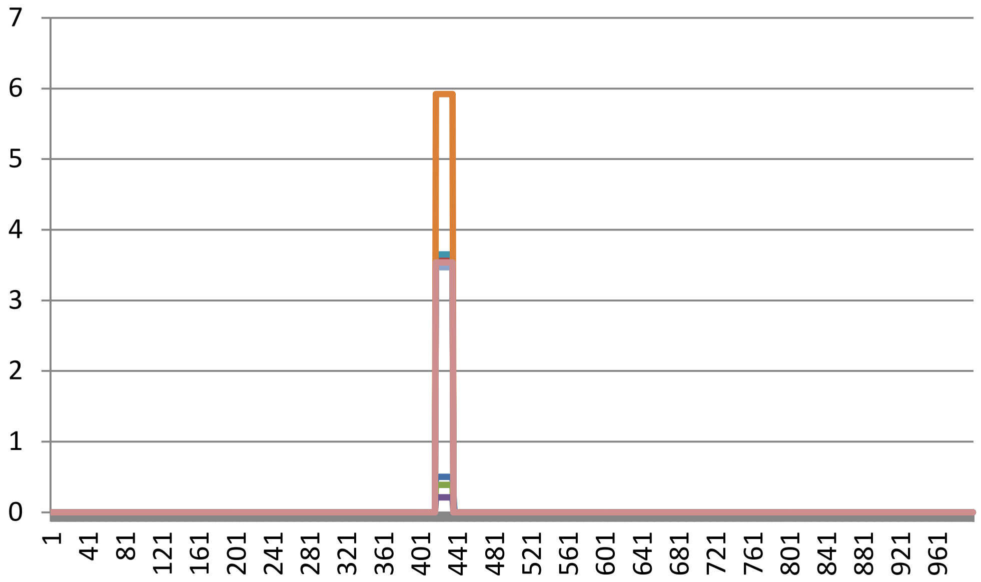

AT1G08035_AGO4

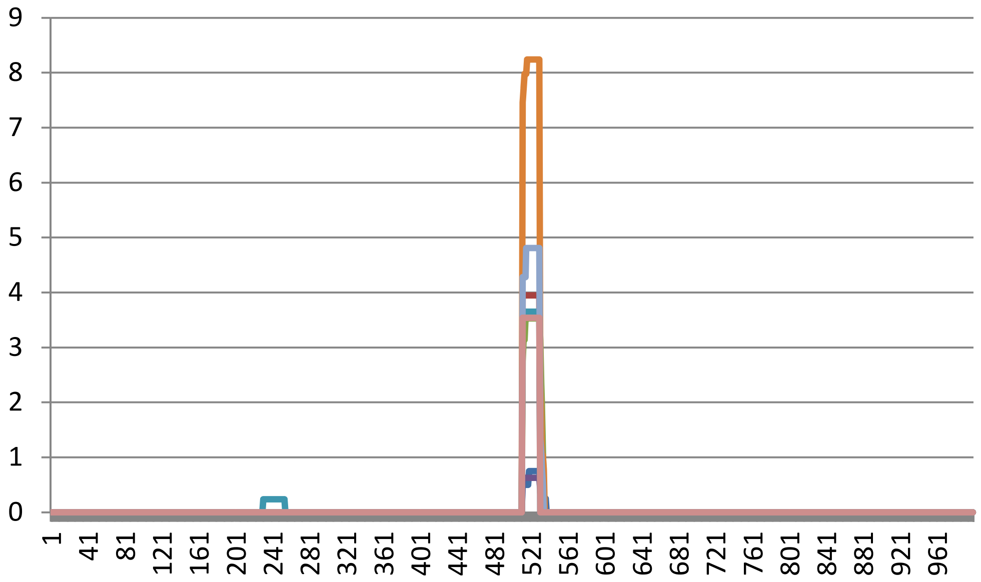

AT1G09060_AGO4

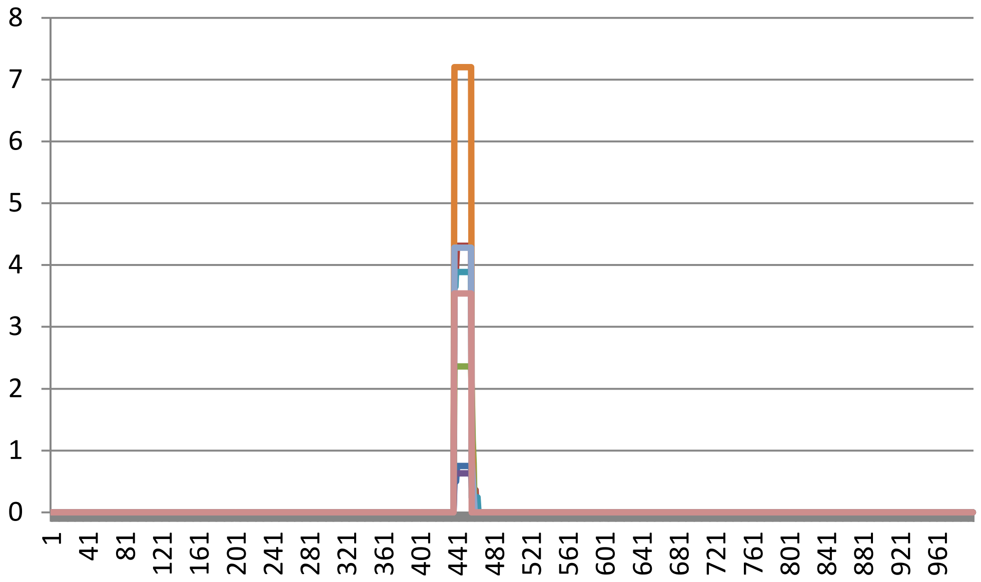

AT1G10820_AGO4

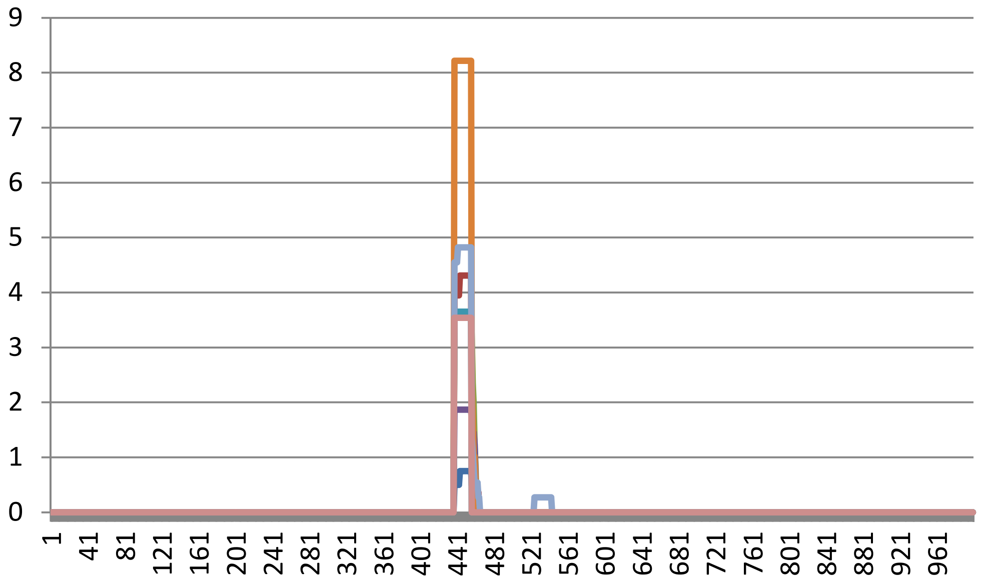

AT1G11020_AGO1 root

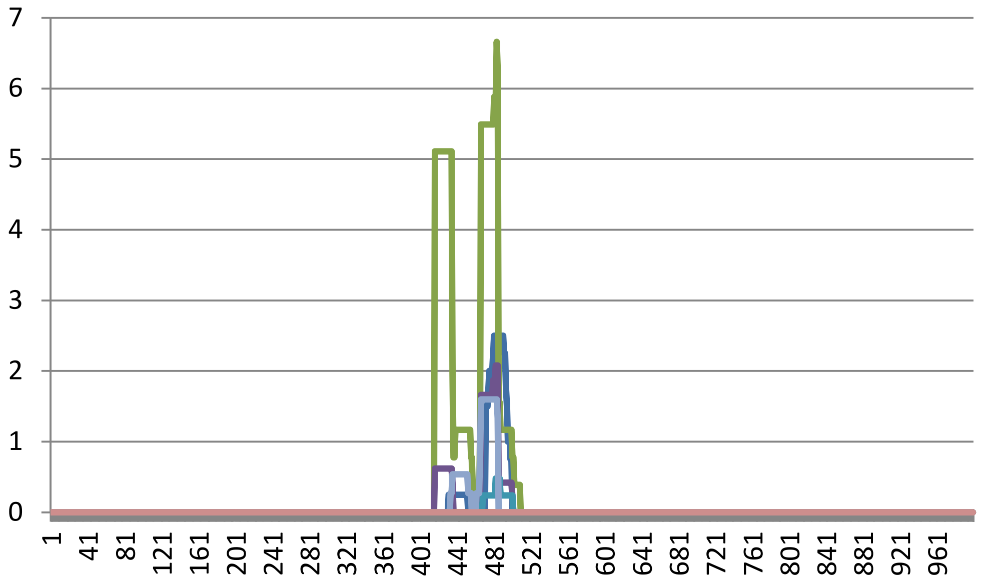

AT1G11785_AGO4 flower

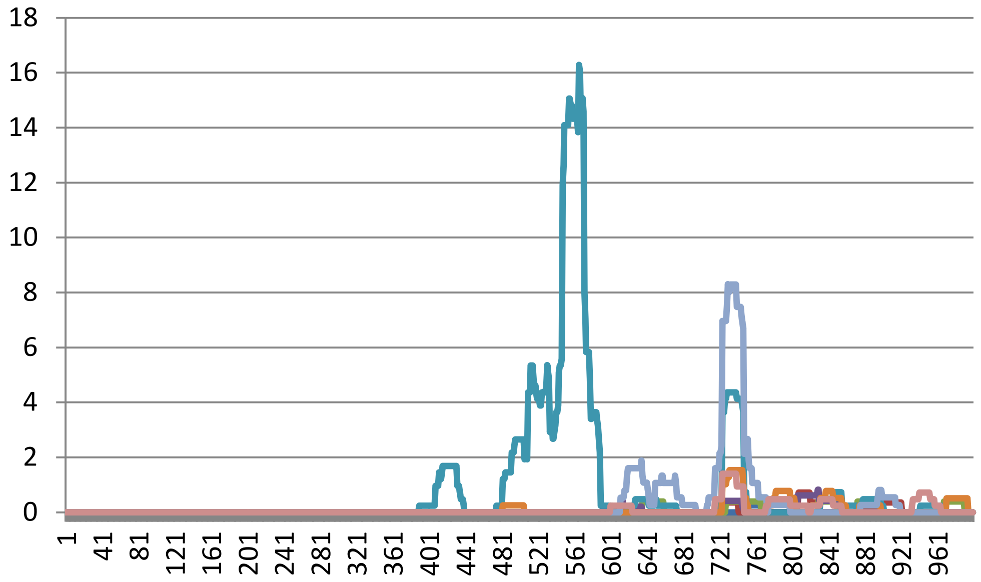

AT1G16760_AGO4

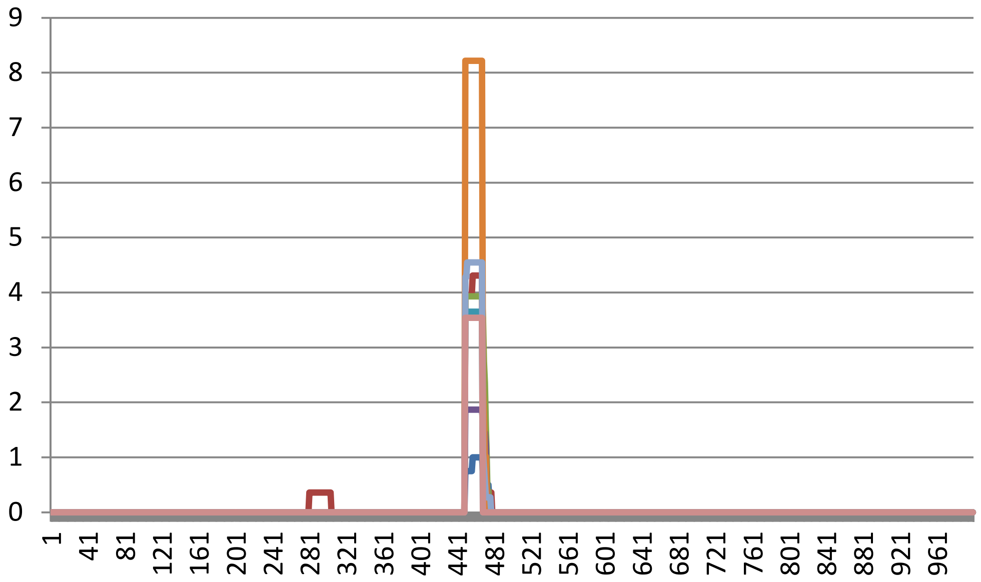

AT1G17720

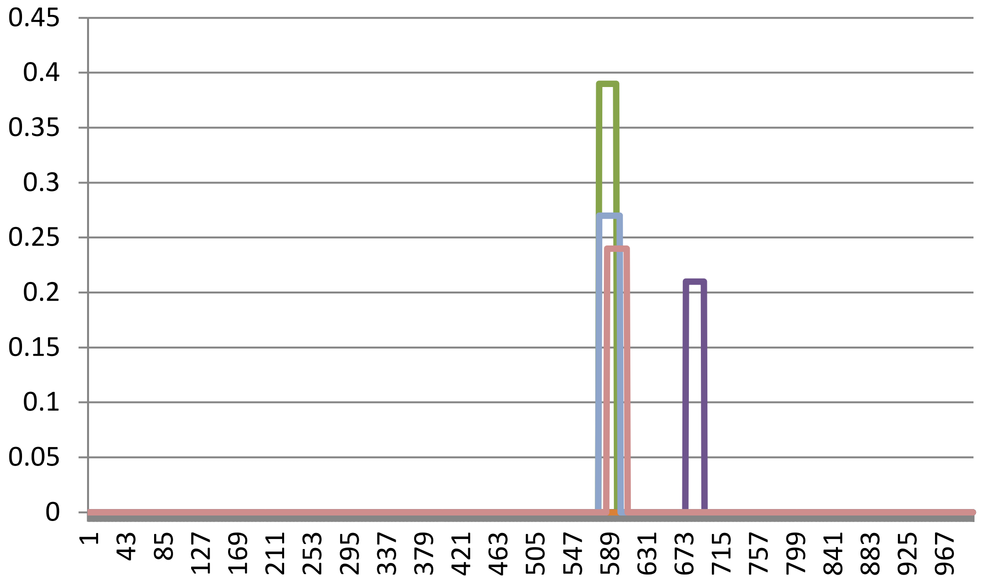

0.45
0.4
0.35
0.3
0.25
0.2
0.15
0.1
0.05
0
1
43
85
127
169
211
253
295
337
379
421
463
505
547
589
631
673
715
757
799
841
883
925
967

AT1G20967

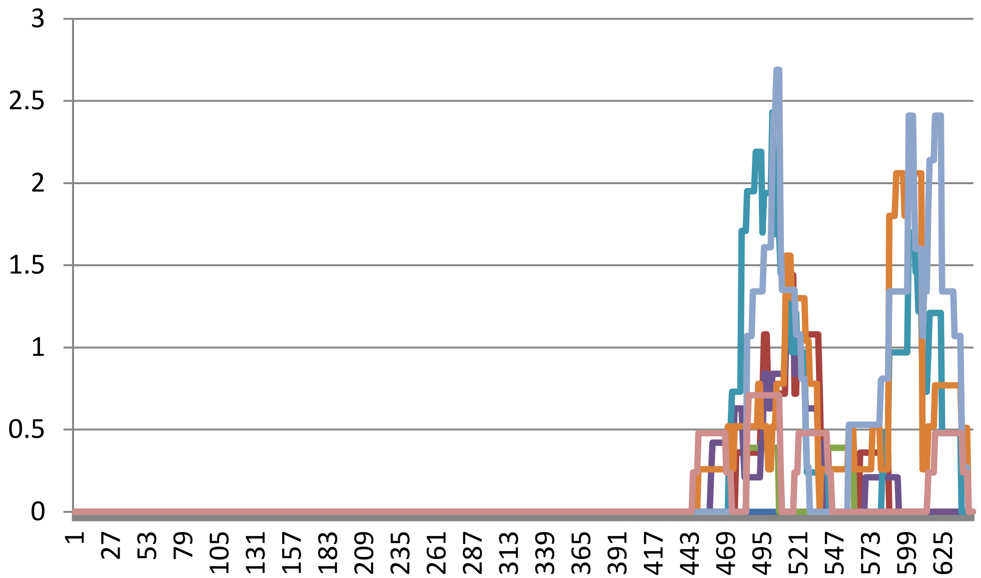

3
2.5
2
1.5
1
0.5
0
1
27
53
79
105
131
157
183
209
235
261
287
313
339
365
391
417
443
469
495
521
547
573
599
625

AT1G24388_AGO4

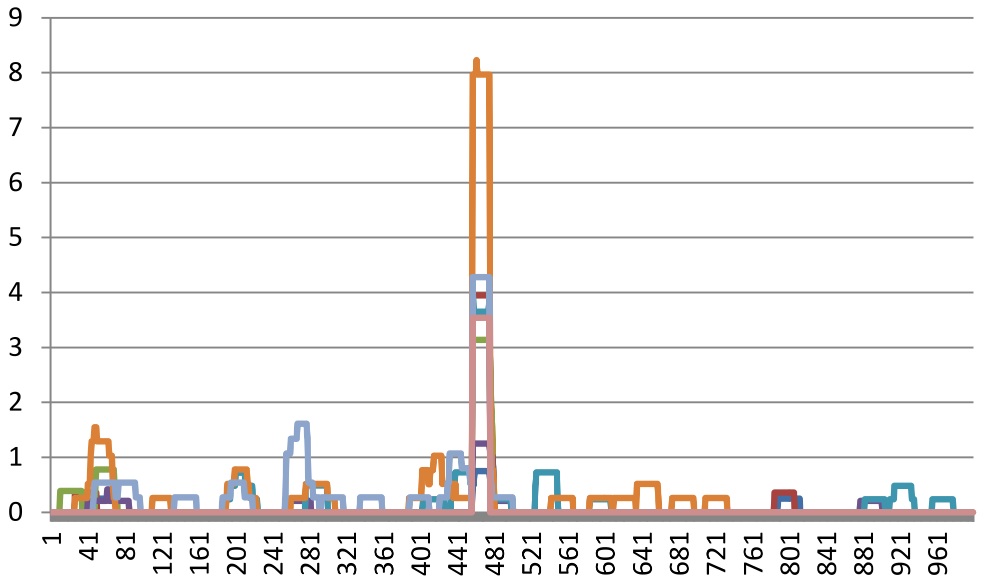

AT1G26640_AGO4

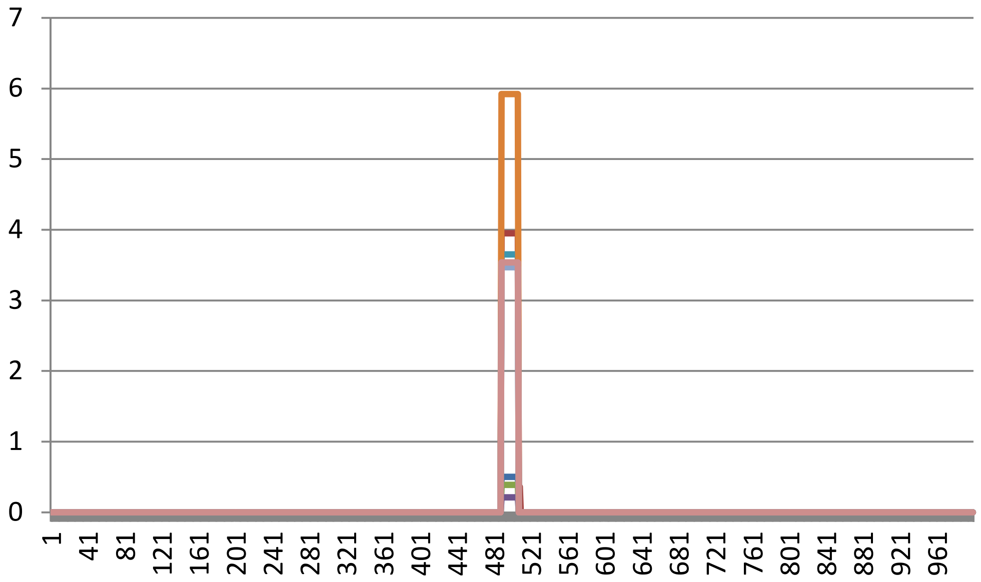

AT1G27160_AGO4

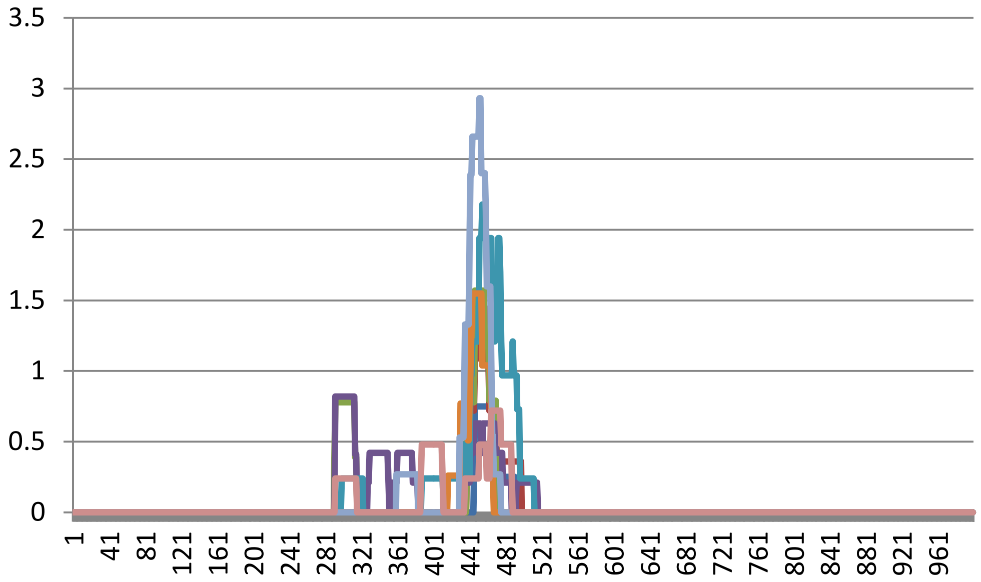

AT1G28281_root

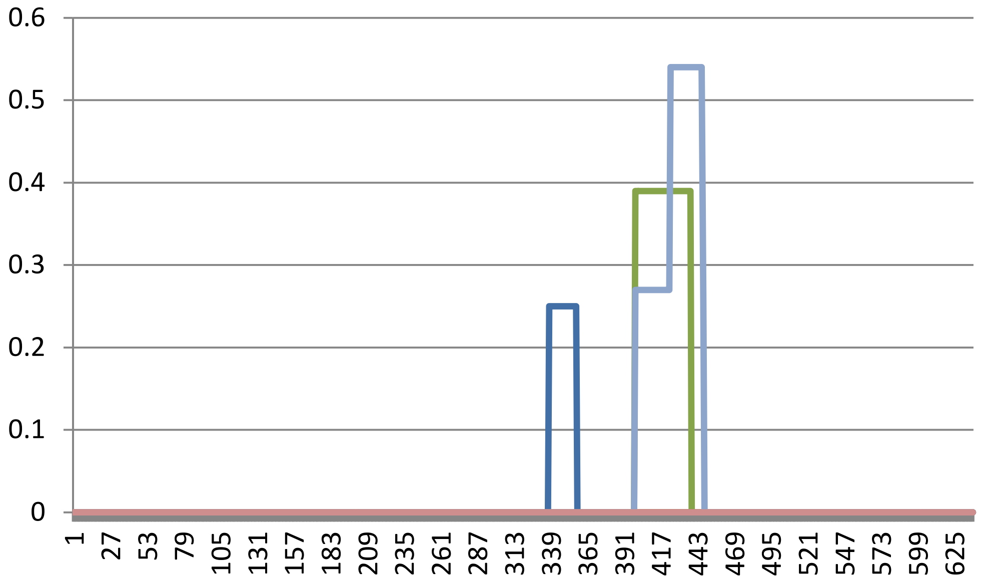

AT1G31960_AGO4

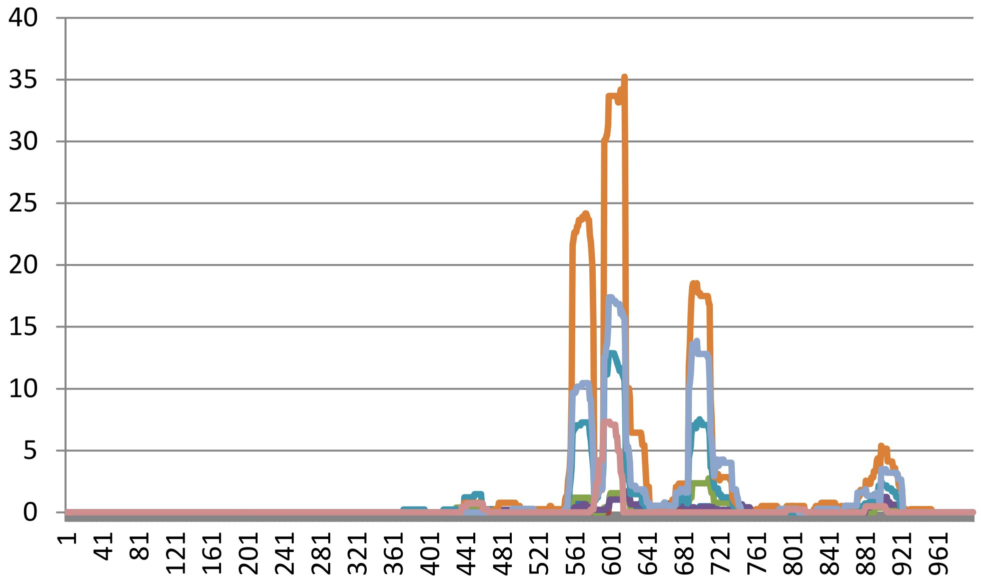

AT1G32610_root

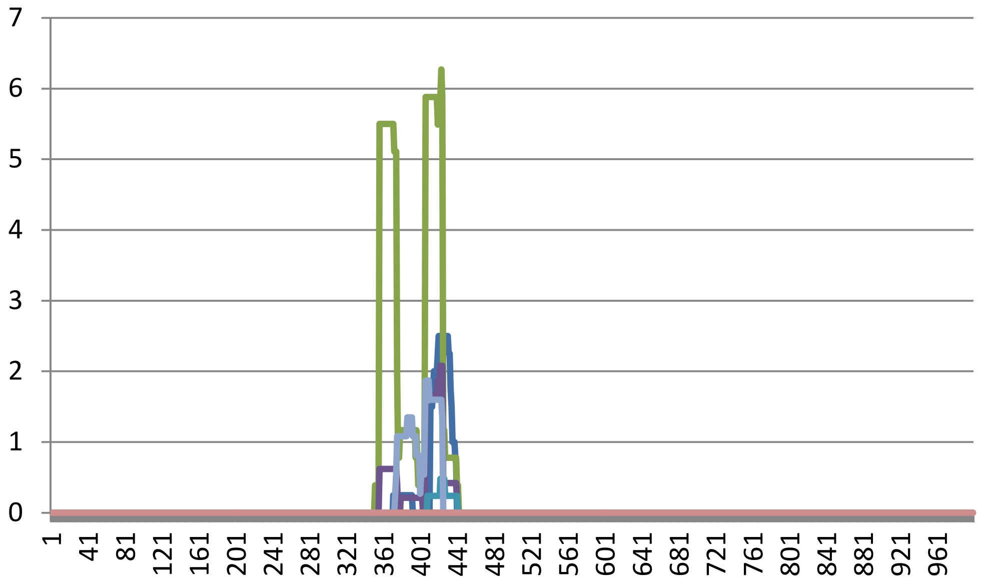

AT1G47280_AGO4

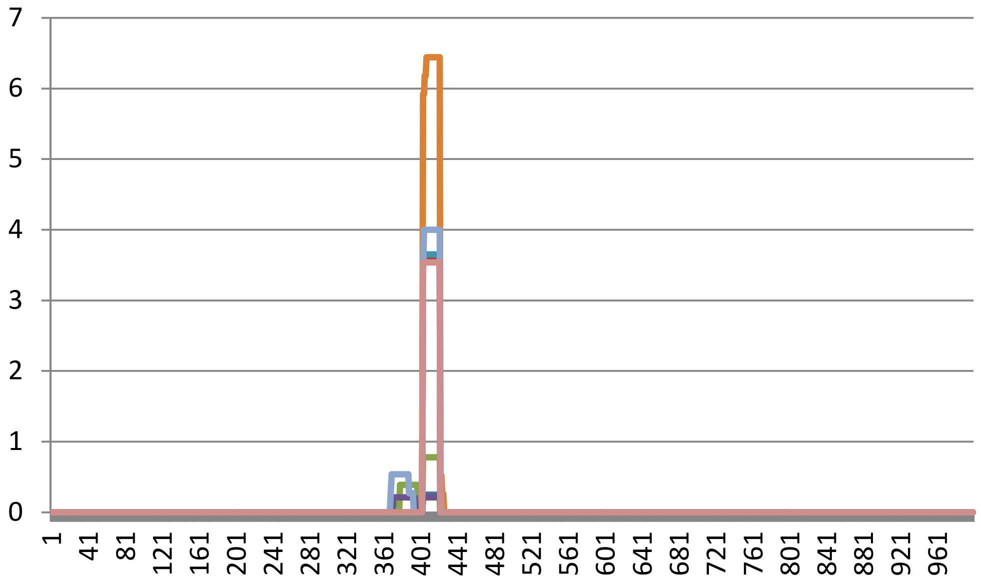

AT1G47765_AGO4

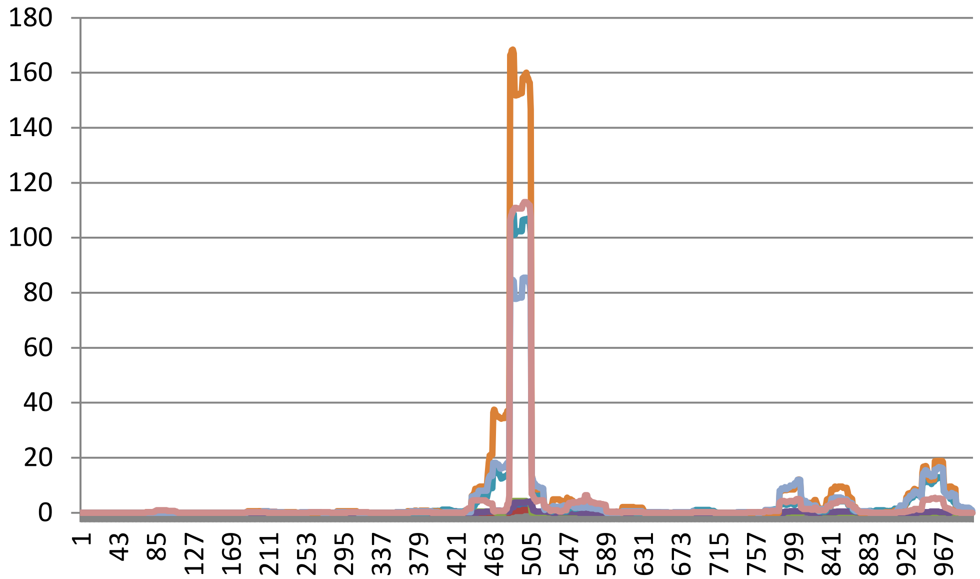

AT1G48660_flower

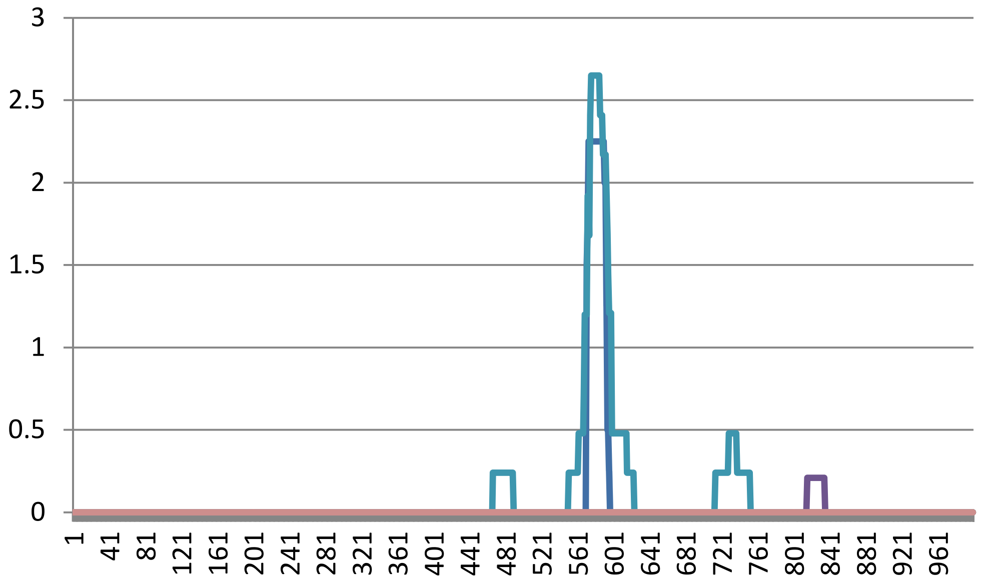

AT1G50160_AGO4

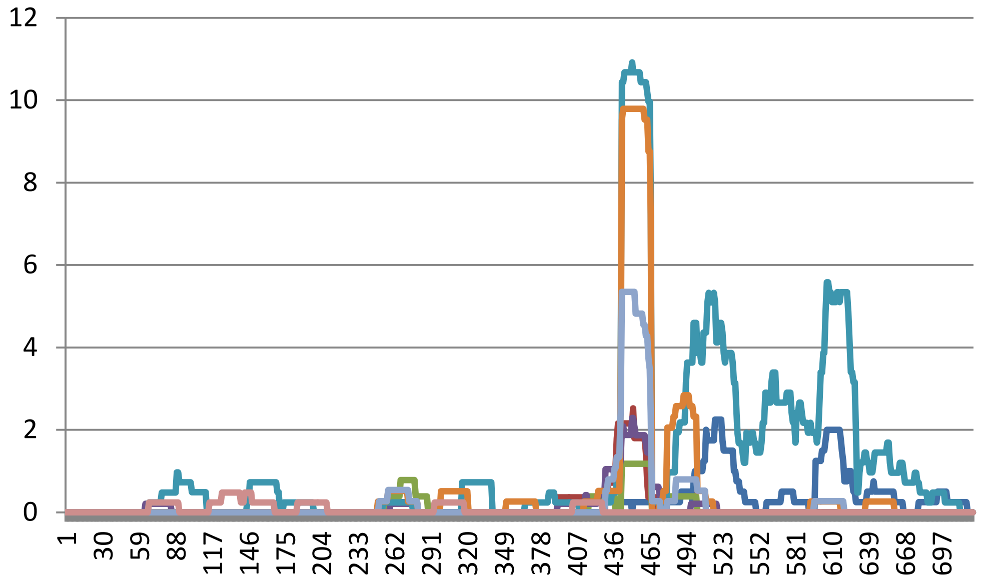

AT1G53400

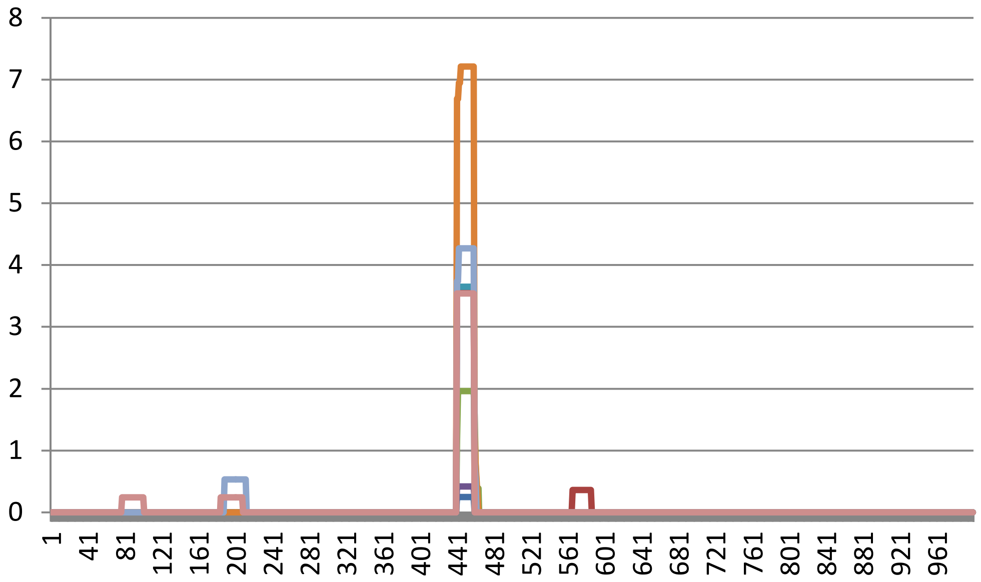

8
7
6
5
4
3
2
1
0
1
41
81
121
161
201
241
281
321
361
401
441
481
521
561
601
641
681
721
761
801
841
881
921
961

AT1G53542_AGO1 root

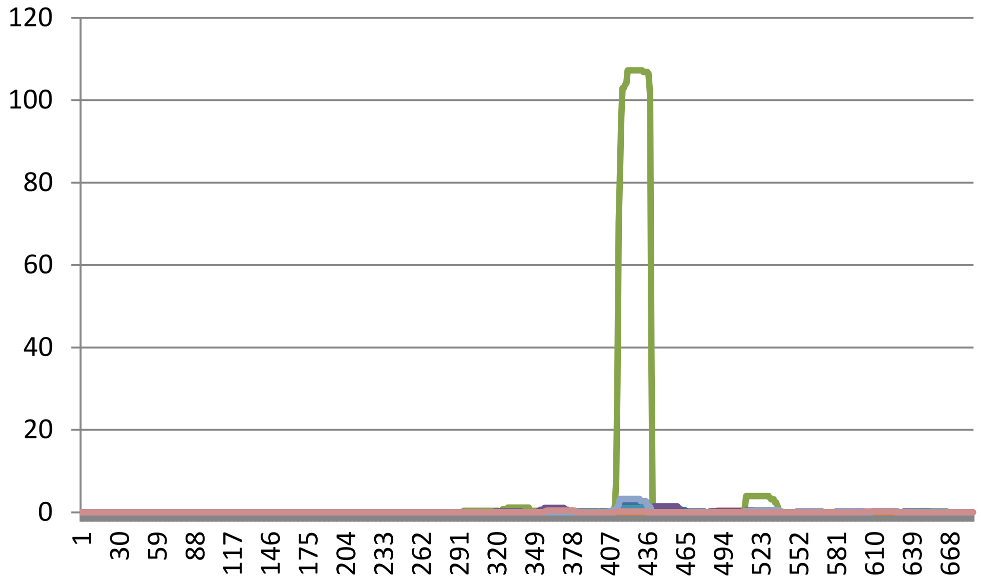

AT1G59680_AGO4

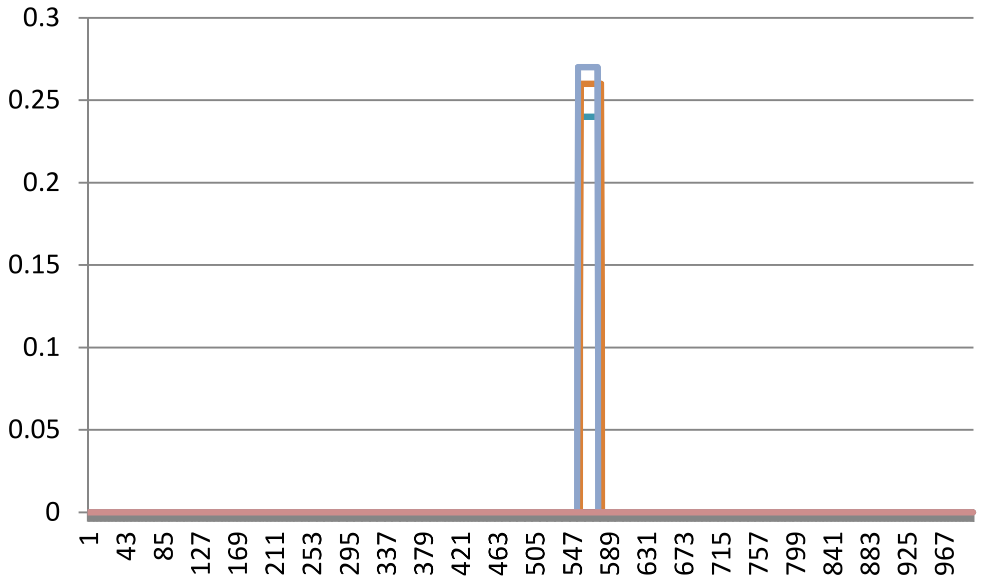

AT1G64130_AGO1 root

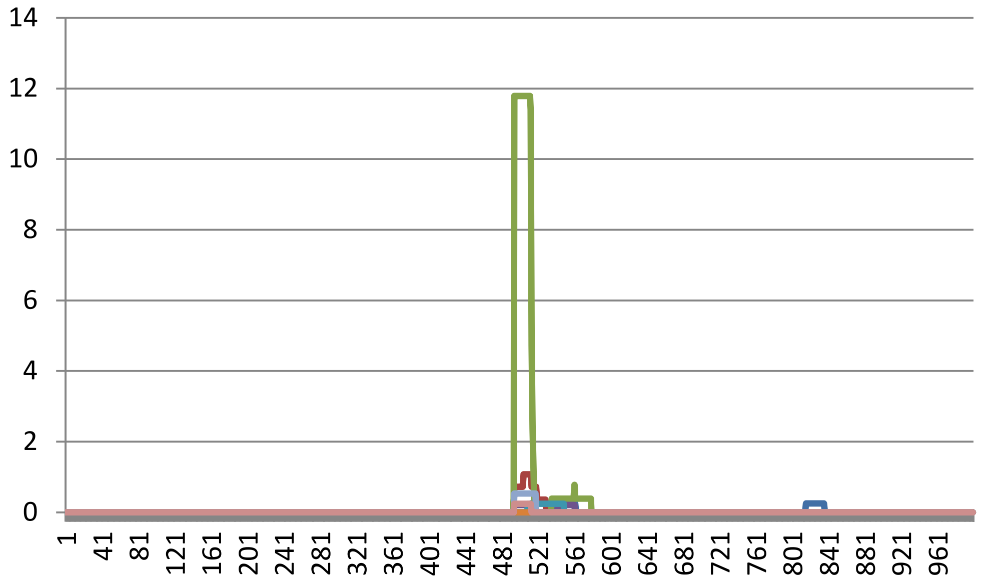

AT1G64770_AGO4

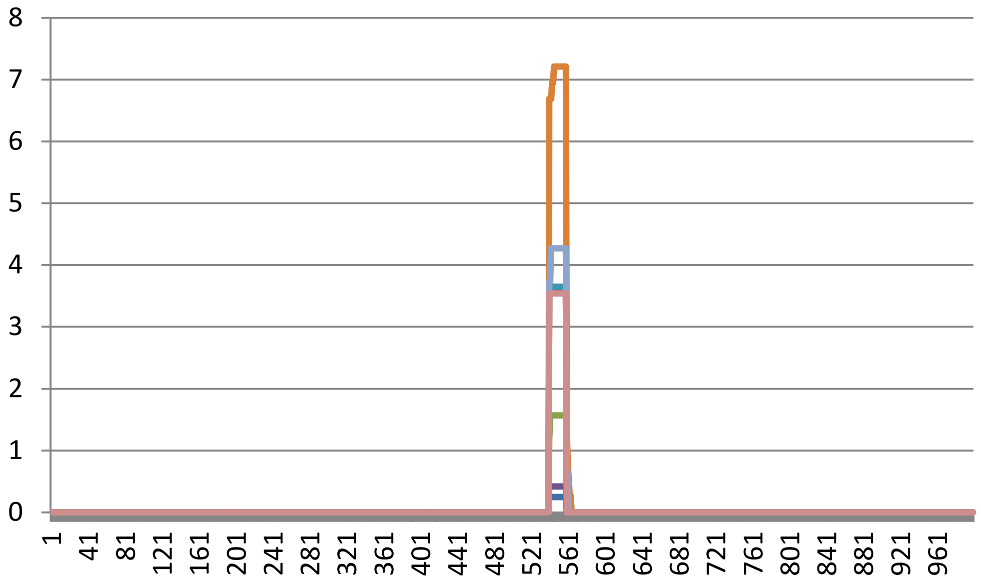

AT1G66553_AGO4

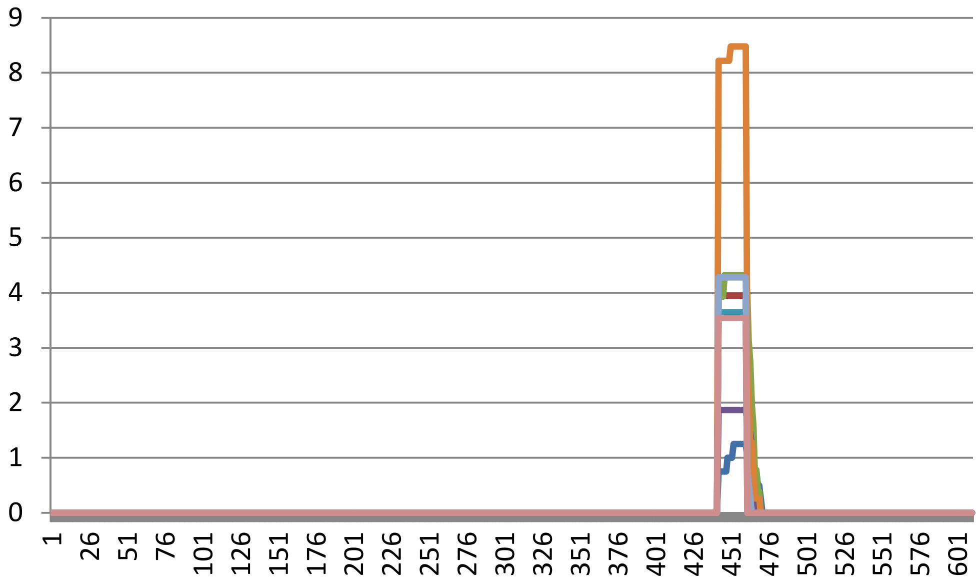

AT1G66740_AGO4

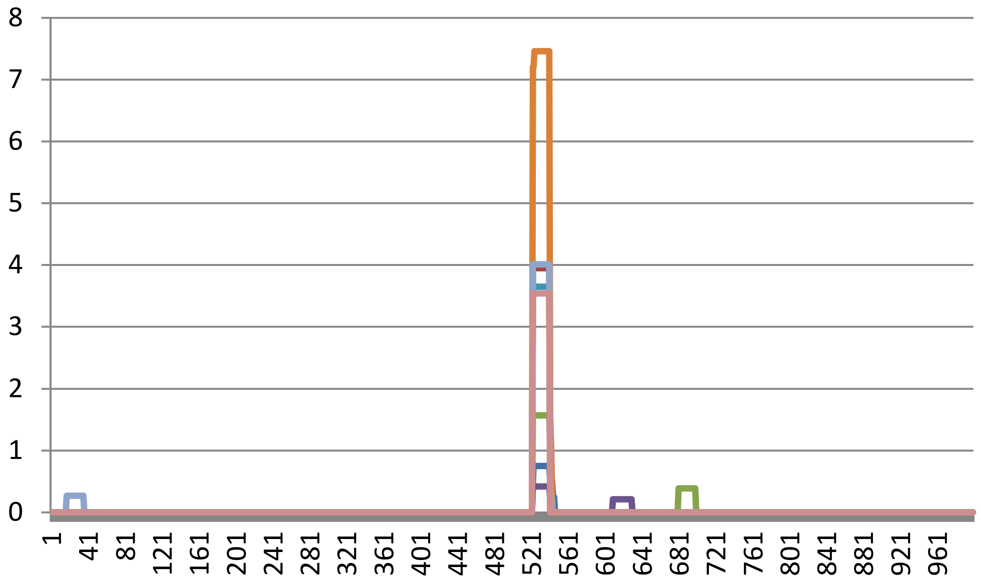

AT1G67450_AGO4

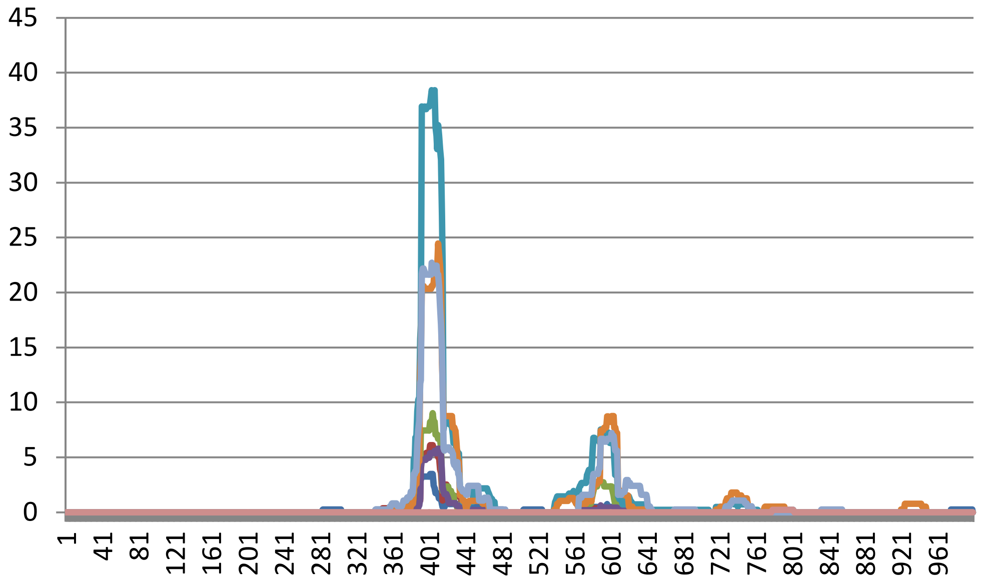

AT1G68830_AGO4

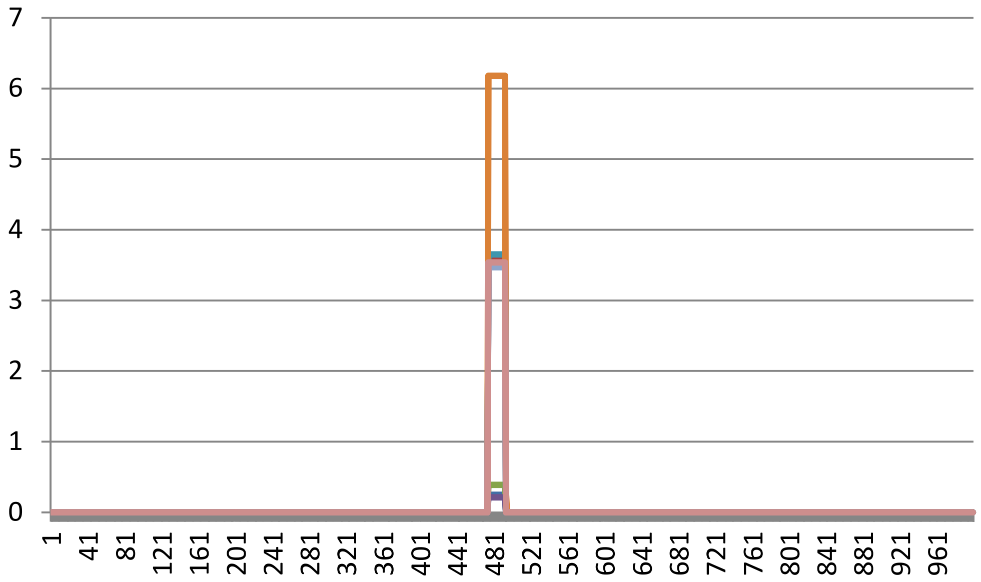

AT1G70650

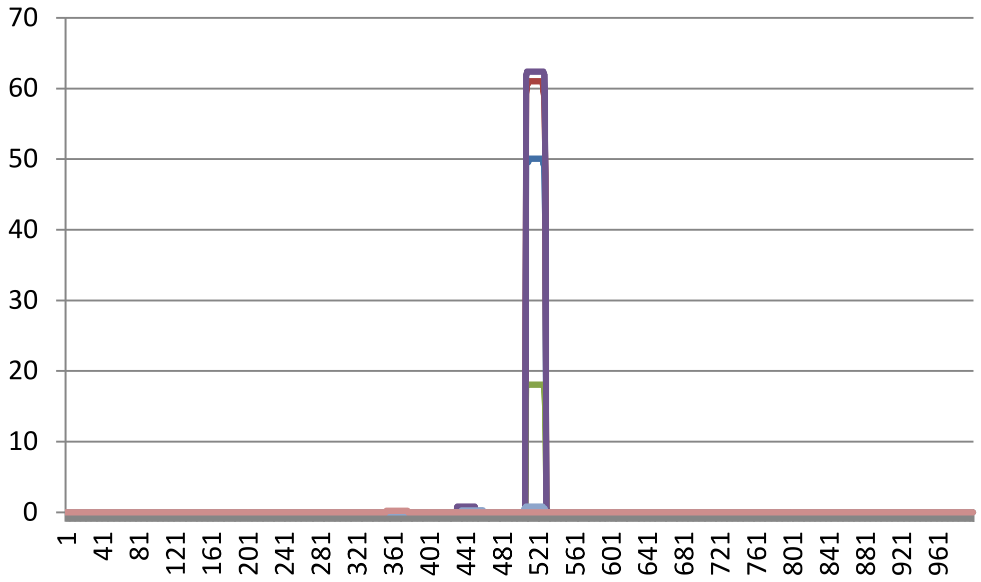

70
60
50
40
30
20
10
0
1
41
81
121
161
201
241
281
321
361
401
441
481
521
561
601
641
681
721
761
801
841
881
921
961

AT1G71820_AGO4

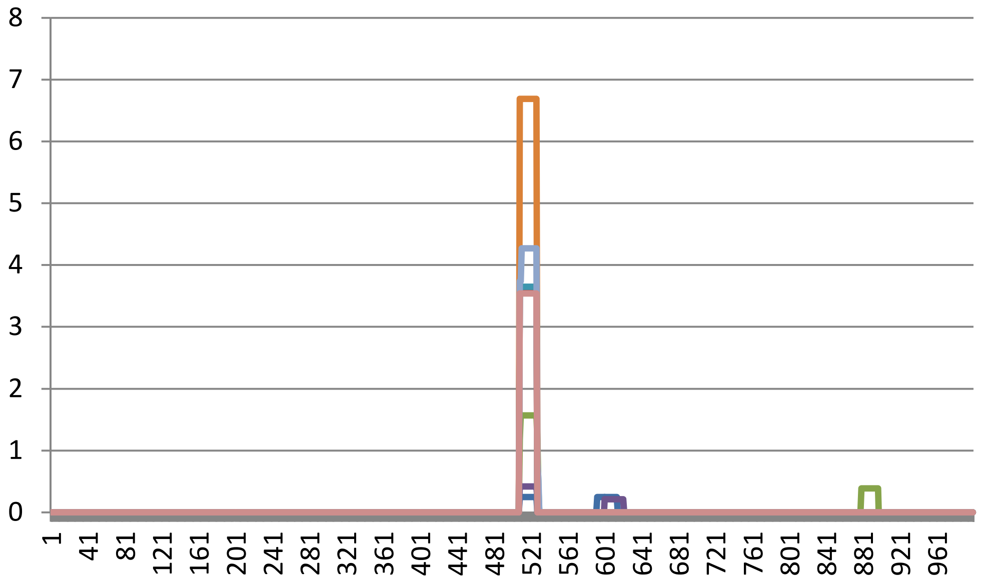

AT1G78820_AGO4

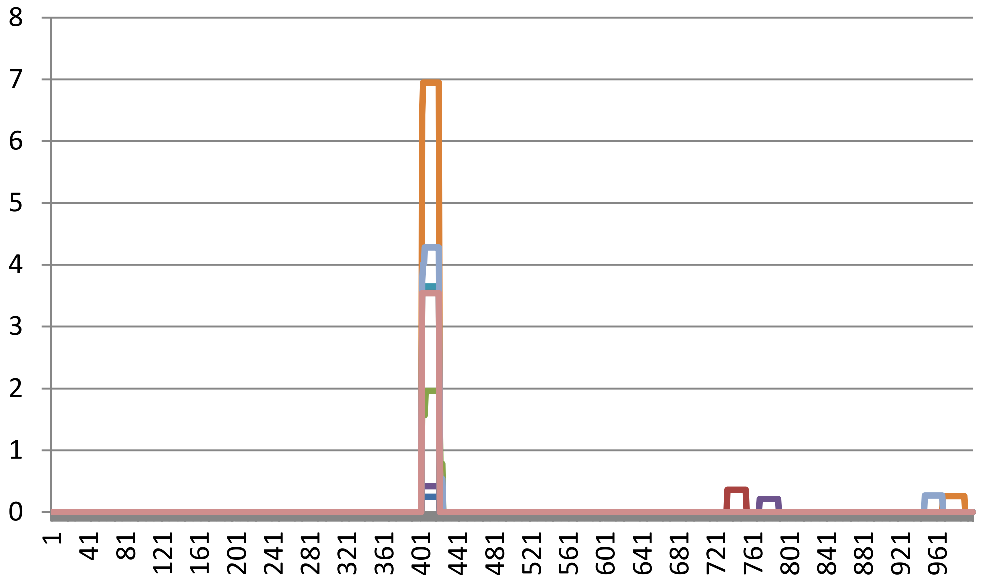

AT1G80490_root

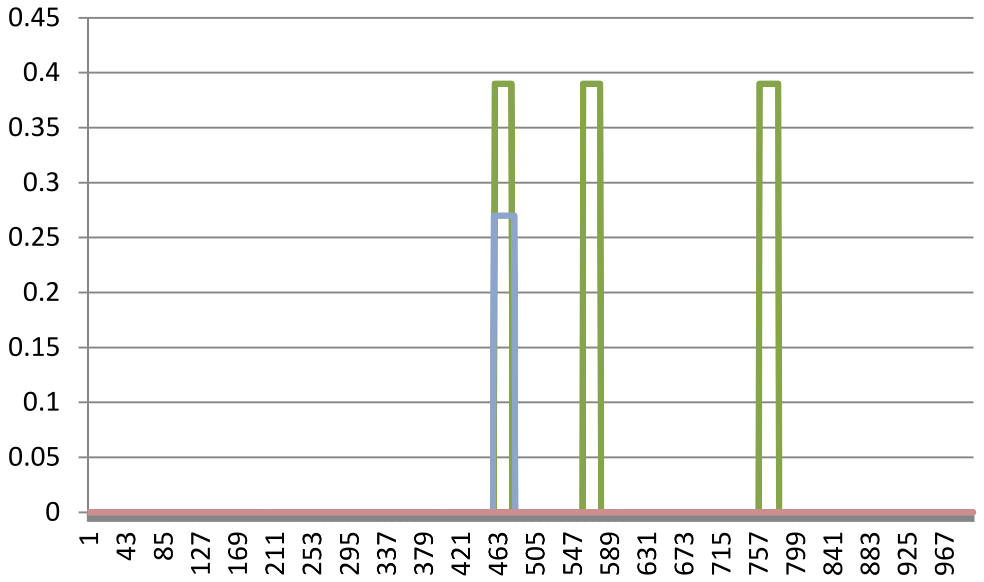

AT2G06020_AGO4

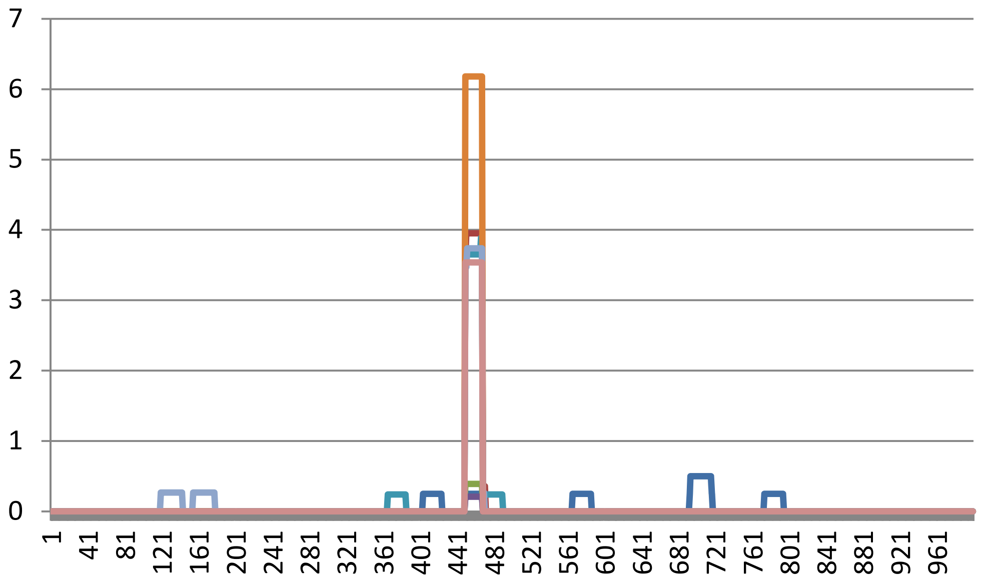

AT2G07708_root

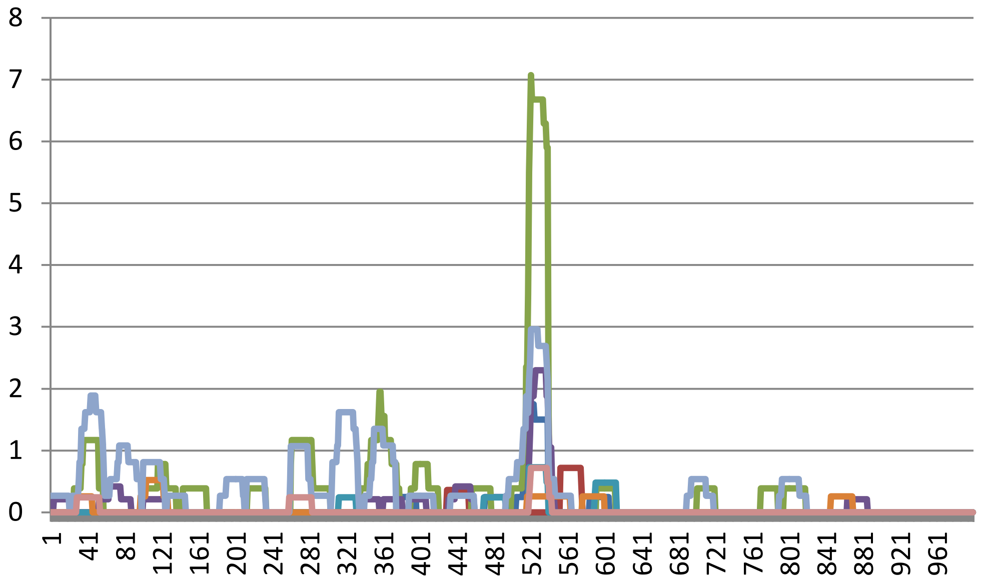

AT2G16090_AGO4

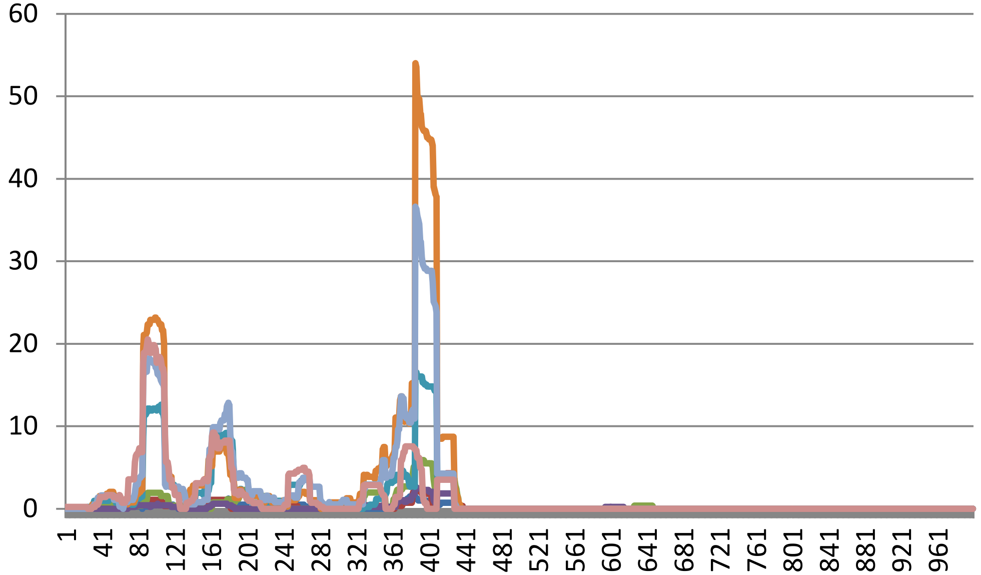

AT2G23950_AGO4

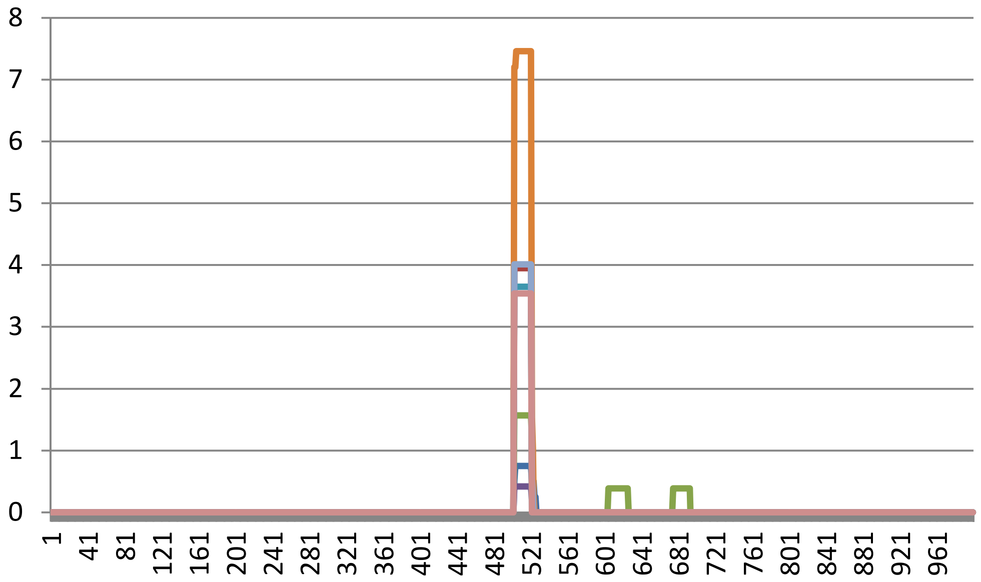

AT2G26740

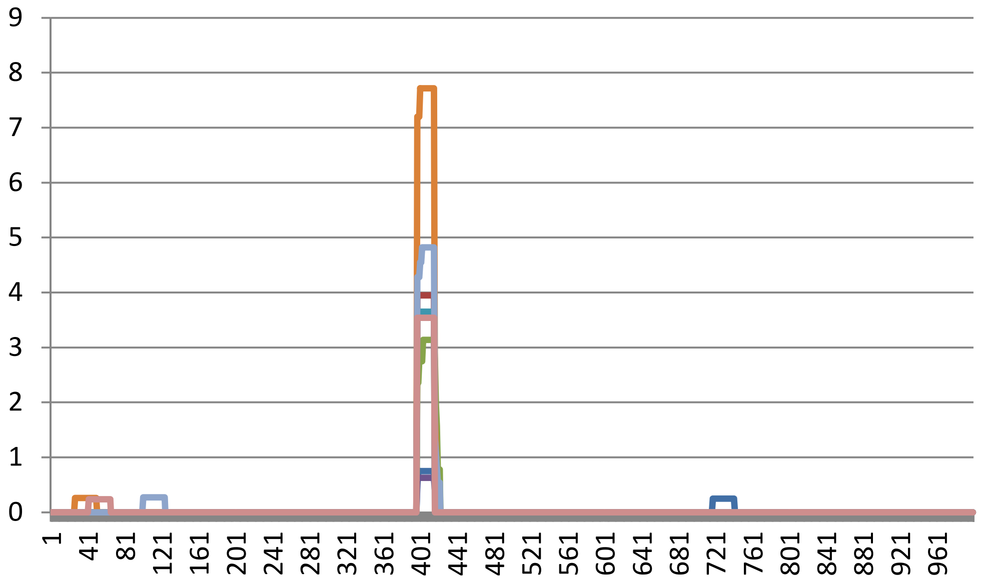

9
8
7
6
5
4
3
2
1
0
1
41
81
121
161
201
241
281
321
361
401
441
481
521
561
601
641
681
721
761
801
841
881
921
961

AT2G27130

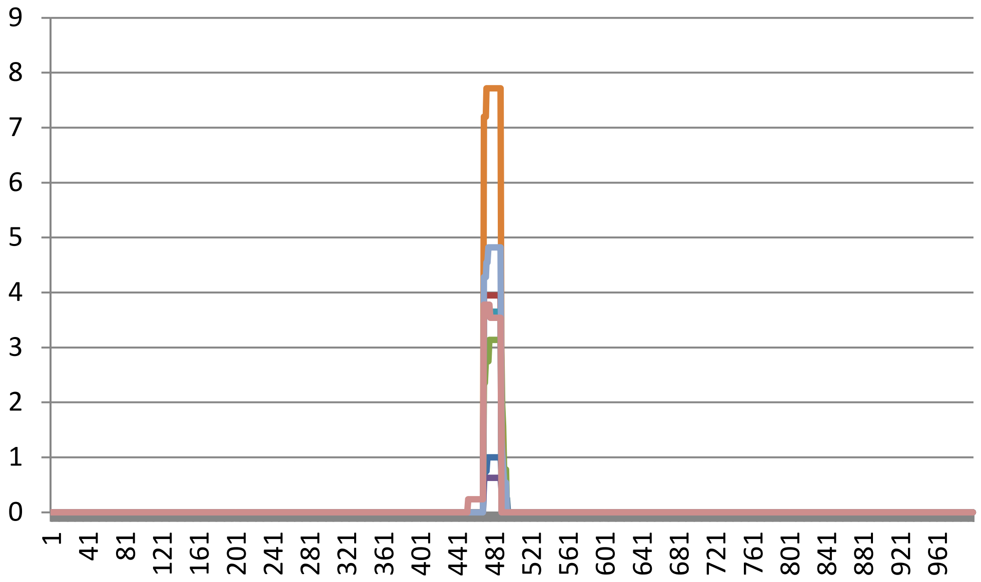

9
8
7
6
5
4
3
2
1
0
1
41
81
121
161
201
241
281
321
361
401
441
481
521
561
601
641
681
721
761
801
841
881
921
961

AT2G28310

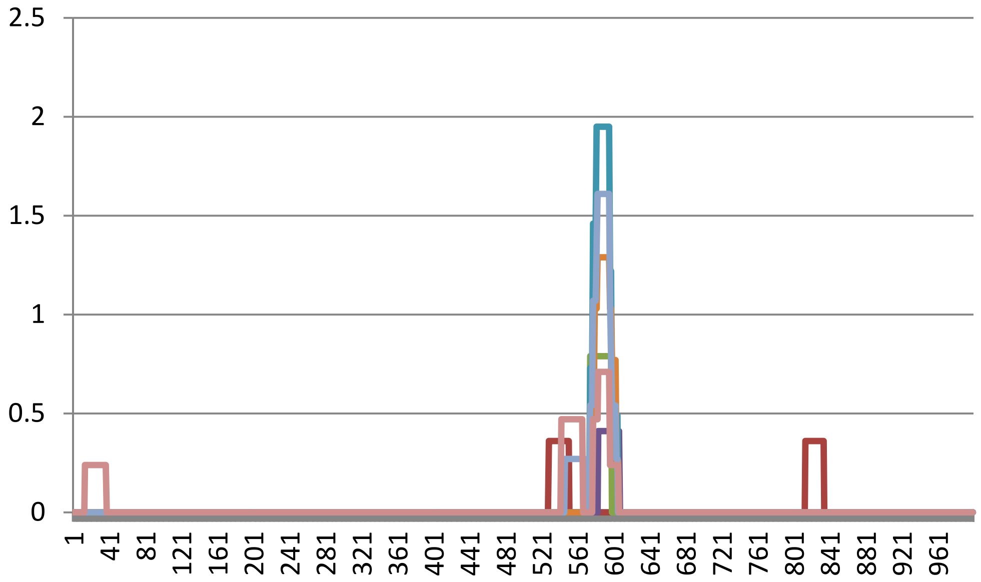

2.5
2
1.5
1
0.5
0
1
41
81
121
161
201
241
281
321
361
401
441
481
521
561
601
641
681
721
761
801
841
881
921
961

AT2G30910

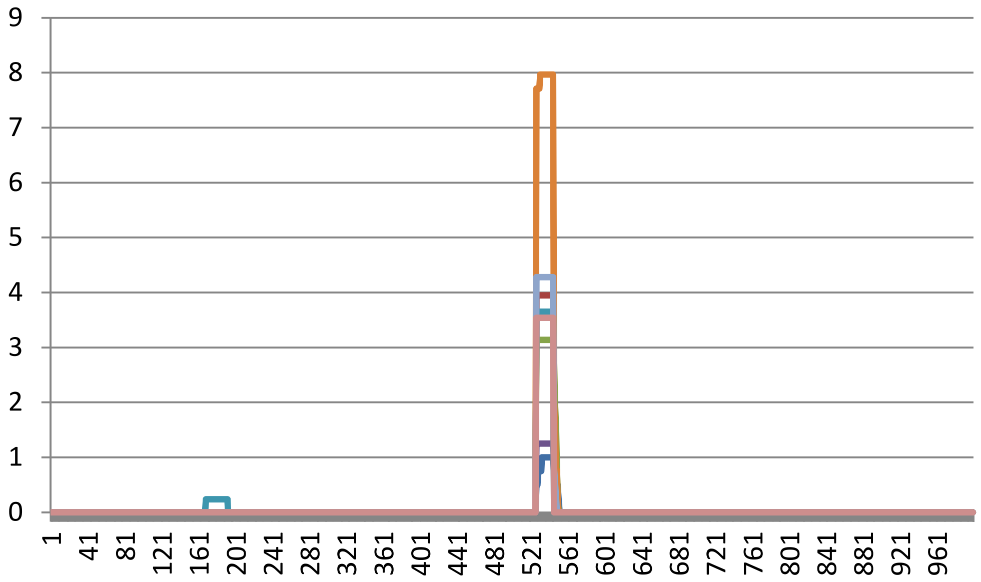

9
8
7
6
5
4
3
2
1
0
1
41
81
121
161
201
241
281
321
361
401
441
481
521
561
601
641
681
721
761
801
841
881
921
961

AT2G30940

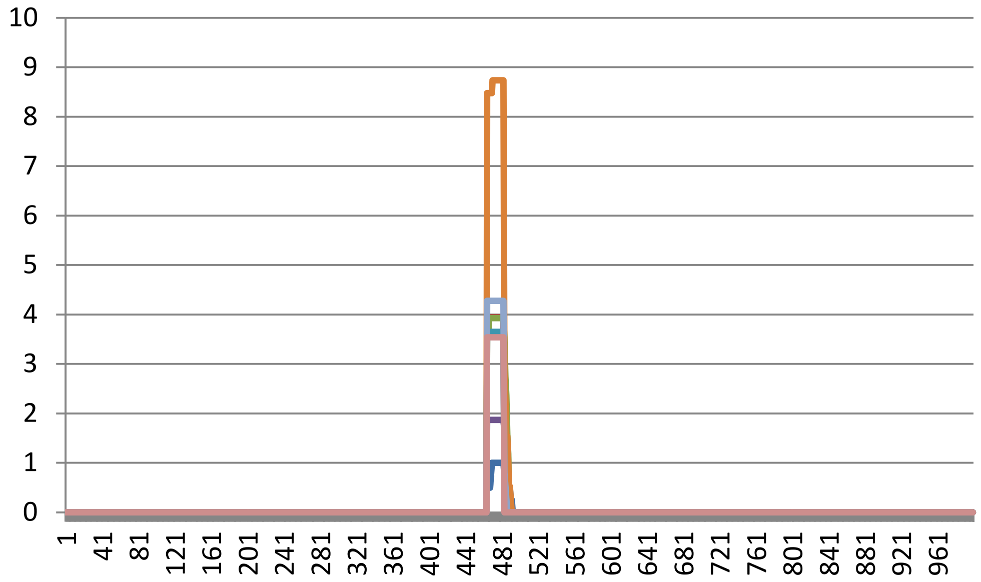

10
9
8
7
6
5
4
3
2
1
0
1
41
81
121
161
201
241
281
321
361
401
441
481
521
561
601
641
681
721
761
801
841
881
921
961

AT2G31230

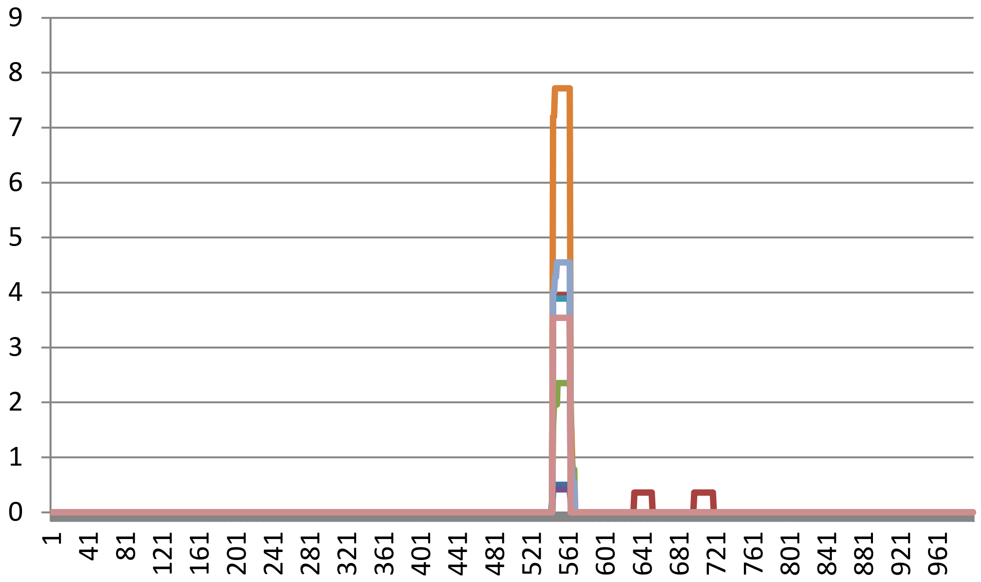

9
8
7
6
5
4
3
2
1
0
1
41
81
121
161
201
241
281
321
361
401
441
481
521
561
601
641
681
721
761
801
841
881
921
961

AT2G34655

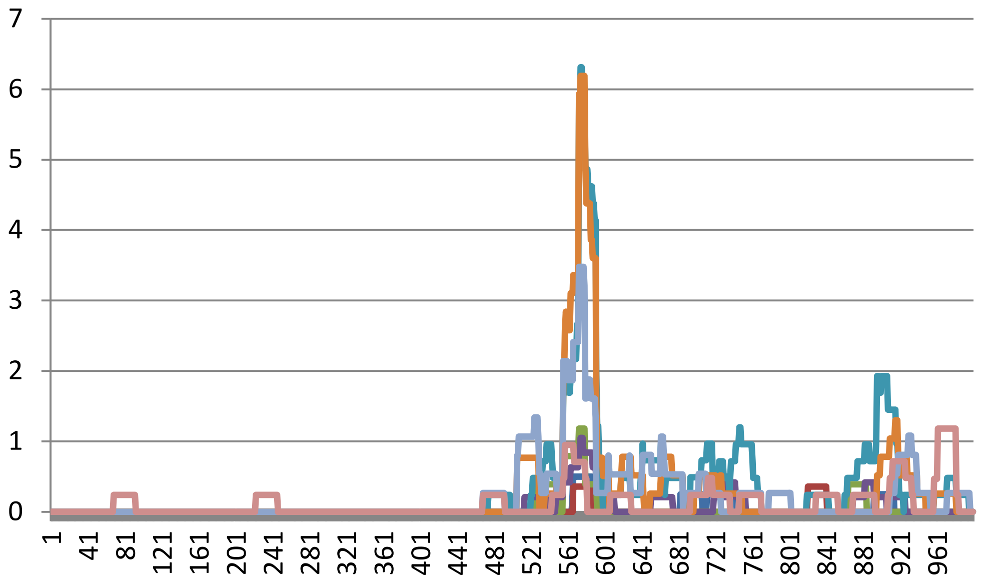

7
6
5
4
3
2
1
0
1
41
81
121
161
201
241
281
321
361
401
441
481
521
561
601
641
681
721
761
801
841
881
921
961

AT2G35650

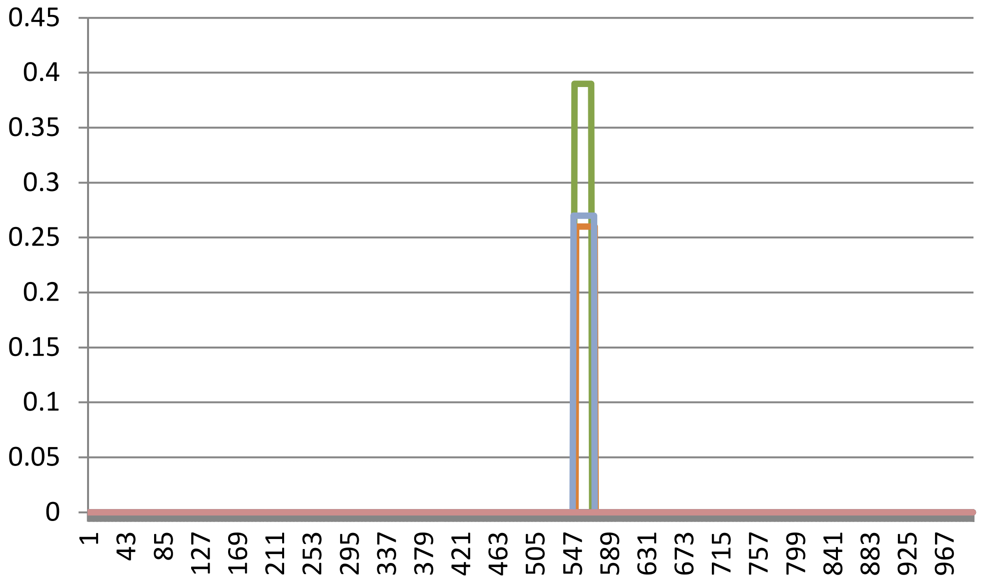
0.45
0.4
0.35
0.3
0.25
0.2
0.15
0.1
0.05
0
1
43
85
127
169
211
253
295
337
379
421
463
505
547
589
631
673
715
757
799
841
883
925
967

AT2G35800

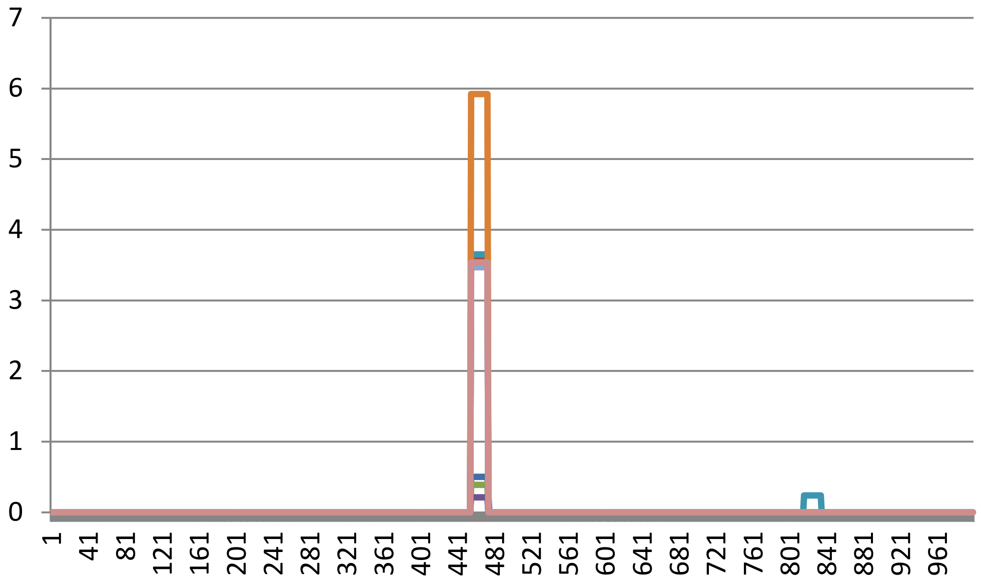

7
6
5
4
3
2
1
0
1
41
81
121
161
201
241
281
321
361
401
441
481
521
561
601
641
681
721
761
801
841
881
921
961

AT2G38380

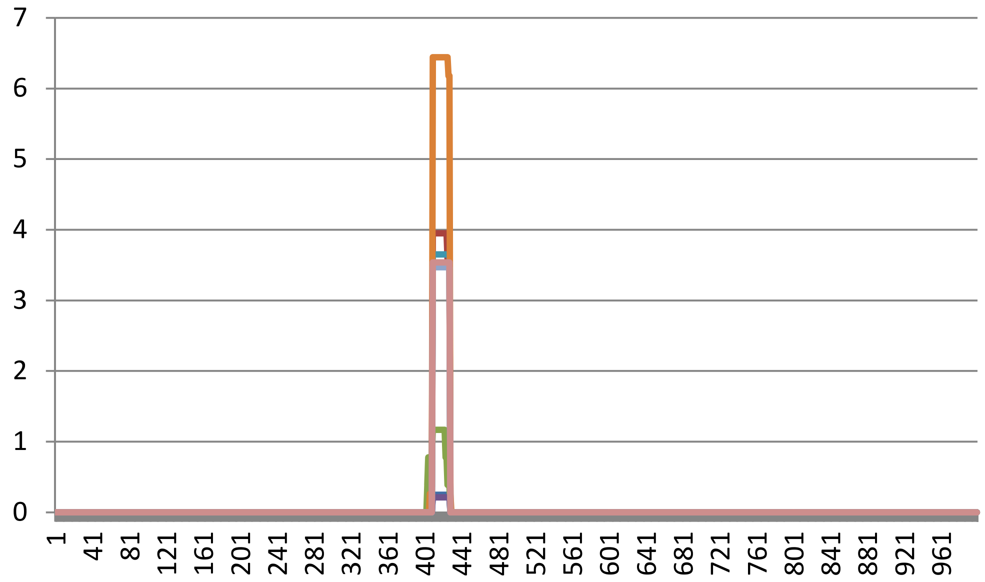

7
6
5
4
3
2
1
0
1
41
81
121
161
201
241
281
321
361
401
441
481
521
561
601
641
681
721
761
801
841
881
921
961

AT2G38630

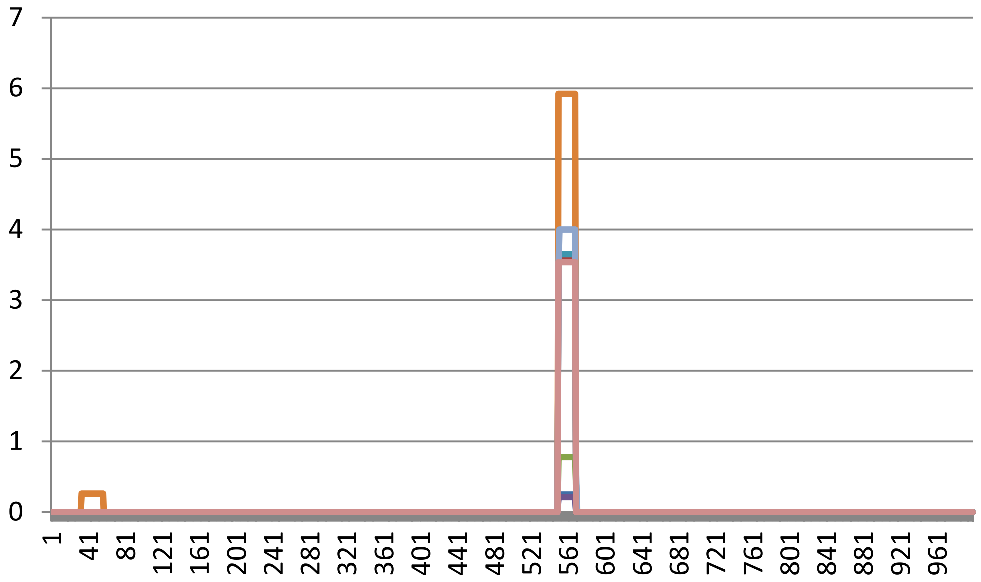

7
6
5
4
3
2
1
0
1
41
81
121
161
201
241
281
321
361
401
441
481
521
561
601
641
681
721
761
801
841
881
921
961

AT2G39450

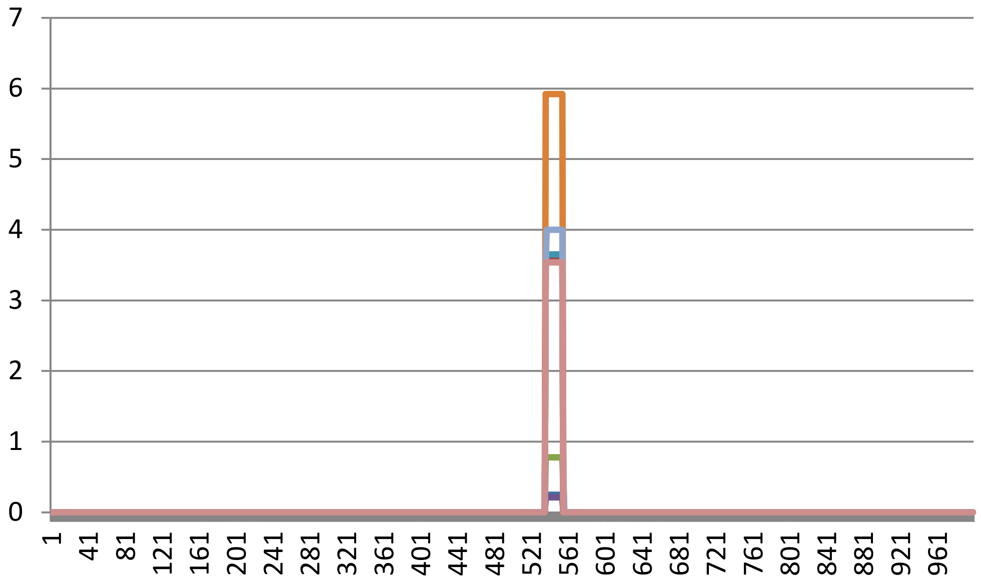

7
6
5
4
3
2
1
0
1
41
81
121
161
201
241
281
321
361
401
441
481
521
561
601
641
681
721
761
801
841
881
921
961

AT2G40004

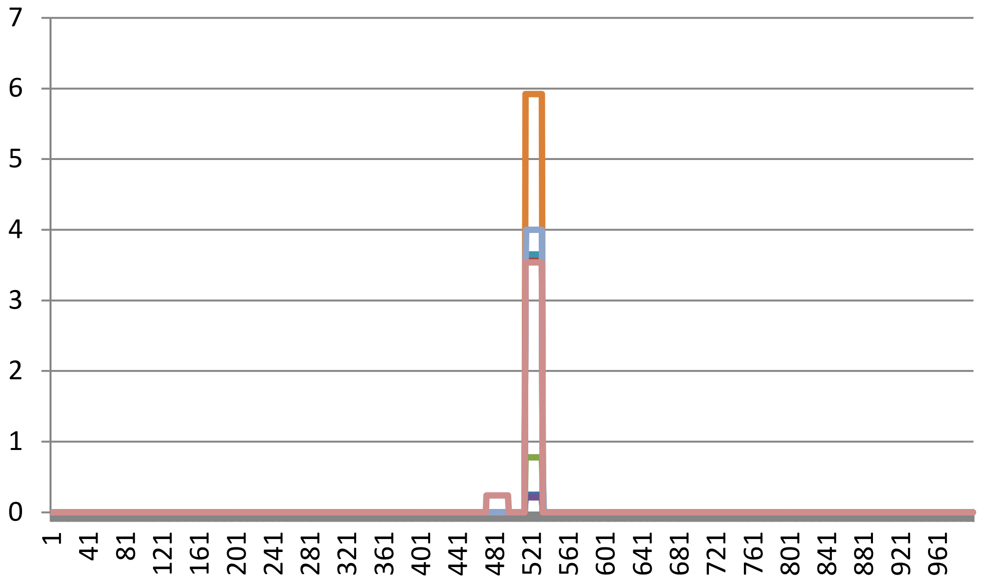

7
6
5
4
3
2
1
0
1
41
81
121
161
201
241
281
321
361
401
441
481
521
561
601
641
681
721
761
801
841
881
921
961

AT2G40510

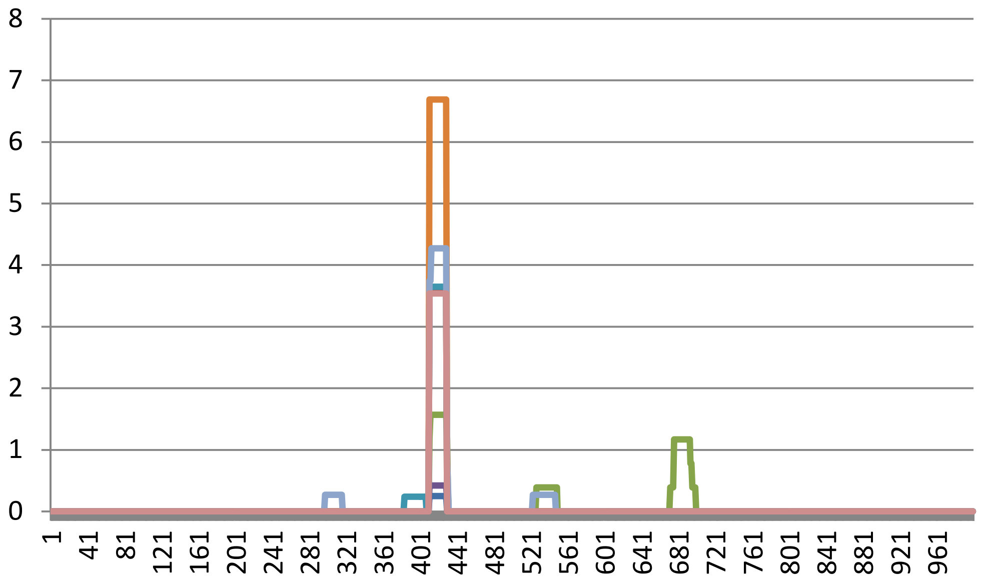

8
7
6
5
4
3
2
1
0
1
41
81
121
161
201
241
281
321
361
401
441
481
521
561
601
641
681
721
761
801
841
881
921
961

AT2G43790

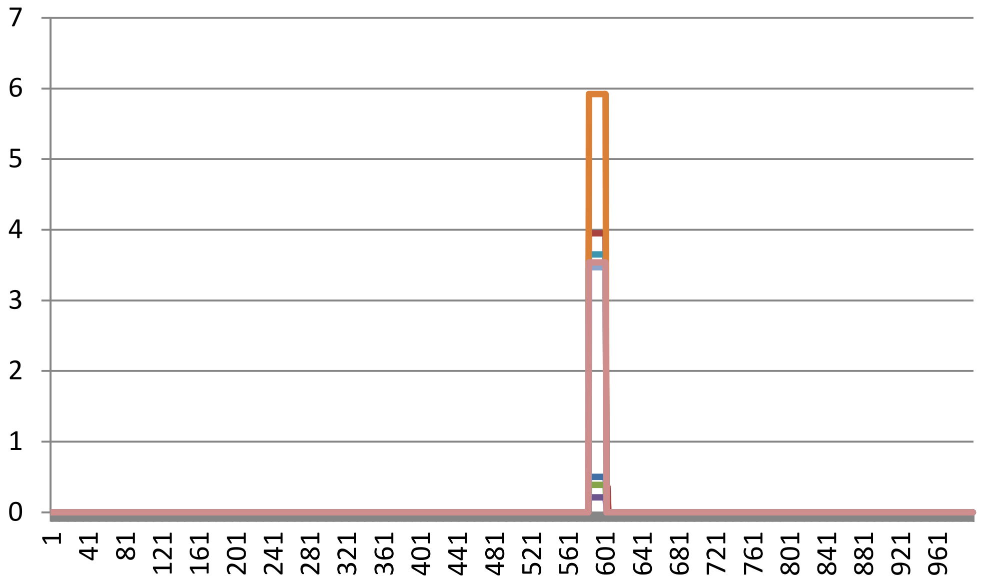

7
6
5
4
3
2
1
0
1
41
81
121
161
201
241
281
321
361
401
441
481
521
561
601
641
681
721
761
801
841
881
921
961

AT2G43865

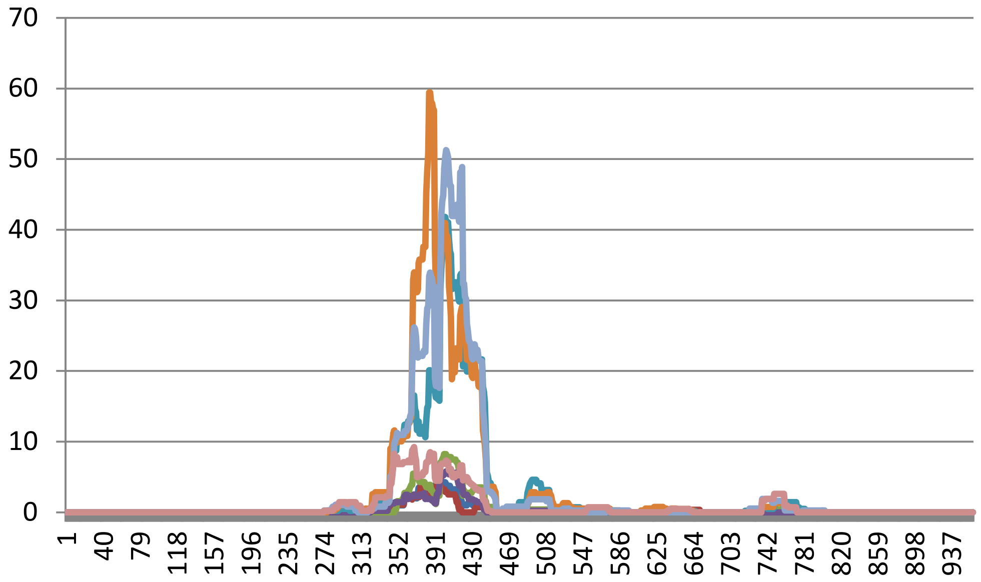

70
60
50
40
30
20
10
0
1
40
79
118
157
196
235
274
313
352
391
430
469
508
547
586
625
664
703
742
781
820
859
898
937

AT2G47050

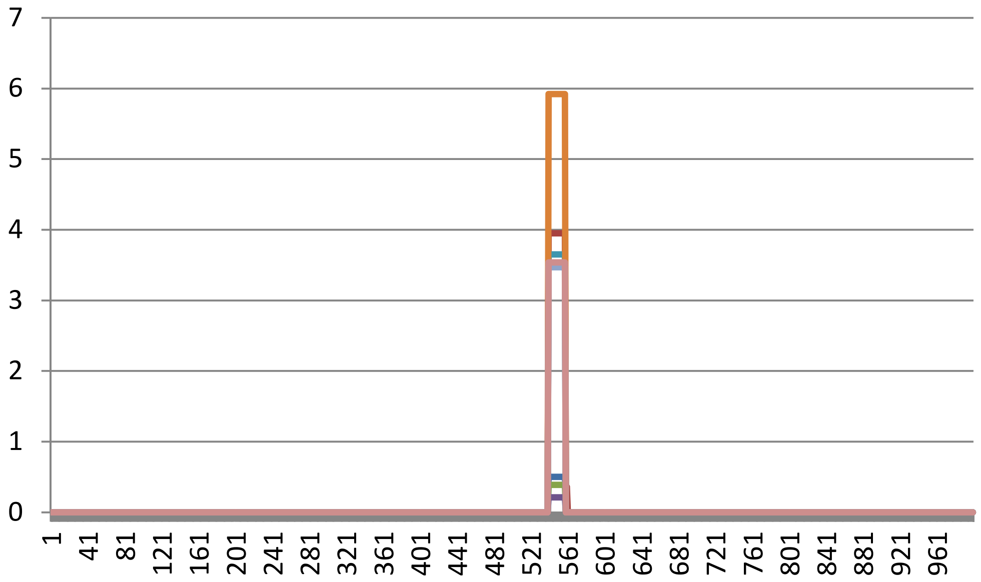

7
6
5
4
3
2
1
0
1
41
81
121
161
201
241
281
321
361
401
441
481
521
561
601
641
681
721
761
801
841
881
921
961

AT2G47570

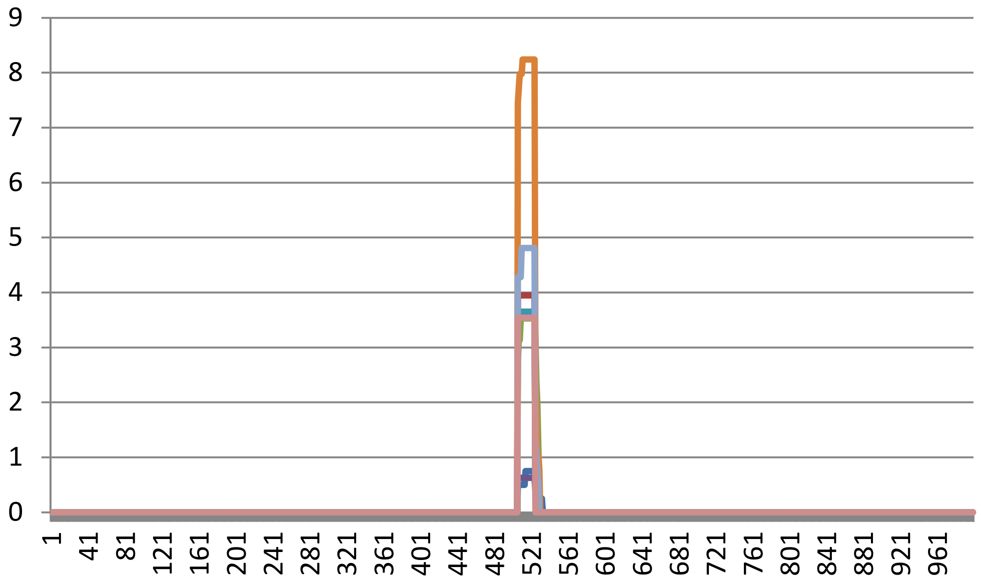

9
8
7
6
5
4
3
2
1
0
1
41
81
121
161
201
241
281
321
361
401
441
481
521
561
601
641
681
721
761
801
841
881
921
961

AT3G04290

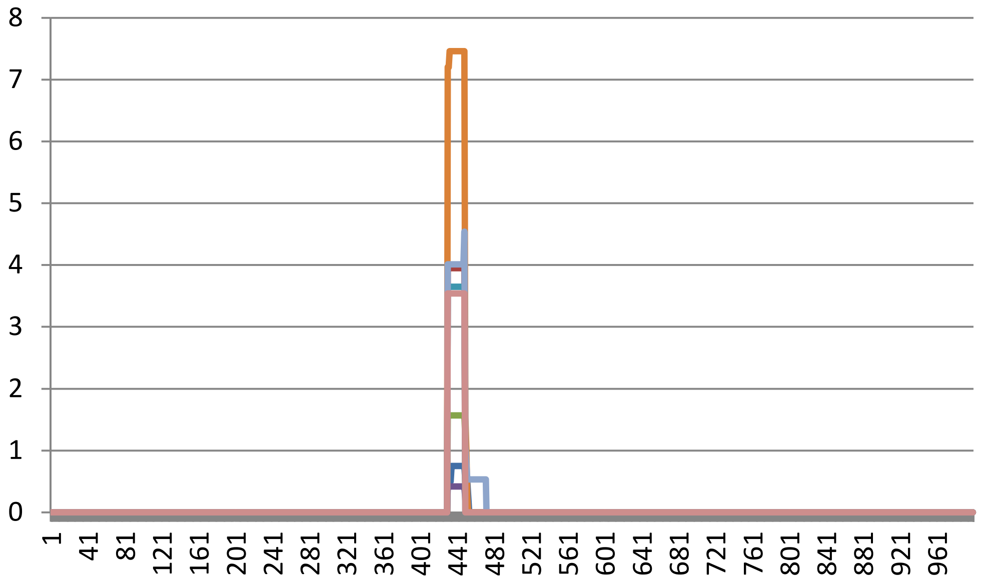

8
7
6
5
4
3
2
1
0
1
41
81
121
161
201
241
281
321
361
401
441
481
521
561
601
641
681
721
761
801
841
881
921
961

AT3G04630

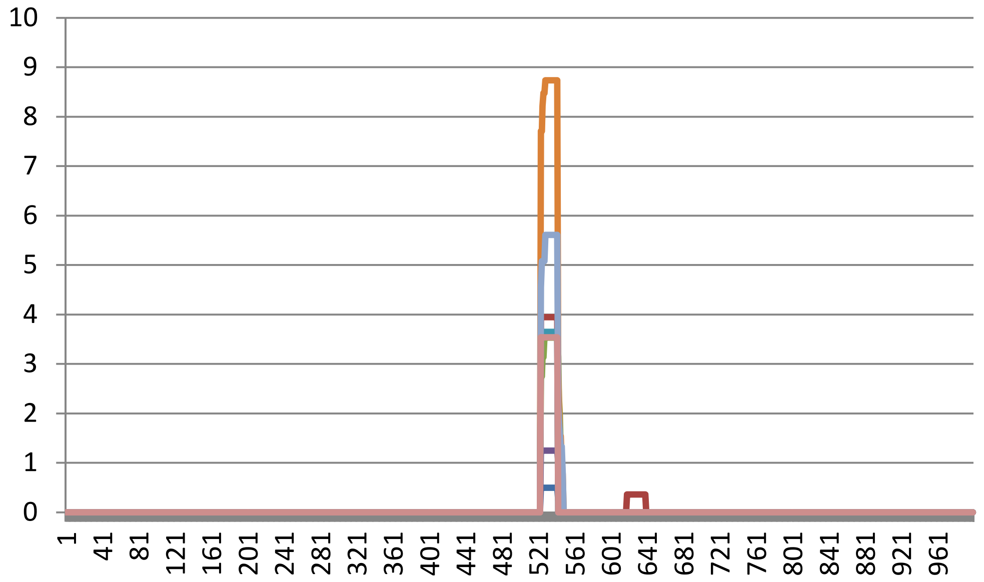
10
9
8
7
6
5
4
3
2
1
0
1
41
81
121
161
201
241
281
321
361
401
441
481
521
561
601
641
681
721
761
801
841
881
921
961

AT3G06490

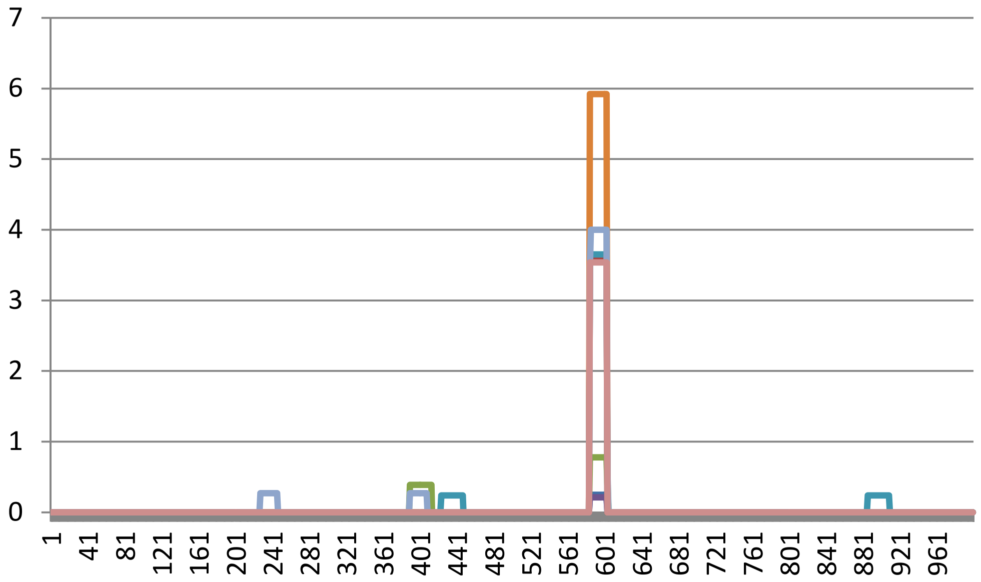

7
6
5
4
3
2
1
0
1
41
81
121
161
201
241
281
321
361
401
441
481
521
561
601
641
681
721
761
801
841
881
921
961

AT3G09980

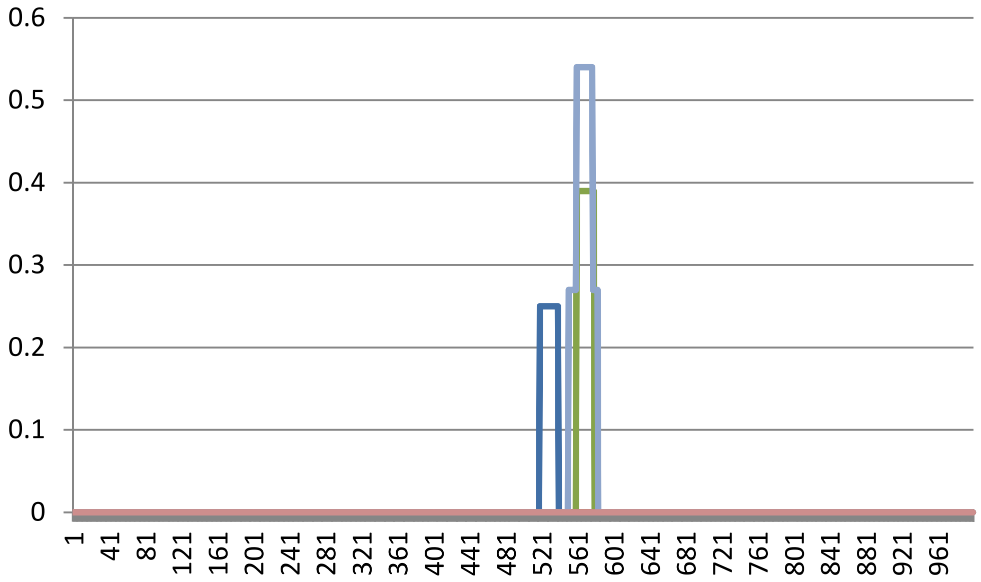

0.6
0.5
0.4
0.3
0.2
0.1
0
1
41
81
121
161
201
241
281
321
361
401
441
481
521
561
601
641
681
721
761
801
841
881
921
961

AT3G10760

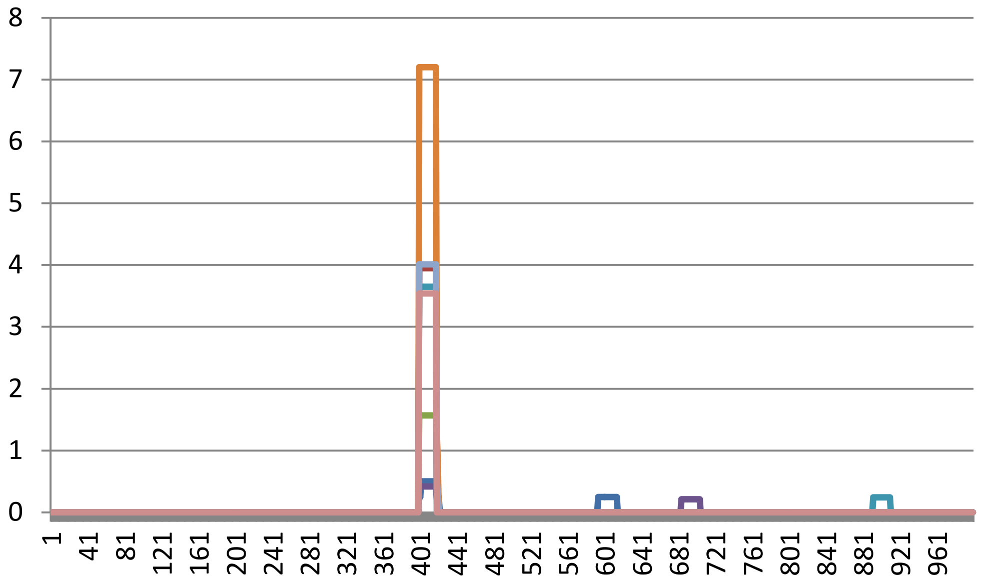

8
7
6
5
4
3
2
1
0
1
41
81
121
161
201
241
281
321
361
401
441
481
521
561
601
641
681
721
761
801
841
881
921
961

AT3G12955

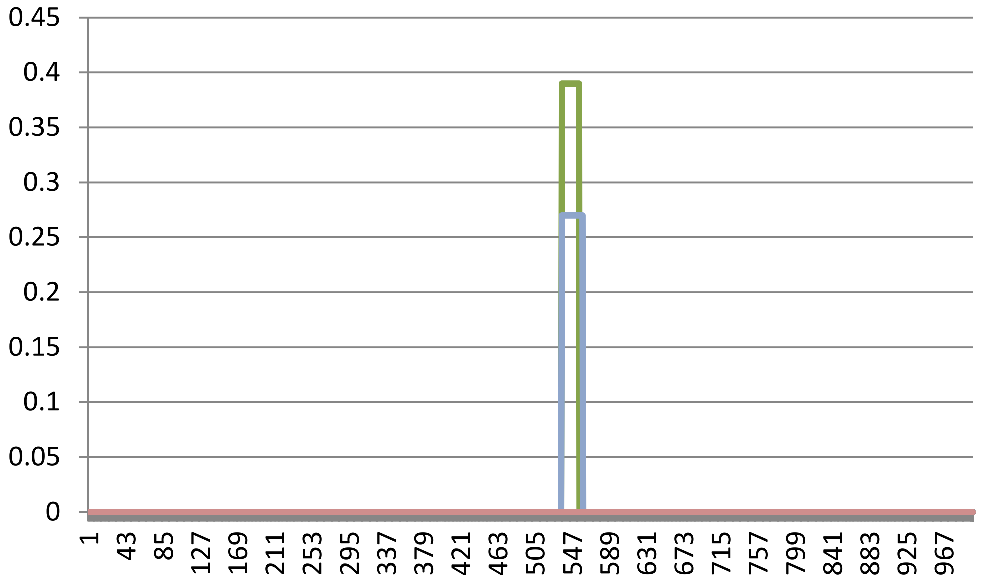

0.45
0.4
0.35
0.3
0.25
0.2
0.15
0.1
0.05
0
1
43
85
127
169
211
253
295
337
379
421
463
505
547
589
631
673
715
757
799
841
883
925
967

AT3G13620

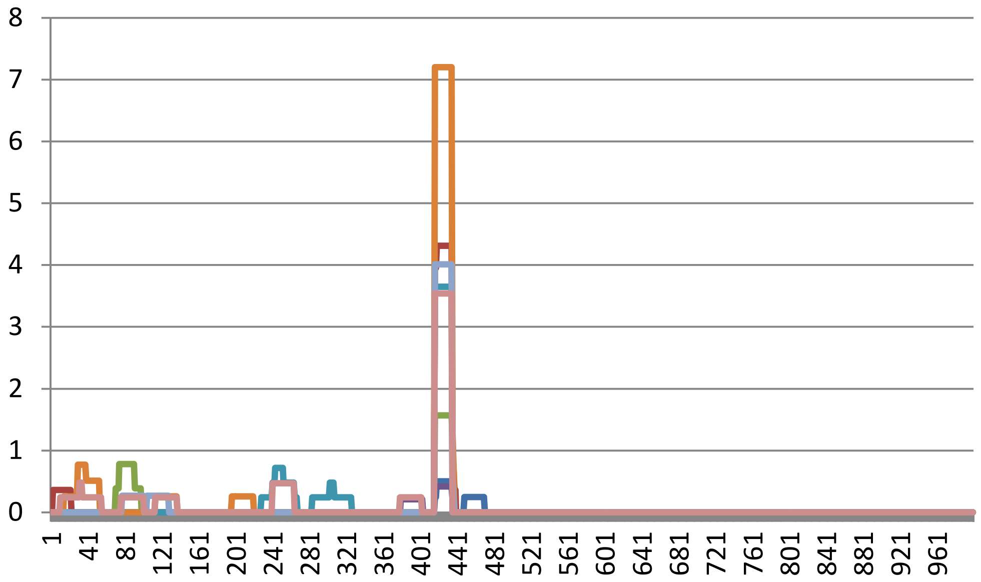

8
7
6
5
4
3
2
1
0
1
41
81
121
161
201
241
281
321
361
401
441
481
521
561
601
641
681
721
761
801
841
881
921
961

AT3G16370

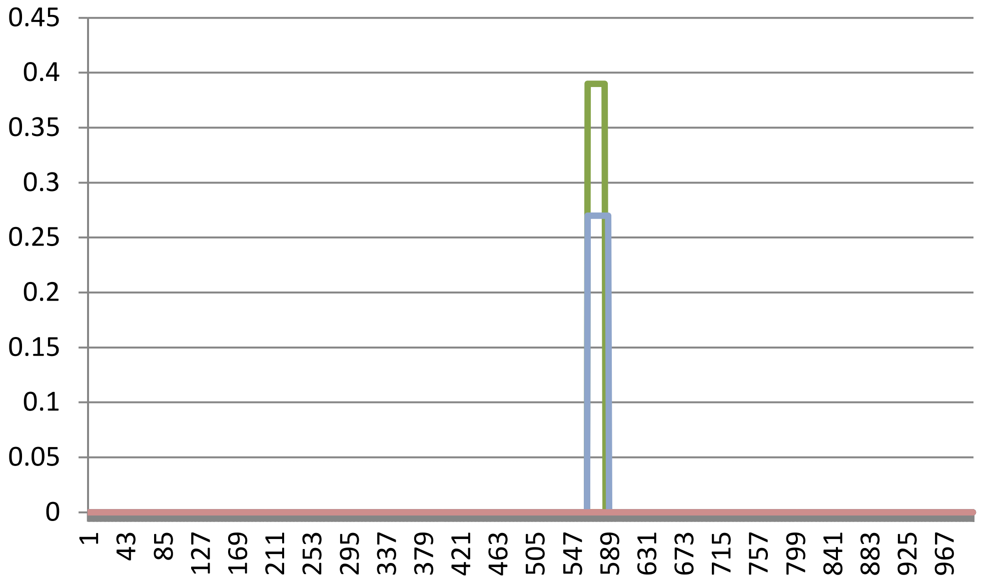
0.45
0.4
0.35
0.3
0.25
0.2
0.15
0.1
0.05
0
1
43
85
127
169
211
253
295
337
379
421
463
505
547
589
631
673
715
757
799
841
883
925
967

AT3G17490

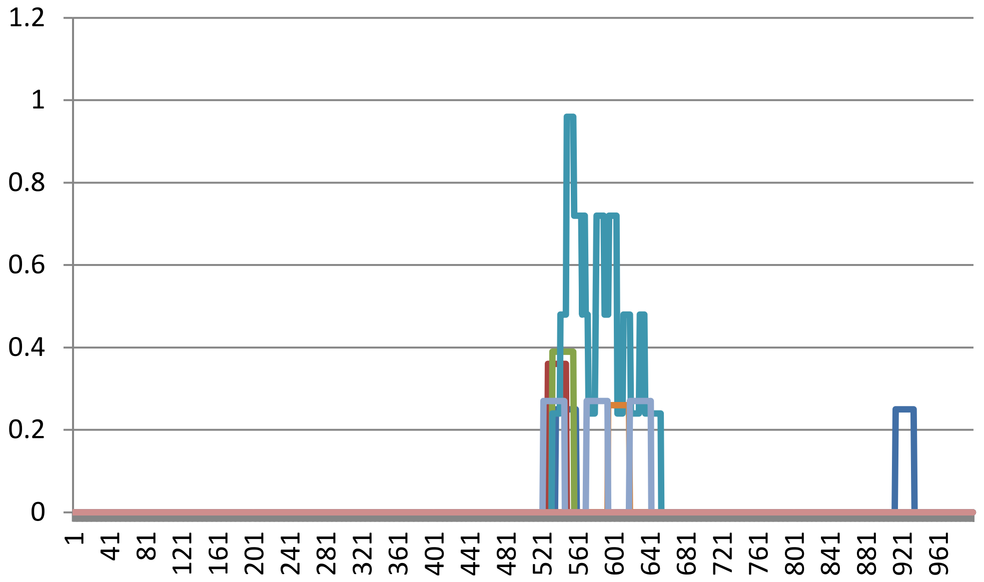

1.2
1
0.8
0.6
0.4
0.2
0
1
41
81
121
161
201
241
281
321
361
401
441
481
521
561
601
641
681
721
761
801
841
881
921
961

AT3G19710

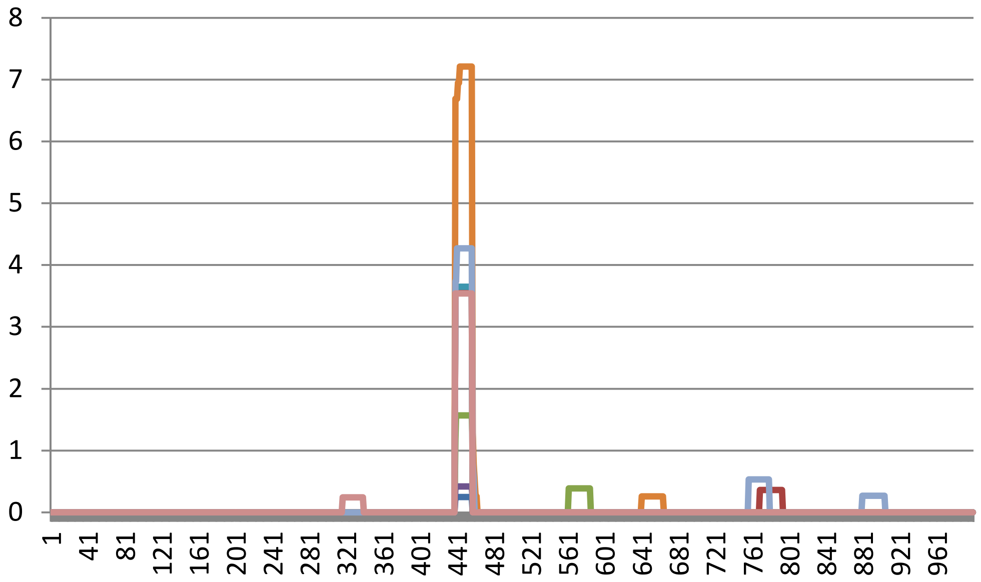

8
7
6
5
4
3
2
1
0
1
41
81
121
161
201
241
281
321
361
401
441
481
521
561
601
641
681
721
761
801
841
881
921
961

AT3G20760_AGO1 root

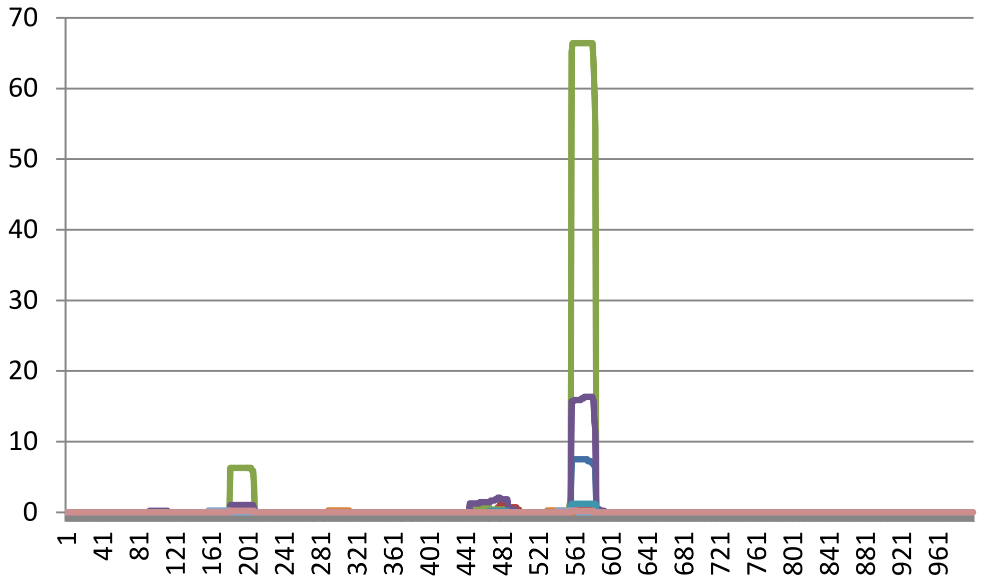

AT3G23040

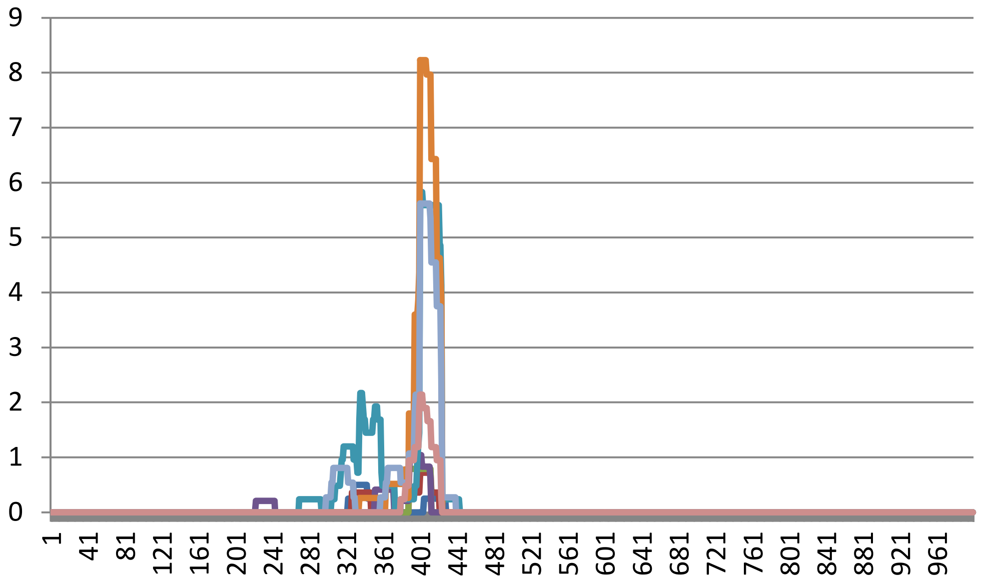

9
8
7
6
5
4
3
2
1
0
1
41
81
121
161
201
241
281
321
361
401
441
481
521
561
601
641
681
721
761
801
841
881
921
961

AT3G24542

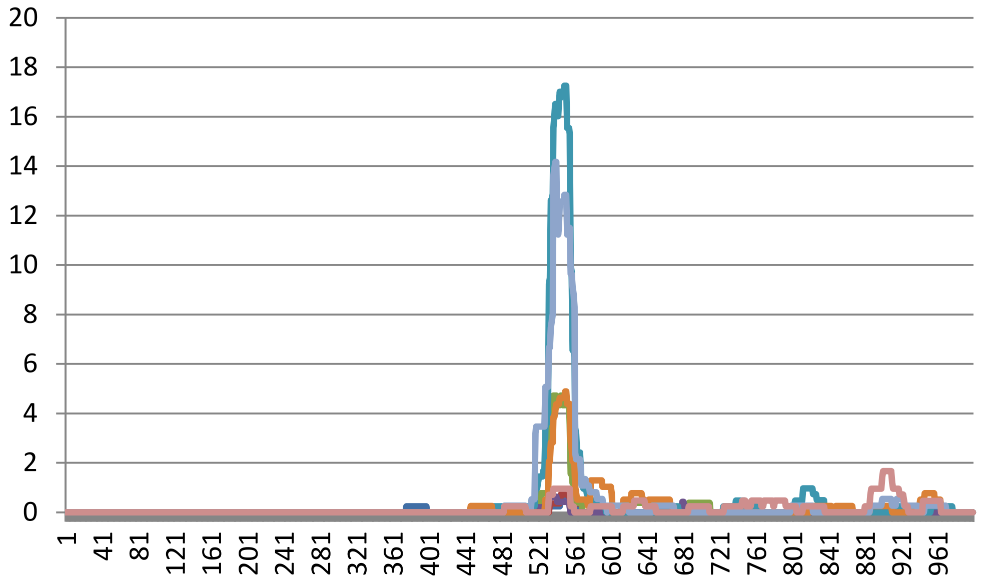

20
18
16
14
12
10
8
6
4
2
0
1
41
81
121
161
201
241
281
321
361
401
441
481
521
561
601
641
681
721
761
801
841
881
921
961

AT3G28840

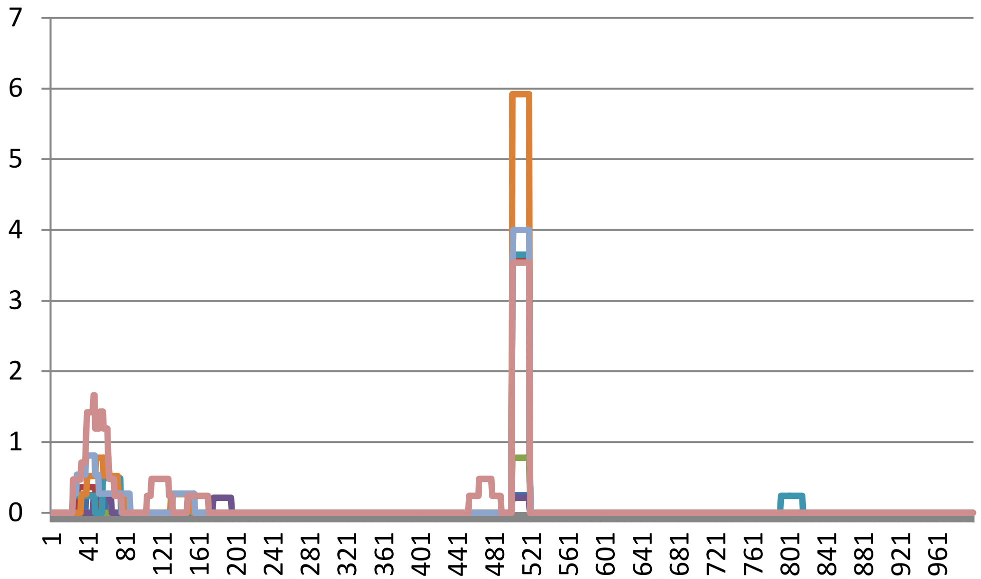

7
6
5
4
3
2
1
0
1
41
81
121
161
201
241
281
321
361
401
441
481
521
561
601
641
681
721
761
801
841
881
921
961

AT3G28980

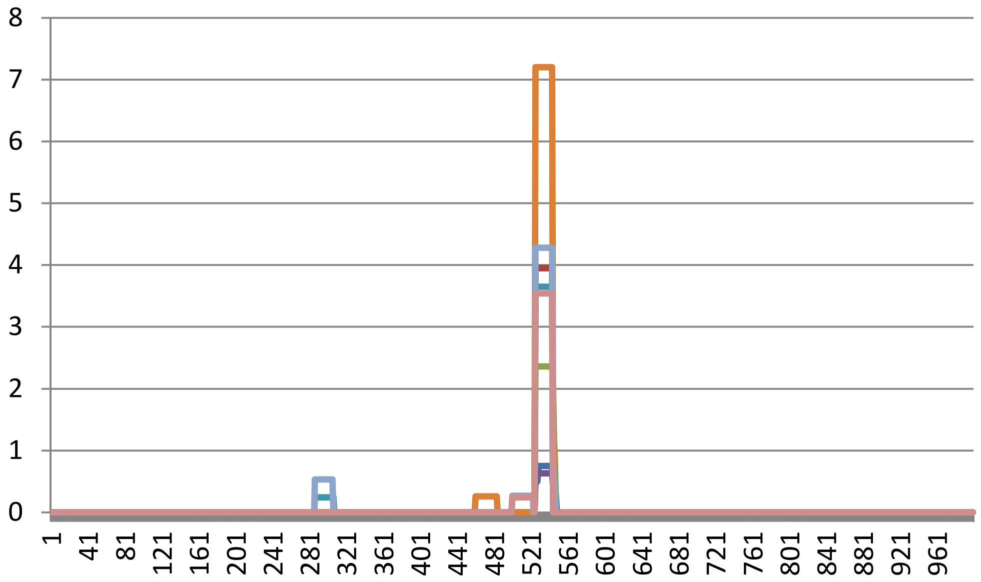

8
7
6
5
4
3
2
1
0
1
41
81
121
161
201
241
281
321
361
401
441
481
521
561
601
641
681
721
761
801
841
881
921
961

AT3G46640

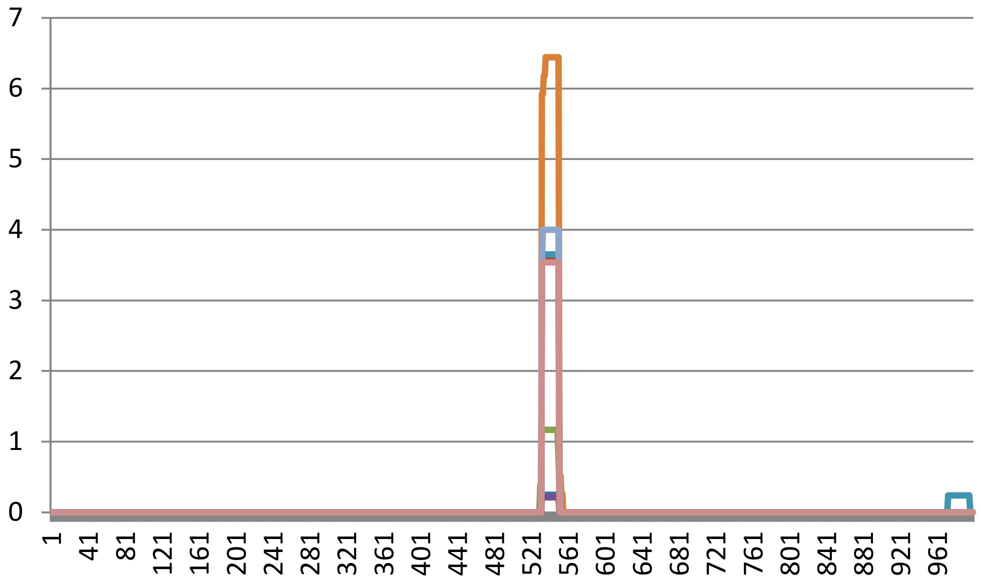

7
6
5
4
3
2
1
0
1
41
81
121
161
201
241
281
321
361
401
441
481
521
561
601
641
681
721
761
801
841
881
921
961

AT3G47030

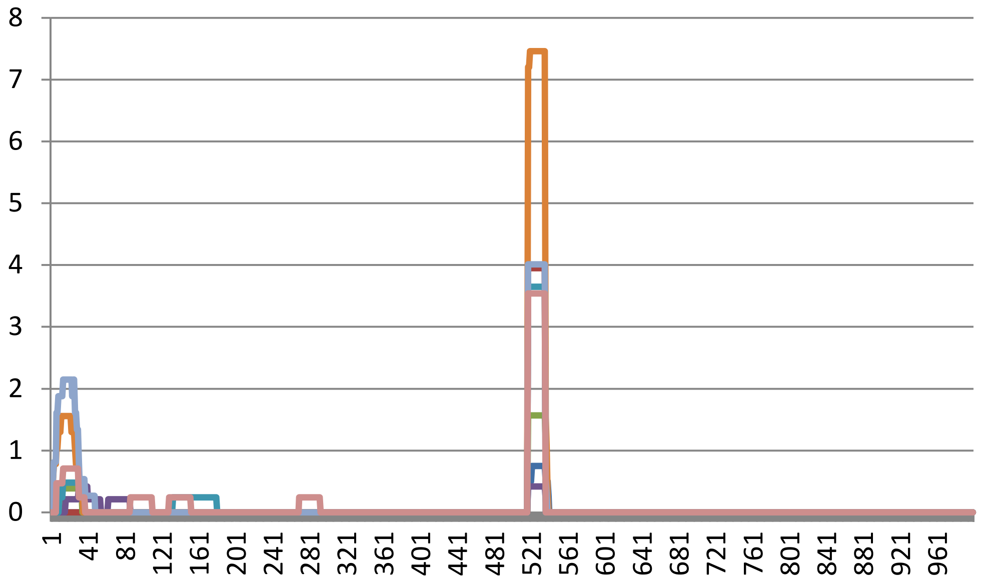

8
7
6
5
4
3
2
1
0
1
41
81
121
161
201
241
281
321
361
401
441
481
521
561
601
641
681
721
761
801
841
881
921
961

AT3G49645

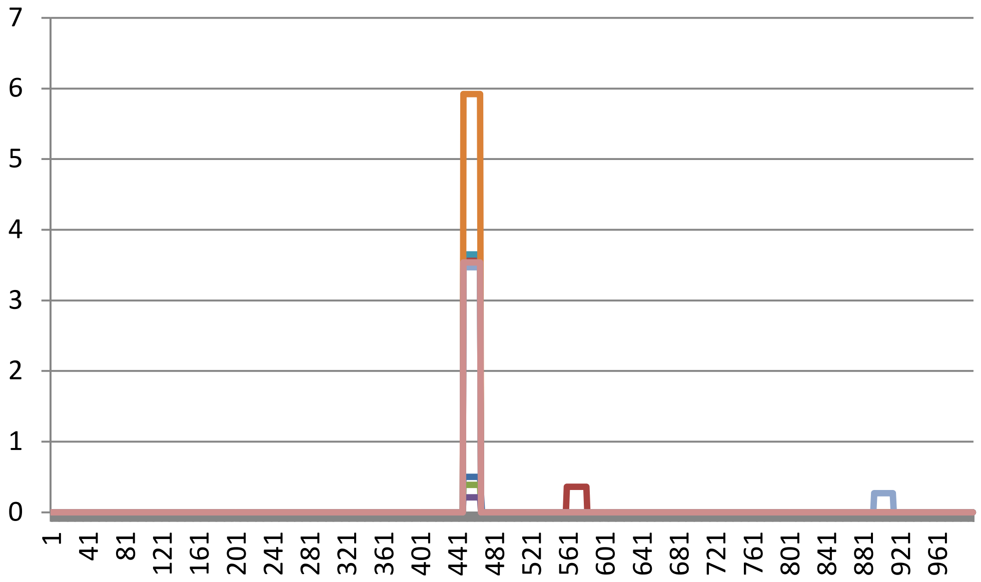

7
6
5
4
3
2
1
0
1
41
81
121
161
201
241
281
321
361
401
441
481
521
561
601
641
681
721
761
801
841
881
921
961

AT3G52570_AGO1 root

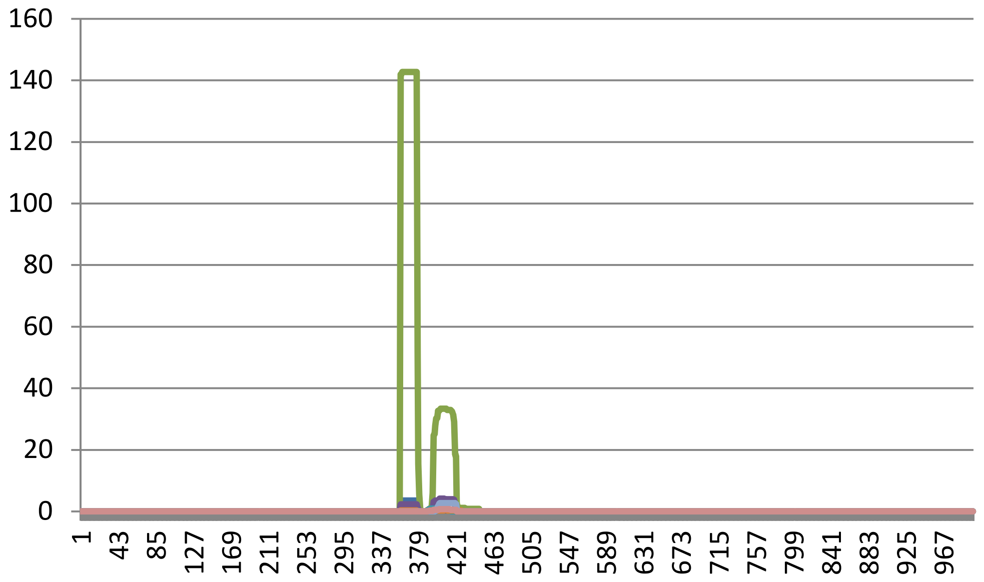

AT3G52700

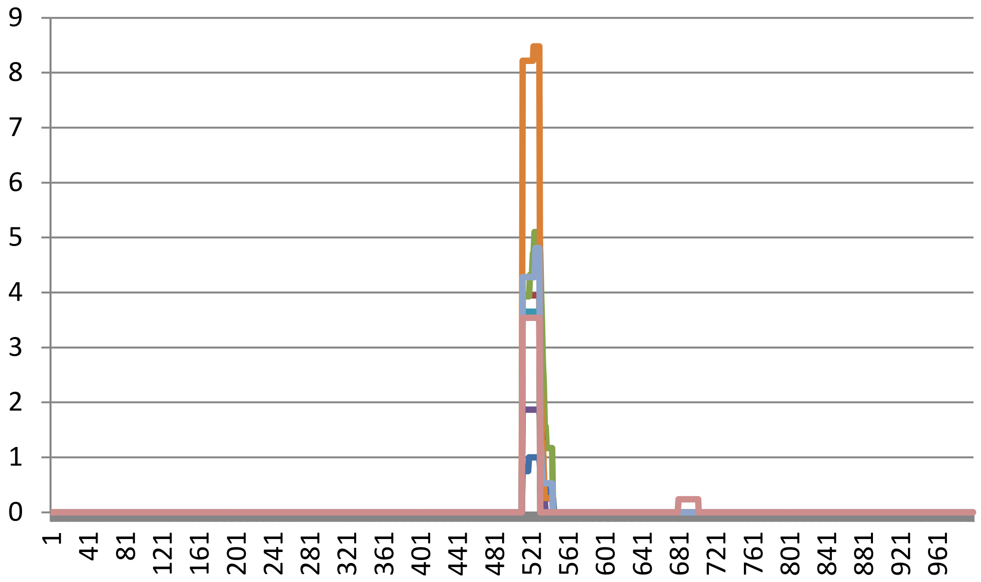

9
8
7
6
5
4
3
2
1
0
1
41
81
121
161
201
241
281
321
361
401
441
481
521
561
601
641
681
721
761
801
841
881
921
961

AT3G53920

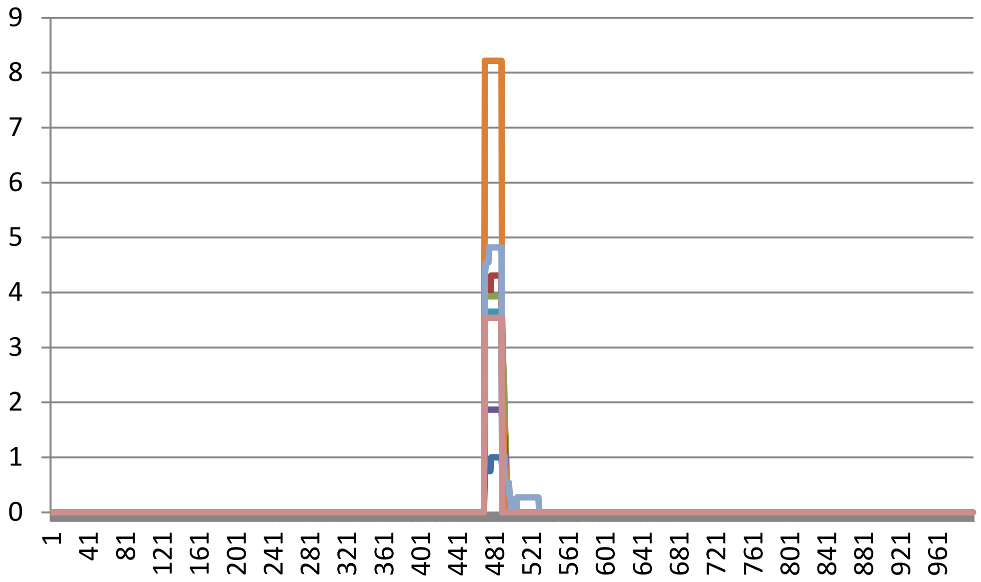

9
8
7
6
5
4
3
2
1
0
1
41
81
121
161
201
241
281
321
361
401
441
481
521
561
601
641
681
721
761
801
841
881
921
961

AT3G54360

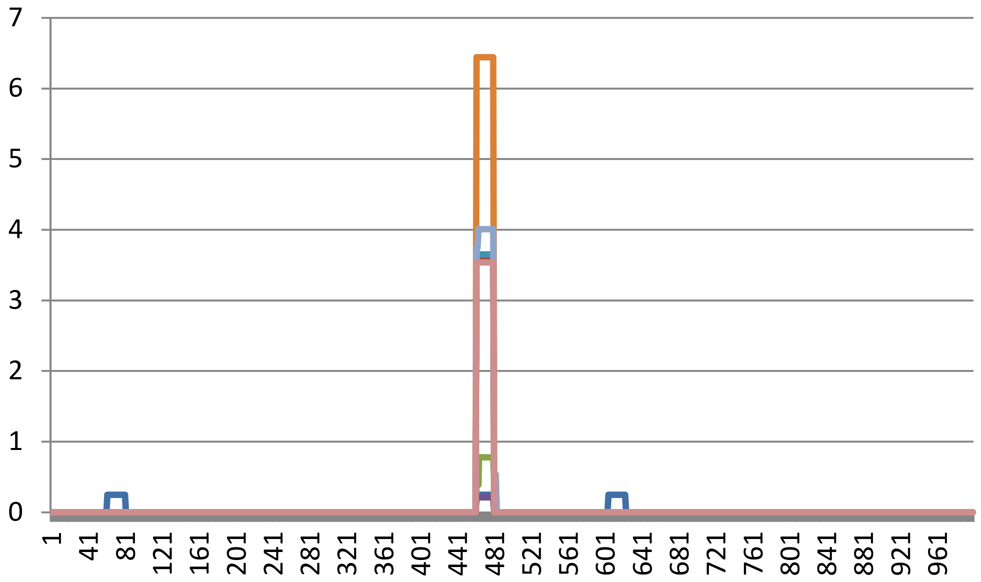

7
6
5
4
3
2
1
0
1
41
81
121
161
201
241
281
321
361
401
441
481
521
561
601
641
681
721
761
801
841
881
921
961

AT3G55020

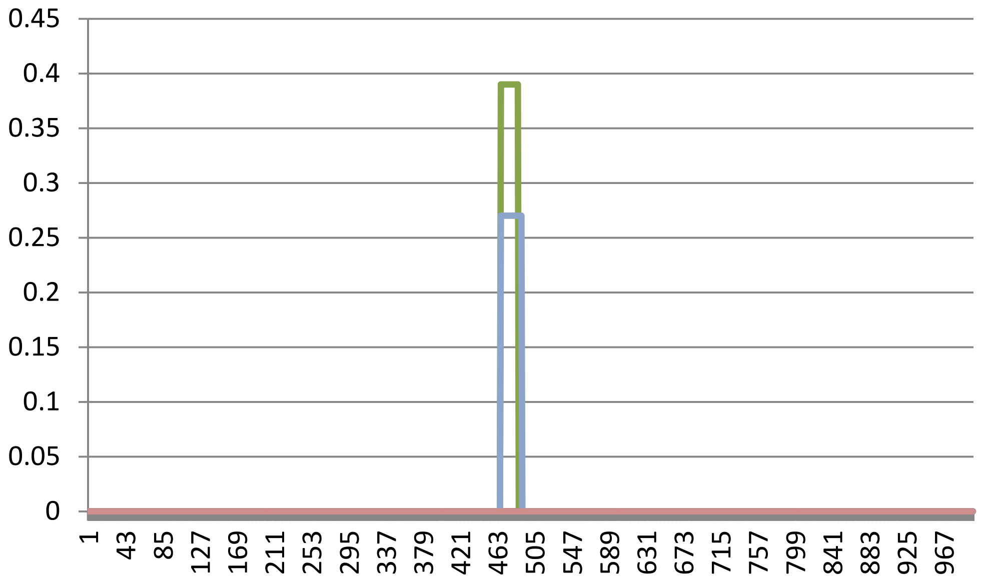

0.45
0.4
0.35
0.3
0.25
0.2
0.15
0.1
0.05
0
1
43
85
127
169
211
253
295
337
379
421
463
505
547
589
631
673
715
757
799
841
883
925
967

AT3G55850_root

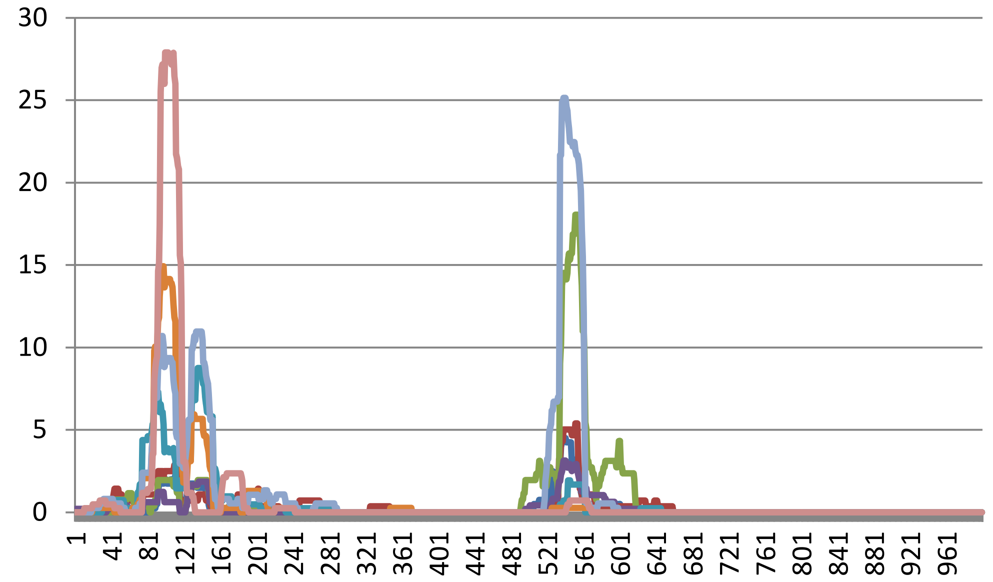

AT3G55860_root

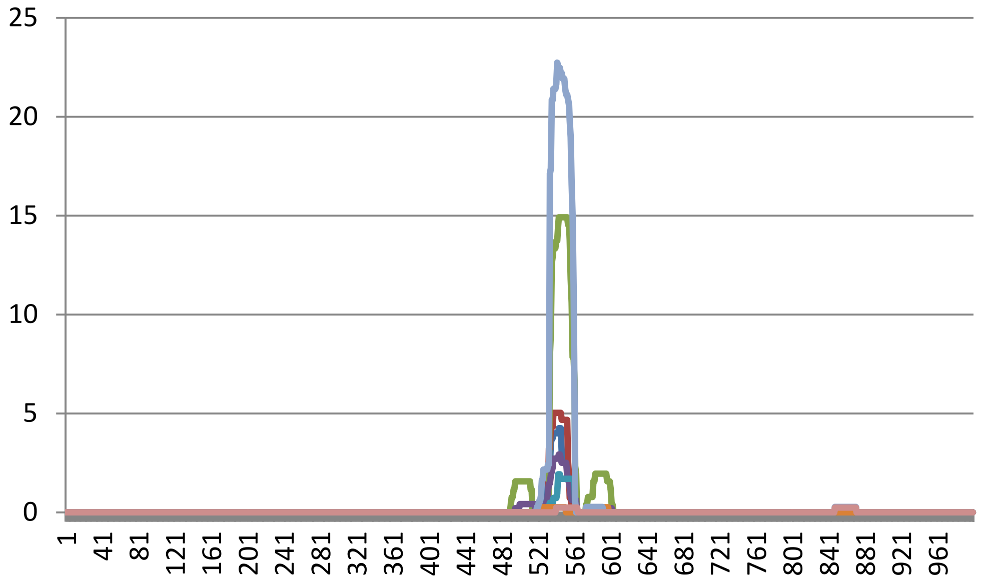

AT3G63470

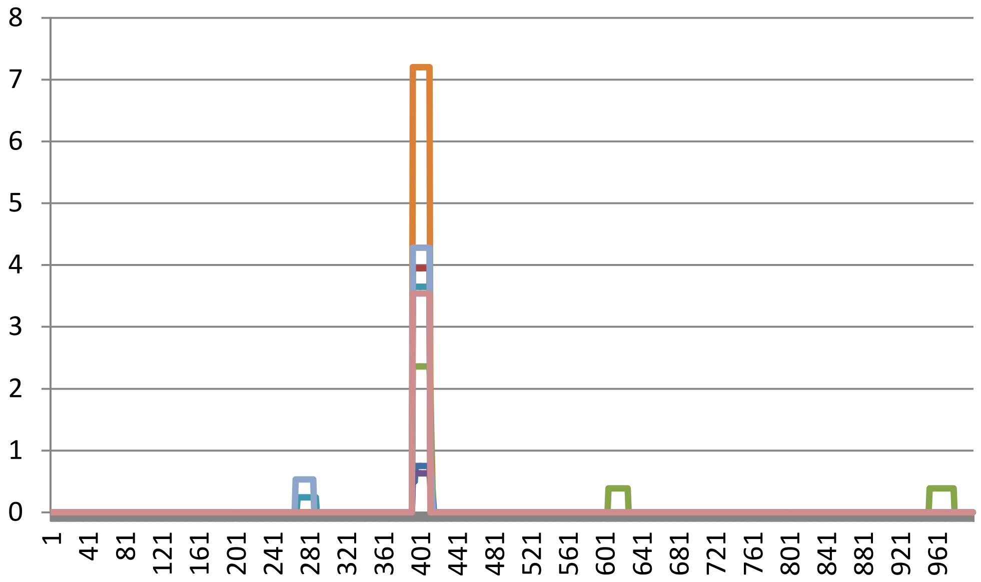

8
7
6
5
4
3
2
1
0
1
41
81
121
161
201
241
281
321
361
401
441
481
521
561
601
641
681
721
761
801
841
881
921
961

AT4G00580

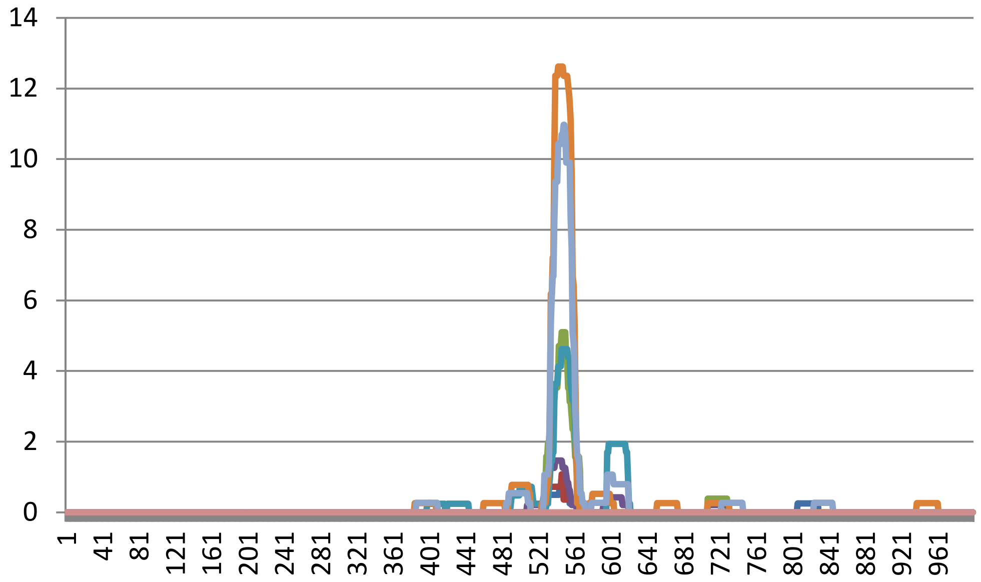

14
12
10
8
6
4
2
0
1
41
81
121
161
201
241
281
321
361
401
441
481
521
561
601
641
681
721
761
801
841
881
921
961

AT4G00950

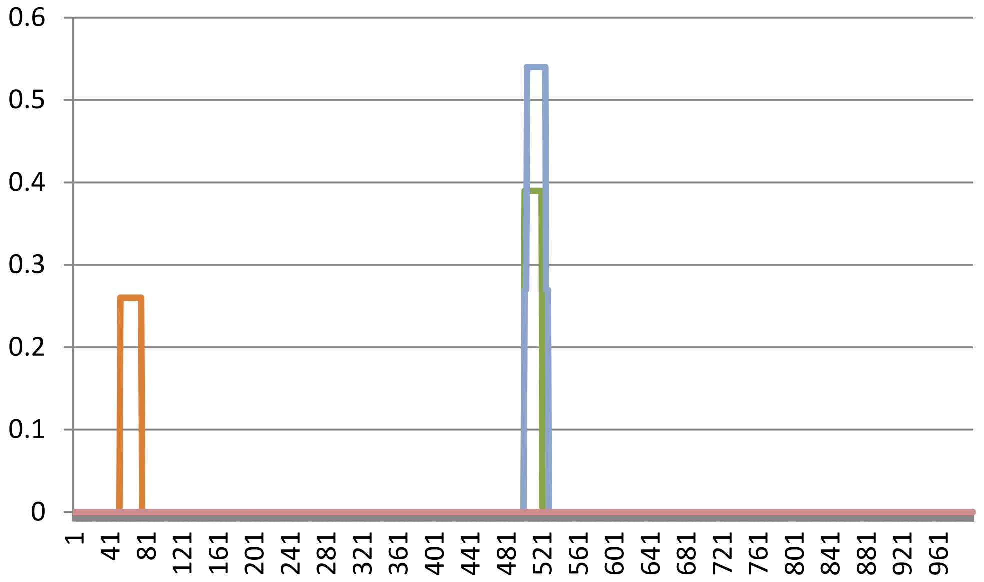

0.6
0.5
0.4
0.3
0.2
0.1
0
1
41
81
121
161
201
241
281
321
361
401
441
481
521
561
601
641
681
721
761
801
841
881
921
961

AT4G03940

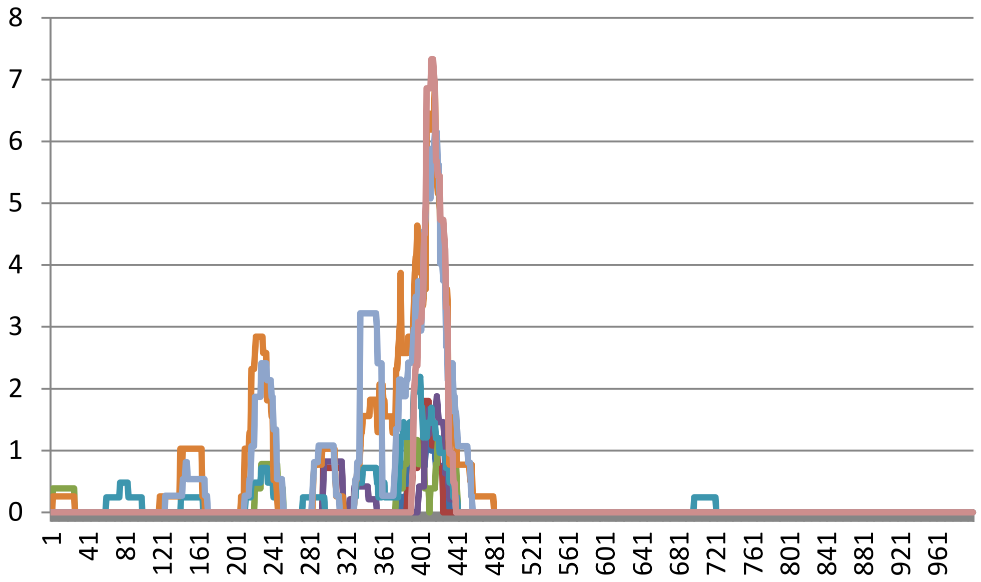

8
7
6
5
4
3
2
1
0
1
41
81
121
161
201
241
281
321
361
401
441
481
521
561
601
641
681
721
761
801
841
881
921
961

AT4G05030

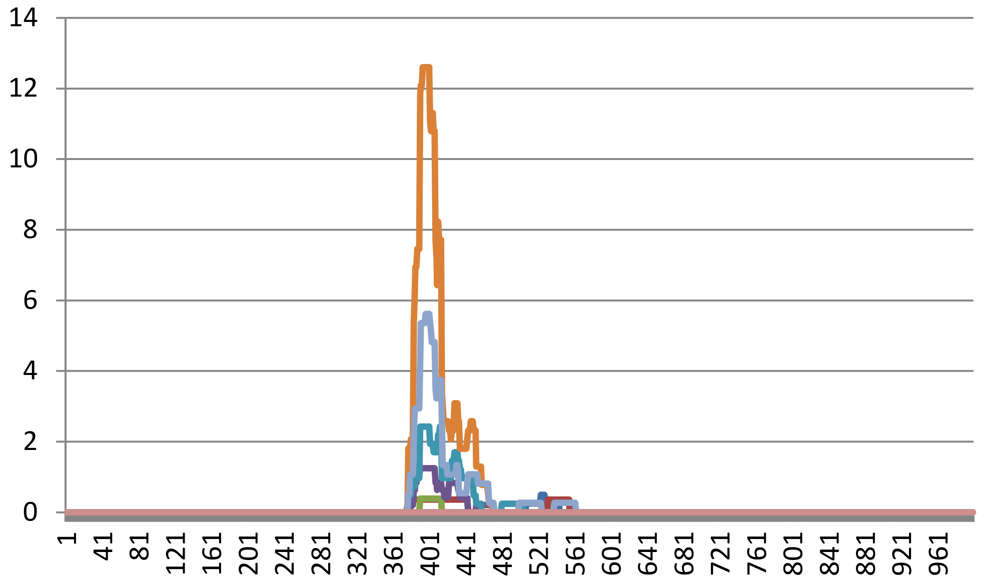

14
12
10
8
6
4
2
0
1
41
81
121
161
201
241
281
321
361
401
441
481
521
561
601
641
681
721
761
801
841
881
921
961

AT4G06740

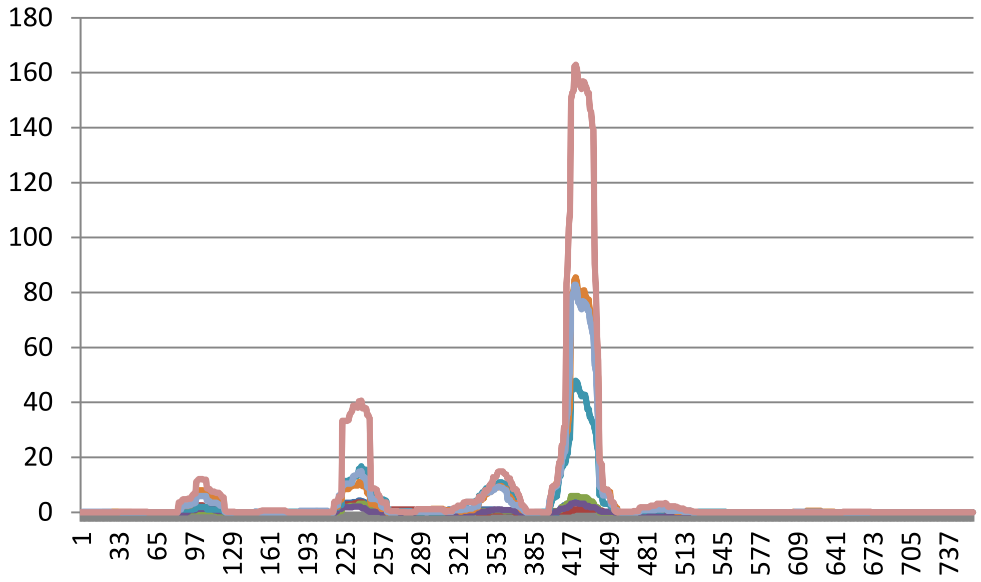

180
160
140
120
100
80
60
40
20
0
1
33
65
97
129
161
193
225
257
289
321
353
385
417
449
481
513
545
577
609
641
673
705
737

AT4G07526

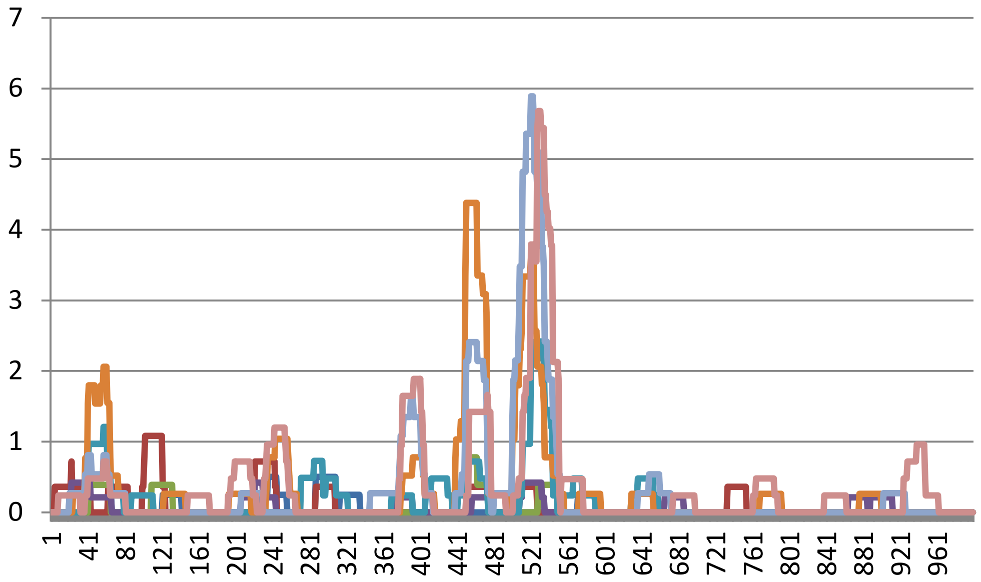

7
6
5
4
3
2
1
0
1
41
81
121
161
201
241
281
321
361
401
441
481
521
561
601
641
681
721
761
801
841
881
921
961

AT4G07960

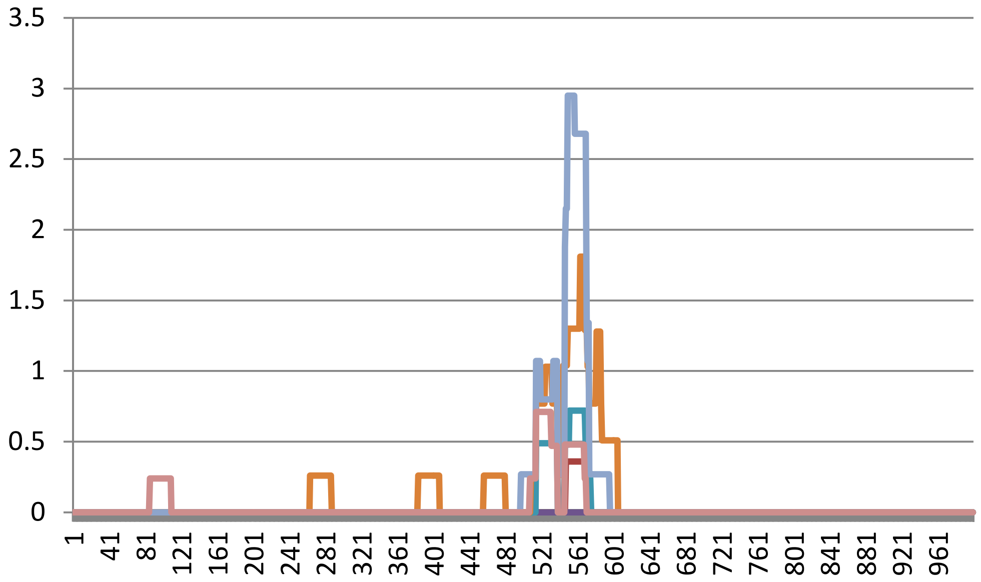

3.5
3
2.5
2
1.5
1
0.5
0
1
41
81
121
161
201
241
281
321
361
401
441
481
521
561
601
641
681
721
761
801
841
881
921
961

AT4G10596

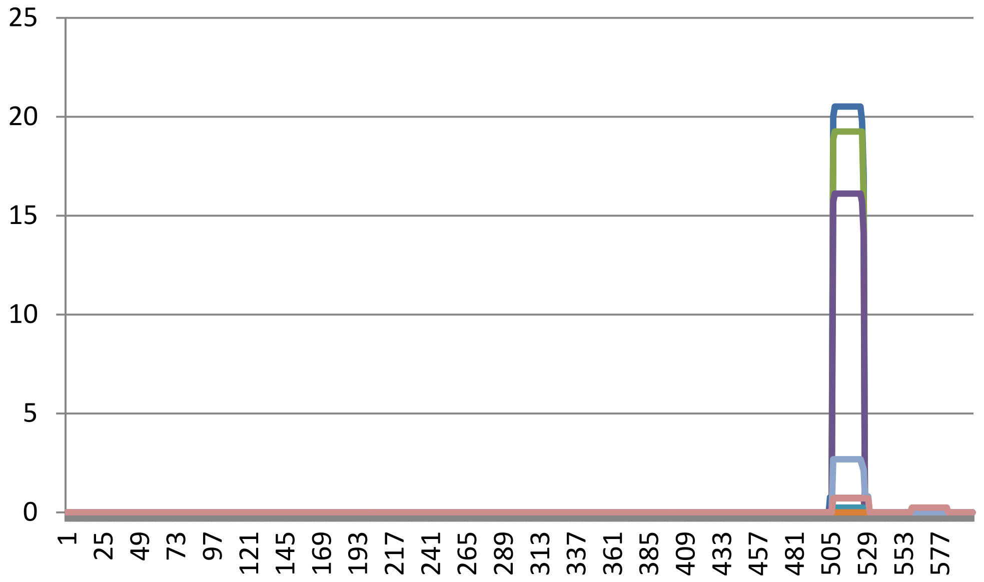

25
20
15
10
5
0
1
25
49
73
97
121
145
169
193
217
241
265
289
313
337
361
385
409
433
457
481
505
529
553
577

AT4G11485

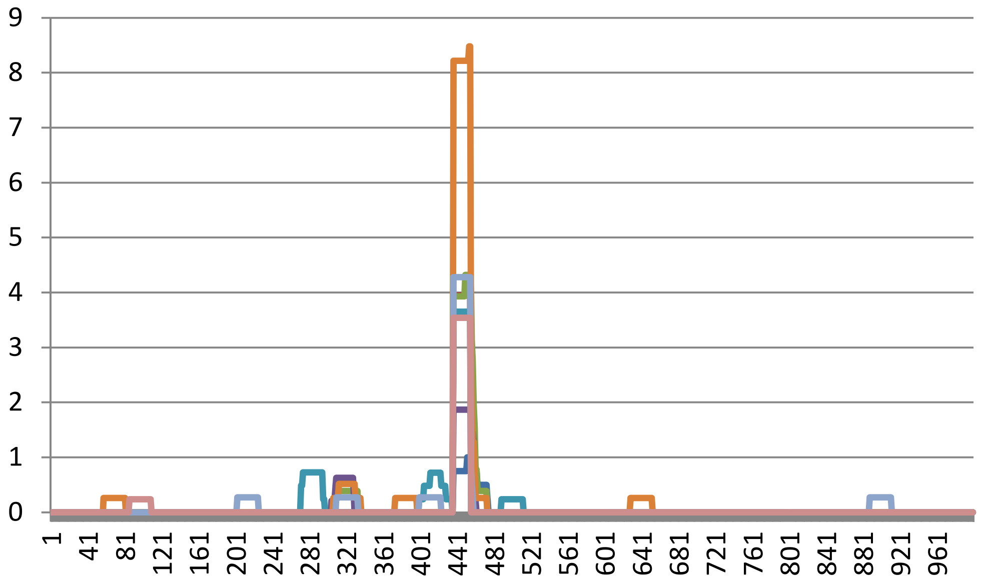

9
8
7
6
5
4
3
2
1
0
1
41
81
121
161
201
241
281
321
361
401
441
481
521
561
601
641
681
721
761
801
841
881
921
961

AT4G13420

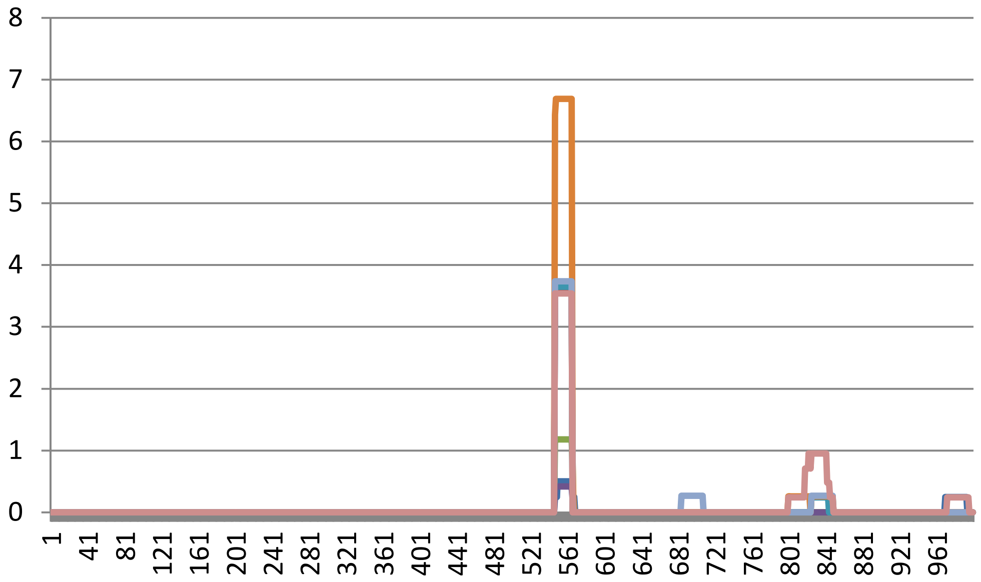

8
7
6
5
4
3
2
1
0
1
41
81
121
161
201
241
281
321
361
401
441
481
521
561
601
641
681
721
761
801
841
881
921
961

AT4G13992

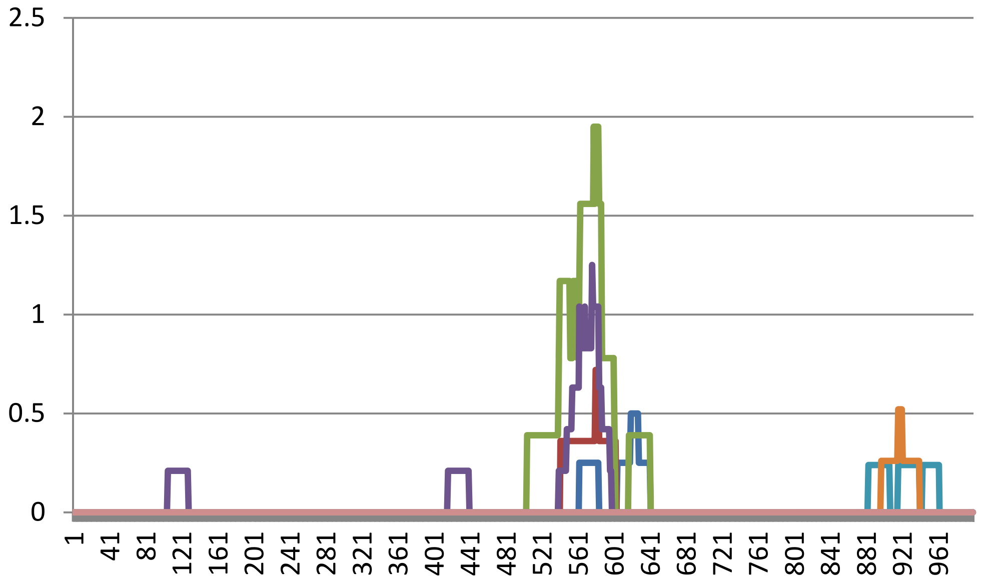

2.5
2
1.5
1
0.5
0
1
41
81
121
161
201
241
281
321
361
401
441
481
521
561
601
641
681
721
761
801
841
881
921
961

AT4G16240

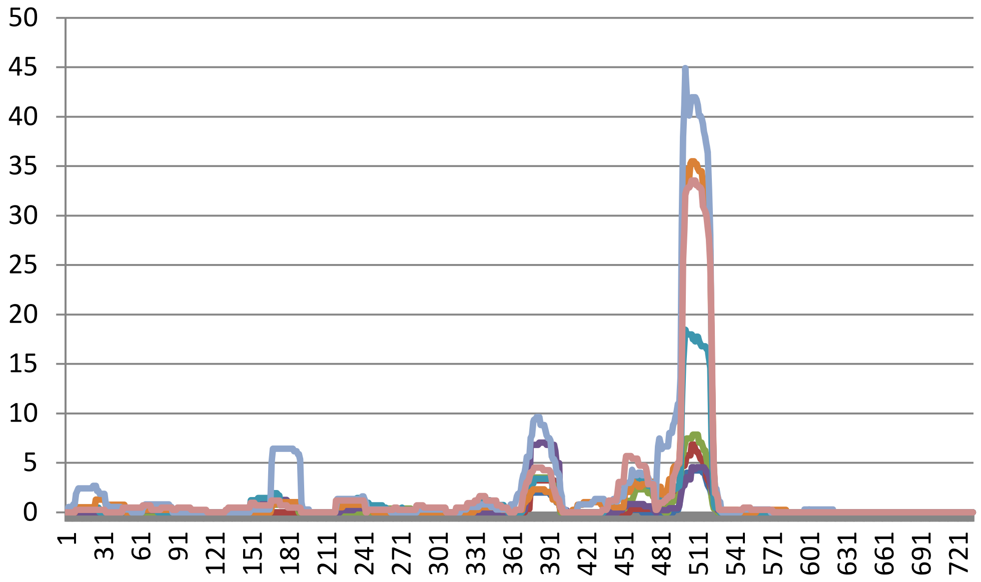

50
45
40
35
30
25
20
15
10
5
0
1
31
61
91
121
151
181
211
241
271
301
331
361
391
421
451
481
511
541
571
601
631
661
691
721

AT4G16790

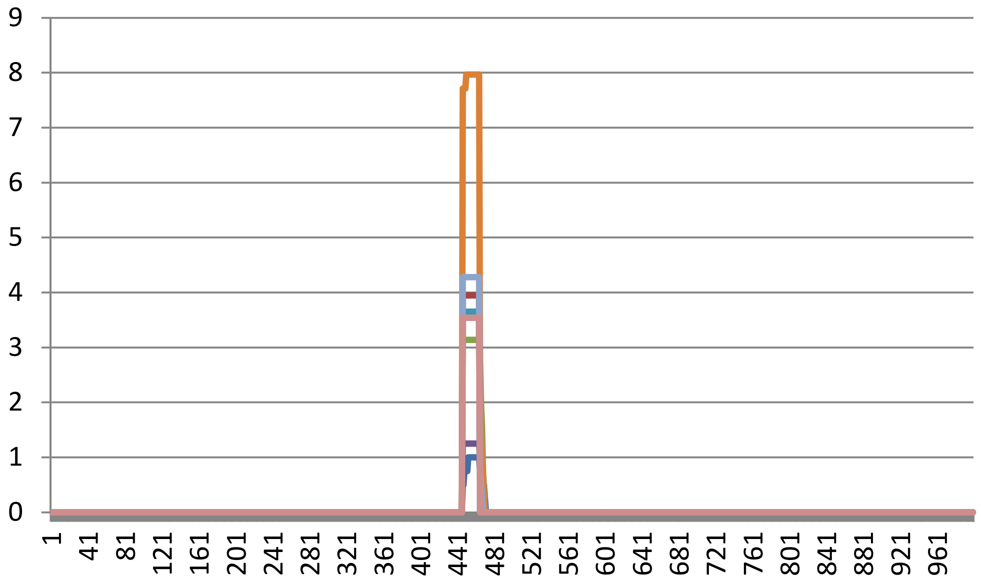

9
8
7
6
5
4
3
2
1
0
1
41
81
121
161
201
241
281
321
361
401
441
481
521
561
601
641
681
721
761
801
841
881
921
961

AT4G21210

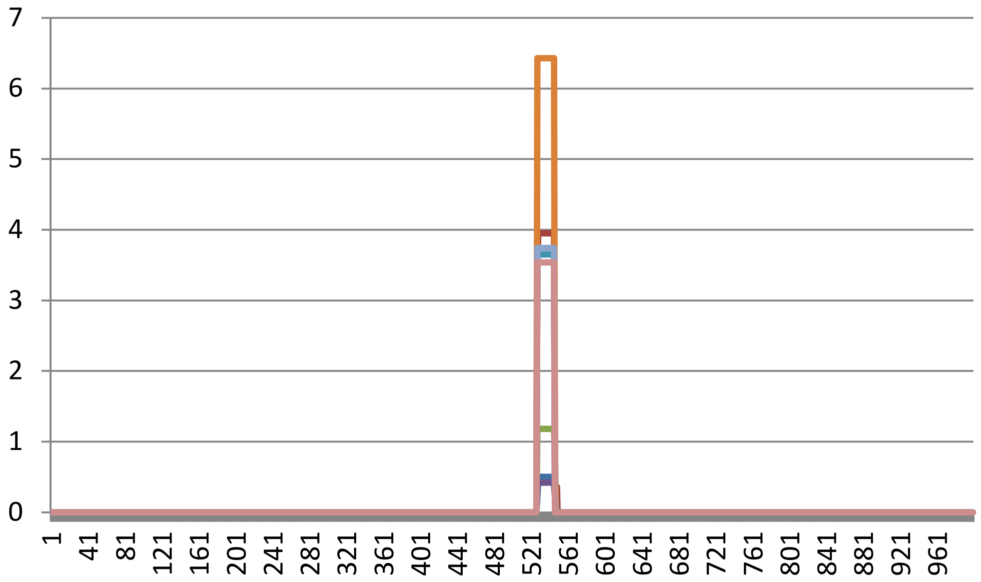

7
6
5
4
3
2
1
0
1
41
81
121
161
201
241
281
321
361
401
441
481
521
561
601
641
681
721
761
801
841
881
921
961

AT4G22150

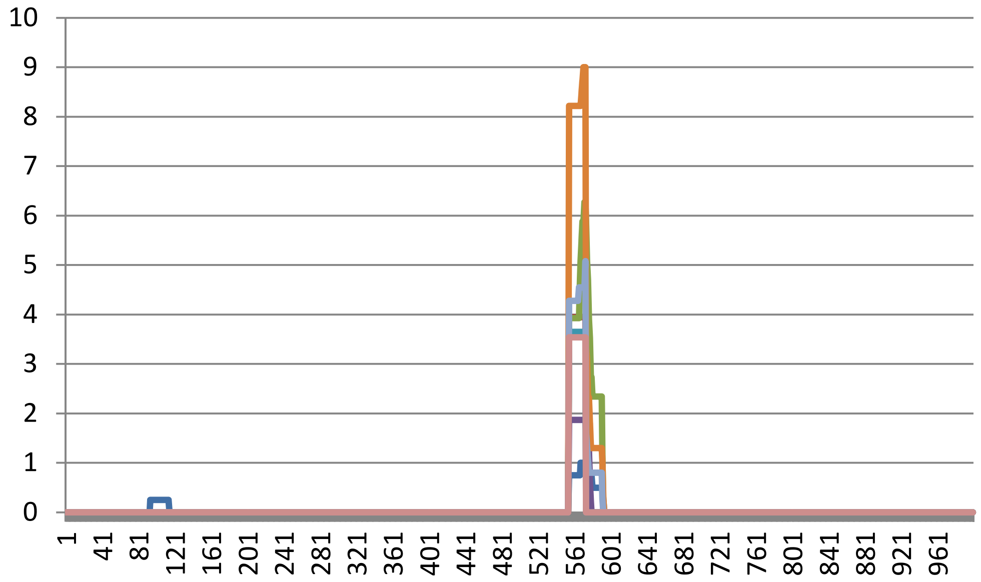
10
9
8
7
6
5
4
3
2
1
0
1
41
81
121
161
201
241
281
321
361
401
441
481
521
561
601
641
681
721
761
801
841
881
921
961

AT4G22890

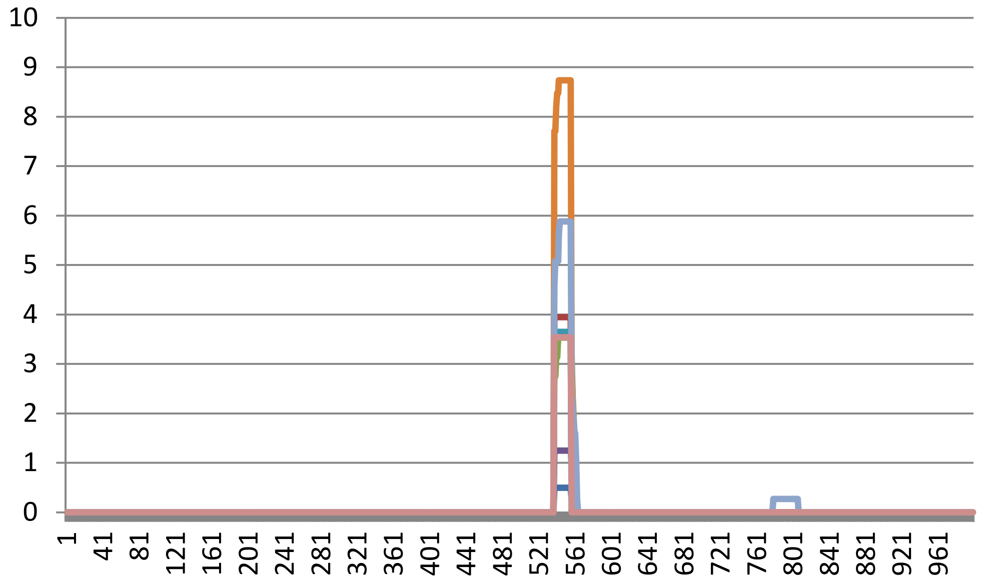

10
9
8
7
6
5
4
3
2
1
0
1
41
81
121
161
201
241
281
321
361
401
441
481
521
561
601
641
681
721
761
801
841
881
921
961

AT4G25240

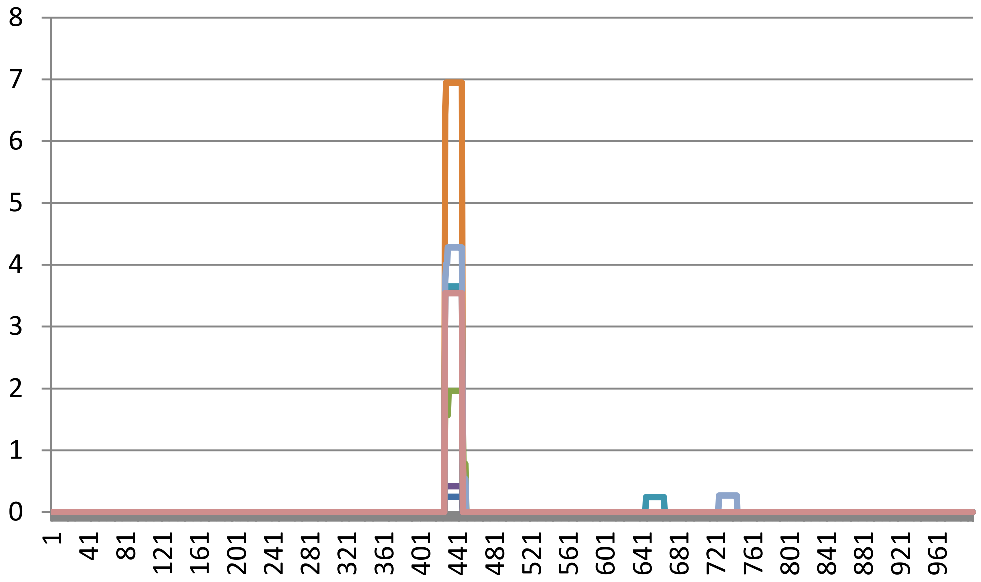

8
7
6
5
4
3
2
1
0
1
41
81
121
161
201
241
281
321
361
401
441
481
521
561
601
641
681
721
761
801
841
881
921
961

AT4G29033

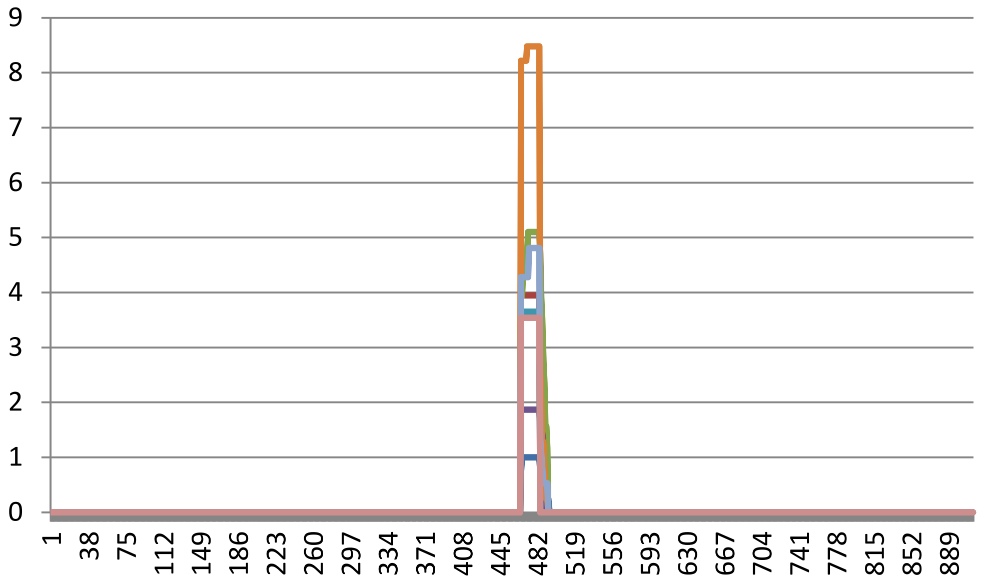

9
8
7
6
5
4
3
2
1
0
1
38
75
112
149
186
223
260
297
334
371
408
445
482
519
556
593
630
667
704
741
778
815
852
889

AT4G31980

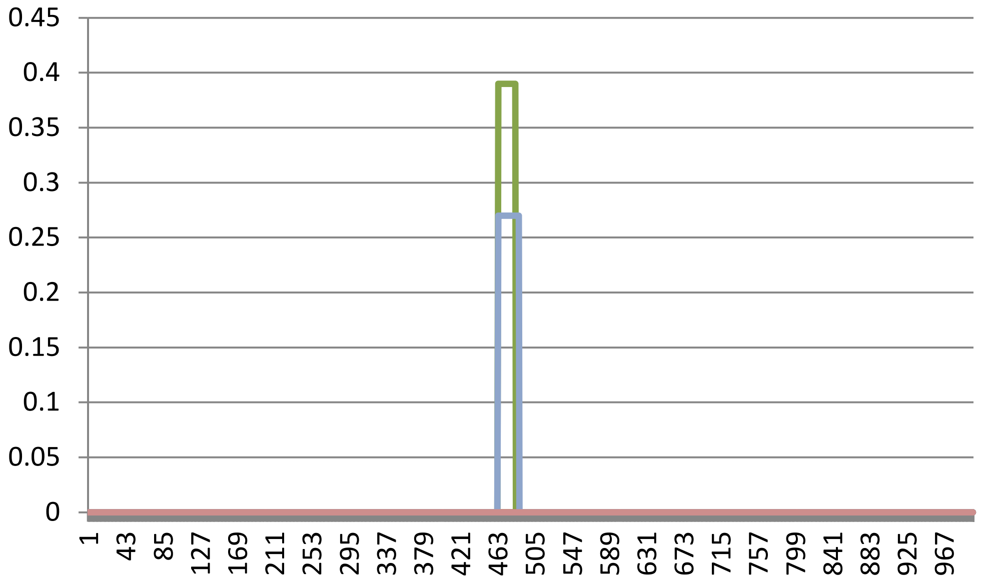

0.45
0.4
0.35
0.3
0.25
0.2
0.15
0.1
0.05
0
1
43
85
127
169
211
253
295
337
379
421
463
505
547
589
631
673
715
757
799
841
883
925
967

AT4G32950

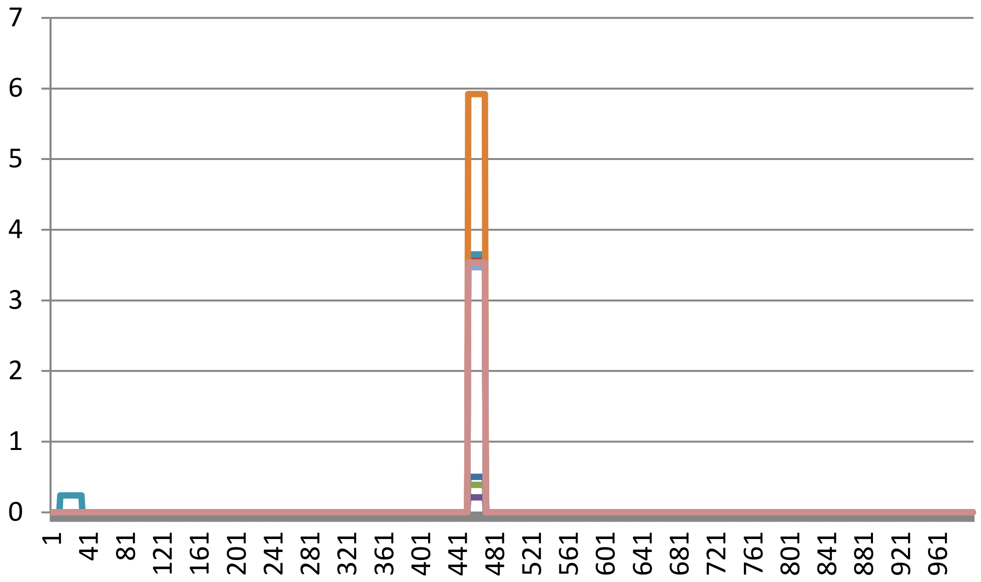

7
6
5
4
3
2
1
0
1
41
81
121
161
201
241
281
321
361
401
441
481
521
561
601
641
681
721
761
801
841
881
921
961

AT4G33840

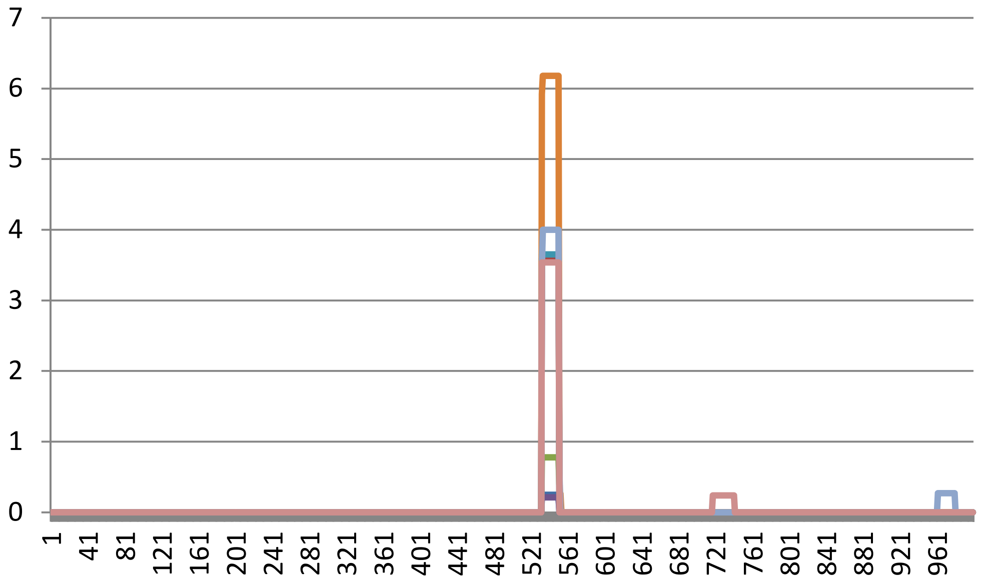

7
6
5
4
3
2
1
0
1
41
81
121
161
201
241
281
321
361
401
441
481
521
561
601
641
681
721
761
801
841
881
921
961

AT5G02700

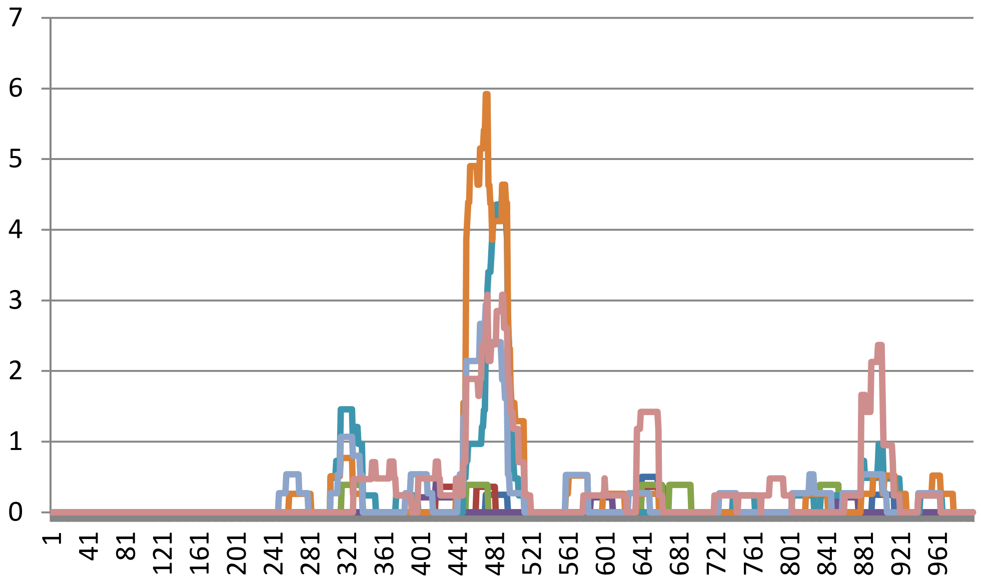

7
6
5
4
3
2
1
0
1
41
81
121
161
201
241
281
321
361
401
441
481
521
561
601
641
681
721
761
801
841
881
921
961

AT5G05950_AGO1 root

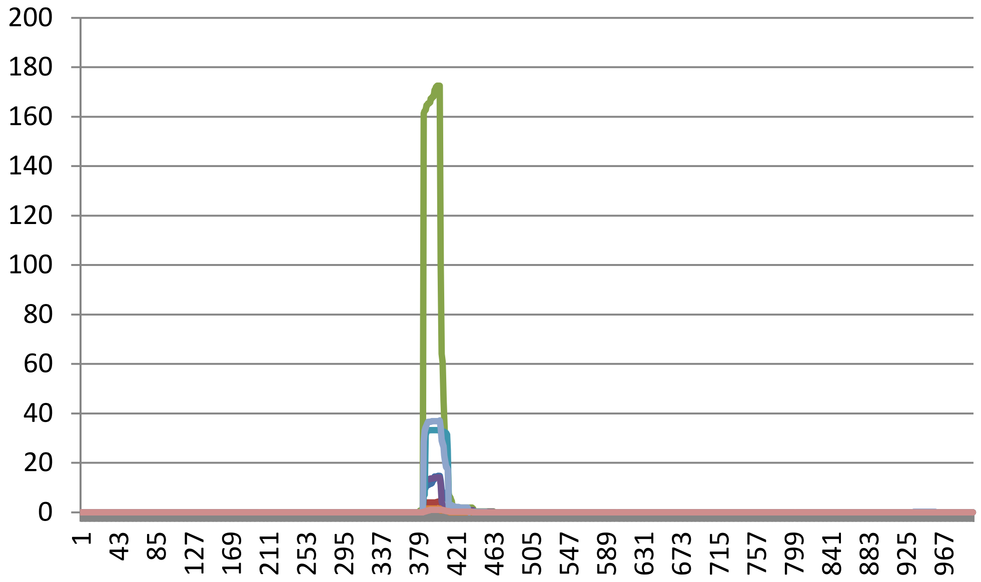

AT5G08550

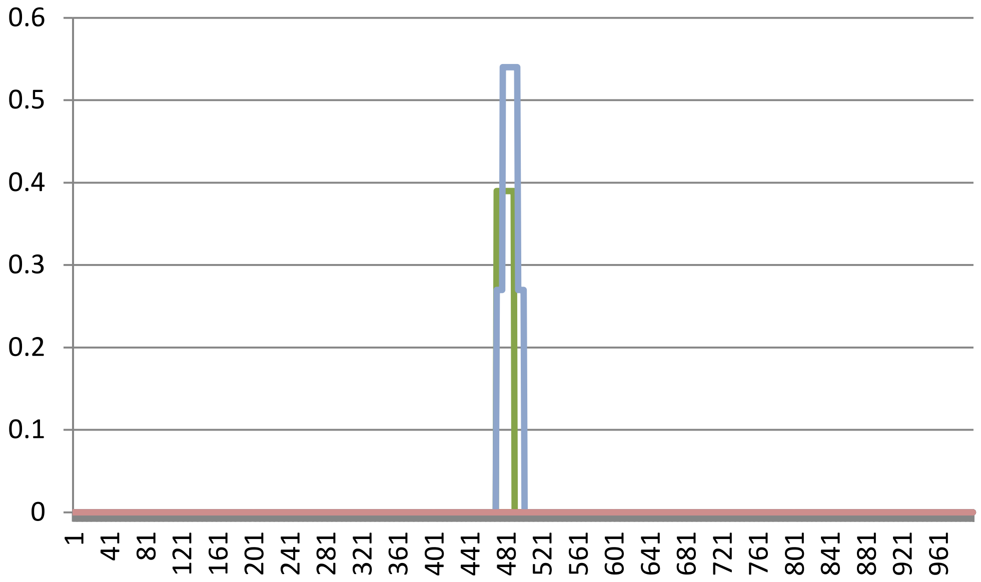

0.6
0.5
0.4
0.3
0.2
0.1
0
1
41
81
121
161
201
241
281
321
361
401
441
481
521
561
601
641
681
721
761
801
841
881
921
961

AT5G10340_leaf

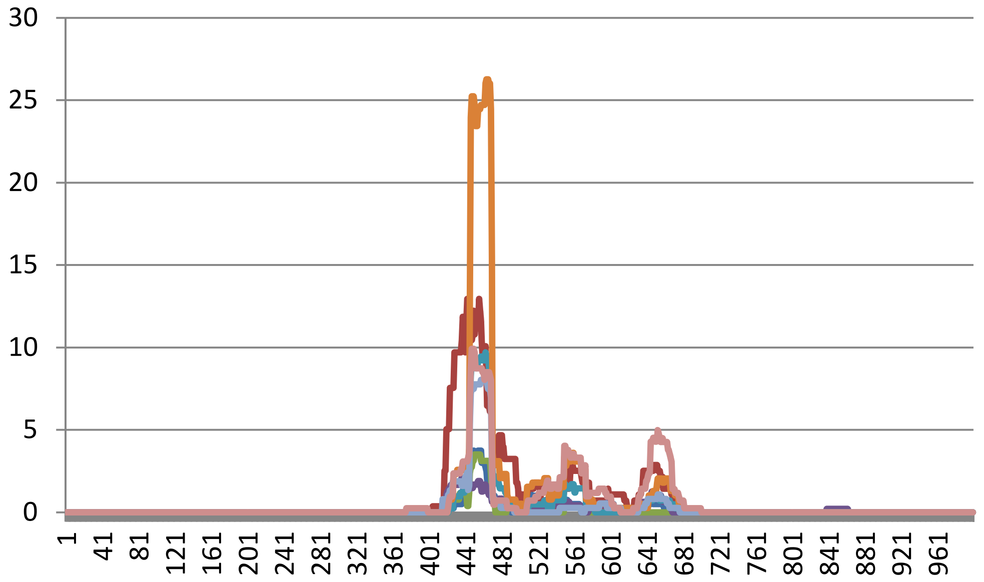

AT5G11510

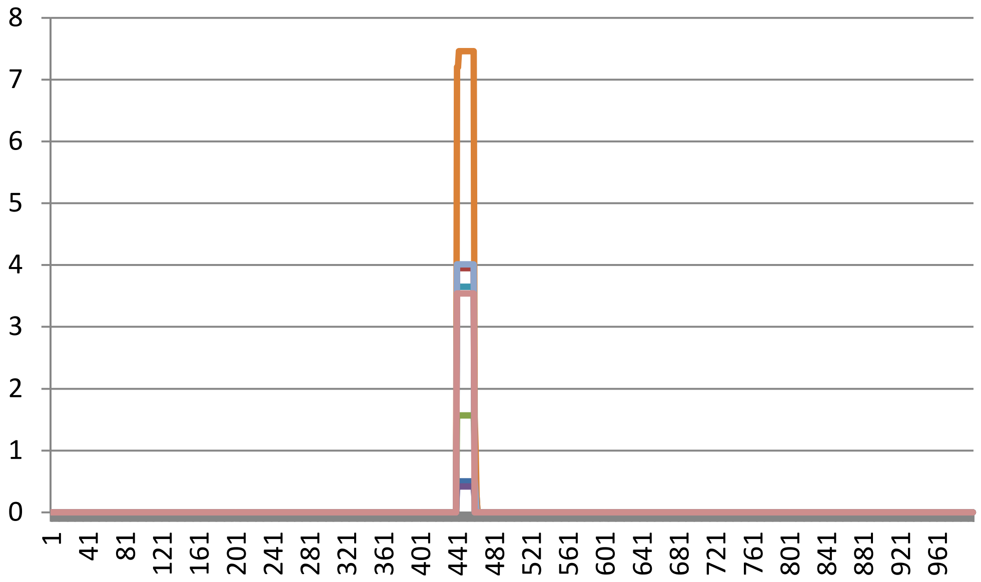

8
7
6
5
4
3
2
1
0
1
41
81
121
161
201
241
281
321
361
401
441
481
521
561
601
641
681
721
761
801
841
881
921
961

AT5G15420

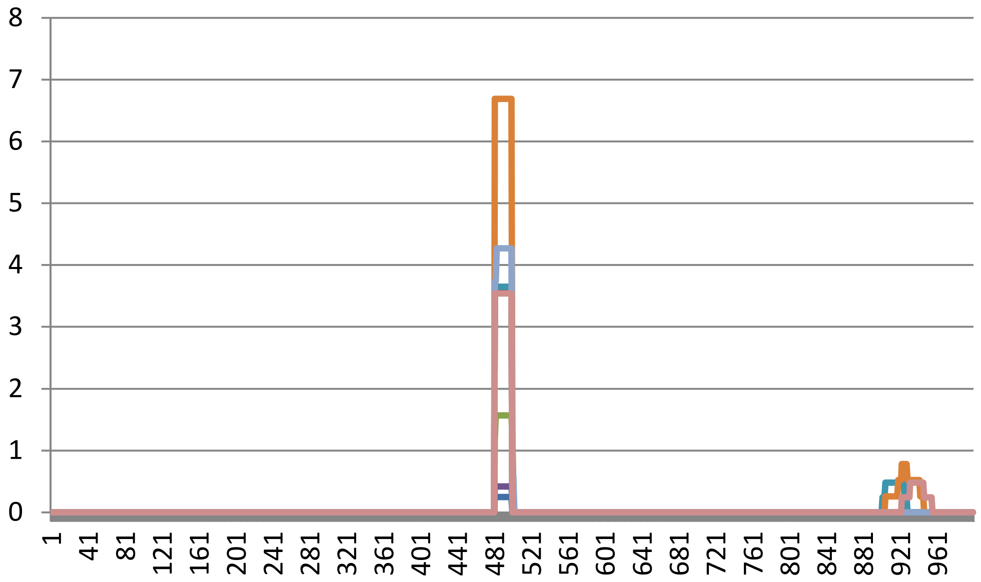

8
7
6
5
4
3
2
1
0
1
41
81
121
161
201
241
281
321
361
401
441
481
521
561
601
641
681
721
761
801
841
881
921
961

AT5G18260

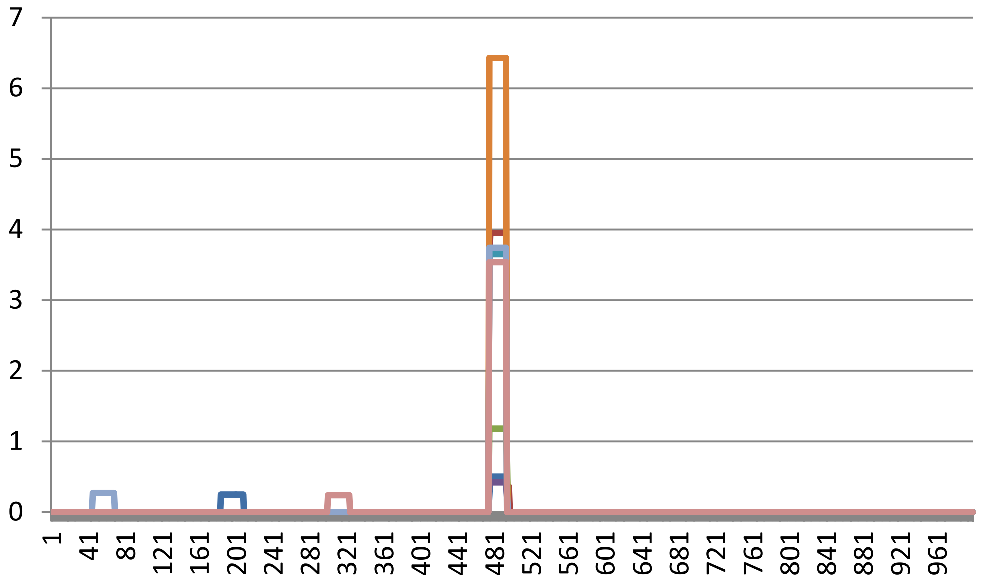

7
6
5
4
3
2
1
0
1
41
81
121
161
201
241
281
321
361
401
441
481
521
561
601
641
681
721
761
801
841
881
921
961

AT5G18610

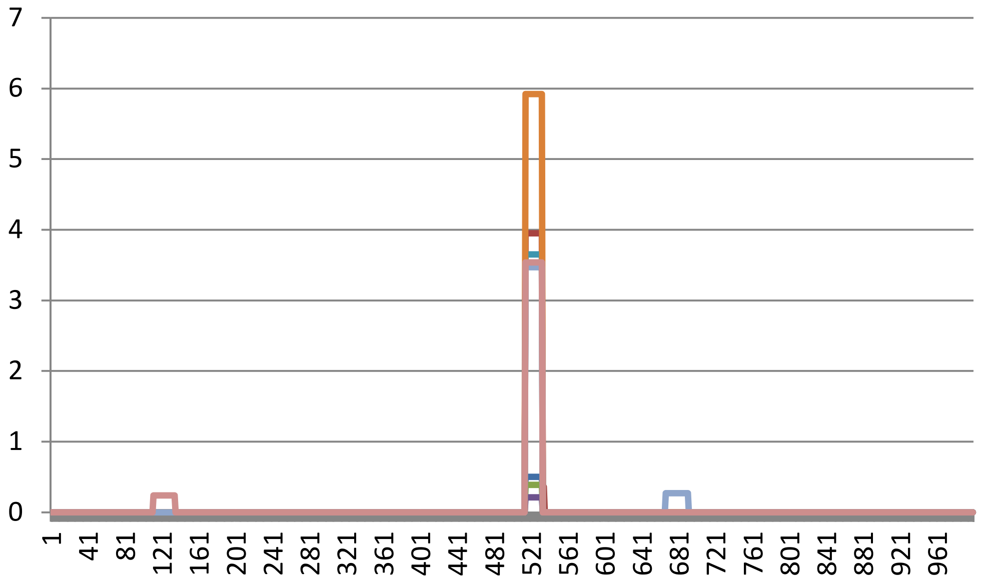

7
6
5
4
3
2
1
0
1
41
81
121
161
201
241
281
321
361
401
441
481
521
561
601
641
681
721
761
801
841
881
921
961

AT5G27870

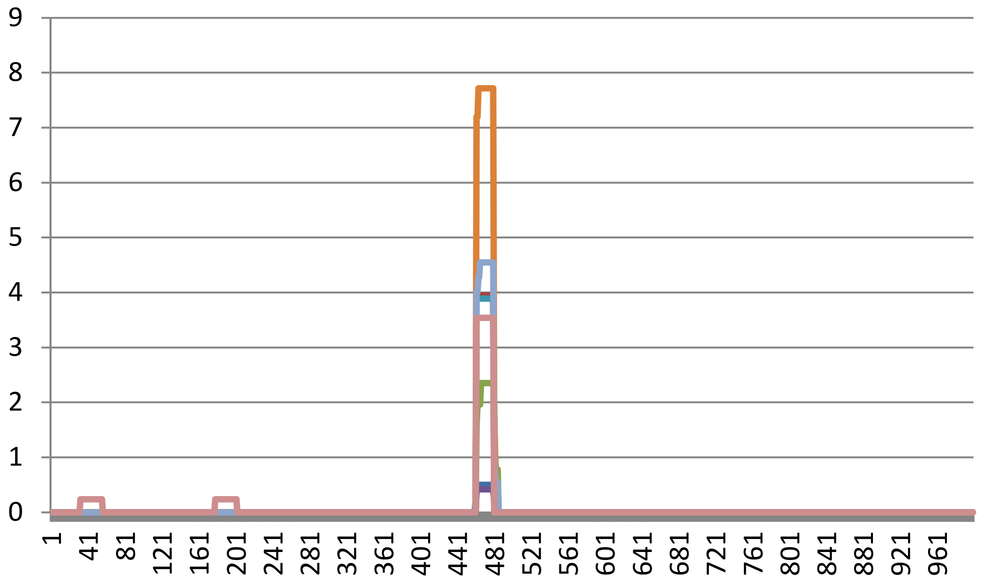

9
8
7
6
5
4
3
2
1
0
1
41
81
121
161
201
241
281
321
361
401
441
481
521
561
601
641
681
721
761
801
841
881
921
961

AT5G28465

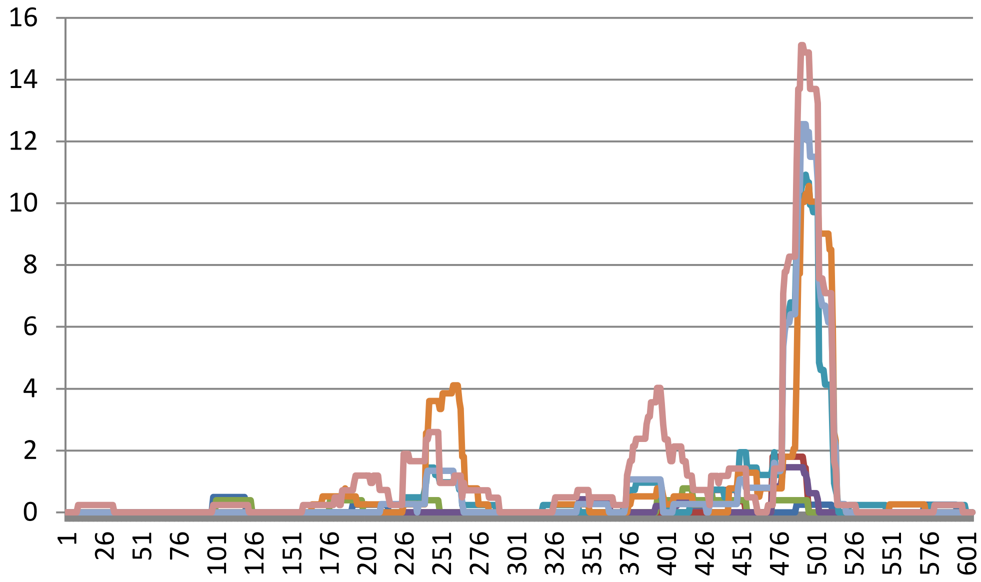

16
14
12
10
8
6
4
2
0
1
26
51
76
101
126
151
176
201
226
251
276
301
326
351
376
401
426
451
476
501
526
551
576
601

AT5G35930

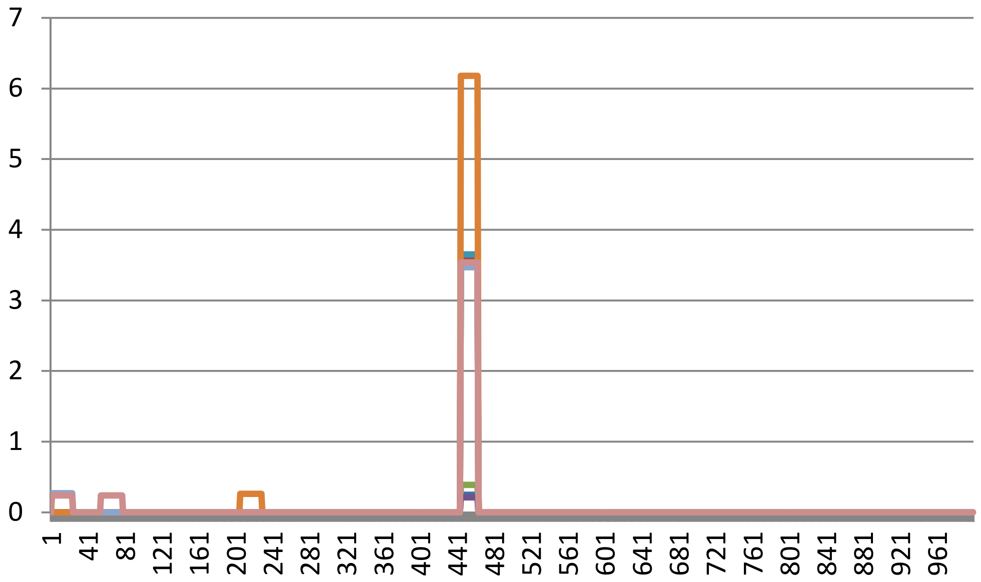

7
6
5
4
3
2
1
0
1
41
81
121
161
201
241
281
321
361
401
441
481
521
561
601
641
681
721
761
801
841
881
921
961

AT5G41330

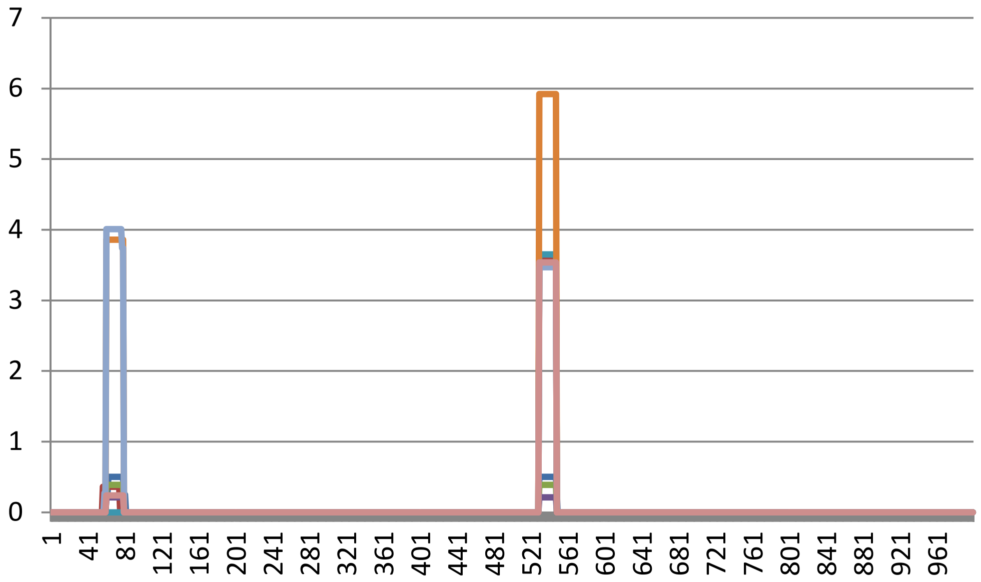

7
6
5
4
3
2
1
0
1
41
81
121
161
201
241
281
321
361
401
441
481
521
561
601
641
681
721
761
801
841
881
921
961

AT5G42620

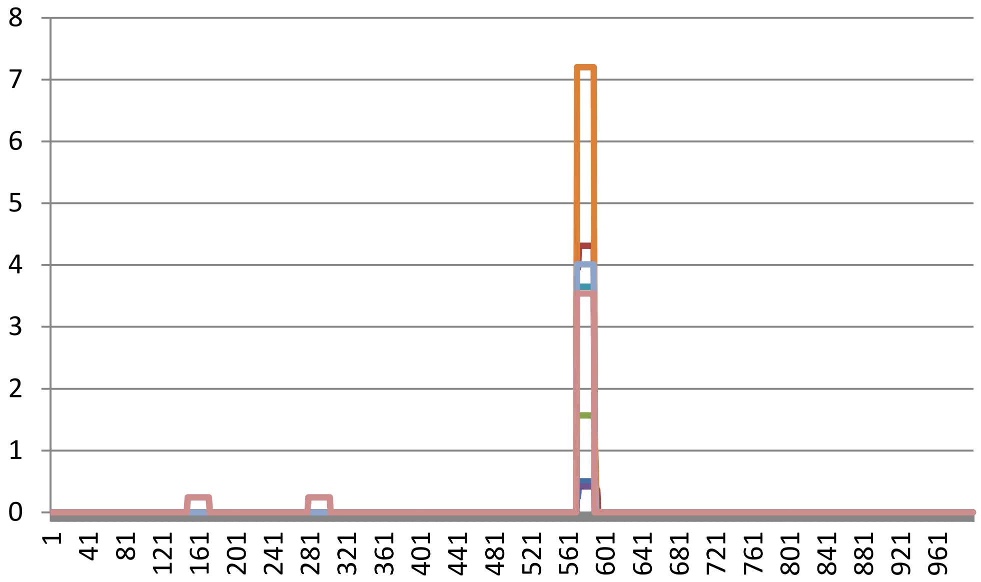

8
7
6
5
4
3
2
1
0
1
41
81
121
161
201
241
281
321
361
401
441
481
521
561
601
641
681
721
761
801
841
881
921
961

AT5G42970

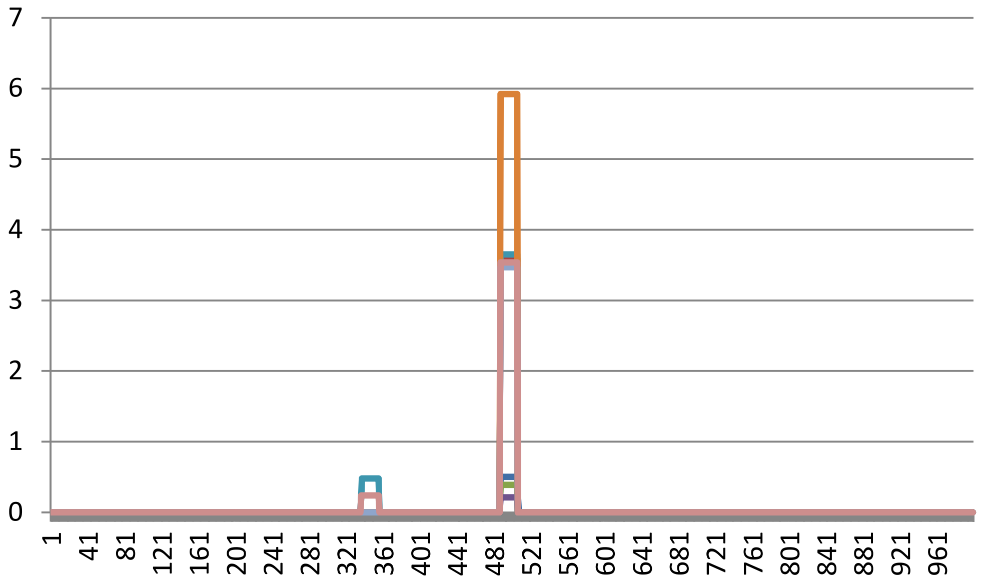

7
6
5
4
3
2
1
0
1
41
81
121
161
201
241
281
321
361
401
441
481
521
561
601
641
681
721
761
801
841
881
921
961

AT5G45428_root

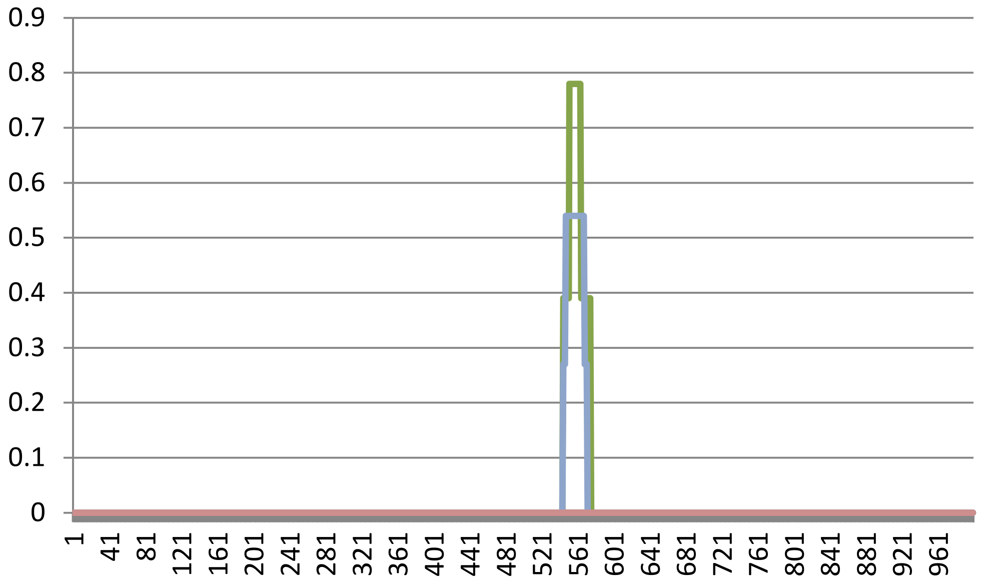

AT5G45430_root

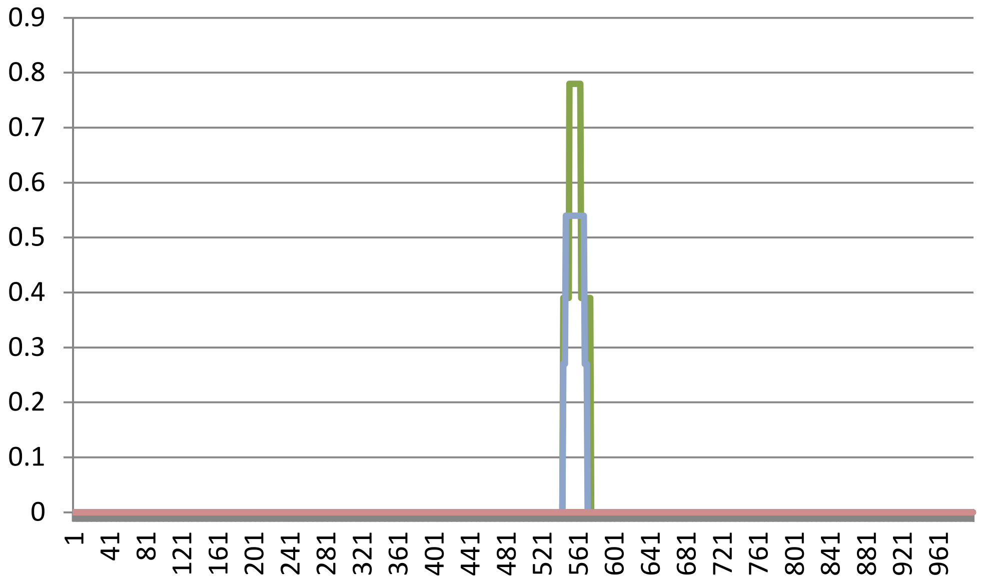

AT5G46930

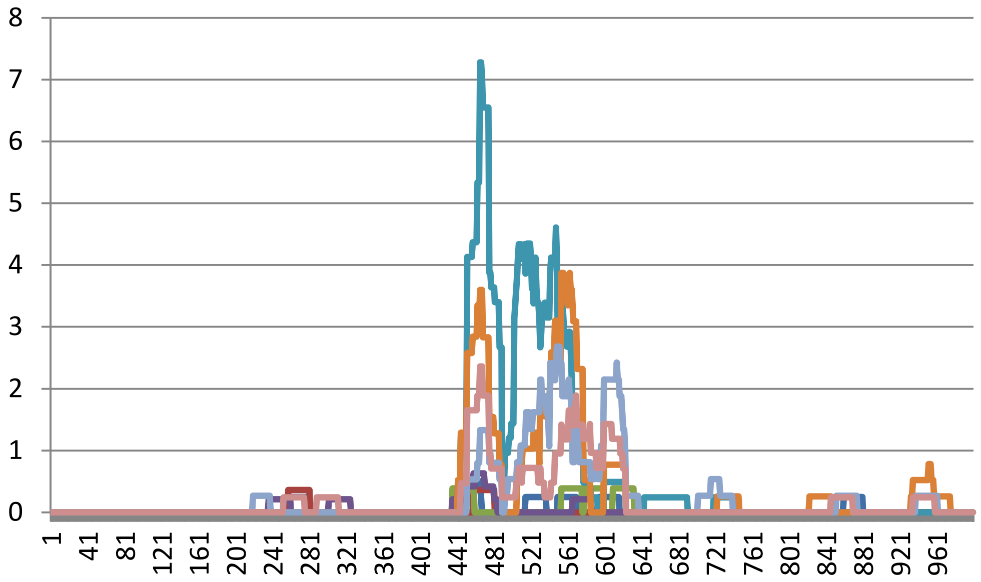

8
7
6
5
4
3
2
1
0
1
41
81
121
161
201
241
281
321
361
401
441
481
521
561
601
641
681
721
761
801
841
881
921
961

AT5G47880

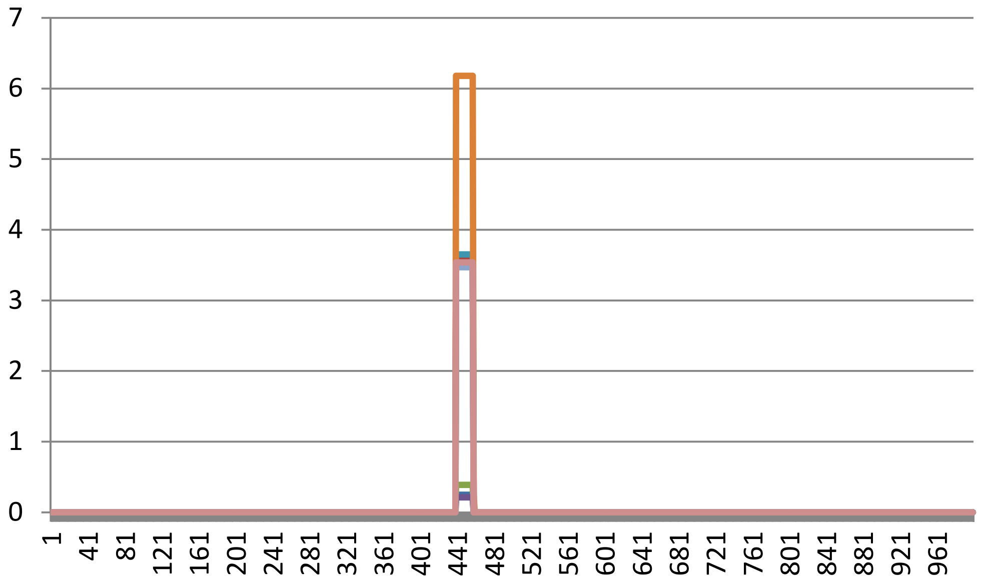

7
6
5
4
3
2
1
0
1
41
81
121
161
201
241
281
321
361
401
441
481
521
561
601
641
681
721
761
801
841
881
921
961

AT5G48280_AGO4 root

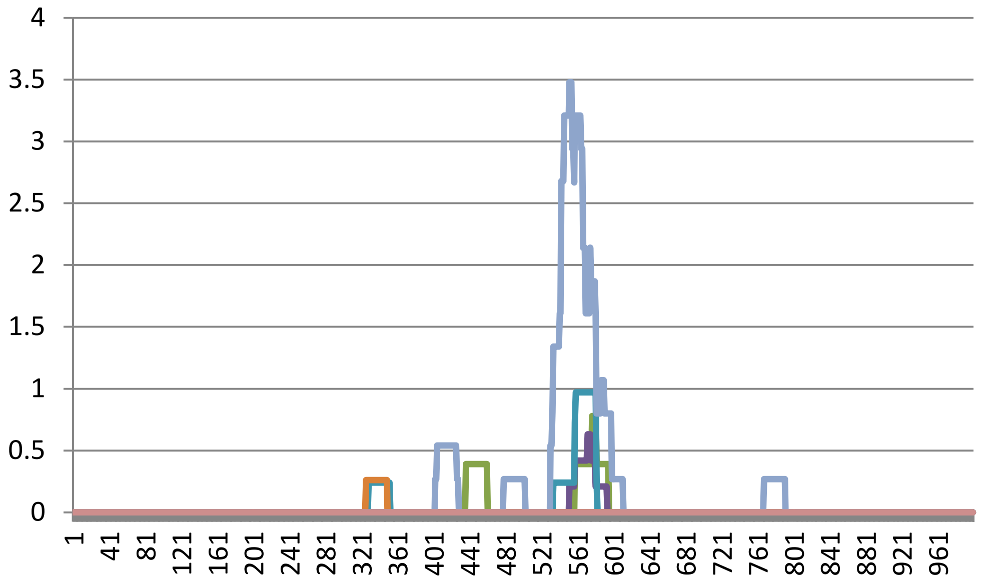

AT5G49240

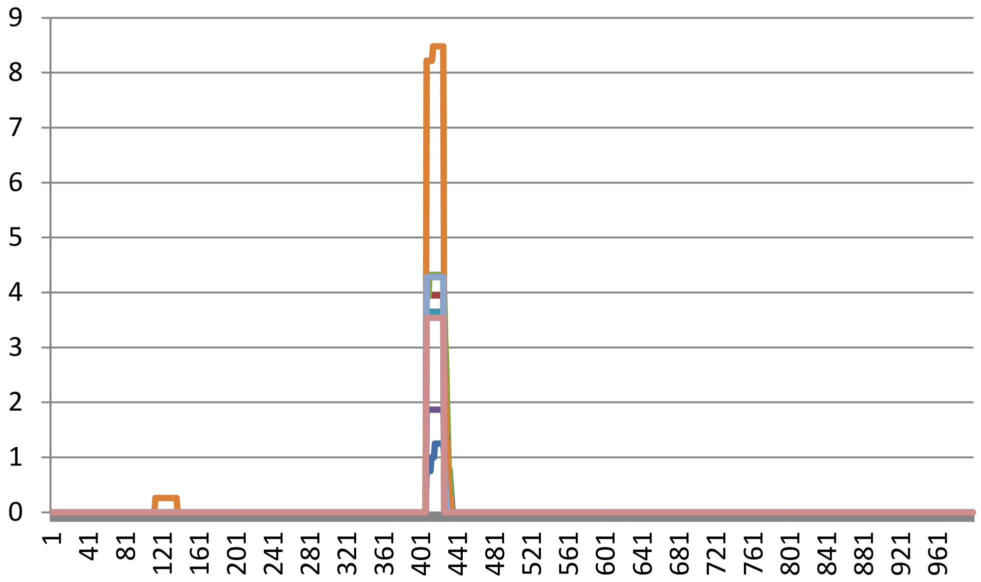

9
8
7
6
5
4
3
2
1
0
1
41
81
121
161
201
241
281
321
361
401
441
481
521
561
601
641
681
721
761
801
841
881
921
961

AT5G61420

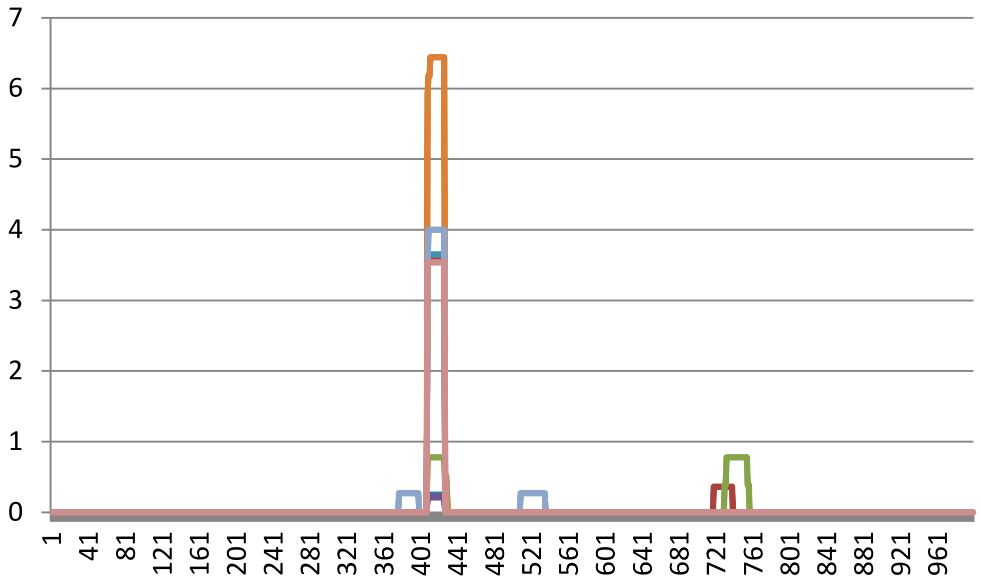
7
6
5
4
3
2
1
0
1
41
81
121
161
201
241
281
321
361
401
441
481
521
561
601
641
681
721
761
801
841
881
921
961

AT5G65050

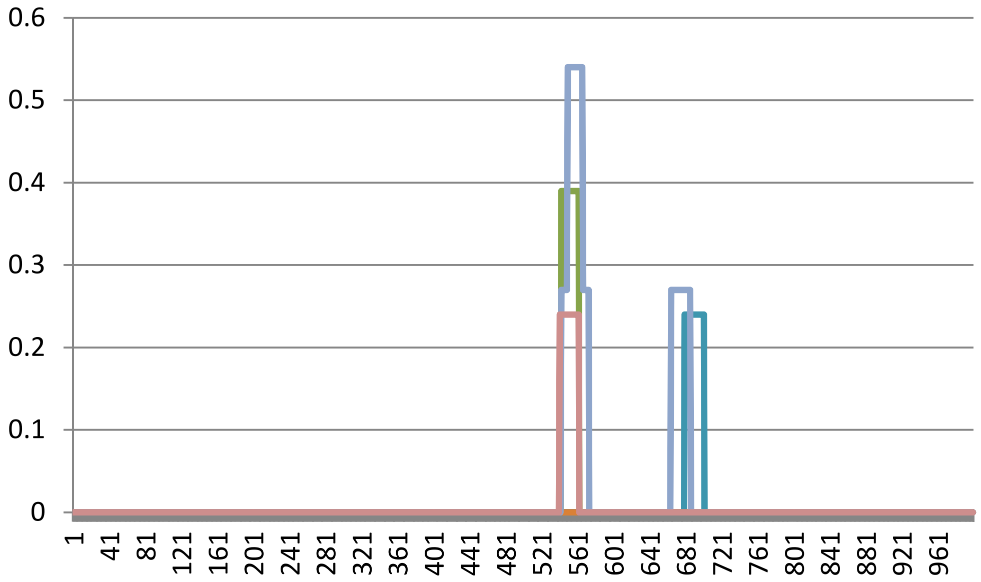

0.6
0.5
0.4
0.3
0.2
0.1
0
1
41
81
121
161
201
241
281
321
361
401
441
481
521
561
601
641
681
721
761
801
841
881
921
961

ATCG00050_AGO1

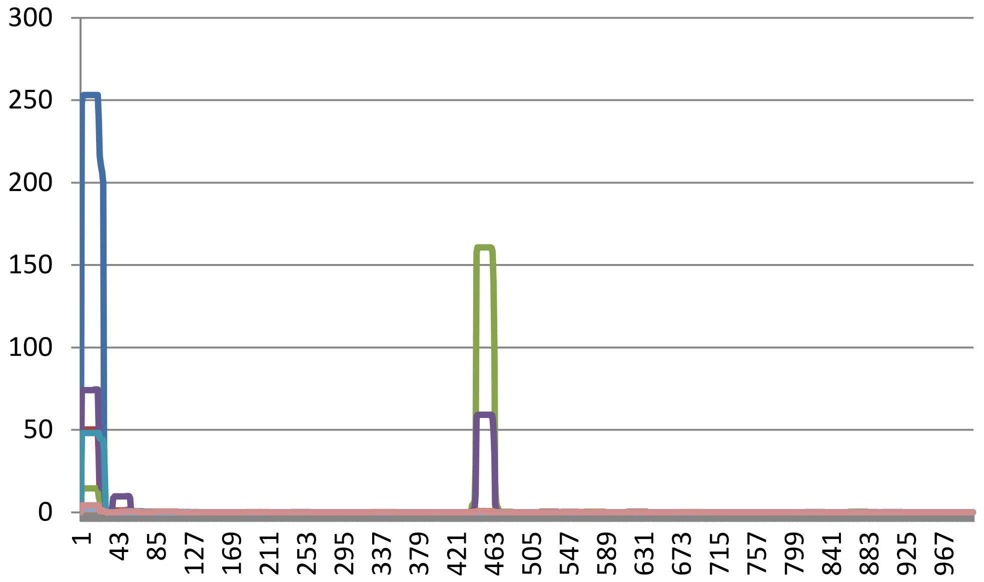

ATCG00065_AGO1

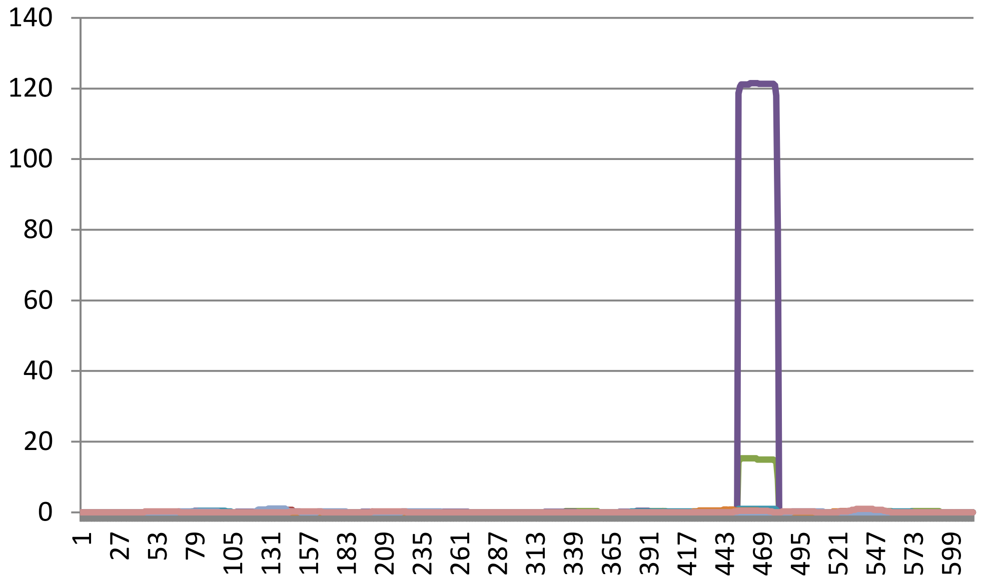

ATCG00120

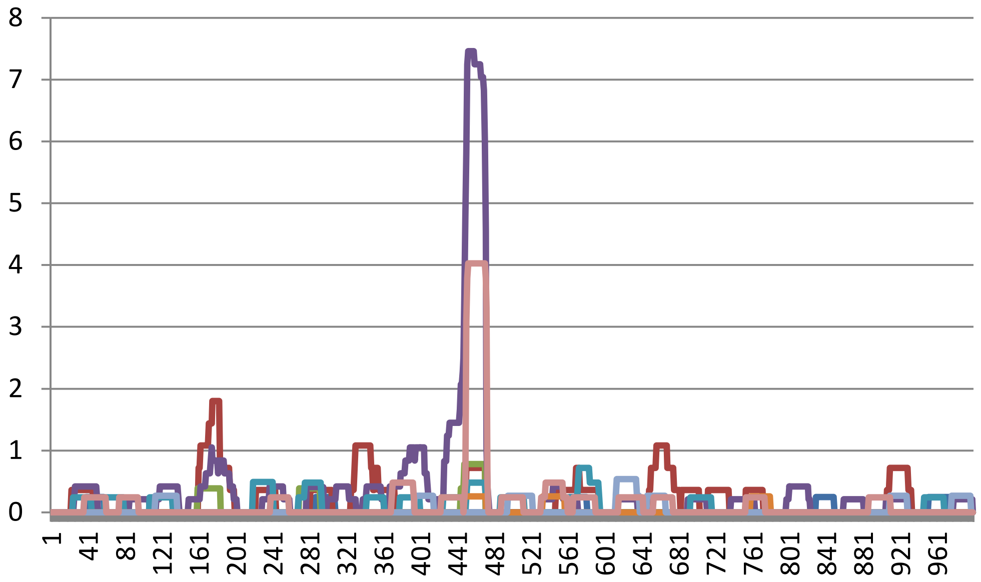

8
7
6
5
4
3
2
1
0
1
41
81
121
161
201
241
281
321
361
401
441
481
521
561
601
641
681
721
761
801
841
881
921
961

ATCG00140

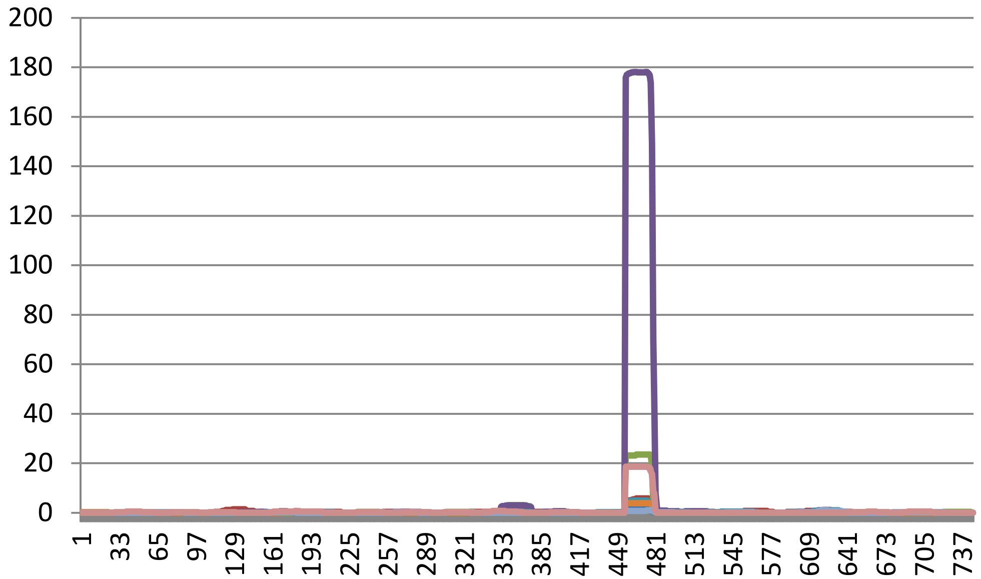

200
180
160
140
120
100
80
60
40
20
0
1
33
65
97
129
161
193
225
257
289
321
353
385
417
449
481
513
545
577
609
641
673
705
737

ATCG00150

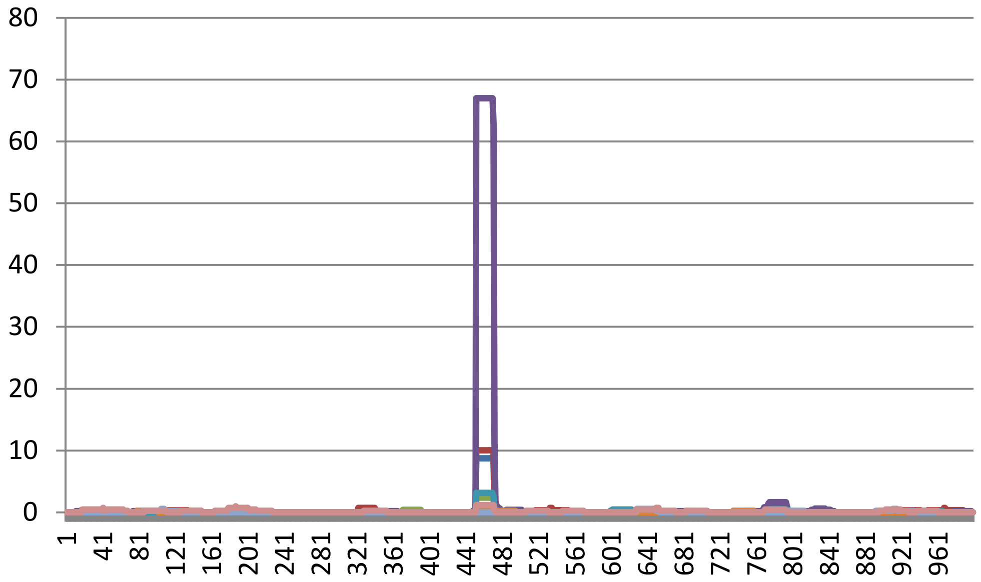

80
70
60
50
40
30
20
10
0
1
41
81
121
161
201
241
281
321
361
401
441
481
521
561
601
641
681
721
761
801
841
881
921
961

ATCG00170

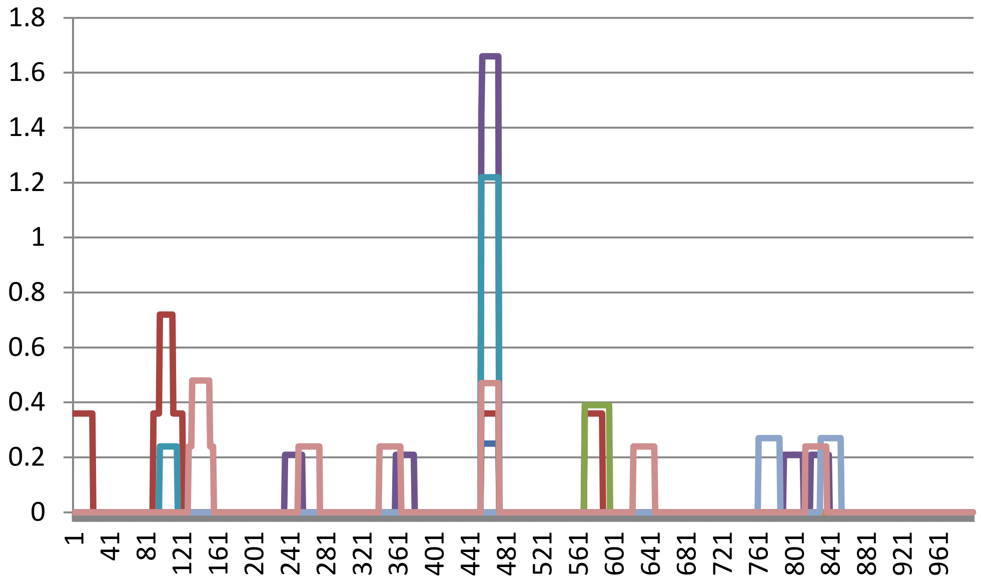
1.8
1.6
1.4
1.2
1
0.8
0.6
0.4
0.2
0
1
41
81
121
161
201
241
281
321
361
401
441
481
521
561
601
641
681
721
761
801
841
881
921
961

ATCG00280_AGO1 seedling

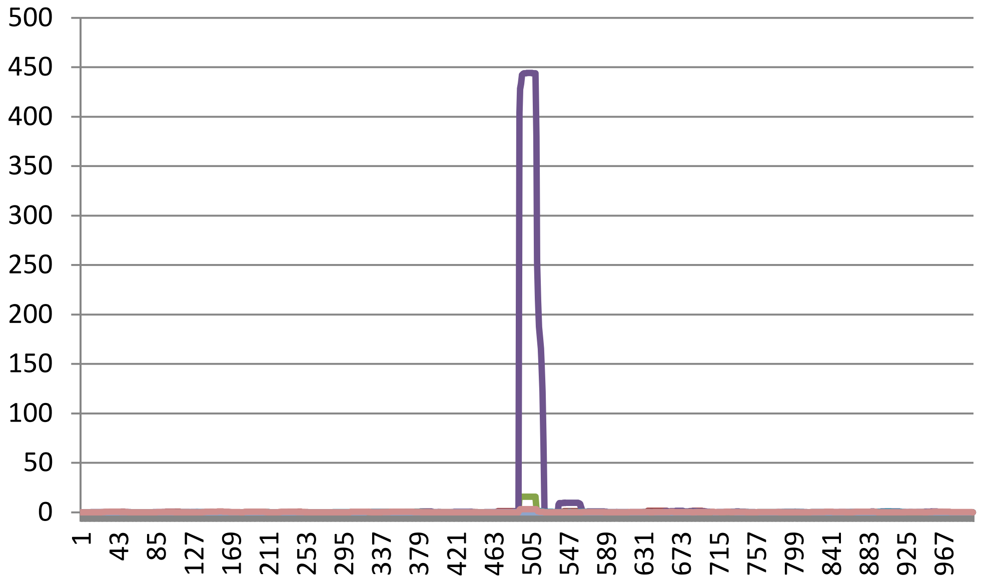

ATCG00500_AGO1

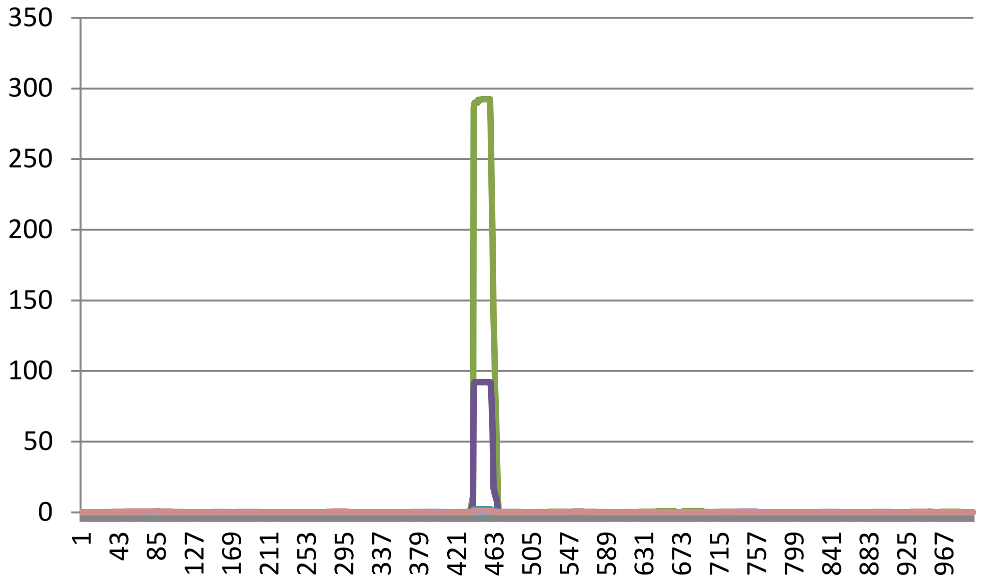

ATCG00540

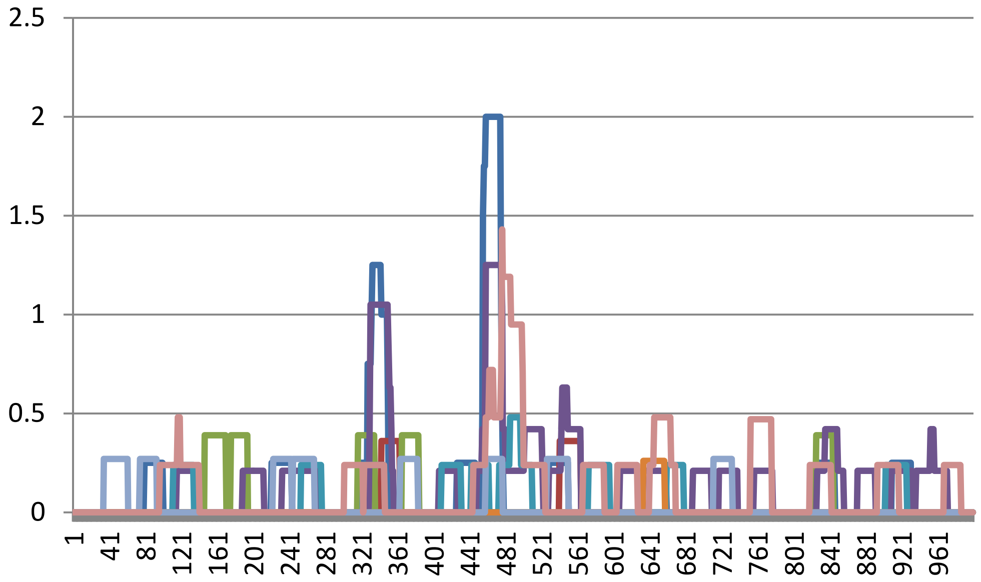

2.5
2
1.5
1
0.5
0
1
41
81
121
161
201
241
281
321
361
401
441
481
521
561
601
641
681
721
761
801
841
881
921
961

ATCG00590

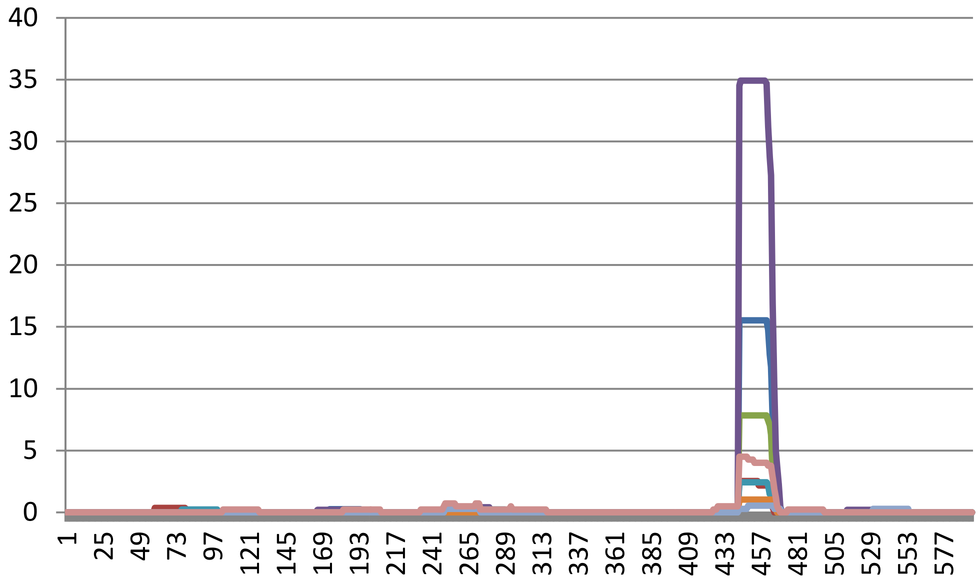
40
35
30
25
20
15
10
5
0
1
25
49
73
97
121
145
169
193
217
241
265
289
313
337
361
385
409
433
457
481
505
529
553
577

ATCG00670

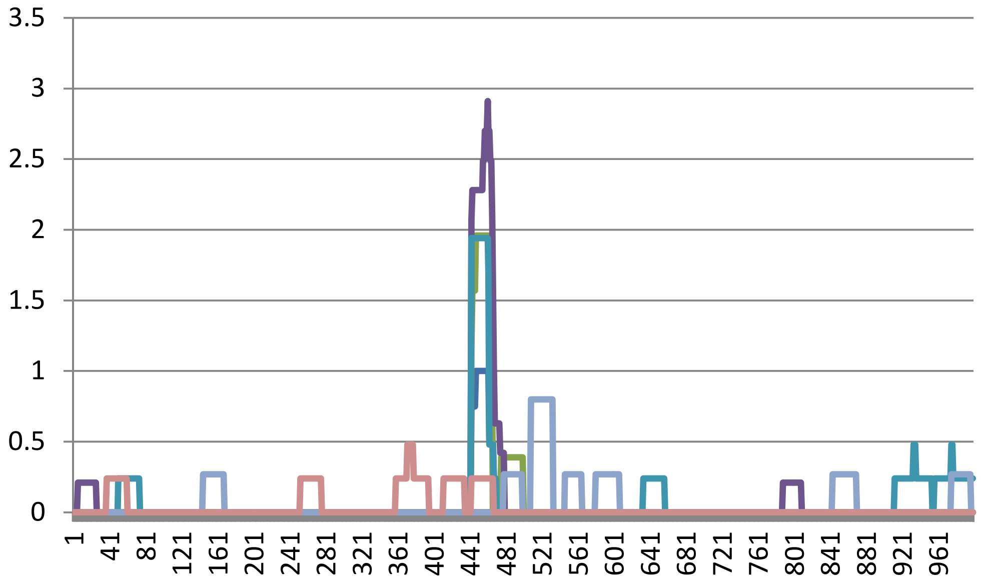

3.5
3
2.5
2
1.5
1
0.5
0
1
41
81
121
161
201
241
281
321
361
401
441
481
521
561
601
641
681
721
761
801
841
881
921
961

ATCG00720

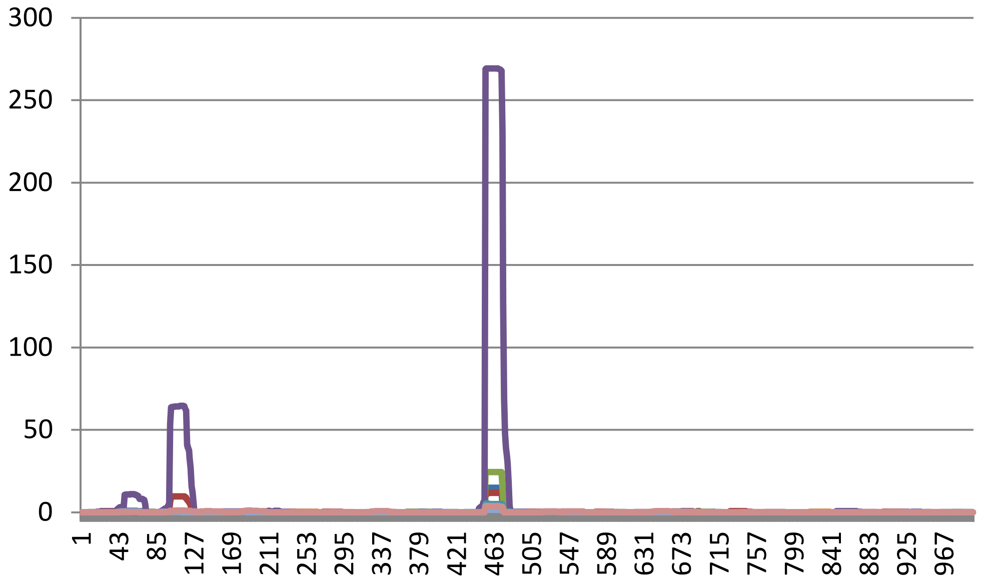
300
250
200
150
100
50
0
1
43
85
127
169
211
253
295
337
379
421
463
505
547
589
631
673
715
757
799
841
883
925
967

ATCG00830

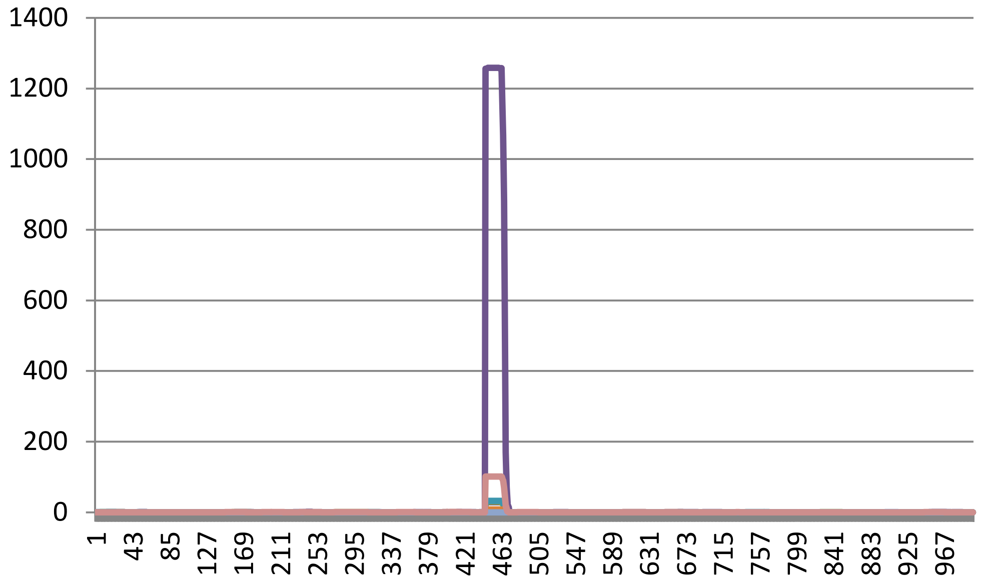

1400
1200
1000
800
600
400
200
0
1
43
85
127
169
211
253
295
337
379
421
463
505
547
589
631
673
715
757
799
841
883
925
967

ATCG00870

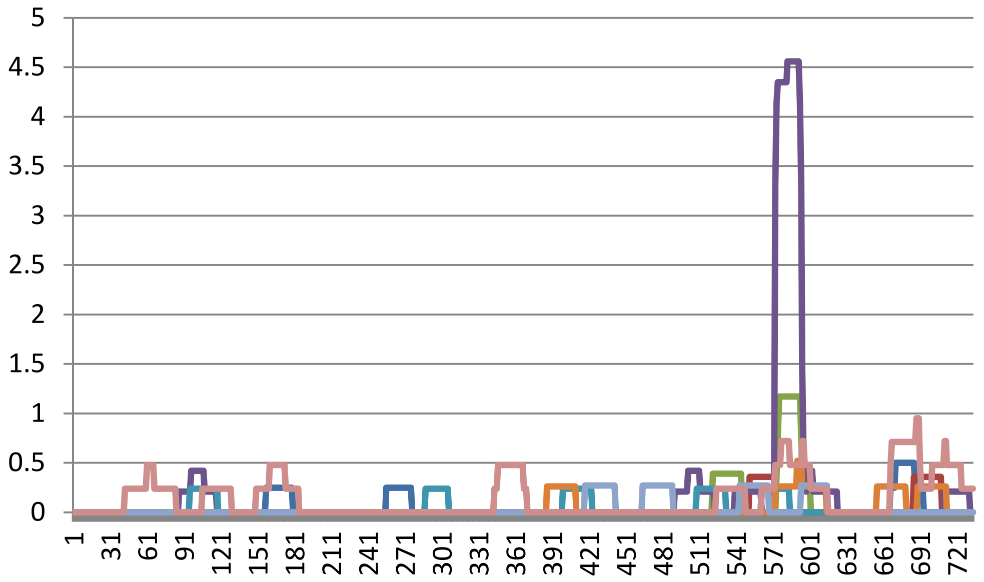

5
4.5
4
3.5
3
2.5
2
1.5
1
0.5
0
1
31
61
91
121
151
181
211
241
271
301
331
361
391
421
451
481
511
541
571
601
631
661
691
721

ATCG01100

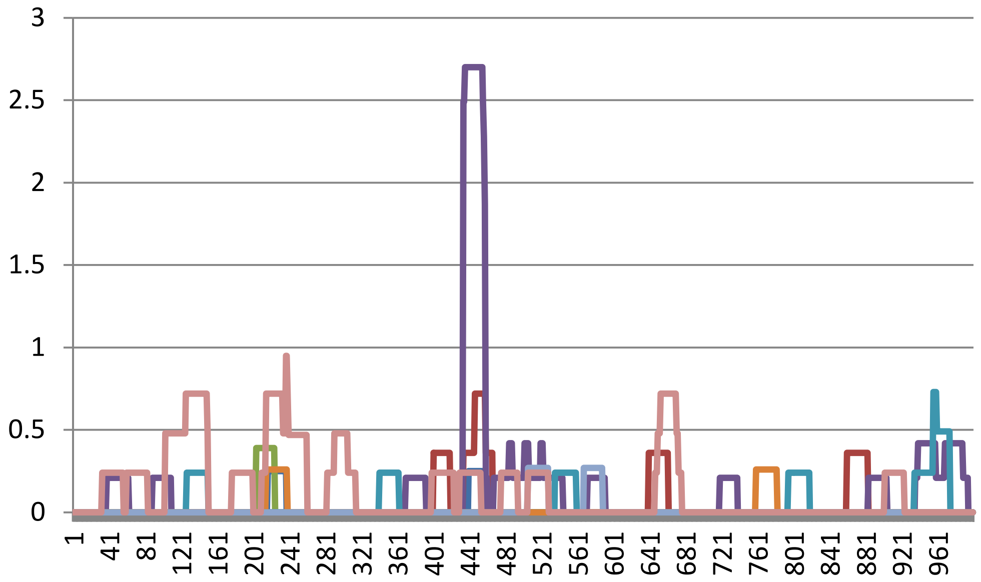

3
2.5
2
1.5
1
0.5
0
1
41
81
121
161
201
241
281
321
361
401
441
481
521
561
601
641
681
721
761
801
841
881
921
961

ATCG01270

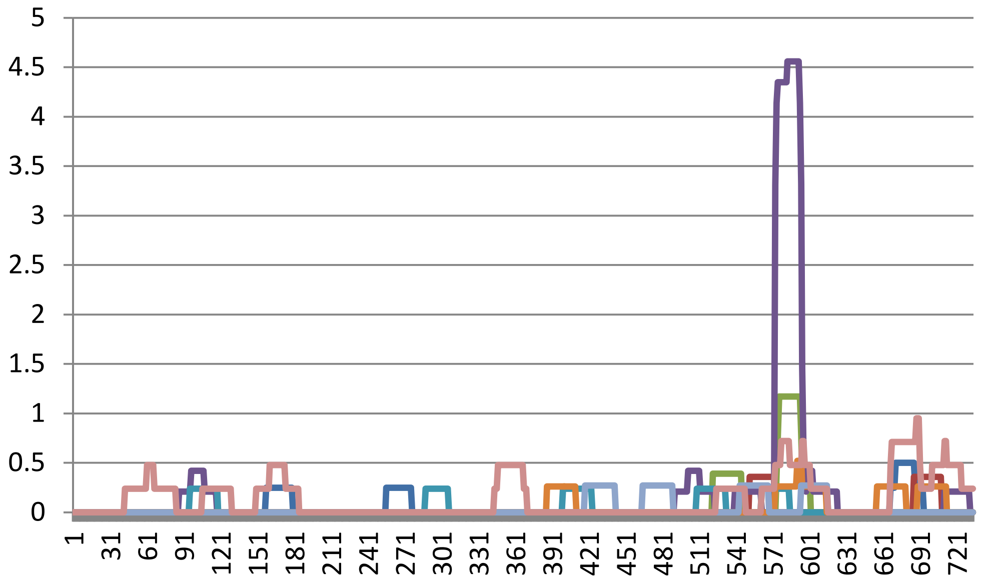

5
4.5
4
3.5
3
2.5
2
1.5
1
0.5
0
1
31
61
91
121
151
181
211
241
271
301
331
361
391
421
451
481
511
541
571
601
631
661
691
721

ATCG01310

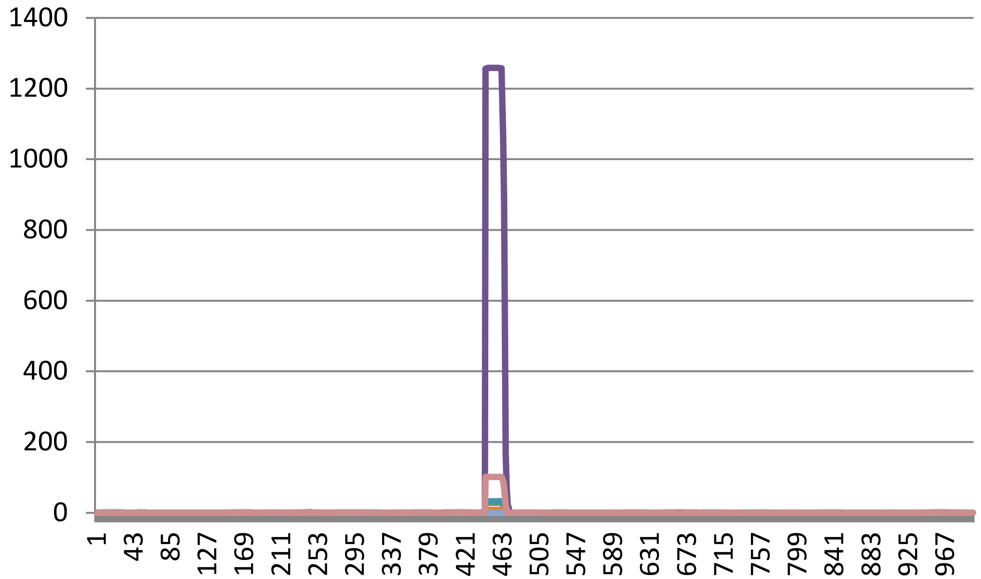

1400
1200
1000
800
600
400
200
0
1
43
85
127
169
211
253
295
337
379
421
463
505
547
589
631
673
715
757
799
841
883
925
967

ATMG00890

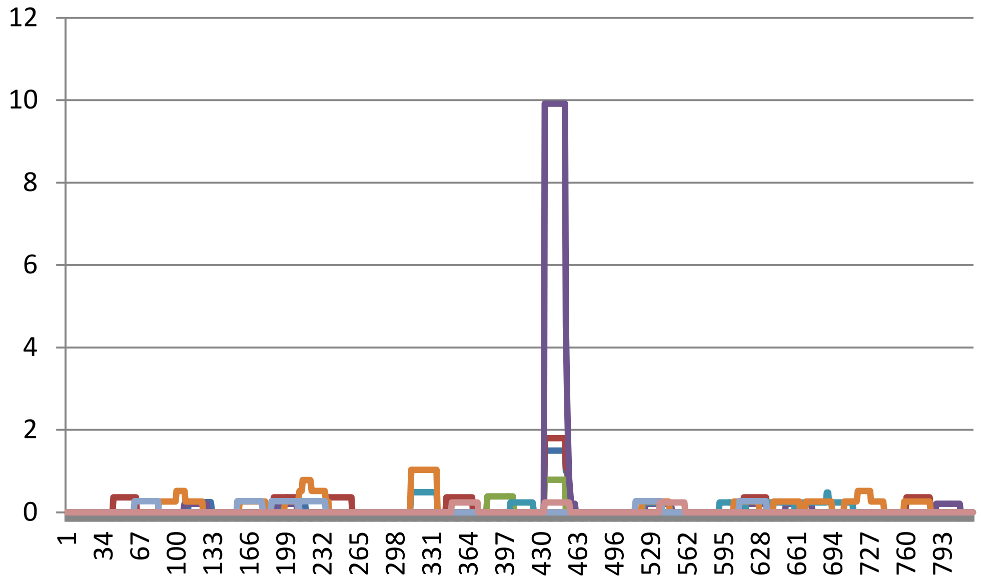

12
10
8
6
4
2
0
1
34
67
100
133
166
199
232
265
298
331
364
397
430
463
496
529
562
595
628
661
694
727
760
793

ATMG01350

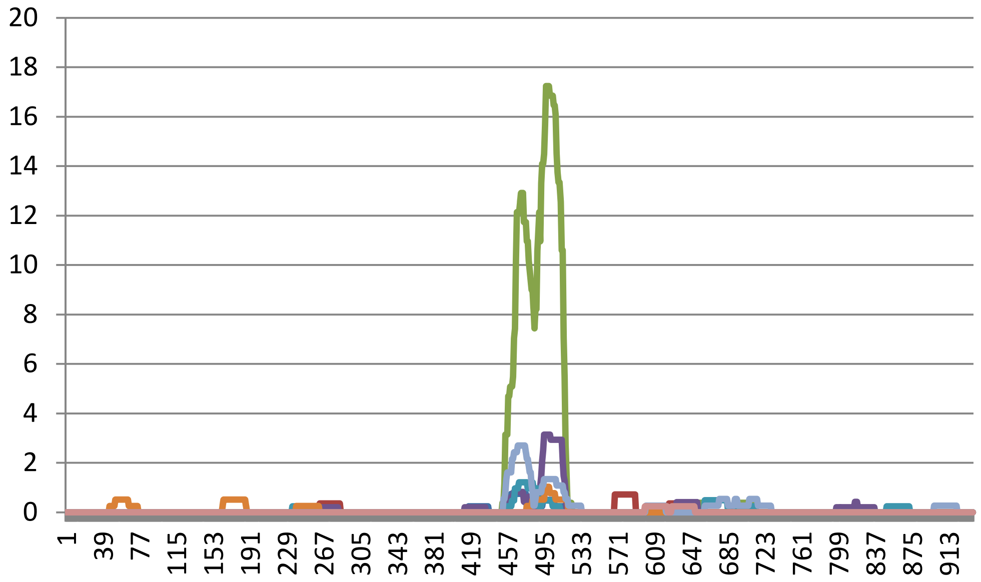

20
18
16
14
12
10
8
6
4
2
0
1
39
77
115
153
191
229
267
305
343
381
419
457
495
533
571
609
647
685
723
761
799
837
875
913
