# Supplementary material for: Transcriptome-Wide Discovery of PASRs (Promoter-Associated Small RNAs) and TASRs (Terminus-Associated Small RNAs) in Arabidopsis thaliana
Source: PLoS One. 2017 Jan 3;12(1):e0169212. doi: 10.1371/journal.pone.0169212 (PMC5207706; doi:10.1371/journal.pone.0169212)

Figure S8 AGO-associated PASR peaks identified on the antisense strands of the protein-coding genes of *Arabidopsis*. For each plot, x axis measures the position of the antisense strand, and y axis measures the abundance (in RPM, reads per million) of sRNAs.

AT1G01073RC

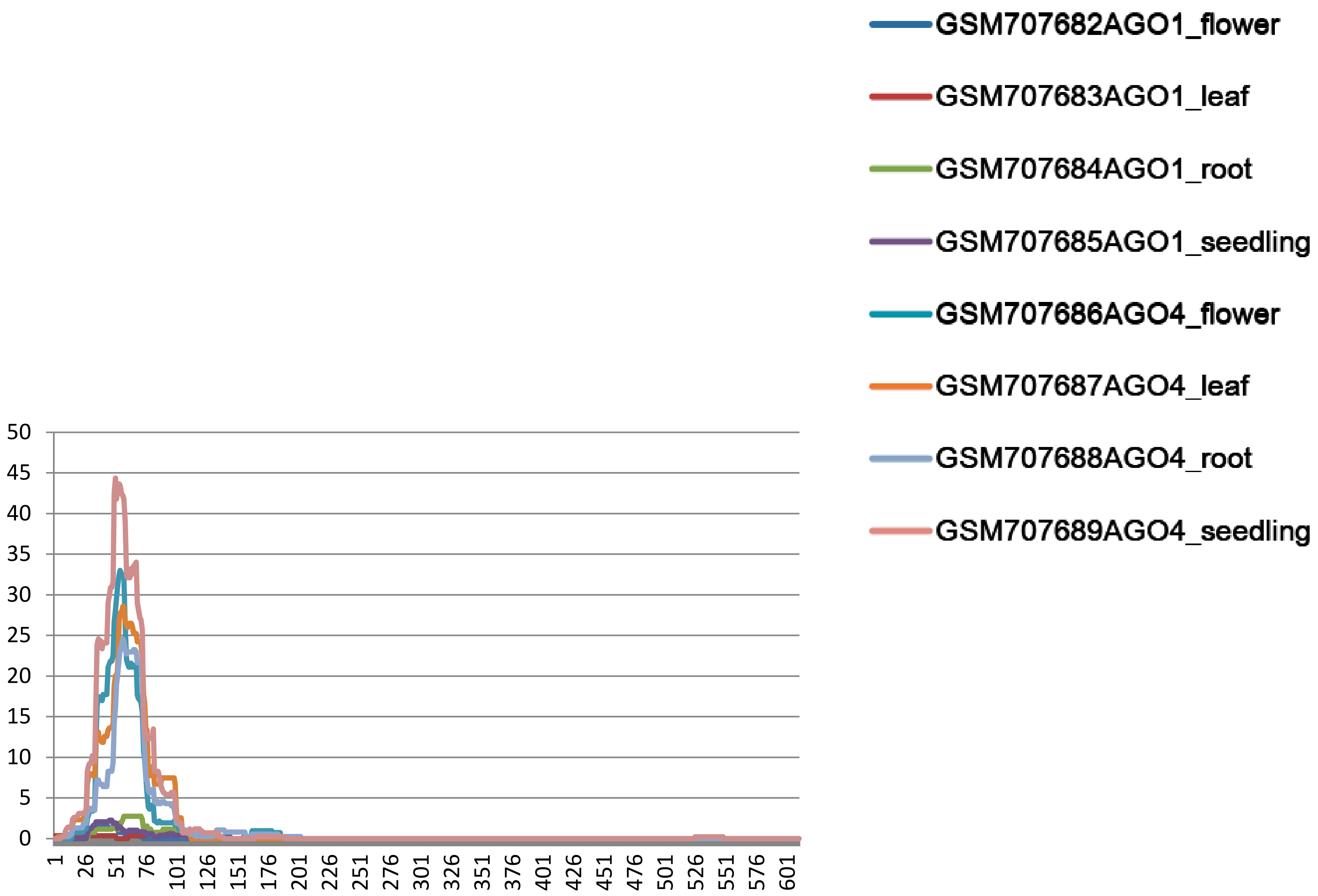

AT1G02475RC

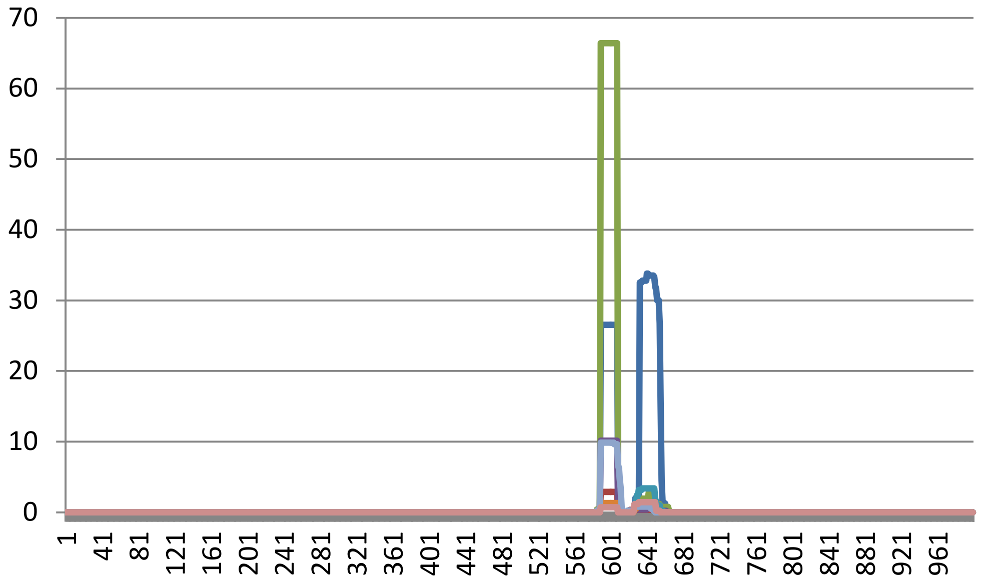

AT1G04310RC

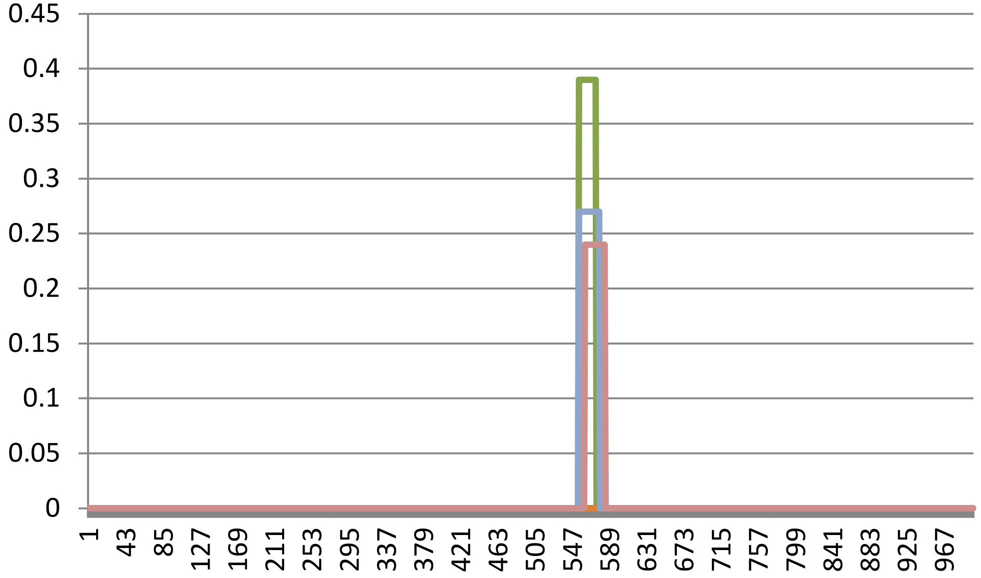

AT1G08940RC

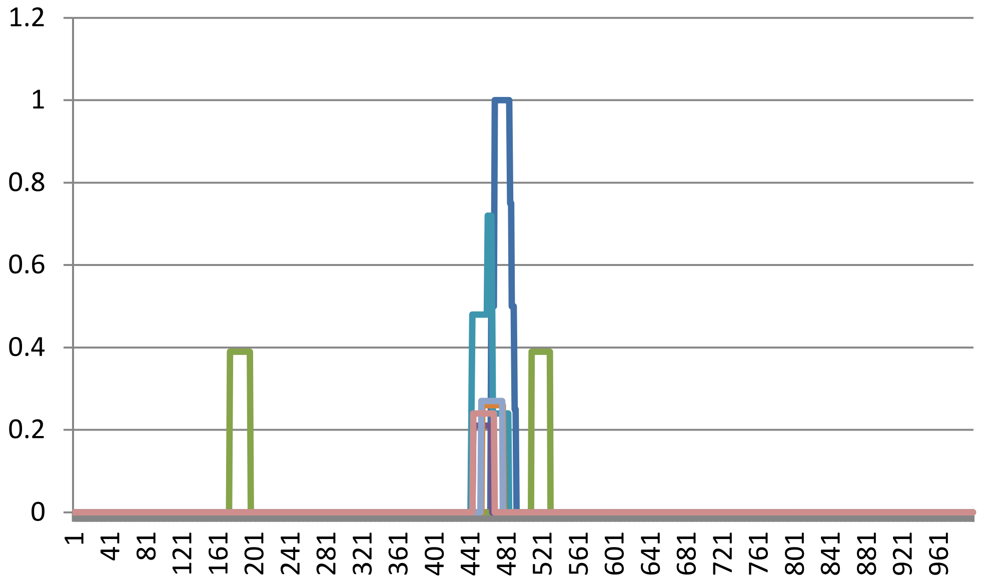

AT1G09290RC

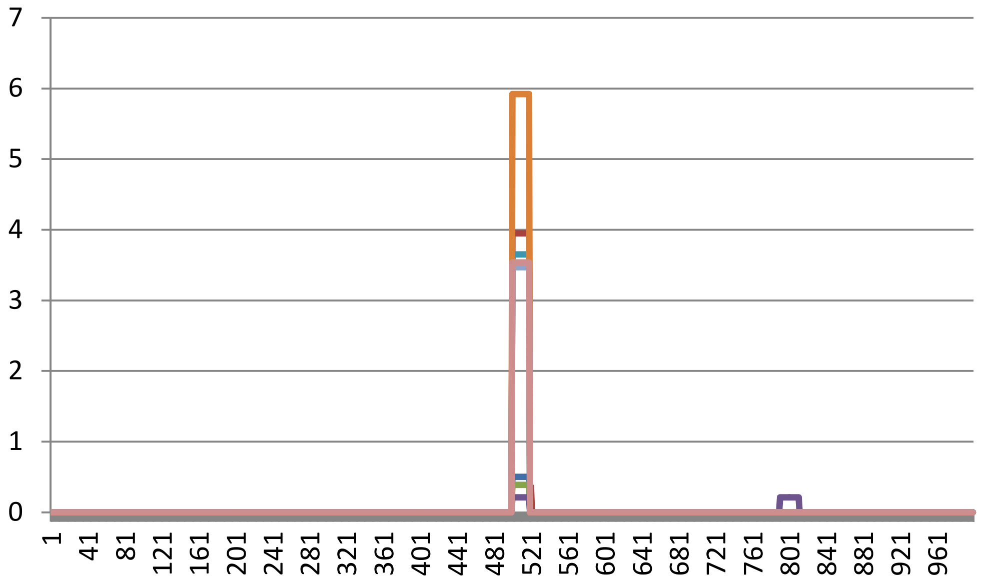

AT1G09910RC

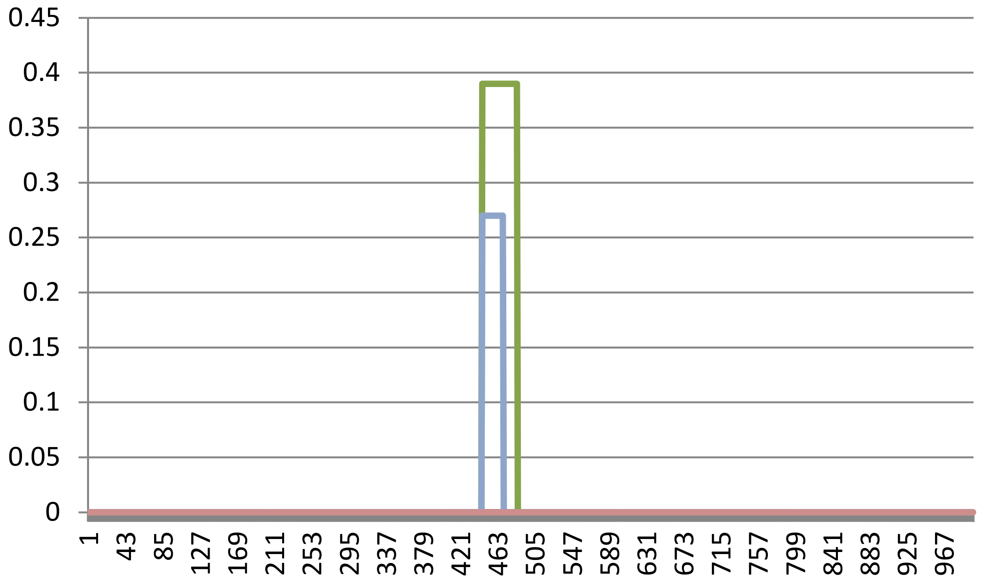

AT1G12870RC

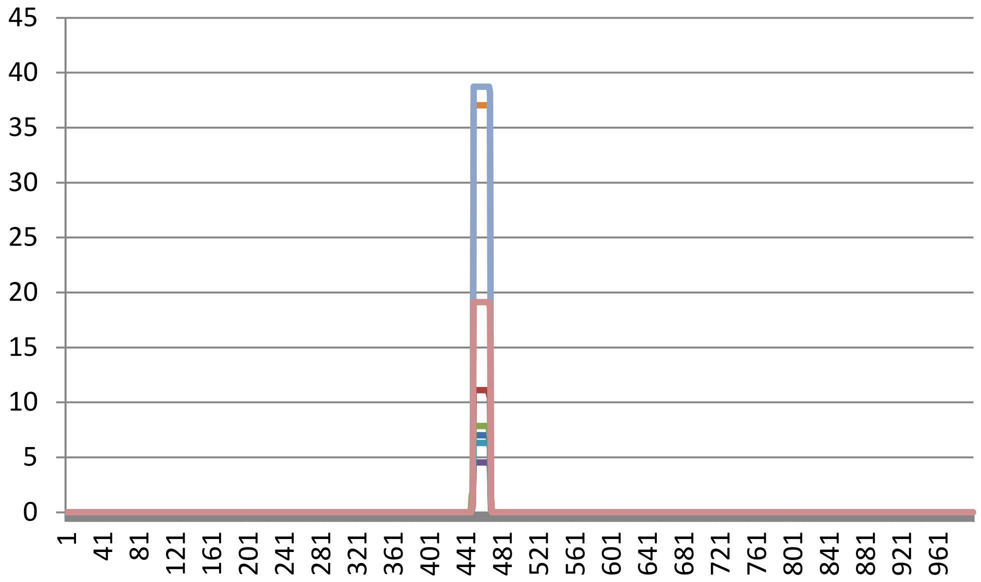

AT1G17680RC_root

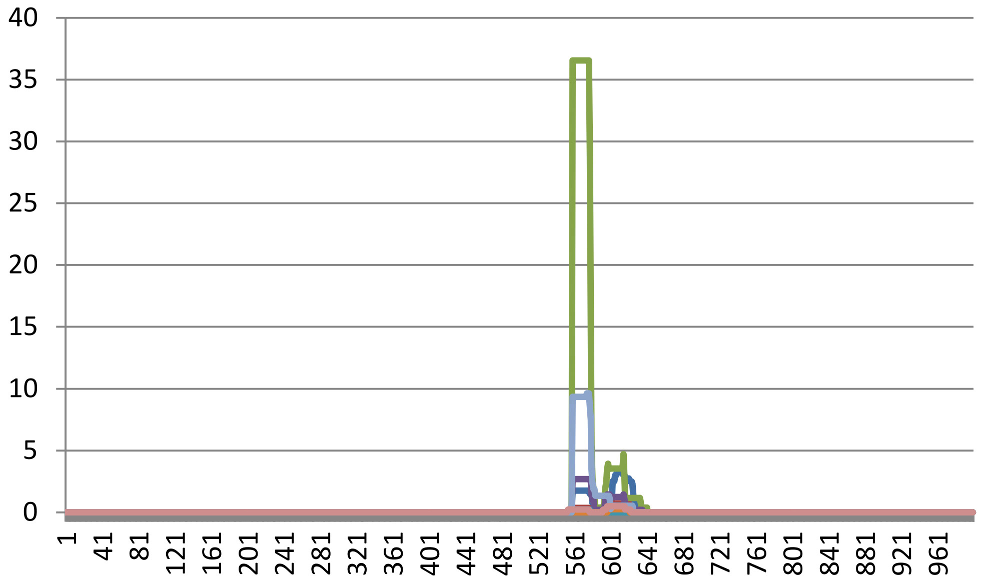

AT1G17830RC

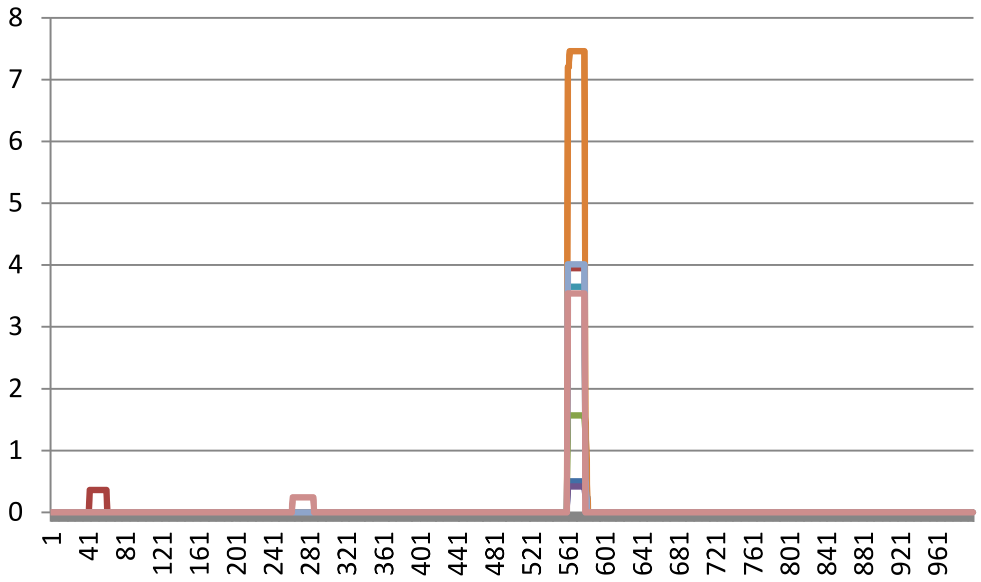

AT1G22067RC_AGO4 flower

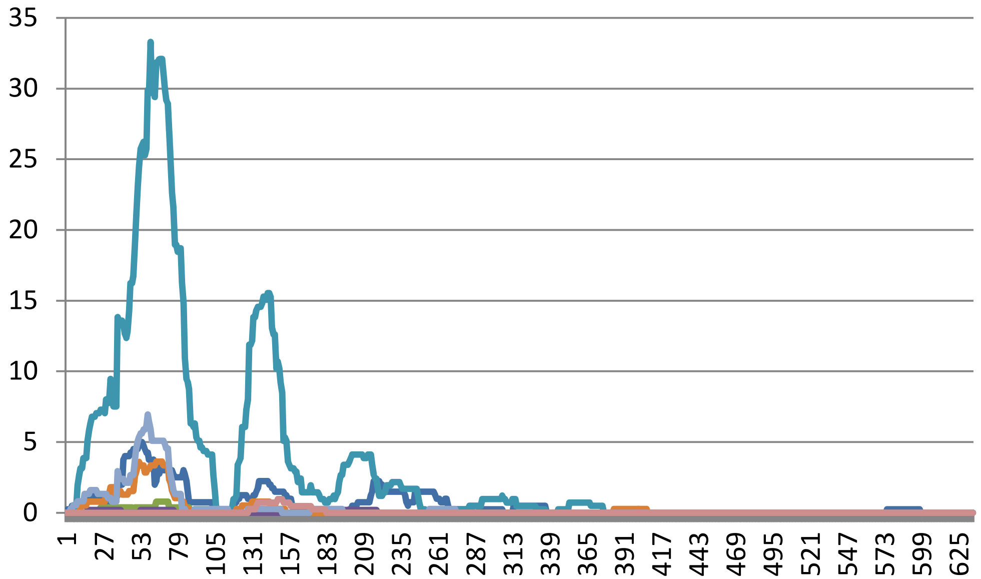

AT1G24625RC

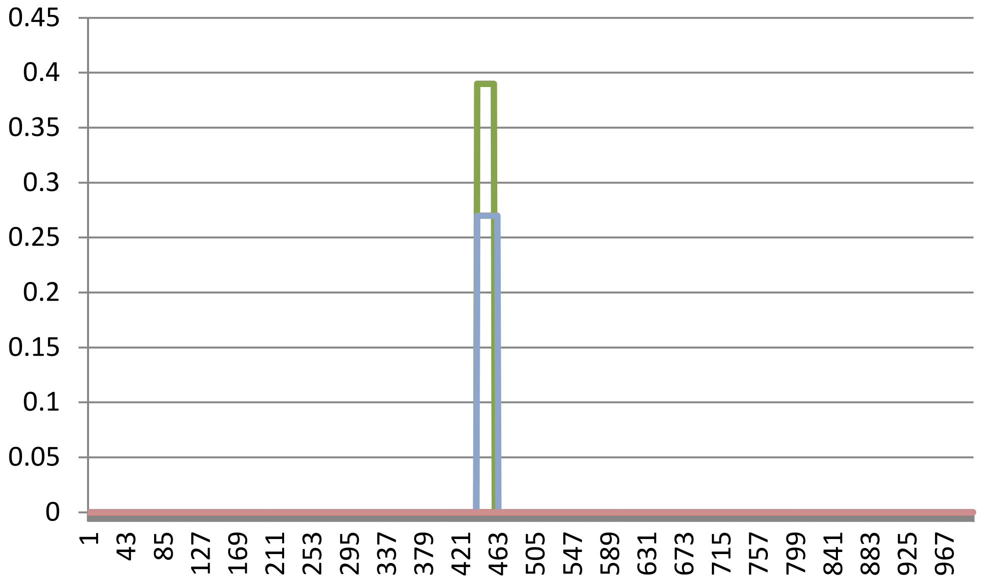

AT1G26110RC

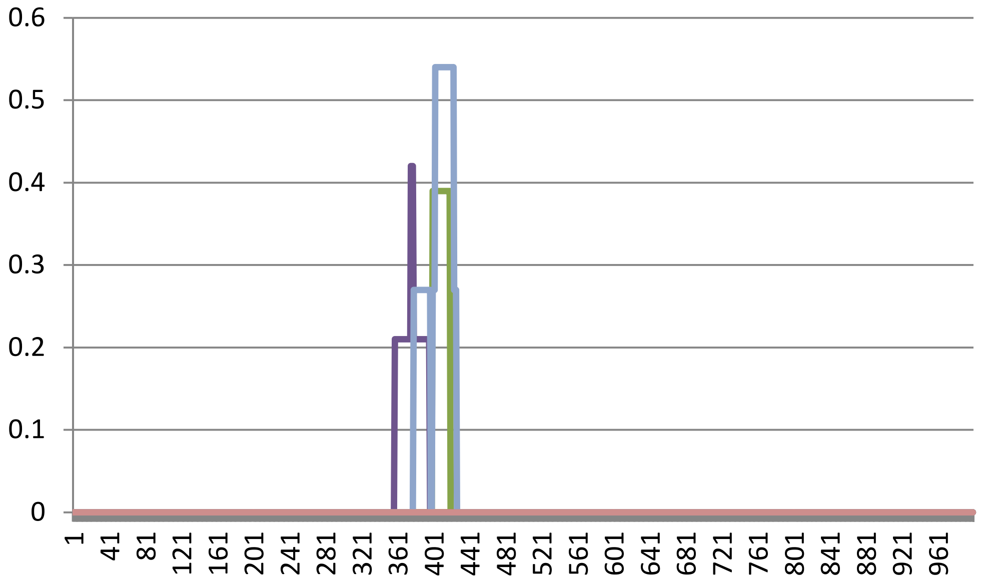

AT1G28290RC

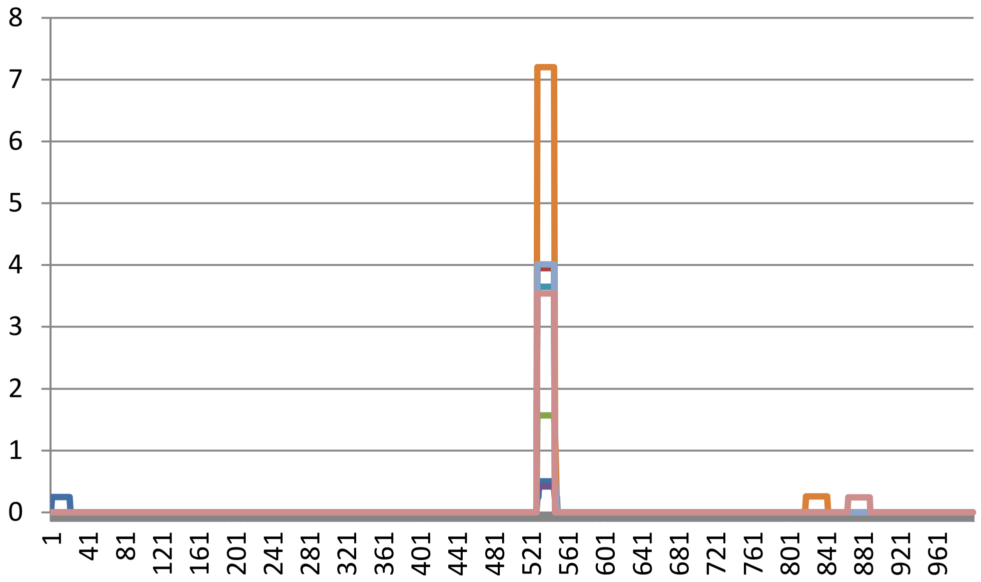

AT1G30370RC

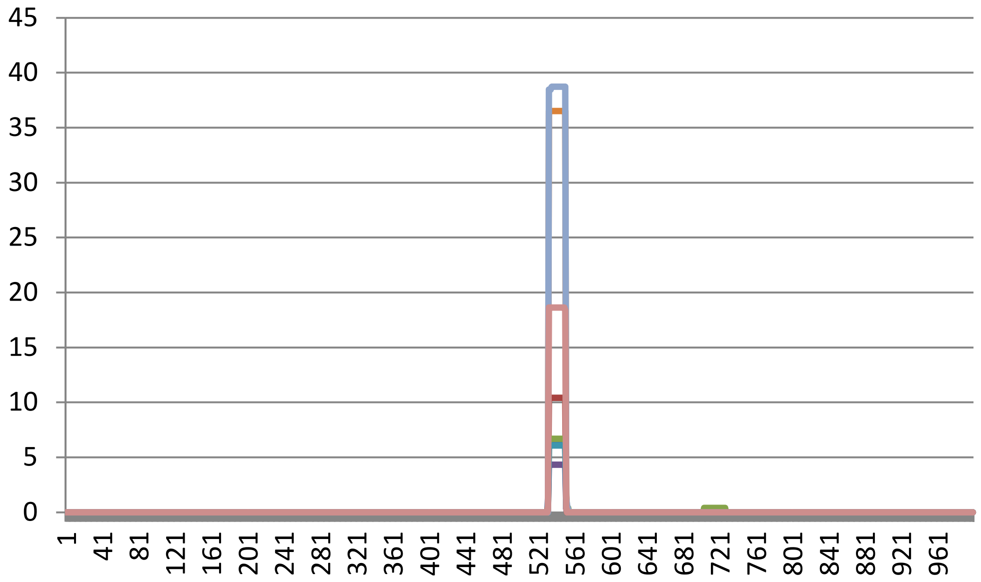

AT1G32630RC

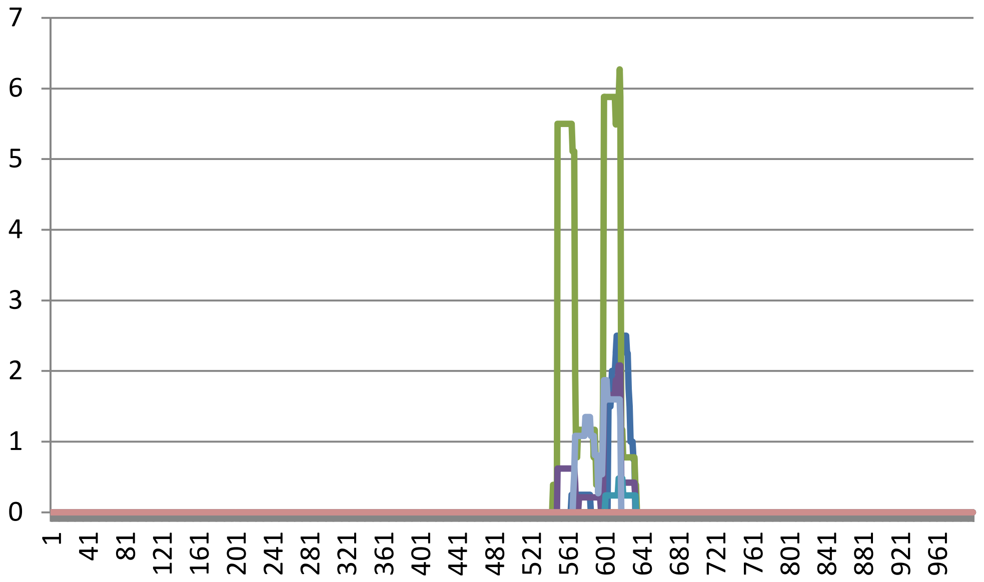

AT1G42540RC

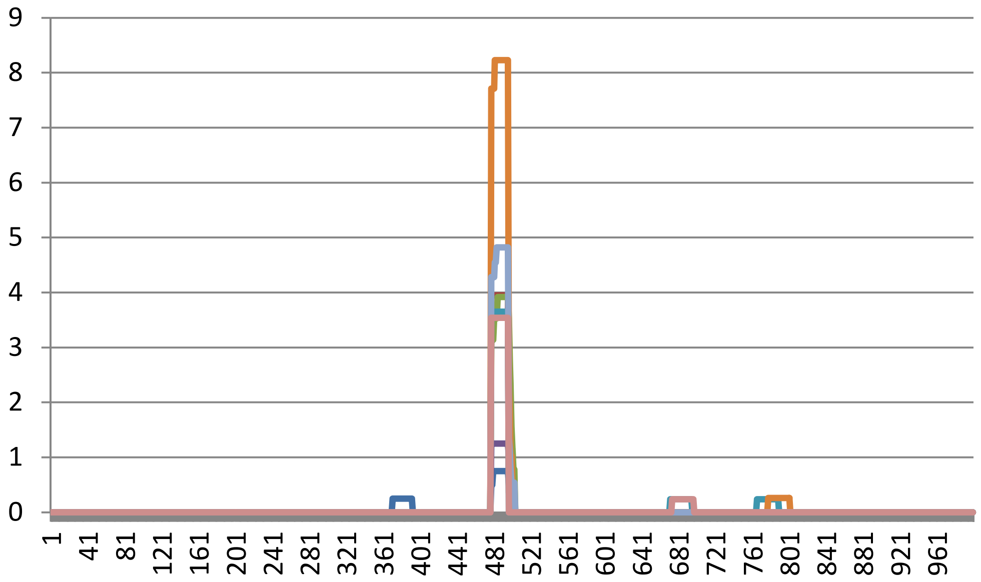

AT1G43624RC

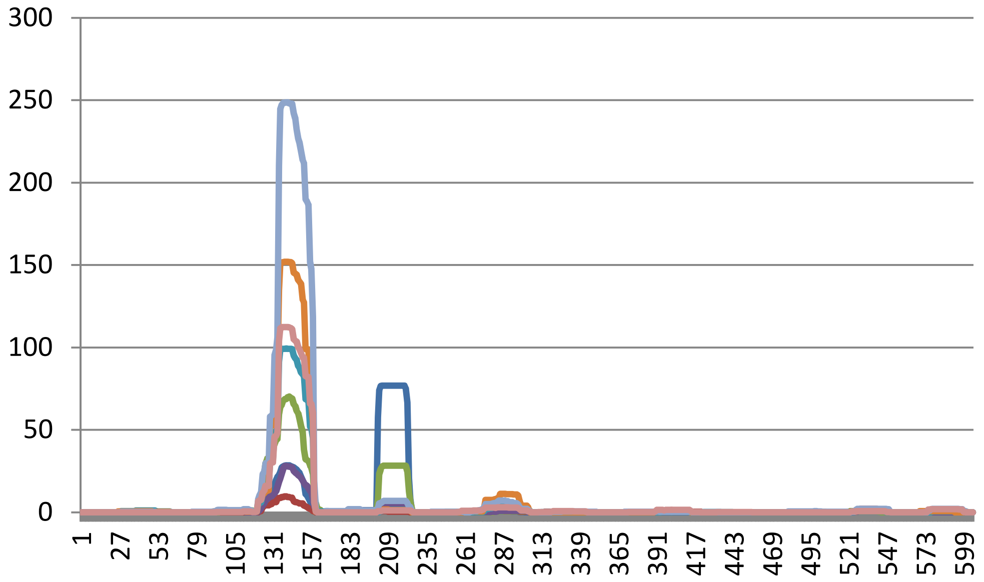

AT1G45233RC

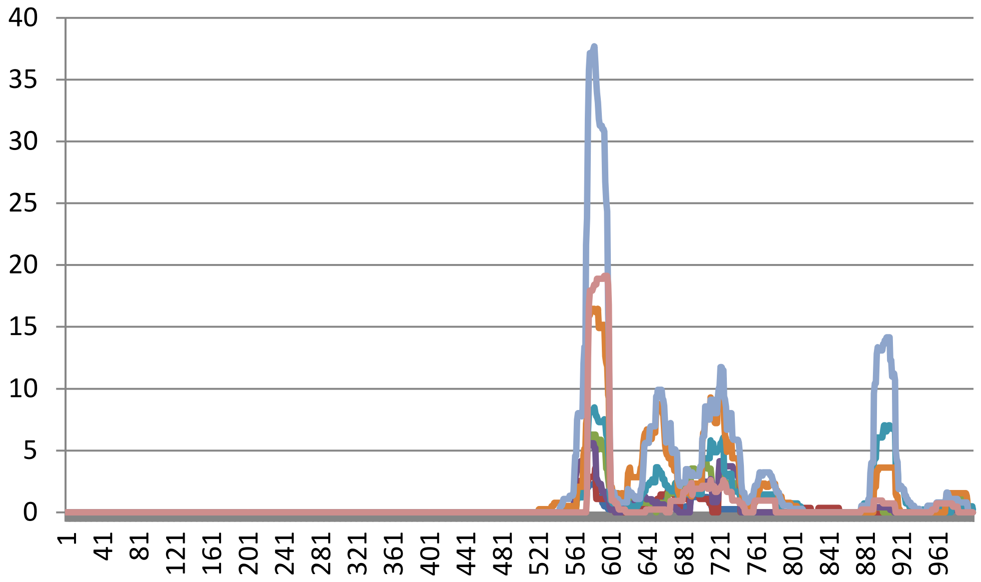

AT1G48598RC

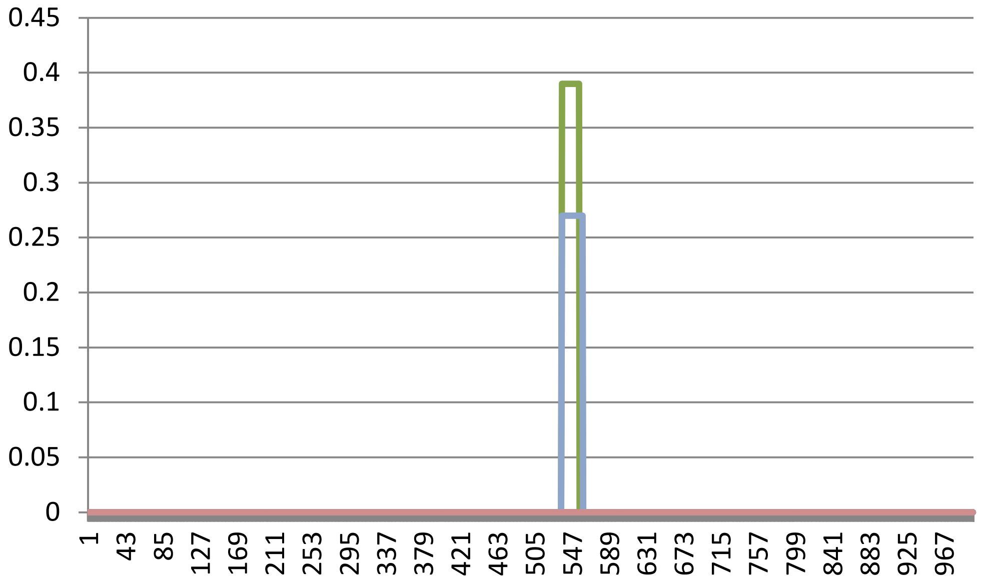

AT1G48600RC

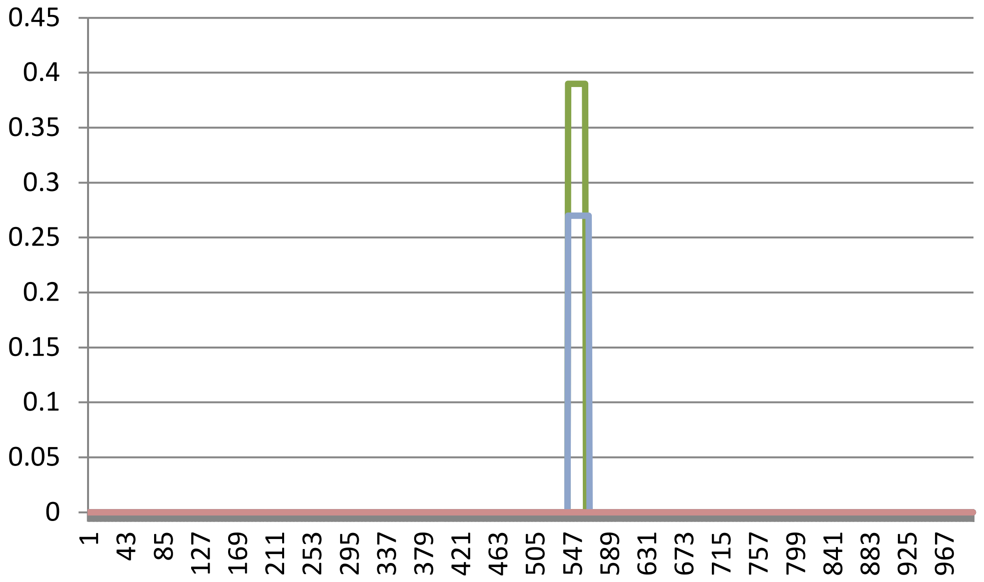

AT1G52618RC

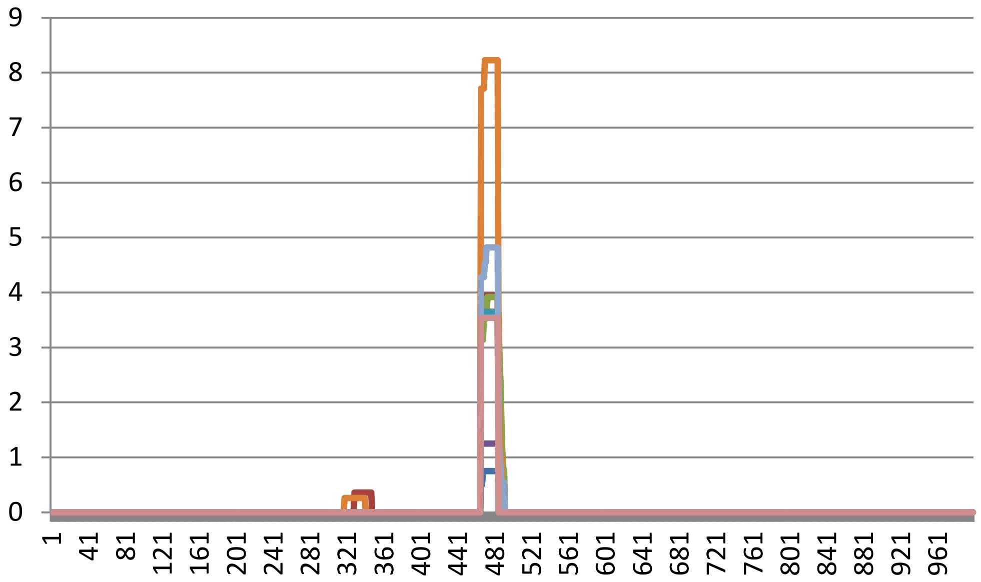

AT1G53541RC_AGO1 root

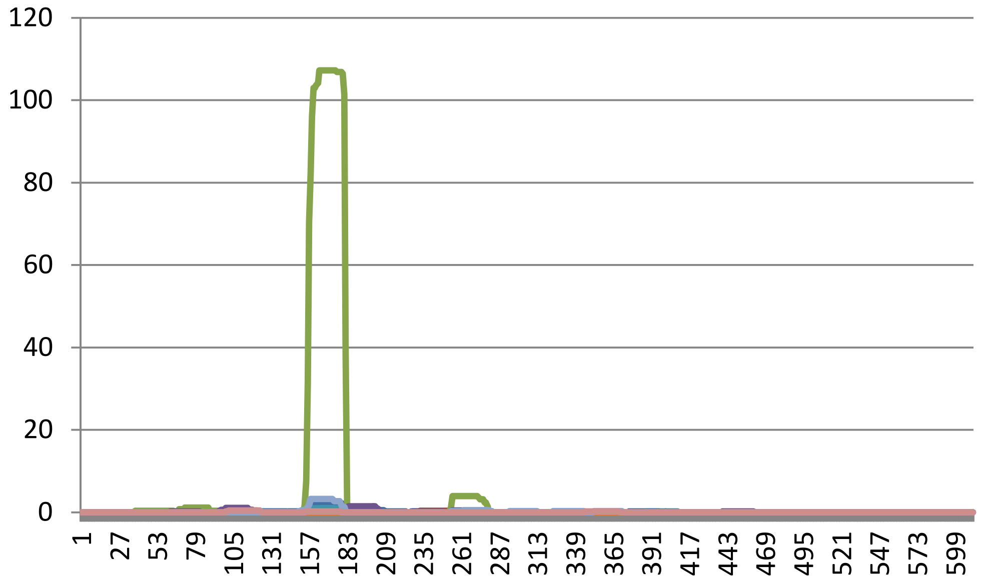

AT1G54030RC

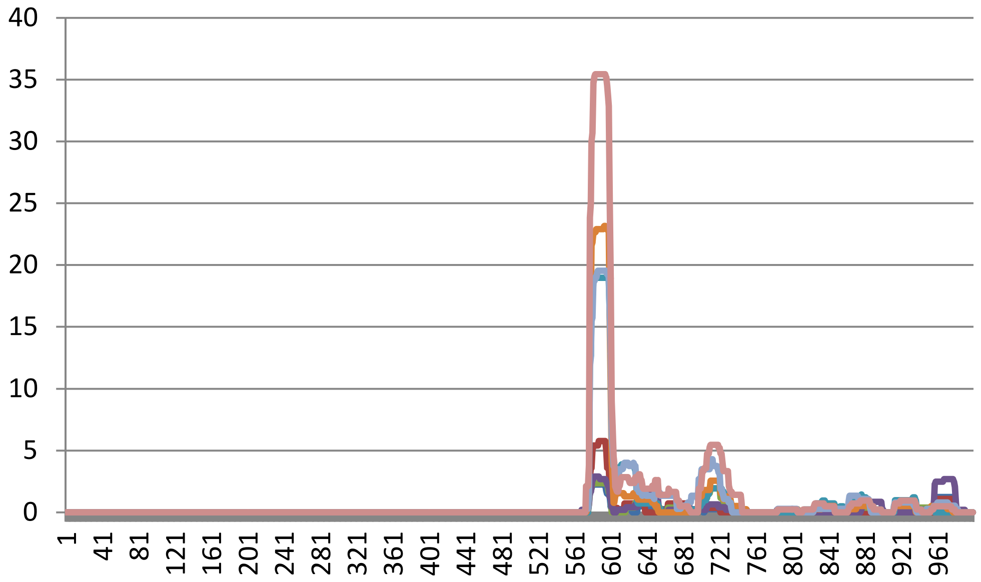

AT1G54775RC_AGO4

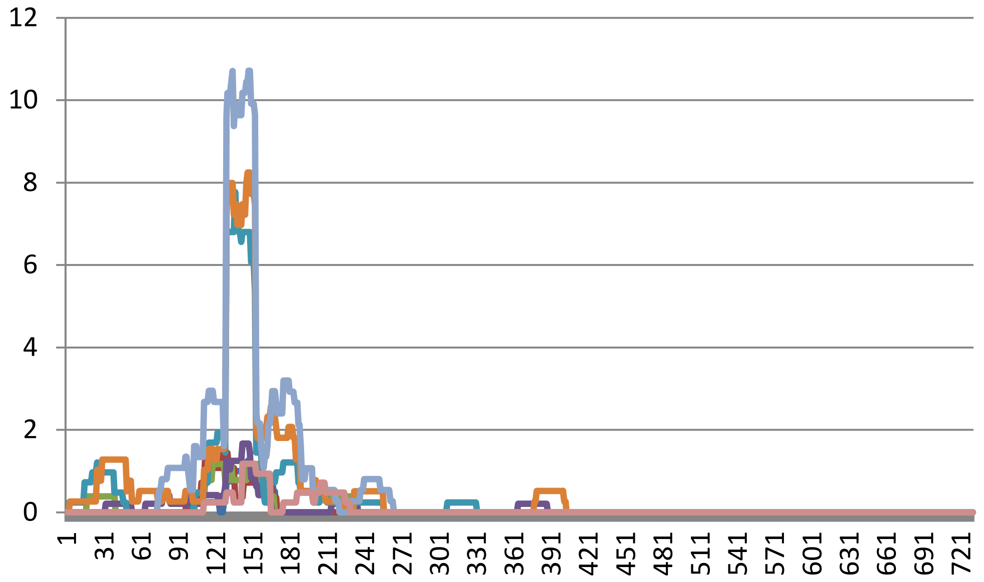

AT1G55300RC

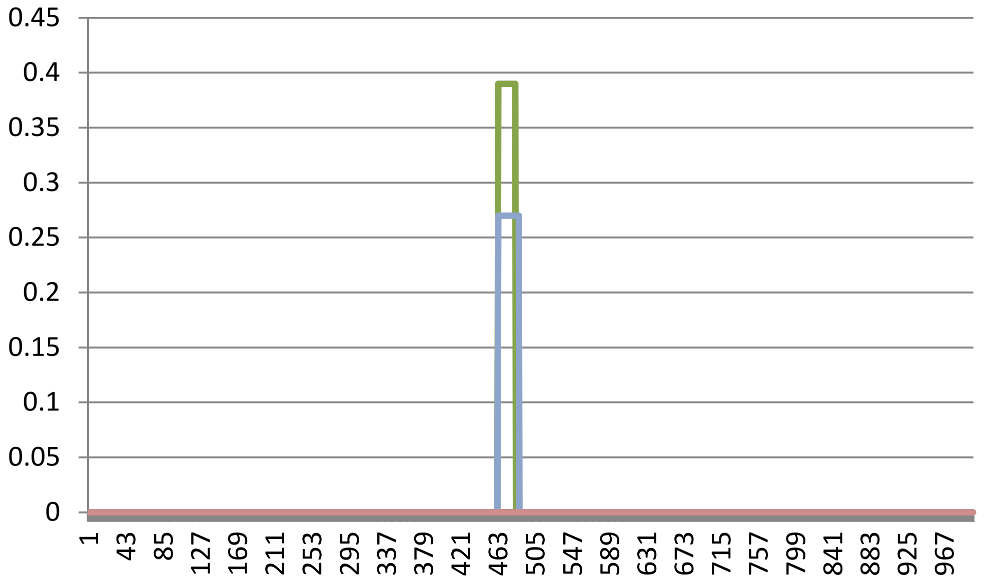

AT1G55680RC

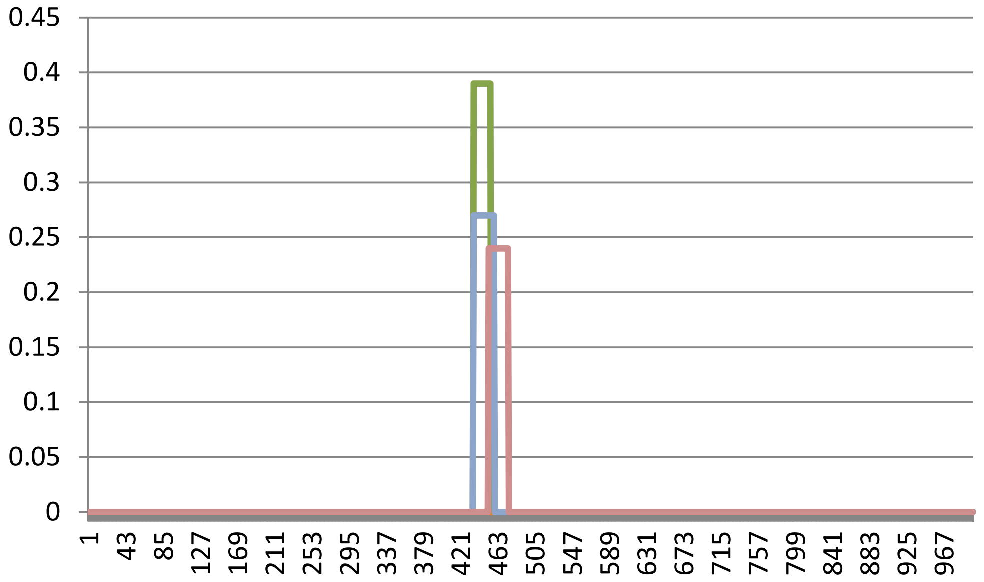

AT1G58245RC

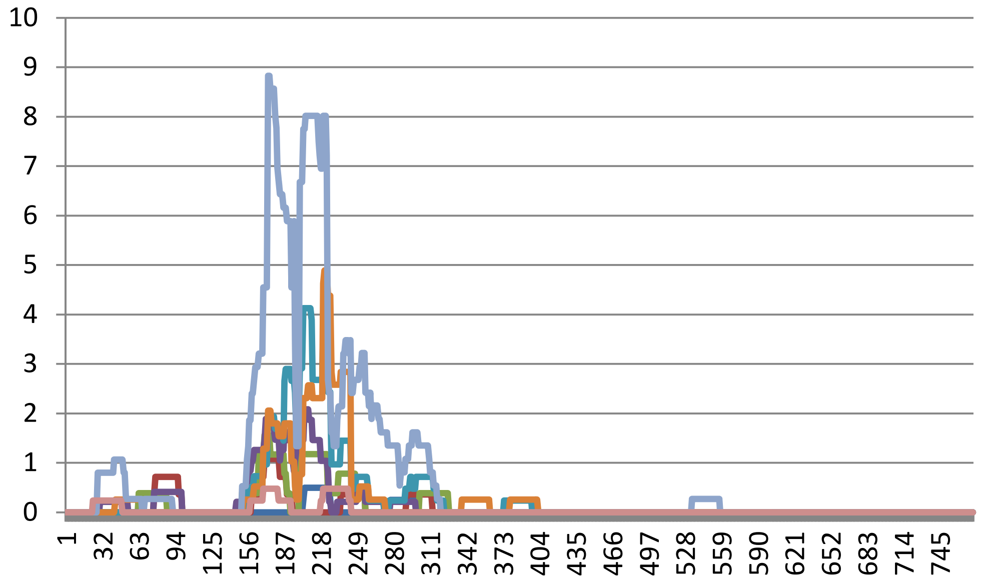

AT1G58248RC

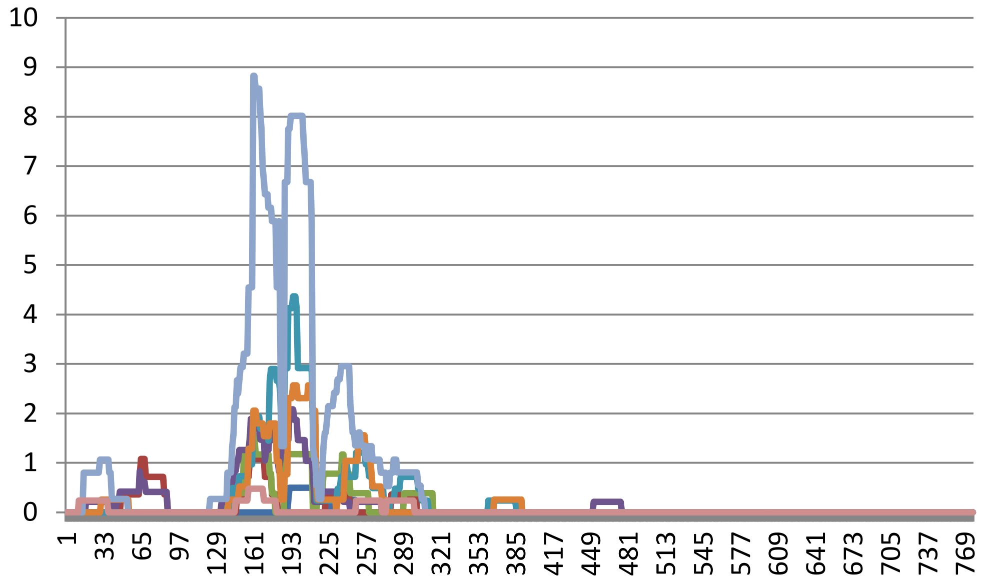

AT1G58390RC

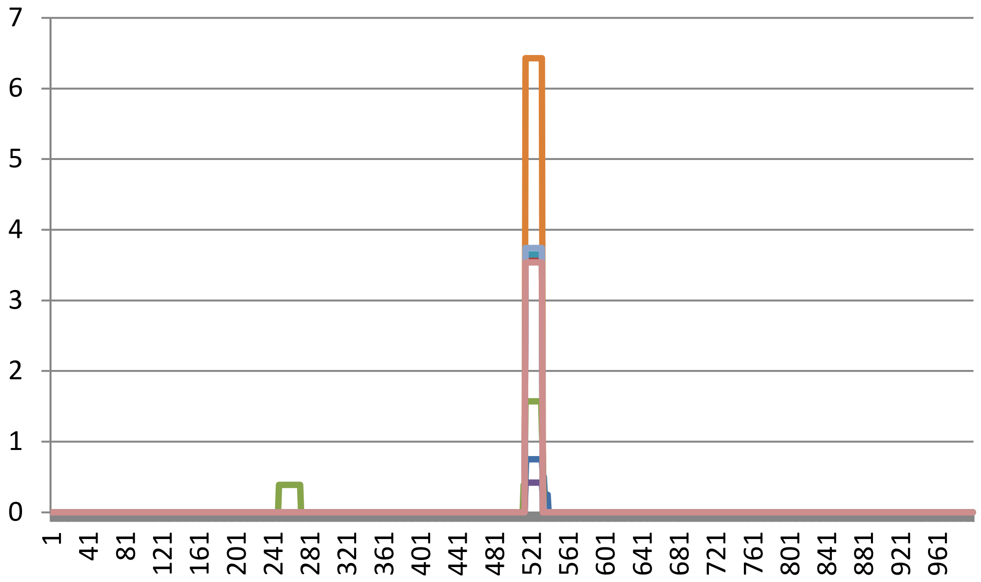

AT1G61030RC_AGO1 flower

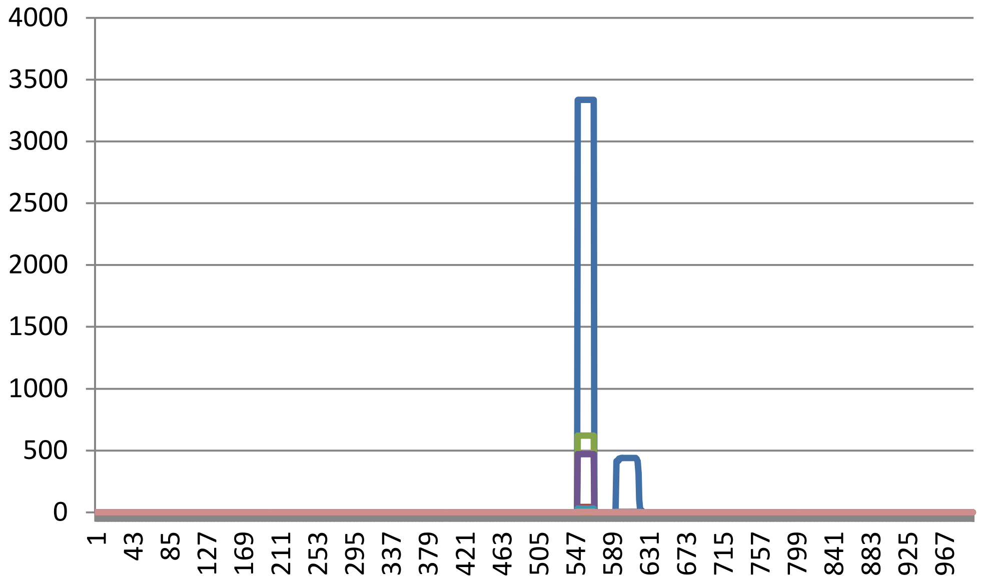

AT1G68945RC

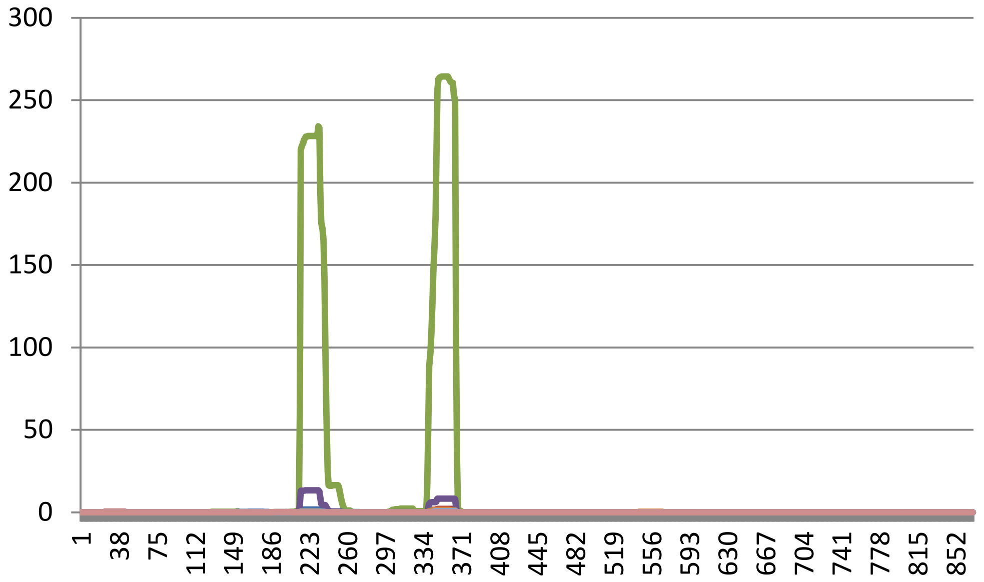

AT1G77950RC

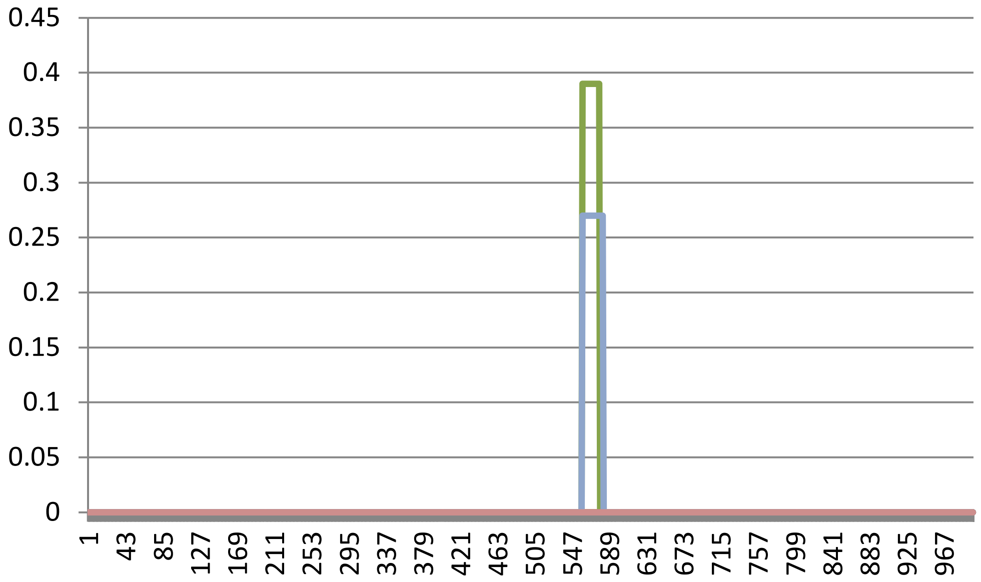

AT1G78420RC

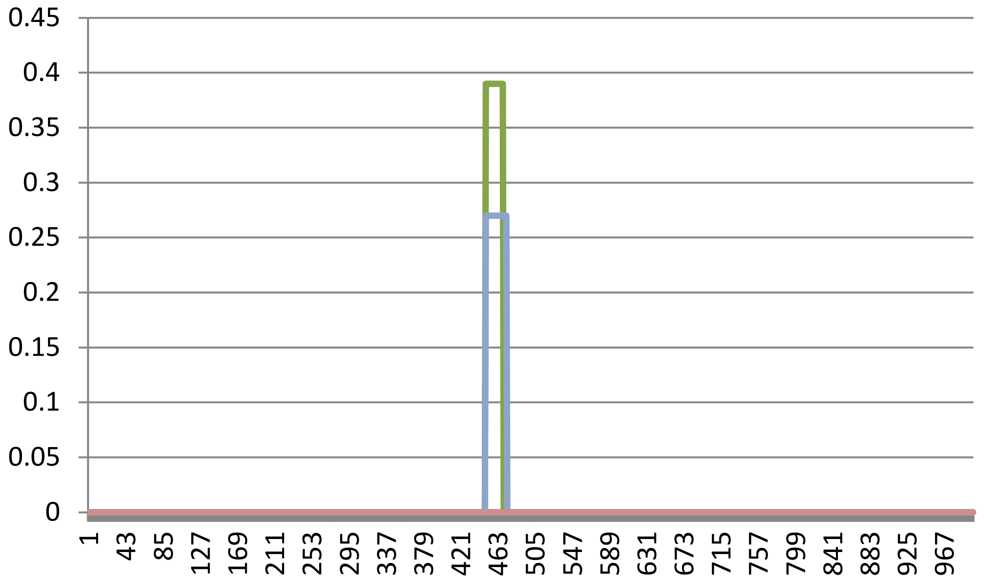

AT1G79990RC

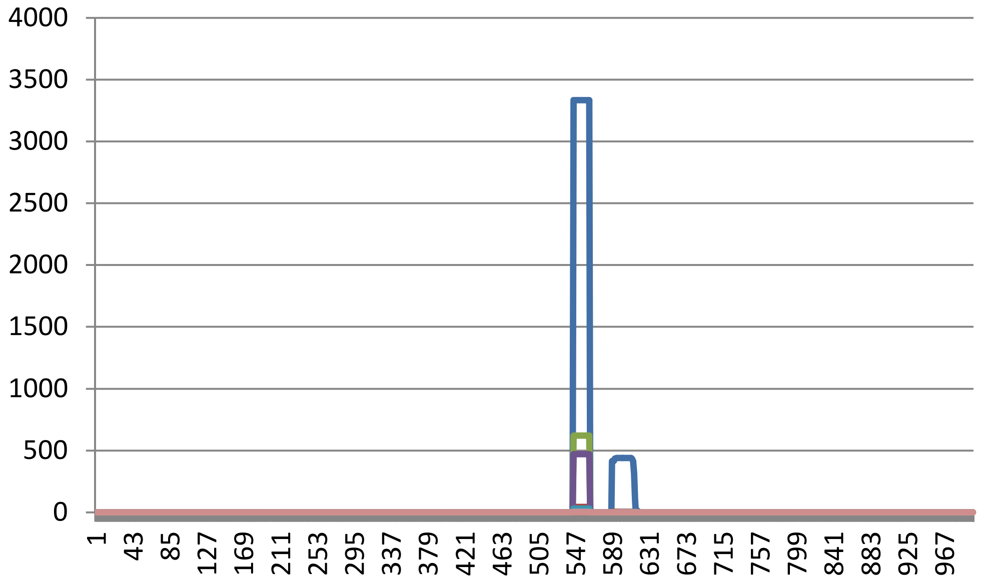

AT2G03667RC

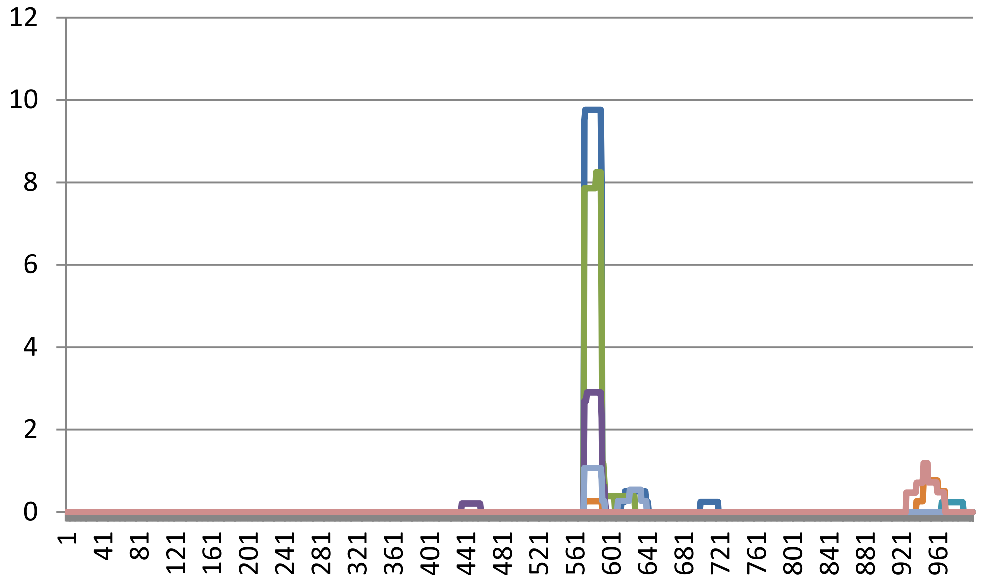

AT2G13540RC_AGO4

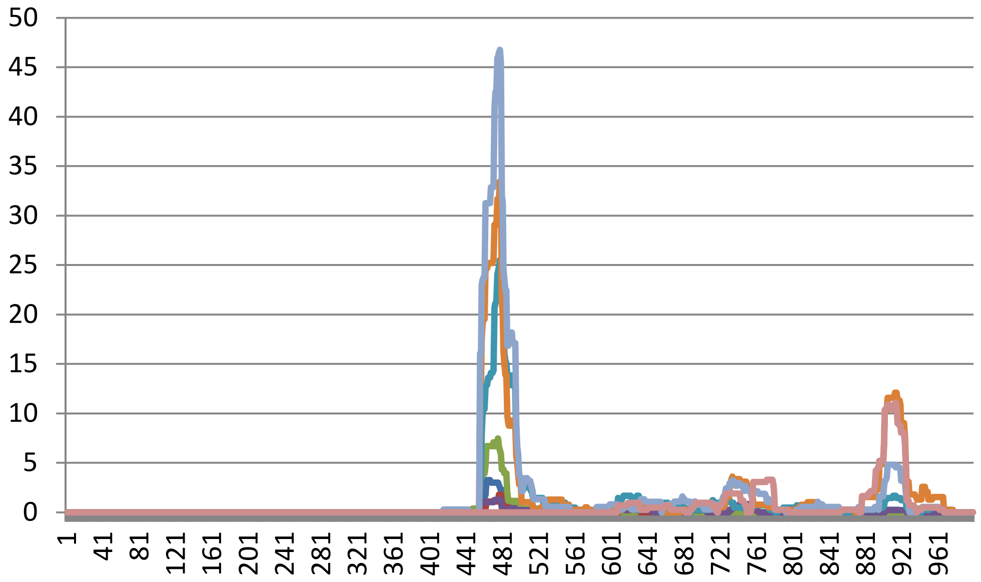

AT2G16365RC

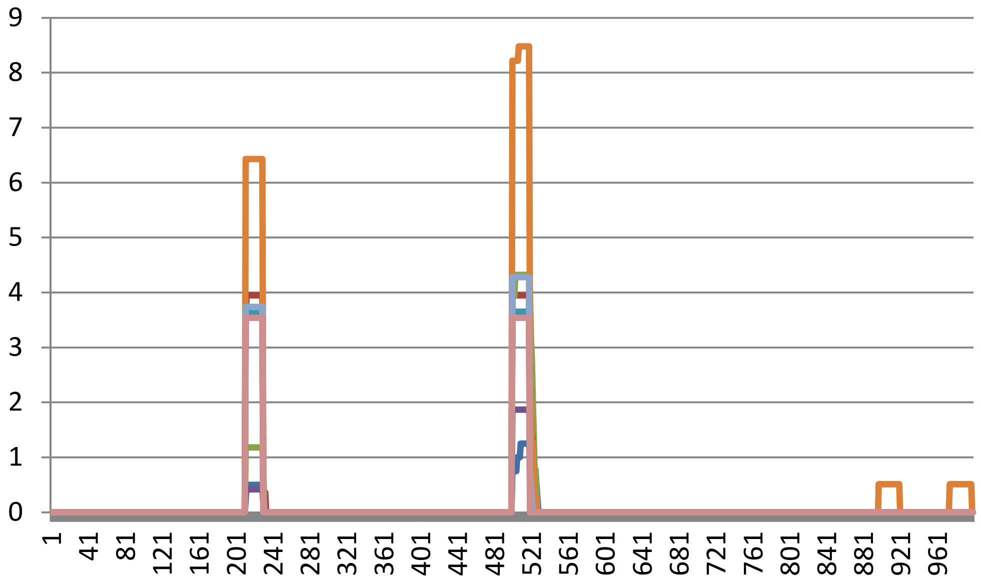

AT2G16640RC

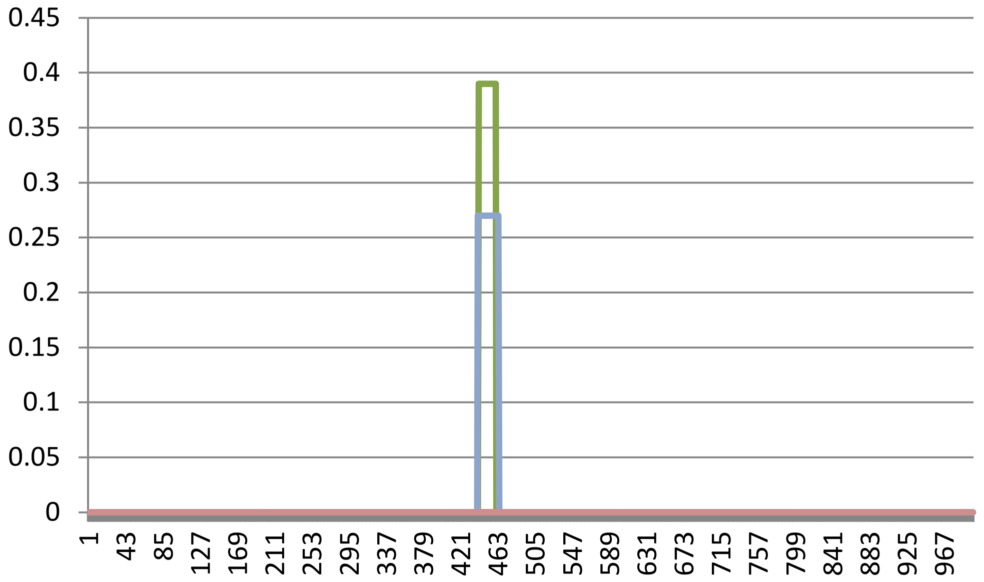

AT2G17510RC_AGO4

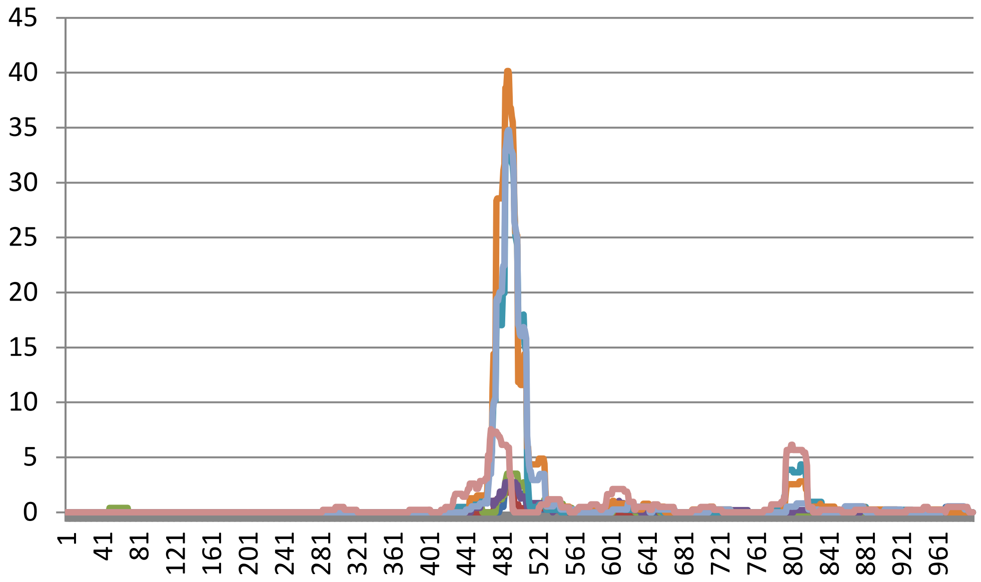

AT2G18870RC

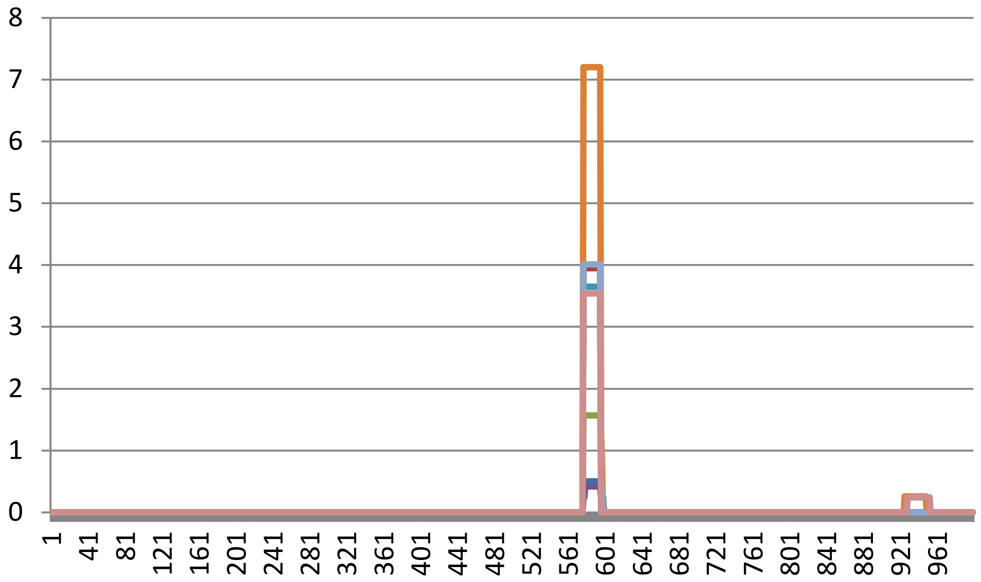

AT2G19270RC

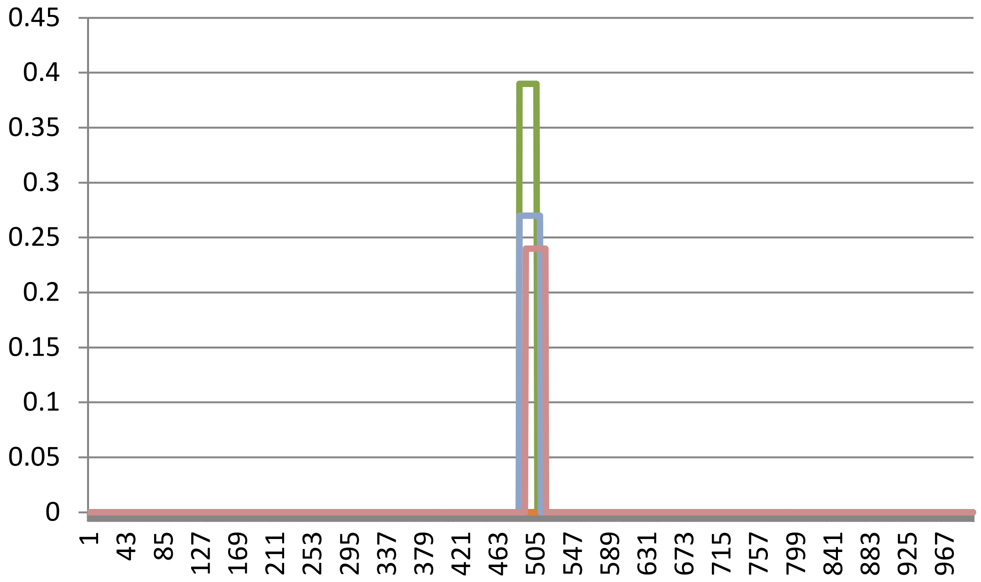

AT2G21420RC

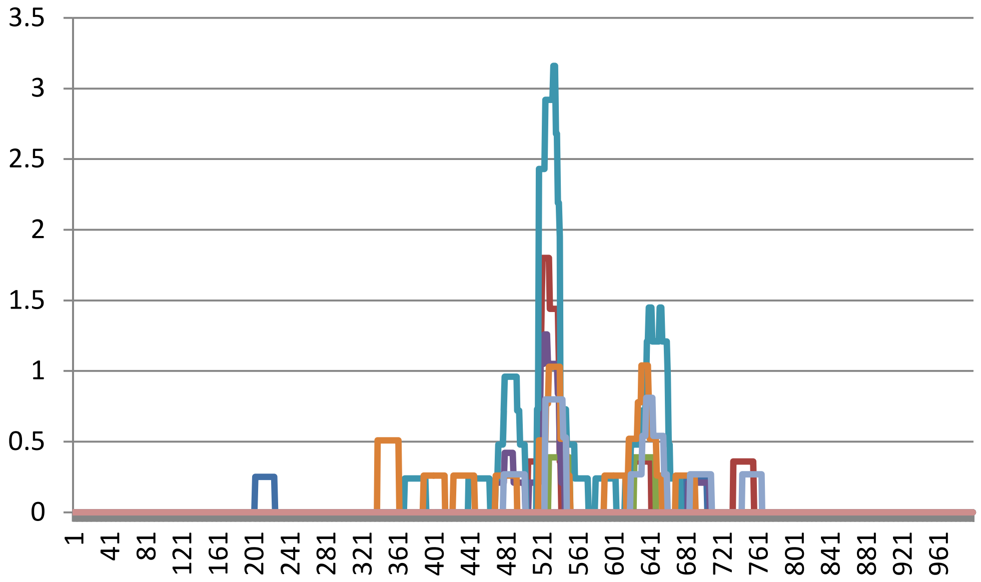

AT2G24670RC_AGO4 flower

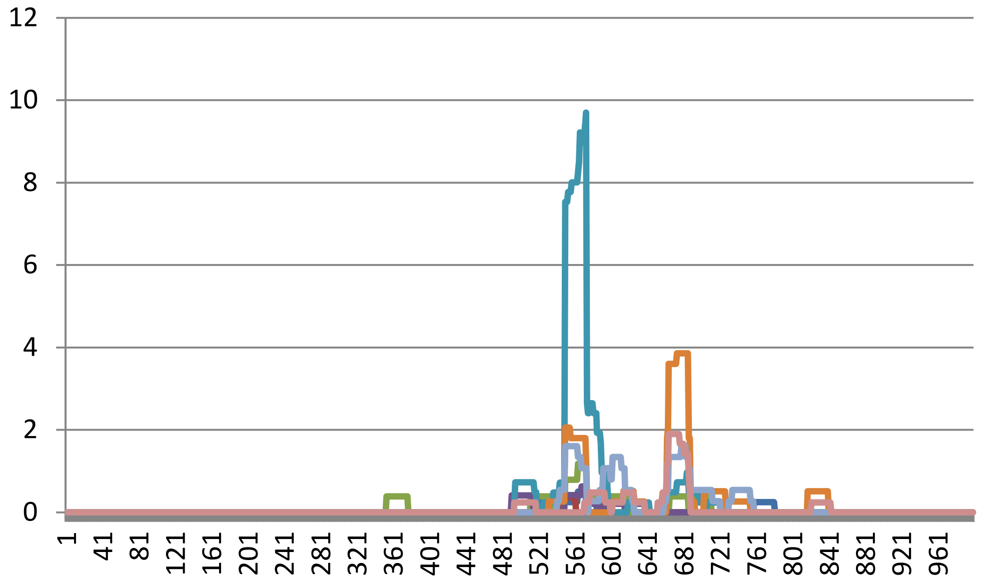

AT2G26210RC

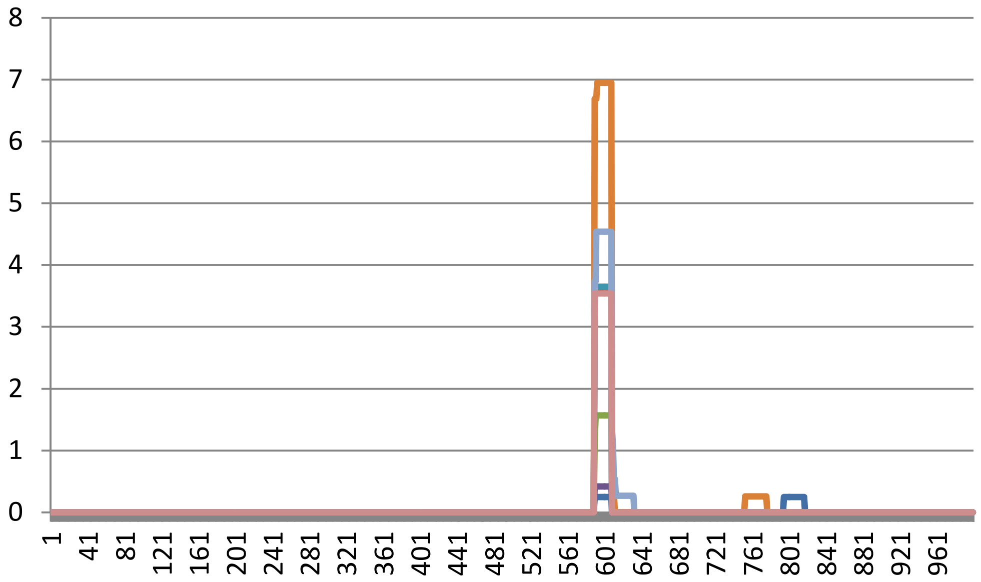

AT2G27050RC

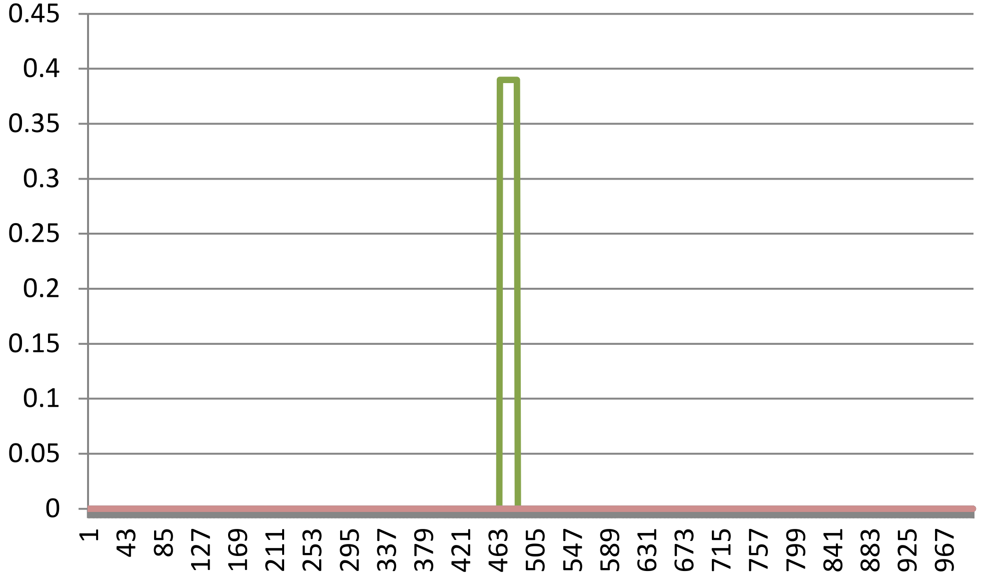

AT2G28710RC

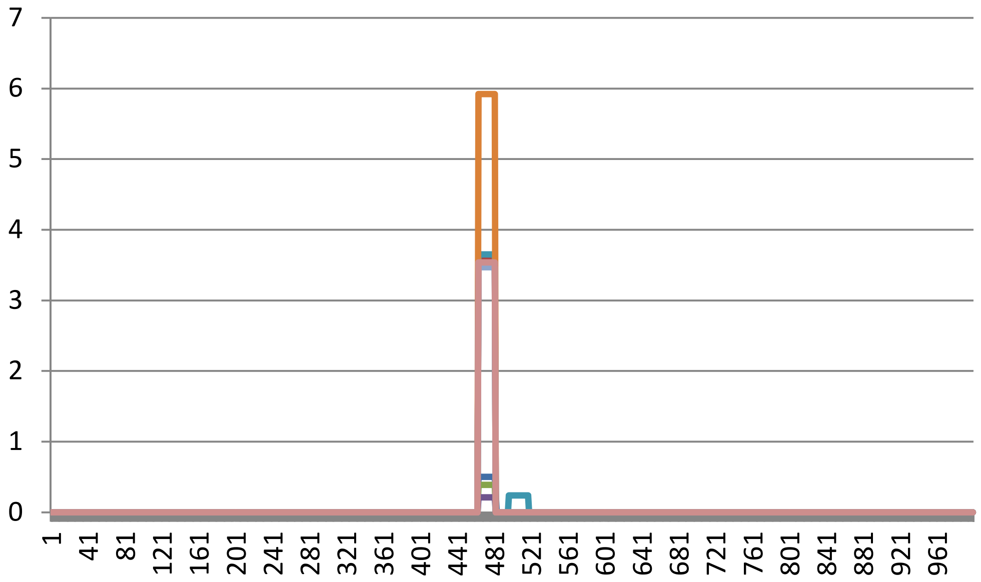

AT2G29460RC

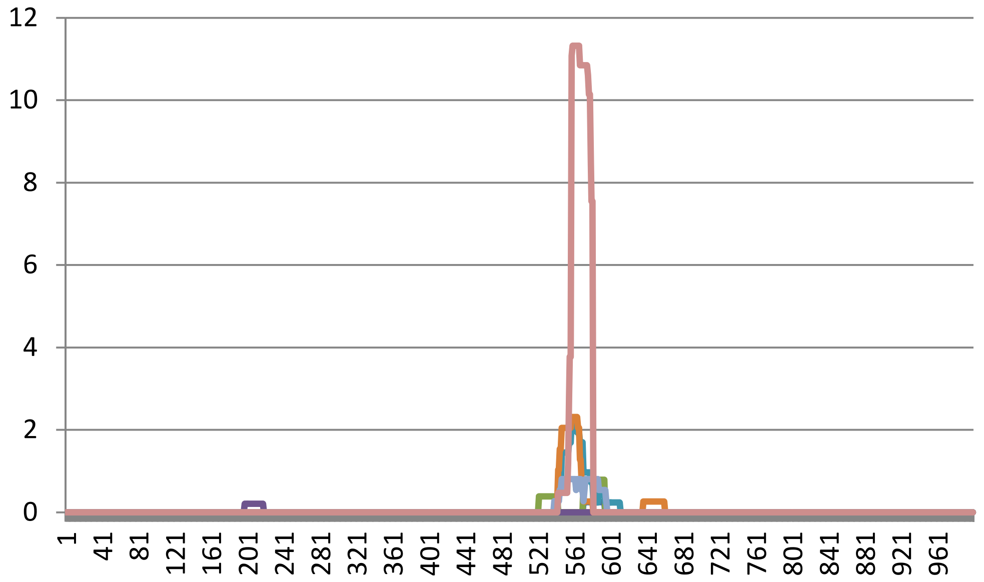

AT2G29605RC

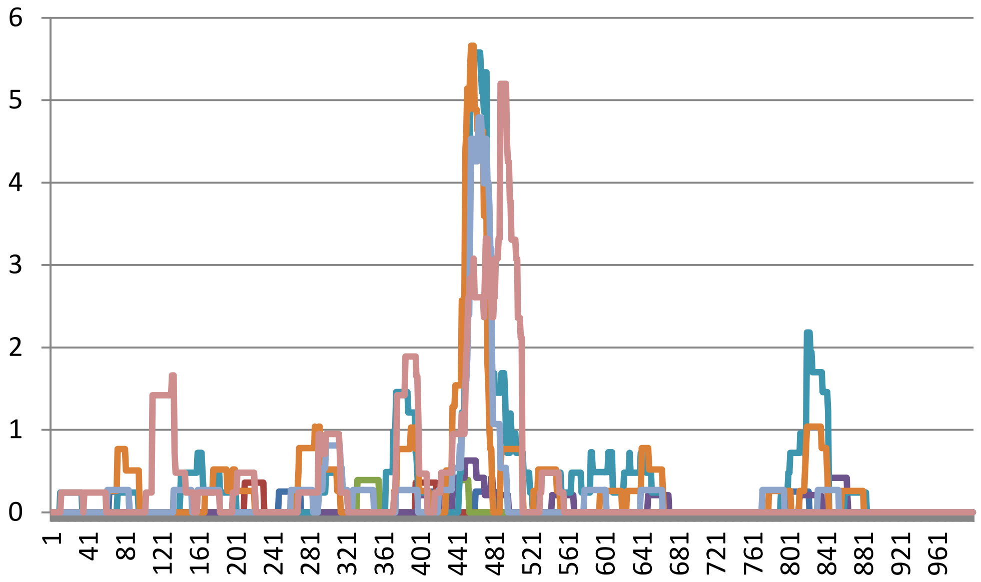

AT2G31305RC

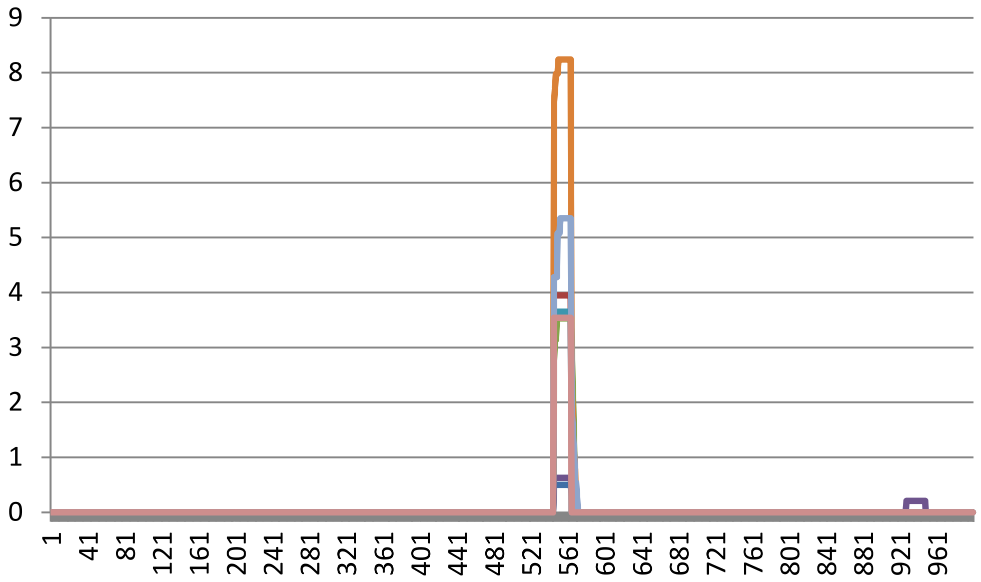

AT2G32410RC

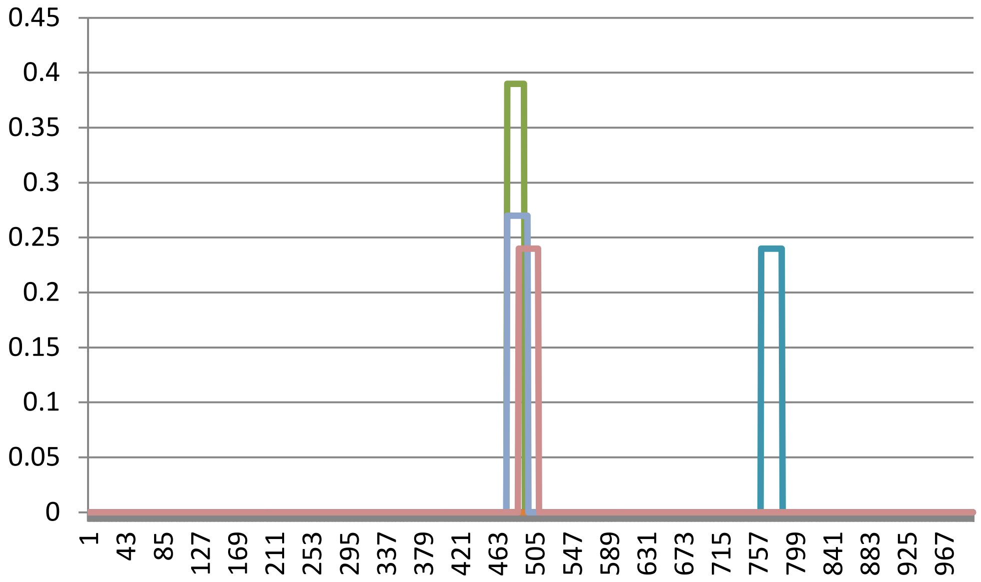

AT2G35830RC

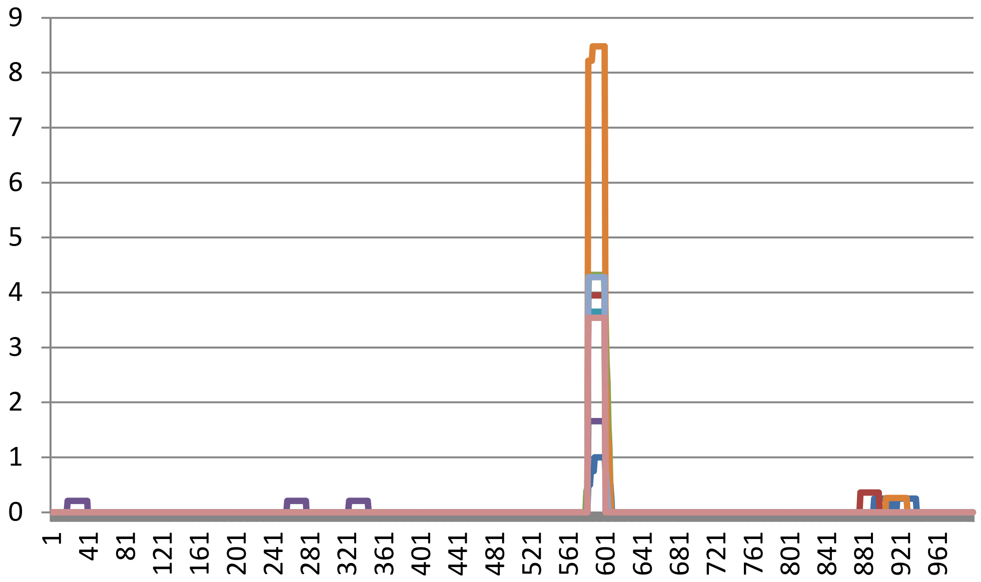

AT2G38025RC_AGO1 root

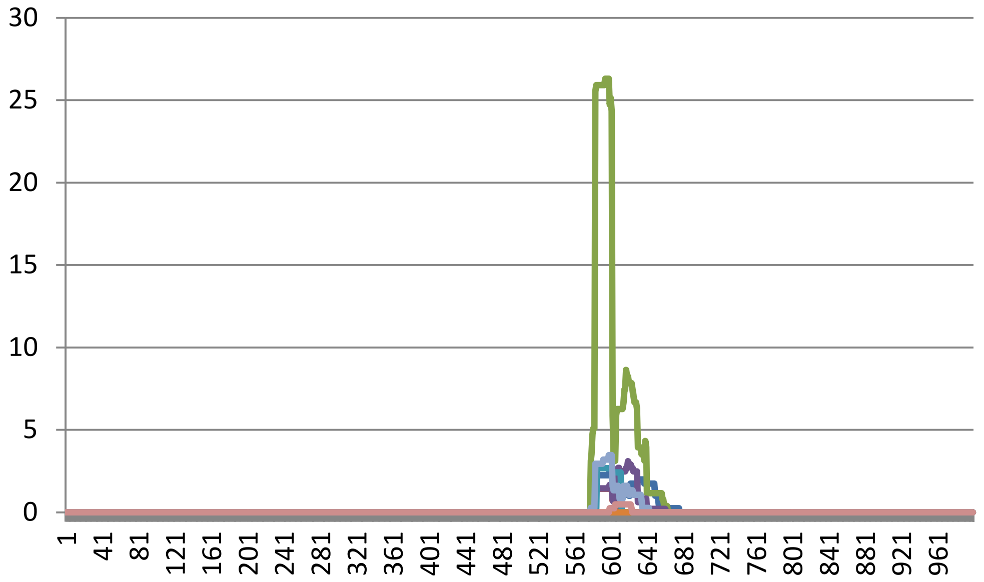

AT2G39310RC

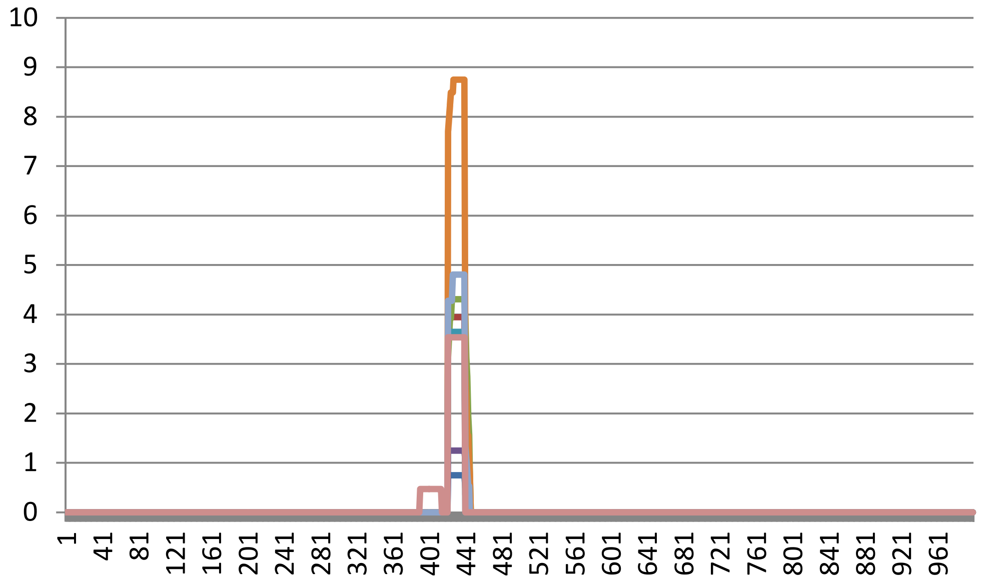

AT2G42880RC

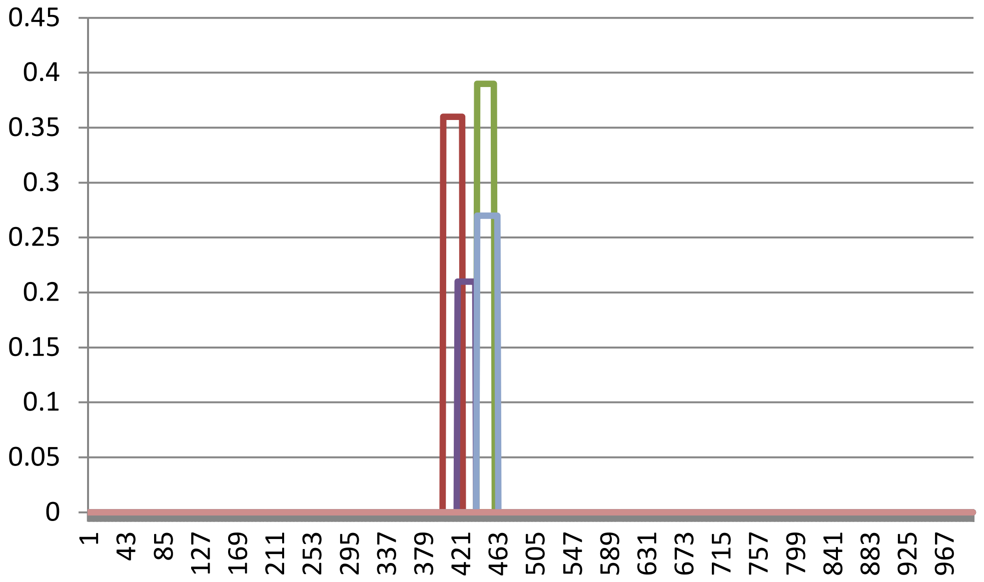

AT2G46920RC

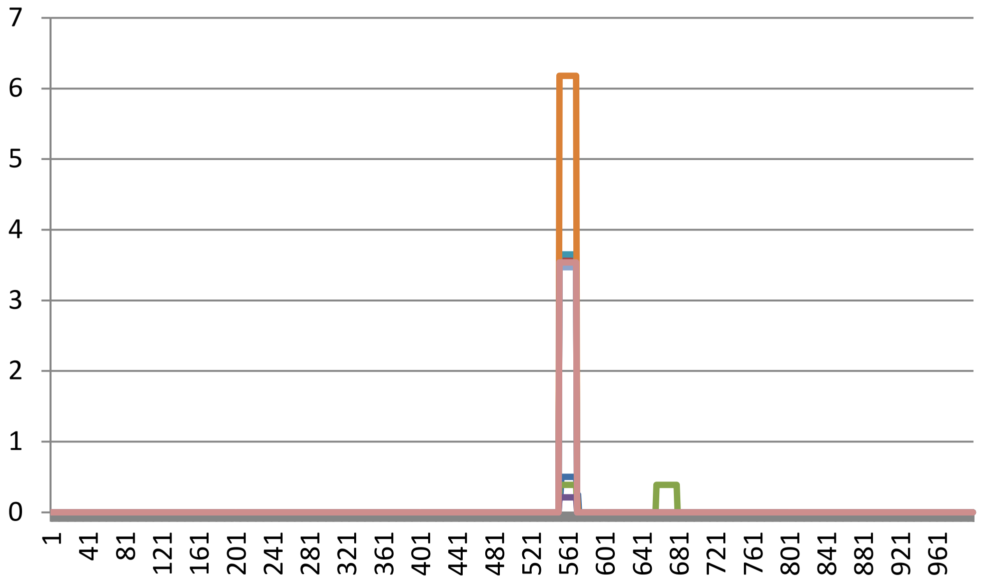

AT2G47090RC_root

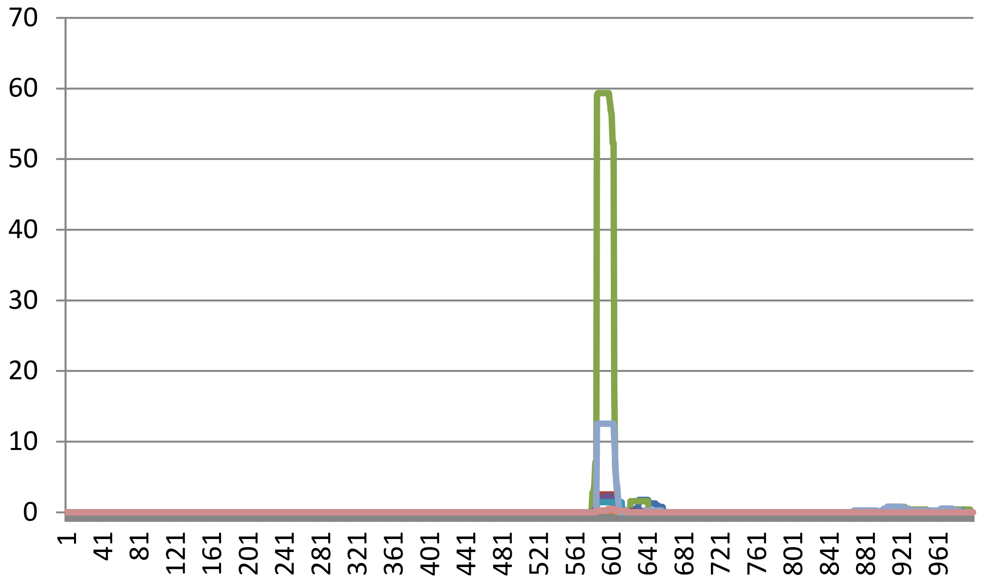

AT3G01890RC

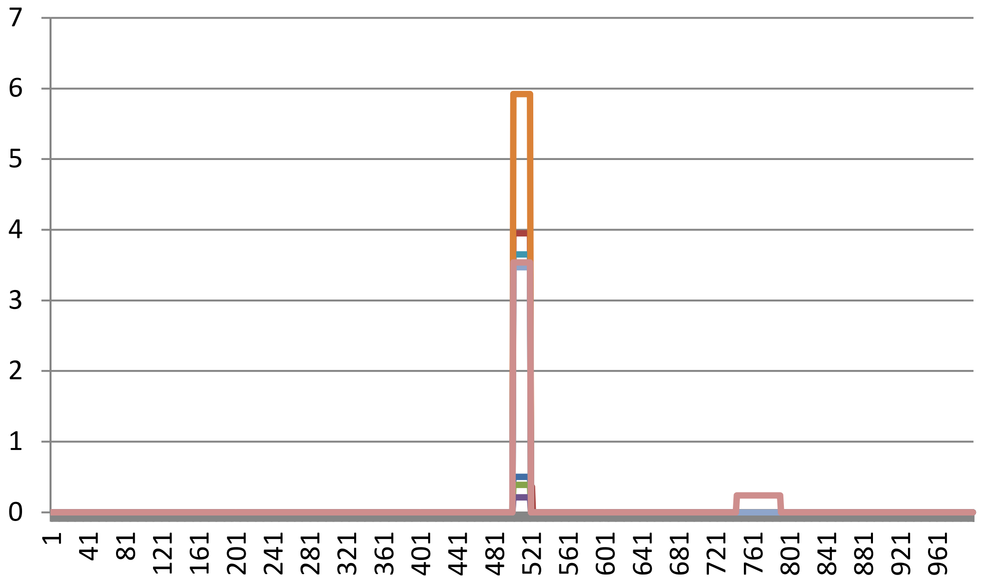

AT3G02110RC

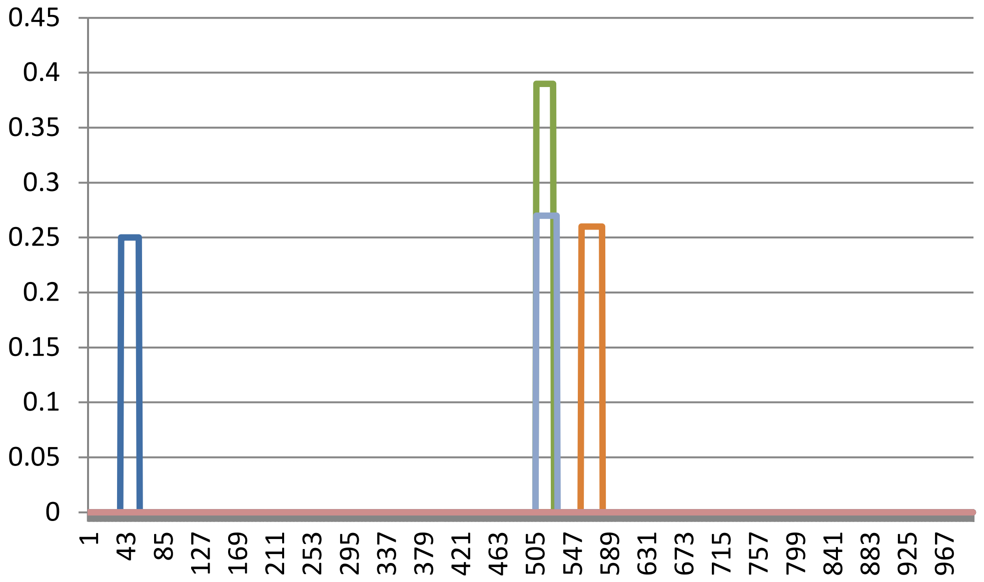

AT3G04740RC

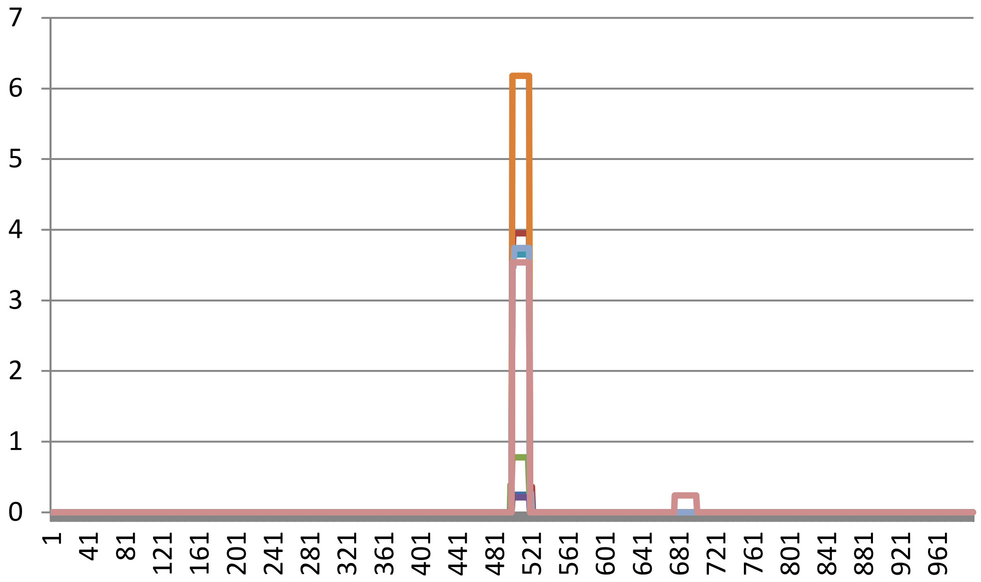

AT3G05320RC

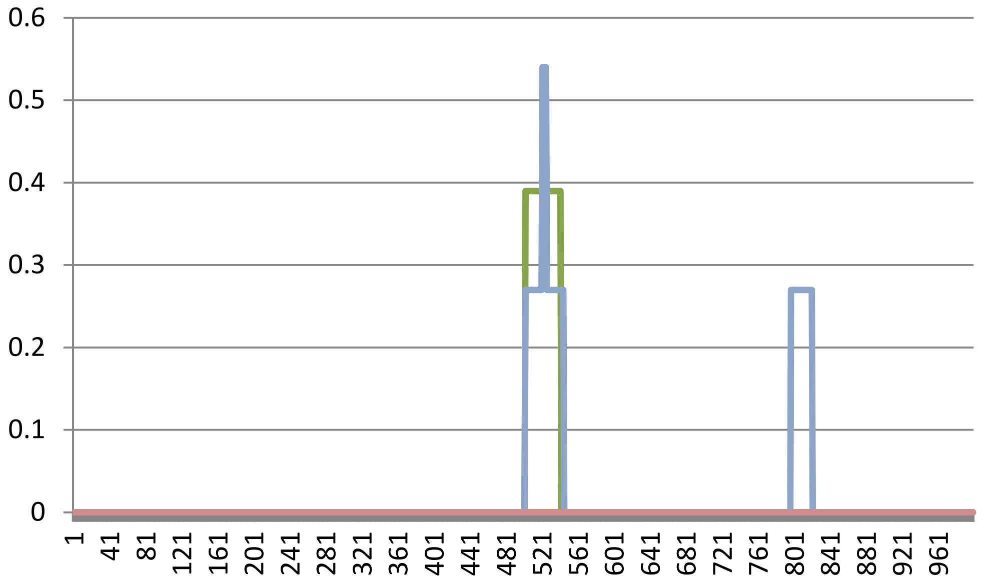

AT3G05710RC

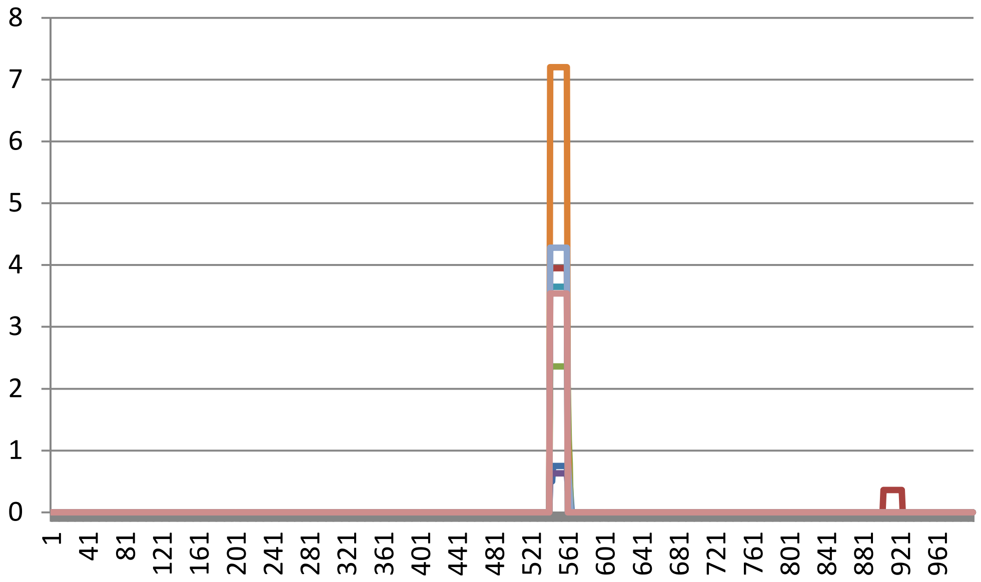

AT3G05760RC

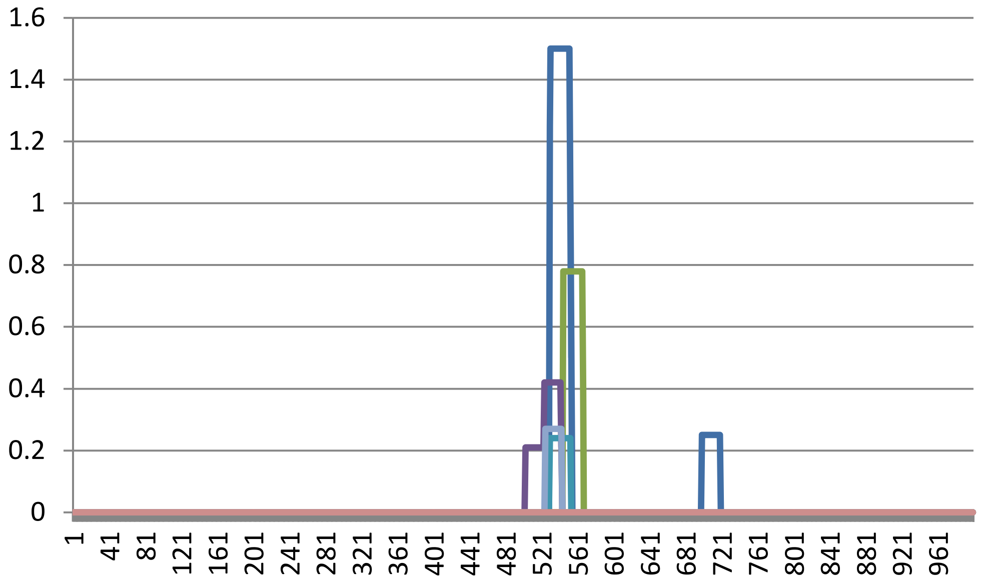

AT3G06110RC_AGO1

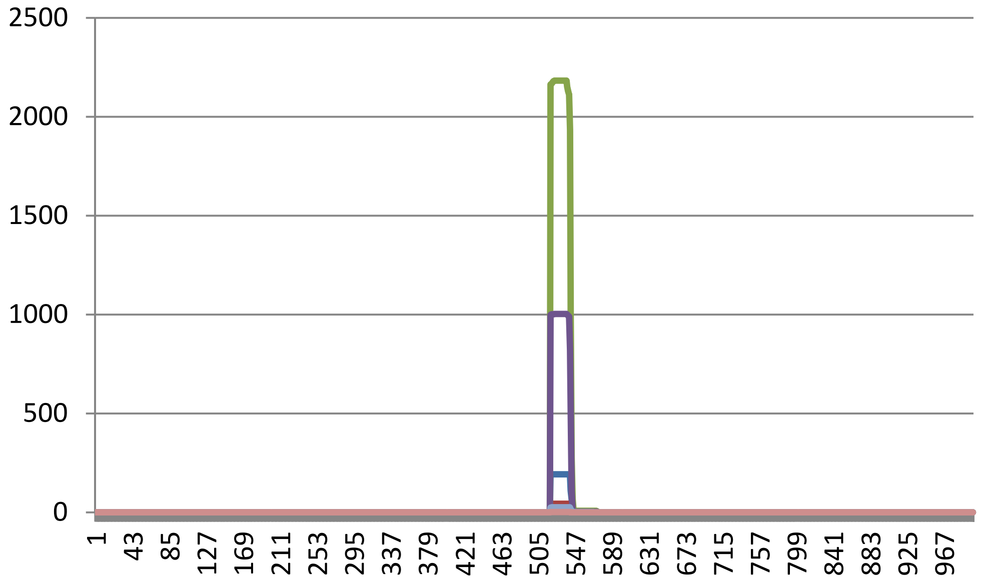

AT3G06490RC

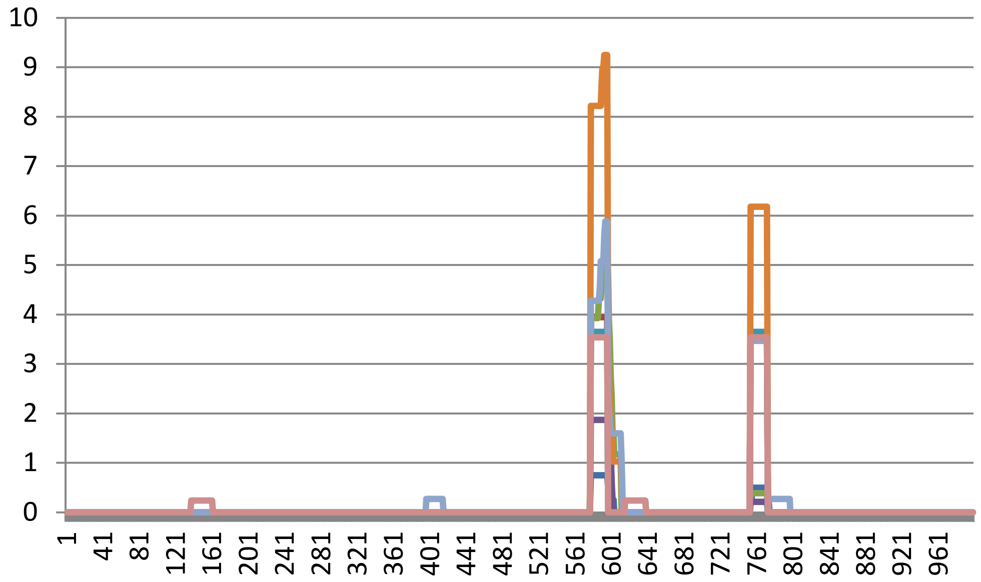

AT3G07180RC

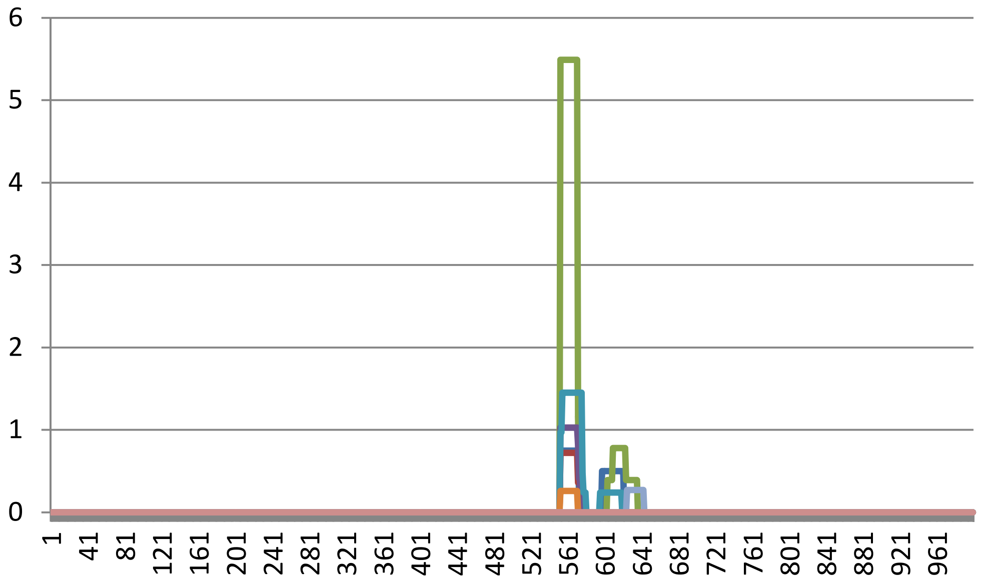

AT3G11760RC

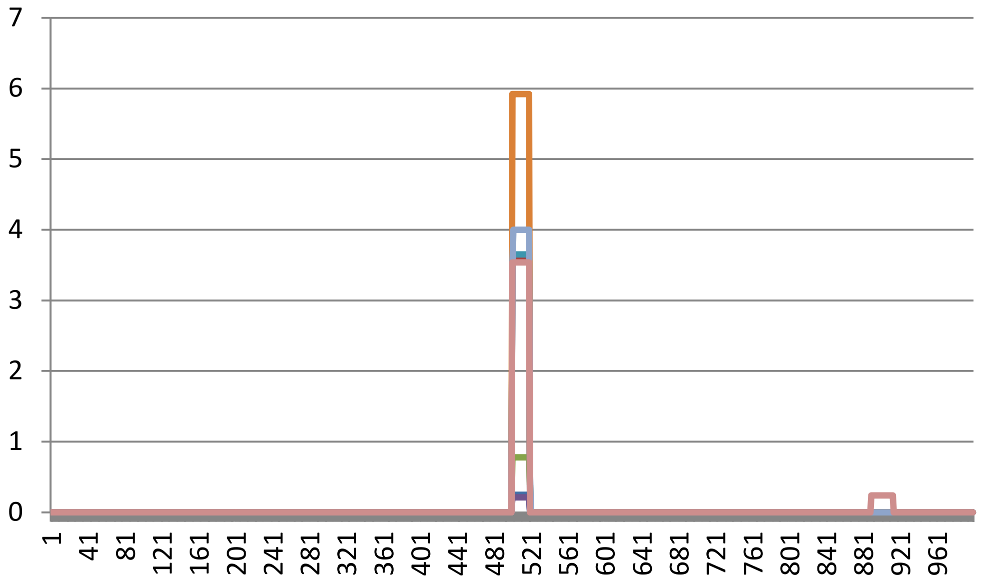

AT3G13440RC

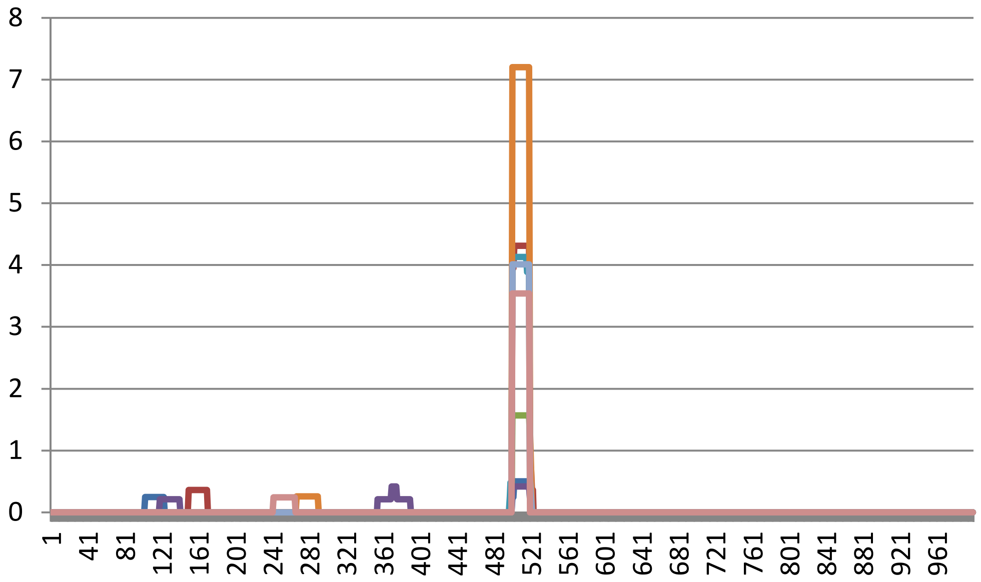

AT3G13480RC

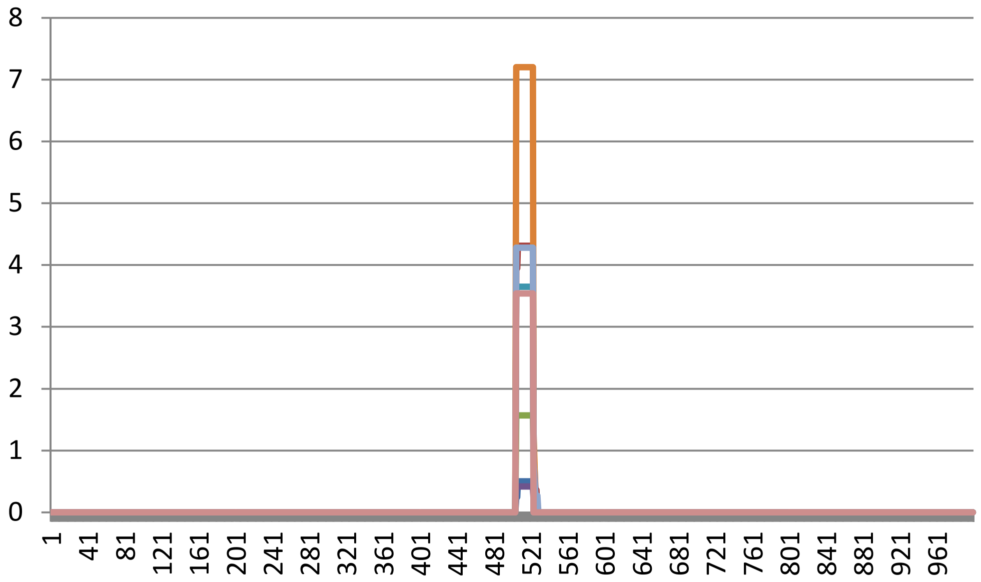

AT3G15580RC

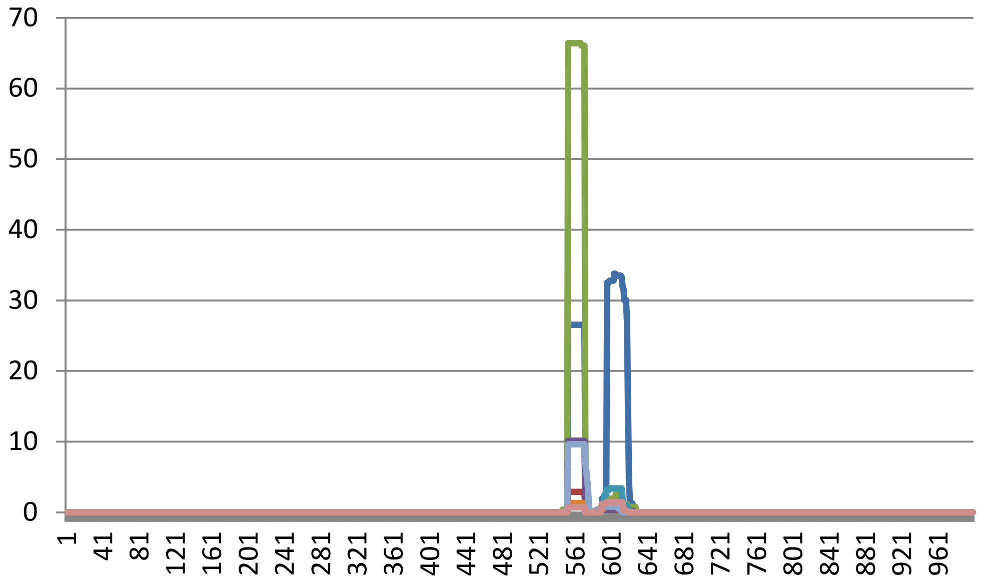

AT3G16230RC_AGO1 flower

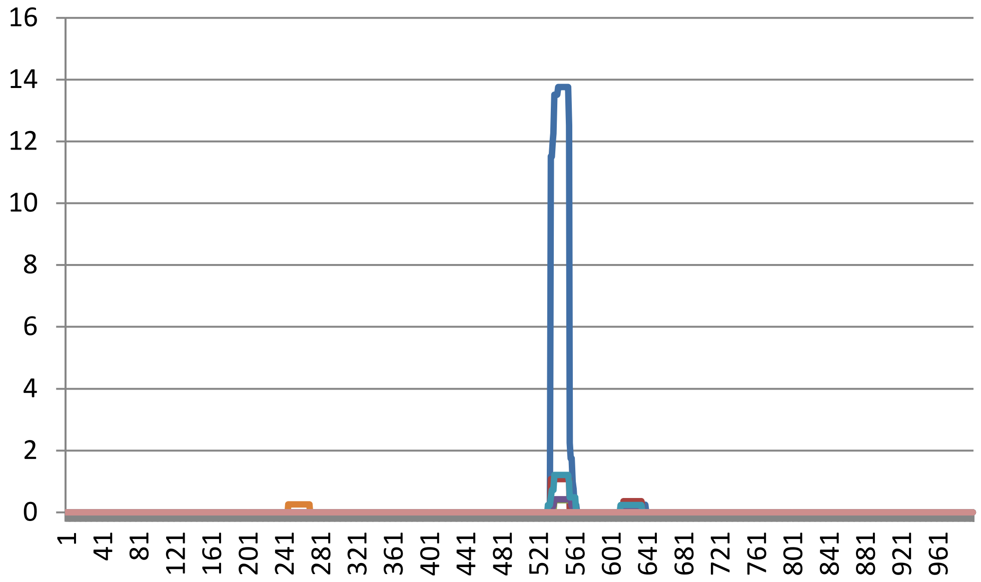

AT3G18820RC_AGO1_root

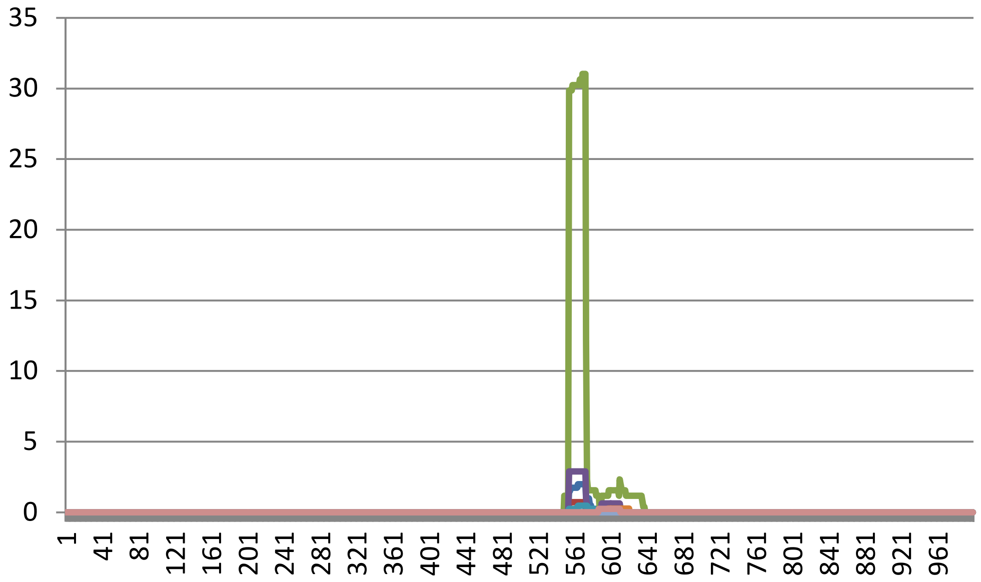

AT3G20090RC

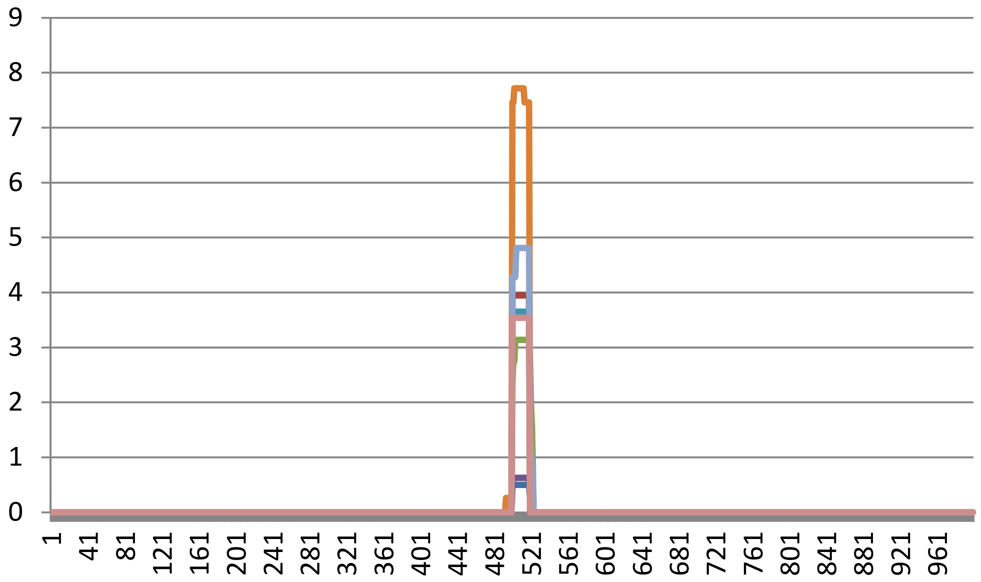

AT3G20290RC

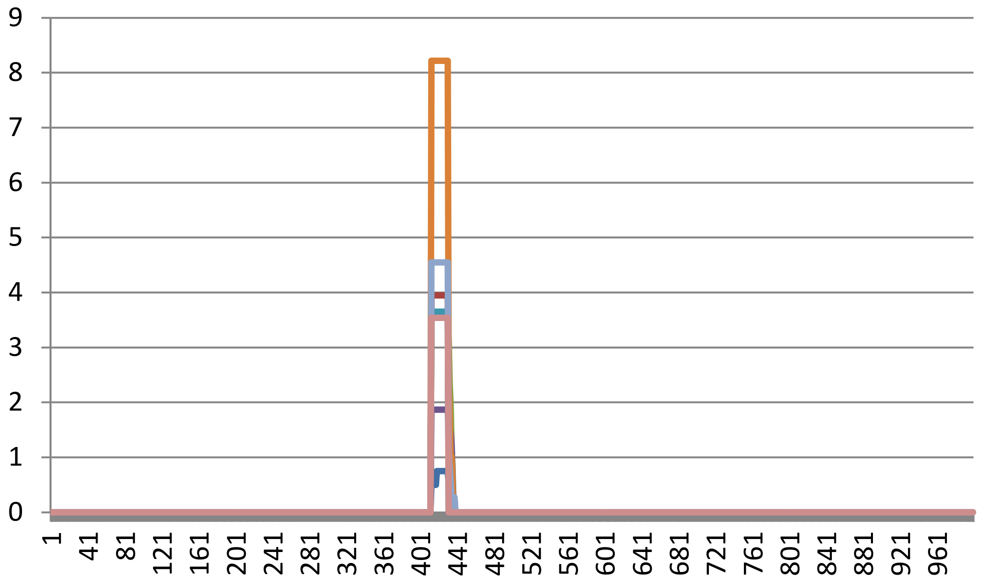

AT3G20720RC

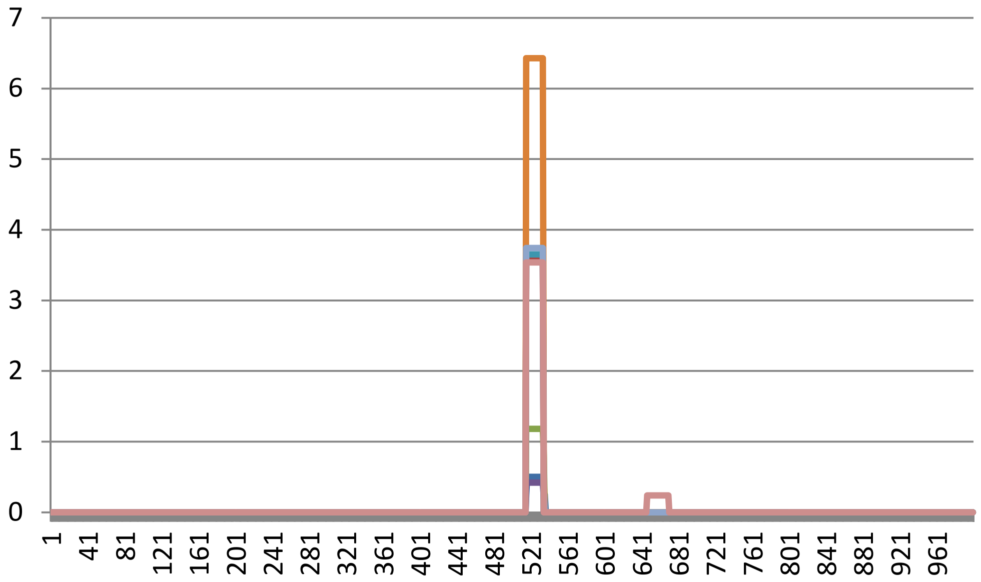

AT3G25585RC

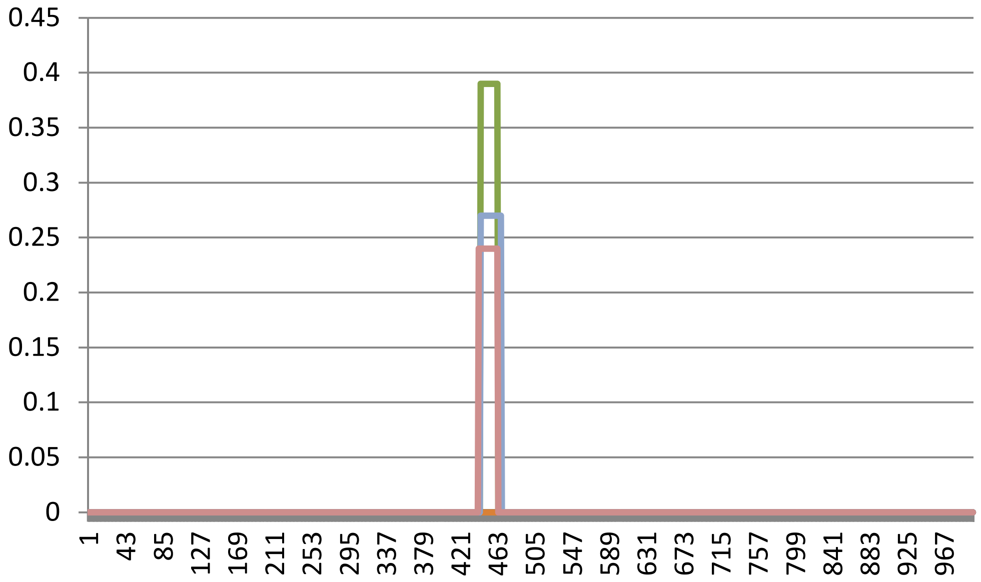

AT3G30290RC

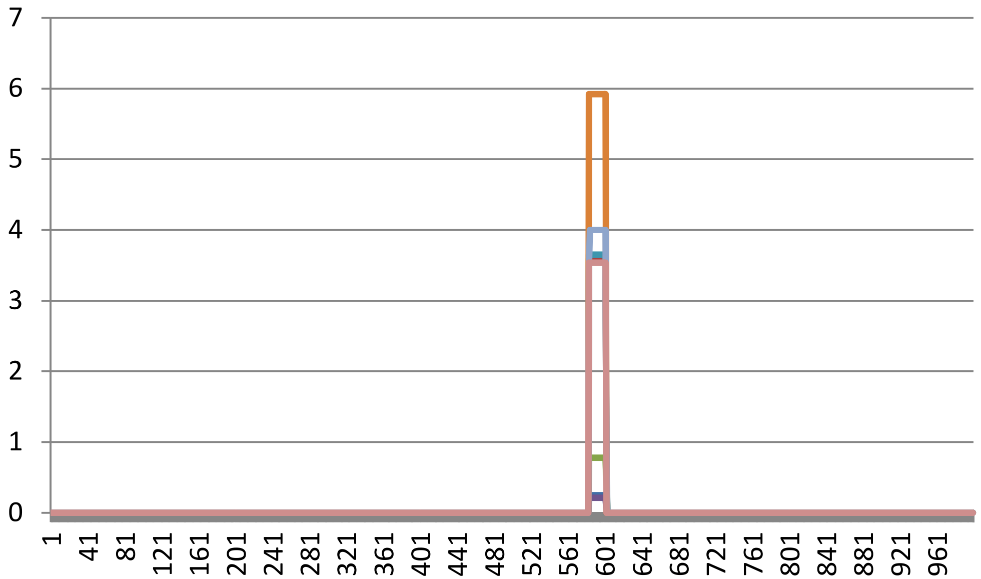

AT3G30580RC

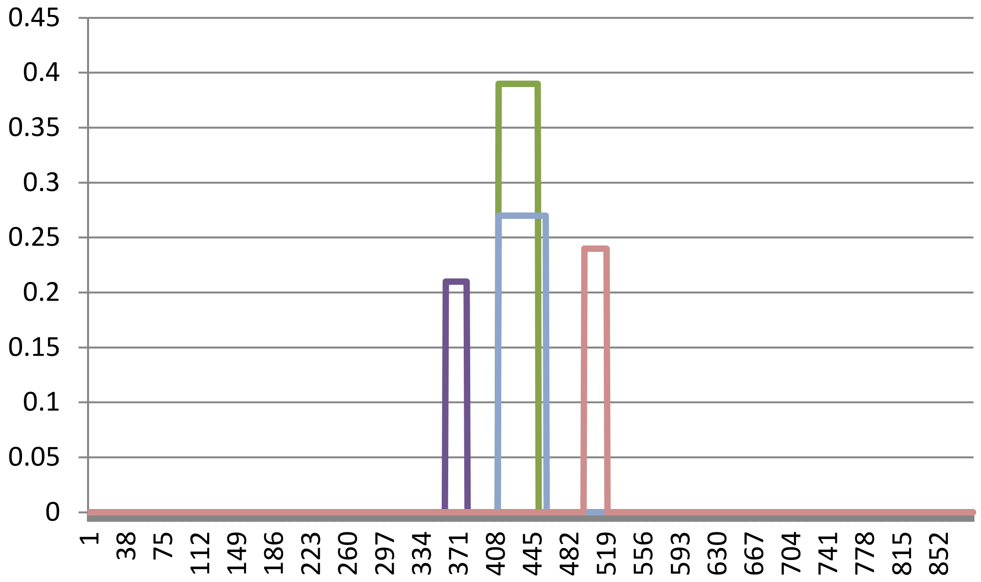

AT3G42550RC_AGO4 flower

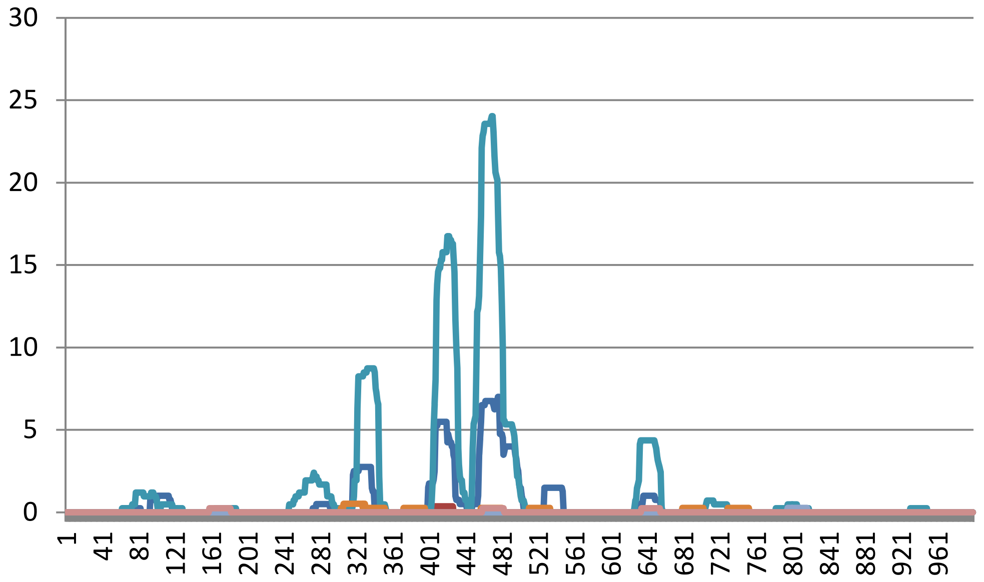

AT3G44020RC

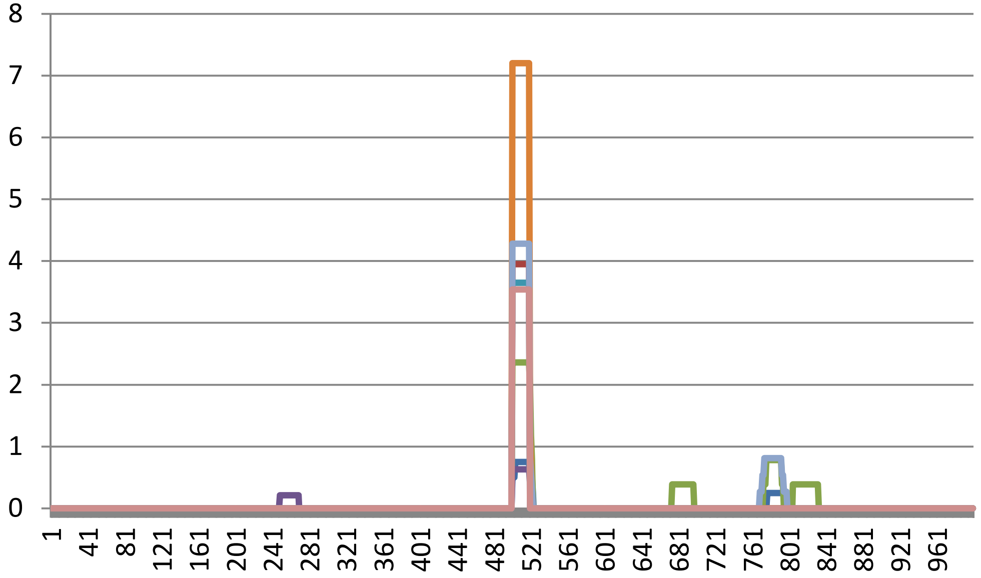

AT3G44230RC

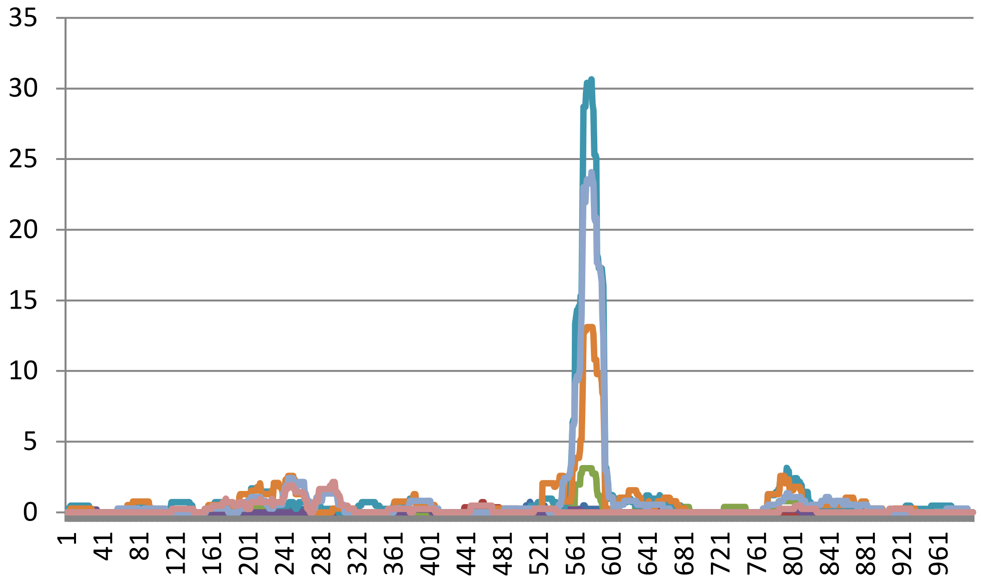

AT3G45190RC

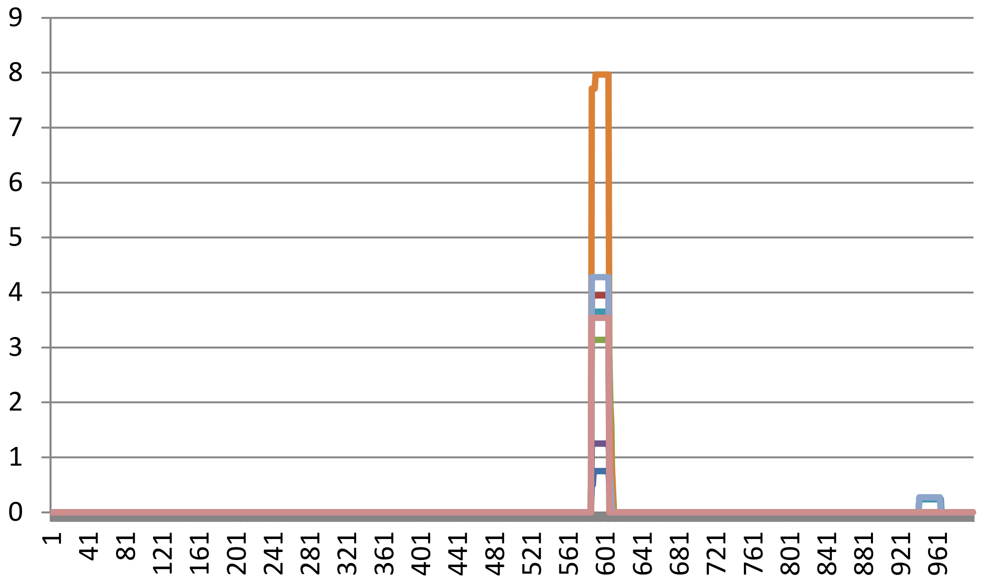

AT3G45800RC

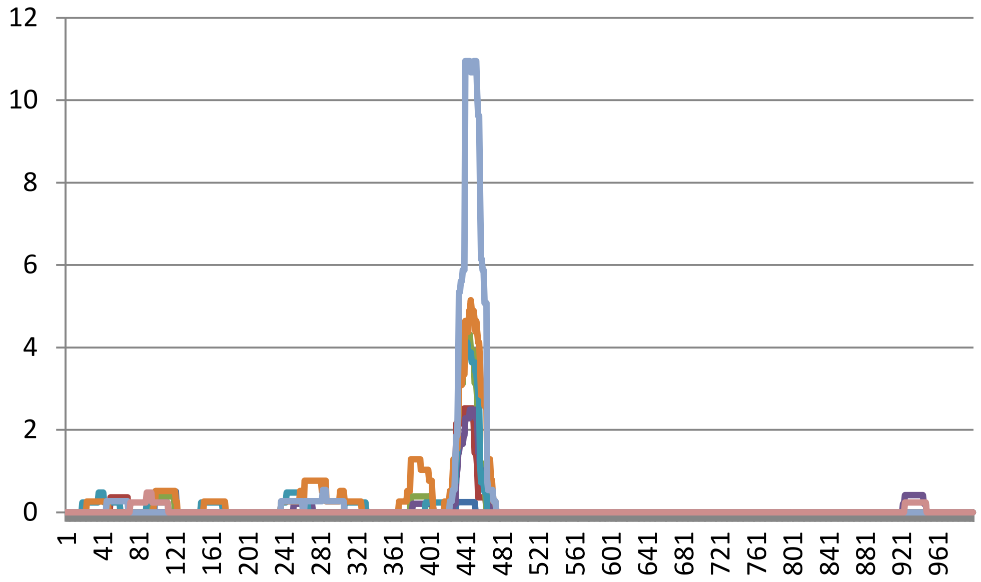

AT3G47600RC

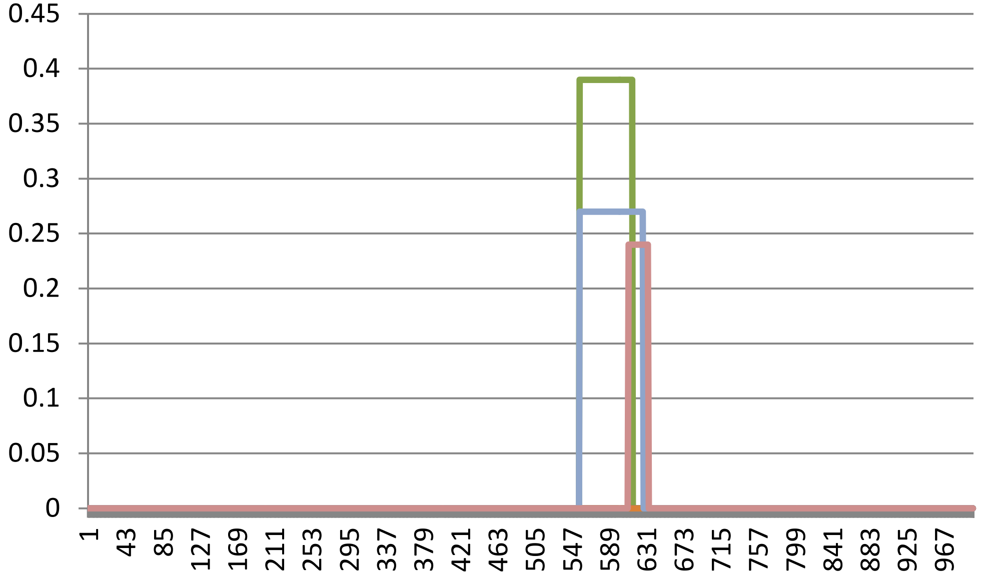

AT3G49520RC

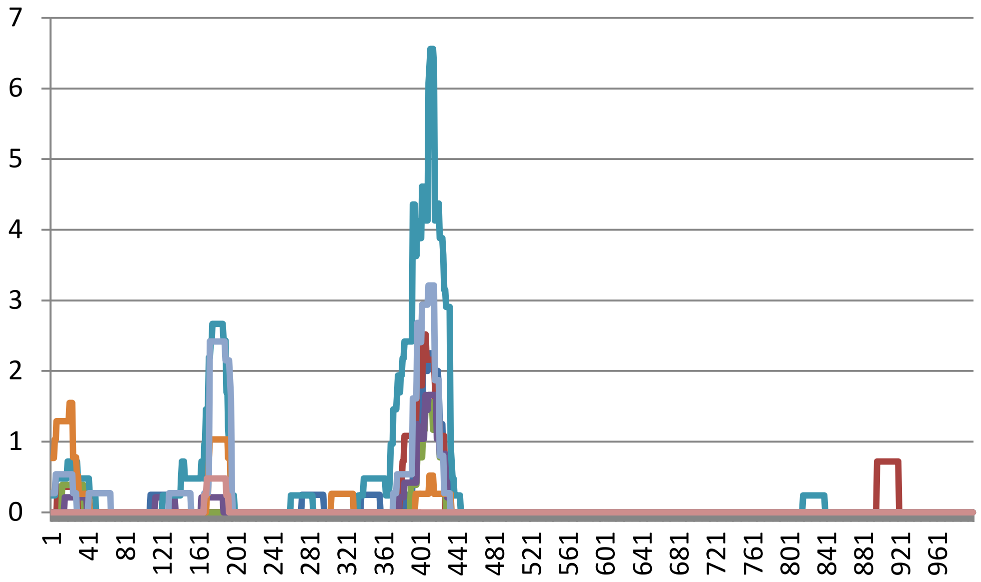

AT3G49920RC

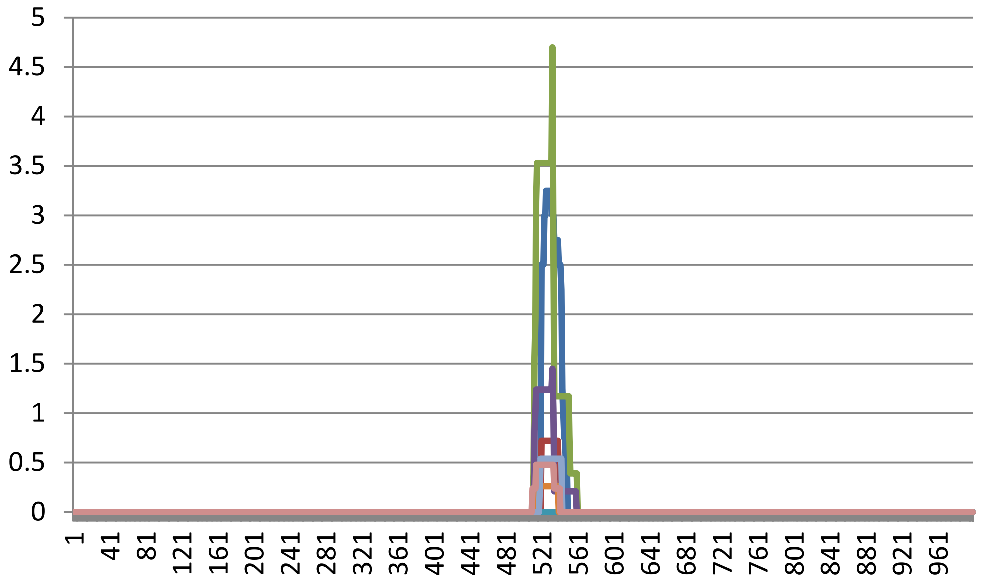

AT3G50370RC

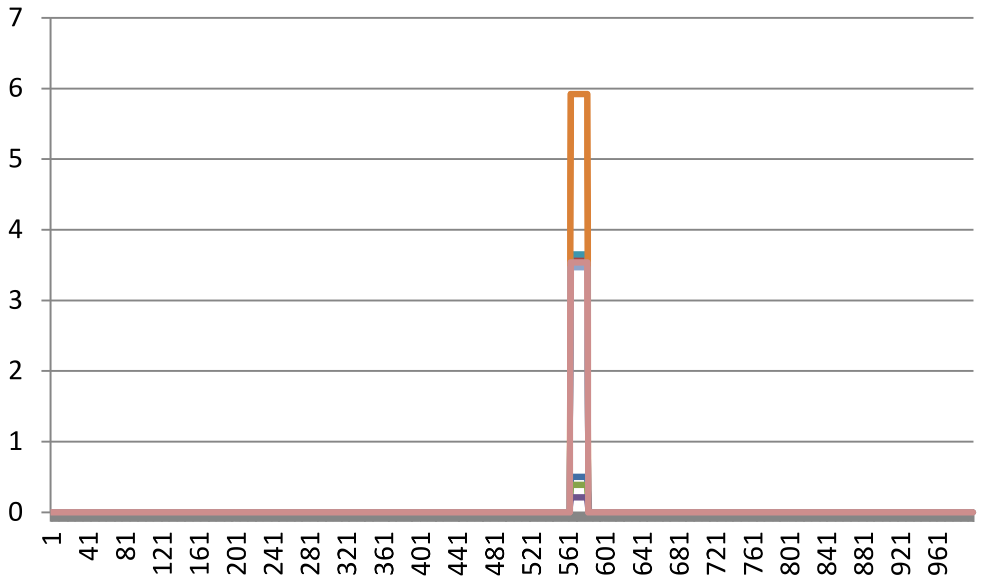

AT3G51140RC

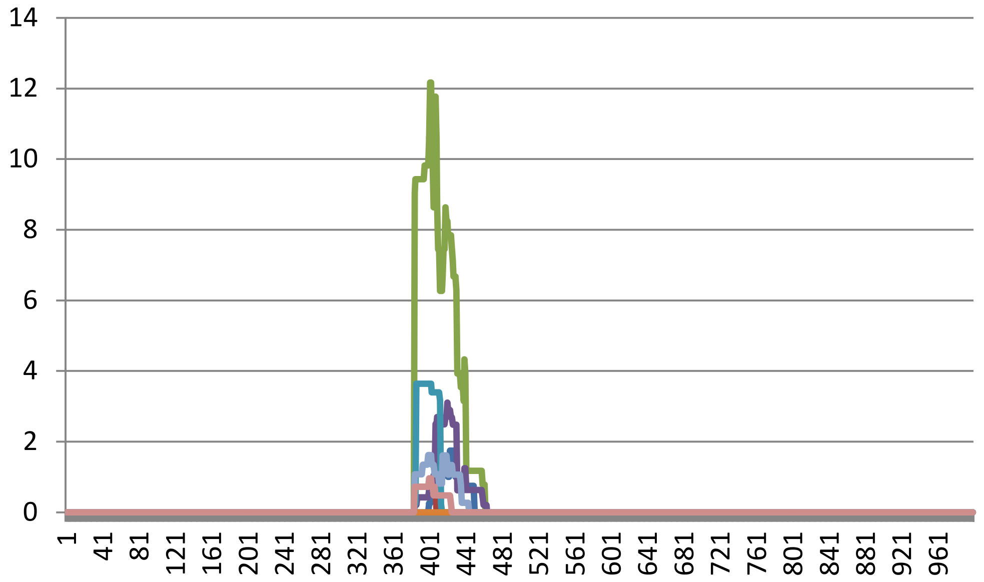

AT3G51270RC

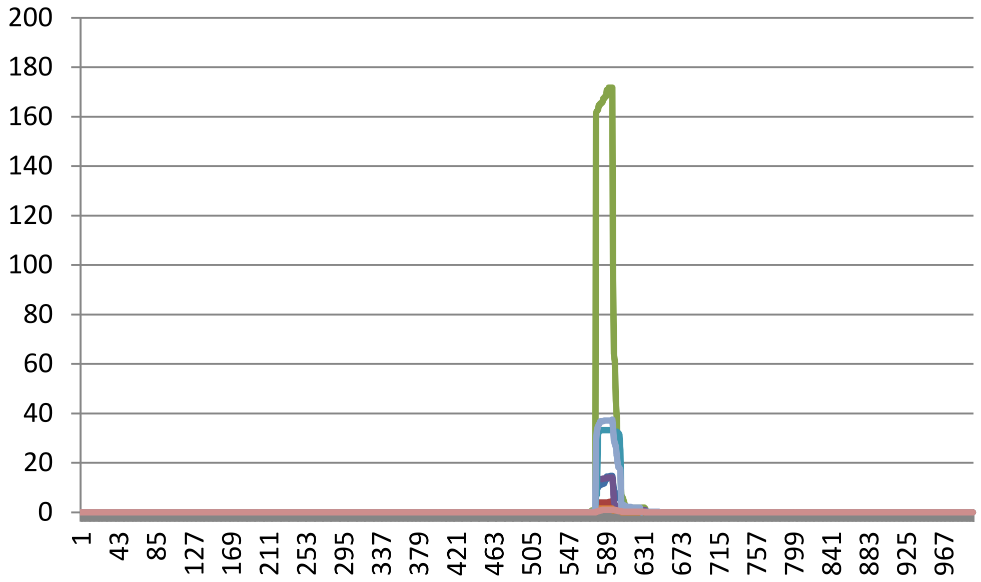

AT3G51390RC

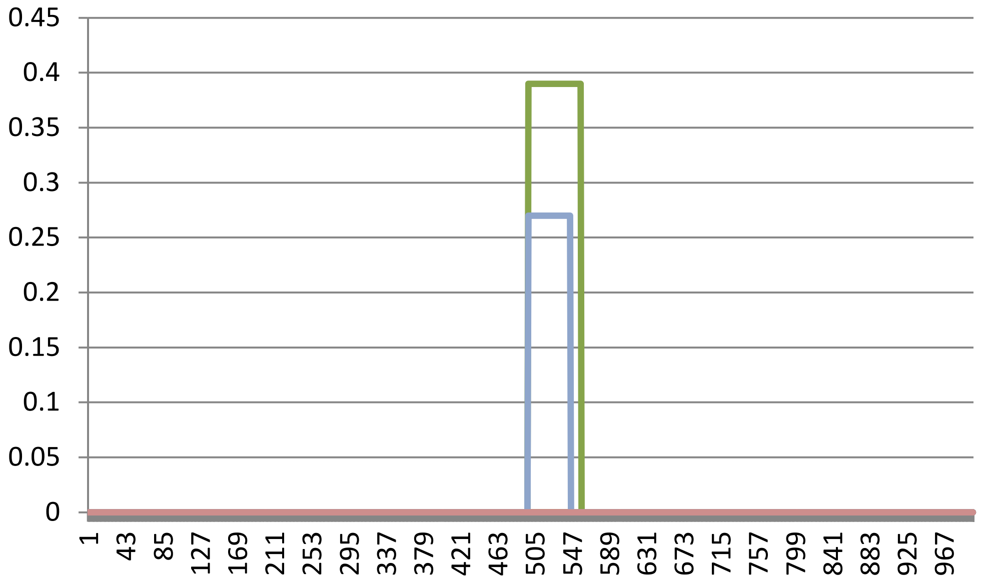

AT3G54960RC

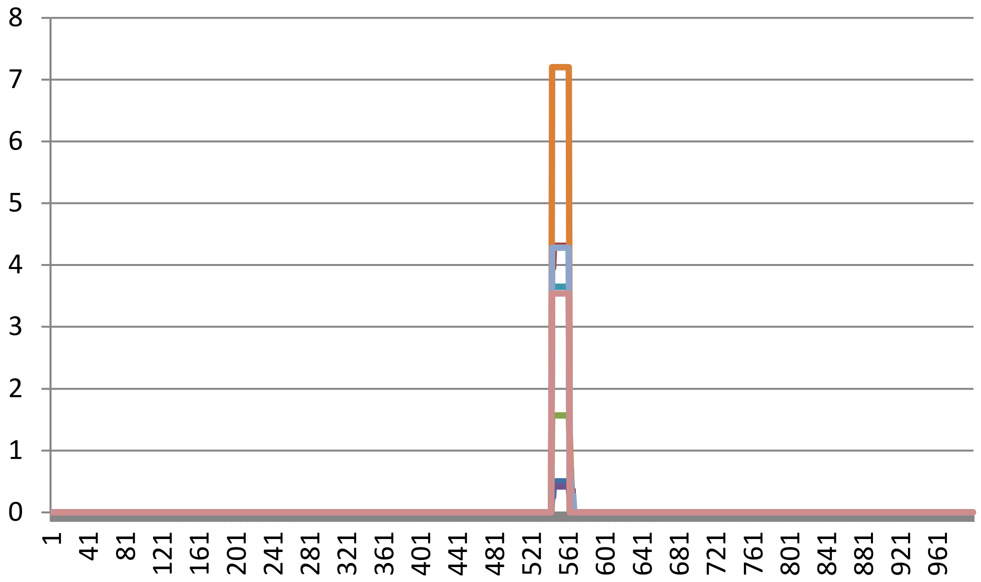

AT3G55370RC

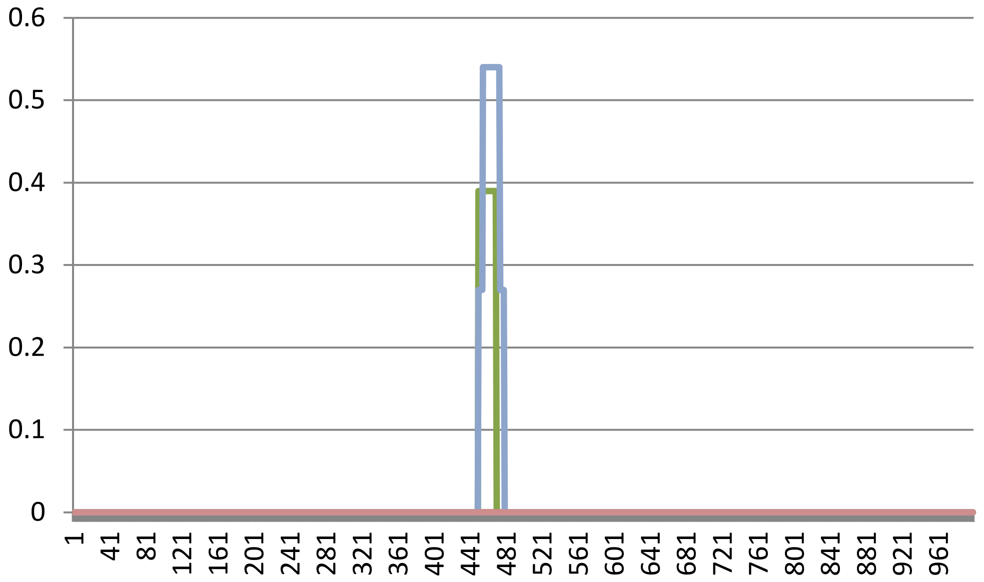

AT3G55740RC

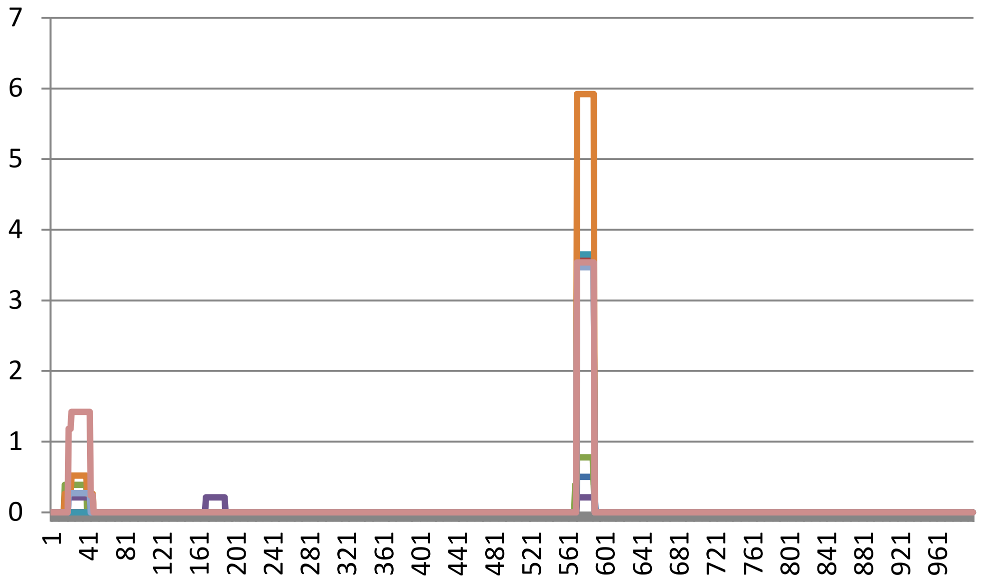

AT3G56450RC

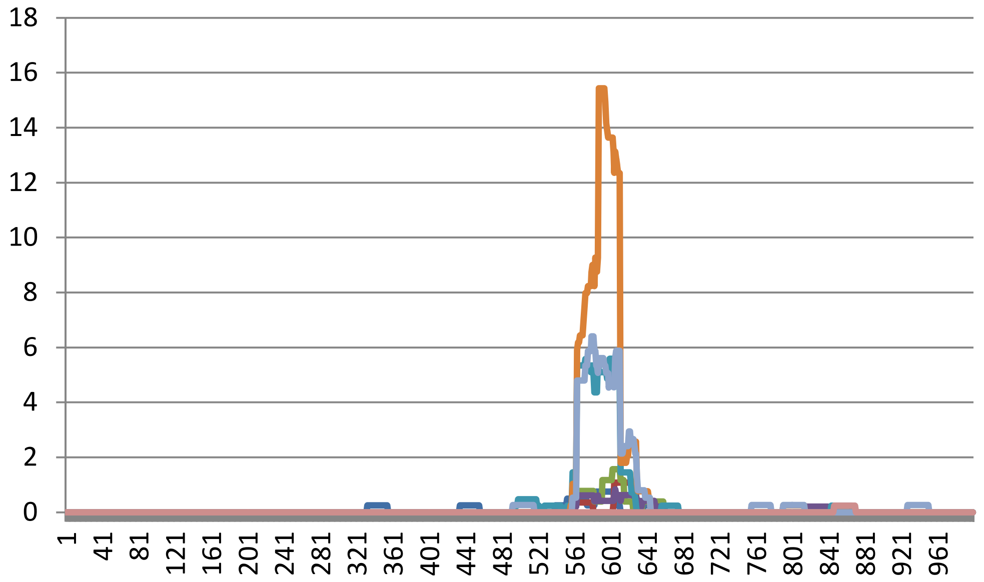

AT3G58820RC

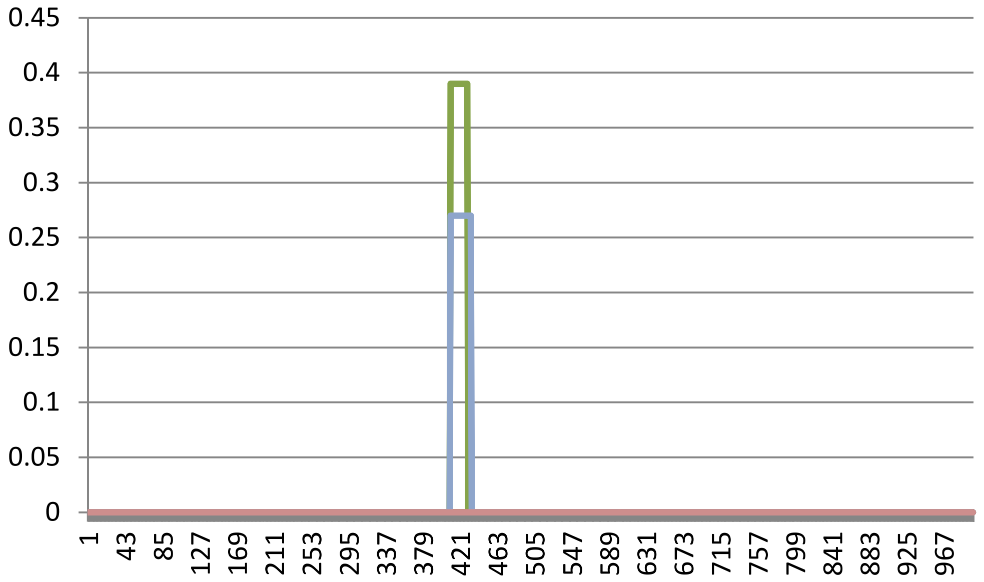

AT3G62290RC

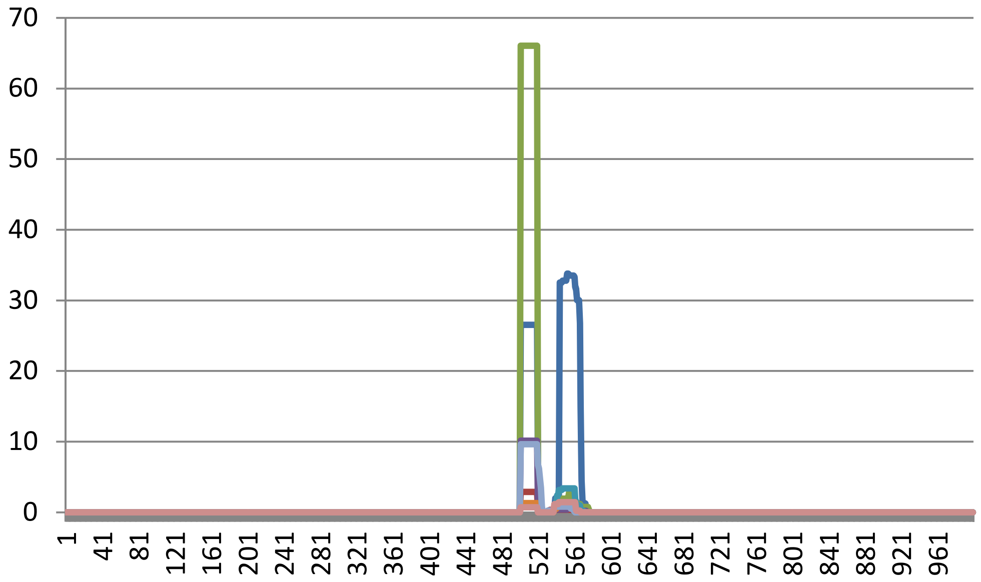

AT4G14810RC_AGO1 flower

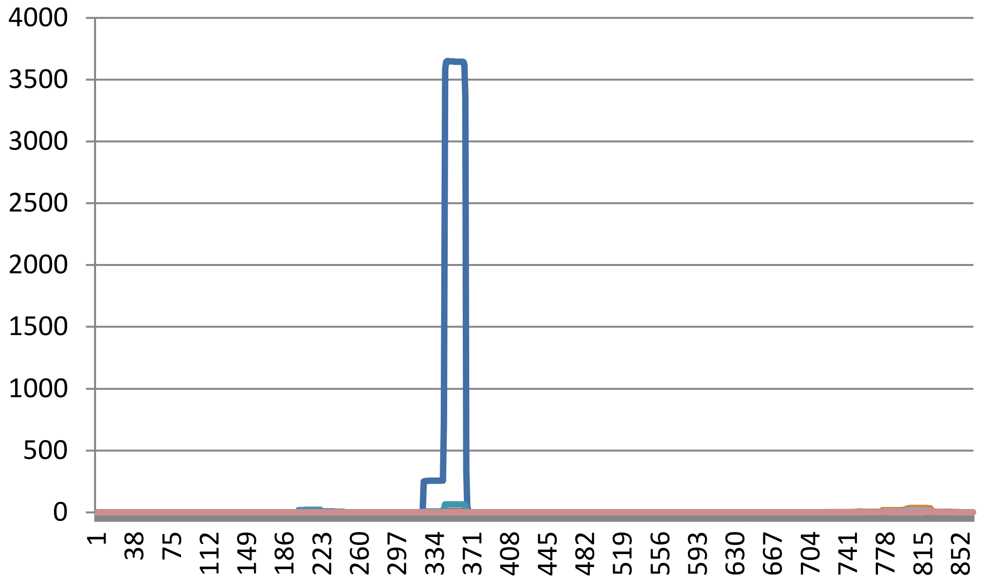

AT4G14910RC

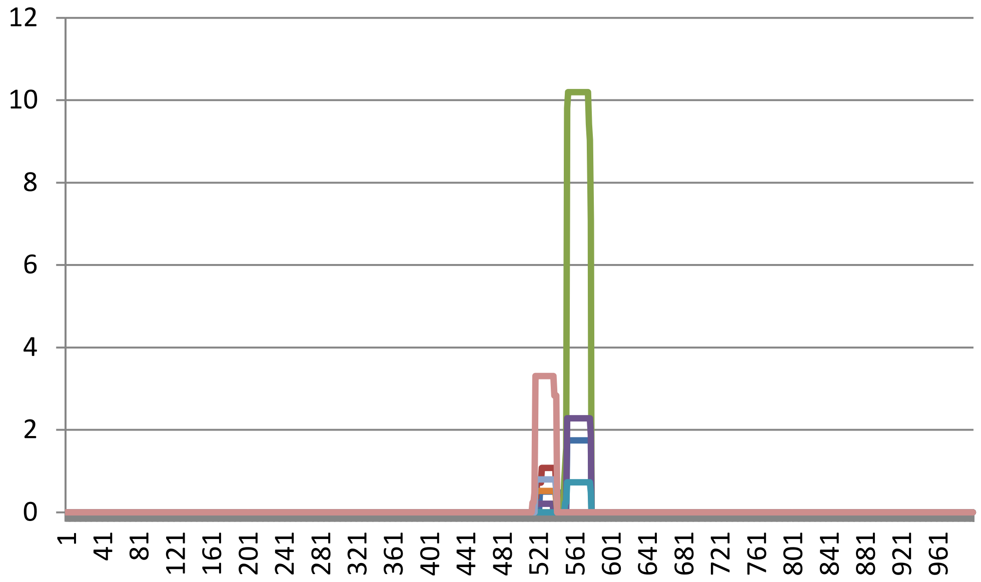

AT4G15820RC

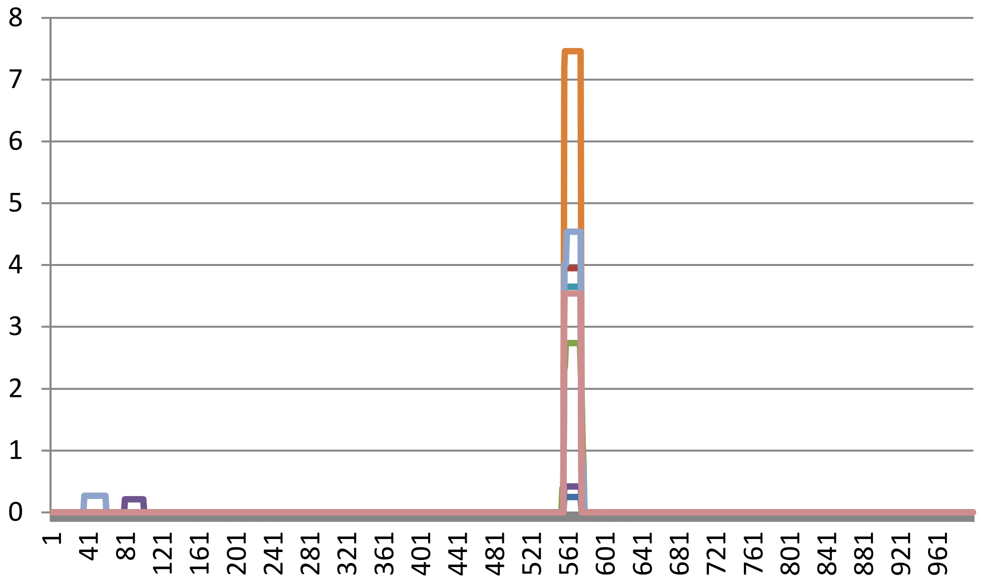

AT4G16460RC_AGO4

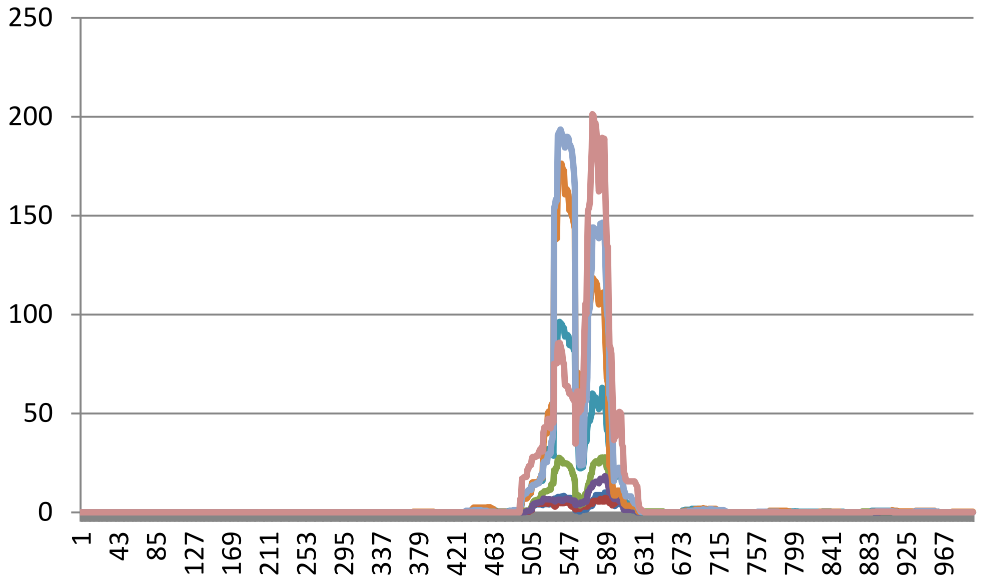

AT4G21160RC

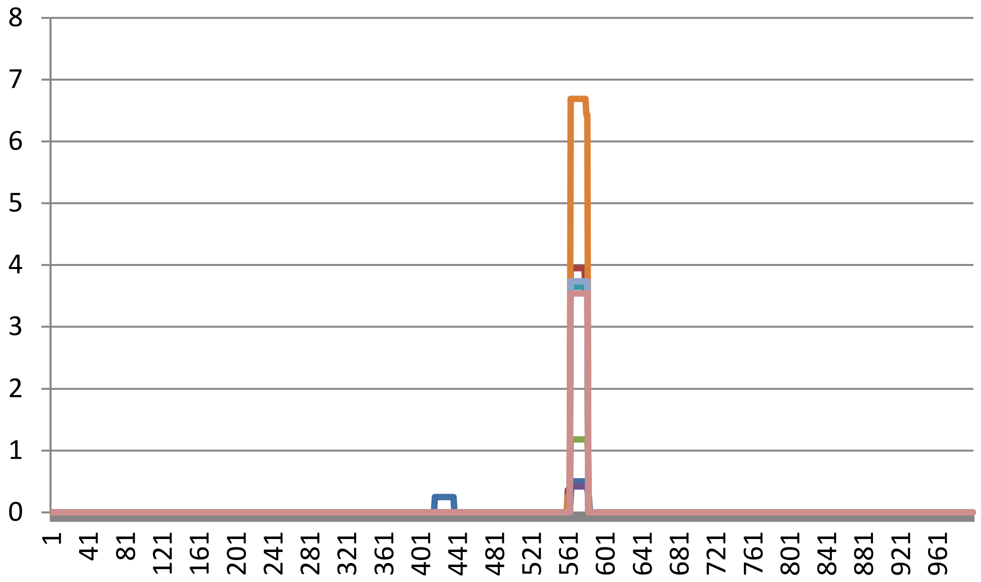

AT4G24026RC_AGO1

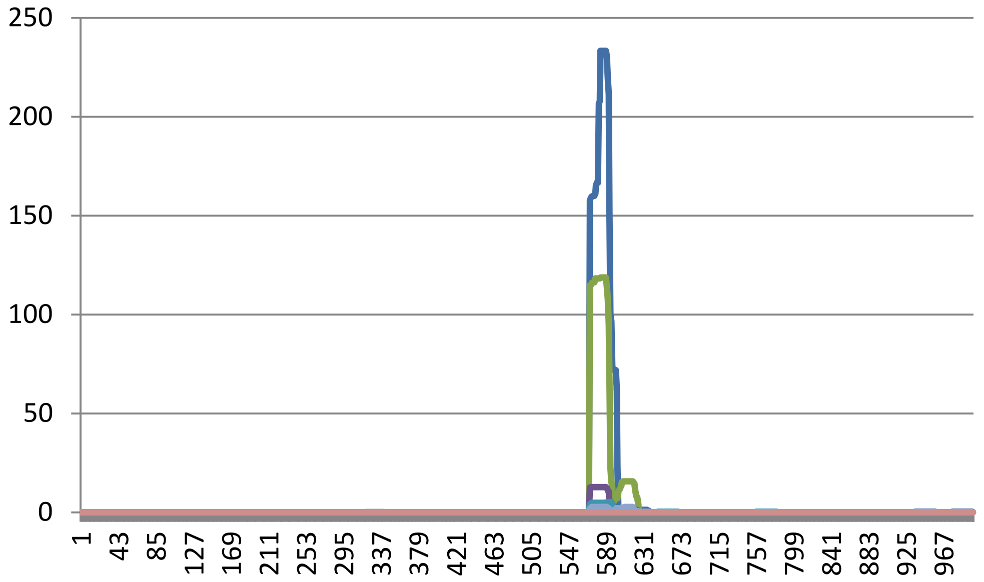

AT4G24060RC

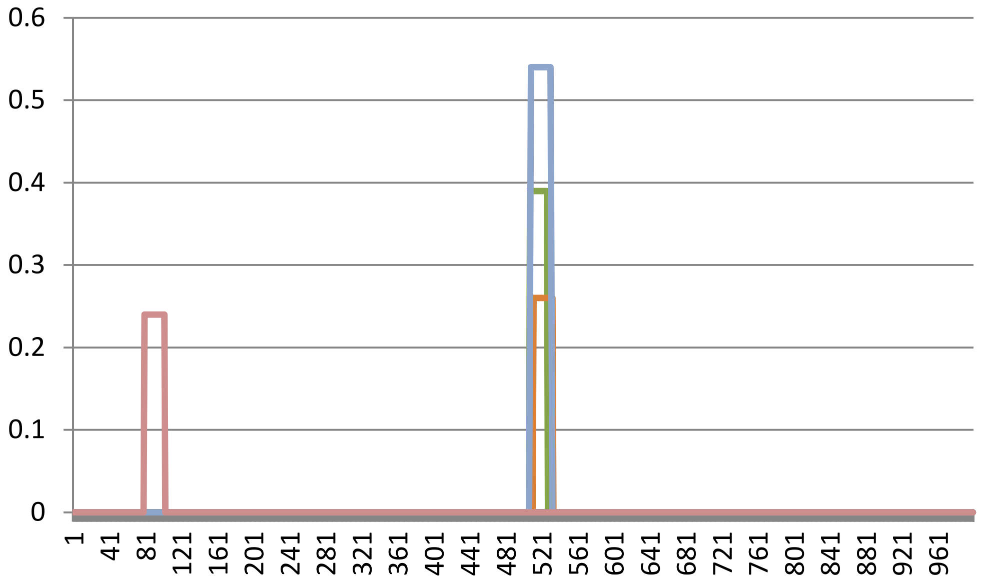

AT4G25870RC

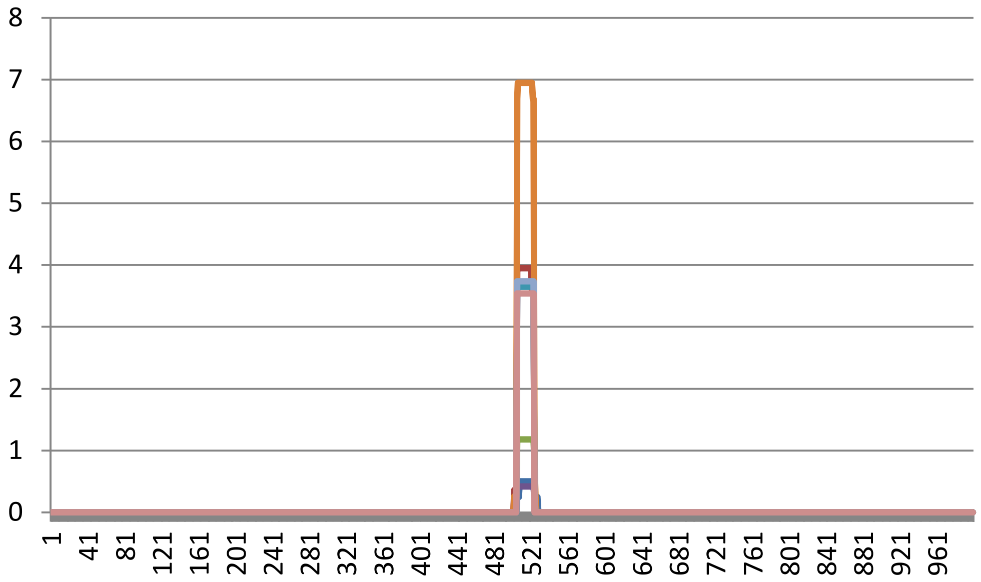

AT4G27390RC

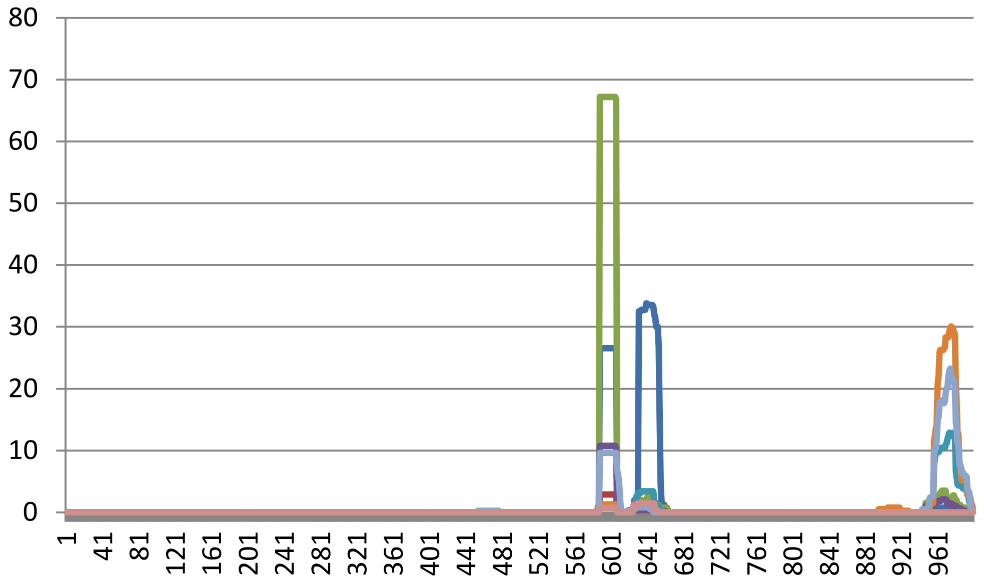

AT4G29360RC

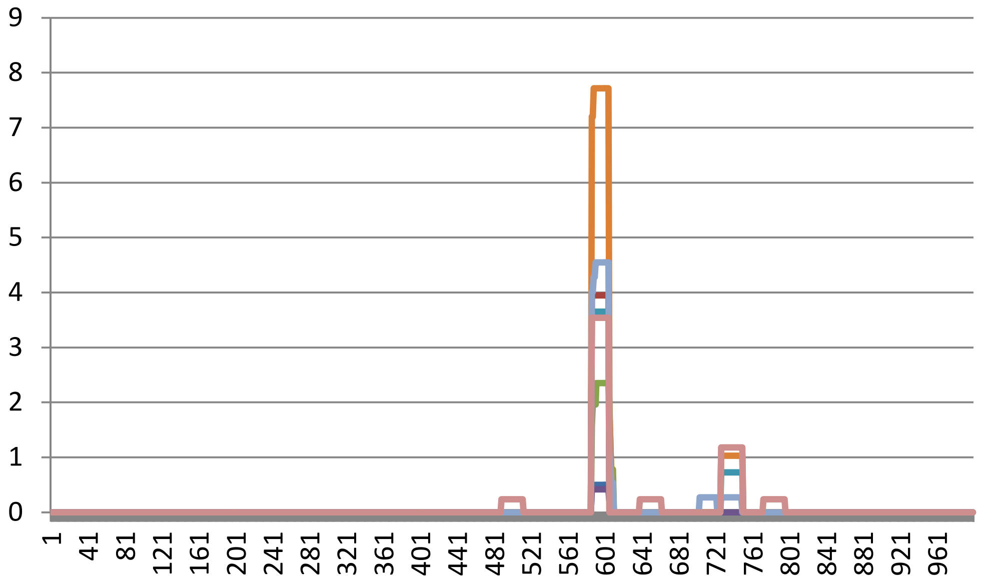

AT4G30110RC

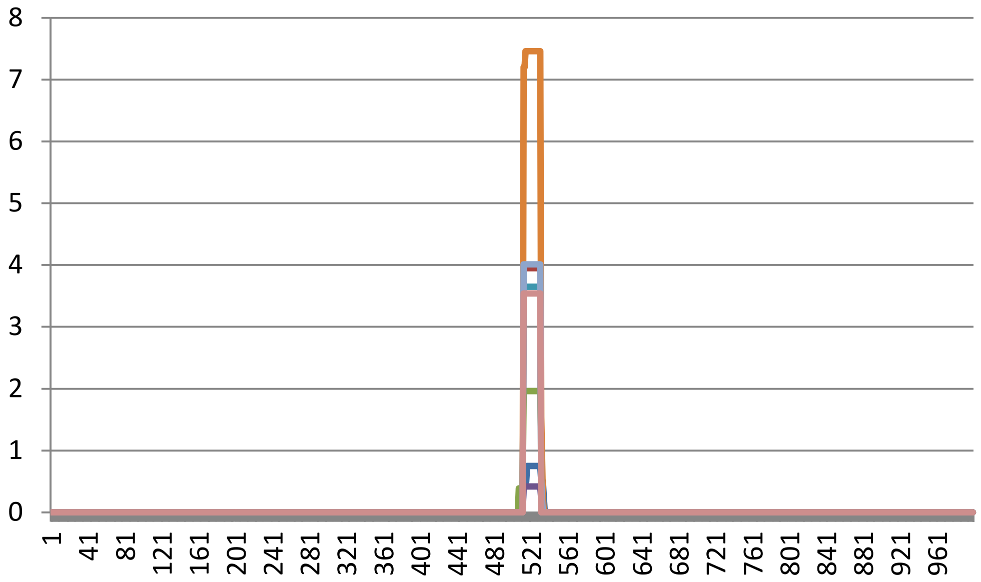

AT5G02820RC_AGO1 root

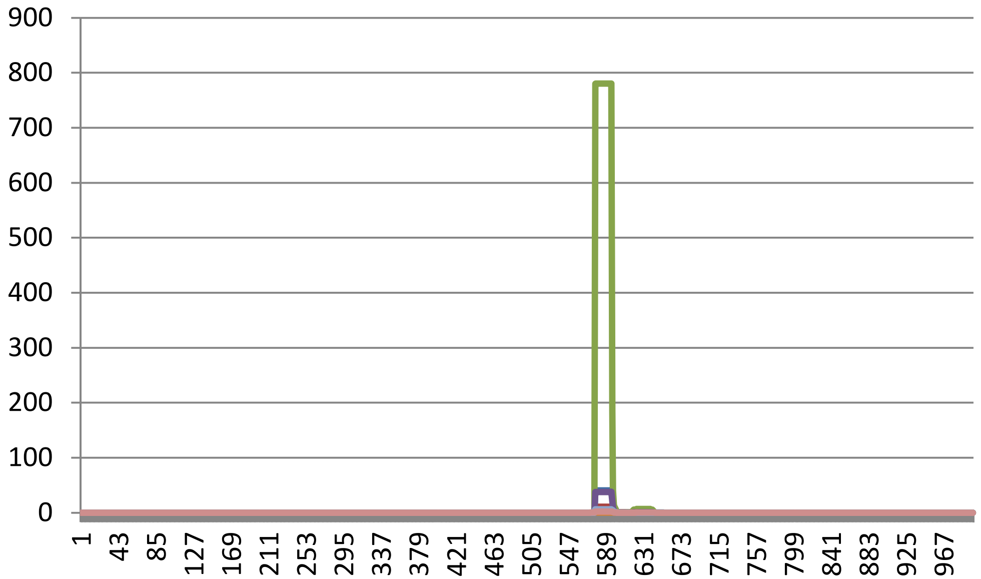

AT5G03455RC

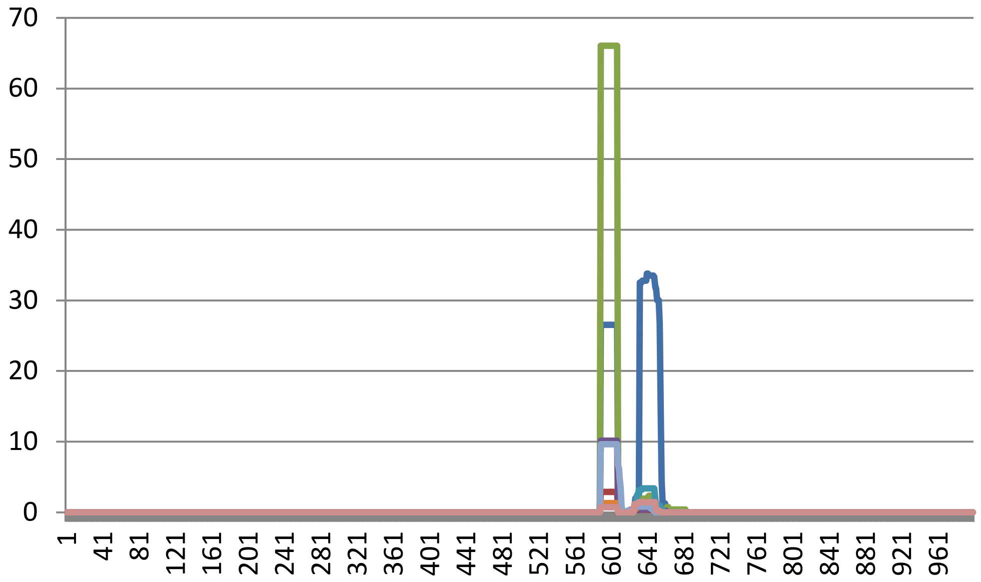

AT5G03570RC

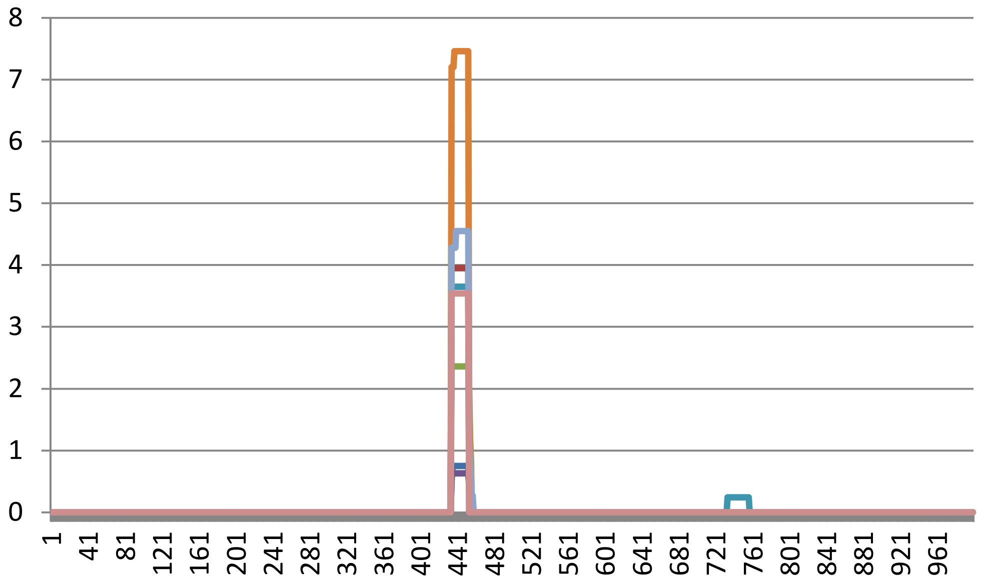

AT5G07140RC_AGO1

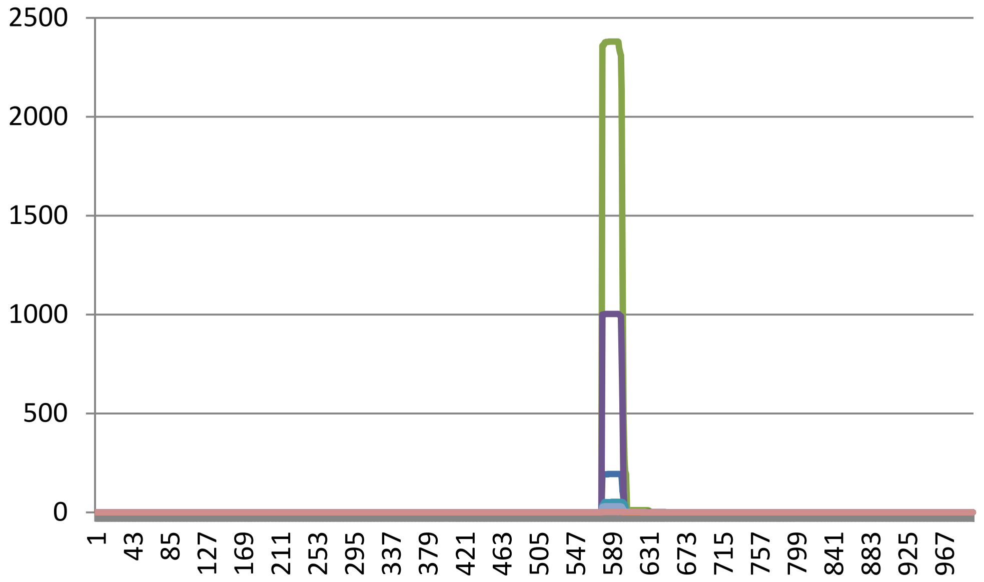

AT5G08430RC

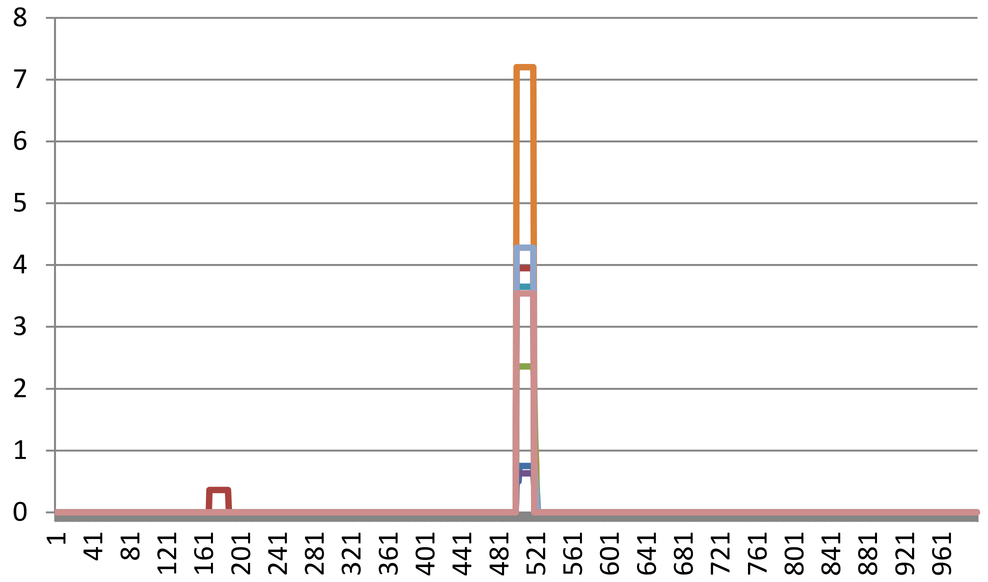

AT5G15170RC

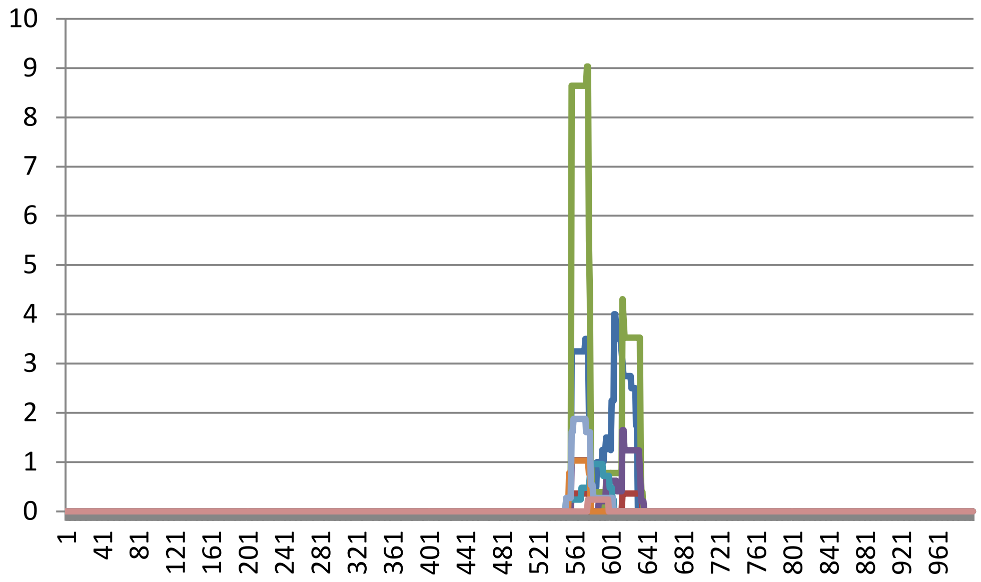

AT5G16280RC

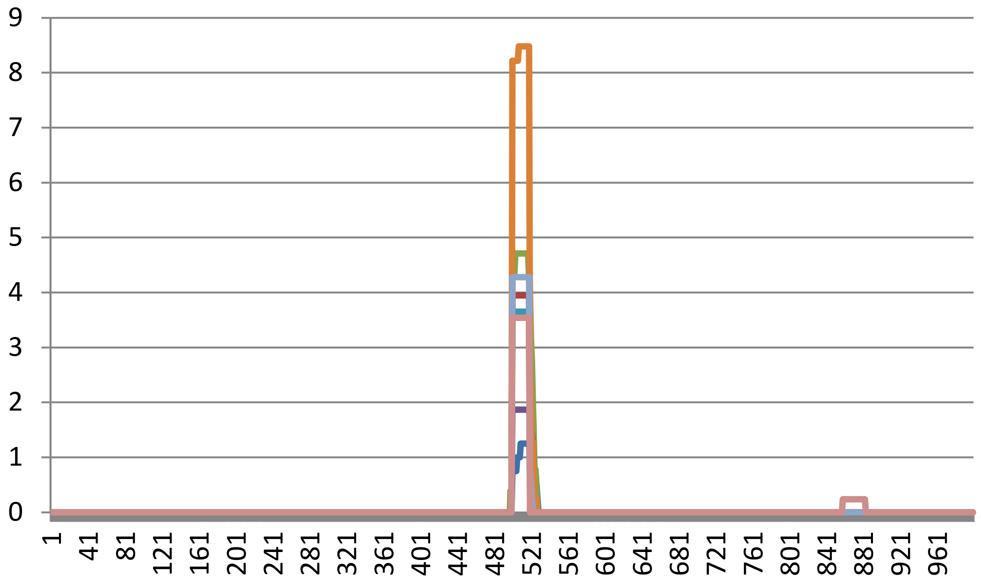

AT5G16520RC

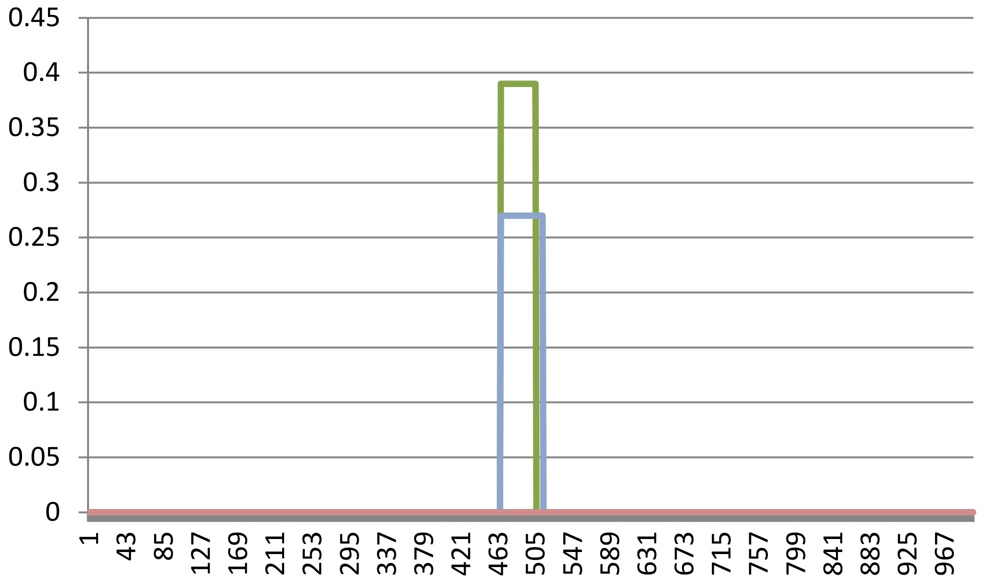

AT5G16720RC

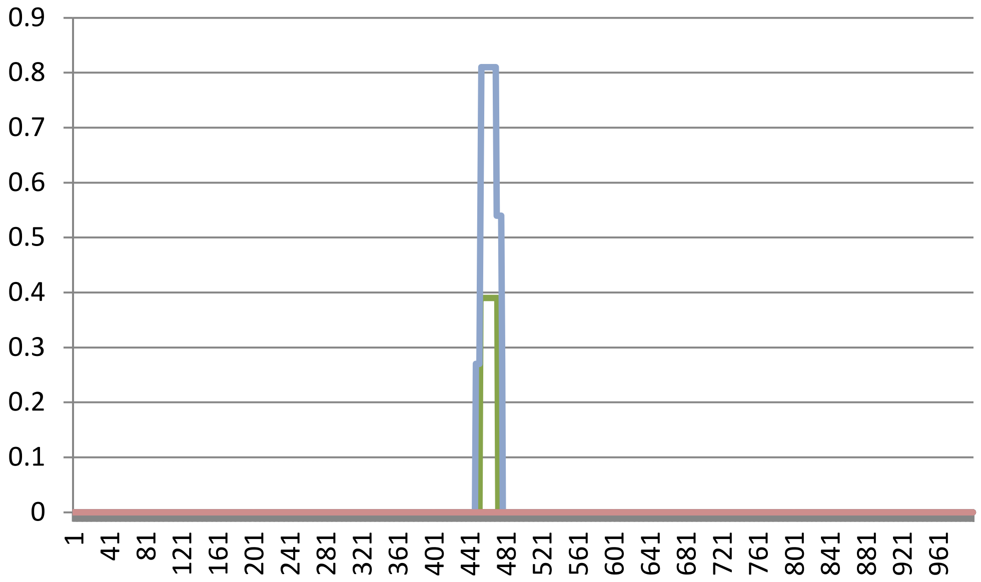

AT5G23580RC

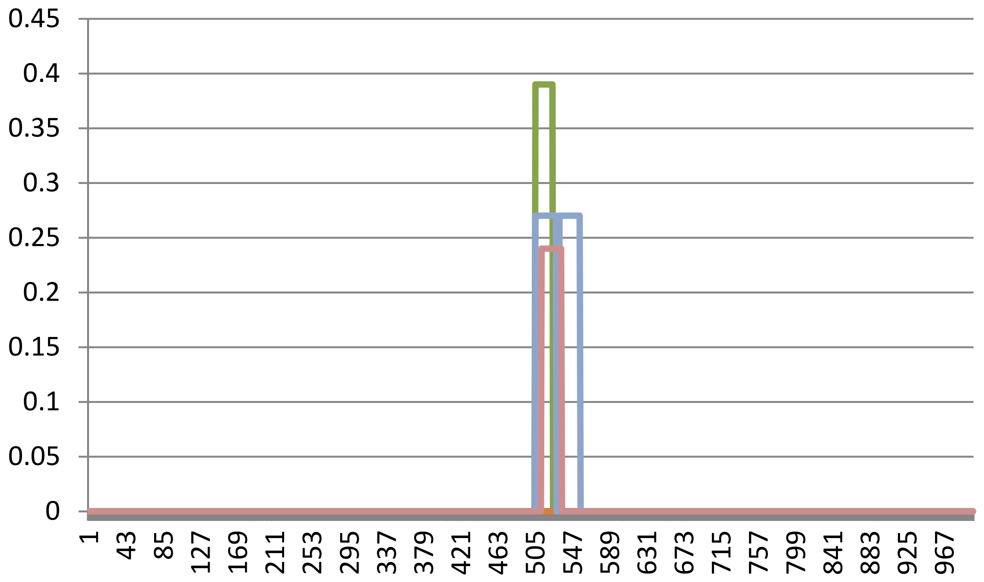

AT5G27720RC_flower&root

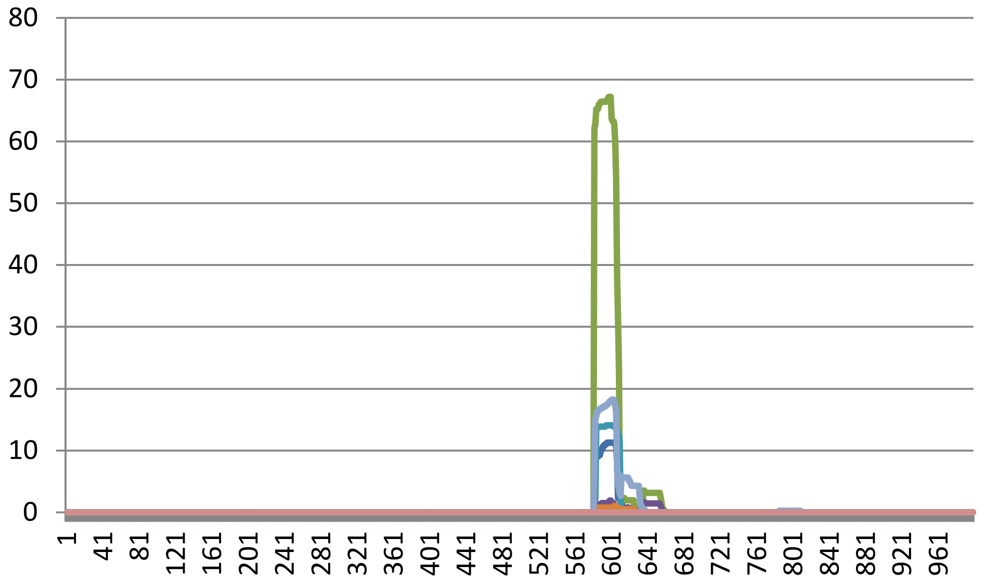

AT5G34581RC

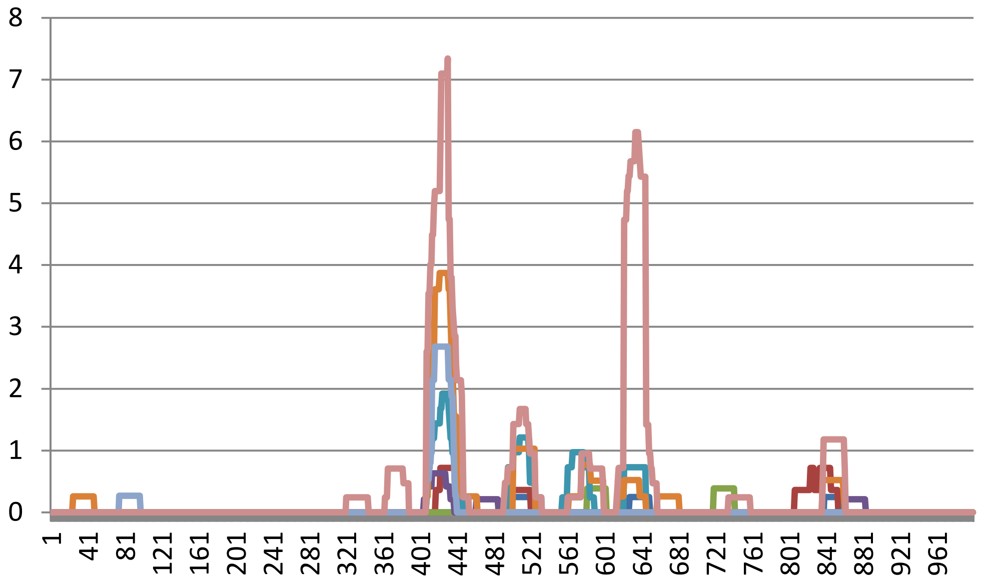

AT5G35526RC_AGO4

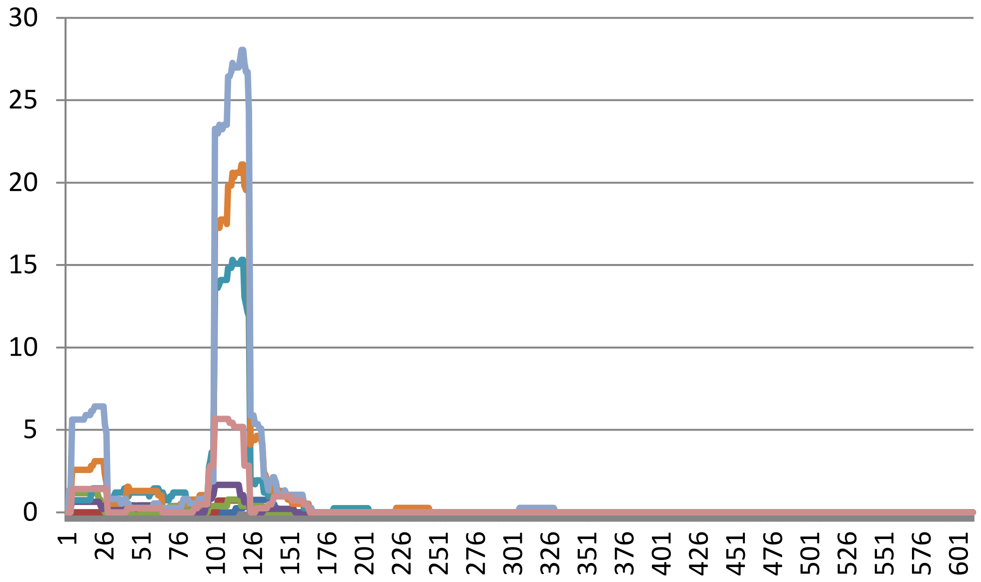

AT5G38270RC

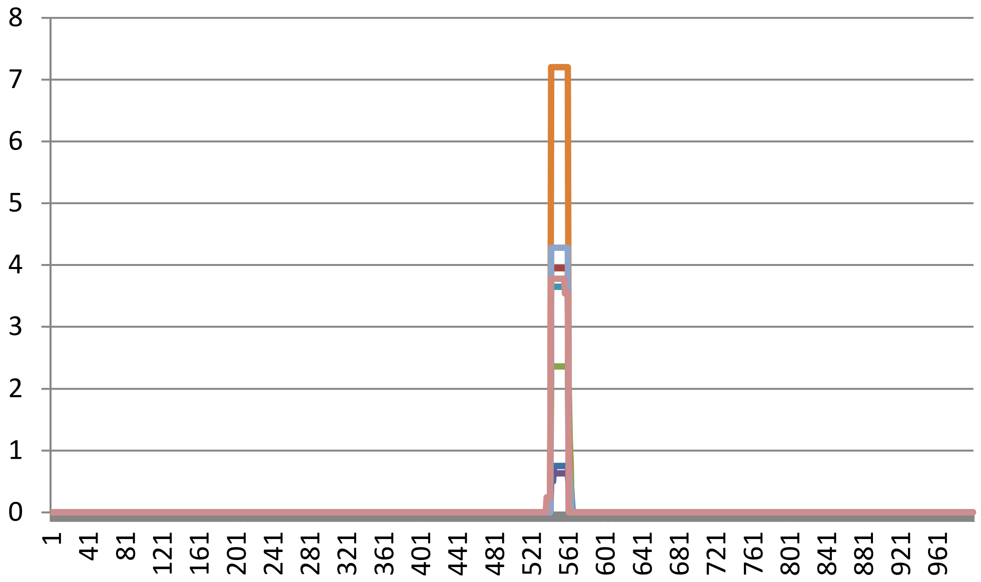

AT5G39560RC

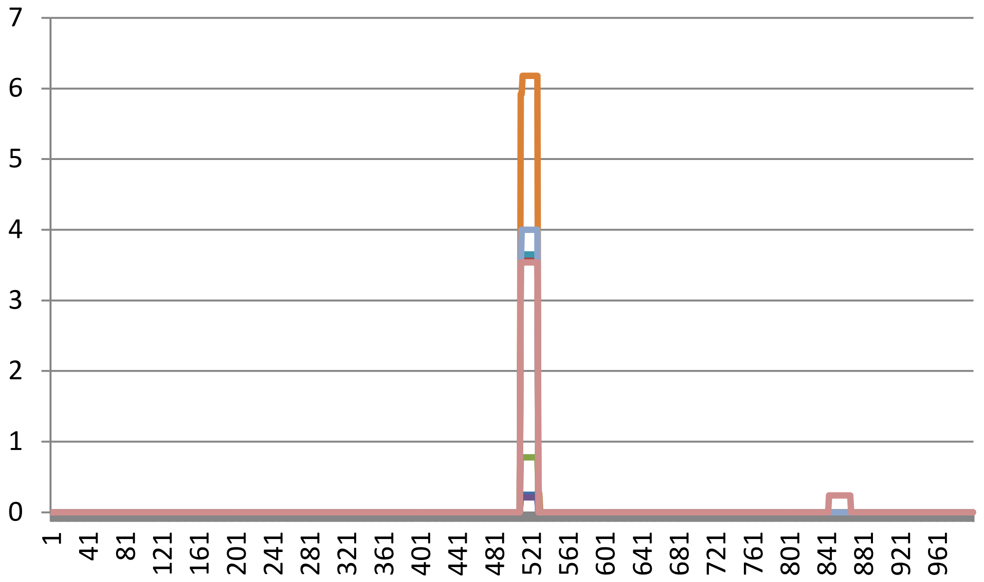

AT5G40330RC

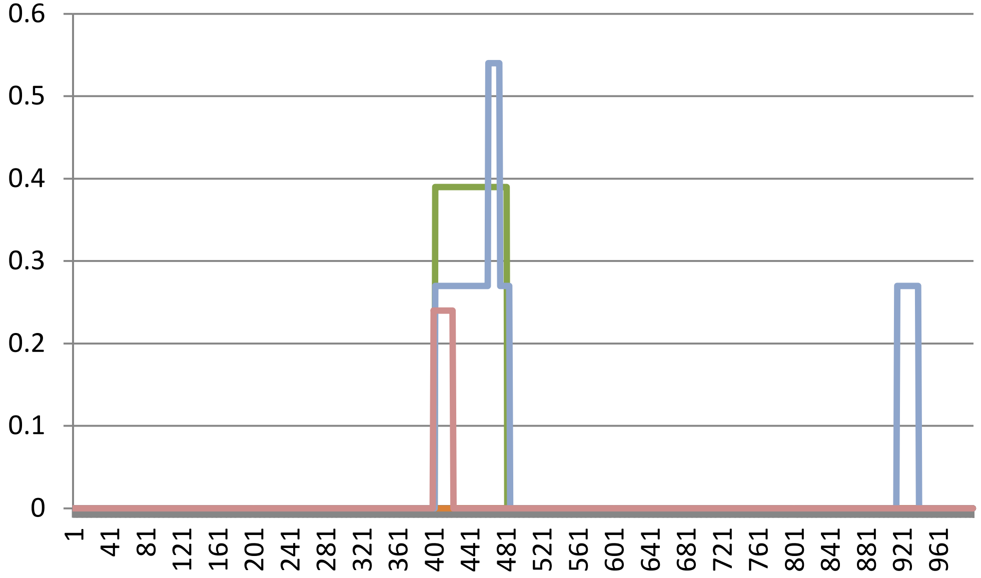

AT5G40820RC

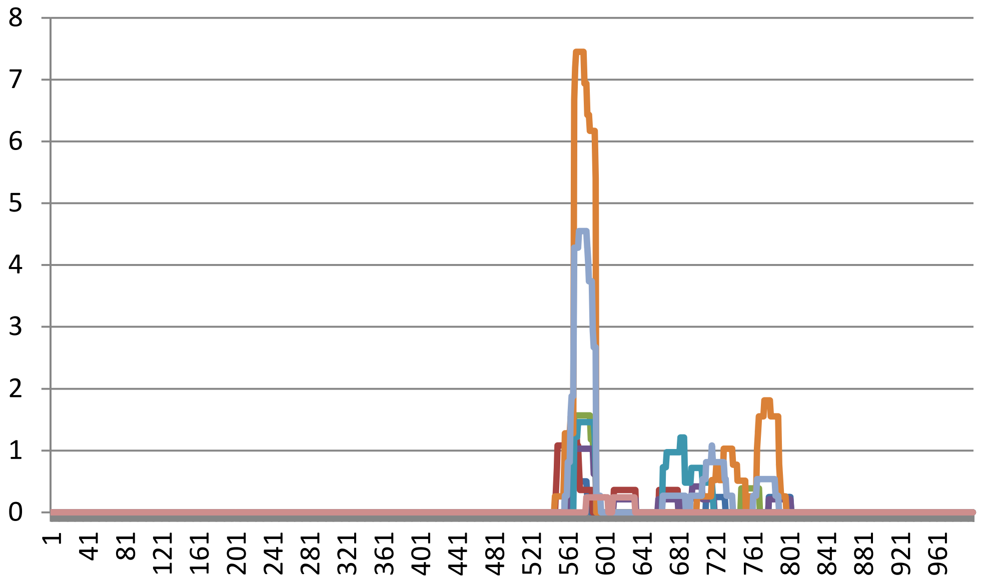

AT5G42635RC

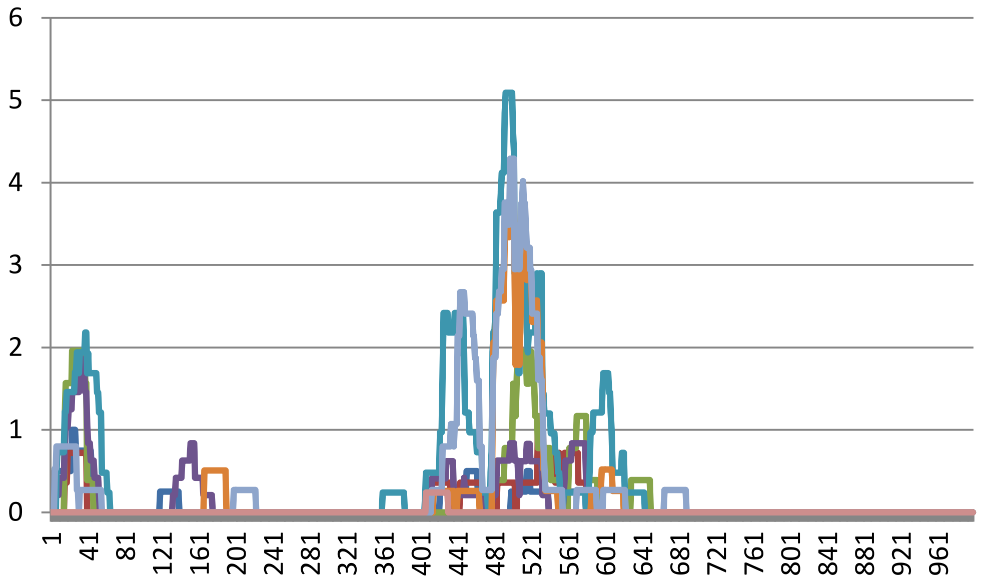

AT5G48830RC_AGO1

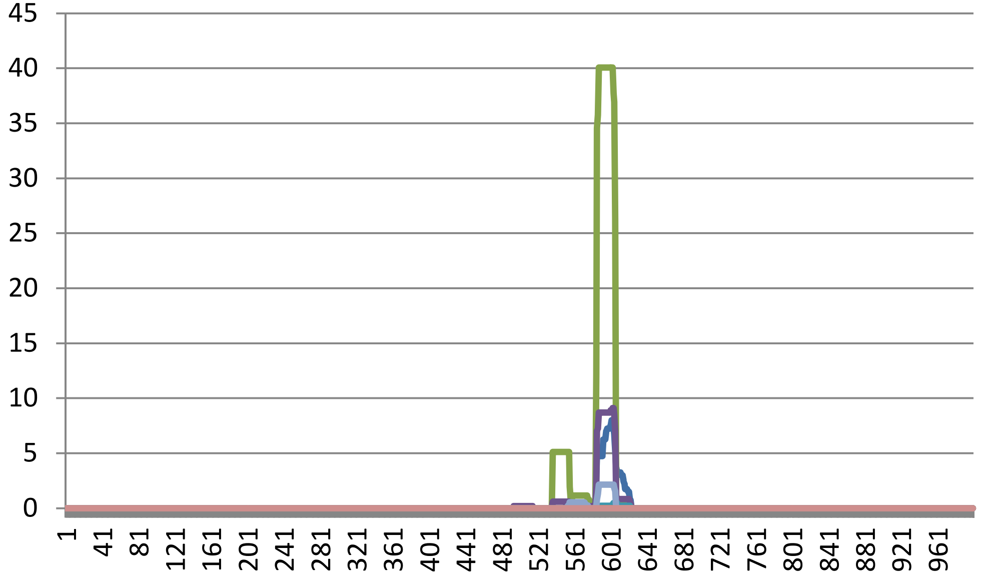

AT5G50530RC

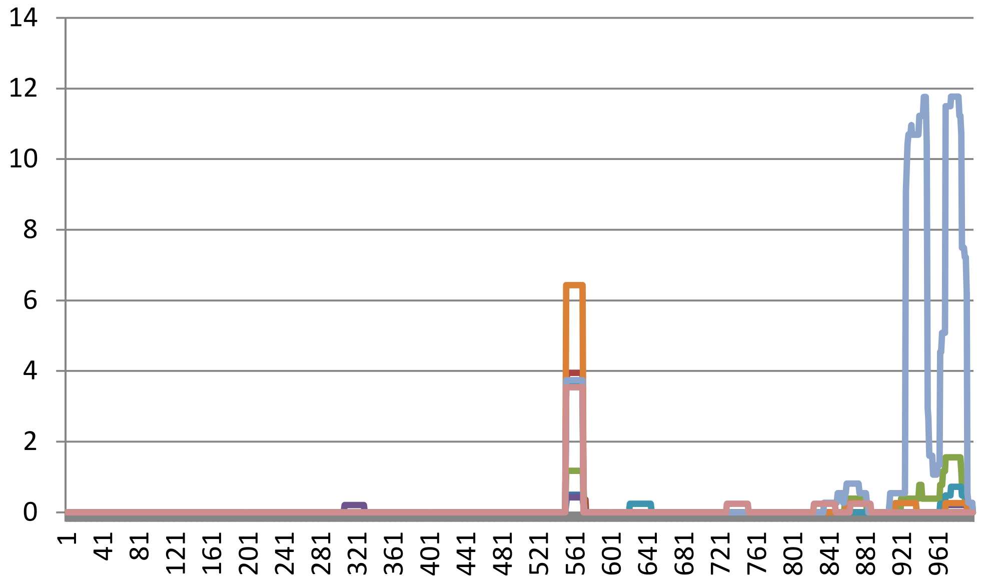

AT5G50640RC

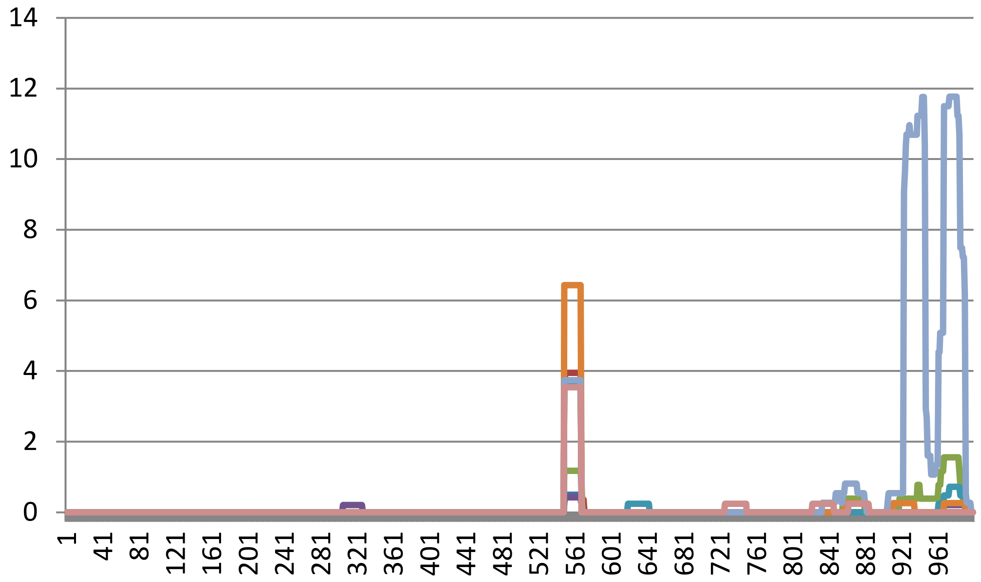

AT5G52690RC

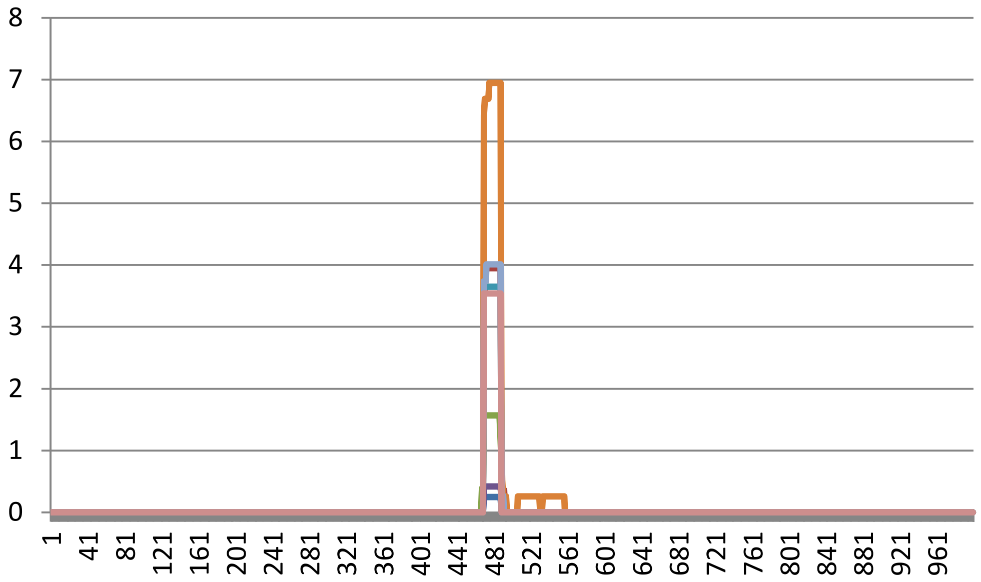

AT5G53120RC

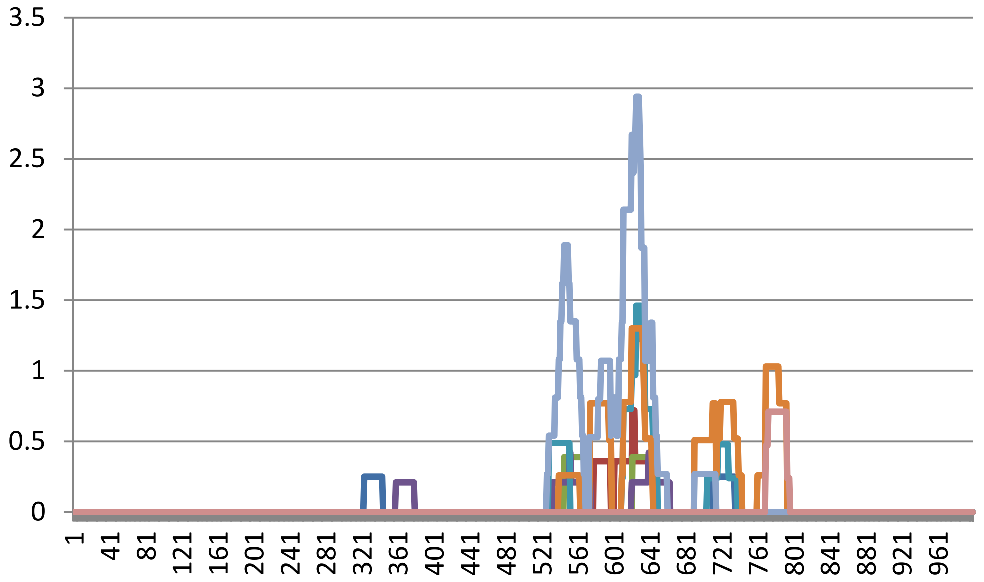

AT5G55480RC

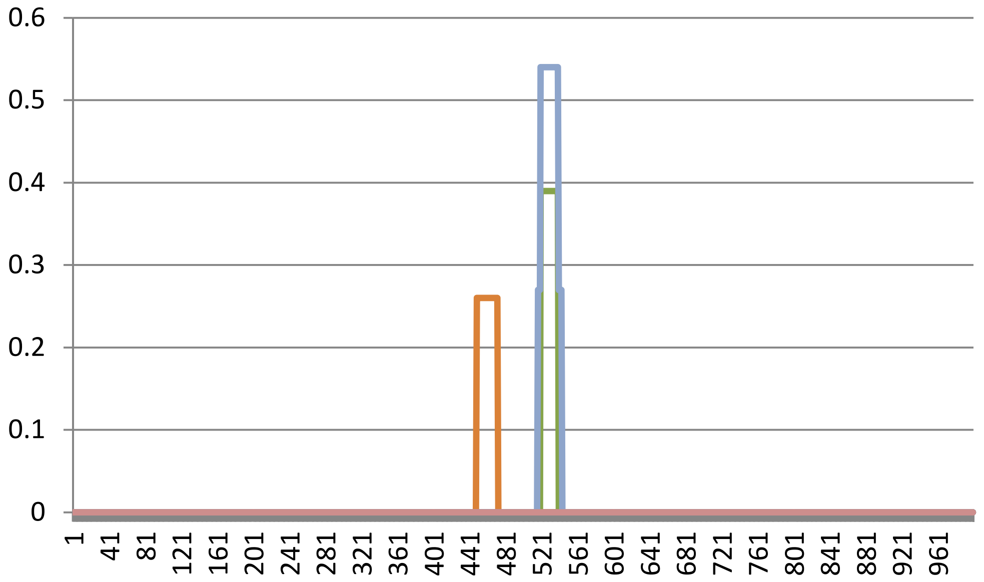

AT5G57655RC

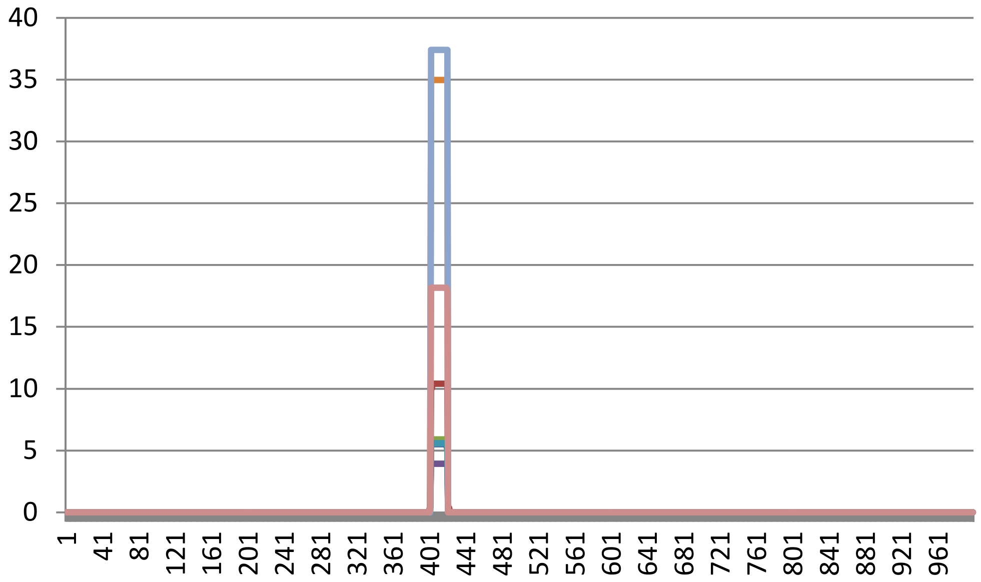

AT5G58375RC

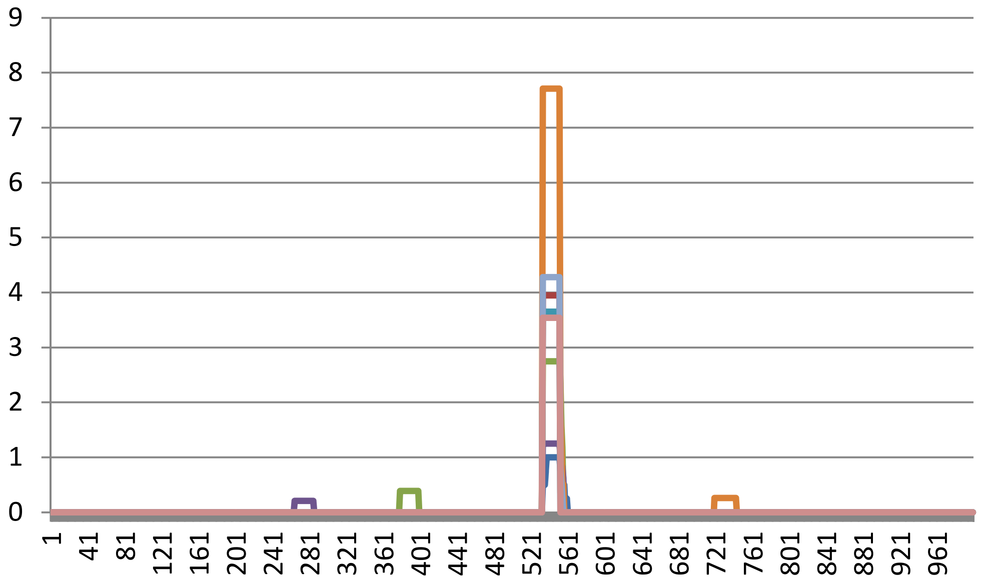

AT5G59030RC

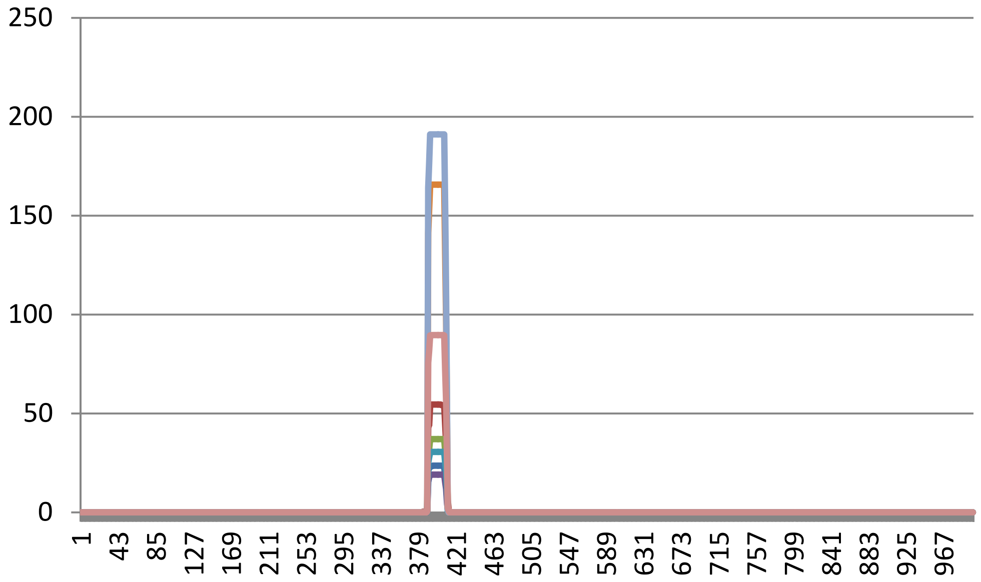

AT5G59950RC_root

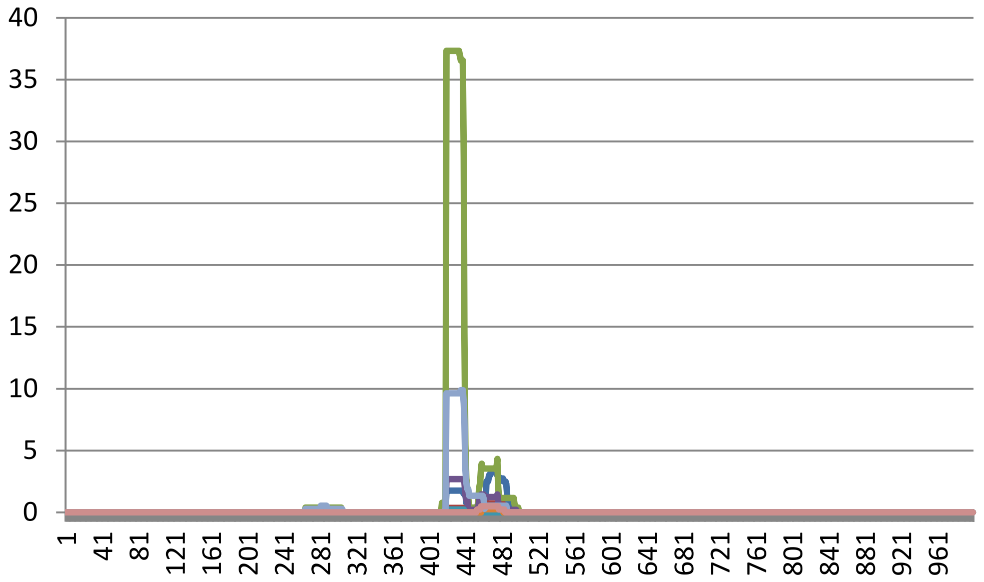

Supplement: S8 Fig — (PDF) [file pone.0169212.s008.pdf]
