# Supplementary material for: Transcriptome-Wide Discovery of PASRs (Promoter-Associated Small RNAs) and TASRs (Terminus-Associated Small RNAs) in Arabidopsis thaliana
Source: PLoS One. 2017 Jan 3;12(1):e0169212. doi: 10.1371/journal.pone.0169212 (PMC5207706; doi:10.1371/journal.pone.0169212)

Figure S9 AGO-associated paired PASR peaks identified on both strands of the protein-coding genes of *Arabidopsis*. For each plot, x axis measures the position on the paired strands, and y axis measures the abundance (in RPM, reads per million) of sRNAs.

AT1G03810

Nucleic acid-binding,
OB-fold-like protein

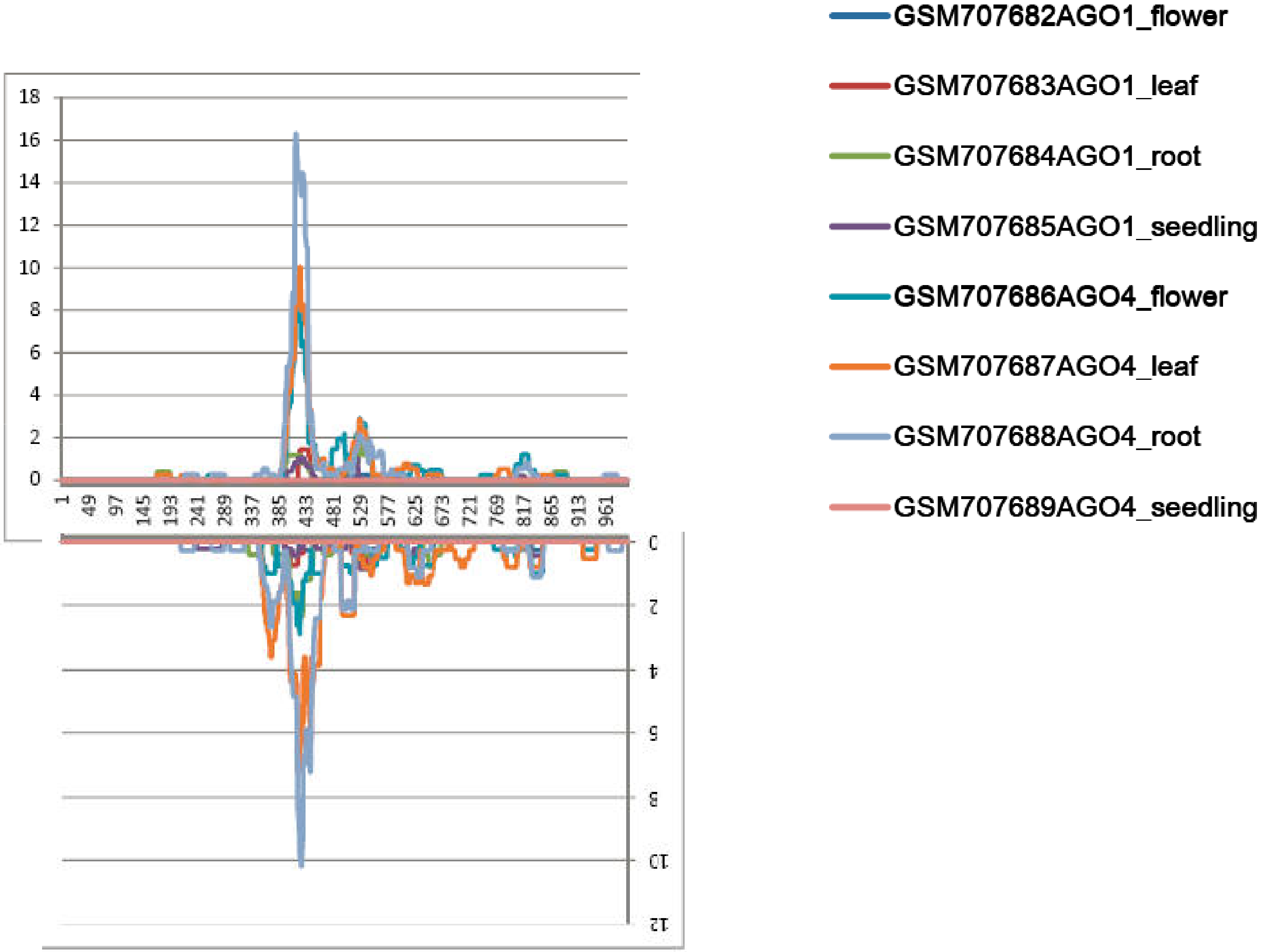

AT1G07660

Histone superfamily protein

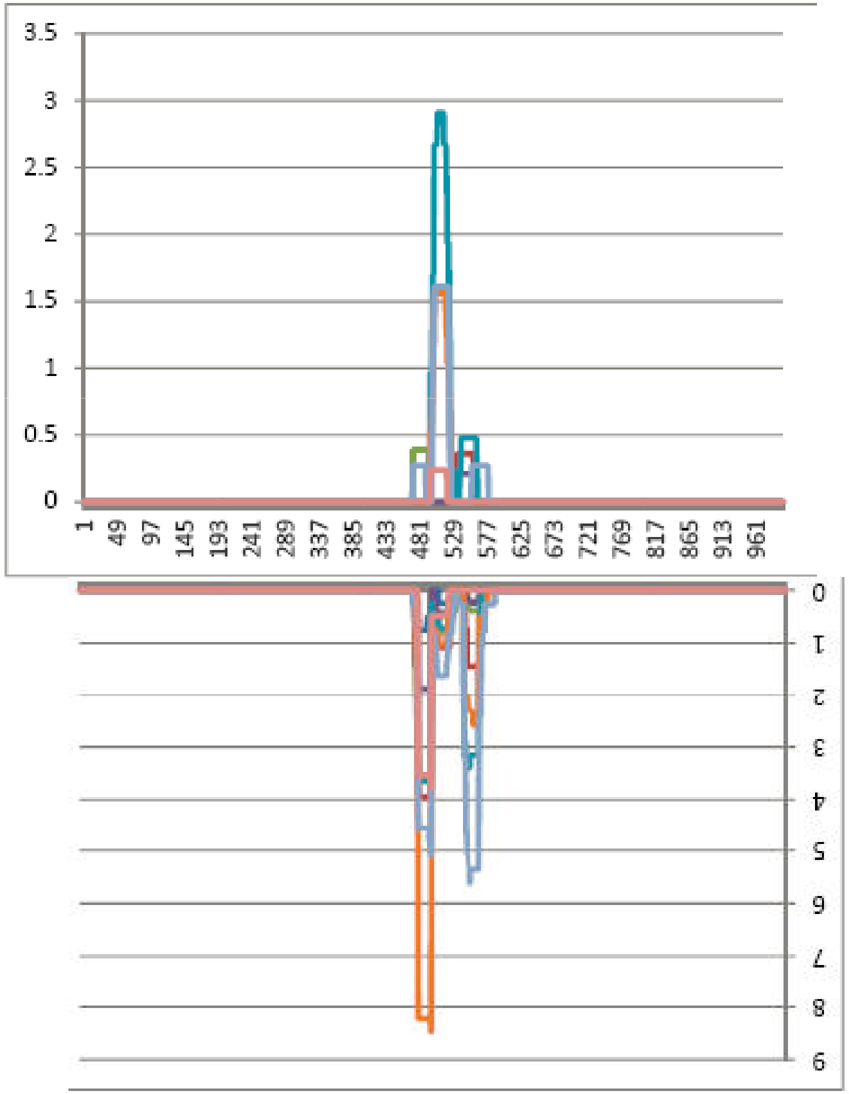

AT1G16820

vacuolar ATP synthase catalytic subunit-related / V-ATPase-related / vacuolar proton pump-related

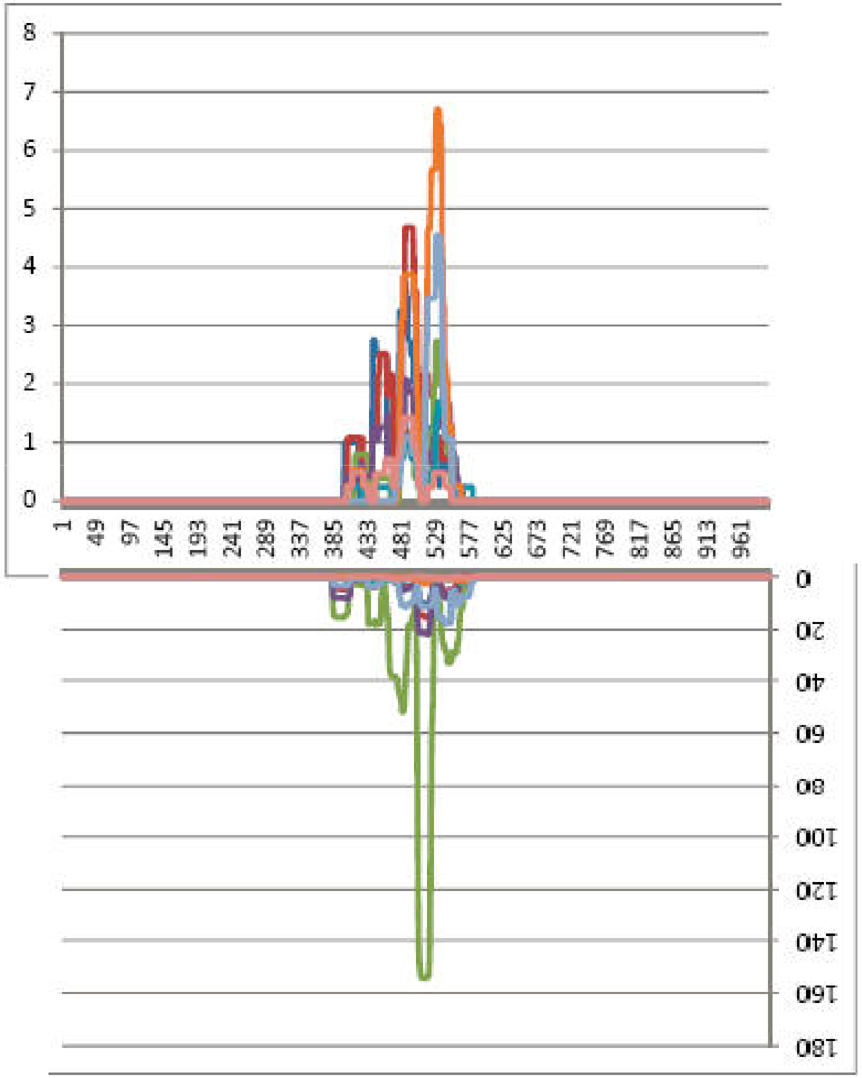

AT1G17270

O-fucosyltransferase family protein

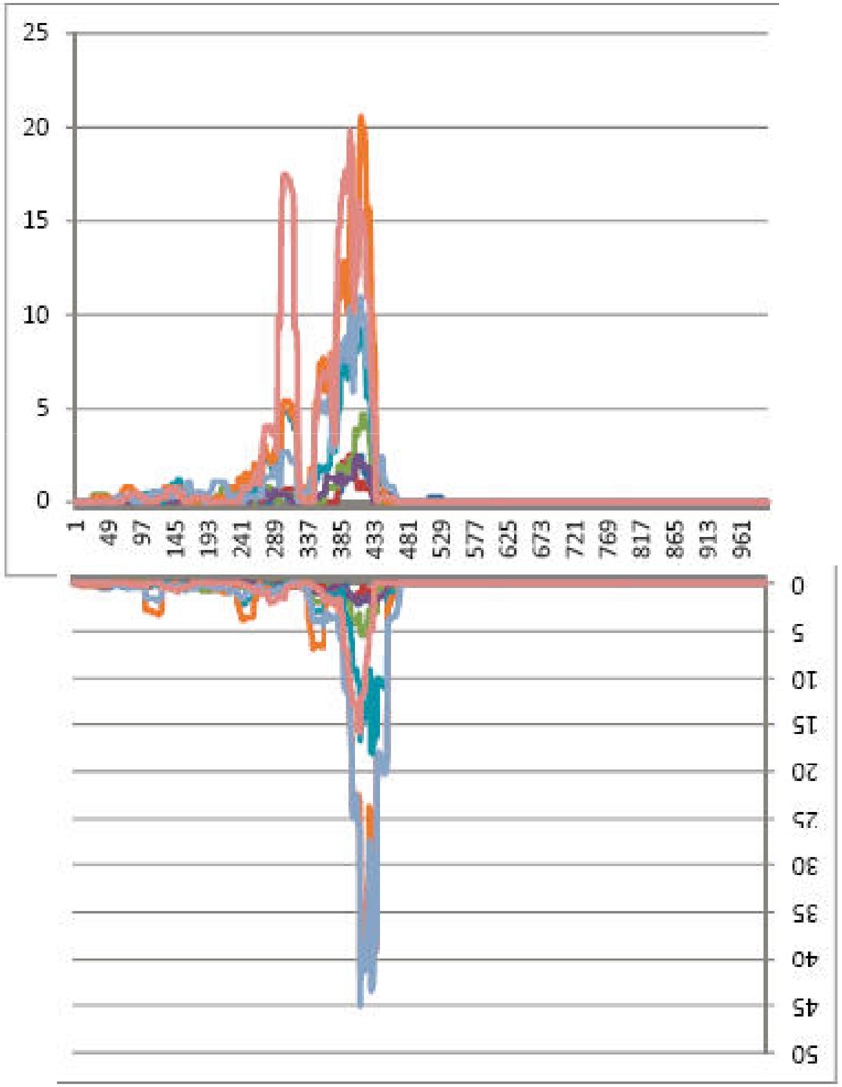

AT1G32140

F-box family protein

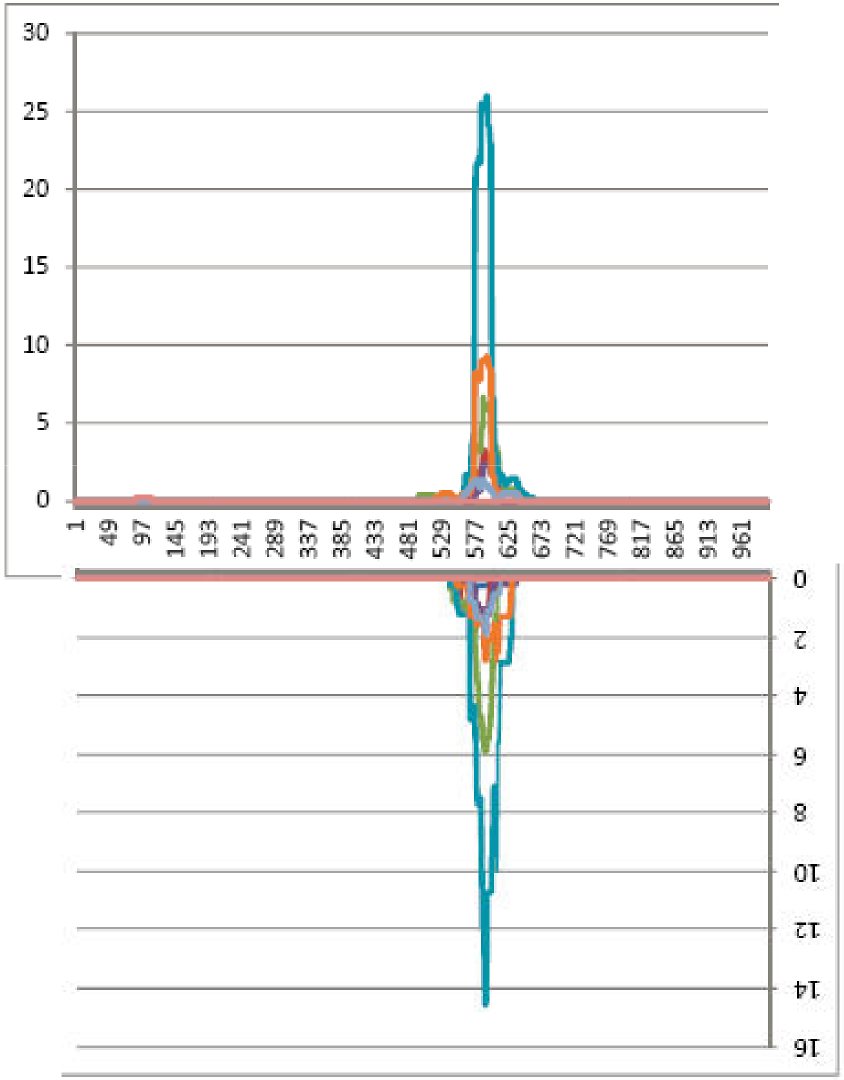

AT1G52110

Mannose-binding lectin superfamily protein

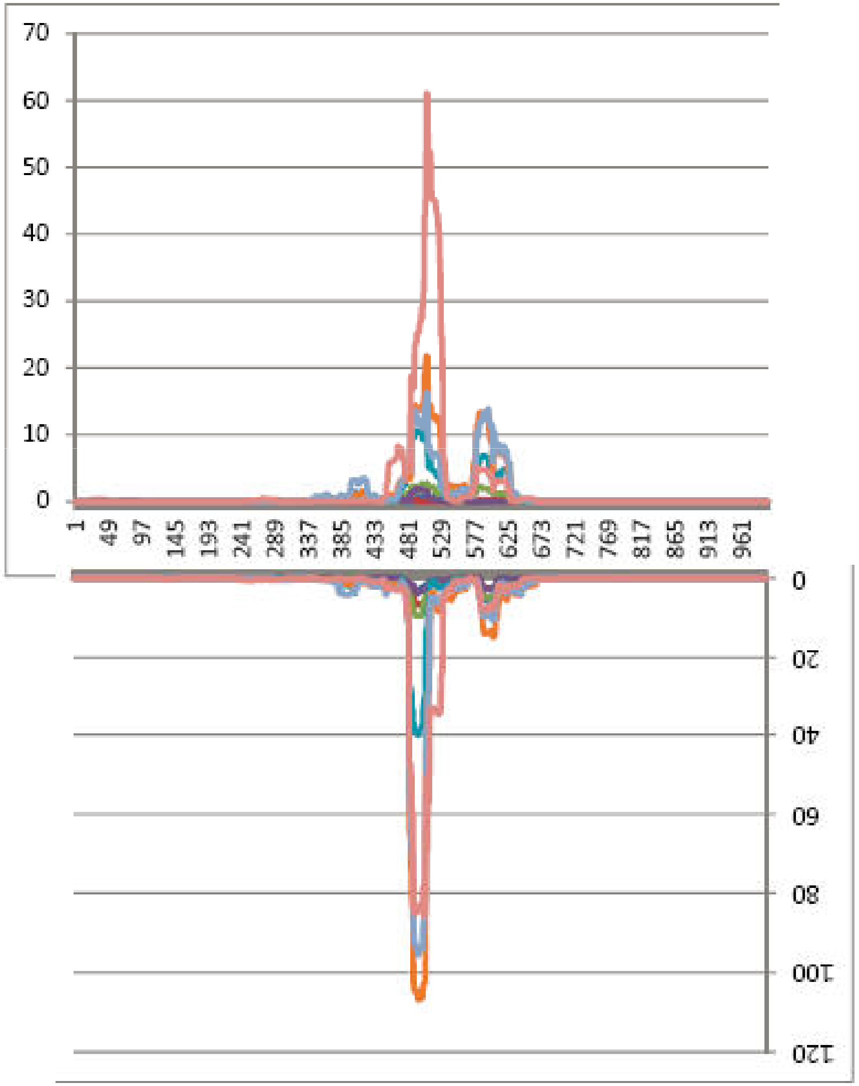

AT1G52940

purple acid phosphatase 5 (PAP5)

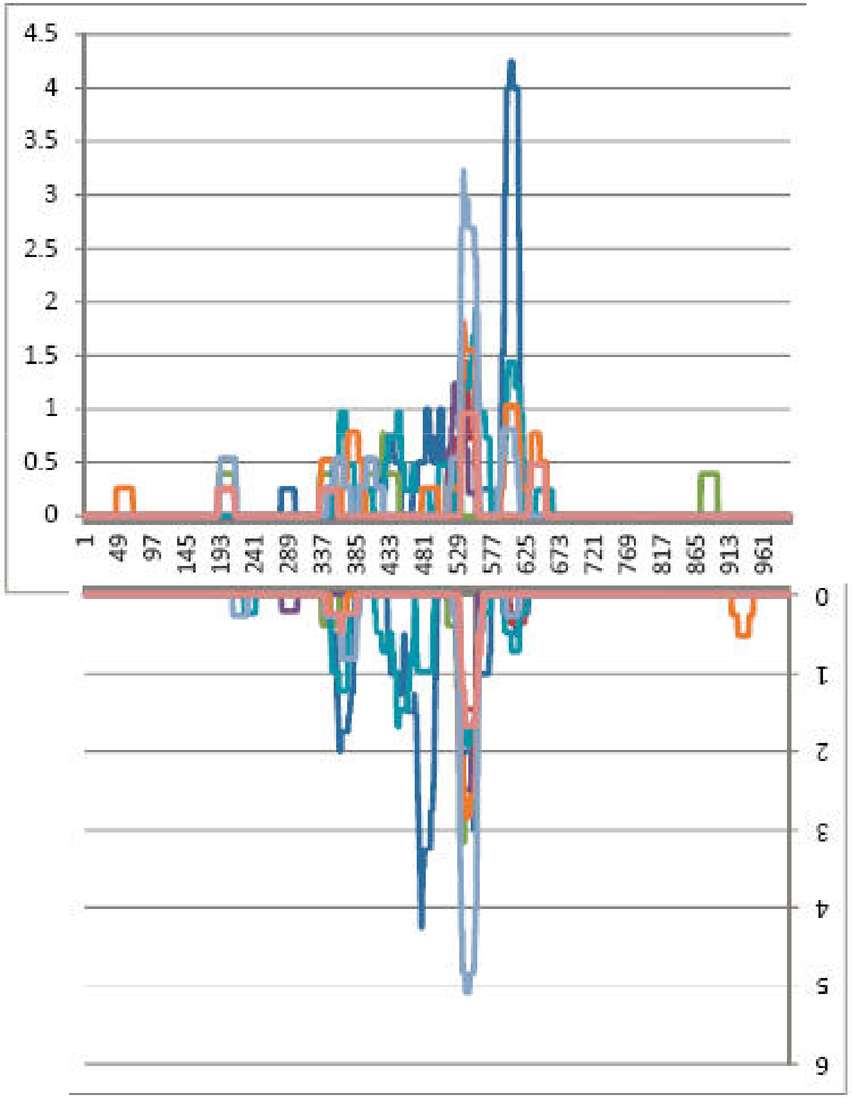

AT1G53265

unknown protein

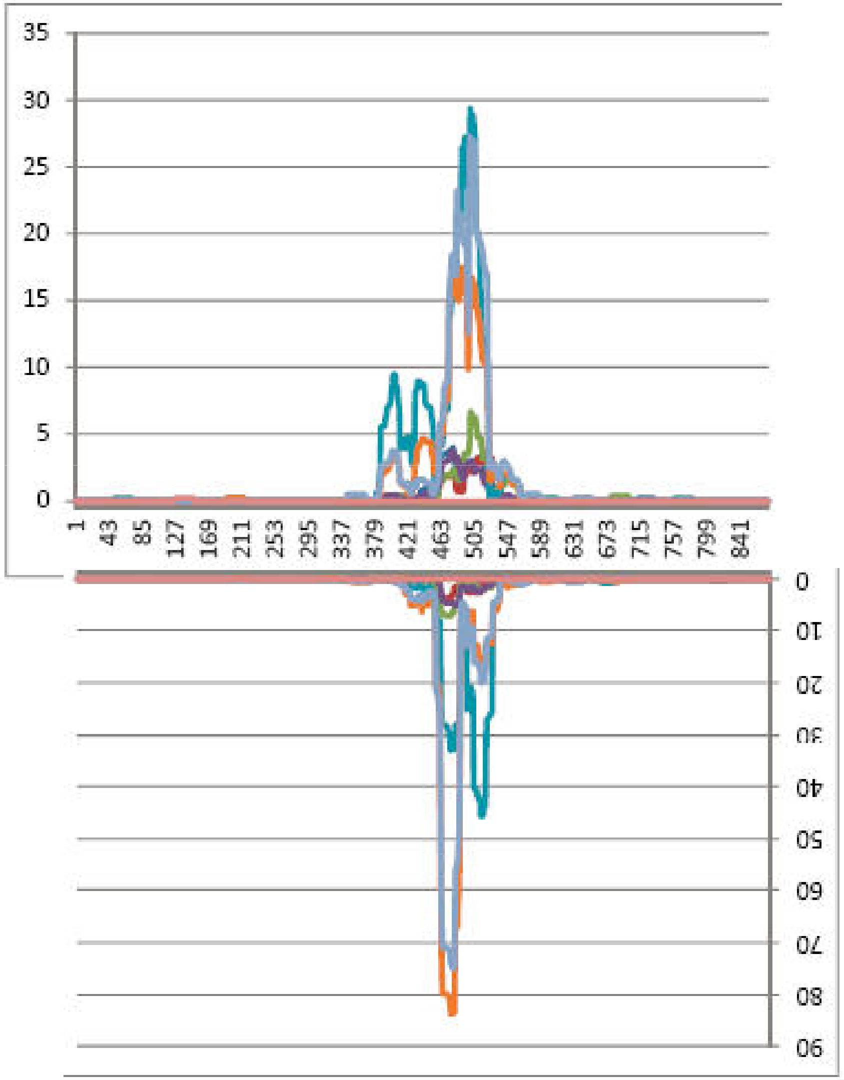

AT1G60720

RNA-directed DNA polymerase (reverse transcriptase)-related family protein

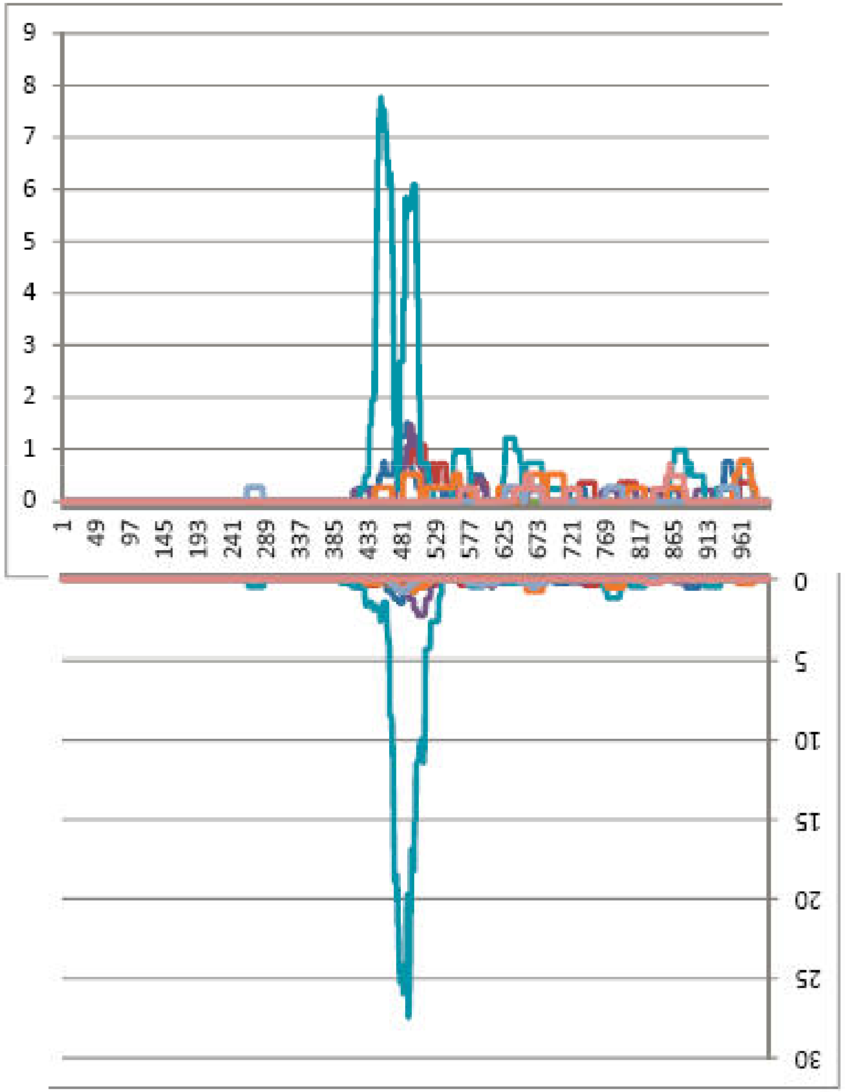

AT1G60940

encodes a member of SNF1-related protein kinases (SnRK2) whose activity is activated by ionic (salt) and non-ionic (mannitol) osmotic stress

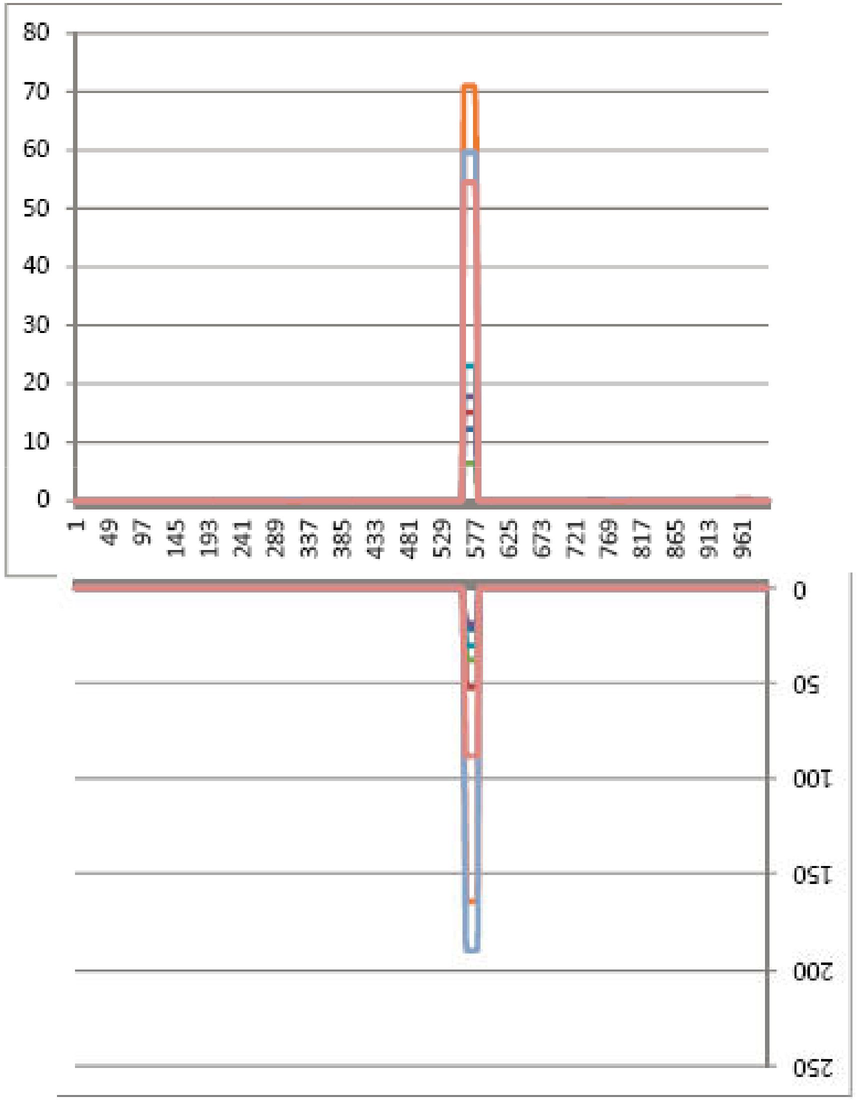

AT1G61820

beta glucosidase 46 (BGLU46)

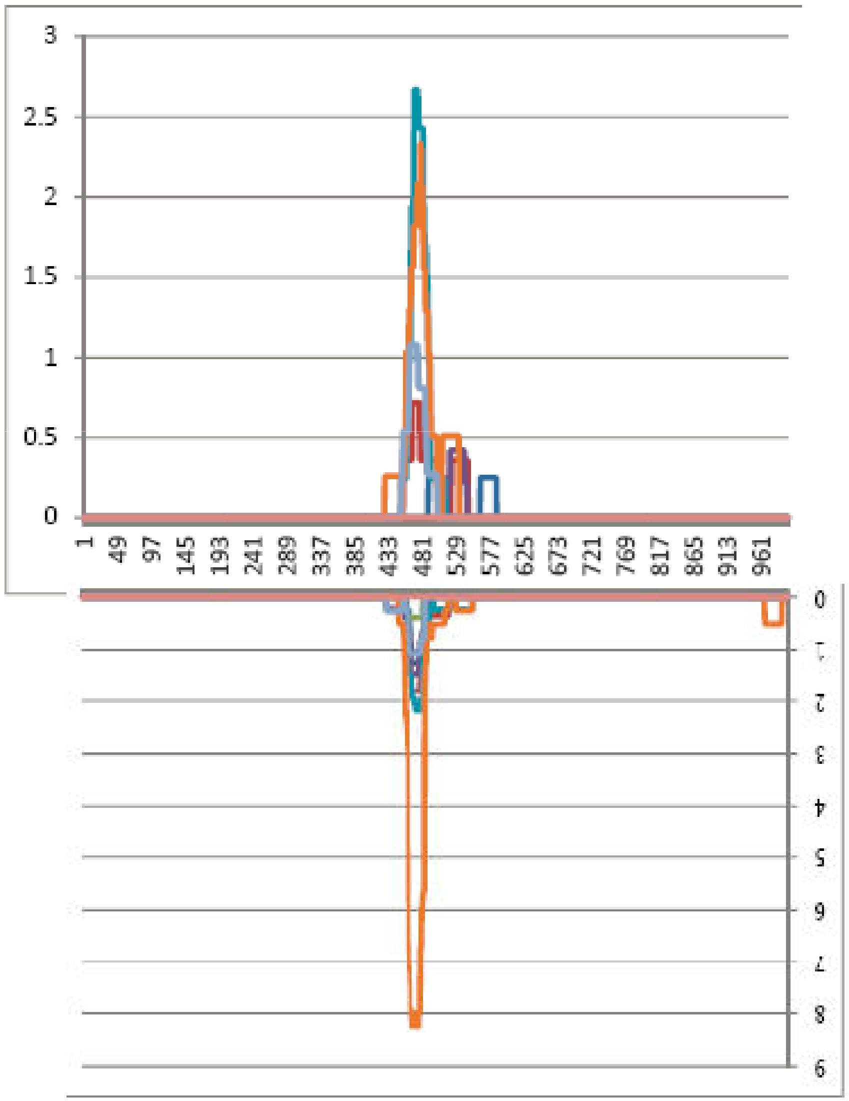

AT1G63210

SPT6L encodes a putative WG/GW-repeat protein involved in the regulation of apical–basal polarity of embryo

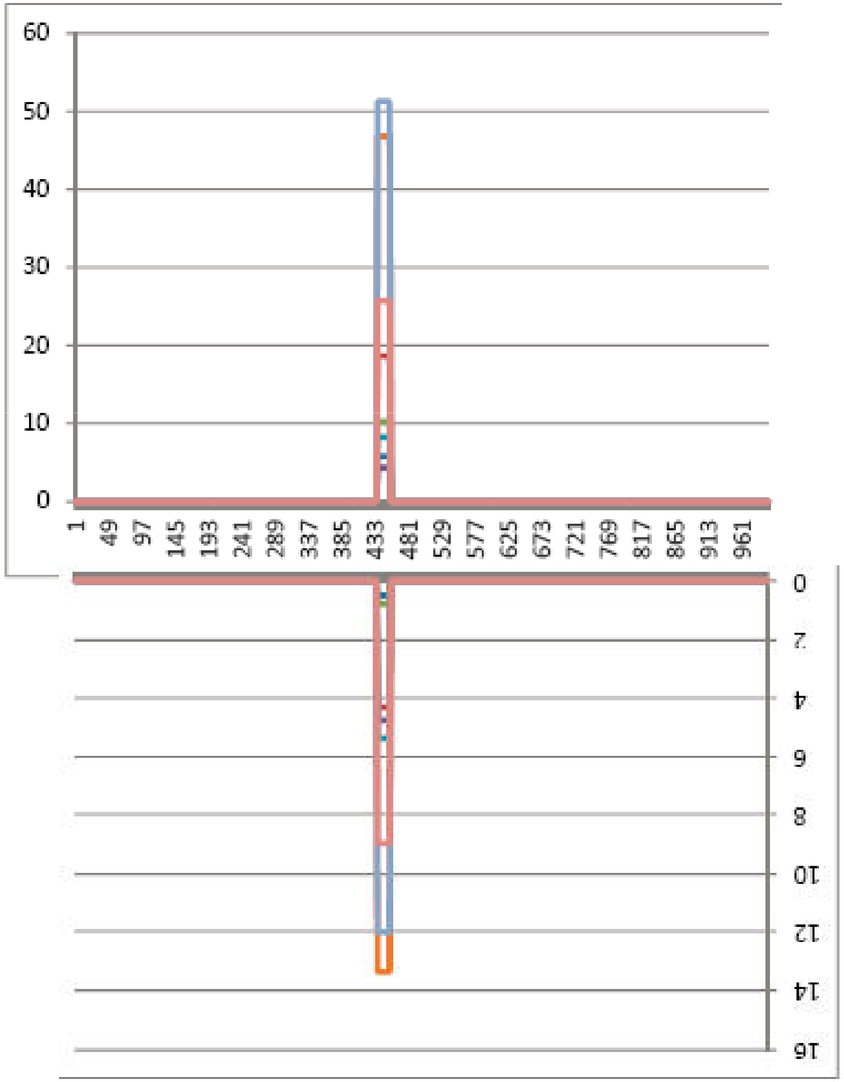

AT1G63800

ubiquitin-conjugating enzyme 5 (UBC5)

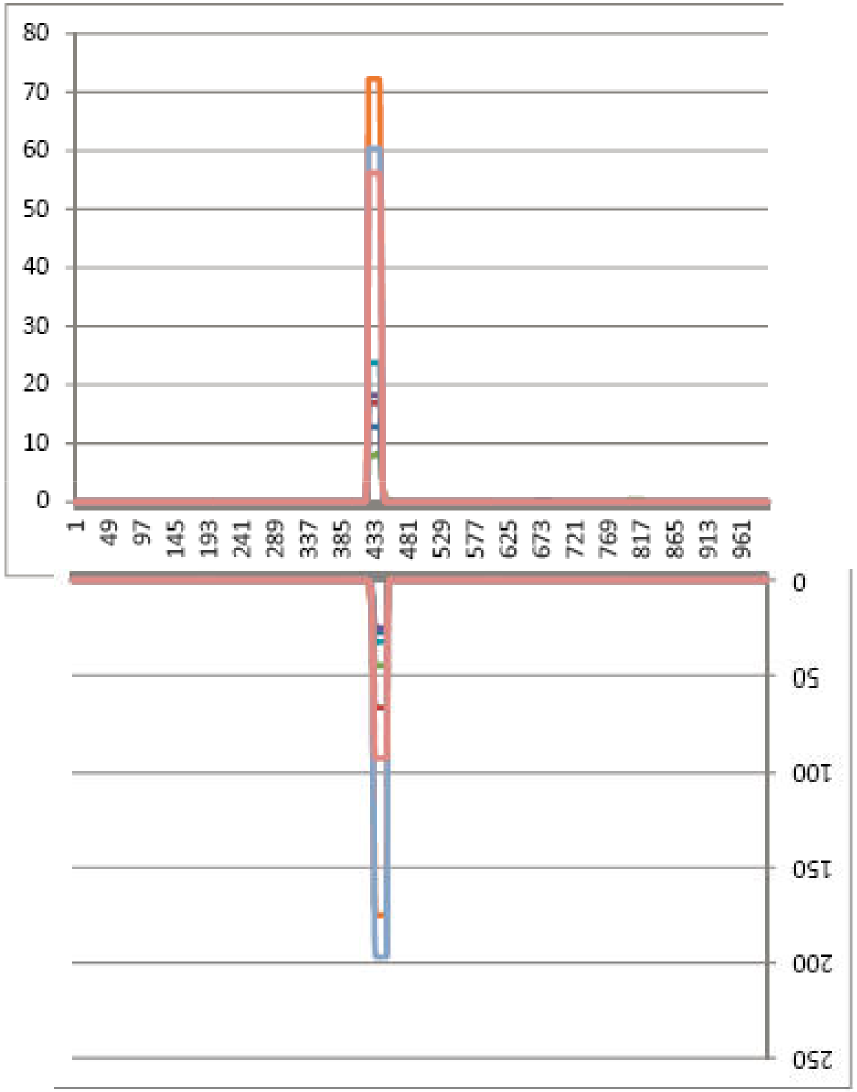

AT1G66290

F-box/RNI-like superfamily protein

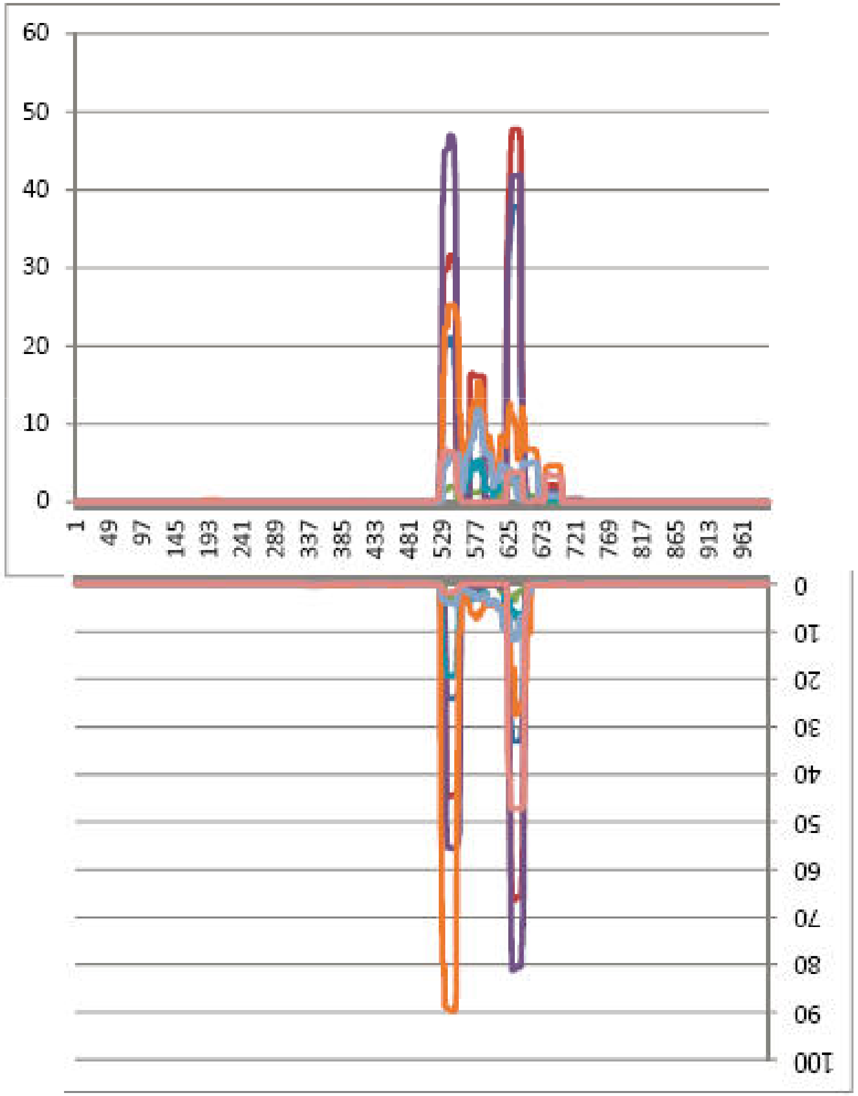

AT1G66490

F-box and associated interaction domains-containing protein

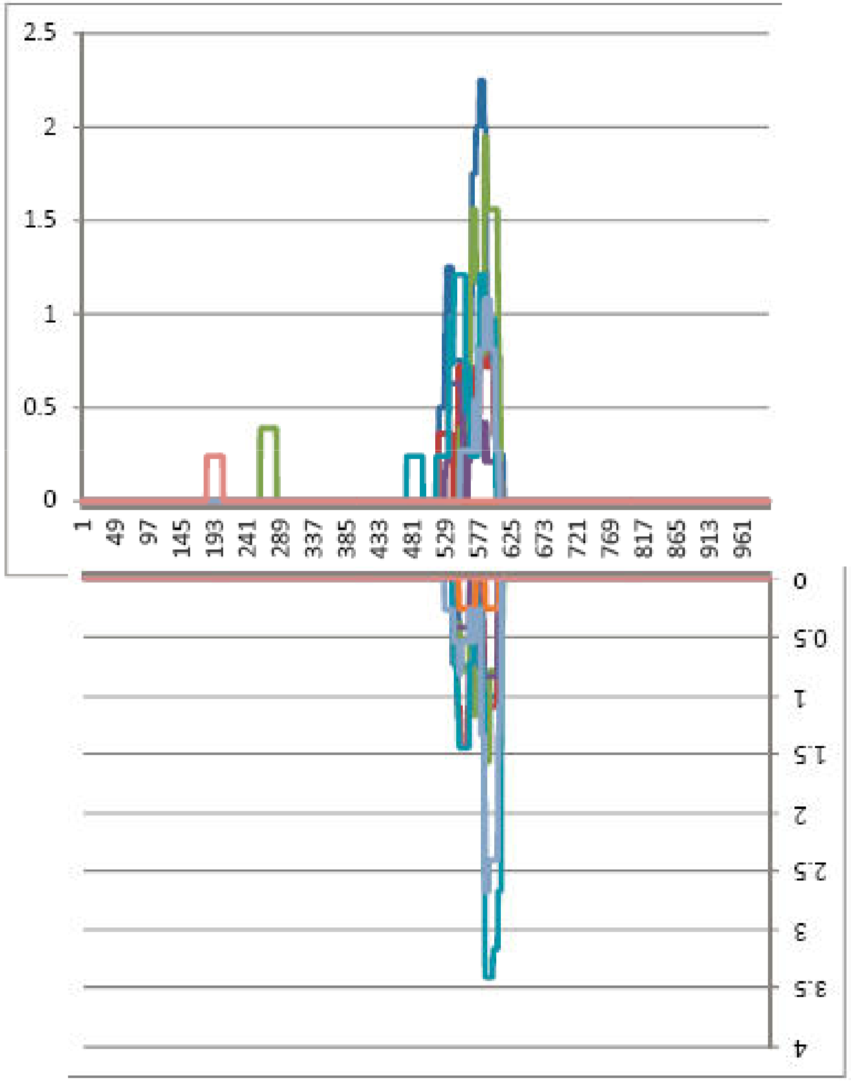

AT1G66640

RNI-like superfamily protein

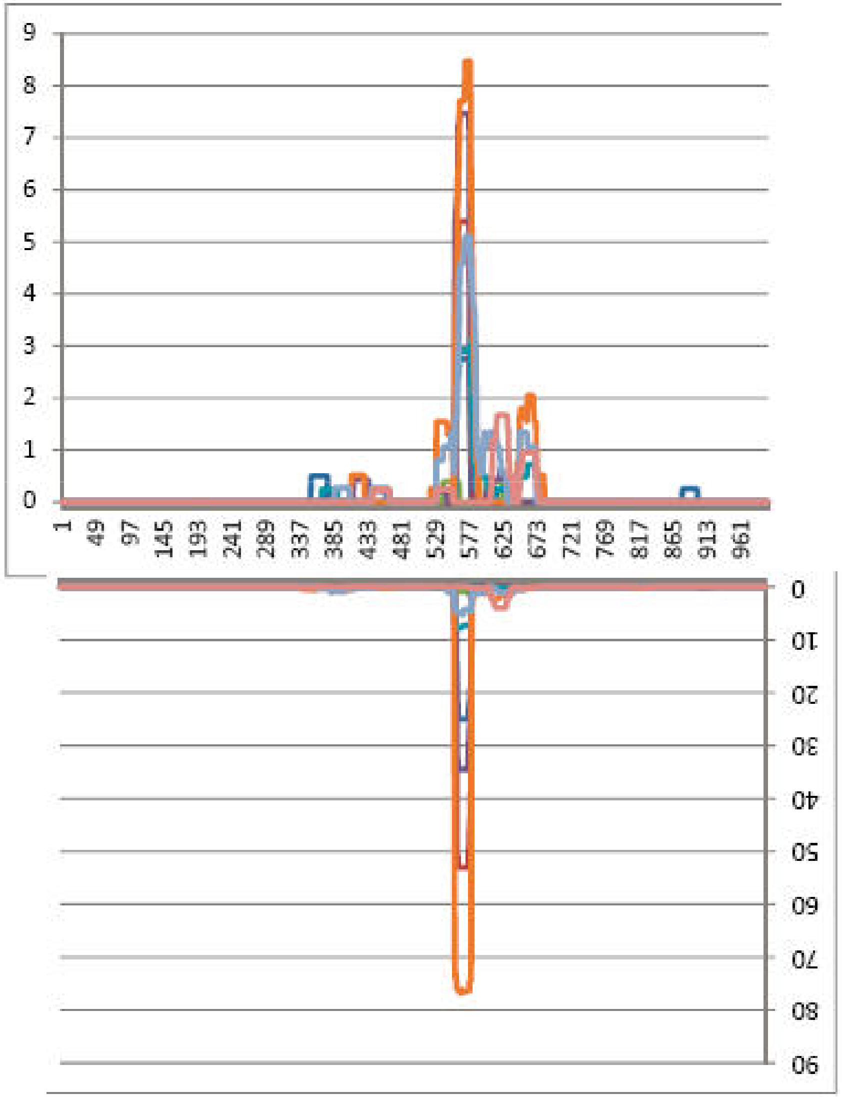

AT1G73710

Pentatricopeptide repeat (PPR) superfamily protein

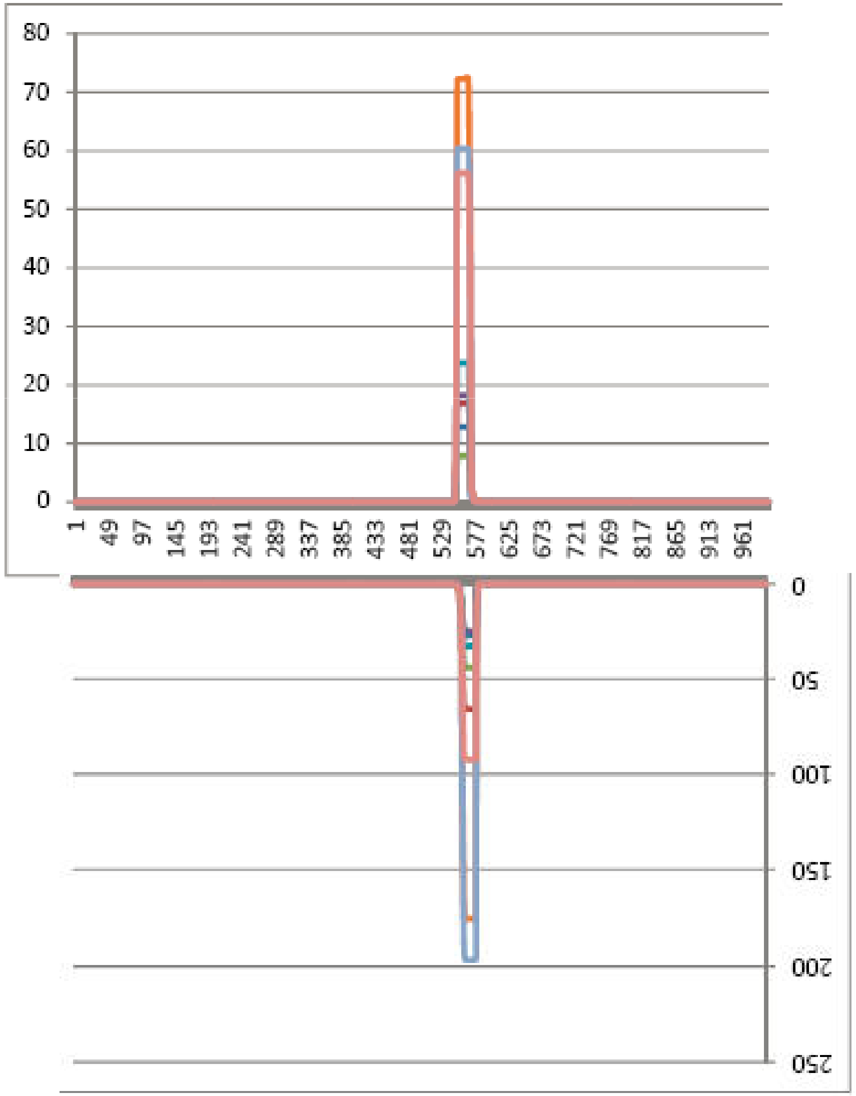

AT1G75050

Pathogenesis-related thaumatin superfamily protein

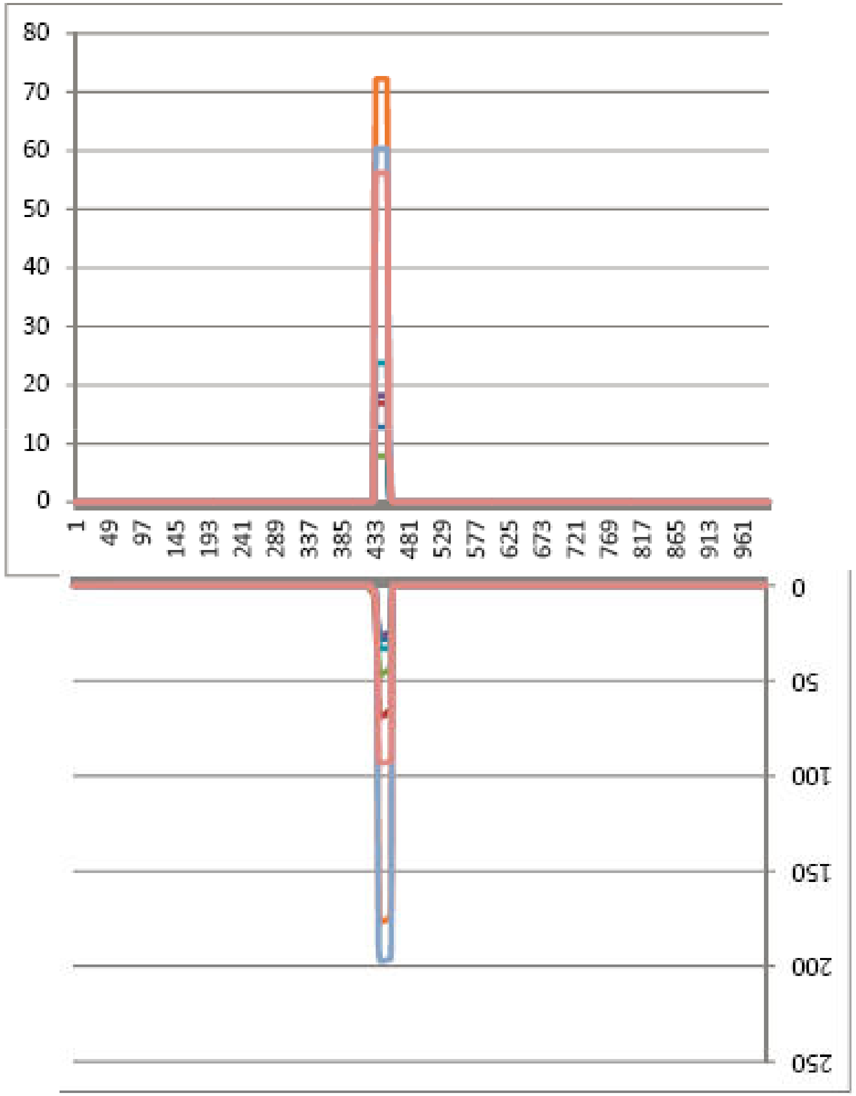

AT1G79490

embryo defective 2217 (EMB2217)

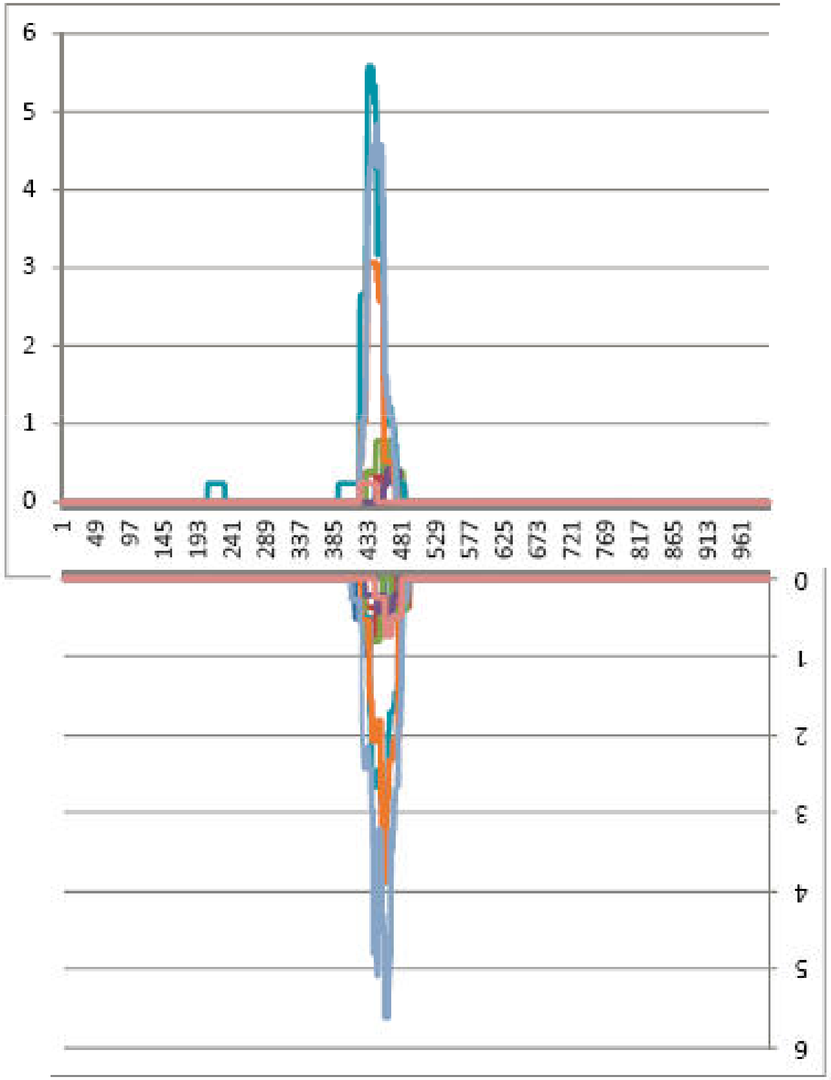

AT1G79800

early nodulin-like protein 7 (ENODL7)

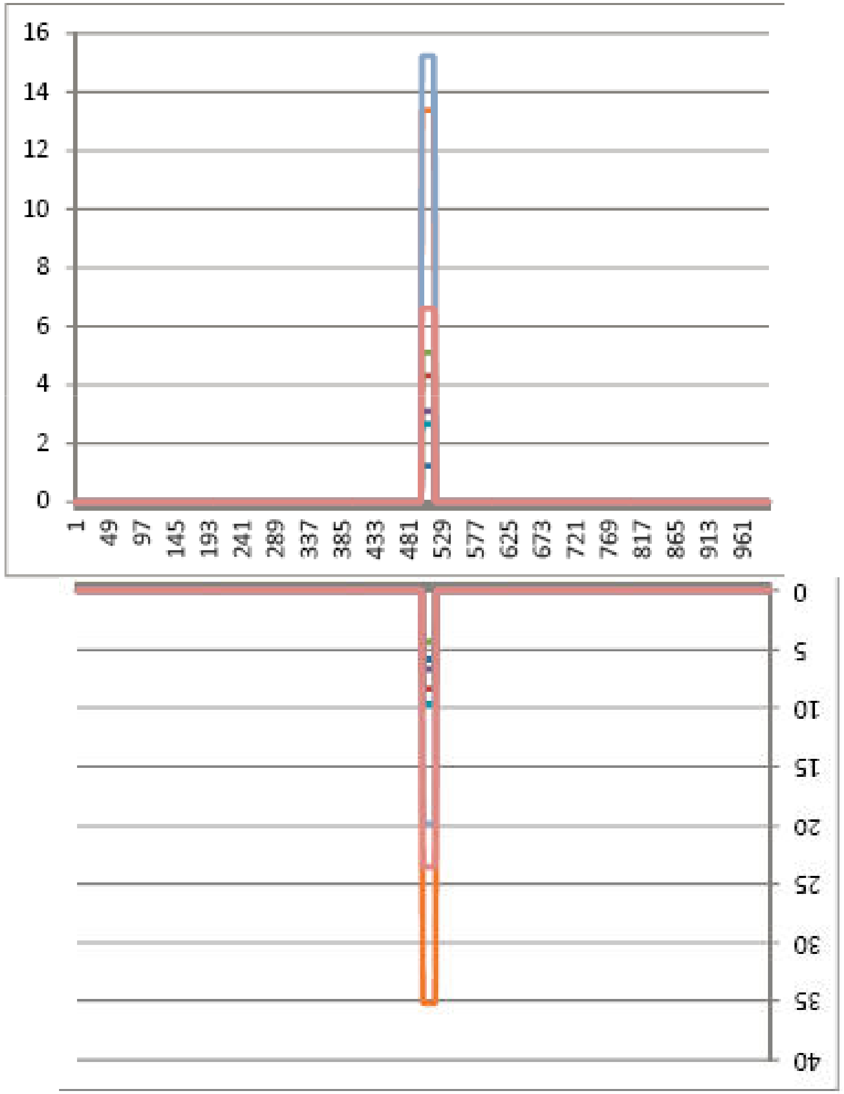

AT2G04620

Cation efflux family protein

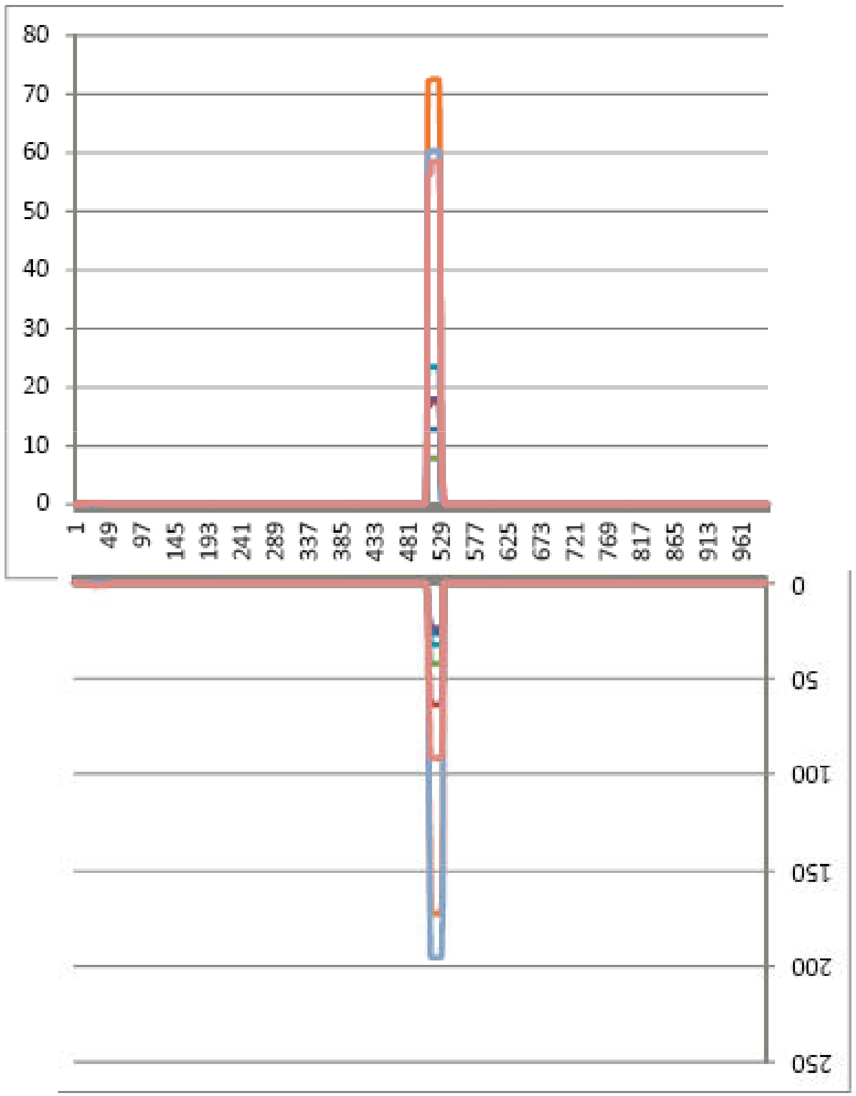

AT2G06850

endoxyloglucan transferase (EXGT-A1) gene

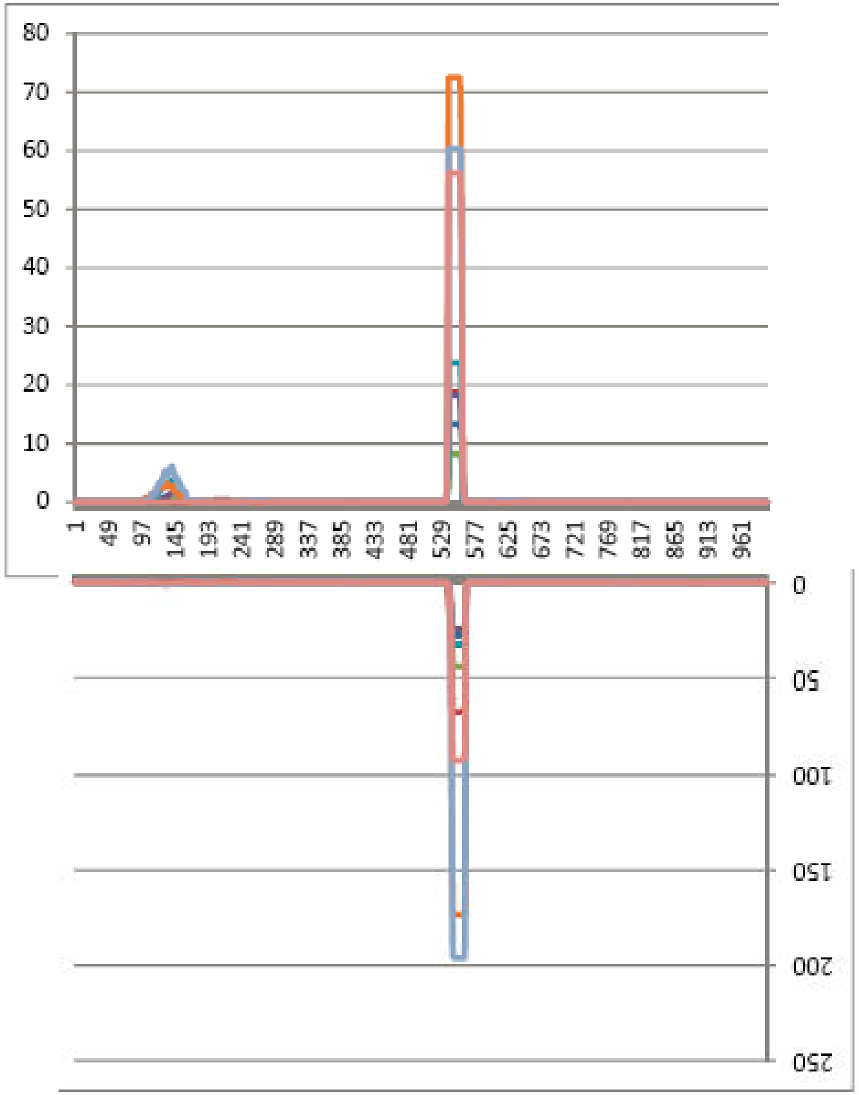

AT2G07000

unknown protein

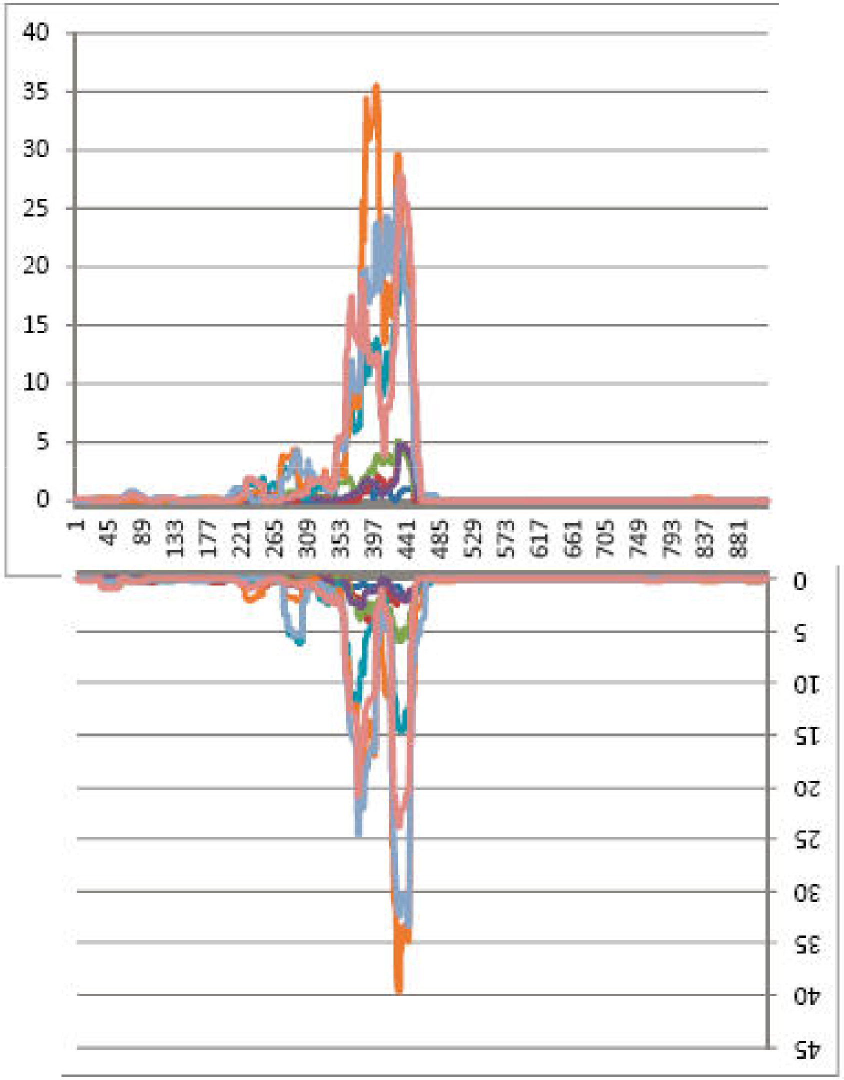

AT2G18600

Ubiquitin-conjugating enzyme family protein

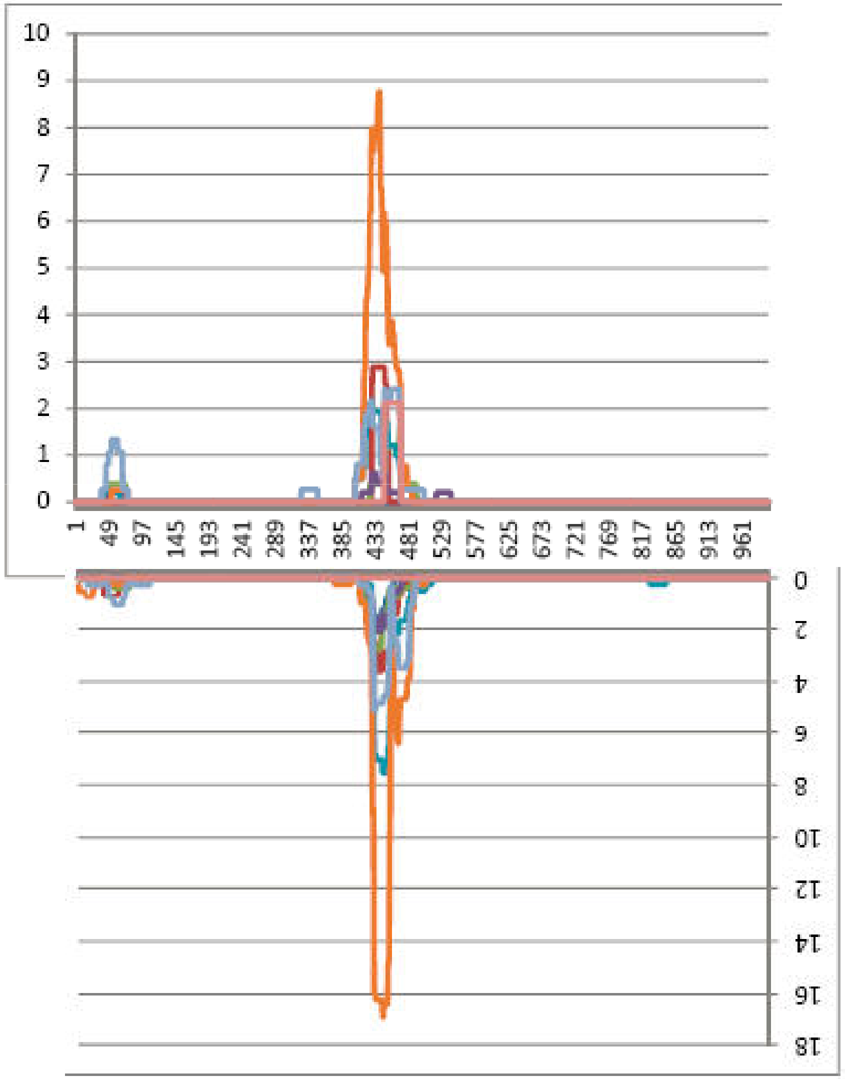

AT2G24010

serine carboxypeptidase-like 23 (scpl23)

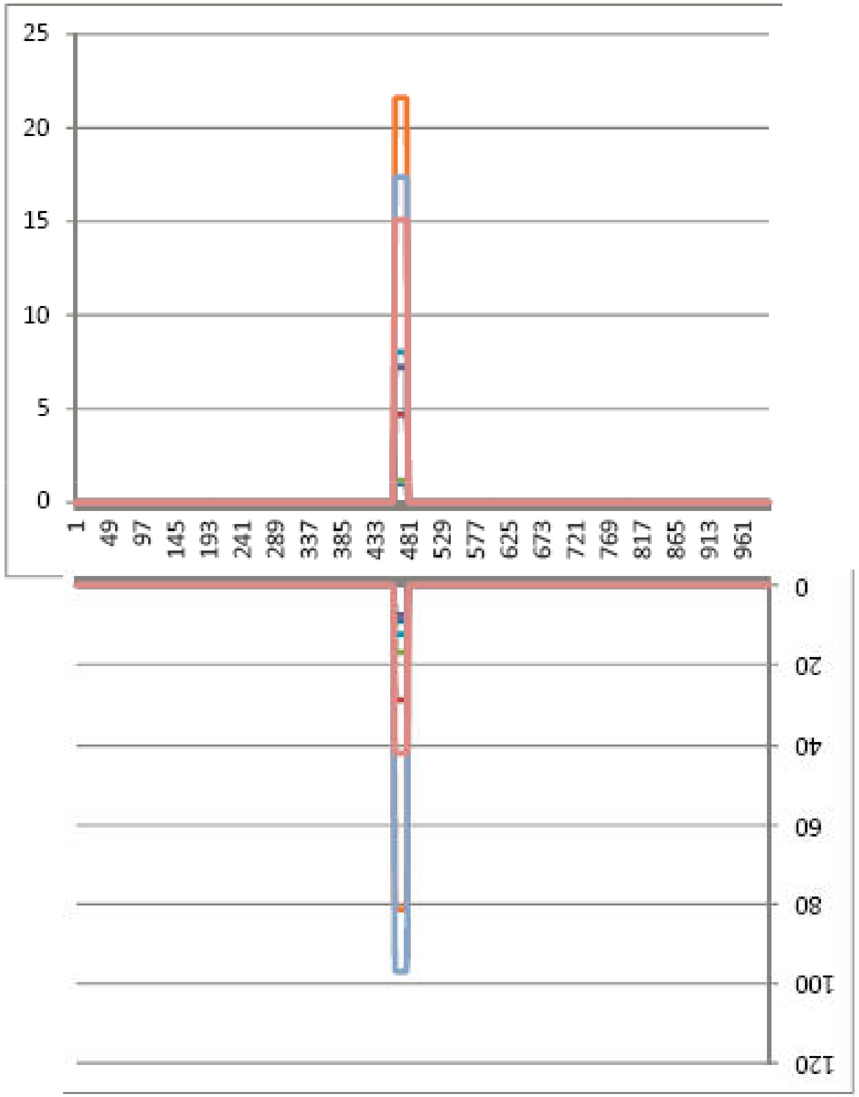

AT2G32130

Plant protein of unknown function (DUF641)

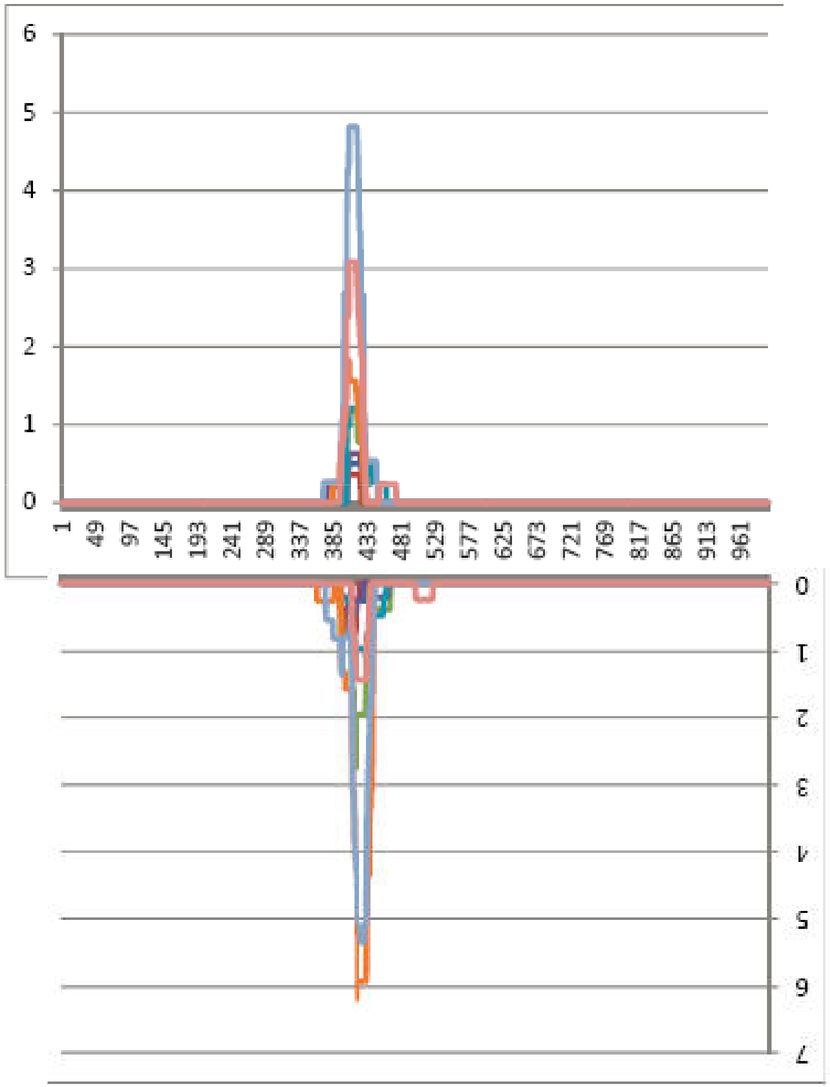

AT2G36460

Aldolase superfamily protein

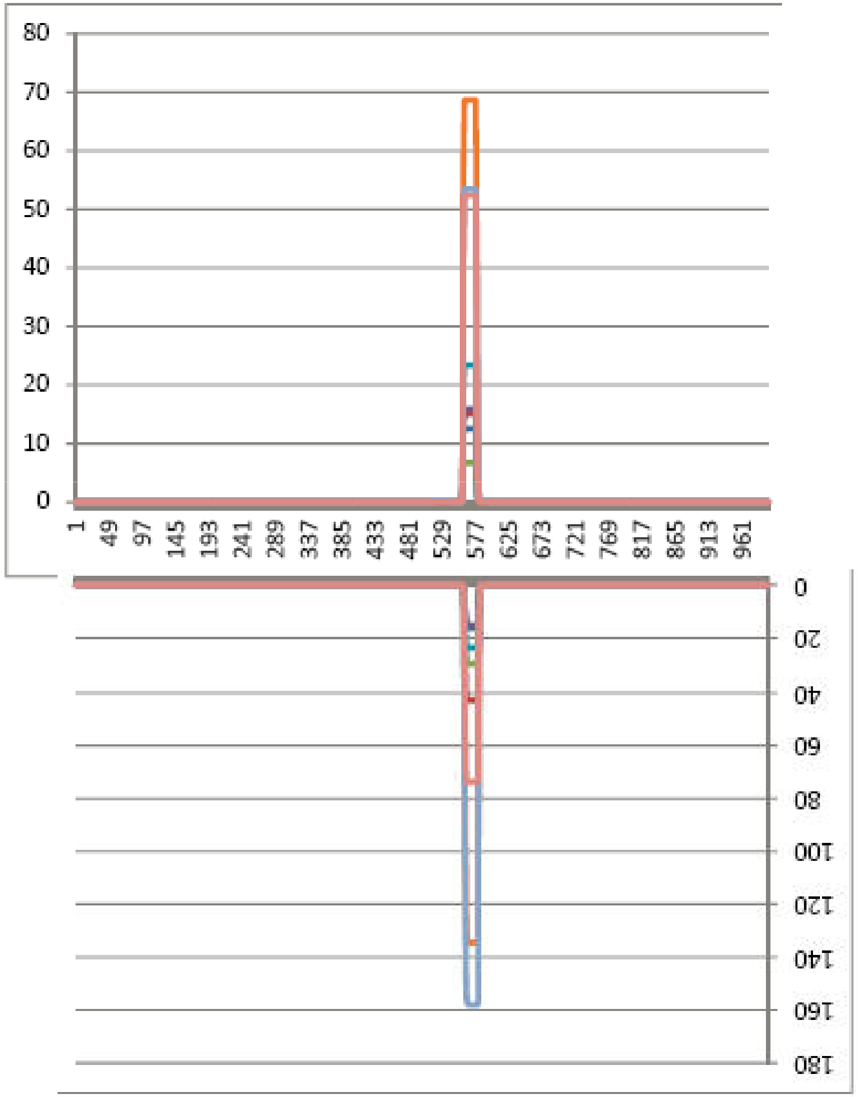

AT3G05770

unknown protein

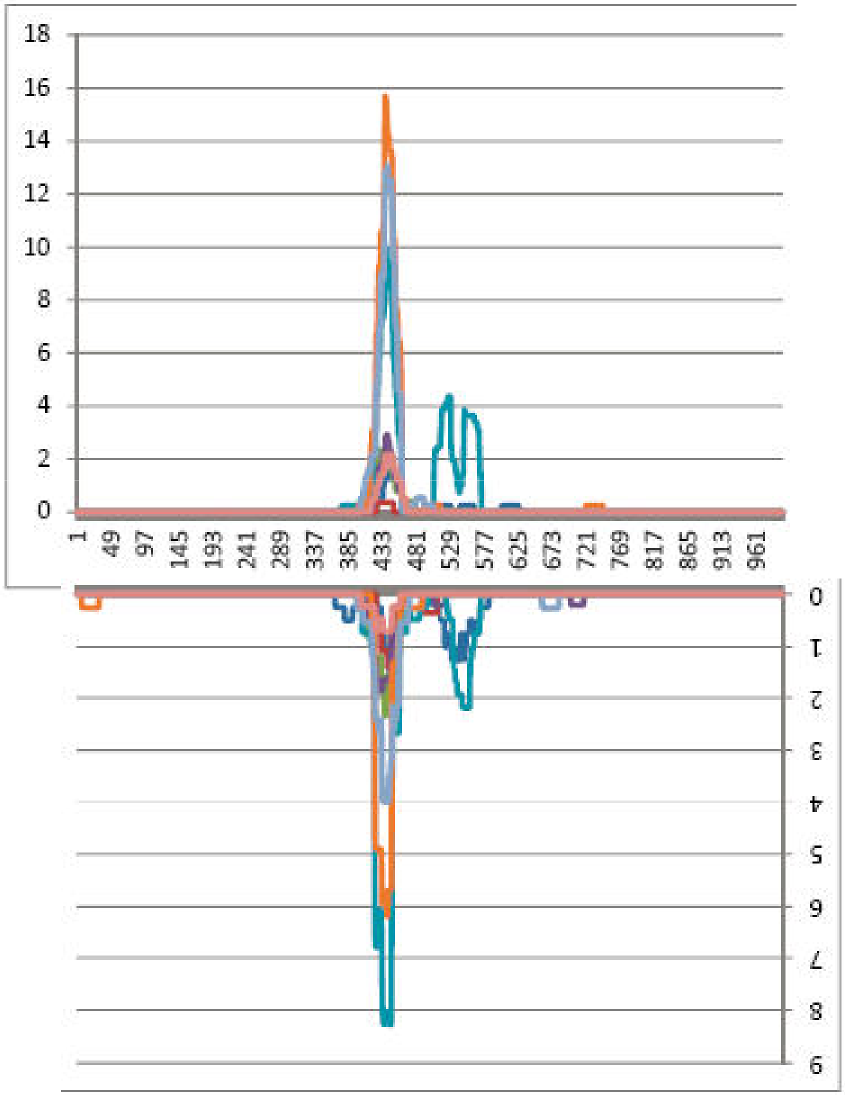

AT3G13857

unknown protein

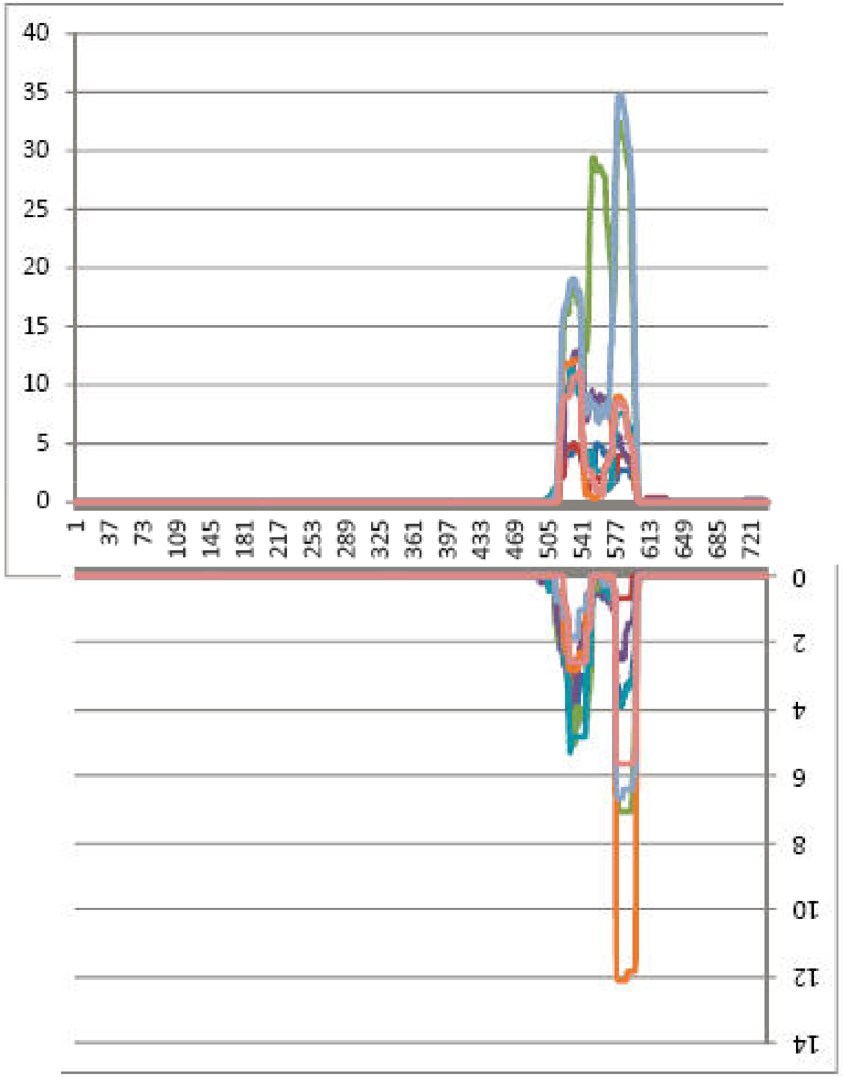

AT3G17500

F-box family protein

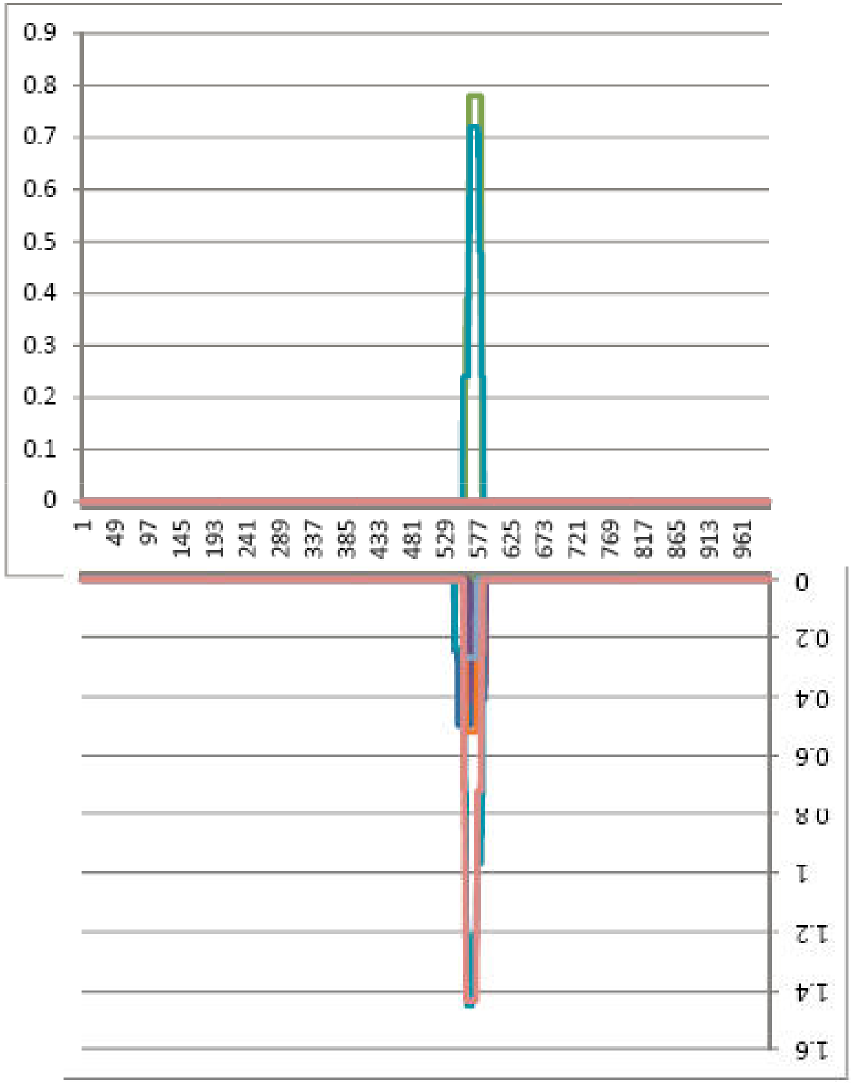

AT3G19880

F-box and associated interaction domains-containing protein

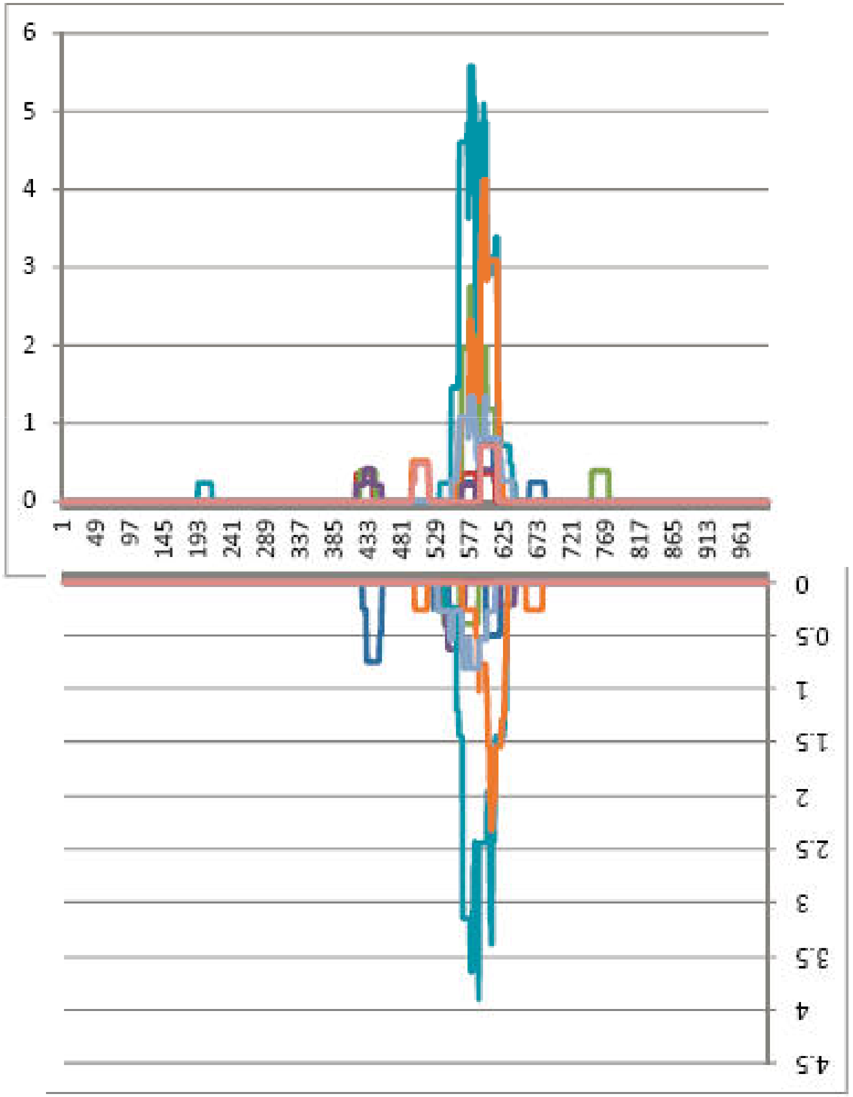

AT3G22350

F-box and associated interaction domains-containing protein

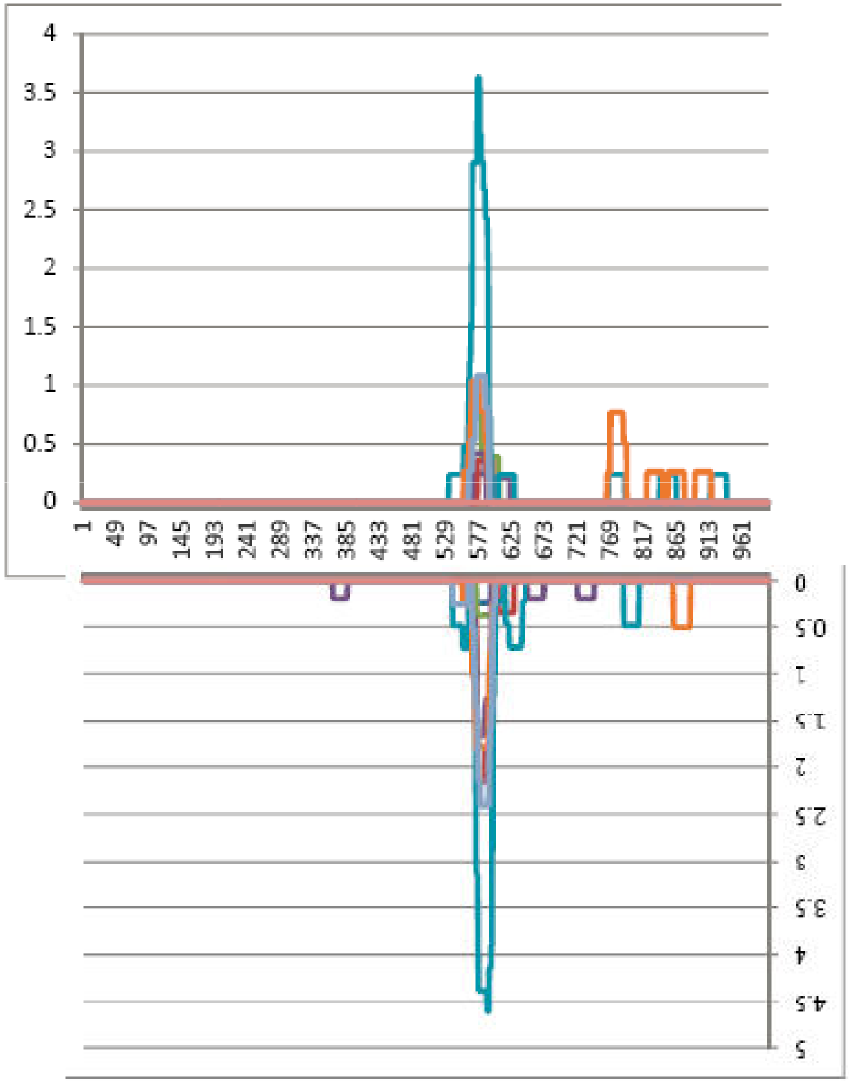

AT3G22730

F-box and associated interaction domains-containing protein

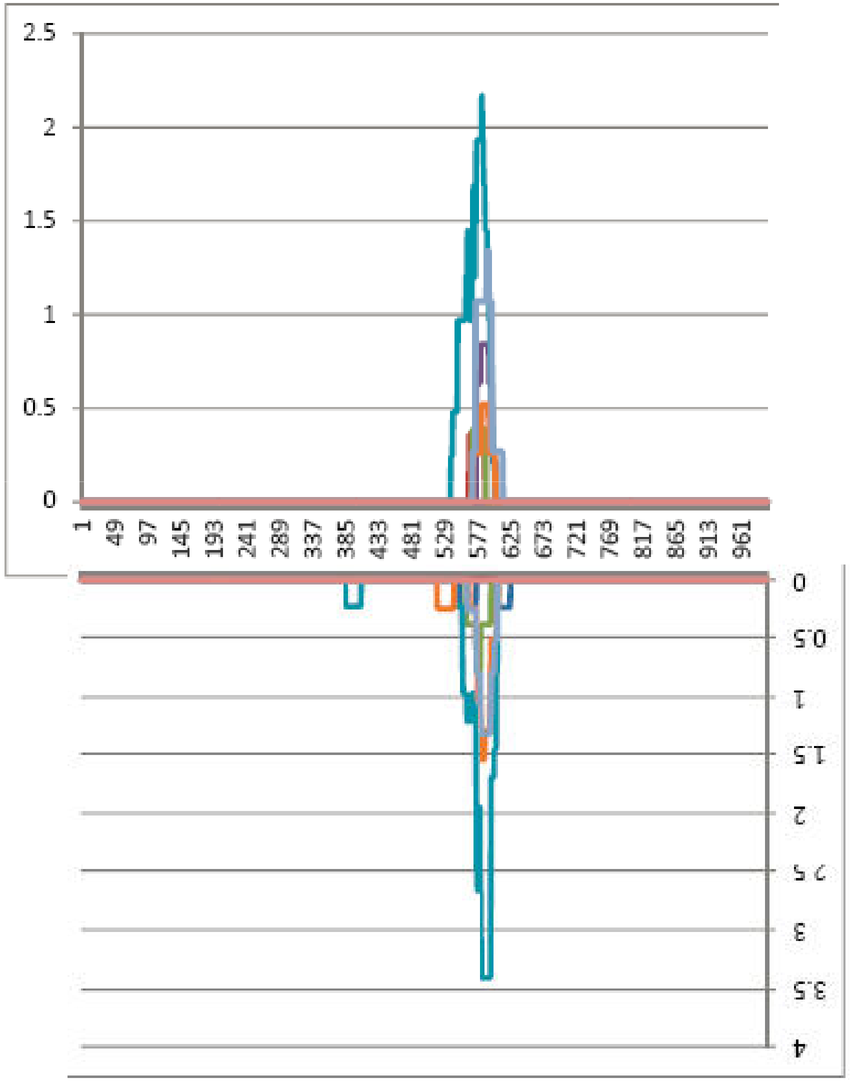

AT3G22770

F-box associated ubiquitination effector family protein

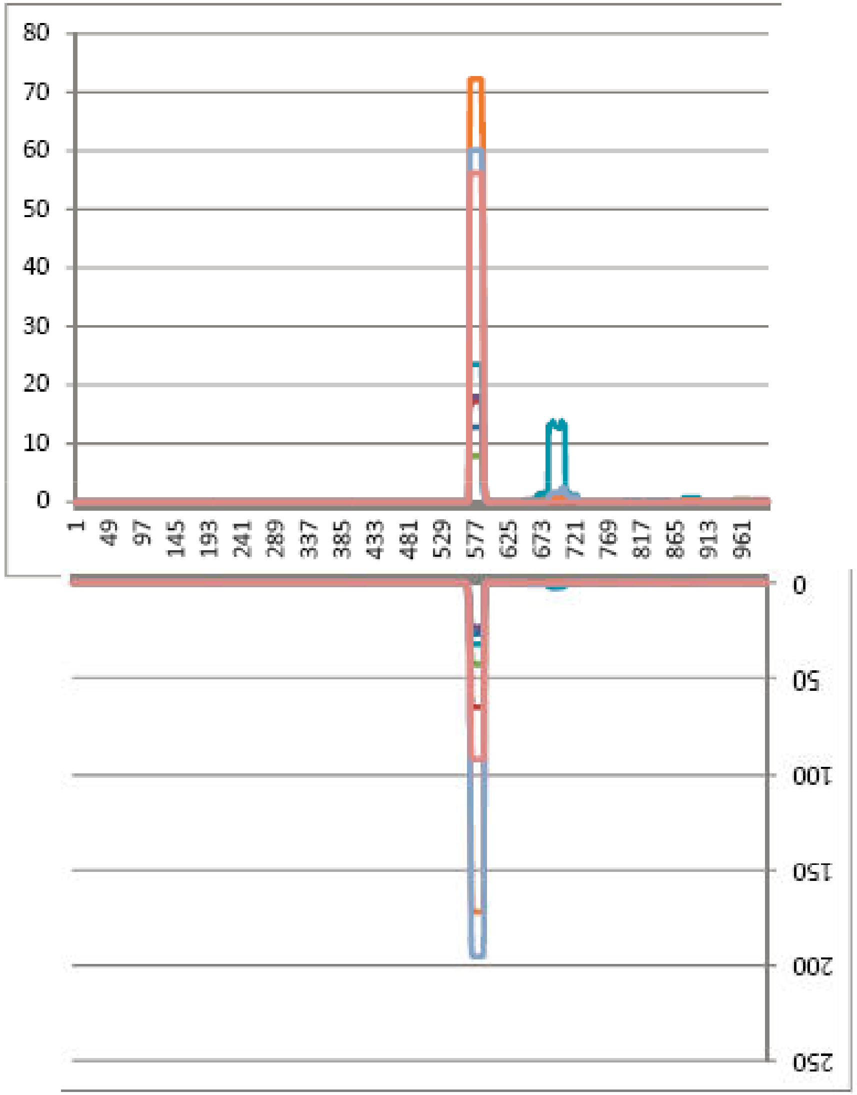

AT3G26616

unknown protein

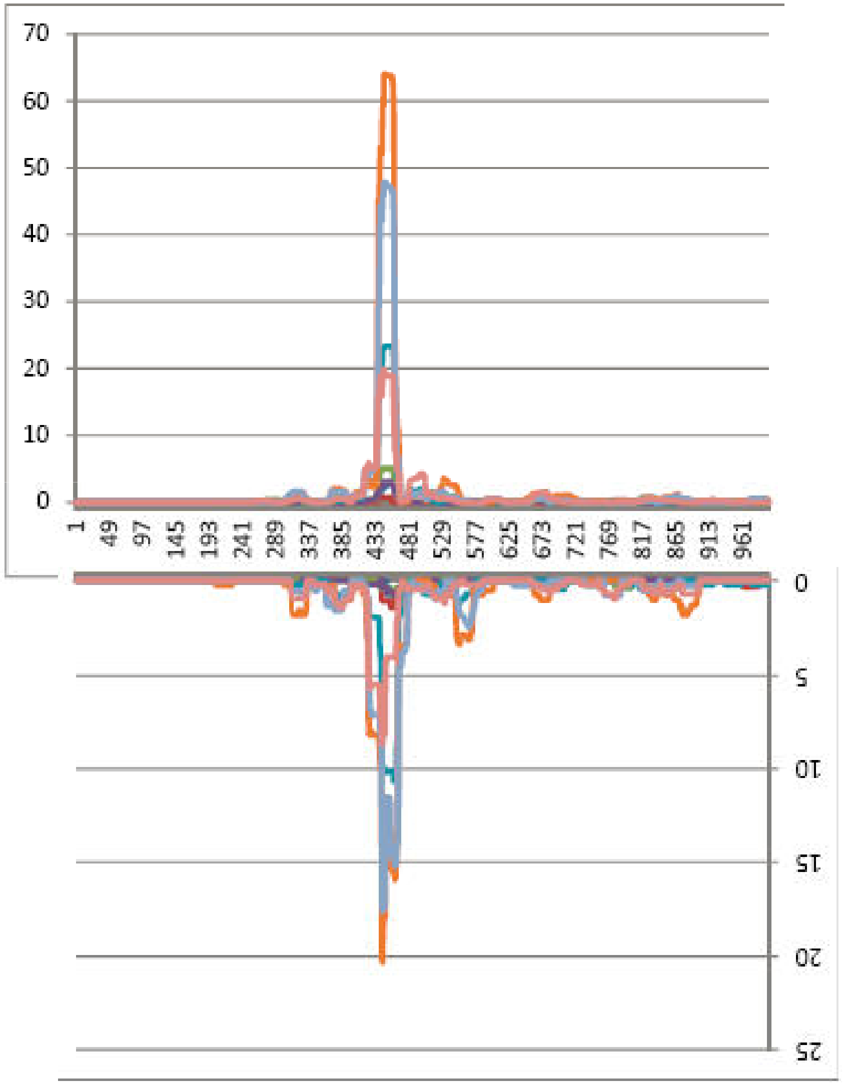

AT3G43270

Plant invertase/pectin methylesterase inhibitor superfamily

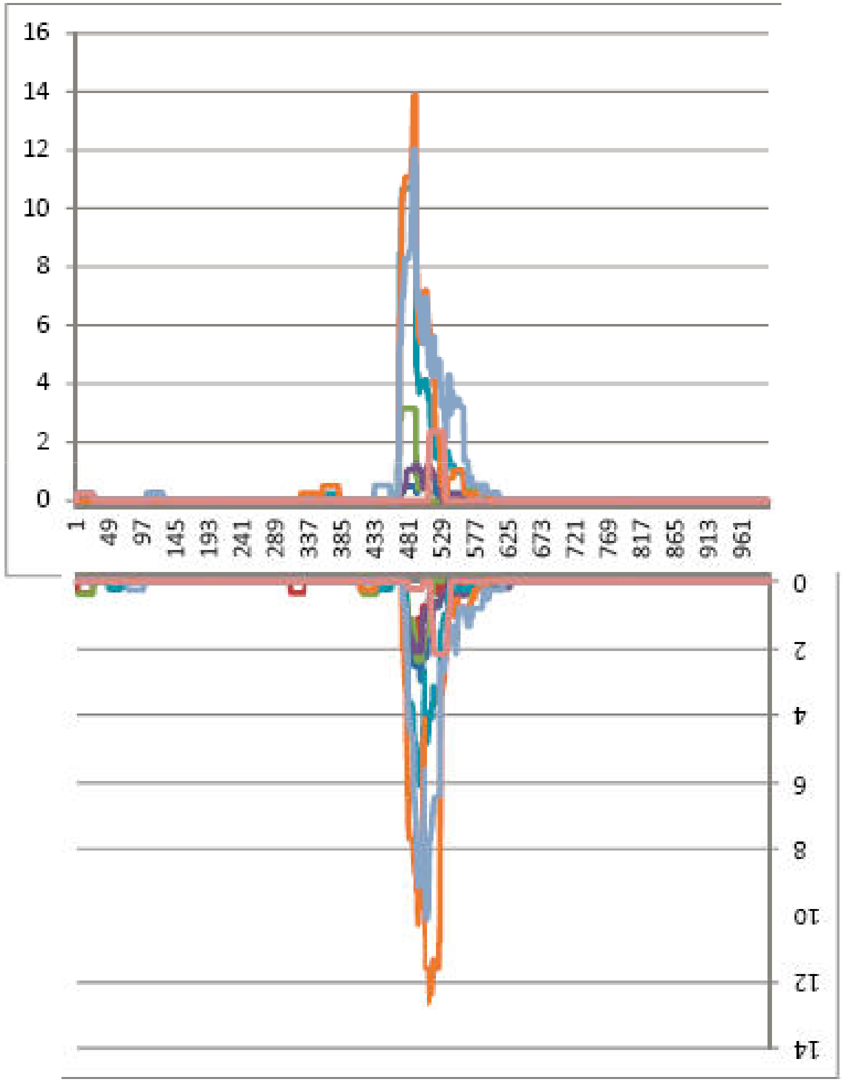

AT3G47300

SELT-like protein precursor (SELT)

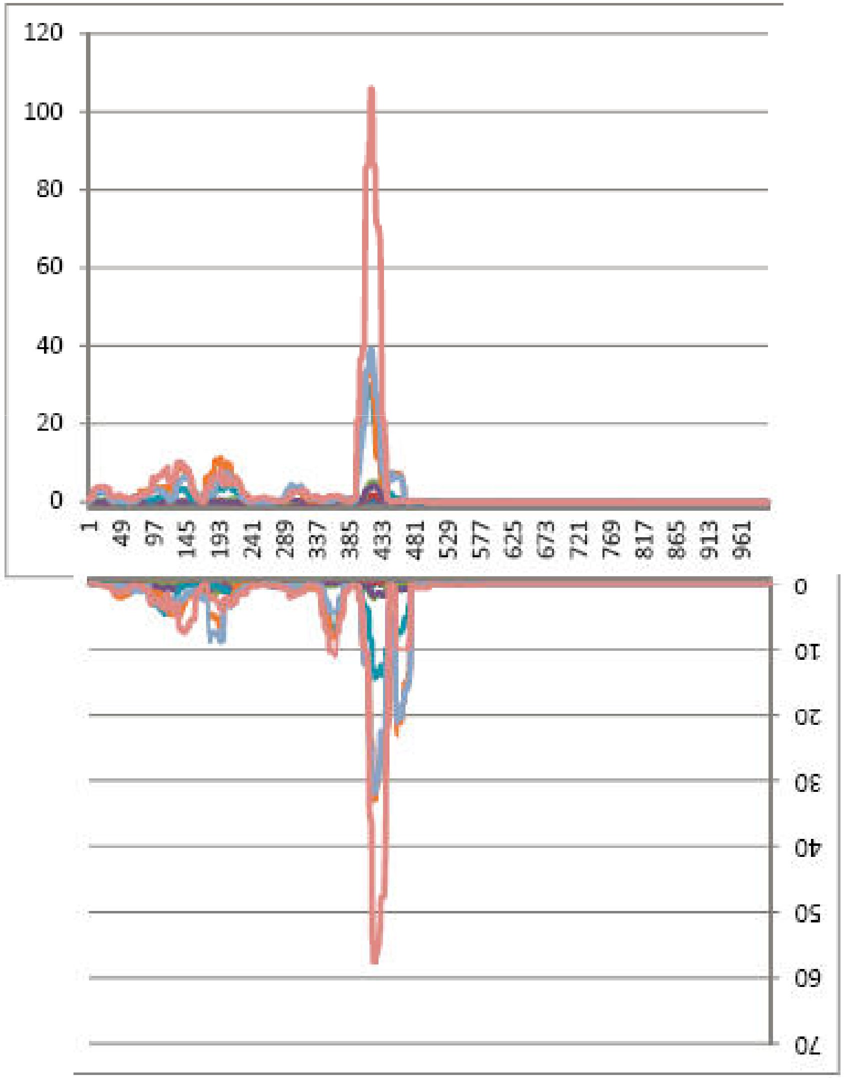

AT3G57770

Protein kinase superfamily protein

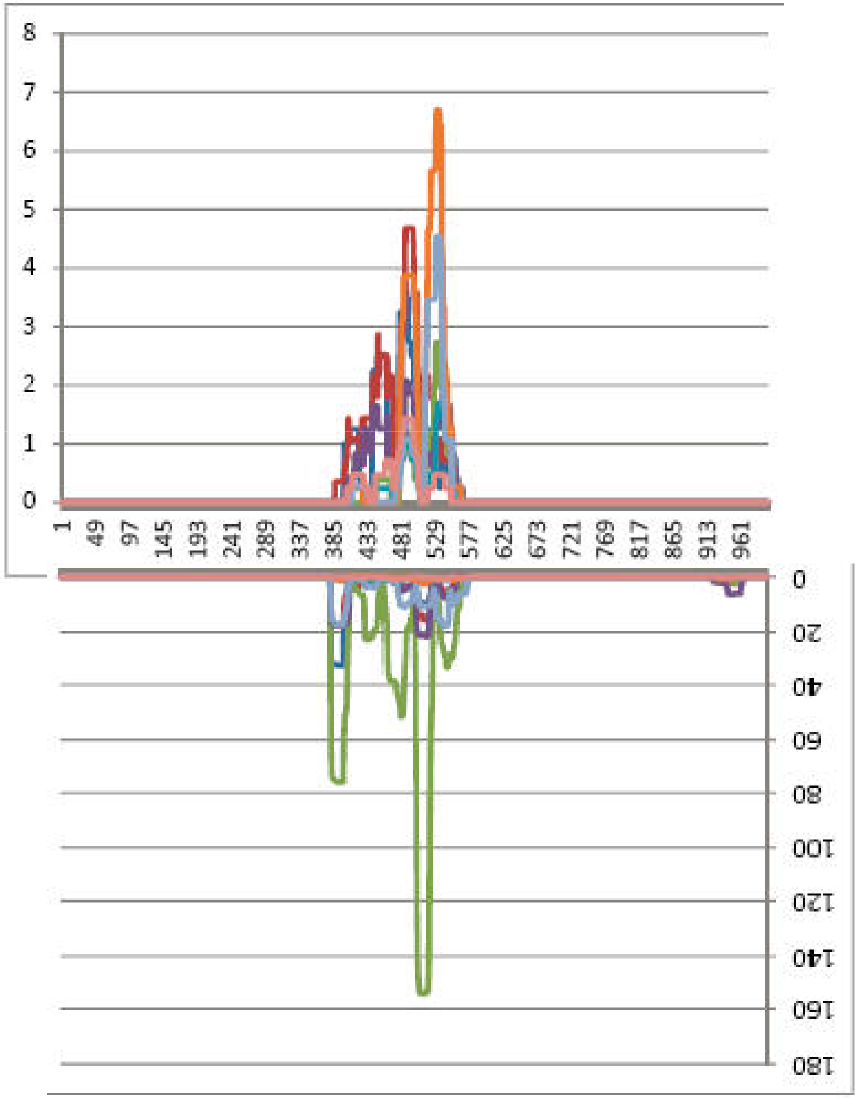

AT3G59220

encodes a cupin-domain containing protein that is similar to pirins which interact with a CCAAT box binding transcription factor. The protein interacts with GPA1 (G protein alpha-subunit) in vitro. Mutants in the gene are affected in germination and early seedling development.

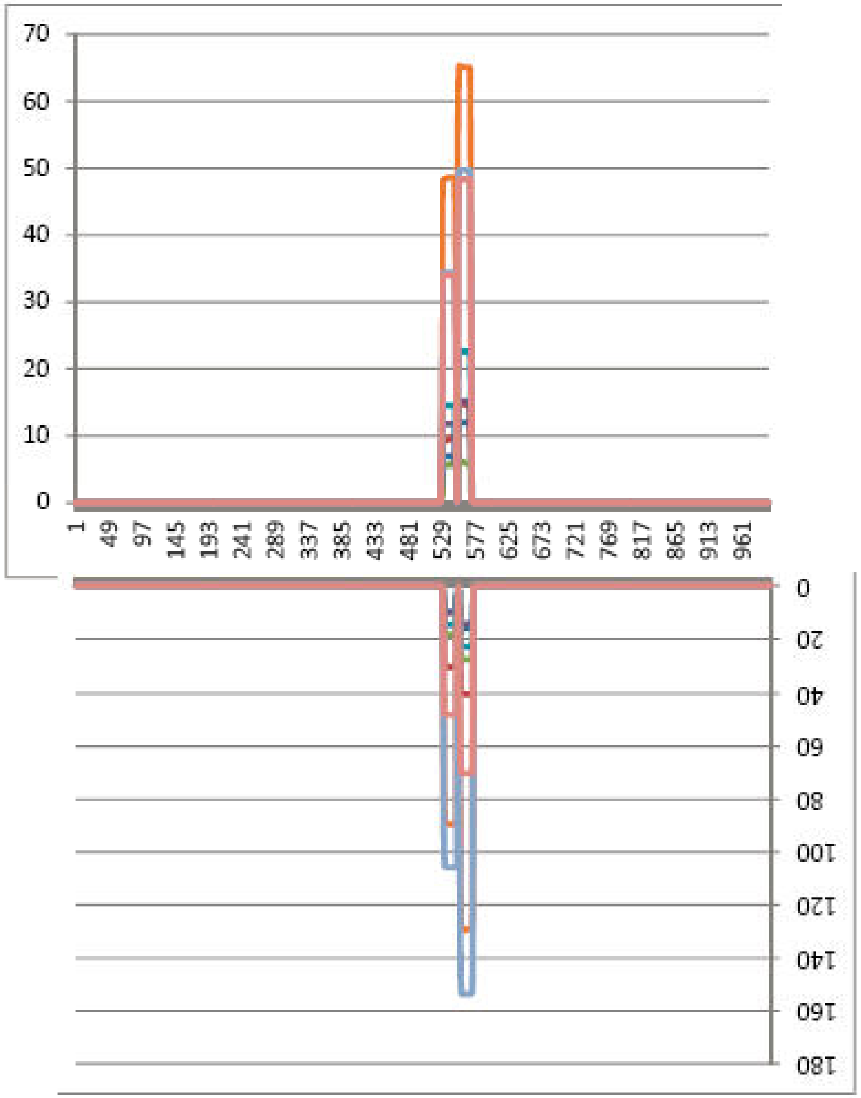

AT3G60790

F-box family protein

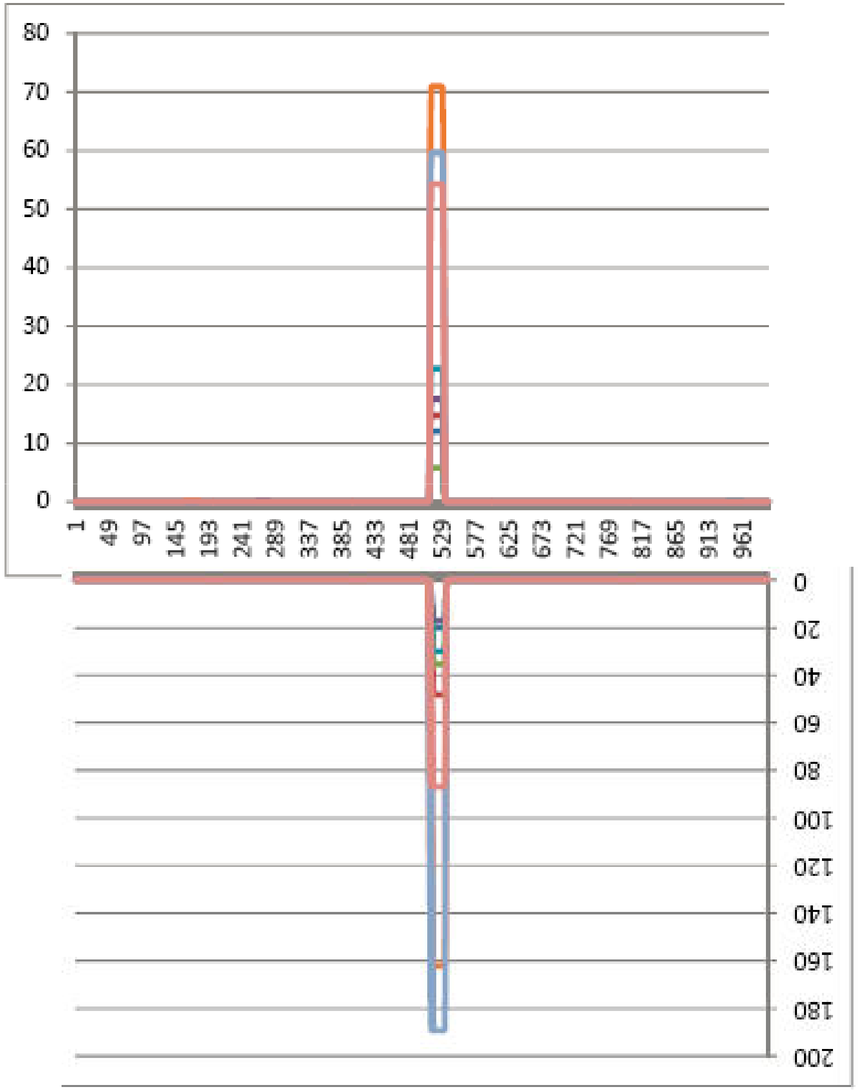

AT4G14130

xyloglucan endotransglycosylase-related protein (XTR7)

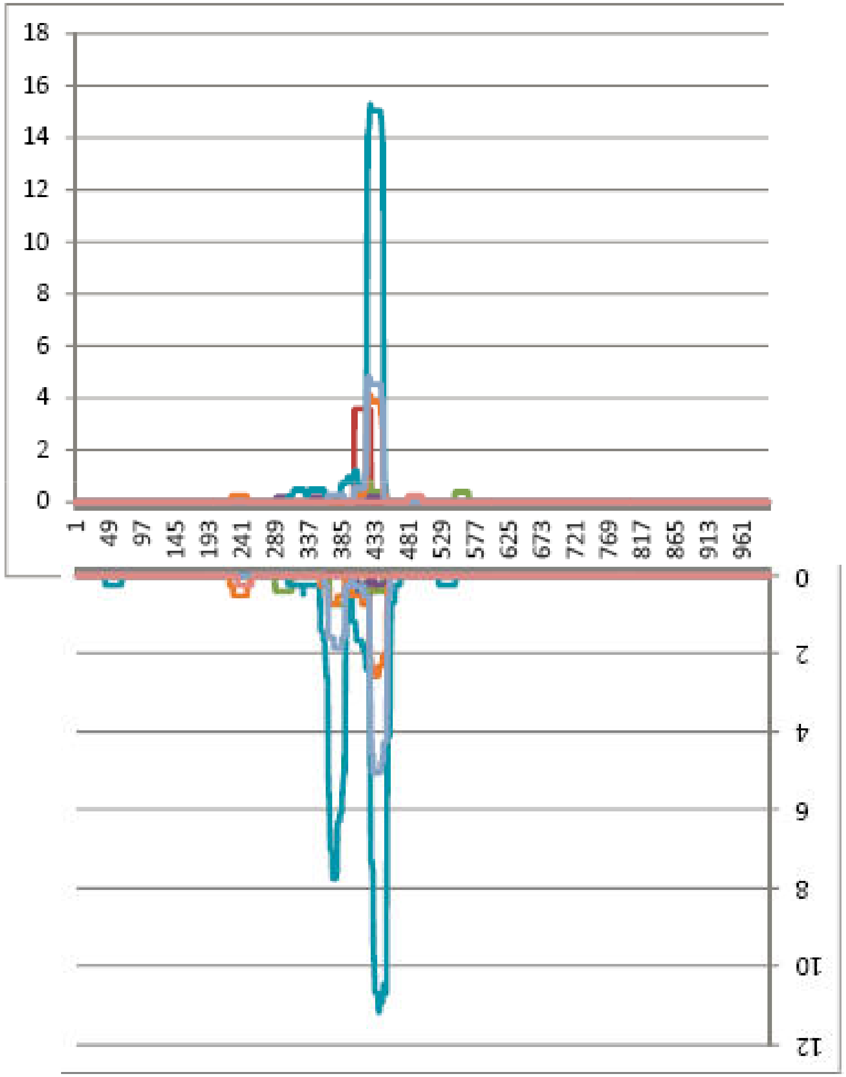

AT4G16640

Matrixin family protein

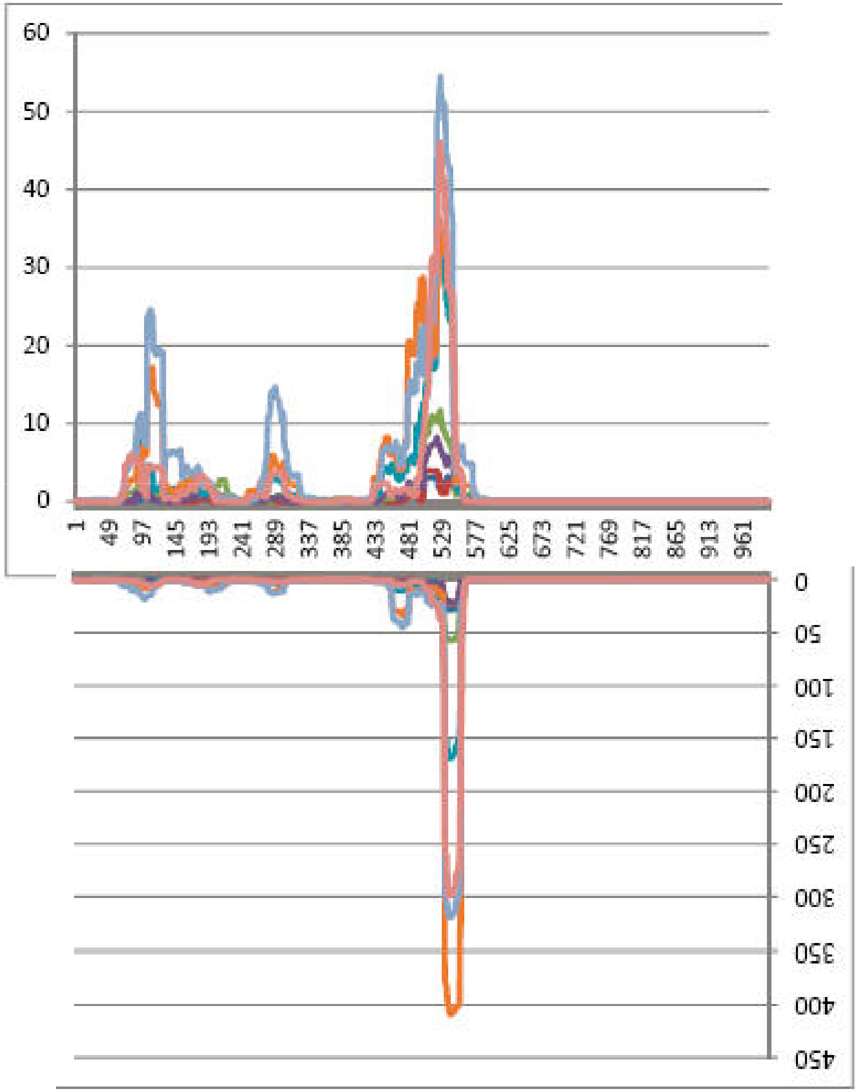

AT4G29090

Ribonuclease H-like superfamily protein

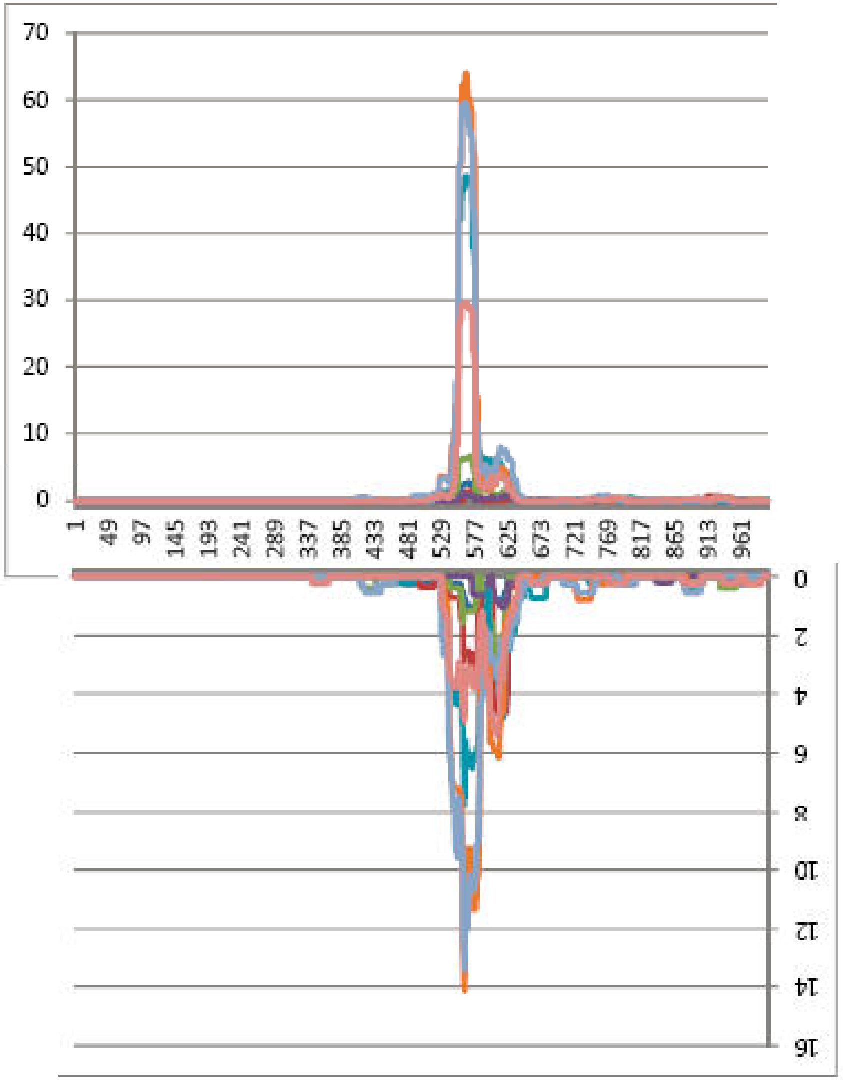

AT5G03340

ATPase, AAA-type, CDC48 protein

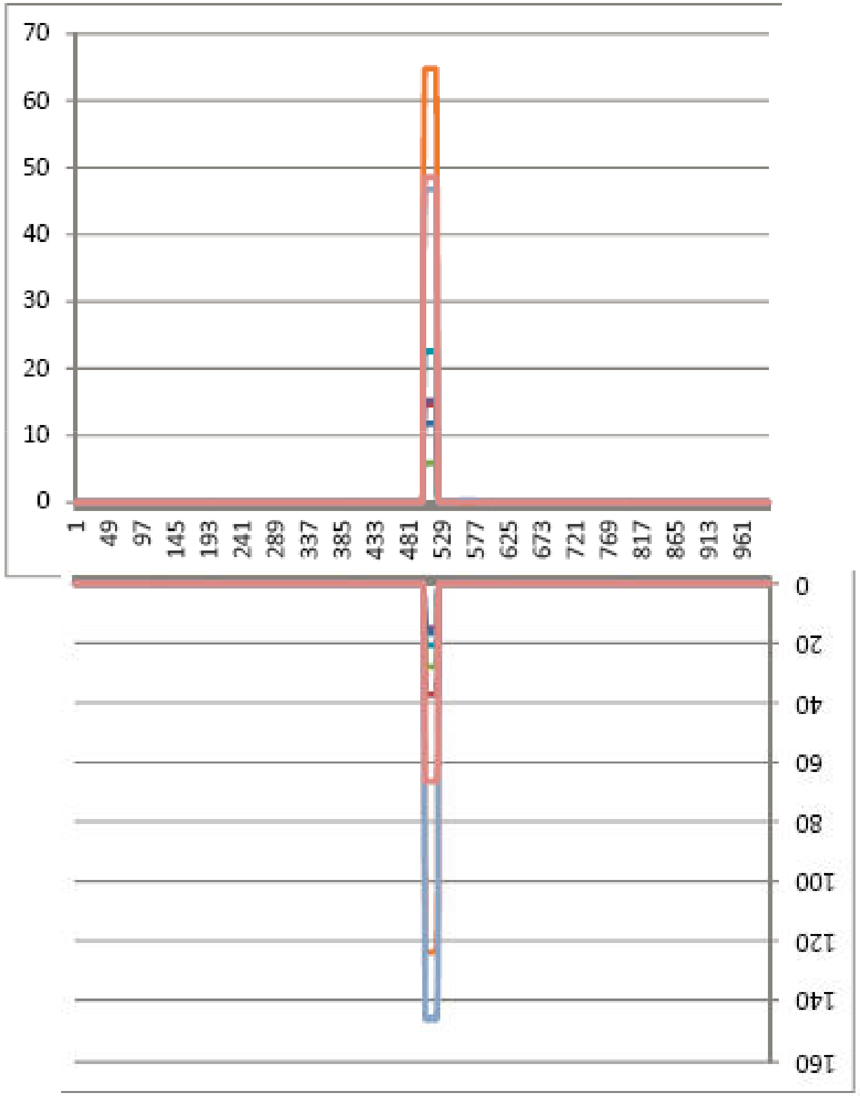

AT5G22590

Leucine Rich Repeat protein family

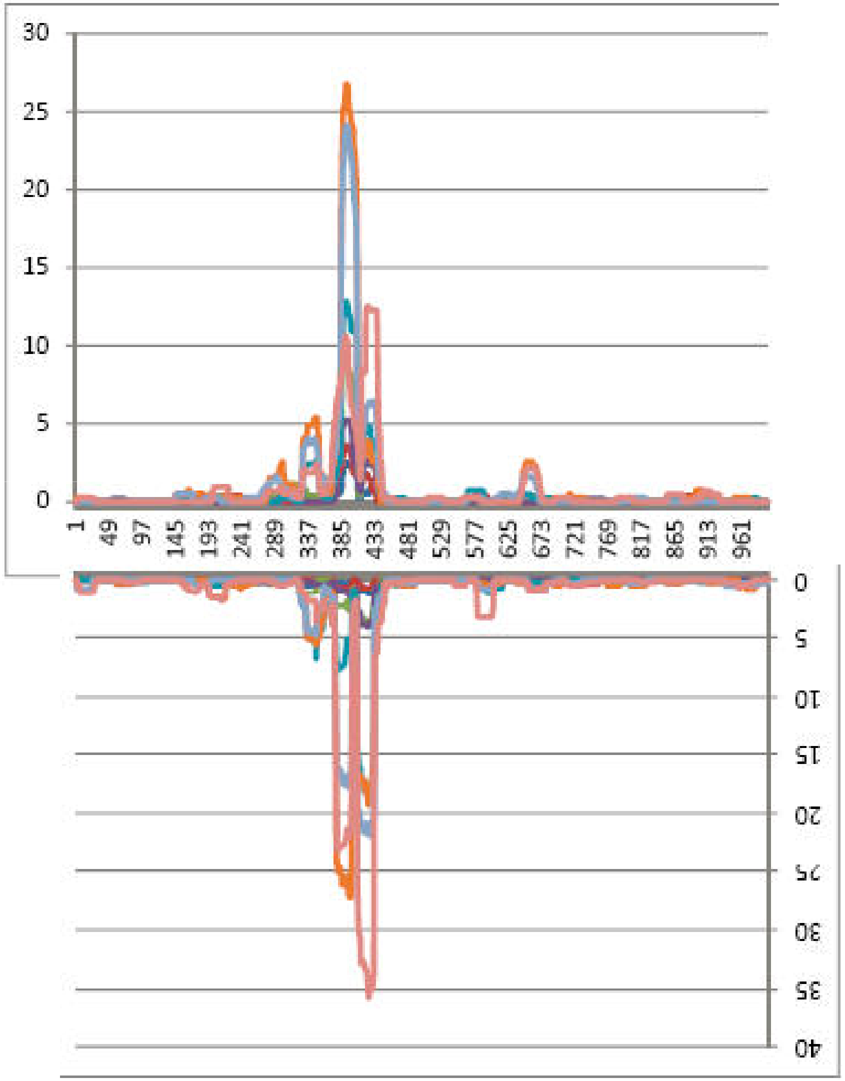

AT5G24240

Phosphatidylinositol 3- and 4-kinase ;Ubiquitin family protein

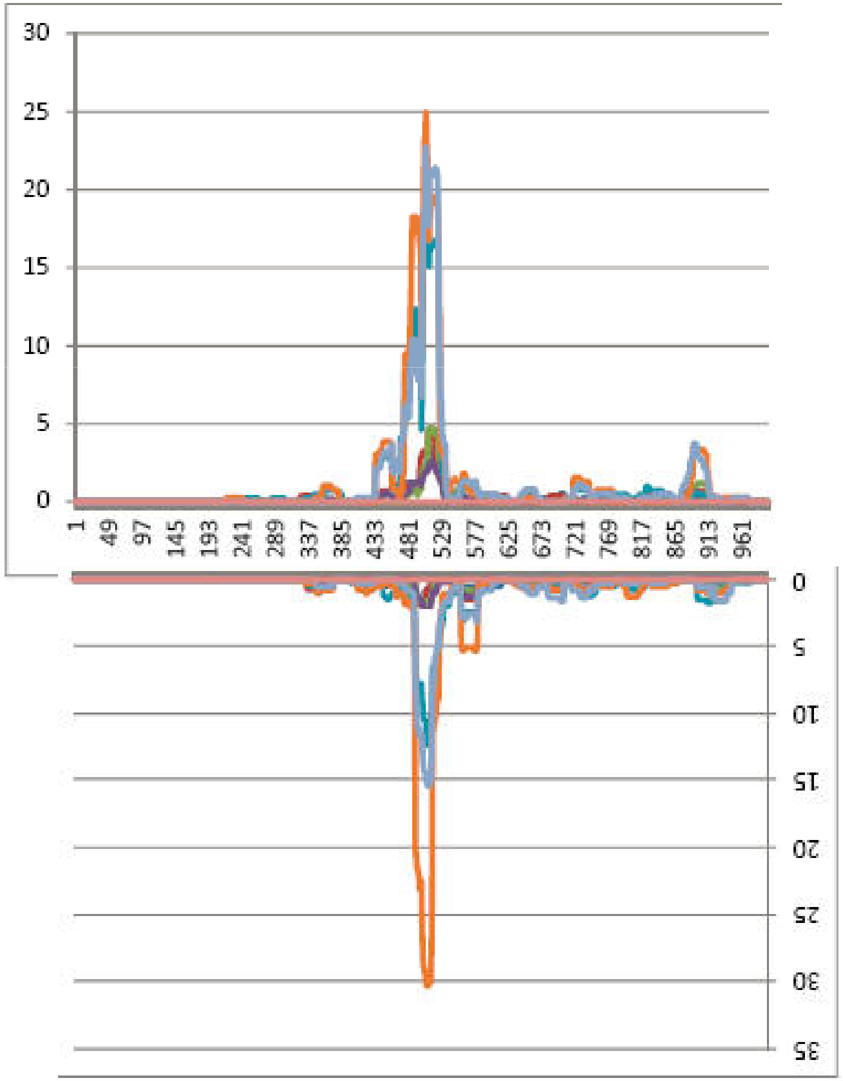

AT5G26700

RmlC-like cupins superfamily protein

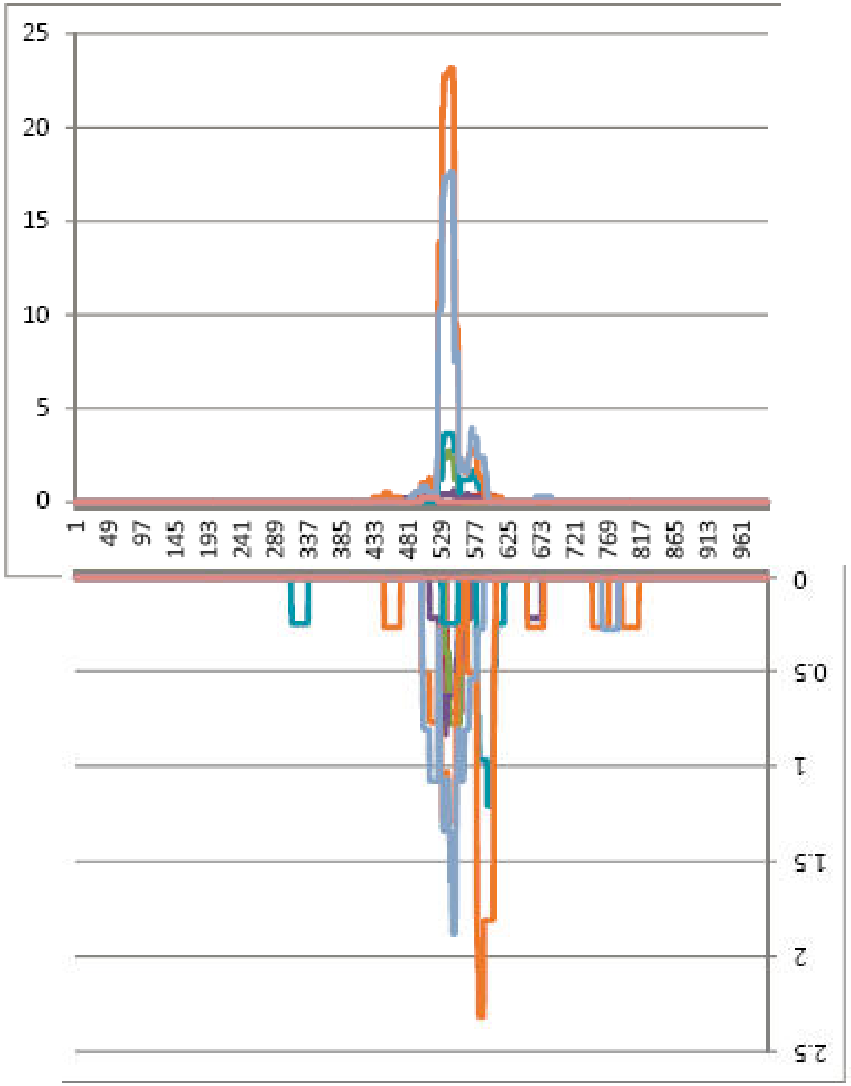

AT5G26840

unknown protein

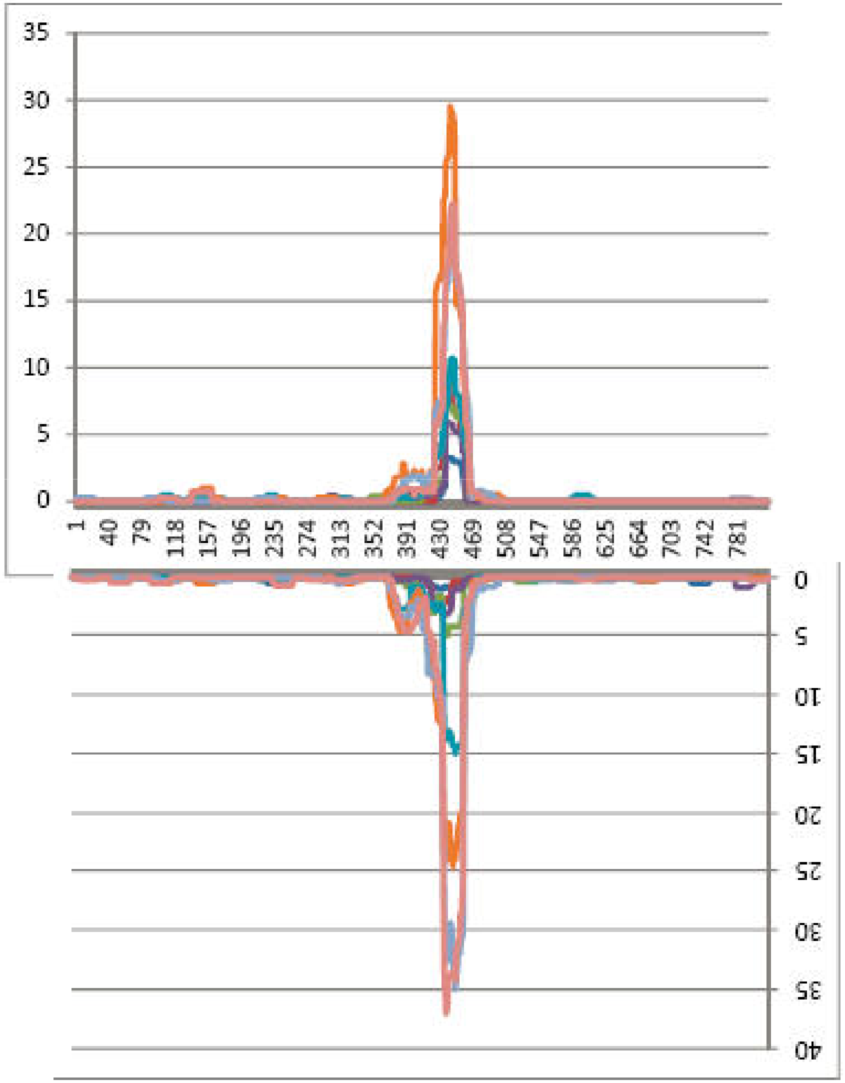

AT5G40320

Cysteine/Histidine-rich
C1 domain family protein

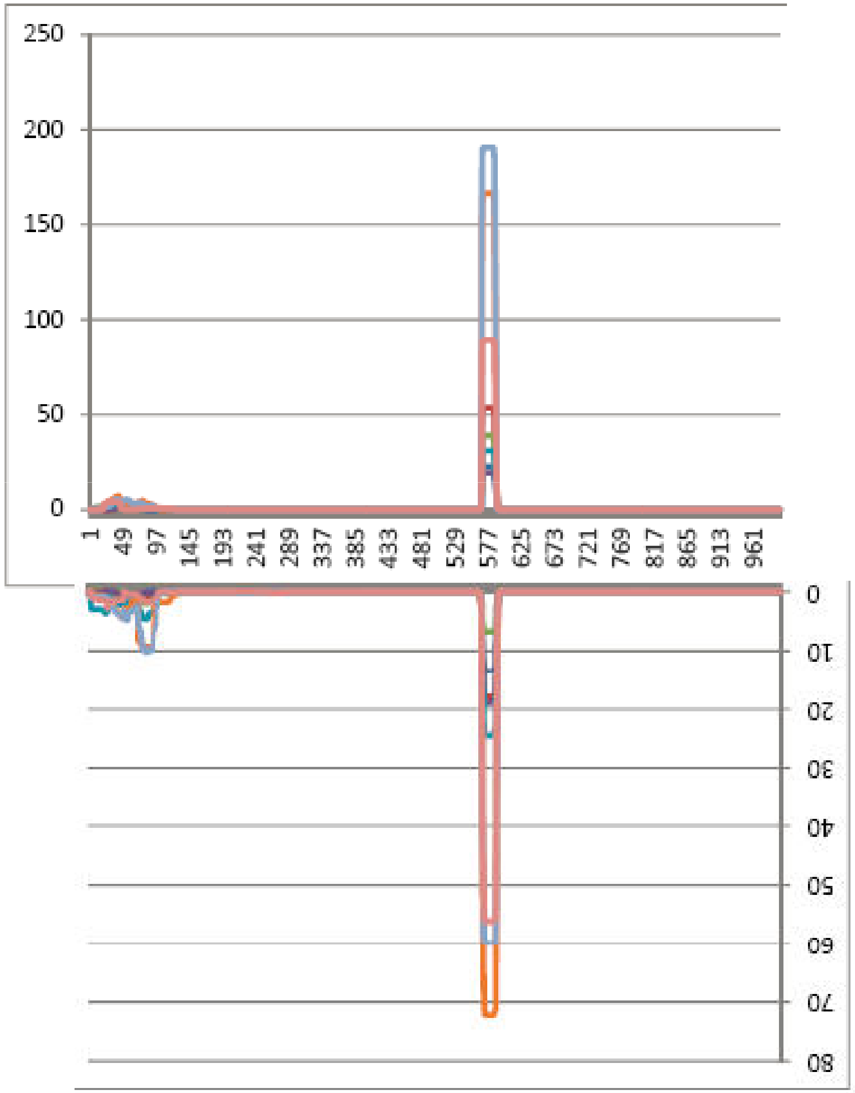

AT5G43500

encodes a protein whose sequence is similar to actin-related proteins (ARPs) in other organisms. Member of nuclear ARP family of genes

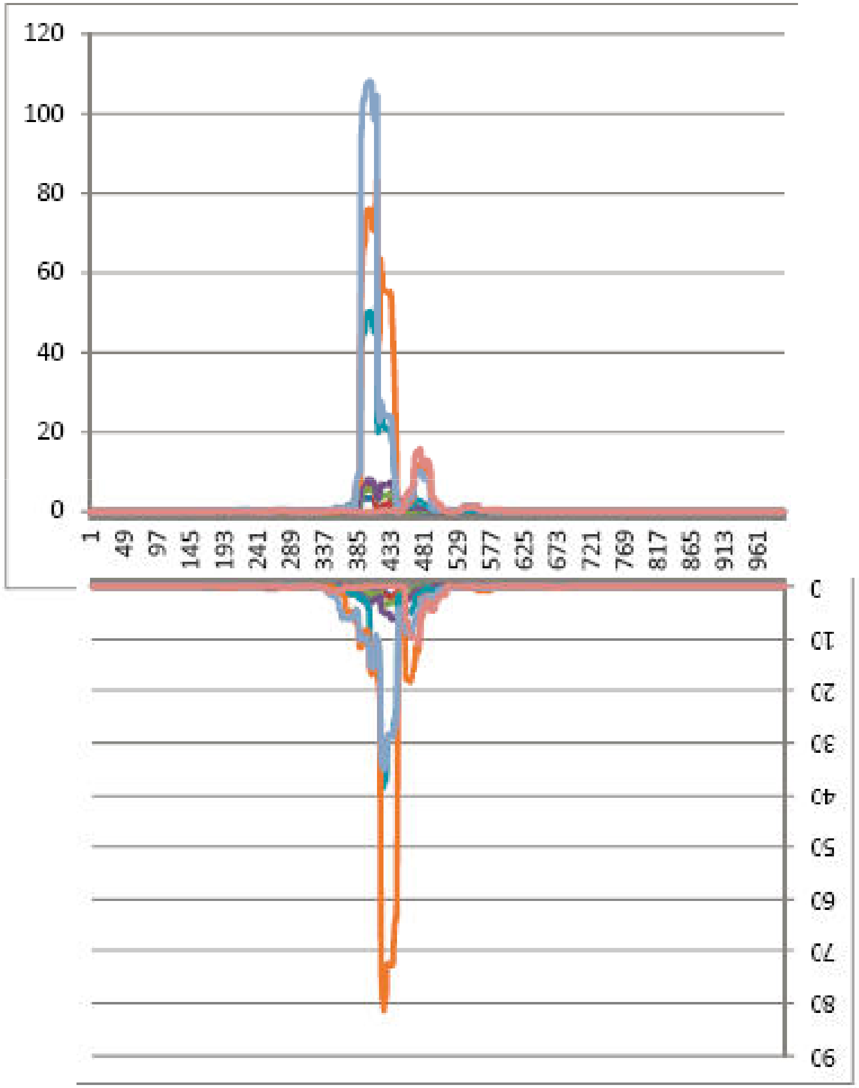

AT5G43810

Encodes Argonaute10, a member of the EIF2C (elongation initiation factor 2c)/ Argonaute class of proteins. Required to establish the central-peripheral organization of the embryo apex. Along with WUS and CLV genes, controls the relative organization of central zone and peripheral zone cells in meristems. Acts in embryonic provascular tissue potentiating WUSCHEL function during meristem development in the embryo. AGO10 specifically sequesters miR166/165 to regulate shoot apical meristem development.

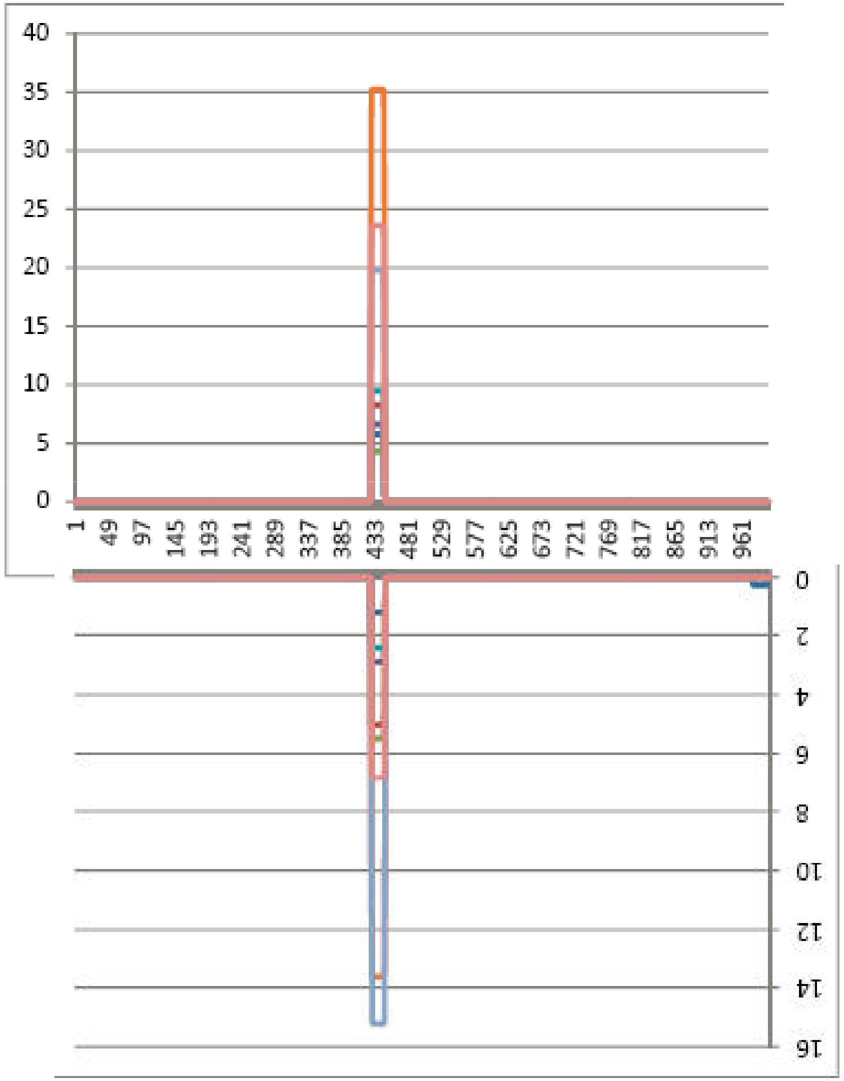

AT5G44930

Encodes a putative arabinosyltransferase that is associated with arabinan biosynthesis and is not redundant with ARAD1. The two glycosyltransferases may function in complexes held together by disulfide bridges.

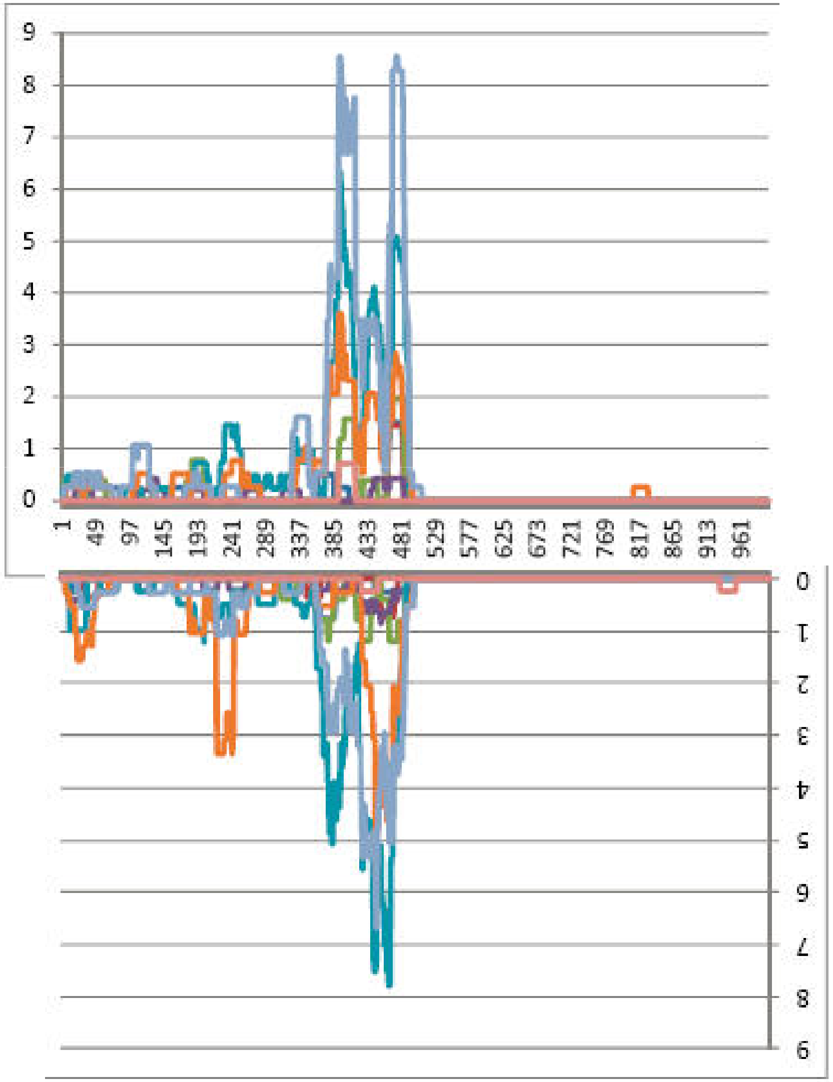

AT5G47260

ATP binding;GTP binding;nucleotide binding;nucleoside-triphosphatases

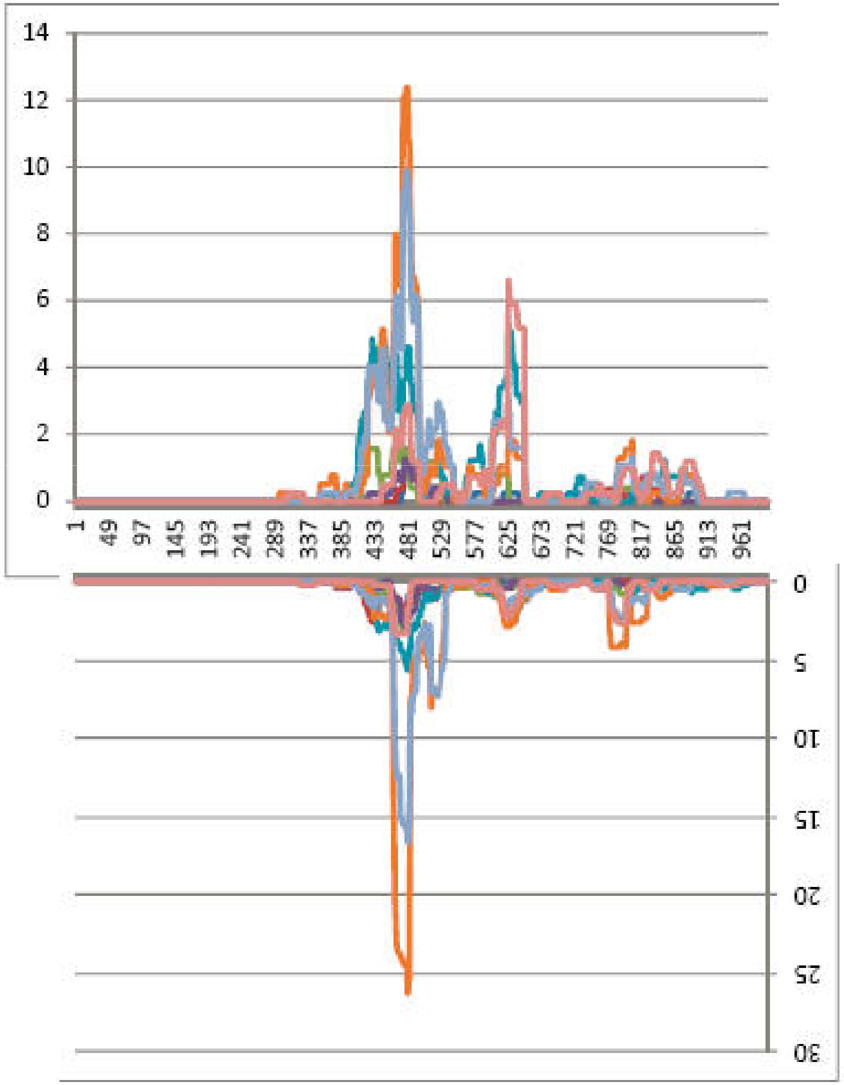

AT5G48000

THALIANOL HYDROXYLASE (THAH). Encodes a member of the CYP708A family of cytochrome P450 enzymes. THAH appears to add a hydroxyl group to the triterpene thalianol. thah1 mutants have an elevated accumulation of thalianol. thah1-1 mutants have longer roots than wild type plants. Thalian-diol and desaturated thalian-diol are lost from the root extracts of thah1-1 mutants. Overexpression of the sequence from At5g48000.1 rescues the thah1-1 mutant phenotype (Field 2008); it is unknown whether the shorter sequences associated with other gene models would provide functional complementation.

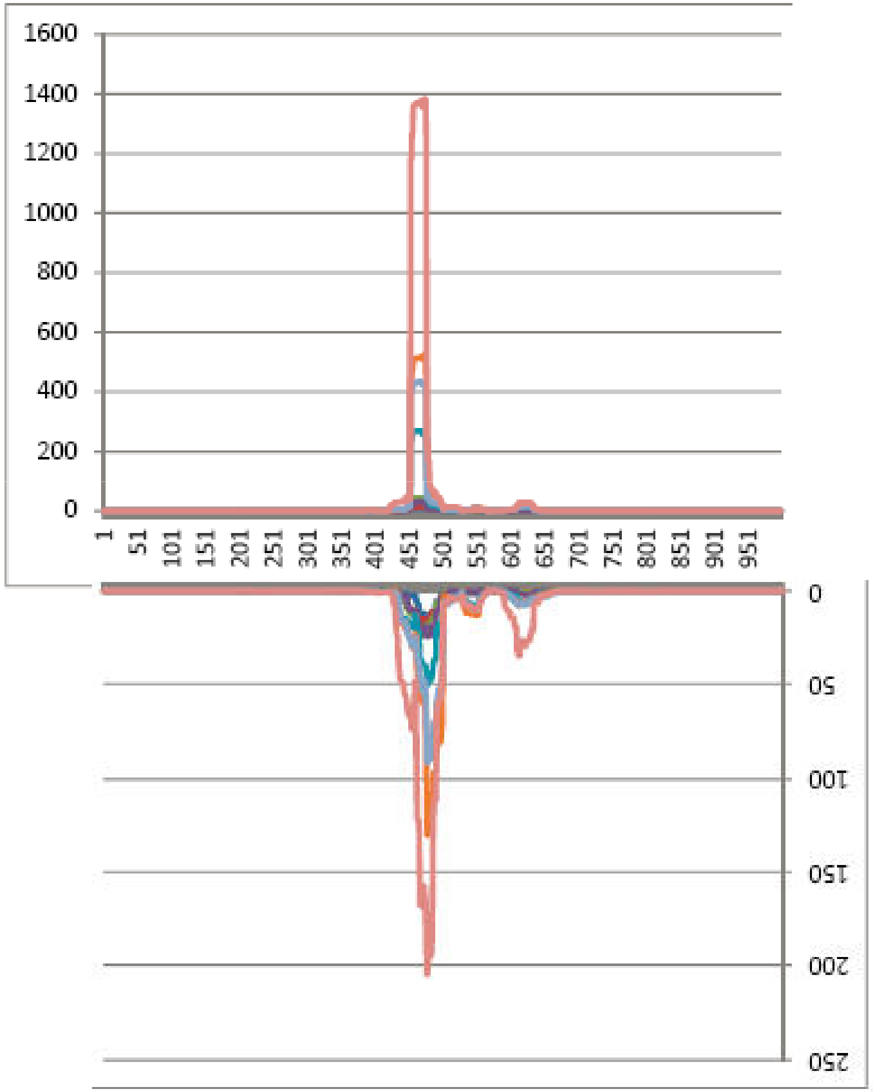

AT5G61510

GroES-like zinc-binding alcohol dehydrogenase family protein

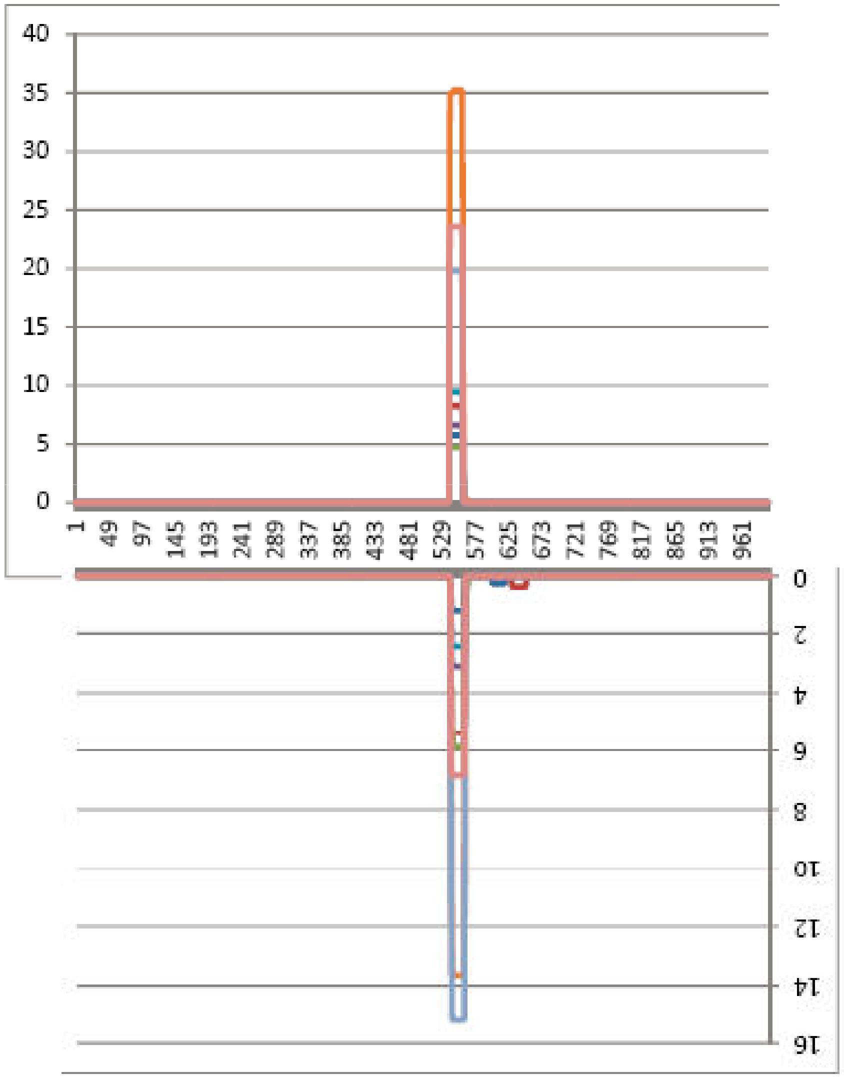

ATCG01120

encodes a chloroplast ribosomal protein S15, a constituent of the small subunit of the ribosomal complex

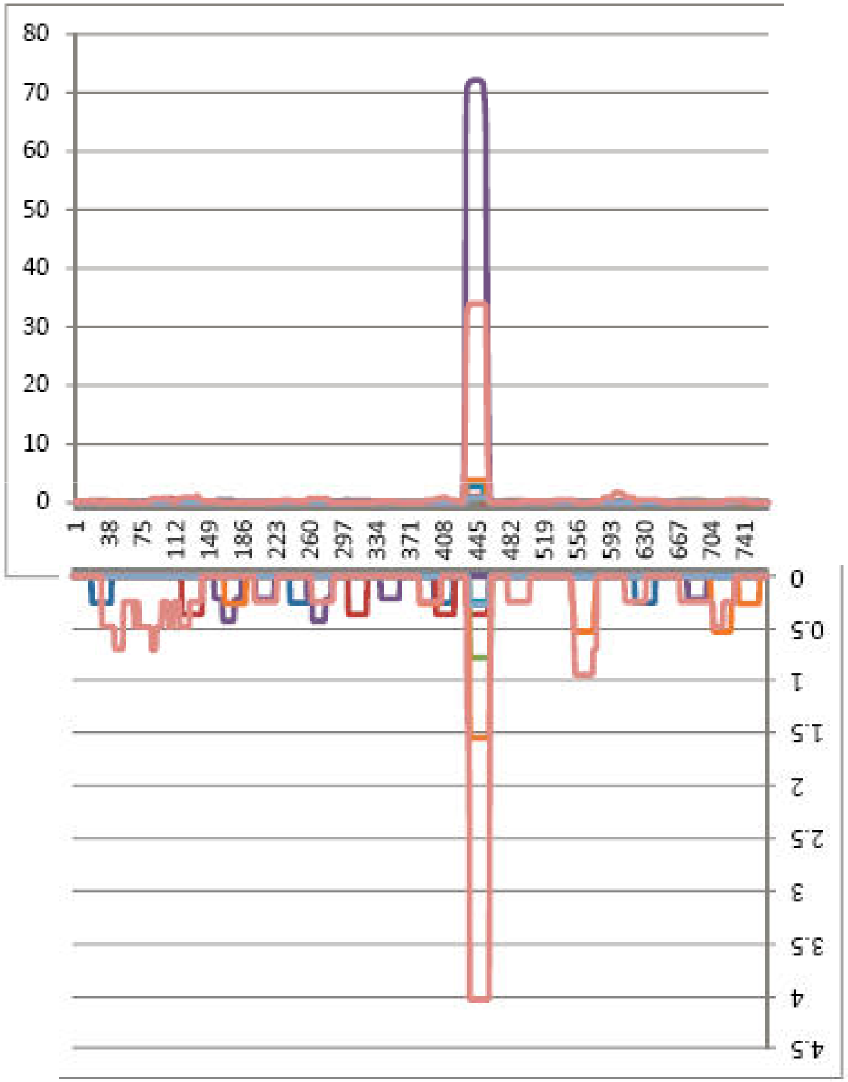

Supplement: S9 Fig — (PDF) [file pone.0169212.s009.pdf]
