# Supplementary material for: Transcriptome-Wide Discovery of PASRs (Promoter-Associated Small RNAs) and TASRs (Terminus-Associated Small RNAs) in Arabidopsis thaliana
Source: PLoS One. 2017 Jan 3;12(1):e0169212. doi: 10.1371/journal.pone.0169212 (PMC5207706; doi:10.1371/journal.pone.0169212)

Figure S10 AGO-associated TASR peaks identified on the sense strands of the protein-coding genes of *Arabidopsis*. For each plot, x axis measures the position of the sense strand, and y axis measures the abundance (in RPM, reads per million) of sRNAs.

AT1G01073

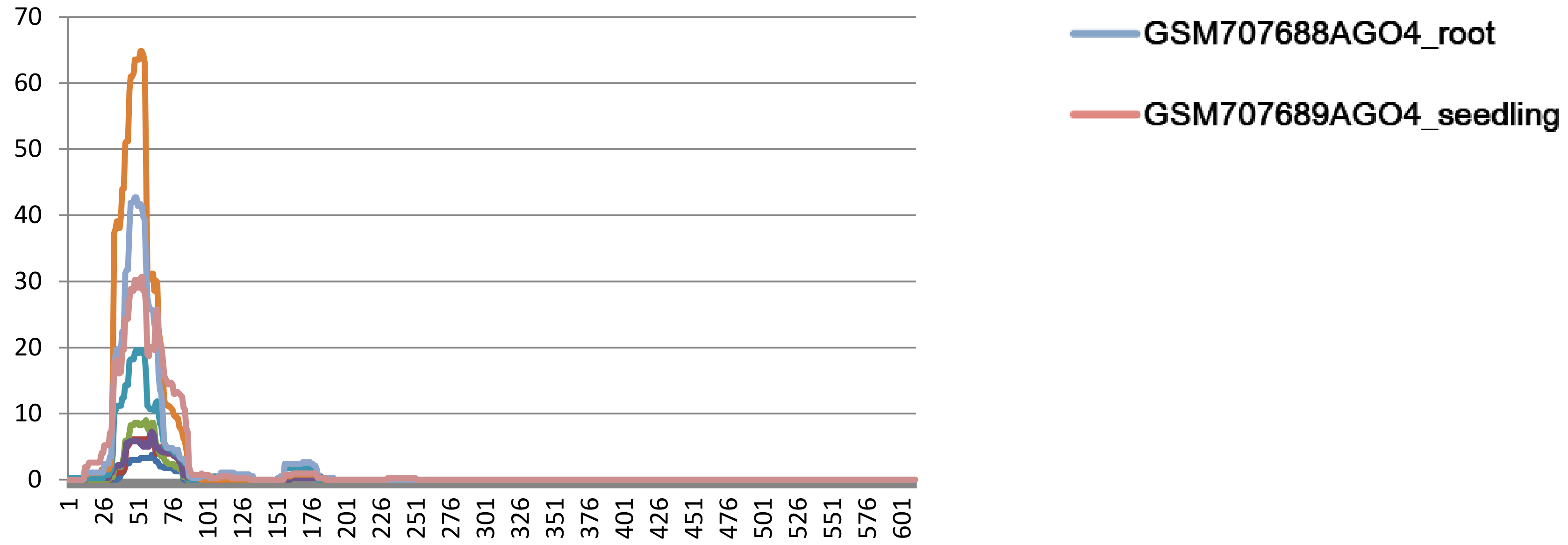

70
60
50
40
30
20
10
0
1
26
51
76
101
126
151
176
201
226
251
276
301
326
351
376
401
426
451
476
501
526
551
576
601
GSM707682AGO1_flower
GSM707683AGO1_leaf
GSM707684AGO1_root
GSM707685AGO1_seedling
GSM707686AGO4_flower
GSM707687AGO4_leaf
GSM707688AGO4_root
GSM707689AGO4_seedling

AT1G01180_AGO4

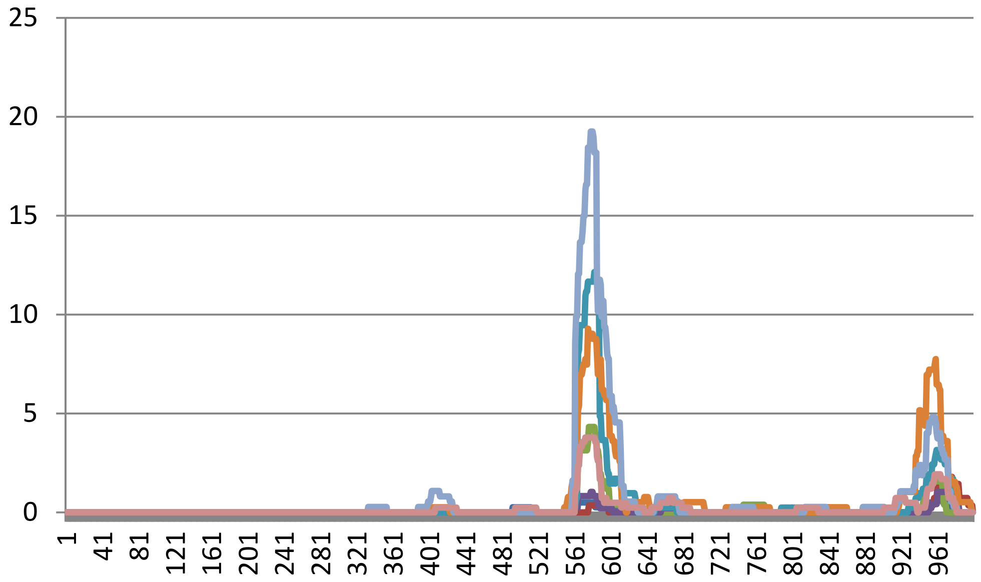

AT1G10000

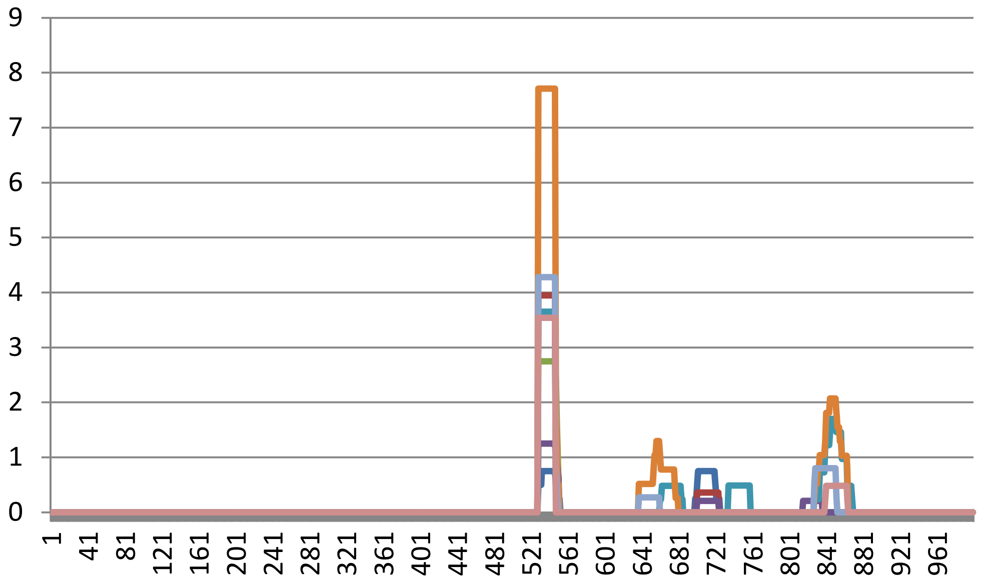

9
8
7
6
5
4
3
2
1
0
1
41
81
121
161
201
241
281
321
361
401
441
481
521
561
601
641
681
721
761
801
841
881
921
961

AT1G12520_root

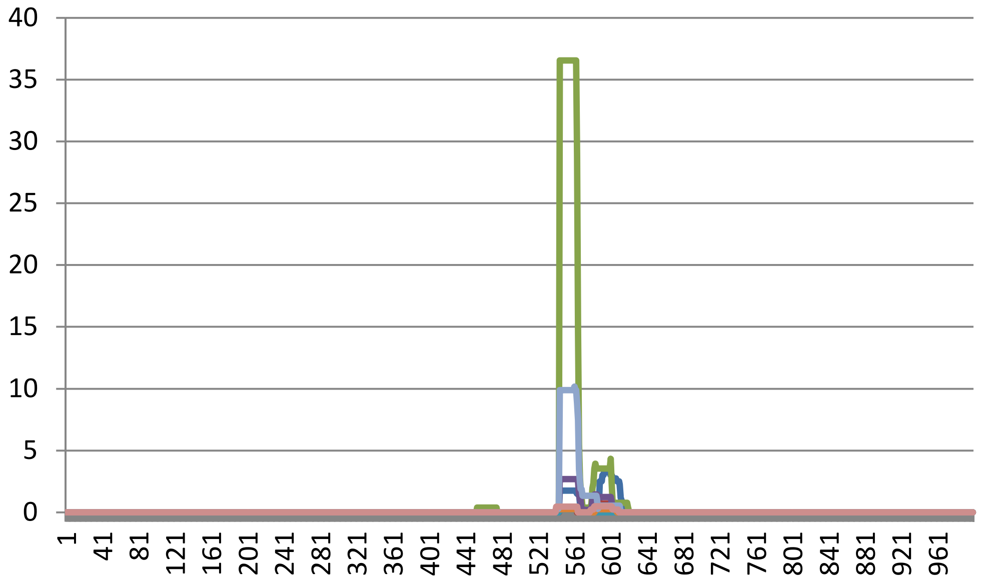

AT1G15130

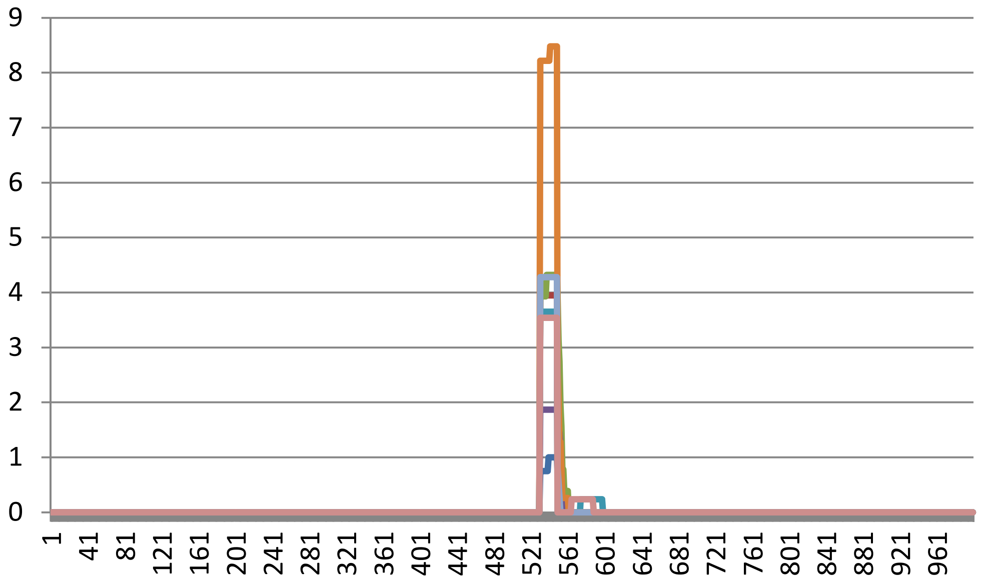

9
8
7
6
5
4
3
2
1
0
1
41
81
121
161
201
241
281
321
361
401
441
481
521
561
601
641
681
721
761
801
841
881
921
961

AT1G16270

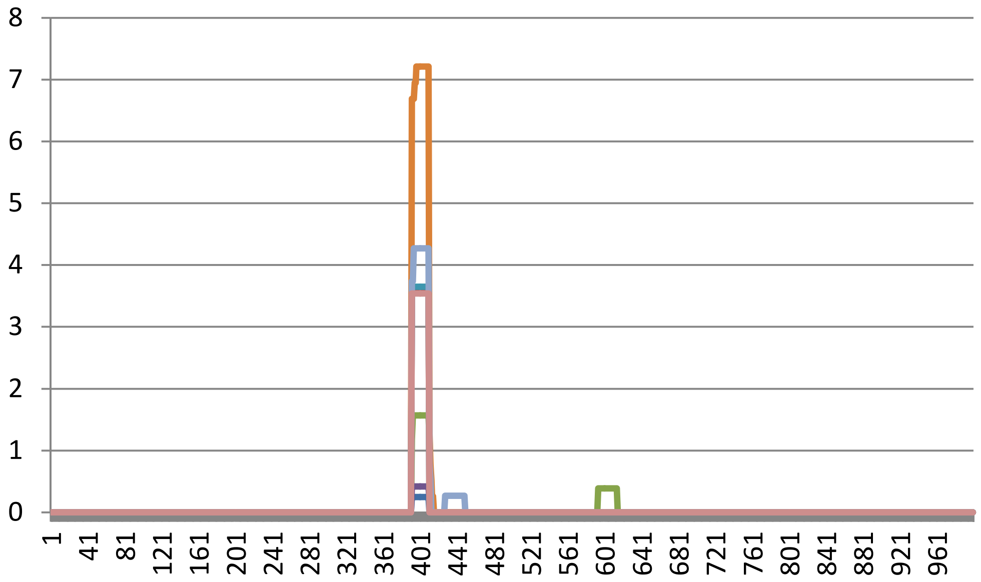

0
1
2
3
4
5
6
7
8
1
41
81
121
161
201
241
281
321
361
401
441
481
521
561
601
641
681
721
761
801
841
881
921
961

AT1G16460_AGO1

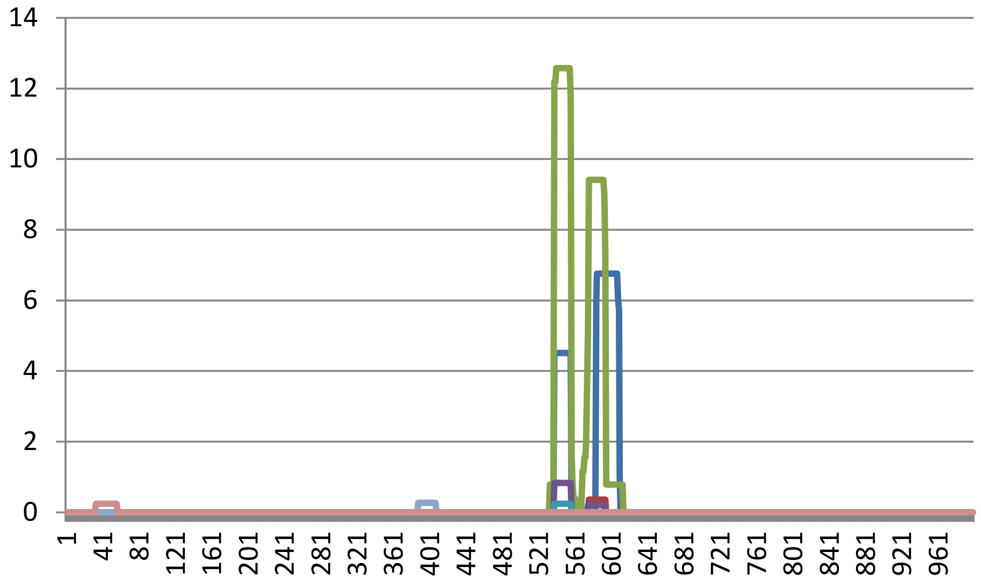

AT1G16610

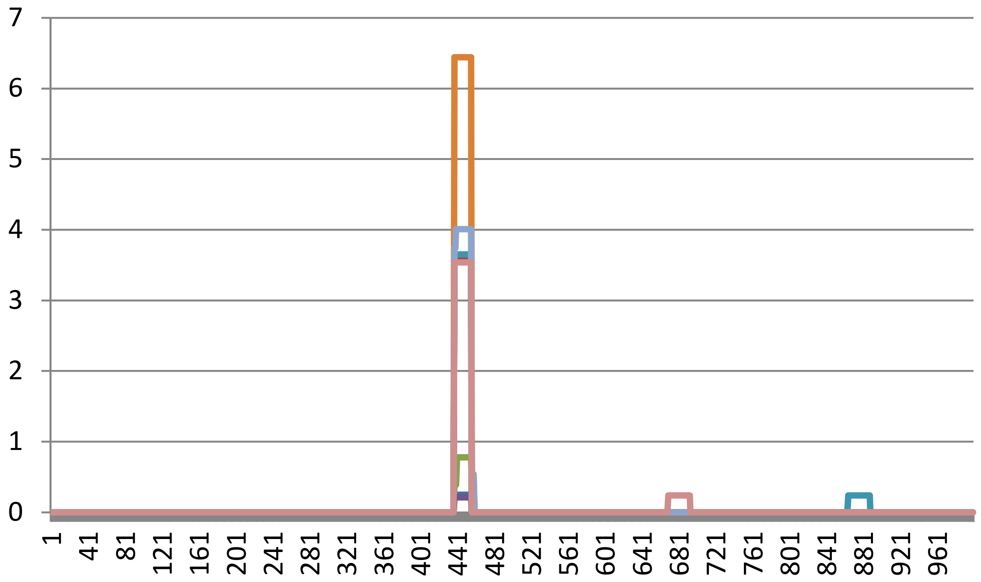

7
6
5
4
3
2
1
0
1
41
81
121
161
201
241
281
321
361
401
441
481
521
561
601
641
681
721
761
801
841
881
921
961

AT1G18420_AGO1 root

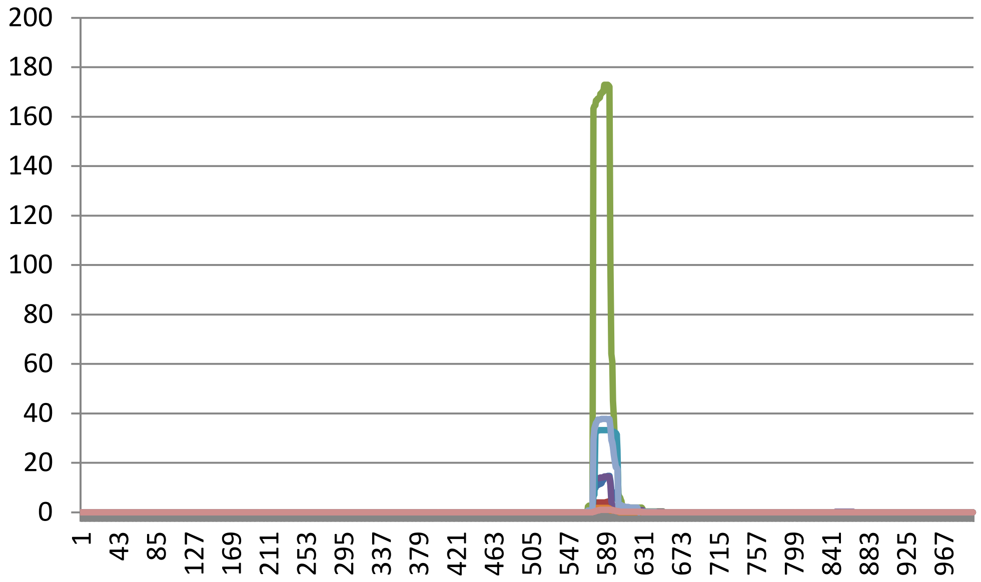

AT1G20830_AGO4

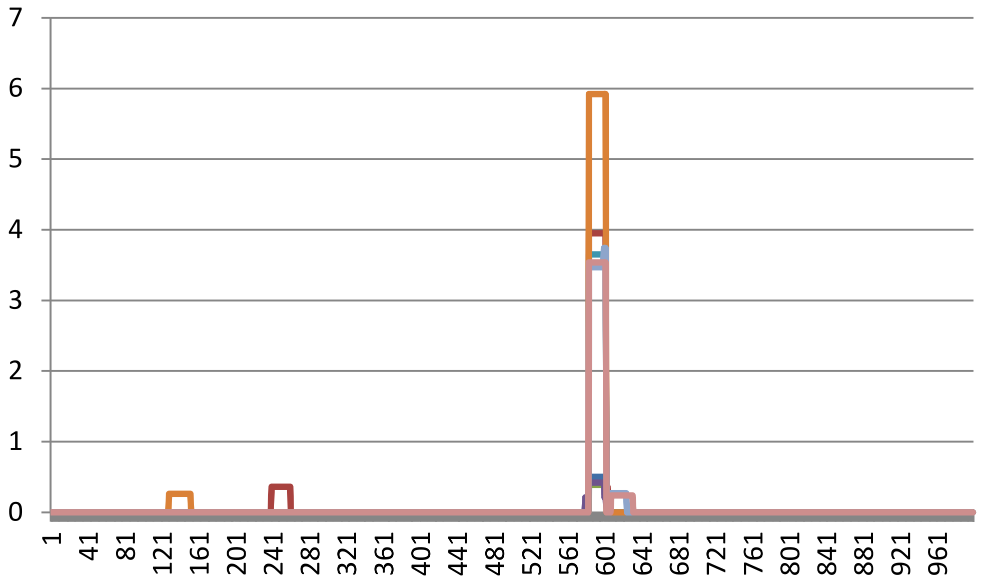

AT1G22720_AGO4 root

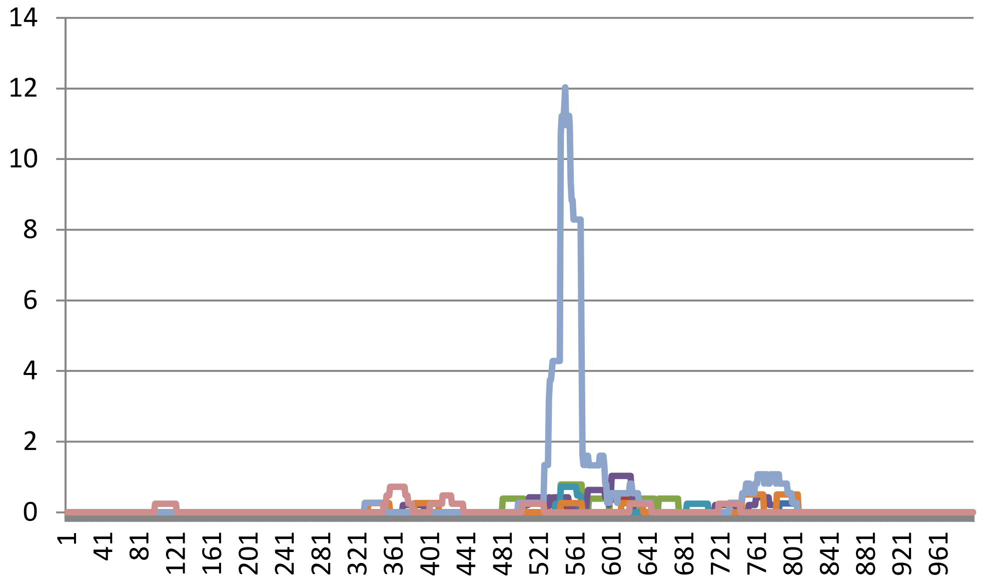

AT1G26400_AGO4

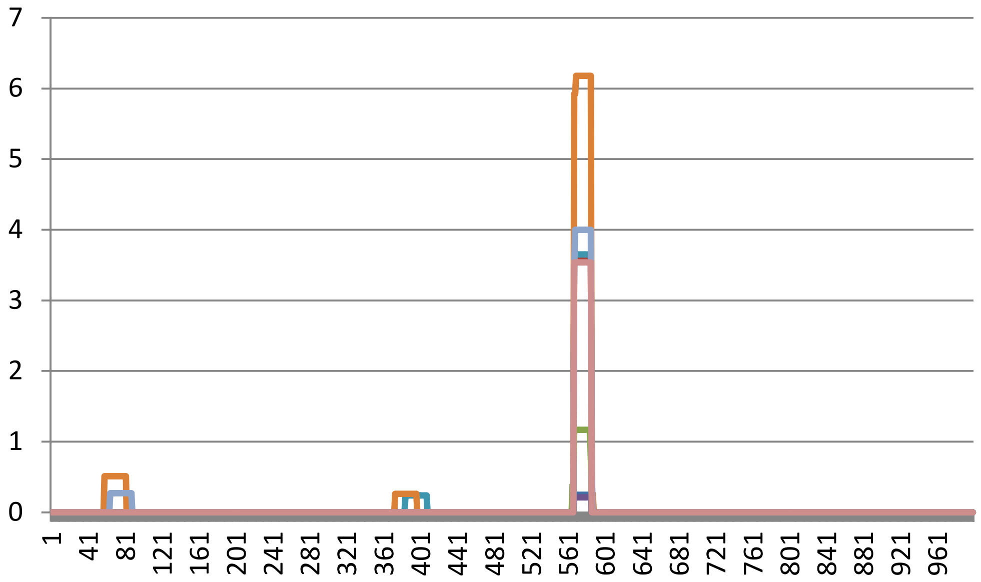

AT1G28140_AGO4

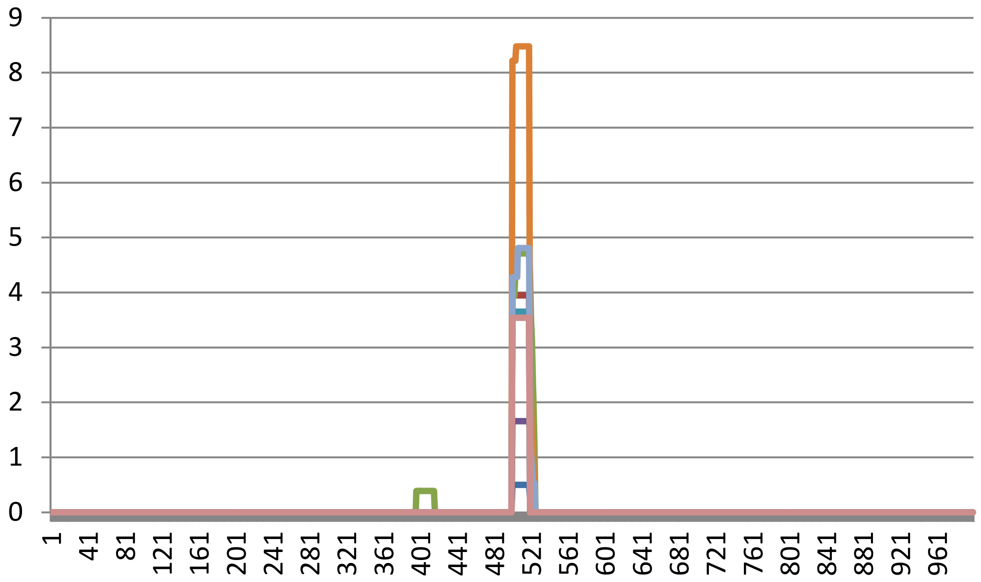

AT1G35710

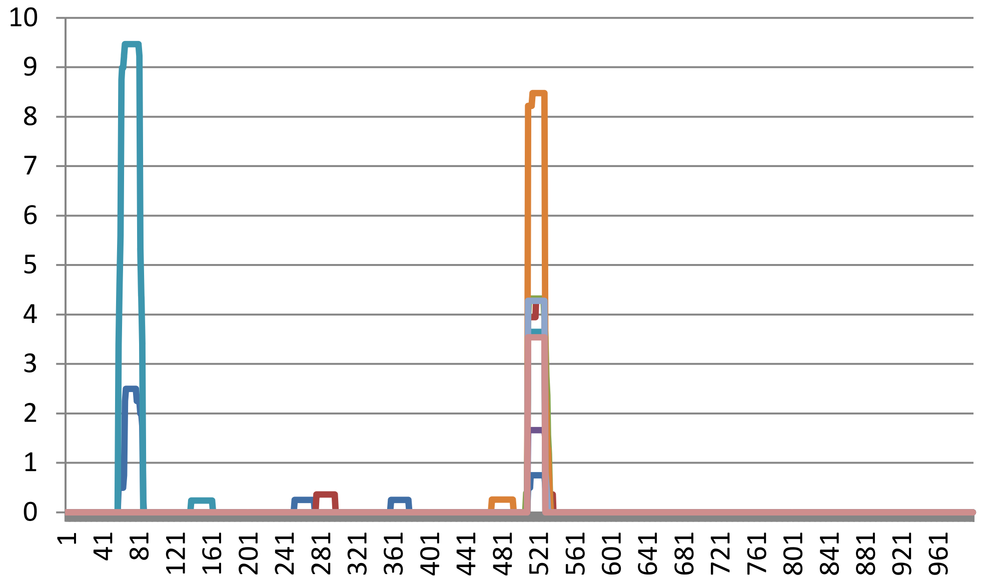
10
9
8
7
6
5
4
3
2
1
0
1
41
81
121
161
201
241
281
321
361
401
441
481
521
561
601
641
681
721
761
801
841
881
921
961

AT1G36640

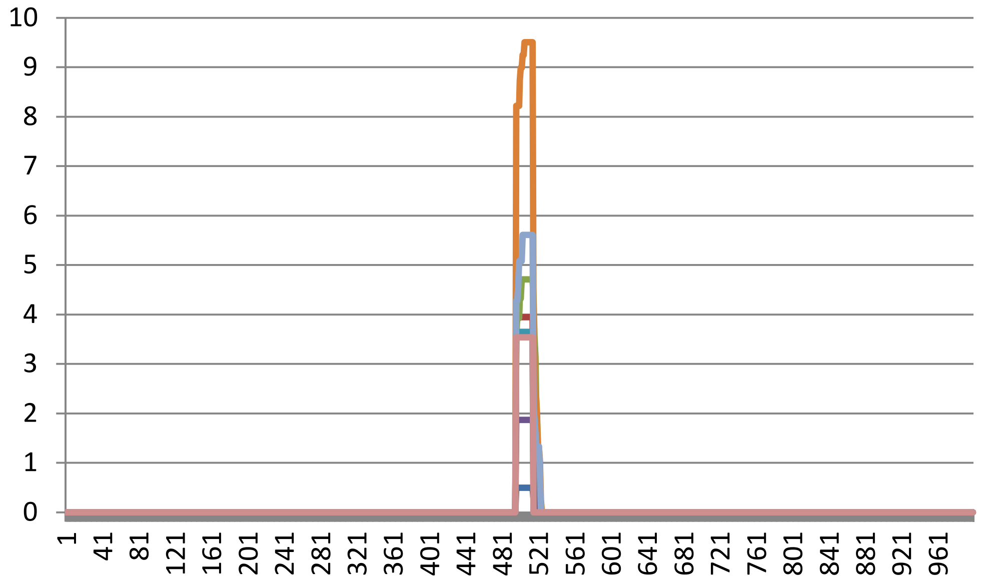

10
9
8
7
6
5
4
3
2
1
0
1
41
81
121
161
201
241
281
321
361
401
441
481
521
561
601
641
681
721
761
801
841
881
921
961

AT1G36950_AGO4

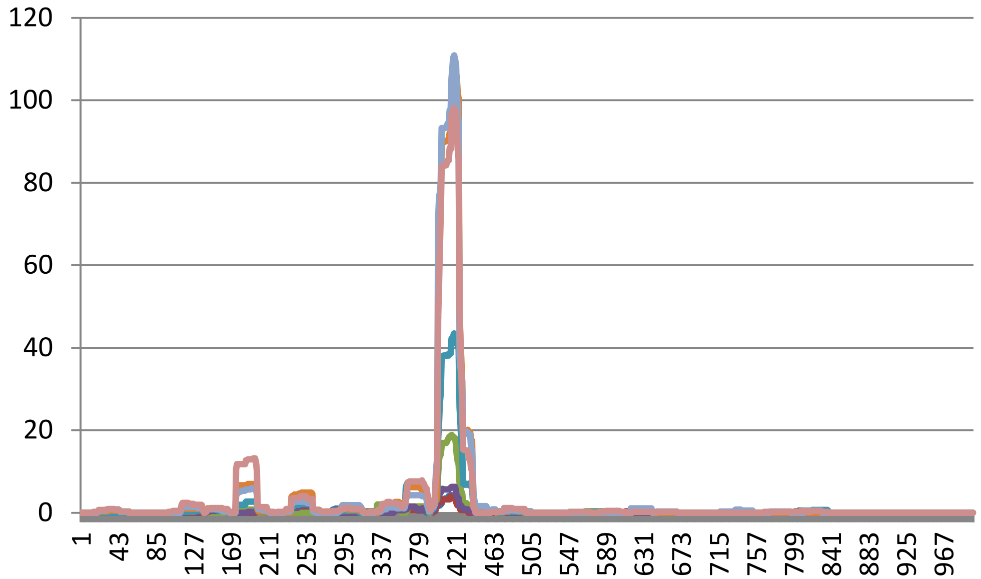

AT1G38790_AGO4

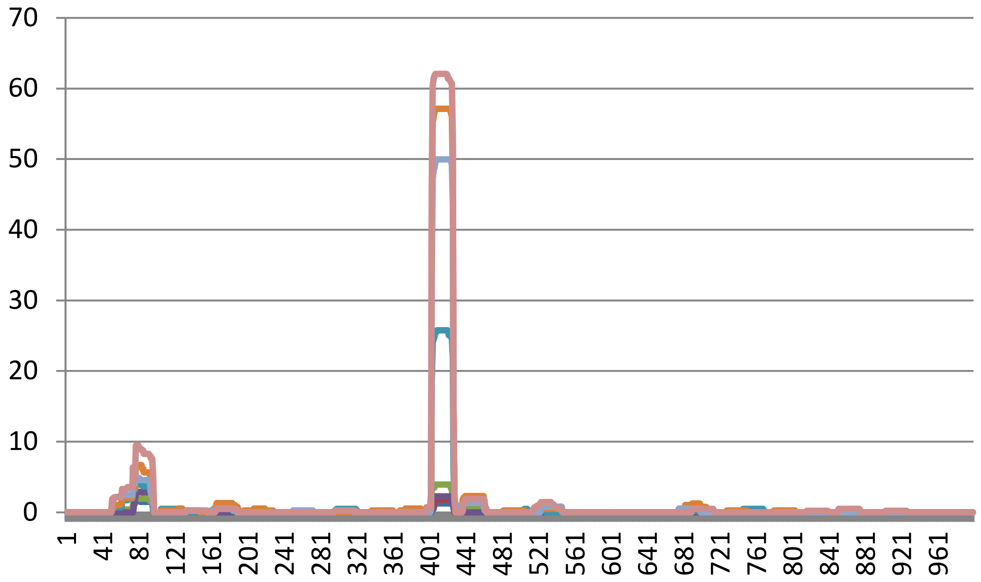

AT1G41820_AGO4 flower

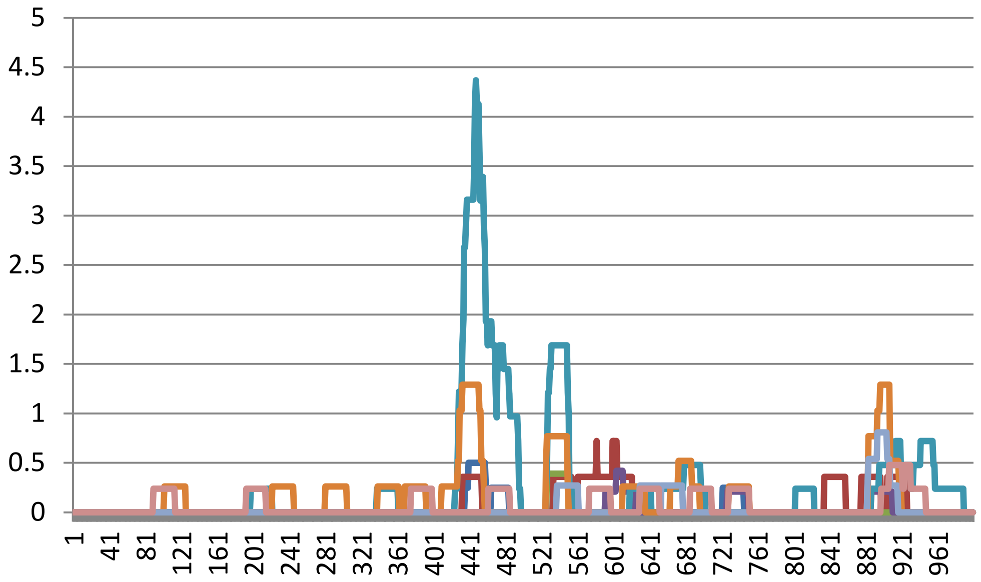

AT1G49700

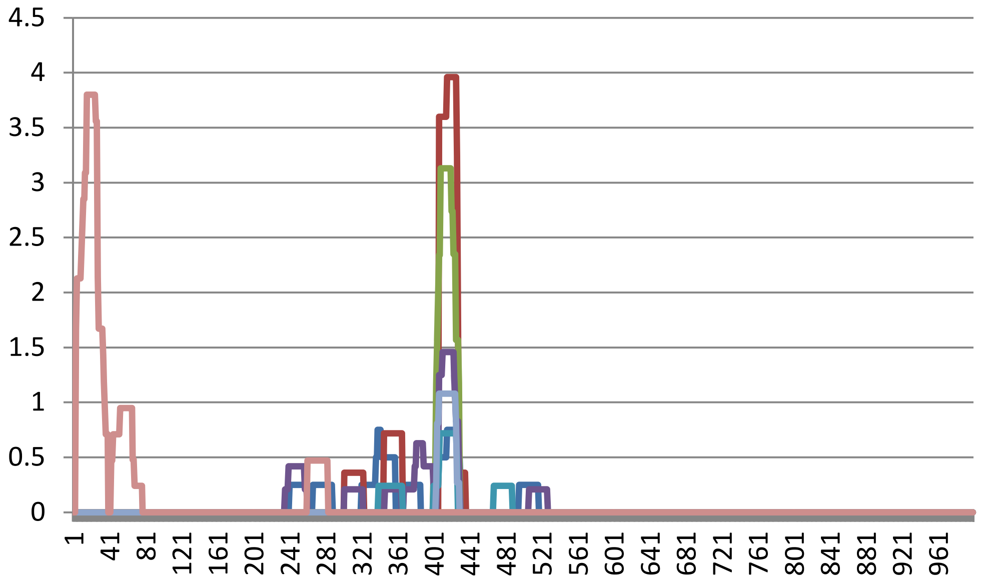

4.5
4
3.5
3
2.5
2
1.5
1
0.5
0
1
41
81
121
161
201
241
281
321
361
401
441
481
521
561
601
641
681
721
761
801
841
881
921
961

AT1G52180

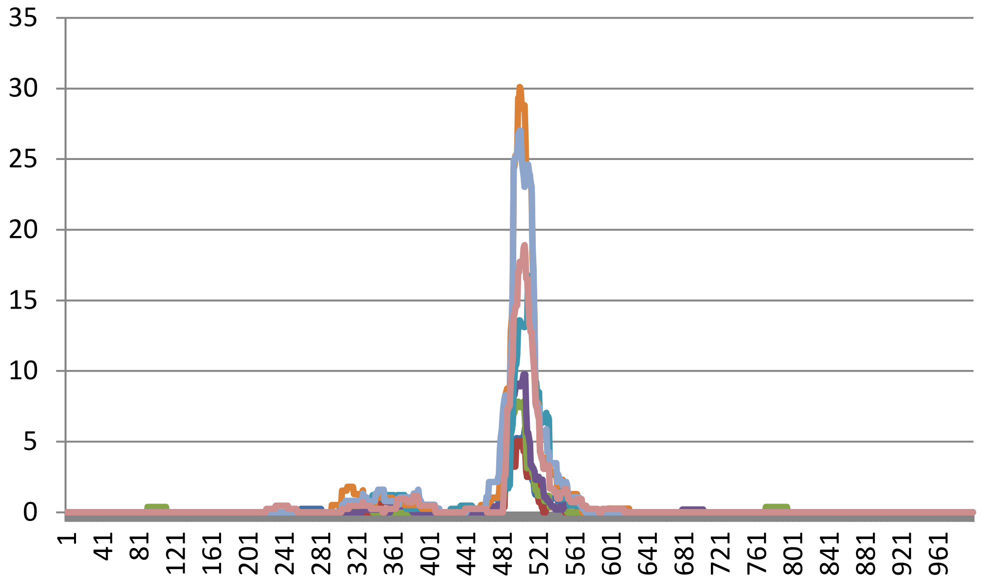

35
30
25
20
15
10
5
0
1
41
81
121
161
201
241
281
321
361
401
441
481
521
561
601
641
681
721
761
801
841
881
921
961

AT1G54000

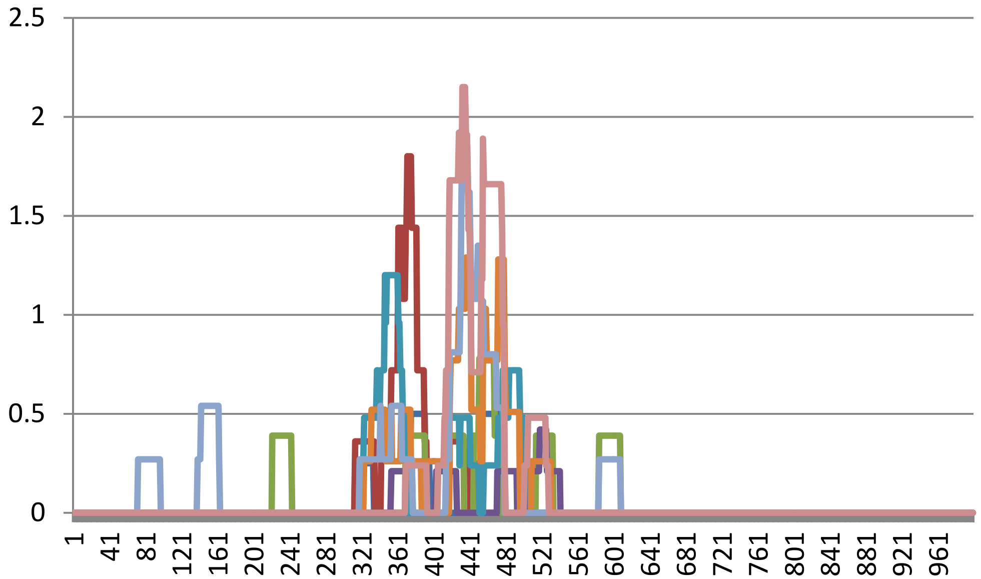

2.5
2
1.5
1
0.5
0
1
41
81
121
161
201
241
281
321
361
401
441
481
521
561
601
641
681
721
761
801
841
881
921
961

AT1G54990

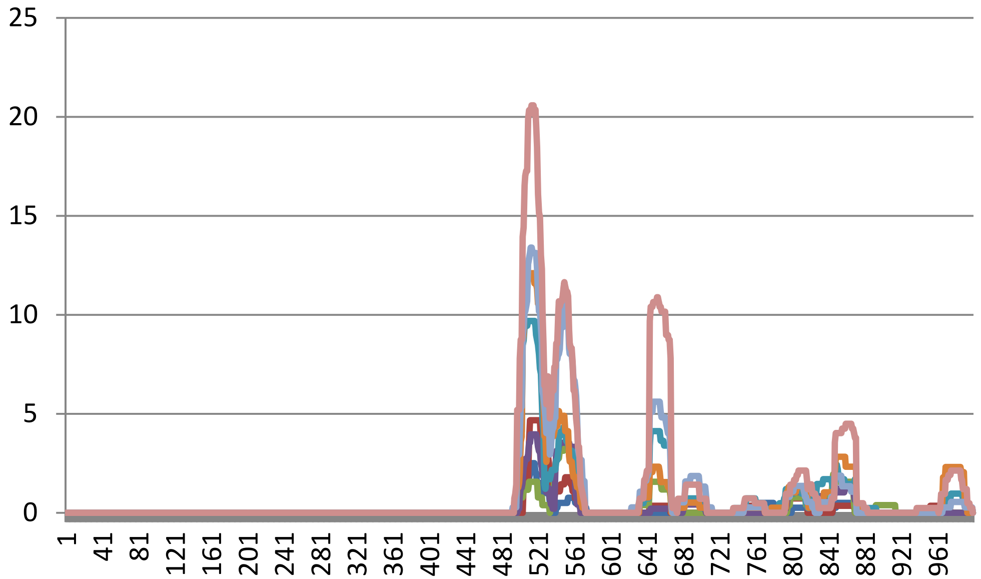

25
20
15
10
5
0
1
41
81
121
161
201
241
281
321
361
401
441
481
521
561
601
641
681
721
761
801
841
881
921
961

AT1G61255

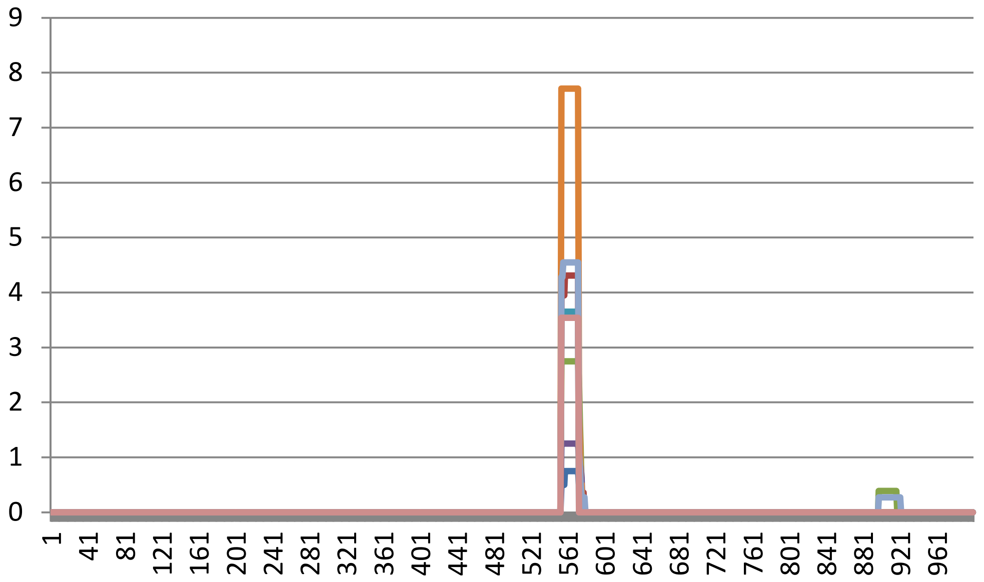
9
8
7
6
5
4
3
2
1
0
1
41
81
121
161
201
241
281
321
361
401
441
481
521
561
601
641
681
721
761
801
841
881
921
961

AT1G61320

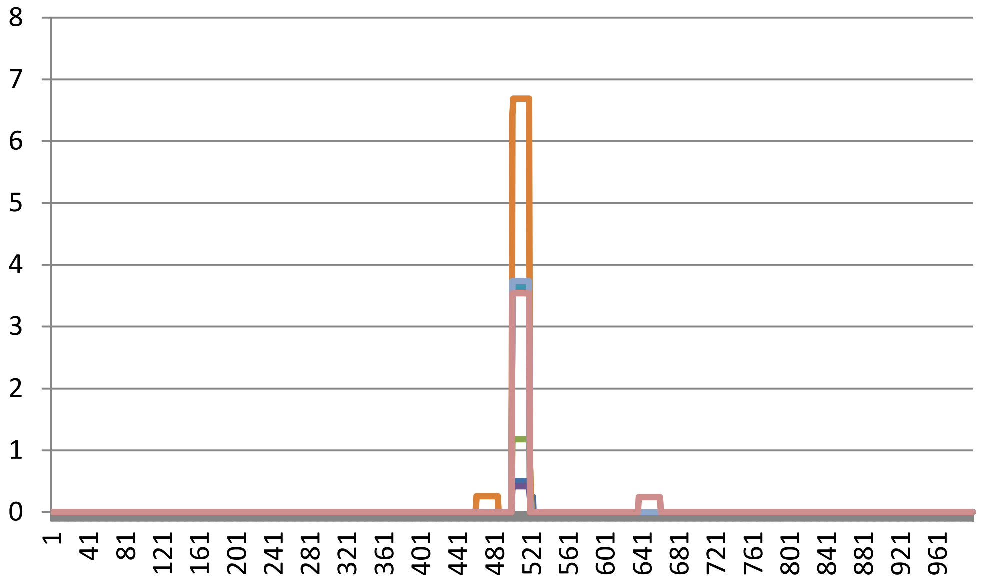

8
7
6
5
4
3
2
1
0
1
41
81
121
161
201
241
281
321
361
401
441
481
521
561
601
641
681
721
761
801
841
881
921
961

AT1G62560

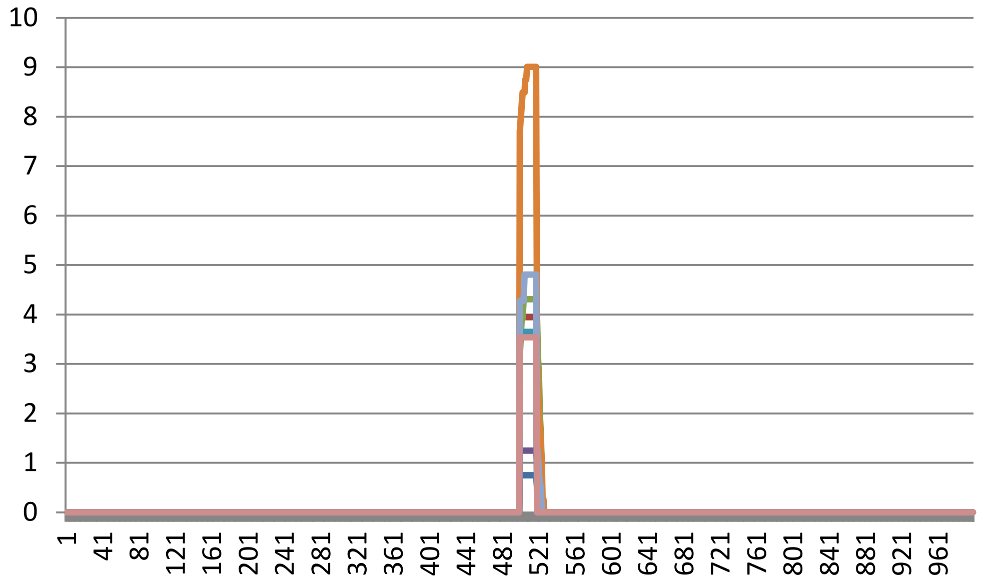

10
9
8
7
6
5
4
3
2
1
0
1
41
81
121
161
201
241
281
321
361
401
441
481
521
561
601
641
681
721
761
801
841
881
921
961

AT1G63520

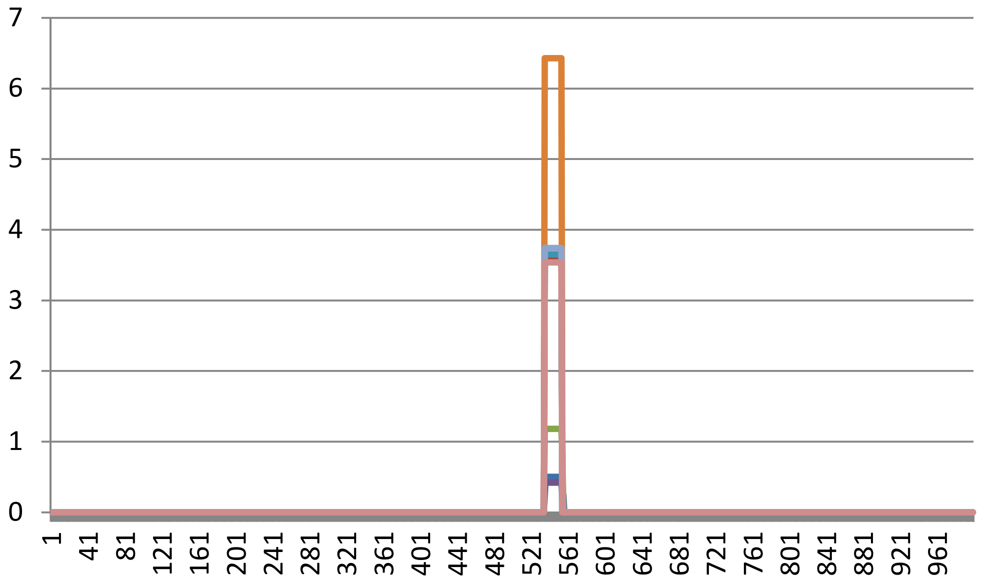

7
6
5
4
3
2
1
0
1
41
81
121
161
201
241
281
321
361
401
441
481
521
561
601
641
681
721
761
801
841
881
921
961

AT1G65860

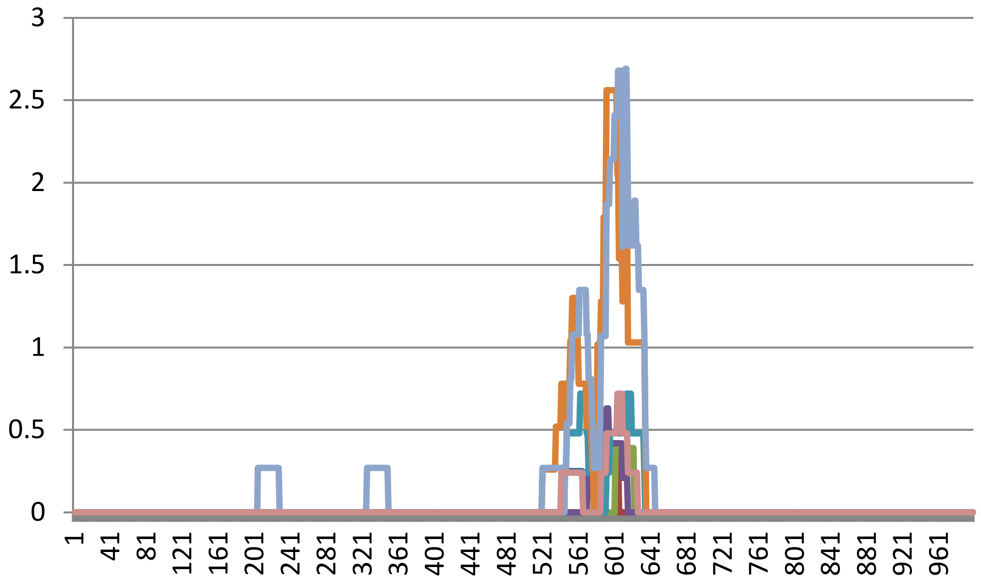

3
2.5
2
1.5
1
0.5
0
1
41
81
121
161
201
241
281
321
361
401
441
481
521
561
601
641
681
721
761
801
841
881
921
961

AT1G69280

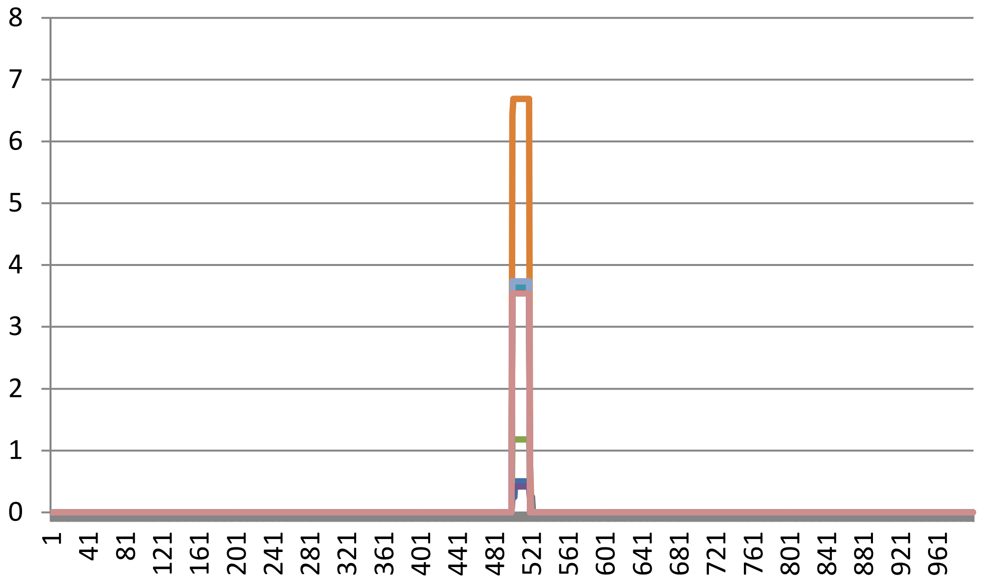

8
7
6
5
4
3
2
1
0
1
41
81
121
161
201
241
281
321
361
401
441
481
521
561
601
641
681
721
761
801
841
881
921
961

AT1G71697_AGO1

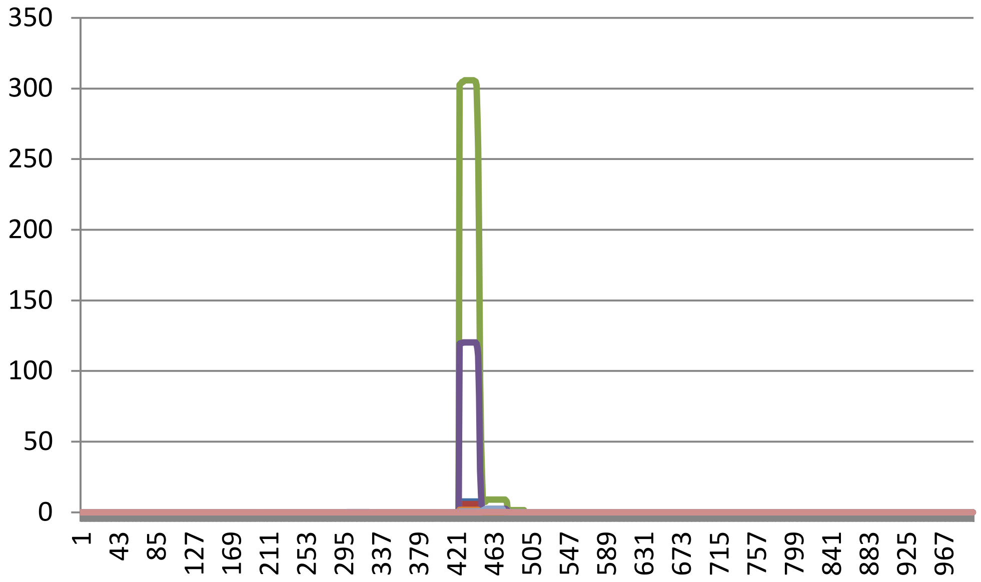

AT1G72140

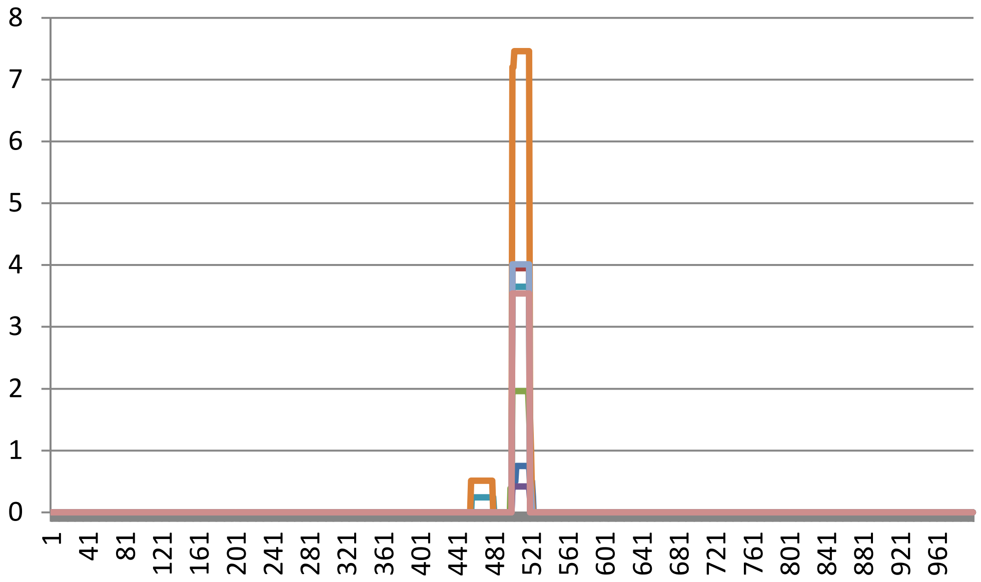

8
7
6
5
4
3
2
1
0
1
41
81
121
161
201
241
281
321
361
401
441
481
521
561
601
641
681
721
761
801
841
881
921
961

AT1G73640

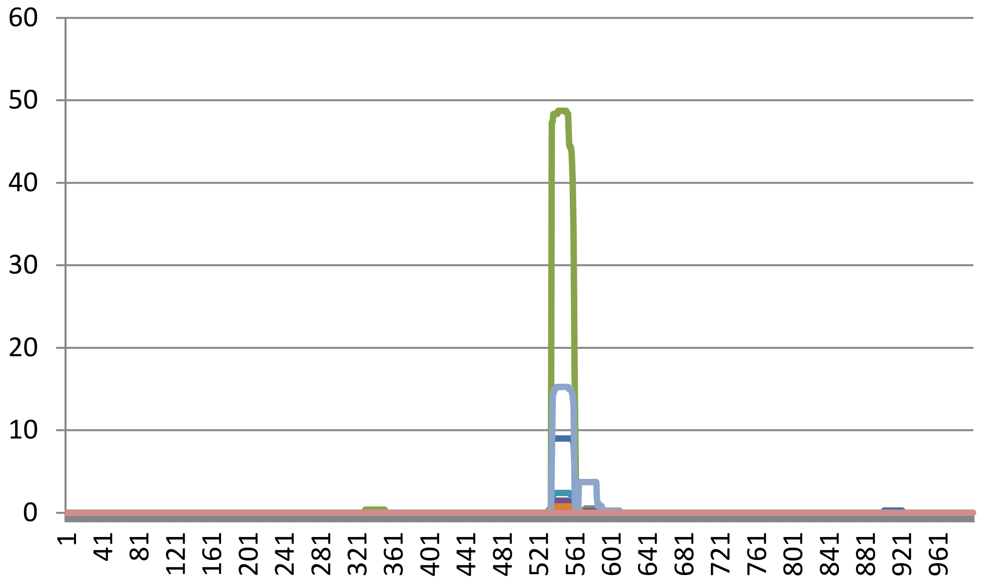

60
50
40
30
20
10
0
1
41
81
121
161
201
241
281
321
361
401
441
481
521
561
601
641
681
721
761
801
841
881
921
961

AT1G79170_AGO4

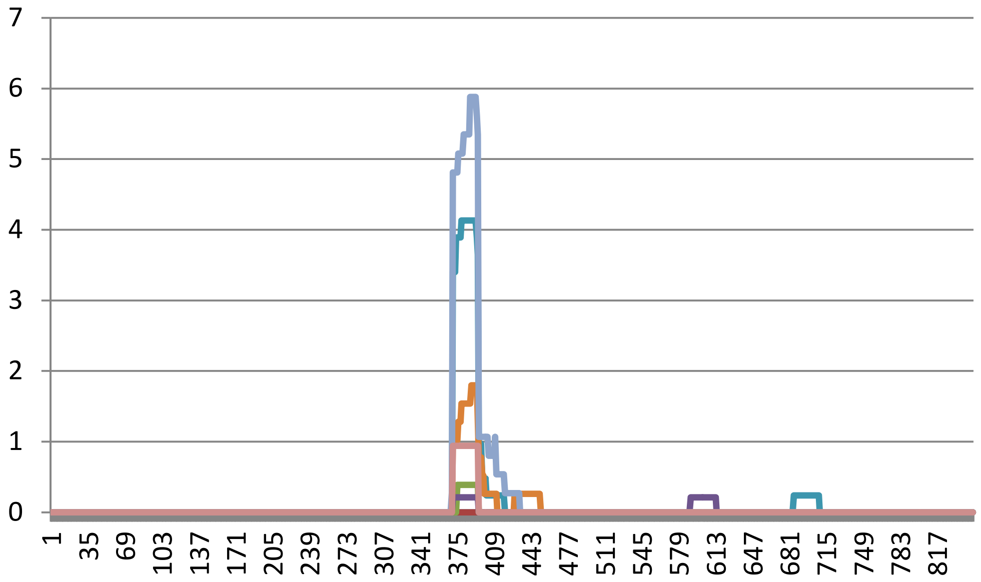

AT1G80690

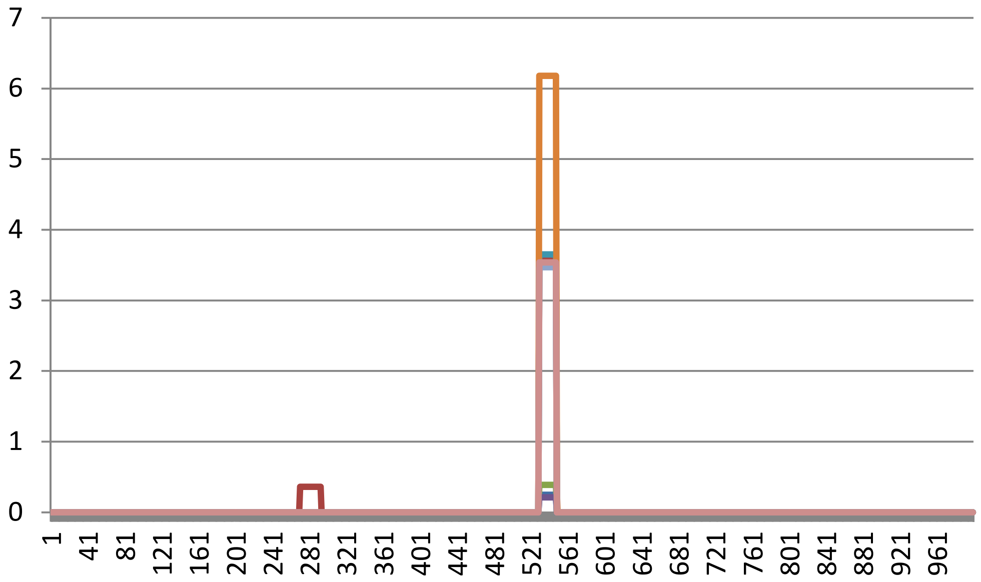

7
6
5
4
3
2
1
0
1
41
81
121
161
201
241
281
321
361
401
441
481
521
561
601
641
681
721
761
801
841
881
921
961

AT2G02390

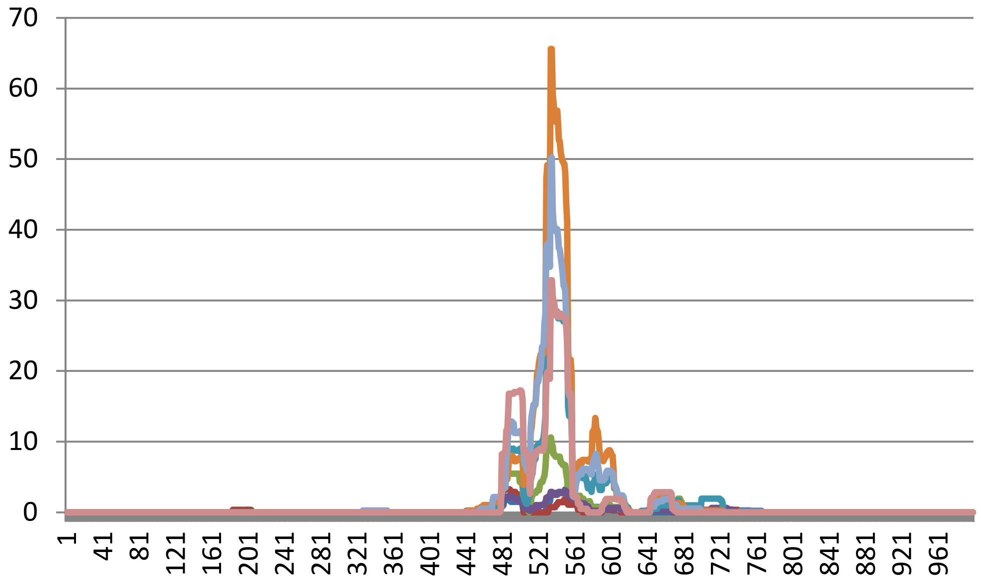

70
60
50
40
30
20
10
0
1
41
81
121
161
201
241
281
321
361
401
441
481
521
561
601
641
681
721
761
801
841
881
921
961

AT2G05540_AGO4

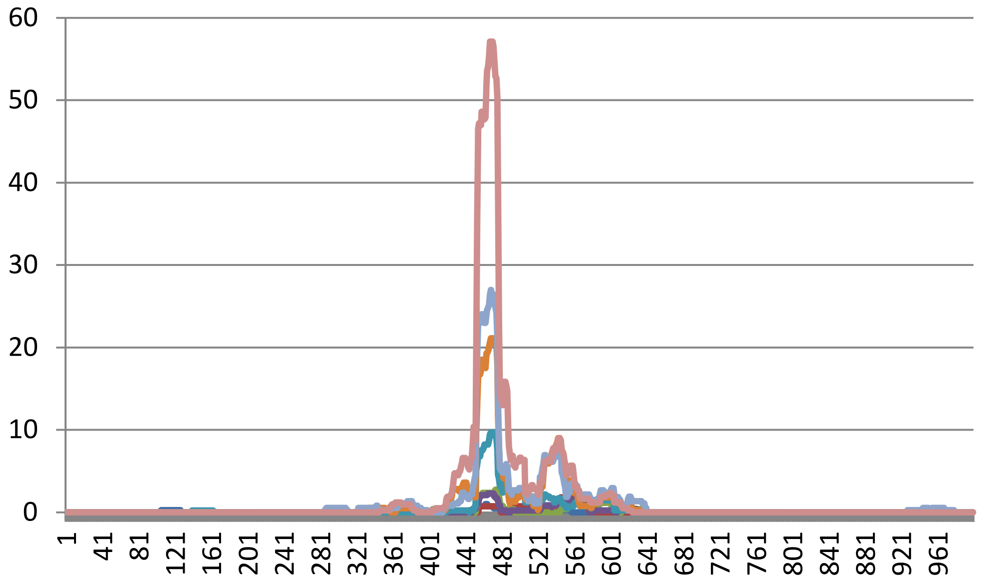

AT2G07771_AGO1

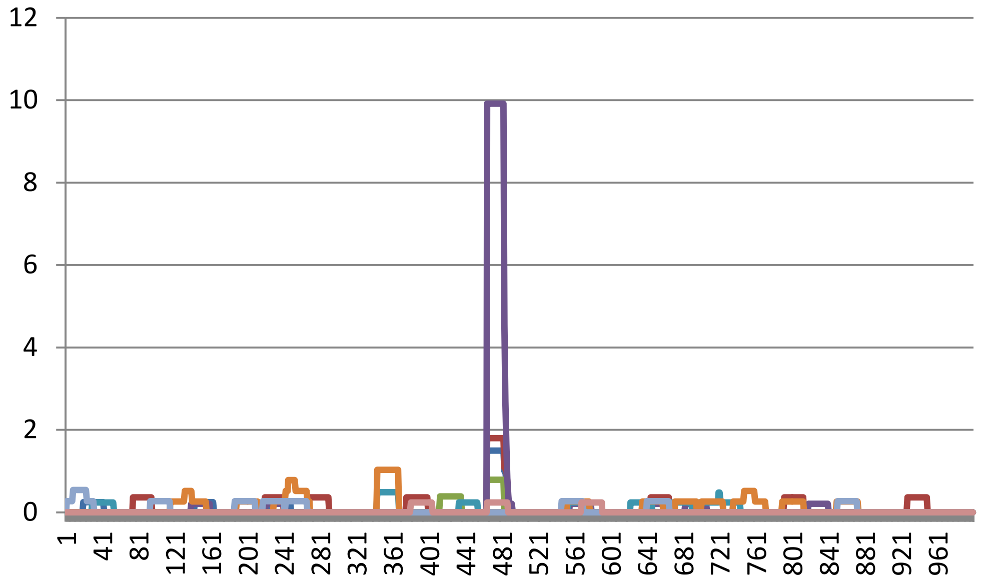

AT2G14247_AGO4

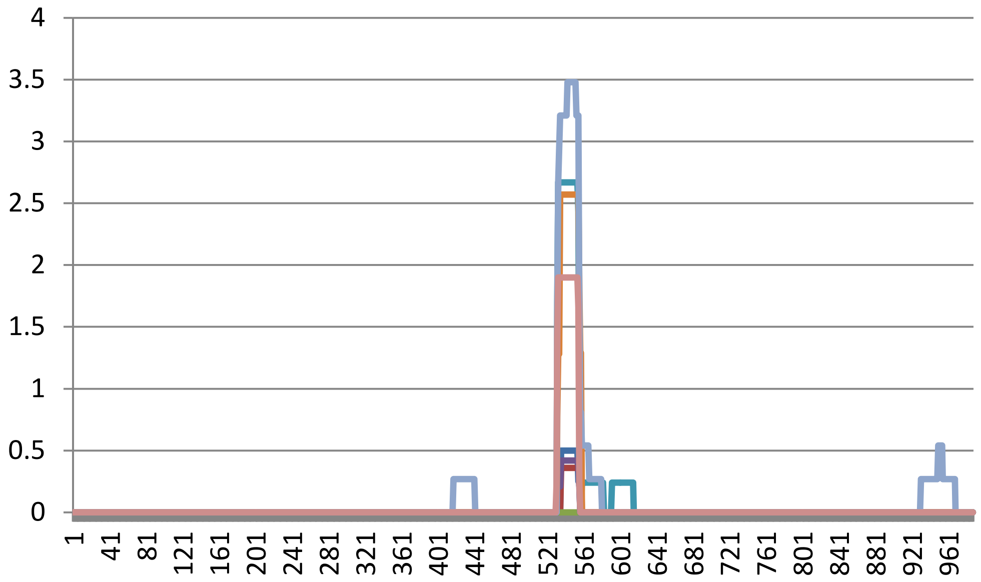

AT2G16030

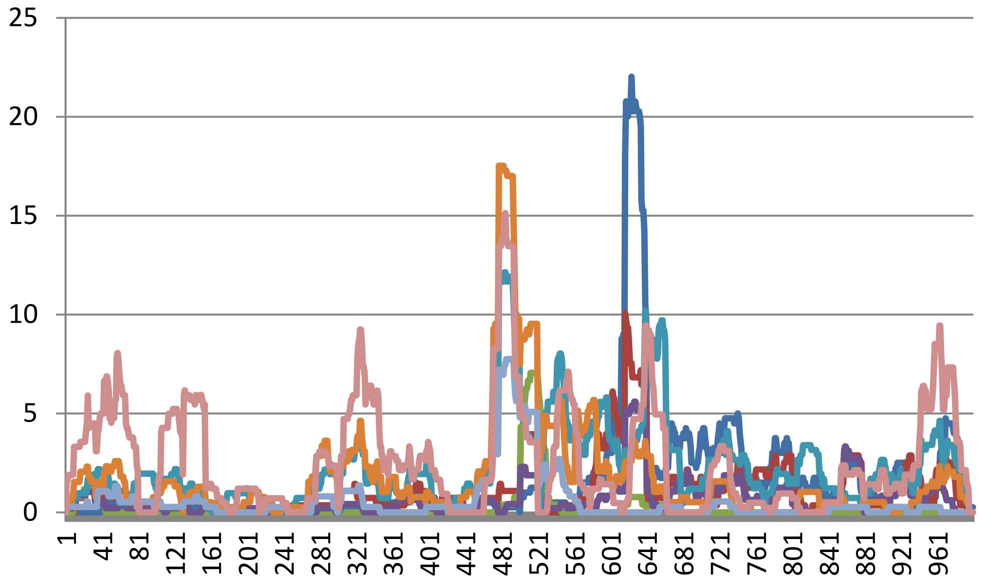

25
20
15
10
5
0
1
41
81
121
161
201
241
281
321
361
401
441
481
521
561
601
641
681
721
761
801
841
881
921
961

AT2G16380

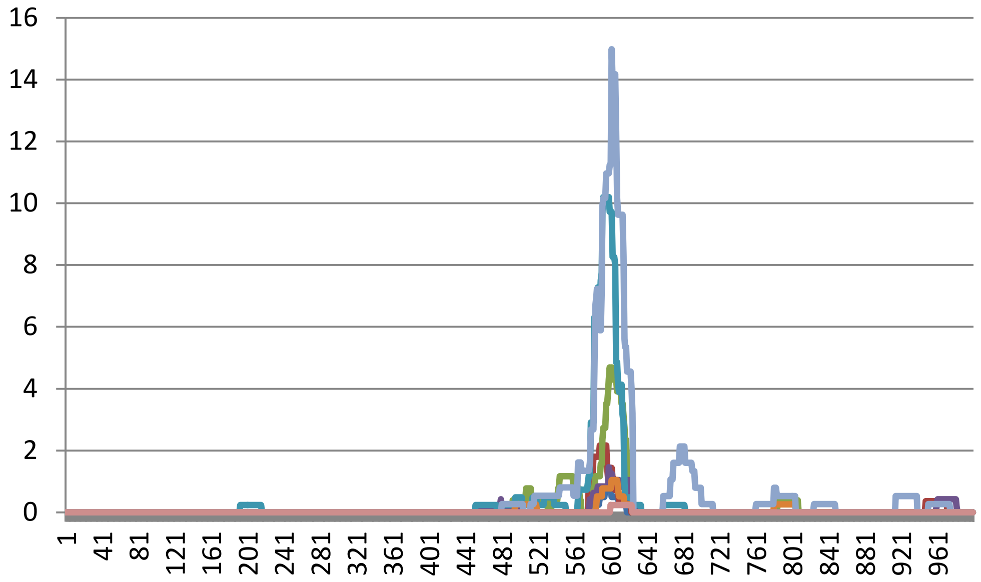

16
14
12
10
8
6
4
2
0
1
41
81
121
161
201
241
281
321
361
401
441
481
521
561
601
641
681
721
761
801
841
881
921
961

AT2G18465_AGO4

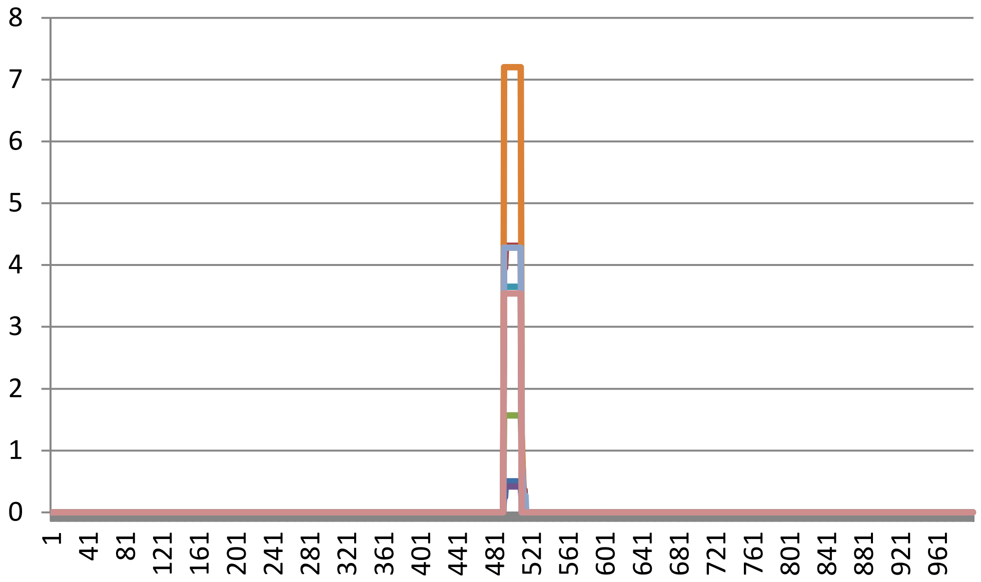

AT2G20190

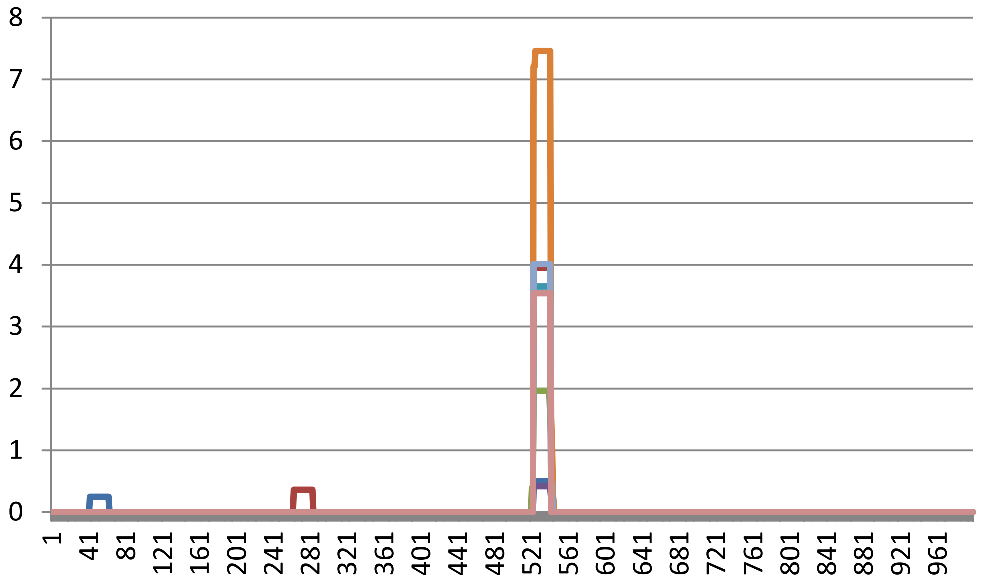

8
7
6
5
4
3
2
1
0
1
41
81
121
161
201
241
281
321
361
401
441
481
521
561
601
641
681
721
761
801
841
881
921
961

AT2G23640

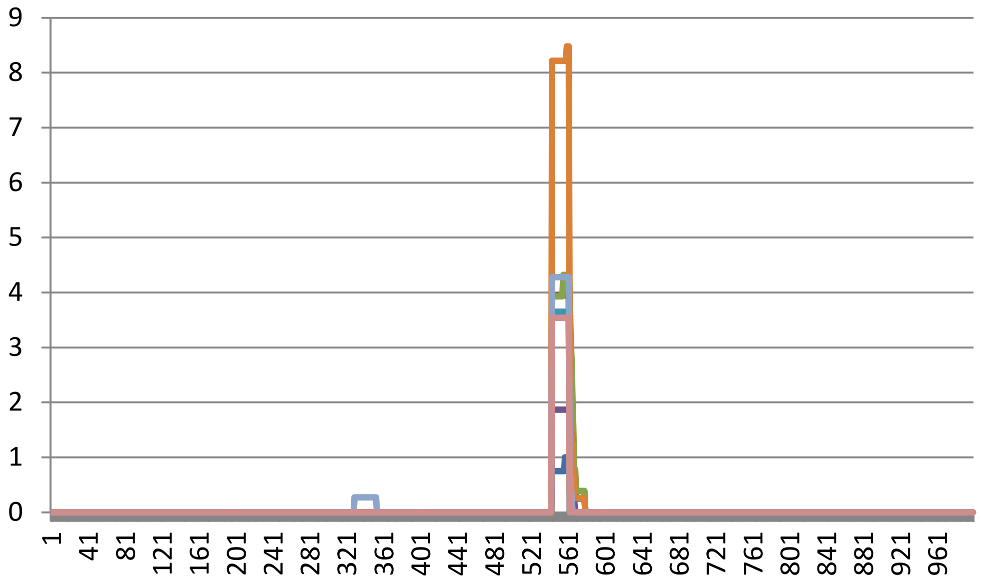
9
8
7
6
5
4
3
2
1
0
1
41
81
121
161
201
241
281
321
361
401
441
481
521
561
601
641
681
721
761
801
841
881
921
961

AT2G23890

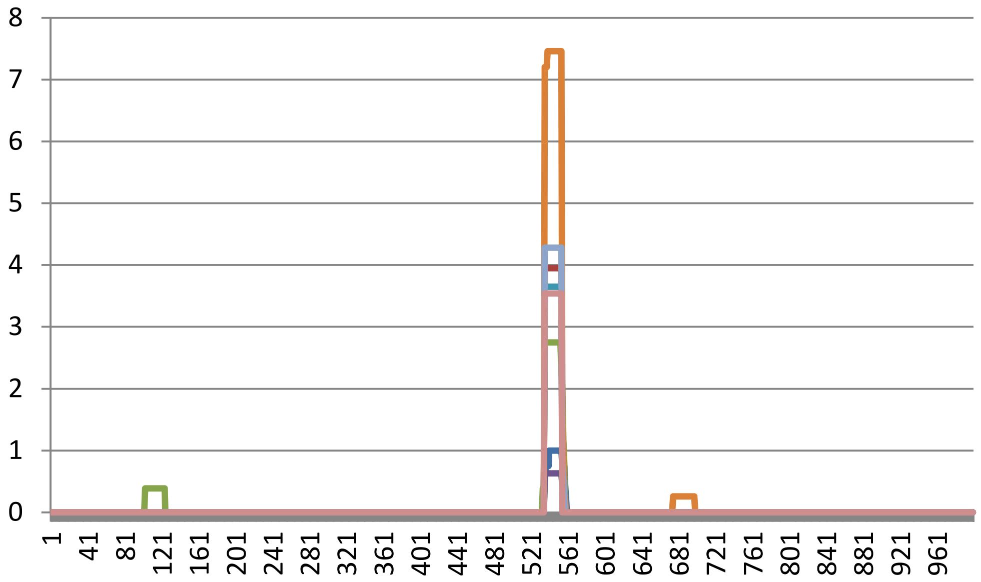

8
7
6
5
4
3
2
1
0
1
41
81
121
161
201
241
281
321
361
401
441
481
521
561
601
641
681
721
761
801
841
881
921
961

AT2G24740

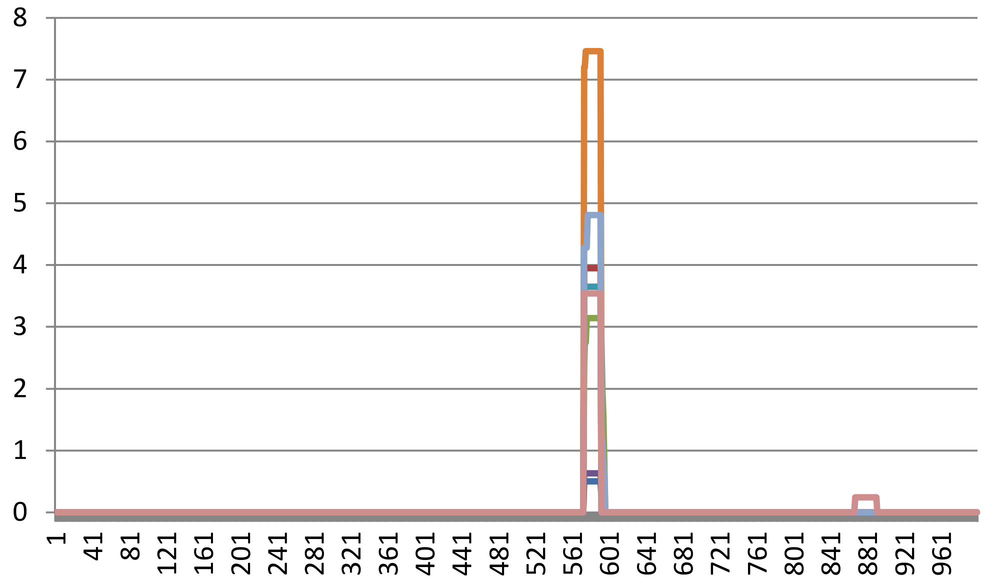

8
7
6
5
4
3
2
1
0
1
41
81
121
161
201
241
281
321
361
401
441
481
521
561
601
641
681
721
761
801
841
881
921
961

AT2G27420

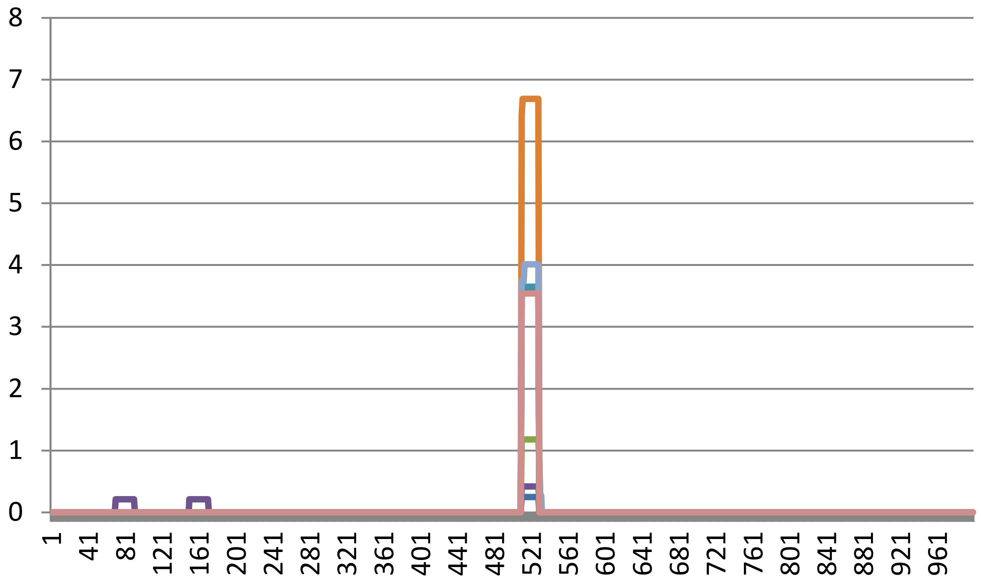

8
7
6
5
4
3
2
1
0
1
41
81
121
161
201
241
281
321
361
401
441
481
521
561
601
641
681
721
761
801
841
881
921
961

AT2G28725_AGO1

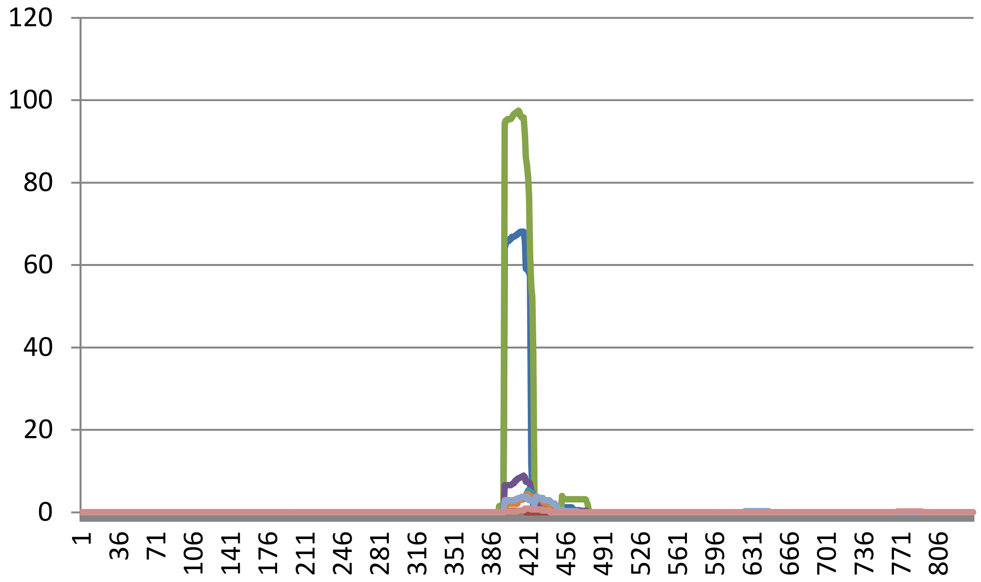

AT2G33150

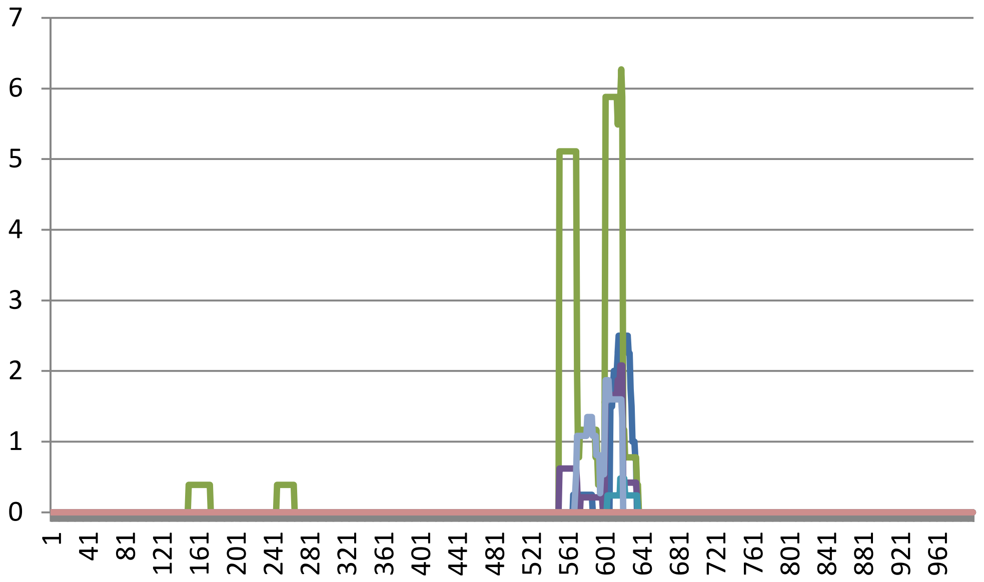

7
6
5
4
3
2
1
0
1
41
81
121
161
201
241
281
321
361
401
441
481
521
561
601
641
681
721
761
801
841
881
921
961

AT2G39630

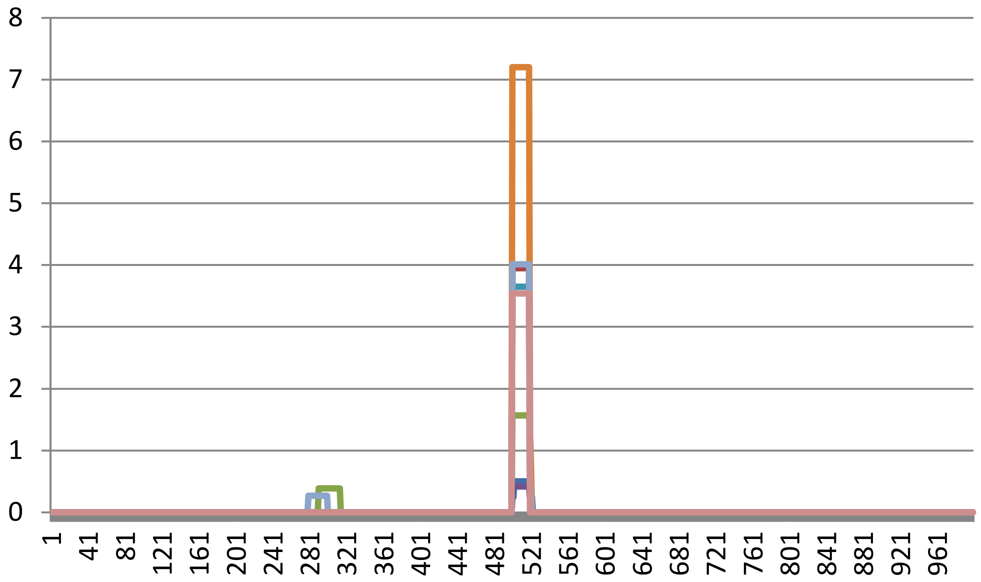

8
7
6
5
4
3
2
1
0
1
41
81
121
161
201
241
281
321
361
401
441
481
521
561
601
641
681
721
761
801
841
881
921
961

AT2G41920_AGO4 flower

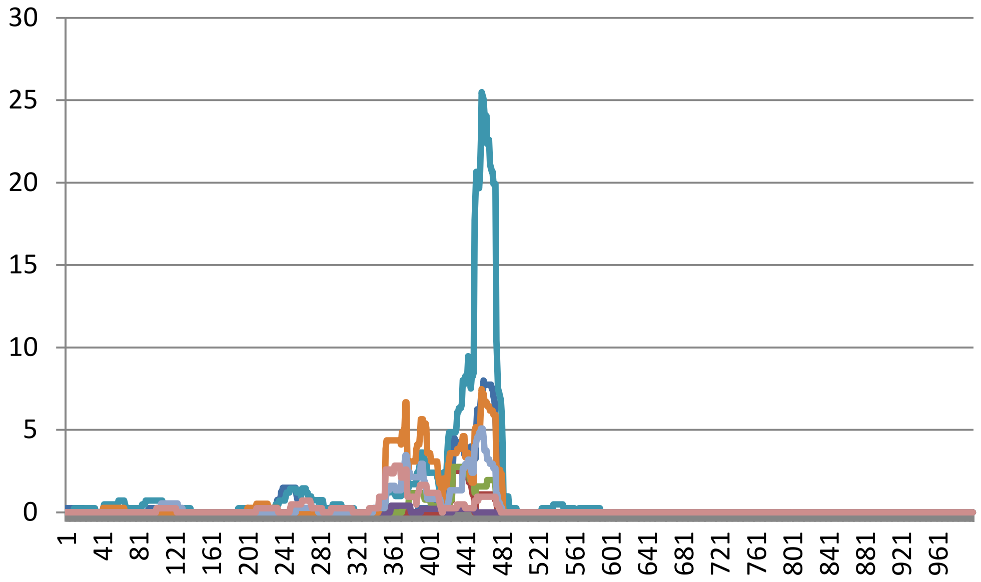

AT2G45010_AGO1 root

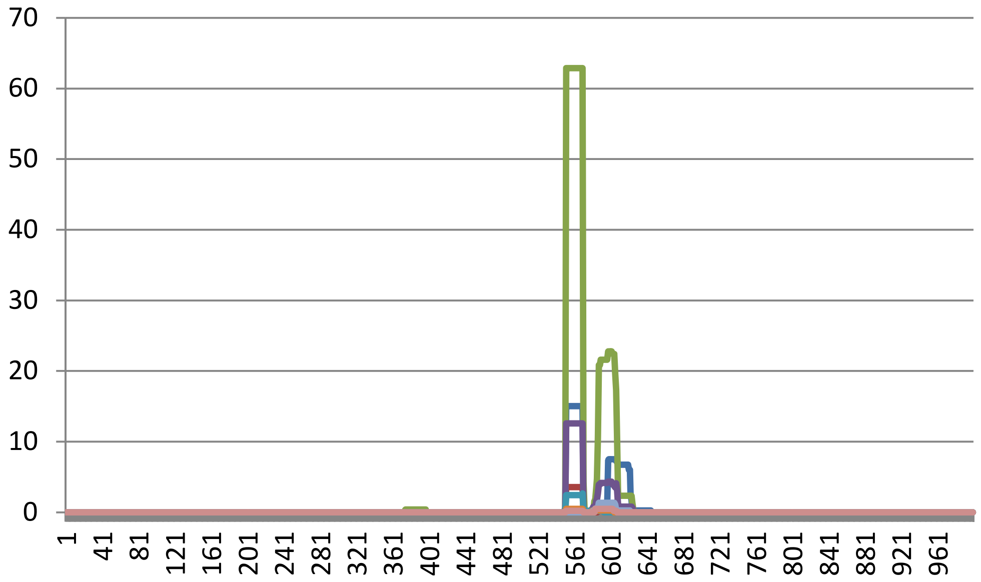

AT3G01240

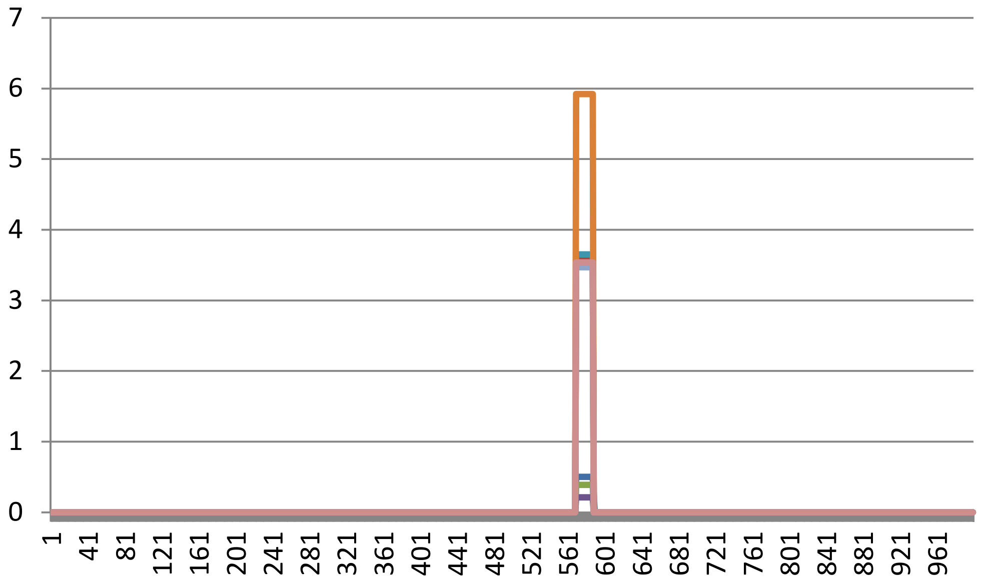

7
6
5
4
3
2
1
0
1
41
81
121
161
201
241
281
321
361
401
441
481
521
561
601
641
681
721
761
801
841
881
921
961

AT3G01700_AGO1 root

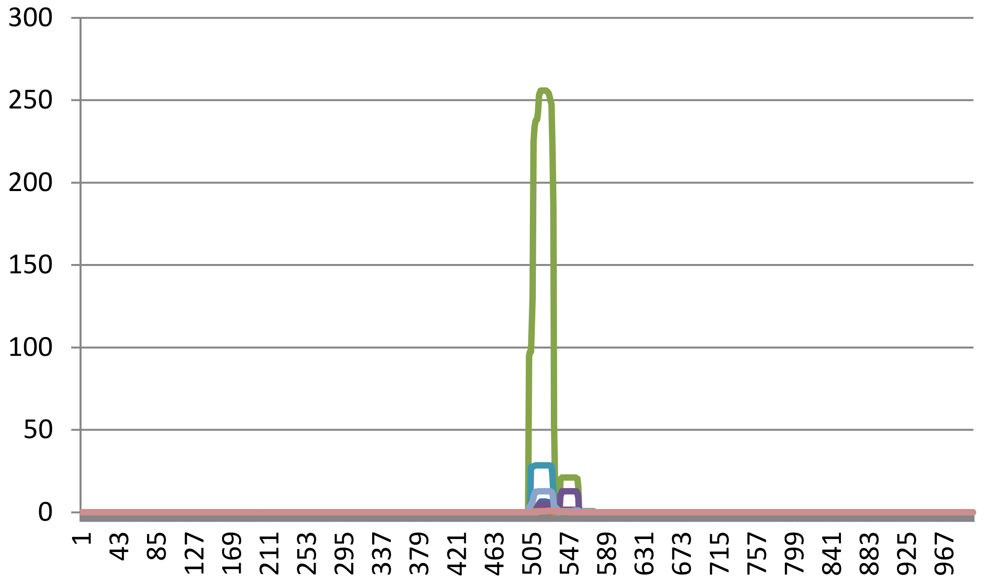

AT3G05660

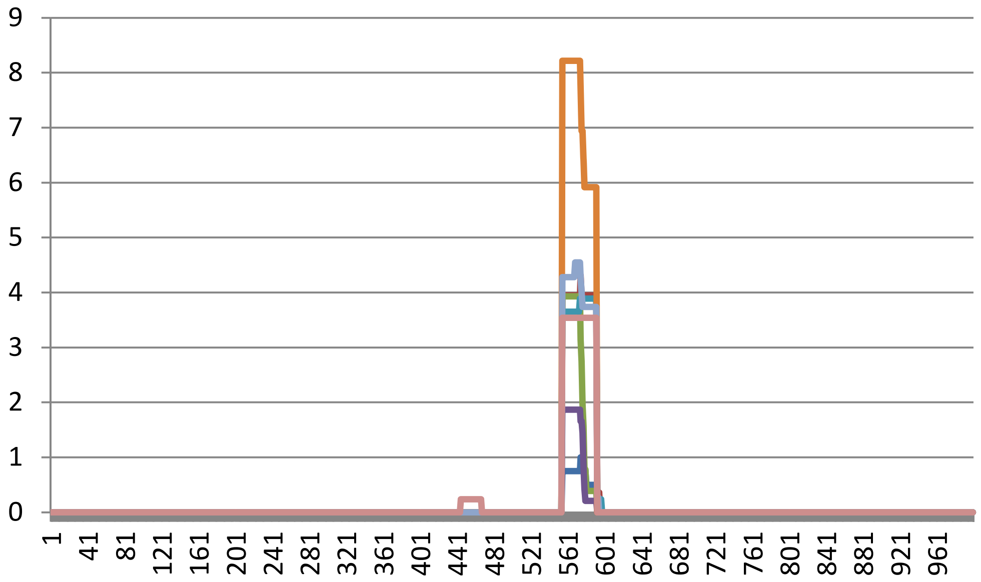

9
8
7
6
5
4
3
2
1
0
1
41
81
121
161
201
241
281
321
361
401
441
481
521
561
601
641
681
721
761
801
841
881
921
961

AT3G16400

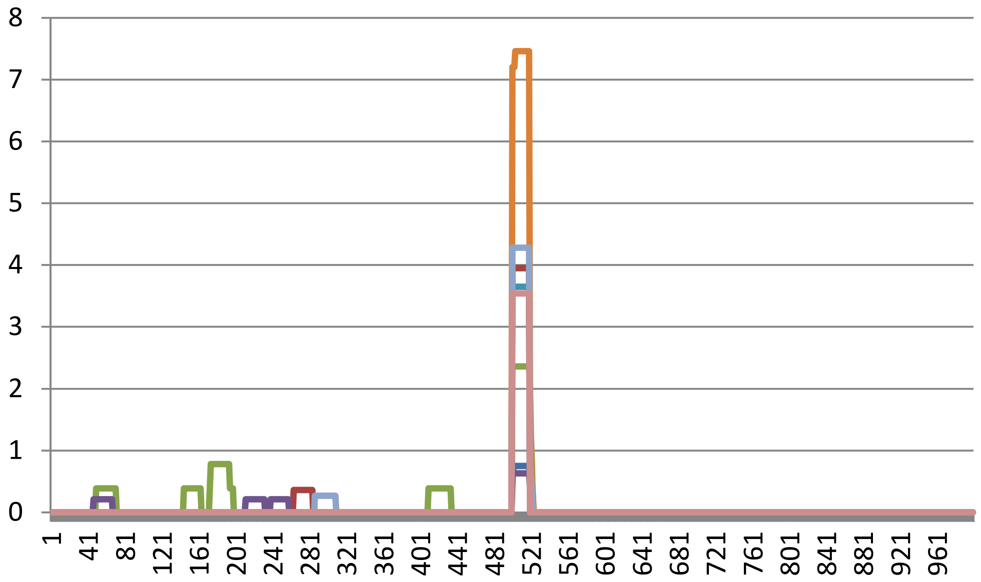

8
7
6
5
4
3
2
1
0
1
41
81
121
161
201
241
281
321
361
401
441
481
521
561
601
641
681
721
761
801
841
881
921
961

AT3G21960

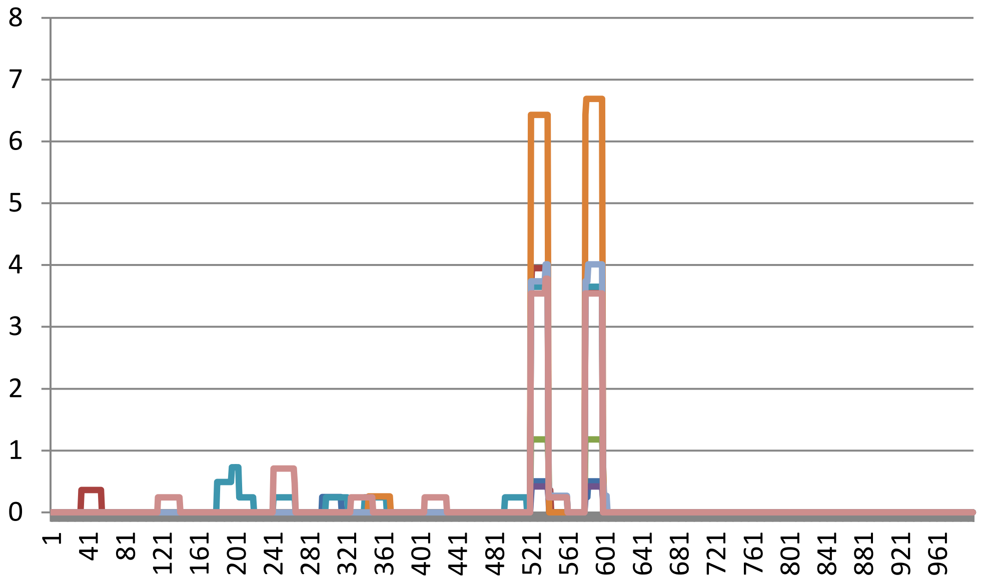

8
7
6
5
4
3
2
1
0
1
41
81
121
161
201
241
281
321
361
401
441
481
521
561
601
641
681
721
761
801
841
881
921
961

AT3G23310

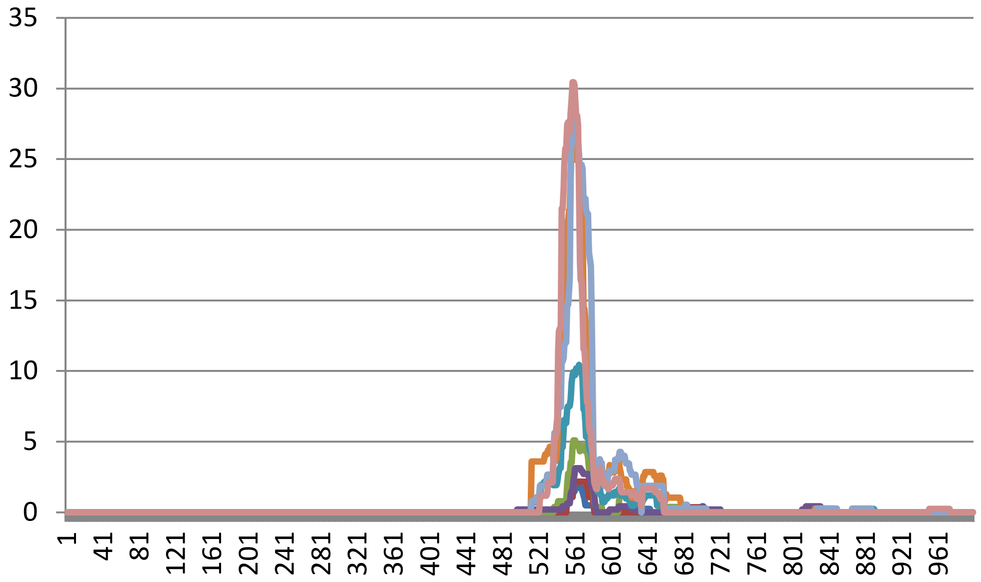
35
30
25
20
15
10
5
0
1
41
81
121
161
201
241
281
321
361
401
441
481
521
561
601
641
681
721
761
801
841
881
921
961

AT3G23590

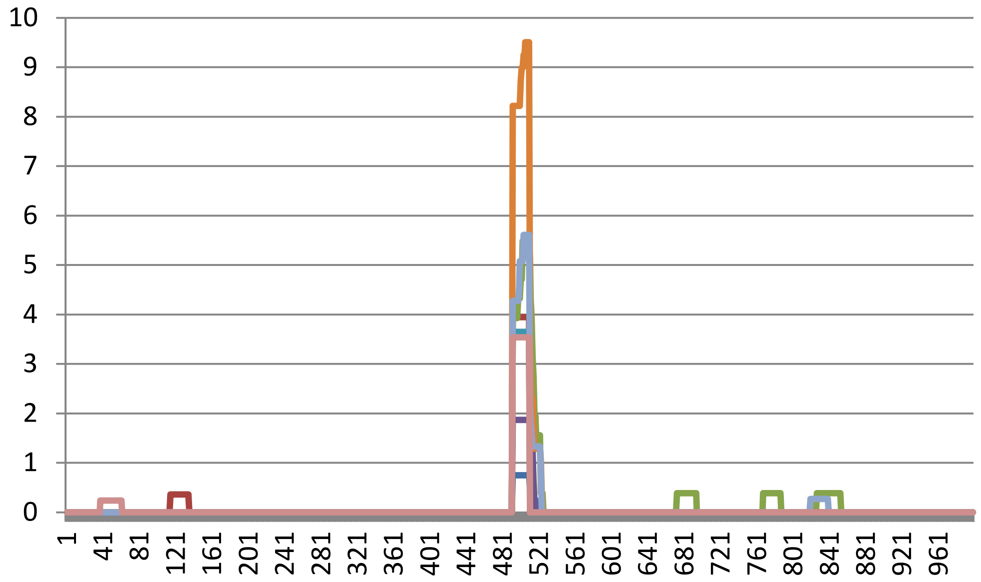
10
9
8
7
6
5
4
3
2
1
0
1
41
81
121
161
201
241
281
321
361
401
441
481
521
561
601
641
681
721
761
801
841
881
921
961

AT3G25585

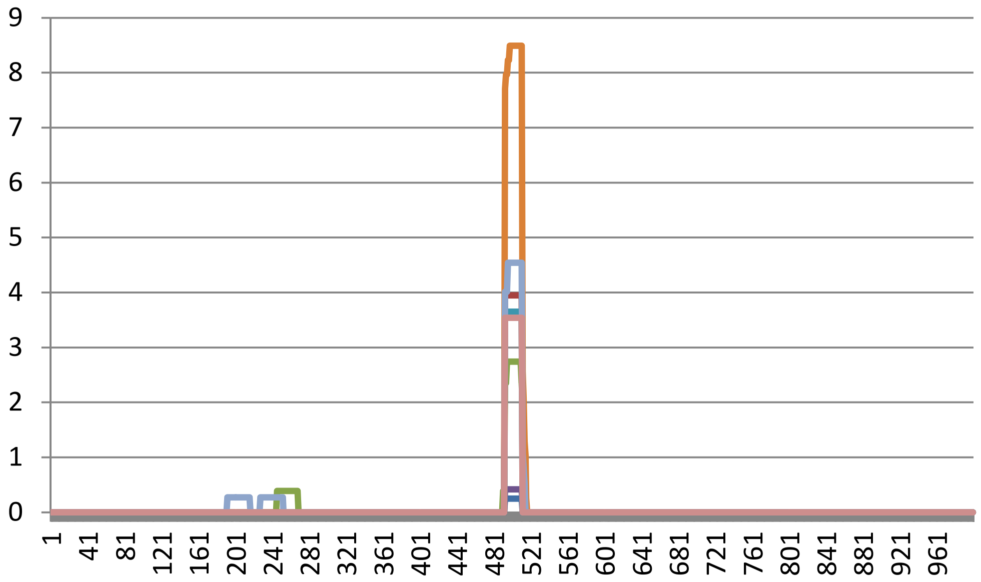

9
8
7
6
5
4
3
2
1
0
1
41
81
121
161
201
241
281
321
361
401
441
481
521
561
601
641
681
721
761
801
841
881
921
961

AT3G27590

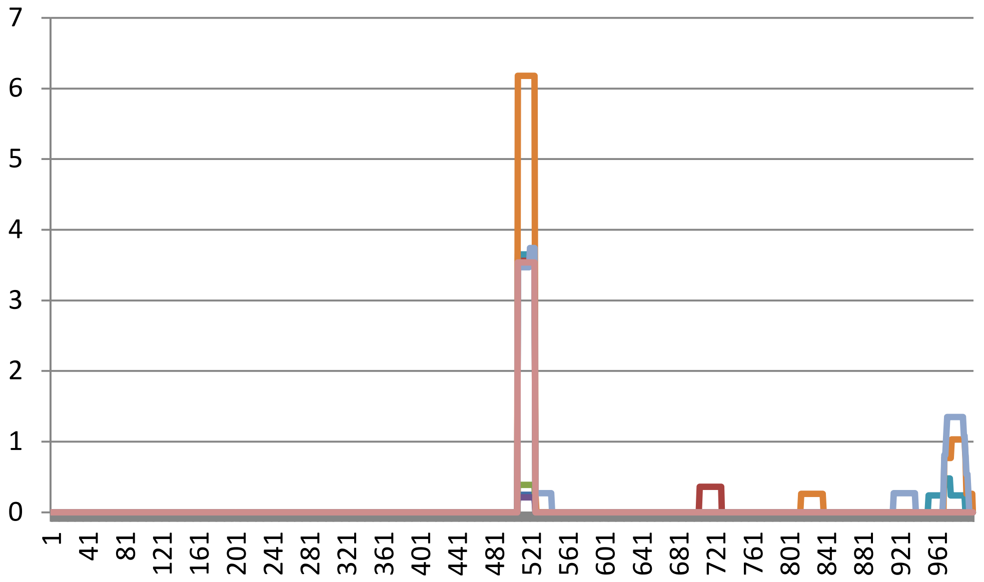

7
6
5
4
3
2
1
0
1
41
81
121
161
201
241
281
321
361
401
441
481
521
561
601
641
681
721
761
801
841
881
921
961

AT3G30340

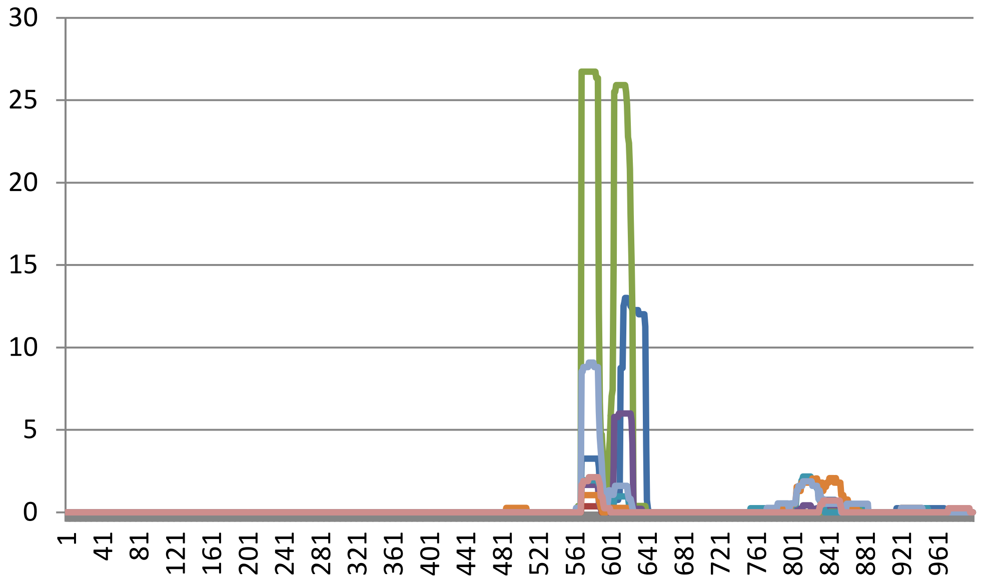

30
25
20
15
10
5
0
1
41
81
121
161
201
241
281
321
361
401
441
481
521
561
601
641
681
721
761
801
841
881
921
961

AT3G30385

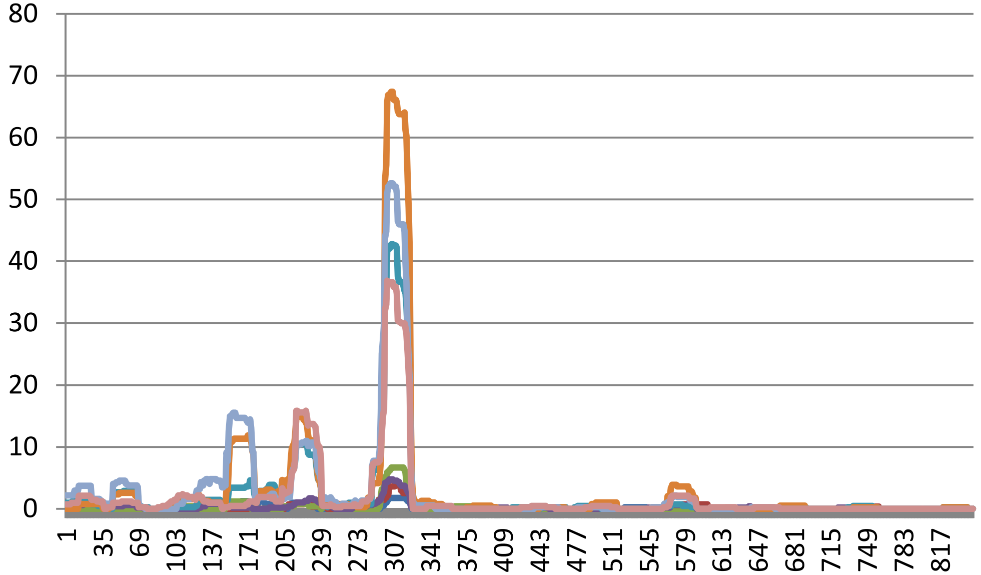

80
70
60
50
40
30
20
10
0
1
35
69
103
137
171
205
239
273
307
341
375
409
443
477
511
545
579
613
647
681
715
749
783
817

AT3G30387

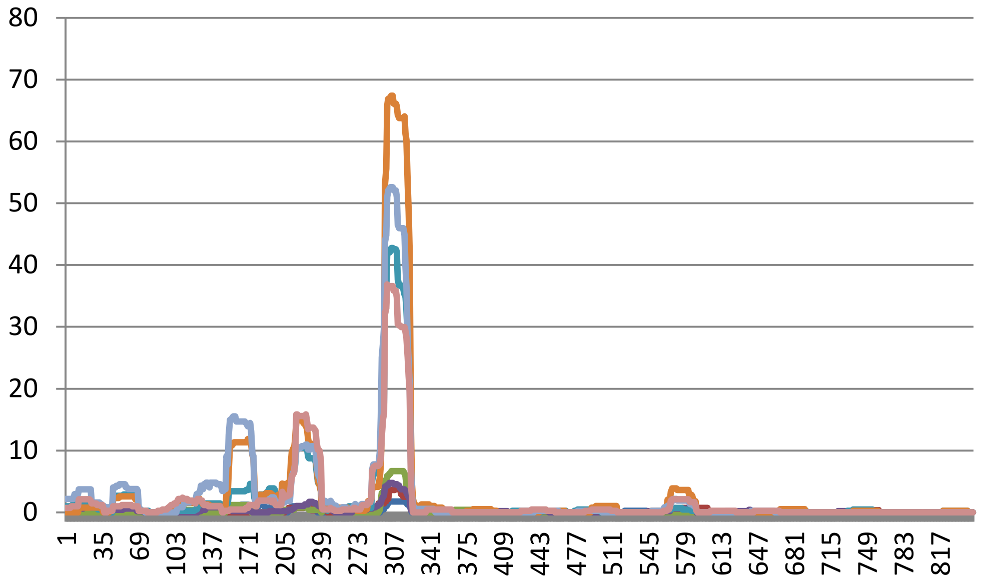
80
70
60
50
40
30
20
10
0
1
35
69
103
137
171
205
239
273
307
341
375
409
443
477
511
545
579
613
647
681
715
749
783
817

AT3G44980

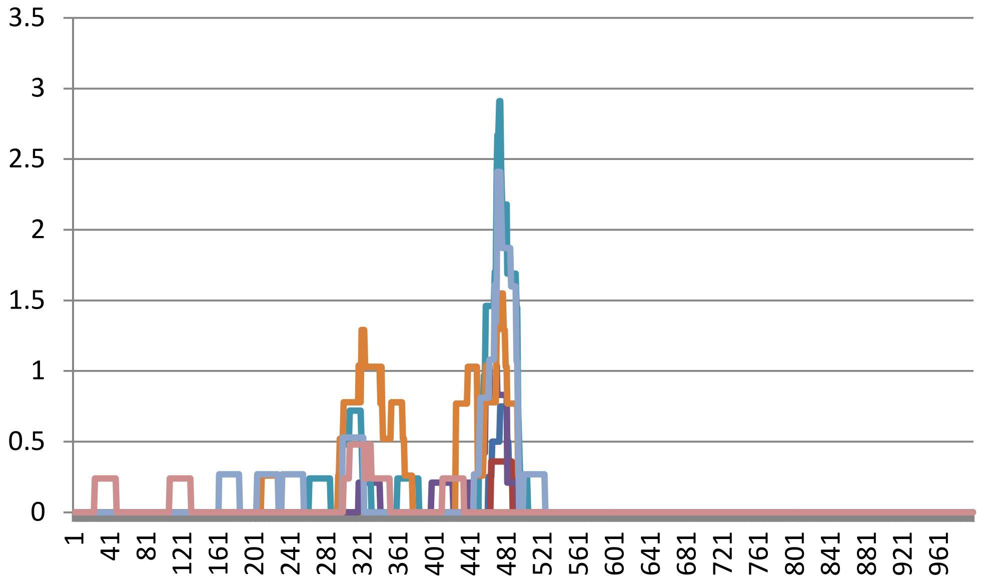

3.5
3
2.5
2
1.5
1
0.5
0
1
41
81
121
161
201
241
281
321
361
401
441
481
521
561
601
641
681
721
761
801
841
881
921
961

AT3G49810

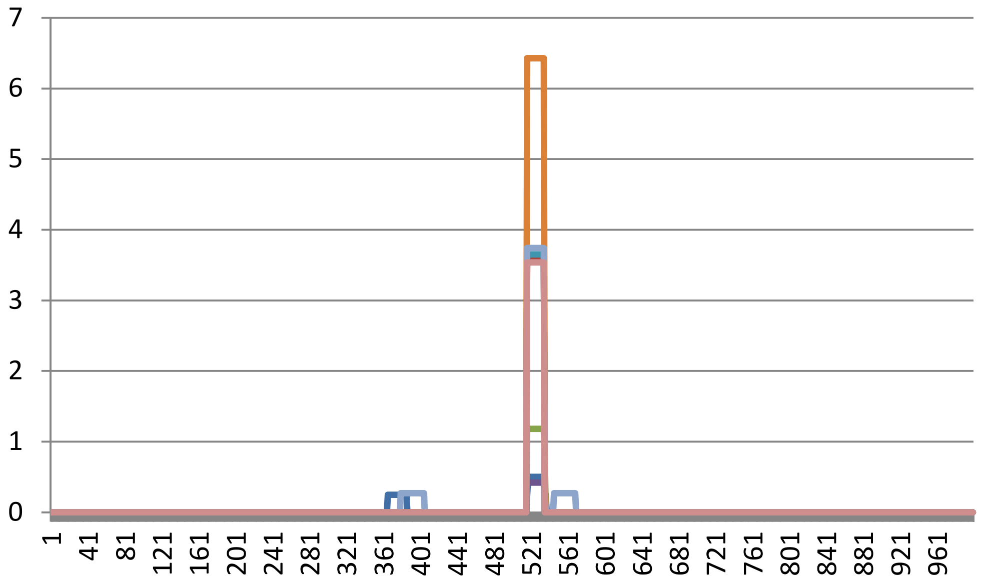

7
6
5
4
3
2
1
0
1
41
81
121
161
201
241
281
321
361
401
441
481
521
561
601
641
681
721
761
801
841
881
921
961

AT3G50510_root

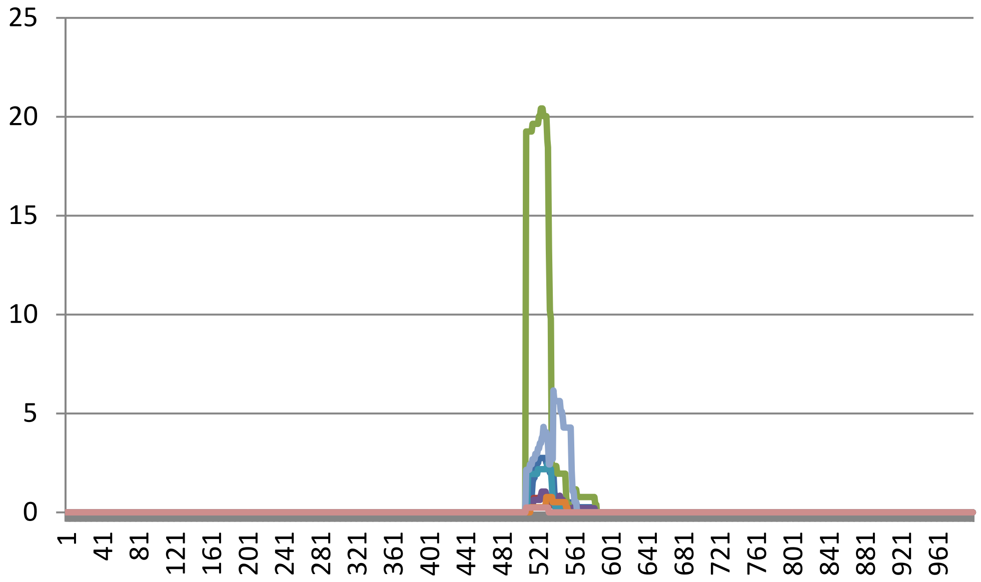

AT3G53280

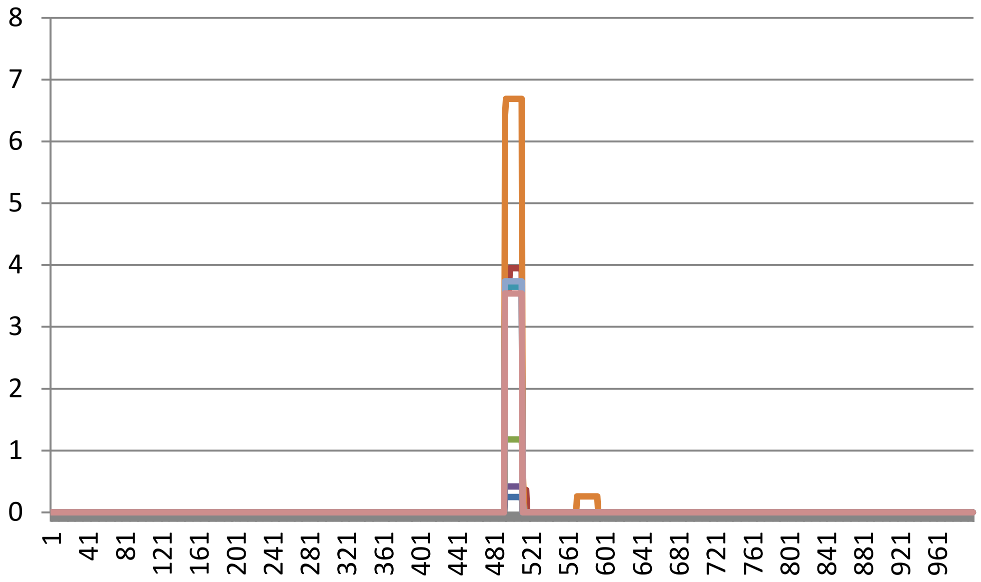

8
7
6
5
4
3
2
1
0
1
41
81
121
161
201
241
281
321
361
401
441
481
521
561
601
641
681
721
761
801
841
881
921
961

AT3G54150

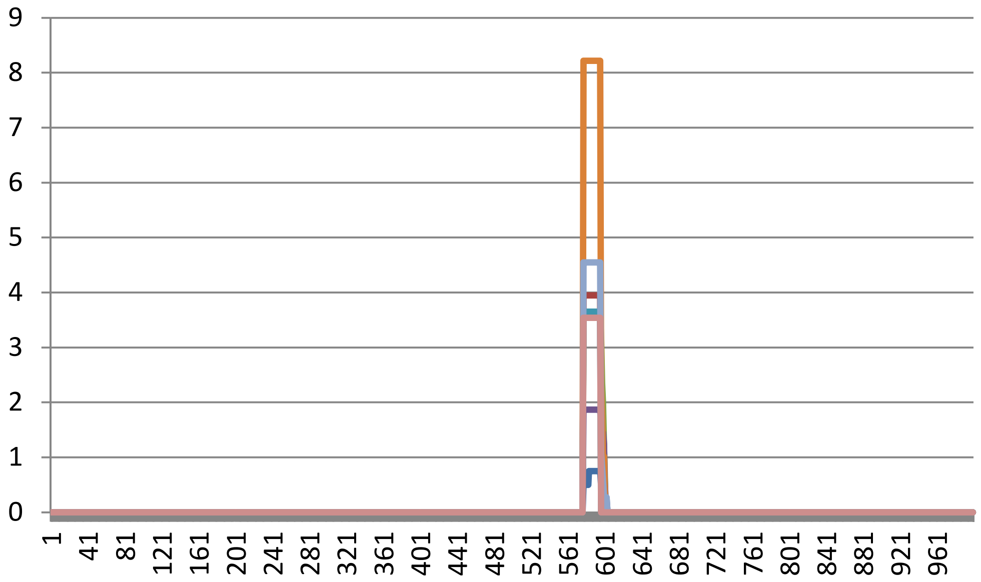

9
8
7
6
5
4
3
2
1
0
1
41
81
121
161
201
241
281
321
361
401
441
481
521
561
601
641
681
721
761
801
841
881
921
961

AT3G54730

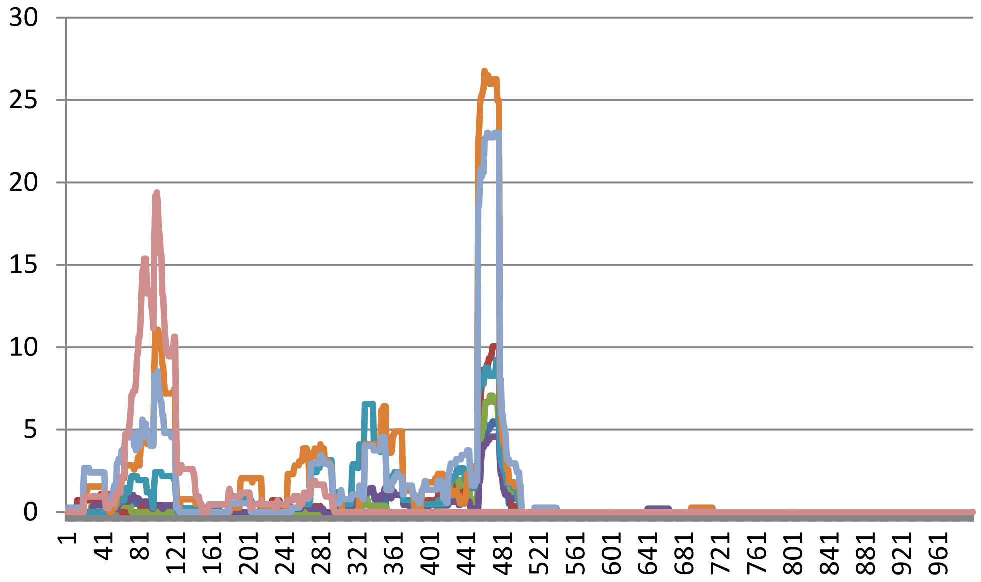

30
25
20
15
10
5
0
1
41
81
121
161
201
241
281
321
361
401
441
481
521
561
601
641
681
721
761
801
841
881
921
961

AT3G59580

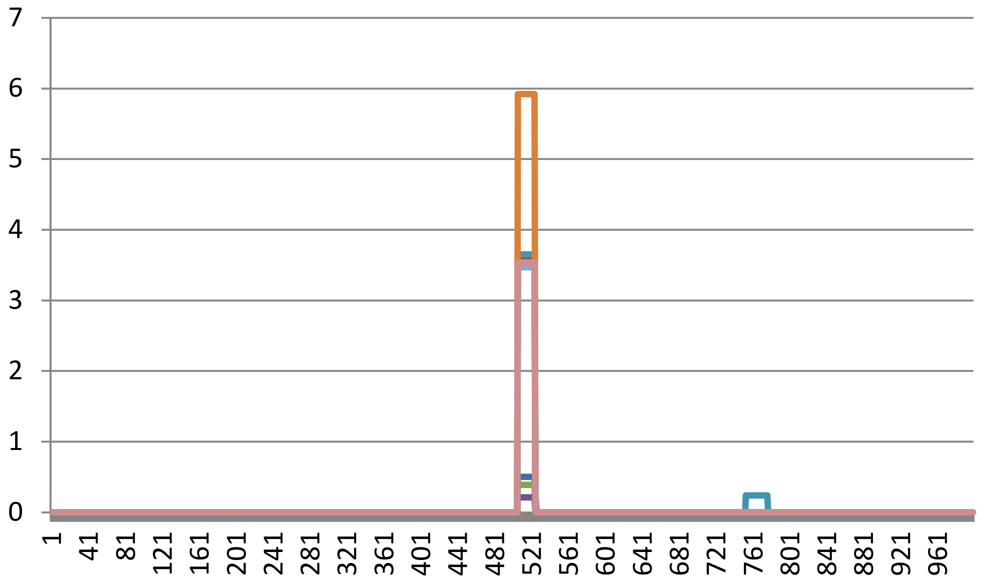

7
6
5
4
3
2
1
0
1
41
81
121
161
201
241
281
321
361
401
441
481
521
561
601
641
681
721
761
801
841
881
921
961

AT4G00080

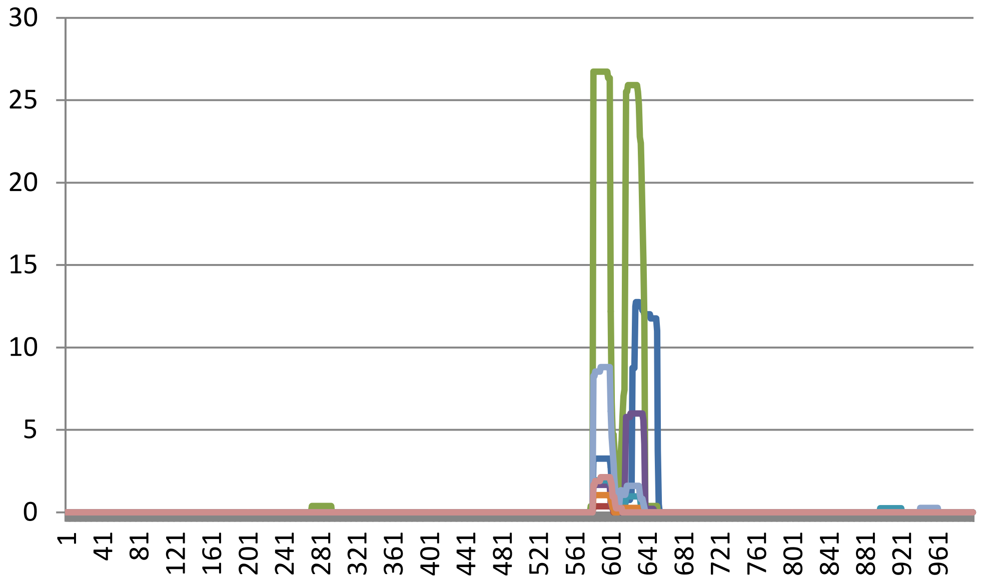

30
25
20
15
10
5
0
1
41
81
121
161
201
241
281
321
361
401
441
481
521
561
601
641
681
721
761
801
841
881
921
961

AT4G01870

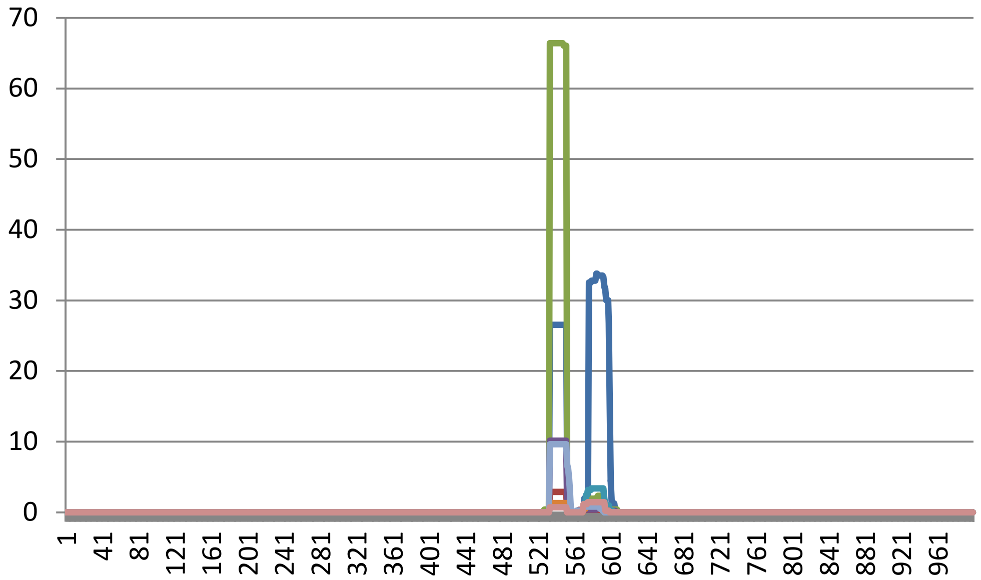
70
60
50
40
30
20
10
0
1
41
81
121
161
201
241
281
321
361
401
441
481
521
561
601
641
681
721
761
801
841
881
921
961

AT4G03165

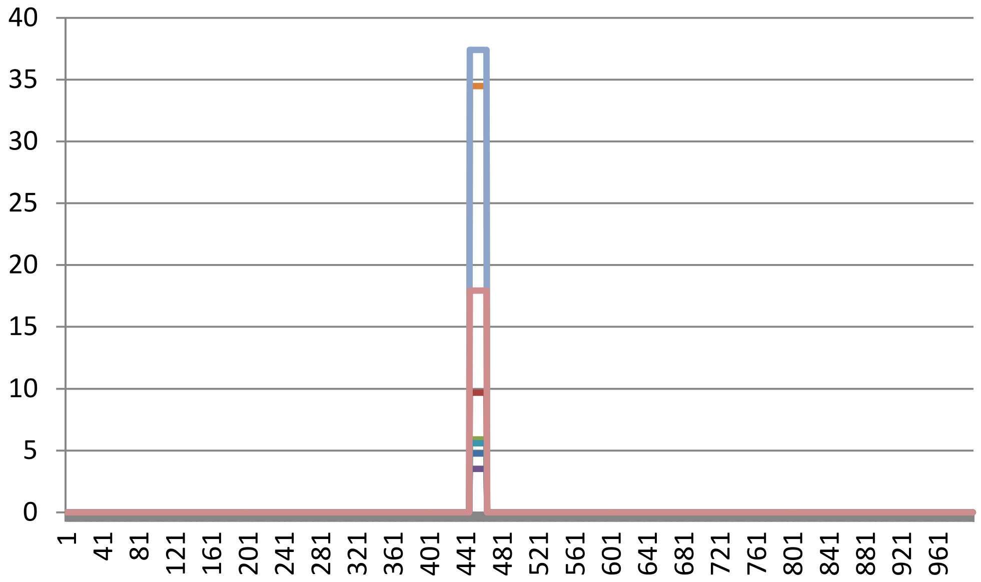

40
35
30
25
20
15
10
5
0
1
41
81
121
161
201
241
281
321
361
401
441
481
521
561
601
641
681
721
761
801
841
881
921
961

AT4G03930

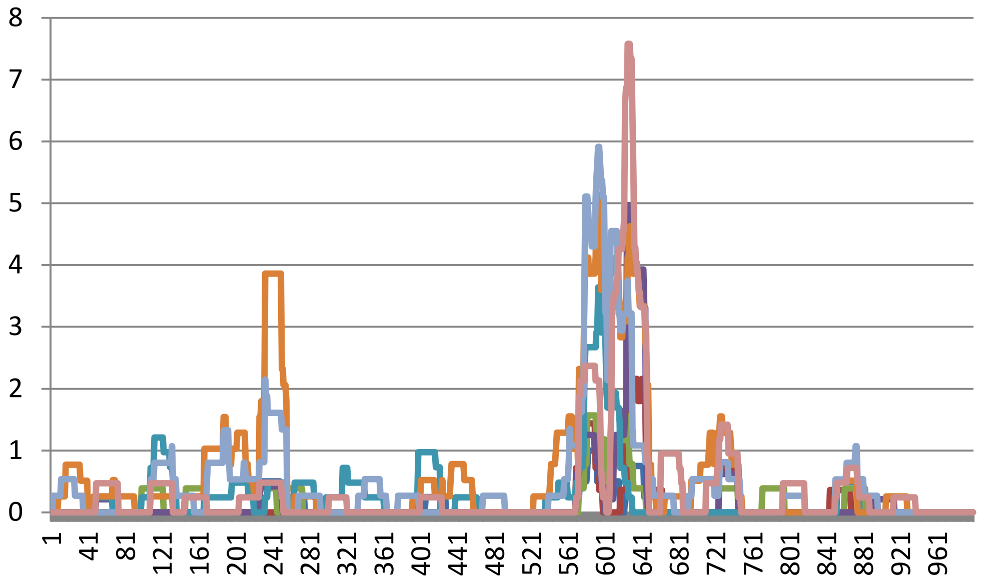

8
7
6
5
4
3
2
1
0
1
41
81
121
161
201
241
281
321
361
401
441
481
521
561
601
641
681
721
761
801
841
881
921
961

AT4G04925

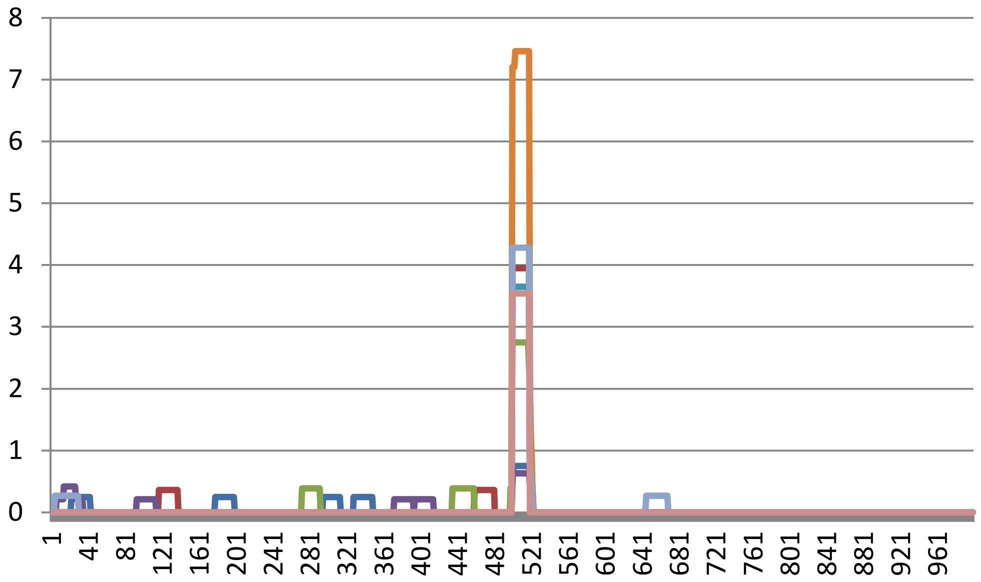

8
7
6
5
4
3
2
1
0
1
41
81
121
161
201
241
281
321
361
401
441
481
521
561
601
641
681
721
761
801
841
881
921
961

AT4G08039

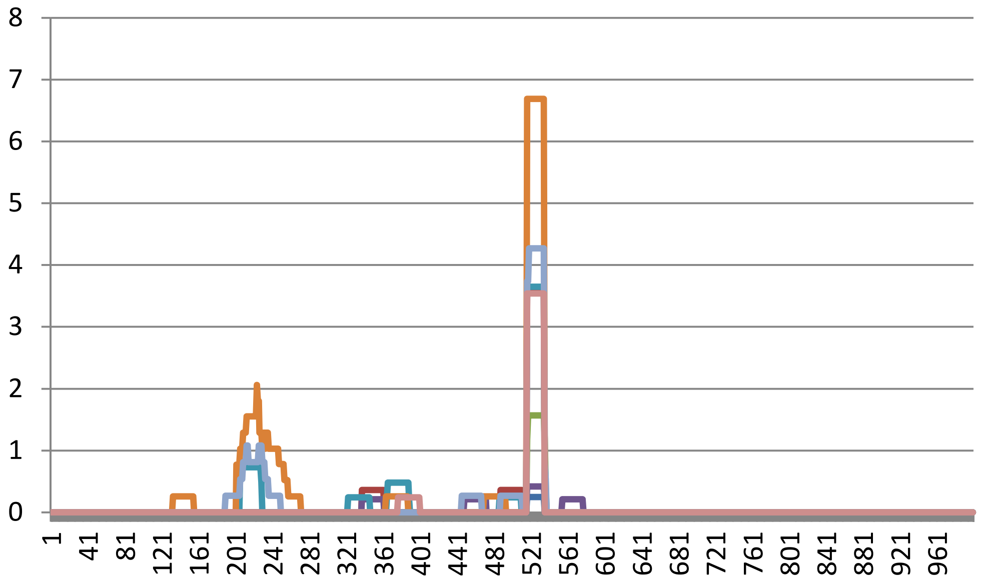

8
7
6
5
4
3
2
1
0
1
41
81
121
161
201
241
281
321
361
401
441
481
521
561
601
641
681
721
761
801
841
881
921
961

AT4G10596

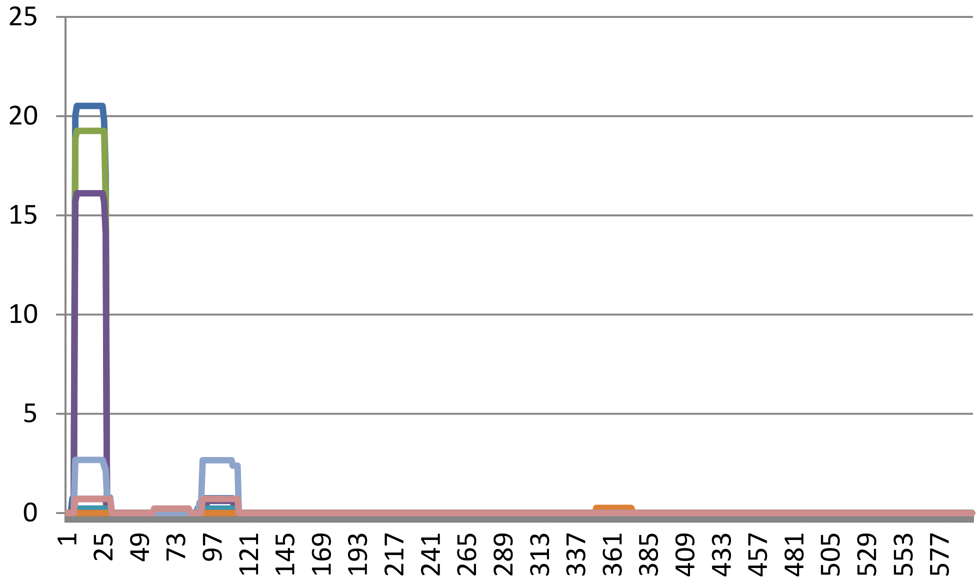
25
20
15
10
5
0
1
25
49
73
97
121
145
169
193
217
241
265
289
313
337
361
385
409
433
457
481
505
529
553
577

AT4G11370

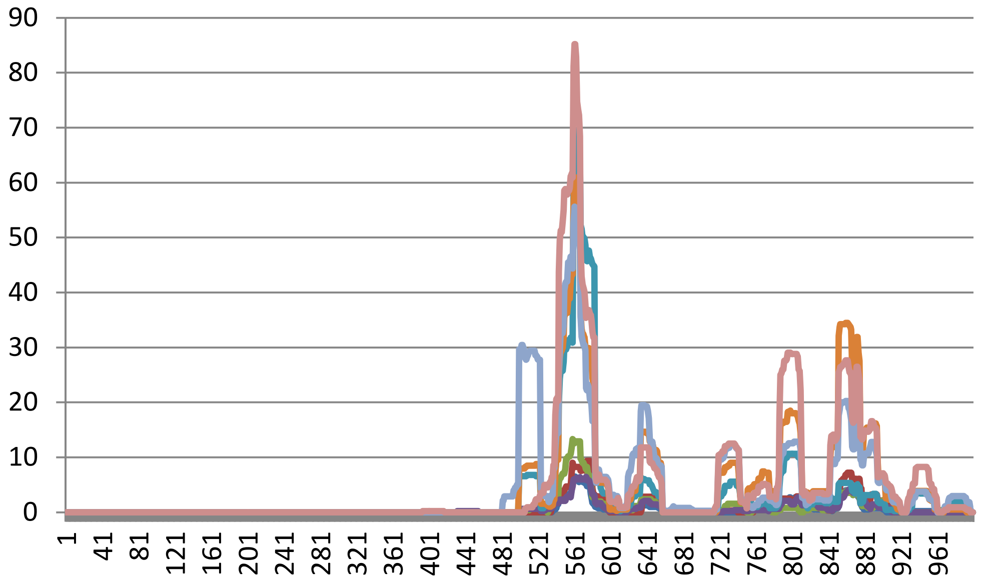

90
80
70
60
50
40
30
20
10
0
1
41
81
121
161
201
241
281
321
361
401
441
481
521
561
601
641
681
721
761
801
841
881
921
961

AT4G11900

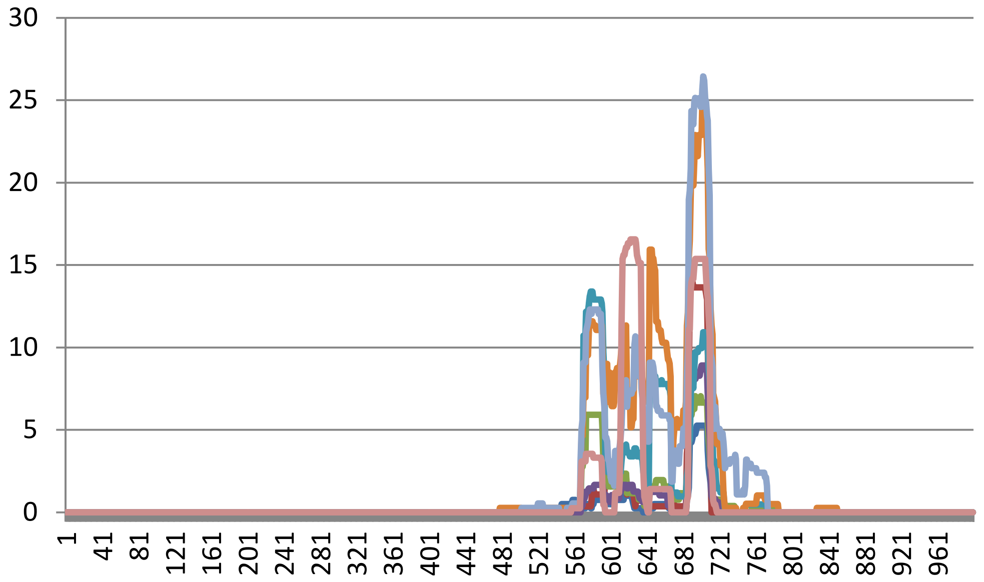

30
25
20
15
10
5
0
1
41
81
121
161
201
241
281
321
361
401
441
481
521
561
601
641
681
721
761
801
841
881
921
961

AT4G12110

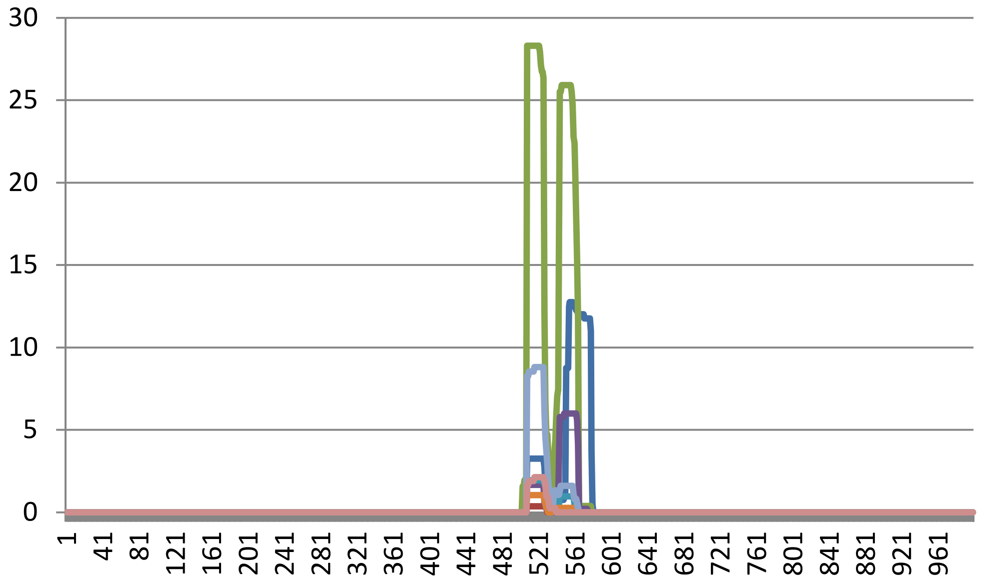
30
25
20
15
10
5
0
1
41
81
121
161
201
241
281
321
361
401
441
481
521
561
601
641
681
721
761
801
841
881
921
961

AT4G19090

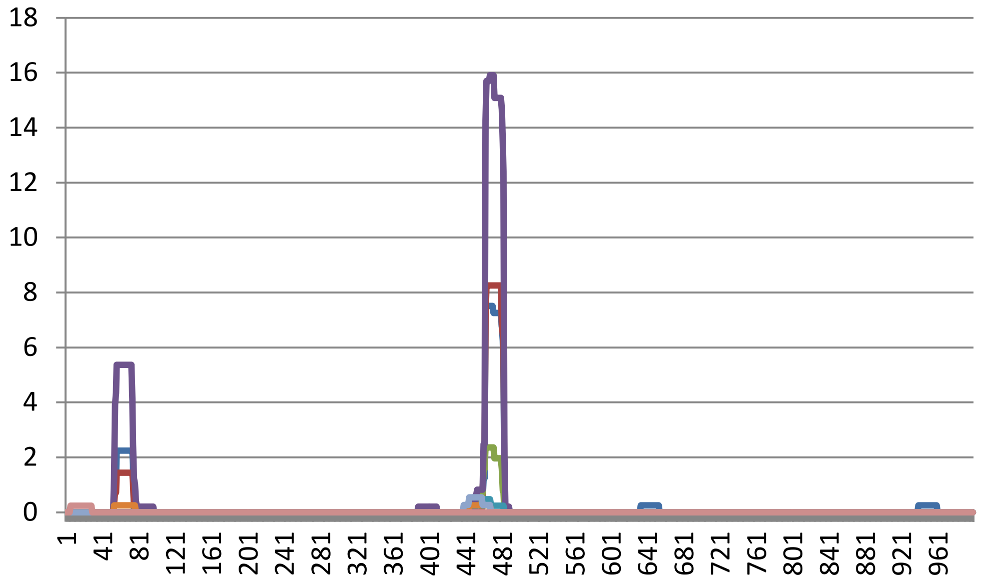

18
16
14
12
10
8
6
4
2
0
1
41
81
121
161
201
241
281
321
361
401
441
481
521
561
601
641
681
721
761
801
841
881
921
961

AT4G22756

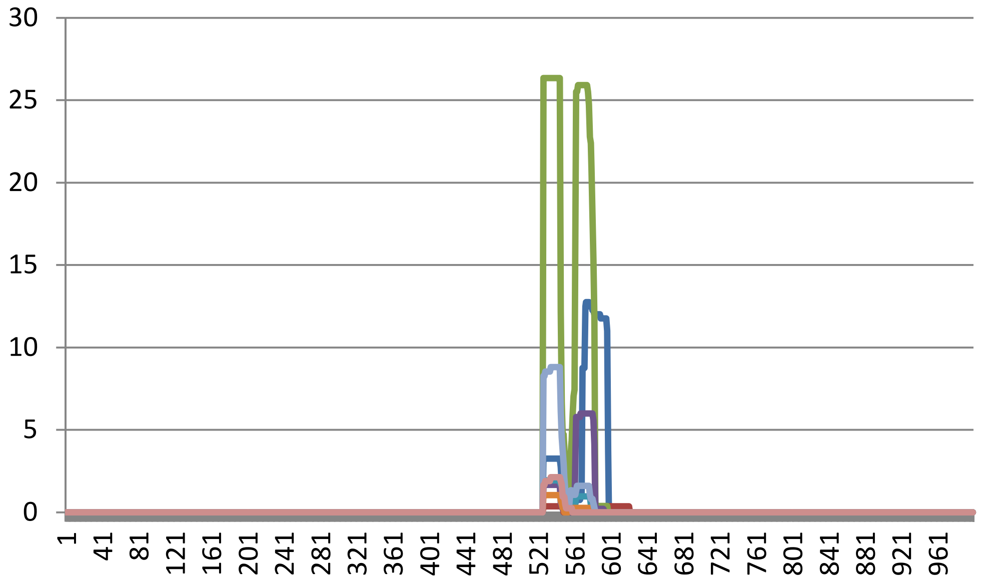

30
25
20
15
10
5
0
1
41
81
121
161
201
241
281
321
361
401
441
481
521
561
601
641
681
721
761
801
841
881
921
961

AT4G24030_AGO1

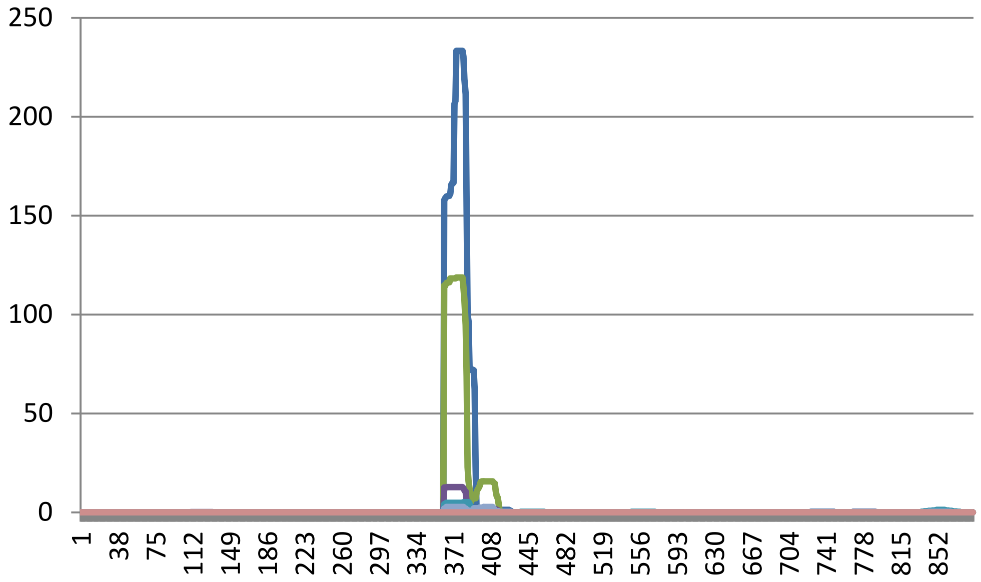

AT4G25590_AGO1 root

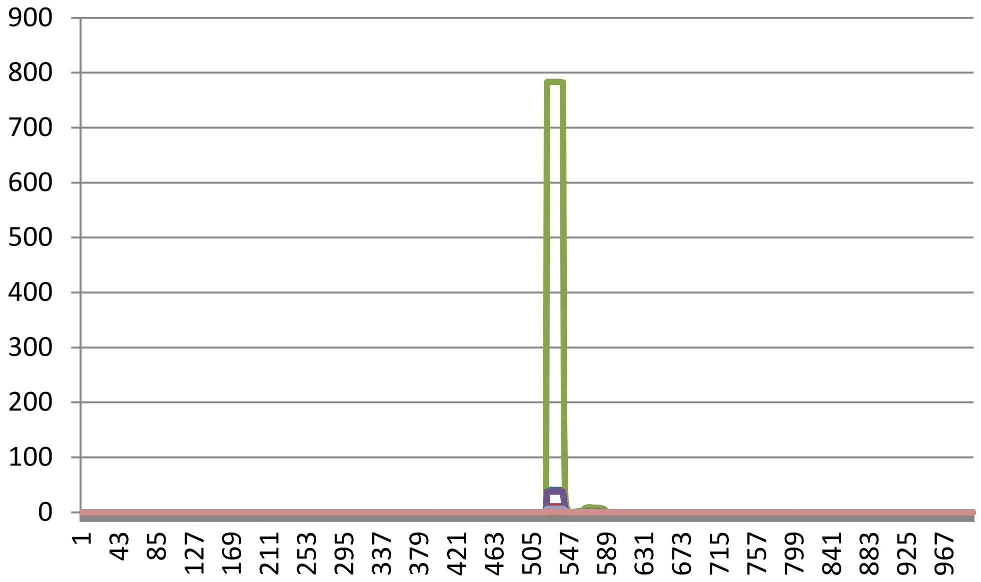

AT4G29200

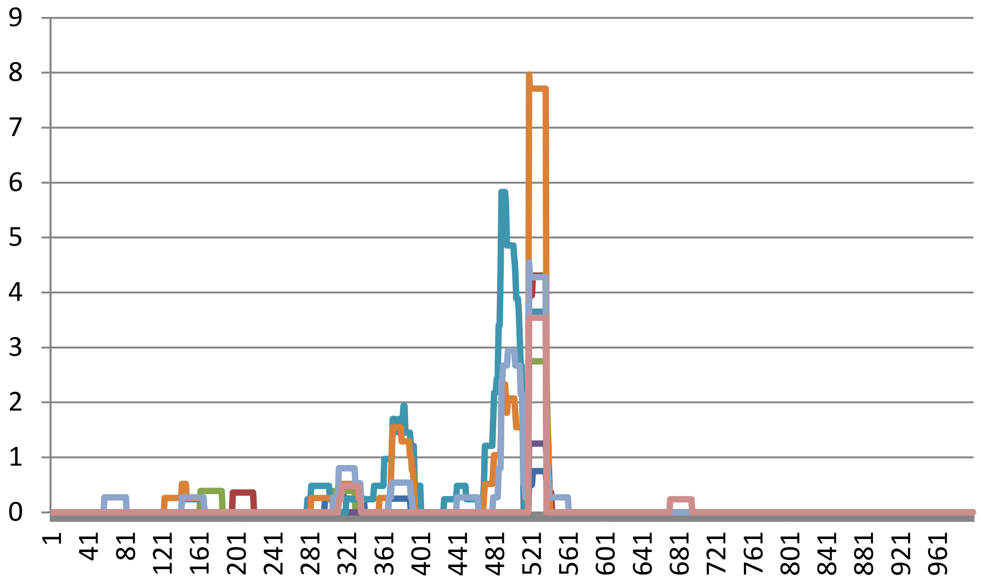

9
8
7
6
5
4
3
2
1
0
1
41
81
121
161
201
241
281
321
361
401
441
481
521
561
601
641
681
721
761
801
841
881
921
961

AT4G30993_AGO1 root

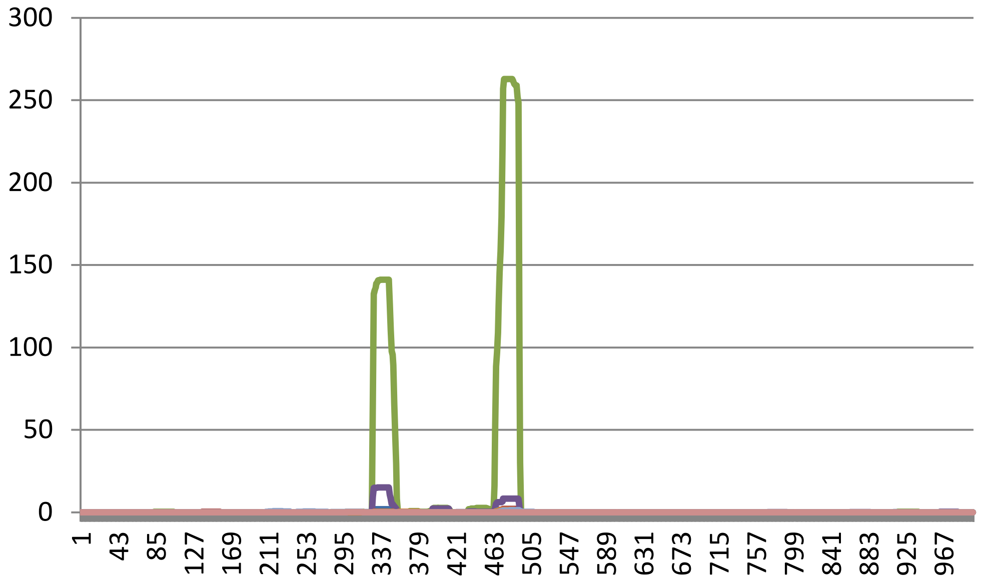

AT4G33820

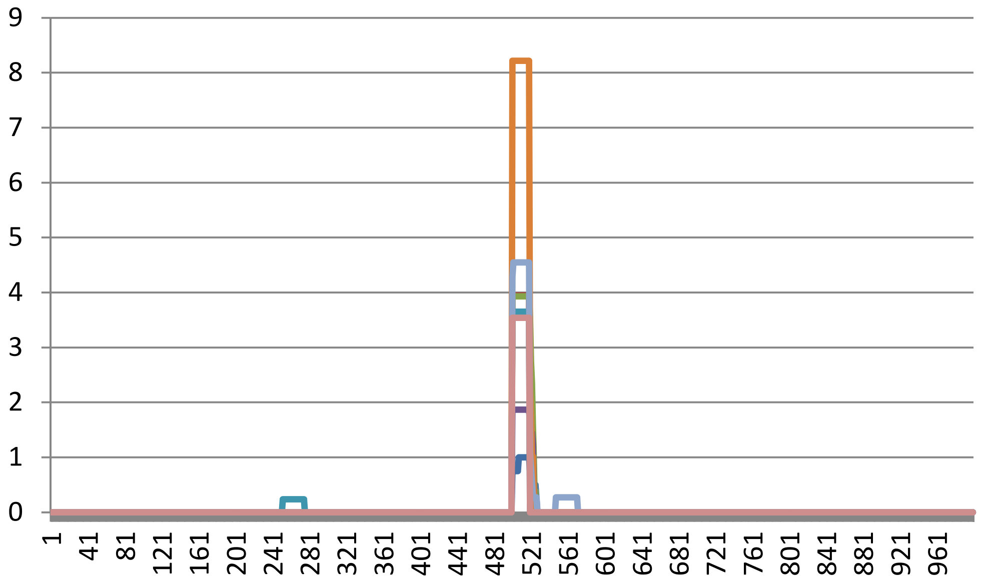

9
8
7
6
5
4
3
2
1
0
1
41
81
121
161
201
241
281
321
361
401
441
481
521
561
601
641
681
721
761
801
841
881
921
961

AT4G34030_AGO1 root

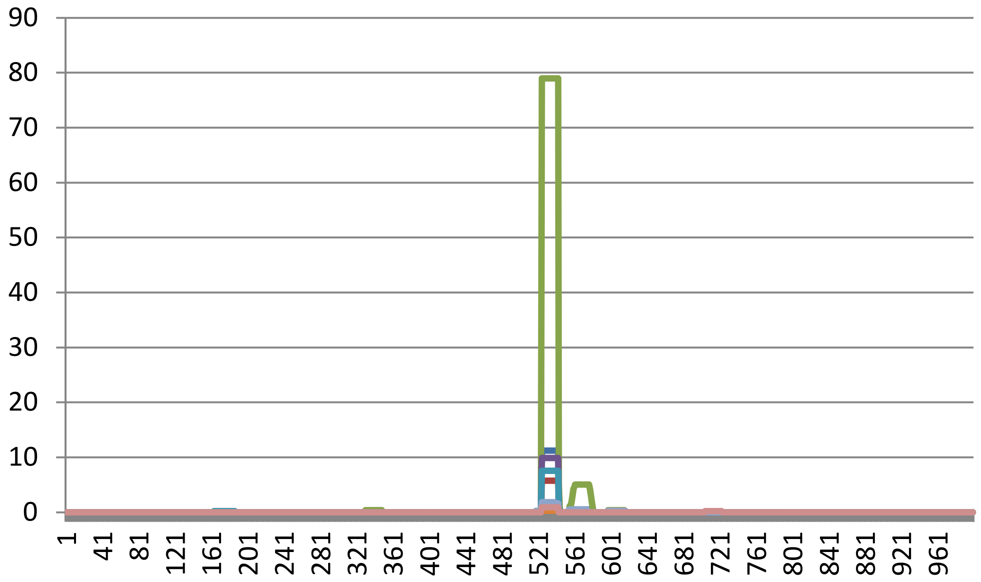

AT4G34400

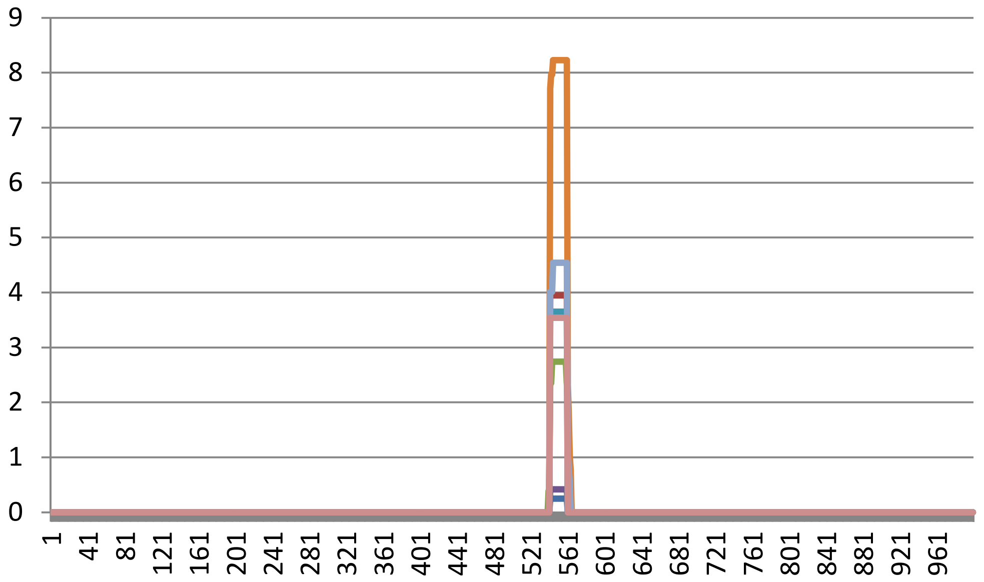

9
8
7
6
5
4
3
2
1
0
1
41
81
121
161
201
241
281
321
361
401
441
481
521
561
601
641
681
721
761
801
841
881
921
961

AT4G36190

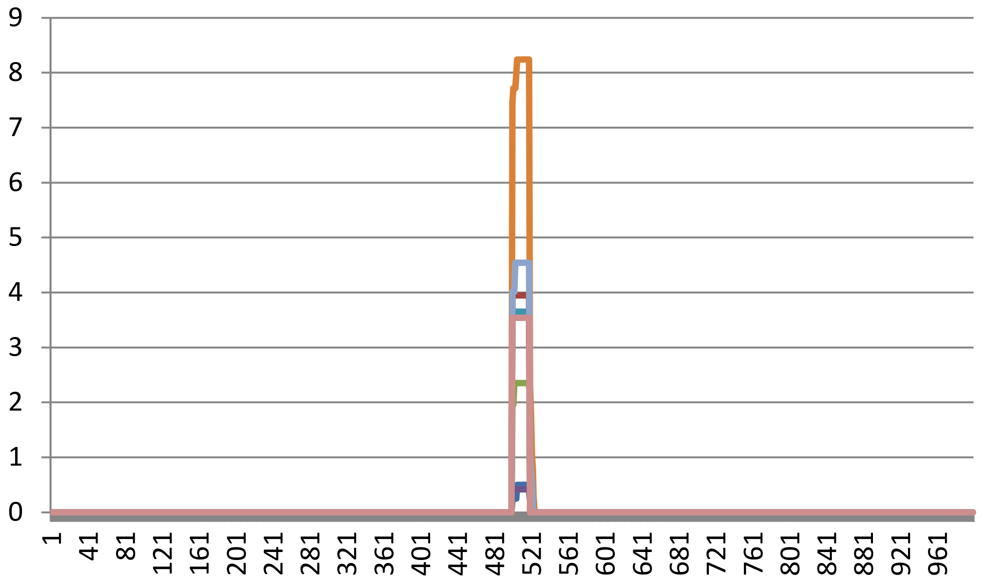

9
8
7
6
5
4
3
2
1
0
1
41
81
121
161
201
241
281
321
361
401
441
481
521
561
601
641
681
721
761
801
841
881
921
961

AT4G37130

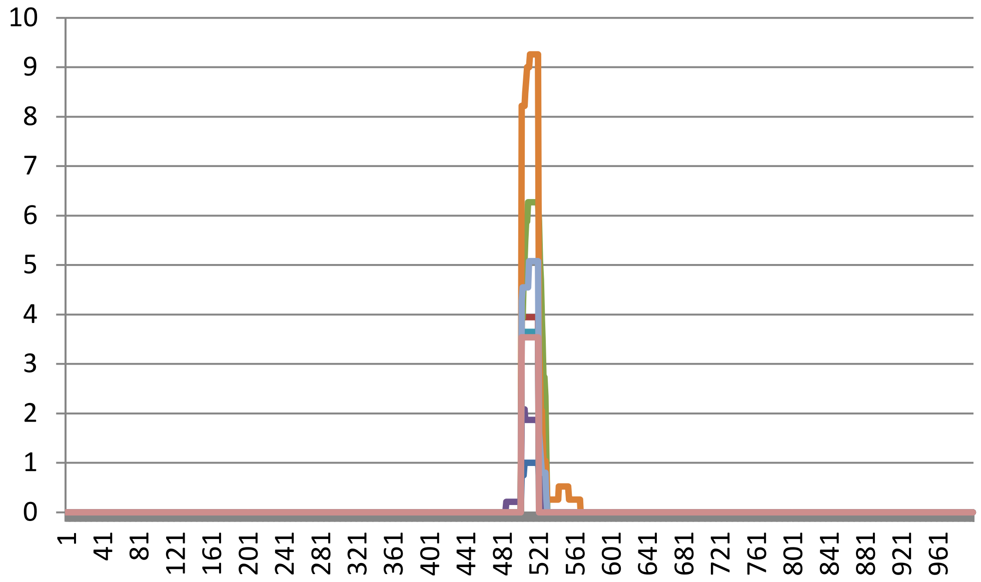

10
9
8
7
6
5
4
3
2
1
0
1
41
81
121
161
201
241
281
321
361
401
441
481
521
561
601
641
681
721
761
801
841
881
921
961

AT4G39130

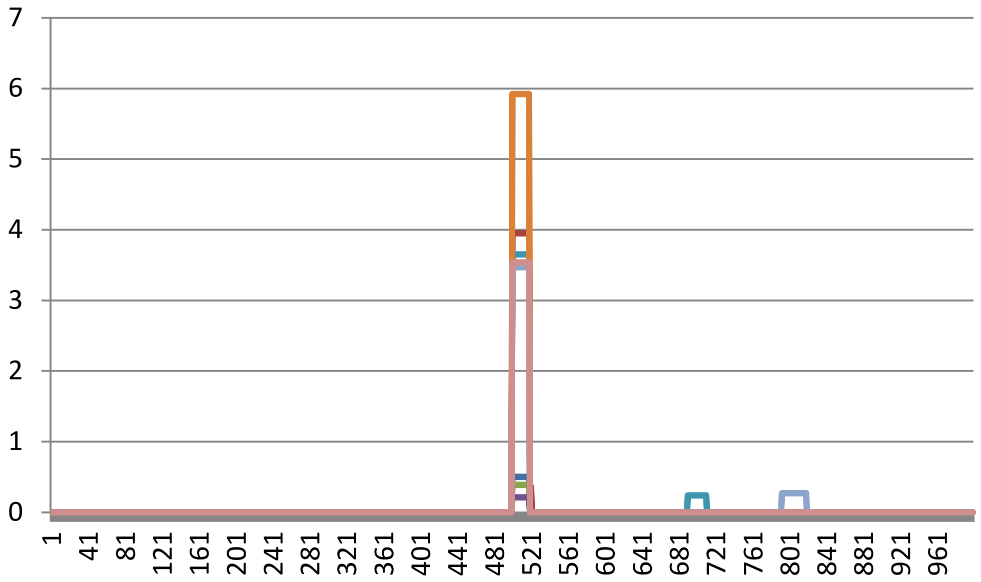

7
6
5
4
3
2
1
0
1
41
81
121
161
201
241
281
321
361
401
441
481
521
561
601
641
681
721
761
801
841
881
921
961

AT4G39980

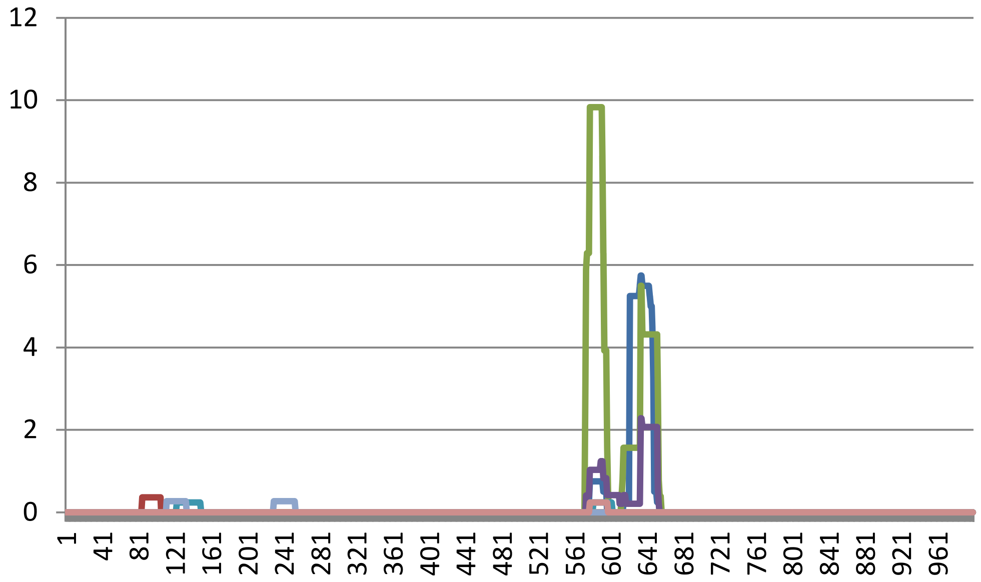

12
10
8
6
4
2
0
1
41
81
121
161
201
241
281
321
361
401
441
481
521
561
601
641
681
721
761
801
841
881
921
961

AT5G01015

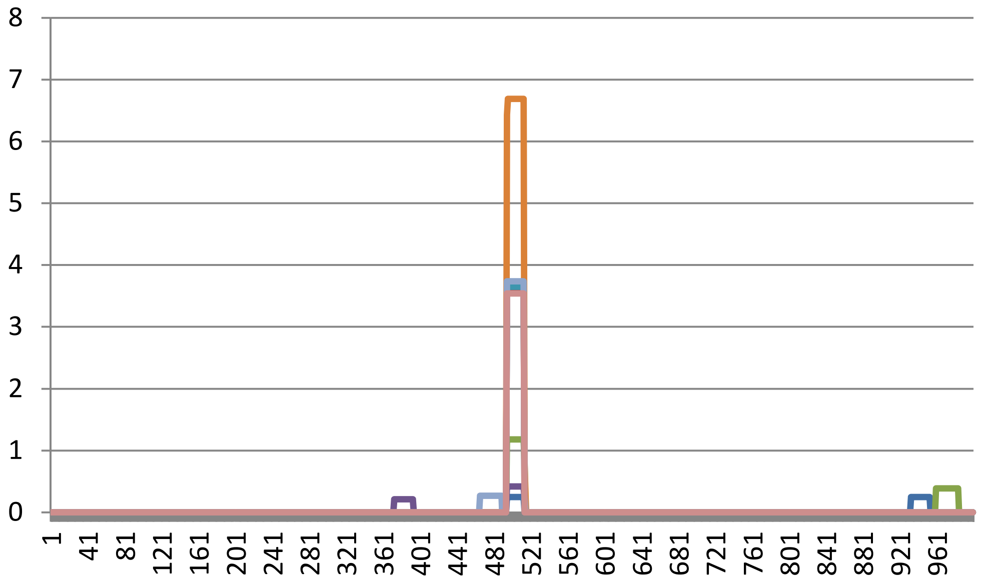

8
7
6
5
4
3
2
1
0
1
41
81
121
161
201
241
281
321
361
401
441
481
521
561
601
641
681
721
761
801
841
881
921
961

AT5G04970

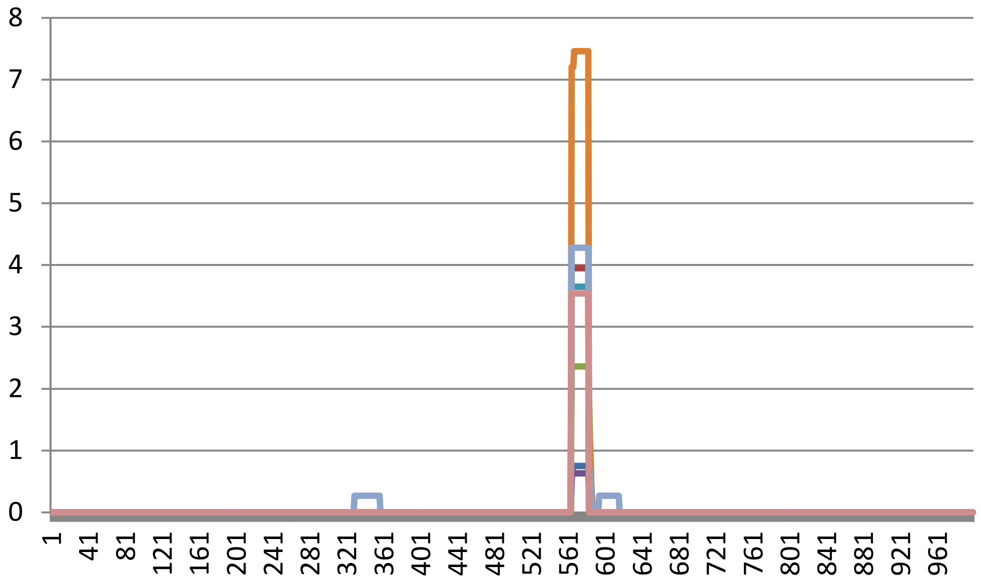

8
7
6
5
4
3
2
1
0
1
41
81
121
161
201
241
281
321
361
401
441
481
521
561
601
641
681
721
761
801
841
881
921
961

AT5G06130_AGO1 root

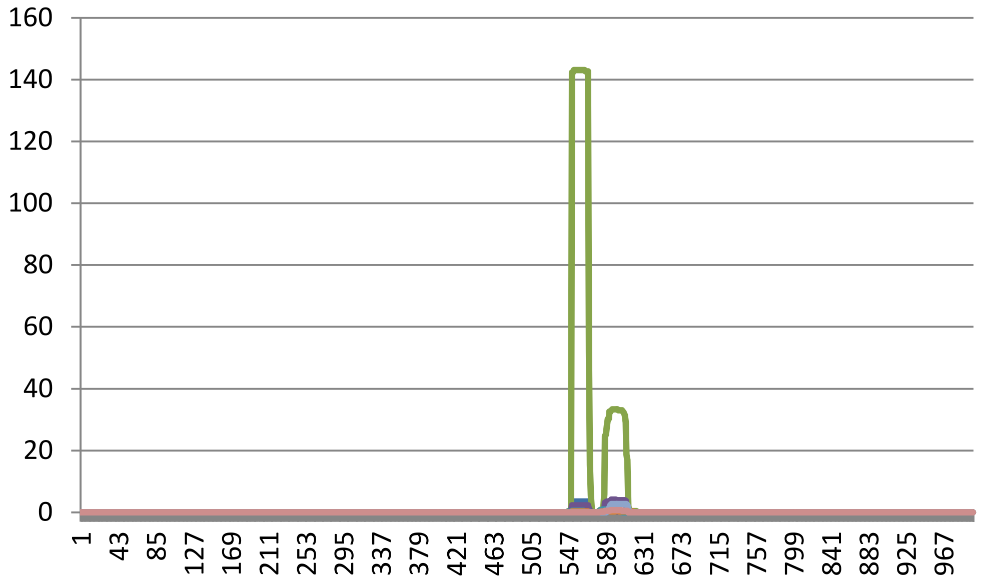

AT5G07140

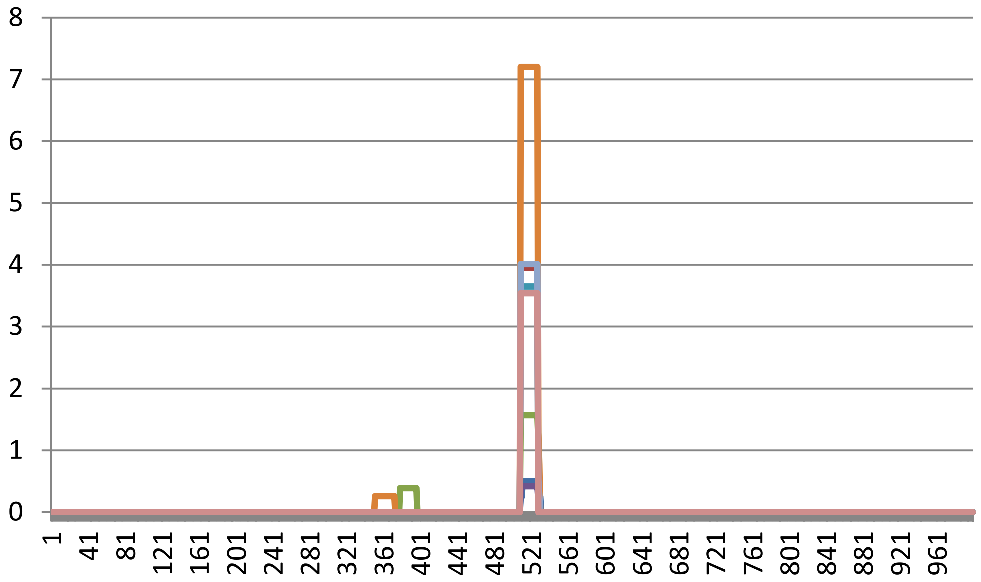

8
7
6
5
4
3
2
1
0
1
41
81
121
161
201
241
281
321
361
401
441
481
521
561
601
641
681
721
761
801
841
881
921
961

AT5G10100

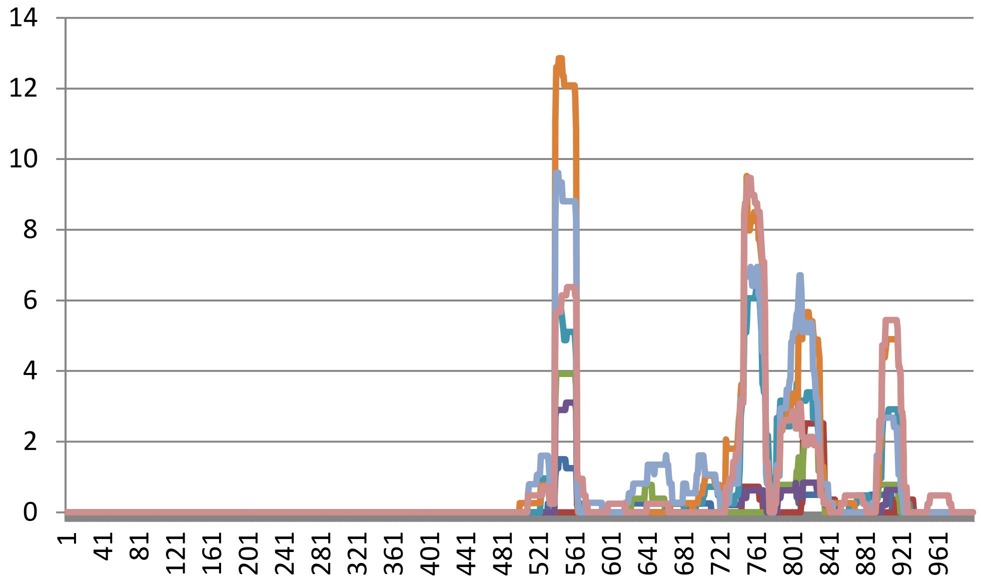

14
12
10
8
6
4
2
0
1
41
81
121
161
201
241
281
321
361
401
441
481
521
561
601
641
681
721
761
801
841
881
921
961

AT5G10660

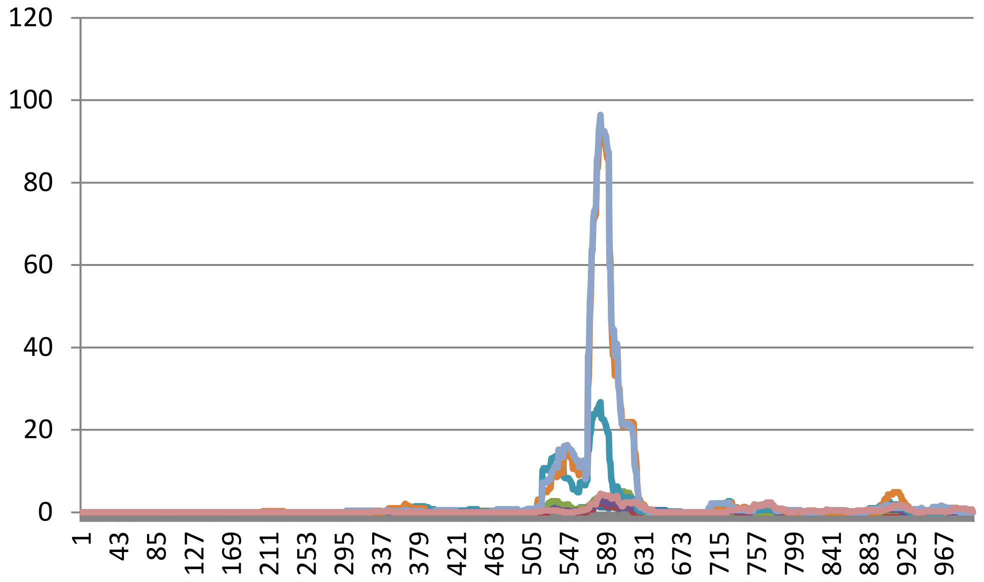

120
100
80
60
40
20
0
1
43
85
127
169
211
253
295
337
379
421
463
505
547
589
631
673
715
757
799
841
883
925
967

AT5G19160

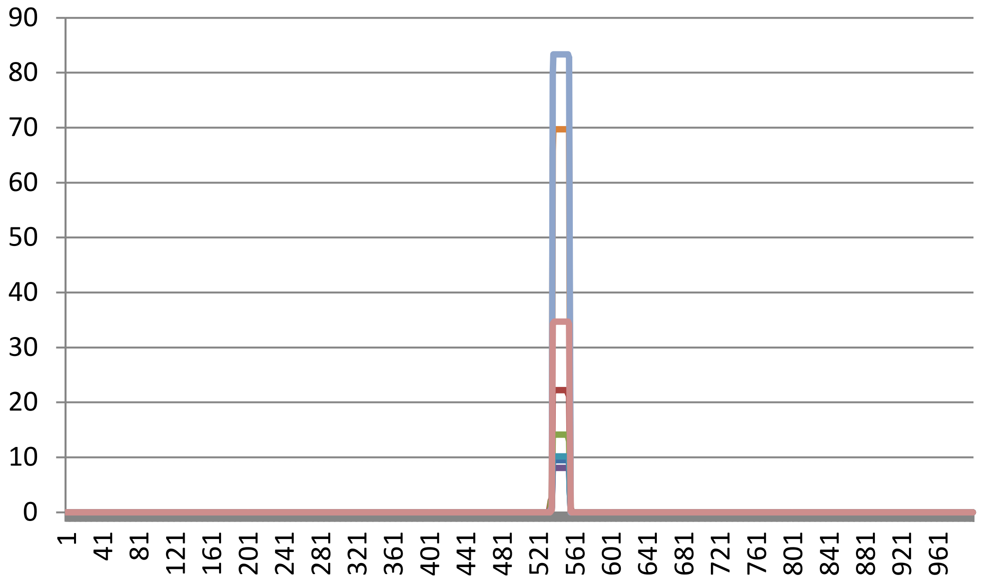

90
80
70
60
50
40
30
20
10
0
1
41
81
121
161
201
241
281
321
361
401
441
481
521
561
601
641
681
721
761
801
841
881
921
961

AT5G19170_AGO4 flower

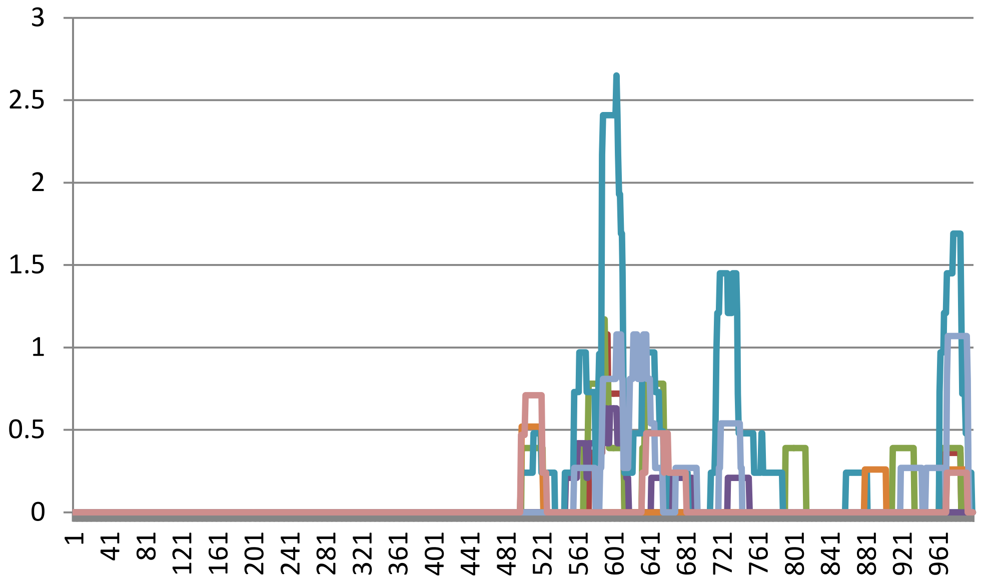

AT5G21125

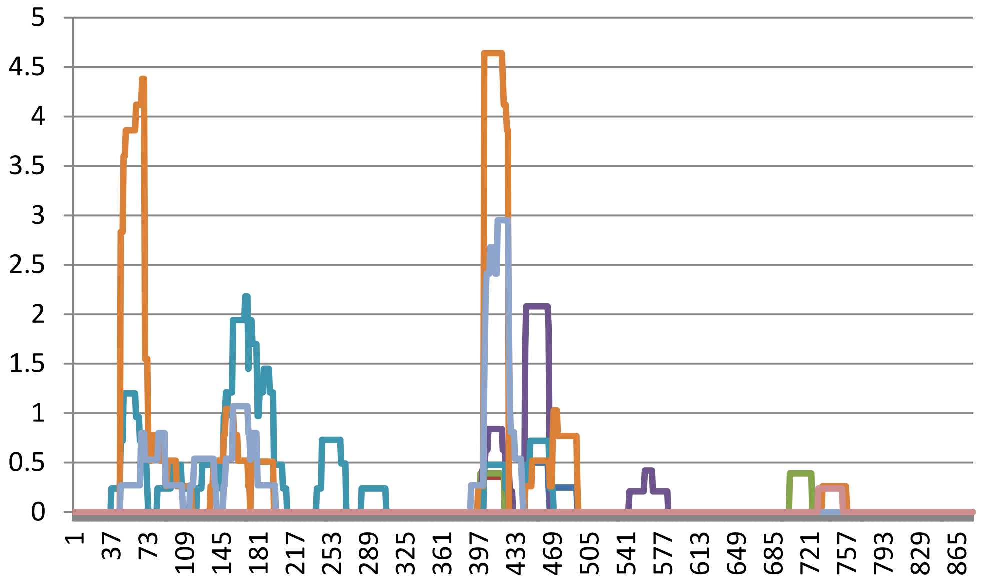
5
4.5
4
3.5
3
2.5
2
1.5
1
0.5
0
1
37
73
109
145
181
217
253
289
325
361
397
433
469
505
541
577
613
649
685
721
757
793
829
865

AT5G26770_AGO4

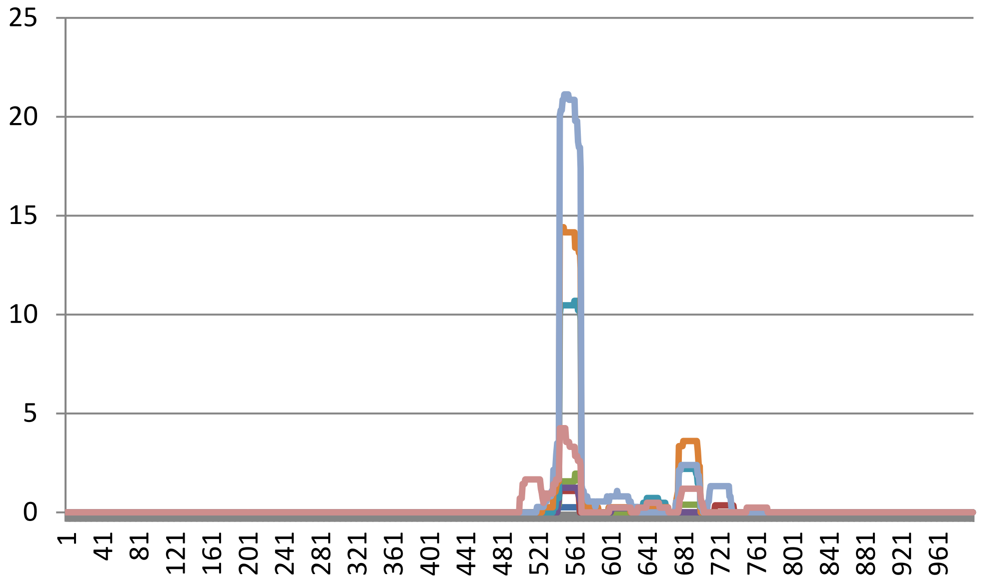

AT5G27870

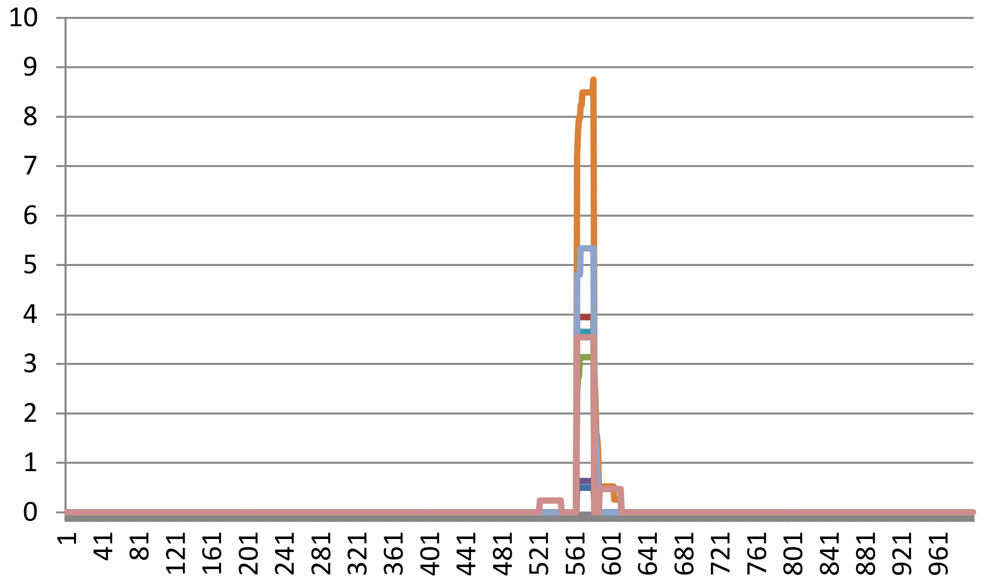
10
9
8
7
6
5
4
3
2
1
0
1
41
81
121
161
201
241
281
321
361
401
441
481
521
561
601
641
681
721
761
801
841
881
921
961

AT5G27880

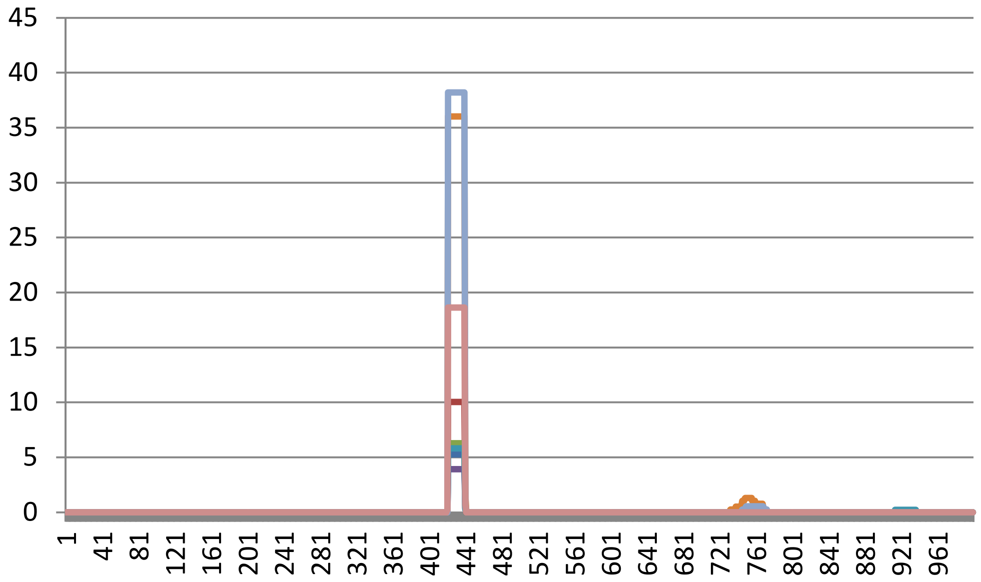

45
40
35
30
25
20
15
10
5
0
1
41
81
121
161
201
241
281
321
361
401
441
481
521
561
601
641
681
721
761
801
841
881
921
961

AT5G36220_root

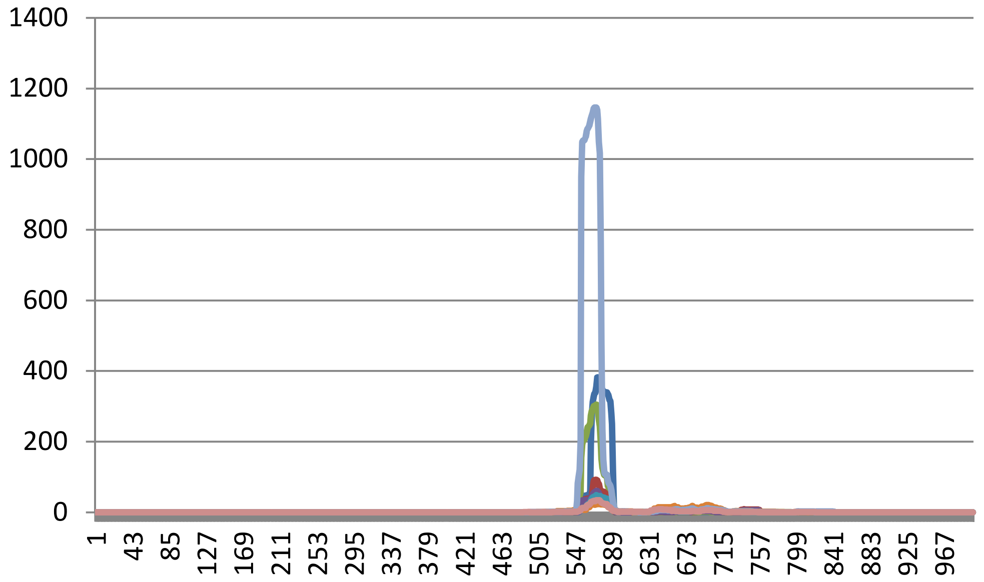

AT5G37440

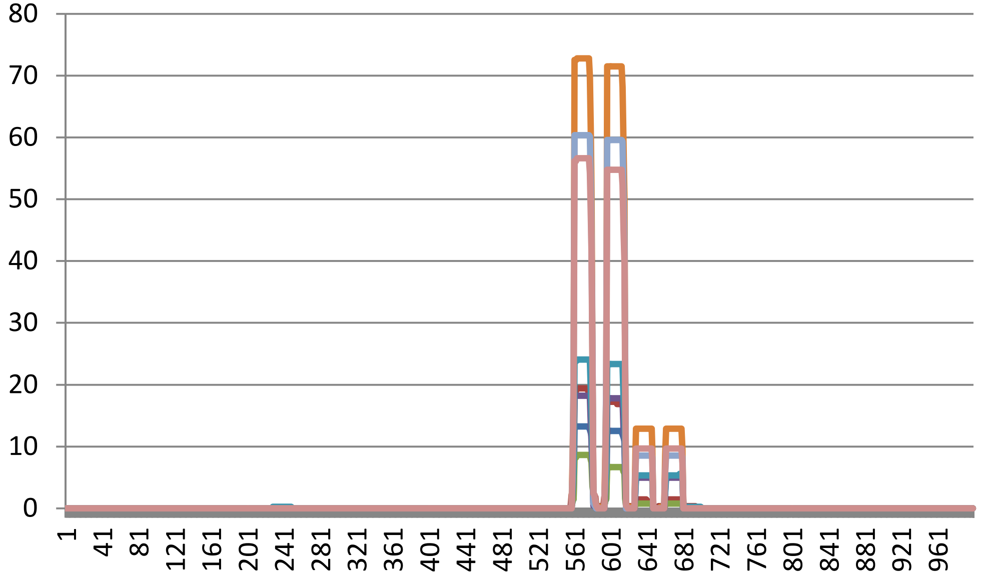

80
70
60
50
40
30
20
10
0
1
41
81
121
161
201
241
281
321
361
401
441
481
521
561
601
641
681
721
761
801
841
881
921
961

AT5G38440

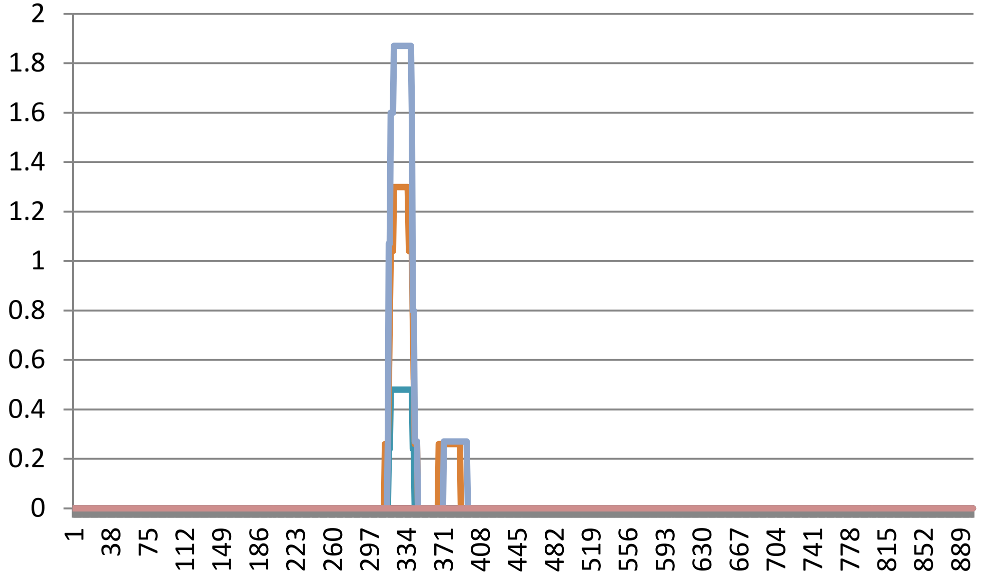
2
1.8
1.6
1.4
1.2
1
0.8
0.6
0.4
0.2
0
1
38
75
112
149
186
223
260
297
334
371
408
445
482
519
556
593
630
667
704
741
778
815
852
889

AT5G39890

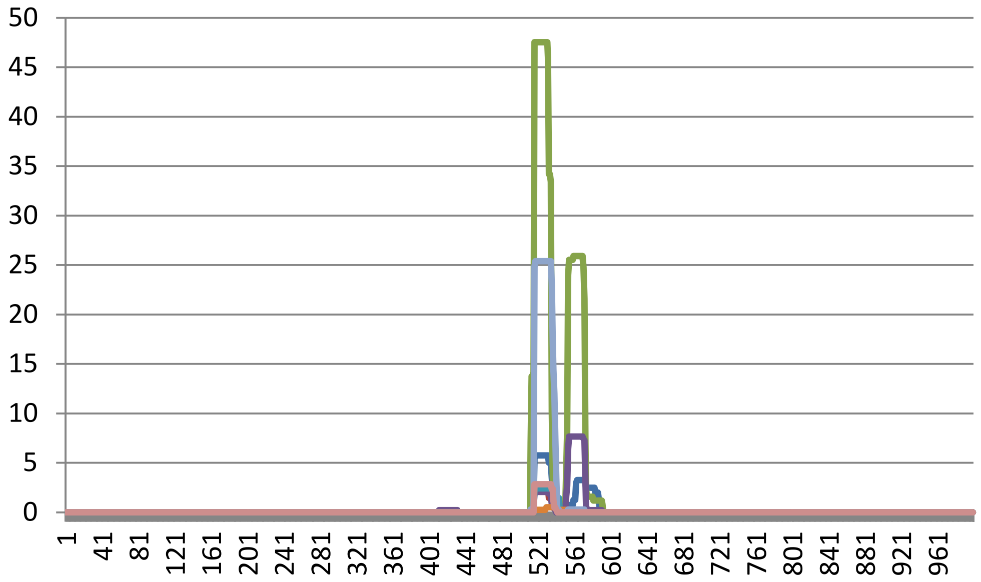

50
45
40
35
30
25
20
15
10
5
0
1
41
81
121
161
201
241
281
321
361
401
441
481
521
561
601
641
681
721
761
801
841
881
921
961

AT5G41150

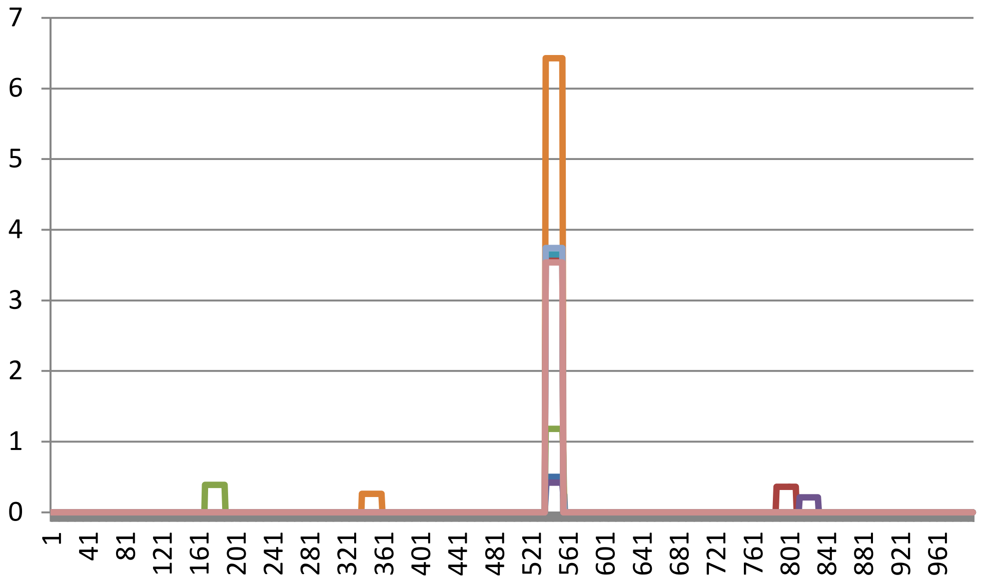

7
6
5
4
3
2
1
0
1
41
81
121
161
201
241
281
321
361
401
441
481
521
561
601
641
681
721
761
801
841
881
921
961

AT5G43285

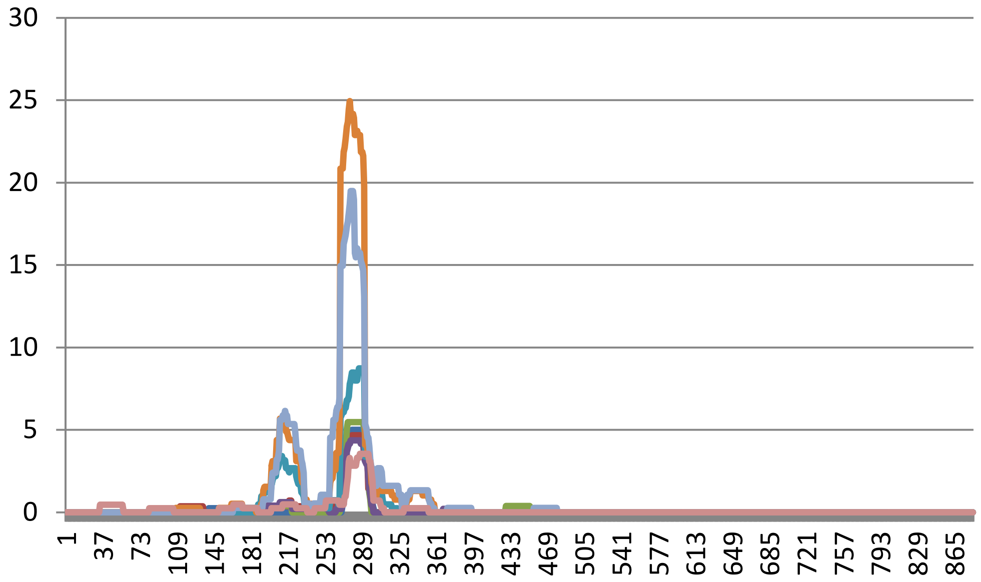

30
25
20
15
10
5
0
1
37
73
109
145
181
217
253
289
325
361
397
433
469
505
541
577
613
649
685
721
757
793
829
865

AT5G44870

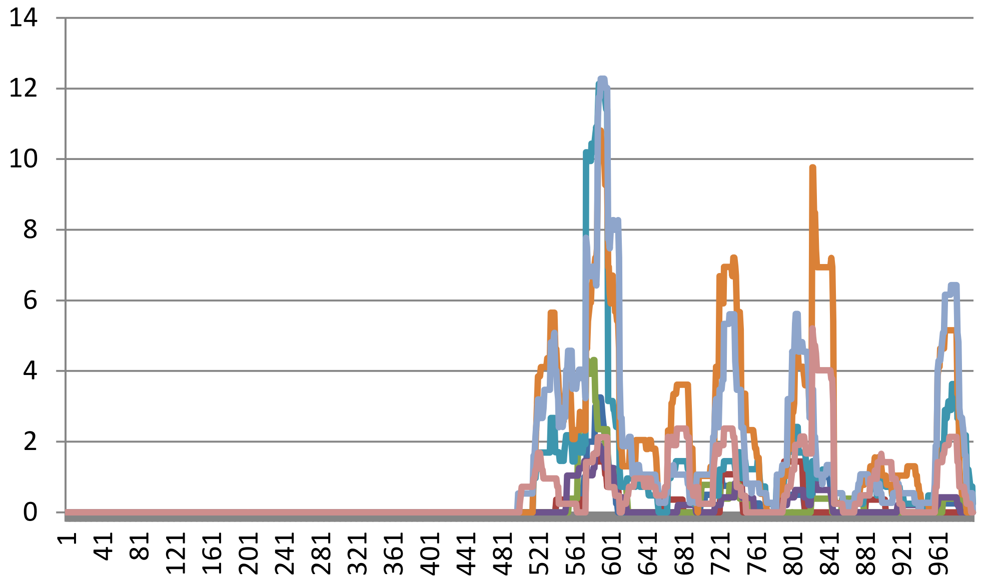

14
12
10
8
6
4
2
0
1
41
81
121
161
201
241
281
321
361
401
441
481
521
561
601
641
681
721
761
801
841
881
921
961

AT5G46750

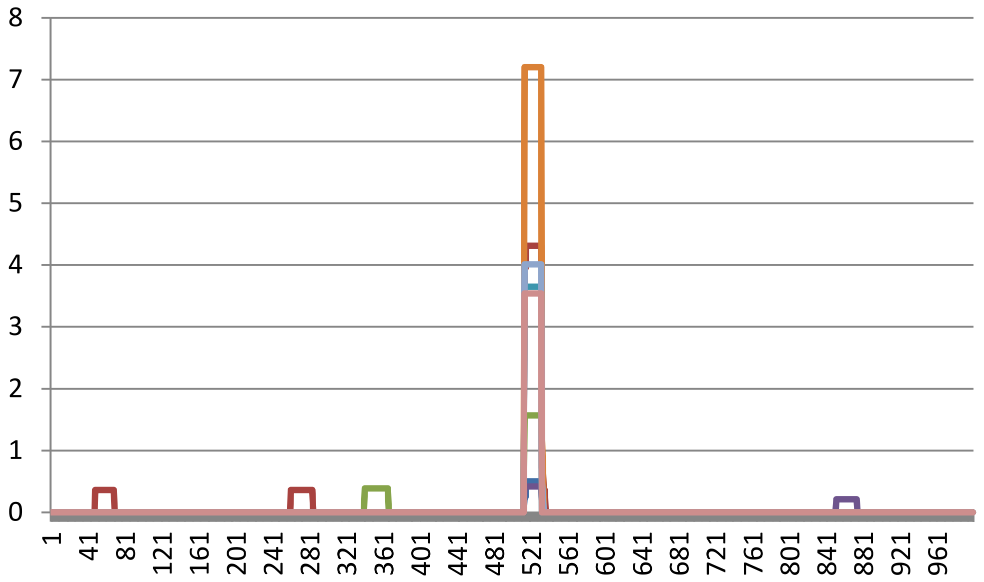

8
7
6
5
4
3
2
1
0
1
41
81
121
161
201
241
281
321
361
401
441
481
521
561
601
641
681
721
761
801
841
881
921
961

AT5G52360_AGO1 root

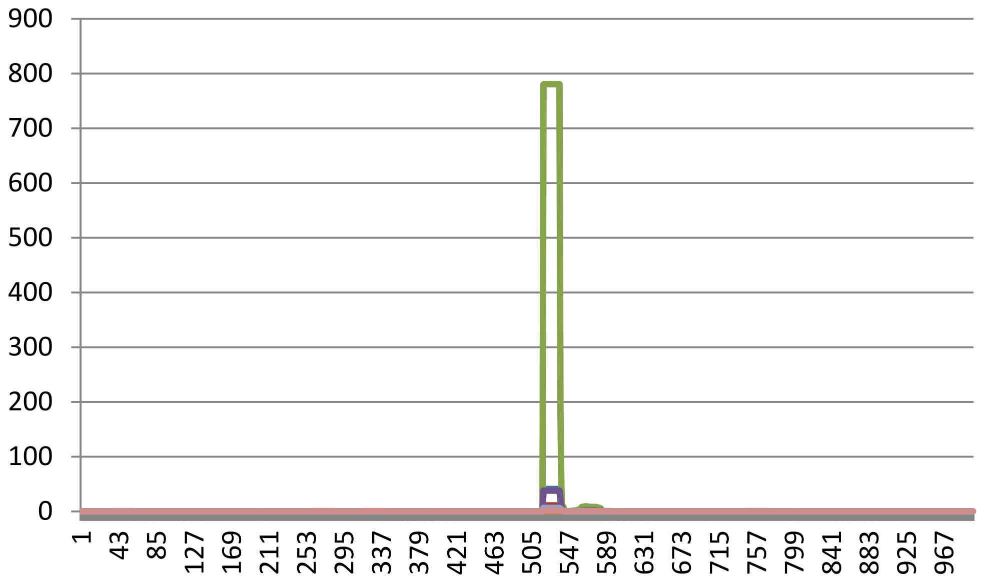

AT5G54070

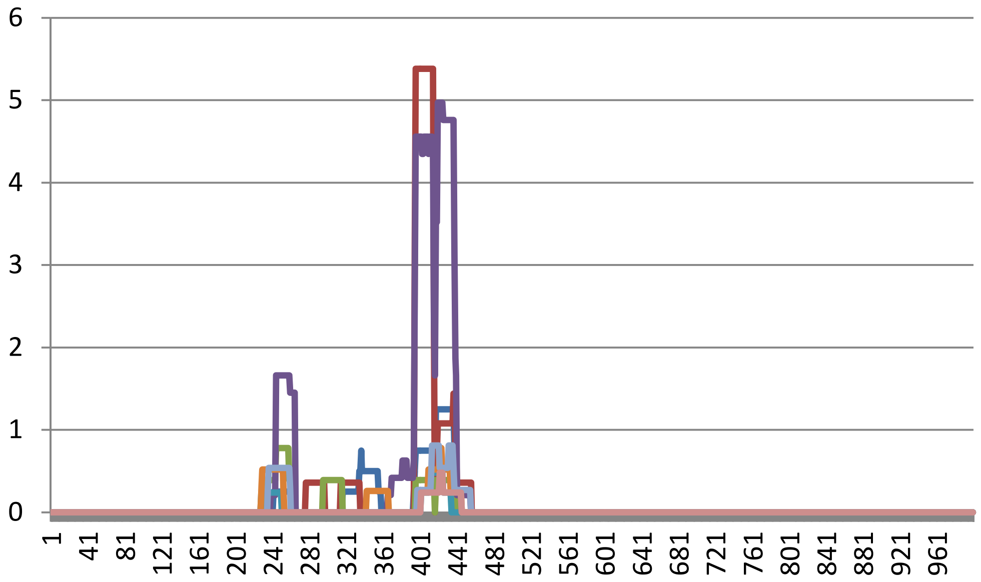

6
5
4
3
2
1
0
1
41
81
121
161
201
241
281
321
361
401
441
481
521
561
601
641
681
721
761
801
841
881
921
961

AT5G54370

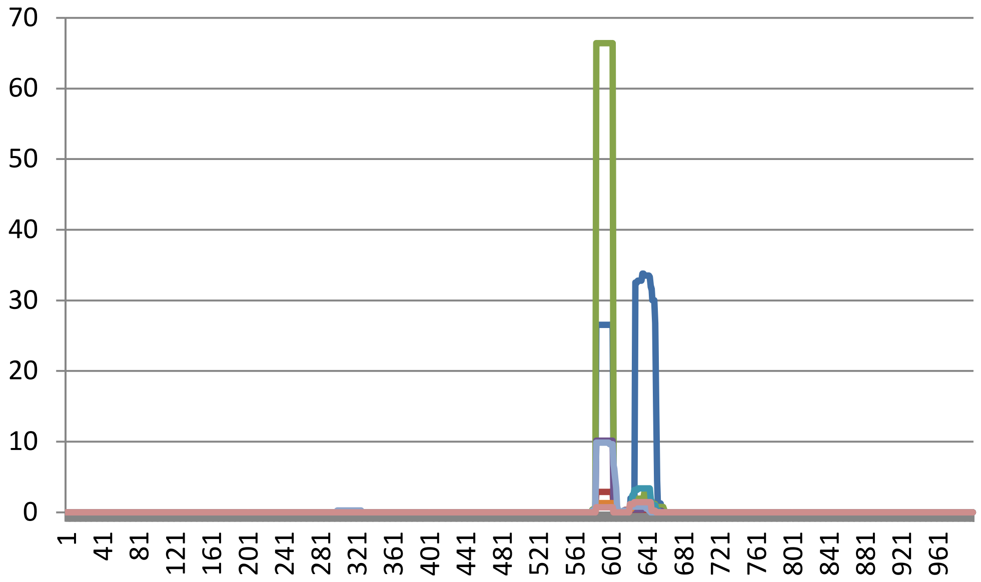

70
60
50
40
30
20
10
0
1
41
81
121
161
201
241
281
321
361
401
441
481
521
561
601
641
681
721
761
801
841
881
921
961

AT5G55110

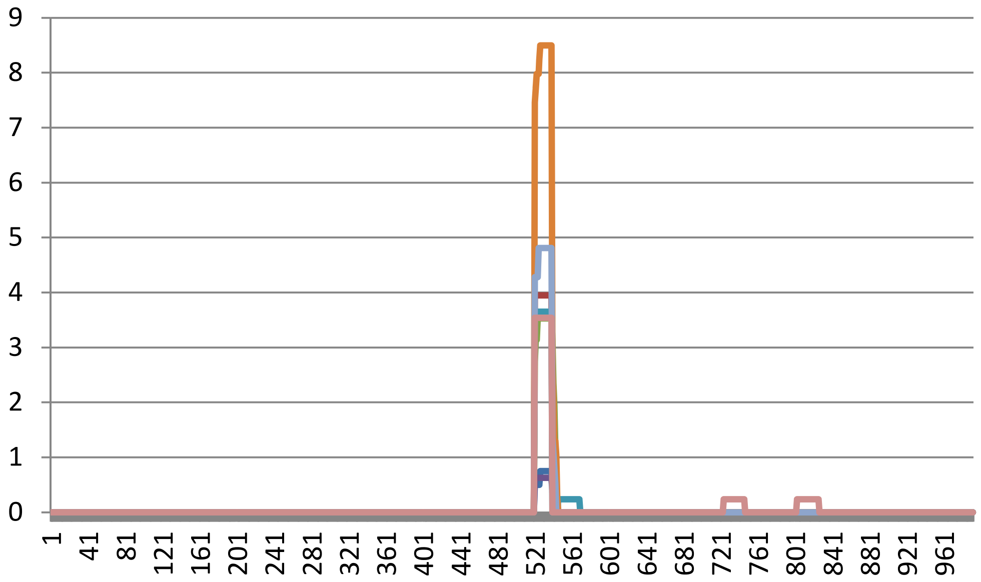

9
8
7
6
5
4
3
2
1
0
1
41
81
121
161
201
241
281
321
361
401
441
481
521
561
601
641
681
721
761
801
841
881
921
961

AT5G60280_AGO1 root

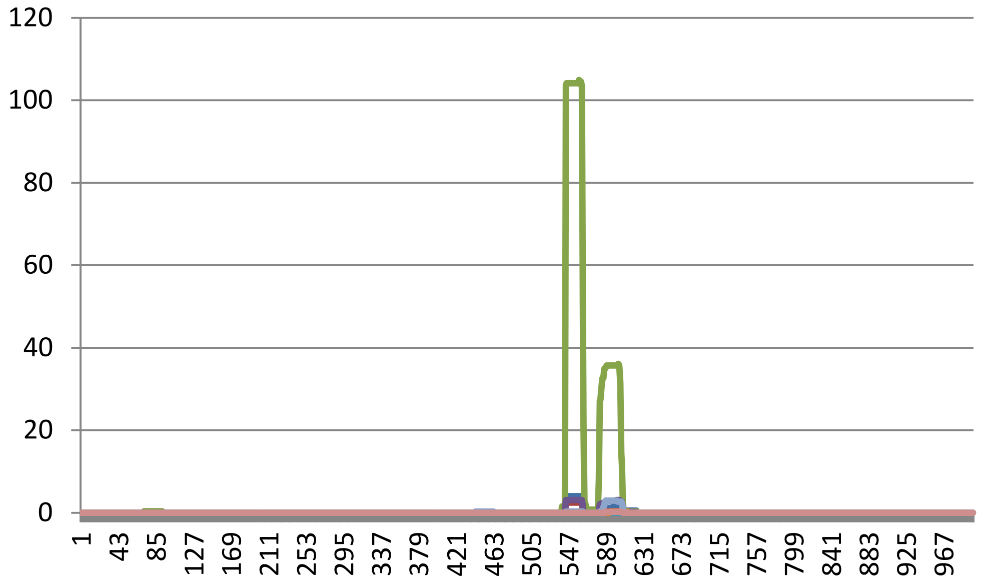

AT5G60548

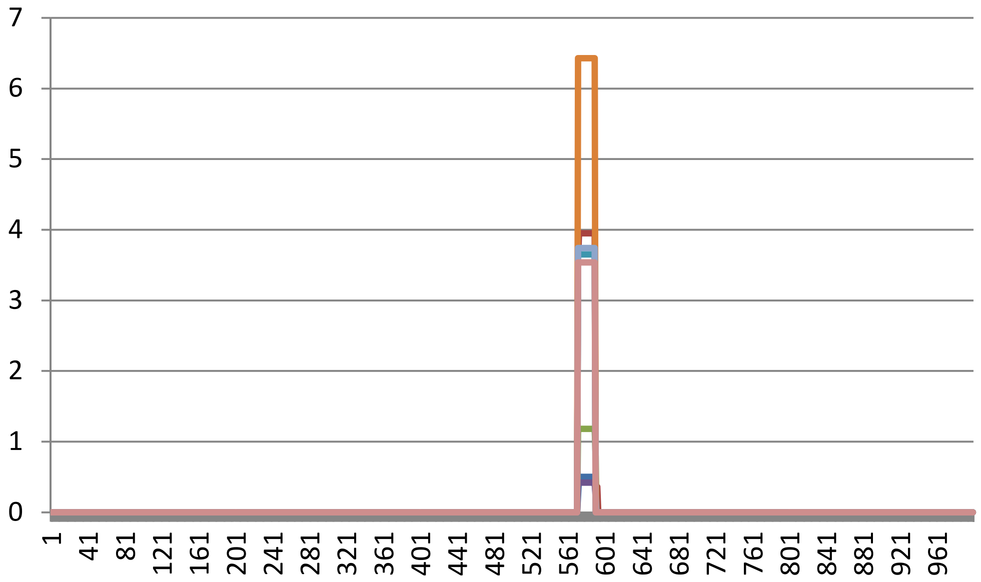

7
6
5
4
3
2
1
0
1
41
81
121
161
201
241
281
321
361
401
441
481
521
561
601
641
681
721
761
801
841
881
921
961

AT5G60550

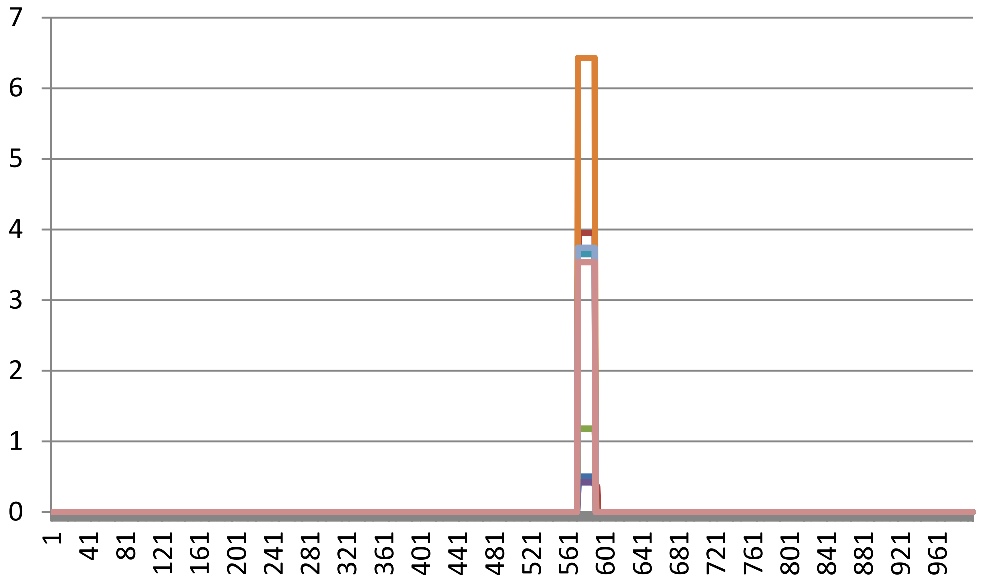

7
6
5
4
3
2
1
0
1
41
81
121
161
201
241
281
321
361
401
441
481
521
561
601
641
681
721
761
801
841
881
921
961

AT5G60553

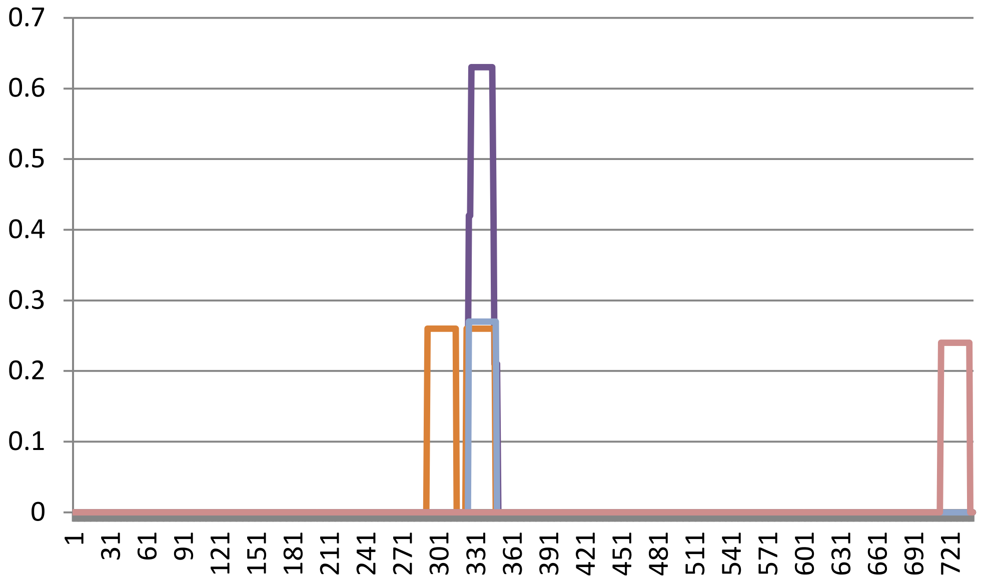
0.7
0.6
0.5
0.4
0.3
0.2
0.1
0
1
31
61
91
121
151
181
211
241
271
301
331
361
391
421
451
481
511
541
571
601
631
661
691
721

AT5G61830

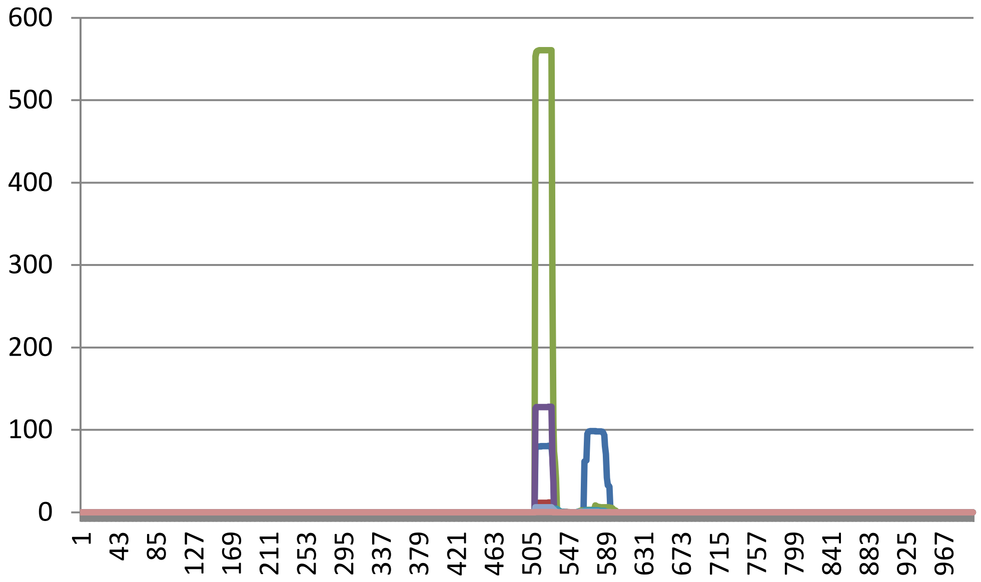

600
500
400
300
200
100
0
1
43
85
127
169
211
253
295
337
379
421
463
505
547
589
631
673
715
757
799
841
883
925
967

AT5G63950

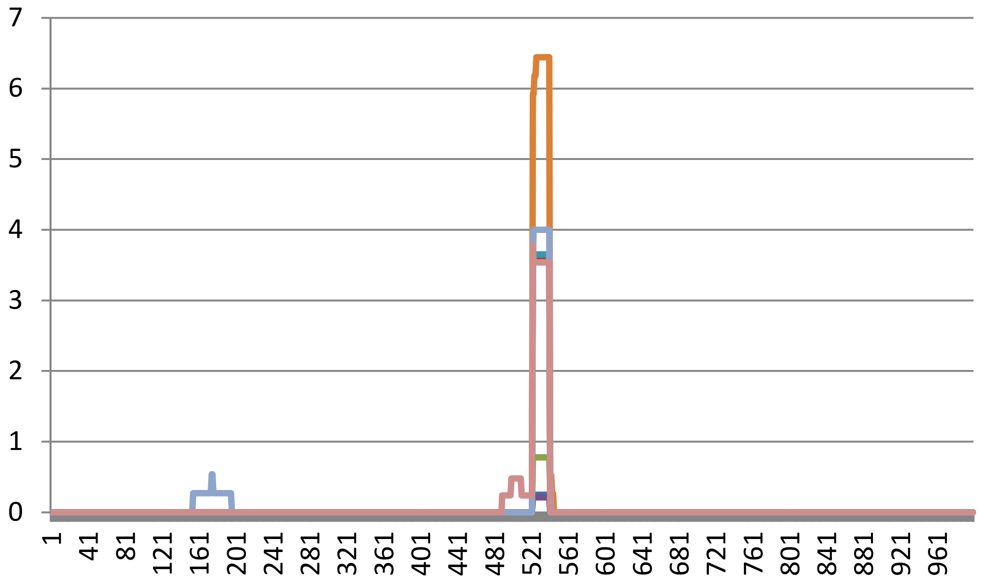

7
6
5
4
3
2
1
0
1
41
81
121
161
201
241
281
321
361
401
441
481
521
561
601
641
681
721
761
801
841
881
921
961

AT5G65430

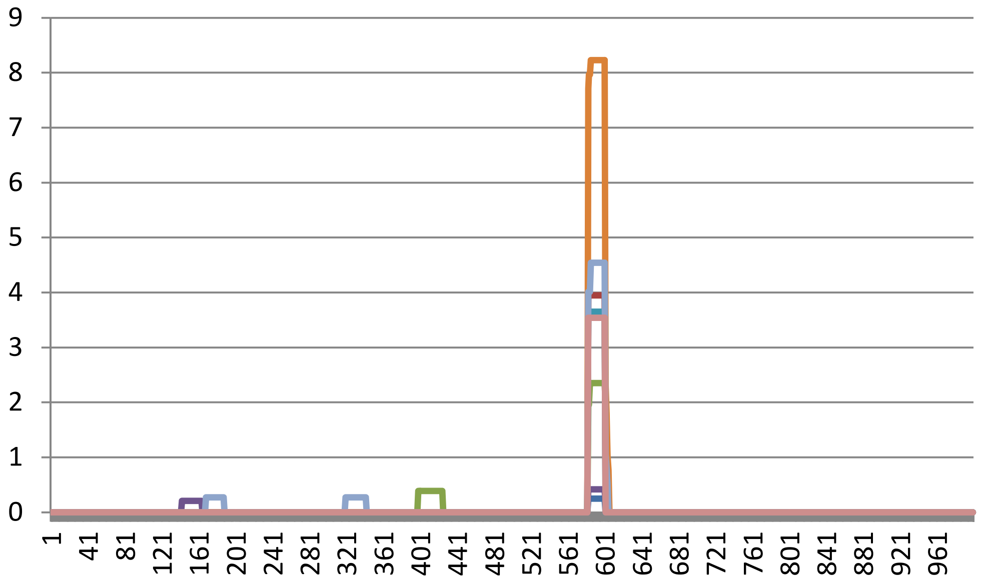

9
8
7
6
5
4
3
2
1
0
1
41
81
121
161
201
241
281
321
361
401
441
481
521
561
601
641
681
721
761
801
841
881
921
961

AT5G66090

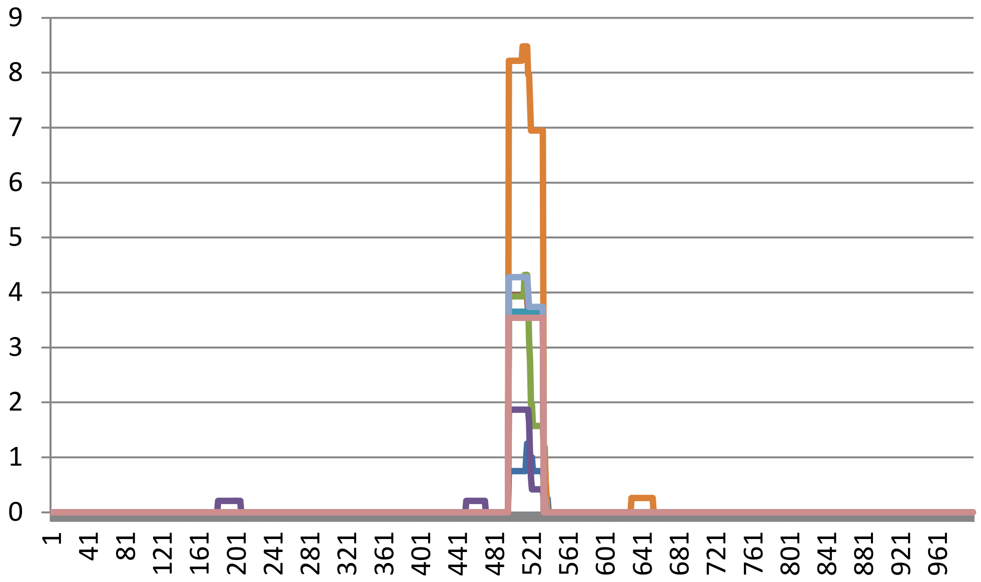

9
8
7
6
5
4
3
2
1
0
1
41
81
121
161
201
241
281
321
361
401
441
481
521
561
601
641
681
721
761
801
841
881
921
961

ATCG00130_seedling

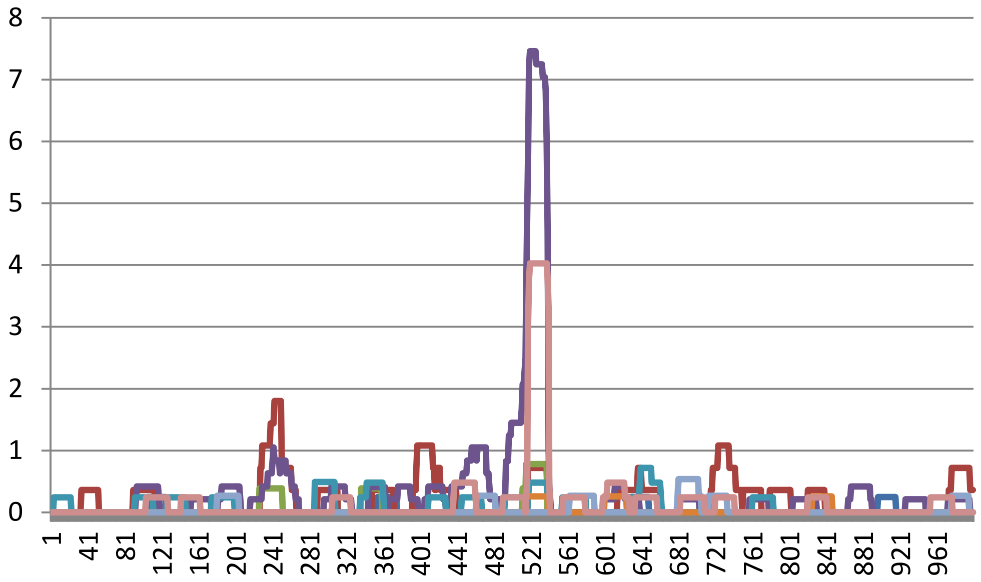

ATCG00270_AGO1 seedling

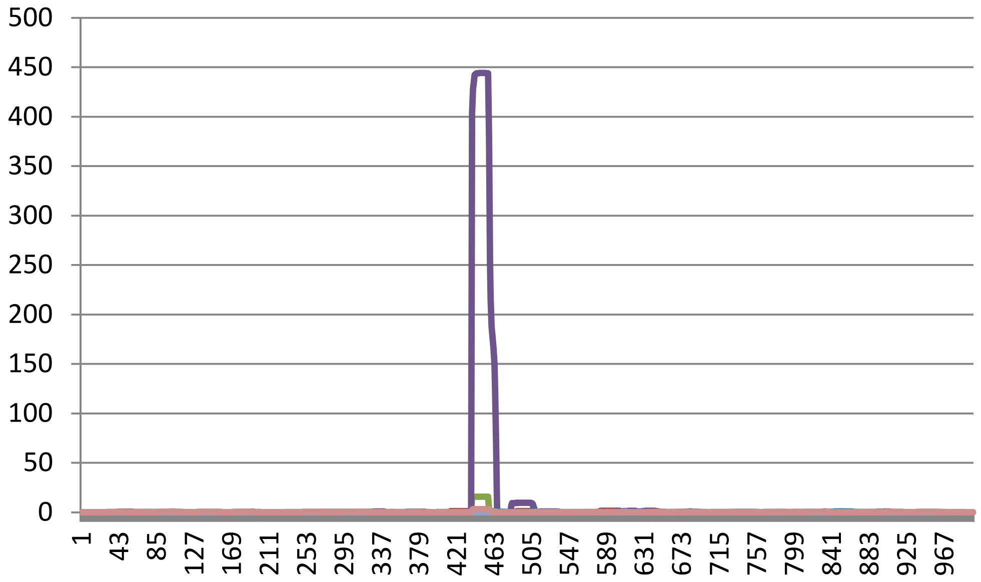

ATCG00420_AGO1 seedling

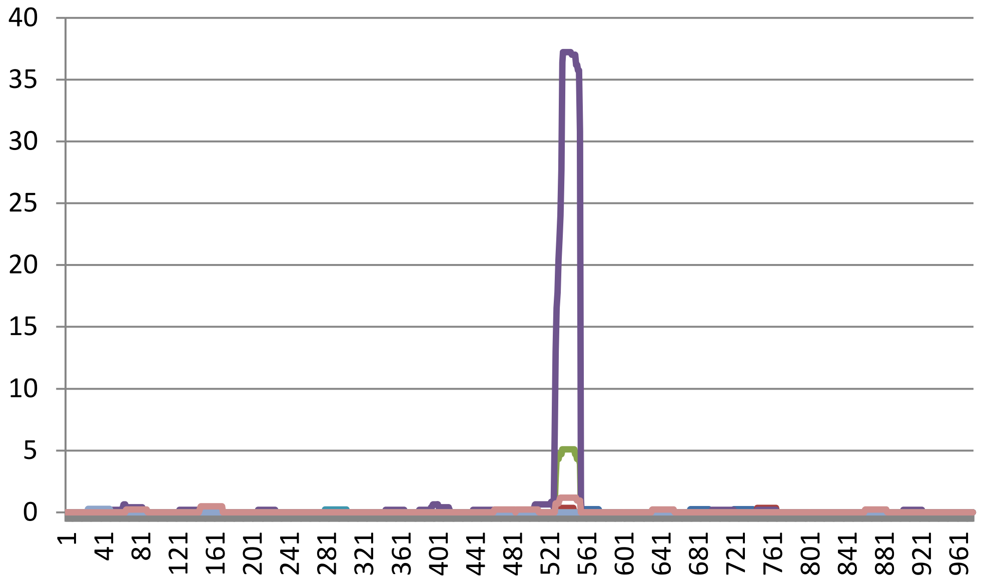

ATCG00840_AGO1 seedling

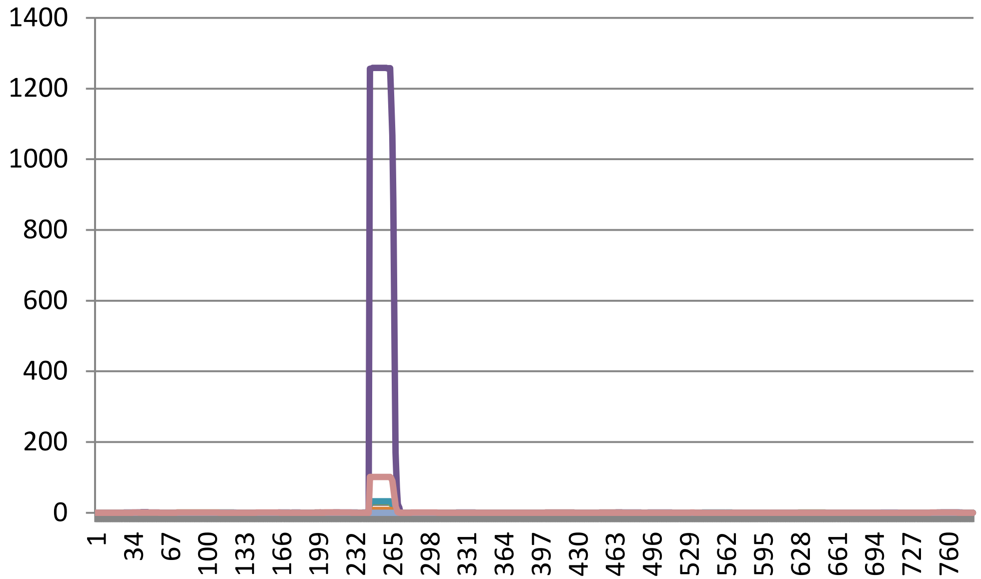

ATCG01110_AGO1 seedling

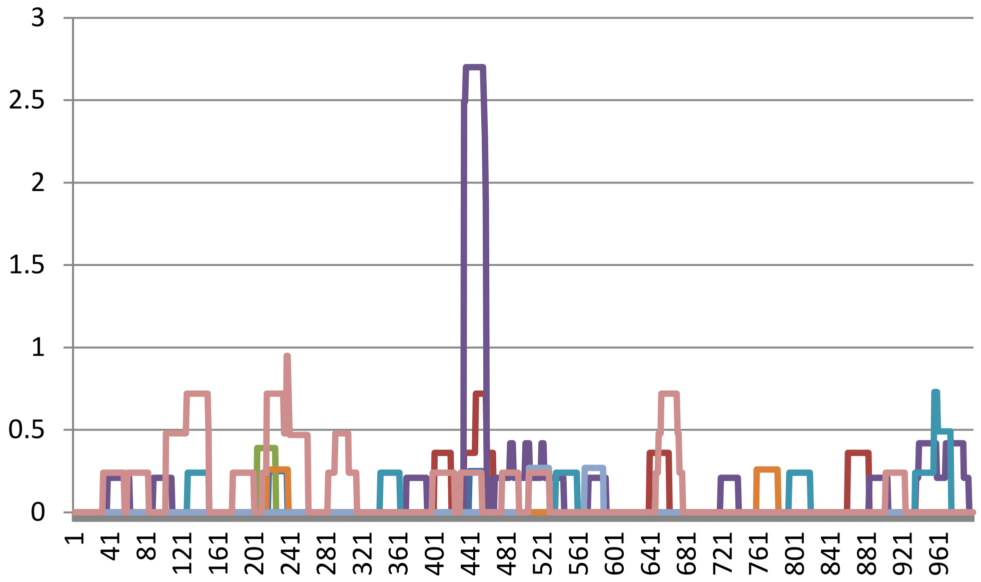

ATCG01300_AGO1 seedling

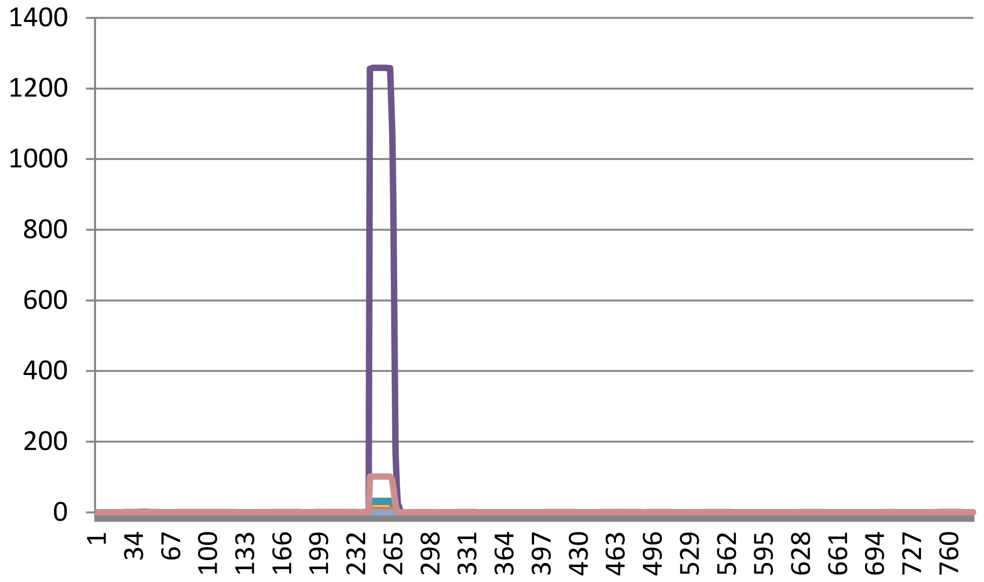

ATMG00060_root

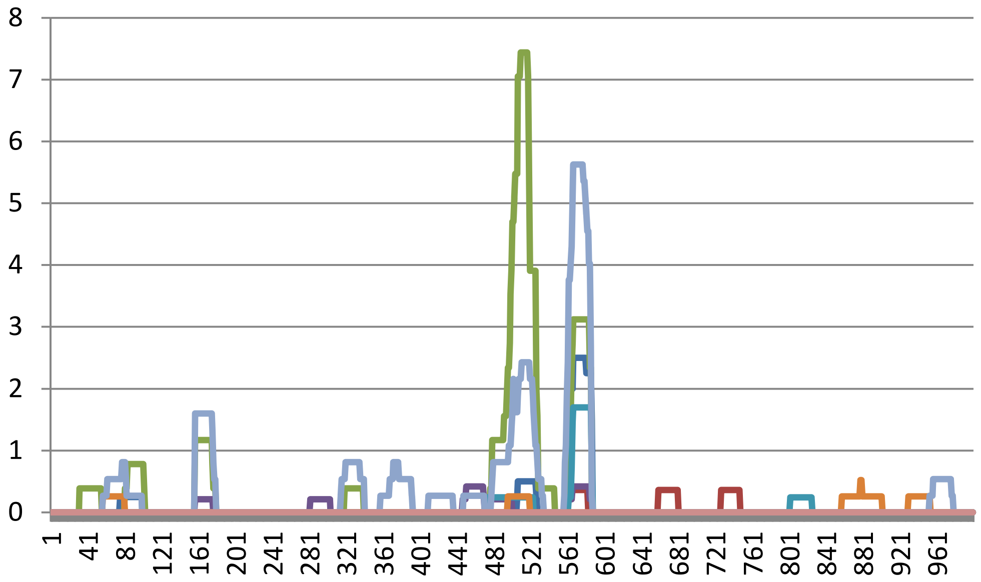

ATMG00070_AGO1 root

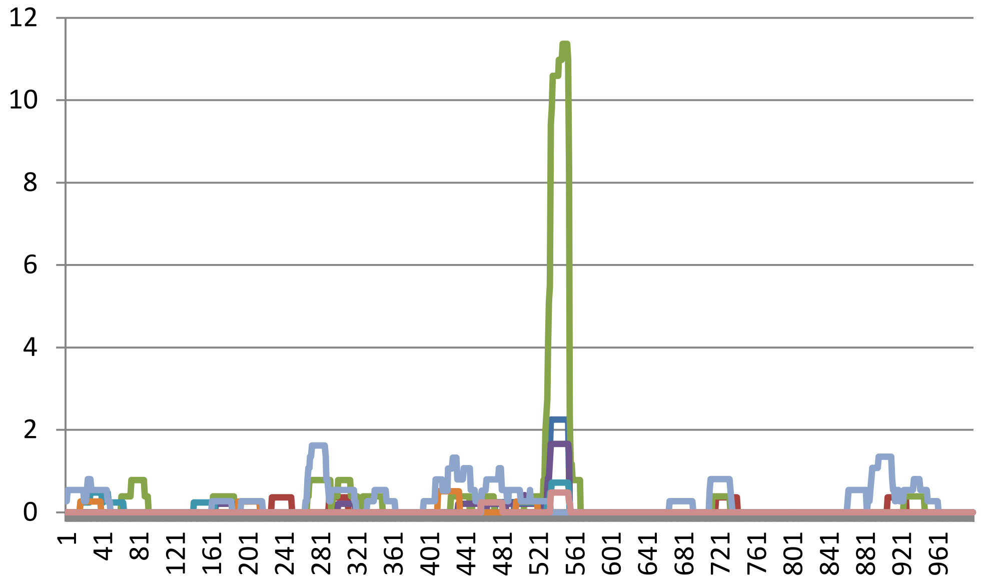

ATMG00110_AGO1 seedling

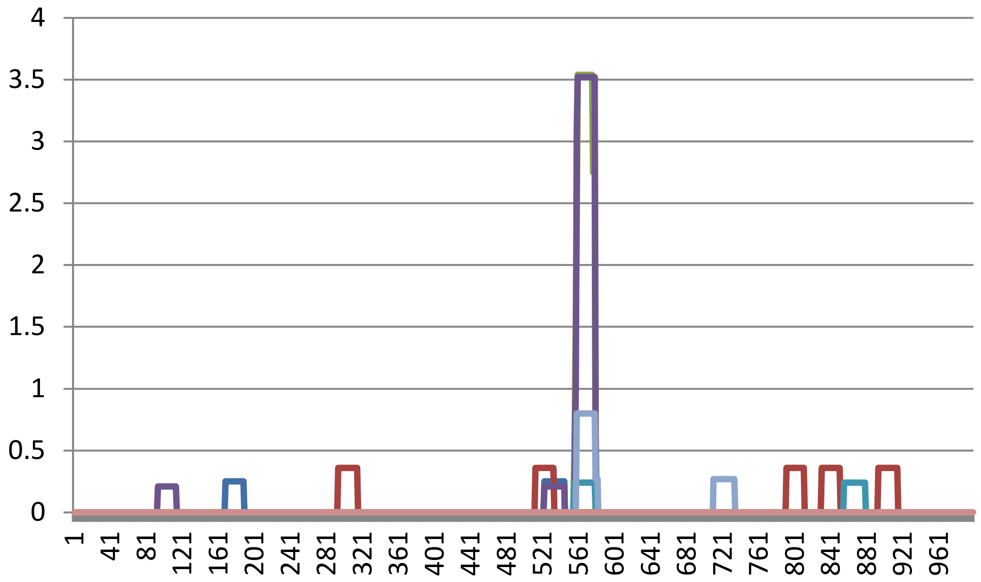

ATMG00270_AGO1

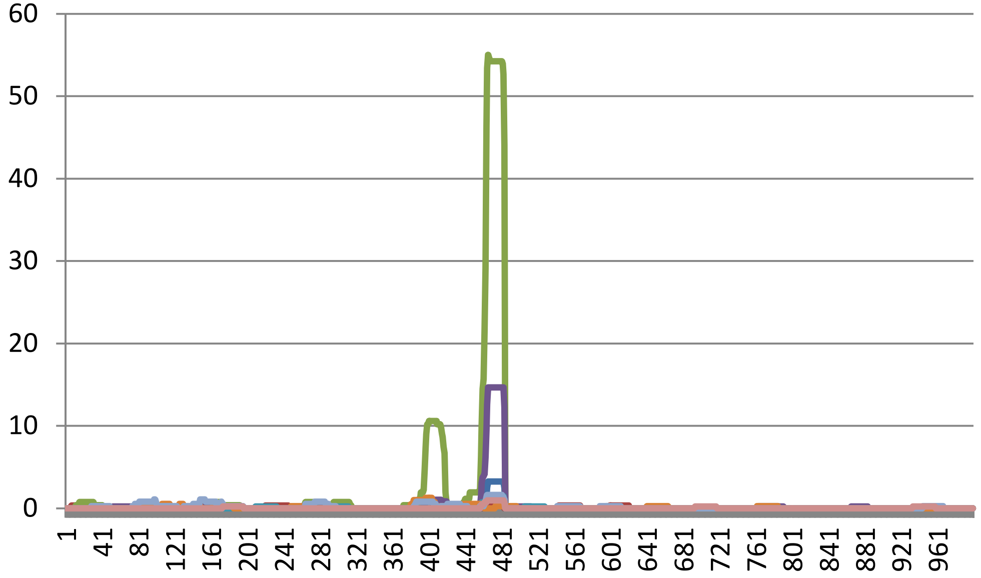

ATMG00510

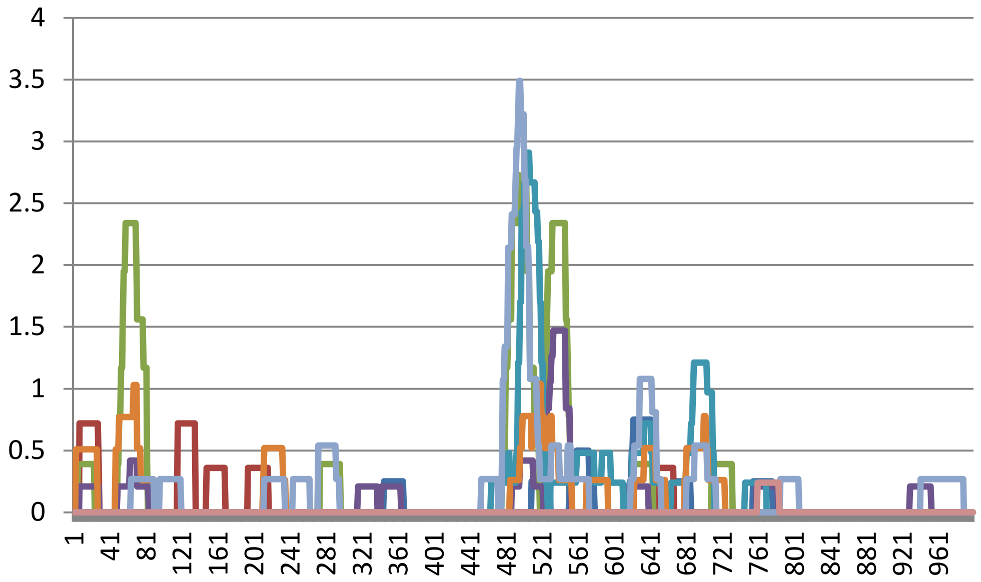

4
3.5
3
2.5
2
1.5
1
0.5
0
1
41
81
121
161
201
241
281
321
361
401
441
481
521
561
601
641
681
721
761
801
841
881
921
961

ATMG00516_AGO1 root

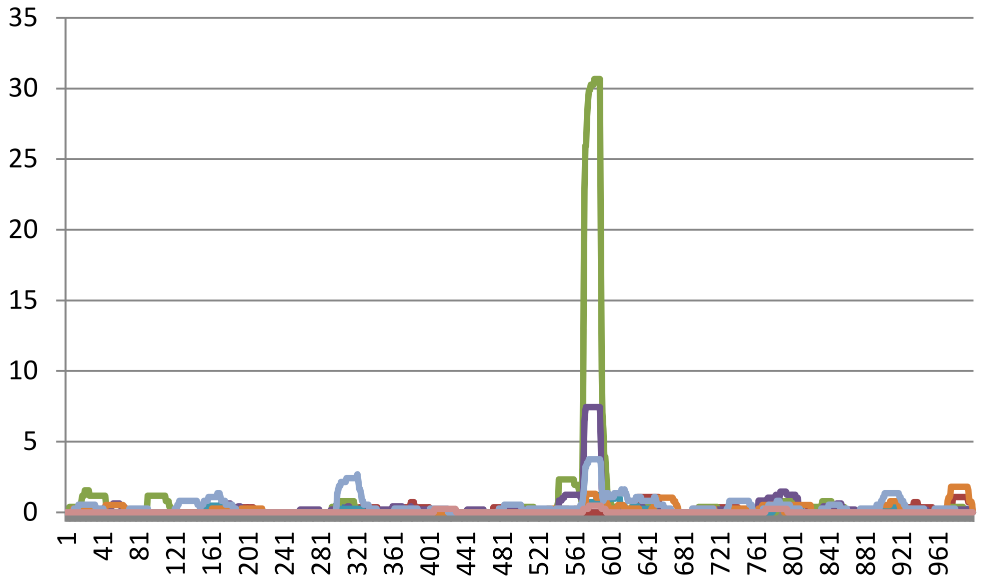

ATMG00570_AGO1

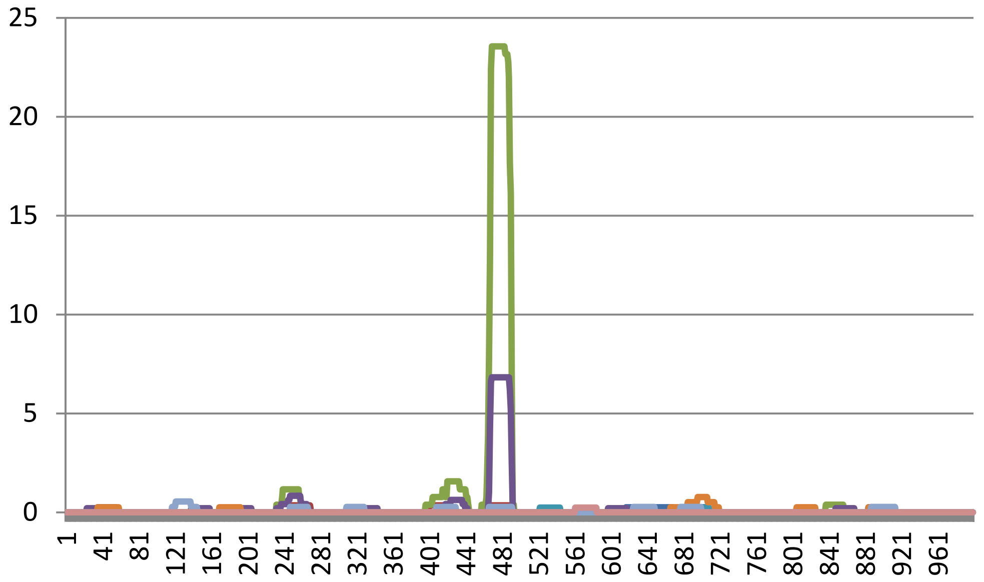

ATMG00580_AGO1

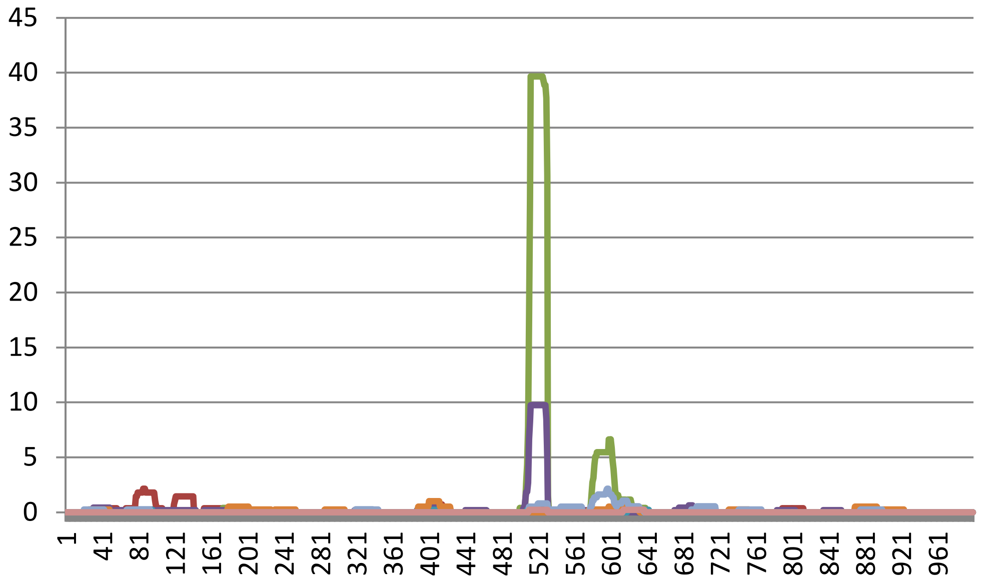

ATMG00640

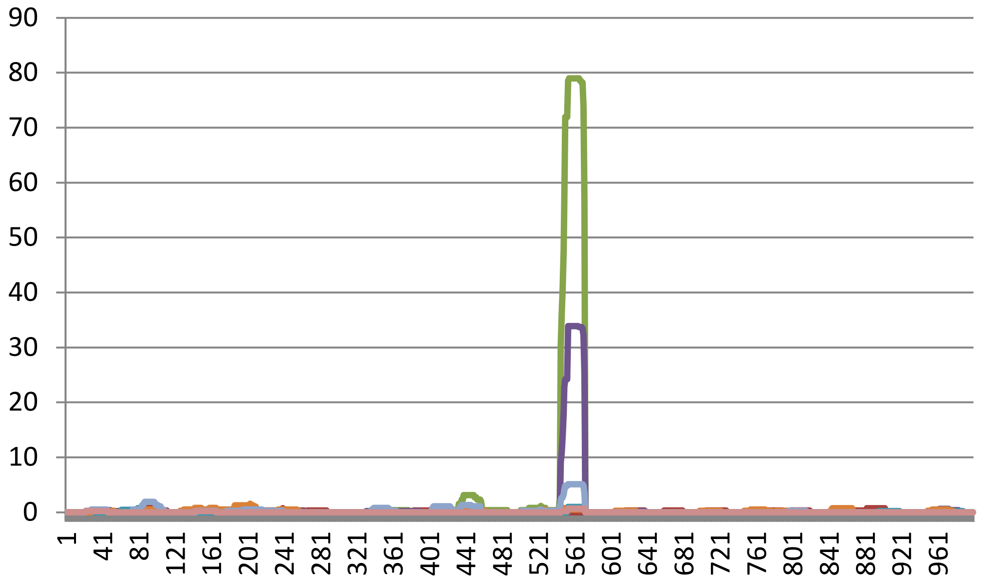

90
80
70
60
50
40
30
20
10
0
1
41
81
121
161
201
241
281
321
361
401
441
481
521
561
601
641
681
721
761
801
841
881
921
961

ATMG00900_AGO1 seedling

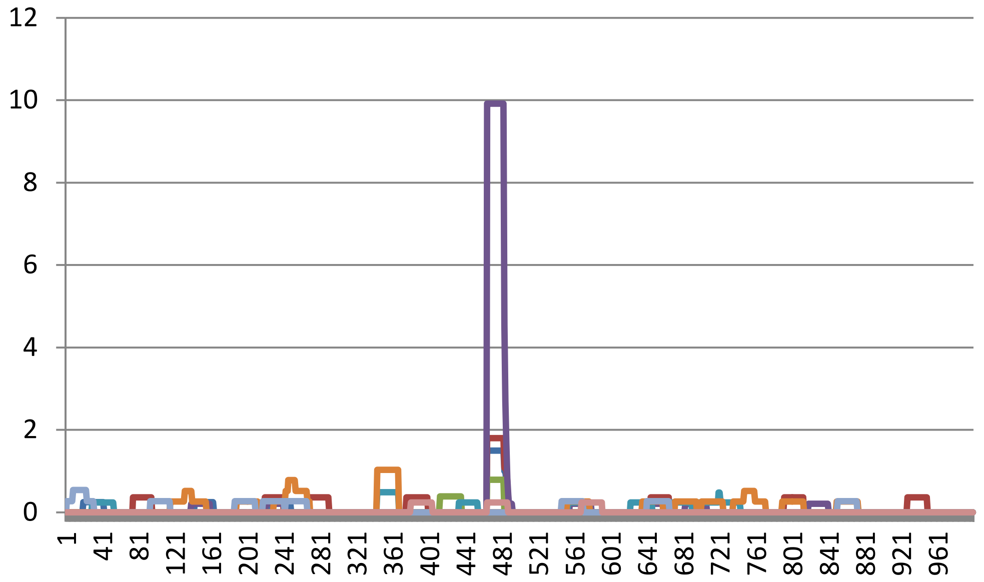

ATMG01320_AGO1

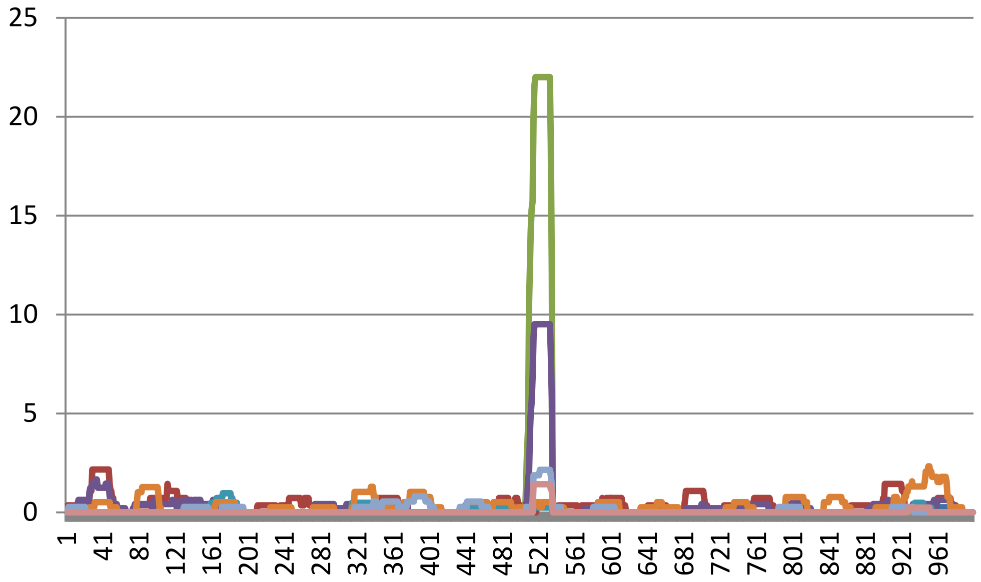

ATMG01360_AGO1

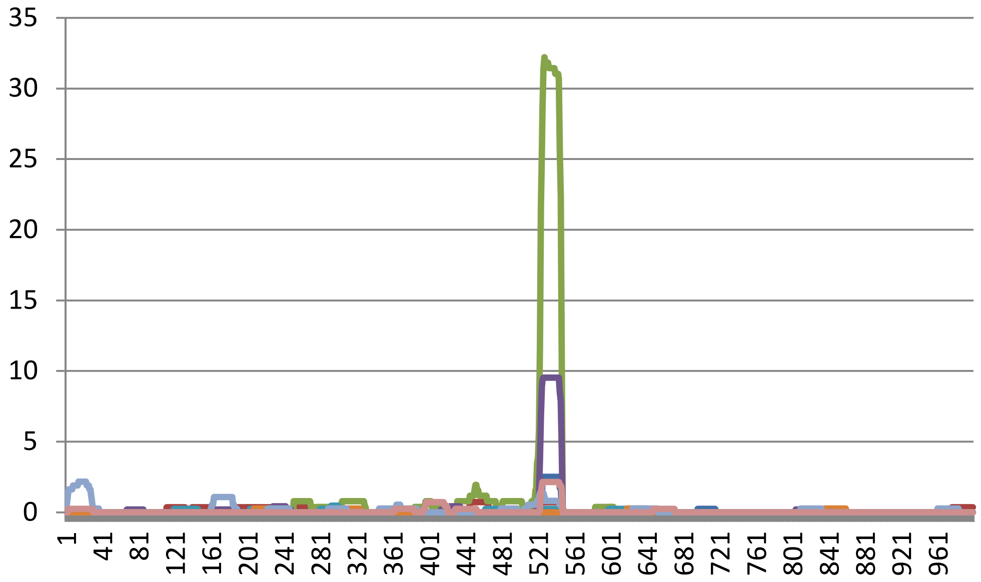

Supplement: S10 Fig — (PDF) [file pone.0169212.s010.pdf]
