# Supplementary material for: Transcriptome-Wide Discovery of PASRs (Promoter-Associated Small RNAs) and TASRs (Terminus-Associated Small RNAs) in Arabidopsis thaliana
Source: PLoS One. 2017 Jan 3;12(1):e0169212. doi: 10.1371/journal.pone.0169212 (PMC5207706; doi:10.1371/journal.pone.0169212)

Figure S11 AGO-associated TASR peaks identified on the antisense strands of the protein-coding genes of *Arabidopsis*. For each plot, x axis measures the position of the antisense strand, and y axis measures the abundance (in RPM, reads per million) of sRNAs.

AT1G01880RC

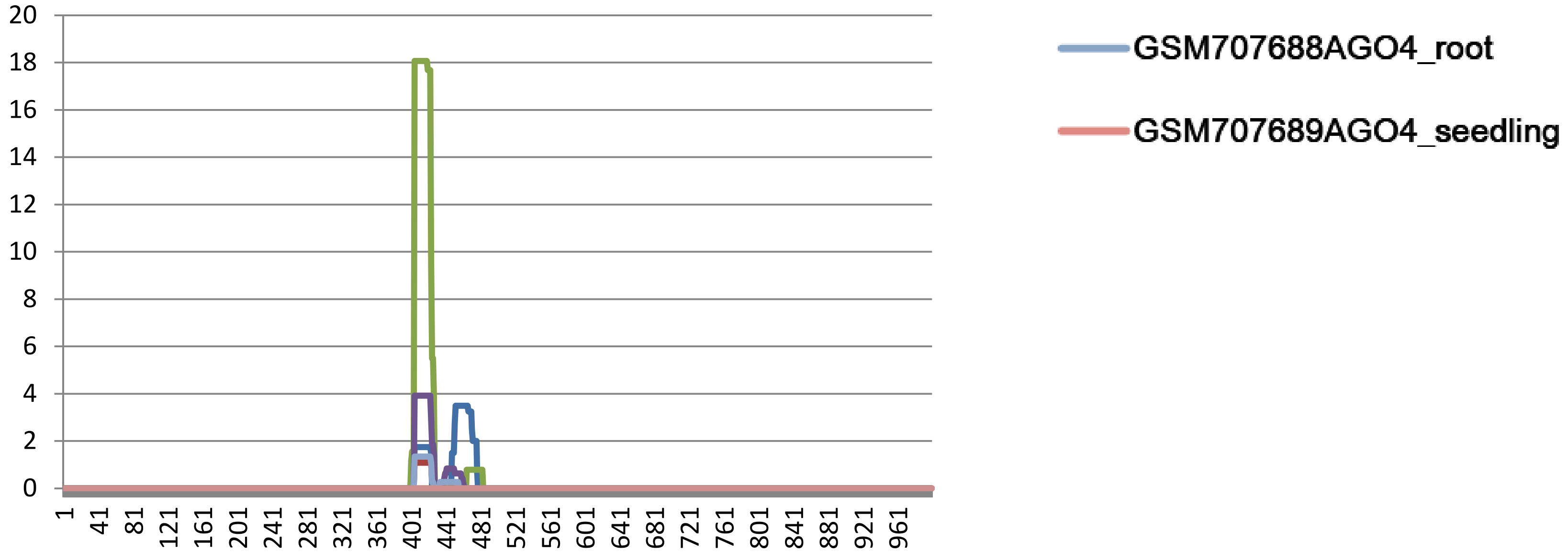

AT1G10095RC

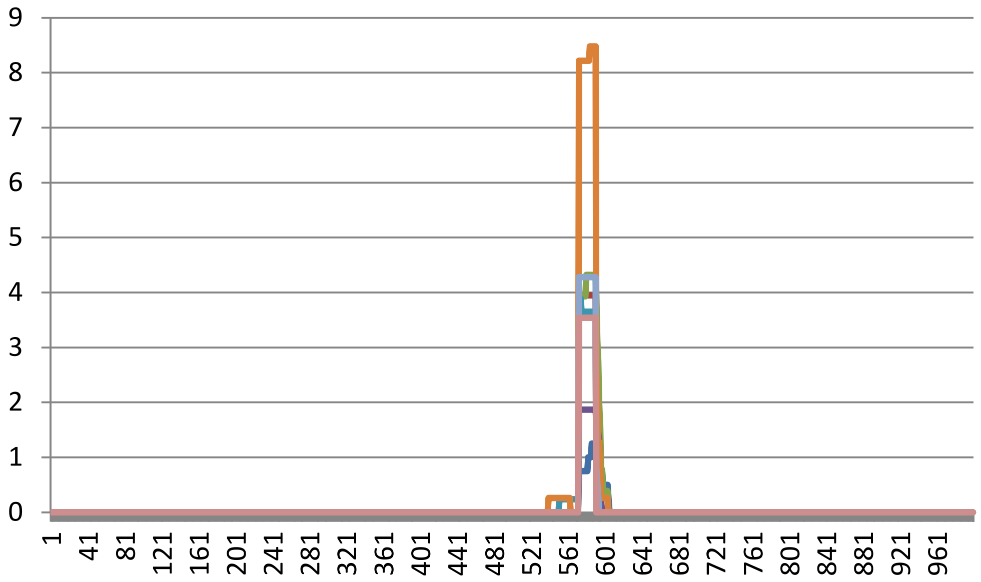

AT1G13860RC

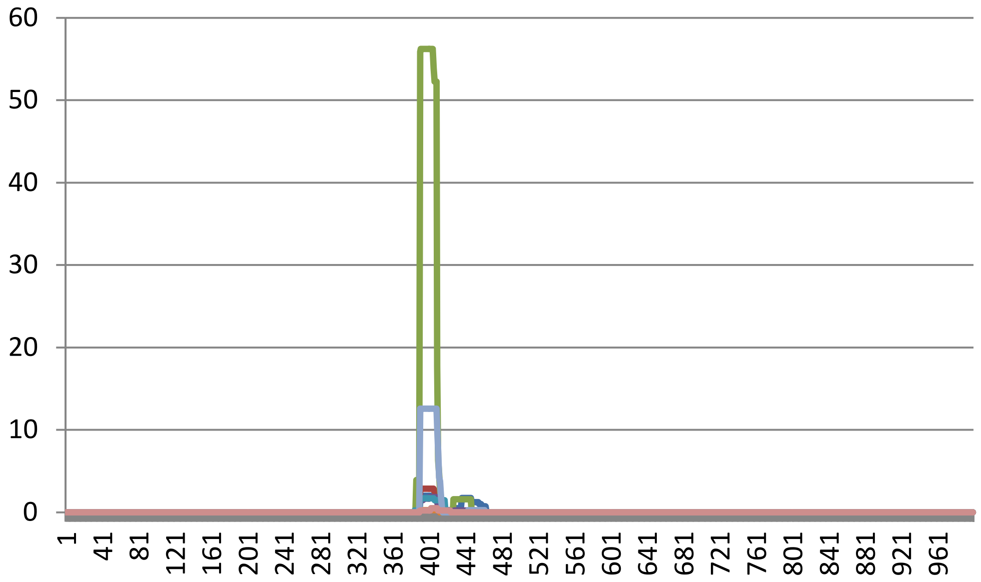

AT1G14890RC

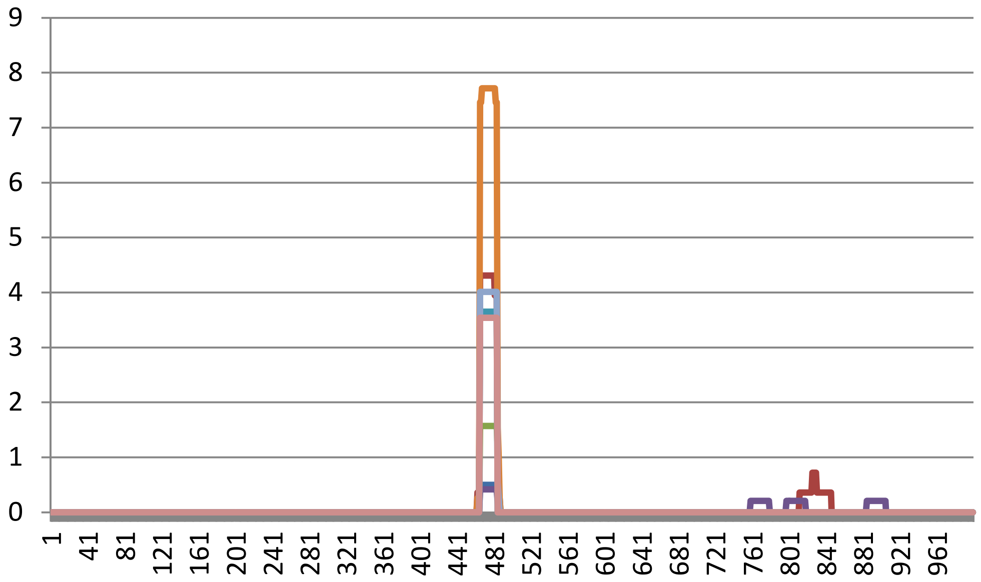

AT1G15080RC

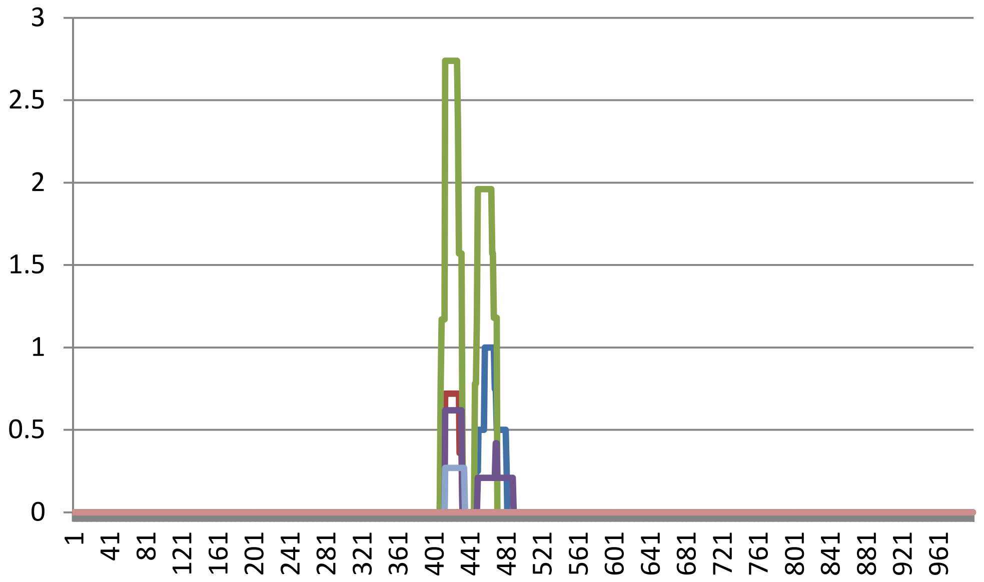

AT1G15125RC

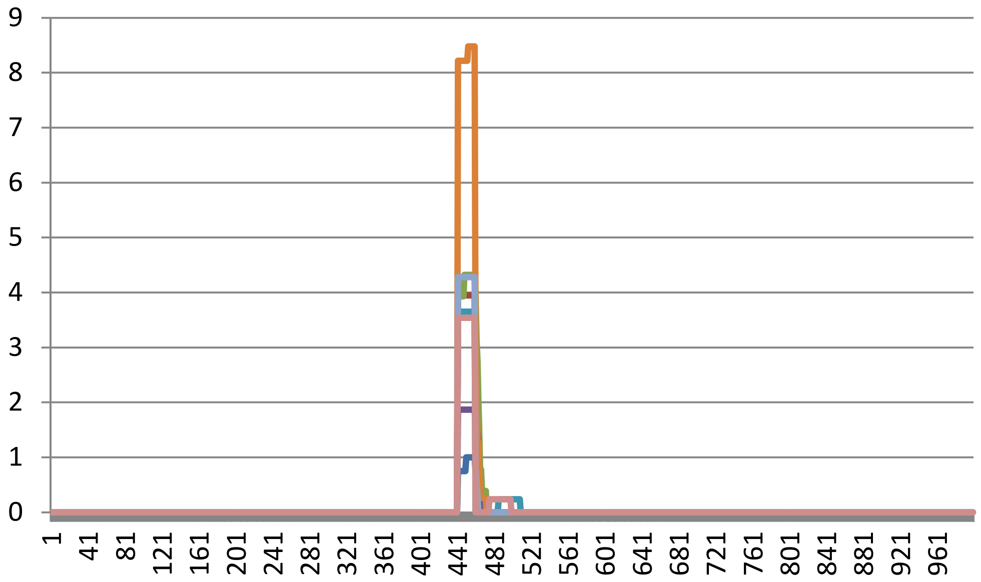

AT1G15530RC

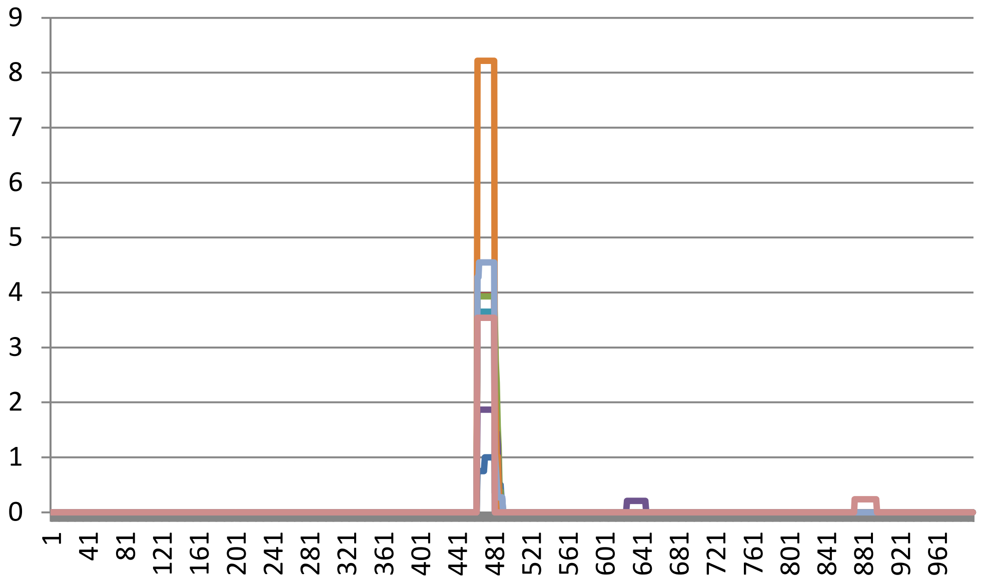

AT1G20290RC

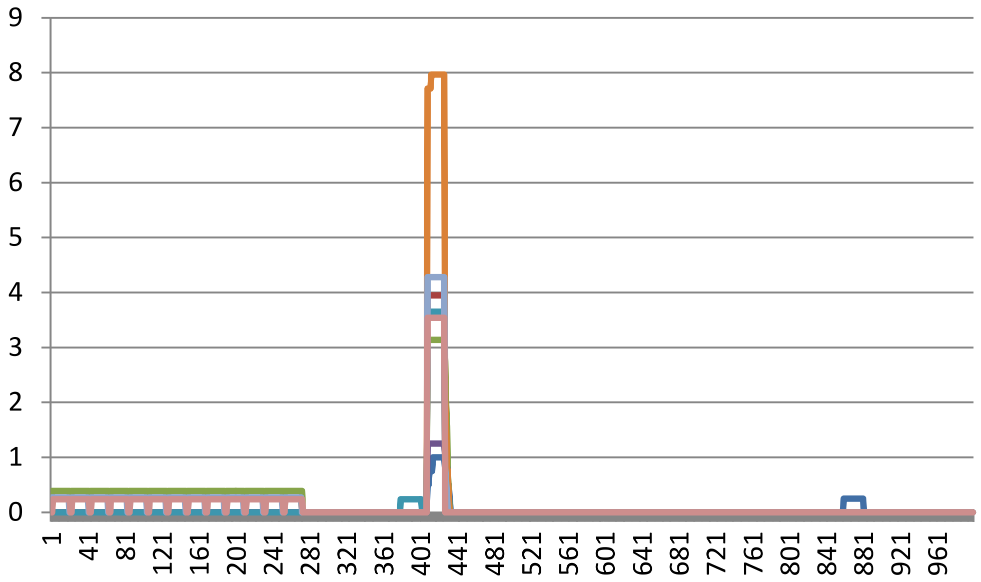

AT1G20840RC

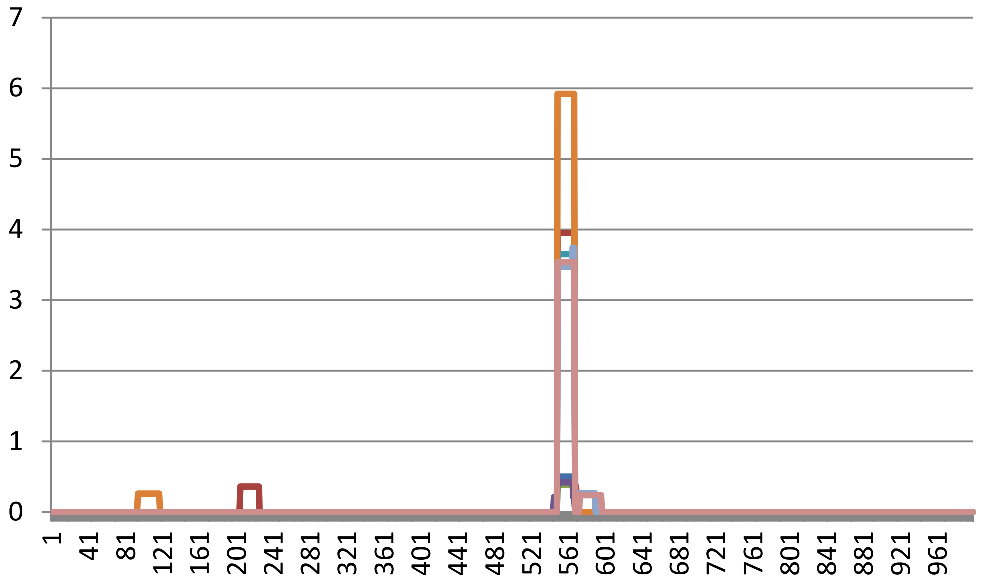

AT1G22360RC

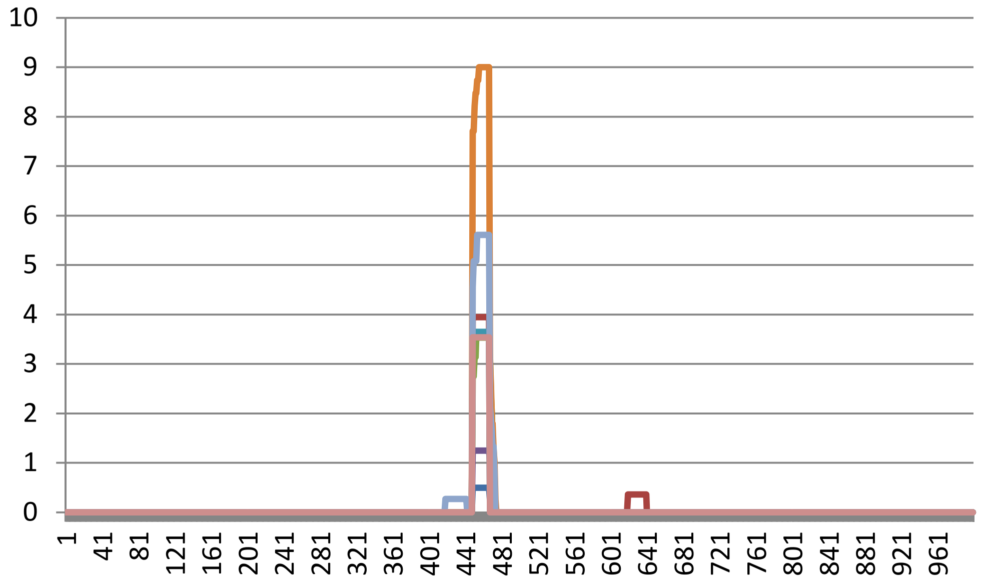

AT1G25375RC

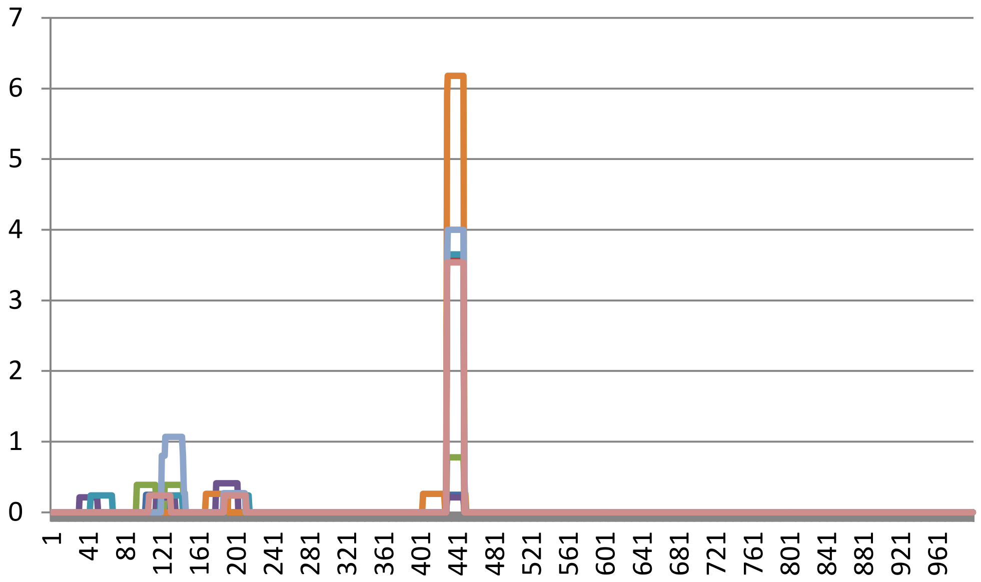

AT1G29660RC

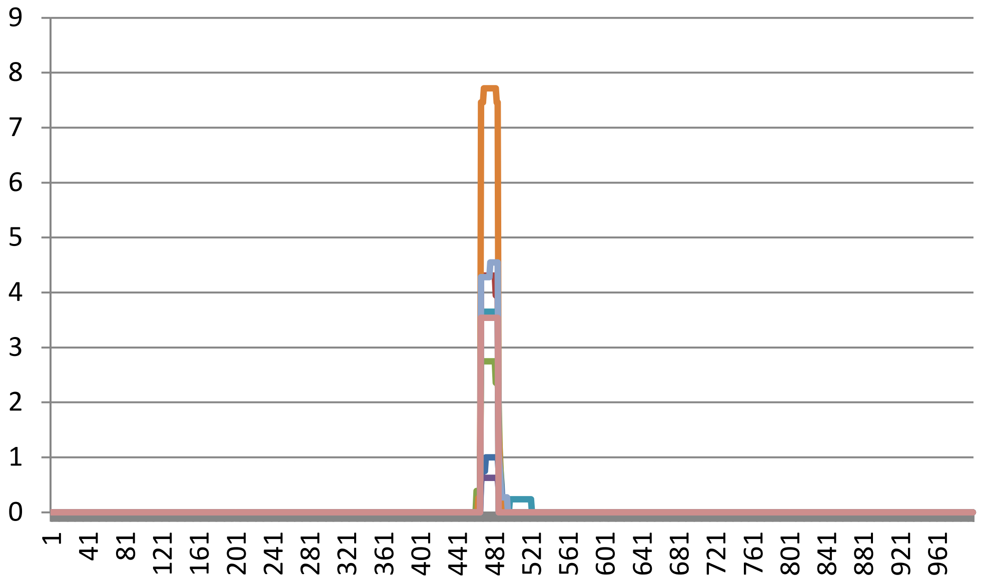

AT1G33680RC

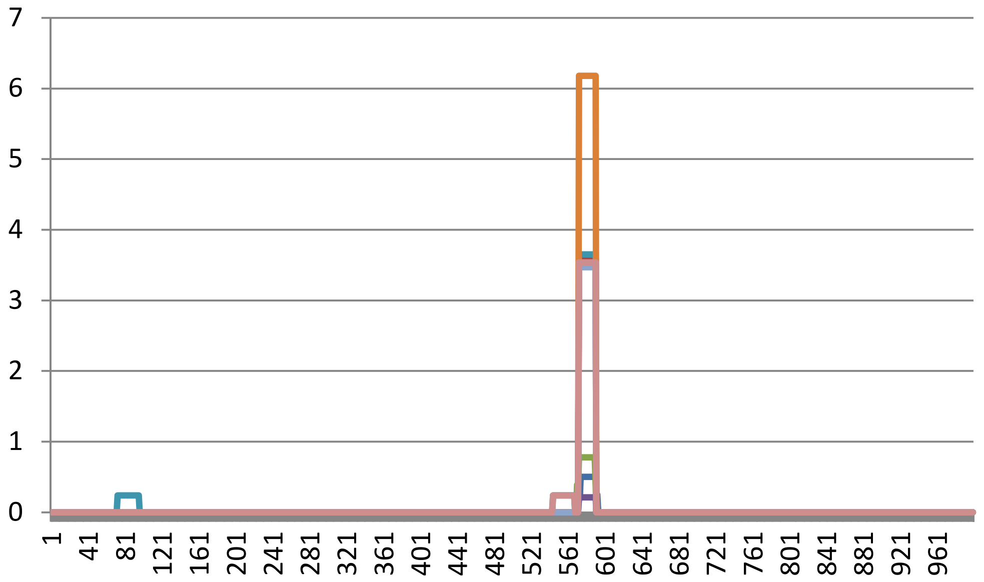

AT1G36310RC

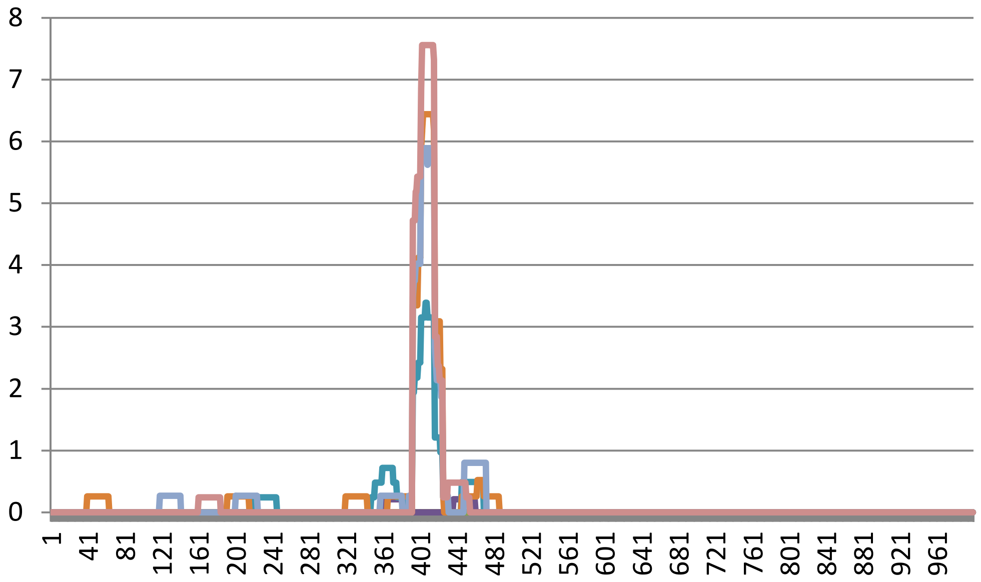

AT1G48990RC

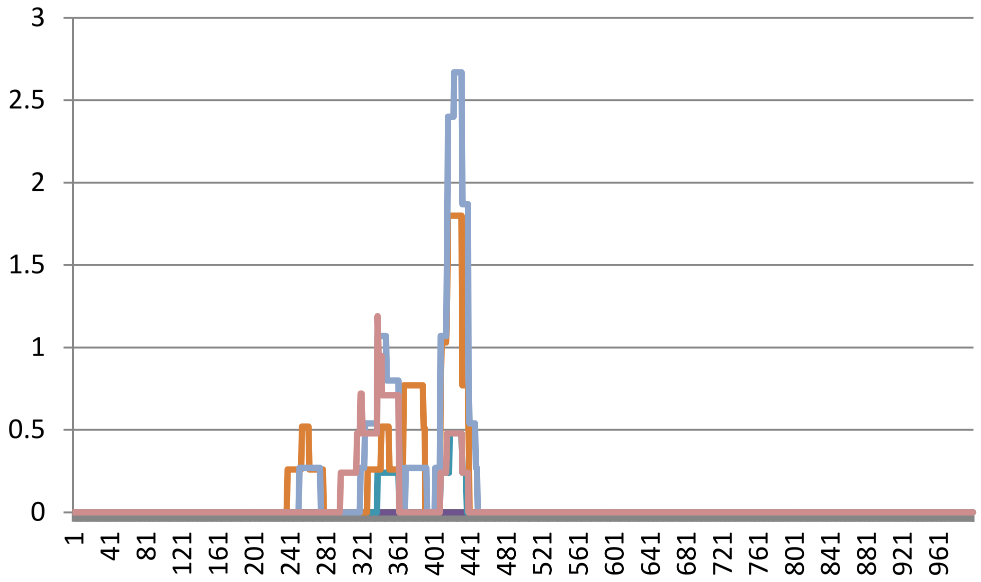

AT1G50110RC_AGO1 root

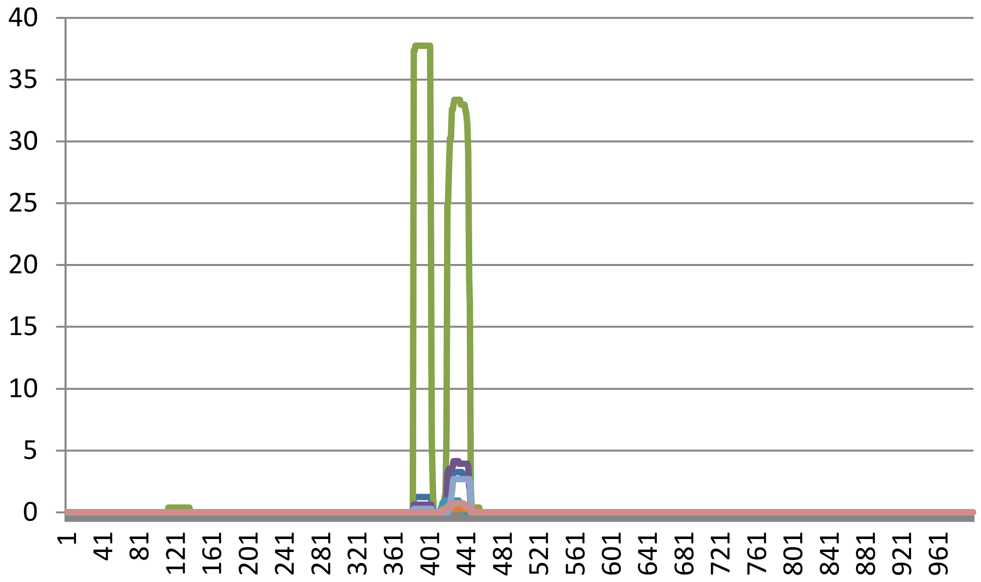

AT1G50970RC

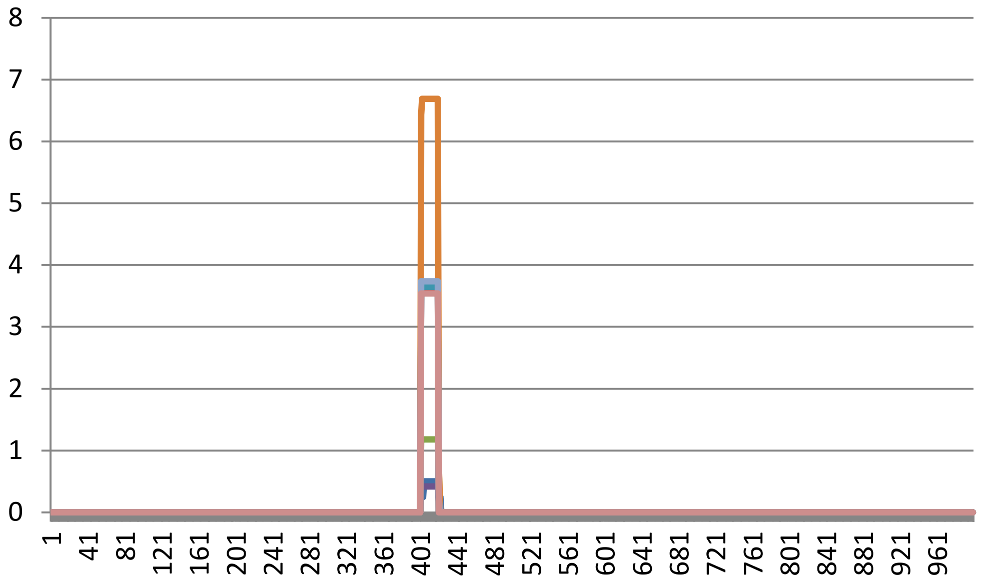

AT1G52160RC

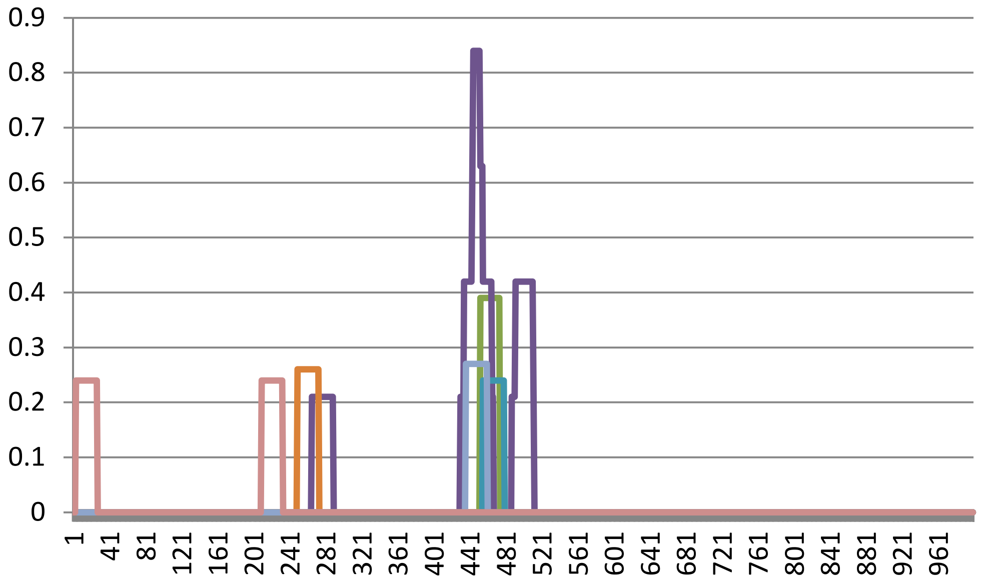

AT1G58025RC

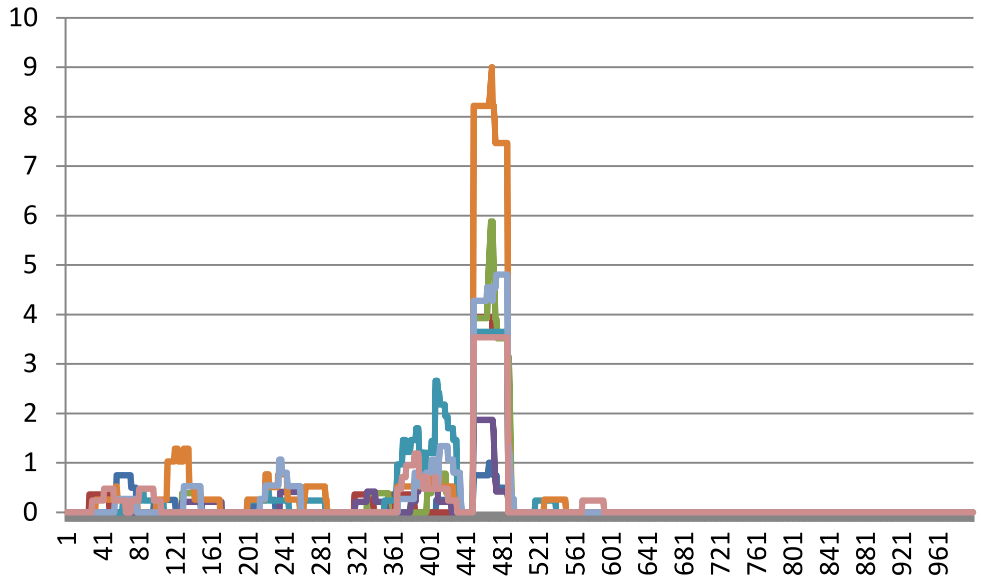

AT1G59835RC

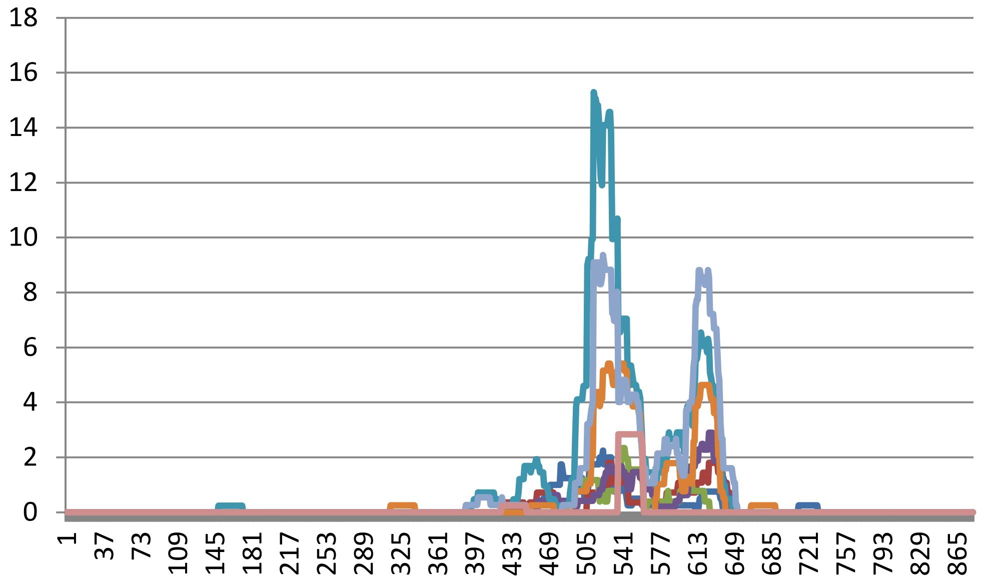

AT1G63522RC

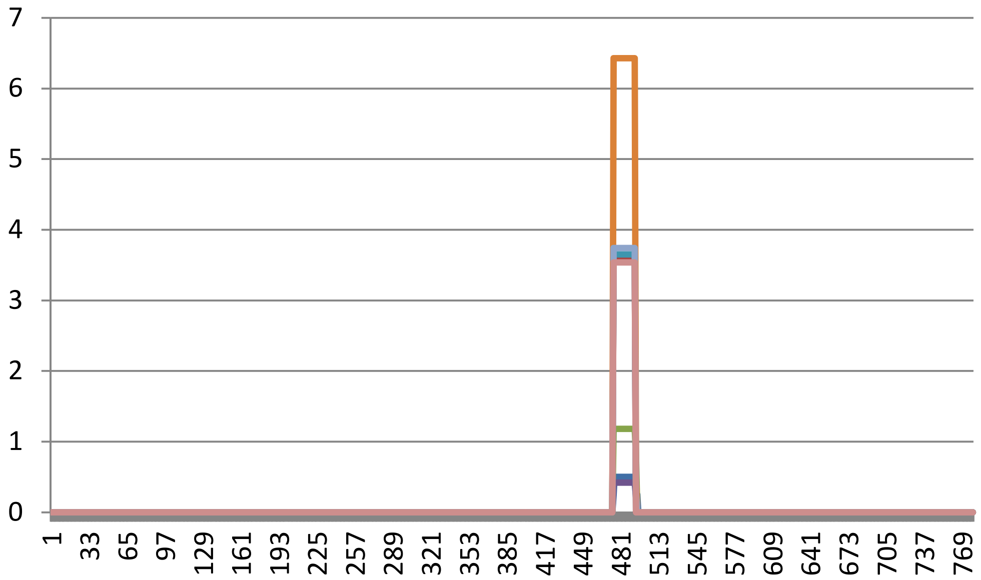

AT1G65210RC

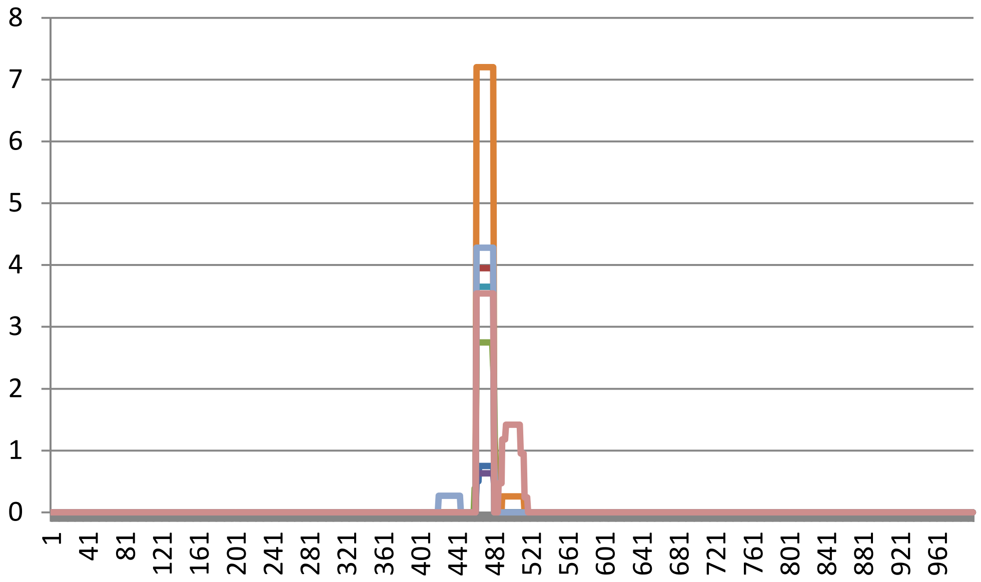

AT1G66540RC

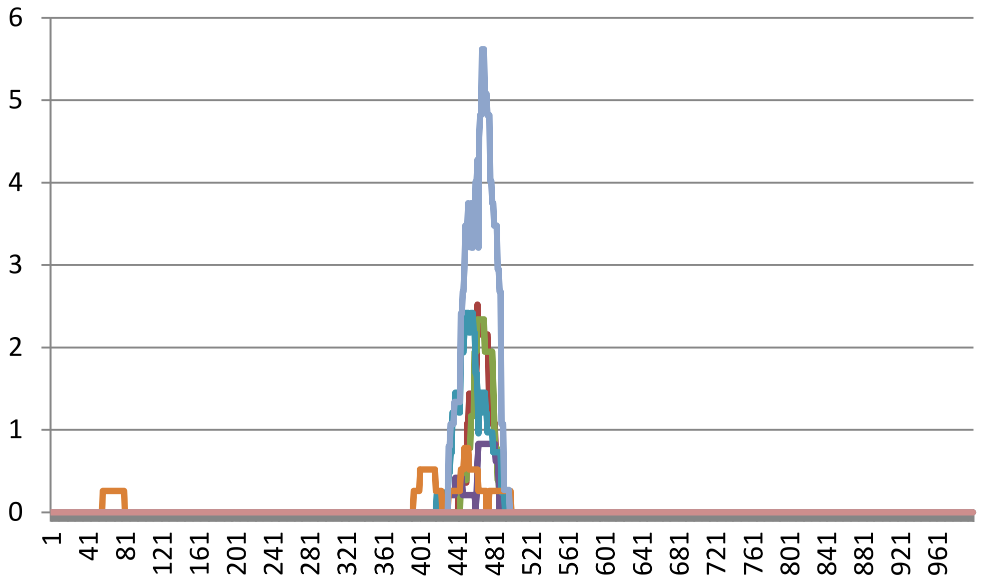

AT1G66570RC

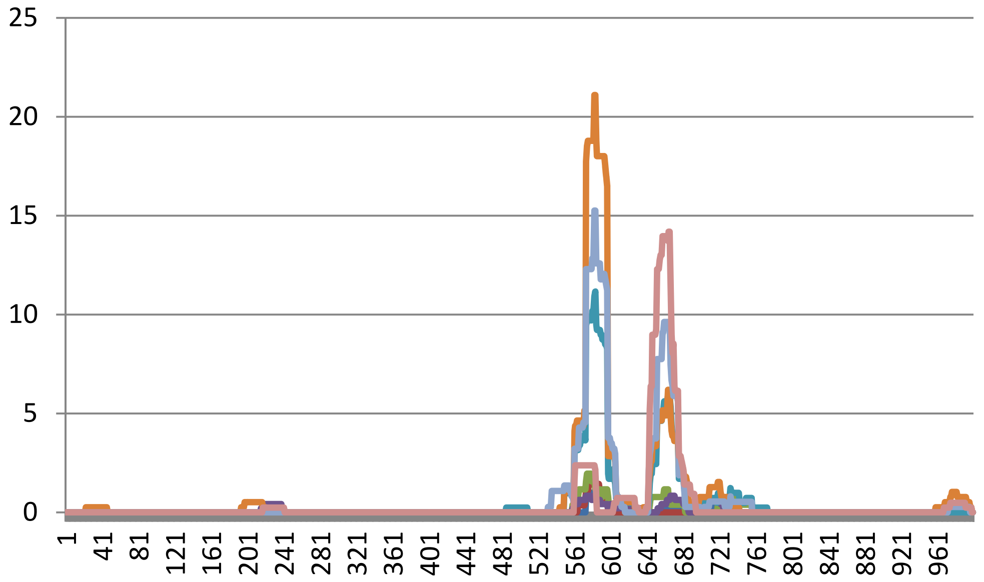

AT1G70040RC

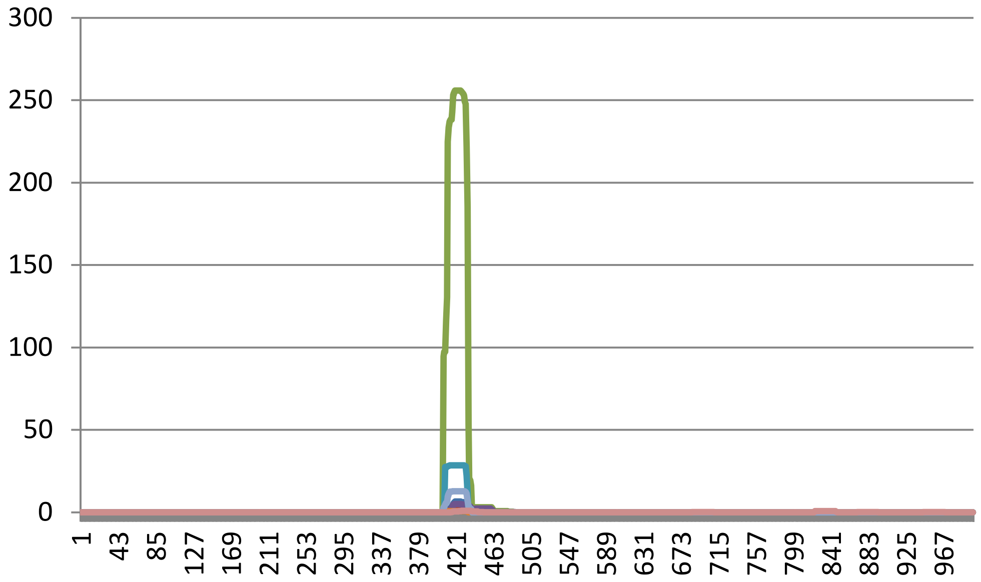

AT1G73360RC

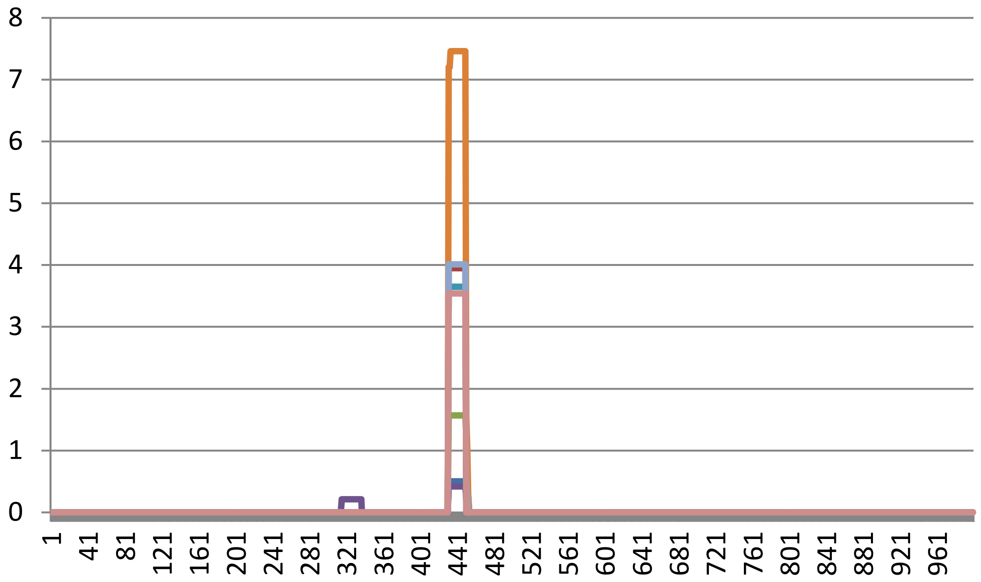

AT1G73650RC

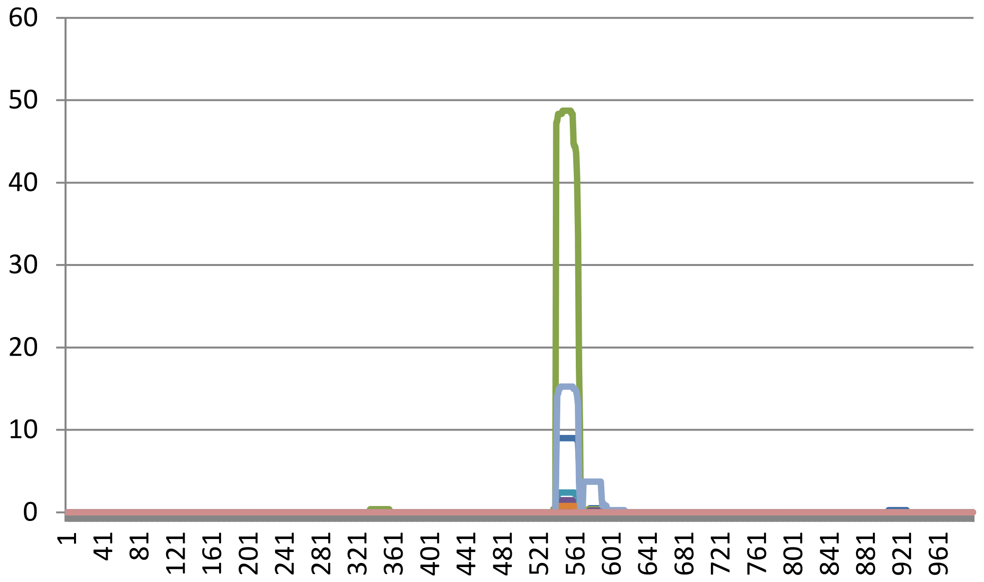

AT1G77450RC

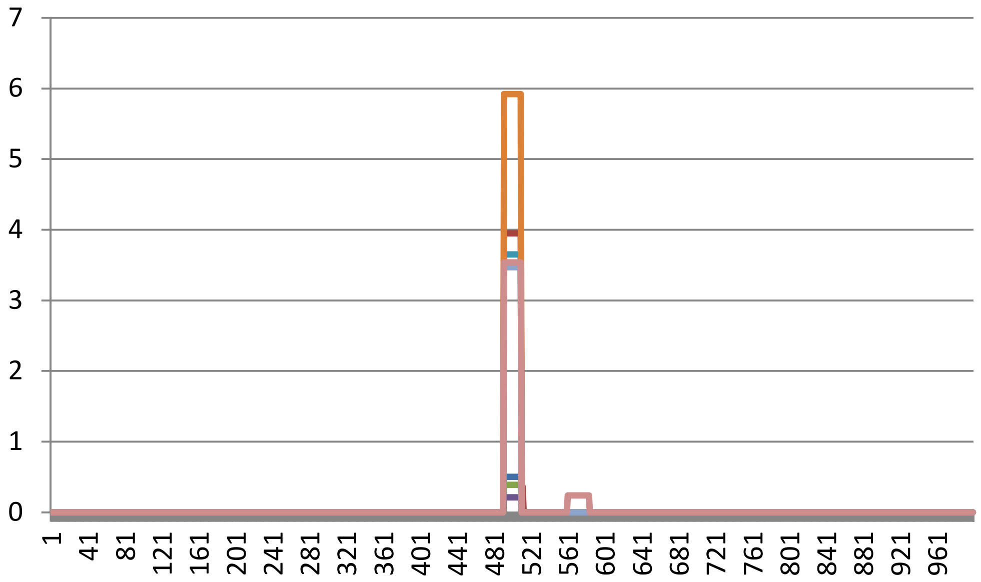

AT2G02400RC

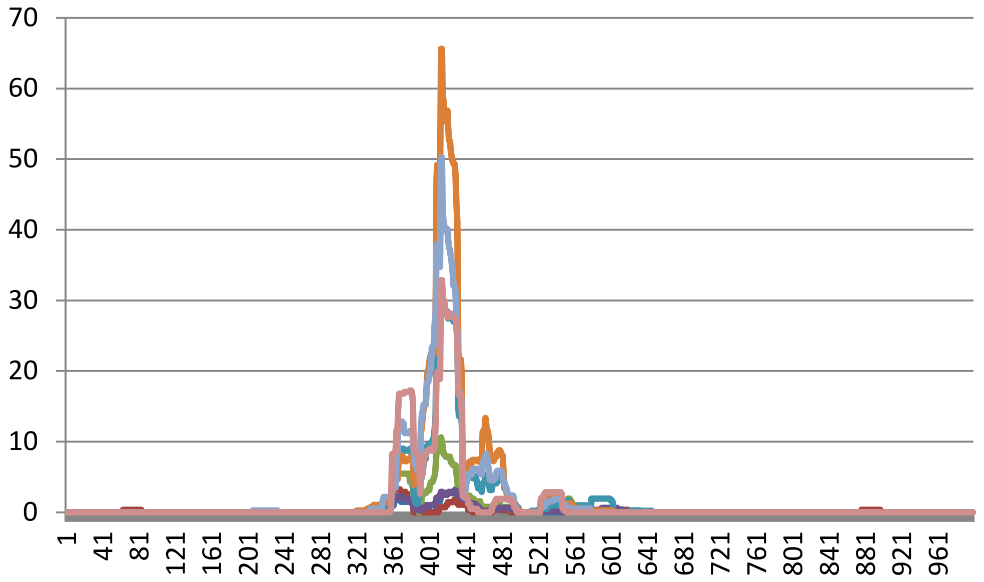

AT2G02520RC_AGO4 seedling

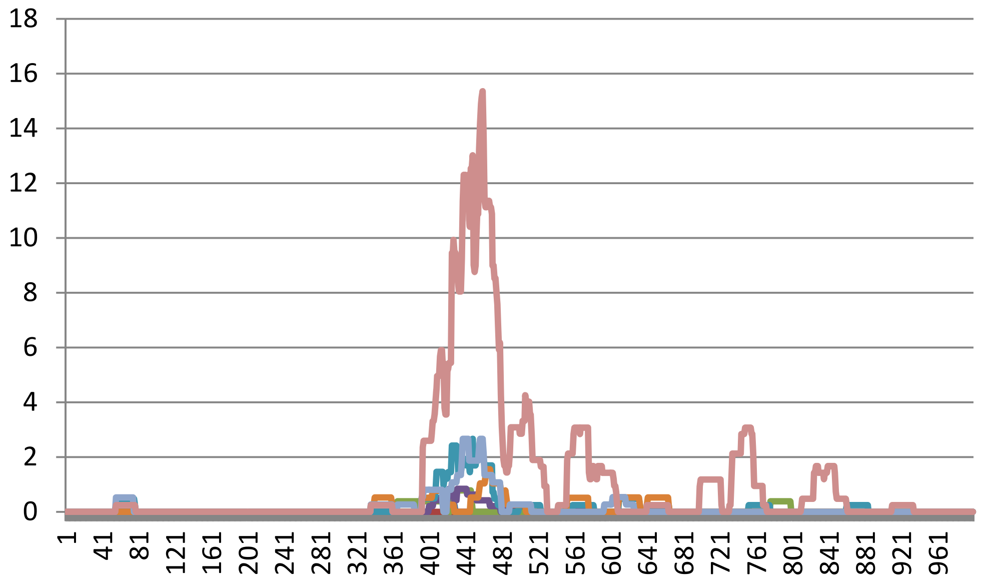

AT2G03370RC

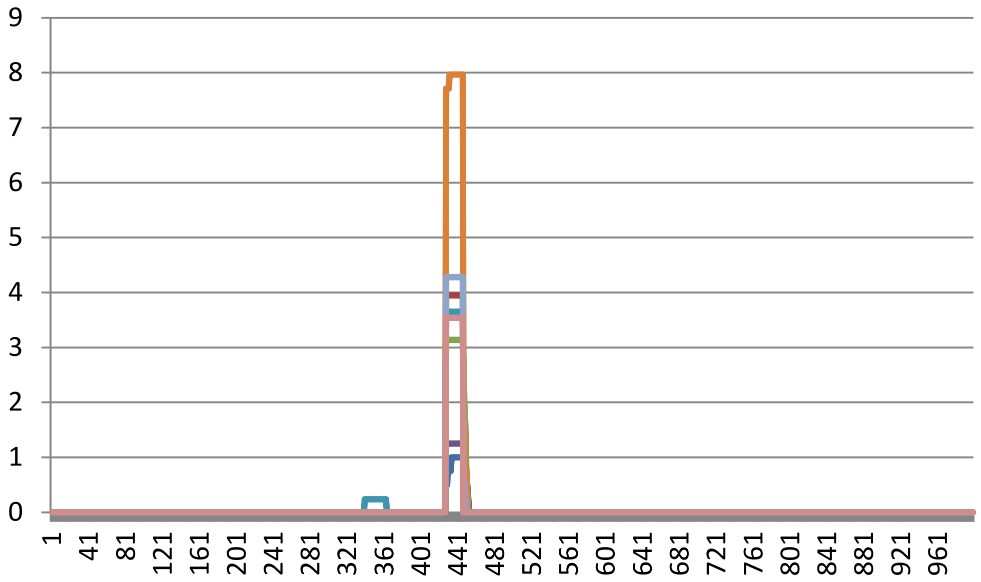

AT2G04090RC

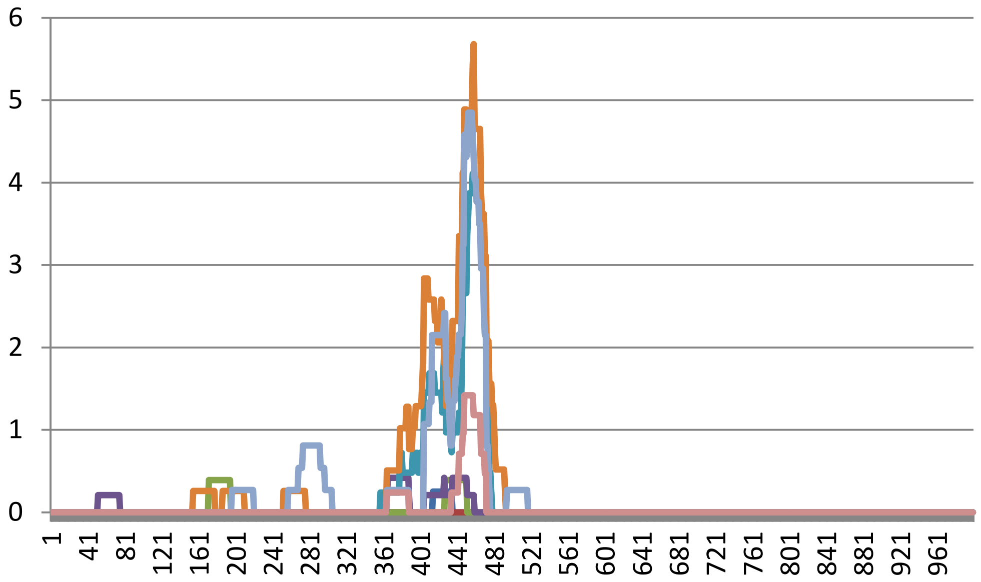

AT2G04115RC

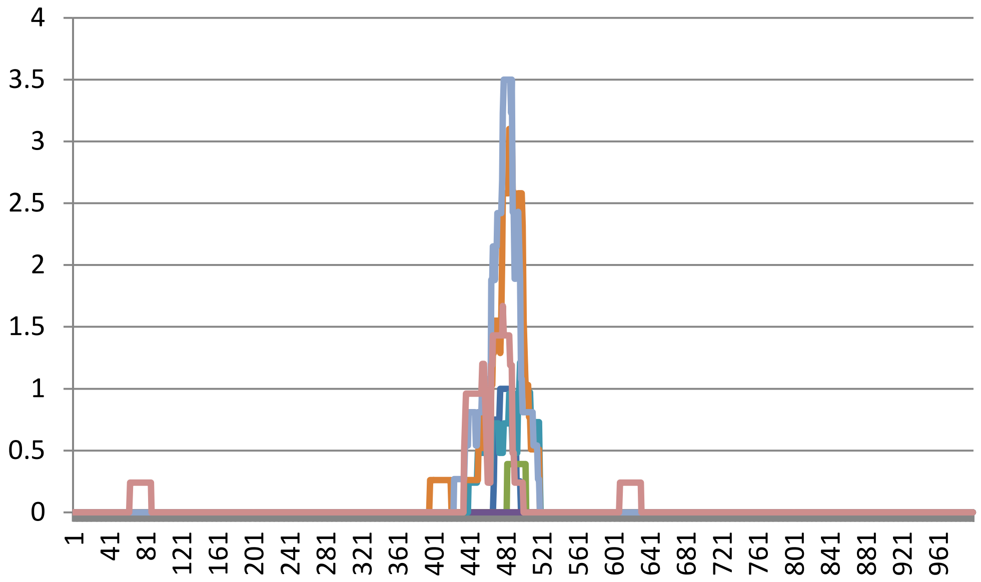

AT2G04830RC

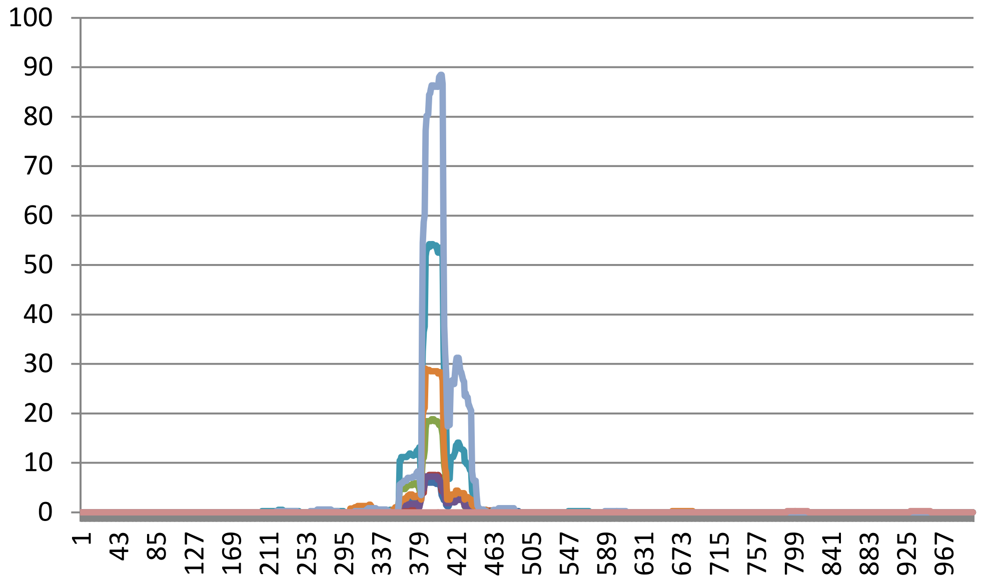

AT2G07360RC

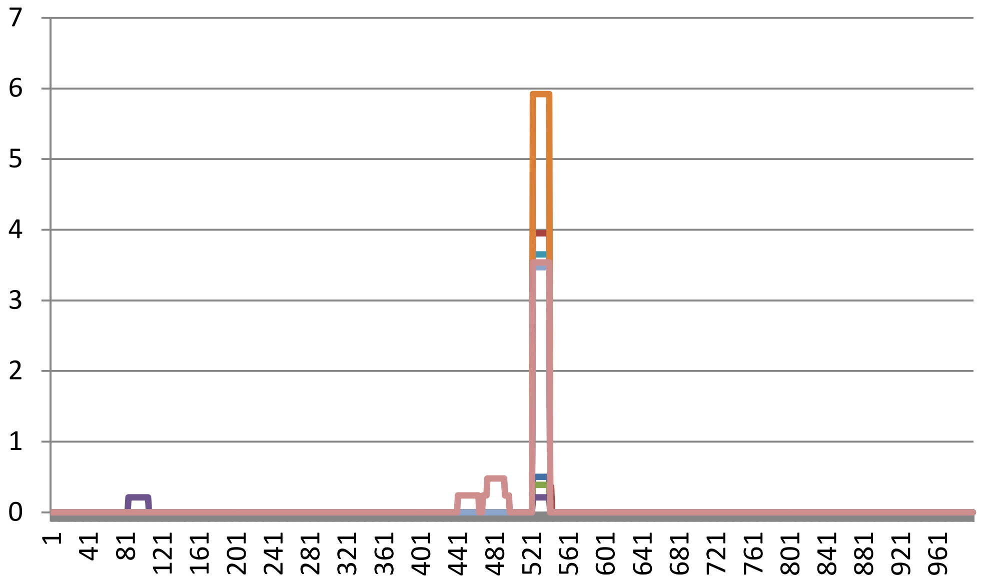

AT2G15535RC

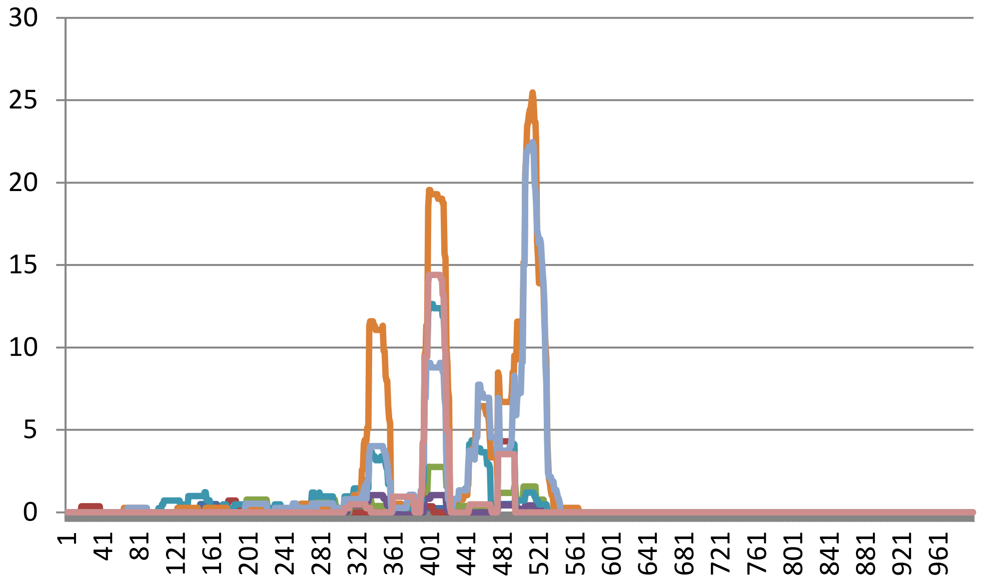

AT2G16870RC

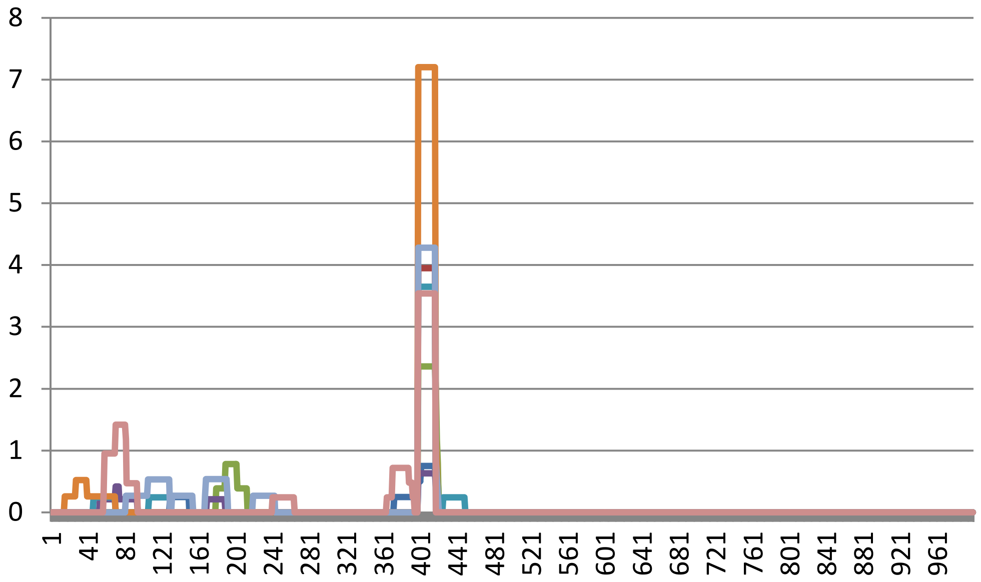

AT2G19360RC

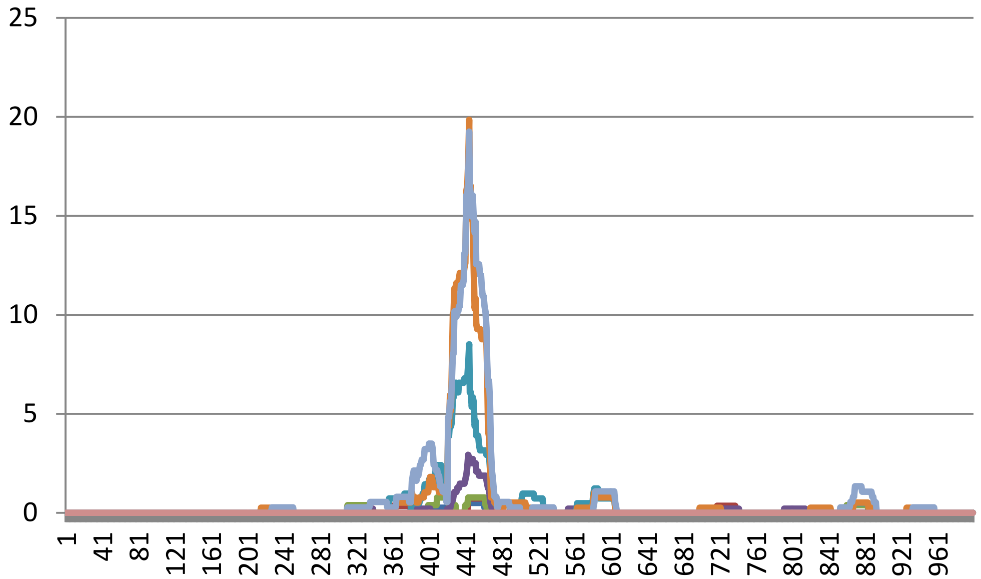

AT2G20580RC

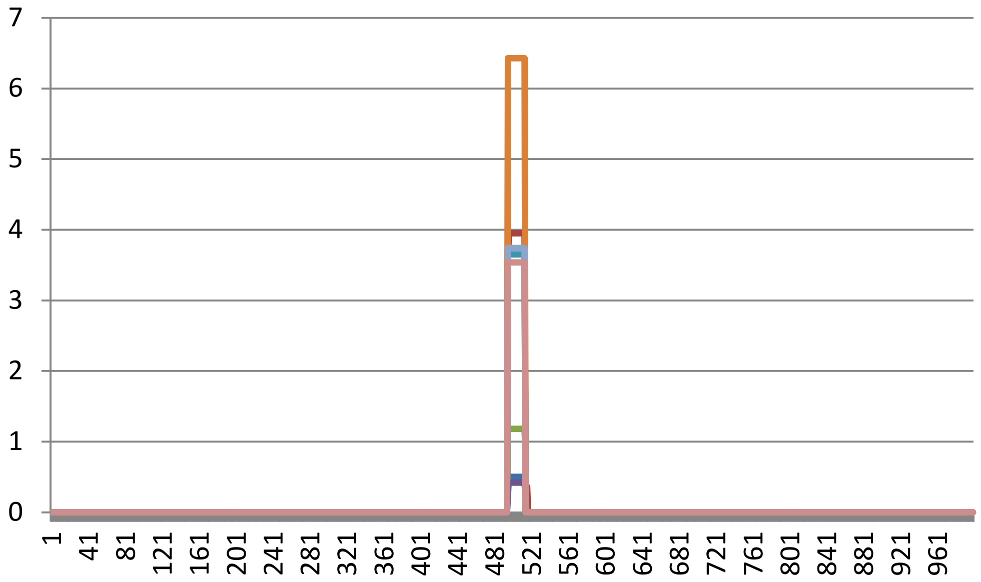

AT2G21370RC

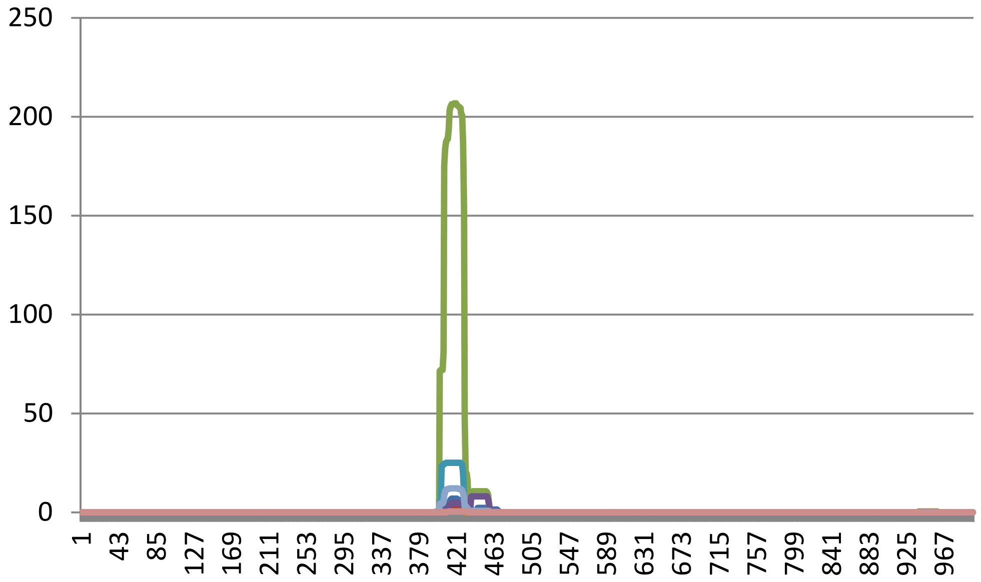

AT2G23770RC

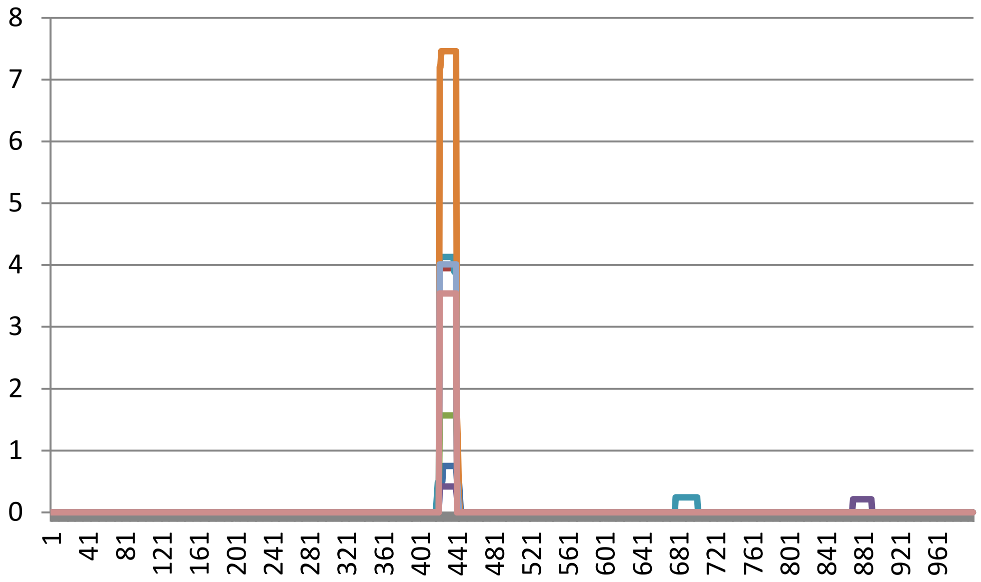

AT2G32310RC

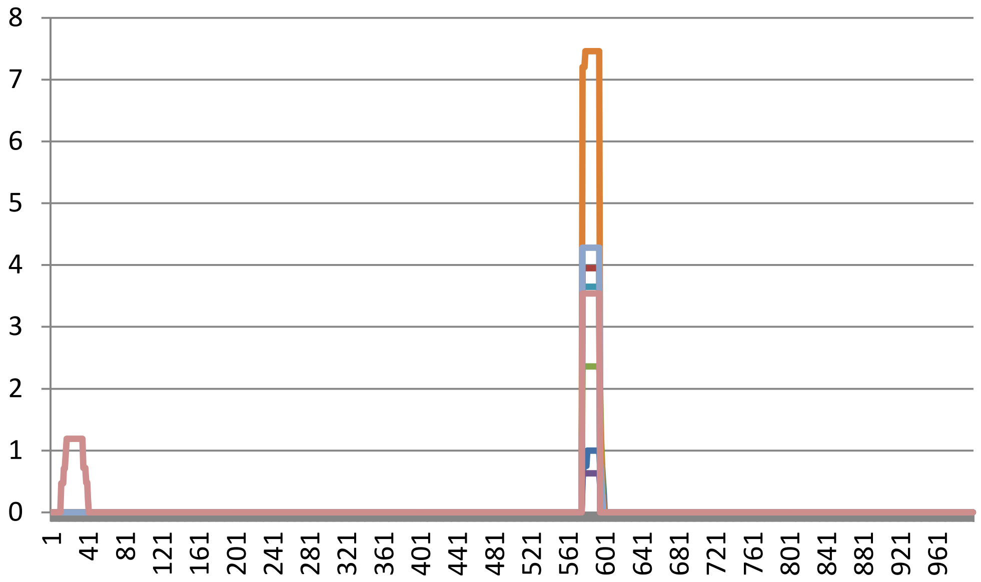

AT2G32785RC

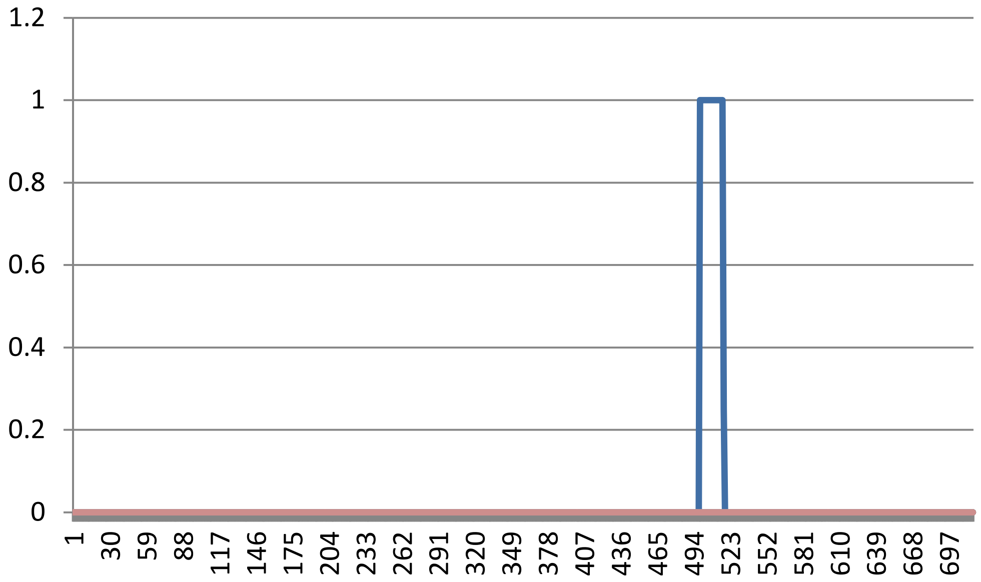

AT2G32790RC

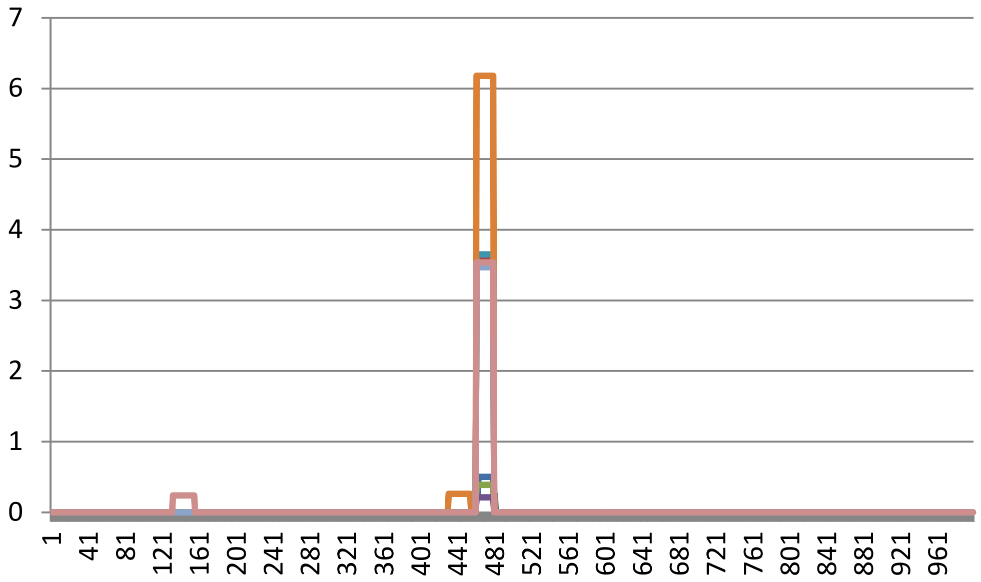

AT2G35250RC

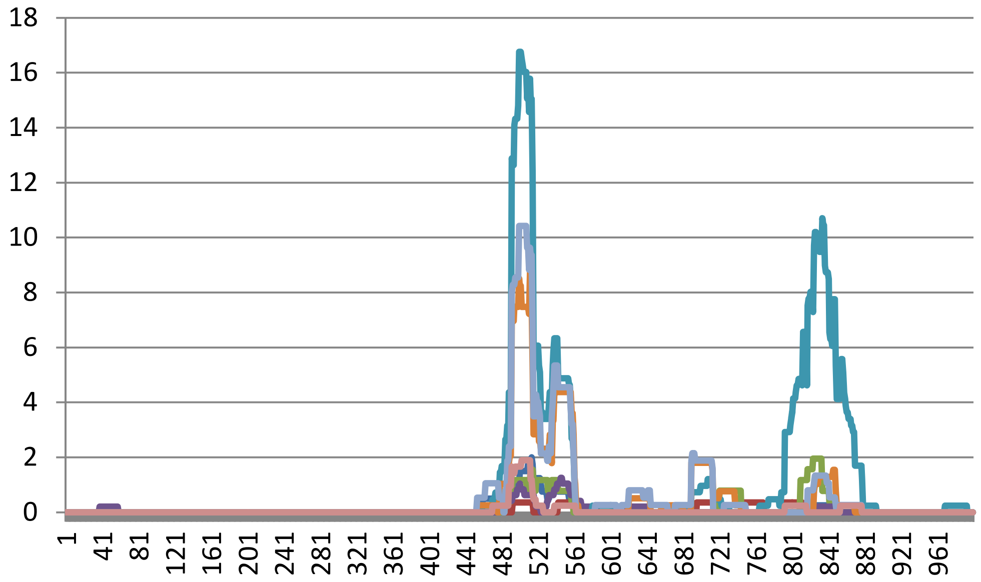

AT2G37780RC

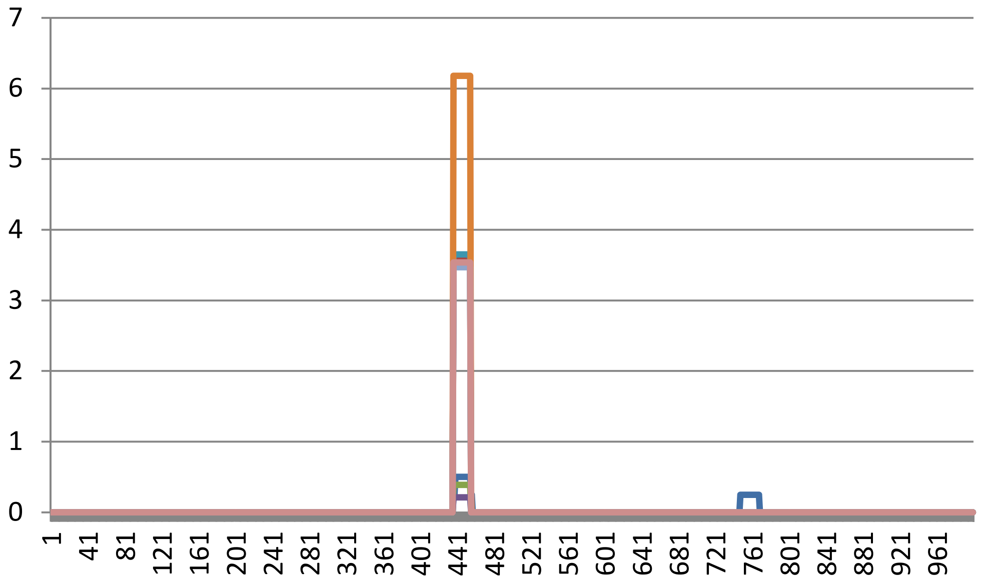

AT2G40920RC

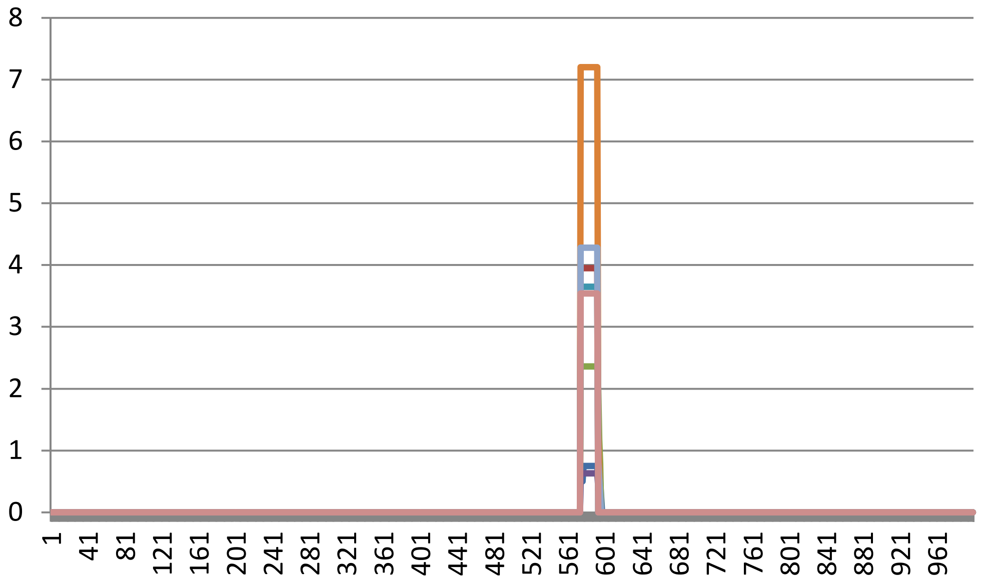

AT2G43580RC

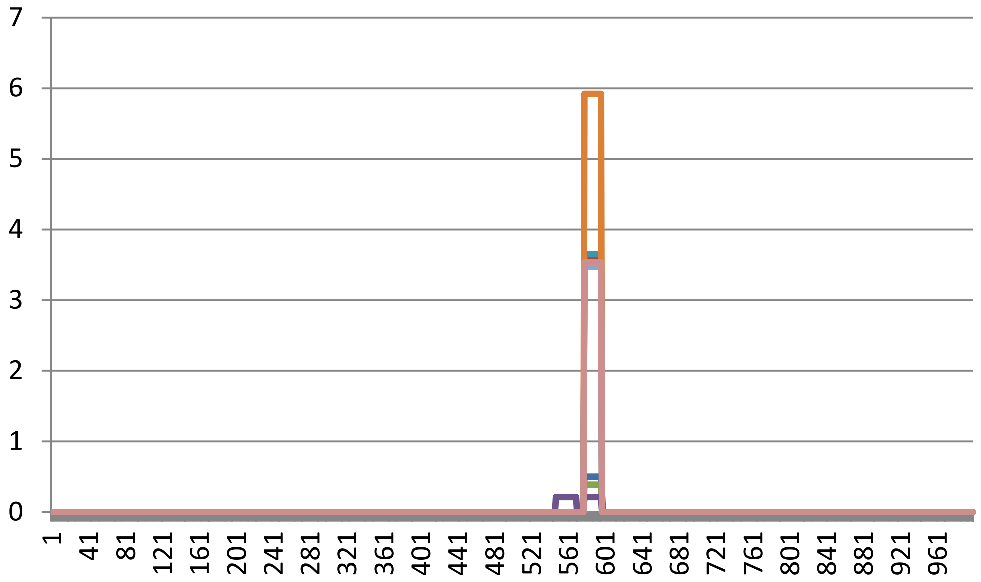

AT2G46460RC

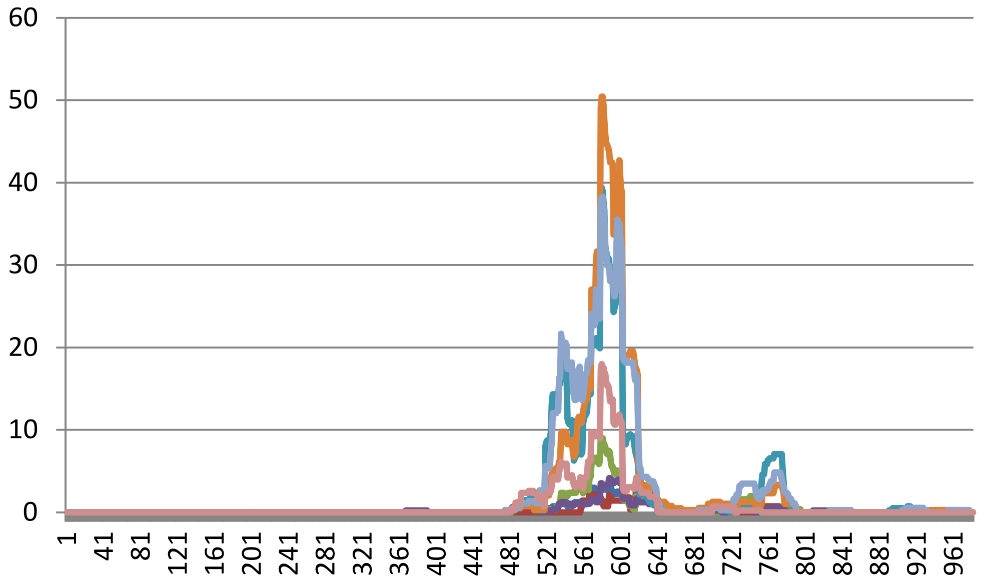

AT2G47660RC

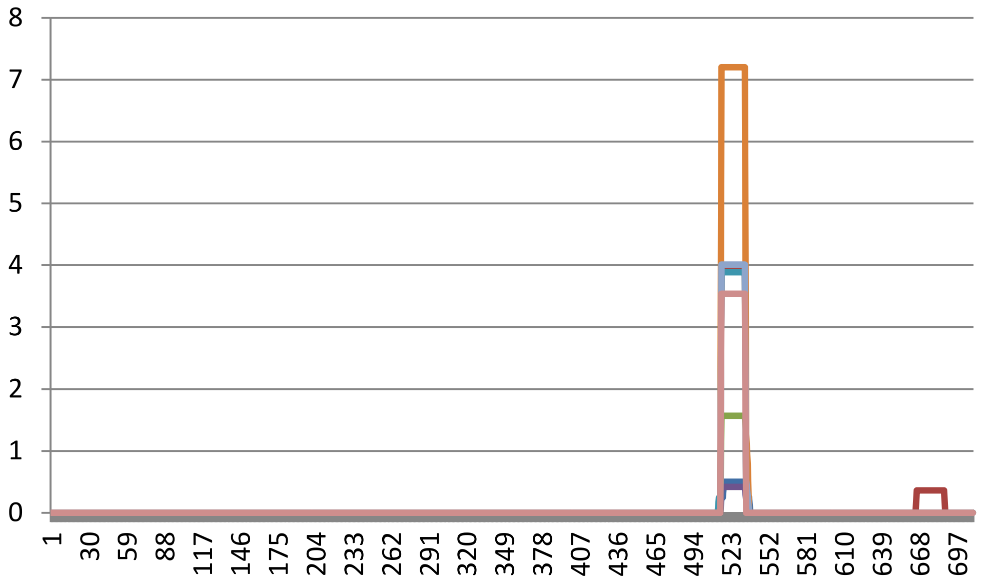

AT2G47750RC

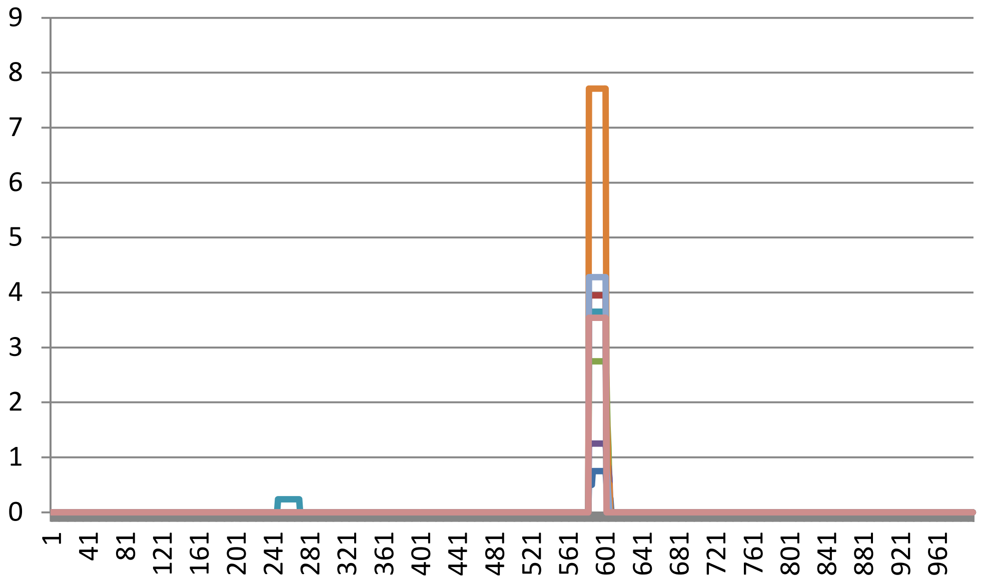

AT3G01710RC

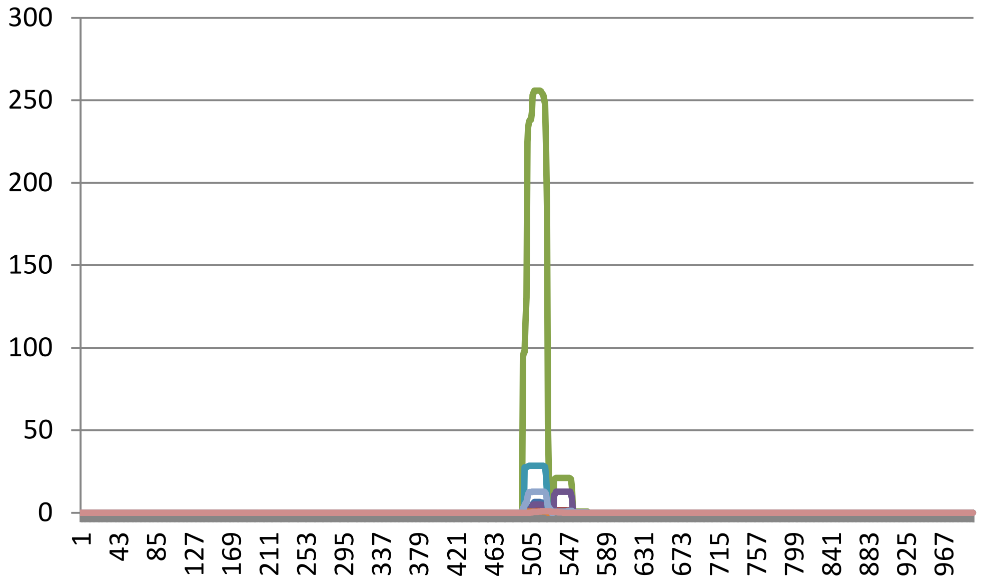

AT3G02350RC

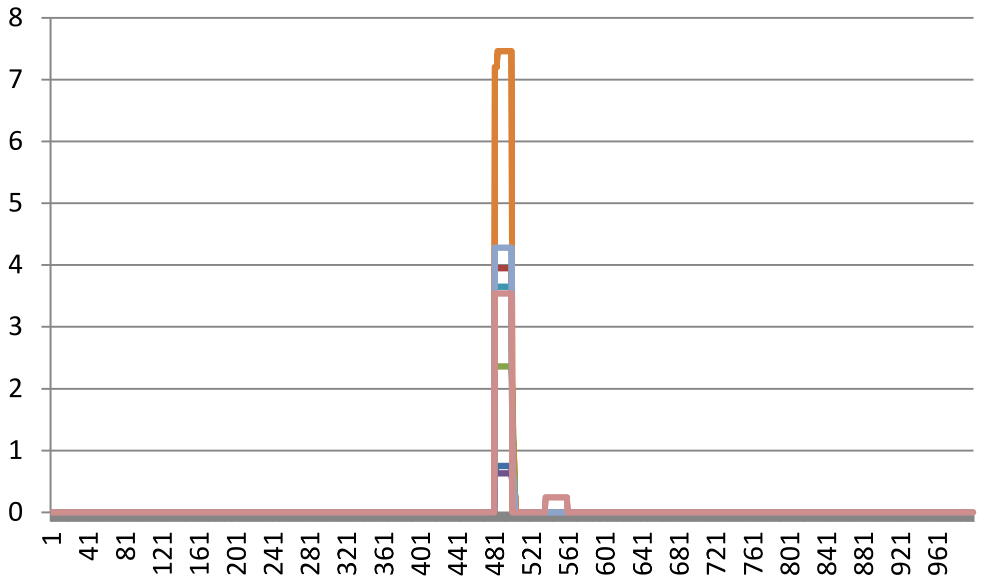

AT3G05520RC

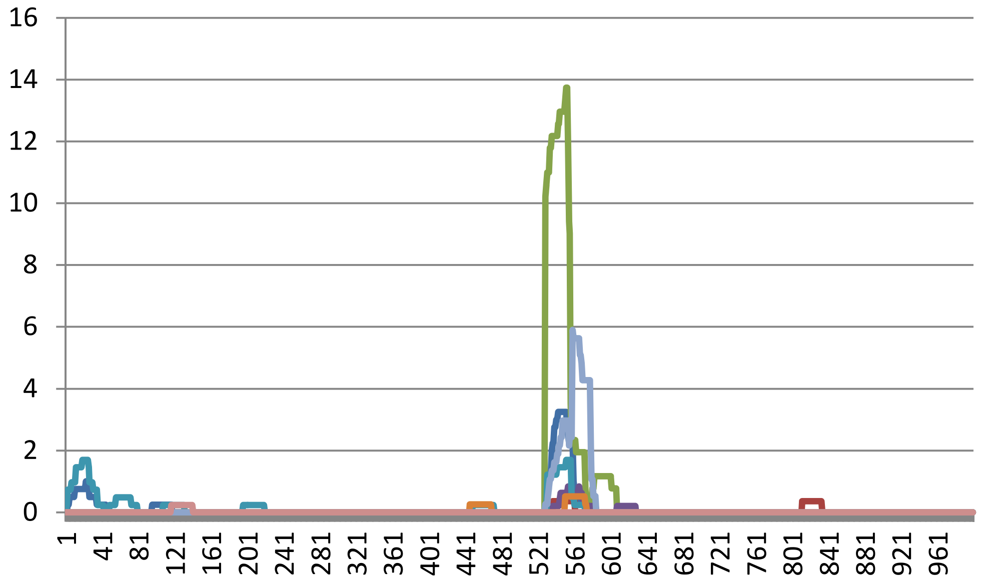

AT3G06660RC

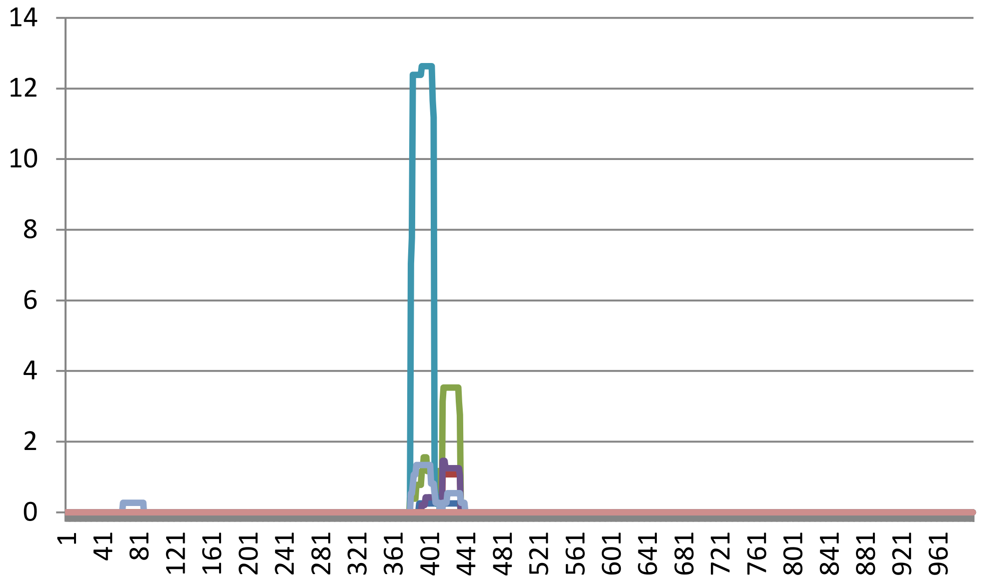

AT3G07050RC

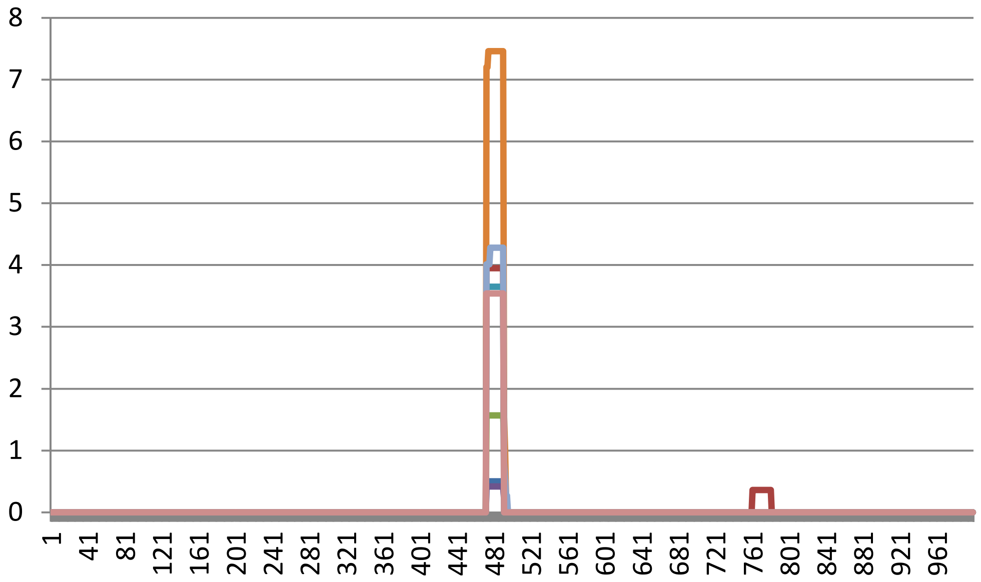

AT3G09510RC

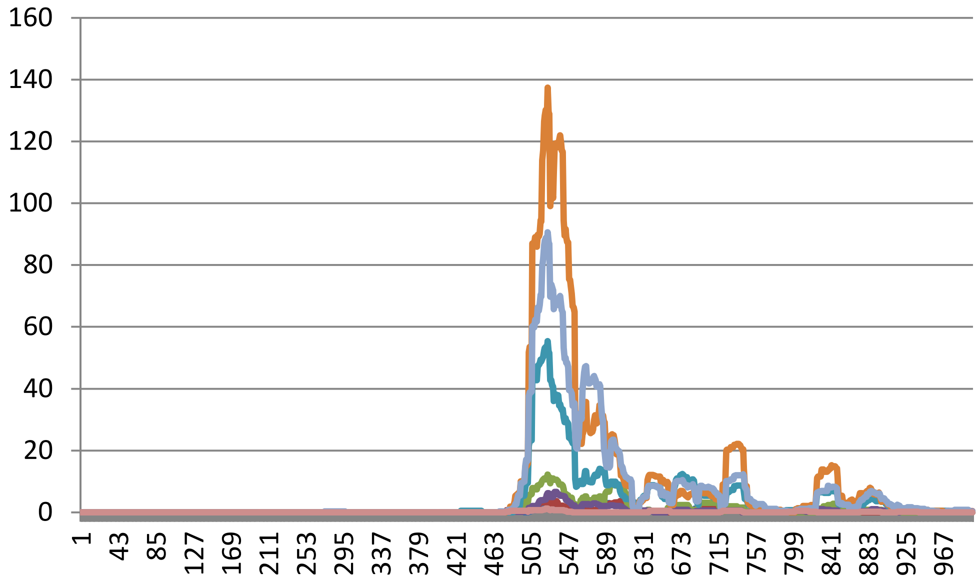

AT3G10090RC

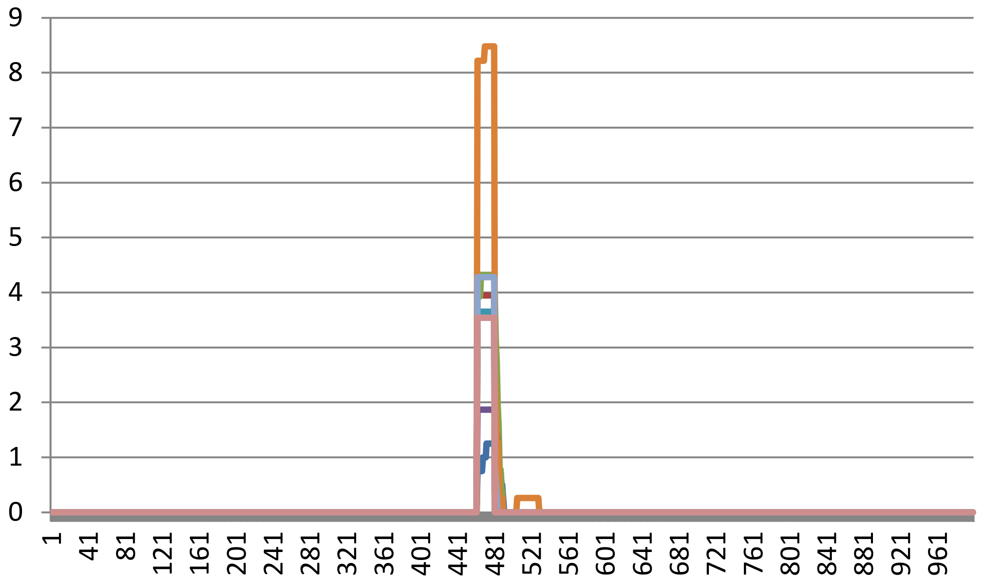

AT3G17890RC_AGO1 root

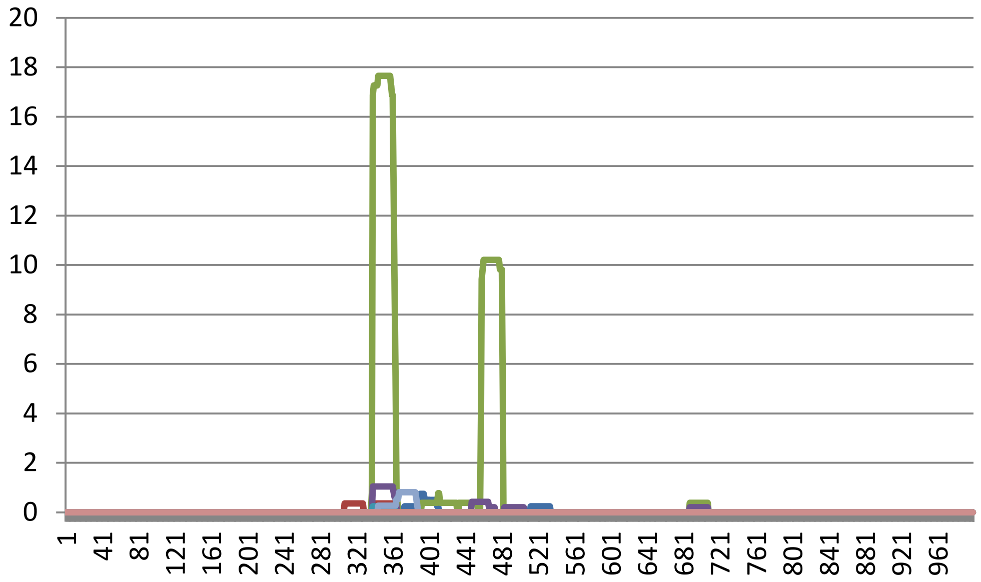

AT3G18770RC

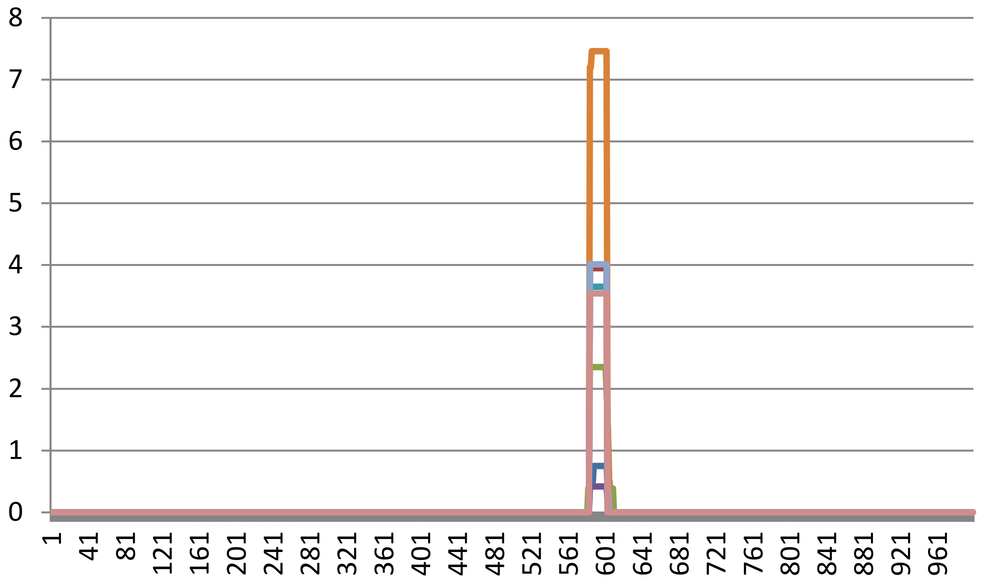

AT3G20640RC

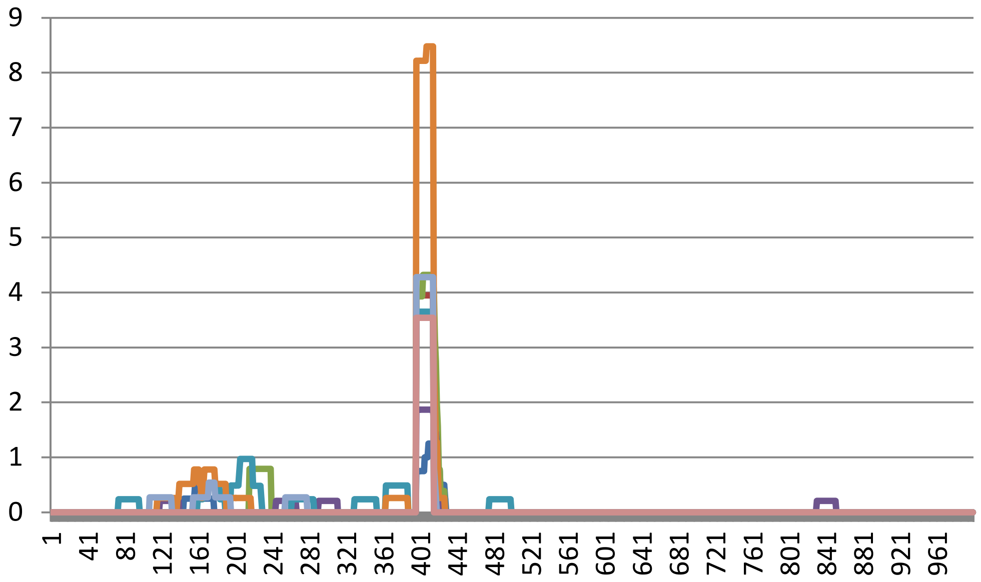

AT3G21730RC

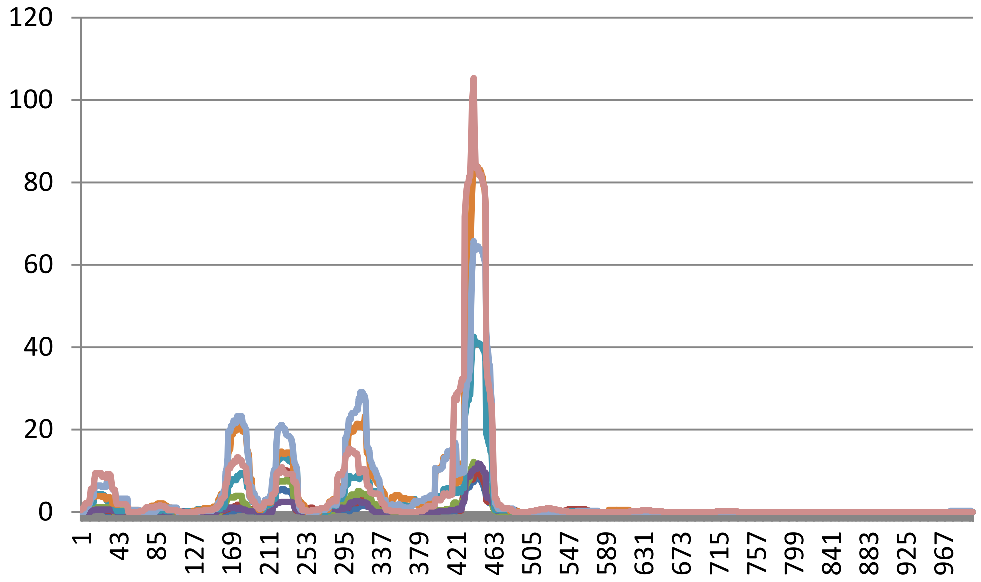

AT3G22050RC

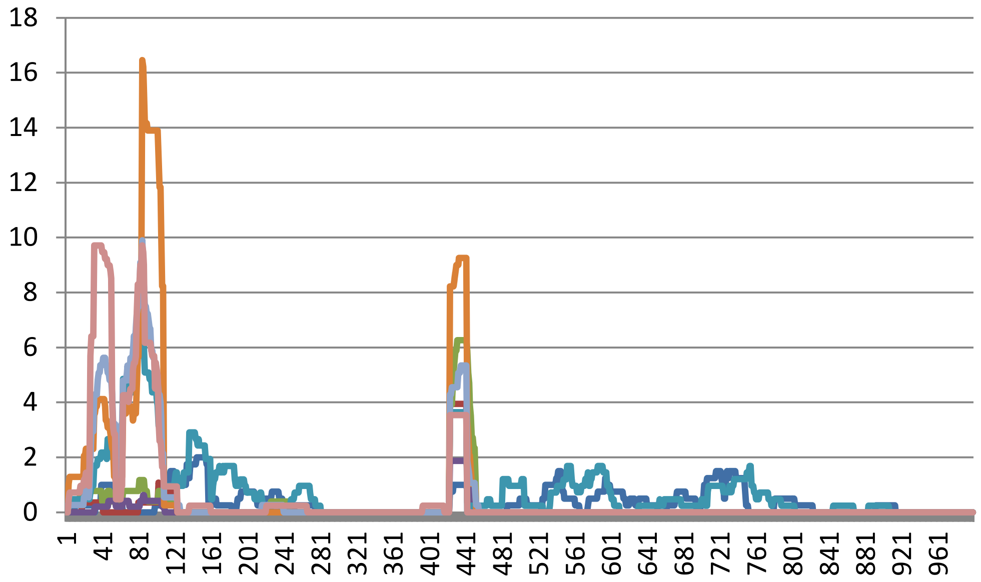

AT3G27040RC

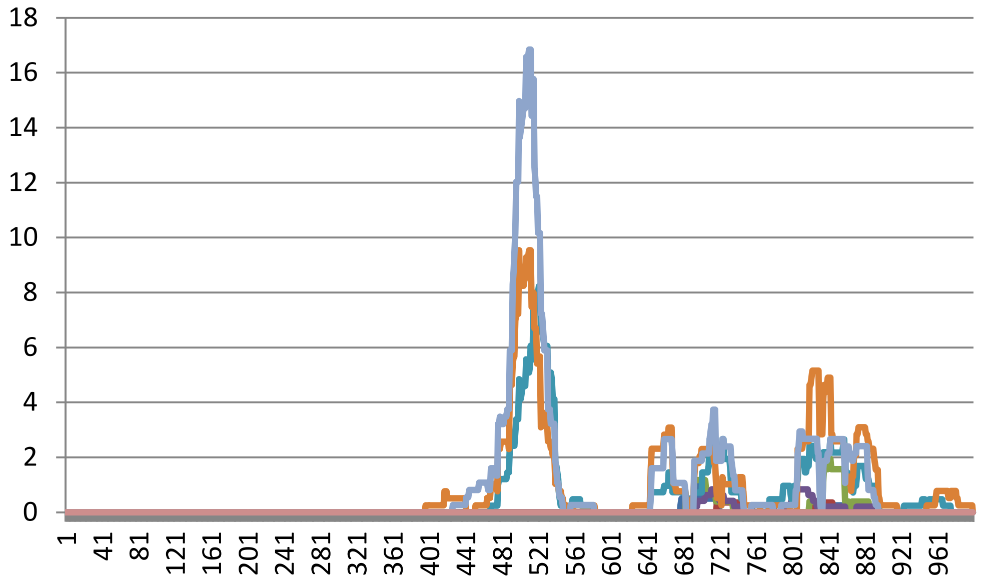

AT3G27510RC

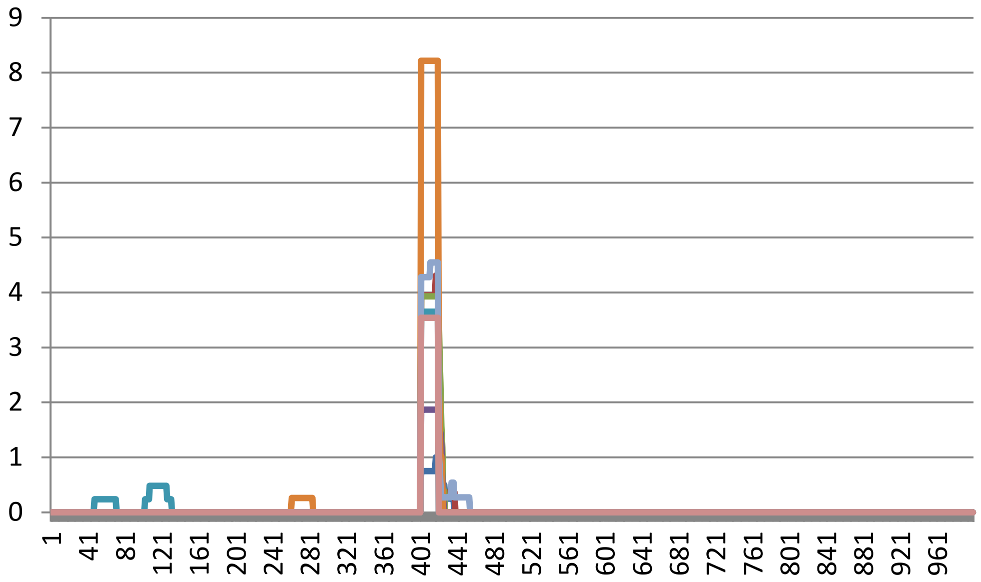

AT3G27560RC

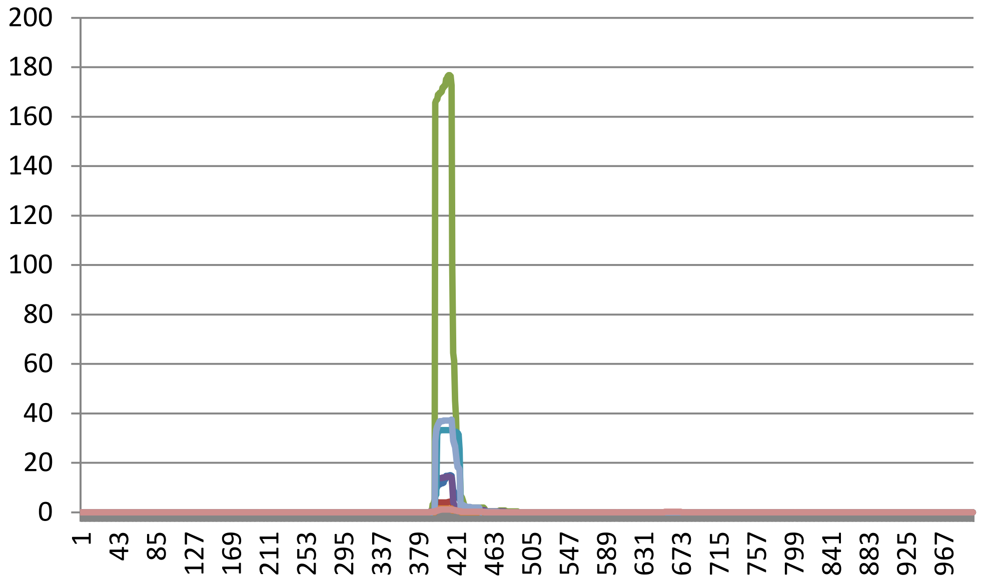

AT3G43153RC

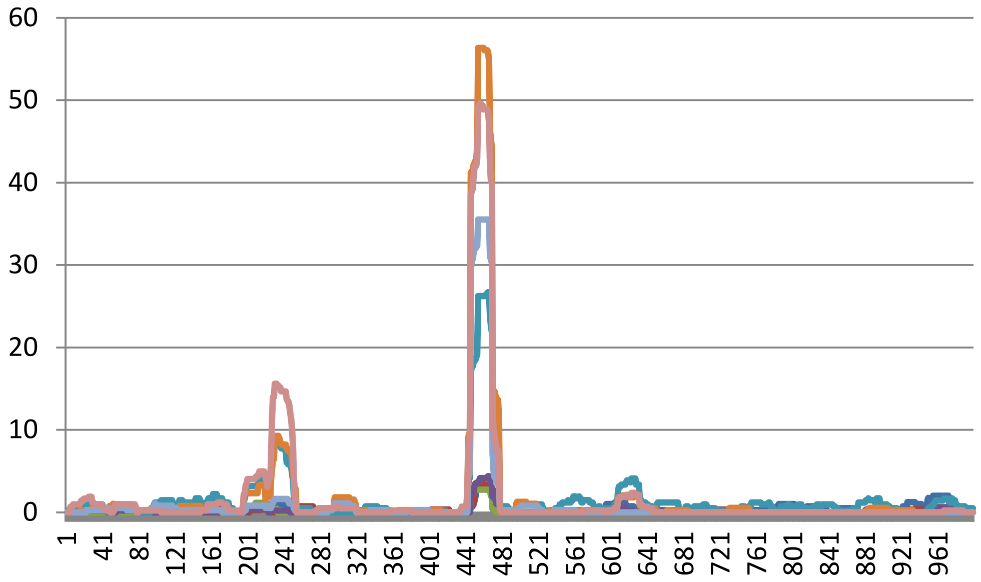

AT3G44210RC

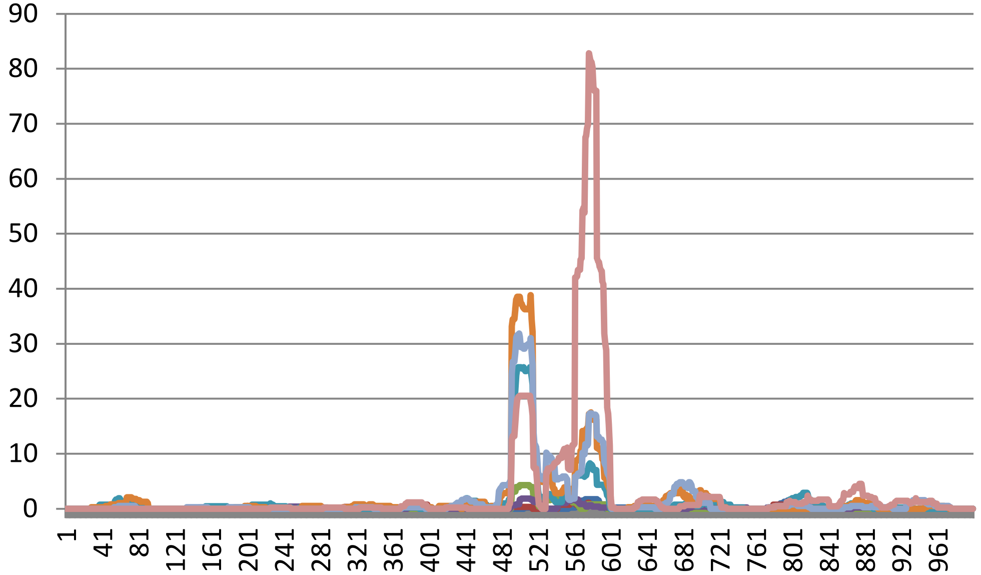

AT3G46270RC

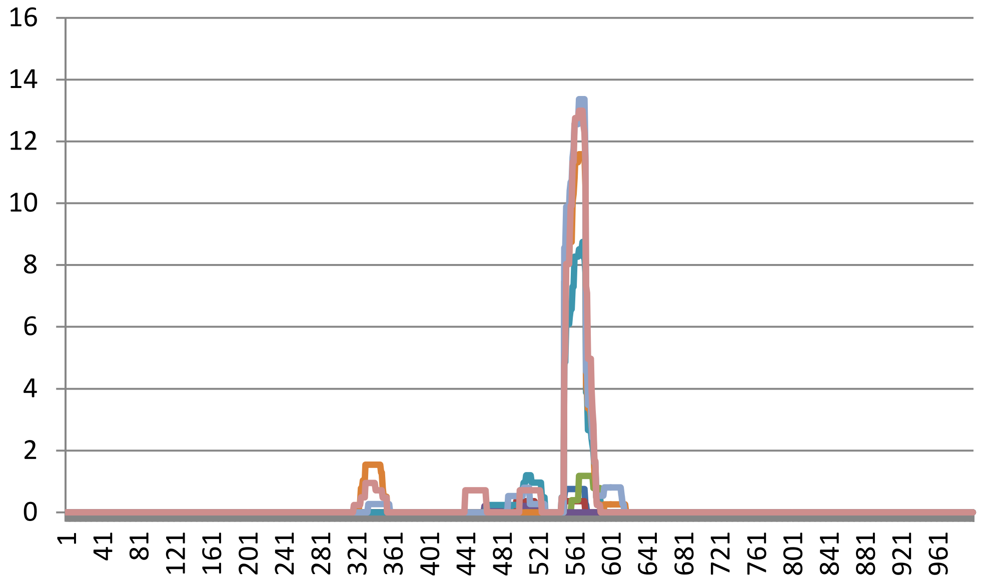

AT3G50090RC

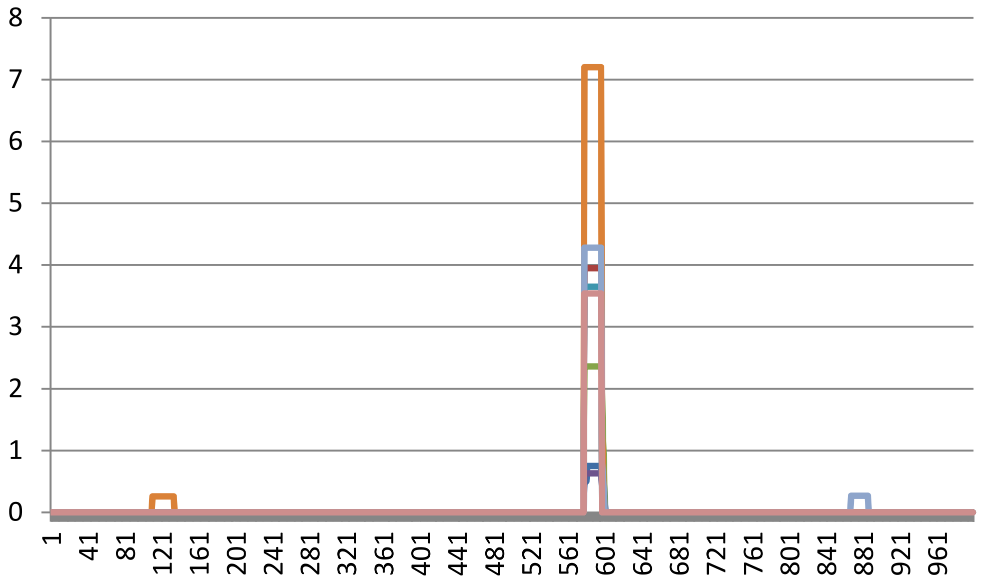

AT3G51760RC

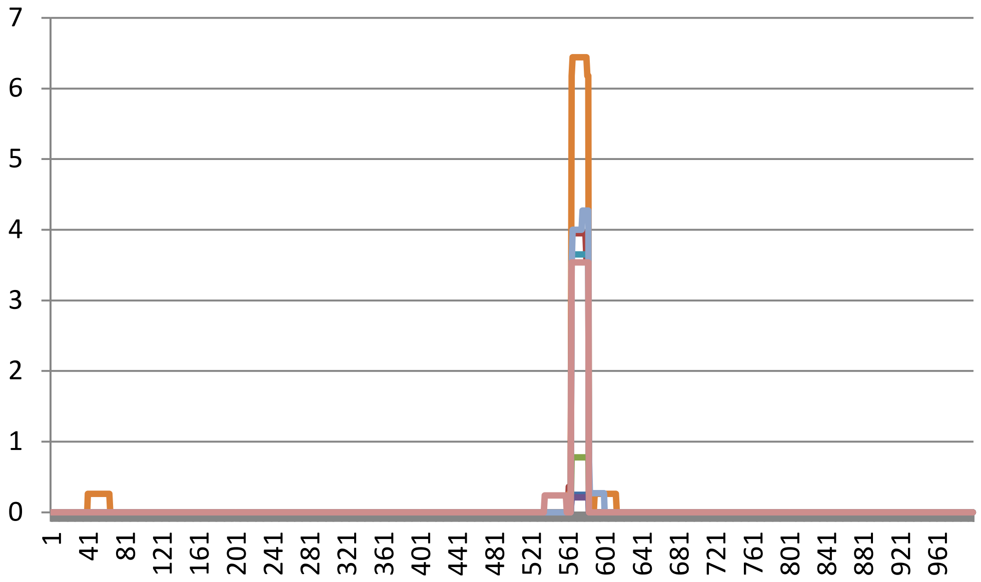

AT3G56380RC

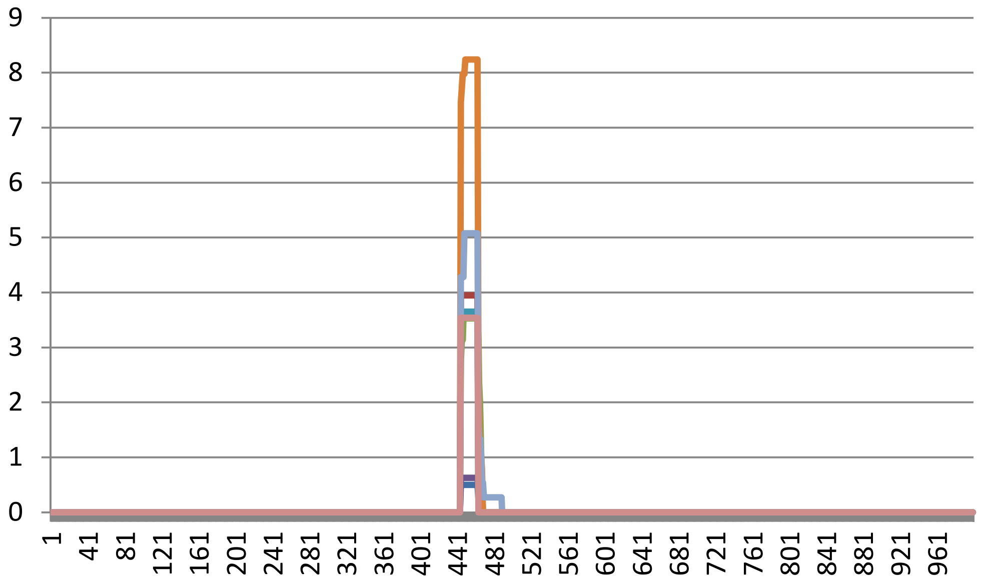

AT3G58170RC

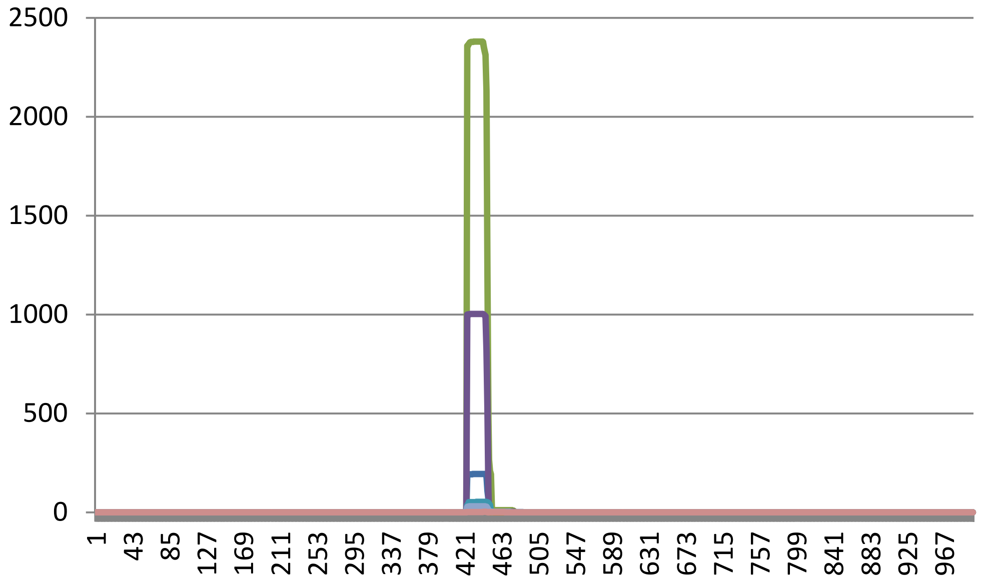

AT3G60550RC

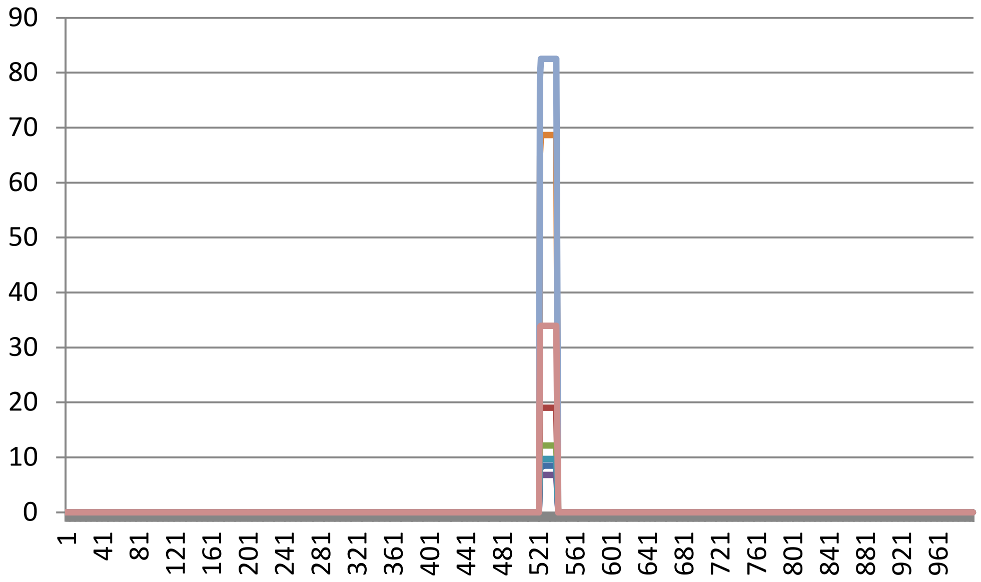

AT4G04510RC

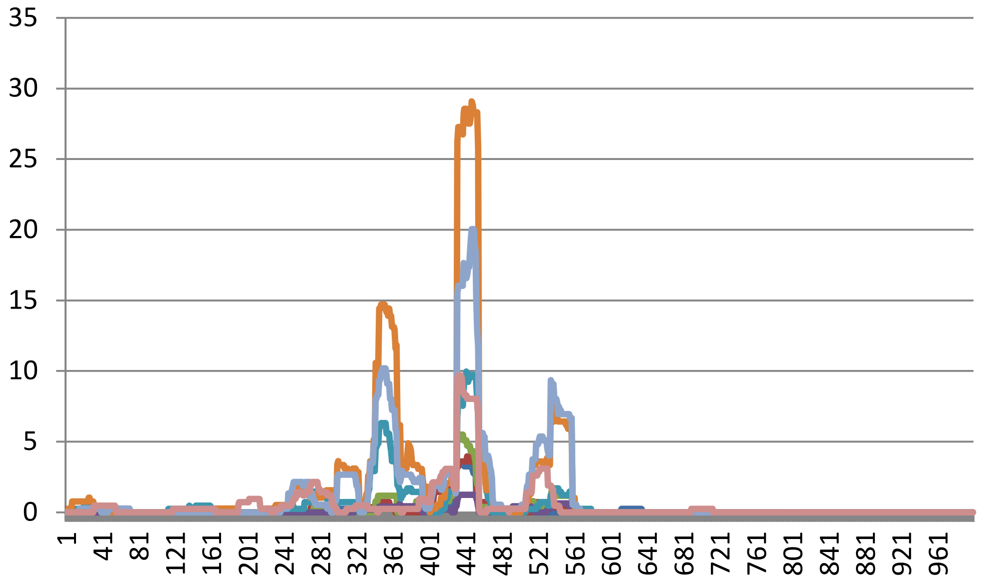

AT4G08300RC

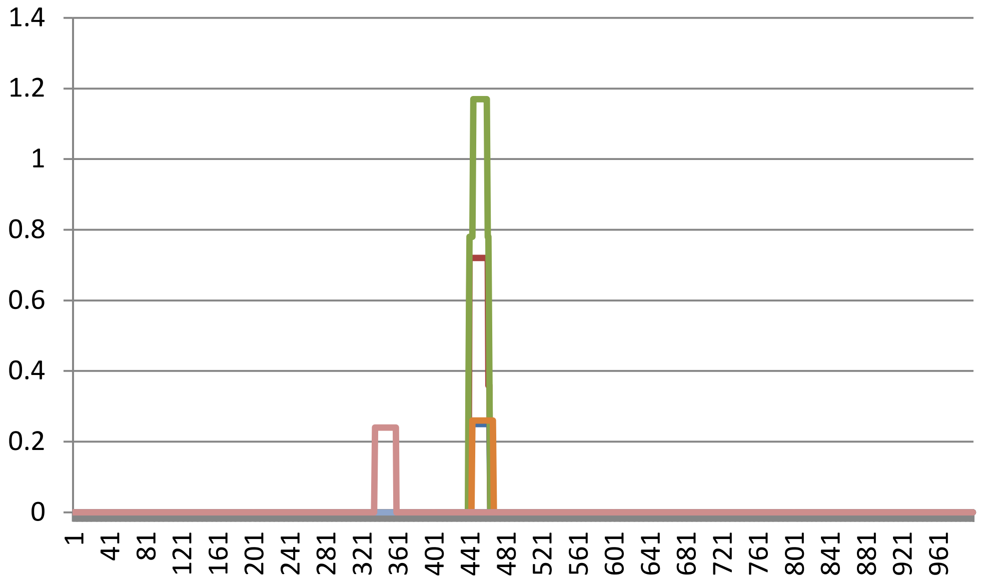

AT4G09770RC

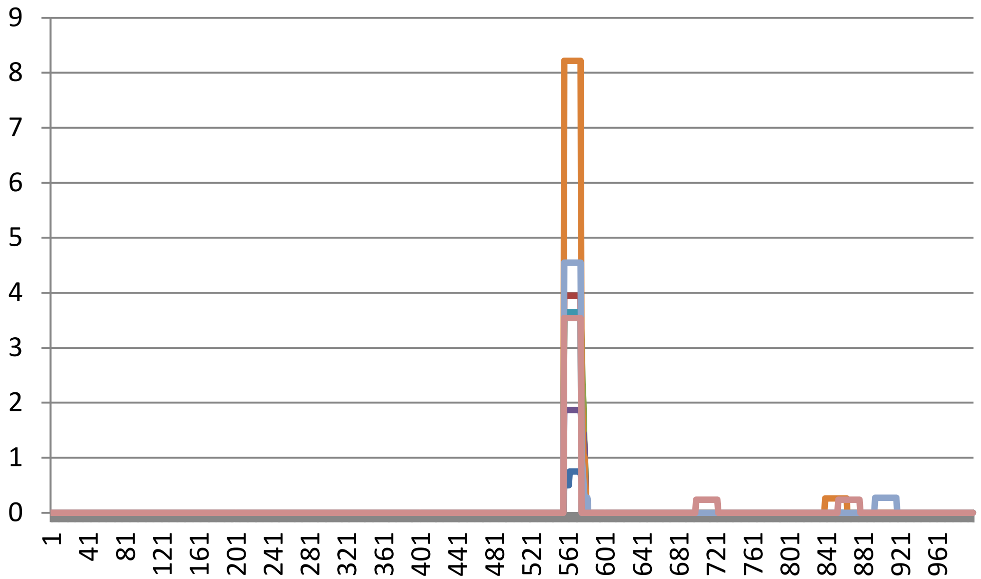

AT4G11040RC

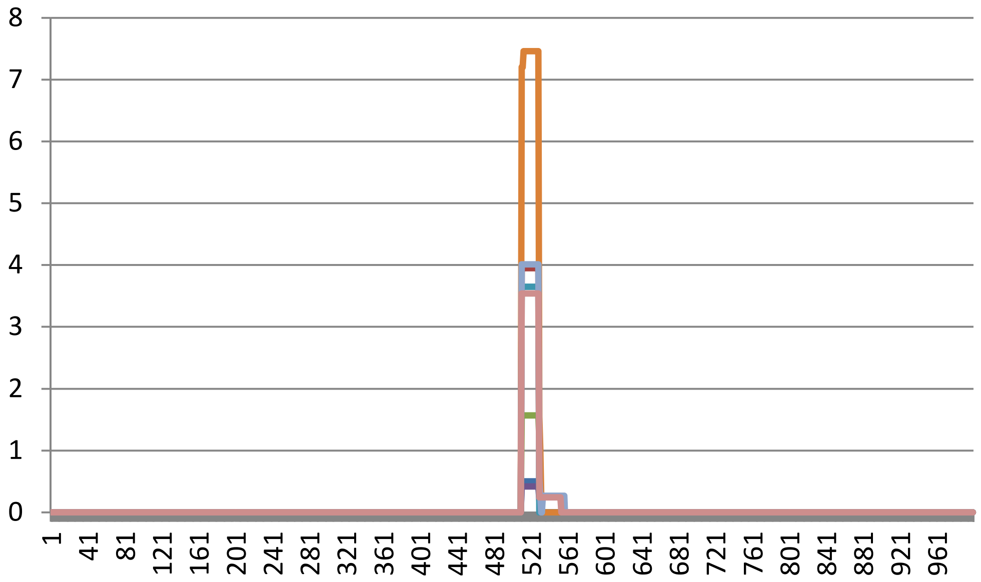

AT4G11950RC

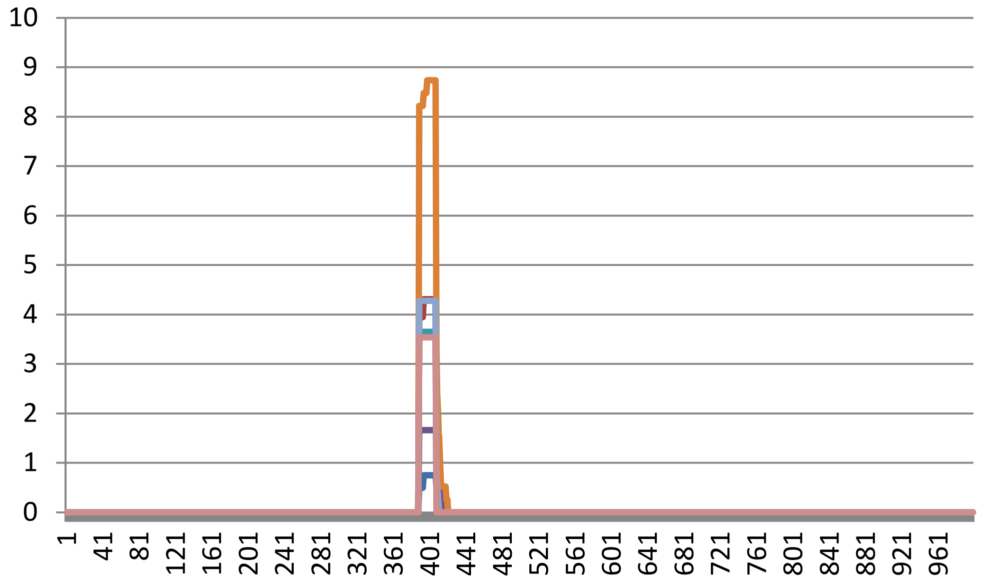

AT4G13261RC

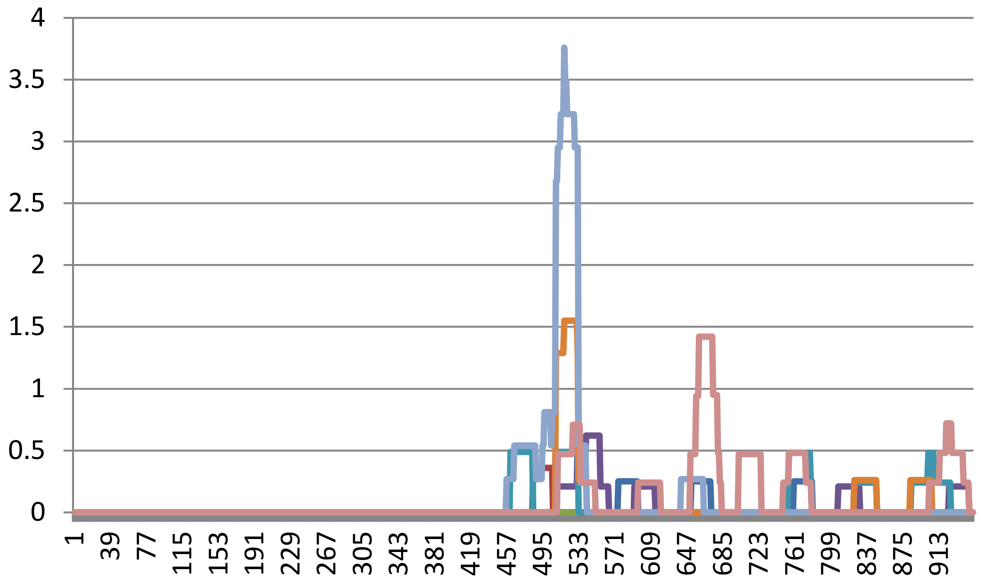

AT4G19330RC

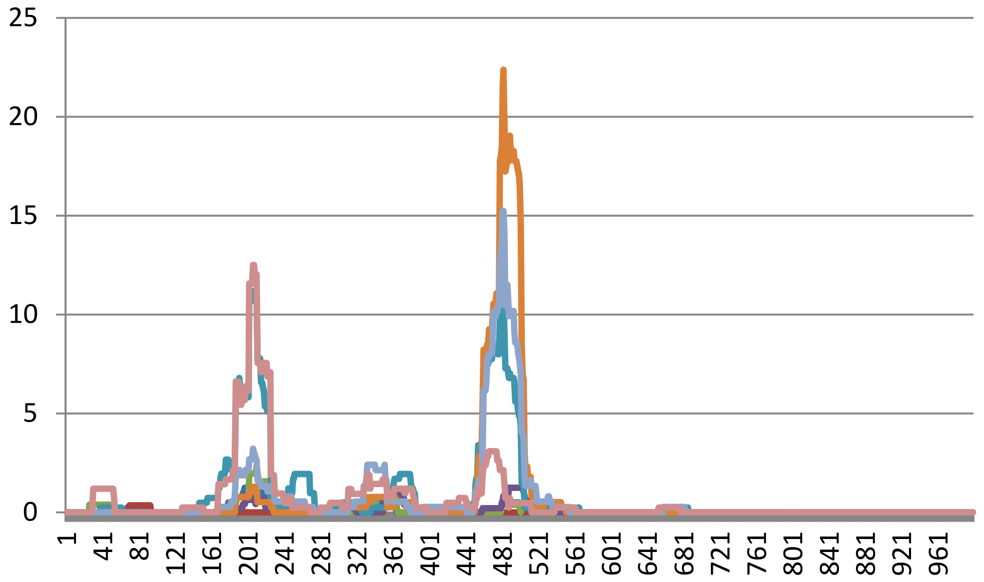

AT4G20110RC

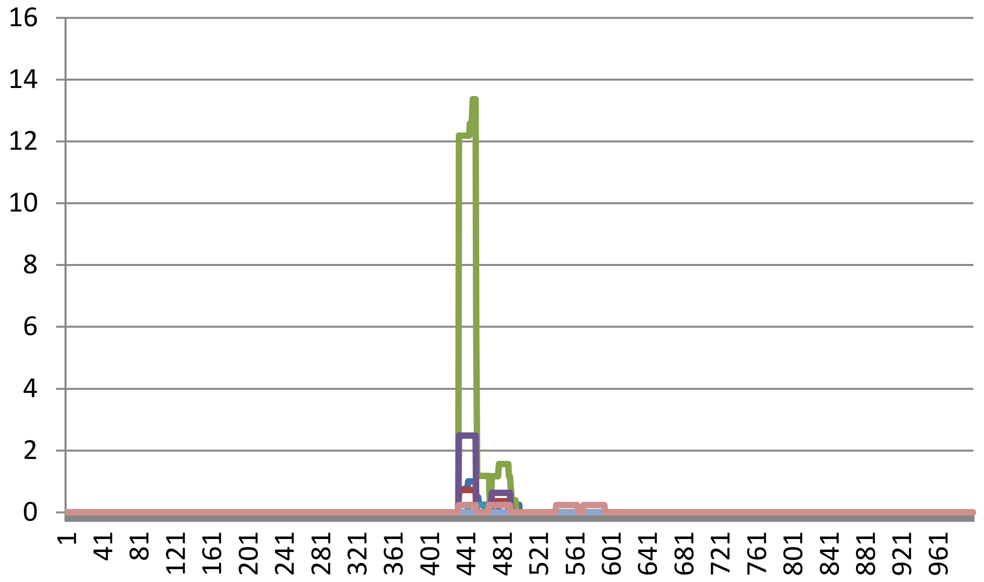

AT4G21585RC

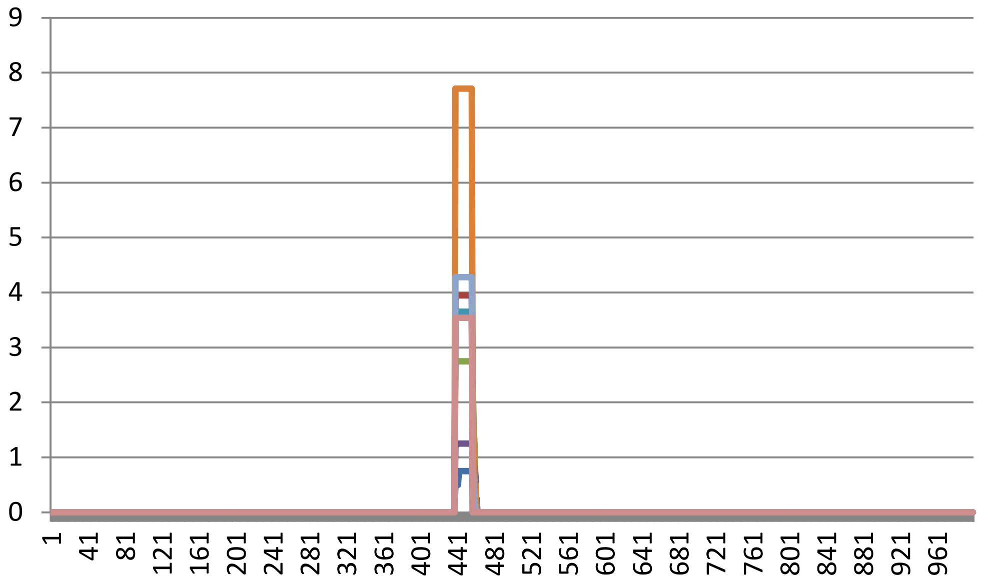

AT4G22790RC

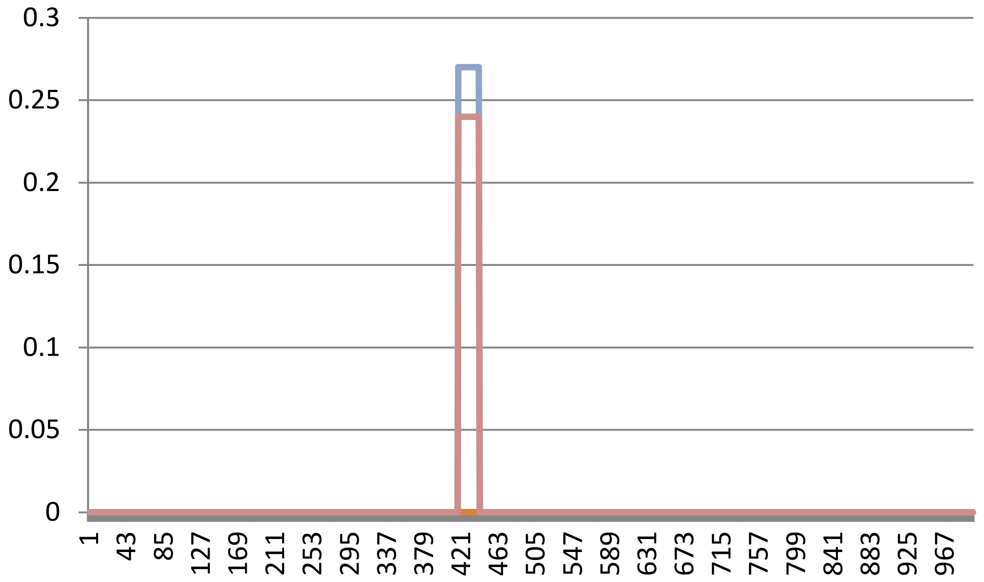

AT4G23120RC

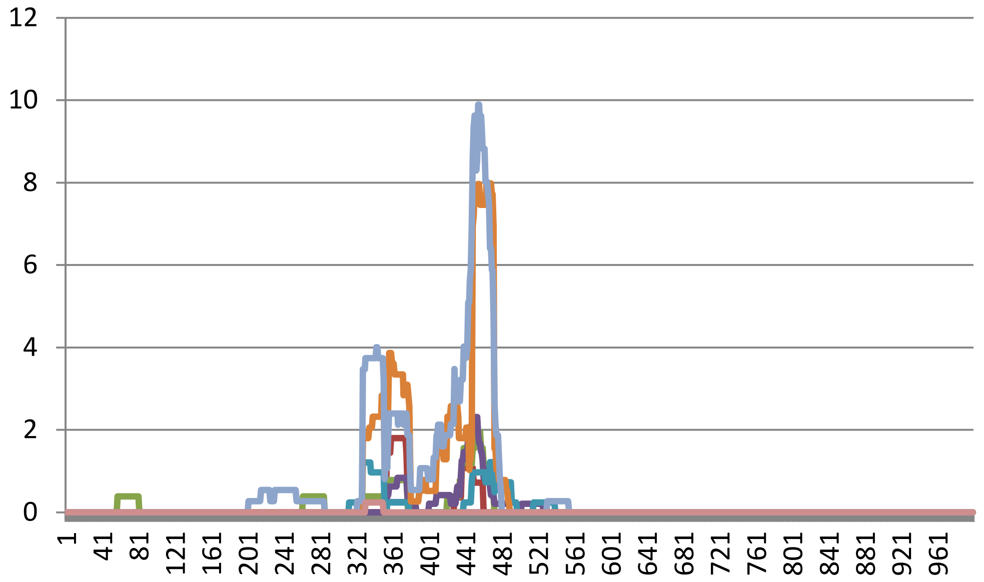

AT4G23640RC

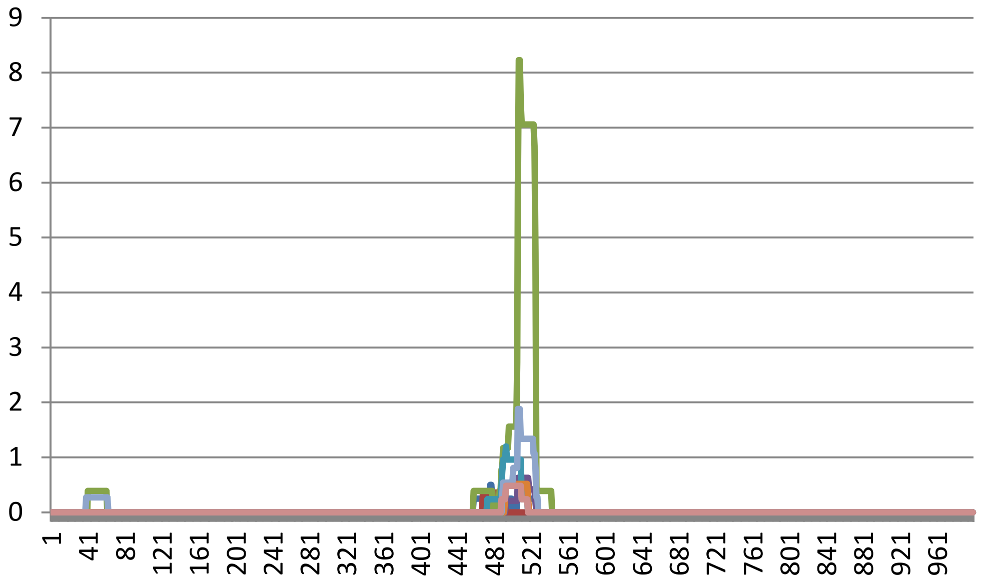

AT4G23730RC

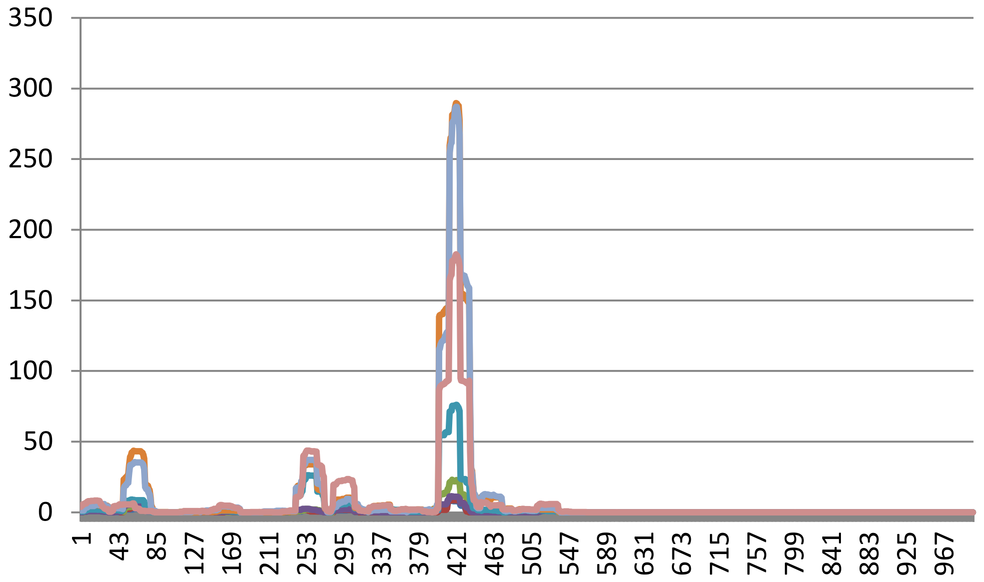

AT4G24440RC

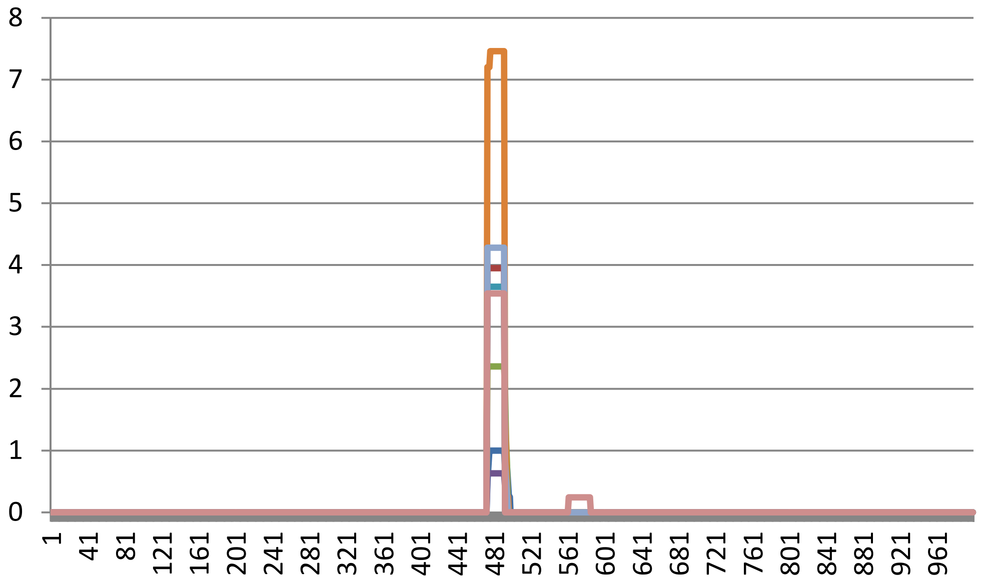

AT4G24644RC

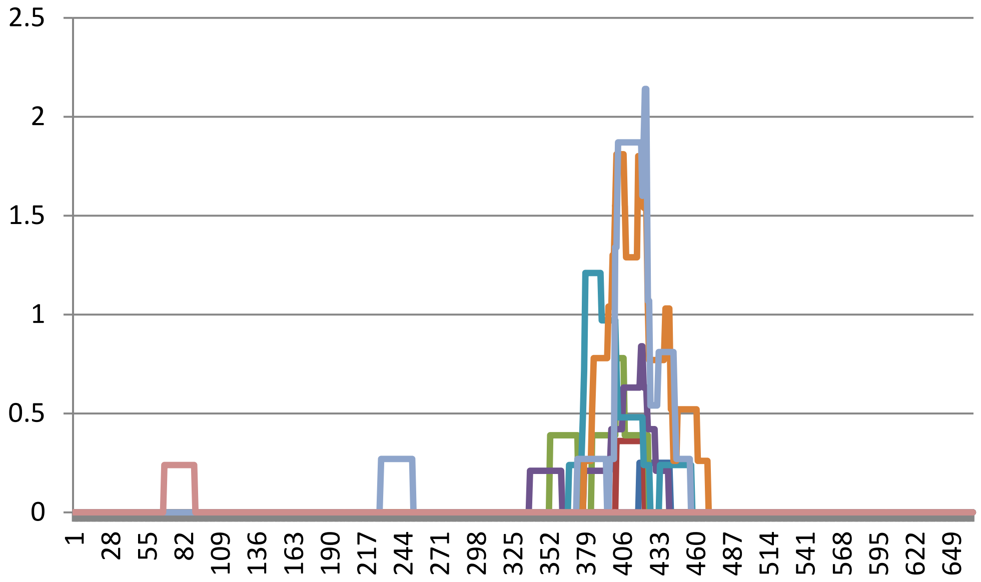

AT4G25580RC_AGO1 root

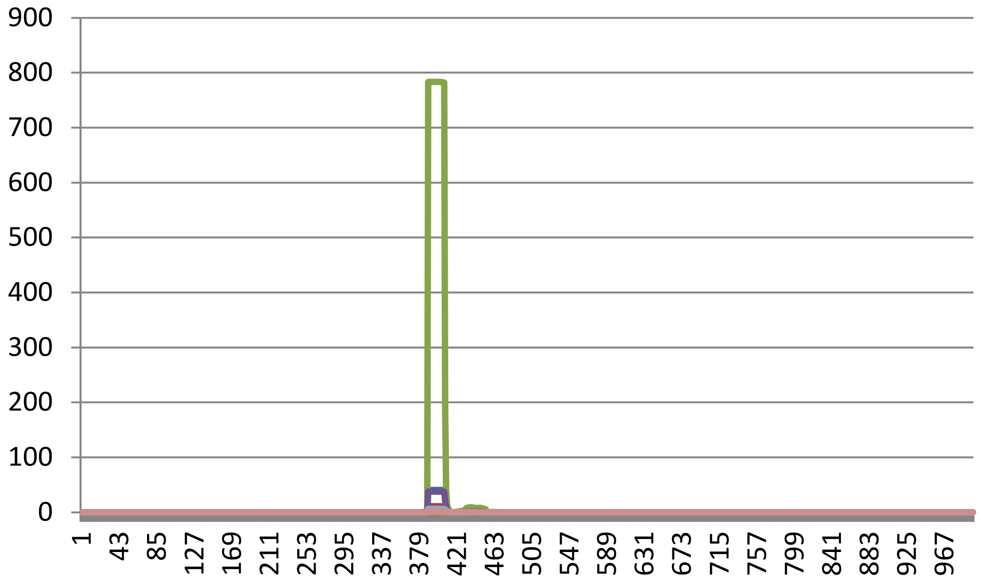

AT4G26380RC

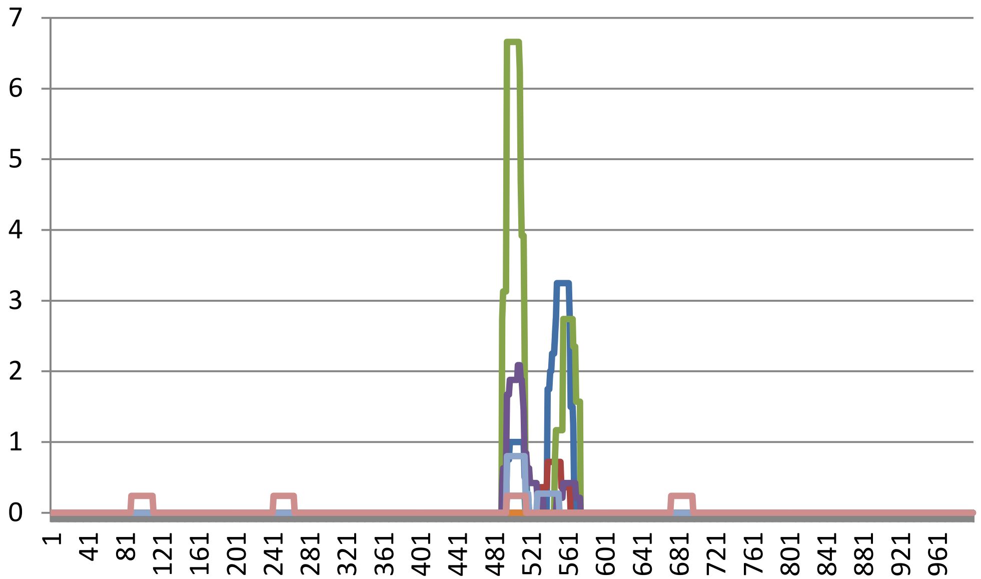

AT4G27870RC

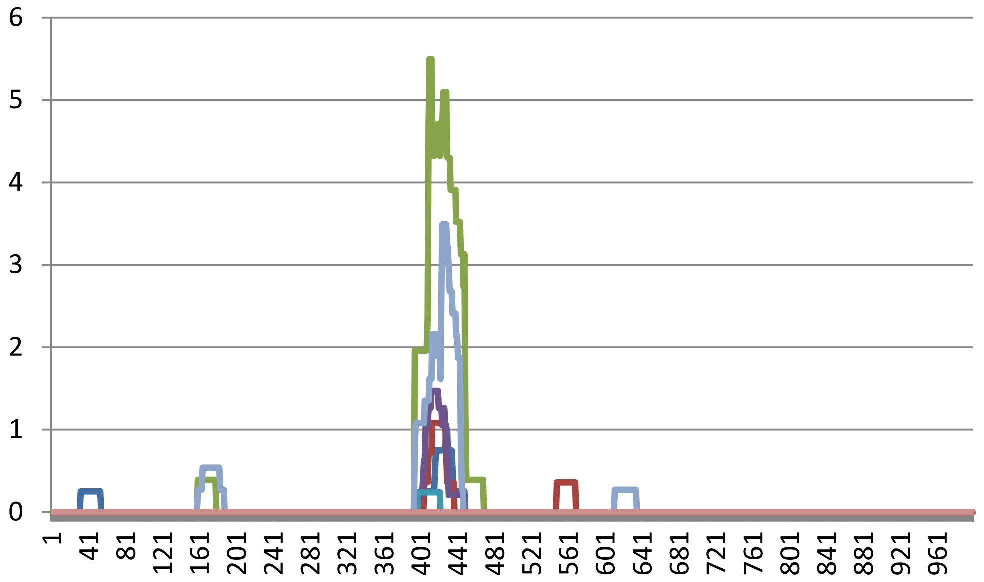

AT4G31270RC

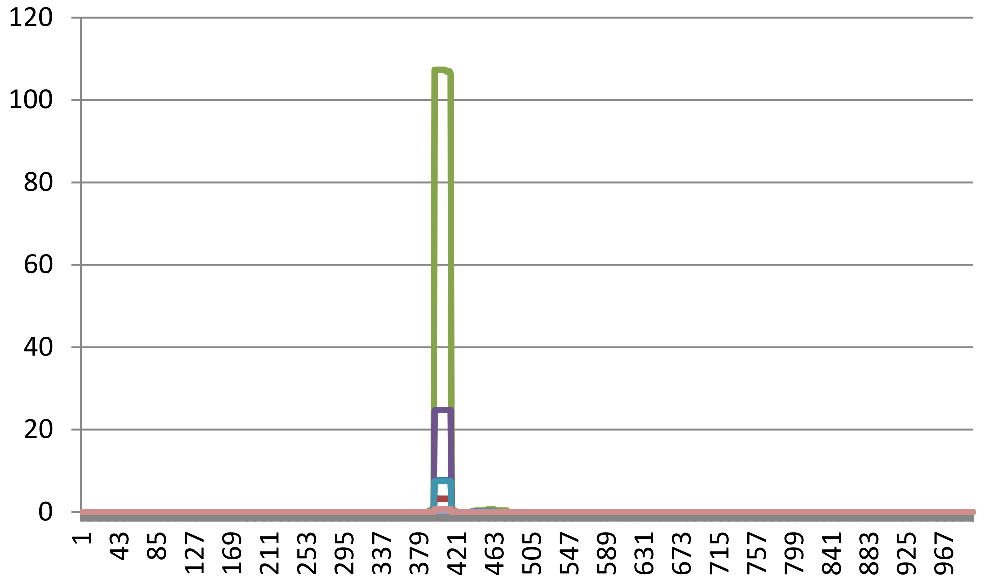

AT4G32200RC

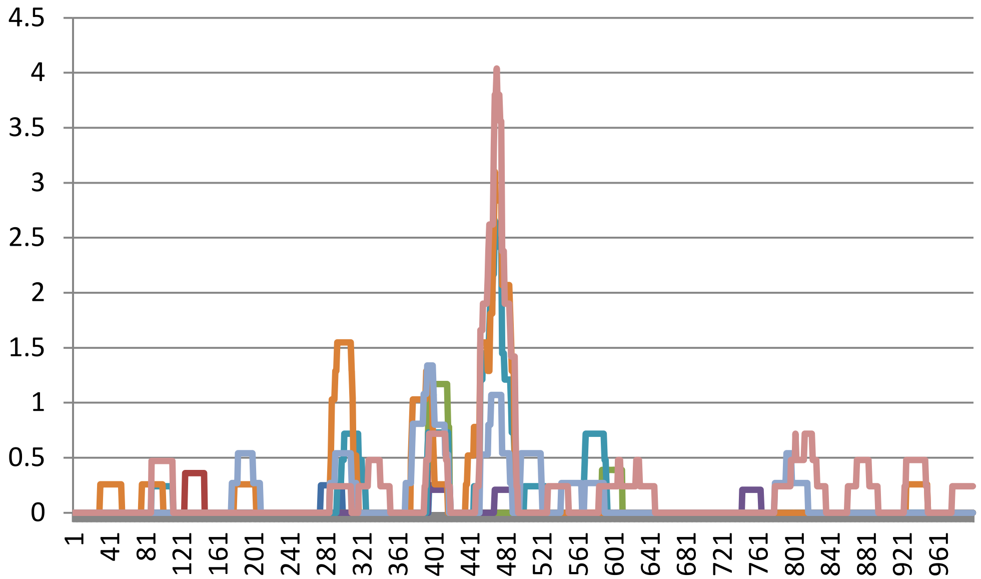

AT4G32860RC

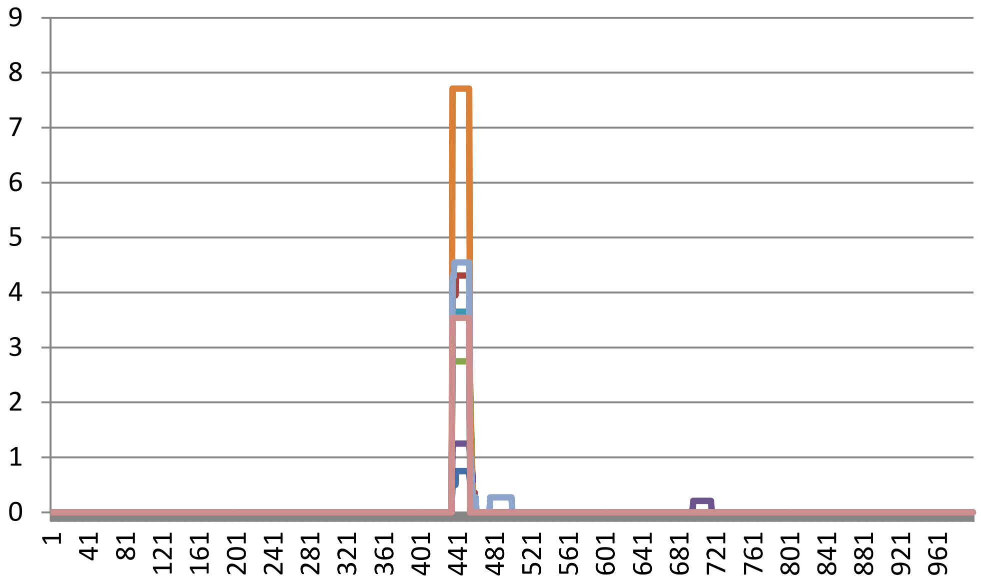

AT4G36925RC

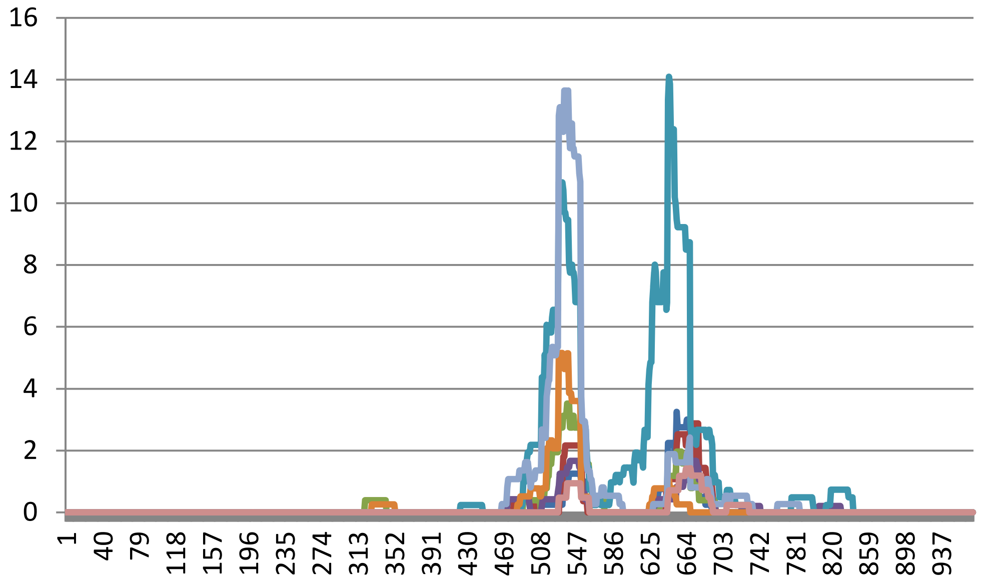

AT4G37140RC

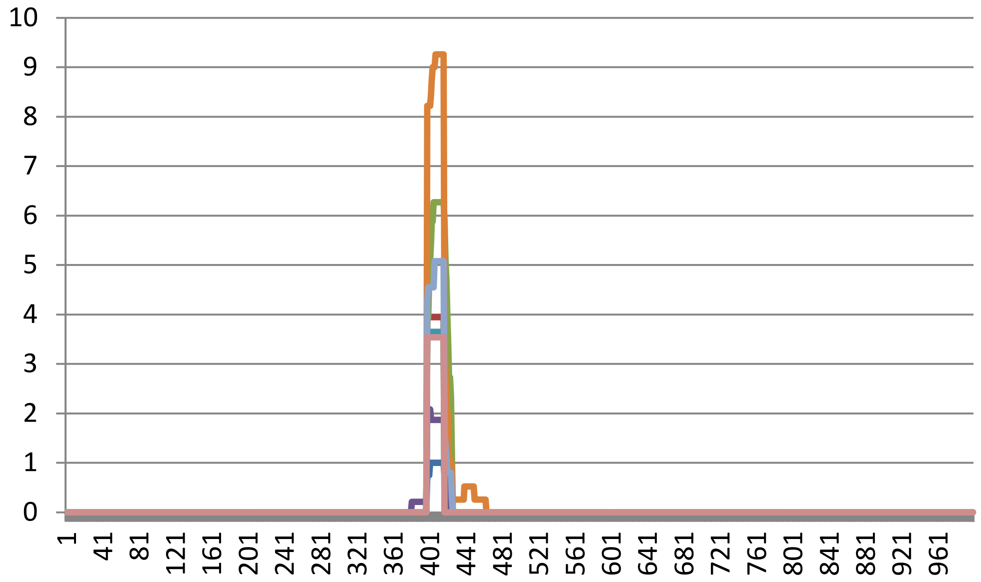

AT4G37180RC

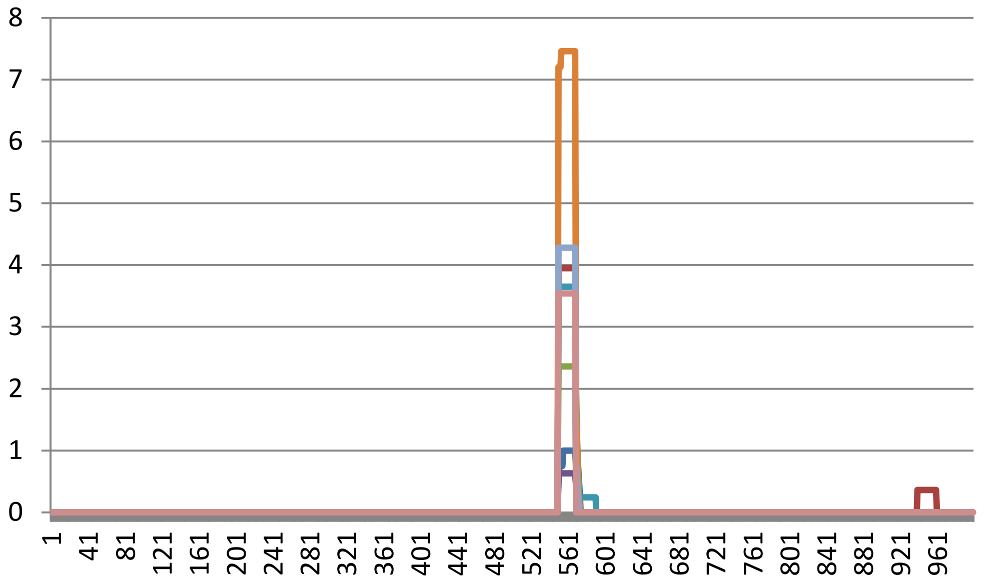

AT4G38930RC

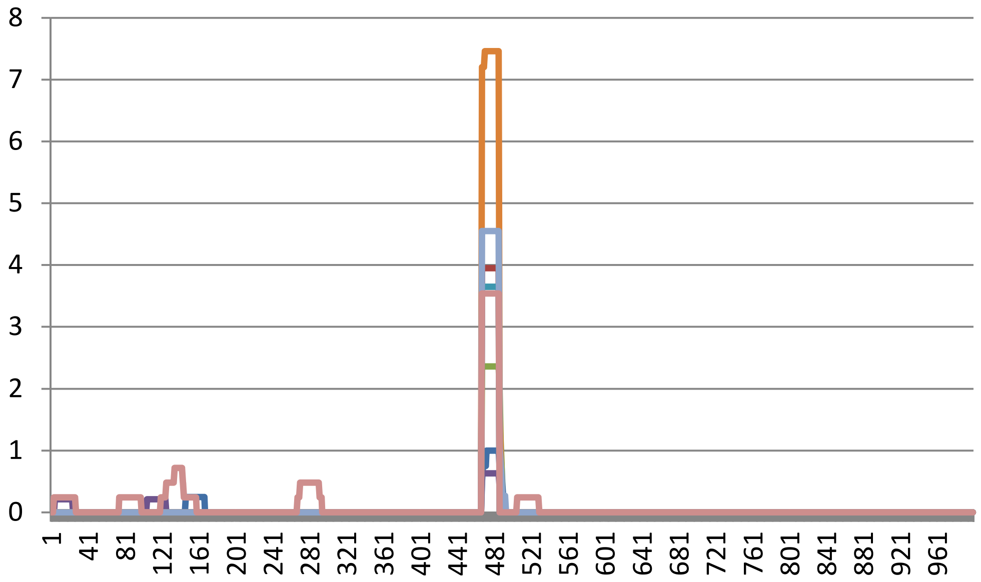

AT4G39756RC

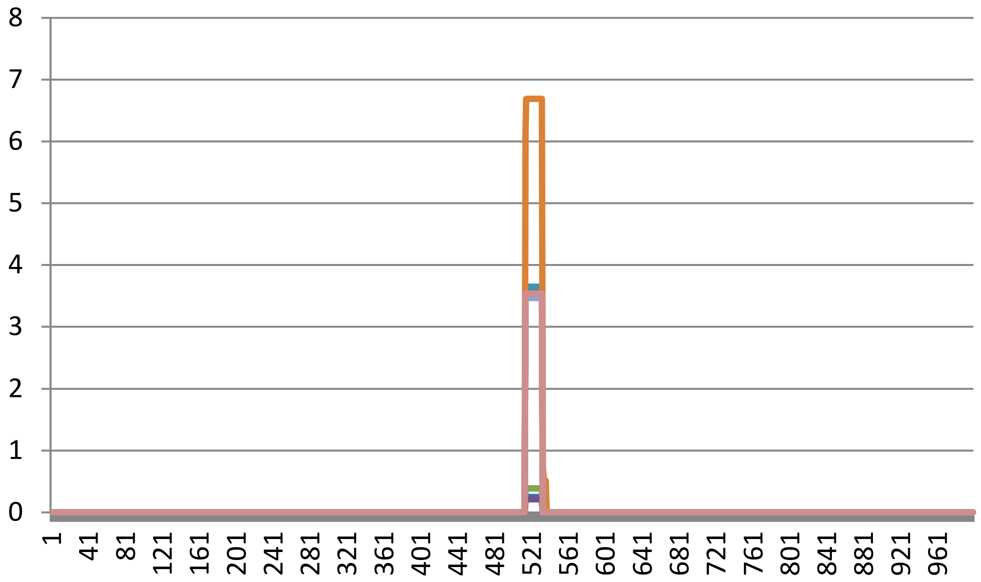

AT5G01090RC

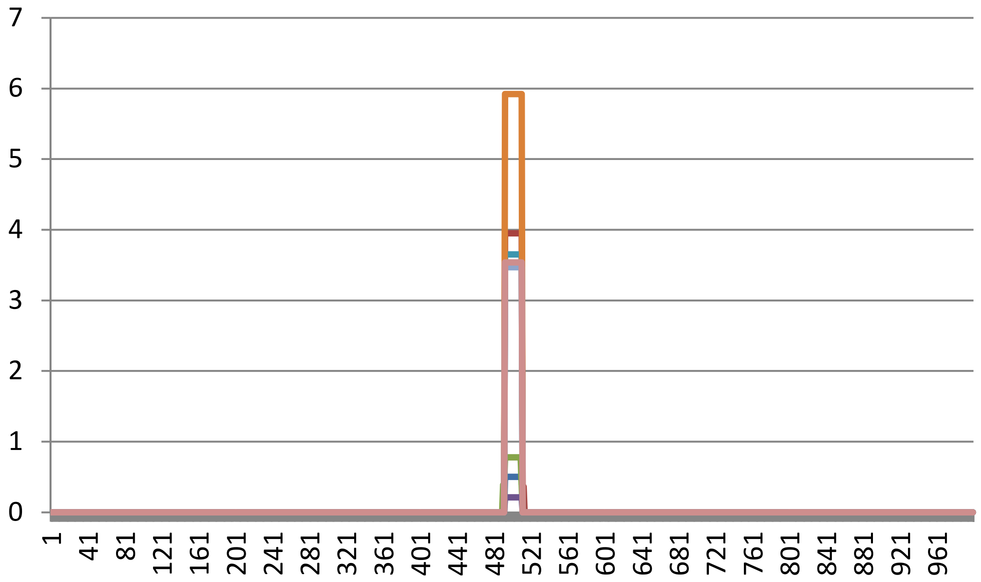

AT5G04960RC

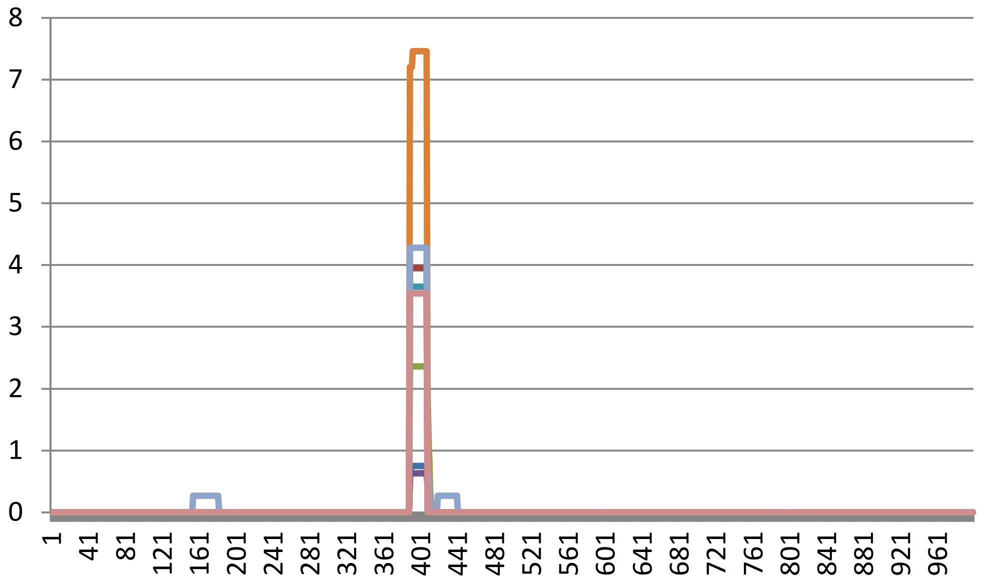

AT5G09660RC

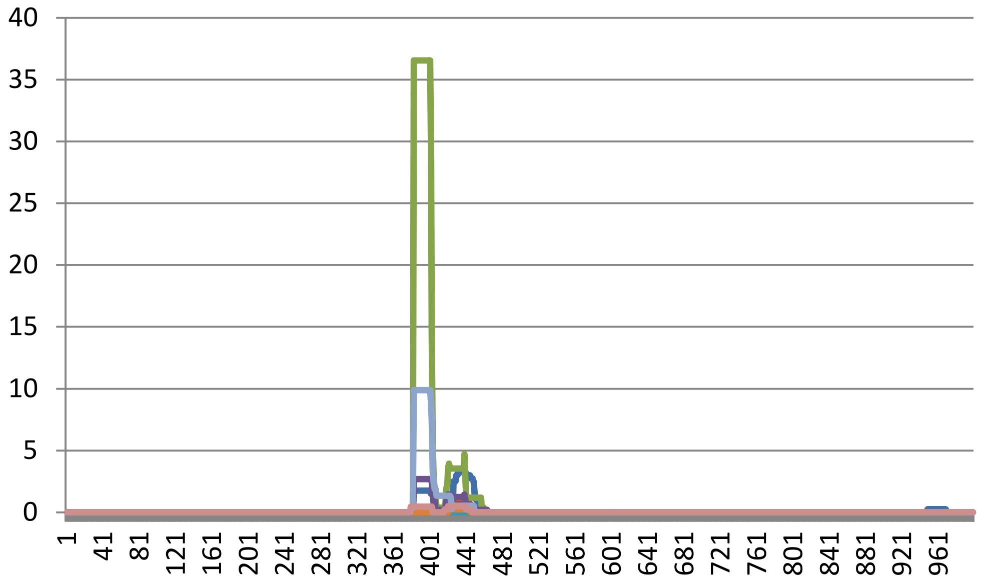

AT5G09910RC

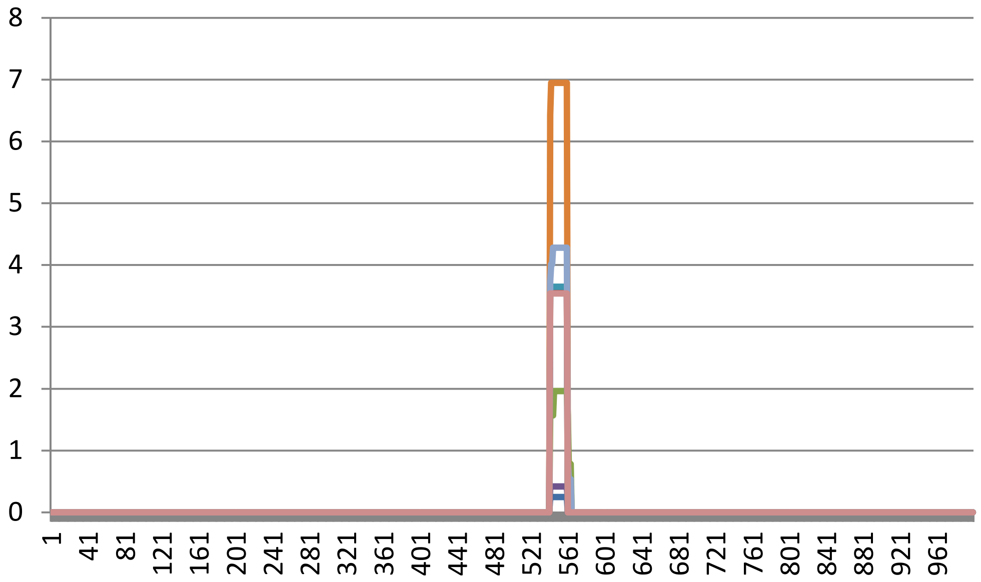

AT5G11590RC

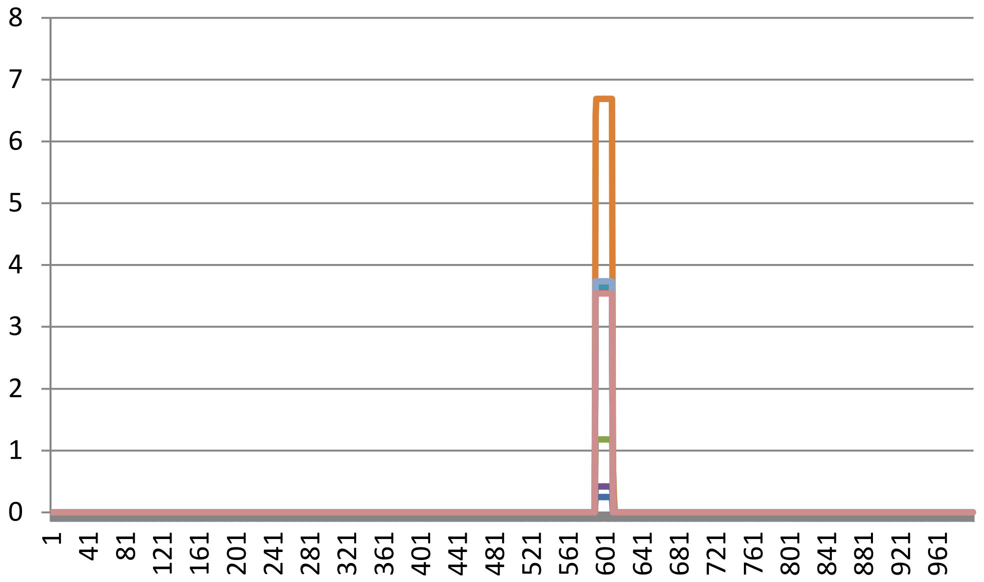

AT5G15270RC

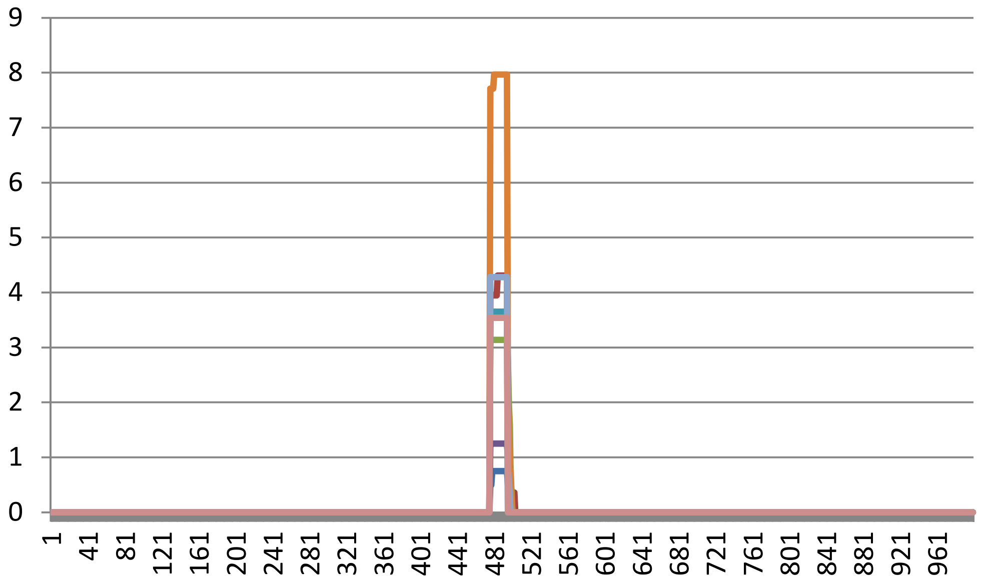

AT5G18610RC

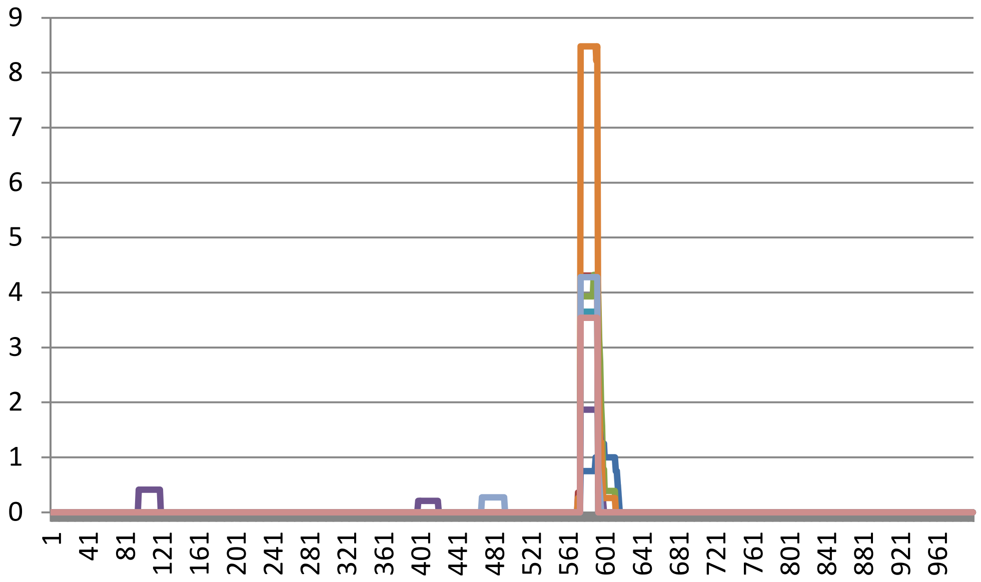

AT5G22170RC

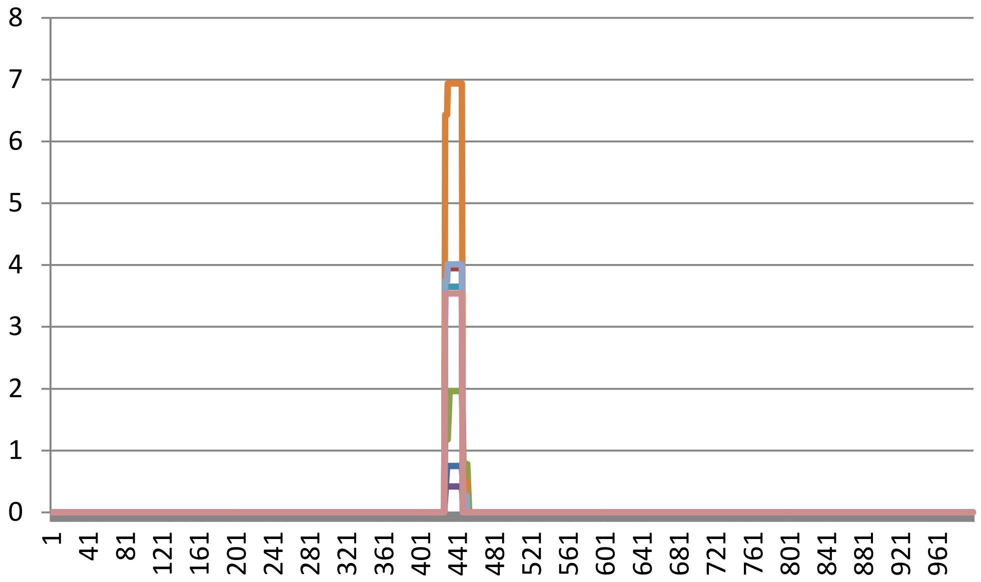

AT5G22320RC_AGO1 root

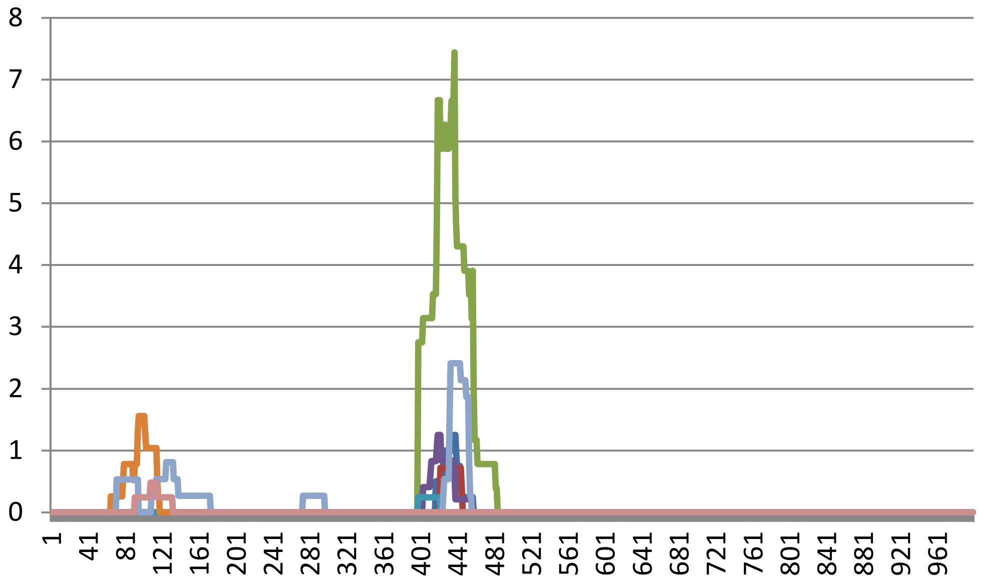

AT5G24593RC

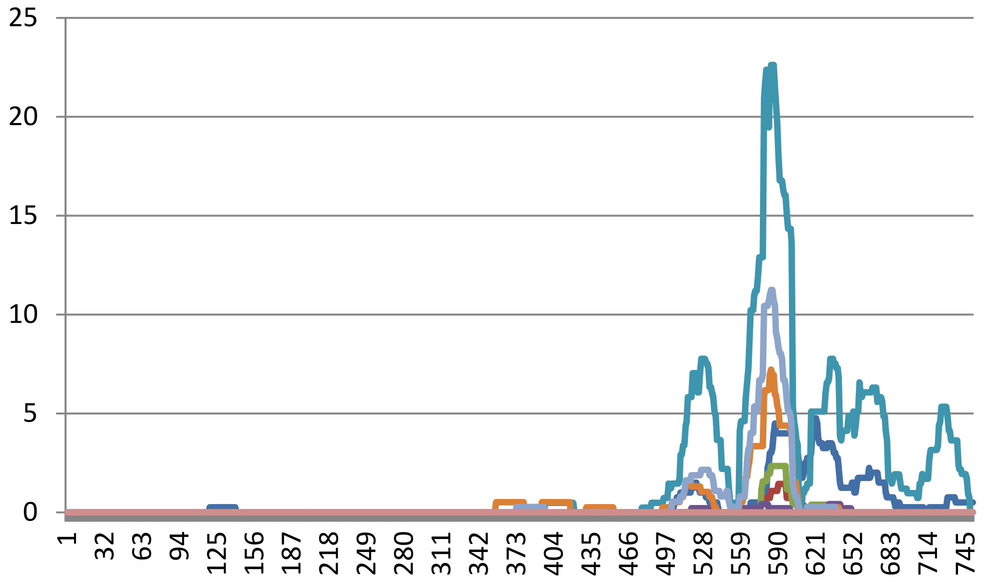

AT5G27710RC

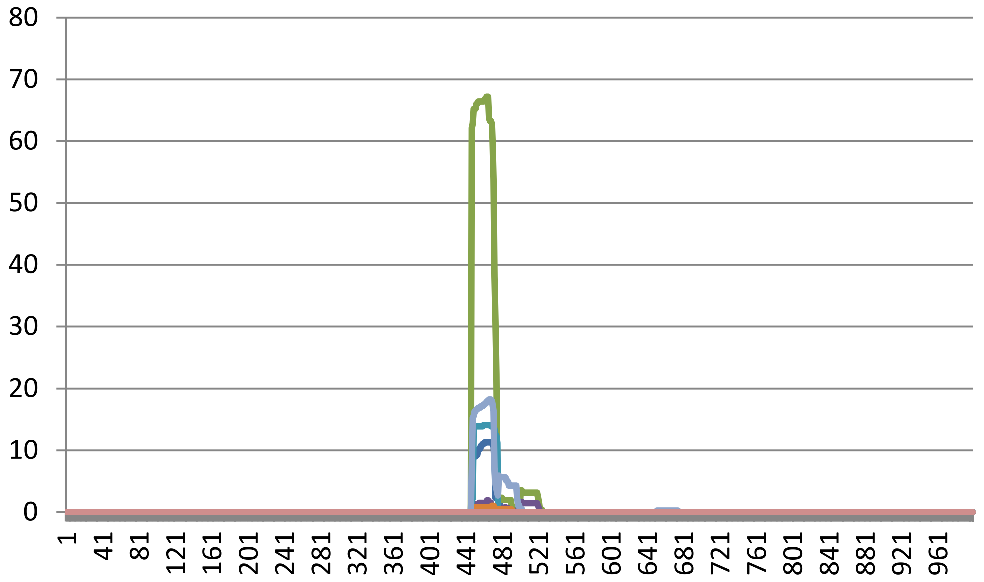

AT5G35526RC

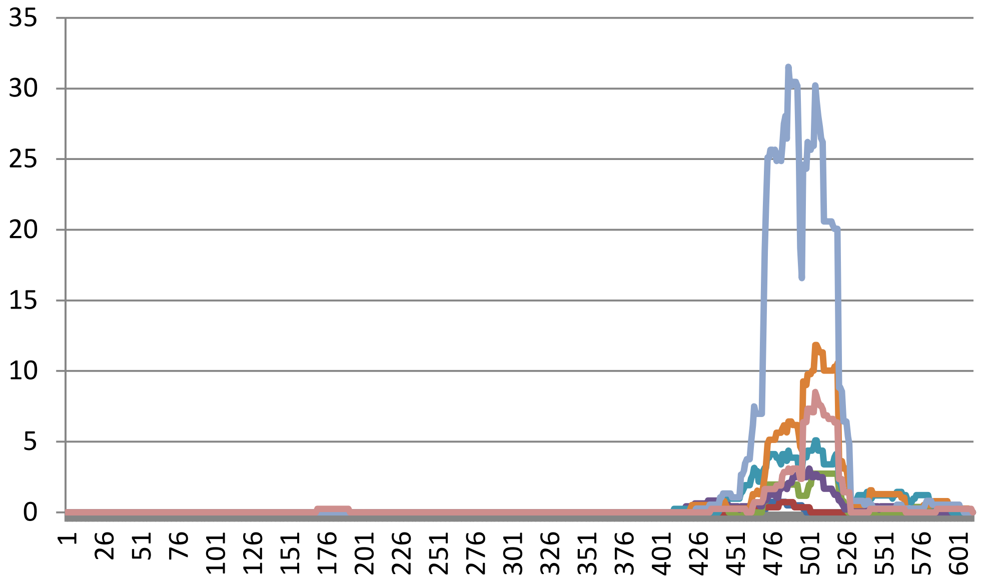

AT5G37980RC

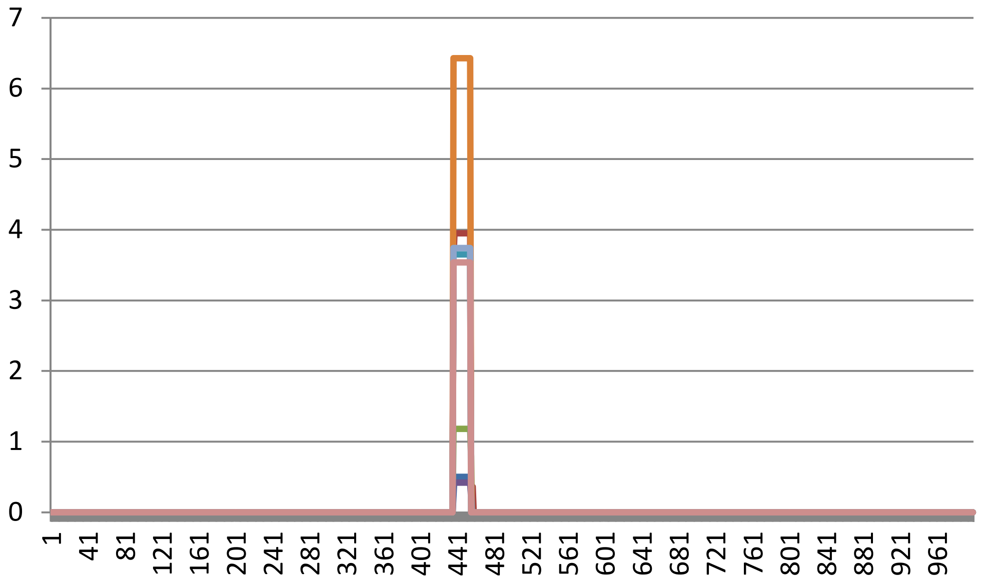

AT5G40540RC

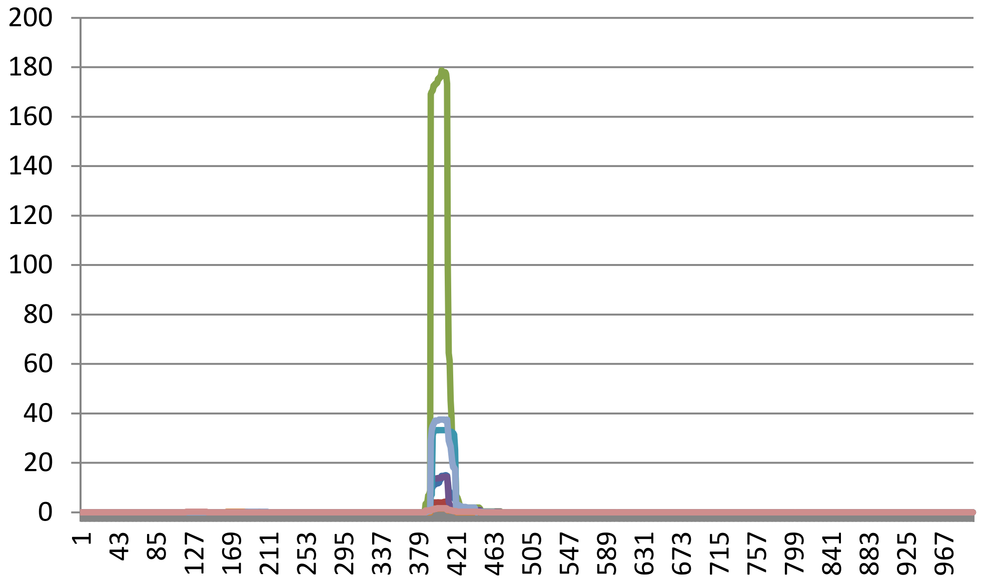

AT5G41140RC

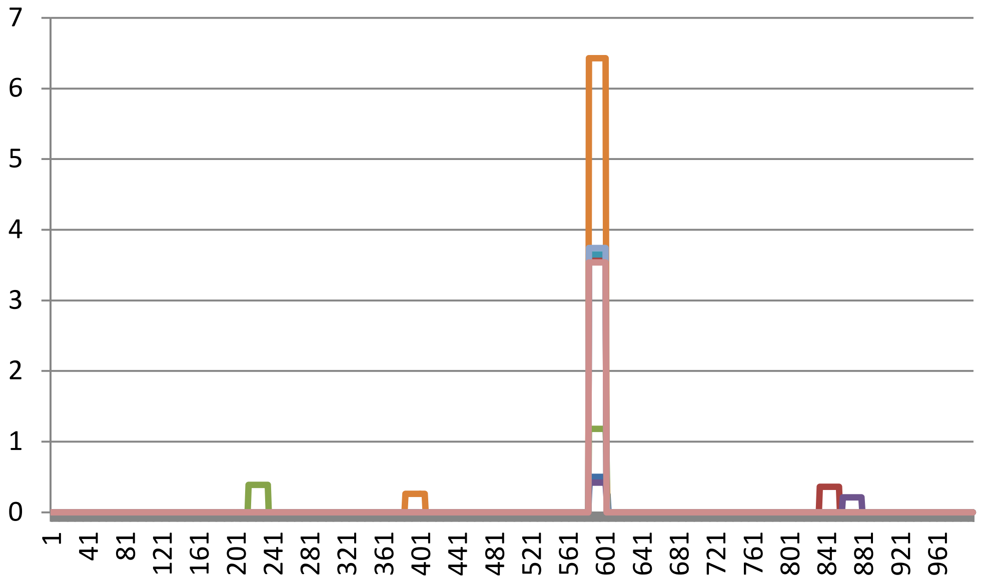

AT5G41900RC

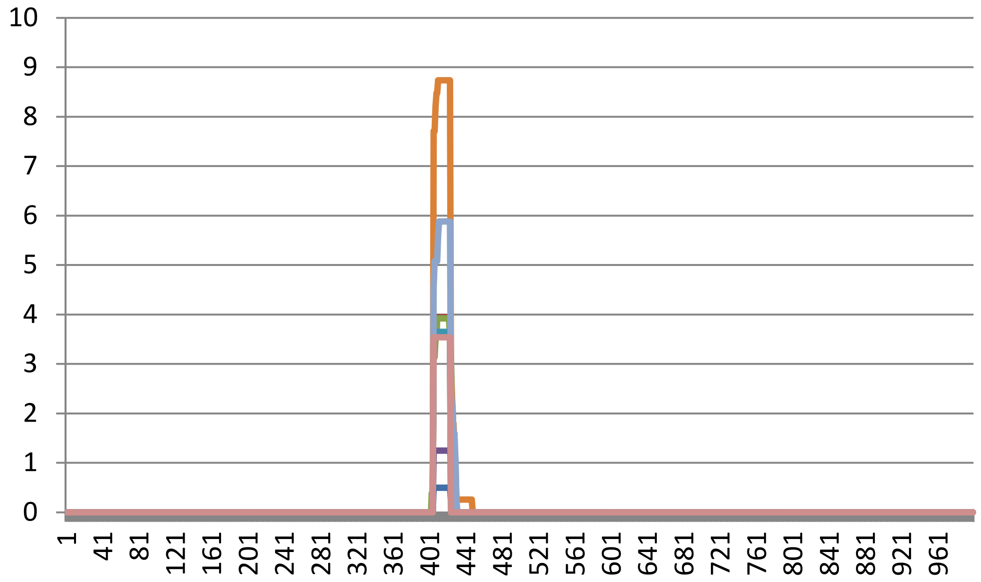

AT5G42100RC

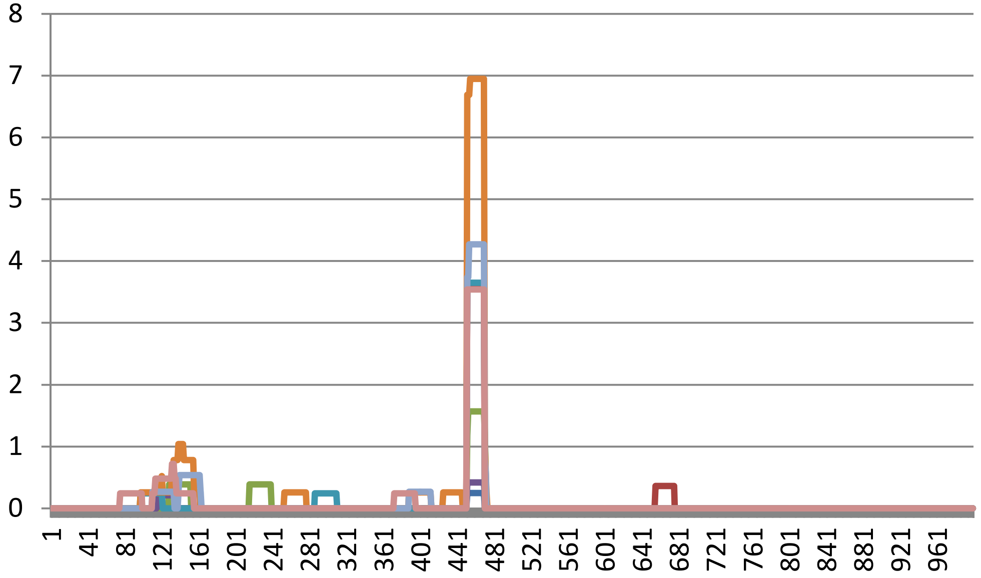

AT5G43513RC

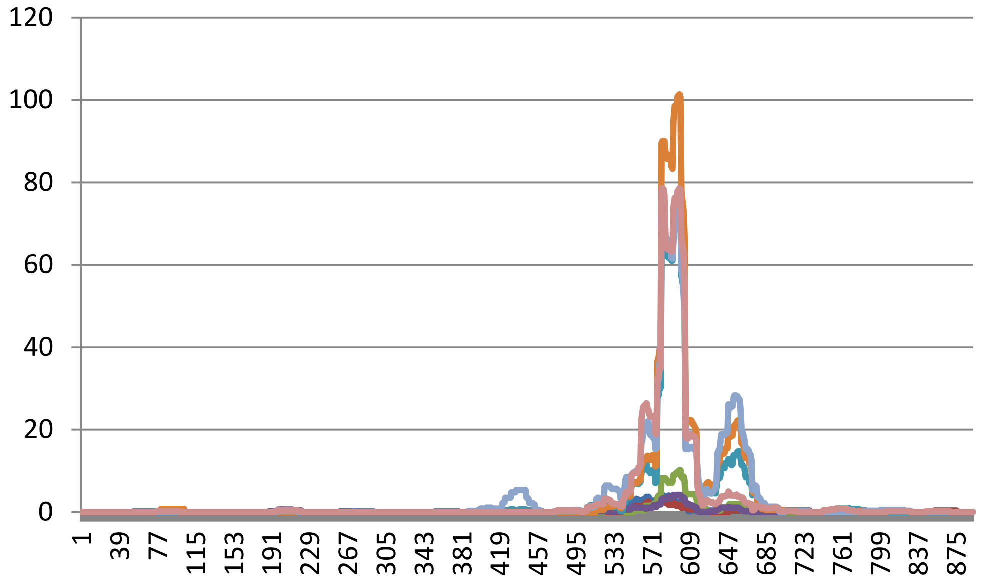

AT5G43610RC

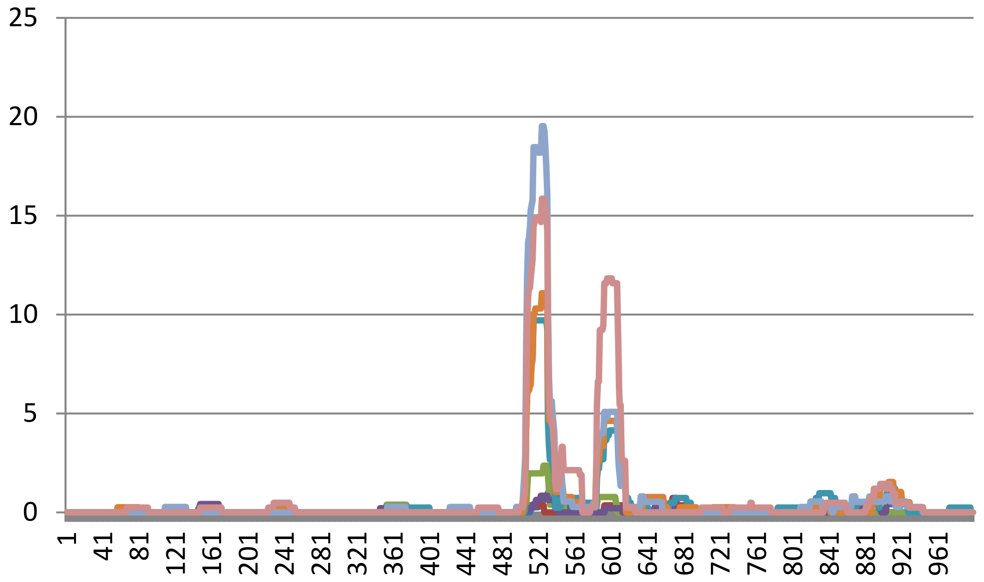

AT5G48605RC

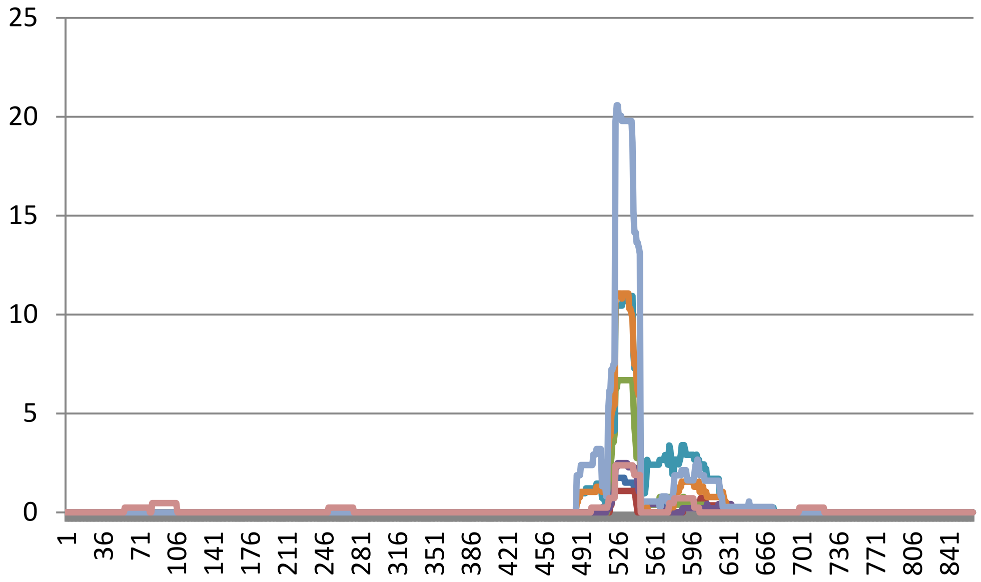

AT5G49900RC

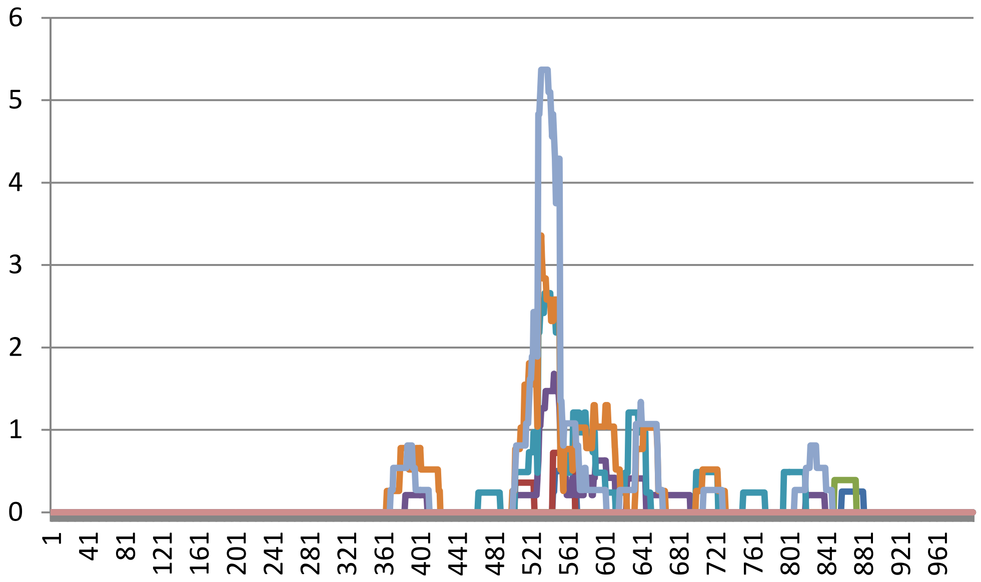

AT5G50880RC

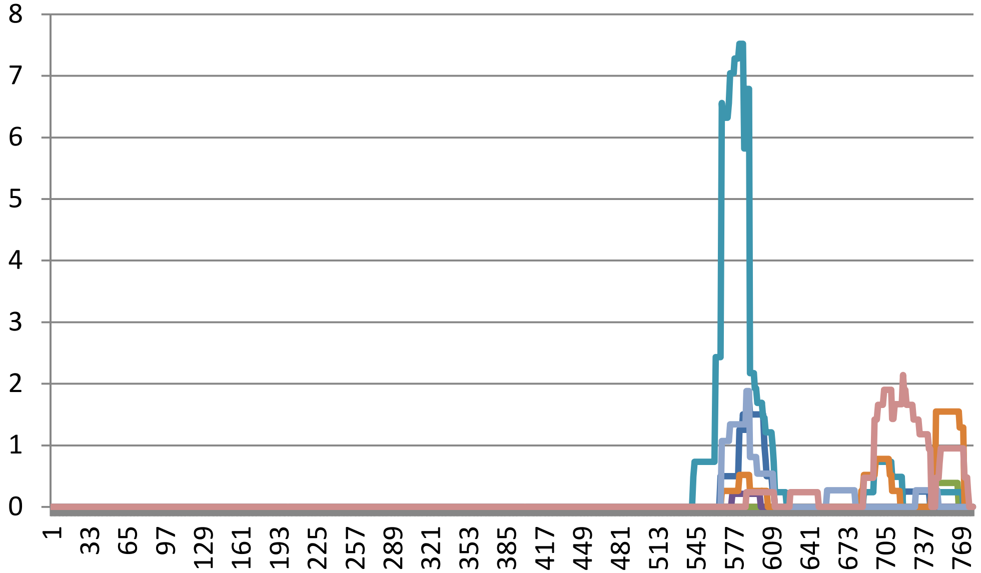

AT5G53742RC

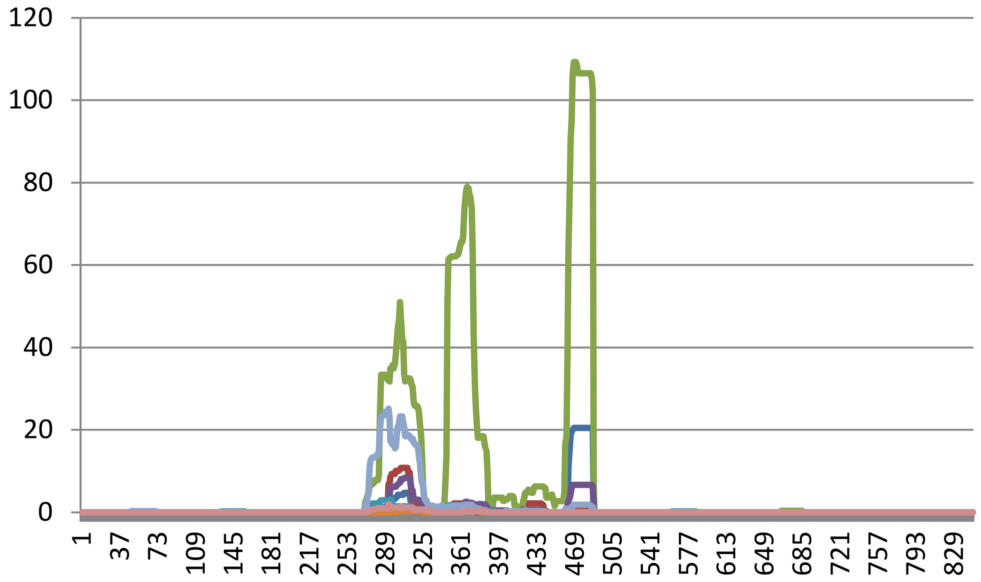

AT5G58390RC

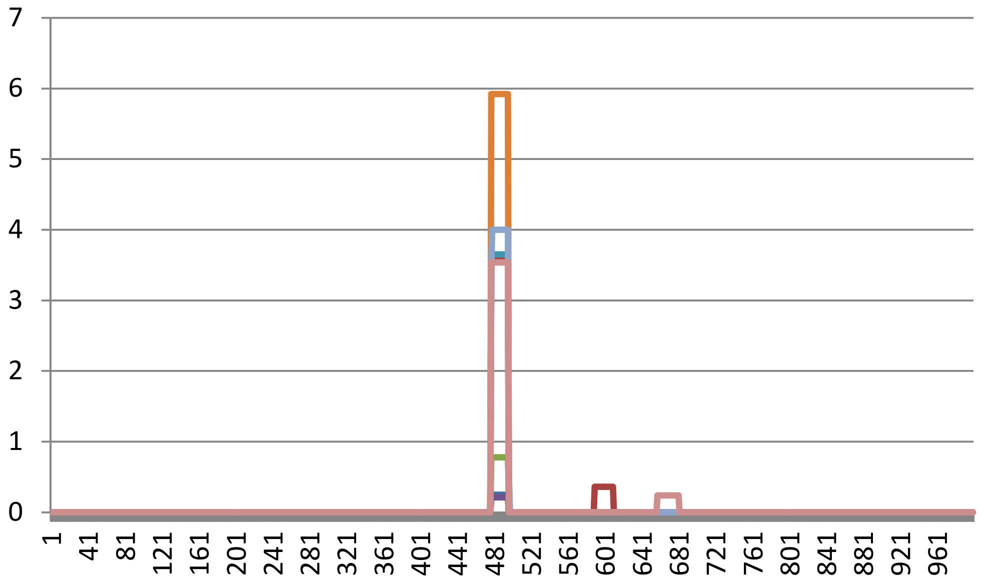

AT5G59060RC

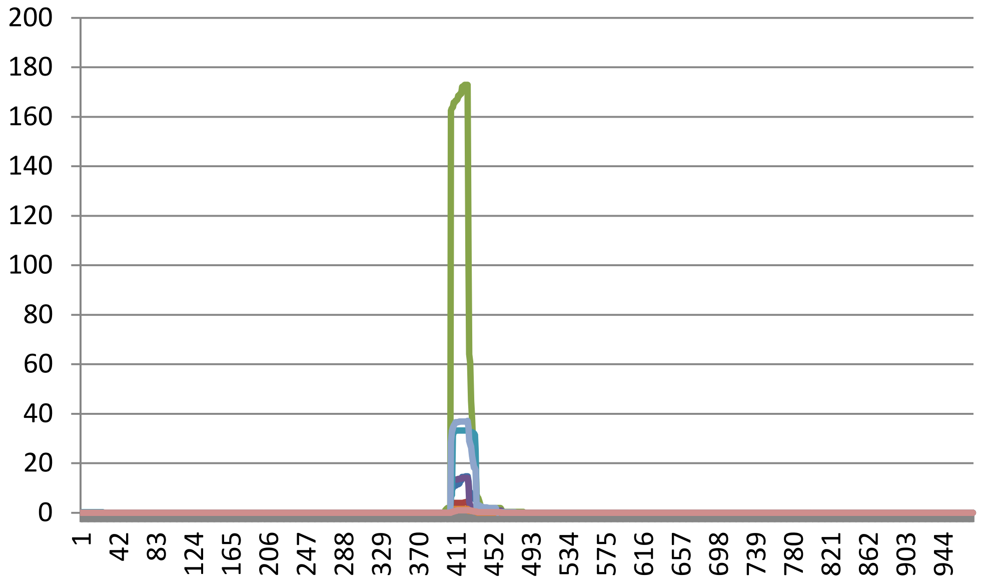

AT5G62280RC

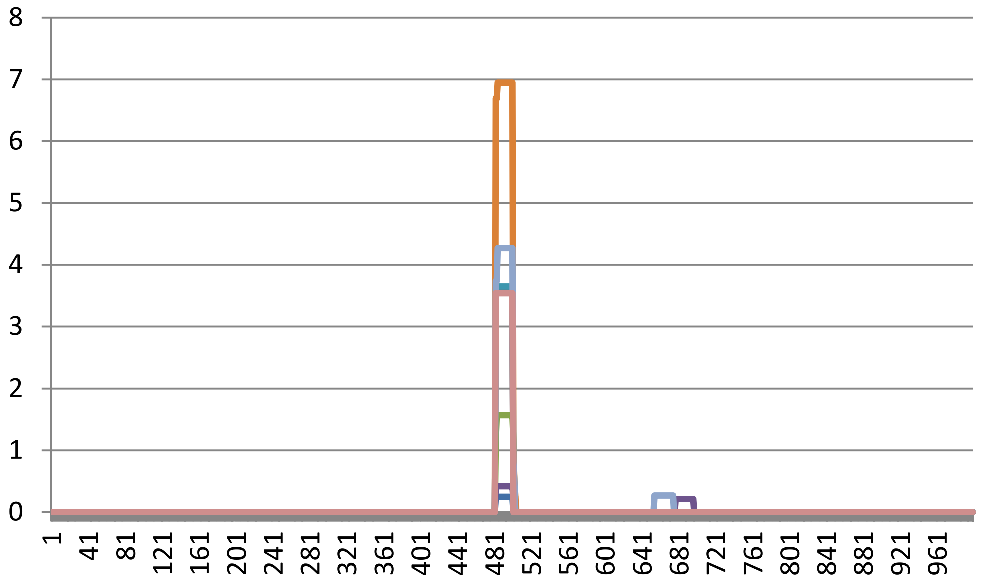

AT5G64860RC

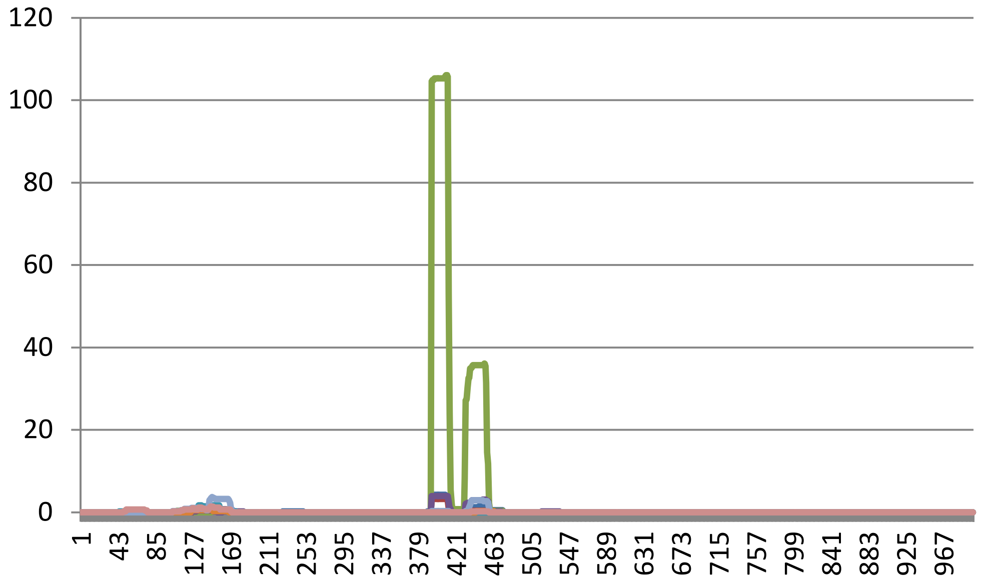

AT5G67190RC

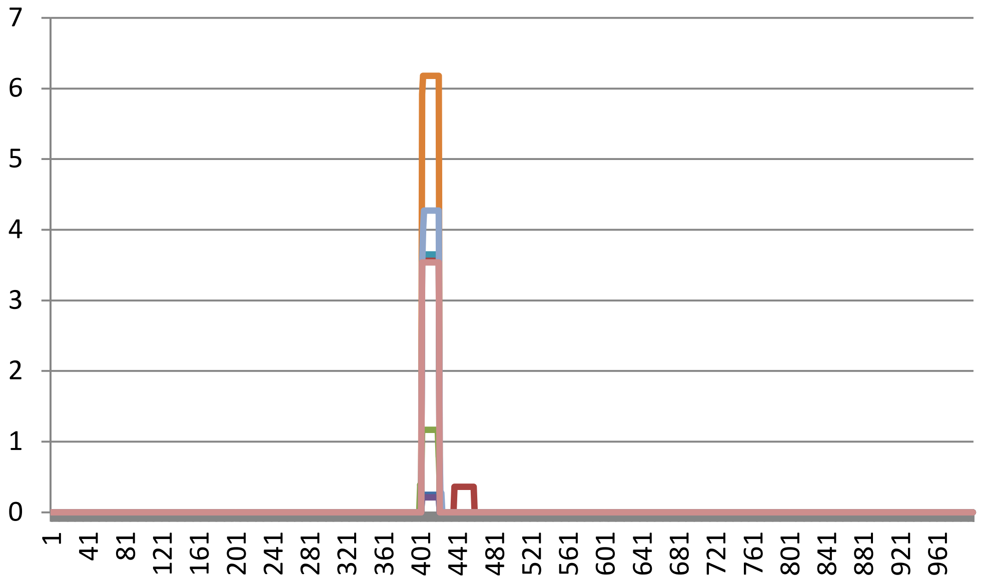

Supplement: S11 Fig — (PDF) [file pone.0169212.s011.pdf]
