# Supplementary material for: Transcriptome-Wide Discovery of PASRs (Promoter-Associated Small RNAs) and TASRs (Terminus-Associated Small RNAs) in Arabidopsis thaliana
Source: PLoS One. 2017 Jan 3;12(1):e0169212. doi: 10.1371/journal.pone.0169212 (PMC5207706; doi:10.1371/journal.pone.0169212)
Supplement: S13 Fig — (PDF) [file pone.0169212.s013.pdf]

Figure S13 DsRNA-seq read-covered PASR peaks identified on the sense strands of the protein-coding genes of *Arabidopsis*. For each plot, x axis measures the position of the sense strand, and y axis measures the abundance (in RPM, reads per million) of sRNAs. The dsRNA-seq read covered region was highlighted in gray background.

AT1G28281

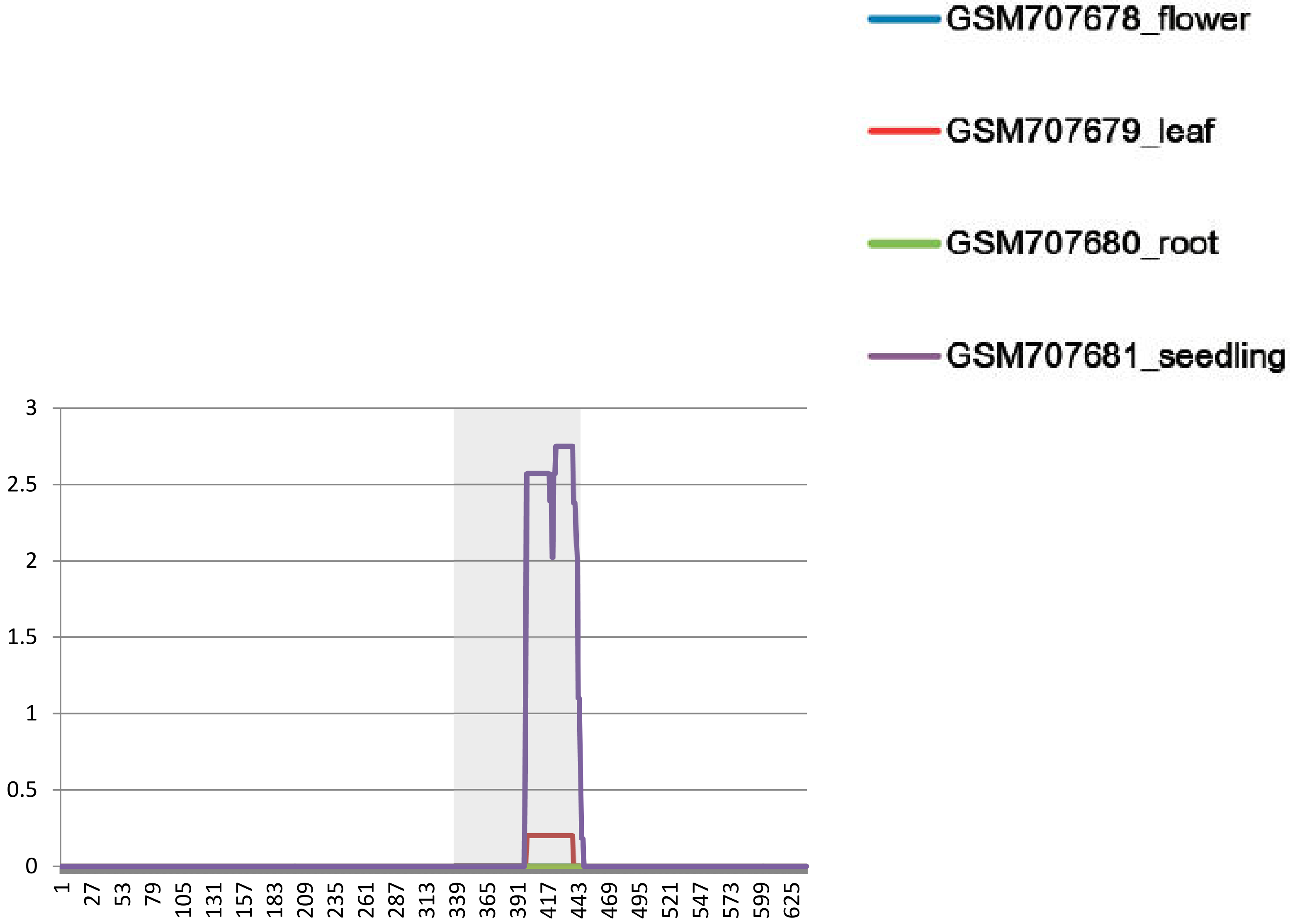

GSM707678_flower
GSM707679_leaf
GSM707680_root
GSM707681_seedling
3
2.5
2
1.5
1
0.5
0
1
27
53
79
105
131
157
183
209
235
261
287
313
339
365
391
417
443
469
495
521
547
573
599
625

AT1G53542

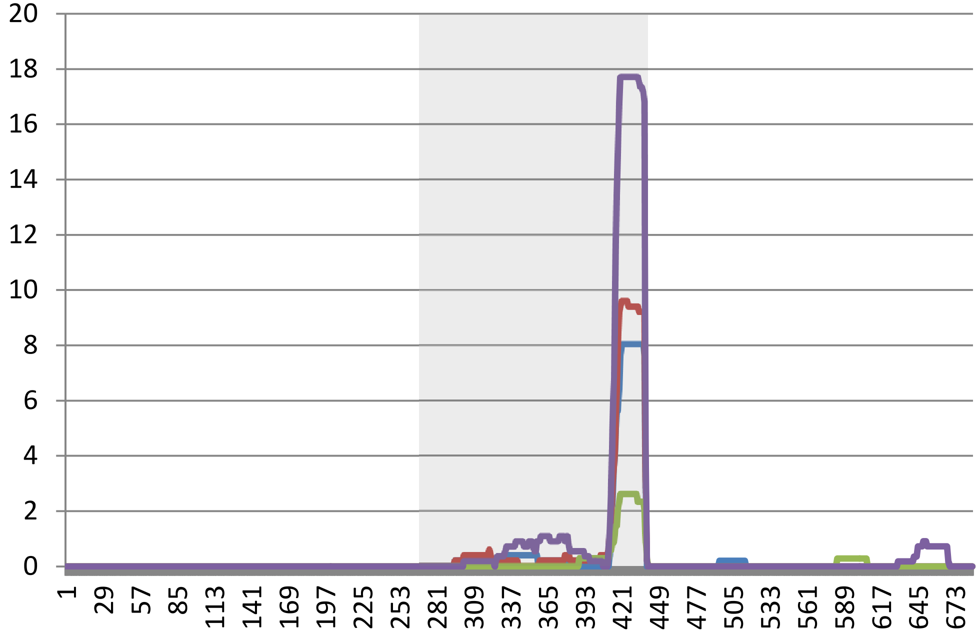

20
18
16
14
12
10
8
6
4
2
0
1
29
57
85
113
141
169
197
225
253
281
309
337
365
393
421
449
477
505
533
561
589
617
645
673

AT1G67450

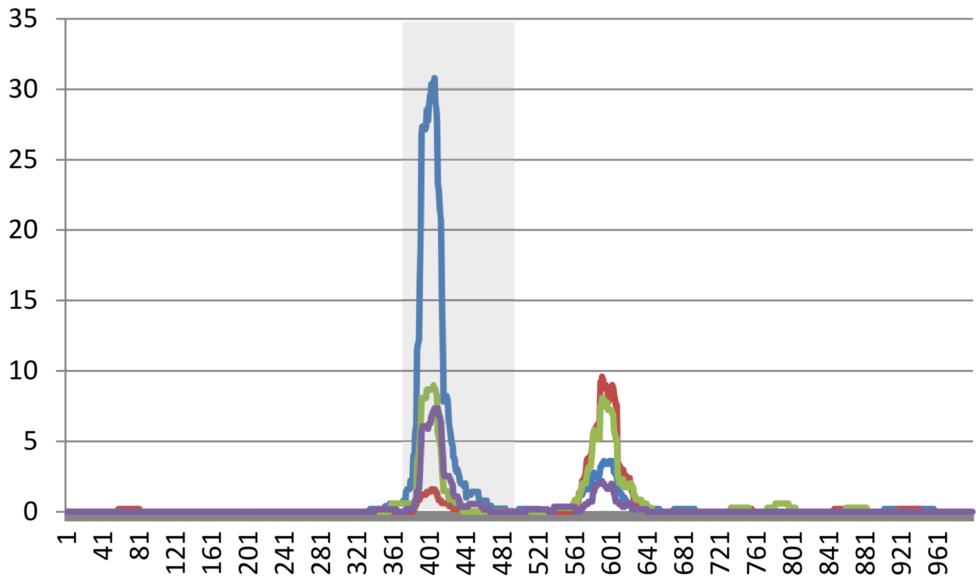

35
30
25
20
15
10
5
0
1
41
81
121
161
201
241
281
321
361
401
441
481
521
561
601
641
681
721
761
801
841
881
921
961

AT2G07708

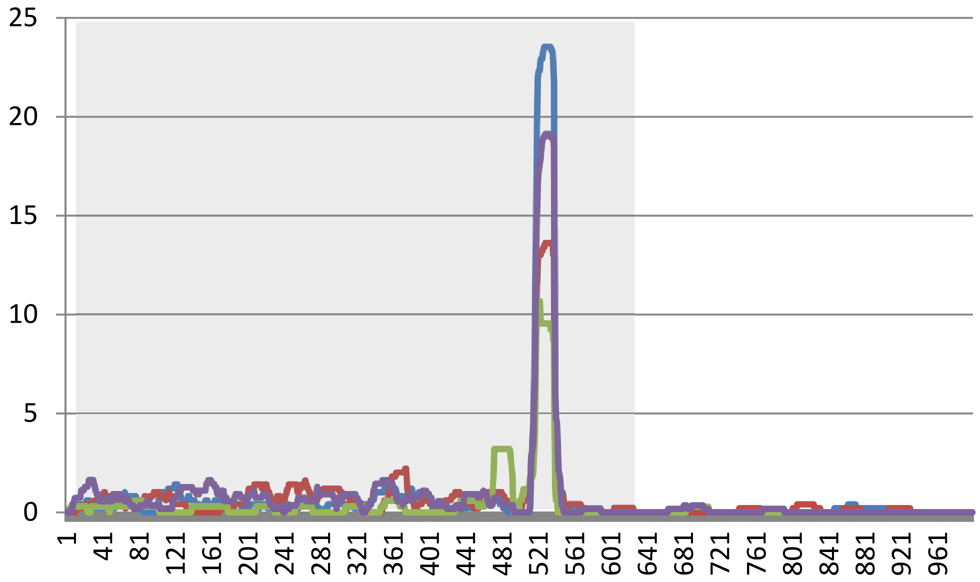

25
20
15
10
5
0
1
41
81
121
161
201
241
281
321
361
401
441
481
521
561
601
641
681
721
761
801
841
881
921
961

AT2G34655

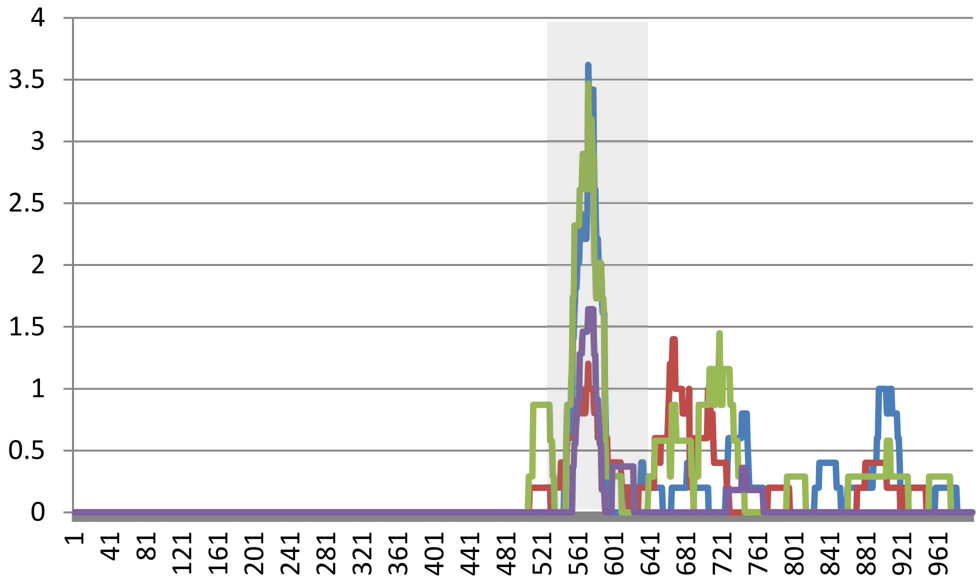

4
3.5
3
2.5
2
1.5
1
0.5
0
1
41
81
121
161
201
241
281
321
361
401
441
481
521
561
601
641
681
721
761
801
841
881
921
961

AT2G43865

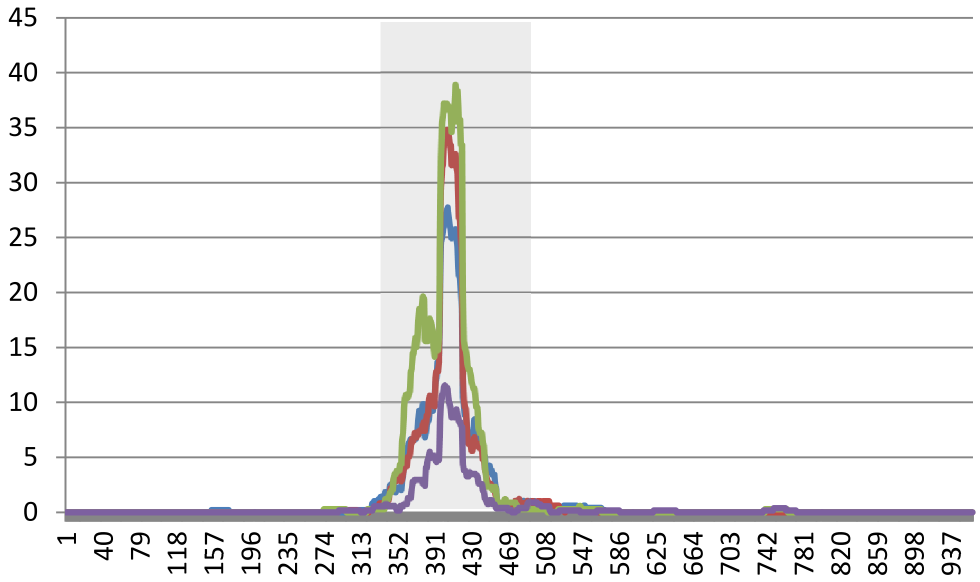

45
40
35
30
25
20
15
10
5
0
1
40
79
118
157
196
235
274
313
352
391
430
469
508
547
586
625
664
703
742
781
820
859
898
937

AT3G03340

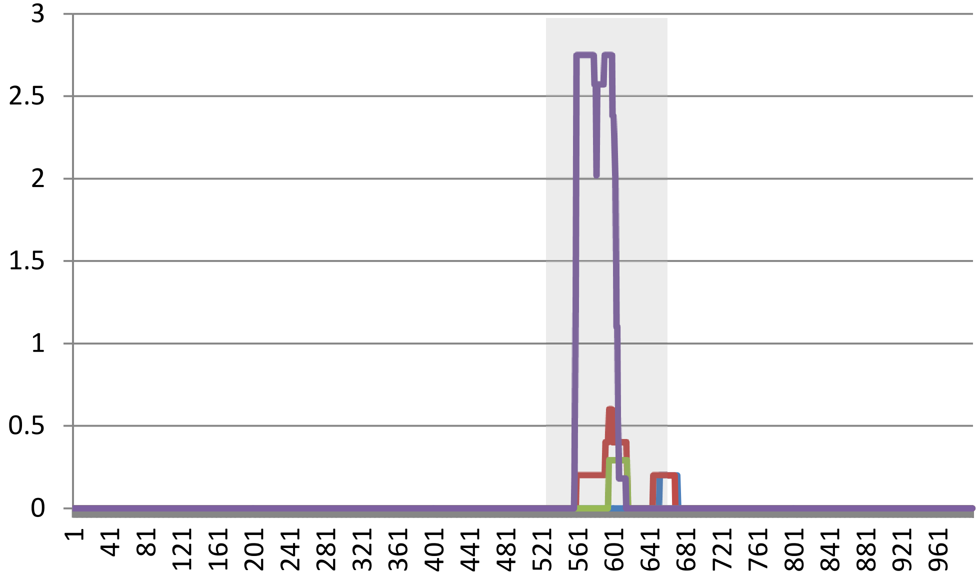

3
2.5
2
1.5
1
0.5
0
1
41
81
121
161
201
241
281
321
361
401
441
481
521
561
601
641
681
721
761
801
841
881
921
961

AT3G20760

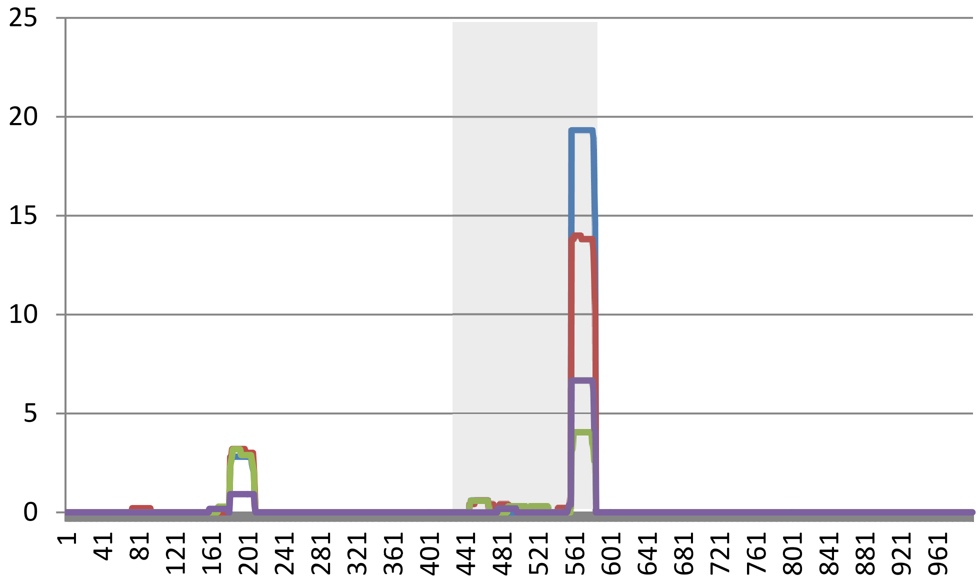

25
20
15
10
5
0
1
41
81
121
161
201
241
281
321
361
401
441
481
521
561
601
641
681
721
761
801
841
881
921
961

AT3G55850

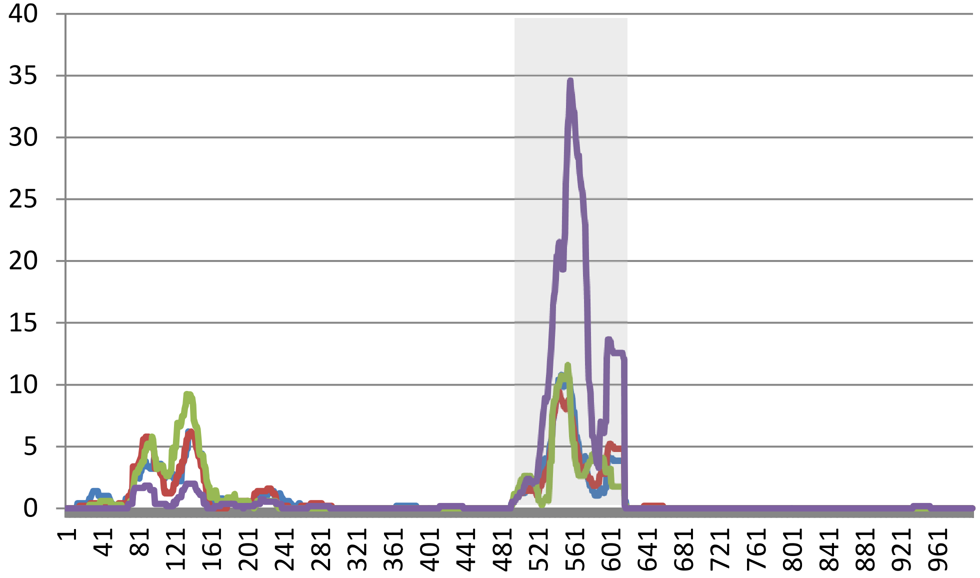

40
35
30
25
20
15
10
5
0
1
41
81
121
161
201
241
281
321
361
401
441
481
521
561
601
641
681
721
761
801
841
881
921
961

AT3G55860

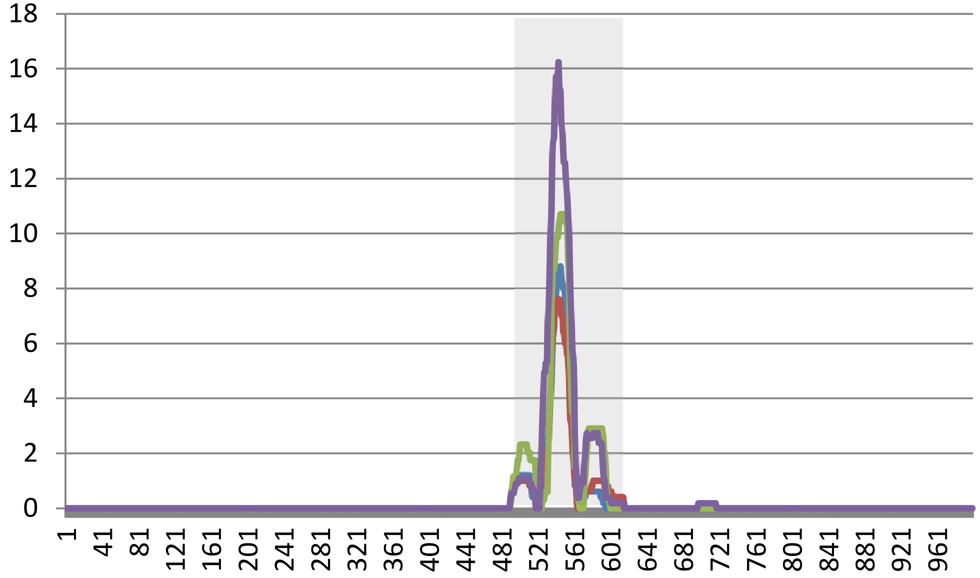

18
16
14
12
10
8
6
4
2
0
1
41
81
121
161
201
241
281
321
361
401
441
481
521
561
601
641
681
721
761
801
841
881
921
961

AT5G10340

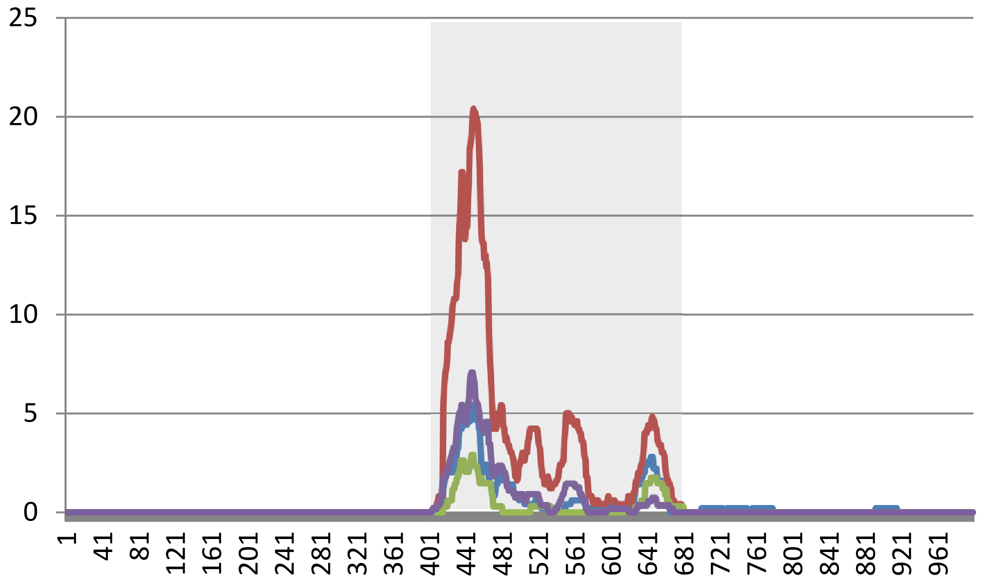

25
20
15
10
5
0
1
41
81
121
161
201
241
281
321
361
401
441
481
521
561
601
641
681
721
761
801
841
881
921
961

ATCG00065

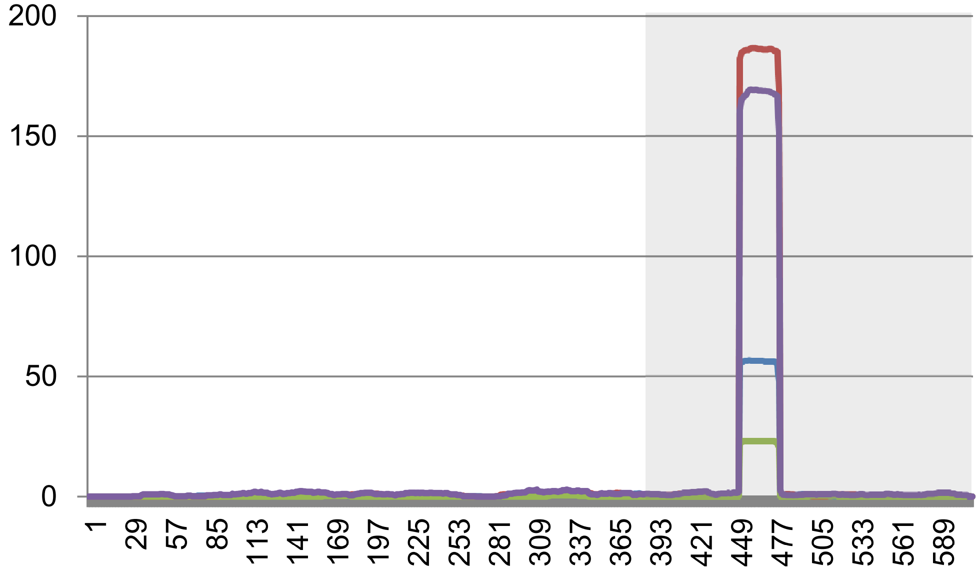
200
150
100
50
0
1
29
57
85
113
141
169
197
225
253
281
309
337
365
393
421
449
477
505
533
561
589

ATCG00120

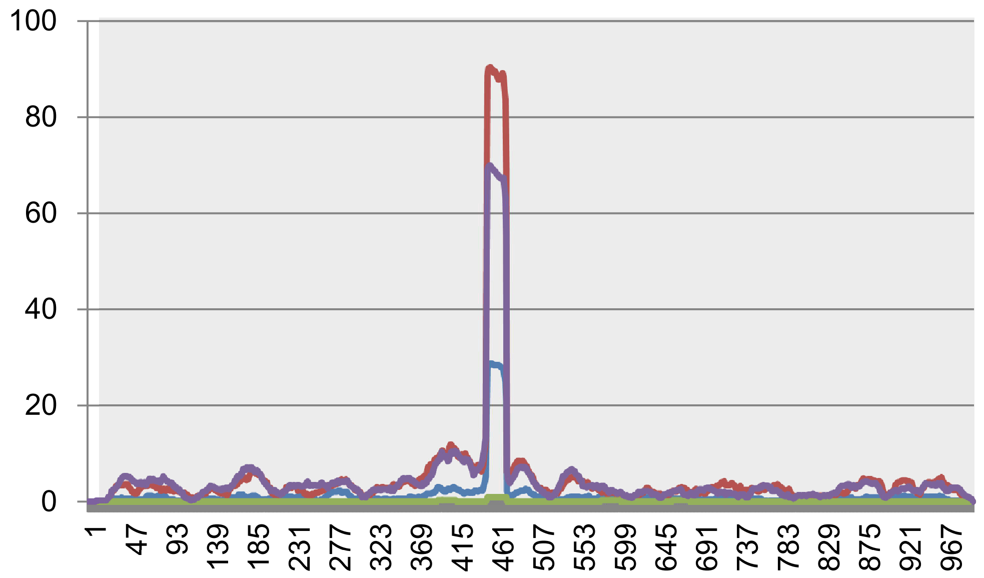

100
80
60
40
20
0
1
47
93
139
185
231
277
323
369
415
461
507
553
599
645
691
737
783
829
875
921
967

ATCG00140

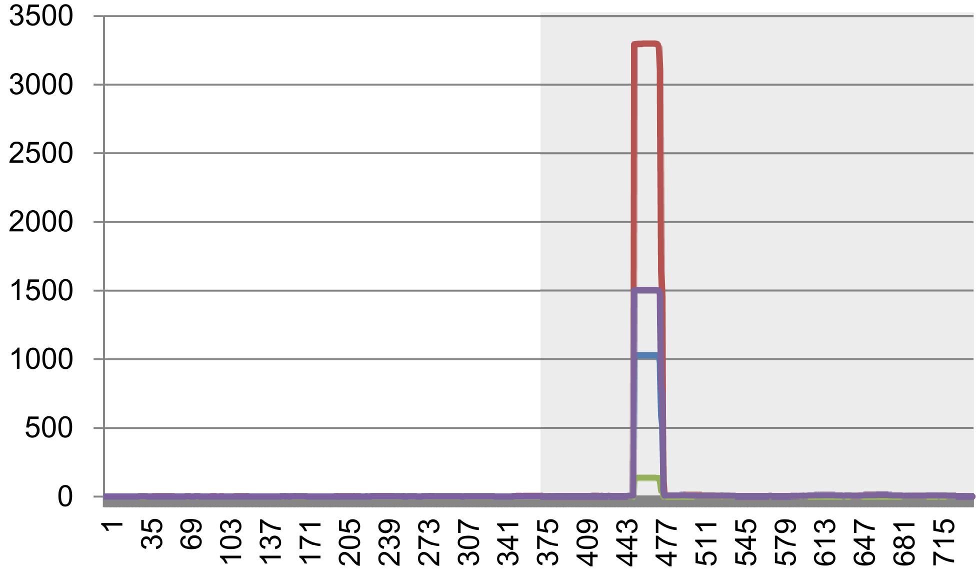

3500
3000
2500
2000
1500
1000
500
0
1
35
69
103
137
171
205
239
273
307
341
375
409
443
477
511
545
579
613
647
681
715

ATCG00150

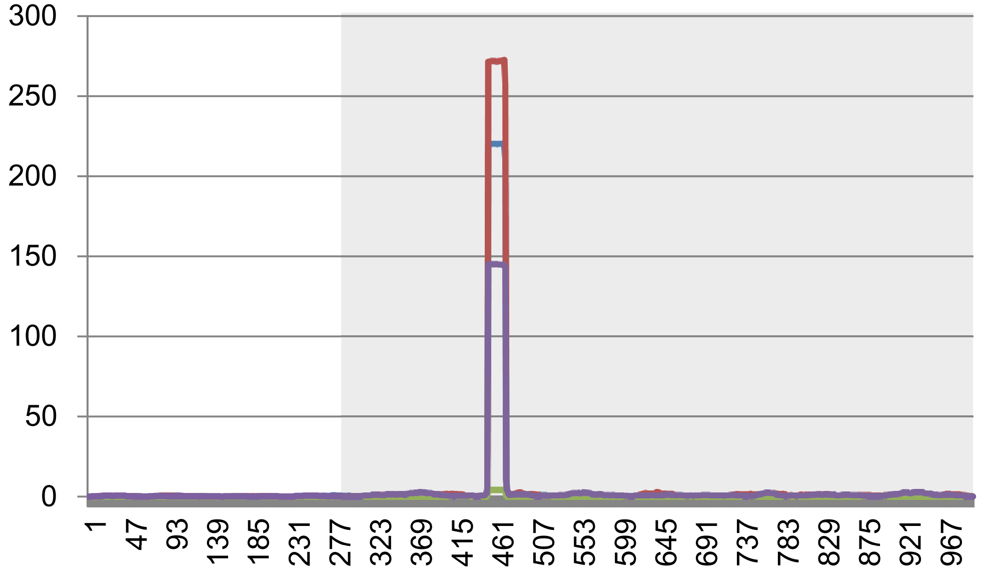
300
250
200
150
100
50
0
1
47
93
139
185
231
277
323
369
415
461
507
553
599
645
691
737
783
829
875
921
967

ATCG00170

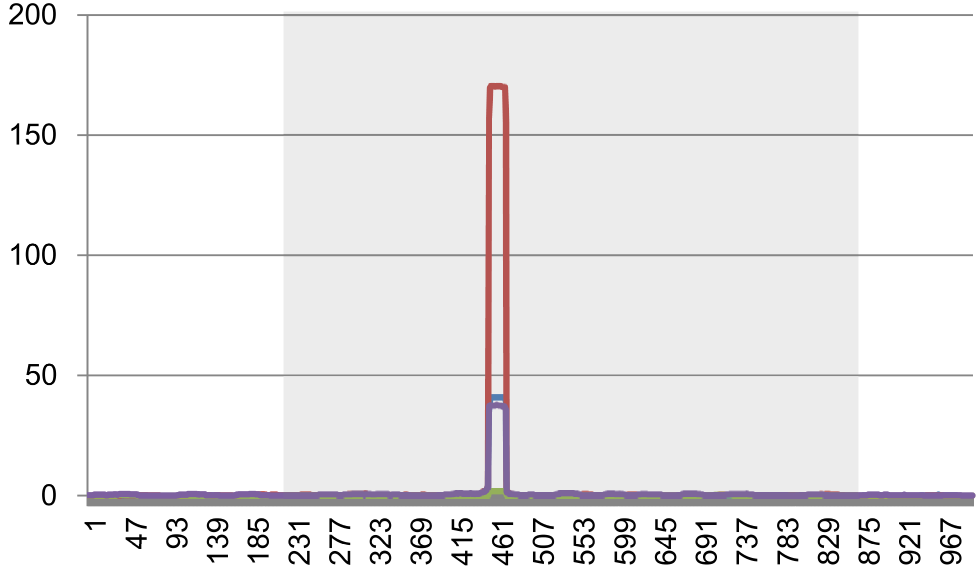

200
150
100
50
0
1
47
93
139
185
231
277
323
369
415
461
507
553
599
645
691
737
783
829
875
921
967

ATCG00280

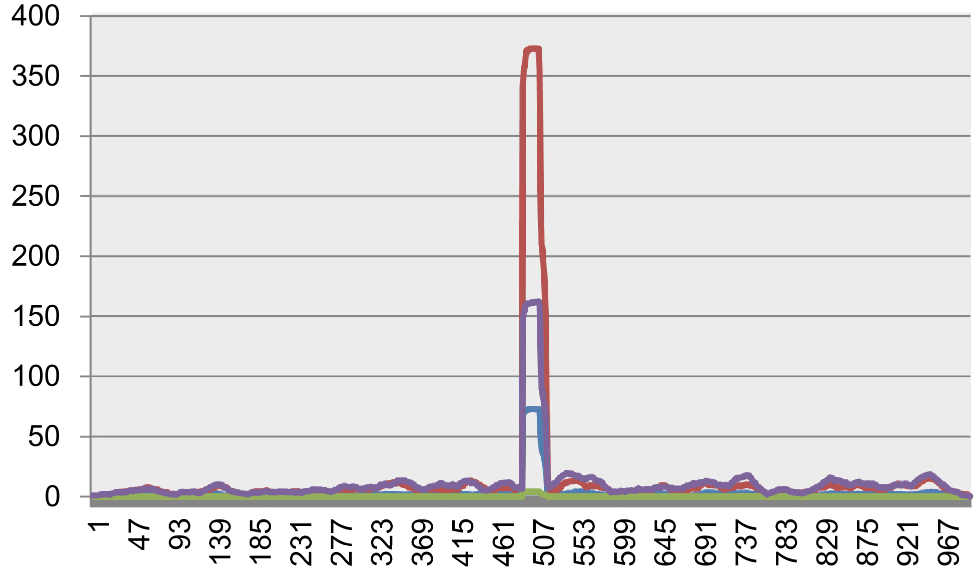

400
350
300
250
200
150
100
50
0
1
47
93
139
185
231
277
323
369
415
461
507
553
599
645
691
737
783
829
875
921
967

ATCG00500

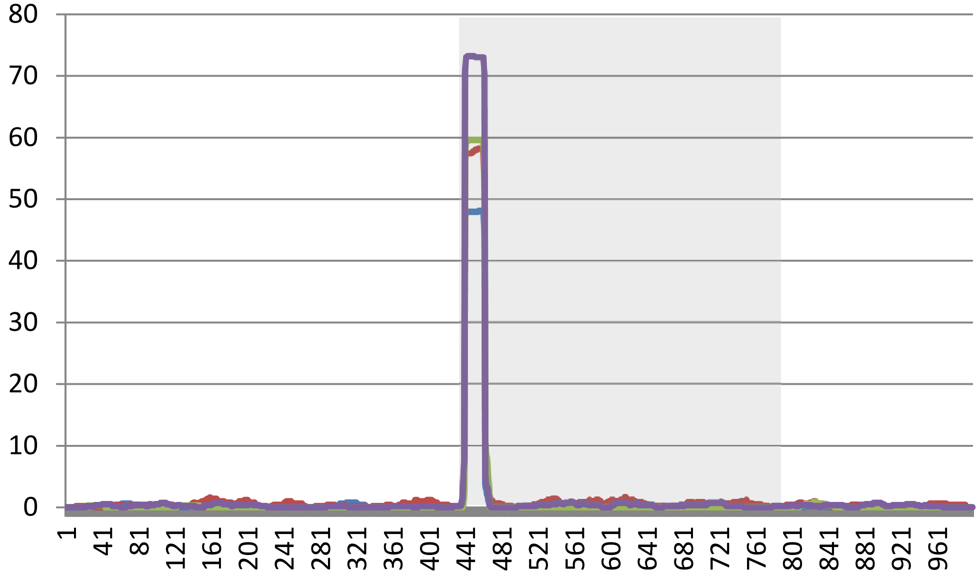

80
70
60
50
40
30
20
10
0
1
41
81
121
161
201
241
281
321
361
401
441
481
521
561
601
641
681
721
761
801
841
881
921
961

ATCG00540

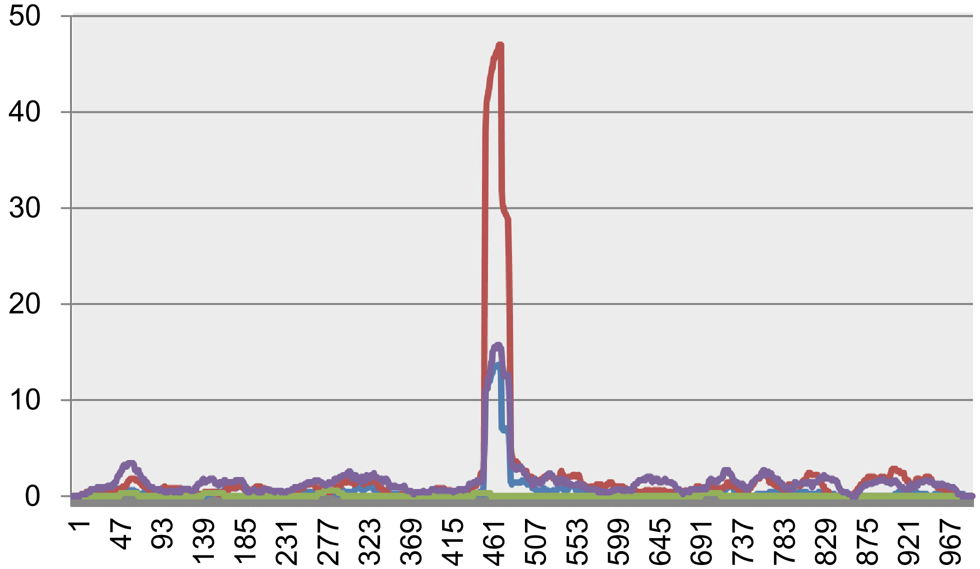

50
40
30
20
10
0
1
47
93
139
185
231
277
323
369
415
461
507
553
599
645
691
737
783
829
875
921
967

ATCG00590

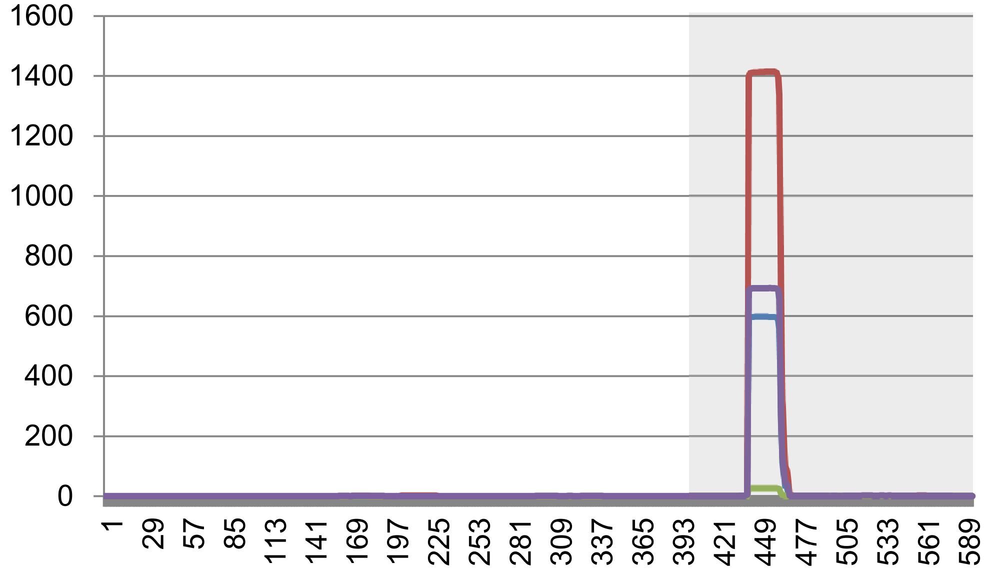
1600
1400
1200
1000
800
600
400
200
0
1
29
57
85
113
141
169
197
225
253
281
309
337
365
393
421
449
477
505
533
561
589

ATCG00670

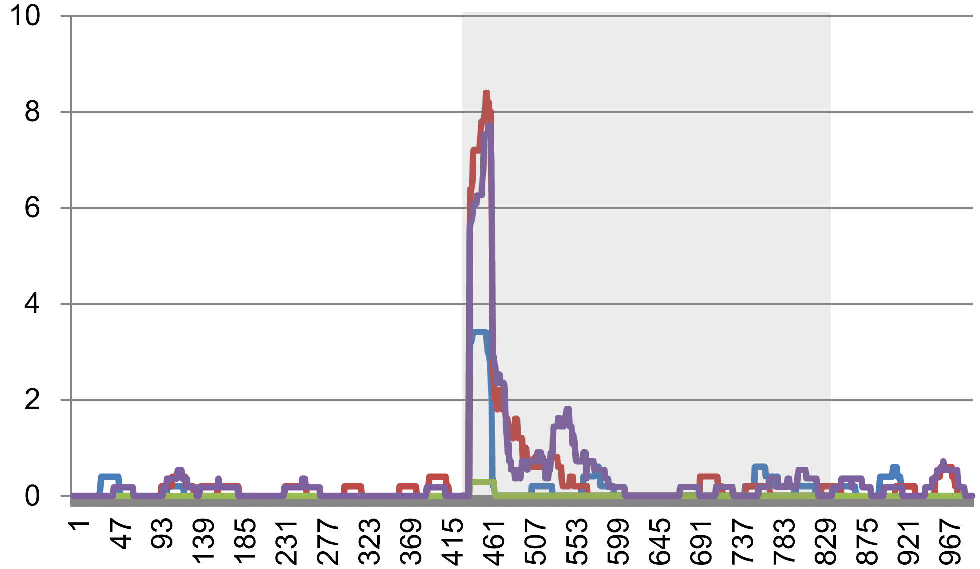

10
8
6
4
2
0
1
47
93
139
185
231
277
323
369
415
461
507
553
599
645
691
737
783
829
875
921
967

ATCG00720

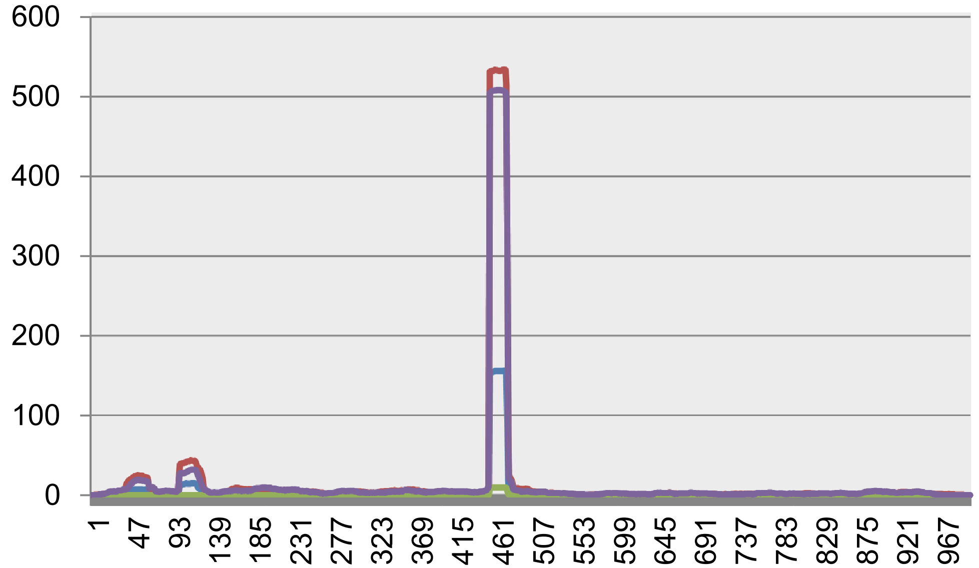

600
500
400
300
200
100
0
1
47
93
139
185
231
277
323
369
415
461
507
553
599
645
691
737
783
829
875
921
967

ATCG00830

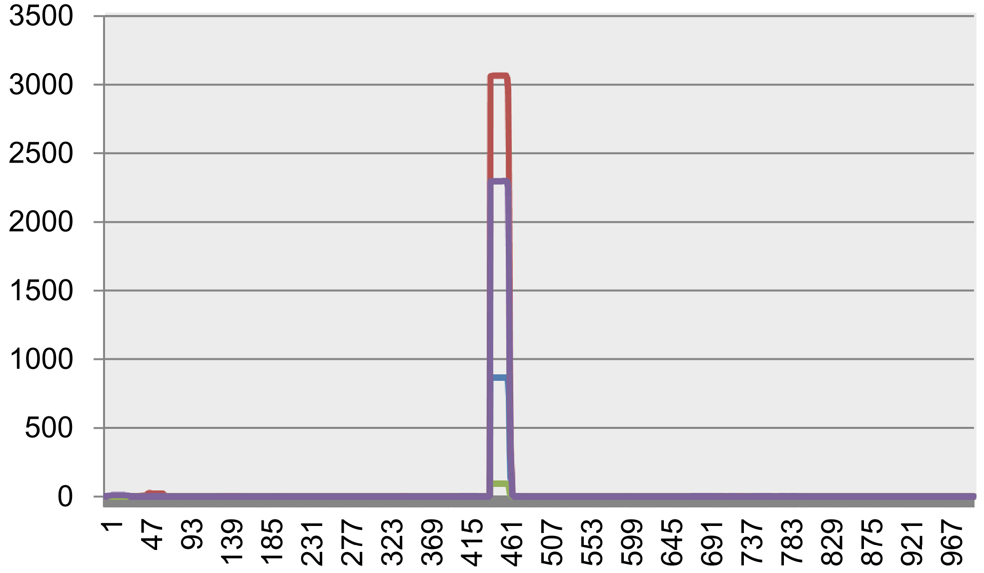

3500
3000
2500
2000
1500
1000
500
0
1
47
93
139
185
231
277
323
369
415
461
507
553
599
645
691
737
783
829
875
921
967

ATCG00870

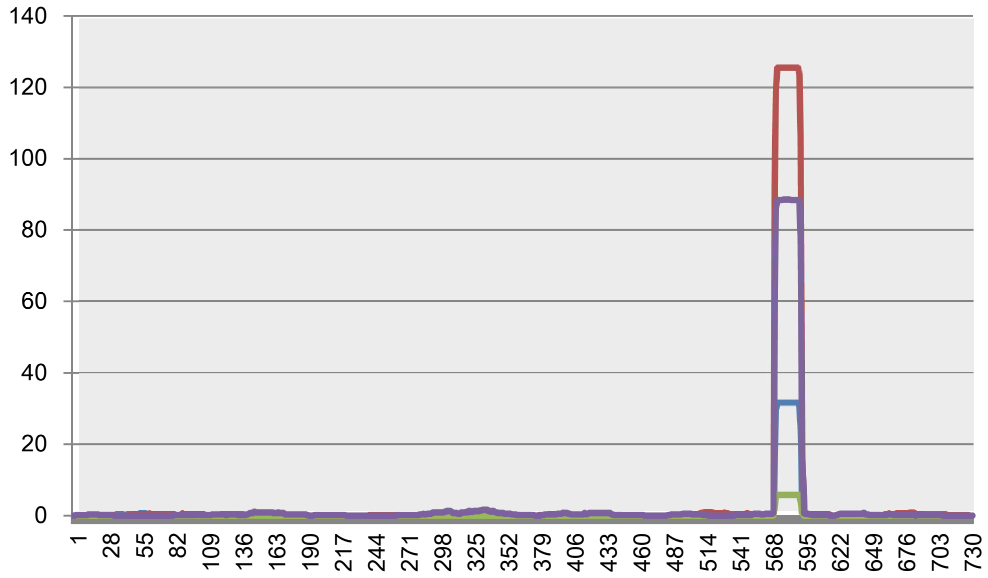

140
120
100
80
60
40
20
0
1
28
55
82
109
136
163
190
217
244
271
298
325
352
379
406
433
460
487
514
541
568
595
622
649
676
703
730

ATCG01100

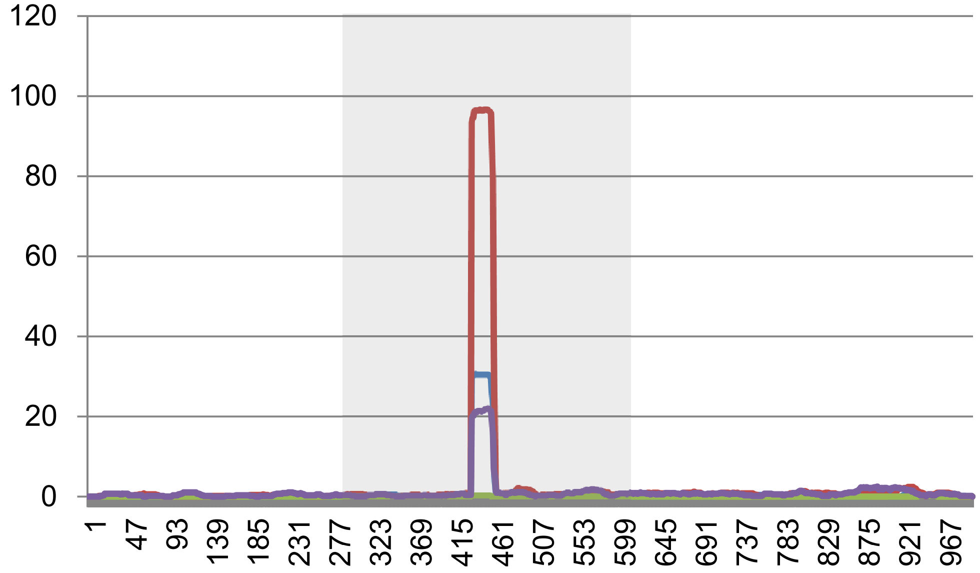

120
100
80
60
40
20
0
1
47
93
139
185
231
277
323
369
415
461
507
553
599
645
691
737
783
829
875
921
967

ATCG01270

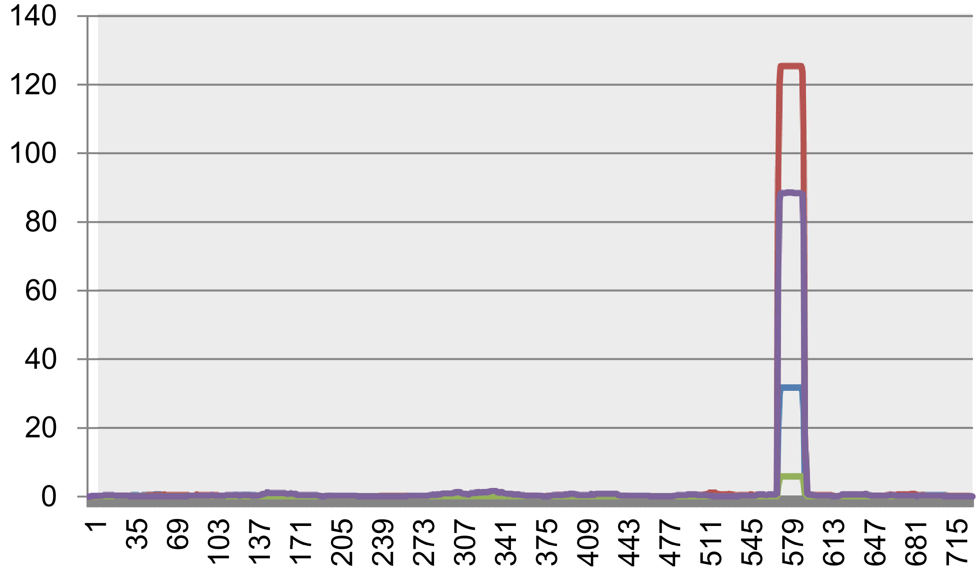
140
120
100
80
60
40
20
0
1
35
69
103
137
171
205
239
273
307
341
375
409
443
477
511
545
579
613
647
681
715

ATCG01310

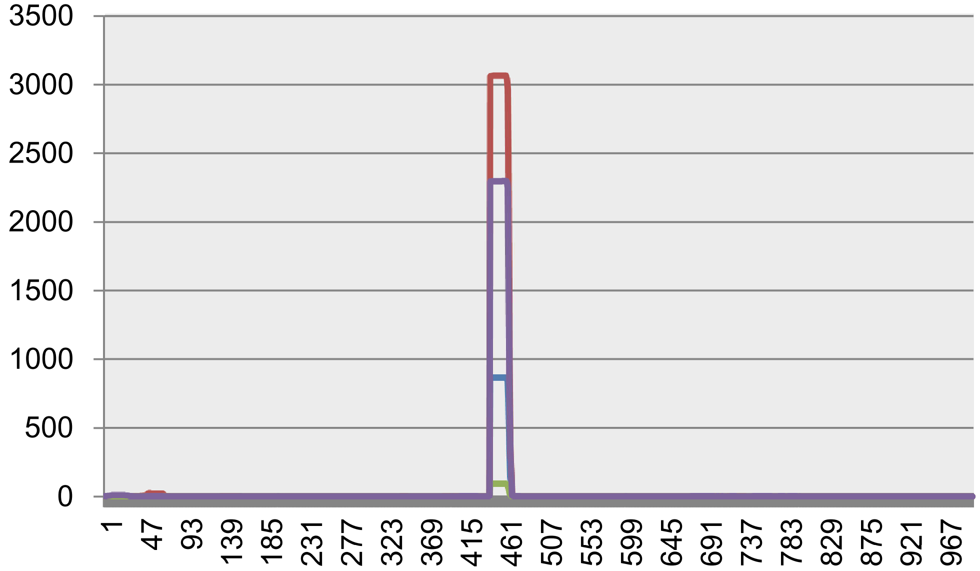
3500
3000
2500
2000
1500
1000
500
0
1
47
93
139
185
231
277
323
369
415
461
507
553
599
645
691
737
783
829
875
921
967

ATMG00570

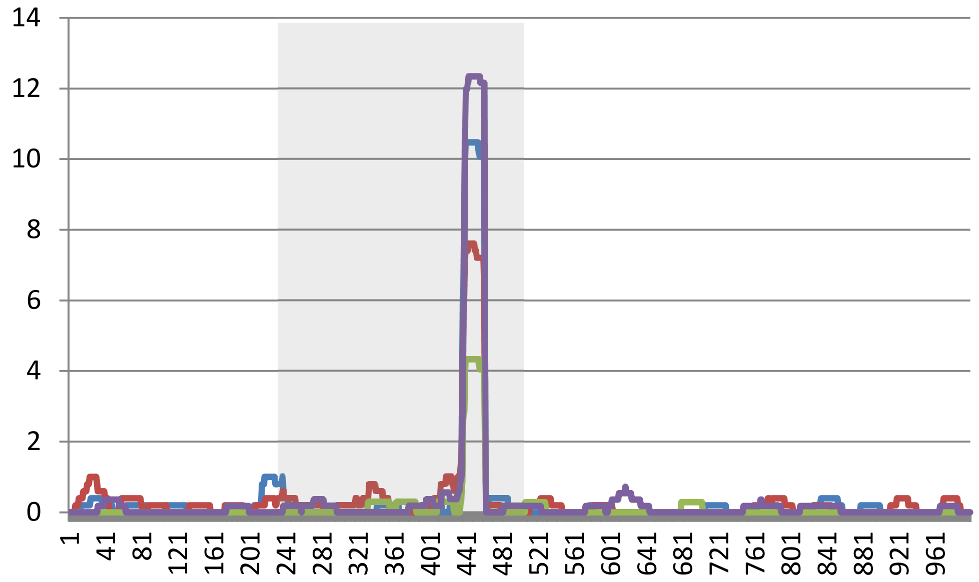

14
12
10
8
6
4
2
0
1
41
81
121
161
201
241
281
321
361
401
441
481
521
561
601
641
681
721
761
801
841
881
921
961

ATMG00690

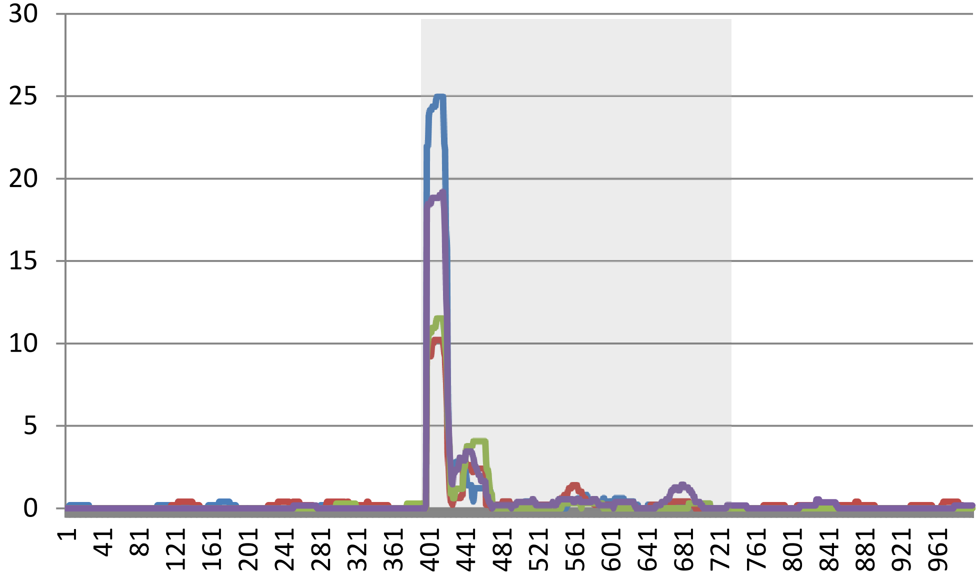

30
25
20
15
10
5
0
1
41
81
121
161
201
241
281
321
361
401
441
481
521
561
601
641
681
721
761
801
841
881
921
961

ATMG01350

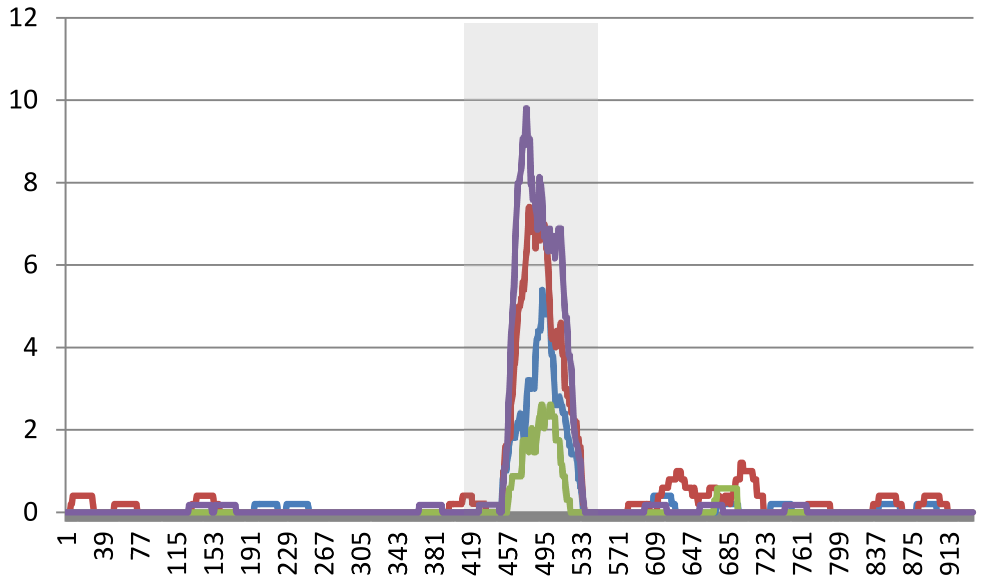

12
10
8
6
4
2
0
1
39
77
115
153
191
229
267
305
343
381
419
457
495
533
571
609
647
685
723
761
799
837
875
913
