# Supplementary material for: Transcriptome-Wide Discovery of PASRs (Promoter-Associated Small RNAs) and TASRs (Terminus-Associated Small RNAs) in Arabidopsis thaliana
Source: PLoS One. 2017 Jan 3;12(1):e0169212. doi: 10.1371/journal.pone.0169212 (PMC5207706; doi:10.1371/journal.pone.0169212)

Figure S14 DsRNA-seq read-covered PASR peaks identified on the antisense strands of the protein-coding genes of *Arabidopsis*. For each plot, x axis measures the position of the antisense strand, and y axis measures the abundance (in RPM, reads per million) of sRNAs. The dsRNA-seq read covered region was highlighted in gray background.

AT1G28280RC

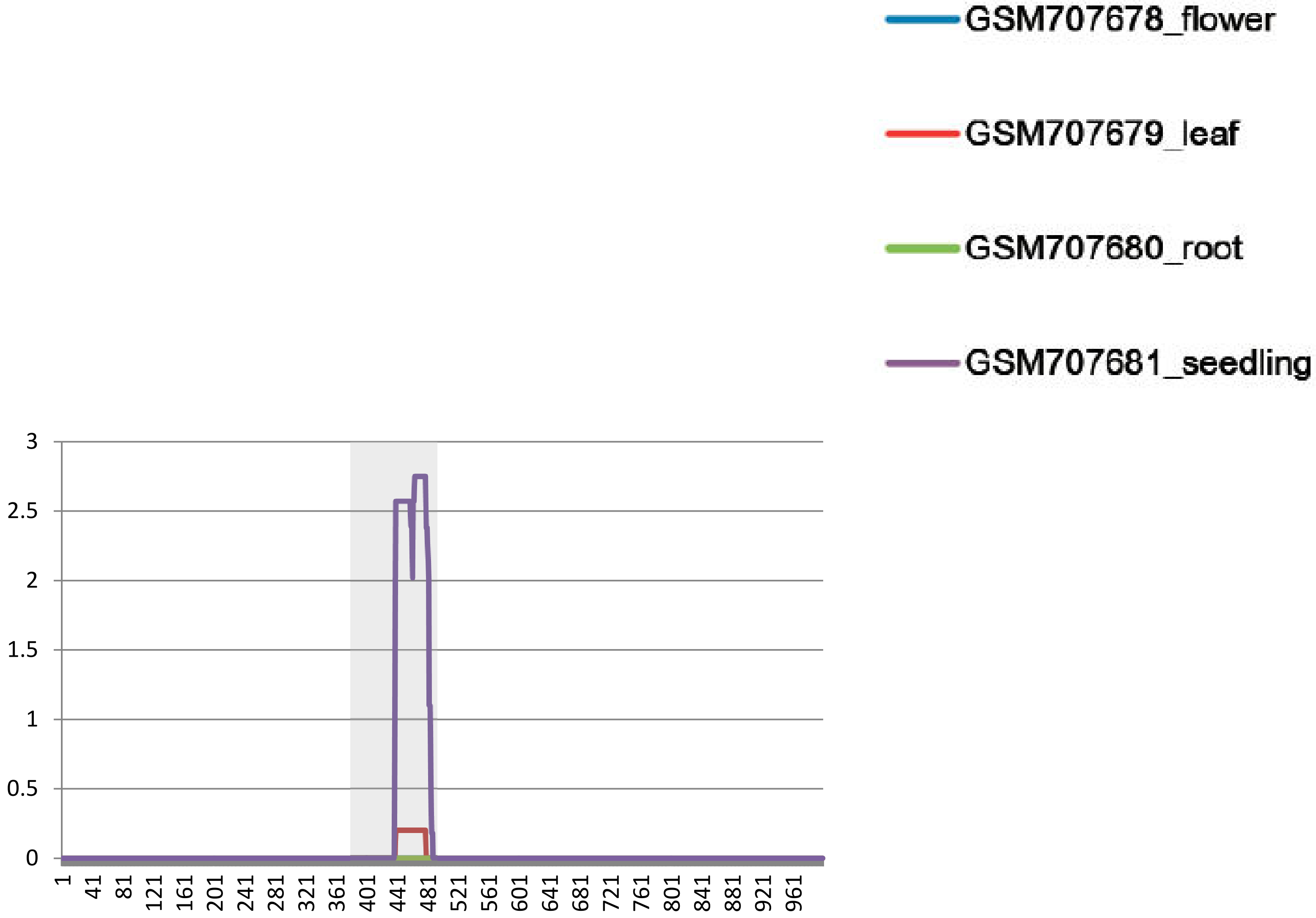

AT1G33350RC

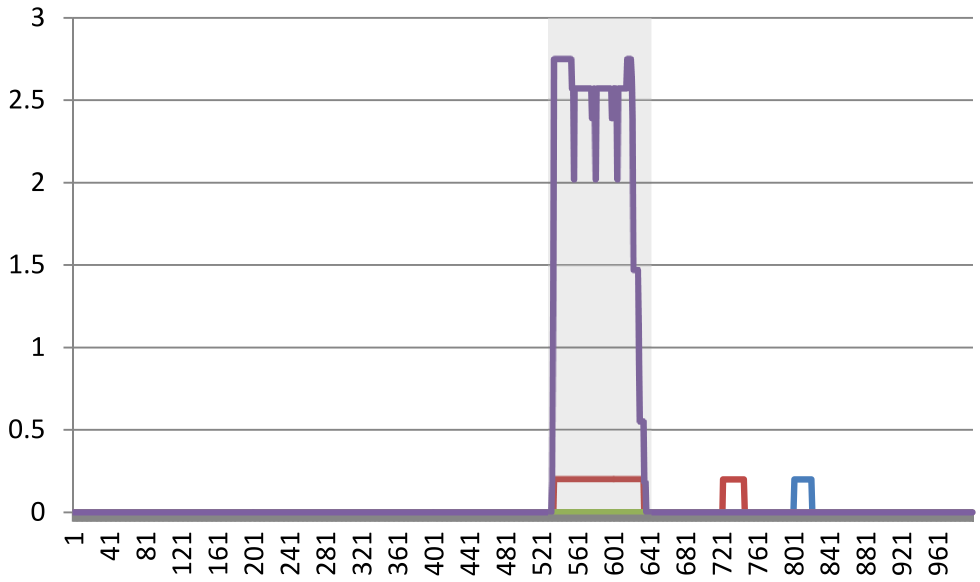

AT1G43624RC

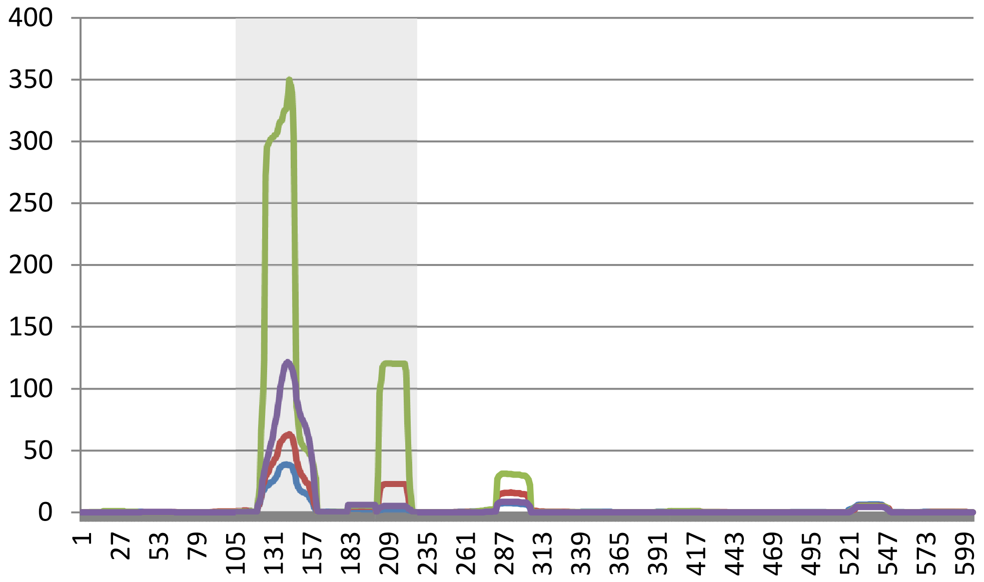

AT1G53541RC

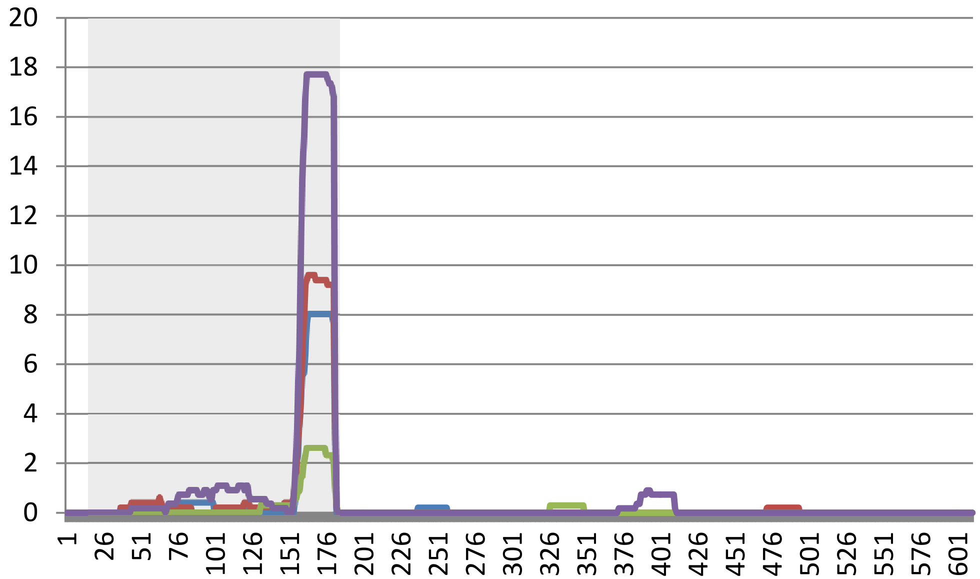

AT1G68945RC

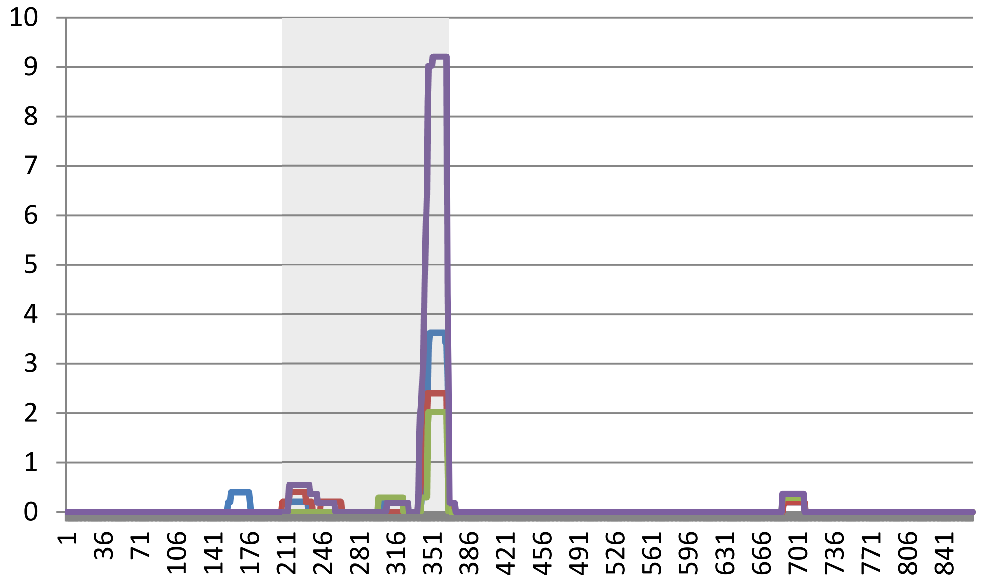

AT3G25460RC

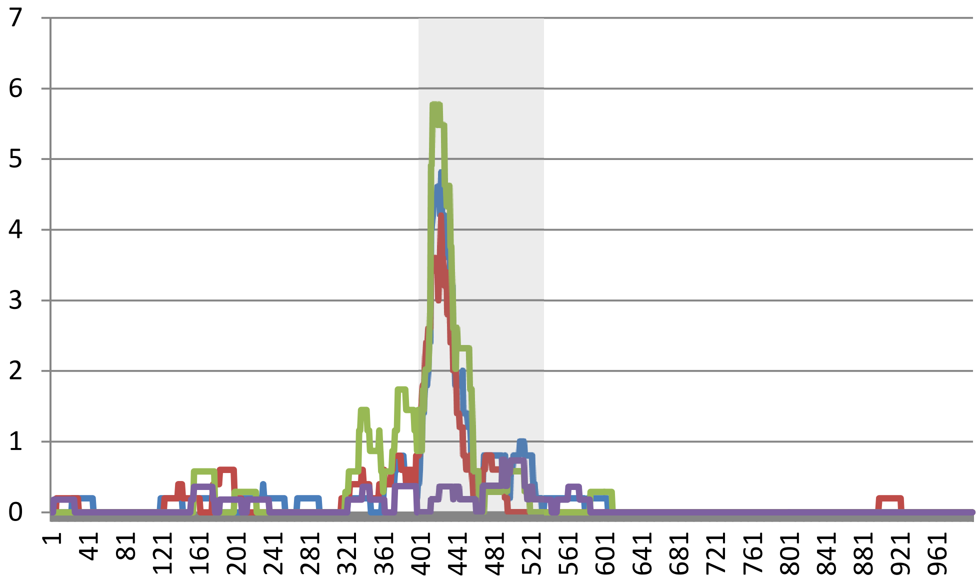

AT4G16460RC

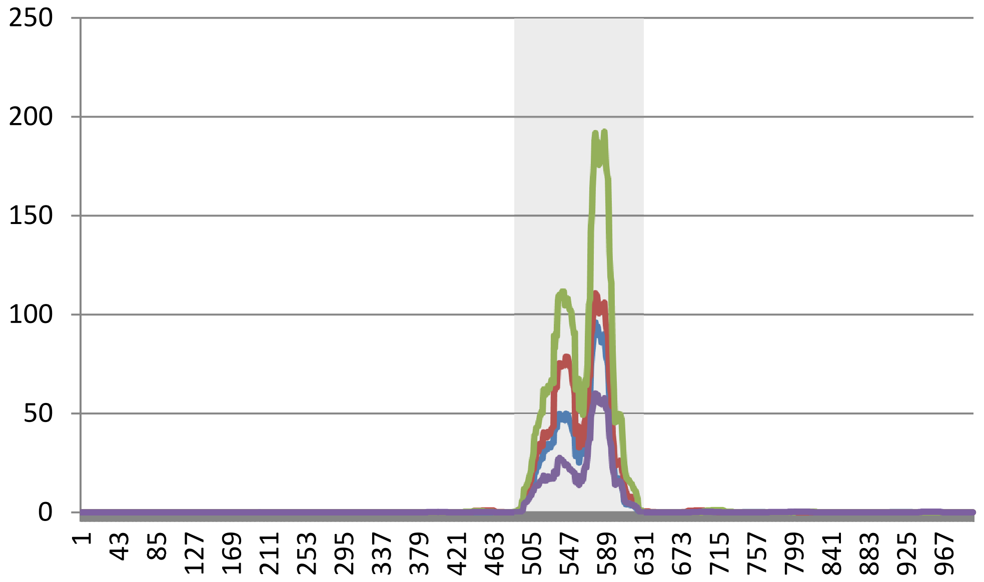

AT4G30740RC

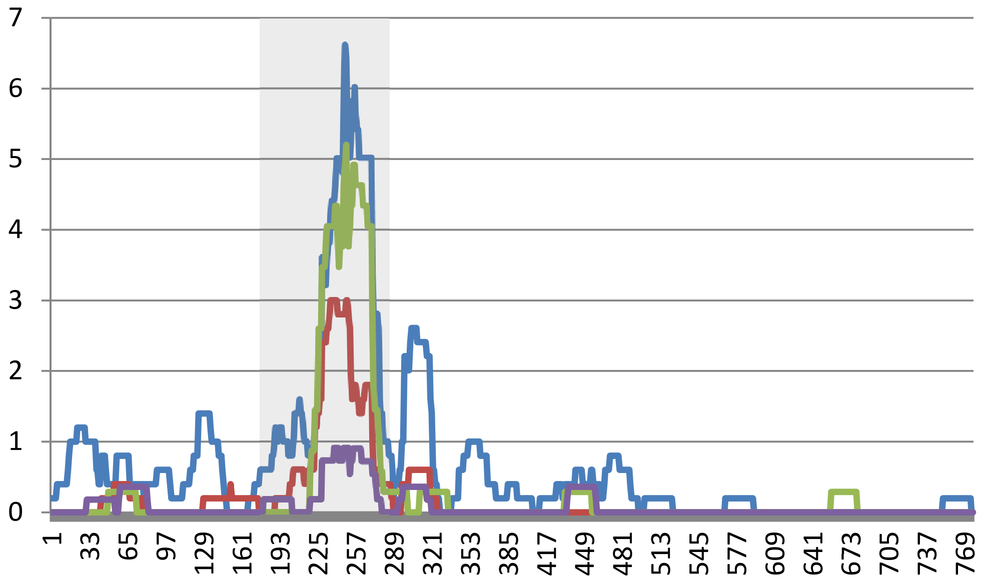

AT4G39900RC

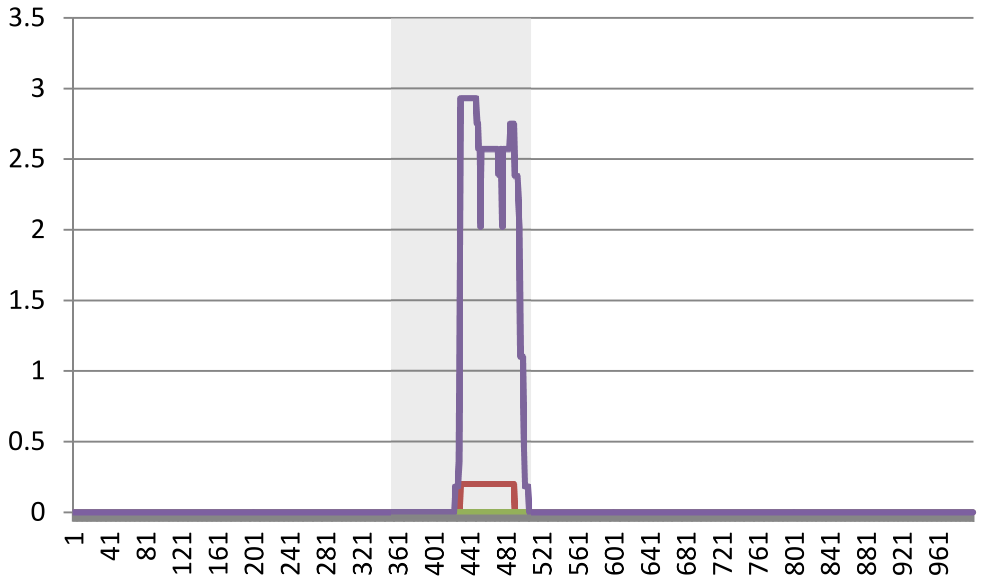

AT5G35526RC

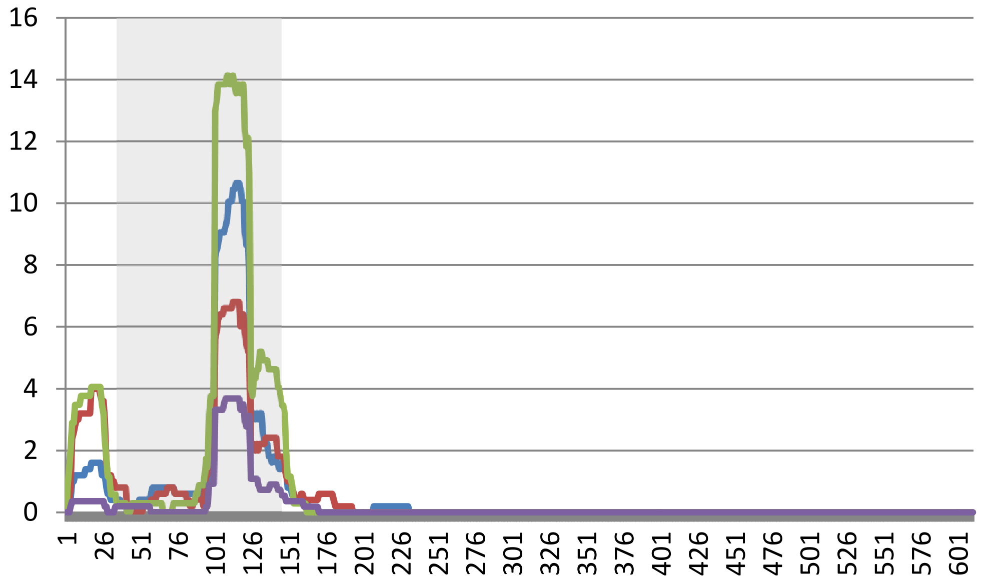

ATCG00790RC

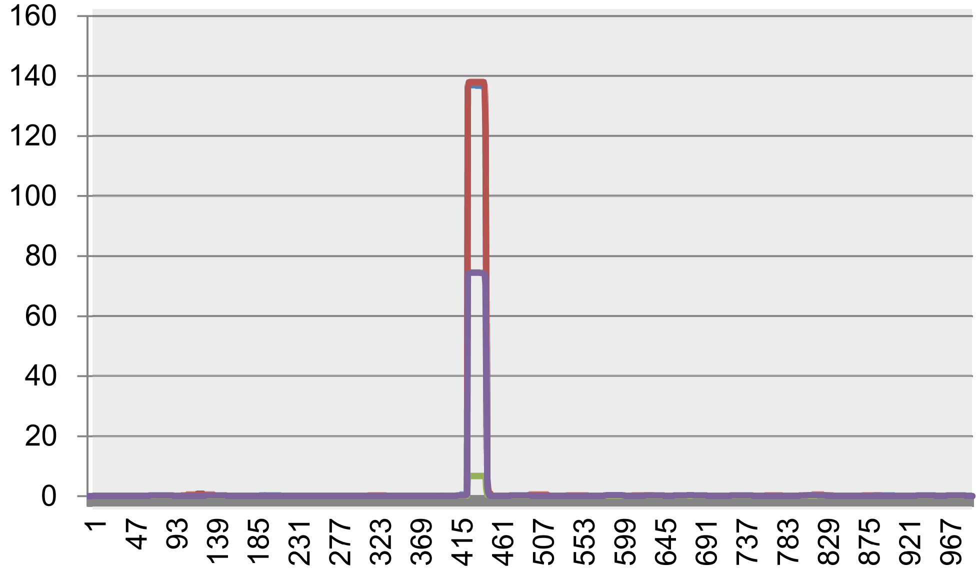

ATCG00840RC

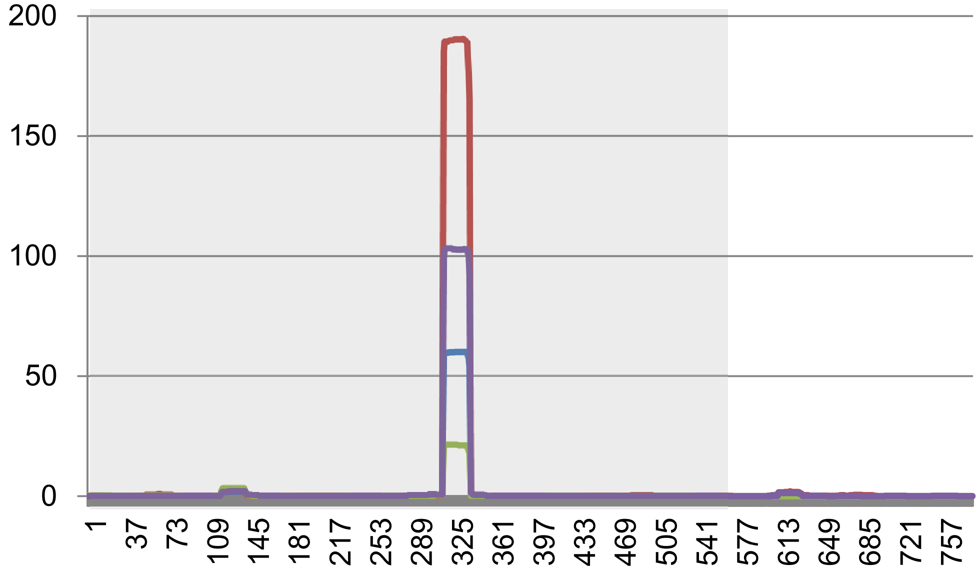

ATCG01300RC

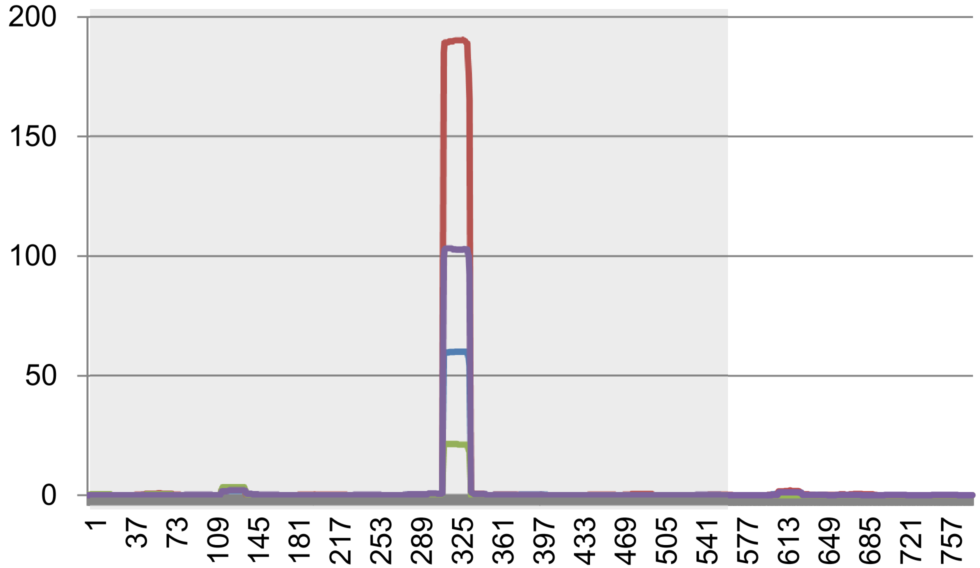

Supplement: S14 Fig — (PDF) [file pone.0169212.s014.pdf]
