# Supplementary material for: Transcriptome-Wide Discovery of PASRs (Promoter-Associated Small RNAs) and TASRs (Terminus-Associated Small RNAs) in Arabidopsis thaliana
Source: PLoS One. 2017 Jan 3;12(1):e0169212. doi: 10.1371/journal.pone.0169212 (PMC5207706; doi:10.1371/journal.pone.0169212)

Figure S15 DsRNA-seq read-covered paired PASR peaks identified on both strands of the protein-coding genes of *Arabidopsis*. For each plot, x axis measures the position of the paired strands, and y axis measures the abundance (in RPM, reads per million) of sRNAs. The dsRNA-seq read covered region was highlighted in gray background.

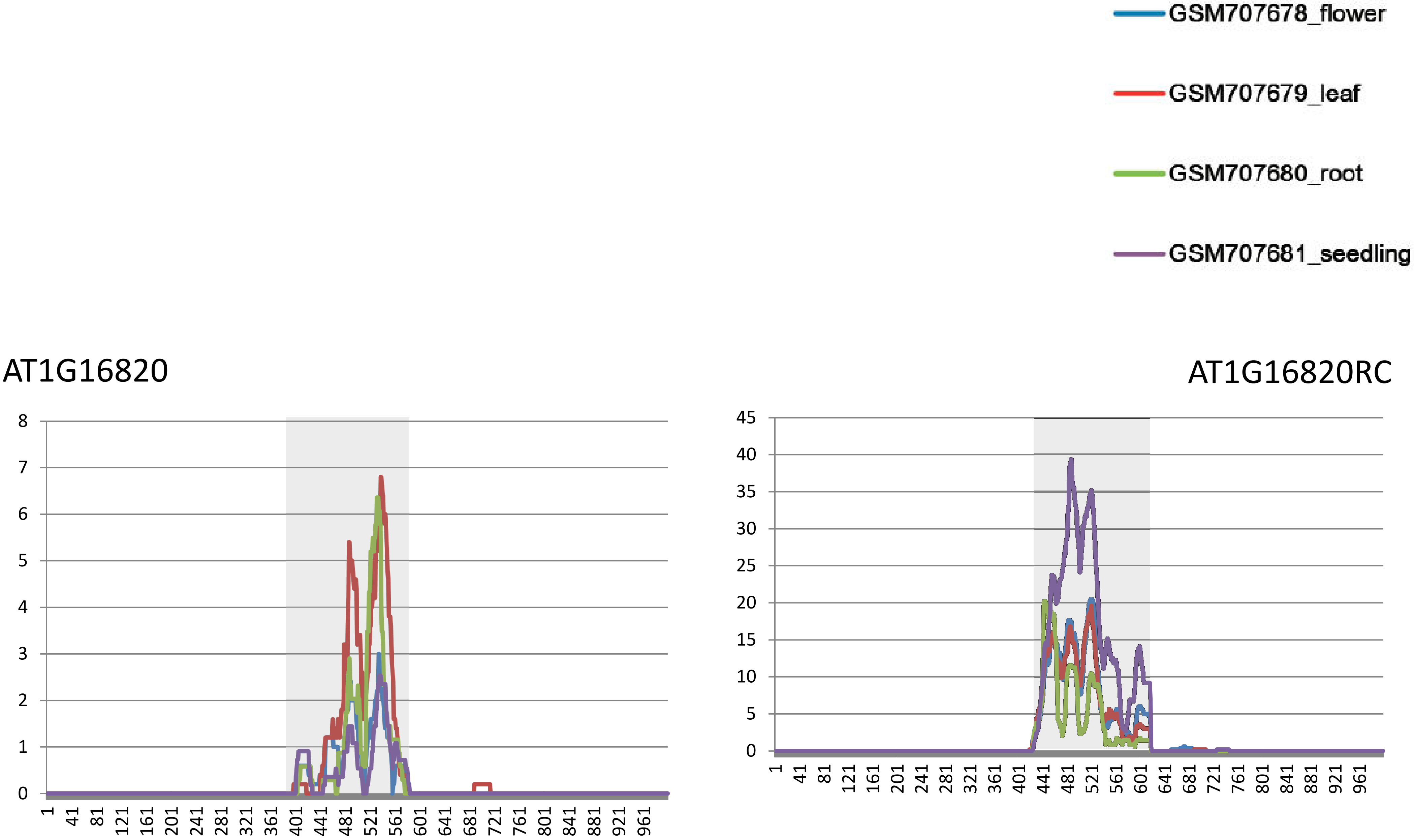
GSM707678_flower
GSM707679_leaf
GSM707680_root
GSM707681_seedling
AT1G16820
AT1G16820RC

AT1G53265

AT1G53265RC

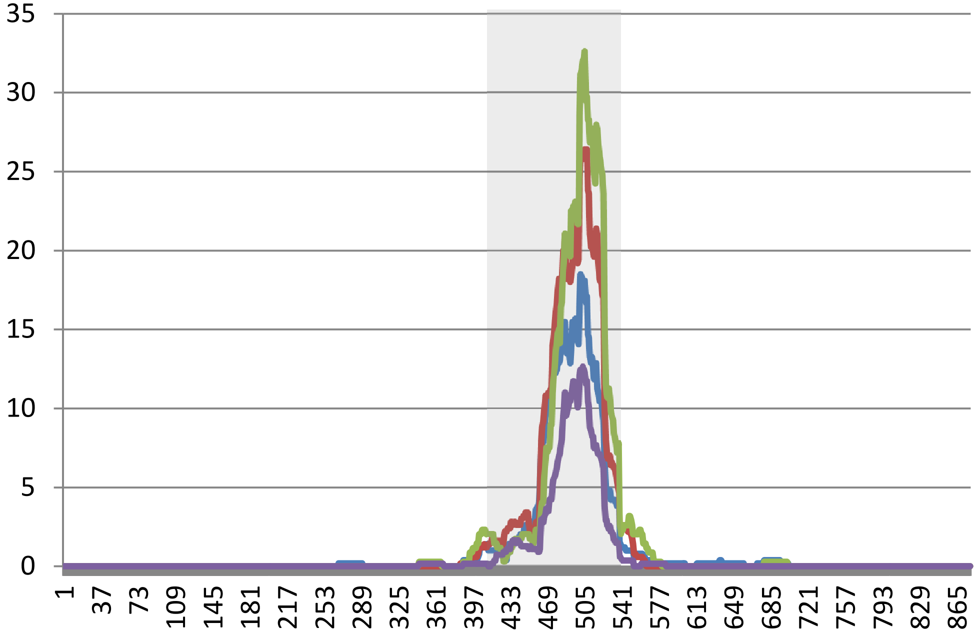

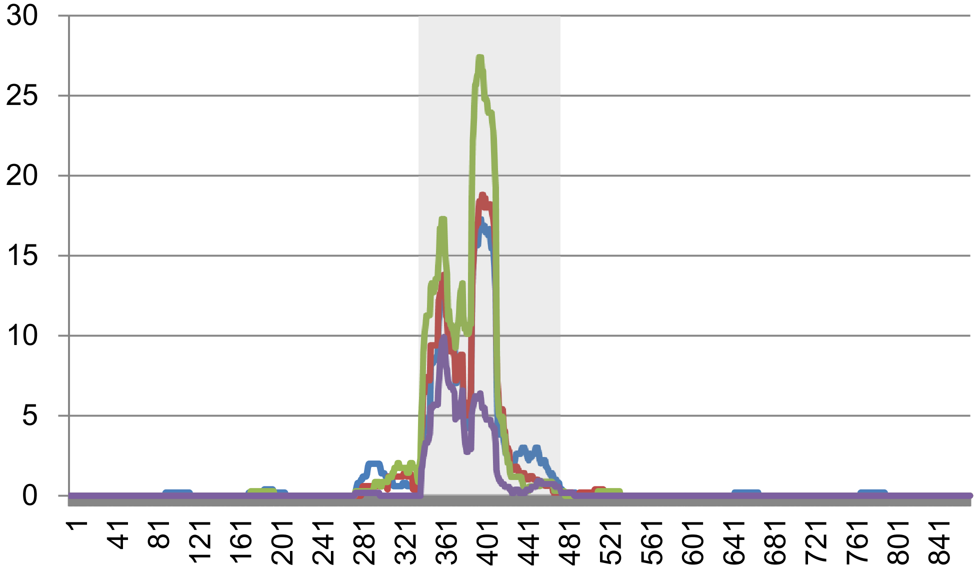

AT3G13857

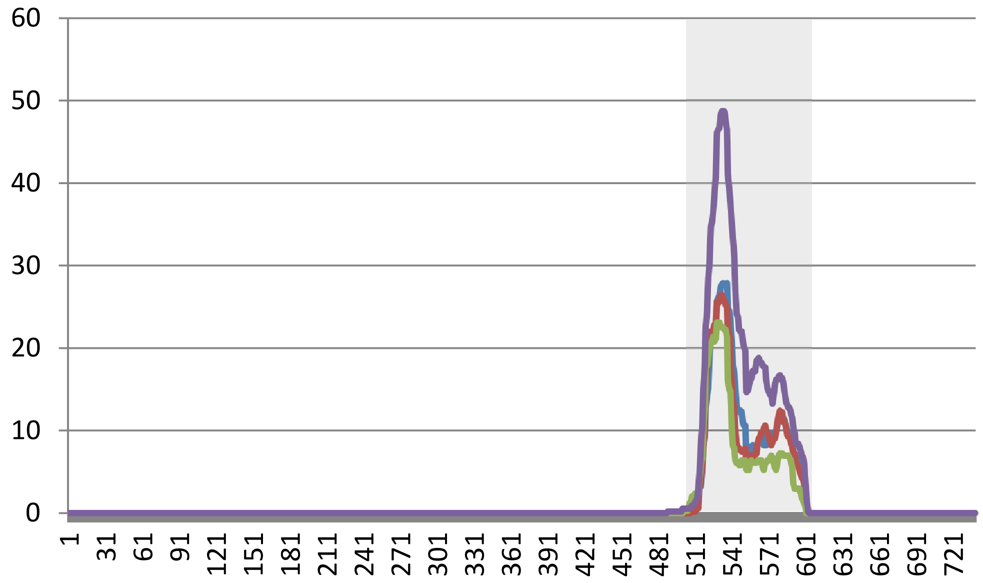

AT3G13857RC

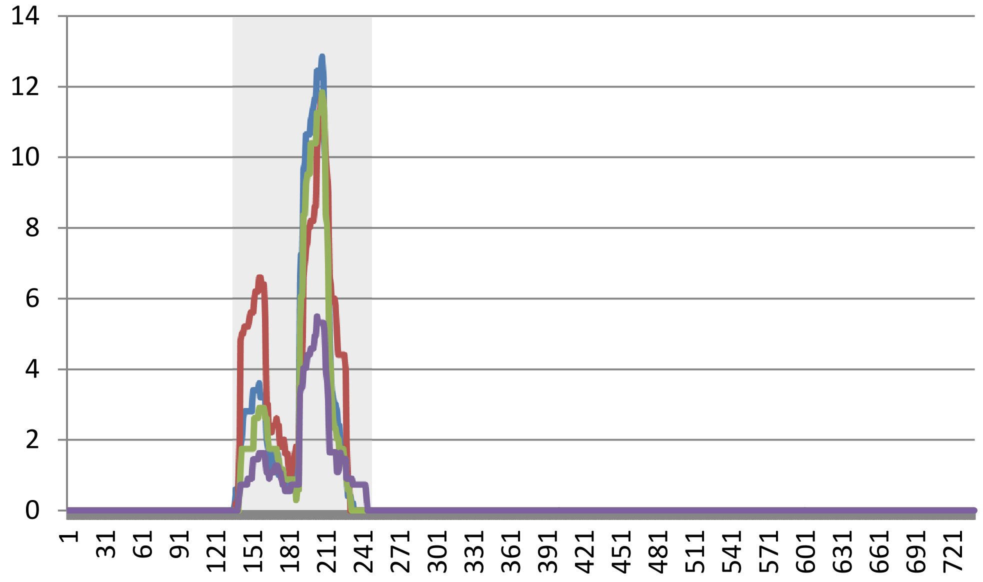

AT3G43270

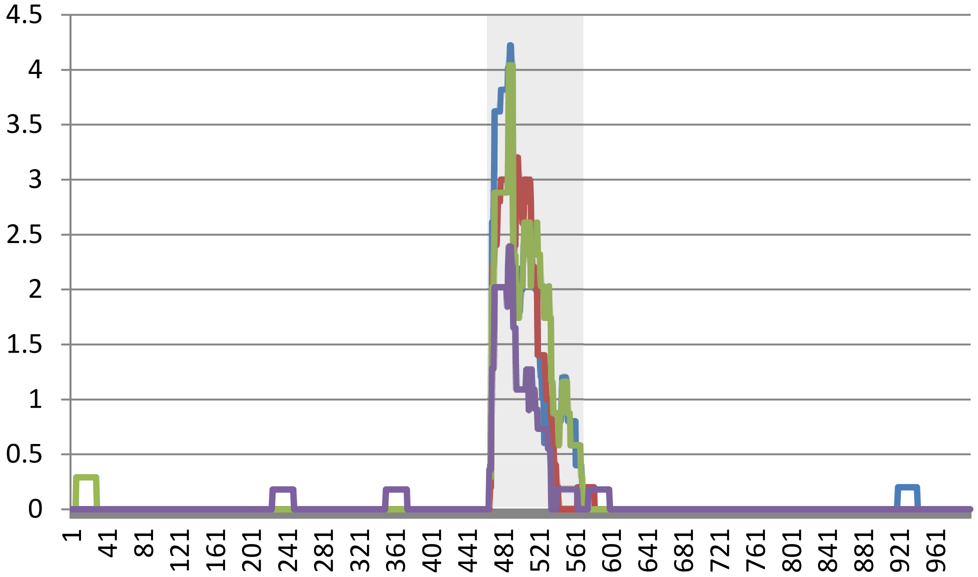

AT3G43270RC

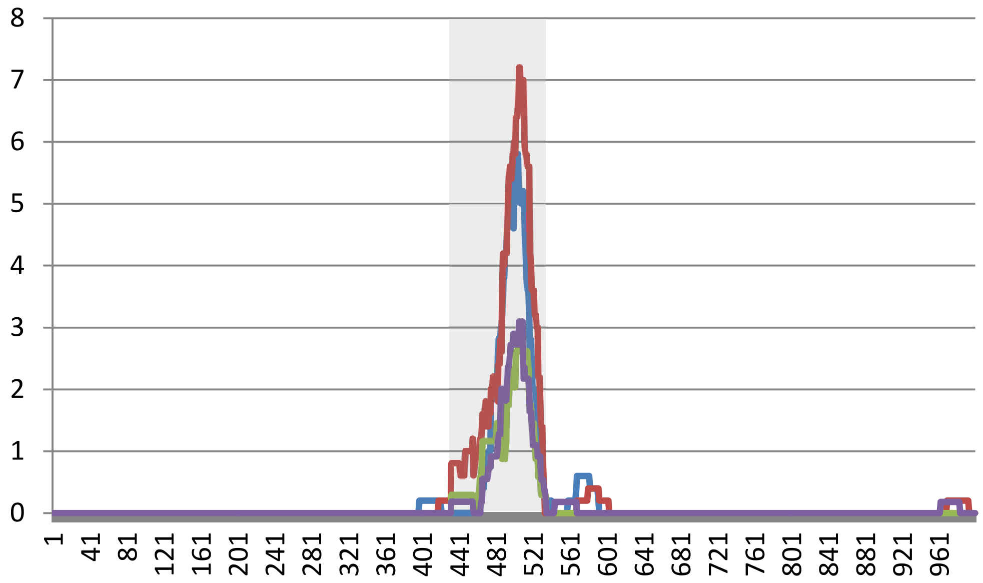

AT3G57770

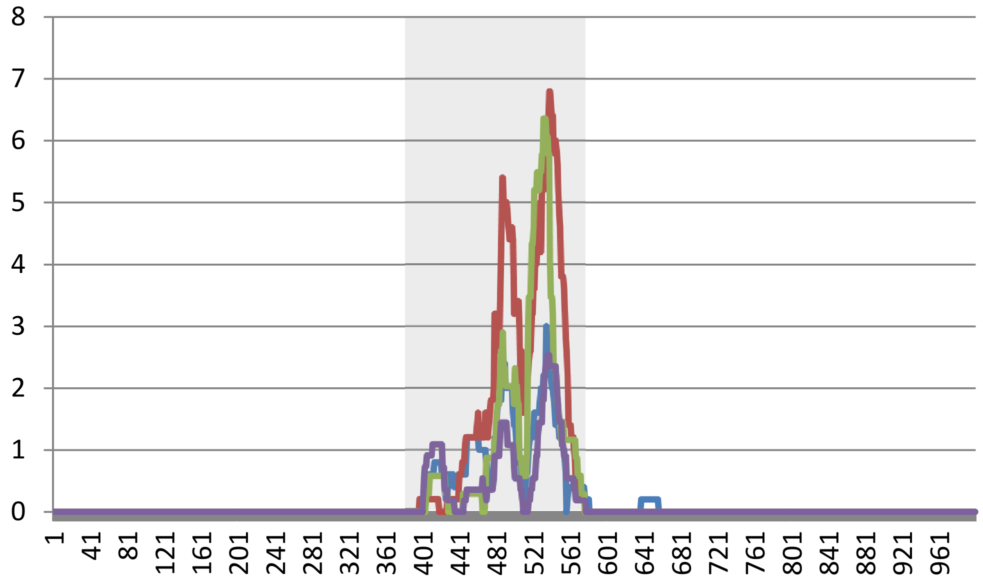

AT3G57770RC

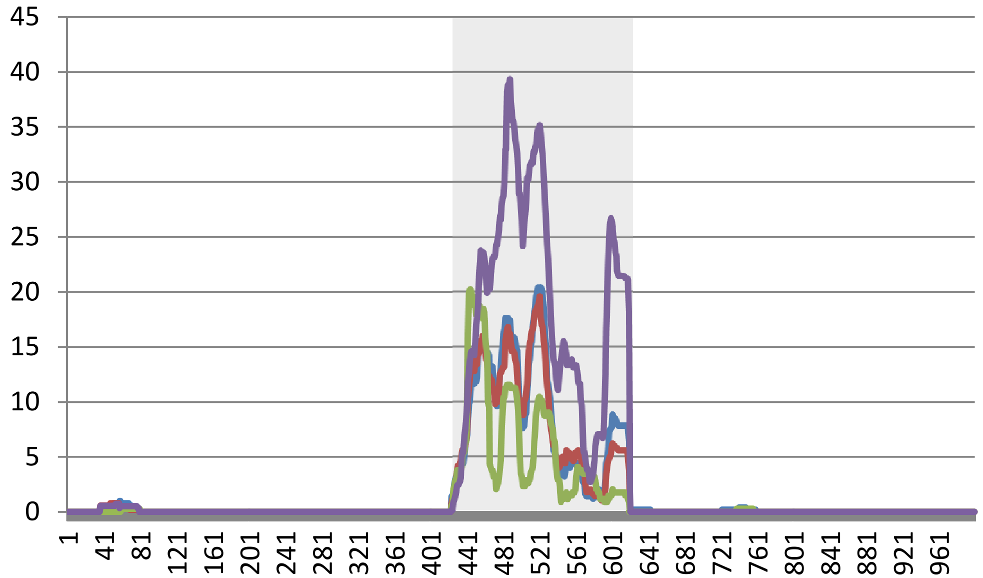

AT4G16640

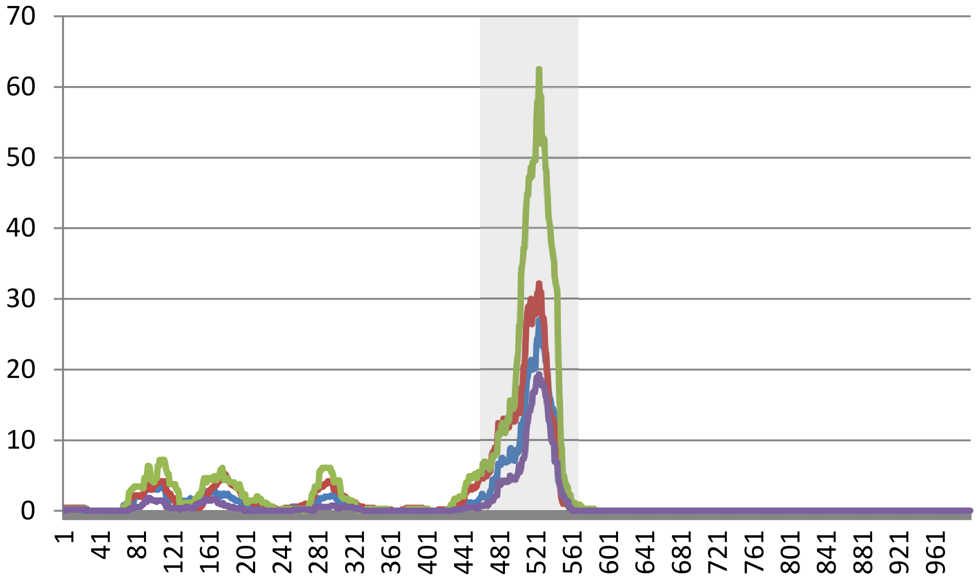

AT4G16640RC

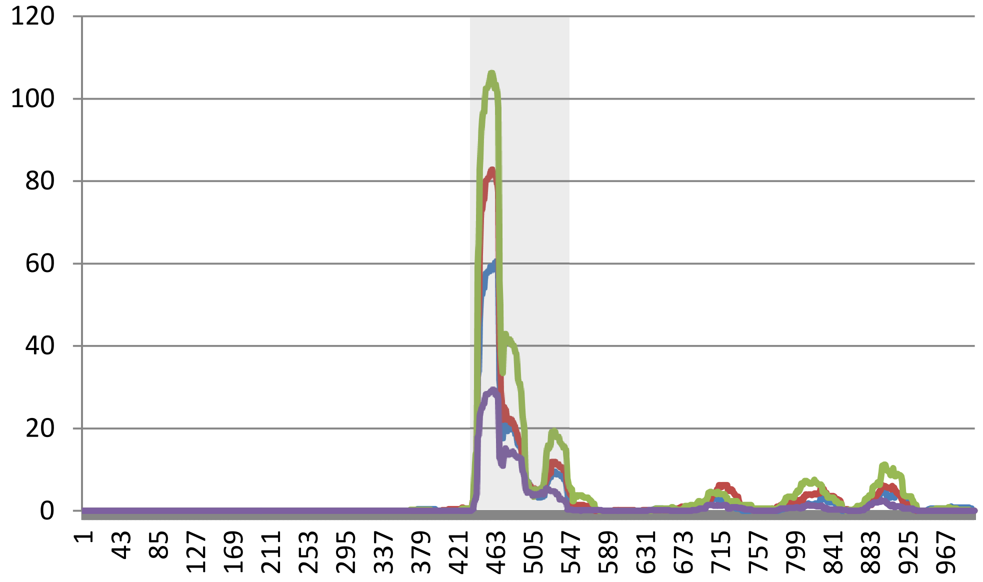

AT5G48000

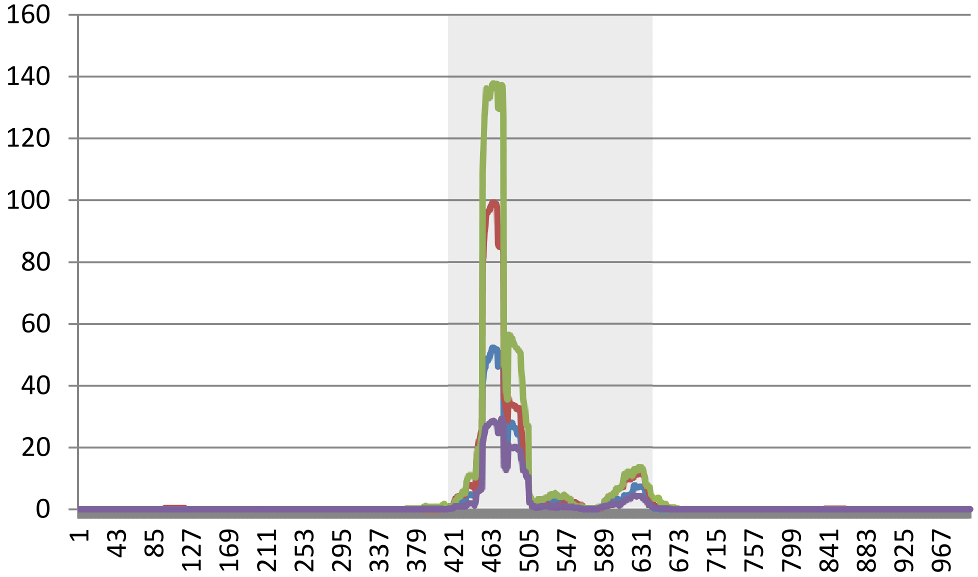

AT5G48000RC

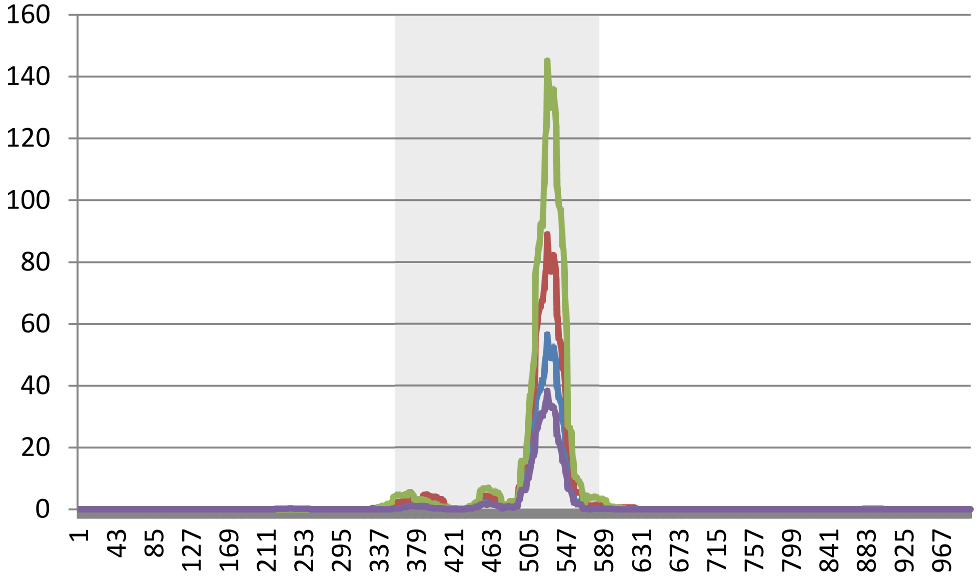

Supplement: S15 Fig — (PDF) [file pone.0169212.s015.pdf]
