# Supplementary material for: Transcriptome-Wide Discovery of PASRs (Promoter-Associated Small RNAs) and TASRs (Terminus-Associated Small RNAs) in Arabidopsis thaliana
Source: PLoS One. 2017 Jan 3;12(1):e0169212. doi: 10.1371/journal.pone.0169212 (PMC5207706; doi:10.1371/journal.pone.0169212)
Supplement: S16 Fig — (PDF) [file pone.0169212.s016.pdf]

Figure S16 DsRNA-seq read-covered TASR peaks identified on the sense strands of the protein-coding genes of *Arabidopsis*. For each plot, x axis measures the position of the sense strand, and y axis measures the abundance (in RPM, reads per million) of sRNAs. The dsRNA-seq read covered region was highlighted in gray background.

AT1G49700

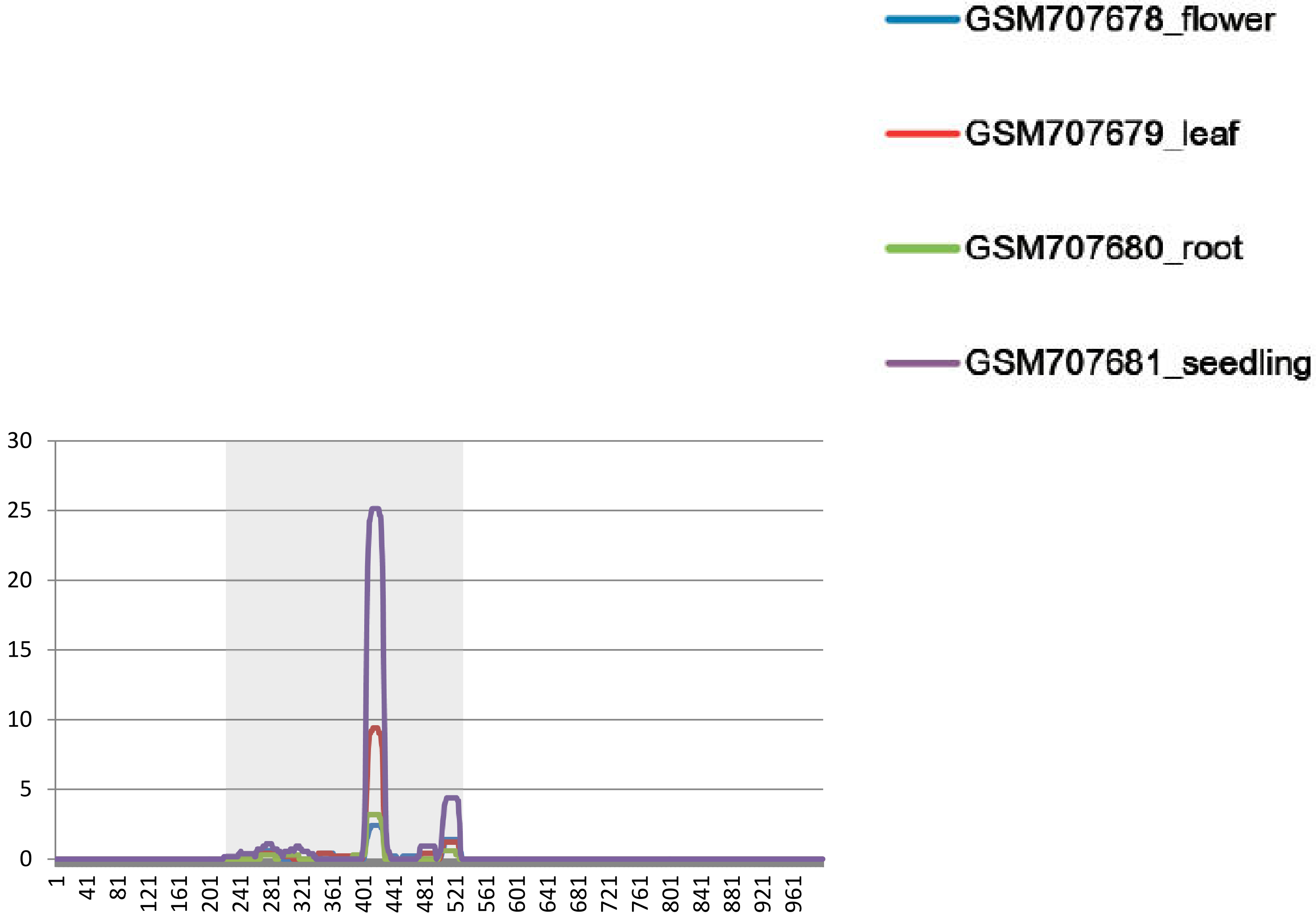
GSM707678_flower
GSM707679_leaf
GSM707680_root
GSM707681_seedling
30
25
20
15
10
5
0
1
41
81
121
161
201
241
281
321
361
401
441
481
521
561
601
641
681
721
761
801
841
881
921
961

AT1G52180

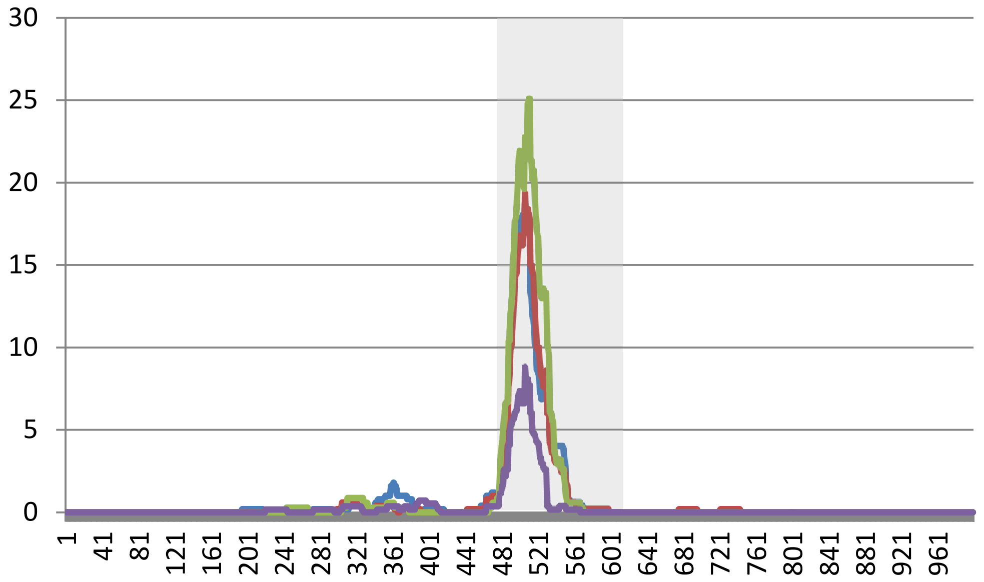
30
25
20
15
10
5
0
1
41
81
121
161
201
241
281
321
361
401
441
481
521
561
601
641
681
721
761
801
841
881
921
961

AT2G02390

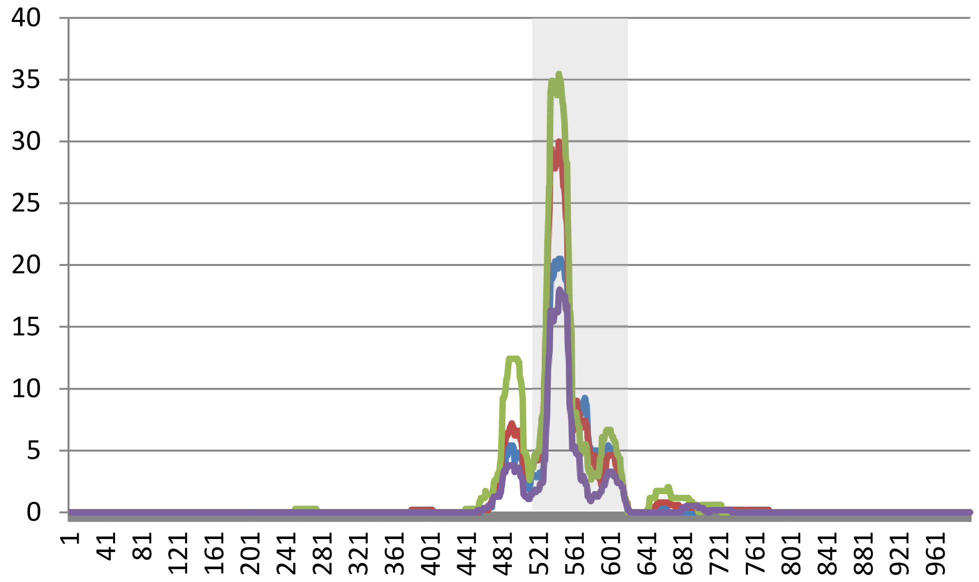

40
35
30
25
20
15
10
5
0
1
41
81
121
161
201
241
281
321
361
401
441
481
521
561
601
641
681
721
761
801
841
881
921
961

AT2G07777

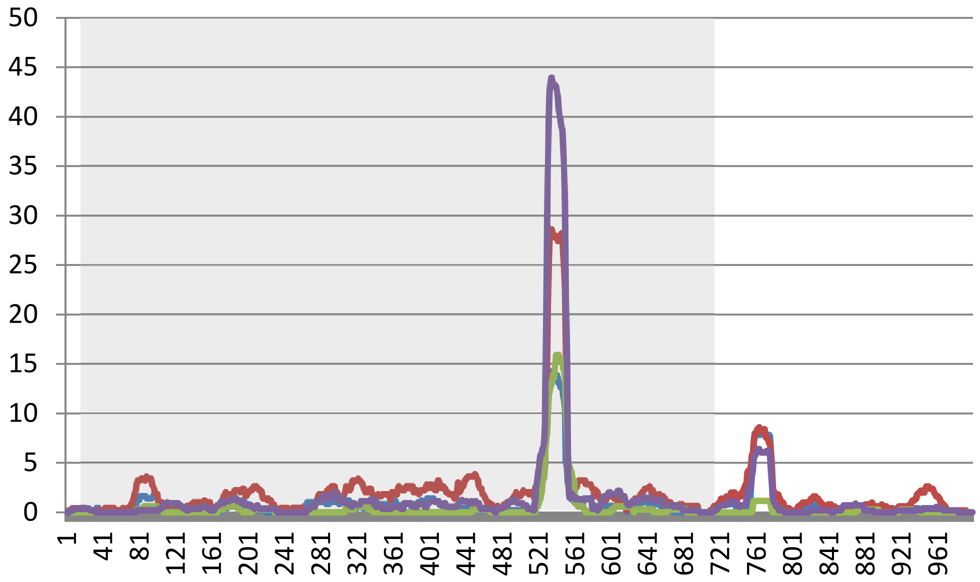

50
45
40
35
30
25
20
15
10
5
0
1
41
81
121
161
201
241
281
321
361
401
441
481
521
561
601
641
681
721
761
801
841
881
921
961

AT3G54730

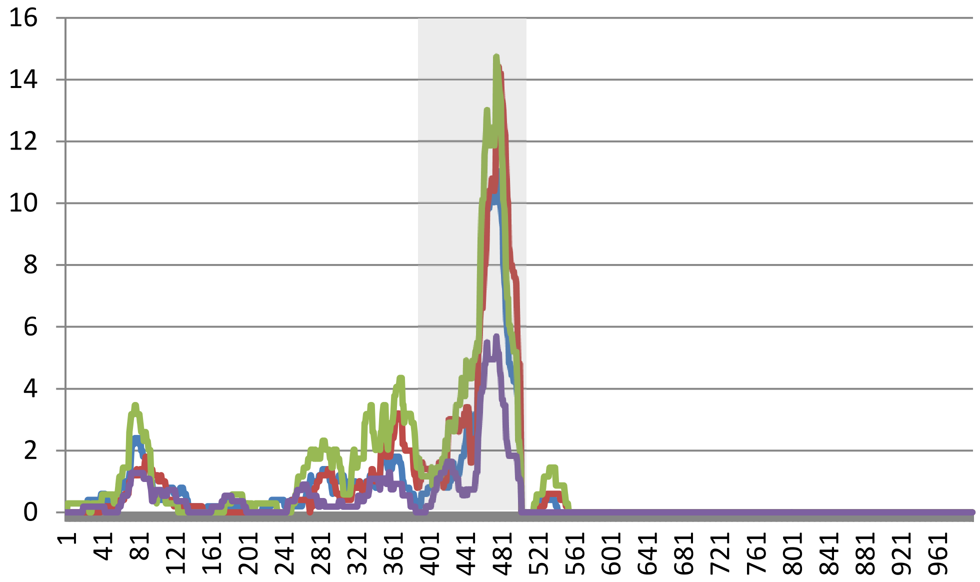

16
14
12
10
8
6
4
2
0
1
41
81
121
161
201
241
281
321
361
401
441
481
521
561
601
641
681
721
761
801
841
881
921
961

AT4G11370

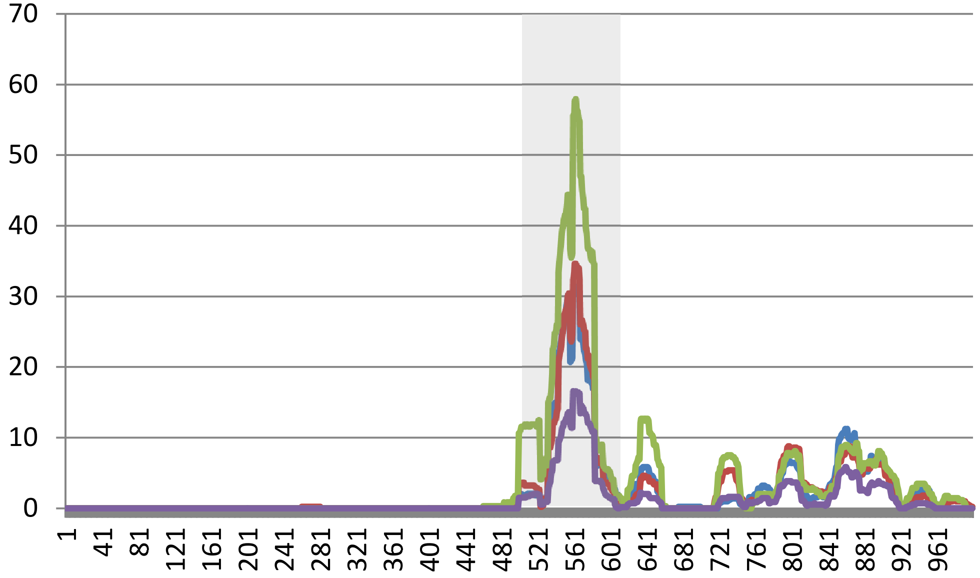

70
60
50
40
30
20
10
0
1
41
81
121
161
201
241
281
321
361
401
441
481
521
561
601
641
681
721
761
801
841
881
921
961

AT4G30993

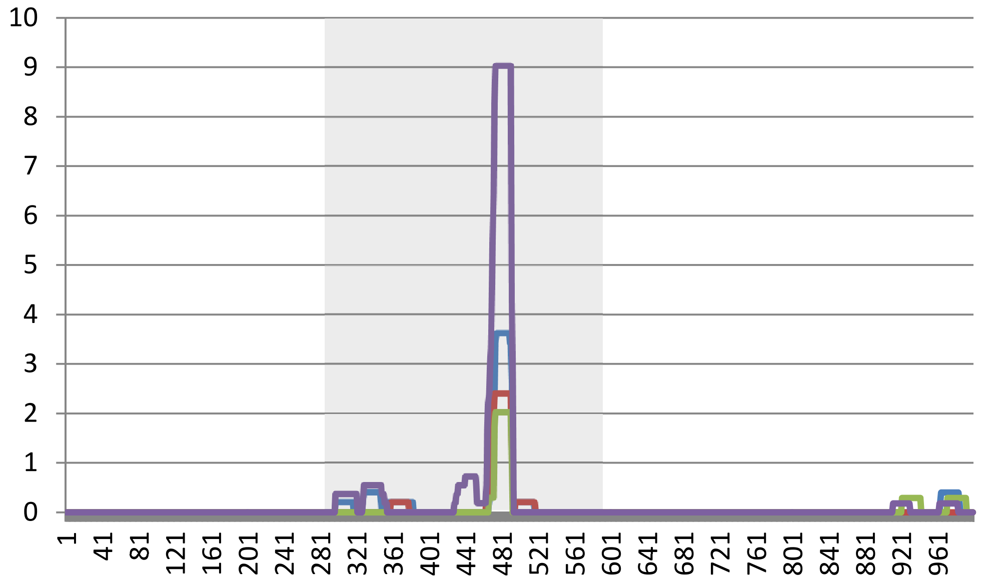

10
9
8
7
6
5
4
3
2
1
0
1
41
81
121
161
201
241
281
321
361
401
441
481
521
561
601
641
681
721
761
801
841
881
921
961

AT5G10660

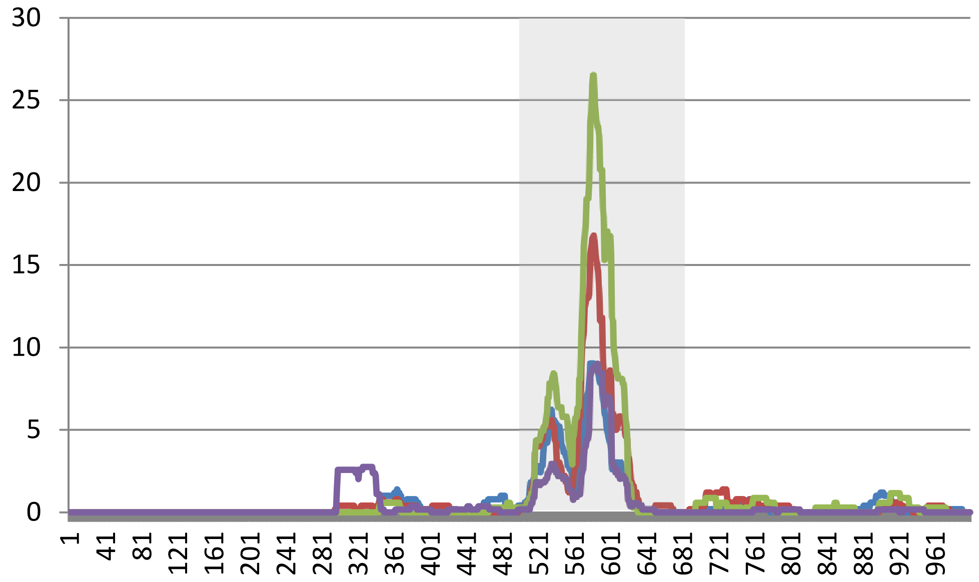

30
25
20
15
10
5
0
1
41
81
121
161
201
241
281
321
361
401
441
481
521
561
601
641
681
721
761
801
841
881
921
961

AT5G27660

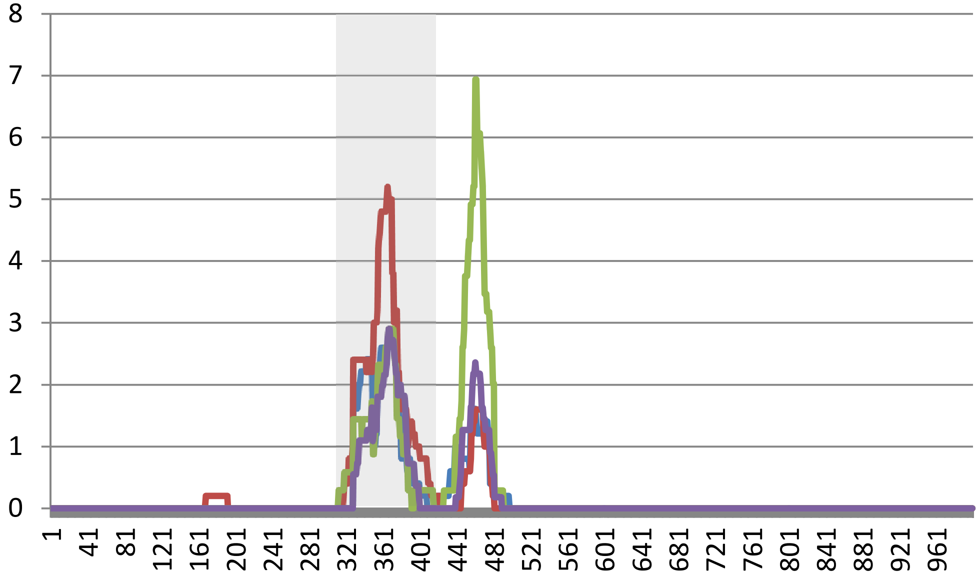

8
7
6
5
4
3
2
1
0
1
41
81
121
161
201
241
281
321
361
401
441
481
521
561
601
641
681
721
761
801
841
881
921
961

AT5G36220

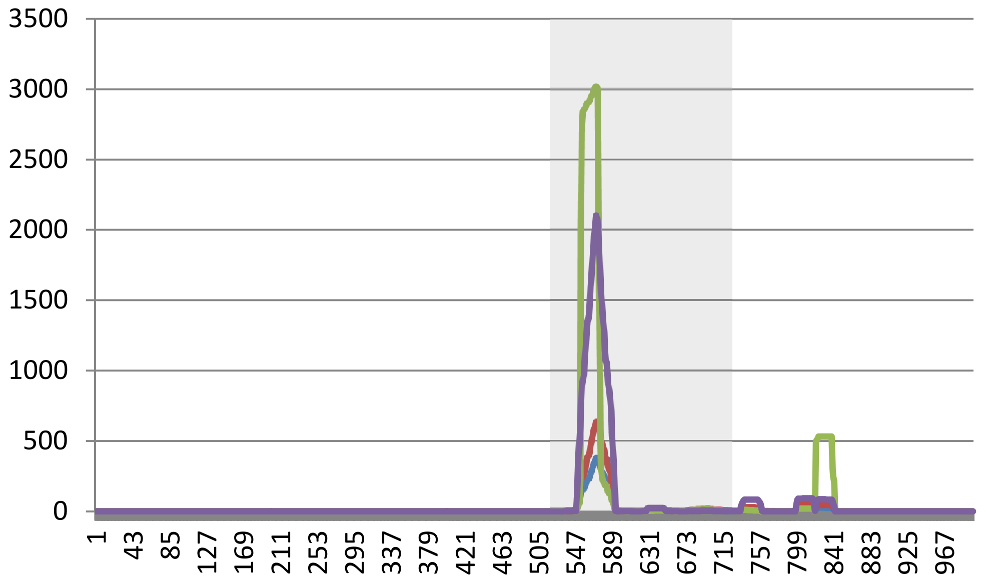

3500
3000
2500
2000
1500
1000
500
0
1
43
85
127
169
211
253
295
337
379
421
463
505
547
589
631
673
715
757
799
841
883
925
967

AT5G54070

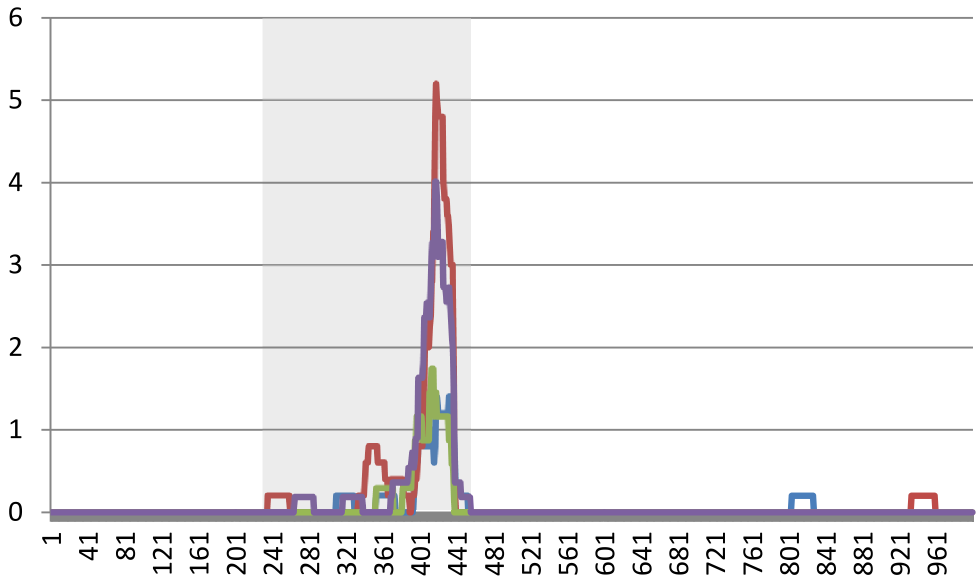

6
5
4
3
2
1
0
1
41
81
121
161
201
241
281
321
361
401
441
481
521
561
601
641
681
721
761
801
841
881
921
961

ATCG00130

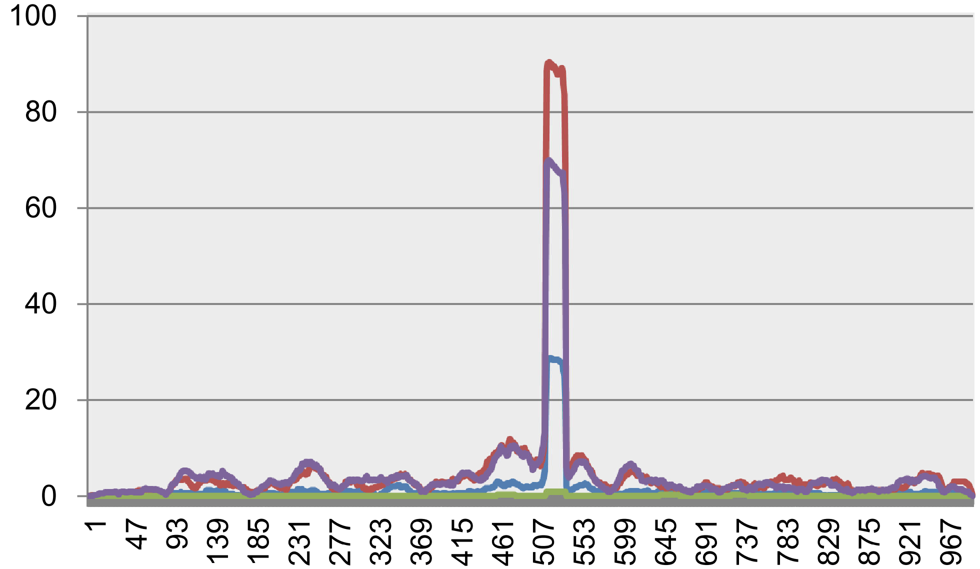

100
80
60
40
20
0
1
47
93
139
185
231
277
323
369
415
461
507
553
599
645
691
737
783
829
875
921
967

ATCG00270

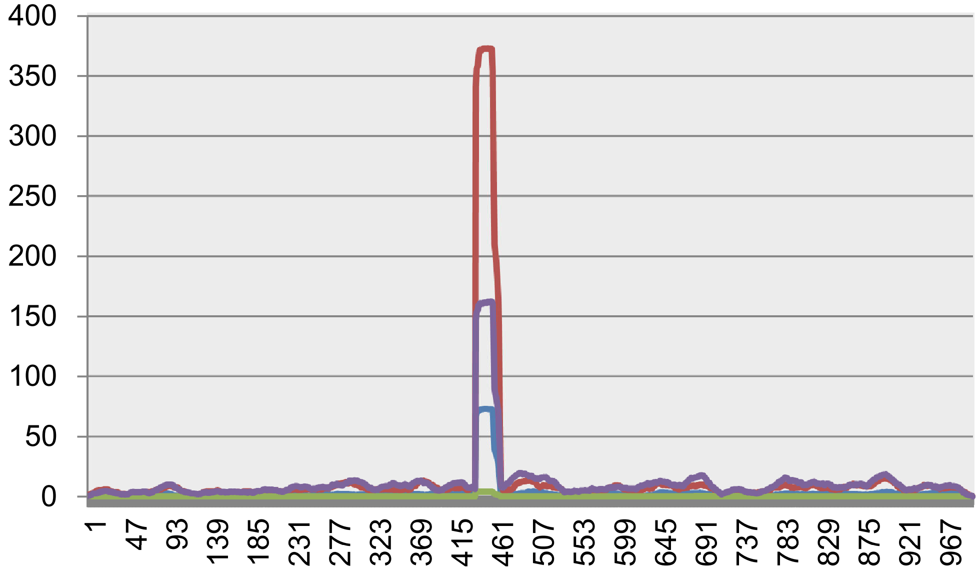
400
350
300
250
200
150
100
50
0
1
47
93
139
185
231
277
323
369
415
461
507
553
599
645
691
737
783
829
875
921
967

ATCG00420

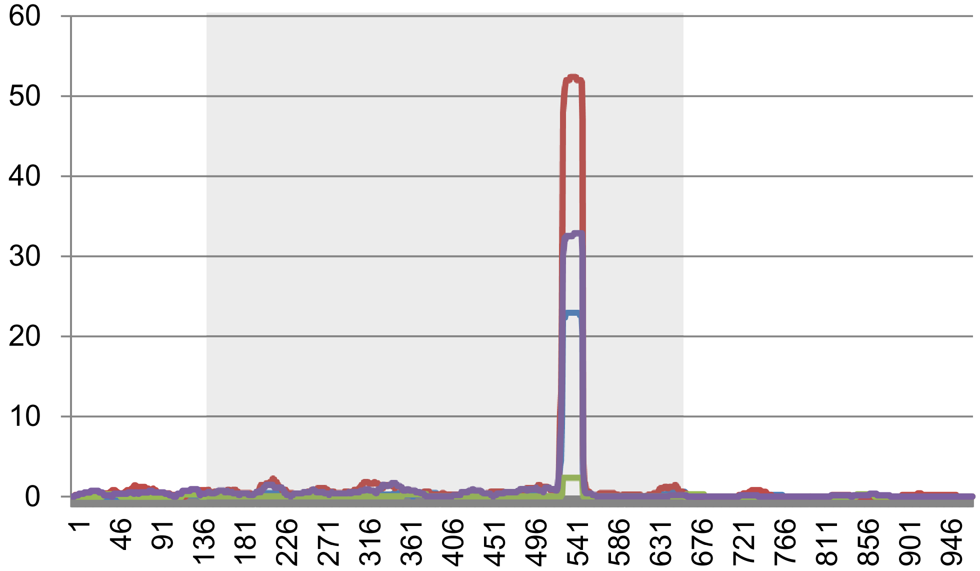

60
50
40
30
20
10
0
1
46
91
136
181
226
271
316
361
406
451
496
541
586
631
676
721
766
811
856
901
946

ATCG00840

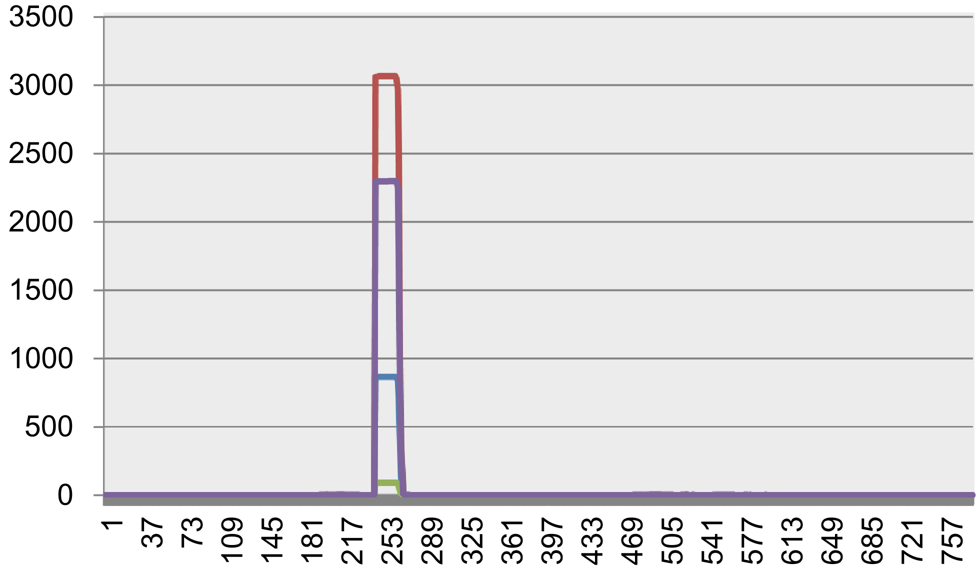

3500
3000
2500
2000
1500
1000
500
0
1
37
73
109
145
181
217
253
289
325
361
397
433
469
505
541
577
613
649
685
721
757

ATCG01110

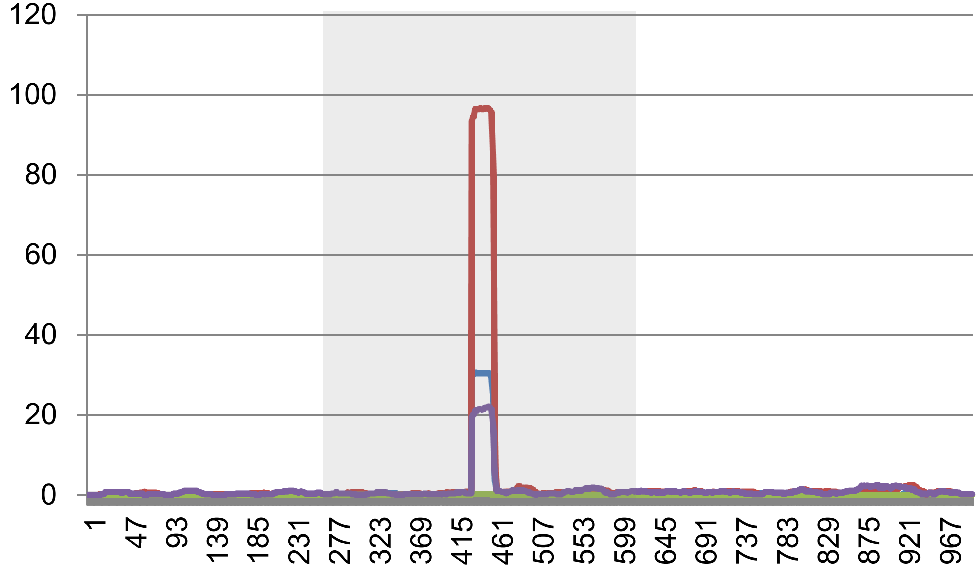

120
100
80
60
40
20
0
1
47
93
139
185
231
277
323
369
415
461
507
553
599
645
691
737
783
829
875
921
967

ATCG01300

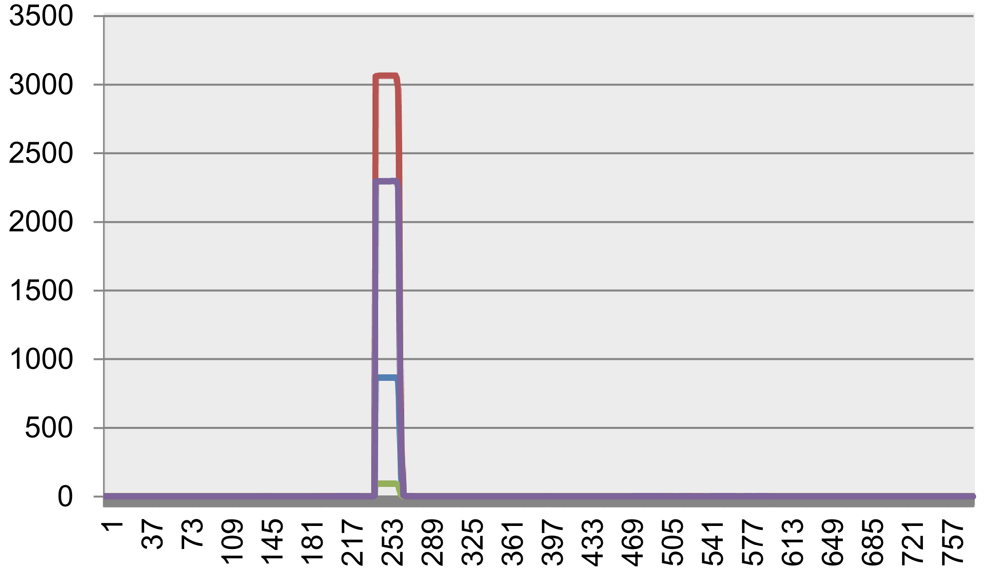
3500
3000
2500
2000
1500
1000
500
0
1
37
73
109
145
181
217
253
289
325
361
397
433
469
505
541
577
613
649
685
721
757

ATMG00060

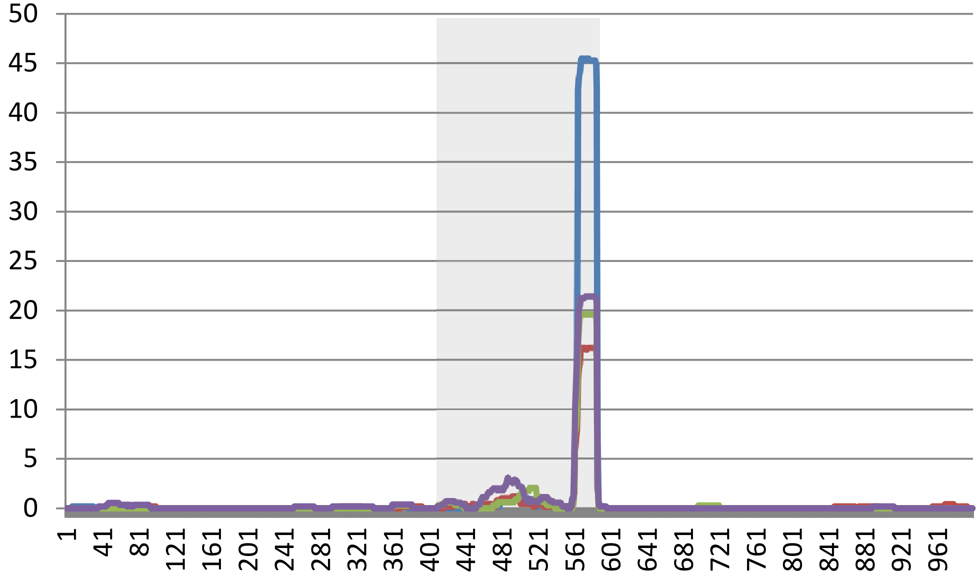

50
45
40
35
30
25
20
15
10
5
0
1
41
81
121
161
201
241
281
321
361
401
441
481
521
561
601
641
681
721
761
801
841
881
921
961

ATMG00070

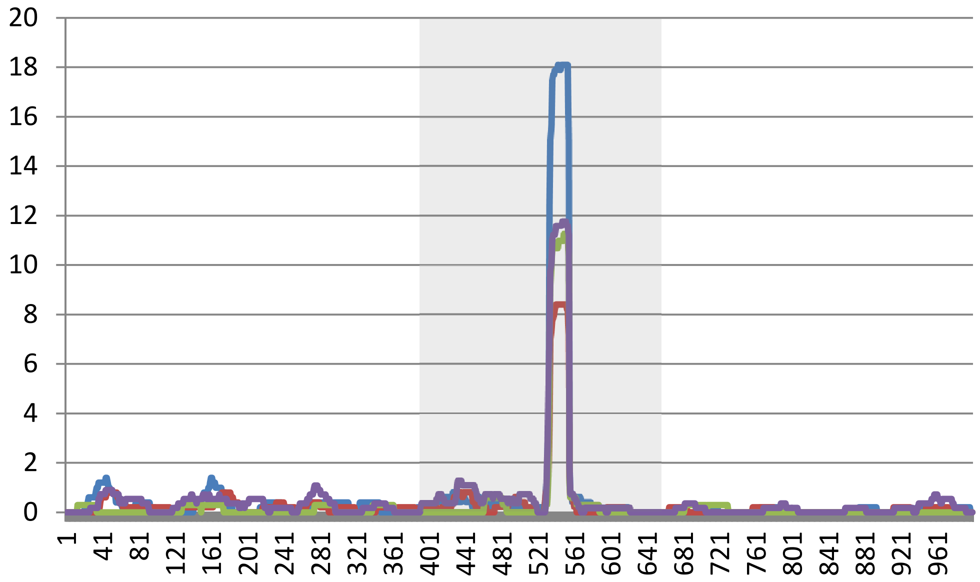

20
18
16
14
12
10
8
6
4
2
0
1
41
81
121
161
201
241
281
321
361
401
441
481
521
561
601
641
681
721
761
801
841
881
921
961

ATMG00080

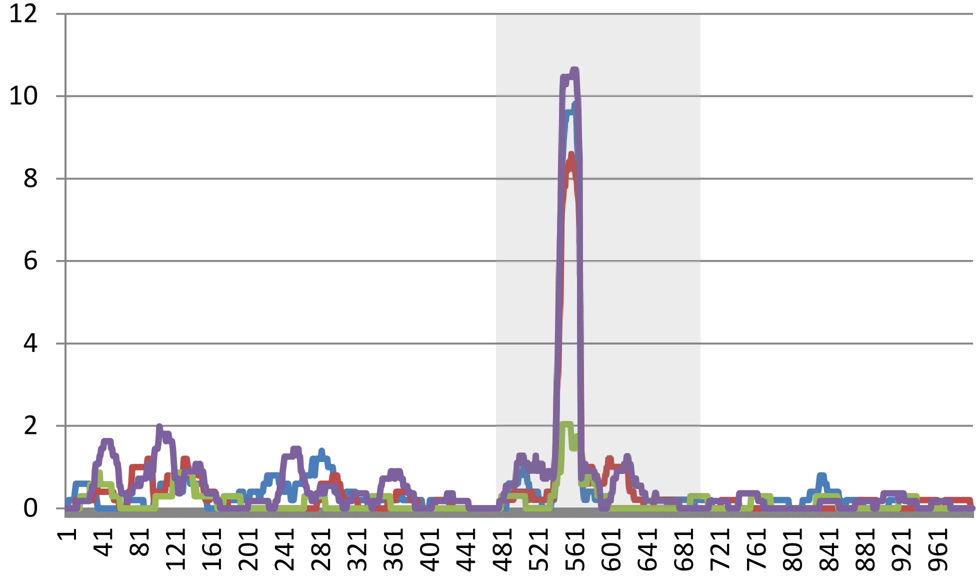

12
10
8
6
4
2
0
1
41
81
121
161
201
241
281
321
361
401
441
481
521
561
601
641
681
721
761
801
841
881
921
961

ATMG00110

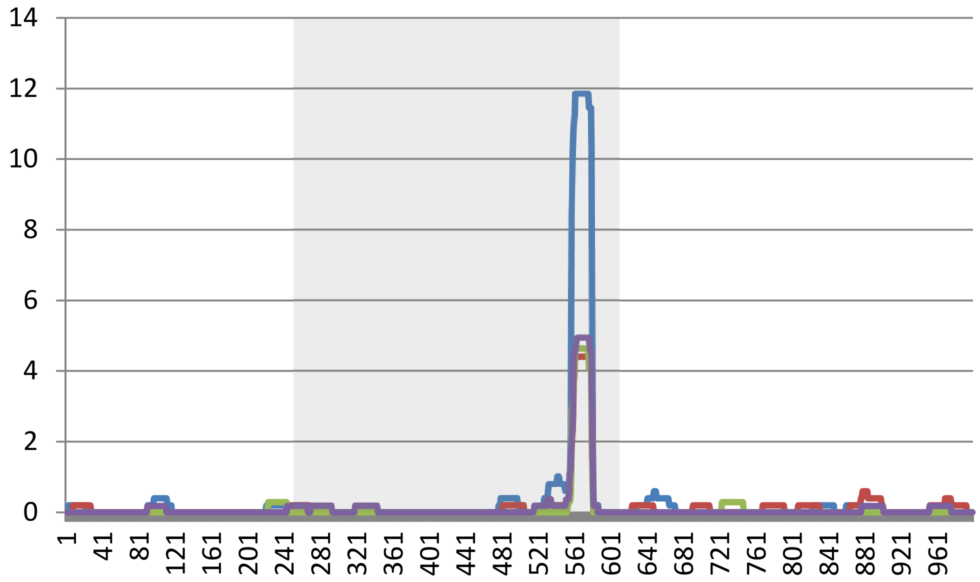
14
12
10
8
6
4
2
0
1
41
81
121
161
201
241
281
321
361
401
441
481
521
561
601
641
681
721
761
801
841
881
921
961

ATMG00270

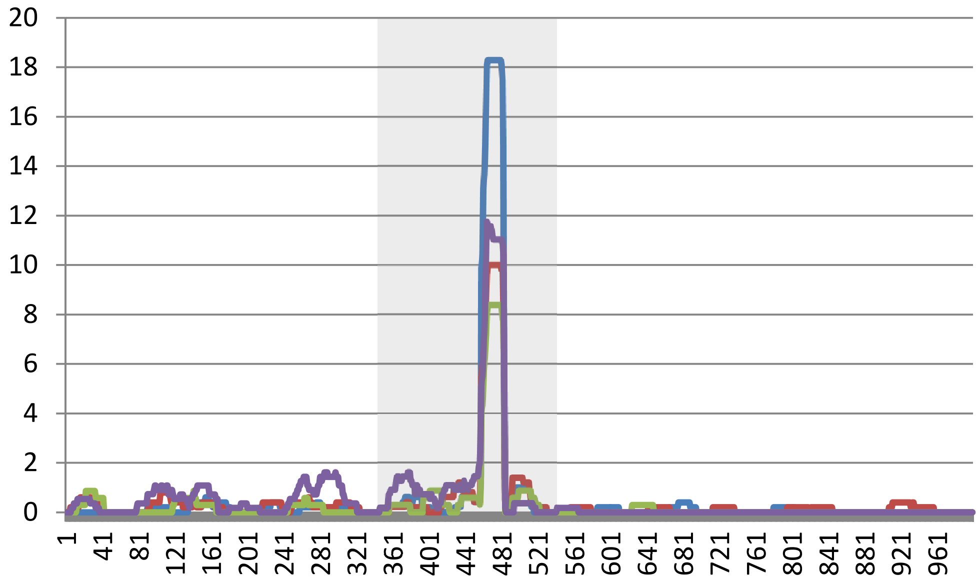

20
18
16
14
12
10
8
6
4
2
0
1
41
81
121
161
201
241
281
321
361
401
441
481
521
561
601
641
681
721
761
801
841
881
921
961

ATMG00510

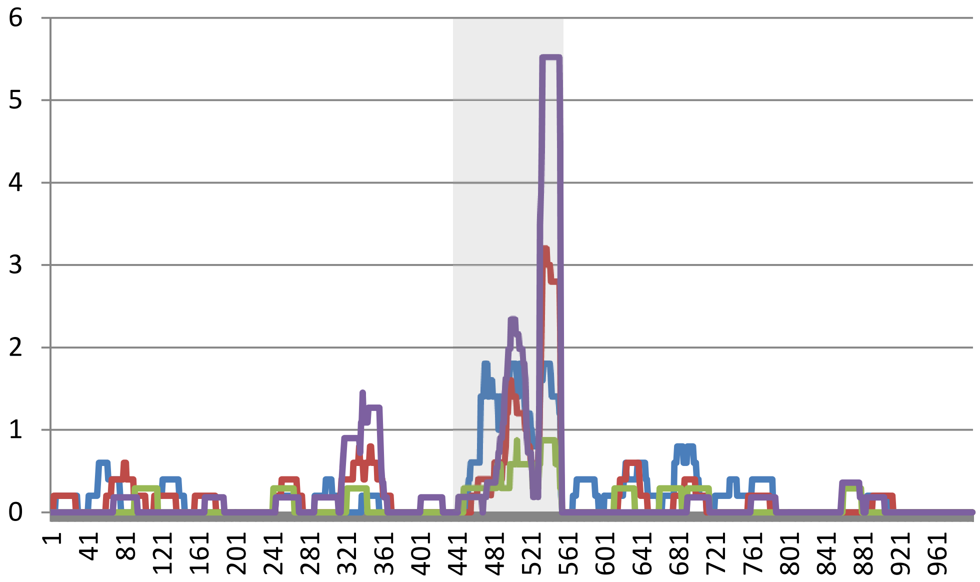

6
5
4
3
2
1
0
1
41
81
121
161
201
241
281
321
361
401
441
481
521
561
601
641
681
721
761
801
841
881
921
961

ATMG00516

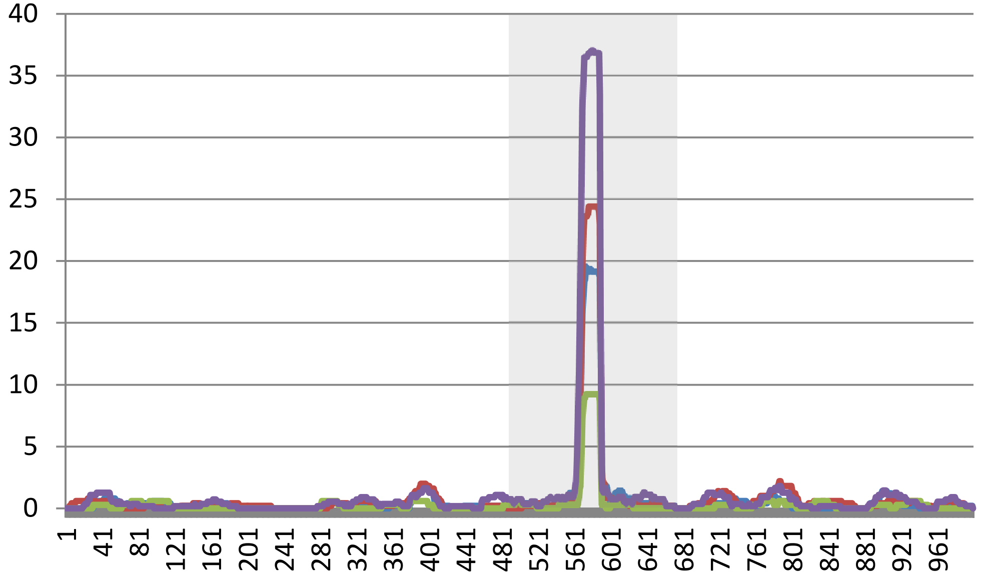

40
35
30
25
20
15
10
5
0
1
41
81
121
161
201
241
281
321
361
401
441
481
521
561
601
641
681
721
761
801
841
881
921
961

ATMG00560

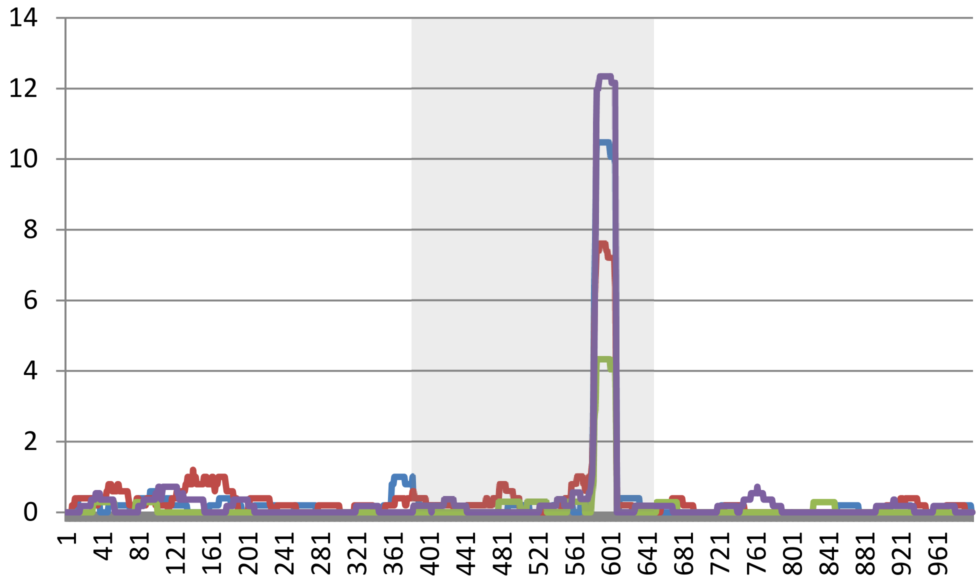

14
12
10
8
6
4
2
0
1
41
81
121
161
201
241
281
321
361
401
441
481
521
561
601
641
681
721
761
801
841
881
921
961

ATMG00570

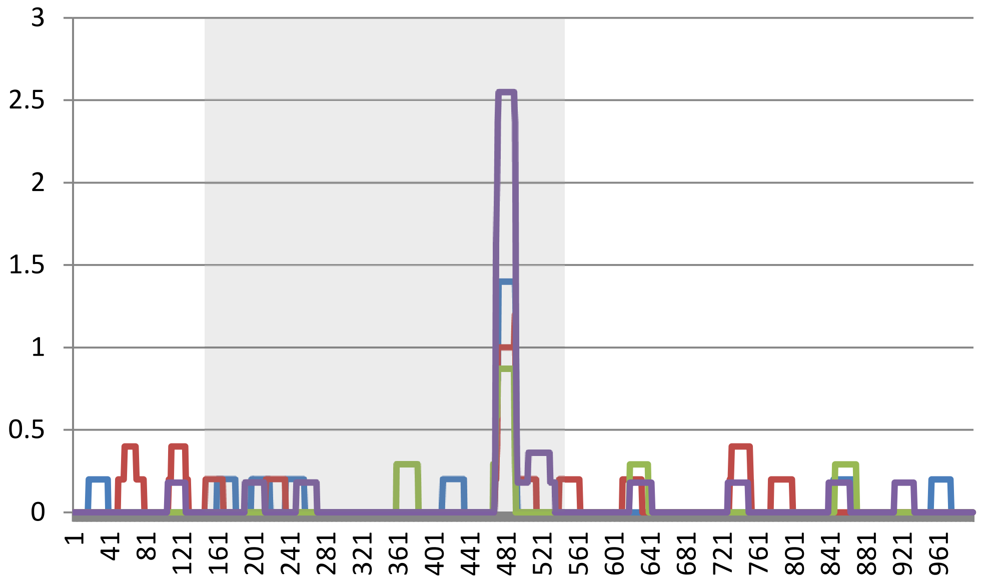

3
2.5
2
1.5
1
0.5
0
1
41
81
121
161
201
241
281
321
361
401
441
481
521
561
601
641
681
721
761
801
841
881
921
961

ATMG00580

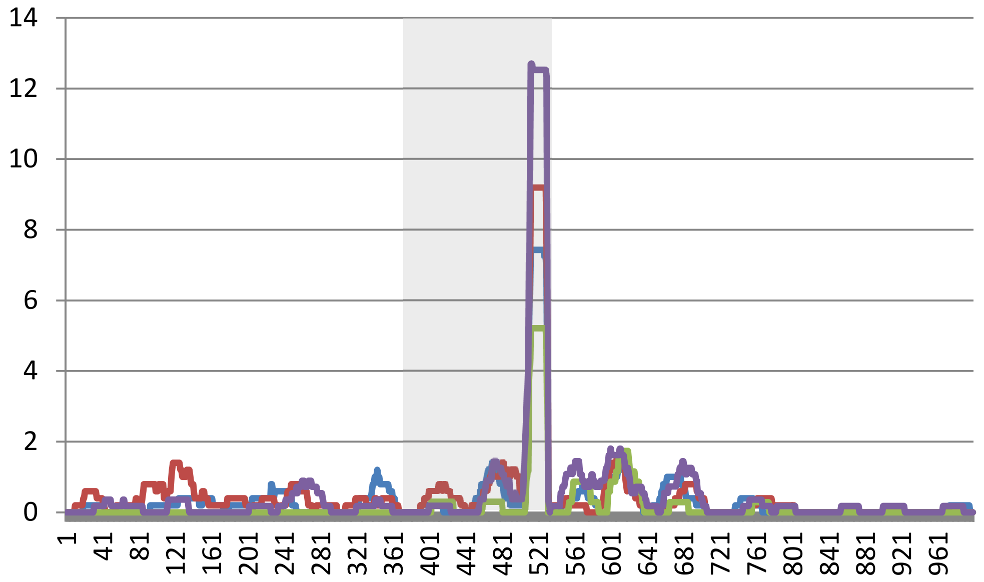

14
12
10
8
6
4
2
0
1
41
81
121
161
201
241
281
321
361
401
441
481
521
561
601
641
681
721
761
801
841
881
921
961

ATMG01090

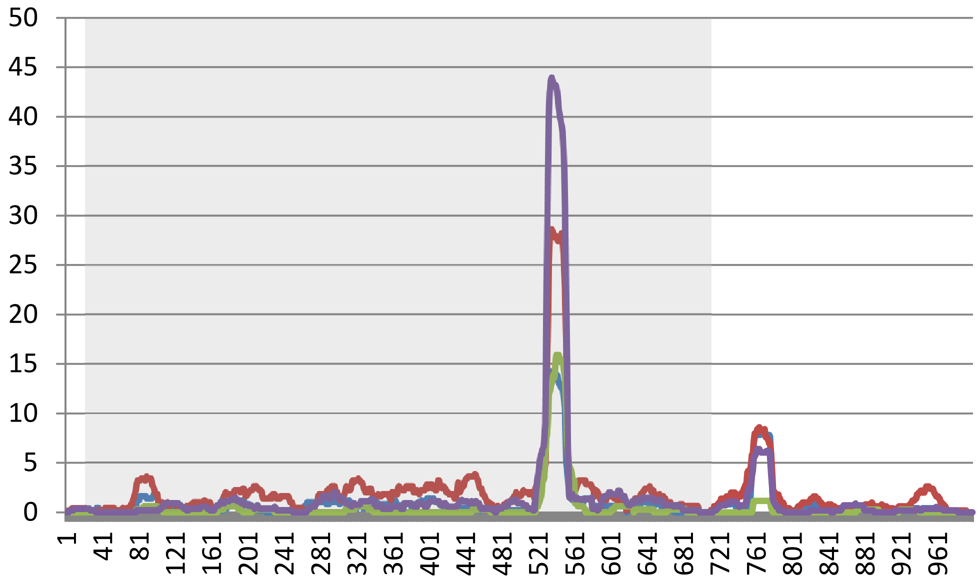

50
45
40
35
30
25
20
15
10
5
0
1
41
81
121
161
201
241
281
321
361
401
441
481
521
561
601
641
681
721
761
801
841
881
921
961

ATMG01360

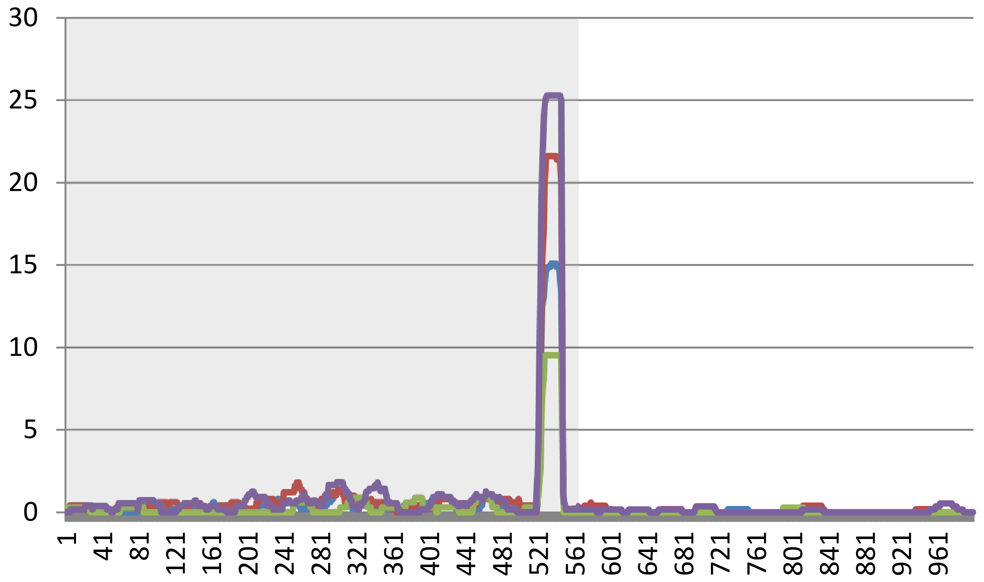
30
25
20
15
10
5
0
1
41
81
121
161
201
241
281
321
361
401
441
481
521
561
601
641
681
721
761
801
841
881
921
961
