# Supplementary material for: Transcriptome-Wide Discovery of PASRs (Promoter-Associated Small RNAs) and TASRs (Terminus-Associated Small RNAs) in Arabidopsis thaliana
Source: PLoS One. 2017 Jan 3;12(1):e0169212. doi: 10.1371/journal.pone.0169212 (PMC5207706; doi:10.1371/journal.pone.0169212)

Figure S17 DsRNA-seq read-covered TASR peaks identified on the antisense strands of the protein-coding genes of *Arabidopsis*. For each plot, x axis measures the position of the antisense strand, and y axis measures the abundance (in RPM, reads per million) of sRNAs. The dsRNA-seq read covered region was highlighted in gray background.

AT1G59835RC

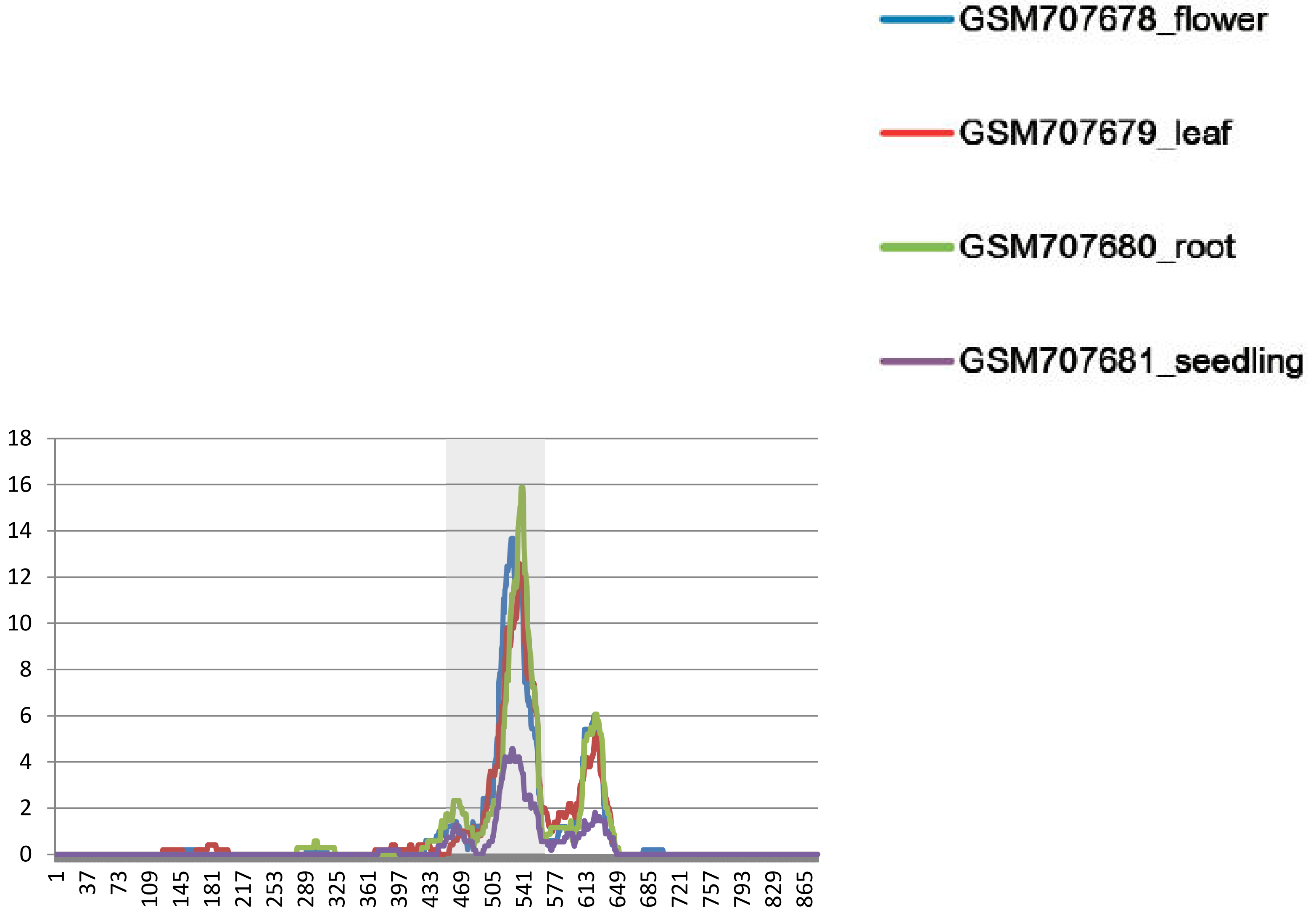

AT2G02400RC

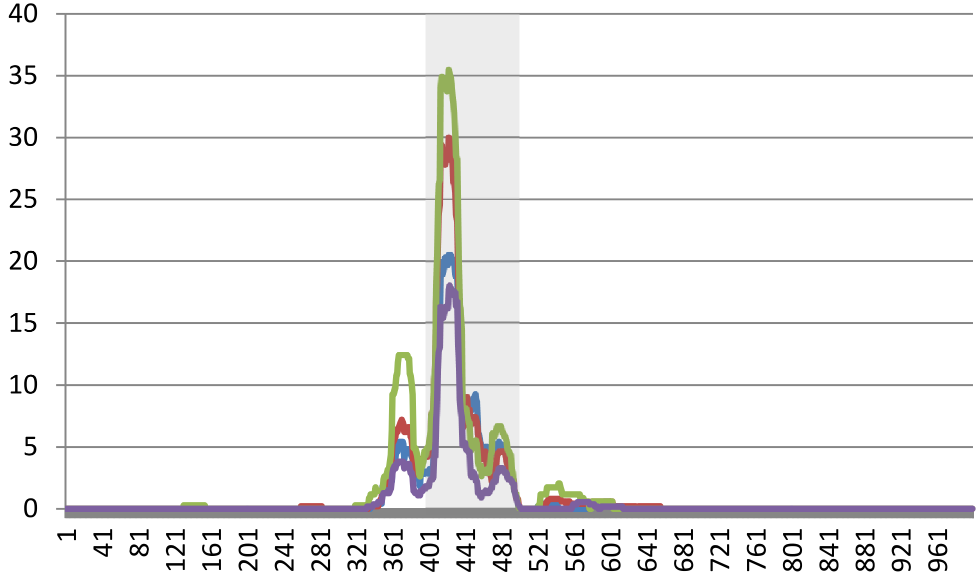

AT3G05520RC

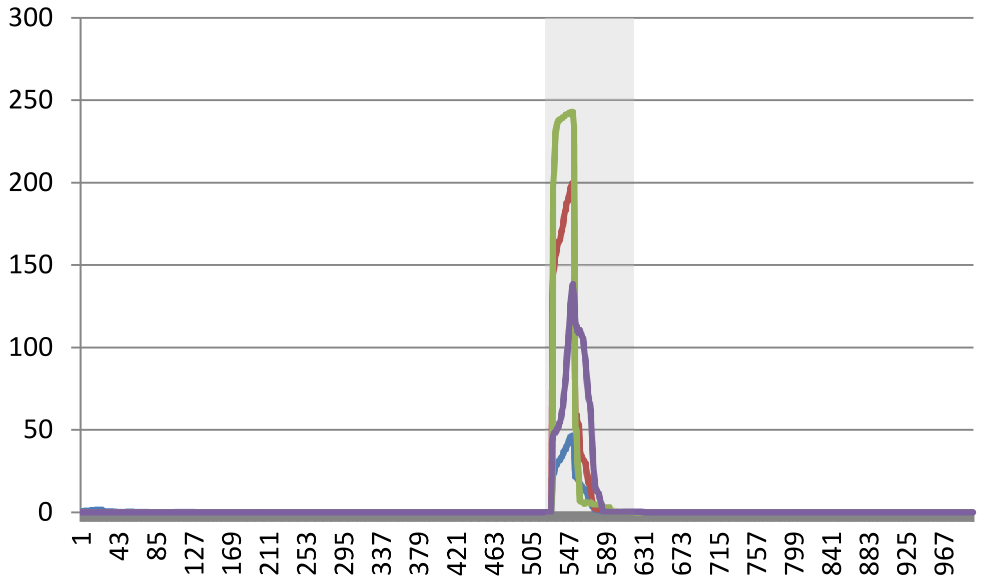

AT3G17890RC

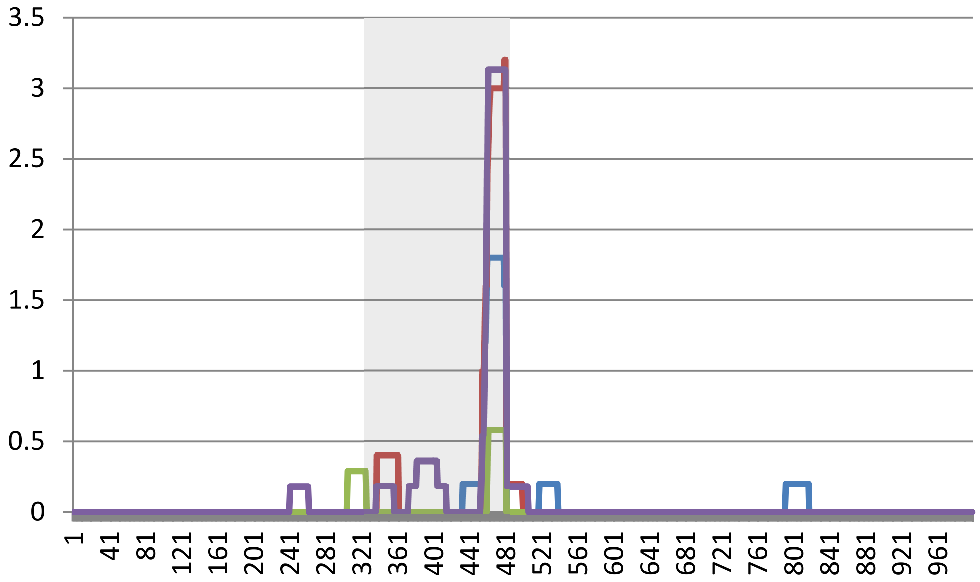

AT3G44210RC

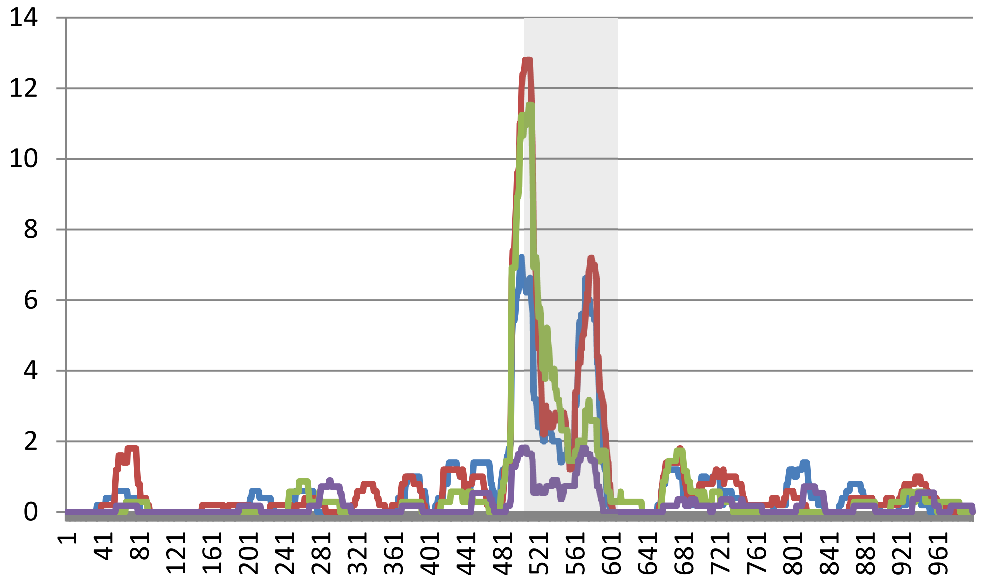

AT5G24593RC

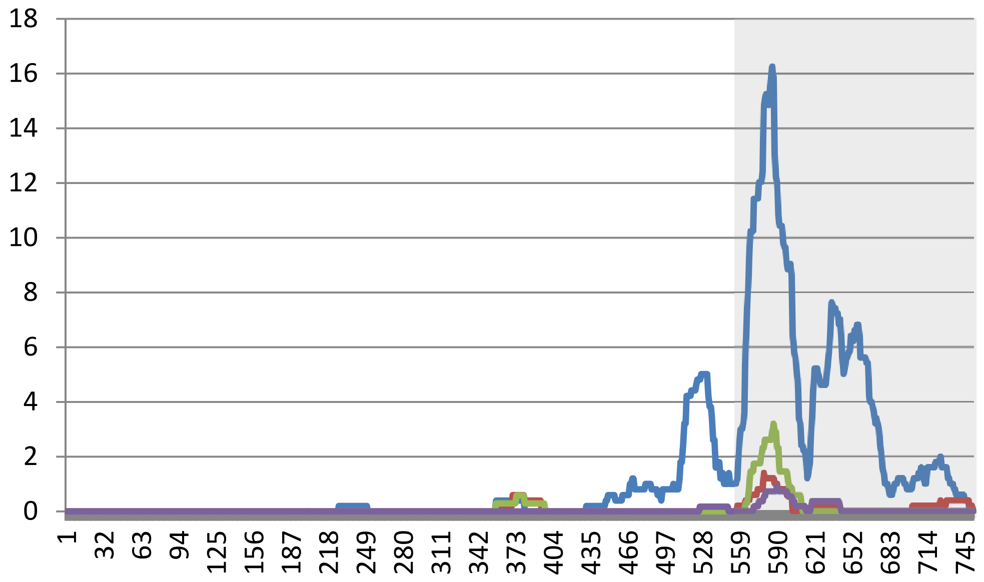

AT5G43513RC

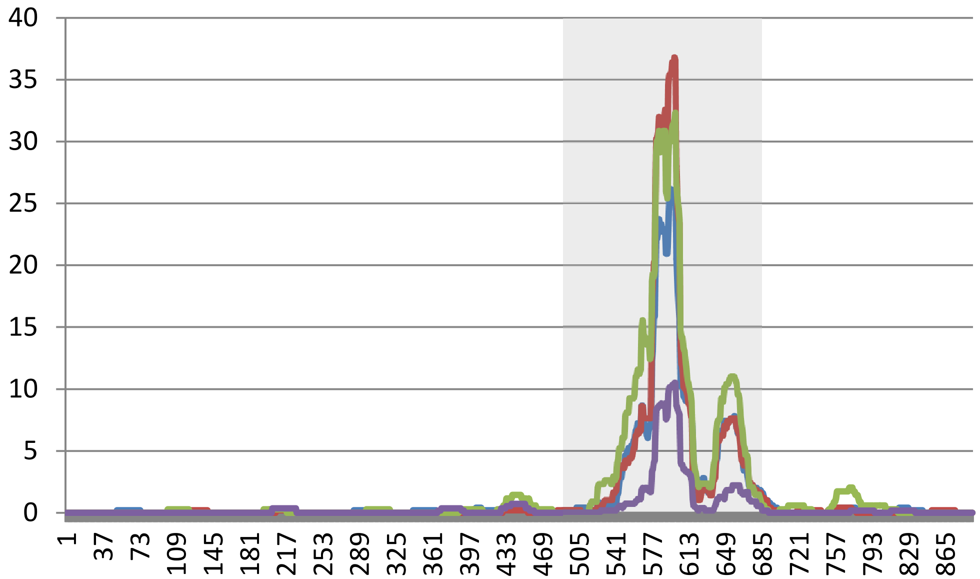

AT5G53742RC

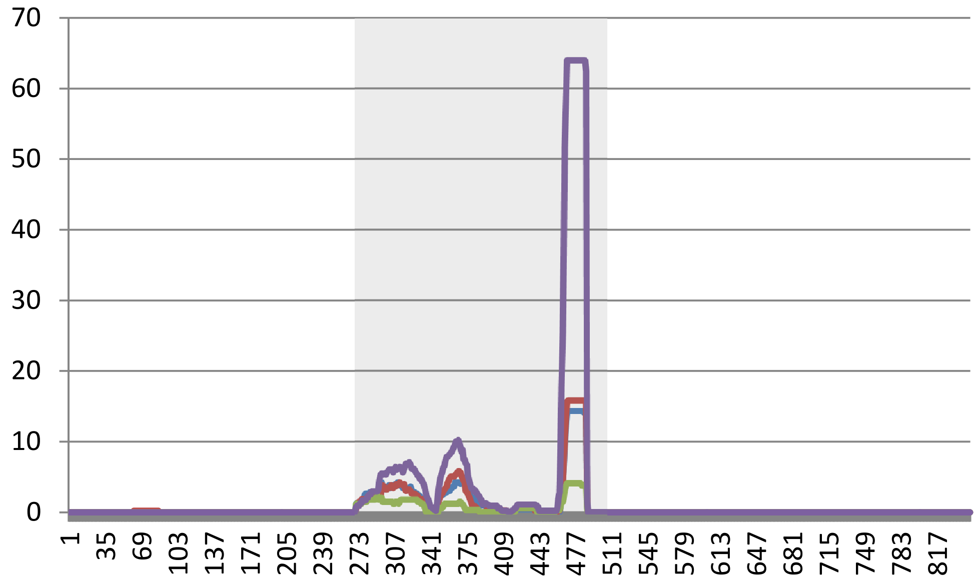

ATMG00810RC

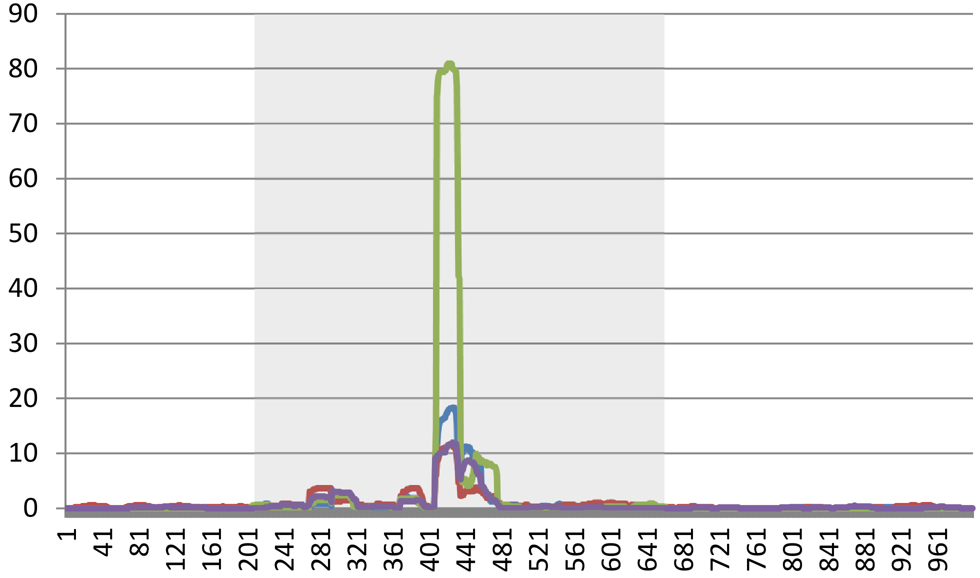

Supplement: S17 Fig — (PDF) [file pone.0169212.s017.pdf]
