# Supplementary material for: Transcriptome-Wide Discovery of PASRs (Promoter-Associated Small RNAs) and TASRs (Terminus-Associated Small RNAs) in Arabidopsis thaliana
Source: PLoS One. 2017 Jan 3;12(1):e0169212. doi: 10.1371/journal.pone.0169212 (PMC5207706; doi:10.1371/journal.pone.0169212)

Figure S18 DsRNA-seq read-covered paired TASR peaks identified on both strands of the protein-coding genes of *Arabidopsis*. For each plot, x axis measures the position on the paired strands, and y axis measures the abundance (in RPM, reads per million) of sRNAs. The dsRNA-seq read covered region was highlighted in gray background.

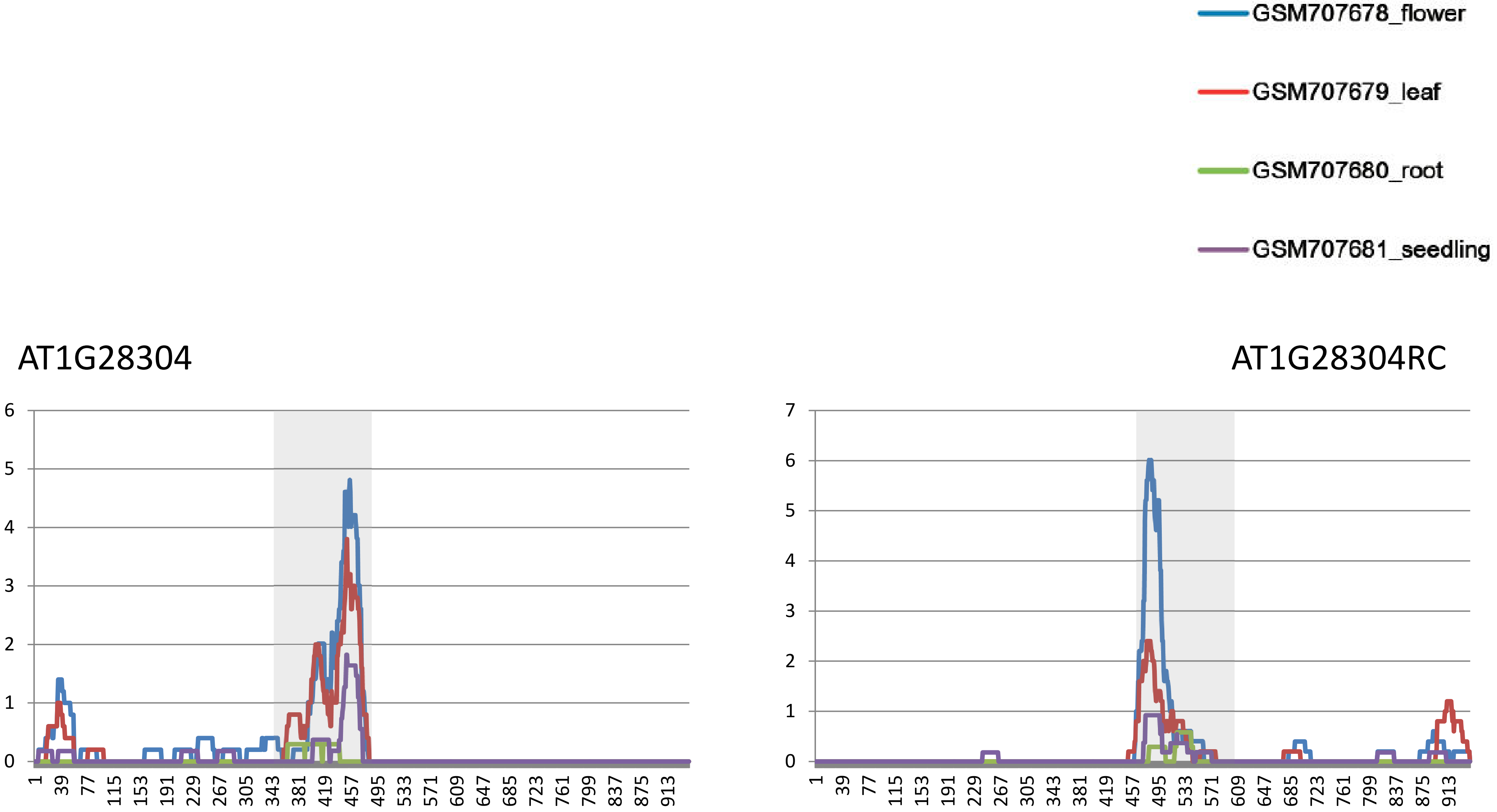
GSM707678_flower
GSM707679_leaf
GSM707680_root
GSM707681_seedling
AT1G28304
AT1G28304RC
0
1
2
3
4
5
6
7
1
39
77
115
153
191
229
267
305
343
381
419
457
495
533
571
609
647
685
723
761
799
837
875
913

AT1G66620

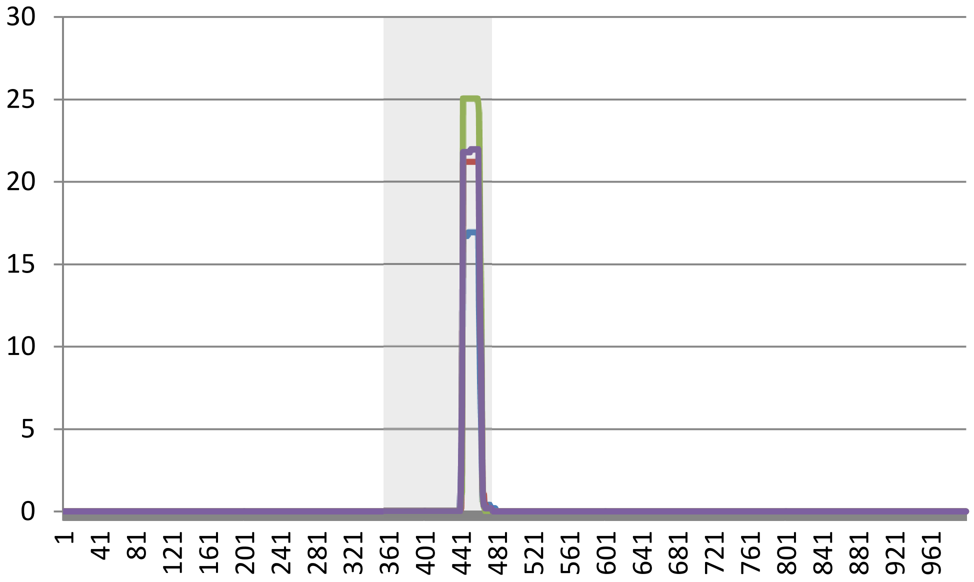

AT1G66620RC

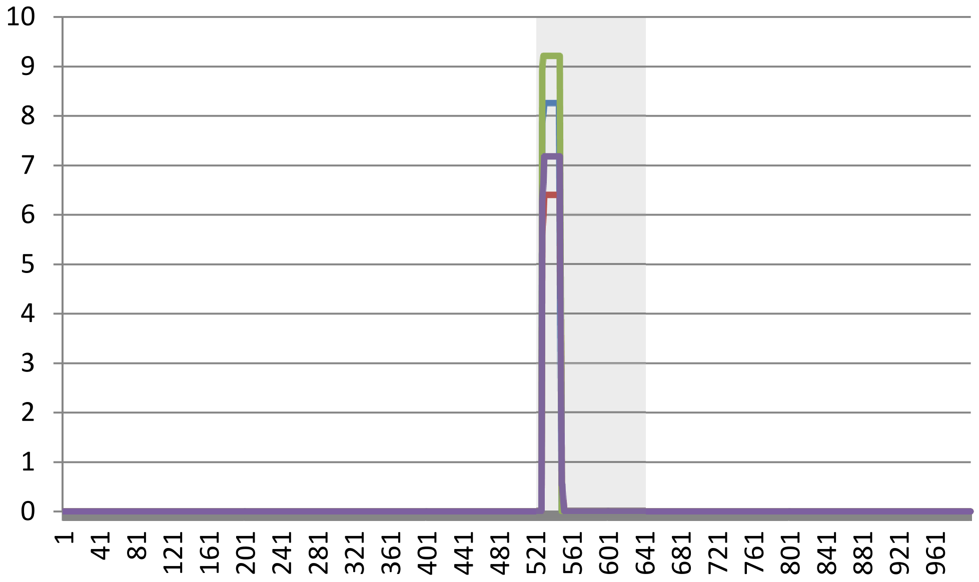

AT3G25130

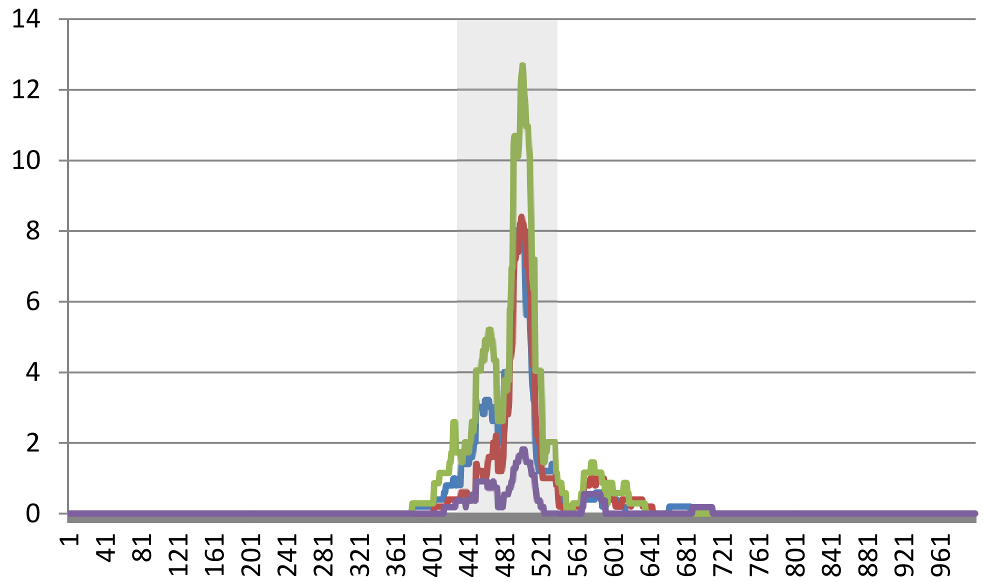

AT3G25130RC

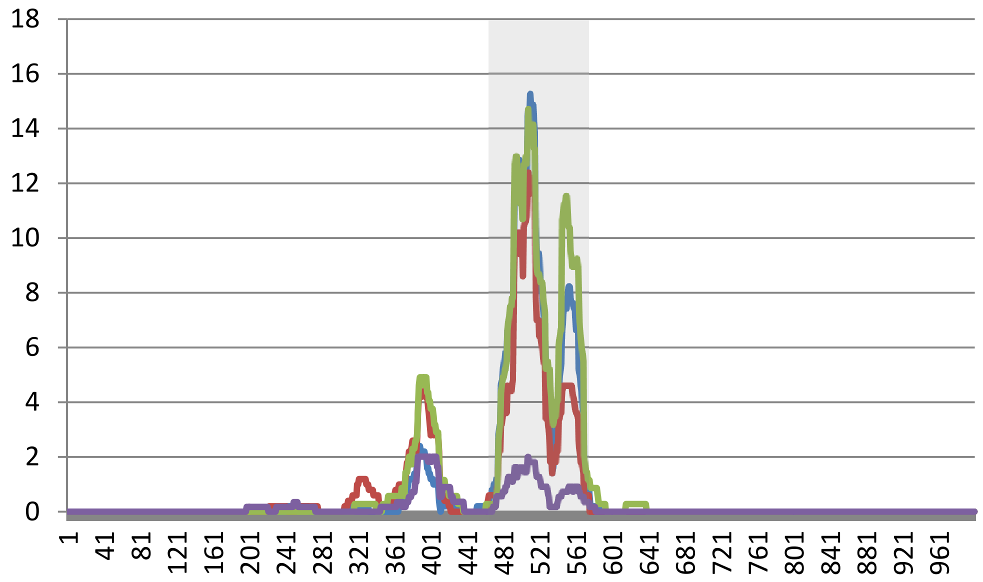

AT3G41762

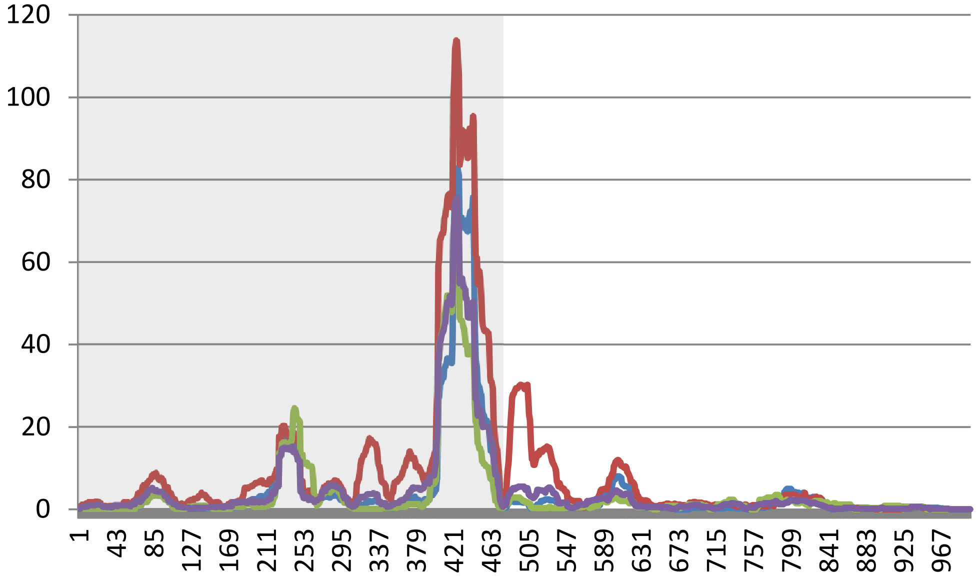

AT3G41762RC

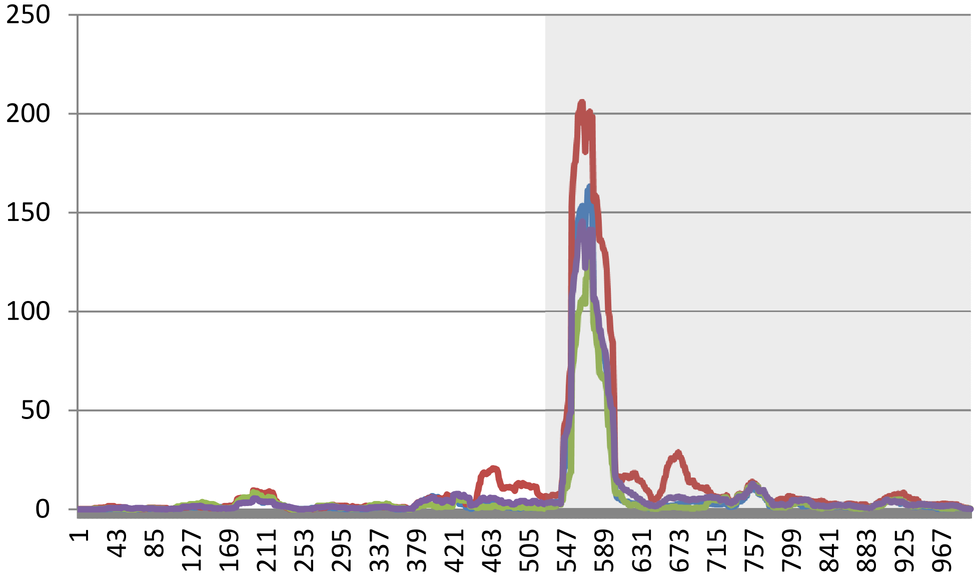

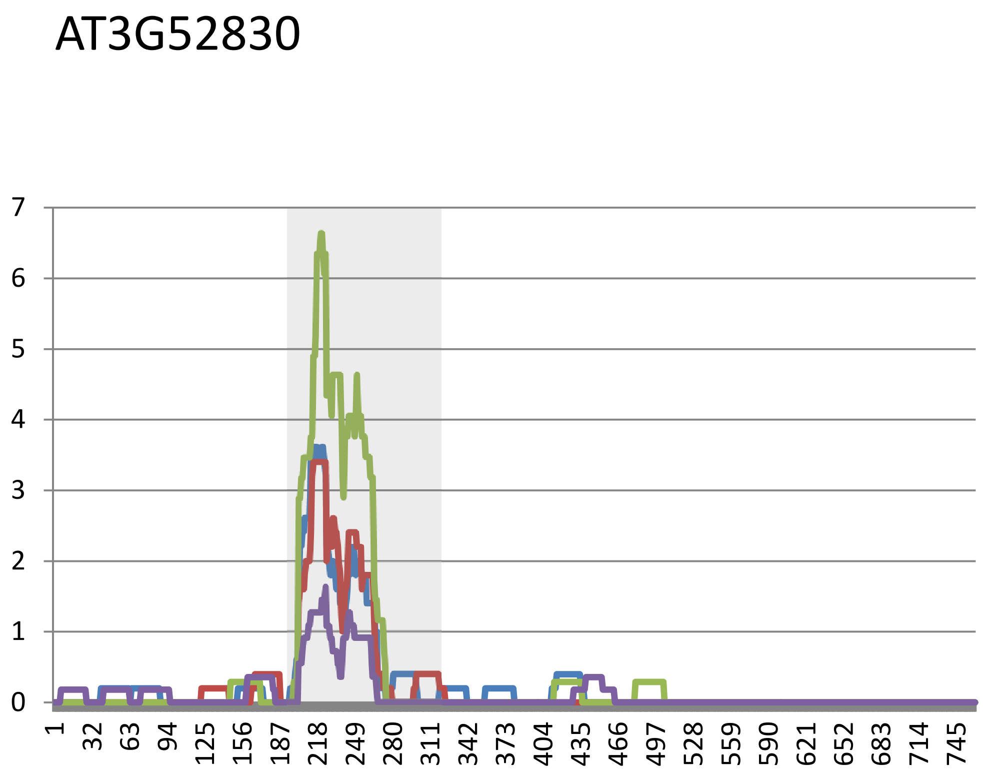
AT3G52830
7
6
5
4
3
2
1
0
1
32
63
94
125
156
187
218
249
280
311
342
373
404
435
466
497
528
559
590
621
652
683
714
745

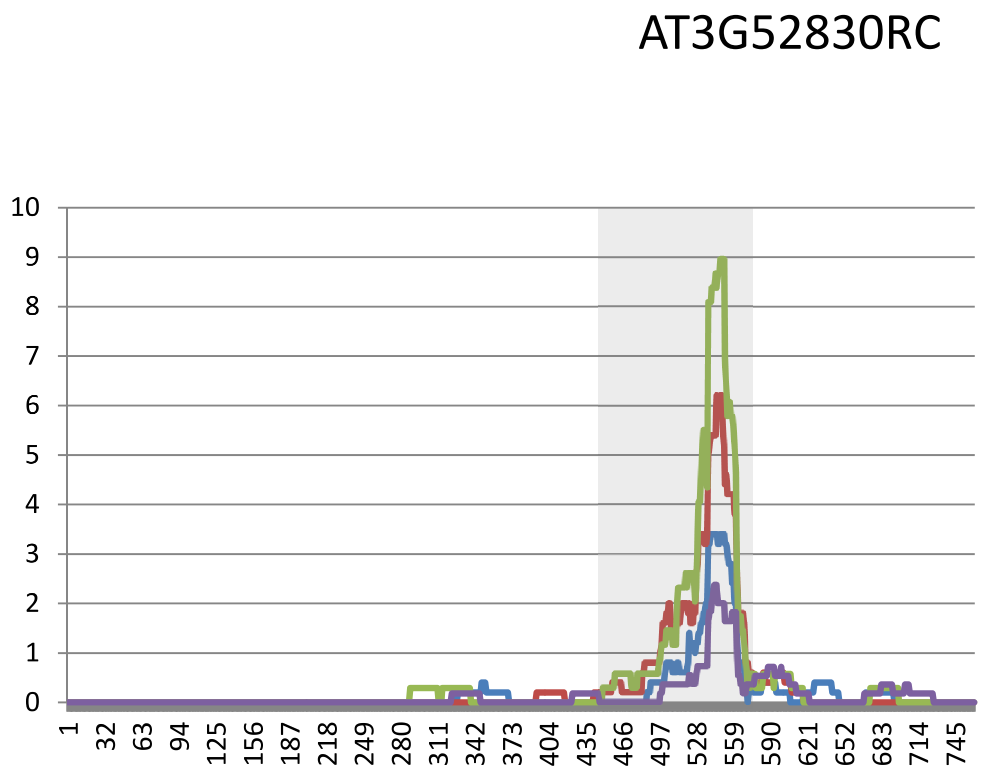
AT3G52830RC
10
9
8
7
6
5
4
3
2
1
0
1
32
63
94
125
156
187
218
249
280
311
342
373
404
435
466
497
528
559
590
621
652
683
714
745

AT4G04030

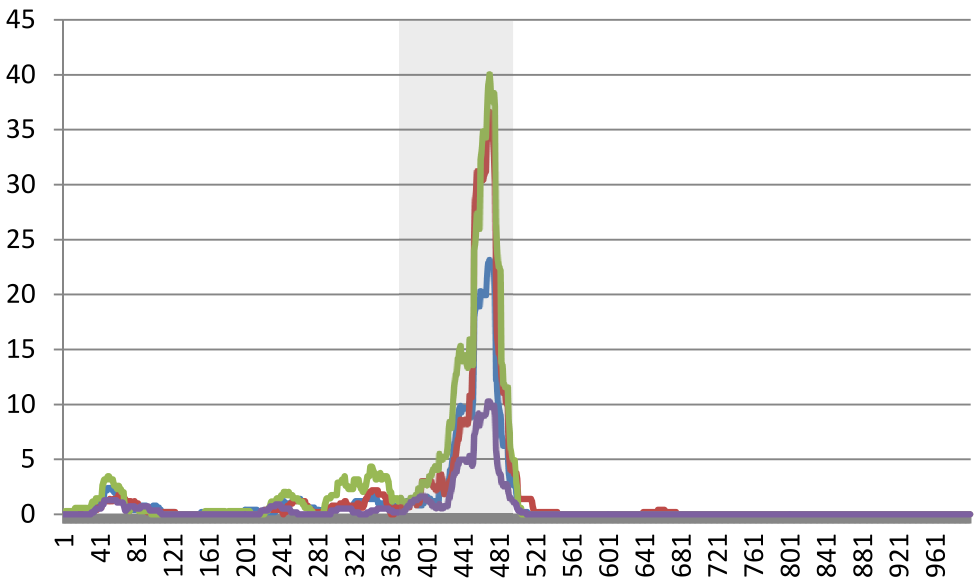

AT4G04030RC

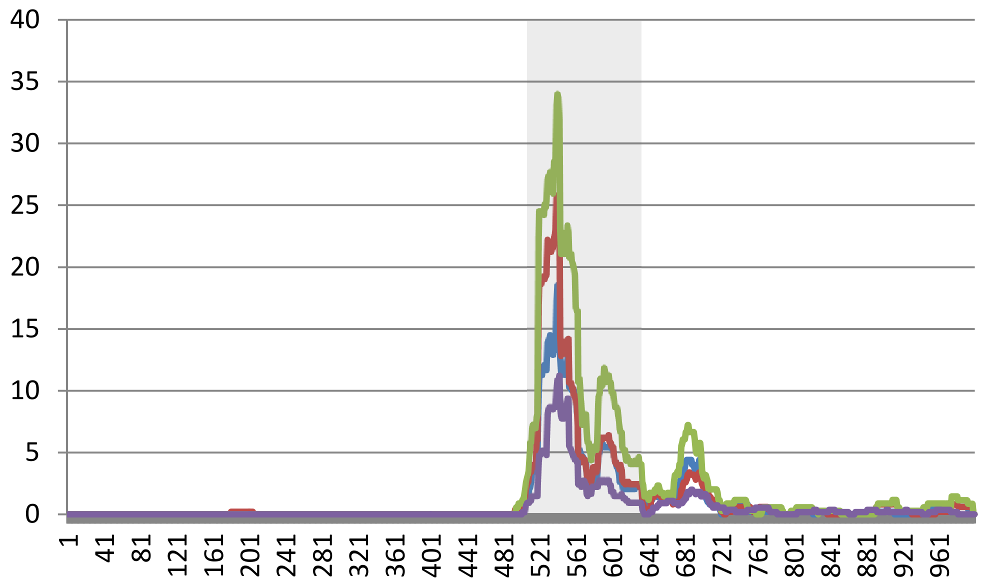

AT4G08160

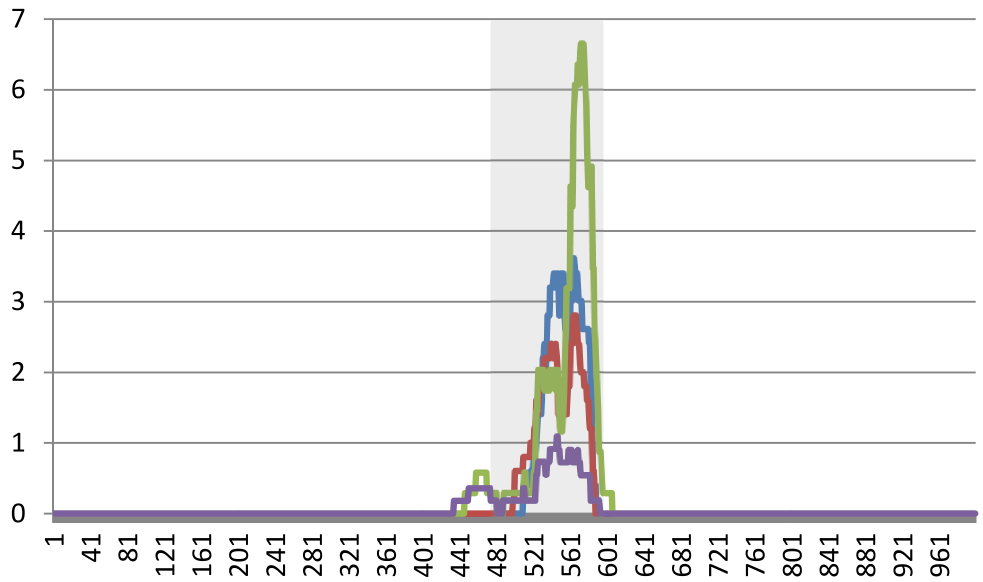

AT4G08160RC

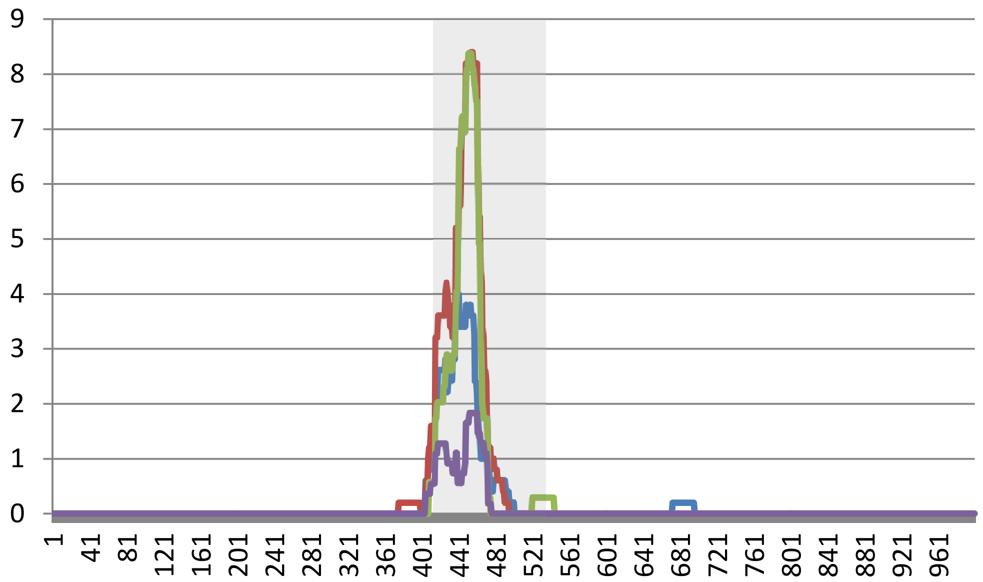

AT4G14365

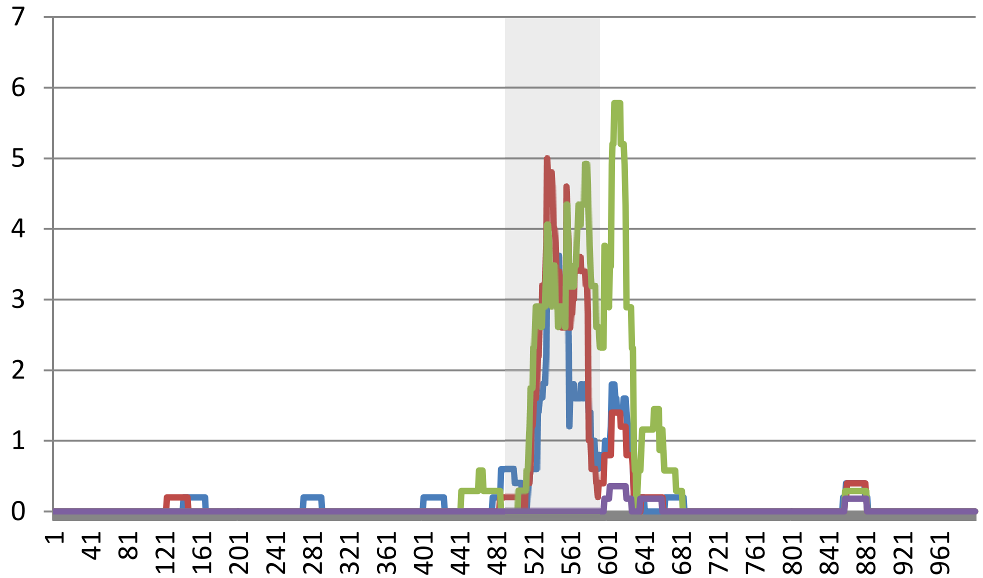

AT4G14365RC

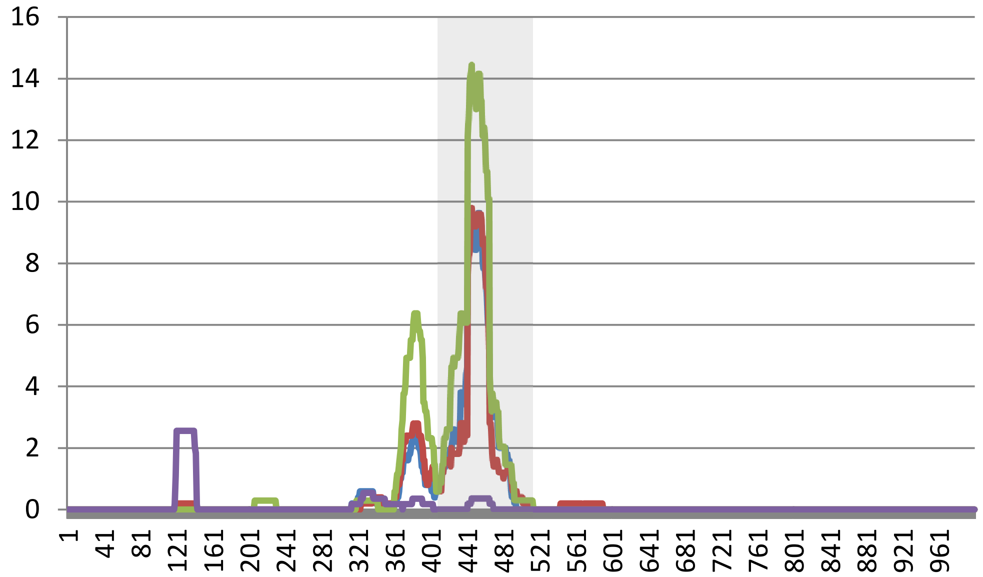

AT5G43525

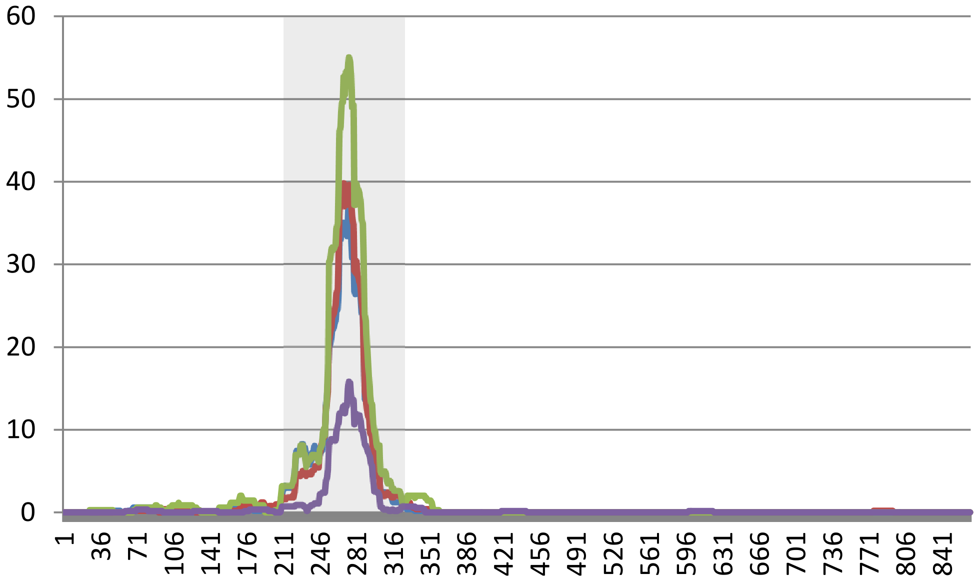

AT5G43525RC

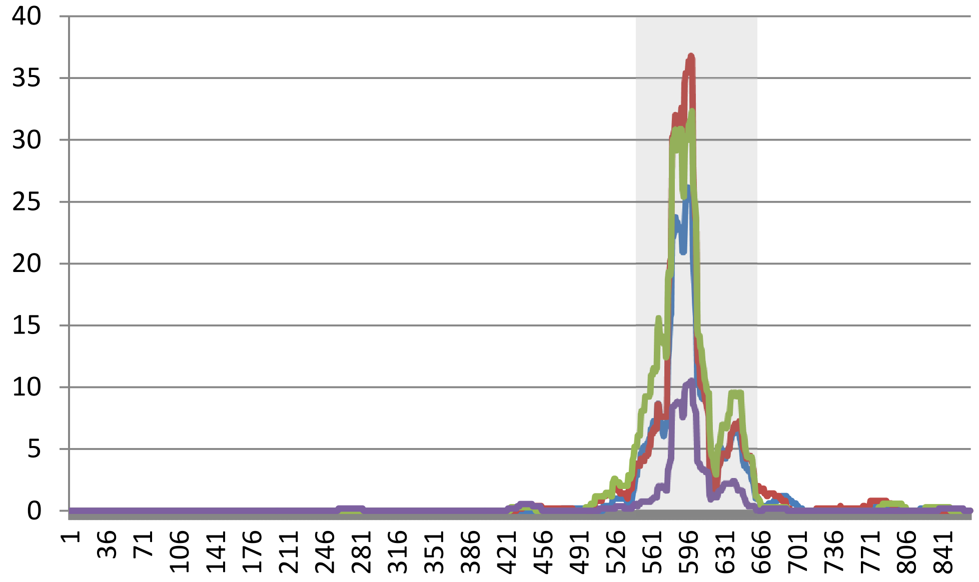

AT5G50480

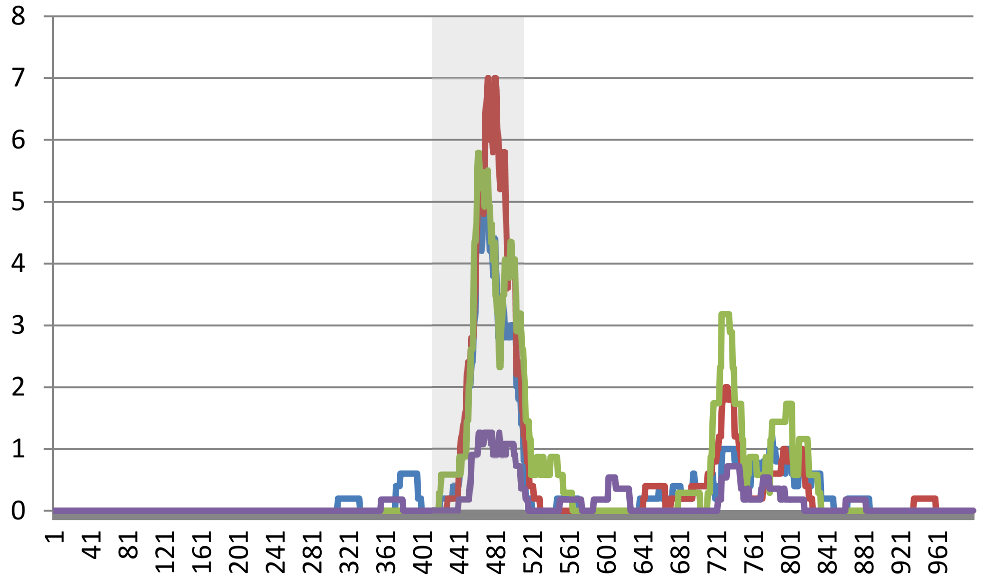

AT5G50480RC

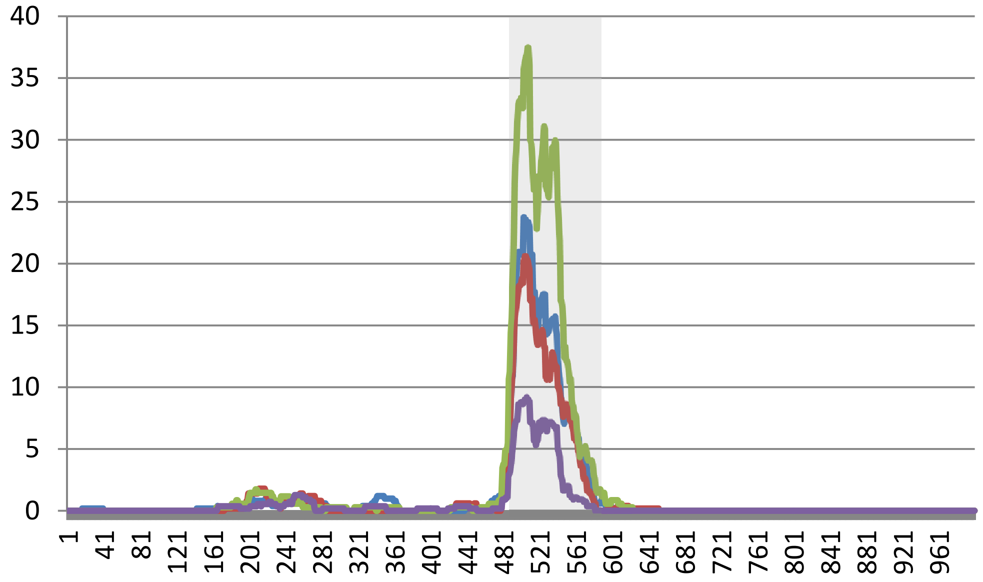

AT5G54410

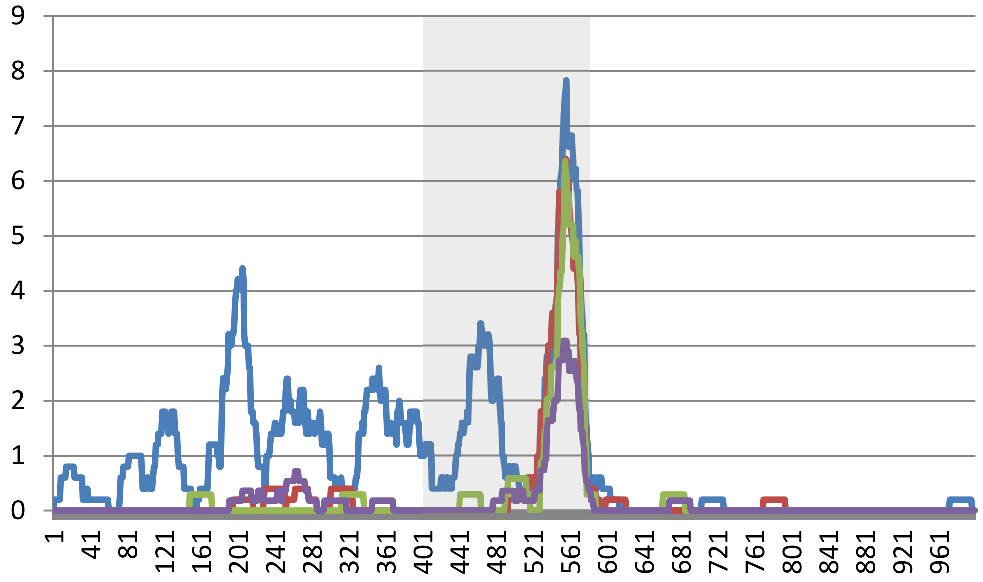

AT5G54410RC

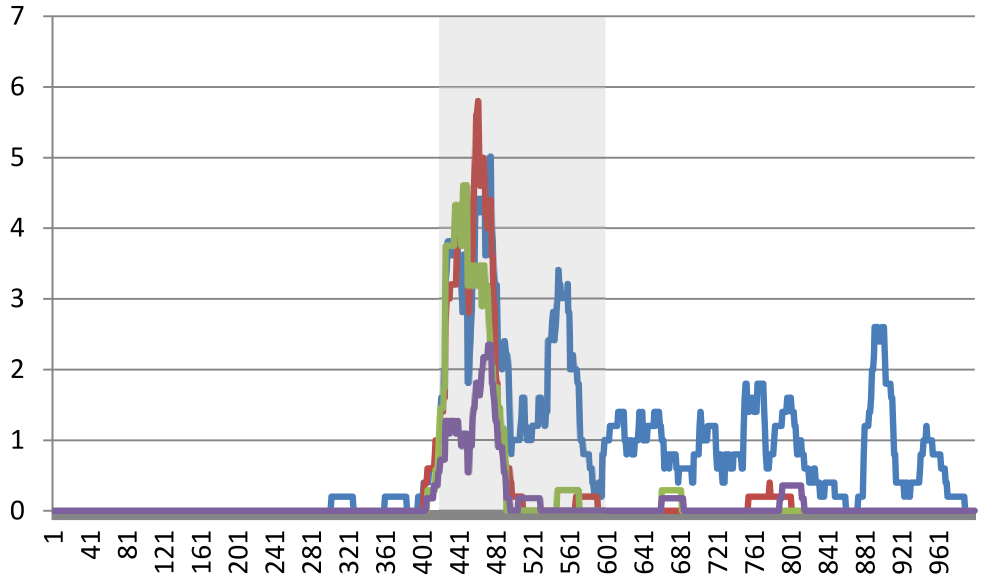

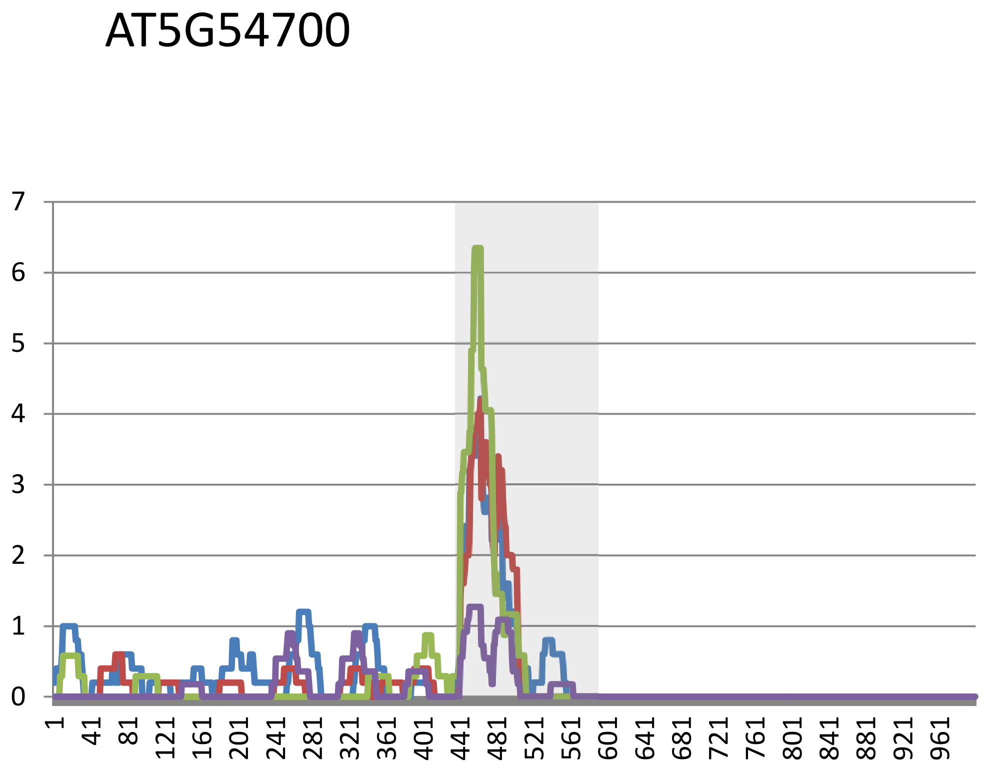
AT5G54700
0
1
2
3
4
5
6
7
1
41
81
121
161
201
241
281
321
361
401
441
481
521
561
601
641
681
721
761
801
841
881
921
961

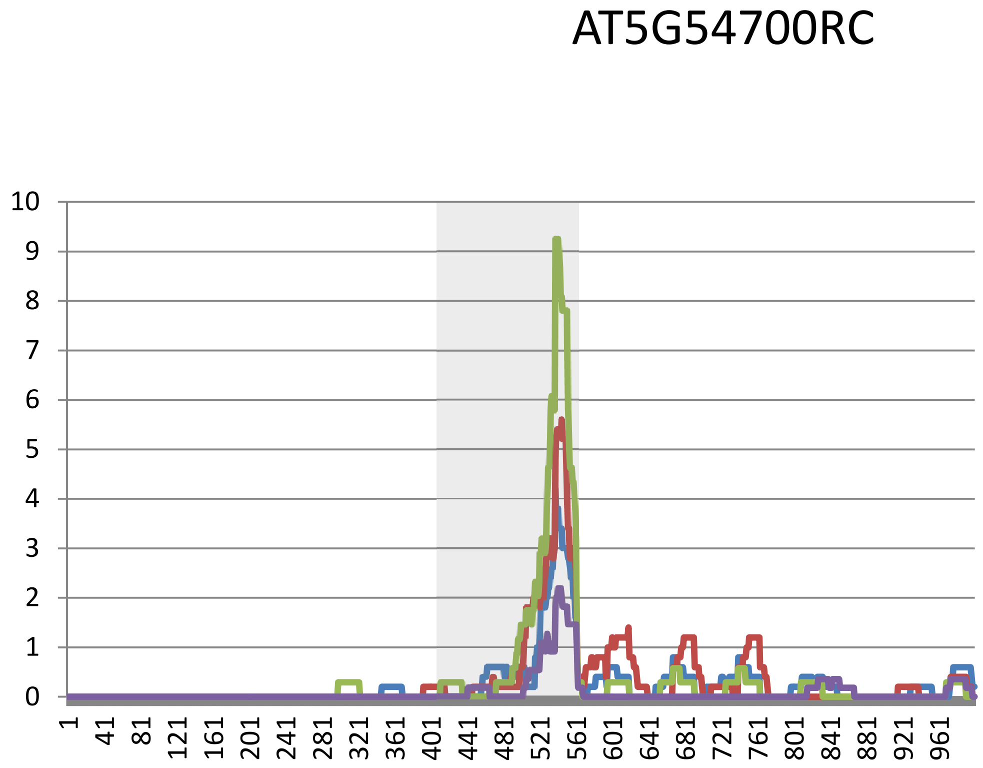
AT5G54700RC
0
1
2
3
4
5
6
7
8
9
10
1
41
81
121
161
201
241
281
321
361
401
441
481
521
561
601
641
681
721
761
801
841
881
921
961

AT5G65005

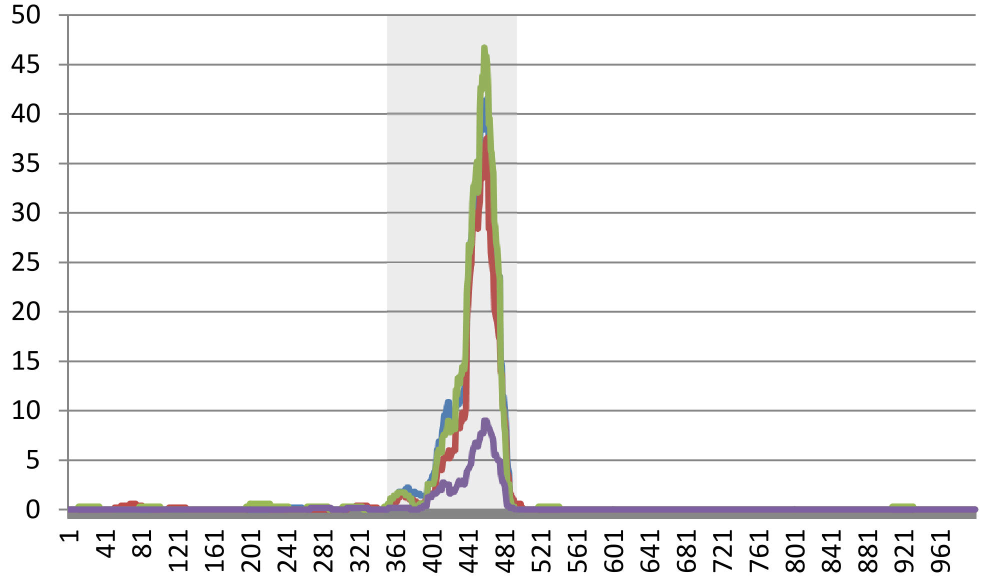

AT5G65005RC

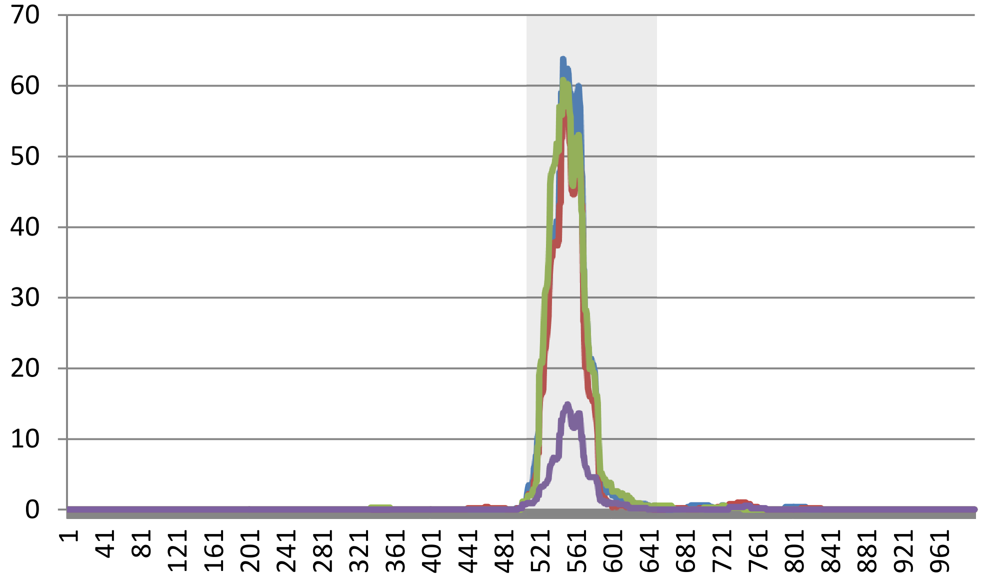

Supplement: S18 Fig — (PDF) [file pone.0169212.s018.pdf]
