# Supplementary material for: Transcriptome-Wide Discovery of PASRs (Promoter-Associated Small RNAs) and TASRs (Terminus-Associated Small RNAs) in Arabidopsis thaliana
Source: PLoS One. 2017 Jan 3;12(1):e0169212. doi: 10.1371/journal.pone.0169212 (PMC5207706; doi:10.1371/journal.pone.0169212)

Figure S19 Results showing the dependence of certain PASR and TASR peaks on the activities of specific DCL(s), RDR(s) and Pol IV, and the loading preference of the PASRs and TASRs into specific AGO(s). PASR_plus: PASR peaks on the sense strands of the protein-coding genes of *Arabidopsis*. PASR_RC: PASR peaks on the antisense strands of the protein-coding genes of *Arabidopsis*. TASR_plus: TASR peaks on the sense strands of the protein-coding genes of *Arabidopsis*. TASR__RC: TASR peaks on the antisense strands of the protein-coding genes of *Arabidopsis*. For each plot, x axis measures the position of the genomic sequence, and y axis measures the abundance (in RPM, reads per million) of sRNAs.

PASR_plus

GSE6682 | GSE14695 | GSE44622 | GSE10180

AT1G53265

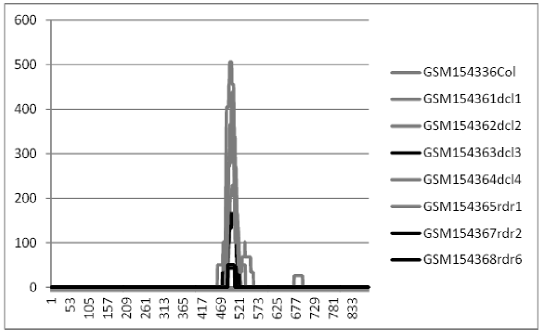

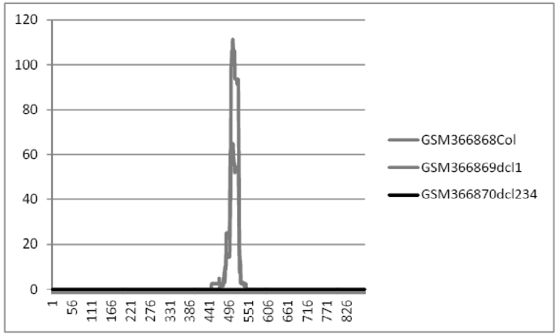

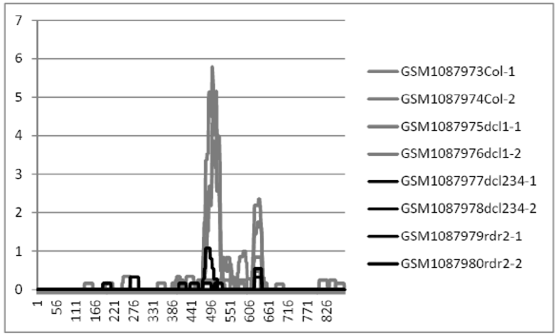

AT4G16640

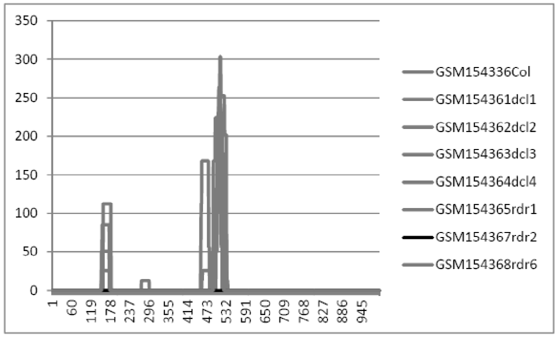

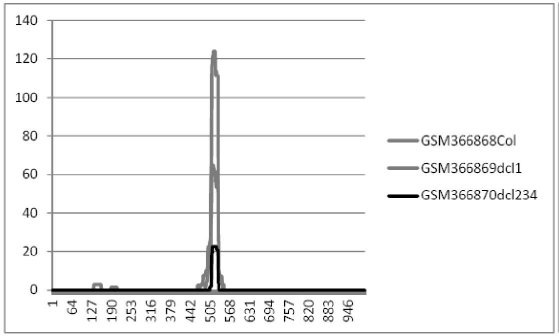

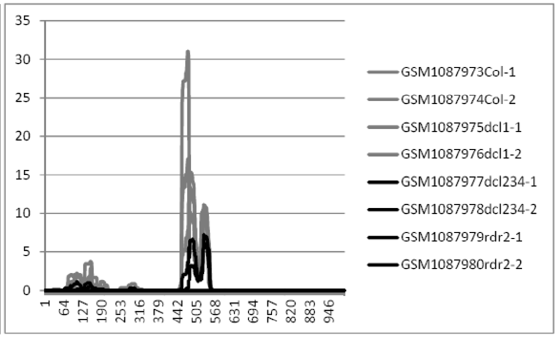

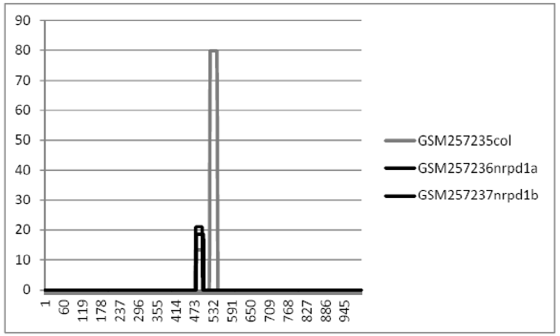

AT5G48000

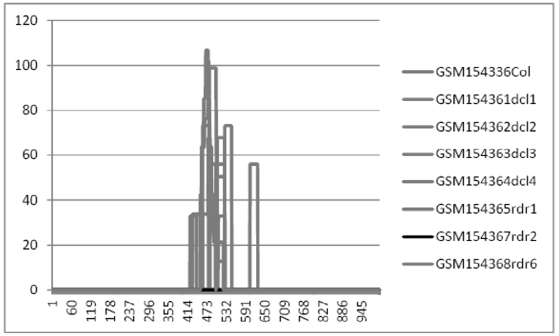

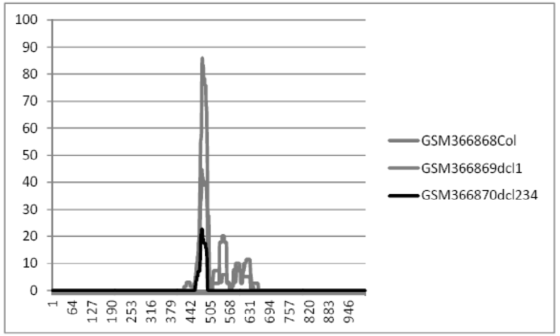

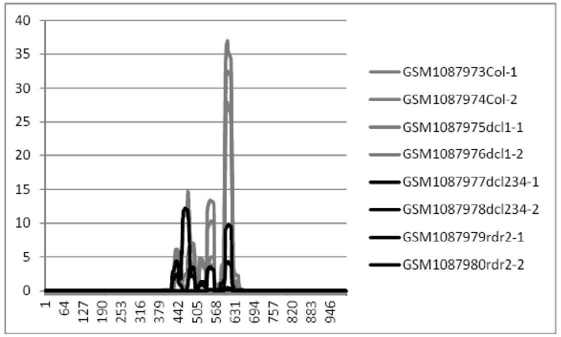

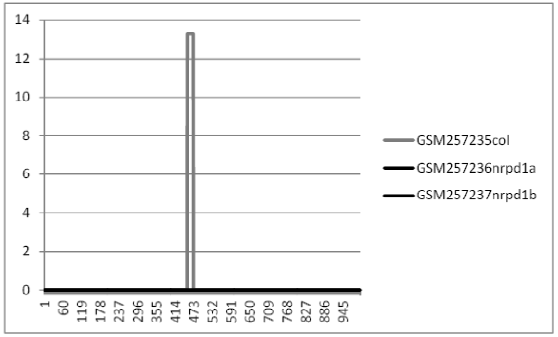

PASR_RC

GSE6682 GSE14695 GSE44622 GSE10180

AT1G53265

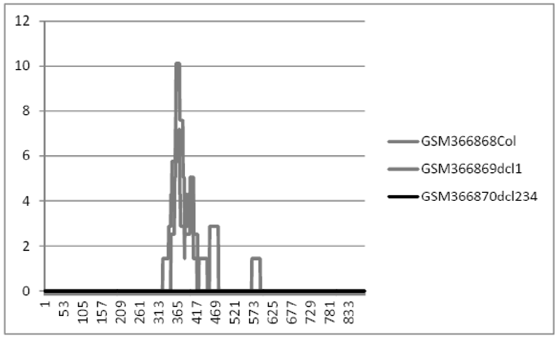

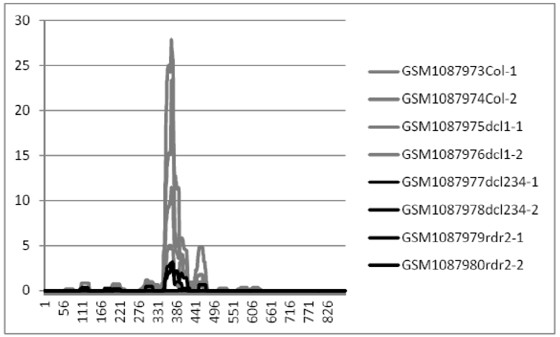

AT3G43270

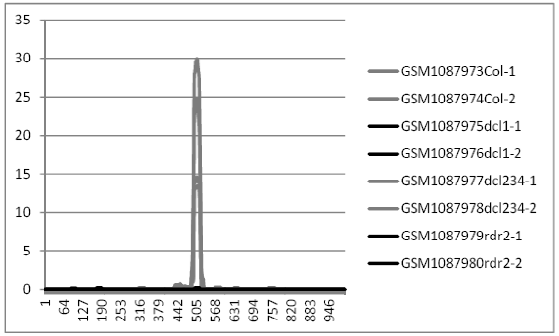

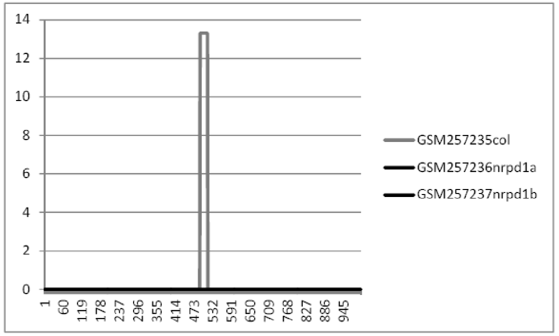

PASR_RC

GSE6682 GSE14695 GSE44622 GSE10180

AT4G16640

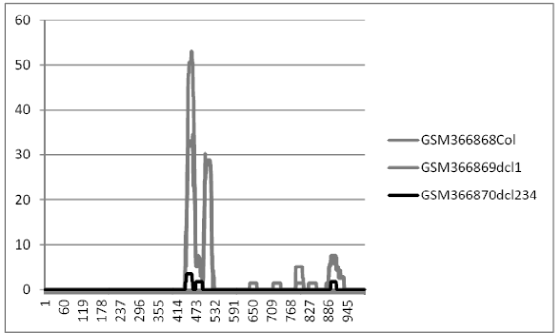

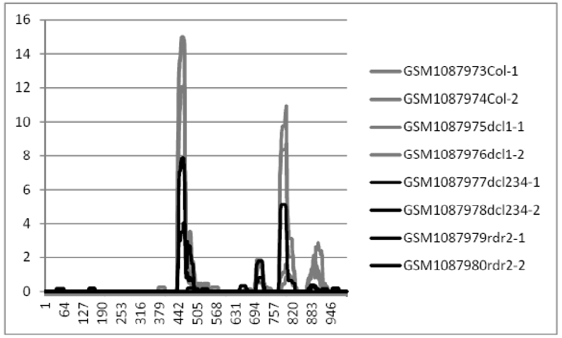

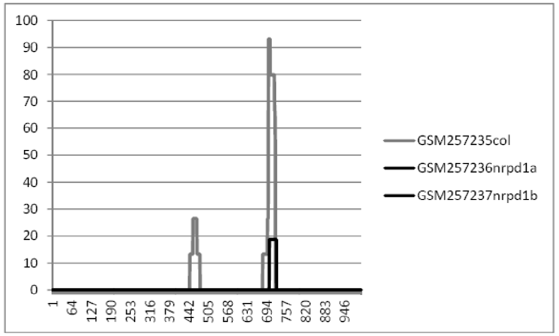

AT5G48000

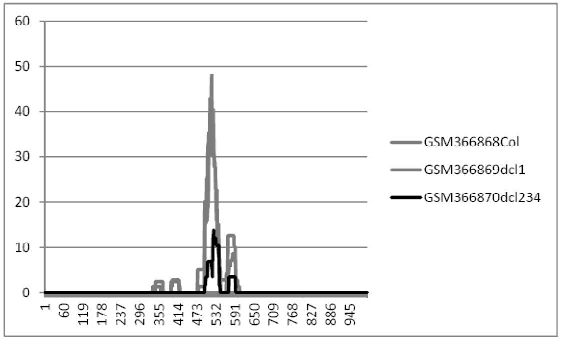

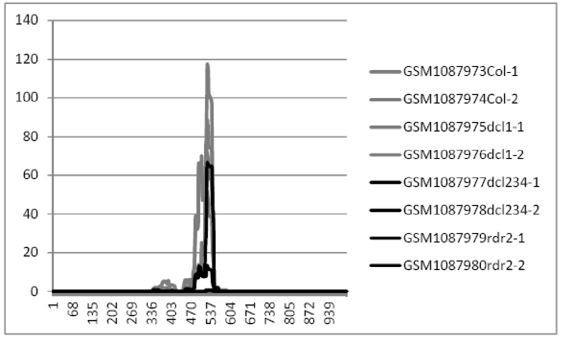

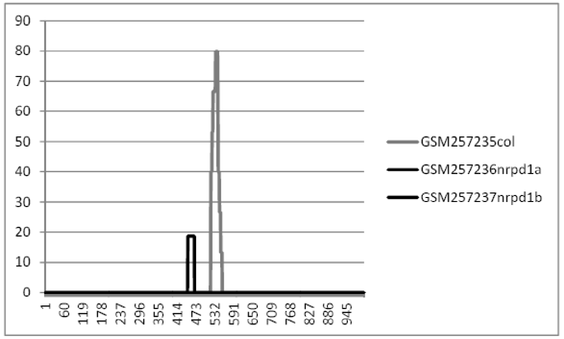

TASR_plus

GSE6682 GSE14695 GSE44622 GSE10180

AT1G28304

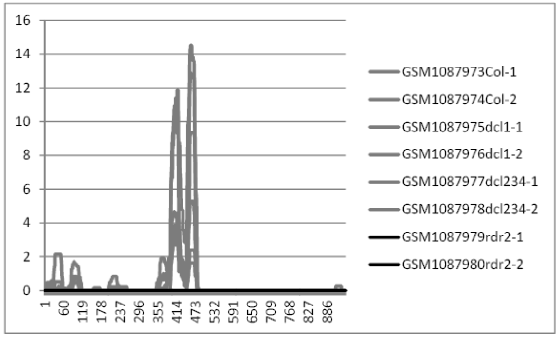

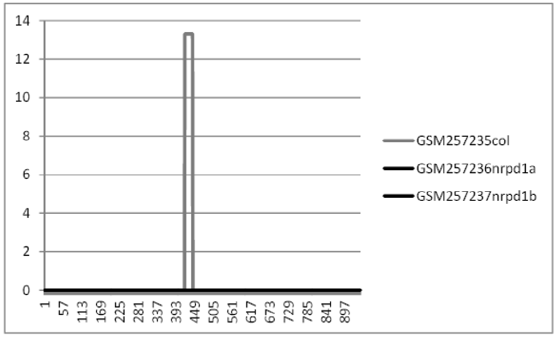

AT3G25130

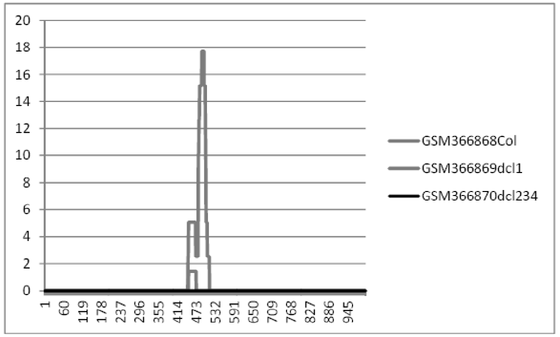

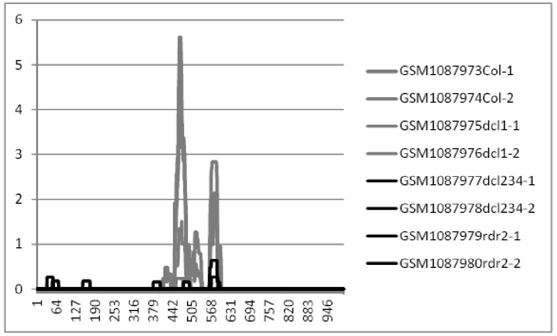

AT3G41762

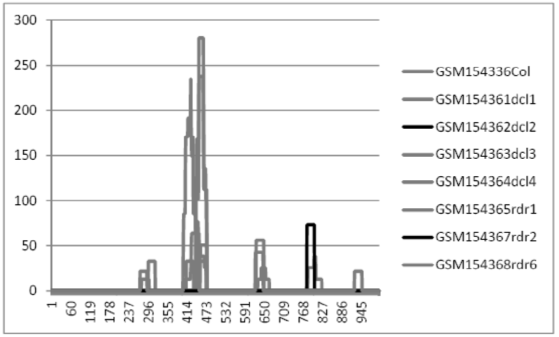

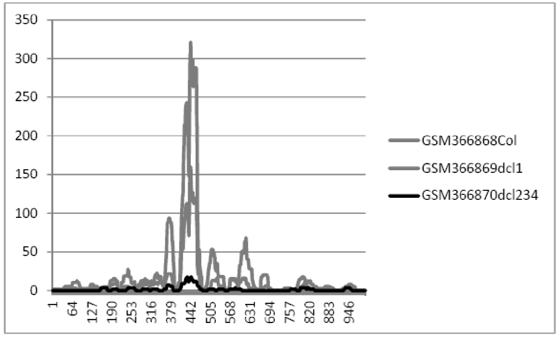

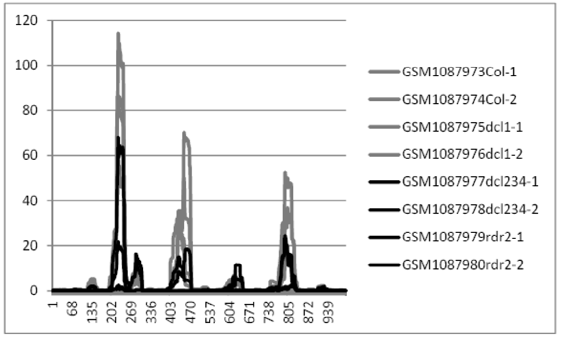

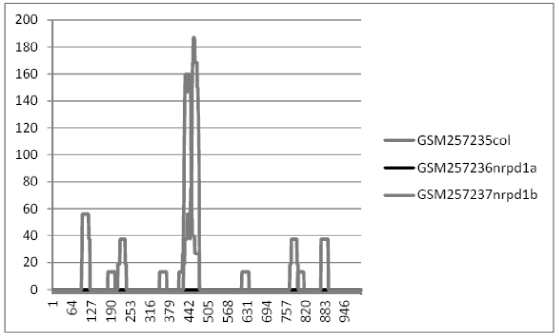

TASR_plus

GSE6682 GSE14695 GSE44622 GSE10180

AT3G52830

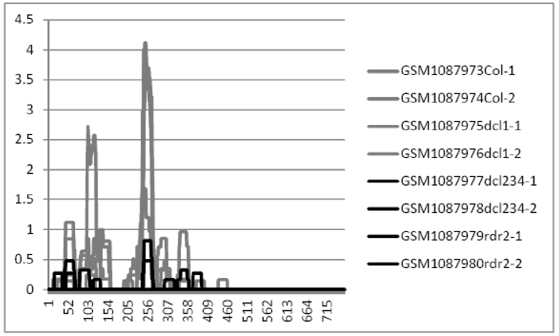

AT4G04030

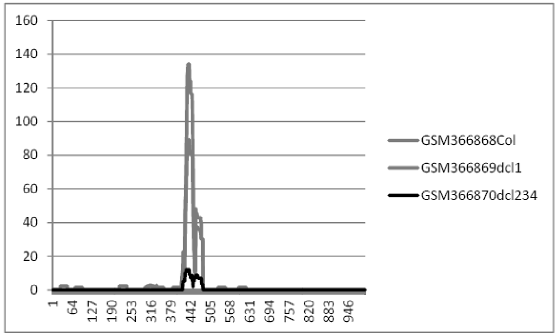

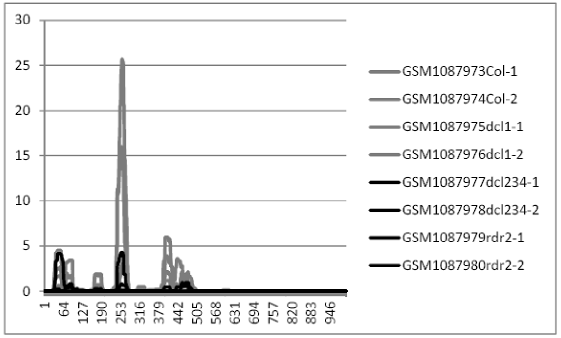

AT4G08160

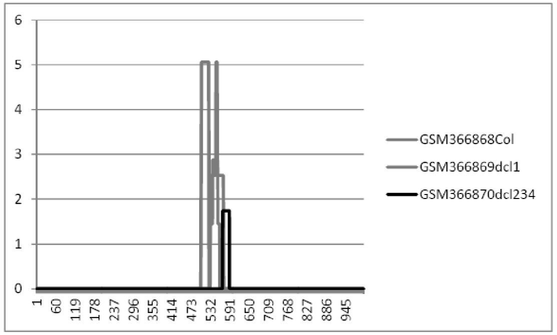

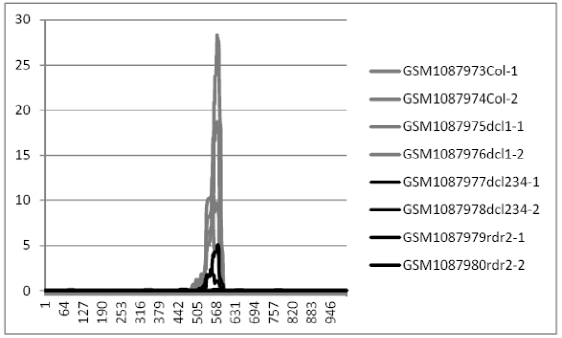

TASR_plus

GSE6682 | GSE14695 | GSE44622 | GSE10180

AT4G14365

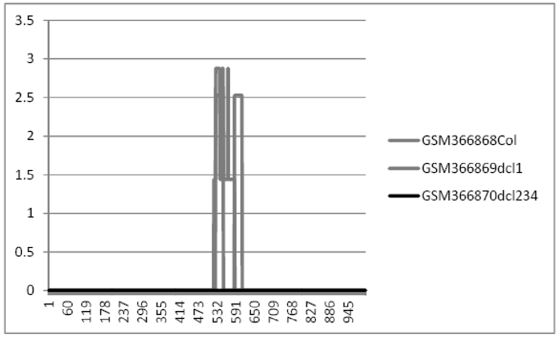

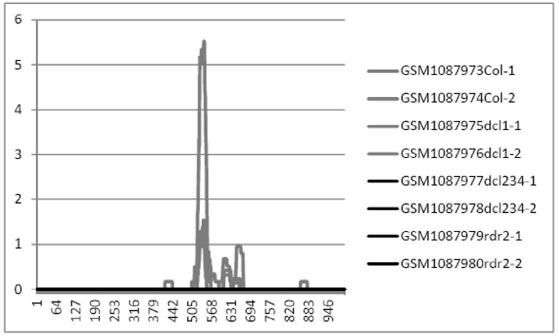

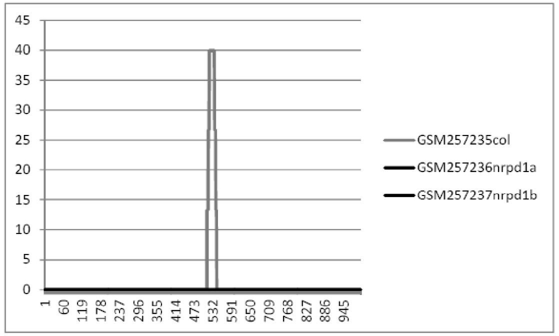

AT5G43525

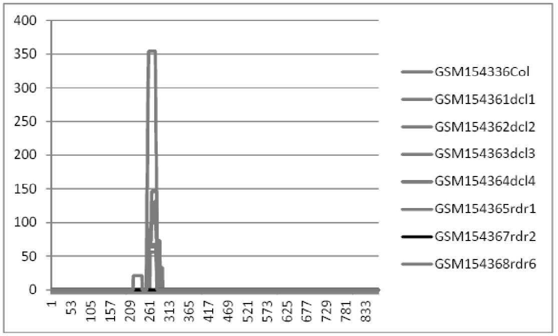

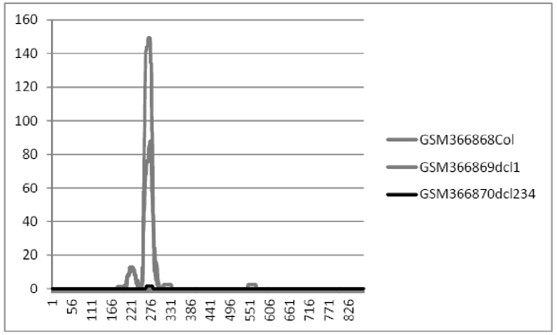

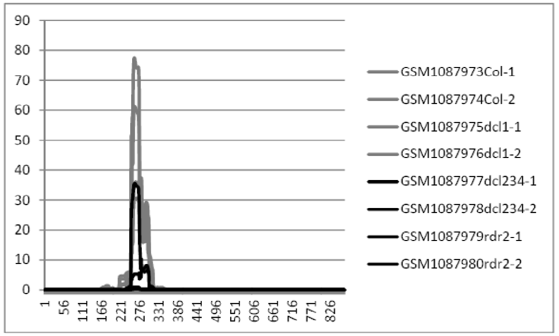

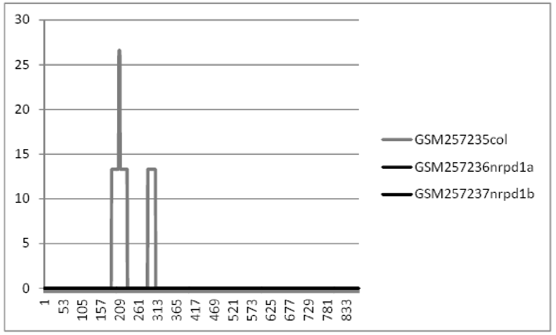

AT5G50480

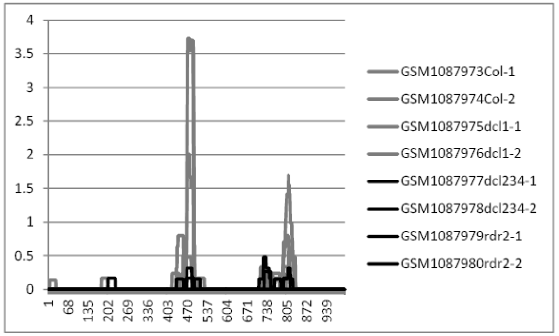

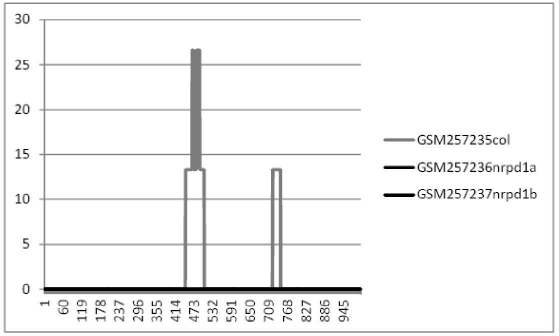

TASR_plus

GSE6682 GSE14695 GSE44622 GSE10180

AT5G54700

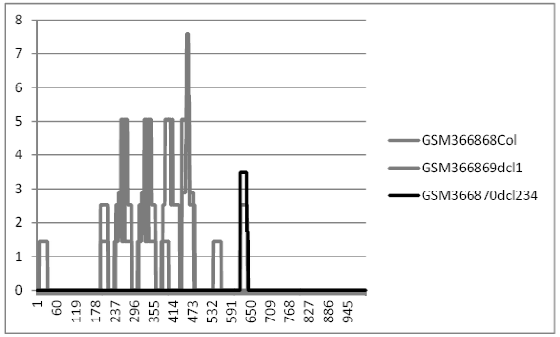

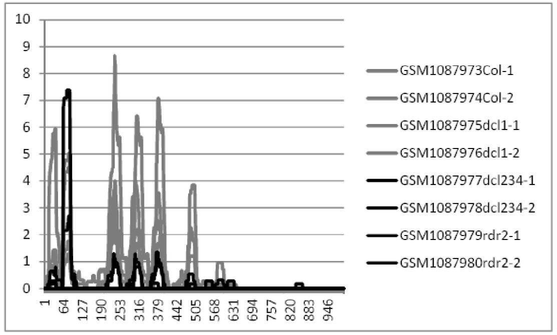

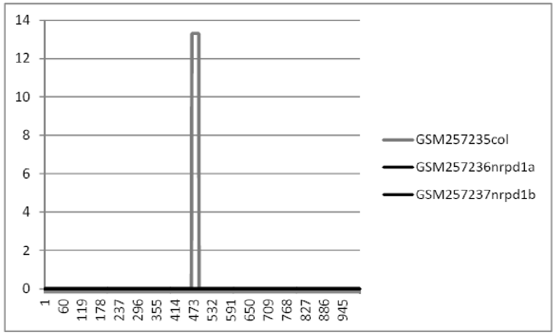

AT5G65005

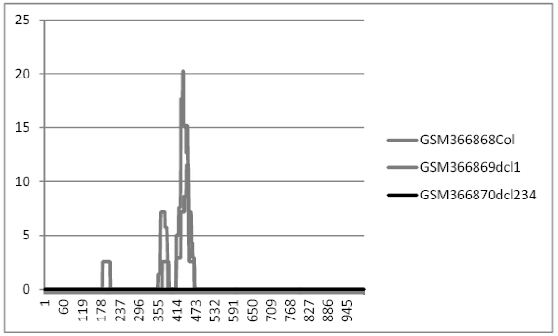

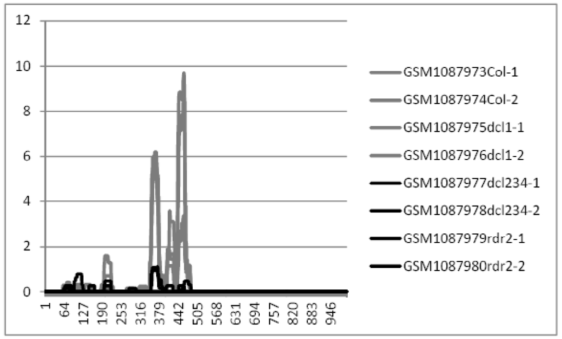

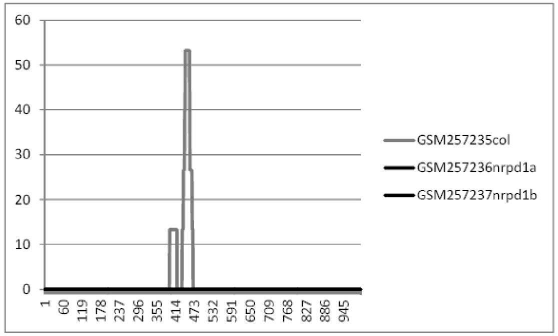

TASR_RC

GSE6682 | GSE14695 | GSE44622 | GSE10180

AT1G28304

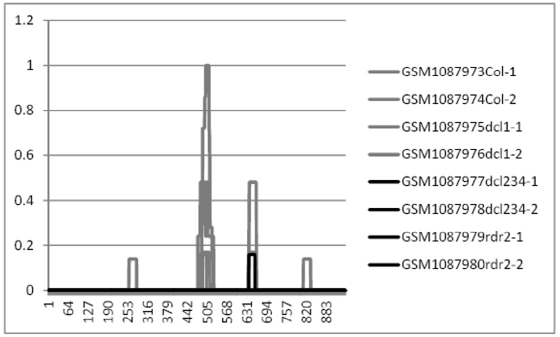

AT3G25130

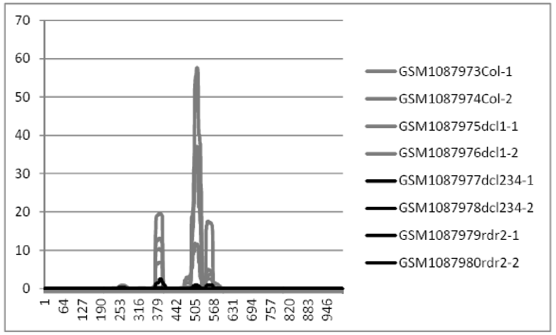

AT3G41762

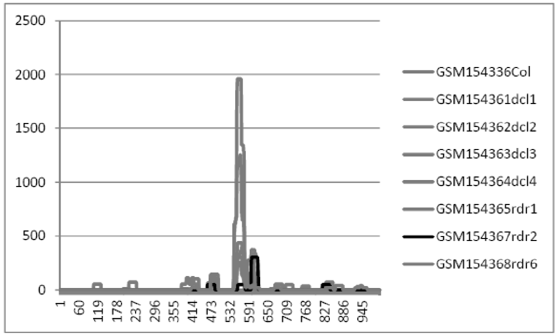

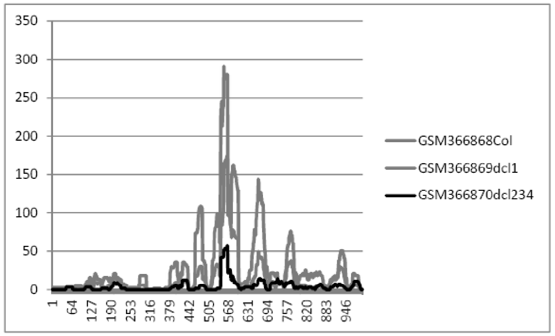

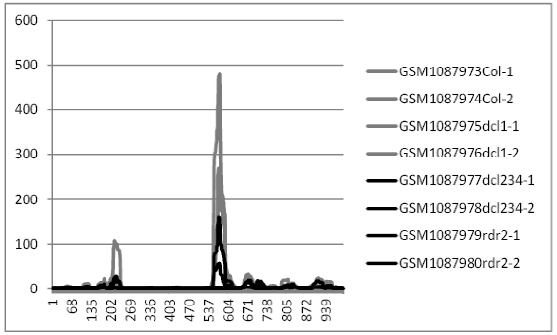

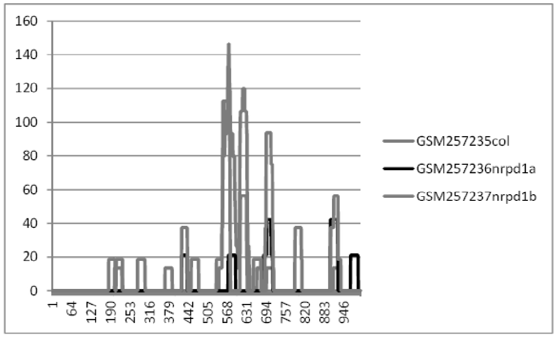

TASR_RC

GSE6682 GSE14695 GSE44622 GSE10180

AT3G52830

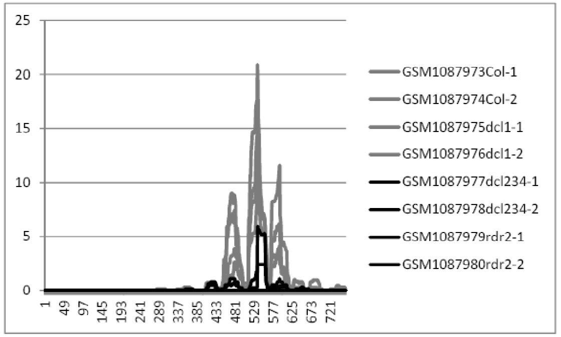

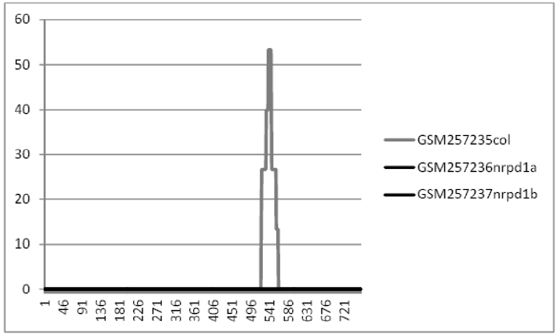

AT4G04030

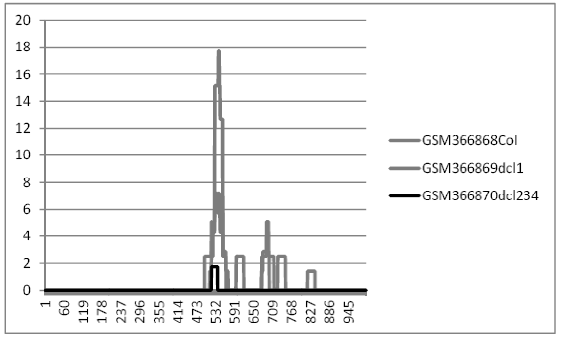

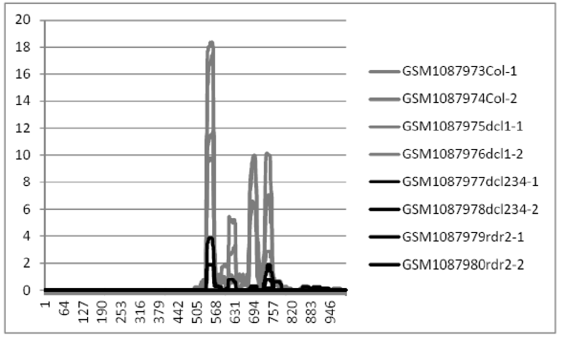

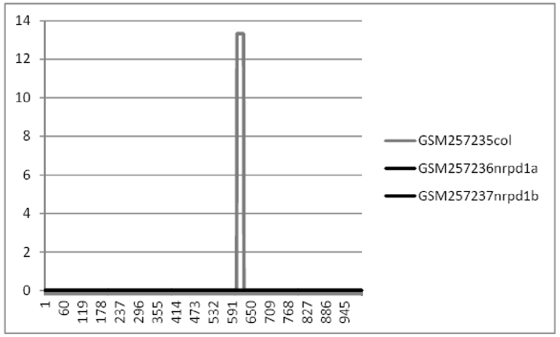

AT4G08160

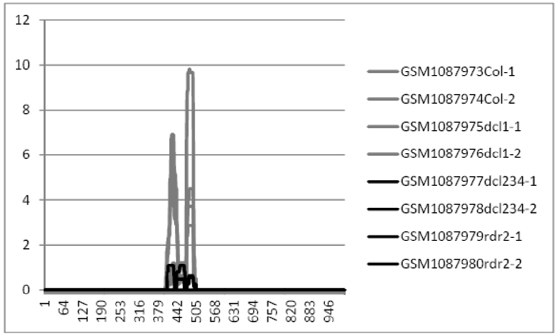

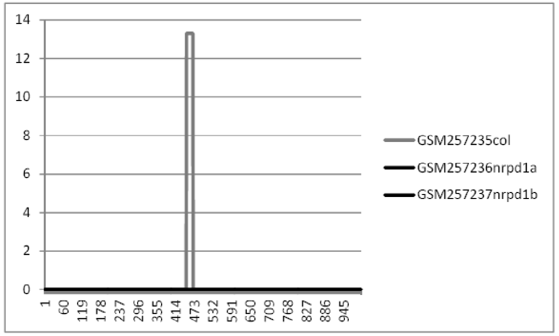

TASR_RC

GSE6682 | GSE14695 | GSE44622 | GSE10180

AT4G14365

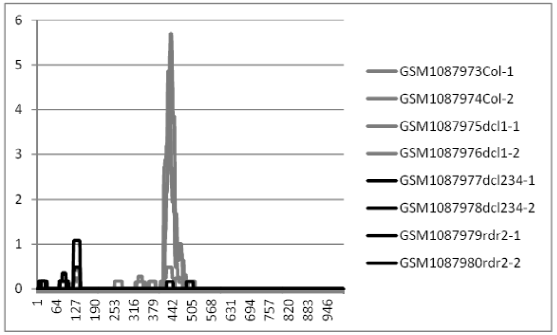

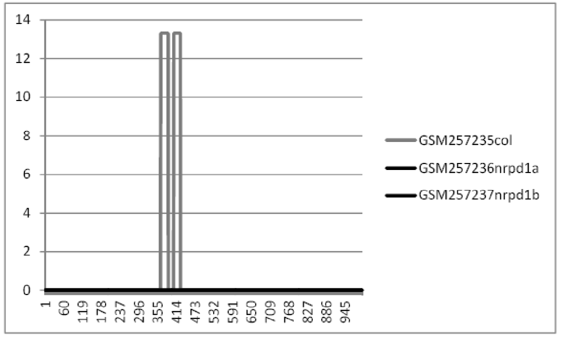

AT5G43525

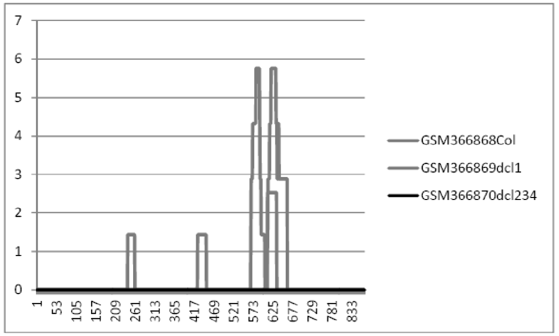

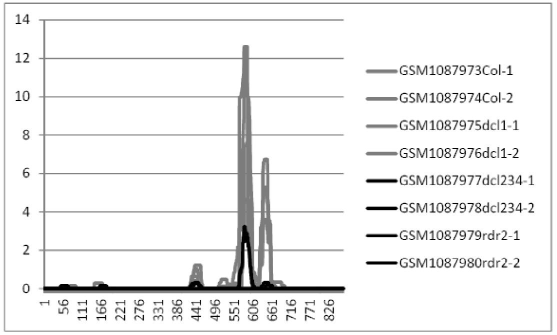

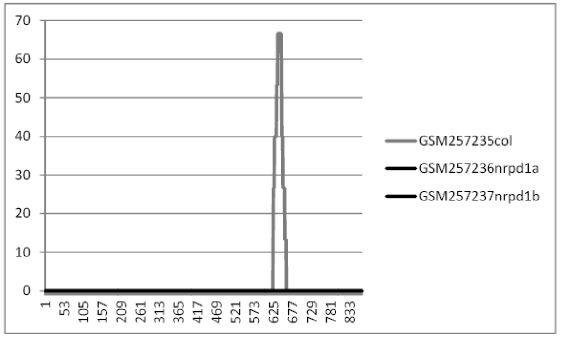

AT5G50480

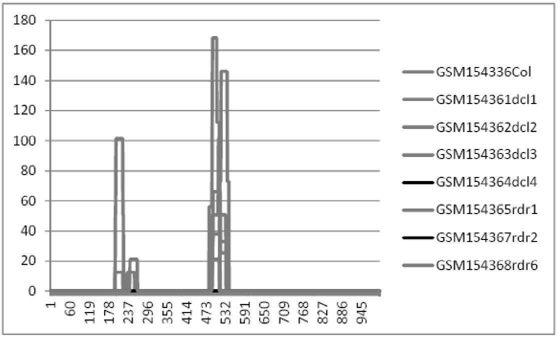

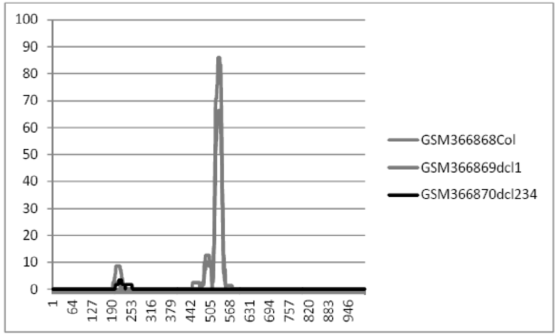

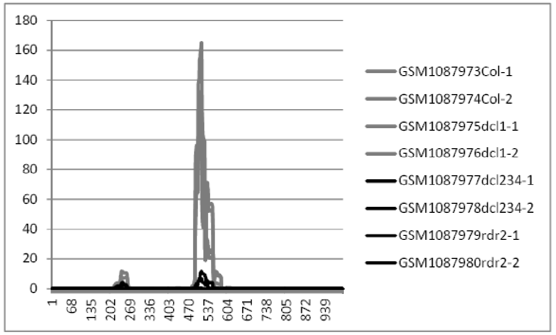

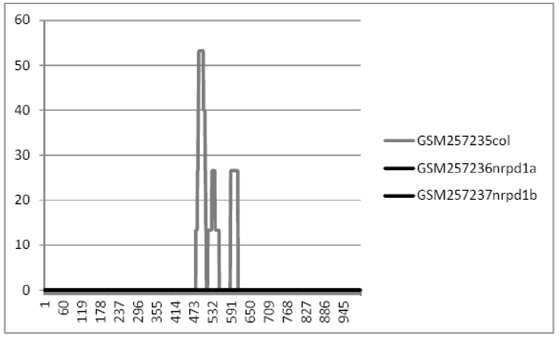

TASR_RC

GSE6682 GSE14695 GSE44622 GSE10180

AT5G54410

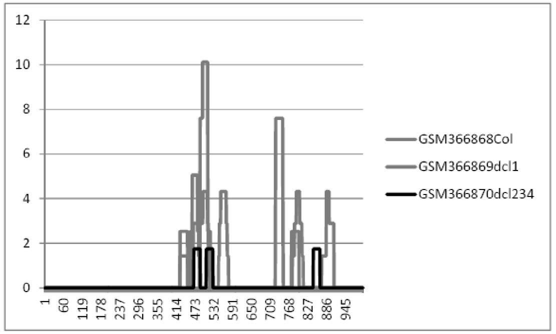

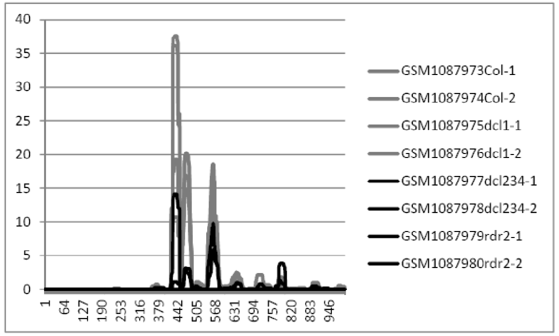

AT5G54700

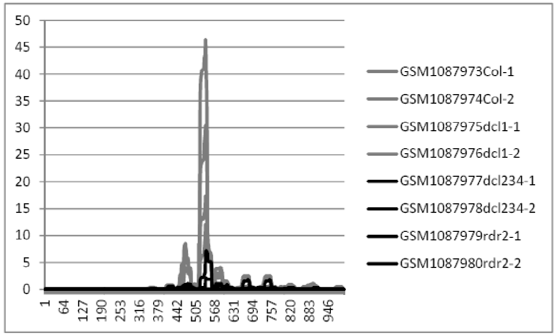

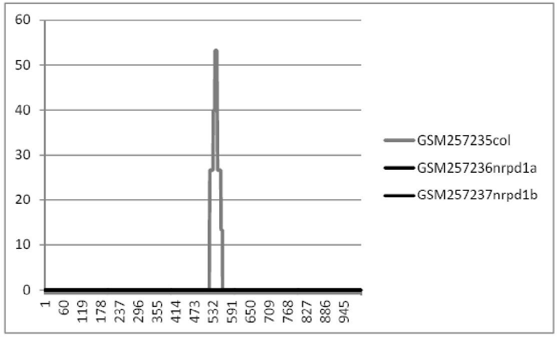

AT5G65005

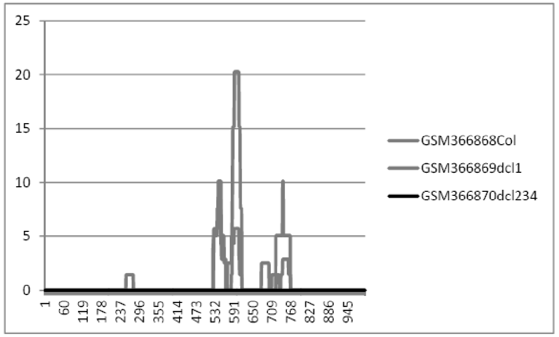

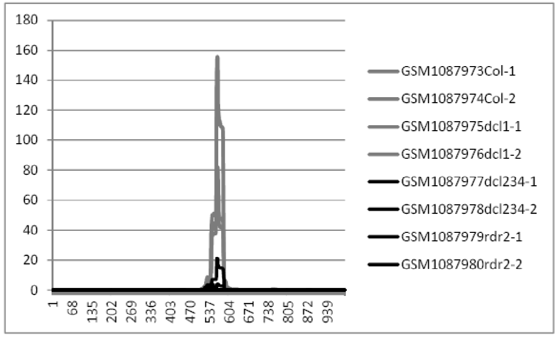

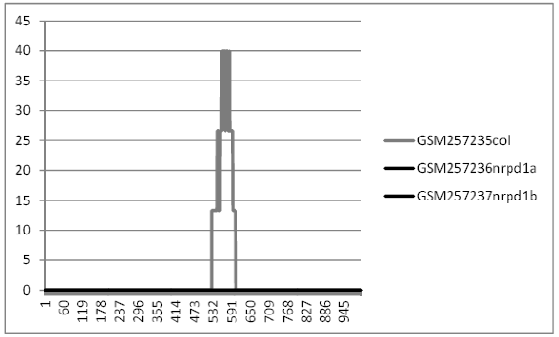

Supplement: S19 Fig — PASR_plus: PASR peaks on the sense strands of the protein-coding genes of Arabidopsis. PASR_RC: PASR peaks on the antisense strands of the protein-coding genes of Arabidopsis. TASR_plus: TASR peaks on the sense strands of the protein-coding genes of Arabidopsis. TASR__RC: TASR peaks on the antisense strands of the protein-coding genes of Arabidopsis. (PDF) [file pone.0169212.s019.pdf]
