# Supplementary material for: Transcriptome-Wide Discovery of PASRs (Promoter-Associated Small RNAs) and TASRs (Terminus-Associated Small RNAs) in Arabidopsis thaliana
Source: PLoS One. 2017 Jan 3;12(1):e0169212. doi: 10.1371/journal.pone.0169212 (PMC5207706; doi:10.1371/journal.pone.0169212)

Figure S20 Results showing the dependence of certain paired PASR and TASR peaks on the activities of specific DCL(s), RDR(s) and Pol IV, and the loading preference of the PASRs and TASRs into specific AGO(s). For each plot, x axis measures the position of the genomic sequence, and y axis measures the abundance (in RPM, reads per million) of sRNAs.

PASR

AT1G53265

Total GSE28591

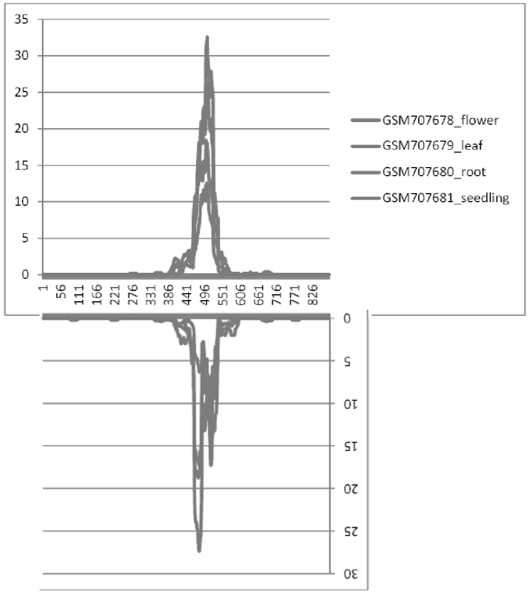

AGO GSE28591

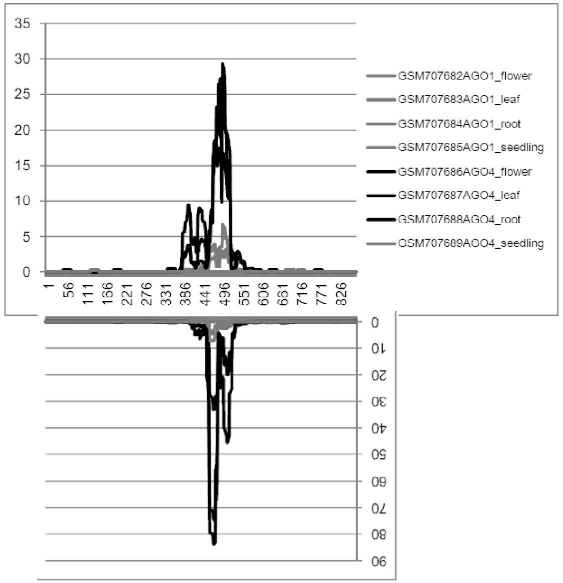

RDR, DCL

GSE6682

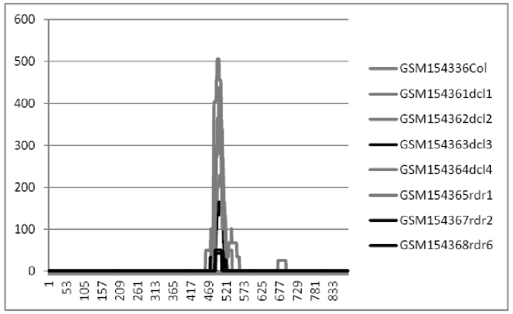

GSE14695

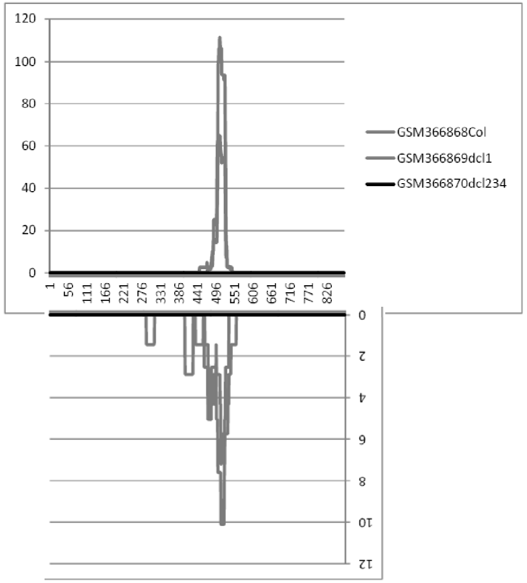

GSE44622

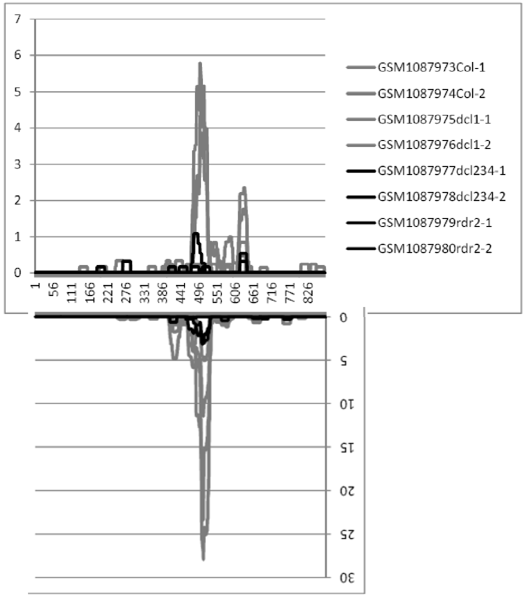

GSE10180

PASR

AT4G16640

Total GSE28591

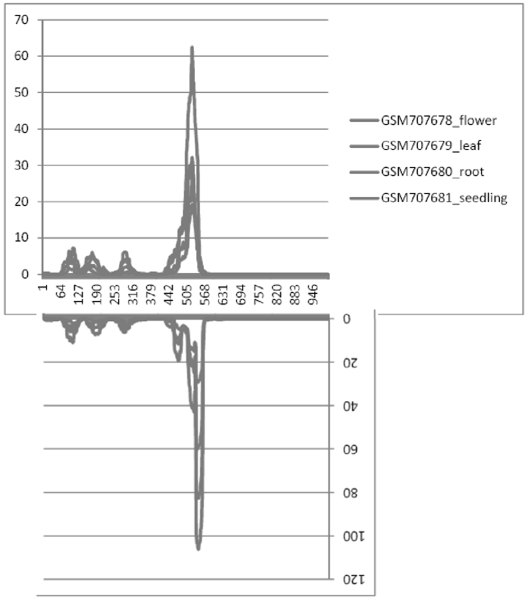

AGO GSE28591

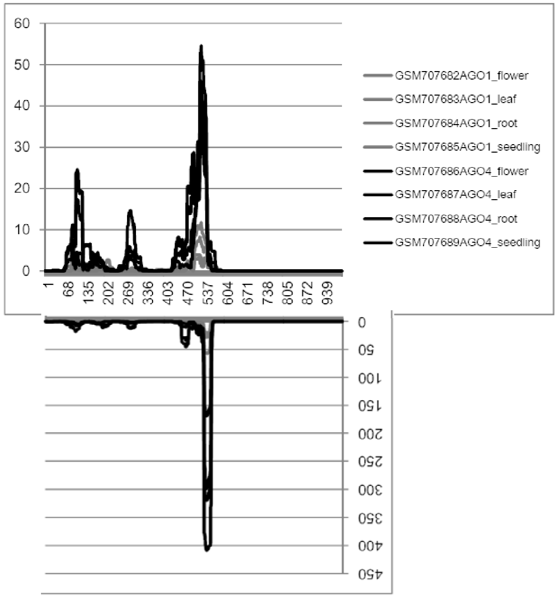

RDR, DCL

GSE6682

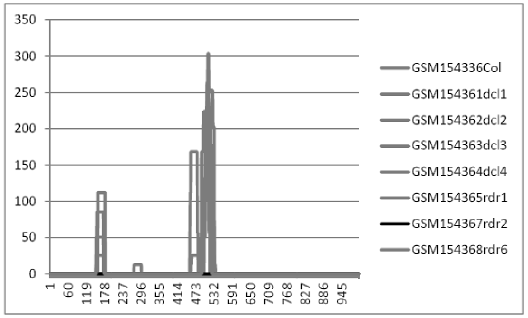

GSE14695

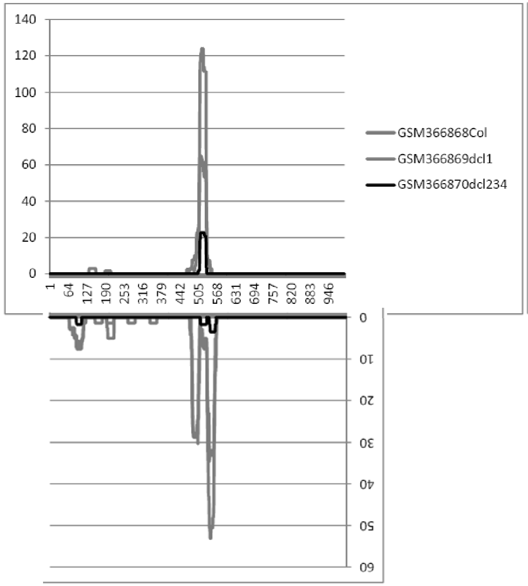

GSE44622

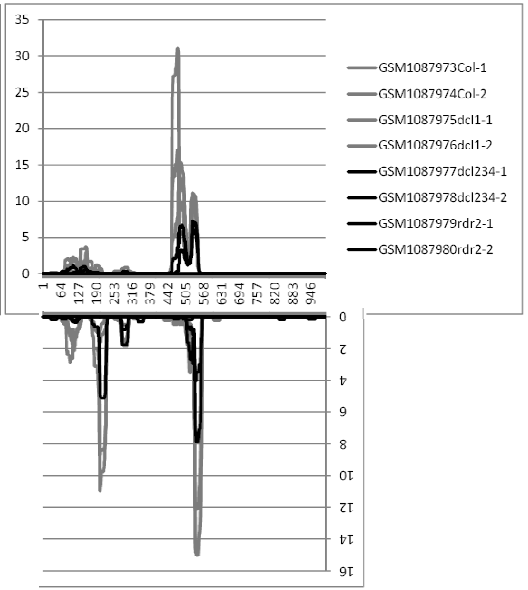

GSE10180

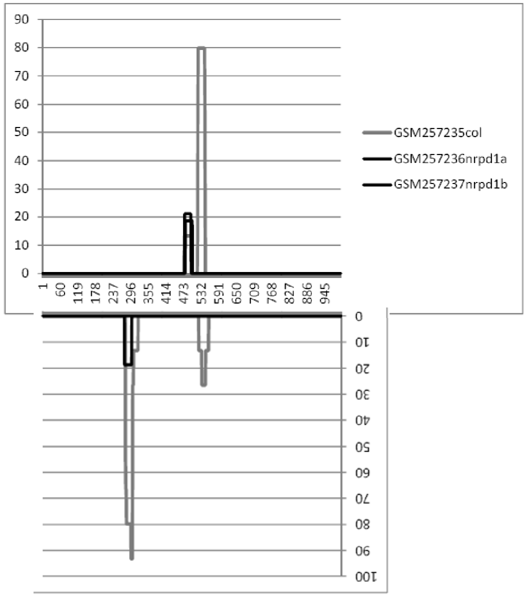

PASR

AT5G48000

Total GSE28591

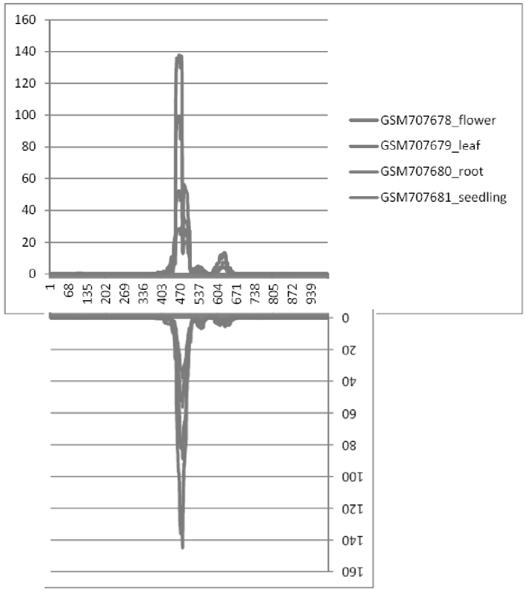

AGO GSE28591

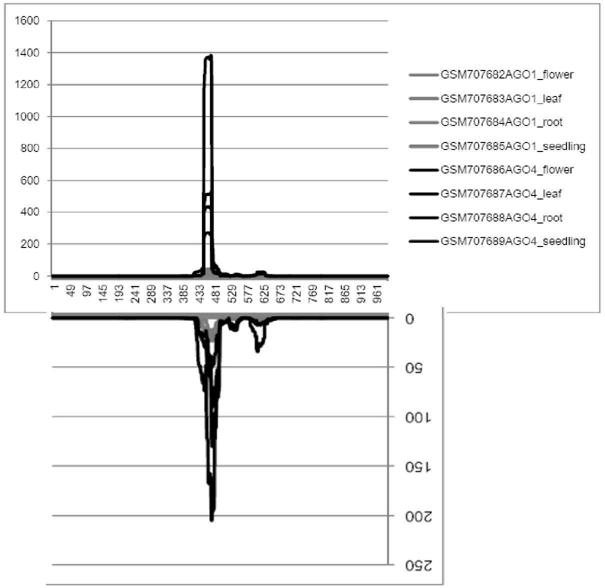

RDR, DCL

GSE6682

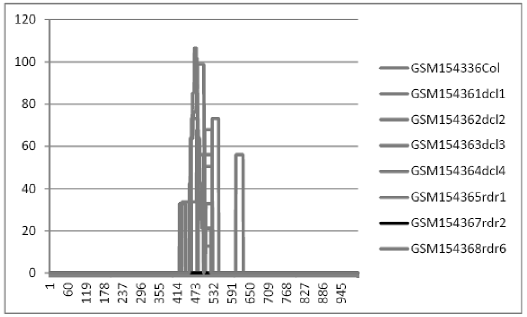

GSE14695

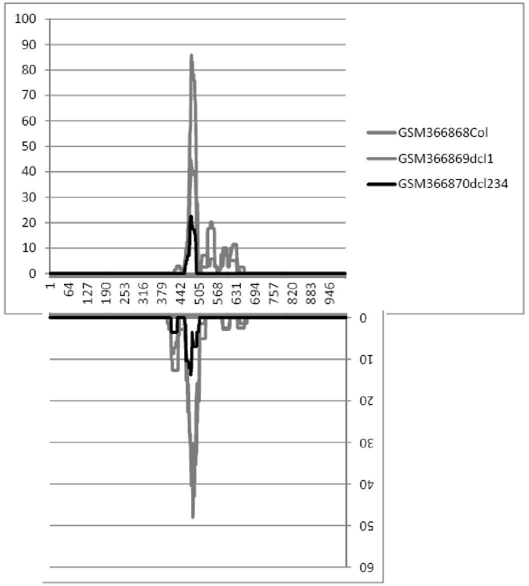

GSE44622

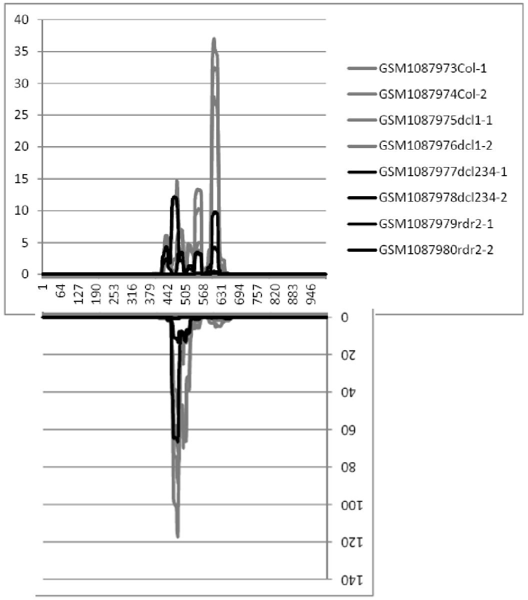

GSE10180

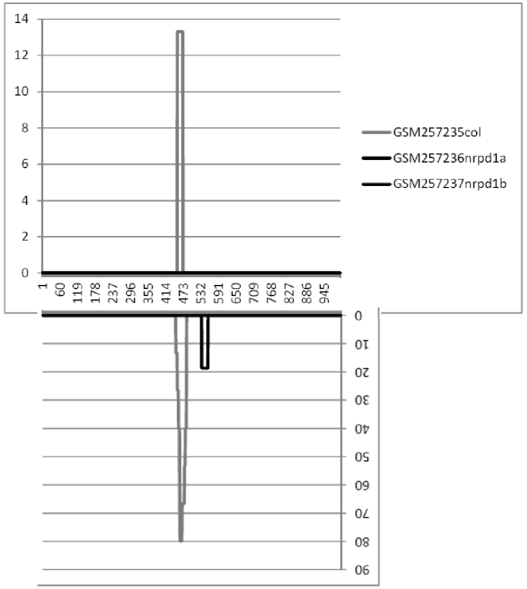

TASR

AT1G28304

Total GSE28591

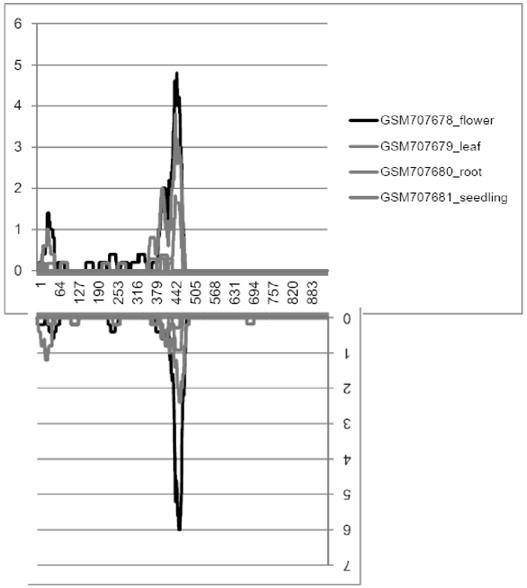

AGO GSE28591

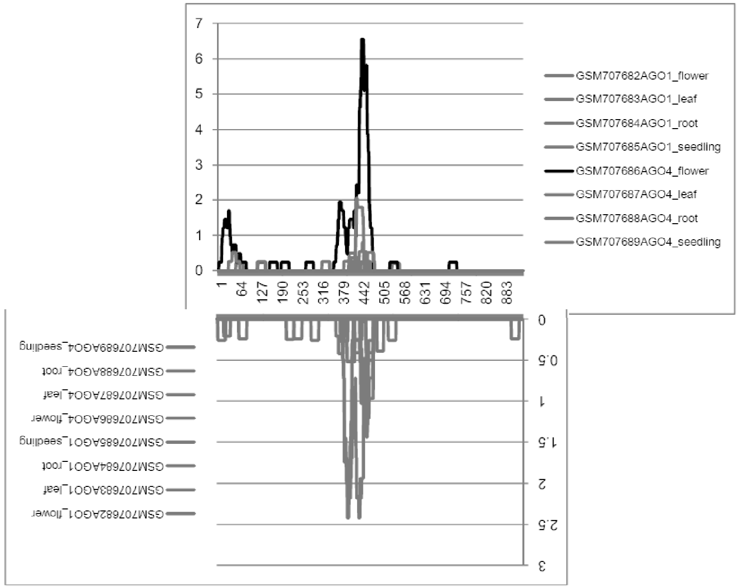

RDR, DCL

GSE6682 GSE14695 GSE44622 GSE10180

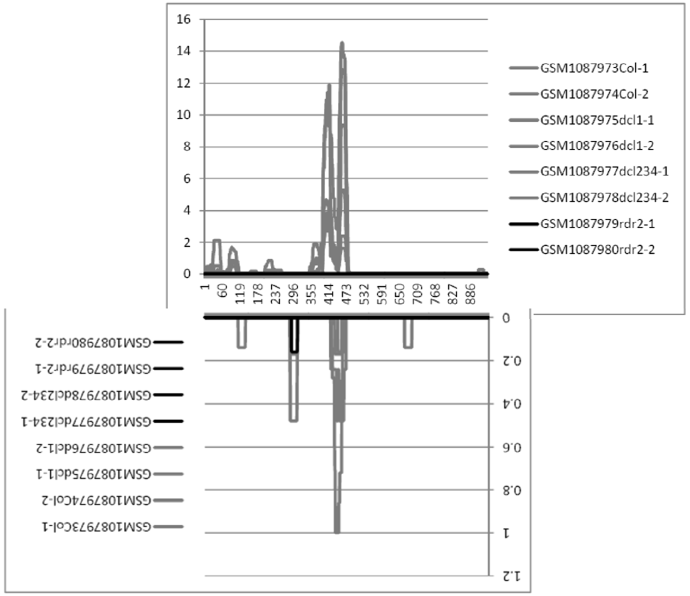

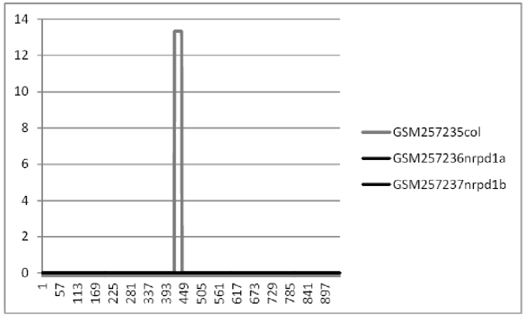

TASR

AT3G25130

Total GSE28591

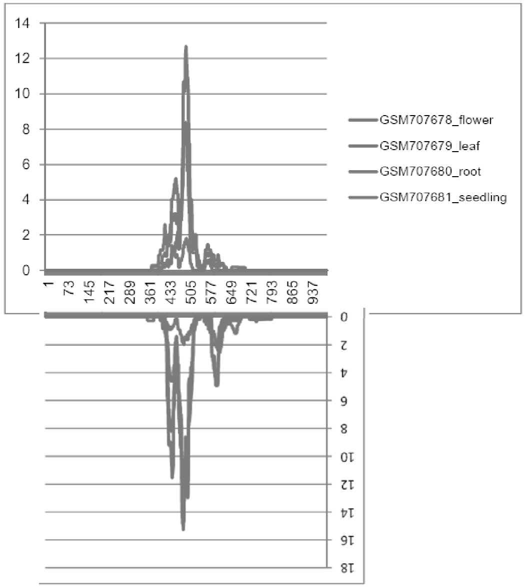

AGO GSE28591

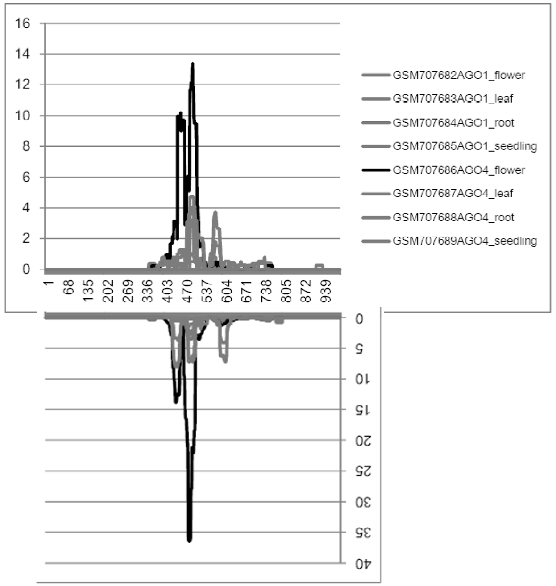

RDR, DCL

GSE6682

GSE14695

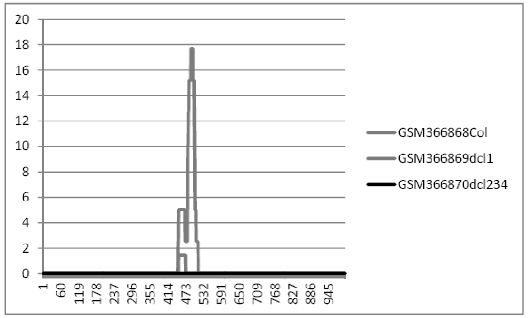

GSE44622

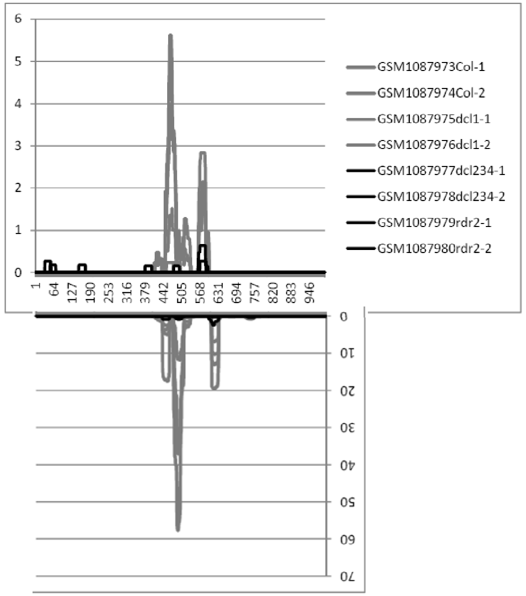

GSE10180

TASR

AT3G41762

Total GSE28591

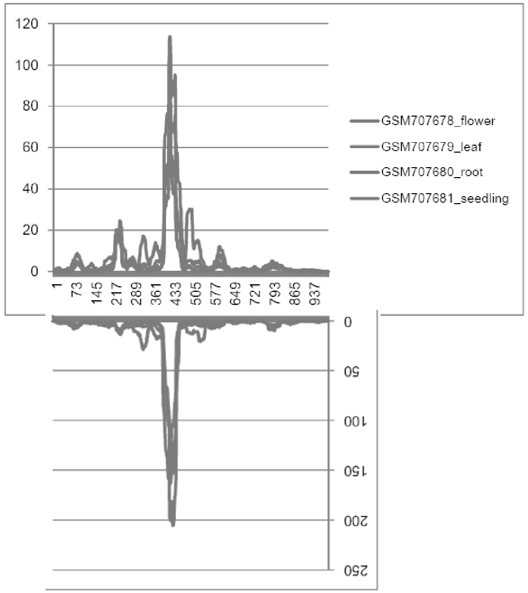

AGO GSE28591

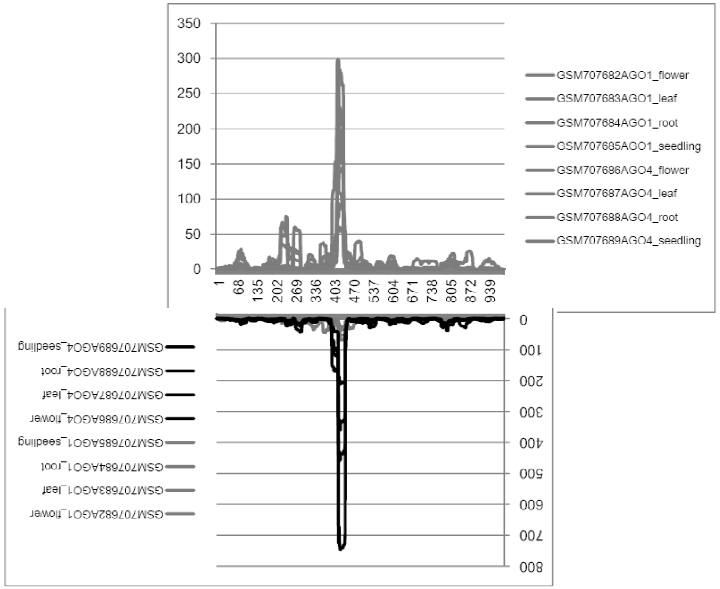

RDR, DCL

GSE6682

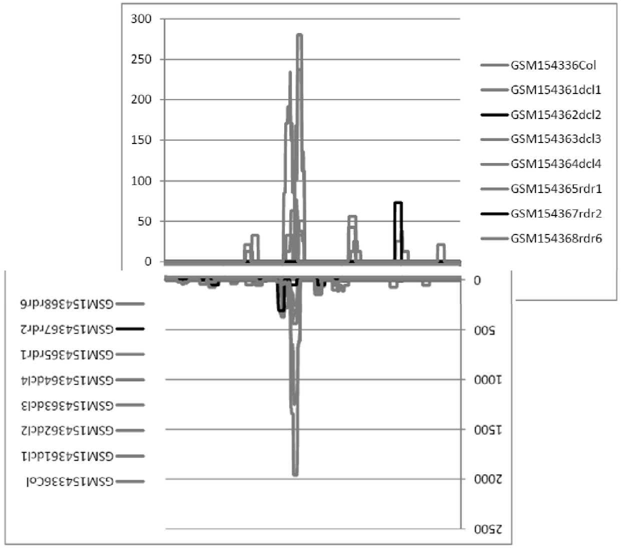

GSE14695

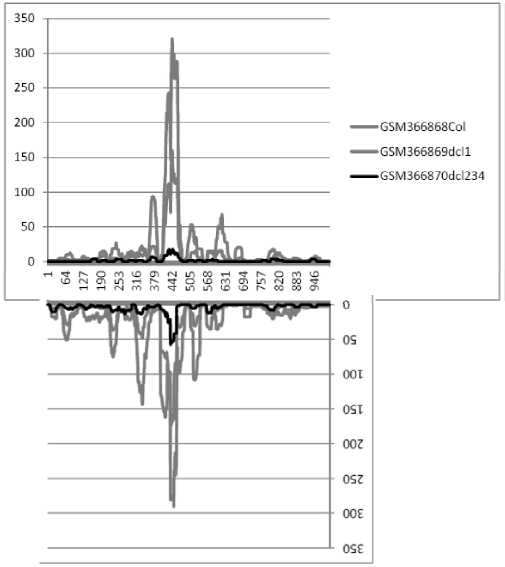

GSE44622

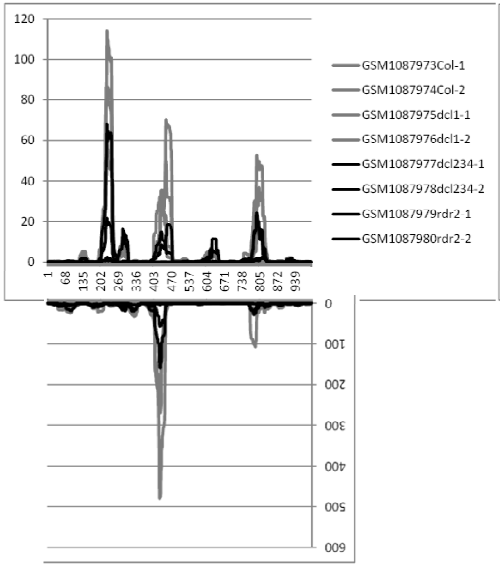

GSE10180

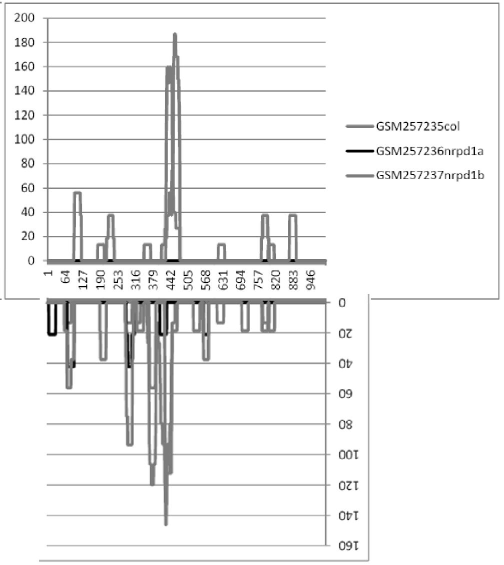

TASR

AT3G52830

Total GSE28591

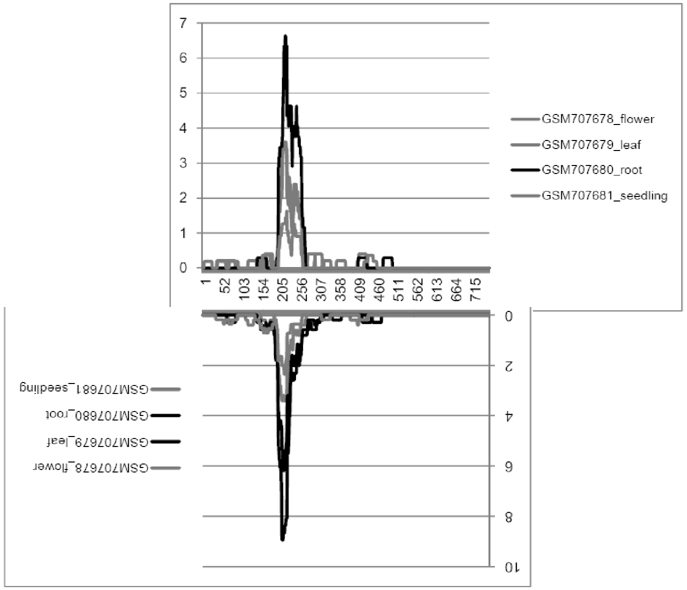

AGO GSE28591

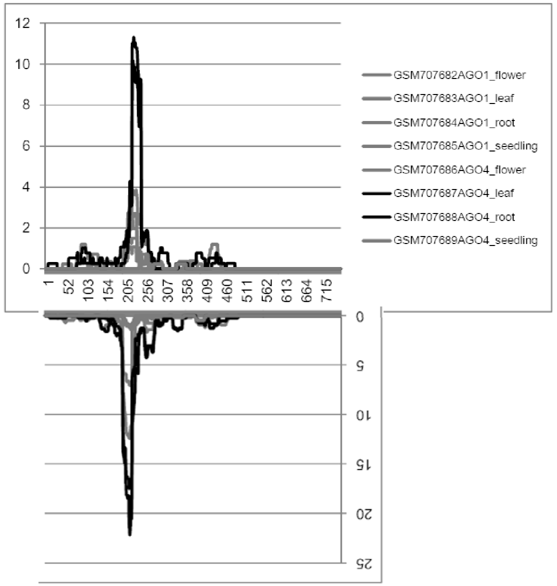

RDR, DCL

GSE6682

GSE14695

GSE44622

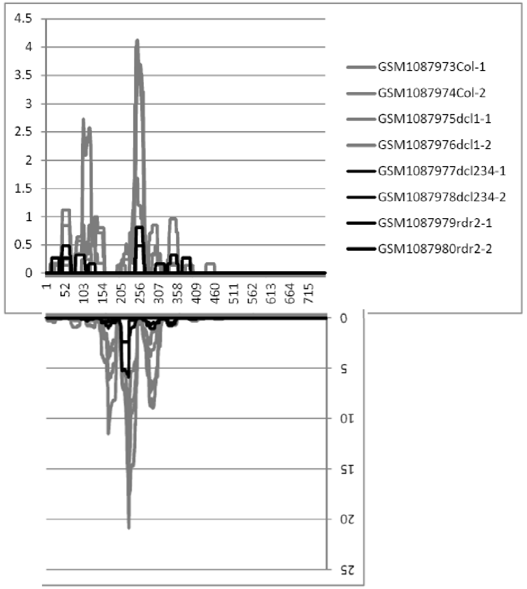

GSE10180

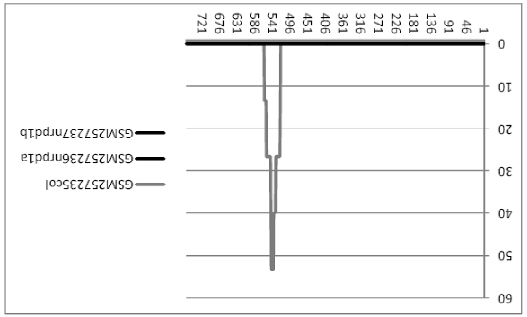

TASR

AT4G04030

Total GSE28591

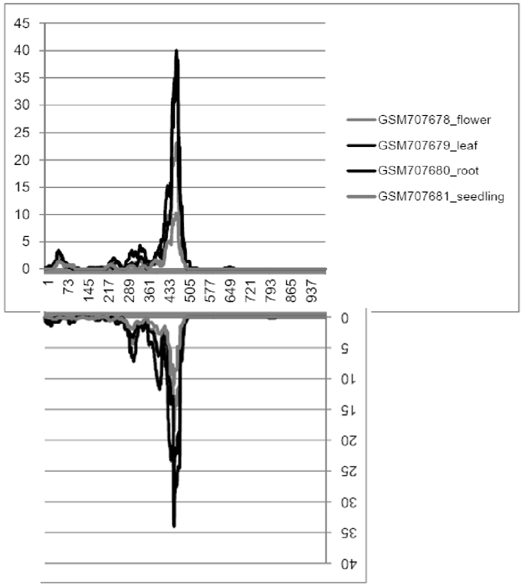

AGO GSE28591

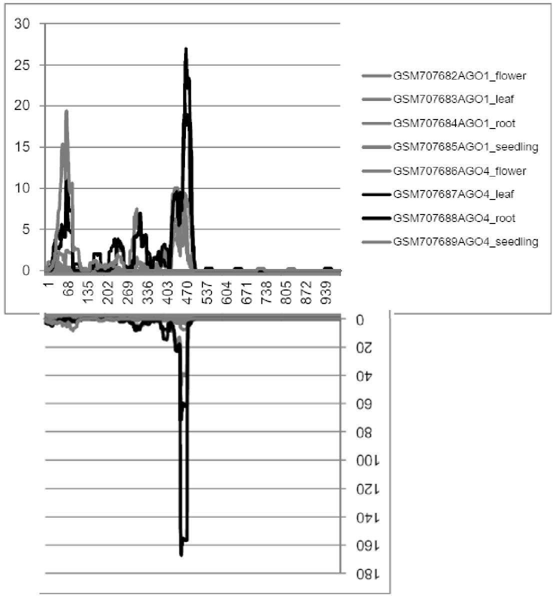

RDR, DCL

GSE6682

GSE14695

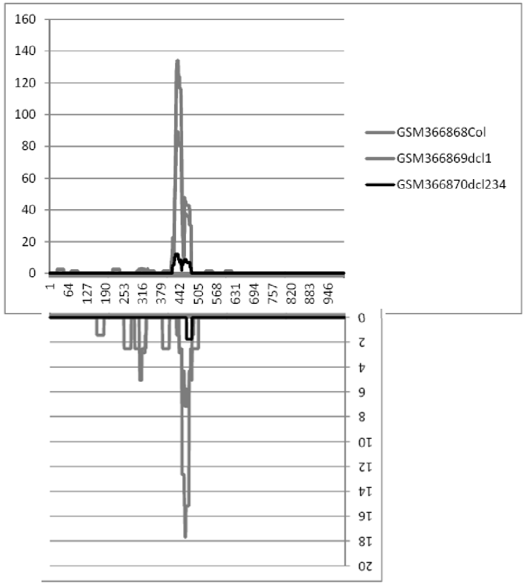

GSE44622

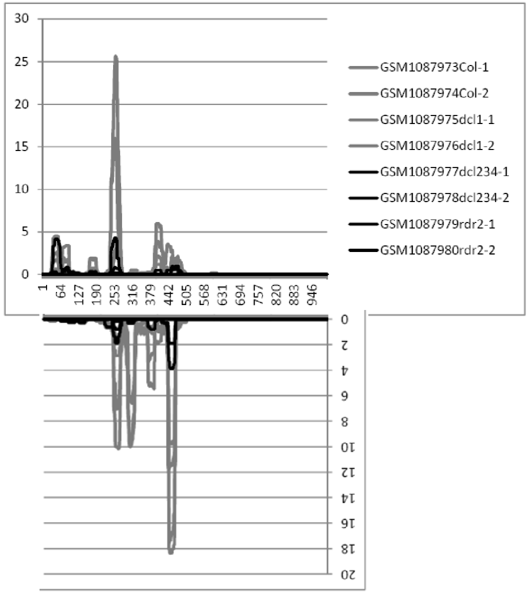

GSE10180

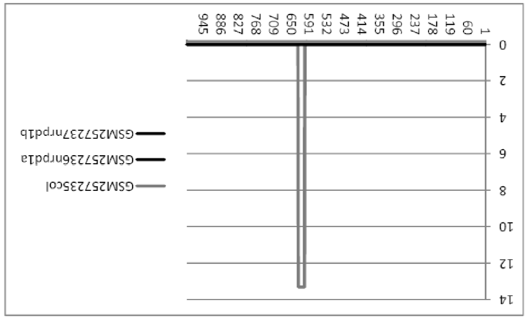

TASR

AT4G08160

Total GSE28591

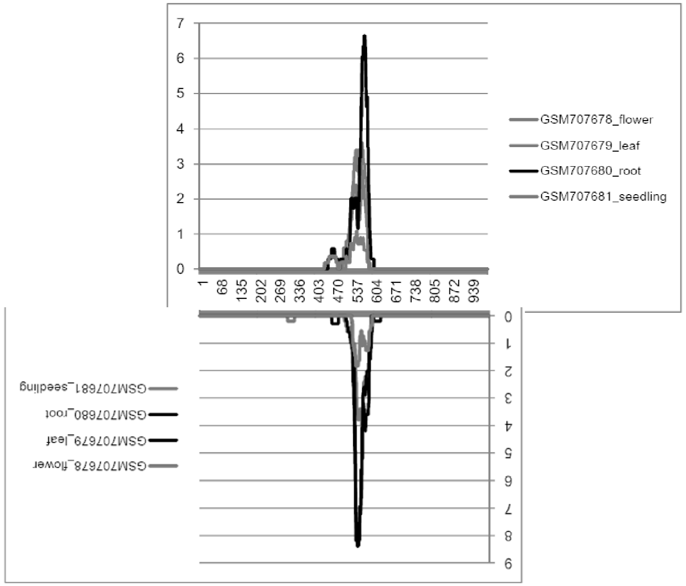

AGO GSE28591

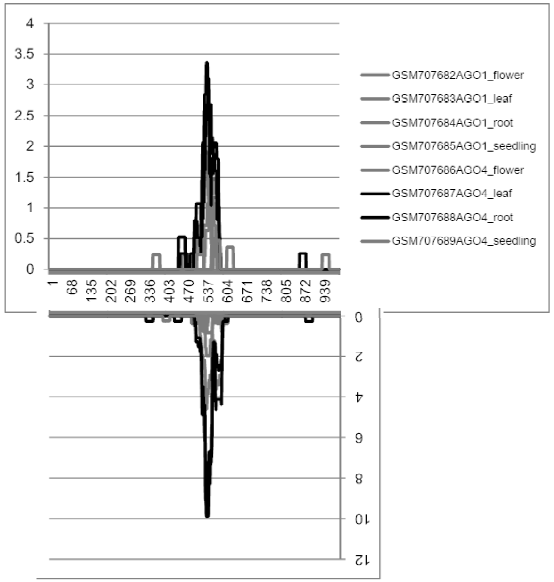

RDR, DCL

GSE6682

GSE14695

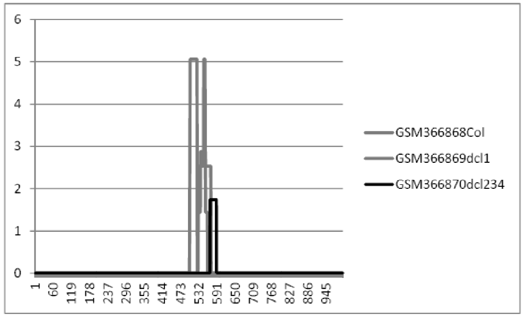

GSE44622

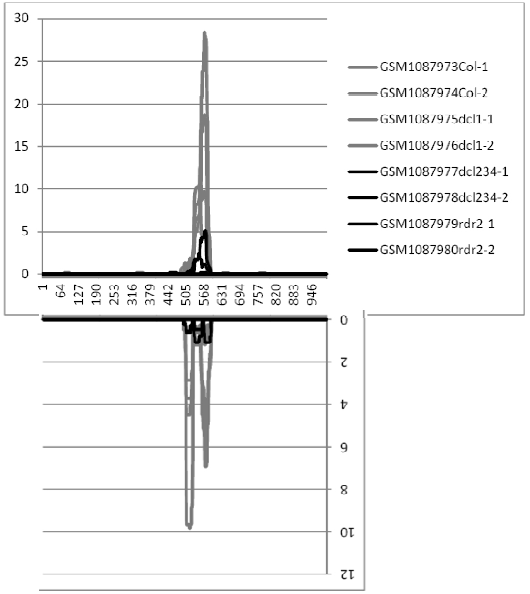

GSE10180

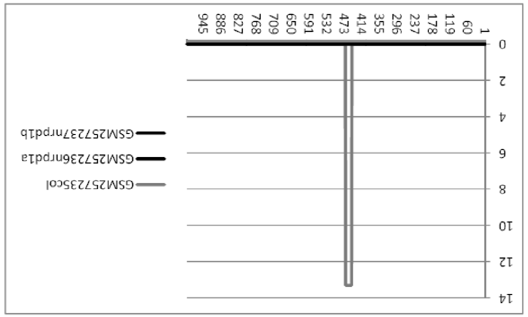

TASR

AT4G14365

Total GSE28591

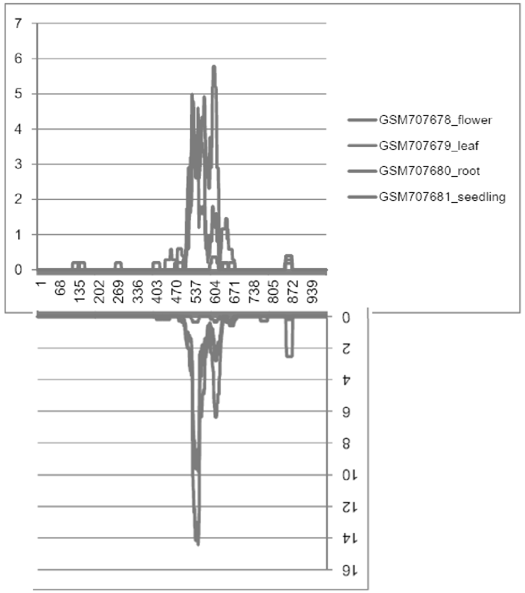

AGO GSE28591

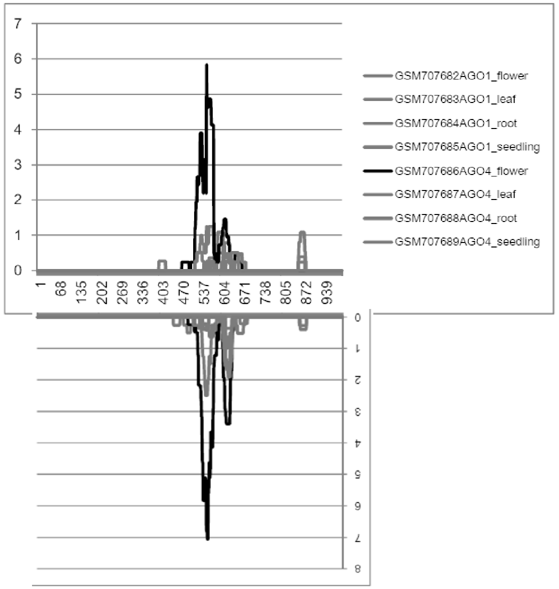

RDR, DCL

GSE6682

GSE14695

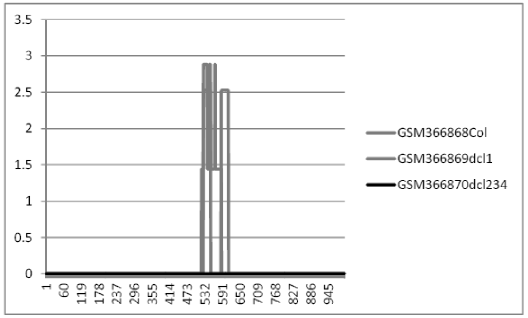

GSE44622

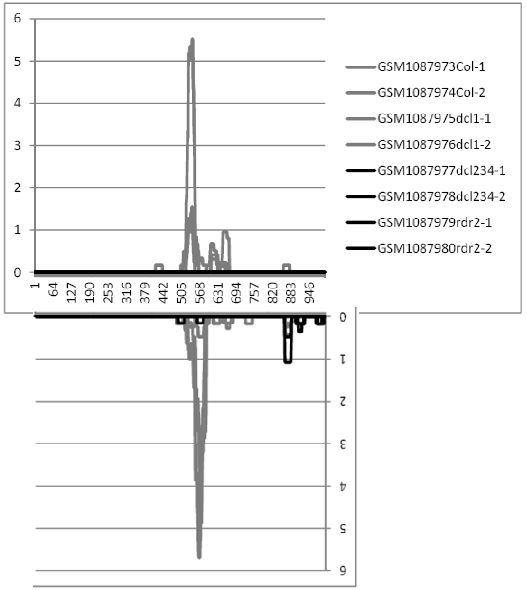

GSE10180

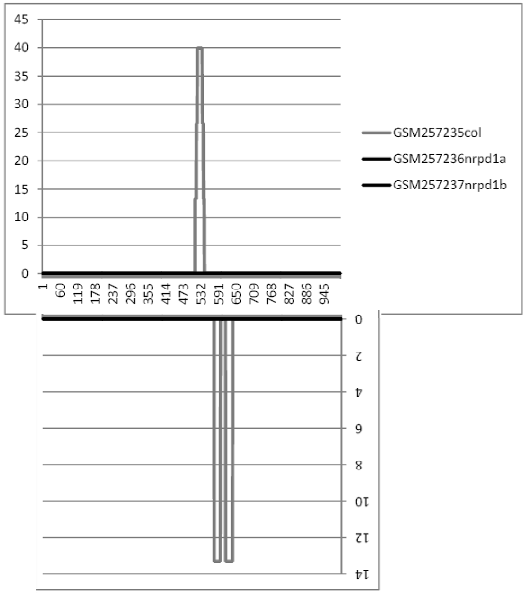

TASR

AT5G43525

Total GSE28591

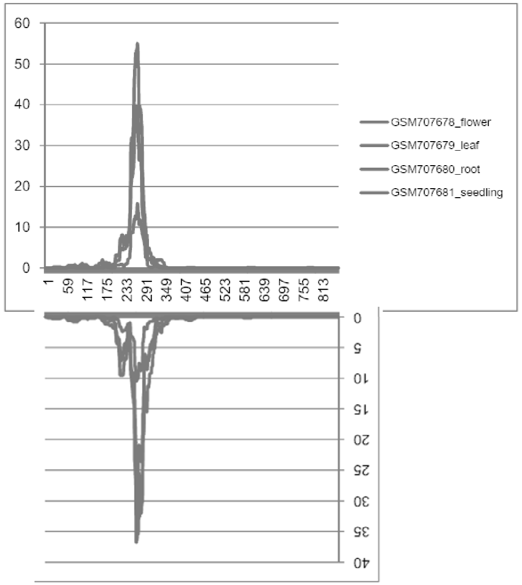

AGO GSE28591

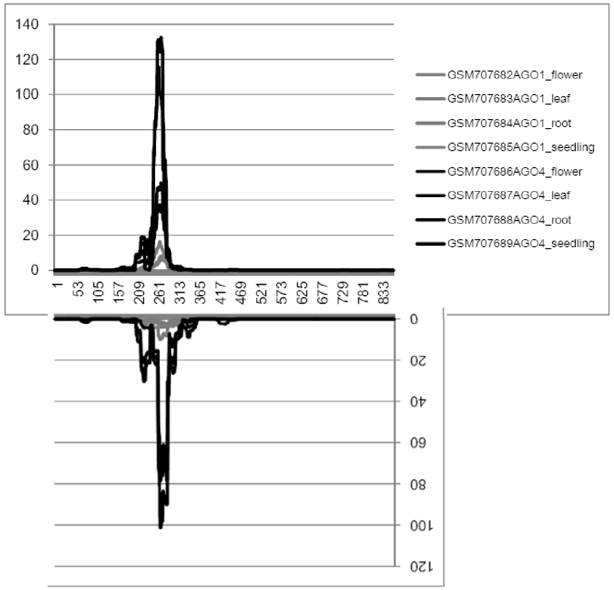

RDR, DCL

GSE6682

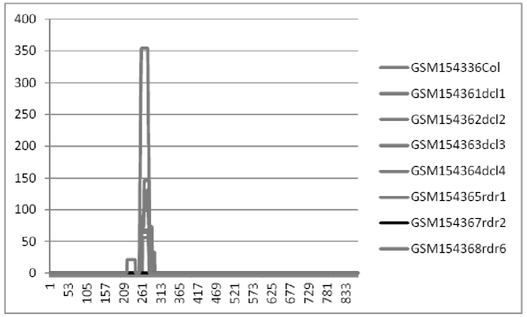

GSE14695

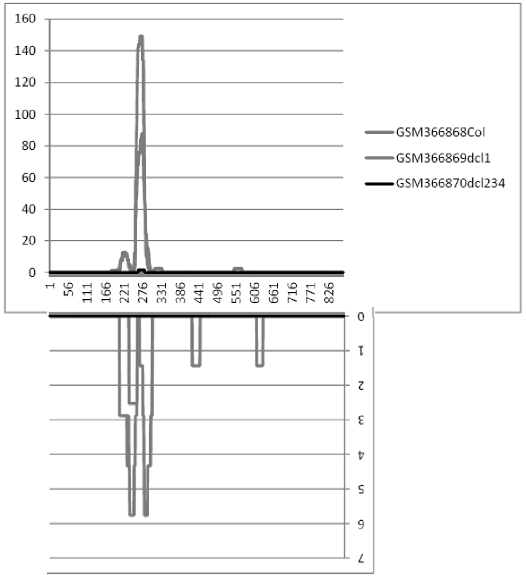

GSE44622

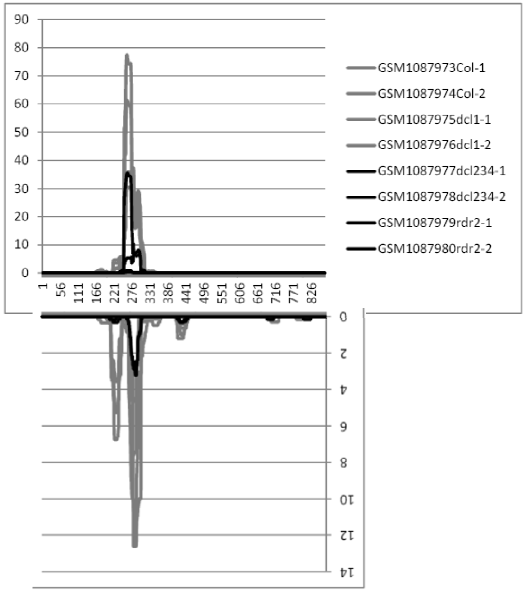

GSE10180

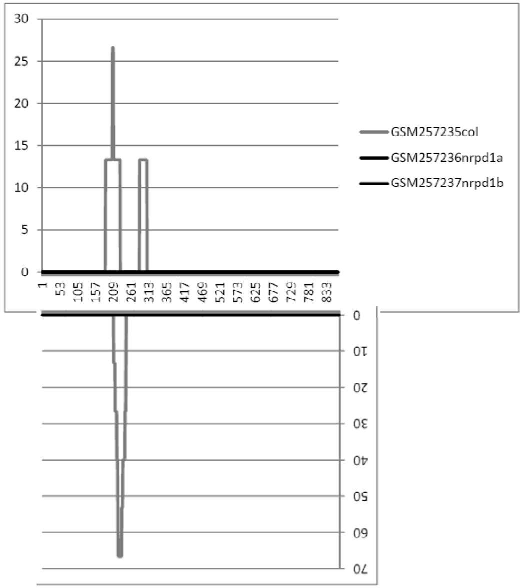

TASR

AT5G50480

Total GSE28591

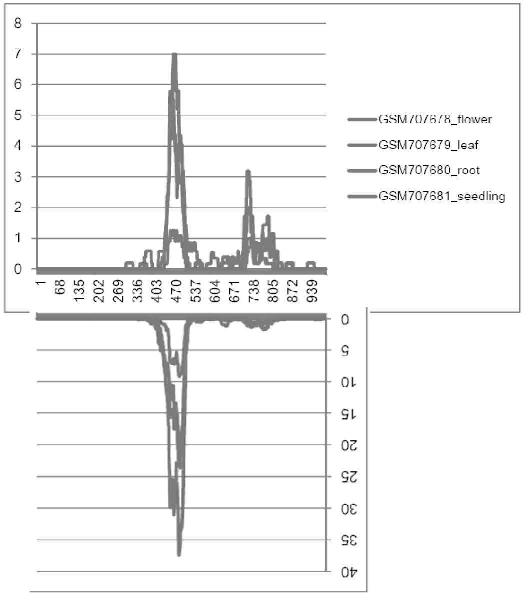

AGO GSE28591

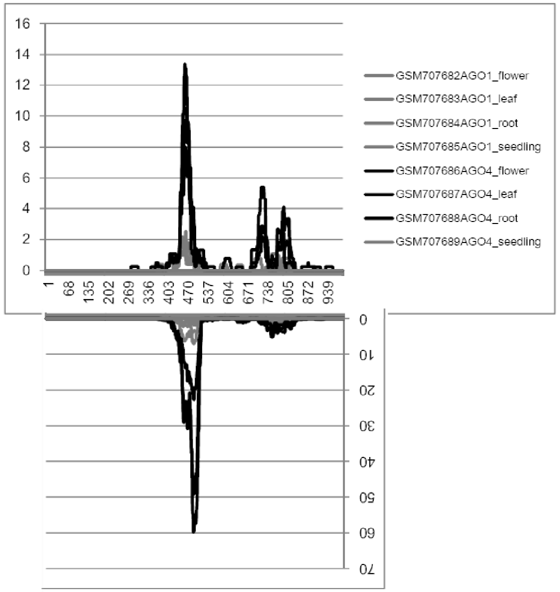

RDR, DCL

GSE6682

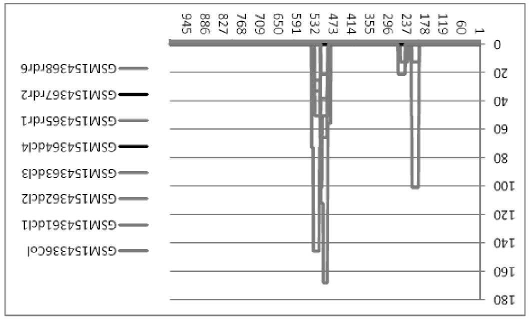

GSE14695

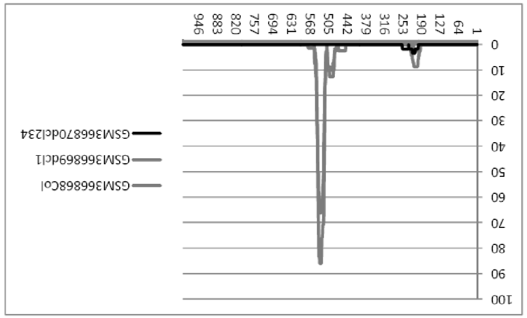

GSE44622

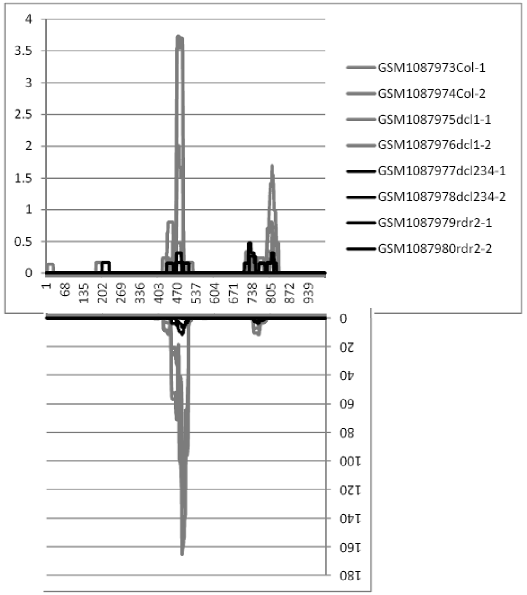

GSE10180

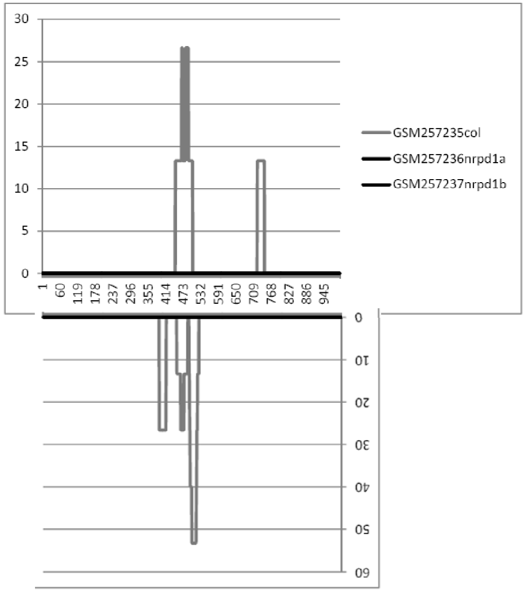

TASR

AT5G54700

Total GSE28591

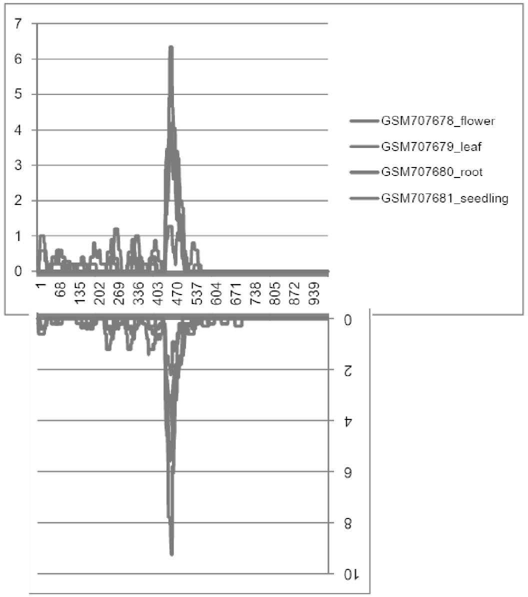

AGO GSE28591

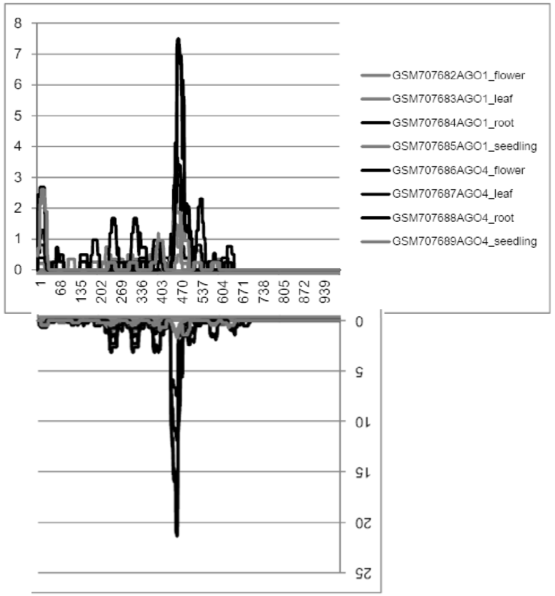

RDR, DCL

GSE6682

GSE14695

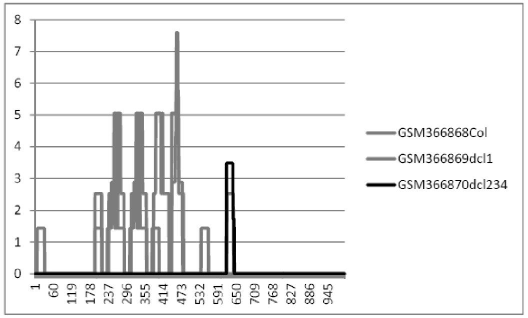

GSE44622

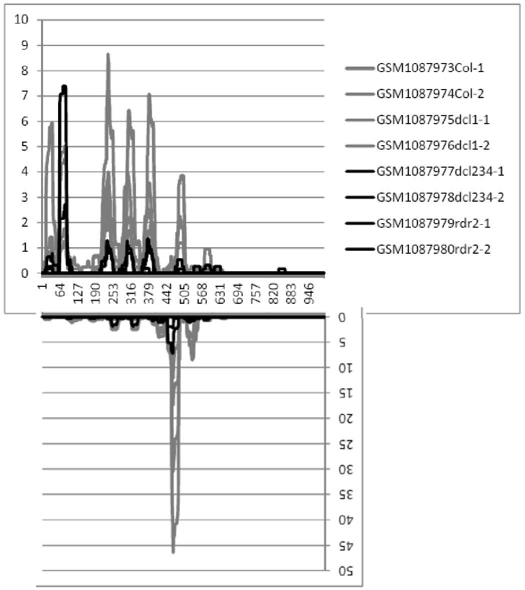

GSE10180

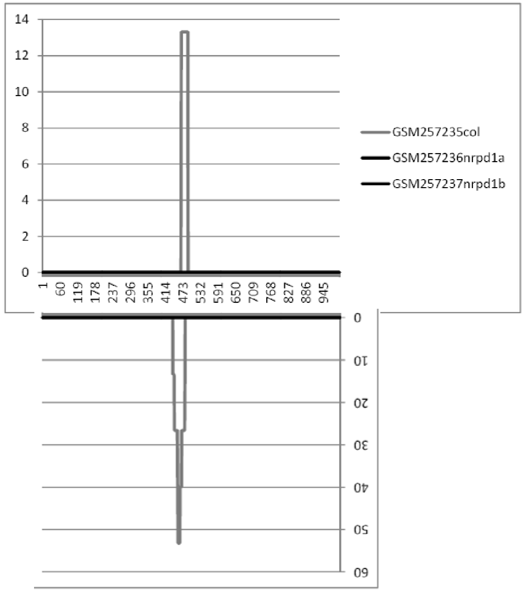

TASR

AT5G65005

Total

GSE28591

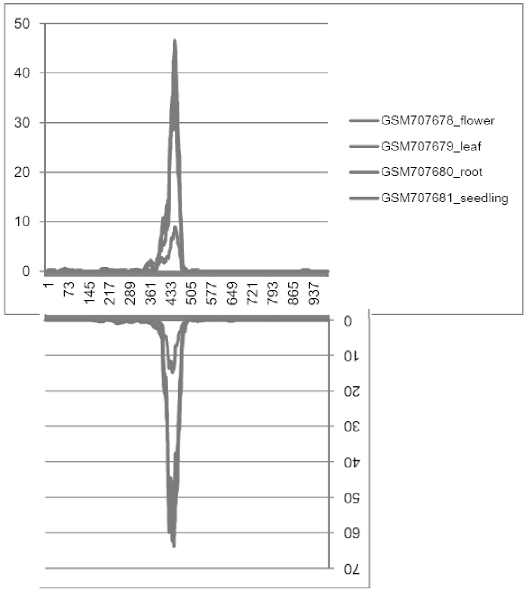

AGO

GSE28591

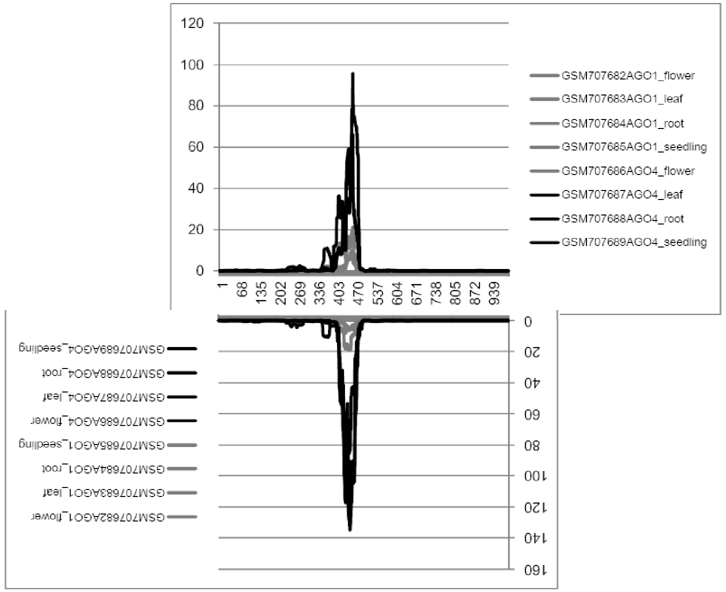

RDR, DCL

GSE6682

GSE14695

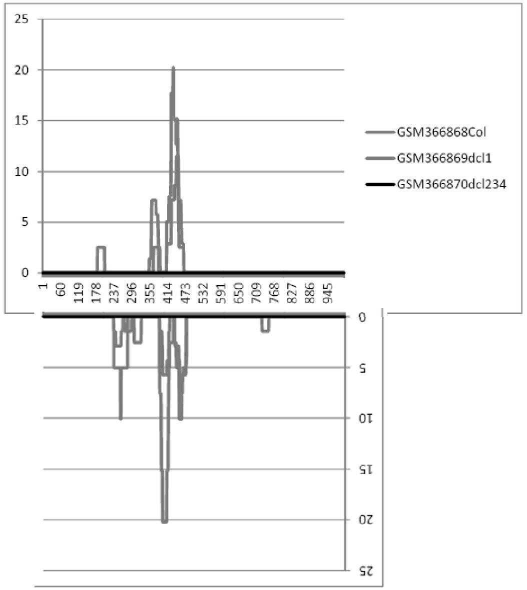

GSE44622

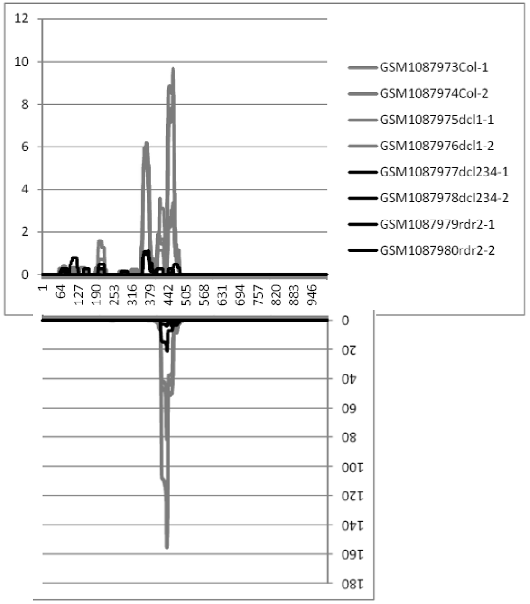

GSE10180

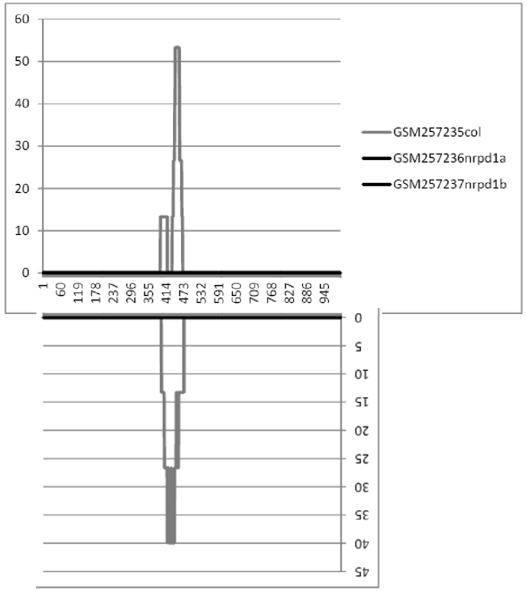

Supplement: S20 Fig — (PDF) [file pone.0169212.s020.pdf]
