# Supplementary material for: Transcriptome-Wide Discovery of PASRs (Promoter-Associated Small RNAs) and TASRs (Terminus-Associated Small RNAs) in Arabidopsis thaliana
Source: PLoS One. 2017 Jan 3;12(1):e0169212. doi: 10.1371/journal.pone.0169212 (PMC5207706; doi:10.1371/journal.pone.0169212)
Supplement: S21 Fig — (PDF) [file pone.0169212.s021.pdf]

Figure S21 Site-specific DNA methylation signals were detected at the genomic positions well corresponding to those of the PASR peaks identified on the sense strands of the protein-coding genes in *Arabidopsis*.

AT1G48660

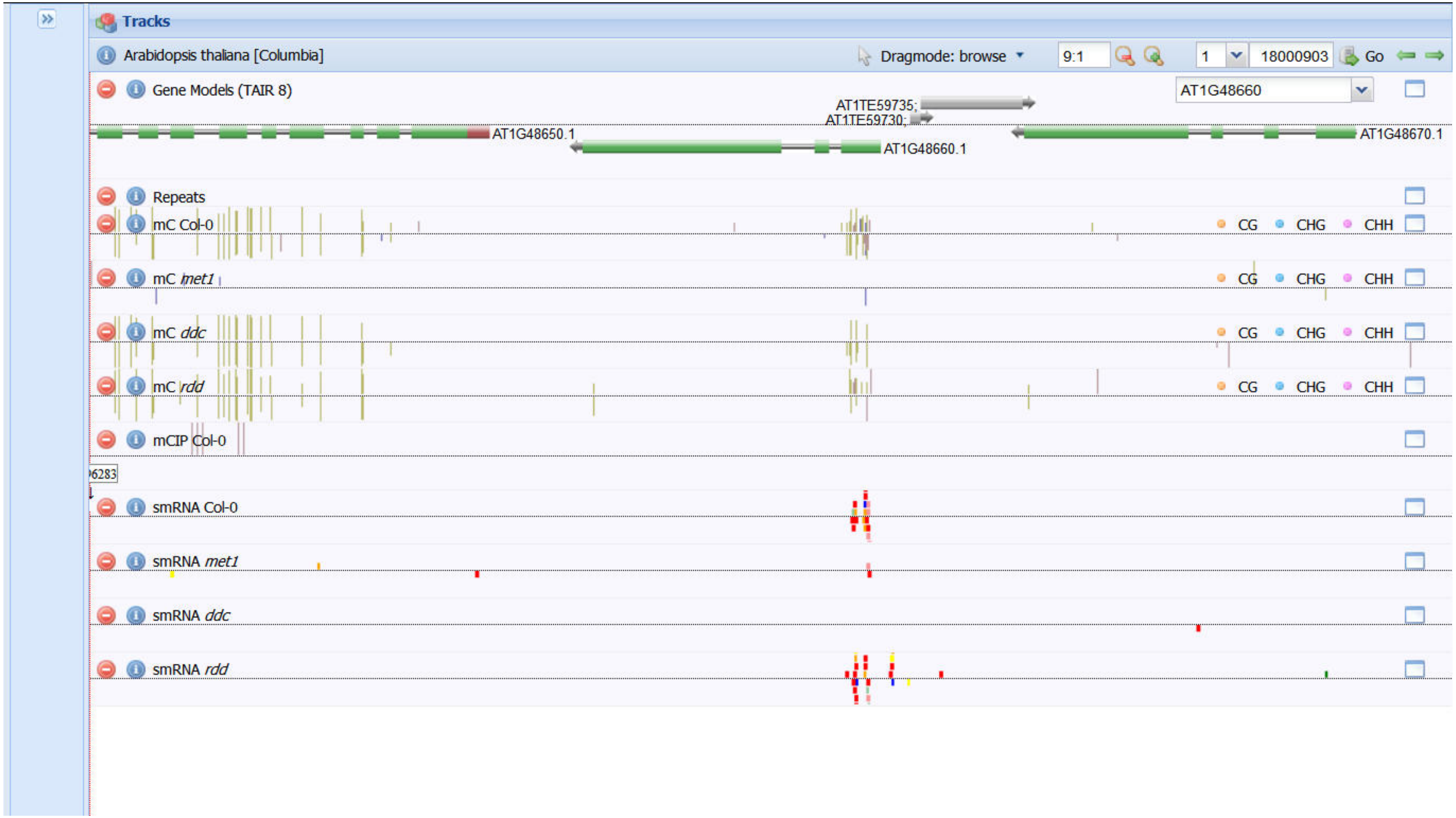
Tracks
Arabidopsis thaliana [Columbia]
Dragmode: browse
9:1
1
18000903
Go
Gene Models (TAIR 8)
AT1G48660
AT1TE59735;
AT1TE59730;
AT1G48650.1
AT1G48660.1
AT1G48670.1
Repeats
mC Col-0
CG
CHG
CHH
mC met1
mC ddc
mC rdd
mCIP Col-0
smRNA Col-0
smRNA met1
smRNA ddc
smRNA rdd

AT1G67450

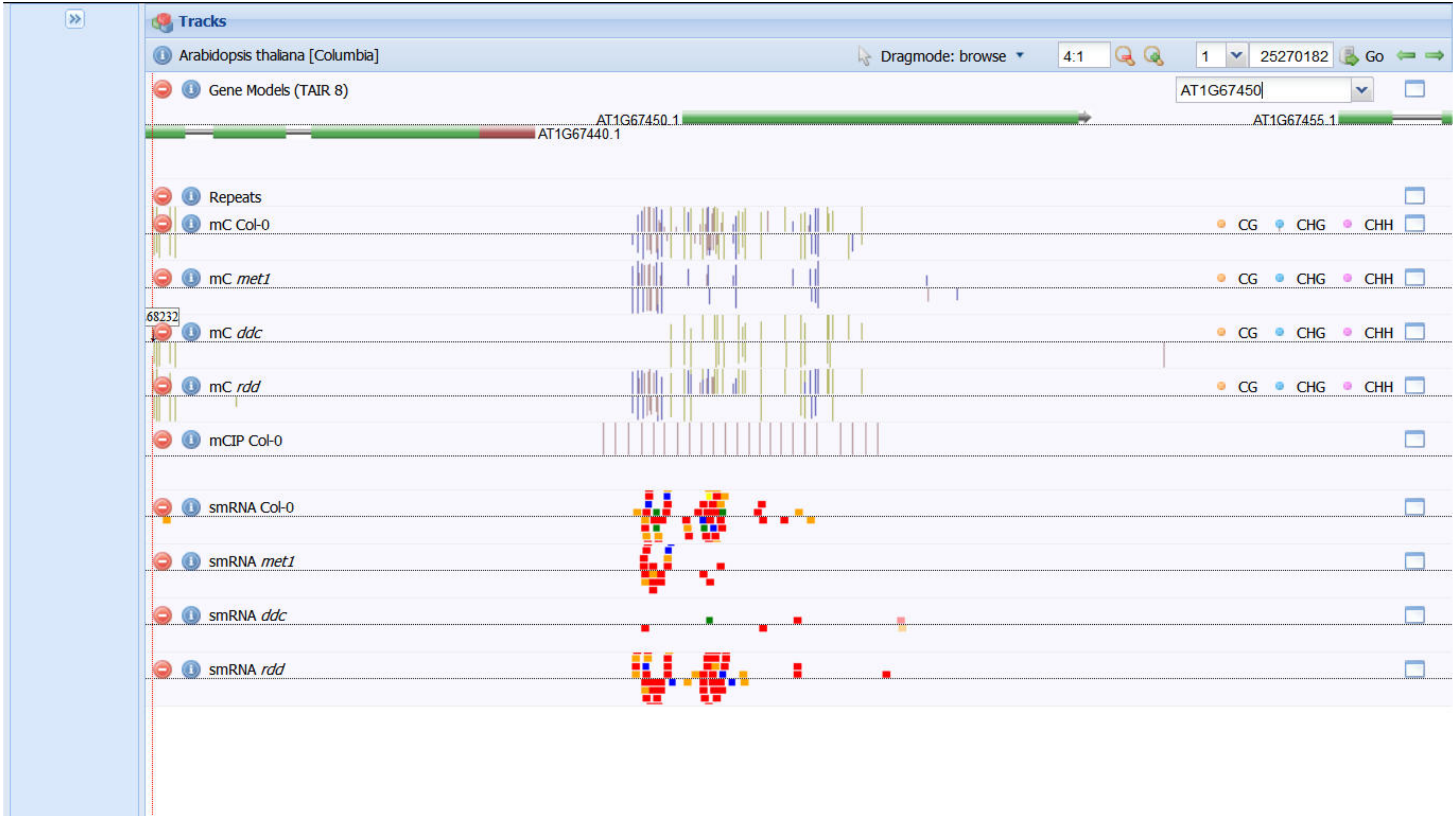
Tracks
Arabidopsis thaliana [Columbia]
Dragmode: browse
4:1
1
25270182
Go
Gene Models (TAIR 8)
AT1G67450
AT1G67450.1
AT1G67440.1
AT1G67455.1
Repeats
mC Col-0
CG CHG CHH
mC met1
68232
mC ddc
mC rdd
mCIP Col-0
smRNA Col-0
smRNA met1
smRNA ddc
smRNA rdd

AT2G36180

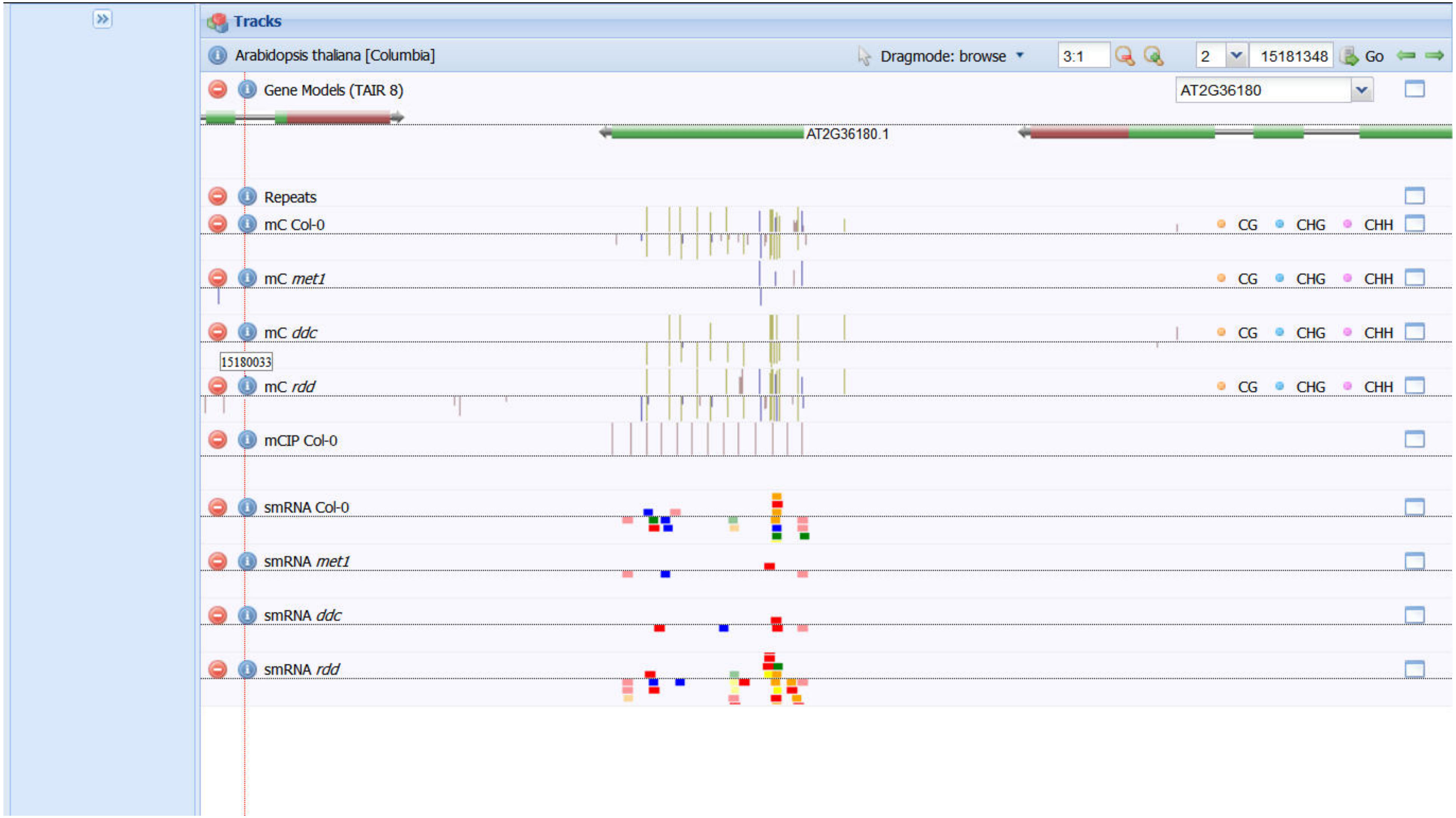

Tracks
Arabidopsis thaliana [Columbia]
Dragmode: browse
3:1
2
15181348
Go
Gene Models (TAIR 8)
AT2G36180
AT2G36180.1
Repeats
mC Col-0
CG
CHG
CHH
mC met1
mC ddc
15180033
mC rdd
mCIP Col-0
smRNA Col-0
smRNA met1
smRNA ddc
smRNA rdd

AT3G17490

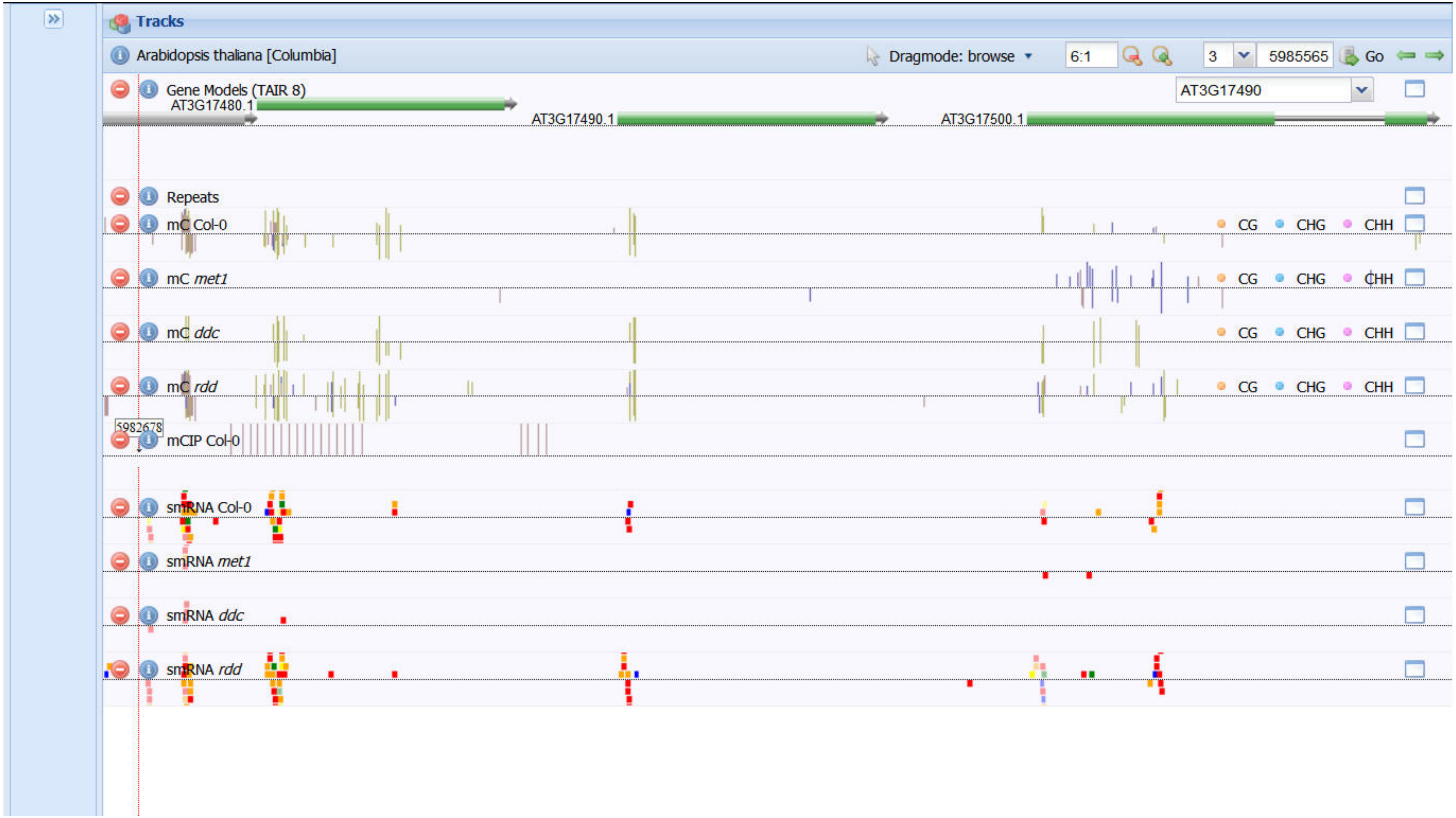
Tracks
Arabidopsis thaliana [Columbia]
Dragmode: browse
6:1
3
5985565
Go
Gene Models (TAIR 8)
AT3G17490
AT3G17480.1
AT3G17490.1
AT3G17500.1
Repeats
mC Col-0
CG
CHG
CHH
mC met1
mC ddc
mC rdd
5982678
mCIP Col-0
smRNA Col-0
smRNA met1
smRNA ddc
smRNA rdd

AT3G22730

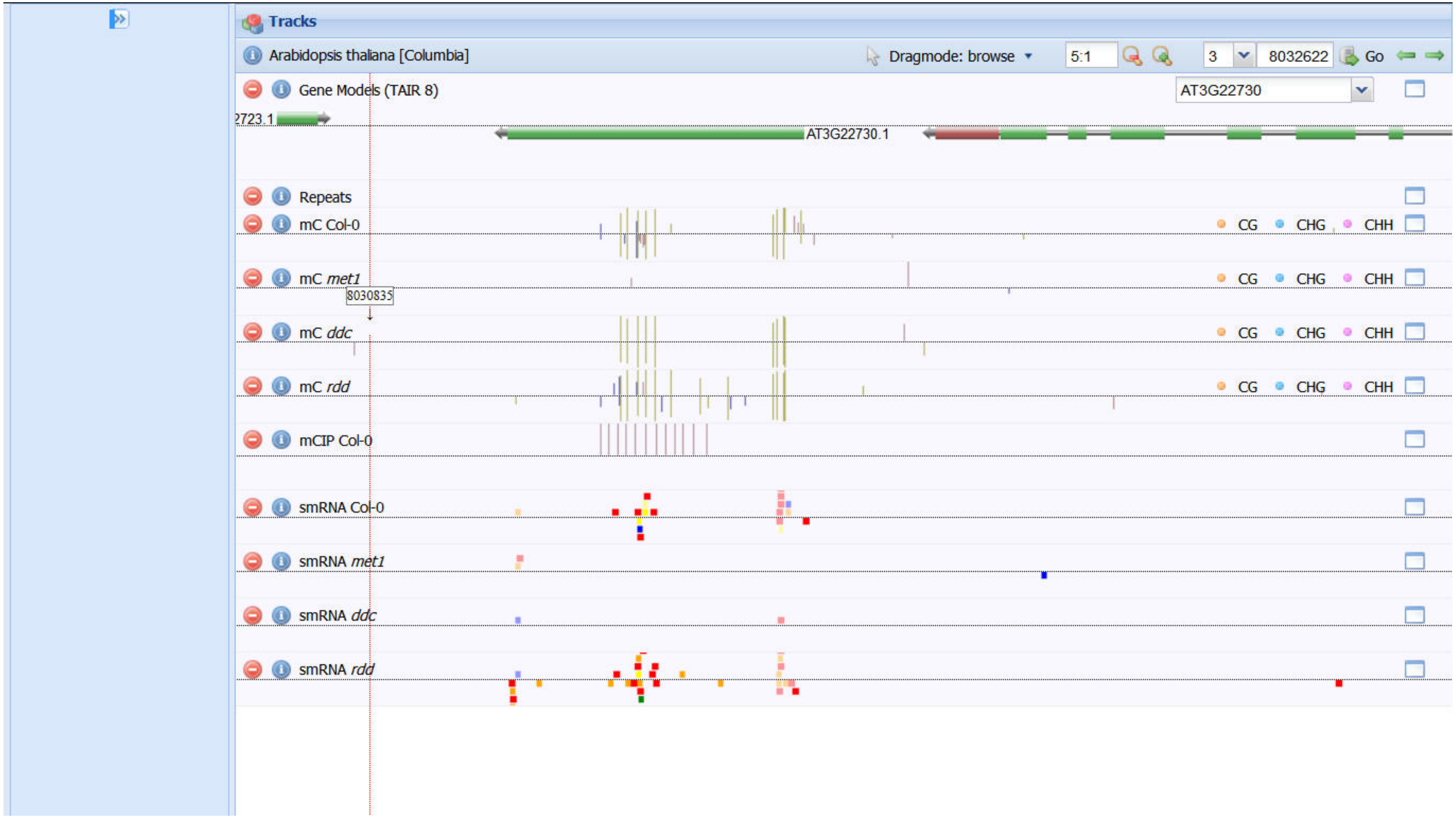
Tracks
Arabidopsis thaliana [Columbia]
Dragmode: browse
5:1
3
8032622
Go
Gene Models (TAIR 8)
AT3G22730
2723.1
AT3G22730.1
Repeats
mC Col-0
CG
CHG
CHH
mC met1
8030835
mC ddc
mC rdd
mCIP Col-0
smRNA Col-0
smRNA met1
smRNA ddc
smRNA rdd

AT3G56450

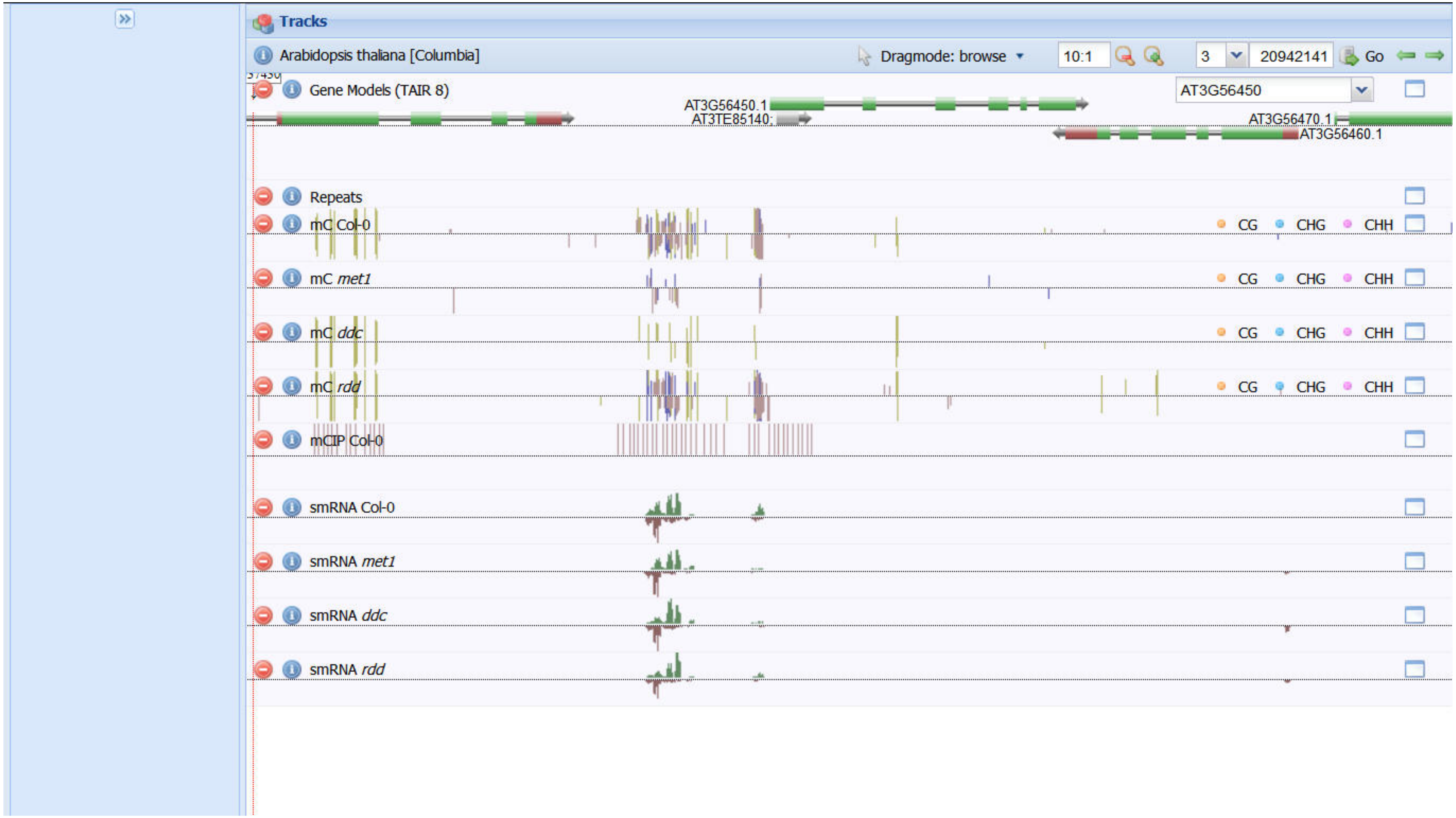

Tracks
Arabidopsis thaliana [Columbia]
Dragmode: browse
10:1
3
20942141
Go
Gene Models (TAIR 8)
AT3G56450
AT3G56450.1
AT3TE85140
AT3G56470.1
AT3G56460.1
Repeats
mC Col-0
mC met1
mC ddc
mC rdd
mCIP Col-0
smRNA Col-0
smRNA met1
smRNA ddc
smRNA rdd
CG
CHG
CHH

AT5G40320

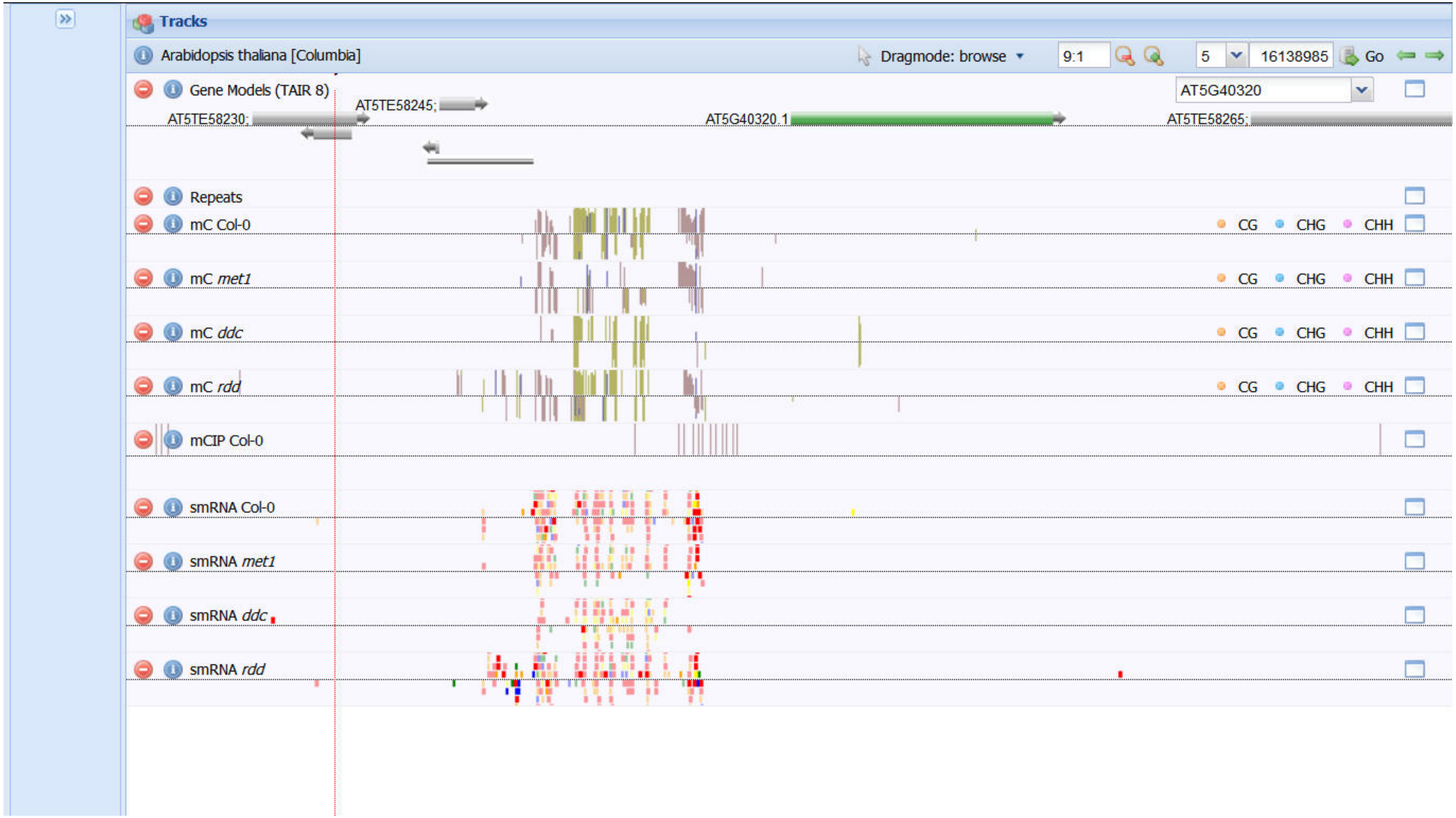
Tracks
Arabidopsis thaliana [Columbia]
Dragmode: browse
9:1
5
16138985
Go
Gene Models (TAIR 8)
AT5G40320
AT5TE58230;
AT5TE58245;
AT5G40320.1
AT5TE58265;
Repeats
mC Col-0
CG
CHG
CHH
mC *met1*
mC *ddc*
mC *rdd*
mCIP Col-0
smRNA Col-0
smRNA *met1*
smRNA *ddc*
smRNA *rdd*
