# Supplementary material for: Transcriptome-Wide Discovery of PASRs (Promoter-Associated Small RNAs) and TASRs (Terminus-Associated Small RNAs) in Arabidopsis thaliana
Source: PLoS One. 2017 Jan 3;12(1):e0169212. doi: 10.1371/journal.pone.0169212 (PMC5207706; doi:10.1371/journal.pone.0169212)
Supplement: S22 Fig — (PDF) [file pone.0169212.s022.pdf]

Figure S22 Site-specific DNA methylation signals were detected at the genomic positions well corresponding to those of the PASR peaks identified on the antisense strands of the protein-coding genes in *Arabidopsis*.

AT2G29460

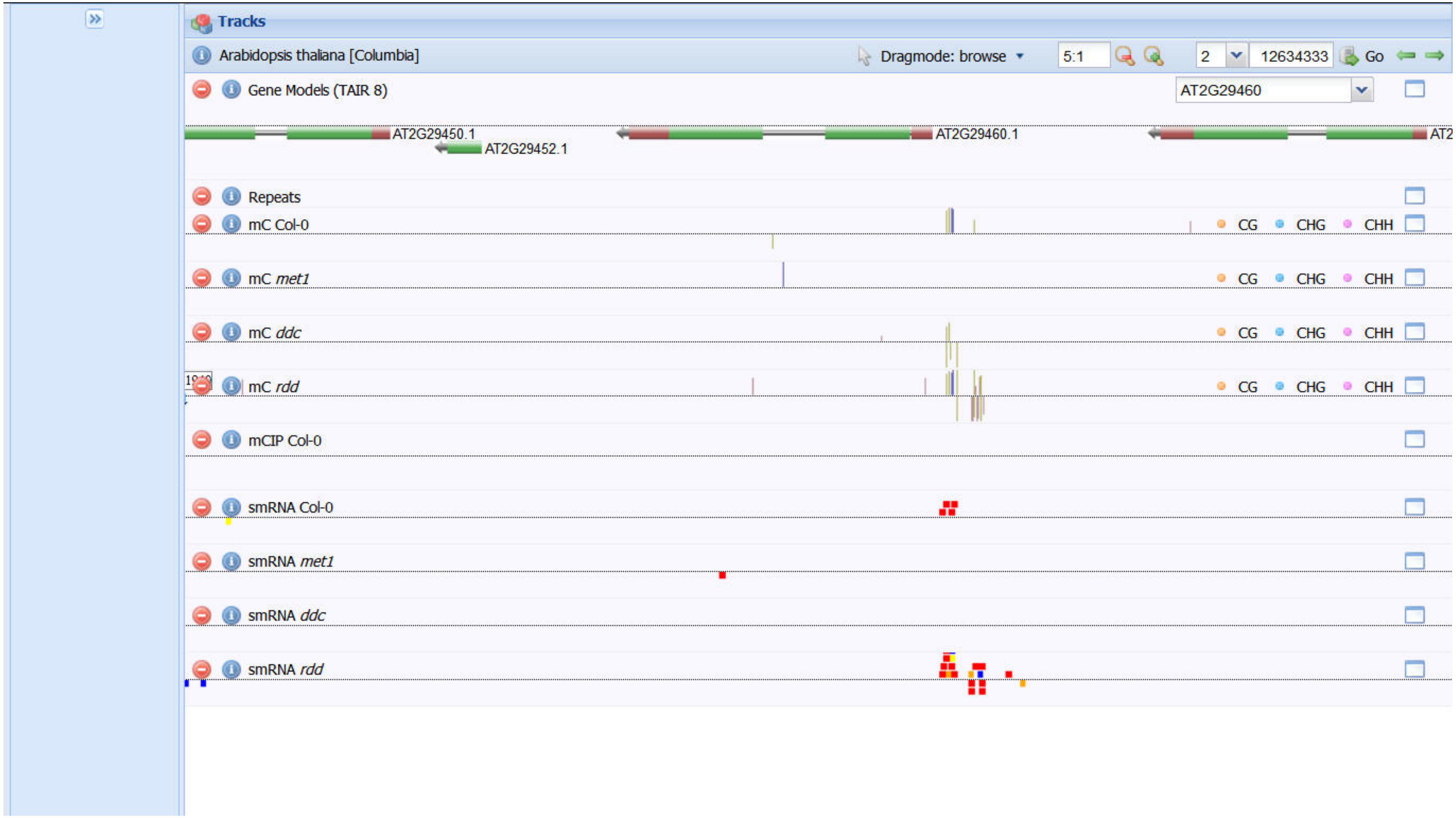
Tracks
Arabidopsis thaliana [Columbia]
Dragmode: browse
5:1
2
12634333
Go
Gene Models (TAIR 8)
AT2G29460
AT2G29450.1
AT2G29452.1
AT2G29460.1
Repeats
mC Col-0
CG
CHG
CHH
mC met1
mC ddc
mC rdd
mCIP Col-0
smRNA Col-0
smRNA met1
smRNA ddc
smRNA rdd
