# Supplementary material for: Transcriptome-Wide Discovery of PASRs (Promoter-Associated Small RNAs) and TASRs (Terminus-Associated Small RNAs) in Arabidopsis thaliana
Source: PLoS One. 2017 Jan 3;12(1):e0169212. doi: 10.1371/journal.pone.0169212 (PMC5207706; doi:10.1371/journal.pone.0169212)
Supplement: S23 Fig — (PDF) [file pone.0169212.s023.pdf]

Figure S23 Site-specific DNA methylation signals were detected at the genomic positions well corresponding to those of the paired PASR peaks identified on both strands of the protein-coding genes in *Arabidopsis*.

AT1G03810

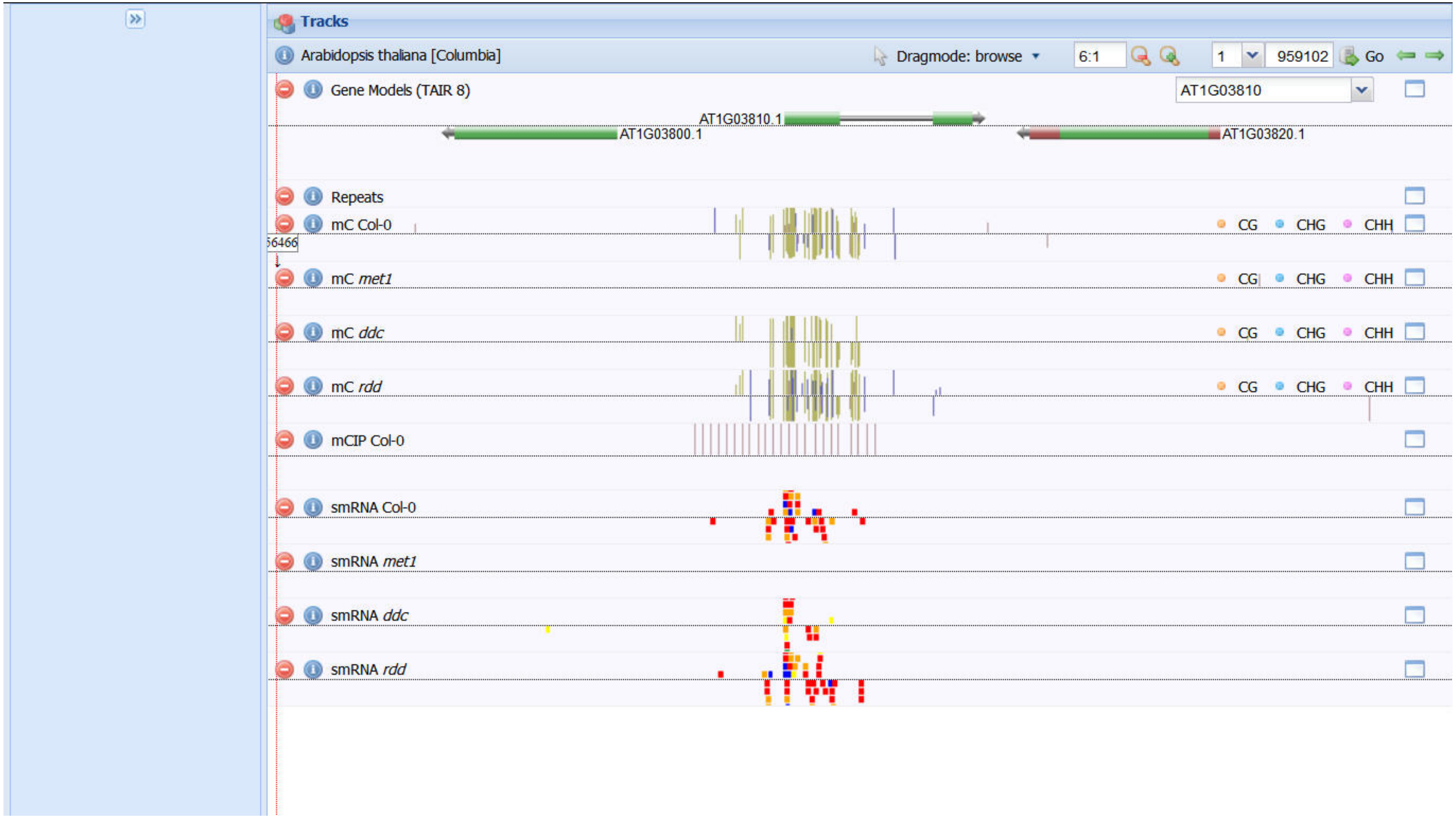
Tracks
Arabidopsis thaliana [Columbia]
Dragmode: browse
6:1
1
959102
Go
Gene Models (TAIR 8)
AT1G03810
AT1G03810.1
AT1G03800.1
AT1G03820.1
Repeats
mC Col-0
CG
CHG
CHH
mC met1
mC ddc
mC rdd
mCIP Col-0
smRNA Col-0
smRNA met1
smRNA ddc
smRNA rdd

AT1G53265

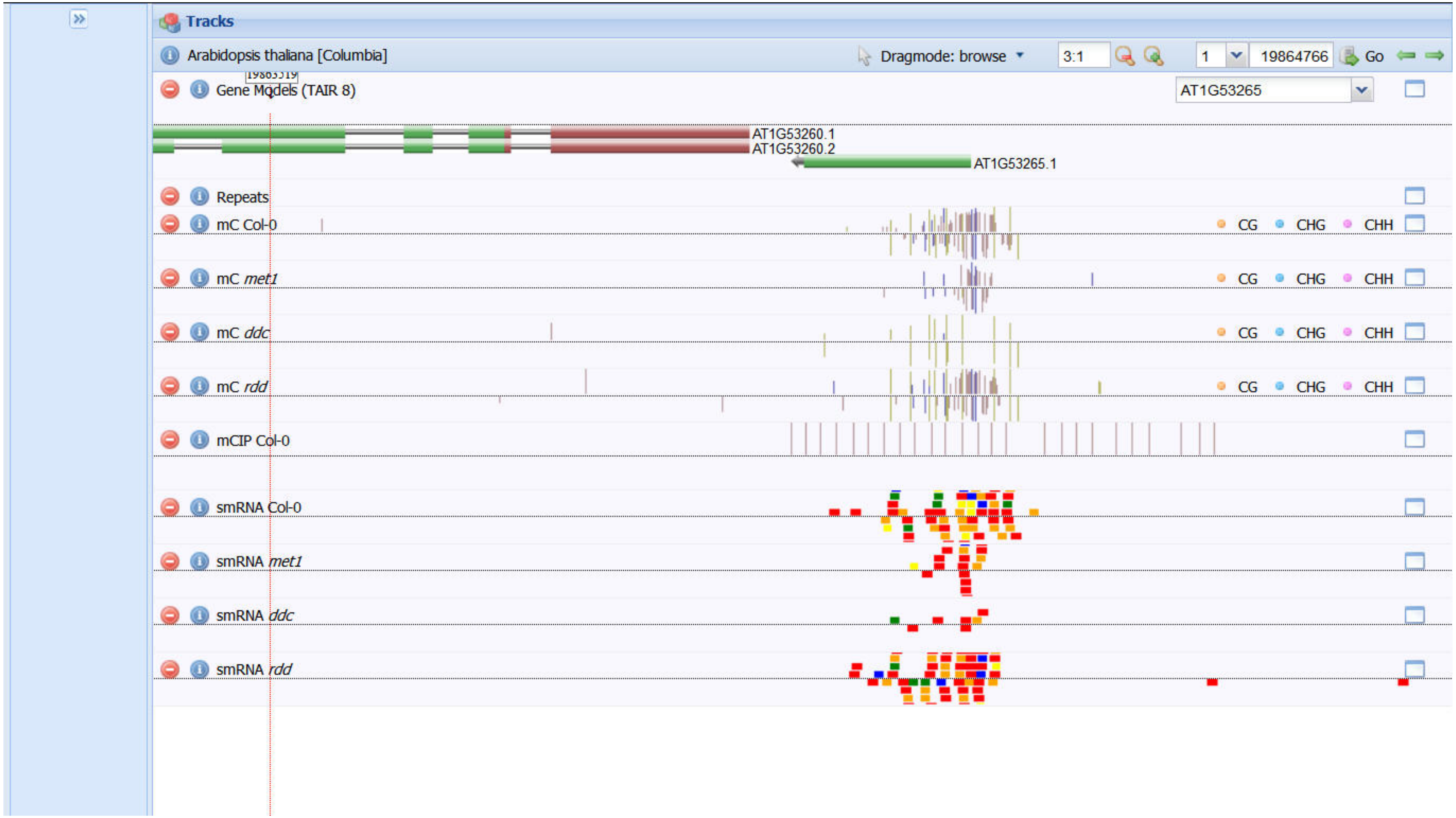
Tracks
Arabidopsis thaliana [Columbia]
Dragmode: browse
3:1
1
19864766
Go
Gene Models (TAIR 8)
AT1G53265
AT1G53260.1
AT1G53260.2
AT1G53265.1
Repeats
mC Col-0
mC met1
mC ddc
mC rdd
CG
CHG
CHH
mCIP Col-0
smRNA Col-0
smRNA met1
smRNA ddc
smRNA rdd

AT1G59680

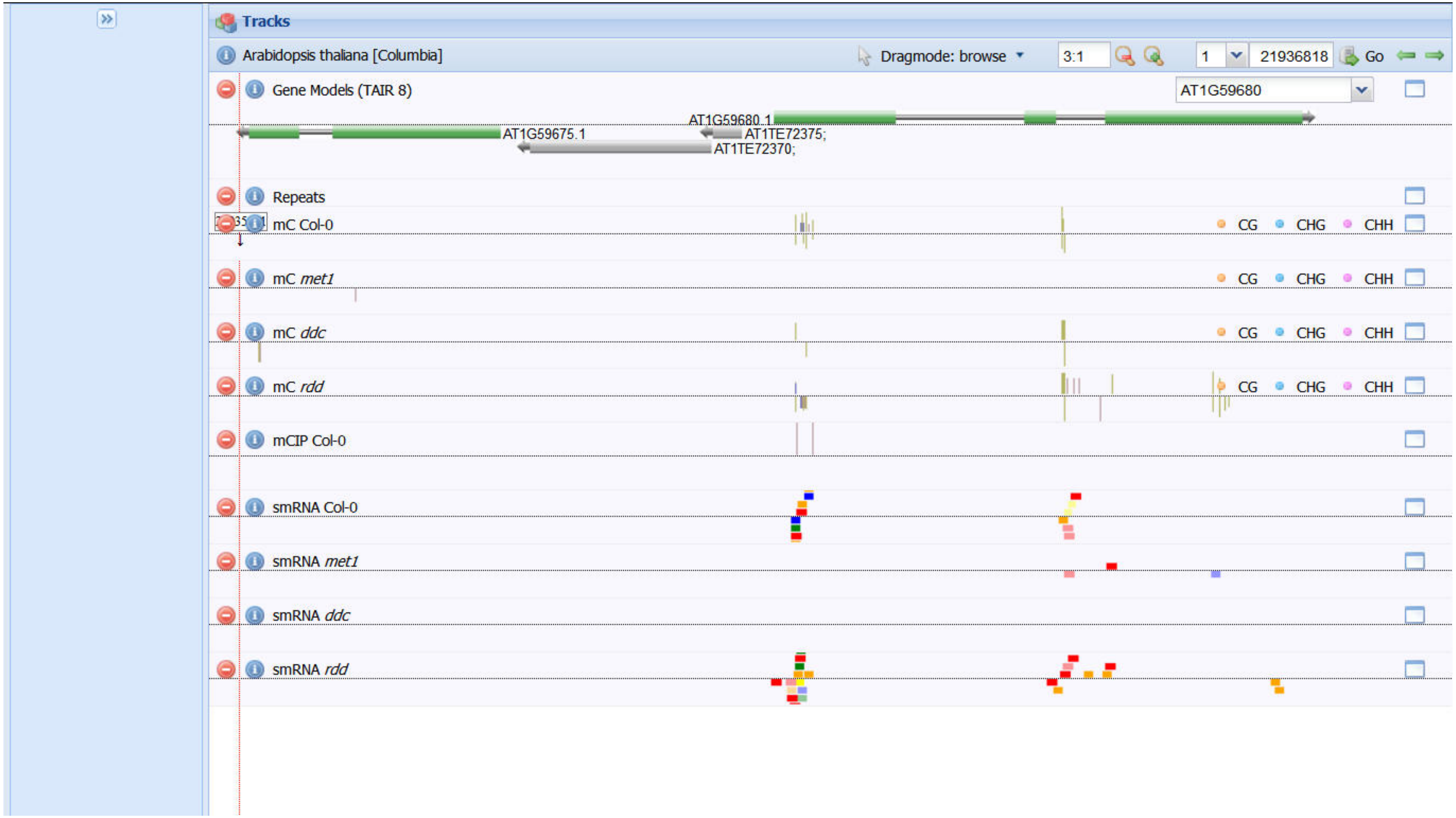

Tracks
Arabidopsis thaliana [Columbia]
Dragmode: browse
3:1
1
21936818
Go
Gene Models (TAIR 8)
AT1G59680
AT1G59680.1
AT1G59675.1
AT1TE72375;
AT1TE72370;
Repeats
mC Col-0
mC met1
mC ddc
mC rdd
CG
CHG
CHH
mCIP Col-0
smRNA Col-0
smRNA met1
smRNA ddc
smRNA rdd

AT1G60720

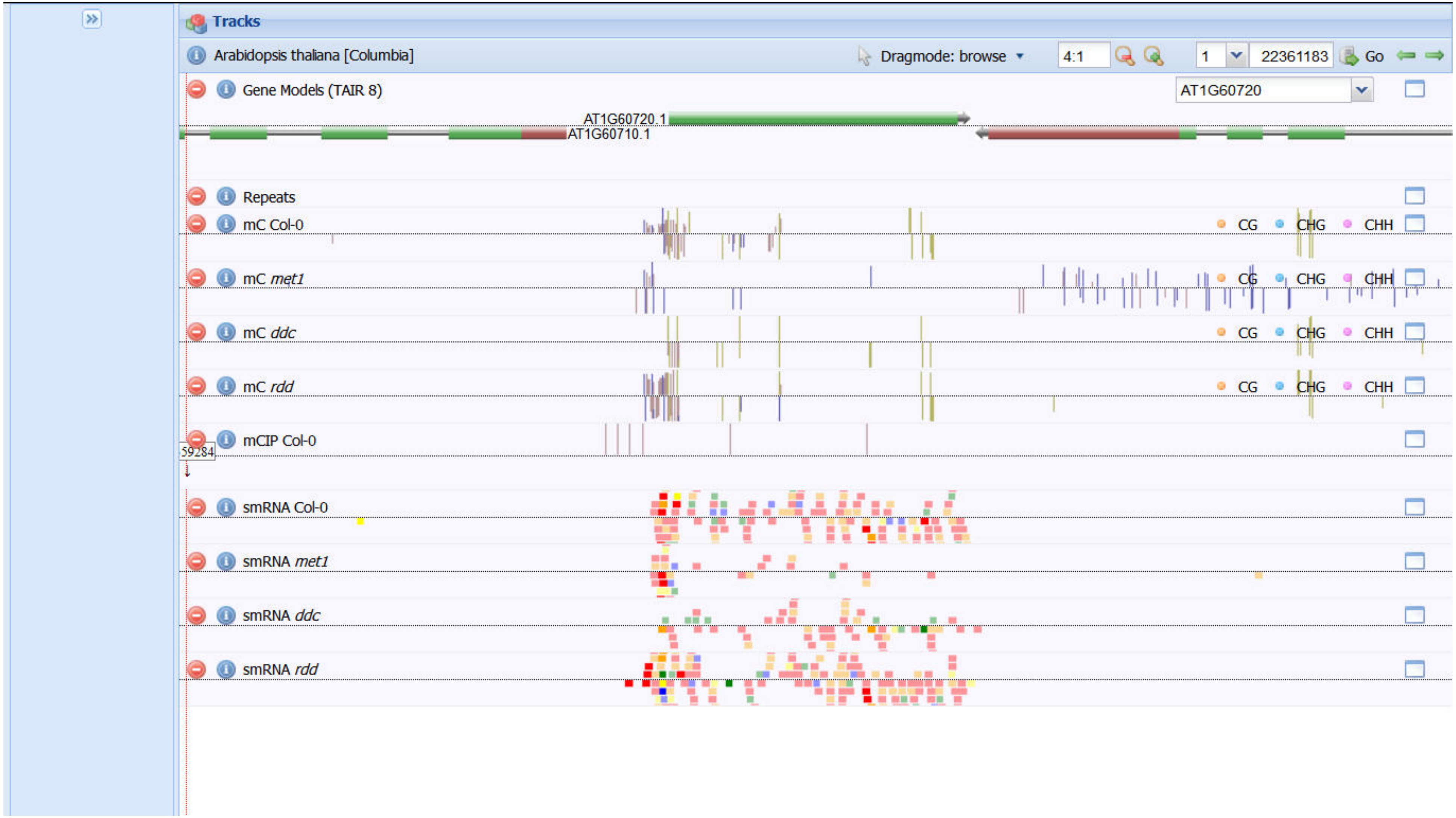

Tracks
Arabidopsis thaliana [Columbia]
Dragmode: browse
4:1
1
22361183
Go
Gene Models (TAIR 8)
AT1G60720
AT1G60720.1
AT1G60710.1
Repeats
mC Col-0
CG
CHG
CHH
mC met1
mC ddc
mC rdd
mCIP Col-0
59284
smRNA Col-0
smRNA met1
smRNA ddc
smRNA rdd

AT1G61820

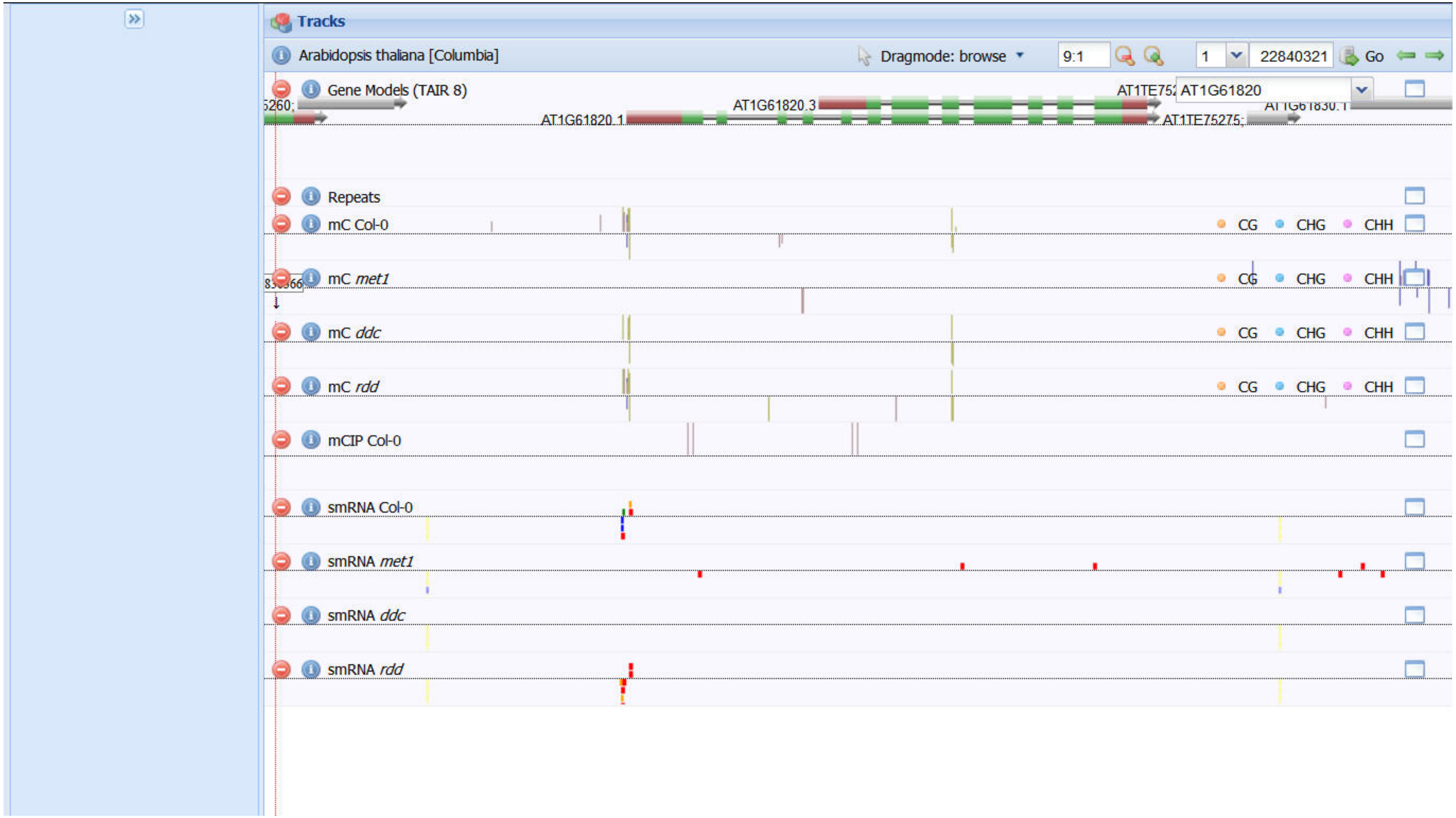

Tracks
Arabidopsis thaliana [Columbia]
Dragmode: browse
9:1
1
22840321
Go
Gene Models (TAIR 8)
AT1G61820.1
AT1G61820.3
AT1G61820
AT1TE75275
Repeats
mC Col-0
mC met1
mC ddc
mC rdd
CG
CHG
CHH
mCIP Col-0
smRNA Col-0
smRNA met1
smRNA ddc
smRNA rdd

AT1G66290

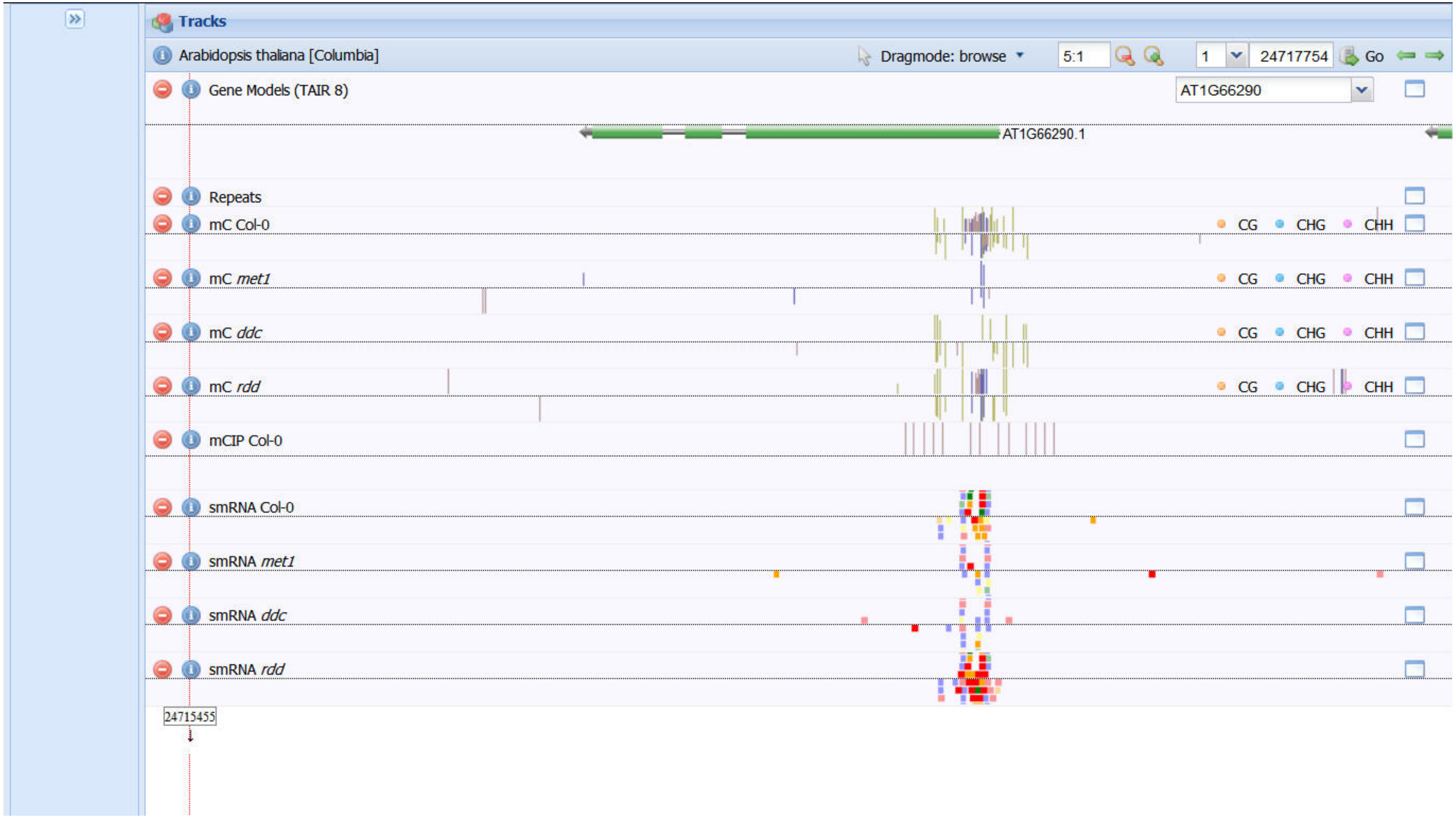

Tracks
Arabidopsis thaliana [Columbia]
Dragmode: browse
5:1
1
24717754
Go
Gene Models (TAIR 8)
AT1G66290
AT1G66290.1
Repeats
mC Col-0
CG
CHG
CHH
mC met1
CG
CHG
CHH
mC ddc
CG
CHG
CHH
mC rdd
CG
CHG
CHH
mCIP Col-0
smRNA Col-0
smRNA met1
smRNA ddc
smRNA rdd
24715455

AT1G66490

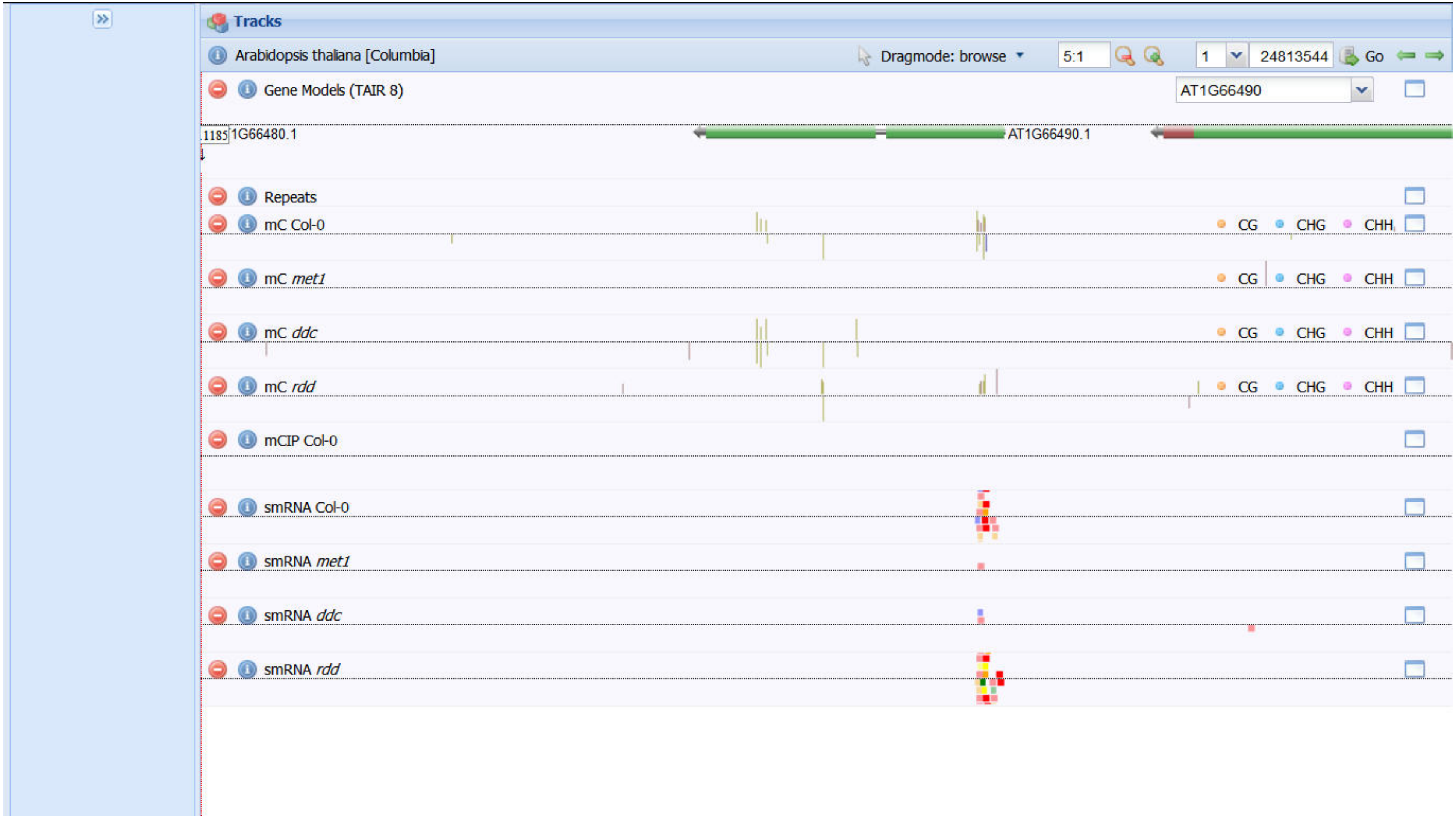

Tracks
Arabidopsis thaliana [Columbia]
Dragmode: browse
5:1
1
24813544
Go
Gene Models (TAIR 8)
AT1G66490
1185 1G66480.1
AT1G66490.1
Repeats
mC Col-0
mC met1
mC ddc
mC rdd
CG
CHG
CHH
mCIP Col-0
smRNA Col-0
smRNA met1
smRNA ddc
smRNA rdd

AT1G66640

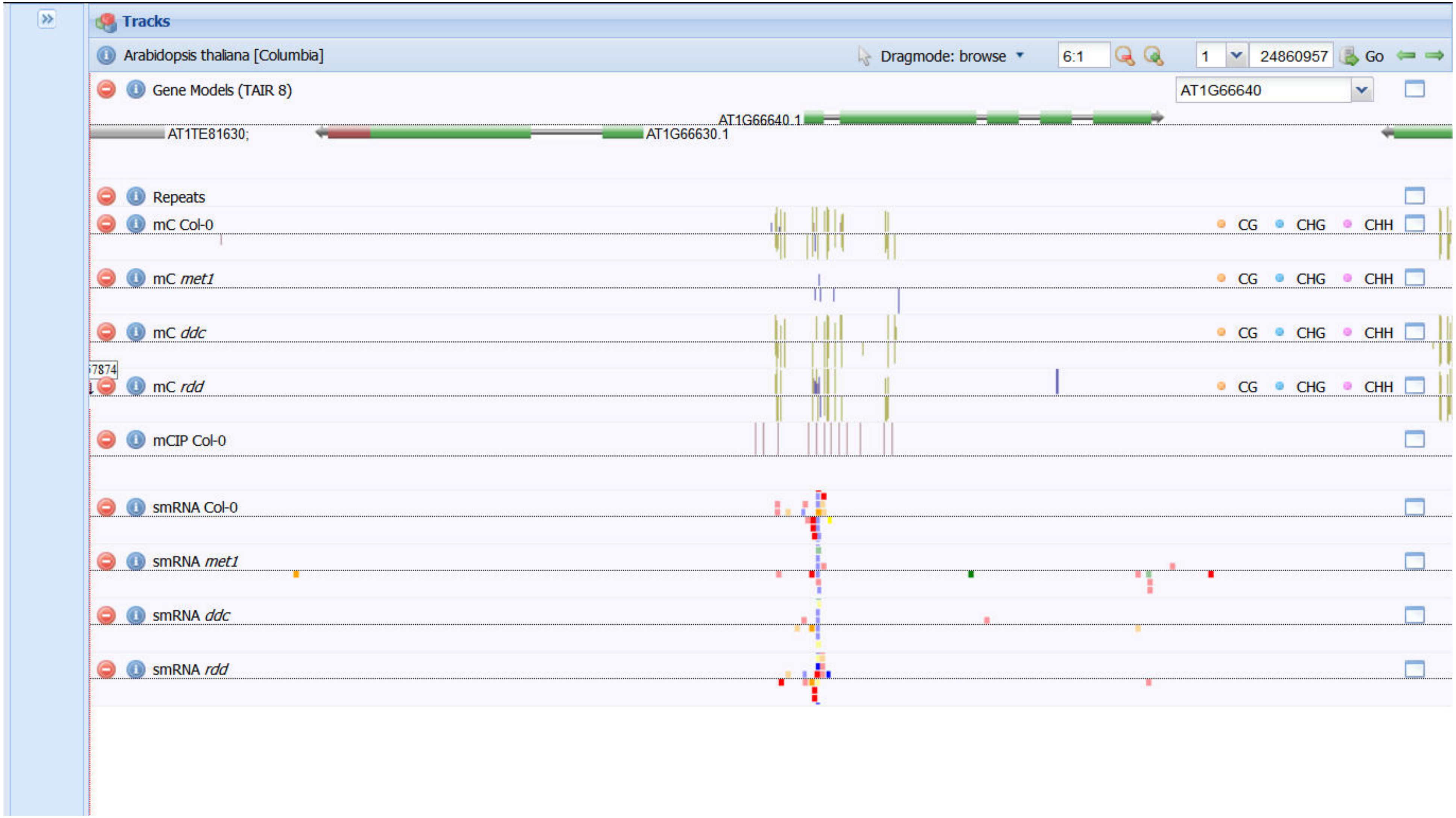

Tracks
Arabidopsis thaliana [Columbia]
Dragmode: browse
6:1
1
24860957
Go
Gene Models (TAIR 8)
AT1G66640
AT1G66640.1
AT1TE81630;
AT1G66630.1
Repeats
mC Col-0
CG
CHG
CHH
mC met1
mC ddc
mC rdd
mCIP Col-0
smRNA Col-0
smRNA met1
smRNA ddc
smRNA rdd

AT3G05770

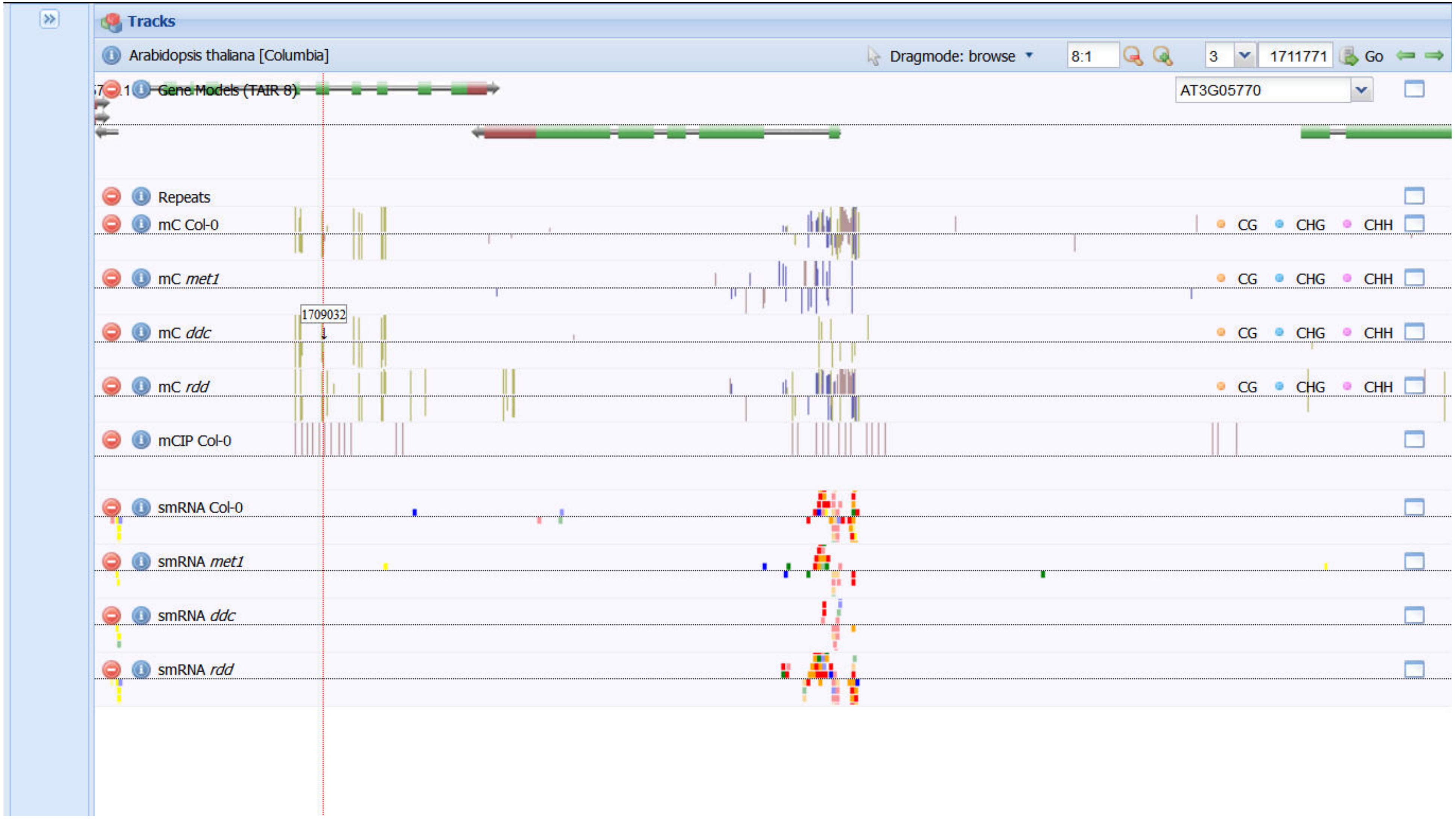

Tracks
Arabidopsis thaliana [Columbia]
Dragmode: browse
8:1
3
1711771
Go
Gene Models (TAIR 8)
AT3G05770
Repeats
mC Col-0
CG
CHG
CHH
mC met1
mC ddc
1709032
mC rdd
mCIP Col-0
smRNA Col-0
smRNA met1
smRNA ddc
smRNA rdd

AT3G19880

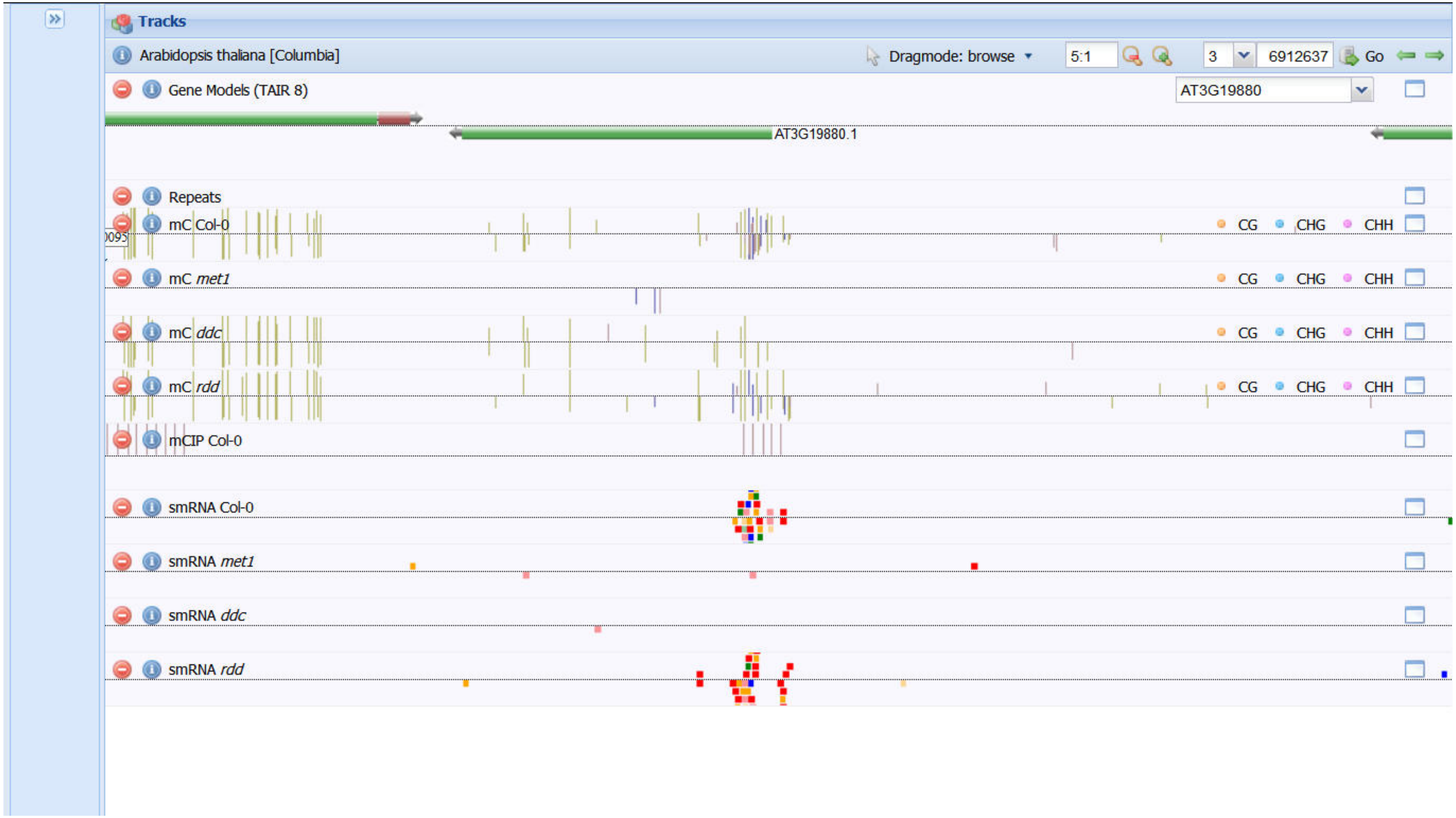

Tracks
Arabidopsis thaliana [Columbia]
Dragmode: browse
5:1
3
6912637
Go
Gene Models (TAIR 8)
AT3G19880
AT3G19880.1
Repeats
mC Col-0
CG CHG CHH
mC met1
CG CHG CHH
mC ddc
CG CHG CHH
mC rdd
CG CHG CHH
mCIP Col-0
smRNA Col-0
smRNA met1
smRNA ddc
smRNA rdd

AT5G24240

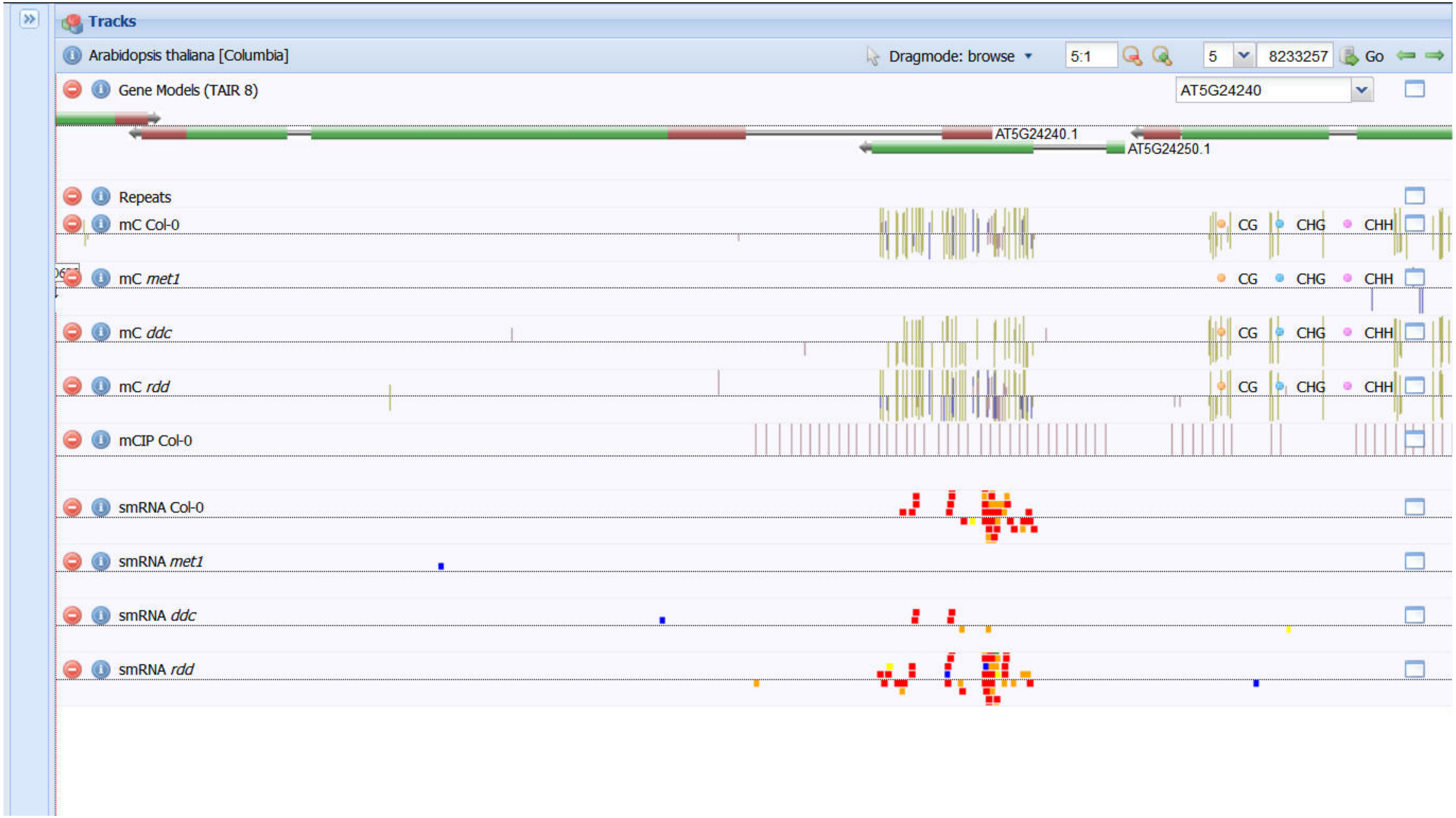

Tracks
Arabidopsis thaliana [Columbia]
Dragmode: browse
5:1
5
8233257
Go
Gene Models (TAIR 8)
AT5G24240
AT5G24240.1
AT5G24250.1
Repeats
mC Col-0
CG
CHG
CHH
mC met1
mC ddc
mC rdd
mCIP Col-0
smRNA Col-0
smRNA met1
smRNA ddc
smRNA rdd

AT5G43500

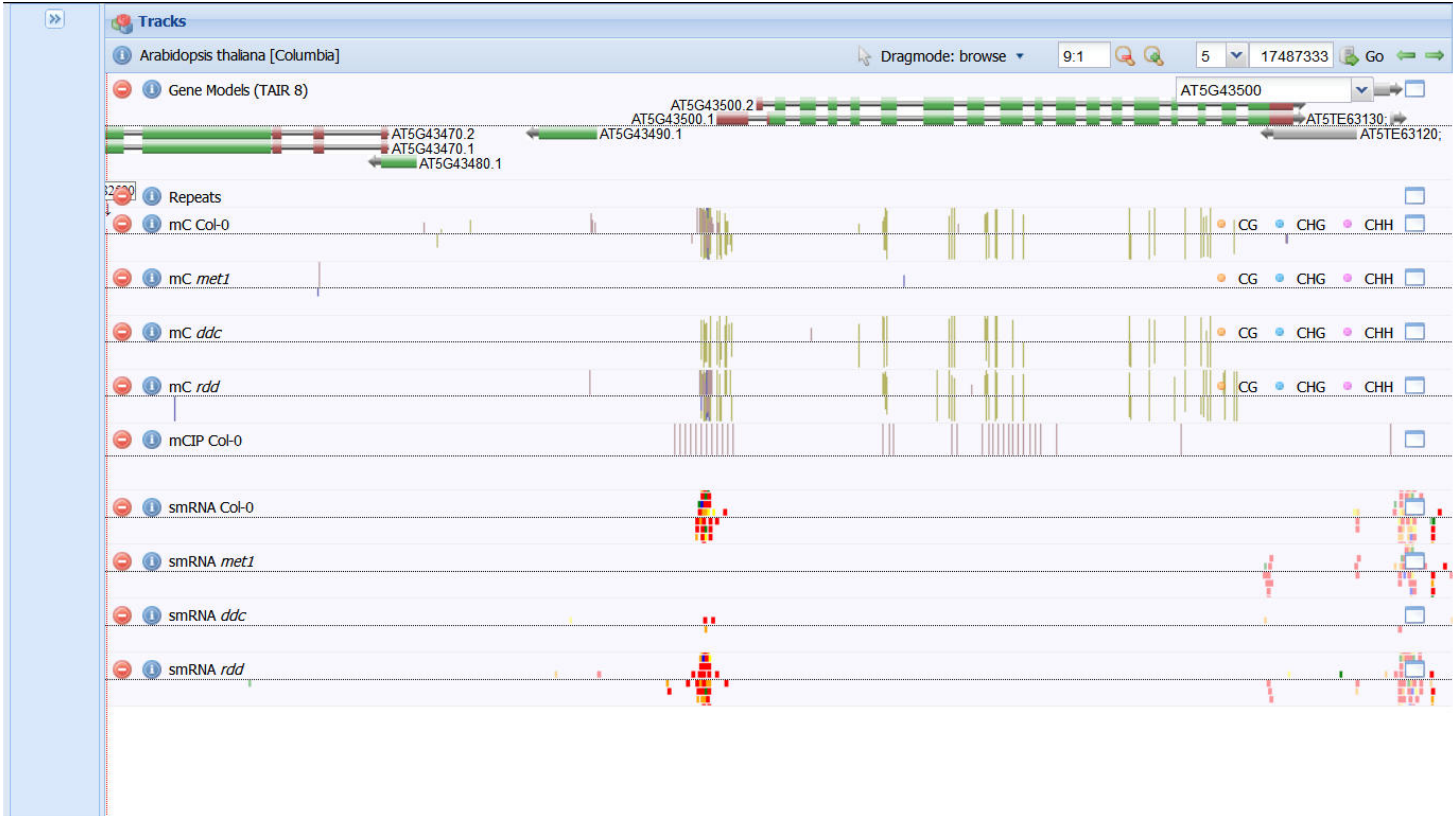
Tracks
Arabidopsis thaliana [Columbia]
Dragmode: browse
9:1
5
17487333
Go
Gene Models (TAIR 8)
AT5G43500
AT5G43500.2
AT5G43500.1
AT5TE63130;
AT5G43470.2
AT5G43470.1
AT5G43490.1
AT5TE63120;
AT5G43480.1
Repeats
mC Col-0
CG CHG CHH
mC met1
CG CHG CHH
mC ddc
CG CHG CHH
mC rdd
CG CHG CHH
mCIP Col-0
smRNA Col-0
smRNA met1
smRNA ddc
smRNA rdd

AT5G48000

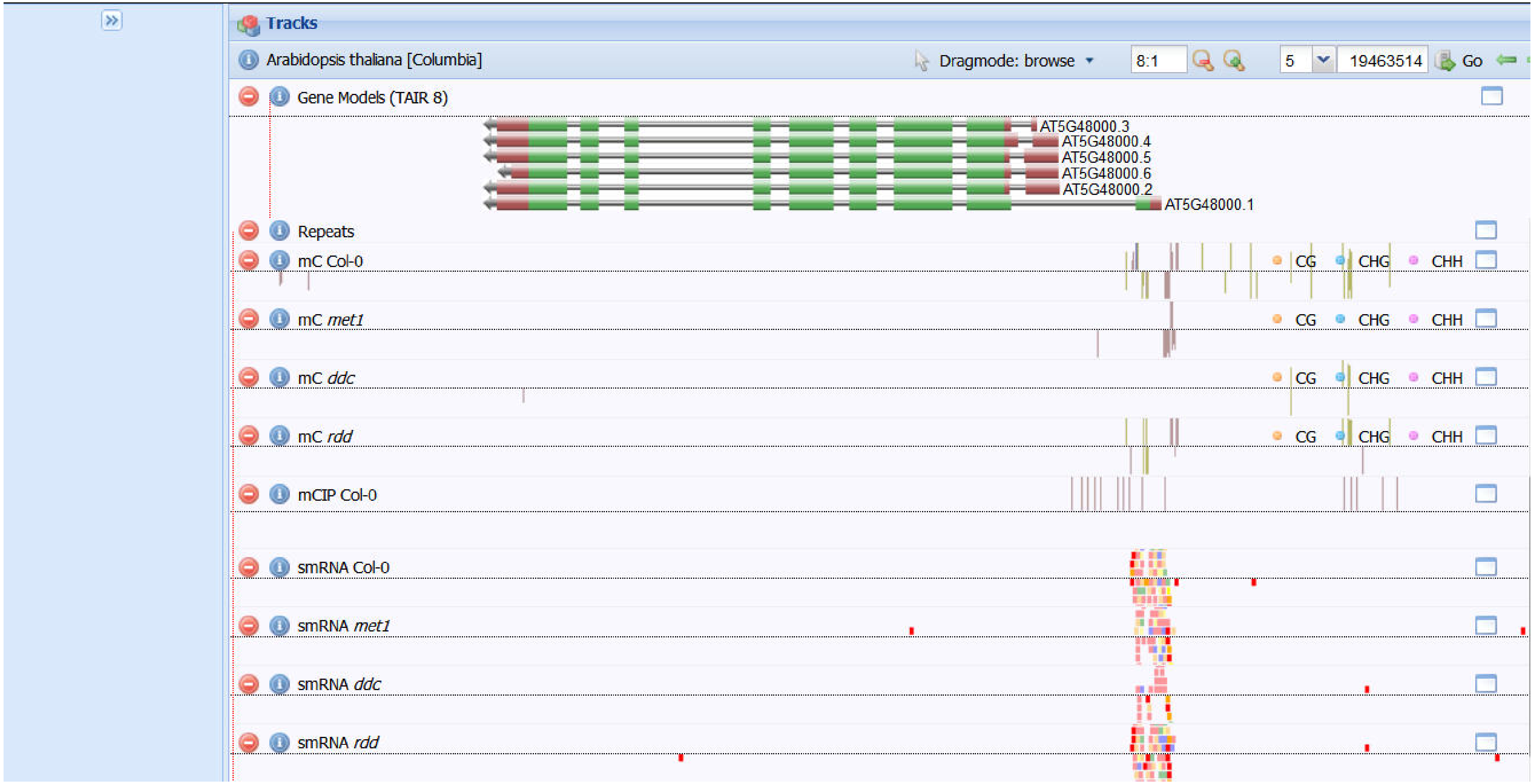
Tracks
Arabidopsis thaliana [Columbia]
Dragmode: browse
8:1
5
19463514
Go
Gene Models (TAIR 8)
AT5G48000.3
AT5G48000.4
AT5G48000.5
AT5G48000.6
AT5G48000.2
AT5G48000.1
Repeats
mC Col-0
CG
CHG
CHH
mC met1
CG
CHG
CHH
mC ddc
CG
CHG
CHH
mC rdd
CG
CHG
CHH
mCIP Col-0
smRNA Col-0
smRNA met1
smRNA ddc
smRNA rdd
