# Supplementary material for: Transcriptome-Wide Discovery of PASRs (Promoter-Associated Small RNAs) and TASRs (Terminus-Associated Small RNAs) in Arabidopsis thaliana
Source: PLoS One. 2017 Jan 3;12(1):e0169212. doi: 10.1371/journal.pone.0169212 (PMC5207706; doi:10.1371/journal.pone.0169212)
Supplement: S24 Fig — (PDF) [file pone.0169212.s024.pdf]

Figure S24 Site-specific DNA methylation signals were detected at the genomic positions well corresponding to those of the TASR peaks identified on the sense strands of the protein-coding genes in *Arabidopsis*.

AT1G52180

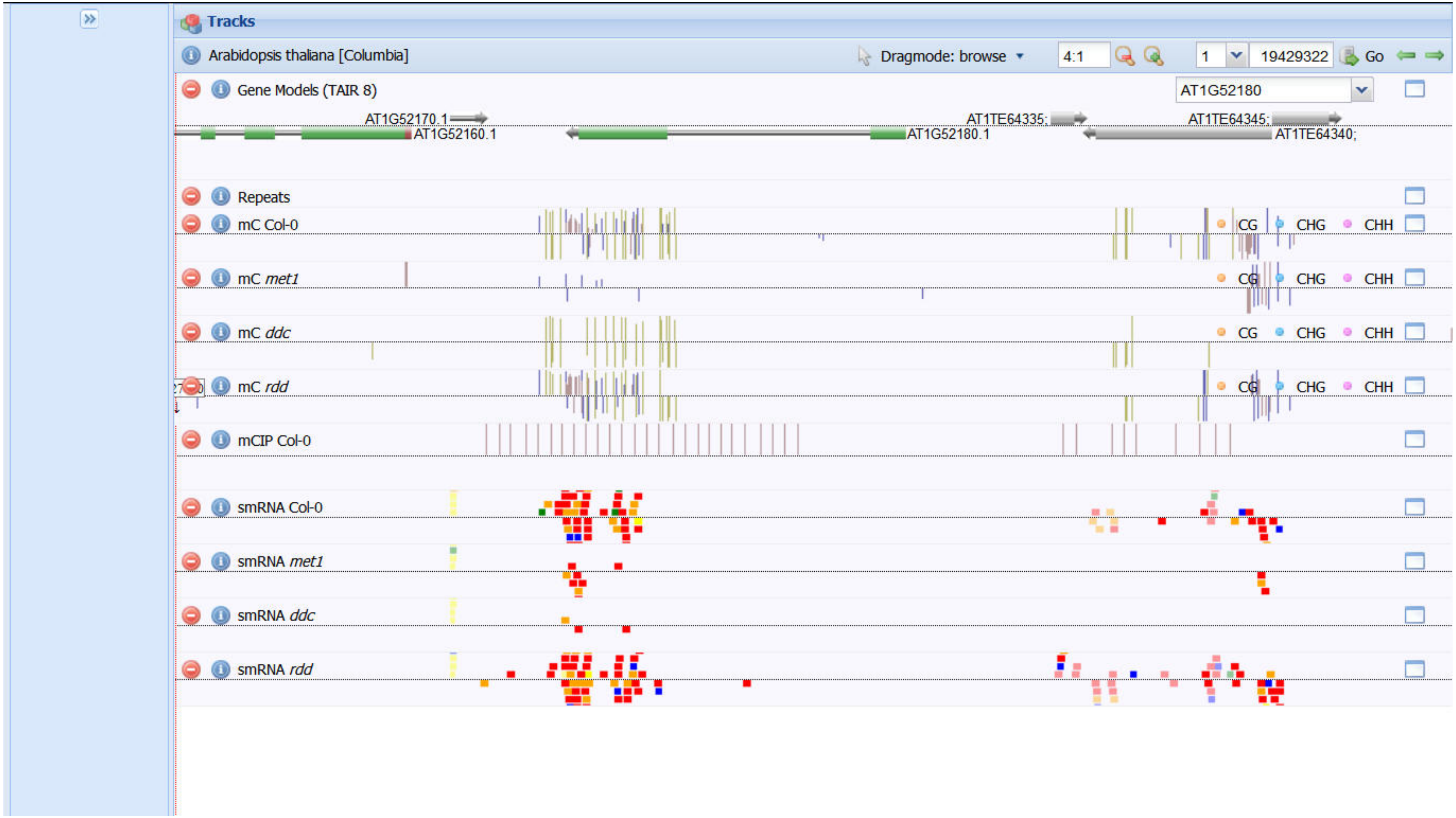
Tracks
Arabidopsis thaliana [Columbia]
Dragmode: browse
4:1
1
19429322
Go
Gene Models (TAIR 8)
AT1G52180
AT1G52170.1
AT1G52160.1
AT1TE64335;
AT1G52180.1
AT1TE64345;
AT1TE64340;
Repeats
mC Col-0
CG
CHG
CHH
mC met1
mC ddc
mC rdd
mCIP Col-0
smRNA Col-0
smRNA met1
smRNA ddc
smRNA rdd

AT5G36220

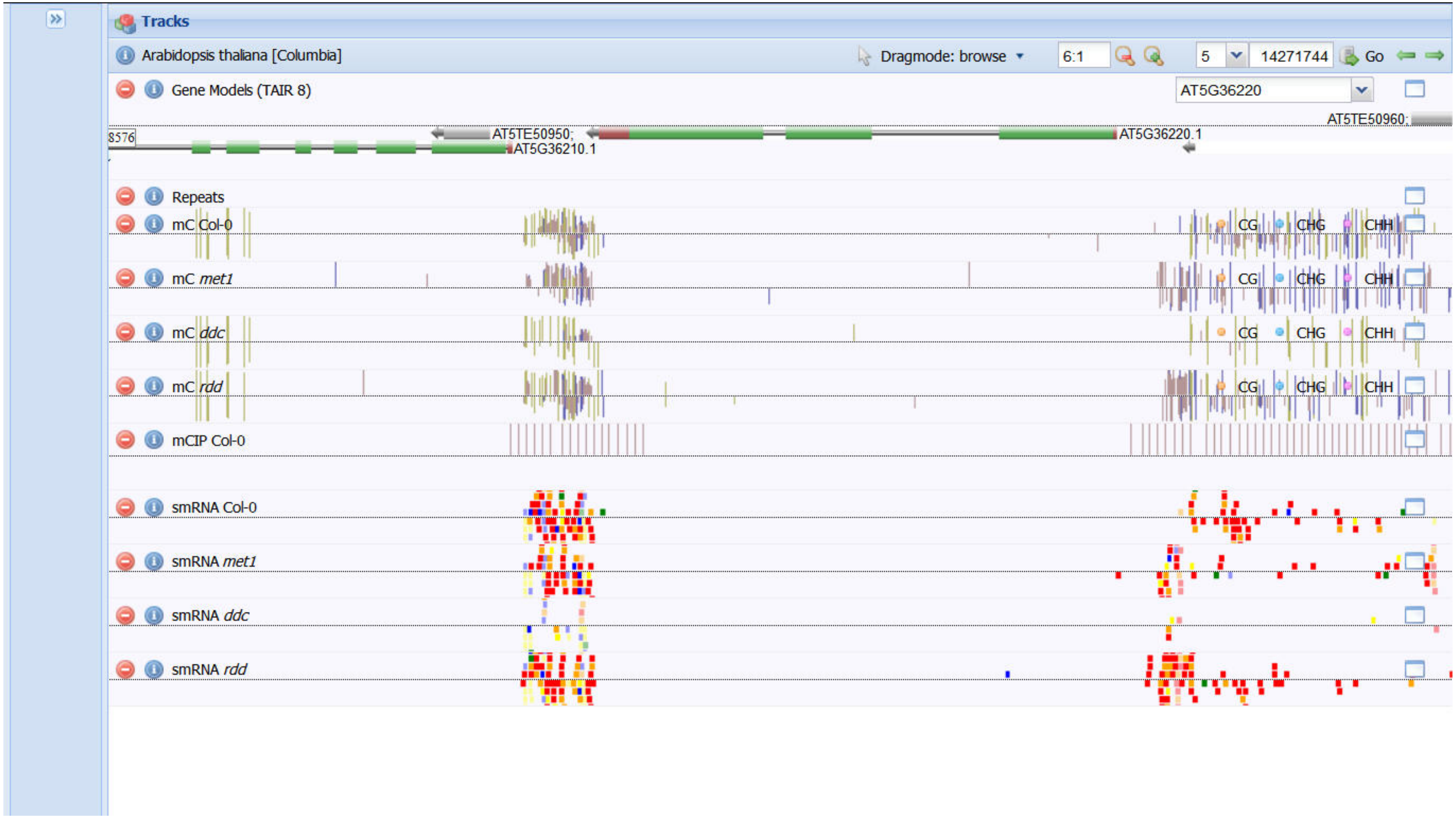

Tracks
Arabidopsis thaliana [Columbia]
Dragmode: browse
6:1
5
14271744
Go
Gene Models (TAIR 8)
AT5G36220
AT5TE50960;
8576
AT5TE50950;
AT5G36210.1
AT5G36220.1
Repeats
mC Col-0
mC met1
mC ddc
mC rdd
CG
CHG
CHH
mCIP Col-0
smRNA Col-0
smRNA met1
smRNA ddc
smRNA rdd

AT5G42203

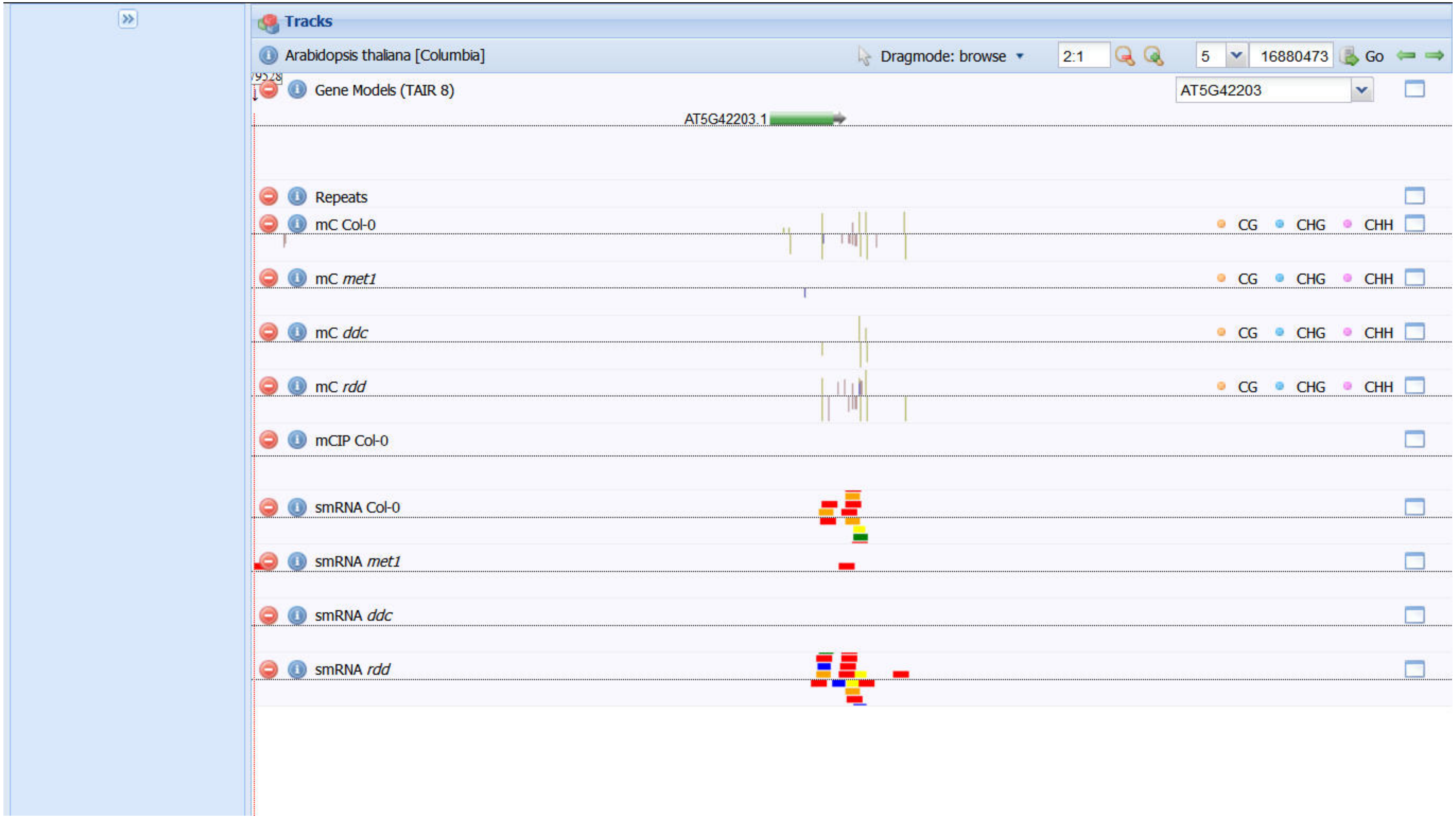

Tracks
Arabidopsis thaliana [Columbia]
Dragmode: browse
2:1
5
16880473
Go
Gene Models (TAIR 8)
AT5G42203
AT5G42203.1
Repeats
mC Col-0
CG CHG CHH
mC met1
CG CHG CHH
mC ddc
CG CHG CHH
mC rdd
CG CHG CHH
mCIP Col-0
smRNA Col-0
smRNA met1
smRNA ddc
smRNA rdd
