# Supplementary material for: Transcriptome-Wide Discovery of PASRs (Promoter-Associated Small RNAs) and TASRs (Terminus-Associated Small RNAs) in Arabidopsis thaliana
Source: PLoS One. 2017 Jan 3;12(1):e0169212. doi: 10.1371/journal.pone.0169212 (PMC5207706; doi:10.1371/journal.pone.0169212)
Supplement: S25 Fig — (PDF) [file pone.0169212.s025.pdf]

Figure S25 Site-specific DNA methylation signals were detected at the genomic positions well corresponding to those of the TASR peaks identified on the antisense strands of the protein-coding genes in *Arabidopsis*.

AT1G59835

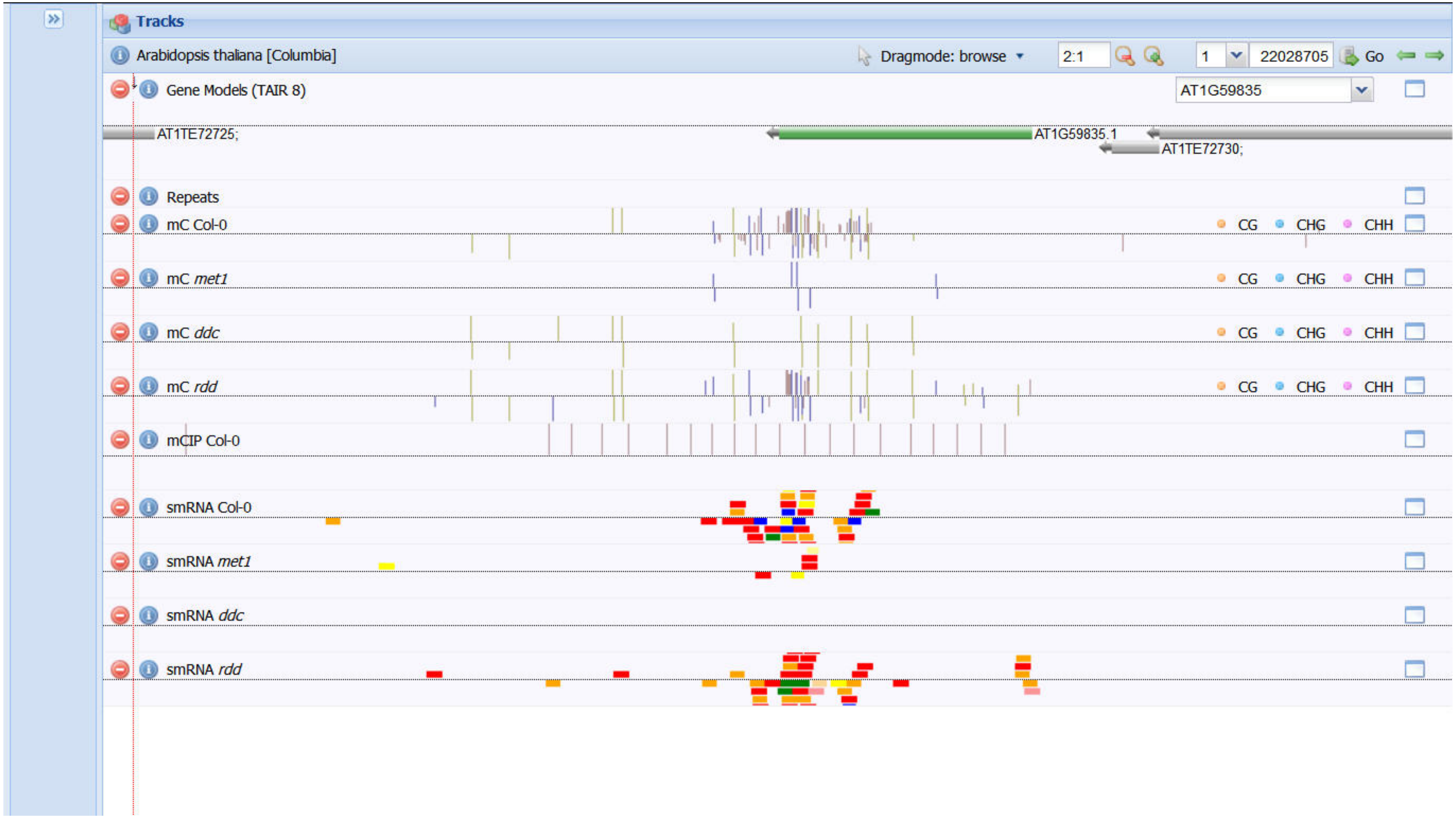
Tracks
Arabidopsis thaliana [Columbia]
Dragmode: browse
2:1
1
22028705
Go
Gene Models (TAIR 8)
AT1G59835
AT1TE72725;
AT1G59835.1
AT1TE72730;
Repeats
mC Col-0
CG
CHG
CHH
mC met1
mC ddc
mC rdd
mCIP Col-0
smRNA Col-0
smRNA met1
smRNA ddc
smRNA rdd

AT1G66540

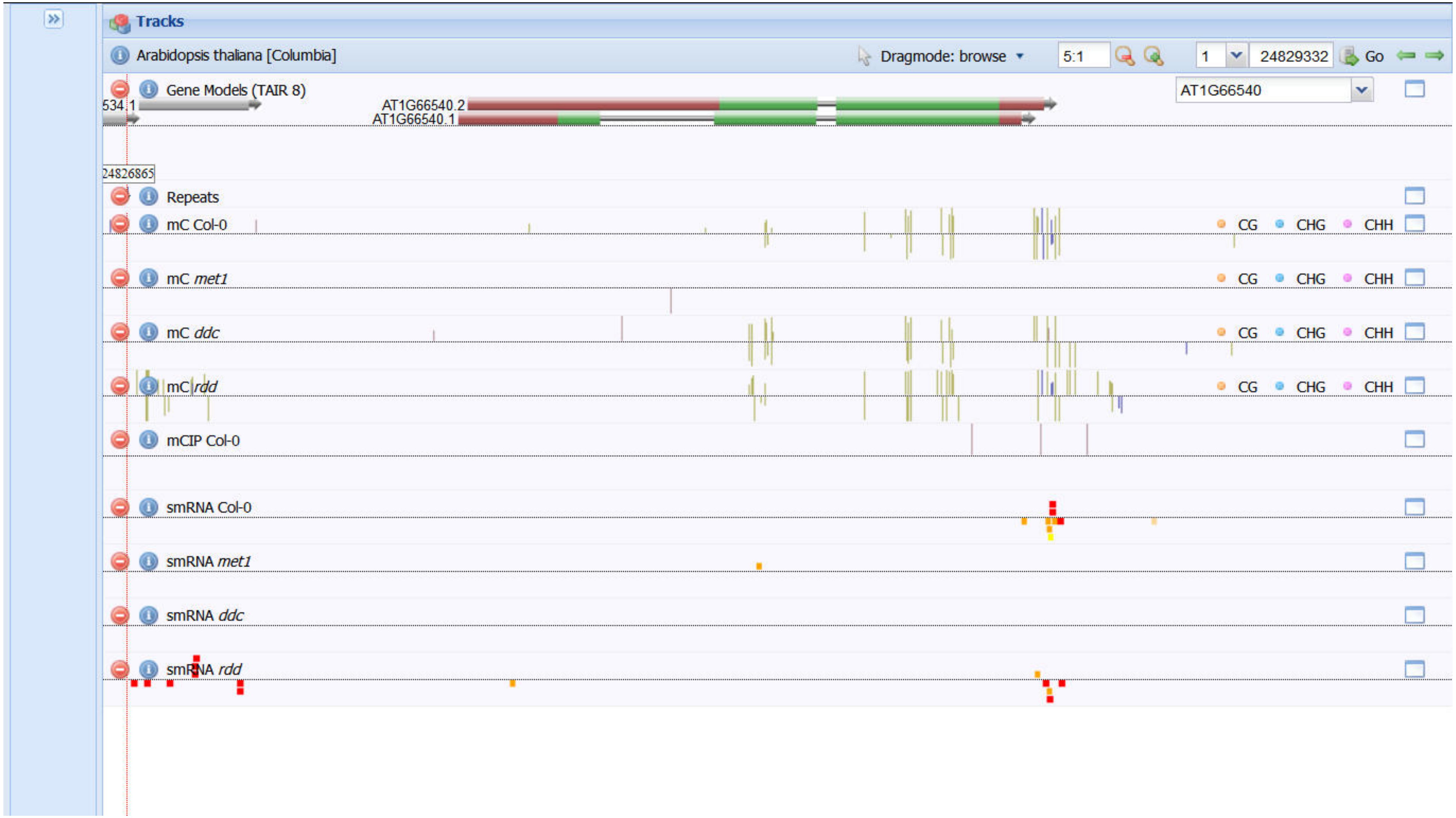

Tracks
Arabidopsis thaliana [Columbia]
Dragmode: browse
5:1
1
24829332
Go
Gene Models (TAIR 8)
AT1G66540
534.1
AT1G66540.2
AT1G66540.1
24826865
Repeats
mC Col-0
CG CHG CHH
mC met1
mC ddc
mC rdd
mCIP Col-0
smRNA Col-0
smRNA met1
smRNA ddc
smRNA rdd

AT2G04090

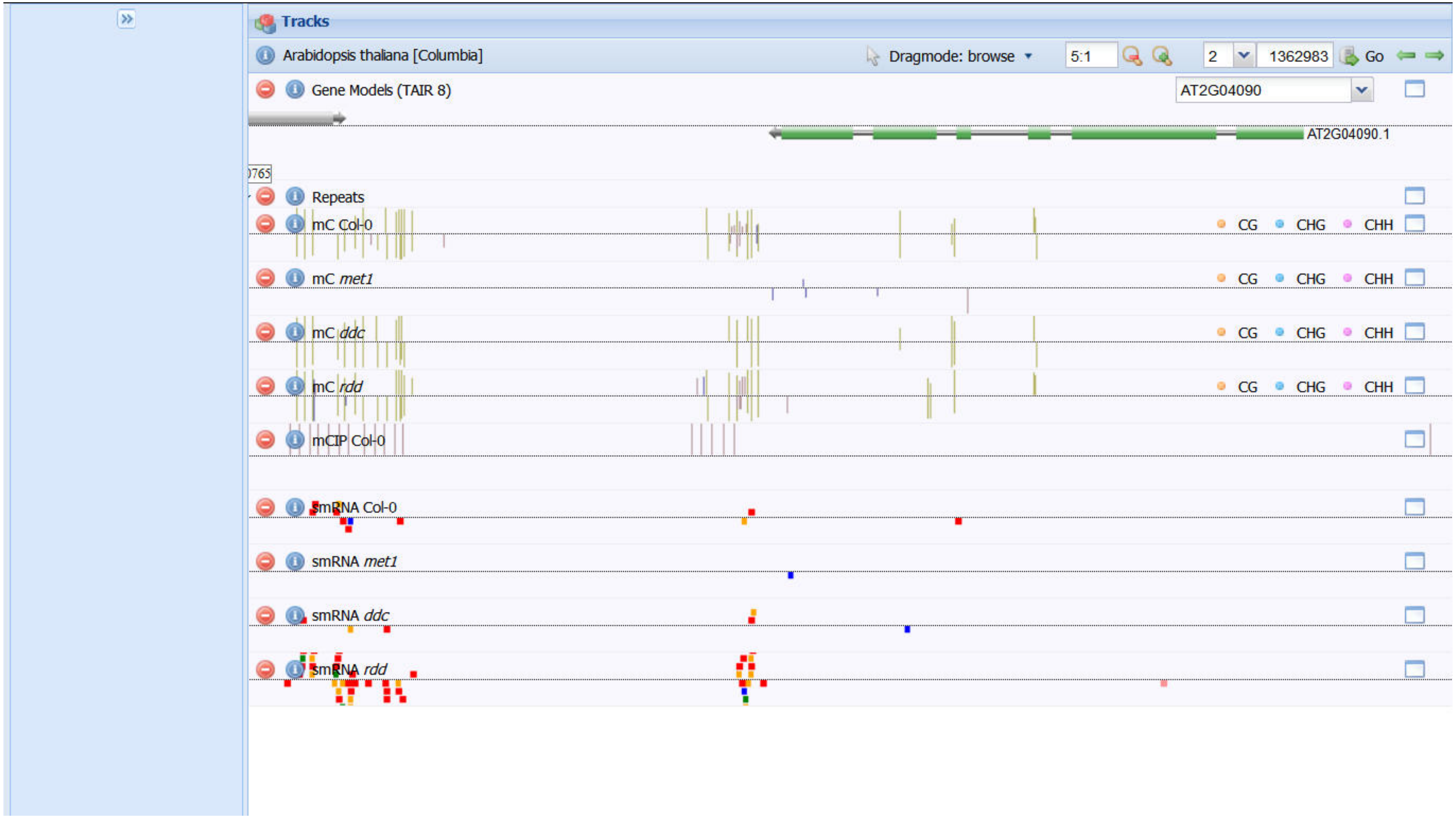

Tracks
Arabidopsis thaliana [Columbia]
Dragmode: browse
5:1
2
1362983
Go
Gene Models (TAIR 8)
AT2G04090
AT2G04090.1
Repeats
mC Col-0
CG CHG CHH
mC *met1*
CG CHG CHH
mC *ddc*
CG CHG CHH
mC *rdd*
CG CHG CHH
mCIP Col-0
smRNA Col-0
smRNA *met1*
smRNA *ddc*
smRNA *rdd*

AT2G04830

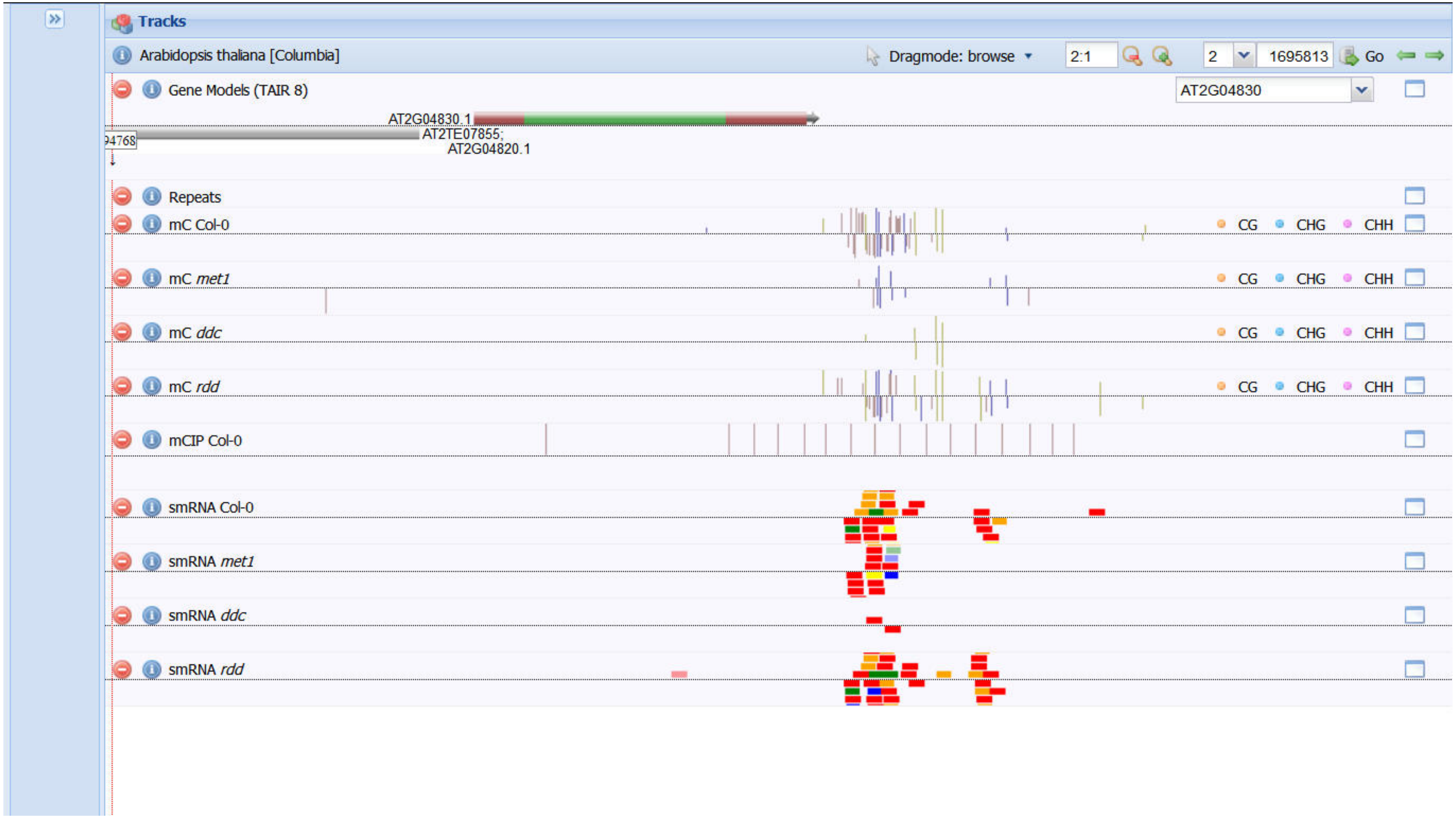

Tracks
Arabidopsis thaliana [Columbia]
Dragmode: browse
2:1
2
1695813
Go
Gene Models (TAIR 8)
AT2G04830
AT2G04830.1
94768
AT2TE07855;
AT2G04820.1
Repeats
mC Col-0
CG
CHG
CHH
mC met1
mC ddc
mC rdd
mCIP Col-0
smRNA Col-0
smRNA met1
smRNA ddc
smRNA rdd

AT5G48605

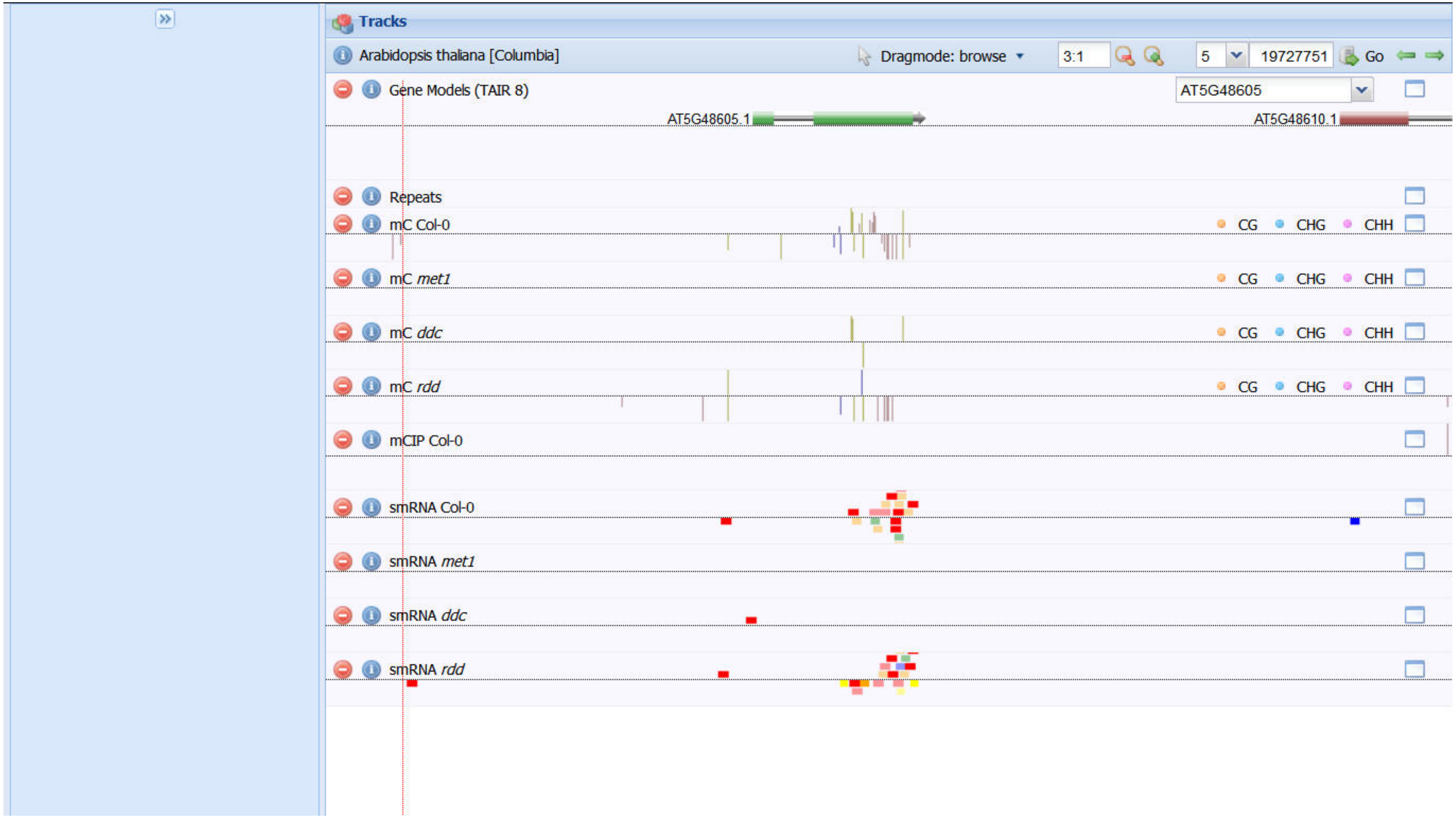
Tracks
Arabidopsis thaliana [Columbia]
Dragmode: browse
3:1
5
19727751
Go
Gene Models (TAIR 8)
AT5G48605
AT5G48605.1
AT5G48610.1
Repeats
mC Col-0
CG
CHG
CHH
mC met1
mC ddc
mC rdd
mCIP Col-0
smRNA Col-0
smRNA met1
smRNA ddc
smRNA rdd
