# Supplementary material for: Transcriptome-Wide Discovery of PASRs (Promoter-Associated Small RNAs) and TASRs (Terminus-Associated Small RNAs) in Arabidopsis thaliana
Source: PLoS One. 2017 Jan 3;12(1):e0169212. doi: 10.1371/journal.pone.0169212 (PMC5207706; doi:10.1371/journal.pone.0169212)
Supplement: S26 Fig — (PDF) [file pone.0169212.s026.pdf]

Figure S26 Site-specific DNA methylation signals were detected at the genomic positions well corresponding to those of the paired TASR peaks identified on both strands of the protein-coding genes in *Arabidopsis*.

AT1G14580

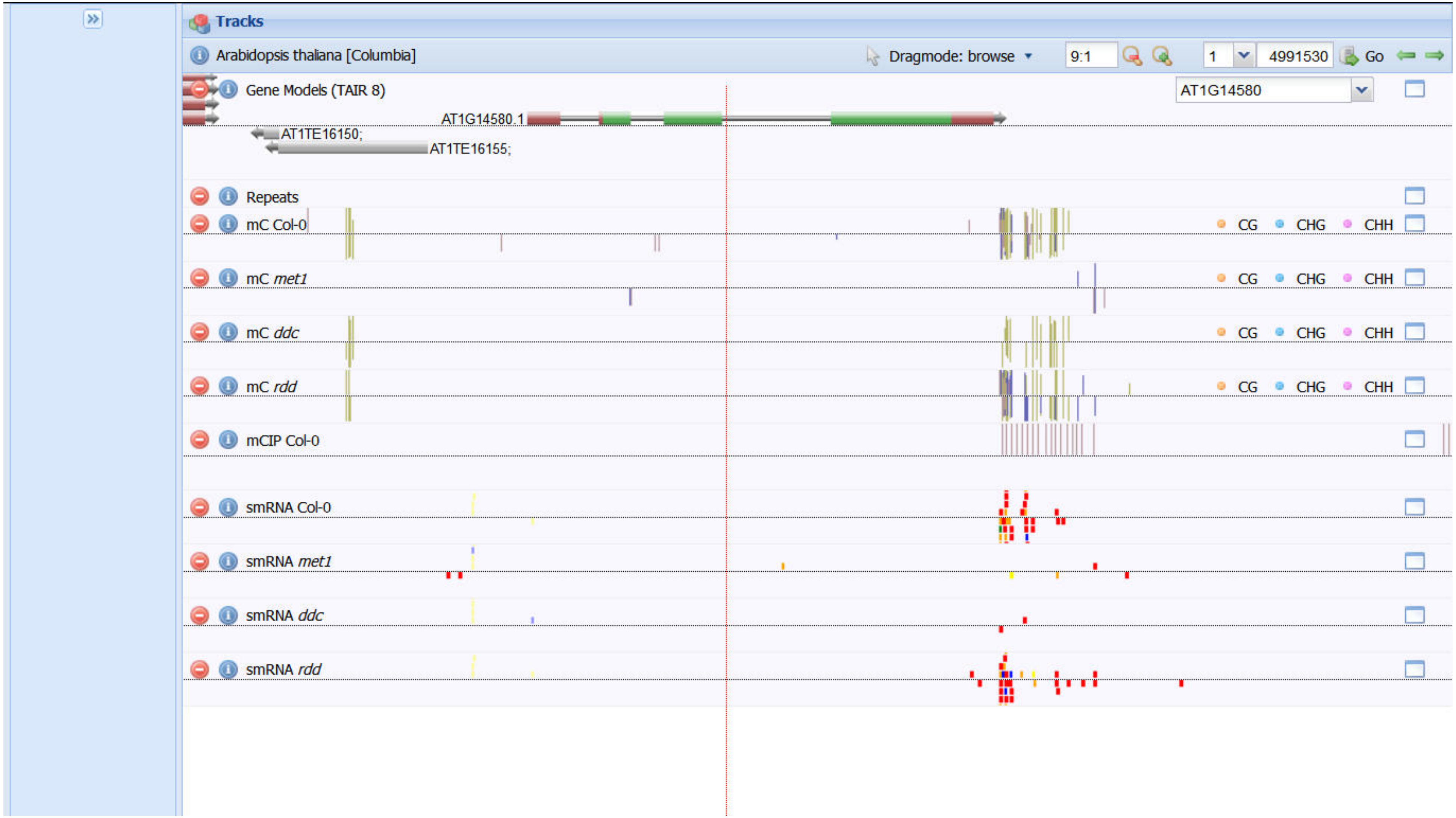

Tracks
Arabidopsis thaliana [Columbia]
Dragmode: browse
9:1
1
4991530
Go
Gene Models (TAIR 8)
AT1G14580
AT1G14580.1
AT1TE16150;
AT1TE16155;
Repeats
mC Col-0
mC met1
mC ddc
mC rdd
CG
CHG
CHH
mCIP Col-0
smRNA Col-0
smRNA met1
smRNA ddc
smRNA rdd

AT1G18770

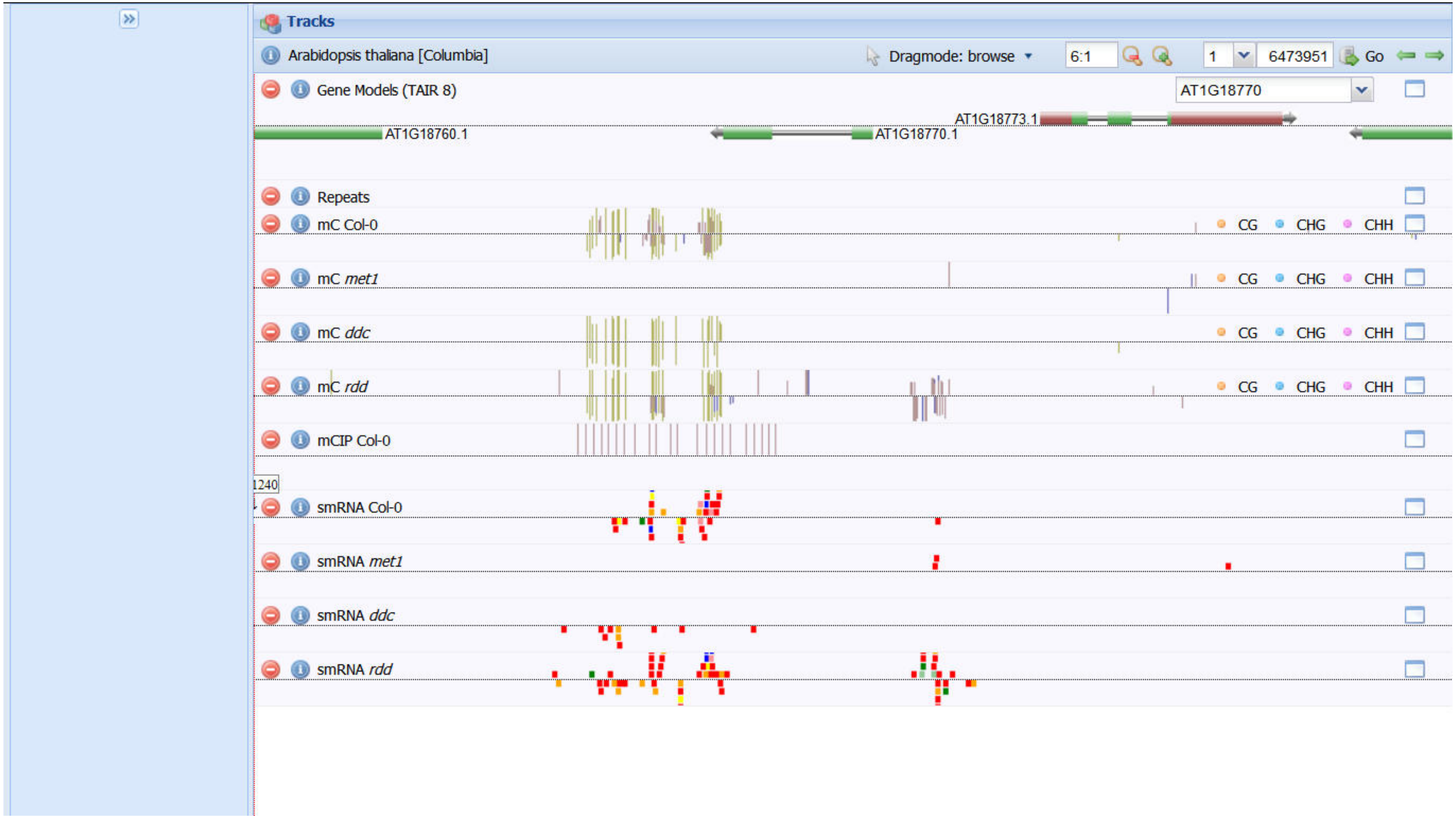

Tracks
Arabidopsis thaliana [Columbia]
Dragmode: browse
6:1
1
6473951
Go
Gene Models (TAIR 8)
AT1G18770
AT1G18773.1
AT1G18760.1
AT1G18770.1
Repeats
mC Col-0
CG
CHG
CHH
mC met1
mC ddc
mC rdd
mCIP Col-0
1240
smRNA Col-0
smRNA met1
smRNA ddc
smRNA rdd

AT1G19830

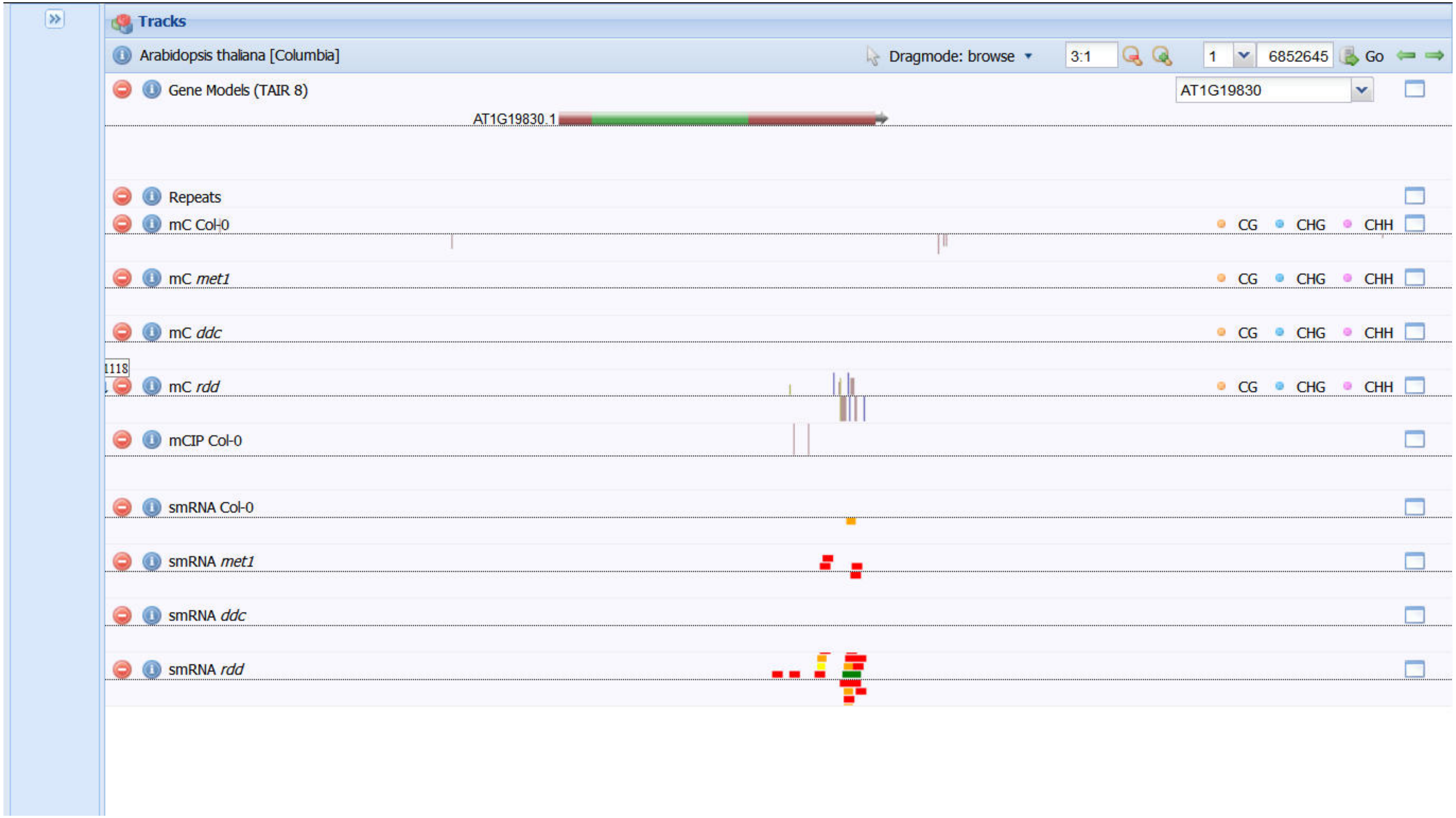
Tracks
Arabidopsis thaliana [Columbia]
Dragmode: browse
3:1
1
6852645
Go
Gene Models (TAIR 8)
AT1G19830
AT1G19830.1
Repeats
mC Col-0
CG CHG CHH
mC met1
CG CHG CHH
mC ddc
CG CHG CHH
1118
mC rdd
CG CHG CHH
mCIP Col-0
smRNA Col-0
smRNA met1
smRNA ddc
smRNA rdd

AT1G26762

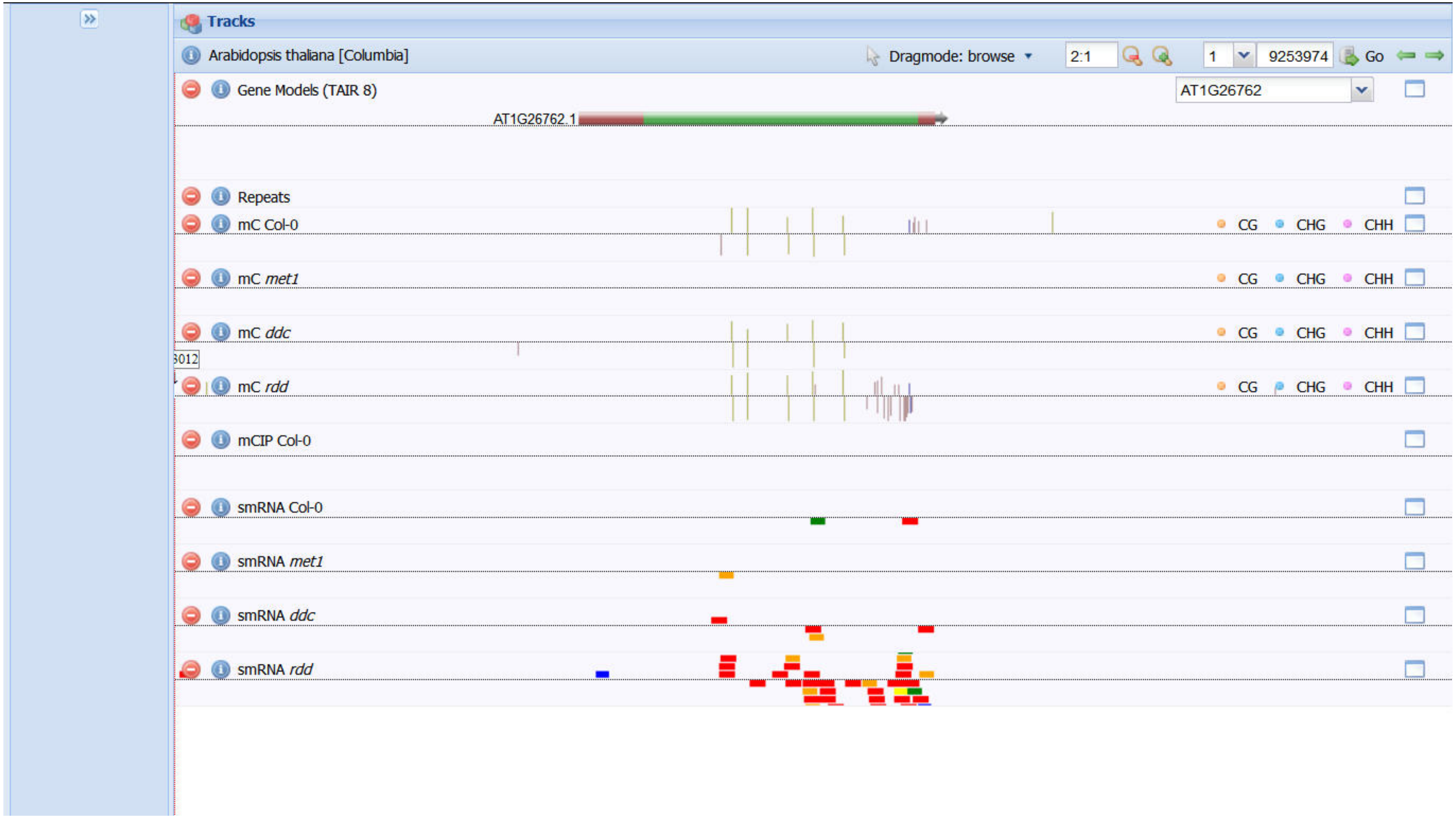

Tracks
Arabidopsis thaliana [Columbia]
Dragmode: browse
2:1
1
9253974
Go
Gene Models (TAIR 8)
AT1G26762
AT1G26762.1
Repeats
mC Col-0
CG CHG CHH
mC met1
CG CHG CHH
mC ddc
CG CHG CHH
mC rdd
CG CHG CHH
mCIP Col-0
smRNA Col-0
smRNA met1
smRNA ddc
smRNA rdd

AT1G51150

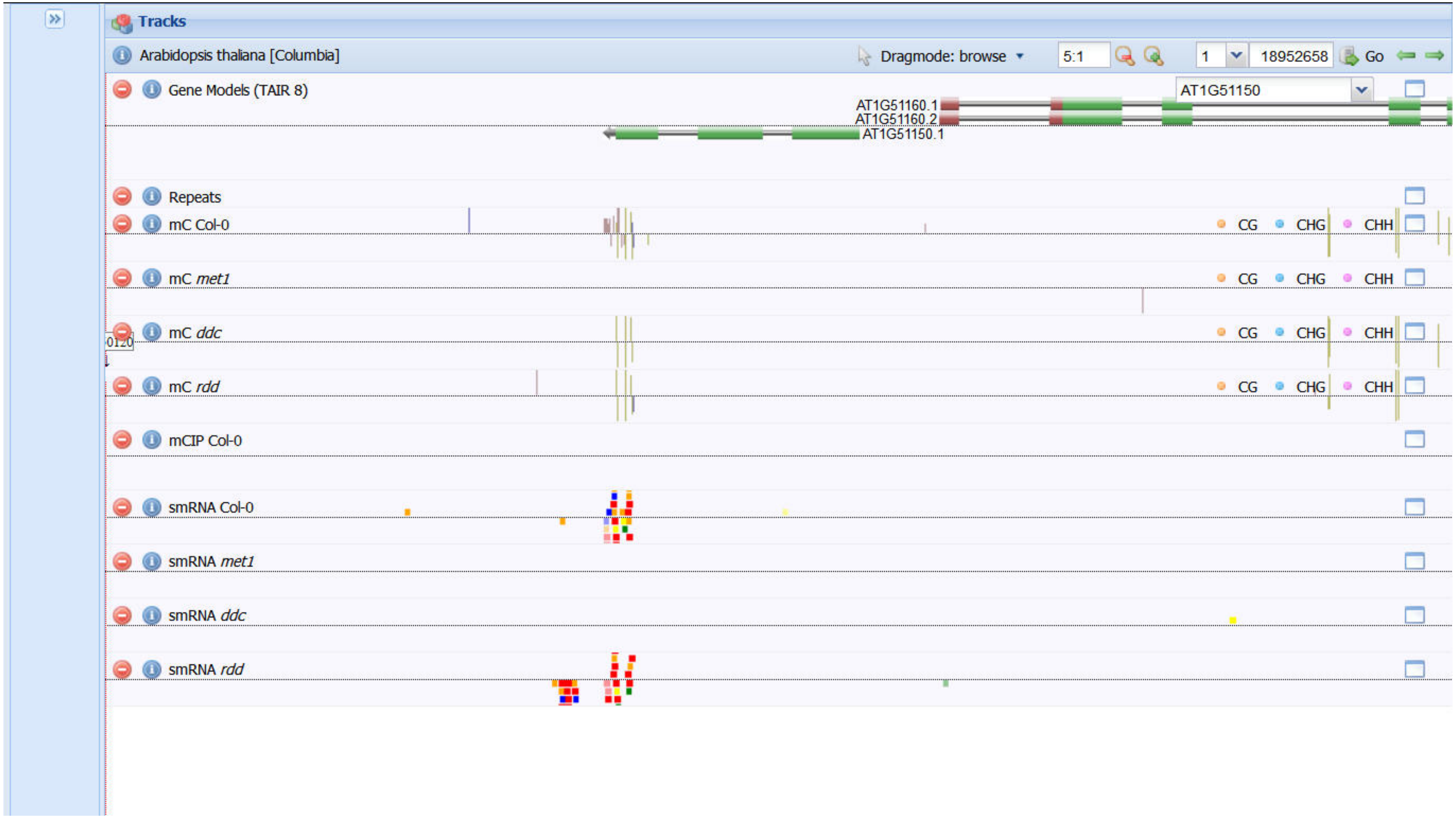

Tracks
Arabidopsis thaliana [Columbia]
Dragmode: browse
5:1
1
18952658
Go
Gene Models (TAIR 8)
AT1G51150
AT1G51160.1
AT1G51160.2
AT1G51150.1
Repeats
mC Col-0
CG
CHG
CHH
mC met1
mC ddc
mC rdd
mCIP Col-0
smRNA Col-0
smRNA met1
smRNA ddc
smRNA rdd

AT1G59885

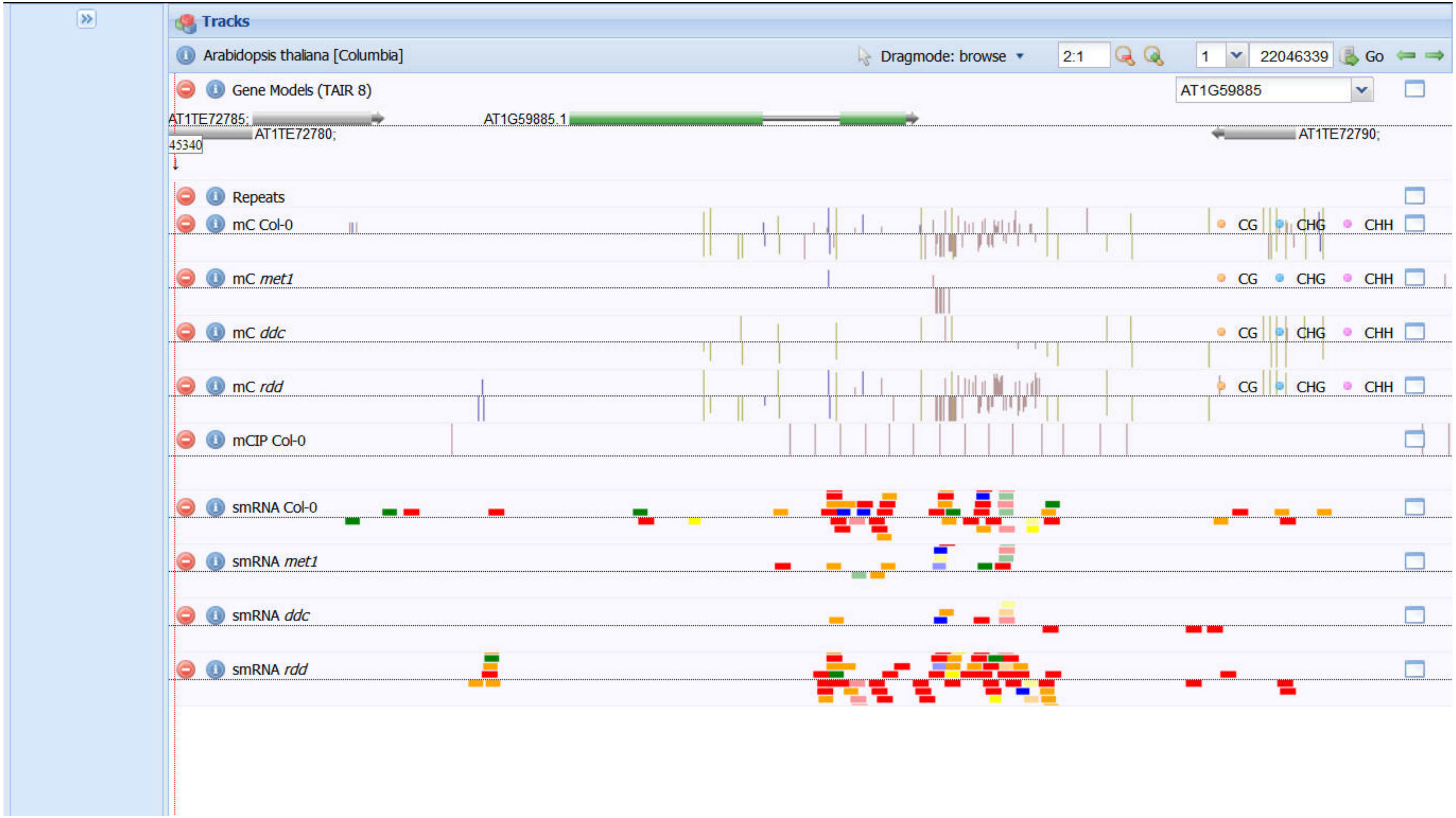

Tracks
Arabidopsis thaliana [Columbia]
Dragmode: browse
2:1
1
22046339
Go
Gene Models (TAIR 8)
AT1G59885
AT1TE72785;
AT1G59885.1
AT1TE72780;
AT1TE72790;
45340
Repeats
mC Col-0
CG
CHG
CHH
mC met1
mC ddc
mC rdd
mCIP Col-0
smRNA Col-0
smRNA met1
smRNA ddc
smRNA rdd

AT1G60986

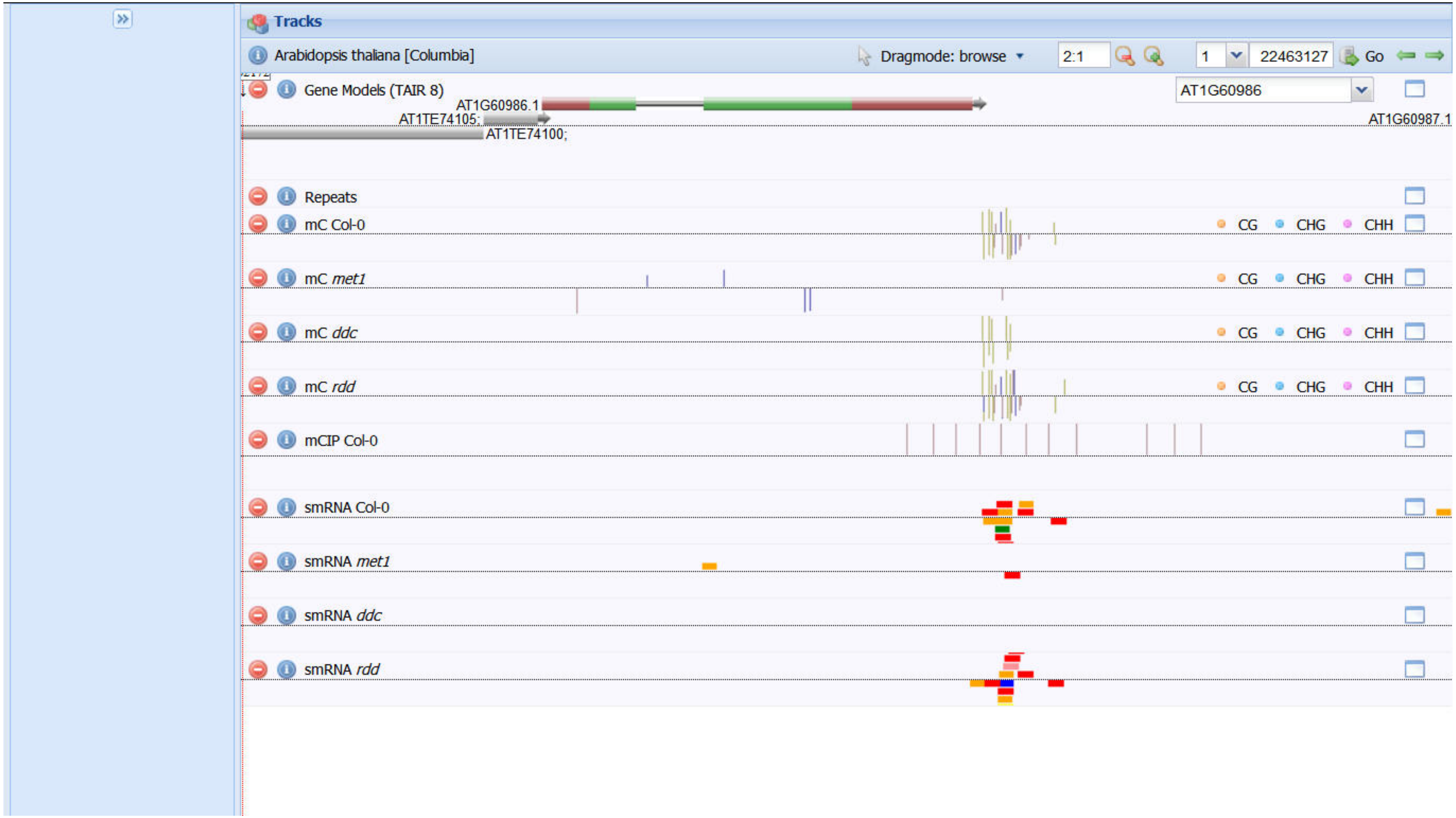
Tracks
Arabidopsis thaliana [Columbia]
Dragmode: browse
2:1
1
22463127
Go
Gene Models (TAIR 8)
AT1G60986
AT1G60986.1
AT1TE74105;
AT1TE74100;
AT1G60987.1
Repeats
mC Col-0
CG CHG CHH
mC met1
CG CHG CHH
mC ddc
CG CHG CHH
mC rdd
CG CHG CHH
mCIP Col-0
smRNA Col-0
smRNA met1
smRNA ddc
smRNA rdd

AT1G68040

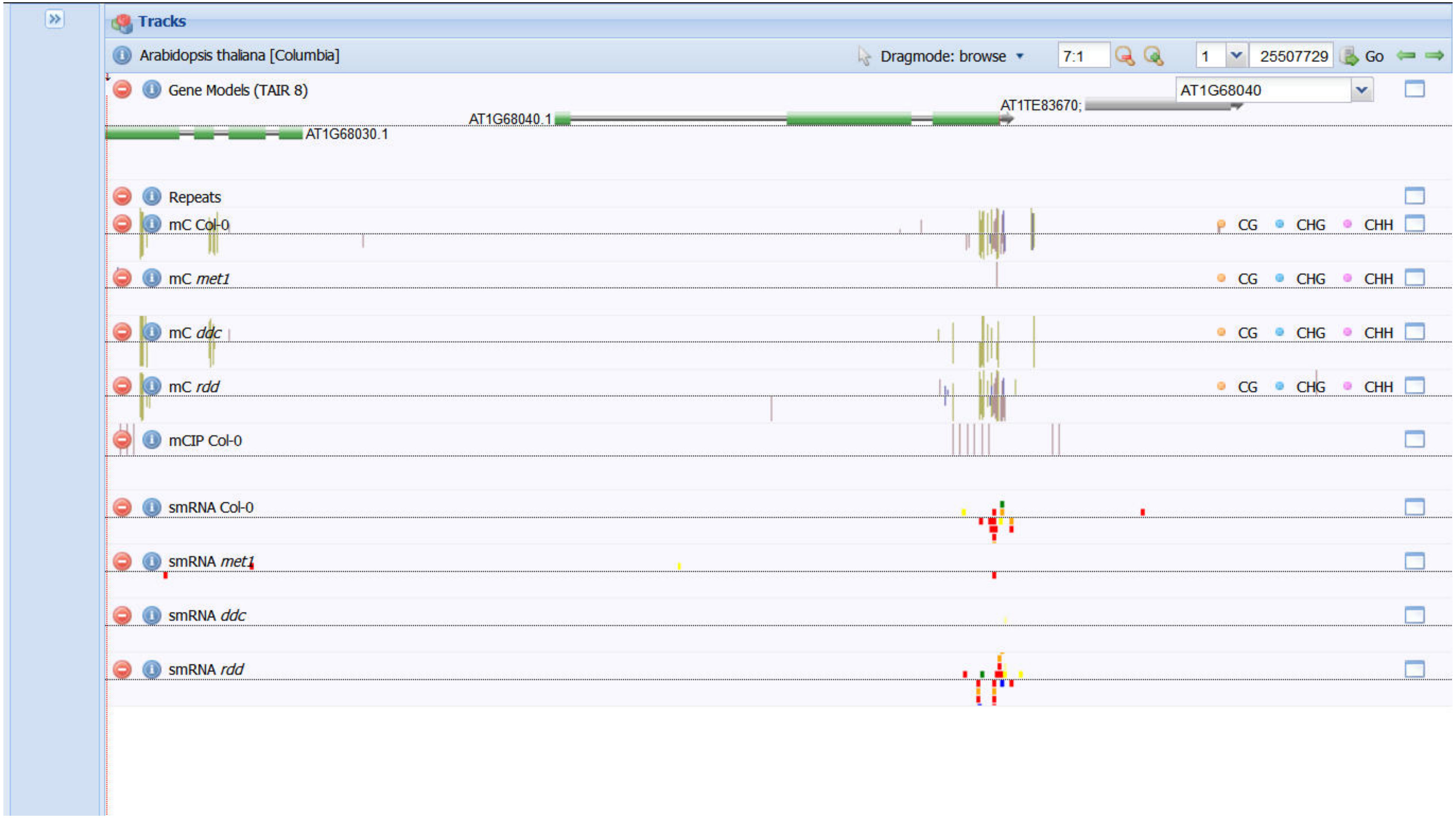
Tracks
Arabidopsis thaliana [Columbia]
Dragmode: browse
7:1
1
25507729
Go
Gene Models (TAIR 8)
AT1G68040
AT1TE83670;
AT1G68040.1
AT1G68030.1
Repeats
mC Col-0
CG CHG CHH
mC met1
CG CHG CHH
mC ddc
CG CHG CHH
mC rdd
CG CHG CHH
mCIP Col-0
smRNA Col-0
smRNA met1
smRNA ddc
smRNA rdd

AT2G22890

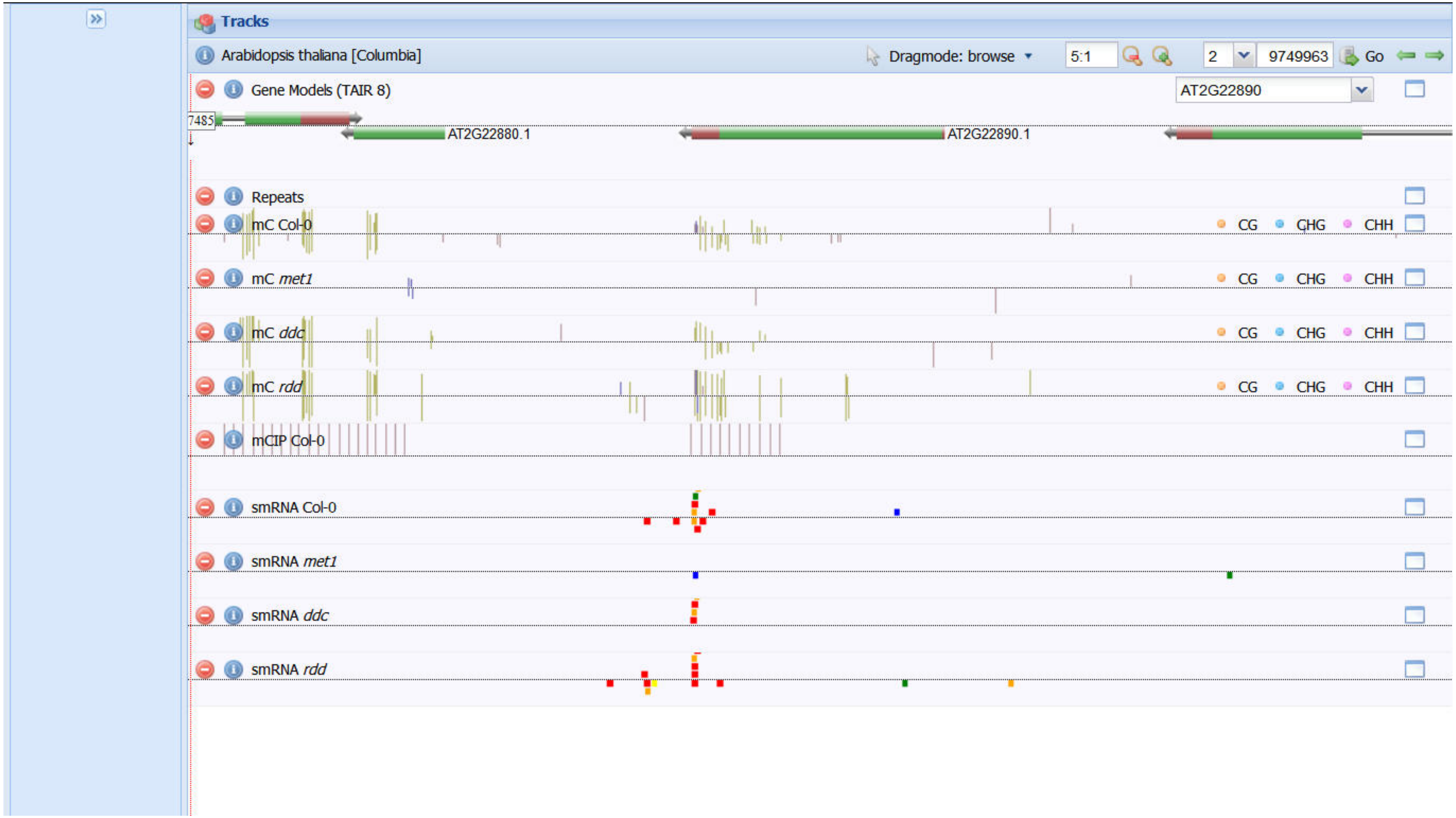

Tracks
Arabidopsis thaliana [Columbia]
Dragmode: browse
5:1
2
9749963
Go
Gene Models (TAIR 8)
AT2G22890
7485
AT2G22880.1
AT2G22890.1
Repeats
mC Col-0
CG CHG CHH
mC met1
CG CHG CHH
mC ddc
CG CHG CHH
mC rdd
CG CHG CHH
mCIP Col-0
smRNA Col-0
smRNA met1
smRNA ddc
smRNA rdd

AT2G35250

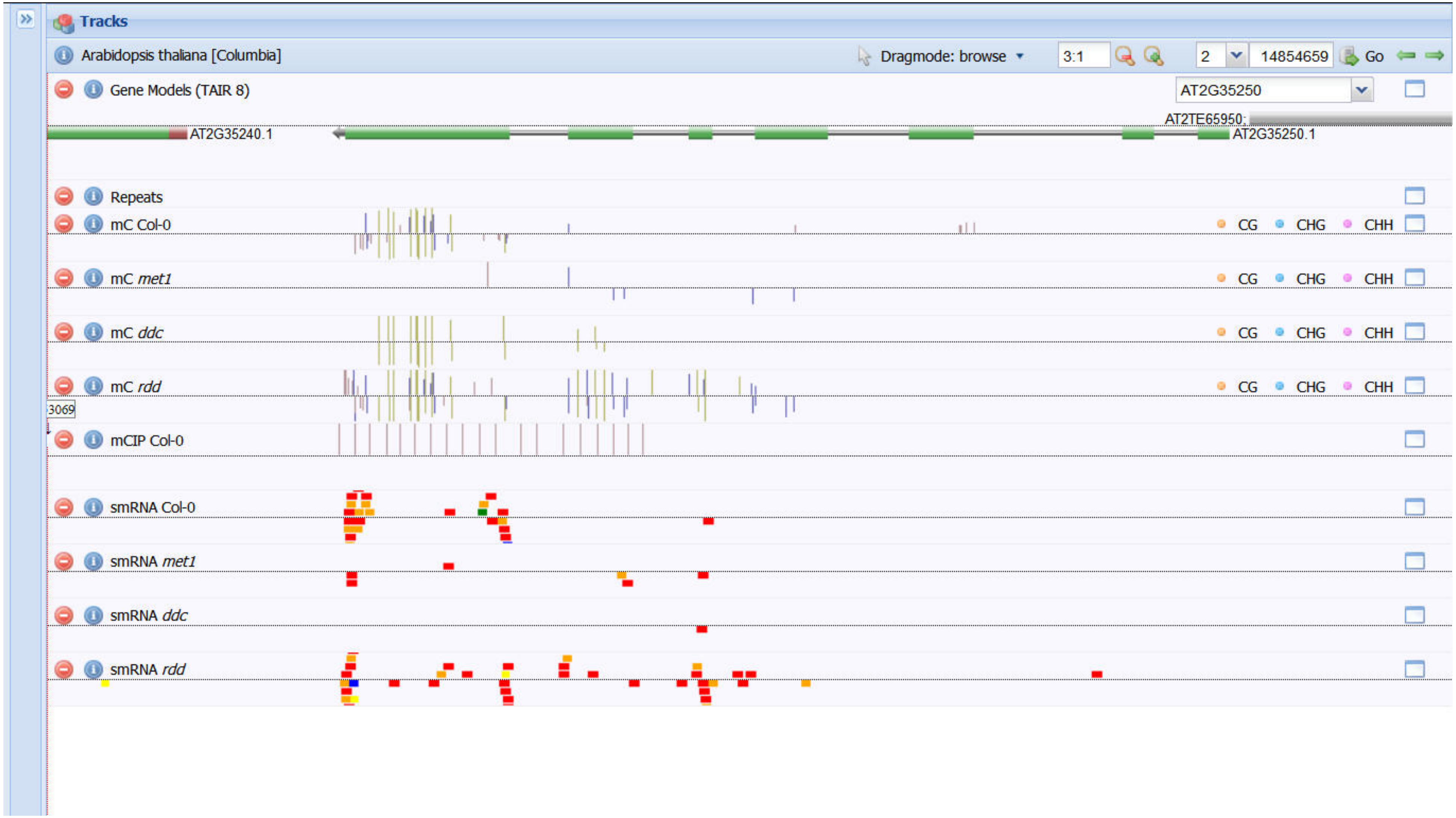

Tracks
Arabidopsis thaliana [Columbia]
Dragmode: browse
3:1
2
14854659
Go
Gene Models (TAIR 8)
AT2G35250
AT2TE65950
AT2G35240.1
AT2G35250.1
Repeats
mC Col-0
CG
CHG
CHH
mC met1
mC ddc
mC rdd
3069
mCIP Col-0
smRNA Col-0
smRNA met1
smRNA ddc
smRNA rdd

AT3G09960

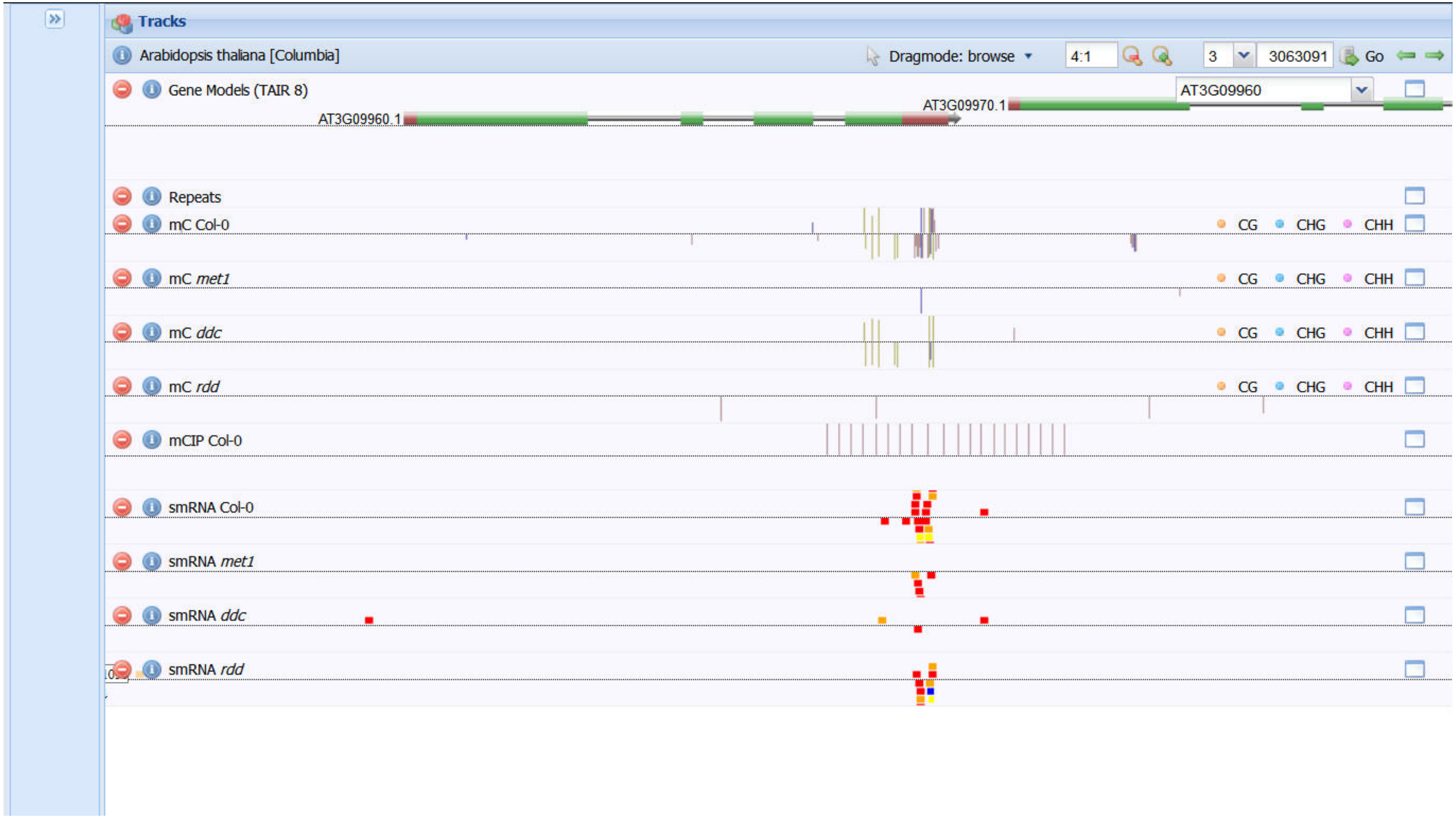

Tracks
Arabidopsis thaliana [Columbia]
Dragmode: browse
4:1
3
3063091
Go
Gene Models (TAIR 8)
AT3G09960
AT3G09970.1
AT3G09960.1
Repeats
mC Col-0
CG
CHG
CHH
mC met1
mC ddc
mC rdd
mCIP Col-0
smRNA Col-0
smRNA met1
smRNA ddc
smRNA rdd

AT3G21870

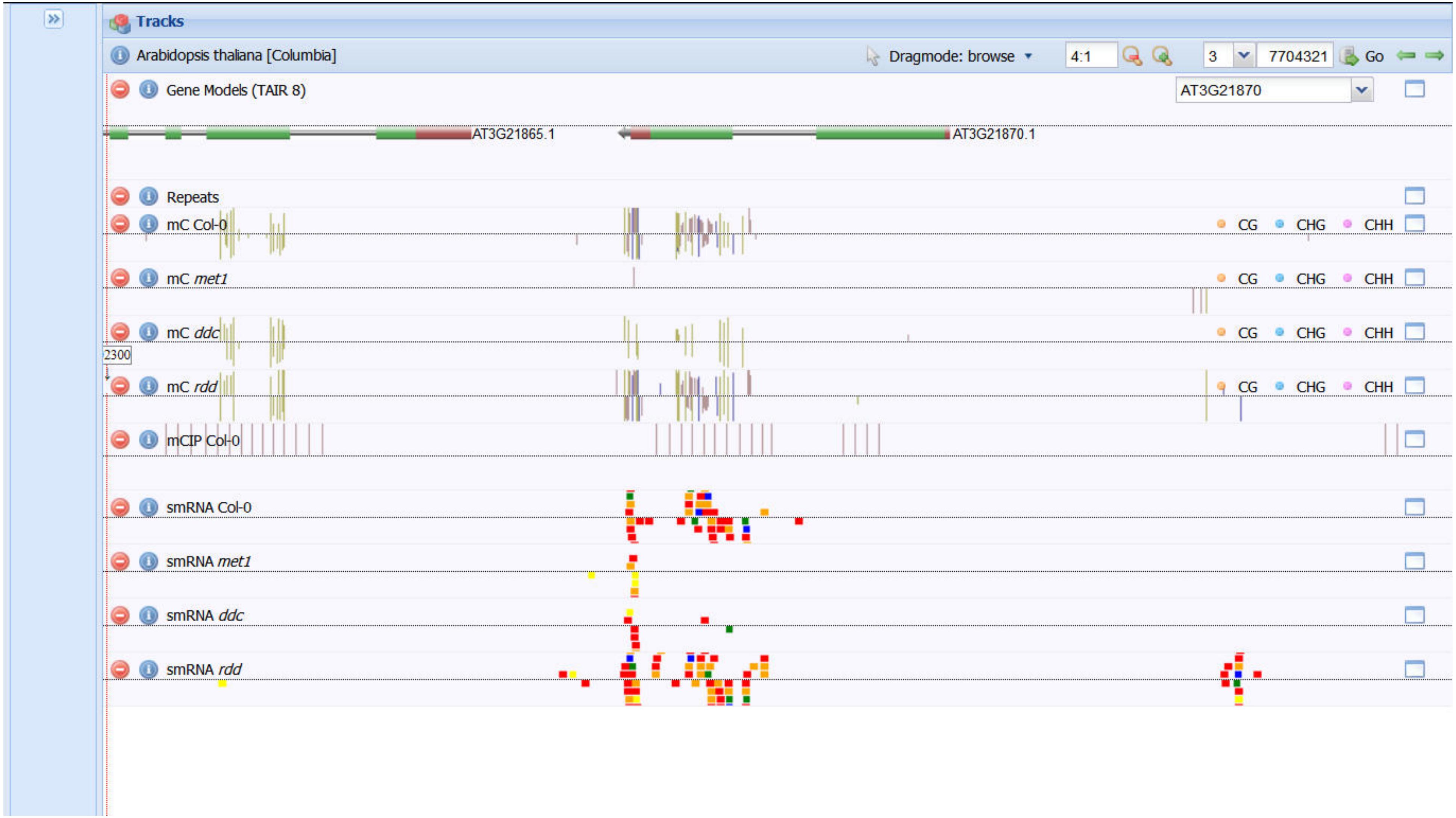

Tracks
Arabidopsis thaliana [Columbia]
Dragmode: browse
4:1
3
7704321
Go
Gene Models (TAIR 8)
AT3G21870
AT3G21865.1
AT3G21870.1
Repeats
mC Col-0
CG CHG CHH
mC met1
mC ddc
2300
mC rdd
mCIP Col-0
smRNA Col-0
smRNA met1
smRNA ddc
smRNA rdd

AT3G25130

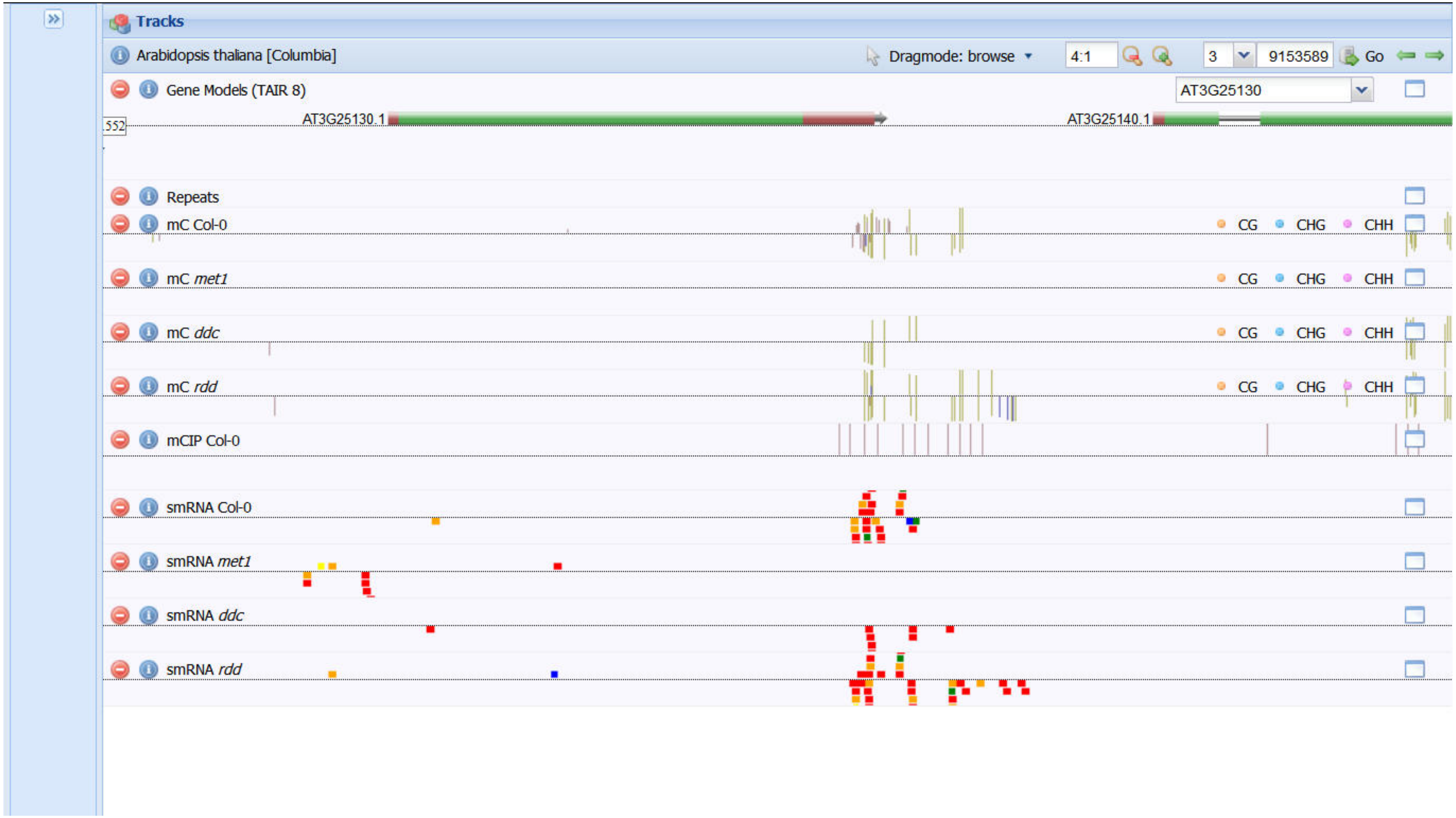

Tracks
Arabidopsis thaliana [Columbia]
Dragmode: browse
4:1
3
9153589
Go
Gene Models (TAIR 8)
AT3G25130
AT3G25130.1
AT3G25140.1
552
Repeats
mC Col-0
CG
CHG
CHH
mC met1
mC ddc
mC rdd
mCIP Col-0
smRNA Col-0
smRNA met1
smRNA ddc
smRNA rdd

AT3G25855

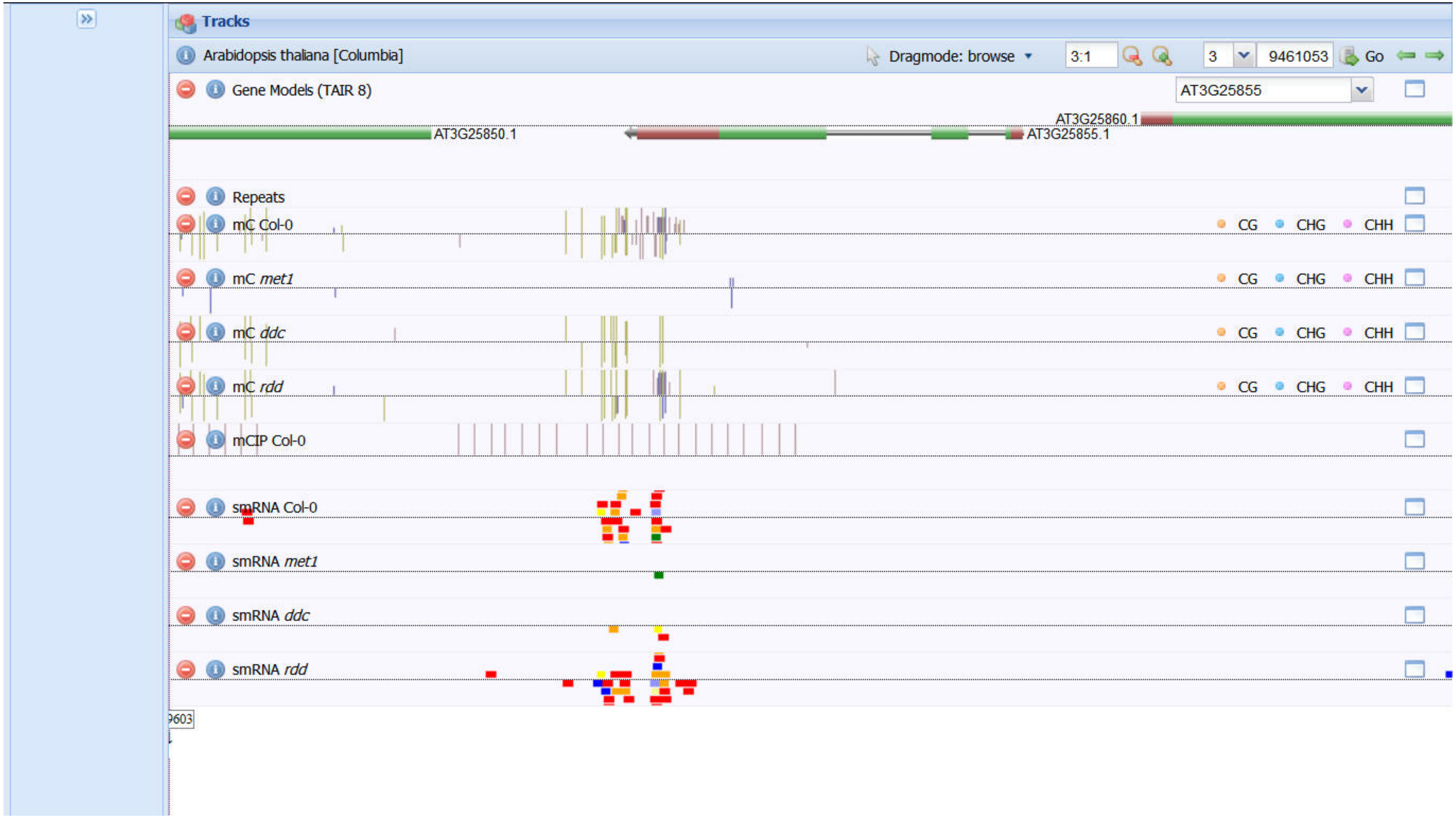

Tracks
Arabidopsis thaliana [Columbia]
Dragmode: browse
3:1
3
9461053
Go
Gene Models (TAIR 8)
AT3G25855
AT3G25860.1
AT3G25850.1
AT3G25855.1
Repeats
mC Col-0
CG
CHG
CHH
mC met1
mC ddc
mC rdd
mCIP Col-0
smRNA Col-0
smRNA met1
smRNA ddc
smRNA rdd

AT3G27250

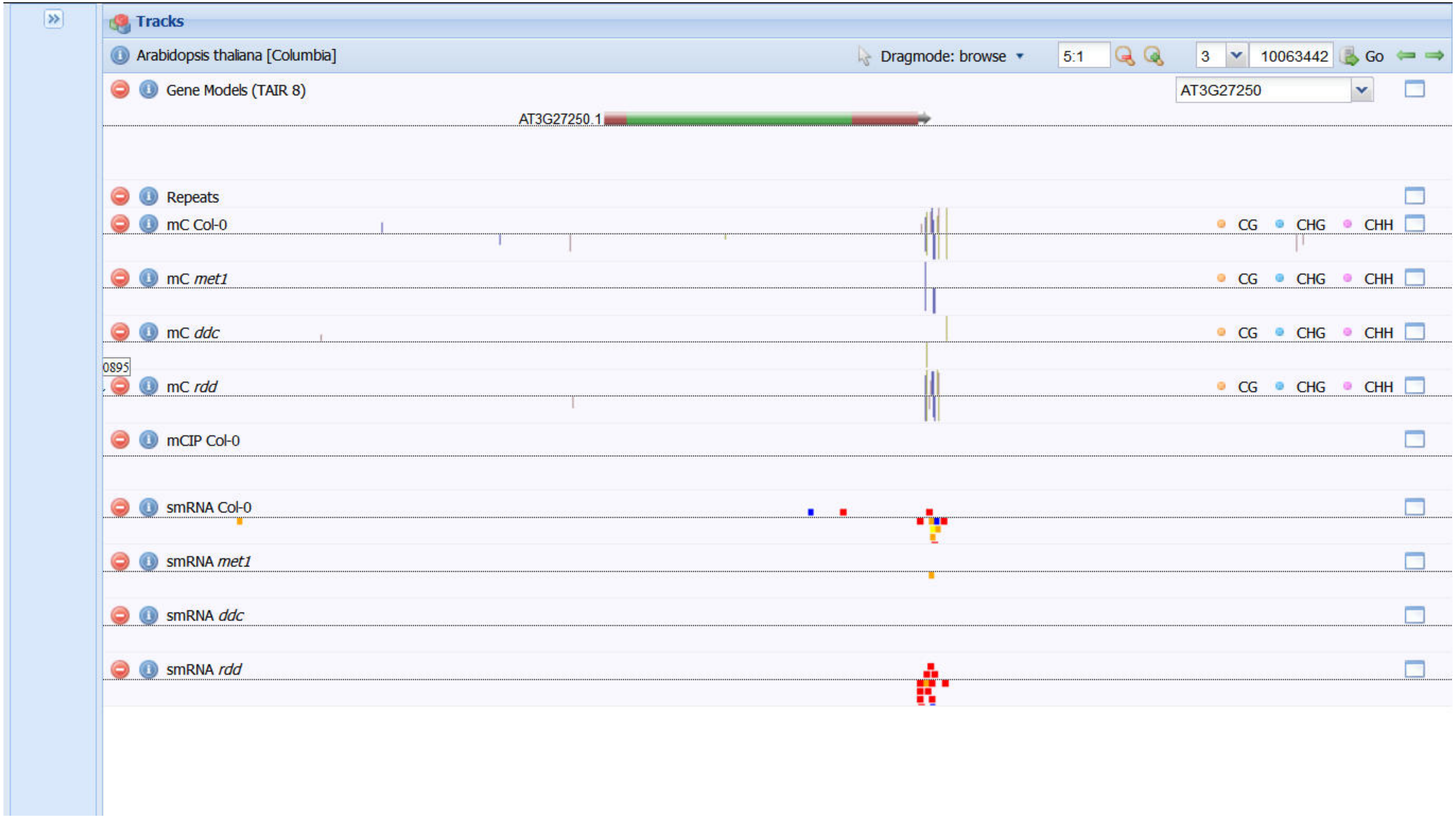

Tracks
Arabidopsis thaliana [Columbia]
Dragmode: browse
5:1
3
10063442
Go
Gene Models (TAIR 8)
AT3G27250
AT3G27250.1
Repeats
mC Col-0
CG CHG CHH
mC met1
mC ddc
mC rdd
0895
mCIP Col-0
smRNA Col-0
smRNA met1
smRNA ddc
smRNA rdd

AT4G14365

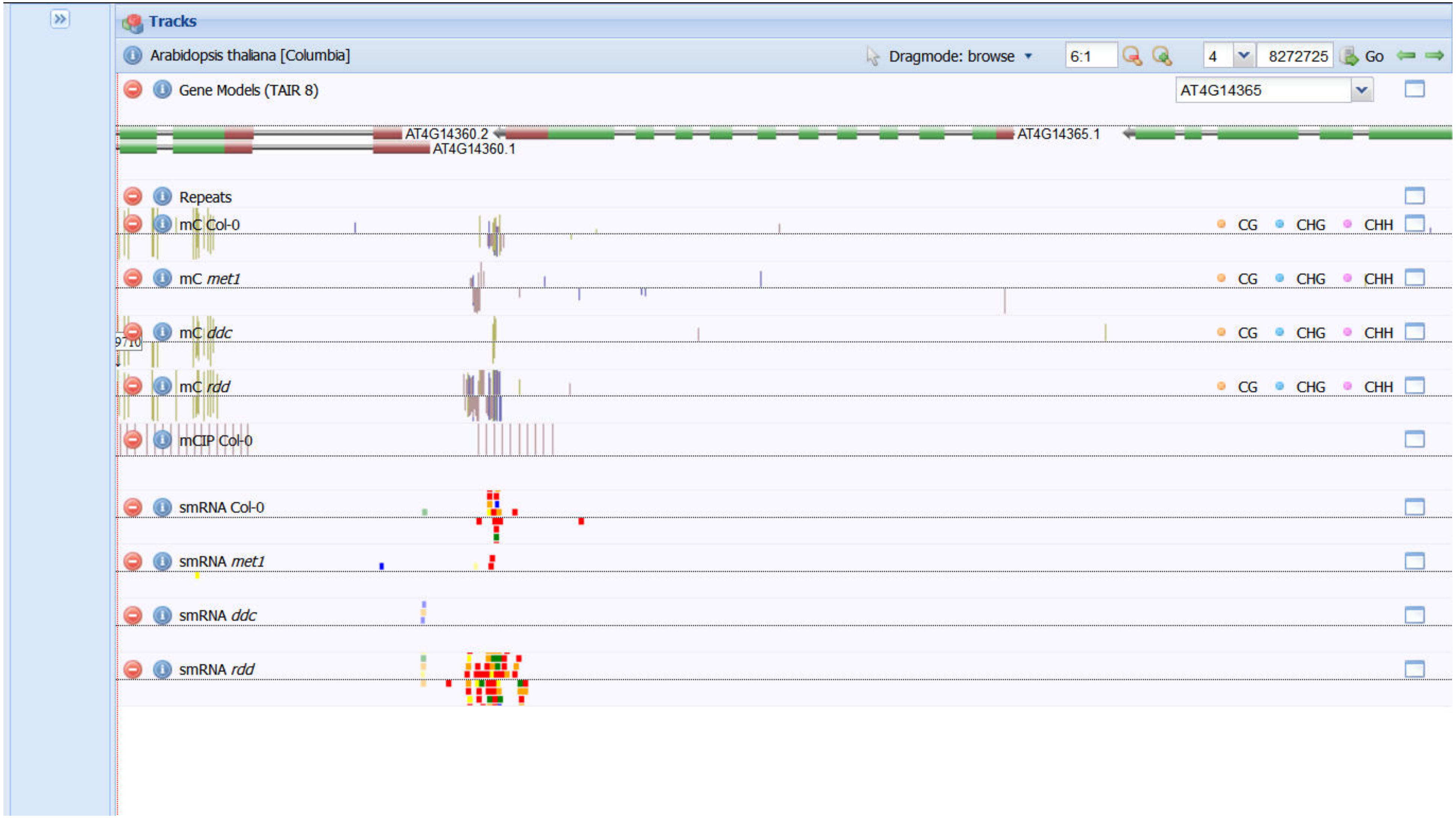

Tracks
Arabidopsis thaliana [Columbia]
Dragmode: browse
6:1
4
8272725
Go
Gene Models (TAIR 8)
AT4G14365
AT4G14360.2
AT4G14360.1
AT4G14365.1
Repeats
mC Col-0
CG CHG CHH
mC met1
CG CHG CHH
mC ddc
CG CHG CHH
mC rdd
CG CHG CHH
mCIP Col-0
smRNA Col-0
smRNA met1
smRNA ddc
smRNA rdd

AT4G14940

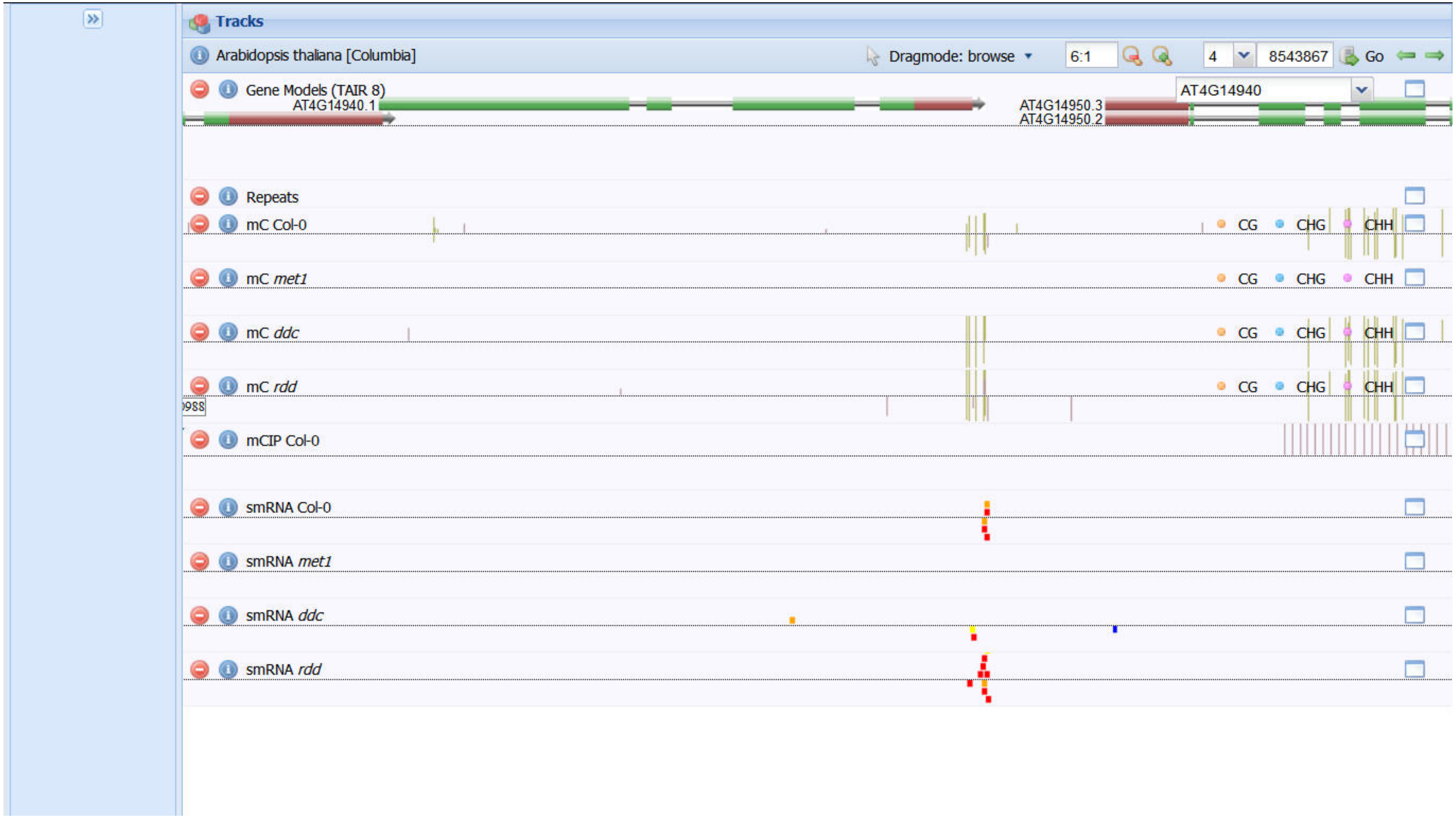

Tracks
Arabidopsis thaliana [Columbia]
Dragmode: browse
6:1
4
8543867
Go
Gene Models (TAIR 8)
AT4G14940
AT4G14940.1
AT4G14950.3
AT4G14950.2
Repeats
mC Col-0
CG
CHG
CHH
mC met1
mC ddc
mC rdd
mCIP Col-0
smRNA Col-0
smRNA met1
smRNA ddc
smRNA rdd

AT4G18690

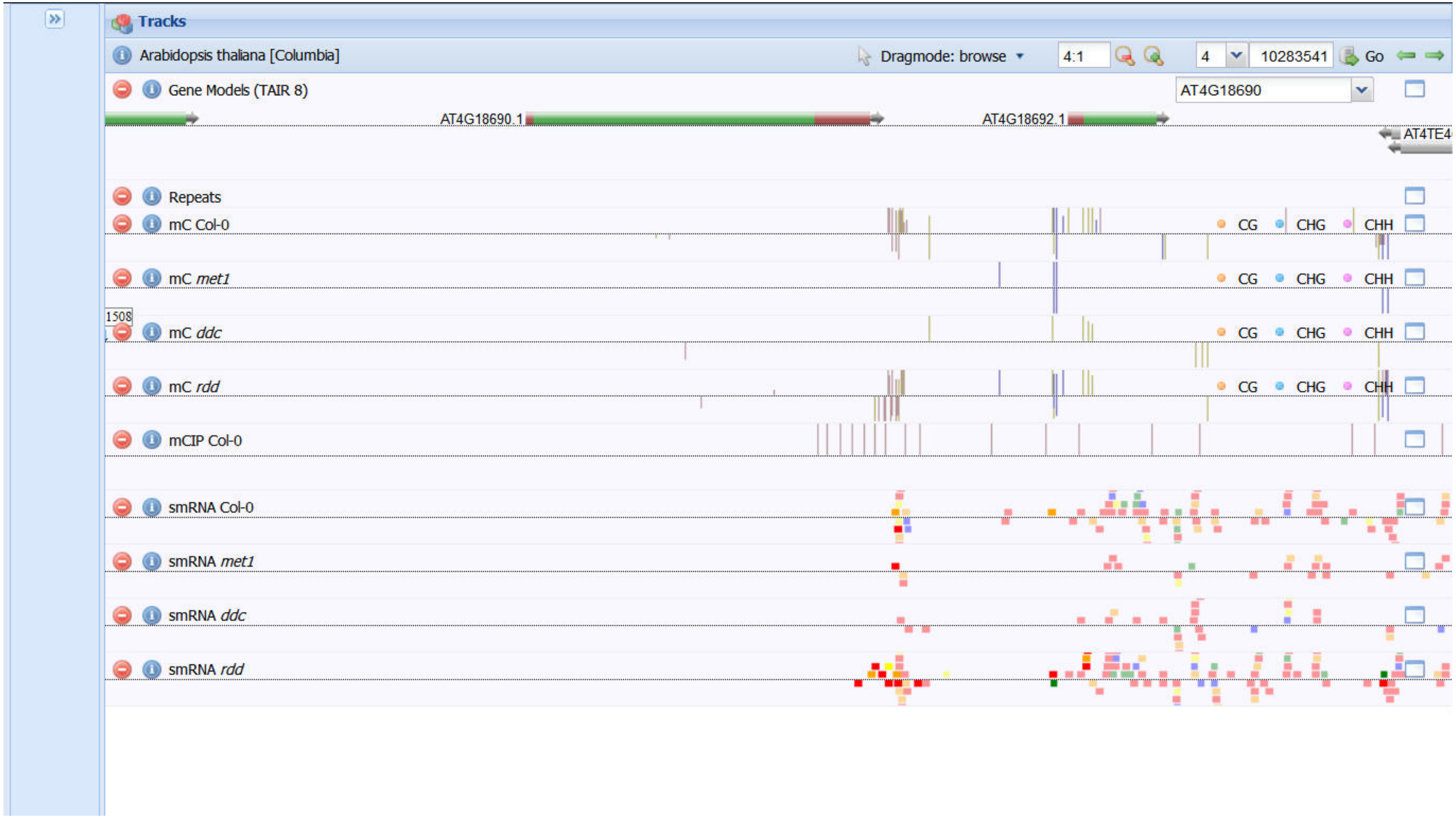

Tracks
Arabidopsis thaliana [Columbia]
Dragmode: browse
4:1
4
10283541
Go
Gene Models (TAIR 8)
AT4G18690
AT4G18690.1
AT4G18692.1
Repeats
mC Col-0
CG
CHG
CHH
mC met1
1508
mC ddc
mC rdd
mCIP Col-0
smRNA Col-0
smRNA met1
smRNA ddc
smRNA rdd

AT4G22650

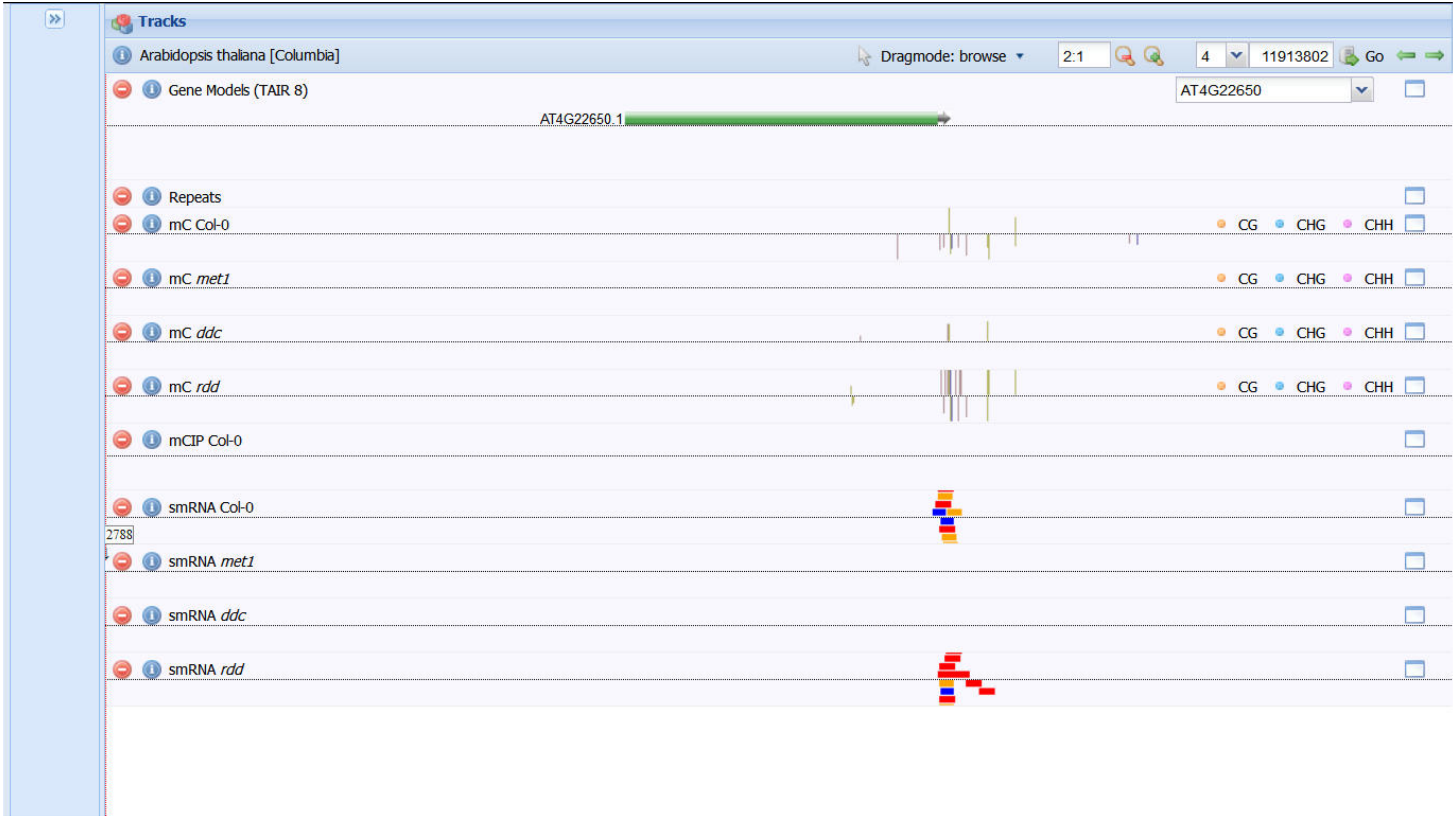

Tracks
Arabidopsis thaliana [Columbia]
Dragmode: browse
2:1
4
11913802
Go
Gene Models (TAIR 8)
AT4G22650
AT4G22650.1
Repeats
mC Col-0
CG CHG CHH
mC met1
CG CHG CHH
mC ddc
CG CHG CHH
mC rdd
CG CHG CHH
mCIP Col-0
smRNA Col-0
2788
smRNA met1
smRNA ddc
smRNA rdd

AT4G28850

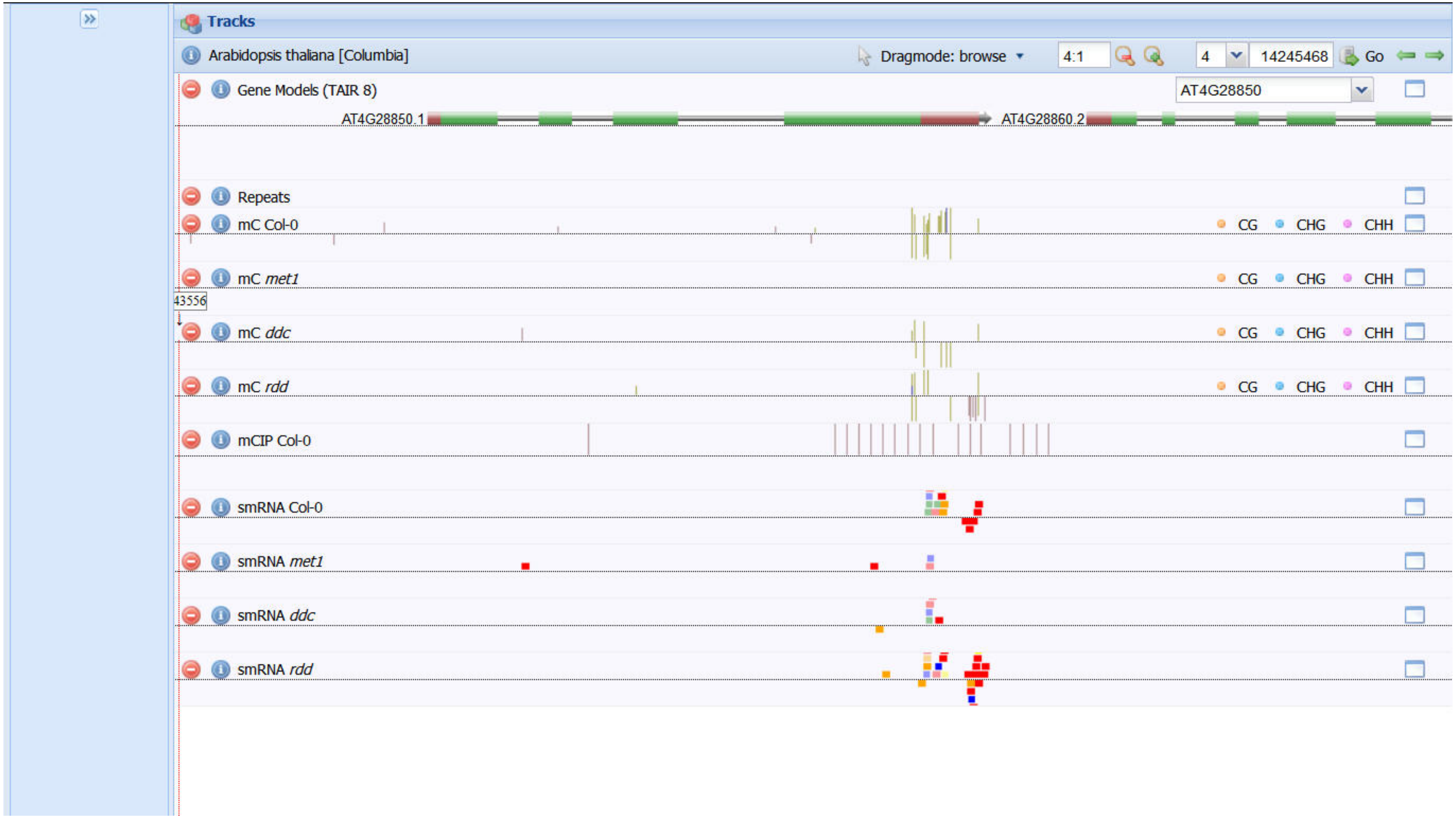

Tracks
Arabidopsis thaliana [Columbia]
Dragmode: browse
4:1
4
14245468
Go
Gene Models (TAIR 8)
AT4G28850
AT4G28850.1
AT4G28860.2
Repeats
mC Col-0
CG
CHG
CHH
mC met1
43556
mC ddc
mC rdd
mCIP Col-0
smRNA Col-0
smRNA met1
smRNA ddc
smRNA rdd

AT4G29290

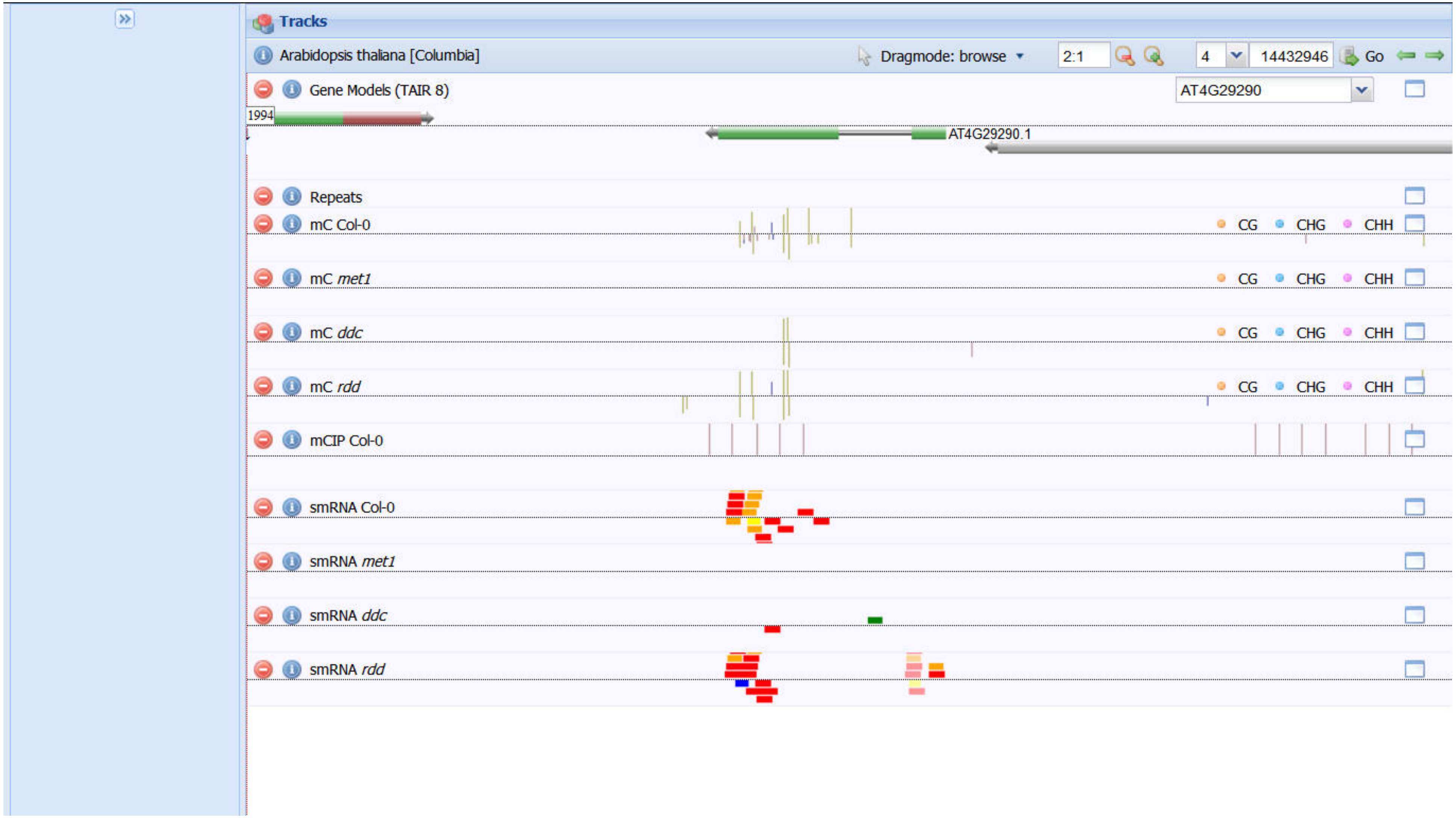
Tracks
Arabidopsis thaliana [Columbia]
Dragmode: browse
2:1
4
14432946
Go
Gene Models (TAIR 8)
AT4G29290
1994
AT4G29290.1
Repeats
mC Col-0
CG
CHG
CHH
mC met1
mC ddc
mC rdd
mCIP Col-0
smRNA Col-0
smRNA met1
smRNA ddc
smRNA rdd

AT4G29305

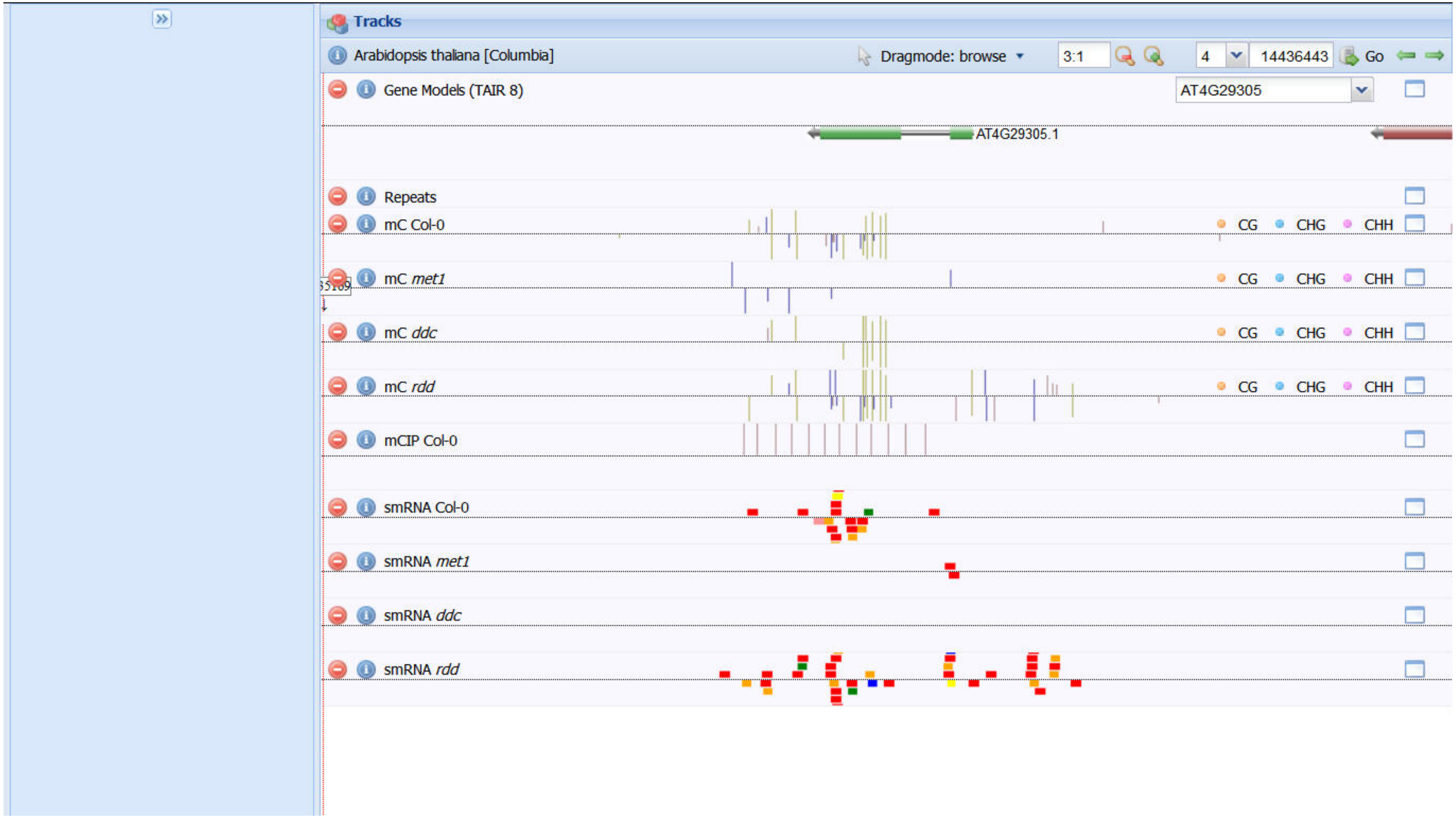

Tracks
Arabidopsis thaliana [Columbia]
Dragmode: browse
3:1
4
14436443
Go
Gene Models (TAIR 8)
AT4G29305
AT4G29305.1
Repeats
mC Col-0
CG
CHG
CHH
mC *met1*
mC *ddc*
mC *rdd*
mCIP Col-0
smRNA Col-0
smRNA *met1*
smRNA *ddc*
smRNA *rdd*

AT4G29740

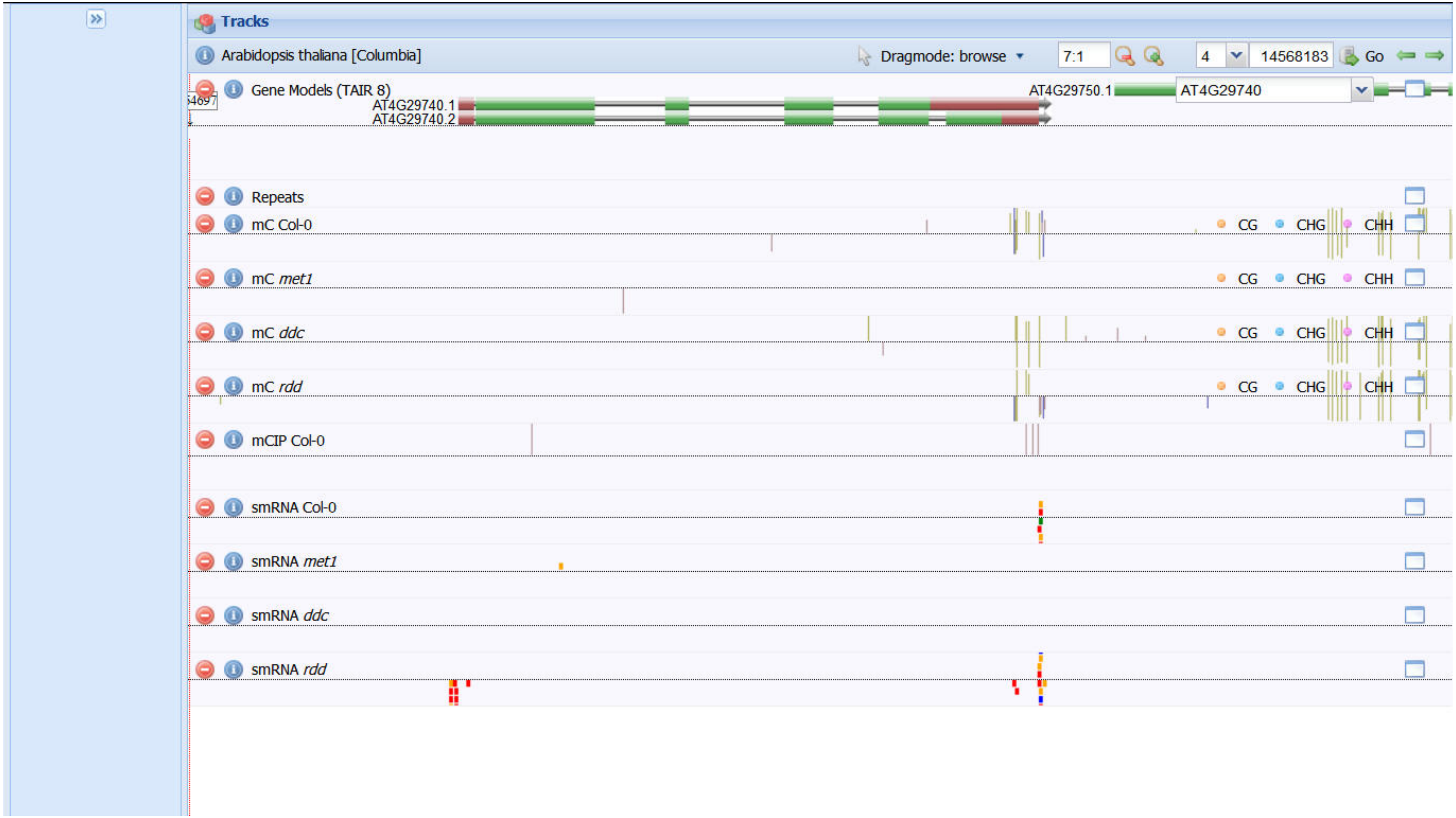

Tracks
Arabidopsis thaliana [Columbia]
Dragmode: browse
7:1
4
14568183
Go
Gene Models (TAIR 8)
AT4G29740.1
AT4G29740.2
AT4G29750.1
AT4G29740
Repeats
mC Col-0
CG
CHG
CHH
mC met1
mC ddc
mC rdd
mCIP Col-0
smRNA Col-0
smRNA met1
smRNA ddc
smRNA rdd

AT5G02990

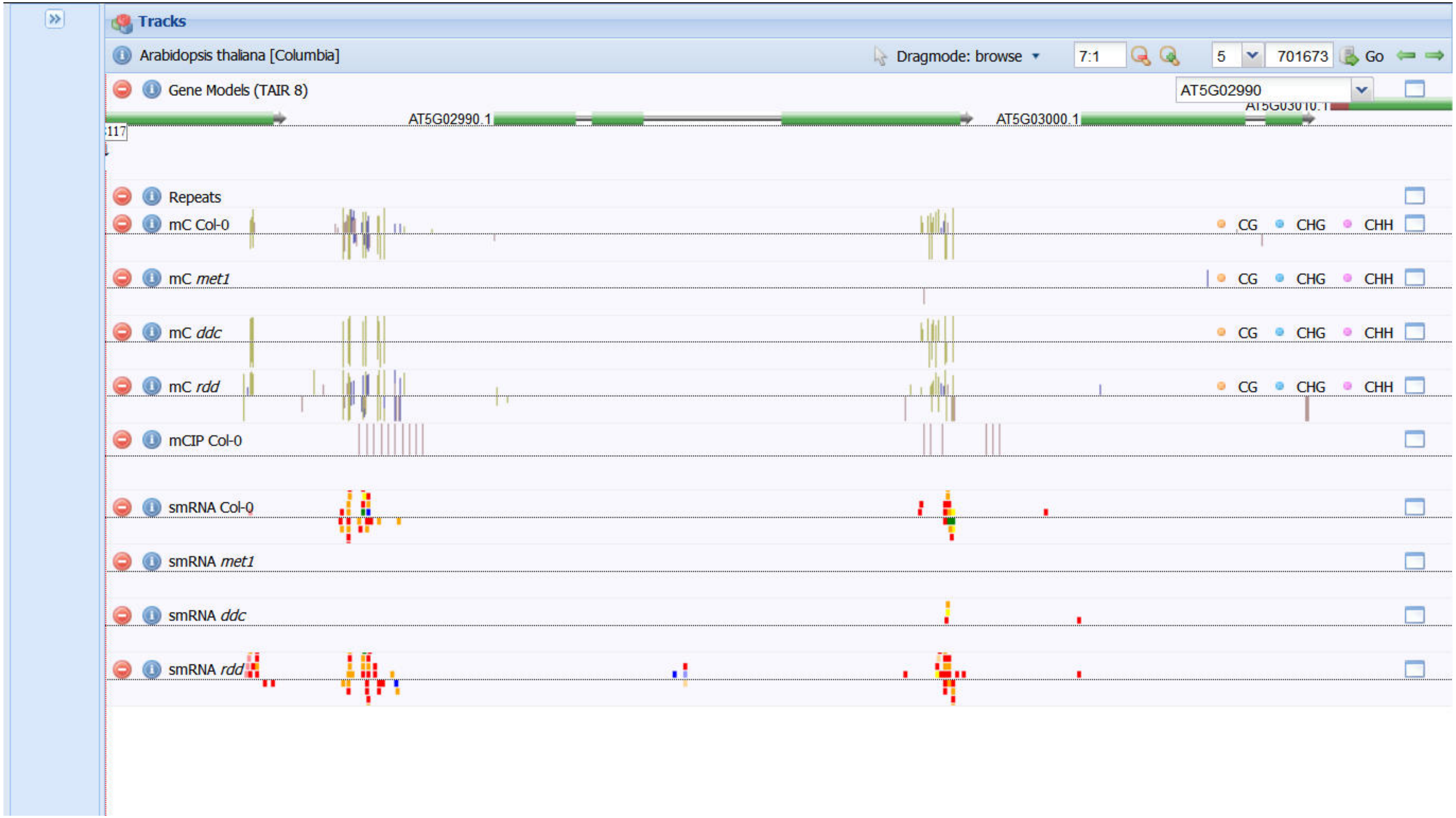
Tracks
Arabidopsis thaliana [Columbia]
Dragmode: browse
7:1
5
701673
Go
Gene Models (TAIR 8)
AT5G02990
AT5G02990.1
AT5G03000.1
AT5G03010.1
Repeats
mC Col-0
mC met1
mC ddc
mC rdd
CG
CHG
CHH
mCIP Col-0
smRNA Col-0
smRNA met1
smRNA ddc
smRNA rdd

AT5G04950

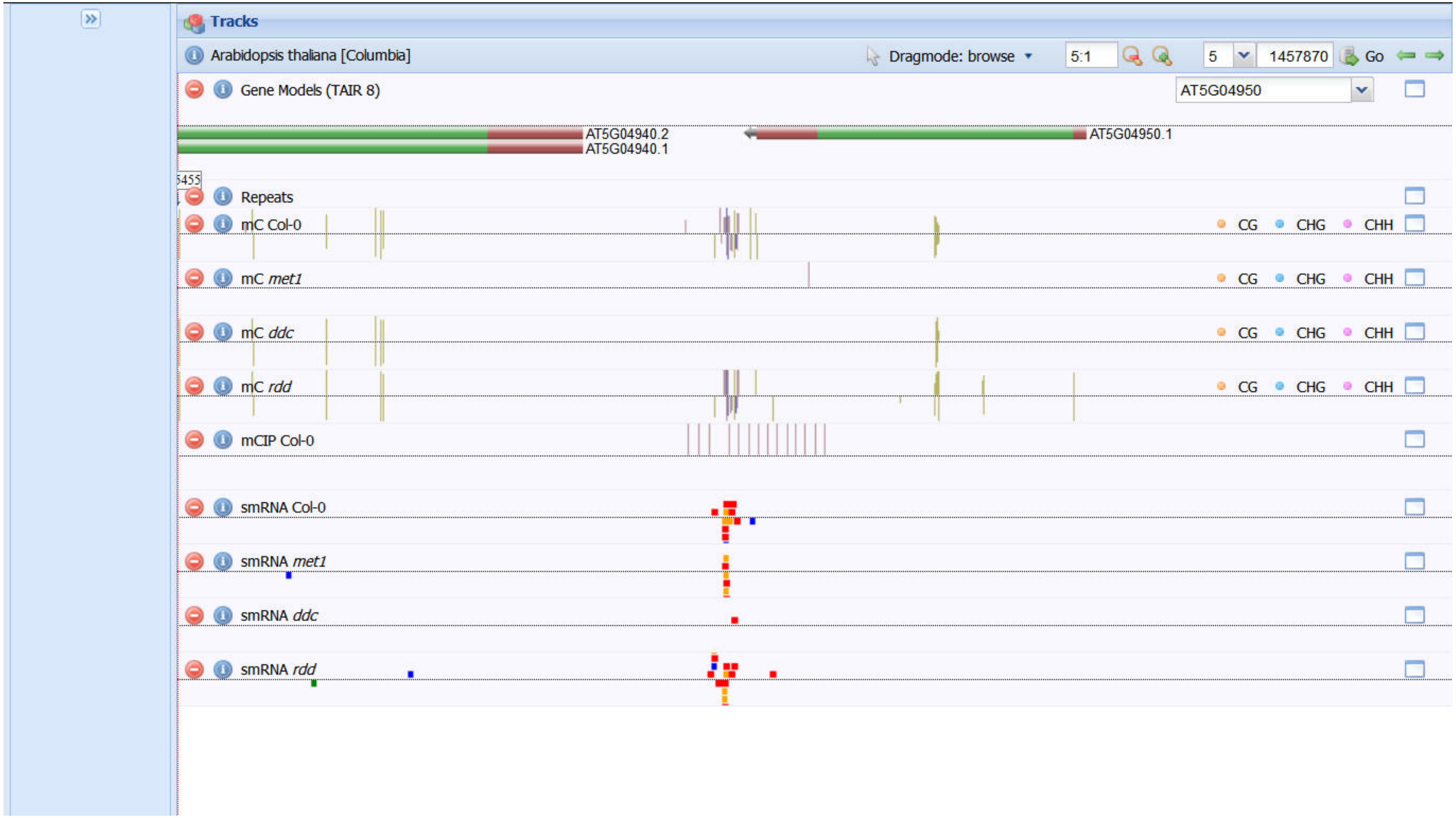
Tracks
Arabidopsis thaliana [Columbia]
Dragmode: browse
5:1
5
1457870
Go
Gene Models (TAIR 8)
AT5G04950
AT5G04940.2
AT5G04940.1
AT5G04950.1
Repeats
mC Col-0
CG
CHG
CHH
mC met1
mC ddc
mC rdd
mCIP Col-0
smRNA Col-0
smRNA met1
smRNA ddc
smRNA rdd

AT5G13825

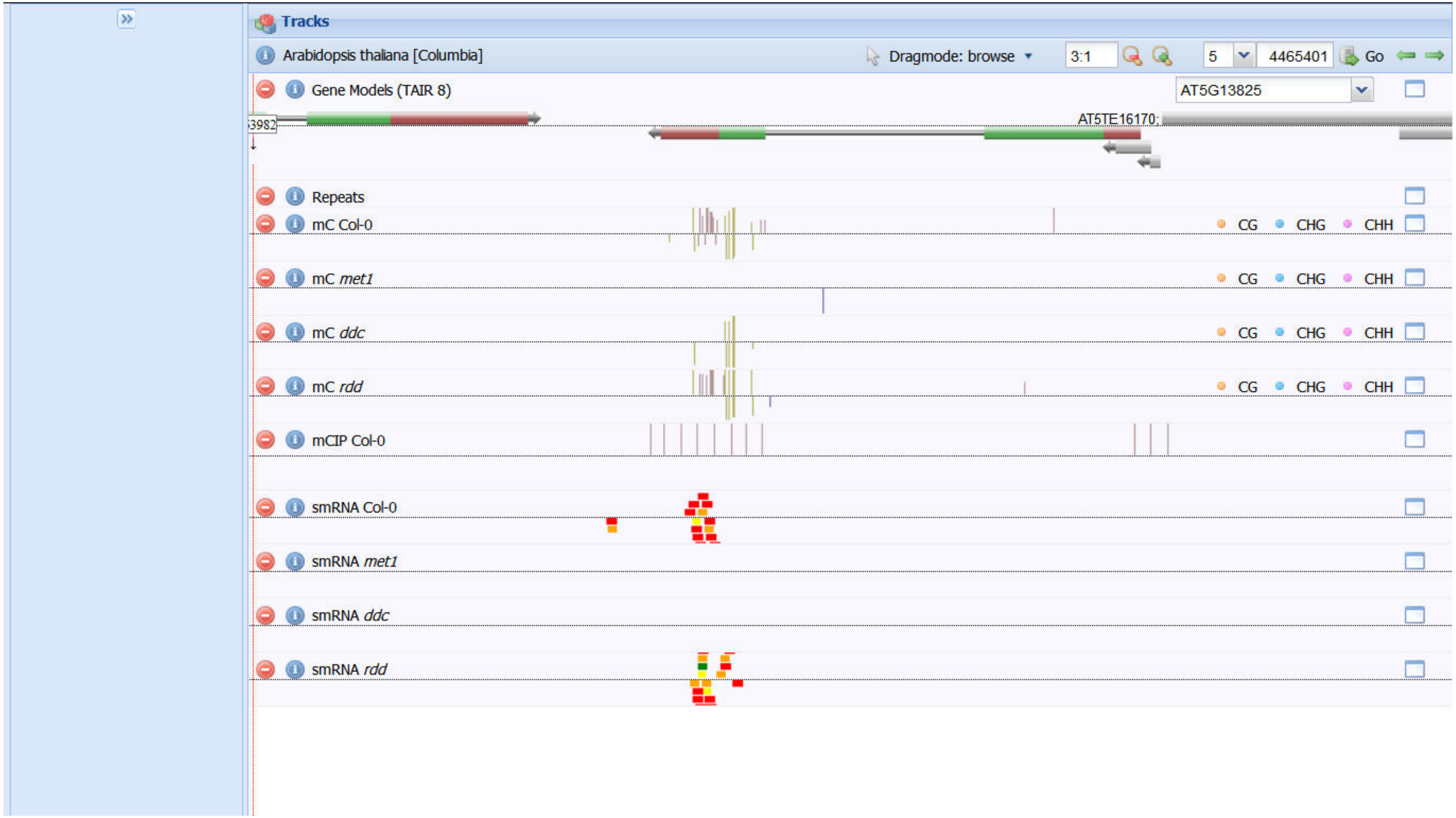
Tracks
Arabidopsis thaliana [Columbia]
Dragmode: browse
3:1
5
4465401
Go
Gene Models (TAIR 8)
AT5G13825
AT5TE16170:
Repeats
mC Col-0
CG CHG CHH
mC met1
CG CHG CHH
mC ddc
CG CHG CHH
mC rdd
CG CHG CHH
mCIP Col-0
smRNA Col-0
smRNA met1
smRNA ddc
smRNA rdd

AT5G24290

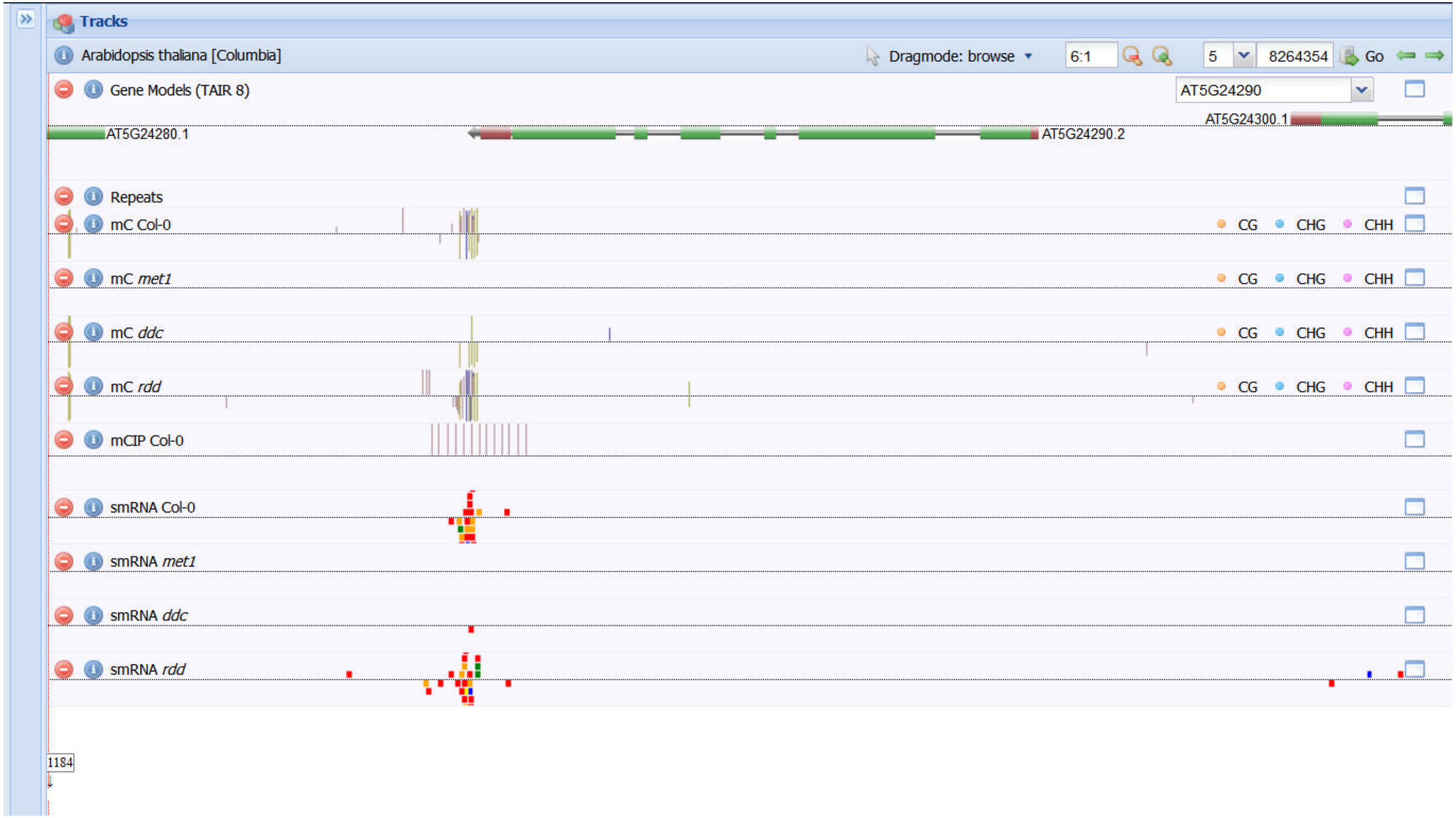
Tracks
Arabidopsis thaliana [Columbia]
Dragmode: browse
6:1
5
8264354
Go
Gene Models (TAIR 8)
AT5G24290
AT5G24280.1
AT5G24290.2
AT5G24300.1
Repeats
mC Col-0
CG CHG CHH
mC met1
CG CHG CHH
mC ddc
CG CHG CHH
mC rdd
CG CHG CHH
mCIP Col-0
smRNA Col-0
smRNA met1
smRNA ddc
smRNA rdd
1184

AT5G39720

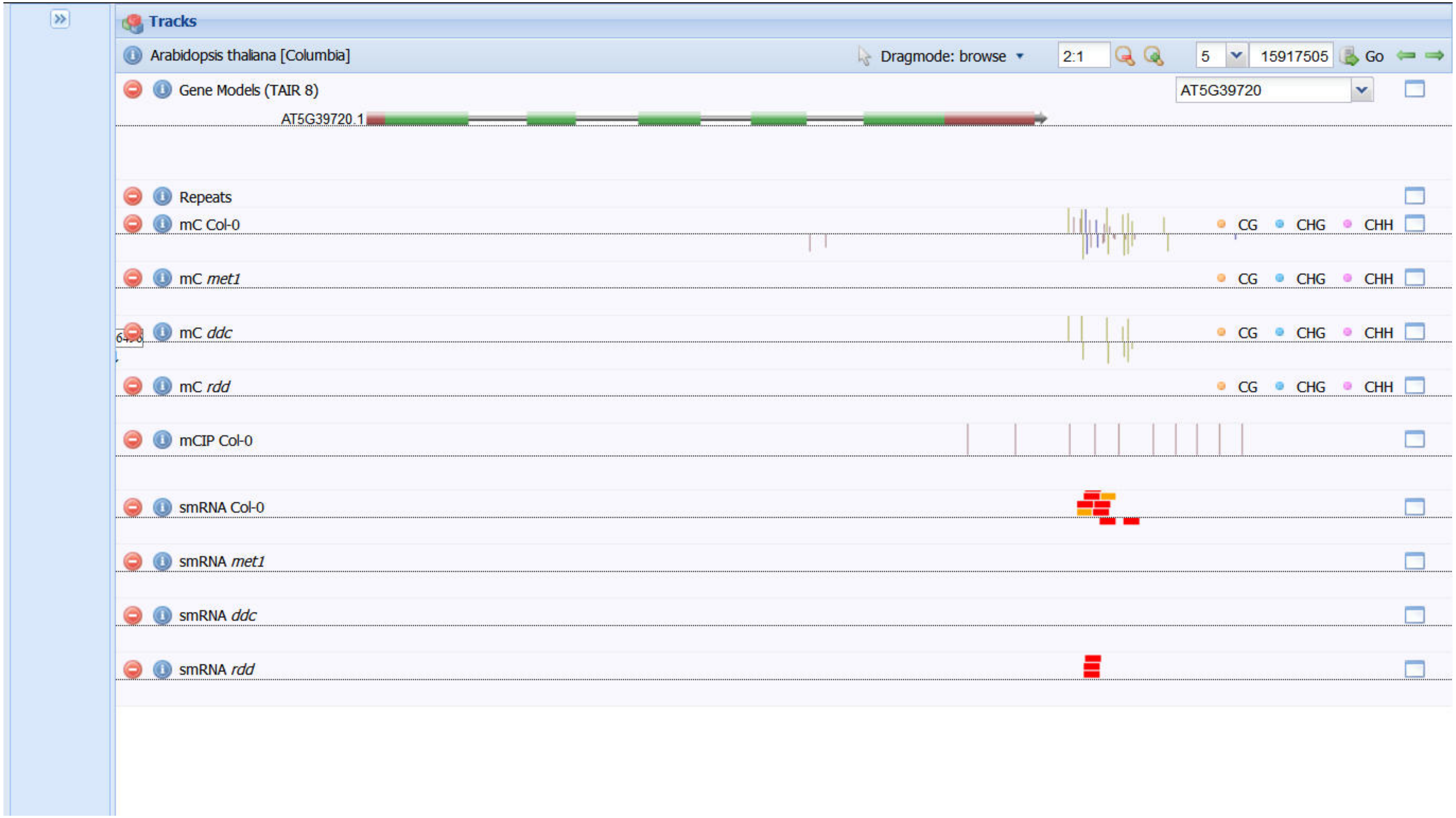

Tracks
Arabidopsis thaliana [Columbia]
Dragmode: browse
2:1
5
15917505
Go
Gene Models (TAIR 8)
AT5G39720
AT5G39720.1
Repeats
mC Col-0
CG
CHG
CHH
mC met1
mC ddc
mC rdd
mCIP Col-0
smRNA Col-0
smRNA met1
smRNA ddc
smRNA rdd

AT5G43755

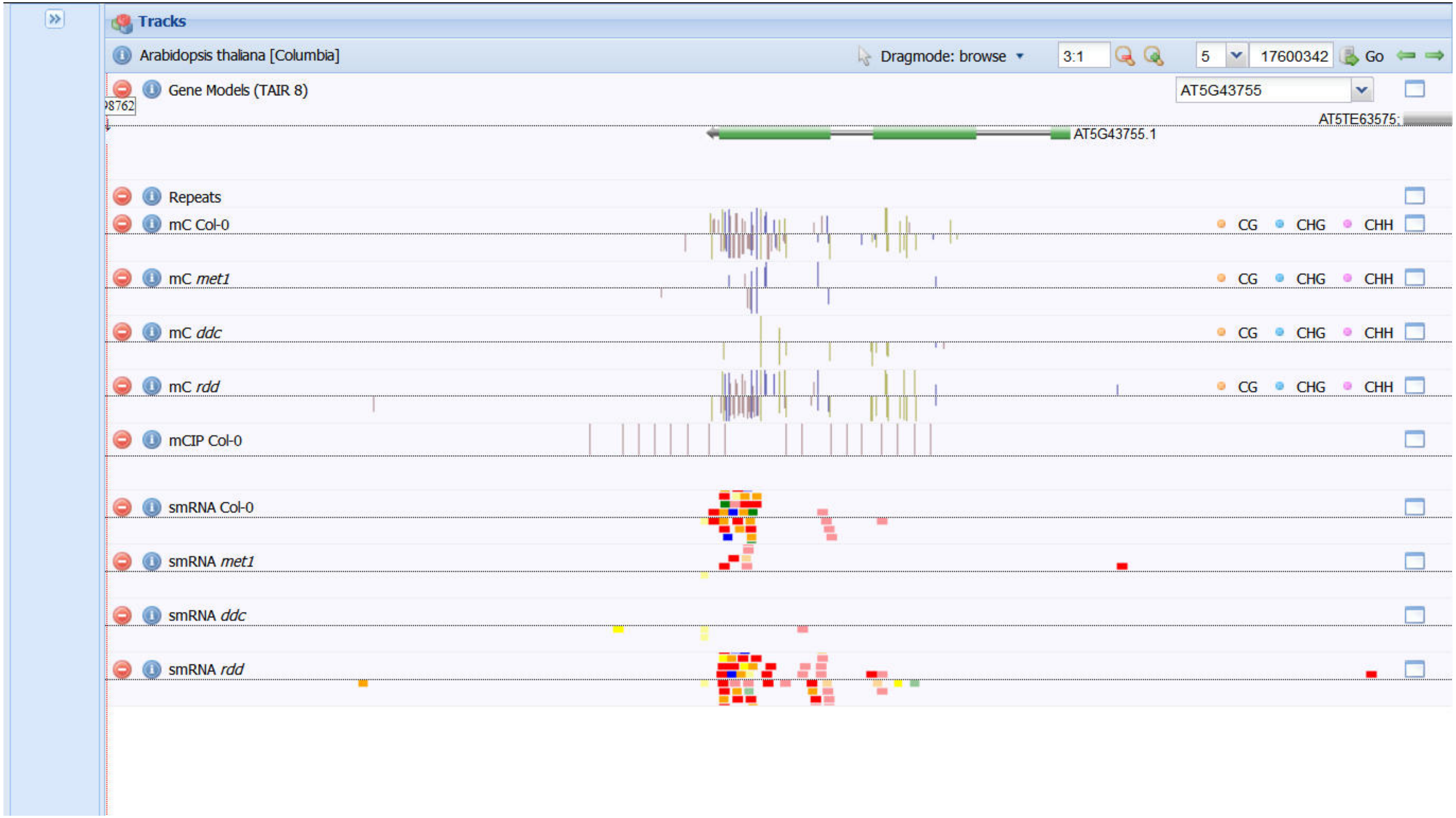
Tracks
Arabidopsis thaliana [Columbia]
Dragmode: browse
3:1
5
17600342
Go
Gene Models (TAIR 8)
AT5G43755
AT5TE63575
AT5G43755.1
Repeats
mC Col-0
mC met1
mC ddc
mC rdd
CG
CHG
CHH
mCIP Col-0
smRNA Col-0
smRNA met1
smRNA ddc
smRNA rdd

AT5G48515

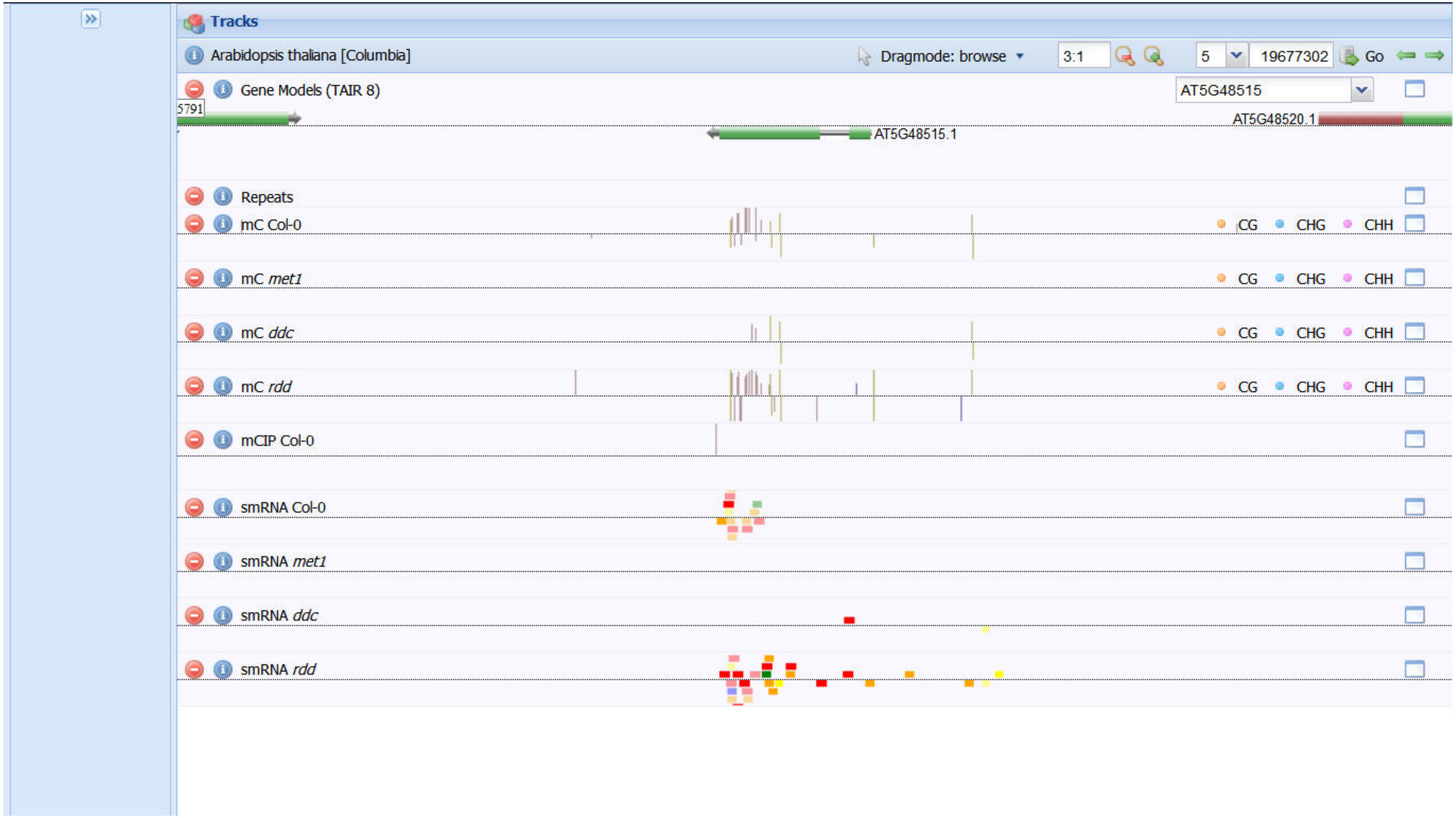

Tracks
Arabidopsis thaliana [Columbia]
Dragmode: browse
3:1
5
19677302
Go
Gene Models (TAIR 8)
AT5G48515
5791
AT5G48520.1
AT5G48515.1
Repeats
mC Col-0
CG CHG CHH
mC *met1*
CG CHG CHH
mC *ddc*
CG CHG CHH
mC *rdd*
CG CHG CHH
mCIP Col-0
smRNA Col-0
smRNA *met1*
smRNA *ddc*
smRNA *rdd*

AT5G50480

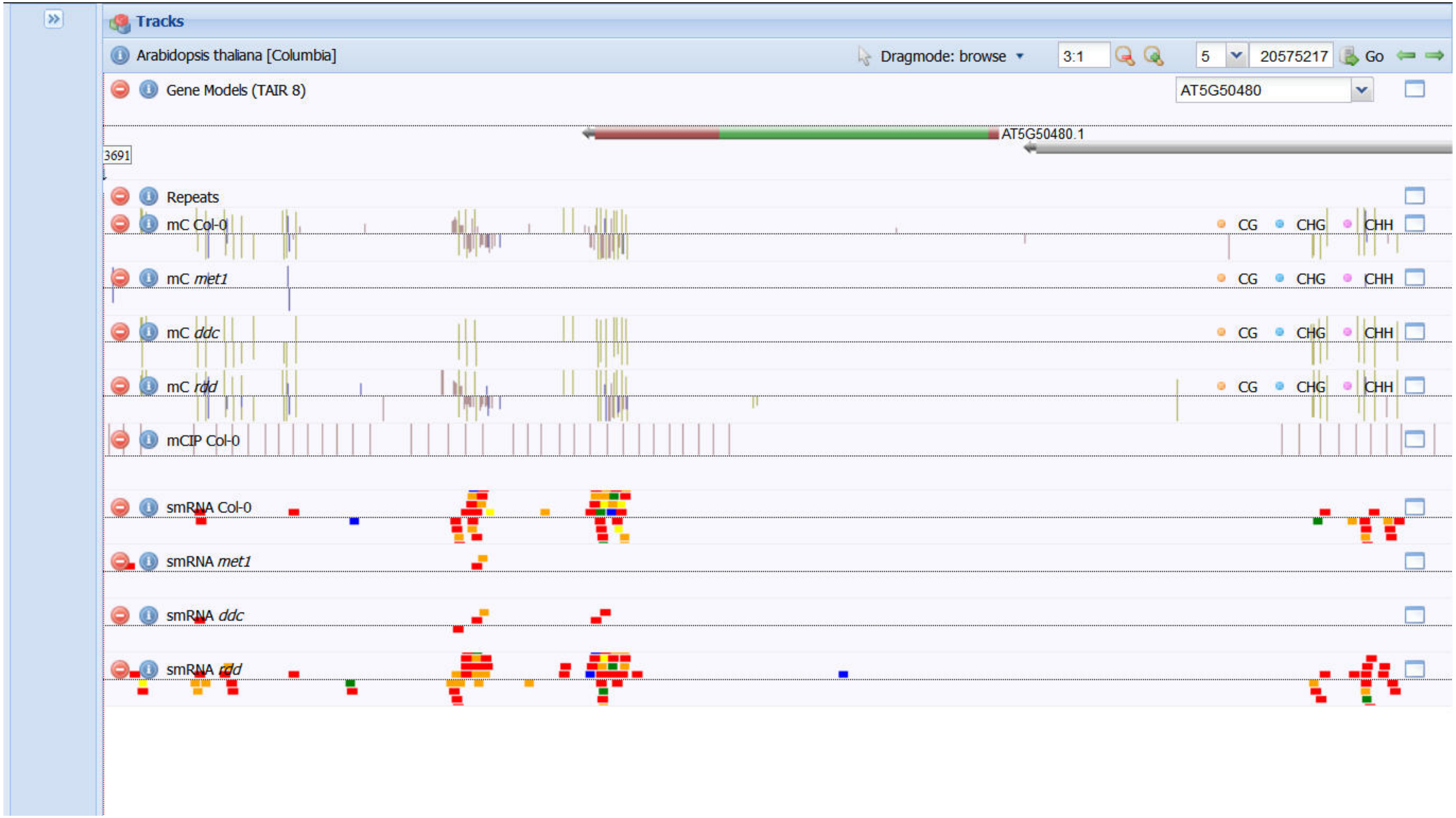

Tracks
Arabidopsis thaliana [Columbia]
Dragmode: browse
3:1
5
20575217
Go
Gene Models (TAIR 8)
AT5G50480
AT5G50480.1
3691
Repeats
mC Col-0
CG
CHG
CHH
mC met1
mC ddc
mC rdd
mCIP Col-0
smRNA Col-0
smRNA met1
smRNA ddc
smRNA rdd

AT5G54700

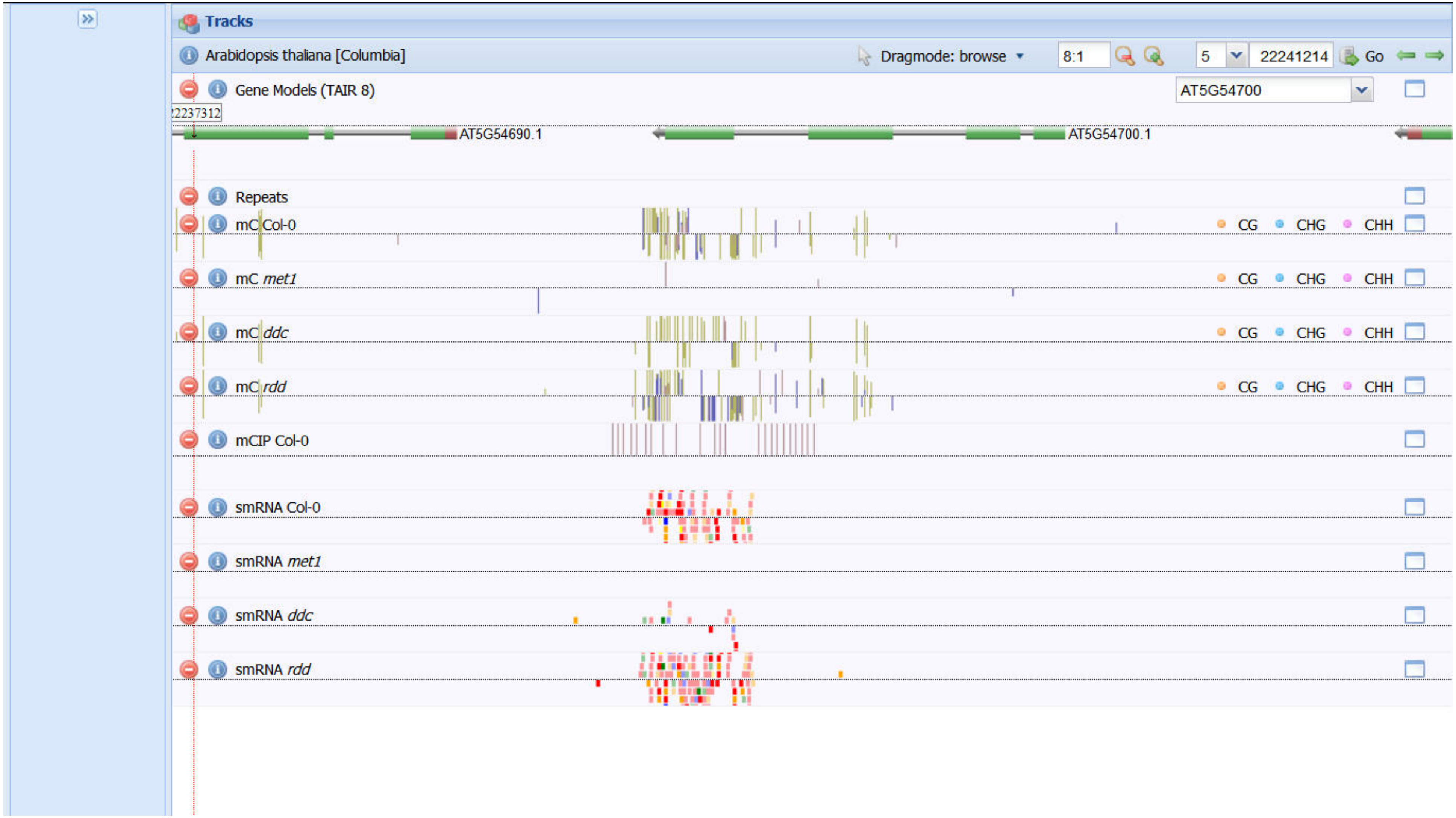

Tracks
Arabidopsis thaliana [Columbia]
Dragmode: browse
8:1
5
22241214
Go
Gene Models (TAIR 8)
AT5G54700
22237312
AT5G54690.1
AT5G54700.1
Repeats
mC Col-0
mC met1
mC ddc
mC rdd
CG
CHG
CHH
mCIP Col-0
smRNA Col-0
smRNA met1
smRNA ddc
smRNA rdd
