# Supplementary material for: Transcriptome-Wide Discovery of PASRs (Promoter-Associated Small RNAs) and TASRs (Terminus-Associated Small RNAs) in Arabidopsis thaliana
Source: PLoS One. 2017 Jan 3;12(1):e0169212. doi: 10.1371/journal.pone.0169212 (PMC5207706; doi:10.1371/journal.pone.0169212)
Supplement: S1 Table — (PDF) [file pone.0169212.s027.pdf]

Table S1 Full list of Argonaute 4 (AGO4)-associated PASR (promoter-associated small RNA) and TASR (termini-associated small RNA) peaks potentially mediating site-specific DNA methylation, and analysis of their biogenesis and action pathways.

	Gene ID	Site-specific methylation[1]	DsRNA-seq coverage[2]	AGO preference[3]	Dependence[4]		
					DCL2/3/4	RDR2/6	Pol IV
PASR peaks_ sense strand	AT1G67450	D[5]	D	AGO4	ND[6]	ND	ND
	AT3G17490	D	ND	AGO4	ND	ND	ND
PASR peaks_ antisense strand	AT2G29460	D	ND	AGO4	ND	ND	ND
Paired PASR peaks	AT1G03810	D	ND	AGO4	ND	ND	ND
	AT1G53265	D	D	AGO4	Repressed in *dcl3* and *dcl234*	Repressed in *rdr2* and *rdr6*	ND
	AT1G60720	D	ND	AGO4	ND	ND	ND
	AT1G61820	D	ND	AGO4	ND	ND	ND
	AT1G66640	D	ND	AGO4	ND	ND	ND
	AT3G05770	D	ND	AGO4	ND	ND	ND
	AT3G19880	D	ND	AGO4	ND	ND	ND
	AT5G24240	D	ND	AGO4	ND	ND	ND
	AT5G43500	D	ND	AGO4	ND	ND	ND
	AT5G48000	D	D	AGO4	Repressed in *dcl234*	Repressed in *rdr2*	Repressed in *nrpd1a* and *nrpd1b*
TASR peaks_ sense strand	AT1G52180	D	D	AGO4	ND	ND	ND
	AT5G36220	D	D	AGO4	ND	ND	ND
TASR peaks_ antisense strand	AT1G59835	D	D	AGO4	ND	ND	ND
	AT1G66540	D	ND	AGO4	ND	ND	ND
	AT2G04090	D	ND	AGO4	ND	ND	ND
	AT2G04830	D	ND	AGO4	ND	ND	ND
	AT5G48605	D	ND	AGO4	ND	ND	ND
Paired TASR peaks	AT1G14580	D	ND	AGO4	ND	ND	ND
	AT1G18770	D	ND	AGO4	ND	ND	ND
	AT1G19830	D	ND	AGO4	ND	ND	ND
	AT1G26762	D	ND	AGO4	ND	ND	ND
	AT1G51150	D	ND	AGO4	ND	ND	ND
	AT1G59885	D	ND	AGO4	ND	ND	ND
	AT1G60986	D	ND	AGO4	ND	ND	ND
	AT1G68040	D	ND	AGO4	ND	ND	ND
	AT3G21870	D	ND	AGO4	ND	ND	ND
	AT3G25130	D	D	AGO4	Repressed in *dcl234*	Repressed in *rdr2*	ND
	AT3G25855	D	ND	AGO4	ND	ND	ND
	AT3G27250	D	ND	AGO4	ND	ND	ND
	AT4G14365	D	D	AGO4	Repressed in *dcl234*	Repressed in *rdr2*	Repressed in *nrpd1a* and *nrpd1b*
	AT4G14940	D	ND	AGO4	ND	ND	ND
	AT4G18690	D	ND	AGO4	ND	ND	ND
	AT4G22650	D	ND	AGO4	ND	ND	ND
	AT4G29305	D	ND	AGO4	ND	ND	ND
	AT4G29740	D	ND	AGO4	ND	ND	ND
	AT5G02990	D	ND	AGO4	ND	ND	ND
	AT5G04950	D	ND	AGO4	ND	ND	ND
	AT5G13825	D	ND	AGO4	ND	ND	ND
	AT5G24290	D	ND	AGO4	ND	ND	ND
	AT5G39720	D	ND	AGO4	ND	ND	ND
	AT5G48515	D	ND	AGO4	ND	ND	ND
	AT5G50480	D	D	AGO4	Repressed in *dcl4* and *dcl234*	Repressed in *rdr2*	Repressed in *nrpd1a* and *nrpd1b*
	AT5G54700	D	D	AGO4	Repressed in *dcl234*	Repressed in *rdr2*	Repressed in *nrpd1a* and *nrpd1b*

[1]Based on the information provided by *Arabidopsis* epigenome maps (http://neomorph.salk.edu/epigenome/epigenome.html), DNA methylation signals were detected on the genomic positions well corresponding to those of the PASR or TASR peaks.

[2]The PASR or TASR peaks were observed to be covered by dsRNA-seq (double-stranded sequencing) reads.

[3]High-throughput sequencing (HTS) data from AGO (Argonaute)-associated small RNA (sRNA) population was utilized, including AGO1 data group and AGO4 data group. A comparison of accumulation levels was made between AGO1 (GSM707682, GSM707683, GSM707684 and GSM707685) and AGO4 (GSM707686, GSM707687, GSM707688 and GSM707689) groups, which facilitated us to deduce the preference of PASRs and TASRs when loading into specific AGO complexes.

[4]Dependence of PASR and TASR biogenesis on the activities of DCL2 (Dicer-like 2), DCL3, DCL4, RDR2 (RNA-dependent RNA

polymerase 2), RDR6, Pol IV (RNA polymerase IV) in *Arabidopsis*. The accumulation of PASRs and TASRs was observed to be repressed in several mutants, such as *dcl3*, *dcl4*, *dcl234* (triple mutant of *DCL2*, *DCL3* and *DCL4*), *rdr2*, *rdr6*, *nrpd1a* (NRPD1 is the subunit of Pol IV), *nrpd1b*.

[5]D: Detected.

[6]ND: Not detected.
