# Supplementary material for: Transcriptome-Wide Discovery of PASRs (Promoter-Associated Small RNAs) and TASRs (Terminus-Associated Small RNAs) in Arabidopsis thaliana
Source: PLoS One. 2017 Jan 3;12(1):e0169212. doi: 10.1371/journal.pone.0169212 (PMC5207706; doi:10.1371/journal.pone.0169212)
Supplement: S2 Table — (PDF) [file pone.0169212.s028.pdf]

Table S2 High-throughput sequencing data sets used in this study.

Data types	GEO accession IDs		Data set descriptions	Reference
sRNA sequencing data	GSE28591	GSM707678	Flowers of wild type plants	[1]
		GSM707679	Leaves of wild type plants	
		GSM707680	Roots of wild type plants	
		GSM707681	Wild type seedlings	
		GSM707682	AGO1-associated sRNAs from flowers of wild type plants	
		GSM707683	AGO1-associated sRNAs from leaves of wild type plants	
		GSM707684	AGO1-associated sRNAs from roots of wild type plants	
		GSM707685	AGO1-associated sRNAs from wild type seedlings	
		GSM707686	AGO4-associated sRNAs from flowers of wild type plants	
		GSM707687	AGO4-associated sRNAs from leaves of wild type plants	
		GSM707688	AGO4-associated sRNAs from roots of wild type plants	
		GSM707689	AGO4-associated sRNAs from wild type seedlings	
	GSE6682	GSM154336	Inflorescences of wild type plants	[2-4]
		GSM154361	Inflorescences of the mutant *dcl1*	
		GSM154362	Inflorescences of the mutant *dcl2*	
		GSM154363	Inflorescences of the mutant *dcl3*	
		GSM154364	Inflorescences of the mutant *dcl4*	
		GSM154365	Inflorescences of the mutant *rdr1*	
		GSM154367	Inflorescences of the mutant *rdr2*	
		GSM154368	Inflorescences of the mutant *rdr6*	
	GSE14695	GSM366868	Whole aerials of wild type plants	[5]
		GSM366869	Whole aerials of the mutant *dcl1*	
		GSM366870	Whole aerials of the triple mutant *dcl2dcl3dcl4*	
	GSE44622	GSM1087973	Flowers of wild type plants, replicate 1	[6]
		GSM1087974	Flowers of wild type plants, replicate 2	
		GSM1087975	Flowers of the mutant *dcl1*, replicate 1	
		GSM1087976	Flowers of the mutant *dcl1*, replicate 2	
		GSM1087977	Flowers of triple mutant *dcl2dcl3dcl4*, replicate 1	
		GSM1087978	Flowers of triple mutant *dcl2dcl3dcl4*, replicate 2	
		GSM1087979	Flowers of the mutant *rdr2*, replicate 1	
		GSM1087980	Flowers of the mutant *rdr2*, replicate 2	
	GSE10180	GSM257235	Flower buds of wild type plants	[7]
		GSM257236	Flower buds of the Pol IV mutant *nrpd1a*	
		GSM257237	Flower buds of the Pol IV mutant *nrpd1b*	
DsRNA-seq data	GSE23439	GSM575243	Ath_col0_dsRNA_1Xribominus	[8]
		GSM575244	Ath_col0_dsRNA_2Xribominus	

[1] Wang H, Zhang X, Liu J, Kiba T et al. Deep sequencing of small RNAs specifically associated with Arabidopsis AGO1 and AGO4 uncovers new AGO functions. Plant J 2011 Jul;67(2):292-304.

[2] Kasschau KD, Fahlgren N, Chapman EJ, Sullivan CM et al. Genome-wide profiling and analysis of Arabidopsis siRNAs. PLoS Biol 2007 Mar;5(3):e57.

[3] Fahlgren N, Howell MD, Kasschau KD, Chapman EJ et al. High-throughput sequencing of Arabidopsis microRNAs: evidence for frequent birth and death of MIRNA genes. PLoS One 2007 Feb 14;2(2):e219.

[4] Howell MD, Fahlgren N, Chapman EJ, Cumbie JS et al. Genome-wide analysis of the RNA-DEPENDENT RNA POLYMERASE6/DICER-LIKE4 pathway in Arabidopsis reveals dependency on miRNA- and tasiRNA-directed targeting. Plant Cell 2007 Mar;19(3):926-42.

[5] Fahlgren N, Sullivan CM, Kasschau KD, Chapman EJ et al. Computational and analytical framework for small RNA profiling by high-throughput sequencing. RNA 2009 May;15(5):992-1002.

[6] Jeong DH, Thatcher SR, Brown RS, Zhai J et al. Comprehensive investigation of microRNAs enhanced by analysis of sequence variants,

expression patterns, ARGONAUTE loading, and target cleavage. Plant Physiol 2013 Jul;162(3):1225-45.
[7] Mosher RA, Schwach F, Studholme D, Baulcombe DC. PolIVb influences RNA-directed DNA methylation independently of its role in siRNA biogenesis. Proc Natl Acad Sci U S A 2008 Feb 26;105(8):3145-50.
[8] Zheng Q, Ryvkin P, Li F, Dragomir I et al. Genome-wide double-stranded RNA sequencing reveals the functional significance of base-paired RNAs in Arabidopsis. PLoS Genet 2010 Sep 30;6(9):e1001141.
